# Benchmarking mutation effect prediction algorithms using functionally validated cancer-related missense mutations

**DOI:** 10.1186/s13059-014-0484-1

**Published:** 2014-10-28

**Authors:** Luciano G Martelotto, Charlotte KY Ng, Maria R De Filippo, Yan Zhang, Salvatore Piscuoglio, Raymond S Lim, Ronglai Shen, Larry Norton, Jorge S Reis-Filho, Britta Weigelt

**Affiliations:** Department of Pathology, Memorial Sloan Kettering Cancer Center, 1275 York Avenue, New York, NY 10065 USA; Department of Epidemiology and Biostatistics, Memorial Sloan Kettering Cancer Center, 1275 York Avenue, New York, NY 10065 USA; Department of Medicine, Memorial Sloan Kettering Cancer Center, 1275 York Avenue, New York, NY 10065 USA

## Abstract

**Background:**

Massively parallel sequencing studies have led to the identification of a large number of mutations present in a minority of cancers of a given site. Hence, methods to identify the likely pathogenic mutations that are worth exploring experimentally and clinically are required. We sought to compare the performance of 15 mutation effect prediction algorithms and their agreement. As a hypothesis-generating aim, we sought to define whether combinations of prediction algorithms would improve the functional effect predictions of specific mutations.

**Results:**

Literature and database mining of single nucleotide variants (SNVs) affecting 15 cancer genes was performed to identify mutations supported by functional evidence or hereditary disease association to be classified either as non-neutral (n = 849) or neutral (n = 140) with respect to their impact on protein function. These SNVs were employed to test the performance of 15 mutation effect prediction algorithms. The accuracy of the prediction algorithms varies considerably. Although all algorithms perform consistently well in terms of positive predictive value, their negative predictive value varies substantially. Cancer-specific mutation effect predictors display no-to-almost perfect agreement in their predictions of these SNVs, whereas the non-cancer-specific predictors showed no-to-moderate agreement. Combinations of predictors modestly improve accuracy and significantly improve negative predictive values.

**Conclusions:**

The information provided by mutation effect predictors is not equivalent. No algorithm is able to predict sufficiently accurately SNVs that should be taken forward for experimental or clinical testing. Combining algorithms aggregates orthogonal information and may result in improvements in the negative predictive value of mutation effect predictions.

**Electronic supplementary material:**

The online version of this article (doi:10.1186/s13059-014-0484-1) contains supplementary material, which is available to authorized users.

## Background

Massively parallel sequencing studies have demonstrated that tumors can be regarded as genetically heterogeneous populations of individual clones that accumulate mutations during the process of tumorigenesis and tumor progression [1]. These mutations, likely the result of genetic instability, may confer a selective growth advantage and be causally implicated in carcinogenesis (that is, driver mutations), or are either selectively neutral (that is, passenger mutations) or deleterious for the cancer cells and eventually purged [2,3].

Recent advances in nucleic acid sequencing technologies now provide the means to explore whole genomes at base-pair resolution [4]. The Cancer Genome Atlas (TCGA), the International Cancer Genome Consortium (ICGC) and endeavors led by individual investigators have demonstrated that the repertoire of genes affected by highly recurrent mutations is limited and that there is a large collection of genes affected by mutations in 1% to 2% of cancers from a given anatomical site [2,4,5]. Although defining driver mutations based on the presence of hotspot mutations and recurrence rates has resulted in the identification of *bona fide* oncogenes and tumor suppressor genes (TSGs) and a partial repertoire of genes significantly mutated in cancer [6-8], this strategy cannot be readily applied to the study of the genes affected by mutations in a minority of tumors of a given anatomical site. In fact, recent studies have demonstrated that some of these mutations are of functional significance and likely constitute *bona fide* drivers, therapeutic targets, or mechanisms of therapy resistance (for example, *ERBB2* and *ESR1* activating mutations in breast cancer) [9-12].

Defining whether a non-hotspot mutation is biologically and/or clinically relevant is by no means a trivial task, in particular for missense mutations, and often laborious functional assays need to be performed [9-12]. Given the vast number of mutations being identified by massively parallel sequencing efforts, finding ways to prioritize which mutations should be subjected to functional analysis is crucial. Computational methods to discern which somatic mutations likely result in amino acid changes that could have biologic implications have been developed [13]. Most of these algorithms rely on the assumption that protein sequences derived from existing living organisms have survived natural selection [14], and many also utilize sequence, structural information, and/or protein annotation (that is, whether a mutation affects an active site, ligand binding domain, disulfide bridges, or protein-protein interactions) to differentiate mutations that result in no or negligible impact on protein function from those that are likely pathogenic. Prediction is feasible because mutations that affect protein function tend to occur at evolutionarily conserved sites [15]. Examples of such computational prediction methods (Additional file 1) are Sorted Intolerant From Tolerant (SIFT) [16], PolyPhen-2 [17], Mutation Assessor [18], CONsensus DELeteriousness score of missense mutations (Condel) [19], Cancer-specific High-throughput Annotation of Somatic Mutations (CHASM) [20], Protein Variation Effect Analyzer (PROVEAN) [14], Functional Analysis Through Hidden Markov Models (FATHMM) [21], Variant Effect Scoring Tool (VEST) [22], MutationTaster [23], Cancer Driver Annotation (CanDrA) [24], and others [25]. Additionally, CHASM, FATHMM, and CanDrA were developed explicitly to differentiate mutations that are likely to constitute cancer drivers from passengers. In particular, FATHMM is a species-independent method, which incorporates pathogenicity weights and is capable of recognizing protein domains (species-independent/evolutionary units) sensitive to missense mutations [21]. CHASM [20] is a machine-learning system trained using the information from the Catalogue Of Somatic Mutations In Cancer (COSMIC) [26] and other cancer-related databases, and utilizes a set of 49 predictive features, including the frequency of a given missense change type in COSMIC. Cancer-Related Analysis of VAriants Toolkit (CRAVAT) is a web-based application for CHASM that provides a simple interface to prioritize genes and variants important for specific cancer tissue types [22]. CanDrA is a support vector machine method that renders predictions from a set of 95 features and scores computed by 10 other prediction algorithms [24]. While most of these predictors are single/independent predictors, Condel and CanDrA make use of scores generated by other algorithms and, therefore, can be considered meta-predictors (Additional file 1).

These predictors provide a fast and inexpensive way to define functional annotation and to predict the effects of mutations, and could theoretically be employed to assist in the selection of mutations that would be worth exploring experimentally and clinically. Different predictors have been designed based on different algorithms (Additional file 1) and, most importantly, were trained using different sets of functional and neutral mutations. As a consequence of the differences in the underlying methodology, these predictors often return dissimilar or even contradictory results [27]. Therefore, we sought to benchmark the performance of 15 mutation effect prediction algorithms comprehensively using a set of missense mutations whose functional effects have been experimentally validated and/or that have been shown to result in early onset breast and ovarian cancer syndrome, Li-Fraumeni syndrome or Li-Fraumeni-like syndrome. To generate a list of neutral and non-neutral mutations, we rigorously compiled a set of mutations in *bona fide* oncogenes, recently described cancer genes and *bona fide* TSGs by mining the literature and mutation databases (see Methods) [28-30]. As a hypothesis-generating aim and using our ‘gold standard’ list of validated mutations, we sought to define whether the mutation effect predictions made by combinations of algorithms would outperform those made by individual predictors or meta-predictors.

## Results

### Categorization of mutations based on functional evidence

We included known missense mutations in six *bona fide* oncogenes (*BRAF*, *KIT*, *PIK3CA*, *KRAS*, *EGFR*, *ERRB2*), six recently described cancer genes (*ESR1*, *DICER1*, *MYOD1*, *IDH1*, *IDH2*, *SF3B1*) and three *bona fide* TSGs (*TP53*, *BRCA1*, *BRCA2*) in this study. We next performed an exhaustive search in the literature and/or existing databases to gather functional evidence for each of the 3,706 mutations compiled for the 15 genes (see Methods; Additional file 2). Given that PolyPhen-2, MutationTaster, CanDrA, and Condel can only define the potential functional impact of single nucleotide variants (SNVs), dinucleotide and trinucleotide changes were excluded from this study. The final dataset employed consists of 3,591 SNVs (Table 1; Additional file 2).

**Table 1:** **Single nucleotide variants included in this study stratified according to the evidence supporting their impact on protein function**

		**Functional categories**		
**Gene**	**Total SNVs (n)**	**Neutral (n)**	**Non-neutral (n)**	**Uncertain (n)**
BRAF	54	0	23	31
BRCA1	505	61	20	424
BRCA2	837	50	12	775
DICER1	81	0	11	70
EGFR	131	0	33	98
ERBB2	75	5	33	37
ESR1	31	0	7	24
IDH1	19	0	1	18
IDH2	15	0	3	12
KIT	89	1	24	64
KRAS	41	0	25	16
MYOD1	11	0	1	10
PIK3CA	139	1	31	107
SF3B1	53	0	7	46
TP53	1,510	22	618	870
Total	3,591	140	849	2,602

A total of 3,591 single nucleotide variants (SNVs) in six *bona fide* oncogenes, six new cancer genes and three *bona fide* tumor suppressor genes were assessed for the evidence supporting a functional role for each of the mutations. These SNVs were classified as non-neutral*,* neutral or uncertain based on direct experimental/functional data in the literature and/or on the basis of causation of Li-Fraumeni syndrome and Li-Fraumeni-like syndrome (for *TP53*) or early onset breast and ovarian cancer syndrome (for *BRCA1* and *BRCA2*), as recorded in dedicated mutation databases [28-30]. For a detailed list, see Additional file 2.

SNVs with experimentally validated effects on the target protein function or proven to be causative of Li-Fraumeni syndrome, Li-Fraumeni-like syndrome, or early onset breast and ovarian cancer syndrome were considered non-neutral, those that have been experimentally validated as non-functional or proven not to be causative of Li-Fraumeni syndrome, Li-Fraumeni-like syndrome, and early onset breast and ovarian cancer syndrome as neutral, and those without definitive experimental validation or considered germline variants of unknown significance were considered uncertain (see Methods).

Using these criteria, 849 SNVs were categorized as non-neutral, 140 SNVs were assigned to the neutral category, and the remaining 2,602 were regarded as uncertain (Table 1; Additional file 2). Of the neutral and non-neutral mutations (n = 989), we collected a median of 28.5 SNVs (range, 23 to 38) for the *bona fide* oncogenes, a median of five SNVs (range, 1 to 11) for the new cancer genes, and a median of 81 SNVs (range, 62 to 640) for the *bona fide* TSGs (Table 1).

### Agreement between mutation effect prediction algorithms

We evaluated 11 single/independent prediction algorithms, namely CHASM (breast), CHASM (lung), CHASM (melanoma) [20], FATHMM (cancer), FATHMM (missense) [21], Mutation Assessor [18], MutationTaster [23], PolyPhen-2 [17], PROVEAN [14], SIFT [16], and VEST [22], and four meta-predictors, namely CanDrA (breast), CanDrA (lung), CanDrA (melanoma) [24], and Condel [19] using the 3,591 SNVs compiled (Additional files 1 and 2). These 15 algorithms returned predictions that were strikingly distinct from one another (Table 2). For instance, VEST predicted 2,465 SNVs to be ‘functional’ and 1,126 to be ‘neutral’, whereas PROVEAN predicted that 1,630 SNVs would be ‘deleterious’ and 1,961 would be ‘neutral’; these discrepancies were also observed when the other mutation effect prediction algorithms were employed. It should be noted that CanDrA appears to be gene- and tissue-specific, as all but one *TP53* SNVs were predicted to be ‘Drivers’ by all CanDrA algorithms (that is, CanDrA breast/lung/melanoma), whereas all *BRCA1* and *BRCA2* SNVs were predicted to be ‘Drivers’ by CanDrA (breast) but almost exclusively ‘Passengers/No-call’ by CanDrA (lung/melanoma) (Additional file 2). To allow comparison between predictors, we converted the calls made by each predictor into ‘neutral’ or ‘non-neutral’ (see Methods).

**Table 2:** **Predictions of 3,591 functionally validated single nucleotide variants by 15 mutation effect prediction algorithms**

**Prediction algorithm**	**Prediction class**	**Functional categories**			**Total**
		**Neutral**	**Non-neutral**	**Uncertain**	
		**(n = 140)**	**(n = 849)**	**(n = 2,602)**	**(n = 3,591)**
CHASM (breast)	Driver	27	764	1,085	1,876
	Passenger	113	85	1,517	1,715
CHASM (lung)	Driver	32	783	1,155	1,970
	Passenger	108	66	1,447	1,621
CHASM (melanoma)	Driver	48	795	1,440	2,283
	Passenger	92	54	1,162	1,308
FATHMM (cancer)	CANCER	71	831	1,655	2,557
	PASSENGER/OTHER	69	18	947	1,034
FATHMM (missense)	Damaging	69	745	1,416	2,230
	Tolerated	71	104	1,167	1,342
	No weights	0	0	19	19
Mutation Assessor	High	2	71	97	170
	Medium	50	579	1,053	1,682
	Low	51	129	919	1,099
	Neutral	37	69	527	633
	N/A	0	1	6	7
MutationTaster	Disease_causing	34	740	1,313	2,087
	Disease_causing_automatic	1	31	4	36
	Polymorphism	99	78	1,285	1,462
	Polymorphism_automatic	6	0	0	6
PolyPhen-2	Probably damaging	40	600	920	1,560
	Possibly damaging	26	115	478	619
	Benign	74	134	1,204	1,412
PROVEAN	Deleterious	43	632	955	1,630
	Neutral	97	217	1,647	1,961
SIFT	Damaging	70	731	1,469	2,270
	Tolerated	70	118	1,133	1,321
VEST	Functional	100	702	1,663	2,465
	Neutral	40	147	939	1,126
CanDrA (breast)	Driver	140	805	2,423	3,368
	Passenger	0	39	140	179
	No-call	0	5	39	44
CanDrA (lung)	Driver	24	767	1,150	1,941
	Passenger	102	59	1,282	1,443
	No-call	14	23	170	207
CanDrA (melanoma)	Driver	28	734	1,147	1,909
	Passenger	97	75	1,260	1,432
	No-call	15	40	195	250
Condel	Deleterious	77	786	1,741	2,604
	Neutral	63	63	861	987

Single nucleotide variants (SNVs) were classified as non-neutral, neutral or uncertain based on functional/experimental data from the literature or mutation databases [28-30]. Each SNV was classified by each of the mutation effect prediction algorithms independently.

**Figure 1:**
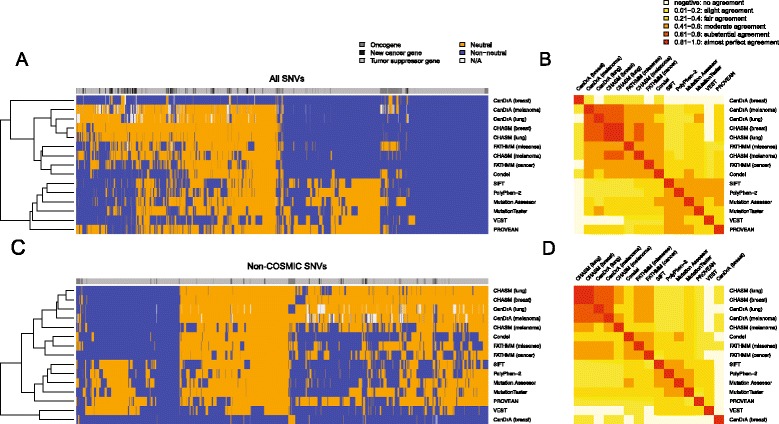
**Inter-rater agreement between 15 mutation effect prediction algorithms.** Hierarchical clustering of the calls made by 15 mutation effect prediction algorithms using **(A)** all 3,591 single nucleotide variants (SNVs) included in this study, and **(C)** the 1,699 SNVs not present in the COSMIC database. The unweighted Cohen’s Kappa coefficient was computed for each pair of predictors using **(B)** all 3,591 SNVs and **(D)** the 1,699 SNVs not present in the COSMIC database. The ranges of unweighted Kappa values and their corresponding colors are indicated in the color key.

To evaluate the inter-rater agreement between prediction methods, we performed unsupervised clustering of the calls made for all 3,591 SNVs by each predictor and calculated pairwise unweighted Cohen’s Kappa coefficients for each pair of predictors. Unsupervised clustering of the results of the mutation effect prediction algorithms revealed two main groups with an additional outlier CanDrA (breast) (Figure 1A). One of the clusters (referred to as ‘Cluster 1’) contained all but one of the cancer-specific predictors, namely CHASM (breast), CHASM (lung), CHASM (melanoma), FATHMM (cancer), CanDrA (lung) and CanDrA (melanoma), and the non-cancer-specific predictor FATHMM (missense) and its related meta-predictor Condel (Figure 1A; Additional file 1). Their pairwise unweighted Kappa coefficients showed fair-to-almost perfect agreement (median unweighted κ = 0.5679, range κ = 0.3861 to 0.9004; Figure 1B; Additional file 3). CanDrA (breast) was the sole cancer-specific predictor that did not belong to this cluster. The best agreement within this group was between CHASM (breast) and CHASM (lung) (κ = 0.9004), which is not surprising considering that they share the underlying prediction engine. The second cluster (referred to as ‘Cluster 2’) was composed of non-cancer-specific mutation effect prediction algorithms, namely Mutation Assessor, MutationTaster, PolyPhen-2, PROVEAN, SIFT, and VEST (Figure 1A). The pairwise agreement between these predictors ranged from fair to moderate (median unweighted κ = 0.4347, range κ = 0.3355 to 0.5662, Figure 1B; Additional file 3). The modest agreement between these predictors was surprising, given that conservation is a feature employed by all algorithms (Additional file 1), and the sole feature employed by Mutation Assessor and SIFT [27]. Overall, the agreement between predictors from distinct clusters ranged from no to fair agreement (median unweighted κ = 0.2062, range κ = -0.0520 to 0.4216, Figure 1B; Additional file 3). CanDrA (breast) was an outlier and displayed no-to-slight agreement with each of the other predictors (Figure 1B; Additional file 3). We repeated the same comparisons employing only the 989 SNVs considered to be non-neutral or neutral on the basis of functional analyses, which revealed similar results, with the exception of CanDrA (breast), which now belongs to Cluster 1 (Additional files 4 and 5).

As the cancer-specific CanDrA, CHASM, and FATHMM (cancer) are all trained using training sets consisting of canonical somatic SNVs and their frequencies, we evaluated the inter-rater agreement of all predictors after excluding from our dataset the SNVs found in the COSMIC database (v68). Of the 3,591 SNVs, 1,699 (47.3%) were not present in COSMIC, of which 297 were experimentally validated as either non-neutral or neutral (Additional files 6 and 7). Akin to the analysis including all SNVs, unsupervised clustering of the predictions made for non-COSMIC SNVs demonstrated that the two main clusters and their compositions were largely maintained (Figure 1C). In this analysis, not only CanDrA (breast) but also VEST emerged as outliers, clustering separately from the two main clusters (Figure 1C). Compared to the Kappa values obtained using all SNVs, when employing only non-COSMIC SNVs, we observed that median Kappa coefficients in Cluster 2 remained largely unchanged, whereas the median unweighted Kappa scores within Cluster 1 decreased from κ = 0.5679 to 0.4558 (Figure 1D; Additional file 3). These data provide evidence to suggest that the agreement between predictors in Cluster 1 is reduced when SNVs present in the COSMIC database were removed given that some mutation effect predictors from Cluster 1 were trained using SNVs included in COSMIC.

It could be hypothesized that the discrepancies in the predictions made by different mutation effect prediction algorithms would predominantly affect SNVs whose classifications are based on predictions of relatively poor confidence. CanDrA (breast), CanDrA (lung), CanDrA (melanoma), PolyPhen-2, and Mutation Assessor have pre-specified categories that identify SNVs whose predictions are based on limited confidence. For the other predictors, we employed a heuristic approach based on the original description of each predictor and additional online sources to define a category of SNVs whose predictions were of poor confidence (see Methods). We classified the 3,591 SNVs included in this study into non-neutral, neutral, and low confidence categories (Additional file 8), and observed that a median of 437 (range, 44 to 1,298) SNVs were classified as of low confidence. CanDrA (breast) classified only 44 SNVs as of low confidence, whereas PROVEAN classified 1,298 in this category. These marked differences may be a mere reflection of the different cutoffs chosen; however, when only the predictors that have a pre-defined low confidence category (that is, CanDrA (breast), CanDrA (lung), CanDrA (melanoma), PolyPhen-2, and Mutation Assessor) were assessed, the number of SNVs classified as such ranged from 44 (CanDrA (breast)) to 1,099 (Mutation Assessor). In fact, only 27 SNVs had a majority vote of low confidence (that is, eight or more predictors classifying a given SNV as of low confidence; Additional file 9). Hierarchical clustering of the predictions for the 3,591 SNVs including a low confidence category revealed a cluster structure similar to that obtained with only non-neutral and neutral categories (that is, Cluster 1 enriched for cancer-specific predictors and Cluster 2 exclusively composed of non-cancer-specific predictors), however PROVEAN clustered in a separate branch from Cluster 2. Noteworthy, the Cohen’s Kappa coefficients were lower than those observed when SNVs were classified into two categories only (that is, as neutral or non-neutral; Additional files 10 and 11). When these analyses were repeated including only the non-COSMIC SNVs, the results of the clustering analysis were similar, however the agreement between predictors was reduced even further (Additional files 10 and 11). By focusing only on the 989 SNVs with functional evidence to classify them as neutral or non-neutral, the addition of a low confidence category resulted again in a similar cluster structure, however PROVEAN was an outlier. This observation was likely due to the fact that 132 of the 989 (13.3%) SNVs were classified as of low confidence by PROVEAN only (Additional file 12). As compared to the Cohen’s Kappa coefficients obtained with two categories, the analysis of agreement between predictors for the classification of these 989 SNVs was generally lower when the low confidence category was included (Additional file 13). Similar results were obtained when the subset of 297 non-COSMIC SNVs were analyzed (Additional files 12 and 13).

### Performance of 15 commonly used mutation effect prediction algorithms

**Figure 2:**
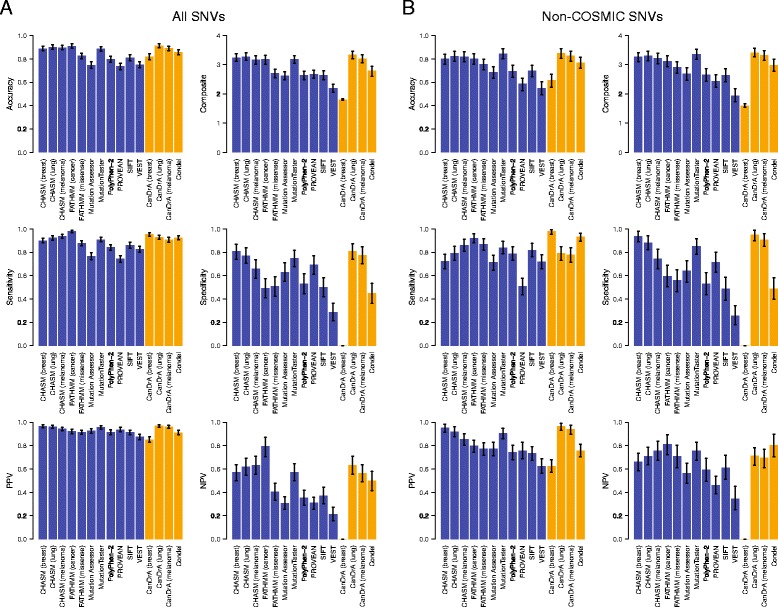
**Performance statistics of mutation effect prediction algorithms.** Based on the prediction results of **(A)** the non-neutral (n = 849) and neutral (n = 140) single nucleotide variants (SNVs) included in this study and **(B)** the non-neutral (n = 188) and neutral (n = 109) SNVs not present in the COSMIC database, the accuracy, sensitivity, specificity, positive predictive value (PPV), negative predictive value (NPV), and composite score for each predictor are plotted. Error bars represent the 95% CIs generated by bootstrapping. Single/independent predictors are shown in blue bars, and meta-predictors are shown in orange bars.

Among the SNVs included in this study, 989 had sufficient functional evidence to support their classification as either non-neutral (n = 849) or neutral (n = 140) with respect to an effect on protein function (Table 1). Hence, the performance of the predictors was assessed using these validated SNVs. Accuracy, specificity, sensitivity, positive predictive value (PPV), negative predictive value (NPV), and composite score were calculated to evaluate the performance of each predictor (see Methods). This analysis revealed that the proportion of SNVs correctly classified by the different predictors varied considerably (median, 85.84%; range, 73.71% to 91.28%; Figure 2A, Table 3). Of the single predictors, FATHMM (cancer) was the most accurate (91.00%, 95% confidence interval (CI) 89.18-92.62%), while PROVEAN was the least accurate (73.71%, 95% CI 70.88-76.64%; Figure 2A, Table 3). The meta-predictors with the highest accuracy (Figure 2A, Table 3) were CanDrA (lung; 91.28%, 95% CI 89.54-93.04%) and CanDrA (melanoma; 88.97%, 95% CI 87.02-90.91%), though their accuracy was not statistically different from that of the best single predictor FATHMM (cancer) in this set of SNVs (*P* >0.05).

**Table 3:** **Performance statistics of mutation effect prediction algorithms using all single nucleotide variants tested functionally (n = 989)**

**Prediction algorithm**	**Accuracy (95% CI)**	**Sensitivity (95% CI)**	**Specificity (95% CI)**	**PPV (95% CI)**	**NPV (95% CI)**	**Composite (95% CI)**
CHASM (breast)	88.68% (86.55-90.70%)	89.99% (87.81-91.92%)	80.71% (73.33-87.12%)	96.59% (95.20-97.73%)	57.07% (50.23-64.58%)	3.2436 (3.1127-3.3669)
CHASM (lung)	90.09% (88.27-91.81%)	92.23% (90.40-94.04%)	77.14% (69.93-83.58%)	96.07% (94.74-97.31%)	62.07% (54.72-69.38%)	3.2751 (3.1418-3.3988)
CHASM (melanoma)	89.69% (87.77-91.51%)	93.64% (92.00-95.37%)	65.71% (57.35-73.51%)	94.31% (92.67-95.76%)	63.01% (55.40-70.92%)	3.1667 (3.0181-3.3079)
FATHMM (cancer)	91.00% (89.18-92.62%)	97.88% (96.87-98.82%)	49.29% (41.08-57.24%)	92.13% (90.26-93.72%)	79.31% (70.59-87.66%)	3.1860 (3.0326-3.3292)
FATHMM (missense)	82.51% (79.98-84.73%)	87.75% (85.46-89.94%)	50.71% (42.98-58.27%)	91.52% (89.53-93.23%)	40.57% (33.53-47.72%)	2.7056 (2.5576-2.8521)
Mutation Assessor	74.70% (71.89-77.51%)	76.65% (73.87-79.65%)	62.86% (54.61-71.21%)	92.59% (90.35-94.55%)	30.77% (25.69-36.70%)	2.6287 (2.4835-2.7842)
MutationTaster	88.57% (86.65-90.60%)	90.81% (88.94-92.84%)	75.00% (66.91-82.00%)	95.66% (94.10-97.00%)	57.38% (50.00-65.14%)	3.1885 (3.0502-3.3300)
PolyPhen-2	79.78% (77.05-82.11%)	84.22% (81.83-86.61%)	52.86% (44.51-61.15%)	91.55% (89.45-93.40%)	35.58% (29.44-42.16%)	2.6420 (2.4895-2.7915)
PROVEAN	73.71% (70.88-76.64%)	74.44% (71.51-77.43%)	69.29% (61.72-76.06%)	93.63% (91.82-95.31%)	30.89% (25.93-36.56%)	2.6825 (2.5505-2.8088)
SIFT	80.99% (78.26-83.42%)	86.10% (83.89-88.45%)	50.00% (41.93-58.40%)	91.26% (89.08-93.15%)	37.23% (30.34-44.28%)	2.6460 (2.4895-2.7968)
VEST	75.03% (72.50-77.76%)	82.69% (80.28-85.24%)	28.57% (21.01-35.40%)	87.53% (85.12-89.75%)	21.39% (15.91-27.57%)	2.2018 (2.0568-2.3395)
CanDrA (breast)	81.81% (79.23-84.26%)	95.38% (93.85-96.81%)	0% (0-0%)	85.19% (82.83-87.30%)	0% (0-0%)	1.8056 (1.7765-1.8340)
CanDrA (lung)	91.28% (89.54-93.04%)	92.86% (91.05-94.62%)	80.95% (73.64-87.32%)	96.97% (95.75-98.03%)	63.35% (56.10-71.01%)	3.3413 (3.2103-3.4747)
CanDrA (melanoma)	88.97% (87.02-90.91%)	90.73% (88.69-92.81%)	77.60% (69.57-84.40%)	96.33% (94.89-97.53%)	56.40% (48.77-63.74%)	3.2105 (3.0705-3.3507)
Condel	85.84% (83.42-88.17%)	92.58% (90.81-94.28%)	45.00% (37.06-53.72%)	91.08% (89.16-92.94%)	50.00% (41.43-59.06%)	2.7866 (2.6222-2.9552)

Based on the prediction results of all non-neutral and neutral single nucleotide variants included in this study, the accuracy, sensitivity, specificity, positive predictive value (PPV), negative predictive value (NPV), and composite score for each predictor were computed. The 95% confidence intervals (CI) generated by bootstrapping are shown in parentheses.

The sensitivity and specificity of the algorithms varied substantially (median, 90.73%; range, 74.44% to 97.88%; median, 62.86%; range, 0% to 80.95%, respectively; Figure 2A, Table 3). The most sensitive single predictor was FATHMM (cancer; 97.88%, 95% CI 96.87-98.82%), which was statistically more sensitive than the most sensitive meta-predictor CanDrA (breast) in this mutation set (95.38%, 95% CI 93.85-96.81%, Figure 2A, Table 3; *P* <0.05). Of the single predictors, CHASM (breast) was the most specific (80.71%, 95% CI 73.33-87.12%) and the most specific meta-predictor was CanDrA (lung; 80.95%, 95% CI 73.64-87.32%), though no statistically significant differences between these were observed in this mutation set (*P* >0.05). Noteworthy, CanDrA (breast) had 0% specificity with the set of SNVs tested, while the other CanDrA predictors (lung/melanoma) achieved >75% specificity, which suggests that the predictions made by CanDrA have a strong dependency on tissue of origin. Although all 15 predictors performed consistently well in terms of PPV (median, 92.59%; range, 85.19% to 96.97%), a dramatic difference in NPV was observed (median, 50.00%; range, 0.00% to 79.31%; Figure 2A, Table 3). In particular, FATHMM (cancer; 79.31%, 95% CI 70.59-87.66%) significantly outperformed all single predictors (*P* <0.05) but CHASM (melanoma; 63.01%, 95% CI 55.40-70.92%) in the set of SNVs tested, and its NPV did not significantly differ from that of the best meta-predictor CanDrA (lung; 63.35%, 95% CI 56.10-71.01%; *P* >0.05; Figure 2A, Table 3).

Intuitively, the best algorithm would have high and balanced values for each of the performance statistics. With this rationale, we calculated a ‘composite score’, ranging from 0 to 4, by summing up the sensitivity, specificity, PPV, and NPV of each predictor as an overall performance indicator for each predictor. The median composite score was 2.7866 (range, 1.8056 to 3.3413; Figure 2A, Table 3). Using this parameter, the best-performing single predictor was CHASM (lung; 3.2751, 95% CI 3.1418-3.3988) but it was not significantly different from the best meta-predictor CanDrA (lung) in this set of SNVs (3.3413, 95% CI 3.2103-3.4747, *P* >0.05, Figure 2A, Table 3).

We next performed the same analysis using only the 297 SNVs not included in the COSMIC database. In this analysis, the median accuracy and sensitivity were 76.77% (range, 54.88% to 84.70%) and 79.26% (range, 51.06% to 97.85%), respectively (Figure 2B, Table 4). The most accurate single and meta-predictors in this context were MutationTaster (84.51%, 95% CI 80.13-88.55%) and CanDrA (lung; 84.70%, 95% CI 80.50-88.65%), respectively (Figure 2B, Table 4). As compared to the analysis including all SNVs, when excluding SNVs present in the COSMIC database, the accuracy of all predictors but Mutation Assessor and MutationTaster were statistically significantly reduced (*P* <0.05). Furthermore, eight of 15 mutation effect prediction algorithms showed statistically significant reduction in sensitivity and 10 of 15 showed a statistically significant reduction in PPV (Figure 2B; Table 4).

**Table 4:** **Performance statistics of mutation effect prediction algorithms using only functionally tested single nucleotide variants not present in the COSMIC database (n = 297)**

**Prediction algorithm**	**Accuracy (95% CI)**	**Sensitivity (95% CI)**	**Specificity (95% CI)**	**PPV (95% CI)**	**NPV (95% CI)**	**Composite (95% CI)**
CHASM (breast)	80.13% (75.42-84.18%)	72.34% (65.70-78.34%)	93.58% (88.89-97.96%)	95.10% (91.27-98.47%)	66.23% (58.62-73.42%)	3.2726 (3.1090-3.4151)
CHASM (lung)	82.49% (78.11-86.53%)	79.26% (73.26-84.95%)	88.07% (81.98-94.18%)	91.98% (87.58-96.20%)	71.11% (63.57-78.74%)	3.3042 (3.1370-3.4665)
CHASM (melanoma)	81.82% (77.44-86.20%)	86.17% (80.93-90.96%)	74.31% (65.71-82.46%)	85.26% (80.21-90.00%)	75.70% (67.29-83.91%)	3.2145 (3.0259-3.3971)
FATHMM (cancer)	80.13% (75.42-84.51%)	92.02% (87.98-95.83%)	59.63% (50.43-69.00%)	79.72% (74.21-84.82%)	81.25% (72.06-89.22%)	3.1263 (2.9242-3.3078)
FATHMM (missense)	75.42% (70.71-79.80%)	86.70% (81.82-91.37%)	55.96% (46.29-64.82%)	77.25% (71.67-82.33%)	70.93% (60.92-80.23%)	2.9085 (2.7014-3.1033)
Mutation Assessor	68.69% (63.64-73.40%)	71.28% (64.77-77.25%)	64.22% (54.46-72.81%)	77.46% (71.26-82.93%)	56.45% (47.82-64.87%)	2.6941 (2.4757-2.8892)
MutationTaster	84.51% (80.13-88.55%)	84.04% (78.53-89.25%)	85.32% (78.18-91.75%)	90.80% (86.05-95.00%)	75.61% (67.42-82.88%)	3.3578 (3.1879-3.5244)
PolyPhen-2	69.36% (64.31-74.41%)	78.72% (73.16-84.53%)	53.21% (43.59-62.50%)	74.37% (68.08-80.21%)	59.18% (49.47-69.01%)	2.6549 (2.4296-2.8672)
PROVEAN	58.59% (52.86-63.64%)	51.06% (43.78-57.73%)	71.56% (62.83-80.00%)	75.59% (68.61-82.81%)	45.88% (38.61-53.76%)	2.4410 (2.2263-2.6521)
SIFT	69.70% (64.65-74.75%)	81.91% (76.09-87.57%)	48.62% (38.89-58.62%)	73.33% (67.31-78.92%)	60.92% (51.14-71.64%)	2.6479 (2.4175-2.8681)
VEST	54.88% (49.49-60.27%)	71.81% (65.80-77.89%)	25.69% (18.01-34.19%)	62.50% (56.34-68.78%)	34.57% (24.99-45.21%)	1.9456 (1.7302-2.1701)
CanDrA (breast)	61.69% (55.89-66.78%)	97.85% (95.50-99.48%)	0% (0-0%)	62.54% (57.04-67.82%)	0% (0-0%)	1.6039 (1.5437-1.6593)
CanDrA (lung)	84.70% (80.50-88.65%)	79.12% (73.21-84.57%)	94.95% (89.81-98.94%)	96.64% (93.29-99.31%)	71.21% (63.41-78.30%)	3.4193 (3.2675-3.5628)
CanDrA (melanoma)	82.61% (77.94-86.64%)	78.09% (71.43-83.82%)	90.82% (85.06-96.00%)	93.92% (89.86-97.33%)	69.53% (61.10-77.05%)	3.3236 (3.1491-3.4780)
Condel	76.77% (72.05-81.48%)	93.09% (89.36-96.32%)	48.62% (39.62-58.33%)	75.76% (70.38-81.03%)	80.30% (70.58-89.55%)	2.9777 (2.7814-3.1877)

Based on the prediction results of non-neutral and neutral single nucleotide variants not present in the COSMIC database, the accuracy, sensitivity, specificity, positive predictive value (PPV), negative predictive value (NPV), and composite score for each predictor were computed. The 95% confidence intervals (CI) generated by bootstrapping are shown in parentheses.

To assess whether the mutation effect prediction algorithms would have different performances when SNVs in *bona fide* oncogenes, *bona fide* TSGs or new cancer genes were considered, we selected from the set of 989 SNVs those found in *bona fide* oncogenes, *bona fide* TSGs or new cancer genes (n = 176, n = 783 or n = 30 SNVs, respectively; Additional file 14). When only SNVs in oncogenes were assessed, FATHMM (cancer) remained the most accurate single predictor (96.59%, 95% CI 93.75-98.86%; Additional files 15 and 16) and CanDrA (lung) remained the most accurate meta-predictor (95.00%, 95% CI 91.36-98.15%; Additional files 15 and 16). When only SNVs affecting TSGs were tested, CHASM (lung) was the most accurate single predictor (92.98%, 95% CI 91.19-94.76%) and CanDrA (melanoma) was the most accurate meta-predictor (93.46%, 95% CI 91.74-95.15%, Additional files 15 and 16). Interestingly, six predictors, namely CHASM (breast), CHASM (melanoma), FATHMM (missense), Mutation Assessor, CanDrA (melanoma), and Condel performed significantly better for SNVs in TSGs as compared to SNVs in oncogenes (*P* <0.05; Additional files 15 and 16). On the other hand, FATHMM (cancer), MutationTaster, PolyPhen-2, PROVEAN, and VEST performed better for SNVs in oncogenes than in TSGs (*P* <0.05). These results suggest that some of the predictors showed preferential performance towards SNVs in either oncogenes or TSGs; alternatively, these differences in performance may stem from the fact that there was a statistically significant difference in the proportion of neutral SNVs in oncogenes as compared to TSGs (Fisher’s exact test, two-tailed, *P* <0.0001). The same comparisons could not be performed for SNVs affecting new cancer genes, as no evidence to support a neutral classification for SNVs affecting these genes was obtained in the literature search, reflecting the relative novelty of these SNVs.

Taken together, these results demonstrate that mutation effect prediction algorithms are not equivalent for the classification of individual SNVs, that the predictions from some algorithms may be tumor tissue dependent, and that some may have a better performance for the identification of neutral than non-neutral SNVs.

### Combination of mutation effect prediction algorithms

To evaluate whether combinations of single predictors would result in an improvement of the predictions made in this dataset, we generated 11,253 combinations by using *n* (*n =2, 3, … 11*) single predictors at a time, with a given mutation being considered non-neutral if at least *p* (*p =1, 2, … 11*) predictors called it non-neutral for all combinations of *n* and *p.* We computed the performance statistics and their confidence intervals for each of these combinations from 1,000 random subsets comprising two-thirds and one-third of the total dataset (referred to as ‘subset 1’ and ‘subset 2’, respectively; Additional file 17). We ranked the combinations based on mean accuracy or based on mean composite score for subsets 1 and 2 separately, and compared their performance to the best-performing single and meta-predictors, respectively. Furthermore, as excluding SNVs not present in the COSMIC database had a significant impact on the performance of many predictors and their pairwise agreement, we also performed the same experiment using only non-COSMIC SNVs (Additional file 18).

**Figure 3:**
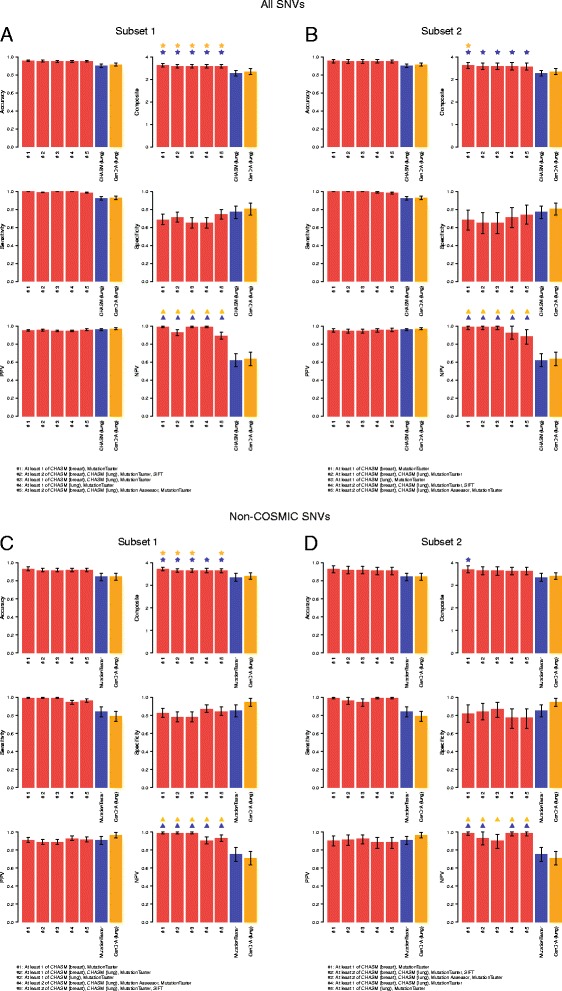
**Performance statistics of the top five mutation effect prediction algorithm combinations as ranked by composite scores.** Prediction results of the non-neutral (n = 849) and neutral (n = 140) single nucleotide variants (SNVs) in the entire dataset **(A, B)** and the non-neutral (n = 188) and neutral (n = 109) SNVs not present in the COSMIC dataset **(C, D)** are shown. Results are ranked according to the composite scores of each mutation effect prediction algorithm combination, and the corresponding accuracy, sensitivity, specificity, positive predictive value (PPV), negative predictive value (NPV), and composite score of the top five prediction algorithm combinations in subset 1 **(A, C)** and subset 2 **(B, D)** are plotted. Error bars represent the 95% CIs generated by 1,000 random samples of subsets 1 and 2. Red bars represent predictor combinations, blue bars single/independent predictors, and orange bars meta-predictors. Blue stars: statistically significant improvement in composite score as compared to that of the best performing single/independent predictor; orange stars: statistically significant improvement in composite score as compared to that of the best performing meta-predictor; blue triangles: statistically significant improvement in NPV as compared to that of the best performing single/independent predictor; orange triangles: statistically significant improvement in NPV as compared to that of the best performing meta-predictor.

Of the 11,253 possible single predictor combinations we evaluated using all SNVs, 1,854 predictor combinations were found to have a numerically higher mean accuracy in subsets 1 and 2 than the most accurate single predictor (that is, FATHMM (cancer)). Six of these combinations were concurrently significantly more accurate than the most accurate single predictor (that is, FATHMM (cancer)) in both subsets (Additional files 19, 20, and 21). When ranking the predictor combinations according to composite score, 1,483 combinations had numerically higher mean composite scores in subsets 1 and 2 than the composite score of the best single predictor (that is, CHASM (lung)); five of these showed statistically significantly higher composite scores than those of CHASM (lung) in both subsets (Figure 3, Additional files 19 and 21). The mutation effect prediction algorithm combination that resulted in a significant increase in both accuracy and composite score as compared to the best single predictor and meta-predictor in both subsets was CHASM (breast) and MutationTaster. This predictor combination called a given SNV non-neutral if at least one of CHASM (breast) and MutationTaster called it non-neutral, and it was ranked first in terms of both accuracy and composite score in subsets 1 and 2 independently (in subset 1: 95.46%, 95% CI 94.54-96.51% and 3.6255, 95% CI 3.5584-3.6982; in subset 2: 95.43%, 95% CI 93.33-97.27%, and 3.6236, 95% CI 3.4841-3.7573, respectively; Additional file 21). Similar observations were made when SNVs found in the COSMIC database were excluded. Only the CHASM (breast) and MutationTaster predictor combination outperformed the most accurate single predictor MutationTaster consistently in both subsets (Figure 3; Additional files 19, 20, and 22).

**Figure 4:**
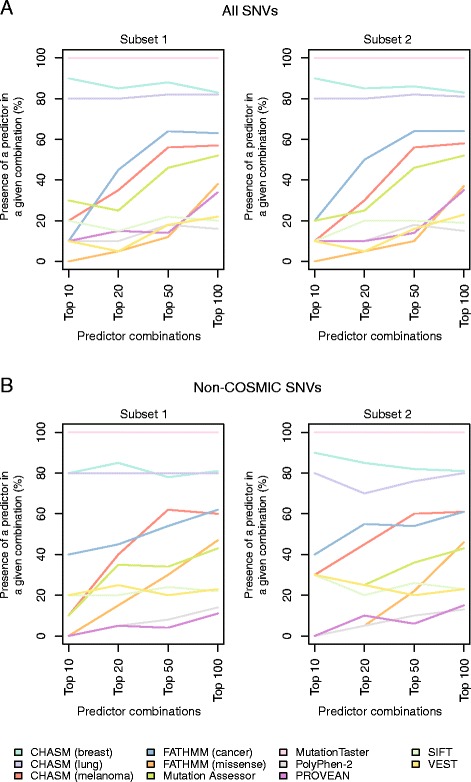
**Recurrence of individual mutation effect prediction algorithms in the top performing mutation effect prediction algorithm combinations ranked by composite score.** The top 10, top 20, top 50, and top 100 combinations of prediction algorithms were defined using the non-neutral (n = 849) and neutral (n = 140) single nucleotide variants (SNVs) included in the entire dataset and ranked according to composite score. The frequency of each single mutation effect predictor present in these top combinations was determined in subset 1 and subset 2 **(A)**. The top 10, top 20, top 50, and top 100 combinations of prediction algorithms were defined using the non-neutral (n = 188) and neutral (n = 109) SNVs not present in the COSMIC database and ranked according to composite score. The frequency of each single mutation effect predictor present in these top combinations was determined in subset 1 and subset 2 **(B)**.

Although mutation effect prediction algorithm combinations had a relatively limited impact on accuracy and composite score, some predictor combinations significantly improved the NPV as compared to the best single and meta-predictor (Figure 3, Additional files 20, 21, and 22). Again, the CHASM (breast) and MutationTaster predictor combination resulted in a significant improvement in NPV as compared to the NPV of the best single predictor or the best meta-predictor in all subsets. When analyzing the top 10, top 20, top 50, and top 100 combinations of mutation effect prediction algorithms, we noted that MutationTaster, CHASM (breast), and CHASM (lung) were consistently present in the top performing predictor combinations in subsets 1 and 2 using the 989 functionally validated SNVs, irrespective of whether the combination predictor performance was ranked according to accuracy or composite score (Figure 4; Additional file 23). When only the non-COSMIC SNVs were included in the analysis, the same mutation effect prediction algorithms were consistently present in the best performing mutation effect prediction algorithm combinations (Figure 4; Additional file 23).

While the most consistently accurate predictor combination called a given mutation non-neutral in this dataset if at least one of CHASM (breast) and MutationTaster called it non-neutral, we also evaluated whether there were optimal combinations of *n* and *p*. In both subsets, for any given *n* (*1 ≤ n ≤4*), the highest accuracy was achieved when *p ≈ 2n* (Additional files 24 and 25 for subset 1; data for subset 2 not shown). Similarly, for any given *p* (*3 ≤ p ≤11*), the optimal *n* was approximately *p/2* (Additional files 24 and 25 for subset 1; data for subset 2 not shown). Similar observations could be made for the results generated by using only the non-COSMIC SNVs (Additional files 26 and 27 for subset 1; data for subset 2 not shown).

Taken together, the combination of mutation effect prediction algorithms resulted in a modest but significant improvement in accuracy and composite score. It should be noted, however, that selected combinations of mutation effect prediction algorithms significantly improved NPV. This information can be instrumental in ruling out SNVs that should not be followed up experimentally and/or clinically, given that SNVs considered neutral by these combinations have a higher probability of being genuinely neutral than those called neutral by single predictors or meta-predictors individually.

## Discussion

Here we demonstrated, by using a set of extensively curated, ‘gold-standard’ list of mutations, that mutation effect prediction algorithms are not equivalent for the classification of individual mutations, and that the agreement between predictors is modest and dependent on the set of mutations and mutation effect type. The agreement between cancer-specific prediction algorithms, which define driver versus passenger mutations, was more consistent than that of non-cancer-specific predictors, which differentiate pathogenic versus non-pathogenic mutations. Furthermore, we observed that the predictions made by some algorithms may be tumor tissue dependent, and that others may have a better performance for the identification of neutral than non-neutral mutations.

A comparative analysis of the functional predictions of 15 commonly used mutation effect prediction algorithms revealed distinct sensitivities, specificities, PPVs and NPVs when a dataset of functionally/experimentally assessed mutations from six *bona fide* oncogenes, six new cancer genes, and three *bona fide* TSGs was tested. For instance, while FATHMM (cancer) had the highest sensitivity (97.88%, 95% CI 96.87-98.82%) in the dataset analyzed, its specificity was limited (49.29%, 95% CI 41.08-57.24%). On the other hand, CHASM (breast) had good specificity (80.71%, 95% CI 73.33-87.12%) but limited NPV (57.07%, 95% CI 50.23-64.58%).

Most of the single predictors and meta-predictors displayed very good PPVs, however their NPVs were found to be relatively low. Using a combination of single prediction algorithms, the combination of CHASM (breast) and MutationTaster resulted in a significant improvement in accuracy as compared to the accuracy obtained with the best single predictor and meta-predictor in the SNV dataset studied, however this increase was modest. Importantly, however, by using mutation effect predictor algorithm combinations, we achieved substantial statistically significant improvements in NPV. Different combinations of individual predictors including CHASM (breast) and MutationTaster were repeatedly found to have a significantly higher NPV than the best single predictor and the best meta-predictor in this dataset, while at least maintaining equivalent accuracy and composite score. In the effort to sift through lists of mutations to identify biologically interesting candidates to take forward for functional experiments, NPV is an often-overlooked measure. A high NPV allows for the exclusion of passenger or neutral alterations with greater confidence, without the risk of losing truly pathogenic mutations called neutral/passenger by a given algorithm.

Our analysis further revealed that some mutation effect prediction algorithms are dependent on the type of gene altered. In particular for the case of CanDrA meta-predictor, as all but one *TP53* SNVs were predicted to be ‘drivers’ by all CanDrA algorithms (that is, CanDrA breast/lung/melanoma), whereas all *BRCA1* and *BRCA2* mutations were predicted to be ‘drivers’ by CanDrA (breast) but almost exclusively ‘passengers/no-call’ by CanDrA (lung/melanoma). This suggests that some predictors are highly tissue-specific and users ought to employ predictors appropriate for the tumor tissue type analyzed.

Our study has several limitations, despite using a set of curated mutations in *bona fide* oncogenes, new cancer genes, and *bona fide* TSGs. First, the dataset we employed has a limited size, and neutral mutations were largely derived from TSGs, in particular *BRCA1* and *BRCA2*, which may have caused biases in the estimation of specificity and NPV. Importantly, however, unlike previous comparisons of mutation effect prediction algorithms, this study has employed rather rigorous standards to define the mutation set to be analyzed, by leveraging functional evidence from an extensive literature search and database mining. Second, mutations may be context-dependent, in that they would only elicit a phenotype under particular circumstances, such as genetic background. Hence, the number of mutations considered of unknown or indeterminate significance was high. Third, a substantial number of neutral and non-neutral mutations was obtained from datasets related to the impact of germline mutations. The actual impact of those mutations when they are found as somatic genetic alterations would require further investigation. Fourth, given that some predictors display distinct performances according to the tumor tissue type and that some SNVs included in this dataset are preferentially found in specific tumor types (for example, *BRCA1* and *BRCA2* mutations are more frequently found in breast and ovarian cancers), this dataset could theoretically favor mutation effect prediction algorithms that are breast cancer-specific. This was not observed, however, given that the best performing single predictors and meta-predictors were not breast cancer-specific. Fifth, the agreement between mutation effect prediction algorithms employing a low confidence category in addition to the neutral and non-neutral categories of SNVs is dependent on the cut-points chosen to define predictions of low confidence. It should be noted, however, that similar results for prediction algorithms with a pre-defined low confidence category were observed as compared to those where a low confidence category was defined employing a heuristic approach based on the original descriptions and online sources of the predictors. Sixth, CHASM (breast/lung/melanoma), FATHMM (cancer/missense), and PolyPhen-2 are algorithms that employed training sets in their development and validation. Given the approach employed to create the ‘gold standard’ dataset used in this study, overlaps between our ‘gold standard’ dataset and the training sets employed for the development of these algorithms were inevitable. To minimize potential biases, we have performed all analyses after the removal of all functionally curated SNVs present in COSMIC; however, even after taking this step, a residual number of SNVs present in the original training sets remained (Additional file 28). When we compared the performance of these mutation effect predictors after the removal of SNVs present in COSMIC or in the original training sets, we either observed no significant differences in their accuracy or a lower accuracy when COSMIC SNVs were removed (Additional file 29). Finally, although experimental validation is informative, it is not often definitive. In particular for neutral effects, it is plausible that the results of such experiments are context dependent (that is, cell line or organism employed and the constellation of mutations already present in a given model). For instance, the true effect of some mutations may be conditioned by the genetic make-up (that is, quantitative trait loci, epistasis) [31,32], be only effective in a particular developmental stage [33], or be species-specific [34]. On the other hand, non-neutral mutation effects do not necessarily imply causality with regards to an organismal level phenotype; many dozens of loss-of-function variants exist in healthy humans [35].

## Conclusions

Our study demonstrates that the challenges researchers face at the time of analyzing massively parallel sequencing data to identify variants for further experimental studies are genuine. The information provided by mutation effect prediction algorithms is not equivalent. None of the algorithms analyzed here was found to deliver optimal accuracy, sensitivity, specificity, PPV, and NPV in the mutation dataset studied. Mutation effect predictors are not equivalent for the classification of individual mutations. The performance of some of these predictors may be dependent on tumor tissue and mutation type (that is, canonical versus non-canonical mutations, neutral versus non-neutral mutations). Combinations of mutation effect predictors, albeit providing only modest but significant improvements in the overall accuracy when compared to individual predictors or meta-predictors, were found to result in substantially improved NPVs without compromising accuracy.

## Materials and methods

### Mutation sets

To standardize the procedure of compiling mutations that can be employed for the benchmarking of mutation effect predictors, mutations affecting six *bona fide* oncogenes (*BRAF*, *KIT*, *PIK3CA*, *KRAS*, *EGFR*, and *ERRB2*), whose mutations preferentially affect kinase domains, six recently described cancer genes (*DICER1*, *ESR1*, *IDH1*, *IDH2*, *MYOD1*, and *SF3B1*), whose mutations do not affect kinase domains, and three *bona fide* TSGs (*TP53*, *BRCA1*, and *BRCA2*) were retrieved from the TCGA Pan-Cancer dataset by Kandoth *et al*. [36] and from studies functionally testing mutations affecting these genes (Additional file 2). In addition, for TSGs, specific databases were employed; for *TP53*, the IARC database [29,37], and for *BRCA1* and *BRCA2*, the Universal Mutation Database (UMD) [28,38,39]. This mining exercise resulted in the identification of 3,706 mutations, of which 3,591 were SNVs (Table 1, Additional file 2). Given that some mutation effect prediction algorithms (that is, PolyPhen-2, MutationTaster, CanDrA, and Condel) do not process dinucleotide or trinucleotide missense mutations, and to have the same number of mutations successfully analyzed by each predictor, we have only included SNVs for the purpose of creating a mutation dataset to benchmark mutation effect predictors. SNVs were also annotated based on their presence in the COSMIC dataset v68 [26].

### Literature search

Literature search was performed by four of the authors (LGM, MRDF, YZ, SP) to identify experimental evidence of functional effects of each mutation. This strategy entailed the use of Boolean logic in combination with search engines such as PubMed, ScienceDirect, Google Scholar, and MEDLINE. Search terms were combined using Boolean (that is, AND, OR) and Adjacency (that is, NEAR) operators to create search statements as well as to narrow and refine the search (for example, *BRAF* AND V600E AND validation/function, *ERBB2* AND mutation NEAR kinase domain AND validation/function). In addition, the references listed in the papers found were also scrutinized to aid the search of additional literature in support of the findings. For TSGs, in addition to literature search, the IARC *TP53* functional assessment dataset [29], UMD-BRCA1, and UMD-BRCA2 mutation databases [28] were employed to ascertain whether specific missense mutations in *TP53* would be causative of Li-Fraumeni or Li-Fraumeni-like syndrome or have been functionally assessed, and mutations in *BRCA1* and *BRCA2* would be causative of early onset breast and ovarian cancer syndrome, respectively (see below).

### Oncogenes and new cancer genes

The six *bona fide* oncogenes, namely *BRAF*, *KIT*, *PIK3CA*, *KRAS*, *EGFR*, and *ERRB2* and the six new cancer genes, namely *DICER1*, *ESR1*, *IDH1*, *IDH2*, *MYOD1*, and *SF3B1* used for the present analysis were selected on the basis of the presence and the absence of a kinase domain, respectively, and the availability of studies investigating functionally the impact of mutations affecting these genes. In this literature search, direct functional evidence to determine whether a mutation was neutral (that is, passenger) or non-neutral (that is, pathogenic) was sought. The functional impact of SNVs affecting these 12 genes was categorized into six groups, namely: (1) Change in kinase, GTPase, or other enzymatic activity (for example, RNase); (2) Effect in response to ligand/substrate, impact on downstream effectors/pathways, or cell proliferation/survival, differentiation, apoptosis; (3) Ability to immortalize or transform human or murine cells (for example, MCF10A, BaF3, NIH3T3 cell lines) and/or anchorage-independent growth; (4) Response to specific chemical/biological compounds, therapeutic agents, or temperature; (5) Tumor growth/induction *in vivo* (for example, xenografts, mouse/fish models), or changes in the rates of progression-free or overall survival in pre-clinical models; and (6) Changes in genome (that is, aneuploidy), epigenome (that is, methylation), transcriptome (that is, splicing), miRNA biogenesis, or DNA/RNA binding affinity (Additional file 2). SNVs affecting these genes were considered non-neutral if there was literature evidence to support their impact on at least one of the mentioned categories. When the functional testing demonstrated no significant impact on the wild-type function of the protein in at least one functional category, and/or no evidence was found for other categories, the SNVs were classified as neutral mutations. SNVs for which no reliable functional evidence or conflicting evidence was found for any of the six categories, were regarded as uncertain.

### Tumor suppressor genes (TSGs)

The IARC datasets ‘TP53 germline mutations and family history’ and ‘Functional assessment of p53 mutant proteins in various experimental assays’ (R17) [29,37] were employed to ascertain whether specific mutations affecting *TP53* would be associated with the development of Li-Fraumeni syndrome or Li-Fraumeni-like syndrome, and/or whether specific *TP53* SNVs would result in conserved wild-type function, loss of function, dominant negative activity, gain of function, and/or temperature sensitivity in various systems and cell lines [29,37]. *TP53* mutations strictly associated with Li-Fraumeni syndrome or Li-Fraumeni-like syndrome, and present in patients without additional germline mutations affecting cancer causing genes (for example, *PTEN*, *BRCA1*, or *BRCA2*), and/or meeting at least one of the above functional levels of evidence were considered non-neutral, whereas those mutations lacking an association with Li-Fraumeni syndrome or Li-Fraumeni-like syndrome or a functional impact were considered neutral. The remaining *TP53* SNVs were considered uncertain. For *BRCA1* and *BRCA2*, the UMD-BRCA1 (4 February 2014 update) and UMD-BRCA2 (22 January 2014 update) mutation databases [28,38,39] were used to collect information on specific *BRCA1* and *BRCA2* mutations. Mutations with validated functional evidence classified as ‘5 - Causal’ in the database were classified as non-neutral in this study, ‘1 - Neutral’ in the database were classified as neutral in this study, and ‘4 - Likely causal’, ‘3 - UV’, and ‘2 - Likely neutral’ in the database were classified as uncertain in this study.

### Assessment of mutation effect predictors

We first tested the set of 3,591 SNVs using 15 prediction algorithms, including 11 single predictors and four meta-predictors or consensus classifiers (Additional file 1) using default settings. The single predictors included PROVEAN (v1.1.3) [14] and SIFT (Ensemble 66) [16] from [40], which used the ‘PROVEAN Human Genome Variants’ and provided both PROVEAN and SIFT results (default prediction cutoff of -2.5 and 0.05, respectively). For PolyPhen-2 (v2.2.2) [17], we employed the Batch Query Data tool from [41], using the ‘HumDiv’ classifier model (default prediction categories). We used the downloadable version of CHASM (v1.0.7) [20] employing a cutoff of 0.3, which was selected as the approximate point of intersection of the distributions of scores for the drivers and passengers from the original study [20]. SNVs with scores ≤0.3 were classified as ‘drivers’ and those with scores >0.3 were classified as ‘passengers’. For CHASM, we selected classifiers specific to breast cancer, melanoma and lung adenocarcinoma, referred to as ‘CHASM (breast)’, ‘CHASM (melanoma)’, and ‘CHASM (lung)’, respectively. We obtained VEST (v3.0) [22] predictions from [42], employing a cutoff of 0.5. For VEST, given that no particular threshold was recommend by the authors, the cutoff for this study was selected to balance false-positive and false-negative rates based on simulations from the original study [22]. SNVs with scores ≥0.5 were considered ‘functional’ and those with scores <0.5 were considered ‘neutral’. We ran Mutation Assessor (release 2) [18] and MutationTaster (24 July 2014 update) [23] from [43,44], respectively, following the instructions for data input. For FATHMM ([45], v2.3) [21], we used both ‘Inherited Disease’ tool (prediction algorithm: weighted; phenotypic associations: disease ontology, default prediction categories; referred to as ‘FATHMM (missense)’) and ‘Cancer’ tool (default prediction threshold -0.75, referred to as ‘FATHMM (cancer)’), as previously described [21].

The meta-predictors tested in this study included Condel (db version 05) [19] and CanDrA (v1.0) [24]. For Condel, we employed the website [46] following instructions for data input (default prediction categories). For CanDrA [24], we downloaded and installed the executable package and associated annotation data files for breast cancer (‘CanDrA (breast)’), lung adenocarcinoma (‘CanDrA (lung)’), and melanoma (‘CanDrA (melanoma)’) from [47] and performed the classifications as described (default prediction categories) [24].

For the purposes of assessing performance and comparing predictors, some of the categories returned by the predictors were merged. For PolyPhen-2, ‘probably damaging’ and ‘possibly damaging’ were considered non-neutral. For Mutation Assessor, ‘high’ and ‘medium’ were considered non-neutral and ‘low’ and ‘neutral’ were considered neutral. For FATHMM (cancer), ‘CANCER’ was considered non-neutral and ‘PASSENGER/OTHER’ was considered neutral. For MutationTaster, ‘disease_causing’ and ‘disease_causing_automatic’ were considered non-neutral and ‘polymorphism’ and ‘polymorphism_automatic’ were considered neutral. For the remaining predictors, ‘damaging’, ‘functional’, ‘driver’, and ‘deleterious’ were considered non-neutral and ‘tolerated’, ‘neutral’, ‘passenger’, and ‘benign’ were considered neutral. For CanDrA, ‘no-call’ was considered equivocal.

We next tested the set of 3,591 SNVs using 15 prediction algorithms by introducing a prediction category of low confidence. CanDrA (breast/lung/melanoma) (‘no-call’), PolyPhen-2 (‘possibly damaging’), and Mutation Assessor (‘low’) have pre-specified categories that identify SNVs whose predictions are based on limited confidence. For the other predictors, we employed a heuristic approach based on the original description of each predictor and additional online sources to define a category of SNVs whose predictions were of low confidence. For CHASM (breast/lung/melanoma), based on the histograms of CHASM scores for driver mutations and passenger mutations described in the original study [20], we called predictions with scores between 0.25 and 0.35 as low confidence. For FATHMM (cancer), based on the interactive prediction threshold graph (online documentation, [48]), prediction scores between -1.65 and 0.1 were considered as low confidence. For FATHMM (missense), based on the distribution of the predicted magnitude of effect for disease-associated and functionally neutral mutations using the weighted method in the original study [21], the region with the highest overlap of the disease-associated and functionally neutral scores (that is, between -2.5 and 0) was classified as low confidence. For MutationTaster, we considered predictions with probabilities of ≤95% as low confidence. For PROVEAN, based on the stringency score thresholds described in the online documentation [49], predictions with scores between -4.1 and -1.3 were considered low confidence. For SIFT, given the lack of reported stringency score thresholds in the original study [16] or in the online documentation, low confidence prediction scores between 0.04 and 0.1 were selected based on the distribution of SIFT scores in our dataset. In brief, these cutoffs were chosen on the basis of a density plot of the SIFT scores generated with the results from our dataset, which displayed a non-parametric distribution (left skewed) with a mode centered around 0.005 and a second much smaller mode centered around 0.999. For VEST, based on the density plots created from VEST score distributions reported in the original study [22], predictions with scores between 0.45 and 0.55 were considered low confidence. For Condel, based on the density of scores for the known neutral and deleterious mutations as reported in the online documentation [50] the region with the highest overlap of the neutral and deleterious mutation scores (that is, between 0.489 and 0.547) was classified as low confidence.

The mutation effect prediction algorithms were assessed using either all SNVs included in this study (n = 3,591) or using only the neutral and non-neutral SNVs (n = 989). In addition, we performed the analyses of these two sets of SNVs by removing either all SNVs present in the COSMIC dataset v68 (n = 1,699 and n = 297, respectively) or by removing all SNVs present in the training sets of CHASM (breast/lung/melanoma), FATHMM (cancer), FATHMM (missense) or PolyPhen-2. For CHASM (breast/lung/melanoma), training sets were retrieved from the drivers.tmps, null.tmps and passengers.tmps of the respective built classifiers [51] (Additional file 28). For FATHMM (cancer) and FATHMM (missense), training sets were retrieved from [52] (Additional file 28). For PolyPhen-2, training sets were retrieved from [53] (Additional file 28).

### Analysis of agreement between mutation effect predictors

We converted the calls made by each predictor into neutral and non-neutral (as described above) and performed hierarchical clustering using complete linkage and Hamming distance metric. We assessed the agreement between the predictors using unweighted Cohen’s Kappa coefficient with 95% confidence intervals (CIs). Inter-rater agreement was tested to determine the agreement between different mutation effect prediction algorithms. We considered Kappa coefficients <0 to be no agreement, between 0.01 and 0.20 to be slight agreement, between 0.21 and 0.40 to be fair agreement, between 0.41 and 0.60 to be moderate agreement, between 0.61 and 0.80 to be substantial agreement, and between 0.81 and 1 to constitute almost-perfect agreement [54].

### Assessment of mutation effect predictor performance

The performance of each predictor was evaluated based on the concordance with our established, functionally validated neutral and non-neutral mutation categories. We evaluated the accuracy, sensitivity, specificity, positive predictive value (PPV), negative predictive value (NPV) for each predictor, and a composite score, ranging from 0 to 4, defined as the sum of sensitivity, specificity, PPV, and NPV. For the analyses performed, sensitivity measures the proportion of experimentally validated non-neutral mutations that were correctly identified, that is,

$$ sensitivity=\frac{\mathrm{the}\ \mathrm{number}\ \mathrm{of}\ non\mathit{\hbox{-}} neutral\ \mathrm{mutations}\ \mathrm{correctly}\ \mathrm{classified}\ \mathrm{a}\mathrm{s}\ non\mathit{\hbox{-}} neutral\ }{\mathrm{the}\ \mathrm{number}\ \mathrm{of}\ \mathrm{a}\mathrm{ll}\ \mathrm{experimentally}\ \mathrm{validated}\ non\mathit{\hbox{-}} neutral\ \mathrm{mutations}} $$

whereas specificity measures the proportion of experimentally validated neutral mutations that were correctly identified, that is,

$$ specificity = \frac{\mathrm{the}\ \mathrm{number}\ \mathrm{of}\  neutral\ \mathrm{mutations}\ \mathrm{correctly}\ \mathrm{classified}\ \mathrm{a}\mathrm{s}\  neutral}{\mathrm{the}\ \mathrm{number}\ \mathrm{of}\ \mathrm{a}\mathrm{ll}\ \mathrm{experimentally}\ \mathrm{validated}\  neutral\ \mathrm{mutations}} $$

PPV is defined as the proportion of mutations predicted to be non-neutral that were experimentally validated as non-neutral, that is,

$$ PPV = \frac{\mathrm{the}\ \mathrm{number}\ \mathrm{o}\mathrm{f}\ non\mathit{\hbox{-}} neutral\ \mathrm{mutations}\ \mathrm{correctly}\ \mathrm{classified}\ \mathrm{a}\mathrm{s}\ non\mathit{\hbox{-}} neutral}{\mathrm{the}\ \mathrm{number}\ \mathrm{o}\mathrm{f}\ \mathrm{mutations}\ \mathrm{predicted}\ \mathrm{t}\mathrm{o}\ \mathrm{be}\ non\mathit{\hbox{-}} neutral} $$

and NPV is the proportion of mutations predicted to be neutral that were experimentally validated as neutral, that is,

$$ NPV = \frac{\mathrm{the}\ \mathrm{number}\ \mathrm{o}\mathrm{f}\  neutral\ \mathrm{mutations}\ \mathrm{correctly}\ \mathrm{classified}\ \mathrm{a}\mathrm{s}\  neutral}{\mathrm{the}\ \mathrm{number}\ \mathrm{o}\mathrm{f}\ \mathrm{mutations}\ \mathrm{predicted}\ \mathrm{t}\mathrm{o}\ \mathrm{be}\  neutral\ } $$

95% CIs for each of these measures were generated by performing resampling with replacement (that is, bootstrapping) for 1,000 iterations. For the CIs generated from bootstrapping, if CIs touch or do not overlap, the difference is considered statistically significant as the significance levels satisfies *P* <0.05 [55]. Briefly, if the two standard errors are *se*_*1*_ and *se*_*2*_, then the standard error of the difference is $\sqrt{s{e_1}^2+s{e_2}^2}$ and the difference between the means is 2(*se*_1_ + *se*_2_), hence the *P* value can be calculated from $z=\frac{2\left(s{e}_1+s{e}_2\right)}{\sqrt{s{e_1}^2+s{e_2}^2}}$. This approach has been shown to be valid for a range of values and successfully employed in a previous study [55].

### Combination of mutation effect predictors

To assess the effect of combining single predictors, we made use of the computed categories of individual predictors. For each possible combination of the 11 single predictors using *n* (*n =2, 3, … 11*) predictors at a time, a mutation would be considered non-neutral if at least *p* (*p =1, 2, … n*) predictors considered it non-neutral. To test the impact of the combinations of predictors, we used a split-sample approach by dividing the dataset of functionally validated mutations randomly into two sub-datasets (that is, subsets), each consisting of two-thirds (‘subset 1’) and one-third (‘subset 2’) of the mutations included in the entire dataset. We computed performance statistics (accuracy, sensitivity, specificity, PPV, NPV, and composite score) for each combination of *n* and *p*, resulting in 11,253 unique combinations. To define the confidence intervals for the predictions, we repeated the splitting of the dataset 1,000 times to create 1,000 random splits of the dataset and computed the performance statistics for each iteration. Separately for subsets 1 and 2, we ranked the predictor combinations based on their accuracy or their composite scores. Differences in the performance statistics were considered statistically significant if their confidence intervals touched or did not overlap (see above).

## Additional files

Additional file 1:
**Overview of computational mutation effect prediction algorithms analyzed in this study.** Prediction algorithms in blue boxes represent single/independent predictors, those in black boxes meta-predictors. Arrows between predictors indicate dependency, such that predictions made by the predictor at the tail of the arrows are integrated by the predictor at the head of the arrows. Predictors are annotated based on the features used in the algorithms. Features are divided into four categories: C: conservation, such as conservation scores and homology search; S: protein structure, such as secondary and tertiary structures and accessible surfaces; A: protein sequence annotation, such as annotation information from Uniprot and Pfam; and F: mutational frequency from databases such as COSMIC, HapMap, and Human Gene Mutation Database. The version of each algorithm included in this study is described in each box. Additional file 2:
**List of all mutations included in this study.** This table lists all 3,706 missense mutations included in the study, their classification using 15 mutation effect prediction algorithms, their experimental functional categories, the evidence supporting the experimental functional classification, and their association with hereditary diseases (*TP53*, *BRCA1*, and *BRCA2*). Additional file 3:
**Inter-rater agreement of mutation effect prediction algorithms as defined by unweighted Cohen’s Kappa coefficients for all 3,591 single nucleotide variants and all 1,699 non-COSMIC single nucleotide variants included in the dataset.**
Additional file 4:
**Inter-rater agreement between 15 mutation effect prediction algorithms for the single nucleotide variants for which functional data are available.** Hierarchical clustering of the calls made by 15 mutation effect prediction algorithms using **(A)** all 989 single nucleotide variants (SNVs) for which functional data are available, and **(C)** the subset of 297 SNVs not present in the COSMIC database. The unweighted Cohen’s Kappa coefficient was computed for each pair of predictors using **(B)** all 989 SNVs and **(D)** the subset of 297 SNVs not present in the COSMIC database. The ranges of unweighted Kappa values and their corresponding colors are indicated in the color key. Additional file 5:
**Inter-rater agreement of mutation effect prediction algorithms as defined by unweighted Cohen’s Kappa coefficients for the 989 single nucleotide variants for which functional data are available and for the subset of these single nucleotide variants (n = 297) that are not in the COSMIC database.**
Additional file 6:
**Summary of the 1,699 single nucleotide variants included in this study not present in the COSMIC database and their experimental functional categories.**
Additional file 7:
**Predictions of single nucleotide variants not present in the COSMIC database (n = 1,699) by 15 mutation effect prediction algorithms.**
Additional file 8:
**Predictions of functionally validated single nucleotide variants by 15 mutation effect prediction algorithms with low confidence prediction categories, for all mutations (n = 3,591) and for mutations not present in the COSMIC database (n = 1,699).**
Additional file 9:
**Number of single nucleotide variants classified as of low confidence by 15 mutation effect prediction algorithms.**
Additional file 10:
**Inter-rater agreement between 15 mutation effect prediction algorithms for all 3,591 single nucleotide variants (SNVs) and all 1,699 non-COSMIC SNVs included in the dataset, when a low confidence category is included.** Hierarchical clustering of the calls (that is, neutral, non-neutral, low confidence) made by 15 mutation effect prediction algorithms using **(A)** all 3,591 SNVs included in this study, and **(C)** the 1,699 SNVs not present in the COSMIC database. The unweighted Cohen’s Kappa coefficient was computed for each pair of predictors using **(B)** all 3,591 SNVs and **(D)** the 1,699 SNVs not present in the COSMIC database. The ranges of unweighted Kappa values and their corresponding colors are indicated in the color key. Additional file 11:
**Inter-rater agreement of mutation effect prediction algorithms as defined by unweighted Cohen’s Kappa coefficients for predictions made for all single nucleotide variants (n = 3,591) and for single nucleotide variants not present in the COSMIC database (n = 1,699) when a low confidence category is included.**
Additional file 12:
**Inter-rater agreement between 15 mutation effect prediction algorithms for single nucleotide variants for which functional data are available, when a low confidence category is included.** Hierarchical clustering of the calls made (that is, neutral, non-neutral, low confidence) by 15 mutation effect prediction algorithms using **(A)** all 989 single nucleotide variants (SNVs) for which functional data are available, and **(C)** the subset of 297 SNVs not present in the COSMIC database. The unweighted Cohen’s Kappa coefficient was computed for each pair of predictors using **(B)** all 989 SNVs and **(D)** the subset of 297 SNVs not present in the COSMIC database. The ranges of unweighted Kappa values and their corresponding colors are indicated in the color key. Additional file 13:
**Inter-rater agreement of mutation effect prediction algorithms as defined by unweighted Cohen’s Kappa coefficients for 989 single nucleotide variants for which functional data are available and for the subset of these single nucleotide variants (n = 297) that are not present in the COSMIC database, when a low confidence category is included.**
Additional file 14:
**Predictions of functionally validated single nucleotide variants in** ***bona fide*** **oncogenes,** ***bona fide*** **tumor suppressor genes, and new cancer genes by 15 mutation effect prediction algorithms (n = 989).**
Additional file 15:
**Performance statistics of mutation effect prediction algorithms using only single nucleotide variants (SNVs) in** ***bona fide*** **oncogenes or in** ***bona fide*** **tumor suppressor genes.** Based on the prediction results of the non-neutral and neutral SNVs (n = 989) in *bona fide* oncogenes (n = 176) **(A)** or in *bona fide* tumor suppressor genes (n = 783) **(B)**, the accuracy, sensitivity, specificity, positive predictive value (PPV), negative predictive value (NPV), and composite score for each predictor are plotted. Error bars represent the 95% confidence intervals generated by bootstrapping. Blue bars represent single/independent predictors, orange bars meta-predictors. Additional file 16:
**Performance statistics and 95% confidence intervals of mutation effect prediction algorithms using only functionally validated single nucleotide variants in** ***bona fide*** **oncogenes or** ***bona fide*** **tumor suppressor genes.**
Additional file 17:
**Performance statistics for mutation effect prediction algorithm combinations using all 989 single nucleotide variants with functional data included in this study.**
Additional file 18:
**Performance statistics for mutation effect prediction algorithm combinations using 297 single nucleotide variants not present in the COSMIC database for which functional data are available.**
Additional file 19:
**Number of mutation effect prediction algorithm combinations that outperform single predictors and meta-predictors.**
Additional file 20:
**Performance statistics of the top five mutation effect prediction algorithm combinations as ranked by accuracy.** Prediction results of the non-neutral (n = 849) and neutral (n = 140) single nucleotide variants (SNVs) in the entire dataset **(A, B)** and the non-neutral (n = 188) and neutral (n = 109) SNVs not present in the COSMIC dataset **(C, D)** are shown. Results are ranked according to the accuracy of each mutation effect prediction algorithm combination, and the accuracy, sensitivity, specificity, positive predictive value (PPV), negative predictive value (NPV), and composite score of the top five prediction algorithm combinations in subset 1 **(A, C)** and subset 2 **(B, D)** are plotted. Error bars represent the 95% confidence intervals generated by 1,000 random samples of subsets 1 and 2. Red bars represent predictor combinations, blue bars single/independent predictors, and orange bars meta-predictors. Blue stars: statistically significant improvement in accuracy as compared to that of the best performing single/independent predictor; orange stars: statistically significant improvement in accuracy as compared to that of the best performing meta-predictor; blue triangles: statistically significant improvement in NPV as compared to that of the best performing single/independent predictor; orange triangles: statistically significant improvement in NPV as compared to that of the best performing meta-predictor. Additional file 21:
**Top five mutation effect prediction algorithm combinations ranked by either accuracy or composite score separately for subsets 1 and 2 using all 989 single nucleotide variants for which functional data are available and the corresponding best performing single and meta-predictors.**
Additional file 22:
**Top five mutation effect prediction algorithm combinations ranked by either accuracy or composite score separately for subsets 1 and 2 using all 297 single nucleotide variants not included in the COSMIC database for which functional data are available and the corresponding best performing single and meta-predictors.**
Additional file 23:
**Recurrence of individual mutation effect prediction algorithms in top performing mutation effect prediction algorithm combinations ranked by accuracy.** The top 10, top 20, top 50, and top 100 combinations of mutation effect prediction algorithms were defined using the non-neutral (n = 849) and neutral (n = 140) single nucleotide variants (SNVs) included the entire dataset and ranked according to accuracy. The frequency of each single mutation effect predictor present in these top combinations was determined in subset 1 and subset 2 **(A)**. The top 10, top 20, top 50, and top 100 combinations of mutation effect prediction algorithms were defined using the non-neutral (n = 188) and neutral (n = 109) SNVs not present in the COSMIC database and ranked according to accuracy. The frequency of each single mutation effect predictor present in these top combinations was determined in subset 1 and subset 2 **(B)**. Additional file 24:
**Accuracy of mutation effect prediction algorithm combinations according to** ***n*** **and** ***p,*** **using all non-neutral and neutral single nucleotide variants (n =989) in this dataset.** Based on the prediction results of 11,253 combinations using all non-neutral and neutral single nucleotide variants included in this study, boxplots showing the accuracy of the combinations were plotted and grouped by *n* with increasing *p* along the x-axis **(A)** and by *p* with increasing *n* along the x-axis **(B)**. Additional file 25:
**Optimal** ***p*** **and** ***n*** **using all 989 functionally defined non-neutral or neutral single nucleotide variants included in this dataset.**
Additional file 26:
**Accuracy of mutation effect prediction algorithm combinations according to** ***n*** **and** ***p*** **, using all non-neutral and neutral single nucleotide variants not included in the COSMIC database (n =297) in this dataset.** Based on the prediction results of 11,253 combinations using all non-neutral and neutral single nucleotide variants included in this study, boxplots showing the accuracy of the combinations were plotted and grouped by *n* with increasing *p* along the x-axis **(A)** and by *p* with increasing *n* along the x-axis **(B)**. Additional file 27:
**Optimal** ***p*** **and** ***n*** **using all 297 functionally defined non-neutral or neutral single nucleotide variants not included in the COSMIC database.**
Additional file 28:
**Residual single nucleotide variants in the set of 989 functionally defined non-neutral and neutral single nucleotide variants and in the subset of 297 single nucleotide variants not present in the COSMIC database.**
Additional file 29:
**Performance statistics of mutation effect prediction algorithms after exclusion of single nucleotide variants (SNVs) present in COSMIC or in the training sets of each mutation effect predictor.** The accuracy, sensitivity, specificity, positive predictive value (PPV), negative predictive value (NPV), and composite score of CHASM (breast), CHASM (lung), CHASM (melanoma), FATHMM (cancer), FATHMM (missense) and PolyPhen-2 using all 989 functionally defined non-neutral or neutral SNVs (red bars), 297 non-COSMIC non-neutral or neutral SNVs (blue bars) and SNVs after exclusion of those found in the training set of each mutation effect predictor (orange bars). Error bars indicate 95% confidence intervals generated by bootstrapping. Y-axis on the left represents the scale of accuracy, sensitivity, specificity, PPV and NPV, whereas the y-axis on the right represents the scale of composite score.

## Abbreviations

CI: Confidence interval
NPV: Negative predictive value
PPV: Positive predictive value
TSG: Tumor suppressor gene
