# Supplementary figures and images for: Benchmarking mutation effect prediction algorithms using functionally validated cancer-related missense mutations
Source: Genome Biol. 2014 Oct 28;15(10):484. doi: 10.1186/s13059-014-0484-1 (PMC4232638; doi:10.1186/s13059-014-0484-1)

# Additional file 1

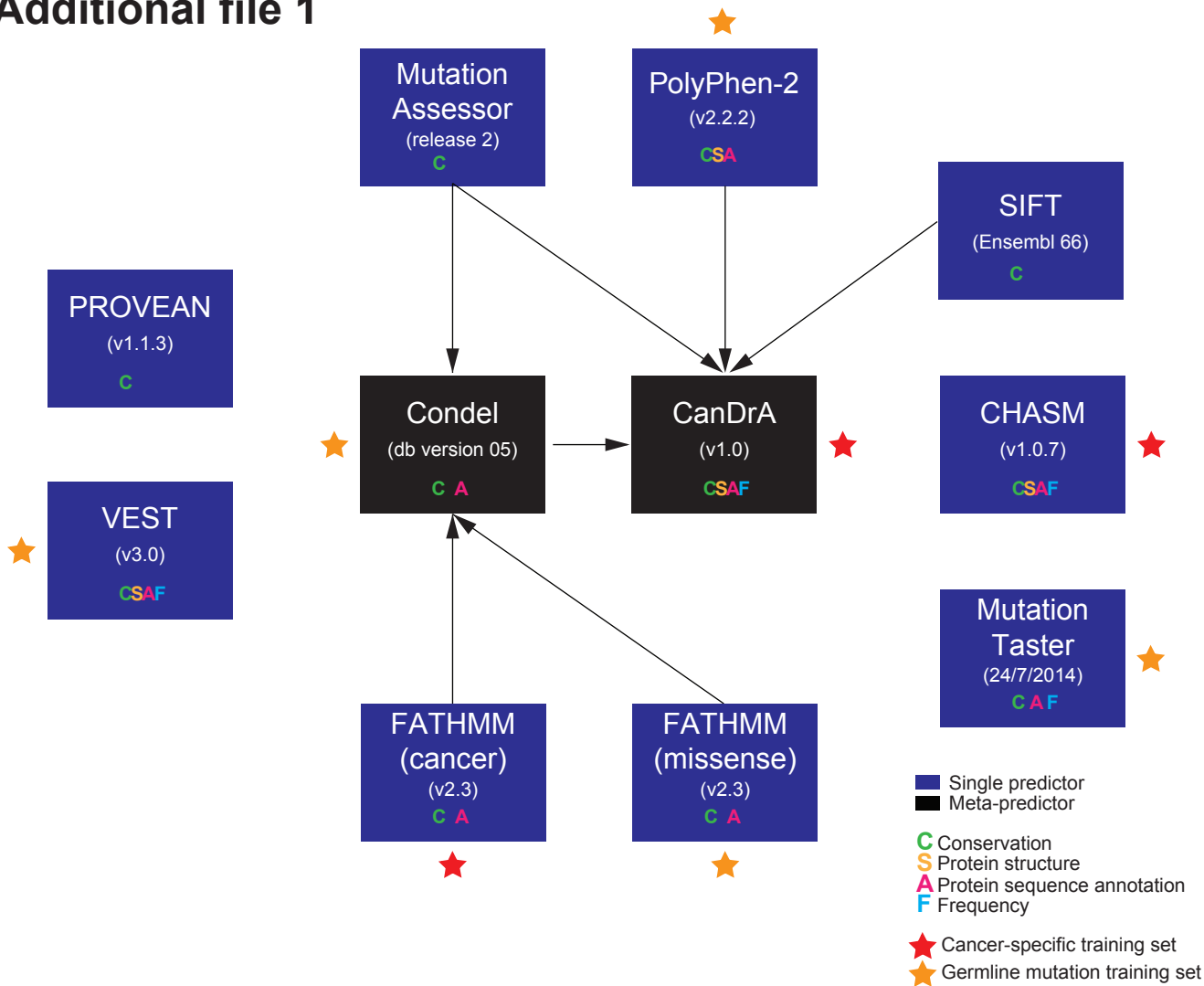

Supplement: Additional file 1: — Overview of computational mutation effect prediction algorithms analyzed in this study. Prediction algorithms in blue boxes represent single/independent predictors, those in black boxes meta-predictors. Arrows between predictors indicate dependency, such that predictions made by the predictor at the tail of the arrows are integrated by the predictor at the head of the arrows. Predictors are annotated based on the features used in the algorithms. Features are divided into four categories: C: conservation, such as conservation scores and homology search; S: protein structure, such as secondary and tertiary structures and accessible surfaces; A: protein sequence annotation, such as annotation information from Uniprot and Pfam; and F: mutational frequency from databases such as COSMIC, HapMap, and Human Gene Mutation Database. The version of each algorithm included in this study is described in each box. [file 13059_2014_484_MOESM1_ESM.pdf]

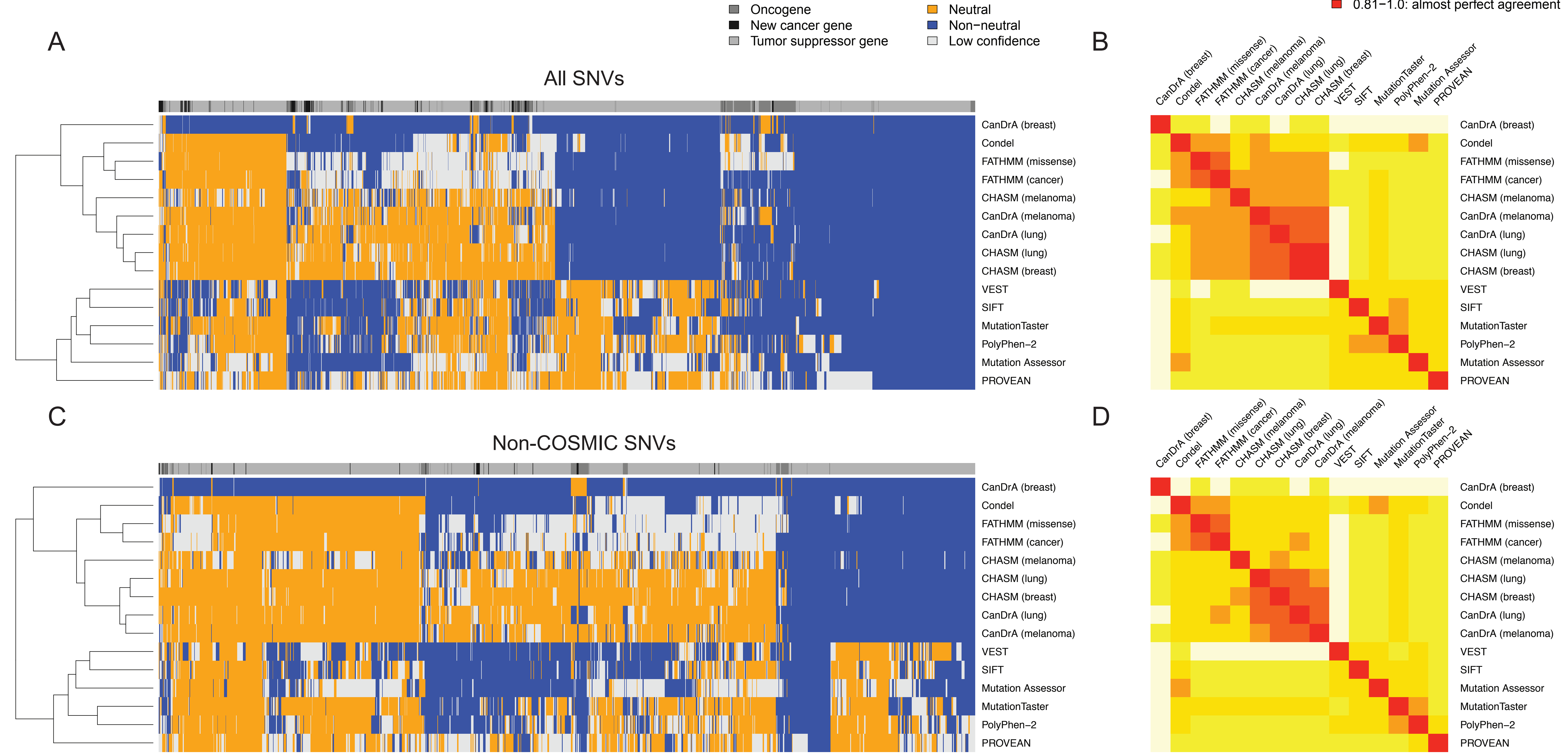

Supplement: Additional file 10: — Inter-rater agreement between 15 mutation effect prediction algorithms for all 3,591 single nucleotide variants (SNVs) and all 1,699 non-COSMIC SNVs included in the dataset, when a low confidence category is included. Hierarchical clustering of the calls (that is, neutral, non-neutral, low confidence) made by 15 mutation effect prediction algorithms using (A) all 3,591 SNVs included in this study, and (C) the 1,699 SNVs not present in the COSMIC database. The unweighted Cohen’s Kappa coefficient was computed for each pair of predictors using (B) all 3,591 SNVs and (D) the 1,699 SNVs not present in the COSMIC database. The ranges of unweighted Kappa values and their corresponding colors are indicated in the color key. [file 13059_2014_484_MOESM10_ESM.pdf]

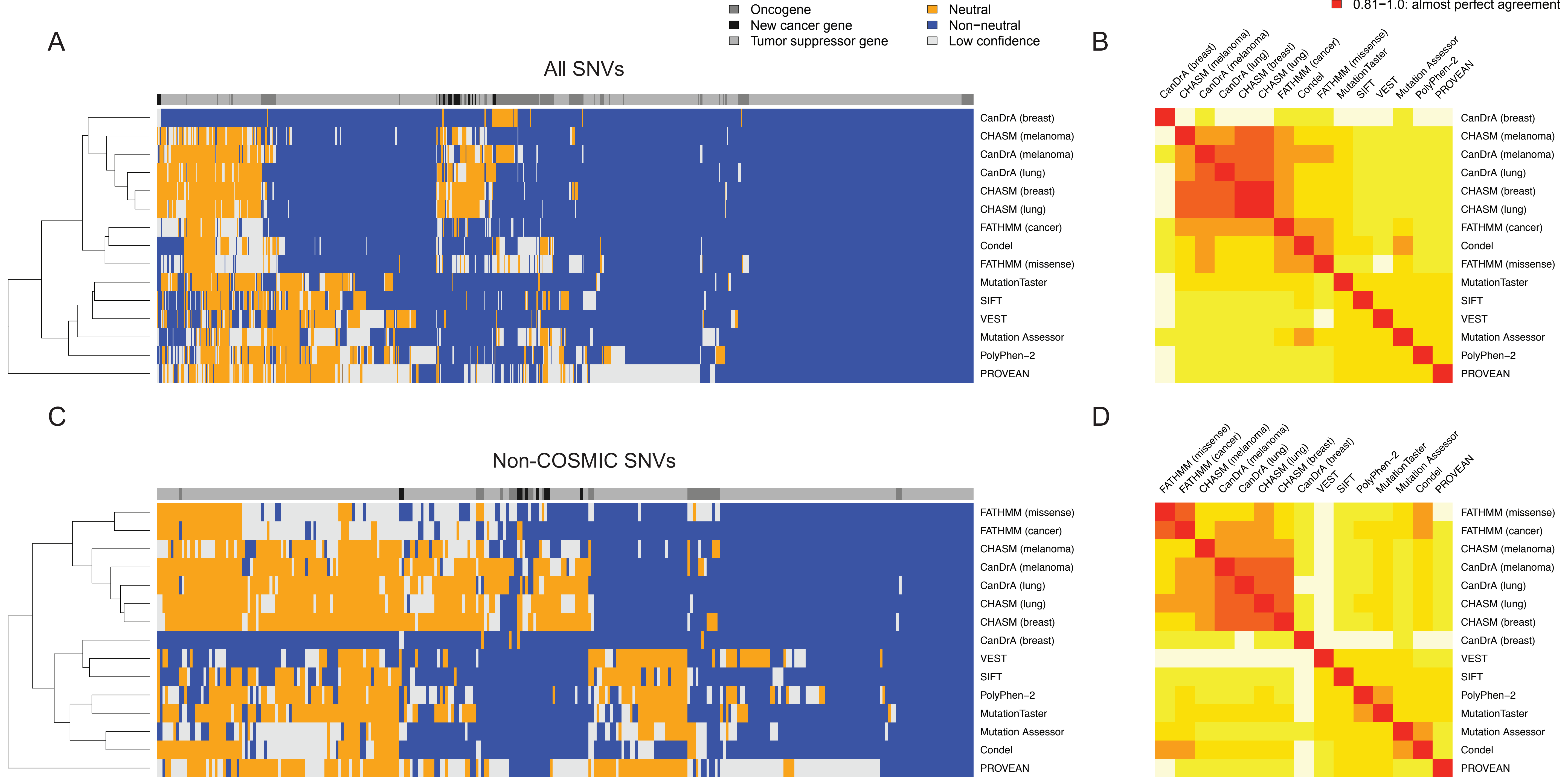

Supplement: Additional file 12: — Inter-rater agreement between 15 mutation effect prediction algorithms for single nucleotide variants for which functional data are available, when a low confidence category is included. Hierarchical clustering of the calls made (that is, neutral, non-neutral, low confidence) by 15 mutation effect prediction algorithms using (A) all 989 single nucleotide variants (SNVs) for which functional data are available, and (C) the subset of 297 SNVs not present in the COSMIC database. The unweighted Cohen’s Kappa coefficient was computed for each pair of predictors using (B) all 989 SNVs and (D) the subset of 297 SNVs not present in the COSMIC database. The ranges of unweighted Kappa values and their corresponding colors are indicated in the color key. [file 13059_2014_484_MOESM12_ESM.pdf]

A

Oncogenes

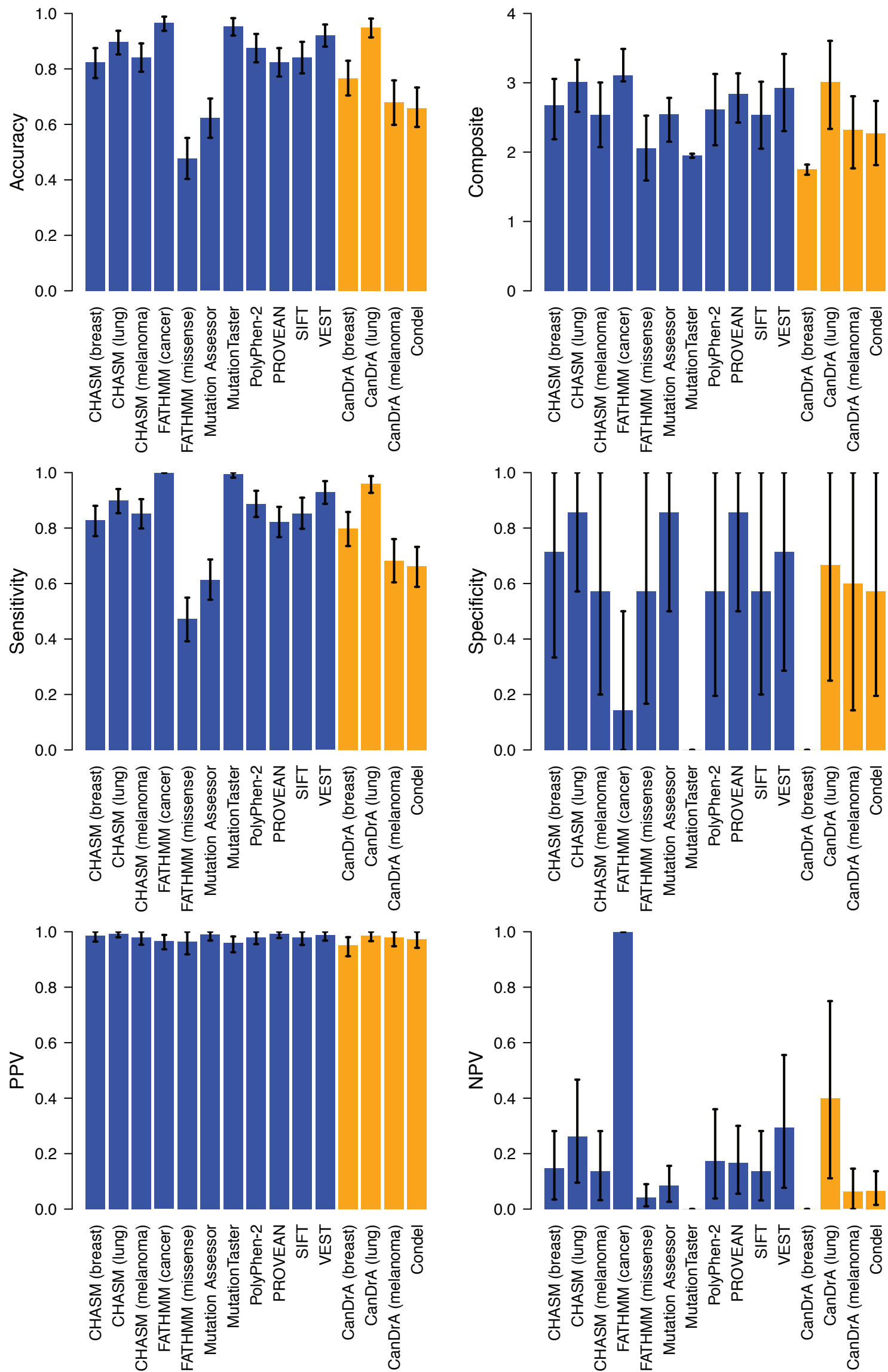

B

Tumor suppressor genes

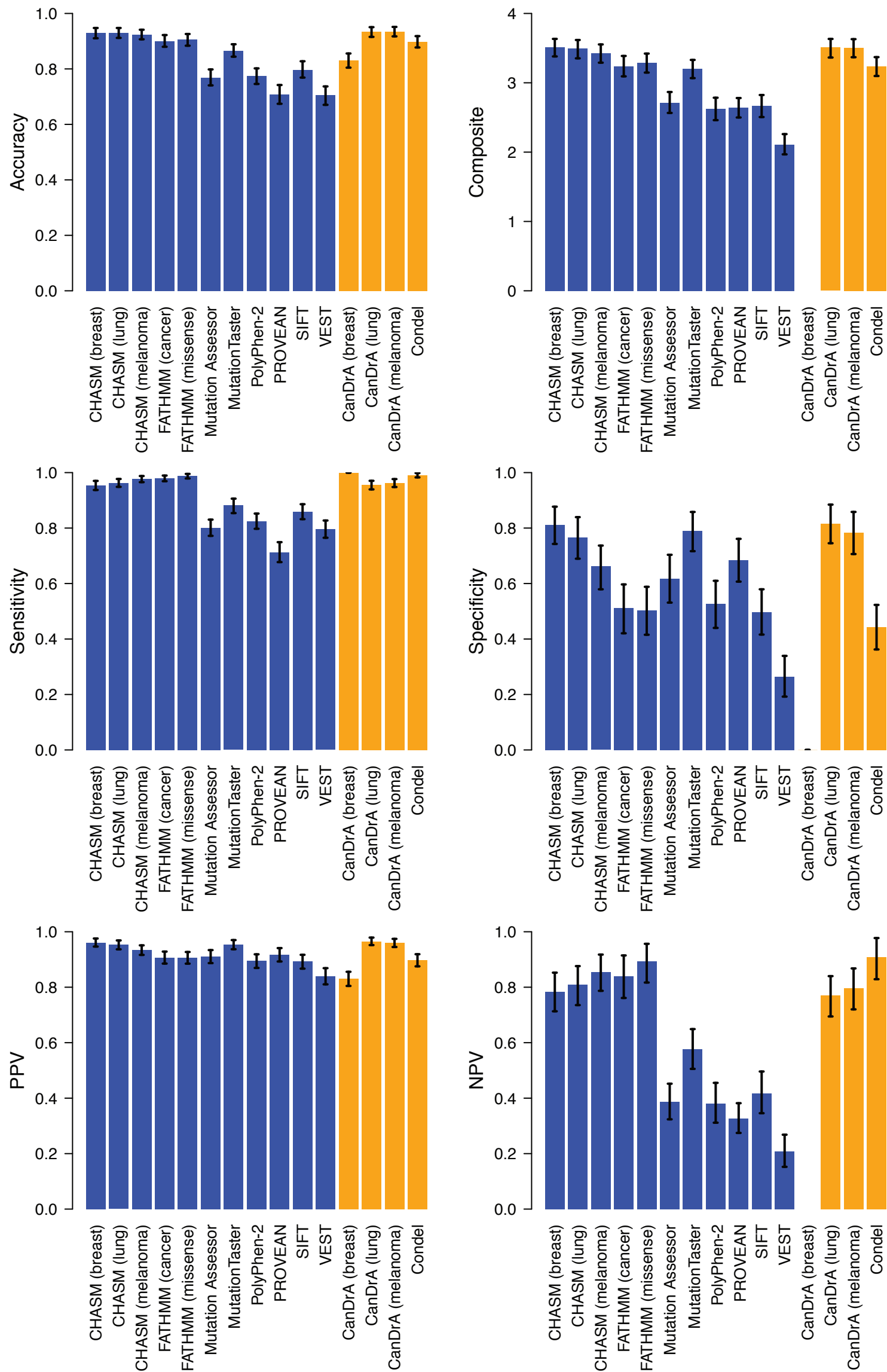

Supplement: Additional file 15: — Performance statistics of mutation effect prediction algorithms using only single nucleotide variants (SNVs) in bona fide oncogenes or in bona fide tumor suppressor genes. Based on the prediction results of the non-neutral and neutral SNVs (n = 989) in bona fide oncogenes (n = 176) (A) or in bona fide tumor suppressor genes (n = 783) (B), the accuracy, sensitivity, specificity, positive predictive value (PPV), negative predictive value (NPV), and composite score for each predictor are plotted. Error bars represent the 95% confidence intervals generated by bootstrapping. Blue bars represent single/independent predictors, orange bars meta-predictors. [file 13059_2014_484_MOESM15_ESM.pdf]

Additional file 20

All SNVs

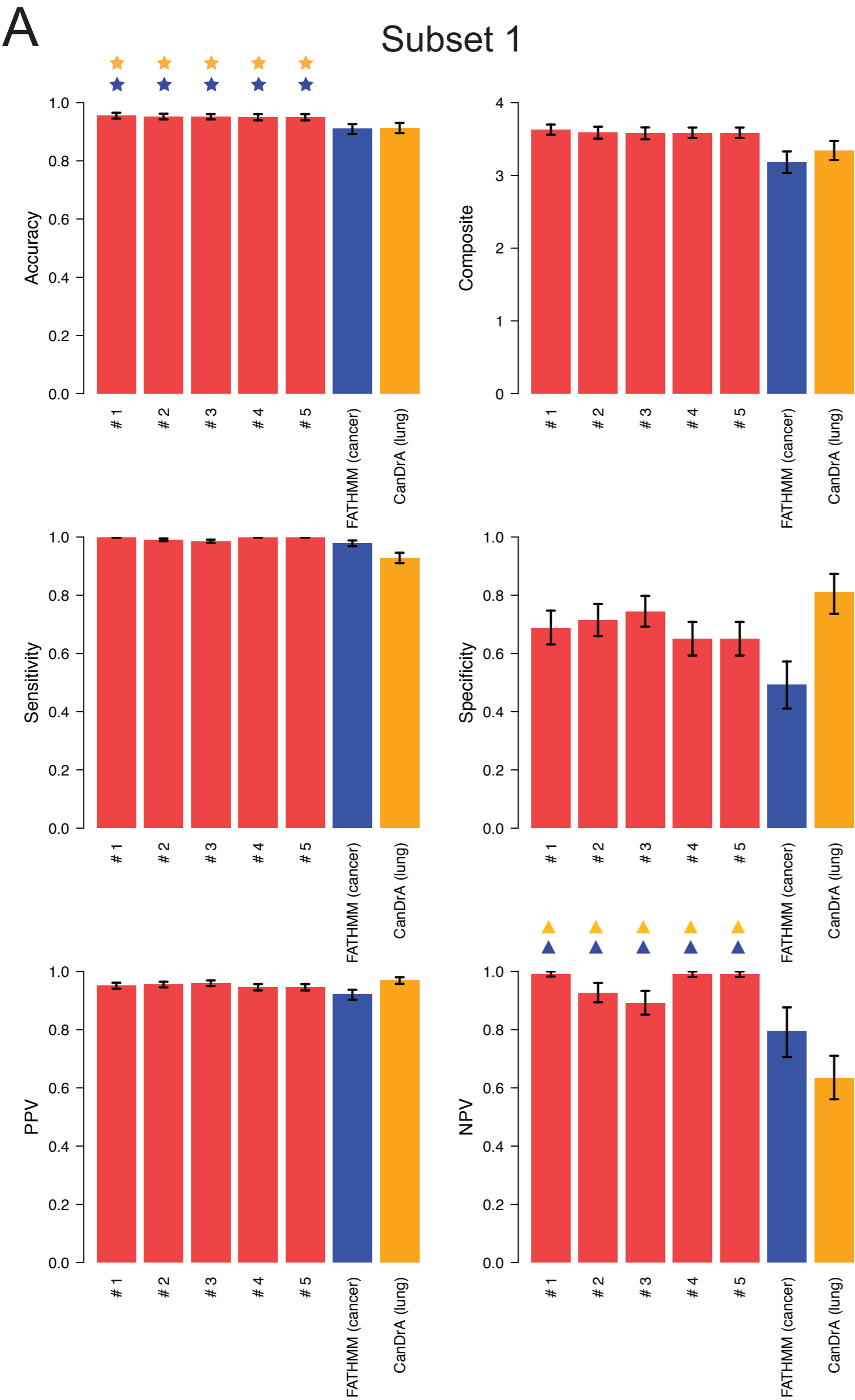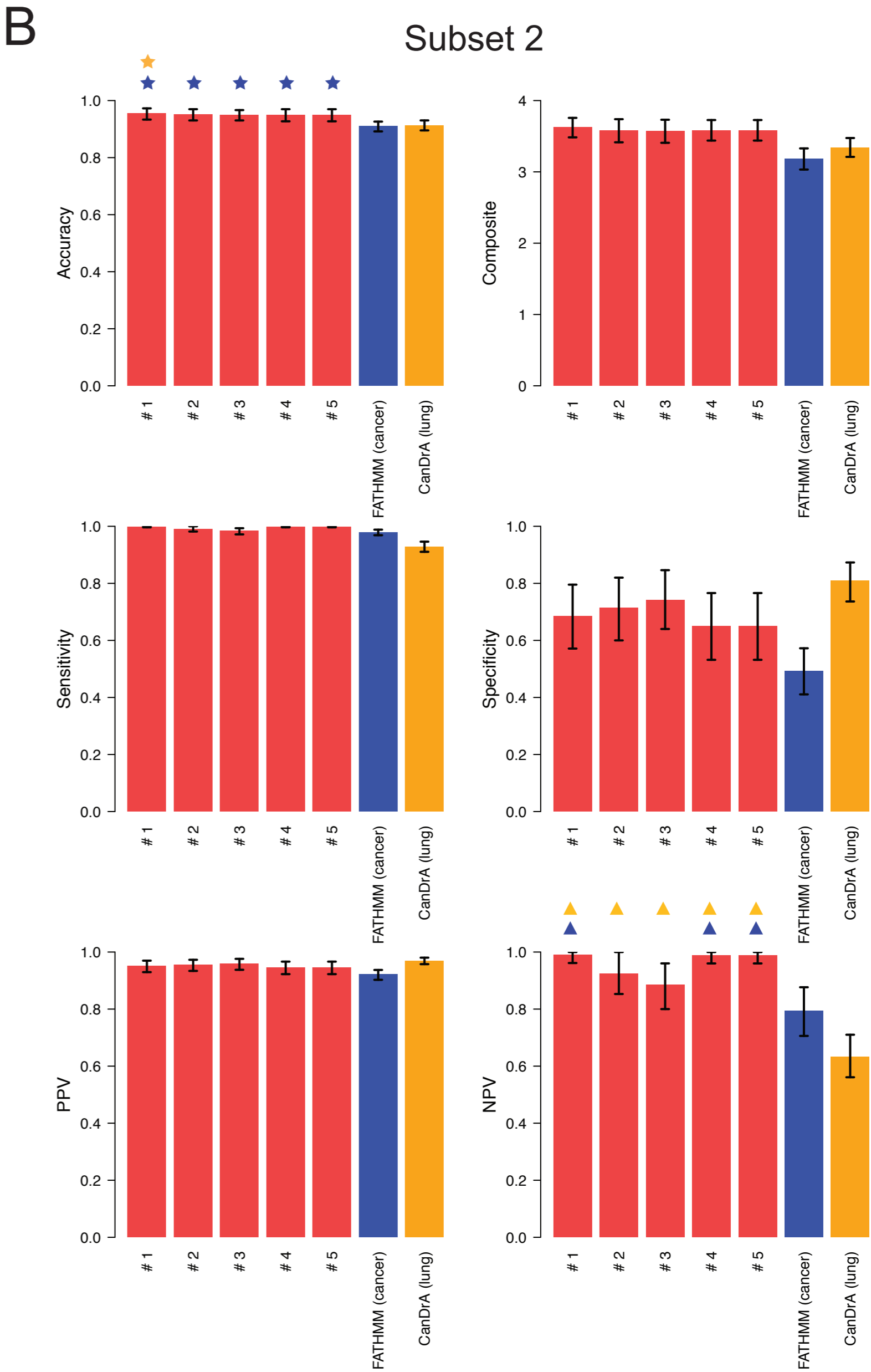

Non-COSMIC SNVs

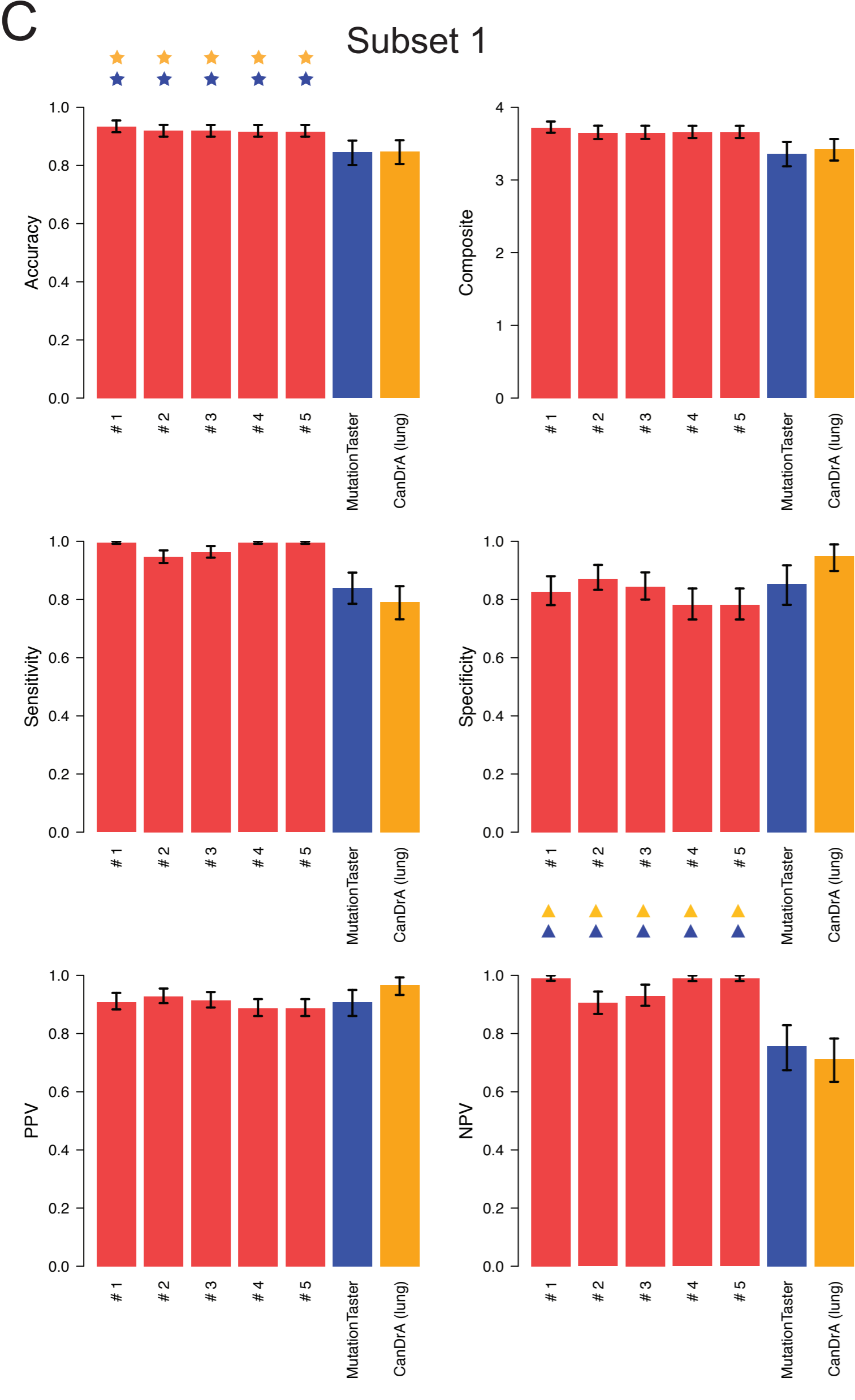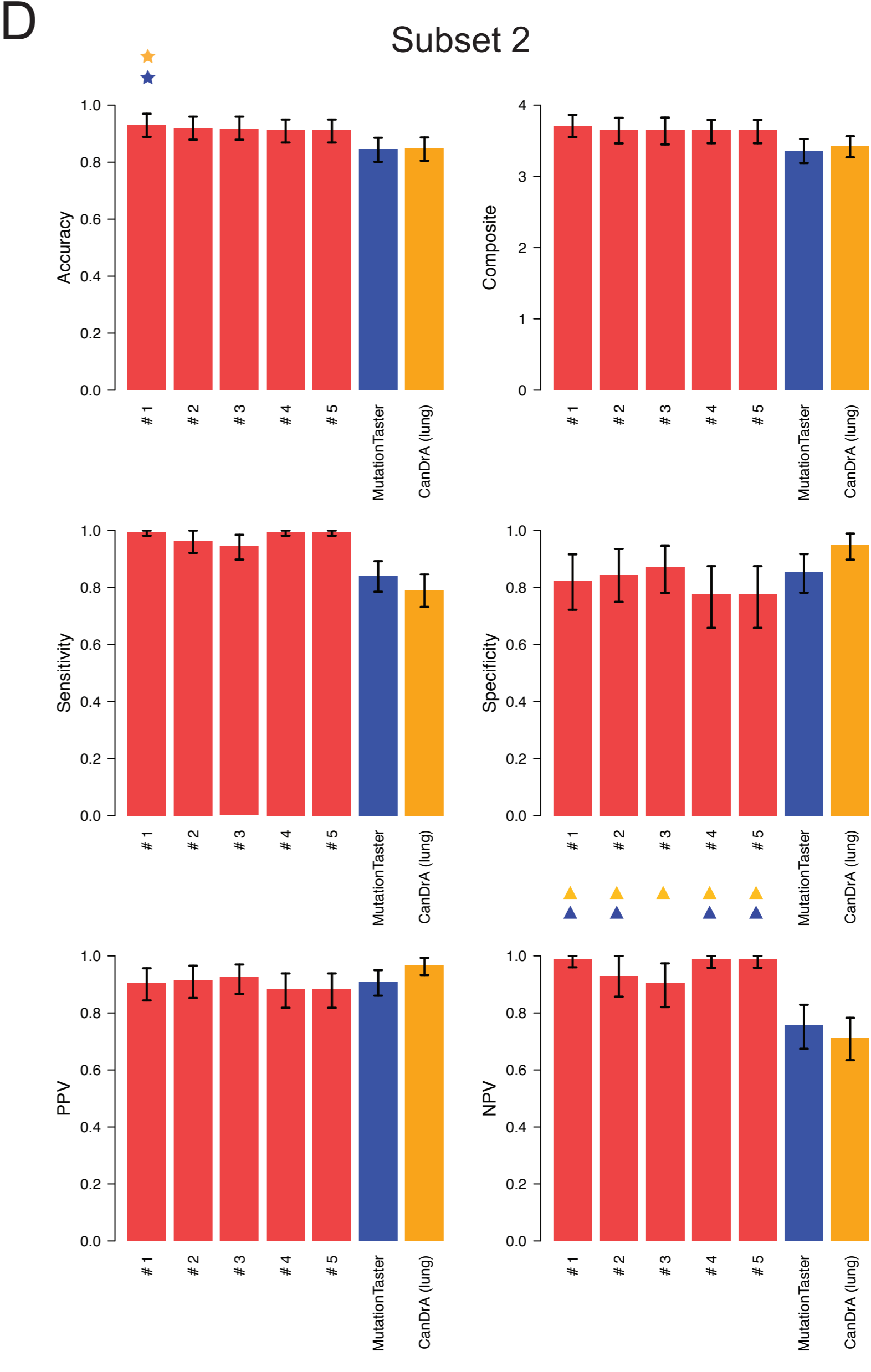

Supplement: Additional file 20: — Performance statistics of the top five mutation effect prediction algorithm combinations as ranked by accuracy. Prediction results of the non-neutral (n = 849) and neutral (n = 140) single nucleotide variants (SNVs) in the entire dataset (A, B) and the non-neutral (n = 188) and neutral (n = 109) SNVs not present in the COSMIC dataset (C, D) are shown. Results are ranked according to the accuracy of each mutation effect prediction algorithm combination, and the accuracy, sensitivity, specificity, positive predictive value (PPV), negative predictive value (NPV), and composite score of the top five prediction algorithm combinations in subset 1 (A, C) and subset 2 (B, D) are plotted. Error bars represent the 95% confidence intervals generated by 1,000 random samples of subsets 1 and 2. Red bars represent predictor combinations, blue bars single/independent predictors, and orange bars meta-predictors. Blue stars: statistically significant improvement in accuracy as compared to that of the best performing single/independent predictor; orange stars: statistically significant improvement in accuracy as compared to that of the best performing meta-predictor; blue triangles: statistically significant improvement in NPV as compared to that of the best performing single/independent predictor; orange triangles: statistically significant improvement in NPV as compared to that of the best performing meta-predictor. [file 13059_2014_484_MOESM20_ESM.pdf]

A

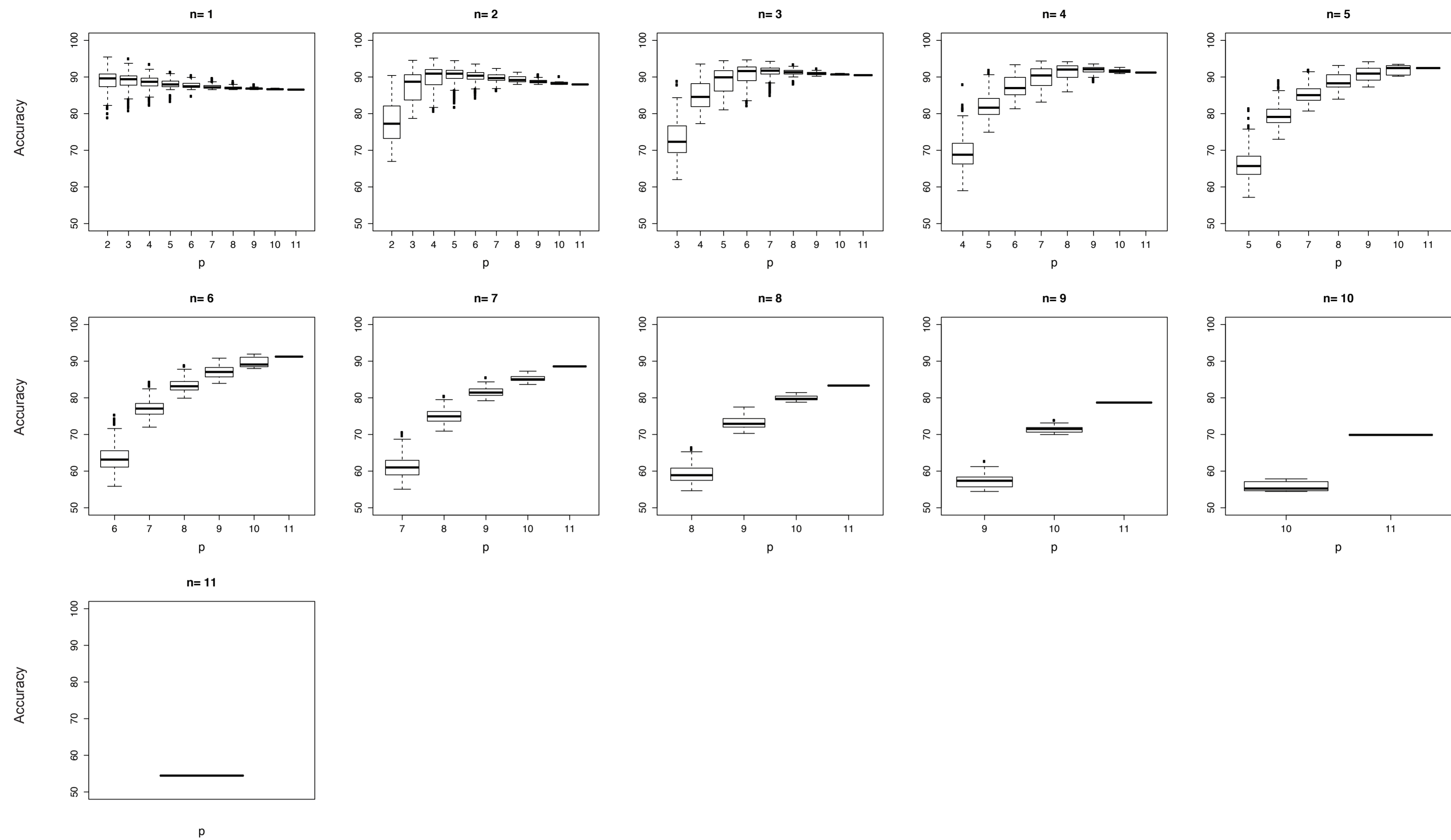

B

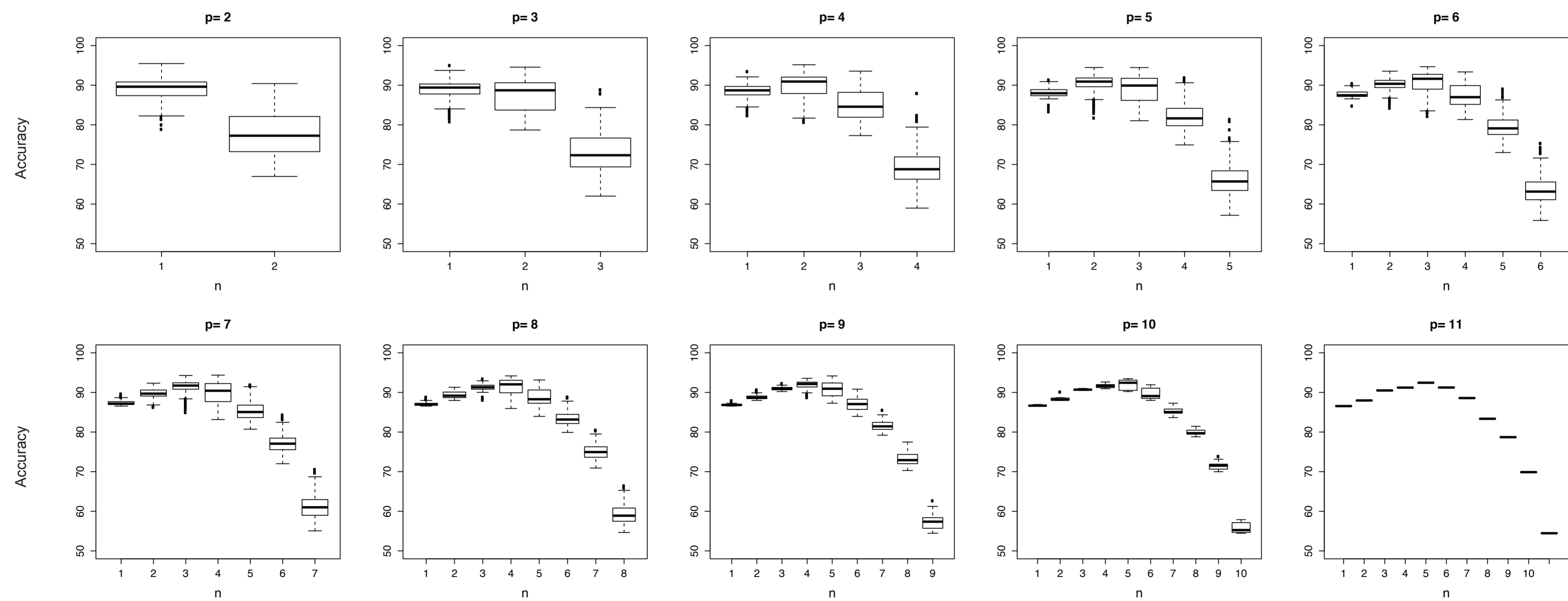

Supplement: Additional file 24: — Accuracy of mutation effect prediction algorithm combinations according to n and p, using all non-neutral and neutral single nucleotide variants (n =989) in this dataset. Based on the prediction results of 11,253 combinations using all non-neutral and neutral single nucleotide variants included in this study, boxplots showing the accuracy of the combinations were plotted and grouped by n with increasing p along the x-axis (A) and by p with increasing n along the x-axis (B). [file 13059_2014_484_MOESM24_ESM.pdf]

A

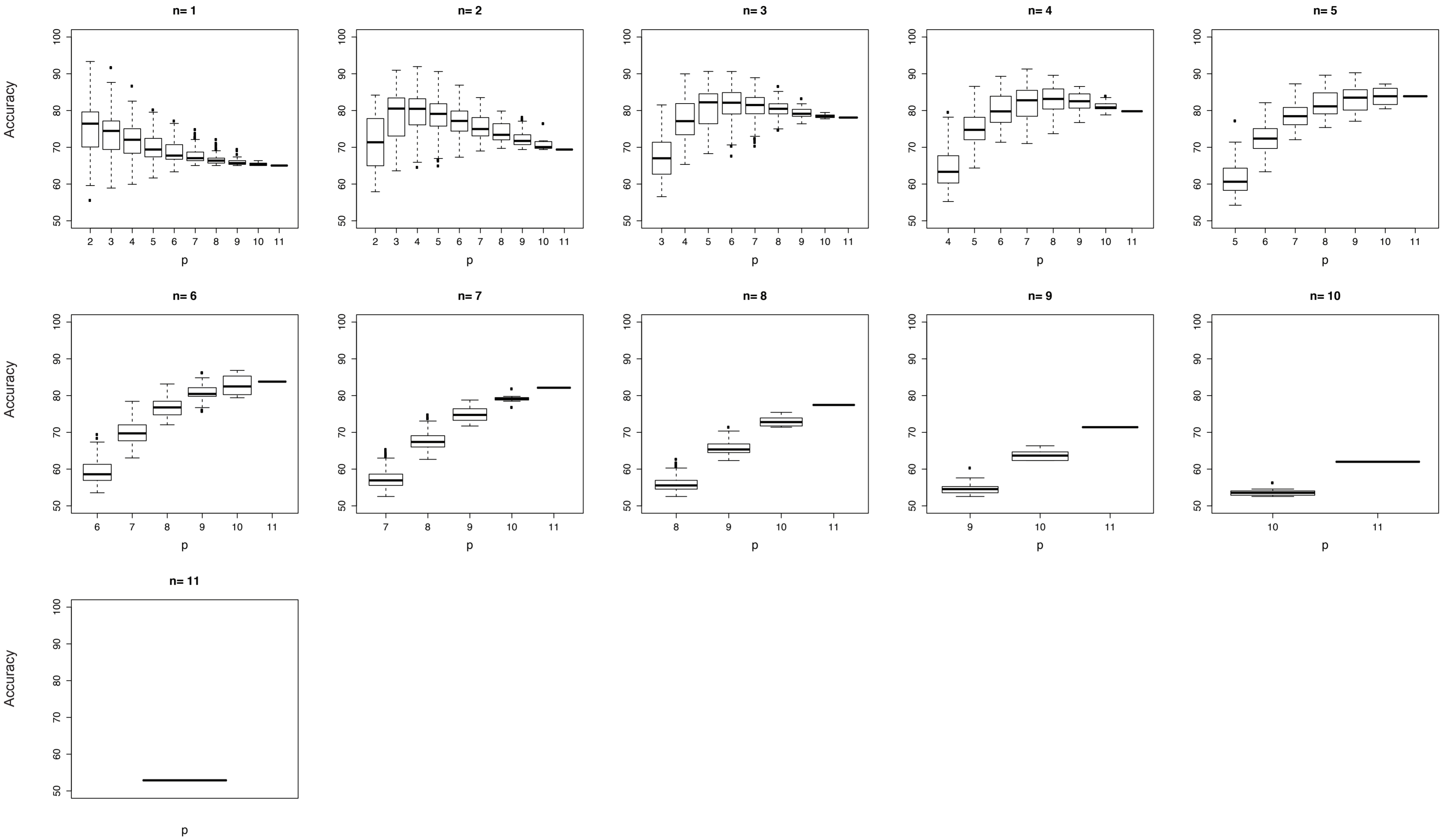

B

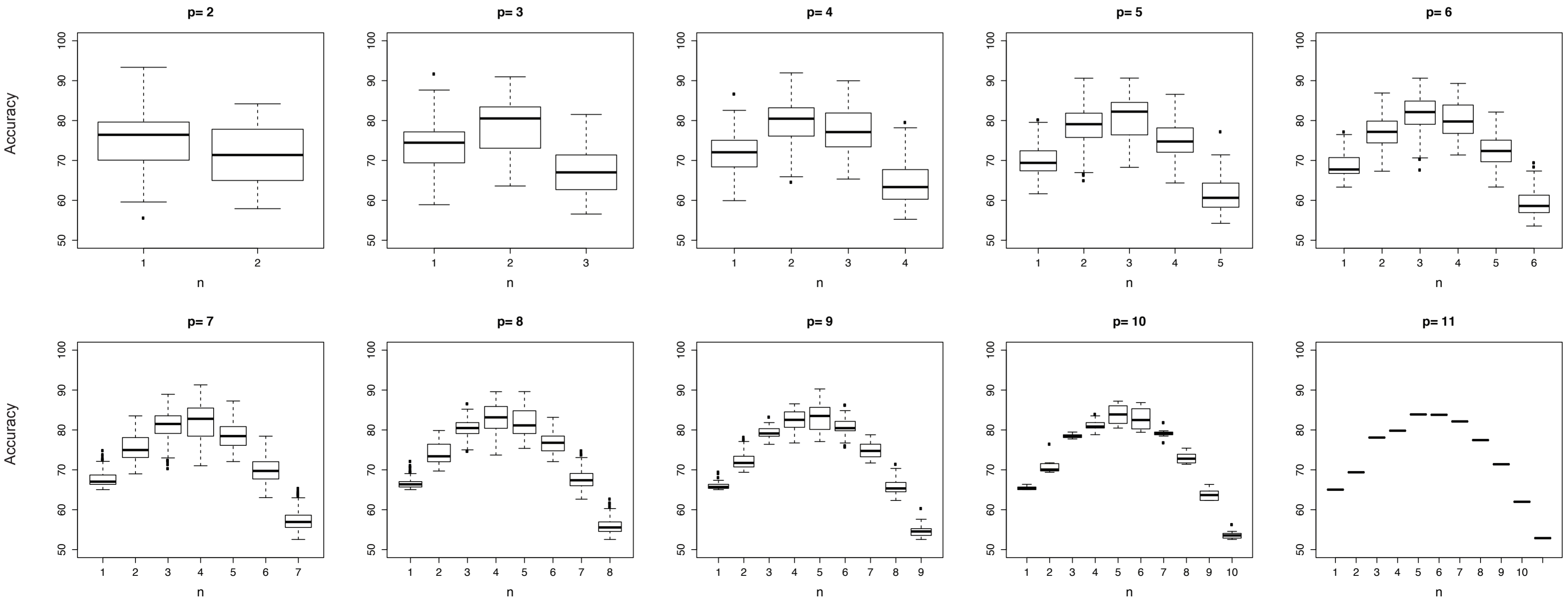

Supplement: Additional file 26: — Accuracy of mutation effect prediction algorithm combinations according to n and p , using all non-neutral and neutral single nucleotide variants not included in the COSMIC database (n =297) in this dataset. Based on the prediction results of 11,253 combinations using all non-neutral and neutral single nucleotide variants included in this study, boxplots showing the accuracy of the combinations were plotted and grouped by n with increasing p along the x-axis (A) and by p with increasing n along the x-axis (B). [file 13059_2014_484_MOESM26_ESM.pdf]

Additional file 29

- All SNVs
- Non-COSMIC SNVs
- Excluding training set

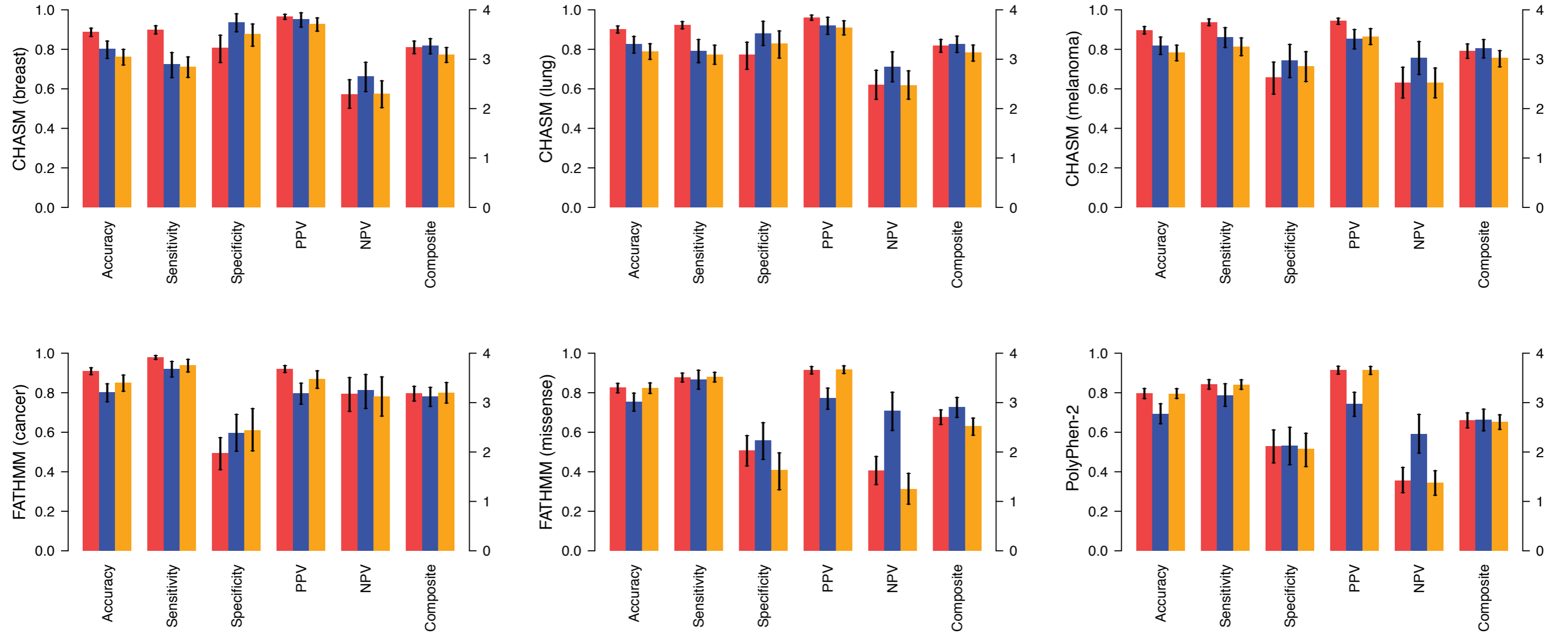

Supplement: Additional file 29: — Performance statistics of mutation effect prediction algorithms after exclusion of single nucleotide variants (SNVs) present in COSMIC or in the training sets of each mutation effect predictor. The accuracy, sensitivity, specificity, positive predictive value (PPV), negative predictive value (NPV), and composite score of CHASM (breast), CHASM (lung), CHASM (melanoma), FATHMM (cancer), FATHMM (missense) and PolyPhen-2 using all 989 functionally defined non-neutral or neutral SNVs (red bars), 297 non-COSMIC non-neutral or neutral SNVs (blue bars) and SNVs after exclusion of those found in the training set of each mutation effect predictor (orange bars). Error bars indicate 95% confidence intervals generated by bootstrapping. Y-axis on the left represents the scale of accuracy, sensitivity, specificity, PPV and NPV, whereas the y-axis on the right represents the scale of composite score. [file 13059_2014_484_MOESM29_ESM.pdf]
