# Supplementary material for: Benchmarking mutation effect prediction algorithms using functionally validated cancer-related missense mutations
Source: Genome Biol. 2014 Oct 28;15(10):484. doi: 10.1186/s13059-014-0484-1 (PMC4232638; doi:10.1186/s13059-014-0484-1)
Supplement: Additional file 3: — Inter-rater agreement of mutation effect prediction algorithms as defined by unweighted Cohen’s Kappa coefficients for all 3,591 single nucleotide variants and all 1,699 non-COSMIC single nucleotide variants included in the dataset. [file 13059_2014_484_MOESM3_ESM.pdf]

**Additional file 3: Inter-rater agreement of mutation effect prediction algorithms as defined by unweighted Cohen's Kappa coefficients for all 3,591 single nucleotide variants and all 1,699 non-COSMIC single nucleotide variants included in the dataset.**

**All single nucleotide variants (n=3,591)**

| *Kappa scores* | CHASM (breast) | CHASM (lung) | CHASM (melanoma) | FATHMM (cancer) | FATHMM (missense) | Mutation Assessor | MutationTaster | PolyPhen-2 | PROVEAN | SIFT | VEST | CanDrA (breast) | CanDrA (lung) | CanDrA (melanoma) | Condel |
|---|---|---|---|---|---|---|---|---|---|---|---|---|---|---|---|
| **CHASM (breast)** | 1 (0.9673-1) | 0.9004 (0.8678-0.9331) | 0.7096 (0.6778-0.7415) | 0.5816 (0.5514-0.6117) | 0.5647 (0.533-0.5965) | 0.2106 (0.178-0.2432) | 0.3694 (0.337-0.4018) | 0.1503 (0.1181-0.1825) | 0.2412 (0.2088-0.2736) | 0.1568 (0.1249-0.1887) | -0.04391 (-0.07474--0.01307) | 0.03265 (0.01766-0.04765) | 0.7537 (0.7238-0.7835) | 0.6795 (0.6502-0.7089) | 0.4168 (0.387-0.4466) |
| **CHASM (lung)** | 0.9004 (0.8678-0.9331) | 1 (0.9673-1) | 0.6654 (0.6332-0.6975) | 0.5858 (0.5551-0.6166) | 0.5754 (0.5434-0.6075) | 0.2193 (0.1867-0.2518) | 0.3916 (0.359-0.4242) | 0.177 (0.1445-0.2095) | 0.2239 (0.1918-0.256) | 0.1643 (0.1321-0.1965) | -0.01766 (-0.04901-0.01368) | 0.03401 (0.01832-0.04969) | 0.744 (0.714-0.7739) | 0.6743 (0.6448-0.7037) | 0.4409 (0.4105-0.4713) |
| **CHASM (melanoma)** | 0.7096 (0.6778-0.7415) | 0.6654 (0.6332-0.6975) | 1 (0.9673-1) | 0.5053 (0.4731-0.5375) | 0.4912 (0.4588-0.5236) | 0.2225 (0.1909-0.2542) | 0.3179 (0.2854-0.3505) | 0.1699 (0.1373-0.2026) | 0.2018 (0.1712-0.2324) | 0.205 (0.1723-0.2377) | 0.03329 (0.0007922-0.06579) | 0.05025 (0.03203-0.06846) | 0.549 (0.5193-0.5786) | 0.5296 (0.5005-0.5587) | 0.4245 (0.3925-0.4565) |
| **FATHMM (cancer)** | 0.5816 (0.5514-0.6117) | 0.5858 (0.5551-0.6166) | 0.5053 (0.4731-0.5375) | 1 (0.9673-1) | 0.4575 (0.4257-0.4893) | 0.258 (0.228-0.2879) | 0.3964 (0.3648-0.4279) | 0.2308 (0.199-0.2626) | 0.2276 (0.1992-0.256) | 0.2003 (0.1681-0.2324) | 0.03411 (0.00146-0.06675) | -0.03483 (-0.05563--0.01404) | 0.5723 (0.5438-0.6008) | 0.5262 (0.4983-0.5541) | 0.5711 (0.5384-0.6038) |
| **FATHMM (missense)** | 0.5647 (0.533-0.5965) | 0.5754 (0.5434-0.6075) | 0.4912 (0.4588-0.5236) | 0.4575 (0.4257-0.4893) | 1 (0.9678-1) | 0.2328 (0.2012-0.2644) | 0.272 (0.2397-0.3044) | 0.1632 (0.1308-0.1957) | 0.1321 (0.1015-0.1628) | 0.1989 (0.1665-0.2313) | 0.00698 (-0.02513-0.03909) | 0.07216 (0.05445-0.08987) | 0.4776 (0.448-0.5072) | 0.5131 (0.4841-0.5422) | 0.5838 (0.5523-0.6154) |
| **Mutation Assessor** | 0.2106 (0.178-0.2432) | 0.2193 (0.1867-0.2518) | 0.2225 (0.1909-0.2542) | 0.258 (0.228-0.2879) | 0.2328 (0.2012-0.2644) | 1 (0.9675-1) | 0.429 (0.3967-0.4612) | 0.518 (0.4859-0.55) | 0.4486 (0.4163-0.481) | 0.5143 (0.4826-0.5461) | 0.355 (0.3243-0.3856) | -0.0189 (-0.03371--0.004092) | 0.1672 (0.1374-0.197) | 0.2074 (0.1782-0.2367) | 0.4216 (0.392-0.4511) |
| **MutationTaster** | 0.3694 (0.337-0.4018) | 0.3916 (0.359-0.4242) | 0.3179 (0.2854-0.3505) | 0.3964 (0.3648-0.4279) | 0.272 (0.2397-0.3044) | 0.429 (0.3967-0.4612) | 1 (0.9673-1) | 0.5074 (0.4747-0.5401) | 0.3575 (0.326-0.389) | 0.4447 (0.4121-0.4773) | 0.3355 (0.3035-0.3675) | -0.08341 (-0.1003--0.06653) | 0.4012 (0.3712-0.4311) | 0.3665 (0.3372-0.3959) | 0.3429 (0.3116-0.3741) |
| **PolyPhen-2** | 0.1503 (0.1181-0.1825) | 0.177 (0.1445-0.2095) | 0.1699 (0.1373-0.2026) | 0.2308 (0.199-0.2626) | 0.1632 (0.1308-0.1957) | 0.518 (0.4859-0.55) | 0.5074 (0.4747-0.5401) | 1 (0.9673-1) | 0.4063 (0.3751-0.4375) | 0.5662 (0.5335-0.5988) | 0.4372 (0.405-0.4694) | -0.04609 (-0.06342--0.02875) | 0.1608 (0.1309-0.1907) | 0.1388 (0.1095-0.1682) | 0.3129 (0.2814-0.3445) |
| **PROVEAN** | 0.2412 (0.2088-0.2736) | 0.2239 (0.1918-0.256) | 0.2018 (0.1712-0.2324) | 0.2276 (0.1992-0.256) | 0.1321 (0.1015-0.1628) | 0.4486 (0.4163-0.481) | 0.3575 (0.326-0.389) | 0.4063 (0.3751-0.4375) | 1 (0.9673-1) | 0.4204 (0.3897-0.4511) | 0.349 (0.3198-0.3782) | -0.04099 (-0.05427--0.02771) | 0.2383 (0.209-0.2675) | 0.2422 (0.2134-0.271) | 0.2257 (0.1978-0.2536) |
| **SIFT** | 0.1568 (0.1249-0.1887) | 0.1643 (0.1321-0.1965) | 0.205 (0.1723-0.2377) | 0.2003 (0.1681-0.2324) | 0.1989 (0.1665-0.2313) | 0.5143 (0.4826-0.5461) | 0.4447 (0.4121-0.4773) | 0.5662 (0.5335-0.5988) | 0.4204 (0.3897-0.4511) | 1 (0.9673-1) | 0.4347 (0.4022-0.4672) | -0.02427 (-0.04237--0.006166) | 0.1343 (0.1046-0.164) | 0.1521 (0.1229-0.1812) | 0.3147 (0.2828-0.3467) |
| **VEST** | -0.04391 (-0.07474--0.01307) | -0.01766 (-0.04901-0.01368) | 0.03329 (0.0007922-0.06579) | 0.03411 (0.00146-0.06675) | 0.00698 (-0.02513-0.03909) | 0.355 (0.3243-0.3856) | 0.3355 (0.3035-0.3675) | 0.4372 (0.405-0.4694) | 0.349 (0.3198-0.3782) | 0.4347 (0.4022-0.4672) | 1 (0.9673-1) | -0.02294 (-0.04282--0.003056) | -0.05196 (-0.08095--0.02297) | -0.039 (-0.06742--0.01058) | 0.1372 (0.1047-0.1698) |
| **CanDrA (breast)** | 0.03265 (0.01766-0.04765) | 0.03401 (0.01832-0.04969) | 0.05025 (0.03203-0.06846) | -0.03483 (-0.05563--0.01404) | 0.07216 (0.05445-0.08987) | -0.0189 (-0.03371--0.004092) | -0.08341 (-0.1003--0.06653) | -0.04609 (-0.06342--0.02875) | -0.04099 (-0.05427--0.02771) | -0.02427 (-0.04237--0.006166) | -0.02294 (-0.04282--0.003056) | 1 (0.9716-1) | -0.03769 (-0.0528--0.02259) | 0.06321 (0.0484-0.07801) | 0.006829 (-0.01445-0.02811) |
| **CanDrA (lung)** | 0.7537 (0.7238-0.7835) | 0.744 (0.714-0.7739) | 0.549 (0.5193-0.5786) | 0.5723 (0.5438-0.6008) | 0.4776 (0.448-0.5072) | 0.1672 (0.1374-0.197) | 0.4012 (0.3712-0.4311) | 0.1608 (0.1309-0.1907) | 0.2383 (0.209-0.2675) | 0.1343 (0.1046-0.164) | -0.05196 (-0.08095--0.02297) | -0.03769 (-0.0528--0.02259) | 1 (0.9718-1) | 0.6826 (0.6547-0.7105) | 0.3861 (0.3579-0.4143) |
| **CanDrA (melanoma)** | 0.6795 (0.6502-0.7089) | 0.6743 (0.6448-0.7037) | 0.5296 (0.5005-0.5587) | 0.5262 (0.4983-0.5541) | 0.5131 (0.4841-0.5422) | 0.2074 (0.1782-0.2367) | 0.3665 (0.3372-0.3959) | 0.1388 (0.1095-0.1682) | 0.2422 (0.2134-0.271) | 0.1521 (0.1229-0.1812) | -0.039 (-0.06742--0.01058) | 0.06321 (0.0484-0.07801) | 0.6826 (0.6547-0.7105) | 1 (0.9724-1) | 0.4314 (0.4037-0.459) |
| **Condel** | 0.4168 (0.387-0.4466) | 0.4409 (0.4105-0.4713) | 0.4245 (0.3925-0.4565) | 0.5711 (0.5384-0.6038) | 0.5838 (0.5523-0.6154) | 0.4216 (0.392-0.4511) | 0.3429 (0.3116-0.3741) | 0.3129 (0.2814-0.3445) | 0.2257 (0.1978-0.2536) | 0.3147 (0.2828-0.3467) | 0.1372 (0.1047-0.1698) | 0.006829 (-0.01445-0.02811) | 0.3861 (0.3579-0.4143) | 0.4314 (0.4037-0.459) | 1 (0.9673-1) |

**All non-COSMIC single nucleotide variants (n=1,699)**

| *Kappa scores* | CHASM (breast) | CHASM (lung) | CHASM (melanoma) | FATHMM (cancer) | FATHMM (missense) | Mutation Assessor | MutationTaster | PolyPhen-2 | PROVEAN | SIFT | VEST | CanDrA (breast) | CanDrA (lung) | CanDrA (melanoma) | Condel |
|---|---|---|---|---|---|---|---|---|---|---|---|---|---|---|---|
| **CHASM (breast)** | 1 (0.9524-1) | 0.8288 (0.7814-0.8762) | 0.544 (0.5007-0.5874) | 0.4636 (0.4223-0.505) | 0.4653 (0.423-0.5076) | 0.1686 (0.1243-0.213) | 0.3411 (0.2951-0.3871) | 0.1143 (0.07259-0.156) | 0.1053 (0.05792-0.1527) | 0.06132 (0.02104-0.1016) | -0.1288 (-0.1635--0.09414) | 0.01178 (0.001478-0.02208) | 0.6887 (0.6467-0.7307) | 0.6539 (0.6121-0.6958) | 0.3095 (0.2719-0.3471) |
| **CHASM (lung)** | 0.8288 (0.7814-0.8762) | 1 (0.9524-1) | 0.4928 (0.448-0.5377) | 0.4633 (0.4202-0.5064) | 0.494 (0.4501-0.5379) | 0.1949 (0.1492-0.2406) | 0.3858 (0.3389-0.4327) | 0.1619 (0.1185-0.2053) | 0.06609 (0.01854-0.1136) | 0.09166 (0.04953-0.1338) | -0.09242 (-0.1292--0.0556) | 0.01682 (0.00558-0.02806) | 0.6704 (0.6281-0.7126) | 0.6451 (0.603-0.6873) | 0.3634 (0.3237-0.403) |
| **CHASM (melanoma)** | 0.544 (0.5007-0.5874) | 0.4928 (0.448-0.5377) | 1 (0.9524-1) | 0.3823 (0.335-0.4296) | 0.3908 (0.3435-0.4381) | 0.1998 (0.1524-0.2471) | 0.307 (0.2603-0.3538) | 0.1681 (0.1207-0.2155) | 0.09887 (0.05412-0.1436) | 0.1632 (0.1162-0.2102) | -0.02155 (-0.06553-0.02243) | 0.02262 (0.006989-0.03826) | 0.3986 (0.3585-0.4386) | 0.4411 (0.4009-0.4812) | 0.3535 (0.3076-0.3993) |
| **FATHMM (cancer)** | 0.4636 (0.4223-0.505) | 0.4633 (0.4202-0.5064) | 0.3823 (0.335-0.4296) | 1 (0.9524-1) | 0.4276 (0.3802-0.4749) | 0.2876 (0.2409-0.3344) | 0.3547 (0.309-0.4004) | 0.237 (0.1895-0.2846) | 0.1602 (0.1172-0.2032) | 0.16 (0.1125-0.2074) | -0.00139 (-0.04693-0.04415) | -0.0292 (-0.04642--0.01199) | 0.4414 (0.4028-0.48) | 0.4247 (0.3859-0.4634) | 0.56 (0.5131-0.6068) |
| **FATHMM (missense)** | 0.4653 (0.423-0.5076) | 0.494 (0.4501-0.5379) | 0.3908 (0.3435-0.4381) | 0.4276 (0.3802-0.4749) | 1 (0.9528-1) | 0.2538 (0.2068-0.3008) | 0.3477 (0.3016-0.3939) | 0.2397 (0.1923-0.287) | 0.03244 (-0.01136-0.07623) | 0.2107 (0.1636-0.2578) | 0.03245 (-0.01218-0.07708) | 0.02211 (0.005766-0.03845) | 0.4004 (0.3611-0.4397) | 0.4482 (0.4088-0.4877) | 0.5888 (0.5426-0.6351) |
| **Mutation Assessor** | 0.1686 (0.1243-0.213) | 0.1949 (0.1492-0.2406) | 0.1998 (0.1524-0.2471) | 0.2876 (0.2409-0.3344) | 0.2538 (0.2068-0.3008) | 1 (0.9526-1) | 0.4386 (0.3914-0.4857) | 0.5017 (0.4548-0.5486) | 0.2972 (0.2516-0.3428) | 0.4784 (0.4321-0.5247) | 0.2508 (0.2081-0.2935) | -0.02195 (-0.03662--0.007285) | 0.1268 (0.08597-0.1675) | 0.1601 (0.1193-0.201) | 0.4477 (0.4029-0.4926) |
| **MutationTaster** | 0.3411 (0.2951-0.3871) | 0.3858 (0.3389-0.4327) | 0.307 (0.2603-0.3538) | 0.3547 (0.309-0.4004) | 0.3477 (0.3016-0.3939) | 0.4386 (0.3914-0.4857) | 1 (0.9524-1) | 0.4759 (0.43-0.5218) | 0.1525 (0.1056-0.1994) | 0.3984 (0.3534-0.4434) | 0.2209 (0.1804-0.2615) | -0.04738 (-0.06057--0.03419) | 0.35 (0.3083-0.3918) | 0.3175 (0.2757-0.3593) | 0.3744 (0.3314-0.4174) |
| **PolyPhen-2** | 0.1143 (0.07259-0.156) | 0.1619 (0.1185-0.2053) | 0.1681 (0.1207-0.2155) | 0.237 (0.1895-0.2846) | 0.2397 (0.1923-0.287) | 0.5017 (0.4548-0.5486) | 0.4759 (0.43-0.5218) | 1 (0.9524-1) | 0.2407 (0.1974-0.284) | 0.5429 (0.4955-0.5903) | 0.3615 (0.3161-0.4068) | -0.03228 (-0.04923--0.01533) | 0.12 (0.08121-0.1589) | 0.1112 (0.07219-0.1502) | 0.3759 (0.3291-0.4226) |
| **PROVEAN** | 0.1053 (0.05792-0.1527) | 0.06609 (0.01854-0.1136) | 0.09887 (0.05412-0.1436) | 0.1602 (0.1172-0.2032) | 0.03244 (-0.01136-0.07623) | 0.2972 (0.2516-0.3428) | 0.1525 (0.1056-0.1994) | 0.2407 (0.1974-0.284) | 1 (0.9524-1) | 0.241 (0.199-0.283) | 0.2012 (0.1645-0.2379) | -0.02958 (-0.04076--0.0184) | 0.1357 (0.09351-0.178) | 0.142 (0.09985-0.1841) | 0.1625 (0.123-0.202) |
| **SIFT** | 0.06132 (0.02104-0.1016) | 0.09166 (0.04953-0.1338) | 0.1632 (0.1162-0.2102) | 0.16 (0.1125-0.2074) | 0.2107 (0.1636-0.2578) | 0.4784 (0.4321-0.5247) | 0.3984 (0.3534-0.4434) | 0.5429 (0.4955-0.5903) | 0.241 (0.199-0.283) | 1 (0.9524-1) | 0.3923 (0.3461-0.4385) | -0.01264 (-0.0307-0.005419) | 0.05247 (0.01475-0.0902) | 0.07799 (0.04008-0.1159) | 0.3438 (0.2966-0.3911) |
| **VEST** | -0.1288 (-0.1635--0.09414) | -0.09242 (-0.1292--0.0556) | -0.02155 (-0.06553-0.02243) | -0.00139 (-0.04693-0.04415) | 0.03245 (-0.01218-0.07708) | 0.2508 (0.2081-0.2935) | 0.2209 (0.1804-0.2615) | 0.3615 (0.3161-0.4068) | 0.2012 (0.1645-0.2379) | 0.3923 (0.3461-0.4385) | 1 (0.9524-1) | -0.01422 (-0.03666-0.00822) | -0.1251 (-0.1581--0.09199) | -0.1022 (-0.1356--0.06892) | 0.1454 (0.09818-0.1925) |
| **CanDrA (breast)** | 0.01178 (0.001478-0.02208) | 0.01682 (0.00558-0.02806) | 0.02262 (0.006989-0.03826) | -0.0292 (-0.04642--0.01199) | 0.02211 (0.005766-0.03845) | -0.02195 (-0.03662--0.007285) | -0.04738 (-0.06057--0.03419) | -0.03228 (-0.04923--0.01533) | -0.02958 (-0.04076--0.0184) | -0.01264 (-0.0307-0.005419) | -0.01422 (-0.03666-0.00822) | 1 (0.9579-1) | -0.04649 (-0.0568--0.03619) | 0.01454 (0.004101-0.02498) | -0.01403 (-0.03414-0.006079) |
| **CanDrA (lung)** | 0.6887 (0.6467-0.7307) | 0.6704 (0.6281-0.7126) | 0.3986 (0.3585-0.4386) | 0.4414 (0.4028-0.48) | 0.4004 (0.3611-0.4397) | 0.1268 (0.08597-0.1675) | 0.35 (0.3083-0.3918) | 0.12 (0.08121-0.1589) | 0.1357 (0.09351-0.178) | 0.05247 (0.01475-0.0902) | -0.1251 (-0.1581--0.09199) | -0.04649 (-0.0568--0.03619) | 1 (0.9605-1) | 0.6563 (0.6169-0.6957) | 0.2986 (0.263-0.3342) |
| **CanDrA (melanoma)** | 0.6539 (0.6121-0.6958) | 0.6451 (0.603-0.6873) | 0.4411 (0.4009-0.4812) | 0.4247 (0.3859-0.4634) | 0.4482 (0.4088-0.4877) | 0.1601 (0.1193-0.201) | 0.3175 (0.2757-0.3593) | 0.1112 (0.07219-0.1502) | 0.142 (0.09985-0.1841) | 0.07799 (0.04008-0.1159) | -0.1022 (-0.1356--0.06892) | 0.01454 (0.004101-0.02498) | 0.6563 (0.6169-0.6957) | 1 (0.9606-1) | 0.3436 (0.3078-0.3794) |
| **Condel** | 0.3095 (0.2719-0.3471) | 0.3634 (0.3237-0.403) | 0.3535 (0.3076-0.3993) | 0.56 (0.5131-0.6068) | 0.5888 (0.5426-0.6351) | 0.4477 (0.4029-0.4926) | 0.3744 (0.3314-0.4174) | 0.3759 (0.3291-0.4226) | 0.1625 (0.123-0.202) | 0.3438 (0.2966-0.3911) | 0.1454 (0.09818-0.1925) | -0.01403 (-0.03414-0.006079) | 0.2986 (0.263-0.3342) | 0.3436 (0.3078-0.3794) | 1 (0.9524-1) |

Cohen's Kappa coefficients and 95% confidence intervals reported for each comparison.
