# Supplementary material for: Benchmarking mutation effect prediction algorithms using functionally validated cancer-related missense mutations
Source: Genome Biol. 2014 Oct 28;15(10):484. doi: 10.1186/s13059-014-0484-1 (PMC4232638; doi:10.1186/s13059-014-0484-1)
Supplement: Additional file 5: — Inter-rater agreement of mutation effect prediction algorithms as defined by unweighted Cohen’s Kappa coefficients for the 989 single nucleotide variants for which functional data are available and for the subset of these single nucleotide variants (n = 297) that are not in the COSMIC database. [file 13059_2014_484_MOESM5_ESM.pdf]

Additional file 5: Inter-rater agreement of mutation effect prediction algorithms as defined by unweighted Cohen's Kappa coefficients for the 989 single nucleotide variants for which functional data are available and for the subset of these single nucleotide variants (n=297) that are not in the COSMIC database.

All single nucleotide variants (n=989)

Kappa scores	CHASM (breast)	CHASM (lung)	CHASM (melanoma)	FATHMM (cancer)	FATHMM (missense)	Mutation Assessor	MutationTaster	PolyPhen-2	PROVEAN	SIFT	VEST	CanDrA (breast)	CanDrA (lung)	CanDrA (melanoma)	Condel
**CHASM (breast)**	1 (0.9377-1)	0.9008 (0.8386-0.9629)	0.7549 (0.6936-0.8161)	0.5403 (0.4844-0.5962)	0.4223 (0.3602-0.4845)	0.1844 (0.124-0.2448)	0.3923 (0.3301-0.4546)	0.1819 (0.1196-0.2442)	0.2855 (0.226-0.3449)	0.1761 (0.1138-0.2384)	0.01652 (-0.04576-0.07881)	-0.01605 (-0.05924-0.02714)	0.7871 (0.7303-0.8439)	0.6465 (0.5917-0.7013)	0.3127 (0.2526-0.3729)
**CHASM (lung)**	0.9008 (0.8386-0.9629)	1 (0.9377-1)	0.7394 (0.6774-0.8014)	0.5616 (0.5039-0.6193)	0.4329 (0.3706-0.4952)	0.1921 (0.133-0.2512)	0.4088 (0.3465-0.4711)	0.2034 (0.1415-0.2653)	0.253 (0.1951-0.3109)	0.1955 (0.1333-0.2578)	0.04133 (-0.02094-0.1036)	-0.02755 (-0.07308-0.01799)	0.7895 (0.7328-0.8462)	0.6372 (0.5828-0.6917)	0.3272 (0.266-0.3885)
**CHASM (melanoma)**	0.7549 (0.6936-0.8161)	0.7394 (0.6774-0.8014)	1 (0.9377-1)	0.5128 (0.4531-0.5726)	0.4394 (0.3774-0.5013)	0.1992 (0.1423-0.2561)	0.4072 (0.3454-0.469)	0.2344 (0.1735-0.2953)	0.2104 (0.155-0.2659)	0.2316 (0.17-0.2932)	0.06044 (-0.001205-0.1221)	-0.04643 (-0.09498-0.002116)	0.6151 (0.5591-0.6712)	0.5447 (0.4913-0.5981)	0.3356 (0.2735-0.3977)
**FATHMM (cancer)**	0.5403 (0.4844-0.5962)	0.5616 (0.5039-0.6193)	0.5128 (0.4531-0.5726)	1 (0.9377-1)	0.3339 (0.2763-0.3916)	0.1842 (0.1352-0.2331)	0.386 (0.329-0.4431)	0.2067 (0.1516-0.2617)	0.2278 (0.1808-0.2748)	0.1857 (0.1291-0.2423)	0.04604 (-0.01067-0.1028)	-0.04286 (-0.09856-0.01284)	0.5067 (0.4553-0.5582)	0.4084 (0.3604-0.4564)	0.4288 (0.3678-0.4898)
**FATHMM (missense)**	0.4223 (0.3602-0.4845)	0.4329 (0.3706-0.4952)	0.4394 (0.3774-0.5013)	0.3339 (0.2763-0.3916)	1 (0.9377-1)	0.2097 (0.1505-0.2688)	0.1952 (0.1329-0.2575)	0.137 (0.07504-0.199)	0.1399 (0.08198-0.1979)	0.1601 (0.09785-0.2224)	-0.007362 (-0.06964-0.05491)	0.1631 (0.1176-0.2085)	0.3602 (0.3035-0.4169)	0.4659 (0.4114-0.5204)	0.5515 (0.4903-0.6127)
**Mutation Assessor**	0.1844 (0.124-0.2448)	0.1921 (0.133-0.2512)	0.1992 (0.1423-0.2561)	0.1842 (0.1352-0.2331)	0.2097 (0.1505-0.2688)	1 (0.9379-1)	0.3956 (0.3359-0.4552)	0.5113 (0.4505-0.5722)	0.4822 (0.4201-0.5443)	0.5001 (0.4402-0.56)	0.3601 (0.3002-0.4199)	0.009606 (-0.02633-0.04554)	0.16 (0.1052-0.2147)	0.2243 (0.1704-0.2781)	0.4176 (0.3628-0.4725)
**MutationTaster**	0.3923 (0.3301-0.4546)	0.4088 (0.3465-0.4711)	0.4072 (0.3454-0.469)	0.386 (0.329-0.4431)	0.1952 (0.1329-0.2575)	0.3956 (0.3359-0.4552)	1 (0.9377-1)	0.5 (0.4379-0.5622)	0.4301 (0.3716-0.4887)	0.4194 (0.3571-0.4817)	0.3816 (0.3193-0.444)	-0.07257 (-0.1172--0.02793)	0.3833 (0.3265-0.4401)	0.298 (0.2434-0.3526)	0.2644 (0.2036-0.3252)
**PolyPhen-2**	0.1819 (0.1196-0.2442)	0.2034 (0.1415-0.2653)	0.2344 (0.1735-0.2953)	0.2067 (0.1516-0.2617)	0.137 (0.07504-0.199)	0.5113 (0.4505-0.5722)	0.5 (0.4379-0.5622)	1 (0.9377-1)	0.4922 (0.4322-0.5522)	0.5204 (0.4582-0.5826)	0.4278 (0.3656-0.49)	-0.04884 (-0.09111--0.006577)	0.183 (0.1263-0.2398)	0.1484 (0.09347-0.2032)	0.2598 (0.2002-0.3194)
**PROVEAN**	0.2855 (0.226-0.3449)	0.253 (0.1951-0.3109)	0.2104 (0.155-0.2659)	0.2278 (0.1808-0.2748)	0.1399 (0.08198-0.1979)	0.4822 (0.4201-0.5443)	0.4301 (0.3716-0.4887)	0.4922 (0.4322-0.5522)	1 (0.9377-1)	0.5243 (0.4655-0.5832)	0.3748 (0.316-0.4336)	-0.06137 (-0.09551--0.02722)	0.2613 (0.2076-0.3151)	0.2445 (0.1914-0.2977)	0.2389 (0.1857-0.292)
**SIFT**	0.1761 (0.1138-0.2384)	0.1955 (0.1333-0.2578)	0.2316 (0.17-0.2932)	0.1857 (0.1291-0.2423)	0.1601 (0.09785-0.2224)	0.5001 (0.4402-0.56)	0.4194 (0.3571-0.4817)	0.5204 (0.4582-0.5826)	0.5243 (0.4655-0.5832)	1 (0.9377-1)	0.4242 (0.3618-0.4865)	-0.0406 (-0.08474-0.003548)	0.144 (0.08721-0.2008)	0.1685 (0.1138-0.2232)	0.2785 (0.2178-0.3391)
**VEST**	0.01652 (-0.04576-0.07881)	0.04133 (-0.02094-0.1036)	0.06044 (-0.001205-0.1221)	0.04604 (-0.01067-0.1028)	-0.007362 (-0.06964-0.05491)	0.3601 (0.3002-0.4199)	0.3816 (0.3193-0.444)	0.4278 (0.3656-0.49)	0.3748 (0.316-0.4336)	0.4242 (0.3618-0.4865)	1 (0.9377-1)	-0.04967 (-0.09391--0.005427)	0.01299 (-0.0438-0.06979)	0.004599 (-0.05009-0.05929)	0.1068 (0.04619-0.1675)
**CanDrA (breast)**	-0.01605 (-0.05924-0.02714)	-0.02755 (-0.07308-0.01799)	-0.04643 (-0.09498-0.002116)	-0.04286 (-0.09856-0.01284)	0.1631 (0.1176-0.2085)	0.009606 (-0.02633-0.04554)	-0.07257 (-0.1172--0.02793)	-0.04884 (-0.09111--0.006577)	-0.06137 (-0.09551--0.02722)	-0.0406 (-0.08474-0.003548)	-0.04967 (-0.09391--0.005427)	1 (0.9428-1)	-0.001521 (-0.04209-0.03905)	0.1648 (0.1274-0.2022)	0.08421 (0.03334-0.1351)
**CanDrA (lung)**	0.7871 (0.7303-0.8439)	0.7895 (0.7328-0.8462)	0.6151 (0.5591-0.6712)	0.5067 (0.4553-0.5582)	0.3602 (0.3035-0.4169)	0.16 (0.1052-0.2147)	0.3833 (0.3265-0.4401)	0.183 (0.1263-0.2398)	0.2613 (0.2076-0.3151)	0.144 (0.08721-0.2008)	0.01299 (-0.0438-0.06979)	-0.001521 (-0.04209-0.03905)	1 (0.9465-1)	0.6051 (0.553-0.6573)	0.2848 (0.2297-0.34)
**CanDrA (melanoma)**	0.6465 (0.5917-0.7013)	0.6372 (0.5828-0.6917)	0.5447 (0.4913-0.5981)	0.4084 (0.3604-0.4564)	0.4659 (0.4114-0.5204)	0.2243 (0.1704-0.2781)	0.298 (0.2434-0.3526)	0.1484 (0.09347-0.2032)	0.2445 (0.1914-0.2977)	0.1685 (0.1138-0.2232)	0.004599 (-0.05009-0.05929)	0.1648 (0.1274-0.2022)	0.6051 (0.553-0.6573)	1 (0.9487-1)	0.3911 (0.3389-0.4432)
**Condel**	0.3127 (0.2526-0.3729)	0.3272 (0.266-0.3885)	0.3356 (0.2735-0.3977)	0.4288 (0.3678-0.4898)	0.5515 (0.4903-0.6127)	0.4176 (0.3628-0.4725)	0.2644 (0.2036-0.3252)	0.2598 (0.2002-0.3194)	0.2389 (0.1857-0.292)	0.2785 (0.2178-0.3391)	0.1068 (0.04619-0.1675)	0.08421 (0.03334-0.1351)	0.2848 (0.2297-0.34)	0.3911 (0.3389-0.4432)	1 (0.9377-1)

All non-COSMIC single nucleotide variants (n=297)

Kappa scores	CHASM (breast)	CHASM (lung)	CHASM (melanoma)	FATHMM (cancer)	FATHMM (missense)	Mutation Assessor	MutationTaster	PolyPhen-2	PROVEAN	SIFT	VEST	CanDrA (breast)	CanDrA (lung)	CanDrA (melanoma)	Condel
**CHASM (breast)**	1 (0.8863-1)	0.8591 (0.7463-0.9719)	0.6468 (0.5387-0.7548)	0.4836 (0.3844-0.5827)	0.4165 (0.315-0.518)	0.1834 (0.07203-0.2949)	0.3643 (0.2531-0.4756)	0.1488 (0.04322-0.2543)	0.2417 (0.1287-0.3548)	0.1444 (0.04255-0.2463)	-0.1589 (-0.2585--0.05938)	0.005263 (-0.02132-0.03185)	0.7768 (0.6723-0.8813)	0.6863 (0.5844-0.7881)	0.2874 (0.1948-0.38)
**CHASM (lung)**	0.8591 (0.7463-0.9719)	1 (0.8863-1)	0.6407 (0.5291-0.7523)	0.515 (0.4101-0.6199)	0.4468 (0.34-0.5537)	0.2275 (0.1141-0.3409)	0.4117 (0.2983-0.525)	0.2148 (0.1049-0.3247)	0.2091 (0.09845-0.3197)	0.2163 (0.1092-0.3234)	-0.1098 (-0.2151--0.004602)	0.01066 (-0.0194-0.04072)	0.7881 (0.6832-0.893)	0.6837 (0.5814-0.786)	0.3404 (0.2412-0.4396)
**CHASM (melanoma)**	0.6468 (0.5387-0.7548)	0.6407 (0.5291-0.7523)	1 (0.8863-1)	0.4666 (0.3554-0.5778)	0.5039 (0.3917-0.6162)	0.2729 (0.16-0.3858)	0.42 (0.307-0.533)	0.2931 (0.1796-0.4066)	0.1263 (0.02241-0.2301)	0.2842 (0.1717-0.3966)	-0.07994 (-0.1914-0.03148)	-0.005509 (-0.04166-0.03064)	0.5588 (0.4566-0.661)	0.5711 (0.4712-0.6709)	0.3862 (0.2787-0.4937)
**FATHMM (cancer)**	0.4836 (0.3844-0.5827)	0.515 (0.4101-0.6199)	0.4666 (0.3554-0.5778)	1 (0.8863-1)	0.4151 (0.3015-0.5286)	0.2566 (0.1488-0.3643)	0.3784 (0.2704-0.4863)	0.2652 (0.1527-0.3778)	0.2425 (0.1489-0.3361)	0.2258 (0.1123-0.3394)	-0.03098 (-0.1447-0.08274)	-0.008326 (-0.05219-0.03554)	0.4953 (0.4-0.5907)	0.443 (0.3496-0.5363)	0.5654 (0.4526-0.6782)
**FATHMM (missense)**	0.4165 (0.315-0.518)	0.4468 (0.34-0.5537)	0.5039 (0.3917-0.6162)	0.4151 (0.3015-0.5286)	1 (0.8863-1)	0.2185 (0.1091-0.3278)	0.2814 (0.1718-0.391)	0.1827 (0.06946-0.2959)	0.0733 (-0.02291-0.1695)	0.16 (0.04625-0.2737)	-0.07419 (-0.1878-0.03945)	0.03484 (-0.00709-0.07677)	0.3788 (0.2815-0.476)	0.4444 (0.3492-0.5396)	0.5957 (0.4837-0.7077)
**Mutation Assessor**	0.1834 (0.07203-0.2949)	0.2275 (0.1141-0.3409)	0.2729 (0.16-0.3858)	0.2566 (0.1488-0.3643)	0.2185 (0.1091-0.3278)	1 (0.8863-1)	0.4664 (0.3526-0.5801)	0.5719 (0.4602-0.6837)	0.3818 (0.2734-0.4902)	0.5302 (0.4206-0.6398)	0.3084 (0.2004-0.4164)	-0.001391 (-0.03368-0.03089)	0.1683 (0.06398-0.2726)	0.1929 (0.09108-0.2947)	0.3773 (0.2746-0.4799)
**MutationTaster**	0.3643 (0.2531-0.4756)	0.4117 (0.2983-0.525)	0.42 (0.307-0.533)	0.3784 (0.2704-0.4863)	0.2814 (0.1718-0.391)	0.4664 (0.3526-0.5801)	1 (0.8863-1)	0.5208 (0.4089-0.6327)	0.2575 (0.1493-0.3657)	0.3765 (0.2667-0.4863)	0.2696 (0.1613-0.3778)	-0.03317 (-0.06567--0.0006718)	0.3937 (0.2895-0.4979)	0.3133 (0.2116-0.4151)	0.3672 (0.2643-0.4702)
**PolyPhen-2**	0.1488 (0.04322-0.2543)	0.2148 (0.1049-0.3247)	0.2931 (0.1796-0.4066)	0.2652 (0.1527-0.3778)	0.1827 (0.06946-0.2959)	0.5719 (0.4602-0.6837)	0.5208 (0.4089-0.6327)	1 (0.8863-1)	0.3197 (0.2188-0.4206)	0.5219 (0.4086-0.6352)	0.3707 (0.2581-0.4834)	-0.03277 (-0.07123-0.005696)	0.1797 (0.07934-0.2801)	0.1324 (0.03423-0.2305)	0.3026 (0.1929-0.4122)
**PROVEAN**	0.2417 (0.1287-0.3548)	0.2091 (0.09845-0.3197)	0.1263 (0.02241-0.2301)	0.2425 (0.1489-0.3361)	0.0733 (-0.02291-0.1695)	0.3818 (0.2734-0.4902)	0.2575 (0.1493-0.3657)	0.3197 (0.2188-0.4206)	1 (0.8863-1)	0.3583 (0.2617-0.455)	0.2102 (0.1162-0.3043)	-0.03362 (-0.05755--0.009682)	0.2582 (0.1554-0.3611)	0.2209 (0.1208-0.3211)	0.2022 (0.1156-0.2888)
**SIFT**	0.1444 (0.04255-0.2463)	0.2163 (0.1092-0.3234)	0.2842 (0.1717-0.3966)	0.2258 (0.1123-0.3394)	0.16 (0.04625-0.2737)	0.5302 (0.4206-0.6398)	0.3765 (0.2667-0.4863)	0.5219 (0.4086-0.6352)	0.3583 (0.2617-0.455)	1 (0.8863-1)	0.3363 (0.2228-0.4499)	-0.02142 (-0.06304-0.0202)	0.1241 (0.02649-0.2216)	0.1476 (0.05215-0.2431)	0.344 (0.2322-0.4558)
**VEST**	-0.1589 (-0.2585--0.05938)	-0.1098 (-0.2151--0.004602)	-0.07994 (-0.1914-0.03148)	-0.03098 (-0.1447-0.08274)	-0.07419 (-0.1878-0.03945)	0.3084 (0.2004-0.4164)	0.2696 (0.1613-0.3778)	0.3707 (0.2581-0.4834)	0.2102 (0.1162-0.3043)	0.3363 (0.2228-0.4499)	1 (0.8863-1)	-0.0205 (-0.06403-0.02304)	-0.1465 (-0.2422--0.05082)	-0.146 (-0.2396--0.05231)	0.05405 (-0.05863-0.1667)
**CanDrA (breast)**	0.005263 (-0.02132-0.03185)	0.01066 (-0.0194-0.04072)	-0.005509 (-0.04166-0.03064)	-0.008326 (-0.05219-0.03554)	0.03484 (-0.00709-0.07677)	-0.001391 (-0.03368-0.03089)	-0.03317 (-0.06567--0.0006718)	-0.03277 (-0.07123-0.005696)	-0.03362 (-0.05755--0.009682)	-0.02142 (-0.06304-0.0202)	-0.0205 (-0.06403-0.02304)	1 (0.9074-1)	-0.01928 (-0.04685-0.008288)	0.02081 (-0.006545-0.04817)	-0.003185 (-0.05226-0.04589)
**CanDrA (lung)**	0.7768 (0.6723-0.8813)	0.7881 (0.6832-0.893)	0.5588 (0.4566-0.661)	0.4953 (0.4-0.5907)	0.3788 (0.2815-0.476)	0.1683 (0.06398-0.2726)	0.3937 (0.2895-0.4979)	0.1797 (0.07934-0.2801)	0.2582 (0.1554-0.3611)	0.1241 (0.02649-0.2216)	-0.1465 (-0.2422--0.05082)	-0.01928 (-0.04685-0.008288)	1 (0.9009-1)	0.6783 (0.5809-0.7756)	0.2884 (0.1986-0.3783)
**CanDrA (melanoma)**	0.6863 (0.5844-0.7881)	0.6837 (0.5814-0.786)	0.5711 (0.4712-0.6709)	0.443 (0.3496-0.5363)	0.4444 (0.3492-0.5396)	0.1929 (0.09108-0.2947)	0.3133 (0.2116-0.4151)	0.1324 (0.03423-0.2305)	0.2209 (0.1208-0.3211)	0.1476 (0.05215-0.2431)	-0.146 (-0.2396--0.05231)	0.02081 (-0.006545-0.04817)	0.6783 (0.5809-0.7756)	1 (0.9041-1)	0.3483 (0.2603-0.4363)
**Condel**	0.2874 (0.1948-0.38)	0.3404 (0.2412-0.4396)	0.3862 (0.2787-0.4937)	0.5654 (0.4526-0.6782)	0.5957 (0.4837-0.7077)	0.3773 (0.2746-0.4799)	0.3672 (0.2643-0.4702)	0.3026 (0.1929-0.4122)	0.2022 (0.1156-0.2888)	0.344 (0.2322-0.4558)	0.05405 (-0.05863-0.1667)	-0.003185 (-0.05226-0.04589)	0.2884 (0.1986-0.3783)	0.3483 (0.2603-0.4363)	1 (0.8863-1)
