# Supplementary material for: Benchmarking mutation effect prediction algorithms using functionally validated cancer-related missense mutations
Source: Genome Biol. 2014 Oct 28;15(10):484. doi: 10.1186/s13059-014-0484-1 (PMC4232638; doi:10.1186/s13059-014-0484-1)
Supplement: Additional file 6: — Summary of the 1,699 single nucleotide variants included in this study not present in the COSMIC database and their experimental functional categories. [file 13059_2014_484_MOESM6_ESM.pdf]

Additional file 6: Summary of the 1,699 single nucleotide variants included in this study not present in the COSMIC database and their experimental functional categories.

		Functional categories		
**Gene**	**Total SNVs (n)**	**Neutral (n)**	**Non-neutral (n)**	**Uncertain (n)**
BRAF	8	0	1	7
BRCA1	453	56	20	377
BRCA2	712	48	12	652
DICER1	13	0	5	8
EGFR	41	0	3	38
ERBB2	27	1	13	13
ESR1	7	0	3	4
IDH1	3	0	0	3
IDH2	1	0	0	1
KIT	15	0	2	13
KRAS	9	0	7	2
MYOD1	4	0	1	3
PIK3CA	13	0	1	12
SF3B1	7	0	0	7
TP53	386	4	120	262
**Total**	1699	109	188	1402
