# Supplementary material for: Benchmarking mutation effect prediction algorithms using functionally validated cancer-related missense mutations
Source: Genome Biol. 2014 Oct 28;15(10):484. doi: 10.1186/s13059-014-0484-1 (PMC4232638; doi:10.1186/s13059-014-0484-1)
Supplement: Additional file 7: — Predictions of single nucleotide variants not present in the COSMIC database (n = 1,699) by 15 mutation effect prediction algorithms. [file 13059_2014_484_MOESM7_ESM.pdf]

Additional file 7: Predictions of single nucleotide variants not present in the COSMIC database (n=1,699) by 15 mutation effect prediction algorithms.

Prediction algorithm	Prediction class	Functional categories			Total (n=1,699)
		Neutral (n=109)	Non-neutral (n=188)	Uncertain (n=1,402)	
**CHASM (breast)**	driver	7	136	332	475
	passenger	102	52	1070	1224
**CHASM (lung)**	driver	13	149	377	539
	passenger	96	39	1025	1160
**CHASM (melanoma)**	driver	28	162	628	818
	passenger	81	26	774	881
**FATHMM (cancer)**	CANCER	44	173	689	906
	PASSENGER/OTHER	65	15	713	793
**FATHMM (missense)**	Damaging	48	163	648	859
	Tolerated	61	25	750	836
	no weights	0	0	4	4
**Mutation Assessor**	high	2	27	37	66
	medium	37	107	551	695
	low	43	35	528	606
	neutral	27	19	284	330
	N/A	0	0	2	2
**MutationTaster**	disease_causing	16	155	491	662
	disease_causing_automatic	0	3	3	6
	polymorphism	89	30	908	1027
	polymorphism_automatic	4	0	0	4
**PolyPhen-2**	probably damaging	28	117	407	552
	possibly damaging	23	30	286	339
	benign	58	40	709	807
**PROVEAN**	Deleterious	31	96	408	535
	Neutral	78	92	994	1164
**SIFT**	Damaging	56	154	740	950
	Tolerated	53	34	662	749
**VEST**	functional	81	135	929	1145
	neutral	28	53	473	554
**CanDrA (breast)**	Driver	109	182	1349	1640
	Passenger	0	4	45	49
	No-call	0	2	8	10
**CanDrA (lung)**	Driver	5	144	324	473
	Passenger	94	38	977	1109
	No-call	10	6	101	117
**CanDrA (melanoma)**	Driver	9	139	335	483
	Passenger	89	39	968	1096
	No-call	11	10	99	120
**Condel**	Deleterious	56	175	817	1048
	Neutral	53	13	585	651
