# Supplementary material for: Benchmarking mutation effect prediction algorithms using functionally validated cancer-related missense mutations
Source: Genome Biol. 2014 Oct 28;15(10):484. doi: 10.1186/s13059-014-0484-1 (PMC4232638; doi:10.1186/s13059-014-0484-1)
Supplement: Additional file 8: — Predictions of functionally validated single nucleotide variants by 15 mutation effect prediction algorithms with low confidence prediction categories, for all mutations (n = 3,591) and for mutations not present in the COSMIC database (n = 1,699). [file 13059_2014_484_MOESM8_ESM.pdf]

Additional file 8: Predictions of functionally validated single nucleotide variants by 15 mutation effect prediction algorithms with low confidence prediction categories, for all mutations (n=3,591) and for mutations not present in the COSMIC database (n=1,699).

ALL SINGLE NUCLEOTIDE VARIANTS (n=3,591)

Prediction algorithm	Prediction class	Functional categories			Total (n=3,591)
		Neutral (n=140)	Non-neutral (n=849)	Uncertain (n=2,602)	
**CHASM (breast)**	Driver	22	754	1013	1789
	Passenger	108	73	1435	1616
	Low confidence	10	22	154	186
**CHASM (lung)**	Driver	24	768	1051	1843
	Passenger	94	55	1323	1472
	Low confidence	22	26	228	276
**CHASM (melanoma)**	Driver	35	768	1215	2018
	Passenger	71	37	900	1008
	Low confidence	34	44	487	565
**FATHMM (cancer)**	CANCER	35	807	1240	2082
	PASSENGER/OTHER	35	9	616	660
	Low confidence	70	33	746	849
**FATHMM (missense)**	Damaging	36	686	1060	1782
	Tolerated	34	28	581	643
	No weights/ Low confidence	70	135	961	1166
**Mutation Assessor**	high	2	71	97	170
	medium	50	579	1053	1682
	neutral	37	69	527	633
	Low confidence	51	129	919	1099
**MutationTaster**	disease_causing	31	721	1153	1905
	disease_causing_automatic	1	30	2	33
	polymorphism	92	69	1171	1332
	polymorphism_automatic	6	0	0	6
	Low confidence	10	29	276	315
**PolyPhen-2**	probably damaging	40	600	920	1560
	benign	74	134	1204	1412
	Low confidence	26	115	478	619
**PROVEAN**	Deleterious	20	433	519	972
	Neutral	74	113	1134	1321
	Low confidence	46	303	949	1298
**SIFT**	Damaging	66	715	1383	2164
	Tolerated	57	92	866	1015
	Low confidence	17	42	353	412
**VEST**	functional	80	679	1465	2224
	neutral	37	126	767	930
	Low confidence	23	44	370	437
**CanDrA (breast)**	Driver	140	805	2423	3368
	Passenger	0	39	140	179
	No-call/ Low confidence	0	5	39	44
**CanDrA (lung)**	Driver	24	767	1150	1941
	Passenger	102	59	1282	1443
	No-call/ Low confidence	14	23	170	207
**CanDrA (melanoma)**	Driver	28	734	1147	1909
	Passenger	97	75	1260	1432
	No-call/ Low confidence	15	40	195	250
**Condel**	Deleterious	54	751	1442	2247
	Neutral	52	32	665	749
	Low confidence	34	66	495	595

ALL SINGLE NUCLEOTIDE VARIANTS NOT PRESENT IN COSMIC (n=1,699)

Prediction algorithm	Prediction class	Functional categories			Total (n=1,699)
		Neutral (n=109)	Non-neutral (n=188)	Uncertain (n=1,402)	
**CHASM (breast)**	Driver	4	134	283	421
	Passenger	97	44	1016	1157
	Low confidence	8	10	103	121
**CHASM (lung)**	Driver	5	139	305	449
	Passenger	83	31	932	1046
	Low confidence	21	18	165	204
**CHASM (melanoma)**	Driver	16	148	458	622
	Passenger	62	19	587	668
	Low confidence	31	21	357	409
**FATHMM (cancer)**	CANCER	14	167	396	577
	PASSENGER/OTHER	34	6	448	488
	Low confidence	61	15	558	634
**FATHMM (missense)**	Damaging	18	150	395	563
	Tolerated	33	8	421	462
	No weights/ Low confidence	58	30	586	674
**Mutation Assessor**	high	2	27	37	66
	medium	37	107	551	695
	neutral	27	19	284	330
	Low confidence	43	35	528	606
**MutationTaster**	disease_causing	13	148	401	562
	disease_causing_automatic	0	2	2	4
	polymorphism	84	26	831	941
	polymorphism_automatic	4	0	0	4
	Low confidence	8	12	168	188
**PolyPhen-2**	probably damaging	28	118	407	553
	benign	58	40	709	807
	Low confidence	23	30	286	339
**PROVEAN**	Deleterious	13	53	196	262
	Neutral	63	50	693	806
	Low confidence	33	85	513	631
**SIFT**	Damaging	53	149	689	891
	Tolerated	45	29	500	574
	Low confidence	11	10	213	234
**VEST**	functional	64	128	807	999
	neutral	26	49	356	431
	Low confidence	19	11	239	269
**CanDrA (breast)**	Driver	109	182	1349	1640
	Passenger	0	4	45	49
	No-call/ Low confidence	0	2	8	10
**CanDrA (lung)**	Driver	5	144	324	473
	Passenger	94	38	977	1109
	No-call/ Low confidence	10	6	101	117
**CanDrA (melanoma)**	Driver	9	139	335	483
	Passenger	89	39	968	1096
	No-call/ Low confidence	11	10	99	120
**Condel**	Deleterious	35	160	628	823
	Neutral	46	6	477	529
	Low confidence	28	22	297	347
