# Supplementary material for: Benchmarking mutation effect prediction algorithms using functionally validated cancer-related missense mutations
Source: Genome Biol. 2014 Oct 28;15(10):484. doi: 10.1186/s13059-014-0484-1 (PMC4232638; doi:10.1186/s13059-014-0484-1)
Supplement: Additional file 9: — Number of single nucleotide variants classified as of low confidence by 15 mutation effect prediction algorithms. [file 13059_2014_484_MOESM9_ESM.pdf]

Additional file 9: Number of single nucleotide variants (SNVs) classified as of low confidence by 15 mutation effect prediction algorithms.

Number of predictors classifying SNV as of low confidence	All SNVs (n=3,591)	All non-COSMIC SNVs (n=1,699)	All SNVs for which functional data are available (n=989)	All non-COSMIC SNVs for which functional data are available (n=297)
**0**	679	170	321	50
**1**	741	287	274	72
**2**	708	334	166	57
**3**	556	308	111	41
**4**	414	259	58	38
**5**	253	164	37	21
**6**	135	97	13	11
**7**	78	58	7	6
**8**	21	17	2	1
**9**	5	4	0	0
**10**	1	1	0	0
**11**	0	0	0	0
**12**	0	0	0	0
**13**	0	0	0	0
**14**	0	0	0	0
**15**	0	0	0	0

SNVs with a majority vote of low confidence: *BRAF* (R95T), *BRCA1* (A1752T, C1697R, D1739G, D1778G, E1419Q, G1738E, G1738V, N417S, N723D, Q380R, R136K, W1782R), *BRCA2* (D3170G, D806H, H2361R, I729M, K1440E, K1440R, L1687P, N2266H, P2762S, V3079I), *ERBB2* (E1114G, L313V, R678Q, G451V)

SNVs with a majority vote of low confidence: *BRCA1* (A1752T, C1697R, D1739G, D1778G, E1419Q, G1738E, G1738V, N417S, N723D, Q380R, R136K, W1782R), *BRCA2* (D3170G, D806H, H2361R, I729M, K1440E, K1440R, L1687P, N2266H, P2762S, V3079I)

SNVs with a majority vote of low confidence: *BRCA1* (N723D), *ERBB2* (R678Q)

SNVs with a majority vote of low confidence: *BRCA1* (N723D)
