# Supplementary material for: Benchmarking mutation effect prediction algorithms using functionally validated cancer-related missense mutations
Source: Genome Biol. 2014 Oct 28;15(10):484. doi: 10.1186/s13059-014-0484-1 (PMC4232638; doi:10.1186/s13059-014-0484-1)
Supplement: Additional file 11: — Inter-rater agreement of mutation effect prediction algorithms as defined by unweighted Cohen’s Kappa coefficients for predictions made for all single nucleotide variants (n = 3,591) and for single nucleotide variants not present in the COSMIC database (n = 1,699) when a low confidence category is included. [file 13059_2014_484_MOESM11_ESM.pdf]

Additional file 11: Inter-rater agreement of mutation effect prediction algorithms as defined by unweighted Cohen's Kappa coefficients for predictions made for all single nucleotide variants (n=3,591) and for mutations not present in the COSMIC database (n=1,699) when a low confidence category is included.

All single nucleotide variants (n=3,591)

Kappa scores	CHASM (breast)	CHASM (lung)	CHASM (melanoma)	FATHMM (cancer)	FATHMM (missense)	Mutation Assessor	MutationTaster	PolyPhen-2	PROVEAN	SIFT	VEST	CanDrA (breast)	CanDrA (lung)	CanDrA (melanoma)	Condel
**CHASM (breast)**	1 (0.9714-1)	0.8257 (0.7978-0.8535)	0.5815 (0.5568-0.6062)	0.4839 (0.4624-0.5054)	0.4319 (0.4119-0.4519)	0.09982 (0.0796-0.12)	0.3174 (0.2901-0.3447)	0.146 (0.1208-0.1711)	0.1603 (0.1403-0.1804)	0.1218 (0.0962-0.1474)	-0.04218 (-0.06718--0.01717)	0.03238 (0.01825-0.04651)	0.7215 (0.6932-0.7498)	0.6575 (0.6295-0.6855)	0.3792 (0.3561-0.4023)
**CHASM (lung)**	0.8257 (0.7978-0.8535)	1 (0.9728-1)	0.5364 (0.5116-0.5611)	0.5162 (0.4941-0.5382)	0.4499 (0.4292-0.4706)	0.1085 (0.08756-0.1294)	0.3225 (0.2956-0.3494)	0.1636 (0.1386-0.1885)	0.1326 (0.1122-0.1529)	0.1307 (0.1052-0.1562)	-0.03255 (-0.0576--0.007497)	0.03119 (0.01686-0.04552)	0.7087 (0.681-0.7363)	0.6474 (0.62-0.6748)	0.388 (0.3646-0.4115)
**CHASM (melanoma)**	0.5815 (0.5568-0.6062)	0.5364 (0.5116-0.5611)	1 (0.9756-1)	0.4434 (0.42-0.4669)	0.3957 (0.3732-0.4182)	0.1503 (0.1276-0.1731)	0.2484 (0.2235-0.2732)	0.1444 (0.1207-0.1681)	0.1469 (0.1263-0.1674)	0.1458 (0.121-0.1705)	0.03251 (0.007897-0.05712)	0.04396 (0.02924-0.05869)	0.4803 (0.4553-0.5053)	0.468 (0.443-0.4929)	0.3504 (0.3264-0.3745)
**FATHMM (cancer)**	0.4839 (0.4624-0.5054)	0.5162 (0.4941-0.5382)	0.4434 (0.42-0.4669)	1 (0.9759-1)	0.659 (0.6353-0.6827)	0.1624 (0.1385-0.1862)	0.2958 (0.2733-0.3182)	0.1871 (0.165-0.2092)	0.1246 (0.1043-0.145)	0.142 (0.1187-0.1652)	0.03025 (0.006848-0.05364)	-0.002514 (-0.01661-0.01158)	0.5236 (0.5015-0.5456)	0.4847 (0.4626-0.5069)	0.5234 (0.4995-0.5472)
**FATHMM (missense)**	0.4319 (0.4119-0.4519)	0.4499 (0.4292-0.4706)	0.3957 (0.3732-0.4182)	0.659 (0.6353-0.6827)	1 (0.9759-1)	0.1562 (0.132-0.1803)	0.196 (0.1749-0.2171)	0.1426 (0.121-0.1643)	0.1145 (0.09315-0.1359)	0.1143 (0.0924-0.1362)	-0.00737 (-0.0294-0.01466)	0.04288 (0.03094-0.05482)	0.4075 (0.3871-0.428)	0.4523 (0.4317-0.473)	0.5737 (0.5509-0.5965)
**Mutation Assessor**	0.09982 (0.0796-0.12)	0.1085 (0.08756-0.1294)	0.1503 (0.1276-0.1731)	0.1624 (0.1385-0.1862)	0.1562 (0.132-0.1803)	1 (0.9758-1)	0.2951 (0.2738-0.3165)	0.3306 (0.3089-0.3523)	0.2467 (0.2255-0.2678)	0.3758 (0.3536-0.3979)	0.2892 (0.2669-0.3115)	-0.006134 (-0.01849-0.006223)	0.09677 (0.07606-0.1175)	0.1239 (0.103-0.1448)	0.4624 (0.4394-0.4854)
**MutationTaster**	0.3174 (0.2901-0.3447)	0.3225 (0.2956-0.3494)	0.2484 (0.2235-0.2732)	0.2958 (0.2733-0.3182)	0.196 (0.1749-0.2171)	0.2951 (0.2738-0.3165)	1 (0.9733-1)	0.4384 (0.4136-0.4631)	0.2485 (0.2282-0.2687)	0.3523 (0.3267-0.378)	0.2886 (0.2634-0.3138)	-0.07059 (-0.08546--0.05573)	0.3681 (0.3408-0.3954)	0.3427 (0.3157-0.3698)	0.3024 (0.2786-0.3262)
**PolyPhen-2**	0.146 (0.1208-0.1711)	0.1636 (0.1386-0.1885)	0.1444 (0.1207-0.1681)	0.1871 (0.165-0.2092)	0.1426 (0.121-0.1643)	0.3306 (0.3089-0.3523)	0.4384 (0.4136-0.4631)	1 (0.9757-1)	0.3218 (0.3-0.3437)	0.4222 (0.3985-0.446)	0.3173 (0.2939-0.3407)	-0.03029 (-0.04246--0.01812)	0.1598 (0.1348-0.1848)	0.1404 (0.1154-0.1653)	0.247 (0.2244-0.2696)
**PROVEAN**	0.1603 (0.1403-0.1804)	0.1326 (0.1122-0.1529)	0.1469 (0.1263-0.1674)	0.1246 (0.1043-0.145)	0.1145 (0.09315-0.1359)	0.2467 (0.2255-0.2678)	0.2485 (0.2282-0.2687)	0.3218 (0.3-0.3437)	1 (0.9767-1)	0.2875 (0.2678-0.3073)	0.2467 (0.2272-0.2662)	-0.0271 (-0.0358--0.0184)	0.1415 (0.1216-0.1613)	0.1514 (0.1314-0.1715)	0.1473 (0.1278-0.1669)
**SIFT**	0.1218 (0.0962-0.1474)	0.1307 (0.1052-0.1562)	0.1458 (0.121-0.1705)	0.142 (0.1187-0.1652)	0.1143 (0.0924-0.1362)	0.3758 (0.3536-0.3979)	0.3523 (0.3267-0.378)	0.4222 (0.3985-0.446)	0.2875 (0.2678-0.3073)	1 (0.9746-1)	0.3653 (0.34-0.3905)	-0.02209 (-0.03814--0.006039)	0.1139 (0.08797-0.1399)	0.1281 (0.1024-0.1539)	0.2565 (0.2321-0.2808)
**VEST**	-0.04218 (-0.06718--0.01717)	-0.03255 (-0.0576--0.007497)	0.03251 (0.007897-0.05712)	0.03025 (0.006848-0.05364)	-0.00737 (-0.0294-0.01466)	0.2892 (0.2669-0.3115)	0.2886 (0.2634-0.3138)	0.3173 (0.2939-0.3407)	0.2467 (0.2272-0.2662)	0.3653 (0.34-0.3905)	1 (0.9749-1)	-0.02656 (-0.0429--0.01022)	-0.05126 (-0.07674--0.02579)	-0.04562 (-0.07092--0.02032)	0.1004 (0.07595-0.1248)
**CanDrA (breast)**	0.03238 (0.01825-0.04651)	0.03119 (0.01686-0.04552)	0.04396 (0.02924-0.05869)	-0.002514 (-0.01661-0.01158)	0.04288 (0.03094-0.05482)	-0.006134 (-0.01849-0.006223)	-0.07059 (-0.08546--0.05573)	-0.03029 (-0.04246--0.01812)	-0.0271 (-0.0358--0.0184)	-0.02209 (-0.03814--0.006039)	-0.02656 (-0.0429--0.01022)	1 (0.9716-1)	-0.03769 (-0.0528--0.02259)	0.06321 (0.0484-0.07801)	0.01523 (-0.0006331-0.03109)
**CanDrA (lung)**	0.7215 (0.6932-0.7498)	0.7087 (0.681-0.7363)	0.4803 (0.4553-0.5053)	0.5236 (0.5015-0.5456)	0.4075 (0.3871-0.428)	0.09677 (0.07606-0.1175)	0.3681 (0.3408-0.3954)	0.1598 (0.1348-0.1848)	0.1415 (0.1216-0.1613)	0.1139 (0.08797-0.1399)	-0.05126 (-0.07674--0.02579)	-0.03769 (-0.0528--0.02259)	1 (0.9718-1)	0.6826 (0.6547-0.7105)	0.371 (0.3472-0.3947)
**CanDrA (melanoma)**	0.6575 (0.6295-0.6855)	0.6474 (0.62-0.6748)	0.468 (0.443-0.4929)	0.4847 (0.4626-0.5069)	0.4523 (0.4317-0.473)	0.1239 (0.103-0.1448)	0.3427 (0.3157-0.3698)	0.1404 (0.1154-0.1653)	0.1514 (0.1314-0.1715)	0.1281 (0.1024-0.1539)	-0.04562 (-0.07092--0.02032)	0.06321 (0.0484-0.07801)	0.6826 (0.6547-0.7105)	1 (0.9724-1)	0.4228 (0.3991-0.4464)
**Condel**	0.3792 (0.3561-0.4023)	0.388 (0.3646-0.4115)	0.3504 (0.3264-0.3745)	0.5234 (0.4995-0.5472)	0.5737 (0.5509-0.5965)	0.4624 (0.4394-0.4854)	0.3024 (0.2786-0.3262)	0.247 (0.2244-0.2696)	0.1473 (0.1278-0.1669)	0.2565 (0.2321-0.2808)	0.1004 (0.07595-0.1248)	0.01523 (-0.0006331-0.03109)	0.371 (0.3472-0.3947)	0.4228 (0.3991-0.4464)	1 (0.9757-1)

All single nucleotide variants not present in COSMIC (n=1,699)

Kappa scores	CHASM (breast)	CHASM (lung)	CHASM (melanoma)	FATHMM (cancer)	FATHMM (missense)	Mutation Assessor	MutationTaster	PolyPhen-2	PROVEAN	SIFT	VEST	CanDrA (breast)	CanDrA (lung)	CanDrA (melanoma)	Condel
**CHASM (breast)**	1 (0.961-1)	0.7177 (0.6805-0.755)	0.4071 (0.3759-0.4383)	0.3492 (0.3227-0.3757)	0.3408 (0.315-0.3665)	0.05586 (0.03179-0.07993)	0.2726 (0.2355-0.3097)	0.0964 (0.06276-0.13)	0.0983 (0.06948-0.1271)	0.04358 (0.01249-0.07468)	-0.117 (-0.1447--0.08926)	0.01394 (0.004343-0.02353)	0.6395 (0.6003-0.6786)	0.6106 (0.5715-0.6497)	0.2821 (0.2525-0.3116)
**CHASM (lung)**	0.7177 (0.6805-0.755)	1 (0.9634-1)	0.3558 (0.3233-0.3884)	0.3966 (0.3679-0.4252)	0.3864 (0.3584-0.4144)	0.06361 (0.03732-0.08991)	0.2783 (0.2417-0.315)	0.1402 (0.1058-0.1746)	0.04989 (0.0191-0.08068)	0.05919 (0.02725-0.09114)	-0.1026 (-0.1315--0.0736)	0.01477 (0.004794-0.02474)	0.6249 (0.5874-0.6625)	0.5999 (0.5623-0.6374)	0.308 (0.2771-0.3389)
**CHASM (melanoma)**	0.4071 (0.3759-0.4383)	0.3558 (0.3233-0.3884)	1 (0.9659-1)	0.3212 (0.2885-0.354)	0.322 (0.2896-0.3544)	0.1357 (0.1039-0.1675)	0.2056 (0.172-0.2392)	0.1208 (0.08684-0.1549)	0.08929 (0.05769-0.1209)	0.08507 (0.0515-0.1186)	-0.02179 (-0.05406-0.01048)	0.02036 (0.0086-0.03212)	0.3199 (0.2879-0.3519)	0.3675 (0.3353-0.3996)	0.2904 (0.2567-0.324)
**FATHMM (cancer)**	0.3492 (0.3227-0.3757)	0.3966 (0.3679-0.4252)	0.3212 (0.2885-0.354)	1 (0.9663-1)	0.7152 (0.6815-0.7489)	0.1357 (0.1024-0.1691)	0.2439 (0.2143-0.2735)	0.1855 (0.1538-0.2172)	0.06128 (0.02956-0.09301)	0.1033 (0.07235-0.1343)	-0.01625 (-0.04689-0.0144)	-0.01669 (-0.02724--0.006151)	0.4006 (0.3734-0.4278)	0.3925 (0.3652-0.4199)	0.5115 (0.4792-0.5437)
**FATHMM (missense)**	0.3408 (0.315-0.3665)	0.3864 (0.3584-0.4144)	0.322 (0.2896-0.3544)	0.7152 (0.6815-0.7489)	1 (0.9662-1)	0.1334 (0.09997-0.1669)	0.2152 (0.1863-0.2441)	0.1634 (0.1322-0.1946)	0.05311 (0.02146-0.08476)	0.09857 (0.06816-0.129)	-0.001707 (-0.03192-0.02851)	0.009013 (-0.001252-0.01928)	0.3218 (0.2954-0.3482)	0.3656 (0.339-0.3922)	0.558 (0.5261-0.5898)
**Mutation Assessor**	0.05586 (0.03179-0.07993)	0.06361 (0.03732-0.08991)	0.1357 (0.1039-0.1675)	0.1357 (0.1024-0.1691)	0.1334 (0.09997-0.1669)	1 (0.9652-1)	0.2533 (0.2256-0.2811)	0.296 (0.2659-0.3261)	0.1487 (0.12-0.1773)	0.3319 (0.3005-0.3633)	0.2376 (0.2054-0.2697)	-0.01122 (-0.02344-0.001005)	0.0625 (0.03758-0.08742)	0.06671 (0.04155-0.09186)	0.4834 (0.4507-0.5161)
**MutationTaster**	0.2726 (0.2355-0.3097)	0.2783 (0.2417-0.315)	0.2056 (0.172-0.2392)	0.2439 (0.2143-0.2735)	0.2152 (0.1863-0.2441)	0.2533 (0.2256-0.2811)	1 (0.9626-1)	0.4273 (0.3922-0.4624)	0.1441 (0.114-0.1743)	0.3028 (0.2687-0.3369)	0.1812 (0.1499-0.2125)	-0.0451 (-0.05662--0.03358)	0.3155 (0.2778-0.3532)	0.2898 (0.252-0.3276)	0.3206 (0.2879-0.3533)
**PolyPhen-2**	0.0964 (0.06276-0.13)	0.1402 (0.1058-0.1746)	0.1208 (0.08684-0.1549)	0.1855 (0.1538-0.2172)	0.1634 (0.1322-0.1946)	0.296 (0.2659-0.3261)	0.4273 (0.3922-0.4624)	1 (0.9653-1)	0.2181 (0.1861-0.2501)	0.3906 (0.3573-0.424)	0.2434 (0.212-0.2747)	-0.02638 (-0.0375--0.01525)	0.1236 (0.0893-0.1578)	0.1116 (0.07718-0.146)	0.2626 (0.2296-0.2956)
**PROVEAN**	0.0983 (0.06948-0.1271)	0.04989 (0.0191-0.08068)	0.08929 (0.05769-0.1209)	0.06128 (0.02956-0.09301)	0.05311 (0.02146-0.08476)	0.1487 (0.12-0.1773)	0.1441 (0.114-0.1743)	0.2181 (0.1861-0.2501)	1 (0.9642-1)	0.1891 (0.1617-0.2165)	0.1512 (0.1254-0.177)	-0.02386 (-0.03139--0.01633)	0.06484 (0.03599-0.09368)	0.06888 (0.03994-0.09781)	0.09164 (0.06291-0.1204)
**SIFT**	0.04358 (0.01249-0.07468)	0.05919 (0.02725-0.09114)	0.08507 (0.0515-0.1186)	0.1033 (0.07235-0.1343)	0.09857 (0.06816-0.129)	0.3319 (0.3005-0.3633)	0.3028 (0.2687-0.3369)	0.3906 (0.3573-0.424)	0.1891 (0.1617-0.2165)	1 (0.9637-1)	0.3249 (0.2895-0.3603)	-0.0146 (-0.03048-0.001269)	0.04071 (0.008402-0.07302)	0.05751 (0.02497-0.09005)	0.2545 (0.2195-0.2895)
**VEST**	-0.117 (-0.1447--0.08926)	-0.1026 (-0.1315--0.0736)	-0.02179 (-0.05406-0.01048)	-0.01625 (-0.04689-0.0144)	-0.001707 (-0.03192-0.02851)	0.2376 (0.2054-0.2697)	0.1812 (0.1499-0.2125)	0.2434 (0.212-0.2747)	0.1512 (0.1254-0.177)	0.3249 (0.2895-0.3603)	1 (0.9645-1)	-0.01432 (-0.03154-0.002891)	-0.1151 (-0.144--0.08609)	-0.1002 (-0.1294--0.07099)	0.1052 (0.07078-0.1397)
**CanDrA (breast)**	0.01394 (0.004343-0.02353)	0.01477 (0.004794-0.02474)	0.02036 (0.0086-0.03212)	-0.01669 (-0.02724--0.006151)	0.009013 (-0.001252-0.01928)	-0.01122 (-0.02344-0.001005)	-0.0451 (-0.05662--0.03358)	-0.02638 (-0.0375--0.01525)	-0.02386 (-0.03139--0.01633)	-0.0146 (-0.03048-0.001269)	-0.01432 (-0.03154-0.002891)	1 (0.9579-1)	-0.04649 (-0.0568--0.03619)	0.01454 (0.004101-0.02498)	-0.01095 (-0.02531-0.003406)
**CanDrA (lung)**	0.6395 (0.6003-0.6786)	0.6249 (0.5874-0.6625)	0.3199 (0.2879-0.3519)	0.4006 (0.3734-0.4278)	0.3218 (0.2954-0.3482)	0.0625 (0.03758-0.08742)	0.3155 (0.2778-0.3532)	0.1236 (0.0893-0.1578)	0.06484 (0.03599-0.09368)	0.04071 (0.008402-0.07302)	-0.1151 (-0.144--0.08609)	-0.04649 (-0.0568--0.03619)	1 (0.9605-1)	0.6563 (0.6169-0.6957)	0.285 (0.2544-0.3156)
**CanDrA (melanoma)**	0.6106 (0.5715-0.6497)	0.5999 (0.5623-0.6374)	0.3675 (0.3353-0.3996)	0.3925 (0.3652-0.4199)	0.3656 (0.339-0.3922)	0.06671 (0.04155-0.09186)	0.2898 (0.252-0.3276)	0.1116 (0.07718-0.146)	0.06888 (0.03994-0.09781)	0.05751 (0.02497-0.09005)	-0.1002 (-0.1294--0.07099)	0.01454 (0.004101-0.02498)	0.6563 (0.6169-0.6957)	1 (0.9606-1)	0.3186 (0.2878-0.3494)
**Condel**	0.2821 (0.2525-0.3116)	0.308 (0.2771-0.3389)	0.2904 (0.2567-0.324)	0.5115 (0.4792-0.5437)	0.558 (0.5261-0.5898)	0.4834 (0.4507-0.5161)	0.3206 (0.2879-0.3533)	0.2626 (0.2296-0.2956)	0.09164 (0.06291-0.1204)	0.2545 (0.2195-0.2895)	0.1052 (0.07078-0.1397)	-0.01095 (-0.02531-0.003406)	0.285 (0.2544-0.3156)	0.3186 (0.2878-0.3494)	1 (0.9654-1)

Cohen's Kappa coefficients and 95% confidence intervals reported for each comparison.
