# Supplementary material for: Benchmarking mutation effect prediction algorithms using functionally validated cancer-related missense mutations
Source: Genome Biol. 2014 Oct 28;15(10):484. doi: 10.1186/s13059-014-0484-1 (PMC4232638; doi:10.1186/s13059-014-0484-1)
Supplement: Additional file 13: — Inter-rater agreement of mutation effect prediction algorithms as defined by unweighted Cohen’s Kappa coefficients for 989 single nucleotide variants for which functional data are available and for the subset of these single nucleotide variants (n = 297) that are not present in the COSMIC database, when a low confidence category is included. [file 13059_2014_484_MOESM13_ESM.pdf]

Additional file 13: Inter-rater agreement of mutation effect prediction algorithms as defined by unweighted Cohen's Kappa coefficients for 989 single nucleotide variants for which functional data are available and for the subset of these single nucleotide variants (n=297) that are not present in the COSMIC database, when a low confidence category is included.

All single nucleotide variants (n=989)

Kappa scores	CHASM (breast)	CHASM (lung)	CHASM (melanoma)	FATHMM (cancer)	FATHMM (missense)	Mutation Assessor	MutationTaster	PolyPhen-2	PROVEAN	SIFT	VEST	CanDrA (breast)	CanDrA (lung)	CanDrA (melanoma)	Condel
**CHASM (breast)**	1 (0.9452-1)	0.8288 (0.776-0.8816)	0.6346 (0.5857-0.6836)	0.4605 (0.4187-0.5022)	0.3449 (0.3051-0.3847)	0.1112 (0.06837-0.1541)	0.3378 (0.2839-0.3918)	0.1468 (0.1005-0.1931)	0.1463 (0.1122-0.1804)	0.147 (0.09506-0.199)	0.02895 (-0.02272-0.08063)	-0.01572 (-0.05553-0.02409)	0.7394 (0.6854-0.7934)	0.6423 (0.5896-0.6951)	0.3122 (0.2661-0.3583)
**CHASM (lung)**	0.8288 (0.776-0.8816)	1 (0.9484-1)	0.6011 (0.5523-0.6499)	0.5051 (0.4615-0.5487)	0.341 (0.2995-0.3826)	0.1295 (0.08596-0.1731)	0.3401 (0.2878-0.3924)	0.1695 (0.1242-0.2148)	0.1293 (0.09503-0.1635)	0.1693 (0.1184-0.2201)	0.04634 (-0.004126-0.09682)	-0.02159 (-0.06141-0.01822)	0.7451 (0.6927-0.7975)	0.6174 (0.5661-0.6686)	0.333 (0.2862-0.3798)
**CHASM (melanoma)**	0.6346 (0.5857-0.6836)	0.6011 (0.5523-0.6499)	1 (0.9518-1)	0.4426 (0.3962-0.489)	0.3445 (0.2999-0.3891)	0.153 (0.1081-0.198)	0.3282 (0.2793-0.3771)	0.1482 (0.1042-0.1921)	0.147 (0.1119-0.1821)	0.1586 (0.11-0.2071)	0.08769 (0.03957-0.1358)	-0.01349 (-0.05142-0.02444)	0.5464 (0.4973-0.5954)	0.5075 (0.4591-0.5558)	0.3026 (0.2551-0.3501)
**FATHMM (cancer)**	0.4605 (0.4187-0.5022)	0.5051 (0.4615-0.5487)	0.4426 (0.3962-0.489)	1 (0.9495-1)	0.4946 (0.447-0.5422)	0.2006 (0.1553-0.2459)	0.2988 (0.2562-0.3414)	0.1432 (0.1033-0.1832)	0.09785 (0.06383-0.1319)	0.1391 (0.09523-0.183)	0.04743 (0.004148-0.09071)	0.04503 (0.009271-0.08078)	0.4878 (0.445-0.5305)	0.4218 (0.3791-0.4645)	0.4404 (0.3924-0.4884)
**FATHMM (missense)**	0.3449 (0.3051-0.3847)	0.341 (0.2995-0.3826)	0.3445 (0.2999-0.3891)	0.4946 (0.447-0.5422)	1 (0.9487-1)	0.2321 (0.1832-0.281)	0.1325 (0.09192-0.173)	0.1053 (0.06156-0.1491)	0.06832 (0.02619-0.1105)	0.119 (0.07655-0.1614)	-0.0004849 (-0.04325-0.04228)	0.09687 (0.07022-0.1235)	0.327 (0.2866-0.3674)	0.4324 (0.3907-0.4742)	0.5631 (0.5164-0.6097)
**Mutation Assessor**	0.1112 (0.06837-0.1541)	0.1295 (0.08596-0.1731)	0.153 (0.1081-0.198)	0.2006 (0.1553-0.2459)	0.2321 (0.1832-0.281)	1 (0.9521-1)	0.3189 (0.2758-0.3621)	0.3383 (0.2931-0.3835)	0.2665 (0.2246-0.3085)	0.3797 (0.3355-0.4239)	0.3175 (0.2729-0.3621)	0.0164 (-0.01152-0.04433)	0.1054 (0.06236-0.1484)	0.1707 (0.1266-0.2147)	0.5132 (0.4673-0.5591)
**MutationTaster**	0.3378 (0.2839-0.3918)	0.3401 (0.2878-0.3924)	0.3282 (0.2793-0.3771)	0.2988 (0.2562-0.3414)	0.1325 (0.09192-0.173)	0.3189 (0.2758-0.3621)	1 (0.9467-1)	0.3999 (0.354-0.4458)	0.2513 (0.2171-0.2855)	0.3619 (0.3103-0.4134)	0.3658 (0.3146-0.417)	-0.06814 (-0.108--0.02829)	0.3625 (0.3091-0.4159)	0.2892 (0.237-0.3413)	0.2535 (0.2071-0.2999)
**PolyPhen-2**	0.1468 (0.1005-0.1931)	0.1695 (0.1242-0.2148)	0.1482 (0.1042-0.1921)	0.1432 (0.1033-0.1832)	0.1053 (0.06156-0.1491)	0.3383 (0.2931-0.3835)	0.3999 (0.354-0.4458)	1 (0.9532-1)	0.3276 (0.286-0.3692)	0.3872 (0.3416-0.4327)	0.3216 (0.2753-0.3678)	-0.03918 (-0.06706--0.01131)	0.1552 (0.1097-0.2008)	0.1237 (0.07728-0.17)	0.1929 (0.1498-0.2361)
**PROVEAN**	0.1463 (0.1122-0.1804)	0.1293 (0.09503-0.1635)	0.147 (0.1119-0.1821)	0.09785 (0.06383-0.1319)	0.06832 (0.02619-0.1105)	0.2665 (0.2246-0.3085)	0.2513 (0.2171-0.2855)	0.3276 (0.286-0.3692)	1 (0.9543-1)	0.2942 (0.2589-0.3294)	0.2673 (0.2309-0.3037)	-0.02739 (-0.04543--0.009356)	0.1238 (0.09005-0.1575)	0.1463 (0.1106-0.1821)	0.1337 (0.09781-0.1697)
**SIFT**	0.147 (0.09506-0.199)	0.1693 (0.1184-0.2201)	0.1586 (0.11-0.2071)	0.1391 (0.09523-0.183)	0.119 (0.07655-0.1614)	0.3797 (0.3355-0.4239)	0.3619 (0.3103-0.4134)	0.3872 (0.3416-0.4327)	0.2942 (0.2589-0.3294)	1 (0.9497-1)	0.3769 (0.3268-0.4269)	-0.0314 (-0.0697-0.006908)	0.121 (0.06944-0.1726)	0.1538 (0.1031-0.2045)	0.2441 (0.1973-0.2909)
**VEST**	0.02895 (-0.02272-0.08063)	0.04634 (-0.004126-0.09682)	0.08769 (0.03957-0.1358)	0.04743 (0.004148-0.09071)	-0.0004849 (-0.04325-0.04228)	0.3175 (0.2729-0.3621)	0.3658 (0.3146-0.417)	0.3216 (0.2753-0.3678)	0.2673 (0.2309-0.3037)	0.3769 (0.3268-0.4269)	1 (0.95-1)	-0.04669 (-0.08319--0.01019)	0.03942 (-0.01174-0.09058)	0.01917 (-0.0314-0.06975)	0.1129 (0.06651-0.1593)
**CanDrA (breast)**	-0.01572 (-0.05553-0.02409)	-0.02159 (-0.06141-0.01822)	-0.01349 (-0.05142-0.02444)	0.04503 (0.009271-0.08078)	0.09687 (0.07022-0.1235)	0.0164 (-0.01152-0.04433)	-0.06814 (-0.108--0.02829)	-0.03918 (-0.06706--0.01131)	-0.02739 (-0.04543--0.009356)	-0.0314 (-0.0697-0.006908)	-0.04669 (-0.08319--0.01019)	1 (0.9428-1)	-0.001521 (-0.04209-0.03905)	0.1648 (0.1274-0.2022)	0.04165 (0.005725-0.07757)
**CanDrA (lung)**	0.7394 (0.6854-0.7934)	0.7451 (0.6927-0.7975)	0.5464 (0.4973-0.5954)	0.4878 (0.445-0.5305)	0.327 (0.2866-0.3674)	0.1054 (0.06236-0.1484)	0.3625 (0.3091-0.4159)	0.1552 (0.1097-0.2008)	0.1238 (0.09005-0.1575)	0.121 (0.06944-0.1726)	0.03942 (-0.01174-0.09058)	-0.001521 (-0.04209-0.03905)	1 (0.9465-1)	0.6051 (0.553-0.6573)	0.3043 (0.2578-0.3508)
**CanDrA (melanoma)**	0.6423 (0.5896-0.6951)	0.6174 (0.5661-0.6686)	0.5075 (0.4591-0.5558)	0.4218 (0.3791-0.4645)	0.4324 (0.3907-0.4742)	0.1707 (0.1266-0.2147)	0.2892 (0.237-0.3413)	0.1237 (0.07728-0.17)	0.1463 (0.1106-0.1821)	0.1538 (0.1031-0.2045)	0.01917 (-0.0314-0.06975)	0.1648 (0.1274-0.2022)	0.6051 (0.553-0.6573)	1 (0.9487-1)	0.4033 (0.3571-0.4496)
**Condel**	0.3122 (0.2661-0.3583)	0.333 (0.2862-0.3798)	0.3026 (0.2551-0.3501)	0.4404 (0.3924-0.4884)	0.5631 (0.5164-0.6097)	0.5132 (0.4673-0.5591)	0.2535 (0.2071-0.2999)	0.1929 (0.1498-0.2361)	0.1337 (0.09781-0.1697)	0.2441 (0.1973-0.2909)	0.1129 (0.06651-0.1593)	0.04165 (0.005725-0.07757)	0.3043 (0.2578-0.3508)	0.4033 (0.3571-0.4496)	1 (0.9521-1)

All single nucleotide variants not present in the COSMIC dataset (n=297)

Kappa scores	CHASM (breast)	CHASM (lung)	CHASM (melanoma)	FATHMM (cancer)	FATHMM (missense)	Mutation Assessor	MutationTaster	PolyPhen-2	PROVEAN	SIFT	VEST	CanDrA (breast)	CanDrA (lung)	CanDrA (melanoma)	Condel
**CHASM (breast)**	1 (0.9022-1)	0.7581 (0.6676-0.8485)	0.5424 (0.4594-0.6254)	0.3925 (0.3243-0.4607)	0.351 (0.2839-0.4181)	0.08972 (0.01976-0.1597)	0.319 (0.2234-0.4146)	0.1528 (0.0675-0.2382)	0.134 (0.06693-0.201)	0.1244 (0.03771-0.211)	-0.1329 (-0.2184--0.0473)	0.004805 (-0.02093-0.03054)	0.7076 (0.6093-0.8058)	0.663 (0.5665-0.7596)	0.2948 (0.2195-0.3701)
**CHASM (lung)**	0.7581 (0.6676-0.8485)	1 (0.9124-1)	0.5044 (0.4206-0.5881)	0.4563 (0.3822-0.5303)	0.4051 (0.3315-0.4786)	0.1254 (0.04997-0.2008)	0.3123 (0.2219-0.4027)	0.2127 (0.1277-0.2977)	0.1171 (0.04674-0.1874)	0.1602 (0.07556-0.2449)	-0.09241 (-0.177--0.007825)	0.00721 (-0.01928-0.0337)	0.7209 (0.6297-0.8121)	0.6308 (0.5404-0.7213)	0.3634 (0.2851-0.4417)
**CHASM (melanoma)**	0.5424 (0.4594-0.6254)	0.5044 (0.4206-0.5881)	1 (0.916-1)	0.3867 (0.3067-0.4667)	0.3808 (0.3014-0.4602)	0.1841 (0.1036-0.2647)	0.3155 (0.2301-0.401)	0.185 (0.1016-0.2684)	0.1486 (0.0799-0.2174)	0.1646 (0.08036-0.2488)	-0.02255 (-0.1071-0.06204)	0.01388 (-0.01549-0.04325)	0.46 (0.3757-0.5443)	0.5133 (0.429-0.5976)	0.3329 (0.2508-0.415)
**FATHMM (cancer)**	0.3925 (0.3243-0.4607)	0.4563 (0.3822-0.5303)	0.3867 (0.3067-0.4667)	1 (0.9137-1)	0.6518 (0.5658-0.7378)	0.2564 (0.1709-0.3419)	0.3002 (0.2273-0.3731)	0.2056 (0.1285-0.2827)	0.06796 (0.002837-0.1331)	0.1549 (0.07802-0.2317)	-0.02335 (-0.102-0.05529)	0.02197 (-0.008916-0.05286)	0.452 (0.3823-0.5218)	0.4082 (0.3375-0.479)	0.4948 (0.4115-0.5781)
**FATHMM (missense)**	0.351 (0.2839-0.4181)	0.4051 (0.3315-0.4786)	0.3808 (0.3014-0.4602)	0.6518 (0.5658-0.7378)	1 (0.9136-1)	0.2456 (0.1602-0.3311)	0.1919 (0.1205-0.2632)	0.1576 (0.08066-0.2345)	-0.004283 (-0.07198-0.06341)	0.1461 (0.07192-0.2203)	-0.01093 (-0.08749-0.06564)	0.001898 (-0.02643-0.03023)	0.3307 (0.2624-0.399)	0.3822 (0.3127-0.4518)	0.5811 (0.4992-0.6629)
**Mutation Assessor**	0.08972 (0.01976-0.1597)	0.1254 (0.04997-0.2008)	0.1841 (0.1036-0.2647)	0.2564 (0.1709-0.3419)	0.2456 (0.1602-0.3311)	1 (0.915-1)	0.3804 (0.3062-0.4546)	0.3438 (0.2656-0.4219)	0.1717 (0.1046-0.2387)	0.3964 (0.3194-0.4734)	0.2817 (0.2029-0.3605)	0.01069 (-0.01891-0.04028)	0.08925 (0.01795-0.1606)	0.1103 (0.03796-0.1826)	0.5562 (0.4735-0.6389)
**MutationTaster**	0.319 (0.2234-0.4146)	0.3123 (0.2219-0.4027)	0.3155 (0.2301-0.401)	0.3002 (0.2273-0.3731)	0.1919 (0.1205-0.2632)	0.3804 (0.3062-0.4546)	1 (0.9038-1)	0.4593 (0.373-0.5455)	0.2218 (0.1566-0.287)	0.3411 (0.2499-0.4323)	0.2911 (0.2016-0.3807)	-0.03227 (-0.06228--0.002267)	0.3822 (0.2851-0.4793)	0.31 (0.2144-0.4056)	0.3279 (0.2477-0.4082)
**PolyPhen-2**	0.1528 (0.0675-0.2382)	0.2127 (0.1277-0.2977)	0.185 (0.1016-0.2684)	0.2056 (0.1285-0.2827)	0.1576 (0.08066-0.2345)	0.3438 (0.2656-0.4219)	0.4593 (0.373-0.5455)	1 (0.9161-1)	0.2871 (0.2155-0.3588)	0.3925 (0.3101-0.4748)	0.2928 (0.2098-0.3758)	-0.03104 (-0.05758--0.004505)	0.188 (0.1019-0.2741)	0.1361 (0.05015-0.2221)	0.2517 (0.1724-0.331)
**PROVEAN**	0.134 (0.06693-0.201)	0.1171 (0.04674-0.1874)	0.1486 (0.0799-0.2174)	0.06796 (0.002837-0.1331)	-0.004283 (-0.07198-0.06341)	0.1717 (0.1046-0.2387)	0.2218 (0.1566-0.287)	0.2871 (0.2155-0.3588)	1 (0.918-1)	0.2394 (0.1808-0.298)	0.1818 (0.1201-0.2435)	-0.01297 (-0.02877-0.002832)	0.1124 (0.04659-0.1783)	0.1307 (0.06376-0.1977)	0.08886 (0.02684-0.1509)
**SIFT**	0.1244 (0.03771-0.211)	0.1602 (0.07556-0.2449)	0.1646 (0.08036-0.2488)	0.1549 (0.07802-0.2317)	0.1461 (0.07192-0.2203)	0.3964 (0.3194-0.4734)	0.3411 (0.2499-0.4323)	0.3925 (0.3101-0.4748)	0.2394 (0.1808-0.298)	1 (0.9066-1)	0.3201 (0.2292-0.411)	-0.021 (-0.05932-0.01731)	0.09057 (0.00119-0.18)	0.1225 (0.03411-0.2109)	0.2967 (0.2125-0.3809)
**VEST**	-0.1329 (-0.2184--0.0473)	-0.09241 (-0.177--0.007825)	-0.02255 (-0.1071-0.06204)	-0.02335 (-0.102-0.05529)	-0.01093 (-0.08749-0.06564)	0.2817 (0.2029-0.3605)	0.2911 (0.2016-0.3807)	0.2928 (0.2098-0.3758)	0.1818 (0.1201-0.2435)	0.3201 (0.2292-0.411)	1 (0.9107-1)	-0.02167 (-0.05719-0.01384)	-0.09819 (-0.1861--0.01031)	-0.1146 (-0.2018--0.02736)	0.1162 (0.03205-0.2004)
**CanDrA (breast)**	0.004805 (-0.02093-0.03054)	0.00721 (-0.01928-0.0337)	0.01388 (-0.01549-0.04325)	0.02197 (-0.008916-0.05286)	0.001898 (-0.02643-0.03023)	0.01069 (-0.01891-0.04028)	-0.03227 (-0.06228--0.002267)	-0.03104 (-0.05758--0.004505)	-0.01297 (-0.02877-0.002832)	-0.021 (-0.05932-0.01731)	-0.02167 (-0.05719-0.01384)	1 (0.9074-1)	-0.01928 (-0.04685-0.008288)	0.02081 (-0.006545-0.04817)	-0.02 (-0.055-0.01501)
**CanDrA (lung)**	0.7076 (0.6093-0.8058)	0.7209 (0.6297-0.8121)	0.46 (0.3757-0.5443)	0.452 (0.3823-0.5218)	0.3307 (0.2624-0.399)	0.08925 (0.01795-0.1606)	0.3822 (0.2851-0.4793)	0.188 (0.1019-0.2741)	0.1124 (0.04659-0.1783)	0.09057 (0.00119-0.18)	-0.09819 (-0.1861--0.01031)	-0.01928 (-0.04685-0.008288)	1 (0.9009-1)	0.6783 (0.5809-0.7756)	0.3078 (0.2304-0.3852)
**CanDrA (melanoma)**	0.663 (0.5665-0.7596)	0.6308 (0.5404-0.7213)	0.5133 (0.429-0.5976)	0.4082 (0.3375-0.479)	0.3822 (0.3127-0.4518)	0.1103 (0.03796-0.1826)	0.31 (0.2144-0.4056)	0.1361 (0.05015-0.2221)	0.1307 (0.06376-0.1977)	0.1225 (0.03411-0.2109)	-0.1146 (-0.2018--0.02736)	0.02081 (-0.006545-0.04817)	0.6783 (0.5809-0.7756)	1 (0.9041-1)	0.3444 (0.2667-0.4221)
**Condel**	0.2948 (0.2195-0.3701)	0.3634 (0.2851-0.4417)	0.3329 (0.2508-0.415)	0.4948 (0.4115-0.5781)	0.5811 (0.4992-0.6629)	0.5562 (0.4735-0.6389)	0.3279 (0.2477-0.4082)	0.2517 (0.1724-0.331)	0.08886 (0.02684-0.1509)	0.2967 (0.2125-0.3809)	0.1162 (0.03205-0.2004)	-0.02 (-0.055-0.01501)	0.3078 (0.2304-0.3852)	0.3444 (0.2667-0.4221)	1 (0.9154-1)

Cohen's Kappa coefficients and 95% confidence intervals reported for each comparison.
