# Supplementary material for: Benchmarking mutation effect prediction algorithms using functionally validated cancer-related missense mutations
Source: Genome Biol. 2014 Oct 28;15(10):484. doi: 10.1186/s13059-014-0484-1 (PMC4232638; doi:10.1186/s13059-014-0484-1)
Supplement: Additional file 14: — Predictions of functionally validated single nucleotide variants in bona fide oncogenes, bona fide tumor suppressor genes, and new cancer genes by 15 mutation effect prediction algorithms (n = 989). [file 13059_2014_484_MOESM14_ESM.pdf]

Additional file 14: Predictions of functionally validated single nucleotide variants in *bona fide* oncogenes, *bona fide* tumor suppressor genes and new cancer genes by 15 mutation effect prediction algorithms (n=989).

ONCOGENES

Predition algorithm	Prediction class	Functional categories	
		Neutral (n)	Non-neutral (n)
CHASM (breast)	driver	2	140
	passenger	5	29
CHASM (lung)	driver	1	152
	passenger	6	17
CHASM (melanoma)	driver	3	144
	passenger	4	25
FATHMM (cancer)	CANCER	6	169
	PASSENGER/OTHER	1	0
FATHMM (missense)	Damaging	3	80
	Tolerated	4	89
	no weights	0	0
Mutation Assessor	high	0	37
	medium	1	66
	low	3	34
	neutral	3	31
	N/A	0	1
MutationTaster	disease_causing	7	159
	disease_causing_automatic	0	9
	polymorphism	0	1
	polymorphism_automatic	0	0
PolyPhen-2	probably damaging	2	128
	possibly damaging	1	22
	benign	4	19
PROVEAN	Deleterious	1	139
	Neutral	6	30
SIFT	Damaging	3	144
	Tolerated	4	25
VEST	functional	2	157
	neutral	5	12
CanDrA (breast)	Driver	7	135
	Passenger	0	34
	No-call	0	0
CanDrA (lung)	Driver	2	148
	Passenger	4	6
	No-call	1	15
CanDrA (melanoma)	Driver	2	97
	Passenger	3	45
	No-call	2	27
Condel	Deleterious	3	112
	Neutral	4	57

TUMOR SUPPRESSOR GENES

Predition algorithm	Prediction class	Functional categories	
		Neutral (n)	Non-neutral (n)
CHASM (breast)	driver	25	620
	passenger	108	30
CHASM (lung)	driver	31	626
	passenger	102	24
CHASM (melanoma)	driver	45	635
	passenger	88	15
FATHMM (cancer)	CANCER	65	637
	PASSENGER/OTHER	68	13
FATHMM (missense)	Damaging	66	642
	Tolerated	67	8
	no weights	0	0
Mutation Assessor	high	2	13
	medium	49	507
	low	48	92
	neutral	34	38
	N/A	0	0
MutationTaster	disease_causing	27	552
	disease_causing_automatic	1	21
	polymorphism	99	77
	polymorphism_automatic	6	0
PolyPhen-2	probably damaging	38	444
	possibly damaging	25	92
	benign	70	114
PROVEAN	Deleterious	42	463
	Neutral	91	187
SIFT	Damaging	67	558
	Tolerated	66	92
VEST	functional	98	517
	neutral	35	133
CanDrA (breast)	Driver	133	650
	Passenger	0	0
	No-call	0	0
CanDrA (lung)	Driver	22	618
	Passenger	98	29
	No-call	13	3
CanDrA (melanoma)	Driver	26	621
	Passenger	94	24
	No-call	13	5
Condel	Deleterious	74	644
	Neutral	59	6

NEW CANCER GENES

Predition algorithm	Prediction class	Functional categories	
		Neutral (n)	Non-neutral (n)
CHASM (breast)	driver	0	4
	passenger	0	26
CHASM (lung)	driver	0	5
	passenger	0	25
CHASM (melanoma)	driver	0	16
	passenger	0	14
FATHMM (cancer)	CANCER	0	25
	PASSENGER/OTHER	0	5
FATHMM (missense)	Damaging	0	23
	Tolerated	0	7
	no weights	0	0
Mutation Assessor	high	0	21
	medium	0	6
	low	0	3
	neutral	0	0
	N/A	0	0
MutationTaster	disease_causing	0	29
	disease_causing_automatic	0	1
	polymorphism	0	0
	polymorphism_automatic	0	0
PolyPhen-2	probably damaging	0	28
	possibly damaging	0	1
	benign	0	1
PROVEAN	Deleterious	0	30
	Neutral	0	0
SIFT	Damaging	0	29
	Tolerated	0	1
VEST	functional	0	28
	neutral	0	2
CanDrA (breast)	Driver	0	20
	Passenger	0	5
	No-call	0	5
CanDrA (lung)	Driver	0	1
	Passenger	0	24
	No-call	0	5
CanDrA (melanoma)	Driver	0	16
	Passenger	0	6
	No-call	0	8
Condel	Deleterious	0	30
	Neutral	0	0
