# Supplementary material for: Benchmarking mutation effect prediction algorithms using functionally validated cancer-related missense mutations
Source: Genome Biol. 2014 Oct 28;15(10):484. doi: 10.1186/s13059-014-0484-1 (PMC4232638; doi:10.1186/s13059-014-0484-1)
Supplement: Additional file 16: — Performance statistics and 95% confidence intervals of mutation effect prediction algorithms using only functionally validated single nucleotide variants in bona fide oncogenes or bona fide tumor suppressor genes. [file 13059_2014_484_MOESM16_ESM.pdf]

Additional file 16: Performance statistics and 95% confidence intervals of mutation effect prediction algorithms using only functionally validated single nucleotide variants in *bona fide* oncogenes or *bona fide* tumor suppressor genes.

ONCOGENES (n=176)

Prediction algorithm	Accuracy (95% CI)	Sensitivity (95% CI)	Specificity (95% CI)	PPV (95% CI)	NPV (95% CI)	Composite (95% CI)
CHASM (breast)	82.39% (76.71%-87.5%)	82.84% (77.11%-88.02%)	71.43% (33.33%-100%)	98.59% (96.45%-100%)	14.706% (3.448%-28.12%)	2.6757 (2.1860-3.0558)
CHASM (lung)	89.77% (85.23%-93.75%)	89.94% (85.38%-94.08%)	85.71% (57.14%-100%)	99.35% (98.01%-100%)	26.087% (9.524%-46.67%)	3.0109 (2.5806-3.3314)
CHASM (melanoma)	84.09% (78.98%-89.2%)	85.21% (79.88%-90.42%)	57.14% (20%-100%)	97.96% (95.33%-100%)	13.793% (3.226%-28.12%)	2.5410 (2.0719-3.0042)
FATHMM (cancer)	96.59% (93.75%-98.86%)	100% (100%-100%)	14.29% (0%-50%)	96.57% (93.68%-98.86%)	100% (100%-100%)	3.1086 (3.0205-3.4885)
FATHMM (missense)	47.73% (40.34%-55.11%)	47.34% (39.18%-54.92%)	57.14% (16.67%-100%)	96.39% (91.86%-100%)	4.301% (1%-8.991%)	2.0517 (1.5906-2.5256)
Mutation Assessor	62.29% (55.17%-69.32%)	61.31% (54.16%-68.67%)	85.71% (50%-100%)	99.04% (96.84%-100%)	8.451% (2.632%-15.58%)	2.5451 (2.1506-2.7822)
MutationTaster	95.45% (92.05%-98.3%)	99.41% (98.19%-100%)	0% (0%-0%)	96% (92.61%-98.3%)	0% (0%-0%)	1.9541 (1.9187-1.9771)
PolyPhen-2	87.5% (82.39%-92.61%)	88.76% (84.02%-93.45%)	57.14% (19.5%-100%)	98.04% (95.54%-100%)	17.391% (3.846%-36%)	2.6133 (2.0994-3.1272)
PROVEAN	82.39% (77.27%-87.51%)	82.25% (76.74%-87.65%)	85.71% (50%-100%)	99.29% (97.76%-100%)	16.667% (5.556%-30.01%)	2.8392 (2.4261-3.1364)
SIFT	84.09% (78.41%-89.77%)	85.21% (79.76%-90.96%)	57.14% (20%-100%)	97.96% (95.27%-100%)	13.793% (3.125%-28.14%)	2.5410 (2.0504-3.0154)
VEST	92.05% (88.05%-96.02%)	92.9% (88.75%-96.93%)	71.43% (28.57%-100%)	98.74% (96.81%-100%)	29.412% (7.692%-55.56%)	2.9248 (2.3037-3.4156)
CanDrA (breast)	76.7% (70.45%-82.95%)	79.88% (73.53%-85.8%)	0% (0%-0%)	95.07% (91.18%-98.04%)	0% (0%-0%)	1.7495 (1.6739-1.8207)
CanDrA (lung)	95% (91.36%-98.15%)	96.1% (92.71%-98.73%)	66.67% (25%-100%)	98.67% (96.62%-100%)	40% (11.11%-75%)	3.0144 (2.3353-3.6049)
CanDrA (melanoma)	68.03% (59.85%-75.86%)	68.31% (60.43%-76.03%)	60% (14.29%-100%)	97.98% (94.79%-100%)	6.25% (0%-14.58%)	2.3254 (1.7661-2.8052)
Condel	65.91% (59.09%-73.3%)	66.27% (58.82%-73.22%)	57.14% (19.5%-100%)	97.39% (94.22%-100%)	6.557% (1.515%-13.64%)	2.2736 (1.8128-2.7380)

TUMOR SUPPRESSOR GENES (n=783)

Prediction algorithm	Accuracy (95% CI)	Sensitivity (95% CI)	Specificity (95% CI)	PPV (95% CI)	NPV (95% CI)	Composite (95% CI)
CHASM (breast)	92.98% (91.06%-94.76%)	95.38% (93.73%-97.01%)	81.2% (74.28%-87.71%)	96.12% (94.66%-97.57%)	78.26% (71.32%-85.25%)	3.5097 (3.3804-3.6330)
CHASM (lung)	92.98% (91.19%-94.76%)	96.31% (94.86%-97.71%)	76.69% (68.96%-83.94%)	95.28% (93.67%-96.87%)	80.95% (73.55%-87.61%)	3.4923 (3.3528-3.6172)
CHASM (melanoma)	92.34% (90.68%-94.13%)	97.69% (96.56%-98.77%)	66.17% (57.93%-73.69%)	93.38% (91.65%-95.1%)	85.44% (78.7%-91.77%)	3.4268 (3.2902-3.5534)
FATHMM (cancer)	90.04% (87.99%-92.21%)	98% (96.9%-98.94%)	51.13% (42.06%-59.68%)	90.74% (88.53%-92.85%)	83.95% (76.12%-91.46%)	3.2382 (3.0930-3.3871)
FATHMM (missense)	90.55% (88.38%-92.59%)	98.77% (97.9%-99.53%)	50.38% (41.54%-58.82%)	90.68% (88.5%-92.71%)	89.33% (81.69%-95.65%)	3.2916 (3.1475-3.4212)
Mutation Assessor	76.88% (74.07%-79.82%)	80% (77.19%-83.05%)	61.65% (53.12%-70.37%)	91.07% (88.7%-93.39%)	38.68% (32.37%-45.21%)	2.7140 (2.5651-2.8672)
MutationTaster	86.59% (84.42%-88.89%)	88.15% (85.4%-90.61%)	78.95% (71.67%-85.81%)	95.34% (93.7%-97%)	57.69% (50.56%-64.9%)	3.2013 (3.0675-3.3297)
PolyPhen-2	77.39% (74.58%-80.2%)	82.46% (79.75%-85.23%)	52.63% (44%-60.98%)	89.48% (86.95%-91.87%)	38.04% (31.15%-45.51%)	2.6262 (2.4610-2.7845)
Provean	70.75% (67.43%-74.2%)	71.23% (67.73%-74.92%)	68.42% (60.69%-76.11%)	91.68% (89.28%-94.11%)	32.73% (27.46%-38.18%)	2.6407 (2.4997-2.7801)
SIFT	79.69% (76.88%-82.76%)	85.85% (83.21%-88.6%)	49.62% (41.6%-57.93%)	89.28% (86.7%-91.69%)	41.77% (34.57%-49.61%)	2.6652 (2.5059-2.8237)
VEST	70.5% (67.05%-73.69%)	79.54% (76.51%-82.73%)	26.32% (19.23%-33.91%)	84.07% (81.04%-86.87%)	20.83% (15.23%-26.82%)	2.1075 (1.9678-2.2599)
CanDrA (breast)	83.01% (80.46%-85.57%)	100% (100%-100%)	0% (0%-0%)	83.01% (80.46%-85.57%)	NA	NA
CanDrA (lung)	93.35% (91.55%-95.06%)	95.52% (93.95%-97.05%)	81.67% (74.54%-88.46%)	96.56% (95.19%-97.9%)	77.17% (69.46%-84%)	3.5091 (3.3645-3.6327)
CanDrA (melanoma)	93.46% (91.74%-95.15%)	96.28% (94.8%-97.66%)	78.33% (70.63%-85.82%)	95.98% (94.5%-97.43%)	79.66% (72.02%-86.78%)	3.5025 (3.3689-3.6294)
Condel	89.78% (87.74%-91.83%)	99.08% (98.27%-99.84%)	44.36% (36.24%-52.3%)	89.69% (87.53%-91.9%)	90.77% (82.89%-97.73%)	3.2390 (3.0983-3.3702)

Based on the prediction results of non-neutral and neutral single nucleotide variants in *bona fide* oncogenes and *bona fide* tumor suppressor genes, the accuracy, sensitivity, specificity, positive predictive value (PPV), negative predictive value (NPV) and composite score for each predictor were computed. The 95% confidence intervals (CI) generated by bootstrapping are shown in parentheses.
