# Supplementary material for: Benchmarking mutation effect prediction algorithms using functionally validated cancer-related missense mutations
Source: Genome Biol. 2014 Oct 28;15(10):484. doi: 10.1186/s13059-014-0484-1 (PMC4232638; doi:10.1186/s13059-014-0484-1)
Supplement: Additional file 18: — Performance statistics for mutation effect prediction algorithm combinations using 297 single nucleotide variants not present in the COSMIC database for which functional data are available. [file 13059_2014_484_MOESM18_ESM.pdf]

Additional file 18: Performance statistics for mutation effect prediction algorithm combinations using 297 single nucleotide variants not present in the COSMIC database for which functional data are available.

Predictor algorithm combination	Accuracy (subset 1)	Sensitivity (subset 1)	Specificity (subset 1)	PPV (subset 1)	NPV (subset 1)	Composite score (subset 1)	Accuracy (subset 2)	Sensitivity (subset 2)	Specificity (subset 2)	PPV (subset 2)	NPV (subset 2)	Composite score (subset 2)
At least 1 of CHASM (breast), CHASM (lung)	82.24% (79.29%-85.35%)	79.41% (75.2%-83.46%)	87.122% (83.099%-91.781%)	91.42% (88.68%-94.64%)	71% (65.48%-76.53%)	3.2895 (3.1716-3.4186)	81.99% (75.76%-87.88%)	78.93% (70.68%-87.04%)	87.229% (77.769%-94.87%)	91.37% (84.62%-96.55%)	70.7% (60%-81.4%)	3.2822 (3.0269-3.5202)
At least 1 of CHASM (breast), CHASM (lung), CHASM (melanoma)	83.24% (80.3%-86.36%)	90.5% (87.6%-93.55%)	70.689% (64.789%-76.812%)	84.22% (80.88%-88.06%)	81.14% (75.81%-86.89%)	3.2655 (3.1358-3.4063)	83.01% (76.77%-88.89%)	90.27% (84.13%-95.72%)	70.522% (58.532%-81.82%)	84.02% (76.47%-90.48%)	80.87% (69.7%-91.43%)	3.2568 (2.9736-3.5148)
At least 1 of CHASM (breast), CHASM (lung), CHASM (melanoma), FATHMM (cancer)	77.46% (74.24%-80.81%)	96.33% (94.49%-98.39%)	44.847% (38.23%-51.429%)	75.12% (71.34%-79.25%)	87.59% (81.58%-94.12%)	3.0388 (2.9067-3.1708)	77.4% (70.71%-83.84%)	96.17% (92.19%-100%)	45.149% (31.704%-58.54%)	75.06% (67.09%-82.5%)	87.28% (74.97%-100%)	3.0366 (2.7704-3.2984)
At least 1 of CHASM (breast), CHASM (lung), CHASM (melanoma), FATHMM (cancer), FATHMM (missense)	73.41% (69.7%-76.77%)	97.93% (96.72%-99.24%)	30.998% (24.658%-37.179%)	71.05% (67.25%-74.86%)	89.67% (83.32%-96.3%)	2.8965 (2.7610-3.0269)	73.38% (66.67%-80.81%)	97.74% (94.74%-100%)	31.529% (19.355%-44.44%)	71.02% (63.22%-78.65%)	89.01% (75%-100%)	2.8930 (2.6312-3.1508)
At least 1 of CHASM (breast), CHASM (lung), CHASM (melanoma), FATHMM (cancer), Mutation Assessor	74.42% (71.21%-78.28%)	98.44% (97.52%-100%)	32.898% (26.667%-39.194%)	71.72% (68%-75.88%)	92.4% (86.96%-100%)	2.9546 (2.8359-3.0768)	74.39% (66.67%-80.81%)	98.34% (95.45%-100%)	33.286% (20.5%-45.72%)	71.67% (63.41%-79.52%)	92.11% (78.57%-100%)	2.9540 (2.6927-3.1853)
At least 1 of CHASM (breast), CHASM (lung), CHASM (melanoma), FATHMM (cancer), MutationTaster	77.84% (74.75%-81.31%)	99.49% (99.16%-100%)	40.393% (33.802%-46.667%)	74.27% (70.59%-78.11%)	97.88% (96%-100%)	3.1202 (3.0195-3.2275)	77.66% (70.71%-83.84%)	99.42% (98.21%-100%)	40.317% (27.778%-52.95%)	74.08% (66.28%-81.61%)	97.61% (91.67%-100%)	3.1143 (2.9107-3.3153)
At least 1 of CHASM (breast), CHASM (lung), CHASM (melanoma), FATHMM (cancer), PolyPhen-2	74.74% (71.72%-78.28%)	98.94% (98.33%-100%)	32.888% (26.56%-39.132%)	71.82% (68.2%-75.72%)	94.74% (90.48%-100%)	2.9840 (2.8682-3.0863)	74.76% (67.68%-80.81%)	98.92% (96.67%-100%)	33.275% (20.687%-45.46%)	71.79% (63.86%-79.07%)	94.72% (83.33%-100%)	2.9870 (2.7642-3.2150)
At least 1 of CHASM (breast), CHASM (lung), CHASM (melanoma), FATHMM (cancer), PROVEAN	74.11% (70.71%-77.78%)	96.85% (95.2%-98.46%)	34.794% (27.937%-41.027%)	71.98% (68.05%-75.88%)	86.44% (79.31%-93.55%)	2.9006 (2.7600-3.0330)	74% (66.67%-80.81%)	96.73% (93.1%-100%)	34.946% (22.222%-48.39%)	71.85% (64.04%-79.76%)	86.12% (70.59%-100%)	2.8965 (2.6015-3.1602)
At least 1 of CHASM (breast), CHASM (lung), CHASM (melanoma), FATHMM (cancer), SIFT	73.74% (70.2%-77.27%)	99.46% (99.16%-100%)	29.259% (23.288%-35.302%)	70.85% (67.23%-74.72%)	96.93% (94.44%-100%)	2.9651 (2.8697-3.0727)	73.74% (66.67%-80.81%)	99.48% (98.25%-100%)	29.57% (16.667%-41.03%)	70.79% (62.92%-78.16%)	97.07% (87.5%-100%)	2.9690 (2.7516-3.1614)
At least 1 of CHASM (breast), CHASM (lung), CHASM (melanoma), FATHMM (cancer), VEST	68.06% (64.63%-72.22%)	99.5% (99.16%-100%)	13.711% (9.088%-18.182%)	66.6% (62.7%-70.81%)	94.08% (87.5%-100%)	2.7389 (2.6155-2.8570)	67.91% (59.6%-74.77%)	99.4% (98.25%-100%)	13.851% (5.263%-23.08%)	66.45% (58.33%-74.19%)	93.09% (66.67%-100%)	2.7282 (2.4017-2.9297)
At least 1 of CHASM (breast), CHASM (lung), CHASM (melanoma), FATHMM (missense)	77.16% (73.74%-80.3%)	95.31% (93.33%-97.6%)	45.749% (38.887%-52.113%)	75.24% (71.34%-79.01%)	84.96% (78.94%-91.67%)	3.0125 (2.8752-3.1480)	77% (70.71%-83.84%)	95% (90.48%-98.53%)	46.039% (33.333%-59.47%)	75.14% (67.49%-82.72%)	84.31% (71.41%-95.45%)	3.0049 (2.7341-3.2700)
At least 1 of CHASM (breast), CHASM (lung), CHASM (melanoma), FATHMM (missense), Mutation Assessor	73.75% (70.2%-77.27%)	97.91% (96.69%-99.24%)	31.955% (25.974%-38.028%)	71.33% (67.42%-75.29%)	89.87% (83.33%-96.43%)	2.9107 (2.7721-3.0400)	73.72% (66.67%-80.81%)	97.78% (94.64%-100%)	32.391% (20%-45.46%)	71.28% (63.33%-79.31%)	89.52% (75%-100%)	2.9097 (2.6313-3.1663)
At least 1 of CHASM (breast), CHASM (lung), CHASM (melanoma), FATHMM (missense), MutationTaster	78.15% (74.75%-81.31%)	99.49% (99.16%-100%)	41.244% (34.845%-47.297%)	74.54% (70.76%-78.36%)	97.92% (96.15%-100%)	3.1320 (3.0349-3.2283)	78.04% (71.72%-84.85%)	99.42% (98.21%-100%)	41.334% (28.947%-54.29%)	74.41% (66.66%-82.14%)	97.66% (90.91%-100%)	3.1283 (2.9283-3.3214)
At least 1 of CHASM (breast), CHASM (lung), CHASM (melanoma), FATHMM (missense), PolyPhen-2	75.05% (71.72%-78.28%)	99.48% (99.16%-100%)	32.805% (26.387%-38.67%)	71.91% (68.23%-75.72%)	97.33% (95%-100%)	3.0153 (2.9098-3.1180)	75.15% (68.69%-81.82%)	99.44% (98.25%-100%)	33.438% (22.222%-45.71%)	71.94% (64.37%-79.52%)	97.21% (88.89%-100%)	3.0204 (2.8055-3.2283)
At least 1 of CHASM (breast), CHASM (lung), CHASM (melanoma), FATHMM (missense), PROVEAN	73.11% (69.7%-76.77%)	96.88% (95.2%-98.48%)	31.986% (25.756%-38.462%)	71.13% (67.46%-75%)	85.55% (77.78%-93.1%)	2.8554 (2.7100-3.0029)	72.98% (65.66%-79.8%)	96.66% (92.86%-100%)	32.269% (18.919%-44.44%)	71.02% (63.22%-78.32%)	84.85% (69.23%-100%)	2.8479 (2.5435-3.1276)
At least 1 of CHASM (breast), CHASM (lung), CHASM (melanoma), FATHMM (missense), SIFT	72.74% (69.19%-76.26%)	99.49% (99.15%-100%)	26.475% (20.27%-32%)	70.06% (66.11%-73.89%)	96.78% (93.75%-100%)	2.9279 (2.8247-3.0269)	72.71% (65.66%-79.8%)	99.43% (98.24%-100%)	26.838% (15.375%-39.47%)	69.99% (62.07%-77.91%)	96.56% (85.71%-100%)	2.9282 (2.7162-3.1313)
At least 1 of CHASM (breast), CHASM (lung), CHASM (melanoma), FATHMM (missense), VEST	68.04% (64.14%-71.72%)	99.5% (99.16%-100%)	13.626% (8.974%-17.911%)	66.58% (62.63%-70.59%)	94.08% (87.5%-100%)	2.7378 (2.6194-2.8459)	67.97% (60.61%-75.76%)	99.4% (98.25%-100%)	13.988% (5.263%-22.51%)	66.48% (58.51%-74.23%)	93.36% (75%-100%)	2.7325 (2.4571-2.9295)
At least 1 of CHASM (breast), CHASM (lung), CHASM (melanoma), Mutation Assessor	78.15% (74.75%-81.31%)	95.76% (93.8%-97.71%)	47.686% (41.327%-54.548%)	75.99% (72.05%-80%)	86.7% (81.39%-92.86%)	3.0614 (2.9296-3.1926)	78.05% (71.72%-84.85%)	95.69% (91.3%-100%)	47.771% (33.333%-61.29%)	75.86% (67.95%-83.75%)	86.65% (72.73%-100%)	3.0598 (2.7842-3.3272)
At least 1 of CHASM (breast), CHASM (lung), CHASM (melanoma), Mutation Assessor, MutationTaster	79.54% (76.26%-82.83%)	99.49% (99.16%-100%)	45.059% (38.748%-52.055%)	75.79% (71.78%-79.75%)	98.09% (96.55%-100%)	3.1844 (3.0861-3.2910)	79.3% (72.73%-85.86%)	99.42% (98.21%-100%)	44.774% (31.429%-58.06%)	75.54% (67.5%-83.53%)	97.85% (92.31%-100%)	3.1759 (2.9638-3.3810)
At least 1 of CHASM (breast), CHASM (lung), CHASM (melanoma), Mutation Assessor, PolyPhen-2	76.45% (73.23%-79.8%)	98.41% (97.52%-100%)	38.473% (31.644%-45.07%)	73.44% (69.59%-77.25%)	93.32% (88.46%-100%)	3.0365 (2.9163-3.1565)	76.4% (69.7%-82.83%)	98.4% (95.31%-100%)	38.628% (25.641%-51.72%)	73.35% (65.48%-80.72%)	93.33% (81.25%-100%)	3.0370 (2.7924-3.2724)
At least 1 of CHASM (breast), CHASM (lung), CHASM (melanoma), Mutation Assessor, PROVEAN	75.12% (71.72%-78.79%)	96.3% (94.44%-98.39%)	38.5% (31.943%-44.74%)	73.03% (69.05%-76.92%)	85.77% (79.31%-93.55%)	2.9361 (2.7947-3.0730)	75% (67.68%-81.82%)	96.21% (92.19%-100%)	38.586% (25.714%-51.35%)	72.89% (64.83%-80.69%)	85.61% (70%-100%)	2.9330 (2.6419-3.2023)
At least 1 of CHASM (breast), CHASM (lung), CHASM (melanoma), Mutation Assessor, SIFT	73.73% (70.2%-77.78%)	97.34% (95.93%-99.19%)	32.914% (26.085%-39.195%)	71.5% (67.46%-75.58%)	87.73% (80.77%-95.83%)	2.8948 (2.7511-3.0381)	73.75% (65.66%-80.81%)	97.34% (93.75%-100%)	33.289% (20.586%-46.16%)	71.46% (63.22%-79.31%)	87.95% (72.73%-100%)	2.9004 (2.6022-3.1662)
At least 1 of CHASM (breast), CHASM (lung), CHASM (melanoma), Mutation Assessor, VEST	68.06% (64.65%-72.22%)	98.92% (98.33%-100%)	14.683% (9.996%-19.118%)	66.72% (62.84%-70.81%)	88.75% (80%-100%)	2.6907 (2.5478-2.8459)	67.93% (59.6%-74.75%)	98.96% (96.67%-100%)	14.642% (5.556%-23.68%)	66.55% (58.51%-74.19%)	88.97% (60%-100%)	2.6913 (2.2871-2.9191)
At least 1 of CHASM (breast), CHASM (lung), CHASM (melanoma), MutationTaster	86.63% (84.34%-89.39%)	99.49% (99.16%-100%)	64.405% (58.227%-71.014%)	82.85% (79.61%-86.62%)	98.66% (97.62%-100%)	3.4541 (3.3628-3.5520)	86.33% (80.81%-90.91%)	99.42% (98.21%-100%)	63.852% (51.22%-75.76%)	82.51% (75%-89.19%)	98.48% (94.74%-100%)	3.4427 (3.2557-3.6250)
At least 1 of CHASM (breast), CHASM (lung), CHASM (melanoma), MutationTaster, PolyPhen-2	80.17% (77.27%-83.33%)	100% (100%-100%)	45.869% (39.023%-52.703%)	76.16% (72.56%-80%)	100% (100%-100%)	3.2203 (3.1268-3.3204)	80.07% (73.74%-85.86%)	100% (100%-100%)	45.86% (32.498%-60%)	76.02% (68.24%-83.13%)	100% (100%-100%)	3.2188 (3.0363-3.4111)
At least 1 of CHASM (breast), CHASM (lung), CHASM (melanoma), MutationTaster, PROVEAN	78.89% (75.76%-81.82%)	99.49% (99.16%-100%)	43.269% (36.613%-50%)	75.2% (71.6%-78.92%)	98.01% (96.3%-100%)	3.1598 (3.0636-3.2609)	78.58% (72.73%-84.85%)	99.42% (98.21%-100%)	42.795% (30.295%-56.11%)	74.89% (67.46%-82.14%)	97.75% (91.67%-100%)	3.1486 (2.9454-3.3429)
At least 1 of CHASM (breast), CHASM (lung), CHASM (melanoma), MutationTaster, SIFT	76.81% (73.74%-80.3%)	100% (100%-100%)	36.713% (30.378%-42.857%)	73.2% (69.41%-77.01%)	100% (100%-100%)	3.0992 (3.0132-3.1905)	76.69% (69.7%-82.83%)	100% (100%-100%)	36.689% (24.324%-48.78%)	73.04% (65.06%-80.46%)	100% (100%-100%)	3.0973 (2.9306-3.2800)
At least 1 of CHASM (breast), CHASM (lung), CHASM (melanoma), MutationTaster, VEST	70.07% (66.65%-73.74%)	100% (100%-100%)	18.322% (13.428%-22.976%)	67.92% (63.98%-71.89%)	100% (100%-100%)	2.8624 (2.7916-2.9319)	69.96% (62.63%-76.79%)	100% (100%-100%)	18.373% (8.565%-28.14%)	67.77% (59.78%-75.56%)	100% (100%-100%)	2.8614 (2.7289-3.0249)
At least 1 of CHASM (breast), CHASM (lung), CHASM (melanoma), PolyPhen-2	79.48% (76.26%-82.83%)	98.41% (97.52%-100%)	46.735% (40%-53.249%)	76.16% (72.56%-79.87%)	94.44% (90.62%-100%)	3.1575 (3.0502-3.2744)	79.43% (72.73%-85.86%)	98.4% (95.31%-100%)	46.868% (34.144%-60.53%)	76.07% (68.74%-83.33%)	94.45% (84.21%-100%)	3.1579 (2.9168-3.3777)
At least 1 of CHASM (breast), CHASM (lung), CHASM (melanoma), PolyPhen-2, PROVEAN	75.8% (72.73%-79.29%)	98.41% (97.52%-100%)	36.695% (30.299%-43.038%)	72.89% (69.05%-76.61%)	93.03% (88.45%-100%)	3.0102 (2.8974-3.1291)	75.67% (68.69%-81.82%)	98.4% (95.31%-100%)	36.643% (24.127%-50%)	72.72% (65.11%-80.24%)	93.03% (80%-100%)	3.0079 (2.7593-3.2198)
At least 1 of CHASM (breast), CHASM (lung), CHASM (melanoma), PolyPhen-2, SIFT	75.1% (71.72%-78.28%)	98.93% (98.32%-100%)	33.91% (27.536%-40%)	72.13% (68.39%-76%)	94.81% (90.91%-100%)	2.9978 (2.8876-3.1215)	75.05% (68.69%-81.82%)	98.95% (96.67%-100%)	34.024% (22.214%-47.06%)	72.02% (63.95%-79.55%)	94.97% (83.33%-100%)	2.9997 (2.7498-3.2101)
At least 1 of CHASM (breast), CHASM (lung), CHASM (melanoma), PolyPhen-2, VEST	69.39% (65.66%-73.23%)	100% (100%-100%)	16.465% (11.536%-21.127%)	67.43% (63.59%-71.35%)	100% (100%-100%)	2.8389 (2.7734-2.9105)	69.29% (61.62%-76.77%)	100% (100%-100%)	16.569% (7.313%-26.67%)	67.29% (59.55%-75%)	100% (100%-100%)	2.8386 (2.7120-2.9856)
At least 1 of CHASM (breast), CHASM (lung), CHASM (melanoma), PROVEAN	77.18% (74.24%-80.81%)	93.66% (91.47%-96.06%)	48.669% (41.667%-55.405%)	75.94% (72.43%-79.62%)	81.62% (75%-88.89%)	2.9989 (2.8604-3.1419)	76.95% (69.7%-82.83%)	93.52% (88.52%-98.31%)	48.475% (34.48%-61.9%)	75.71% (68%-82.67%)	81.3% (66.67%-94.44%)	2.9899 (2.6870-3.2582)
At least 1 of CHASM (breast), CHASM (lung), CHASM (melanoma), PROVEAN, SIFT	74.43% (71.2%-77.78%)	97.88% (96.72%-99.23%)	33.878% (27.394%-40%)	71.91% (68.05%-75.72%)	90.22% (84.62%-96.55%)	2.9389 (2.8152-3.0640)	74.37% (67.68%-80.83%)	97.86% (94.83%-100%)	34.044% (21.212%-46.15%)	71.8% (64.19%-79.52%)	90.27% (75%-100%)	2.9398 (2.6614-3.1784)
At least 1 of CHASM (breast), CHASM (lung), CHASM (melanoma), PROVEAN, VEST	67.73% (64.14%-71.21%)	98.96% (98.33%-100%)	13.733% (9.091%-18.056%)	66.48% (62.57%-70.43%)	88.48% (77.78%-100%)	2.6766 (2.5288-2.8231)	67.56% (60.61%-74.75%)	98.88% (96.67%-100%)	13.79% (5.402%-22.58%)	66.31% (58.51%-74.19%)	87.76% (60%-100%)	2.6678 (2.3267-2.9124)
At least 1 of CHASM (breast), CHASM (lung), CHASM (melanoma), SIFT	[illegible]	[illegible]	[illegible]	[illegible]	[illegible]	[illegible]	[illegible]	[illegible]	[illegible]	[illegible]	[illegible]	[illegible]
At least 1 of CHASM (breast), CHASM (lung), CHASM (melanoma), SIFT, VEST	[illegible]	[illegible]	[illegible]	[illegible]	[illegible]	[illegible]	[illegible]	[illegible]	[illegible]	[illegible]	[illegible]	[illegible]
At least 1 of CHASM (breast), CHASM (lung), CHASM (melanoma), VEST	[illegible]	[illegible]	[illegible]	[illegible]	[illegible]	[illegible]	[illegible]	[illegible]	[illegible]	[illegible]	[illegible]	[illegible]
At least 1 of CHASM (breast), CHASM (lung), FATHMM (cancer)	[illegible]	[illegible]	[illegible]	[illegible]	[illegible]	[illegible]	[illegible]	[illegible]	[illegible]	[illegible]	[illegible]	[illegible]
At least 1 of CHASM (breast), CHASM (lung), FATHMM (cancer), FATHMM (missense)	[illegible]	[illegible]	[illegible]	[illegible]	[illegible]	[illegible]	[illegible]	[illegible]	[illegible]	[illegible]	[illegible]	[illegible]
At least 1 of CHASM (breast), CHASM (lung), FATHMM (cancer), FATHMM (missense), Mutation Assessor	[illegible]	[illegible]	[illegible]	[illegible]	[illegible]	[illegible]	[illegible]	[illegible]	[illegible]	[illegible]	[illegible]	[illegible]
At least 1 of CHASM (breast), CHASM (lung), FATHMM (cancer), FATHMM (missense), MutationTaster	[illegible]	[illegible]	[illegible]	[illegible]	[illegible]	[illegible]	[illegible]	[illegible]	[illegible]	[illegible]	[illegible]	[illegible]
At least 1 of CHASM (breast), CHASM (lung), FATHMM (cancer), FATHMM (missense), PolyPhen-2	[illegible]	[illegible]	[illegible]	[illegible]	[illegible]	[illegible]	[illegible]	[illegible]	[illegible]	[illegible]	[illegible]	[illegible]
At least 1 of CHASM (breast), CHASM (lung), FATHMM (cancer), FATHMM (missense), PROVEAN	[illegible]	[illegible]	[illegible]	[illegible]	[illegible]	[illegible]	[illegible]	[illegible]	[illegible]	[illegible]	[illegible]	[illegible]
At least 1 of CHASM (breast), CHASM (lung), FATHMM (cancer), FATHMM (missense), SIFT	[illegible]	[illegible]	[illegible]	[illegible]	[illegible]	[illegible]	[illegible]	[illegible]	[illegible]	[illegible]	[illegible]	[illegible]
At least 1 of CHASM (breast), CHASM (lung), FATHMM (cancer), FATHMM (missense), VEST	[illegible]	[illegible]	[illegible]	[illegible]	[illegible]	[illegible]	[illegible]	[illegible]	[illegible]	[illegible]	[illegible]	[illegible]
At least 1 of CHASM (breast), CHASM (lung), FATHMM (cancer), Mutation Assessor	[illegible]	[illegible]	[illegible]	[illegible]	[illegible]	[illegible]	[illegible]	[illegible]	[illegible]	[illegible]	[illegible]	[illegible]
At least 1 of CHASM (breast), CHASM (lung), FATHMM (cancer), Mutation Assessor, MutationTaster	[illegible]	[illegible]	[illegible]	[illegible]	[illegible]	[illegible]	[illegible]	[illegible]	[illegible]	[illegible]	[illegible]	[illegible]
At least 1 of CHASM (breast), CHASM (lung), FATHMM (cancer), Mutation Assessor, PolyPhen-2	[illegible]	[illegible]	[illegible]	[illegible]	[illegible]	[illegible]	[illegible]	[illegible]	[illegible]	[illegible]	[illegible]	[illegible]
At least 1 of CHASM (breast), CHASM (lung), FATHMM (cancer), Mutation Assessor, PROVEAN	[illegible]	[illegible]	[illegible]	[illegible]	[illegible]	[illegible]	[illegible]	[illegible]	[illegible]	[illegible]	[illegible]	[illegible]
At least 1 of CHASM (breast), CHASM (lung), FATHMM (cancer), Mutation Assessor, SIFT	[illegible]	[illegible]	[illegible]	[illegible]	[illegible]	[illegible]	[illegible]	[illegible]	[illegible]	[illegible]	[illegible]	[illegible]
At least 1 of CHASM (breast), CHASM (lung), FATHMM (cancer), Mutation Assessor, VEST	[illegible]	[illegible]	[illegible]	[illegible]	[illegible]	[illegible]	[illegible]	[illegible]	[illegible]	[illegible]	[illegible]	[illegible]
At least 1 of CHASM (breast), CHASM (lung), FATHMM (cancer), MutationTaster	[illegible]	[illegible]	[illegible]	[illegible]	[illegible]	[illegible]	[illegible]	[illegible]	[illegible]	[illegible]	[illegible]	[illegible]
At least 1 of CHASM (breast), CHASM (lung), FATHMM (cancer), MutationTaster, PolyPhen-2	[illegible]	[illegible]	[illegible]	[illegible]	[illegible]	[illegible]	[illegible]	[illegible]	[illegible]	[illegible]	[illegible]	[illegible]
At least 1 of CHASM (breast), CHASM (lung), FATHMM (cancer), MutationTaster, PROVEAN	[illegible]	[illegible]	[illegible]	[illegible]	[illegible]	[illegible]	[illegible]	[illegible]	[illegible]	[illegible]	[illegible]	[illegible]
At least 1 of CHASM (breast), CHASM (lung), FATHMM (cancer), MutationTaster, SIFT	[illegible]	[illegible]	[illegible]	[illegible]	[illegible]	[illegible]	[illegible]	[illegible]	[illegible]	[illegible]	[illegible]	[illegible]
At least 1 of CHASM (breast), CHASM (lung), FATHMM (cancer), MutationTaster, VEST	[illegible]	[illegible]	[illegible]	[illegible]	[illegible]	[illegible]	[illegible]	[illegible]	[illegible]	[illegible]	[illegible]	[illegible]
At least 1 of CHASM (breast), CHASM (lung), FATHMM (cancer), PolyPhen-2	[illegible]	[illegible]	[illegible]	[illegible]	[illegible]	[illegible]	[illegible]	[illegible]	[illegible]	[illegible]	[illegible]	[illegible]
At least 1 of CHASM (breast), CHASM (lung), FATHMM (cancer), PolyPhen-2, PROVEAN	[illegible]	[illegible]	[illegible]	[illegible]	[illegible]	[illegible]	[illegible]	[illegible]	[illegible]	[illegible]	[illegible]	[illegible]
At least 1 of CHASM (breast), CHASM (lung), FATHMM (cancer), PolyPhen-2, SIFT	[illegible]	[illegible]	[illegible]	[illegible]	[illegible]	[illegible]	[illegible]	[illegible]	[illegible]	[illegible]	[illegible]	[illegible]
At least 1 of CHASM (breast), CHASM (lung), FATHMM (cancer), PolyPhen-2, VEST	[illegible]	[illegible]	[illegible]	[illegible]	[illegible]	[illegible]	[illegible]	[illegible]	[illegible]	[illegible]	[illegible]	[illegible]
At least 1 of CHASM (breast), CHASM (lung), FATHMM (cancer), PROVEAN	[illegible]	[illegible]	[illegible]	[illegible]	[illegible]	[illegible]	[illegible]	[illegible]	[illegible]	[illegible]	[illegible]	[illegible]
At least 1 of CHASM (breast), CHASM (lung), FATHMM (cancer), PROVEAN, SIFT	[illegible]	[illegible]	[illegible]	[illegible]	[illegible]	[illegible]	[illegible]	[illegible]	[illegible]	[illegible]	[illegible]	[illegible]
At least 1 of CHASM (breast), CHASM (lung), FATHMM (cancer), PROVEAN, VEST	[illegible]	[illegible]	[illegible]	[illegible]	[illegible]	[illegible]	[illegible]	[illegible]	[illegible]	[illegible]	[illegible]	[illegible]
At least 1 of CHASM (breast), CHASM (lung), FATHMM (cancer), SIFT	[illegible]	[illegible]	[illegible]	[illegible]	[illegible]	[illegible]	[illegible]	[illegible]	[illegible]	[illegible]	[illegible]	[illegible]
At least 1 of CHASM (breast), CHASM (lung), FATHMM (cancer), SIFT, VEST	[illegible]	[illegible]	[illegible]	[illegible]	[illegible]	[illegible]	[illegible]	[illegible]	[illegible]	[illegible]	[illegible]	[illegible]
At least 1 of CHASM (breast), CHASM (lung), FATHMM (cancer), VEST	[illegible]	[illegible]	[illegible]	[illegible]	[illegible]	[illegible]	[illegible]	[illegible]	[illegible]	[illegible]	[illegible]	[illegible]
At least 1 of CHASM (breast), CHASM (lung), FATHMM (missense)	[illegible]	[illegible]	[illegible]	[illegible]	[illegible]	[illegible]	[illegible]	[illegible]	[illegible]	[illegible]	[illegible]	[illegible]
At least 1 of CHASM (breast), CHASM (lung), FATHMM (missense), Mutation Assessor	[illegible]	[illegible]	[illegible]	[illegible]	[illegible]	[illegible]	[illegible]	[illegible]	[illegible]	[illegible]	[illegible]	[illegible]
At least 1 of CHASM (breast), CHASM (lung), FATHMM (missense), Mutation Assessor, MutationTaster	[illegible]	[illegible]	[illegible]	[illegible]	[illegible]	[illegible]	[illegible]	[illegible]	[illegible]	[illegible]	[illegible]	[illegible]
At least 1 of CHASM (breast), CHASM (lung), FATHMM (missense), Mutation Assessor, PolyPhen-2	[illegible]	[illegible]	[illegible]	[illegible]	[illegible]	[illegible]	[illegible]	[illegible]	[illegible]	[illegible]	[illegible]	[illegible]
At least 1 of CHASM (breast), CHASM (lung), FATHMM (missense), Mutation Assessor, PROVEAN	[illegible]	[illegible]	[illegible]	[illegible]	[illegible]	[illegible]	[illegible]	[illegible]	[illegible]	[illegible]	[illegible]	[illegible]
At least 1 of CHASM (breast), CHASM (lung), FATHMM (missense), Mutation Assessor, SIFT	[illegible]	[illegible]	[illegible]	[illegible]	[illegible]	[illegible]	[illegible]	[illegible]	[illegible]	[illegible]	[illegible]	[illegible]
At least 1 of CHASM (breast), CHASM (lung), FATHMM (missense), Mutation Assessor, VEST	[illegible]	[illegible]	[illegible]	[illegible]	[illegible]	[illegible]	[illegible]	[illegible]	[illegible]	[illegible]	[illegible]	[illegible]
At least 1 of CHASM (breast), CHASM (lung), FATHMM (missense), MutationTaster	[illegible]	[illegible]	[illegible]	[illegible]	[illegible]	[illegible]	[illegible]	[illegible]	[illegible]	[illegible]	[illegible]	[illegible]
At least 1 of CHASM (breast), CHASM (lung), FATHMM (missense), MutationTaster, PolyPhen-2	[illegible]	[illegible]	[illegible]	[illegible]	[illegible]	[illegible]	[illegible]	[illegible]	[illegible]	[illegible]	[illegible]	[illegible]
At least 1 of CHASM (breast), CHASM (lung), FATHMM (missense), MutationTaster, PROVEAN	[illegible]	[illegible]	[illegible]	[illegible]	[illegible]	[illegible]	[illegible]	[illegible]	[illegible]	[illegible]	[illegible]	[illegible]
At least 1 of CHASM (breast), CHASM (lung), FATHMM (missense), MutationTaster, SIFT	[illegible]	[illegible]	[illegible]	[illegible]	[illegible]	[illegible]	[illegible]	[illegible]	[illegible]	[illegible]	[illegible]	[illegible]
At least 1 of CHASM (breast), CHASM (lung), FATHMM (missense), MutationTaster, VEST	[illegible]	[illegible]	[illegible]	[illegible]	[illegible]	[illegible]	[illegible]	[illegible]	[illegible]	[illegible]	[illegible]	[illegible]
At least 1 of CHASM (breast), CHASM (lung), FATHMM (missense), PolyPhen-2	[illegible]	[illegible]	[illegible]	[illegible]	[illegible]	[illegible]	[illegible]	[illegible]	[illegible]	[illegible]	[illegible]	[illegible]
At least 1 of CHASM (breast), CHASM (lung), FATHMM (missense), PolyPhen-2, PROVEAN	[illegible]	[illegible]	[illegible]	[illegible]	[illegible]	[illegible]	[illegible]	[illegible]	[illegible]	[illegible]	[illegible]	[illegible]
At least 1 of CHASM (breast), CHASM (lung), FATHMM (missense), PolyPhen-2, SIFT	[illegible]	[illegible]	[illegible]	[illegible]	[illegible]	[illegible]	[illegible]	[illegible]	[illegible]	[illegible]	[illegible]	[illegible]
At least 1 of CHASM (breast), CHASM (lung), FATHMM (missense), PolyPhen-2, VEST	[illegible]	[illegible]	[illegible]	[illegible]	[illegible]	[illegible]	[illegible]	[illegible]	[illegible]	[illegible]	[illegible]	[illegible]
At least 1 of CHASM (breast), CHASM (lung), FATHMM (missense), PROVEAN	[illegible]	[illegible]	[illegible]	[illegible]	[illegible]	[illegible]	[illegible]	[illegible]	[illegible]	[illegible]	[illegible]	[illegible]
At least 1 of CHASM (breast), CHASM (lung), FATHMM (missense), PROVEAN, SIFT	[illegible]	[illegible]	[illegible]	[illegible]	[illegible]	[illegible]	[illegible]	[illegible]	[illegible]	[illegible]	[illegible]	[illegible]
At least 1 of CHASM (breast), CHASM (lung), FATHMM (missense), PROVEAN, VEST	[illegible]	[illegible]	[illegible]	[illegible]	[illegible]	[illegible]	[illegible]	[illegible]	[illegible]	[illegible]	[illegible]	[illegible]
At least 1 of CHASM (breast), CHASM (lung), FATHMM (missense), SIFT	[illegible]	[illegible]	[illegible]	[illegible]	[illegible]	[illegible]	[illegible]	[illegible]	[illegible]	[illegible]	[illegible]	[illegible]
At least 1 of CHASM (breast), CHASM (lung), FATHMM (missense), SIFT, VEST	[illegible]	[illegible]	[illegible]	[illegible]	[illegible]	[illegible]	[illegible]	[illegible]	[illegible]	[illegible]	[illegible]	[illegible]
At least 1 of CHASM (breast), CHASM (lung), FATHMM (missense), VEST	[illegible]	[illegible]	[illegible]	[illegible]	[illegible]	[illegible]	[illegible]	[illegible]	[illegible]	[illegible]	[illegible]	[illegible]
At least 1 of CHASM (breast), CHASM (lung), Mutation Assessor	[illegible]	[illegible]	[illegible]	[illegible]	[illegible]	[illegible]	[illegible]	[illegible]	[illegible]	[illegible]	[illegible]	[illegible]
At least 1 of CHASM (breast), CHASM (lung), Mutation Assessor, MutationTaster	[illegible]	[illegible]	[illegible]	[illegible]	[illegible]	[illegible]	[illegible]	[illegible]	[illegible]	[illegible]	[illegible]	[illegible]
At least 1 of CHASM (breast), CHASM (lung), Mutation Assessor, MutationTaster, PolyPhen-2	[illegible]	[illegible]	[illegible]	[illegible]	[illegible]	[illegible]	[illegible]	[illegible]	[illegible]	[illegible]	[illegible]	[illegible]
At least 1 of CHASM (breast), CHASM (lung), Mutation Assessor, MutationTaster, PROVEAN	[illegible]	[illegible]	[illegible]	[illegible]	[illegible]	[illegible]	[illegible]	[illegible]	[illegible]	[illegible]	[illegible]	[illegible]
At least 1 of CHASM (breast), CHASM (lung), Mutation Assessor, MutationTaster, SIFT	[illegible]	[illegible]	[illegible]	[illegible]	[illegible]	[illegible]	[illegible]	[illegible]	[illegible]	[illegible]	[illegible]	[illegible]
At least 1 of CHASM (breast), CHASM (lung), Mutation Assessor, MutationTaster, VEST	[illegible]	[illegible]	[illegible]	[illegible]	[illegible]	[illegible]	[illegible]	[illegible]	[illegible]	[illegible]	[illegible]	[illegible]
At least 1 of CHASM (breast), CHASM (lung), Mutation Assessor, PolyPhen-2	[illegible]	[illegible]	[illegible]	[illegible]	[illegible]	[illegible]	[illegible]	[illegible]	[illegible]	[illegible]	[illegible]	[illegible]
At least 1 of CHASM (breast), CHASM (lung), Mutation Assessor, PolyPhen-2, PROVEAN	[illegible]	[illegible]	[illegible]	[illegible]	[illegible]	[illegible]	[illegible]	[illegible]	[illegible]	[illegible]	[illegible]	[illegible]
At least 1 of CHASM (breast), CHASM (lung), Mutation Assessor, PolyPhen-2, SIFT	[illegible]	[illegible]	[illegible]	[illegible]	[illegible]	[illegible]	[illegible]	[illegible]	[illegible]	[illegible]	[illegible]	[illegible]
At least 1 of CHASM (breast), CHASM (lung), Mutation Assessor, PolyPhen-2, VEST	[illegible]	[illegible]	[illegible]	[illegible]	[illegible]	[illegible]	[illegible]	[illegible]	[illegible]	[illegible]	[illegible]	[illegible]
At least 1 of CHASM (breast), CHASM (lung), Mutation Assessor, PROVEAN	[illegible]	[illegible]	[illegible]	[illegible]	[illegible]	[illegible]	[illegible]	[illegible]	[illegible]	[illegible]	[illegible]	[illegible]
At least 1 of CHASM (breast), CHASM (lung), Mutation Assessor, PROVEAN, SIFT	[illegible]	[illegible]	[illegible]	[illegible]	[illegible]	[illegible]	[illegible]	[illegible]	[illegible]	[illegible]	[illegible]	[illegible]
At least 1 of CHASM (breast), CHASM (lung), Mutation Assessor, PROVEAN, VEST	[illegible]	[illegible]	[illegible]	[illegible]	[illegible]	[illegible]	[illegible]	[illegible]	[illegible]	[illegible]	[illegible]	[illegible]
At least 1 of CHASM (breast), CHASM (lung), Mutation Assessor, SIFT	[illegible]	[illegible]	[illegible]	[illegible]	[illegible]	[illegible]	[illegible]	[illegible]	[illegible]	[illegible]	[illegible]	[illegible]
At least 1 of CHASM (breast), CHASM (lung), Mutation Assessor, SIFT, VEST	[illegible]	[illegible]	[illegible]	[illegible]	[illegible]	[illegible]	[illegible]	[illegible]	[illegible]	[illegible]	[illegible]	[illegible]
At least 1 of CHASM (breast), CHASM (lung), Mutation Assessor, VEST	[illegible]	[illegible]	[illegible]	[illegible]	[illegible]	[illegible]	[illegible]	[illegible]	[illegible]	[illegible]	[illegible]	[illegible]
At least 1 of CHASM (breast), CHASM (lung), MutationTaster	[illegible]	[illegible]	[illegible]	[illegible]	[illegible]	[illegible]	[illegible]	[illegible]	[illegible]	[illegible]	[illegible]	[illegible]
At least 1 of CHASM (breast), CHASM (lung), MutationTaster, PolyPhen-2	[illegible]	[illegible]	[illegible]	[illegible]	[illegible]	[illegible]	[illegible]	[illegible]	[illegible]	[illegible]	[illegible]	[illegible]
At least 1 of CHASM (breast), CHASM (lung), MutationTaster, PolyPhen-2, PROVEAN	[illegible]	[illegible]	[illegible]	[illegible]	[illegible]	[illegible]	[illegible]	[illegible]	[illegible]	[illegible]	[illegible]	[illegible]
At least 1 of CHASM (breast), CHASM (lung), MutationTaster, PolyPhen-2, SIFT	[illegible]	[illegible]	[illegible]	[illegible]	[illegible]	[illegible]	[illegible]	[illegible]	[illegible]	[illegible]	[illegible]	[illegible]
At least 1 of CHASM (breast), CHASM (lung), MutationTaster, PolyPhen-2, VEST	[illegible]	[illegible]	[illegible]	[illegible]	[illegible]	[illegible]	[illegible]	[illegible]	[illegible]	[illegible]	[illegible]	[illegible]
At least 1 of CHASM (breast), CHASM (lung), MutationTaster, PROVEAN	[illegible]	[illegible]	[illegible]	[illegible]	[illegible]	[illegible]	[illegible]	[illegible]	[illegible]	[illegible]	[illegible]	[illegible]
At least 1 of CHASM (breast), CHASM (lung), MutationTaster, PROVEAN, SIFT	[illegible]	[illegible]	[illegible]	[illegible]	[illegible]	[illegible]	[illegible]	[illegible]	[illegible]	[illegible]	[illegible]	[illegible]
At least 1 of CHASM (breast), CHASM (lung), MutationTaster, PROVEAN, VEST	[illegible]	[illegible]	[illegible]	[illegible]	[illegible]	[illegible]	[illegible]	[illegible]	[illegible]	[illegible]	[illegible]	[illegible]
At least 1 of CHASM (breast), CHASM (lung), MutationTaster, SIFT	[illegible]	[illegible]	[illegible]	[illegible]	[illegible]	[illegible]	[illegible]	[illegible]	[illegible]	[illegible]	[illegible]	[illegible]
At least 1 of CHASM (breast), CHASM (lung), MutationTaster, SIFT, VEST	[illegible]	[illegible]	[illegible]	[illegible]	[illegible]	[illegible]	[illegible]	[illegible]	[illegible]	[illegible]	[illegible]	[illegible]
At least 1 of CHASM (breast), CHASM (lung), MutationTaster, VEST	[illegible]	[illegible]	[illegible]	[illegible]	[illegible]	[illegible]	[illegible]	[illegible]	[illegible]	[illegible]	[illegible]	[illegible]
At least 1 of CHASM (breast), CHASM (lung), PolyPhen-2	[illegible]	[illegible]	[illegible]	[illegible]	[illegible]	[illegible]	[illegible]	[illegible]	[illegible]	[illegible]	[illegible]	[illegible]
At least 1 of CHASM (breast), CHASM (lung), PolyPhen-2, PROVEAN	[illegible]	[illegible]	[illegible]	[illegible]	[illegible]	[illegible]	[illegible]	[illegible]	[illegible]	[illegible]	[illegible]	[illegible]
At least 1 of CHASM (breast), CHASM (lung), PolyPhen-2, PROVEAN, SIFT	[illegible]	[illegible]	[illegible]	[illegible]	[illegible]	[illegible]	[illegible]	[illegible]	[illegible]	[illegible]	[illegible]	[illegible]
At least 1 of CHASM (breast), CHASM (lung), PolyPhen-2, PROVEAN, VEST	[illegible]	[illegible]	[illegible]	[illegible]	[illegible]	[illegible]	[illegible]	[illegible]	[illegible]	[illegible]	[illegible]	[illegible]
At least 1 of CHASM (breast), CHASM (lung), PolyPhen-2, SIFT	[illegible]	[illegible]	[illegible]	[illegible]	[illegible]	[illegible]	[illegible]	[illegible]	[illegible]	[illegible]	[illegible]	[illegible]
At least 1 of CHASM (breast), CHASM (lung), PolyPhen-2, SIFT, VEST	[illegible]	[illegible]	[illegible]	[illegible]	[illegible]	[illegible]	[illegible]	[illegible]	[illegible]	[illegible]	[illegible]	[illegible]
At least 1 of CHASM (breast), CHASM (lung), PolyPhen-2, VEST	[illegible]	[illegible]	[illegible]	[illegible]	[illegible]	[illegible]	[illegible]	[illegible]	[illegible]	[illegible]	[illegible]	[illegible]
At least 1 of CHASM (breast), CHASM (lung), PROVEAN	[illegible]	[illegible]	[illegible]	[illegible]	[illegible]	[illegible]	[illegible]	[illegible]	[illegible]	[illegible]	[illegible]	[illegible]
At least 1 of CHASM (breast), CHASM (lung), PROVEAN, SIFT	[illegible]	[illegible]	[illegible]	[illegible]	[illegible]	[illegible]	[illegible]	[illegible]	[illegible]	[illegible]	[illegible]	[illegible]
At least 1 of CHASM (breast), CHASM (lung), PROVEAN, SIFT, VEST	[illegible]	[illegible]	[illegible]	[illegible]	[illegible]	[illegible]	[illegible]	[illegible]	[illegible]	[illegible]	[illegible]	[illegible]
At least 1 of CHASM (breast), CHASM (lung), PROVEAN, VEST	[illegible]	[illegible]	[illegible]	[illegible]	[illegible]	[illegible]	[illegible]	[illegible]	[illegible]	[illegible]	[illegible]	[illegible]
At least 1 of CHASM (breast), CHASM (lung), SIFT	[illegible]	[illegible]	[illegible]	[illegible]	[illegible]	[illegible]	[illegible]	[illegible]	[illegible]	[illegible]	[illegible]	[illegible]
At least 1 of CHASM (breast), CHASM (lung), SIFT, VEST	[illegible]	[illegible]	[illegible]	[illegible]	[illegible]	[illegible]	[illegible]	[illegible]	[illegible]	[illegible]	[illegible]	[illegible]
At least 1 of CHASM (breast), CHASM (lung), VEST	[illegible]	[illegible]	[illegible]	[illegible]	[illegible]	[illegible]	[illegible]	[illegible]	[illegible]	[illegible]	[illegible]	[illegible]
At least 1 of CHASM (breast), CHASM (melanoma)	[illegible]	[illegible]	[illegible]	[illegible]	[illegible]	[illegible]	[illegible]	[illegible]	[illegible]	[illegible]	[illegible]	[illegible]
At least 1 of CHASM (breast), CHASM (melanoma), FATHMM (cancer)	[illegible]	[illegible]	[illegible]	[illegible]	[illegible]	[illegible]	[illegible]	[illegible]	[illegible]	[illegible]	[illegible]	[illegible]
At least 1 of CHASM (breast), CHASM (melanoma), FATHMM (cancer), FATHMM (missense)	[illegible]	[illegible]	[illegible]	[illegible]	[illegible]	[illegible]	[illegible]	[illegible]	[illegible]	[illegible]	[illegible]	[illegible]
At least 1 of CHASM (breast), CHASM (melanoma), FATHMM (cancer), FATHMM (missense), Mutation Assessor	[illegible]	[illegible]	[illegible]	[illegible]	[illegible]	[illegible]	[illegible]	[illegible]	[illegible]	[illegible]	[illegible]	[illegible]
At least 1 of CHASM (breast), CHASM (melanoma), FATHMM (cancer), FATHMM (missense), MutationTaster	[illegible]	[illegible]	[illegible]	[illegible]	[illegible]	[illegible]	[illegible]	[illegible]	[illegible]	[illegible]	[illegible]	[illegible]
At least 1 of CHASM (breast), CHASM (melanoma), FATHMM (cancer), FATHMM (missense), PolyPhen-2	[illegible]	[illegible]	[illegible]	[illegible]	[illegible]	[illegible]	[illegible]	[illegible]	[illegible]	[illegible]	[illegible]	[illegible]
At least 1 of CHASM (breast), CHASM (melanoma), FATHMM (cancer), FATHMM (missense), PROVEAN	[illegible]	[illegible]	[illegible]	[illegible]	[illegible]	[illegible]	[illegible]	[illegible]	[illegible]	[illegible]	[illegible]	[illegible]
At least 1 of CHASM (breast), CHASM (melanoma), FATHMM (cancer), FATHMM (missense), SIFT	[illegible]	[illegible]	[illegible]	[illegible]	[illegible]	[illegible]	[illegible]	[illegible]	[illegible]	[illegible]	[illegible]	[illegible]
At least 1 of CHASM (breast), CHASM (melanoma), FATHMM (cancer), FATHMM (missense), VEST	[illegible]	[illegible]	[illegible]	[illegible]	[illegible]	[illegible]	[illegible]	[illegible]	[illegible]	[illegible]	[illegible]	[illegible]
At least 1 of CHASM (breast), CHASM (melanoma), FATHMM (cancer), Mutation Assessor	[illegible]	[illegible]	[illegible]	[illegible]	[illegible]	[illegible]	[illegible]	[illegible]	[illegible]	[illegible]	[illegible]	[illegible]
At least 1 of CHASM (breast), CHASM (melanoma), FATHMM (cancer), Mutation Assessor, MutationTaster	[illegible]	[illegible]	[illegible]	[illegible]	[illegible]	[illegible]	[illegible]	[illegible]	[illegible]	[illegible]	[illegible]	[illegible]
At least 1 of CHASM (breast), CHASM (melanoma), FATHMM (cancer), Mutation Assessor, PolyPhen-2	[illegible]	[illegible]	[illegible]	[illegible]	[illegible]	[illegible]	[illegible]	[illegible]	[illegible]	[illegible]	[illegible]	[illegible]
At least 1 of CHASM (breast), CHASM (melanoma), FATHMM (cancer), Mutation Assessor, PROVEAN	[illegible]	[illegible]	[illegible]	[illegible]	[illegible]	[illegible]	[illegible]	[illegible]	[illegible]	[illegible]	[illegible]	[illegible]
At least 1 of CHASM (breast), CHASM (melanoma), FATHMM (cancer), Mutation Assessor, SIFT	[illegible]	[illegible]	[illegible]	[illegible]	[illegible]	[illegible]	[illegible]	[illegible]	[illegible]	[illegible]	[illegible]	[illegible]
At least 1 of CHASM (breast), CHASM (melanoma), FATHMM (cancer), Mutation Assessor, VEST	[illegible]	[illegible]	[illegible]	[illegible]	[illegible]	[illegible]	[illegible]	[illegible]	[illegible]	[illegible]	[illegible]	[illegible]
At least 1 of CHASM (breast), CHASM (melanoma), FATHMM (cancer), MutationTaster	[illegible]	[illegible]	[illegible]	[illegible]	[illegible]	[illegible]	[illegible]	[illegible]	[illegible]	[illegible]	[illegible]	[illegible]
At least 1 of CHASM (breast), CHASM (melanoma), FATHMM (cancer), MutationTaster, PolyPhen-2	[illegible]	[illegible]	[illegible]	[illegible]	[illegible]	[illegible]	[illegible]	[illegible]	[illegible]	[illegible]	[illegible]	[illegible]
At least 1 of CHASM (breast), CHASM (melanoma), FATHMM (cancer), MutationTaster, PROVEAN	[illegible]	[illegible]	[illegible]	[illegible]	[illegible]	[illegible]	[illegible]	[illegible]	[illegible]	[illegible]	[illegible]	[illegible]
At least 1 of CHASM (breast), CHASM (melanoma), FATHMM (cancer), MutationTaster, SIFT	[illegible]	[illegible]	[illegible]	[illegible]	[illegible]	[illegible]	[illegible]	[illegible]	[illegible]	[illegible]	[illegible]	[illegible]
At least 1 of CHASM (breast), CHASM (melanoma), FATHMM (cancer), MutationTaster, VEST	[illegible]	[illegible]	[illegible]	[illegible]	[illegible]	[illegible]	[illegible]	[illegible]	[illegible]	[illegible]	[illegible]	[illegible]
At least 1 of CHASM (breast), CHASM (melanoma), FATHMM (cancer), PolyPhen-2	[illegible]	[illegible]	[illegible]	[illegible]	[illegible]	[illegible]	[illegible]	[illegible]	[illegible]	[illegible]	[illegible]	[illegible]
At least 1 of CHASM (breast), CHASM (melanoma), FATHMM (cancer), PolyPhen-2, PROVEAN	[illegible]	[illegible]	[illegible]	[illegible]	[illegible]	[illegible]	[illegible]	[illegible]	[illegible]	[illegible]	[illegible]	[illegible]
At least 1 of CHASM (breast), CHASM (melanoma), FATHMM (cancer), PolyPhen-2, SIFT	[illegible]	[illegible]	[illegible]	[illegible]	[illegible]	[illegible]	[illegible]	[illegible]	[illegible]	[illegible]	[illegible]	[illegible]
At least 1 of CHASM (breast), CHASM (melanoma), FATHMM (cancer), PolyPhen-2, VEST	[illegible]	[illegible]	[illegible]	[illegible]	[illegible]	[illegible]	[illegible]	[illegible]	[illegible]	[illegible]	[illegible]	[illegible]
At least 1 of CHASM (breast), CHASM (melanoma), FATHMM (cancer), PROVEAN	[illegible]	[illegible]	[illegible]	[illegible]	[illegible]	[illegible]	[illegible]	[illegible]	[illegible]	[illegible]	[illegible]	[illegible]
At least 1 of CHASM (breast), CHASM (melanoma), FATHMM (cancer), PROVEAN, SIFT	[illegible]	[illegible]	[illegible]	[illegible]	[illegible]	[illegible]	[illegible]	[illegible]	[illegible]	[illegible]	[illegible]	[illegible]
At least 1 of CHASM (breast), CHASM (melanoma), FATHMM (cancer), PROVEAN, VEST	[illegible]	[illegible]	[illegible]	[illegible]	[illegible]	[illegible]	[illegible]	[illegible]	[illegible]	[illegible]	[illegible]	[illegible]
At least 1 of CHASM (breast), CHASM (melanoma), FATHMM (cancer), SIFT	[illegible]	[illegible]	[illegible]	[illegible]	[illegible]	[illegible]	[illegible]	[illegible]	[illegible]	[illegible]	[illegible]	[illegible]
At least 1 of CHASM (breast), CHASM (melanoma), FATHMM (cancer), SIFT, VEST	[illegible]	[illegible]	[illegible]	[illegible]	[illegible]	[illegible]	[illegible]	[illegible]	[illegible]	[illegible]	[illegible]	[illegible]
At least 1 of CHASM (breast), CHASM (melanoma), FATHMM (cancer), VEST	[illegible]	[illegible]	[illegible]	[illegible]	[illegible]	[illegible]	[illegible]	[illegible]	[illegible]	[illegible]	[illegible]	[illegible]
At least 1 of CHASM (breast), CHASM (melanoma), FATHMM (missense)	[illegible]	[illegible]	[illegible]	[illegible]	[illegible]	[illegible]	[illegible]	[illegible]	[illegible]	[illegible]	[illegible]	[illegible]
At least 1 of CHASM (breast), CHASM (melanoma), FATHMM (missense), Mutation Assessor	[illegible]	[illegible]	[illegible]	[illegible]	[illegible]	[illegible]	[illegible]	[illegible]	[illegible]	[illegible]	[illegible]	[illegible]
At least 1 of CHASM (breast), CHASM (melanoma), FATHMM (missense), Mutation Assessor, MutationTaster	[illegible]	[illegible]	[illegible]	[illegible]	[illegible]	[illegible]	[illegible]	[illegible]	[illegible]	[illegible]	[illegible]	[illegible]
At least 1 of CHASM (breast), CHASM (melanoma), FATHMM (missense), Mutation Assessor, PolyPhen-2	[illegible]	[illegible]	[illegible]	[illegible]	[illegible]	[illegible]	[illegible]	[illegible]	[illegible]	[illegible]	[illegible]	[illegible]
At least 1 of CHASM (breast), CHASM (melanoma), FATHMM (missense), Mutation Assessor, PROVEAN	[illegible]	[illegible]	[illegible]	[illegible]	[illegible]	[illegible]	[illegible]	[illegible]	[illegible]	[illegible]	[illegible]	[illegible]
At least 1 of CHASM (breast), CHASM (melanoma), FATHMM (missense), Mutation Assessor, SIFT	[illegible]	[illegible]	[illegible]	[illegible]	[illegible]	[illegible]	[illegible]	[illegible]	[illegible]	[illegible]	[illegible]	[illegible]
At least 1 of CHASM (breast), CHASM (melanoma), FATHMM (missense), Mutation Assessor, VEST	[illegible]	[illegible]	[illegible]	[illegible]	[illegible]	[illegible]	[illegible]	[illegible]	[illegible]	[illegible]	[illegible]	[illegible]
At least 1 of CHASM (breast), CHASM (melanoma), FATHMM (missense), MutationTaster	[illegible]	[illegible]	[illegible]	[illegible]	[illegible]	[illegible]	[illegible]	[illegible]	[illegible]	[illegible]	[illegible]	[illegible]
At least 1 of CHASM (breast), CHASM (melanoma), FATHMM (missense), MutationTaster, PolyPhen-2	[illegible]	[illegible]	[illegible]	[illegible]	[illegible]	[illegible]	[illegible]	[illegible]	[illegible]	[illegible]	[illegible]	[illegible]
At least 1 of CHASM (breast), CHASM (melanoma), FATHMM (missense), MutationTaster, PROVEAN	[illegible]	[illegible]	[illegible]	[illegible]	[illegible]	[illegible]	[illegible]	[illegible]	[illegible]	[illegible]	[illegible]	[illegible]
At least 1 of CHASM (breast), CHASM (melanoma), FATHMM (missense), MutationTaster, SIFT	[illegible]	[illegible]	[illegible]	[illegible]	[illegible]	[illegible]	[illegible]	[illegible]	[illegible]	[illegible]	[illegible]	[illegible]
At least 1 of CHASM (breast), CHASM (melanoma), FATHMM (missense), MutationTaster, VEST	[illegible]	[illegible]	[illegible]	[illegible]	[illegible]	[illegible]	[illegible]	[illegible]	[illegible]	[illegible]	[illegible]	[illegible]
At least 1 of CHASM (breast), CHASM (melanoma), FATHMM (missense), PolyPhen-2	[illegible]	[illegible]	[illegible]	[illegible]	[illegible]	[illegible]	[illegible]	[illegible]	[illegible]	[illegible]	[illegible]	[illegible]
At least 1 of CHASM (breast), CHASM (melanoma), FATHMM (missense), PolyPhen-2, PROVEAN	[illegible]	[illegible]	[illegible]	[illegible]	[illegible]	[illegible]	[illegible]	[illegible]	[illegible]	[illegible]	[illegible]	[illegible]
At least 1 of CHASM (breast), CHASM (melanoma), FATHMM (missense), PolyPhen-2, SIFT	[illegible]	[illegible]	[illegible]	[illegible]	[illegible]	[illegible]	[illegible]	[illegible]	[illegible]	[illegible]	[illegible]	[illegible]
At least 1 of CHASM (breast), CHASM (melanoma), FATHMM (missense), PolyPhen-2, VEST	[illegible]	[illegible]	[illegible]	[illegible]	[illegible]	[illegible]	[illegible]	[illegible]	[illegible]	[illegible]	[illegible]	[illegible]
At least 1 of CHASM (breast), CHASM (melanoma), FATHMM (missense), PROVEAN	[illegible]	[illegible]	[illegible]	[illegible]	[illegible]	[illegible]	[illegible]	[illegible]	[illegible]	[illegible]	[illegible]	[illegible]
At least 1 of CHASM (breast), CHASM (melanoma), FATHMM (missense), PROVEAN, SIFT	[illegible]	[illegible]	[illegible]	[illegible]	[illegible]	[illegible]	[illegible]	[illegible]	[illegible]	[illegible]	[illegible]	[illegible]
At least 1 of CHASM (breast), CHASM (melanoma), FATHMM (missense), PROVEAN, VEST	[illegible]	[illegible]	[illegible]	[illegible]	[illegible]	[illegible]	[illegible]	[illegible]	[illegible]	[illegible]	[illegible]	[illegible]
At least 1 of CHASM (breast), CHASM (melanoma), FATHMM (missense), SIFT	[illegible]	[illegible]	[illegible]	[illegible]	[illegible]	[illegible]	[illegible]	[illegible]	[illegible]	[illegible]	[illegible]	[illegible]
At least 1 of CHASM (breast), CHASM (melanoma), FATHMM (missense), SIFT, VEST	[illegible]	[illegible]	[illegible]	[illegible]	[illegible]	[illegible]	[illegible]	[illegible]	[illegible]	[illegible]	[illegible]	[illegible]
At least 1 of CHASM (breast), CHASM (melanoma), FATHMM (missense), VEST	[illegible]	[illegible]	[illegible]	[illegible]	[illegible]	[illegible]	[illegible]	[illegible]	[illegible]	[illegible]	[illegible]	[illegible]
At least 1 of CHASM (breast), CHASM (melanoma), Mutation Assessor	[illegible]	[illegible]	[illegible]	[illegible]	[illegible]	[illegible]	[illegible]	[illegible]	[illegible]	[illegible]	[illegible]	[illegible]
At least 1 of CHASM (breast), CHASM (melanoma), Mutation Assessor, MutationTaster	[illegible]	[illegible]	[illegible]	[illegible]	[illegible]	[illegible]	[illegible]	[illegible]	[illegible]	[illegible]	[illegible]	[illegible]
At least 1 of CHASM (breast), CHASM (melanoma), Mutation Assessor, MutationTaster, PolyPhen-2	[illegible]	[illegible]	[illegible]	[illegible]	[illegible]	[illegible]	[illegible]	[illegible]	[illegible]	[illegible]	[illegible]	[illegible]
At least 1 of CHASM (breast), CHASM (melanoma), Mutation Assessor, MutationTaster, PROVEAN	[illegible]	[illegible]	[illegible]	[illegible]	[illegible]	[illegible]	[illegible]	[illegible]	[illegible]	[illegible]	[illegible]	[illegible]
At least 1 of CHASM (breast), CHASM (melanoma), Mutation Assessor, MutationTaster, SIFT	[illegible]	[illegible]	[illegible]	[illegible]	[illegible]	[illegible]	[illegible]	[illegible]	[illegible]	[illegible]	[illegible]	[illegible]
At least 1 of CHASM (breast), CHASM (melanoma), Mutation Assessor, MutationTaster, VEST	[illegible]	[illegible]	[illegible]	[illegible]	[illegible]	[illegible]	[illegible]	[illegible]	[illegible]	[illegible]	[illegible]	[illegible]
At least 1 of CHASM (breast), CHASM (melanoma), Mutation Assessor, PolyPhen-2	[illegible]	[illegible]	[illegible]	[illegible]	[illegible]	[illegible]	[illegible]	[illegible]	[illegible]	[illegible]	[illegible]	[illegible]
At least 1 of CHASM (breast), CHASM (melanoma), Mutation Assessor, PolyPhen-2, PROVEAN	[illegible]	[illegible]	[illegible]	[illegible]	[illegible]	[illegible]	[illegible]	[illegible]	[illegible]	[illegible]	[illegible]	[illegible]
At least 1 of CHASM (breast), CHASM (melanoma), Mutation Assessor, PolyPhen-2, SIFT	[illegible]	[illegible]	[illegible]	[illegible]	[illegible]	[illegible]	[illegible]	[illegible]	[illegible]	[illegible]	[illegible]	[illegible]
At least 1 of CHASM (breast), CHASM (melanoma), Mutation Assessor, PolyPhen-2, VEST	[illegible]	[illegible]	[illegible]	[illegible]	[illegible]	[illegible]	[illegible]	[illegible]	[illegible]	[illegible]	[illegible]	[illegible]
At least 1 of CHASM (breast), CHASM (melanoma), Mutation Assessor, PROVEAN	[illegible]	[illegible]	[illegible]	[illegible]	[illegible]	[illegible]	[illegible]	[illegible]	[illegible]	[illegible]	[illegible]	[illegible]
At least 1 of CHASM (breast), CHASM (melanoma), Mutation Assessor, PROVEAN, SIFT	[illegible]	[illegible]	[illegible]	[illegible]	[illegible]	[illegible]	[illegible]	[illegible]	[illegible]	[illegible]	[illegible]	[illegible]
At least 1 of CHASM (breast), CHASM (melanoma), Mutation Assessor, PROVEAN, VEST	[illegible]	[illegible]	[illegible]	[illegible]	[illegible]	[illegible]	[illegible]	[illegible]	[illegible]	[illegible]	[illegible]	[illegible]
At least 1 of CHASM (breast), CHASM (melanoma), Mutation Assessor, SIFT	[illegible]	[illegible]	[illegible]	[illegible]	[illegible]	[illegible]	[illegible]	[illegible]	[illegible]	[illegible]	[illegible]	[illegible]
At least 1 of CHASM (breast), CHASM (melanoma), Mutation Assessor, SIFT, VEST	[illegible]	[illegible]	[illegible]	[illegible]	[illegible]	[illegible]	[illegible]	[illegible]	[illegible]	[illegible]	[illegible]	[illegible]
At least 1 of CHASM (breast), CHASM (melanoma), Mutation Assessor, VEST	[illegible]	[illegible]	[illegible]	[illegible]	[illegible]	[illegible]	[illegible]	[illegible]	[illegible]	[illegible]	[illegible]	[illegible]
At least 1 of CHASM (breast), CHASM (melanoma), MutationTaster	[illegible]	[illegible]	[illegible]	[illegible]	[illegible]	[illegible]	[illegible]	[illegible]	[illegible]	[illegible]	[illegible]	[illegible]
At least 1 of CHASM (breast), CHASM (melanoma), MutationTaster, PolyPhen-2	[illegible]	[illegible]	[illegible]	[illegible]	[illegible]	[illegible]	[illegible]	[illegible]	[illegible]	[illegible]	[illegible]	[illegible]
At least 1 of CHASM (breast), CHASM (melanoma), MutationTaster, PolyPhen-2, PROVEAN	[illegible]	[illegible]	[illegible]	[illegible]	[illegible]	[illegible]	[illegible]	[illegible]	[illegible]	[illegible]	[illegible]	[illegible]
At least 1 of CHASM (breast), CHASM (melanoma), MutationTaster, PolyPhen-2, SIFT	[illegible]	[illegible]	[illegible]	[illegible]	[illegible]	[illegible]	[illegible]	[illegible]	[illegible]	[illegible]	[illegible]	[illegible]
At least 1 of CHASM (breast), CHASM (melanoma), MutationTaster, PolyPhen-2, VEST	[illegible]	[illegible]	[illegible]	[illegible]	[illegible]	[illegible]	[illegible]	[illegible]	[illegible]	[illegible]	[illegible]	[illegible]
At least 1 of CHASM (breast), CHASM (melanoma), MutationTaster, PROVEAN	[illegible]	[illegible]	[illegible]	[illegible]	[illegible]	[illegible]	[illegible]	[illegible]	[illegible]	[illegible]	[illegible]	[illegible]
At least 1 of CHASM (breast), CHASM (melanoma), MutationTaster, PROVEAN, SIFT	[illegible]	[illegible]	[illegible]	[illegible]	[illegible]	[illegible]	[illegible]	[illegible]	[illegible]	[illegible]	[illegible]	[illegible]
At least 1 of CHASM (breast), CHASM (melanoma), MutationTaster, PROVEAN, VEST	[illegible]	[illegible]	[illegible]	[illegible]	[illegible]	[illegible]	[illegible]	[illegible]	[illegible]	[illegible]	[illegible]	[illegible]
At least 1 of CHASM (breast), CHASM (melanoma), MutationTaster, SIFT	[illegible]	[illegible]	[illegible]	[illegible]	[illegible]	[illegible]	[illegible]	[illegible]	[illegible]	[illegible]	[illegible]	[illegible]
At least 1 of CHASM (breast), CHASM (melanoma), MutationTaster, SIFT, VEST	[illegible]	[illegible]	[illegible]	[illegible]	[illegible]	[illegible]	[illegible]	[illegible]	[illegible]	[illegible]	[illegible]	[illegible]
At least 1 of CHASM (breast), CHASM (melanoma), MutationTaster, VEST	[illegible]	[illegible]	[illegible]	[illegible]	[illegible]	[illegible]	[illegible]	[illegible]	[illegible]	[illegible]	[illegible]	[illegible]
At least 1 of CHASM (breast), CHASM (melanoma), PolyPhen-2	[illegible]	[illegible]	[illegible]	[illegible]	[illegible]	[illegible]	[illegible]	[illegible]	[illegible]	[illegible]	[illegible]	[illegible]
At least 1 of CHASM (breast), CHASM (melanoma), PolyPhen-2, PROVEAN	[illegible]	[illegible]	[illegible]	[illegible]	[illegible]	[illegible]	[illegible]	[illegible]	[illegible]	[illegible]	[illegible]	[illegible]
At least 1 of CHASM (breast), CHASM (melanoma), PolyPhen-2, PROVEAN, SIFT	[illegible]	[illegible]	[illegible]	[illegible]	[illegible]	[illegible]	[illegible]	[illegible]	[illegible]	[illegible]	[illegible]	[illegible]
At least 1 of CHASM (breast), CHASM (melanoma), PolyPhen-2, PROVEAN, VEST	[illegible]	[illegible]	[illegible]	[illegible]	[illegible]	[illegible]	[illegible]	[illegible]	[illegible]	[illegible]	[illegible]	[illegible]
At least 1 of CHASM (breast), CHASM (melanoma), PolyPhen-2, SIFT	[illegible]	[illegible]	[illegible]	[illegible]	[illegible]	[illegible]	[illegible]	[illegible]	[illegible]	[illegible]	[illegible]	[illegible]
At least 1 of CHASM (breast), CHASM (melanoma), PolyPhen-2, SIFT, VEST	[illegible]	[illegible]	[illegible]	[illegible]	[illegible]	[illegible]	[illegible]	[illegible]	[illegible]	[illegible]	[illegible]	[illegible]
At least 1 of CHASM (breast), CHASM (melanoma), PolyPhen-2, VEST	[illegible]	[illegible]	[illegible]	[illegible]	[illegible]	[illegible]	[illegible]	[illegible]	[illegible]	[illegible]	[illegible]	[illegible]
At least 1 of CHASM (breast), CHASM (melanoma), PROVEAN	[illegible]	[illegible]	[illegible]	[illegible]	[illegible]	[illegible]	[illegible]	[illegible]	[illegible]	[illegible]	[illegible]	[illegible]
At least 1 of CHASM (breast), CHASM (melanoma), PROVEAN, SIFT	[illegible]	[illegible]	[illegible]	[illegible]	[illegible]	[illegible]	[illegible]	[illegible]	[illegible]	[illegible]	[illegible]	[illegible]
At least 1 of CHASM (breast), CHASM (melanoma), PROVEAN, SIFT, VEST	[illegible]	[illegible]	[illegible]	[illegible]	[illegible]	[illegible]	[illegible]	[illegible]	[illegible]	[illegible]	[illegible]	[illegible]
At least 1 of CHASM (breast), CHASM (melanoma), PROVEAN, VEST	[illegible]	[illegible]	[illegible]	[illegible]	[illegible]	[illegible]	[illegible]	[illegible]	[illegible]	[illegible]	[illegible]	[illegible]
At least 1 of CHASM (breast), CHASM (melanoma), SIFT	[illegible]	[illegible]	[illegible]	[illegible]	[illegible]	[illegible]	[illegible]	[illegible]	[illegible]	[illegible]	[illegible]	[illegible]
At least 1 of CHASM (breast), CHASM (melanoma), SIFT, VEST	[illegible]	[illegible]	[illegible]	[illegible]	[illegible]	[illegible]	[illegible]	[illegible]	[illegible]	[illegible]	[illegible]	[illegible]
At least 1 of CHASM (breast), CHASM (melanoma), VEST	[illegible]	[illegible]	[illegible]	[illegible]	[illegible]	[illegible]	[illegible]	[illegible]	[illegible]	[illegible]	[illegible]	[illegible]
At least 1 of CHASM (breast), FATHMM (cancer)	[illegible]	[illegible]	[illegible]	[illegible]	[illegible]	[illegible]	[illegible]	[illegible]	[illegible]	[illegible]	[illegible]	[illegible]
At least 1 of CHASM (breast), FATHMM (cancer), FATHMM (missense)	[illegible]	[illegible]	[illegible]	[illegible]	[illegible]	[illegible]	[illegible]	[illegible]	[illegible]	[illegible]	[illegible]	[illegible]
At least 1 of CHASM (breast), FATHMM (cancer), FATHMM (missense), Mutation Assessor	[illegible]	[illegible]	[illegible]	[illegible]	[illegible]	[illegible]	[illegible]	[illegible]	[illegible]	[illegible]	[illegible]	[illegible]
At least 1 of CHASM (breast), FATHMM (cancer), FATHMM (missense), Mutation Assessor, MutationTaster	[illegible]	[illegible]	[illegible]	[illegible]	[illegible]	[illegible]	[illegible]	[illegible]	[illegible]	[illegible]	[illegible]	[illegible]
At least 1 of CHASM (breast), FATHMM (cancer), FATHMM (missense), Mutation Assessor, PolyPhen-2	[illegible]	[illegible]	[illegible]	[illegible]	[illegible]	[illegible]	[illegible]	[illegible]	[illegible]	[illegible]	[illegible]	[illegible]
At least 1 of CHASM (breast), FATHMM (cancer), FATHMM (missense), Mutation Assessor, PROVEAN	[illegible]	[illegible]	[illegible]	[illegible]	[illegible]	[illegible]	[illegible]	[illegible]	[illegible]	[illegible]	[illegible]	[illegible]
At least 1 of CHASM (breast), FATHMM (cancer), FATHMM (missense), Mutation Assessor, SIFT	[illegible]	[illegible]	[illegible]	[illegible]	[illegible]	[illegible]	[illegible]	[illegible]	[illegible]	[illegible]	[illegible]	[illegible]
At least 1 of CHASM (breast), FATHMM (cancer), FATHMM (missense), Mutation Assessor, VEST	[illegible]	[illegible]	[illegible]	[illegible]	[illegible]	[illegible]	[illegible]	[illegible]	[illegible]	[illegible]	[illegible]	[illegible]
At least 1 of CHASM (breast), FATHMM (cancer), FATHMM (missense), MutationTaster	[illegible]	[illegible]	[illegible]	[illegible]	[illegible]	[illegible]	[illegible]	[illegible]	[illegible]	[illegible]	[illegible]	[illegible]
At least 1 of CHASM (breast), FATHMM (cancer), FATHMM (missense), MutationTaster, PolyPhen-2	[illegible]	[illegible]	[illegible]	[illegible]	[illegible]	[illegible]	[illegible]	[illegible]	[illegible]	[illegible]	[illegible]	[illegible]
At least 1 of CHASM (breast), FATHMM (cancer), FATHMM (missense), MutationTaster, PROVEAN	[illegible]	[illegible]	[illegible]	[illegible]	[illegible]	[illegible]	[illegible]	[illegible]	[illegible]	[illegible]	[illegible]	[illegible]
At least 1 of CHASM (breast), FATHMM (cancer), FATHMM (missense), MutationTaster, SIFT	[illegible]	[illegible]	[illegible]	[illegible]	[illegible]	[illegible]	[illegible]	[illegible]	[illegible]	[illegible]	[illegible]	[illegible]
At least 1 of CHASM (breast), FATHMM (cancer), FATHMM (missense), MutationTaster, VEST	[illegible]	[illegible]	[illegible]	[illegible]	[illegible]	[illegible]	[illegible]	[illegible]	[illegible]	[illegible]	[illegible]	[illegible]
At least 1 of CHASM (breast), FATHMM (cancer), FATHMM (missense), PolyPhen-2	[illegible]	[illegible]	[illegible]	[illegible]	[illegible]	[illegible]	[illegible]	[illegible]	[illegible]	[illegible]	[illegible]	[illegible]
At least 1 of CHASM (breast), FATHMM (cancer), FATHMM (missense), PolyPhen-2, PROVEAN	[illegible]	[illegible]	[illegible]	[illegible]	[illegible]	[illegible]	[illegible]	[illegible]	[illegible]	[illegible]	[illegible]	[illegible]
At least 1 of CHASM (breast), FATHMM (cancer), FATHMM (missense), PolyPhen-2, SIFT	[illegible]	[illegible]	[illegible]	[illegible]	[illegible]	[illegible]	[illegible]	[illegible]	[illegible]	[illegible]	[illegible]	[illegible]
At least 1 of CHASM (breast), FATHMM (cancer), FATHMM (missense), PolyPhen-2, VEST	[illegible]	[illegible]	[illegible]	[illegible]	[illegible]	[illegible]	[illegible]	[illegible]	[illegible]	[illegible]	[illegible]	[illegible]
At least 1 of CHASM (breast), FATHMM (cancer), FATHMM (missense), PROVEAN	[illegible]	[illegible]	[illegible]	[illegible]	[illegible]	[illegible]	[illegible]	[illegible]	[illegible]	[illegible]	[illegible]	[illegible]
At least 1 of CHASM (breast), FATHMM (cancer), FATHMM (missense), PROVEAN, SIFT	[illegible]	[illegible]	[illegible]	[illegible]	[illegible]	[illegible]	[illegible]	[illegible]	[illegible]	[illegible]	[illegible]	[illegible]
At least 1 of CHASM (breast), FATHMM (cancer), FATHMM (missense), PROVEAN, VEST	[illegible]	[illegible]	[illegible]	[illegible]	[illegible]	[illegible]	[illegible]	[illegible]	[illegible]	[illegible]	[illegible]	[illegible]
At least 1 of CHASM (breast), FATHMM (cancer), FATHMM (missense), SIFT	[illegible]	[illegible]	[illegible]	[illegible]	[illegible]	[illegible]	[illegible]	[illegible]	[illegible]	[illegible]	[illegible]	[illegible]
At least 1 of CHASM (breast), FATHMM (cancer), FATHMM (missense), SIFT, VEST	[illegible]	[illegible]	[illegible]	[illegible]	[illegible]	[illegible]	[illegible]	[illegible]	[illegible]	[illegible]	[illegible]	[illegible]
At least 1 of CHASM (breast), FATHMM (cancer), FATHMM (missense), VEST	[illegible]	[illegible]	[illegible]	[illegible]	[illegible]	[illegible]	[illegible]	[illegible]	[illegible]	[illegible]	[illegible]	[illegible]
At least 1 of CHASM (breast), FATHMM (cancer), Mutation Assessor	[illegible]	[illegible]	[illegible]	[illegible]	[illegible]	[illegible]	[illegible]	[illegible]	[illegible]	[illegible]	[illegible]	[illegible]
At least 1 of CHASM (breast), FATHMM (cancer), Mutation Assessor, MutationTaster	[illegible]	[illegible]	[illegible]	[illegible]	[illegible]	[illegible]	[illegible]	[illegible]	[illegible]	[illegible]	[illegible]	[illegible]

At least 1 of CHASM (breast), FATHMM (cancer), Mutation Assessor, MutationTaster, PolyPhen-2	74.08% (70.71%-77.78%)	100% (100%-100%)	29.271% (23.288%-35.294%)	70.97% (67.22%-75%)	100% (100%-100%)	3.0024 (2.9233-3.0942)	74.06% (66.67%-80.81%)	100% (100%-100%)	29.523% (17.494%-41.18%)	70.89% (62.79%-78.41%)	100% (100%-100%)	3.0041 (2.8325-3.1687)
At least 1 of CHASM (breast), FATHMM (cancer), Mutation Assessor, MutationTaster, PROVEAN	75.48% (72.22%-78.79%)	99.49% (99.16%-100%)	33.959% (27.999%-40.006%)	72.26% (68.42%-76.03%)	97.48% (95.24%-100%)	3.0319 (2.9292-3.1382)	75.3% (68.69%-81.82%)	99.42% (98.21%-100%)	33.903% (21.208%-45.95%)	72.08% (64.37%-79.55%)	97.15% (88.89%-100%)	3.0255 (2.8138-3.2256)
At least 1 of CHASM (breast), FATHMM (cancer), Mutation Assessor, MutationTaster, SIFT	72.09% (68.69%-75.76%)	100% (100%-100%)	23.849% (18.053%-29.335%)	69.42% (65.57%-73.48%)	100% (100%-100%)	2.9327 (2.8518-3.0146)	71.97% (64.65%-78.79%)	100% (100%-100%)	23.891% (13.509%-35.48%)	69.27% (61.29%-76.92%)	100% (100%-100%)	2.9316 (2.7805-3.0989)
At least 1 of CHASM (breast), FATHMM (cancer), Mutation Assessor, MutationTaster, VEST	68.39% (64.65%-72.22%)	100% (100%-100%)	13.726% (9.208%-18.31%)	66.71% (62.9%-70.75%)	100% (100%-100%)	2.8044 (2.7436-2.8776)	68.28% (60.61%-75.76%)	100% (100%-100%)	13.828% (5.128%-22.86%)	66.57% (58.51%-74.19%)	100% (100%-100%)	2.8045 (2.6722-2.9324)
At least 1 of CHASM (breast), FATHMM (cancer), Mutation Assessor, PolyPhen-2	73.73% (70.2%-77.27%)	98.94% (98.33%-100%)	30.137% (24.236%-36.364%)	71.01% (67.23%-75.14%)	94.28% (89.99%-100%)	2.9437 (2.8308-3.0582)	73.75% (66.67%-80.81%)	98.92% (96.67%-100%)	30.531% (17.946%-42.5%)	70.96% (62.92%-78.57%)	94.27% (81.82%-100%)	2.9468 (2.6977-3.1529)
At least 1 of CHASM (breast), FATHMM (cancer), Mutation Assessor, PolyPhen-2, PROVEAN	72.07% (68.69%-75.76%)	98.94% (98.33%-100%)	25.591% (20.253%-31.25%)	69.69% (65.93%-73.6%)	93.34% (88.24%-100%)	2.8757 (2.7637-2.9939)	72.02% (64.65%-78.79%)	98.92% (96.67%-100%)	25.838% (14.276%-37.14%)	69.6% (61.54%-77.28%)	93.3% (77.78%-100%)	2.8765 (2.6092-3.0937)
At least 1 of CHASM (breast), FATHMM (cancer), Mutation Assessor, PolyPhen-2, SIFT	71.07% (67.66%-74.75%)	99.46% (99.16%-100%)	21.965% (16.438%-27.539%)	68.79% (65.03%-72.78%)	95.95% (92.31%-100%)	2.8616 (2.7548-2.9749)	71% (63.64%-77.8%)	99.48% (98.25%-100%)	22.131% (11.427%-33.33%)	68.68% (60.67%-76.4%)	96.13% (83.33%-100%)	2.8641 (2.6206-3.0622)
At least 1 of CHASM (breast), FATHMM (cancer), Mutation Assessor, PolyPhen-2, VEST	67.71% (64.14%-71.72%)	100% (100%-100%)	11.881% (7.35%-16.176%)	66.24% (62.43%-70.21%)	100% (100%-100%)	2.7812 (2.7206-2.8536)	67.61% (59.6%-74.75%)	100% (100%-100%)	11.998% (3.223%-20.59%)	66.11% (58.06%-73.68%)	100% (100%-100%)	2.7819 (2.6565-2.9124)
At least 1 of CHASM (breast), FATHMM (cancer), Mutation Assessor, PROVEAN	76.11% (72.73%-79.29%)	98.44% (97.52%-100%)	37.503% (31.168%-43.478%)	73.14% (69.36%-76.97%)	93.28% (88.46%-100%)	3.0236 (2.9059-3.1384)	76.06% (69.7%-82.83%)	98.34% (95.45%-100%)	37.82% (25.714%-50%)	73.07% (65.48%-80.73%)	93% (81.25%-100%)	3.0223 (2.7818-3.2423)
At least 1 of CHASM (breast), FATHMM (cancer), Mutation Assessor, PROVEAN, SIFT	71.72% (68.18%-75.25%)	99.46% (99.16%-100%)	23.738% (18.31%-29.577%)	69.28% (65.38%-73.18%)	96.24% (92.86%-100%)	2.8872 (2.7866-2.9869)	71.72% (64.65%-78.79%)	99.48% (98.25%-100%)	24.075% (12.5%-34.49%)	69.21% (61.36%-76.93%)	96.4% (85.65%-100%)	2.8916 (2.6660-3.0861)
At least 1 of CHASM (breast), FATHMM (cancer), Mutation Assessor, PROVEAN, VEST	67.39% (63.64%-71.21%)	100% (100%-100%)	10.998% (6.757%-15.068%)	66.02% (62.11%-70%)	100% (100%-100%)	2.7702 (2.7081-2.8348)	67.25% (59.6%-74.75%)	100% (100%-100%)	11.039% (2.857%-20%)	65.86% (57.89%-73.47%)	100% (100%-100%)	2.7696 (2.6467-2.9018)
At least 1 of CHASM (breast), FATHMM (cancer), Mutation Assessor, SIFT	73.05% (69.7%-76.77%)	99.46% (99.16%-100%)	27.392% (21.622%-33.343%)	70.31% (66.66%-74.16%)	96.72% (94.12%-100%)	2.9389 (2.8374-3.0514)	73.08% (65.66%-79.8%)	99.48% (98.25%-100%)	27.808% (16.216%-39.4%)	70.28% (62.49%-77.91%)	96.86% (87.5%-100%)	2.9442 (2.7164-3.1335)
At least 1 of CHASM (breast), FATHMM (cancer), Mutation Assessor, SIFT, VEST	66.75% (63.13%-70.71%)	100% (100%-100%)	9.254% (5.479%-12.857%)	65.58% (61.7%-69.63%)	100% (100%-100%)	2.7483 (2.6932-2.8130)	66.5% (58.59%-73.74%)	100% (100%-100%)	9.016% (2.498%-16.67%)	65.35% (57.29%-72.92%)	100% (100%-100%)	2.7449 (2.6270-2.8596)
At least 1 of CHASM (breast), FATHMM (cancer), Mutation Assessor, VEST	68.39% (64.65%-72.22%)	100% (100%-100%)	13.726% (9.208%-18.31%)	66.71% (62.9%-70.75%)	100% (100%-100%)	2.8044 (2.7436-2.8776)	68.28% (60.61%-75.76%)	100% (100%-100%)	13.828% (5.128%-22.86%)	66.57% (58.51%-74.19%)	100% (100%-100%)	2.8045 (2.6722-2.9324)
At least 1 of CHASM (breast), FATHMM (cancer), MutationTaster	82.52% (79.29%-85.86%)	99.49% (99.16%-100%)	53.184% (46.374%-59.459%)	78.61% (75%-82.47%)	98.38% (97.06%-100%)	3.2966 (3.1964-3.3963)	82.43% (75.76%-88.89%)	99.42% (98.21%-100%)	53.277% (40%-66.67%)	78.5% (70.89%-85.9%)	98.17% (93.74%-100%)	3.2937 (3.0989-3.4993)
At least 1 of CHASM (breast), FATHMM (cancer), MutationTaster, PolyPhen-2	76.43% (73.23%-79.81%)	100% (100%-100%)	35.682% (29.729%-41.936%)	72.89% (69.28%-76.75%)	100% (100%-100%)	3.0857 (3.0061-3.1785)	76.43% (69.67%-82.83%)	100% (100%-100%)	35.963% (23.525%-47.51%)	72.83% (65.06%-80%)	100% (100%-100%)	3.0879 (2.9171-3.2623)
At least 1 of CHASM (breast), FATHMM (cancer), MutationTaster, PolyPhen-2, PROVEAN	73.77% (70.2%-77.27%)	100% (100%-100%)	28.402% (22.854%-34.211%)	70.72% (67.05%-74.43%)	100% (100%-100%)	2.9912 (2.9140-3.0727)	73.68% (66.67%-80.81%)	100% (100%-100%)	28.476% (16.667%-40.01%)	70.59% (63.09%-77.91%)	100% (100%-100%)	2.9906 (2.8325-3.1532)
At least 1 of CHASM (breast), FATHMM (cancer), MutationTaster, PolyPhen-2, SIFT	72.77% (69.19%-76.77%)	100% (100%-100%)	25.689% (19.696%-31.944%)	69.94% (66.29%-73.89%)	100% (100%-100%)	2.9563 (2.8775-3.0433)	72.64% (64.65%-79.8%)	100% (100%-100%)	25.706% (14.286%-37.21%)	69.79% (61.63%-77.27%)	100% (100%-100%)	2.9549 (2.7947-3.1276)
At least 1 of CHASM (breast), FATHMM (cancer), MutationTaster, PolyPhen-2, VEST	68.71% (65.15%-72.73%)	100% (100%-100%)	14.593% (9.589%-19.231%)	66.94% (63.1%-70.9%)	100% (100%-100%)	2.8153 (2.7438-2.8885)	68.65% (60.61%-75.76%)	100% (100%-100%)	14.834% (5.714%-25%)	66.84% (58.88%-74.47%)	100% (100%-100%)	2.8169 (2.6815-2.9683)
At least 1 of CHASM (breast), FATHMM (cancer), MutationTaster, PROVEAN	78.17% (74.75%-81.31%)	99.49% (99.16%-100%)	41.285% (34.919%-47.368%)	74.55% (70.83%-78.16%)	97.92% (96.15%-100%)	3.1325 (3.0338-3.2337)	78.01% (71.72%-84.85%)	99.42% (98.21%-100%)	41.256% (28.571%-54.29%)	74.39% (67.07%-81.82%)	97.66% (91.67%-100%)	3.1272 (2.9356-3.3319)
At least 1 of CHASM (breast), FATHMM (cancer), MutationTaster, PROVEAN, SIFT	72.45% (69.19%-75.76%)	100% (100%-100%)	24.802% (19.113%-30.435%)	69.69% (65.92%-73.48%)	100% (100%-100%)	2.9449 (2.8695-3.0245)	72.28% (65.66%-78.79%)	100% (100%-100%)	24.681% (13.152%-36.36%)	69.5% (61.8%-76.92%)	100% (100%-100%)	2.9418 (2.7947-3.1086)
At least 1 of CHASM (breast), FATHMM (cancer), MutationTaster, PROVEAN, VEST	68.05% (64.14%-71.72%)	100% (100%-100%)	12.796% (8.333%-17.106%)	66.47% (62.63%-70.43%)	100% (100%-100%)	2.7927 (2.7276-2.8613)	67.95% (60.61%-75.76%)	100% (100%-100%)	12.939% (4.875%-21.88%)	66.34% (58.33%-73.96%)	100% (100%-100%)	2.7931 (2.6698-2.9419)
At least 1 of CHASM (breast), FATHMM (cancer), MutationTaster, SIFT	74.12% (70.71%-77.78%)	100% (100%-100%)	29.37% (23.074%-35.211%)	71% (67.24%-75%)	100% (100%-100%)	3.0037 (2.9217-3.0900)	73.99% (66.67%-80.81%)	100% (100%-100%)	29.35% (17.497%-41.38%)	70.83% (63.04%-78.16%)	100% (100%-100%)	3.0018 (2.8453-3.1765)
At least 1 of CHASM (breast), FATHMM (cancer), MutationTaster, SIFT, VEST	67.74% (64.14%-71.72%)	100% (100%-100%)	11.965% (7.246%-16.131%)	66.26% (62.37%-70.37%)	100% (100%-100%)	2.7823 (2.7206-2.8536)	67.55% (59.6%-74.75%)	100% (100%-100%)	11.852% (2.941%-20.59%)	66.07% (57.89%-73.91%)	100% (100%-100%)	2.7800 (2.6531-2.9145)
At least 1 of CHASM (breast), FATHMM (cancer), MutationTaster, VEST	69.72% (66.16%-73.74%)	100% (100%-100%)	17.365% (12.329%-22.388%)	67.66% (63.78%-71.81%)	100% (100%-100%)	2.8503 (2.7811-2.9310)	69.65% (61.62%-76.77%)	100% (100%-100%)	17.567% (7.687%-27.5%)	67.55% (59.14%-75.27%)	100% (100%-100%)	2.8513 (2.7081-2.9976)
At least 1 of CHASM (breast), FATHMM (cancer), PolyPhen-2	75.74% (72.73%-79.29%)	98.41% (97.5%-100%)	36.549% (30.299%-42.667%)	72.84% (69.19%-76.79%)	93.02% (88.46%-100%)	3.0081 (2.8891-3.1230)	75.78% (68.69%-81.82%)	98.39% (95.52%-100%)	36.972% (24.242%-48.72%)	72.82% (65.11%-80.01%)	93.07% (81.25%-100%)	3.0125 (2.7570-3.2405)
At least 1 of CHASM (breast), FATHMM (cancer), PolyPhen-2, PROVEAN	73.08% (69.7%-76.77%)	98.41% (97.5%-100%)	29.268% (23.288%-35.135%)	70.64% (66.86%-74.44%)	91.43% (85.71%-100%)	2.8975 (2.7809-3.0195)	73.03% (65.66%-79.8%)	98.39% (95.52%-100%)	29.485% (17.494%-41.18%)	70.54% (62.92%-78.16%)	91.47% (76.92%-100%)	2.8989 (2.6334-3.1249)
At least 1 of CHASM (breast), FATHMM (cancer), PolyPhen-2, PROVEAN, SIFT	71.08% (67.68%-74.75%)	99.46% (99.16%-100%)	21.988% (16.438%-27.536%)	68.8% (65.17%-72.68%)	95.96% (92.31%-100%)	2.8621 (2.7548-2.9676)	70.98% (63.64%-77.78%)	99.48% (98.25%-100%)	22.046% (10.808%-33.33%)	68.66% (60.87%-75.83%)	96.13% (83.33%-100%)	2.8631 (2.6395-3.0575)
At least 1 of CHASM (breast), FATHMM (cancer), PolyPhen-2, PROVEAN, VEST	67.71% (64.14%-71.22%)	100% (100%-100%)	11.871% (7.46%-16.176%)	66.24% (62.5%-70.21%)	100% (100%-100%)	2.7811 (2.7162-2.8496)	67.61% (60.58%-74.75%)	100% (100%-100%)	12.024% (3.125%-21.06%)	66.11% (58.24%-73.47%)	100% (100%-100%)	2.7818 (2.6581-2.9247)
At least 1 of CHASM (breast), FATHMM (cancer), PolyPhen-2, SIFT	72.75% (69.19%-76.26%)	99.46% (99.16%-100%)	26.556% (20.507%-32.394%)	70.07% (66.47%-74.03%)	96.63% (93.75%-100%)	2.9272 (2.8247-3.0309)	72.69% (65.66%-79.8%)	99.48% (98.25%-100%)	26.714% (15.148%-38.71%)	69.96% (61.9%-77.5%)	96.77% (87.5%-100%)	2.9292 (2.7183-3.1276)
At least 1 of CHASM (breast), FATHMM (cancer), PolyPhen-2, SIFT, VEST	67.4% (63.64%-71.21%)	100% (100%-100%)	11.04% (6.849%-15.002%)	66.03% (62.23%-70.16%)	100% (100%-100%)	2.7707 (2.7116-2.8422)	67.21% (59.6%-74.75%)	100% (100%-100%)	10.938% (2.778%-19.44%)	65.84% (57.45%-73.4%)	100% (100%-100%)	2.7688 (2.6411-2.9007)
At least 1 of CHASM (breast), FATHMM (cancer), PolyPhen-2, VEST	68.71% (65.15%-72.73%)	100% (100%-100%)	14.593% (9.589%-19.231%)	66.94% (63.1%-70.9%)	100% (100%-100%)	2.8153 (2.7438-2.8885)	68.65% (60.61%-75.76%)	100% (100%-100%)	14.834% (5.714%-25%)	66.84% (58.88%-74.47%)	100% (100%-100%)	2.8169 (2.6815-2.9683)
At least 1 of CHASM (breast), FATHMM (cancer), PROVEAN	76.42% (73.22%-79.8%)	93.62% (91.34%-96.09%)	46.657% (40.253%-52.857%)	75.22% (71.34%-78.95%)	80.89% (74.29%-87.8%)	2.9638 (2.8168-3.1099)	76.46% (69.7%-82.85%)	93.6% (88.4%-98.36%)	47.007% (33.333%-60%)	75.2% (67.85%-83.12%)	81.04% (66.67%-94.74%)	2.9684 (2.6823-3.2589)
At least 1 of CHASM (breast), FATHMM (cancer), PROVEAN, SIFT	73.41% (70.2%-76.77%)	99.46% (99.16%-100%)	28.346% (22.368%-34.617%)	70.59% (67.03%-74.3%)	96.84% (94.12%-100%)	2.9524 (2.8548-3.0535)	73.39% (66.67%-79.8%)	99.48% (98.25%-100%)	28.598% (16.216%-40.01%)	70.51% (63.04%-77.91%)	96.97% (87.5%-100%)	2.9556 (2.7470-3.1451)
At least 1 of CHASM (breast), FATHMM (cancer), PROVEAN, SIFT, VEST	67.07% (63.13%-71.21%)	100% (100%-100%)	10.135% (5.882%-14.085%)	65.8% (61.9%-69.79%)	100% (100%-100%)	2.7594 (2.7019-2.8237)	66.87% (58.59%-74.75%)	100% (100%-100%)	10.003% (2.63%-18.18%)	65.6% (57.73%-73.4%)	100% (100%-100%)	2.7574 (2.6399-2.8847)
At least 1 of CHASM (breast), FATHMM (cancer), PROVEAN, VEST	67.73% (64.13%-71.72%)	99.5% (99.16%-100%)	12.796% (8.333%-17.106%)	66.36% (62.43%-70.37%)	93.69% (85.71%-100%)	2.7235 (2.5934-2.8459)	67.57% (59.6%-74.77%)	99.4% (98.25%-100%)	12.939% (4.875%-21.88%)	66.21% (58.24%-73.96%)	92.69% (66.67%-100%)	2.7127 (2.3644-2.9247)
At least 1 of CHASM (breast), FATHMM (cancer), SIFT	75.08% (71.72%-78.28%)	99.46% (99.16%-100%)	32.913% (26.919%-39.132%)	71.94% (68.39%-75.72%)	97.26% (95%-100%)	3.0158 (2.9189-3.1201)	75.1% (68.69%-81.82%)	99.48% (98.25%-100%)	33.266% (20.588%-45.46%)	71.89% (64.29%-79.07%)	97.38% (90%-100%)	3.0201 (2.8090-3.2125)
At least 1 of CHASM (breast), FATHMM (cancer), SIFT, VEST	67.74% (64.14%-71.72%)	100% (100%-100%)	11.965% (7.246%-16.131%)	66.26% (62.37%-70.37%)	100% (100%-100%)	2.7823 (2.7206-2.8536)	67.55% (59.6%-74.75%)	100% (100%-100%)	11.852% (2.941%-20.59%)	66.07% (57.89%-73.91%)	100% (100%-100%)	2.7800 (2.6531-2.9145)
At least 1 of CHASM (breast), FATHMM (cancer), VEST	69.4% (65.66%-73.23%)	99.5% (99.16%-100%)	17.365% (12.329%-22.388%)	67.55% (63.64%-71.74%)	95.28% (90%-100%)	2.7970 (2.6938-2.9053)	69.28% (61.62%-76.77%)	99.4% (98.25%-100%)	17.567% (7.687%-27.5%)	67.42% (59.14%-75.27%)	94.57% (79.75%-100%)	2.7898 (2.5136-2.9833)
At least 1 of CHASM (breast), FATHMM (missense)	78.13% (75.25%-81.31%)	91.59% (88.89%-94.53%)	54.838% (47.823%-61.431%)	77.82% (74%-81.51%)	79.05% (72.73%-85.42%)	3.0330 (2.8839-3.1726)	78.08% (71.72%-83.84%)	91.26% (85.25%-96.77%)	55.357% (41.659%-68.75%)	77.84% (70.42%-85.33%)	78.72% (66.67%-91.3%)	3.0318 (2.7514-3.3082)
At least 1 of CHASM (breast), FATHMM (missense), Mutation Assessor	75.41% (71.72%-78.79%)	97.38% (95.9%-99.2%)	37.393% (31.429%-43.662%)	72.9% (69.09%-76.79%)	89.22% (83.33%-96.3%)	2.9689 (2.8372-3.0976)	75.45% (68.69%-82.83%)	97.25% (93.65%-100%)	38.008% (25.625%-50%)	72.92% (65%-80.49%)	89% (76.46%-100%)	2.9717 (2.7083-3.2312)
At least 1 of CHASM (breast), FATHMM (missense), Mutation Assessor, MutationTaster	75.77% (72.22%-79.29%)	99.49% (99.16%-100%)	34.745% (28.767%-40.845%)	72.5% (68.64%-76.33%)	97.53% (95.45%-100%)	3.0427 (2.9464-3.1458)	75.74% (68.69%-82.83%)	99.42% (98.21%-100%)	35.065% (22.579%-47.22%)	72.43% (64.71%-80.22%)	97.24% (90%-100%)	3.0416 (2.8229-3.2403)
At least 1 of CHASM (breast), FATHMM (missense), Mutation Assessor, MutationTaster, PolyPhen-2	73.38% (70.19%-76.77%)	100% (100%-100%)	27.353% (21.622%-32.877%)	70.42% (66.48%-74.18%)	100% (100%-100%)	2.9777 (2.8975-3.0622)	73.44% (66.67%-79.82%)	100% (100%-100%)	27.834% (16.216%-39.47%)	70.4% (62.5%-78.26%)	100% (100%-100%)	2.9823 (2.8288-3.1508)
At least 1 of CHASM (breast), FATHMM (missense), Mutation Assessor, MutationTaster, PROVEAN	72.75% (69.19%-76.26%)	99.49% (99.16%-100%)	26.503% (20.833%-32.394%)	70.07% (66.1%-74.01%)	96.78% (93.75%-100%)	2.9285 (2.8289-3.0345)	72.68% (65.66%-79.8%)	99.42% (98.21%-100%)	26.763% (14.695%-37.94%)	69.97% (61.9%-77.78%)	96.38% (85.71%-100%)	2.9253 (2.6818-3.1156)
At least 1 of CHASM (breast), FATHMM (missense), Mutation Assessor, MutationTaster, SIFT	70.74% (67.17%-74.24%)	100% (100%-100%)	20.144% (14.492%-25.352%)	68.41% (64.52%-72.43%)	100% (100%-100%)	2.8855 (2.8087-2.9592)	70.65% (63.64%-77.78%)	100% (100%-100%)	20.278% (10%-31.25%)	68.28% (60.22%-76.4%)	100% (100%-100%)	2.8856 (2.7417-3.0459)
At least 1 of CHASM (breast), FATHMM (missense), Mutation Assessor, MutationTaster, VEST	68.02% (64.65%-71.72%)	100% (100%-100%)	12.716% (8.195%-17.144%)	66.46% (62.57%-70.53%)	100% (100%-100%)	2.7917 (2.7285-2.8570)	68% (60.61%-74.75%)	100% (100%-100%)	13.047% (3.448%-22.22%)	66.38% (58.16%-74.19%)	100% (100%-100%)	2.7945 (2.6706-2.9295)
At least 1 of CHASM (breast), FATHMM (missense), Mutation Assessor, PolyPhen-2	73.03% (69.7%-76.77%)	98.95% (98.32%-100%)	28.22% (21.918%-33.824%)	70.45% (66.67%-74.3%)	93.96% (89.47%-100%)	2.9157 (2.8018-3.0345)	73.12% (65.66%-79.8%)	98.91% (96.67%-100%)	28.843% (17.852%-41.38%)	70.46% (62.5%-78.02%)	93.96% (80%-100%)	2.9217 (2.6707-3.1383)
At least 1 of CHASM (breast), FATHMM (missense), Mutation Assessor, PolyPhen-2, PROVEAN	71.03% (67.68%-74.75%)	98.95% (98.32%-100%)	22.749% (16.896%-28.571%)	68.89% (64.97%-72.78%)	92.6% (86.67%-100%)	2.8320 (2.7102-2.9584)	71.07% (63.64%-77.78%)	98.91% (96.67%-100%)	23.255% (11.761%-35%)	68.87% (61.11%-76.34%)	92.57% (75%-100%)	2.8361 (2.5390-3.0565)
At least 1 of CHASM (breast), FATHMM (missense), Mutation Assessor, PolyPhen-2, SIFT	70.72% (67.17%-74.24%)	99.47% (99.16%-100%)	21.01% (15.382%-26.391%)	68.53% (64.67%-72.53%)	95.81% (91.67%-100%)	2.8482 (2.7392-2.9563)	70.68% (63.64%-77.78%)	99.47% (98.28%-100%)	21.286% (10.811%-32.52%)	68.44% (60.43%-76.4%)	95.91% (83.33%-100%)	2.8510 (2.6213-3.0446)
At least 1 of CHASM (breast), FATHMM (missense), Mutation Assessor, PolyPhen-2, VEST	67.34% (63.64%-71.21%)	100% (100%-100%)	10.866% (6.667%-14.865%)	65.99% (62.11%-70%)	100% (100%-100%)	2.7685 (2.7081-2.8341)	67.34% (59.6%-74.75%)	100% (100%-100%)	11.242% (2.857%-19.52%)	65.92% (57.89%-73.68%)	100% (100%-100%)	2.7722 (2.6566-2.9007)
At least 1 of CHASM (breast), FATHMM (missense), Mutation Assessor, PROVEAN	72.39% (68.69%-75.77%)	97.38% (95.9%-99.2%)	29.152% (23.074%-35.213%)	70.39% (66.47%-74.29%)	86.56% (79.16%-95.24%)	2.8348 (2.6920-2.9757)	72.4% (65.63%-79.2%)	97.25% (93.65%-100%)	29.705% (17.239%-41.68%)	70.37% (62.35%-78.16%)	86.28% (69.23%-100%)	2.8360 (2.5210-3.1163)
At least 1 of CHASM (breast), FATHMM (missense), Mutation Assessor, PROVEAN, SIFT	69.7% (65.66%-73.23%)	98.95% (98.32%-100%)	19.114% (13.043%-24.006%)	67.9% (63.93%-71.82%)	91.36% (84.58%-100%)	2.7733 (2.6345-2.9003)	69.69% (62.63%-77.78%)	98.89% (96.67%-100%)	19.543% (9.67%-30.95%)	67.84% (60.22%-75.82%)	91.16% (75%-100%)	2.7743 (2.4844-3.0048)
At least 1 of CHASM (breast), FATHMM (missense), Mutation Assessor, PROVEAN, VEST	66.34% (62.63%-70.2%)	100% (100%-100%)	8.122% (4.167%-11.594%)	65.3% (61.46%-69.39%)	100% (100%-100%)	2.7342 (2.6799-2.7949)	66.32% (58.59%-73.74%)	100% (100%-100%)	8.485% (2.439%-16.22%)	65.22% (57.14%-72.92%)	100% (100%-100%)	2.7386 (2.6270-2.8566)
At least 1 of CHASM (breast), FATHMM (missense), Mutation Assessor, SIFT	71.05% (67.17%-74.75%)	98.95% (98.32%-100%)	22.792% (16.901%-28.17%)	68.91% (64.97%-72.73%)	92.66% (86.67%-100%)	2.8331 (2.7102-2.9501)	71.04% (63.64%-78.79%)	98.89% (96.67%-100%)	23.22% (12.5%-35.29%)	68.85% (60.92%-76.92%)	92.5% (77.78%-100%)	2.8347 (2.5848-3.0515)
At least 1 of CHASM (breast), FATHMM (missense), Mutation Assessor, SIFT, VEST	66.38% (62.63%-70.2%)	100% (100%-100%)	8.237% (4.284%-11.598%)	65.33% (61.34%-69.43%)	100% (100%-100%)	2.7357 (2.6773-2.7949)	66.23% (58.59%-73.74%)	100% (100%-100%)	8.264% (2.326%-15.79%)	65.17% (56.84%-73.2%)	100% (100%-100%)	2.7360 (2.6270-2.8605)
At least 1 of CHASM (breast), FATHMM (missense), Mutation Assessor, VEST	68.02% (64.65%-71.72%)	100% (100%-100%)	12.716% (8.195%-17.144%)	66.46% (62.57%-70.53%)	100% (100%-100%)	2.7917 (2.7285-2.8570)	68% (60.61%-74.75%)	100% (100%-100%)	13.047% (3.448%-22.22%)	66.38% (58.16%-74.19%)	100% (100%-100%)	2.7945 (2.6706-2.9295)
At least 1 of CHASM (breast), FATHMM (missense), MutationTaster	81.15% (78.28%-83.84%)	99.49% (99.16%-100%)	49.416% (42.619%-55.845%)	77.28% (73.75%-80.73%)	98.26% (96.77%-100%)	3.2444 (3.1469-3.3397)	81.14% (75.76%-86.87%)	99.42% (98.21%-100%)	49.733% (36.842%-63.34%)	77.25% (70%-84.34%)	98.05% (92.86%-100%)	3.2445 (3.0458-3.4486)
At least 1 of CHASM (breast), FATHMM (missense), MutationTaster, PolyPhen-2	75.72% (72.73%-79.29%)	100% (100%-100%)	33.736% (27.394%-39.443%)	72.29% (68.64%-76.03%)	100% (100%-100%)	3.0603 (2.9754-3.1457)	75.83% (68.69%-81.82%)	100% (100%-100%)	34.333% (22.857%-47.06%)	72.32% (64.77%-79.76%)	100% (100%-100%)	3.0666 (2.9129-3.2519)
At least 1 of CHASM (breast), FATHMM (missense), MutationTaster, PolyPhen-2, PROVEAN	72.38% (69.19%-75.76%)	100% (100%-100%)	24.607% (18.919%-30.14%)	69.64% (65.92%-73.34%)	100% (100%-100%)	2.9424 (2.8640-3.0245)	72.42% (65.66%-78.79%)	100% (100%-100%)	25.047% (13.509%-36.37%)	69.6% (62.1%-77.02%)	100% (100%-100%)	2.9465 (2.7990-3.1156)
At least 1 of CHASM (breast), FATHMM (missense), MutationTaster, PolyPhen-2, SIFT	72.07% (68.69%-75.76%)	100% (100%-100%)	23.782% (17.904%-29.168%)	69.4% (65.56%-73.33%)	100% (100%-100%)	2.9319 (2.8551-3.0133)	72.02% (64.65%-78.79%)	100% (100%-100%)	24.014% (13.158%-35.72%)	69.31% (61.36%-76.93%)	100% (100%-100%)	2.9332 (2.7844-3.1002)
At least 1 of CHASM (breast), FATHMM (missense), MutationTaster, PolyPhen-2, VEST	68.34% (64.65%-72.22%)	100% (100%-100%)	13.578% (8.955%-17.724%)	66.68% (62.77%-70.68%)	100% (100%-100%)	2.8025 (2.7373-2.8690)	68.38% (60.61%-75.76%)	100% (100%-100%)	14.078% (5.552%-23.33%)	66.64% (58.51%-74.47%)	100% (100%-100%)	2.8072 (2.6856-2.9500)
At least 1 of CHASM (breast), FATHMM (missense), MutationTaster, PROVEAN	75.42% (72.22%-78.79%)	99.49% (99.16%-100%)	33.787% (27.629%-40%)	72.21% (68.42%-75.88%)	97.46% (95.24%-100%)	3.0295 (2.9291-3.1276)	75.42% (68.69%-81.82%)	99.42% (98.21%-100%)	34.197% (21.212%-45.95%)	72.17% (64.63%-79.55%)	97.17% (89.97%-100%)	3.0296 (2.8237-3.2258)
At least 1 of CHASM (breast), FATHMM (missense), MutationTaster, PROVEAN, SIFT	71.07% (67.17%-74.75%)	100% (100%-100%)	21.031% (15.584%-26.158%)	68.65% (64.8%-72.53%)	100% (100%-100%)	2.8968 (2.8239-2.9683)	71% (63.64%-78.79%)	100% (100%-100%)	21.204% (10.804%-31.83%)	68.54% (60.87%-76.14%)	100% (100%-100%)	2.8974 (2.7653-3.0622)
At least 1 of CHASM (breast), FATHMM (missense), MutationTaster, PROVEAN, VEST	67% (63.13%-70.71%)	100% (100%-100%)	9.921% (5.714%-13.514%)	65.75% (61.9%-69.63%)	100% (100%-100%)	2.7567 (2.6957-2.8160)	67.02% (59.6%-74.75%)	100% (100%-100%)	10.385% (2.857%-18.75%)	65.7% (57.89%-73.4%)	100% (100%-100%)	2.7612 (2.6525-2.8959)
At least 1 of CHASM (breast), FATHMM (missense), MutationTaster, SIFT	72.75% (69.19%-76.26%)	100% (100%-100%)	25.622% (19.48%-30.986%)	69.92% (66.1%-73.77%)	100% (100%-100%)	2.9554 (2.8776-3.0332)	72.69% (65.66%-79.8%)	100% (100%-100%)	25.817% (14.706%-37.84%)	69.82% (61.7%-77.28%)	100% (100%-100%)	2.9563 (2.8019-3.1224)
At least 1 of CHASM (breast), FATHMM (missense), MutationTaster, SIFT, VEST	67.37% (63.64%-71.21%)	100% (100%-100%)	10.949% (6.849%-14.865%)	66.01% (61.98%-70.16%)	100% (100%-100%)	2.7695 (2.7081-2.8341)	67.28% (59.6%-74.75%)	100% (100%-100%)	11.1% (2.941%-19.36%)	65.88% (57.73%-73.96%)	100% (100%-100%)	2.7701 (2.6488-2.9079)
At least 1 of CHASM (breast), FATHMM (missense), MutationTaster, VEST	69.36% (65.66%-73.23%)	100% (100%-100%)	16.377% (11.538%-20.899%)	67.4% (63.59%-71.35%)	100% (100%-100%)	2.8378 (2.7685-2.9080)	69.36% (61.62%-76.77%)	100% (100%-100%)	16.733% (6.891%-26.47%)	67.34% (59.34%-74.74%)	100% (100%-100%)	2.8407 (2.7120-2.9869)
At least 1 of CHASM (breast), FATHMM (missense), PolyPhen-2	75.37% (72.22%-78.79%)	98.95% (98.32%-100%)	34.603% (28.358%-40.541%)	72.35% (68.79%-76.16%)	95.02% (91.3%-100%)	3.0092 (2.8990-3.1210)	75.52% (68.69%-81.82%)	98.91% (96.67%-100%)	35.341% (24.13%-48.28%)	72.42% (64.77%-79.76%)	95.02% (83.33%-100%)	3.0168 (2.7880-3.2403)
At least 1 of CHASM (breast), FATHMM (missense), PolyPhen-2, PROVEAN	72.03% (68.69%-75.76%)	98.95% (98.32%-100%)	25.473% (19.692%-31.081%)	69.66% (65.93%-73.48%)	93.34% (88.24%-100%)	2.8742 (2.7514-2.9938)	72.1% (64.65%-78.79%)	98.91% (96.67%-100%)	26.055% (14.625%-37.94%)	69.66% (62.1%-77.17%)	93.32% (77.78%-100%)	2.8795 (2.6235-3.1078)
At least 1 of CHASM (breast), FATHMM (missense), PolyPhen-2, PROVEAN, SIFT	70.37% (66.67%-73.74%)	99.47% (99.16%-100%)	20.057% (14.102%-25%)	68.27% (64.32%-72.04%)	95.61% (90.91%-100%)	2.8341 (2.7185-2.9407)	70.37% (63.64%-77.78%)	99.47% (98.28%-100%)	20.409% (10.336%-31.58%)	68.2% (60.44%-76.09%)	95.71% (85.45%-100%)	2.8379 (2.5793-3.0441)
At least 1 of CHASM (breast), FATHMM (missense), PolyPhen-2, PROVEAN, VEST	67% (63.13%-70.71%)	100% (100%-100%)	9.921% (5.714%-13.514%)	65.75% (61.9%-69.63%)	100% (100%-100%)	2.7567 (2.6957-2.8160)	67.02% (59.6%-74.75%)	100% (100%-100%)	10.385% (2.857%-18.75%)	65.7% (57.89%-73.4%)	100% (100%-100%)	2.7612 (2.6525-2.8959)
At least 1 of CHASM (breast), FATHMM (missense), PolyPhen-2, SIFT	72.05% (68.69%-75.76%)	99.47% (99.16%-100%)	24.648% (18.571%-30%)	69.53% (65.71%-73.45%)	96.41% (93.32%-100%)	2.9006 (2.7966-3.0048)	72.06% (64.65%-78.79%)	99.47% (98.28%-100%)	25.022% (14.286%-37.15%)	69.48% (61.54%-77.18%)	96.49% (85.71%-100%)	2.9046 (2.6818-3.1001)
At least 1 of CHASM (breast), FATHMM (missense), PolyPhen-2, SIFT, VEST	67.37% (63.64%-71.21%)	100% (100%-100%)	10.949% (6.849%-14.865%)	66.01% (61.98%-70.16%)	100% (100%-100%)	2.7695 (2.7081-2.8341)	67.28% (59.6%-74.75%)	100% (100%-100%)	11.1% (2.941%-19.36%)	65.88% (57.73%-73.96%)	100% (100%-100%)	2.7701 (2.6488-2.9079)
At least 1 of CHASM (breast), FATHMM (missense), PolyPhen-2, VEST	68.34% (64.65%-72.22%)	100% (100%-100%)	13.578% (8.955%-17.724%)	66.68% (62.77%-70.68%)	100% (100%-100%)	2.8025 (2.7373-2.8690)	68.38% (60.61%-75.76%)	100% (100%-100%)	14.078% (5.552%-23.33%)	66.64% (58.51%-74.47%)	100% (100%-100%)	2.8072 (2.6856-2.9500)
At least 1 of CHASM (breast), FATHMM (missense), PROVEAN	74.41% (70.71%-77.78%)	94.75% (92.56%-96.99%)	39.208% (32.431%-45.836%)	72.94% (69.18%-76.79%)	81.21% (74.06%-89.29%)	2.8811 (2.7291-3.0299)	74.42% (67.68%-81.82%)	94.53% (89.65%-98.51%)	39.822% (26.316%-52.79%)	72.96% (65.38%-80.49%)	80.91% (64.71%-94.44%)	2.8822 (2.5744-3.1885)
At least 1 of CHASM (breast), FATHMM (missense), PROVEAN, SIFT	71.72% (68.18%-75.25%)	98.95% (98.32%-100%)	24.624% (18.418%-30.14%)	69.42% (65.54%-73.08%)	93.16% (87.5%-100%)	2.8616 (2.7413-2.9771)	71.7% (64.65%-78.79%)	98.89% (96.67%-100%)	24.995% (13.333%-36.84%)	69.36% (61.63%-77.11%)	92.94% (77.78%-100%)	2.8618 (2.6047-3.0861)
At least 1 of CHASM (breast), FATHMM (missense), PROVEAN, SIFT, VEST	66.36% (62.63%-70.2%)	100% (100%-100%)	8.183% (4.225%-11.538%)	65.32% (61.46%-69.27%)	100% (100%-100%)	2.7350 (2.6773-2.7941)	66.27% (58.59%-74.75%)	100% (100%-100%)	8.367% (2.498%-15.79%)	65.19% (57.14%-72.92%)	100% (100%-100%)	2.7371 (2.6292-2.8596)
At least 1 of CHASM (breast), FATHMM (missense), PROVEAN, VEST	66.68% (63.13%-70.2%)	99.5% (99.16%-100%)	9.921% (5.714%-13.514%)	65.64% (61.78%-69.48%)	92.07% (80%-100%)	2.6713 (2.5195-2.7987)	66.64% (59.6%-73.74%)	99.4% (98.25%-100%)	10.385% (2.857%-18.75%)	65.56% (57.61%-73.2%)	91.26% (66.67%-100%)	2.6663 (2.3267-2.8754)
At least 1 of CHASM (breast), FATHMM (missense), SIFT	73.4% (70.2%-76.77%)	98.95% (98.32%-100%)	29.215% (22.854%-34.85%)	70.74% (66.86%-74.58%)	94.18% (89.47%-100%)	2.9309 (2.8125-3.0411)	73.39% (66.67%-79.8%)	98.89% (96.67%-100%)	29.608% (17.643%-42.86%)	70.69% (62.92%-78.26%)	94% (81.82%-100%)	2.9319 (2.6940-3.1615)
At least 1 of CHASM (breast), FATHMM (missense), SIFT, VEST	67.37% (63.64%-71.21%)	100% (100%-100%)	10.949% (6.849%-14.865%)	66.01% (61.98%-70.16%)	100% (100%-100%)	2.7695 (2.7081-2.8341)	67.28% (59.6%-74.75%)	100% (100%-100%)	11.1% (2.941%-19.36%)	65.88% (57.73%-73.96%)	100% (100%-100%)	2.7701 (2.6488-2.9079)
At least 1 of CHASM (breast), FATHMM (missense), VEST	69.04% (65.66%-72.73%)	99.5% (99.16%-100%)	16.377% (11.538%-20.899%)	67.29% (63.44%-71.35%)	95.03% (90%-100%)	2.7820 (2.6723-2.8885)	68.98% (61.62%-75.76%)	99.4% (98.25%-100%)	16.733% (6.891%-26.47%)	67.2% (59.14%-74.74%)	94.36% (75%-100%)	2.7770 (2.5148-2.9833)
At least 1 of CHASM (breast), Mutation Assessor	82.84% (79.8%-85.86%)	94.71% (92.56%-96.9%)	62.317% (55.881%-68.919%)	81.29% (77.62%-85%)	87.22% (82.35%-92.6%)	3.2554 (3.1337-3.3794)	82.8% (76.77%-88.89%)	94.6% (89.83%-98.51%)	62.549% (50%-75%)	81.24% (73.68%-88.75%)	87.15% (76%-96.43%)	3.2555 (3.0011-3.4975)
At least 1 of CHASM (breast), Mutation Assessor, MutationTaster	84.24% (81.31%-87.37%)	99.49% (99.16%-100%)	57.871% (51.35%-64.62%)	80.33% (76.73%-83.95%)	98.51% (97.3%-100%)	3.3619 (3.2613-3.4639)	84.05% (77.78%-89.9%)	99.42% (98.21%-100%)	57.686% (44.737%-71.05%)	80.12% (72.6%-87.18%)	98.31% (94.12%-100%)	3.3554 (3.1547-3.5723)
At least 1 of CHASM (breast), Mutation Assessor, MutationTaster, PolyPhen-2	78.82% (75.76%-82.32%)	100% (100%-100%)	42.194% (35.714%-48.571%)	74.94% (71.17%-78.82%)	100% (100%-100%)	3.1714 (3.0829-3.2664)	78.73% (71.72%-84.85%)	100% (100%-100%)	42.219% (29.268%-55%)	74.81% (67.06%-82.23%)	100% (100%-100%)	3.1703 (2.9973-3.3484)
At least 1 of CHASM (breast), Mutation Assessor, MutationTaster, PolyPhen-2, PROVEAN	76.48% (73.23%-79.8%)	100% (100%-100%)	35.812% (29.41%-42.031%)	72.93% (69.01%-76.88%)	100% (100%-100%)	3.0874 (3.0035-3.1810)	76.33% (69.7%-82.83%)	100% (100%-100%)	35.692% (23.327%-48.39%)	72.74% (64.77%-80.46%)	100% (100%-100%)	3.0844 (2.9146-3.2537)
At least 1 of CHASM (breast), Mutation Assessor, MutationTaster, PolyPhen-2, SIFT	74.12% (70.71%-77.78%)	100% (100%-100%)	29.367% (22.857%-35.296%)	71% (67.05%-75%)	100% (100%-100%)	3.0036 (2.9217-3.0901)	73.99% (66.67%-80.81%)	100% (100%-100%)	29.356% (17.647%-41.94%)	70.84% (62.79%-78.65%)	100% (100%-100%)	3.0019 (2.8384-3.1784)
At least 1 of CHASM (breast), Mutation Assessor, MutationTaster, PolyPhen-2, VEST	68.72% (65.15%-72.73%)	100% (100%-100%)	14.634% (9.859%-19.119%)	66.95% (63.1%-70.97%)	100% (100%-100%)	2.8158 (2.7503-2.8915)	68.62% (60.61%-75.76%)	100% (100%-100%)	14.736% (5.556%-24.24%)	66.81% (58.95%-74.23%)	100% (100%-100%)	2.8156 (2.6816-2.9496)
At least 1 of CHASM (breast), Mutation Assessor, MutationTaster, PROVEAN	80.54% (77.27%-83.84%)	99.49% (99.16%-100%)	47.767% (41.333%-54.795%)	76.71% (72.89%-80.37%)	98.19% (96.67%-100%)	3.2216 (3.1290-3.3290)	80.34% (73.74%-86.87%)	99.42% (98.21%-100%)	47.586% (34.211%-61.11%)	76.5% (69.05%-83.95%)	97.96% (92.86%-100%)	3.2146 (3.0059-3.4155)
At least 1 of CHASM (breast), Mutation Assessor, MutationTaster, PROVEAN, SIFT	73.78% (70.2%-77.27%)	100% (100%-100%)	28.436% (22.497%-34.288%)	70.73% (66.86%-74.59%)	100% (100%-100%)	2.9916 (2.9124-3.0745)	73.66% (66.67%-80.81%)	100% (100%-100%)	28.452% (16.667%-40.64%)	70.57% (63.04%-78.16%)	100% (100%-100%)	2.9903 (2.8325-3.1687)
At least 1 of CHASM (breast), Mutation Assessor, MutationTaster, PROVEAN, VEST	68.05% (64.65%-71.72%)	100% (100%-100%)	12.816% (8.219%-17.143%)	66.48% (62.63%-70.37%)	100% (100%-100%)	2.7930 (2.7276-2.8631)	67.93% (60.61%-74.75%)	100% (100%-100%)	12.893% (3.568%-21.96%)	66.33% (58.51%-73.96%)	100% (100%-100%)	2.7925 (2.6652-2.9323)
At least 1 of CHASM (breast), Mutation Assessor, MutationTaster, SIFT	75.46% (72.22%-78.79%)	100% (100%-100%)	33.025% (26.667%-39.194%)	72.08% (68.21%-75.86%)	100% (100%-100%)	3.0510 (2.9656-3.1435)	75.35% (68.69%-81.82%)	100% (100%-100%)	33.069% (20%-46.16%)	71.94% (63.95%-79.76%)	100% (100%-100%)	3.0500 (2.8805-3.2403)
At least 1 of CHASM (breast), Mutation Assessor, MutationTaster, SIFT, VEST	67.76% (64.14%-71.72%)	100% (100%-100%)	12.007% (7.576%-16.176%)	66.27% (62.43%-70.22%)	100% (100%-100%)	2.7828 (2.7211-2.8536)	67.52% (59.6%-74.75%)	100% (100%-100%)	11.754% (3.028%-20.46%)	66.04% (58.06%-73.47%)	100% (100%-100%)	2.7783 (2.6531-2.9124)
At least 1 of CHASM (breast), Mutation Assessor, MutationTaster, VEST	69.74% (66.16%-73.74%)	100% (100%-100%)	17.41% (12.496%-22.222%)	67.67% (63.78%-71.74%)	100% (100%-100%)	2.8509 (2.7844-2.9274)	69.62% (61.62%-76.77%)	100% (100%-100%)	17.455% (7.405%-27.03%)	67.52% (59.55%-75.26%)	100% (100%-100%)	2.8498 (2.7140-2.9873)
At least 1 of CHASM (breast), Mutation Assessor, PolyPhen-2	77.79% (74.75%-81.31%)	97.88% (96.69%-99.23%)	43.06% (36.483%-49.398%)	74.83% (70.99%-78.82%)	92.15% (87.5%-97.22%)	3.0791 (2.9562-3.2015)	77.75% (70.71%-83.84%)	97.86% (94.83%-100%)	43.227% (30.549%-55.88%)	74.74% (66.67%-82.15%)	92.19% (80%-100%)	3.0801 (2.8328-3.3201)
At least 1 of CHASM (breast), Mutation Assessor, PolyPhen-2, PROVEAN	75.46% (72.22%-78.79%)	97.88% (96.69%-99.23%)	36.678% (30.303%-43.038%)	72.77% (68.86%-76.75%)	90.91% (85.71%-96.88%)	2.9824 (2.8567-3.1133)	75.35% (68.69%-81.82%)	97.86% (94.83%-100%)	36.7% (24.322%-50%)	72.63% (64.71%-80.25%)	90.92% (76.92%-100%)	2.9811 (2.7014-3.2197)
At least 1 of CHASM (breast), Mutation Assessor, PolyPhen-2, PROVEAN, SIFT	71.74% (68.18%-75.25%)	98.4% (97.52%-100%)	25.644% (19.443%-31.429%)	69.59% (65.71%-73.45%)	90.24% (83.33%-100%)	2.8387 (2.6993-2.9683)	71.67% (64.65%-78.79%)	98.42% (95.31%-100%)	25.748% (14.634%-38.24%)	69.47% (61.79%-77.02%)	90.48% (72.73%-100%)	2.8412 (2.5552-3.0861)
At least 1 of CHASM (breast), Mutation Assessor, PolyPhen-2, PROVEAN, VEST	67.72% (64.14%-71.22%)	100% (100%-100%)	11.89% (7.35%-16.176%)	66.24% (62.43%-70.21%)	100% (100%-100%)	2.7813 (2.7180-2.8497)	67.6% (60.58%-74.75%)	100% (100%-100%)	11.978% (3.226%-20.59%)	66.1% (58.24%-73.68%)	100% (100%-100%)	2.7813 (2.6582-2.9171)
At least 1 of CHASM (breast), Mutation Assessor, PolyPhen-2, SIFT	73.42% (69.7%-76.77%)	98.4% (97.52%-100%)	30.233% (23.68%-36.486%)	70.92% (67.05%-74.86%)	91.6% (85.71%-100%)	2.9115 (2.7809-3.0418)	73.36% (66.67%-80.81%)	98.42% (95.31%-100%)	30.365% (18.605%-43.59%)	70.81% (62.79%-78.57%)	91.81% (76.92%-100%)	2.9140 (2.6407-3.1474)
At least 1 of CHASM (breast), Mutation Assessor, PolyPhen-2, SIFT, VEST	67.42% (63.64%-71.21%)	100% (100%-100%)	11.081% (6.944%-15.068%)	66.04% (62.23%-70.16%)	100% (100%-100%)	2.7712 (2.7110-2.8385)	67.18% (59.6%-74.75%)	100% (100%-100%)	10.839% (2.857%-19.06%)	65.81% (57.61%-73.41%)	100% (100%-100%)	2.7671 (2.6449-2.9007)
At least 1 of CHASM (breast), Mutation Assessor, PolyPhen-2, VEST	68.72% (65.15%-72.73%)	100% (100%-100%)	14.634% (9.859%-19.119%)	66.95% (63.1%-70.97%)	100% (100%-100%)	2.8158 (2.7503-2.8915)	68.62% (60.61%-75.76%)	100% (100%-100%)	14.736% (5.556%-24.24%)	66.81% (58.95%-74.23%)	100% (100%-100%)	2.8156 (2.6816-2.9496)
At least 1 of CHASM (breast), Mutation Assessor, PROVEAN	79.81% (76.77%-82.83%)	95.77% (93.7%-97.64%)	52.213% (45.83%-58.97%)	77.6% (73.87%-81.29%)	87.73% (82.22%-93.33%)	3.1332 (3.0017-3.2627)	79.77% (73.74%-85.86%)	95.68% (91.8%-100%)	52.449% (38.889%-65.72%)	77.54% (70.13%-85.33%)	87.64% (75.98%-100%)	3.1331 (2.8554-3.3882)
At least 1 of CHASM (breast), Mutation Assessor, PROVEAN, SIFT	73.73% (70.2%-77.27%)	97.35% (95.9%-99.18%)	32.882% (26.761%-39.395%)	71.49% (67.47%-75.44%)	87.74% (80.77%-95.65%)	2.8946 (2.7612-3.0331)	73.76% (66.67%-80.81%)	97.33% (93.94%-100%)	33.315% (21.05%-45.45%)	71.46% (63.22%-79.32%)	87.86% (72.73%-100%)	2.8996 (2.5929-3.1674)
At least 1 of CHASM (breast), Mutation Assessor, PROVEAN, SIFT, VEST	67.07% (63.6%-70.71%)	99.46% (99.16%-100%)	11.057% (6.944%-15.068%)	65.91% (62.11%-69.85%)	92.28% (83.33%-100%)	2.6871 (2.5584-2.8160)	66.87% (59.6%-73.74%)	99.48% (98.25%-100%)	10.904% (2.778%-18.92%)	65.71% (57.73%-73.4%)	92.52% (66.67%-100%)	2.6864 (2.3279-2.8749)
At least 1 of CHASM (breast), Mutation Assessor, PROVEAN, VEST	68.04% (64.65%-71.72%)	99.46% (99.16%-100%)	13.718% (9.231%-18.293%)	66.59% (62.77%-70.53%)	93.66% (87.5%-100%)	2.7343 (2.6155-2.8493)	67.95% (60.61%-74.75%)	99.48% (98.25%-100%)	13.839% (5.128%-22.86%)	66.46% (58.51%-73.96%)	94% (74.58%-100%)	2.7379 (2.4313-2.9295)
At least 1 of CHASM (breast), Mutation Assessor, SIFT	75.06% (71.72%-78.79%)	96.81% (95.16%-98.45%)	37.471% (31.079%-44.286%)	72.8% (68.9%-76.65%)	87.15% (80.65%-93.75%)	2.9423 (2.8024-3.0854)	75.13% (67.68%-81.82%)	96.81% (93.22%-100%)	37.932% (24.985%-51.29%)	72.79% (64.7%-80.72%)	87.36% (73.33%-100%)	2.9490 (2.6448-3.2150)
At least 1 of CHASM (breast), Mutation Assessor, SIFT, VEST	67.41% (63.64%-71.21%)	98.92% (98.33%-100%)	12.909% (8.219%-16.928%)	66.26% (62.43%-70.37%)	87.43% (77.78%-100%)	2.6552 (2.5067-2.8139)	67.21% (59.6%-74.75%)	98.96% (96.67%-100%)	12.7% (4.997%-21.88%)	66.05% (57.89%-73.68%)	87.71% (60%-100%)	2.6542 (2.2605-2.8974)
At least 1 of CHASM (breast), Mutation Assessor, VEST	69.39% (65.66%-73.23%)	98.92% (98.33%-100%)	18.313% (13.333%-23.191%)	67.68% (63.78%-71.74%)	90.76% (83.33%-100%)	2.7567 (2.6231-2.8942)	69.31% (61.62%-76.77%)	98.96% (96.67%-100%)	18.401% (8.105%-27.78%)	67.55% (59.34%-75.53%)	90.99% (66.67%-100%)	2.7590 (2.4312-2.9856)
At least 1 of CHASM (breast), MutationTaster	93.34% (91.41%-95.45%)	99.49% (99.16%-100%)	82.718% (78.082%-88.001%)	90.87% (88.32%-93.99%)	98.95% (98.15%-100%)	3.7203 (3.6502-3.8042)	93.11% (88.89%-96.97%)	99.42% (98.21%-100%)	82.288% (72.214%-91.67%)	90.59% (84.38%-95.65%)	98.81% (96%-100%)	3.7111 (3.5515-3.8634)
At least 1 of CHASM (breast), MutationTaster, PolyPhen-2	81.85% (79.29%-84.85%)	100% (100%-100%)	50.457% (43.833%-57.143%)	77.73% (74.21%-81.48%)	100% (100%-100%)	3.2818 (3.1914-3.3811)	81.76% (75.76%-86.87%)	100% (100%-100%)	50.458% (37.155%-63.89%)	77.6% (70%-84.42%)	100% (100%-100%)	3.2806 (3.0999-3.4642)
At least 1 of CHASM (breast), MutationTaster, PolyPhen-2, PROVEAN	78.17% (75.24%-81.31%)	100% (100%-100%)	40.416% (33.802%-46.575%)	74.37% (70.66%-78.04%)	100% (100%-100%)	3.1479 (3.0618-3.2403)	78.01% (71.72%-83.86%)	100% (100%-100%)	40.233% (27.778%-52.94%)	74.17% (66.67%-81.93%)	100% (100%-100%)	3.1440 (2.9782-3.3319)
At least 1 of CHASM (breast), MutationTaster, PolyPhen-2, PROVEAN, SIFT	73.79% (70.2%-77.27%)	100% (100%-100%)	28.455% (22.365%-33.847%)	70.73% (67.04%-74.44%)	100% (100%-100%)	2.9919 (2.9062-3.0723)	73.64% (66.67%-80.81%)	100% (100%-100%)	28.387% (17.647%-41.18%)	70.56% (62.92%-78.16%)	100% (100%-100%)	2.9895 (2.8325-3.1687)
At least 1 of CHASM (breast), MutationTaster, PolyPhen-2, PROVEAN, VEST	68.72% (65.15%-72.22%)	100% (100%-100%)	14.616% (9.589%-19.118%)	66.94% (63.1%-70.81%)	100% (100%-100%)	2.8156 (2.7474-2.8857)	68.63% (61.62%-75.76%)	100% (100%-100%)	14.773% (5.714%-24.39%)	66.82% (59.13%-74.47%)	100% (100%-100%)	2.8160 (2.6867-2.9649)
At least 1 of CHASM (breast), MutationTaster, PolyPhen-2, SIFT	75.8% (72.22%-79.29%)	100% (100%-100%)	33.957% (27.632%-40%)	72.36% (68.6%-76.27%)	100% (100%-100%)	3.0632 (2.9749-3.1458)	75.67% (68.69%-82.83%)	100% (100%-100%)	33.939% (22.222%-46.88%)	72.2% (64.29%-79.77%)	100% (100%-100%)	3.0614 (2.8975-3.2517)
At least 1 of CHASM (breast), MutationTaster, PolyPhen-2, SIFT, VEST	68.41% (64.65%-72.22%)	100% (100%-100%)	13.792% (8.974%-18.056%)	66.73% (62.77%-70.74%)	100% (100%-100%)	2.8052 (2.7407-2.8768)	68.23% (60.61%-75.76%)	100% (100%-100%)	13.675% (5.128%-23.33%)	66.54% (58.51%-74.47%)	100% (100%-100%)	2.8021 (2.6706-2.9470)
At least 1 of CHASM (breast), MutationTaster, PolyPhen-2, VEST	70.06% (66.67%-73.74%)	100% (100%-100%)	18.273% (13.042%-23.077%)	67.9% (64.13%-71.74%)	100% (100%-100%)	2.8618 (2.7899-2.9397)	69.99% (62.63%-76.77%)	100% (100%-100%)	18.467% (8.571%-29.03%)	67.8% (59.99%-75.29%)	100% (100%-100%)	2.8626 (2.7253-3.0249)
At least 1 of CHASM (breast), MutationTaster, PROVEAN	84.24% (81.31%-86.87%)	99.49% (99.16%-100%)	57.864% (51.351%-64.386%)	80.33% (76.87%-83.97%)	98.5% (97.3%-100%)	3.3619 (3.2613-3.4639)	84.05% (78.79%-89.9%)	99.42% (98.21%-100%)	57.643% (44.444%-70.59%)	80.11% (72.73%-87.01%)	98.31% (94.12%-100%)	3.3549 (3.1600-3.5652)
At least 1 of CHASM (breast), MutationTaster, PROVEAN, SIFT	75.8% (72.73%-78.79%)	100% (100%-100%)	33.954% (27.941%-40%)	72.36% (68.64%-76.14%)	100% (100%-100%)	3.0631 (2.9826-3.1529)	75.67% (69.7%-81.82%)	100% (100%-100%)	33.902% (21.212%-46.15%)	72.19% (64.83%-79.52%)	100% (100%-100%)	3.0610 (2.8955-3.2403)
At least 1 of CHASM (breast), MutationTaster, PROVEAN, SIFT, VEST	67.74% (64.14%-71.72%)	100% (100%-100%)	11.953% (7.499%-16%)	66.26% (62.43%-70.16%)	100% (100%-100%)	2.7821 (2.7206-2.8493)	67.55% (59.6%-74.75%)	100% (100%-100%)	11.857% (3.226%-20.46%)	66.07% (58.06%-73.63%)	100% (100%-100%)	2.7801 (2.6597-2.9145)
At least 1 of CHASM (breast), MutationTaster, PROVEAN, VEST	69.05% (65.15%-72.73%)	100% (100%-100%)	15.542% (10.39%-20.29%)	67.18% (63.3%-70.97%)	100% (100%-100%)	2.8273 (2.7571-2.8997)	68.96% (61.62%-76.77%)	100% (100%-100%)	15.687% (6.447%-25.82%)	67.06% (59.34%-74.74%)	100% (100%-100%)	2.8275 (2.6951-2.9833)
At least 1 of CHASM (breast), MutationTaster, SIFT	78.15% (74.75%-81.31%)	100% (100%-100%)	40.361% (34.211%-46.578%)	74.35% (70.66%-78.11%)	100% (100%-100%)	3.1471 (3.0639-3.2405)	78.05% (71.72%-84.85%)	100% (100%-100%)	40.393% (27.778%-52.78%)	74.21% (66.67%-81.49%)	100% (100%-100%)	3.1461 (2.9760-3.3354)
At least 1 of CHASM (breast), MutationTaster, SIFT, VEST	68.75% (65.15%-72.73%)	100% (100%-100%)	14.718% (10%-19.119%)	66.97% (63.04%-70.97%)	100% (100%-100%)	2.8169 (2.7503-2.8915)	68.56% (60.61%-75.76%)	100% (100%-100%)	14.59% (5.714%-25%)	66.77% (58.76%-74.5%)	100% (100%-100%)	2.8136 (2.6773-2.9597)
At least 1 of CHASM (breast), MutationTaster, VEST	71.76% (68.18%-75.76%)	100% (100%-100%)	22.926% (17.497%-28.378%)	69.17% (65.38%-73.19%)	100% (100%-100%)	2.9209 (2.8472-3.0048)	71.63% (63.64%-78.79%)	100% (100%-100%)	22.939% (11.429%-34.21%)	69.01% (60.92%-76.6%)	100% (100%-100%)	2.9195 (2.7712-3.0866)
At least 1 of CHASM (breast), PolyPhen-2	80.14% (77.27%-83.33%)	96.81% (95.16%-98.45%)	51.323% (44.928%-58.209%)	77.47% (73.89%-81.41%)	90.31% (85.71%-95.56%)	3.1591 (3.0397-3.2913)	80.12% (73.74%-85.86%)	96.8% (93.22%-100%)	51.467% (38.456%-64.12%)	77.39% (69.62%-84.53%)	90.4% (78.94%-100%)	3.1606 (2.9017-3.3985)
At least 1 of CHASM (breast), PolyPhen-2, PROVEAN	76.47% (73.23%-79.8%)	96.81% (95.16%-98.45%)	41.283% (34.783%-47.828%)	74.03% (70.3%-77.71%)	88.23% (82.35%-94.59%)	3.0035 (2.8791-3.1355)	76.36% (69.7%-82.83%)	96.8% (93.22%-100%)	41.241% (28.571%-54.06%)	73.88% (66.28%-81.48%)	88.3% (74.97%-100%)	3.0022 (2.7174-3.2519)
At least 1 of CHASM (breast), PolyPhen-2, PROVEAN, SIFT	73.09% (69.7%-76.77%)	98.4% (97.52%-100%)	29.321% (22.857%-34.852%)	70.65% (66.86%-74.3%)	91.36% (85%-100%)	2.8973 (2.7651-3.0270)	73.01% (65.66%-79.8%)	98.42% (95.31%-100%)	29.395% (18.176%-42.44%)	70.53% (63.1%-78.16%)	91.56% (76.92%-100%)	2.8989 (2.6230-3.1334)
At least 1 of CHASM (breast), PolyPhen-2, PROVEAN, SIFT, VEST	67.4% (63.64%-71.21%)	100% (100%-100%)	11.027% (6.847%-14.866%)	66.03% (62.23%-70%)	100% (100%-100%)	2.7705 (2.7081-2.8385)	67.22% (59.6%-74.75%)	100% (100%-100%)	10.943% (2.857%-19.51%)	65.84% (57.89%-73.4%)	100% (100%-100%)	2.7689 (2.6467-2.9060)
At least 1 of CHASM (breast), PolyPhen-2, PROVEAN, VEST	68.72% (65.15%-72.22%)	100% (100%-100%)	14.616% (9.589%-19.118%)	66.94% (63.1%-70.81%)	100% (100%-100%)	2.8156 (2.7474-2.8857)	68.63% (61.62%-75.76%)	100% (100%-100%)	14.773% (5.714%-24.39%)	66.82% (59.13%-74.47%)	100% (100%-100%)	2.8160 (2.6867-2.9649)
At least 1 of CHASM (breast), PolyPhen-2, SIFT	75.1% (71.72%-78.3%)	98.4% (97.52%-100%)	34.824% (28.358%-40.847%)	72.3% (68.45%-76.16%)	92.62% (87.5%-100%)	2.9815 (2.8598-3.1046)	75.05% (68.66%-81.82%)	98.42% (95.31%-100%)	34.948% (23.525%-48.48%)	72.19% (64.29%-79.76%)	92.8% (80%-100%)	2.9835 (2.7361-3.2126)
At least 1 of CHASM (breast), PolyPhen-2, SIFT, VEST	68.41% (64.65%-72.22%)	100% (100%-100%)	13.792% (8.974%-18.056%)	66.73% (62.77%-70.74%)	100% (100%-100%)	2.8052 (2.7407-2.8768)	68.23% (60.61%-75.76%)	100% (100%-100%)	13.675% (5.128%-23.33%)	66.54% (58.51%-74.47%)	100% (100%-100%)	2.8021 (2.6706-2.9470)
At least 1 of CHASM (breast), PolyPhen-2, VEST	70.06% (66.67%-73.74%)	100% (100%-100%)	18.273% (13.042%-23.077%)	67.9% (64.13%-71.74%)	100% (100%-100%)	2.8618 (2.7899-2.9397)	69.99% (62.63%-76.77%)	100% (100%-100%)	18.467% (8.571%-29.03%)	67.8% (59.99%-75.29%)	100% (100%-100%)	2.8626 (2.7253-3.0249)
At least 1 of CHASM (breast), PROVEAN	76.79% (73.23%-79.81%)	82.52% (78.51%-86.41%)	66.875% (60.868%-73.242%)	81.16% (77.34%-84.85%)	68.88% (62.5%-75.34%)	2.9943 (2.8511-3.1367)	76.72% (70.68%-83.84%)	82.29% (74.14%-90.28%)	67.108% (54.539%-78.79%)	81.12% (73.68%-88.89%)	68.86% (55.88%-82.35%)	2.9938 (2.7001-3.2897)
At least 1 of CHASM (breast), PROVEAN, SIFT	76.1% (72.73%-79.29%)	97.35% (95.9%-99.18%)	39.344% (32.876%-46.053%)	73.51% (69.75%-77.3%)	89.54% (83.87%-96.3%)	2.9975 (2.8636-3.1279)	76.09% (69.7%-82.83%)	97.33% (93.94%-100%)	39.614% (25.714%-51.64%)	73.45% (65.88%-80.95%)	89.58% (76.92%-100%)	2.9997 (2.7158-3.2555)
At least 1 of CHASM (breast), PROVEAN, SIFT, VEST	67.73% (64.14%-71.72%)	99.46% (99.16%-100%)	12.855% (8.448%-16.901%)	66.37% (62.5%-70.21%)	93.26% (85.71%-100%)	2.7195 (2.6000-2.8386)	67.57% (59.6%-74.75%)	99.48% (98.25%-100%)	12.803% (4.759%-21.43%)	66.19% (58.51%-73.91%)	93.46% (66.67%-100%)	2.7197 (2.3644-2.9124)
At least 1 of CHASM (breast), PROVEAN, VEST	68.73% (65.15%-72.73%)	98.96% (98.33%-100%)	16.444% (11.268%-21.429%)	67.19% (63.33%-71.2%)	90.17% (81.82%-100%)	2.7277 (2.5936-2.8613)	68.61% (60.61%-75.76%)	98.88% (96.67%-100%)	16.634% (6.897%-27.03%)	67.06% (59.14%-75%)	89.65% (66.67%-100%)	2.7224 (2.4138-2.9500)
At least 1 of CHASM (breast), SIFT	78.1% (74.75%-81.31%)	96.81% (95.16%-98.45%)	45.751% (39.188%-52.778%)	75.52% (71.87%-79.37%)	89.23% (83.77%-94.74%)	3.0731 (2.9423-3.2074)	78.14% (71.72%-84.85%)	96.81% (93.22%-3.2074)	46.105% (32.432%-58.97%)	75.5% (67.9%-82.89%)	89.36% (77.78%-100%)	3.0777 (2.8000-3.3290)
At least 1 of CHASM (breast), SIFT, VEST	68.4% (64.65%-72.22%)	98.92% (98.33%-100%)	15.621% (10.769%-20.27%)	66.96% (63.04%-70.97%)	89.35% (80%-100%)	2.7086 (2.5635-2.8493)	68.25% (60.61%-75.76%)	98.96% (96.67%-100%)	15.536% (6.452%-25.64%)	66.79% (58.88%-74.47%)	89.64% (66.67%-100%)	2.7093 (2.3767-2.9470)
At least 1 of CHASM (breast), VEST	70.76% (67.17%-74.24%)	97.9% (96.72%-99.23%)	23.828% (18.182%-28.989%)	68.96% (65.17%-73.03%)	86.77% (78.95%-95.24%)	2.7746 (2.6354-2.9140)	70.61% (63.64%-77.78%)	97.82% (94.83%-100%)	23.885% (13.329%-35.14%)	68.8% (60.67%-76.47%)	86.39% (66.67%-100%)	2.7690 (2.4538-3.0245)
At least 1 of CHASM (lung), CHASM (melanoma)	83.24% (80.3%-86.36%)	90.5% (87.6%-93.55%)	70.689% (64.789%-76.812%)	84.22% (80.88%-88.06%)	81.14% (75.81%-86.89%)	3.2655 (3.1358-3.4063)	83.01% (76.77%-88.89%)	90.27% (84.13%-95.72%)	70.522% (58.532%-81.82%)	84.02% (76.47%-90.48%)	80.87% (69.7%-91.43%)	3.2568 (2.9736-3.5148)
At least 1 of CHASM (lung), CHASM (melanoma), FATHMM (cancer)	77.46% (74.24%-80.81%)	96.33% (94.49%-98.39%)	44.847% (38.23%-51.429%)	75.12% (71.34%-79.25%)	87.59% (81.58%-94.12%)	3.0388 (2.9067-3.1708)	77.4% (70.71%-83.84%)	96.17% (92.19%-100%)	45.149% (31.704%-58.54%)	75.06% (67.09%-82.5%)	87.28% (74.97%-100%)	3.0366 (2.7704-3.2984)
At least 1 of CHASM (lung), CHASM (melanoma), FATHMM (cancer), FATHMM (missense)	73.41% (69.7%-76.77%)	97.93% (96.72%-99.24%)	30.998% (24.658%-37.179%)	71.05% (67.27%-75.05%)	89.67% (83.32%-96.3%)	2.8965 (2.7610-3.0269)	73.38% (66.67%-80.81%)	97.74% (94.74%-100%)	31.529% (19.355%-44.44%)	71.02% (63.22%-78.65%)	89.01% (75%-100%)	2.8930 (2.6312-3.1508)
At least 1 of CHASM (lung), CHASM (melanoma), FATHMM (cancer), FATHMM (missense), Mutation Assessor	70.72% (67.17%-74.75%)	98.97% (98.33%-100%)	21.854% (15.999%-27.397%)	68.65% (64.86%-72.63%)	92.46% (85.71%-100%)	2.8194 (2.6862-2.9472)	70.69% (62.63%-77.78%)	98.86% (96.67%-100%)	22.318% (11.101%-34.15%)	68.6% (60.47%-76.14%)	91.95% (75%-100%)	2.8173 (2.5470-3.0446)
At least 1 of CHASM (lung), CHASM (melanoma), FATHMM (cancer), FATHMM (missense), MutationTaster	73.09% (69.7%-76.77%)	99.49% (99.16%-100%)	27.439% (21.333%-33.333%)	70.33% (66.48%-74.3%)	96.9% (93.75%-100%)	2.9417 (2.8374-3.0455)	73.01% (65.66%-79.8%)	99.42% (98.21%-100%)	27.672% (16.655%-40.54%)	70.23% (62.22%-77.78%)	96.54% (87.5%-100%)	2.9387 (2.7094-3.1382)
At least 1 of CHASM (lung), CHASM (melanoma), FATHMM (cancer), FATHMM (missense), PolyPhen-2	71.35% (68.17%-75.25%)	99.48% (99.16%-100%)	22.713% (16.896%-27.782%)	69% (65.36%-72.93%)	96.19% (92.31%-100%)	2.8738 (2.7647-2.9762)	71.44% (63.64%-77.8%)	99.44% (98.25%-100%)	23.35% (12.903%-34.48%)	69.01% (60.92%-76.35%)	96.05% (83.33%-100%)	2.8785 (2.6380-3.0844)
At least 1 of CHASM (lung), CHASM (melanoma), FATHMM (cancer), FATHMM (missense), PROVEAN	70.74% (67.17%-74.24%)	97.93% (96.72%-99.24%)	23.708% (17.647%-29.335%)	68.94% (65.14%-72.73%)	86.89% (78.57%-95.24%)	2.7747 (2.6301-2.9189)	70.64% (63.64%-77.78%)	97.74% (94.74%-100%)	24.071% (12.903%-35.9%)	68.85% (61.17%-76.67%)	86.01% (66.67%-100%)	2.7667 (2.4544-3.0370)
At least 1 of CHASM (lung), CHASM (melanoma), FATHMM (cancer), FATHMM (missense), SIFT	69.68% (66.16%-73.23%)	100% (100%-100%)	17.263% (11.94%-22.059%)	67.64% (63.78%-71.66%)	100% (100%-100%)	2.8490 (2.7777-2.9223)	69.72% (62.63%-76.77%)	100% (100%-100%)	17.756% (8.108%-28.57%)	67.6% (59.57%-75.27%)	100% (100%-100%)	2.8536 (2.7169-3.0048)
At least 1 of CHASM (lung), CHASM (melanoma), FATHMM (cancer), FATHMM (missense), VEST	66.68% (63.13%-70.71%)	99.5% (99.16%-100%)	9.941% (5.714%-13.638%)	65.64% (61.78%-69.79%)	92.06% (80%-100%)	2.6714 (2.5195-2.7987)	66.63% (58.59%-74.75%)	99.4% (98.25%-100%)	10.361% (2.941%-18.75%)	65.56% (57.29%-73.4%)	91.14% (66.67%-100%)	2.6652 (2.3159-2.8754)
At least 1 of CHASM (lung), CHASM (melanoma), FATHMM (cancer), Mutation Assessor	74.42% (71.21%-78.28%)	98.44% (97.52%-100%)	32.898% (26.667%-39.194%)	71.72% (68%-75.88%)	92.4% (86.96%-100%)	2.9546 (2.8359-3.0768)	74.39% (66.67%-80.81%)	98.34% (95.45%-100%)	33.286% (20.5%-45.72%)	71.67% (63.41%-79.52%)	92.11% (78.57%-100%)	2.9540 (2.6927-3.1853)
At least 1 of CHASM (lung), CHASM (melanoma), FATHMM (cancer), Mutation Assessor, MutationTaster	74.13% (70.71%-77.78%)	99.49% (99.16%-100%)	30.272% (24.323%-36.235%)	71.16% (67.41%-75.28%)	97.19% (94.74%-100%)	2.9811 (2.8806-3.0919)	73.97% (66.67%-80.81%)	99.42% (98.21%-100%)	30.289% (18.742%-42.31%)	70.99% (62.78%-78.82%)	96.84% (87.5%-100%)	2.9754 (2.7497-3.1688)
At least 1 of CHASM (lung), CHASM (melanoma), FATHMM (cancer), Mutation Assessor, PolyPhen-2	72.39% (68.69%-75.77%)	98.94% (98.33%-100%)	26.476% (20.548%-32.394%)	69.94% (66.11%-74.01%)	93.54% (88.24%-100%)	2.8890 (2.7668-3.0057)	72.39% (65.63%-79.8%)	98.92% (96.67%-100%)	26.835% (15.148%-38.89%)	69.88% (61.63%-77.53%)	93.53% (80%-100%)	2.8916 (2.6405-3.1110)
At least 1 of CHASM (lung), CHASM (melanoma), FATHMM (cancer), Mutation Assessor, PROVEAN	72.42% (68.69%-76.26%)	98.44% (97.52%-100%)	27.432% (21.33%-33.333%)	70.11% (66.11%-74.14%)	91.03% (85%-100%)	2.8700 (2.7456-2.9902)	72.33% (64.65%-79.8%)	98.34% (95.45%-100%)	27.682% (16.129%-40%)	70% (62.07%-77.78%)	90.67% (75%-100%)	2.8669 (2.6114-3.0861)
At least 1 of CHASM (lung), CHASM (melanoma), FATHMM (cancer), Mutation Assessor, SIFT	71.71% (68.18%-75.25%)	99.46% (99.16%-100%)	23.738% (17.91%-29.577%)	69.28% (65.36%-73.19%)	96.24% (92.86%-100%)	2.8872 (2.7865-2.9960)	71.72% (64.65%-78.79%)	99.48% (98.25%-100%)	24.111% (12.5%-35.48%)	69.22% (61.36%-77.18%)	96.4% (85.71%-100%)	2.8921 (2.6505-3.0867)
At least 1 of CHASM (lung), CHASM (melanoma), FATHMM (cancer), Mutation Assessor, VEST	67.39% (63.64%-71.21%)	100% (100%-100%)	10.999% (6.849%-15.068%)	66.02% (62.11%-70.16%)	100% (100%-100%)	2.7702 (2.7116-2.8385)	67.24% (59.6%-74.75%)	100% (100%-100%)	11.015% (2.778%-19.44%)	65.86% (57.72%-73.47%)	100% (100%-100%)	2.7694 (2.6467-2.8957)
At least 1 of CHASM (lung), CHASM (melanoma), FATHMM (cancer), MutationTaster	77.84% (74.75%-81.31%)	99.49% (99.16%-100%)	40.393% (33.802%-46.667%)	74.27% (70.59%-78.11%)	97.88% (96%-100%)	3.1202 (3.0195-3.2275)	77.66% (70.71%-83.84%)	99.42% (98.21%-100%)	40.317% (27.778%-52.95%)	74.08% (66.28%-81.61%)	97.61% (91.67%-100%)	3.1143 (2.9107-3.3153)
At least 1 of CHASM (lung), CHASM (melanoma), FATHMM (cancer), MutationTaster, PolyPhen-2	75.09% (71.72%-78.79%)	100% (100%-100%)	32.022% (26.027%-37.883%)	71.78% (68.18%-75.72%)	100% (100%-100%)	3.0380 (2.9586-3.1268)	75.07% (67.68%-81.82%)	100% (100%-100%)	32.267% (20%-44.44%)	71.7% (63.73%-79.07%)	100% (100%-100%)	3.0397 (2.8785-3.2101)
At least 1 of CHASM (lung), CHASM (melanoma), FATHMM (cancer), MutationTaster, PROVEAN	74.15% (70.71%-77.78%)	99.49% (99.16%-100%)	30.339% (23.944%-36%)	71.18% (67.43%-75%)	97.19% (94.74%-100%)	2.9820 (2.8815-3.0861)	73.91% (66.67%-80.81%)	99.42% (98.21%-100%)	30.114% (18.176%-42.86%)	70.94% (63.33%-78.41%)	96.83% (87.5%-100%)	2.9731 (2.7517-3.1614)
At least 1 of CHASM (lung), CHASM (melanoma), FATHMM (cancer), MutationTaster, SIFT	73.11% (69.7%-76.77%)	100% (100%-100%)	26.633% (21.125%-32.394%)	70.21% (66.66%-74.16%)	100% (100%-100%)	2.9684 (2.8892-3.0546)	72.96% (65.66%-79.8%)	100% (100%-100%)	26.573% (15.148%-38.24%)	70.03% (61.95%-77.28%)	100% (100%-100%)	2.9660 (2.8091-3.1382)
At least 1 of CHASM (lung), CHASM (melanoma), FATHMM (cancer), MutationTaster, VEST	68.38% (64.65%-72.22%)	100% (100%-100%)	13.711% (9.088%-18.182%)	66.71% (62.9%-70.9%)	100% (100%-100%)	2.8042 (2.7395-2.8776)	68.29% (60.61%-75.76%)	100% (100%-100%)	13.851% (5.263%-23.08%)	66.58% (58.33%-74.19%)	100% (100%-100%)	2.8046 (2.6699-2.9473)
At least 1 of CHASM (lung), CHASM (melanoma), FATHMM (cancer), PolyPhen-2	74.74% (71.72%-78.28%)	98.94% (98.33%-100%)	32.888% (26.56%-39.132%)	71.82% (68.2%-75.72%)	94.74% (90.48%-100%)	2.9840 (2.8682-3.0863)	74.76% (67.68%-80.81%)	98.92% (96.67%-100%)	33.275% (20.687%-45.46%)	71.79% (63.86%-79.07%)	94.72% (83.33%-100%)	2.9870 (2.7642-3.2150)
At least 1 of CHASM (lung), CHASM (melanoma), FATHMM (cancer), PolyPhen-2, PROVEAN	72.08% (68.69%-75.76%)	98.94% (98.33%-100%)	25.607% (19.718%-31.081%)	69.7% (65.92%-73.48%)	93.35% (88.24%-100%)	2.8760 (2.7582-2.9952)	72.01% (64.65%-78.79%)	98.92% (96.67%-100%)	25.788% (14.286%-37.14%)	69.59% (61.8%-77.02%)	93.3% (77.78%-100%)	2.8759 (2.6380-3.0881)
At least 1 of CHASM (lung), CHASM (melanoma), FATHMM (cancer), PolyPhen-2, SIFT	72.41% (69.19%-76.26%)	99.46% (99.16%-100%)	25.643% (19.736%-31.148%)	69.81% (66.11%-73.86%)	96.51% (93.33%-100%)	2.9143 (2.8120-3.0215)	72.35% (64.65%-79.8%)	99.48% (98.25%-100%)	25.791% (14.625%-37.14%)	69.7% (61.36%-77.17%)	96.64% (85.71%-100%)	2.9161 (2.6895-3.1156)
At least 1 of CHASM (lung), CHASM (melanoma), FATHMM (cancer), PolyPhen-2, VEST	68.04% (64.65%-71.73%)	100% (100%-100%)	12.785% (8.219%-16.927%)	66.47% (62.63%-70.59%)	100% (100%-100%)	2.7926 (2.7308-2.8613)	67.95% (60.58%-74.75%)	100% (100%-100%)	12.937% (5%-22.22%)	66.35% (58.33%-73.96%)	100% (100%-100%)	2.7932 (2.6693-2.9295)
At least 1 of CHASM (lung), CHASM (melanoma), FATHMM (cancer), PROVEAN	74.11% (70.71%-77.78%)	96.85% (95.2%-98.46%)	34.794% (27.937%-41.027%)	71.98% (68.05%-75.88%)	86.44% (79.31%-93.55%)	2.9006 (2.7600-3.0330)	74% (66.67%-80.81%)	96.73% (93.1%-100%)	34.946% (22.222%-48.39%)	71.85% (64.04%-79.76%)	86.12% (70.59%-100%)	2.8965 (2.6015-3.1602)
At least 1 of CHASM (lung), CHASM (melanoma), FATHMM (cancer), PROVEAN, SIFT	72.07% (68.69%-75.76%)	99.46% (99.16%-100%)	24.692% (19.177%-30.435%)	69.55% (65.92%-73.34%)	96.39% (93.33%-100%)	2.9009 (2.7984-2.9992)	72.03% (64.65%-78.79%)	99.48% (98.25%-100%)	24.901% (13.786%-36.37%)	69.45% (61.95%-76.93%)	96.56% (85.71%-100%)	2.9038 (2.6977-3.0937)
At least 1 of CHASM (lung), CHASM (melanoma), FATHMM (cancer), PROVEAN, VEST	67.07% (63.13%-70.71%)	99.5% (99.16%-100%)	10.988% (6.942%-15.068%)	65.9% (62.03%-69.85%)	92.74% (83.33%-100%)	2.6913 (2.5551-2.8179)	66.88% (59.6%-74.75%)	99.4% (98.25%-100%)	11.041% (2.857%-19.35%)	65.73% (57.73%-73.41%)	91.42% (66.67%-100%)	2.6764 (2.3237-2.8915)
At least 1 of CHASM (lung), CHASM (melanoma), FATHMM (cancer), SIFT	73.74% (70.2%-77.27%)	99.46% (99.16%-100%)	29.259% (23.288%-35.302%)	70.85% (67.23%-74.72%)	96.93% (94.44%-100%)	2.9651 (2.8697-3.0727)	73.74% (66.67%-80.81%)	99.48% (98.25%-100%)	29.57% (16.667%-41.03%)	70.79% (62.92%-78.16%)	97.07% (87.5%-100%)	2.9690 (2.7516-3.1614)
At least 1 of CHASM (lung), CHASM (melanoma), FATHMM (cancer), SIFT, VEST	67.74% (64.14%-71.72%)	100% (100%-100%)	11.965% (7.246%-16.131%)	66.26% (62.37%-70.37%)	100% (100%-100%)	2.7823 (2.7206-2.8536)	67.55% (59.6%-74.75%)	100% (100%-100%)	11.852% (2.941%-20.59%)	66.07% (57.89%-73.91%)	100% (100%-100%)	2.7800 (2.6531-2.9145)
At least 1 of CHASM (lung), CHASM (melanoma), FATHMM (cancer), VEST	68.06% (64.63%-72.22%)	99.5% (99.16%-100%)	13.711% (9.088%-18.182%)	66.6% (62.7%-70.81%)	94.08% (87.5%-100%)	2.7389 (2.6155-2.8570)	67.91% (59.6%-74.77%)	99.4% (98.25%-100%)	13.851% (5.263%-23.08%)	66.45% (58.33%-74.19%)	93.09% (66.67%-100%)	2.7282 (2.4017-2.9297)
At least 1 of CHASM (lung), CHASM (melanoma), FATHMM (missense)	77.16% (73.74%-80.3%)	95.31% (93.33%-97.6%)	45.749% (38.887%-52.113%)	75.24% (71.34%-79.01%)	84.96% (78.94%-91.67%)	3.0125 (2.8752-3.1480)	77% (70.71%-83.84%)	95% (90.48%-98.53%)	46.039% (33.333%-59.47%)	75.14% (67.49%-82.75%)	84.31% (71.41%-95.45%)	3.0049 (2.7341-3.2700)
At least 1 of CHASM (lung), CHASM (melanoma), FATHMM (missense), Mutation Assessor	73.75% (70.2%-77.27%)	97.91% (96.69%-99.24%)	31.955% (25.974%-38.028%)	71.33% (67.42%-75.2%)	89.87% (83.33%-96.43%)	2.9107 (2.7721-3.0400)	73.72% (66.67%-80.81%)	97.78% (94.64%-100%)	32.391% (20%-45.46%)	71.28% (63.33%-79.31%)	89.52% (75%-100%)	2.9097 (2.6313-3.1663)
At least 1 of CHASM (lung), CHASM (melanoma), FATHMM (missense), Mutation Assessor, MutationTaster	74.11% (70.71%-77.78%)	99.49% (99.16%-100%)	30.224% (24.631%-36.232%)	71.14% (67.24%-75.14%)	97.18% (94.74%-100%)	2.9804 (2.8824-3.0829)	74% (66.67%-80.81%)	99.42% (98.21%-100%)	30.368% (18.421%-42.42%)	71.02% (62.92%-78.83%)	96.82% (87.5%-100%)	2.9763 (2.7516-3.1748)
At least 1 of CHASM (lung), CHASM (melanoma), FATHMM (missense), Mutation Assessor, PolyPhen-2	72.71% (69.19%-76.26%)	99.48% (99.16%-100%)	26.422% (20.541%-32.354%)	70.04% (66.11%-74.01%)	96.7% (93.75%-100%)	2.9265 (2.8236-3.0315)	72.76% (65.66%-79.8%)	99.44% (98.25%-100%)	26.939% (15.785%-38.71%)	70.02% (62.07%-77.78%)	96.55% (85.71%-100%)	2.9296 (2.7163-3.1313)
At least 1 of CHASM (lung), CHASM (melanoma), FATHMM (missense), Mutation Assessor, PROVEAN	71.07% (67.66%-74.75%)	97.91% (96.69%-99.24%)	24.632% (18.664%-30.435%)	69.2% (65.19%-73.18%)	87.22% (78.95%-95.45%)	2.7897 (2.6389-2.9485)	71% (63.64%-77.78%)	97.78% (94.64%-100%)	25.004% (13.333%-37.14%)	69.11% (61.11%-77.18%)	86.77% (66.67%-100%)	2.7867 (2.4457-3.0576)
At least 1 of CHASM (lung), CHASM (melanoma), FATHMM (missense), Mutation Assessor, SIFT	70.71% (67.17%-74.75%)	99.49% (99.15%-100%)	20.962% (15.385%-26.156%)	68.52% (64.48%-72.43%)	95.95% (91.67%-100%)	2.8492 (2.7392-2.9537)	70.69% (62.63%-77.78%)	99.43% (98.24%-100%)	21.377% (11.11%-32.5%)	68.46% (60.44%-76.67%)	95.71% (83.33%-100%)	2.8497 (2.6230-3.0565)
At least 1 of CHASM (lung), CHASM (melanoma), FATHMM (missense), Mutation Assessor, VEST	67.36% (63.64%-71.21%)	100% (100%-100%)	10.914% (6.849%-14.865%)	66% (62.03%-70%)	100% (100%-100%)	2.7691 (2.7081-2.8311)	67.3% (59.6%-74.75%)	100% (100%-100%)	11.152% (2.857%-19.44%)	65.89% (57.89%-73.91%)	100% (100%-100%)	2.7708 (2.6573-2.9007)
At least 1 of CHASM (lung), CHASM (melanoma), FATHMM (missense), MutationTaster	78.15% (74.75%-81.31%)	99.49% (99.16%-100%)	41.244% (34.845%-47.297%)	74.54% (70.76%-78.36%)	97.92% (96.15%-100%)	3.1320 (3.0349-3.2283)	78.04% (71.72%-84.85%)	99.42% (98.21%-100%)	41.334% (28.947%-54.29%)	74.41% (66.66%-82.14%)	97.66% (90.91%-100%)	3.1283 (2.9283-3.3214)
At least 1 of CHASM (lung), CHASM (melanoma), FATHMM (missense), MutationTaster, PolyPhen-2	75.06% (71.72%-78.28%)	100% (100%-100%)	31.939% (25.713%-37.838%)	71.75% (68.05%-75.44%)	100% (100%-100%)	3.0369 (2.9545-3.1268)	75.13% (68.69%-81.82%)	100% (100%-100%)	32.429% (21.05%-44.83%)	71.75% (64.29%-79.07%)	100% (100%-100%)	3.0418 (2.8805-3.2156)
At least 1 of CHASM (lung), CHASM (melanoma), FATHMM (missense), MutationTaster, PROVEAN	73.11% (69.7%-76.77%)	99.49% (99.16%-100%)	27.482% (21.739%-33.333%)	70.35% (66.48%-74.16%)	96.9% (94.12%-100%)	2.9422 (2.8494-3.0416)	72.98% (65.66%-79.8%)	99.42% (98.21%-100%)	27.563% (15.613%-38.89%)	70.2% (62.22%-78.03%)	96.5% (87.5%-100%)	2.9369 (2.7193-3.1157)
At least 1 of CHASM (lung), CHASM (melanoma), FATHMM (missense), MutationTaster, SIFT	72.08% (68.69%-75.76%)	100% (100%-100%)	23.799% (18.053%-28.986%)	69.41% (65.56%-73.37%)	100% (100%-100%)	2.9321 (2.8551-3.0146)	72.01% (64.65%-78.79%)	100% (100%-100%)	23.966% (13.329%-35.29%)	69.3% (61.36%-76.75%)	100% (100%-100%)	2.9326 (2.7844-3.1001)
At least 1 of CHASM (lung), CHASM (melanoma), FATHMM (missense), MutationTaster, VEST	68.35% (64.65%-72.22%)	100% (100%-100%)	13.626% (8.974%-17.911%)	66.69% (62.77%-70.68%)	100% (100%-100%)	2.8031 (2.7407-2.8696)	68.34% (60.61%-75.76%)	100% (100%-100%)	13.988% (5.263%-22.51%)	66.62% (58.51%-74.47%)	100% (100%-100%)	2.8061 (2.6825-2.9422)
At least 1 of CHASM (lung), CHASM (melanoma), FATHMM (missense), PolyPhen-2	75.05% (71.72%-78.28%)	99.48% (99.16%-100%)	32.805% (26.387%-38.67%)	71.91% (68.23%-75.72%)	97.33% (95%-100%)	3.0153 (2.9098-3.1180)	75.15% (68.69%-81.82%)	99.44% (98.25%-100%)	33.438% (22.222%-45.71%)	71.94% (64.37%-79.52%)	97.21% (88.89%-100%)	3.0204 (2.8055-3.2283)
At least 1 of CHASM (lung), CHASM (melanoma), FATHMM (missense), PolyPhen-2, PROVEAN	71.71% (68.18%-75.25%)	99.48% (99.16%-100%)	23.676% (17.808%-29.167%)	69.27% (65.54%-73.08%)	96.34% (92.86%-100%)	2.8876 (2.7811-2.9939)	71.74% (64.65%-78.79%)	99.44% (98.25%-100%)	24.152% (13.152%-35.9%)	69.23% (61.54%-76.92%)	96.17% (83.33%-100%)	2.8900 (2.6686-3.0937)
At least 1 of CHASM (lung), CHASM (melanoma), FATHMM (missense), PolyPhen-2, SIFT	72.05% (68.69%-75.76%)	100% (100%-100%)	23.735% (17.948%-29.167%)	69.39% (65.56%-73.37%)	100% (100%-100%)	2.9313 (2.8551-3.0062)	72.05% (64.65%-78.79%)	100% (100%-100%)	24.099% (13.514%-35.71%)	69.33% (61.29%-76.93%)	100% (100%-100%)	2.9343 (2.7862-3.1001)
At least 1 of CHASM (lung), CHASM (melanoma), FATHMM (missense), PolyPhen-2, VEST	68.01% (64.14%-71.72%)	100% (100%-100%)	12.694% (8.219%-16.883%)	66.45% (62.57%-70.53%)	100% (100%-100%)	2.7914 (2.7276-2.8572)	68.02% (60.61%-75.76%)	100% (100%-100%)	13.099% (5%-22.22%)	66.39% (58.33%-74.19%)	100% (100%-100%)	2.7951 (2.6723-2.9319)
At least 1 of CHASM (lung), CHASM (melanoma), FATHMM (missense), PROVEAN	73.11% (69.7%-76.77%)	96.88% (95.2%-98.48%)	31.986% (25.756%-38.462%)	71.13% (67.46%-75%)	85.55% (77.78%-93.1%)	2.8554 (2.7100-3.0029)	72.98% (65.66%-79.8%)	96.66% (92.86%-100%)	32.269% (18.919%-44.44%)	71.02% (63.22%-78.32%)	84.85% (69.23%-100%)	2.8479 (2.5435-3.1276)
At least 1 of CHASM (lung), CHASM (melanoma), FATHMM (missense), PROVEAN, SIFT	71.06% (67.17%-74.75%)	99.49% (99.15%-100%)	21.884% (15.871%-27.027%)	68.77% (64.86%-72.68%)	96.12% (92.31%-100%)	2.8626 (2.7493-2.9767)	71.02% (63.64%-77.78%)	99.43% (98.24%-100%)	22.225% (11.111%-34.09%)	68.7% (60.92%-76.35%)	95.87% (83.33%-100%)	2.8621 (2.6271-3.0667)
At least 1 of CHASM (lung), CHASM (melanoma), FATHMM (missense), PROVEAN, VEST	66.35% (62.63%-70.2%)	99.5% (99.16%-100%)	9.036% (5.193%-12.676%)	65.41% (61.46%-69.43%)	91.37% (80%-100%)	2.6532 (2.4967-2.7847)	66.28% (58.59%-73.74%)	99.4% (98.25%-100%)	9.405% (2.632%-17.24%)	65.32% (57.29%-73.2%)	90.43% (50%-100%)	2.6461 (2.2292-2.8599)
At least 1 of CHASM (lung), CHASM (melanoma), FATHMM (missense), SIFT	72.74% (69.19%-76.26%)	99.49% (99.15%-100%)	26.475% (20.27%-32%)	70.06% (66.11%-73.89%)	96.78% (93.75%-100%)	2.9279 (2.8247-3.0269)	72.71% (65.66%-79.8%)	99.43% (98.24%-100%)	26.838% (15.375%-39.47%)	69.99% (62.07%-77.91%)	96.56% (85.71%-100%)	2.9282 (2.7162-3.1313)
At least 1 of CHASM (lung), CHASM (melanoma), FATHMM (missense), SIFT, VEST	67.37% (63.64%-71.21%)	100% (100%-100%)	10.949% (6.849%-14.865%)	66.01% (61.98%-70.16%)	100% (100%-100%)	2.7695 (2.7081-2.8341)	67.28% (59.6%-74.75%)	100% (100%-100%)	11.1% (2.941%-19.36%)	65.88% (57.73%-73.96%)	100% (100%-100%)	2.7701 (2.6488-2.9079)
At least 1 of CHASM (lung), CHASM (melanoma), FATHMM (missense), VEST	68.04% (64.14%-71.72%)	99.5% (99.16%-100%)	13.626% (8.974%-17.911%)	66.58% (62.63%-70.59%)	94.08% (87.5%-100%)	2.7378 (2.6194-2.8459)	67.97% (60.61%-75.76%)	99.4% (98.25%-100%)	13.988% (5.263%-22.51%)	66.48% (58.51%-74.23%)	93.36% (75%-100%)	2.7325 (2.4571-2.9295)
At least 1 of CHASM (lung), CHASM (melanoma), Mutation Assessor	78.15% (74.75%-81.31%)	95.76% (93.8%-97.71%)	47.686% (41.327%-54.548%)	75.99% (72.05%-80%)	86.7% (81.39%-92.86%)	3.0614 (2.9296-3.1926)	78.05% (71.72%-84.85%)	95.69% (91.3%-100%)	47.771% (33.333%-61.29%)	75.86% (67.95%-83.75%)	86.65% (72.73%-100%)	3.0598 (2.7842-3.3272)
At least 1 of CHASM (lung), CHASM (melanoma), Mutation Assessor, MutationTaster	79.54% (76.26%-82.83%)	99.49% (99.16%-100%)	45.059% (38.748%-52.055%)	75.79% (71.78%-79.75%)	98.09% (96.55%-100%)	3.1844 (3.0861-3.2910)	79.3% (72.73%-85.86%)	99.42% (98.21%-100%)	44.774% (31.429%-58.06%)	75.54% (67.5%-83.53%)	97.85% (92.31%-100%)	3.1759 (2.9638-3.3810)
At least 1 of CHASM (lung), CHASM (melanoma), Mutation Assessor, MutationTaster, PolyPhen-2	77.14% (73.74%-80.3%)	100% (100%-100%)	37.606% (31.081%-43.836%)	73.48% (69.64%-77.33%)	100% (100%-100%)	3.1109 (3.0209-3.2035)	77.04% (70.71%-83.84%)	100% (100%-100%)	37.62% (25%-51.29%)	73.34% (65.85%-80.9%)	100% (100%-100%)	3.1096 (2.9381-3.2986)
At least 1 of CHASM (lung), CHASM (melanoma), Mutation Assessor, MutationTaster, PROVEAN	76.18% (73.23%-79.8%)	99.49% (99.16%-100%)	35.874% (29.167%-42.031%)	72.84% (68.96%-76.75%)	97.62% (95.45%-100%)	3.0583 (2.9662-3.1600)	75.92% (68.69%-82.83%)	99.42% (98.21%-100%)	35.589% (23.071%-48.57%)	72.59% (64.37%-80.23%)	97.3% (90%-100%)	3.0490 (2.8377-3.2403)
At least 1 of CHASM (lung), CHASM (melanoma), Mutation Assessor, MutationTaster, SIFT	74.45% (71.2%-78.28%)	100% (100%-100%)	30.288% (23.944%-36.238%)	71.27% (67.41%-75.43%)	100% (100%-100%)	3.0155 (2.9316-3.1078)	74.33% (66.67%-80.83%)	100% (100%-100%)	30.292% (18.6%-42.86%)	71.11% (62.92%-79.07%)	100% (100%-100%)	3.0140 (2.8476-3.1997)
At least 1 of CHASM (lung), CHASM (melanoma), Mutation Assessor, MutationTaster, VEST	68.74% (65.15%-72.73%)	100% (100%-100%)	14.683% (9.996%-19.118%)	66.96% (63.1%-70.97%)	100% (100%-100%)	2.8164 (2.7540-2.8885)	68.58% (60.61%-75.76%)	100% (100%-100%)	14.642% (5.556%-23.68%)	66.79% (58.95%-74.23%)	100% (100%-100%)	2.8143 (2.6825-2.9500)
At least 1 of CHASM (lung), CHASM (melanoma), Mutation Assessor, PolyPhen-2	76.45% (73.23%-79.8%)	98.41% (97.52%-100%)	38.473% (31.644%-45.07%)	73.44% (69.59%-77.25%)	93.32% (88.46%-100%)	3.0365 (2.9163-3.1565)	76.4% (69.7%-82.83%)	98.4% (95.31%-100%)	38.628% (25.641%-51.72%)	73.35% (65.48%-80.72%)	93.33% (81.25%-100%)	3.0370 (2.7924-3.2724)
At least 1 of CHASM (lung), CHASM (melanoma), Mutation Assessor, PolyPhen-2, PROVEAN	74.11% (70.71%-77.78%)	98.41% (97.52%-100%)	32.09% (25.675%-38.028%)	71.48% (67.46%-75.43%)	92.11% (86.96%-100%)	2.9408 (2.8202-3.0622)	74% (66.67%-80.81%)	98.4% (95.31%-100%)	32.101% (20%-44.45%)	71.33% (63.53%-79.08%)	92.11% (76.92%-100%)	2.9393 (2.6704-3.1676)
At least 1 of CHASM (lung), CHASM (melanoma), Mutation Assessor, PolyPhen-2, SIFT	73.42% (69.7%-77.27%)	98.93% (98.32%-100%)	29.32% (22.857%-35.39%)	70.76% (66.86%-74.72%)	94.05% (89.47%-100%)	2.9306 (2.8127-3.0518)	73.36% (65.66%-80.81%)	98.95% (96.67%-100%)	29.441% (17.643%-42.11%)	70.65% (62.5%-78.57%)	94.24% (80%-100%)	2.9328 (2.6746-3.1383)
At least 1 of CHASM (lung), CHASM (melanoma), Mutation Assessor, PolyPhen-2, VEST	68.06% (64.65%-71.72%)	100% (100%-100%)	12.826% (8.333%-17.335%)	66.48% (62.7%-70.43%)	100% (100%-100%)	2.7931 (2.7332-2.8631)	67.92% (60.61%-74.75%)	100% (100%-100%)	12.838% (3.843%-21.62%)	66.32% (58.33%-73.69%)	100% (100%-100%)	2.7920 (2.6688-2.9252)
At least 1 of CHASM (lung), CHASM (melanoma), Mutation Assessor, PROVEAN	75.12% (71.72%-78.79%)	96.3% (94.44%-98.39%)	38.5% (31.943%-44.74%)	73.03% (69.05%-76.92%)	85.77% (79.31%-93.55%)	2.9361 (2.7947-3.0730)	75% (67.68%-81.82%)	96.21% (92.19%-100%)	38.586% (25.714%-51.35%)	72.89% (64.83%-80.69%)	85.61% (70%-100%)	2.9330 (2.6419-3.2023)
At least 1 of CHASM (lung), CHASM (melanoma), Mutation Assessor, PROVEAN, SIFT	72.39% (68.69%-76.26%)	97.88% (96.72%-99.23%)	28.326% (21.917%-33.824%)	70.25% (66.1%-74.16%)	88.52% (81.82%-96%)	2.8497 (2.7151-2.9873)	72.39% (64.65%-79.8%)	97.86% (94.83%-100%)	28.673% (17.141%-41.03%)	70.19% (62.22%-78.17%)	88.64% (71.43%-100%)	2.8536 (2.5553-3.1110)
At least 1 of CHASM (lung), CHASM (melanoma), Mutation Assessor, PROVEAN, VEST	67.05% (63.64%-70.72%)	99.46% (99.16%-100%)	11.007% (7.04%-14.865%)	65.9% (62.03%-69.84%)	92.24% (83.33%-100%)	2.6861 (2.5613-2.8118)	66.91% (59.57%-73.74%)	99.48% (98.25%-100%)	10.995% (2.857%-18.75%)	65.73% (57.89%-73.4%)	92.54% (66.67%-100%)	2.6879 (2.3159-2.8800)
At least 1 of CHASM (lung), CHASM (melanoma), Mutation Assessor, SIFT	73.73% (70.2%-77.78%)	97.34% (95.93%-99.19%)	32.914% (26.085%-39.195%)	71.5% (67.46%-75.58%)	87.73% (80.77%-95.83%)	2.8948 (2.7511-3.0381)	73.75% (65.66%-80.81%)	97.34% (93.75%-100%)	33.289% (20.586%-46.16%)	71.46% (63.22%-79.31%)	87.95% (72.73%-100%)	2.9004 (2.6022-3.1662)
At least 1 of CHASM (lung), CHASM (melanoma), Mutation Assessor, SIFT, VEST	67.08% (63.64%-70.72%)	98.92% (98.33%-100%)	12.007% (7.576%-16.176%)	66.03% (62.16%-70.05%)	86.62% (75%-100%)	2.6358 (2.4767-2.8021)	66.86% (59.57%-74.75%)	98.96% (96.67%-100%)	11.754% (3.028%-20.46%)	65.81% (57.61%-73.4%)	86.8% (50%-100%)	2.6334 (2.2085-2.8847)
At least 1 of CHASM (lung), CHASM (melanoma), Mutation Assessor, VEST	68.06% (64.65%-72.22%)	98.92% (98.33%-100%)	14.683% (9.996%-19.118%)	66.72% (62.84%-70.81%)	88.75% (80%-100%)	2.6907 (2.5478-2.8459)	67.93% (59.6%-74.75%)	98.96% (96.67%-100%)	14.642% (5.556%-23.68%)	66.55% (58.51%-74.19%)	88.97% (60%-100%)	2.6913 (2.2871-2.9191)
At least 1 of CHASM (lung), CHASM (melanoma), MutationTaster	86.63% (84.34%-89.39%)	99.49% (99.16%-100%)	64.405% (58.227%-71.014%)	82.85% (79.61%-86.62%)	98.66% (97.62%-100%)	3.4541 (3.3628-3.5520)	86.33% (80.81%-90.91%)	99.42% (98.21%-100%)	63.852% (51.22%-75.76%)	82.51% (75%-89.19%)	98.48% (94.74%-100%)	3.4427 (3.2557-3.6250)
At least 1 of CHASM (lung), CHASM (melanoma), MutationTaster, PolyPhen-2	80.17% (77.27%-83.33%)	100% (100%-100%)	45.869% (39.023%-52.703%)	76.16% (72.56%-80%)	100% (100%-100%)	3.2203 (3.1268-3.3204)	80.07% (73.74%-85.86%)	100% (100%-100%)	45.86% (32.498%-60%)	76.02% (68.24%-83.13%)	100% (100%-100%)	3.2188 (3.0363-3.4111)
At least 1 of CHASM (lung), CHASM (melanoma), MutationTaster, PolyPhen-2, PROVEAN	76.49% (73.23%-79.8%)	100% (100%-100%)	35.829% (29.577%-41.892%)	72.93% (69.27%-76.7%)	100% (100%-100%)	3.0876 (3.0018-3.1785)	76.32% (69.7%-82.83%)	100% (100%-100%)	35.634% (23.684%-48.57%)	72.73% (65.42%-80.24%)	100% (100%-100%)	3.0836 (2.9167-3.2665)
At least 1 of CHASM (lung), CHASM (melanoma), MutationTaster, PolyPhen-2, SIFT	75.46% (72.22%-78.79%)	100% (100%-100%)	33.044% (26.866%-39.13%)	72.08% (68.23%-76.14%)	100% (100%-100%)	3.0513 (2.9669-3.1382)	75.33% (68.69%-81.82%)	100% (100%-100%)	33.016% (21.208%-45.71%)	71.92% (63.64%-79.55%)	100% (100%-100%)	3.0494 (2.8806-3.2282)
At least 1 of CHASM (lung), CHASM (melanoma), MutationTaster, PolyPhen-2, VEST	69.39% (65.66%-73.23%)	100% (100%-100%)	16.465% (11.536%-21.127%)	67.43% (63.59%-71.35%)	100% (100%-100%)	2.8389 (2.7734-2.9105)	69.29% (61.62%-76.77%)	100% (100%-100%)	16.569% (7.313%-26.67%)	67.29% (59.55%-75%)	100% (100%-100%)	2.8386 (2.7120-2.9856)
At least 1 of CHASM (lung), CHASM (melanoma), MutationTaster, PROVEAN	78.89% (75.76%-81.82%)	99.49% (99.16%-100%)	43.269% (36.613%-50%)	75.2% (71.6%-78.92%)	98.01% (96.3%-100%)	3.1598 (3.0636-3.2609)	78.58% (72.73%-84.85%)	99.42% (98.21%-100%)	42.795% (30.295%-56.11%)	74.89% (67.46%-82.14%)	97.75% (91.67%-100%)	3.1486 (2.9454-3.3429)
At least 1 of CHASM (lung), CHASM (melanoma), MutationTaster, PROVEAN, SIFT	74.46% (71.21%-77.78%)	100% (100%-100%)	30.307% (24.323%-36.111%)	71.27% (67.43%-75%)	100% (100%-100%)	3.0158 (2.9319-3.1001)	74.31% (67.68%-80.81%)	100% (100%-100%)	30.198% (18.182%-42.11%)	71.09% (63.52%-78.82%)	100% (100%-100%)	3.0128 (2.8631-3.1905)
At least 1 of CHASM (lung), CHASM (melanoma), MutationTaster, PROVEAN, VEST	68.39% (64.65%-72.22%)	100% (100%-100%)	13.733% (9.091%-18.056%)	66.72% (62.9%-70.68%)	100% (100%-100%)	2.8045 (2.7373-2.8690)	68.27% (60.61%-75.76%)	100% (100%-100%)	13.79% (5.402%-22.58%)	66.56% (58.69%-74.19%)	100% (100%-100%)	2.8040 (2.6825-2.9496)
At least 1 of CHASM (lung), CHASM (melanoma), MutationTaster, SIFT	76.81% (73.74%-80.3%)	100% (100%-100%)	36.713% (30.378%-42.857%)	73.2% (69.41%-77.01%)	100% (100%-100%)	3.0992 (3.0132-3.1905)	76.69% (69.7%-82.83%)	100% (100%-100%)	36.689% (24.324%-48.78%)	73.04% (65.06%-80.46%)	100% (100%-100%)	3.0973 (2.9306-3.2800)
At least 1 of CHASM (lung), CHASM (melanoma), MutationTaster, SIFT, VEST	68.75% (65.15%-72.73%)	100% (100%-100%)	14.718% (10%-19.119%)	66.97% (63.04%-70.97%)	100% (100%-100%)	2.8169 (2.7503-2.8915)	68.56% (60.61%-75.76%)	100% (100%-100%)	14.59% (5.714%-25%)	66.77% (58.76%-74.5%)	100% (100%-100%)	2.8136 (2.6773-2.9597)
At least 1 of CHASM (lung), CHASM (melanoma), MutationTaster, VEST	70.07% (66.65%-73.74%)	100% (100%-100%)	18.322% (13.428%-22.976%)	67.92% (63.98%-71.89%)	100% (100%-100%)	2.8624 (2.7916-2.9319)	69.96% (62.63%-76.79%)	100% (100%-100%)	18.373% (8.565%-28.14%)	67.77% (59.78%-75.56%)	100% (100%-100%)	2.8614 (2.7289-3.0249)
At least 1 of CHASM (lung), CHASM (melanoma), PolyPhen-2	79.48% (76.26%-82.83%)	98.41% (97.52%-100%)	46.735% (40%-53.249%)	76.16% (72.56%-79.87%)	94.44% (90.62%-100%)	3.1575 (3.0502-3.2744)	79.43% (72.73%-85.86%)	98.4% (95.31%-100%)	46.868% (34.144%-60.53%)	76.07% (68.74%-83.33%)	94.45% (84.21%-100%)	3.1579 (2.9168-3.3777)
At least 1 of CHASM (lung), CHASM (melanoma), PolyPhen-2, PROVEAN	75.8% (72.73%-79.29%)	98.41% (97.52%-100%)	36.695% (30.299%-43.038%)	72.89% (69.05%-76.61%)	93.03% (88.45%-100%)	3.0102 (2.8974-3.1291)	75.67% (68.69%-81.82%)	98.4% (95.31%-100%)	36.643% (24.127%-50%)	72.72% (65.11%-80.24%)	93.03% (80%-100%)	3.0079 (2.7593-3.2198)
At least 1 of CHASM (lung), CHASM (melanoma), PolyPhen-2, PROVEAN, SIFT	73.09% (69.7%-76.77%)	98.93% (98.32%-100%)	28.408% (22.077%-34.211%)	70.5% (66.67%-74.3%)	93.88% (88.89%-100%)	2.9171 (2.8101-3.0345)	73.01% (65.66%-79.8%)	98.95% (96.67%-100%)	28.472% (17.239%-41.18%)	70.37% (62.5%-77.91%)	94.07% (80%-100%)	2.9186 (2.6570-3.1259)
At least 1 of CHASM (lung), CHASM (melanoma), PolyPhen-2, PROVEAN, VEST	68.05% (64.65%-71.72%)	100% (100%-100%)	12.807% (8.219%-16.901%)	66.48% (62.7%-70.37%)	100% (100%-100%)	2.7929 (2.7301-2.8631)	67.93% (60.61%-74.75%)	100% (100%-100%)	12.875% (4.878%-22.22%)	66.33% (58.51%-73.91%)	100% (100%-100%)	2.7925 (2.6815-2.9323)
At least 1 of CHASM (lung), CHASM (melanoma), PolyPhen-2, SIFT	75.1% (71.72%-78.28%)	98.93% (98.32%-100%)	33.91% (27.536%-40%)	72.13% (68.39%-76%)	94.81% (90.91%-100%)	2.9978 (2.8876-3.1215)	75.05% (68.69%-81.82%)	98.95% (96.67%-100%)	34.024% (22.214%-47.06%)	72.02% (63.95%-79.55%)	94.97% (83.33%-100%)	2.9997 (2.7498-3.2101)
At least 1 of CHASM (lung), CHASM (melanoma), PolyPhen-2, SIFT, VEST	68.41% (64.65%-72.22%)	100% (100%-100%)	13.792% (8.974%-18.056%)	66.73% (62.77%-70.74%)	100% (100%-100%)	2.8052 (2.7407-2.8768)	68.23% (60.61%-75.76%)	100% (100%-100%)	13.675% (5.128%-23.33%)	66.54% (58.51%-74.47%)	100% (100%-100%)	2.8021 (2.6706-2.9470)
At least 1 of CHASM (lung), CHASM (melanoma), PolyPhen-2, VEST	69.39% (65.66%-73.23%)	100% (100%-100%)	16.465% (11.536%-21.127%)	67.43% (63.59%-71.35%)	100% (100%-100%)	2.8389 (2.7734-2.9105)	69.29% (61.62%-76.77%)	100% (100%-100%)	16.569% (7.313%-26.67%)	67.29% (59.55%-75%)	100% (100%-100%)	2.8386 (2.7120-2.9856)
At least 1 of CHASM (lung), CHASM (melanoma), PROVEAN	77.18% (74.24%-80.81%)	93.66% (91.47%-96.06%)	48.669% (41.667%-55.405%)	75.94% (72.43%-79.62%)	81.62% (75%-88.89%)	2.9989 (2.8604-3.1419)	76.95% (69.7%-82.83%)	93.52% (88.52%-98.31%)	48.475% (34.48%-61.9%)	75.71% (68%-82.67%)	81.3% (66.67%-94.44%)	2.9899 (2.6870-3.2582)
At least 1 of CHASM (lung), CHASM (melanoma), PROVEAN, SIFT	74.43% (71.2%-77.78%)	97.88% (96.72%-99.23%)	33.878% (27.394%-40%)	71.91% (68.05%-75.72%)	90.22% (84.62%-96.55%)	2.9389 (2.8152-3.0640)	74.37% (67.68%-80.83%)	97.86% (94.83%-100%)	34.044% (21.212%-46.15%)	71.8% (64.19%-79.52%)	90.27% (75%-100%)	2.9398 (2.6614-3.1784)
At least 1 of CHASM (lung), CHASM (melanoma), PROVEAN, SIFT, VEST	67.4% (63.64%-71.21%)	99.46% (99.16%-100%)	11.953% (7.499%-16%)	66.14% (62.36%-70%)	92.79% (85.71%-100%)	2.7035 (2.5763-2.8275)	67.23% (59.6%-74.75%)	99.48% (98.25%-100%)	11.857% (3.226%-20.46%)	65.95% (58.06%-73.47%)	92.92% (66.67%-100%)	2.7027 (2.3522-2.8980)
At least 1 of CHASM (lung), CHASM (melanoma), PROVEAN, VEST	67.73% (64.14%-71.21%)	98.96% (98.33%-100%)	13.733% (9.091%-18.056%)	66.48% (62.57%-70.43%)	88.48% (77.78%-100%)	2.6766 (2.5288-2.8231)	67.56% (60.61%-74.75%)	98.88% (96.67%-100%)	13.79% (5.402%-22.58%)	66.31% (58.51%-74.19%)	87.76% (60%-100%)	2.6678 (2.3267-2.9124)
At least 1 of CHASM (lung), CHASM (melanoma), SIFT	76.43% (73.23%-79.8%)	97.34% (95.93%-99.19%)	40.285% (34.201%-46.667%)	73.81% (70%-77.51%)	89.75% (83.87%-96.67%)	3.0118 (2.8885-3.1384)	76.43% (69.7%-82.83%)	97.34% (93.75%-100%)	40.535% (27.586%-53.34%)	73.74% (66.25%-81.18%)	89.9% (77.26%-100%)	3.0152 (2.7547-3.2516)
At least 1 of CHASM (lung), CHASM (melanoma), SIFT, VEST	68.07% (64.14%-71.73%)	98.92% (98.33%-100%)	14.718% (10%-19.119%)	66.73% (62.76%-70.81%)	88.78% (80%-100%)	2.6915 (2.5477-2.8441)	67.9% (60.58%-75.76%)	98.96% (96.67%-100%)	14.59% (5.714%-25%)	66.54% (58.51%-74.45%)	89.03% (60%-100%)	2.6912 (2.3140-2.9295)
At least 1 of CHASM (lung), CHASM (melanoma), VEST	69.07% (65.15%-72.73%)	98.42% (97.52%-100%)	18.322% (13.428%-22.976%)	67.57% (63.59%-71.58%)	87.05% (76.92%-100%)	2.7136 (2.5606-2.8582)	68.92% (61.62%-76.77%)	98.37% (95.38%-100%)	18.373% (8.565%-28.14%)	67.41% (59.55%-75.28%)	86.58% (66.67%-100%)	2.7073 (2.3601-2.9677)
At least 1 of CHASM (lung), FATHMM (cancer)	79.78% (76.26%-82.84%)	93.67% (91.41%-96.09%)	55.765% (49.296%-62.652%)	78.55% (74.82%-82.52%)	83.59% (77.78%-89.58%)	3.1158 (2.9790-3.2509)	79.83% (73.71%-86.87%)	93.5% (88.33%-98.33%)	56.362% (42.498%-70%)	78.62% (70.88%-86.12%)	83.45% (71.41%-95.45%)	3.1193 (2.8447-3.3947)
At least 1 of CHASM (lung), FATHMM (cancer), FATHMM (missense)	75.06% (71.72%-78.28%)	96.86% (95.2%-98.48%)	37.343% (30.553%-43.662%)	72.78% (69.05%-76.51%)	87.32% (80.65%-93.94%)	2.9430 (2.8024-3.0747)	75.14% (68.69%-81.82%)	96.69% (92.86%-100%)	38.1% (25.567%-51.52%)	72.84% (65.11%-80.49%)	87.03% (73.33%-100%)	2.9467 (2.6775-3.2065)
At least 1 of CHASM (lung), FATHMM (cancer), FATHMM (missense), Mutation Assessor	72.38% (69.18%-76.26%)	98.97% (98.33%-100%)	26.407% (20.27%-32.354%)	69.93% (66.11%-73.86%)	93.68% (88.24%-100%)	2.8899 (2.7683-3.0142)	72.4% (64.65%-78.81%)	98.86% (96.67%-100%)	26.966% (14.706%-39.47%)	69.91% (62.07%-77.42%)	93.23% (80%-100%)	2.8897 (2.6505-3.1156)
At least 1 of CHASM (lung), FATHMM (cancer), FATHMM (missense), Mutation Assessor, MutationTaster	71.74% (68.18%-75.76%)	99.49% (99.16%-100%)	23.758% (17.808%-29.577%)	69.29% (65.54%-73.33%)	96.43% (92.86%-100%)	2.8897 (2.7865-2.9964)	71.67% (64.65%-78.79%)	99.42% (98.21%-100%)	24.023% (13.158%-35.45%)	69.19% (60.87%-76.67%)	96% (83.33%-100%)	2.8867 (2.6421-3.0835)
At least 1 of CHASM (lung), FATHMM (cancer), FATHMM (missense), Mutation Assessor, PolyPhen-2	70.01% (66.65%-73.74%)	99.48% (99.16%-100%)	19.051% (13.511%-24.286%)	68% (64.32%-72.05%)	95.48% (90.91%-100%)	2.8201 (2.7118-2.9275)	70.08% (62.63%-76.79%)	99.44% (98.25%-100%)	19.66% (9.368%-29.74%)	68% (59.57%-75.56%)	95.35% (80%-100%)	2.8244 (2.5688-3.0157)
At least 1 of CHASM (lung), FATHMM (cancer), FATHMM (missense), Mutation Assessor, PROVEAN	70.38% (66.67%-74.24%)	98.97% (98.33%-100%)	20.933% (15.183%-26.087%)	68.4% (64.6%-72.22%)	92.15% (85.71%-100%)	2.8045 (2.6692-2.9321)	70.35% (62.63%-77.78%)	98.86% (96.67%-100%)	21.386% (10.808%-32.43%)	68.34% (60.46%-76.09%)	91.58% (75%-100%)	2.8017 (2.5250-3.0312)
At least 1 of CHASM (lung), FATHMM (cancer), FATHMM (missense), Mutation Assessor, SIFT	68.68% (65.15%-72.23%)	100% (100%-100%)	14.524% (9.208%-18.919%)	66.92% (63.1%-70.9%)	100% (100%-100%)	2.8144 (2.7471-2.8857)	68.7% (61.59%-75.76%)	100% (100%-100%)	14.987% (5.878%-25.02%)	66.87% (58.95%-74.47%)	100% (100%-100%)	2.8189 (2.6924-2.9651)
At least 1 of CHASM (lung), FATHMM (cancer), FATHMM (missense), Mutation Assessor, VEST	66.67% (63.13%-70.71%)	99.5% (99.16%-100%)	9.032% (5.195%-12.701%)	65.53% (61.66%-69.64%)	90.88% (80%-100%)	2.7457 (2.6896-2.8109)	66.66% (58.59%-74.75%)	100% (100%-100%)	9.42% (2.5%-17.5%)	65.46% (57.29%-73.2%)	100% (100%-100%)	2.7498 (2.6292-2.8748)
At least 1 of CHASM (lung), FATHMM (cancer), FATHMM (missense), MutationTaster	75.08% (71.72%-78.79%)	99.49% (99.16%-100%)	32.866% (26.087%-39.13%)	71.93% (68.36%-75.73%)	97.4% (95%-100%)	3.0168 (2.9095-3.1223)	75.09% (67.68%-81.82%)	99.42% (98.21%-100%)	33.324% (21.053%-46.35%)	71.9% (64.19%-79.07%)	97.11% (88.89%-100%)	3.0175 (2.8123-3.2268)
At least 1 of CHASM (lung), FATHMM (cancer), FATHMM (missense), MutationTaster, PolyPhen-2	72.02% (68.69%-75.76%)	100% (100%-100%)	23.644% (17.91%-28.793%)	69.37% (65.73%-73.37%)	100% (100%-100%)	2.9301 (2.8551-3.0133)	72.12% (64.65%-78.79%)	100% (100%-100%)	24.245% (13.509%-35.3%)	69.38% (61.36%-76.67%)	100% (100%-100%)	2.9362 (2.7844-3.1001)
At least 1 of CHASM (lung), FATHMM (cancer), FATHMM (missense), MutationTaster, PROVEAN	72.07% (68.69%-75.76%)	99.49% (99.16%-100%)	24.658% (18.919%-30.137%)	69.54% (65.91%-73.34%)	96.56% (92.86%-100%)	2.9025 (2.7996-3.0060)	72.01% (64.65%-78.79%)	99.42% (98.21%-100%)	24.95% (13.889%-36.37%)	69.45% (61.79%-76.75%)	96.18% (85.71%-100%)	2.9000 (2.6768-3.1002)
At least 1 of CHASM (lung), FATHMM (cancer), FATHMM (missense), MutationTaster, SIFT	69.38% (65.66%-73.23%)	100% (100%-100%)	16.444% (11.268%-21.333%)	67.42% (63.64%-71.66%)	100% (100%-100%)	2.8386 (2.7665-2.9124)	69.32% (61.62%-76.77%)	100% (100%-100%)	16.657% (7.405%-27.27%)	67.31% (58.95%-75%)	100% (100%-100%)	2.8397 (2.7050-3.0019)
At least 1 of CHASM (lung), FATHMM (cancer), FATHMM (missense), MutationTaster, VEST	67.66% (64.14%-71.72%)	100% (100%-100%)	11.743% (7.246%-15.789%)	66.21% (62.43%-70.31%)	100% (100%-100%)	2.7795 (2.7162-2.8493)	67.71% (59.6%-74.75%)	100% (100%-100%)	12.256% (3.331%-21.21%)	66.17% (57.89%-73.68%)	100% (100%-100%)	2.7847 (2.6582-2.9180)
At least 1 of CHASM (lung), FATHMM (cancer), FATHMM (missense), PolyPhen-2	72.01% (68.69%-75.76%)	99.48% (99.16%-100%)	24.51% (18.919%-29.73%)	69.5% (65.75%-73.6%)	96.46% (93.33%-100%)	2.8995 (2.7954-2.9960)	72.14% (64.65%-78.79%)	99.44% (98.25%-100%)	25.253% (14.286%-36.11%)	69.54% (61.53%-77.18%)	96.33% (85.71%-100%)	2.9057 (2.6818-3.1051)
At least 1 of CHASM (lung), FATHMM (cancer), FATHMM (missense), PolyPhen-2, PROVEAN	69.68% (66.16%-73.23%)	99.48% (99.16%-100%)	18.14% (12.984%-23.188%)	67.76% (64.17%-71.74%)	95.26% (90%-100%)	2.8064 (2.6939-2.9145)	69.73% (62.63%-76.77%)	99.44% (98.25%-100%)	18.706% (8.817%-29.03%)	67.74% (59.78%-75.01%)	95.11% (80%-100%)	2.8100 (2.5373-3.0053)
At least 1 of CHASM (lung), FATHMM (cancer), FATHMM (missense), PolyPhen-2, SIFT	69.36% (65.66%-73.23%)	100% (100%-100%)	16.38% (10.975%-21.127%)	67.4% (63.49%-71.51%)	100% (100%-100%)	2.8378 (2.7653-2.9124)	69.36% (61.62%-76.77%)	100% (100%-100%)	16.789% (7.692%-27.5%)	67.35% (58.95%-75%)	100% (100%-100%)	2.8414 (2.7080-3.0019)
At least 1 of CHASM (lung), FATHMM (cancer), FATHMM (missense), PolyPhen-2, VEST	67.33% (63.64%-71.21%)	100% (100%-100%)	10.825% (6.579%-14.493%)	65.98% (62.18%-70.06%)	100% (100%-100%)	2.7680 (2.7080-2.8312)	67.37% (59.6%-74.75%)	100% (100%-100%)	11.341% (3.125%-20%)	65.94% (57.6%-73.47%)	100% (100%-100%)	2.7731 (2.6568-2.9079)
At least 1 of CHASM (lung), FATHMM (cancer), FATHMM (missense), PROVEAN	72.05% (68.69%-75.25%)	96.86% (95.2%-98.48%)	29.134% (22.973%-35.135%)	70.27% (66.47%-74.1%)	84.29% (76%-92.61%)	2.8056 (2.6562-2.9506)	72.06% (65.66%-78.79%)	96.69% (92.86%-100%)	29.727% (17.494%-42.51%)	70.26% (62.5%-77.78%)	83.93% (66.67%-100%)	2.8061 (2.5035-3.0746)
At least 1 of CHASM (lung), FATHMM (cancer), FATHMM (missense), PROVEAN, SIFT	69.02% (65.66%-72.73%)	100% (100%-100%)	15.436% (9.996%-20.007%)	67.16% (63.39%-70.97%)	100% (100%-100%)	2.8259 (2.7568-2.8957)	69.04% (61.62%-75.76%)	100% (100%-100%)	15.869% (6.452%-26.32%)	67.11% (59.34%-74.74%)	100% (100%-100%)	2.8298 (2.7019-2.9857)
At least 1 of CHASM (lung), FATHMM (cancer), FATHMM (missense), PROVEAN, VEST	66.01% (62.12%-69.7%)	99.5% (99.16%-100%)	8.103% (4.225%-11.431%)	65.18% (61.21%-69.11%)	90.47% (75%-100%)	2.6325 (2.4514-2.7743)	65.96% (58.59%-73.74%)	99.4% (98.25%-100%)	8.531% (2.5%-15.91%)	65.1% (56.99%-72.73%)	89.49% (50%-100%)	2.6256 (2.1624-2.8536)
At least 1 of CHASM (lung), FATHMM (cancer), FATHMM (missense), SIFT	70.35% (66.67%-73.74%)	100% (100%-100%)	19.093% (13.43%-23.945%)	68.12% (64.24%-72.05%)	100% (100%-100%)	2.8722 (2.7986-2.9486)	70.4% (63.64%-77.78%)	100% (100%-100%)	19.599% (10%-30.95%)	68.1% (60%-75.82%)	100% (100%-100%)	2.8770 (2.7396-3.0416)

At least 1 of CHASM (lung), FATHMM (cancer), FATHMM (missense), SIFT, VEST	66.36% (62.63%-70.2%)	100% (100%-100%)	8.196% (4.286%-11.594%)	65.32% (61.46%-69.43%)	100% (100%-100%)	2.7352 (2.6799-2.7972)	66.27% (58.59%-73.74%)	100% (100%-100%)	8.362% (2.12%-15.79%)	65.19% (56.84%-72.92%)	100% (100%-100%)	2.7376 (2.6270-2.8596)
[illegible]	[illegible]	[illegible]	[illegible]	[illegible]	[illegible]	[illegible]	[illegible]	[illegible]	[illegible]	[illegible]	[illegible]	[illegible]

At least 1 of CHASM (melanoma), Mutation Assessor, MutationTaster, PolyPhen-2, SIFT	73.78% (70.2%-77.27%)	100% (100%-100%)	28.454% (21.913%-34.288%)	70.73% (66.85%-74.72%)	100% (100%-100%)	2.9918 (2.9062-3.0786)	73.65% (66.67%-80.81%)	100% (100%-100%)	28.433% (17.063%-41.03%)	70.57% (62.35%-78.26%)	100% (100%-100%)	2.9900 (2.8287-3.1667)
At least 1 of CHASM (melanoma), Mutation Assessor, MutationTaster, PolyPhen-2, VEST	68.06% (64.65%-71.72%)	100% (100%-100%)	12.826% (8.333%-17.335%)	66.48% (62.7%-70.43%)	100% (100%-100%)	2.7931 (2.7332-2.8631)	67.92% (60.61%-74.75%)	100% (100%-100%)	12.838% (3.843%-21.62%)	66.32% (58.33%-73.69%)	100% (100%-100%)	2.7920 (2.6688-2.9252)
At least 1 of CHASM (melanoma), Mutation Assessor, MutationTaster, PROVEAN	77.19% (73.74%-80.81%)	99.49% (99.16%-100%)	38.636% (31.884%-45.313%)	73.71% (69.79%-77.78%)	97.78% (95.83%-100%)	3.0961 (2.9993-3.2007)	76.93% (69.7%-83.84%)	99.42% (98.21%-100%)	38.312% (25%-52.38%)	73.45% (65.48%-81.4%)	97.48% (90.91%-100%)	3.0866 (2.8794-3.2899)
At least 1 of CHASM (melanoma), Mutation Assessor, MutationTaster, PROVEAN, SIFT	72.77% (69.19%-76.26%)	100% (100%-100%)	25.699% (19.993%-31.25%)	69.94% (65.92%-73.89%)	100% (100%-100%)	2.9564 (2.8797-3.0416)	72.63% (65.66%-79.8%)	100% (100%-100%)	25.675% (14.634%-37.22%)	69.78% (61.8%-77.43%)	100% (100%-100%)	2.9545 (2.7990-3.1276)
At least 1 of CHASM (melanoma), Mutation Assessor, MutationTaster, PROVEAN, VEST	67.39% (63.64%-71.21%)	100% (100%-100%)	11.007% (7.04%-14.865%)	66.02% (62.11%-70%)	100% (100%-100%)	2.7703 (2.7143-2.8348)	67.24% (59.6%-74.75%)	100% (100%-100%)	10.995% (2.857%-18.75%)	65.85% (57.89%-73.47%)	100% (100%-100%)	2.7690 (2.6531-2.8922)
At least 1 of CHASM (melanoma), Mutation Assessor, MutationTaster, SIFT	74.45% (71.2%-78.28%)	100% (100%-100%)	30.288% (23.944%-36.238%)	71.27% (67.41%-75.43%)	100% (100%-100%)	3.0155 (2.9316-3.1078)	74.33% (66.67%-80.83%)	100% (100%-100%)	30.292% (18.6%-42.86%)	71.11% (62.92%-79.07%)	100% (100%-100%)	3.0140 (2.8476-3.1997)
At least 1 of CHASM (melanoma), Mutation Assessor, MutationTaster, SIFT, VEST	67.76% (64.14%-71.72%)	100% (100%-100%)	12.007% (7.576%-16.176%)	66.27% (62.43%-70.22%)	100% (100%-100%)	2.7828 (2.7211-2.8536)	67.52% (59.6%-74.75%)	100% (100%-100%)	11.754% (3.028%-20.46%)	66.04% (58.06%-73.47%)	100% (100%-100%)	2.7783 (2.6531-2.9124)
At least 1 of CHASM (melanoma), Mutation Assessor, MutationTaster, VEST	68.74% (65.15%-72.73%)	100% (100%-100%)	14.683% (9.996%-19.118%)	66.96% (63.1%-70.97%)	100% (100%-100%)	2.8164 (2.7540-2.8885)	68.58% (60.61%-75.76%)	100% (100%-100%)	14.642% (5.556%-23.68%)	66.79% (58.95%-74.23%)	100% (100%-100%)	2.8143 (2.6825-2.9500)
At least 1 of CHASM (melanoma), Mutation Assessor, PolyPhen-2	76.79% (73.23%-79.81%)	98.41% (97.52%-100%)	39.412% (32.836%-45.717%)	73.74% (69.94%-77.51%)	93.47% (88.89%-100%)	3.0504 (2.9290-3.1707)	76.72% (70.68%-83.84%)	98.4% (95.31%-100%)	39.503% (27.027%-52.5%)	73.63% (65.85%-81.11%)	93.48% (81.25%-100%)	3.0500 (2.8004-3.2815)
At least 1 of CHASM (melanoma), Mutation Assessor, PolyPhen-2, PROVEAN	74.46% (71.21%-77.78%)	98.41% (97.52%-100%)	33.029% (26.665%-38.967%)	71.76% (67.82%-75.72%)	92.32% (86.96%-100%)	2.9551 (2.8366-3.0749)	74.32% (67.68%-80.81%)	98.4% (95.31%-100%)	32.976% (20.513%-45.95%)	71.59% (63.63%-79.55%)	92.3% (78.55%-100%)	2.9527 (2.6939-3.1765)
At least 1 of CHASM (melanoma), Mutation Assessor, PolyPhen-2, PROVEAN, SIFT	71.74% (68.18%-75.25%)	98.93% (98.32%-100%)	24.731% (18.667%-30.263%)	69.44% (65.54%-73.3%)	93.03% (87.5%-100%)	2.8613 (2.7419-2.9873)	71.67% (64.65%-78.79%)	98.95% (96.67%-100%)	24.825% (14.277%-37.21%)	69.32% (61.62%-77.01%)	93.27% (77.78%-100%)	2.8637 (2.5830-3.0785)
At least 1 of CHASM (melanoma), Mutation Assessor, PolyPhen-2, PROVEAN, VEST	67.05% (63.64%-70.71%)	100% (100%-100%)	10.081% (5.882%-13.699%)	65.79% (61.98%-69.79%)	100% (100%-100%)	2.7587 (2.7039-2.8202)	66.9% (59.6%-73.74%)	100% (100%-100%)	10.081% (2.778%-17.95%)	65.62% (57.6%-73.2%)	100% (100%-100%)	2.7579 (2.6467-2.8764)
At least 1 of CHASM (melanoma), Mutation Assessor, PolyPhen-2, SIFT	73.42% (69.7%-77.27%)	98.93% (98.32%-100%)	29.32% (22.857%-35.39%)	70.76% (66.86%-74.72%)	94.05% (89.47%-100%)	2.9306 (2.8127-3.0518)	73.36% (65.66%-80.81%)	98.95% (96.67%-100%)	29.441% (17.643%-42.11%)	70.65% (62.5%-78.57%)	94.24% (80%-100%)	2.9328 (2.6746-3.1383)
At least 1 of CHASM (melanoma), Mutation Assessor, PolyPhen-2, SIFT, VEST	67.42% (63.64%-71.21%)	100% (100%-100%)	11.081% (6.944%-15.068%)	66.04% (62.23%-70.16%)	100% (100%-100%)	2.7712 (2.7110-2.8385)	67.18% (59.6%-74.75%)	100% (100%-100%)	10.839% (2.857%-19.06%)	65.81% (57.61%-73.41%)	100% (100%-100%)	2.7671 (2.6449-2.9007)
At least 1 of CHASM (melanoma), Mutation Assessor, PolyPhen-2, VEST	68.06% (64.65%-71.72%)	100% (100%-100%)	12.826% (8.333%-17.335%)	66.48% (62.7%-70.43%)	100% (100%-100%)	2.7931 (2.7332-2.8631)	67.92% (60.61%-74.75%)	100% (100%-100%)	12.838% (3.843%-21.62%)	66.32% (58.33%-73.69%)	100% (100%-100%)	2.7920 (2.6688-2.9252)
At least 1 of CHASM (melanoma), Mutation Assessor, PROVEAN	76.14% (72.73%-79.8%)	96.3% (94.44%-98.39%)	41.262% (34.659%-47.692%)	73.92% (69.88%-77.85%)	86.6% (80.56%-93.94%)	2.9809 (2.8497-3.1159)	76.01% (68.69%-82.83%)	96.21% (92.19%-100%)	41.309% (28.125%-55%)	73.78% (65.85%-81.82%)	86.44% (71.43%-100%)	2.9774 (2.6869-3.2258)
At least 1 of CHASM (melanoma), Mutation Assessor, PROVEAN, SIFT	72.39% (68.69%-76.26%)	97.88% (96.72%-99.23%)	28.326% (21.917%-33.824%)	70.25% (66.1%-74.16%)	88.52% (81.82%-96%)	2.8497 (2.7151-2.9873)	72.39% (64.65%-79.8%)	97.86% (94.83%-100%)	28.673% (17.141%-41.03%)	70.19% (62.22%-78.17%)	88.64% (71.43%-100%)	2.8536 (2.5553-3.1110)
At least 1 of CHASM (melanoma), Mutation Assessor, PROVEAN, SIFT, VEST	66.74% (63.13%-70.71%)	99.46% (99.16%-100%)	10.154% (6.023%-13.889%)	65.68% (61.85%-69.68%)	91.66% (83.33%-100%)	2.6696 (2.5273-2.8020)	66.52% (58.59%-73.74%)	99.48% (98.25%-100%)	9.957% (2.703%-17.65%)	65.47% (57.45%-73.12%)	91.78% (50%-100%)	2.6675 (2.2315-2.8596)
At least 1 of CHASM (melanoma), Mutation Assessor, PROVEAN, VEST	67.05% (63.64%-70.72%)	99.46% (99.16%-100%)	11.007% (7.04%-14.865%)	65.9% (62.03%-69.84%)	92.24% (83.33%-100%)	2.6861 (2.5613-2.8118)	66.91% (59.57%-73.74%)	99.48% (98.25%-100%)	10.995% (2.857%-18.75%)	65.73% (57.89%-73.47%)	92.54% (66.67%-100%)	2.6879 (2.3159-2.8800)
At least 1 of CHASM (melanoma), Mutation Assessor, SIFT	73.38% (69.7%-77.27%)	96.79% (95.2%-98.46%)	32.914% (26.085%-39.195%)	71.38% (67.27%-75.44%)	85.56% (78.57%-93.33%)	2.8665 (2.7277-3.0134)	73.44% (65.66%-80.81%)	96.85% (93.1%-100%)	33.289% (20.586%-46.16%)	71.35% (63.22%-79.31%)	86.03% (70%-100%)	2.8751 (2.5684-3.1416)
At least 1 of CHASM (melanoma), Mutation Assessor, SIFT, VEST	67.08% (63.64%-70.72%)	98.92% (98.33%-100%)	12.007% (7.576%-16.176%)	66.03% (62.16%-70.05%)	86.62% (75%-100%)	2.6358 (2.4767-2.8021)	66.86% (59.57%-73.74%)	98.96% (96.67%-100%)	11.754% (3.028%-20.46%)	65.81% (57.61%-73.4%)	86.8% (50%-100%)	2.6334 (2.2085-2.8847)
At least 1 of CHASM (melanoma), Mutation Assessor, VEST	68.06% (64.65%-72.22%)	98.92% (98.33%-100%)	14.683% (9.996%-19.118%)	66.72% (62.84%-70.81%)	88.75% (80%-100%)	2.6907 (2.5478-2.8459)	67.93% (59.6%-74.75%)	98.96% (96.67%-100%)	14.642% (5.556%-23.68%)	66.55% (58.51%-74.19%)	88.97% (60%-100%)	2.6913 (2.2871-2.9191)
At least 1 of CHASM (melanoma), MutationTaster	87.65% (85.35%-90.4%)	99.49% (99.16%-100%)	67.167% (61.111%-73.848%)	83.97% (80.82%-87.59%)	98.71% (97.73%-100%)	3.4934 (3.4035-3.5921)	87.33% (81.82%-91.92%)	99.42% (98.21%-100%)	66.575% (53.65%-78.39%)	83.62% (76.32%-90%)	98.54% (95%-100%)	3.4816 (3.2898-3.6652)
At least 1 of CHASM (melanoma), MutationTaster, PolyPhen-2	80.51% (77.77%-83.84%)	100% (100%-100%)	46.808% (40.238%-53.251%)	76.47% (72.89%-80.25%)	100% (100%-100%)	3.2328 (3.1418-3.3319)	80.39% (73.74%-85.88%)	100% (100%-100%)	46.735% (34.286%-60.47%)	76.31% (68.75%-83.55%)	100% (100%-100%)	3.2305 (3.0459-3.4205)
At least 1 of CHASM (melanoma), MutationTaster, PolyPhen-2, PROVEAN	76.83% (73.74%-79.8%)	100% (100%-100%)	36.768% (30.435%-42.857%)	73.22% (69.41%-76.88%)	100% (100%-100%)	3.0999 (3.0141-3.1905)	76.64% (70.71%-82.83%)	100% (100%-100%)	36.509% (25%-50%)	73% (65.48%-80.49%)	100% (100%-100%)	3.0951 (2.9306-3.2800)
At least 1 of CHASM (melanoma), MutationTaster, PolyPhen-2, PROVEAN, SIFT	73.45% (70.2%-76.77%)	100% (100%-100%)	27.542% (21.212%-32.906%)	70.47% (66.67%-74.3%)	100% (100%-100%)	2.9801 (2.8974-3.0622)	73.3% (66.67%-79.8%)	100% (100%-100%)	27.463% (16.214%-39.48%)	70.29% (62.5%-77.78%)	100% (100%-100%)	2.9775 (2.8290-3.1529)
At least 1 of CHASM (melanoma), MutationTaster, PolyPhen-2, PROVEAN, VEST	68.05% (64.65%-71.72%)	100% (100%-100%)	12.807% (8.219%-16.901%)	66.48% (62.7%-70.37%)	100% (100%-100%)	2.7929 (2.7301-2.8537)	67.93% (60.61%-74.75%)	100% (100%-100%)	12.875% (4.878%-22.22%)	66.33% (58.51%-73.91%)	100% (100%-100%)	2.7925 (2.6815-2.9319)
At least 1 of CHASM (melanoma), MutationTaster, PolyPhen-2, SIFT	75.46% (72.22%-78.79%)	100% (100%-100%)	33.044% (26.866%-39.13%)	72.08% (68.23%-76.14%)	100% (100%-100%)	3.0513 (2.9669-3.1382)	75.33% (68.69%-81.82%)	100% (100%-100%)	33.016% (21.208%-45.71%)	71.92% (63.64%-79.55%)	100% (100%-100%)	3.0494 (2.8806-3.2282)
At least 1 of CHASM (melanoma), MutationTaster, PolyPhen-2, SIFT, VEST	68.41% (64.65%-72.22%)	100% (100%-100%)	13.792% (8.974%-18.056%)	66.73% (62.77%-70.74%)	100% (100%-100%)	2.8052 (2.7407-2.8768)	68.23% (60.61%-75.76%)	100% (100%-100%)	13.675% (5.128%-23.33%)	66.54% (58.51%-74.47%)	100% (100%-100%)	2.8021 (2.6706-2.9470)
At least 1 of CHASM (melanoma), MutationTaster, PolyPhen-2, VEST	69.39% (65.66%-73.23%)	100% (100%-100%)	16.465% (11.536%-21.127%)	67.43% (63.59%-71.35%)	100% (100%-100%)	2.8389 (2.7734-2.9105)	69.29% (61.62%-76.77%)	100% (100%-100%)	16.569% (7.313%-26.67%)	67.29% (59.55%-75%)	100% (100%-100%)	2.8386 (2.7120-2.9856)
At least 1 of CHASM (melanoma), MutationTaster, PROVEAN	79.9% (76.77%-83.33%)	99.49% (99.16%-100%)	46.032% (39.436%-52.705%)	76.12% (72.39%-79.88%)	98.13% (96.55%-100%)	3.1977 (3.1034-3.2999)	79.59% (72.73%-85.86%)	99.42% (98.21%-100%)	45.518% (32.351%-58.54%)	75.8% (68%-83.53%)	97.87% (92.31%-100%)	3.1861 (2.9868-3.3816)
At least 1 of CHASM (melanoma), MutationTaster, PROVEAN, SIFT	74.46% (71.21%-77.78%)	100% (100%-100%)	30.307% (24.323%-36.111%)	71.27% (67.43%-75%)	100% (100%-100%)	3.0158 (2.9319-3.1001)	74.31% (67.68%-80.81%)	100% (100%-100%)	30.198% (18.182%-42.11%)	71.09% (63.52%-78.82%)	100% (100%-100%)	3.0128 (2.8631-3.1905)
At least 1 of CHASM (melanoma), MutationTaster, PROVEAN, SIFT, VEST	67.74% (64.14%-71.72%)	100% (100%-100%)	11.953% (7.499%-16%)	66.26% (62.43%-70.16%)	100% (100%-100%)	2.7821 (2.7206-2.8493)	67.55% (59.6%-74.75%)	100% (100%-100%)	11.857% (3.226%-20.46%)	66.07% (58.06%-73.63%)	100% (100%-100%)	2.7801 (2.6597-2.9145)
At least 1 of CHASM (melanoma), MutationTaster, PROVEAN, VEST	68.39% (64.65%-72.22%)	100% (100%-100%)	13.733% (9.091%-18.056%)	66.72% (62.9%-70.68%)	100% (100%-100%)	2.8045 (2.7373-2.8696)	68.27% (60.61%-75.76%)	100% (100%-100%)	13.79% (5.402%-22.58%)	66.56% (58.69%-74.19%)	100% (100%-100%)	2.8040 (2.6825-2.9496)
At least 1 of CHASM (melanoma), MutationTaster, SIFT	76.81% (73.74%-80.3%)	100% (100%-100%)	36.713% (30.378%-42.857%)	73.2% (69.41%-77.01%)	100% (100%-100%)	3.0992 (3.0132-3.1905)	76.69% (69.7%-82.83%)	100% (100%-100%)	36.689% (24.324%-48.78%)	73.04% (65.06%-80.46%)	100% (100%-100%)	3.0973 (2.9306-3.2800)
At least 1 of CHASM (melanoma), MutationTaster, SIFT, VEST	68.75% (65.15%-72.73%)	100% (100%-100%)	14.718% (10%-19.119%)	66.97% (63.04%-70.97%)	100% (100%-100%)	2.8169 (2.7503-2.8915)	68.56% (60.61%-75.76%)	100% (100%-100%)	14.59% (5.714%-25%)	66.77% (58.76%-74.5%)	100% (100%-100%)	2.8136 (2.6773-2.9597)
At least 1 of CHASM (melanoma), MutationTaster, VEST	70.07% (66.65%-73.74%)	100% (100%-100%)	18.322% (13.428%-22.976%)	67.92% (63.98%-71.89%)	100% (100%-100%)	2.8624 (2.7916-2.9319)	69.96% (62.63%-76.79%)	100% (100%-100%)	18.373% (8.565%-28.14%)	67.77% (59.78%-75.56%)	100% (100%-100%)	2.8614 (2.7289-3.0249)
At least 1 of CHASM (melanoma), PolyPhen-2	79.82% (76.77%-83.33%)	98.41% (97.52%-100%)	47.674% (41.096%-54.286%)	76.48% (72.84%-80.25%)	94.55% (90.91%-100%)	3.1711 (3.0623-3.2903)	79.75% (72.73%-85.86%)	98.4% (95.31%-100%)	47.743% (35.132%-61.11%)	76.37% (69.04%-83.54%)	94.55% (84.21%-100%)	3.1706 (2.9222-3.3867)
At least 1 of CHASM (melanoma), PolyPhen-2, PROVEAN	76.14% (72.73%-79.29%)	98.41% (97.52%-100%)	37.634% (31.343%-43.664%)	73.18% (69.41%-76.97%)	93.19% (88.88%-100%)	3.0242 (2.9118-3.1429)	76% (69.7%-82.83%)	98.4% (95.31%-100%)	37.518% (25%-50%)	73% (65.42%-80.49%)	93.18% (80%-100%)	3.0209 (2.7613-3.2406)
At least 1 of CHASM (melanoma), PolyPhen-2, PROVEAN, SIFT	73.09% (69.7%-76.77%)	98.93% (98.32%-100%)	28.408% (22.077%-34.211%)	70.5% (66.67%-74.3%)	93.88% (88.89%-100%)	2.9171 (2.8101-3.0345)	73.01% (65.66%-79.8%)	98.95% (96.67%-100%)	28.472% (17.239%-41.18%)	70.37% (62.5%-77.91%)	94.07% (80%-100%)	2.9186 (2.6570-3.1259)
At least 1 of CHASM (melanoma), PolyPhen-2, PROVEAN, SIFT, VEST	67.4% (63.64%-71.21%)	100% (100%-100%)	11.027% (6.847%-14.866%)	66.03% (62.23%-70%)	100% (100%-100%)	2.7705 (2.7081-2.8385)	67.22% (59.6%-74.75%)	100% (100%-100%)	10.943% (2.857%-19.51%)	65.84% (57.89%-73.4%)	100% (100%-100%)	2.7689 (2.6467-2.9060)
At least 1 of CHASM (melanoma), PolyPhen-2, PROVEAN, VEST	68.05% (64.65%-71.72%)	100% (100%-100%)	12.807% (8.219%-16.901%)	66.48% (62.7%-70.37%)	100% (100%-100%)	2.7929 (2.7301-2.8537)	67.93% (60.61%-74.75%)	100% (100%-100%)	12.875% (4.878%-22.22%)	66.33% (58.51%-73.91%)	100% (100%-100%)	2.7925 (2.6815-2.9319)
At least 1 of CHASM (melanoma), PolyPhen-2, SIFT	75.1% (71.72%-78.28%)	98.93% (98.32%-100%)	33.91% (27.536%-40%)	72.13% (68.39%-76%)	94.81% (90.91%-100%)	2.9978 (2.8876-3.1215)	75.05% (68.69%-81.82%)	98.95% (96.67%-100%)	34.024% (22.214%-47.06%)	72.02% (63.95%-79.55%)	94.97% (83.33%-100%)	2.9997 (2.7498-3.2101)
At least 1 of CHASM (melanoma), PolyPhen-2, SIFT, VEST	68.41% (64.65%-72.22%)	100% (100%-100%)	13.792% (8.974%-18.056%)	66.73% (62.77%-70.74%)	100% (100%-100%)	2.8052 (2.7407-2.8768)	68.23% (60.61%-75.76%)	100% (100%-100%)	13.675% (5.128%-23.33%)	66.54% (58.51%-74.47%)	100% (100%-100%)	2.8021 (2.6706-2.9470)
At least 1 of CHASM (melanoma), PolyPhen-2, VEST	69.39% (65.66%-73.23%)	100% (100%-100%)	16.465% (11.536%-21.127%)	67.43% (63.59%-71.35%)	100% (100%-100%)	2.8389 (2.7734-2.9105)	69.29% (61.62%-76.77%)	100% (100%-100%)	16.569% (7.313%-26.67%)	67.29% (59.55%-75%)	100% (100%-100%)	2.8386 (2.7120-2.9856)
At least 1 of CHASM (melanoma), PROVEAN	77.16% (74.24%-80.81%)	92.03% (89.51%-94.96%)	51.431% (44.591%-58.108%)	76.62% (73.02%-80.54%)	78.87% (72.34%-86.05%)	2.9896 (2.8399-3.1451)	76.99% (69.7%-82.83%)	91.99% (86.36%-96.92%)	51.198% (37.143%-64.71%)	76.4% (68.75%-83.34%)	78.82% (63.16%-92%)	2.9841 (2.6693-3.2708)
At least 1 of CHASM (melanoma), PROVEAN, SIFT	74.43% (71.2%-77.78%)	97.88% (96.72%-99.23%)	33.878% (27.394%-40%)	71.91% (68.05%-75.72%)	90.22% (84.62%-96.55%)	2.9389 (2.8152-3.0640)	74.37% (67.68%-80.83%)	97.86% (94.83%-100%)	34.044% (21.212%-46.15%)	71.8% (64.19%-79.52%)	90.27% (75%-100%)	2.9398 (2.6614-3.1784)
At least 1 of CHASM (melanoma), PROVEAN, SIFT, VEST	67.4% (63.64%-71.21%)	99.46% (99.16%-100%)	11.953% (7.499%-16%)	66.14% (62.36%-70%)	92.79% (85.71%-100%)	2.7035 (2.5763-2.8275)	67.23% (59.6%-74.75%)	99.48% (98.25%-100%)	11.857% (3.226%-20.46%)	65.95% (58.06%-73.47%)	92.92% (66.67%-100%)	2.7027 (2.3522-2.8980)
At least 1 of CHASM (melanoma), PROVEAN, VEST	67.73% (64.14%-71.21%)	98.96% (98.33%-100%)	13.733% (9.091%-18.056%)	66.48% (62.57%-70.43%)	88.48% (77.78%-100%)	2.6766 (2.5288-2.8231)	67.56% (60.61%-74.75%)	98.88% (96.67%-100%)	13.79% (5.402%-22.58%)	66.31% (58.51%-74.19%)	87.76% (60%-100%)	2.6678 (2.3267-2.9124)
At least 1 of CHASM (melanoma), SIFT	76.08% (72.73%-79.29%)	96.79% (95.2%-98.46%)	40.285% (34.201%-46.667%)	73.7% (69.88%-77.44%)	87.89% (81.82%-94.12%)	2.9866 (2.8604-3.1179)	76.12% (69.7%-82.83%)	96.85% (93.1%-100%)	40.535% (27.586%-53.34%)	73.64% (66.25%-81.02%)	88.25% (75%-100%)	2.9928 (2.7221-3.2403)
At least 1 of CHASM (melanoma), SIFT, VEST	68.07% (64.14%-71.73%)	98.92% (98.33%-100%)	14.718% (10%-19.119%)	66.73% (62.76%-70.81%)	88.78% (80%-100%)	2.6915 (2.5477-2.8441)	67.9% (60.58%-75.76%)	98.96% (96.67%-100%)	14.59% (5.714%-25%)	66.54% (58.51%-74.45%)	89.03% (60%-100%)	2.6912 (2.3140-2.9295)
At least 1 of CHASM (melanoma), VEST	68.74% (65.15%-72.23%)	97.9% (96.72%-99.23%)	18.322% (13.428%-22.976%)	67.45% (63.59%-71.43%)	83.46% (72.73%-94.12%)	2.6713 (2.5075-2.8266)	68.58% (61.59%-75.76%)	97.82% (94.83%-100%)	18.373% (8.565%-28.14%)	67.29% (59.34%-75.27%)	82.98% (60%-100%)	2.6646 (2.3140-2.9496)
At least 1 of FATHMM (cancer), FATHMM (missense)	75.39% (71.72%-78.79%)	96.86% (95.2%-98.48%)	38.264% (31.339%-44.602%)	73.07% (69.33%-76.88%)	87.58% (80.77%-93.94%)	2.9578 (2.8110-3.0893)	75.47% (68.69%-82.83%)	96.69% (92.86%-100%)	39.014% (26.306%-52.5%)	73.13% (65.48%-80.72%)	87.29% (73.33%-100%)	2.9614 (2.6939-3.2114)
At least 1 of FATHMM (cancer), FATHMM (missense), Mutation Assessor	72.38% (69.18%-76.26%)	98.97% (98.33%-100%)	26.407% (20.27%-32.354%)	69.93% (66.11%-73.86%)	93.68% (88.24%-100%)	2.8899 (2.7683-3.0142)	72.4% (64.65%-78.81%)	98.86% (96.67%-100%)	26.966% (14.706%-39.47%)	69.91% (62.07%-77.42%)	93.23% (80%-100%)	2.8897 (2.6505-3.1156)
At least 1 of FATHMM (cancer), FATHMM (missense), Mutation Assessor, MutationTaster	71.74% (68.18%-75.76%)	99.49% (99.16%-100%)	23.758% (17.808%-29.577%)	69.29% (65.54%-73.33%)	96.43% (92.86%-100%)	2.8897 (2.7865-2.9964)	71.67% (63.64%-78.79%)	99.42% (98.21%-100%)	24.023% (13.158%-35.49%)	69.19% (60.87%-76.67%)	96% (83.33%-100%)	2.8867 (2.6421-3.0835)
At least 1 of FATHMM (cancer), FATHMM (missense), Mutation Assessor, MutationTaster, PolyPhen-2	70.02% (66.67%-73.75%)	100% (100%-100%)	18.184% (12.856%-23.188%)	67.88% (64.13%-72.04%)	100% (100%-100%)	2.8607 (2.7916-2.9407)	70.06% (62.6%-76.77%)	100% (100%-100%)	18.651% (8.571%-29.03%)	67.85% (59.57%-75.53%)	100% (100%-100%)	2.8652 (2.7169-3.0063)
At least 1 of FATHMM (cancer), FATHMM (missense), Mutation Assessor, MutationTaster, PROVEAN	69.74% (66.16%-73.74%)	99.49% (99.16%-100%)	18.285% (12.857%-23.378%)	67.8% (63.93%-71.74%)	95.41% (90.91%-100%)	2.8098 (2.6939-2.9223)	69.62% (61.62%-76.77%)	99.42% (98.21%-100%)	18.444% (8.328%-29.03%)	67.66% (59.78%-75.29%)	94.82% (80%-100%)	2.8037 (2.5373-3.0048)
At least 1 of FATHMM (cancer), FATHMM (missense), Mutation Assessor, MutationTaster, SIFT	67.71% (64.14%-71.72%)	100% (100%-100%)	11.876% (7.143%-16%)	66.24% (62.43%-70.37%)	100% (100%-100%)	2.7812 (2.7169-2.8493)	67.61% (59.6%-74.75%)	100% (100%-100%)	12.044% (3.123%-21.05%)	66.12% (58.06%-73.47%)	100% (100%-100%)	2.7820 (2.6582-2.9225)
At least 1 of FATHMM (cancer), FATHMM (missense), Mutation Assessor, MutationTaster, VEST	66.67% (63.13%-70.71%)	100% (100%-100%)	9.032% (5.195%-12.701%)	65.53% (61.66%-69.64%)	100% (100%-100%)	2.7457 (2.6896-2.8109)	66.66% (58.59%-73.74%)	100% (100%-100%)	9.42% (2.5%-17.5%)	65.46% (57.29%-73.2%)	100% (100%-100%)	2.7498 (2.6292-2.8748)
At least 1 of FATHMM (cancer), FATHMM (missense), Mutation Assessor, PolyPhen-2	70.01% (66.65%-73.74%)	99.48% (99.16%-100%)	19.051% (13.511%-24.286%)	68% (64.32%-72.05%)	95.48% (90.91%-100%)	2.8201 (2.7118-2.9275)	70.08% (62.63%-76.79%)	99.44% (98.25%-100%)	19.66% (9.368%-29.74%)	68% (59.57%-75.56%)	95.35% (80%-100%)	2.8244 (2.5688-3.0157)
At least 1 of FATHMM (cancer), FATHMM (missense), Mutation Assessor, PolyPhen-2, PROVEAN	68.68% (65.15%-72.22%)	99.48% (99.16%-100%)	15.416% (10.294%-20.27%)	67.04% (63.3%-71.06%)	94.47% (88.89%-100%)	2.7640 (2.6400-2.8793)	68.7% (61.62%-75.76%)	99.44% (98.25%-100%)	15.905% (5.714%-25.71%)	66.99% (58.89%-74.47%)	94.3% (75%-100%)	2.7664 (2.4700-2.9649)
At least 1 of FATHMM (cancer), FATHMM (missense), Mutation Assessor, PolyPhen-2, SIFT	68.03% (64.63%-72.22%)	100% (100%-100%)	12.742% (7.689%-17.143%)	66.46% (62.63%-70.53%)	100% (100%-100%)	2.7920 (2.7284-2.8596)	67.99% (59.6%-74.77%)	100% (100%-100%)	13.053% (4.528%-22.51%)	66.37% (58.33%-73.96%)	100% (100%-100%)	2.7946 (2.6699-2.9295)
At least 1 of FATHMM (cancer), FATHMM (missense), Mutation Assessor, PolyPhen-2, VEST	66.33% (62.63%-70.2%)	100% (100%-100%)	8.114% (4.286%-11.598%)	65.3% (61.46%-69.43%)	100% (100%-100%)	2.7342 (2.6815-2.7950)	66.32% (58.59%-73.74%)	100% (100%-100%)	8.505% (2.381%-15.79%)	65.23% (56.84%-72.92%)	100% (100%-100%)	2.7391 (2.6270-2.8536)
At least 1 of FATHMM (cancer), FATHMM (missense), Mutation Assessor, PROVEAN	70.38% (66.67%-74.24%)	98.97% (98.33%-100%)	20.933% (15.183%-26.087%)	68.4% (64.6%-72.22%)	92.15% (85.71%-100%)	2.8045 (2.6692-2.9321)	70.35% (62.63%-77.78%)	98.86% (96.67%-100%)	21.386% (10.808%-32.43%)	68.34% (60.46%-76.09%)	91.58% (75%-100%)	2.8017 (2.5250-3.0312)
At least 1 of FATHMM (cancer), FATHMM (missense), Mutation Assessor, PROVEAN, SIFT	67.68% (64.14%-71.22%)	100% (100%-100%)	11.781% (7.042%-15.789%)	66.22% (62.43%-70.16%)	100% (100%-100%)	2.7800 (2.7144-2.8416)	67.68% (60.58%-74.75%)	100% (100%-100%)	12.194% (3.448%-21.88%)	66.16% (58.16%-73.68%)	100% (100%-100%)	2.7841 (2.6698-2.9247)
At least 1 of FATHMM (cancer), FATHMM (missense), Mutation Assessor, PROVEAN, VEST	65.67% (62.12%-69.7%)	100% (100%-100%)	6.304% (2.817%-9.377%)	64.86% (61.14%-68.91%)	100% (100%-100%)	2.7118 (2.6597-2.7660)	65.63% (57.58%-72.73%)	100% (100%-100%)	6.631% (0%-13.16%)	64.77% (56.7%-72.16%)	100% (100%-100%)	2.7168 (2.6162-2.8289)
At least 1 of FATHMM (cancer), FATHMM (missense), Mutation Assessor, SIFT	68.68% (65.15%-72.23%)	100% (100%-100%)	14.524% (9.208%-18.919%)	66.92% (63.1%-70.9%)	100% (100%-100%)	2.8144 (2.7471-2.8857)	68.7% (61.59%-75.76%)	100% (100%-100%)	14.987% (5.878%-25.02%)	66.87% (58.95%-74.47%)	100% (100%-100%)	2.8189 (2.6924-2.9651)
At least 1 of FATHMM (cancer), FATHMM (missense), Mutation Assessor, SIFT, VEST	65.37% (61.62%-69.19%)	100% (100%-100%)	5.484% (2.631%-8.219%)	64.66% (60.82%-68.72%)	100% (100%-100%)	2.7017 (2.6535-2.7534)	65.22% (57.58%-72.73%)	100% (100%-100%)	5.526% (0%-12.12%)	64.5% (56.25%-72.17%)	100% (100%-100%)	2.7048 (2.6054-2.8051)
At least 1 of FATHMM (cancer), FATHMM (missense), Mutation Assessor, VEST	66.67% (63.13%-70.71%)	100% (100%-100%)	9.032% (5.195%-12.701%)	65.53% (61.66%-69.64%)	100% (100%-100%)	2.7457 (2.6896-2.8109)	66.66% (58.59%-73.74%)	100% (100%-100%)	9.42% (2.5%-17.5%)	65.46% (57.29%-73.2%)	100% (100%-100%)	2.7498 (2.6292-2.8748)
At least 1 of FATHMM (cancer), FATHMM (missense), MutationTaster	75.08% (71.72%-78.79%)	99.49% (99.16%-100%)	32.866% (26.087%-39.13%)	71.93% (68.36%-75.73%)	97.4% (95%-100%)	3.0168 (2.9095-3.1223)	75.09% (67.68%-81.82%)	99.42% (98.21%-100%)	33.324% (21.053%-46.35%)	71.9% (64.19%-79.07%)	97.11% (88.89%-100%)	3.0175 (2.8123-3.2268)
At least 1 of FATHMM (cancer), FATHMM (missense), MutationTaster, PolyPhen-2	72.02% (68.69%-75.76%)	100% (100%-100%)	23.644% (17.91%-28.793%)	69.37% (65.73%-73.37%)	100% (100%-100%)	2.9301 (2.8551-3.0133)	72.12% (64.65%-78.79%)	100% (100%-100%)	24.245% (13.509%-35.3%)	69.38% (61.36%-76.67%)	100% (100%-100%)	2.9362 (2.7844-3.1001)
At least 1 of FATHMM (cancer), FATHMM (missense), MutationTaster, PolyPhen-2, PROVEAN	69.69% (66.16%-73.23%)	100% (100%-100%)	17.274% (12.117%-22.222%)	67.64% (63.98%-71.58%)	100% (100%-100%)	2.8491 (2.7782-2.9236)	69.71% (62.63%-76.77%)	100% (100%-100%)	17.697% (8.108%-28.13%)	67.59% (59.57%-75%)	100% (100%-100%)	2.8529 (2.7120-3.0048)
At least 1 of FATHMM (cancer), FATHMM (missense), MutationTaster, PolyPhen-2, SIFT	69.04% (65.15%-73.23%)	100% (100%-100%)	15.514% (10.141%-20.27%)	67.18% (63.44%-71.35%)	100% (100%-100%)	2.8269 (2.7557-2.9040)	68.99% (60.61%-76.77%)	100% (100%-100%)	15.781% (5.882%-25.82%)	67.08% (58.94%-74.73%)	100% (100%-100%)	2.8286 (2.6896-2.9827)
At least 1 of FATHMM (cancer), FATHMM (missense), MutationTaster, PolyPhen-2, VEST	67.33% (63.64%-71.21%)	100% (100%-100%)	10.825% (6.579%-14.493%)	65.98% (62.18%-70.06%)	100% (100%-100%)	2.7680 (2.7080-2.8312)	67.37% (59.6%-74.75%)	100% (100%-100%)	11.341% (3.125%-20%)	65.94% (57.6%-73.47%)	100% (100%-100%)	2.7731 (2.6568-2.9079)
At least 1 of FATHMM (cancer), FATHMM (missense), MutationTaster, PROVEAN	72.07% (68.69%-75.76%)	99.49% (99.16%-100%)	24.658% (18.919%-30.137%)	69.54% (65.91%-73.34%)	96.56% (92.86%-100%)	2.9025 (2.7996-3.0060)	72.01% (64.65%-78.79%)	99.42% (98.21%-100%)	24.95% (13.889%-36.37%)	69.45% (61.79%-76.75%)	96.18% (85.71%-100%)	2.9000 (2.6768-3.1002)
At least 1 of FATHMM (cancer), FATHMM (missense), MutationTaster, PROVEAN, SIFT	68.05% (64.65%-71.72%)	100% (100%-100%)	12.788% (8.219%-17.143%)	66.47% (62.77%-70.53%)	100% (100%-100%)	2.7926 (2.7276-2.8596)	67.95% (60.61%-74.75%)	100% (100%-100%)	12.927% (4.878%-22.22%)	66.34% (58.33%-73.69%)	100% (100%-100%)	2.7930 (2.6699-2.9320)
At least 1 of FATHMM (cancer), FATHMM (missense), MutationTaster, PROVEAN, VEST	66.33% (62.63%-70.2%)	100% (100%-100%)	8.103% (4.225%-11.431%)	65.3% (61.46%-69.27%)	100% (100%-100%)	2.7340 (2.6773-2.7949)	66.33% (58.59%-73.74%)	100% (100%-100%)	8.531% (2.5%-15.91%)	65.23% (57.28%-72.92%)	100% (100%-100%)	2.7387 (2.6270-2.8611)
At least 1 of FATHMM (cancer), FATHMM (missense), MutationTaster, SIFT	69.38% (65.66%-73.23%)	100% (100%-100%)	16.444% (11.268%-21.333%)	67.42% (63.64%-71.66%)	100% (100%-100%)	2.8386 (2.7665-2.9124)	69.32% (61.62%-76.77%)	100% (100%-100%)	16.657% (7.405%-27.27%)	67.31% (58.95%-75%)	100% (100%-100%)	2.8397 (2.7050-3.0019)
At least 1 of FATHMM (cancer), FATHMM (missense), MutationTaster, SIFT, VEST	66.36% (62.63%-70.2%)	100% (100%-100%)	8.196% (4.286%-11.594%)	65.32% (61.46%-69.43%)	100% (100%-100%)	2.7352 (2.6799-2.7972)	66.27% (58.59%-73.74%)	100% (100%-100%)	8.362% (2.12%-15.79%)	65.19% (56.84%-72.92%)	100% (100%-100%)	2.7376 (2.6270-2.8596)
At least 1 of FATHMM (cancer), FATHMM (missense), MutationTaster, VEST	67.66% (64.14%-71.72%)	100% (100%-100%)	11.743% (7.246%-15.789%)	66.21% (62.43%-70.31%)	100% (100%-100%)	2.7795 (2.7162-2.8493)	67.71% (59.6%-74.75%)	100% (100%-100%)	12.256% (3.331%-21.21%)	66.17% (57.89%-73.68%)	100% (100%-100%)	2.7847 (2.6582-2.9180)
At least 1 of FATHMM (cancer), FATHMM (missense), PolyPhen-2	72.01% (68.69%-75.76%)	99.48% (99.16%-100%)	24.51% (18.919%-29.73%)	69.5% (65.75%-73.6%)	96.46% (93.33%-100%)	2.8995 (2.7954-2.9960)	72.14% (64.65%-78.79%)	99.44% (98.25%-100%)	25.253% (14.286%-36.11%)	69.54% (61.53%-77.18%)	96.33% (85.71%-100%)	2.9057 (2.6818-3.1051)
At least 1 of FATHMM (cancer), FATHMM (missense), PolyPhen-2, PROVEAN	69.68% (66.16%-73.23%)	99.48% (99.16%-100%)	18.14% (12.984%-23.188%)	67.76% (64.17%-71.74%)	95.26% (90%-100%)	2.8064 (2.6939-2.9145)	69.73% (62.63%-76.77%)	99.44% (98.25%-100%)	18.706% (8.817%-29.03%)	67.74% (59.78%-75.01%)	95.11% (80%-100%)	2.8100 (2.5373-3.0053)
At least 1 of FATHMM (cancer), FATHMM (missense), PolyPhen-2, PROVEAN, SIFT	68.02% (64.65%-71.72%)	100% (100%-100%)	12.724% (7.576%-17.105%)	66.46% (62.77%-70.53%)	100% (100%-100%)	2.7918 (2.7244-2.8596)	68% (60.61%-74.75%)	100% (100%-100%)	13.06% (4.997%-23.08%)	66.38% (58.33%-73.91%)	100% (100%-100%)	2.7944 (2.6699-2.9322)
At least 1 of FATHMM (cancer), FATHMM (missense), PolyPhen-2, PROVEAN, VEST	66.33% (62.63%-70.2%)	100% (100%-100%)	8.103% (4.225%-11.431%)	65.3% (61.46%-69.27%)	100% (100%-100%)	2.7340 (2.6773-2.7949)	66.33% (58.59%-73.74%)	100% (100%-100%)	8.531% (2.5%-15.91%)	65.23% (57.28%-72.92%)	100% (100%-100%)	2.7387 (2.6270-2.8611)
At least 1 of FATHMM (cancer), FATHMM (missense), PolyPhen-2, SIFT	69.36% (65.66%-73.23%)	100% (100%-100%)	16.38% (10.975%-21.127%)	67.4% (63.49%-71.51%)	100% (100%-100%)	2.8378 (2.7653-2.9124)	69.36% (61.62%-76.77%)	100% (100%-100%)	16.789% (7.692%-27.5%)	67.35% (58.95%-75%)	100% (100%-100%)	2.8414 (2.7080-3.0019)
At least 1 of FATHMM (cancer), FATHMM (missense), PolyPhen-2, SIFT, VEST	66.36% (62.63%-70.2%)	100% (100%-100%)	8.196% (4.286%-11.594%)	65.32% (61.46%-69.43%)	100% (100%-100%)	2.7352 (2.6799-2.7972)	66.27% (58.59%-73.74%)	100% (100%-100%)	8.362% (2.12%-15.79%)	65.19% (56.84%-72.92%)	100% (100%-100%)	2.7376 (2.6270-2.8596)
At least 1 of FATHMM (cancer), FATHMM (missense), PolyPhen-2, VEST	67.33% (63.64%-71.21%)	100% (100%-100%)	10.825% (6.579%-14.493%)	65.98% (62.18%-70.06%)	100% (100%-100%)	2.7680 (2.7080-2.8312)	67.37% (59.6%-74.75%)	100% (100%-100%)	11.341% (3.125%-20%)	65.94% (57.6%-73.47%)	100% (100%-100%)	2.7731 (2.6568-2.9079)
At least 1 of FATHMM (cancer), FATHMM (missense), PROVEAN	72.39% (68.69%-75.76%)	96.86% (95.2%-98.48%)	30.056% (23.527%-36.232%)	70.54% (66.67%-74.27%)	84.7% (76.92%-92.87%)	2.8216 (2.6771-2.9680)	72.39% (65.66%-79.8%)	96.69% (92.86%-100%)	30.641% (18.421%-43.24%)	70.53% (62.96%-78.41%)	84.32% (66.67%-100%)	2.8219 (2.5210-3.0890)
At least 1 of FATHMM (cancer), FATHMM (missense), PROVEAN, SIFT	69.02% (65.66%-72.73%)	100% (100%-100%)	15.436% (9.996%-20.007%)	67.16% (63.39%-70.97%)	100% (100%-100%)	2.8259 (2.7568-2.8957)	69.04% (61.62%-75.76%)	100% (100%-100%)	15.869% (6.452%-26.32%)	67.11% (59.34%-74.74%)	100% (100%-100%)	2.8298 (2.7019-2.9857)
At least 1 of FATHMM (cancer), FATHMM (missense), PROVEAN, SIFT, VEST	65.69% (62.12%-69.7%)	100% (100%-100%)	6.365% (2.817%-9.459%)	64.87% (61.13%-68.91%)	100% (100%-100%)	2.7124 (2.6595-2.7660)	65.59% (57.58%-72.73%)	100% (100%-100%)	6.513% (0%-13.16%)	64.74% (56.7%-72.17%)	100% (100%-100%)	2.7158 (2.6162-2.8303)
At least 1 of FATHMM (cancer), FATHMM (missense), PROVEAN, VEST	66.01% (62.12%-69.7%)	99.5% (99.16%-100%)	8.103% (4.225%-11.431%)	65.18% (61.26%-69.11%)	90.47% (75%-100%)	2.6325 (2.4514-2.7743)	65.96% (58.59%-73.74%)	99.4% (98.25%-100%)	8.531% (2.5%-15.91%)	65.1% (56.99%-72.73%)	89.45% (50%-100%)	2.6256 (2.1624-2.8536)
At least 1 of FATHMM (cancer), FATHMM (missense), SIFT	70.35% (66.67%-73.74%)	100% (100%-100%)	19.093% (13.43%-23.945%)	68.12% (64.24%-72.05%)	100% (100%-100%)	2.8722 (2.7986-2.9486)	70.4% (63.64%-77.78%)	100% (100%-100%)	19.599% (10%-30.95%)	68.1% (60%-75.82%)	100% (100%-100%)	2.8770 (2.7396-3.0416)
At least 1 of FATHMM (cancer), FATHMM (missense), SIFT, VEST	66.36% (62.63%-70.2%)	100% (100%-100%)	8.196% (4.286%-11.594%)	65.32% (61.46%-69.43%)	100% (100%-100%)	2.7352 (2.6799-2.7972)	66.27% (58.59%-73.74%)	100% (100%-100%)	8.362% (2.12%-15.79%)	65.19% (56.84%-72.92%)	100% (100%-100%)	2.7376 (2.6270-2.8596)
At least 1 of FATHMM (cancer), FATHMM (missense), VEST	67.35% (63.64%-71.21%)	99.5% (99.16%-100%)	11.743% (7.246%-15.789%)	66.09% (62.3%-70.21%)	93.21% (85.71%-100%)	2.7055 (2.5741-2.8275)	67.33% (59.6%-74.75%)	99.4% (98.25%-100%)	12.256% (3.331%-21.21%)	66.04% (57.61%-73.63%)	92.49% (66.67%-100%)	2.7023 (2.3644-2.9014)
At least 1 of FATHMM (cancer), Mutation Assessor	78.45% (75.25%-81.82%)	98.44% (97.52%-100%)	43.887% (37.333%-50%)	75.21% (71.52%-79.14%)	94.2% (90%-100%)	3.1172 (2.9957-3.2276)	78.46% (71.72%-84.85%)	98.34% (95.45%-100%)	44.339% (31.429%-57.5%)	75.19% (67.47%-82.72%)	93.97% (84.21%-100%)	3.1184 (2.8934-3.3362)
At least 1 of FATHMM (cancer), Mutation Assessor, MutationTaster	77.82% (74.75%-81.31%)	99.49% (99.16%-100%)	40.344% (33.846%-46.753%)	74.25% (70.55%-78.18%)	97.87% (96%-100%)	3.1196 (3.0201-3.2258)	77.7% (70.71%-83.84%)	99.42% (98.21%-100%)	40.422% (27.5%-52.95%)	74.11% (66.25%-81.25%)	97.6% (91.67%-100%)	3.1156 (2.9042-3.3163)
At least 1 of FATHMM (cancer), Mutation Assessor, MutationTaster, PolyPhen-2	74.08% (70.71%-77.78%)	100% (100%-100%)	29.271% (23.288%-35.294%)	70.97% (67.22%-75%)	100% (100%-100%)	3.0024 (2.9233-3.0942)	74.06% (66.67%-80.81%)	100% (100%-100%)	29.523% (17.494%-41.18%)	70.89% (62.79%-78.41%)	100% (100%-100%)	3.0041 (2.8325-3.1687)
At least 1 of FATHMM (cancer), Mutation Assessor, MutationTaster, PolyPhen-2, PROVEAN	72.42% (69.19%-76.26%)	100% (100%-100%)	24.725% (19.229%-30.306%)	69.67% (65.92%-73.48%)	100% (100%-100%)	2.9439 (2.8711-3.0257)	72.33% (64.65%-78.79%)	100% (100%-100%)	24.83% (13.514%-35.9%)	69.54% (61.63%-76.92%)	100% (100%-100%)	2.9437 (2.7905-3.1051)
At least 1 of FATHMM (cancer), Mutation Assessor, MutationTaster, PolyPhen-2, SIFT	71.09% (67.68%-74.75%)	100% (100%-100%)	21.099% (15.49%-26.471%)	68.67% (64.86%-72.83%)	100% (100%-100%)	2.8976 (2.8212-2.9777)	70.96% (63.64%-77.78%)	100% (100%-100%)	21.123% (10.71%-32.26%)	68.51% (60%-76.09%)	100% (100%-100%)	2.8963 (2.7425-3.0576)
At least 1 of FATHMM (cancer), Mutation Assessor, MutationTaster, PolyPhen-2, VEST	67.71% (64.14%-71.72%)	100% (100%-100%)	11.881% (7.35%-16.176%)	66.24% (62.43%-70.21%)	100% (100%-100%)	2.7812 (2.7206-2.8536)	67.61% (59.6%-74.75%)	100% (100%-100%)	11.998% (3.223%-20.59%)	66.11% (58.06%-73.68%)	100% (100%-100%)	2.7819 (2.6565-2.9124)
At least 1 of FATHMM (cancer), Mutation Assessor, MutationTaster, PROVEAN	75.48% (72.22%-78.79%)	99.49% (99.16%-100%)	33.959% (27.999%-40.006%)	72.26% (68.42%-76.03%)	97.48% (95.24%-100%)	3.0319 (2.9292-3.1382)	75.3% (68.69%-81.82%)	99.42% (98.21%-100%)	33.903% (21.208%-45.95%)	72.08% (64.37%-79.55%)	97.15% (88.89%-100%)	3.0255 (2.8138-3.2256)
At least 1 of FATHMM (cancer), Mutation Assessor, MutationTaster, PROVEAN, SIFT	70.76% (67.17%-74.24%)	100% (100%-100%)	20.195% (14.865%-25.352%)	68.42% (64.64%-72.43%)	100% (100%-100%)	2.8861 (2.8138-2.9591)	70.61% (63.64%-77.78%)	100% (100%-100%)	20.158% (9.297%-30.77%)	68.25% (60.21%-75.82%)	100% (100%-100%)	2.8842 (2.7471-3.0446)
At least 1 of FATHMM (cancer), Mutation Assessor, MutationTaster, PROVEAN, VEST	67.39% (63.64%-71.21%)	100% (100%-100%)	10.998% (6.757%-15.068%)	66.02% (62.11%-70%)	100% (100%-100%)	2.7702 (2.7081-2.8348)	67.25% (59.6%-74.75%)	100% (100%-100%)	11.039% (2.857%-20%)	65.86% (57.89%-73.47%)	100% (100%-100%)	2.7696 (2.6467-2.9018)
At least 1 of FATHMM (cancer), Mutation Assessor, MutationTaster, SIFT	72.09% (68.69%-75.76%)	100% (100%-100%)	23.849% (18.053%-29.335%)	69.42% (65.57%-73.48%)	100% (100%-100%)	2.9327 (2.8518-3.0146)	71.97% (64.65%-78.79%)	100% (100%-100%)	23.891% (13.509%-35.48%)	69.27% (61.29%-76.92%)	100% (100%-100%)	2.9316 (2.7805-3.0989)
At least 1 of FATHMM (cancer), Mutation Assessor, MutationTaster, SIFT, VEST	66.75% (63.13%-70.71%)	100% (100%-100%)	9.254% (5.479%-12.857%)	65.58% (61.7%-69.63%)	100% (100%-100%)	2.7483 (2.6932-2.8130)	66.5% (58.59%-73.74%)	100% (100%-100%)	9.016% (2.498%-16.67%)	65.35% (57.29%-72.92%)	100% (100%-100%)	2.7449 (2.6270-2.8596)
At least 1 of FATHMM (cancer), Mutation Assessor, MutationTaster, VEST	68.39% (64.65%-72.22%)	100% (100%-100%)	13.726% (9.208%-18.31%)	66.71% (62.9%-70.75%)	100% (100%-100%)	2.8044 (2.7436-2.8776)	68.28% (60.61%-75.76%)	100% (100%-100%)	13.828% (5.128%-22.86%)	66.57% (58.51%-74.19%)	100% (100%-100%)	2.8045 (2.6722-2.9324)
At least 1 of FATHMM (cancer), Mutation Assessor, PolyPhen-2	73.73% (70.2%-77.27%)	98.94% (98.33%-100%)	30.137% (24.236%-36.364%)	71.01% (67.23%-75.14%)	94.28% (89.99%-100%)	2.9437 (2.8308-3.0582)	73.75% (66.67%-80.81%)	98.92% (96.67%-100%)	30.531% (17.946%-42.5%)	70.96% (62.92%-78.57%)	94.27% (81.82%-100%)	2.9468 (2.6977-3.1529)
At least 1 of FATHMM (cancer), Mutation Assessor, PolyPhen-2, PROVEAN	72.07% (68.69%-75.76%)	98.94% (98.33%-100%)	25.591% (20.253%-31.25%)	69.69% (65.93%-73.6%)	93.34% (88.24%-100%)	2.8757 (2.7637-2.9939)	72.02% (64.65%-78.79%)	98.92% (96.67%-100%)	25.838% (14.276%-37.14%)	69.6% (61.54%-77.28%)	93.3% (77.78%-100%)	2.8765 (2.6092-3.0937)
At least 1 of FATHMM (cancer), Mutation Assessor, PolyPhen-2, PROVEAN, SIFT	69.73% (66.16%-73.24%)	99.46% (99.16%-100%)	18.311% (13.333%-23.188%)	67.8% (63.98%-71.89%)	95.18% (90.91%-100%)	2.8075 (2.7011-2.9186)	69.63% (62.6%-76.77%)	99.48% (98.25%-100%)	18.399% (8.333%-28.58%)	67.66% (59.34%-75.28%)	95.41% (80%-100%)	2.8095 (2.5623-3.0053)
At least 1 of FATHMM (cancer), Mutation Assessor, PolyPhen-2, PROVEAN, VEST	67.05% (63.64%-70.71%)	100% (100%-100%)	10.072% (5.968%-13.889%)	65.79% (62.03%-69.79%)	100% (100%-100%)	2.7586 (2.6990-2.8237)	66.91% (59.6%-73.74%)	100% (100%-100%)	10.124% (2.703%-18.18%)	65.63% (57.73%-73.12%)	100% (100%-100%)	2.7588 (2.6461-2.8847)
At least 1 of FATHMM (cancer), Mutation Assessor, PolyPhen-2, SIFT	71.07% (67.66%-74.75%)	99.46% (99.16%-100%)	21.965% (16.438%-27.539%)	68.79% (65.03%-72.78%)	95.95% (92.31%-100%)	2.8616 (2.7548-2.9749)	71% (63.64%-77.8%)	99.48% (98.25%-100%)	22.131% (11.427%-33.33%)	68.68% (60.67%-76.4%)	96.13% (83.33%-100%)	2.8641 (2.6206-3.0622)
At least 1 of FATHMM (cancer), Mutation Assessor, PolyPhen-2, SIFT, VEST	66.41% (62.63%-70.2%)	100% (100%-100%)	8.328% (4.348%-11.769%)	65.35% (61.54%-69.43%)	100% (100%-100%)	2.7368 (2.6834-2.7987)	66.17% (58.59%-73.74%)	100% (100%-100%)	8.101% (0%-15.38%)	65.13% (56.99%-72.73%)	100% (100%-100%)	2.7342 (2.6238-2.8448)
At least 1 of FATHMM (cancer), Mutation Assessor, PolyPhen-2, VEST	67.71% (64.14%-71.72%)	100% (100%-100%)	11.881% (7.35%-16.176%)	66.24% (62.43%-70.21%)	100% (100%-100%)	2.7812 (2.7206-2.8536)	67.61% (59.6%-74.75%)	100% (100%-100%)	11.998% (3.223%-20.59%)	66.11% (58.06%-73.68%)	100% (100%-100%)	2.7819 (2.6565-2.9124)
At least 1 of FATHMM (cancer), Mutation Assessor, PROVEAN	76.11% (72.73%-79.29%)	98.44% (97.52%-100%)	37.503% (31.168%-43.478%)	73.14% (69.36%-76.97%)	93.28% (88.46%-100%)	3.0236 (2.9059-3.1384)	76.06% (69.7%-82.83%)	98.34% (95.45%-100%)	37.82% (25.714%-50%)	73.07% (65.48%-80.73%)	93% (81.25%-100%)	3.0223 (2.7818-3.2423)
At least 1 of FATHMM (cancer), Mutation Assessor, PROVEAN, SIFT	71.72% (68.18%-75.25%)	99.46% (99.16%-100%)	23.738% (18.31%-29.577%)	69.28% (65.38%-73.18%)	96.24% (92.86%-100%)	2.8872 (2.7866-2.9869)	71.72% (64.65%-78.79%)	99.48% (98.25%-100%)	24.075% (12.5%-34.49%)	69.21% (61.36%-76.93%)	96.4% (85.65%-100%)	2.8916 (2.6660-3.0861)
At least 1 of FATHMM (cancer), Mutation Assessor, PROVEAN, SIFT, VEST	66.41% (62.63%-70.21%)	100% (100%-100%)	8.336% (4.348%-11.765%)	65.35% (61.46%-69.43%)	100% (100%-100%)	2.7369 (2.6815-2.7972)	66.17% (58.56%-73.74%)	100% (100%-100%)	8.103% (2.167%-15.22%)	65.13% (57.14%-72.92%)	100% (100%-100%)	2.7340 (2.6238-2.8536)
At least 1 of FATHMM (cancer), Mutation Assessor, PROVEAN, VEST	67.39% (63.64%-71.21%)	100% (100%-100%)	10.998% (6.757%-15.068%)	66.02% (62.11%-70%)	100% (100%-100%)	2.7702 (2.7081-2.8348)	67.25% (59.6%-74.75%)	100% (100%-100%)	11.039% (2.857%-20%)	65.86% (57.89%-73.47%)	100% (100%-100%)	2.7696 (2.6467-2.9018)
At least 1 of FATHMM (cancer), Mutation Assessor, SIFT	73.05% (69.7%-76.77%)	99.46% (99.16%-100%)	27.392% (21.622%-33.343%)	70.31% (66.66%-74.16%)	96.72% (94.12%-100%)	2.9389 (2.8374-3.0514)	73.08% (65.66%-79.8%)	99.48% (98.25%-100%)	27.808% (16.216%-39.4%)	70.28% (62.49%-77.91%)	96.86% (87.5%-100%)	2.9442 (2.7164-3.1335)
At least 1 of FATHMM (cancer), Mutation Assessor, SIFT, VEST	66.75% (63.13%-70.71%)	100% (100%-100%)	9.254% (5.479%-12.857%)	65.58% (61.7%-69.63%)	100% (100%-100%)	2.7483 (2.6932-2.8130)	66.5% (58.59%-73.74%)	100% (100%-100%)	9.016% (2.498%-16.67%)	65.35% (57.29%-72.92%)	100% (100%-100%)	2.7449 (2.6270-2.8596)
At least 1 of FATHMM (cancer), Mutation Assessor, VEST	68.39% (64.65%-72.22%)	100% (100%-100%)	13.726% (9.208%-18.31%)	66.71% (62.9%-70.75%)	100% (100%-100%)	2.8044 (2.7436-2.8776)	68.28% (60.61%-75.76%)	100% (100%-100%)	13.828% (5.128%-22.86%)	66.57% (58.51%-74.19%)	100% (100%-100%)	2.8045 (2.6722-2.9324)
At least 1 of FATHMM (cancer), MutationTaster	82.52% (79.29%-85.86%)	99.49% (99.16%-100%)	53.184% (46.374%-59.459%)	78.61% (75%-82.47%)	98.38% (97.06%-100%)	3.2966 (3.1964-3.3963)	82.43% (75.76%-88.89%)	99.42% (98.21%-100%)	53.277% (40%-66.67%)	78.5% (70.89%-85.9%)	98.17% (93.74%-100%)	3.2937 (3.0989-3.4993)
At least 1 of FATHMM (cancer), MutationTaster, PolyPhen-2	76.43% (73.23%-79.81%)	100% (100%-100%)	35.682% (29.729%-41.936%)	72.89% (69.28%-76.75%)	100% (100%-100%)	3.0857 (3.0061-3.1785)	76.43% (69.67%-82.83%)	100% (100%-100%)	35.963% (23.525%-47.51%)	72.83% (65.06%-80%)	100% (100%-100%)	3.0879 (2.9171-3.2623)
At least 1 of FATHMM (cancer), MutationTaster, PolyPhen-2, PROVEAN	73.77% (70.2%-77.27%)	100% (100%-100%)	28.402% (22.854%-34.211%)	70.72% (67.05%-74.43%)	100% (100%-100%)	2.9912 (2.9140-3.0727)	73.68% (66.67%-80.81%)	100% (100%-100%)	28.476% (16.667%-40.01%)	70.59% (63.09%-77.91%)	100% (100%-100%)	2.9906 (2.8325-3.1532)
At least 1 of FATHMM (cancer), MutationTaster, PolyPhen-2, PROVEAN, SIFT	71.1% (67.68%-74.75%)	100% (100%-100%)	21.122% (15.714%-26.471%)	68.67% (65%-72.53%)	100% (100%-100%)	2.8979 (2.8265-2.9741)	70.93% (63.64%-77.78%)	100% (100%-100%)	21.037% (10.256%-31.58%)	68.49% (60.67%-75.82%)	100% (100%-100%)	2.8955 (2.7527-3.0451)
At least 1 of FATHMM (cancer), MutationTaster, PolyPhen-2, PROVEAN, VEST	67.71% (64.14%-71.22%)	100% (100%-100%)	11.871% (7.46%-16.176%)	66.24% (62.5%-70.21%)	100% (100%-100%)	2.7811 (2.7162-2.8496)	67.61% (60.58%-74.75%)	100% (100%-100%)	12.024% (3.125%-21.06%)	66.11% (58.24%-73.47%)	100% (100%-100%)	2.7818 (2.6581-2.9247)
At least 1 of FATHMM (cancer), MutationTaster, PolyPhen-2, SIFT	72.77% (69.19%-76.77%)	100% (100%-100%)	25.689% (19.696%-31.944%)	69.94% (66.29%-73.89%)	100% (100%-100%)	2.9563 (2.8775-3.0433)	72.64% (64.65%-79.8%)	100% (100%-100%)	25.706% (14.286%-37.21%)	69.79% (61.63%-77.27%)	100% (100%-100%)	2.9549 (2.7947-3.1276)
At least 1 of FATHMM (cancer), MutationTaster, PolyPhen-2, SIFT, VEST	67.4% (63.64%-71.21%)	100% (100%-100%)	11.04% (6.849%-15.002%)	66.03% (62.23%-70.16%)	100% (100%-100%)	2.7707 (2.7116-2.8422)	67.21% (59.6%-74.75%)	100% (100%-100%)	10.938% (2.778%-19.44%)	65.84% (57.45%-73.4%)	100% (100%-100%)	2.7688 (2.6411-2.9007)
At least 1 of FATHMM (cancer), MutationTaster, PolyPhen-2, VEST	68.71% (65.15%-72.73%)	100% (100%-100%)	14.593% (9.589%-19.231%)	66.94% (63.1%-70.9%)	100% (100%-100%)	2.8153 (2.7438-2.8885)	68.65% (60.61%-75.76%)	100% (100%-100%)	14.834% (5.714%-25%)	66.84% (58.88%-74.47%)	100% (100%-100%)	2.8169 (2.6815-2.9683)
At least 1 of FATHMM (cancer), MutationTaster, PROVEAN	78.17% (74.75%-81.31%)	99.49% (99.16%-100%)	41.285% (34.919%-47.368%)	74.55% (70.83%-78.16%)	97.92% (96.15%-100%)	3.1325 (3.0338-3.2337)	78.01% (71.72%-84.85%)	99.42% (98.21%-100%)	41.256% (28.571%-54.29%)	74.39% (67.07%-81.82%)	97.66% (91.67%-100%)	3.1272 (2.9356-3.3319)
At least 1 of FATHMM (cancer), MutationTaster, PROVEAN, SIFT	72.45% (69.19%-75.76%)	100% (100%-100%)	24.802% (19.113%-30.435%)	69.69% (65.92%-73.48%)	100% (100%-100%)	2.9449 (2.8695-3.0245)	72.28% (65.66%-78.79%)	100% (100%-100%)	24.681% (13.152%-36.36%)	69.5% (61.8%-76.92%)	100% (100%-100%)	2.9418 (2.7947-3.1086)
At least 1 of FATHMM (cancer), MutationTaster, PROVEAN, SIFT, VEST	67.07% (63.13%-71.21%)	100% (100%-100%)	10.135% (5.882%-14.085%)	65.8% (61.9%-69.79%)	100% (100%-100%)	2.7594 (2.7019-2.8237)	66.87% (58.59%-74.75%)	100% (100%-100%)	10.003% (2.63%-18.18%)	65.6% (57.73%-73.4%)	100% (100%-100%)	2.7574 (2.6399-2.8847)
At least 1 of FATHMM (cancer), MutationTaster, PROVEAN, VEST	68.05% (64.14%-71.72%)	100% (100%-100%)	12.796% (8.333%-17.106%)	66.47% (62.63%-70.43%)	100% (100%-100%)	2.7927 (2.7276-2.8613)	67.95% (60.61%-75.76%)	100% (100%-100%)	12.939% (4.875%-21.88%)	66.34% (58.33%-73.96%)	100% (100%-100%)	2.7931 (2.6698-2.9419)
At least 1 of FATHMM (cancer), MutationTaster, SIFT	74.12% (70.71%-77.78%)	100% (100%-100%)	29.37% (23.074%-35.211%)	71% (67.24%-75%)	100% (100%-100%)	3.0037 (2.9217-3.0900)	73.99% (66.67%-80.81%)	100% (100%-100%)	29.35% (17.497%-41.38%)	70.83% (63.04%-78.16%)	100% (100%-100%)	3.0018 (2.8453-3.1765)
At least 1 of FATHMM (cancer), MutationTaster, SIFT, VEST	67.74% (64.14%-71.72%)	100% (100%-100%)	11.965% (7.246%-16.131%)	66.26% (62.37%-70.37%)	100% (100%-100%)	2.7823 (2.7206-2.8536)	67.55% (59.6%-74.75%)	100% (100%-100%)	11.852% (2.941%-20.59%)	66.07% (57.89%-73.91%)	100% (100%-100%)	2.7800 (2.6531-2.9145)
At least 1 of FATHMM (cancer), MutationTaster, VEST	69.72% (66.16%-73.74%)	100% (100%-100%)	17.365% (12.329%-22.388%)	67.66% (63.78%-71.81%)	100% (100%-100%)	2.8503 (2.7811-2.9310)	69.65% (61.62%-76.77%)	100% (100%-100%)	17.567% (7.687%-27.5%)	67.55% (59.14%-75.27%)	100% (100%-100%)	2.8513 (2.7081-2.9976)
At least 1 of FATHMM (cancer), PolyPhen-2	75.74% (72.73%-79.29%)	98.41% (97.5%-100%)	36.549% (30.299%-42.667%)	72.84% (69.19%-76.79%)	93.02% (88.46%-100%)	3.0081 (2.8891-3.1230)	75.78% (68.69%-81.82%)	98.39% (95.52%-100%)	36.972% (24.242%-48.72%)	72.82% (65.11%-80.01%)	93.07% (81.25%-100%)	3.0125 (2.7570-3.2405)
At least 1 of FATHMM (cancer), PolyPhen-2, PROVEAN	73.08% (69.7%-76.77%)	98.41% (97.5%-100%)	29.268% (23.288%-35.135%)	70.64% (66.86%-74.44%)	91.43% (85.71%-100%)	2.8975 (2.7809-3.0195)	73.03% (65.66%-79.8%)	98.39% (95.52%-100%)	29.485% (17.494%-41.18%)	70.54% (62.92%-78.16%)	91.47% (76.92%-100%)	2.8989 (2.6334-3.1249)
At least 1 of FATHMM (cancer), PolyPhen-2, PROVEAN, SIFT	71.08% (67.68%-74.75%)	99.46% (99.16%-100%)	21.988% (16.438%-27.536%)	68.8% (65.17%-72.68%)	95.96% (92.31%-100%)	2.8621 (2.7548-2.9676)	70.98% (63.64%-77.78%)	99.48% (98.25%-100%)	22.046% (10.808%-33.33%)	68.66% (60.87%-75.83%)	96.13% (83.33%-100%)	2.8631 (2.6395-3.0575)
At least 1 of FATHMM (cancer), PolyPhen-2, PROVEAN, SIFT, VEST	66.73% (63.13%-70.71%)	100% (100%-100%)	9.209% (5.332%-12.862%)	65.57% (61.78%-69.48%)	100% (100%-100%)	2.7478 (2.6919-2.8130)	66.53% (58.59%-73.74%)	100% (100%-100%)	9.089% (2.439%-17.07%)	65.37% (57.44%-72.92%)	100% (100%-100%)	2.7464 (2.6292-2.8690)
At least 1 of FATHMM (cancer), PolyPhen-2, PROVEAN, VEST	67.71% (64.14%-71.22%)	100% (100%-100%)	11.871% (7.46%-16.176%)	66.24% (62.5%-70.21%)	100% (100%-100%)	2.7811 (2.7162-2.8496)	67.61% (60.58%-74.75%)	100% (100%-100%)	12.024% (3.125%-21.06%)	66.11% (58.24%-73.47%)	100% (100%-100%)	2.7818 (2.6581-2.9247)
At least 1 of FATHMM (cancer), PolyPhen-2, SIFT	72.75% (69.19%-76.26%)	99.46% (99.16%-100%)	26.556% (20.507%-32.394%)	70.07% (66.47%-74.03%)	96.63% (93.75%-100%)	2.9272 (2.8247-3.0309)	72.69% (65.66%-79.8%)	99.48% (98.25%-100%)	26.714% (15.148%-38.71%)	69.96% (61.9%-77.27%)	96.77% (87.5%-100%)	2.9292 (2.7183-3.1276)
At least 1 of FATHMM (cancer), PolyPhen-2, SIFT, VEST	67.4% (63.64%-71.21%)	100% (100%-100%)	11.04% (6.849%-15.002%)	66.03% (62.23%-70.16%)	100% (100%-100%)	2.7707 (2.7116-2.8422)	67.21% (59.6%-74.75%)	100% (100%-100%)	10.938% (2.778%-19.44%)	65.84% (57.45%-73.4%)	100% (100%-100%)	2.7688 (2.6411-2.9007)
At least 1 of FATHMM (cancer), PolyPhen-2, VEST	68.71% (65.15%-72.73%)	100% (100%-100%)	14.593% (9.589%-19.231%)	66.94% (63.1%-70.9%)	100% (100%-100%)	2.8153 (2.7438-2.8885)	68.65% (60.61%-75.76%)	100% (100%-100%)	14.834% (5.714%-25%)	66.84% (58.88%-74.47%)	100% (100%-100%)	2.8169 (2.6815-2.9683)
At least 1 of FATHMM (cancer), PROVEAN	76.43% (73.23%-79.8%)	93.11% (90.77%-95.83%)	47.578% (41.333%-54.054%)	75.44% (71.52%-79.2%)	79.97% (72.97%-86.96%)	2.9609 (2.8166-3.1029)	76.44% (69.7%-82.83%)	93.03% (87.88%-98.25%)	47.921% (34.211%-61.3%)	75.41% (67.95%-83.33%)	80.02% (65.22%-94.12%)	2.9638 (2.6672-3.2416)
At least 1 of FATHMM (cancer), PROVEAN, SIFT	73.41% (70.2%-76.77%)	99.46% (99.16%-100%)	28.346% (22.368%-34.617%)	70.59% (67.03%-74.3%)	96.84% (94.12%-100%)	2.9524 (2.8548-3.0535)	73.39% (66.67%-79.8%)	99.48% (98.25%-100%)	28.598% (16.216%-40.01%)	70.51% (63.04%-77.91%)	96.97% (87.5%-100%)	2.9556 (2.7470-3.1451)
At least 1 of FATHMM (cancer), PROVEAN, SIFT, VEST	67.07% (63.13%-71.21%)	100% (100%-100%)	10.135% (5.882%-14.085%)	65.8% (61.9%-69.79%)	100% (100%-100%)	2.7594 (2.7019-2.8237)	66.87% (58.59%-74.75%)	100% (100%-100%)	10.003% (2.63%-18.18%)	65.6% (57.73%-73.4%)	100% (100%-100%)	2.7574 (2.6399-2.8847)
At least 1 of FATHMM (cancer), PROVEAN, VEST	67.73% (64.13%-71.72%)	99.5% (99.16%-100%)	12.796% (8.333%-17.106%)	66.36% (62.43%-70.37%)	93.69% (85.71%-100%)	2.7235 (2.5934-2.8459)	67.57% (59.6%-74.77%)	99.4% (98.25%-100%)	12.939% (4.875%-21.88%)	66.21% (58.24%-73.96%)	92.69% (66.67%-100%)	2.7127 (2.3644-2.9247)
At least 1 of FATHMM (cancer), SIFT	75.08% (71.72%-78.28%)	99.46% (99.16%-100%)	32.913% (26.919%-39.132%)	71.94% (68.39%-75.72%)	97.26% (95%-100%)	3.0158 (2.9189-3.1201)	75.1% (68.69%-81.82%)	99.48% (98.25%-100%)	33.266% (20.588%-45.46%)	71.89% (64.29%-79.07%)	97.38% (90%-100%)	3.0201 (2.8090-3.2125)
At least 1 of FATHMM (cancer), SIFT, VEST	67.74% (64.14%-71.72%)	100% (100%-100%)	11.965% (7.246%-16.131%)	66.26% (62.37%-70.37%)	100% (100%-100%)	2.7823 (2.7206-2.8536)	67.55% (59.6%-74.75%)	100% (100%-100%)	11.852% (2.941%-20.59%)	66.07% (57.89%-73.91%)	100% (100%-100%)	2.7800 (2.6531-2.9145)
At least 1 of FATHMM (cancer), VEST	69.4% (65.66%-73.23%)	99.5% (99.16%-100%)	17.365% (12.329%-22.388%)	67.55% (63.64%-71.74%)	95.28% (90%-100%)	2.7970 (2.6938-2.9053)	69.28% (61.62%-76.77%)	99.4% (98.25%-100%)	17.567% (7.687%-27.5%)	67.42% (59.14%-75.27%)	94.57% (79.75%-100%)	2.7898 (2.5136-2.9833)
At least 1 of FATHMM (missense), Mutation Assessor	73.73% (70.2%-77.27%)	94.73% (92.62%-96.88%)	37.393% (31.429%-43.662%)	72.35% (68.52%-76.33%)	80.43% (73.33%-88.89%)	2.8491 (2.7017-2.9990)	73.76% (66.67%-80.81%)	94.56% (90%-98.48%)	38.008% (25.625%-50%)	72.36% (64.29%-80.01%)	80.36% (63.64%-94.44%)	2.8529 (2.5348-3.1404)
At least 1 of FATHMM (missense), Mutation Assessor, MutationTaster	75.77% (72.22%-79.29%)	99.49% (99.16%-100%)	34.745% (28.767%-40.845%)	72.5% (68.64%-76.33%)	97.53% (95.45%-100%)	3.0427 (2.9464-3.1458)	75.74% (68.69%-82.83%)	99.42% (98.21%-100%)	35.065% (22.579%-47.22%)	72.43% (64.71%-80.22%)	97.24% (90%-100%)	3.0416 (2.8229-3.2403)
At least 1 of FATHMM (missense), Mutation Assessor, MutationTaster, PolyPhen-2	73.38% (70.19%-76.77%)	100% (100%-100%)	27.353% (21.622%-32.877%)	70.42% (66.48%-74.18%)	100% (100%-100%)	2.9777 (2.8975-3.0622)	73.44% (66.67%-79.82%)	100% (100%-100%)	27.834% (16.216%-39.47%)	70.4% (62.5%-78.26%)	100% (100%-100%)	2.9823 (2.8288-3.1508)
At least 1 of FATHMM (missense), Mutation Assessor, MutationTaster, PolyPhen-2, PROVEAN	71.38% (67.68%-74.75%)	100% (100%-100%)	21.882% (16.216%-27.536%)	68.88% (65%-72.73%)	100% (100%-100%)	2.9076 (2.8305-2.9869)	71.38% (64.65%-78.79%)	100% (100%-100%)	22.247% (11.427%-33.35%)	68.82% (61.11%-76.6%)	100% (100%-100%)	2.9107 (2.7655-3.0724)
At least 1 of FATHMM (missense), Mutation Assessor, MutationTaster, PolyPhen-2, SIFT	70.74% (67.17%-74.24%)	100% (100%-100%)	20.144% (14.492%-25.352%)	68.41% (64.52%-72.43%)	100% (100%-100%)	2.8855 (2.8087-2.9592)	70.65% (63.64%-77.78%)	100% (100%-100%)	20.278% (10%-31.25%)	68.28% (60.22%-76.4%)	100% (100%-100%)	2.8856 (2.7417-3.0459)
At least 1 of FATHMM (missense), Mutation Assessor, MutationTaster, PolyPhen-2, VEST	67.34% (63.64%-71.21%)	100% (100%-100%)	10.866% (6.667%-14.865%)	65.99% (62.11%-70%)	100% (100%-100%)	2.7685 (2.7081-2.8341)	67.34% (59.6%-74.75%)	100% (100%-100%)	11.242% (2.857%-19.52%)	65.92% (57.89%-73.68%)	100% (100%-100%)	2.7722 (2.6566-2.9007)
At least 1 of FATHMM (missense), Mutation Assessor, MutationTaster, PROVEAN	72.75% (69.19%-76.26%)	99.49% (99.16%-100%)	26.503% (20.833%-32.394%)	70.07% (66.1%-74.01%)	96.78% (93.75%-100%)	2.9285 (2.8289-3.0345)	72.68% (65.66%-79.8%)	99.42% (98.21%-100%)	26.763% (14.695%-37.94%)	69.97% (61.9%-77.78%)	96.38% (85.71%-100%)	2.9253 (2.6818-3.1156)
At least 1 of FATHMM (missense), Mutation Assessor, MutationTaster, PROVEAN, SIFT	69.39% (65.66%-72.74%)	100% (100%-100%)	16.466% (11.429%-21.215%)	67.43% (63.44%-71.28%)	100% (100%-100%)	2.8389 (2.7702-2.9124)	69.3% (62.6%-76.77%)	100% (100%-100%)	16.601% (7.135%-26.47%)	67.3% (59.34%-75.27%)	100% (100%-100%)	2.8390 (2.7050-2.9869)
At least 1 of FATHMM (missense), Mutation Assessor, MutationTaster, PROVEAN, VEST	66.34% (62.63%-70.2%)	100% (100%-100%)	8.122% (4.167%-11.594%)	65.3% (61.46%-69.39%)	100% (100%-100%)	2.7342 (2.6799-2.7949)	66.32% (58.59%-73.74%)	100% (100%-100%)	8.485% (2.439%-16.22%)	65.22% (57.14%-72.92%)	100% (100%-100%)	2.7386 (2.6270-2.8596)
At least 1 of FATHMM (missense), Mutation Assessor, MutationTaster, SIFT	70.74% (67.17%-74.24%)	100% (100%-100%)	20.144% (14.492%-25.352%)	68.41% (64.52%-72.43%)	100% (100%-100%)	2.8855 (2.8087-2.9592)	70.65% (63.64%-77.78%)	100% (100%-100%)	20.278% (10%-31.25%)	68.28% (60.22%-76.4%)	100% (100%-100%)	2.8856 (2.7417-3.0459)
At least 1 of FATHMM (missense), Mutation Assessor, MutationTaster, SIFT, VEST	66.38% (62.63%-70.2%)	100% (100%-100%)	8.237% (4.284%-11.598%)	65.33% (61.34%-69.43%)	100% (100%-100%)	2.7357 (2.6773-2.7949)	66.23% (58.59%-73.74%)	100% (100%-100%)	8.264% (2.326%-15.79%)	65.17% (56.84%-73.2%)	100% (100%-100%)	2.7360 (2.6270-2.8605)
At least 1 of FATHMM (missense), Mutation Assessor, MutationTaster, VEST	68.02% (64.65%-71.72%)	100% (100%-100%)	12.716% (8.195%-17.144%)	66.46% (62.57%-70.53%)	100% (100%-100%)	2.7917 (2.7285-2.8570)	68% (60.61%-74.75%)	100% (100%-100%)	13.047% (3.448%-22.22%)	66.38% (58.16%-74.19%)	100% (100%-100%)	2.7945 (2.6706-2.9295)
At least 1 of FATHMM (missense), Mutation Assessor, PolyPhen-2	73.03% (69.7%-76.77%)	98.95% (98.32%-100%)	28.22% (21.918%-33.824%)	70.45% (66.67%-74.3%)	93.96% (89.47%-100%)	2.9157 (2.8018-3.0345)	73.12% (65.66%-79.8%)	98.91% (96.67%-100%)	28.843% (17.852%-41.38%)	70.46% (62.5%-78.02%)	93.96% (80%-100%)	2.9217 (2.6707-3.1383)
At least 1 of FATHMM (missense), Mutation Assessor, PolyPhen-2, PROVEAN	71.03% (67.68%-74.75%)	98.95% (98.32%-100%)	22.749% (16.896%-28.571%)	68.89% (64.97%-72.78%)	92.6% (86.67%-100%)	2.8320 (2.7102-2.9584)	71.07% (63.64%-77.78%)	98.91% (96.67%-100%)	23.255% (11.761%-35%)	68.87% (61.11%-76.34%)	92.57% (75%-100%)	2.8361 (2.5390-3.0565)
At least 1 of FATHMM (missense), Mutation Assessor, PolyPhen-2, PROVEAN, SIFT	69.37% (65.66%-73.23%)	99.47% (99.16%-100%)	17.332% (11.94%-22.368%)	67.54% (63.58%-71.43%)	94.96% (90%-100%)	2.7930 (2.6794-2.9040)	69.33% (61.62%-76.77%)	99.47% (98.28%-100%)	17.609% (7.895%-28.21%)	67.45% (59.55%-75.53%)	95.05% (80%-100%)	2.7958 (2.5228-2.9964)
At least 1 of FATHMM (missense), Mutation Assessor, PolyPhen-2, PROVEAN, VEST	66.34% (62.63%-70.2%)	100% (100%-100%)	8.122% (4.167%-11.594%)	65.3% (61.46%-69.39%)	100% (100%-100%)	2.7342 (2.6799-2.7949)	66.32% (58.59%-73.74%)	100% (100%-100%)	8.485% (2.439%-16.22%)	65.22% (57.14%-72.92%)	100% (100%-100%)	2.7386 (2.6270-2.8596)
At least 1 of FATHMM (missense), Mutation Assessor, PolyPhen-2, SIFT	70.72% (67.17%-74.24%)	99.47% (99.16%-100%)	21.01% (15.382%-26.391%)	68.53% (64.67%-72.53%)	95.81% (91.67%-100%)	2.8482 (2.7392-2.9563)	70.68% (63.64%-77.78%)	99.47% (98.28%-100%)	21.286% (10.811%-32.52%)	68.44% (60.43%-76.4%)	95.91% (83.33%-100%)	2.8510 (2.6213-3.0446)
At least 1 of FATHMM (missense), Mutation Assessor, PolyPhen-2, SIFT, VEST	66.38% (62.63%-70.2%)	100% (100%-100%)	8.237% (4.284%-11.598%)	65.33% (61.34%-69.43%)	100% (100%-100%)	2.7357 (2.6773-2.7949)	66.23% (58.59%-73.74%)	100% (100%-100%)	8.264% (2.326%-15.79%)	65.17% (56.84%-73.2%)	100% (100%-100%)	2.7360 (2.6270-2.8605)
At least 1 of FATHMM (missense), Mutation Assessor, PolyPhen-2, VEST	67.34% (63.64%-71.21%)	100% (100%-100%)	10.866% (6.667%-14.865%)	65.99% (62.11%-70%)	100% (100%-100%)	2.7685 (2.7081-2.8341)	67.34% (59.6%-74.75%)	100% (100%-100%)	11.242% (2.857%-19.52%)	65.92% (57.89%-73.68%)	100% (100%-100%)	2.7722 (2.6566-2.9007)
At least 1 of FATHMM (missense), Mutation Assessor, PROVEAN	72.05% (68.67%-75.76%)	96.85% (95.12%-98.45%)	29.152% (23.074%-35.213%)	70.27% (66.29%-74.14%)	84.26% (76%-92.31%)	2.8054 (2.6556-2.9503)	72.06% (64.65%-78.81%)	96.71% (93.22%-100%)	29.705% (17.239%-41.68%)	70.25% (62.35%-77.91%)	84.04% (66.67%-100%)	2.8070 (2.4859-3.1000)
At least 1 of FATHMM (missense), Mutation Assessor, PROVEAN, SIFT	69.37% (65.66%-73.23%)	98.42% (97.5%-100%)	19.114% (13.043%-24.006%)	67.78% (63.74%-71.67%)	87.51% (78.57%-100%)	2.7283 (2.5754-2.8726)	69.35% (61.62%-76.77%)	98.36% (95.59%-100%)	19.543% (9.67%-30.95%)	67.72% (60%-75.59%)	87.39% (66.67%-100%)	2.7301 (2.4164-2.9871)
At least 1 of FATHMM (missense), Mutation Assessor, PROVEAN, SIFT, VEST	65.7% (61.62%-69.7%)	100% (100%-100%)	6.384% (2.857%-9.459%)	64.88% (60.94%-68.88%)	100% (100%-100%)	2.7126 (2.6595-2.7702)	65.57% (57.58%-73.74%)	100% (100%-100%)	6.467% (0%-12.91%)	64.73% (56.38%-72.63%)	100% (100%-100%)	2.7150 (2.6162-2.8308)
At least 1 of FATHMM (missense), Mutation Assessor, PROVEAN, VEST	66.34% (62.63%-70.2%)	100% (100%-100%)	8.122% (4.167%-11.594%)	65.3% (61.46%-69.39%)	100% (100%-100%)	2.7342 (2.6799-2.7949)	66.32% (58.59%-73.74%)	100% (100%-100%)	8.485% (2.439%-16.22%)	65.22% (57.14%-72.92%)	100% (100%-100%)	2.7386 (2.6270-2.8596)
At least 1 of FATHMM (missense), Mutation Assessor, SIFT	70.71% (67.16%-74.24%)	98.42% (97.5%-100%)	22.792% (16.901%-28.17%)	68.79% (64.8%-72.68%)	89.33% (81.25%-100%)	2.7933 (2.6570-2.9347)	70.7% (63.64%-77.8%)	98.36% (95.59%-100%)	23.22% (12.5%-35.29%)	68.73% (60.92%-76.75%)	89.23% (71.43%-100%)	2.7954 (2.4998-3.0416)
At least 1 of FATHMM (missense), Mutation Assessor, SIFT, VEST	66.38% (62.63%-70.2%)	100% (100%-100%)	8.237% (4.284%-11.598%)	65.33% (61.34%-69.43%)	100% (100%-100%)	2.7357 (2.6773-2.7949)	66.23% (58.59%-73.74%)	100% (100%-100%)	8.264% (2.326%-15.79%)	65.17% (56.84%-73.2%)	100% (100%-100%)	2.7360 (2.6270-2.8605)
At least 1 of FATHMM (missense), Mutation Assessor, VEST	68.02% (64.65%-71.72%)	100% (100%-100%)	12.716% (8.195%-17.144%)	66.46% (62.57%-70.53%)	100% (100%-100%)	2.7917 (2.7285-2.8570)	68% (60.61%-74.75%)	100% (100%-100%)	13.047% (3.448%-22.22%)	66.38% (58.16%-74.19%)	100% (100%-100%)	2.7945 (2.6706-2.9295)
At least 1 of FATHMM (missense), MutationTaster	81.15% (78.28%-83.84%)	99.49% (99.16%-100%)	49.416% (42.619%-55.845%)	77.28% (73.75%-80.73%)	98.26% (96.77%-100%)	3.2444 (3.1469-3.3391)	81.14% (75.76%-86.87%)	99.42% (98.21%-100%)	49.733% (36.842%-63.34%)	77.25% (70%-84.34%)	98.05% (92.86%-100%)	3.2445 (3.0458-3.4486)
At least 1 of FATHMM (missense), MutationTaster, PolyPhen-2	75.72% (72.73%-79.29%)	100% (100%-100%)	33.736% (27.394%-39.443%)	72.29% (68.64%-76.03%)	100% (100%-100%)	3.0603 (2.9754-3.1457)	75.83% (68.69%-81.82%)	100% (100%-100%)	34.333% (22.857%-47.06%)	72.32% (64.77%-79.76%)	100% (100%-100%)	3.0666 (2.9129-3.2519)
At least 1 of FATHMM (missense), MutationTaster, PolyPhen-2, PROVEAN	72.38% (69.19%-75.76%)	100% (100%-100%)	24.607% (18.919%-30.14%)	69.64% (65.92%-73.34%)	100% (100%-100%)	2.9424 (2.8640-3.0245)	72.42% (65.66%-78.79%)	100% (100%-100%)	25.047% (13.509%-36.37%)	69.6% (62.1%-77.02%)	100% (100%-100%)	2.9465 (2.7990-3.1156)
At least 1 of FATHMM (missense), MutationTaster, PolyPhen-2, PROVEAN, SIFT	70.39% (66.67%-73.74%)	100% (100%-100%)	19.191% (13.749%-24.007%)	68.15% (64.28%-71.89%)	100% (100%-100%)	2.8734 (2.8003-2.9486)	70.33% (63.64%-77.78%)	100% (100%-100%)	19.401% (9.668%-30.23%)	68.04% (60.23%-75.83%)	100% (100%-100%)	2.8744 (2.7396-3.0261)
At least 1 of FATHMM (missense), MutationTaster, PolyPhen-2, PROVEAN, VEST	67% (63.13%-70.71%)	100% (100%-100%)	9.921% (5.714%-13.514%)	65.75% (61.9%-69.63%)	100% (100%-100%)	2.7567 (2.6957-2.8160)	67.02% (59.6%-74.75%)	100% (100%-100%)	10.385% (2.857%-18.75%)	65.7% (57.89%-73.4%)	100% (100%-100%)	2.7612 (2.6525-2.8959)
At least 1 of FATHMM (missense), MutationTaster, PolyPhen-2, SIFT	72.07% (68.69%-75.76%)	100% (100%-100%)	23.782% (17.904%-29.168%)	69.4% (65.56%-73.33%)	100% (100%-100%)	2.9319 (2.8551-3.0133)	72.02% (64.65%-78.79%)	100% (100%-100%)	24.014% (13.158%-35.72%)	69.31% (61.36%-76.93%)	100% (100%-100%)	2.9332 (2.7844-3.1002)
At least 1 of FATHMM (missense), MutationTaster, PolyPhen-2, SIFT, VEST	67.37% (63.64%-71.21%)	100% (100%-100%)	10.949% (6.849%-14.865%)	66.01% (61.98%-70.16%)	100% (100%-100%)	2.7695 (2.7081-2.8341)	67.28% (59.6%-74.75%)	100% (100%-100%)	11.1% (2.941%-19.36%)	65.88% (57.73%-73.96%)	100% (100%-100%)	2.7701 (2.6488-2.9079)
At least 1 of FATHMM (missense), MutationTaster, PolyPhen-2, VEST	68.34% (64.65%-72.22%)	100% (100%-100%)	13.578% (8.955%-17.724%)	66.68% (62.77%-70.68%)	100% (100%-100%)	2.8025 (2.7373-2.8690)	68.38% (60.61%-75.76%)	100% (100%-100%)	14.078% (5.552%-23.33%)	66.64% (58.51%-74.47%)	100% (100%-100%)	2.8072 (2.6856-2.9500)
At least 1 of FATHMM (missense), MutationTaster, PROVEAN	75.42% (72.22%-78.79%)	99.49% (99.16%-100%)	33.787% (27.629%-40%)	72.21% (68.42%-75.88%)	97.46% (95.24%-100%)	3.0295 (2.9291-3.1276)	75.42% (68.69%-81.82%)	99.42% (98.21%-100%)	34.197% (21.212%-45.95%)	72.17% (64.63%-79.55%)	97.17% (89.97%-100%)	3.0296 (2.8237-3.2258)
At least 1 of FATHMM (missense), MutationTaster, PROVEAN, SIFT	71.07% (67.17%-74.75%)	100% (100%-100%)	21.031% (15.584%-26.158%)	68.65% (64.8%-72.53%)	100% (100%-100%)	2.8968 (2.8239-2.9683)	71% (63.64%-78.79%)	100% (100%-100%)	21.204% (10.804%-31.83%)	68.54% (60.87%-76.14%)	100% (100%-100%)	2.8974 (2.7653-3.0622)
At least 1 of FATHMM (missense), MutationTaster, PROVEAN, SIFT, VEST	66.36% (62.63%-70.2%)	100% (100%-100%)	8.183% (4.225%-11.538%)	65.32% (61.46%-69.27%)	100% (100%-100%)	2.7350 (2.6773-2.7917)	66.27% (58.59%-73.74%)	100% (100%-100%)	8.367% (2.498%-15.79%)	65.19% (57.14%-72.92%)	100% (100%-100%)	2.7371 (2.6292-2.8596)
At least 1 of FATHMM (missense), MutationTaster, PROVEAN, VEST	67% (63.13%-70.71%)	100% (100%-100%)	9.921% (5.714%-13.514%)	65.75% (61.9%-69.63%)	100% (100%-100%)	2.7567 (2.6957-2.8160)	67.02% (59.6%-74.75%)	100% (100%-100%)	10.385% (2.857%-18.75%)	65.7% (57.89%-73.4%)	100% (100%-100%)	2.7612 (2.6525-2.8959)
At least 1 of FATHMM (missense), MutationTaster, SIFT	72.75% (69.19%-76.26%)	100% (100%-100%)	25.622% (19.48%-30.986%)	69.92% (66.1%-73.77%)	100% (100%-100%)	2.9554 (2.8776-3.0332)	72.69% (65.66%-79.8%)	100% (100%-100%)	25.817% (14.706%-37.84%)	69.82% (61.7%-77.28%)	100% (100%-100%)	2.9563 (2.8019-3.1224)
At least 1 of FATHMM (missense), MutationTaster, SIFT, VEST	67.37% (63.64%-71.21%)	100% (100%-100%)	10.949% (6.849%-14.865%)	66.01% (61.98%-70.16%)	100% (100%-100%)	2.7695 (2.7081-2.8341)	67.28% (59.6%-74.75%)	100% (100%-100%)	11.1% (2.941%-19.36%)	65.88% (57.73%-73.96%)	100% (100%-100%)	2.7701 (2.6488-2.9079)
At least 1 of FATHMM (missense), MutationTaster, VEST	69.36% (65.66%-73.23%)	100% (100%-100%)	16.377% (11.538%-20.899%)	67.4% (63.59%-71.35%)	100% (100%-100%)	2.8378 (2.7685-2.9080)	69.36% (61.62%-76.77%)	100% (100%-100%)	16.733% (6.891%-26.47%)	67.34% (59.34%-74.74%)	100% (100%-100%)	2.8407 (2.7120-2.9869)
At least 1 of FATHMM (missense), PolyPhen-2	75.37% (72.22%-78.79%)	98.95% (98.32%-100%)	34.603% (28.358%-40.541%)	72.35% (68.79%-76.16%)	95.02% (91.3%-100%)	3.0092 (2.8990-3.1210)	75.52% (68.69%-81.82%)	98.91% (96.67%-100%)	35.341% (24.13%-48.28%)	72.42% (64.77%-79.76%)	95.02% (83.33%-100%)	3.0168 (2.7880-3.2403)
At least 1 of FATHMM (missense), PolyPhen-2, PROVEAN	72.03% (68.69%-75.76%)	98.95% (98.32%-100%)	25.473% (19.692%-31.081%)	69.66% (65.93%-73.48%)	93.34% (88.24%-100%)	2.8742 (2.7514-2.9938)	72.1% (64.65%-78.79%)	98.91% (96.67%-100%)	26.055% (14.625%-37.94%)	69.66% (62.1%-77.17%)	93.32% (77.78%-100%)	2.8795 (2.6235-3.1078)
At least 1 of FATHMM (missense), PolyPhen-2, PROVEAN, SIFT	70.37% (66.67%-73.74%)	99.47% (99.16%-100%)	20.057% (14.102%-25%)	68.27% (64.32%-72.04%)	95.61% (90.91%-100%)	2.8341 (2.7185-2.9407)	70.37% (63.64%-77.78%)	99.47% (98.28%-100%)	20.409% (10.336%-31.58%)	68.2% (60.44%-76.09%)	95.71% (80%-100%)	2.8379 (2.5793-3.0441)
At least 1 of FATHMM (missense), PolyPhen-2, PROVEAN, SIFT, VEST	66.36% (62.63%-70.2%)	100% (100%-100%)	8.183% (4.225%-11.538%)	65.32% (61.46%-69.27%)	100% (100%-100%)	2.7350 (2.6773-2.7917)	66.27% (58.59%-73.74%)	100% (100%-100%)	8.367% (2.498%-15.79%)	65.19% (57.14%-72.92%)	100% (100%-100%)	2.7371 (2.6292-2.8596)
At least 1 of FATHMM (missense), PolyPhen-2, PROVEAN, VEST	67% (63.13%-70.71%)	100% (100%-100%)	9.921% (5.714%-13.514%)	65.75% (61.9%-69.63%)	100% (100%-100%)	2.7567 (2.6957-2.8160)	67.02% (59.6%-74.75%)	100% (100%-100%)	10.385% (2.857%-18.75%)	65.7% (57.89%-73.4%)	100% (100%-100%)	2.7612 (2.6525-2.8959)
At least 1 of FATHMM (missense), PolyPhen-2, SIFT	72.05% (68.69%-75.76%)	99.47% (99.16%-100%)	24.648% (18.571%-30%)	69.53% (65.71%-73.45%)	96.41% (93.32%-100%)	2.9006 (2.7966-3.0048)	72.06% (64.65%-78.79%)	99.47% (98.28%-100%)	25.022% (14.286%-37.15%)	69.48% (61.54%-77.18%)	96.49% (85.71%-100%)	2.9046 (2.6818-3.1001)
At least 1 of FATHMM (missense), PolyPhen-2, SIFT, VEST	67.37% (63.64%-71.21%)	100% (100%-100%)	10.949% (6.849%-14.865%)	66.01% (61.98%-70.16%)	100% (100%-100%)	2.7695 (2.7081-2.8341)	67.28% (59.6%-74.75%)	100% (100%-100%)	11.1% (2.941%-19.36%)	65.88% (57.73%-73.96%)	100% (100%-100%)	2.7701 (2.6488-2.9079)
At least 1 of FATHMM (missense), PolyPhen-2, VEST	68.34% (64.65%-72.22%)	100% (100%-100%)	13.578% (8.955%-17.724%)	66.68% (62.77%-70.68%)	100% (100%-100%)	2.8025 (2.7373-2.8690)	68.38% (60.61%-75.76%)	100% (100%-100%)	14.078% (5.552%-23.33%)	66.64% (58.51%-74.47%)	100% (100%-100%)	2.8072 (2.6856-2.9500)
At least 1 of FATHMM (missense), PROVEAN	74.41% (70.71%-77.78%)	94.22% (91.93%-96.77%)	40.129% (33.755%-46.575%)	73.13% (69.46%-76.93%)	80.06% (72.22%-87.88%)	2.8754 (2.7271-3.0340)	74.42% (67.68%-81.82%)	93.99% (88.89%-98.44%)	40.736% (27.273%-54.29%)	73.15% (65.48%-80.77%)	79.79% (62.5%-94.13%)	2.8766 (2.5574-3.1748)
At least 1 of FATHMM (missense), PROVEAN, SIFT	71.39% (67.68%-74.76%)	98.42% (97.5%-100%)	24.624% (18.418%-30.14%)	69.31% (65.36%-73.03%)	90.02% (82.35%-100%)	2.8237 (2.6907-2.9584)	71.37% (64.62%-78.79%)	98.36% (95.59%-100%)	24.995% (13.333%-36.84%)	69.24% (61.63%-77.01%)	89.83% (72.73%-100%)	2.8242 (2.5397-3.0667)
At least 1 of FATHMM (missense), PROVEAN, SIFT, VEST	66.36% (62.63%-70.2%)	100% (100%-100%)	8.183% (4.225%-11.538%)	65.32% (61.46%-69.27%)	100% (100%-100%)	2.7350 (2.6773-2.7917)	66.27% (58.59%-73.74%)	100% (100%-100%)	8.367% (2.498%-15.79%)	65.19% (57.14%-72.92%)	100% (100%-100%)	2.7371 (2.6292-2.8596)
At least 1 of FATHMM (missense), PROVEAN, VEST	66.68% (63.13%-70.2%)	99.5% (99.16%-100%)	9.921% (5.714%-13.514%)	65.64% (61.78%-69.48%)	92.07% (80%-100%)	2.6713 (2.5195-2.7987)	66.64% (59.6%-73.74%)	99.4% (98.25%-100%)	10.385% (2.857%-18.75%)	65.56% (57.61%-73.2%)	91.26% (66.67%-100%)	2.6663 (2.3267-2.8754)
At least 1 of FATHMM (missense), SIFT	73.07% (69.7%-76.77%)	98.42% (97.5%-100%)	29.215% (22.854%-34.85%)	70.63% (66.67%-74.43%)	91.47% (85.71%-100%)	2.8973 (2.7685-3.0245)	73.06% (65.66%-79.8%)	98.36% (95.59%-100%)	29.608% (17.643%-42.86%)	70.57% (62.79%-78.16%)	91.33% (76.92%-100%)	2.8987 (2.6396-3.1529)
At least 1 of FATHMM (missense), SIFT, VEST	67.37% (63.64%-71.21%)	100% (100%-100%)	10.949% (6.849%-14.865%)	66.01% (61.98%-70.16%)	100% (100%-100%)	2.7695 (2.7081-2.8341)	67.28% (59.6%-74.75%)	100% (100%-100%)	11.1% (2.941%-19.36%)	65.88% (57.73%-73.96%)	100% (100%-100%)	2.7701 (2.6488-2.9079)
At least 1 of FATHMM (missense), VEST	69.04% (65.66%-72.73%)	99.5% (99.16%-100%)	16.377% (11.538%-20.899%)	67.29% (63.44%-71.35%)	95.03% (90%-100%)	2.7820 (2.6723-2.8885)	68.98% (61.62%-75.76%)	99.4% (98.25%-100%)	16.733% (6.891%-26.47%)	67.2% (59.14%-74.74%)	94.36% (75%-100%)	2.7770 (2.5232-2.9833)
At least 1 of Mutation Assessor, MutationTaster	78.46% (75.25%-81.82%)	89.31% (86.4%-92.56%)	59.695% (53.327%-66.667%)	79.3% (75.36%-83.1%)	76.37% (70.37%-82.69%)	3.0467 (2.9091-3.1868)	78.43% (71.72%-84.85%)	89.45% (83.33%-95.24%)	59.55% (46.426%-72.42%)	79.13% (71.43%-86.76%)	76.7% (62.96%-88.46%)	3.0482 (2.7585-3.3182)
At least 1 of Mutation Assessor, MutationTaster, PolyPhen-2	73.38% (69.7%-76.77%)	90.35% (87.6%-93.5%)	44.018% (37.329%-50.015%)	73.62% (69.42%-77.63%)	72.52% (65.31%-80.49%)	2.8052 (2.6628-2.9587)	73.45% (66.67%-80.81%)	90.56% (84.37%-96.37%)	44.082% (31.25%-57.89%)	73.54% (65.75%-81.82%)	73.13% (57.14%-87.51%)	2.8131 (2.4919-3.1066)
At least 1 of Mutation Assessor, MutationTaster, PolyPhen-2, PROVEAN	71.04% (67.66%-74.75%)	90.35% (87.6%-93.5%)	37.636% (31.081%-43.664%)	71.47% (67.09%-75.47%)	69.29% (61.54%-78.06%)	2.6876 (2.5377-2.8433)	71.05% (63.64%-77.78%)	90.56% (84.37%-96.37%)	37.555% (25.625%-51.35%)	71.34% (63.29%-80%)	69.88% (52.38%-85.71%)	2.6932 (2.3710-2.9918)
At least 1 of Mutation Assessor, MutationTaster, PolyPhen-2, PROVEAN, SIFT	69.02% (65.15%-72.73%)	93.55% (91.27%-96.09%)	26.602% (20%-32%)	68.79% (64.53%-72.68%)	70.48% (60.87%-80.77%)	2.5942 (2.4241-2.7577)	69.03% (61.62%-76.77%)	93.74% (88.33%-98.39%)	26.603% (15.789%-39.02%)	68.67% (60.71%-76.83%)	71.35% (50%-90.91%)	2.6036 (2.2611-2.9280)
At least 1 of Mutation Assessor, MutationTaster, PolyPhen-2, PROVEAN, VEST	62.27% (58.59%-66.16%)	90.35% (87.6%-93.5%)	13.714% (8.696%-18.182%)	64.42% (60.23%-68.54%)	45.12% (33.33%-57.89%)	2.1361 (1.9496-2.3180)	62.32% (54.55%-69.7%)	90.56% (84.37%-96.37%)	13.842% (5.263%-23.53%)	64.33% (56.18%-72.53%)	46.1% (20%-70%)	2.1483 (1.7537-2.4943)
At least 1 of Mutation Assessor, MutationTaster, PolyPhen-2, SIFT	70.7% (67.17%-74.24%)	93.55% (91.27%-96.09%)	31.191% (24.658%-36.99%)	70.16% (66.06%-74.25%)	73.68% (65.38%-83.33%)	2.6858 (2.5261-2.8375)	70.72% (63.64%-77.78%)	93.74% (88.33%-98.39%)	31.219% (20%-44.44%)	70.05% (61.9%-78.31%)	74.5% (57.12%-92.31%)	2.6950 (2.3690-3.0041)
At least 1 of Mutation Assessor, MutationTaster, PolyPhen-2, SIFT, VEST	64% (60.1%-67.69%)	93.55% (91.27%-96.09%)	12.905% (8.219%-17.333%)	65% (60.99%-69.1%)	53.67% (38.46%-66.67%)	2.2513 (2.0456-2.4342)	63.91% (56.54%-71.72%)	93.74% (88.33%-98.39%)	12.702% (3.996%-22.22%)	64.82% (56.67%-72.73%)	54.28% (25%-83.33%)	2.2554 (1.8532-2.6420)
At least 1 of Mutation Assessor, MutationTaster, PolyPhen-2, VEST	63.28% (59.6%-67.17%)	90.35% (87.6%-93.5%)	16.459% (11.111%-21.212%)	65.16% (61.05%-69.27%)	49.66% (37.5%-61.55%)	2.2164 (2.0273-2.3941)	63.34% (55.56%-70.71%)	90.56% (84.37%-96.37%)	16.599% (7.407%-27.03%)	65.08% (56.82%-73.26%)	50.59% (25%-73.37%)	2.2282 (1.8668-2.5846)
At least 1 of Mutation Assessor, MutationTaster, PROVEAN	75.1% (71.72%-78.79%)	89.85% (86.92%-92.91%)	49.591% (43.051%-56.338%)	75.5% (71.33%-79.31%)	73.85% (67.31%-81.25%)	2.8879 (2.7501-3.0489)	75.06% (67.68%-81.82%)	89.98% (83.61%-95.52%)	49.449% (36.095%-62.86%)	75.33% (67.57%-83.57%)	74.2% (59.08%-87.51%)	2.8896 (2.5846-3.1622)
At least 1 of Mutation Assessor, MutationTaster, PROVEAN, SIFT	70.36% (66.67%-73.74%)	93.55% (91.27%-96.09%)	30.26% (24%-36.486%)	69.87% (65.66%-73.66%)	73.09% (64.29%-83.33%)	2.6677 (2.5098-2.8222)	70.39% (63.64%-77.78%)	93.74% (88.33%-98.39%)	30.315% (18.182%-43.48%)	69.77% (62.19%-77.91%)	73.92% (54.55%-92.31%)	2.6775 (2.3497-2.9762)
At least 1 of Mutation Assessor, MutationTaster, PROVEAN, SIFT, VEST	63.66% (59.6%-67.68%)	93.55% (91.27%-96.09%)	11.978% (7.57%-15.942%)	64.76% (60.79%-68.85%)	51.83% (37.5%-64.71%)	2.2212 (2.0296-2.4039)	63.58% (55.56%-71.72%)	93.74% (88.33%-98.39%)	11.82% (3.125%-21.05%)	64.59% (56.25%-72.34%)	52.54% (22.17%-83.33%)	2.2269 (1.8273-2.6183)
At least 1 of Mutation Assessor, MutationTaster, PROVEAN, VEST	62.61% (59.09%-66.67%)	90.35% (87.6%-93.5%)	14.64% (9.722%-19.118%)	64.67% (60.47%-68.75%)	46.75% (34.78%-59.09%)	2.1641 (1.9807-2.3471)	62.65% (54.55%-69.7%)	90.56% (84.37%-96.37%)	14.756% (5.556%-25%)	64.58% (56.38%-72.73%)	47.69% (22.17%-71.43%)	2.1758 (1.7843-2.5301)
At least 1 of Mutation Assessor, MutationTaster, SIFT	72.04% (68.18%-75.76%)	93.55% (91.27%-96.09%)	34.849% (28.354%-41.096%)	71.29% (67.27%-75.16%)	75.78% (67.74%-84.85%)	2.7546 (2.5986-2.9022)	72.08% (64.65%-79.8%)	93.74% (88.33%-98.39%)	34.932% (22.724%-48.49%)	71.19% (63.41%-79.31%)	76.56% (60%-93.33%)	2.7642 (2.4519-3.0736)
At least 1 of Mutation Assessor, MutationTaster, SIFT, VEST	64.34% (60.61%-68.18%)	93.55% (91.27%-96.09%)	13.831% (9.088%-18.185%)	65.24% (61.2%-69.44%)	55.4% (41.18%-68.75%)	2.2802 (2.0855-2.4584)	64.25% (56.57%-71.72%)	93.74% (88.33%-98.39%)	13.617% (5.26%-23.34%)	65.06% (56.67%-72.83%)	56% (28.54%-85.71%)	2.2842 (1.8933-2.6515)
At least 1 of Mutation Assessor, MutationTaster, VEST	63.96% (60.1%-67.68%)	89.82% (86.89%-92.97%)	19.235% (13.846%-24.244%)	65.79% (61.63%-69.94%)	52.23% (40.91%-64%)	2.2707 (2.0960-2.4423)	64% (56.57%-71.72%)	90.02% (83.61%-95.52%)	19.318% (9.375%-29.42%)	65.69% (57.32%-73.86%)	53.01% (28.57%-75%)	2.2805 (1.9209-2.6256)
At least 1 of Mutation Assessor, PolyPhen-2	69% (65.66%-72.73%)	82.95% (79.34%-86.89%)	44.885% (38.158%-51.43%)	72.24% (67.86%-76.51%)	60.35% (52.83%-68%)	2.6042 (2.4428-2.7634)	69.07% (61.62%-75.76%)	83.03% (75%-90.48%)	45.09% (31.707%-58.82%)	72.18% (63.51%-80.77%)	60.77% (44.83%-75%)	2.6108 (2.2914-2.9309)
At least 1 of Mutation Assessor, PolyPhen-2, PROVEAN	67.67% (64.14%-71.21%)	84.53% (81.15%-88.28%)	38.502% (31.999%-44.742%)	70.38% (66%-74.67%)	59% (51.11%-67.35%)	2.5242 (2.3611-2.6831)	67.7% (60.61%-74.75%)	84.66% (76.67%-91.67%)	38.563% (26.316%-51.63%)	70.28% (61.84%-79.23%)	59.44% (42.86%-75%)	2.5294 (2.2009-2.8422)
At least 1 of Mutation Assessor, PolyPhen-2, PROVEAN, SIFT	66.65% (62.63%-70.71%)	89.31% (86.29%-92.42%)	27.468% (20.779%-32.877%)	68.04% (63.8%-72.12%)	59.77% (50%-68.75%)	2.4460 (2.2655-2.6118)	66.69% (58.59%-74.75%)	89.45% (83.08%-95.38%)	27.611% (16.667%-40.62%)	67.96% (59.76%-76.47%)	60.43% (41.67%-80%)	2.4545 (2.0999-2.7974)
At least 1 of Mutation Assessor, PolyPhen-2, PROVEAN, SIFT, VEST	61.65% (57.58%-65.66%)	90.91% (88.1%-93.89%)	11.053% (6.755%-14.865%)	63.86% (59.67%-68.13%)	41.3% (27.78%-52.95%)	2.0713 (1.8821-2.2556)	61.54% (53.54%-69.7%)	91.03% (84.75%-96.77%)	10.906% (2.941%-19.58%)	63.68% (55.42%-71.74%)	41.52% (14.29%-66.75%)	2.0714 (1.6975-2.4623)
At least 1 of Mutation Assessor, PolyPhen-2, PROVEAN, VEST	59.93% (56.06%-63.65%)	86.65% (83.47%-90.32%)	13.714% (8.696%-18.182%)	63.46% (59.28%-67.65%)	37.26% (25%-48.15%)	2.0108 (1.8377-2.1864)	59.95% (52.5%-67.68%)	86.8% (79.37%-93.11%)	13.842% (5.263%-23.53%)	63.35% (55%-71.76%)	37.91% (15.36%-61.54%)	2.0190 (1.6759-2.3601)
At least 1 of Mutation Assessor, PolyPhen-2, SIFT	68.33% (64.65%-72.22%)	89.31% (86.29%-92.42%)	32.057% (25.333%-38.028%)	69.45% (65.22%-73.55%)	63.43% (54.55%-72.22%)	2.5424 (2.3772-2.7139)	68.38% (60.61%-75.76%)	89.45% (83.08%-95.38%)	32.228% (20.588%-46.16%)	69.37% (61.25%-77.91%)	64.07% (46.67%-81.26%)	2.5511 (2.2068-2.8756)
At least 1 of Mutation Assessor, PolyPhen-2, SIFT, VEST	62.33% (58.59%-66.16%)	90.91% (88.1%-93.89%)	12.905% (8.219%-17.333%)	64.35% (60.12%-68.54%)	45.1% (31.58%-57.14%)	2.1327 (1.9475-2.3139)	62.21% (54.55%-69.7%)	91.03% (84.75%-96.77%)	12.702% (3.996%-22.22%)	64.15% (55.68%-72.41%)	45.25% (20%-72.73%)	2.1314 (1.7601-2.5079)
At least 1 of Mutation Assessor, PolyPhen-2, VEST	60.61% (57.07%-64.65%)	86.14% (82.94%-89.6%)	16.459% (11.111%-21.212%)	64.07% (60%-68.24%)	40.7% (29.63%-51.72%)	2.0737 (1.8882-2.2452)	60.6% (52.53%-67.68%)	86.22% (78.95%-92.76%)	16.599% (7.407%-27.03%)	63.95% (55.43%-71.95%)	41.21% (20%-62.5%)	2.0798 (1.7470-2.4290)
At least 1 of Mutation Assessor, PROVEAN	69.34% (65.66%-73.23%)	78.18% (74.42%-82.65%)	54.037% (47.824%-60.811%)	74.63% (70.08%-78.91%)	58.89% (52.54%-65.76%)	2.6573 (2.5025-2.8143)	69.41% (61.62%-76.77%)	78.19% (69.08%-85.94%)	54.312% (40%-67.57%)	74.59% (66.15%-83.58%)	59.17% (45.15%-72.23%)	2.6627 (2.3388-2.9815)
At least 1 of Mutation Assessor, PROVEAN, SIFT	66.63% (62.63%-70.2%)	85.09% (81.6%-88.71%)	34.706% (28.378%-41.176%)	69.26% (64.86%-73.47%)	57.37% (48.84%-65.12%)	2.4643 (2.3003-2.6240)	66.74% (59.6%-74.75%)	85.13% (77.78%-91.94%)	35.178% (22.222%-48.28%)	69.26% (60.81%-77.94%)	57.93% (42.11%-75%)	2.4749 (2.1272-2.7974)
At least 1 of Mutation Assessor, PROVEAN, SIFT, VEST	60.98% (57.07%-65.15%)	88.8% (85.82%-92.13%)	12.881% (8.571%-16.884%)	63.8% (59.54%-68%)	39.96% (28.57%-50%)	2.0544 (1.8801-2.2237)	60.86% (52.53%-68.69%)	88.88% (81.97%-95.08%)	12.767% (5%-21.62%)	63.61% (55.17%-71.91%)	40.14% (16.67%-63.64%)	2.0540 (1.7062-2.4135)
At least 1 of Mutation Assessor, PROVEAN, VEST	58.91% (55.05%-62.63%)	83.99% (80.65%-87.71%)	15.543% (10.769%-20.29%)	63.23% (59.04%-67.46%)	35.95% (25.81%-45.17%)	1.9871 (1.8275-2.1508)	58.95% (51.52%-66.67%)	84.14% (76.66%-90.91%)	15.702% (6.245%-25.71%)	63.14% (54.76%-71.59%)	36.58% (16.67%-57.14%)	1.9956 (1.6778-2.3200)
At least 1 of Mutation Assessor, SIFT	67.62% (63.64%-71.21%)	84% (80.47%-87.7%)	39.295% (32.835%-45.946%)	70.52% (66.19%-74.83%)	58.67% (51.06%-66%)	2.5249 (2.3604-2.6875)	67.79% (60.61%-75.76%)	84.11% (76.27%-91.05%)	39.795% (26.829%-52.94%)	70.56% (62.02%-79.22%)	59.31% (44%-75%)	2.5378 (2.2068-2.8650)
At least 1 of Mutation Assessor, SIFT, VEST	61.32% (57.56%-65.15%)	88.26% (85.12%-91.41%)	14.733% (9.859%-19.178%)	64.15% (59.88%-68.42%)	42.06% (30.76%-52.17%)	2.0921 (1.9191-2.2612)	61.2% (53.54%-68.71%)	88.36% (81.96%-94.45%)	14.563% (5.556%-23.68%)	63.96% (55.56%-72.22%)	42.18% (20%-66.67%)	2.0906 (1.7464-2.4524)
At least 1 of Mutation Assessor, VEST	59.59% (55.56%-63.64%)	82.41% (78.79%-86.26%)	20.137% (14.861%-25.352%)	64.08% (59.87%-68.52%)	39.83% (30.3%-48.57%)	2.0646 (1.8944-2.2229)	59.61% (51.52%-67.68%)	82.51% (74.63%-89.55%)	20.265% (10%-30.56%)	63.98% (55.29%-72.51%)	40.29% (21.73%-58.82%)	2.0704 (1.7494-2.4095)
At least 1 of MutationTaster, PolyPhen-2	75.73% (72.22%-79.29%)	89.29% (86.29%-92.62%)	52.281% (45.33%-58.824%)	76.39% (72.41%-80.27%)	73.84% (67.35%-80.77%)	2.9179 (2.7764-3.0656)	75.82% (68.69%-82.83%)	89.5% (83.04%-95.31%)	52.321% (39.391%-65.85%)	76.31% (68.49%-84%)	74.41% (60%-87.51%)	2.9254 (2.6305-3.2072)
At least 1 of MutationTaster, PolyPhen-2, PROVEAN	72.05% (68.18%-75.76%)	89.29% (86.29%-92.62%)	42.241% (35.897%-48.053%)	72.77% (68.39%-76.67%)	69.51% (62.22%-77.5%)	2.7381 (2.5832-2.8934)	72.06% (64.65%-79.8%)	89.5% (83.04%-95.31%)	42.096% (29.624%-55.56%)	72.62% (64.86%-81.18%)	70.04% (52.94%-85%)	2.7427 (2.4338-3.0469)
At least 1 of MutationTaster, PolyPhen-2, PROVEAN, SIFT	69.69% (66.15%-73.24%)	92.48% (90%-95.28%)	30.279% (23.529%-36%)	69.64% (65.64%-73.49%)	69.97% (60.71%-80%)	2.6237 (2.4538-2.7918)	69.71% (62.6%-76.79%)	92.69% (86.89%-98.15%)	30.25% (18.736%-43.33%)	69.52% (61.73%-77.65%)	70.76% (50%-88.89%)	2.6321 (2.3031-2.9497)
At least 1 of MutationTaster, PolyPhen-2, PROVEAN, SIFT, VEST	63.31% (59.6%-67.17%)	92.48% (90%-95.28%)	12.851% (8.451%-17.143%)	64.73% (60.56%-68.72%)	49.72% (36.84%-63.16%)	2.1978 (2.0150-2.3779)	63.29% (55.56%-70.71%)	92.69% (86.89%-98.15%)	12.806% (4.759%-21.88%)	64.59% (56.81%-72.83%)	50.51% (22.22%-80%)	2.2060 (1.8167-2.5899)
At least 1 of MutationTaster, PolyPhen-2, PROVEAN, VEST	62.6% (58.59%-66.16%)	89.29% (86.29%-92.62%)	16.44% (11.111%-21.127%)	64.88% (60.69%-68.93%)	47.01% (35.7%-59.26%)	2.1762 (1.9939-2.3614)	62.69% (55.56%-70.71%)	89.5% (83.04%-95.31%)	16.636% (6.452%-27.03%)	64.82% (56.78%-73.03%)	48.04% (23.06%-71.43%)	2.1900 (1.8208-2.5478)
At least 1 of MutationTaster, PolyPhen-2, SIFT	71.71% (68.18%-75.25%)	92.48% (90%-95.28%)	35.782% (29.412%-41.892%)	71.35% (67.3%-75.46%)	73.37% (65.71%-81.82%)	2.7298 (2.5752-2.8789)	71.74% (64.65%-78.79%)	92.69% (86.89%-98.15%)	35.803% (24.24%-48.78%)	71.24% (63.09%-79.27%)	74.14% (57.14%-90.49%)	2.7387 (2.4334-3.0341)
At least 1 of MutationTaster, PolyPhen-2, SIFT, VEST	64.32% (60.61%-68.18%)	92.48% (90%-95.28%)	15.617% (10.602%-20.29%)	65.46% (61.36%-69.54%)	54.59% (42.86%-66.67%)	2.2814 (2.1024-2.4561)	64.29% (56.57%-71.72%)	92.69% (86.89%-98.15%)	15.538% (5.882%-25.81%)	65.32% (57.3%-73.33%)	55.39% (28.57%-81.82%)	2.2893 (1.9076-2.6573)
At least 1 of MutationTaster, PolyPhen-2, VEST	63.94% (60.1%-67.68%)	89.29% (86.29%-92.62%)	20.098% (14.701%-25.352%)	65.9% (61.76%-70.06%)	52.03% (41.94%-62.96%)	2.2731 (2.0984-2.4454)	64.05% (56.57%-71.72%)	89.5% (83.04%-95.31%)	20.33% (10.343%-31.43%)	65.85% (57.64%-74.12%)	53.05% (30.77%-75%)	2.2873 (1.9337-2.6304)
At least 1 of MutationTaster, PROVEAN	77.45% (74.24%-80.81%)	87.17% (84.03%-90.63%)	60.629% (54.054%-67.165%)	79.29% (75.36%-83.22%)	73.21% (67.17%-79.66%)	3.0029 (2.8587-3.1518)	77.43% (70.71%-83.84%)	87.35% (80%-93.55%)	60.378% (47.365%-72.98%)	79.1% (71.21%-86.77%)	73.54% (60%-85.29%)	3.0037 (2.7004-3.2850)
At least 1 of MutationTaster, PROVEAN, SIFT	71.71% (68.18%-75.25%)	91.95% (89.39%-94.92%)	36.718% (30.378%-43.056%)	71.53% (67.53%-75.32%)	72.51% (64.71%-81.08%)	2.7271 (2.5782-2.8770)	71.73% (64.65%-78.79%)	92.16% (86.36%-96.97%)	36.637% (24.127%-50%)	71.4% (64.1%-79.31%)	73.18% (55.56%-88.89%)	2.7337 (2.4085-3.0300)
At least 1 of MutationTaster, PROVEAN, SIFT, VEST	63.31% (59.6%-67.17%)	91.95% (89.39%-94.92%)	13.777% (9.231%-18.056%)	64.84% (60.67%-68.85%)	49.75% (37.5%-62.5%)	2.2031 (2.0256-2.3859)	63.29% (55.56%-70.71%)	92.16% (86.36%-96.97%)	13.72% (5.402%-22.86%)	64.7% (56.67%-73.03%)	50.53% (24.93%-77.78%)	2.2111 (1.8227-2.5747)
At least 1 of MutationTaster, PROVEAN, VEST	61.92% (58.07%-65.67%)	87.68% (84.68%-91.13%)	17.366% (12.158%-22.368%)	64.72% (60.59%-68.93%)	44.9% (34.48%-56%)	2.1467 (1.9820-2.3229)	62.03% (54.52%-69.72%)	87.93% (80.95%-93.85%)	17.55% (7.687%-27.78%)	64.67% (56.1%-72.94%)	45.85% (23.08%-66.71%)	2.1600 (1.7951-2.5150)
At least 1 of MutationTaster, SIFT	74.06% (70.71%-77.27%)	91.95% (89.39%-94.92%)	43.125% (36.486%-50%)	73.65% (69.68%-77.42%)	75.61% (68.42%-83.33%)	2.8433 (2.6949-2.9888)	74.11% (67.68%-80.81%)	92.16% (86.36%-96.97%)	43.128% (30.227%-56.11%)	73.54% (65.82%-81.43%)	76.28% (61.11%-90.48%)	2.8511 (2.5579-3.1353)
At least 1 of MutationTaster, SIFT, VEST	64.32% (60.61%-68.18%)	91.95% (89.39%-94.92%)	16.543% (11.389%-21.212%)	65.58% (61.36%-69.77%)	54.31% (43.48%-66.67%)	2.2838 (2.1128-2.4647)	64.29% (56.57%-71.72%)	92.16% (86.36%-96.97%)	16.453% (6.891%-26.67%)	65.43% (57.14%-73.63%)	55.08% (30%-78.61%)	2.2913 (1.9201-2.6355)
At least 1 of MutationTaster, VEST	63.94% (60.1%-67.68%)	86.61% (83.46%-90.32%)	24.75% (19.177%-30.305%)	66.56% (62.26%-70.91%)	51.68% (42.42%-61.54%)	2.2959 (2.1329-2.4672)	64.03% (56.57%-71.72%)	86.88% (79.37%-93.44%)	24.802% (13.333%-36.36%)	66.47% (57.65%-74.68%)	52.45% (33.33%-71.43%)	2.3060 (1.9626-2.6374)

At least 1 of PolyPhen-2, PROVEAN	67.66% (64.14%-71.22%)	81.86% (78.12%-85.61%)	43.107% (36.617%-49.276%)	71.33% (67.12%-75.36%)	57.88% (50.88%-64.91%)	2.5417 (2.3866-2.7007)	67.71% (60.58%-74.75%)	82.01% (74.07%-89.23%)	43.104% (30.556%-56.67%)	71.22% (63.01%-80.01%)	58.27% (42.85%-72.73%)	2.5461 (2.2312-2.8539)
At least 1 of PolyPhen-2, PROVEAN, SIFT	67.32% (63.64%-71.21%)	88.24% (85%-91.6%)	31.146% (24.358%-36.986%)	68.91% (64.78%-72.79%)	60.5% (51.28%-68.97%)	2.4879 (2.3163-2.6506)	67.37% (59.6%-74.75%)	88.4% (81.43%-95%)	31.258% (19.442%-44.74%)	68.82% (60.96%-77.22%)	61.1% (43.74%-78.95%)	2.4957 (2.1623-2.8459)
At least 1 of PolyPhen-2, PROVEAN, SIFT, VEST	61.64% (57.58%-65.66%)	89.84% (86.82%-93.02%)	12.851% (8.451%-17.143%)	64.06% (59.78%-68.16%)	42.25% (30.43%-53.85%)	2.0901 (1.9198-2.2758)	61.58% (53.54%-69.7%)	89.98% (83.33%-95.65%)	12.806% (4.759%-21.88%)	63.91% (55.68%-72.22%)	42.7% (16.67%-66.67%)	2.0940 (1.7387-2.4735)
At least 1 of PolyPhen-2, PROVEAN, VEST	60.25% (56.57%-64.14%)	85.58% (82.35%-89.34%)	16.44% (11.111%-21.127%)	63.91% (59.76%-68.07%)	39.73% (29.17%-50%)	2.0566 (1.8834-2.2343)	60.31% (52.53%-67.68%)	85.74% (78.33%-92.19%)	16.636% (6.452%-27.03%)	63.84% (55.42%-72.09%)	40.48% (16.67%-61.54%)	2.0669 (1.7165-2.4164)
At least 1 of PolyPhen-2, SIFT	69.34% (65.66%-73.23%)	88.24% (85%-91.6%)	36.648% (29.87%-42.671%)	70.66% (66.47%-74.68%)	64.32% (56.1%-72.22%)	2.5987 (2.4336-2.7541)	69.41% (61.62%-76.77%)	88.4% (81.43%-95%)	36.811% (24.324%-50%)	70.59% (62.66%-78.75%)	64.93% (47.37%-80.95%)	2.6073 (2.2805-2.9216)
At least 1 of PolyPhen-2, SIFT, VEST	62.65% (59.08%-66.67%)	89.84% (86.82%-93.02%)	15.617% (10.602%-20.29%)	64.8% (60.59%-68.89%)	47.07% (35.98%-58.33%)	2.1733 (1.9920-2.3504)	62.58% (54.55%-69.72%)	89.98% (83.33%-95.65%)	15.538% (5.882%-25.81%)	64.64% (56.32%-72.94%)	47.49% (22.22%-70%)	2.1765 (1.8043-2.5455)
At least 1 of PolyPhen-2, VEST	61.26% (57.58%-65.15%)	85.07% (81.75%-88.64%)	20.098% (14.701%-25.352%)	64.8% (60.64%-69.05%)	43.77% (34.29%-53.33%)	2.1373 (1.9607-2.3069)	61.31% (53.54%-68.69%)	85.17% (77.59%-91.94%)	20.33% (10.343%-31.43%)	64.72% (56.24%-72.84%)	44.41% (23.53%-63.65%)	2.1463 (1.8003-2.4820)
At least 1 of PROVEAN, SIFT	67.99% (64.14%-71.72%)	82.95% (79.16%-86.67%)	42.109% (35.523%-48.685%)	71.25% (66.9%-75.5%)	58.81% (51.02%-65.46%)	2.5512 (2.3933-2.7096)	68.05% (60.61%-75.76%)	83.01% (75.41%-90.48%)	42.348% (28.205%-55.26%)	71.19% (63.01%-80%)	59.17% (44.44%-74.77%)	2.5572 (2.2242-2.8738)
At least 1 of PROVEAN, SIFT, VEST	60.63% (56.57%-64.65%)	87.2% (84%-90.63%)	14.679% (10.145%-19.403%)	63.86% (59.54%-68.02%)	39.86% (29.62%-50%)	2.0560 (1.9038-2.2246)	60.57% (52.53%-68.69%)	87.3% (80%-93.75%)	14.666% (5.556%-23.68%)	63.71% (55.29%-72.42%)	40.21% (18.18%-62.5%)	2.0589 (1.7109-2.3914)
At least 1 of PROVEAN, VEST	55.56% (51.52%-59.6%)	77.12% (73.08%-81.4%)	18.268% (13.043%-23.288%)	62% (57.6%-66.46%)	31.59% (23.25%-40%)	1.8898 (1.7359-2.0432)	55.55% (47.47%-63.64%)	77.13% (67.86%-85.25%)	18.497% (8.328%-29.41%)	61.89% (52.78%-70.89%)	32.01% (15%-48.05%)	1.8953 (1.5928-2.2067)
At least 1 of SIFT, VEST	61.3% (57.56%-65.16%)	86.66% (83.33%-90.08%)	17.445% (12.496%-22.222%)	64.48% (60.12%-68.79%)	43.05% (33.33%-53.33%)	2.1162 (1.9419-2.2797)	61.24% (53.51%-68.71%)	86.78% (79.66%-93.44%)	17.399% (7.895%-27.78%)	64.32% (55.81%-72.95%)	43.37% (22.2%-64.29%)	2.1187 (1.7694-2.4708)
At least 2 of CHASM (breast), CHASM (lung)	80.53% (77.27%-83.84%)	72.48% (67.97%-77.17%)	94.458% (91.667%-97.333%)	95.77% (93.68%-97.98%)	66.5% (61.17%-71.7%)	3.2920 (3.1827-3.4031)	80.35% (73.74%-86.87%)	72.06% (62.9%-81.03%)	94.56% (88.563%-100%)	95.79% (90.74%-100%)	66.36% (56%-77.09%)	3.2877 (3.0724-3.5155)
At least 2 of CHASM (breast), CHASM (lung), CHASM (melanoma)	81.89% (78.79%-84.85%)	76.73% (72.44%-80.99%)	90.818% (87.143%-94.667%)	93.53% (91.09%-96.23%)	69.3% (63.83%-74.5%)	3.3038 (3.1968-3.4199)	81.66% (75.76%-87.88%)	76.31% (67.74%-85.25%)	90.829% (82.857%-97.44%)	93.46% (87.71%-98.11%)	69.09% (58.33%-79.6%)	3.2968 (3.0590-3.5145)
At least 2 of CHASM (breast), CHASM (lung), CHASM (melanoma), FATHMM (cancer)	85.9% (83.33%-88.89%)	87.29% (84.03%-90.55%)	83.483% (78.873%-88.732%)	90.14% (87.4%-93.39%)	79.17% (74.03%-84.21%)	3.4008 (3.2883-3.5244)	85.78% (79.8%-90.91%)	87.1% (80.33%-93.55%)	83.488% (73.168%-92.5%)	90.05% (83.6%-95.45%)	79.06% (68.42%-89.19%)	3.3970 (3.1550-3.6247)
At least 2 of CHASM (breast), CHASM (lung), CHASM (melanoma), FATHMM (cancer), FATHMM (missense)	82.86% (79.8%-85.86%)	92.63% (90%-95.28%)	65.942% (59.994%-72.222%)	82.47% (79.26%-86.01%)	83.81% (78.57%-89.29%)	3.2485 (3.1251-3.3849)	82.77% (76.77%-88.89%)	92.38% (86.89%-97.1%)	66.23% (54.036%-78.38%)	82.45% (75%-89.04%)	83.54% (72.72%-93.75%)	3.2460 (2.9764-3.5006)
At least 2 of CHASM (breast), CHASM (lung), CHASM (melanoma), FATHMM (cancer), Mutation Assessor	84.88% (81.82%-87.88%)	93.65% (91.27%-96.06%)	69.71% (63.636%-75.676%)	84.24% (80.85%-87.77%)	86.41% (81.67%-91.53%)	3.3401 (3.2157-3.4608)	84.78% (78.79%-90.91%)	93.53% (88.52%-98.39%)	69.773% (58.065%-82.15%)	84.14% (77.02%-91.04%)	86.3% (75.86%-96.43%)	3.3375 (3.0857-3.5830)
At least 2 of CHASM (breast), CHASM (lung), CHASM (melanoma), FATHMM (cancer), MutationTaster	90.61% (88.38%-93.43%)	96.33% (94.49%-98.39%)	80.737% (75.713%-86.301%)	89.63% (86.95%-92.7%)	92.71% (89.06%-96.72%)	3.5940 (3.4975-3.7067)	90.49% (84.85%-94.95%)	96.17% (92.19%-100%)	80.73% (70.264%-90.92%)	89.54% (83.1%-95.31%)	92.48% (84.85%-100%)	3.5893 (3.3710-3.7848)
At least 2 of CHASM (breast), CHASM (lung), CHASM (melanoma), FATHMM (cancer), PolyPhen-2	83.2% (80.3%-86.36%)	95.26% (93.22%-97.58%)	62.355% (55.996%-68.75%)	81.4% (77.85%-85.14%)	88.38% (83.33%-93.75%)	3.2738 (3.1571-3.4054)	83.09% (76.77%-88.89%)	95.12% (90.62%-100%)	62.438% (50%-75%)	81.29% (73.68%-88.24%)	88.17% (77.27%-100%)	3.2703 (3.0043-3.5048)
At least 2 of CHASM (breast), CHASM (lung), CHASM (melanoma), FATHMM (cancer), PROVEAN	82.84% (79.8%-86.36%)	90.46% (87.6%-93.55%)	69.671% (63.765%-76.119%)	83.76% (80.15%-87.6%)	80.85% (75.41%-86.67%)	3.2474 (3.1125-3.3869)	82.8% (75.76%-88.89%)	90.35% (84.13%-95.59%)	69.825% (56.241%-81.82%)	83.71% (75.76%-90.91%)	80.83% (68.97%-91.43%)	3.2471 (2.9653-3.5148)
At least 2 of CHASM (breast), CHASM (lung), CHASM (melanoma), FATHMM (cancer), SIFT	81.16% (78.27%-84.34%)	94.2% (91.87%-96.75%)	58.612% (52.055%-64.939%)	79.74% (76.19%-83.56%)	85.39% (79.59%-91.67%)	3.1794 (3.0461-3.3141)	81.11% (74.75%-86.89%)	94.04% (89.06%-98.46%)	58.897% (45.714%-71.43%)	79.7% (71.83%-86.84%)	85.22% (73.08%-95.84%)	3.1786 (2.9015-3.4376)
At least 2 of CHASM (breast), CHASM (lung), CHASM (melanoma), FATHMM (cancer), VEST	79.81% (76.77%-82.83%)	95.25% (93.13%-97.56%)	53.114% (46.269%-59.46%)	77.84% (74.19%-81.76%)	86.61% (80.95%-92.86%)	3.1281 (2.9950-3.2572)	79.77% (73.74%-85.86%)	95.14% (90.74%-100%)	53.386% (40%-67.5%)	77.79% (70.13%-85.14%)	86.48% (73.91%-100%)	3.1279 (2.8523-3.3992)
At least 2 of CHASM (breast), CHASM (lung), CHASM (melanoma), FATHMM (missense)	84.89% (82.32%-87.88%)	87.85% (84.55%-91.13%)	79.778% (74.323%-85.138%)	88.25% (85.37%-91.67%)	79.15% (73.91%-84.51%)	3.3503 (3.2327-3.4766)	84.76% (78.79%-89.9%)	87.59% (80.95%-94.03%)	79.841% (68.571%-90%)	88.19% (81.53%-94.03%)	78.96% (68.42%-89.47%)	3.3458 (3.1018-3.5786)
At least 2 of CHASM (breast), CHASM (lung), CHASM (melanoma), FATHMM (missense), Mutation Assessor	83.21% (80.3%-86.36%)	92.63% (90%-95.2%)	66.918% (60.563%-73.611%)	82.88% (79.41%-86.57%)	84.02% (78.69%-89.47%)	3.2645 (3.1374-3.3920)	83.07% (76.77%-88.89%)	92.38% (87.1%-97.06%)	67.036% (54.054%-79.5%)	82.8% (75.35%-90%)	83.71% (72.41%-93.75%)	3.2592 (2.9987-3.5047)
At least 2 of CHASM (breast), CHASM (lung), CHASM (melanoma), FATHMM (missense), MutationTaster	88.64% (86.36%-91.41%)	95.31% (93.33%-97.6%)	77.081% (71.429%-82.857%)	87.8% (84.96%-90.91%)	90.49% (86.66%-94.83%)	3.5068 (3.4028-3.6192)	88.38% (82.83%-92.93%)	95% (90.48%-98.53%)	76.956% (65.714%-87.51%)	87.64% (81.35%-93.65%)	89.99% (81.48%-97.06%)	3.4959 (3.2732-3.7039)
At least 2 of CHASM (breast), CHASM (lung), CHASM (melanoma), FATHMM (missense), PolyPhen-2	81.9% (79.28%-84.86%)	93.71% (91.41%-96.12%)	61.476% (54.795%-68.001%)	80.8% (77.4%-84.51%)	84.98% (79.63%-90.57%)	3.2096 (3.0812-3.3455)	81.65% (75.73%-86.89%)	93.42% (88.14%-98.33%)	61.373% (48.387%-75%)	80.6% (73.33%-87.5%)	84.49% (72.73%-95.66%)	3.1988 (2.9253-3.4455)
At least 2 of CHASM (breast), CHASM (lung), CHASM (melanoma), FATHMM (missense), PROVEAN	82.53% (79.8%-85.86%)	89.97% (87.2%-93.08%)	69.653% (63.633%-75.676%)	83.68% (80.43%-87.41%)	80.08% (74.6%-86.15%)	3.2338 (3.1029-3.3678)	82.41% (75.76%-87.88%)	89.72% (83.33%-95.24%)	69.797% (57.895%-82.05%)	83.62% (76.19%-90.17%)	79.84% (67.74%-90.62%)	3.2298 (2.9653-3.4891)
At least 2 of CHASM (breast), CHASM (lung), CHASM (melanoma), FATHMM (missense), SIFT	81.52% (78.79%-84.85%)	92.63% (90%-95.2%)	62.298% (55.714%-68.921%)	80.95% (77.21%-84.62%)	83.03% (77.19%-88.89%)	3.1891 (3.0582-3.3309)	81.4% (74.75%-86.87%)	92.38% (87.1%-97.06%)	62.506% (48.779%-75.02%)	80.88% (73.53%-88.06%)	82.72% (70.37%-93.34%)	3.1849 (2.8952-3.4363)
At least 2 of CHASM (breast), CHASM (lung), CHASM (melanoma), FATHMM (missense), VEST	79.2% (75.76%-82.32%)	94.23% (92%-96.77%)	53.197% (46.749%-60%)	77.69% (74%-81.58%)	84.23% (78.57%-90.48%)	3.0935 (2.9561-3.2299)	78.97% (72.73%-85.86%)	93.96% (89.06%-98.41%)	53.169% (39.391%-66.67%)	77.51% (69.74%-84.72%)	83.7% (70.83%-95.45%)	3.0833 (2.8167-3.3494)
At least 2 of CHASM (breast), CHASM (lung), CHASM (melanoma), Mutation Assessor	87.26% (84.84%-89.9%)	89.45% (86.4%-92.68%)	83.478% (78.871%-88.314%)	90.35% (87.61%-93.39%)	82.07% (77.03%-87.01%)	3.4534 (3.3451-3.5684)	87.09% (81.82%-91.94%)	89.18% (83.05%-95.31%)	83.485% (73.684%-92.59%)	90.26% (84.12%-95.52%)	81.83% (71.88%-91.67%)	3.4475 (3.2206-3.6645)
At least 2 of CHASM (breast), CHASM (lung), CHASM (melanoma), Mutation Assessor, MutationTaster	89.26% (86.87%-91.92%)	95.76% (93.8%-97.71%)	78.001% (72.857%-83.562%)	88.27% (85.42%-91.49%)	91.43% (87.72%-95.38%)	3.5346 (3.4364-3.6419)	89.16% (83.84%-93.94%)	95.69% (91.3%-100%)	77.946% (66.646%-88.24%)	88.15% (81.54%-93.85%)	91.38% (82.76%-100%)	3.5318 (3.3193-3.7358)
At least 2 of CHASM (breast), CHASM (lung), CHASM (melanoma), Mutation Assessor, PolyPhen-2	82.18% (79.29%-85.35%)	95.23% (93.27%-97.58%)	59.61% (53.425%-66.177%)	80.3% (76.55%-84.14%)	87.87% (82.98%-93.62%)	3.2301 (3.1100-3.3565)	82.11% (75.76%-87.88%)	95.16% (90.62%-98.55%)	59.707% (46.87%-71.88%)	80.2% (72.72%-87.5%)	87.84% (76.19%-96.67%)	3.2291 (2.9811-3.4728)
At least 2 of CHASM (breast), CHASM (lung), CHASM (melanoma), Mutation Assessor, PROVEAN	84.22% (81.31%-86.87%)	92.59% (90%-95.2%)	69.725% (63.886%-75.362%)	84.1% (80.67%-87.5%)	84.49% (79.36%-90.16%)	3.3091 (3.1889-3.4337)	84.09% (78.79%-89.9%)	92.46% (87.1%-97.06%)	69.707% (58.06%-81.4%)	83.97% (77.42%-90.54%)	84.37% (73.08%-94.12%)	3.3051 (3.0508-3.5442)
At least 2 of CHASM (breast), CHASM (lung), CHASM (melanoma), Mutation Assessor, SIFT	81.85% (78.79%-85.35%)	95.23% (93.27%-97.58%)	58.698% (52.563%-65.219%)	79.95% (76.31%-83.66%)	87.7% (82.69%-93.33%)	3.2157 (3.0859-3.3484)	81.76% (74.75%-87.88%)	95.16% (90.62%-98.55%)	58.746% (45.946%-71.8%)	79.83% (72.37%-87.18%)	87.65% (76.19%-96.67%)	3.2139 (2.9568-3.4815)
At least 2 of CHASM (breast), CHASM (lung), CHASM (melanoma), Mutation Assessor, VEST	80.49% (77.27%-83.84%)	95.26% (93.39%-97.56%)	54.954% (48.57%-61.538%)	78.52% (74.83%-82.47%)	87.04% (82.05%-93.18%)	3.1578 (3.0298-3.2872)	80.43% (73.74%-86.87%)	95.1% (90.62%-98.51%)	55.261% (41.176%-67.74%)	78.47% (70.42%-85.92%)	86.82% (74.07%-96%)	3.1565 (2.8973-3.4080)
At least 2 of CHASM (breast), CHASM (lung), CHASM (melanoma), MutationTaster	89.28% (86.87%-91.92%)	90.5% (87.6%-93.55%)	87.16% (83.093%-92%)	92.42% (90%-95.24%)	84.14% (79.71%-88.75%)	3.5422 (3.4474-3.6566)	89.13% (83.84%-93.94%)	90.27% (84.13%-95.72%)	87.143% (77.143%-94.87%)	92.34% (86.36%-96.88%)	83.94% (74.36%-92.86%)	3.5368 (3.3110-3.7313)
At least 2 of CHASM (breast), CHASM (lung), CHASM (melanoma), MutationTaster, PolyPhen-2	87.28% (84.85%-89.9%)	97.9% (96.72%-99.24%)	68.932% (63.014%-75.325%)	84.49% (81.25%-88.11%)	95% (92.15%-98.25%)	3.4632 (3.3637-3.5646)	87.05% (81.82%-91.92%)	97.82% (94.64%-100%)	68.556% (56.25%-80.56%)	84.22% (77.03%-90.67%)	94.83% (86.96%-100%)	3.4542 (3.2437-3.6444)
At least 2 of CHASM (breast), CHASM (lung), CHASM (melanoma), MutationTaster, PROVEAN	89.6% (87.37%-91.92%)	93.66% (91.47%-96.06%)	82.558% (77.778%-87.671%)	90.28% (87.59%-93.13%)	88.29% (84.29%-92.75%)	3.5478 (3.4479-3.6562)	89.49% (84.85%-93.94%)	93.52% (88.52%-98.31%)	82.565% (72.222%-92.11%)	90.2% (84.38%-95.52%)	88.12% (78.78%-96.67%)	3.5441 (3.3275-3.7401)
At least 2 of CHASM (breast), CHASM (lung), CHASM (melanoma), MutationTaster, SIFT	87.26% (84.85%-89.91%)	96.83% (95.12%-98.49%)	70.709% (64.784%-76.812%)	85.11% (82.07%-88.65%)	92.81% (89.29%-96.61%)	3.4546 (3.3508-3.5717)	87.1% (81.79%-91.92%)	96.76% (92.85%-100%)	70.489% (58.327%-82.06%)	84.91% (77.92%-91.18%)	92.73% (84%-100%)	3.4490 (3.2260-3.6536)
At least 2 of CHASM (breast), CHASM (lung), CHASM (melanoma), MutationTaster, VEST	87.32% (84.85%-89.9%)	97.91% (96.72%-99.22%)	69.008% (63.014%-75.37%)	84.52% (81.33%-88.11%)	95.04% (92.16%-98.21%)	3.4648 (3.3636-3.5714)	86.98% (81.82%-91.92%)	97.79% (94.92%-100%)	68.418% (55.548%-80%)	84.15% (76.47%-90.54%)	94.76% (86.96%-100%)	3.4512 (3.2434-3.6552)
At least 2 of CHASM (breast), CHASM (lung), CHASM (melanoma), PolyPhen-2	83.57% (80.3%-86.87%)	88.9% (85.71%-92.06%)	74.35% (68.825%-80.282%)	85.7% (82.35%-89.39%)	79.49% (74.13%-85.07%)	3.2844 (3.1513-3.4241)	83.37% (76.77%-89.9%)	88.67% (82.14%-95.08%)	74.219% (62.848%-84.86%)	85.51% (78.08%-92.31%)	79.29% (67.65%-90.33%)	3.2769 (2.9967-3.5442)
At least 2 of CHASM (breast), CHASM (lung), CHASM (melanoma), PolyPhen-2, PROVEAN	81.19% (78.28%-84.34%)	92.07% (89.43%-94.82%)	62.37% (55.714%-68.921%)	80.88% (77.3%-84.72%)	81.97% (76.47%-88.14%)	3.1729 (3.0371-3.3188)	81.06% (74.75%-86.87%)	91.92% (86.36%-96.92%)	62.396% (48.649%-75%)	80.76% (73.13%-88.06%)	81.84% (68.18%-92.6%)	3.1691 (2.8706-3.4329)
At least 2 of CHASM (breast), CHASM (lung), CHASM (melanoma), PolyPhen-2, SIFT	80.8% (77.78%-83.84%)	96.29% (94.4%-98.39%)	54.023% (47.826%-60.811%)	78.36% (74.84%-82.17%)	89.38% (84.61%-95.12%)	3.1805 (3.0565-3.3036)	80.82% (74.75%-86.87%)	96.25% (92.19%-100%)	54.302% (41.173%-66.67%)	78.33% (70.66%-85.14%)	89.43% (78.26%-100%)	3.1831 (2.9388-3.4293)
At least 2 of CHASM (breast), CHASM (lung), CHASM (melanoma), PolyPhen-2, VEST	79.82% (76.77%-83.33%)	96.83% (95.2%-98.44%)	50.402% (43.662%-57.143%)	77.14% (73.54%-81.05%)	90.19% (85.37%-95.35%)	3.1456 (3.0241-3.2783)	79.76% (72.73%-85.86%)	96.76% (93.33%-100%)	50.569% (37.143%-63.16%)	77.06% (69.23%-84.15%)	90.06% (78.95%-100%)	3.1445 (2.8795-3.3838)
At least 2 of CHASM (breast), CHASM (lung), CHASM (melanoma), PROVEAN	83.2% (80.3%-86.36%)	81.48% (77.5%-85.6%)	86.166% (81.707%-90.667%)	91.06% (88.23%-93.97%)	72.91% (67.5%-78.41%)	3.3161 (3.2016-3.4445)	83.1% (76.77%-88.89%)	81.17% (72.88%-89.07%)	86.364% (77.143%-94.74%)	91.09% (84.62%-96.55%)	72.81% (61.9%-84.09%)	3.3143 (3.0590-3.5468)
At least 2 of CHASM (breast), CHASM (lung), CHASM (melanoma), PROVEAN, SIFT	80.52% (77.27%-83.84%)	92.59% (90%-95.2%)	59.628% (53.333%-66.216%)	79.86% (76.39%-83.57%)	82.32% (76.6%-88.89%)	3.1440 (3.0128-3.2878)	80.38% (73.74%-86.87%)	92.46% (87.1%-97.06%)	59.622% (46.322%-72.22%)	79.72% (72.22%-86.67%)	82.17% (68.97%-93.1%)	3.1397 (2.8559-3.4019)
At least 2 of CHASM (breast), CHASM (lung), CHASM (melanoma), PROVEAN, VEST	79.51% (76.26%-82.83%)	93.13% (90.76%-95.83%)	55.97% (49.315%-62.5%)	78.53% (74.83%-82.39%)	82.48% (76.78%-88.89%)	3.1011 (2.9673-3.2400)	79.36% (72.73%-85.86%)	93% (87.5%-98.25%)	55.902% (42.848%-68.75%)	78.36% (70.51%-85.53%)	82.3% (69.23%-93.76%)	3.0956 (2.8117-3.3612)
At least 2 of CHASM (breast), CHASM (lung), CHASM (melanoma), SIFT	84.57% (81.82%-87.37%)	89.97% (86.92%-92.97%)	75.241% (69.863%-81.252%)	86.27% (82.96%-89.76%)	81.27% (76.12%-86.57%)	3.3274 (3.2065-3.4554)	84.39% (78.79%-89.9%)	89.73% (83.33%-95.46%)	75.179% (63.889%-86.11%)	86.12% (79.1%-92.65%)	81.04% (70%-91.18%)	3.3207 (3.0661-3.5622)
At least 2 of CHASM (breast), CHASM (lung), CHASM (melanoma), SIFT, VEST	77.77% (74.75%-81.31%)	96.84% (95.2%-98.46%)	44.792% (38.571%-51.429%)	75.2% (71.52%-79.04%)	89.12% (83.33%-94.87%)	3.0596 (2.9412-3.1896)	77.8% (70.71%-83.84%)	96.75% (93.1%-100%)	45.264% (32.132%-58.14%)	75.2% (67.47%-82.43%)	89.02% (76.47%-100%)	3.0624 (2.8000-3.2976)
At least 2 of CHASM (breast), CHASM (lung), CHASM (melanoma), VEST	84.93% (82.32%-87.88%)	89.97% (86.99%-92.97%)	76.195% (71.014%-82.051%)	86.73% (83.72%-90.3%)	81.47% (76.47%-86.76%)	3.3436 (3.2224-3.4715)	84.69% (78.79%-89.9%)	89.72% (83.33%-95.39%)	76.035% (64.516%-86.67%)	86.54% (79.37%-92.54%)	81.19% (70.37%-91.18%)	3.3349 (3.0781-3.5747)
At least 2 of CHASM (breast), CHASM (lung), FATHMM (cancer)	82.57% (79.8%-85.86%)	78.33% (74.22%-82.44%)	89.913% (86.111%-94.118%)	93.07% (90.48%-96.15%)	70.58% (65.26%-75.87%)	3.3189 (3.2098-3.4442)	82.33% (75.76%-87.88%)	77.91% (69.64%-86.15%)	89.896% (81.579%-97.3%)	92.97% (87.04%-98.11%)	70.34% (59.57%-81.25%)	3.3112 (3.0644-3.5271)
At least 2 of CHASM (breast), CHASM (lung), FATHMM (cancer), FATHMM (missense)	83.87% (80.81%-86.87%)	90.52% (87.6%-93.7%)	72.374% (66.655%-78.67%)	85% (81.68%-88.64%)	81.54% (76.19%-86.96%)	3.2943 (3.1693-3.4191)	83.77% (77.78%-89.9%)	90.22% (83.82%-96.43%)	72.649% (60%-84.38%)	85% (77.94%-91.67%)	81.25% (70.58%-91.89%)	3.2912 (3.0415-3.5370)
At least 2 of CHASM (breast), CHASM (lung), FATHMM (cancer), FATHMM (missense), Mutation Assessor	82.79% (79.8%-85.86%)	94.74% (92.56%-96.88%)	62.133% (55.879%-68.919%)	81.22% (77.85%-84.93%)	87.23% (82%-92.31%)	3.2533 (3.1297-3.3837)	82.9% (76.77%-88.89%)	94.55% (90%-98.51%)	62.879% (50%-75.02%)	81.38% (73.97%-88.41%)	87.08% (76.92%-96.43%)	3.2590 (2.9950-3.5035)
At least 2 of CHASM (breast), CHASM (lung), FATHMM (cancer), FATHMM (missense), MutationTaster	87.22% (84.85%-89.9%)	96.86% (95.2%-98.48%)	70.534% (64.384%-76.391%)	85.04% (81.88%-88.28%)	92.86% (89.09%-96.61%)	3.4530 (3.3462-3.5637)	87.18% (81.82%-91.92%)	96.69% (92.86%-100%)	70.826% (58.333%-82.5%)	85.06% (78.57%-91.43%)	92.6% (84.38%-100%)	3.4518 (3.2274-3.6637)
At least 2 of CHASM (breast), CHASM (lung), FATHMM (cancer), FATHMM (missense), PolyPhen-2	81.81% (78.79%-84.85%)	95.26% (93.33%-97.58%)	58.535% (51.936%-65.219%)	79.89% (76.43%-83.54%)	87.73% (82.35%-93.48%)	3.2142 (3.0886-3.3455)	81.84% (75.76%-87.88%)	95.1% (90.62%-98.53%)	59.031% (45.708%-72.23%)	79.95% (72.6%-87.01%)	87.57% (76.92%-96.55%)	3.2165 (2.9607-3.4640)
At least 2 of CHASM (breast), CHASM (lung), FATHMM (cancer), FATHMM (missense), PROVEAN	81.45% (78.28%-84.36%)	93.12% (90.68%-95.93%)	61.262% (54.795%-68.057%)	80.61% (77.08%-84.4%)	83.75% (78%-89.48%)	3.1874 (3.0565-3.3250)	81.54% (75.73%-87.88%)	93% (87.69%-98.25%)	61.839% (47.5%-74.29%)	80.71% (72.97%-87.67%)	83.74% (72%-95.25%)	3.1929 (2.9112-3.4540)
At least 2 of CHASM (breast), CHASM (lung), FATHMM (cancer), FATHMM (missense), SIFT	82.49% (79.29%-85.35%)	95.28% (93.22%-97.58%)	60.371% (54.054%-67.106%)	80.61% (77.18%-84.35%)	88.1% (82.98%-93.75%)	3.2437 (3.1234-3.3675)	82.49% (76.77%-88.89%)	95.06% (90.62%-98.57%)	60.863% (47.497%-72.73%)	80.66% (73.23%-87.5%)	87.81% (76.92%-96.55%)	3.2439 (2.9888-3.4925)
At least 2 of CHASM (breast), CHASM (lung), FATHMM (cancer), FATHMM (missense), VEST	78.81% (75.76%-82.32%)	96.86% (95.2%-98.48%)	47.598% (40.58%-54.933%)	76.17% (72.67%-80.13%)	89.77% (84.21%-95.12%)	3.1040 (2.9715-3.2402)	78.74% (71.72%-84.85%)	96.69% (92.86%-100%)	47.888% (33.333%-61.54%)	76.11% (68.35%-83.12%)	89.4% (77.78%-100%)	3.1010 (2.8395-3.3530)
At least 2 of CHASM (breast), CHASM (lung), FATHMM (cancer), Mutation Assessor	84.53% (81.31%-87.37%)	89.95% (86.99%-93.02%)	75.173% (69.444%-80.887%)	86.23% (82.95%-89.84%)	81.23% (75.81%-86.57%)	3.3258 (3.1974-3.4503)	84.47% (78.79%-90.91%)	89.77% (83.33%-95.52%)	75.367% (64.516%-86.84%)	86.2% (78.87%-92.65%)	81.12% (70.59%-91.18%)	3.3246 (3.0755-3.5883)
At least 2 of CHASM (breast), CHASM (lung), FATHMM (cancer), Mutation Assessor, MutationTaster	88.91% (86.36%-91.41%)	98.44% (97.52%-100%)	72.442% (66.667%-78.125%)	86.06% (82.98%-89.59%)	96.4% (94.12%-100%)	3.5334 (3.4371-3.6307)	88.85% (83.84%-93.94%)	98.34% (95.45%-100%)	72.586% (61.29%-84.21%)	86.01% (78.79%-92.21%)	96.22% (90%-100%)	3.5316 (3.3422-3.7303)
At least 2 of CHASM (breast), CHASM (lung), FATHMM (cancer), Mutation Assessor, PolyPhen-2	83.5% (80.8%-86.36%)	96.84% (95.2%-98.48%)	60.444% (54.283%-66.667%)	80.89% (77.18%-84.46%)	91.71% (87.5%-96.08%)	3.2987 (3.1863-3.4142)	83.51% (77.78%-88.91%)	96.75% (92.98%-100%)	60.781% (48.569%-73.53%)	80.88% (73.61%-88.16%)	91.63% (81.48%-100%)	3.3004 (3.0677-3.5304)
At least 2 of CHASM (breast), CHASM (lung), FATHMM (cancer), Mutation Assessor, PROVEAN	82.48% (79.29%-85.86%)	92.05% (89.43%-94.7%)	65.938% (59.994%-71.831%)	82.37% (78.87%-85.92%)	82.75% (77.35%-88.24%)	3.2311 (3.1057-3.3661)	82.51% (75.76%-88.89%)	91.95% (86.15%-96.92%)	66.284% (54.28%-78.79%)	82.39% (75.32%-89.55%)	82.78% (71.43%-93.33%)	3.2341 (2.9626-3.4923)
At least 2 of CHASM (breast), CHASM (lung), FATHMM (cancer), Mutation Assessor, SIFT	81.79% (78.79%-84.85%)	95.78% (93.75%-97.67%)	57.61% (51.389%-63.889%)	79.62% (76.03%-83.34%)	88.76% (83.72%-93.88%)	3.2177 (3.0944-3.3433)	81.87% (75.76%-87.88%)	95.67% (91.52%-100%)	58.169% (45.161%-70.28%)	79.69% (72.15%-86.67%)	88.71% (77.77%-100%)	3.2223 (2.9705-3.4586)
At least 2 of CHASM (breast), CHASM (lung), FATHMM (cancer), Mutation Assessor, VEST	79.44% (76.26%-82.83%)	96.86% (95.16%-98.45%)	49.323% (43.028%-55.714%)	76.77% (73.25%-80.75%)	90.08% (85%-95.12%)	3.1303 (3.0053-3.2479)	79.5% (72.73%-85.86%)	96.7% (93.22%-100%)	49.984% (37.14%-63.64%)	76.83% (68.92%-84%)	89.85% (78.94%-100%)	3.1337 (2.8751-3.3816)
At least 2 of CHASM (breast), CHASM (lung), FATHMM (cancer), MutationTaster	90.96% (88.89%-93.43%)	93.67% (91.41%-96.09%)	86.277% (82.089%-91.026%)	92.19% (89.92%-95.08%)	88.75% (84.72%-93.06%)	3.6088 (3.5173-3.7129)	90.8% (85.86%-94.95%)	93.5% (88.33%-98.33%)	86.167% (76.917%-94.59%)	92.05% (86.2%-96.77%)	88.54% (80%-96.97%)	3.6026 (3.3970-3.7835)
At least 2 of CHASM (breast), CHASM (lung), FATHMM (cancer), MutationTaster, PolyPhen-2	86.23% (83.84%-88.9%)	97.9% (96.72%-99.22%)	66.046% (59.994%-72.154%)	83.29% (79.85%-86.99%)	94.8% (91.67%-98.11%)	3.4204 (3.3174-3.5303)	86.13% (80.78%-90.91%)	97.81% (94.92%-100%)	66.087% (54.28%-78.38%)	83.19% (75.71%-90%)	94.64% (86.96%-100%)	3.4172 (3.2031-3.6197)
At least 2 of CHASM (breast), CHASM (lung), FATHMM (cancer), MutationTaster, PROVEAN	86.87% (84.34%-89.9%)	94.71% (92.62%-96.9%)	73.323% (67.532%-79.412%)	85.99% (82.86%-89.44%)	88.91% (84.74%-93.44%)	3.4294 (3.3198-3.5501)	86.86% (80.81%-91.92%)	94.61% (89.83%-98.48%)	73.543% (62.069%-85%)	85.98% (79.1%-92.31%)	88.85% (79.31%-96.77%)	3.4298 (3.1891-3.6458)
At least 2 of CHASM (breast), CHASM (lung), FATHMM (cancer), MutationTaster, SIFT	86.82% (84.34%-89.39%)	98.96% (98.33%-100%)	65.842% (59.722%-71.878%)	83.36% (80.14%-86.93%)	97.33% (95.45%-100%)	3.4549 (3.3601-3.5571)	86.96% (81.82%-91.92%)	98.9% (96.61%-100%)	66.484% (54.28%-78.95%)	83.5% (76.31%-90.14%)	97.23% (91.3%-100%)	3.4612 (3.2621-3.6554)
At least 2 of CHASM (breast), CHASM (lung), FATHMM (cancer), MutationTaster, VEST	83.22% (80.3%-86.36%)	98.99% (98.33%-100%)	55.954% (49.296%-62.323%)	79.53% (75.97%-83.33%)	96.98% (94.74%-100%)	3.3146 (3.2131-3.4206)	83.05% (76.77%-88.89%)	98.82% (96.67%-100%)	55.983% (42.498%-68.58%)	79.39% (71.62%-86.59%)	96.54% (90%-100%)	3.3074 (3.1000-3.5067)
At least 2 of CHASM (breast), CHASM (lung), FATHMM (cancer), PolyPhen-2	84.19% (81.31%-87.37%)	92.07% (89.6%-95.12%)	70.551% (64.471%-76.812%)	84.39% (80.88%-88.24%)	83.73% (78.57%-89.23%)	3.3074 (3.1862-3.4366)	84.15% (77.78%-89.9%)	91.92% (85.94%-96.83%)	70.829% (57.895%-82.87%)	84.39% (76.71%-91.43%)	83.61% (72.73%-93.75%)	3.3075 (3.0414-3.5541)
At least 2 of CHASM (breast), CHASM (lung), FATHMM (cancer), PolyPhen-2, PROVEAN	82.14% (78.79%-85.35%)	93.11% (90.77%-95.97%)	63.173% (56.944%-69.866%)	81.38% (77.62%-85.11%)	84.14% (78.85%-90.38%)	3.2180 (3.0902-3.3551)	82.18% (75.76%-88.89%)	93.03% (87.5%-97.24%)	63.557% (50%-76.47%)	81.42% (73.91%-88.74%)	84.16% (72.41%-94.47%)	3.2216 (2.9465-3.4729)
At least 2 of CHASM (breast), CHASM (lung), FATHMM (cancer), PolyPhen-2, SIFT	79.75% (76.77%-82.83%)	97.34% (95.93%-99.2%)	49.32% (42.857%-55.409%)	76.86% (73.01%-80.63%)	91.49% (86.84%-97.37%)	3.1501 (3.0385-3.2700)	79.9% (73.74%-85.86%)	97.33% (93.65%-100%)	49.969% (37.5%-62.86%)	76.95% (69.51%-84.42%)	91.64% (80%-100%)	3.1588 (2.9162-3.3910)
At least 2 of CHASM (breast), CHASM (lung), FATHMM (cancer), PolyPhen-2, VEST	77.45% (74.24%-80.81%)	97.91% (96.69%-99.22%)	42.062% (35.714%-48.529%)	74.5% (70.99%-78.4%)	92.1% (87.49%-97.14%)	3.0657 (2.9530-3.1855)	77.43% (70.71%-83.84%)	97.79% (94.92%-100%)	42.461% (29.032%-55%)	74.47% (66.66%-81.48%)	91.85% (80%-100%)	3.0657 (2.8113-3.2885)
At least 2 of CHASM (breast), CHASM (lung), FATHMM (cancer), PROVEAN	81.19% (77.78%-84.34%)	84.15% (80.45%-88%)	76.074% (70.311%-81.821%)	85.88% (82.5%-89.52%)	73.52% (67.57%-79.27%)	3.1962 (3.0573-3.3330)	81.05% (74.75%-87.88%)	83.82% (76.18%-91.38%)	76.281% (64.516%-87.88%)	85.85% (78.33%-92.73%)	73.32% (61.11%-85.29%)	3.1927 (2.9211-3.4665)
At least 2 of CHASM (breast), CHASM (lung), FATHMM (cancer), PROVEAN, SIFT	80.78% (77.27%-84.34%)	92.59% (90.08%-95.31%)	60.346% (53.945%-67.568%)	80.15% (76.39%-83.92%)	82.49% (76.92%-88.68%)	3.1558 (3.0260-3.2977)	80.87% (73.74%-87.88%)	92.46% (86.76%-97.06%)	60.939% (47.054%-74.08%)	80.25% (72.46%-87.5%)	82.49% (70.37%-93.1%)	3.1614 (2.8713-3.4283)
At least 2 of CHASM (breast), CHASM (lung), FATHMM (cancer), PROVEAN, VEST	78.75% (75.76%-81.83%)	94.17% (91.94%-96.75%)	52.082% (44.869%-58.91%)	77.27% (73.51%-81.05%)	83.79% (77.78%-90%)	3.0731 (2.9360-3.2107)	78.86% (72.7%-84.85%)	94.09% (89.23%-98.44%)	52.664% (38.71%-66.67%)	77.34% (69.44%-84.62%)	83.86% (70.83%-95.65%)	3.0796 (2.7961-3.3529)
At least 2 of CHASM (breast), CHASM (lung), FATHMM (cancer), SIFT	83.14% (79.8%-86.36%)	91.01% (88.28%-94.26%)	69.518% (63.768%-75.723%)	83.77% (80.45%-87.41%)	81.74% (76.27%-87.33%)	3.2604 (3.1260-3.3981)	83.22% (76.77%-89.9%)	90.83% (84.61%-96.55%)	70.113% (57.5%-81.25%)	83.91% (76.66%-90.41%)	81.68% (70.97%-92.32%)	3.2654 (2.9888-3.5282)
At least 2 of CHASM (breast), CHASM (lung), FATHMM (cancer), SIFT, VEST	77.06% (73.74%-80.3%)	97.89% (96.72%-99.22%)	41.053% (34.328%-47.826%)	74.17% (70.55%-78.05%)	91.83% (86.67%-97.06%)	3.0494 (2.9330-3.1729)	77.19% (70.71%-83.84%)	97.84% (94.92%-100%)	41.752% (28.571%-54.84%)	74.24% (66.67%-81.48%)	91.85% (80%-100%)	3.0569 (2.8124-3.2927)
At least 2 of CHASM (breast), CHASM (lung), FATHMM (cancer), VEST	81.47% (78.28%-84.85%)	92.59% (90.08%-95.39%)	62.228% (55.556%-69.014%)	80.91% (77.3%-84.78%)	82.94% (77.36%-88.89%)	3.1867 (3.0489-3.3236)	81.51% (74.75%-87.88%)	92.46% (86.67%-97.02%)	62.68% (48.647%-75.76%)	80.96% (73.13%-88.06%)	82.89% (71.43%-93.55%)	3.1900 (2.9048-3.4729)
At least 2 of CHASM (breast), CHASM (lung), FATHMM (missense)	81.22% (77.78%-84.34%)	77.3% (72.87%-81.6%)	88.007% (84.211%-92.43%)	91.76% (88.89%-95%)	69.16% (63.63%-74.45%)	3.2622 (3.1424-3.3875)	80.99% (74.75%-87.88%)	76.77% (68.25%-85.49%)	88.199% (78.947%-96.78%)	91.77% (85.42%-97.92%)	68.89% (58.14%-80.01%)	3.2563 (3.0064-3.4988)
At least 2 of CHASM (breast), CHASM (lung), FATHMM (missense), Mutation Assessor	85.55% (82.83%-88.38%)	91.04% (88.28%-94.22%)	76.043% (70.311%-82.051%)	86.79% (83.72%-90.23%)	83.08% (78.12%-88.53%)	3.3696 (3.2562-3.5041)	85.47% (79.8%-90.91%)	90.77% (84.62%-96.61%)	76.331% (64.516%-87.18%)	86.82% (80%-92.99%)	82.86% (72.41%-93.33%)	3.3678 (3.0962-3.5836)
At least 2 of CHASM (breast), CHASM (lung), FATHMM (missense), Mutation Assessor, MutationTaster	87.89% (85.35%-90.4%)	97.38% (95.9%-99.2%)	71.472% (65.713%-77.143%)	85.51% (82.39%-88.89%)	94.05% (90.74%-98.11%)	3.4842 (3.3774-3.5924)	87.86% (82.83%-92.93%)	97.25% (93.65%-100%)	71.727% (60%-83.33%)	85.51% (78.79%-91.78%)	93.85% (86.36%-100%)	3.4834 (3.2659-3.6969)
At least 2 of CHASM (breast), CHASM (lung), FATHMM (missense), Mutation Assessor, PolyPhen-2	81.49% (78.28%-84.85%)	96.31% (94.4%-98.39%)	55.865% (48.718%-62.319%)	79.05% (75.33%-82.69%)	89.76% (85.11%-95.45%)	3.2099 (3.0830-3.3377)	81.46% (74.75%-87.88%)	96.2% (92.19%-100%)	56.143% (43.243%-70%)	79.01% (71.43%-86.3%)	89.63% (78.26%-100%)	3.2098 (2.9540-3.4657)
At least 2 of CHASM (breast), CHASM (lung), FATHMM (missense), Mutation Assessor, PROVEAN	83.82% (80.81%-86.87%)	93.66% (91.34%-96.06%)	66.792% (60.563%-73.239%)	82.99% (79.56%-86.33%)	85.91% (81.03%-91.23%)	3.2935 (3.1707-3.4227)	83.87% (77.78%-89.9%)	93.51% (88.52%-98.36%)	67.3% (54.286%-79.49%)	83.08% (76.12%-90%)	85.81% (75%-96%)	3.2970 (3.0397-3.5335)
At least 2 of CHASM (breast), CHASM (lung), FATHMM (missense), Mutation Assessor, SIFT	81.12% (77.78%-84.34%)	95.23% (93.27%-97.58%)	56.695% (50.633%-63.38%)	79.18% (75.5%-82.78%)	87.32% (82.22%-93.19%)	3.1842 (3.0585-3.3131)	81.2% (74.75%-87.88%)	95.16% (90.62%-98.55%)	57.224% (43.899%-70.01%)	79.24% (72%-86.67%)	87.37% (75%-96.56%)	3.1899 (2.9297-3.4410)
At least 2 of CHASM (breast), CHASM (lung), FATHMM (missense), Mutation Assessor, VEST	77.47% (74.24%-80.81%)	95.8% (93.7%-97.67%)	45.765% (39.7%-52.383%)	75.33% (71.6%-79.25%)	86.32% (80.49%-92.68%)	3.0323 (2.8957-3.1654)	77.38% (70.71%-83.84%)	95.61% (91.53%-100%)	46.078% (32.43%-58.63%)	75.26% (67.11%-82.72%)	85.98% (72.22%-100%)	3.0293 (2.7531-3.3036)
At least 2 of CHASM (breast), CHASM (lung), FATHMM (missense), MutationTaster	90.31% (88.38%-92.93%)	93.71% (91.27%-96.09%)	84.421% (79.994%-89.335%)	91.23% (88.72%-94.08%)	88.59% (84.72%-92.76%)	3.5795 (3.4859-3.6821)	90.09% (84.85%-93.94%)	93.42% (88.33%-98.39%)	84.344% (74.286%-93.55%)	91.11% (85.24%-96.37%)	88.22% (79.49%-96.67%)	3.5709 (3.3660-3.7531)
At least 2 of CHASM (breast), CHASM (lung), FATHMM (missense), MutationTaster, PolyPhen-2	85.92% (83.33%-88.89%)	98.44% (97.52%-100%)	64.251% (57.971%-70.594%)	82.64% (79.33%-86.27%)	95.98% (93.33%-100%)	3.4131 (3.3117-3.5192)	85.75% (79.8%-90.91%)	98.33% (95.31%-100%)	64.119% (51.429%-76.67%)	82.47% (75%-89.19%)	95.74% (88.89%-100%)	3.4066 (3.1927-3.6051)
At least 2 of CHASM (breast), CHASM (lung), FATHMM (missense), MutationTaster, PROVEAN	87.92% (85.35%-90.4%)	95.27% (93.33%-97.58%)	75.197% (69.442%-80.887%)	86.92% (83.82%-90.3%)	90.2% (86.15%-94.74%)	3.4759 (3.3656-3.5819)	87.79% (82.83%-92.93%)	95.08% (90.62%-98.53%)	75.235% (64.097%-86.67%)	86.84% (80.3%-92.86%)	89.93% (81.25%-97.14%)	3.4708 (3.2554-3.6881)
At least 2 of CHASM (breast), CHASM (lung), FATHMM (missense), MutationTaster, SIFT	86.54% (83.84%-89.39%)	98.45% (97.52%-100%)	65.947% (59.493%-72.222%)	83.33% (80%-86.67%)	96.09% (93.48%-100%)	3.4382 (3.3334-3.5438)	86.51% (80.81%-91.92%)	98.31% (95.24%-100%)	66.216% (52.941%-78.95%)	83.33% (76.71%-90%)	95.83% (88.89%-100%)	3.4369 (3.2219-3.6385)
At least 2 of CHASM (breast), CHASM (lung), FATHMM (missense), MutationTaster, VEST	82.9% (79.8%-85.86%)	98.99% (98.33%-100%)	55.079% (48.684%-61.649%)	79.21% (75.8%-82.89%)	96.94% (94.59%-100%)	3.3022 (3.2016-3.4069)	82.68% (76.77%-88.89%)	98.82% (96.67%-100%)	54.968% (41.929%-68.43%)	79.02% (71.43%-85.72%)	96.47% (89.47%-100%)	3.2928 (3.0897-3.4976)
At least 2 of CHASM (breast), CHASM (lung), FATHMM (missense), PolyPhen-2	83.23% (80.3%-86.36%)	91.57% (88.8%-94.4%)	68.806% (62.162%-75.362%)	83.55% (80.15%-87.31%)	82.53% (77.19%-87.93%)	3.2645 (3.1374-3.3988)	83.03% (76.77%-88.89%)	91.31% (85.71%-96.78%)	68.736% (55.556%-80.66%)	83.39% (75.76%-90.28%)	82.2% (71.42%-92.86%)	3.2564 (2.9928-3.4925)
At least 2 of CHASM (breast), CHASM (lung), FATHMM (missense), PolyPhen-2, PROVEAN	81.51% (78.79%-84.85%)	93.13% (90.7%-95.93%)	61.395% (54.791%-68.001%)	80.67% (77.21%-84.35%)	83.8% (78.18%-89.8%)	3.1900 (3.0599-3.3217)	81.43% (74.75%-87.88%)	92.97% (87.5%-98.25%)	61.547% (48.569%-74.43%)	80.6% (73.24%-87.67%)	83.63% (72.22%-94.74%)	3.1874 (2.9328-3.4364)
At least 2 of CHASM (breast), CHASM (lung), FATHMM (missense), PolyPhen-2, SIFT	79.77% (76.26%-82.83%)	97.36% (95.93%-99.19%)	49.346% (42.646%-56%)	76.87% (73.2%-80.39%)	91.55% (86.84%-97.37%)	3.1513 (3.0274-3.2676)	79.85% (73.74%-86.87%)	97.29% (93.75%-100%)	49.852% (36.111%-62.87%)	76.91% (69.73%-84%)	91.49% (80%-100%)	3.1554 (2.9188-3.3964)
At least 2 of CHASM (breast), CHASM (lung), FATHMM (missense), PolyPhen-2, VEST	77.8% (74.75%-80.82%)	98.45% (97.5%-100%)	42.097% (35.818%-48.53%)	74.62% (71.25%-78.57%)	94.01% (90%-100%)	3.0918 (2.9756-3.2015)	77.73% (71.69%-83.84%)	98.31% (95.31%-100%)	42.384% (29.624%-55.17%)	74.54% (66.67%-81.48%)	93.62% (81.82%-100%)	3.0886 (2.8596-3.3200)
At least 2 of CHASM (breast), CHASM (lung), FATHMM (missense), PROVEAN	81.89% (78.79%-85.35%)	84.19% (80.49%-87.9%)	77.899% (72.464%-83.333%)	86.82% (83.61%-90.32%)	74.03% (68.42%-79.71%)	3.2294 (3.0993-3.3653)	81.68% (74.75%-87.88%)	83.73% (76.27%-91.18%)	78.099% (66.667%-88.89%)	86.79% (79.69%-93.44%)	73.7% (62.16%-84.85%)	3.2232 (2.9548-3.4702)
At least 2 of CHASM (breast), CHASM (lung), FATHMM (missense), PROVEAN, SIFT	80.13% (76.77%-83.33%)	93.66% (91.34%-96.06%)	56.719% (50%-63.158%)	78.91% (75.18%-82.88%)	83.81% (78.18%-90%)	3.1311 (2.9945-3.2661)	80.14% (73.74%-86.87%)	93.51% (88.52%-98.36%)	57.171% (42.857%-70.59%)	78.94% (71.01%-86.49%)	83.69% (71.43%-95.45%)	3.1331 (2.8613-3.4032)
At least 2 of CHASM (breast), CHASM (lung), FATHMM (missense), PROVEAN, VEST	78.83% (75.76%-82.32%)	94.74% (92.56%-96.88%)	51.303% (44.775%-58.574%)	77.09% (73.55%-80.8%)	84.93% (79.07%-91.3%)	3.0806 (2.9463-3.2146)	78.71% (71.72%-84.85%)	94.56% (90%-98.51%)	51.449% (37.491%-64.29%)	76.99% (69.44%-84.21%)	84.63% (72%-95.46%)	3.0763 (2.8002-3.3485)
At least 2 of CHASM (breast), CHASM (lung), FATHMM (missense), SIFT	83.51% (80.81%-86.36%)	91.56% (88.8%-94.4%)	69.573% (63.633%-75.721%)	83.88% (80.45%-87.41%)	82.67% (77.42%-88.53%)	3.2769 (3.1518-3.4142)	83.49% (77.78%-89.9%)	91.33% (85.48%-96.78%)	69.971% (57.143%-82.35%)	83.94% (76.81%-90.77%)	82.49% (71.43%-93.33%)	3.2774 (2.9945-3.5217)
At least 2 of CHASM (breast), CHASM (lung), FATHMM (missense), SIFT, VEST	76.11% (72.73%-79.29%)	97.38% (95.93%-99.2%)	39.316% (32.857%-45.946%)	73.51% (69.88%-77.25%)	89.66% (83.87%-96.67%)	2.9986 (2.8656-3.1219)	76.07% (69.7%-82.83%)	97.26% (93.65%-100%)	39.678% (26.313%-52.78%)	73.46% (65.85%-80.49%)	89.39% (75%-100%)	2.9979 (2.7308-3.2614)
At least 2 of CHASM (breast), CHASM (lung), FATHMM (missense), VEST	81.19% (78.28%-84.34%)	92.63% (90.08%-95.24%)	61.403% (54.795%-68.003%)	80.58% (76.92%-84.29%)	82.82% (77.36%-88.46%)	3.1744 (3.0418-3.3071)	81.05% (74.75%-86.87%)	92.38% (87.1%-97.06%)	61.539% (48.387%-75%)	80.49% (73.24%-87.84%)	82.5% (70.37%-93.11%)	3.1691 (2.9055-3.4348)
At least 2 of CHASM (breast), CHASM (lung), Mutation Assessor	83.93% (81.31%-86.87%)	79.41% (75.2%-83.46%)	91.726% (88.235%-95.654%)	94.32% (91.96%-97.12%)	72.05% (66.67%-77.53%)	3.3750 (3.2691-3.4913)	83.66% (77.78%-88.89%)	78.93% (70.68%-87.04%)	91.768% (84.211%-97.67%)	94.27% (88.89%-98.31%)	71.74% (61.36%-82.36%)	3.3671 (3.1400-3.5798)
At least 2 of CHASM (breast), CHASM (lung), Mutation Assessor, MutationTaster	91.95% (89.9%-93.95%)	94.71% (92.56%-96.9%)	87.164% (83.333%-91.895%)	92.73% (90.48%-95.49%)	90.52% (86.76%-94.44%)	3.6512 (3.5626-3.7463)	91.86% (87.85%-95.96%)	94.6% (89.83%-98.51%)	87.151% (78.123%-94.59%)	92.66% (86.66%-96.97%)	90.43% (82.05%-97.37%)	3.6484 (3.4479-3.8254)
At least 2 of CHASM (breast), CHASM (lung), Mutation Assessor, MutationTaster, PolyPhen-2	85.24% (82.32%-87.88%)	97.37% (95.93%-99.2%)	64.262% (58.328%-70.59%)	82.49% (79.17%-85.91%)	93.39% (89.58%-97.96%)	3.3751 (3.2635-3.4853)	85.08% (79.8%-90.91%)	97.28% (93.65%-100%)	64.173% (51.282%-75.87%)	82.32% (75.36%-89.04%)	93.23% (84%-100%)	3.3701 (3.1509-3.5895)
At least 2 of CHASM (breast), CHASM (lung), Mutation Assessor, MutationTaster, PROVEAN	87.21% (84.85%-89.9%)	95.77% (93.7%-97.64%)	72.414% (66.667%-78.127%)	85.72% (82.61%-89.13%)	90.84% (86.67%-95%)	3.4475 (3.3370-3.5548)	87.19% (81.82%-91.92%)	95.68% (91.8%-100%)	72.606% (61.111%-84.62%)	85.7% (79.1%-92.19%)	90.76% (82.13%-100%)	3.4475 (3.2230-3.6675)
At least 2 of CHASM (breast), CHASM (lung), Mutation Assessor, MutationTaster, SIFT	85.18% (82.32%-87.88%)	96.3% (94.4%-98.39%)	65.963% (59.755%-72.063%)	83.03% (79.59%-86.52%)	91.16% (86.96%-96.08%)	3.3644 (3.2499-3.4856)	85.19% (79.8%-90.91%)	96.23% (92.19%-100%)	66.249% (54.286%-78.79%)	83.02% (76.06%-90.14%)	91.12% (81.81%-100%)	3.3661 (3.1341-3.5996)
At least 2 of CHASM (breast), CHASM (lung), Mutation Assessor, MutationTaster, VEST	84.22% (81.31%-87.37%)	98.41% (97.5%-100%)	59.685% (53.425%-66.217%)	80.84% (77.27%-84.56%)	95.61% (92.86%-100%)	3.3455 (3.2426-3.4556)	84.09% (77.78%-89.9%)	98.38% (95.31%-100%)	59.578% (46.429%-72.1%)	80.67% (73.23%-87.84%)	95.57% (87.5%-100%)	3.3420 (3.1243-3.5431)
At least 2 of CHASM (breast), CHASM (lung), Mutation Assessor, PolyPhen-2	84.19% (81.31%-86.88%)	93.65% (91.2%-96.12%)	67.842% (62.117%-73.772%)	83.42% (80%-86.86%)	86.08% (81.03%-91.38%)	3.3099 (3.1814-3.4316)	84.15% (78.76%-89.9%)	93.54% (88.24%-98.41%)	68.028% (55.874%-80.49%)	83.37% (76.39%-90.28%)	86.05% (75%-96.43%)	3.3099 (3.0586-3.5626)
At least 2 of CHASM (breast), CHASM (lung), Mutation Assessor, PolyPhen-2, PROVEAN	82.16% (78.79%-85.35%)	94.71% (92.5%-96.88%)	60.462% (54.283%-66.667%)	80.55% (77.03%-84.21%)	86.86% (81.63%-92.31%)	3.2257 (3.0973-3.3530)	82.15% (75.76%-88.89%)	94.62% (90%-98.53%)	60.757% (47.727%-74.19%)	80.52% (73.24%-87.5%)	86.85% (75%-96.43%)	3.2275 (2.9495-3.4875)
At least 2 of CHASM (breast), CHASM (lung), Mutation Assessor, PolyPhen-2, SIFT	80.1% (76.77%-83.33%)	96.29% (94.4%-98.39%)	52.108% (45.9%-58.578%)	77.66% (74.02%-81.41%)	89.03% (83.72%-95%)	3.1508 (3.0178-3.2847)	80.21% (73.74%-86.87%)	96.25% (92.19%-100%)	52.686% (40.539%-65.71%)	77.72% (70.13%-84.93%)	89.12% (77.27%-100%)	3.1578 (2.8801-3.4240)
At least 2 of CHASM (breast), CHASM (lung), Mutation Assessor, PolyPhen-2, VEST	79.11% (75.76%-82.32%)	96.8% (95.12%-98.45%)	48.512% (42.25%-54.931%)	76.47% (72.78%-80.13%)	89.76% (84.62%-95.24%)	3.1155 (2.9887-3.2474)	79.16% (72.73%-85.86%)	96.82% (93.22%-100%)	48.845% (35.714%-61.76%)	76.46% (68.82%-83.95%)	89.95% (77.27%-100%)	3.1208 (2.8501-3.3676)
At least 2 of CHASM (breast), CHASM (lung), Mutation Assessor, PROVEAN	81.88% (78.79%-84.85%)	85.22% (81.45%-89.06%)	76.092% (70.149%-81.579%)	86.04% (82.54%-89.34%)	74.86% (68.92%-80.77%)	3.2221 (3.0889-3.3606)	81.7% (75.76%-87.88%)	84.87% (77.05%-92.31%)	76.232% (65.209%-87.88%)	85.97% (79.31%-92.86%)	74.63% (63.04%-86.21%)	3.2170 (2.9478-3.4832)
At least 2 of CHASM (breast), CHASM (lung), Mutation Assessor, PROVEAN, SIFT	82.17% (79.29%-85.35%)	95.23% (93.27%-97.58%)	59.575% (53.03%-66.198%)	80.29% (76.67%-83.78%)	87.85% (82.98%-93.48%)	3.2295 (3.1029-3.3621)	82.13% (75.76%-87.88%)	95.16% (90.62%-98.55%)	59.739% (46.426%-72.73%)	80.22% (73.13%-87.67%)	87.82% (77.26%-96.78%)	3.2294 (2.9665-3.4873)
At least 2 of CHASM (breast), CHASM (lung), Mutation Assessor, PROVEAN, VEST	80.15% (76.77%-83.33%)	94.73% (92.62%-96.9%)	54.929% (48.611%-60.874%)	78.42% (74.67%-82.19%)	85.79% (80.49%-91.49%)	3.1387 (3.0105-3.2661)	80.1% (73.74%-86.87%)	94.56% (89.85%-98.48%)	55.267% (42.424%-67.57%)	78.38% (70.67%-86.16%)	85.59% (73.68%-95.66%)	3.1381 (2.8681-3.3957)
At least 2 of CHASM (breast), CHASM (lung), Mutation Assessor, SIFT	84.53% (81.31%-87.37%)	94.71% (92.56%-96.9%)	66.908% (60.87%-72.86%)	83.19% (79.72%-86.71%)	87.99% (83.33%-92.98%)	3.3280 (3.2046-3.4540)	84.48% (78.79%-90.91%)	94.6% (89.83%-98.51%)	67.098% (55.263%-79.31%)	83.14% (76.06%-90.14%)	87.91% (77.27%-96.67%)	3.3275 (3.0735-3.5755)
At least 2 of CHASM (breast), CHASM (lung), Mutation Assessor, SIFT, VEST	77.7% (74.24%-81.31%)	96.81% (95.16%-98.45%)	44.671% (38.459%-51.389%)	75.15% (71.34%-79.02%)	89% (83.32%-94.59%)	3.0563 (2.9326-3.2031)	77.93% (70.71%-84.85%)	96.81% (93.22%-100%)	45.556% (32.258%-58.82%)	75.31% (67.46%-83.12%)	89.26% (77.26%-100%)	3.0693 (2.7840-3.3159)
At least 2 of CHASM (breast), CHASM (lung), Mutation Assessor, VEST	82.86% (79.8%-85.86%)	94.21% (91.93%-96.75%)	63.228% (56.941%-69.333%)	81.58% (77.93%-85.33%)	86.35% (81.48%-91.84%)	3.2537 (3.1314-3.3837)	82.77% (76.77%-88.89%)	94.01% (89.09%-98.44%)	63.494% (51.429%-76.48%)	81.53% (73.91%-88.74%)	86.1% (75%-96.15%)	3.2513 (2.9959-3.5034)
At least 2 of CHASM (breast), CHASM (lung), MutationTaster	83.93% (81.31%-86.87%)	79.41% (75.2%-83.46%)	91.726% (88.235%-95.654%)	94.32% (91.96%-97.12%)	72.05% (66.67%-77.53%)	3.3750 (3.2691-3.4913)	83.66% (77.78%-88.89%)	78.93% (70.68%-87.04%)	91.768% (84.211%-97.67%)	94.27% (88.89%-98.31%)	71.74% (61.36%-82.36%)	3.3671 (3.1400-3.5798)
At least 2 of CHASM (breast), CHASM (lung), MutationTaster, PolyPhen-2	90.64% (88.38%-93.43%)	96.3% (94.44%-98.4%)	80.846% (76.056%-86.301%)	89.68% (86.96%-92.81%)	92.68% (89.06%-96.72%)	3.5950 (3.4993-3.6990)	90.44% (84.85%-94.95%)	96.22% (92.06%-100%)	80.536% (69.444%-89.74%)	89.44% (83.08%-94.52%)	92.57% (84.61%-100%)	3.5877 (3.3846-3.7809)
At least 2 of CHASM (breast), CHASM (lung), MutationTaster, PolyPhen-2, PROVEAN	85.21% (82.32%-88.38%)	96.3% (94.44%-98.4%)	66.033% (60.267%-72.464%)	83.06% (79.71%-86.81%)	91.18% (87.04%-96%)	3.3657 (3.2532-3.4835)	85.14% (78.79%-90.91%)	96.22% (92.06%-100%)	66.116% (53.841%-77.79%)	82.97% (75.68%-89.86%)	91.1% (80.95%-100%)	3.3641 (3.1303-3.5870)
At least 2 of CHASM (breast), CHASM (lung), MutationTaster, PolyPhen-2, SIFT	82.48% (79.8%-85.37%)	98.4% (97.52%-100%)	54.945% (48.571%-61.538%)	79.06% (75.66%-82.78%)	95.2% (92.11%-100%)	3.2760 (3.1666-3.3854)	82.52% (76.74%-87.88%)	98.42% (95.31%-100%)	55.23% (42.298%-67.74%)	79.05% (71.6%-85.9%)	95.31% (86.67%-100%)	3.2800 (3.0677-3.4975)
At least 2 of CHASM (breast), CHASM (lung), MutationTaster, PolyPhen-2, VEST	82.87% (80.3%-85.86%)	100% (100%-100%)	53.243% (46.872%-60%)	78.71% (75.16%-82.53%)	100% (100%-100%)	3.3196 (3.2345-3.4205)	82.75% (76.77%-88.89%)	100% (100%-100%)	53.153% (39.474%-65.52%)	78.55% (71.05%-85.71%)	100% (100%-100%)	3.3170 (3.1300-3.4958)
At least 2 of CHASM (breast), CHASM (lung), MutationTaster, PROVEAN	87.95% (85.35%-90.4%)	86.28% (82.81%-89.85%)	90.841% (87.319%-94.667%)	94.22% (92.04%-96.64%)	79.29% (74.12%-84.53%)	3.5063 (3.4047-3.6133)	87.74% (82.83%-92.93%)	85.95% (78.79%-93.1%)	90.777% (83.323%-97.44%)	94.12% (88.71%-98.31%)	79.03% (68.42%-89.36%)	3.4987 (3.2799-3.7079)
At least 2 of CHASM (breast), CHASM (lung), MutationTaster, PROVEAN, SIFT	85.53% (82.83%-88.38%)	96.84% (95.16%-98.45%)	65.973% (59.994%-72.226%)	83.11% (79.71%-86.77%)	92.35% (88.46%-96.36%)	3.3827 (3.2716-3.5003)	85.51% (79.8%-90.91%)	96.75% (93.22%-100%)	66.221% (53.645%-78.79%)	83.09% (76%-90.14%)	92.23% (83.33%-100%)	3.3829 (3.1564-3.6074)
At least 2 of CHASM (breast), CHASM (lung), MutationTaster, PROVEAN, VEST	84.93% (82.32%-87.88%)	98.45% (97.52%-100%)	61.547% (55.224%-68.001%)	81.57% (78.23%-85.03%)	95.84% (93.02%-100%)	3.3741 (3.2782-3.4812)	84.69% (78.79%-89.9%)	98.31% (95.38%-100%)	61.302% (48.482%-73.33%)	81.34% (74.32%-88%)	95.49% (88%-100%)	3.3643 (3.1483-3.5582)
At least 2 of CHASM (breast), CHASM (lung), MutationTaster, SIFT	91.95% (89.9%-93.94%)	96.3% (94.4%-98.39%)	84.419% (80%-89.339%)	91.44% (88.98%-94.29%)	92.96% (89.55%-96.83%)	3.6512 (3.5640-3.7456)	91.87% (87.88%-95.96%)	96.23% (92.19%-100%)	84.375% (74.982%-93.55%)	91.35% (85.25%-96.55%)	92.9% (85.7%-100%)	3.6485 (3.4633-3.8216)
At least 2 of CHASM (breast), CHASM (lung), MutationTaster, SIFT, VEST	80.47% (77.27%-83.84%)	98.92% (98.33%-100%)	48.556% (42.25%-55.074%)	76.87% (73.33%-80.63%)	96.3% (93.75%-100%)	3.2066 (3.1014-3.3180)	80.48% (73.74%-87.87%)	98.96% (96.67%-100%)	48.775% (36.106%-61.12%)	76.82% (69.13%-83.95%)	96.47% (88.24%-100%)	3.2102 (2.9907-3.4202)
At least 2 of CHASM (breast), CHASM (lung), MutationTaster, VEST	90.99% (88.89%-93.43%)	97.91% (96.72%-99.22%)	79.016% (74.286%-84.618%)	88.97% (86.15%-92.2%)	95.64% (93.1%-98.41%)	3.6154 (3.5249-3.7195)	90.75% (85.86%-94.95%)	97.79% (94.92%-100%)	78.696% (67.74%-87.88%)	88.72% (81.96%-94.2%)	95.42% (88.89%-100%)	3.6062 (3.4097-3.7848)
At least 2 of CHASM (breast), CHASM (lung), PolyPhen-2	82.92% (79.8%-85.86%)	79.41% (75.2%-83.46%)	88.988% (85.29%-93.336%)	92.57% (89.91%-95.5%)	71.43% (66.28%-77.01%)	3.3240 (3.2157-3.4451)	82.64% (76.77%-88.89%)	78.93% (70.68%-87.04%)	89.007% (80.554%-97.06%)	92.48% (86.2%-98.08%)	71.12% (60.47%-81.64%)	3.3153 (3.0724-3.5336)
At least 2 of CHASM (breast), CHASM (lung), PolyPhen-2, PROVEAN	81.52% (78.28%-84.85%)	86.28% (82.81%-89.85%)	73.29% (67.557%-79.71%)	84.81% (81.25%-88.71%)	75.54% (69.86%-81.69%)	3.1992 (3.0597-3.3409)	81.41% (74.75%-87.88%)	85.95% (78.79%-93.1%)	73.597% (61.29%-85%)	84.82% (77.42%-91.67%)	75.33% (63.41%-87.1%)	3.1969 (2.9110-3.4623)
At least 2 of CHASM (breast), CHASM (lung), PolyPhen-2, PROVEAN, SIFT	80.47% (77.27%-83.84%)	95.76% (93.7%-97.73%)	54.027% (47.434%-60.564%)	78.27% (74.68%-82.12%)	88.05% (82.93%-93.75%)	3.1610 (3.0311-3.2996)	80.48% (73.74%-86.87%)	95.71% (91.07%-100%)	54.322% (41.461%-67.5%)	78.24% (70.51%-85.53%)	88.08% (76.19%-100%)	3.1634 (2.8747-3.4086)
At least 2 of CHASM (breast), CHASM (lung), PolyPhen-2, PROVEAN, VEST	77.13% (73.74%-80.3%)	95.77% (93.89%-97.73%)	44.902% (38.462%-51.351%)	75.03% (71.34%-78.98%)	86.01% (80%-92.86%)	3.0172 (2.8865-3.1541)	77.04% (70.71%-83.84%)	95.69% (91.07%-100%)	45.018% (32.498%-57.9%)	74.92% (67.46%-82.28%)	85.92% (71.43%-100%)	3.0154 (2.7306-3.2700)
At least 2 of CHASM (breast), CHASM (lung), PolyPhen-2, SIFT	82.12% (79.29%-85.35%)	95.22% (93.28%-97.58%)	59.47% (52.857%-65.763%)	80.25% (76.67%-83.89%)	87.8% (82.98%-93.62%)	3.2273 (3.1029-3.3671)	82.22% (75.76%-88.89%)	95.19% (90.62%-98.55%)	59.933% (47.054%-72.73%)	80.3% (72.86%-86.96%)	87.92% (76.92%-96.55%)	3.2334 (2.9480-3.4873)
At least 2 of CHASM (breast), CHASM (lung), PolyPhen-2, SIFT, VEST	76.73% (73.23%-80.3%)	97.32% (95.93%-99.2%)	41.133% (34.286%-47.503%)	74.08% (70.3%-78.05%)	89.87% (83.87%-96.77%)	3.0241 (2.8852-3.1576)	76.84% (69.7%-83.84%)	97.38% (93.65%-100%)	41.6% (29.412%-55%)	74.1% (66.24%-81.71%)	90.24% (77.78%-100%)	3.0332 (2.7614-3.2898)
At least 2 of CHASM (breast), CHASM (lung), PolyPhen-2, VEST	80.49% (77.27%-83.84%)	95.23% (93.33%-97.6%)	55.012% (48.571%-61.765%)	78.54% (74.83%-82.5%)	86.97% (81.63%-93.19%)	3.1576 (3.0298-3.2928)	80.42% (73.74%-86.87%)	95.17% (90.48%-98.53%)	55.107% (41.667%-66.69%)	78.43% (70.83%-85.53%)	86.93% (75%-96.15%)	3.1564 (2.8791-3.4080)
At least 2 of CHASM (breast), CHASM (lung), PROVEAN	81.87% (78.79%-84.85%)	75.66% (71.2%-79.85%)	92.613% (89.394%-96.053%)	94.65% (92.31%-97.17%)	68.76% (63.54%-74.23%)	3.3168 (3.2122-3.4349)	81.71% (75.76%-87.88%)	75.27% (66.67%-84.21%)	92.743% (85.364%-100%)	94.68% (88.89%-100%)	68.62% (57.99%-79.25%)	3.3131 (3.0777-3.5283)
At least 2 of CHASM (breast), CHASM (lung), PROVEAN, SIFT	79.5% (76.26%-82.83%)	85.74% (82.11%-89.43%)	68.705% (62.667%-74.648%)	82.57% (78.85%-86.36%)	73.59% (67.16%-80%)	3.1060 (2.9645-3.2507)	79.39% (72.73%-85.86%)	85.43% (78.12%-92.76%)	68.998% (57.133%-81.82%)	82.54% (74.99%-90.16%)	73.42% (60.71%-85.72%)	3.1038 (2.8055-3.3830)
At least 2 of CHASM (breast), CHASM (lung), PROVEAN, SIFT, VEST	77.43% (74.24%-80.81%)	96.31% (94.44%-98.39%)	44.784% (38.356%-51.389%)	75.1% (71.34%-78.88%)	87.52% (81.25%-94.12%)	3.0371 (2.9068-3.1729)	77.46% (70.71%-83.84%)	96.21% (92.18%-100%)	45.263% (32.351%-57.89%)	75.1% (67.5%-82.36%)	87.44% (75%-100%)	3.0401 (2.7532-3.2985)
At least 2 of CHASM (breast), CHASM (lung), PROVEAN, VEST	79.52% (76.26%-82.83%)	85.74% (82.11%-89.43%)	68.768% (62.662%-75%)	82.6% (78.91%-86.36%)	73.61% (67.19%-80.01%)	3.1071 (2.9709-3.2547)	79.34% (72.73%-85.86%)	85.43% (78.12%-92.76%)	68.845% (56.757%-80.65%)	82.48% (74.6%-89.55%)	73.38% (60.59%-85.72%)	3.1013 (2.8166-3.3711)
At least 2 of CHASM (breast), CHASM (lung), SIFT	82.57% (79.8%-85.86%)	79.41% (75.2%-83.46%)	88.036% (84.207%-92.107%)	91.98% (89.28%-94.74%)	71.21% (65.55%-76.77%)	3.3064 (3.1914-3.4315)	82.33% (75.76%-87.88%)	78.93% (70.68%-87.04%)	88.143% (79.412%-96.88%)	91.94% (85.71%-97.92%)	70.91% (60.42%-81.64%)	3.2992 (3.0464-3.5320)
At least 2 of CHASM (breast), CHASM (lung), SIFT, VEST	80.45% (77.77%-83.84%)	96.31% (94.44%-98.39%)	53.044% (46.667%-59.747%)	78% (74.51%-81.82%)	89.26% (84.09%-95.12%)	3.1661 (3.0431-3.2927)	80.51% (73.74%-85.88%)	96.21% (92.18%-100%)	53.54% (40%-65.81%)	78.03% (70.51%-85%)	89.19% (78.26%-100%)	3.1697 (2.9055-3.4080)
At least 2 of CHASM (breast), CHASM (lung), VEST	81.91% (78.79%-84.86%)	78.89% (74.8%-83.07%)	87.122% (83.099%-91.781%)	91.37% (88.57%-94.59%)	70.47% (65.11%-76.09%)	3.2785 (3.1632-3.4044)	81.64% (75.73%-87.88%)	78.38% (69.84%-86.77%)	87.229% (77.769%-94.87%)	91.32% (84.48%-96.55%)	70.16% (59.52%-81.25%)	3.2709 (3.0181-3.5043)
At least 2 of CHASM (breast), CHASM (melanoma)	79.17% (75.76%-82.34%)	70.85% (66.38%-75.59%)	93.547% (90.411%-97.183%)	95% (92.55%-97.8%)	64.99% (59.62%-70.37%)	3.2438 (3.1290-3.3603)	79.04% (72.7%-85.86%)	70.53% (60.94%-79.42%)	93.62% (86.842%-100%)	95% (89.58%-100%)	64.93% (54.16%-75.93%)	3.2408 (3.0156-3.4711)
At least 2 of CHASM (breast), CHASM (melanoma), FATHMM (cancer)	85.21% (82.32%-87.89%)	84.62% (80.99%-88.52%)	86.218% (81.818%-91.304%)	91.39% (88.79%-94.6%)	76.44% (71.25%-81.93%)	3.3867 (3.2765-3.5056)	85.13% (79.77%-90.91%)	84.46% (76.92%-91.43%)	86.249% (76.667%-94.74%)	91.34% (85.18%-96.55%)	76.39% (65.21%-86.84%)	3.3845 (3.1457-3.5985)
At least 2 of CHASM (breast), CHASM (melanoma), FATHMM (cancer), FATHMM (missense)	82.86% (79.8%-85.86%)	91.03% (88.23%-93.75%)	68.704% (62.667%-74.691%)	83.42% (80.14%-87.02%)	81.59% (76.56%-87.1%)	3.2475 (3.1203-3.3753)	82.77% (76.77%-88.89%)	90.79% (85.25%-96.61%)	68.953% (56.25%-81.09%)	83.4% (76.12%-90.17%)	81.37% (70%-92.31%)	3.2451 (2.9903-3.5042)
At least 2 of CHASM (breast), CHASM (melanoma), FATHMM (cancer), FATHMM (missense), Mutation Assessor	82.83% (79.8%-85.86%)	96.34% (94.49%-98.43%)	59.458% (53.028%-65.753%)	80.43% (76.87%-84.03%)	90.39% (86%-95.65%)	3.2662 (3.1456-3.3888)	82.83% (76.77%-88.89%)	96.14% (91.8%-100%)	59.939% (47.054%-72.98%)	80.47% (72.97%-87.85%)	90.06% (80%-100%)	3.2661 (3.0150-3.5097)
At least 2 of CHASM (breast), CHASM (melanoma), FATHMM (cancer), FATHMM (missense), MutationTaster	86.22% (83.84%-88.89%)	97.93% (96.72%-99.24%)	65.947% (59.722%-72.059%)	83.26% (80.14%-86.67%)	94.87% (91.67%-98.15%)	3.4201 (3.3149-3.5269)	86.15% (80.81%-90.91%)	97.74% (94.74%-100%)	66.211% (53.328%-78.38%)	83.25% (76.39%-89.71%)	94.47% (86.96%-100%)	3.4168 (3.1961-3.6172)
At least 2 of CHASM (breast), CHASM (melanoma), FATHMM (cancer), FATHMM (missense), PolyPhen-2	81.5% (78.28%-84.85%)	97.4% (95.93%-99.19%)	54.003% (47.222%-60.563%)	78.55% (75%-82.24%)	92.31% (88.09%-97.5%)	3.2227 (3.1061-3.3420)	81.44% (74.75%-87.88%)	97.22% (93.85%-100%)	54.32% (41.379%-68.29%)	78.52% (71.42%-85.9%)	91.92% (81.82%-100%)	3.2198 (2.9746-3.4456)
At least 2 of CHASM (breast), CHASM (melanoma), FATHMM (cancer), FATHMM (missense), PROVEAN	80.79% (77.78%-83.84%)	93.65% (91.27%-96.09%)	58.55% (51.89%-64.874%)	79.62% (76%-83.44%)	84.21% (78.43%-90.2%)	3.1602 (3.0219-3.2957)	80.84% (74.75%-86.87%)	93.55% (88.4%-98.39%)	58.98% (45.714%-72.23%)	79.67% (71.83%-87.18%)	84.22% (72%-96%)	3.1642 (2.8911-3.4438)
At least 2 of CHASM (breast), CHASM (melanoma), FATHMM (cancer), FATHMM (missense), SIFT	81.19% (78.28%-84.34%)	96.88% (95.16%-98.5%)	54.038% (47.826%-60.811%)	78.47% (74.83%-82.24%)	90.94% (86.36%-95.65%)	3.2034 (3.0785-3.3275)	81.06% (74.75%-86.87%)	96.64% (92.73%-100%)	54.268% (40.623%-66.69%)	78.39% (70.89%-85.71%)	90.42% (80%-100%)	3.1973 (2.9480-3.4415)
At least 2 of CHASM (breast), CHASM (melanoma), FATHMM (cancer), FATHMM (missense), VEST	76.8% (73.74%-80.3%)	97.93% (96.72%-99.24%)	40.255% (33.784%-47.297%)	73.92% (70.3%-77.78%)	91.86% (86.67%-97.06%)	3.0397 (2.9182-3.1613)	76.7% (69.7%-82.83%)	97.74% (94.74%-100%)	40.541% (26.316%-54.05%)	73.84% (65.88%-81.25%)	91.26% (78.94%-100%)	3.0338 (2.7763-3.2748)
At least 2 of CHASM (breast), CHASM (melanoma), FATHMM (cancer), Mutation Assessor	85.19% (82.32%-87.88%)	93.11% (90.55%-95.87%)	71.51% (65.714%-77.612%)	84.96% (81.56%-88.41%)	85.72% (80.95%-90.91%)	3.3530 (3.2314-3.4783)	85.17% (79.8%-90.91%)	93.03% (87.5%-98.36%)	71.656% (59.986%-83.78%)	84.92% (77.77%-91.55%)	85.73% (75%-96.3%)	3.3534 (3.0975-3.6074)
At least 2 of CHASM (breast), CHASM (melanoma), FATHMM (cancer), Mutation Assessor, MutationTaster	87.56% (84.85%-90.4%)	98.44% (97.52%-100%)	68.765% (63.01%-74.675%)	84.49% (81.29%-87.84%)	96.22% (93.75%-100%)	3.4791 (3.3796-3.5777)	87.5% (81.82%-92.93%)	98.34% (95.45%-100%)	68.903% (57.143%-80.65%)	84.43% (77.46%-90.79%)	96.03% (89.47%-100%)	3.4771 (3.2766-3.6712)
At least 2 of CHASM (breast), CHASM (melanoma), FATHMM (cancer), Mutation Assessor, PolyPhen-2	82.51% (79.79%-85.86%)	97.9% (96.72%-99.24%)	55.905% (49.367%-62.691%)	79.33% (75.68%-83.23%)	93.91% (90.24%-97.83%)	3.2704 (3.1605-3.3850)	82.45% (75.76%-87.9%)	97.82% (94.64%-100%)	56.094% (42.498%-69.05%)	79.26% (71.6%-86.49%)	93.75% (84.21%-100%)	3.2692 (3.0357-3.4823)
At least 2 of CHASM (breast), CHASM (melanoma), FATHMM (cancer), Mutation Assessor, PROVEAN	82.49% (79.8%-85.35%)	94.17% (91.94%-96.75%)	62.285% (55.714%-68.919%)	81.19% (77.78%-84.67%)	86.07% (81.13%-92%)	3.2371 (3.1135-3.3637)	82.5% (76.77%-87.88%)	94.11% (89.23%-98.44%)	62.561% (49.968%-75.68%)	81.18% (73.97%-88.07%)	86.1% (74.07%-96.15%)	3.2395 (2.9845-3.4859)
At least 2 of CHASM (breast), CHASM (melanoma), FATHMM (cancer), Mutation Assessor, SIFT	80.49% (77.27%-83.84%)	96.31% (94.4%-98.4%)	53.126% (46.573%-59.42%)	78.03% (74.49%-81.82%)	89.28% (84.44%-95.24%)	3.1675 (3.0468-3.2927)	80.44% (73.74%-86.87%)	96.2% (92.06%-100%)	53.372% (40.623%-65.87%)	77.97% (70.51%-84.93%)	89.17% (77.27%-100%)	3.1671 (2.9111-3.4081)
At least 2 of CHASM (breast), CHASM (melanoma), FATHMM (cancer), Mutation Assessor, VEST	77.45% (74.24%-80.81%)	96.86% (95.16%-98.45%)	43.898% (37.349%-50%)	74.91% (71.26%-78.88%)	88.99% (83.78%-94.6%)	3.0465 (2.9193-3.1769)	77.42% (70.71%-83.84%)	96.7% (93.22%-100%)	44.315% (31.429%-57.5%)	74.87% (67.04%-82.15%)	88.69% (76.47%-100%)	3.0458 (2.7817-3.2898)
At least 2 of CHASM (breast), CHASM (melanoma), FATHMM (cancer), MutationTaster	90.93% (88.89%-93.43%)	95.78% (93.75%-97.64%)	82.537% (77.778%-88.001%)	90.46% (87.79%-93.57%)	91.87% (88.06%-95.52%)	3.6065 (3.5080-3.7110)	90.87% (85.86%-94.95%)	95.68% (91.8%-100%)	82.613% (72.214%-91.89%)	90.43% (84.06%-95.59%)	91.76% (84%-100%)	3.6048 (3.3992-3.7950)
At least 2 of CHASM (breast), CHASM (melanoma), FATHMM (cancer), MutationTaster, PolyPhen-2	84.24% (81.31%-87.37%)	98.44% (97.52%-100%)	59.704% (53.326%-66.216%)	80.86% (77.33%-84.62%)	95.67% (92.86%-100%)	3.3466 (3.2413-3.4602)	84.04% (77.78%-89.9%)	98.34% (95.45%-100%)	59.492% (46.667%-72.23%)	80.64% (73.33%-87.81%)	95.44% (88%-100%)	3.3391 (3.1156-3.5459)
At least 2 of CHASM (breast), CHASM (melanoma), FATHMM (cancer), MutationTaster, PROVEAN	86.52% (83.84%-89.39%)	96.3% (94.44%-98.36%)	69.609% (63.377%-76.056%)	84.57% (81.21%-88.36%)	91.58% (87.5%-96.16%)	3.4205 (3.3084-3.5387)	86.56% (80.81%-91.92%)	96.23% (92.42%-100%)	69.941% (57.143%-82.35%)	84.61% (77.46%-91.18%)	91.53% (82.6%-100%)	3.4231 (3.1847-3.6393)
At least 2 of CHASM (breast), CHASM (melanoma), FATHMM (cancer), MutationTaster, SIFT	83.84% (80.81%-86.87%)	98.96% (98.33%-100%)	57.714% (51.388%-64.286%)	80.18% (76.73%-83.66%)	96.97% (94.87%-100%)	3.3382 (3.2344-3.4443)	83.83% (77.78%-89.9%)	98.9% (96.61%-100%)	57.961% (45.157%-70.59%)	80.14% (72.84%-87.32%)	96.84% (90%-100%)	3.3384 (3.1385-3.5401)
At least 2 of CHASM (breast), CHASM (melanoma), FATHMM (cancer), MutationTaster, VEST	81.23% (78.28%-84.34%)	98.99% (98.33%-100%)	50.535% (44.155%-56.949%)	77.58% (74.02%-81.53%)	96.67% (94.12%-100%)	3.2377 (3.1369-3.3480)	80.97% (74.75%-86.87%)	98.82% (96.67%-100%)	50.308% (37.5%-62.86%)	77.34% (69.51%-84.53%)	96.16% (88.89%-100%)	3.2263 (3.0163-3.4251)
At least 2 of CHASM (breast), CHASM (melanoma), FATHMM (cancer), PolyPhen-2	83.2% (80.3%-86.36%)	94.71% (92.5%-96.88%)	63.293% (56.962%-69.622%)	81.69% (78.23%-85.42%)	87.37% (82.35%-92.6%)	3.2706 (3.1542-3.4028)	83.1% (76.77%-88.89%)	94.62% (90%-98.53%)	63.315% (50%-75.68%)	81.57% (74.32%-88.57%)	87.29% (76.19%-96.55%)	3.2680 (2.9928-3.5069)
At least 2 of CHASM (breast), CHASM (melanoma), FATHMM (cancer), PolyPhen-2, PROVEAN	81.5% (78.28%-84.85%)	95.23% (93.08%-97.56%)	57.76% (51.351%-64.388%)	79.58% (75.81%-83.22%)	87.51% (82.35%-93.48%)	3.2008 (3.0768-3.3321)	81.44% (74.75%-87.88%)	95.18% (90.62%-100%)	57.861% (44.444%-70.97%)	79.5% (72.22%-87.01%)	87.5% (76%-100%)	3.2003 (2.9310-3.4466)
At least 2 of CHASM (breast), CHASM (melanoma), FATHMM (cancer), PolyPhen-2, SIFT	79.44% (76.26%-82.32%)	98.41% (97.52%-100%)	46.625% (40.541%-52.941%)	76.12% (72.39%-79.87%)	94.43% (90.91%-100%)	3.1559 (3.0501-3.2668)	79.51% (73.74%-85.86%)	98.4% (95.31%-100%)	47.087% (34.286%-60%)	76.14% (68.67%-83.75%)	94.49% (84.21%-100%)	3.1611 (2.9409-3.3816)
At least 2 of CHASM (breast), CHASM (melanoma), FATHMM (cancer), PolyPhen-2, VEST	77.12% (73.74%-80.3%)	98.44% (97.5%-100%)	40.242% (34.247%-46.778%)	74.01% (70.41%-78.11%)	93.73% (89.29%-100%)	3.0643 (2.9506-3.1762)	77.08% (70.71%-83.84%)	98.32% (95.45%-100%)	40.595% (27.578%-52.78%)	73.96% (65.88%-81.18%)	93.38% (81.82%-100%)	3.0626 (2.8291-3.2867)
At least 2 of CHASM (breast), CHASM (melanoma), FATHMM (cancer), PROVEAN	82.48% (79.29%-85.86%)	88.84% (85.83%-92.07%)	71.471% (65.332%-77.778%)	84.34% (80.85%-88.15%)	78.74% (72.97%-84.62%)	3.2339 (3.0993-3.3756)	82.52% (75.76%-88.89%)	88.8% (82.14%-94.92%)	71.709% (58.818%-83.33%)	84.35% (76.92%-91.18%)	78.86% (66.67%-89.66%)	3.2372 (2.9526-3.4966)
At least 2 of CHASM (breast), CHASM (melanoma), FATHMM (cancer), PROVEAN, SIFT	79.82% (76.77%-82.83%)	94.71% (92.56%-96.97%)	54.078% (47.367%-60.87%)	78.1% (74.5%-82%)	85.54% (80%-91.67%)	3.1242 (2.9927-3.2572)	79.75% (73.74%-85.86%)	94.62% (89.7%-98.51%)	54.206% (40%-67.5%)	78.01% (70.27%-85.33%)	85.45% (72.22%-95.84%)	3.1228 (2.8447-3.3830)
At least 2 of CHASM (breast), CHASM (melanoma), FATHMM (cancer), PROVEAN, VEST	76.78% (73.74%-80.3%)	95.76% (93.75%-97.69%)	43.947% (37.139%-50.65%)	74.71% (70.99%-78.53%)	85.7% (79.41%-92.11%)	3.0012 (2.8676-3.1347)	76.75% (69.7%-82.83%)	95.71% (91.38%-100%)	44.155% (30.764%-57.14%)	74.64% (66.67%-81.93%)	85.73% (71.43%-100%)	3.0023 (2.7129-3.2699)
At least 2 of CHASM (breast), CHASM (melanoma), FATHMM (cancer), SIFT	81.84% (78.79%-84.85%)	93.65% (91.2%-96.15%)	61.404% (54.93%-67.606%)	80.75% (77.24%-84.4%)	84.84% (79.59%-90.74%)	3.2064 (3.0776-3.3358)	81.78% (75.76%-87.88%)	93.54% (88.24%-98.41%)	61.563% (48.571%-74.29%)	80.69% (73.61%-87.69%)	84.78% (73.08%-95.83%)	3.2057 (2.9465-3.4706)
At least 2 of CHASM (breast), CHASM (melanoma), FATHMM (cancer), SIFT, VEST	74.39% (71.21%-77.78%)	97.89% (96.72%-99.22%)	33.757% (27.778%-40.278%)	71.87% (68.23%-75.72%)	90.24% (84%-96.55%)	2.9375 (2.8108-3.0667)	74.45% (67.68%-80.81%)	97.84% (94.92%-100%)	34.307% (21.044%-45.95%)	71.88% (64.29%-79.07%)	90.25% (75%-100%)	2.9427 (2.6661-3.1853)
At least 2 of CHASM (breast), CHASM (melanoma), FATHMM (cancer), VEST	79.82% (76.77%-82.83%)	94.17% (91.74%-96.72%)	54.99% (48%-61.765%)	78.34% (74.67%-82.19%)	84.52% (79.07%-90.48%)	3.1203 (2.9837-3.2517)	79.76% (73.74%-85.86%)	94.09% (89.23%-98.44%)	55.139% (41.935%-68.42%)	78.26% (70.13%-85.53%)	84.49% (72.71%-95.65%)	3.1198 (2.8444-3.3910)
At least 2 of CHASM (breast), CHASM (melanoma), FATHMM (missense)	83.53% (80.81%-86.36%)	84.07% (80.62%-88%)	82.589% (77.778%-87.671%)	89.31% (86.32%-92.5%)	74.99% (69.74%-80.77%)	3.3095 (3.1878-3.4324)	83.45% (77.78%-88.89%)	83.98% (76.19%-91.23%)	82.471% (72.222%-92.11%)	89.18% (82.81%-95%)	75.01% (63.16%-85.71%)	3.3065 (3.0564-3.5439)
At least 2 of CHASM (breast), CHASM (melanoma), FATHMM (missense), Mutation Assessor	83.24% (80.3%-86.36%)	91.58% (88.8%-94.4%)	68.794% (62.5%-75.325%)	83.54% (79.86%-87.12%)	82.55% (77.42%-88.14%)	3.2647 (3.1378-3.3843)	83.02% (76.77%-88.89%)	91.28% (85.51%-96.78%)	68.789% (55.556%-81.82%)	83.4% (76.12%-90.63%)	82.17% (71.42%-92.59%)	3.2564 (3.0101-3.4975)
At least 2 of CHASM (breast), CHASM (melanoma), FATHMM (missense), Mutation Assessor, MutationTaster	85.57% (82.83%-88.38%)	97.91% (96.69%-99.24%)	64.209% (57.971%-70.588%)	82.55% (79.19%-86.18%)	94.69% (91.49%-98.08%)	3.3936 (3.2844-3.5044)	85.43% (79.8%-90.91%)	97.78% (94.64%-100%)	64.213% (51.513%-76.67%)	82.43% (75.34%-89.19%)	94.44% (86.36%-100%)	3.3886 (3.1654-3.6073)
At least 2 of CHASM (breast), CHASM (melanoma), FATHMM (missense), Mutation Assessor, PolyPhen-2	80.81% (77.78%-83.84%)	96.31% (94.4%-98.39%)	54.011% (47.295%-60.811%)	78.36% (74.66%-81.94%)	89.45% (84.62%-95.24%)	3.1813 (3.0560-3.3086)	80.8% (74.75%-86.87%)	96.2% (92.19%-100%)	54.326% (40.625%-67.5%)	78.33% (71.05%-85.71%)	89.33% (77.78%-100%)	3.1819 (2.9187-3.4239)
At least 2 of CHASM (breast), CHASM (melanoma), FATHMM (missense), Mutation Assessor, PROVEAN	81.53% (78.28%-84.34%)	94.21% (91.94%-96.77%)	59.574% (52.857%-66.198%)	80.12% (76.51%-83.68%)	85.63% (80.39%-91.67%)	3.1954 (3.0679-3.3185)	81.39% (75.76%-87.88%)	94.01% (89.06%-98.44%)	59.693% (45.714%-72.98%)	80.02% (72.6%-87.5%)	85.33% (73.08%-95.83%)	3.1905 (2.9248-3.4510)
At least 2 of CHASM (breast), CHASM (melanoma), FATHMM (missense), Mutation Assessor, SIFT	79.44% (76.26%-82.83%)	95.23% (93.27%-97.58%)	52.136% (45.714%-58.904%)	77.48% (73.82%-81.29%)	86.36% (80.84%-92.68%)	3.1121 (2.9835-3.2425)	79.49% (72.73%-85.86%)	95.16% (90.62%-98.55%)	52.576% (38.71%-64.87%)	77.5% (69.86%-85%)	86.4% (73.68%-96.43%)	3.1164 (2.8478-3.3876)
At least 2 of CHASM (breast), CHASM (melanoma), FATHMM (missense), Mutation Assessor, VEST	76.11% (72.73%-79.31%)	96.34% (94.49%-98.39%)	41.125% (34.937%-47.826%)	73.88% (70%-77.78%)	86.66% (80.56%-93.94%)	2.9800 (2.8496-3.1159)	76.07% (69.67%-82.83%)	96.15% (92.15%-100%)	41.582% (28.56%-54.29%)	73.85% (65.82%-81.25%)	86.28% (71.43%-100%)	2.9786 (2.6901-3.2469)
At least 2 of CHASM (breast), CHASM (melanoma), FATHMM (missense), MutationTaster	88.66% (86.36%-90.91%)	94.26% (92%-96.75%)	78.958% (73.846%-84.376%)	88.57% (85.82%-91.73%)	88.85% (84.75%-93.34%)	3.5064 (3.3975-3.6145)	88.34% (83.84%-92.93%)	93.9% (89.23%-98.41%)	78.709% (67.852%-88.89%)	88.36% (81.97%-93.94%)	88.28% (79.31%-96.67%)	3.4925 (3.2736-3.7035)
At least 2 of CHASM (breast), CHASM (melanoma), FATHMM (missense), MutationTaster, PolyPhen-2	84.57% (81.82%-87.37%)	98.97% (98.33%-100%)	59.663% (53.425%-66.198%)	80.93% (77.48%-84.62%)	97.11% (95%-100%)	3.3667 (3.2688-3.4734)	84.39% (78.79%-89.9%)	98.86% (96.67%-100%)	59.52% (46.667%-72.22%)	80.74% (73.42%-87.5%)	96.84% (90.48%-100%)	3.3596 (3.1509-3.5526)
At least 2 of CHASM (breast), CHASM (melanoma), FATHMM (missense), MutationTaster, PROVEAN	86.6% (83.84%-89.39%)	96.35% (94.44%-98.39%)	69.718% (63.636%-75.642%)	84.62% (81.56%-88.03%)	91.72% (87.76%-96.23%)	3.4241 (3.3096-3.5347)	86.4% (80.81%-91.92%)	96.11% (92.19%-100%)	69.657% (57.576%-81.26%)	84.49% (77.63%-90.77%)	91.28% (82.61%-100%)	3.4153 (3.1847-3.6447)
At least 2 of CHASM (breast), CHASM (melanoma), FATHMM (missense), MutationTaster, SIFT	84.53% (81.82%-87.37%)	98.98% (98.32%-100%)	59.544% (53.125%-65.789%)	80.88% (77.42%-84.31%)	97.13% (95%-100%)	3.3653 (3.2675-3.4733)	84.47% (78.79%-89.9%)	98.85% (96.61%-100%)	59.748% (46.87%-72.23%)	80.83% (73.68%-88%)	96.84% (90%-100%)	3.3627 (3.1547-3.5630)
At least 2 of CHASM (breast), CHASM (melanoma), FATHMM (missense), MutationTaster, VEST	80.89% (77.78%-83.84%)	98.99% (98.33%-100%)	49.578% (43.051%-56.004%)	77.24% (73.62%-81.01%)	96.61% (93.94%-100%)	3.2242 (3.1230-3.3236)	80.65% (74.75%-86.87%)	98.82% (96.67%-100%)	49.433% (37.032%-62.86%)	77.03% (69.51%-83.96%)	96.09% (88.24%-100%)	3.2138 (2.9996-3.4205)
At least 2 of CHASM (breast), CHASM (melanoma), FATHMM (missense), PolyPhen-2	81.91% (78.79%-84.85%)	92.66% (90.08%-95.28%)	63.301% (56.757%-69.863%)	81.37% (77.94%-84.93%)	83.31% (77.96%-88.89%)	3.2064 (3.0723-3.3435)	81.64% (75.76%-87.88%)	92.32% (86.89%-97.01%)	63.217% (50%-76.32%)	81.18% (73.91%-88.06%)	82.78% (71.43%-93.1%)	3.1950 (2.9206-3.4506)
At least 2 of CHASM (breast), CHASM (melanoma), FATHMM (missense), PolyPhen-2, PROVEAN	81.2% (78.28%-84.34%)	94.75% (92.68%-96.92%)	57.757% (51.351%-64.103%)	79.51% (76%-82.91%)	86.43% (81.24%-92.16%)	3.1845 (3.0561-3.3085)	81.03% (74.75%-86.87%)	94.53% (89.65%-98.46%)	57.794% (45.153%-71.44%)	79.38% (72.22%-86.67%)	86.05% (73.91%-96%)	3.1775 (2.9112-3.4399)
At least 2 of CHASM (breast), CHASM (melanoma), FATHMM (missense), PolyPhen-2, SIFT	78.78% (75.76%-81.82%)	97.36% (95.93%-99.19%)	46.634% (39.994%-53.03%)	75.93% (72.22%-79.4%)	91.1% (86.11%-97.22%)	3.1103 (2.9818-3.2344)	78.8% (72.73%-84.85%)	97.29% (93.75%-100%)	47.026% (34.286%-60.98%)	75.92% (68.42%-83.13%)	91.03% (80%-100%)	3.1126 (2.8524-3.3675)
At least 2 of CHASM (breast), CHASM (melanoma), FATHMM (missense), PolyPhen-2, VEST	76.78% (73.23%-80.3%)	98.98% (98.32%-100%)	38.393% (32.096%-44.29%)	73.53% (70.06%-77.38%)	95.61% (92.3%-100%)	3.0651 (2.9510-3.1732)	76.74% (69.7%-83.84%)	98.85% (96.67%-100%)	38.777% (26.667%-51.62%)	73.48% (65.48%-80.49%)	95.13% (85.71%-100%)	3.0623 (2.8414-3.2800)
At least 2 of CHASM (breast), CHASM (melanoma), FATHMM (missense), PROVEAN	82.19% (79.29%-85.35%)	88.34% (85.12%-91.8%)	71.53% (65.753%-77.465%)	84.29% (81.06%-87.79%)	78.01% (72.46%-83.88%)	3.2218 (3.0930-3.3607)	82.09% (75.76%-87.88%)	88.2% (81.54%-94.83%)	71.551% (59.459%-83.33%)	84.2% (77.27%-90.91%)	77.95% (66.64%-89.66%)	3.2191 (2.9392-3.4792)
At least 2 of CHASM (breast), CHASM (melanoma), FATHMM (missense), PROVEAN, SIFT	78.82% (75.76%-82.32%)	94.21% (91.94%-96.77%)	52.197% (45.714%-58.904%)	77.31% (73.68%-81.05%)	83.92% (78.05%-90.48%)	3.0764 (2.9425-3.2152)	78.72% (71.72%-84.85%)	94.01% (89.06%-98.44%)	52.441% (38.889%-64.87%)	77.24% (69.44%-84.42%)	83.61% (70%-95.45%)	3.0730 (2.7816-3.3454)
At least 2 of CHASM (breast), CHASM (melanoma), FATHMM (missense), PROVEAN, VEST	76.48% (73.23%-79.8%)	95.82% (93.84%-97.69%)	43.043% (36.483%-50%)	74.42% (70.81%-78.32%)	85.62% (79.41%-92.31%)	2.9890 (2.8431-3.1283)	76.32% (69.7%-82.83%)	95.59% (91.38%-100%)	43.186% (29.933%-55.56%)	74.29% (66.25%-81.48%)	85.09% (70.59%-100%)	2.9815 (2.6901-3.2538)
At least 2 of CHASM (breast), CHASM (melanoma), FATHMM (missense), SIFT	81.18% (78.28%-84.34%)	91.58% (88.8%-94.4%)	63.184% (56.756%-69.842%)	81.14% (77.53%-84.67%)	81.28% (75.86%-87.27%)	3.1719 (3.0422-3.3083)	81.07% (74.75%-86.87%)	91.28% (85.51%-96.78%)	63.476% (50%-75.77%)	81.11% (73.85%-88.41%)	80.95% (69.23%-92.01%)	3.1681 (2.8794-3.4251)
At least 2 of CHASM (breast), CHASM (melanoma), FATHMM (missense), SIFT, VEST	74.1% (70.71%-77.78%)	97.91% (96.69%-99.23%)	32.922% (27.027%-39.19%)	71.62% (67.88%-75.58%)	90.11% (83.33%-96.55%)	2.9256 (2.7904-3.0603)	74.03% (66.67%-80.81%)	97.79% (94.83%-100%)	33.216% (20.69%-45.45%)	71.53% (63.73%-78.82%)	89.77% (75%-100%)	2.9231 (2.6405-3.1689)
At least 2 of CHASM (breast), CHASM (melanoma), FATHMM (missense), VEST	78.53% (75.25%-81.82%)	92.66% (90.15%-95.24%)	54.083% (47.495%-60.812%)	77.73% (74%-81.7%)	81% (75%-87.51%)	3.0547 (2.9120-3.1879)	78.29% (71.72%-84.85%)	92.32% (87.03%-97.01%)	54.139% (40.464%-66.69%)	77.57% (69.86%-84.81%)	80.43% (66.67%-92.31%)	3.0445 (2.7684-3.3255)
At least 2 of CHASM (breast), CHASM (melanoma), Mutation Assessor	86.23% (83.84%-88.89%)	86.22% (82.68%-89.63%)	86.255% (81.944%-90.792%)	91.56% (88.98%-94.55%)	78.35% (73.33%-83.33%)	3.4238 (3.3195-3.5343)	86.12% (80.81%-90.91%)	86.07% (79.3%-93.1%)	86.178% (77.412%-94.59%)	91.45% (85.45%-96.61%)	78.3% (67.65%-88.65%)	3.4200 (3.1958-3.6294)
At least 2 of CHASM (breast), CHASM (melanoma), Mutation Assessor, MutationTaster	89.93% (87.88%-92.42%)	95.76% (93.8%-97.71%)	79.843% (75%-85.294%)	89.15% (86.33%-92.42%)	91.61% (88.06%-95.52%)	3.5636 (3.4704-3.6667)	89.83% (84.85%-93.94%)	95.69% (91.3%-100%)	79.762% (69.048%-89.2%)	89.02% (82.75%-94.52%)	91.56% (82.85%-100%)	3.5604 (3.3505-3.7455)
At least 2 of CHASM (breast), CHASM (melanoma), Mutation Assessor, MutationTaster, PolyPhen-2	84.23% (81.31%-87.37%)	97.9% (96.72%-99.24%)	60.599% (54.409%-67.124%)	81.12% (77.63%-84.67%)	94.35% (90.91%-98%)	3.3397 (3.2350-3.4583)	84.06% (77.78%-89.9%)	97.82% (94.64%-100%)	60.468% (47.368%-72.5%)	80.93% (73.42%-88%)	94.18% (85.7%-100%)	3.3339 (3.0937-3.5391)
At least 2 of CHASM (breast), CHASM (melanoma), Mutation Assessor, MutationTaster, PROVEAN	85.56% (82.83%-88.38%)	96.3% (94.44%-98.39%)	66.975% (60.976%-73.077%)	83.45% (80.13%-86.81%)	91.3% (87.23%-96.15%)	3.3802 (3.2718-3.4941)	85.45% (79.8%-90.91%)	96.21% (92.19%-100%)	66.974% (55.168%-78.8%)	83.32% (76.62%-89.88%)	91.19% (81.48%-100%)	3.3770 (3.1403-3.6006)
At least 2 of CHASM (breast), CHASM (melanoma), Mutation Assessor, MutationTaster, SIFT	83.19% (80.3%-86.36%)	96.83% (95.12%-98.49%)	59.611% (53.521%-66.176%)	80.56% (76.97%-84.21%)	91.58% (87.23%-96.08%)	3.2859 (3.1738-3.4126)	83.11% (76.77%-88.89%)	96.76% (92.85%-100%)	59.668% (46.875%-72.22%)	80.46% (73.17%-87.67%)	91.51% (81.81%-100%)	3.2840 (3.0474-3.5035)
At least 2 of CHASM (breast), CHASM (melanoma), Mutation Assessor, MutationTaster, VEST	82.21% (79.29%-85.35%)	98.41% (97.5%-100%)	54.187% (47.759%-60.811%)	78.79% (75.17%-82.89%)	95.19% (92.11%-100%)	3.2658 (3.1595-3.3783)	82.05% (75.76%-87.88%)	98.38% (95.31%-100%)	54.04% (41.176%-67.5%)	78.6% (70.66%-85.71%)	95.15% (86.36%-100%)	3.2617 (3.0410-3.4754)
At least 2 of CHASM (breast), CHASM (melanoma), Mutation Assessor, PolyPhen-2	83.19% (80.3%-86.36%)	95.23% (93.27%-97.58%)	62.36% (56.164%-68.663%)	81.39% (77.78%-85.24%)	88.34% (83.67%-93.75%)	3.2732 (3.1554-3.3950)	83.12% (76.77%-88.89%)	95.16% (90.62%-98.55%)	62.457% (50%-74.36%)	81.3% (73.91%-88.32%)	88.32% (77.26%-96.78%)	3.2723 (3.0301-3.5082)
At least 2 of CHASM (breast), CHASM (melanoma), Mutation Assessor, PolyPhen-2, PROVEAN	81.16% (78.28%-84.34%)	95.77% (93.7%-97.64%)	55.898% (49.315%-62.319%)	78.97% (75.17%-82.7%)	88.45% (83.33%-93.75%)	3.1909 (3.0633-3.3138)	81.11% (74.75%-86.87%)	95.68% (91.8%-100%)	56.101% (42.498%-68.58%)	78.9% (71.25%-86.25%)	88.37% (77.27%-100%)	3.1906 (2.9336-3.4394)
At least 2 of CHASM (breast), CHASM (melanoma), Mutation Assessor, PolyPhen-2, SIFT	78.78% (75.25%-82.32%)	96.29% (94.4%-98.39%)	48.51% (42.254%-54.937%)	76.38% (72.43%-80.13%)	88.31% (82.86%-94.6%)	3.0949 (2.9679-3.2272)	78.8% (71.72%-85.86%)	96.25% (92.19%-100%)	48.858% (35.897%-61.3%)	76.35% (68.67%-83.96%)	88.38% (76.46%-100%)	3.0984 (2.8265-3.3645)
At least 2 of CHASM (breast), CHASM (melanoma), Mutation Assessor, PolyPhen-2, VEST	78.78% (75.76%-82.32%)	97.33% (95.9%-99.19%)	46.717% (40%-53.126%)	75.95% (72.22%-79.76%)	91.01% (86.11%-97.22%)	3.1101 (2.9887-3.2352)	78.79% (71.72%-85.86%)	97.36% (93.85%-100%)	46.935% (33.333%-59.54%)	75.89% (67.95%-83.33%)	91.17% (78.94%-100%)	3.1135 (2.8486-3.3434)
At least 2 of CHASM (breast), CHASM (melanoma), Mutation Assessor, PROVEAN	83.86% (81.31%-86.87%)	90.96% (88.19%-93.94%)	71.567% (65.753%-77.465%)	84.69% (81.4%-88.03%)	82.09% (76.67%-87.88%)	3.2931 (3.1728-3.4218)	83.8% (77.78%-89.9%)	90.94% (85.18%-96.72%)	71.522% (60.513%-82.86%)	84.57% (77.78%-91.05%)	82.17% (70.96%-93.1%)	3.2920 (3.0396-3.5329)
At least 2 of CHASM (breast), CHASM (melanoma), Mutation Assessor, PROVEAN, SIFT	80.85% (77.78%-83.84%)	95.23% (93.27%-97.58%)	55.96% (49.333%-62.667%)	78.9% (75.16%-82.5%)	87.17% (81.82%-93.02%)	3.1727 (3.0444-3.2995)	80.73% (74.75%-86.87%)	95.16% (90.62%-98.55%)	55.939% (42.416%-69.24%)	78.75% (71.62%-86.25%)	87.11% (75%-96.43%)	3.1697 (2.9054-3.4195)
At least 2 of CHASM (breast), CHASM (melanoma), Mutation Assessor, PROVEAN, VEST	78.82% (75.76%-81.83%)	95.26% (93.39%-97.56%)	50.375% (44.156%-56.758%)	76.85% (73.08%-80.51%)	86.03% (80.49%-92.68%)	3.0852 (2.9665-3.2237)	78.73% (72.7%-84.85%)	95.1% (90.62%-98.51%)	50.594% (37.493%-63.16%)	76.76% (68.92%-84.15%)	85.77% (72.73%-95.65%)	3.0823 (2.8031-3.3188)
At least 2 of CHASM (breast), CHASM (melanoma), Mutation Assessor, SIFT	82.85% (79.8%-85.86%)	94.68% (92.62%-96.9%)	62.375% (56.338%-68.921%)	81.31% (77.71%-84.83%)	87.16% (82.35%-92.59%)	3.2554 (3.1271-3.3838)	82.79% (76.77%-88.89%)	94.66% (89.83%-98.48%)	62.383% (50%-75.68%)	81.2% (73.91%-88.57%)	87.24% (75.86%-96.43%)	3.2549 (3.0063-3.5154)
At least 2 of CHASM (breast), CHASM (melanoma), Mutation Assessor, SIFT, VEST	76.04% (72.73%-79.8%)	97.34% (95.93%-99.19%)	39.206% (32.827%-45.948%)	73.46% (69.7%-77.38%)	89.49% (83.87%-96.55%)	2.9950 (2.8677-3.1303)	76.21% (68.69%-82.83%)	97.34% (93.75%-100%)	39.959% (26.313%-52.78%)	73.55% (65.43%-81.02%)	89.75% (76.47%-100%)	3.0061 (2.7174-3.2423)
At least 2 of CHASM (breast), CHASM (melanoma), Mutation Assessor, VEST	81.17% (78.28%-84.34%)	94.74% (92.62%-96.92%)	57.716% (51.388%-64.384%)	79.48% (75.84%-83.44%)	86.39% (81.4%-92%)	3.1833 (3.0543-3.3181)	81.09% (74.75%-86.87%)	94.55% (89.83%-98.48%)	57.984% (44.73%-70.59%)	79.42% (71.62%-86.67%)	86.14% (74.98%-95.83%)	3.1810 (2.9040-3.4269)
At least 2 of CHASM (breast), CHASM (melanoma), MutationTaster	88.59% (86.36%-90.92%)	87.82% (84.55%-91.13%)	89.937% (86.301%-94.286%)	93.78% (91.6%-96.52%)	81.02% (76.25%-85.72%)	3.5256 (3.4257-3.6354)	88.47% (83.81%-92.93%)	87.65% (80.95%-94.03%)	89.836% (81.579%-97.3%)	93.67% (88.46%-98.25%)	80.93% (71.04%-90.7%)	3.5209 (3.3018-3.7157)
At least 2 of CHASM (breast), CHASM (melanoma), MutationTaster, PolyPhen-2	88.29% (85.86%-90.91%)	97.9% (96.72%-99.24%)	71.682% (65.714%-77.923%)	85.67% (82.55%-89.12%)	95.18% (92.31%-98.31%)	3.5043 (3.4064-3.6078)	88.06% (82.83%-92.93%)	97.82% (94.64%-100%)	71.306% (58.824%-82.93%)	85.4% (78.57%-91.67%)	95.02% (87.49%-100%)	3.4954 (3.2870-3.6879)
At least 2 of CHASM (breast), CHASM (melanoma), MutationTaster, PolyPhen-2, PROVEAN	84.23% (81.31%-87.37%)	97.9% (96.72%-99.24%)	60.587% (54.164%-67.533%)	81.11% (77.71%-84.87%)	94.35% (90.91%-98%)	3.3395 (3.2354-3.4583)	84.07% (77.78%-89.9%)	97.82% (94.64%-100%)	60.475% (47.368%-72.98%)	80.95% (73.33%-87.5%)	94.18% (85.71%-100%)	3.3341 (3.0877-3.5459)
At least 2 of CHASM (breast), CHASM (melanoma), MutationTaster, PolyPhen-2, SIFT	81.83% (79.29%-84.85%)	98.93% (98.32%-100%)	52.257% (45.946%-58.571%)	78.18% (74.84%-81.87%)	96.58% (94.29%-100%)	3.2594 (3.1605-3.3624)	81.8% (75.76%-86.87%)	98.95% (96.67%-100%)	52.34% (39.987%-64.71%)	78.08% (70.73%-84.81%)	96.69% (88.89%-100%)	3.2606 (3.0506-3.4531)
At least 2 of CHASM (breast), CHASM (melanoma), MutationTaster, PolyPhen-2, VEST	81.85% (78.79%-84.85%)	100% (100%-100%)	50.474% (43.901%-57.334%)	77.73% (74.19%-81.53%)	100% (100%-100%)	3.2821 (3.1914-3.3811)	81.75% (75.76%-87.88%)	100% (100%-100%)	50.436% (36.665%-63.64%)	77.58% (69.99%-84.71%)	100% (100%-100%)	3.2802 (3.0881-3.4678)
At least 2 of CHASM (breast), CHASM (melanoma), MutationTaster, PROVEAN	89.24% (86.87%-91.92%)	92.03% (89.51%-94.96%)	84.4% (80%-89.233%)	91.07% (88.55%-93.98%)	85.97% (81.54%-90.91%)	3.5348 (3.4330-3.6446)	89.2% (83.84%-93.94%)	91.99% (86.36%-96.92%)	84.38% (74.984%-93.34%)	91% (85.25%-96.55%)	85.99% (75.86%-94.59%)	3.5336 (3.3134-3.7318)
At least 2 of CHASM (breast), CHASM (melanoma), MutationTaster, PROVEAN, SIFT	83.55% (80.81%-86.36%)	97.37% (95.93%-99.2%)	59.657% (53.333%-65.753%)	80.67% (77.4%-84.42%)	92.92% (89.13%-97.78%)	3.3062 (3.2030-3.4208)	83.4% (77.78%-88.89%)	97.28% (93.65%-100%)	59.559% (47.054%-72.1%)	80.5% (73.24%-87.01%)	92.77% (83.33%-100%)	3.3011 (3.0745-3.5134)
At least 2 of CHASM (breast), CHASM (melanoma), MutationTaster, PROVEAN, VEST	81.59% (78.79%-84.36%)	98.45% (97.52%-100%)	52.429% (45.83%-58.671%)	78.16% (74.53%-81.82%)	95.15% (91.89%-100%)	3.2419 (3.1406-3.3525)	81.27% (75.73%-86.87%)	98.31% (95.38%-100%)	51.998% (39.282%-65.13%)	77.85% (70.27%-85%)	94.73% (85.71%-100%)	3.2288 (3.0009-3.4399)
At least 2 of CHASM (breast), CHASM (melanoma), MutationTaster, SIFT	88.61% (86.36%-91.41%)	96.83% (95.12%-98.49%)	74.386% (68.919%-79.73%)	86.73% (83.69%-90.21%)	93.14% (89.66%-96.77%)	3.5109 (3.4144-3.6253)	88.44% (82.83%-92.93%)	96.76% (92.85%-100%)	74.125% (62.857%-85.3%)	86.53% (79.73%-92.65%)	93.06% (84.62%-100%)	3.5048 (3.2879-3.6970)
At least 2 of CHASM (breast), CHASM (melanoma), MutationTaster, SIFT, VEST	77.45% (74.24%-80.81%)	98.92% (98.33%-100%)	40.318% (34.247%-46.479%)	74.13% (70.41%-77.91%)	95.58% (92.59%-100%)	3.0896 (2.9838-3.2027)	77.43% (70.71%-83.84%)	98.96% (96.67%-100%)	40.472% (28.205%-52.78%)	74.04% (66.25%-81.4%)	95.78% (85.71%-100%)	3.0925 (2.8603-3.3063)
At least 2 of CHASM (breast), CHASM (melanoma), MutationTaster, VEST	88% (85.35%-90.91%)	97.39% (95.93%-99.19%)	71.77% (65.789%-78.205%)	85.64% (82.52%-89.29%)	94.08% (90.91%-98.15%)	3.4888 (3.3827-3.5953)	87.64% (81.82%-92.93%)	97.24% (93.75%-100%)	71.14% (58.532%-82.94%)	85.26% (77.77%-91.55%)	93.77% (86.21%-100%)	3.4741 (3.2554-3.6828)
At least 2 of CHASM (breast), CHASM (melanoma), PolyPhen-2	83.22% (80.3%-86.36%)	86.22% (82.79%-89.63%)	78.035% (72.599%-83.828%)	87.16% (84.03%-90.76%)	76.61% (71.05%-82.05%)	3.2802 (3.1562-3.4057)	83.05% (76.77%-88.89%)	86.06% (79.1%-93.22%)	77.846% (66.648%-88.58%)	86.96% (79.69%-93.44%)	76.52% (65.62%-88.24%)	3.2739 (3.0207-3.5223)
At least 2 of CHASM (breast), CHASM (melanoma), PolyPhen-2, PROVEAN	81.16% (78.28%-84.34%)	90.44% (87.59%-93.55%)	65.121% (58.664%-71.795%)	81.76% (78.26%-85.62%)	79.75% (73.84%-85.94%)	3.1707 (3.0397-3.3065)	81.11% (74.75%-86.87%)	90.4% (84.13%-96.61%)	65.146% (51.849%-77.51%)	81.67% (73.91%-88.57%)	79.84% (66.67%-92.01%)	3.1704 (2.8935-3.4257)
At least 2 of CHASM (breast), CHASM (melanoma), PolyPhen-2, PROVEAN, SIFT	79.49% (76.26%-82.83%)	96.83% (95.12%-98.45%)	49.519% (43.418%-55.885%)	76.83% (73.08%-80.5%)	90.03% (85%-95.12%)	3.1320 (3.0086-3.2499)	79.4% (72.73%-85.86%)	96.77% (93.22%-100%)	49.566% (36.838%-62.16%)	76.7% (69.33%-83.95%)	89.95% (78.95%-100%)	3.1299 (2.8911-3.3810)
At least 2 of CHASM (breast), CHASM (melanoma), PolyPhen-2, PROVEAN, VEST	77.83% (74.75%-81.31%)	97.37% (95.93%-99.19%)	44.026% (37.5%-50%)	75.05% (71.34%-79.01%)	90.64% (85.29%-96.97%)	3.0708 (2.9477-3.1923)	77.68% (70.71%-83.84%)	97.28% (93.75%-100%)	44.024% (31.429%-57.14%)	74.89% (66.67%-82.28%)	90.41% (78.57%-100%)	3.0660 (2.8053-3.2977)
At least 2 of CHASM (breast), CHASM (melanoma), PolyPhen-2, SIFT	81.15% (78.28%-84.34%)	96.29% (94.4%-98.39%)	54.962% (48.649%-61.429%)	78.71% (75.17%-82.52%)	89.55% (84.78%-95.12%)	3.1950 (3.0741-3.3117)	81.14% (74.75%-86.87%)	96.25% (92.19%-100%)	55.178% (42.495%-67.74%)	78.66% (70.83%-85.53%)	89.58% (78.95%-100%)	3.1966 (2.9541-3.4358)
At least 2 of CHASM (breast), CHASM (melanoma), PolyPhen-2, SIFT, VEST	75.74% (72.71%-79.29%)	97.85% (96.72%-99.22%)	37.509% (31.343%-43.592%)	73.03% (69.28%-77.06%)	90.98% (85.7%-96.77%)	2.9937 (2.8743-3.1191)	75.79% (68.69%-81.84%)	97.91% (94.92%-100%)	37.832% (25.714%-50%)	72.99% (64.7%-80.46%)	91.34% (78.57%-100%)	3.0008 (2.7485-3.2282)
At least 2 of CHASM (breast), CHASM (melanoma), PolyPhen-2, VEST	79.83% (76.77%-83.33%)	96.31% (94.44%-98.39%)	51.341% (44.928%-58.212%)	77.38% (73.68%-81.29%)	88.93% (83.72%-95%)	3.1396 (3.0119-3.2700)	79.74% (72.73%-85.86%)	96.22% (92.18%-100%)	51.444% (38.089%-63.89%)	77.27% (69.62%-84.62%)	88.77% (76.47%-100%)	3.1370 (2.8727-3.3838)
At least 2 of CHASM (breast), CHASM (melanoma), PROVEAN	82.53% (79.29%-85.86%)	78.3% (74.01%-82.54%)	89.829% (86.107%-94.203%)	93.02% (90.38%-96.04%)	70.54% (65.22%-76.04%)	3.3169 (3.1958-3.4324)	82.42% (75.76%-88.89%)	77.95% (69.23%-86.67%)	90.033% (81.812%-97.3%)	93.08% (87.27%-98.15%)	70.43% (60%-81.25%)	3.3150 (3.0802-3.5628)
At least 2 of CHASM (breast), CHASM (melanoma), PROVEAN, SIFT	80.83% (77.78%-84.34%)	90.96% (88.19%-93.94%)	63.305% (57.143%-69.565%)	81.08% (77.7%-84.78%)	80.21% (74.14%-86.44%)	3.1556 (3.0234-3.2989)	80.76% (73.74%-86.87%)	90.94% (85.18%-96.72%)	63.259% (50%-76.32%)	80.95% (73.43%-87.84%)	80.28% (67.72%-92.31%)	3.1542 (2.8615-3.4225)
At least 2 of CHASM (breast), CHASM (melanoma), PROVEAN, SIFT, VEST	75.77% (72.22%-79.29%)	96.84% (95.2%-98.46%)	39.327% (33.322%-45.588%)	73.4% (69.7%-77.25%)	87.8% (81.25%-94.29%)	2.9737 (2.8375-3.1016)	75.74% (68.69%-82.83%)	96.75% (93.1%-100%)	39.656% (27.267%-52.5%)	73.34% (65.82%-80.72%)	87.67% (73.33%-100%)	2.9742 (2.6994-3.2270)
At least 2 of CHASM (breast), CHASM (melanoma), PROVEAN, VEST	79.16% (76.26%-82.32%)	90.97% (88.28%-93.85%)	58.732% (52.172%-65.152%)	79.22% (75.71%-83%)	79% (73.08%-85.45%)	3.0792 (2.9473-3.2204)	79.05% (72.73%-85.86%)	90.93% (85.18%-96.61%)	58.624% (45.232%-71.43%)	79.05% (71.25%-86.11%)	79.03% (65.22%-90.91%)	3.0763 (2.7925-3.3359)
At least 2 of CHASM (breast), CHASM (melanoma), SIFT	84.22% (81.31%-87.37%)	86.74% (83.33%-90.25%)	79.852% (74.286%-85.526%)	88.16% (84.92%-91.45%)	77.69% (72.46%-82.86%)	3.3244 (3.1995-3.4528)	84.09% (77.78%-89.9%)	86.62% (79.69%-93.33%)	79.692% (68.421%-90.24%)	88% (81.03%-94.64%)	77.65% (66.67%-88.38%)	3.3197 (3.0529-3.5729)
At least 2 of CHASM (breast), CHASM (melanoma), SIFT, VEST	77.43% (74.24%-80.81%)	96.31% (94.49%-98.39%)	44.792% (38.571%-51.429%)	75.1% (71.43%-78.92%)	87.54% (81.57%-94.29%)	3.0375 (2.9084-3.1654)	77.45% (70.71%-83.84%)	96.2% (92.19%-100%)	45.264% (32.132%-58.14%)	75.1% (67.47%-82.28%)	87.42% (73.91%-100%)	3.0398 (2.7814-3.2848)
At least 2 of CHASM (breast), CHASM (melanoma), VEST	84.91% (82.32%-87.88%)	87.82% (84.55%-91.13%)	79.892% (74.627%-85.135%)	88.31% (85.37%-91.54%)	79.14% (74.02%-84.29%)	3.3515 (3.2384-3.4766)	84.72% (78.79%-89.9%)	87.65% (80.95%-94.03%)	79.635% (68.564%-90%)	88.09% (81.03%-94.13%)	79% (67.74%-89.66%)	3.3438 (3.0909-3.5747)
At least 2 of CHASM (breast), FATHMM (cancer)	80.21% (77.27%-83.33%)	71.96% (67.24%-76.42%)	94.469% (91.549%-97.333%)	95.74% (93.62%-97.96%)	66.09% (60.61%-71.29%)	3.2826 (3.1777-3.3944)	79.99% (73.74%-85.86%)	71.5% (62.3%-80.88%)	94.535% (88.235%-100%)	95.74% (90.7%-100%)	65.9% (55.17%-76.92%)	3.2768 (3.0514-3.4857)
At least 2 of CHASM (breast), FATHMM (cancer), FATHMM (missense)	83.53% (80.3%-86.36%)	87.31% (84%-90.63%)	76.972% (71.831%-82.895%)	86.77% (83.58%-90.24%)	77.83% (72.46%-83.1%)	3.2888 (3.1608-3.4126)	83.45% (77.78%-89.9%)	87.05% (80%-93.65%)	77.2% (65%-87.5%)	86.78% (79.66%-92.99%)	77.67% (67.5%-87.89%)	3.2870 (3.0397-3.5333)
At least 2 of CHASM (breast), FATHMM (cancer), FATHMM (missense), Mutation Assessor	84.14% (81.31%-87.37%)	94.74% (92.56%-96.88%)	65.81% (59.74%-72.222%)	82.73% (79.31%-86.49%)	87.86% (82.97%-92.73%)	3.3114 (3.1875-3.4351)	84.24% (77.78%-89.9%)	94.55% (90%-98.51%)	66.515% (54.054%-78.58%)	82.9% (75.64%-89.55%)	87.71% (77.78%-96.67%)	3.3168 (3.0674-3.5606)
At least 2 of CHASM (breast), FATHMM (cancer), FATHMM (missense), Mutation Assessor, MutationTaster	85.82% (83.33%-88.4%)	98.97% (98.33%-100%)	63.087% (56.944%-69.231%)	82.26% (79.05%-85.91%)	97.26% (95.24%-100%)	3.4158 (3.3179-3.5136)	85.93% (80.78%-90.91%)	98.86% (96.67%-100%)	63.706% (51.429%-75.77%)	82.38% (75.32%-88.89%)	97.04% (90.91%-100%)	3.4199 (3.2246-3.6138)
At least 2 of CHASM (breast), FATHMM (cancer), FATHMM (missense), Mutation Assessor, PolyPhen-2	81.13% (78.28%-84.34%)	97.9% (96.69%-99.22%)	52.121% (45.452%-58.337%)	77.95% (74.34%-81.53%)	93.51% (89.47%-97.67%)	3.2148 (3.0934-3.3281)	81.17% (74.75%-87.88%)	97.8% (94.92%-100%)	52.614% (40%-65.81%)	77.98% (70.67%-85.33%)	93.33% (83.33%-100%)	3.2174 (2.9829-3.4510)
At least 2 of CHASM (breast), FATHMM (cancer), FATHMM (missense), Mutation Assessor, PROVEAN	81.79% (78.79%-84.85%)	94.74% (92.56%-96.88%)	59.396% (52.939%-66.197%)	80.14% (76.67%-83.78%)	86.72% (81.25%-92%)	3.2100 (3.0774-3.3404)	81.87% (75.76%-87.88%)	94.55% (90%-98.51%)	60.059% (46.422%-72.98%)	80.26% (72.86%-87.18%)	86.56% (76%-96.3%)	3.2143 (2.9563-3.4822)
At least 2 of CHASM (breast), FATHMM (cancer), FATHMM (missense), Mutation Assessor, SIFT	80.8% (77.78%-83.84%)	97.39% (95.9%-99.2%)	52.111% (45.714%-58.904%)	77.86% (74.36%-81.53%)	92.04% (87.8%-97.44%)	3.1940 (3.0768-3.3099)	80.82% (74.75%-86.87%)	97.23% (93.65%-100%)	52.632% (39.474%-65.71%)	77.89% (70.51%-85%)	91.76% (81.82%-100%)	3.1951 (2.9570-3.4269)
At least 2 of CHASM (breast), FATHMM (cancer), FATHMM (missense), Mutation Assessor, VEST	76.13% (72.73%-79.8%)	98.47% (97.52%-100%)	37.497% (31.319%-43.756%)	73.15% (69.46%-77.01%)	93.41% (88.89%-100%)	3.0253 (2.9071-3.1451)	76.02% (68.69%-82.83%)	98.27% (95.38%-100%)	37.837% (25%-50%)	73.07% (65.17%-80.46%)	92.68% (81.25%-100%)	3.0185 (2.7761-3.2403)
At least 2 of CHASM (breast), FATHMM (cancer), FATHMM (missense), MutationTaster	88.57% (86.36%-90.91%)	96.86% (95.2%-98.48%)	74.211% (68.655%-80.282%)	86.66% (83.69%-90%)	93.19% (89.65%-96.77%)	3.5093 (3.4059-3.6092)	88.52% (83.84%-92.93%)	96.69% (92.86%-100%)	74.462% (62.162%-85.19%)	86.68% (79.73%-92.75%)	92.94% (85%-100%)	3.5078 (3.3024-3.7094)
At least 2 of CHASM (breast), FATHMM (cancer), FATHMM (missense), MutationTaster, PolyPhen-2	83.18% (80.3%-86.36%)	98.97% (98.33%-100%)	55.864% (49.275%-62.319%)	79.5% (75.97%-83.33%)	96.92% (94.59%-100%)	3.3125 (3.2085-3.4138)	83.14% (76.77%-88.89%)	98.86% (96.67%-100%)	56.138% (42.857%-69.23%)	79.46% (71.83%-86.49%)	96.65% (90%-100%)	3.3111 (3.1022-3.5152)
At least 2 of CHASM (breast), FATHMM (cancer), FATHMM (missense), MutationTaster, PROVEAN	84.49% (81.82%-87.37%)	96.86% (95.2%-98.48%)	63.098% (56.517%-69.333%)	81.95% (78.43%-85.53%)	92.09% (87.76%-96.23%)	3.3400 (3.2233-3.4573)	84.55% (78.79%-89.9%)	96.69% (92.86%-100%)	63.652% (50%-77.14%)	82.05% (74.64%-89.04%)	91.83% (82.61%-100%)	3.3423 (3.1099-3.5702)
At least 2 of CHASM (breast), FATHMM (cancer), FATHMM (missense), MutationTaster, SIFT	84.15% (81.31%-86.87%)	99.49% (99.16%-100%)	57.618% (51.351%-63.768%)	80.23% (76.97%-83.85%)	98.5% (97.3%-100%)	3.3584 (3.2618-3.4532)	84.22% (78.79%-89.9%)	99.42% (98.21%-100%)	58.12% (45.714%-70.59%)	80.3% (72.84%-87.01%)	98.32% (94.12%-100%)	3.3616 (3.1784-3.5576)
At least 2 of CHASM (breast), FATHMM (cancer), FATHMM (missense), MutationTaster, VEST	78.52% (75.25%-81.82%)	98.99% (98.33%-100%)	43.121% (36.842%-50%)	75.06% (71.43%-78.88%)	96.12% (93.1%-100%)	3.1329 (3.0239-3.2409)	78.32% (71.72%-84.85%)	98.82% (96.67%-100%)	43.112% (29.991%-56.1%)	74.88% (67.43%-82.14%)	95.54% (86.67%-100%)	3.1236 (2.9042-3.3346)
At least 2 of CHASM (breast), FATHMM (cancer), FATHMM (missense), PolyPhen-2	82.15% (79.29%-85.35%)	95.26% (93.33%-97.58%)	59.474% (52.778%-66.197%)	80.26% (76.67%-83.92%)	87.9% (82.61%-93.48%)	3.2290 (3.1047-3.3567)	82.16% (75.76%-88.89%)	95.1% (90.62%-98.53%)	59.906% (46.422%-72.97%)	80.29% (72.86%-87.5%)	87.74% (76.92%-96.55%)	3.2304 (2.9877-3.4771)
At least 2 of CHASM (breast), FATHMM (cancer), FATHMM (missense), PolyPhen-2, PROVEAN	80.13% (76.77%-83.33%)	95.26% (93.33%-97.58%)	53.965% (46.753%-60.564%)	78.16% (74.32%-81.82%)	86.83% (80.95%-93.03%)	3.1421 (3.0158-3.2747)	80.14% (73.74%-86.87%)	95.1% (90.62%-98.53%)	54.403% (41.173%-68.57%)	78.18% (70.67%-85.9%)	86.65% (75%-96.3%)	3.1433 (2.8795-3.3948)
At least 2 of CHASM (breast), FATHMM (cancer), FATHMM (missense), PolyPhen-2, SIFT	79.09% (75.76%-82.32%)	98.41% (97.5%-100%)	45.666% (39.13%-51.621%)	75.8% (72.02%-79.5%)	94.34% (90.62%-100%)	3.1422 (3.0328-3.2551)	79.19% (72.73%-85.86%)	98.38% (95.45%-100%)	46.222% (34.126%-60%)	75.85% (68.67%-83.53%)	94.37% (84.61%-100%)	3.1482 (2.9212-3.3675)
At least 2 of CHASM (breast), FATHMM (cancer), FATHMM (missense), PolyPhen-2, VEST	74.42% (71.21%-77.79%)	98.98% (98.32%-100%)	31.949% (25.756%-37.879%)	71.55% (68%-75.57%)	94.77% (90.48%-100%)	2.9724 (2.8588-3.0830)	74.39% (67.65%-80.81%)	98.85% (96.67%-100%)	32.4% (20%-44.74%)	71.51% (63.63%-78.66%)	94.21% (81.82%-100%)	2.9696 (2.7348-3.1853)

At least 2 of CHASM (breast), FATHMM (cancer), FATHMM (missense), PROVEAN	82.12% (79.28%-85.35%)	91.51% (88.89%-94.4%)	65.86% (59.42%-72.309%)	82.26% (78.72%-86.01%)	81.78% (75.86%-87.72%)	3.2141 (3.0739-3.3525)	82.23% (75.76%-87.9%)	91.43% (85.71%-96.77%)	66.39% (52.774%-78.38%)	82.37% (74.655%-89.47%)	81.89% (69.99%-93.33%)	3.2208 (2.9437-3.4892)
At least 2 of CHASM (breast), FATHMM (cancer), FATHMM (missense), PROVEAN, SIFT	80.48% (77.27%-83.64%)	95.28% (93.22%-97.58%)	54.888% (48.529%-61.842%)	78.51% (74.84%-82.28%)	87.07% (81.63%-93.02%)	3.1574 (3.0209-3.2884)	80.44% (73.74%-86.87%)	95.06% (90.62%-98.57%)	55.309% (41.147%-68.58%)	78.51% (70.89%-85.71%)	86.73% (74.97%-96.3%)	3.1561 (2.8895-3.4206)
At least 2 of CHASM (breast), FATHMM (cancer), FATHMM (missense), PROVEAN, VEST	75.77% (72.22%-79.29%)	96.86% (95.2%-98.48%)	39.287% (32.432%-46.055%)	73.4% (69.7%-77.11%)	87.87% (81.48%-94.45%)	2.9741 (2.8372-3.1089)	75.73% (68.69%-82.83%)	96.69% (92.86%-100%)	39.714% (25.714%-54.29%)	73.36% (65.82%-80.68%)	87.47% (73.68%-100%)	2.9725 (2.6925-3.2316)
At least 2 of CHASM (breast), FATHMM (cancer), FATHMM (missense), SIFT	82.33% (79.8%-85.86%)	95.28% (93.22%-97.58%)	61.286% (55.072%-67.902%)	80.66% (77.12%-84.62%)	88.26% (83.33%-93.88%)	3.2580 (3.1254-3.3848)	82.26% (75.76%-88.89%)	95.06% (90.62%-98.57%)	61.77% (48.568%-74.2%)	80.69% (73.33%-87.84%)	87.96% (77.27%-97.67%)	3.2584 (3.0020-3.5113)
At least 2 of CHASM (breast), FATHMM (cancer), FATHMM (missense), SIFT, VEST	73.73% (70.2%-77.27%)	99.5% (99.16%-100%)	29.163% (23.074%-35.135%)	70.83% (67.23%-74.58%)	97.13% (94.44%-100%)	2.9663 (2.7657-3.0679)	73.76% (66.67%-80.81%)	99.4% (98.25%-100%)	29.721% (17.239%-42.11%)	70.83% (63.33%-78.03%)	96.71% (87.5%-100%)	2.9666 (2.7512-3.1561)
At least 2 of CHASM (breast), FATHMM (cancer), FATHMM (missense), VEST	78.81% (75.76%-82.32%)	96.86% (95.2%-98.48%)	47.598% (40.58%-54.933%)	76.17% (72.67%-80.13%)	89.77% (84.21%-95.12%)	3.1040 (2.9715-3.2402)	78.74% (71.72%-84.85%)	96.69% (92.86%-100%)	47.888% (33.333%-61.54%)	76.11% (68.35%-83.12%)	89.4% (77.78%-100%)	3.1010 (2.8395-3.3530)
At least 2 of CHASM (breast), FATHMM (cancer), Mutation Assessor	84.51% (81.31%-87.37%)	88.86% (85.83%-92.19%)	76.985% (71.233%-82.609%)	86.97% (83.72%-90.48%)	80% (74.65%-85.71%)	3.3282 (3.2029-3.4570)	84.51% (78.79%-90.91%)	88.75% (81.97%-95%)	77.239% (66.645%-88.89%)	86.99% (80%-93.55%)	80.02% (69.23%-90.91%)	3.3300 (3.0693-3.5883)
At least 2 of CHASM (breast), FATHMM (cancer), Mutation Assessor, MutationTaster	89.57% (87.37%-91.92%)	98.44% (97.52%-100%)	74.255% (68.491%-80.303%)	86.86% (83.82%-90.41%)	96.49% (94.23%-100%)	3.5603 (3.4663-3.6580)	89.54% (84.85%-93.94%)	98.34% (95.45%-100%)	74.458% (63.153%-86.21%)	86.84% (79.73%-92.86%)	96.31% (90.31%-100%)	3.5595 (3.3667-3.7464)
At least 2 of CHASM (breast), FATHMM (cancer), Mutation Assessor, MutationTaster, PolyPhen-2	84.55% (81.82%-87.37%)	98.44% (97.52%-100%)	60.546% (54.406%-67.143%)	81.18% (77.74%-84.72%)	95.72% (92.86%-100%)	3.3546 (3.2532-3.4649)	84.43% (78.79%-89.9%)	98.34% (95.45%-100%)	60.566% (47.487%-72.97%)	81.05% (73.97%-88%)	95.5% (87.5%-100%)	3.3546 (3.1404-3.5655)
At least 2 of CHASM (breast), FATHMM (cancer), Mutation Assessor, MutationTaster, PROVEAN	86.19% (83.33%-88.89%)	98.44% (97.52%-100%)	65.021% (59.155%-71.429%)	82.95% (79.45%-86.36%)	96.01% (93.33%-100%)	3.4242 (3.3272-3.5269)	86.2% (80.81%-91.92%)	98.34% (95.45%-100%)	65.376% (52.632%-77.78%)	82.97% (76%-90.14%)	95.83% (88.89%-100%)	3.4251 (3.2195-3.6184)
At least 2 of CHASM (breast), FATHMM (cancer), Mutation Assessor, MutationTaster, SIFT	84.16% (81.31%-87.37%)	98.96% (98.33%-100%)	58.584% (52.632%-65.217%)	80.51% (77.12%-84.11%)	97.01% (94.87%-100%)	3.3506 (3.2587-3.4556)	84.2% (77.78%-89.9%)	98.92% (96.61%-100%)	58.975% (45.946%-71.83%)	80.53% (73.42%-87.35%)	96.9% (90.48%-100%)	3.3529 (3.1422-3.5492)
At least 2 of CHASM (breast), FATHMM (cancer), Mutation Assessor, MutationTaster, VEST	80.5% (77.77%-83.84%)	99.49% (99.16%-100%)	47.668% (41.094%-54.167%)	76.67% (73.01%-80.49%)	98.19% (96.67%-100%)	3.2203 (3.1295-3.3204)	80.41% (73.74%-85.88%)	99.42% (98.21%-100%)	47.788% (33.333%-62.5%)	76.56% (68.79%-83.95%)	97.97% (92.86%-100%)	3.2174 (3.0218-3.4112)
[illegible]	[illegible]	[illegible]	[illegible]	[illegible]	[illegible]	[illegible]	[illegible]	[illegible]	[illegible]	[illegible]	[illegible]	[illegible]

At least 2 of CHASM (lung), FATHMM (cancer), FATHMM (missense), PolyPhen-2	81.81% (78.79%-84.85%)	95.26% (93.33%-97.58%)	58.535% (51.9365%-65.219%)	79.89% (76.43%-83.54%)	87.73% (82.35%-93.48%)	3.2142 (3.0886-3.3455)	81.84% (75.76%-87.88%)	95.1% (90.62%-98.53%)	59.031% (45.708%-72.23%)	79.95% (72.6%-87.01%)	87.57% (76.92%-96.55%)	3.2165 (2.9607-3.4640)
[illegible]	[illegible]	[illegible]	[illegible]	[illegible]	[illegible]	[illegible]	[illegible]	[illegible]	[illegible]	[illegible]	[illegible]	[illegible]

At least 2 of CHASM (melanoma), FATHMM (missense), PROVEAN, VEST	76.48% (73.23%-79.8%)	95.82% (93.84%-97.69%)	43.043% (36.483%-50%)	74.42% (70.81%-78.32%)	85.62% (79.41%-92.31%)	2.9890 (2.8431-3.1283)	76.32% (69.7%-82.83%)	95.59% (91.38%-100%)	43.186% (29.993%-55.56%)	74.29% (66.25%-81.48%)	85.09% (70.59%-100%)	2.9815 (2.6901-3.2538)
At least 2 of CHASM (melanoma), FATHMM (missense), SIFT	79.82% (76.77%-83.33%)	89.42% (86.36%-92.62%)	63.184% (56.756%-69.842%)	80.77% (77.04%-84.29%)	77.56% (71.7%-83.64%)	3.1094 (2.9740-3.2545)	79.76% (72.73%-85.86%)	89.21% (83.04%-95.24%)	63.476% (50%-75.77%)	80.75% (73.53%-88.06%)	77.45% (65.38%-89.29%)	3.1090 (2.8161-3.3805)
At least 2 of CHASM (melanoma), FATHMM (missense), SIFT, VEST	74.1% (70.71%-77.78%)	97.91% (96.69%-99.23%)	32.922% (27.027%-39.19%)	71.62% (67.88%-75.58%)	90.11% (83.33%-96.55%)	2.9256 (2.7904-3.0603)	74.03% (66.67%-80.81%)	97.79% (94.83%-100%)	33.216% (20.69%-45.45%)	71.53% (63.73%-78.82%)	89.77% (75%-100%)	2.9231 (2.6405-3.1689)
At least 2 of CHASM (melanoma), FATHMM (missense), VEST	77.85% (74.73%-81.31%)	91.58% (88.89%-94.36%)	54.083% (47.495%-60.812%)	77.52% (73.76%-81.51%)	78.8% (72.55%-85.42%)	3.0199 (2.8691-3.1595)	77.64% (70.71%-83.86%)	91.28% (85.71%-96.77%)	54.139% (40.464%-66.69%)	77.37% (69.44%-84.72%)	78.36% (64.28%-91.3%)	3.0115 (2.7287-3.2940)
At least 2 of CHASM (melanoma), Mutation Assessor	71.71% (68.18%-75.25%)	62.24% (57.25%-67.2%)	88.079% (84.058%-92.754%)	90.03% (86.42%-94.05%)	57.43% (52.17%-62.73%)	2.9779 (2.8565-3.1087)	71.72% (64.65%-78.79%)	62.19% (52.38%-72.58%)	88.041% (78.947%-96.67%)	89.93% (82.22%-97.5%)	57.58% (47.06%-68.53%)	2.9773 (2.7100-3.2274)
At least 2 of CHASM (melanoma), Mutation Assessor, MutationTaster	83.8% (81.31%-86.87%)	85.04% (81.51%-88.71%)	81.667% (76.809%-86.958%)	88.91% (85.84%-92.19%)	75.95% (70.66%-81.26%)	3.3156 (3.2004-3.4420)	83.91% (77.78%-88.89%)	85.22% (77.78%-92.19%)	81.625% (71.429%-91.43%)	88.83% (82.09%-94.92%)	76.33% (64.7%-86.49%)	3.3201 (3.0607-3.5504)
At least 2 of CHASM (melanoma), Mutation Assessor, MutationTaster, PolyPhen-2	78.79% (75.76%-81.82%)	88.26% (85.24%-91.48%)	62.424% (56.164%-68.919%)	80.24% (76.47%-84.17%)	75.46% (69.35%-81.82%)	3.0637 (2.9260-3.2096)	78.78% (72.73%-84.85%)	88.37% (81.67%-94.65%)	62.331% (50%-75%)	80.09% (72.22%-87.33%)	75.76% (62.5%-87.88%)	3.0655 (2.7628-3.3424)
At least 2 of CHASM (melanoma), Mutation Assessor, MutationTaster, PolyPhen-2, PROVEAN	76.42% (73.23%-79.8%)	88.26% (85.24%-91.48%)	55.962% (49.296%-62.319%)	77.6% (73.53%-81.56%)	73.37% (66.67%-80.4%)	2.9519 (2.8033-3.1009)	76.45% (69.7%-82.83%)	88.37% (81.67%-94.65%)	55.975% (42.857%-69.05%)	77.5% (69.44%-85.53%)	73.72% (60%-86.96%)	2.9557 (2.6560-3.2536)
At least 2 of CHASM (melanoma), Mutation Assessor, MutationTaster, PolyPhen-2, SIFT	76.39% (72.73%-79.8%)	92.48% (90%-95.31%)	48.574% (42.029%-54.933%)	75.66% (71.81%-79.47%)	78.89% (72.09%-86.12%)	2.9561 (2.8091-3.0980)	76.51% (69.7%-83.84%)	92.69% (86.76%-98.15%)	48.732% (35.893%-62.51%)	75.62% (68%-83.12%)	79.57% (65%-93.76%)	2.9662 (2.6822-3.2421)
At least 2 of CHASM (melanoma), Mutation Assessor, MutationTaster, PolyPhen-2, VEST	74.72% (71.21%-77.79%)	90.35% (87.6%-93.5%)	47.675% (41.174%-53.846%)	74.91% (70.86%-78.95%)	74.09% (67.35%-81.4%)	2.8703 (2.7283-3.0207)	74.81% (68.66%-81.82%)	90.56% (84.37%-96.37%)	47.79% (35.135%-61.29%)	74.84% (66.67%-82.9%)	74.7% (59.98%-88.24%)	2.8789 (2.5743-3.1566)
At least 2 of CHASM (melanoma), Mutation Assessor, MutationTaster, PROVEAN	80.12% (77.27%-83.33%)	86.66% (83.19%-90.15%)	68.799% (62.856%-74.691%)	82.76% (79.07%-86.37%)	74.9% (69.12%-80.96%)	3.1312 (2.9933-3.2783)	80.17% (73.74%-85.86%)	86.77% (79.62%-93.44%)	68.837% (56.757%-81.08%)	82.68% (75.38%-89.71%)	75.22% (62.85%-86.85%)	3.1351 (2.8367-3.4021)
At least 2 of CHASM (melanoma), Mutation Assessor, MutationTaster, PROVEAN, SIFT	77.77% (74.75%-80.81%)	90.92% (88.24%-93.99%)	55.02% (48.529%-61.844%)	77.75% (73.79%-81.69%)	77.81% (71.7%-84.91%)	3.0150 (2.8800-3.1518)	77.8% (71.72%-83.84%)	91.02% (85%-96.61%)	55.088% (42.094%-68.57%)	77.67% (69.74%-85.34%)	78.18% (64.7%-90.49%)	3.0196 (2.7439-3.2861)
At least 2 of CHASM (melanoma), Mutation Assessor, MutationTaster, PROVEAN, VEST	74.42% (71.21%-77.78%)	89.31% (86.29%-92.37%)	48.67% (42.254%-55.556%)	75.05% (70.95%-79.02%)	72.47% (65.91%-80%)	2.8550 (2.7134-3.0087)	74.39% (67.68%-80.81%)	89.46% (83.04%-95.31%)	48.514% (35.294%-61.77%)	74.89% (66.67%-82.89%)	72.85% (56.67%-86.36%)	2.8570 (2.5396-3.1404)
At least 2 of CHASM (melanoma), Mutation Assessor, MutationTaster, SIFT	79.43% (76.26%-82.83%)	89.83% (86.92%-92.91%)	61.435% (55.262%-68.116%)	80.11% (76.42%-83.94%)	77.75% (71.93%-84.21%)	3.0913 (2.9534-3.2287)	79.53% (72.73%-85.86%)	90.01% (83.87%-95.52%)	61.532% (48.571%-74.29%)	80.06% (72.46%-87.33%)	78.23% (65.38%-90.01%)	3.0983 (2.8127-3.3622)
At least 2 of CHASM (melanoma), Mutation Assessor, MutationTaster, SIFT, VEST	72.33% (68.69%-75.77%)	92.47% (90%-95.2%)	37.501% (31.167%-43.662%)	71.89% (67.9%-76.1%)	74.25% (66.67%-82.35%)	2.7612 (2.6094-2.9065)	72.51% (65.63%-79.8%)	92.7% (87.3%-98.18%)	37.879% (25.714%-51.43%)	71.91% (63.29%-80.01%)	75.22% (58.81%-91.67%)	2.7771 (2.4707-3.0788)
At least 2 of CHASM (melanoma), Mutation Assessor, MutationTaster, VEST	76.43% (73.23%-79.8%)	88.24% (85.27%-91.74%)	56.011% (49.275%-62.667%)	77.62% (73.76%-81.69%)	73.36% (67.24%-80.36%)	2.9523 (2.8085-3.0979)	76.44% (69.7%-82.83%)	88.41% (81.67%-94.12%)	55.903% (42.857%-69.05%)	77.47% (69.33%-85.08%)	73.77% (59.38%-86.21%)	2.9555 (2.6612-3.2421)
At least 2 of CHASM (melanoma), Mutation Assessor, PolyPhen-2	74.05% (70.69%-77.78%)	79.75% (75.81%-83.72%)	64.184% (57.745%-70.372%)	79.38% (75.2%-83.47%)	64.71% (58.44%-71.02%)	2.8802 (2.7362-3.0281)	74.13% (66.67%-80.83%)	79.84% (71.88%-87.72%)	64.32% (51.613%-76.67%)	79.32% (70.97%-87.5%)	65.04% (51.61%-77.78%)	2.8852 (2.5837-3.1832)
At least 2 of CHASM (melanoma), Mutation Assessor, PolyPhen-2, PROVEAN	73.37% (70.19%-76.77%)	82.42% (78.74%-86.29%)	57.722% (51.282%-64.288%)	77.12% (72.93%-81.2%)	65.51% (59.02%-72.31%)	2.8277 (2.6807-2.9760)	73.46% (66.67%-79.82%)	82.48% (74.58%-89.83%)	57.964% (45.157%-71.05%)	77.09% (68.75%-85.08%)	65.85% (51.85%-78.57%)	2.8339 (2.5354-3.1286)
At least 2 of CHASM (melanoma), Mutation Assessor, PolyPhen-2, PROVEAN, SIFT	73.04% (69.19%-76.77%)	87.74% (84.55%-90.99%)	47.627% (40.845%-53.731%)	74.34% (70%-78.38%)	69.2% (62.22%-76.09%)	2.7891 (2.6326-2.9410)	73.11% (65.66%-80.81%)	87.8% (81.13%-93.94%)	47.874% (35.294%-61.12%)	74.29% (66.22%-82.86%)	69.6% (55.56%-83.33%)	2.7956 (2.4890-3.1064)
At least 2 of CHASM (melanoma), Mutation Assessor, PolyPhen-2, PROVEAN, VEST	69.33% (65.66%-72.73%)	84.52% (80.99%-88.24%)	43.058% (36.483%-49.276%)	71.96% (67.81%-76.19%)	61.67% (54.34%-69.23%)	2.6121 (2.4586-2.7600)	69.42% (62.63%-76.77%)	84.67% (77.05%-91.8%)	43.203% (30.764%-56.25%)	71.9% (63.64%-80.26%)	62.18% (46.15%-77.27%)	2.6196 (2.3175-2.9289)
At least 2 of CHASM (melanoma), Mutation Assessor, PolyPhen-2, SIFT	73.34% (69.7%-76.77%)	86.65% (83.33%-90%)	50.334% (43.66%-56.757%)	75.1% (70.92%-79.17%)	68.56% (61.82%-75.01%)	2.8065 (2.6533-2.9579)	73.51% (66.67%-80.81%)	86.79% (80%-93.44%)	50.721% (37.931%-63.89%)	75.13% (67.12%-83.34%)	69.12% (55.17%-82.61%)	2.8176 (2.5041-3.1194)
At least 2 of CHASM (melanoma), Mutation Assessor, PolyPhen-2, SIFT, VEST	69.62% (65.66%-73.23%)	88.76% (85.71%-92%)	36.527% (30.137%-42.667%)	70.74% (66.46%-75%)	65.29% (56.66%-73.17%)	2.6132 (2.4507-2.7667)	69.84% (62.63%-77.78%)	88.95% (82.35%-95.16%)	37.054% (25%-50%)	70.8% (62.16%-79.02%)	66.18% (50%-83.33%)	2.6297 (2.3022-2.9554)
[illegible]	[illegible]	[illegible]	[illegible]	[illegible]	[illegible]	[illegible]	[illegible]	[illegible]	[illegible]	[illegible]	[illegible]	[illegible]

At least 2 of MutationTaster, PolyPhen-2, VEST	73.06% (69.7%-76.26%)	82.39% (78.69%-86.4%)	56.934% (50.704%-63.514%)	76.78% (72.73%-81.02%)	65.16% (58.46%-71.67%)	2.8127 (2.6662-2.9656)	73.07% (66.67%-79.8%)	82.54% (74.58%-90%)	56.793% (43.331%-69.7%)	76.62% (67.99%-84.62%)	65.49% (51.61%-78.58%)	2.8144 (2.5041-3.1025)
At least 2 of MutationTaster, PROVEAN	65.67% (62.12%-69.19%)	47.91% (43.18%-53.03%)	96.354% (94.203%-98.667%)	95.78% (93.22%-98.46%)	51.69% (46.67%-56.52%)	2.9174 (2.8124-3.0229)	65.64% (58.59%-72.73%)	47.77% (37.49%-57.58%)	96.276% (91.176%-100%)	95.65% (89.66%-100%)	51.79% (42.03%-61.76%)	2.9148 (2.7009-3.1282)
At least 2 of MutationTaster, PROVEAN, SIFT	76.07% (72.73%-79.29%)	78.18% (74.38%-82.17%)	72.426% (66.667%-78.574%)	83.06% (79.13%-87.16%)	65.74% (59.76%-71.43%)	2.9940 (2.8571-3.1369)	76.14% (69.7%-82.83%)	78.2% (70%-85.94%)	72.581% (60%-83.88%)	83.03% (75%-90.91%)	65.97% (54.29%-77.14%)	2.9978 (2.7120-3.2729)
At least 2 of MutationTaster, PROVEAN, SIFT, VEST	68.66% (65.15%-72.22%)	82.93% (79.39%-86.83%)	43.975% (37.5%-50.65%)	71.9% (67.83%-76.03%)	59.83% (52.08%-67.31%)	2.5864 (2.4275-2.7355)	68.75% (61.62%-75.76%)	83.07% (75%-90.16%)	44.146% (31.028%-57.14%)	71.85% (63.51%-80%)	60.3% (45.44%-75.02%)	2.5936 (2.2865-2.9005)
At least 2 of MutationTaster, PROVEAN, VEST	73.12% (69.7%-76.77%)	75.54% (71.32%-80%)	68.915% (62.667%-75.342%)	80.78% (76.86%-84.96%)	61.97% (56.1%-68.24%)	2.8721 (2.7240-3.0255)	72.96% (65.66%-79.8%)	75.49% (67.16%-83.61%)	68.576% (55.556%-81.09%)	80.48% (71.92%-88.52%)	61.98% (50%-74.36%)	2.8653 (2.5677-3.1510)
At least 2 of MutationTaster, SIFT	80.11% (77.27%-83.33%)	73.88% (69.77%-78.33%)	90.873% (87.342%-94.872%)	93.33% (90.82%-96.23%)	66.8% (61.61%-71.85%)	3.2489 (3.1464-3.3640)	80.18% (73.74%-85.86%)	74.02% (65.08%-82.26%)	90.735% (82.855%-97.3%)	93.2% (86.96%-98.04%)	67.05% (56.52%-77.08%)	3.2500 (3.0175-3.4564)
At least 2 of MutationTaster, SIFT, VEST	70.66% (67.17%-74.24%)	81.32% (77.6%-85.59%)	52.235% (45.946%-58.667%)	74.64% (70.59%-78.91%)	61.79% (54.55%-68.86%)	2.6999 (2.5497-2.8524)	70.8% (63.64%-77.78%)	81.5% (72.88%-88.89%)	52.423% (39.987%-64.87%)	74.6% (66.1%-82.61%)	62.29% (48.27%-75.76%)	2.7081 (2.4076-2.9992)
At least 2 of MutationTaster, VEST	75.47% (72.22%-78.79%)	69.16% (64.52%-73.81%)	86.384% (82.192%-91.176%)	89.77% (86.36%-93.69%)	61.83% (56.31%-67.62%)	3.0715 (2.9482-3.2014)	75.32% (68.69%-81.82%)	69.11% (59.65%-78.33%)	85.969% (77.143%-94.29%)	89.4% (81.63%-95.92%)	61.86% (50.86%-72.23%)	3.0634 (2.8087-3.3118)
At least 2 of PolyPhen-2, PROVEAN	60.25% (56.06%-64.14%)	47.93% (42.86%-53.04%)	81.541% (76.189%-86.486%)	81.78% (76.47%-86.96%)	47.53% (42.27%-52.42%)	2.5878 (2.4432-2.7334)	60.31% (52.53%-68.69%)	47.72% (37.28%-57.97%)	81.857% (71.873%-91.9%)	81.86% (71.05%-92.31%)	47.71% (37.5%-58.33%)	2.5914 (2.3034-2.8858)
At least 2 of PolyPhen-2, PROVEAN, SIFT	70.34% (66.67%-73.75%)	76.57% (72.52%-80.49%)	59.572% (53.026%-66.216%)	76.61% (72.22%-81.1%)	59.52% (53.33%-65.39%)	2.7226 (2.5683-2.8665)	70.42% (63.61%-77.78%)	76.63% (68.51%-84.51%)	59.747% (46.87%-72.97%)	76.56% (68.18%-85.3%)	59.8% (47.06%-72.73%)	2.7273 (2.4286-3.0228)
At least 2 of PolyPhen-2, PROVEAN, SIFT, VEST	65.94% (62.63%-69.7%)	81.33% (77.69%-85.37%)	39.329% (32.876%-45.333%)	69.86% (65.75%-74%)	54.92% (47.17%-62.5%)	2.4544 (2.3013-2.6089)	66.1% (58.59%-72.73%)	81.48% (73.33%-88.89%)	39.682% (27.584%-52.78%)	69.86% (61.19%-78.21%)	55.53% (39.29%-71.43%)	2.4655 (2.1513-2.7742)
At least 2 of PolyPhen-2, PROVEAN, VEST	63.99% (60.1%-67.68%)	72.89% (68.75%-77.24%)	48.593% (42.308%-54.841%)	71.03% (66.67%-75.4%)	50.9% (44.12%-57.14%)	2.4340 (2.2855-2.5886)	63.94% (56.57%-71.72%)	72.82% (64.06%-81.25%)	48.659% (36.111%-61.54%)	70.88% (61.9%-80%)	51.05% (37.5%-63.89%)	2.4341 (2.1282-2.7357)
At least 2 of PolyPhen-2, SIFT	69.63% (66.16%-73.23%)	72.29% (68.22%-76.8%)	65.015% (58.904%-71.233%)	78.13% (73.91%-82.46%)	57.57% (51.28%-63.43%)	2.7302 (2.5874-2.8833)	69.83% (62.63%-76.77%)	72.41% (63.49%-80.4%)	65.359% (51.613%-77.78%)	78.2% (69.84%-86.46%)	57.98% (45.95%-69.77%)	2.7394 (2.4391-3.0249)
At least 2 of PolyPhen-2, SIFT, VEST	66.6% (62.63%-70.2%)	80.28% (76.61%-84.21%)	42.957% (36.364%-49.296%)	70.87% (66.67%-75.18%)	55.74% (48%-63.16%)	2.4985 (2.3405-2.6472)	66.79% (59.6%-74.75%)	80.39% (72.41%-87.88%)	43.463% (31.424%-57.15%)	70.92% (61.84%-79.22%)	56.35% (41.67%-70.97%)	2.5112 (2.2093-2.8317)
At least 2 of PolyPhen-2, VEST	62.95% (59.09%-66.67%)	65.41% (60.8%-70.25%)	58.703% (52.055%-65.217%)	73.25% (68.69%-78.07%)	49.53% (43.68%-55.56%)	2.4689 (2.3085-2.6204)	62.98% (55.56%-70.71%)	65.44% (55.93%-74.24%)	58.748% (45.714%-72.22%)	73.13% (63.16%-82.46%)	49.75% (37.13%-61.77%)	2.4707 (2.1691-2.7910)
At least 2 of PROVEAN, SIFT	60.25% (56.57%-64.14%)	50.03% (45.08%-54.92%)	77.907% (72.464%-83.562%)	79.66% (74.42%-84.72%)	47.41% (42.15%-52.46%)	2.5501 (2.4105-2.6906)	60.31% (52.53%-67.68%)	49.91% (39.66%-60%)	78.123% (66.667%-88.89%)	79.65% (69.69%-89.74%)	47.61% (37.29%-58.07%)	2.5530 (2.2633-2.8419)
At least 2 of PROVEAN, SIFT, VEST	63.6% (60.1%-67.17%)	72.34% (68.25%-76.61%)	48.463% (41.791%-55.405%)	70.82% (66.39%-75.19%)	50.33% (43.48%-57.14%)	2.4195 (2.2624-2.5762)	63.72% (56.57%-70.71%)	72.32% (63.79%-80.65%)	48.912% (35.135%-62.5%)	70.84% (62.12%-79.7%)	50.69% (37.83%-63.33%)	2.4276 (2.1164-2.7297)
At least 2 of PROVEAN, VEST	57.93% (54.04%-61.63%)	45.8% (40.65%-50.76%)	78.885% (73.682%-84.512%)	78.95% (73.61%-84.62%)	45.7% (40.65%-50.77%)	2.4935 (2.3446-2.6416)	57.88% (50.48%-65.66%)	45.6% (35.93%-55.56%)	78.883% (66.667%-89.47%)	78.76% (67.57%-89.47%)	45.8% (35.59%-55.94%)	2.4904 (2.1934-2.7920)
At least 2 of SIFT, VEST	63.25% (59.6%-66.68%)	67.01% (62.4%-71.65%)	56.723% (50.633%-63.52%)	72.8% (68.33%-77.5%)	49.86% (43.53%-55.95%)	2.4640 (2.3159-2.6190)	63.41% (56.54%-70.71%)	67.01% (57.81%-76.19%)	57.188% (43.746%-70.59%)	72.86% (63.16%-81.67%)	50.25% (38.09%-62.79%)	2.4731 (2.1691-2.7723)
At least 3 of CHASM (breast), CHASM (lung), CHASM (melanoma)	79.5% (76.26%-82.83%)	70.85% (66.38%-75.59%)	94.458% (91.667%-97.333%)	95.67% (93.48%-97.94%)	65.21% (60%-70.54%)	3.2619 (3.1548-3.3714)	79.38% (72.73%-85.86%)	70.53% (60.94%-79.42%)	94.56% (88.563%-100%)	95.7% (90.56%-100%)	65.16% (54.24%-76%)	3.2595 (3.0409-3.4780)
At least 3 of CHASM (breast), CHASM (lung), CHASM (melanoma), FATHMM (cancer)	81.89% (78.79%-84.86%)	76.19% (71.79%-80.49%)	91.733% (88.406%-95.775%)	94.1% (91.67%-97%)	69.03% (63.74%-74.44%)	3.3105 (3.2036-3.4255)	81.67% (75.73%-87.88%)	75.79% (67.19%-84.21%)	91.743% (83.871%-97.56%)	94.04% (88.46%-98.22%)	68.83% (58.13%-79.55%)	3.3040 (3.0744-3.5181)
At least 3 of CHASM (breast), CHASM (lung), CHASM (melanoma), FATHMM (cancer), FATHMM (missense)	85.91% (83.33%-88.89%)	85.71% (82.26%-89.34%)	86.228% (81.941%-91.304%)	91.5% (88.98%-94.64%)	77.74% (72.72%-83.12%)	3.4118 (3.3037-3.5346)	85.76% (79.8%-90.91%)	85.47% (78.12%-92.43%)	86.236% (76.471%-94.74%)	91.43% (85.45%-96.55%)	77.59% (67.42%-87.5%)	3.4072 (3.1648-3.6270)
At least 3 of CHASM (breast), CHASM (lung), CHASM (melanoma), FATHMM (cancer), Mutation Assessor	86.56% (83.84%-89.39%)	86.24% (82.81%-89.77%)	87.112% (82.895%-91.667%)	92.05% (89.6%-94.92%)	78.56% (73.42%-84%)	3.4396 (3.3319-3.5520)	86.47% (80.81%-91.92%)	86.01% (78.69%-92.98%)	87.225% (78.567%-94.87%)	92.04% (86.21%-96.77%)	78.44% (68.56%-88.89%)	3.4371 (3.2153-3.6484)
At least 3 of CHASM (breast), CHASM (lung), CHASM (melanoma), FATHMM (cancer), MutationTaster	87.92% (85.35%-90.4%)	87.29% (84.03%-90.55%)	88.992% (85.071%-93.243%)	93.2% (90.91%-95.87%)	80.21% (75.31%-85.06%)	3.4969 (3.3959-3.6074)	87.8% (82.83%-92.93%)	87.1% (80.33%-93.55%)	88.976% (80.488%-96.97%)	93.13% (87.3%-98.15%)	80.1% (70.27%-89.74%)	3.4930 (3.2737-3.6888)
At least 3 of CHASM (breast), CHASM (lung), CHASM (melanoma), FATHMM (cancer), PolyPhen-2	85.22% (82.32%-88.38%)	86.23% (82.68%-89.76%)	83.483% (78.873%-88.732%)	90.03% (87.29%-93.33%)	77.81% (72.5%-83.12%)	3.3755 (3.2596-3.4986)	85.11% (78.79%-90.91%)	86.03% (78.94%-93.1%)	83.488% (73.168%-92.5%)	89.94% (83.33%-95.39%)	77.72% (66.67%-88.24%)	3.3719 (3.1153-3.6053)
At least 3 of CHASM (breast), CHASM (lung), CHASM (melanoma), FATHMM (cancer), PROVEAN	83.54% (80.81%-86.87%)	80.42% (76.47%-84.43%)	88.926% (84.932%-93.058%)	92.62% (90.18%-95.45%)	72.44% (67.05%-77.78%)	3.3441 (3.2308-3.4688)	83.42% (76.77%-88.89%)	80.09% (71.87%-87.88%)	89.096% (80.643%-97.22%)	92.65% (86.95%-98.04%)	72.31% (60.98%-82.99%)	3.3415 (3.0918-3.5728)
At least 3 of CHASM (breast), CHASM (lung), CHASM (melanoma), FATHMM (cancer), SIFT	86.56% (83.84%-89.39%)	86.76% (83.33%-90.37%)	86.21% (81.69%-91.176%)	91.58% (88.98%-94.69%)	79.03% (73.97%-84.15%)	3.4358 (3.3317-3.5508)	86.47% (80.81%-91.92%)	86.56% (79.37%-93.22%)	86.275% (76.471%-94.74%)	91.55% (85.48%-96.67%)	78.94% (68.29%-89.47%)	3.4332 (3.1978-3.6462)
At least 3 of CHASM (breast), CHASM (lung), CHASM (melanoma), FATHMM (cancer), VEST	86.24% (83.84%-88.89%)	86.77% (83.47%-90.16%)	85.334% (80.822%-90.411%)	91.1% (88.49%-94.22%)	78.87% (73.75%-84.15%)	3.4206 (3.3149-3.5412)	86.1% (80.81%-90.91%)	86.55% (79.69%-93.22%)	85.285% (75.595%-94.29%)	90.99% (84.75%-96.49%)	78.73% (68.29%-88.9%)	3.4156 (3.1687-3.6276)
At least 3 of CHASM (breast), CHASM (lung), CHASM (melanoma), FATHMM (missense)	80.54% (77.27%-83.84%)	74.59% (70%-78.86%)	90.818% (87.143%-94.667%)	93.35% (90.82%-96.12%)	67.39% (62.1%-72.63%)	3.2615 (3.1516-3.3828)	80.34% (73.74%-86.87%)	74.22% (65.57%-83.33%)	90.829% (82.857%-97.44%)	93.28% (87.23%-98.08%)	67.25% (56.52%-78.27%)	3.2558 (3.0116-3.4851)
At least 3 of CHASM (breast), CHASM (lung), CHASM (melanoma), FATHMM (missense), Mutation Assessor	87.26% (84.85%-89.9%)	87.33% (84%-90.77%)	87.129% (82.895%-91.667%)	92.15% (89.66%-94.96%)	79.91% (75%-85.14%)	3.4651 (3.3609-3.5759)	87.1% (81.82%-91.92%)	87.03% (80.3%-93.65%)	87.187% (78.372%-94.87%)	92.1% (86.44%-96.88%)	79.7% (69.99%-89.75%)	3.4602 (3.2340-3.6708)
At least 3 of CHASM (breast), CHASM (lung), CHASM (melanoma), FATHMM (missense), MutationTaster	87.93% (85.35%-90.4%)	87.85% (84.55%-91.13%)	88.077% (84.058%-92.86%)	92.72% (90.27%-95.73%)	80.74% (75.95%-85.71%)	3.4939 (3.3959-3.6044)	87.77% (82.83%-92.93%)	87.59% (80.95%-94.03%)	88.063% (78.947%-95.57%)	92.64% (86.79%-97%)	80.55% (70.27%-90.25%)	3.4884 (3.2636-3.6893)
At least 3 of CHASM (breast), CHASM (lung), CHASM (melanoma), FATHMM (missense), PolyPhen-2	84.9% (82.32%-87.88%)	86.78% (83.33%-90.17%)	81.641% (76.469%-86.842%)	89.1% (86.21%-92.31%)	78.13% (72.86%-84%)	3.3565 (3.2363-3.4797)	84.75% (78.79%-89.9%)	86.53% (79.66%-93.34%)	81.634% (71.041%-91.43%)	89% (82.46%-95%)	77.96% (66.67%-88.58%)	3.3513 (3.1057-3.5853)
At least 3 of CHASM (breast), CHASM (lung), CHASM (melanoma), FATHMM (missense), PROVEAN	83.55% (80.81%-86.36%)	80.95% (76.98%-85.04%)	88.033% (84.058%-92.54%)	92.13% (89.52%-95.29%)	72.78% (67.42%-78.31%)	3.3389 (3.2277-3.4550)	83.41% (77.78%-88.89%)	80.63% (72.41%-88.71%)	88.13% (78.947%-96.78%)	92.11% (85.96%-97.92%)	72.64% (61.9%-83.72%)	3.3350 (3.1041-3.5566)
At least 3 of CHASM (breast), CHASM (lung), CHASM (melanoma), FATHMM (missense), SIFT	85.56% (82.83%-88.38%)	87.85% (84.55%-91.13%)	81.59% (76.712%-86.887%)	89.19% (86.32%-92.42%)	79.52% (74.62%-84.93%)	3.3815 (3.2675-3.5025)	85.45% (79.8%-90.91%)	87.59% (80.95%-94.03%)	81.727% (71.053%-91.43%)	89.18% (82.54%-95.08%)	79.35% (68.96%-89.74%)	3.3784 (3.1384-3.6064)
At least 3 of CHASM (breast), CHASM (lung), CHASM (melanoma), FATHMM (missense), VEST	85.57% (82.83%-88.38%)	87.32% (84%-90.98%)	82.532% (77.778%-87.5%)	89.63% (86.82%-92.68%)	79.02% (73.97%-84.42%)	3.3850 (3.2710-3.5008)	85.43% (79.8%-90.91%)	87.04% (79.99%-93.65%)	82.608% (72.727%-91.9%)	89.58% (83.33%-95.16%)	78.82% (67.65%-89.19%)	3.3806 (3.1432-3.6067)
At least 3 of CHASM (breast), CHASM (lung), CHASM (melanoma), Mutation Assessor	82.22% (79.29%-85.35%)	76.18% (71.77%-80.32%)	92.66% (89.189%-96%)	94.72% (92.38%-97.12%)	69.23% (63.83%-74.49%)	3.3280 (3.2223-3.4390)	82.02% (75.76%-87.88%)	75.82% (67.21%-84.21%)	92.645% (85.294%-100%)	94.66% (89.36%-100%)	69.07% (58.7%-79.59%)	3.3218 (3.0965-3.5332)
At least 3 of CHASM (breast), CHASM (lung), CHASM (melanoma), Mutation Assessor, MutationTaster	89.95% (87.88%-92.42%)	89.45% (86.4%-92.68%)	90.821% (87.179%-94.668%)	94.4% (92.37%-96.75%)	83.28% (78.66%-88%)	3.5794 (3.4832-3.6827)	89.79% (84.85%-93.94%)	89.18% (83.05%-95.31%)	90.825% (82.925%-97.44%)	94.35% (89.09%-98.33%)	83.05% (73.52%-92.11%)	3.5740 (3.3709-3.7572)
At least 3 of CHASM (breast), CHASM (lung), CHASM (melanoma), Mutation Assessor, PolyPhen-2	86.92% (84.34%-89.9%)	88.38% (85.12%-91.48%)	84.405% (79.746%-89.19%)	90.74% (88.1%-93.8%)	80.78% (75.64%-85.9%)	3.4430 (3.3329-3.5622)	86.76% (80.81%-91.92%)	88.12% (81.53%-94.92%)	84.388% (75%-93.03%)	90.64% (84.48%-95.66%)	80.58% (70.26%-91.18%)	3.4373 (3.2106-3.6633)
At least 3 of CHASM (breast), CHASM (lung), CHASM (melanoma), Mutation Assessor, PROVEAN	83.88% (81.31%-86.87%)	80.96% (76.92%-85%)	88.926% (85.071%-93.151%)	92.67% (90.27%-95.41%)	72.99% (67.44%-78.31%)	3.3554 (3.2495-3.4758)	83.75% (77.78%-88.89%)	80.62% (72.46%-88.71%)	89.096% (80.556%-97.14%)	92.7% (87.27%-98.04%)	72.85% (62.16%-83.72%)	3.3526 (3.1119-3.5599)
At least 3 of CHASM (breast), CHASM (lung), CHASM (melanoma), Mutation Assessor, SIFT	87.58% (84.85%-90.4%)	88.9% (85.83%-92.07%)	85.303% (80.882%-89.707%)	91.27% (88.62%-94.17%)	81.63% (76.71%-86.67%)	3.4711 (3.3638-3.5805)	87.46% (81.82%-92.93%)	88.68% (82.26%-95.08%)	85.326% (76.079%-94.29%)	91.21% (85.45%-96.61%)	81.48% (71.43%-91.43%)	3.4670 (3.2474-3.6799)
At least 3 of CHASM (breast), CHASM (lung), CHASM (melanoma), Mutation Assessor, VEST	87.62% (85.35%-90.4%)	88.92% (85.83%-92.13%)	85.354% (81.079%-90.278%)	91.3% (88.79%-94.35%)	81.68% (76.71%-86.42%)	3.4726 (3.3703-3.5876)	87.39% (81.82%-91.92%)	88.63% (82.25%-95.08%)	85.238% (75.676%-94.29%)	91.16% (84.85%-96.55%)	81.41% (71.43%-91.43%)	3.4644 (3.2247-3.6670)
At least 3 of CHASM (breast), CHASM (lung), CHASM (melanoma), MutationTaster	82.57% (79.8%-85.86%)	76.73% (72.44%-80.99%)	92.66% (89.189%-96%)	94.76% (92.45%-97.14%)	69.73% (64.28%-75%)	3.3388 (3.2341-3.4536)	82.33% (75.76%-87.88%)	76.31% (67.74%-85.25%)	92.645% (85.294%-100%)	94.69% (89.36%-100%)	69.51% (59.09%-80.01%)	3.3315 (3.1073-3.5424)
At least 3 of CHASM (breast), CHASM (lung), CHASM (melanoma), MutationTaster, PolyPhen-2	88.6% (86.36%-91.41%)	88.9% (85.71%-92.06%)	88.087% (84.058%-92.647%)	92.81% (90.43%-95.76%)	82.12% (77.33%-87.01%)	3.5191 (3.4151-3.6335)	88.45% (82.83%-92.93%)	88.67% (82.14%-95.08%)	88.046% (78.947%-95.57%)	92.71% (86.67%-97.88%)	81.96% (71.43%-91.89%)	3.5139 (3.2902-3.7178)
At least 3 of CHASM (breast), CHASM (lung), CHASM (melanoma), MutationTaster, PROVEAN	85.24% (82.82%-88.38%)	81.48% (77.5%-85.6%)	91.726% (88.235%-95.654%)	94.45% (92.23%-97.17%)	74.13% (68.89%-79.35%)	3.4179 (3.3196-3.5319)	85.08% (78.79%-89.92%)	81.17% (72.88%-89.07%)	91.768% (84.211%-97.67%)	94.42% (89.09%-98.33%)	73.99% (63.41%-84.78%)	3.4135 (3.1859-3.6094)
At least 3 of CHASM (breast), CHASM (lung), CHASM (melanoma), MutationTaster, SIFT	89.94% (87.88%-92.42%)	89.97% (86.92%-92.97%)	89.894% (86.111%-94.286%)	93.9% (91.8%-96.64%)	83.83% (79.22%-88.46%)	3.5759 (3.4812-3.6806)	89.81% (84.85%-93.94%)	89.73% (83.33%-95.46%)	89.922% (81.25%-97.3%)	93.86% (88.52%-98.28%)	83.64% (74.29%-92.5%)	3.5716 (3.3661-3.7531)
At least 3 of CHASM (breast), CHASM (lung), CHASM (melanoma), MutationTaster, VEST	89.27% (86.87%-91.92%)	89.97% (86.99%-92.97%)	88.063% (84.058%-93.056%)	92.87% (90.55%-95.8%)	83.56% (78.95%-88.31%)	3.5446 (3.4485-3.6571)	89.13% (83.84%-93.94%)	89.72% (83.33%-95.39%)	88.09% (78.567%-95.35%)	92.82% (87.03%-97.06%)	83.34% (73.53%-92.11%)	3.5397 (3.3166-3.7294)
At least 3 of CHASM (breast), CHASM (lung), CHASM (melanoma), PolyPhen-2	82.23% (79.29%-85.35%)	76.73% (72.44%-80.99%)	91.746% (88.155%-95.716%)	94.14% (91.59%-97.03%)	69.52% (64%-74.75%)	3.3214 (3.2121-3.4390)	82% (75.76%-87.88%)	76.31% (67.74%-85.25%)	91.732% (84.211%-100%)	94.06% (88.24%-100%)	69.3% (59.08%-80%)	3.3140 (3.0801-3.5332)
At least 3 of CHASM (breast), CHASM (lung), CHASM (melanoma), PolyPhen-2, PROVEAN	83.54% (80.81%-86.87%)	81.48% (77.5%-85.6%)	87.093% (82.665%-91.667%)	91.61% (88.89%-94.55%)	73.12% (67.78%-78.41%)	3.3330 (3.2175-3.4576)	83.43% (76.77%-88.89%)	81.17% (72.88%-89.07%)	87.267% (78.372%-95.12%)	91.62% (85.71%-96.72%)	73.01% (62.5%-84.09%)	3.3308 (3.0819-3.5561)
At least 3 of CHASM (breast), CHASM (lung), CHASM (melanoma), PolyPhen-2, SIFT	84.9% (82.32%-87.88%)	88.9% (85.71%-92.06%)	77.988% (72.603%-83.562%)	87.47% (84.33%-90.7%)	80.26% (75%-85.71%)	3.3462 (3.2217-3.4780)	84.74% (78.79%-89.9%)	88.67% (82.14%-95.08%)	77.952% (67.479%-88.89%)	87.35% (80.64%-93.75%)	80.08% (68.57%-90.63%)	3.3405 (3.0826-3.5797)
At least 3 of CHASM (breast), CHASM (lung), CHASM (melanoma), PolyPhen-2, VEST	84.59% (81.82%-87.88%)	88.37% (85.12%-91.87%)	78.047% (72.464%-83.583%)	87.44% (84.38%-91.13%)	79.53% (74.32%-85%)	3.3339 (3.2143-3.4621)	84.35% (77.78%-89.9%)	88.13% (81.13%-94.83%)	77.833% (67.373%-88.57%)	87.22% (79.71%-93.55%)	79.3% (67.74%-90%)	3.3248 (3.0640-3.5655)
At least 3 of CHASM (breast), CHASM (lung), CHASM (melanoma), PROVEAN	81.55% (78.28%-84.85%)	74.61% (70%-79.03%)	93.547% (90.411%-97.183%)	95.24% (93%-97.92%)	68.06% (62.77%-73.47%)	3.3145 (3.2025-3.4247)	81.35% (74.75%-87.88%)	74.18% (65.08%-83.61%)	93.62% (86.842%-100%)	95.23% (90.2%-100%)	67.88% (57.14%-78.44%)	3.3092 (3.0901-3.5330)
At least 3 of CHASM (breast), CHASM (lung), CHASM (melanoma), PROVEAN, SIFT	83.53% (80.81%-86.87%)	81.48% (77.5%-85.6%)	87.08% (82.852%-91.667%)	91.6% (88.89%-94.55%)	73.12% (67.78%-78.57%)	3.3328 (3.2175-3.4600)	83.44% (76.77%-88.89%)	81.17% (72.88%-89.07%)	87.278% (78.375%-95.35%)	91.64% (85.71%-96.77%)	73.01% (62.49%-84.09%)	3.3310 (3.0775-3.5560)
At least 3 of CHASM (breast), CHASM (lung), CHASM (melanoma), PROVEAN, VEST	83.89% (81.3%-86.87%)	80.95% (76.98%-85.04%)	88.961% (85.134%-93.245%)	92.69% (90.19%-95.58%)	72.99% (67.7%-78.41%)	3.3559 (3.2466-3.4697)	83.74% (77.78%-88.91%)	80.63% (72.13%-88.71%)	89.033% (80.476%-97.14%)	92.66% (86.53%-98.04%)	72.84% (61.9%-83.79%)	3.3516 (3.1245-3.5747)
At least 3 of CHASM (breast), CHASM (lung), CHASM (melanoma), SIFT	81.88% (78.79%-84.85%)	76.18% (71.77%-80.32%)	91.733% (88.406%-95.775%)	94.1% (91.75%-97%)	69.02% (63.72%-74.45%)	3.3103 (3.2068-3.4246)	81.69% (75.76%-87.88%)	75.82% (67.21%-84.21%)	91.743% (83.871%-97.56%)	94.04% (88.46%-98.25%)	68.86% (58.14%-79.25%)	3.3045 (3.0802-3.5180)
At least 3 of CHASM (breast), CHASM (lung), CHASM (melanoma), SIFT, VEST	85.93% (83.33%-88.89%)	89.44% (86.4%-92.74%)	79.844% (74.667%-85.247%)	88.47% (85.38%-91.81%)	81.4% (76.47%-86.42%)	3.3915 (3.2776-3.5199)	85.72% (79.8%-90.91%)	89.19% (82.54%-95.24%)	79.745% (69.231%-90%)	88.32% (81.54%-94.65%)	81.15% (70.59%-91.18%)	3.3840 (3.1301-3.6179)
At least 3 of CHASM (breast), CHASM (lung), CHASM (melanoma), VEST	81.89% (78.79%-84.85%)	76.73% (72.44%-80.99%)	90.818% (87.143%-94.667%)	93.53% (91.09%-96.23%)	69.3% (63.83%-74.5%)	3.3038 (3.1968-3.4199)	81.66% (75.76%-87.88%)	76.31% (67.74%-85.25%)	90.829% (82.857%-97.44%)	93.46% (87.71%-98.11%)	69.09% (58.33%-79.6%)	3.2968 (3.0590-3.5145)
At least 3 of CHASM (breast), CHASM (lung), FATHMM (cancer)	80.54% (77.27%-83.84%)	71.96% (67.24%-76.42%)	95.379% (92.857%-98.592%)	96.42% (94.56%-98.91%)	66.3% (60.95%-71.43%)	3.3006 (3.1966-3.4130)	80.33% (73.74%-86.87%)	71.5% (62.3%-80.88%)	95.474% (89.474%-100%)	96.45% (91.67%-100%)	66.12% (55.77%-76.93%)	3.2954 (3.0732-3.5122)
At least 3 of CHASM (breast), CHASM (lung), FATHMM (cancer), FATHMM (missense)	81.89% (78.79%-84.85%)	76.21% (71.77%-80.49%)	91.72% (88.406%-95.775%)	94.09% (91.75%-97.03%)	69.04% (63.63%-74.47%)	3.3106 (3.1971-3.4277)	81.66% (75.76%-87.88%)	75.76% (67.19%-84.48%)	91.78% (83.871%-97.56%)	94.05% (88.24%-98.21%)	68.82% (58.33%-80%)	3.3041 (3.0665-3.5330)
At least 3 of CHASM (breast), CHASM (lung), FATHMM (cancer), FATHMM (missense), Mutation Assessor	86.26% (83.84%-88.89%)	88.38% (85.04%-91.74%)	82.567% (77.918%-87.838%)	89.76% (86.99%-92.86%)	80.44% (75.34%-85.53%)	3.4116 (3.3020-3.5319)	86.07% (80.81%-90.91%)	88.1% (81.35%-94.92%)	82.552% (72.214%-91.89%)	89.66% (83.05%-95.16%)	80.21% (70%-90.33%)	3.4053 (3.1591-3.6214)
At least 3 of CHASM (breast), CHASM (lung), FATHMM (cancer), FATHMM (missense), MutationTaster	89.64% (87.37%-91.92%)	90.52% (87.6%-93.7%)	88.107% (84.207%-92.537%)	92.94% (90.55%-95.87%)	84.32% (79.73%-88.89%)	3.5588 (3.4643-3.6631)	89.41% (84.85%-93.94%)	90.22% (83.82%-96.43%)	87.985% (78.788%-95.57%)	92.8% (86.89%-97.22%)	84.01% (74.42%-93.11%)	3.5502 (3.3330-3.7429)
At least 3 of CHASM (breast), CHASM (lung), FATHMM (cancer), FATHMM (missense), PolyPhen-2	84.91% (82.32%-87.88%)	89.45% (86.44%-92.62%)	77.068% (71.617%-82.895%)	87.09% (83.97%-90.63%)	80.86% (75.71%-86.15%)	3.3447 (3.2290-3.4713)	84.72% (78.79%-89.9%)	89.17% (82.81%-95.16%)	77.029% (64.997%-88.24%)	86.96% (79.99%-93.33%)	80.58% (70.27%-90.91%)	3.3374 (3.0768-3.5708)
At least 3 of CHASM (breast), CHASM (lung), FATHMM (cancer), FATHMM (missense), PROVEAN	83.25% (80.3%-86.36%)	82.06% (78.33%-86.05%)	85.303% (80.822%-90%)	90.62% (87.83%-93.75%)	73.34% (67.86%-78.82%)	3.3132 (3.1922-3.4336)	82.99% (76.77%-88.89%)	81.6% (73.43%-89.47%)	85.328% (75.756%-94.44%)	90.53% (84.31%-96.36%)	73.01% (61.76%-83.79%)	3.3047 (3.0618-3.5487)
At least 3 of CHASM (breast), CHASM (lung), FATHMM (cancer), FATHMM (missense), SIFT	84.18% (81.31%-87.37%)	88.9% (85.83%-92.19%)	76.004% (70.586%-82.192%)	86.5% (83.33%-90.08%)	79.85% (74.29%-85.29%)	3.3126 (3.1909-3.4421)	84.16% (77.78%-89.9%)	88.66% (81.97%-95.08%)	76.392% (64.276%-87.1%)	86.58% (79.69%-92.65%)	79.72% (68.75%-90.62%)	3.3135 (3.0484-3.5597)
At least 3 of CHASM (breast), CHASM (lung), FATHMM (cancer), FATHMM (missense), VEST	83.86% (80.81%-86.87%)	89.44% (86.4%-92.68%)	74.204% (68.116%-80.282%)	85.71% (82.4%-89.32%)	80.26% (74.63%-85.72%)	3.2961 (3.1690-3.4259)	83.79% (77.78%-89.9%)	89.18% (82.76%-95.24%)	74.49% (62.5%-85.71%)	85.72% (78.57%-92.31%)	80.08% (68.75%-90.62%)	3.2948 (3.0342-3.5385)
At least 3 of CHASM (breast), CHASM (lung), FATHMM (cancer), Mutation Assessor	83.24% (80.3%-86.36%)	78.33% (74.22%-82.44%)	91.726% (88.235%-95.654%)	94.24% (91.92%-97.09%)	71% (65.59%-76.35%)	3.3529 (3.2467-3.4700)	83.02% (76.77%-88.89%)	77.91% (69.64%-86.15%)	91.768% (84.211%-97.67%)	94.2% (88.68%-98.28%)	70.77% (60%-81.4%)	3.3465 (3.1148-3.5626)
At least 3 of CHASM (breast), CHASM (lung), FATHMM (cancer), Mutation Assessor, MutationTaster	89.6% (87.37%-92.42%)	89.95% (86.99%-93.02%)	88.992% (85.332%-93.06%)	93.39% (91.27%-96%)	83.67% (79.16%-88.41%)	3.5600 (3.4603-3.6676)	89.49% (83.84%-93.94%)	89.77% (83.33%-95.52%)	88.985% (80.645%-96.77%)	93.32% (87.69%-98.08%)	83.55% (74.29%-92.5%)	3.5562 (3.3423-3.7519)
At least 3 of CHASM (breast), CHASM (lung), FATHMM (cancer), Mutation Assessor, PolyPhen-2	85.21% (82.32%-87.88%)	89.42% (86.4%-92.74%)	77.951% (72.603%-83.784%)	87.51% (84.38%-91.06%)	80.99% (75.71%-86.37%)	3.3587 (3.2333-3.4821)	85.13% (79.8%-90.91%)	89.24% (82.54%-95.24%)	78.068% (67.5%-88.89%)	87.46% (80.33%-93.75%)	80.9% (70.37%-91.18%)	3.3566 (3.1112-3.6119)
At least 3 of CHASM (breast), CHASM (lung), FATHMM (cancer), Mutation Assessor, PROVEAN	82.55% (79.29%-85.35%)	83.09% (79.2%-87.1%)	81.606% (76.469%-86.567%)	88.65% (85.34%-92.11%)	73.63% (67.86%-79.31%)	3.2697 (3.1336-3.3914)	82.38% (76.77%-88.89%)	82.74% (74.99%-90.48%)	81.734% (72.222%-92.11%)	88.6% (81.82%-95.08%)	73.42% (61.7%-85.37%)	3.2650 (3.0126-3.5403)
At least 3 of CHASM (breast), CHASM (lung), FATHMM (cancer), Mutation Assessor, SIFT	85.55% (82.32%-88.38%)	89.95% (86.99%-93.02%)	77.932% (72.222%-83.562%)	87.57% (84.38%-90.98%)	81.77% (76.56%-87.1%)	3.3723 (3.2491-3.4993)	85.47% (79.8%-91.92%)	89.77% (83.33%-95.52%)	78.081% (67.5%-89.19%)	87.54% (80.95%-93.85%)	81.66% (71.05%-91.43%)	3.3704 (3.1161-3.6213)
At least 3 of CHASM (breast), CHASM (lung), FATHMM (cancer), Mutation Assessor, VEST	85.22% (82.32%-87.88%)	89.95% (86.99%-93.02%)	77.049% (71.424%-82.609%)	87.14% (84%-90.55%)	81.6% (76.31%-86.77%)	3.3574 (3.2339-3.4779)	85.11% (79.8%-90.91%)	89.77% (83.33%-95.52%)	77.111% (65.789%-88.58%)	87.05% (80.27%-93.44%)	81.47% (70.83%-91.43%)	3.3540 (3.1074-3.6116)
At least 3 of CHASM (breast), CHASM (lung), FATHMM (cancer), MutationTaster	83.24% (80.3%-86.36%)	78.33% (74.22%-82.44%)	91.726% (88.235%-95.654%)	94.24% (91.92%-97.09%)	71% (65.59%-76.35%)	3.3529 (3.2467-3.4700)	83.02% (76.77%-88.89%)	77.91% (69.64%-86.15%)	91.768% (84.211%-97.67%)	94.2% (88.68%-98.28%)	70.77% (60%-81.4%)	3.3465 (3.1148-3.5626)
At least 3 of CHASM (breast), CHASM (lung), FATHMM (cancer), MutationTaster, PolyPhen-2	90.29% (87.88%-92.93%)	92.07% (89.6%-95.12%)	87.204% (83.117%-91.67%)	92.56% (90.24%-95.28%)	86.42% (81.94%-91.05%)	3.5825 (3.4877-3.6926)	90.13% (84.85%-94.95%)	91.92% (85.94%-96.83%)	87.069% (78.116%-94.74%)	92.41% (86.66%-96.88%)	86.26% (76.92%-94.74%)	3.5766 (3.3596-3.7680)
At least 3 of CHASM (breast), CHASM (lung), FATHMM (cancer), MutationTaster, PROVEAN	86.6% (83.84%-89.39%)	84.15% (80.45%-88%)	90.841% (87.319%-94.667%)	94.08% (91.82%-96.55%)	76.83% (71.59%-82.14%)	3.4590 (3.3522-3.5710)	86.39% (80.81%-91.92%)	83.82% (76.18%-91.38%)	90.777% (83.323%-97.44%)	93.98% (88.52%-98.28%)	76.59% (65.85%-87.24%)	3.4516 (3.2203-3.6686)
At least 3 of CHASM (breast), CHASM (lung), FATHMM (cancer), MutationTaster, SIFT	89.96% (87.88%-92.42%)	91.01% (88.28%-94.26%)	88.124% (84.058%-92.647%)	92.98% (90.68%-95.87%)	85.02% (80.55%-89.86%)	3.5714 (3.4748-3.6790)	89.78% (84.85%-93.94%)	90.83% (84.61%-96.55%)	87.962% (79.412%-95.24%)	92.83% (87.1%-96.97%)	84.85% (75.68%-93.75%)	3.5648 (3.3507-3.7535)
At least 3 of CHASM (breast), CHASM (lung), FATHMM (cancer), MutationTaster, VEST	90.61% (88.38%-92.93%)	92.59% (90.08%-95.39%)	87.179% (83.099%-91.892%)	92.58% (90.32%-95.35%)	87.2% (83.1%-91.78%)	3.5955 (3.5005-3.6981)	90.5% (85.86%-94.95%)	92.46% (86.67%-97.02%)	87.113% (78.042%-94.74%)	92.48% (86.66%-98.08%)	87.09% (77.78%-95%)	3.5914 (3.3888-3.7835)
At least 3 of CHASM (breast), CHASM (lung), FATHMM (cancer), PolyPhen-2	82.91% (80.3%-85.86%)	78.33% (74.22%-82.44%)	90.84% (87.143%-94.668%)	93.66% (91.18%-96.3%)	70.8% (65.59%-76.14%)	3.3363 (3.2298-3.4530)	82.66% (76.77%-88.89%)	77.91% (69.64%-86.15%)	90.798% (82.752%-97.5%)	93.55% (87.76%-98.18%)	70.55% (60%-81.4%)	3.3281 (3.0864-3.5447)
At least 3 of CHASM (breast), CHASM (lung), FATHMM (cancer), PolyPhen-2, PROVEAN	82.54% (79.29%-85.86%)	84.15% (80.45%-88%)	79.761% (74.286%-85.294%)	87.79% (84.55%-91.34%)	74.43% (68.75%-80.23%)	3.2613 (3.1256-3.3941)	82.39% (75.76%-88.89%)	83.82% (76.18%-91.38%)	79.922% (69.046%-91.18%)	87.75% (80.36%-94.64%)	74.22% (62.16%-86.05%)	3.2571 (2.9942-3.5317)
At least 3 of CHASM (breast), CHASM (lung), FATHMM (cancer), PolyPhen-2, SIFT	85.19% (82.32%-87.88%)	90.48% (87.6%-93.65%)	76.036% (70.769%-81.944%)	86.72% (83.46%-90.08%)	82.21% (77.05%-87.88%)	3.3544 (3.2299-3.4822)	85.18% (79.8%-90.91%)	90.3% (83.93%-95.71%)	76.348% (64.865%-87.1%)	86.76% (80%-93.33%)	82.12% (71.43%-91.67%)	3.3554 (3.1023-3.6017)
At least 3 of CHASM (breast), CHASM (lung), FATHMM (cancer), PolyPhen-2, VEST	84.52% (81.82%-87.37%)	90.99% (88.28%-94.12%)	73.334% (67.143%-79.71%)	85.51% (82.09%-89.15%)	82.49% (77.42%-88.06%)	3.3232 (3.2003-3.4535)	84.49% (78.79%-89.9%)	90.88% (84.37%-96.55%)	73.515% (60.712%-85.29%)	85.48% (78.33%-92.31%)	82.44% (71.43%-92.86%)	3.3232 (3.0620-3.5749)
At least 3 of CHASM (breast), CHASM (lung), FATHMM (cancer), PROVEAN	81.88% (78.79%-84.85%)	75.14% (70.63%-79.53%)	93.534% (90.541%-97.184%)	95.26% (93%-97.92%)	68.52% (63.27%-74%)	3.3245 (3.2198-3.4424)	81.69% (75.76%-87.88%)	74.71% (66.03%-83.61%)	93.658% (86.479%-100%)	95.29% (90.38%-100%)	68.34% (57.44%-79.17%)	3.3199 (3.0851-3.5281)
At least 3 of CHASM (breast), CHASM (lung), FATHMM (cancer), PROVEAN, SIFT	81.52% (78.28%-84.85%)	83.61% (79.84%-87.5%)	77.914% (72.464%-83.122%)	86.75% (83.47%-90.08%)	73.34% (67.53%-79.45%)	3.2161 (3.0777-3.3491)	81.39% (74.75%-87.88%)	83.3% (75.38%-91.18%)	78.104% (66.667%-88.89%)	86.72% (79.69%-93.22%)	73.18% (60.98%-85.3%)	3.2130 (2.9384-3.4934)
At least 3 of CHASM (breast), CHASM (lung), FATHMM (cancer), PROVEAN, VEST	81.88% (78.79%-84.86%)	83.61% (79.84%-87.5%)	78.89% (73.437%-84.211%)	87.26% (84.03%-90.6%)	73.58% (67.94%-79.49%)	3.2334 (3.0969-3.3665)	81.69% (75.73%-87.88%)	83.3% (75.38%-91.18%)	78.904% (67.647%-89.47%)	87.14% (80%-93.55%)	73.38% (61.36%-85.37%)	3.2272 (2.9564-3.4975)
At least 3 of CHASM (breast), CHASM (lung), FATHMM (cancer), SIFT	82.57% (79.8%-85.86%)	78.33% (74.22%-82.44%)	89.913% (86.111%-94.118%)	93.07% (90.48%-96.15%)	70.58% (65.26%-75.87%)	3.3189 (3.2098-3.4442)	82.33% (75.76%-87.88%)	77.91% (69.64%-86.15%)	89.896% (81.579%-97.3%)	92.97% (87.04%-98.11%)	70.34% (59.57%-81.25%)	3.3112 (3.0644-3.5271)
At least 3 of CHASM (breast), CHASM (lung), FATHMM (cancer), SIFT, VEST	84.17% (81.31%-87.37%)	91.01% (88.28%-94.26%)	72.326% (66.197%-78.873%)	85.05% (81.82%-88.55%)	82.32% (76.92%-87.88%)	3.3070 (3.1784-3.4415)	84.19% (77.78%-89.9%)	90.83% (84.61%-96.55%)	72.747% (60%-84.38%)	85.12% (78.12%-91.67%)	82.23% (71.86%-92.86%)	3.3093 (3.0434-3.5702)
At least 3 of CHASM (breast), CHASM (lung), FATHMM (cancer), VEST	82.24% (79.29%-85.35%)	77.8% (73.6%-81.9%)	89.913% (86.111%-94.118%)	93.02% (90.47%-96.12%)	70.08% (64.77%-75.53%)	3.3082 (3.1975-3.4332)	81.98% (75.76%-87.88%)	77.36% (68.75%-85.49%)	89.896% (81.579%-97.3%)	92.92% (86.96%-98.08%)	69.83% (59.18%-80.85%)	3.3001 (3.0508-3.5221)
At least 3 of CHASM (breast), CHASM (lung), FATHMM (missense)	77.85% (74.75%-81.31%)	67.72% (62.99%-72.44%)	95.379% (92.857%-98.592%)	96.2% (94.19%-98.85%)	63.08% (57.69%-68.27%)	3.2238 (3.1137-3.3302)	77.63% (70.71%-84.85%)	67.22% (57.38%-76.67%)	95.474% (89.474%-100%)	96.22% (91.11%-100%)	62.93% (52.73%-73.34%)	3.2184 (3.0075-3.4414)
At least 3 of CHASM (breast), CHASM (lung), FATHMM (missense), Mutation Assessor	80.91% (77.78%-83.84%)	74.65% (70.09%-79.03%)	91.726% (88.235%-95.654%)	93.97% (91.51%-96.94%)	67.67% (62.24%-72.92%)	3.2801 (3.1673-3.3971)	80.61% (74.75%-86.87%)	74.09% (65.57%-83.08%)	91.768% (84.211%-97.67%)	93.92% (88.09%-98.25%)	67.38% (56.52%-78%)	3.2716 (3.0385-3.5008)
At least 3 of CHASM (breast), CHASM (lung), FATHMM (missense), Mutation Assessor, MutationTaster	90.64% (88.38%-92.93%)	91.04% (88.28%-94.22%)	89.937% (86.301%-94.032%)	93.99% (92%-96.67%)	85.32% (81.01%-90.14%)	3.6029 (3.5132-3.6984)	90.44% (85.86%-94.95%)	90.77% (84.62%-96.61%)	89.834% (81.818%-97.22%)	93.88% (88.52%-98.25%)	85.06% (76.19%-94.12%)	3.5954 (3.3974-3.7809)
At least 3 of CHASM (breast), CHASM (lung), FATHMM (missense), Mutation Assessor, PolyPhen-2	86.24% (83.84%-88.89%)	89.97% (87.02%-93.08%)	79.789% (74.286%-85.135%)	88.5% (85.6%-91.74%)	82.16% (77.14%-87.3%)	3.4043 (3.2966-3.5244)	86.1% (80.81%-90.91%)	89.71% (83.33%-95.38%)	79.849% (68.75%-90.01%)	88.43% (81.66%-94.64%)	81.94% (71.88%-91.67%)	3.3994 (3.1584-3.6089)
At least 3 of CHASM (breast), CHASM (lung), FATHMM (missense), Mutation Assessor, PROVEAN	82.93% (79.8%-85.86%)	82.6% (78.57%-86.61%)	83.48% (78.571%-88.41%)	89.64% (86.67%-92.86%)	73.52% (67.86%-78.95%)	3.2923 (3.1608-3.4143)	82.63% (76.77%-88.89%)	82.12% (74.14%-90.16%)	83.466% (73.516%-93.33%)	89.52% (83.02%-95.84%)	73.16% (62.16%-84.62%)	3.2826 (3.0260-3.5308)
At least 3 of CHASM (breast), CHASM (lung), FATHMM (missense), Mutation Assessor, SIFT	86.23% (83.84%-89.39%)	90.51% (87.6%-93.55%)	78.815% (73.844%-84.512%)	88.08% (85.16%-91.41%)	82.78% (77.92%-88.06%)	3.4018 (3.2933-3.5337)	86.13% (79.8%-90.91%)	90.24% (84.37%-95.79%)	79.029% (67.645%-88.9%)	88.09% (81.54%-93.85%)	82.55% (72.09%-92.5%)	3.3990 (3.1371-3.6123)
At least 3 of CHASM (breast), CHASM (lung), FATHMM (missense), Mutation Assessor, VEST	86.23% (83.84%-88.89%)	91.04% (88.28%-94.22%)	77.898% (72.603%-83.333%)	87.69% (84.62%-91.05%)	83.42% (78.57%-88.89%)	3.4005 (3.2920-3.5223)	86.13% (80.81%-90.91%)	90.77% (84.62%-96.61%)	78.128% (66.667%-88.89%)	87.7% (80.95%-93.94%)	83.19% (72.97%-93.33%)	3.3979 (3.1517-3.6117)
At least 3 of CHASM (breast), CHASM (lung), FATHMM (missense), MutationTaster	82.59% (79.29%-85.86%)	77.3% (72.87%-81.6%)	91.726% (88.235%-95.654%)	94.17% (91.75%-97.03%)	70.03% (64.51%-75.27%)	3.3322 (3.2222-3.4491)	82.3% (75.76%-88.89%)	76.77% (68.25%-85.49%)	91.768% (84.211%-97.67%)	94.12% (88.63%-98.31%)	69.73% (59.09%-80.85%)	3.3240 (3.0948-3.5467)
At least 3 of CHASM (breast), CHASM (lung), FATHMM (missense), MutationTaster, PolyPhen-2	89.64% (87.37%-92.42%)	91.57% (88.8%-94.4%)	86.287% (81.944%-90.792%)	92.03% (89.68%-95.08%)	85.56% (81.08%-90.14%)	3.5544 (3.4562-3.6631)	89.41% (83.84%-93.94%)	91.31% (85.71%-96.78%)	86.122% (76.471%-94.74%)	91.87% (85.71%-96.72%)	85.27% (76.32%-94.29%)	3.5457 (3.3307-3.7427)
At least 3 of CHASM (breast), CHASM (lung), FATHMM (missense), MutationTaster, PROVEAN	86.63% (83.84%-89.39%)	84.19% (80.49%-87.9%)	90.841% (87.319%-94.667%)	94.08% (91.82%-96.55%)	76.87% (71.76%-82.05%)	3.4598 (3.3553-3.5692)	86.34% (80.81%-91.92%)	83.73% (76.27%-91.18%)	90.777% (83.323%-97.44%)	93.98% (88.46%-98.28%)	76.51% (65.91%-86.67%)	3.4499 (3.2352-3.6653)
At least 3 of CHASM (breast), CHASM (lung), FATHMM (missense), MutationTaster, SIFT	89.94% (87.88%-92.42%)	91.56% (88.8%-94.4%)	87.133% (83.099%-91.67%)	92.48% (90.16%-95.28%)	85.66% (81.33%-90.41%)	3.5684 (3.4737-3.6756)	89.82% (84.85%-93.94%)	91.33% (85.48%-96.78%)	87.178% (77.778%-94.74%)	92.44% (86.44%-96.88%)	85.46% (76.19%-94.29%)	3.5641 (3.3527-3.7483)
At least 3 of CHASM (breast), CHASM (lung), FATHMM (missense), MutationTaster, VEST	89.96% (87.88%-92.42%)	92.63% (90.08%-95.24%)	85.323% (80.822%-90.144%)	91.61% (89.06%-94.57%)	87.02% (82.86%-91.43%)	3.5658 (3.4696-3.6737)	89.78% (84.85%-93.94%)	92.38% (87.1%-97.06%)	85.291% (75.676%-94.29%)	91.52% (85.25%-96.72%)	86.74% (77.5%-94.74%)	3.5593 (3.3495-3.7479)
At least 3 of CHASM (breast), CHASM (lung), FATHMM (missense), PolyPhen-2	81.91% (78.79%-84.85%)	77.3% (72.87%-81.6%)	89.873% (86.111%-93.752%)	92.95% (90.38%-96.12%)	69.6% (64.04%-74.74%)	3.2973 (3.1838-3.4168)	81.64% (75.76%-87.88%)	76.77% (68.25%-85.49%)	89.977% (81.476%-97.3%)	92.92% (86.66%-98.08%)	69.31% (58.82%-80.44%)	3.2899 (3.0489-3.5202)
At least 3 of CHASM (breast), CHASM (lung), FATHMM (missense), PolyPhen-2, PROVEAN	82.91% (80.29%-85.86%)	84.19% (80.49%-87.9%)	80.676% (75.362%-85.719%)	88.28% (85.12%-91.8%)	74.7% (69.23%-80.23%)	3.2785 (3.1550-3.4066)	82.67% (76.77%-87.9%)	83.73% (76.27%-91.18%)	80.816% (70.588%-91.18%)	88.24% (81.36%-94.83%)	74.36% (63.16%-85.3%)	3.2714 (3.0146-3.5082)
At least 3 of CHASM (breast), CHASM (lung), FATHMM (missense), PolyPhen-2, SIFT	84.55% (81.82%-87.37%)	90.49% (87.59%-93.55%)	74.252% (68.571%-80%)	85.87% (82.44%-89.47%)	81.88% (76.81%-87.3%)	3.3250 (3.2058-3.4549)	84.44% (78.79%-89.9%)	90.27% (84.13%-95.72%)	74.394% (62.5%-85.71%)	85.83% (78.69%-92.65%)	81.71% (70.59%-91.67%)	3.3220 (3.0661-3.5597)
At least 3 of CHASM (breast), CHASM (lung), FATHMM (missense), PolyPhen-2, VEST	83.56% (80.81%-86.87%)	90.49% (87.59%-93.55%)	71.562% (65.663%-77.781%)	84.62% (81.34%-88.37%)	81.33% (76.12%-86.89%)	3.2801 (3.1579-3.4110)	83.38% (76.77%-88.89%)	90.27% (84.13%-95.72%)	71.499% (58.818%-83.33%)	84.48% (76.91%-91.18%)	81.1% (69.99%-91.43%)	3.2736 (3.0050-3.5205)
At least 3 of CHASM (breast), CHASM (lung), FATHMM (missense), PROVEAN	80.86% (77.78%-83.84%)	73.53% (68.96%-77.95%)	93.534% (90.541%-97.184%)	95.16% (92.86%-97.89%)	67.15% (61.9%-72.22%)	3.2937 (3.1823-3.4124)	80.7% (74.75%-86.87%)	73.13% (64.06%-82.46%)	93.658% (86.479%-100%)	95.19% (90%-100%)	67.03% (56.86%-77.78%)	3.2901 (3.0515-3.5140)
At least 3 of CHASM (breast), CHASM (lung), FATHMM (missense), PROVEAN, SIFT	82.21% (79.29%-85.35%)	83.12% (79.23%-87.1%)	80.63% (75.676%-85.714%)	88.13% (85%-91.53%)	73.43% (67.5%-79.02%)	3.2530 (3.1267-3.3801)	82.04% (75.76%-87.88%)	82.68% (74.99%-90.32%)	80.891% (70.58%-91.18%)	88.15% (81.13%-94.44%)	73.17% (61.54%-84.62%)	3.2488 (2.9903-3.4940)
At least 3 of CHASM (breast), CHASM (lung), FATHMM (missense), PROVEAN, VEST	81.89% (78.79%-84.85%)	83.65% (79.83%-87.5%)	78.827% (73.609%-84.212%)	87.23% (84.03%-90.52%)	73.61% (67.95%-79.37%)	3.2332 (3.1045-3.3653)	81.68% (75.76%-87.88%)	83.21% (75.41%-90.77%)	79.002% (67.647%-89.74%)	87.19% (79.69%-93.65%)	73.32% (61.36%-84.62%)	3.2272 (2.9557-3.4793)
At least 3 of CHASM (breast), CHASM (lung), FATHMM (missense), SIFT	81.22% (77.78%-84.34%)	76.77% (72.44%-80.95%)	88.922% (85.135%-93.058%)	92.29% (89.52%-95.33%)	68.88% (63.44%-73.96%)	3.2687 (3.1509-3.3945)	80.99% (74.75%-87.88%)	76.24% (67.8%-85.07%)	89.113% (80.645%-97.06%)	92.31% (86%-98.04%)	68.62% (58.14%-80%)	3.2628 (3.0155-3.5099)
At least 3 of CHASM (breast), CHASM (lung), FATHMM (missense), SIFT, VEST	84.53% (81.82%-87.37%)	91.56% (88.8%-94.4%)	72.352% (66.667%-78.261%)	85.14% (81.75%-88.81%)	83.22% (78.12%-88.71%)	3.3227 (3.1980-3.4549)	84.48% (78.79%-89.9%)	91.33% (85.48%-96.78%)	72.687% (60.712%-84.62%)	85.17% (77.94%-91.94%)	83.04% (72.41%-93.34%)	3.3223 (3.0626-3.5535)
At least 3 of CHASM (breast), CHASM (lung), FATHMM (missense), VEST	80.89% (77.78%-84.34%)	76.77% (72.44%-81.06%)	88.007% (84.211%-92.43%)	91.71% (88.79%-95%)	68.67% (62.92%-74.12%)	3.2516 (3.1296-3.3767)	80.64% (73.74%-86.87%)	76.23% (67.74%-85%)	88.199% (78.947%-96.78%)	91.72% (85.19%-97.92%)	68.38% (57.78%-79.55%)	3.2453 (2.9876-3.4918)
At least 3 of CHASM (breast), CHASM (lung), Mutation Assessor	66.35% (62.63%-70.2%)	49.03% (43.94%-54.2%)	96.282% (94.284%-98.667%)	95.8% (93.44%-98.48%)	52.21% (47.32%-56.74%)	2.9333 (2.8353-3.0415)	66.29% (58.59%-73.74%)	48.72% (38.1%-58.83%)	96.423% (91.176%-100%)	95.89% (90%-100%)	52.29% (42.86%-62.32%)	2.9333 (2.7223-3.1409)
At least 3 of CHASM (breast), CHASM (lung), Mutation Assessor, MutationTaster	78.15% (74.75%-81.31%)	69.24% (64.46%-74.02%)	93.55% (90.537%-97.102%)	94.89% (92.47%-97.78%)	63.76% (58.25%-68.81%)	3.2143 (3.1063-3.3271)	78.05% (71.72%-84.85%)	68.95% (59.37%-78.47%)	93.631% (86.842%-100%)	94.89% (89.13%-100%)	63.75% (53.57%-74.15%)	3.2122 (2.9879-3.4344)
At least 3 of CHASM (breast), CHASM (lung), Mutation Assessor, MutationTaster, PolyPhen-2	85.83% (83.33%-88.89%)	84% (80.49%-87.88%)	88.988% (85.29%-93.243%)	92.95% (90.52%-95.76%)	76.29% (71.43%-81.48%)	3.4223 (3.3157-3.5370)	85.92% (79.8%-90.91%)	84.1% (75.86%-90.91%)	89.014% (80.488%-97.06%)	92.91% (86.88%-98.15%)	76.57% (65.85%-86.36%)	3.4260 (3.1851-3.6341)
At least 3 of CHASM (breast), CHASM (lung), Mutation Assessor, MutationTaster, PROVEAN	81.84% (78.79%-84.85%)	75.57% (71.09%-80%)	92.665% (89.394%-96.105%)	94.68% (92.39%-97.17%)	68.7% (63.11%-73.79%)	3.3162 (3.2054-3.4229)	81.78% (75.76%-87.88%)	75.43% (66.13%-84.38%)	92.64% (85.366%-100%)	94.62% (88.89%-100%)	68.74% (58.33%-80%)	3.3143 (3.0964-3.5361)
At least 3 of CHASM (breast), CHASM (lung), Mutation Assessor, MutationTaster, SIFT	88.88% (86.36%-91.41%)	88.27% (85.16%-91.67%)	89.933% (86.301%-94.288%)	93.81% (91.53%-96.55%)	81.6% (76.92%-86.59%)	3.5361 (3.4404-3.6397)	88.91% (83.84%-93.94%)	88.34% (81.25%-94.37%)	89.863% (81.081%-97.22%)	93.73% (88.33%-98.31%)	81.83% (71.43%-91.11%)	3.5376 (3.3331-3.7215)
At least 3 of CHASM (breast), CHASM (lung), Mutation Assessor, MutationTaster, VEST	85.85% (83.33%-88.89%)	84.04% (80.51%-87.81%)	88.988% (85.29%-93.243%)	92.95% (90.52%-95.76%)	76.33% (71.26%-81.48%)	3.4230 (3.3195-3.5329)	85.87% (79.8%-90.91%)	84.03% (76.19%-90.91%)	89.014% (80.488%-97.06%)	92.91% (86.88%-98.15%)	76.49% (65.91%-86.36%)	3.4244 (3.2064-3.6306)
At least 3 of CHASM (breast), CHASM (lung), Mutation Assessor, PolyPhen-2	75.14% (71.72%-78.79%)	64.48% (59.66%-69.35%)	93.55% (90.537%-97.102%)	94.53% (92.05%-97.65%)	60.37% (55.14%-65.45%)	3.1293 (3.0228-3.2421)	74.98% (67.68%-81.82%)	64.1% (54.1%-73.85%)	93.631% (86.842%-100%)	94.52% (88.1%-100%)	60.32% (50%-70.7%)	3.1257 (2.9068-3.3347)
At least 3 of CHASM (breast), CHASM (lung), Mutation Assessor, PolyPhen-2, PROVEAN	75.43% (71.72%-78.79%)	71.86% (67.21%-76.42%)	81.574% (76.389%-86.957%)	87.09% (83.5%-90.82%)	62.64% (57.14%-68.04%)	3.0317 (2.8942-3.1658)	75.41% (68.69%-82.83%)	71.67% (62.69%-81.03%)	81.794% (71.429%-91.9%)	87.1% (79.66%-94.23%)	62.73% (51.91%-73.91%)	3.0329 (2.7591-3.3077)
At least 3 of CHASM (breast), CHASM (lung), Mutation Assessor, PolyPhen-2, SIFT	80.79% (77.78%-83.84%)	84.56% (80.95%-88.19%)	74.271% (68.655%-80%)	85.03% (81.85%-88.71%)	73.57% (68.05%-79.17%)	3.1743 (3.0341-3.3061)	80.84% (74.75%-86.87%)	84.58% (77.05%-91.67%)	74.395% (63.333%-85.71%)	84.99% (77.78%-92.06%)	73.79% (62.5%-84.62%)	3.1776 (2.9091-3.4523)
At least 3 of CHASM (breast), CHASM (lung), Mutation Assessor, PolyPhen-2, VEST	76.45% (73.23%-79.8%)	79.28% (75.38%-83.46%)	71.543% (65.753%-77.465%)	82.8% (78.86%-86.78%)	66.64% (61.03%-72.37%)	3.0027 (2.8629-3.1418)	76.4% (69.7%-82.83%)	79.17% (70.68%-86.89%)	71.644% (60%-83.33%)	82.71% (74.6%-90.48%)	66.73% (55%-78.05%)	3.0025 (2.7237-3.2820)
At least 3 of CHASM (breast), CHASM (lung), Mutation Assessor, PROVEAN	71.4% (68.18%-74.75%)	58.07% (52.89%-63.11%)	94.437% (91.667%-97.333%)	94.75% (92.21%-97.53%)	56.57% (51.64%-61.29%)	3.0382 (2.9360-3.1452)	71.35% (64.65%-77.78%)	57.78% (47.54%-67.75%)	94.607% (88.569%-100%)	94.83% (88.57%-100%)	56.63% (46.87%-66.67%)	3.0385 (2.8223-3.2432)
At least 3 of CHASM (breast), CHASM (lung), Mutation Assessor, PROVEAN, SIFT	75.44% (72.22%-78.79%)	72.96% (68.55%-77.42%)	79.734% (74.358%-84.932%)	86.16% (82.35%-90%)	63.04% (57.3%-68.48%)	3.0189 (2.8841-3.1544)	75.37% (68.69%-81.82%)	72.67% (63.33%-81.25%)	79.971% (69.691%-90.32%)	86.16% (78.43%-93.75%)	63.04% (52.27%-73.91%)	3.0184 (2.7502-3.2947)
At least 3 of CHASM (breast), CHASM (lung), Mutation Assessor, PROVEAN, VEST	73.76% (70.2%-77.27%)	69.74% (65.08%-74.38%)	80.706% (75.714%-85.915%)	86.2% (82.35%-90.2%)	60.67% (55.17%-66.03%)	2.9733 (2.8330-3.1101)	73.69% (66.67%-80.81%)	69.53% (60.32%-78.88%)	80.785% (70.368%-91.18%)	86.12% (78%-94%)	60.71% (49.98%-72%)	2.9715 (2.7056-3.2516)
At least 3 of CHASM (breast), CHASM (lung), Mutation Assessor, SIFT	76.48% (73.23%-79.8%)	66.61% (61.98%-71.2%)	93.55% (90.537%-97.102%)	94.69% (92.22%-97.7%)	61.84% (56.6%-66.96%)	3.1668 (3.0622-3.2842)	76.33% (69.7%-82.83%)	66.23% (56.66%-75%)	93.631% (86.842%-100%)	94.69% (88.64%-100%)	61.77% (51.02%-72%)	3.1632 (2.9308-3.3704)
At least 3 of CHASM (breast), CHASM (lung), Mutation Assessor, SIFT, VEST	79.15% (75.76%-82.32%)	83.55% (80%-87.4%)	71.536% (65.569%-77.473%)	83.54% (79.84%-87.2%)	71.56% (66.2%-77.64%)	3.1018 (2.9626-3.2385)	79.07% (72.73%-85.86%)	83.41% (75.41%-90.32%)	71.608% (59.986%-83.78%)	83.44% (76.11%-90.91%)	71.56% (59.52%-82.5%)	3.1001 (2.8181-3.3661)
At least 3 of CHASM (breast), CHASM (lung), Mutation Assessor, VEST	73.8% (70.2%-77.27%)	62.37% (57.48%-67.2%)	93.55% (90.537%-97.102%)	94.35% (91.86%-97.56%)	58.98% (53.91%-64.04%)	3.0926 (2.9867-3.2047)	73.62% (66.67%-80.81%)	61.93% (52.38%-72.13%)	93.631% (86.842%-100%)	94.34% (88.09%-100%)	58.91% (49.09%-68.97%)	3.0881 (2.8564-3.3023)
At least 3 of CHASM (breast), CHASM (lung), MutationTaster	71.71% (67.68%-75.25%)	56.95% (51.94%-62.2%)	97.222% (95.588%-100%)	97.26% (95.65%-100%)	56.64% (51.59%-61.6%)	3.0807 (2.9805-3.1859)	71.73% (64.65%-79.8%)	56.83% (45.97%-67.21%)	97.294% (92.303%-100%)	97.3% (92.85%-100%)	56.78% (46.77%-67.24%)	3.0821 (2.8795-3.2998)
At least 3 of CHASM (breast), CHASM (lung), MutationTaster, PolyPhen-2	77.81% (74.24%-81.31%)	68.7% (63.79%-73.39%)	93.55% (90.537%-97.102%)	94.85% (92.47%-97.78%)	63.35% (57.94%-68.52%)	3.2045 (3.0944-3.3163)	77.72% (70.71%-84.85%)	68.43% (59.01%-77.78%)	93.631% (86.842%-100%)	94.85% (89.12%-100%)	63.36% (52.83%-73.81%)	3.2027 (2.9839-3.4231)
At least 3 of CHASM (breast), CHASM (lung), MutationTaster, PolyPhen-2, PROVEAN	81.83% (78.79%-84.85%)	75.56% (71.09%-80.15%)	92.665% (89.394%-96.105%)	94.68% (92.39%-97.17%)	68.69% (63.33%-73.96%)	3.3160 (3.2054-3.4261)	81.79% (75.76%-87.88%)	75.45% (66.1%-84.49%)	92.64% (85.366%-100%)	94.62% (88.89%-100%)	68.76% (58%-80%)	3.3148 (3.0944-3.5361)
At least 3 of CHASM (breast), CHASM (lung), MutationTaster, PolyPhen-2, SIFT	87.53% (84.85%-90.4%)	87.19% (83.87%-90.62%)	88.11% (84.058%-92.857%)	92.69% (90.18%-95.61%)	79.92% (75%-84.81%)	3.4791 (3.3703-3.5900)	87.57% (81.82%-92.93%)	87.3% (80%-93.75%)	88.015% (79.409%-95.46%)	92.58% (86.79%-96.97%)	80.18% (69.77%-89.48%)	3.4807 (3.2623-3.6888)
At least 3 of CHASM (breast), CHASM (lung), MutationTaster, PolyPhen-2, VEST	83.85% (80.81%-86.87%)	84.01% (80.49%-87.9%)	83.573% (78.946%-88.575%)	89.83% (86.89%-93.16%)	75.14% (69.88%-80.49%)	3.3256 (3.2087-3.4437)	83.82% (77.78%-89.9%)	84.09% (75.93%-91.04%)	83.345% (73.329%-92.11%)	89.63% (83.01%-95.16%)	75.34% (64.1%-85.37%)	3.3241 (3.0965-3.5525)
At least 3 of CHASM (breast), CHASM (lung), MutationTaster, PROVEAN	75.08% (71.72%-78.79%)	63.34% (58.27%-68.22%)	95.377% (92.958%-98.529%)	95.95% (93.75%-98.77%)	60.08% (54.87%-65%)	3.1474 (3.0430-3.2563)	75.09% (67.68%-81.82%)	63.2% (53.12%-73.14%)	95.478% (90%-100%)	95.99% (90.7%-100%)	60.2% (50%-70.5%)	3.1488 (2.9385-3.3680)
At least 3 of CHASM (breast), CHASM (lung), MutationTaster, PROVEAN, SIFT	83.52% (80.8%-86.36%)	77.68% (73.23%-82.26%)	93.605% (90.539%-97.102%)	95.46% (93.33%-98%)	70.82% (65.59%-75.79%)	3.3756 (3.2626-3.4849)	83.46% (77.78%-88.91%)	77.59% (68.33%-86.44%)	93.512% (86.667%-100%)	95.36% (90.56%-100%)	70.89% (60.46%-81.49%)	3.3734 (3.1549-3.5967)
At least 3 of CHASM (breast), CHASM (lung), MutationTaster, PROVEAN, VEST	80.47% (77.27%-83.33%)	73.41% (68.91%-78.23%)	92.665% (89.394%-96.105%)	94.54% (92.22%-97.09%)	66.85% (61.54%-71.88%)	3.2746 (3.1643-3.3858)	80.47% (74.75%-86.87%)	73.36% (64.06%-82.46%)	92.64% (85.366%-100%)	94.48% (88.68%-100%)	66.98% (56.25%-77.78%)	3.2746 (3.0567-3.4970)
At least 3 of CHASM (breast), CHASM (lung), MutationTaster, SIFT	79.84% (76.77%-83.33%)	71.36% (66.67%-76.19%)	94.49% (91.667%-97.333%)	95.72% (93.48%-97.96%)	65.62% (60.37%-70.59%)	3.2719 (3.1661-3.3813)	79.72% (72.73%-85.86%)	71.09% (61.29%-80.33%)	94.503% (88.235%-100%)	95.68% (90.48%-100%)	65.59% (55.36%-75.94%)	3.2686 (3.0494-3.4868)
At least 3 of CHASM (breast), CHASM (lung), MutationTaster, SIFT, VEST	87.2% (84.85%-89.9%)	87.74% (84.5%-90.91%)	86.244% (81.941%-91.026%)	91.68% (89.08%-94.78%)	80.28% (75.32%-84.93%)	3.4595 (3.3510-3.5684)	87.23% (81.82%-91.92%)	87.79% (81.36%-94.03%)	86.238% (76.923%-94.45%)	91.62% (85.71%-96.72%)	80.47% (70.73%-90.25%)	3.4612 (3.2449-3.6684)
At least 3 of CHASM (breast), CHASM (lung), MutationTaster, VEST	75.78% (72.22%-79.29%)	65.5% (60.63%-70.54%)	93.55% (90.537%-97.102%)	94.61% (92.13%-97.65%)	61.06% (55.85%-66.07%)	3.1472 (3.0409-3.2567)	75.72% (68.69%-82.83%)	65.26% (54.83%-75%)	93.631% (86.842%-100%)	94.62% (88.37%-100%)	61.11% (50.91%-71.67%)	3.1461 (2.9303-3.3580)
At least 3 of CHASM (breast), CHASM (lung), PolyPhen-2	69.71% (66.16%-73.23%)	54.34% (49.58%-59.84%)	96.282% (94.284%-98.667%)	96.19% (94.12%-98.63%)	54.95% (50%-59.54%)	3.0176 (2.9149-3.1196)	69.67% (62.63%-76.77%)	54.07% (43.54%-63.8%)	96.423% (91.176%-100%)	96.29% (90.91%-100%)	55.03% (45.9%-65.08%)	3.0181 (2.8178-3.2200)
At least 3 of CHASM (breast), CHASM (lung), PolyPhen-2, PROVEAN	73.07% (69.7%-76.26%)	60.71% (55.56%-65.39%)	94.437% (91.667%-97.333%)	94.96% (92.5%-97.62%)	58.16% (53.1%-63.03%)	3.0827 (2.9757-3.1872)	73.06% (66.67%-79.8%)	60.48% (50.79%-70.49%)	94.607% (88.569%-100%)	95.06% (88.89%-100%)	58.25% (48.44%-68.52%)	3.0840 (2.8736-3.3006)
At least 3 of CHASM (breast), CHASM (lung), PolyPhen-2, PROVEAN, SIFT	76.08% (72.73%-79.29%)	75.58% (71.07%-80%)	76.934% (71.429%-82.863%)	85% (81.2%-88.89%)	64.57% (58.42%-70.11%)	3.0208 (2.8819-3.1591)	76.12% (69.7%-82.83%)	75.4% (66.13%-84.48%)	77.312% (65.714%-88.24%)	85.08% (77.35%-92.59%)	64.71% (53.49%-76.19%)	3.0250 (2.7503-3.3023)
At least 3 of CHASM (breast), CHASM (lung), PolyPhen-2, PROVEAN, VEST	74.08% (70.2%-77.27%)	71.32% (66.67%-75.97%)	78.841% (73.333%-84.507%)	85.35% (81.48%-89.53%)	61.39% (55.68%-67.01%)	2.9690 (2.8274-3.1023)	74.07% (67.68%-80.81%)	71.17% (61.9%-80.65%)	79.017% (67.647%-90%)	85.34% (76.92%-93.34%)	61.5% (50%-72.73%)	2.9703 (2.7056-3.2581)
At least 3 of CHASM (breast), CHASM (lung), PolyPhen-2, SIFT	77.49% (74.24%-80.81%)	69.26% (64.51%-73.77%)	91.727% (88.31%-95.588%)	93.53% (90.91%-96.67%)	63.32% (58.1%-68.27%)	3.1783 (3.0638-3.2901)	77.34% (70.71%-83.84%)	68.9% (59.37%-78.33%)	91.783% (84.375%-97.67%)	93.48% (87.49%-98.11%)	63.25% (53.06%-73.69%)	3.1742 (2.9548-3.4056)
At least 3 of CHASM (breast), CHASM (lung), PolyPhen-2, SIFT, VEST	77.41% (74.24%-80.81%)	84.56% (81.15%-88.19%)	65.021% (58.904%-71.233%)	80.69% (76.85%-84.5%)	70.9% (65.07%-76.92%)	3.0118 (2.8727-3.1523)	77.51% (70.71%-83.84%)	84.57% (77.05%-91.43%)	65.354% (52.778%-77.78%)	80.73% (73.01%-88.24%)	71.18% (59.26%-82.86%)	3.0183 (2.7320-3.2990)
At least 3 of CHASM (breast), CHASM (lung), PolyPhen-2, VEST	73.8% (70.2%-77.27%)	63.96% (59.01%-68.8%)	90.812% (87.324%-94.668%)	92.32% (89.41%-95.61%)	59.3% (54.2%-64.15%)	3.0640 (2.9478-3.1784)	73.62% (66.67%-80.81%)	63.55% (53.97%-73.34%)	90.87% (82.752%-97.37%)	92.25% (85%-97.78%)	59.23% (49.06%-69.81%)	3.0591 (2.8212-3.2893)
At least 3 of CHASM (breast), CHASM (lung), PROVEAN	62.33% (58.59%-65.67%)	41.08% (36.22%-45.9%)	99.084% (98.485%-100%)	98.73% (97.83%-100%)	49.3% (44.44%-53.64%)	2.8819 (2.7961-2.9660)	62.2% (55.53%-69.7%)	40.7% (31.03%-50.85%)	99.083% (96.774%-100%)	98.71% (95.24%-100%)	49.33% (40.57%-58.91%)	2.8782 (2.7092-3.0570)
At least 3 of CHASM (breast), CHASM (lung), PROVEAN, SIFT	73.77% (70.2%-77.27%)	61.27% (56.35%-66.4%)	95.377% (92.958%-98.529%)	95.82% (93.51%-98.72%)	58.75% (53.78%-63.64%)	3.1121 (3.0092-3.2282)	73.68% (66.67%-80.81%)	60.95% (50.73%-70.84%)	95.478% (90%-100%)	95.85% (90.48%-100%)	58.77% (49.06%-68.86%)	3.1104 (2.8910-3.3239)
At least 3 of CHASM (breast), CHASM (lung), PROVEAN, SIFT, VEST	74.09% (70.71%-77.27%)	73.47% (69.05%-78.05%)	75.148% (69.333%-81.081%)	83.64% (79.8%-87.61%)	62.1% (56.04%-67.82%)	2.9435 (2.7985-3.0850)	74.05% (67.68%-80.81%)	73.25% (63.93%-82.09%)	75.38% (63.631%-87.1%)	83.61% (75.51%-91.67%)	62.16% (50%-73.81%)	2.9440 (2.6569-3.2344)
At least 3 of CHASM (breast), CHASM (lung), PROVEAN, VEST	68.37% (64.65%-71.72%)	53.29% (47.97%-58.33%)	94.437% (91.667%-97.333%)	94.31% (91.43%-97.33%)	53.9% (48.85%-58.6%)	2.9594 (2.8472-3.0681)	68.31% (61.62%-75.76%)	52.97% (43.1%-63.5%)	94.607% (88.569%-100%)	94.4% (87.5%-100%)	53.97% (44.26%-63.77%)	2.9594 (2.7339-3.1857)
At least 3 of CHASM (breast), CHASM (lung), SIFT	72.08% (68.69%-75.76%)	57.54% (52.54%-62.7%)	97.222% (95.588%-100%)	97.28% (95.65%-100%)	56.97% (52.03%-61.79%)	3.0902 (2.9930-3.1897)	72% (64.65%-78.79%)	57.25% (46.43%-67.22%)	97.294% (92.303%-100%)	97.32% (92.68%-100%)	57.01% (47.54%-67.19%)	3.0887 (2.8877-3.2944)
At least 3 of CHASM (breast), CHASM (lung), SIFT, VEST	75.14% (71.72%-78.79%)	66.62% (62.01%-71.32%)	89.861% (86.11%-94.03%)	91.91% (89%-95.4%)	60.89% (55.66%-66.04%)	3.0928 (2.9781-3.2155)	74.97% (67.68%-81.82%)	66.2% (56.25%-75.41%)	90.006% (82.045%-97.3%)	91.9% (85.1%-97.78%)	60.81% (50.91%-71.15%)	3.0892 (2.8438-3.3228)
At least 3 of CHASM (breast), CHASM (lung), VEST	64.67% (61.1%-68.18%)	46.39% (41.27%-51.26%)	96.282% (94.284%-98.667%)	95.57% (93.1%-98.41%)	50.95% (46.04%-55.32%)	2.8919 (2.7898-2.9888)	64.6% (57.58%-71.74%)	46.04% (36.07%-56.14%)	96.423% (91.176%-100%)	95.68% (89.29%-100%)	51.02% (41.66%-60.66%)	2.8916 (2.6973-3.0993)
At least 3 of CHASM (breast), CHASM (melanoma), FATHMM (cancer)	79.18% (75.76%-82.34%)	70.33% (65.62%-75%)	94.469% (91.549%-97.333%)	95.65% (93.48%-97.92%)	64.81% (59.43%-69.92%)	3.2526 (3.1457-3.3683)	79.02% (72.7%-85.86%)	69.97% (60.66%-79.1%)	94.535% (88.235%-100%)	95.65% (90.48%-100%)	64.71% (54%-75.47%)	3.2487 (3.0156-3.4693)
At least 3 of CHASM (breast), CHASM (melanoma), FATHMM (cancer), FATHMM (missense)	84.53% (81.82%-87.37%)	81.95% (78.29%-86.05%)	88.998% (85.134%-93.336%)	92.8% (90.29%-95.65%)	74.04% (68.75%-79.55%)	3.3778 (3.2622-3.4991)	84.47% (78.79%-89.9%)	81.83% (73.77%-89.4%)	88.94% (79.412%-96.97%)	92.71% (86.66%-98.08%)	74.06% (63.04%-84.44%)	3.3755 (3.1336-3.5952)
At least 3 of CHASM (breast), CHASM (melanoma), FATHMM (cancer), FATHMM (missense), Mutation Assessor	84.88% (82.32%-87.88%)	88.89% (85.71%-92%)	77.951% (72.599%-83.562%)	87.46% (84.42%-90.91%)	80.24% (75%-85.51%)	3.3453 (3.2247-3.4705)	84.78% (78.79%-89.9%)	88.69% (82.45%-95.08%)	78.018% (66.667%-88.57%)	87.39% (80.59%-93.55%)	80.12% (69.7%-90.32%)	3.3422 (3.0926-3.5805)
At least 3 of CHASM (breast), CHASM (melanoma), FATHMM (cancer), FATHMM (missense), MutationTaster	89.29% (86.87%-91.92%)	91.03% (88.23%-93.75%)	86.258% (82.049%-90.909%)	91.97% (89.6%-95%)	84.77% (80.3%-89.23%)	3.5403 (3.4458-3.6487)	89.1% (83.84%-93.94%)	90.79% (85.25%-96.61%)	86.158% (76.316%-94.45%)	91.86% (86.15%-96.72%)	84.52% (75.61%-93.75%)	3.5333 (3.3182-3.7333)
At least 3 of CHASM (breast), CHASM (melanoma), FATHMM (cancer), FATHMM (missense), PolyPhen-2	83.56% (80.81%-86.36%)	89.43% (86.4%-92.59%)	73.383% (67.646%-79.688%)	85.32% (82.09%-88.89%)	80.07% (74.63%-85.51%)	3.2820 (3.1524-3.4068)	83.39% (77.78%-88.89%)	89.2% (83.05%-95.24%)	73.376% (61.362%-84.62%)	85.2% (78.12%-91.55%)	79.86% (68.97%-90.91%)	3.2764 (3.0261-3.5283)
At least 3 of CHASM (breast), CHASM (melanoma), FATHMM (cancer), FATHMM (missense), PROVEAN	84.2% (81.31%-86.87%)	86.22% (82.9%-89.76%)	80.693% (75.758%-85.915%)	88.54% (85.47%-91.94%)	77.2% (71.79%-82.72%)	3.3265 (3.1981-3.4486)	84.13% (78.79%-89.9%)	86.06% (78.69%-92.65%)	80.778% (70.583%-90.62%)	88.49% (81.67%-94.92%)	77.16% (65.91%-87.81%)	3.3249 (3.0811-3.5730)
At least 3 of CHASM (breast), CHASM (melanoma), FATHMM (cancer), FATHMM (missense), SIFT	83.16% (80.3%-86.36%)	88.89% (85.71%-92%)	73.251% (67.567%-79.173%)	85.18% (82.03%-88.72%)	79.23% (73.91%-84.62%)	3.2655 (3.1403-3.3942)	83.17% (76.77%-88.89%)	88.69% (82.45%-95.08%)	73.617% (61.111%-85%)	85.25% (77.97%-91.53%)	79.17% (68%-90.25%)	3.2673 (3.0050-3.5111)
At least 3 of CHASM (breast), CHASM (melanoma), FATHMM (cancer), FATHMM (missense), VEST	83.21% (80.3%-86.36%)	89.43% (86.29%-92.62%)	72.432% (66.667%-78.875%)	84.87% (81.54%-88.64%)	79.86% (74.6%-85.71%)	3.2659 (3.1361-3.3916)	83.08% (76.77%-88.89%)	89.21% (82.81%-95.24%)	72.518% (59.459%-84.62%)	84.8% (77.42%-91.38%)	79.68% (68.18%-89.66%)	3.2621 (3.0053-3.5267)
At least 3 of CHASM (breast), CHASM (melanoma), FATHMM (cancer), Mutation Assessor	85.21% (82.32%-88.38%)	83.02% (79.37%-86.89%)	88.974% (85.134%-93.151%)	92.87% (90.48%-95.61%)	75.2% (70%-80.68%)	3.4006 (3.2962-3.5203)	85.14% (78.79%-90.91%)	82.87% (75.38%-90%)	89.005% (80.645%-97.06%)	92.83% (87.04%-98.08%)	75.2% (64.81%-85.42%)	3.3990 (3.1660-3.6116)
At least 3 of CHASM (breast), CHASM (melanoma), FATHMM (cancer), Mutation Assessor, MutationTaster	90.25% (87.88%-92.93%)	93.11% (90.55%-95.87%)	85.307% (80.822%-90.141%)	91.64% (89.23%-94.62%)	87.75% (83.33%-92.43%)	3.5779 (3.4779-3.6832)	90.21% (84.85%-94.95%)	93.03% (87.5%-98.36%)	85.342% (75.758%-94.29%)	91.59% (85.48%-96.72%)	87.74% (78.38%-96.88%)	3.5771 (3.3654-3.7809)
At least 3 of CHASM (breast), CHASM (melanoma), FATHMM (cancer), Mutation Assessor, PolyPhen-2	85.54% (82.83%-88.38%)	92.04% (89.26%-95%)	74.288% (68.75%-80%)	86.09% (82.69%-89.47%)	84.38% (79.36%-89.71%)	3.3680 (3.2432-3.4919)	85.49% (79.8%-90.91%)	91.97% (86.21%-97.01%)	74.358% (63.15%-85.71%)	86.02% (79.03%-92.31%)	84.4% (73.33%-94.12%)	3.3675 (3.1186-3.6135)
At least 3 of CHASM (breast), CHASM (melanoma), FATHMM (cancer), Mutation Assessor, PROVEAN	84.51% (81.31%-87.37%)	87.77% (84.62%-91.2%)	78.864% (73.416%-84.288%)	87.77% (84.8%-91.06%)	78.86% (73.33%-84.62%)	3.3327 (3.2049-3.4581)	84.52% (78.79%-90.91%)	87.75% (80.95%-94.03%)	78.961% (68.568%-89.47%)	87.74% (80.95%-93.75%)	78.99% (67.42%-89.66%)	3.3344 (3.0782-3.5822)
At least 3 of CHASM (breast), CHASM (melanoma), FATHMM (cancer), Mutation Assessor, SIFT	85.52% (82.83%-88.38%)	92.02% (89.26%-95.08%)	74.262% (68.569%-80%)	86.08% (82.84%-89.47%)	84.35% (79.31%-90%)	3.3671 (3.2423-3.4871)	85.53% (79.8%-91.92%)	92% (85.94%-97.06%)	74.39% (62.857%-86.1%)	86.05% (79.31%-92.65%)	84.46% (73.33%-94.29%)	3.3690 (3.1185-3.6135)
At least 3 of CHASM (breast), CHASM (melanoma), FATHMM (cancer), Mutation Assessor, VEST	85.87% (83.33%-88.89%)	92.58% (89.92%-95.28%)	74.267% (68.493%-80%)	86.15% (82.98%-89.47%)	85.28% (80.59%-90.48%)	3.3827 (3.2645-3.5031)	85.83% (79.8%-90.91%)	92.49% (87.04%-98.28%)	74.4% (62.857%-86.11%)	86.11% (79.1%-92.54%)	85.26% (75%-94.76%)	3.3826 (3.1331-3.6164)
At least 3 of CHASM (breast), CHASM (melanoma), FATHMM (cancer), MutationTaster	86.91% (84.34%-89.9%)	84.62% (80.99%-88.52%)	90.854% (87.32%-94.668%)	94.12% (91.96%-96.55%)	77.37% (72.29%-82.76%)	3.4696 (3.3706-3.5832)	86.79% (80.81%-91.92%)	84.46% (76.92%-91.43%)	90.755% (82.857%-97.44%)	94.01% (88.68%-98.25%)	77.3% (66.67%-87.18%)	3.4652 (3.2442-3.6684)
At least 3 of CHASM (breast), CHASM (melanoma), FATHMM (cancer), MutationTaster, PolyPhen-2	90.59% (88.38%-92.93%)	94.71% (92.5%-96.88%)	83.464% (78.873%-88.571%)	90.83% (88.23%-93.89%)	90.12% (86.15%-94.29%)	3.5912 (3.4922-3.6963)	90.54% (85.86%-94.95%)	94.6% (90%-98.53%)	83.515% (73.529%-92.5%)	90.79% (84.62%-95.78%)	90.06% (81.25%-97.3%)	3.5899 (3.3759-3.7859)
At least 3 of CHASM (breast), CHASM (melanoma), FATHMM (cancer), MutationTaster, PROVEAN	88.23% (85.86%-90.91%)	88.84% (85.83%-92.07%)	87.162% (83.099%-91.549%)	92.29% (90%-95.12%)	81.87% (77.03%-87.14%)	3.5016 (3.4006-3.6157)	88.19% (82.83%-92.93%)	88.8% (82.14%-94.42%)	87.118% (78.125%-94.87%)	92.21% (86.21%-96.72%)	81.93% (71.43%-91.43%)	3.5006 (3.2635-3.7045)
At least 3 of CHASM (breast), CHASM (melanoma), FATHMM (cancer), MutationTaster, SIFT	90.6% (88.38%-92.93%)	93.65% (91.2%-96.15%)	85.309% (80.882%-90.141%)	91.68% (89.34%-94.53%)	88.6% (84.5%-93.06%)	3.5925 (3.4966-3.6967)	90.53% (85.86%-94.95%)	93.54% (88.24%-98.41%)	85.308% (75.756%-93.94%)	91.63% (85.94%-96.67%)	88.53% (79.41%-97.06%)	3.5901 (3.3812-3.7835)
At least 3 of CHASM (breast), CHASM (melanoma), FATHMM (cancer), MutationTaster, VEST	90.26% (87.88%-92.93%)	94.17% (91.74%-96.72%)	83.486% (79.165%-88.732%)	90.79% (88.19%-93.89%)	89.23% (85.25%-93.55%)	3.5769 (3.4702-3.6841)	90.19% (84.85%-93.94%)	94.09% (89.23%-98.51%)	83.463% (73.52%-92.11%)	90.72% (84.48%-95.59%)	89.18% (80.65%-97.06%)	3.5746 (3.3593-3.7835)
At least 3 of CHASM (breast), CHASM (melanoma), FATHMM (cancer), PolyPhen-2	84.88% (82.32%-87.88%)	83.56% (80%-87.4%)	87.145% (82.857%-91.892%)	91.83% (89.25%-94.87%)	75.41% (70.11%-80.77%)	3.3794 (3.2681-3.4988)	84.79% (78.79%-89.9%)	83.4% (75.41%-90.32%)	87.151% (77.771%-95.12%)	91.77% (85.41%-96.72%)	75.38% (64.28%-86.05%)	3.3769 (3.1431-3.5924)
At least 3 of CHASM (breast), CHASM (melanoma), FATHMM (cancer), PolyPhen-2, PROVEAN	83.15% (80.3%-86.36%)	87.77% (84.55%-91.2%)	75.158% (69.565%-81.429%)	85.94% (82.64%-89.84%)	78.05% (72.31%-84%)	3.2692 (3.1418-3.4056)	83.19% (76.77%-88.89%)	87.74% (80.95%-94.03%)	75.349% (63.158%-86.11%)	85.94% (78.12%-92.42%)	78.18% (66.67%-89.47%)	3.2721 (3.0015-3.5266)
At least 3 of CHASM (breast), CHASM (melanoma), FATHMM (cancer), PolyPhen-2, SIFT	83.87% (80.81%-86.87%)	92.58% (89.92%-95.31%)	68.791% (62.667%-75.008%)	83.69% (80.29%-87.15%)	84.3% (78.95%-90%)	3.2936 (3.1692-3.4313)	83.78% (77.78%-89.9%)	92.48% (86.79%-97.29%)	68.82% (55.882%-80.66%)	83.58% (76.39%-90.48%)	84.24% (72.4%-94.75%)	3.2912 (3.0102-3.5498)
At least 3 of CHASM (breast), CHASM (melanoma), FATHMM (cancer), PolyPhen-2, VEST	83.2% (80.3%-86.36%)	93.1% (90.47%-95.94%)	66.071% (59.722%-72.606%)	82.59% (79.1%-86.39%)	84.72% (79.63%-90.38%)	3.2648 (3.1395-3.4028)	83.1% (76.77%-88.89%)	93.04% (87.5%-98.39%)	66.023% (53.12%-78.95%)	82.45% (75%-89.33%)	84.7% (73.07%-96%)	3.2621 (2.9889-3.5111)
At least 3 of CHASM (breast), CHASM (melanoma), FATHMM (cancer), PROVEAN	82.89% (79.8%-85.86%)	77.27% (72.88%-81.6%)	92.596% (89.189%-96.001%)	94.75% (92.38%-97.2%)	70.2% (64.89%-75.53%)	3.3482 (3.2348-3.4615)	82.71% (76.77%-88.89%)	76.83% (68.18%-85.72%)	92.765% (85.294%-100%)	94.81% (89.36%-100%)	70.02% (59.08%-80.85%)	3.3442 (3.1148-3.5653)
At least 3 of CHASM (breast), CHASM (melanoma), FATHMM (cancer), PROVEAN, SIFT	83.14% (80.3%-86.36%)	87.77% (84.62%-91.2%)	75.123% (69.562%-80.822%)	85.92% (82.71%-89.43%)	78.04% (72.46%-83.87%)	3.2685 (3.1434-3.4054)	83.22% (76.77%-88.89%)	87.75% (80.95%-94.03%)	75.405% (64.097%-86.49%)	85.97% (79.24%-92.31%)	78.22% (65.71%-88.9%)	3.2734 (2.9985-3.5282)
At least 3 of CHASM (breast), CHASM (melanoma), FATHMM (cancer), PROVEAN, VEST	83.49% (80.8%-86.36%)	87.78% (84.68%-91.2%)	76.086% (70.423%-82.092%)	86.39% (83.21%-90.08%)	78.26% (72.86%-84%)	3.2851 (3.1560-3.4210)	83.52% (77.78%-88.91%)	87.74% (80.36%-93.85%)	76.246% (64.51%-87.18%)	86.38% (79.31%-92.99%)	78.39% (66.65%-88.89%)	3.2875 (3.0160-3.5370)
At least 3 of CHASM (breast), CHASM (melanoma), FATHMM (cancer), SIFT	85.19% (82.32%-87.88%)	83.54% (80%-87.4%)	88.031% (83.784%-92.311%)	92.35% (89.83%-95.37%)	75.58% (70.24%-80.96%)	3.3950 (3.2801-3.5125)	85.17% (79.8%-90.91%)	83.43% (75.47%-90.32%)	88.122% (79.485%-96.88%)	92.35% (86.54%-98%)	75.62% (65.11%-86.05%)	3.3952 (3.1517-3.6247)
At least 3 of CHASM (breast), CHASM (melanoma), FATHMM (cancer), SIFT, VEST	83.85% (80.81%-86.87%)	93.12% (90.55%-95.87%)	67.819% (61.644%-73.913%)	83.34% (80%-86.99%)	85.09% (80%-90.57%)	3.2938 (3.1679-3.4175)	83.81% (77.78%-89.9%)	93% (87.69%-98.36%)	68.009% (55.882%-80%)	83.31% (75.86%-89.86%)	85.01% (74.07%-96%)	3.2932 (3.0414-3.5442)
At least 3 of CHASM (breast), CHASM (melanoma), FATHMM (cancer), VEST	85.89% (83.33%-88.89%)	84.62% (80.99%-88.52%)	88.07% (84.058%-92.857%)	92.47% (89.91%-95.54%)	76.82% (71.79%-82.28%)	3.4198 (3.3149-3.5313)	85.79% (79.8%-90.91%)	84.46% (76.92%-91.43%)	88.045% (78.788%-96.67%)	92.4% (86.44%-98.08%)	76.77% (65.85%-86.96%)	3.4167 (3.1945-3.6307)
At least 3 of CHASM (breast), CHASM (melanoma), FATHMM (missense)	77.17% (73.74%-80.32%)	67.17% (62.31%-72.13%)	94.469% (91.549%-97.333%)	95.45% (93.18%-97.85%)	62.46% (57.01%-67.86%)	3.1955 (3.0847-3.3034)	76.97% (70.68%-83.84%)	66.72% (56.92%-76.47%)	94.535% (88.235%-100%)	95.45% (89.79%-100%)	62.34% (51.92%-73.21%)	3.1905 (2.9754-3.4161)
At least 3 of CHASM (breast), CHASM (melanoma), FATHMM (missense), Mutation Assessor	83.85% (80.81%-86.87%)	80.35% (76.19%-84.5%)	89.905% (86.111%-94.286%)	93.23% (90.83%-96.15%)	72.58% (67.39%-77.91%)	3.3606 (3.2457-3.4711)	83.81% (77.78%-89.9%)	80.25% (72.05%-88.52%)	89.879% (81.579%-97.3%)	93.17% (87.5%-98.11%)	72.64% (62.21%-82.99%)	3.3593 (3.1409-3.5919)
At least 3 of CHASM (breast), CHASM (melanoma), FATHMM (missense), Mutation Assessor, MutationTaster	89.98% (87.88%-92.42%)	91.58% (88.8%-94.4%)	87.184% (83.328%-91.781%)	92.51% (90.24%-95.16%)	85.7% (81.43%-90.28%)	3.5699 (3.4770-3.6663)	89.75% (84.85%-93.94%)	91.28% (85.51%-96.78%)	87.065% (77.778%-94.74%)	92.38% (86.79%-96.83%)	85.37% (76.18%-94.12%)	3.5610 (3.3636-3.7438)
At least 3 of CHASM (breast), CHASM (melanoma), FATHMM (missense), Mutation Assessor, PolyPhen-2	84.6% (81.82%-87.37%)	91.58% (88.8%-94.4%)	72.525% (66.667%-78.264%)	85.22% (81.95%-88.81%)	83.3% (78.33%-88.52%)	3.3262 (3.2029-3.4435)	84.33% (78.79%-89.9%)	91.28% (85.51%-96.78%)	72.351% (60.513%-83.78%)	85% (77.61%-91.67%)	82.9% (72.41%-93.1%)	3.3154 (3.0741-3.5569)
At least 3 of CHASM (breast), CHASM (melanoma), FATHMM (missense), Mutation Assessor, PROVEAN	85.21% (82.32%-87.88%)	87.27% (83.87%-90.55%)	81.627% (76.562%-86.958%)	89.15% (86.29%-92.37%)	78.77% (73.61%-83.95%)	3.3681 (3.2545-3.4821)	85.15% (79.8%-90.91%)	87.15% (80.39%-93.75%)	81.672% (71.429%-91.67%)	89.09% (82.46%-95%)	78.77% (68.57%-89.19%)	3.3668 (3.1331-3.5926)
At least 3 of CHASM (breast), CHASM (melanoma), FATHMM (missense), Mutation Assessor, SIFT	84.57% (81.82%-87.37%)	90.5% (87.59%-93.5%)	74.288% (68.571%-80.264%)	85.89% (82.61%-89.31%)	81.9% (76.81%-87.1%)	3.3259 (3.2041-3.4521)	84.4% (78.79%-89.9%)	90.25% (84.38%-96.43%)	74.302% (62.061%-85.71%)	85.79% (79.1%-92.42%)	81.65% (70.97%-92%)	3.3199 (3.0637-3.5569)
At least 3 of CHASM (breast), CHASM (melanoma), FATHMM (missense), Mutation Assessor, VEST	84.26% (81.82%-86.87%)	91.06% (88.19%-93.89%)	72.497% (67.105%-78.667%)	85.13% (81.82%-88.46%)	82.43% (77.42%-87.5%)	3.3112 (3.1917-3.4295)	84% (78.79%-88.89%)	90.74% (85%-96.67%)	72.399% (60%-83.79%)	84.96% (78.12%-91.55%)	82.03% (71.43%-92.59%)	3.3012 (3.0626-3.5362)
At least 3 of CHASM (breast), CHASM (melanoma), FATHMM (missense), MutationTaster	86.56% (83.84%-89.39%)	84.07% (80.62%-88%)	90.854% (87.32%-94.668%)	94.08% (91.89%-96.55%)	76.74% (71.91%-81.93%)	3.4574 (3.3554-3.5716)	86.48% (80.81%-91.92%)	83.98% (76.19%-91.23%)	90.755% (82.857%-97.44%)	93.97% (88.67%-98.25%)	76.77% (65.85%-86.67%)	3.4548 (3.2290-3.6653)
At least 3 of CHASM (breast), CHASM (melanoma), FATHMM (missense), MutationTaster, PolyPhen-2	88.32% (85.86%-90.91%)	92.66% (90.08%-95.28%)	80.809% (75.676%-86.111%)	89.31% (86.61%-92.42%)	86.44% (81.97%-91.18%)	3.4921 (3.3856-3.6015)	88% (82.83%-92.93%)	92.32% (86.89%-97.01%)	80.53% (69.697%-90%)	89.08% (82.54%-94.45%)	85.96% (76.47%-94.44%)	3.4789 (3.2537-3.6967)
At least 3 of CHASM (breast), CHASM (melanoma), FATHMM (missense), MutationTaster, PROVEAN	87.92% (85.35%-90.4%)	88.34% (85.12%-91.8%)	87.184% (83.328%-91.781%)	92.26% (90%-95%)	81.22% (76.32%-86.25%)	3.4901 (3.3899-3.5973)	87.8% (82.83%-92.93%)	88.2% (81.54%-94.83%)	87.065% (77.778%-94.74%)	92.14% (86.54%-96.72%)	81.15% (71.05%-90.92%)	3.4855 (3.2799-3.6967)
At least 3 of CHASM (breast), CHASM (melanoma), FATHMM (missense), MutationTaster, SIFT	88.29% (85.86%-90.91%)	91.58% (88.8%-94.4%)	82.594% (77.778%-87.839%)	90.1% (87.4%-93.28%)	85.03% (80.56%-89.71%)	3.4931 (3.3852-3.6066)	88.06% (82.83%-92.93%)	91.28% (85.51%-96.78%)	82.457% (71.429%-91.89%)	89.95% (83.87%-95.31%)	84.67% (75%-93.75%)	3.4836 (3.2613-3.6931)
At least 3 of CHASM (breast), CHASM (melanoma), FATHMM (missense), MutationTaster, VEST	88.32% (85.86%-90.91%)	92.66% (90.15%-95.24%)	80.81% (75.676%-86.301%)	89.31% (86.51%-92.48%)	86.43% (82.09%-91.18%)	3.4921 (3.3784-3.6052)	88% (82.83%-92.93%)	92.32% (87.03%-97.01%)	80.53% (69.697%-90.7%)	89.08% (82.61%-94.83%)	85.96% (76.46%-94.29%)	3.4789 (3.2538-3.6931)
At least 3 of CHASM (breast), CHASM (melanoma), FATHMM (missense), PolyPhen-2	83.53% (80.81%-86.36%)	83% (79.17%-86.89%)	84.441% (79.71%-89.189%)	90.22% (87.29%-93.22%)	74.18% (68.97%-79.75%)	3.3184 (3.1921-3.4398)	83.44% (77.78%-88.89%)	82.92% (75%-90.48%)	84.291% (74.286%-93.94%)	90.07% (83.93%-96.16%)	74.22% (63.16%-85%)	3.3150 (3.0739-3.5629)
At least 3 of CHASM (breast), CHASM (melanoma), FATHMM (missense), PolyPhen-2, PROVEAN	82.52% (79.8%-85.35%)	87.27% (84%-90.55%)	74.292% (68.831%-80.282%)	85.45% (82.17%-89.06%)	77.15% (71.6%-82.67%)	3.2416 (3.1179-3.3690)	82.44% (76.77%-88.89%)	87.14% (80.33%-93.75%)	74.31% (62.5%-85.71%)	85.36% (77.97%-92.19%)	77.12% (65.79%-88.24%)	3.2393 (2.9864-3.4923)
At least 3 of CHASM (breast), CHASM (melanoma), FATHMM (missense), PolyPhen-2, SIFT	83.23% (80.3%-86.36%)	91.58% (88.8%-94.4%)	68.762% (63.01%-75%)	83.53% (80.15%-87.05%)	82.54% (77.36%-87.93%)	3.2641 (3.1359-3.3890)	83.04% (76.77%-88.89%)	91.28% (85.51%-96.78%)	68.836% (56.663%-80.56%)	83.42% (76.12%-90.28%)	82.18% (71.43%-92.86%)	3.2572 (3.0086-3.5134)
At least 3 of CHASM (breast), CHASM (melanoma), FATHMM (missense), PolyPhen-2, VEST	82.26% (79.29%-85.35%)	91.06% (88.19%-93.89%)	67.018% (60.87%-73.333%)	82.68% (79.14%-86.43%)	81.26% (75.86%-86.89%)	3.2202 (3.0911-3.3533)	81.95% (75.76%-87.88%)	90.74% (85%-96.67%)	66.815% (54.28%-78.79%)	82.45% (75%-89.66%)	80.81% (69.56%-92%)	3.2081 (2.9354-3.4585)
At least 3 of CHASM (breast), CHASM (melanoma), FATHMM (missense), PROVEAN	81.86% (78.79%-84.85%)	75.62% (71.09%-80.31%)	92.618% (89.189%-95.892%)	94.66% (92.16%-97.06%)	68.73% (63.27%-73.96%)	3.3163 (3.2038-3.4247)	81.74% (75.76%-87.88%)	75.33% (65.62%-84.21%)	92.712% (86.101%-100%)	94.68% (89.58%-100%)	68.67% (58.14%-79.59%)	3.3139 (3.0977-3.5466)
At least 3 of CHASM (breast), CHASM (melanoma), FATHMM (missense), PROVEAN, SIFT	83.17% (80.3%-86.36%)	87.27% (83.87%-90.55%)	76.072% (70.423%-81.821%)	86.32% (83.2%-89.76%)	77.56% (72.22%-83.08%)	3.2722 (3.1473-3.4054)	83.15% (76.77%-88.89%)	87.15% (80.39%-93.75%)	76.228% (64.861%-86.84%)	86.31% (79.41%-92.65%)	77.58% (66.67%-88.57%)	3.2727 (3.0022-3.5232)
At least 3 of CHASM (breast), CHASM (melanoma), FATHMM (missense), PROVEAN, VEST	82.86% (79.8%-85.86%)	87.28% (84%-90.55%)	75.224% (69.562%-80.884%)	85.9% (82.4%-89.31%)	77.38% (72%-83.33%)	3.2578 (3.1274-3.3852)	82.76% (76.77%-88.89%)	87.14% (80.33%-93.65%)	75.18% (63.636%-86.49%)	85.79% (78.69%-92.54%)	77.33% (65.62%-88.57%)	3.2544 (2.9985-3.5112)
At least 3 of CHASM (breast), CHASM (melanoma), FATHMM (missense), SIFT	83.84% (81.31%-86.87%)	82.99% (79.23%-86.99%)	85.316% (80.816%-90.411%)	90.72% (87.9%-93.86%)	74.36% (69.05%-79.78%)	3.3339 (3.2194-3.4550)	83.83% (77.78%-89.9%)	82.95% (75%-90.32%)	85.271% (75%-94.44%)	90.65% (84.37%-96.43%)	74.47% (63.41%-85.11%)	3.3334 (3.0962-3.5544)
At least 3 of CHASM (breast), CHASM (melanoma), FATHMM (missense), SIFT, VEST	82.53% (79.8%-85.86%)	91.06% (88.19%-93.89%)	67.765% (61.429%-73.613%)	83.01% (79.56%-86.52%)	81.43% (75.92%-87.1%)	3.2327 (3.1078-3.3694)	82.42% (75.76%-88.89%)	90.74% (85%-96.67%)	68.063% (55.997%-80%)	83% (75.76%-89.86%)	81.1% (69.22%-92%)	3.2290 (2.9468-3.4758)
At least 3 of CHASM (breast), CHASM (melanoma), FATHMM (missense), VEST	84.54% (81.82%-87.37%)	84.07% (80.62%-88%)	85.344% (80.556%-90.004%)	90.84% (88.03%-93.86%)	75.6% (70.45%-81.25%)	3.3586 (3.2369-3.4798)	84.46% (78.79%-89.9%)	83.98% (76.19%-91.23%)	85.238% (75.758%-94.59%)	90.73% (84.62%-96.49%)	75.63% (64.29%-86.05%)	3.3558 (3.1163-3.5900)
At least 3 of CHASM (breast), CHASM (melanoma), Mutation Assessor	65.33% (61.62%-69.19%)	47.96% (42.74%-53.18%)	95.372% (92.955%-98.571%)	94.71% (91.93%-98.39%)	51.45% (46.38%-56.12%)	2.8949 (2.7928-3.0087)	65.29% (57.58%-72.73%)	47.69% (37.7%-57.81%)	95.484% (89.651%-100%)	94.77% (87.88%-100%)	51.54% (41.79%-61.67%)	2.8948 (2.6681-3.1031)
At least 3 of CHASM (breast), CHASM (melanoma), Mutation Assessor, MutationTaster	82.47% (79.29%-85.35%)	76.04% (71.54%-80.65%)	93.581% (90.537%-97.183%)	95.34% (93.14%-97.98%)	69.32% (64%-74.49%)	3.3428 (3.2436-3.4556)	82.54% (76.77%-87.9%)	76.09% (66.67%-84.75%)	93.565% (86.838%-100%)	95.31% (90%-100%)	69.54% (59.26%-80.39%)	3.3450 (3.1204-3.5495)
At least 3 of CHASM (breast), CHASM (melanoma), Mutation Assessor, MutationTaster, PolyPhen-2	84.15% (81.31%-86.87%)	85.59% (82.17%-89.08%)	81.667% (76.809%-86.958%)	88.97% (85.95%-92.24%)	76.63% (71.43%-81.93%)	3.3286 (3.2158-3.4495)	84.22% (78.79%-89.9%)	85.72% (78.33%-92.42%)	81.625% (71.429%-91.43%)	88.89% (82.26%-94.92%)	76.94% (65.71%-87.18%)	3.3317 (3.0830-3.5580)
At least 3 of CHASM (breast), CHASM (melanoma), Mutation Assessor, MutationTaster, PROVEAN	84.12% (81.31%-86.87%)	81.32% (77.23%-85.16%)	88.961% (85.135%-93.056%)	92.72% (90.2%-95.5%)	73.37% (68.18%-78.67%)	3.3637 (3.2525-3.4794)	84.28% (78.79%-89.9%)	81.49% (73.33%-89.48%)	89.025% (80.643%-97.14%)	92.73% (87.04%-98.04%)	73.73% (62.86%-84.09%)	3.3698 (3.1434-3.5927)
At least 3 of CHASM (breast), CHASM (melanoma), Mutation Assessor, MutationTaster, SIFT	87.18% (84.85%-89.9%)	88.23% (85.25%-91.6%)	85.341% (80.822%-90.144%)	91.23% (88.52%-94.12%)	80.76% (76%-85.9%)	3.4557 (3.3493-3.5629)	87.26% (81.82%-91.92%)	88.41% (81.54%-94.37%)	85.266% (75%-94.29%)	91.15% (85.45%-96.61%)	81.11% (70.96%-90.48%)	3.4594 (3.2399-3.6661)
At least 3 of CHASM (breast), CHASM (melanoma), Mutation Assessor, MutationTaster, VEST	84.52% (81.82%-87.37%)	84.56% (81.1%-88.19%)	84.446% (80%-89.335%)	90.38% (87.38%-93.55%)	75.99% (70.89%-81.34%)	3.3538 (3.2443-3.4798)	84.49% (78.79%-89.9%)	84.58% (77.04%-91.43%)	84.324% (74.984%-93.94%)	90.24% (83.93%-96.43%)	76.14% (64.86%-86.36%)	3.3528 (3.1078-3.5685)
At least 3 of CHASM (breast), CHASM (melanoma), Mutation Assessor, PolyPhen-2	76.77% (73.74%-80.3%)	70.22% (65.32%-74.82%)	88.079% (84.058%-92.754%)	91.06% (87.88%-94.62%)	63.11% (57.69%-68.27%)	3.1246 (3.0123-3.2567)	76.77% (69.7%-82.83%)	70.18% (60.6%-79.7%)	88.041% (78.947%-96.67%)	90.97% (84.09%-97.78%)	63.26% (52.94%-74%)	3.1245 (2.8620-3.3568)
At least 3 of CHASM (breast), CHASM (melanoma), Mutation Assessor, PolyPhen-2, PROVEAN	76.73% (73.72%-80.3%)	76.54% (72.31%-80.83%)	77.05% (71.795%-82.353%)	85.22% (81.58%-88.89%)	65.52% (59.76%-71.26%)	3.0433 (2.9095-3.1834)	76.84% (69.7%-82.83%)	76.67% (68.25%-85.08%)	77.084% (65.788%-88.24%)	85.16% (77.78%-92.59%)	65.84% (54.28%-76.93%)	3.0476 (2.7712-3.3109)
At least 3 of CHASM (breast), CHASM (melanoma), Mutation Assessor, PolyPhen-2, SIFT	80.11% (76.77%-83.33%)	85.6% (82.17%-89.15%)	70.607% (64.286%-76.712%)	83.43% (79.84%-86.99%)	73.93% (68.18%-79.71%)	3.1357 (3.0004-3.2636)	80.19% (73.74%-86.87%)	85.7% (78.33%-92.31%)	70.702% (58.614%-82.86%)	83.39% (76.19%-90.32%)	74.26% (62.96%-85.3%)	3.1405 (2.8814-3.4050)
At least 3 of CHASM (breast), CHASM (melanoma), Mutation Assessor, PolyPhen-2, VEST	75.09% (71.72%-78.28%)	80.35% (76.61%-84.25%)	66.005% (60%-72.309%)	80.34% (76.47%-84.38%)	66.01% (60.27%-72.22%)	2.9270 (2.7798-3.0704)	75.07% (68.69%-81.82%)	80.24% (72.13%-87.72%)	66.182% (53.846%-78.79%)	80.27% (71.93%-87.88%)	66.13% (53.85%-78.05%)	2.9282 (2.6377-3.2218)
At least 3 of CHASM (breast), CHASM (melanoma), Mutation Assessor, PROVEAN	71.71% (68.18%-75.25%)	59.64% (54.62%-64.57%)	92.572% (89.189%-96.053%)	93.28% (90.36%-96.34%)	57.02% (51.72%-61.86%)	3.0252 (2.9165-3.1452)	71.73% (64.65%-78.79%)	59.41% (49.15%-68.86%)	92.811% (85.714%-100%)	93.43% (86.84%-100%)	57.13% (47.45%-67.25%)	3.0278 (2.7867-3.2450)
At least 3 of CHASM (breast), CHASM (melanoma), Mutation Assessor, PROVEAN, SIFT	77.41% (74.24%-80.81%)	78.16% (73.98%-82.4%)	76.12% (70.423%-81.435%)	84.98% (81.41%-88.6%)	66.84% (60.98%-72.83%)	3.0610 (2.9237-3.2017)	77.5% (70.71%-83.84%)	78.24% (70%-86.44%)	76.188% (65.625%-87.5%)	84.93% (77.77%-92.19%)	67.13% (55.81%-78.57%)	3.0649 (2.7844-3.3442)
At least 3 of CHASM (breast), CHASM (melanoma), Mutation Assessor, PROVEAN, VEST	75.06% (71.72%-78.28%)	74.97% (70.63%-79.23%)	75.223% (69.564%-80.771%)	83.95% (80.37%-87.83%)	63.48% (57.95%-69.23%)	2.9762 (2.8367-3.1130)	75.13% (68.69%-81.82%)	75.05% (66.15%-83.61%)	75.237% (64.701%-86.49%)	83.86% (76.27%-91.08%)	63.74% (51.98%-75.56%)	2.9789 (2.7143-3.2572)
At least 3 of CHASM (breast), CHASM (melanoma), Mutation Assessor, SIFT	79.47% (76.26%-82.83%)	73.96% (69.49%-78.69%)	88.989% (85.134%-93.245%)	92.07% (89.11%-95.15%)	66.4% (61%-71.58%)	3.2142 (3.0950-3.3430)	79.44% (72.73%-85.86%)	73.87% (64.52%-82.81%)	88.968% (80%-97.06%)	92.01% (85.41%-98%)	66.49% (55.81%-76.93%)	3.2134 (2.9580-3.4506)
At least 3 of CHASM (breast), CHASM (melanoma), Mutation Assessor, SIFT, VEST	77.45% (74.24%-80.81%)	83% (79.51%-86.78%)	67.853% (61.972%-73.98%)	81.7% (77.95%-85.39%)	69.77% (63.89%-75.77%)	3.0232 (2.8899-3.1631)	77.42% (70.71%-83.84%)	82.92% (75.38%-90%)	67.953% (55.556%-80%)	81.62% (74.19%-89.06%)	69.88% (57.14%-81.25%)	3.0237 (2.7293-3.2946)
At least 3 of CHASM (breast), CHASM (melanoma), Mutation Assessor, VEST	77.12% (74.23%-80.32%)	69.7% (65%-74.25%)	89.955% (86.301%-94.366%)	92.31% (89.32%-95.7%)	63.2% (58.16%-68.42%)	3.1516 (3.0399-3.2762)	77.06% (70.68%-82.85%)	69.62% (60.32%-78.95%)	89.794% (81.392%-97.22%)	92.14% (85.71%-97.96%)	63.28% (52.83%-73.69%)	3.1483 (2.9015-3.3693)
At least 3 of CHASM (breast), CHASM (melanoma), MutationTaster	70.34% (66.16%-74.24%)	55.32% (50.37%-60.63%)	96.311% (94.286%-98.667%)	96.29% (94.12%-98.68%)	55.49% (50.41%-60.47%)	3.0342 (2.9240-3.1375)	70.42% (62.63%-78.79%)	55.3% (44.77%-65.67%)	96.355% (91.176%-100%)	96.31% (90.62%-100%)	55.69% (45.76%-66.07%)	3.0365 (2.8270-3.2574)
At least 3 of CHASM (breast), CHASM (melanoma), MutationTaster, PolyPhen-2	81.46% (78.28%-84.85%)	75.5% (71.2%-80%)	91.761% (88.312%-95.714%)	94.06% (91.67%-96.97%)	68.42% (63.37%-73.53%)	3.2975 (3.1864-3.4168)	81.52% (74.75%-87.88%)	75.56% (66.18%-84.13%)	91.699% (84.211%-97.62%)	93.99% (88.24%-98.18%)	68.64% (57.69%-78.95%)	3.2989 (3.0598-3.5261)
At least 3 of CHASM (breast), CHASM (melanoma), MutationTaster, PolyPhen-2, PROVEAN	82.11% (79.29%-85.35%)	79.72% (75.61%-84.03%)	86.224% (81.815%-90.667%)	90.92% (88.03%-93.86%)	71.1% (65.85%-76.62%)	3.2796 (3.1671-3.4025)	82.25% (75.76%-87.88%)	79.9% (71.64%-87.88%)	86.243% (77.143%-94.74%)	90.89% (84.9%-96.49%)	71.46% (59.99%-81.82%)	3.2849 (3.0501-3.5109)
At least 3 of CHASM (breast), CHASM (melanoma), MutationTaster, PolyPhen-2, SIFT	84.85% (82.32%-87.88%)	88.26% (85.25%-91.54%)	78.946% (73.611%-84.51%)	87.88% (84.8%-91.47%)	79.55% (74.29%-84.72%)	3.3464 (3.2299-3.4723)	84.85% (78.79%-89.9%)	88.36% (81.67%-94.12%)	78.788% (67.5%-89.2%)	87.74% (80.65%-93.94%)	79.8% (68.75%-89.66%)	3.3468 (3.1039-3.5798)
At least 3 of CHASM (breast), CHASM (melanoma), MutationTaster, PolyPhen-2, VEST	81.87% (78.79%-84.85%)	85.08% (81.75%-88.71%)	76.301% (70.833%-82.09%)	86.12% (82.64%-89.6%)	74.74% (69.56%-80.49%)	3.2224 (3.0941-3.3464)	81.72% (75.76%-87.88%)	85.14% (77.78%-91.8%)	75.838% (64.286%-87.18%)	85.81% (78.69%-92.73%)	74.84% (63.16%-85.29%)	3.2162 (2.9583-3.4704)
At least 3 of CHASM (breast), CHASM (melanoma), MutationTaster, PROVEAN	77.1% (73.74%-80.3%)	65.98% (60.98%-70.97%)	96.311% (94.286%-98.667%)	96.87% (95.12%-98.88%)	62.09% (56.76%-66.97%)	3.2125 (3.1122-3.3214)	77.12% (70.71%-83.84%)	65.88% (55.93%-75.72%)	96.355% (91.176%-100%)	96.88% (92.31%-100%)	62.22% (51.92%-73.02%)	3.2134 (2.9943-3.4208)
At least 3 of CHASM (breast), CHASM (melanoma), MutationTaster, PROVEAN, SIFT	85.14% (82.32%-87.88%)	82.91% (79.07%-86.78%)	88.983% (84.932%-93.243%)	92.87% (90.35%-95.58%)	75.08% (69.77%-80.01%)	3.3984 (3.2868-3.5126)	85.28% (79.8%-90.91%)	83.1% (75%-90.77%)	88.981% (80.556%-97.22%)	92.84% (87.27%-98.15%)	75.44% (64.58%-86.49%)	3.4035 (3.1747-3.6315)
At least 3 of CHASM (breast), CHASM (melanoma), MutationTaster, PROVEAN, VEST	82.78% (79.8%-85.86%)	78.65% (74.59%-82.81%)	89.921% (86.111%-94.03%)	93.1% (90.57%-96.08%)	70.9% (65.88%-76.41%)	3.3257 (3.2100-3.4338)	82.92% (76.77%-88.89%)	78.86% (70.31%-87.1%)	89.858% (82.051%-97.22%)	93.04% (87.72%-98.11%)	71.25% (60.42%-81.4%)	3.3300 (3.1040-3.5529)
At least 3 of CHASM (breast), CHASM (melanoma), MutationTaster, SIFT	84.49% (81.82%-87.37%)	78.68% (74.6%-82.95%)	94.52% (91.667%-97.368%)	96.13% (94.12%-98.15%)	71.95% (66.67%-77.08%)	3.4128 (3.3164-3.5139)	84.56% (78.79%-89.9%)	78.78% (70.17%-87.1%)	94.436% (88.232%-100%)	96.05% (91.3%-100%)	72.18% (61.9%-82.7%)	3.4146 (3.2127-3.6127)
At least 3 of CHASM (breast), CHASM (melanoma), MutationTaster, SIFT, VEST	85.54% (82.83%-88.38%)	87.75% (84.68%-91.13%)	81.716% (76.809%-86.957%)	89.25% (86.09%-92.5%)	79.42% (74.36%-84.81%)	3.3813 (3.2667-3.5008)	85.48% (79.8%-90.91%)	87.78% (81.24%-93.94%)	81.5% (71.419%-91.43%)	89.07% (82.81%-95.24%)	79.55% (68.89%-89.47%)	3.3791 (3.1455-3.6099)
At least 3 of CHASM (breast), CHASM (melanoma), MutationTaster, VEST	81.11% (78.28%-84.34%)	74.43% (70.23%-79.03%)	92.664% (89.394%-96%)	94.61% (92.23%-97.09%)	67.7% (62.61%-72.92%)	3.2939 (3.1928-3.4049)	81.21% (74.75%-86.87%)	74.53% (65.08%-83.05%)	92.645% (85.714%-100%)	94.57% (89.13%-100%)	67.95% (57.45%-78%)	3.2970 (3.0814-3.5025)
At least 3 of CHASM (breast), CHASM (melanoma), PolyPhen-2	68.35% (64.65%-72.22%)	52.71% (47.62%-58.06%)	95.372% (92.955%-98.571%)	95.17% (92.54%-98.51%)	53.84% (48.84%-58.46%)	2.9709 (2.8666-3.0772)	68.36% (60.61%-75.76%)	52.54% (41.94%-62.71%)	95.484% (89.651%-100%)	95.24% (88.89%-100%)	53.97% (44.61%-63.93%)	2.9723 (2.7646-3.1848)
At least 3 of CHASM (breast), CHASM (melanoma), PolyPhen-2, PROVEAN	73.73% (70.2%-77.27%)	63.35% (58.33%-68.22%)	91.654% (88.154%-95.775%)	92.93% (89.89%-96.39%)	59.12% (54.05%-64.15%)	3.0705 (2.9592-3.1892)	73.76% (66.67%-80.81%)	63.17% (53.03%-73.02%)	91.896% (84.211%-100%)	93.06% (86.48%-100%)	59.25% (49.09%-69.49%)	3.0738 (2.8348-3.3024)
At least 3 of CHASM (breast), CHASM (melanoma), PolyPhen-2, PROVEAN, SIFT	77.06% (73.74%-80.3%)	79.74% (75.6%-83.87%)	72.403% (66.197%-78.378%)	83.33% (79.66%-87.16%)	67.4% (61.33%-73.42%)	3.0287 (2.8903-3.1755)	77.2% (70.71%-83.84%)	79.85% (71.64%-87.88%)	72.595% (60.526%-84.85%)	83.35% (75.76%-90.91%)	67.77% (55.56%-78.95%)	3.0357 (2.7329-3.3159)
At least 3 of CHASM (breast), CHASM (melanoma), PolyPhen-2, PROVEAN, VEST	72.69% (69.19%-76.26%)	74.95% (70.73%-79.2%)	68.779% (62.5%-75.325%)	80.59% (76.52%-84.75%)	61.36% (55.29%-67.5%)	2.8568 (2.7200-3.0054)	72.8% (65.66%-79.8%)	75.08% (66.67%-83.87%)	68.845% (55.556%-81.09%)	80.54% (71.93%-88.53%)	61.69% (49.97%-73.33%)	2.8614 (2.5572-3.1410)
At least 3 of CHASM (breast), CHASM (melanoma), PolyPhen-2, SIFT	78.78% (75.76%-81.82%)	75.51% (71.31%-79.84%)	84.412% (79.682%-89.613%)	89.34% (86.11%-93.07%)	66.6% (61.18%-71.91%)	3.1586 (3.0372-3.2897)	78.81% (72.73%-84.85%)	75.54% (66.67%-83.87%)	84.355% (73.91%-94.12%)	89.25% (82.14%-96%)	66.79% (56.09%-77.28%)	3.1594 (2.9054-3.3998)
At least 3 of CHASM (breast), CHASM (melanoma), PolyPhen-2, SIFT, VEST	76.07% (72.73%-79.29%)	85.1% (81.74%-88.52%)	60.429% (53.846%-66.667%)	78.81% (75%-82.74%)	70.12% (63.79%-76.12%)	2.9446 (2.8043-3.0800)	76.15% (69.7%-82.83%)	85.09% (77.97%-91.94%)	60.774% (48.384%-74.19%)	78.82% (71.01%-86.84%)	70.37% (57.69%-82.15%)	2.9506 (2.6719-3.2207)
At least 3 of CHASM (breast), CHASM (melanoma), PolyPhen-2, VEST	75.79% (72.73%-79.29%)	71.29% (66.67%-75.78%)	83.556% (78.571%-88.571%)	88.23% (84.76%-92.08%)	62.73% (57.29%-68.04%)	3.0580 (2.9355-3.1937)	75.7% (68.69%-81.82%)	71.23% (62.3%-80.33%)	83.324% (73.329%-93.02%)	88.01% (80%-94.83%)	62.8% (52.08%-73.47%)	3.0536 (2.7730-3.3033)
At least 3 of CHASM (breast), CHASM (melanoma), PROVEAN	60.97% (57.07%-64.65%)	39.45% (34.43%-44.26%)	98.173% (97.015%-100%)	97.39% (95.65%-100%)	48.39% (43.45%-52.74%)	2.8341 (2.7425-2.9233)	60.89% (53.54%-68.69%)	39.17% (29.73%-49.18%)	98.144% (94.118%-100%)	97.32% (91.3%-100%)	48.46% (39.44%-58.33%)	2.8309 (2.6427-3.0161)
At least 3 of CHASM (breast), CHASM (melanoma), PROVEAN, SIFT	74.08% (70.71%-77.27%)	63.36% (58.59%-68.6%)	92.593% (89.333%-96.053%)	93.67% (90.7%-96.63%)	59.38% (54.29%-64.6%)	3.0900 (2.9756-3.2003)	74.07% (67.68%-80.81%)	63.14% (52.46%-72.88%)	92.767% (85.294%-100%)	93.76% (87.03%-100%)	59.46% (49.15%-69.65%)	3.0913 (2.8522-3.3197)
At least 3 of CHASM (breast), CHASM (melanoma), PROVEAN, SIFT, VEST	74.73% (71.21%-78.28%)	78.17% (74.16%-82.4%)	68.761% (62.857%-75%)	81.23% (77.5%-85.13%)	64.56% (58.66%-70.52%)	2.9273 (2.7862-3.0770)	74.79% (67.68%-81.82%)	78.21% (69.64%-86.21%)	68.87% (56.748%-80.01%)	81.18% (73.21%-88.52%)	64.81% (52.26%-76.47%)	2.9307 (2.6333-3.2170)
At least 3 of CHASM (breast), CHASM (melanoma), PROVEAN, VEST	70.39% (66.67%-73.74%)	56.46% (51.16%-61.42%)	94.448% (91.667%-97.368%)	94.63% (92%-97.44%)	55.65% (50.82%-60.47%)	3.0118 (2.9098-3.1205)	70.34% (63.64%-77.78%)	56.2% (45.9%-66.67%)	94.564% (88.095%-100%)	94.69% (88.57%-100%)	55.71% (45.76%-65.46%)	3.0117 (2.7829-3.2154)
At least 3 of CHASM (breast), CHASM (melanoma), SIFT	71.06% (67.68%-74.75%)	56.46% (51.54%-61.6%)	96.311% (94.286%-98.667%)	96.36% (94.2%-98.72%)	56.13% (51.2%-60.8%)	3.0526 (2.9537-3.1586)	71% (63.64%-77.78%)	56.21% (45.61%-66.67%)	96.355% (91.176%-100%)	96.36% (90.62%-100%)	56.19% (46.55%-66.17%)	3.0512 (2.8424-3.2634)
At least 3 of CHASM (breast), CHASM (melanoma), SIFT, VEST	78.8% (75.76%-81.82%)	74.47% (70.16%-78.91%)	86.28% (81.818%-91.026%)	90.38% (87.25%-94%)	66.15% (61.05%-71.58%)	3.1728 (3.0575-3.2917)	78.76% (72.73%-84.85%)	74.44% (65.57%-82.82%)	86.121% (76.316%-94.6%)	90.23% (83.33%-96.3%)	66.25% (55.32%-76.2%)	3.1705 (2.9277-3.3998)
At least 3 of CHASM (breast), CHASM (melanoma), VEST	63.31% (59.6%-67.17%)	44.76% (39.65%-50%)	95.372% (92.955%-98.571%)	94.36% (91.23%-98.25%)	49.96% (45.07%-54.41%)	2.8445 (2.7375-2.9492)	63.29% (55.56%-70.71%)	44.51% (34.38%-54.84%)	95.484% (89.651%-100%)	94.43% (87.49%-100%)	50.07% (40.84%-60.01%)	2.8450 (2.6331-3.0569)
At least 3 of CHASM (breast), FATHMM (cancer), FATHMM (missense)	77.19% (73.74%-80.81%)	67.2% (62.31%-72%)	94.469% (91.549%-97.333%)	95.46% (93.18%-97.83%)	62.49% (56.88%-67.63%)	3.1961 (3.0847-3.3034)	76.93% (69.7%-83.84%)	66.66% (56.92%-76.06%)	94.535% (88.235%-100%)	95.44% (90%-100%)	62.29% (51.79%-73.21%)	3.1893 (2.9726-3.4123)
At least 3 of CHASM (breast), FATHMM (cancer), FATHMM (missense), Mutation Assessor	83.23% (80.3%-86.36%)	82.53% (78.46%-86.29%)	84.415% (79.994%-89.335%)	90.16% (87.27%-93.39%)	73.66% (68.29%-78.89%)	3.3076 (3.1848-3.4211)	83.04% (76.77%-88.89%)	82.25% (74.6%-90.14%)	84.368% (74.286%-93.11%)	90.04% (83.33%-95.92%)	73.5% (63.26%-84.21%)	3.3016 (3.0691-3.5584)
At least 3 of CHASM (breast), FATHMM (cancer), FATHMM (missense), Mutation Assessor, MutationTaster	89.94% (87.37%-92.42%)	94.74% (92.56%-96.88%)	81.646% (76.809%-86.767%)	89.92% (87.12%-93.02%)	89.98% (85.94%-94.03%)	3.5629 (3.4615-3.6726)	89.81% (84.85%-94.95%)	94.55% (90%-98.51%)	81.659% (71.417%-91.18%)	89.84% (83.58%-95.39%)	89.76% (81.25%-97.14%)	3.5582 (3.3432-3.7715)
At least 3 of CHASM (breast), FATHMM (cancer), FATHMM (missense), Mutation Assessor, PolyPhen-2	85.53% (82.83%-88.38%)	93.67% (91.34%-96.09%)	71.441% (65.714%-77.635%)	85.01% (81.68%-88.72%)	86.72% (81.82%-91.67%)	3.3684 (3.2466-3.4844)	85.51% (79.8%-90.91%)	93.5% (88.33%-98.36%)	71.779% (60%-83.34%)	85.05% (77.94%-91.78%)	86.58% (76.67%-96.3%)	3.3691 (3.1374-3.6116)
At least 3 of CHASM (breast), FATHMM (cancer), FATHMM (missense), Mutation Assessor, PROVEAN	84.83% (82.32%-87.88%)	89.91% (86.92%-93.08%)	76.03% (70.27%-81.818%)	86.64% (83.33%-90.15%)	81.34% (76.12%-86.57%)	3.3392 (3.2214-3.4623)	84.89% (78.79%-89.9%)	89.85% (83.33%-95.52%)	76.362% (64.856%-87.88%)	86.71% (80%-93.45%)	81.45% (70.96%-91.67%)	3.3438 (3.0920-3.5786)
At least 3 of CHASM (breast), FATHMM (cancer), FATHMM (missense), Mutation Assessor, SIFT	84.48% (81.82%-87.37%)	92.59% (90.08%-95.24%)	70.428% (64.384%-76.923%)	84.41% (81.02%-87.97%)	84.62% (80%-89.83%)	3.3205 (3.2014-3.4436)	84.58% (78.79%-89.9%)	92.46% (87.03%-97.02%)	71.028% (58.06%-82.37%)	84.57% (77.46%-91.38%)	84.61% (74.98%-93.55%)	3.3266 (3.0636-3.5708)
At least 3 of CHASM (breast), FATHMM (cancer), FATHMM (missense), Mutation Assessor, VEST	84.48% (81.81%-87.37%)	93.66% (91.27%-96.09%)	68.589% (62.662%-75.009%)	83.76% (80.43%-87.5%)	86.23% (81.36%-91.38%)	3.3224 (3.1899-3.4441)	84.58% (78.79%-89.92%)	93.52% (88.33%-98.39%)	69.214% (56.755%-80.56%)	83.91% (76.31%-90.41%)	86.19% (76%-96.43%)	3.3283 (3.0664-3.5871)
At least 3 of CHASM (breast), FATHMM (cancer), FATHMM (missense), MutationTaster	88.28% (85.86%-90.91%)	87.31% (84%-90.63%)	89.956% (86.301%-94.205%)	93.76% (91.52%-96.55%)	80.4% (75.64%-85.37%)	3.5143 (3.4061-3.6241)	88.08% (82.83%-92.93%)	87.05% (80%-93.65%)	89.802% (81.579%-97.22%)	93.61% (88.13%-98.25%)	80.19% (70.45%-89.74%)	3.5065 (3.2873-3.7147)
At least 3 of CHASM (breast), FATHMM (cancer), FATHMM (missense), MutationTaster, PolyPhen-2	89.27% (86.87%-91.92%)	95.26% (93.33%-97.58%)	78.893% (73.611%-84.376%)	88.64% (85.82%-91.91%)	90.6% (86.57%-95.08%)	3.5340 (3.4306-3.6420)	89.14% (83.84%-93.94%)	95.1% (90.62%-98.53%)	78.87% (67.74%-89.19%)	88.55% (82.19%-94.2%)	90.4% (81.81%-97.3%)	3.5292 (3.3163-3.7318)
At least 3 of CHASM (breast), FATHMM (cancer), FATHMM (missense), MutationTaster, PROVEAN	88.92% (86.36%-91.41%)	91.51% (88.89%-94.4%)	84.423% (79.994%-89.335%)	91.04% (88.43%-94.16%)	85.2% (80.56%-90.28%)	3.5217 (3.4139-3.6307)	88.83% (83.84%-93.94%)	91.43% (85.71%-96.77%)	84.34% (74.283%-93.34%)	90.93% (84.74%-96.43%)	85.18% (75%-94.59%)	3.5188 (3.3058-3.7368)
At least 3 of CHASM (breast), FATHMM (cancer), FATHMM (missense), MutationTaster, SIFT	88.9% (86.36%-91.41%)	95.28% (93.22%-97.58%)	77.864% (72.464%-83.784%)	88.16% (85.4%-91.49%)	90.52% (86.44%-95.08%)	3.5183 (3.4165-3.6304)	88.86% (83.84%-93.94%)	95.06% (90.62%-98.57%)	78.168% (65.789%-88.57%)	88.22% (81.54%-93.85%)	90.25% (81.81%-97.3%)	3.5170 (3.2868-3.7227)

At least 3 of CHASM (breast), FATHMM (cancer), FATHMM (missense), MutationTaster, VEST	88.91% (86.36%-91.41%)	96.86% (95.2%-98.48%)	75.138% (69.695%-81.159%)	87.08% (84.17%-90.41%)	93.27% (89.66%-96.77%)	3.5235 (3.4225-3.6250)	88.85% (83.84%-93.94%)	96.69% (92.86%-100%)	75.357% (63.15%-86.67%)	87.09% (80.56%-92.86%)	93.01% (85.29%-100%)	3.5215 (3.3254-3.7261)
At least 3 of CHASM (breast), FATHMM (cancer), FATHMM (missense), PolyPhen-2	84.57% (81.82%-87.37%)	86.24% (82.93%-89.76%)	81.654% (76.812%-87.016%)	89.05% (86.09%-92.44%)	77.45% (72.22%-82.67%)	3.3439 (3.2204-3.4630)	84.4% (78.79%-89.9%)	86% (78.69%-92.54%)	81.607% (70.588%-91.43%)	88.93% (82.14%-94.92%)	77.27% (66.67%-87.5%)	3.3381 (3.0975-3.5786)
At least 3 of CHASM (breast), FATHMM (cancer), FATHMM (missense), PolyPhen-2, PROVEAN	84.5% (81.82%-87.37%)	90.44% (87.69%-93.44%)	74.212% (68.571%-80.282%)	85.85% (82.68%-89.47%)	81.8% (76.56%-87.5%)	3.3230 (3.2006-3.4449)	84.54% (78.79%-89.9%)	90.38% (84.21%-96.09%)	74.474% (63.15%-85.29%)	85.88% (78.69%-92.31%)	81.88% (70.96%-92.31%)	3.3260 (3.0739-3.5635)
At least 3 of CHASM (breast), FATHMM (cancer), FATHMM (missense), PolyPhen-2, SIFT	83.5% (80.81%-86.36%)	94.21% (92%-96.77%)	64.974% (59.089%-71.233%)	82.31% (78.91%-86.01%)	86.66% (81.48%-92.16%)	3.2816 (3.1640-3.4147)	83.5% (77.78%-88.89%)	94.01% (89.06%-98.41%)	65.424% (52.632%-77.42%)	82.36% (75.32%-89.05%)	86.45% (75.86%-96.3%)	3.2825 (3.0235-3.5148)
At least 3 of CHASM (breast), FATHMM (cancer), FATHMM (missense), PolyPhen-2, VEST	83.86% (80.81%-86.87%)	95.26% (93.33%-97.58%)	64.136% (57.746%-70.593%)	82.12% (78.77%-85.82%)	88.68% (83.93%-94%)	3.3020 (3.1810-3.4269)	83.8% (77.78%-89.9%)	95.1% (90.62%-98.53%)	64.357% (51.348%-77.14%)	82.09% (74.65%-88.89%)	88.48% (78.26%-96.77%)	3.3003 (3.0595-3.5336)
At least 3 of CHASM (breast), FATHMM (cancer), FATHMM (missense), PROVEAN	80.88% (77.78%-83.84%)	77.27% (72.95%-81.67%)	87.117% (82.812%-91.667%)	91.21% (88.29%-94.34%)	68.92% (63.33%-74.19%)	3.2452 (3.1152-3.3668)	80.66% (74.75%-86.87%)	76.82% (67.86%-85.94%)	87.205% (77.778%-95%)	91.17% (84.78%-96.61%)	68.69% (57.5%-80.01%)	3.2389 (2.9982-3.4919)
At least 3 of CHASM (breast), FATHMM (cancer), FATHMM (missense), PROVEAN, SIFT	82.44% (79.79%-85.35%)	89.91% (86.92%-93.08%)	69.516% (63.38%-76.056%)	83.61% (80.15%-87.5%)	79.94% (73.85%-85.48%)	3.2298 (3.0992-3.3665)	82.59% (76.77%-87.9%)	89.85% (83.33%-95.52%)	70.103% (57.143%-82.05%)	83.77% (76.06%-90.67%)	80.13% (68.96%-91.18%)	3.2385 (2.9553-3.4920)
At least 3 of CHASM (breast), FATHMM (cancer), FATHMM (missense), PROVEAN, VEST	83.82% (80.81%-86.87%)	90.97% (88.28%-93.95%)	71.433% (65.276%-77.465%)	84.63% (81.2%-88.41%)	82.08% (76.56%-87.69%)	3.2912 (3.1612-3.4243)	83.88% (77.78%-89.9%)	90.91% (84.85%-96.67%)	71.756% (58.971%-83.33%)	84.69% (77.02%-91.43%)	82.18% (70.58%-93.33%)	3.2953 (3.0337-3.5422)
At least 3 of CHASM (breast), FATHMM (cancer), FATHMM (missense), SIFT	83.17% (80.3%-86.36%)	85.17% (81.51%-88.8%)	79.688% (74.647%-85.507%)	87.88% (84.87%-91.45%)	75.66% (70.15%-81.25%)	3.2841 (3.1583-3.4117)	83.16% (76.77%-88.89%)	84.96% (77.78%-92.06%)	80.029% (68.418%-90.25%)	87.97% (81.03%-93.94%)	75.64% (64.86%-86.36%)	3.2859 (3.0277-3.5283)
At least 3 of CHASM (breast), FATHMM (cancer), FATHMM (missense), SIFT, VEST	83.84% (80.81%-86.87%)	94.2% (91.87%-96.83%)	65.921% (59.458%-72.73%)	82.7% (79.33%-86.4%)	86.81% (81.82%-92.16%)	3.2964 (3.1611-3.4325)	83.83% (77.78%-89.9%)	94.02% (88.71%-98.46%)	66.283% (52.628%-78.79%)	82.73% (75.34%-89.48%)	86.63% (76%-96.43%)	3.2966 (3.0238-3.5487)
At least 3 of CHASM (breast), FATHMM (cancer), FATHMM (missense), VEST	83.19% (80.3%-86.36%)	85.71% (82.11%-89.23%)	78.802% (73.527%-84.618%)	87.49% (84.43%-90.98%)	76.14% (70.67%-81.58%)	3.2814 (3.1474-3.4077)	83.12% (76.77%-88.89%)	85.47% (78.26%-92.31%)	79.041% (67.568%-89.47%)	87.52% (80.65%-93.65%)	76.05% (64.86%-86.5%)	3.2807 (3.0254-3.5371)
At least 3 of CHASM (breast), FATHMM (cancer), Mutation Assessor	66.03% (62.12%-69.7%)	48.52% (43.51%-53.6%)	96.293% (94.203%-98.667%)	95.77% (93.44%-98.46%)	51.96% (47.06%-56.62%)	2.9254 (2.8278-3.0374)	65.93% (58.59%-73.74%)	48.16% (37.7%-58.62%)	96.398% (91.176%-100%)	95.83% (90%-100%)	52.01% (42.46%-62.12%)	2.9240 (2.7009-3.1285)
At least 3 of CHASM (breast), FATHMM (cancer), Mutation Assessor, MutationTaster	83.13% (80.3%-86.36%)	78.69% (74.4%-82.81%)	90.816% (87.324%-94.522%)	93.67% (91.35%-96.33%)	71.14% (65.93%-76.29%)	3.3431 (3.2286-3.4621)	83.23% (76.77%-88.89%)	78.77% (70.31%-87.5%)	90.849% (82.927%-97.37%)	93.65% (87.93%-98.15%)	71.4% (60.86%-81.83%)	3.3467 (3.1143-3.5728)
At least 3 of CHASM (breast), FATHMM (cancer), Mutation Assessor, MutationTaster, PolyPhen-2	83.8% (80.81%-86.87%)	87.19% (83.9%-90.62%)	77.93% (72.603%-83.583%)	87.23% (84%-90.84%)	77.87% (72.72%-83.33%)	3.3022 (3.1804-3.4302)	83.92% (77.78%-89.9%)	87.31% (80%-93.85%)	78.121% (67.422%-88.89%)	87.24% (80.33%-93.65%)	78.22% (67.57%-88.89%)	3.3089 (3.0564-3.5562)
At least 3 of CHASM (breast), FATHMM (cancer), Mutation Assessor, MutationTaster, PROVEAN	81.42% (78.28%-84.34%)	81.32% (77.24%-85.19%)	81.582% (76.118%-86.842%)	88.41% (85.09%-91.96%)	71.64% (66.25%-76.92%)	3.2295 (3.1071-3.3592)	81.61% (75.76%-87.88%)	81.49% (73.33%-89.47%)	81.805% (71.429%-92.11%)	88.48% (81.66%-95%)	72.05% (61.11%-83.33%)	3.2383 (2.9786-3.4794)
At least 3 of CHASM (breast), FATHMM (cancer), Mutation Assessor, MutationTaster, SIFT	85.47% (82.83%-88.38%)	89.33% (86.32%-92.5%)	78.796% (73.604%-84.375%)	87.93% (84.73%-91.34%)	81.03% (75.99%-86.16%)	3.3708 (3.2469-3.4859)	85.63% (79.8%-90.91%)	89.41% (83.01%-95.31%)	79.134% (68.421%-90.01%)	88.02% (81.25%-94.74%)	81.35% (70.59%-91.18%)	3.3791 (3.1373-3.6136)
At least 3 of CHASM (breast), FATHMM (cancer), Mutation Assessor, MutationTaster, VEST	83.81% (80.81%-86.87%)	86.68% (83.58%-90.15%)	78.858% (73.418%-84.51%)	87.63% (84.3%-91.2%)	77.4% (72.05%-82.72%)	3.3057 (3.1811-3.4274)	83.89% (77.78%-89.9%)	86.73% (79.69%-93.22%)	79.013% (67.742%-89.75%)	87.63% (80.36%-94.03%)	77.63% (66.67%-87.81%)	3.3100 (3.0643-3.5570)
At least 3 of CHASM (breast), FATHMM (cancer), Mutation Assessor, PolyPhen-2	76.06% (72.73%-79.8%)	73.4% (69.05%-77.95%)	80.66% (75.362%-86.111%)	86.77% (83.18%-90.83%)	63.68% (58.24%-69.23%)	3.0451 (2.9112-3.1846)	76.16% (68.69%-82.83%)	73.39% (63.93%-82.09%)	80.901% (70.27%-91.67%)	86.82% (78.43%-94.23%)	63.93% (52.94%-74.52%)	3.0504 (2.7683-3.3132)
At least 3 of CHASM (breast), FATHMM (cancer), Mutation Assessor, PolyPhen-2, PROVEAN	76.35% (73.23%-79.8%)	77.08% (73.08%-81.25%)	75.074% (69.444%-81.159%)	84.24% (80.51%-88.39%)	65.45% (60%-71.08%)	3.0184 (2.8893-3.1596)	76.6% (69.7%-82.83%)	77.21% (68.33%-85.49%)	75.541% (63.636%-86.85%)	84.41% (76.67%-91.84%)	65.88% (54.55%-76.92%)	3.0304 (2.7503-3.2820)
At least 3 of CHASM (breast), FATHMM (cancer), Mutation Assessor, PolyPhen-2, SIFT	79.43% (76.26%-82.32%)	86.16% (82.64%-89.57%)	67.788% (61.903%-74.286%)	82.22% (78.57%-85.94%)	73.91% (68.18%-79.71%)	3.1008 (2.9692-3.2393)	79.53% (73.74%-85.86%)	86.17% (79.31%-92.65%)	68.104% (55.548%-80.01%)	82.25% (74.67%-89.4%)	74.19% (62.16%-85.3%)	3.1071 (2.8204-3.3813)
At least 3 of CHASM (breast), FATHMM (cancer), Mutation Assessor, PolyPhen-2, VEST	76.76% (73.74%-80.3%)	81.4% (77.78%-85.12%)	68.737% (62.5%-74.648%)	81.82% (78.15%-85.71%)	68.12% (62.5%-73.97%)	3.0007 (2.8602-3.1369)	76.79% (69.7%-82.83%)	81.34% (73.58%-88.74%)	68.971% (57.133%-80.77%)	81.79% (74.14%-89.23%)	68.29% (55.88%-79.49%)	3.0040 (2.7210-3.2843)
[illegible]	[illegible]	[illegible]	[illegible]	[illegible]	[illegible]	[illegible]	[illegible]	[illegible]	[illegible]	[illegible]	[illegible]	[illegible]

At least 3 of CHASM (lung), FATHMM (cancer), FATHMM (missense), PROVEAN	82.59% (79.8%-85.86%)	81.02% (77.17%-85.04%)	85.303% (80.822%-90%)	90.51% (87.72%-93.69%)	72.22% (66.67%-77.53%)	3.2904 (3.1640-3.4125)	82.3% (75.76%-87.88%)	80.51% (72.13%-88.24%)	85.328% (75.756%-94.44%)	90.41% (84%-96.3%)	71.86% (60.53%-83.33%)	3.2810 (3.0339-3.5198)
At least 3 of CHASM (lung), FATHMM (cancer), FATHMM (missense), PROVEAN, SIFT	82.45% (79.29%-85.37%)	91.52% (88.89%-94.35%)	66.754% (60.52%-73.239%)	82.64% (79.1%-86.43%)	81.99% (76.36%-87.51%)	3.2290 (3.1014-3.3659)	82.58% (76.74%-88.89%)	91.42% (85.71%-96.77%)	67.381% (54.28%-79.31%)	82.79% (75.34%-89.71%)	82.07% (70.97%-93.1%)	3.2366 (2.9548-3.4874)
At least 3 of CHASM (lung), FATHMM (cancer), FATHMM (missense), PROVEAN, VEST	83.49% (80.8%-86.87%)	92.58% (90%-95.28%)	67.756% (61.536%-74.648%)	83.24% (79.86%-87.22%)	84.09% (78.69%-89.39%)	3.2767 (3.1539-3.4122)	83.53% (76.77%-88.91%)	92.48% (86.88%-97.06%)	68.12% (55.261%-80%)	83.28% (75.68%-90%)	84.09% (73.08%-93.94%)	3.2797 (2.9955-3.5218)
At least 3 of CHASM (lung), FATHMM (cancer), FATHMM (missense), SIFT	84.18% (81.31%-87.37%)	88.37% (85.04%-91.8%)	76.926% (71.83%-83.099%)	86.88% (83.72%-90.4%)	79.29% (73.61%-84.72%)	3.3147 (3.1949-3.4425)	84.16% (77.78%-89.9%)	88.12% (81.35%-94.74%)	77.306% (65.517%-87.5%)	86.96% (80%-93.44%)	79.16% (67.74%-89.48%)	3.3156 (3.0603-3.5635)
At least 3 of CHASM (lung), FATHMM (cancer), FATHMM (missense), SIFT, VEST	83.51% (80.3%-86.87%)	94.2% (91.87%-96.83%)	65.006% (58.902%-71.796%)	82.32% (78.87%-86.13%)	86.65% (81.48%-92%)	3.2818 (3.1476-3.4177)	83.49% (76.77%-89.9%)	94.02% (88.71%-98.46%)	65.37% (51.721%-77.28%)	82.34% (74.65%-89.04%)	86.47% (76%-96.43%)	3.2820 (3.0019-3.5421)
At least 3 of CHASM (lung), FATHMM (cancer), FATHMM (missense), VEST	84.2% (81.31%-87.37%)	89.44% (86.4%-92.68%)	75.125% (69.333%-81.159%)	86.15% (82.92%-89.68%)	80.46% (75%-85.94%)	3.3117 (3.1847-3.4438)	84.13% (77.78%-89.9%)	89.18% (82.76%-95.24%)	75.404% (63.631%-86.67%)	86.16% (78.79%-92.54%)	80.28% (69.23%-90.62%)	3.3103 (3.0456-3.5599)
At least 3 of CHASM (lung), FATHMM (cancer), Mutation Assessor	69.4% (65.66%-73.23%)	54.37% (49.59%-59.51%)	95.382% (92.857%-98.611%)	95.31% (92.75%-98.57%)	54.73% (49.62%-59.84%)	2.9979 (2.8982-3.1059)	69.28% (61.62%-76.77%)	54.01% (43.94%-63.93%)	95.485% (89.182%-100%)	95.34% (88.89%-100%)	54.75% (44.93%-64.62%)	2.9959 (2.7774-3.1927)
At least 3 of CHASM (lung), FATHMM (cancer), Mutation Assessor, MutationTaster	83.82% (80.81%-86.87%)	79.77% (75.61%-84.25%)	90.816% (87.324%-94.522%)	93.75% (91.43%-96.4%)	72.2% (67.02%-77.17%)	3.3654 (3.2590-3.4761)	83.87% (77.78%-89.9%)	79.79% (70.49%-87.93%)	90.849% (82.927%-97.37%)	93.72% (88%-98.18%)	72.4% (62%-82.93%)	3.3676 (3.1455-3.5798)
At least 3 of CHASM (lung), FATHMM (cancer), Mutation Assessor, MutationTaster, PolyPhen-2	83.47% (80.3%-86.36%)	87.19% (83.9%-90.62%)	77.044% (71.831%-82.67%)	86.78% (83.33%-90.4%)	77.68% (72.46%-83.1%)	3.2869 (3.1656-3.4140)	83.56% (77.78%-89.9%)	87.31% (80%-93.85%)	77.151% (65.852%-87.51%)	86.75% (79.45%-93.55%)	78.01% (67.42%-88.89%)	3.2921 (3.0411-3.5390)
At least 3 of CHASM (lung), FATHMM (cancer), Mutation Assessor, MutationTaster, PROVEAN	82.1% (79.29%-85.35%)	82.4% (78.63%-86.4%)	81.582% (76.118%-86.842%)	88.55% (85.22%-92.11%)	72.84% (67.47%-78.05%)	3.2537 (3.1288-3.3785)	82.26% (75.76%-87.88%)	82.51% (74.57%-90.16%)	81.805% (71.429%-92.11%)	88.61% (81.82%-95.08%)	73.18% (62.49%-84.21%)	3.2610 (3.0081-3.5008)
At least 3 of CHASM (lung), FATHMM (cancer), Mutation Assessor, MutationTaster, SIFT	84.81% (81.82%-87.37%)	89.33% (86.32%-92.5%)	76.983% (71.622%-82.609%)	87.03% (83.72%-90.55%)	80.67% (75.38%-85.73%)	3.3401 (3.2204-3.4643)	84.94% (79.8%-90.91%)	89.41% (83.01%-95.31%)	77.262% (65.714%-87.88%)	87.08% (80%-93.65%)	80.99% (70.27%-91.18%)	3.3474 (3.0896-3.5884)
At least 3 of CHASM (lung), FATHMM (cancer), Mutation Assessor, MutationTaster, VEST	83.15% (80.3%-85.86%)	86.68% (83.58%-90.15%)	77.045% (71.429%-82.667%)	86.71% (83.33%-90.23%)	76.99% (71.64%-82.43%)	3.2743 (3.1471-3.4024)	83.2% (77.78%-88.89%)	86.73% (79.69%-93.22%)	77.14% (65.712%-88.24%)	86.67% (79.65%-93.55%)	77.22% (65.79%-87.5%)	3.2776 (3.0226-3.5325)
At least 3 of CHASM (lung), FATHMM (cancer), Mutation Assessor, PolyPhen-2	76.42% (73.23%-79.8%)	74.48% (70.25%-79.03%)	79.775% (74.358%-85.139%)	86.42% (82.86%-90.29%)	64.39% (58.82%-69.77%)	3.0507 (2.9178-3.1899)	76.45% (69.7%-82.83%)	74.41% (65.57%-83.05%)	79.931% (68.885%-90.33%)	86.4% (78.18%-93.76%)	64.55% (53.65%-75.61%)	3.0529 (2.7618-3.3181)
At least 3 of CHASM (lung), FATHMM (cancer), Mutation Assessor, PolyPhen-2, PROVEAN	76.71% (73.74%-79.8%)	78.16% (74.38%-82.26%)	74.188% (68.421%-80.282%)	83.96% (80.17%-87.96%)	66.27% (60.71%-71.83%)	3.0258 (2.8881-3.1629)	76.89% (70.71%-82.83%)	78.22% (69.69%-86.36%)	74.571% (62.848%-86.11%)	84.07% (76.27%-91.67%)	66.62% (55.26%-77.5%)	3.0348 (2.7554-3.3040)
At least 3 of CHASM (lung), FATHMM (cancer), Mutation Assessor, PolyPhen-2, SIFT	78.75% (75.76%-81.82%)	86.16% (82.64%-89.57%)	65.924% (60%-72.224%)	81.38% (77.69%-85.16%)	73.37% (67.69%-79.31%)	3.0684 (2.9283-3.2103)	78.87% (72.73%-84.85%)	86.17% (79.31%-92.65%)	66.324% (53.846%-78.05%)	81.44% (74.19%-89.06%)	73.68% (62.07%-85.19%)	3.0762 (2.7884-3.3494)
At least 3 of CHASM (lung), FATHMM (cancer), Mutation Assessor, PolyPhen-2, VEST	75.74% (72.22%-78.79%)	81.4% (77.78%-85.12%)	65.957% (60%-71.878%)	80.52% (76.42%-84.38%)	67.22% (61.43%-73.14%)	2.9509 (2.8100-3.0868)	75.79% (69.7%-82.83%)	81.34% (73.58%-88.74%)	66.277% (54.286%-78.79%)	80.52% (72.58%-88.53%)	67.43% (55.16%-78.79%)	2.9557 (2.6747-3.2441)
At least 3 of CHASM (lung), FATHMM (cancer), Mutation Assessor, PROVEAN	72.08% (68.69%-75.76%)	64.98% (60.32%-69.84%)	84.352% (79.219%-89.189%)	87.77% (83.87%-91.67%)	58.21% (52.83%-63.21%)	2.9532 (2.8138-3.0850)	72% (64.65%-78.79%)	64.69% (55%-74.19%)	84.512% (74.286%-94.59%)	87.75% (79.99%-95.65%)	58.23% (47.83%-69.09%)	2.9518 (2.6802-3.2230)
At least 3 of CHASM (lung), FATHMM (cancer), Mutation Assessor, PROVEAN, SIFT	76.4% (73.23%-79.8%)	79.25% (75.21%-83.47%)	71.466% (65.714%-78.082%)	82.76% (79.03%-86.67%)	66.58% (60.56%-72.37%)	3.0006 (2.8581-3.1455)	76.49% (69.7%-82.83%)	79.24% (70.77%-87.04%)	71.743% (58.818%-83.33%)	82.79% (74.6%-90.32%)	66.82% (55.26%-78.05%)	3.0059 (2.7186-3.2875)
At least 3 of CHASM (lung), FATHMM (cancer), Mutation Assessor, PROVEAN, VEST	74.7% (71.21%-78.28%)	76.04% (72%-80.33%)	72.398% (66.667%-78.67%)	82.64% (78.7%-86.79%)	63.6% (57.83%-69.34%)	2.9468 (2.8078-3.0905)	74.83% (67.68%-81.82%)	76.09% (67.21%-84.13%)	72.654% (60.712%-84.38%)	82.67% (74.14%-90.39%)	63.91% (52.5%-75.61%)	2.9532 (2.6652-3.2294)
At least 3 of CHASM (lung), FATHMM (cancer), Mutation Assessor, SIFT	78.1% (74.75%-81.31%)	77.14% (73.02%-81.48%)	79.757% (74.598%-85.333%)	86.82% (83.33%-90.48%)	66.87% (61.25%-72.37%)	3.1059 (2.9725-3.2445)	78.14% (71.72%-84.85%)	77.07% (67.8%-85.25%)	79.944% (68.746%-90.62%)	86.82% (79.54%-94.12%)	67.02% (56.1%-78.05%)	3.1086 (2.8291-3.3779)
At least 3 of CHASM (lung), FATHMM (cancer), Mutation Assessor, SIFT, VEST	76.07% (72.73%-79.29%)	84.61% (81.15%-88.15%)	61.294% (55.072%-67.606%)	79.08% (75.18%-83.09%)	69.74% (63.63%-76.19%)	2.9472 (2.8021-3.0916)	76.14% (69.7%-82.83%)	84.47% (77.05%-91.43%)	61.824% (48.571%-73.69%)	79.14% (71.01%-86.96%)	69.89% (56.75%-82.14%)	2.9533 (2.6510-3.2370)
At least 3 of CHASM (lung), FATHMM (cancer), Mutation Assessor, VEST	75.43% (72.22%-78.79%)	73.43% (69.23%-77.86%)	78.873% (73.527%-84.507%)	85.73% (81.82%-89.63%)	63.2% (57.61%-68.54%)	3.0123 (2.8747-3.1507)	75.41% (68.69%-81.82%)	73.32% (64.06%-82.09%)	78.975% (67.647%-90%)	85.67% (77.19%-93.11%)	63.3% (52.38%-74.36%)	3.0126 (2.7374-3.2895)
At least 3 of CHASM (lung), FATHMM (cancer), MutationTaster	74.76% (71.21%-78.28%)	62.29% (57.25%-67.46%)	96.322% (94.203%-98.685%)	96.69% (94.74%-98.82%)	59.63% (54.38%-64.66%)	3.1493 (3.0500-3.2564)	74.73% (67.68%-81.82%)	62.12% (51.67%-72.41%)	96.356% (91.111%-100%)	96.69% (91.67%-100%)	59.72% (50%-70.59%)	3.1488 (2.9404-3.3600)
At least 3 of CHASM (lung), FATHMM (cancer), MutationTaster, PolyPhen-2	84.17% (81.31%-87.37%)	81.36% (77.42%-85.6%)	89.028% (85.075%-93.243%)	92.76% (90.2%-95.58%)	73.42% (68.24%-78.48%)	3.3657 (3.2495-3.4826)	84.19% (77.78%-89.9%)	81.42% (72.3%-89.06%)	88.932% (80.643%-97.06%)	92.64% (86.54%-98.08%)	73.62% (63.63%-84.09%)	3.3662 (3.1250-3.5966)
At least 3 of CHASM (lung), FATHMM (cancer), MutationTaster, PolyPhen-2, PROVEAN	81.09% (78.28%-84.34%)	82.4% (78.57%-86.51%)	78.834% (73.609%-84.288%)	87.06% (83.87%-90.76%)	72.15% (67.07%-77.78%)	3.2044 (3.0817-3.3355)	81.25% (74.75%-86.87%)	82.53% (73.77%-90.16%)	79.047% (68.568%-89.2%)	87.1% (79.66%-93.44%)	72.53% (61.11%-83.72%)	3.2121 (2.9450-3.4572)
At least 3 of CHASM (lung), FATHMM (cancer), MutationTaster, PolyPhen-2, SIFT	83.77% (80.81%-86.36%)	89.32% (86.26%-92.5%)	74.185% (68.657%-80%)	85.68% (82.4%-89.31%)	80.07% (74.99%-85.51%)	3.2925 (3.1721-3.4159)	83.97% (78.79%-89.9%)	89.44% (83.05%-95.38%)	74.582% (63.158%-85.71%)	85.78% (78.69%-92.31%)	80.48% (69.44%-90.91%)	3.3028 (3.0434-3.5440)
At least 3 of CHASM (lung), FATHMM (cancer), MutationTaster, PolyPhen-2, VEST	80.48% (77.27%-83.84%)	86.69% (83.33%-90.08%)	69.751% (63.997%-76.001%)	83.21% (79.39%-87.02%)	75.19% (69.84%-80.88%)	3.1483 (3.0224-3.2824)	80.45% (73.74%-86.87%)	86.71% (79.69%-93.44%)	69.689% (57.5%-81.82%)	83.07% (75.41%-90.48%)	75.37% (63.64%-86.49%)	3.1484 (2.8738-3.4088)
At least 3 of CHASM (lung), FATHMM (cancer), MutationTaster, PROVEAN	79.82% (76.26%-82.83%)	71.31% (66.67%-76.16%)	94.526% (91.667%-97.436%)	95.75% (93.55%-97.98%)	65.59% (60.19%-70.75%)	3.2717 (3.1622-3.3764)	79.75% (73.74%-86.87%)	71.19% (61.19%-80.3%)	94.426% (88.235%-100%)	95.64% (90.38%-100%)	65.65% (55.1%-76.47%)	3.2690 (3.0614-3.4922)
At least 3 of CHASM (lung), FATHMM (cancer), MutationTaster, PROVEAN, SIFT	82.8% (79.8%-85.86%)	84.54% (80.99%-88.37%)	79.774% (74.627%-85.295%)	87.84% (84.75%-91.34%)	74.91% (69.33%-80.26%)	3.2707 (3.1465-3.3995)	82.89% (76.77%-88.89%)	84.62% (76.67%-91.81%)	79.895% (68.75%-89.75%)	87.84% (80.82%-93.88%)	75.21% (64.71%-86.37%)	3.2757 (3.0191-3.5176)
At least 3 of CHASM (lung), FATHMM (cancer), MutationTaster, PROVEAN, VEST	80.76% (77.78%-83.84%)	81.85% (78.29%-85.94%)	78.865% (73.527%-84.507%)	87% (83.76%-90.68%)	71.54% (66.25%-77.11%)	3.1926 (3.0610-3.3265)	80.91% (74.75%-86.87%)	82.02% (73.58%-89.1%)	78.975% (68.421%-89.48%)	87% (79.69%-93.33%)	71.93% (60.53%-82.86%)	3.1992 (2.9354-3.4577)
At least 3 of CHASM (lung), FATHMM (cancer), MutationTaster, SIFT	85.87% (83.33%-88.89%)	82.96% (79.23%-87.02%)	90.888% (87.324%-94.807%)	94.03% (91.67%-96.58%)	75.53% (70.24%-80.49%)	3.4340 (3.3242-3.5509)	85.84% (79.8%-90.91%)	82.99% (75%-90.33%)	90.696% (82.5%-97.37%)	93.87% (88.24%-98.28%)	75.68% (65.85%-85.72%)	3.4324 (3.1998-3.6463)
At least 3 of CHASM (lung), FATHMM (cancer), MutationTaster, SIFT, VEST	82.75% (79.8%-85.86%)	89.32% (86.44%-92.37%)	71.377% (65.714%-77.468%)	84.36% (80.95%-87.97%)	79.46% (74.13%-84.85%)	3.2452 (3.1164-3.3704)	82.98% (76.77%-88.89%)	89.42% (83.33%-95.16%)	71.928% (59.986%-83.33%)	84.53% (77.46%-91.18%)	79.87% (69.22%-90.33%)	3.2575 (2.9992-3.5111)
At least 3 of CHASM (lung), FATHMM (cancer), MutationTaster, VEST	82.8% (79.8%-85.86%)	79.2% (75.2%-83.47%)	89.004% (85.294%-93.338%)	92.56% (89.91%-95.54%)	71.23% (65.91%-76.41%)	3.3200 (3.2034-3.4391)	82.89% (76.77%-88.89%)	79.34% (70.69%-87.3%)	88.976% (80.554%-96.97%)	92.5% (86.27%-98.08%)	71.53% (61.1%-82.5%)	3.3234 (3.0920-3.5561)
At least 3 of CHASM (lung), FATHMM (cancer), PolyPhen-2	72.43% (68.69%-76.26%)	59.67% (54.83%-64.84%)	94.496% (91.667%-97.403%)	94.93% (92.31%-97.59%)	57.54% (52.5%-62.4%)	3.0664 (2.9638-3.1737)	72.3% (64.65%-79.8%)	59.36% (49.21%-69.23%)	94.515% (88.232%-100%)	94.87% (88.57%-100%)	57.54% (48.07%-67.8%)	3.0628 (2.8446-3.2723)
At least 3 of CHASM (lung), FATHMM (cancer), PolyPhen-2, PROVEAN	73.75% (70.2%-77.27%)	68.68% (64.06%-73.44%)	82.507% (77.465%-87.5%)	87.16% (83.49%-91.09%)	60.38% (54.73%-65.62%)	2.9872 (2.8492-3.1182)	73.72% (66.67%-80.81%)	68.47% (58.62%-78.19%)	82.699% (72.5%-92.5%)	87.16% (79.17%-94.23%)	60.45% (50%-71.74%)	2.9878 (2.7223-3.2622)
At least 3 of CHASM (lung), FATHMM (cancer), PolyPhen-2, PROVEAN, SIFT	77.73% (74.24%-80.82%)	81.9% (78.22%-85.94%)	70.512% (64.384%-76.812%)	82.77% (78.86%-86.62%)	69.27% (63.51%-75%)	3.0445 (2.9063-3.1835)	77.87% (71.69%-84.85%)	81.91% (73.33%-89.55%)	70.884% (57.895%-83.33%)	82.84% (75.38%-90.48%)	69.57% (58.33%-81.08%)	3.0521 (2.7812-3.3304)
At least 3 of CHASM (lung), FATHMM (cancer), PolyPhen-2, PROVEAN, VEST	74.68% (71.21%-78.28%)	78.15% (74.42%-82.4%)	68.68% (62.5%-75.325%)	81.18% (77.05%-85.35%)	64.51% (58.67%-70.27%)	2.9253 (2.7817-3.0696)	74.88% (67.68%-81.82%)	78.25% (69.81%-85.72%)	69.069% (55.882%-81.09%)	81.27% (72.88%-89.83%)	64.92% (53.12%-76.32%)	2.9351 (2.6345-3.2232)
At least 3 of CHASM (lung), FATHMM (cancer), PolyPhen-2, SIFT	79.42% (76.26%-82.83%)	80.32% (76.56%-84.5%)	77.86% (72.464%-83.339%)	86.25% (82.76%-89.74%)	69.6% (64%-75%)	3.1403 (3.0055-3.2809)	79.53% (72.73%-85.86%)	80.28% (71.43%-88.14%)	78.211% (67.479%-88.9%)	86.35% (79.25%-93.22%)	69.83% (59.09%-80.57%)	3.1466 (2.8680-3.4109)
At least 3 of CHASM (lung), FATHMM (cancer), PolyPhen-2, SIFT, VEST	74.33% (70.71%-77.78%)	85.61% (82.17%-89.06%)	54.82% (48.484%-60.87%)	76.61% (72.73%-80.58%)	68.79% (62.71%-75%)	2.8583 (2.7091-2.9999)	74.57% (67.68%-81.82%)	85.67% (78.57%-92.43%)	55.491% (43.331%-68.75%)	76.75% (68.57%-84.93%)	69.33% (56.25%-82.61%)	2.8724 (2.5925-3.1577)
At least 3 of CHASM (lung), FATHMM (cancer), PolyPhen-2, VEST	75.73% (72.22%-79.29%)	76.06% (72%-80.47%)	75.159% (69.444%-81.159%)	84.11% (80.36%-88.18%)	64.49% (58.82%-70.11%)	2.9982 (2.8628-3.1380)	75.81% (68.69%-82.83%)	76.05% (67.21%-84.38%)	75.378% (62.857%-87.1%)	84.12% (75.93%-91.38%)	64.71% (53.65%-76.19%)	3.0026 (2.7197-3.2762)
At least 3 of CHASM (lung), FATHMM (cancer), PROVEAN	64.01% (60.1%-67.68%)	44.26% (39.34%-49.21%)	98.149% (97.015%-100%)	97.63% (96.08%-100%)	50.45% (45.59%-54.8%)	2.9049 (2.8140-2.9982)	63.91% (56.57%-71.72%)	43.91% (33.87%-53.85%)	98.206% (94.282%-100%)	97.66% (92.31%-100%)	50.5% (41.54%-60.01%)	2.9027 (2.7142-3.0902)
At least 3 of CHASM (lung), FATHMM (cancer), PROVEAN, SIFT	73.43% (69.7%-76.77%)	68.7% (64.06%-73.44%)	81.599% (76.316%-86.961%)	86.59% (82.65%-90.72%)	60.13% (54.54%-65.62%)	2.9702 (2.8349-3.1044)	73.34% (66.67%-80.81%)	68.42% (58.73%-77.78%)	81.753% (71.053%-91.89%)	86.55% (78.43%-93.88%)	60.14% (49.05%-71.43%)	2.9685 (2.6934-3.2372)
At least 3 of CHASM (lung), FATHMM (cancer), PROVEAN, SIFT, VEST	75.02% (71.72%-78.28%)	79.79% (75.83%-83.85%)	66.77% (60.605%-73.239%)	80.59% (76.74%-84.87%)	65.64% (59.21%-71.62%)	2.9279 (2.7828-3.0719)	75.21% (68.69%-81.82%)	79.76% (70.77%-87.5%)	67.359% (54.831%-79.41%)	80.75% (72.72%-88.52%)	65.99% (54.05%-77.78%)	2.9386 (2.6460-3.2247)
At least 3 of CHASM (lung), FATHMM (cancer), PROVEAN, VEST	68.72% (65.14%-72.22%)	61.25% (56.2%-66.13%)	81.636% (76.623%-86.957%)	85.22% (80.9%-89.54%)	54.93% (49.5%-60.19%)	2.8304 (2.6833-2.9696)	68.61% (61.62%-75.78%)	60.98% (50.85%-70.91%)	81.682% (71.053%-91.67%)	85.1% (76.19%-93.33%)	54.95% (44.44%-66.04%)	2.8271 (2.5453-3.1103)
At least 3 of CHASM (lung), FATHMM (cancer), SIFT	74.46% (71.21%-77.78%)	62.87% (58.27%-67.72%)	94.509% (91.667%-97.469%)	95.19% (92.68%-97.75%)	59.55% (54.46%-64.6%)	3.1211 (3.0172-3.2300)	74.3% (67.68%-80.81%)	62.53% (52.54%-71.88%)	94.484% (88.095%-100%)	95.09% (89.19%-100%)	59.5% (50%-69.64%)	3.1161 (2.8965-3.3254)
At least 3 of CHASM (lung), FATHMM (cancer), SIFT, VEST	77.07% (73.74%-80.81%)	78.75% (74.6%-82.93%)	74.15% (68.571%-80.282%)	84.05% (80.36%-88.14%)	66.87% (60.81%-72.46%)	3.0381 (2.8979-3.1862)	77.18% (69.7%-83.84%)	78.65% (69.81%-87.1%)	74.611% (62.5%-85.3%)	84.17% (76.36%-91.38%)	67.07% (55.55%-78.57%)	3.0451 (2.7504-3.3246)
At least 3 of CHASM (lung), FATHMM (cancer), VEST	66.72% (63.13%-70.2%)	51.2% (46.28%-56.3%)	93.569% (90.541%-97.183%)	93.22% (90.14%-97.1%)	52.58% (47.62%-57.15%)	2.9057 (2.7972-3.0178)	66.55% (59.6%-73.74%)	50.78% (40.62%-60.56%)	93.612% (86.842%-100%)	93.16% (85.29%-100%)	52.56% (43.08%-62.69%)	2.9012 (2.6759-3.1238)
At least 3 of CHASM (lung), FATHMM (missense), Mutation Assessor	67.73% (64.14%-71.21%)	51.74% (46.4%-56.69%)	95.382% (92.857%-98.611%)	95.09% (92.42%-98.53%)	53.34% (48.44%-58.07%)	2.9555 (2.8581-3.0630)	67.56% (60.61%-74.75%)	51.27% (40.98%-61.67%)	95.485% (89.182%-100%)	95.11% (88.24%-100%)	53.31% (44.29%-63.08%)	2.9519 (2.7384-3.1428)
At least 3 of CHASM (lung), FATHMM (missense), Mutation Assessor, MutationTaster	83.18% (80.3%-86.36%)	78.21% (74.01%-82.4%)	91.761% (88.406%-95.652%)	94.26% (92%-97.09%)	70.9% (65.59%-76%)	3.3513 (3.2496-3.4618)	83.13% (76.77%-88.89%)	78.12% (69.84%-86.15%)	91.697% (84.375%-97.5%)	94.17% (88.46%-98.22%)	70.97% (60.86%-81.25%)	3.3496 (3.1288-3.5599)
At least 3 of CHASM (lung), FATHMM (missense), Mutation Assessor, MutationTaster, PolyPhen-2	83.14% (80.3%-85.86%)	86.67% (83.33%-90.17%)	77.042% (71.429%-82.667%)	86.71% (83.33%-90.15%)	76.97% (71.64%-82.61%)	3.2740 (3.1409-3.4025)	83.21% (77.78%-88.89%)	86.76% (79.66%-93.55%)	77.109% (65.854%-87.88%)	86.67% (79.65%-93.34%)	77.26% (65.62%-88.24%)	3.2779 (3.0224-3.5330)
At least 3 of CHASM (lung), FATHMM (missense), Mutation Assessor, MutationTaster, PROVEAN	83.83% (81.31%-86.87%)	83.49% (79.84%-87.4%)	84.4% (79.729%-89.479%)	90.25% (87.27%-93.46%)	74.73% (69.51%-79.71%)	3.3286 (3.2154-3.4528)	83.86% (77.78%-89.9%)	83.53% (75.92%-90.63%)	84.385% (74.359%-93.75%)	90.19% (84%-96.36%)	74.93% (64.86%-85.37%)	3.3304 (3.0873-3.5500)
At least 3 of CHASM (lung), FATHMM (missense), Mutation Assessor, MutationTaster, SIFT	84.8% (81.82%-87.88%)	88.25% (85.25%-91.54%)	78.811% (73.846%-84.722%)	87.81% (84.68%-91.06%)	79.52% (74.28%-84.93%)	3.3439 (3.2224-3.4701)	84.95% (78.79%-90.91%)	88.37% (81.54%-94.29%)	79.058% (67.645%-88.9%)	87.87% (81.25%-94.03%)	79.87% (68.75%-89.74%)	3.3516 (3.1077-3.5925)
At least 3 of CHASM (lung), FATHMM (missense), Mutation Assessor, MutationTaster, VEST	82.12% (78.79%-84.85%)	85.63% (82.17%-89.23%)	76.054% (70.27%-81.944%)	86.08% (82.54%-89.68%)	75.38% (70%-80.88%)	3.2313 (3.0913-3.3531)	82.22% (76.77%-88.89%)	85.64% (78.33%-92.31%)	76.334% (64.997%-87.19%)	86.12% (78.87%-92.86%)	75.62% (63.64%-86.49%)	3.2372 (2.9945-3.5005)
At least 3 of CHASM (lung), FATHMM (missense), Mutation Assessor, PolyPhen-2	75.78% (72.73%-79.29%)	72.39% (68.03%-76.86%)	81.613% (76.71%-86.842%)	87.19% (83.63%-91%)	63.1% (57.61%-68.69%)	3.0429 (2.9112-3.1725)	75.72% (68.69%-81.82%)	72.2% (63.33%-80.65%)	81.713% (71.053%-91.67%)	87.14% (79.63%-94.23%)	63.15% (52.08%-74.42%)	3.0421 (2.7785-3.2984)
At least 3 of CHASM (lung), FATHMM (missense), Mutation Assessor, PolyPhen-2, PROVEAN	78.06% (74.75%-81.31%)	78.71% (75%-82.71%)	76.932% (71.233%-82.609%)	85.51% (81.98%-89.38%)	67.64% (62.03%-73.08%)	3.0879 (2.9499-3.2156)	78.21% (71.72%-84.85%)	78.71% (70.37%-86.36%)	77.319% (65.788%-88.57%)	85.62% (78.18%-92.86%)	67.92% (57.12%-78.95%)	3.0957 (2.8312-3.3628)
At least 3 of CHASM (lung), FATHMM (missense), Mutation Assessor, PolyPhen-2, SIFT	78.42% (75.25%-81.82%)	85.62% (82.17%-89.26%)	65.968% (59.459%-72.222%)	81.31% (77.61%-85.16%)	72.63% (66.67%-78.69%)	3.0552 (2.9144-3.1957)	78.51% (71.72%-84.85%)	85.66% (78.69%-92.31%)	66.224% (52.941%-78.39%)	81.31% (73.77%-88.89%)	72.93% (61.11%-84.01%)	3.0612 (2.7789-3.3436)
At least 3 of CHASM (lung), FATHMM (missense), Mutation Assessor, PolyPhen-2, VEST	73.74% (70.2%-76.77%)	80.87% (77.05%-84.68%)	61.394% (55.22%-67.647%)	78.36% (74.26%-82.23%)	64.99% (58.72%-71.24%)	2.8562 (2.7164-2.9965)	73.74% (67.68%-80.81%)	80.79% (73.02%-88.14%)	61.612% (48.647%-73.7%)	78.31% (70.49%-86.15%)	65.14% (51.72%-77.78%)	2.8584 (2.5678-3.1518)
At least 3 of CHASM (lung), FATHMM (missense), Mutation Assessor, PROVEAN	71.78% (68.18%-75.25%)	63.42% (58.68%-68.29%)	86.225% (81.687%-90.789%)	88.84% (85.23%-92.77%)	57.69% (52.29%-62.83%)	2.9617 (2.8376-3.0937)	71.59% (64.65%-78.79%)	63.02% (53.12%-72.14%)	86.243% (76.471%-94.74%)	88.73% (80.95%-95.75%)	57.61% (47.27%-68.52%)	2.9559 (2.6944-3.1961)
At least 3 of CHASM (lung), FATHMM (missense), Mutation Assessor, PROVEAN, SIFT	77.74% (74.24%-81.31%)	80.87% (76.98%-85%)	72.308% (66.667%-78.378%)	83.47% (79.66%-87.39%)	68.61% (62.86%-74.36%)	3.0526 (2.9103-3.1995)	77.86% (70.71%-84.85%)	80.79% (72.41%-87.88%)	72.798% (60%-84.38%)	83.6% (75.81%-91.38%)	68.83% (57.13%-79.56%)	3.0602 (2.7708-3.3384)
At least 3 of CHASM (lung), FATHMM (missense), Mutation Assessor, PROVEAN, VEST	75.03% (71.72%-78.28%)	77.12% (73.08%-81.4%)	71.406% (65.713%-77.465%)	82.34% (78.45%-86.32%)	64.35% (58.54%-70.37%)	2.9522 (2.8099-3.0941)	75.19% (68.69%-81.82%)	77.12% (68.52%-84.85%)	71.844% (59.372%-83.33%)	82.46% (74.58%-90%)	64.65% (52.63%-75.68%)	2.9607 (2.6720-3.2374)
At least 3 of CHASM (lung), FATHMM (missense), Mutation Assessor, SIFT	77.78% (74.75%-81.31%)	76.11% (72.03%-80.31%)	80.639% (75.675%-86.301%)	87.18% (83.76%-90.99%)	66.13% (60.46%-71.59%)	3.1007 (2.9772-3.2392)	77.78% (70.71%-83.84%)	75.93% (67.21%-84.06%)	80.892% (69.691%-90.62%)	87.23% (80%-94.34%)	66.2% (54.99%-77.55%)	3.1025 (2.8252-3.3528)
At least 3 of CHASM (lung), FATHMM (missense), Mutation Assessor, SIFT, VEST	75.06% (71.72%-78.3%)	83.54% (79.84%-87.4%)	60.392% (54.164%-66.667%)	78.48% (74.44%-82.4%)	67.97% (61.9%-74.29%)	2.9038 (2.7553-3.0520)	75.13% (68.66%-81.82%)	83.43% (75.76%-90.33%)	60.855% (47.054%-72.97%)	78.52% (70.96%-86.57%)	68.15% (55.16%-80%)	2.9096 (2.6120-3.2090)
At least 3 of CHASM (lung), FATHMM (missense), Mutation Assessor, VEST	74.75% (71.72%-78.28%)	71.87% (67.5%-76.3%)	79.722% (74.62%-85.294%)	85.97% (82.18%-89.81%)	62.11% (56.67%-67.71%)	2.9968 (2.8614-3.1285)	74.73% (67.68%-81.82%)	71.64% (62.5%-80.3%)	79.991% (68.571%-90.33%)	86.01% (78.26%-93.49%)	62.17% (51.06%-72.92%)	2.9982 (2.7378-3.2697)
At least 3 of CHASM (lung), FATHMM (missense), MutationTaster	71.42% (67.68%-74.76%)	57.01% (52.03%-62.21%)	96.322% (94.203%-98.685%)	96.4% (94.29%-98.72%)	56.45% (51.49%-61.24%)	3.0618 (2.9612-3.1680)	71.31% (64.62%-78.79%)	56.7% (45.76%-66.67%)	96.356% (91.111%-100%)	96.38% (90.9%-100%)	56.47% (46.87%-66.68%)	3.0591 (2.8522-3.2638)
At least 3 of CHASM (lung), FATHMM (missense), MutationTaster, PolyPhen-2	83.52% (80.81%-86.36%)	80.86% (76.92%-84.8%)	88.111% (84.057%-92.647%)	92.16% (89.42%-95.33%)	72.7% (67.44%-77.91%)	3.3383 (3.2254-3.4522)	83.47% (77.78%-89.9%)	80.81% (72.88%-88.52%)	87.985% (79.412%-96.88%)	92.03% (85.71%-98%)	72.8% (62.5%-83.72%)	3.3363 (3.1058-3.5630)
At least 3 of CHASM (lung), FATHMM (missense), MutationTaster, PolyPhen-2, PROVEAN	81.46% (78.28%-84.36%)	82.42% (78.63%-86.26%)	79.799% (74.647%-85.294%)	87.59% (84.35%-91.13%)	72.42% (66.67%-77.78%)	3.2223 (3.0967-3.3509)	81.52% (75.73%-87.88%)	82.48% (74.58%-90.14%)	79.815% (68.571%-90%)	87.53% (80.36%-94.23%)	72.66% (61.54%-83.34%)	3.2248 (2.9655-3.4702)
At least 3 of CHASM (lung), FATHMM (missense), MutationTaster, PolyPhen-2, SIFT	83.15% (80.3%-85.86%)	89.34% (86.32%-92.43%)	72.45% (66.197%-78.378%)	84.87% (81.4%-88.32%)	79.72% (74.14%-85.07%)	3.2638 (3.1297-3.3873)	83.19% (77.78%-88.89%)	89.4% (83.04%-95.38%)	72.502% (60%-84.38%)	84.81% (77.78%-91.67%)	79.96% (68.97%-90.62%)	3.2667 (3.0216-3.5284)
At least 3 of CHASM (lung), FATHMM (missense), MutationTaster, PolyPhen-2, VEST	80.16% (76.77%-83.33%)	87.23% (84.03%-90.68%)	67.929% (61.644%-74.242%)	82.46% (78.79%-86.26%)	75.47% (69.99%-81.25%)	3.1309 (2.9900-3.2644)	80.09% (73.74%-86.87%)	87.24% (80%-93.75%)	67.799% (55.172%-80.01%)	82.3% (74.63%-89.55%)	75.6% (64.28%-87.18%)	3.1293 (2.8483-3.4020)
At least 3 of CHASM (lung), FATHMM (missense), MutationTaster, PROVEAN	79.84% (76.77%-83.33%)	71.33% (66.67%-76.19%)	94.526% (91.667%-97.436%)	95.75% (93.55%-97.96%)	65.61% (60.2%-70.71%)	3.2722 (3.1707-3.3825)	79.72% (72.73%-85.86%)	71.13% (61.4%-80.33%)	94.426% (88.235%-100%)	95.64% (90.47%-100%)	65.61% (55.32%-76.01%)	3.2680 (3.0556-3.4697)
At least 3 of CHASM (lung), FATHMM (missense), MutationTaster, PROVEAN, SIFT	84.14% (81.31%-86.87%)	85.08% (81.45%-88.8%)	82.489% (77.918%-87.838%)	89.37% (86.44%-92.5%)	76.18% (70.83%-81.33%)	3.3312 (3.2081-3.4503)	84.25% (78.79%-89.9%)	85.14% (77.36%-92.16%)	82.682% (72.414%-91.9%)	89.42% (83.05%-95.16%)	76.45% (65.79%-87.5%)	3.3369 (3.0920-3.5702)
At least 3 of CHASM (lung), FATHMM (missense), MutationTaster, PROVEAN, VEST	81.11% (77.78%-84.34%)	82.41% (78.62%-86.29%)	78.851% (73.522%-84.418%)	87.08% (83.76%-90.68%)	72.17% (66.67%-77.63%)	3.2052 (3.0734-3.3354)	81.21% (74.75%-87.88%)	82.49% (74.58%-90.16%)	78.947% (67.742%-89.47%)	87.07% (79.99%-93.45%)	72.45% (61.54%-84.21%)	3.2096 (2.9423-3.4699)
At least 3 of CHASM (lung), FATHMM (missense), MutationTaster, SIFT	85.52% (82.83%-88.38%)	82.98% (79.2%-86.82%)	89.897% (86.111%-94.203%)	93.42% (90.99%-96.3%)	75.34% (70.24%-80.46%)	3.4164 (3.3114-3.5234)	85.53% (79.8%-90.91%)	82.96% (75%-90.77%)	89.913% (81.25%-97.3%)	93.39% (87.93%-98.22%)	75.49% (65.77%-86.05%)	3.4175 (3.2064-3.6316)
At least 3 of CHASM (lung), FATHMM (missense), MutationTaster, SIFT, VEST	82.46% (79.29%-85.35%)	88.82% (85.71%-92.07%)	71.453% (65.217%-77.333%)	84.33% (80.77%-87.88%)	78.71% (73.02%-84.13%)	3.2330 (3.0972-3.3533)	82.56% (76.77%-88.89%)	88.84% (82.14%-95%)	71.741% (60%-83.33%)	84.37% (77.05%-91.18%)	78.95% (68.28%-89.66%)	3.2391 (2.9943-3.5081)
At least 3 of CHASM (lung), FATHMM (missense), MutationTaster, VEST	82.14% (79.29%-84.85%)	79.24% (75.2%-83.33%)	87.147% (82.894%-91.667%)	91.42% (88.68%-94.5%)	70.83% (65.59%-76.14%)	3.2865 (3.1758-3.4021)	82.18% (76.77%-87.88%)	79.26% (71.01%-87.33%)	87.154% (78.125%-95.14%)	91.38% (85.11%-96.67%)	71.03% (60.52%-81.83%)	3.2882 (3.0561-3.5142)
At least 3 of CHASM (lung), FATHMM (missense), PolyPhen-2	68.74% (65.15%-72.22%)	54.4% (49.21%-59.68%)	93.53% (90.411%-97.183%)	93.56% (90.54%-97.22%)	54.26% (49.23%-58.87%)	2.9574 (2.8487-3.0739)	68.58% (61.62%-75.76%)	53.94% (43.75%-64.29%)	93.693% (86.667%-100%)	93.59% (86.48%-100%)	54.25% (45%-63.94%)	2.9547 (2.7290-3.1860)
At least 3 of CHASM (lung), FATHMM (missense), PolyPhen-2, PROVEAN	74.1% (70.71%-77.78%)	68.7% (64.06%-73.55%)	83.422% (78.459%-88.406%)	87.75% (84.16%-91.51%)	60.66% (55.1%-65.98%)	3.0054 (2.8731-3.1364)	74.02% (66.67%-80.81%)	68.41% (58.72%-77.97%)	83.594% (73.684%-93.55%)	87.74% (80.39%-95.24%)	60.67% (50%-71.43%)	3.0042 (2.7497-3.2756)
At least 3 of CHASM (lung), FATHMM (missense), PolyPhen-2, PROVEAN, SIFT	77.06% (73.74%-80.3%)	81.91% (78.22%-85.84%)	68.671% (62.319%-75%)	81.89% (78.05%-85.71%)	68.71% (62.69%-74.29%)	3.0118 (2.8693-3.1543)	77.19% (70.71%-83.84%)	81.89% (73.68%-89.4%)	69.069% (56.755%-81.58%)	81.97% (74.19%-89.83%)	68.99% (57.69%-80.56%)	3.0192 (2.7385-3.3016)
At least 3 of CHASM (lung), FATHMM (missense), PolyPhen-2, PROVEAN, VEST	74.4% (71.21%-77.78%)	78.18% (74.4%-82.22%)	67.846% (61.111%-74.324%)	80.79% (76.86%-84.82%)	64.26% (58.11%-70.13%)	2.9107 (2.7656-3.0560)	74.44% (67.68%-80.81%)	78.2% (69.84%-85.94%)	67.925% (54.539%-80.56%)	80.73% (73.01%-88.34%)	64.47% (52.5%-76.47%)	2.9132 (2.6239-3.2045)
At least 3 of CHASM (lung), FATHMM (missense), PolyPhen-2, SIFT	78.44% (75.25%-81.82%)	79.8% (75.81%-83.72%)	76.076% (70.667%-81.944%)	85.23% (81.58%-89.08%)	68.55% (62.67%-74.07%)	3.0966 (2.9631-3.2383)	78.46% (71.72%-84.85%)	79.71% (71.67%-87.69%)	76.257% (64.706%-87.5%)	85.22% (77.36%-92.65%)	68.68% (57.5%-80%)	3.0987 (2.8182-3.3711)
At least 3 of CHASM (lung), FATHMM (missense), PolyPhen-2, SIFT, VEST	75.38% (72.22%-78.79%)	85.63% (82.31%-89.06%)	57.649% (51.428%-63.936%)	77.76% (73.76%-81.62%)	69.88% (63.49%-75.93%)	2.9092 (2.7629-3.0456)	75.5% (68.69%-81.82%)	85.63% (78.57%-92.19%)	58.043% (45.161%-70.97%)	77.8% (70%-85.71%)	70.19% (57.14%-83.33%)	2.9166 (2.6443-3.2059)
At least 3 of CHASM (lung), FATHMM (missense), PolyPhen-2, VEST	74.77% (71.21%-77.79%)	75.56% (71.43%-80%)	73.387% (67.143%-79.106%)	83.08% (79.28%-87.27%)	63.46% (57.47%-68.97%)	2.9549 (2.8140-3.0896)	74.7% (68.66%-81.82%)	75.44% (66.67%-83.93%)	73.362% (61.765%-85.01%)	82.96% (74.58%-90.63%)	63.52% (52.17%-75.51%)	2.9527 (2.6696-3.2385)
At least 3 of CHASM (lung), FATHMM (missense), PROVEAN	60.32% (56.57%-63.65%)	38.43% (33.59%-43.31%)	98.149% (97.015%-100%)	97.29% (95.45%-100%)	47.97% (43.15%-52.09%)	2.8184 (2.7280-2.9097)	60.18% (53.51%-67.68%)	38% (27.87%-47.69%)	98.206% (94.282%-100%)	97.31% (90.91%-100%)	47.99% (39.44%-57.69%)	2.8151 (2.6276-2.9963)
At least 3 of CHASM (lung), FATHMM (missense), PROVEAN, SIFT	74.78% (71.21%-78.28%)	69.26% (64.41%-73.85%)	84.315% (79.73%-89.554%)	88.42% (85.11%-92.23%)	61.34% (55.88%-66.35%)	3.0333 (2.9062-3.1623)	74.68% (67.68%-81.82%)	68.89% (59.32%-77.97%)	84.54% (74.286%-93.94%)	88.46% (81.08%-95.56%)	61.3% (50.98%-71.7%)	3.0319 (2.7696-3.2813)
At least 3 of CHASM (lung), FATHMM (missense), PROVEAN, SIFT, VEST	74.38% (70.71%-77.78%)	80.33% (76.42%-84.25%)	64.086% (57.971%-70.515%)	79.46% (75.59%-83.46%)	65.33% (58.82%-71.24%)	2.8920 (2.7433-3.0401)	74.47% (67.68%-81.82%)	80.28% (72.22%-87.88%)	64.462% (51.429%-76.47%)	79.5% (71.43%-87.3%)	65.57% (52.94%-77.78%)	2.8980 (2.6031-3.1945)
At least 3 of CHASM (lung), FATHMM (missense), PROVEAN, VEST	68.72% (65.15%-72.22%)	61.28% (56.2%-66.39%)	81.572% (76.71%-86.667%)	85.18% (81.39%-89.29%)	54.92% (49.51%-60.19%)	2.8296 (2.6903-2.9644)	68.62% (61.62%-75.76%)	60.92% (50.79%-71.19%)	81.779% (70.962%-91.67%)	85.16% (76.74%-93.02%)	54.95% (44.23%-66.04%)	2.8281 (2.5527-3.1060)
At least 3 of CHASM (lung), FATHMM (missense), SIFT	71.1% (67.68%-74.75%)	58.13% (53.12%-62.99%)	93.518% (90.623%-97.101%)	93.94% (90.91%-97.37%)	56.36% (51.56%-61.16%)	3.0194 (2.9102-3.1341)	70.94% (63.64%-77.78%)	57.65% (47.62%-67.74%)	93.701% (87.177%-100%)	94% (87.5%-100%)	56.32% (46.67%-66.1%)	3.0167 (2.7801-3.2434)
At least 3 of CHASM (lung), FATHMM (missense), SIFT, VEST	77.09% (73.74%-80.3%)	78.76% (74.6%-82.93%)	74.177% (68.18%-80%)	84.06% (80.34%-88.03%)	66.89% (60.76%-72.6%)	3.0389 (2.9003-3.1842)	77.14% (70.71%-83.84%)	78.61% (70.31%-86.89%)	74.55% (62.5%-86.11%)	84.14% (76.27%-91.53%)	67.02% (55%-78.58%)	3.0432 (2.7522-3.3229)
At least 3 of CHASM (lung), FATHMM (missense), VEST	62.68% (59.09%-66.16%)	45.92% (40.8%-50.82%)	91.663% (88.403%-95.835%)	90.49% (86.56%-95.08%)	49.5% (44.69%-54.07%)	2.7758 (2.6577-2.8952)	62.51% (55.56%-69.7%)	45.37% (35.38%-55.56%)	91.916% (83.773%-97.78%)	90.57% (81.82%-97.5%)	49.5% (40.58%-59.32%)	2.7735 (2.5345-3.0029)
At least 3 of CHASM (lung), Mutation Assessor, MutationTaster	67.03% (63.13%-70.71%)	50.62% (45.45%-55.65%)	95.4% (92.955%-98.571%)	95% (92.31%-98.46%)	52.77% (48%-57.58%)	2.9380 (2.8359-3.0427)	66.95% (59.6%-74.75%)	50.33% (40.62%-60.32%)	95.442% (89.474%-100%)	94.98% (88.46%-100%)	52.82% (43.1%-62.5%)	2.9357 (2.7235-3.1395)
At least 3 of CHASM (lung), Mutation Assessor, MutationTaster, PolyPhen-2	81.47% (78.28%-84.85%)	77.11% (72.95%-81.45%)	88.988% (85.29%-93.243%)	92.37% (89.79%-95.46%)	69.22% (64.13%-74.45%)	3.2769 (3.1649-3.3995)	81.51% (74.75%-87.88%)	77.13% (68.42%-85.51%)	89.014% (80.488%-97.06%)	92.32% (85.71%-97.96%)	69.42% (58.7%-79.49%)	3.2788 (3.0390-3.5070)
At least 3 of CHASM (lung), Mutation Assessor, MutationTaster, PolyPhen-2, PROVEAN	79.41% (76.26%-82.83%)	80.28% (76.42%-83.97%)	77.897% (72.603%-83.562%)	86.26% (82.79%-89.92%)	69.56% (64.1%-75%)	3.1401 (3.0061-3.2718)	79.56% (72.73%-85.86%)	80.36% (72.58%-87.93%)	78.168% (67.5%-88.89%)	86.32% (78.95%-93.22%)	69.89% (58.69%-80.65%)	3.1474 (2.8764-3.4085)
At least 3 of CHASM (lung), Mutation Assessor, MutationTaster, PolyPhen-2, SIFT	79.09% (75.76%-82.32%)	82.43% (78.69%-86.4%)	73.326% (67.606%-79.226%)	84.23% (80.51%-87.81%)	70.7% (65.21%-76.54%)	3.1069 (2.9702-3.2475)	79.19% (72.73%-85.86%)	82.46% (74.6%-89.4%)	73.546% (62.061%-84.85%)	84.24% (76.92%-91.67%)	70.98% (59.37%-81.82%)	3.1122 (2.8286-3.3778)
At least 3 of CHASM (lung), Mutation Assessor, MutationTaster, PolyPhen-2, VEST	76.44% (73.23%-79.8%)	80.83% (76.92%-84.8%)	68.865% (63.014%-75%)	81.77% (77.87%-85.95%)	67.51% (61.64%-73.91%)	2.9898 (2.8511-3.1289)	76.41% (69.7%-82.83%)	80.88% (72.72%-88.33%)	68.736% (56.748%-80.49%)	81.59% (73.68%-89.07%)	67.7% (55.17%-79.41%)	2.9891 (2.7073-3.2662)
At least 3 of CHASM (lung), Mutation Assessor, MutationTaster, PROVEAN	75.12% (71.72%-78.79%)	64.42% (59.67%-69.36%)	93.605% (90.539%-97.102%)	94.57% (92.13%-97.62%)	60.34% (55.36%-65.22%)	3.1293 (3.0247-3.2434)	75.02% (67.68%-81.82%)	64.22% (54.23%-73.85%)	93.512% (86.667%-100%)	94.44% (88.37%-100%)	60.36% (50%-70.59%)	3.1254 (2.9036-3.3440)
At least 3 of CHASM (lung), Mutation Assessor, MutationTaster, PROVEAN, SIFT	78.09% (74.75%-81.31%)	78.18% (74.36%-82.26%)	77.941% (72.727%-83.562%)	85.97% (82.14%-89.6%)	67.38% (61.73%-72.94%)	3.0946 (2.9635-3.2330)	78.16% (71.72%-84.85%)	78.2% (69.84%-85.94%)	78.065% (66.667%-89.19%)	85.94% (78.57%-93.11%)	67.59% (56.1%-78.79%)	3.0979 (2.8204-3.3660)
At least 3 of CHASM (lung), Mutation Assessor, MutationTaster, PROVEAN, VEST	76.41% (73.23%-79.8%)	76.05% (72.03%-80.47%)	77.029% (71.642%-82.61%)	85.12% (81.25%-89.17%)	65.04% (59.52%-70.74%)	3.0324 (2.8996-3.1731)	76.47% (69.7%-82.83%)	76.07% (67.24%-84.13%)	77.159% (66.667%-87.88%)	85.09% (76.92%-92.73%)	65.26% (53.84%-76.09%)	3.0357 (2.7524-3.3021)
At least 3 of CHASM (lung), Mutation Assessor, MutationTaster, SIFT	81.82% (79.28%-84.85%)	76.57% (72.36%-80.8%)	90.873% (87.342%-94.872%)	93.55% (91.17%-96.36%)	69.17% (64%-74.45%)	3.3016 (3.1971-3.4163)	81.82% (75.76%-86.89%)	76.61% (67.8%-84.75%)	90.735% (82.855%-97.3%)	93.41% (87.04%-98.08%)	69.33% (58.69%-79.55%)	3.3009 (3.0651-3.5099)
At least 3 of CHASM (lung), Mutation Assessor, MutationTaster, SIFT, VEST	76.75% (73.23%-79.8%)	80.3% (76.42%-84.21%)	70.591% (64.787%-76.473%)	82.52% (78.69%-86.32%)	67.46% (61.84%-73.68%)	3.0087 (2.8654-3.1491)	76.81% (70.71%-83.84%)	80.33% (72.41%-87.94%)	70.76% (58.824%-82.51%)	82.49% (74.6%-90.16%)	67.71% (55.26%-78.95%)	3.0128 (2.7222-3.3021)
At least 3 of CHASM (lung), Mutation Assessor, MutationTaster, VEST	79.8% (76.77%-82.83%)	74.48% (70.4%-78.86%)	88.988% (85.29%-93.243%)	92.12% (89.42%-95.28%)	66.85% (61.61%-72%)	3.2244 (3.1080-3.3417)	79.79% (73.74%-85.86%)	74.41% (65.62%-82.76%)	89.014% (80.488%-97.06%)	92.06% (85.42%-97.92%)	66.97% (56.51%-76.92%)	3.2246 (2.9876-3.4586)
At least 3 of CHASM (lung), Mutation Assessor, PolyPhen-2	68.04% (64.63%-71.72%)	52.76% (47.54%-57.86%)	94.461% (91.549%-97.403%)	94.27% (91.43%-97.3%)	53.63% (48.8%-58.4%)	2.9513 (2.8465-3.0605)	67.95% (60.61%-74.77%)	52.42% (41.67%-62.9%)	94.57% (87.879%-100%)	94.3% (87.5%-100%)	53.67% (44.26%-63.49%)	2.9496 (2.7361-3.1589)
At least 3 of CHASM (lung), Mutation Assessor, PolyPhen-2, PROVEAN	70.72% (67.17%-74.24%)	64.44% (59.83%-69.47%)	81.574% (76.389%-86.957%)	85.81% (81.72%-89.9%)	57.02% (51.43%-62.38%)	2.8885 (2.7519-3.0275)	70.67% (63.64%-77.78%)	64.17% (54.1%-73.85%)	81.794% (71.429%-91.9%)	85.81% (77.55%-93.88%)	57.08% (46.29%-68%)	2.8885 (2.6143-3.1574)
At least 3 of CHASM (lung), Mutation Assessor, PolyPhen-2, PROVEAN, SIFT	73.03% (69.7%-76.26%)	76.58% (72.66%-80.65%)	66.899% (60.806%-73.239%)	80% (76.03%-84.03%)	62.29% (56.1%-68.5%)	2.8576 (2.7124-3.0036)	73.13% (66.67%-79.8%)	76.61% (68.33%-84.75%)	67.121% (54.831%-79.41%)	79.99% (71.92%-87.93%)	62.58% (50%-75%)	2.8630 (2.5715-3.1505)
At least 3 of CHASM (lung), Mutation Assessor, PolyPhen-2, PROVEAN, VEST	72.38% (68.69%-75.76%)	76.61% (72.65%-80.83%)	65.049% (58.904%-71.233%)	79.12% (75%-83.34%)	61.67% (56%-67.95%)	2.8245 (2.6789-2.9626)	72.42% (65.66%-79.8%)	76.54% (68.18%-84.75%)	65.361% (53.328%-77.78%)	79.11% (70.59%-87.1%)	61.88% (50%-73.53%)	2.8288 (2.5396-3.1119)
At least 3 of CHASM (lung), Mutation Assessor, PolyPhen-2, SIFT	74.05% (70.2%-77.78%)	73.92% (69.6%-78.51%)	74.271% (68.655%-80%)	83.24% (79.28%-87.16%)	62.23% (56.32%-67.9%)	2.9366 (2.7871-3.0773)	74.11% (66.67%-81.82%)	73.94% (64.99%-82.54%)	74.395% (63.333%-85.71%)	83.2% (75.44%-91.08%)	62.46% (50.99%-73.81%)	2.9399 (2.6568-3.2273)
At least 3 of CHASM (lung), Mutation Assessor, PolyPhen-2, SIFT, VEST	71.33% (67.68%-74.75%)	79.78% (76.03%-83.61%)	56.709% (50%-63.015%)	76.11% (71.77%-80.16%)	61.86% (55.38%-68.66%)	2.7446 (2.5977-2.8950)	71.49% (64.65%-78.79%)	79.79% (72.13%-87.1%)	57.248% (44.737%-70.27%)	76.19% (67.69%-84.85%)	62.26% (48.57%-75%)	2.7549 (2.4379-3.0475)
At least 3 of CHASM (lung), Mutation Assessor, PolyPhen-2, VEST	73.41% (69.7%-76.77%)	74.49% (70.25%-78.91%)	71.543% (65.753%-77.465%)	81.9% (77.78%-86.09%)	61.86% (56.1%-67.9%)	2.8979 (2.7503-3.0393)	73.38% (66.67%-80.81%)	74.4% (65.57%-82.76%)	71.644% (60%-83.33%)	81.8% (73.43%-90%)	62% (50%-73.81%)	2.8983 (2.6096-3.1906)
At least 3 of CHASM (lung), Mutation Assessor, PROVEAN	60.3% (56.57%-64.14%)	38.4% (33.6%-43.31%)	98.149% (97.015%-100%)	97.28% (95.45%-100%)	47.96% (43.33%-52.41%)	2.8180 (2.7284-2.9117)	60.21% (52.53%-67.68%)	38.06% (28.33%-47.62%)	98.206% (94.282%-100%)	97.31% (91.29%-100%)	48.02% (39.18%-57.53%)	2.8159 (2.6265-2.9943)
At least 3 of CHASM (lung), Mutation Assessor, PROVEAN, SIFT	70.71% (67.17%-74.24%)	64.94% (60.15%-69.77%)	80.674% (75.362%-85.915%)	85.32% (81.25%-89.47%)	57.1% (51.49%-62.5%)	2.8803 (2.7412-3.0179)	70.7% (63.64%-77.78%)	64.76% (55.37%-74.25%)	80.842% (71.053%-91.18%)	85.29% (77.08%-93.48%)	57.2% (46.34%-68%)	2.8810 (2.6003-3.1621)
At least 3 of CHASM (lung), Mutation Assessor, PROVEAN, SIFT, VEST	72.37% (68.69%-75.76%)	76.06% (72.03%-80.45%)	65.982% (59.722%-72.059%)	79.45% (75.21%-83.34%)	61.45% (55.56%-67.57%)	2.8294 (2.6741-2.9657)	72.43% (65.66%-79.8%)	76.05% (67.69%-84.13%)	66.199% (54.054%-78.57%)	79.42% (71.19%-87.72%)	61.68% (48.89%-73.34%)	2.8334 (2.5569-3.1410)
At least 3 of CHASM (lung), Mutation Assessor, PROVEAN, VEST	67.73% (64.14%-71.21%)	60.21% (55.28%-65.29%)	80.706% (75.714%-85.915%)	84.36% (80%-88.89%)	53.98% (48.62%-59.46%)	2.7927 (2.6510-2.9314)	67.58% (60.61%-74.75%)	59.85% (49.25%-69.5%)	80.785% (70.368%-91.18%)	84.23% (75%-93.02%)	53.97% (43.33%-64.41%)	2.7884 (2.5115-3.0730)
At least 3 of CHASM (lung), Mutation Assessor, SIFT	69.07% (65.66%-72.73%)	53.84% (48.78%-59.06%)	95.4% (92.955%-98.571%)	95.29% (92.75%-98.55%)	54.45% (49.61%-59.2%)	2.9897 (2.8856-3.0991)	68.93% (61.62%-75.76%)	53.47% (43.33%-63.64%)	95.442% (89.474%-100%)	95.25% (88.89%-100%)	54.45% (44.93%-64.18%)	2.9861 (2.7522-3.1917)
At least 3 of CHASM (lung), Mutation Assessor, SIFT, VEST	73.07% (69.7%-76.77%)	73.95% (69.67%-78.3%)	71.536% (65.569%-77.473%)	81.79% (77.68%-85.71%)	61.36% (55.56%-67.42%)	2.8863 (2.7400-3.0267)	73.06% (65.66%-79.8%)	73.89% (65.08%-82.26%)	71.608% (59.986%-83.78%)	81.69% (73.58%-89.66%)	61.5% (50%-73.17%)	2.8869 (2.6079-3.1791)
At least 3 of CHASM (lung), Mutation Assessor, VEST	63.68% (60.1%-67.68%)	45.87% (40.94%-51.18%)	94.461% (91.549%-97.403%)	93.47% (90.32%-96.92%)	50.23% (45.39%-54.82%)	2.8403 (2.7357-2.9524)	63.55% (55.56%-70.71%)	45.47% (35.18%-55.74%)	94.57% (87.879%-100%)	93.49% (85.71%-100%)	50.26% (41.09%-60%)	2.8379 (2.6184-3.0453)
At least 3 of CHASM (lung), MutationTaster, PolyPhen-2	70.73% (67.17%-74.24%)	56.46% (51.3%-61.48%)	95.4% (92.955%-98.571%)	95.5% (93.06%-98.61%)	55.9% (50.81%-60.69%)	3.0326 (2.9292-3.1424)	70.66% (63.64%-77.78%)	56.2% (46.03%-66.21%)	95.442% (89.474%-100%)	95.48% (89.74%-100%)	55.95% (46.55%-66.13%)	3.0307 (2.8182-3.2430)
At least 3 of CHASM (lung), MutationTaster, PolyPhen-2, PROVEAN	78.47% (75.25%-81.82%)	69.71% (65.12%-74.4%)	93.605% (90.539%-97.102%)	94.96% (92.55%-97.78%)	64.13% (58.88%-69.16%)	3.2241 (3.1147-3.3337)	78.41% (71.72%-84.85%)	69.59% (60.32%-78.95%)	93.512% (86.667%-100%)	94.85% (89.47%-100%)	64.19% (54.16%-75%)	3.2214 (2.9959-3.4465)
At least 3 of CHASM (lung), MutationTaster, PolyPhen-2, PROVEAN, SIFT	79.09% (75.76%-82.32%)	81.36% (77.69%-85.25%)	75.142% (69.562%-81.162%)	84.98% (81.36%-88.79%)	69.99% (64.47%-75.32%)	3.1147 (2.9773-3.2516)	79.2% (72.73%-86.87%)	81.4% (73.43%-88.71%)	75.406% (62.857%-86.67%)	85.02% (77.59%-92.43%)	70.26% (59.38%-80.95%)	3.1209 (2.8393-3.3870)
At least 3 of CHASM (lung), MutationTaster, PolyPhen-2, PROVEAN, VEST	76.76% (73.72%-80.3%)	79.23% (75.41%-83.2%)	72.487% (66.667%-78.691%)	83.27% (79.63%-87.29%)	66.88% (61.04%-72.5%)	3.0187 (2.8802-3.1562)	76.78% (69.7%-82.85%)	79.28% (71.19%-86.89%)	72.483% (60.526%-84.21%)	83.17% (75.44%-90.74%)	67.09% (55.56%-78.38%)	3.0202 (2.7471-3.2946)
At least 3 of CHASM (lung), MutationTaster, PolyPhen-2, SIFT	83.84% (81.31%-86.87%)	80.82% (77.16%-84.75%)	89.05% (85.294%-93.338%)	92.73% (90.2%-95.65%)	72.87% (67.86%-77.78%)	3.3547 (3.2458-3.4718)	83.84% (77.78%-88.89%)	80.88% (73.13%-88.14%)	88.887% (80%-96.88%)	92.57% (86.54%-98.04%)	73.05% (62.79%-82.98%)	3.3539 (3.1240-3.5707)
At least 3 of CHASM (lung), MutationTaster, PolyPhen-2, SIFT, VEST	75.36% (72.22%-78.79%)	83.43% (80.15%-87.22%)	61.399% (54.795%-68.056%)	78.89% (75%-82.97%)	68.19% (61.9%-74.6%)	2.9191 (2.7757-3.0597)	75.54% (68.69%-81.82%)	83.65% (76.11%-90.32%)	61.597% (48.649%-74.36%)	78.89% (70.77%-86.36%)	68.69% (55.88%-80.56%)	2.9283 (2.6300-3.2143)
At least 3 of CHASM (lung), MutationTaster, PolyPhen-2, VEST	79.48% (76.26%-82.83%)	77.12% (73.01%-81.2%)	83.573% (78.946%-88.575%)	89.02% (85.96%-92.52%)	67.87% (62.5%-73.26%)	3.1758 (3.0534-3.3051)	79.42% (72.73%-85.86%)	77.13% (68.74%-85.46%)	83.345% (73.329%-92.11%)	88.8% (81.48%-94.83%)	68% (56.82%-78.43%)	3.1727 (2.9178-3.4114)
At least 3 of CHASM (lung), MutationTaster, PROVEAN	61.98% (58.08%-65.66%)	41.05% (36.15%-46.22%)	98.149% (97.015%-100%)	97.46% (95.74%-100%)	49.06% (44.29%-53.24%)	2.8571 (2.7658-2.9515)	61.91% (54.55%-69.7%)	40.75% (30.36%-50.77%)	98.206% (94.282%-100%)	97.49% (91.67%-100%)	49.13% (40.54%-58.67%)	2.8557 (2.6712-3.0453)
At least 3 of CHASM (lung), MutationTaster, PROVEAN, SIFT	76.8% (73.74%-80.3%)	67.08% (62.4%-71.67%)	93.605% (90.539%-97.102%)	94.77% (92.31%-97.73%)	62.18% (57.14%-66.99%)	3.1764 (3.0737-3.2894)	76.7% (69.7%-82.83%)	66.88% (57.63%-76.19%)	93.512% (86.667%-100%)	94.65% (88.89%-100%)	62.2% (51.85%-72.22%)	3.1724 (2.9430-3.3811)
At least 3 of CHASM (lung), MutationTaster, PROVEAN, SIFT, VEST	77.1% (73.74%-80.3%)	79.26% (75.42%-83.33%)	73.356% (67.606%-79.452%)	83.72% (80%-87.5%)	67.17% (61.33%-72.94%)	3.0350 (2.8979-3.1673)	77.11% (70.71%-83.84%)	79.22% (71.19%-86.89%)	73.474% (61.111%-85.01%)	83.67% (75.86%-91.07%)	67.31% (55%-78.38%)	3.0368 (2.7631-3.3038)
At least 3 of CHASM (lung), MutationTaster, PROVEAN, VEST	74.11% (70.71%-77.78%)	62.83% (58.01%-67.75%)	93.605% (90.539%-97.102%)	94.44% (91.95%-97.53%)	59.29% (54.2%-64.6%)	3.1016 (2.9875-3.2155)	74.01% (66.67%-80.81%)	62.62% (52.46%-72.31%)	93.512% (86.667%-100%)	94.31% (88.24%-100%)	59.31% (49.09%-69.09%)	3.0975 (2.8656-3.3273)
At least 3 of CHASM (lung), MutationTaster, SIFT	71.07% (67.68%-74.75%)	57% (51.94%-62.1%)	95.4% (92.955%-98.571%)	95.54% (93.15%-98.61%)	56.2% (51.22%-61.02%)	3.0414 (2.9411-3.1452)	70.99% (63.64%-77.78%)	56.72% (46.67%-66.68%)	95.442% (89.474%-100%)	95.51% (89.66%-100%)	56.23% (46.43%-66.17%)	3.0391 (2.8336-3.2434)
At least 3 of CHASM (lung), MutationTaster, SIFT, VEST	81.49% (78.79%-84.34%)	78.19% (74.36%-82.35%)	87.184% (83.099%-91.667%)	91.34% (88.57%-94.5%)	69.81% (64.71%-75.28%)	3.2653 (3.1523-3.3827)	81.47% (75.76%-87.88%)	78.17% (69.84%-85.94%)	87.109% (78.123%-94.87%)	91.23% (84.62%-96.49%)	69.93% (59.18%-79.6%)	3.2643 (3.0300-3.4891)
At least 3 of CHASM (lung), MutationTaster, VEST	67.06% (63.13%-70.71%)	50.66% (45.67%-55.81%)	95.4% (92.955%-98.571%)	95.01% (92.31%-98.46%)	52.79% (47.76%-57.47%)	2.9386 (2.8359-3.0456)	66.9% (59.6%-74.75%)	50.25% (40.29%-60.35%)	95.442% (89.474%-100%)	94.97% (88.57%-100%)	52.78% (43.28%-63.08%)	2.9344 (2.7205-3.1565)
At least 3 of CHASM (lung), PolyPhen-2, PROVEAN	61.99% (58.08%-65.66%)	41.07% (36.07%-45.97%)	98.149% (97.015%-100%)	97.45% (95.74%-100%)	49.06% (44.21%-53.52%)	2.8574 (2.7641-2.9494)	61.88% (54.55%-69.7%)	40.7% (30.77%-50.79%)	98.206% (94.282%-100%)	97.47% (91.67%-100%)	49.11% (40%-58.67%)	2.8549 (2.6668-3.0441)
At least 3 of CHASM (lung), PolyPhen-2, PROVEAN, SIFT	70.7% (67.17%-74.24%)	66.55% (61.98%-71.2%)	77.874% (72.464%-83.562%)	83.87% (79.8%-88.17%)	57.38% (51.58%-62.86%)	2.8567 (2.7130-2.9987)	70.72% (63.64%-77.78%)	66.34% (57.35%-75.45%)	78.183% (66.667%-88.89%)	83.92% (75.5%-92%)	57.5% (46.34%-68.75%)	2.8595 (2.5658-3.1425)
At least 3 of CHASM (lung), PolyPhen-2, PROVEAN, SIFT, VEST	70.33% (66.67%-73.74%)	78.19% (74.4%-82.11%)	56.743% (50%-63.239%)	75.76% (71.65%-80%)	60.07% (53.62%-66.22%)	2.7076 (2.5596-2.8545)	70.45% (63.64%-77.78%)	78.18% (70%-85.71%)	57.146% (43.899%-70.37%)	75.79% (67.19%-83.87%)	60.39% (47.22%-72.73%)	2.7150 (2.4101-3.0065)
At least 3 of CHASM (lung), PolyPhen-2, PROVEAN, VEST	68.71% (65.15%-72.22%)	62.85% (57.98%-67.55%)	78.841% (73.333%-84.507%)	83.7% (79.54%-88.42%)	55.1% (49.52%-60.58%)	2.8050 (2.6626-2.9514)	68.63% (61.62%-75.76%)	62.56% (53.02%-72.31%)	79.017% (67.647%-90%)	83.65% (74%-92.5%)	55.15% (44%-66.1%)	2.8038 (2.5116-3.0930)
At least 3 of CHASM (lung), PolyPhen-2, SIFT	70.08% (66.67%-73.74%)	56.49% (51.24%-61.6%)	93.577% (90.411%-97.102%)	93.82% (90.79%-97.3%)	55.44% (50.41%-60.32%)	2.9932 (2.8837-3.1026)	69.94% (62.63%-76.77%)	56.14% (45.9%-66.17%)	93.594% (87.097%-100%)	93.74% (86.84%-100%)	55.43% (45.76%-65.57%)	2.9891 (2.7667-3.2145)
At least 3 of CHASM (lung), PolyPhen-2, SIFT, VEST	73.04% (69.7%-76.28%)	77.68% (73.81%-81.6%)	65.021% (58.904%-71.233%)	79.34% (75.41%-83.33%)	62.75% (56.52%-68.83%)	2.8478 (2.6945-2.9903)	73.11% (66.64%-79.8%)	77.6% (69.64%-85.48%)	65.354% (52.778%-77.78%)	79.35% (71.19%-87.31%)	62.95% (51.19%-75%)	2.8526 (2.5569-3.1554)
At least 3 of CHASM (lung), PolyPhen-2, VEST	65.03% (61.6%-68.69%)	49.59% (44.54%-54.84%)	91.723% (88.406%-95.654%)	91.19% (87.69%-95.52%)	51.28% (46.38%-55.73%)	2.8378 (2.7263-2.9643)	64.89% (57.58%-71.74%)	49.2% (38.98%-59.32%)	91.809% (84.202%-97.5%)	91.13% (82.14%-97.3%)	51.29% (42.19%-61.43%)	2.8343 (2.5812-3.0558)
At least 3 of CHASM (lung), PROVEAN, SIFT	63.66% (60.09%-67.17%)	43.71% (38.76%-48.46%)	98.149% (97.015%-100%)	97.61% (96%-100%)	50.21% (45.32%-54.61%)	2.8967 (2.8046-2.9883)	63.59% (56.57%-70.73%)	43.41% (33.33%-53.33%)	98.206% (94.282%-100%)	97.63% (92%-100%)	50.28% (41.18%-60%)	2.8953 (2.7034-3.0828)
At least 3 of CHASM (lung), PROVEAN, SIFT, VEST	67.02% (63.13%-70.71%)	61.77% (56.91%-66.67%)	76.088% (70.311%-82.192%)	81.71% (77.17%-86.02%)	53.51% (47.96%-58.88%)	2.7307 (2.5868-2.8795)	66.97% (59.6%-74.75%)	61.53% (51.56%-71.19%)	76.252% (64.5%-87.51%)	81.63% (72.22%-90.7%)	53.59% (42.86%-64.81%)	2.7300 (2.4310-3.0273)
At least 3 of CHASM (lung), PROVEAN, VEST	60.98% (57.07%-64.65%)	39.48% (34.43%-44.26%)	98.149% (97.015%-100%)	97.36% (95.65%-100%)	48.4% (43.54%-52.74%)	2.8338 (2.7425-2.9239)	60.87% (53.54%-68.69%)	39.1% (29.4%-49.23%)	98.206% (94.282%-100%)	97.38% (91.3%-100%)	48.44% (39.47%-57.89%)	2.8313 (2.6445-3.0183)
At least 3 of CHASM (lung), SIFT, VEST	65.36% (61.62%-69.19%)	50.12% (44.96%-55.28%)	91.711% (88.235%-95.588%)	91.26% (87.67%-95.46%)	51.54% (46.56%-56.06%)	2.8463 (2.7287-2.9679)	65.23% (57.58%-72.73%)	49.73% (39.68%-60%)	91.817% (84.615%-97.73%)	91.22% (82.85%-97.5%)	51.55% (42.37%-61.54%)	2.8432 (2.5958-3.0749)
At least 3 of CHASM (melanoma), FATHMM (cancer), FATHMM (missense)	81.53% (78.28%-84.85%)	77.2% (72.95%-81.6%)	88.998% (85.134%-93.336%)	92.39% (89.81%-95.41%)	69.3% (63.72%-75%)	3.2789 (3.1577-3.4024)	81.39% (74.75%-87.88%)	76.97% (68.18%-85.25%)	88.94% (79.412%-96.97%)	92.29% (85.71%-97.96%)	69.24% (58%-79.55%)	3.2744 (3.0249-3.5202)
At least 3 of CHASM (melanoma), FATHMM (cancer), FATHMM (missense), Mutation Assessor	82.51% (79.29%-85.35%)	84.61% (80.95%-88.28%)	78.872% (73.907%-84.507%)	87.38% (84.17%-90.98%)	74.78% (69.23%-80.01%)	3.2565 (3.1274-3.3873)	82.45% (76.77%-88.89%)	84.48% (77.05%-91.8%)	78.932% (67.645%-89.47%)	87.32% (80%-93.55%)	74.8% (64.1%-86.05%)	3.2553 (2.9991-3.5038)
At least 3 of CHASM (melanoma), FATHMM (cancer), FATHMM (missense), Mutation Assessor, MutationTaster	89.26% (86.87%-91.92%)	96.34% (94.49%-98.43%)	77.006% (71.831%-82.899%)	87.87% (84.96%-91.24%)	92.42% (88.89%-96.61%)	3.5363 (3.4397-3.6446)	89.16% (83.84%-93.94%)	96.14% (91.8%-100%)	77.17% (65.789%-87.18%)	87.84% (81.16%-93.65%)	92.11% (83.87%-100%)	3.5326 (3.3164-3.7333)
At least 3 of CHASM (melanoma), FATHMM (cancer), FATHMM (missense), Mutation Assessor, PolyPhen-2	84.21% (81.31%-87.37%)	95.27% (93.39%-97.56%)	65.086% (58.904%-71.062%)	82.51% (78.95%-86.11%)	88.85% (84.31%-94%)	3.3173 (3.1989-3.4391)	84.1% (77.78%-89.9%)	95.08% (90.74%-98.51%)	65.218% (53.125%-77.14%)	82.43% (75%-89.71%)	88.56% (78.26%-96.67%)	3.3129 (3.0722-3.5500)
At least 3 of CHASM (melanoma), FATHMM (cancer), FATHMM (missense), Mutation Assessor, PROVEAN	84.5% (81.82%-87.37%)	91.51% (88.89%-94.35%)	72.364% (66.216%-78.261%)	85.13% (81.68%-88.64%)	83.14% (77.78%-88.71%)	3.3215 (3.1947-3.4449)	84.54% (78.79%-89.9%)	91.44% (85.51%-96.77%)	72.679% (60.606%-84.85%)	85.18% (78.12%-92.19%)	83.2% (72.73%-93.75%)	3.3250 (3.0763-3.5748)
At least 3 of CHASM (melanoma), FATHMM (cancer), FATHMM (missense), Mutation Assessor, SIFT	82.48% (79.29%-85.35%)	93.12% (90.6%-95.83%)	64.088% (57.811%-70.27%)	81.77% (78.17%-85.42%)	84.34% (79.16%-89.59%)	3.2331 (3.1093-3.3661)	82.51% (76.77%-88.89%)	93.01% (87.5%-98.31%)	64.431% (51.429%-77.14%)	81.79% (74.29%-88.89%)	84.31% (73.08%-95.46%)	3.2354 (2.9606-3.4884)
At least 3 of CHASM (melanoma), FATHMM (cancer), FATHMM (missense), Mutation Assessor, VEST	82.49% (79.29%-85.86%)	95.26% (93.33%-97.58%)	60.382% (53.623%-66.667%)	80.61% (76.92%-84.31%)	88.07% (83.33%-93.75%)	3.2433 (3.1191-3.3739)	82.5% (75.76%-88.89%)	95.1% (90.48%-98.53%)	60.856% (48.567%-73.68%)	80.66% (73.01%-87.88%)	87.88% (76.92%-96.43%)	3.2449 (2.9730-3.4987)
At least 3 of CHASM (melanoma), FATHMM (cancer), FATHMM (missense), MutationTaster	88.94% (86.36%-91.41%)	89.95% (86.99%-92.86%)	87.179% (83.099%-91.892%)	92.39% (90%-95.12%)	83.39% (78.75%-88.06%)	3.5291 (3.4276-3.6431)	88.78% (83.84%-93.94%)	89.75% (83.87%-95.52%)	87.073% (77.143%-94.87%)	92.27% (86.67%-96.83%)	83.22% (73.81%-92.68%)	3.5232 (3.2979-3.7368)
At least 3 of CHASM (melanoma), FATHMM (cancer), FATHMM (missense), MutationTaster, PolyPhen-2	87.59% (85.34%-90.4%)	97.4% (95.93%-99.19%)	70.626% (64.787%-76.812%)	85.15% (82.07%-88.51%)	94.02% (90.57%-98.11%)	3.4719 (3.3704-3.5828)	87.44% (81.82%-91.94%)	97.22% (93.85%-100%)	70.633% (58.971%-82.35%)	85.04% (78.08%-91.43%)	93.67% (85.71%-100%)	3.4656 (3.2437-3.6675)
At least 3 of CHASM (melanoma), FATHMM (cancer), FATHMM (missense), MutationTaster, PROVEAN	88.9% (86.36%-91.41%)	93.65% (91.27%-96.09%)	80.693% (75.758%-85.915%)	89.35% (86.4%-92.54%)	88.02% (83.58%-92.42%)	3.5171 (3.4069-3.6261)	88.86% (83.84%-93.94%)	93.55% (88.4%-98.39%)	80.778% (70.583%-90.62%)	89.32% (83.08%-95.31%)	87.98% (78.94%-96.88%)	3.5162 (3.3015-3.7401)
At least 3 of CHASM (melanoma), FATHMM (cancer), FATHMM (missense), MutationTaster, SIFT	88.23% (85.86%-90.91%)	96.88% (95.16%-98.5%)	73.251% (67.567%-79.173%)	86.24% (83.33%-89.63%)	93.16% (89.47%-96.77%)	3.4953 (3.3935-3.6064)	88.19% (82.83%-92.93%)	96.64% (92.73%-100%)	73.617% (61.111%-85%)	86.3% (79.41%-92.42%)	92.77% (84.61%-100%)	3.4933 (3.2687-3.6970)
At least 3 of CHASM (melanoma), FATHMM (cancer), FATHMM (missense), MutationTaster, VEST	87.58% (85.35%-90.4%)	97.93% (96.72%-99.24%)	69.675% (63.636%-76%)	84.82% (81.69%-88.32%)	95.13% (92%-98.25%)	3.4755 (3.3747-3.5804)	87.46% (81.82%-91.92%)	97.74% (94.74%-100%)	69.776% (57.143%-81.82%)	84.75% (77.63%-90.91%)	94.74% (87.5%-100%)	3.4701 (3.2590-3.6644)
At least 3 of CHASM (melanoma), FATHMM (cancer), FATHMM (missense), PolyPhen-2	83.21% (80.3%-86.36%)	88.35% (85.12%-91.48%)	74.304% (68.655%-80.282%)	85.6% (82.4%-89.23%)	78.69% (73.23%-84.29%)	3.2695 (3.1433-3.4024)	83.07% (76.77%-88.89%)	88.17% (81.54%-94.92%)	74.29% (62.5%-84.85%)	85.48% (78.18%-91.94%)	78.56% (67.74%-89.66%)	3.2650 (3.0050-3.5266)
At least 3 of CHASM (melanoma), FATHMM (cancer), FATHMM (missense), PolyPhen-2, PROVEAN	83.17% (80.3%-86.36%)	92.04% (89.39%-94.74%)	67.821% (61.644%-74.195%)	83.18% (79.71%-86.96%)	83.15% (77.77%-88.52%)	3.2620 (3.1277-3.3891)	83.15% (76.77%-88.89%)	91.96% (86.21%-96.97%)	67.995% (55.263%-80%)	83.15% (75.71%-90%)	83.17% (71.88%-93.75%)	3.2627 (2.9945-3.5294)
At least 3 of CHASM (melanoma), FATHMM (cancer), FATHMM (missense), PolyPhen-2, SIFT	81.85% (78.79%-84.86%)	95.82% (94.02%-97.67%)	57.686% (50.769%-64.104%)	79.66% (76.03%-83.34%)	88.87% (84.09%-94%)	3.2203 (3.0978-3.3462)	81.76% (75.73%-87.88%)	95.59% (91.52%-98.59%)	57.977% (44.828%-71.43%)	79.62% (72.06%-86.67%)	88.47% (76.19%-96.78%)	3.2165 (2.9530-3.4458)
At least 3 of CHASM (melanoma), FATHMM (cancer), FATHMM (missense), PolyPhen-2, VEST	83.2% (80.3%-86.36%)	97.4% (95.93%-99.19%)	58.631% (52.174%-64.935%)	80.28% (76.82%-84%)	92.88% (88.89%-97.73%)	3.2919 (3.1828-3.4085)	83.1% (76.77%-88.89%)	97.22% (93.85%-100%)	58.852% (46.332%-71.05%)	80.22% (72.83%-87.14%)	92.5% (83.33%-100%)	3.2879 (3.0516-3.5059)
At least 3 of CHASM (melanoma), FATHMM (cancer), FATHMM (missense), PROVEAN	82.85% (79.8%-85.86%)	84.09% (80.62%-87.91%)	80.693% (75.758%-85.915%)	88.28% (85.12%-91.74%)	74.58% (69.12%-80%)	3.2765 (3.1487-3.4037)	82.78% (76.77%-88.89%)	83.93% (76.27%-91.18%)	80.778% (70.583%-90.62%)	88.23% (81.36%-94.74%)	74.56% (63.64%-85.3%)	3.2750 (3.0197-3.5207)
At least 3 of CHASM (melanoma), FATHMM (cancer), FATHMM (missense), PROVEAN, SIFT	81.79% (78.79%-84.85%)	91.51% (88.89%-94.35%)	64.959% (58.664%-71.647%)	81.87% (78.17%-85.61%)	81.57% (75.92%-87.51%)	3.1991 (3.0657-3.3322)	81.88% (75.76%-87.88%)	91.44% (85.51%-96.77%)	65.443% (52.481%-78.05%)	81.97% (74.36%-89.19%)	81.68% (70.36%-92.86%)	3.2053 (2.9371-3.4640)
At least 3 of CHASM (melanoma), FATHMM (cancer), FATHMM (missense), PROVEAN, VEST	82.48% (79.29%-85.35%)	93.11% (90.55%-95.93%)	64.108% (57.746%-70.314%)	81.77% (78.1%-85.4%)	84.33% (78.95%-90.38%)	3.2332 (3.0985-3.3663)	82.51% (76.77%-88.89%)	93.03% (87.69%-98.36%)	64.395% (51.721%-76.75%)	81.78% (74.28%-89.04%)	84.36% (73.08%-95.83%)	3.2356 (2.9792-3.5012)
At least 3 of CHASM (melanoma), FATHMM (cancer), FATHMM (missense), SIFT	82.13% (79.29%-85.35%)	86.73% (83.33%-90.32%)	74.172% (68.182%-80%)	85.31% (82.11%-88.81%)	76.38% (70.83%-82.09%)	3.2260 (3.1025-3.3622)	82.2% (75.76%-87.88%)	86.63% (79.41%-93.44%)	74.531% (62.061%-85.71%)	85.39% (78.33%-92.06%)	76.5% (65%-87.11%)	3.2305 (2.9467-3.4839)
At least 3 of CHASM (melanoma), FATHMM (cancer), FATHMM (missense), SIFT, VEST	81.51% (78.28%-84.85%)	95.81% (93.85%-98.33%)	56.772% (50.684%-63.239%)	79.31% (75.68%-83.11%)	88.68% (83.72%-95%)	3.2057 (3.0826-3.3370)	81.43% (74.75%-87.88%)	95.61% (90.91%-100%)	57.052% (44.73%-69.7%)	79.26% (71.43%-86.25%)	88.35% (76.46%-100%)	3.2027 (2.9288-3.4392)
At least 3 of CHASM (melanoma), FATHMM (cancer), FATHMM (missense), VEST	82.86% (79.8%-85.86%)	88.35% (85.04%-91.67%)	73.354% (67.568%-79.73%)	85.15% (81.82%-88.72%)	78.47% (73.13%-84.21%)	3.2532 (3.1220-3.3817)	82.76% (76.77%-88.89%)	88.17% (81.36%-94.92%)	73.432% (61.286%-84.85%)	85.08% (77.78%-91.81%)	78.38% (66.67%-88.89%)	3.2506 (2.9814-3.5207)
At least 3 of CHASM (melanoma), FATHMM (cancer), Mutation Assessor	70.7% (67.17%-74.24%)	58.54% (53.49%-63.5%)	91.719% (88.406%-95.716%)	92.44% (89.47%-96.25%)	56.13% (51.19%-61.29%)	2.9882 (2.8772-3.1137)	70.72% (63.64%-77.78%)	58.43% (48.33%-69.12%)	91.782% (84.211%-97.56%)	92.43% (84.62%-97.73%)	56.28% (46.43%-66.67%)	2.9892 (2.7334-3.2153)
At least 3 of CHASM (melanoma), FATHMM (cancer), Mutation Assessor, MutationTaster	84.12% (81.31%-86.87%)	82.38% (78.69%-86.29%)	87.132% (82.894%-91.667%)	91.71% (88.99%-94.83%)	74.09% (68.89%-79.31%)	3.3532 (3.2432-3.4688)	84.28% (78.79%-89.9%)	82.56% (74.63%-89.83%)	87.205% (78.123%-95%)	91.71% (85.42%-96.72%)	74.47% (63.41%-84.62%)	3.3595 (3.1193-3.5853)
At least 3 of CHASM (melanoma), FATHMM (cancer), Mutation Assessor, MutationTaster, PolyPhen-2	82.8% (79.8%-85.86%)	88.26% (85.24%-91.48%)	73.367% (67.646%-79.227%)	85.14% (81.68%-88.8%)	78.32% (72.85%-84.13%)	3.2509 (3.1295-3.3817)	82.88% (76.77%-88.89%)	88.37% (81.67%-94.65%)	73.468% (61.753%-85.19%)	85.1% (77.78%-91.8%)	78.65% (67.48%-89.29%)	3.2559 (2.9874-3.4941)
At least 3 of CHASM (melanoma), FATHMM (cancer), Mutation Assessor, MutationTaster, PROVEAN	82.44% (79.29%-85.35%)	84.52% (80.67%-88.19%)	78.841% (73.611%-84.507%)	87.35% (83.9%-90.91%)	74.66% (68.92%-80.29%)	3.2537 (3.1335-3.3847)	82.59% (76.77%-88.89%)	84.66% (77.05%-92.19%)	79.029% (68.421%-89.47%)	87.38% (80.33%-93.85%)	75.03% (63.63%-85.72%)	3.2610 (2.9903-3.4940)
At least 3 of CHASM (melanoma), FATHMM (cancer), Mutation Assessor, MutationTaster, SIFT	83.12% (80.3%-85.86%)	89.31% (86.29%-92.5%)	72.411% (66.667%-78.378%)	84.84% (81.4%-88.46%)	79.67% (74.19%-85.08%)	3.2623 (3.1378-3.3917)	83.26% (77.78%-88.89%)	89.45% (83.07%-95.31%)	72.624% (60.526%-83.88%)	84.86% (77.61%-91.67%)	80.08% (68.75%-90.91%)	3.2701 (3.0159-3.5150)
At least 3 of CHASM (melanoma), FATHMM (cancer), Mutation Assessor, MutationTaster, VEST	81.46% (78.28%-84.34%)	86.68% (83.58%-90.15%)	72.444% (67.094%-78.38%)	84.47% (80.83%-88.19%)	75.88% (70.27%-81.54%)	3.1947 (3.0653-3.3240)	81.52% (75.76%-87.88%)	86.73% (79.69%-93.22%)	72.563% (61.108%-83.88%)	84.42% (76.92%-91.31%)	76.13% (64.52%-87.1%)	3.1984 (2.9299-3.4587)
At least 3 of CHASM (melanoma), FATHMM (cancer), Mutation Assessor, PolyPhen-2	76.4% (73.23%-79.8%)	76.56% (72.22%-80.83%)	76.112% (70.423%-81.818%)	84.71% (81.03%-88.62%)	65.25% (59.52%-71.09%)	3.0264 (2.8893-3.1669)	76.5% (69.7%-82.83%)	76.64% (68.25%-85.08%)	76.221% (65%-87.5%)	84.68% (76.79%-92%)	65.54% (53.84%-76.74%)	3.0309 (2.7502-3.3023)
At least 3 of CHASM (melanoma), FATHMM (cancer), Mutation Assessor, PolyPhen-2, PROVEAN	76.38% (73.23%-79.8%)	79.75% (75.81%-83.74%)	70.535% (64.61%-76.628%)	82.39% (78.76%-86.15%)	66.83% (61.04%-72.84%)	2.9951 (2.8615-3.1410)	76.54% (69.7%-82.83%)	79.84% (71.67%-87.88%)	70.848% (58.607%-82.5%)	82.45% (75%-89.83%)	67.18% (54.54%-78.95%)	3.0032 (2.7181-3.2737)
At least 3 of CHASM (melanoma), FATHMM (cancer), Mutation Assessor, PolyPhen-2, SIFT	77.41% (74.24%-80.81%)	86.14% (82.68%-89.57%)	62.308% (56.162%-68.919%)	79.8% (75.94%-83.82%)	72.23% (66.13%-78.19%)	3.0048 (2.8693-3.1490)	77.5% (70.71%-83.84%)	86.21% (79.1%-92.73%)	62.55% (50%-75%)	79.79% (71.88%-87.69%)	72.57% (60%-84.62%)	3.0112 (2.7085-3.2879)
At least 3 of CHASM (melanoma), FATHMM (cancer), Mutation Assessor, PolyPhen-2, VEST	74.42% (71.21%-77.78%)	82.46% (78.91%-86.29%)	60.506% (54.409%-66.679%)	78.31% (74.07%-82.31%)	66.61% (60.32%-73.02%)	2.8789 (2.7294-3.0282)	74.4% (67.68%-81.81%)	82.4% (74.6%-89.48%)	60.656% (47.356%-73.33%)	78.22% (70.31%-86.57%)	66.76% (53.12%-79.31%)	2.8804 (2.5861-3.1695)
At least 3 of CHASM (melanoma), FATHMM (cancer), Mutation Assessor, PROVEAN	73.69% (70.2%-77.27%)	69.11% (64.39%-73.6%)	81.61% (76.389%-87.147%)	86.66% (82.83%-90.72%)	60.45% (55%-65.69%)	2.9783 (2.8483-3.1178)	73.82% (66.67%-80.81%)	69.2% (60%-78.46%)	81.738% (71.053%-92.5%)	86.66% (78.43%-94.23%)	60.73% (50%-71.44%)	2.9833 (2.6979-3.2461)
At least 3 of CHASM (melanoma), FATHMM (cancer), Mutation Assessor, PROVEAN, SIFT	75.72% (72.22%-78.79%)	80.29% (76.27%-84.25%)	67.805% (61.765%-74.286%)	81.18% (77.42%-85.04%)	66.55% (60.56%-72.46%)	2.9582 (2.8171-3.0941)	75.84% (69.7%-82.83%)	80.36% (72.13%-87.88%)	68.039% (55.261%-80%)	81.19% (73.44%-88.89%)	66.88% (55.17%-78.79%)	2.9646 (2.6946-3.2508)
At least 3 of CHASM (melanoma), FATHMM (cancer), Mutation Assessor, PROVEAN, VEST	75.04% (71.72%-78.28%)	78.15% (74.38%-82.44%)	69.637% (63.889%-75.765%)	81.65% (77.78%-85.6%)	64.83% (58.97%-70.89%)	2.9428 (2.8007-3.0792)	75.18% (68.69%-81.82%)	78.25% (69.49%-86.15%)	69.889% (57.491%-81.25%)	81.68% (74%-89.2%)	65.18% (52.63%-76.92%)	2.9499 (2.6754-3.2330)
At least 3 of CHASM (melanoma), FATHMM (cancer), Mutation Assessor, SIFT	78.42% (75.25%-81.82%)	79.77% (75.83%-83.74%)	76.086% (70.42%-81.432%)	85.22% (81.58%-88.98%)	68.51% (62.79%-74.33%)	3.0958 (2.9664-3.2292)	78.51% (71.72%-84.85%)	79.81% (71.7%-87.3%)	76.253% (65%-87.5%)	85.23% (77.78%-92.46%)	68.76% (57.5%-79.55%)	3.1005 (2.8366-3.3629)
At least 3 of CHASM (melanoma), FATHMM (cancer), Mutation Assessor, SIFT, VEST	75.41% (72.22%-78.79%)	84.6% (81.25%-88.19%)	59.511% (52.941%-65.832%)	78.32% (74.6%-82.35%)	69.09% (62.5%-75.81%)	2.9151 (2.7709-3.0603)	75.45% (68.69%-81.82%)	84.51% (77.05%-91.43%)	59.884% (46.51%-72.42%)	78.33% (70.31%-85.71%)	69.27% (56.24%-82.35%)	2.9199 (2.6213-3.2089)
At least 3 of CHASM (melanoma), FATHMM (cancer), Mutation Assessor, VEST	75.73% (72.22%-79.29%)	75.51% (71.43%-80%)	76.092% (70.423%-81.818%)	84.52% (80.55%-88.5%)	64.25% (58.62%-69.88%)	3.0038 (2.8635-3.1396)	75.82% (68.69%-82.83%)	75.55% (66.67%-83.61%)	76.263% (65%-87.5%)	84.52% (76.47%-92%)	64.51% (53.33%-75.61%)	3.0084 (2.7338-3.2886)
At least 3 of CHASM (melanoma), FATHMM (cancer), MutationTaster	77.06% (73.74%-80.81%)	66.96% (61.98%-72%)	94.539% (91.664%-97.437%)	95.5% (93.26%-97.87%)	62.33% (57.01%-67.62%)	3.1932 (3.0865-3.3097)	77.18% (69.7%-83.84%)	67.14% (57.14%-77.2%)	94.404% (87.879%-100%)	95.37% (90%-100%)	62.62% (52%-73.22%)	3.1953 (2.9679-3.4032)
At least 3 of CHASM (melanoma), FATHMM (cancer), MutationTaster, PolyPhen-2	84.47% (81.82%-87.37%)	83.99% (80.33%-87.81%)	85.288% (80.822%-90.141%)	90.8% (87.96%-93.91%)	75.5% (70.37%-80.77%)	3.3558 (3.2412-3.4772)	84.6% (78.79%-89.9%)	84.13% (76.27%-91.43%)	85.379% (75.676%-94.44%)	90.8% (84.48%-96.49%)	75.82% (65.7%-86.11%)	3.3612 (3.1163-3.5880)
At least 3 of CHASM (melanoma), FATHMM (cancer), MutationTaster, PolyPhen-2, PROVEAN	81.07% (78.28%-84.34%)	84.51% (80.95%-88.24%)	75.121% (69.694%-81.159%)	85.45% (81.97%-89.15%)	73.72% (68.05%-79.45%)	3.1881 (3.0607-3.3285)	81.29% (74.75%-86.87%)	84.68% (77.05%-91.94%)	75.444% (63.158%-87.1%)	85.55% (78.12%-92.42%)	74.16% (62.86%-85.71%)	3.1983 (2.9182-3.4560)
At least 3 of CHASM (melanoma), FATHMM (cancer), MutationTaster, PolyPhen-2, SIFT	81.79% (78.79%-84.85%)	90.38% (87.6%-93.55%)	66.926% (61.107%-73.137%)	82.53% (78.95%-86.16%)	80.11% (74.55%-85.96%)	3.1995 (3.0678-3.3284)	81.88% (75.76%-87.88%)	90.5% (84.37%-96.49%)	67.081% (54.049%-79.31%)	82.51% (75%-89.71%)	80.48% (68.75%-91.67%)	3.2058 (2.9354-3.4539)
At least 3 of CHASM (melanoma), FATHMM (cancer), MutationTaster, PolyPhen-2, VEST	78.82% (75.76%-82.32%)	87.22% (84%-90.4%)	64.306% (58.108%-71.014%)	80.86% (77.04%-84.8%)	74.43% (68.85%-80.33%)	3.0682 (2.9376-3.2096)	78.72% (71.72%-84.85%)	87.25% (80.33%-93.65%)	64.063% (51.429%-77.14%)	80.64% (72.46%-88.06%)	74.55% (62.07%-86.21%)	3.0650 (2.7748-3.3245)
At least 3 of CHASM (melanoma), FATHMM (cancer), MutationTaster, PROVEAN	81.44% (78.79%-84.85%)	76% (71.77%-80.49%)	90.848% (87.324%-94.668%)	93.49% (91%-96.23%)	68.65% (63.33%-73.96%)	3.2899 (3.1848-3.4107)	81.56% (74.75%-86.87%)	76.17% (67.15%-84.62%)	90.767% (82.857%-97.37%)	93.4% (87.5%-98.11%)	68.96% (58.14%-79.6%)	3.2930 (3.0456-3.5098)
At least 3 of CHASM (melanoma), FATHMM (cancer), MutationTaster, PROVEAN, SIFT	83.12% (80.3%-85.86%)	86.66% (83.33%-90.32%)	76.985% (71.43%-82.671%)	86.69% (83.33%-90.16%)	76.95% (71.43%-82.54%)	3.2728 (3.1514-3.3995)	83.26% (77.78%-88.89%)	86.78% (79.66%-93.55%)	77.193% (65.714%-87.5%)	86.73% (79.73%-93.22%)	77.3% (66.67%-88.24%)	3.2800 (3.0227-3.5175)
At least 3 of CHASM (melanoma), FATHMM (cancer), MutationTaster, PROVEAN, VEST	80.4% (77.27%-83.33%)	83.44% (79.84%-87.3%)	75.146% (69.118%-81.159%)	85.3% (81.75%-89.08%)	72.4% (67.11%-78.08%)	3.1629 (3.0324-3.2945)	80.61% (74.75%-86.87%)	83.64% (75.81%-90.74%)	75.395% (63.883%-87.1%)	85.37% (77.78%-92.42%)	72.86% (61.53%-84.38%)	3.1727 (2.8954-3.4398)
At least 3 of CHASM (melanoma), FATHMM (cancer), MutationTaster, SIFT	86.16% (83.84%-88.89%)	85.05% (81.51%-88.8%)	88.073% (84.058%-92.65%)	92.5% (90.09%-95.45%)	77.31% (72.09%-82.35%)	3.4293 (3.3231-3.5354)	86.27% (80.81%-90.91%)	85.21% (77.78%-92.07%)	88.043% (79.31%-96.77%)	92.46% (86.88%-98%)	77.64% (67.64%-87.88%)	3.4335 (3.2170-3.6389)
At least 3 of CHASM (melanoma), FATHMM (cancer), MutationTaster, SIFT, VEST	80.43% (77.27%-83.84%)	89.32% (86.44%-92.37%)	65.051% (58.904%-71.622%)	81.55% (77.94%-85.27%)	77.9% (72.13%-83.87%)	3.1383 (3.0069-3.2729)	80.55% (73.74%-86.87%)	89.42% (83.33%-95.16%)	65.324% (52.5%-77.78%)	81.56% (74.24%-88.57%)	78.28% (66.67%-89.29%)	3.1459 (2.8781-3.4050)
At least 3 of CHASM (melanoma), FATHMM (cancer), MutationTaster, VEST	82.44% (79.29%-85.35%)	80.78% (76.92%-84.85%)	85.31% (81.081%-90.278%)	90.48% (87.61%-93.75%)	71.97% (66.67%-77.39%)	3.2855 (3.1646-3.4079)	82.59% (76.77%-88.89%)	80.97% (72.41%-88.58%)	85.326% (75.61%-94.29%)	90.45% (84%-96.3%)	72.32% (61.36%-82.5%)	3.2908 (3.0502-3.5336)
At least 3 of CHASM (melanoma), FATHMM (cancer), PolyPhen-2	73.03% (69.7%-76.77%)	63.28% (58.4%-68.55%)	89.891% (85.937%-94.366%)	91.54% (88.46%-95.29%)	58.61% (53.57%-63.89%)	3.0332 (2.9176-3.1672)	73.13% (65.66%-79.8%)	63.31% (52.83%-73.22%)	89.928% (81.579%-97.3%)	91.52% (84.09%-97.67%)	58.83% (48.33%-69.09%)	3.0359 (2.7712-3.2700)
At least 3 of CHASM (melanoma), FATHMM (cancer), PolyPhen-2, PROVEAN	74.36% (71.21%-77.78%)	72.31% (68.03%-76.61%)	77.903% (72.464%-83.587%)	84.98% (81.19%-89.08%)	61.93% (56.32%-67.42%)	2.9712 (2.8381-3.1078)	74.51% (67.68%-80.81%)	72.39% (63.33%-81.36%)	78.127% (66.667%-88.58%)	85.03% (76.79%-92.59%)	62.25% (51.06%-73.47%)	2.9780 (2.6973-3.2380)
At least 3 of CHASM (melanoma), FATHMM (cancer), PolyPhen-2, PROVEAN, SIFT	76.76% (73.23%-79.81%)	83.49% (79.84%-87.31%)	65.113% (58.904%-71.642%)	80.54% (76.69%-84.5%)	69.53% (63.64%-75.38%)	2.9867 (2.8523-3.1289)	76.78% (70.68%-83.84%)	83.53% (75.75%-90.74%)	65.174% (51.719%-77.79%)	80.46% (72.73%-88.33%)	69.77% (57.56%-81.26%)	2.9893 (2.7066-3.2631)
At least 3 of CHASM (melanoma), FATHMM (cancer), PolyPhen-2, PROVEAN, VEST	73.03% (69.7%-76.26%)	80.27% (76.61%-84.35%)	60.519% (54.167%-66.667%)	77.85% (73.64%-81.95%)	63.95% (57.58%-70.15%)	2.8259 (2.6770-2.9707)	73.12% (66.67%-79.8%)	80.4% (72.22%-87.89%)	60.608% (48.384%-73.33%)	77.79% (69.49%-85.94%)	64.3% (50%-76.48%)	2.8310 (2.5282-3.1286)
At least 3 of CHASM (melanoma), FATHMM (cancer), PolyPhen-2, SIFT	77.76% (74.75%-81.31%)	81.88% (78.05%-85.6%)	70.616% (64.555%-76.812%)	82.81% (79.07%-86.78%)	69.27% (63.85%-75%)	3.0458 (2.9143-3.1889)	77.82% (70.71%-83.84%)	81.96% (74.19%-89.39%)	70.683% (58.333%-82.76%)	82.76% (75%-90.33%)	69.56% (57.58%-81.25%)	3.0497 (2.7614-3.3159)
At least 3 of CHASM (melanoma), FATHMM (cancer), PolyPhen-2, SIFT, VEST	73.67% (70.2%-77.27%)	86.67% (83.33%-90.08%)	51.185% (44.926%-57.334%)	75.43% (71.43%-79.45%)	68.97% (62.22%-75.86%)	2.8226 (2.6802-2.9591)	73.86% (66.67%-80.81%)	86.74% (79.69%-93.22%)	51.742% (38.889%-64.52%)	75.51% (67.14%-84.06%)	69.48% (56%-83.33%)	2.8347 (2.5498-3.1221)
At least 3 of CHASM (melanoma), FATHMM (cancer), PolyPhen-2, VEST	74.41% (71.21%-77.78%)	78.17% (74.22%-82.4%)	67.895% (61.765%-74.324%)	80.81% (76.86%-85%)	64.27% (58.33%-70.27%)	2.9115 (2.7757-3.0521)	74.42% (67.68%-80.81%)	78.21% (69.84%-86.36%)	67.886% (54.756%-80.01%)	80.69% (72.31%-88.53%)	64.47% (51.61%-76.19%)	2.9126 (2.6171-3.1948)
At least 3 of CHASM (melanoma), FATHMM (cancer), PROVEAN	63.65% (60.1%-67.17%)	44.75% (39.67%-49.61%)	96.301% (94.203%-98.701%)	95.44% (92.86%-98.36%)	50.2% (45.39%-54.61%)	2.8670 (2.7704-2.9699)	63.62% (56.57%-70.71%)	44.5% (34.43%-54.69%)	96.374% (90.904%-100%)	95.48% (88.89%-100%)	50.29% (40.91%-60.01%)	2.8665 (2.6538-3.0554)
At least 3 of CHASM (melanoma), FATHMM (cancer), PROVEAN, SIFT	74.7% (71.21%-78.28%)	72.31% (67.74%-76.99%)	78.809% (73.438%-84.507%)	85.51% (81.63%-89.47%)	62.21% (56.67%-67.48%)	2.9884 (2.8565-3.1286)	74.85% (67.68%-81.82%)	72.37% (63.07%-81.25%)	79.054% (68.421%-89.66%)	85.58% (77.59%-93.11%)	62.52% (52.08%-73.34%)	2.9952 (2.7186-3.2582)
At least 3 of CHASM (melanoma), FATHMM (cancer), PROVEAN, SIFT, VEST	74.38% (71.21%-77.78%)	81.9% (78.12%-85.84%)	61.367% (55.405%-67.606%)	78.57% (74.8%-82.71%)	66.24% (59.7%-72.58%)	2.8808 (2.7370-3.0209)	74.47% (67.68%-81.82%)	81.92% (73.77%-89.29%)	61.653% (48.571%-74.08%)	78.57% (70.14%-86.15%)	66.52% (53.33%-79.31%)	2.8866 (2.6015-3.1715)
At least 3 of CHASM (melanoma), FATHMM (cancer), PROVEAN, VEST	70.34% (66.67%-73.74%)	65.42% (60.83%-70.4%)	78.832% (73.435%-84.932%)	84.24% (80.21%-88.66%)	56.87% (51.09%-62.5%)	2.8535 (2.7109-2.9908)	70.44% (63.64%-77.78%)	65.42% (55.4%-74.58%)	79.023% (67.643%-90%)	84.26% (75.46%-92.11%)	57.11% (46%-68%)	2.8581 (2.5806-3.1378)
At least 3 of CHASM (melanoma), FATHMM (cancer), SIFT	76.42% (73.23%-79.8%)	67.56% (62.79%-72.31%)	91.716% (88.312%-95.652%)	93.38% (90.7%-96.63%)	62.05% (56.76%-67.29%)	3.1471 (3.0362-3.2614)	76.46% (69.7%-82.83%)	67.51% (57.63%-76.8%)	91.771% (84.611%-97.68%)	93.38% (86.96%-98.08%)	62.21% (51.85%-72.73%)	3.1488 (2.9211-3.3683)
At least 3 of CHASM (melanoma), FATHMM (cancer), SIFT, VEST	76.4% (73.23%-79.8%)	80.31% (76.38%-84.25%)	69.644% (63.636%-76%)	82.06% (78.29%-86.09%)	67.16% (61.25%-72.98%)	2.9918 (2.8563-3.1289)	76.49% (69.7%-82.83%)	80.32% (72.22%-87.93%)	69.872% (57.574%-81.82%)	82.07% (73.77%-89.66%)	67.41% (55.55%-79.41%)	2.9968 (2.7129-3.2661)
At least 3 of CHASM (melanoma), FATHMM (cancer), VEST	69.01% (65.15%-72.73%)	56.39% (51.56%-61.54%)	90.815% (87.324%-94.742%)	91.4% (88.06%-95.12%)	54.64% (49.21%-59.66%)	2.9324 (2.8159-3.0595)	69.05% (61.62%-76.77%)	56.35% (46.27%-66.15%)	90.822% (82.848%-97.37%)	91.36% (83.78%-97.56%)	54.8% (44.83%-65.15%)	2.9333 (2.6878-3.1628)
At least 3 of CHASM (melanoma), FATHMM (missense), Mutation Assessor	71.38% (67.68%-74.76%)	59.07% (53.9%-63.91%)	92.651% (89.189%-96.054%)	93.29% (90.24%-96.43%)	56.7% (51.35%-61.61%)	3.0171 (2.9034-3.1313)	71.38% (64.62%-78.79%)	58.96% (48.48%-69.49%)	92.657% (85.283%-100%)	93.26% (86.48%-100%)	56.82% (47.17%-67.24%)	3.0169 (2.7898-3.2450)
At least 3 of CHASM (melanoma), FATHMM (missense), Mutation Assessor, MutationTaster	81.49% (78.79%-84.34%)	77.13% (72.95%-81.4%)	89.008% (85.135%-93.333%)	92.39% (89.72%-95.33%)	69.24% (64%-74.49%)	3.2777 (3.1682-3.3882)	81.47% (75.76%-86.87%)	77.09% (68.24%-85.25%)	88.928% (80%-97.06%)	92.29% (86.2%-98%)	69.36% (58.54%-80%)	3.2767 (3.0502-3.5008)
At least 3 of CHASM (melanoma), FATHMM (missense), Mutation Assessor, MutationTaster, PolyPhen-2	80.48% (77.27%-83.84%)	86.67% (83.33%-90.17%)	69.764% (63.768%-75.813%)	83.21% (79.55%-86.92%)	75.17% (69.56%-81.08%)	3.1481 (3.0172-3.2841)	80.46% (73.74%-86.87%)	86.76% (79.66%-93.55%)	69.638% (57.5%-81.09%)	83.06% (75.71%-90.28%)	75.42% (63.33%-86.68%)	3.1487 (2.8796-3.4085)
At least 3 of CHASM (melanoma), FATHMM (missense), Mutation Assessor, MutationTaster, PROVEAN	83.5% (80.81%-86.36%)	84.04% (80.33%-87.71%)	82.548% (77.778%-87.504%)	89.28% (86.29%-92.56%)	74.95% (69.62%-80.23%)	3.3081 (3.1904-3.4335)	83.51% (77.78%-88.89%)	84.03% (76.27%-91.23%)	82.589% (72.722%-91.89%)	89.23% (82.76%-95%)	75.1% (64.7%-85.37%)	3.3095 (3.0586-3.5439)
At least 3 of CHASM (melanoma), FATHMM (missense), Mutation Assessor, MutationTaster, SIFT	82.46% (79.29%-85.35%)	87.7% (84.61%-91.06%)	73.382% (67.524%-79.487%)	85.07% (81.6%-88.71%)	77.54% (71.88%-83.1%)	3.2370 (3.1117-3.3678)	82.55% (76.77%-88.89%)	87.87% (81.03%-93.94%)	73.384% (61.286%-85.3%)	85.01% (77.78%-92.06%)	77.93% (66.67%-88.24%)	3.2419 (2.9735-3.5006)
At least 3 of CHASM (melanoma), FATHMM (missense), Mutation Assessor, MutationTaster, VEST	79.81% (76.77%-82.83%)	86.16% (82.93%-89.66%)	68.834% (63.01%-74.605%)	82.7% (78.95%-86.37%)	74.21% (68.57%-80.01%)	3.1190 (2.9828-3.2580)	79.77% (73.74%-85.86%)	86.17% (78.94%-92.86%)	68.739% (55.548%-80.95%)	82.55% (75.35%-89.86%)	74.36% (61.29%-85.72%)	3.1182 (2.8362-3.3830)
At least 3 of CHASM (melanoma), FATHMM (missense), Mutation Assessor, PolyPhen-2	73.1% (69.7%-76.77%)	72.37% (67.93%-76.69%)	74.35% (68.75%-80.264%)	82.99% (79.05%-87.16%)	60.88% (55.17%-66.67%)	2.9060 (2.7628-3.0437)	72.99% (65.66%-79.8%)	72.24% (63.23%-80.95%)	74.214% (62.5%-85.3%)	82.78% (74.07%-90.74%)	60.9% (48.98%-72.51%)	2.9014 (2.6254-3.1833)
At least 3 of CHASM (melanoma), FATHMM (missense), Mutation Assessor, PolyPhen-2, PROVEAN	76.44% (73.22%-79.8%)	80.33% (76.42%-84.5%)	69.699% (63.633%-75.677%)	82.09% (78.33%-85.95%)	67.21% (61.11%-73.33%)	2.9933 (2.8439-3.1321)	76.41% (69.7%-82.85%)	80.27% (71.87%-87.88%)	69.756% (57.143%-82.05%)	82% (74.19%-89.66%)	67.32% (54.83%-78.95%)	2.9934 (2.7128-3.2870)
At least 3 of CHASM (melanoma), FATHMM (missense), Mutation Assessor, PolyPhen-2, SIFT	76.73% (73.23%-79.8%)	85.07% (81.6%-88.71%)	62.309% (55.844%-68.574%)	79.6% (75.57%-83.58%)	70.71% (64.52%-76.67%)	2.9768 (2.8357-3.1086)	76.84% (70.71%-83.84%)	85.16% (77.61%-91.94%)	62.513% (50%-75%)	79.59% (71.64%-87.33%)	71.08% (58.82%-82.36%)	2.9834 (2.7124-3.2627)
At least 3 of CHASM (melanoma), FATHMM (missense), Mutation Assessor, PolyPhen-2, VEST	73.05% (70.19%-76.26%)	80.87% (77.05%-84.68%)	59.53% (53.12%-66.197%)	77.55% (73.64%-81.75%)	64.28% (57.75%-71.01%)	2.8224 (2.6786-2.9675)	73.08% (66.67%-78.81%)	80.79% (73.02%-88.14%)	59.832% (46.661%-72.51%)	77.53% (69.23%-85.3%)	64.47% (51.28%-77.14%)	2.8262 (2.5276-3.0966)
At least 3 of CHASM (melanoma), FATHMM (missense), Mutation Assessor, PROVEAN	73.03% (69.7%-76.26%)	66.46% (61.67%-70.97%)	84.372% (79.994%-89.042%)	88.03% (84.52%-91.75%)	59.27% (53.92%-64.71%)	2.9813 (2.8510-3.1088)	73.13% (66.67%-79.8%)	66.51% (56.92%-76.12%)	84.449% (75.673%-93.75%)	88.01% (80.84%-95.35%)	59.51% (49.05%-70.37%)	2.9848 (2.7273-3.2457)
At least 3 of CHASM (melanoma), FATHMM (missense), Mutation Assessor, PROVEAN, SIFT	76.09% (72.73%-79.29%)	80.87% (77.12%-84.8%)	67.812% (61.429%-74.286%)	81.29% (77.5%-85.08%)	67.21% (61.11%-73.61%)	2.9718 (2.8284-3.1217)	76.11% (69.7%-82.83%)	80.79% (72.58%-88.14%)	68.006% (55.548%-80.56%)	81.26% (73.77%-88.89%)	67.35% (55.55%-78.95%)	2.9741 (2.6827-3.2659)
At least 3 of CHASM (melanoma), FATHMM (missense), Mutation Assessor, PROVEAN, VEST	73.39% (70.2%-76.77%)	77.67% (73.77%-81.82%)	65.984% (59.993%-72.467%)	79.79% (75.86%-83.62%)	63.09% (57.14%-69.41%)	2.8653 (2.7236-3.0036)	73.42% (66.67%-79.8%)	77.61% (68.96%-85.25%)	66.167% (52.941%-78.12%)	79.75% (71.66%-87.72%)	63.26% (50%-75.02%)	2.8678 (2.5830-3.1473)
At least 3 of CHASM (melanoma), FATHMM (missense), Mutation Assessor, SIFT	75.78% (72.73%-79.29%)	75.58% (71.43%-79.67%)	76.112% (70.588%-82.052%)	84.55% (80.87%-88.5%)	64.32% (58.53%-69.88%)	3.0056 (2.8742-3.1406)	75.71% (68.69%-81.82%)	75.4% (67.27%-83.61%)	76.165% (64.514%-87.18%)	84.47% (76.59%-92.16%)	64.33% (52.63%-76.09%)	3.0037 (2.7288-3.2613)
At least 3 of CHASM (melanoma), FATHMM (missense), Mutation Assessor, SIFT, VEST	73.35% (69.7%-76.77%)	82.99% (79.36%-86.67%)	56.666% (50.667%-63.384%)	76.81% (72.79%-80.88%)	65.83% (59.09%-72.47%)	2.8229 (2.6792-2.9627)	73.51% (66.67%-80.81%)	82.93% (75.41%-90.14%)	57.281% (43.895%-70.27%)	76.92% (68.66%-84.93%)	66.16% (53.12%-78.79%)	2.8329 (2.5394-3.1062)
At least 3 of CHASM (melanoma), FATHMM (missense), Mutation Assessor, VEST	71.76% (68.18%-75.25%)	70.27% (65.83%-74.8%)	74.321% (68.748%-80.556%)	82.55% (78.3%-86.61%)	59.11% (53.33%-64.84%)	2.8625 (2.7217-3.0044)	71.64% (64.65%-78.79%)	70.08% (60.71%-78.79%)	74.262% (61.765%-85.3%)	82.38% (74%-90.74%)	59.11% (46.94%-70.21%)	2.8582 (2.5705-3.1442)
At least 3 of CHASM (melanoma), FATHMM (missense), MutationTaster	75.39% (72.21%-79.29%)	64.31% (59.16%-69.36%)	94.539% (91.664%-97.437%)	95.32% (92.94%-97.75%)	60.51% (55.36%-65.74%)	3.1467 (3.0441-3.2578)	75.49% (67.68%-81.84%)	64.45% (53.97%-75%)	94.404% (87.879%-100%)	95.19% (89.74%-100%)	60.76% (50.88%-71.15%)	3.1480 (2.9348-3.3605)
At least 3 of CHASM (melanoma), FATHMM (missense), MutationTaster, PolyPhen-2	81.52% (78.79%-84.34%)	80.87% (76.98%-84.85%)	82.634% (77.922%-88.062%)	88.96% (85.84%-92.37%)	71.42% (65.85%-76.83%)	3.2388 (3.1204-3.3628)	81.4% (75.76%-86.87%)	80.79% (72.72%-88.33%)	82.393% (71.795%-91.89%)	88.75% (81.97%-94.74%)	71.44% (60.46%-82.5%)	3.2337 (2.9786-3.4675)
At least 3 of CHASM (melanoma), FATHMM (missense), MutationTaster, PolyPhen-2, PROVEAN	80.48% (77.27%-83.84%)	84.04% (80.33%-87.9%)	74.305% (68.657%-80%)	84.98% (81.6%-88.62%)	72.92% (67.12%-78.67%)	3.1624 (3.0311-3.2947)	80.46% (73.74%-86.87%)	84.03% (76.56%-91.53%)	74.28% (62.857%-85.71%)	84.88% (77.42%-91.94%)	73.06% (61.11%-84.38%)	3.1625 (2.8792-3.4259)
At least 3 of CHASM (melanoma), FATHMM (missense), MutationTaster, PolyPhen-2, SIFT	81.14% (78.28%-83.84%)	89.34% (86.32%-92.43%)	66.946% (60.563%-72.973%)	82.38% (78.79%-86.13%)	78.42% (72.73%-84.21%)	3.1708 (3.0399-3.2947)	81.16% (75.76%-86.87%)	89.4% (83.04%-95.38%)	66.971% (54.839%-79.49%)	82.3% (75%-89.48%)	78.67% (67.44%-89.66%)	3.1733 (2.9294-3.4308)
At least 3 of CHASM (melanoma), FATHMM (missense), MutationTaster, PolyPhen-2, VEST	79.5% (76.26%-82.83%)	87.76% (84.68%-91.06%)	65.204% (59.155%-71.622%)	81.35% (77.54%-85.27%)	75.49% (69.64%-81.54%)	3.0980 (2.9653-3.2384)	79.39% (72.73%-85.86%)	87.77% (81.24%-93.94%)	64.981% (52.5%-76.92%)	81.14% (73.43%-88.71%)	75.6% (63.15%-87.11%)	3.0949 (2.8189-3.3563)
At least 3 of CHASM (melanoma), FATHMM (missense), MutationTaster, PROVEAN	80.44% (77.27%-83.84%)	74.41% (69.84%-78.99%)	90.869% (87.324%-94.667%)	93.38% (90.72%-96.08%)	67.26% (62.11%-72.45%)	3.2591 (3.1485-3.3743)	80.53% (73.74%-86.87%)	74.56% (65.45%-83.87%)	90.714% (82.759%-97.37%)	93.25% (87.5%-98.11%)	67.53% (57.14%-78.26%)	3.2606 (3.0315-3.4845)
At least 3 of CHASM (melanoma), FATHMM (missense), MutationTaster, PROVEAN, SIFT	82.82% (80.29%-85.86%)	85.63% (82.17%-89.23%)	77.934% (72.603%-83.562%)	87.04% (83.62%-90.4%)	75.83% (70.27%-81.43%)	3.2643 (3.1444-3.3888)	82.85% (76.77%-87.9%)	85.63% (78.12%-92.43%)	78.017% (66.667%-88.89%)	87.01% (80.36%-93.55%)	76% (65.37%-86.84%)	3.2667 (3.0061-3.5067)
At least 3 of CHASM (melanoma), FATHMM (missense), MutationTaster, PROVEAN, VEST	80.14% (77.27%-83.33%)	83.49% (79.84%-87.2%)	74.333% (68.493%-80.282%)	84.91% (81.45%-88.71%)	72.26% (66.67%-78.08%)	3.1500 (3.0212-3.2869)	80.12% (73.74%-86.85%)	83.52% (75.92%-90.63%)	74.203% (61.765%-85.71%)	84.77% (77.19%-91.8%)	72.41% (60.6%-83.78%)	3.1491 (2.8705-3.4090)
At least 3 of CHASM (melanoma), FATHMM (missense), MutationTaster, SIFT	82.84% (79.8%-85.86%)	81.37% (77.42%-85.48%)	85.358% (80.881%-90.283%)	90.58% (87.74%-93.81%)	72.61% (67.44%-77.92%)	3.2992 (3.1853-3.4185)	82.81% (76.77%-88.89%)	81.38% (73.33%-89.29%)	85.191% (75.756%-94.29%)	90.44% (84.21%-96.3%)	72.73% (62.06%-83.33%)	3.2975 (3.0563-3.5226)
At least 3 of CHASM (melanoma), FATHMM (missense), MutationTaster, SIFT, VEST	80.45% (77.27%-83.33%)	89.35% (86.36%-92.62%)	65.047% (58.973%-71.254%)	81.55% (78.1%-85.3%)	77.94% (72.13%-83.87%)	3.1389 (3.0016-3.2610)	80.52% (74.75%-86.87%)	89.37% (83.05%-95.31%)	65.251% (52.628%-77.5%)	81.55% (74.24%-88.41%)	78.2% (66.67%-90%)	3.1437 (2.8895-3.4106)
At least 3 of CHASM (melanoma), FATHMM (missense), MutationTaster, VEST	79.83% (76.77%-83.33%)	78.19% (73.98%-82.4%)	82.634% (77.608%-88%)	88.62% (85.45%-92.18%)	68.67% (63.29%-74.12%)	3.1812 (3.0594-3.3081)	79.74% (72.73%-85.86%)	78.16% (69.49%-86.15%)	82.393% (71.795%-92.11%)	88.42% (81.25%-94.65%)	68.75% (57.49%-79.17%)	3.1772 (2.9210-3.4189)
At least 3 of CHASM (melanoma), FATHMM (missense), PolyPhen-2	70.36% (66.67%-74.24%)	60.63% (55.74%-65.6%)	87.186% (82.856%-91.781%)	89.11% (85.56%-93.02%)	56.15% (50.89%-61.26%)	2.9308 (2.8056-3.0589)	70.38% (62.63%-77.78%)	60.63% (50.82%-70.78%)	87.068% (77.778%-95%)	88.96% (81.25%-95.56%)	56.31% (45.76%-66.67%)	2.9296 (2.6718-3.1741)
At least 3 of CHASM (melanoma), FATHMM (missense), PolyPhen-2, PROVEAN	73.03% (69.7%-76.26%)	70.71% (66.14%-75.21%)	77.038% (71.429%-82.438%)	84.19% (80.37%-88.35%)	60.34% (54.73%-65.59%)	2.9227 (2.7835-3.0632)	73.12% (66.67%-79.8%)	70.78% (61.67%-80%)	77.088% (65.788%-88.1%)	84.14% (76%-92%)	60.6% (50%-72.1%)	2.9261 (2.6366-3.1998)
At least 3 of CHASM (melanoma), FATHMM (missense), PolyPhen-2, PROVEAN, SIFT	76.76% (73.23%-79.8%)	83.53% (80%-87.2%)	65.048% (58.75%-71.429%)	80.52% (76.69%-84.21%)	69.55% (63.63%-75.71%)	2.9865 (2.8440-3.1225)	76.78% (70.71%-83.84%)	83.45% (75.86%-90.32%)	65.276% (51.613%-77.79%)	80.5% (72.88%-88.33%)	69.7% (58.06%-81.08%)	2.9893 (2.7197-3.2723)
At least 3 of CHASM (melanoma), FATHMM (missense), PolyPhen-2, PROVEAN, VEST	73.41% (69.7%-76.77%)	79.79% (76.03%-83.97%)	62.363% (55.882%-68.832%)	78.57% (74.6%-82.64%)	64.09% (57.57%-70.59%)	2.8482 (2.6984-2.9939)	73.38% (66.67%-80.81%)	79.75% (71.42%-87.5%)	62.379% (50%-75.61%)	78.45% (70.31%-86.54%)	64.22% (51.19%-76.93%)	2.8480 (2.5427-3.1555)
At least 3 of CHASM (melanoma), FATHMM (missense), PolyPhen-2, SIFT	76.09% (72.73%-79.29%)	79.27% (75%-83.33%)	70.586% (64.615%-76.389%)	82.33% (78.4%-86.18%)	66.33% (60.75%-72.16%)	2.9852 (2.8468-3.1222)	76.09% (69.7%-82.83%)	79.19% (71.01%-87.5%)	70.699% (58.694%-82.86%)	82.28% (74.58%-90.32%)	66.46% (54.55%-78.38%)	2.9863 (2.7101-3.2582)
At least 3 of CHASM (melanoma), FATHMM (missense), PolyPhen-2, SIFT, VEST	73.36% (69.7%-76.77%)	85.63% (82.31%-89.06%)	52.128% (45.711%-58.824%)	75.57% (71.53%-79.58%)	67.72% (61.11%-74.47%)	2.8105 (2.6539-2.9538)	73.48% (66.67%-80.81%)	85.63% (78.57%-92.19%)	52.567% (40%-64.87%)	75.6% (67.6%-83.59%)	68.08% (54.55%-81.48%)	2.8189 (2.5383-3.1269)
At least 3 of CHASM (melanoma), FATHMM (missense), PolyPhen-2, VEST	72.78% (69.19%-76.26%)	75.05% (70.73%-79.51%)	68.842% (62.853%-75%)	80.64% (76.58%-84.82%)	61.47% (55.68%-67.53%)	2.8601 (2.7120-3.0033)	72.62% (65.66%-79.8%)	74.87% (66.06%-83.33%)	68.679% (55.556%-80.56%)	80.42% (72%-88.34%)	61.43% (48.84%-72.92%)	2.8539 (2.5601-3.1539)
At least 3 of CHASM (melanoma), FATHMM (missense), PROVEAN	61.31% (57.58%-65.15%)	41.05% (36.22%-45.9%)	96.322% (94.203%-98.684%)	95.09% (92.16%-98.21%)	48.59% (43.62%-52.86%)	2.8105 (2.7099-2.9156)	61.22% (53.54%-68.69%)	40.73% (30.77%-50.79%)	96.322% (90.909%-100%)	95.04% (88%-100%)	48.64% (39.73%-58.57%)	2.8074 (2.5983-3.0099)
At least 3 of CHASM (melanoma), FATHMM (missense), PROVEAN, SIFT	74.71% (71.21%-77.79%)	71.78% (67.19%-76.38%)	79.758% (74.358%-85.076%)	85.99% (82.35%-89.72%)	62.04% (56.25%-67.42%)	2.9957 (2.8618-3.1347)	74.82% (68.66%-81.82%)	71.83% (62.5%-80.95%)	79.877% (69.224%-90.48%)	86% (78.72%-93.48%)	62.3% (51.16%-73.47%)	3.0001 (2.7255-3.2738)
At least 3 of CHASM (melanoma), FATHMM (missense), PROVEAN, SIFT, VEST	72.71% (69.68%-75.77%)	80.88% (76.98%-84.73%)	58.559% (52.499%-64.789%)	77.14% (73.28%-81.06%)	63.92% (57.58%-70.15%)	2.8049 (2.6574-2.9483)	72.77% (66.64%-78.81%)	80.77% (72.85%-88.52%)	58.975% (46.149%-70.97%)	77.17% (69.23%-84.62%)	64.12% (51.52%-76.67%)	2.8104 (2.5187-3.1039)
At least 3 of CHASM (melanoma), FATHMM (missense), PROVEAN, VEST	69.01% (65.15%-72.73%)	63.83% (59.16%-68.75%)	77.969% (72.6%-83.333%)	83.36% (79.17%-87.63%)	55.49% (49.99%-60.82%)	2.8065 (2.6636-2.9453)	69.04% (61.62%-76.77%)	63.81% (53.97%-73.77%)	77.958% (66.667%-88.89%)	83.26% (74.51%-91.67%)	55.66% (44.9%-66.67%)	2.8069 (2.5186-3.0906)
At least 3 of CHASM (melanoma), FATHMM (missense), SIFT	74.42% (71.21%-77.78%)	65.97% (61.2%-70.73%)	89.001% (84.929%-93.333%)	91.22% (88.17%-94.57%)	60.2% (55%-65.38%)	3.0639 (2.9444-3.1886)	74.4% (67.68%-80.81%)	65.9% (56.25%-75.41%)	88.92% (79.412%-97.22%)	91.11% (84.31%-97.62%)	60.3% (50%-70.84%)	3.0623 (2.8118-3.3011)
At least 3 of CHASM (melanoma), FATHMM (missense), SIFT, VEST	74.06% (70.71%-77.27%)	76.63% (72.58%-80.8%)	69.59% (63.636%-75.676%)	81.34% (77.48%-85.37%)	63.27% (57.5%-69.05%)	2.9083 (2.7693-3.0550)	74.1% (67.68%-80.81%)	76.49% (68.18%-84.48%)	69.926% (57.576%-81.58%)	81.38% (73.6%-89.29%)	63.41% (51.35%-75%)	2.9120 (2.6230-3.1928)
At least 3 of CHASM (melanoma), FATHMM (missense), VEST	66.33% (62.63%-70.2%)	53.74% (48.74%-58.68%)	88.089% (83.824%-92.647%)	88.65% (84.72%-92.86%)	52.41% (47.15%-57.26%)	2.8289 (2.7033-2.9634)	66.33% (58.59%-73.74%)	53.66% (43.94%-63.93%)	88.015% (78.571%-96.78%)	88.52% (79.55%-96.77%)	52.53% (42.42%-62.69%)	2.8272 (2.5607-3.0749)
At least 3 of CHASM (melanoma), Mutation Assessor, MutationTaster	70.69% (66.67%-74.24%)	56.9% (51.93%-61.9%)	94.52% (91.667%-97.368%)	94.73% (92%-97.5%)	55.91% (50.83%-60.87%)	3.0206 (2.9160-3.1314)	70.75% (63.64%-78.79%)	56.93% (46.87%-67.19%)	94.436% (88.232%-100%)	94.62% (88.37%-100%)	56.1% (46.29%-66.13%)	3.0209 (2.7919-3.2421)
At least 3 of CHASM (melanoma), Mutation Assessor, MutationTaster, PolyPhen-2	79.44% (76.26%-82.83%)	78.15% (74.02%-82.44%)	81.667% (76.809%-86.958%)	88.05% (84.76%-91.53%)	68.38% (63.04%-74.12%)	3.1624 (3.0396-3.3006)	79.5% (72.73%-85.86%)	78.25% (69.64%-86.36%)	81.625% (71.429%-91.43%)	87.95% (80.33%-94.55%)	68.65% (57.14%-79.49%)	3.1647 (2.8962-3.4172)
At least 3 of CHASM (melanoma), Mutation Assessor, MutationTaster, PolyPhen-2, PROVEAN	78.09% (74.75%-81.31%)	81.35% (77.68%-85.25%)	72.458% (66.667%-78.378%)	83.62% (79.83%-87.4%)	69.21% (63.41%-75%)	3.0664 (2.9270-3.2080)	78.15% (71.72%-84.85%)	81.42% (73.43%-88.74%)	72.535% (60.526%-83.87%)	83.56% (75.75%-90.91%)	69.48% (57.14%-80.56%)	3.0700 (2.7850-3.3436)
At least 3 of CHASM (melanoma), Mutation Assessor, MutationTaster, PolyPhen-2, SIFT	76.74% (73.23%-79.8%)	81.88% (78.03%-85.72%)	67.843% (61.903%-74.195%)	81.49% (77.6%-85.39%)	68.41% (62.86%-74.63%)	2.9961 (2.8559-3.1355)	76.83% (70.71%-83.84%)	81.97% (74.19%-89.23%)	67.988% (55.164%-80%)	81.46% (73.43%-89.06%)	68.73% (56.24%-80.8%)	3.0014 (2.7053-3.2655)
At least 3 of CHASM (melanoma), Mutation Assessor, MutationTaster, PolyPhen-2, VEST	74.76% (71.21%-78.28%)	81.36% (77.6%-85.37%)	63.342% (56.578%-70%)	79.32% (75.38%-83.46%)	66.28% (60%-73.13%)	2.9030 (2.7529-3.0546)	74.73% (67.68%-81.82%)	81.41% (73.44%-88.89%)	63.247% (50%-75.61%)	79.16% (70.77%-86.67%)	66.48% (54.17%-78.79%)	2.9031 (2.5950-3.2004)
At least 3 of CHASM (melanoma), Mutation Assessor, MutationTaster, PROVEAN	77.05% (73.74%-80.81%)	69.61% (65.12%-74.19%)	89.901% (86.111%-94.118%)	92.26% (89.47%-95.61%)	63.11% (57.84%-68.57%)	3.1489 (3.0302-3.2708)	77.21% (69.7%-83.84%)	69.79% (60.65%-78.95%)	89.896% (82.051%-97.3%)	92.23% (85.99%-97.87%)	63.42% (52.83%-73.77%)	3.1534 (2.9105-3.3909)
At least 3 of CHASM (melanoma), Mutation Assessor, MutationTaster, PROVEAN, SIFT	76.75% (73.74%-80.3%)	77.63% (73.73%-81.82%)	75.231% (69.444%-80.827%)	84.42% (80.53%-88.24%)	66.04% (60.22%-71.76%)	3.0332 (2.9001-3.1730)	76.8% (69.7%-82.83%)	77.7% (69.35%-85.48%)	75.225% (64.097%-86.84%)	84.32% (76.78%-91.67%)	66.27% (54.35%-77.78%)	3.0352 (2.7521-3.3024)
At least 3 of CHASM (melanoma), Mutation Assessor, MutationTaster, PROVEAN, VEST	74.73% (71.21%-78.28%)	76.58% (72.58%-81.1%)	71.533% (65.852%-77.465%)	82.3% (78.45%-86.21%)	63.86% (58.23%-69.77%)	2.9427 (2.8007-3.0848)	74.78% (67.68%-81.82%)	76.6% (67.69%-84.85%)	71.635% (59.986%-83.33%)	82.23% (74%-89.83%)	64.08% (52.07%-75%)	2.9455 (2.6568-3.2163)
At least 3 of CHASM (melanoma), Mutation Assessor, MutationTaster, SIFT	80.46% (77.77%-83.33%)	77.09% (72.95%-81.25%)	86.281% (82.086%-91.026%)	90.67% (87.74%-93.81%)	68.54% (63.44%-73.96%)	3.2258 (3.1121-3.3482)	80.49% (74.75%-85.88%)	77.17% (68.75%-85.25%)	86.138% (77.498%-94.44%)	90.53% (84%-96.3%)	68.74% (57.77%-79.55%)	3.2258 (2.9787-3.4554)

At least 3 of CHASM (melanoma), Mutation Assessor, MutationTaster, SIFT, VEST	75.05% (71.72%-78.28%)	80.28% (76.67%-84.43%)	65.992% (60.274%-72.464%)	80.32% (76.42%-84.26%)	65.94% (60%-72.37%)	2.9254 (2.7916-3.0719)	75.15% (68.69%-81.82%)	80.37% (72.12%-87.88%)	66.186% (53.12%-77.42%)	80.3% (72.58%-87.69%)	66.28% (52.94%-77.78%)	2.9313 (2.6376-3.2045)
At least 3 of CHASM (melanoma), Mutation Assessor, MutationTaster, VEST	78.12% (75.25%-81.31%)	74.46% (70.25%-78.87%)	84.446% (80%-89.335%)	89.22% (85.85%-92.79%)	65.66% (60.44%-71.28%)	3.1379 (3.0176-3.2636)	78.1% (71.72%-83.84%)	74.47% (65.57%-82.81%)	84.324% (74.984%-93.94%)	89.06% (81.67%-96.08%)	65.81% (54.35%-76.19%)	3.1366 (2.8816-3.3784)
At least 3 of CHASM (melanoma), Mutation Assessor, PolyPhen-2	69.01% (65.66%-72.73%)	57.97% (52.94%-62.88%)	88.079% (84.058%-92.754%)	89.37% (85.52%-93.51%)	54.79% (49.57%-59.66%)	2.9021 (2.7772-3.0316)	69.06% (61.62%-75.76%)	57.97% (48.2%-68.18%)	88.041% (78.947%-96.67%)	89.27% (80.95%-97.22%)	54.97% (45%-65.38%)	2.9025 (2.6438-3.1510)
At least 3 of CHASM (melanoma), Mutation Assessor, PolyPhen-2, PROVEAN	71.68% (68.18%-75.25%)	68.57% (64%-73.39%)	77.05% (71.795%-82.353%)	83.78% (79.79%-87.88%)	58.64% (53.12%-64.21%)	2.8805 (2.7432-3.0158)	71.79% (64.65%-78.79%)	68.68% (59.37%-77.78%)	77.084% (65.788%-88.24%)	83.71% (75.61%-91.84%)	58.92% (47.82%-70.21%)	2.8840 (2.6144-3.1587)
At least 3 of CHASM (melanoma), Mutation Assessor, PolyPhen-2, PROVEAN, SIFT	72.35% (68.69%-75.76%)	77.09% (73.17%-81.25%)	64.154% (57.971%-70.671%)	78.81% (74.79%-82.93%)	61.83% (55.71%-68.06%)	2.8188 (2.6760-2.9674)	72.46% (65.66%-79.8%)	77.18% (68.42%-84.85%)	64.344% (51.429%-76.93%)	78.79% (70.49%-86.89%)	62.15% (49.97%-74.38%)	2.8246 (2.5295-3.1168)
At least 3 of CHASM (melanoma), Mutation Assessor, PolyPhen-2, PROVEAN, VEST	71.37% (67.68%-74.75%)	77.68% (73.77%-81.75%)	60.47% (53.846%-67.124%)	77.26% (73.02%-81.4%)	61.04% (54.93%-67.57%)	2.7645 (2.6126-2.9086)	71.39% (64.65%-78.79%)	77.6% (69.23%-85.48%)	60.736% (47.059%-73.17%)	77.22% (68.51%-85.71%)	61.23% (48.26%-73.18%)	2.7678 (2.4769-3.0530)
At least 3 of CHASM (melanoma), Mutation Assessor, PolyPhen-2, SIFT	73.72% (70.2%-77.27%)	75.51% (71.31%-80%)	70.607% (64.286%-76.712%)	81.62% (77.68%-85.59%)	62.51% (56.63%-68.83%)	2.9026 (2.7527-3.0481)	73.78% (66.67%-80.81%)	75.55% (66.18%-83.93%)	70.702% (58.614%-82.86%)	81.57% (73.33%-89.47%)	62.75% (50%-74.43%)	2.9057 (2.6000-3.1998)
At least 3 of CHASM (melanoma), Mutation Assessor, PolyPhen-2, SIFT, VEST	70.32% (66.67%-73.74%)	79.23% (75.4%-83.33%)	54.904% (48.485%-61.196%)	75.23% (70.89%-79.26%)	60.45% (53.73%-67.19%)	2.6982 (2.5529-2.8472)	70.48% (63.64%-77.78%)	79.29% (71.19%-86.89%)	55.349% (42.105%-69.24%)	75.28% (66.67%-83.82%)	60.89% (47.06%-74.07%)	2.7081 (2.4064-2.9937)
At least 3 of CHASM (melanoma), Mutation Assessor, PolyPhen-2, VEST	71.71% (68.18%-75.25%)	75% (70.87%-79.68%)	66.005% (60%-72.309%)	79.23% (75.21%-83.47%)	60.43% (54.43%-67.09%)	2.8067 (2.6580-2.9558)	71.74% (64.65%-78.79%)	74.97% (66.1%-83.33%)	66.182% (53.846%-78.79%)	79.17% (70.18%-87.04%)	60.65% (47.5%-72.23%)	2.8098 (2.5081-3.1118)
At least 3 of CHASM (melanoma), Mutation Assessor, PROVEAN	59.95% (56.06%-63.64%)	39.45% (34.64%-44.35%)	95.373% (92.857%-98.551%)	93.65% (90.2%-97.87%)	47.67% (42.95%-52.08%)	2.7615 (2.6575-2.8731)	59.91% (52.53%-67.68%)	39.15% (29.09%-49.02%)	95.471% (89.655%-100%)	93.71% (85.71%-100%)	47.76% (38.57%-57.36%)	2.7609 (2.5315-2.9670)
At least 3 of CHASM (melanoma), Mutation Assessor, PROVEAN, SIFT	73.03% (69.7%-76.26%)	70.69% (66.14%-75%)	77.06% (71.617%-82.435%)	84.2% (80.2%-88.18%)	60.32% (54.64%-65.93%)	2.9227 (2.7872-3.0602)	73.14% (66.67%-79.8%)	70.83% (62.06%-79.7%)	77.059% (66.667%-87.88%)	84.12% (76.19%-92.16%)	60.61% (48.84%-71.75%)	2.9262 (2.6486-3.2068)
At least 3 of CHASM (melanoma), Mutation Assessor, PROVEAN, SIFT, VEST	71.02% (67.68%-74.24%)	75.51% (71.54%-79.82%)	63.257% (57.352%-69.863%)	78.04% (73.91%-82.12%)	59.9% (53.75%-66.22%)	2.7671 (2.6193-2.9091)	71.08% (64.65%-77.78%)	75.55% (66.67%-83.61%)	63.381% (51.134%-75.87%)	77.97% (69.49%-86.21%)	60.15% (47.5%-72.1%)	2.7706 (2.4768-3.0565)
At least 3 of CHASM (melanoma), Mutation Assessor, PROVEAN, VEST	68.68% (65.15%-72.73%)	64.89% (60%-69.67%)	75.223% (69.564%-80.771%)	81.91% (77.78%-86.11%)	55.34% (50%-61%)	2.7735 (2.6300-2.9164)	68.71% (60.61%-75.76%)	64.88% (54.83%-74.24%)	75.237% (64.701%-86.49%)	81.79% (73.07%-90%)	55.53% (43.75%-66.02%)	2.7744 (2.4890-3.0652)
At least 3 of CHASM (melanoma), Mutation Assessor, SIFT	71.04% (67.66%-74.75%)	60.11% (55.2%-65.04%)	89.929% (86.3%-94.203%)	91.17% (87.65%-95%)	56.59% (51.64%-61.6%)	2.9780 (2.8579-3.1014)	71.05% (63.64%-77.8%)	60.07% (50%-69.84%)	89.84% (81.818%-97.3%)	91.04% (83.72%-97.67%)	56.73% (46.55%-67.24%)	2.9767 (2.7330-3.2231)
At least 3 of CHASM (melanoma), Mutation Assessor, SIFT, VEST	71.71% (68.18%-75.25%)	73.94% (69.67%-78.23%)	67.853% (61.972%-73.98%)	79.91% (75.86%-84.03%)	60.09% (54.32%-66.25%)	2.8180 (2.6763-2.9605)	71.72% (64.65%-78.79%)	73.9% (65.08%-82.54%)	67.953% (55.556%-80%)	79.82% (71.43%-87.93%)	60.27% (47.62%-71.79%)	2.8194 (2.5206-3.1031)
At least 3 of CHASM (melanoma), Mutation Assessor, VEST	66.34% (62.63%-70.2%)	52.67% (47.5%-57.6%)	89.955% (86.301%-94.366%)	90.07% (86.25%-94.44%)	52.36% (47.29%-57.39%)	2.8506 (2.7357-2.9765)	66.32% (58.59%-73.74%)	52.61% (42.37%-62.72%)	89.794% (81.392%-97.22%)	89.87% (81.25%-97.37%)	52.48% (42.37%-62.32%)	2.8475 (2.5959-3.0748)
At least 3 of CHASM (melanoma), MutationTaster, PolyPhen-2	73.02% (69.7%-76.77%)	61.64% (56.66%-66.93%)	92.701% (89.472%-96.104%)	93.59% (90.8%-96.59%)	58.29% (53.21%-63.16%)	3.0623 (2.9532-3.1756)	73.14% (65.66%-79.8%)	61.8% (51.55%-71.88%)	92.571% (85.415%-100%)	93.46% (86.67%-100%)	58.55% (49%-69.09%)	3.0639 (2.8218-3.2806)
At least 3 of CHASM (melanoma), MutationTaster, PolyPhen-2, PROVEAN	78.75% (75.76%-81.82%)	73.87% (69.67%-78.23%)	87.164% (82.857%-91.549%)	90.87% (87.76%-94.06%)	65.87% (60.44%-71.28%)	3.1777 (3.0633-3.3055)	78.87% (72.73%-84.85%)	74.04% (65.57%-82.82%)	87.115% (78.372%-94.87%)	90.8% (84.31%-96.43%)	66.16% (55.32%-76.79%)	3.1811 (2.9301-3.4114)
At least 3 of CHASM (melanoma), MutationTaster, PolyPhen-2, PROVEAN, SIFT	77.77% (74.73%-81.31%)	82.43% (78.86%-86.36%)	69.695% (63.768%-75.952%)	82.47% (78.86%-86.29%)	69.64% (63.89%-75.36%)	3.0424 (2.9035-3.1835)	77.8% (70.71%-83.86%)	82.46% (74.58%-89.4%)	69.766% (57.143%-81.82%)	82.4% (74.63%-89.83%)	69.86% (57.89%-81.08%)	3.0448 (2.7574-3.3138)
At least 3 of CHASM (melanoma), MutationTaster, PolyPhen-2, PROVEAN, VEST	74.43% (71.21%-77.78%)	80.83% (77.12%-84.85%)	63.343% (56.94%-69.698%)	79.22% (75.21%-83.33%)	65.65% (59.42%-71.88%)	2.8905 (2.7414-3.0399)	74.38% (67.68%-80.81%)	80.87% (72.46%-88.33%)	63.227% (50%-75.76%)	79.05% (71.21%-86.57%)	65.82% (52.78%-78.38%)	2.8897 (2.5839-3.1790)
At least 3 of CHASM (melanoma), MutationTaster, PolyPhen-2, SIFT	80.81% (77.78%-83.84%)	81.34% (77.78%-85.22%)	79.885% (74.666%-85.508%)	87.49% (84.3%-91.3%)	71.23% (66.23%-76.74%)	3.1995 (3.0748-3.3318)	80.8% (74.75%-86.87%)	81.44% (73.77%-88.71%)	79.659% (68.568%-89.66%)	87.31% (80%-93.88%)	71.45% (60%-81.82%)	3.1986 (2.9332-3.4481)
At least 3 of CHASM (melanoma), MutationTaster, PolyPhen-2, SIFT, VEST	74.02% (70.71%-77.29%)	83.96% (80.65%-87.88%)	56.821% (50%-63.517%)	77.08% (73.13%-81.16%)	67.21% (60.71%-73.85%)	2.8507 (2.7022-3.0013)	74.18% (67.65%-80.81%)	84.18% (76.56%-90.7%)	56.99% (44.118%-70.97%)	77.05% (68.66%-85.34%)	67.74% (53.84%-80.02%)	2.8596 (2.5471-3.1384)
At least 3 of CHASM (melanoma), MutationTaster, PolyPhen-2, VEST	77.5% (74.24%-80.81%)	78.19% (74.22%-82.35%)	76.301% (70.833%-82.09%)	85.08% (81.31%-88.89%)	66.92% (61.53%-72.84%)	3.0650 (2.9276-3.2020)	77.33% (70.71%-83.84%)	78.18% (69.86%-86.36%)	75.838% (64.286%-87.18%)	84.74% (76.92%-92.16%)	66.95% (54.99%-78.26%)	3.0571 (2.7702-3.3339)
At least 3 of CHASM (melanoma), MutationTaster, PROVEAN	62.63% (58.59%-66.17%)	42.07% (37.09%-47.2%)	98.173% (97.015%-100%)	97.55% (95.92%-100%)	49.5% (44.68%-54.11%)	2.8729 (2.7823-2.9657)	62.62% (55.53%-70.71%)	41.9% (31.25%-51.62%)	98.144% (94.118%-100%)	97.49% (92%-100%)	49.61% (40.3%-59.42%)	2.8714 (2.6834-3.0569)
At least 3 of CHASM (melanoma), MutationTaster, PROVEAN, SIFT	78.07% (74.75%-81.31%)	71.75% (67.21%-76.03%)	88.983% (84.932%-93.243%)	91.85% (88.89%-95%)	64.56% (59.41%-69.91%)	3.1715 (3.0576-3.2930)	78.2% (71.72%-84.85%)	71.89% (63.49%-80.56%)	88.981% (80.556%-97.22%)	91.81% (85.36%-97.92%)	64.85% (54%-75.44%)	3.1753 (2.9284-3.4023)
At least 3 of CHASM (melanoma), MutationTaster, PROVEAN, SIFT, VEST	75.09% (72.22%-78.28%)	79.79% (75.97%-83.87%)	66.956% (61.111%-73.442%)	80.68% (76.8%-84.5%)	65.71% (60%-71.83%)	2.9314 (2.7988-3.0702)	75.07% (68.69%-80.81%)	79.75% (71.43%-87.5%)	66.988% (54.054%-79.08%)	80.57% (72.41%-88.52%)	65.84% (53.57%-77.14%)	2.9315 (2.6500-3.2004)
At least 3 of CHASM (melanoma), MutationTaster, PROVEAN, VEST	76.42% (73.23%-79.8%)	68.06% (63.57%-72.8%)	90.861% (87.324%-94.667%)	92.8% (90%-95.7%)	62.2% (57.01%-67.65%)	3.1392 (3.0200-3.2593)	76.46% (69.7%-82.83%)	68.12% (58.46%-77.05%)	90.73% (82.857%-97.37%)	92.67% (86.49%-97.92%)	62.38% (50.98%-72.42%)	3.1389 (2.9007-3.3834)
At least 3 of CHASM (melanoma), MutationTaster, SIFT	75.06% (71.72%-78.28%)	63.79% (59.05%-68.55%)	94.52% (91.667%-97.368%)	95.27% (92.85%-97.73%)	60.16% (55.14%-65.1%)	3.1374 (3.0391-3.2436)	75.14% (68.69%-81.82%)	63.88% (54.23%-73.34%)	94.436% (88.232%-100%)	95.18% (89.74%-100%)	60.37% (50%-70.69%)	3.1386 (2.9169-3.3368)
At least 3 of CHASM (melanoma), MutationTaster, SIFT, VEST	79.49% (76.26%-82.83%)	77.65% (73.73%-82.03%)	82.655% (77.778%-87.672%)	88.56% (85.19%-92.18%)	68.14% (62.92%-73.68%)	3.1701 (3.0490-3.3051)	79.41% (72.73%-85.86%)	77.66% (68.85%-85.71%)	82.372% (71.795%-92.11%)	88.33% (81.13%-94.74%)	68.25% (56.1%-78.57%)	3.1661 (2.8923-3.4115)
At least 3 of CHASM (melanoma), MutationTaster, VEST	71.02% (67.68%-74.75%)	57.96% (53.12%-62.9%)	93.603% (90.537%-97.102%)	94% (91.14%-97.33%)	56.29% (51.26%-61.42%)	3.0186 (2.9097-3.1411)	71.08% (63.64%-77.78%)	57.99% (48.39%-67.74%)	93.517% (86.838%-100%)	93.9% (87.18%-100%)	56.47% (46.15%-66.67%)	3.0188 (2.7896-3.2420)
At least 3 of CHASM (melanoma), PolyPhen-2, PROVEAN	61.28% (57.58%-65.15%)	42.09% (37.01%-46.83%)	94.455% (91.667%-97.368%)	92.93% (89.29%-96.77%)	48.54% (43.45%-53.15%)	2.7801 (2.6622-2.8924)	61.28% (53.54%-68.69%)	41.85% (32.13%-52.17%)	94.556% (88.232%-100%)	92.98% (84.85%-100%)	48.65% (39.47%-58.83%)	2.7804 (2.5530-3.0029)
At least 3 of CHASM (melanoma), PolyPhen-2, PROVEAN, SIFT	71.33% (67.68%-74.75%)	70.16% (65.83%-74.4%)	73.343% (67.605%-79.168%)	81.99% (77.88%-86.11%)	58.7% (52.81%-64.52%)	2.8419 (2.6951-2.9912)	71.48% (64.65%-78.79%)	70.3% (61.9%-79.37%)	73.466% (61.29%-85.29%)	81.99% (73.33%-90.38%)	59.02% (47.49%-70.46%)	2.8477 (2.5468-3.1437)
At least 3 of CHASM (melanoma), PolyPhen-2, PROVEAN, SIFT, VEST	70.35% (66.67%-73.74%)	79.26% (75.4%-83.08%)	54.946% (48.557%-61.644%)	75.26% (70.99%-79.26%)	60.5% (53.97%-67.16%)	2.6996 (2.5511-2.8444)	70.41% (63.64%-77.78%)	79.24% (71.42%-87.1%)	55.234% (41.935%-68.19%)	75.23% (67.19%-83.58%)	60.76% (47.99%-73.53%)	2.7047 (2.4100-2.9992)
At least 3 of CHASM (melanoma), PolyPhen-2, PROVEAN, VEST	67.33% (63.64%-71.21%)	66.49% (61.9%-71.2%)	68.779% (62.5%-75.325%)	78.64% (74.29%-83.18%)	54.27% (48.45%-60.2%)	2.6818 (2.5354-2.8361)	67.36% (59.6%-74.75%)	66.47% (56.72%-75.76%)	68.845% (55.556%-81.09%)	78.56% (69.23%-87.04%)	54.46% (42.49%-65.31%)	2.6833 (2.3746-2.9719)
At least 3 of CHASM (melanoma), PolyPhen-2, SIFT	70.7% (67.16%-74.24%)	62.22% (57.36%-66.94%)	85.352% (80.881%-90.411%)	88.02% (84.14%-92.13%)	56.64% (51.38%-61.69%)	2.9223 (2.7855-3.0681)	70.72% (63.64%-77.78%)	62.24% (52.46%-72.13%)	85.227% (75%-94.44%)	87.86% (79.49%-95.56%)	56.81% (46.43%-67.8%)	2.9214 (2.6313-3.1932)
At least 3 of CHASM (melanoma), PolyPhen-2, SIFT, VEST	71.36% (68.18%-74.75%)	77.67% (73.73%-81.89%)	60.429% (53.846%-66.667%)	77.24% (73.08%-81.3%)	61.01% (54.93%-67.2%)	2.7635 (2.6129-2.9093)	71.43% (64.65%-77.78%)	77.62% (69.09%-85.71%)	60.774% (48.384%-74.19%)	77.25% (68.96%-85.92%)	61.26% (48.48%-73.53%)	2.7691 (2.4707-3.0547)
At least 3 of CHASM (melanoma), PolyPhen-2, VEST	65.65% (62.12%-69.19%)	55.29% (50.38%-60%)	83.556% (78.571%-88.571%)	85.33% (81.08%-90%)	51.94% (46.96%-56.9%)	2.7612 (2.6329-2.9001)	65.67% (58.59%-72.73%)	55.35% (45.9%-65.15%)	83.324% (73.329%-93.02%)	85.09% (75.59%-93.48%)	52.09% (42.3%-62.07%)	2.7585 (2.4893-3.0117)
At least 3 of CHASM (melanoma), PROVEAN, SIFT	63.3% (59.6%-67.17%)	45.27% (40.17%-50.39%)	94.455% (91.667%-97.368%)	93.39% (90%-96.97%)	49.95% (45%-54.55%)	2.8307 (2.7204-2.9426)	63.3% (55.56%-70.71%)	45.06% (34.54%-55.39%)	94.556% (88.232%-100%)	93.45% (85.71%-100%)	50.07% (40.62%-60%)	2.8314 (2.6061-3.0460)
At least 3 of CHASM (melanoma), PROVEAN, SIFT, VEST	67.31% (63.64%-71.21%)	65.92% (61.34%-70.54%)	69.701% (63.889%-75.765%)	79% (74.76%-83.5%)	54.19% (48.77%-60.2%)	2.6882 (2.5383-2.8384)	67.4% (59.6%-74.75%)	66% (56.25%-75.01%)	69.741% (57.5%-81.4%)	78.92% (70%-87.5%)	54.44% (42.5%-65.91%)	2.6911 (2.3959-2.9954)
At least 3 of CHASM (melanoma), PROVEAN, VEST	61.63% (58.08%-65.15%)	41.02% (36.13%-45.9%)	97.25% (95.588%-100%)	96.28% (93.88%-100%)	48.81% (43.88%-53.43%)	2.8336 (2.7350-2.9310)	61.59% (54.55%-68.69%)	40.8% (31.14%-50.77%)	97.224% (92.105%-100%)	96.22% (89.47%-100%)	48.9% (39.74%-58.57%)	2.8314 (2.6280-3.0229)
At least 3 of CHASM (melanoma), SIFT, VEST	68.36% (64.65%-71.73%)	57.44% (52.46%-62.4%)	87.22% (83.098%-91.892%)	88.61% (84.81%-92.68%)	54.24% (49.11%-59.35%)	2.8750 (2.7468-3.0011)	68.34% (61.59%-75.76%)	57.43% (47.62%-67.21%)	86.993% (77.143%-94.87%)	88.37% (80%-95.65%)	54.35% (44.07%-64.52%)	2.8714 (2.6185-3.1320)
At least 3 of FATHMM (cancer), FATHMM (missense), Mutation Assessor	69.06% (65.66%-72.73%)	59.11% (54.03%-63.78%)	86.239% (81.69%-90.909%)	88.14% (84.34%-92.5%)	54.95% (49.57%-59.84%)	2.8844 (2.7593-3.0172)	68.96% (61.62%-75.76%)	58.88% (49.12%-68.85%)	86.232% (76.471%-94.74%)	88.02% (79.49%-95.45%)	54.99% (45.28%-65.38%)	2.8811 (2.6184-3.1302)
At least 3 of FATHMM (cancer), FATHMM (missense), Mutation Assessor, MutationTaster	82.82% (79.8%-85.86%)	81.91% (78.05%-85.83%)	84.392% (80%-89.394%)	90.07% (87.04%-93.5%)	72.96% (67.86%-78.21%)	3.2934 (3.1726-3.4176)	82.84% (76.77%-88.89%)	81.9% (73.68%-89.55%)	84.437% (74.286%-93.75%)	90.03% (83.02%-96.23%)	73.13% (62.49%-83.78%)	3.2949 (3.0343-3.5391)
At least 3 of FATHMM (cancer), FATHMM (missense), Mutation Assessor, MutationTaster, PolyPhen-2	81.77% (78.79%-84.85%)	88.26% (85.04%-91.41%)	70.541% (64.789%-76.812%)	83.82% (80.31%-87.41%)	77.66% (72.13%-83.34%)	3.2028 (3.0769-3.3342)	81.92% (75.76%-87.88%)	88.36% (81.96%-95%)	70.834% (58.966%-82.5%)	83.87% (76.56%-90.78%)	78.02% (65.79%-89.19%)	3.2108 (2.9299-3.4566)
At least 3 of FATHMM (cancer), FATHMM (missense), Mutation Assessor, MutationTaster, PROVEAN	81.43% (78.28%-84.34%)	84.56% (80.99%-88.19%)	76.007% (70.267%-81.821%)	85.9% (82.35%-89.6%)	74.01% (68.57%-79.41%)	3.2048 (3.0695-3.3372)	81.58% (75.76%-87.88%)	84.58% (76.78%-91.81%)	76.43% (64.997%-88.1%)	86.02% (78.69%-92.98%)	74.3% (62.86%-85.71%)	3.2133 (2.9451-3.4875)
At least 3 of FATHMM (cancer), FATHMM (missense), Mutation Assessor, MutationTaster, SIFT	82.41% (79.29%-85.35%)	90.41% (87.6%-93.5%)	68.577% (62.687%-75%)	83.26% (79.7%-86.86%)	80.54% (75%-86.21%)	3.2279 (3.0976-3.3608)	82.65% (76.77%-88.89%)	90.44% (84.38%-96.3%)	69.262% (56.748%-80.56%)	83.47% (76.19%-90.28%)	80.87% (69.23%-91.67%)	3.2404 (2.9657-3.4975)
At least 3 of FATHMM (cancer), FATHMM (missense), Mutation Assessor, MutationTaster, VEST	80.42% (77.27%-83.33%)	88.29% (85.25%-91.41%)	66.788% (60.807%-73.081%)	82.13% (78.63%-86.05%)	76.74% (70.77%-82.54%)	3.1396 (3.0046-3.2685)	80.58% (74.75%-86.87%)	88.29% (81.67%-94.12%)	67.323% (54.539%-79.31%)	82.26% (74.63%-89.39%)	77.03% (65.52%-88.01%)	3.1491 (2.8794-3.4116)
At least 3 of FATHMM (cancer), FATHMM (missense), Mutation Assessor, PolyPhen-2	75.39% (72.22%-78.79%)	76.09% (72%-80.31%)	74.186% (68.569%-80.52%)	83.6% (79.81%-87.72%)	64.21% (58.54%-70.24%)	2.9808 (2.8440-3.1163)	75.47% (68.69%-81.82%)	75.99% (67.21%-84.06%)	74.556% (62.5%-86.11%)	83.67% (75.8%-91.38%)	64.41% (52.38%-75.61%)	2.9862 (2.7198-3.2564)
At least 3 of FATHMM (cancer), FATHMM (missense), Mutation Assessor, PolyPhen-2, PROVEAN	77.06% (73.74%-80.3%)	79.79% (76.07%-83.61%)	72.331% (66.216%-78.378%)	83.29% (79.34%-87.4%)	67.42% (61.54%-73.17%)	3.0283 (2.8895-3.1579)	77.2% (70.71%-83.84%)	79.76% (71.67%-87.5%)	72.762% (60.526%-85%)	83.4% (75.75%-91.08%)	67.69% (55.88%-79.49%)	3.0362 (2.7747-3.3246)
At least 3 of FATHMM (cancer), FATHMM (missense), Mutation Assessor, PolyPhen-2, SIFT	77.07% (73.74%-80.32%)	87.24% (84.03%-90.76%)	59.474% (53.422%-66.13%)	78.82% (75%-82.61%)	72.95% (66.67%-79.32%)	2.9848 (2.8420-3.1315)	77.18% (70.68%-83.84%)	87.2% (80%-93.55%)	59.942% (47.222%-72.51%)	78.88% (71.23%-86.36%)	73.22% (60.87%-85.19%)	2.9924 (2.6998-3.2870)
At least 3 of FATHMM (cancer), FATHMM (missense), Mutation Assessor, PolyPhen-2, VEST	73.38% (70.2%-76.77%)	83.54% (80.16%-87.32%)	55.804% (49.275%-62.165%)	76.57% (72.59%-80.6%)	66.23% (59.32%-72.88%)	2.8214 (2.6698-2.9645)	73.44% (66.67%-79.8%)	83.43% (75.41%-90.33%)	56.281% (43.333%-69.7%)	76.6% (68.57%-84.85%)	66.43% (52.17%-78.96%)	2.8274 (2.5314-3.1292)
At least 3 of FATHMM (cancer), FATHMM (missense), Mutation Assessor, PROVEAN	73.02% (69.7%-76.26%)	70.21% (65.87%-74.8%)	77.854% (72.222%-83.562%)	84.58% (80.58%-88.78%)	60.18% (54.44%-65.62%)	2.9283 (2.7905-3.0695)	73.16% (66.67%-79.8%)	70.18% (60.94%-78.79%)	78.225% (66.667%-89.2%)	84.69% (76.09%-92.45%)	60.44% (48.98%-71.16%)	2.9354 (2.6498-3.2198)
At least 3 of FATHMM (cancer), FATHMM (missense), Mutation Assessor, PROVEAN, SIFT	77.06% (73.74%-80.32%)	81.41% (77.78%-85.5%)	69.519% (63.514%-76.056%)	82.2% (78.4%-86.18%)	68.38% (62.31%-74.29%)	3.0151 (2.8779-3.1607)	77.2% (70.68%-83.84%)	81.31% (73.02%-88.33%)	70.109% (57.143%-81.48%)	82.36% (74.24%-89.83%)	68.61% (57.14%-80%)	3.0239 (2.7347-3.3021)
At least 3 of FATHMM (cancer), FATHMM (missense), Mutation Assessor, PROVEAN, VEST	73.67% (70.2%-77.27%)	78.2% (74.19%-82.4%)	65.824% (59.724%-72.464%)	79.82% (75.67%-84.25%)	63.58% (57.33%-69.74%)	2.8742 (2.7276-3.0231)	73.88% (66.67%-80.81%)	78.16% (69.49%-85.94%)	66.492% (52.941%-78.38%)	80.01% (71.43%-88.14%)	63.94% (51.35%-76.19%)	2.8860 (2.5861-3.1730)
At least 3 of FATHMM (cancer), FATHMM (missense), Mutation Assessor, SIFT	76.36% (73.23%-79.8%)	78.2% (74.38%-82.26%)	73.174% (67.523%-79.71%)	83.45% (79.65%-87.6%)	66% (59.76%-71.63%)	3.0082 (2.8702-3.1509)	76.57% (69.7%-82.83%)	78.15% (69.84%-85.72%)	73.806% (60.526%-85.37%)	83.67% (75.41%-91.23%)	66.3% (54.76%-77.78%)	3.0193 (2.7236-3.2903)
At least 3 of FATHMM (cancer), FATHMM (missense), Mutation Assessor, SIFT, VEST	74.39% (70.71%-77.78%)	84.62% (81.15%-88.19%)	56.694% (50.649%-63.079%)	77.16% (73.19%-81.29%)	68.07% (61.54%-74.59%)	2.8654 (2.7200-3.0100)	74.45% (67.68%-81.82%)	84.46% (77.05%-91.31%)	57.249% (44.444%-69.7%)	77.21% (69.12%-85.07%)	68.23% (53.83%-80.77%)	2.8715 (2.5715-3.1551)
At least 3 of FATHMM (cancer), FATHMM (missense), Mutation Assessor, VEST	73.34% (69.7%-76.77%)	74.49% (70.4%-78.91%)	71.335% (65.67%-77.778%)	81.8% (77.78%-86.09%)	61.79% (55.7%-67.9%)	2.8942 (2.7473-3.0376)	73.52% (66.67%-80.81%)	74.39% (65.62%-82.46%)	71.992% (59.986%-83.33%)	82.01% (73.21%-90.2%)	62.07% (50%-73.34%)	2.9046 (2.6211-3.1879)
At least 3 of FATHMM (cancer), FATHMM (missense), MutationTaster	76.12% (72.73%-79.29%)	66.51% (61.72%-71.32%)	92.72% (89.706%-96.001%)	94.05% (91.49%-96.77%)	61.56% (56.31%-66.37%)	3.1484 (3.0363-3.2604)	76.05% (69.7%-82.83%)	66.42% (56.6%-75.81%)	92.537% (85.294%-97.62%)	93.86% (87.8%-98.08%)	61.63% (51.65%-72%)	3.1444 (2.9250-3.3710)
At least 3 of FATHMM (cancer), FATHMM (missense), MutationTaster, PolyPhen-2	83.49% (80.81%-86.87%)	84.55% (80.95%-88.24%)	81.638% (76.712%-86.767%)	88.84% (85.83%-92.37%)	75.35% (70%-80.56%)	3.3038 (3.1821-3.4326)	83.53% (76.77%-88.89%)	84.61% (76.92%-91.94%)	81.647% (71.053%-91.67%)	88.79% (81.81%-94.92%)	75.58% (64.86%-86.49%)	3.3062 (3.0411-3.5469)
At least 3 of FATHMM (cancer), FATHMM (missense), MutationTaster, PolyPhen-2, PROVEAN	80.76% (77.78%-83.84%)	84.55% (80.95%-88.24%)	74.196% (68.116%-80.007%)	85% (81.51%-88.71%)	73.53% (67.65%-79.17%)	3.1727 (3.0452-3.3111)	80.91% (74.75%-86.87%)	84.61% (76.92%-91.94%)	74.515% (62.855%-86.21%)	85.07% (77.78%-92.07%)	73.85% (62.49%-85.3%)	3.1805 (2.9053-3.4460)
At least 3 of FATHMM (cancer), FATHMM (missense), MutationTaster, PolyPhen-2, SIFT	80.4% (77.27%-83.33%)	90.39% (87.5%-93.55%)	63.13% (56.919%-69.336%)	80.91% (77.3%-84.56%)	79.17% (73.33%-85.42%)	3.1360 (3.0012-3.2662)	80.61% (74.75%-86.87%)	90.49% (84.13%-96.67%)	63.63% (51.427%-75.76%)	81.02% (73.53%-88.24%)	79.63% (66.67%-91.43%)	3.1477 (2.8754-3.4195)
At least 3 of FATHMM (cancer), FATHMM (missense), MutationTaster, PolyPhen-2, VEST	78.45% (75.25%-81.31%)	87.76% (84.68%-91.06%)	62.35% (56.059%-68.75%)	80.12% (76.34%-84.06%)	74.65% (68.42%-80.7%)	3.0488 (2.9081-3.1784)	78.46% (72.73%-84.85%)	87.77% (81.24%-93.94%)	62.452% (50%-75%)	80.04% (72.36%-87.7%)	74.86% (62.49%-86.84%)	3.0512 (2.7850-3.3345)
At least 3 of FATHMM (cancer), FATHMM (missense), MutationTaster, PROVEAN	81.46% (78.28%-84.34%)	78.14% (73.98%-82.54%)	87.187% (83.071%-91.67%)	91.34% (88.46%-94.5%)	69.77% (64.52%-75%)	3.2644 (3.1430-3.3818)	81.52% (75.76%-87.88%)	78.26% (69.35%-86.21%)	87.075% (78.116%-95%)	91.23% (84.75%-96.55%)	70.03% (59.57%-80.39%)	3.2660 (3.0269-3.5095)
At least 3 of FATHMM (cancer), FATHMM (missense), MutationTaster, PROVEAN, SIFT	81.09% (77.78%-84.34%)	86.7% (83.21%-90.32%)	71.378% (65.672%-78.087%)	83.97% (80.31%-87.7%)	75.64% (70%-81.43%)	3.1768 (3.0415-3.3091)	81.26% (74.75%-87.88%)	86.69% (79.31%-93.65%)	71.892% (58.327%-83.33%)	84.12% (76.92%-91.18%)	75.93% (64.28%-87.88%)	3.1862 (2.9237-3.4516)
At least 3 of FATHMM (cancer), FATHMM (missense), MutationTaster, PROVEAN, VEST	79.41% (75.76%-82.32%)	84.54% (80.99%-88.28%)	70.514% (64.381%-77.217%)	83.22% (79.51%-87.1%)	72.51% (66.23%-78.26%)	3.1079 (2.9710-3.2454)	79.57% (73.74%-86.87%)	84.63% (77.05%-91.8%)	70.85% (57.776%-82.93%)	83.3% (75.76%-90.63%)	72.88% (61.53%-84.62%)	3.1165 (2.8367-3.3952)
At least 3 of FATHMM (cancer), FATHMM (missense), MutationTaster, SIFT	84.82% (81.82%-87.88%)	86.7% (83.21%-90.32%)	81.549% (76.809%-87.328%)	89.05% (86.06%-92.44%)	78.01% (72.73%-83.33%)	3.3530 (3.2328-3.4784)	84.91% (78.79%-90.91%)	86.69% (79.31%-93.65%)	81.817% (70.27%-91.43%)	89.13% (82.45%-95.16%)	78.21% (68.42%-89.19%)	3.3585 (3.1038-3.5945)
At least 3 of FATHMM (cancer), FATHMM (missense), MutationTaster, SIFT, VEST	80.77% (77.78%-83.84%)	90.94% (88.13%-93.8%)	63.175% (56.757%-70.27%)	81.03% (77.46%-85%)	80.13% (74.07%-86.01%)	3.1527 (3.0100-3.2859)	80.89% (74.75%-86.87%)	90.99% (85%-96.72%)	63.543% (50%-76.32%)	81.07% (73.33%-88.24%)	80.46% (69.23%-92.31%)	3.1606 (2.8952-3.4349)
At least 3 of FATHMM (cancer), FATHMM (missense), MutationTaster, VEST	81.43% (78.28%-84.34%)	83.47% (79.7%-87.4%)	77.884% (72.464%-83.333%)	86.72% (83.33%-90.24%)	73.16% (67.6%-78.95%)	3.2123 (3.0754-3.3378)	81.59% (75.76%-87.88%)	83.57% (75.44%-91.05%)	78.134% (66.667%-88.57%)	86.79% (79.63%-93.44%)	73.51% (61.9%-85.29%)	3.2201 (2.9658-3.4780)
At least 3 of FATHMM (cancer), FATHMM (missense), PolyPhen-2	70.4% (66.67%-73.74%)	62.83% (58.06%-67.74%)	83.478% (78.748%-88.611%)	86.81% (82.98%-91.11%)	56.5% (50.96%-61.4%)	2.8961 (2.7656-3.0219)	70.31% (63.64%-77.78%)	62.61% (53.02%-72.31%)	83.47% (73.529%-93.1%)	86.69% (78.56%-94.12%)	56.53% (46.43%-67.24%)	2.8929 (2.6416-3.1509)
At least 3 of FATHMM (cancer), FATHMM (missense), PolyPhen-2, PROVEAN	75.03% (71.72%-78.28%)	74.44% (70.33%-78.99%)	76.036% (70.423%-81.69%)	84.31% (80.73%-88.39%)	63.25% (57.65%-68.82%)	2.9803 (2.8472-3.1103)	75.19% (68.69%-81.82%)	74.49% (65.57%-83.05%)	76.338% (65.625%-86.85%)	84.39% (76.46%-91.49%)	63.56% (52.17%-74.47%)	2.9878 (2.7198-3.2521)
At least 3 of FATHMM (cancer), FATHMM (missense), PolyPhen-2, PROVEAN, SIFT	76.4% (73.23%-79.8%)	83.52% (79.84%-87.4%)	64.056% (57.745%-70.423%)	80.07% (76.15%-84.09%)	69.22% (62.9%-75.34%)	2.9688 (2.8224-3.1078)	76.5% (69.7%-82.83%)	83.45% (75.44%-90.74%)	64.521% (51.719%-77.14%)	80.15% (72.31%-87.88%)	69.46% (57.56%-81.82%)	2.9758 (2.6954-3.2582)
At least 3 of FATHMM (cancer), FATHMM (missense), PolyPhen-2, PROVEAN, VEST	73.36% (69.7%-76.77%)	80.84% (77.05%-84.68%)	60.416% (53.519%-66.678%)	77.93% (73.81%-81.82%)	64.59% (58.06%-71.01%)	2.8378 (2.6854-2.9794)	73.48% (66.67%-80.81%)	80.85% (73.02%-88.71%)	60.788% (47.497%-74.29%)	77.97% (69.84%-86.44%)	64.9% (52%-77.43%)	2.8451 (2.5650-3.1533)
At least 3 of FATHMM (cancer), FATHMM (missense), PolyPhen-2, SIFT	77.74% (74.75%-80.81%)	83.52% (79.84%-87.4%)	67.72% (61.644%-74.079%)	81.73% (77.95%-85.6%)	70.4% (64.52%-76.32%)	3.0338 (2.8954-3.1710)	77.86% (71.72%-83.84%)	83.45% (75.44%-90.74%)	68.202% (55.263%-80%)	81.84% (74.19%-89.23%)	70.63% (58.82%-82.36%)	3.0411 (2.7702-3.3240)
At least 3 of FATHMM (cancer), FATHMM (missense), PolyPhen-2, SIFT, VEST	74.02% (70.71%-77.27%)	87.76% (84.73%-91.2%)	50.258% (43.587%-56.717%)	75.31% (71.33%-79.19%)	70.37% (63.46%-77.08%)	2.8370 (2.6839-2.9772)	74.18% (67.68%-80.81%)	87.77% (80.95%-93.94%)	50.833% (37.5%-63.89%)	75.39% (67.52%-83.78%)	70.81% (57.13%-84.62%)	2.8479 (2.5756-3.1519)
At least 3 of FATHMM (cancer), FATHMM (missense), PolyPhen-2, VEST	75.41% (71.72%-78.79%)	80.33% (76.51%-84.3%)	66.881% (60.805%-73.239%)	80.75% (76.8%-84.8%)	66.29% (60%-72.47%)	2.9425 (2.7929-3.0755)	75.45% (68.69%-82.83%)	80.27% (72.31%-88.06%)	67.135% (54.286%-80%)	80.75% (72.72%-88.71%)	66.47% (54.28%-78.58%)	2.9463 (2.6583-3.2443)
At least 3 of FATHMM (cancer), FATHMM (missense), PROVEAN	60.33% (56.57%-64.14%)	42.69% (37.8%-47.69%)	90.821% (87.013%-94.595%)	88.94% (84.48%-93.55%)	47.82% (42.75%-52.21%)	2.7027 (2.5774-2.8308)	60.15% (52.53%-67.68%)	42.25% (31.74%-52.46%)	90.815% (82.857%-97.5%)	88.75% (78.56%-96.88%)	47.81% (38.46%-57.54%)	2.6963 (2.4322-2.9514)
At least 3 of FATHMM (cancer), FATHMM (missense), PROVEAN, SIFT	74.35% (71.21%-77.78%)	75.53% (71.43%-80%)	72.28% (66.197%-78.873%)	82.5% (78.57%-86.61%)	63.08% (57.14%-68.75%)	2.9339 (2.7981-3.0769)	74.54% (67.68%-80.81%)	75.51% (66.15%-83.87%)	72.838% (60%-84.38%)	82.68% (74.19%-90.32%)	63.41% (52.5%-75.01%)	2.9443 (2.6554-3.2130)
At least 3 of FATHMM (cancer), FATHMM (missense), PROVEAN, SIFT, VEST	74.72% (71.21%-78.28%)	81.95% (78.23%-85.83%)	62.205% (55.714%-68.576%)	78.95% (75%-82.84%)	66.59% (60.29%-72.86%)	2.8968 (2.7438-3.0450)	74.81% (67.68%-81.82%)	81.83% (73.85%-89.39%)	62.711% (50%-75.68%)	79.02% (70.97%-86.96%)	66.79% (53.57%-79.41%)	2.9035 (2.6112-3.2110)
At least 3 of FATHMM (cancer), FATHMM (missense), PROVEAN, VEST	69.99% (66.16%-73.74%)	68.09% (63.2%-72.81%)	73.257% (67.558%-79.73%)	81.49% (77.36%-86%)	57.04% (51.09%-62.75%)	2.7987 (2.6494-2.9472)	70.13% (62.63%-77.78%)	68.05% (58.33%-77.59%)	73.62% (60.712%-85.3%)	81.58% (72.34%-89.8%)	57.32% (46%-69.05%)	2.8057 (2.5110-3.1029)
At least 3 of FATHMM (cancer), FATHMM (missense), SIFT	72.04% (68.69%-75.76%)	66.01% (61.11%-70.63%)	82.452% (77.612%-88.062%)	86.68% (82.98%-91.01%)	58.39% (52.69%-63.46%)	2.9353 (2.8070-3.0707)	72.07% (64.65%-78.79%)	65.81% (56.25%-75.36%)	82.764% (71.053%-92.11%)	86.78% (78.72%-94%)	58.51% (48.15%-69.57%)	2.9387 (2.6557-3.1950)
At least 3 of FATHMM (cancer), FATHMM (missense), SIFT, VEST	76.74% (73.23%-80.3%)	81.41% (77.58%-85.27%)	68.667% (62.161%-75.342%)	81.79% (78.07%-85.83%)	68.11% (61.97%-73.97%)	2.9998 (2.8471-3.1463)	76.82% (69.7%-83.84%)	81.31% (73.33%-88.89%)	69.06% (55.263%-82.35%)	81.86% (73.77%-89.47%)	68.28% (56.25%-80.01%)	3.0051 (2.7245-3.3140)
At least 3 of FATHMM (cancer), FATHMM (missense), VEST	63.98% (60.1%-67.68%)	54.34% (49.21%-59.09%)	80.626% (75.675%-86.158%)	82.91% (78.75%-87.5%)	50.53% (45%-55.56%)	2.6841 (2.5479-2.8177)	63.96% (56.57%-71.72%)	54.05% (44.26%-64.07%)	80.904% (69.697%-91.11%)	82.96% (73.68%-91.67%)	50.63% (40.68%-61.54%)	2.6853 (2.4186-2.9583)
At least 3 of FATHMM (cancer), Mutation Assessor, MutationTaster	71.69% (68.17%-75.25%)	59.56% (54.62%-64.46%)	92.677% (89.55%-96.055%)	93.36% (90.41%-96.55%)	56.99% (52.07%-61.74%)	3.0259 (2.9112-3.1436)	71.76% (64.65%-78.81%)	59.59% (50%-69.57%)	92.634% (85.294%-100%)	93.27% (86.49%-100%)	57.19% (47.37%-67.8%)	3.0269 (2.7972-3.2684)
At least 3 of FATHMM (cancer), Mutation Assessor, MutationTaster, PolyPhen-2	79.43% (76.26%-82.83%)	80.3% (76.51%-84.38%)	77.93% (72.603%-83.583%)	86.28% (82.76%-90.18%)	69.59% (64.2%-75.32%)	3.1410 (3.0140-3.2762)	79.52% (72.73%-85.86%)	80.34% (72.06%-87.88%)	78.121% (67.422%-88.89%)	86.28% (78.69%-93.22%)	69.85% (58.14%-80.49%)	3.1459 (2.8776-3.4025)
At least 3 of FATHMM (cancer), Mutation Assessor, MutationTaster, PolyPhen-2, PROVEAN	78.73% (75.76%-81.82%)	82.42% (78.79%-86.29%)	72.343% (66.667%-78.383%)	83.74% (80%-87.7%)	70.41% (64.79%-76.32%)	3.0891 (2.9555-3.2256)	78.91% (72.73%-84.85%)	82.49% (74.6%-90%)	72.76% (60.526%-84.38%)	83.85% (76.11%-91.23%)	70.78% (58.97%-82.05%)	3.0989 (2.8229-3.3723)
At least 3 of FATHMM (cancer), Mutation Assessor, MutationTaster, PolyPhen-2, SIFT	77.74% (74.24%-80.81%)	84.02% (80.64%-87.88%)	66.886% (61.035%-72.973%)	81.44% (77.52%-85.37%)	70.77% (65%-77.03%)	3.0311 (2.8913-3.1716)	77.85% (71.72%-84.85%)	84.07% (76.46%-90.91%)	67.158% (54.286%-79.41%)	81.45% (73.84%-89.07%)	71.09% (58.33%-82.87%)	3.0377 (2.7495-3.3237)
At least 3 of FATHMM (cancer), Mutation Assessor, MutationTaster, PolyPhen-2, VEST	76.44% (73.23%-79.8%)	82.97% (79.36%-86.72%)	65.157% (59.155%-71.433%)	80.45% (76.56%-84.38%)	68.88% (62.86%-75.42%)	2.9746 (2.8356-3.1189)	76.4% (69.7%-82.83%)	82.98% (75%-90.32%)	65.116% (52.632%-76.93%)	80.31% (72.58%-87.88%)	69.04% (56.24%-80.66%)	2.9744 (2.6792-3.2539)
At least 3 of FATHMM (cancer), Mutation Assessor, MutationTaster, PROVEAN	74.7% (71.21%-78.28%)	69.64% (65.25%-74.22%)	83.443% (78.571%-88.732%)	87.91% (84.37%-91.92%)	61.39% (55.91%-66.67%)	3.0238 (2.8930-3.1559)	74.84% (67.68%-81.82%)	69.73% (60.34%-78.57%)	83.591% (73.684%-93.75%)	87.93% (79.99%-95.45%)	61.67% (51.02%-72.55%)	3.0291 (2.7699-3.2933)
At least 3 of FATHMM (cancer), Mutation Assessor, MutationTaster, PROVEAN, SIFT	76.4% (73.22%-79.8%)	79.26% (75.41%-83.46%)	71.445% (65.714%-77.781%)	82.76% (78.81%-86.78%)	66.58% (60.53%-72.37%)	3.0004 (2.8537-3.1427)	76.5% (69.7%-82.85%)	79.23% (70.77%-86.67%)	71.804% (58.818%-83.72%)	82.82% (74.6%-90.32%)	66.8% (54.84%-78.05%)	3.0065 (2.7229-3.2901)
At least 3 of FATHMM (cancer), Mutation Assessor, MutationTaster, PROVEAN, VEST	76.39% (73.23%-79.8%)	78.71% (74.8%-82.93%)	72.382% (66.667%-78.378%)	83.13% (79.31%-87.29%)	66.29% (60.49%-72.22%)	3.0051 (2.8665-3.1418)	76.5% (69.7%-82.83%)	78.72% (70.31%-86.67%)	72.692% (60.604%-83.87%)	83.17% (74.6%-90.64%)	66.56% (54.76%-78.57%)	3.0115 (2.7388-3.2723)
At least 3 of FATHMM (cancer), Mutation Assessor, MutationTaster, SIFT	78.41% (75.25%-81.82%)	77.64% (73.6%-81.74%)	79.736% (74.647%-85.507%)	86.88% (83.33%-90.66%)	67.34% (61.8%-72.84%)	3.1160 (2.9867-3.2488)	78.54% (71.72%-84.85%)	77.68% (69.23%-85.71%)	80.005% (69.439%-90.32%)	86.94% (79.62%-94.12%)	67.62% (56.52%-78.73%)	3.1224 (2.8424-3.3810)
At least 3 of FATHMM (cancer), Mutation Assessor, MutationTaster, SIFT, VEST	75.03% (71.72%-78.28%)	81.88% (78.33%-85.83%)	63.171% (57.138%-69.565%)	79.35% (75.41%-83.34%)	66.85% (60.87%-73.24%)	2.9126 (2.7715-3.0520)	75.2% (68.69%-81.82%)	81.96% (73.84%-88.89%)	63.577% (51.427%-75.68%)	79.42% (71.42%-86.96%)	67.25% (54.05%-79.49%)	2.9221 (2.6377-3.2030)
At least 3 of FATHMM (cancer), Mutation Assessor, MutationTaster, VEST	77.76% (74.24%-80.81%)	77.13% (73.17%-81.3%)	78.858% (73.418%-84.51%)	86.31% (82.61%-90.18%)	66.6% (61.18%-72.23%)	3.0890 (2.9535-3.2199)	77.81% (71.72%-84.85%)	77.11% (68.52%-85.08%)	79.013% (67.742%-89.75%)	86.3% (78.57%-93.22%)	66.79% (55.56%-77.78%)	3.0921 (2.8204-3.3551)
At least 3 of FATHMM (cancer), Mutation Assessor, PolyPhen-2	68.65% (65.15%-72.23%)	61.16% (56.41%-66.09%)	81.582% (76.623%-87.144%)	85.16% (81.11%-89.78%)	54.85% (49.56%-59.83%)	2.8276 (2.6888-2.9672)	68.77% (61.59%-75.76%)	61.16% (51.46%-70.42%)	81.816% (71.053%-92.11%)	85.22% (76.08%-93.48%)	55.1% (44.9%-65.45%)	2.8329 (2.5546-3.1060)
At least 3 of FATHMM (cancer), Mutation Assessor, PolyPhen-2, PROVEAN	71.65% (68.18%-75.25%)	69.14% (64.75%-74.02%)	75.995% (70.267%-81.818%)	83.28% (79.21%-87.62%)	58.75% (53.19%-64.77%)	2.8716 (2.7341-3.0116)	71.84% (64.65%-78.79%)	69.15% (59.38%-77.78%)	76.455% (64.861%-87.81%)	83.44% (75.43%-91.67%)	59.07% (47.73%-70.37%)	2.8811 (2.6030-3.1516)
At least 3 of FATHMM (cancer), Mutation Assessor, PolyPhen-2, PROVEAN, SIFT	72.71% (69.19%-76.26%)	78.19% (74.4%-82.11%)	63.227% (57.143%-69.565%)	78.62% (74.57%-82.79%)	62.64% (56.52%-68.66%)	2.8267 (2.6801-2.9731)	72.76% (65.66%-79.8%)	78.17% (70%-85.71%)	63.472% (51.189%-75.77%)	78.59% (70.31%-86.89%)	62.87% (50%-75.02%)	2.8310 (2.5339-3.1270)
At least 3 of FATHMM (cancer), Mutation Assessor, PolyPhen-2, PROVEAN, VEST	73.06% (69.7%-76.26%)	78.74% (75%-82.79%)	63.234% (57.333%-69.444%)	78.73% (74.78%-82.81%)	63.24% (57.35%-69.57%)	2.8395 (2.6960-2.9846)	73.07% (66.67%-79.8%)	78.67% (70.31%-86.45%)	63.462% (51.351%-75.76%)	78.68% (70.18%-86.89%)	63.41% (50%-75%)	2.8422 (2.5506-3.1257)
At least 3 of FATHMM (cancer), Mutation Assessor, PolyPhen-2, SIFT	72.69% (69.19%-76.26%)	75.52% (71.43%-80%)	67.788% (61.903%-74.286%)	80.21% (76.07%-84.48%)	61.56% (55.7%-67.57%)	2.8508 (2.7063-2.9975)	72.8% (65.66%-79.8%)	75.53% (66.67%-83.62%)	68.104% (55.548%-80.01%)	80.24% (72.13%-88.33%)	61.85% (50%-73.68%)	2.8573 (2.5568-3.1533)
At least 3 of FATHMM (cancer), Mutation Assessor, PolyPhen-2, SIFT, VEST	69.95% (66.16%-73.23%)	80.29% (76.42%-84.25%)	52.067% (45.708%-58.333%)	74.33% (70.23%-78.42%)	60.44% (53.57%-67.21%)	2.6714 (2.5158-2.8174)	70.2% (63.64%-77.78%)	80.36% (72.31%-87.88%)	52.749% (40%-65.62%)	74.47% (66.18%-82.61%)	61.01% (46.67%-74.29%)	2.6859 (2.3884-2.9992)
At least 3 of FATHMM (cancer), Mutation Assessor, PolyPhen-2, VEST	73.72% (70.2%-77.27%)	76.6% (72.44%-80.87%)	68.737% (62.5%-74.648%)	80.9% (76.92%-85%)	62.95% (57.14%-69.33%)	2.8919 (2.7444-3.0343)	73.77% (66.67%-80.81%)	76.56% (68.17%-84.75%)	68.971% (57.133%-80.77%)	80.87% (72.41%-88.71%)	63.17% (50%-74.36%)	2.8958 (2.6076-3.1792)
At least 3 of FATHMM (cancer), Mutation Assessor, PROVEAN	59.27% (55.56%-63.14%)	43.17% (38.4%-48.25%)	87.119% (82.609%-91.67%)	85.28% (80%-90.62%)	46.99% (42.11%-51.54%)	2.6256 (2.4901-2.7641)	59.23% (51.49%-66.67%)	42.89% (32.79%-52.64%)	87.233% (78.042%-96.88%)	85.2% (73.9%-96.43%)	47.09% (37.7%-57.14%)	2.6242 (2.3373-2.8981)
At least 3 of FATHMM (cancer), Mutation Assessor, PROVEAN, SIFT	71.65% (68.18%-75.25%)	69.63% (65.12%-74.38%)	75.14% (69.565%-81.431%)	82.89% (78.64%-87.16%)	58.86% (53.12%-64.37%)	2.8652 (2.7234-3.0087)	71.84% (64.65%-78.79%)	69.75% (60.31%-78.57%)	75.401% (62.857%-86.49%)	82.95% (74.57%-90.91%)	59.21% (47.91%-70.37%)	2.8732 (2.5868-3.1567)
At least 3 of FATHMM (cancer), Mutation Assessor, PROVEAN, SIFT, VEST	70.67% (67.17%-74.24%)	76.61% (72.73%-80.6%)	60.391% (53.968%-66.667%)	76.98% (72.8%-81.15%)	59.88% (53.62%-66.2%)	2.7386 (2.5812-2.8780)	70.79% (63.64%-77.78%)	76.56% (68.32%-84.48%)	60.875% (48.381%-73.53%)	77.04% (68.66%-85.48%)	60.18% (48.14%-71.79%)	2.7466 (2.4618-3.0605)
At least 3 of FATHMM (cancer), Mutation Assessor, PROVEAN, VEST	68.65% (65.14%-72.22%)	64.9% (60.32%-69.75%)	75.132% (69.694%-81.159%)	81.85% (77.32%-86.54%)	55.32% (49.5%-61%)	2.7721 (2.6300-2.9180)	68.75% (61.62%-75.78%)	64.84% (55.22%-74.19%)	75.44% (63.631%-86.49%)	81.89% (72.73%-90.57%)	55.56% (44.44%-66.67%)	2.7773 (2.4827-3.0668)
At least 3 of FATHMM (cancer), Mutation Assessor, SIFT	70.34% (66.67%-74.24%)	62.77% (58.06%-67.72%)	83.43% (78.462%-88.893%)	86.75% (82.76%-91.11%)	56.44% (50.94%-61.77%)	2.8940 (2.7590-3.0321)	70.42% (62.63%-77.78%)	62.73% (53.02%-71.93%)	83.602% (72.967%-93.75%)	86.78% (78.26%-94.87%)	56.65% (46%-67.27%)	2.8976 (2.6143-3.1726)
At least 3 of FATHMM (cancer), Mutation Assessor, SIFT, VEST	70.67% (67.17%-74.24%)	75.01% (70.83%-79.2%)	63.171% (57.138%-69.565%)	77.88% (73.73%-82.12%)	59.38% (53.24%-65.75%)	2.7544 (2.6084-2.8987)	70.77% (63.64%-77.78%)	74.95% (66.15%-83.1%)	63.577% (51.427%-75.68%)	77.91% (69.09%-85.96%)	59.66% (47.5%-71.43%)	2.7610 (2.4724-3.0530)
At least 3 of FATHMM (cancer), Mutation Assessor, VEST	64.63% (60.61%-68.69%)	54.81% (49.62%-59.84%)	81.607% (76.389%-87.324%)	83.74% (79.07%-88.75%)	51.08% (45.87%-56.3%)	2.7124 (2.5667-2.8657)	64.69% (56.57%-72.73%)	54.73% (44.64%-64.29%)	81.761% (71.053%-92.11%)	83.73% (73.81%-92.86%)	51.27% (41.18%-61.54%)	2.7149 (2.4062-3.0132)
At least 3 of FATHMM (cancer), MutationTaster, PolyPhen-2	76.41% (72.73%-79.8%)	67.52% (63.08%-72.31%)	91.775% (88.571%-95.775%)	93.41% (90.8%-96.7%)	62.04% (56.73%-67%)	3.1474 (3.0334-3.2634)	76.48% (69.7%-83.84%)	67.61% (57.81%-76.67%)	91.688% (84.211%-97.5%)	93.3% (86.67%-97.96%)	62.26% (52.08%-72.88%)	3.1486 (2.9107-3.3792)
At least 3 of FATHMM (cancer), MutationTaster, PolyPhen-2, PROVEAN	77.38% (74.24%-80.81%)	74.95% (70.94%-79.13%)	81.581% (76.389%-86.957%)	87.55% (84.16%-91.45%)	65.32% (60.23%-70.65%)	3.0940 (2.9692-3.2215)	77.57% (70.71%-83.84%)	75.09% (66.65%-83.33%)	81.802% (71.429%-91.89%)	87.62% (79.66%-94.44%)	65.68% (55.1%-75.62%)	3.1019 (2.8442-3.3563)
At least 3 of FATHMM (cancer), MutationTaster, PolyPhen-2, PROVEAN, SIFT	78.07% (74.75%-81.31%)	82.95% (79.53%-86.72%)	69.641% (64%-75.714%)	82.53% (78.62%-86.36%)	70.26% (64.79%-76.06%)	3.0538 (2.9207-3.2003)	78.19% (71.72%-84.85%)	83.02% (75.38%-90.16%)	69.907% (57.143%-81.58%)	82.55% (74.99%-90.17%)	70.59% (58.82%-82.35%)	3.0606 (2.7636-3.3354)
At least 3 of FATHMM (cancer), MutationTaster, PolyPhen-2, PROVEAN, VEST	75.76% (72.73%-79.29%)	81.37% (77.69%-85.25%)	66.052% (60.267%-72.312%)	80.56% (76.74%-84.68%)	67.22% (61.19%-73.24%)	2.9521 (2.8081-3.0887)	75.75% (68.69%-81.82%)	81.38% (73.43%-88.89%)	66.073% (54.054%-77.78%)	80.45% (72.41%-88.24%)	67.41% (55.26%-78.95%)	2.9531 (2.6769-3.2348)
At least 3 of FATHMM (cancer), MutationTaster, PolyPhen-2, SIFT	80.77% (77.78%-83.84%)	82.95% (79.53%-86.72%)	76.989% (71.826%-82.671%)	86.17% (82.79%-89.85%)	72.31% (67.09%-77.63%)	3.1842 (3.0570-3.3121)	80.89% (74.75%-86.87%)	83.02% (75.38%-90.16%)	77.234% (65.788%-87.5%)	86.21% (78.46%-93.1%)	72.62% (61.76%-83.72%)	3.1908 (2.9279-3.4457)
At least 3 of FATHMM (cancer), MutationTaster, PolyPhen-2, SIFT, VEST	73.68% (70.2%-77.27%)	85.58% (82.35%-89.17%)	53.094% (46.476%-59.155%)	75.93% (71.94%-80%)	68.04% (61.54%-74.58%)	2.8264 (2.6719-2.9697)	73.86% (66.67%-80.81%)	85.74% (78.46%-92.19%)	53.425% (41.173%-66.67%)	75.95% (68%-83.82%)	68.6% (55%-81.48%)	2.8372 (2.5443-3.1304)
At least 3 of FATHMM (cancer), MutationTaster, PolyPhen-2, VEST	77.13% (73.74%-80.3%)	79.79% (75.81%-83.74%)	72.53% (67.105%-78.571%)	83.39% (79.64%-87.4%)	67.49% (61.73%-73.08%)	3.0321 (2.8949-3.1653)	77.05% (70.71%-83.84%)	79.75% (71.43%-87.5%)	72.383% (60%-83.87%)	83.2% (75%-90.63%)	67.58% (56.09%-79.31%)	3.0292 (2.7631-3.3018)
At least 3 of FATHMM (cancer), MutationTaster, PROVEAN	65.01% (61.11%-68.69%)	46.88% (42.06%-52.11%)	96.354% (94.203%-98.667%)	95.69% (93.1%-98.44%)	51.2% (46.1%-55.88%)	2.9012 (2.7939-3.0053)	64.93% (57.58%-72.73%)	46.66% (36.07%-56.53%)	96.276% (91.176%-100%)	95.55% (89.47%-100%)	51.26% (41.79%-61.43%)	2.8974 (2.6972-3.1070)
At least 3 of FATHMM (cancer), MutationTaster, PROVEAN, SIFT	76.06% (72.73%-79.29%)	72.33% (67.8%-76.8%)	82.508% (77.465%-87.842%)	87.73% (84.26%-91.67%)	63.29% (58.06%-68.63%)	3.0586 (2.9333-3.1898)	76.16% (69.7%-82.83%)	72.34% (63.08%-80.95%)	82.681% (71.786%-92.5%)	87.76% (80%-94.45%)	63.52% (53.19%-74%)	3.0630 (2.8044-3.3126)
At least 3 of FATHMM (cancer), MutationTaster, PROVEAN, SIFT, VEST	76.04% (72.73%-79.29%)	81.38% (77.86%-85.48%)	66.814% (60.868%-73.239%)	80.91% (77.17%-85.04%)	67.48% (61.19%-73.24%)	2.9659 (2.8227-3.1020)	76.19% (69.7%-82.83%)	81.37% (73.43%-88.89%)	67.301% (54.996%-79.41%)	81.02% (73.02%-88.71%)	67.79% (56.24%-79.56%)	2.9748 (2.6997-3.2571)
At least 3 of FATHMM (cancer), MutationTaster, PROVEAN, VEST	74.38% (70.71%-77.79%)	69.66% (65.32%-74.38%)	82.539% (77.465%-87.879%)	87.34% (83.67%-91.43%)	61.14% (55.67%-66.67%)	3.0068 (2.8702-3.1474)	74.47% (67.65%-81.82%)	69.7% (60%-78.13%)	82.633% (71.429%-92.31%)	87.32% (78.72%-94.23%)	61.38% (50%-72.1%)	3.0103 (2.7237-3.2826)
At least 3 of FATHMM (cancer), MutationTaster, SIFT	76.76% (73.23%-79.8%)	67% (62.39%-71.78%)	93.622% (90.541%-97.183%)	94.78% (92.22%-97.75%)	62.13% (57.01%-67%)	3.1753 (3.0673-3.2803)	76.79% (70.71%-83.84%)	67.05% (57.14%-76.19%)	93.483% (86.667%-100%)	94.64% (88.64%-100%)	62.31% (52.17%-72.88%)	3.1748 (2.9641-3.3902)
At least 3 of FATHMM (cancer), MutationTaster, SIFT, VEST	77.73% (74.75%-80.81%)	79.77% (75.83%-84%)	74.194% (68.829%-80.282%)	84.24% (80.67%-88.18%)	67.96% (62.33%-73.97%)	3.0617 (2.9243-3.1905)	77.87% (71.72%-83.84%)	79.8% (71.43%-87.3%)	74.553% (62.493%-85.37%)	84.32% (76.36%-91.53%)	68.26% (56.41%-79.08%)	3.0693 (2.8145-3.3354)
At least 3 of FATHMM (cancer), MutationTaster, VEST	73.39% (69.7%-77.27%)	62.24% (57.48%-67.18%)	92.677% (89.55%-96.055%)	93.62% (90.91%-96.63%)	58.67% (53.27%-63.89%)	3.0721 (2.9577-3.1879)	73.41% (65.66%-80.81%)	62.21% (52.38%-71.22%)	92.634% (85.294%-100%)	93.53% (86.96%-100%)	58.82% (48.15%-69.64%)	3.0719 (2.8469-3.3165)
At least 3 of FATHMM (cancer), PolyPhen-2, PROVEAN	61.28% (57.07%-65.15%)	46.89% (41.79%-52%)	86.16% (81.579%-90.667%)	85.42% (80.6%-90.63%)	48.41% (43.31%-53.29%)	2.6688 (2.5316-2.8082)	61.27% (53.54%-69.7%)	46.61% (36.07%-57.14%)	86.391% (77.143%-95%)	85.44% (75%-94.45%)	48.53% (38.79%-58.73%)	2.6697 (2.3840-2.9457)
At least 3 of FATHMM (cancer), PolyPhen-2, PROVEAN, SIFT	72.36% (68.69%-75.76%)	71.27% (66.94%-75.78%)	74.238% (68.419%-80.822%)	82.71% (78.57%-87.16%)	59.9% (54.12%-65.52%)	2.8812 (2.7416-3.0230)	72.45% (65.66%-79.8%)	71.27% (61.9%-79.69%)	74.45% (61.765%-86.11%)	82.72% (74.07%-91.07%)	60.14% (49.97%-71.12%)	2.8859 (2.6040-3.1650)
At least 3 of FATHMM (cancer), PolyPhen-2, PROVEAN, SIFT, VEST	69.97% (66.67%-73.23%)	79.78% (76%-83.6%)	53.007% (46.377%-59.155%)	74.59% (70.5%-78.63%)	60.25% (53.7%-66.68%)	2.6762 (2.5279-2.8157)	70.16% (63.64%-76.77%)	79.8% (71.67%-87.33%)	53.604% (41.016%-65.71%)	74.69% (66.67%-82.81%)	60.72% (46.66%-74.19%)	2.6881 (2.3970-2.9805)
At least 3 of FATHMM (cancer), PolyPhen-2, PROVEAN, VEST	69.66% (66.16%-73.23%)	68.09% (63.63%-72.73%)	72.381% (66.667%-78.574%)	81% (76.7%-85.71%)	56.74% (51.06%-62.22%)	2.7820 (2.6422-2.9274)	69.77% (62.63%-76.77%)	68.07% (58.46%-76.92%)	72.676% (60%-84.38%)	81.04% (71.43%-89.8%)	57% (45.83%-68.63%)	2.7878 (2.5045-3.0646)
At least 3 of FATHMM (cancer), PolyPhen-2, SIFT	71.68% (68.67%-75.25%)	65.95% (61.54%-71.07%)	81.586% (76.389%-87.016%)	86.1% (82.29%-90.22%)	58.09% (52.77%-63.37%)	2.9172 (2.7937-3.0504)	71.78% (64.65%-77.8%)	65.94% (56.05%-75%)	81.777% (71.053%-91.67%)	86.13% (78.05%-93.88%)	58.31% (48%-68.75%)	2.9216 (2.6559-3.1735)
At least 3 of FATHMM (cancer), PolyPhen-2, SIFT, VEST	70.65% (67.17%-74.24%)	78.72% (75%-82.76%)	56.674% (50.666%-62.687%)	75.85% (71.76%-80%)	60.64% (54.38%-67.14%)	2.7189 (2.5655-2.8611)	70.83% (63.64%-77.78%)	78.71% (70.77%-86.37%)	57.279% (45%-70.27%)	75.96% (67.69%-84.38%)	61.04% (47.22%-73.69%)	2.7299 (2.4464-3.0292)
At least 3 of FATHMM (cancer), PolyPhen-2, VEST	66.64% (63.13%-70.71%)	59.57% (54.92%-64.57%)	78.859% (73.611%-84.507%)	82.97% (78.82%-87.78%)	53.01% (47.52%-58.1%)	2.7440 (2.6040-2.8976)	66.72% (58.59%-73.74%)	59.57% (49.21%-68.42%)	78.986% (68.282%-89.66%)	82.94% (73.33%-91.43%)	53.23% (43.09%-64%)	2.7473 (2.4545-3.0280)
At least 3 of FATHMM (cancer), PROVEAN, SIFT	62.28% (58.59%-66.16%)	48.99% (43.94%-53.97%)	85.239% (80.556%-90.141%)	85.16% (80.3%-90.14%)	49.15% (44.16%-53.97%)	2.6854 (2.5470-2.8331)	62.32% (54.55%-69.7%)	48.8% (38.71%-59.02%)	85.475% (75.758%-94.59%)	85.21% (75%-94.45%)	49.31% (39.68%-59.32%)	2.6880 (2.3867-2.9612)
At least 3 of FATHMM (cancer), PROVEAN, SIFT, VEST	67.96% (64.65%-71.72%)	66.49% (62.1%-71.1%)	70.508% (64.384%-76.817%)	79.58% (75.26%-84.26%)	54.88% (48.94%-60.44%)	2.7146 (2.5668-2.8642)	68.11% (60.61%-74.75%)	66.47% (56.92%-75.36%)	70.898% (58.333%-82.51%)	79.67% (70.21%-88.24%)	55.19% (44%-66.67%)	2.7223 (2.4283-3.0113)
At least 3 of FATHMM (cancer), PROVEAN, VEST	59.96% (56.06%-64.14%)	44.76% (39.67%-49.61%)	86.216% (81.333%-90.667%)	84.88% (80%-90.17%)	47.44% (42.31%-52.31%)	2.6330 (2.4920-2.7757)	59.89% (51.52%-67.68%)	44.49% (34.43%-54.42%)	86.275% (76.919%-95%)	84.75% (74.06%-94.29%)	47.52% (38.03%-57.82%)	2.6304 (2.3359-2.9117)
At least 3 of FATHMM (cancer), SIFT, VEST	66.63% (63.13%-70.71%)	60.12% (55.38%-64.85%)	77.888% (72.603%-84.001%)	82.46% (78.16%-87.21%)	53.04% (47.12%-58.25%)	2.7351 (2.5938-2.8832)	66.73% (58.59%-73.74%)	60.06% (50%-69.49%)	78.149% (65.714%-88.58%)	82.51% (73.16%-91.11%)	53.26% (42.31%-64.15%)	2.7398 (2.4438-3.0182)
At least 3 of FATHMM (missense), Mutation Assessor, MutationTaster	71.07% (67.68%-74.24%)	58.02% (52.89%-62.9%)	93.622% (90.541%-97.183%)	94.02% (91.25%-97.4%)	56.33% (51.28%-61.21%)	3.0200 (2.9160-3.1310)	70.99% (64.65%-77.78%)	57.87% (48.32%-68.34%)	93.483% (86.667%-100%)	93.85% (87.18%-100%)	56.39% (46.77%-66.13%)	3.0159 (2.7898-3.2253)
At least 3 of FATHMM (missense), Mutation Assessor, MutationTaster, PolyPhen-2	77.78% (74.75%-81.31%)	77.13% (73.13%-81.67%)	78.894% (73.913%-84.286%)	86.34% (82.73%-90.09%)	66.61% (61.11%-72.5%)	3.0897 (2.9547-3.2261)	77.78% (70.71%-83.84%)	77.1% (68.42%-85%)	78.9% (68.293%-88.89%)	86.24% (78.43%-93.33%)	66.76% (54.76%-77.27%)	3.0900 (2.8119-3.3538)
At least 3 of FATHMM (missense), Mutation Assessor, MutationTaster, PolyPhen-2, PROVEAN	78.74% (75.76%-81.82%)	81.36% (77.69%-85.27%)	74.213% (68.829%-80%)	84.51% (80.99%-88.33%)	69.72% (64.28%-75.64%)	3.0980 (2.9655-3.2308)	78.88% (72.73%-84.85%)	81.41% (73.44%-88.89%)	74.507% (63.158%-85.71%)	84.56% (76.92%-91.67%)	70.03% (58.14%-81.26%)	3.1050 (2.8362-3.3694)
At least 3 of FATHMM (missense), Mutation Assessor, MutationTaster, PolyPhen-2, SIFT	77.08% (73.74%-80.3%)	82.97% (79.37%-86.92%)	66.892% (60.606%-73.244%)	81.25% (77.6%-85.04%)	69.44% (63.77%-75.76%)	3.0055 (2.8654-3.1416)	77.15% (70.71%-83.84%)	82.97% (75.38%-89.86%)	67.115% (54.539%-78.79%)	81.24% (73.68%-88.74%)	69.67% (56.76%-81.25%)	3.0099 (2.7287-3.2843)
At least 3 of FATHMM (missense), Mutation Assessor, MutationTaster, PolyPhen-2, VEST	75.09% (71.72%-78.28%)	82.98% (79.34%-86.96%)	61.461% (55.221%-67.901%)	78.82% (74.82%-82.84%)	67.62% (61.43%-74.19%)	2.9088 (2.7655-3.0542)	75.06% (68.69%-81.82%)	82.97% (74.99%-90.17%)	61.479% (48.267%-73.69%)	78.7% (70.59%-86.36%)	67.78% (54.83%-80.56%)	2.9093 (2.6146-3.1939)
At least 3 of FATHMM (missense), Mutation Assessor, MutationTaster, PROVEAN	76.1% (72.73%-79.29%)	70.22% (65.67%-74.8%)	86.261% (81.941%-91.178%)	89.84% (86.41%-93.41%)	62.62% (57.28%-68%)	3.0894 (2.9646-3.2141)	76.07% (69.7%-82.83%)	70.16% (61.01%-78.79%)	86.171% (76.923%-94.44%)	89.71% (82.69%-96.15%)	62.72% (52%-73.08%)	3.0876 (2.8374-3.3389)
At least 3 of FATHMM (missense), Mutation Assessor, MutationTaster, PROVEAN, SIFT	77.07% (73.74%-80.32%)	78.72% (74.8%-82.81%)	74.223% (68.657%-80.282%)	84.08% (80.17%-87.9%)	66.85% (60.98%-72.62%)	3.0387 (2.9007-3.1789)	77.17% (70.68%-83.84%)	78.72% (70%-86.44%)	74.491% (62.162%-86.05%)	84.11% (76.56%-91.8%)	67.09% (55%-78.38%)	3.0441 (2.7631-3.3145)
At least 3 of FATHMM (missense), Mutation Assessor, MutationTaster, PROVEAN, VEST	75.05% (71.72%-78.28%)	77.13% (73.17%-81.3%)	71.456% (65.333%-77.465%)	82.37% (78.57%-86.4%)	64.37% (58.44%-70.37%)	2.9533 (2.8092-3.0971)	75.14% (68.69%-81.82%)	77.1% (68.33%-85.25%)	71.756% (60%-83.33%)	82.4% (74.19%-89.86%)	64.61% (52.63%-76.33%)	2.9586 (2.6576-3.2450)
At least 3 of FATHMM (missense), Mutation Assessor, MutationTaster, SIFT	77.76% (74.75%-80.81%)	74.98% (70.73%-79.51%)	82.554% (77.941%-87.882%)	88.14% (84.47%-91.84%)	65.61% (60.24%-71.43%)	3.1128 (2.9856-3.2449)	77.82% (71.72%-83.84%)	75.02% (66.1%-83.1%)	82.585% (71.429%-92.69%)	88.09% (80.77%-94.92%)	65.82% (53.99%-76.2%)	3.1152 (2.8471-3.3660)
At least 3 of FATHMM (missense), Mutation Assessor, MutationTaster, SIFT, VEST	73.34% (69.7%-76.77%)	80.84% (77.16%-84.92%)	60.353% (53.846%-67.124%)	77.91% (74.01%-82.03%)	64.56% (58.32%-71.43%)	2.8366 (2.6851-2.9850)	73.52% (66.67%-80.81%)	80.84% (72.88%-88.14%)	60.916% (46.149%-72.73%)	78.02% (70%-86.11%)	64.94% (51.51%-76.92%)	2.8472 (2.5467-3.1465)
At least 3 of FATHMM (missense), Mutation Assessor, MutationTaster, VEST	75.75% (72.22%-78.79%)	73.43% (69.53%-77.87%)	79.772% (74.286%-85.294%)	86.26% (82.41%-90.27%)	63.45% (57.83%-69.01%)	3.0290 (2.8879-3.1573)	75.76% (69.7%-82.83%)	73.33% (64.7%-81.25%)	79.903% (68.956%-90%)	86.23% (78%-93.34%)	63.58% (52.27%-74.47%)	3.0304 (2.7631-3.3062)
At least 3 of FATHMM (missense), Mutation Assessor, PolyPhen-2	68.37% (64.65%-71.73%)	59.09% (53.97%-63.78%)	84.386% (79.71%-89.189%)	86.75% (82.76%-90.91%)	54.4% (49.11%-59.32%)	2.8463 (2.7190-2.9781)	68.32% (61.59%-75.76%)	58.9% (49.21%-68.86%)	84.418% (74.357%-93.55%)	86.65% (78.26%-94.6%)	54.48% (44.64%-64.52%)	2.8445 (2.5785-3.0978)
At least 3 of FATHMM (missense), Mutation Assessor, PolyPhen-2, PROVEAN	73.04% (69.7%-76.77%)	69.18% (64.71%-73.91%)	79.705% (74.242%-84.851%)	85.5% (81.73%-89.36%)	59.93% (54.35%-65.59%)	2.9432 (2.8026-3.0760)	73.11% (65.66%-79.8%)	69.05% (59.38%-77.78%)	80.025% (69.444%-90.25%)	85.57% (77.36%-93.1%)	60.1% (49.09%-70.84%)	2.9475 (2.6796-3.2262)
At least 3 of FATHMM (missense), Mutation Assessor, PolyPhen-2, PROVEAN, SIFT	73.03% (69.7%-76.26%)	78.18% (74.36%-82.58%)	64.119% (57.575%-70.669%)	79.03% (74.99%-83.05%)	62.96% (56.72%-69.01%)	2.8428 (2.6942-2.9908)	73.13% (66.67%-79.8%)	78.19% (69.23%-85.94%)	64.408% (51.429%-77.42%)	79.04% (71.19%-87.1%)	63.23% (50%-75.76%)	2.8487 (2.5621-3.1454)
At least 3 of FATHMM (missense), Mutation Assessor, PolyPhen-2, PROVEAN, VEST	70.7% (67.17%-74.24%)	77.69% (73.77%-81.74%)	58.62% (52.113%-64.865%)	76.45% (72.36%-80.92%)	60.31% (53.97%-66.67%)	2.7307 (2.5770-2.8767)	70.71% (63.64%-77.78%)	77.58% (69.23%-85.48%)	58.897% (45.714%-71.05%)	76.4% (67.69%-84.38%)	60.48% (47.06%-72.97%)	2.7336 (2.4395-3.0338)
At least 3 of FATHMM (missense), Mutation Assessor, PolyPhen-2, SIFT	72.02% (68.67%-75.76%)	73.39% (69.1%-77.95%)	69.635% (63.747%-75.758%)	80.69% (76.57%-84.82%)	60.22% (54.22%-66.28%)	2.8394 (2.6871-2.9831)	72.12% (64.65%-78.81%)	73.41% (64.28%-81.97%)	69.885% (57.491%-81.82%)	80.71% (72.72%-88.68%)	60.48% (48.57%-72.34%)	2.8448 (2.5510-3.1437)
At least 3 of FATHMM (missense), Mutation Assessor, PolyPhen-2, SIFT, VEST	68.27% (64.65%-71.72%)	79.25% (75.38%-83.34%)	49.29% (42.62%-55.714%)	72.99% (68.88%-77.37%)	57.87% (50.77%-65.08%)	2.5939 (2.4368-2.7427)	68.5% (61.62%-75.76%)	79.25% (71.19%-87.1%)	50.038% (37.5%-62.86%)	73.13% (64.51%-81.43%)	58.41% (44.12%-72.41%)	2.6083 (2.3008-2.9125)
At least 3 of FATHMM (missense), Mutation Assessor, PolyPhen-2, VEST	70.03% (66.16%-73.74%)	73.43% (69.17%-78.23%)	64.146% (57.745%-70.372%)	77.98% (73.68%-82.35%)	58.27% (51.95%-64.64%)	2.7382 (2.5833-2.8869)	70.04% (62.63%-77.78%)	73.33% (64.18%-81.82%)	64.359% (51.61%-76.75%)	77.92% (68.97%-86.44%)	58.43% (45.65%-70.46%)	2.7404 (2.4417-3.0521)
At least 3 of FATHMM (missense), Mutation Assessor, PROVEAN	57.28% (53.03%-61.11%)	38.94% (34.38%-43.7%)	88.993% (84.848%-93.058%)	85.95% (80.7%-91.38%)	45.74% (40.85%-50.35%)	2.5962 (2.4640-2.7304)	57.15% (49.49%-65.66%)	38.58% (28.81%-48.28%)	88.965% (80.643%-97.22%)	85.73% (74.07%-96.43%)	45.77% (36.92%-55.56%)	2.5904 (2.3175-2.8581)
At least 3 of FATHMM (missense), Mutation Assessor, PROVEAN, SIFT	72.69% (69.19%-76.26%)	70.21% (65.67%-74.8%)	76.973% (71.622%-82.895%)	84.06% (80%-88.24%)	59.91% (54.35%-65.52%)	2.9115 (2.7684-3.0467)	72.8% (65.66%-79.8%)	70.19% (61.19%-79.03%)	77.24% (65.378%-87.89%)	84.11% (75.92%-92.31%)	60.14% (48.89%-71.11%)	2.9167 (2.6465-3.1888)
At least 3 of FATHMM (missense), Mutation Assessor, PROVEAN, SIFT, VEST	69.64% (66.16%-73.23%)	76.06% (72.03%-80.45%)	58.511% (52.174%-65.217%)	76.02% (71.88%-80.3%)	58.56% (52.05%-65.22%)	2.6916 (2.5340-2.8413)	69.82% (62.63%-76.77%)	76.05% (67.69%-84.13%)	59.089% (45.455%-71.8%)	76.14% (67.8%-84.38%)	58.94% (45.71%-71.43%)	2.7021 (2.3922-3.0162)
At least 3 of FATHMM (missense), Mutation Assessor, PROVEAN, VEST	69.36% (65.66%-73.23%)	65.48% (60.8%-70%)	76.046% (70.423%-81.821%)	82.54% (78.12%-86.73%)	56.03% (50.5%-61.7%)	2.8009 (2.6519-2.9489)	69.36% (61.62%-76.77%)	65.28% (55.88%-74.24%)	76.327% (64.865%-87.5%)	82.55% (73.58%-91.49%)	56.15% (44.68%-66.67%)	2.8031 (2.5055-3.1075)
At least 3 of FATHMM (missense), Mutation Assessor, SIFT	70.06% (66.67%-73.74%)	61.24% (56.2%-66.13%)	85.304% (80.881%-90.141%)	87.82% (83.91%-91.96%)	56% (50.89%-60.91%)	2.9035 (2.7760-3.0348)	69.98% (62.63%-76.77%)	61% (51.56%-70.77%)	85.334% (75.659%-94.12%)	87.73% (79.41%-95.45%)	56.04% (46%-66.1%)	2.9010 (2.6173-3.1450)
At least 3 of FATHMM (missense), Mutation Assessor, SIFT, VEST	68.63% (64.65%-72.22%)	72.35% (68.07%-76.8%)	62.193% (56.059%-68.919%)	76.79% (72.5%-81.2%)	56.53% (50%-62.82%)	2.6787 (2.5244-2.8337)	68.79% (61.62%-76.77%)	72.3% (63.33%-80.7%)	62.739% (48.571%-75%)	76.91% (68.33%-85.46%)	56.87% (43.59%-69.23%)	2.6881 (2.3743-2.9946)
At least 3 of FATHMM (missense), Mutation Assessor, VEST	64.67% (60.61%-68.69%)	53.27% (48%-58.33%)	84.362% (79.71%-89.189%)	85.49% (81.25%-90.14%)	51.08% (45.61%-56.04%)	2.7420 (2.6022-2.8757)	64.61% (56.57%-72.73%)	53.01% (42.85%-63.33%)	84.474% (74.286%-93.55%)	85.42% (75.76%-93.75%)	51.16% (41.07%-61.77%)	2.7406 (2.4746-3.0177)
At least 3 of FATHMM (missense), MutationTaster, PolyPhen-2	72.77% (69.19%-76.26%)	61.74% (56.91%-66.42%)	91.824% (88.567%-95.714%)	92.89% (90%-96.34%)	58.13% (53.15%-63.06%)	3.0458 (2.9324-3.1592)	72.64% (65.66%-79.8%)	61.59% (51.67%-71.21%)	91.562% (84.211%-97.5%)	92.62% (86.05%-97.78%)	58.14% (48.33%-68%)	3.0391 (2.8083-3.2700)
At least 3 of FATHMM (missense), MutationTaster, PolyPhen-2, PROVEAN	78.45% (75.25%-81.82%)	75.52% (71.43%-79.84%)	83.512% (78.871%-88.732%)	88.79% (85.32%-92.38%)	66.37% (60.87%-71.59%)	3.1419 (3.0145-3.2658)	78.44% (71.72%-84.85%)	75.52% (66.67%-83.61%)	83.392% (72.973%-93.1%)	88.66% (81.48%-95.35%)	66.51% (55.56%-77.29%)	3.1408 (2.8889-3.3959)
At least 3 of FATHMM (missense), MutationTaster, PolyPhen-2, PROVEAN, SIFT	77.76% (74.75%-80.81%)	82.44% (78.81%-86.18%)	69.652% (63.768%-75.676%)	82.45% (78.69%-86.15%)	69.64% (64%-75.71%)	3.0418 (2.9091-3.1727)	77.82% (71.72%-83.84%)	82.44% (75%-89.55%)	69.846% (57.491%-81.82%)	82.44% (75%-90%)	69.87% (57.89%-81.09%)	3.0459 (2.7720-3.3164)
At least 3 of FATHMM (missense), MutationTaster, PolyPhen-2, PROVEAN, VEST	75.77% (72.22%-79.29%)	81.91% (78.23%-85.61%)	65.141% (59.155%-71.233%)	80.25% (76.42%-84.38%)	67.56% (61.64%-73.34%)	2.9486 (2.8095-3.0874)	75.73% (68.69%-82.83%)	81.9% (73.85%-89.23%)	65.11% (52.937%-77.15%)	80.12% (71.87%-87.5%)	67.71% (55.16%-80.01%)	2.9484 (2.6575-3.2295)
At least 3 of FATHMM (missense), MutationTaster, PolyPhen-2, SIFT	80.47% (77.27%-83.84%)	82.44% (78.81%-86.18%)	77.057% (71.233%-82.858%)	86.14% (82.76%-89.69%)	71.73% (66.23%-77.5%)	3.1737 (3.0419-3.3119)	80.47% (73.74%-86.87%)	82.44% (75%-89.55%)	77.035% (64.861%-88.37%)	86.05% (78.95%-93.1%)	71.89% (60.51%-82.51%)	3.1741 (2.8949-3.4332)
At least 3 of FATHMM (missense), MutationTaster, PolyPhen-2, SIFT, VEST	74.02% (70.71%-77.78%)	85.58% (82.26%-89.06%)	54.02% (47.297%-60.611%)	76.29% (72.53%-80.42%)	68.43% (61.82%-75%)	2.8432 (2.6922-2.9908)	74.18% (66.67%-80.81%)	85.73% (78.33%-92.19%)	54.314% (41.379%-67.5%)	76.31% (68.05%-84%)	68.94% (56%-82.14%)	2.8529 (2.5592-3.1468)
At least 3 of FATHMM (missense), MutationTaster, PolyPhen-2, VEST	76.8% (73.23%-79.8%)	80.33% (76.56%-84.38%)	70.681% (64.555%-76.623%)	82.57% (78.69%-86.51%)	67.52% (61.84%-73.42%)	3.0110 (2.8654-3.1503)	76.71% (70.71%-83.84%)	80.28% (72.13%-88.14%)	70.547% (58.06%-82.05%)	82.39% (74.58%-90%)	67.59% (55.26%-79.41%)	3.0080 (2.7300-3.2995)
At least 3 of FATHMM (missense), MutationTaster, PROVEAN	60.99% (57.07%-64.65%)	40.53% (35.94%-45.38%)	96.354% (94.203%-98.667%)	95.05% (92%-98.21%)	48.37% (43.54%-52.78%)	2.8031 (2.6947-2.9023)	60.86% (53.54%-68.69%)	40.2% (30.35%-49.28%)	96.276% (91.176%-100%)	94.88% (88%-100%)	48.4% (39.39%-57.89%)	2.7976 (2.5947-3.0148)
At least 3 of FATHMM (missense), MutationTaster, PROVEAN, SIFT	78.79% (75.76%-81.83%)	74.48% (70.25%-78.86%)	86.214% (81.815%-91.026%)	90.33% (87.04%-93.62%)	66.15% (60.67%-71.58%)	3.1718 (3.0528-3.3011)	78.79% (72.7%-84.85%)	74.41% (65.57%-82.81%)	86.252% (76.923%-94.6%)	90.29% (83.67%-96.36%)	66.26% (55.55%-76.6%)	3.1722 (2.9110-3.4138)
At least 3 of FATHMM (missense), MutationTaster, PROVEAN, SIFT, VEST	74.4% (70.71%-77.78%)	80.34% (76.42%-84.38%)	64.102% (57.971%-70.833%)	79.47% (75.57%-83.34%)	65.35% (58.82%-71.84%)	2.8926 (2.7444-3.0401)	74.44% (67.68%-81.82%)	80.25% (72.13%-88.14%)	64.408% (51.35%-76.67%)	79.47% (71.43%-87.5%)	65.51% (52.77%-77.5%)	2.8964 (2.6059-3.2000)
At least 3 of FATHMM (missense), MutationTaster, PROVEAN, VEST	75.78% (72.22%-79.29%)	70.77% (66.13%-75.4%)	84.431% (79.682%-89.189%)	88.72% (85.23%-92.38%)	62.56% (57.14%-68.27%)	3.0648 (2.9274-3.2013)	75.71% (68.69%-82.83%)	70.66% (61.02%-79.69%)	84.302% (74.286%-93.94%)	88.56% (81.39%-95.74%)	62.61% (51.06%-73.08%)	3.0613 (2.7855-3.3298)
At least 3 of FATHMM (missense), MutationTaster, SIFT	74.1% (70.71%-77.27%)	62.8% (57.85%-67.48%)	93.622% (90.541%-97.183%)	94.45% (91.86%-97.62%)	59.27% (54.24%-64.1%)	3.1015 (2.9960-3.2047)	74.03% (67.68%-80.81%)	62.67% (53.33%-72.42%)	93.483% (86.667%-100%)	94.3% (87.8%-100%)	59.33% (49.18%-69.64%)	3.0978 (2.8789-3.3080)
At least 3 of FATHMM (missense), MutationTaster, SIFT, VEST	77.08% (73.74%-80.3%)	78.73% (74.8%-82.93%)	74.193% (68.571%-80.007%)	84.07% (80.17%-88.03%)	66.86% (61.25%-73.17%)	3.0386 (2.8960-3.1798)	77.16% (70.71%-83.84%)	78.68% (70.17%-86.44%)	74.496% (61.765%-85.72%)	84.13% (76.27%-91.81%)	67.06% (54.54%-78.05%)	3.0436 (2.7533-3.3309)
At least 3 of FATHMM (missense), MutationTaster, VEST	70.1% (66.67%-73.74%)	57% (52%-62.02%)	92.727% (89.333%-96%)	93.13% (90.14%-96.2%)	55.5% (50.42%-60.5%)	2.9836 (2.8758-3.1018)	69.91% (62.63%-76.77%)	56.71% (46.67%-66.67%)	92.508% (85.706%-100%)	92.87% (86.11%-100%)	55.46% (45.31%-65.46%)	2.9754 (2.7397-3.1879)
At least 3 of FATHMM (missense), PolyPhen-2, PROVEAN	57.95% (54.04%-61.62%)	40.54% (35.61%-45.32%)	88.041% (83.562%-92.108%)	85.43% (80.33%-90.48%)	46.13% (41.01%-50.71%)	2.6015 (2.4761-2.7353)	57.83% (50.51%-65.66%)	40.15% (30.16%-50%)	88.106% (80%-97.14%)	85.29% (74.07%-96.3%)	46.17% (36.76%-56.72%)	2.5972 (2.3276-2.8469)
At least 3 of FATHMM (missense), PolyPhen-2, PROVEAN, SIFT	73.37% (69.7%-76.77%)	72.89% (68.75%-77.24%)	74.199% (68.116%-80.282%)	83.01% (78.84%-87.04%)	61.28% (55.29%-67.05%)	2.9137 (2.7702-3.0475)	73.46% (66.67%-80.81%)	72.82% (64.06%-81.54%)	74.515% (63.153%-86.49%)	83.07% (74.55%-91.38%)	61.48% (50%-73.17%)	2.9189 (2.6499-3.2017)
At least 3 of FATHMM (missense), PolyPhen-2, PROVEAN, SIFT, VEST	68.65% (65.15%-72.22%)	79.26% (75.41%-83.08%)	50.292% (43.478%-56.522%)	73.38% (69.4%-77.61%)	58.38% (51.56%-65.08%)	2.6132 (2.4583-2.7587)	68.75% (61.62%-75.76%)	79.22% (71.18%-86.89%)	50.737% (37.838%-62.86%)	73.41% (65.22%-81.25%)	58.71% (45.16%-72.41%)	2.6208 (2.3155-2.9234)
At least 3 of FATHMM (missense), PolyPhen-2, PROVEAN, VEST	69.71% (66.16%-73.24%)	68.66% (64.1%-73.39%)	71.519% (65.276%-77.465%)	80.66% (76.47%-84.76%)	56.89% (51.09%-62.63%)	2.7772 (2.6333-2.9252)	69.66% (62.6%-76.77%)	68.5% (59.37%-77.27%)	71.587% (59.248%-83.33%)	80.56% (71.92%-88.64%)	56.96% (45.83%-68.18%)	2.7761 (2.4750-3.0639)
At least 3 of FATHMM (missense), PolyPhen-2, SIFT	68.7% (65.15%-72.22%)	61.22% (56.45%-66.12%)	81.604% (76.71%-86.957%)	85.2% (80.9%-89.53%)	54.89% (49.52%-60%)	2.8292 (2.6927-2.9770)	68.67% (61.62%-75.76%)	61.03% (51.52%-70.49%)	81.704% (70.728%-91.43%)	85.15% (76.74%-93.62%)	54.98% (44.64%-65.47%)	2.8286 (2.5317-3.0993)
At least 3 of FATHMM (missense), PolyPhen-2, SIFT, VEST	70.68% (67.17%-74.24%)	78.21% (74.4%-82.26%)	57.649% (51.428%-63.936%)	76.15% (72.09%-80.3%)	60.47% (53.85%-67.14%)	2.7249 (2.5668-2.8683)	70.75% (63.64%-77.78%)	78.13% (70%-85.94%)	58.043% (45.161%-70.97%)	76.17% (67.86%-84.62%)	60.71% (47.22%-73.69%)	2.7305 (2.4430-3.0387)
At least 3 of FATHMM (missense), PolyPhen-2, VEST	62.32% (58.59%-66.16%)	53.79% (48.78%-58.4%)	77.06% (71.424%-82.437%)	80.22% (75.61%-85.06%)	49.09% (43.75%-54.13%)	2.6016 (2.4608-2.7513)	62.22% (54.55%-69.7%)	53.55% (44.07%-63.08%)	77.024% (65.714%-88.1%)	80.02% (69.99%-89.19%)	49.13% (38.98%-59.33%)	2.5973 (2.2965-2.8785)
At least 3 of FATHMM (missense), PROVEAN, SIFT	59.61% (56.06%-63.13%)	42.64% (37.88%-47.29%)	88.946% (84.848%-93.058%)	86.97% (82.26%-91.94%)	47.28% (42.1%-51.82%)	2.6584 (2.5420-2.7846)	59.56% (52.53%-66.67%)	42.34% (32.73%-52.12%)	89.045% (80.556%-97.22%)	86.93% (76.67%-96.67%)	47.36% (38.1%-57.75%)	2.6568 (2.3908-2.9066)
At least 3 of FATHMM (missense), PROVEAN, SIFT, VEST	68.32% (64.65%-72.22%)	68.64% (64.17%-73.17%)	67.747% (61.644%-74.324%)	78.63% (74.14%-83.04%)	55.54% (49.41%-61.54%)	2.7056 (2.5476-2.8572)	68.42% (60.61%-75.76%)	68.54% (59.32%-77.14%)	68.131% (55%-80.56%)	78.69% (69.64%-87.5%)	55.78% (44%-67.5%)	2.7114 (2.4018-3.0220)
At least 3 of FATHMM (missense), PROVEAN, VEST	56.61% (52.53%-60.61%)	38.41% (33.33%-43.18%)	88.058% (83.582%-92.424%)	84.76% (79.62%-90%)	45.26% (40.29%-50%)	2.5650 (2.4255-2.7047)	56.48% (48.48%-64.65%)	38.03% (28.57%-47.62%)	88.07% (79.409%-97.06%)	84.55% (72.73%-96%)	45.3% (36%-55.08%)	2.5595 (2.2773-2.8419)
At least 3 of FATHMM (missense), SIFT, VEST	63.96% (60.1%-67.68%)	55.92% (50.79%-60.8%)	77.838% (72.222%-83.784%)	81.36% (76.74%-86.21%)	50.53% (44.86%-55.83%)	2.6565 (2.5168-2.8110)	64% (56.57%-71.72%)	55.67% (45.9%-65.64%)	78.219% (65.517%-89.2%)	81.45% (71.43%-91.18%)	50.69% (40%-61.29%)	2.6603 (2.3587-2.9361)
At least 3 of Mutation Assessor, MutationTaster, PolyPhen-2	72.69% (69.19%-76.26%)	62.78% (58.2%-67.77%)	89.928% (86.301%-94.122%)	91.5% (88.51%-95.29%)	58.25% (53.09%-63.46%)	3.0239 (2.9054-3.1475)	72.76% (65.66%-79.8%)	62.84% (52.46%-71.88%)	89.886% (81.081%-97.22%)	91.41% (84.09%-97.62%)	58.5% (48.15%-68.42%)	3.0264 (2.7692-3.2638)
At least 3 of Mutation Assessor, MutationTaster, PolyPhen-2, PROVEAN	74.38% (71.2%-77.78%)	71.79% (67.42%-76.04%)	78.837% (73.684%-84.507%)	85.43% (81.63%-89.32%)	61.78% (56.38%-67.39%)	2.9784 (2.8453-3.1193)	74.48% (67.68%-80.83%)	71.81% (62.96%-80.65%)	79.039% (67.742%-89.66%)	85.45% (77.19%-92.98%)	62.03% (51.06%-72.73%)	2.9833 (2.7004-3.2450)
At least 3 of Mutation Assessor, MutationTaster, PolyPhen-2, PROVEAN, SIFT	74.72% (71.21%-78.28%)	78.19% (74.36%-82.31%)	68.717% (62.662%-75%)	81.21% (77.12%-85.25%)	64.56% (58.67%-70.83%)	2.9268 (2.7877-3.0717)	74.8% (67.68%-81.82%)	78.18% (69.84%-85.94%)	68.992% (55.882%-80.96%)	81.22% (73.02%-89.07%)	64.79% (52.5%-76.48%)	2.9318 (2.6381-3.2159)
At least 3 of Mutation Assessor, MutationTaster, PolyPhen-2, PROVEAN, VEST	73.05% (69.7%-76.28%)	77.64% (73.77%-81.74%)	65.11% (59.015%-71.622%)	79.37% (75.2%-83.61%)	62.74% (56.58%-68.92%)	2.8486 (2.6960-2.9894)	73.1% (66.64%-79.8%)	77.69% (68.97%-85.71%)	65.213% (52.632%-77.42%)	79.3% (70.77%-87.1%)	63% (50%-75.68%)	2.8520 (2.5626-3.1467)
At least 3 of Mutation Assessor, MutationTaster, PolyPhen-2, SIFT	75.39% (71.72%-78.79%)	74.99% (70.83%-79.53%)	76.089% (70.732%-81.944%)	84.43% (80.37%-88.39%)	63.76% (57.83%-69.42%)	2.9926 (2.8472-3.1334)	75.47% (68.69%-82.83%)	75% (65.62%-83.33%)	76.266% (64.861%-87.1%)	84.41% (76.47%-91.94%)	64% (52.27%-75%)	2.9968 (2.7072-3.2870)
At least 3 of Mutation Assessor, MutationTaster, PolyPhen-2, SIFT, VEST	71.34% (67.68%-74.75%)	79.75% (76%-83.87%)	56.813% (50%-63.16%)	76.15% (72.09%-80.15%)	61.86% (55.56%-68.5%)	2.7457 (2.5977-2.9009)	71.45% (64.65%-78.79%)	79.86% (71.43%-87.5%)	57.031% (44.436%-70.27%)	76.12% (68.11%-84.38%)	62.25% (48.57%-75.68%)	2.7526 (2.4431-3.0419)
At least 3 of Mutation Assessor, MutationTaster, PolyPhen-2, VEST	74.07% (70.71%-77.27%)	75.49% (71.21%-79.84%)	71.603% (65.714%-77.612%)	82.13% (78.07%-86.33%)	62.82% (56.98%-68.48%)	2.9205 (2.7724-3.0640)	74.09% (67.68%-80.81%)	75.6% (67.19%-83.82%)	71.497% (59.986%-82.86%)	81.97% (73.77%-89.66%)	63.06% (51.22%-74.43%)	2.9213 (2.6324-3.2150)
At least 3 of Mutation Assessor, MutationTaster, PROVEAN	61.62% (57.58%-65.66%)	41.54% (36.44%-46.34%)	96.354% (94.203%-98.667%)	95.17% (92.31%-98.25%)	48.8% (43.97%-53.57%)	2.8186 (2.7141-2.9263)	61.6% (53.54%-69.7%)	41.38% (31.5%-51.56%)	96.276% (91.176%-100%)	95.01% (88%-100%)	48.9% (39.47%-58.67%)	2.8157 (2.5965-3.0288)
At least 3 of Mutation Assessor, MutationTaster, PROVEAN, SIFT	75.04% (71.72%-78.28%)	73.35% (69.05%-77.44%)	77.941% (72.727%-83.562%)	85.18% (81.31%-88.99%)	62.85% (57.3%-68.54%)	2.9932 (2.8572-3.1363)	75.18% (68.69%-81.82%)	73.47% (64.91%-81.97%)	78.065% (66.667%-89.19%)	85.17% (77.08%-92.73%)	63.16% (52.07%-73.92%)	2.9987 (2.7101-3.2737)
At least 3 of Mutation Assessor, MutationTaster, PROVEAN, SIFT, VEST	73.69% (70.2%-77.27%)	76.02% (72%-80.32%)	69.666% (63.886%-75.758%)	81.25% (77.12%-85.25%)	62.69% (56.96%-68.75%)	2.8962 (2.7548-3.0326)	73.82% (66.67%-80.81%)	76.13% (68.11%-84.13%)	69.847% (57.576%-81.58%)	81.24% (73.08%-89.23%)	63.02% (51.08%-74.42%)	2.9024 (2.6261-3.1852)
At least 3 of Mutation Assessor, MutationTaster, PROVEAN, VEST	72.41% (68.69%-76.26%)	69.2% (64.75%-73.64%)	77.968% (72.603%-83.562%)	84.44% (80.55%-88.68%)	59.41% (54.02%-64.95%)	2.9102 (2.7668-3.0535)	72.35% (64.65%-79.8%)	69.03% (60%-77.78%)	78.03% (66.667%-88.89%)	84.33% (75.55%-92.31%)	59.48% (47.92%-70%)	2.9087 (2.6198-3.2025)
At least 3 of Mutation Assessor, MutationTaster, SIFT	74.73% (71.21%-78.28%)	65.39% (60.66%-70.25%)	90.873% (87.342%-94.872%)	92.53% (89.69%-95.83%)	60.3% (55.14%-65.46%)	3.0909 (2.9750-3.2139)	74.78% (67.68%-81.82%)	65.47% (55.56%-74.29%)	90.735% (82.855%-97.3%)	92.38% (85.29%-97.83%)	60.49% (50%-70.69%)	3.0907 (2.8486-3.3251)
At least 3 of Mutation Assessor, MutationTaster, SIFT, VEST	74.72% (71.21%-77.78%)	74.42% (70.09%-78.86%)	75.22% (69.511%-81.083%)	83.85% (80%-87.85%)	62.97% (57.14%-68.61%)	2.9647 (2.8215-3.0993)	74.81% (67.68%-81.82%)	74.54% (66.07%-83.05%)	75.257% (63.15%-86.49%)	83.77% (75.47%-91.23%)	63.27% (51.35%-75%)	2.9684 (2.6865-3.2519)
At least 3 of Mutation Assessor, MutationTaster, VEST	70.04% (66.67%-73.74%)	58.54% (53.54%-63.41%)	89.928% (86.301%-94.122%)	90.94% (87.67%-95%)	55.64% (50.83%-60.83%)	2.9505 (2.8327-3.0836)	70.02% (62.63%-77.78%)	58.44% (48.48%-68.34%)	89.886% (81.081%-97.22%)	90.82% (82.61%-97.44%)	55.76% (45.58%-65.58%)	2.9491 (2.6879-3.1871)
At least 3 of Mutation Assessor, PolyPhen-2, PROVEAN	58.24% (54.55%-62.12%)	42.6% (37.6%-47.66%)	85.263% (80.303%-89.856%)	83.33% (78.12%-88.89%)	46.21% (41.35%-50.79%)	2.5740 (2.4325-2.7121)	58.27% (50.51%-65.66%)	42.43% (32.2%-52.38%)	85.43% (76.316%-94.6%)	83.31% (72%-93.75%)	46.37% (37.09%-56.45%)	2.5753 (2.2864-2.8546)
At least 3 of Mutation Assessor, PolyPhen-2, PROVEAN, SIFT	71.01% (67.68%-74.75%)	71.78% (67.46%-76.3%)	69.662% (63.768%-76.056%)	80.36% (76.11%-84.68%)	58.81% (52.94%-64.77%)	2.8060 (2.6582-2.9533)	71.12% (63.64%-77.78%)	71.84% (62.9%-80.33%)	69.84% (57.143%-81.58%)	80.34% (71.7%-88.89%)	59.09% (47.06%-70.73%)	2.8111 (2.5172-3.1078)
At least 3 of Mutation Assessor, PolyPhen-2, PROVEAN, SIFT, VEST	69.64% (66.16%-73.23%)	77.1% (73.23%-81.25%)	56.739% (50%-63.014%)	75.5% (71.21%-79.84%)	58.9% (52.31%-65.22%)	2.6824 (2.5335-2.8327)	69.81% (62.63%-76.77%)	77.16% (68.65%-84.85%)	57.185% (44.118%-70.27%)	75.55% (67.17%-84.06%)	59.31% (46.87%-71.88%)	2.6920 (2.3884-2.9932)
At least 3 of Mutation Assessor, PolyPhen-2, PROVEAN, VEST	67.33% (63.64%-71.21%)	67.06% (62.5%-71.67%)	67.787% (62.121%-74.078%)	78.25% (73.83%-83.02%)	54.34% (48.89%-60.22%)	2.6744 (2.5285-2.8234)	67.36% (59.6%-74.75%)	66.91% (57.38%-75.76%)	68.122% (55.556%-80%)	78.25% (69.09%-87.04%)	54.53% (42.86%-65.12%)	2.6781 (2.3710-2.9870)
At least 3 of Mutation Assessor, PolyPhen-2, SIFT	70% (66.67%-73.74%)	65.93% (61.48%-70.64%)	77.034% (71.831%-82.667%)	83.23% (78.95%-87.5%)	56.67% (51.52%-62.14%)	2.8286 (2.6874-2.9695)	70.09% (62.63%-77.78%)	65.98% (56.45%-74.6%)	77.114% (65.789%-87.51%)	83.17% (74.51%-91.67%)	56.91% (45.65%-67.35%)	2.8317 (2.5438-3.1167)
At least 3 of Mutation Assessor, PolyPhen-2, SIFT, VEST	69.29% (65.66%-72.73%)	74.95% (70.77%-79.53%)	59.49% (53.422%-65.714%)	76.18% (71.79%-80.51%)	57.87% (51.43%-64%)	2.6849 (2.5331-2.8341)	69.51% (62.63%-76.77%)	75.08% (66.1%-83.08%)	59.939% (47.497%-72.73%)	76.27% (67.24%-85%)	58.36% (45.71%-70.59%)	2.6964 (2.3932-2.9932)
At least 3 of Mutation Assessor, PolyPhen-2, VEST	63.63% (60.1%-67.68%)	57.47% (52.54%-62.5%)	74.28% (68.571%-80.264%)	79.43% (74.71%-84.34%)	50.25% (45.1%-55.77%)	2.6142 (2.4675-2.7695)	63.65% (55.56%-70.71%)	57.38% (46.87%-66.67%)	74.405% (62.848%-85.71%)	79.34% (69.56%-88.47%)	50.42% (39.21%-60.35%)	2.6155 (2.3081-2.9128)
At least 3 of Mutation Assessor, PROVEAN, SIFT	58.58% (54.55%-62.63%)	44.21% (39.23%-49.21%)	83.422% (78.261%-88.407%)	82.18% (76.81%-87.3%)	46.37% (41.35%-51.18%)	2.5618 (2.4251-2.6960)	58.6% (50.51%-66.67%)	44.01% (33.93%-54.39%)	83.606% (74.064%-94.12%)	82.16% (71.43%-93.1%)	46.52% (36.92%-56.67%)	2.5629 (2.2918-2.8439)
At least 3 of Mutation Assessor, PROVEAN, SIFT, VEST	69.37% (65.66%-72.74%)	68.65% (64.12%-73.23%)	70.611% (64.789%-76.715%)	80.15% (75.73%-84.32%)	56.57% (51.09%-62.22%)	2.7599 (2.6166-2.9016)	69.33% (62.6%-76.77%)	68.51% (59.32%-77.47%)	70.695% (58.818%-82.36%)	80.04% (71.43%-88.64%)	56.67% (45.23%-67.44%)	2.7592 (2.4670-3.0480)
At least 3 of Mutation Assessor, PROVEAN, VEST	56.57% (52.53%-61.11%)	40.48% (35.66%-45.19%)	84.394% (79.412%-89.041%)	81.76% (76.18%-87.27%)	45.05% (40.15%-50%)	2.5169 (2.3760-2.6602)	56.56% (47.47%-64.65%)	40.31% (30.36%-50%)	84.421% (75%-94.12%)	81.6% (70.37%-93.33%)	45.18% (35.71%-55.22%)	2.5150 (2.2269-2.7949)
At least 3 of Mutation Assessor, SIFT, VEST	66.02% (62.61%-70.2%)	60.14% (55.37%-65.12%)	76.165% (70.769%-81.693%)	81.35% (76.92%-85.87%)	52.5% (47.17%-57.9%)	2.7015 (2.5579-2.8477)	65.95% (57.58%-72.75%)	60.01% (50%-69.36%)	76.105% (64.865%-87.1%)	81.15% (72.09%-90.25%)	52.58% (41.67%-63.46%)	2.6984 (2.3910-2.9816)
At least 3 of MutationTaster, PolyPhen-2, PROVEAN	63.65% (59.6%-67.17%)	44.73% (39.69%-49.61%)	96.354% (94.203%-98.667%)	95.49% (92.73%-98.36%)	50.21% (45.25%-54.82%)	2.8678 (2.7576-2.9746)	63.61% (56.57%-71.72%)	44.56% (34.38%-54.55%)	96.276% (91.176%-100%)	95.35% (88.89%-100%)	50.3% (40.85%-60.56%)	2.8648 (2.6507-3.0829)
At least 3 of MutationTaster, PolyPhen-2, PROVEAN, SIFT	75.72% (72.22%-79.29%)	74.99% (70.97%-79.07%)	76.965% (71.642%-82.863%)	84.91% (81.13%-88.89%)	64.02% (58.53%-69.51%)	3.0089 (2.8733-3.1548)	75.84% (68.69%-82.83%)	75% (66.67%-83.1%)	77.254% (65.622%-88.24%)	84.98% (76.92%-92.46%)	64.28% (52.63%-75.56%)	3.0151 (2.7236-3.2832)
At least 3 of MutationTaster, PolyPhen-2, PROVEAN, SIFT, VEST	71.68% (68.18%-75.25%)	80.26% (76.38%-84.35%)	56.842% (50%-63.517%)	76.28% (72.14%-80.6%)	62.47% (55.88%-69.12%)	2.7585 (2.6039-2.9042)	71.79% (64.65%-78.79%)	80.44% (72.31%-88.06%)	56.951% (43.243%-70.28%)	76.23% (67.69%-84.51%)	62.89% (50%-75.87%)	2.7651 (2.4714-3.0627)
At least 3 of MutationTaster, PolyPhen-2, PROVEAN, VEST	73.09% (69.7%-76.77%)	71.31% (66.94%-75.76%)	76.164% (70.512%-81.945%)	83.8% (79.81%-88.07%)	60.55% (55.05%-66.28%)	2.9183 (2.7824-3.0694)	73.01% (65.66%-79.8%)	71.19% (62.06%-79.69%)	76.115% (64.516%-87.18%)	83.64% (75%-91.67%)	60.61% (48.84%-71.74%)	2.9156 (2.6145-3.1839)
At least 3 of MutationTaster, PolyPhen-2, SIFT	76.07% (72.73%-79.29%)	67.51% (63.08%-72.58%)	90.873% (87.342%-94.872%)	92.75% (90%-95.92%)	61.8% (56.73%-66.97%)	3.1292 (3.0189-3.2501)	76.14% (68.69%-82.83%)	67.62% (57.63%-76.67%)	90.735% (82.855%-97.3%)	92.6% (85.42%-97.87%)	62.02% (51.77%-72.23%)	3.1297 (2.8945-3.3524)
At least 3 of MutationTaster, PolyPhen-2, SIFT, VEST	73.37% (70.2%-76.77%)	78.14% (74.22%-82.4%)	65.12% (58.973%-71.831%)	79.48% (75.59%-83.74%)	63.27% (57.14%-69.74%)	2.8600 (2.7125-3.0106)	73.46% (66.67%-79.8%)	78.29% (69.86%-86.36%)	65.159% (51.61%-77.78%)	79.41% (70.91%-87.5%)	63.61% (50%-75.77%)	2.8646 (2.5594-3.1517)
At least 3 of MutationTaster, PolyPhen-2, VEST	71.74% (68.18%-75.25%)	62.77% (57.98%-67.72%)	87.251% (83.099%-91.781%)	89.48% (86.05%-93.41%)	57.54% (52.29%-62.93%)	2.9704 (2.8464-3.0994)	71.68% (64.65%-78.79%)	62.75% (53.22%-72.14%)	86.978% (77.778%-94.6%)	89.2% (81.24%-95.65%)	57.64% (46.94%-67.93%)	2.9657 (2.7083-3.2143)
At least 3 of MutationTaster, PROVEAN, SIFT	64.98% (61.11%-68.69%)	46.83% (41.86%-52%)	96.354% (94.203%-98.667%)	95.69% (93.1%-98.41%)	51.17% (46.32%-55.89%)	2.9004 (2.7927-3.0051)	65% (57.58%-72.73%)	46.75% (36.36%-56.86%)	96.276% (91.176%-100%)	95.56% (89.65%-100%)	51.31% (41.67%-61.11%)	2.8990 (2.6972-3.1167)
At least 3 of MutationTaster, PROVEAN, SIFT, VEST	73.07% (69.7%-76.77%)	70.76% (66.39%-75.21%)	77.045% (71.642%-82.857%)	84.2% (80.2%-88.42%)	60.38% (55.17%-65.96%)	2.9239 (2.7835-3.0645)	73.06% (65.66%-79.8%)	70.68% (61.9%-79.11%)	77.099% (65.709%-87.5%)	84.11% (75.85%-92%)	60.5% (48.93%-71.16%)	2.9239 (2.6400-3.1972)
At least 3 of MutationTaster, PROVEAN, VEST	62.98% (59.09%-66.67%)	43.67% (38.52%-48.49%)	96.354% (94.203%-98.667%)	95.39% (92.59%-98.31%)	49.73% (44.85%-54.41%)	2.8514 (2.7437-2.9543)	62.94% (55.56%-70.71%)	43.49% (33.9%-53.45%)	96.276% (91.176%-100%)	95.24% (88.89%-100%)	49.82% (40.3%-59.73%)	2.8483 (2.6423-3.0652)
At least 3 of MutationTaster, SIFT, VEST	74.08% (70.71%-77.78%)	64.37% (59.68%-69.35%)	90.873% (87.342%-94.872%)	92.42% (89.53%-95.7%)	59.6% (54.46%-64.76%)	3.0726 (2.9598-3.1911)	74.06% (66.67%-80.81%)	64.33% (54.1%-73.77%)	90.735% (82.855%-97.3%)	92.25% (85.1%-97.78%)	59.7% (49.06%-70%)	3.0701 (2.8306-3.2996)
At least 3 of PolyPhen-2, PROVEAN, SIFT	59.89% (56.06%-63.64%)	46.84% (41.54%-51.94%)	82.446% (77.143%-87.671%)	82.19% (77.03%-87.33%)	47.28% (42.18%-52.27%)	2.5876 (2.4430-2.7312)	60.01% (52.53%-67.68%)	46.71% (36.23%-56.92%)	82.795% (72.222%-92.6%)	82.33% (71.79%-92.59%)	47.51% (37.87%-57.82%)	2.5934 (2.3118-2.8807)
At least 3 of PolyPhen-2, PROVEAN, SIFT, VEST	66.33% (62.63%-69.7%)	69.71% (65.32%-74.02%)	60.464% (54.052%-67.123%)	75.3% (70.69%-80%)	53.58% (47.56%-59.49%)	2.5905 (2.4405-2.7361)	66.34% (59.6%-73.74%)	69.59% (60.71%-78.13%)	60.708% (47.365%-73.53%)	75.24% (66.15%-84.49%)	53.75% (41.67%-65.32%)	2.5929 (2.3021-2.8932)
At least 3 of PolyPhen-2, PROVEAN, VEST	58.57% (54.55%-62.63%)	43.13% (38.1%-47.93%)	85.268% (80.597%-90.141%)	83.5% (78.12%-88.71%)	46.44% (41.35%-51.45%)	2.5834 (2.4463-2.7247)	58.61% (50.51%-66.67%)	42.97% (33.33%-52.86%)	85.414% (75.859%-94.44%)	83.47% (72.73%-93.75%)	46.6% (36.76%-56.72%)	2.5846 (2.2993-2.8615)
At least 3 of PolyPhen-2, SIFT, VEST	64.61% (61.11%-68.69%)	62.22% (57.48%-67.19%)	68.742% (62.856%-75%)	77.48% (72.73%-82.24%)	51.27% (45.54%-56.82%)	2.5972 (2.4525-2.7585)	64.71% (56.57%-71.72%)	62.24% (52.38%-71.67%)	68.916% (56.25%-80.56%)	77.45% (67.92%-86.67%)	51.53% (40.43%-62.97%)	2.6013 (2.2865-2.8874)
At least 3 of PROVEAN, SIFT, VEST	58.91% (55.05%-62.63%)	45.25% (40%-50%)	82.526% (77.465%-87.671%)	81.74% (76.47%-87.14%)	46.58% (41.46%-51.54%)	2.5610 (2.4266-2.7043)	58.94% (51.52%-66.67%)	45.11% (35.59%-55.36%)	82.641% (72.493%-92.11%)	81.67% (70.59%-91.67%)	46.73% (36.66%-56.93%)	2.5615 (2.2688-2.8469)
At least 4 of CHASM (breast), CHASM (lung), CHASM (melanoma), FATHMM (cancer)	79.51% (76.26%-82.83%)	70.33% (65.62%-75%)	95.379% (92.857%-98.592%)	96.34% (94.44%-98.89%)	65.03% (59.8%-70.1%)	3.2708 (3.1654-3.3795)	79.36% (72.73%-85.86%)	69.97% (60.66%-79.1%)	95.474% (89.474%-100%)	96.37% (91.49%-100%)	64.94% (54.54%-75.86%)	3.2675 (3.0504-3.4834)
At least 4 of CHASM (breast), CHASM (lung), CHASM (melanoma), FATHMM (cancer), FATHMM (missense)	80.87% (77.78%-84.34%)	74.05% (69.42%-78.69%)	92.654% (89.394%-96%)	94.57% (92.22%-97.03%)	67.37% (62.1%-72.82%)	3.2865 (3.1795-3.4037)	80.69% (73.74%-86.87%)	73.7% (64.61%-82.54%)	92.657% (85.714%-100%)	94.52% (89.13%-100%)	67.25% (56.52%-78.26%)	3.2812 (3.0426-3.4991)
At least 4 of CHASM (breast), CHASM (lung), CHASM (melanoma), FATHMM (cancer), Mutation Assessor	81.88% (78.79%-84.85%)	75.64% (71.54%-80%)	92.66% (89.189%-96%)	94.69% (92.31%-97.12%)	68.75% (63.37%-74%)	3.3175 (3.2118-3.4332)	81.69% (75.76%-87.88%)	75.3% (66.67%-83.61%)	92.645% (85.294%-100%)	94.62% (89.36%-100%)	68.61% (57.69%-79.59%)	3.3117 (3.0814-3.5283)
At least 4 of CHASM (breast), CHASM (lung), CHASM (melanoma), FATHMM (cancer), MutationTaster	82.23% (79.29%-85.35%)	76.19% (71.79%-80.49%)	92.66% (89.189%-96%)	94.72% (92.38%-97.14%)	69.24% (63.92%-74.52%)	3.3282 (3.2223-3.4422)	82.01% (75.76%-87.88%)	75.79% (67.19%-84.21%)	92.645% (85.294%-100%)	94.65% (89.36%-100%)	69.04% (58.54%-79.64%)	3.3213 (3.0920-3.5344)
At least 4 of CHASM (breast), CHASM (lung), CHASM (melanoma), FATHMM (cancer), PolyPhen-2	82.23% (79.29%-85.35%)	76.19% (71.79%-80.49%)	92.66% (89.189%-96%)	94.72% (92.38%-97.14%)	69.24% (63.92%-74.52%)	3.3282 (3.2223-3.4422)	82.01% (75.76%-87.88%)	75.79% (67.19%-84.21%)	92.645% (85.294%-100%)	94.65% (89.36%-100%)	69.04% (58.54%-79.64%)	3.3213 (3.0920-3.5344)
At least 4 of CHASM (breast), CHASM (lung), CHASM (melanoma), FATHMM (cancer), PROVEAN	81.56% (78.28%-84.85%)	74.09% (69.42%-78.51%)	94.469% (91.549%-97.333%)	95.86% (93.75%-98%)	67.83% (62.63%-73.08%)	3.3225 (3.2155-3.4303)	81.33% (74.75%-87.88%)	73.62% (64.91%-82.46%)	94.535% (88.235%-100%)	95.86% (91.11%-100%)	67.62% (57.14%-78.18%)	3.3163 (3.0993-3.5334)
At least 4 of CHASM (breast), CHASM (lung), CHASM (melanoma), FATHMM (cancer), SIFT	81.54% (78.79%-84.85%)	75.64% (71.54%-80%)	91.733% (88.406%-95.775%)	94.06% (91.67%-96.97%)	68.54% (63.16%-73.74%)	3.2997 (3.1950-3.4143)	81.36% (74.75%-86.87%)	75.3% (66.67%-83.61%)	91.743% (83.871%-97.56%)	94% (88.37%-98.21%)	68.4% (57.45%-79.25%)	3.2943 (3.0685-3.5037)
At least 4 of CHASM (breast), CHASM (lung), CHASM (melanoma), FATHMM (cancer), VEST	81.89% (78.79%-84.86%)	76.19% (71.79%-80.49%)	91.733% (88.406%-95.775%)	94.1% (91.67%-97%)	69.03% (63.74%-74.44%)	3.3105 (3.2036-3.4255)	81.67% (75.73%-87.88%)	75.79% (67.19%-84.21%)	91.743% (83.871%-97.56%)	94.04% (88.46%-98.22%)	68.83% (58.13%-79.55%)	3.3040 (3.0744-3.5181)
At least 4 of CHASM (breast), CHASM (lung), CHASM (melanoma), FATHMM (missense)	77.51% (74.24%-80.81%)	67.17% (62.31%-72.13%)	95.379% (92.857%-98.592%)	96.17% (94.12%-98.84%)	62.69% (57.28%-67.93%)	3.2141 (3.1018-3.3235)	77.31% (70.71%-83.84%)	66.72% (56.92%-76.47%)	95.474% (89.474%-100%)	96.2% (91.11%-100%)	62.58% (51.92%-73.22%)	3.2097 (2.9931-3.4339)
At least 4 of CHASM (breast), CHASM (lung), CHASM (melanoma), FATHMM (missense), Mutation Assessor	79.18% (76.25%-82.34%)	71.39% (66.67%-76.15%)	92.66% (89.189%-96%)	94.39% (91.84%-96.97%)	65.19% (59.62%-70.48%)	3.2363 (3.1291-3.3527)	79.01% (72.7%-84.87%)	71.04% (61.76%-80.4%)	92.645% (85.294%-100%)	94.31% (88.63%-100%)	65.09% (54.34%-75.51%)	3.2310 (3.0055-3.4505)
At least 4 of CHASM (breast), CHASM (lung), CHASM (melanoma), FATHMM (missense), MutationTaster	81.21% (78.28%-84.34%)	74.59% (70%-78.86%)	92.66% (89.189%-96%)	94.62% (92.22%-97.06%)	67.83% (62.5%-73.12%)	3.2970 (3.1920-3.4151)	81.01% (74.75%-86.87%)	74.22% (65.57%-83.33%)	92.645% (85.294%-100%)	94.55% (89.13%-100%)	67.68% (57.45%-79.07%)	3.2910 (3.0614-3.5035)
At least 4 of CHASM (breast), CHASM (lung), CHASM (melanoma), FATHMM (missense), PolyPhen-2	80.87% (77.78%-84.34%)	74.59% (70%-78.86%)	91.746% (88.155%-95.716%)	93.98% (91.4%-96.94%)	67.62% (62.36%-72.83%)	3.2793 (3.1673-3.3989)	80.67% (73.74%-86.87%)	74.22% (65.57%-83.33%)	91.732% (84.211%-100%)	93.9% (88.09%-100%)	67.47% (57.14%-78.73%)	3.2732 (3.0330-3.4991)
At least 4 of CHASM (breast), CHASM (lung), CHASM (melanoma), FATHMM (missense), PROVEAN	80.19% (76.77%-83.33%)	71.93% (67.48%-76.56%)	94.469% (91.549%-97.333%)	95.74% (93.55%-97.96%)	66.06% (60.74%-71.3%)	3.2820 (3.1740-3.3936)	80.02% (73.74%-86.87%)	71.55% (62.29%-80.4%)	94.535% (88.235%-100%)	95.74% (90.69%-100%)	65.95% (55.36%-76.6%)	3.2779 (3.0603-3.4955)
At least 4 of CHASM (breast), CHASM (lung), CHASM (melanoma), FATHMM (missense), SIFT	80.19% (77.27%-83.33%)	73.5% (69.1%-77.95%)	91.733% (88.406%-95.775%)	93.89% (91.4%-96.88%)	66.69% (61.38%-72%)	3.2583 (3.1452-3.3740)	80.03% (73.74%-85.86%)	73.19% (64.29%-81.97%)	91.743% (83.871%-97.56%)	93.84% (88%-98.21%)	66.61% (56.25%-77.55%)	3.2537 (3.0239-3.4762)
At least 4 of CHASM (breast), CHASM (lung), CHASM (melanoma), FATHMM (missense), VEST	80.54% (77.27%-83.84%)	74.59% (70%-78.86%)	90.818% (87.143%-94.667%)	93.35% (90.82%-96.12%)	67.39% (62.1%-72.63%)	3.2615 (3.1516-3.3828)	80.34% (73.74%-86.87%)	74.22% (65.57%-83.33%)	90.829% (82.857%-97.44%)	93.28% (87.23%-98.08%)	67.25% (56.52%-78.27%)	3.2558 (3.0116-3.4851)
At least 4 of CHASM (breast), CHASM (lung), CHASM (melanoma), Mutation Assessor	65.67% (62.11%-69.19%)	47.96% (42.74%-53.18%)	96.282% (94.284%-98.667%)	95.71% (93.33%-98.46%)	51.69% (46.67%-56.25%)	2.9164 (2.8168-3.0173)	65.64% (58.59%-72.75%)	47.69% (37.7%-57.81%)	96.423% (91.176%-100%)	95.81% (89.66%-100%)	51.79% (42.62%-61.76%)	2.9170 (2.7146-3.1193)
At least 4 of CHASM (breast), CHASM (lung), CHASM (melanoma), Mutation Assessor, MutationTaster	76.44% (73.23%-79.8%)	66% (61.11%-70.87%)	94.485% (91.667%-97.368%)	95.39% (93.02%-97.78%)	61.65% (56.25%-66.67%)	3.1753 (3.0710-3.2879)	76.41% (69.7%-82.83%)	65.84% (55.93%-75.72%)	94.508% (88.09%-100%)	95.36% (90%-100%)	61.73% (50.94%-72.42%)	3.1744 (2.9509-3.3902)
At least 4 of CHASM (breast), CHASM (lung), CHASM (melanoma), Mutation Assessor, PolyPhen-2	73.43% (70.19%-76.77%)	61.25% (56.35%-66.14%)	94.485% (91.667%-97.368%)	95.05% (92.59%-97.62%)	58.51% (53.51%-63.33%)	3.0930 (2.9883-3.2011)	73.34% (66.67%-79.82%)	60.99% (50.82%-71.19%)	94.508% (88.09%-100%)	95.02% (89.19%-100%)	58.54% (48.44%-68.97%)	3.0905 (2.8752-3.3044)
At least 4 of CHASM (breast), CHASM (lung), CHASM (melanoma), Mutation Assessor, PROVEAN	70.72% (67.17%-74.24%)	56.47% (51.28%-61.48%)	95.372% (92.955%-98.571%)	95.47% (93.06%-98.61%)	55.89% (50.83%-60.63%)	3.0320 (2.9291-3.1331)	70.67% (63.64%-77.78%)	56.19% (45.61%-66.13%)	95.484% (89.651%-100%)	95.53% (89.47%-100%)	55.95% (46.03%-66.13%)	3.0315 (2.8250-3.2420)
At least 4 of CHASM (breast), CHASM (lung), CHASM (melanoma), Mutation Assessor, SIFT	75.13% (71.72%-78.79%)	63.92% (58.91%-68.8%)	94.485% (91.667%-97.368%)	95.25% (92.77%-97.73%)	60.24% (55.04%-65.46%)	3.1389 (3.0418-3.2554)	75% (67.68%-81.82%)	63.62% (53.96%-73.44%)	94.508% (88.09%-100%)	95.21% (89.47%-100%)	60.22% (49.98%-70.59%)	3.1355 (2.9053-3.3390)
At least 4 of CHASM (breast), CHASM (lung), CHASM (melanoma), Mutation Assessor, VEST	72.43% (69.19%-75.76%)	59.67% (54.61%-64.8%)	94.485% (91.667%-97.368%)	94.92% (92.31%-97.56%)	57.53% (52.54%-62.5%)	3.0661 (2.9627-3.1733)	72.32% (65.66%-78.79%)	59.37% (49.18%-69.23%)	94.508% (88.09%-100%)	94.89% (88.89%-100%)	57.55% (47.27%-67.27%)	3.0631 (2.8446-3.2720)
At least 4 of CHASM (breast), CHASM (lung), CHASM (melanoma), MutationTaster	70.68% (66.67%-74.24%)	55.32% (50.37%-60.63%)	97.222% (95.588%-100%)	97.18% (95.52%-100%)	55.73% (50.79%-60.63%)	3.0545 (2.9487-3.1591)	70.77% (63.64%-78.79%)	55.3% (44.77%-65.67%)	97.294% (92.303%-100%)	97.22% (92.59%-100%)	55.93% (46.03%-66.13%)	3.0574 (2.8538-3.2723)
At least 4 of CHASM (breast), CHASM (lung), CHASM (melanoma), MutationTaster, PolyPhen-2	76.45% (73.23%-79.8%)	66.02% (61.24%-71.07%)	94.485% (91.667%-97.368%)	95.39% (93.1%-97.78%)	61.66% (56.48%-66.67%)	3.1755 (3.0711-3.2879)	76.39% (69.7%-82.83%)	65.82% (55.74%-75.76%)	94.508% (88.09%-100%)	95.36% (90%-100%)	61.71% (50.98%-72.22%)	3.1740 (2.9502-3.3902)
At least 4 of CHASM (breast), CHASM (lung), CHASM (melanoma), MutationTaster, PROVEAN	74.76% (71.21%-78.28%)	62.28% (57.5%-67.21%)	96.311% (94.286%-98.667%)	96.69% (94.81%-98.82%)	59.63% (54.54%-64.66%)	3.1491 (3.0487-3.2573)	74.73% (67.68%-81.82%)	62.11% (52.46%-71.83%)	96.355% (91.176%-100%)	96.7% (91.67%-100%)	59.72% (50%-70.18%)	3.1488 (2.9342-3.3600)
At least 4 of CHASM (breast), CHASM (lung), CHASM (melanoma), MutationTaster, SIFT	78.13% (74.75%-81.33%)	68.13% (63.33%-72.95%)	95.425% (92.958%-98.592%)	96.26% (94.19%-98.86%)	63.39% (58.18%-68.63%)	3.2321 (3.1286-3.3407)	78.08% (71.69%-84.85%)	67.98% (58.06%-77.42%)	95.38% (89.469%-100%)	96.19% (91.11%-100%)	63.45% (53.06%-74.07%)	3.2300 (3.0169-3.4432)
At least 4 of CHASM (breast), CHASM (lung), CHASM (melanoma), MutationTaster, VEST	74.76% (71.21%-78.28%)	63.34% (58.4%-68.55%)	94.485% (91.667%-97.368%)	95.21% (92.77%-97.7%)	59.85% (54.62%-64.91%)	3.1288 (3.0182-3.2377)	74.73% (67.68%-81.82%)	63.19% (52.63%-73.24%)	94.508% (88.09%-100%)	95.19% (89.47%-100%)	59.94% (50%-70.59%)	3.1283 (2.9068-3.3543)
At least 4 of CHASM (breast), CHASM (lung), CHASM (melanoma), PolyPhen-2	68.68% (64.65%-72.22%)	52.71% (47.62%-58.06%)	96.282% (94.284%-98.667%)	96.08% (93.85%-98.59%)	54.08% (49.21%-58.78%)	2.9915 (2.8887-3.0934)	68.7% (61.62%-76.77%)	52.54% (41.94%-62.71%)	96.423% (91.176%-100%)	96.18% (90.62%-100%)	54.21% (44.99%-64.41%)	2.9936 (2.7920-3.2116)
At least 4 of CHASM (breast), CHASM (lung), CHASM (melanoma), PolyPhen-2, PROVEAN	72.74% (69.19%-76.26%)	59.65% (54.62%-64.75%)	95.372% (92.955%-98.571%)	95.71% (93.33%-98.68%)	57.76% (52.89%-62.6%)	3.0849 (2.9811-3.1875)	72.69% (65.66%-79.8%)	59.39% (49.23%-69.49%)	95.484% (89.651%-100%)	95.76% (90.24%-100%)	57.81% (48.21%-67.86%)	3.0845 (2.8776-3.2983)

At least 4 of CHASM (breast), CHASM (lung), CHASM (melanoma), PolyPhen-2, SIFT	76.46% (73.23%-79.8%)	66.03% (61.16%-70.73%)	94.485% (91.667%-97.368%)	95.39% (93.02%-97.78%)	61.66% (56.6%-66.67%)	3.1757 (3.0687-3.2834)	76.38% (69.7%-82.83%)	65.79% (56.06%-75.41%)	94.508% (88.09%-100%)	95.36% (90%-100%)	61.69% (51.6%-72%)	3.1735 (2.9582-3.3908)
At least 4 of CHASM (breast), CHASM (lung), CHASM (melanoma), PolyPhen-2, VEST	73.44% (70.2%-76.77%)	61.8% (56.8%-66.67%)	93.57% (90.537%-97.184%)	94.32% (91.67%-97.56%)	58.62% (53.72%-63.48%)	3.0831 (2.9757-3.1928)	73.31% (66.67%-79.8%)	61.48% (51.67%-71.43%)	93.595% (86.662%-100%)	94.27% (87.8%-100%)	58.61% (48.21%-68.85%)	3.0795 (2.8499-3.2964)
At least 4 of CHASM (breast), CHASM (lung), CHASM (melanoma), PROVEAN	61.3% (57.58%-65.15%)	39.45% (34.43%-44.26%)	99.084% (98.148%-100%)	98.67% (97.33%-100%)	48.62% (43.75%-52.98%)	2.8583 (2.7711-2.9411)	61.23% (53.54%-68.69%)	39.17% (29.73%-49.18%)	99.083% (96.774%-100%)	98.66% (95.24%-100%)	48.69% (39.74%-58.44%)	2.8560 (2.6907-3.0180)
At least 4 of CHASM (breast), CHASM (lung), CHASM (melanoma), PROVEAN, SIFT	73.1% (69.7%-76.77%)	59.66% (54.84%-64.8%)	96.311% (94.286%-98.667%)	96.55% (94.52%-98.78%)	58% (52.99%-62.91%)	3.1053 (3.0071-3.2132)	73% (66.66%-79.8%)	59.37% (49.53%-69.07%)	96.306% (91.176%-100%)	96.55% (91.18%-100%)	58.02% (49.21%-66.28%)	3.1029 (2.8910-3.3008)
At least 4 of CHASM (breast), CHASM (lung), CHASM (melanoma), PROVEAN, VEST	68.38% (64.65%-71.72%)	52.77% (47.5%-57.85%)	95.372% (92.955%-98.571%)	95.17% (92.64%-98.53%)	53.87% (49.19%-58.79%)	2.9718 (2.8656-3.0741)	68.29% (61.62%-75.76%)	52.42% (42.37%-62.72%)	95.484% (89.651%-100%)	95.23% (88.89%-100%)	53.91% (44.07%-63.64%)	2.9705 (2.7667-3.1877)
At least 4 of CHASM (breast), CHASM (lung), CHASM (melanoma), SIFT	71.4% (67.68%-74.75%)	56.46% (51.54%-61.6%)	97.222% (95.588%-100%)	97.23% (95.59%-100%)	56.36% (51.26%-61.11%)	3.0727 (2.9735-3.1757)	71.35% (64.65%-78.79%)	56.21% (45.61%-66.67%)	97.294% (92.303%-100%)	97.27% (92.68%-100%)	56.43% (46.66%-66.67%)	3.0720 (2.8670-3.2703)
At least 4 of CHASM (breast), CHASM (lung), CHASM (melanoma), SIFT, VEST	74.78% (71.72%-78.28%)	63.92% (59.05%-68.85%)	93.557% (90.537%-97.183%)	94.49% (91.86%-97.67%)	59.99% (54.95%-65.18%)	3.1196 (3.0170-3.2388)	74.68% (67.68%-80.81%)	63.64% (53.57%-73.13%)	93.600% (86.842%-100%)	94.47% (87.8%-100%)	60% (49.15%-70.18%)	3.1171 (2.8765-3.3254)
At least 4 of CHASM (breast), CHASM (lung), CHASM (melanoma), VEST	63.64% (59.6%-67.17%)	44.76% (39.65%-50%)	96.282% (94.284%-98.667%)	95.42% (92.88%-98.39%)	50.2% (45.32%-54.55%)	2.8666 (2.7646-2.9680)	63.63% (56.57%-71.72%)	44.51% (34.38%-54.84%)	96.423% (91.176%-100%)	95.53% (88.57%-100%)	50.32% (40.91%-60.27%)	2.8678 (2.6622-3.0732)
At least 4 of CHASM (breast), CHASM (lung), FATHMM (cancer), FATHMM (missense)	77.53% (74.24%-80.81%)	67.2% (62.31%-72%)	95.379% (92.857%-97.802%)	96.18% (94.12%-98.02%)	62.71% (57.72%-67.63%)	3.2147 (3.1115-3.3214)	77.27% (70.71%-83.84%)	66.66% (56.92%-76.06%)	95.474% (89.474%-100%)	96.19% (90.91%-100%)	62.53% (51.92%-73.22%)	3.2085 (2.9924-3.4196)
At least 4 of CHASM (breast), CHASM (lung), FATHMM (cancer), FATHMM (missense), Mutation Assessor	80.56% (77.27%-83.84%)	73.56% (68.8%-77.95%)	92.647% (89.392%-96.053%)	94.53% (92.16%-97.09%)	66.96% (61.46%-72.2%)	3.2770 (3.1678-3.3930)	80.3% (73.74%-86.87%)	73.07% (63.93%-82.54%)	92.682% (85.766%-100%)	94.48% (88.68%-100%)	66.74% (56.24%-77.55%)	3.2698 (3.0399-3.4918)
At least 4 of CHASM (breast), CHASM (lung), FATHMM (cancer), FATHMM (missense), MutationTaster	82.23% (79.29%-85.35%)	76.21% (71.77%-80.49%)	92.647% (89.392%-96.053%)	94.71% (92.45%-97.17%)	69.26% (63.83%-74.51%)	3.3283 (3.2188-3.4468)	82% (75.76%-87.88%)	75.76% (67.19%-84.48%)	92.682% (85.766%-100%)	94.67% (89.13%-100%)	69.03% (58.7%-80.01%)	3.3214 (3.0817-3.5423)
At least 4 of CHASM (breast), CHASM (lung), FATHMM (cancer), FATHMM (missense), PolyPhen-2	82.23% (79.29%-85.35%)	76.21% (71.77%-80.49%)	92.647% (89.392%-96.053%)	94.71% (92.45%-97.17%)	69.26% (63.83%-74.51%)	3.3283 (3.2188-3.4468)	82% (75.76%-87.88%)	75.76% (67.19%-84.48%)	92.682% (85.766%-100%)	94.67% (89.13%-100%)	69.03% (58.7%-80.01%)	3.3214 (3.0817-3.5423)
At least 4 of CHASM (breast), CHASM (lung), FATHMM (cancer), FATHMM (missense), PROVEAN	80.54% (77.27%-83.84%)	73.02% (68.5%-77.43%)	93.534% (90.541%-97.184%)	95.13% (92.73%-97.87%)	66.72% (61.38%-71.96%)	3.2840 (3.1763-3.3992)	80.34% (73.74%-86.87%)	72.57% (63.63%-81.67%)	93.658% (86.479%-100%)	95.15% (89.8%-100%)	66.56% (56.6%-77.36%)	3.2795 (3.0454-3.4956)
At least 4 of CHASM (breast), CHASM (lung), FATHMM (cancer), FATHMM (missense), SIFT	81.56% (78.28%-84.85%)	75.68% (71.18%-80%)	91.72% (88.406%-95.775%)	94.05% (91.67%-97%)	68.57% (63.16%-73.83%)	3.3001 (3.1920-3.4163)	81.33% (74.75%-87.88%)	75.22% (66.67%-84.13%)	91.78% (83.871%-97.56%)	94.01% (88.23%-98.18%)	68.33% (57.78%-79.55%)	3.2936 (3.0481-3.5145)
At least 4 of CHASM (breast), CHASM (lung), FATHMM (cancer), FATHMM (missense), VEST	81.56% (78.28%-84.85%)	75.69% (71.32%-80%)	91.72% (88.406%-95.775%)	94.05% (91.67%-97%)	68.57% (63.27%-73.83%)	3.3003 (3.1888-3.4206)	81.32% (74.75%-87.88%)	75.21% (66.67%-84.21%)	91.78% (83.871%-97.56%)	94.01% (88.23%-98.18%)	68.34% (57.78%-79.55%)	3.2934 (3.0489-3.5181)
At least 4 of CHASM (breast), CHASM (lung), FATHMM (cancer), Mutation Assessor	66.36% (62.63%-70.2%)	48.52% (43.51%-53.6%)	97.233% (95.588%-100%)	96.78% (94.83%-100%)	52.2% (47.16%-56.72%)	2.9470 (2.8518-3.0480)	66.27% (58.59%-73.74%)	48.16% (37.76%-58.62%)	97.337% (92.5%-100%)	96.88% (91.17%-100%)	52.25% (42.86%-62.32%)	2.9463 (2.7438-3.1420)
At least 4 of CHASM (breast), CHASM (lung), FATHMM (cancer), Mutation Assessor, MutationTaster	77.48% (74.23%-80.81%)	68.15% (63.56%-72.87%)	93.93% (90.537%-97.102%)	94.81% (92.45%-97.37%)	62.95% (57.55%-68.14%)	3.1946 (3.0825-3.3072)	77.41% (70.71%-83.84%)	67.93% (58.33%-77.42%)	93.931% (88.235%-100%)	94.81% (89.09%-100%)	62.97% (52.88%-72.26%)	3.1937 (2.9660-3.4194)
At least 4 of CHASM (breast), CHASM (lung), FATHMM (cancer), Mutation Assessor, PolyPhen-2	74.45% (71.21%-77.78%)	63.4% (58.68%-68.33%)	93.555% (90.537%-97.102%)	94.44% (91.86%-97.59%)	59.65% (54.46%-64.66%)	3.1103 (3.0045-3.2169)	74.34% (67.68%-80.81%)	63.08% (53.33%-72.88%)	93.631% (86.842%-100%)	94.44% (88.1%-100%)	59.64% (49.21%-69.85%)	3.1079 (2.8945-3.3297)
At least 4 of CHASM (breast), CHASM (lung), FATHMM (cancer), Mutation Assessor, PROVEAN	71.41% (68.18%-75.25%)	57.55% (52.71%-62.6%)	95.358% (92.857%-98.592%)	95.54% (93.15%-98.63%)	56.51% (51.61%-61.29%)	3.0496 (2.9513-3.1599)	71.33% (63.64%-77.78%)	57.22% (47.37%-67.61%)	95.521% (89.474%-100%)	95.63% (89.66%-100%)	56.54% (46.67%-66.67%)	3.0491 (2.8286-3.2451)
At least 4 of CHASM (breast), CHASM (lung), FATHMM (cancer), Mutation Assessor, SIFT	75.79% (72.73%-79.29%)	65.52% (60.83%-70.4%)	93.555% (90.537%-97.102%)	94.61% (92.13%-97.67%)	61.08% (55.85%-66.09%)	3.1476 (3.0413-3.2609)	75.69% (68.69%-82.83%)	65.21% (55.56%-74.19%)	93.631% (86.842%-100%)	94.61% (88.64%-100%)	61.07% (50.88%-71.43%)	3.1452 (2.9066-3.3563)
At least 4 of CHASM (breast), CHASM (lung), FATHMM (cancer), Mutation Assessor, VEST	73.11% (69.7%-76.77%)	61.29% (56.45%-66.14%)	93.555% (90.537%-97.102%)	94.26% (91.67%-97.53%)	58.29% (53.22%-63.49%)	3.0739 (2.9642-3.1888)	72.97% (65.66%-79.8%)	60.92% (50.79%-70.69%)	93.631% (86.842%-100%)	94.25% (87.5%-100%)	58.26% (48.21%-68.42%)	3.0706 (2.8479-3.2929)
At least 4 of CHASM (breast), CHASM (lung), FATHMM (cancer), MutationTaster	71.72% (67.68%-75.25%)	56.44% (51.53%-61.6%)	98.148% (96.154%-100%)	98.13% (97.02%-100%)	56.58% (51.56%-61.54%)	3.0929 (3.0006-3.1927)	71.71% (64.65%-78.79%)	56.22% (46.05%-66.67%)	98.189% (94.444%-100%)	98.19% (94.29%-100%)	56.69% (46.77%-67.24%)	3.0935 (2.8911-3.2998)
At least 4 of CHASM (breast), CHASM (lung), FATHMM (cancer), MutationTaster, PolyPhen-2	77.12% (73.74%-80.81%)	67.61% (62.71%-72.5%)	93.55% (90.537%-97.102%)	94.77% (92.31%-97.73%)	62.56% (57.27%-67.86%)	3.1849 (3.0743-3.2983)	77.07% (69.7%-83.84%)	67.41% (57.81%-77.19%)	93.631% (86.842%-100%)	94.78% (88.89%-100%)	62.62% (52.44%-71.21%)	3.1844 (2.9598-3.4078)
At least 4 of CHASM (breast), CHASM (lung), FATHMM (cancer), MutationTaster, PROVEAN	75.09% (71.72%-78.3%)	62.82% (57.85%-67.74%)	96.298% (94.203%-98.684%)	96.7% (94.81%-98.84%)	59.97% (54.7%-64.96%)	3.1579 (3.0590-3.2668)	75.07% (68.69%-81.82%)	62.54% (52.54%-72.46%)	96.392% (90.909%-100%)	96.75% (91.89%-100%)	60.08% (50%-70.59%)	3.1585 (2.9540-3.3783)
At least 4 of CHASM (breast), CHASM (lung), FATHMM (cancer), MutationTaster, SIFT	79.15% (75.76%-82.32%)	70.27% (65.62%-75%)	94.49% (91.667%-97.333%)	95.68% (93.41%-97.94%)	64.79% (59.24%-69.81%)	3.2520 (3.1455-3.3632)	79.07% (72.73%-85.86%)	70.05% (60.66%-79.37%)	94.558% (88.235%-100%)	95.62% (90.2%-100%)	64.8% (54.55%-74.47%)	3.2500 (3.0284-3.4674)
At least 4 of CHASM (breast), CHASM (lung), FATHMM (cancer), MutationTaster, VEST	75.09% (71.72%-78.79%)	64.41% (59.5%-69.75%)	93.55% (90.537%-97.102%)	94.52% (92.05%-97.62%)	60.32% (55.08%-65.39%)	3.1281 (3.0176-3.2409)	75.07% (67.68%-81.82%)	64.24% (53.45%-73.77%)	93.631% (86.842%-100%)	94.54% (88.1%-100%)	60.42% (50%-70.91%)	3.1283 (2.9075-3.3396)
At least 4 of CHASM (breast), CHASM (lung), FATHMM (cancer), PolyPhen-2	69.72% (66.16%-73.23%)	53.82% (48.84%-59.2%)	97.203% (95.588%-100%)	97.08% (95.38%-100%)	54.67% (49.59%-59.44%)	3.0301 (2.9324-3.1313)	69.65% (62.63%-76.77%)	53.51% (42.85%-64.18%)	97.337% (92.5%-100%)	97.19% (92.11%-100%)	54.96% (45.31%-65.08%)	3.0299 (2.8339-3.2388)
At least 4 of CHASM (breast), CHASM (lung), FATHMM (cancer), PolyPhen-2, PROVEAN	73.08% (69.7%-76.26%)	60.19% (55.24%-65.05%)	95.288% (92.857%-98.592%)	95.61% (93.51%-98.58%)	58.08% (52.94%-62.91%)	3.0935 (2.9908-3.2014)	73.04% (66.67%-79.8%)	59.92% (50%-70.18%)	95.521% (89.474%-100%)	95.82% (90.32%-100%)	58.14% (48.33%-68.33%)	3.0941 (2.8809-3.3005)
At least 4 of CHASM (breast), CHASM (lung), FATHMM (cancer), PolyPhen-2, SIFT	77.15% (73.74%-80.3%)	68.17% (63.41%-72.76%)	92.664% (89.392%-96.003%)	94.14% (91.49%-96.84%)	62.74% (57.53%-67.65%)	3.1771 (3.0713-3.2861)	77.02% (70.71%-83.84%)	67.89% (58.33%-77.05%)	92.661% (85.714%-100%)	94.06% (88%-100%)	62.72% (52.73%-73.21%)	3.1732 (2.9572-3.3876)
At least 4 of CHASM (breast), CHASM (lung), FATHMM (cancer), PolyPhen-2, VEST	73.49% (70.2%-77.27%)	62.87% (57.85%-67.74%)	92.664% (89.392%-96.003%)	93.81% (90.91%-96.63%)	59.07% (53.78%-64.23%)	3.0828 (2.9723-3.1881)	73.32% (66.67%-80.81%)	62.59% (52.54%-72.58%)	92.661% (85.714%-100%)	93.58% (87.18%-100%)	59.04% (48.08%-69.23%)	3.0782 (2.8703-3.3024)
At least 4 of CHASM (breast), CHASM (lung), FATHMM (cancer), PROVEAN	62.33% (58.59%-65.67%)	41.08% (36.22%-45.9%)	99.084% (98.485%-100%)	98.73% (97.83%-100%)	49.3% (44.44%-53.64%)	2.8819 (2.7961-2.9660)	62.2% (55.53%-69.7%)	40.7% (31.03%-50.85%)	99.083% (96.774%-100%)	98.71% (95.24%-100%)	49.33% (40.57%-58.91%)	2.8782 (2.7092-3.0570)
At least 4 of CHASM (breast), CHASM (lung), FATHMM (cancer), PROVEAN, SIFT	73.78% (70.2%-77.27%)	60.75% (55.81%-65.89%)	96.298% (94.203%-98.684%)	96.59% (94.59%-98.81%)	58.66% (53.78%-63.64%)	3.1230 (3.0232-3.2295)	73.66% (66.67%-80.81%)	60.39% (50%-70.49%)	96.392% (90.909%-100%)	96.63% (91.42%-100%)	58.65% (49.04%-68.34%)	3.1206 (2.9049-3.3254)
At least 4 of CHASM (breast), CHASM (lung), FATHMM (cancer), PROVEAN, VEST	68.38% (64.65%-71.72%)	52.78% (47.54%-57.72%)	95.358% (92.857%-98.592%)	95.16% (92.54%-98.31%)	53.87% (48.85%-58.56%)	2.9717 (2.8602-3.0806)	68.29% (60.61%-75.76%)	52.41% (42.62%-62.72%)	95.521% (89.474%-100%)	95.26% (88.89%-100%)	53.91% (44.29%-63.95%)	2.9710 (2.7516-3.1865)
At least 4 of CHASM (breast), CHASM (lung), FATHMM (cancer), SIFT	72.09% (68.69%-75.76%)	57.02% (51.97%-62.2%)	98.143% (97.015%-100%)	98.15% (97.01%-100%)	56.91% (51.97%-61.8%)	3.1002 (3.0133-3.2014)	71.98% (64.65%-78.79%)	56.69% (46.03%-66.68%)	98.209% (94.118%-100%)	98.19% (94.29%-100%)	56.92% (47.69%-66.67%)	3.1001 (2.9091-3.2904)
At least 4 of CHASM (breast), CHASM (lung), FATHMM (cancer), SIFT, VEST	75.14% (71.72%-78.79%)	65.54% (60.66%-70.4%)	91.737% (88.406%-95.652%)	93.2% (90.36%-96.63%)	60.62% (55.56%-65.71%)	3.1110 (3.0011-3.2294)	74.98% (67.68%-81.82%)	65.18% (55.38%-75.41%)	91.759% (83.871%-97.62%)	93.13% (86.05%-98%)	60.57% (50.77%-70.69%)	3.1004 (2.8703-3.3005)
At least 4 of CHASM (breast), CHASM (lung), FATHMM (cancer), VEST	64.68% (60.61%-68.18%)	45.87% (40.83%-50.83%)	97.209% (95.588%-100%)	96.6% (94.58%-100%)	50.95% (46.12%-55.92%)	2.9062 (2.8113-2.9984)	64.58% (57.58%-71.72%)	45.49% (35.48%-55.74%)	97.337% (92.5%-100%)	96.74% (91.67%-100%)	50.99% (41.86%-60.81%)	2.9053 (2.7255-3.1021)
At least 4 of CHASM (breast), CHASM (lung), FATHMM (missense), Mutation Assessor	65.35% (61.62%-69.19%)	46.92% (41.88%-52.03%)	97.203% (95.588%-100%)	96.67% (94.64%-100%)	51.44% (46.55%-56.9%)	2.9223 (2.8274-3.0204)	65.26% (57.58%-72.73%)	46.56% (36.36%-56.45%)	97.337% (92.5%-100%)	96.78% (91.17%-100%)	51.49% (42.65%-61.54%)	2.9212 (2.7235-3.1137)
At least 4 of CHASM (breast), CHASM (lung), FATHMM (missense), Mutation Assessor, MutationTaster	75.13% (71.72%-78.79%)	64.47% (59.5%-69.42%)	93.555% (90.537%-97.102%)	94.5% (92%-97.62%)	60.36% (54.95%-65.64%)	3.1291 (3.0209-3.2434)	75% (67.68%-81.82%)	64.13% (53.97%-73.77%)	93.631% (86.842%-100%)	94.52% (88.1%-100%)	60.34% (49.9%-70.92%)	3.1261 (2.8965-3.3399)
At least 4 of CHASM (breast), CHASM (lung), FATHMM (missense), Mutation Assessor, PolyPhen-2	72.12% (68.69%-75.76%)	59.72% (54.69%-64.8%)	93.55% (90.537%-97.102%)	94.12% (91.38%-97.44%)	57.32% (52.14%-62.07%)	3.0471 (2.9441-3.1605)	71.93% (64.65%-78.79%)	59.26% (49.18%-69.12%)	93.631% (86.842%-100%)	94.1% (87.18%-100%)	57.26% (47.37%-67.74%)	3.0425 (2.8149-3.2490)
At least 4 of CHASM (breast), CHASM (lung), FATHMM (missense), Mutation Assessor, PROVEAN	69.38% (66.16%-73.23%)	54.35% (49.22%-59.5%)	95.358% (92.857%-98.592%)	95.29% (92.78%-98.55%)	54.72% (49.59%-59.98%)	2.9972 (2.8963-3.1000)	69.32% (62.63%-75.76%)	54.03% (43.74%-64.06%)	95.521% (89.474%-100%)	95.39% (89.19%-100%)	54.77% (45.45%-64.41%)	2.9971 (2.7920-3.1967)
At least 4 of CHASM (breast), CHASM (lung), FATHMM (missense), Mutation Assessor, SIFT	73.8% (70.2%-77.27%)	62.37% (57.14%-67.42%)	93.55% (90.537%-97.102%)	94.35% (91.76%-97.56%)	58.98% (53.96%-64.11%)	3.0926 (2.9868-3.2036)	73.62% (66.67%-80.81%)	61.89% (52.54%-71.64%)	93.631% (86.842%-100%)	94.34% (88.09%-100%)	58.91% (49.09%-69.09%)	3.0878 (2.8530-3.3045)
At least 4 of CHASM (breast), CHASM (lung), FATHMM (missense), Mutation Assessor, VEST	70.78% (67.17%-74.24%)	57.61% (52.38%-62.7%)	93.55% (90.537%-97.102%)	93.91% (91.14%-97.3%)	56.07% (51.2%-60.81%)	3.0114 (2.9054-3.1336)	70.56% (63.64%-77.78%)	57.12% (46.66%-67.21%)	93.631% (86.842%-100%)	93.89% (86.66%-100%)	55.98% (46.28%-66.1%)	3.0060 (2.7666-3.2174)
At least 4 of CHASM (breast), CHASM (lung), FATHMM (missense), MutationTaster	69.03% (65.15%-72.73%)	52.19% (47.11%-57.6%)	98.143% (97.015%-100%)	97.99% (96.72%-100%)	54.28% (49.29%-59.06%)	3.0260 (2.9289-3.1282)	69.01% (62.62%-76.77%)	51.99% (41.38%-62.3%)	98.209% (94.118%-100%)	98.04% (93.75%-100%)	54.34% (44.78%-63.64%)	3.0252 (2.8182-3.2198)
At least 4 of CHASM (breast), CHASM (lung), FATHMM (missense), MutationTaster, PolyPhen-2	76.47% (73.23%-79.8%)	66.58% (61.76%-71.43%)	93.555% (90.537%-97.102%)	94.69% (92.22%-97.73%)	61.82% (56.48%-67.27%)	3.1665 (3.0563-3.2780)	76.36% (69.7%-82.83%)	66.28% (56.25%-75.71%)	93.631% (86.842%-100%)	94.67% (88.89%-100%)	61.81% (51.56%-71.43%)	3.1642 (2.9459-3.3784)
At least 4 of CHASM (breast), CHASM (lung), FATHMM (missense), MutationTaster, PROVEAN	74.07% (70.71%-77.27%)	61.21% (56.1%-66.41%)	96.298% (94.203%-98.684%)	96.62% (94.67%-98.8%)	58.95% (53.73%-64.11%)	3.1308 (3.0233-3.2315)	74.08% (67.68%-80.81%)	61.07% (50.82%-70.77%)	96.392% (90.909%-100%)	96.68% (91.67%-100%)	59.07% (49.12%-69.49%)	3.1321 (2.9319-3.3327)
At least 4 of CHASM (breast), CHASM (lung), FATHMM (missense), MutationTaster, SIFT	78.16% (74.75%-81.31%)	68.71% (63.93%-73.77%)	94.49% (91.667%-97.333%)	95.61% (93.33%-97.87%)	63.66% (58.41%-68.47%)	3.2236 (3.1163-3.3325)	78.02% (71.69%-84.85%)	68.4% (58.33%-77.97%)	94.558% (88.235%-100%)	95.52% (90.24%-100%)	63.55% (53.46%-74.14%)	3.2198 (3.0078-3.4313)
At least 4 of CHASM (breast), CHASM (lung), FATHMM (missense), MutationTaster, VEST	74.44% (71.21%-77.78%)	63.38% (58.65%-68.55%)	93.555% (90.537%-97.102%)	94.44% (91.89%-97.59%)	59.64% (54.37%-64.55%)	3.1101 (3.0017-3.2169)	74.36% (67.68%-80.81%)	63.11% (52.46%-72.88%)	93.631% (86.842%-100%)	94.45% (88.1%-100%)	59.67% (50%-70.18%)	3.1085 (2.8942-3.3277)
At least 4 of CHASM (breast), CHASM (lung), FATHMM (missense), PolyPhen-2	67.03% (63.13%-70.71%)	49.58% (44.35%-54.48%)	97.203% (95.588%-100%)	96.84% (95%-100%)	52.72% (47.74%-57.59%)	2.9634 (2.8623-3.0697)	66.95% (59.6%-74.75%)	49.23% (39.06%-59.65%)	97.337% (92.5%-100%)	96.95% (91.66%-100%)	52.77% (43.66%-62.69%)	2.9628 (2.7484-3.1679)
At least 4 of CHASM (breast), CHASM (lung), FATHMM (missense), PolyPhen-2, PROVEAN	72.06% (68.69%-75.25%)	58.59% (53.54%-63.78%)	95.358% (92.857%-98.592%)	95.53% (93.33%-98.62%)	57.11% (52.1%-61.99%)	3.0670 (2.9633-3.1764)	72.04% (65.66%-78.79%)	58.29% (47.62%-68.33%)	95.521% (89.474%-100%)	95.72% (90%-100%)	57.2% (47.54%-66.67%)	3.0679 (2.8534-3.2718)
At least 4 of CHASM (breast), CHASM (lung), FATHMM (missense), PolyPhen-2, SIFT	76.14% (72.73%-79.8%)	66.61% (61.67%-71.43%)	92.612% (89.333%-96.053%)	93.97% (91.36%-96.81%)	61.6% (56.19%-66.37%)	3.1498 (3.0379-3.2654)	76% (69.7%-82.83%)	66.22% (56.14%-75.86%)	92.753% (85.714%-100%)	93.99% (87.5%-100%)	61.55% (51.85%-71.93%)	3.1451 (2.9221-3.3657)
At least 4 of CHASM (breast), CHASM (lung), FATHMM (missense), PolyPhen-2, VEST	72.78% (69.19%-76.26%)	61.84% (56.91%-66.82%)	91.698% (88.46%-95.714%)	92.79% (89.87%-96.3%)	58.16% (53.13%-63.63%)	3.0448 (2.9327-3.1582)	72.62% (65.66%-79.8%)	61.4% (51.56%-71.2%)	91.84% (84.085%-97.62%)	92.79% (85.37%-97.87%)	58.1% (48.15%-68.34%)	3.0415 (2.8157-3.2727)
At least 4 of CHASM (breast), CHASM (lung), FATHMM (missense), PROVEAN	59.65% (55.56%-63.13%)	36.85% (32.23%-41.6%)	99.084% (98.485%-100%)	98.59% (97.56%-100%)	47.57% (42.76%-51.67%)	2.8116 (2.7245-2.8966)	59.48% (52.53%-66.67%)	36.39% (26.66%-46.16%)	99.083% (96.774%-100%)	98.56% (94.74%-100%)	47.58% (38.67%-57.14%)	2.8161 (2.6428-2.9919)
At least 4 of CHASM (breast), CHASM (lung), FATHMM (missense), PROVEAN, SIFT	73.1% (69.7%-76.77%)	59.67% (54.68%-64.8%)	96.298% (94.203%-98.684%)	96.54% (94.51%-98.8%)	58% (52.99%-62.7%)	3.1051 (3.0015-3.2136)	73% (65.66%-79.8%)	59.35% (49.15%-69.44%)	96.392% (90.909%-100%)	96.58% (91.42%-100%)	58.02% (48.33%-67.92%)	3.1034 (2.9048-3.3000)
At least 4 of CHASM (breast), CHASM (lung), FATHMM (missense), PROVEAN, VEST	67.36% (63.64%-70.71%)	51.17% (45.83%-56.35%)	95.358% (92.857%-98.592%)	95.02% (92.42%-98.46%)	53.04% (48.12%-57.66%)	2.9458 (2.8421-3.0532)	67.3% (60.61%-74.75%)	50.8% (40.98%-61.29%)	95.521% (89.474%-100%)	95.12% (88.57%-100%)	53.1% (43.55%-62.5%)	2.9458 (2.7397-3.1560)
At least 4 of CHASM (breast), CHASM (lung), FATHMM (missense), SIFT	69.74% (66.16%-73.23%)	53.31% (48.36%-58.4%)	98.143% (97.015%-100%)	98.03% (96.77%-100%)	54.87% (50%-59.54%)	3.0434 (2.9529-3.1424)	69.62% (62.63%-76.77%)	52.94% (42.42%-62.72%)	98.209% (94.118%-100%)	98.07% (93.94%-100%)	54.87% (45.71%-64.62%)	3.0409 (2.8473-3.2408)
At least 4 of CHASM (breast), CHASM (lung), FATHMM (missense), SIFT, VEST	73.79% (70.2%-77.27%)	63.97% (59.02%-69.05%)	90.746% (87.143%-94.521%)	92.28% (89.29%-95.56%)	59.3% (54.13%-64.59%)	3.0629 (2.9467-3.1825)	73.64% (66.67%-80.81%)	63.51% (53.22%-73.33%)	90.976% (83.333%-97.44%)	92.34% (85.37%-97.87%)	59.24% (49.12%-69.65%)	3.0607 (2.8132-3.2797)
At least 4 of CHASM (breast), CHASM (lung), FATHMM (missense), VEST	61.99% (58.08%-65.66%)	41.6% (36.51%-46.72%)	97.203% (95.588%-100%)	96.35% (94%-100%)	49.06% (44.34%-53.42%)	2.8415 (2.7457-2.9365)	61.87% (54.55%-69.7%)	41.2% (30.77%-51.56%)	97.337% (92.5%-100%)	96.42% (90.91%-100%)	49.1% (39.99%-58.5%)	2.8403 (2.6527-3.0362)
At least 4 of CHASM (breast), CHASM (lung), Mutation Assessor, MutationTaster	63.3% (59.09%-67.17%)	43.69% (38.46%-48.8%)	97.222% (95.588%-100%)	96.45% (94.23%-100%)	49.96% (45.07%-54.61%)	2.8732 (2.7792-2.9765)	63.29% (55.56%-71.72%)	43.47% (33.33%-54.24%)	97.294% (92.303%-100%)	96.5% (90.48%-100%)	50.08% (40.62%-59.7%)	2.8733 (2.6668-3.0686)
At least 4 of CHASM (breast), CHASM (lung), Mutation Assessor, MutationTaster, PolyPhen-2	74.12% (70.71%-77.27%)	62.88% (57.85%-67.97%)	93.555% (90.537%-97.102%)	94.4% (91.86%-97.59%)	59.31% (54.46%-64.44%)	3.1014 (2.9934-3.2132)	73.98% (67.68%-80.81%)	62.51% (52.63%-72.46%)	93.631% (86.842%-100%)	94.39% (88.1%-100%)	59.28% (49.18%-69.35%)	3.0982 (2.8789-3.3206)
At least 4 of CHASM (breast), CHASM (lung), Mutation Assessor, MutationTaster, PROVEAN	69.71% (66.16%-73.23%)	54.86% (49.57%-59.85%)	95.377% (92.958%-98.571%)	95.3% (92.75%-98.57%)	55% (50%-59.67%)	3.0058 (2.9028-3.1178)	69.68% (62.63%-76.77%)	54.62% (44.44%-65.08%)	95.484% (89.651%-100%)	95.39% (89.47%-100%)	55.09% (45.9%-65.15%)	3.0057 (2.7994-3.2152)
At least 4 of CHASM (breast), CHASM (lung), Mutation Assessor, MutationTaster, SIFT	73.45% (69.7%-76.77%)	61.2% (56.35%-66.4%)	94.49% (91.667%-97.333%)	95.06% (92.59%-97.65%)	58.53% (53.33%-63.64%)	3.0934 (2.9808-3.2015)	73.31% (66.67%-80.81%)	60.94% (50.79%-70.97%)	94.503% (88.235%-100%)	95% (88.89%-100%)	58.51% (48.38%-69.09%)	3.0894 (2.8749-3.3169)
At least 4 of CHASM (breast), CHASM (lung), Mutation Assessor, MutationTaster, VEST	71.76% (68.18%-75.25%)	59.16% (54.18%-64.06%)	93.55% (90.537%-97.102%)	94.07% (91.46%-97.4%)	56.98% (51.72%-61.9%)	3.0376 (2.9314-3.1453)	71.63% (64.65%-78.79%)	58.79% (49.18%-68.85%)	93.631% (86.842%-100%)	94.06% (87.5%-100%)	56.97% (47.27%-67.24%)	3.0345 (2.8158-3.2473)
At least 4 of CHASM (breast), CHASM (lung), Mutation Assessor, PolyPhen-2	64.32% (60.61%-68.18%)	45.83% (40.8%-51.18%)	96.282% (94.203%-98.667%)	95.52% (92.98%-98.53%)	50.69% (45.83%-55.47%)	2.8832 (2.7850-2.9825)	64.29% (56.57%-71.72%)	45.56% (35.29%-55.74%)	96.423% (91.176%-100%)	95.62% (89.29%-100%)	50.79% (41.54%-60.66%)	2.8840 (2.6678-3.0841)
At least 4 of CHASM (breast), CHASM (lung), Mutation Assessor, PolyPhen-2, PROVEAN	69.38% (65.66%-73.23%)	54.89% (50%-60%)	94.437% (91.667%-97.333%)	94.49% (91.78%-97.4%)	54.77% (49.99%-59.59%)	2.9855 (2.8818-3.0909)	69.32% (62.63%-76.77%)	54.57% (44.26%-65.08%)	94.607% (88.889%-100%)	94.54% (87.8%-100%)	54.82% (45.16%-64.82%)	2.9854 (2.7619-3.1879)
At least 4 of CHASM (breast), CHASM (lung), Mutation Assessor, PolyPhen-2, SIFT	72.1% (68.69%-75.76%)	59.69% (54.84%-64.8%)	93.555% (90.537%-97.102%)	94.11% (91.57%-97.47%)	57.31% (52.16%-62.3%)	3.0466 (2.9416-3.1602)	71.96% (64.65%-78.79%)	59.32% (49.21%-69.35%)	93.631% (86.842%-100%)	94.1% (87.5%-100%)	57.29% (47.37%-67.8%)	3.0434 (2.8150-3.2609)
At least 4 of CHASM (breast), CHASM (lung), Mutation Assessor, PolyPhen-2, VEST	72.44% (68.69%-76.26%)	60.23% (55.04%-65.55%)	93.555% (90.537%-97.102%)	94.16% (91.55%-97.4%)	57.64% (52.63%-62.6%)	3.0559 (2.9480-3.1721)	72.28% (64.65%-79.8%)	59.83% (50%-69.85%)	93.631% (86.842%-100%)	94.15% (87.5%-100%)	57.6% (47.54%-67.87%)	3.0521 (2.8280-3.2720)
At least 4 of CHASM (breast), CHASM (lung), Mutation Assessor, PROVEAN	58.63% (54.55%-62.63%)	35.22% (30.51%-40%)	99.084% (98.485%-100%)	98.52% (97.44%-100%)	46.94% (42.21%-51.32%)	2.7977 (2.7129-2.8868)	58.51% (50.51%-66.67%)	34.85% (25.39%-44.45%)	99.083% (96.774%-100%)	98.49% (94.44%-100%)	46.98% (38.16%-56.41%)	2.7941 (2.6126-2.9704)
At least 4 of CHASM (breast), CHASM (lung), Mutation Assessor, PROVEAN, SIFT	70.04% (66.16%-73.74%)	55.38% (50%-60.48%)	95.377% (92.958%-98.582%)	95.39% (92.86%-98.59%)	55.3% (50.4%-60%)	3.0144 (2.9097-3.1206)	70.02% (62.63%-76.77%)	55.14% (44.82%-65.57%)	95.478% (89.474%-100%)	95.43% (89.29%-100%)	55.42% (45.76%-65.08%)	3.0148 (2.7948-3.2199)
At least 4 of CHASM (breast), CHASM (lung), Mutation Assessor, PROVEAN, VEST	66.37% (62.63%-69.7%)	50.13% (44.8%-55.04%)	94.437% (91.667%-97.333%)	93.97% (91.07%-97.18%)	52.27% (47.56%-57.26%)	2.9081 (2.8011-3.0118)	66.25% (59.6%-73.74%)	49.71% (39.71%-60.32%)	94.607% (88.589%-100%)	94.05% (86.67%-100%)	52.3% (42.85%-61.9%)	2.9066 (2.6803-3.1297)
At least 4 of CHASM (breast), CHASM (lung), Mutation Assessor, SIFT	65.34% (61.62%-69.19%)	46.9% (41.8%-52%)	97.222% (95.588%-100%)	96.69% (94.64%-100%)	51.43% (46.56%-56.25%)	2.9224 (2.8230-3.0253)	65.28% (57.58%-72.73%)	46.6% (36.66%-56.67%)	97.294% (92.303%-100%)	96.76% (91.18%-100%)	51.5% (42.42%-61.11%)	2.9212 (2.7222-3.1168)
At least 4 of CHASM (breast), CHASM (lung), Mutation Assessor, SIFT, VEST	71.42% (67.68%-75.25%)	58.62% (53.49%-63.93%)	93.555% (90.537%-97.102%)	94.01% (91.3%-97.44%)	56.67% (51.64%-61.7%)	3.0285 (2.9193-3.1446)	71.29% (63.64%-78.79%)	58.26% (48.37%-68.33%)	93.631% (86.842%-100%)	94% (87.1%-100%)	56.66% (46.15%-66.67%)	3.0256 (2.7980-3.2473)
At least 4 of CHASM (breast), CHASM (lung), Mutation Assessor, VEST	60.28% (56.06%-64.14%)	39.46% (34.35%-44.8%)	96.282% (94.203%-98.667%)	94.83% (91.8%-98.15%)	47.91% (43.2%-52.48%)	2.7849 (2.6838-2.8880)	60.24% (53.54%-68.69%)	39.15% (28.57%-50%)	96.423% (91.176%-100%)	94.95% (87.5%-100%)	48.01% (38.89%-57.53%)	2.7853 (2.5785-2.9916)
At least 4 of CHASM (breast), CHASM (lung), MutationTaster, PolyPhen-2	67.01% (63.13%-70.71%)	49.53% (44.26%-54.69%)	97.222% (95.588%-100%)	96.86% (94.92%-100%)	52.7% (47.76%-57.55%)	2.9631 (2.8636-3.0675)	66.95% (59.6%-73.74%)	49.34% (38.98%-59.7%)	97.294% (92.303%-100%)	96.91% (91.67%-100%)	52.81% (43.77%-62.71%)	2.9635 (2.7623-3.1605)
At least 4 of CHASM (breast), CHASM (lung), MutationTaster, PolyPhen-2, PROVEAN	72.39% (68.69%-75.76%)	59.1% (53.97%-63.65%)	95.377% (92.958%-98.582%)	95.67% (93.33%-98.67%)	57.42% (52.46%-62.28%)	3.0757 (2.9722-3.1852)	72.38% (65.66%-78.79%)	58.91% (49.09%-69.23%)	95.478% (90%-100%)	95.72% (90.24%-100%)	57.53% (48.14%-67.12%)	3.0763 (2.8655-3.2944)
At least 4 of CHASM (breast), CHASM (lung), MutationTaster, PolyPhen-2, SIFT	76.14% (72.73%-79.8%)	65.53% (60.34%-70.4%)	94.49% (91.667%-97.333%)	95.36% (92.98%-97.78%)	61.32% (56.14%-66.52%)	3.1670 (3.0593-3.2806)	76.01% (69.7%-82.83%)	65.18% (55.56%-74.63%)	94.503% (88.235%-100%)	95.31% (89.74%-100%)	61.29% (51.61%-71.91%)	3.1629 (2.9358-3.3946)
At least 4 of CHASM (breast), CHASM (lung), MutationTaster, PolyPhen-2, VEST	73.11% (69.7%-76.26%)	61.28% (56.1%-66.13%)	93.555% (90.537%-97.102%)	94.21% (91.76%-97.44%)	58.29% (53.1%-63.26%)	3.0738 (2.9619-3.1893)	72.96% (65.66%-79.8%)	60.92% (50.79%-70.97%)	93.631% (86.842%-100%)	94.2% (87.8%-100%)	58.27% (48.08%-68.42%)	3.0709 (2.8469-3.2927)
At least 4 of CHASM (breast), CHASM (lung), MutationTaster, PROVEAN	60.3% (56.06%-64.14%)	37.87% (33.06%-42.86%)	99.084% (98.485%-100%)	98.62% (97.62%-100%)	47.98% (43.22%-52.32%)	2.8356 (2.7468-2.9221)	60.2% (52.53%-68.69%)	37.54% (27.42%-47.83%)	99.083% (96.774%-100%)	98.6% (95%-100%)	48.03% (39.24%-57.65%)	2.8326 (2.6663-3.0163)
At least 4 of CHASM (breast), CHASM (lung), MutationTaster, PROVEAN, SIFT	72.06% (68.18%-75.76%)	57.5% (52.64%-62.1%)	96.298% (94.203%-98.684%)	96.54% (94.44%-98.8%)	57.1% (51.9%-61.94%)	3.0496 (2.9485-3.1537)	71.96% (64.65%-78.79%)	57.18% (47.19%-66.67%)	96.392% (90.909%-100%)	96.55% (91.18%-100%)	57.17% (47.62%-67.27%)	3.0460 (2.8372-3.2430)
At least 4 of CHASM (breast), CHASM (lung), MutationTaster, PROVEAN, VEST	68.04% (64.14%-71.72%)	52.22% (46.77%-57.48%)	95.377% (92.958%-98.582%)	95.13% (92.42%-98.51%)	53.59% (48.8%-58.34%)	2.9632 (2.8581-3.0684)	67.96% (60.61%-75.76%)	51.91% (41.43%-62.5%)	95.478% (90%-100%)	95.17% (88.89%-100%)	53.65% (44.03%-64.64%)	2.9621 (2.7438-3.1775)
At least 4 of CHASM (breast), CHASM (lung), MutationTaster, SIFT	67.35% (63.64%-70.71%)	50.06% (44.83%-55.2%)	97.222% (95.588%-100%)	96.89% (95%-100%)	52.96% (47.97%-57.66%)	2.9714 (2.8738-3.0712)	67.33% (60.61%-74.75%)	49.86% (39.28%-60.32%)	97.294% (92.303%-100%)	96.93% (91.67%-100%)	53.07% (43.74%-62.32%)	2.9715 (2.7705-3.1741)
At least 4 of CHASM (breast), CHASM (lung), MutationTaster, SIFT, VEST	73.46% (70.2%-76.77%)	61.28% (56.25%-66.13%)	94.49% (91.667%-97.333%)	95.09% (92.55%-97.67%)	58.53% (53.25%-63.3%)	3.0936 (2.9878-3.2018)	73.29% (66.67%-79.8%)	60.92% (50.88%-70.97%)	94.503% (88.235%-100%)	95% (89.19%-100%)	58.49% (48.39%-68.52%)	3.0891 (2.8712-3.3020)
At least 4 of CHASM (breast), CHASM (lung), MutationTaster, VEST	63.66% (59.6%-67.17%)	44.25% (38.93%-49.59%)	97.222% (95.588%-100%)	96.5% (94.34%-100%)	50.22% (45.39%-54.84%)	2.8819 (2.7842-2.9823)	63.58% (56.57%-70.71%)	43.93% (33.33%-54.39%)	97.294% (92.303%-100%)	96.54% (90.62%-100%)	50.28% (40.91%-60.27%)	2.8805 (2.6783-3.0812)
At least 4 of CHASM (breast), CHASM (lung), PolyPhen-2, PROVEAN	60.32% (56.57%-64.14%)	37.89% (32.82%-42.86%)	99.084% (98.485%-100%)	98.62% (97.62%-100%)	47.99% (43.18%-52.48%)	2.8358 (2.7501-2.9220)	60.18% (52.53%-67.68%)	37.49% (27.27%-47.46%)	99.083% (96.774%-100%)	98.59% (94.74%-100%)	48.02% (39.29%-57.54%)	2.8319 (2.6663-3.0062)
At least 4 of CHASM (breast), CHASM (lung), PolyPhen-2, PROVEAN, SIFT	70.73% (67.17%-74.24%)	56.47% (51.55%-61.42%)	95.377% (92.958%-98.582%)	95.48% (92.96%-98.59%)	55.99% (50.83%-60.84%)	3.0322 (2.9342-3.1433)	70.66% (63.64%-77.78%)	56.19% (46.03%-66.67%)	95.478% (90%-100%)	95.51% (89.66%-100%)	55.95% (46.43%-65.65%)	3.0312 (2.8181-3.2430)
At least 4 of CHASM (breast), CHASM (lung), PolyPhen-2, PROVEAN, VEST	67.71% (64.14%-71.21%)	52.25% (46.72%-57.14%)	94.437% (91.667%-97.333%)	94.2% (91.3%-97.26%)	53.35% (48.47%-57.89%)	2.9424 (2.8359-3.0509)	67.61% (60.61%-74.75%)	51.86% (42.03%-62.32%)	94.607% (88.235%-100%)	94.29% (87.49%-100%)	53.39% (44.55%-63.16%)	2.9414 (2.7204-3.1495)
At least 4 of CHASM (breast), CHASM (lung), PolyPhen-2, SIFT	67.02% (63.13%-70.71%)	49.55% (44.09%-54.84%)	97.222% (95.588%-100%)	96.87% (94.92%-100%)	52.71% (47.73%-57.27%)	2.9635 (2.8627-3.0630)	66.98% (59.6%-74.75%)	49.32% (39.28%-59.65%)	97.294% (92.303%-100%)	96.96% (91.42%-100%)	52.8% (43.55%-61.9%)	2.9625 (2.7620-3.1651)
At least 4 of CHASM (breast), CHASM (lung), PolyPhen-2, SIFT, VEST	72.8% (69.19%-76.26%)	61.85% (56.91%-66.93%)	91.727% (88.31%-95.588%)	92.81% (89.87%-96.3%)	58.17% (53.15%-63.07%)	3.0455 (2.9312-3.1579)	72.59% (65.66%-79.8%)	61.39% (51.47%-71.19%)	91.783% (84.375%-97.67%)	92.75% (85.71%-97.92%)	58.08% (48.15%-67.67%)	3.0400 (2.8183-3.2711)
At least 4 of CHASM (breast), CHASM (lung), PolyPhen-2, VEST	62.64% (58.59%-66.17%)	43.18% (38.1%-48.33%)	96.282% (94.284%-98.667%)	95.26% (92.59%-98.31%)	49.17% (44.39%-53.85%)	2.8422 (2.7402-2.9418)	62.6% (55.56%-69.7%)	42.88% (32.84%-53.23%)	96.423% (91.176%-100%)	95.37% (88.46%-100%)	49.59% (40.32%-59.15%)	2.8427 (2.6361-3.0513)
At least 4 of CHASM (breast), CHASM (lung), PROVEAN, SIFT	61.98% (58.08%-65.66%)	40.53% (35.66%-45.24%)	99.084% (98.485%-100%)	98.71% (97.78%-100%)	49% (44.09%-53.46%)	2.8733 (2.7864-2.9613)	61.89% (54.55%-69.7%)	40.21% (30%-50%)	99.083% (96.774%-100%)	98.69% (95.24%-100%)	49.05% (40.26%-58.23%)	2.8701 (2.6926-3.0374)
At least 4 of CHASM (breast), CHASM (lung), PROVEAN, SIFT, VEST	67.37% (63.64%-70.71%)	51.17% (45.83%-56.6%)	95.377% (92.958%-98.582%)	95.03% (92.31%-98.51%)	53.04% (48.17%-57.95%)	2.9462 (2.8424-3.0532)	67.28% (60.61%-74.75%)	50.83% (40.96%-61.29%)	95.478% (90%-100%)	95.09% (88.57%-100%)	53.09% (43.52%-62.32%)	2.9448 (2.7255-3.1559)
At least 4 of CHASM (breast), CHASM (lung), PROVEAN, VEST	59.64% (55.56%-63.64%)	36.82% (32.03%-41.41%)	99.084% (98.485%-100%)	98.59% (97.5%-100%)	47.56% (42.86%-52.48%)	2.8119 (2.7243-2.8969)	59.51% (51.52%-67.68%)	36.48% (26.76%-46.88%)	99.083% (96.774%-100%)	98.56% (94.74%-100%)	47.6% (38.67%-57.14%)	2.8108 (2.6467-2.9912)
At least 4 of CHASM (breast), CHASM (lung), SIFT, VEST	63.32% (59.09%-67.17%)	44.24% (38.98%-49.21%)	96.282% (94.284%-98.667%)	95.39% (92.73%-98.39%)	49.9% (45.07%-54.52%)	2.8576 (2.7553-2.9587)	63.26% (56.57%-70.71%)	43.91% (32.81%-54.1%)	96.423% (91.176%-100%)	95.5% (88.89%-100%)	50.05% (40.62%-59.68%)	2.8587 (2.6443-3.0693)
At least 4 of CHASM (breast), CHASM (melanoma), FATHMM (cancer), FATHMM (missense)	78.84% (73.23%-80.3%)	66.65% (61.9%-71.43%)	94.45% (91.549%-97.333%)	95.42% (93.13%-97.83%)	62.1% (56.6%-67.16%)	3.1863 (3.0807-3.2973)	76.61% (69.7%-83.84%)	66.16% (56.14%-75.45%)	94.535% (88.235%-100%)	95.41% (89.79%-100%)	61.95% (50.94%-72.88%)	3.1805 (2.9611-3.4035)
At least 4 of CHASM (breast), CHASM (melanoma), FATHMM (cancer), FATHMM (missense), Mutation Assessor	83.19% (80.3%-86.36%)	78.23% (74.07%-82.46%)	91.725% (88.406%-95.775%)	94.2% (92.08%-96.97%)	70.91% (65.67%-76.29%)	3.3515 (3.2427-3.4667)	83.12% (76.77%-88.89%)	78.1% (69.49%-85.57%)	91.669% (84.383%-97.62%)	94.18% (88.89%-98.18%)	70.82% (60.47%-81.25%)	3.3492 (3.1209-3.5676)
At least 4 of CHASM (breast), CHASM (melanoma), FATHMM (cancer), FATHMM (missense), MutationTaster	85.55% (82.83%-88.89%)	81.95% (78.29%-85.96%)	91.725% (88.406%-95.775%)	94.51% (92.45%-97.12%)	74.62% (69.32%-80%)	3.4286 (3.3219-3.5487)	85.45% (78.79%-90.91%)	81.83% (73.77%-89.4%)	91.669% (84.383%-97.62%)	94.4% (88.89%-98.25%)	74.64% (63.64%-84.78%)	3.4254 (3.1881-3.6388)
At least 4 of CHASM (breast), CHASM (melanoma), FATHMM (cancer), FATHMM (missense), PolyPhen-2	84.54% (81.82%-87.37%)	81.41% (77.58%-85.48%)	89.926% (86.111%-94.366%)	93.32% (90.91%-96.3%)	73.67% (68.39%-78.76%)	3.3834 (3.2683-3.5033)	84.46% (78.79%-89.9%)	81.31% (72.13%-88.89%)	89.843% (80.643%-97.37%)	93.23% (87.49%-98.18%)	73.7% (62.5%-84.21%)	3.3808 (3.1455-3.6046)
At least 4 of CHASM (breast), CHASM (melanoma), FATHMM (cancer), FATHMM (missense), PROVEAN	81.54% (78.28%-84.85%)	74.59% (70%-78.79%)	92.637% (89.333%-96.053%)	94.24% (92.06%-97.18%)	68.05% (62.63%-73.53%)	3.3020 (3.1933-3.4150)	81.43% (74.75%-87.88%)	74.24% (65.08%-82.81%)	92.706% (85.714%-100%)	94.21% (88.89%-100%)	67.92% (57.78%-78.26%)	3.2980 (3.0727-3.5236)
At least 4 of CHASM (breast), CHASM (melanoma), FATHMM (cancer), FATHMM (missense), SIFT	84.18% (81.31%-87.37%)	80.87% (77.05%-85.04%)	89.901% (86.111%-94.366%)	93.27% (90.8%-96.23%)	73.13% (67.86%-78.65%)	3.3714 (3.2556-3.4932)	84.09% (78.79%-89.9%)	80.8% (72.31%-88.33%)	89.887% (81.579%-97.3%)	93.22% (87.1%-98.15%)	73.2% (62.5%-83.33%)	3.3711 (3.1322-3.5980)
At least 4 of CHASM (breast), CHASM (melanoma), FATHMM (cancer), FATHMM (missense), VEST	84.87% (81.82%-87.88%)	81.95% (78.29%-86.02%)	89.901% (86.111%-94.366%)	93.35% (90.99%-96.3%)	74.23% (68.99%-79.78%)	3.3943 (3.2843-3.5139)	84.81% (78.79%-90.91%)	81.83% (73.77%-89.29%)	89.887% (81.579%-97.3%)	93.3% (87.72%-98.15%)	74.26% (63.27%-84.62%)	3.3928 (3.1520-3.6247)
At least 4 of CHASM (breast), CHASM (melanoma), FATHMM (cancer), Mutation Assessor	65.34% (61.62%-69.19%)	47.44% (42.37%-52.76%)	96.293% (94.203%-98.684%)	95.66% (93.1%-98.51%)	51.51% (46.61%-56.19%)	2.9086 (2.8107-3.0062)	65.3% (58.59%-72.73%)	47.13% (36.21%-57.89%)	96.381% (90.909%-100%)	95.75% (89.19%-100%)	51.51% (42.19%-60.87%)	2.9079 (2.7073-3.1051)
At least 4 of CHASM (breast), CHASM (melanoma), FATHMM (cancer), Mutation Assessor, MutationTaster	80.45% (77.27%-83.84%)	72.65% (68.46%-77.6%)	93.583% (90.541%-97.183%)	95.12% (92.75%-97.92%)	66.52% (61.07%-71.84%)	3.2856 (3.1803-3.3967)	80.52% (73.74%-86.87%)	72.9% (63.64%-82.26%)	93.565% (86.838%-100%)	95.11% (89.58%-100%)	66.63% (56.52%-77.55%)	3.2839 (3.0604-3.4970)
At least 4 of CHASM (breast), CHASM (melanoma), FATHMM (cancer), Mutation Assessor, PolyPhen-2	76.08% (72.73%-79.8%)	67.58% (62.99%-72.44%)	90.708% (87.143%-94.596%)	92.7% (89.89%-95.96%)	61.82% (56.45%-67.17%)	3.1287 (3.0177-3.2463)	75.98% (68.69%-82.83%)	67.33% (57.38%-76.67%)	90.888% (82.755%-97.67%)	92.74% (86.54%-97.92%)	61.84% (51.67%-71.92%)	3.1279 (2.8936-3.3512)
At least 4 of CHASM (breast), CHASM (melanoma), FATHMM (cancer), Mutation Assessor, PROVEAN	71.73% (68.18%-75.25%)	58.61% (53.54%-63.78%)	94.421% (91.549%-97.368%)	94.78% (92.21%-97.53%)	56.89% (51.67%-61.9%)	3.0470 (2.9464-3.1599)	71.72% (64.65%-78.79%)	58.29% (48.33%-68.34%)	94.628% (87.879%-100%)	94.92% (88.89%-100%)	56.94% (47.27%-66.67%)	3.0477 (2.8199-3.2459)
At least 4 of CHASM (breast), CHASM (melanoma), FATHMM (cancer), Mutation Assessor, SIFT	78.44% (75.25%-81.82%)	70.77% (66.39%-75.81%)	91.708% (88.406%-95.652%)	93.53% (91.01%-96.74%)	64.47% (59.02%-70%)	3.2060 (3.0984-3.3248)	78.47% (71.72%-84.85%)	70.97% (61.02%-79.71%)	91.795% (84.611%-97.73%)	93.67% (87.8%-98.2%)	64.6% (54.17%-74.58%)	3.2074 (2.9666-3.4207)
At least 4 of CHASM (breast), CHASM (melanoma), FATHMM (cancer), Mutation Assessor, VEST	75.76% (72.22%-79.29%)	66.51% (61.72%-71.2%)	91.748% (88.406%-95.775%)	93.31% (90.53%-96.51%)	61.16% (56.18%-66.41%)	3.1366 (3.0248-3.2470)	75.72% (68.69%-82.83%)	66.31% (56.72%-75.81%)	91.728% (84.211%-97.67%)	93.24% (86.96%-98.21%)	61.19% (51.02%-71.43%)	3.1345 (2.9133-3.3648)
At least 4 of CHASM (breast), CHASM (melanoma), FATHMM (cancer), MutationTaster	70.35% (66.67%-73.75%)	54.81% (49.61%-60%)	97.233% (95.588%-100%)	97.16% (95.45%-100%)	55.45% (50.4%-60.33%)	3.0465 (2.9464-3.1499)	70.4% (63.61%-77.78%)	54.74% (44.26%-65%)	97.337% (92.5%-100%)	97.19% (92.5%-100%)	55.61% (46.15%-66.1%)	3.0480 (2.8402-3.2478)
At least 4 of CHASM (breast), CHASM (melanoma), FATHMM (cancer), MutationTaster, PolyPhen-2	80.12% (76.77%-83.33%)	72.85% (68.29%-77.6%)	92.676% (89.394%-96.053%)	94.5% (92.07%-97.03%)	66.28% (60.82%-71.62%)	3.2641 (3.1586-3.3807)	80.06% (72.73%-86.87%)	72.63% (63.49%-81.82%)	92.818% (85.294%-100%)	94.53% (88.89%-100%)	66.59% (55.56%-77.08%)	3.2654 (3.0504-3.4834)
At least 4 of CHASM (breast), CHASM (melanoma), FATHMM (cancer), MutationTaster, PROVEAN	76.78% (73.23%-80.3%)	64.95% (60.16%-70%)	96.274% (94.203%-98.667%)	96.68% (94.74%-98.85%)	61.46% (56.25%-66.67%)	3.2018 (3.1016-3.3035)	76.79% (70.71%-83.84%)	64.78% (54.55%-74.63%)	96.378% (90.625%-100%)	96.71% (91.43%-100%)	61.53% (51.79%-71.93%)	3.2022 (2.9990-3.4218)
At least 4 of CHASM (breast), CHASM (melanoma), FATHMM (cancer), MutationTaster, SIFT	82.47% (79.29%-85.86%)	75.49% (71.31%-80.16%)	94.458% (91.667%-97.368%)	95.97% (93.88%-98.11%)	69.05% (63.92%-74.23%)	3.3533 (3.2450-3.4598)	82.54% (75.76%-87.88%)	75.59% (66.18%-84.38%)	94.436% (88.235%-100%)	95.94% (90.91%-100%)	69.29% (59.09%-79.59%)	3.3521 (3.1336-3.5595)
At least 4 of CHASM (breast), CHASM (melanoma), FATHMM (cancer), MutationTaster, VEST	79.43% (76.26%-82.83%)	71.23% (66.41%-75.78%)	93.581% (90.541%-97.183%)	95.05% (92.78%-97.8%)	65.3% (60.22%-70.71%)	3.2526 (3.1487-3.3628)	79.53% (72.73%-85.86%)	71.4% (61.81%-80.6%)	93.565% (86.838%-100%)	95.01% (89.13%-100%)	65.46% (54.35%-75.47%)	3.2505 (3.0271-3.4808)
At least 4 of CHASM (breast), CHASM (melanoma), FATHMM (cancer), PolyPhen-2	68.36% (64.65%-72.22%)	52.19% (46.97%-57.5%)	96.293% (94.203%-98.667%)	96.06% (93.85%-98.55%)	53.81% (48.84%-58.54%)	2.9836 (2.8857-3.0863)	68.34% (60.61%-75.76%)	51.96% (41.37%-62.3%)	96.381% (90.909%-100%)	96.13% (90.91%-100%)	53.91% (44.26%-63.64%)	2.9842 (2.7855-3.1807)
At least 4 of CHASM (breast), CHASM (melanoma), FATHMM (cancer), PolyPhen-2, PROVEAN	74.08% (70.71%-77.78%)	62.32% (57.26%-67.21%)	94.421% (91.549%-97.368%)	95.09% (92.68%-97.67%)	59.17% (53.88%-64.19%)	3.1099 (3.0032-3.2202)	74.06% (66.67%-81.82%)	62.05% (51.78%-71.88%)	94.628% (87.879%-100%)	95.24% (89.47%-100%)	59.24% (49.12%-69.85%)	3.1119 (2.8795-3.3281)
At least 4 of CHASM (breast), CHASM (melanoma), FATHMM (cancer), PolyPhen-2, SIFT	79.43% (76.26%-82.83%)	72.76% (68.18%-77.31%)	90.682% (87.143%-94.595%)	93.18% (90.62%-96.04%)	65.54% (60%-71.02%)	3.2265 (3.1177-3.3444)	79.35% (72.73%-85.86%)	72.88% (63.83%-81.81%)	90.721% (82.758%-97.62%)	93.22% (87.27%-98.04%)	66.15% (55.56%-76.6%)	3.2305 (2.9870-3.4611)
At least 4 of CHASM (breast), CHASM (melanoma), FATHMM (cancer), PolyPhen-2, VEST	76.76% (73.74%-80.3%)	68.63% (64.06%-73.4%)	90.821% (87.324%-95.312%)	92.82% (90.11%-96.39%)	62.61% (57.4%-68.18%)	3.1488 (3.0400-3.2675)	76.73% (69.7%-83.84%)	68.57% (58.73%-77.78%)	90.853% (82.496%-97.37%)	92.77% (86.36%-97.92%)	62.74% (52.47%-73.47%)	3.1489 (2.9081-3.3790)
At least 4 of CHASM (breast), CHASM (melanoma), FATHMM (cancer), PROVEAN	60.97% (57.07%-64.65%)	39.45% (34.43%-44.26%)	98.12% (97.015%-100%)	97.39% (95.65%-100%)	48.49% (44.03%-52.88%)	2.8341 (2.7455-2.9233)	60.89% (53.54%-68.69%)	39.17% (29.13%-49.21%)	98.144% (94.118%-100%)	97.32% (91.3%-100%)	48.6% (39.44%-58.23%)	2.8309 (2.6627-3.0153)
At least 4 of CHASM (breast), CHASM (melanoma), FATHMM (cancer), PROVEAN, SIFT	74.43% (70.71%-77.78%)	62.32% (57.14%-67.46%)	95.376% (92.857%-98.611%)	95.97% (93.75%-98.75%)	59.42% (54.31%-64.4%)	3.1298 (3.0302-3.2373)	74.39% (66.67%-81.82%)	62.14% (52.17%-71.88%)	95.388% (88.888%-100%)	95.96% (90.63%-100%)	59.44% (49.09%-69.49%)	3.1267 (2.9219-3.3437)
At least 4 of CHASM (breast), CHASM (melanoma), FATHMM (cancer), PROVEAN, VEST	70.07% (66.67%-73.74%)	55.43% (50.39%-60.48%)	95.376% (92.857%-98.611%)	95.4% (92.86%-98.55%)	55.31% (50.29%-60.94%)	3.0150 (2.9169-3.1200)	69.97% (62.63%-77.78%)	55.08% (45.16%-65.44%)	95.478% (89.474%-100%)	95.47% (89.66%-100%)	55.33% (45.61%-65.15%)	3.0135 (2.7948-3.2154)
At least 4 of CHASM (breast), CHASM (melanoma), FATHMM (cancer), SIFT	71.07% (67.68%-74.75%)	54.98% (50%-60.16%)	99.084% (98.485%-100%)	99.06% (98.44%-100%)	56.07% (51.16%-61.04%)	3.0957 (3.0055-3.1929)	71.01% (63.64%-77.78%)	54.68% (44.64%-65.15%)	99.083% (96.774%-100%)	99.06% (95.83%-100%)	56.16% (46.67%-66.2%)	3.0945 (2.9063-3.2910)
At least 4 of CHASM (breast), CHASM (melanoma), FATHMM (cancer), SIFT, VEST	78.44% (75.25%-81.82%)	71.28% (66.92%-76%)	90.804% (87.324%-94.667%)	93.06% (90.42%-95.96%)	64.65% (59.43%-69.91%)	3.1979 (3.0845-3.3175)	78.48% (71.72%-84.85%)	71.25% (61.43%-80.65%)	90.835% (82.752%-97.44%)	93.05% (86.96%-98.15%)	64.81% (54.56%-74.51%)	3.1994 (2.9601-3.4215)
At least 4 of CHASM (breast), CHASM (melanoma), FATHMM (cancer), VEST	63.32% (59.6%-67.17%)	44.24% (38.98%-49.21%)	96.293% (94.203%-98.684%)	95.39% (92.73%-98.39%)	49.9% (45.06%-54.37%)	2.8589 (2.7662-2.9540)	63.27% (55.56%-70.71%)	43.95% (33.85%-54.55%)	96.398% (91.176%-100%)	95.47% (88.89%-100%)	50.03% (40.62%-59.46%)	2.8592 (2.6575-3.0564)
At least 4 of CHASM (breast), CHASM (melanoma), FATHMM (missense), Mutation Assessor	65.02% (61.11%-68.69%)	46.92% (41.88%-52.03%)	96.293% (94.203%-98.684%)	95.59% (93.02%-98.39%)	51.22% (46.3%-55.8%)	2.9000 (2.8025-3.0000)	64.96% (57.58%-72.73%)	46.6% (36.36%-56.92%)	96.381% (90.909%-100%)	95.67% (89.66%-100%)	51.23% (41.79%-61.02%)	2.8982 (2.6921-3.1066)
At least 4 of CHASM (breast), CHASM (melanoma), FATHMM (missense), Mutation Assessor, MutationTaster	75.79% (72.25%-82.32%)	70.17% (65.28%-75.2%)	93.581% (90.537%-97.183%)	94.98% (92.55%-97.8%)	64.47% (59%-69.73%)	3.2320 (3.1255-3.3516)	78.86% (72.73%-85.86%)	70.27% (60.29%-80%)	93.565% (86.838%-100%)	94.94% (89.36%-100%)	64.74% (54.39%-75.51%)	3.2351 (3.0056-3.4558)
At least 4 of CHASM (breast), CHASM (melanoma), FATHMM (missense), Mutation Assessor, PolyPhen-2	74.39% (70.71%-77.78%)	65.38% (60.43%-69.92%)	90.676% (87.143%-94.595%)	92.53% (89.58%-95.83%)	60.22% (54.92%-65.45%)	3.0860 (2.9756-3.2064)	74.29% (67.68%-80.81%)	65.05% (55.17%-74.65%)	90.777% (82.608%-97.5%)	92.55% (85.72%-97.92%)	60.16% (50%-70.59%)	3.0849 (2.8483-3.3073)
At least 4 of CHASM (breast), CHASM (melanoma), FATHMM (missense), Mutation Assessor, PROVEAN	69.7% (66.16%-73.23%)	55.38% (50%-60.48%)	94.421% (91.549%-97.368%)	94.61% (92.11%-97.5%)	55.43% (50.26%-60.48%)	2.9979 (2.8955-3.1021)	69.66% (62.63%-76.77%)	55.06% (45.31%-65.08%)	94.628% (87.879%-100%)	94.77% (88.57%-100%)	55.44% (45.9%-65.52%)	2.9976 (2.7780-3.2016)
At least 4 of CHASM (breast), CHASM (melanoma), FATHMM (missense), Mutation Assessor, SIFT	77.09% (73.74%-80.3%)	68.62% (63.79%-73.6%)	91.708% (88.235%-95.588%)	93.49% (90.8%-96.67%)	62.84% (57.52%-67.89%)	3.1668 (3.0554-3.2810)	77.1% (70.71%-83.84%)	68.58% (59.02%-78.18%)	91.742% (84.211%-97.62%)	93.46% (87.21%-98.11%)	62.99% (52.94%-73.08%)	3.1678 (2.9332-3.3935)
At least 4 of CHASM (breast), CHASM (melanoma), FATHMM (missense), Mutation Assessor, VEST	74.46% (70.71%-77.78%)	63.83% (58.87%-68.8%)	92.612% (89.333%-96.053%)	93.9% (91.3%-96.94%)	59.62% (54.55%-65%)	3.1047 (2.9920-3.2208)	74.44% (67.68%-80.81%)	63.7% (53.03%-73.44%)	92.615% (85.714%-100%)	93.88% (88.1%-100%)	59.8% (49.12%-70.59%)	3.1003 (2.8768-3.3191)
At least 4 of CHASM (breast), CHASM (melanoma), FATHMM (missense), MutationTaster	68.35% (64.65%-72.22%)	51.64% (46.46%-56.92%)	97.233% (95.588%-100%)	97% (95%-100%)	53.79% (48.8%-58.62%)	2.9972 (2.8972-3.0988)	68.36% (61.62%-75.76%)	51.46% (41.69%-61.54%)	97.269% (92.308%-100%)	97.02% (91.89%-100%)	53.89% (44.12%-63.16%)	2.9967 (2.7930-3.1928)
At least 4 of CHASM (breast), CHASM (melanoma), FATHMM (missense), MutationTaster, PolyPhen-2	79.76% (76.77%-83.33%)	72.28% (67.72%-77.17%)	92.676% (89.394%-96.053%)	94.47% (92%-97.16%)	65.92% (60.78%-71.36%)	3.2535 (3.1502-3.3727)	79.87% (72.73%-85.86%)	72.4% (62.15%-81.16%)	92.818% (85.294%-100%)	94.39% (88.64%-100%)	66.21% (55.54%-76.6%)	3.2564 (3.0203-3.4686)
At least 4 of CHASM (breast), CHASM (melanoma), FATHMM (missense), MutationTaster, PROVEAN	75.74% (72.22%-79.29%)	63.3% (58.19%-68.22%)	96.274% (94.203%-98.667%)	96.62% (94.74%-98.81%)	60.43% (55.36%-65.61%)	3.1746 (3.0740-3.2814)	75.71% (68.69%-82.83%)	63.05% (52.54%-72.86%)	96.378% (90.625%-100%)	96.66% (91.18%-100%)	60.49% (50.82%-70.59%)	3.1729 (2.9706-3.3781)
At least 4 of CHASM (breast), CHASM (melanoma), FATHMM (missense), MutationTaster, SIFT	82.11% (79.29%-85.35%)	74.93% (70.16%-79.54%)	94.452% (91.667%-97.368%)	95.94% (93.81%-98.11%)	68.6% (63.27%-73.68%)	3.3434 (3.2354-3.4552)	82.2% (75.76%-88.89%)	75.11% (66.1%-84.48%)	94.436% (88.235%-100%)	95.91% (91.07%-100%)	68.88% (58.54%-79.17%)	3.3429 (3.1335-3.5438)
At least 4 of CHASM (breast), CHASM (melanoma), FATHMM (missense), MutationTaster, VEST	79.07% (75.76%-82.32%)	70.68% (65.89%-75.4%)	93.581% (90.541%-97.183%)	95.01% (92.69%-97.87%)	64.89% (59.69%-70.21%)	3.2413 (3.1374-3.3517)	79.23% (72.73%-85.86%)	70.86% (61.02%-80.33%)	93.565% (86.838%-100%)	94.98% (89.8%-100%)	65.19% (55.17%-75.51%)	3.2419 (3.0278-3.4558)
At least 4 of CHASM (breast), CHASM (melanoma), FATHMM (missense), PolyPhen-2	66.35% (62.63%-70.2%)	49.03% (44.09%-54.33%)	96.293% (94.203%-98.684%)	95.81% (93.65%-98.46%)	52.21% (47.37%-56.96%)	2.9353 (2.8370-3.0353)	66.34% (59.6%-73.74%)	48.8% (38.89%-58.73%)	96.381% (90.909%-100%)	95.88% (90.32%-100%)	52.3% (43.08%-61.9%)	2.9350 (2.7255-3.1351)
At least 4 of CHASM (breast), CHASM (melanoma), FATHMM (missense), PolyPhen-2, PROVEAN	73.06% (69.7%-76.77%)	60.67% (55.65%-65.6%)	94.421% (91.549%-97.368%)	94.96% (92.41%-97.65%)	58.15% (53.06%-63.37%)	3.0857 (2.9776-3.1960)	73.06% (65.66%-79.8%)	60.54% (49.18%-70.18%)	94.628% (87.879%-100%)	95.12% (88.89%-100%)	58.28% (48.19%-68.42%)	3.0847 (2.8642-3.3029)
At least 4 of CHASM (breast), CHASM (melanoma), FATHMM (missense), PolyPhen-2, SIFT	78.09% (74.75%-81.31%)	71.28% (66.67%-75.78%)	89.903% (86.111%-94.366%)	92.4% (89.67%-95.7%)	64.27% (58.86%-69.68%)	3.1919 (3.0739-3.3098)	78.07% (71.72%-84.85%)	70.94% (60.94%-79.69%)	90.052% (81.25%-97.44%)	92.45% (86.27%-98.04%)	64.38% (54.36%-74.47%)	3.1914 (2.9544-3.4215)
At least 4 of CHASM (breast), CHASM (melanoma), FATHMM (missense), PolyPhen-2, VEST	75.41% (72.22%-78.79%)	68.07% (63.33%-72.8%)	88.095% (83.824%-92.65%)	90.82% (87.5%-94.23%)	61.47% (56.12%-66.67%)	3.0846 (2.9606-3.2098)	75.44% (68.69%-82.83%)	67.89% (58.49%-77.78%)	88.003% (78.947%-96.78%)	90.71% (83.72%-97.62%)	61.61% (51.02%-72.45%)	3.0846 (2.8211-3.3333)
At least 4 of CHASM (breast), CHASM (melanoma), FATHMM (missense), PROVEAN	58.97% (55.05%-62.63%)	36.3% (31.45%-40.99%)	98.12% (97.015%-100%)	97.17% (95.24%-100%)	47.13% (42.22%-51.65%)	2.7877 (2.6997-2.8801)	58.82% (51.52%-66.67%)	35.9% (26.31%-45.9%)	98.144% (94.118%-100%)	97.09% (92.86%-100%)	47.15% (38.36%-56.34%)	2.7827 (2.6078-2.9627)
At least 4 of CHASM (breast), CHASM (melanoma), FATHMM (missense), PROVEAN, SIFT	73.74% (70.2%-77.27%)	61.21% (56.15%-66.13%)	95.376% (92.857%-98.611%)	95.88% (93.51%-98.72%)	58.81% (53.85%-63.76%)	3.1114 (3.0047-3.2174)	73.64% (66.67%-80.81%)	60.92% (51.06%-70.59%)	95.548% (89.474%-100%)	95.95% (90.91%-100%)	58.82% (48.33%-68.68%)	3.1110 (2.8938-3.3154)
At least 4 of CHASM (breast), CHASM (melanoma), FATHMM (missense), PROVEAN, VEST	69.03% (65.66%-72.73%)	53.78% (48.03%-58.91%)	95.376% (92.857%-98.611%)	95.27% (92.75%-98.53%)	54.43% (49.39%-59.3%)	2.9883 (2.8856-3.0993)	68.92% (62.63%-75.76%)	53.42% (43.08%-63.49%)	95.478% (89.474%-100%)	95.34% (89.29%-100%)	54.41% (44.83%-64.37%)	2.9891 (2.7667-3.1994)
At least 4 of CHASM (breast), CHASM (melanoma), FATHMM (missense), SIFT	69.4% (65.66%-72.74%)	53.31% (48.36%-58.4%)	97.233% (95.588%-100%)	97.09% (95.24%-100%)	54.43% (49.19%-59.26%)	3.0224 (2.9240-3.1219)	69.35% (62.63%-76.77%)	52.94% (42.62%-63.08%)	97.337% (92.5%-100%)	97.2% (92.5%-100%)	54.51% (45.23%-64.06%)	3.0194 (2.8149-3.2169)
At least 4 of CHASM (breast), CHASM (melanoma), FATHMM (missense), SIFT, VEST	77.42% (74.24%-80.81%)	70.2% (65.89%-75.4%)	89.903% (86.111%-94.366%)	92.27% (89.47%-95.45%)	63.47% (58.22%-68.87%)	3.1694 (3.0518-3.2917)	77.49% (70.71%-83.84%)	70.29% (61.02%-79.69%)	89.933% (81.818%-97.06%)	92.26% (85.71%-97.96%)	63.58% (53.19%-73.91%)	3.1716 (2.9217-3.4060)
At least 4 of CHASM (breast), CHASM (melanoma), FATHMM (missense), VEST	61.31% (57.07%-65.15%)	41.08% (35.71%-46.03%)	96.293% (94.203%-98.684%)	95.05% (92.16%-98.15%)	48.53% (43.71%-53.43%)	2.8100 (2.7090-2.9111)	61.22% (53.54%-68.69%)	40.7% (30.3%-50.85%)	96.423% (91.176%-100%)	95.13% (88.24%-100%)	48.63% (39.13%-57.89%)	2.8082 (2.6062-3.0125)
At least 4 of CHASM (breast), CHASM (melanoma), Mutation Assessor, MutationTaster	62.29% (58.08%-66.16%)	42.61% (37.5%-47.62%)	96.311% (94.286%-98.667%)	95.21% (92.67%-98.39%)	49.18% (44.44%-53.91%)	2.8342 (2.7296-2.9328)	62.29% (54.55%-69.7%)	42.37% (32.26%-52.46%)	96.388% (91.176%-100%)	95.13% (88.89%-100%)	49.25% (39.71%-58.82%)	2.8339 (2.6236-3.0377)
At least 4 of CHASM (breast), CHASM (melanoma), Mutation Assessor, MutationTaster, PolyPhen-2	74.76% (71.21%-78.28%)	64.95% (60%-69.84%)	91.728% (88.406%-95.588%)	93.25% (90.43%-96.63%)	60.43% (55.11%-65.38%)	3.0942 (2.9829-3.2067)	74.66% (67.68%-81.82%)	64.64% (55.07%-74.14%)	91.783% (84.385%-97.67%)	93.22% (86.96%-98.08%)	60.47% (50%-70.59%)	3.0933 (2.8544-3.3137)
At least 4 of CHASM (breast), CHASM (melanoma), Mutation Assessor, MutationTaster, PROVEAN	71.05% (67.68%-74.75%)	56.43% (51.2%-61.48%)	96.311% (94.286%-98.667%)	96.36% (94.29%-98.76%)	56.19% (51.12%-61.35%)	3.0574 (2.9558-3.1557)	71.03% (63.64%-77.78%)	56.25% (46.48%-66.15%)	96.355% (90.909%-100%)	96.37% (91.18%-100%)	56.21% (46.77%-66.13%)	3.0519 (2.8463-3.2496)
At least 4 of CHASM (breast), CHASM (melanoma), Mutation Assessor, MutationTaster, SIFT	76.45% (72.73%-79.8%)	66.58% (61.6%-71.54%)	94.458% (91.667%-97.368%)	95.48% (93.1%-97.92%)	61.88% (56.25%-67.16%)	3.1627 (3.0534-3.2713)	76.39% (69.7%-82.83%)	66.4% (56.45%-76.56%)	94.436% (88.235%-100%)	95.43% (89.58%-100%)	61.98% (51.72%-72.34%)	3.1610 (2.9324-3.3863)
At least 4 of CHASM (breast), CHASM (melanoma), Mutation Assessor, MutationTaster, VEST	74.09% (70.71%-77.27%)	62.86% (57.75%-68%)	93.581% (90.541%-97.183%)	94.54% (91.95%-97.59%)	59.22% (54.17%-64.71%)	3.0976 (2.9875-3.2086)	74.04% (66.67%-80.81%)	62.6% (52.94%-72.58%)	93.565% (86.838%-100%)	94.52% (88.37%-100%)	59.32% (49.12%-69.81%)	3.0960 (2.8715-3.3227)
At least 4 of CHASM (breast), CHASM (melanoma), Mutation Assessor, PolyPhen-2	63.99% (59.6%-67.68%)	46.34% (41.38%-51.59%)	95.376% (92.857%-98.611%)	94.7% (91.67%-97.96%)	50.72% (45.83%-55.56%)	2.8767 (2.7777-2.9791)	63.94% (56.57%-70.71%)	46.09% (35.71%-56.06%)	95.478% (89.474%-100%)	94.78% (88.24%-100%)	50.79% (41.27%-60.32%)	2.8762 (2.6609-3.0878)
At least 4 of CHASM (breast), CHASM (melanoma), Mutation Assessor, PolyPhen-2, PROVEAN	69.7% (66.16%-73.23%)	56.49% (51.56%-61.82%)	92.612% (89.333%-96.053%)	93.02% (90.24%-96.34%)	55.11% (50%-60.15%)	2.9722 (2.8637-3.0846)	69.63% (62.63%-76.77%)	56.23% (46.03%-65.67%)	92.648% (85.714%-100%)	93.07% (86.49%-100%)	55.13% (45.45%-65.15%)	2.9705 (2.7432-3.1920)
At least 4 of CHASM (breast), CHASM (melanoma), Mutation Assessor, PolyPhen-2, SIFT	74.06% (70.71%-77.27%)	65.33% (60.66%-70.31%)	89.903% (86.111%-94.366%)	91.98% (89.01%-95.45%)	59.87% (54.84%-65.17%)	3.0517 (2.9379-3.1674)	73.99% (66.67%-80.81%)	65.16% (55.07%-74.24%)	89.934% (81.818%-97.06%)	91.95% (85.11%-97.92%)	59.9% (49.15%-70.15%)	3.0491 (2.7981-3.2863)
At least 4 of CHASM (breast), CHASM (melanoma), Mutation Assessor, PolyPhen-2, VEST	70.71% (67.17%-74.24%)	59.54% (54.39%-64.55%)	90.791% (87.143%-94.667%)	91.88% (88.89%-95.31%)	56.1% (50.82%-61.33%)	2.9652 (2.8545-3.0838)	70.7% (63.64%-77.78%)	59.32% (49.21%-69.7%)	90.887% (82.758%-97.37%)	91.89% (84.78%-97.67%)	56.31% (46.55%-66.67%)	2.9682 (2.7297-3.2010)
At least 4 of CHASM (breast), CHASM (melanoma), Mutation Assessor, PROVEAN	57.61% (53.54%-61.62%)	34.15% (29.41%-38.71%)	98.137% (97.015%-100%)	97% (94.87%-100%)	46.3% (41.61%-50.65%)	2.7562 (2.6614-2.8492)	57.59% (50.51%-65.66%)	33.82% (24.56%-43.75%)	98.144% (94.118%-100%)	96.9% (90%-100%)	46.35% (37.66%-55.56%)	2.7522 (2.5611-2.9437)
At least 4 of CHASM (breast), CHASM (melanoma), Mutation Assessor, PROVEAN, SIFT	70.71% (67.17%-74.24%)	57.51% (52.38%-62.7%)	93.512% (90.411%-97.143%)	93.88% (91.25%-97.22%)	56% (50.88%-61.2%)	3.0035 (2.8961-3.1101)	70.65% (63.64%-77.78%)	57.29% (46.77%-67.19%)	93.571% (87.5%-100%)	93.98% (88.57%-100%)	56.14% (46.3%-66.67%)	3.0026 (2.7867-3.2249)
At least 4 of CHASM (breast), CHASM (melanoma), Mutation Assessor, PROVEAN, VEST	67.03% (63.64%-70.71%)	52.23% (47.15%-57.35%)	93.581% (90.541%-97.183%)	93.52% (90.54%-96.92%)	53.08% (48.12%-57.81%)	2.9090 (2.8025-3.0171)	66.96% (59.6%-73.74%)	51.9% (41.54%-61.9%)	93.571% (86.842%-100%)	93.54% (86.84%-100%)	53.13% (43.84%-62.89%)	2.9080 (2.6839-3.1378)
At least 4 of CHASM (breast), CHASM (melanoma), Mutation Assessor, SIFT	64.33% (60.61%-68.18%)	45.82% (40.74%-50.81%)	97.212% (95.312%-100%)	96.84% (94.64%-100%)	50.86% (45.84%-55.62%)	2.8838 (2.7805-2.9882)	64.28% (56.57%-71.72%)	45.46% (34.72%-55.22%)	97.337% (92.5%-100%)	96.9% (91.17%-100%)	50.78% (41.4%-60.61%)	2.8808 (2.6756-3.0822)
At least 4 of CHASM (breast), CHASM (melanoma), Mutation Assessor, SIFT, VEST	75.43% (72.22%-78.79%)	66.59% (61.72%-71.43%)	91.726% (88.406%-95.588%)	93.3% (90.74%-96.59%)	61.02% (55.88%-66.07%)	3.1216 (3.0075-3.2410)	75.32% (68.69%-82.83%)	66.22% (56.45%-75.76%)	91.783% (84.385%-97.67%)	93.24% (86.96%-98.21%)	61.05% (50.91%-71.25%)	3.1192 (2.8744-3.3458)
At least 4 of CHASM (breast), CHASM (melanoma), Mutation Assessor, VEST	59.27% (55.05%-63.13%)	38.39% (33.33%-43.44%)	96.282% (94.203%-98.667%)	94.68% (91.49%-97.92%)	47.32% (42.71%-51.93%)	2.7647 (2.6684-2.8651)	59.23% (51.52%-66.67%)	38.1% (27.87%-48.39%)	96.423% (91.176%-100%)	94.85% (87.5%-100%)	47.42% (38.36%-56.58%)	2.7661 (2.5591-2.9693)
At least 4 of CHASM (breast), CHASM (melanoma), MutationTaster, PolyPhen-2	65.64% (61.62%-69.19%)	47.9% (42.52%-52.76%)	96.311% (94.286%-98.667%)	95.72% (93.2%-98.46%)	51.67% (46.82%-56.62%)	2.9162 (2.8167-3.0241)	65.69% (58.59%-72.73%)	47.81% (37.7%-58.06%)	96.355% (90.909%-100%)	95.65% (89.47%-100%)	51.82% (42.47%-61.18%)	2.9159 (2.7133-3.1230)
At least 4 of CHASM (breast), CHASM (melanoma), MutationTaster, PolyPhen-2, PROVEAN	71.4% (67.68%-74.75%)	58.06% (53.07%-63.2%)	95.413% (92.958%-98.571%)	95.55% (93.06%-98.63%)	56.63% (51.68%-61.57%)	3.0461 (2.9452-3.1555)	71.39% (64.65%-78.79%)	57.84% (47.62%-68.85%)	95.417% (89.474%-100%)	95.58% (89.47%-100%)	56.63% (46.88%-66.67%)	3.0445 (2.8259-3.2587)
At least 4 of CHASM (breast), CHASM (melanoma), MutationTaster, PolyPhen-2, SIFT	80.12% (76.77%-83.33%)	72.75% (68.33%-77.05%)	93.549% (90.411%-97.143%)	95.08% (92.59%-97.78%)	66.29% (60.9%-71.7%)	3.2802 (3.1690-3.3943)	80.15% (73.74%-86.87%)	72.91% (63.79%-81.54%)	93.565% (86.838%-100%)	95.06% (89.8%-100%)	66.48% (56.25%-76.6%)	3.2807 (3.0447-3.5035)
At least 4 of CHASM (breast), CHASM (melanoma), MutationTaster, PolyPhen-2, VEST	72.4% (68.69%-76.26%)	61.27% (56.56%-66.41%)	91.711% (88.406%-95.588%)	92.79% (89.87%-96.3%)	57.86% (52.73%-63.24%)	3.0235 (2.9123-3.1414)	72.32% (65.66%-79.8%)	60.95% (50.77%-71.43%)	91.783% (84.385%-97.67%)	92.8% (86.05%-98%)	57.83% (47.06%-67.8%)	3.0219 (2.7860-3.2604)
At least 4 of CHASM (breast), CHASM (melanoma), MutationTaster, PROVEAN	58.27% (54.04%-62.12%)	35.2% (30.2%-39.84%)	98.173% (97.015%-100%)	97.11% (95.12%-100%)	46.76% (42.06%-51.25%)	2.7719 (2.6805-2.8651)	58.28% (50.51%-65.66%)	34.99% (25.45%-44.44%)	98.144% (94.118%-100%)	97.05% (91.67%-100%)	46.84% (37.68%-56.16%)	2.7696 (2.5839-2.9566)
At least 4 of CHASM (breast), CHASM (melanoma), MutationTaster, PROVEAN, SIFT	72.73% (69.19%-76.26%)	59.09% (54.1%-64%)	96.311% (94.286%-98.667%)	96.52% (94.37%-98.8%)	57.65% (52.73%-62.58%)	3.0958 (2.9981-3.2000)	72.71% (65.66%-79.8%)	58.89% (48.89%-69.23%)	96.355% (90.909%-100%)	96.54% (91.42%-100%)	57.75% (47.54%-67.16%)	3.0954 (2.8889-3.3041)
At least 4 of CHASM (breast), CHASM (melanoma), MutationTaster, PROVEAN, VEST	68.04% (64.14%-71.72%)	52.2% (47.11%-57.5%)	95.413% (92.958%-98.571%)	95.16% (92.54%-98.48%)	53.61% (48.48%-58.37%)	2.9637 (2.8536-3.0681)	67.99% (60.61%-74.75%)	51.91% (41.38%-62.12%)	95.417% (89.474%-100%)	95.18% (88.57%-100%)	53.62% (44.29%-63.38%)	2.9603 (2.7406-3.1698)
At least 4 of CHASM (breast), CHASM (melanoma), MutationTaster, SIFT	70.39% (66.67%-73.74%)	55.37% (50.41%-60.16%)	96.311% (94.286%-98.667%)	96.3% (94.24%-98.8%)	55.53% (50.52%-60.34%)	3.0248 (2.9245-3.1256)	70.37% (63.64%-77.78%)	55.14% (45.16%-65.08%)	96.355% (90.909%-100%)	96.37% (91.43%-100%)	55.59% (45.69%-65.52%)	3.0240 (2.8401-3.2195)
At least 4 of CHASM (breast), CHASM (melanoma), MutationTaster, SIFT, VEST	76.44% (73.23%-79.8%)	68.15% (63.2%-73.02%)	90.804% (87.324%-94.667%)	92.59% (89.86%-95.79%)	62.23% (56.78%-67.62%)	3.1232 (3.0101-3.2409)	76.44% (69.7%-82.83%)	68.1% (57.97%-76.79%)	90.835% (82.752%-97.44%)	92.54% (86.27%-98%)	62.33% (52%-72.92%)	3.1224 (2.8874-3.3503)
At least 4 of CHASM (breast), CHASM (melanoma), MutationTaster, VEST	62.3% (58.59%-66.16%)	42.63% (37.9%-47.62%)	96.311% (94.286%-98.667%)	95.17% (92.31%-98.31%)	49.17% (44.44%-53.91%)	2.8350 (2.7401-2.9307)	62.3% (54.55%-69.7%)	42.38% (31.82%-52.46%)	96.388% (91.176%-100%)	95.16% (88.23%-100%)	49.25% (39.44%-58.44%)	2.8343 (2.6339-3.0433)
At least 4 of CHASM (breast), CHASM (melanoma), PolyPhen-2, PROVEAN	58.95% (55.05%-62.63%)	36.26% (31.2%-41.09%)	98.173% (97.015%-100%)	97.17% (95.24%-100%)	47.11% (42.18%-51.53%)	2.7872 (2.6908-2.8817)	58.87% (51.52%-66.67%)	35.96% (26.23%-45.9%)	98.144% (94.118%-100%)	97.09% (91.67%-100%)	47.16% (38.1%-56.96%)	2.7836 (2.5898-2.9767)
At least 4 of CHASM (breast), CHASM (melanoma), PolyPhen-2, PROVEAN, SIFT	71.04% (67.68%-74.24%)	58.56% (53.6%-63.64%)	93.531% (90.411%-97.143%)	94.06% (91.25%-97.26%)	56.3% (51.16%-61.2%)	3.0123 (2.9107-3.1206)	71.01% (63.64%-78.79%)	58.32% (48.34%-68.85%)	93.605% (86.838%-100%)	94.1% (88.24%-100%)	56.44% (46.67%-66.67%)	3.0092 (2.7941-3.2261)
At least 4 of CHASM (breast), CHASM (melanoma), PolyPhen-2, PROVEAN, VEST	67.02% (63.13%-70.71%)	52.2% (47.01%-57.26%)	93.581% (90.541%-97.183%)	93.5% (90.41%-96.92%)	53.06% (48.21%-57.81%)	2.9075 (2.8021-3.0136)	66.94% (59.6%-74.75%)	51.95% (41.38%-62.3%)	93.605% (86.838%-100%)	93.56% (86.84%-100%)	53.17% (43.59%-62.87%)	2.9080 (2.6793-3.1312)
At least 4 of CHASM (breast), CHASM (melanoma), PolyPhen-2, SIFT	66.01% (62.12%-69.7%)	48.48% (43.31%-53.66%)	96.311% (94.286%-98.667%)	95.81% (93.55%-98.46%)	51.95% (47.01%-56.77%)	2.9252 (2.8178-3.0328)	65.98% (58.59%-73.74%)	48.24% (37.59%-59.02%)	96.388% (91.176%-100%)	95.93% (90.32%-100%)	52.05% (42.47%-61.9%)	2.9242 (2.7208-3.1329)
At least 4 of CHASM (breast), CHASM (melanoma), PolyPhen-2, SIFT, VEST	75.44% (72.22%-78.79%)	68.05% (63.2%-72.73%)	88.148% (83.929%-92.647%)	90.86% (87.77%-94.38%)	61.44% (56.03%-66.67%)	3.0844 (2.9597-3.2069)	75.38% (68.69%-82.83%)	67.7% (58.06%-77.42%)	88.172% (79.412%-96.23%)	90.84% (84.09%-97.5%)	61.47% (50.94%-71.43%)	3.0826 (2.8298-3.3366)
At least 4 of CHASM (breast), CHASM (melanoma), PolyPhen-2, VEST	61.3% (57.07%-64.65%)	41.06% (35.77%-46.15%)	96.311% (94.286%-98.667%)	95.08% (92.31%-98.21%)	48.52% (43.44%-53.23%)	2.8091 (2.7126-2.9060)	61.25% (53.54%-68.69%)	40.78% (30.43%-51.67%)	96.388% (91.176%-100%)	95.15% (88.24%-100%)	48.62% (39.13%-58.82%)	2.8091 (2.5993-3.0107)
At least 4 of CHASM (breast), CHASM (melanoma), PROVEAN, SIFT	60.97% (57.07%-64.65%)	39.44% (34.4%-44.26%)	98.173% (97.015%-100%)	97.38% (95.56%-100%)	48.49% (43.94%-52.8%)	2.8346 (2.7437-2.9233)	60.88% (53.54%-68.69%)	39.11% (28.99%-49.21%)	98.144% (94.118%-100%)	97.31% (92.31%-100%)	48.53% (39.71%-57.97%)	2.8300 (2.6556-3.0146)
At least 4 of CHASM (breast), CHASM (melanoma), PROVEAN, SIFT, VEST	67.69% (63.64%-71.21%)	53.39% (48.03%-58.59%)	93.581% (90.541%-97.183%)	93.67% (90.79%-96.97%)	53.55% (48.39%-58.22%)	2.9243 (2.8121-3.0276)	67.63% (60.61%-74.75%)	53.08% (42.37%-63.49%)	93.565% (86.838%-100%)	93.69% (87.18%-100%)	53.62% (44.29%-63.08%)	2.9236 (2.6999-3.1493)
At least 4 of CHASM (breast), CHASM (melanoma), PROVEAN, VEST	58.28% (54.04%-62.12%)	35.23% (30.4%-40%)	98.173% (97.015%-100%)	97.11% (95.12%-100%)	46.77% (42.04%-51.32%)	2.7720 (2.6806-2.8651)	58.2% (50.51%-65.66%)	34.89% (25.81%-44.44%)	98.144% (94.118%-100%)	97.03% (91.67%-100%)	46.79% (38.57%-56.34%)	2.7679 (2.5898-2.9556)
At least 4 of CHASM (breast), CHASM (melanoma), SIFT, VEST	62.31% (58.59%-66.16%)	42.64% (37.4%-47.62%)	96.311% (94.286%-98.667%)	95.17% (92.42%-98.25%)	49.18% (44.43%-53.85%)	2.8354 (2.7356-2.9364)	62.25% (54.55%-69.7%)	42.33% (32.31%-52.54%)	96.388% (91.176%-100%)	95.2% (88.46%-100%)	49.25% (40%-58.97%)	2.8345 (2.6329-3.0423)
At least 4 of CHASM (breast), FATHMM (cancer), FATHMM (missense), Mutation Assessor	69.72% (66.16%-73.23%)	53.3% (48.03%-58.5%)	98.143% (97.015%-100%)	98.01% (96.67%-100%)	54.8% (49.8%-59.49%)	3.0425 (2.9421-3.1395)	69.62% (62.63%-76.77%)	52.96% (42.47%-63.08%)	98.209% (94.118%-100%)	98.09% (94.12%-100%)	54.88% (45.33%-64.41%)	3.0411 (2.8486-3.2488)
At least 4 of CHASM (breast), FATHMM (cancer), FATHMM (missense), Mutation Assessor, MutationTaster	80.87% (77.78%-84.34%)	71.05% (66.39%-75.86%)	97.17% (95.161%-100%)	97.63% (96.15%-100%)	66.49% (61.05%-71.79%)	3.3476 (3.2495-3.4484)	80.77% (73.74%-86.87%)	70.73% (61.29%-80.33%)	97.126% (92.308%-100%)	97.67% (93.22%-100%)	66.47% (55.77%-76.6%)	3.3418 (3.1386-3.5478)
At least 4 of CHASM (breast), FATHMM (cancer), FATHMM (missense), Mutation Assessor, PolyPhen-2	76.81% (73.23%-80.3%)	64.89% (60%-69.84%)	96.29% (94.203%-98.667%)	96.61% (94.68%-98.85%)	61.56% (56.31%-66.96%)	3.2011 (3.1009-3.3124)	76.74% (69.7%-83.84%)	64.6% (54.55%-74.6%)	96.354% (90.909%-100%)	96.63% (91.67%-100%)	61.6% (51.61%-71.93%)	3.1990 (2.9909-3.4098)
At least 4 of CHASM (breast), FATHMM (cancer), FATHMM (missense), Mutation Assessor, PROVEAN	74.44% (71.21%-77.78%)	59.86% (54.89%-65.08%)	98.143% (97.015%-100%)	98.2% (97.1%-100%)	59.45% (54.45%-64.59%)	3.1837 (3.0925-3.2855)	74.36% (67.68%-81.82%)	59.56% (49.15%-69.69%)	98.209% (94.118%-100%)	98.25% (94.74%-100%)	59.5% (49.09%-69.7%)	3.1806 (2.9846-3.3788)
At least 4 of CHASM (breast), FATHMM (cancer), FATHMM (missense), Mutation Assessor, SIFT	79.52% (76.26%-82.83%)	69.2% (64.43%-73.95%)	96.269% (94.203%-98.667%)	96.88% (95.05%-98.95%)	64.86% (59.65%-70.1%)	3.2758 (3.1727-3.3861)	79.36% (72.73%-85.86%)	68.78% (58.73%-78.13%)	96.286% (90.909%-100%)	96.86% (92.31%-100%)	64.79% (54.17%-75%)	3.2709 (3.0675-3.4911)
At least 4 of CHASM (breast), FATHMM (cancer), FATHMM (missense), Mutation Assessor, VEST	76.81% (73.74%-80.3%)	64.95% (60%-69.84%)	96.269% (94.203%-98.667%)	96.65% (94.74%-98.84%)	61.51% (56.2%-66.67%)	3.2014 (3.0964-3.3123)	76.73% (69.7%-83.84%)	64.67% (54.84%-74.63%)	96.286% (90.909%-100%)	96.6% (91.67%-100%)	61.63% (51.52%-71.74%)	3.1983 (2.9881-3.4035)
At least 4 of CHASM (breast), FATHMM (cancer), FATHMM (missense), MutationTaster	73.44% (69.7%-76.77%)	57.07% (52%-62.2%)	99.089% (98.485%-100%)	99.11% (98.41%-100%)	58.34% (53.26%-63.24%)	3.1499 (3.0568-3.2445)	73.31% (66.67%-80.81%)	56.71% (46.43%-66.67%)	99.083% (96.774%-100%)	99.1% (96.97%-100%)	58.37% (48.44%-68.18%)	3.1453 (2.9610-3.3389)
At least 4 of CHASM (breast), FATHMM (cancer), FATHMM (missense), MutationTaster, PolyPhen-2	81.49% (78.28%-84.85%)	75.53% (71.2%-80%)	91.74% (88.571%-95.588%)	94.08% (91.84%-96.94%)	68.45% (63.03%-73.68%)	3.2984 (3.1890-3.4145)	81.34% (74.75%-87.88%)	75.11% (66.1%-83.87%)	91.665% (84.211%-97.67%)	94.06% (88.24%-98.39%)	68.3% (57.78%-79.07%)	3.2970 (3.0589-3.5252)
At least 4 of CHASM (breast), FATHMM (cancer), FATHMM (missense), MutationTaster, PROVEAN	83.51% (80.3%-86.36%)	75.63% (71.25%-80.43%)	97.115% (95.161%-100%)	97.86% (96.41%-100%)	70.02% (64.58%-74.77%)	3.4203 (3.3186-3.5238)	83.41% (76.77%-89.9%)	75.26% (65.62%-83.61%)	97.126% (92.308%-100%)	97.86% (94.12%-100%)	69.92% (59.18%-79.59%)	3.4178 (3.2111-3.6187)
At least 4 of CHASM (breast), FATHMM (cancer), FATHMM (missense), MutationTaster, SIFT	83.17% (80.3%-86.36%)	77.12% (72.86%-81.6%)	93.555% (90.537%-97.102%)	95.29% (93.33%-97.89%)	70.36% (65.15%-75.51%)	3.3638 (3.2604-3.4725)	83.04% (77.78%-88.89%)	76.72% (67.8%-85.48%)	93.631% (86.842%-100%)	95.3% (90.57%-100%)	70.29% (59.62%-79.59%)	3.3616 (3.1520-3.5678)
At least 4 of CHASM (breast), FATHMM (cancer), FATHMM (missense), MutationTaster, VEST	80.83% (77.78%-84.34%)	73.43% (68.97%-77.95%)	93.555% (90.537%-97.102%)	95.05% (92.93%-97.67%)	67.1% (61.86%-72.73%)	3.2905 (3.1839-3.4002)	80.7% (73.74%-86.87%)	73.07% (64.15%-81.82%)	93.631% (86.842%-100%)	95.06% (90%-100%)	67.06% (57.14%-77.55%)	3.2881 (3.0758-3.5060)
At least 4 of CHASM (breast), FATHMM (cancer), FATHMM (missense), PolyPhen-2	72.07% (68.18%-75.76%)	56.51% (51.24%-61.67%)	97.219% (95.588%-100%)	97.02% (95.12%-100%)	56.77% (51.76%-61.79%)	3.0734 (2.9736-3.1774)	71.98% (64.65%-79.8%)	56.19% (45.61%-66.67%)	97.337% (92.5%-100%)	97.09% (92.31%-100%)	56.86% (46.38%-66.67%)	3.0715 (2.8680-3.2777)
At least 4 of CHASM (breast), FATHMM (cancer), FATHMM (missense), PolyPhen-2, PROVEAN	78.79% (75.76%-82.32%)	66.55% (61.97%-71.43%)	98.143% (97.015%-100%)	98.38% (97.3%-100%)	64.29% (59.02%-69.27%)	3.2880 (3.1937-3.3885)	78.69% (72.73%-85.86%)	66.2% (56.51%-75.81%)	98.209% (94.118%-100%)	98.45% (95.24%-100%)	64.44% (54.01%-74.47%)	3.2861 (3.0929-3.4954)
At least 4 of CHASM (breast), FATHMM (cancer), FATHMM (missense), PolyPhen-2, SIFT	80.14% (77.27%-83.33%)	72.32% (67.74%-77.25%)	92.664% (89.394%-96.053%)	94.31% (91.92%-97.14%)	66.54% (61.2%-71.57%)	3.2610 (3.1543-3.3771)	80.01% (72.73%-86.87%)	71.95% (62.9%-80.88%)	92.757% (85.714%-100%)	94.25% (88.89%-100%)	66.55% (55.77%-77.08%)	3.2573 (3.0348-3.4700)
At least 4 of CHASM (breast), FATHMM (cancer), FATHMM (missense), PolyPhen-2, VEST	77.13% (73.74%-80.3%)	67.57% (62.59%-72.36%)	92.664% (89.394%-96.053%)	94.03% (91.57%-97.03%)	62.79% (57.47%-68.04%)	3.1796 (3.0688-3.2905)	77.06% (70.71%-83.84%)	67.24% (57.58%-76.67%)	92.757% (85.714%-100%)	93.98% (88.46%-100%)	62.83% (52.73%-72.73%)	3.1773 (2.9497-3.3926)
At least 4 of CHASM (breast), FATHMM (cancer), FATHMM (missense), PROVEAN	62.66% (58.59%-66.16%)	41.07% (36.07%-46.03%)	99.084% (98.485%-100%)	98.74% (97.83%-100%)	49.32% (44.54%-53.72%)	2.8824 (2.7961-2.9689)	62.2% (54.55%-69.7%)	40.67% (30.51%-50.85%)	99.083% (96.774%-100%)	98.71% (95.24%-100%)	49.36% (40.51%-58.57%)	2.8781 (2.7058-3.0570)
At least 4 of CHASM (breast), FATHMM (cancer), FATHMM (missense), PROVEAN, SIFT	76.13% (72.73%-79.29%)	62.84% (57.98%-67.97%)	98.143% (97.015%-100%)	98.27% (97.26%-100%)	61.34% (56.11%-66.3%)	3.2235 (3.1346-3.3251)	76.02% (69.7%-82.83%)	62.5% (52.46%-72.13%)	98.209% (94.118%-100%)	98.3% (95.24%-100%)	61.42% (51.02%-71.43%)	3.2210 (3.0299-3.4134)
At least 4 of CHASM (breast), FATHMM (cancer), FATHMM (missense), PROVEAN, VEST	73.1% (69.7%-76.77%)	57.65% (52.71%-62.7%)	98.143% (97.015%-100%)	98.17% (96.95%-100%)	58.06% (53.18%-62.83%)	3.1457 (3.0515-3.2418)	72.98% (65.66%-79.8%)	57.31% (47.46%-67.21%)	98.209% (94.118%-100%)	98.21% (94.44%-100%)	58.12% (48.57%-68.12%)	3.1432 (2.9474-3.3434)
At least 4 of CHASM (breast), FATHMM (cancer), FATHMM (missense), SIFT	70.08% (66.16%-73.74%)	52.7% (47.62%-57.85%)	98.143% (97.015%-100%)	97.98% (96.63%-100%)	54.51% (49.62%-59.53%)	3.0286 (2.9340-3.1322)	69.23% (62.63%-76.77%)	52.36% (42.19%-62.71%)	98.209% (94.118%-100%)	98.04% (93.94%-100%)	54.59% (45.16%-63.89%)	3.0267 (2.8330-3.2253)
At least 4 of CHASM (breast), FATHMM (cancer), FATHMM (missense), SIFT, VEST	78.43% (75.25%-81.82%)	69.2% (64.55%-74.14%)	93.555% (90.537%-97.102%)	94.85% (92.55%-97.75%)	64.08% (58.65%-69.27%)	3.2217 (3.1129-3.3332)	78.34% (71.72%-84.85%)	68.89% (59.38%-78.31%)	93.631% (86.842%-100%)	94.86% (89.47%-100%)	64.07% (53.85%-74.47%)	3.2193 (2.9991-3.4253)
At least 4 of CHASM (breast), FATHMM (cancer), FATHMM (missense), VEST	63.98% (59.6%-67.68%)	44.68% (39.52%-50%)	98.143% (97.015%-100%)	97.73% (96.15%-100%)	50.67% (45.6%-55.41%)	2.9027 (2.8097-2.9942)	63.88% (56.57%-71.72%)	44.32% (33.85%-54.93%)	98.144% (94.118%-100%)	97.72% (93.33%-100%)	50.74% (41.67%-60.53%)	2.8994 (2.7139-3.0922)
At least 4 of CHASM (breast), FATHMM (cancer), Mutation Assessor, MutationTaster	61.99% (58.08%-65.66%)	40.84% (35.77%-46.09%)	99.084% (98.148%-100%)	98.73% (97.78%-100%)	49.21% (44.3%-53.79%)	2.8801 (2.7935-2.9671)	61.89% (54.55%-69.7%)	40.49% (30.16%-50.82%)	99.083% (96.774%-100%)	98.71% (95.24%-100%)	49.24% (40.28%-58.33%)	2.8780 (2.7054-3.0588)
At least 4 of CHASM (breast), FATHMM (cancer), Mutation Assessor, MutationTaster, PolyPhen-2	78.78% (75.76%-82.32%)	71.86% (67.44%-76.56%)	90.816% (87.324%-94.667%)	93.1% (90.53%-96.04%)	65% (60%-71.2%)	3.2078 (3.0947-3.3299)	78.71% (72.73%-84.85%)	71.62% (62.36%-80.95%)	90.849% (82.927%-97.5%)	93.1% (87.04%-98.18%)	65.1% (55.1%-75.62%)	3.2099 (2.9643-3.4369)
At least 4 of CHASM (breast), FATHMM (cancer), Mutation Assessor, MutationTaster, PROVEAN	72.41% (68.69%-75.76%)	59.1% (53.85%-64.17%)	95.413% (92.958%-98.571%)	95.71% (93.33%-98.61%)	57.44% (52.24%-62.01%)	3.0766 (2.9728-3.1804)	72.35% (65.66%-79.8%)	58.83% (48.48%-69.01%)	95.402% (89.655%-100%)	95.69% (89.74%-100%)	57.51% (47.83%-67.16%)	3.0742 (2.8613-3.2836)
At least 4 of CHASM (breast), FATHMM (cancer), Mutation Assessor, MutationTaster, SIFT	79.12% (76.26%-82.32%)	70.73% (66.12%-75.61%)	93.581% (90.541%-97.183%)	95% (92.68%-97.8%)	65.05% (59.66%-70.37%)	3.2434 (3.1393-3.3542)	79.05% (72.73%-85.86%)	70.43% (61.4%-80%)	93.565% (86.838%-100%)	94.97% (89.8%-100%)	65.15% (55.1%-75%)	3.2417 (3.0202-3.4650)
At least 4 of CHASM (breast), FATHMM (cancer), Mutation Assessor, MutationTaster, VEST	76.75% (73.23%-79.8%)	68.61% (63.64%-73.04%)	90.816% (87.324%-94.667%)	92.68% (89.9%-95.83%)	62.59% (57.27%-67.74%)	3.1483 (3.0343-3.2654)	76.68% (69.7%-83.84%)	68.33% (58.46%-77.94%)	90.849% (82.927%-97.5%)	92.69% (86.27%-98.04%)	62.73% (52.17%-72.92%)	3.1487 (2.9123-3.3771)
At least 4 of CHASM (breast), FATHMM (cancer), Mutation Assessor, PolyPhen-2	63.67% (59.6%-67.17%)	44.66% (39.52%-49.59%)	97.195% (95.312%-100%)	96.65% (94.44%-100%)	50.3% (45.53%-55.02%)	2.8878 (2.7920-2.9846)	63.58% (56.57%-70.71%)	44.25% (34.38%-54.24%)	97.297% (92.308%-100%)	96.79% (91.17%-100%)	50.36% (41.18%-60%)	2.8863 (2.6862-3.0835)
At least 4 of CHASM (breast), FATHMM (cancer), Mutation Assessor, PolyPhen-2, PROVEAN	69.39% (65.66%-73.23%)	55.9% (50.79%-61.11%)	92.676% (89.394%-96.053%)	93.21% (90.41%-96.45%)	54.94% (49.59%-59.85%)	2.9731 (2.8621-3.0808)	69.32% (62.63%-76.77%)	55.57% (45.31%-66.13%)	92.818% (85.294%-100%)	93.31% (86.84%-100%)	55.09% (45.57%-65.15%)	2.9726 (2.7504-3.1933)
At least 4 of CHASM (breast), FATHMM (cancer), Mutation Assessor, PolyPhen-2, SIFT	74.04% (70.71%-77.78%)	65.33% (60.48%-70.4%)	89.891% (86.111%-94.366%)	91.97% (89.01%-95.45%)	59.87% (54.62%-65.22%)	3.0512 (2.9341-3.1663)	73.93% (66.67%-80.81%)	64.98% (55.38%-74.6%)	89.963% (80.769%-97.22%)	91.98% (85.42%-97.62%)	59.91% (49.15%-70.37%)	3.0498 (2.8026-3.2918)
At least 4 of CHASM (breast), FATHMM (cancer), Mutation Assessor, PolyPhen-2, VEST	72.4% (68.69%-75.76%)	62.26% (57.26%-67.46%)	90.816% (87.324%-94.667%)	92.28% (89.33%-95.77%)	57.76% (52.42%-63.03%)	3.0133 (2.9002-3.1323)	72.3% (65.66%-79.8%)	61.91% (51.85%-71.43%)	90.835% (83.333%-97.5%)	92.32% (85.71%-97.87%)	57.8% (47.37%-68.42%)	3.0094 (2.7716-3.2427)
At least 4 of CHASM (breast), FATHMM (cancer), Mutation Assessor, PROVEAN	58.95% (55.05%-63.13%)	36.28% (31.4%-41.27%)	98.136% (97.015%-100%)	97.17% (95.12%-100%)	47.12% (42.42%-51.94%)	2.7874 (2.6971-2.8803)	58.91% (51.52%-66.67%)	36% (26.03%-46.03%)	98.1% (94.444%-100%)	97.16% (92.86%-100%)	47.17% (38.25%-56.34%)	2.7869 (2.6036-2.9705)
At least 4 of CHASM (breast), FATHMM (cancer), Mutation Assessor, PROVEAN, SIFT	69.69% (66.16%-73.23%)	55.93% (50.85%-61.24%)	93.581% (90.541%-97.183%)	93.71% (90.99%-97.1%)	55.11% (50%-60.31%)	2.9734 (2.8609-3.0785)	69.63% (62.63%-76.77%)	55.59% (45.16%-65.67%)	93.565% (86.838%-100%)	93.77% (86.84%-100%)	55.24% (45.07%-65.08%)	2.9722 (2.7457-3.1906)
At least 4 of CHASM (breast), FATHMM (cancer), Mutation Assessor, PROVEAN, VEST	65.68% (61.62%-69.19%)	49.6% (44.35%-54.84%)	93.581% (90.541%-97.183%)	93.29% (90.14%-96.88%)	52.15% (47.11%-57.09%)	2.8768 (2.7625-2.9877)	65.61% (58.59%-72.73%)	49.23% (39.04%-59.68%)	93.565% (86.838%-100%)	93.31% (86.11%-100%)	52.18% (42.67%-62.03%)	2.8753 (2.6494-3.0961)
At least 4 of CHASM (breast), FATHMM (cancer), Mutation Assessor, SIFT	65.01% (61.11%-68.69%)	46.31% (40.98%-51.28%)	98.119% (97.015%-100%)	97.7% (96.15%-100%)	51.3% (46.09%-55.99%)	2.9300 (2.8347-3.0249)	64.96% (57.58%-72.73%)	45.97% (36.36%-56.45%)	98.1% (94.444%-100%)	97.69% (92.86%-100%)	51.35% (42.11%-61.04%)	2.9283 (2.7433-3.1240)
At least 4 of CHASM (breast), FATHMM (cancer), Mutation Assessor, SIFT, VEST	73.71% (70.2%-77.27%)	64.26% (59.37%-69.44%)	90.816% (87.324%-94.667%)	92.37% (89.53%-95.65%)	59.36% (53.96%-64.52%)	3.0667 (2.9537-3.1838)	73.66% (66.67%-80.81%)	63.99% (54.24%-73.44%)	90.835% (83.333%-97.5%)	92.36% (85.71%-97.96%)	59.43% (49.09%-69.23%)	3.0650 (2.8238-3.2909)
At least 4 of CHASM (breast), FATHMM (cancer), Mutation Assessor, VEST	59.96% (56.06%-63.64%)	38.38% (33.33%-43.42%)	98.136% (97.015%-100%)	97.31% (95.35%-100%)	47.76% (42.86%-52.17%)	2.8127 (2.7224-2.9032)	59.91% (52.53%-67.68%)	38.04% (27.69%-47.83%)	98.1% (94.444%-100%)	97.28% (92.86%-100%)	47.79% (38.96%-57.14%)	2.8115 (2.6348-2.9961)
At least 4 of CHASM (breast), FATHMM (cancer), MutationTaster, PolyPhen-2	66.68% (62.63%-70.2%)	49.01% (43.7%-54.24%)	97.233% (95.588%-100%)	96.88% (94.92%-100%)	52.47% (47.61%-57.38%)	2.9576 (2.8544-3.0565)	66.61% (59.6%-73.74%)	48.69% (38.24%-59.38%)	97.337% (92.5%-100%)	97% (91.89%-100%)	52.55% (43.33%-62.03%)	2.9564 (2.7549-3.1570)
At least 4 of CHASM (breast), FATHMM (cancer), MutationTaster, PolyPhen-2, PROVEAN	73.4% (69.7%-76.77%)	61.22% (56.15%-66.4%)	94.437% (91.667%-97.333%)	94.99% (92.5%-97.67%)	58.49% (53.28%-63.53%)	3.0944 (2.9870-3.2016)	73.31% (66.67%-80.81%)	60.93% (50.82%-70.97%)	94.536% (88.235%-100%)	95.03% (89.19%-100%)	58.5% (48.39%-68.33%)	3.0907 (2.8661-3.3059)
At least 4 of CHASM (breast), FATHMM (cancer), MutationTaster, PolyPhen-2, SIFT	81.81% (78.79%-84.85%)	75.51% (71.31%-80%)	92.701% (89.609%-96.104%)	94.7% (92.55%-97.17%)	68.65% (63.54%-73.96%)	3.3156 (3.2129-3.4398)	81.8% (75.76%-87.88%)	75.55% (66.18%-83.93%)	92.569% (85.294%-97.67%)	94.58% (89.13%-98.36%)	68.87% (58.7%-79.07%)	3.3135 (3.0837-3.5254)
At least 4 of CHASM (breast), FATHMM (cancer), MutationTaster, PolyPhen-2, VEST	78.76% (75.76%-82.32%)	71.77% (66.93%-76.38%)	90.816% (87.324%-94.667%)	93.04% (90.43%-96%)	64.98% (59.69%-70.53%)	3.2074 (3.0958-3.3308)	78.78% (71.72%-84.85%)	71.86% (62.5%-80.88%)	90.655% (82.759%-97.37%)	93.01% (87.27%-98.08%)	65.19% (54.72%-75.56%)	3.2081 (2.9658-3.4350)
At least 4 of CHASM (breast), FATHMM (cancer), MutationTaster, PROVEAN	59.3% (55.05%-63.13%)	36.83% (31.71%-42.06%)	98.166% (97.015%-100%)	97.23% (95.24%-100%)	47.33% (42.36%-52.07%)	2.7957 (2.6993-2.8937)	59.28% (52.53%-66.67%)	36.55% (26.67%-46.77%)	98.1% (94.444%-100%)	97.22% (92.31%-100%)	47.41% (38.57%-56.41%)	2.7953 (2.6145-2.9839)
At least 4 of CHASM (breast), FATHMM (cancer), MutationTaster, PROVEAN, SIFT	71.73% (67.68%-75.25%)	57.56% (52.38%-62.5%)	95.376% (92.857%-98.611%)	95.58% (93.06%-98.65%)	56.89% (51.64%-61.7%)	3.0474 (2.9451-3.1530)	71.66% (64.65%-78.79%)	57.2% (47.76%-67.19%)	95.548% (89.474%-100%)	95.68% (90%-100%)	56.97% (47.37%-66.15%)	3.0461 (2.8404-3.2416)
At least 4 of CHASM (breast), FATHMM (cancer), MutationTaster, PROVEAN, VEST	68.03% (64.14%-71.72%)	52.2% (47.01%-57.35%)	95.376% (92.857%-98.611%)	95.14% (92.5%-98.5%)	53.62% (48.59%-58.55%)	2.9623 (2.8551-3.0700)	67.97% (60.61%-74.75%)	51.87% (41.94%-62.3%)	95.548% (89.474%-100%)	95.25% (88.89%-100%)	53.7% (44.44%-63.16%)	2.9619 (2.7365-3.1758)
At least 4 of CHASM (breast), FATHMM (cancer), MutationTaster, SIFT	69.05% (65.66%-72.73%)	52.16% (46.76%-57.38%)	98.119% (97.015%-100%)	97.95% (96.63%-100%)	54.18% (49.35%-59.06%)	3.0158 (2.9201-3.1135)	69.03% (61.62%-75.76%)	51.87% (41.43%-61.9%)	98.144% (94.118%-100%)	98.01% (93.94%-100%)	54.31% (44.78%-64.62%)	3.0158 (2.8238-3.2157)
At least 4 of CHASM (breast), FATHMM (cancer), MutationTaster, SIFT, VEST	76.43% (73.23%-79.8%)	67.03% (62.1%-71.88%)	92.631% (89.333%-96.053%)	94.11% (91.58%-97.03%)	61.93% (56.52%-67.26%)	3.1644 (3.0561-3.2784)	76.38% (69.7%-83.84%)	66.73% (57.38%-76.27%)	92.691% (85.714%-100%)	94.12% (88.37%-100%)	61.99% (51.92%-72.73%)	3.1633 (2.9324-3.3941)
At least 4 of CHASM (breast), FATHMM (cancer), MutationTaster, VEST	62.66% (58.59%-66.67%)	42.61% (37.5%-47.69%)	98.136% (97.015%-100%)	97.62% (95.83%-100%)	49.88% (45.19%-54.69%)	2.8766 (2.7839-2.9712)	62.62% (55.56%-69.7%)	42.28% (32.31%-52.38%)	98.1% (94.444%-100%)	97.59% (92.86%-100%)	49.94% (40.91%-59.21%)	2.8755 (2.6861-3.0665)
At least 4 of CHASM (breast), FATHMM (cancer), PolyPhen-2, PROVEAN	58.62% (54.55%-62.63%)	35.76% (30.83%-40.62%)	98.136% (97.015%-100%)	97.13% (95.12%-100%)	46.92% (42.27%-51.68%)	2.7792 (2.6897-2.8715)	58.56% (51.52%-65.66%)	35.43% (25.81%-45.45%)	98.1% (94.444%-100%)	97.08% (91.67%-100%)	46.96% (38.46%-56.34%)	2.7781 (2.6016-2.9646)
At least 4 of CHASM (breast), FATHMM (cancer), PolyPhen-2, PROVEAN, SIFT	70.03% (66.16%-73.74%)	56.44% (51.22%-61.6%)	93.581% (90.541%-97.183%)	93.75% (90.91%-96.97%)	55.38% (50.14%-60.12%)	2.9812 (2.8745-3.0932)	70.01% (63.64%-76.77%)	56.17% (46.38%-66.17%)	93.565% (86.838%-100%)	93.79% (86.84%-100%)	55.43% (45.59%-65.67%)	2.9831 (2.7652-3.1997)
At least 4 of CHASM (breast), FATHMM (cancer), PolyPhen-2, PROVEAN, VEST	66.35% (62.63%-70.2%)	50.65% (45.53%-56.06%)	93.581% (90.541%-97.183%)	93.4% (90.32%-96.97%)	52.63% (47.31%-57.42%)	2.8924 (2.7825-3.0019)	66.34% (59.6%-73.74%)	50.26% (40.3%-60.66%)	93.565% (86.838%-100%)	93.39% (86.49%-100%)	52.61% (43.06%-62.16%)	2.8929 (2.6676-3.1050)
At least 4 of CHASM (breast), FATHMM (cancer), PolyPhen-2, SIFT	65.01% (61.11%-68.69%)	46.82% (41.48%-51.97%)	97.233% (95.588%-100%)	96.71% (94.64%-100%)	51.29% (46.33%-56.03%)	2.9176 (2.8215-3.0187)	64.98% (57.58%-72.73%)	46.6% (36.07%-56.72%)	97.337% (92.5%-100%)	96.81% (91.43%-100%)	51.38% (42.03%-60.76%)	2.9172 (2.7104-3.1163)
At least 4 of CHASM (breast), FATHMM (cancer), PolyPhen-2, SIFT, VEST	75.41% (71.72%-78.79%)	68.04% (63.08%-72.66%)	88.095% (83.824%-92.65%)	90.83% (87.64%-94.38%)	61.45% (55.98%-66.67%)	3.0835 (2.9567-3.2138)	75.34% (68.69%-81.82%)	67.73% (58.33%-77.05%)	88.1% (78.947%-95.74%)	90.83% (84.09%-97.5%)	61.49% (50.98%-71.93%)	3.0830 (2.8097-3.3426)
At least 4 of CHASM (breast), FATHMM (cancer), PolyPhen-2, VEST	61.33% (57.58%-65.15%)	41.09% (35.94%-46.28%)	96.311% (94.286%-98.667%)	95.08% (92.31%-98.28%)	48.54% (43.75%-53.36%)	2.8103 (2.7130-2.9125)	61.32% (53.54%-68.69%)	40.83% (30.16%-51.56%)	96.388% (91.176%-100%)	95.18% (88.46%-100%)	48.66% (39.44%-58.33%)	2.8099 (2.6044-3.0154)
At least 4 of CHASM (breast), FATHMM (cancer), PROVEAN, SIFT	59.96% (55.56%-63.64%)	37.85% (32.52%-42.86%)	98.166% (97.015%-100%)	97.31% (95.24%-100%)	47.77% (43.05%-52.33%)	2.8119 (2.7211-2.9060)	59.91% (52.53%-67.68%)	37.57% (27.69%-47.83%)	98.1% (94.444%-100%)	97.26% (92.31%-100%)	47.85% (38.89%-57.14%)	2.8121 (2.6291-2.9961)
At least 4 of CHASM (breast), FATHMM (cancer), PROVEAN, SIFT, VEST	67.01% (63.13%-70.71%)	51.66% (46.22%-57.03%)	94.437% (91.667%-97.333%)	94.29% (91.55%-97.3%)	53.02% (47.92%-57.93%)	2.9246 (2.8172-3.0350)	66.97% (60.61%-73.74%)	51.34% (40.98%-61.9%)	94.597% (88.235%-100%)	94.38% (88.57%-100%)	53.1% (43.21%-62.67%)	2.9240 (2.7127-3.1405)
At least 4 of CHASM (breast), FATHMM (cancer), PROVEAN, VEST	57.95% (54.04%-61.62%)	34.7% (29.84%-39.84%)	98.136% (97.015%-100%)	97.04% (94.87%-100%)	46.53% (41.88%-51.08%)	2.7636 (2.6739-2.8540)	57.91% (50.51%-64.65%)	34.41% (24.59%-44.07%)	98.1% (94.444%-100%)	97.01% (91.3%-100%)	46.61% (38.36%-55.84%)	2.7628 (2.5854-2.9448)
At least 4 of CHASM (breast), FATHMM (cancer), SIFT, VEST	62.33% (58.08%-66.16%)	42.1% (36.97%-47.2%)	98.136% (97.015%-100%)	97.59% (95.92%-100%)	49.69% (44.87%-54.43%)	2.8686 (2.7765-2.9628)	62.29% (54.55%-69.7%)	41.72% (31.15%-52.31%)	98.1% (94.444%-100%)	97.57% (92.31%-100%)	49.77% (40.54%-58.97%)	2.8671 (2.6785-3.0559)
At least 4 of CHASM (breast), FATHMM (missense), Mutation Assessor, MutationTaster	61.64% (57.58%-65.66%)	40.31% (35.21%-45.39%)	99.084% (98.485%-100%)	98.74% (97.83%-100%)	49.01% (44.3%-53.57%)	2.8719 (2.7854-2.9603)	61.55% (54.55%-68.69%)	39.96% (30.16%-50.82%)	99.083% (96.774%-100%)	98.7% (95.24%-100%)	49.06% (40.26%-58.11%)	2.8705 (2.6974-3.0477)
At least 4 of CHASM (breast), FATHMM (missense), Mutation Assessor, MutationTaster, PolyPhen-2	77.14% (74.24%-80.81%)	68.67% (64.03%-73.33%)	91.761% (88.406%-95.588%)	93.51% (90.91%-96.55%)	62.83% (57.94%-67.92%)	3.1799 (3.0690-3.2956)	77.08% (70.71%-83.84%)	68.41% (59.02%-77.61%)	91.687% (83.871%-97.62%)	93.47% (87.04%-98.15%)	62.9% (52.94%-73.47%)	3.1768 (2.9401-3.4019)
At least 4 of CHASM (breast), FATHMM (missense), Mutation Assessor, MutationTaster, PROVEAN	70.06% (66.67%-73.74%)	55.38% (50.37%-60.48%)	95.413% (92.958%-98.571%)	95.37% (92.96%-98.53%)	55.27% (50%-60.33%)	3.0127 (2.9096-3.1212)	69.99% (62.63%-76.77%)	55.07% (44.78%-65.52%)	95.402% (89.655%-100%)	95.4% (89.19%-100%)	55.36% (45.9%-65.08%)	3.0113 (2.7979-3.2174)
At least 4 of CHASM (breast), FATHMM (missense), Mutation Assessor, MutationTaster, SIFT	78.11% (74.75%-81.31%)	69.19% (64.23%-73.91%)	93.581% (90.541%-97.183%)	94.92% (92.63%-97.73%)	63.91% (58.86%-69.23%)	3.2157 (3.1059-3.3249)	78.04% (71.72%-84.85%)	68.92% (59.38%-78.69%)	93.565% (86.838%-100%)	94.91% (89.66%-100%)	63.99% (53.85%-74.51%)	3.2138 (2.9834-3.4252)
At least 4 of CHASM (breast), FATHMM (missense), Mutation Assessor, MutationTaster, VEST	75.76% (72.22%-79.29%)	67.08% (62.5%-72.13%)	90.816% (87.324%-94.667%)	92.55% (89.69%-95.74%)	61.47% (56.36%-66.67%)	3.1202 (3.0071-3.2338)	75.68% (68.69%-82.83%)	66.78% (56.67%-76.12%)	90.849% (82.927%-97.5%)	92.55% (86%-98.04%)	61.53% (51.35%-71.7%)	3.1193 (2.8807-3.3491)
At least 4 of CHASM (breast), FATHMM (missense), Mutation Assessor, PolyPhen-2	62.66% (58.59%-66.67%)	43.13% (37.93%-48.31%)	97.195% (95.312%-100%)	96.5% (94.23%-100%)	49.68% (44.77%-54.55%)	2.8652 (2.7707-2.9621)	62.59% (55.56%-69.7%)	42.76% (32.81%-53.33%)	97.297% (92.308%-100%)	96.65% (91.17%-100%)	49.74% (40.85%-59.21%)	2.8642 (2.6688-3.0649)
At least 4 of CHASM (breast), FATHMM (missense), Mutation Assessor, PolyPhen-2, PROVEAN	68.04% (64.65%-71.72%)	53.8% (48.39%-58.99%)	92.676% (89.394%-96.053%)	93.02% (90%-96.3%)	53.97% (48.89%-58.82%)	2.9428 (2.8352-3.0506)	67.94% (60.61%-75.76%)	53.48% (43.33%-63.49%)	92.818% (85.294%-100%)	93.13% (86.11%-100%)	54.04% (44.12%-64.06%)	2.9416 (2.7119-3.1533)
At least 4 of CHASM (breast), FATHMM (missense), Mutation Assessor, PolyPhen-2, SIFT	72.4% (68.69%-76.26%)	62.77% (57.85%-67.77%)	89.891% (86.111%-94.366%)	91.64% (88.57%-95.18%)	58.48% (53.3%-63.64%)	3.0138 (2.8973-3.1357)	72.28% (65.66%-79.8%)	62.4% (52.24%-72.13%)	89.963% (80.769%-97.22%)	91.63% (84.78%-97.73%)	58.5% (48.15%-69.01%)	3.0116 (2.7573-3.2436)
At least 4 of CHASM (breast), FATHMM (missense), Mutation Assessor, PolyPhen-2, VEST	70.7% (66.67%-74.24%)	59.6% (54.24%-64.57%)	90.816% (87.324%-94.667%)	91.91% (88.89%-95.35%)	56.34% (51.14%-61.67%)	2.9743 (2.8560-3.0941)	70.63% (63.64%-77.78%)	59.28% (49.09%-69.84%)	90.835% (83.333%-97.5%)	91.94% (85.11%-97.78%)	56.39% (46.3%-66.67%)	2.9721 (2.7355-3.2072)
At least 4 of CHASM (breast), FATHMM (missense), Mutation Assessor, PROVEAN	57.94% (53.54%-61.62%)	34.74% (29.84%-39.68%)	98.136% (97.015%-100%)	97.04% (94.87%-100%)	46.54% (41.56%-50.98%)	2.7644 (2.6735-2.8589)	57.87% (50.51%-64.65%)	34.41% (25%-44.44%)	98.1% (94.444%-100%)	97.01% (91.3%-100%)	46.6% (38.03%-55.56%)	2.7629 (2.5801-2.9433)
At least 4 of CHASM (breast), FATHMM (missense), Mutation Assessor, PROVEAN, SIFT	68.35% (64.65%-72.22%)	53.36% (48.03%-58.4%)	93.581% (90.541%-97.183%)	93.49% (90.54%-97.01%)	54.15% (49.3%-59.09%)	2.9349 (2.8245-3.0449)	68.28% (60.61%-75.76%)	53.03% (42.86%-62.9%)	93.565% (86.838%-100%)	93.52% (86.49%-100%)	54.22% (44.44%-64.18%)	2.9343 (2.7091-3.1574)
At least 4 of CHASM (breast), FATHMM (missense), Mutation Assessor, PROVEAN, VEST	64.68% (60.61%-68.69%)	48.05% (42.52%-53.04%)	93.581% (90.541%-97.183%)	93.13% (89.86%-96.77%)	51.47% (46.24%-56.31%)	2.8492 (2.7447-2.9561)	64.62% (56.57%-71.72%)	47.73% (37.5%-58.06%)	93.565% (86.838%-100%)	93.14% (85.71%-100%)	51.55% (42.03%-61.43%)	2.8485 (2.6202-3.0723)
At least 4 of CHASM (breast), FATHMM (missense), Mutation Assessor, SIFT	63.67% (59.6%-67.68%)	44.29% (39.2%-49.59%)	98.119% (97.015%-100%)	97.6% (95.92%-100%)	50.33% (45.45%-55.08%)	2.8930 (2.8005-2.9888)	63.61% (55.56%-70.71%)	43.92% (33.33%-54.24%)	98.1% (94.444%-100%)	97.6% (92.86%-100%)	50.38% (41.1%-59.74%)	2.8917 (2.7048-3.0875)
At least 4 of CHASM (breast), FATHMM (missense), Mutation Assessor, SIFT, VEST	72.7% (69.19%-76.26%)	62.73% (57.72%-67.84%)	90.816% (87.324%-94.667%)	92.2% (89.29%-95.65%)	58.51% (53.33%-63.75%)	3.0416 (2.9246-3.1591)	72.62% (65.66%-79.8%)	62.41% (52.24%-72.13%)	90.835% (83.333%-97.5%)	92.2% (85.42%-97.92%)	58.55% (48.15%-68.85%)	3.0395 (2.8039-3.2823)
At least 4 of CHASM (breast), FATHMM (missense), Mutation Assessor, VEST	58.61% (54.55%-62.63%)	36.34% (31.5%-41.27%)	98.136% (97.015%-100%)	97.14% (95.12%-100%)	46.92% (42.35%-51.71%)	2.7795 (2.6881-2.8728)	58.56% (51.52%-65.66%)	36.03% (26.09%-46.15%)	98.1% (94.444%-100%)	97.13% (92.31%-100%)	46.98% (38.36%-55.84%)	2.7788 (2.6027-2.9641)
At least 4 of CHASM (breast), FATHMM (missense), MutationTaster, PolyPhen-2	65.01% (61.11%-68.69%)	46.8% (41.6%-51.97%)	97.233% (95.588%-100%)	96.71% (94.74%-100%)	51.3% (46.33%-56.12%)	2.9176 (2.8203-3.0152)	64.94% (57.58%-72.73%)	46.49% (36.07%-57.14%)	97.337% (92.5%-100%)	96.84% (91.42%-100%)	51.34% (42.03%-60.53%)	2.9158 (2.7161-3.1133)
At least 4 of CHASM (breast), FATHMM (missense), MutationTaster, PolyPhen-2, PROVEAN	72.07% (68.18%-75.76%)	59.13% (54.1%-64.23%)	94.437% (91.667%-97.333%)	94.87% (92.31%-97.62%)	57.55% (52.38%-62.79%)	3.0630 (2.9553-3.1717)	71.98% (64.65%-78.79%)	58.81% (48.44%-69.12%)	94.536% (88.235%-100%)	94.94% (88.89%-100%)	57.6% (47.62%-67.69%)	3.0594 (2.8445-3.2742)
At least 4 of CHASM (breast), FATHMM (missense), MutationTaster, PolyPhen-2, SIFT	80.84% (77.78%-83.84%)	73.94% (69.29%-78.46%)	92.701% (89.609%-96.104%)	94.62% (92.31%-97.2%)	67.31% (62.14%-72.73%)	3.2920 (3.1826-3.4100)	80.79% (74.75%-86.87%)	73.95% (64.91%-82.81%)	92.569% (85.294%-97.67%)	94.49% (88.89%-98.31%)	67.51% (56.86%-77.55%)	3.2887 (3.0678-3.5014)
At least 4 of CHASM (breast), FATHMM (missense), MutationTaster, PolyPhen-2, VEST	77.43% (74.24%-80.81%)	69.67% (64.66%-74.4%)	90.816% (87.324%-94.667%)	92.9% (90.11%-95.92%)	63.25% (58.02%-68.5%)	3.1752 (3.0599-3.2925)	77.45% (70.71%-84.85%)	69.75% (60%-79.03%)	90.655% (82.759%-97.37%)	92.86% (86.54%-98.04%)	63.46% (52.94%-73.91%)	3.1759 (2.9405-3.3945)

At least 4 of CHASM (breast), FATHMM (missense), MutationTaster, PROVEAN	57.29% (53.03%-61.11%)	33.64% (29.03%-38.4%)	98.173% (97.015%-100%)	96.96% (94.87%-100%)	46.11% (41.4%-50.33%)	2.7488 (2.6540-2.8403)	57.14% (49.49%-65.66%)	33.23% (23.81%-42.65%)	98.144% (94.118%-100%)	96.86% (90%-100%)	46.14% (37.33%-55.56%)	2.7437 (2.5529-2.9401)
At least 4 of CHASM (breast), FATHMM (missense), MutationTaster, PROVEAN, SIFT	73.08% (69.7%-76.77%)	60.16% (55%-65.22%)	95.413% (92.955%-98.53%)	95.78% (93.42%-98.68%)	58.08% (53.1%-62.61%)	3.0943 (2.9908-3.2008)	73.03% (65.66%-79.8%)	59.96% (49.25%-70.15%)	95.402% (89.655%-100%)	95.73% (90.32%-100%)	58.14% (48.28%-67.93%)	3.0923 (2.8753-3.2992)
At least 4 of CHASM (breast), FATHMM (missense), MutationTaster, PROVEAN, VEST	69.72% (66.16%-73.23%)	54.86% (49.59%-60.17%)	95.413% (92.955%-98.53%)	95.39% (92.85%-98.53%)	55% (50%-59.84%)	3.0066 (2.8977-3.1165)	69.65% (62.63%-76.77%)	54.62% (43.93%-64.91%)	95.402% (89.655%-100%)	95.33% (89.66%-100%)	55.06% (45.31%-64.91%)	3.0041 (2.7932-3.2169)
At least 4 of CHASM (breast), FATHMM (missense), MutationTaster, SIFT	64.67% (61.11%-68.69%)	45.83% (40.5%-50.82%)	97.233% (95.588%-100%)	96.63% (94.55%-100%)	50.94% (46.04%-55.63%)	2.9063 (2.8034-3.0139)	64.6% (56.57%-71.72%)	45.55% (35.29%-55.88%)	97.269% (92.303%-100%)	96.64% (90.9%-100%)	51% (41.54%-60.88%)	2.9046 (2.6999-3.1137)
At least 4 of CHASM (breast), FATHMM (missense), MutationTaster, SIFT, VEST	78.82% (75.76%-81.82%)	70.78% (66.66%-75.2%)	92.701% (89.609%-96%)	94.37% (92.05%-96.97%)	64.73% (59.8%-69.9%)	3.2259 (3.1160-3.3385)	78.73% (72.73%-84.85%)	70.63% (61.53%-79.03%)	92.569% (85.294%-97.78%)	94.23% (88.46%-98.28%)	64.76% (54.35%-74.55%)	3.2219 (2.9941-3.4443)
At least 4 of CHASM (breast), FATHMM (missense), MutationTaster, VEST	60.65% (56.57%-64.15%)	39.49% (34.42%-44.36%)	97.233% (95.588%-100%)	96.11% (93.62%-100%)	48.17% (43.54%-52.74%)	2.8100 (2.7129-2.9115)	60.52% (53.51%-68.69%)	39.09% (29.5%-49.15%)	97.269% (92.303%-100%)	96.11% (89.65%-100%)	48.2% (38.89%-57.75%)	2.8068 (2.6027-3.0051)
At least 4 of CHASM (breast), FATHMM (missense), PolyPhen-2, PROVEAN	57.3% (53.54%-61.11%)	33.66% (28.8%-38.58%)	98.173% (97.015%-100%)	96.96% (94.87%-100%)	46.12% (41.33%-50.33%)	2.7491 (2.6548-2.8428)	57.11% (49.49%-64.65%)	33.19% (23.33%-43.33%)	98.144% (94.118%-100%)	96.85% (89.99%-100%)	46.12% (37.5%-55.7%)	2.7430 (2.5507-2.9292)
At least 4 of CHASM (breast), FATHMM (missense), PolyPhen-2, PROVEAN, SIFT	70.04% (66.65%-73.74%)	59.63% (54.55%-64.57%)	88.006% (83.562%-92.426%)	89.59% (86.05%-93.42%)	55.77% (50.85%-60.87%)	2.9300 (2.8051-3.0520)	70.03% (62.63%-76.79%)	59.43% (49.18%-69.49%)	88.17% (79.301%-97.06%)	89.64% (81.81%-97.22%)	55.88% (45.76%-66.07%)	2.9311 (2.6834-3.1742)
At least 4 of CHASM (breast), FATHMM (missense), PolyPhen-2, PROVEAN, VEST	66.69% (63.12%-70.2%)	54.88% (49.6%-60.16%)	87.089% (82.609%-91.549%)	88.03% (84.06%-92.11%)	52.75% (47.5%-57.52%)	2.8275 (2.7010-2.9504)	66.62% (59.6%-73.76%)	54.57% (44.07%-64.91%)	87.263% (78.378%-95.46%)	88.04% (80%-95.46%)	52.82% (42.85%-63.33%)	2.8269 (2.5746-3.0780)
At least 4 of CHASM (breast), FATHMM (missense), PolyPhen-2, SIFT	64.35% (60.61%-68.18%)	45.32% (40%-50.4%)	97.233% (95.588%-100%)	96.59% (94.44%-100%)	50.7% (45.86%-55.3%)	2.8985 (2.8016-2.9991)	64.24% (56.57%-71.72%)	44.97% (34.43%-55.71%)	97.269% (92.303%-100%)	96.6% (90.9%-100%)	50.74% (41.67%-60.56%)	2.8958 (2.6907-3.0929)
At least 4 of CHASM (breast), FATHMM (missense), PolyPhen-2, SIFT, VEST	74.09% (70.71%-77.78%)	70.28% (65.87%-74.8%)	80.67% (75.713%-86.115%)	86.28% (82.57%-90.2%)	61.08% (55.43%-66.35%)	2.9831 (2.8492-3.1244)	74.04% (66.67%-80.81%)	70.05% (60.93%-78.79%)	80.821% (69.994%-90.7%)	86.26% (78.26%-93.48%)	61.12% (50%-72%)	2.9825 (2.7004-3.2554)
At least 4 of CHASM (breast), FATHMM (missense), PolyPhen-2, VEST	59.63% (55.54%-63.13%)	38.42% (33.08%-43.41%)	96.293% (94.203%-98.667%)	94.72% (91.67%-98.08%)	47.49% (42.67%-51.92%)	2.7692 (2.6636-2.8701)	59.53% (52.53%-67.7%)	38.04% (28.12%-48.44%)	96.398% (91.176%-100%)	94.81% (87.5%-100%)	47.55% (38.66%-57.33%)	2.7679 (2.5611-2.9748)
At least 4 of CHASM (breast), FATHMM (missense), PROVEAN, SIFT	58.97% (55.04%-62.63%)	36.3% (31.45%-40.94%)	98.173% (97.015%-100%)	97.17% (95.24%-100%)	47.13% (42.21%-51.61%)	2.7877 (2.6957-2.8815)	58.82% (51.52%-66.69%)	35.9% (26.31%-45.9%)	98.144% (94.118%-100%)	97.09% (90.48%-100%)	47.15% (38.36%-56.79%)	2.7827 (2.5878-2.9704)
At least 4 of CHASM (breast), FATHMM (missense), PROVEAN, SIFT, VEST	67.02% (63.13%-70.71%)	54.33% (49.15%-59.52%)	88.933% (84.929%-93.243%)	89.47% (85.51%-93.33%)	52.97% (47.97%-57.76%)	2.8570 (2.7330-2.9858)	66.98% (59.6%-74.75%)	54.07% (43.55%-64.19%)	89.073% (80%-97.14%)	89.49% (81.58%-97.22%)	53.06% (43.33%-63.16%)	2.8569 (2.6066-3.1050)
At least 4 of CHASM (breast), FATHMM (missense), PROVEAN, VEST	56.63% (52.53%-60.1%)	32.6% (27.56%-37.4%)	98.173% (97.015%-100%)	96.86% (94.74%-100%)	45.72% (41.02%-50%)	2.7335 (2.6393-2.8308)	56.44% (49.49%-64.65%)	32.13% (22.38%-42.11%)	98.144% (94.118%-100%)	96.75% (90%-100%)	45.73% (37.18%-55.26%)	2.7276 (2.5294-2.9200)
At least 4 of CHASM (breast), FATHMM (missense), SIFT, VEST	60.65% (56.57%-64.14%)	39.48% (34.39%-44.63%)	97.233% (95.588%-100%)	96.11% (93.62%-100%)	48.17% (43.45%-52.67%)	2.8099 (2.7129-2.9118)	60.53% (53.54%-68.69%)	39.1% (28.84%-50%)	97.269% (92.303%-100%)	96.11% (89.29%-100%)	48.21% (38.96%-57.9%)	2.8069 (2.5996-3.0100)
At least 4 of CHASM (breast), Mutation Assessor, MutationTaster, PolyPhen-2	61.62% (57.58%-65.66%)	41.55% (36.29%-46.72%)	96.311% (94.286%-98.667%)	95.12% (92.16%-98.28%)	48.8% (44.14%-53.42%)	2.8177 (2.7122-2.9223)	61.61% (53.54%-69.7%)	41.36% (31.14%-51.56%)	96.355% (91.176%-100%)	95.12% (87.5%-100%)	48.92% (39.68%-58.33%)	2.8175 (2.6049-3.0228)
At least 4 of CHASM (breast), Mutation Assessor, MutationTaster, PolyPhen-2, PROVEAN	71.72% (68.18%-75.25%)	58.55% (53.54%-63.78%)	94.492% (91.667%-97.297%)	94.84% (92.21%-97.53%)	56.87% (52.07%-61.67%)	3.0474 (2.9416-3.1591)	71.71% (64.65%-78.79%)	58.41% (47.54%-68.63%)	94.488% (88.571%-100%)	94.78% (88.57%-100%)	56.97% (47.16%-66.67%)	3.0465 (2.8249-3.2711)
At least 4 of CHASM (breast), Mutation Assessor, MutationTaster, PolyPhen-2, SIFT	79.46% (76.26%-82.83%)	72.86% (68.5%-77.42%)	90.873% (87.342%-94.872%)	93.24% (90.72%-96.23%)	65.94% (60.82%-71.16%)	3.2292 (3.1213-3.3477)	79.46% (72.73%-85.86%)	72.88% (63.63%-81.69%)	90.735% (82.855%-97.3%)	93.09% (86.48%-98%)	66.1% (55.55%-76.6%)	3.2281 (2.9789-3.4475)
At least 4 of CHASM (breast), Mutation Assessor, MutationTaster, PolyPhen-2, VEST	78.11% (74.75%-81.31%)	71.82% (67.45%-76.3%)	88.988% (85.29%-93.243%)	91.85% (89.01%-95.15%)	64.62% (59.57%-69.89%)	3.1728 (3.0548-3.2951)	78.12% (71.72%-84.85%)	71.76% (62.9%-80.33%)	89.014% (80.488%-97.06%)	91.79% (84.44%-97.83%)	64.76% (54.16%-74.56%)	3.1732 (2.9263-3.4080)
At least 4 of CHASM (breast), Mutation Assessor, MutationTaster, PROVEAN	56.6% (52.53%-60.61%)	32.55% (27.78%-37.1%)	98.173% (97.015%-100%)	96.85% (94.59%-100%)	45.71% (41.06%-50.01%)	2.7329 (2.6393-2.8252)	56.5% (48.48%-64.65%)	32.22% (22.95%-42.11%)	98.144% (94.118%-100%)	96.75% (89.99%-100%)	45.77% (36.98%-55.08%)	2.7289 (2.5417-2.9205)
At least 4 of CHASM (breast), Mutation Assessor, MutationTaster, PROVEAN, SIFT	72.38% (68.69%-75.76%)	59.58% (54.4%-64.52%)	94.492% (91.667%-97.297%)	94.92% (92.31%-97.56%)	57.48% (52.59%-62.3%)	3.0648 (2.9619-3.1728)	72.42% (65.66%-79.8%)	59.53% (49.21%-69.85%)	94.488% (88.571%-100%)	94.88% (88.89%-100%)	57.64% (47.37%-67.28%)	3.0654 (2.8446-3.2746)
At least 4 of CHASM (breast), Mutation Assessor, MutationTaster, PROVEAN, VEST	69.73% (66.16%-73.74%)	55.4% (50.4%-60.61%)	94.492% (91.667%-97.297%)	94.56% (91.78%-97.4%)	55.06% (50%-60.16%)	2.9951 (2.8915-3.1117)	69.64% (61.62%-76.77%)	55.13% (44.61%-65%)	94.488% (88.571%-100%)	94.49% (87.88%-100%)	55.11% (45%-64.91%)	2.9922 (2.7699-3.2133)
At least 4 of CHASM (breast), Mutation Assessor, MutationTaster, SIFT	62.64% (58.59%-66.67%)	43.16% (38.16%-48.03%)	96.311% (94.286%-98.667%)	95.29% (92.45%-98.33%)	49.49% (44.76%-54.17%)	2.8425 (2.7410-2.9479)	62.61% (54.55%-70.71%)	42.93% (32.79%-53.34%)	96.355% (91.176%-100%)	95.29% (88%-100%)	49.59% (40%-59.16%)	2.8417 (2.6295-3.0453)
At least 4 of CHASM (breast), Mutation Assessor, MutationTaster, SIFT, VEST	78.8% (75.76%-81.83%)	71.81% (67.21%-76.42%)	90.873% (87.342%-94.872%)	93.15% (90.62%-96.19%)	65.09% (60%-70.3%)	3.2093 (3.0984-3.3285)	78.77% (72.7%-84.85%)	71.78% (62.96%-80.65%)	90.735% (82.855%-97.3%)	93% (86.27%-97.96%)	65.2% (54.72%-75.52%)	3.2070 (2.9683-3.4346)
At least 4 of CHASM (breast), Mutation Assessor, MutationTaster, VEST	58.94% (54.55%-62.63%)	37.33% (31.97%-42.62%)	96.311% (94.286%-98.667%)	94.59% (91.3%-98.08%)	47.06% (42.36%-51.63%)	2.7529 (2.6459-2.8604)	58.89% (51.52%-67.68%)	37.04% (26.86%-48.28%)	96.355% (91.176%-100%)	94.58% (86.94%-100%)	47.15% (37.84%-56.76%)	2.7512 (2.5313-2.9704)
At least 4 of CHASM (breast), Mutation Assessor, PolyPhen-2, PROVEAN	57.28% (53.03%-61.11%)	33.62% (28.68%-38.17%)	98.173% (97.015%-100%)	96.95% (94.87%-100%)	46.1% (41.4%-50.35%)	2.7485 (2.6577-2.8463)	57.17% (49.49%-65.66%)	33.27% (24.14%-42.86%)	98.144% (94.118%-100%)	96.85% (90%-100%)	46.15% (37.5%-55.41%)	2.7442 (2.5547-2.9286)
At least 4 of CHASM (breast), Mutation Assessor, PolyPhen-2, PROVEAN, SIFT	68.32% (64.65%-71.73%)	59.07% (54.03%-64.29%)	84.306% (79.452%-88.893%)	86.68% (82.61%-90.7%)	54.37% (49.11%-59.29%)	2.8443 (2.7032-2.9773)	68.4% (61.59%-75.76%)	58.95% (48.44%-69.36%)	84.58% (75%-94.29%)	86.77% (78.38%-95.12%)	54.56% (44.07%-65.09%)	2.8486 (2.5869-3.1249)
At least 4 of CHASM (breast), Mutation Assessor, PolyPhen-2, PROVEAN, VEST	65.33% (61.62%-69.19%)	54.33% (49.21%-59.52%)	84.338% (79.71%-89.041%)	85.71% (81.33%-90.12%)	51.65% (46.49%-56.67%)	2.7602 (2.6173-2.9126)	65.3% (57.58%-72.73%)	54.07% (43.75%-64.41%)	84.522% (75%-94.29%)	85.69% (76.32%-94.59%)	51.75% (41.38%-61.82%)	2.7604 (2.4695-3.0462)
At least 4 of CHASM (breast), Mutation Assessor, PolyPhen-2, SIFT	63.65% (59.6%-67.68%)	44.76% (39.83%-50%)	96.311% (94.286%-98.667%)	95.45% (92.86%-98.36%)	50.21% (45.32%-54.82%)	2.8674 (2.7638-2.9773)	63.6% (55.56%-71.72%)	44.49% (33.87%-54.55%)	96.355% (91.176%-100%)	95.45% (88.89%-100%)	50.29% (40.91%-59.72%)	2.8659 (2.6508-3.0748)
At least 4 of CHASM (breast), Mutation Assessor, PolyPhen-2, SIFT, VEST	73.74% (70.2%-77.27%)	71.28% (66.67%-75.97%)	77.971% (72.727%-83.333%)	84.83% (80.81%-88.99%)	61.1% (55.43%-67.02%)	2.9518 (2.8114-3.0913)	73.74% (66.67%-80.81%)	71.23% (61.9%-80.3%)	77.995% (66.667%-88.9%)	84.73% (76.36%-92.46%)	61.24% (49.97%-72.34%)	2.9520 (2.6605-3.2295)
At least 4 of CHASM (breast), Mutation Assessor, PolyPhen-2, VEST	58.6% (54.55%-62.63%)	37.32% (31.93%-42.4%)	95.372% (92.955%-98.571%)	93.31% (89.8%-97.96%)	46.81% (42.11%-51.33%)	2.7281 (2.6143-2.8395)	58.57% (50.51%-66.67%)	37.04% (26.78%-47.69%)	95.484% (89.651%-100%)	93.38% (84.62%-100%)	46.92% (37.84%-56.34%)	2.7282 (2.5023-2.9532)
At least 4 of CHASM (breast), Mutation Assessor, PROVEAN, SIFT	58.29% (54.04%-62.12%)	35.22% (30.51%-40%)	98.173% (97.015%-100%)	97.09% (95.12%-100%)	46.71% (42%-51.01%)	2.7719 (2.6797-2.8675)	58.16% (50.51%-66.67%)	34.85% (25.39%-44.45%)	98.144% (94.118%-100%)	96.99% (90.46%-100%)	46.75% (37.97%-56%)	2.7673 (2.5722-2.9550)
At least 4 of CHASM (breast), Mutation Assessor, PROVEAN, SIFT, VEST	65.33% (61.62%-69.19%)	54.85% (49.61%-59.84%)	83.437% (78.459%-88.406%)	85.13% (80.82%-89.61%)	51.67% (46.55%-57.02%)	2.7509 (2.6164-2.8917)	65.3% (57.58%-72.73%)	54.62% (44.44%-65.08%)	83.57% (74.064%-93.55%)	85.07% (75.68%-94.13%)	51.77% (41.07%-62.07%)	2.7503 (2.4661-3.0200)
At least 4 of CHASM (breast), Mutation Assessor, PROVEAN, VEST	56.26% (52.02%-60.1%)	32.02% (27.34%-36.64%)	98.173% (97.015%-100%)	96.81% (94.59%-100%)	45.51% (40.79%-50%)	2.7250 (2.6323-2.8201)	56.17% (48.48%-64.65%)	31.7% (22.22%-41.27%)	98.144% (94.118%-100%)	96.7% (89.47%-100%)	45.57% (36.71%-54.88%)	2.7211 (2.5282-2.9118)
At least 4 of CHASM (breast), Mutation Assessor, SIFT, VEST	59.96% (56.06%-63.64%)	38.93% (34.11%-44.09%)	96.311% (94.286%-98.667%)	94.8% (91.67%-98.15%)	47.7% (42.96%-52.35%)	2.7775 (2.6739-2.8873)	59.88% (52.53%-67.68%)	38.61% (28.35%-49.12%)	96.355% (91.176%-100%)	94.79% (86.96%-100%)	47.77% (38.36%-57.14%)	2.7753 (2.5532-2.9765)
At least 4 of CHASM (breast), MutationTaster, PolyPhen-2, PROVEAN	57.95% (54.04%-61.62%)	34.69% (29.84%-39.54%)	98.173% (97.015%-100%)	97.04% (95%-100%)	46.5% (41.87%-50.97%)	2.7641 (2.6696-2.8567)	57.83% (50.51%-65.66%)	34.33% (24.55%-43.95%)	98.144% (94.118%-100%)	96.94% (90.46%-100%)	46.55% (37.66%-56%)	2.7597 (2.5676-2.9516)
At least 4 of CHASM (breast), MutationTaster, PolyPhen-2, PROVEAN, SIFT	73.73% (70.2%-77.27%)	61.72% (56.59%-66.4%)	94.492% (91.667%-97.297%)	95.09% (92.59%-97.62%)	58.81% (53.78%-63.55%)	3.1011 (3.0007-3.2122)	73.75% (66.67%-80.81%)	61.64% (52.05%-71.88%)	94.488% (88.571%-100%)	95.05% (89.47%-100%)	58.94% (49.06%-68.97%)	3.1012 (2.8794-3.3087)
At least 4 of CHASM (breast), MutationTaster, PolyPhen-2, PROVEAN, VEST	71.06% (67.17%-74.75%)	57.5% (52.5%-62.6%)	94.492% (91.667%-97.297%)	94.75% (92.11%-97.5%)	56.26% (51.2%-61.29%)	3.0300 (2.9190-3.1446)	71.01% (63.64%-78.79%)	57.31% (47.37%-67.65%)	94.488% (88.571%-100%)	94.69% (88.24%-100%)	56.34% (46.15%-66.07%)	3.0283 (2.8027-3.2478)
At least 4 of CHASM (breast), MutationTaster, PolyPhen-2, SIFT	63.98% (60.1%-67.68%)	45.28% (40.16%-50.4%)	96.311% (94.286%-98.667%)	95.5% (92.86%-98.41%)	50.44% (45.59%-54.89%)	2.8753 (2.7701-2.9845)	63.96% (56.57%-71.72%)	45.07% (34.38%-55.17%)	96.355% (91.176%-100%)	95.5% (88.46%-100%)	50.55% (41.27%-60.3%)	2.8748 (2.6621-3.0859)
At least 4 of CHASM (breast), MutationTaster, PolyPhen-2, SIFT, VEST	80.5% (77.78%-83.84%)	74.5% (70.4%-78.91%)	90.873% (87.342%-94.872%)	93.38% (90.91%-96.3%)	67.33% (62.24%-72.55%)	3.2609 (3.1544-3.3823)	80.41% (73.74%-85.86%)	74.38% (65.67%-82.76%)	90.735% (82.855%-97.3%)	93.22% (86.66%-98.04%)	67.36% (56.25%-77.36%)	3.2570 (3.0130-3.4691)
At least 4 of CHASM (breast), MutationTaster, PolyPhen-2, VEST	61.3% (57.07%-65.15%)	41.04% (35.77%-46.22%)	96.311% (94.286%-98.667%)	95.06% (92.16%-98.25%)	48.58% (43.84%-53.06%)	2.8100 (2.7053-2.9168)	61.24% (53.54%-69.7%)	40.77% (30.51%-51.43%)	96.355% (91.176%-100%)	95.06% (87.99%-100%)	48.67% (39.71%-58.46%)	2.8086 (2.5918-3.0215)
At least 4 of CHASM (breast), MutationTaster, PROVEAN, SIFT	59.62% (55.56%-63.13%)	37.32% (32.54%-42.4%)	98.173% (97.015%-100%)	97.25% (95.35%-100%)	47.53% (42.76%-51.95%)	2.8027 (2.7073-2.8938)	59.55% (52.53%-67.68%)	37.04% (26.98%-46.88%)	98.144% (94.118%-100%)	97.17% (90.91%-100%)	47.6% (38.67%-57.33%)	2.7995 (2.6113-2.9917)
At least 4 of CHASM (breast), MutationTaster, PROVEAN, SIFT, VEST	70.37% (67.16%-74.24%)	56.42% (51.22%-61.29%)	94.492% (91.667%-97.297%)	94.65% (92%-97.44%)	55.64% (50.77%-60.8%)	3.0120 (2.9066-3.1301)	70.36% (62.63%-76.79%)	56.28% (45.76%-66.19%)	94.488% (88.571%-100%)	94.6% (88.24%-100%)	55.74% (45.45%-65.57%)	3.0111 (2.7773-3.2199)
At least 4 of CHASM (breast), MutationTaster, PROVEAN, VEST	57.95% (54.04%-61.62%)	34.69% (29.84%-39.2%)	98.173% (97.015%-100%)	97.04% (95%-100%)	46.51% (41.67%-50.97%)	2.7641 (2.6696-2.8580)	57.83% (50.51%-65.66%)	34.33% (24.99%-44.07%)	98.144% (94.118%-100%)	96.95% (90%-100%)	46.55% (37.97%-56.25%)	2.7597 (2.5674-2.9562)
At least 4 of CHASM (breast), MutationTaster, SIFT, VEST	61.63% (57.58%-65.16%)	41.58% (36%-46.83%)	96.311% (94.286%-98.667%)	95.12% (92.16%-98.28%)	48.81% (43.97%-53.29%)	2.8182 (2.7142-2.9220)	61.58% (54.52%-69.7%)	41.3% (30.51%-52.31%)	96.355% (91.176%-100%)	95.11% (88%-100%)	48.89% (39.71%-58.67%)	2.8166 (2.6061-3.0248)
At least 4 of CHASM (breast), PolyPhen-2, PROVEAN, SIFT	59.63% (55.56%-63.13%)	37.34% (32.26%-42.31%)	98.173% (97.015%-100%)	97.25% (95.35%-100%)	47.54% (42.75%-51.97%)	2.8030 (2.7074-2.8984)	59.52% (52.53%-67.68%)	37% (27.12%-46.88%)	98.144% (94.118%-100%)	97.16% (90.91%-100%)	47.58% (38.66%-56.96%)	2.7988 (2.6027-2.9886)
At least 4 of CHASM (breast), PolyPhen-2, PROVEAN, SIFT, VEST	66.66% (62.63%-70.71%)	56.45% (51.24%-61.29%)	84.311% (79.705%-89.194%)	86.15% (82.05%-90.59%)	52.82% (47.46%-57.89%)	2.7973 (2.6615-2.9455)	66.68% (58.59%-74.75%)	56.23% (46.03%-66.14%)	84.564% (74.984%-93.75%)	86.21% (76.92%-94.45%)	52.96% (42.62%-63.79%)	2.7996 (2.5031-3.0778)
At least 4 of CHASM (breast), PolyPhen-2, PROVEAN, VEST	57.61% (53.54%-61.12%)	34.15% (29.17%-38.84%)	98.173% (97.015%-100%)	97% (94.87%-100%)	46.3% (41.45%-50.64%)	2.7562 (2.6560-2.8492)	57.5% (50.48%-65.66%)	33.81% (24.61%-43.64%)	98.144% (94.118%-100%)	96.9% (90%-100%)	46.35% (37.66%-55.84%)	2.7520 (2.5605-2.9513)
At least 4 of CHASM (breast), PolyPhen-2, SIFT, VEST	60.96% (57.07%-64.65%)	40.5% (35.2%-45.67%)	96.311% (94.286%-98.667%)	95% (92%-98.25%)	48.35% (43.54%-52.7%)	2.8017 (2.6915-2.9115)	60.92% (53.54%-68.69%)	40.25% (29.69%-50.79%)	96.355% (91.176%-100%)	94.99% (87.5%-100%)	48.45% (39.7%-58.33%)	2.8005 (2.5769-3.0229)
At least 4 of CHASM (breast), PROVEAN, SIFT, VEST	58.96% (55.05%-62.63%)	36.27% (31.4%-40.98%)	98.173% (97.015%-100%)	97.17% (95.24%-100%)	47.12% (42.28%-51.35%)	2.7874 (2.6948-2.8815)	58.85% (51.52%-66.67%)	35.94% (26.56%-45.9%)	98.144% (94.118%-100%)	97.08% (90.48%-100%)	47.17% (38.15%-56.76%)	2.7834 (2.5893-2.9791)
At least 4 of CHASM (lung), CHASM (melanoma), FATHMM (cancer), FATHMM (missense)	78.19% (74.75%-81.31%)	69.3% (64.51%-74.02%)	93.565% (90.537%-97.183%)	94.9% (92.55%-97.78%)	63.8% (58.33%-68.93%)	3.2157 (3.1063-3.3241)	77.96% (71.72%-84.85%)	68.84% (59.02%-78.12%)	93.596% (86.842%-100%)	94.86% (89.36%-100%)	63.64% (53.06%-74.15%)	3.2093 (2.9896-3.4331)
At least 4 of CHASM (lung), CHASM (melanoma), FATHMM (cancer), FATHMM (missense), Mutation Assessor	84.21% (81.31%-87.37%)	80.92% (76.8%-85.04%)	89.892% (86.111%-94.286%)	93.26% (90.99%-96.15%)	73.16% (67.86%-78.57%)	3.3723 (3.2629-3.4846)	84.11% (77.78%-89.9%)	80.69% (72.13%-88.71%)	89.917% (81.476%-97.3%)	93.22% (87.76%-98.08%)	73.11% (62.5%-83.72%)	3.3694 (3.1476-3.5901)
At least 4 of CHASM (lung), CHASM (melanoma), FATHMM (cancer), FATHMM (missense), MutationTaster	86.57% (83.84%-89.39%)	84.64% (80.99%-88.53%)	89.913% (86.297%-94.288%)	93.55% (91.3%-96.4%)	77.2% (72.29%-82.5%)	3.4530 (3.3497-3.5700)	86.45% (80.81%-91.92%)	84.43% (76.67%-91.67%)	89.89% (81.577%-97.22%)	93.47% (87.72%-98.18%)	77.12% (65.96%-87.18%)	3.4491 (3.2153-3.6632)
At least 4 of CHASM (lung), CHASM (melanoma), FATHMM (cancer), FATHMM (missense), PolyPhen-2	85.22% (82.32%-88.38%)	84.1% (80.33%-87.9%)	87.149% (83.077%-91.892%)	91.88% (89.38%-94.83%)	76.03% (70.93%-81.4%)	3.3916 (3.2800-3.5174)	85.11% (78.79%-90.91%)	83.9% (76.27%-91.38%)	87.15% (77.491%-95%)	91.81% (85.71%-96.61%)	75.96% (65.11%-86.36%)	3.3882 (3.1429-3.6164)
At least 4 of CHASM (lung), CHASM (melanoma), FATHMM (cancer), FATHMM (missense), PROVEAN	82.55% (79.79%-85.86%)	77.77% (73.39%-81.97%)	90.796% (87.324%-94.595%)	93.6% (91.09%-96.23%)	70.27% (64.89%-75.53%)	3.3243 (3.2126-3.4415)	82.38% (75.76%-87.9%)	77.42% (68.85%-85.97%)	90.86% (82.857%-97.37%)	93.57% (88.13%-98.18%)	70.12% (59.57%-80.95%)	3.3197 (3.0879-3.5440)
At least 4 of CHASM (lung), CHASM (melanoma), FATHMM (cancer), FATHMM (missense), SIFT	85.54% (82.83%-88.38%)	83.56% (80%-87.5%)	88.966% (84.932%-93.245%)	92.91% (90.57%-95.65%)	75.79% (70.59%-81.25%)	3.4122 (3.3076-3.5298)	85.48% (79.8%-90.91%)	83.4% (75.41%-90.48%)	89.01% (80%-97.06%)	92.88% (87.04%-98.08%)	75.79% (65.11%-85.71%)	3.4108 (3.1754-3.6243)
At least 4 of CHASM (lung), CHASM (melanoma), FATHMM (cancer), FATHMM (missense), VEST	85.56% (82.83%-88.4%)	84.11% (80.62%-88%)	88.051% (84.058%-92.754%)	92.41% (90.08%-95.45%)	76.23% (71.08%-81.82%)	3.4080 (3.2973-3.5269)	85.45% (79.77%-90.91%)	83.89% (76.19%-91.04%)	88.096% (79.412%-95.46%)	92.37% (86.54%-96.78%)	76.14% (65.12%-86.36%)	3.4049 (3.1584-3.6282)
At least 4 of CHASM (lung), CHASM (melanoma), FATHMM (cancer), Mutation Assessor	67.71% (64.14%-71.21%)	51.16% (46.03%-56.35%)	96.317% (94.203%-98.667%)	96% (93.75%-98.57%)	53.28% (48.46%-58.02%)	2.9676 (2.8720-3.0704)	67.62% (60.61%-74.75%)	50.85% (40.74%-60.94%)	96.362% (91.176%-100%)	96% (90%-100%)	53.33% (43.75%-63.24%)	2.9654 (2.7612-3.1603)
At least 4 of CHASM (lung), CHASM (melanoma), FATHMM (cancer), Mutation Assessor, MutationTaster	81.8% (78.79%-84.85%)	75.52% (71.2%-80.15%)	92.646% (89.548%-96.001%)	94.67% (92.38%-97.14%)	68.64% (63.36%-73.74%)	3.3147 (3.2097-3.4296)	81.86% (75.76%-87.88%)	75.54% (66.13%-84.38%)	92.688% (85.366%-100%)	94.66% (89.13%-100%)	68.86% (58.49%-79.6%)	3.3174 (3.0900-3.5283)
At least 4 of CHASM (lung), CHASM (melanoma), FATHMM (cancer), Mutation Assessor, PolyPhen-2	77.43% (74.24%-80.81%)	70.23% (65.57%-75%)	89.864% (85.915%-94.203%)	92.29% (89.47%-95.7%)	63.58% (58.09%-68.87%)	3.1597 (3.0474-3.2810)	77.47% (70.71%-83.84%)	70.15% (60.66%-79.37%)	89.991% (82.141%-97.37%)	92.32% (85.71%-97.92%)	63.75% (53.33%-74.51%)	3.1622 (2.9135-3.3892)
At least 4 of CHASM (lung), CHASM (melanoma), FATHMM (cancer), Mutation Assessor, PROVEAN	72.74% (69.19%-76.26%)	61.25% (56.35%-66.14%)	92.599% (89.333%-96.053%)	93.47% (90.59%-96.55%)	58.02% (52.68%-62.83%)	3.0533 (2.9445-3.1722)	72.71% (65.66%-79.8%)	60.99% (50.85%-70.77%)	92.776% (85.714%-100%)	93.54% (86.84%-100%)	58.09% (48.15%-68.53%)	3.0540 (2.8195-3.2718)
At least 4 of CHASM (lung), CHASM (melanoma), FATHMM (cancer), Mutation Assessor, SIFT	79.45% (76.26%-82.83%)	73.44% (69.1%-78.12%)	89.846% (86.111%-94.118%)	92.6% (89.9%-95.92%)	66.18% (60.82%-71.43%)	3.2205 (3.1083-3.3426)	79.48% (72.73%-85.86%)	73.31% (63.49%-81.95%)	90.016% (82.22%-97.3%)	92.65% (86.27%-98%)	66.29% (55.56%-76.92%)	3.2227 (2.9797-3.4469)
At least 4 of CHASM (lung), CHASM (melanoma), FATHMM (cancer), Mutation Assessor, VEST	76.44% (73.23%-79.8%)	68.65% (63.84%-73.48%)	89.885% (86.111%-94.205%)	92.15% (89.16%-95.6%)	62.38% (57.14%-67.62%)	3.1307 (3.0172-3.2501)	76.42% (69.7%-82.83%)	68.52% (58.73%-77.97%)	89.939% (81.579%-97.3%)	92.13% (85.1%-97.92%)	62.49% (51.92%-73.08%)	3.1307 (2.8994-3.3605)
At least 4 of CHASM (lung), CHASM (melanoma), FATHMM (cancer), MutationTaster	72.72% (69.19%-76.28%)	58.53% (53.43%-63.85%)	97.256% (95.588%-100%)	97.36% (95.77%-100%)	57.56% (52.42%-62.61%)	3.1070 (3.0101-3.2150)	72.74% (65.63%-79.8%)	58.47% (48.21%-68.97%)	97.233% (92.105%-100%)	97.31% (92.5%-100%)	57.71% (47.69%-68.25%)	3.1073 (2.9004-3.3061)
At least 4 of CHASM (lung), CHASM (melanoma), FATHMM (cancer), MutationTaster, PolyPhen-2	81.46% (78.28%-84.85%)	75.51% (70.97%-79.84%)	91.743% (88.406%-95.652%)	94.05% (91.75%-97%)	68.43% (63.04%-73.69%)	3.2974 (3.1897-3.4184)	81.52% (74.75%-87.88%)	75.54% (66.67%-84.49%)	91.741% (84.375%-97.56%)	94.01% (87.76%-98.18%)	68.63% (57.69%-79.59%)	3.2992 (3.0602-3.5202)
At least 4 of CHASM (lung), CHASM (melanoma), FATHMM (cancer), MutationTaster, PROVEAN	77.78% (74.24%-80.82%)	67.59% (62.88%-72.58%)	95.411% (92.958%-98.571%)	96.22% (94.18%-98.88%)	63% (57.76%-67.93%)	3.2222 (3.1187-3.3240)	77.77% (71.69%-84.85%)	67.46% (57.63%-76.81%)	95.417% (89.474%-100%)	96.19% (90.7%-100%)	63.1% (53.06%-74%)	3.2217 (3.0189-3.4339)
At least 4 of CHASM (lung), CHASM (melanoma), FATHMM (cancer), MutationTaster, SIFT	83.48% (80.81%-86.36%)	78.16% (73.98%-82.44%)	92.658% (89.55%-96%)	94.85% (92.59%-97.22%)	71.05% (65.62%-76.09%)	3.3672 (3.2672-3.4758)	83.55% (77.78%-88.89%)	78.23% (69.35%-86.36%)	92.657% (85.714%-100%)	94.82% (89.58%-100%)	71.29% (61.36%-81.64%)	3.3699 (3.1554-3.5728)
At least 4 of CHASM (lung), CHASM (melanoma), FATHMM (cancer), MutationTaster, VEST	80.1% (76.77%-83.33%)	73.38% (68.85%-77.69%)	91.719% (88.406%-95.833%)	93.87% (91.49%-96.94%)	66.59% (61.39%-71.72%)	3.2556 (3.1472-3.3685)	80.2% (73.74%-86.87%)	73.43% (64.51%-82.46%)	91.785% (83.784%-97.56%)	93.89% (87.5%-98.11%)	66.83% (56.52%-77.55%)	3.2594 (3.0300-3.4805)
At least 4 of CHASM (lung), CHASM (melanoma), FATHMM (cancer), PolyPhen-2	70.72% (67.17%-74.24%)	55.91% (50.82%-61.29%)	96.317% (94.203%-98.667%)	96.33% (94.28%-98.68%)	55.82% (50.81%-60.63%)	3.0438 (2.9431-3.1452)	70.68% (63.64%-77.78%)	55.71% (45.31%-66.07%)	96.362% (91.176%-100%)	96.33% (90.91%-100%)	55.91% (46.66%-65.68%)	3.0431 (2.8402-3.2451)
At least 4 of CHASM (lung), CHASM (melanoma), FATHMM (cancer), PolyPhen-2, PROVEAN	75.09% (71.72%-78.79%)	64.96% (60.16%-69.84%)	92.599% (89.333%-96.053%)	93.82% (91.11%-96.74%)	60.45% (55.26%-65.45%)	3.1182 (3.0105-3.2398)	75.08% (67.68%-81.82%)	64.75% (55%-74.58%)	92.776% (85.714%-100%)	93.89% (87.5%-100%)	60.54% (50%-71.15%)	3.1196 (2.8760-3.3367)
At least 4 of CHASM (lung), CHASM (melanoma), FATHMM (cancer), PolyPhen-2, SIFT	80.11% (77.26%-83.33%)	75.52% (71.21%-79.84%)	88.035% (84%-92.54%)	91.61% (88.79%-95.15%)	67.54% (62.36%-72.73%)	3.2270 (3.1162-3.3493)	80.18% (73.74%-85.88%)	75.52% (66.67%-84.13%)	88.138% (80%-96.78%)	91.62% (84.31%-97.83%)	67.75% (57.45%-78.26%)	3.2302 (2.9888-3.4528)
At least 4 of CHASM (lung), CHASM (melanoma), FATHMM (cancer), PolyPhen-2, VEST	76.78% (73.74%-80.3%)	70.77% (66.11%-75.21%)	87.159% (83.098%-91.892%)	90.5% (87.5%-94.17%)	63.3% (57.84%-68.63%)	3.1174 (2.9983-3.2423)	76.74% (69.7%-82.83%)	70.66% (61.67%-80.3%)	87.148% (78.123%-95%)	90.42% (83.02%-96.15%)	63.4% (52.83%-74.51%)	3.1162 (2.8651-3.3524)
At least 4 of CHASM (lung), CHASM (melanoma), FATHMM (cancer), PROVEAN	62.65% (58.59%-66.16%)	41.58% (36.59%-46.46%)	99.084% (98.485%-100%)	98.74% (97.87%-100%)	49.52% (44.59%-53.74%)	2.8892 (2.8018-2.9725)	62.58% (55.56%-70.71%)	41.29% (31.15%-51.61%)	99.083% (96.774%-100%)	98.73% (95.45%-100%)	49.58% (40.58%-59.42%)	2.8867 (2.7258-3.0671)
At least 4 of CHASM (lung), CHASM (melanoma), FATHMM (cancer), PROVEAN, SIFT	75.1% (71.72%-78.79%)	64.97% (60.15%-69.84%)	92.612% (89.474%-95.947%)	93.83% (91.01%-96.67%)	60.46% (55.36%-65.74%)	3.1187 (3.0105-3.2319)	75.05% (67.68%-81.82%)	64.72% (54.84%-74.24%)	92.745% (85.714%-100%)	93.87% (87.5%-100%)	60.51% (50.85%-70.69%)	3.1184 (2.9007-3.3369)
At least 4 of CHASM (lung), CHASM (melanoma), FATHMM (cancer), PROVEAN, VEST	70.4% (66.67%-74.24%)	57.54% (52.46%-62.4%)	92.621% (89.474%-96.053%)	93.1% (90.14%-96.3%)	55.79% (50.82%-60.5%)	2.9905 (2.8794-3.1119)	70.31% (62.63%-77.78%)	57.23% (47.46%-67.16%)	92.723% (85.294%-100%)	93.13% (86.11%-100%)	55.82% (46.03%-66.1%)	2.9890 (2.7494-3.2155)
[illegible]	[illegible]	[illegible]	[illegible]	[illegible]	[illegible]	[illegible]	[illegible]	[illegible]	[illegible]	[illegible]	[illegible]	[illegible]

At least 4 of CHASM (lung), MutationTaster, PolyPhen-2, VEST	65.03% (61.11%-68.69%)	47.46% (42.28%-52.71%)	95.4% (92.955%-98.571%)	94.69% (91.8%-98.33%)	51.22% (46.32%-55.73%)	2.8876 (2.7818-2.9990)	64.9% (57.58%-72.73%)	47.09% (36.51%-56.93%)	95.442% (89.474%-100%)	94.64% (87.88%-100%)	51.25% (41.93%-61.43%)	2.8843 (2.6557-3.1031)
At least 4 of CHASM (lung), MutationTaster, PROVEAN, SIFT	61.63% (57.58%-65.16%)	40.5% (35.43%-45.67%)	98.149% (97.015%-100%)	97.42% (95.65%-100%)	48.82% (44%-53.1%)	2.8490 (2.7556-2.9451)	61.59% (54.52%-69.7%)	40.25% (29.69%-50%)	98.206% (94.282%-100%)	97.46% (91.3%-100%)	48.92% (40.28%-58.67%)	2.8483 (2.6642-3.0425)
At least 4 of CHASM (lung), MutationTaster, PROVEAN, SIFT, VEST	72.76% (69.19%-76.26%)	60.7% (56%-65.63%)	93.605% (90.539%-97.102%)	94.26% (91.56%-97.47%)	57.94% (52.94%-63.2%)	3.0650 (2.9583-3.1732)	72.66% (65.66%-79.8%)	60.48% (50.77%-69.84%)	93.512% (86.667%-100%)	94.12% (87.88%-100%)	57.96% (47.46%-67.93%)	3.0607 (2.8384-3.2760)
At least 4 of CHASM (lung), MutationTaster, PROVEAN, VEST	59.63% (55.56%-63.64%)	37.35% (32.28%-41.99%)	98.149% (97.015%-100%)	97.21% (95.35%-100%)	47.53% (42.85%-51.95%)	2.8024 (2.7073-2.8958)	59.53% (51.52%-67.68%)	36.99% (27.27%-47.37%)	98.206% (94.282%-100%)	97.23% (90.91%-100%)	47.6% (38.67%-57.15%)	2.8003 (2.6171-2.9916)
At least 4 of CHASM (lung), MutationTaster, SIFT, VEST	65.36% (61.62%-69.19%)	47.99% (42.98%-53.12%)	95.4% (92.955%-98.571%)	94.74% (91.94%-98.36%)	51.47% (46.67%-56.06%)	2.8960 (2.7934-3.0047)	65.23% (57.58%-72.73%)	47.62% (37.5%-57.63%)	95.442% (89.474%-100%)	94.7% (87.88%-100%)	51.49% (42.18%-61.19%)	2.8925 (2.6718-3.1032)
At least 4 of CHASM (lung), PolyPhen-2, PROVEAN, SIFT	61.64% (58.08%-65.15%)	40.52% (35.34%-45.31%)	98.149% (97.015%-100%)	97.42% (95.74%-100%)	48.83% (43.97%-53.2%)	2.8492 (2.7558-2.9390)	61.57% (54.55%-68.69%)	40.21% (30.36%-50%)	98.206% (94.282%-100%)	97.44% (91.67%-100%)	48.9% (40%-58.67%)	2.8476 (2.6636-3.0380)
At least 4 of CHASM (lung), PolyPhen-2, PROVEAN, SIFT, VEST	68.38% (64.65%-72.22%)	60.73% (56%-65.6%)	81.601% (76.71%-86.957%)	85.09% (80.68%-89.53%)	54.58% (49.09%-59.8%)	2.8199 (2.6807-2.9598)	68.29% (60.61%-75.76%)	60.43% (50.77%-70%)	81.741% (70.968%-91.67%)	85.03% (76.19%-93.62%)	54.61% (44%-65.47%)	2.8180 (2.5291-3.0993)
At least 4 of CHASM (lung), PolyPhen-2, PROVEAN, VEST	59.29% (55.56%-63.13%)	36.81% (32%-41.6%)	98.149% (97.015%-100%)	97.17% (95.24%-100%)	47.32% (42.46%-51.66%)	2.7944 (2.7031-2.8917)	59.21% (51.52%-66.67%)	36.47% (26.98%-46.15%)	98.206% (94.282%-100%)	97.19% (90.48%-100%)	47.39% (38.57%-56.96%)	2.7925 (2.5988-2.9766)
At least 4 of CHASM (lung), PolyPhen-2, SIFT, VEST	64.02% (60.1%-67.69%)	46.92% (41.88%-52.04%)	93.577% (90.411%-97.102%)	92.65% (89.06%-96.83%)	50.48% (45.59%-55%)	2.8363 (2.7227-2.9544)	63.89% (56.54%-71.72%)	46.57% (35.94%-56.45%)	93.594% (87.097%-100%)	92.55% (84.2%-100%)	50.51% (41.27%-60.61%)	2.8322 (2.5898-3.0677)
At least 4 of CHASM (lung), PROVEAN, SIFT, VEST	60.63% (56.57%-64.14%)	38.93% (33.86%-43.65%)	98.149% (97.015%-100%)	97.32% (95.56%-100%)	48.17% (43.36%-52.41%)	2.8257 (2.7311-2.9175)	60.56% (53.54%-68.69%)	38.61% (29.03%-48.53%)	98.206% (94.282%-100%)	97.34% (91.29%-100%)	48.24% (39.44%-57.69%)	2.8240 (2.6393-3.0133)
At least 4 of CHASM (melanoma), FATHMM (cancer), FATHMM (missense), Mutation Assessor	70.05% (66.16%-73.74%)	56.44% (51.2%-61.29%)	93.579% (90.411%-97.26%)	93.83% (90.91%-97.33%)	55.4% (50.41%-60.33%)	2.9925 (2.8856-3.1041)	70% (62.63%-77.78%)	56.25% (46.66%-66.67%)	93.559% (86.111%-100%)	93.76% (86.49%-100%)	55.48% (46.03%-65.46%)	2.9906 (2.7620-3.2122)
At least 4 of CHASM (melanoma), FATHMM (cancer), FATHMM (missense), Mutation Assessor, MutationTaster	80.46% (77.27%-83.33%)	74.43% (69.92%-78.87%)	90.857% (87.324%-94.737%)	93.37% (90.82%-96.23%)	67.28% (62%-72.16%)	3.2594 (3.1483-3.3668)	80.5% (74.75%-86.87%)	74.5% (65.57%-83.33%)	90.745% (82.5%-97.37%)	93.27% (87.23%-98.08%)	67.49% (56.86%-77.78%)	3.2601 (3.0473-3.4835)
At least 4 of CHASM (melanoma), FATHMM (cancer), FATHMM (missense), Mutation Assessor, PolyPhen-2	74.06% (70.71%-77.27%)	69.15% (64.41%-73.55%)	82.551% (77.778%-87.838%)	87.27% (83.67%-91.09%)	60.75% (55.21%-66.03%)	2.9971 (2.8678-3.1285)	74.1% (67.68%-80.81%)	69.12% (60.32%-78.69%)	82.593% (71.795%-92.11%)	87.21% (79.54%-94.23%)	60.92% (50%-72.34%)	2.9984 (2.7349-3.2591)
At least 4 of CHASM (melanoma), FATHMM (cancer), FATHMM (missense), Mutation Assessor, PROVEAN	72.04% (68.69%-75.25%)	63.83% (58.97%-68.5%)	86.225% (81.944%-90.77%)	88.9% (85.54%-92.68%)	57.96% (52.38%-63.06%)	2.9691 (2.8438-3.1002)	72.09% (65.66%-78.79%)	63.81% (54.1%-73.53%)	86.255% (77.143%-94.59%)	88.85% (81.25%-95.65%)	58.14% (47.82%-69.1%)	2.9705 (2.7021-3.2174)
At least 4 of CHASM (melanoma), FATHMM (cancer), FATHMM (missense), Mutation Assessor, SIFT	76.75% (73.74%-79.8%)	72.89% (68.29%-77.35%)	83.414% (78.873%-88.406%)	88.37% (85%-91.92%)	64.02% (58.42%-69.23%)	3.0870 (2.9602-3.2151)	76.81% (70.71%-82.83%)	72.82% (63.63%-81.82%)	83.608% (73.525%-92.86%)	88.42% (80.95%-94.93%)	64.2% (53.66%-75.56%)	3.0904 (2.8349-3.3426)
At least 4 of CHASM (melanoma), FATHMM (cancer), FATHMM (missense), Mutation Assessor, VEST	73.74% (70.2%-77.27%)	67.57% (62.9%-72.18%)	84.403% (79.682%-89.333%)	88.23% (84.85%-92%)	60.08% (54.46%-65.35%)	3.0028 (2.8795-3.1256)	73.73% (66.67%-80.81%)	67.49% (58.06%-76.92%)	84.389% (74.194%-93.94%)	88.14% (80.7%-95.45%)	60.2% (50%-70.91%)	3.0022 (2.7556-3.2488)
At least 4 of CHASM (melanoma), FATHMM (cancer), FATHMM (missense), MutationTaster	73.72% (70.2%-77.27%)	61.67% (56.49%-66.93%)	94.539% (91.664%-97.437%)	95.13% (92.68%-97.7%)	58.79% (53.51%-63.79%)	3.1013 (2.9975-3.2139)	73.78% (66.67%-80.81%)	61.74% (50.85%-72.13%)	94.404% (87.879%-100%)	94.99% (89.18%-100%)	58.99% (49.01%-69.64%)	3.1012 (2.8776-3.3083)
At least 4 of CHASM (melanoma), FATHMM (cancer), FATHMM (missense), MutationTaster, PolyPhen-2	82.15% (79.29%-85.35%)	77.64% (73.39%-82.03%)	89.93% (86.111%-94.366%)	93.03% (90.48%-96.15%)	69.94% (64.7%-75%)	3.3054 (3.1960-3.4246)	82.17% (75.76%-87.88%)	77.67% (68.65%-86.15%)	89.838% (81.081%-97.3%)	92.93% (86.66%-98.08%)	70.12% (60%-80.85%)	3.3056 (3.0724-3.5209)
At least 4 of CHASM (melanoma), FATHMM (cancer), FATHMM (missense), MutationTaster, PROVEAN	79.11% (75.76%-82.32%)	71.77% (66.95%-76.92%)	91.797% (88.406%-95.716%)	93.8% (91.3%-96.81%)	65.29% (60%-70.59%)	3.2266 (3.1167-3.3491)	79.15% (72.73%-85.86%)	71.86% (61.53%-81.36%)	91.617% (84.202%-97.5%)	93.65% (87.5%-98.11%)	65.5% (54.9%-75.93%)	3.2263 (2.9841-3.4470)
At least 4 of CHASM (melanoma), FATHMM (cancer), FATHMM (missense), MutationTaster, SIFT	82.81% (79.8%-85.86%)	78.68% (74.22%-83.07%)	89.943% (86.111%-94.288%)	93.12% (90.65%-96.04%)	70.93% (65.56%-76.09%)	3.3268 (3.2188-3.4433)	82.86% (76.77%-88.89%)	78.79% (70.18%-87.93%)	89.807% (81.081%-97.22%)	93.01% (87.27%-98.08%)	71.19% (60.87%-82.23%)	3.3280 (3.0977-3.5503)
At least 4 of CHASM (melanoma), FATHMM (cancer), FATHMM (missense), MutationTaster, VEST	80.11% (77.27%-83.33%)	74.96% (70.25%-79.34%)	89.003% (85.134%-93.427%)	92.18% (89.42%-95.33%)	67.28% (62%-72.63%)	3.2343 (3.1200-3.3514)	80.18% (73.74%-85.86%)	75.05% (66.1%-84.06%)	88.936% (79.487%-97.06%)	92.11% (85.71%-97.96%)	67.52% (56.86%-78.05%)	3.2362 (2.9972-3.4588)
At least 4 of CHASM (melanoma), FATHMM (cancer), FATHMM (missense), PolyPhen-2	70.7% (67.17%-74.24%)	58.53% (53.6%-63.41%)	91.75% (88.235%-95.946%)	92.47% (89.47%-96.25%)	56.13% (50.91%-61.02%)	2.9887 (2.8752-3.1161)	70.71% (63.64%-77.78%)	58.45% (48.43%-68.34%)	91.706% (82.925%-97.67%)	92.38% (84.62%-97.73%)	56.26% (46.55%-66.13%)	2.9880 (2.7384-3.2145)
At least 4 of CHASM (melanoma), FATHMM (cancer), FATHMM (missense), PolyPhen-2, PROVEAN	74.05% (70.71%-77.29%)	68.61% (63.93%-73.55%)	83.445% (78.873%-88.571%)	87.76% (84.37%-91.67%)	60.59% (55.21%-65.98%)	3.0039 (2.8734-3.1388)	74.13% (67.65%-80.81%)	68.61% (58.46%-78.13%)	83.543% (73.684%-92.5%)	87.74% (80%-94.34%)	60.81% (50%-71.74%)	3.0071 (2.7341-3.2591)
At least 4 of CHASM (melanoma), FATHMM (cancer), FATHMM (missense), PolyPhen-2, SIFT	77.74% (74.24%-80.82%)	76.04% (71.32%-80.47%)	80.675% (75.676%-85.915%)	87.19% (83.65%-90.91%)	66.08% (60.46%-71.59%)	3.0998 (2.9708-3.2354)	77.84% (71.69%-84.85%)	76.07% (67.19%-85.25%)	80.817% (70.264%-90.91%)	87.2% (79.99%-94.12%)	66.34% (55.55%-77.27%)	3.1043 (2.8337-3.3618)
At least 4 of CHASM (melanoma), FATHMM (cancer), FATHMM (missense), PolyPhen-2, VEST	74.09% (70.71%-77.78%)	71.82% (67.44%-76.34%)	78.005% (72.464%-83.784%)	84.96% (81.25%-88.89%)	61.55% (55.91%-67.05%)	2.9634 (2.8253-3.0979)	74.04% (66.67%-80.81%)	71.75% (62.68%-80.77%)	77.906% (66.667%-88.58%)	84.8% (76.59%-92.16%)	61.65% (51.02%-72.92%)	2.9611 (2.6877-3.2337)
At least 4 of CHASM (melanoma), FATHMM (cancer), FATHMM (missense), PROVEAN	61.32% (57.58%-65.15%)	40.53% (35.6%-45.38%)	97.25% (95.588%-100%)	96.23% (93.88%-100%)	48.61% (43.83%-53.02%)	2.8263 (2.7354-2.9221)	61.2% (53.54%-68.69%)	40.17% (30.36%-50.02%)	97.224% (92.105%-100%)	96.17% (89.47%-100%)	48.64% (39.73%-58.57%)	2.8220 (2.6268-3.0148)
At least 4 of CHASM (melanoma), FATHMM (cancer), FATHMM (missense), PROVEAN, SIFT	75.06% (71.72%-78.28%)	69.14% (64.57%-73.81%)	85.264% (80.644%-90%)	89.03% (85.71%-92.56%)	61.51% (56%-66.67%)	3.0495 (2.9246-3.1726)	75.14% (68.69%-81.82%)	69.13% (59.38%-78.69%)	85.397% (76.471%-94.45%)	89.06% (81.62%-95.74%)	61.73% (51.78%-72.98%)	3.0531 (2.8031-3.2930)
At least 4 of CHASM (melanoma), FATHMM (cancer), FATHMM (missense), PROVEAN, VEST	70.03% (66.16%-73.74%)	61.19% (56.35%-66.13%)	85.296% (80.765%-90.281%)	87.8% (83.91%-92.05%)	55.97% (50.49%-61.02%)	2.9025 (2.7727-3.0348)	70.05% (62.63%-77.78%)	61.11% (50.85%-70.77%)	85.34% (75%-94.44%)	87.75% (79.59%-95.35%)	56.12% (45.83%-66.67%)	2.9031 (2.6416-3.1605)
At least 4 of CHASM (melanoma), FATHMM (cancer), FATHMM (missense), SIFT	74.09% (70.71%-77.78%)	63.34% (58.27%-68.03%)	92.665% (89.472%-96.207%)	93.73% (90.91%-96.63%)	59.38% (54.24%-64.29%)	3.0911 (2.9827-3.2046)	74.04% (66.67%-80.81%)	63.19% (53.84%-73.02%)	92.621% (84.848%-100%)	93.65% (87.23%-100%)	59.45% (50%-69.81%)	3.0891 (2.8638-3.3061)
At least 4 of CHASM (melanoma), FATHMM (cancer), FATHMM (missense), SIFT, VEST	76.4% (73.23%-79.8%)	73.94% (69.35%-78.4%)	80.627% (75.362%-86.491%)	86.85% (83.33%-90.83%)	64.15% (58.24%-69.48%)	3.0556 (2.9246-3.1971)	76.5% (69.7%-82.83%)	73.9% (64.62%-83.06%)	80.906% (69.439%-91.18%)	86.94% (79.58%-93.88%)	64.38% (53.65%-76.26%)	3.0613 (2.7814-3.3246)
At least 4 of CHASM (melanoma), FATHMM (cancer), FATHMM (missense), VEST	66% (62.12%-69.7%)	51.11% (45.9%-56%)	91.725% (88.403%-95.778%)	91.45% (88%-95.59%)	52.04% (46.92%-56.91%)	2.8632 (2.7448-2.9812)	65.99% (58.59%-73.74%)	50.95% (40.98%-61.02%)	91.75% (83.711%-97.56%)	91.41% (83.33%-97.5%)	52.15% (42.42%-62.5%)	2.8626 (2.6190-3.1021)
At least 4 of CHASM (melanoma), FATHMM (cancer), Mutation Assessor, MutationTaster	68.67% (65.15%-72.22%)	53.19% (47.93%-58.27%)	95.442% (92.958%-98.592%)	95.28% (92.65%-98.53%)	54.11% (49.22%-58.91%)	2.9802 (2.8724-3.0938)	68.71% (61.62%-75.76%)	53.18% (42.85%-63.77%)	95.351% (89.283%-100%)	95.16% (88.89%-100%)	54.27% (44.44%-64.07%)	2.9796 (2.7521-3.1935)
At least 4 of CHASM (melanoma), FATHMM (cancer), Mutation Assessor, MutationTaster, PolyPhen-2	79.42% (76.26%-82.83%)	74.96% (70.63%-79.39%)	87.132% (82.894%-91.667%)	90.96% (88.07%-94.29%)	66.8% (61.54%-72.05%)	3.1986 (3.0815-3.3267)	79.54% (72.73%-85.86%)	75.06% (66.07%-83.87%)	87.205% (78.123%-95%)	90.95% (84%-96.49%)	67.09% (56.52%-77.78%)	3.2031 (2.9451-3.4415)
At least 4 of CHASM (melanoma), FATHMM (cancer), Mutation Assessor, MutationTaster, PROVEAN	75.38% (72.22%-79.29%)	65.9% (61.11%-70.5%)	91.75% (88.406%-95.652%)	93.25% (90.53%-96.63%)	60.88% (55.75%-66.04%)	3.1178 (3.0009-3.2400)	75.51% (67.68%-81.82%)	66.04% (56.6%-75.39%)	91.713% (84.615%-97.56%)	93.19% (86.36%-98%)	61.15% (50.94%-71.7%)	3.1209 (2.8801-3.3563)
At least 4 of CHASM (melanoma), FATHMM (cancer), Mutation Assessor, MutationTaster, SIFT	80.45% (77.27%-83.84%)	74.43% (70.08%-78.74%)	90.829% (87.143%-94.522%)	93.35% (90.82%-96.08%)	67.26% (61.96%-72.34%)	3.2588 (3.1443-3.3746)	80.52% (73.74%-86.87%)	74.51% (66.03%-82.86%)	90.799% (83.333%-97.44%)	93.29% (87.5%-98.08%)	67.49% (57.14%-78%)	3.2609 (3.0330-3.4836)
At least 4 of CHASM (melanoma), FATHMM (cancer), Mutation Assessor, MutationTaster, VEST	78.42% (75.25%-81.82%)	72.3% (68.03%-76.74%)	88.983% (84.929%-93.243%)	91.9% (89.11%-95.05%)	65.01% (60%-70.41%)	3.1819 (3.0694-3.3011)	78.52% (71.72%-84.85%)	72.4% (63.33%-80.96%)	89.002% (80.554%-97.22%)	91.86% (85.36%-97.87%)	65.27% (54.9%-75.51%)	3.1853 (2.9565-3.4167)
At least 4 of CHASM (melanoma), FATHMM (cancer), Mutation Assessor, PolyPhen-2	68.33% (64.65%-72.22%)	54.8% (49.61%-59.84%)	91.719% (88.406%-95.716%)	91.96% (88.88%-95.95%)	53.99% (49.18%-58.87%)	2.9247 (2.8098-3.0506)	68.39% (60.61%-75.76%)	54.74% (44.64%-64.71%)	91.782% (84.211%-97.56%)	91.96% (83.77%-97.67%)	54.17% (44.62%-64.15%)	2.9265 (2.6676-3.1575)
At least 4 of CHASM (melanoma), FATHMM (cancer), Mutation Assessor, PolyPhen-2, PROVEAN	71.68% (68.18%-75.25%)	65.4% (60.83%-70.4%)	82.534% (77.465%-87.671%)	86.62% (82.76%-90.72%)	57.97% (52.53%-63.37%)	2.9252 (2.7939-3.0615)	71.8% (64.65%-78.79%)	65.46% (55.22%-74.58%)	82.643% (72.222%-92.59%)	86.61% (78%-94.24%)	58.24% (47.06%-69.09%)	2.9295 (2.6472-3.1929)
At least 4 of CHASM (melanoma), FATHMM (cancer), Mutation Assessor, PolyPhen-2, SIFT	74.72% (71.21%-78.28%)	72.32% (67.77%-76.8%)	78.864% (73.433%-84.058%)	85.54% (81.82%-89.32%)	62.23% (56.52%-67.86%)	2.9896 (2.8535-3.1256)	74.8% (67.68%-81.82%)	72.36% (63.49%-81.16%)	78.955% (68.571%-89.74%)	85.51% (77.78%-92.86%)	62.46% (51.02%-73.33%)	2.9929 (2.7200-3.2622)
At least 4 of CHASM (melanoma), FATHMM (cancer), Mutation Assessor, PolyPhen-2, VEST	74.38% (71.21%-78.28%)	72.31% (67.97%-77.17%)	77.942% (72.368%-83.583%)	85% (81.31%-89%)	61.95% (56.52%-67.74%)	2.9720 (2.8378-3.1147)	74.48% (66.67%-80.81%)	72.38% (62.68%-81.25%)	78.07% (67.5%-89.47%)	84.99% (76.79%-92.45%)	62.22% (51.06%-72.92%)	2.9766 (2.6872-3.2362)
At least 4 of CHASM (melanoma), FATHMM (cancer), Mutation Assessor, PROVEAN	59.96% (56.06%-63.64%)	38.93% (34.13%-43.65%)	96.301% (94.203%-98.701%)	94.79% (91.67%-98.15%)	47.7% (43.06%-52.12%)	2.7773 (2.6786-2.8941)	59.88% (52.53%-67.68%)	38.59% (28.81%-48.28%)	96.374% (90.904%-100%)	94.82% (86.96%-100%)	47.77% (38.67%-57.14%)	2.7755 (2.5420-2.9704)
At least 4 of CHASM (melanoma), FATHMM (cancer), Mutation Assessor, PROVEAN, SIFT	73.35% (69.7%-76.77%)	66.98% (62.31%-71.54%)	84.365% (79.451%-89.189%)	88.1% (84.46%-92%)	59.64% (54.25%-64.9%)	2.9909 (2.8547-3.1179)	73.5% (66.67%-80.81%)	67.08% (57.62%-76.47%)	84.476% (74.286%-94.44%)	88.12% (80.43%-95.65%)	59.92% (49.06%-70.83%)	2.9959 (2.7374-3.2640)
At least 4 of CHASM (melanoma), FATHMM (cancer), Mutation Assessor, PROVEAN, VEST	69.01% (65.15%-72.74%)	61.18% (56.25%-66.13%)	82.538% (77.333%-87.671%)	85.83% (81.61%-90.32%)	55.15% (50%-60.38%)	2.8469 (2.7078-2.9934)	69.06% (61.59%-76.77%)	61.13% (51.55%-70.97%)	82.641% (72.409%-93.34%)	85.79% (76.19%-94.29%)	55.34% (44.82%-65.91%)	2.8490 (2.5579-3.1378)
At least 4 of CHASM (melanoma), FATHMM (cancer), Mutation Assessor, SIFT	70.03% (66.16%-73.74%)	56.4% (51.2%-61.42%)	93.569% (90.539%-97.183%)	93.82% (90.91%-97.33%)	55.38% (50.41%-60.31%)	2.9917 (2.8810-3.1044)	70.05% (62.63%-77.78%)	56.31% (46.03%-66.18%)	93.581% (86.667%-100%)	93.78% (86.84%-100%)	55.52% (45.45%-65.57%)	2.9919 (2.7641-3.2154)
At least 4 of CHASM (melanoma), FATHMM (cancer), Mutation Assessor, SIFT, VEST	74.04% (70.71%-77.29%)	71.78% (67.46%-76.23%)	77.941% (72.365%-83.562%)	84.91% (81.13%-88.89%)	61.5% (55.81%-67.03%)	2.9613 (2.8254-3.0960)	74.14% (67.65%-80.81%)	71.84% (62.71%-80.6%)	78.05% (67.5%-89.19%)	84.89% (77.08%-92.45%)	61.75% (51.06%-72.92%)	2.9653 (2.6883-3.2379)
At least 4 of CHASM (melanoma), FATHMM (cancer), Mutation Assessor, VEST	64.98% (61.11%-68.69%)	48.96% (43.59%-54.27%)	92.668% (89.333%-96.105%)	92.03% (88.57%-95.83%)	51.22% (46.27%-56.15%)	2.8489 (2.7351-2.9732)	64.99% (57.58%-72.73%)	48.86% (38.33%-59.02%)	92.632% (84.996%-100%)	91.94% (83.33%-100%)	51.35% (41.27%-61.43%)	2.8478 (2.5909-3.0748)
At least 4 of CHASM (melanoma), FATHMM (cancer), MutationTaster, PolyPhen-2	71.69% (68.18%-75.25%)	58.47% (53.33%-63.71%)	94.539% (91.664%-97.437%)	94.87% (92.31%-97.56%)	56.83% (51.97%-61.6%)	3.0471 (2.9455-3.1592)	71.78% (64.65%-78.79%)	58.58% (48.33%-69.02%)	94.404% (87.879%-100%)	94.72% (88.24%-100%)	57.05% (47.54%-67.24%)	3.0476 (2.8207-3.2533)
At least 4 of CHASM (melanoma), FATHMM (cancer), MutationTaster, PolyPhen-2, PROVEAN	78.08% (74.75%-81.31%)	70.7% (66.14%-75.2%)	90.848% (87.324%-94.668%)	93.03% (90.52%-95.96%)	64.2% (59.22%-69.23%)	3.1878 (3.0759-3.3086)	78.18% (71.72%-84.85%)	70.82% (61.66%-79.7%)	90.767% (82.857%-97.37%)	92.94% (86.67%-97.92%)	64.45% (54.16%-75%)	3.1897 (2.9550-3.4161)
At least 4 of CHASM (melanoma), FATHMM (cancer), MutationTaster, PolyPhen-2, SIFT	82.13% (79.29%-85.35%)	78.15% (74.02%-82.4%)	89% (85.135%-93.151%)	92.47% (89.91%-95.33%)	70.2% (65.17%-75.28%)	3.2982 (3.1891-3.4213)	82.21% (75.76%-87.88%)	78.25% (69.69%-86.44%)	88.945% (80.556%-97.14%)	92.4% (86.44%-97.92%)	70.45% (60.37%-80.86%)	3.3005 (3.0602-3.5184)
At least 4 of CHASM (melanoma), FATHMM (cancer), MutationTaster, PolyPhen-2, VEST	79.43% (76.26%-82.34%)	75.5% (71.31%-80%)	86.237% (81.944%-91.183%)	90.46% (87.5%-93.88%)	67.06% (61.46%-72.63%)	3.1925 (3.0670-3.3177)	79.51% (73.71%-85.86%)	75.58% (66.67%-84.38%)	86.229% (76.471%-94.74%)	90.4% (83.67%-96.23%)	67.3% (56.25%-78.72%)	3.1952 (2.9450-3.4427)
At least 4 of CHASM (melanoma), FATHMM (cancer), MutationTaster, PROVEAN	62.3% (58.59%-66.16%)	41.55% (36.64%-46.77%)	98.173% (97.015%-100%)	97.52% (95.83%-100%)	49.28% (44.52%-53.8%)	2.8652 (2.7723-2.9589)	62.27% (54.55%-69.7%)	41.34% (31.03%-50.88%)	98.144% (94.118%-100%)	97.46% (92%-100%)	49.37% (40.27%-59.16%)	2.8631 (2.6722-3.0492)
At least 4 of CHASM (melanoma), FATHMM (cancer), MutationTaster, PROVEAN, SIFT	76.73% (73.74%-80.3%)	68.04% (63.28%-72.58%)	91.75% (88.406%-95.652%)	93.45% (90.8%-96.74%)	62.41% (57.27%-67.59%)	3.1565 (3.0434-3.2768)	76.84% (69.7%-82.83%)	68.14% (58.92%-77.27%)	91.713% (84.615%-97.56%)	93.39% (86.67%-98.04%)	62.66% (52.17%-72.74%)	3.1590 (2.9250-3.3859)
At least 4 of CHASM (melanoma), FATHMM (cancer), MutationTaster, PROVEAN, VEST	74.74% (71.72%-78.28%)	64.35% (59.69%-69.53%)	92.699% (89.333%-96%)	93.84% (91.14%-96.74%)	60.06% (54.87%-65.49%)	3.1096 (2.9996-3.2277)	74.76% (67.68%-80.81%)	64.37% (54.1%-73.68%)	92.563% (85.366%-100%)	93.7% (87.18%-100%)	60.22% (49.18%-70.18%)	3.1085 (2.8752-3.3286)
At least 4 of CHASM (melanoma), FATHMM (cancer), MutationTaster, SIFT	73.04% (69.7%-76.26%)	60.09% (55.12%-64.8%)	95.442% (92.958%-98.592%)	95.8% (93.51%-98.7%)	58.04% (52.89%-62.73%)	3.0936 (2.9908-3.2018)	73.1% (66.67%-79.8%)	60.12% (50.79%-69.84%)	95.351% (89.283%-100%)	95.69% (90%-100%)	58.22% (49.04%-68.42%)	3.0939 (2.8760-3.2996)
At least 4 of CHASM (melanoma), FATHMM (cancer), MutationTaster, SIFT, VEST	80.45% (77.27%-83.84%)	74.97% (70.87%-79.23%)	89.924% (86.153%-94.203%)	92.79% (90.19%-95.92%)	67.51% (62.24%-73.2%)	3.2520 (3.1395-3.3722)	80.51% (73.74%-86.87%)	75.04% (66.07%-83.58%)	89.839% (82.051%-97.3%)	92.71% (86.79%-98%)	67.71% (56.6%-78.26%)	3.2530 (3.0130-3.4824)
At least 4 of CHASM (melanoma), FATHMM (cancer), MutationTaster, VEST	69.35% (65.66%-73.23%)	54.26% (49.22%-59.35%)	95.442% (92.958%-98.592%)	95.37% (92.86%-98.55%)	54.68% (49.61%-59.68%)	2.9975 (2.8915-3.1040)	69.38% (61.62%-76.77%)	54.24% (44.06%-64.41%)	95.351% (89.283%-100%)	95.25% (89.28%-100%)	54.84% (45%-65.08%)	2.9968 (2.7867-3.2144)
At least 4 of CHASM (melanoma), FATHMM (cancer), PolyPhen-2, PROVEAN	61.63% (57.58%-65.15%)	41.57% (36.43%-46.4%)	96.301% (94.203%-98.701%)	95.11% (92.31%-98.28%)	48.8% (43.92%-53.47%)	2.8178 (2.7122-2.9178)	61.59% (54.55%-69.7%)	41.3% (31.48%-51.61%)	96.374% (90.904%-100%)	95.14% (88%-100%)	48.89% (39.73%-58.83%)	2.8170 (2.6051-3.0248)
At least 4 of CHASM (melanoma), FATHMM (cancer), PolyPhen-2, PROVEAN, SIFT	72.33% (68.69%-75.76%)	66.98% (62.39%-71.65%)	81.561% (76.189%-87.147%)	86.27% (82.29%-90.22%)	58.82% (53.46%-64.22%)	2.9363 (2.8044-3.0735)	72.52% (65.66%-79.8%)	67.08% (57.62%-75.87%)	81.81% (71.053%-92.11%)	86.36% (78.38%-94%)	59.15% (48.71%-70.37%)	2.9440 (2.6745-3.2026)
At least 4 of CHASM (melanoma), FATHMM (cancer), PolyPhen-2, PROVEAN, VEST	70.01% (66.67%-74.24%)	63.31% (58.59%-68.51%)	81.584% (76.469%-86.961%)	85.6% (81.61%-90%)	56.25% (50.96%-61.82%)	2.8675 (2.7311-3.0116)	70.08% (61.62%-76.77%)	63.25% (53.12%-72.73%)	81.78% (71.429%-91.9%)	85.63% (76.59%-93.62%)	56.46% (45.45%-66.67%)	2.8712 (2.5919-3.1402)
At least 4 of CHASM (melanoma), FATHMM (cancer), PolyPhen-2, SIFT	71.36% (67.68%-74.76%)	59.04% (54.24%-64.07%)	92.643% (89.331%-95.946%)	93.28% (90.36%-96.39%)	56.68% (51.64%-61.54%)	3.0164 (2.9038-3.1321)	71.42% (64.62%-78.79%)	59.02% (48.48%-68.75%)	92.673% (85.714%-100%)	93.26% (86.48%-100%)	56.85% (47.46%-67.24%)	3.0181 (2.7892-3.2463)
At least 4 of CHASM (melanoma), FATHMM (cancer), PolyPhen-2, SIFT, VEST	74.41% (71.21%-77.78%)	74.47% (70.16%-78.86%)	74.296% (68.35%-80.282%)	83.36% (79.44%-87.39%)	62.72% (57.14%-68.54%)	2.9485 (2.8145-3.0957)	74.42% (67.68%-80.81%)	74.45% (65.45%-82.81%)	74.337% (62.5%-86.21%)	83.28% (75.44%-91.31%)	62.88% (50%-73.81%)	2.9495 (2.6414-3.2249)
At least 4 of CHASM (melanoma), FATHMM (cancer), PolyPhen-2, VEST	66.64% (63.13%-70.2%)	52.12% (47.15%-57.36%)	91.742% (88.406%-95.776%)	91.61% (88.24%-95.65%)	52.56% (47.58%-57.25%)	2.8804 (2.7647-3.0036)	66.72% (59.6%-73.74%)	52.12% (41.89%-62.12%)	91.725% (83.782%-97.56%)	91.56% (83.87%-97.5%)	52.75% (43.33%-62.9%)	2.8815 (2.6461-3.1264)
At least 4 of CHASM (melanoma), FATHMM (cancer), PROVEAN, SIFT	63.65% (60.1%-67.17%)	44.75% (39.67%-49.61%)	96.301% (94.203%-98.701%)	95.44% (92.86%-98.36%)	50.2% (45.39%-54.61%)	2.8670 (2.7704-2.9699)	63.62% (56.57%-70.71%)	44.5% (34.43%-54.69%)	96.374% (90.904%-100%)	95.48% (88.89%-100%)	50.29% (40.91%-60.01%)	2.8665 (2.6538-3.0554)
At least 4 of CHASM (melanoma), FATHMM (cancer), PROVEAN, SIFT, VEST	69.32% (65.66%-73.23%)	62.21% (57.38%-67.18%)	81.586% (76.622%-87.324%)	85.38% (81.52%-89.89%)	55.53% (50%-60.91%)	2.8471 (2.7166-2.9851)	69.45% (61.62%-76.77%)	62.25% (52.46%-71.67%)	81.761% (70.583%-91.67%)	85.42% (76.47%-93.18%)	55.79% (45.1%-66.67%)	2.8522 (2.5786-3.1162)
At least 4 of CHASM (melanoma), FATHMM (cancer), PROVEAN, VEST	61.3% (57.58%-65.15%)	40.5% (35.54%-45.31%)	97.25% (95.588%-100%)	96.23% (93.88%-100%)	48.6% (43.71%-53.15%)	2.8258 (2.7288-2.9236)	61.24% (53.54%-68.69%)	40.24% (30.88%-50%)	97.224% (92.105%-100%)	96.16% (89.47%-100%)	48.67% (39.71%-58.44%)	2.8229 (2.6251-3.0220)
At least 4 of CHASM (melanoma), FATHMM (cancer), SIFT, VEST	68% (64.14%-71.72%)	53.73% (48.78%-58.73%)	92.665% (89.472%-96.207%)	92.69% (89.39%-96.1%)	53.67% (48.44%-58.46%)	2.9276 (2.8103-3.0451)	68.03% (60.61%-75.76%)	53.67% (43.75%-63.49%)	92.621% (84.848%-100%)	92.61% (85.37%-100%)	53.81% (43.86%-64.41%)	2.9271 (2.7015-3.1602)
At least 4 of CHASM (melanoma), FATHMM (missense), Mutation Assessor, MutationTaster	69.01% (65.15%-72.73%)	53.72% (48.39%-58.73%)	95.442% (92.958%-98.592%)	95.33% (92.75%-98.55%)	54.4% (49.59%-59.2%)	2.9889 (2.8854-3.0977)	69.05% (61.62%-76.77%)	53.71% (43.33%-64.41%)	95.351% (89.283%-100%)	95.21% (88.89%-100%)	54.55% (44.92%-64.41%)	2.9882 (2.7690-3.1940)
At least 4 of CHASM (melanoma), FATHMM (missense), Mutation Assessor, MutationTaster, PolyPhen-2	77.46% (74.24%-80.81%)	70.77% (66.4%-75.41%)	89.008% (85.135%-93.333%)	91.76% (88.78%-94.95%)	63.79% (58.76%-69.15%)	3.1533 (3.0385-3.2712)	77.4% (70.71%-83.84%)	70.65% (61.54%-79.37%)	88.928% (80%-97.06%)	91.65% (84.91%-97.87%)	63.85% (53.19%-74.36%)	3.1508 (2.9145-3.3779)
At least 4 of CHASM (melanoma), FATHMM (missense), Mutation Assessor, MutationTaster, PROVEAN	73.71% (70.2%-77.27%)	63.25% (58.54%-67.97%)	91.771% (88.235%-95.716%)	93.01% (90%-96.25%)	59.09% (53.98%-64.04%)	3.0712 (2.9582-3.1865)	73.79% (66.67%-80.81%)	63.35% (53.97%-72.42%)	91.661% (84.211%-97.62%)	92.9% (86.36%-97.96%)	59.31% (49.12%-69.24%)	3.0722 (2.8468-3.2998)
At least 4 of CHASM (melanoma), FATHMM (missense), Mutation Assessor, MutationTaster, SIFT	77.8% (74.75%-80.81%)	70.25% (65.62%-74.6%)	90.85% (87.324%-94.596%)	93% (90.32%-95.92%)	63.85% (58.82%-68.93%)	3.1794 (3.0669-3.2984)	77.73% (71.72%-83.84%)	70.11% (61.02%-78.79%)	90.746% (82.927%-97.37%)	92.88% (86.53%-97.96%)	63.88% (53.45%-73.92%)	3.1761 (2.9375-3.4010)
At least 4 of CHASM (melanoma), FATHMM (missense), Mutation Assessor, MutationTaster, VEST	75.77% (72.73%-79.29%)	67.05% (62.59%-71.65%)	90.838% (87.324%-94.668%)	92.68% (89.8%-95.79%)	61.46% (56.36%-66.67%)	3.1203 (3.0077-3.2393)	75.73% (68.69%-81.82%)	66.93% (57.57%-76.19%)	90.777% (82.5%-97.44%)	92.59% (86.05%-97.92%)	61.53% (50.98%-71.44%)	3.1182 (2.8789-3.3413)
At least 4 of CHASM (melanoma), FATHMM (missense), Mutation Assessor, PolyPhen-2	68.67% (65.15%-72.22%)	54.8% (49.59%-59.84%)	92.651% (89.189%-96.054%)	92.81% (89.47%-96.25%)	54.24% (49.18%-58.87%)	2.9450 (2.8309-3.0601)	68.72% (61.62%-75.76%)	54.74% (44.64%-65.15%)	92.657% (85.283%-100%)	92.77% (85.18%-100%)	54.4% (45.16%-64.06%)	2.9457 (2.7128-3.1681)
At least 4 of CHASM (melanoma), FATHMM (missense), Mutation Assessor, PolyPhen-2, PROVEAN	70.34% (66.67%-73.74%)	62.21% (57.37%-66.94%)	84.372% (79.994%-89.042%)	87.32% (83.7%-91.3%)	56.36% (51.3%-61.61%)	2.9026 (2.7758-3.0331)	70.44% (63.64%-77.78%)	62.24% (53.12%-71.67%)	84.449% (75.673%-93.75%)	87.3% (79.16%-95.12%)	56.58% (46.29%-67.24%)	2.9057 (2.6474-3.1674)
At least 4 of CHASM (melanoma), FATHMM (missense), Mutation Assessor, PolyPhen-2, SIFT	72.42% (69.19%-75.76%)	68.67% (64.12%-73.23%)	78.891% (73.682%-84.418%)	84.91% (81%-88.89%)	59.28% (53.68%-64.95%)	2.9175 (2.7841-3.0571)	72.34% (65.66%-78.79%)	68.49% (59.38%-77.42%)	78.88% (68.285%-89.2%)	84.78% (76.74%-92.68%)	59.32% (47.92%-70%)	2.9147 (2.6335-3.1799)
At least 4 of CHASM (melanoma), FATHMM (missense), Mutation Assessor, PolyPhen-2, VEST	71.09% (67.68%-74.75%)	68.13% (63.36%-72.8%)	76.201% (71.011%-82.052%)	83.19% (79%-87.62%)	58.03% (52.33%-63.74%)	2.8555 (2.7131-2.9989)	70.95% (63.64%-77.78%)	67.97% (58.72%-77.27%)	76.011% (64.516%-86.67%)	82.95% (74.07%-91.08%)	58.03% (46.51%-69.39%)	2.8496 (2.5712-3.1337)
At least 4 of CHASM (melanoma), FATHMM (missense), Mutation Assessor, PROVEAN	58.96% (55.05%-62.63%)	37.35% (32.79%-42.02%)	96.322% (94.203%-98.684%)	94.62% (91.48%-98.04%)	47.07% (42.25%-51.37%)	2.7536 (2.6541-2.8625)	58.84% (51.52%-66.67%)	36.97% (27.27%-46.43%)	96.322% (90.909%-100%)	94.56% (86.96%-100%)	47.1% (38.35%-56.76%)	2.7496 (2.5278-2.9462)
At least 4 of CHASM (melanoma), FATHMM (missense), Mutation Assessor, PROVEAN, SIFT	72.36% (68.69%-75.76%)	64.33% (59.68%-68.85%)	86.217% (81.944%-90.77%)	88.98% (85.39%-92.78%)	58.3% (53.09%-63.54%)	2.9782 (2.8508-3.1014)	72.46% (65.66%-79.8%)	64.39% (54.99%-73.85%)	86.26% (77.143%-94.59%)	88.95% (81.48%-95.74%)	58.53% (47.92%-69.23%)	2.9813 (2.7353-3.2388)
At least 4 of CHASM (melanoma), FATHMM (missense), Mutation Assessor, PROVEAN, VEST	68.35% (64.65%-72.22%)	58.53% (53.33%-63.49%)	85.321% (80.882%-90.141%)	87.33% (83.33%-91.57%)	54.33% (49.06%-59.29%)	2.8552 (2.7186-2.9876)	68.35% (60.61%-75.76%)	58.44% (48.33%-68.34%)	85.3% (75.862%-94.12%)	87.22% (78.78%-95%)	54.46% (44.44%-65%)	2.8543 (2.5888-3.1278)
At least 4 of CHASM (melanoma), FATHMM (missense), Mutation Assessor, SIFT	70.37% (66.67%-73.74%)	56.94% (51.59%-61.79%)	93.591% (90.411%-97.183%)	93.89% (90.91%-97.3%)	55.69% (50.81%-60.48%)	3.0011 (2.8915-3.1073)	70.37% (63.64%-77.78%)	56.84% (46.87%-67.25%)	93.528% (86.486%-100%)	93.8% (87.18%-100%)	55.8% (46.03%-66.07%)	2.9998 (2.7857-3.2174)
At least 4 of CHASM (melanoma), FATHMM (missense), Mutation Assessor, SIFT, VEST	71.75% (68.18%-75.25%)	67.6% (62.99%-72.22%)	78.913% (73.682%-84.507%)	84.72% (80.81%-88.68%)	58.48% (52.88%-64%)	2.8971 (2.7640-3.0373)	71.65% (64.65%-78.79%)	67.43% (58.33%-76.92%)	78.828% (67.545%-89.47%)	84.55% (76.36%-92.59%)	58.5% (46.94%-69.57%)	2.8932 (2.6059-3.1567)
At least 4 of CHASM (melanoma), FATHMM (missense), Mutation Assessor, VEST	65.65% (61.62%-69.7%)	49.5% (44.07%-54.76%)	93.579% (90.411%-97.26%)	93.03% (89.83%-96.92%)	51.73% (46.67%-56.43%)	2.8783 (2.7645-2.9941)	65.66% (57.58%-73.74%)	49.39% (39.06%-60.35%)	93.559% (86.111%-100%)	92.97% (85.19%-100%)	51.86% (42.42%-61.76%)	2.8778 (2.6392-3.1027)
At least 4 of CHASM (melanoma), FATHMM (missense), MutationTaster, PolyPhen-2	70.01% (66.65%-73.74%)	55.82% (50.78%-60.8%)	94.539% (91.664%-97.437%)	94.65% (91.89%-97.44%)	55.31% (50.41%-60.16%)	3.0031 (2.8980-3.1158)	70.08% (62.63%-76.79%)	55.89% (45.9%-66.21%)	94.404% (87.879%-100%)	94.49% (88.09%-100%)	55.51% (45.76%-65.52%)	3.0029 (2.7898-3.2145)
At least 4 of CHASM (melanoma), FATHMM (missense), MutationTaster, PolyPhen-2, PROVEAN	77.08% (73.74%-80.3%)	69.1% (64.52%-73.6%)	90.869% (87.324%-94.667%)	92.9% (90.11%-95.83%)	62.98% (57.8%-67.96%)	3.1585 (3.0458-3.2764)	77.15% (70.71%-83.84%)	69.21% (60%-78.34%)	90.714% (82.759%-97.37%)	92.77% (86.67%-97.96%)	63.2% (52.83%-73.92%)	3.1589 (2.9220-3.3837)
At least 4 of CHASM (melanoma), FATHMM (missense), MutationTaster, PolyPhen-2, SIFT	79.82% (76.77%-82.84%)	75.54% (71.31%-79.83%)	87.209% (82.857%-91.895%)	91.09% (88.12%-94.34%)	67.34% (62.22%-72.53%)	3.2118 (3.0999-3.3368)	79.75% (73.71%-85.86%)	75.48% (66.67%-83.93%)	87.012% (77.143%-94.87%)	90.91% (84%-96.43%)	67.42% (57.14%-77.55%)	3.2082 (2.9580-3.4320)
At least 4 of CHASM (melanoma), FATHMM (missense), MutationTaster, PolyPhen-2, VEST	76.48% (73.23%-79.8%)	72.37% (67.97%-76.67%)	83.561% (78.571%-89.041%)	88.39% (85.05%-92.08%)	63.63% (58.24%-69.07%)	3.0796 (2.9536-3.2054)	76.34% (69.7%-82.83%)	72.24% (63.49%-81.25%)	83.295% (72.222%-92.5%)	88.15% (80.85%-94.45%)	63.62% (52.27%-74.42%)	3.0732 (2.8126-3.3202)
At least 4 of CHASM (melanoma), FATHMM (missense), MutationTaster, PROVEAN	59.96% (56.06%-63.64%)	37.85% (33.06%-42.86%)	98.173% (97.015%-100%)	97.29% (95.45%-100%)	47.74% (43.04%-52.26%)	2.8105 (2.7210-2.9023)	59.89% (52.53%-67.68%)	37.57% (27.42%-47.28%)	98.144% (94.118%-100%)	97.21% (91.3%-100%)	47.81% (38.67%-57.53%)	2.8074 (2.6197-2.9919)
At least 4 of CHASM (melanoma), FATHMM (missense), MutationTaster, PROVEAN, SIFT	76.41% (73.23%-79.8%)	67.51% (62.9%-71.88%)	91.771% (88.235%-95.716%)	93.42% (90.59%-96.51%)	62.03% (57.01%-66.98%)	3.1474 (3.0378-3.2642)	76.48% (69.7%-82.83%)	67.6% (58.93%-77.05%)	91.661% (84.211%-97.62%)	93.32% (87.18%-98.08%)	62.25% (52.27%-72.22%)	3.1483 (2.9165-3.3657)
At least 4 of CHASM (melanoma), FATHMM (missense), MutationTaster, PROVEAN, VEST	73.73% (70.2%-77.27%)	62.76% (57.76%-67.8%)	92.699% (89.333%-96%)	93.7% (90.91%-96.63%)	59.01% (54.05%-64.17%)	3.0817 (2.9710-3.2011)	73.75% (66.67%-80.81%)	62.76% (52.46%-72.88%)	92.563% (85.366%-100%)	93.56% (86.95%-100%)	59.16% (48.43%-69.23%)	3.0804 (2.8446-3.3023)
At least 4 of CHASM (melanoma), FATHMM (missense), MutationTaster, SIFT	72.04% (68.69%-75.25%)	58.5% (53.33%-63.41%)	95.442% (92.958%-98.592%)	95.69% (93.33%-98.67%)	57.08% (52.07%-61.91%)	3.0672 (2.9642-3.1715)	72.08% (65.66%-78.79%)	58.5% (49.15%-68.75%)	95.351% (89.283%-100%)	95.58% (89.99%-100%)	57.25% (47.54%-67.28%)	3.0668 (2.8670-3.2723)
At least 4 of CHASM (melanoma), FATHMM (missense), MutationTaster, SIFT, VEST	77.46% (74.24%-80.81%)	71.3% (66.67%-75.61%)	88.112% (83.824%-92.754%)	91.21% (88.17%-94.62%)	63.97% (58.76%-69.08%)	3.1459 (3.0252-3.2671)	77.4% (70.71%-83.84%)	71.2% (62.5%-80.33%)	87.958% (78.788%-96.77%)	91.06% (84.44%-97.67%)	64.03% (53.06%-74.47%)	3.1425 (2.8992-3.3792)
At least 4 of CHASM (melanoma), FATHMM (missense), MutationTaster, VEST	67.67% (64.14%-71.21%)	51.61% (46.4%-56.67%)	95.442% (92.958%-98.592%)	95.14% (92.54%-98.48%)	53.29% (48.44%-58.2%)	2.9548 (2.8518-3.0614)	67.69% (60.61%-74.75%)	51.55% (41.78%-61.9%)	95.351% (89.283%-100%)	95.02% (88.57%-100%)	53.42% (43.66%-63.16%)	2.9534 (2.7462-3.1638)
At least 4 of CHASM (melanoma), FATHMM (missense), PolyPhen-2, PROVEAN	59.29% (55.54%-63.13%)	37.87% (32.8%-42.62%)	96.322% (94.203%-98.684%)	94.69% (91.49%-98.08%)	47.27% (42.38%-51.68%)	2.7615 (2.6582-2.8682)	59.19% (51.52%-66.69%)	37.53% (27.87%-47.37%)	96.322% (90.909%-100%)	94.63% (86.96%-100%)	47.32% (38.57%-57.5%)	2.7581 (2.5430-2.9603)
At least 4 of CHASM (melanoma), FATHMM (missense), PolyPhen-2, PROVEAN, SIFT	71.67% (68.18%-75.25%)	65.92% (61.42%-70.31%)	81.609% (76.712%-86.845%)	86.11% (82.18%-90%)	58.07% (52.53%-63.3%)	2.9170 (2.7803-3.0481)	71.81% (64.65%-78.79%)	66.01% (57.14%-75.01%)	81.698% (71.053%-91.67%)	86.11% (78.26%-93.88%)	58.33% (47.92%-69.23%)	2.9215 (2.6483-3.1961)
At least 4 of CHASM (melanoma), FATHMM (missense), PolyPhen-2, PROVEAN, VEST	68.35% (64.65%-72.22%)	61.72% (56.78%-66.67%)	79.808% (74.648%-85.002%)	84.09% (80%-88.42%)	54.66% (49.04%-59.81%)	2.8027 (2.6659-2.9421)	68.35% (60.61%-75.76%)	61.64% (52.23%-71.22%)	79.8% (68.96%-90%)	83.98% (75%-92.31%)	54.79% (44.64%-65.46%)	2.8022 (2.5149-3.0770)
At least 4 of CHASM (melanoma), FATHMM (missense), PolyPhen-2, SIFT	69.36% (65.66%-73.23%)	56.92% (52.06%-61.74%)	90.852% (86.957%-94.805%)	91.5% (87.95%-95.12%)	54.95% (50%-59.83%)	2.9423 (2.8191-3.0609)	69.36% (61.62%-76.77%)	56.87% (47.54%-66.67%)	90.74% (82.051%-97.56%)	91.36% (83.87%-97.67%)	55.07% (45.28%-65%)	2.9403 (2.7013-3.1848)
At least 4 of CHASM (melanoma), FATHMM (missense), PolyPhen-2, SIFT, VEST	72.75% (69.19%-76.26%)	71.86% (67.44%-76.2%)	74.28% (68.182%-80.282%)	82.85% (78.9%-86.84%)	60.42% (54.44%-65.93%)	2.8941 (2.7546-3.0384)	72.68% (65.66%-79.8%)	71.68% (62.9%-80.36%)	74.329% (62.065%-85.3%)	82.75% (74.58%-90.91%)	60.46% (48.84%-71.79%)	2.8922 (2.5943-3.1706)
At least 4 of CHASM (melanoma), FATHMM (missense), PolyPhen-2, VEST	63.96% (60.1%-67.68%)	49.47% (44.35%-54.55%)	89.016% (84.932%-93.333%)	88.62% (84.51%-93.06%)	50.47% (45.6%-55.29%)	2.7757 (2.6513-2.9035)	63.99% (56.57%-71.72%)	49.44% (39.34%-59.16%)	88.917% (80%-97.22%)	88.48% (79.31%-96.88%)	50.61% (41.17%-60.32%)	2.7744 (2.5264-3.0176)
At least 4 of CHASM (melanoma), FATHMM (missense), PROVEAN, SIFT	61.31% (57.58%-65.15%)	41.05% (36.22%-45.9%)	96.322% (94.203%-98.684%)	95.09% (92.16%-98.21%)	48.59% (43.62%-52.86%)	2.8105 (2.7099-2.9156)	61.22% (53.54%-68.69%)	40.73% (30.77%-50.79%)	96.322% (90.909%-100%)	95.04% (88%-100%)	48.64% (39.73%-58.57%)	2.8074 (2.5983-3.0099)
At least 4 of CHASM (melanoma), FATHMM (missense), PROVEAN, SIFT, VEST	69.33% (65.66%-72.73%)	61.68% (56.78%-66.67%)	82.549% (77.778%-88%)	85.94% (81.82%-90.32%)	55.47% (50%-60.72%)	2.8565 (2.7187-2.9916)	69.41% (62.63%-76.77%)	61.71% (51.56%-71.43%)	82.561% (71.429%-91.89%)	85.88% (77.49%-93.88%)	55.68% (44.83%-66.67%)	2.8583 (2.5811-3.1391)
At least 4 of CHASM (melanoma), FATHMM (missense), PROVEAN, VEST	58.96% (55.05%-62.63%)	36.8% (31.75%-41.41%)	97.25% (95.588%-100%)	95.87% (93.33%-100%)	47.09% (42.18%-51.63%)	2.7701 (2.6739-2.8683)	58.86% (51.52%-66.67%)	36.47% (27.12%-46.48%)	97.224% (92.105%-100%)	95.79% (88.24%-100%)	47.14% (38.16%-56.94%)	2.7663 (2.5605-2.9658)
At least 4 of CHASM (melanoma), FATHMM (missense), SIFT, VEST	66.34% (62.12%-70.2%)	52.15% (47.06%-57.15%)	90.853% (86.957%-94.873%)	90.8% (86.96%-94.74%)	52.34% (47.15%-57.14%)	2.8613 (2.7376-2.9908)	66.32% (58.59%-74.75%)	52.06% (41.93%-62.3%)	90.74% (82.141%-97.67%)	90.64% (82.5%-97.56%)	52.44% (42.85%-62.5%)	2.8588 (2.5961-3.1031)
At least 4 of CHASM (melanoma), Mutation Assessor, MutationTaster, PolyPhen-2	68.66% (65.15%-72.22%)	53.69% (48.8%-58.54%)	94.52% (91.667%-97.368%)	94.43% (91.55%-97.33%)	54.14% (49.21%-58.82%)	2.9678 (2.8600-3.0802)	68.75% (61.62%-75.76%)	53.76% (43.33%-63.8%)	94.436% (88.232%-100%)	94.32% (87.5%-100%)	54.34% (45%-64.41%)	2.9687 (2.7419-3.1877)
At least 4 of CHASM (melanoma), Mutation Assessor, MutationTaster, PolyPhen-2, PROVEAN	75.04% (71.72%-78.79%)	66.43% (61.6%-71.3%)	89.901% (86.111%-94.118%)	91.92% (89.01%-95.45%)	60.77% (55.66%-66.02%)	3.0902 (2.9716-3.2118)	75.18% (67.68%-81.82%)	66.58% (57.14%-75.81%)	89.896% (82.051%-97.3%)	91.88% (85.37%-97.78%)	61.05% (50.91%-71.15%)	3.0940 (2.8508-3.3306)
At least 4 of CHASM (melanoma), Mutation Assessor, MutationTaster, PolyPhen-2, SIFT	78.78% (75.76%-81.82%)	74.44% (70%-79.03%)	86.281% (82.086%-91.026%)	90.37% (87.38%-93.58%)	66.13% (61%-71.58%)	3.1723 (3.0518-3.2989)	78.79% (72.73%-84.85%)	74.49% (65.08%-83.05%)	86.138% (77.498%-94.44%)	90.22% (83.33%-96.15%)	66.31% (55.32%-76.79%)	3.1716 (2.9200-3.4111)
At least 4 of CHASM (melanoma), Mutation Assessor, MutationTaster, PolyPhen-2, VEST	77.11% (73.74%-80.32%)	72.86% (68.33%-77.42%)	84.446% (80%-89.335%)	89.01% (85.56%-92.66%)	64.28% (58.95%-70%)	3.1059 (2.9844-3.2394)	77.1% (70.68%-83.84%)	72.88% (63.64%-81.82%)	84.324% (74.984%-93.94%)	88.85% (81.25%-95.92%)	64.44% (52.93%-75%)	3.1050 (2.8424-3.3434)
At least 4 of CHASM (melanoma), Mutation Assessor, MutationTaster, PROVEAN	59.28% (55.56%-63.13%)	36.78% (32%-41.67%)	98.173% (97.015%-100%)	97.21% (95.35%-100%)	47.32% (42.48%-52%)	2.7948 (2.7051-2.8873)	59.22% (51.52%-66.67%)	36.52% (26.47%-46.03%)	98.144% (94.118%-100%)	97.13% (90.91%-100%)	47.4% (38.16%-56.94%)	2.7919 (2.6021-2.9704)
At least 4 of CHASM (melanoma), Mutation Assessor, MutationTaster, PROVEAN, SIFT	76.72% (73.72%-80.3%)	69.08% (64.57%-73.44%)	89.901% (86.111%-94.118%)	92.21% (89.36%-95.6%)	62.71% (57.57%-67.96%)	3.1390 (3.0200-3.2593)	76.87% (69.7%-82.85%)	69.25% (60%-78.46%)	89.896% (82.051%-97.3%)	92.17% (85.71%-97.87%)	63.01% (52.73%-73.22%)	3.1433 (2.9040-3.3750)
At least 4 of CHASM (melanoma), Mutation Assessor, MutationTaster, PROVEAN, VEST	74.4% (70.71%-77.78%)	64.35% (59.5%-69.17%)	91.778% (88.406%-95.714%)	93.12% (90.36%-96.43%)	59.82% (54.72%-65.14%)	3.0907 (2.9732-3.2133)	74.42% (67.68%-81.82%)	64.36% (54.54%-73.85%)	91.649% (84.371%-97.56%)	92.98% (86.36%-97.92%)	59.98% (49.15%-70.37%)	3.0897 (2.8467-3.3186)
At least 4 of CHASM (melanoma), Mutation Assessor, MutationTaster, SIFT	70.35% (66.67%-73.75%)	56.37% (51.2%-61.29%)	94.52% (91.667%-97.368%)	94.68% (91.89%-97.47%)	55.61% (50.79%-60.47%)	3.0118 (2.9075-3.1203)	70.41% (63.61%-77.78%)	56.39% (46.66%-66.67%)	94.436% (88.232%-100%)	94.57% (88.23%-100%)	55.79% (46.03%-65.72%)	3.0119 (2.7897-3.2191)
At least 4 of CHASM (melanoma), Mutation Assessor, MutationTaster, SIFT, VEST	78.13% (75.25%-81.31%)	72.33% (67.97%-76.8%)	88.158% (84.058%-92.537%)	91.35% (88.24%-94.74%)	64.82% (59.61%-70%)	3.1666 (3.0524-3.2917)	78.07% (71.72%-83.84%)	72.33% (63.49%-81.25%)	87.891% (79.412%-96.3%)	91.11% (84.31%-97.56%)	64.92% (54.35%-75.56%)	3.1626 (2.9144-3.3945)
At least 4 of CHASM (melanoma), Mutation Assessor, MutationTaster, VEST	66.66% (62.63%-70.2%)	50.54% (45.37%-55.46%)	94.52% (91.667%-97.368%)	94.1% (91.04%-97.14%)	52.5% (47.65%-57.38%)	2.9166 (2.8091-3.0270)	66.69% (59.6%-74.75%)	50.51% (40.57%-60.66%)	94.436% (88.232%-100%)	93.98% (87.1%-100%)	52.65% (42.85%-62.72%)	2.9157 (2.6877-3.1332)
At least 4 of CHASM (melanoma), Mutation Assessor, PolyPhen-2, PROVEAN	58.93% (55.05%-62.63%)	37.85% (33.08%-42.63%)	95.373% (92.857%-98.551%)	93.4% (89.8%-97.78%)	47.02% (42.18%-51.66%)	2.7364 (2.6299-2.8431)	58.91% (51.52%-66.67%)	37.57% (27.59%-47.28%)	95.471% (89.655%-100%)	93.45% (85.71%-100%)	47.12% (38.16%-56.76%)	2.7362 (2.5218-2.9424)
At least 4 of CHASM (melanoma), Mutation Assessor, PolyPhen-2, PROVEAN, SIFT	72.02% (68.69%-75.76%)	67.51% (62.9%-72.36%)	79.805% (74.648%-84.851%)	85.25% (81.25%-89.11%)	58.69% (53.26%-64.29%)	2.9125 (2.7739-3.0531)	72.13% (64.65%-78.79%)	67.62% (58.46%-76.57%)	79.821% (69.231%-90.32%)	85.18% (77.08%-93.33%)	58.95% (47.91%-70%)	2.9156 (2.6329-3.1906)
At least 4 of CHASM (melanoma), Mutation Assessor, PolyPhen-2, PROVEAN, VEST	67.99% (64.14%-71.72%)	62.22% (57.57%-67.19%)	77.968% (72.599%-83.117%)	83% (78.72%-87.23%)	54.41% (49.06%-60.19%)	2.7759 (2.6362-2.9221)	68.06% (60.61%-75.76%)	62.24% (52.46%-71.67%)	77.999% (67.497%-88.57%)	82.91% (74.36%-91.3%)	54.63% (43.18%-64.81%)	2.7777 (2.4867-3.0551)
At least 4 of CHASM (melanoma), Mutation Assessor, PolyPhen-2, SIFT	69.01% (65.15%-72.73%)	56.91% (52.03%-61.72%)	89.929% (86.3%-94.203%)	90.72% (87.18%-94.74%)	54.69% (49.6%-59.35%)	2.9224 (2.8025-3.0447)	69.05% (61.62%-76.77%)	56.9% (46.67%-66.67%)	89.84% (81.818%-97.3%)	90.58% (82.5%-97.5%)	54.85% (45%-64.91%)	2.9217 (2.6765-3.1641)
At least 4 of CHASM (melanoma), Mutation Assessor, PolyPhen-2, SIFT, VEST	72.38% (68.69%-76.26%)	72.87% (68.37%-77.5%)	71.526% (65.385%-77.42%)	81.57% (77.27%-85.83%)	60.39% (54.88%-66.67%)	2.8636 (2.7192-3.0107)	72.41% (64.65%-79.8%)	72.85% (63.76%-81.54%)	71.618% (60%-83.78%)	81.49% (72.88%-89.66%)	60.58% (48.72%-71.8%)	2.8654 (2.5677-3.1566)
At least 4 of CHASM (melanoma), Mutation Assessor, PolyPhen-2, VEST	64.31% (60.61%-68.18%)	49.47% (44.44%-54.47%)	89.955% (86.301%-94.366%)	89.49% (85.5%-94.03%)	50.73% (45.73%-55.31%)	2.7964 (2.6776-2.9217)	64.32% (56.57%-71.72%)	49.45% (39.29%-59.32%)	89.794% (81.392%-97.22%)	89.28% (80%-97.22%)	50.86% (41.26%-60.61%)	2.7938 (2.5346-3.0297)
At least 4 of CHASM (melanoma), Mutation Assessor, PROVEAN, SIFT	59.95% (56.06%-63.64%)	39.45% (34.64%-44.35%)	95.373% (92.857%-98.551%)	93.65% (90.2%-97.87%)	47.67% (42.95%-52.08%)	2.7615 (2.6575-2.8731)	59.91% (52.53%-67.68%)	39.15% (29.09%-49.02%)	95.471% (89.655%-100%)	93.71% (85.71%-100%)	47.76% (38.57%-57.36%)	2.7609 (2.5315-2.9670)
At least 4 of CHASM (melanoma), Mutation Assessor, PROVEAN, SIFT, VEST	69.35% (65.66%-73.23%)	64.36% (59.69%-69.11%)	77.978% (72.367%-83.099%)	83.48% (79.17%-87.63%)	55.85% (50.5%-61.46%)	2.8167 (2.6779-2.9581)	69.38% (61.62%-76.77%)	64.34% (54.69%-73.92%)	77.975% (67.647%-89.19%)	83.36% (75%-91.49%)	56.03% (44.23%-66.67%)	2.8171 (2.5350-3.0965)
At least 4 of CHASM (melanoma), Mutation Assessor, PROVEAN, VEST	58.6% (54.55%-62.63%)	36.24% (31.5%-40.95%)	97.25% (95.588%-100%)	95.8% (93.18%-100%)	46.87% (42.11%-51.37%)	2.7617 (2.6662-2.8656)	58.55% (50.51%-66.67%)	36% (26.46%-45.32%)	97.224% (92.105%-100%)	95.73% (88.46%-100%)	46.95% (37.8%-56.26%)	2.7590 (2.5422-2.9462)
At least 4 of CHASM (melanoma), Mutation Assessor, SIFT, VEST	66.68% (63.13%-70.2%)	52.14% (47.01%-57.14%)	91.805% (88.403%-95.716%)	91.67% (88.16%-95.65%)	52.59% (47.62%-57.38%)	2.8821 (2.7706-3.0036)	66.64% (59.6%-73.74%)	52.07% (41.82%-62.3%)	91.593% (84.205%-97.67%)	91.43% (83.78%-97.62%)	52.69% (42.62%-62.69%)	2.8778 (2.6320-3.1031)
At least 4 of CHASM (melanoma), MutationTaster, PolyPhen-2, PROVEAN	60.61% (56.57%-64.14%)	38.88% (33.87%-43.85%)	98.173% (97.015%-100%)	97.35% (95.56%-100%)	48.16% (43.33%-52.63%)	2.8257 (2.7284-2.9166)	60.59% (53.54%-68.69%)	38.69% (28.57%-48.5%)	98.144% (94.118%-100%)	97.29% (91.66%-100%)	48.26% (39.13%-58.11%)	2.8239 (2.6382-3.0183)
At least 4 of CHASM (melanoma), MutationTaster, PolyPhen-2, PROVEAN, SIFT	76.38% (73.23%-79.8%)	69.09% (64.57%-73.49%)	88.983% (84.932%-93.243%)	91.56% (88.54%-94.79%)	62.47% (57.28%-67.89%)	3.1210 (3.0028-3.2424)	76.53% (69.7%-82.83%)	69.25% (60.66%-78.26%)	88.981% (80.556%-97.22%)	91.52% (84.78%-97.78%)	62.77% (52%-73.08%)	3.1253 (2.8787-3.3617)
At least 4 of CHASM (melanoma), MutationTaster, PolyPhen-2, PROVEAN, VEST	74.74% (71.21%-78.28%)	65.42% (60.9%-70.4%)	90.861% (87.324%-94.667%)	92.53% (89.66%-95.51%)	60.31% (55.17%-65.77%)	3.0912 (2.9722-3.2143)	74.75% (67.68%-81.82%)	65.42% (55.74%-74.24%)	90.73% (82.857%-97.37%)	92.39% (86.11%-97.83%)	60.45% (49.15%-70.38%)	3.0899 (2.8451-3.3273)
At least 4 of CHASM (melanoma), MutationTaster, PolyPhen-2, SIFT	71.35% (67.68%-74.75%)	57.94% (52.94%-62.79%)	94.52% (91.667%-97.368%)	94.82% (92.21%-97.53%)	56.52% (51.61%-61.29%)	3.0379 (2.9335-3.1446)	71.45% (64.65%-78.79%)	58.03% (48.27%-67.8%)	94.436% (88.232%-100%)	94.72% (88.57%-100%)	56.73% (47.46%-66.67%)	3.0392 (2.8182-3.2470)
At least 4 of CHASM (melanoma), MutationTaster, PolyPhen-2, SIFT, VEST	78.16% (75.25%-81.31%)	75.02% (70.77%-79.2%)	83.583% (78.871%-88.611%)	88.77% (85.56%-92.38%)	65.93% (60.64%-71.58%)	3.1330 (3.0098-3.2645)	78.03% (71.72%-83.84%)	74.95% (66.67%-83.34%)	83.274% (73.329%-93.03%)	88.5% (81.35%-95.35%)	65.95% (54.54%-76.5%)	3.1267 (2.8559-3.3818)
At least 4 of CHASM (melanoma), MutationTaster, PolyPhen-2, VEST	67.98% (64.14%-71.72%)	53.16% (48.03%-58.07%)	93.603% (90.537%-97.102%)	93.5% (90.41%-97.14%)	53.61% (48.76%-58.4%)	2.9387 (2.8341-3.0507)	68.08% (60.61%-75.76%)	53.24% (43.33%-63.64%)	93.517% (86.838%-100%)	93.39% (86.48%-100%)	53.82% (44.06%-64.07%)	2.9397 (2.7128-3.1559)
At least 4 of CHASM (melanoma), MutationTaster, PROVEAN, SIFT	62.63% (58.59%-66.17%)	42.07% (37.09%-47.2%)	98.173% (97.015%-100%)	97.55% (95.92%-100%)	49.5% (44.68%-54.11%)	2.8729 (2.7823-2.9657)	62.62% (55.53%-70.71%)	41.9% (31.25%-51.62%)	98.144% (94.118%-100%)	97.49% (92%-100%)	49.61% (40.3%-59.42%)	2.8714 (2.6834-3.0569)
At least 4 of CHASM (melanoma), MutationTaster, PROVEAN, SIFT, VEST	74.72% (71.21%-78.28%)	64.86% (60.15%-69.6%)	91.778% (88.406%-95.714%)	93.17% (90.48%-96.47%)	60.16% (55.17%-65.49%)	3.0997 (2.9855-3.2164)	74.79% (67.68%-81.82%)	64.95% (55.35%-74.19%)	91.649% (84.371%-97.56%)	93.04% (86.66%-97.92%)	60.37% (50%-70.37%)	3.1002 (2.8665-3.3325)
At least 4 of CHASM (melanoma), MutationTaster, PROVEAN, VEST	60.61% (57.07%-64.65%)	38.89% (34.11%-43.75%)	98.173% (97.015%-100%)	97.36% (95.56%-100%)	48.16% (43.45%-52.7%)	2.8258 (2.7326-2.9202)	60.59% (52.53%-67.68%)	38.69% (29.03%-48.48%)	98.144% (94.118%-100%)	97.29% (91.3%-100%)	48.26% (39.19%-57.9%)	2.8239 (2.6305-3.0132)
At least 4 of CHASM (melanoma), MutationTaster, SIFT, VEST	69.68% (66.16%-73.23%)	55.31% (50.39%-60.47%)	94.52% (91.667%-97.368%)	94.58% (91.78%-97.37%)	55.02% (50%-59.69%)	2.9943 (2.8897-3.1024)	69.73% (62.63%-76.77%)	55.32% (45.31%-65.52%)	94.436% (88.232%-100%)	94.48% (88.24%-100%)	55.19% (45.45%-65.16%)	2.9942 (2.7786-3.2122)
At least 4 of CHASM (melanoma), PolyPhen-2, PROVEAN, SIFT	61.28% (57.58%-65.15%)	42.09% (37.01%-46.83%)	94.455% (91.667%-97.368%)	92.93% (89.29%-96.77%)	48.54% (43.45%-53.15%)	2.7801 (2.6622-2.8924)	61.28% (53.54%-68.69%)	41.85% (32.13%-52.17%)	94.556% (88.232%-100%)	92.98% (84.85%-100%)	48.65% (39.47%-58.83%)	2.7804 (2.5530-3.0029)
At least 4 of CHASM (melanoma), PolyPhen-2, PROVEAN, SIFT, VEST	67.65% (63.64%-71.21%)	63.81% (59.01%-68.6%)	74.275% (68.748%-80%)	81.09% (76.92%-85.57%)	54.27% (48.93%-60%)	2.7346 (2.5895-2.8822)	67.73% (60.61%-75.76%)	63.84% (54.1%-73.34%)	74.355% (62.5%-85.71%)	81.04% (71.93%-89.8%)	54.5% (42.86%-65.31%)	2.7373 (2.4305-3.0308)
At least 4 of CHASM (melanoma), PolyPhen-2, PROVEAN, VEST	59.93% (56.06%-63.64%)	38.35% (33.33%-42.98%)	97.25% (95.588%-100%)	96.02% (93.48%-100%)	47.71% (42.76%-52.06%)	2.7933 (2.6955-2.8916)	59.93% (52.53%-67.68%)	38.17% (29.03%-48.39%)	97.224% (92.105%-100%)	95.96% (88.89%-100%)	47.81% (38.96%-57.54%)	2.7917 (2.5989-2.9887)
At least 4 of CHASM (melanoma), PolyPhen-2, SIFT, VEST	65.99% (62.12%-69.7%)	53.17% (48.31%-58.27%)	88.147% (84.058%-92.857%)	88.58% (84.51%-93.06%)	52.12% (47.11%-56.78%)	2.8201 (2.6888-2.9477)	66% (58.59%-73.74%)	53.21% (43.33%-62.91%)	87.895% (78.782%-96.88%)	88.32% (79.49%-96.88%)	52.26% (42.62%-62.07%)	2.8168 (2.5584-3.0752)
At least 4 of CHASM (melanoma), PROVEAN, SIFT, VEST	61.63% (58.08%-65.15%)	41.02% (36.13%-45.9%)	97.25% (95.588%-100%)	96.28% (93.88%-100%)	48.81% (43.88%-53.43%)	2.8336 (2.7350-2.9310)	61.59% (54.55%-68.69%)	40.8% (31.14%-50.77%)	97.224% (92.105%-100%)	96.22% (89.47%-100%)	48.9% (39.74%-58.57%)	2.8314 (2.6280-3.0229)
At least 4 of FATHMM (cancer), FATHMM (missense), Mutation Assessor, MutationTaster	68.37% (64.65%-71.73%)	53.76% (48.44%-58.41%)	93.622% (90.541%-97.183%)	93.58% (90.54%-97.18%)	53.94% (48.87%-58.6%)	2.9490 (2.8409-3.0612)	68.31% (61.59%-75.76%)	53.62% (43.75%-64.29%)	93.483% (86.667%-100%)	93.39% (86.11%-100%)	54.01% (44.61%-63.94%)	2.9451 (2.7166-3.1602)
At least 4 of FATHMM (cancer), FATHMM (missense), Mutation Assessor, MutationTaster, PolyPhen-2	78.46% (75.25%-81.82%)	74.49% (70.25%-78.91%)	85.319% (80.764%-90.278%)	89.77% (86.67%-93.4%)	65.92% (60.44%-71.43%)	3.1549 (3.0395-3.2805)	78.44% (71.72%-84.85%)	74.4% (65.51%-82.76%)	85.331% (75.676%-94.59%)	89.69% (82.14%-96%)	66.03% (55.1%-76.74%)	3.1545 (2.9020-3.3835)
At least 4 of FATHMM (cancer), FATHMM (missense), Mutation Assessor, MutationTaster, PROVEAN	75.08% (71.72%-78.28%)	67% (62.39%-71.65%)	89.041% (84.928%-93.245%)	91.36% (88.24%-94.68%)	60.95% (55.56%-66.06%)	3.0835 (2.9671-3.1972)	75.09% (68.69%-81.82%)	67.03% (57.41%-76.27%)	88.879% (80%-97.14%)	91.2% (84.31%-97.73%)	61.1% (50.88%-71.7%)	3.0820 (2.8423-3.3255)
At least 4 of FATHMM (cancer), FATHMM (missense), Mutation Assessor, MutationTaster, SIFT	78.45% (75.25%-81.82%)	72.87% (68.59%-77.31%)	88.084% (84.211%-92.86%)	91.36% (88.35%-94.9%)	65.25% (60%-70.33%)	3.1756 (3.0602-3.2921)	78.46% (71.72%-84.85%)	72.86% (63.93%-81.43%)	88.042% (78.943%-96.55%)	91.28% (84.48%-97.78%)	65.4% (54.9%-75.56%)	3.1758 (2.9332-3.4027)
At least 4 of FATHMM (cancer), FATHMM (missense), Mutation Assessor, MutationTaster, VEST	76.76% (73.74%-79.8%)	71.28% (67.17%-75.81%)	86.222% (81.69%-90.912%)	89.94% (86.73%-93.69%)	63.45% (58.16%-69%)	3.1090 (2.9906-3.2307)	76.79% (70.71%-82.83%)	71.24% (62.29%-79.69%)	86.278% (76.923%-94.74%)	89.91% (82.69%-96.08%)	63.61% (52.94%-74%)	3.1103 (2.8654-3.3515)
At least 4 of FATHMM (cancer), FATHMM (missense), Mutation Assessor, PolyPhen-2	67.03% (63.13%-70.71%)	55.37% (50.4%-60.16%)	87.166% (82.812%-92%)	88.18% (84.21%-92.5%)	53.04% (48.21%-57.8%)	2.8376 (2.7091-2.9612)	66.96% (59.6%-74.75%)	55.19% (45.45%-65.08%)	87.126% (77.778%-95.25%)	88.05% (79.48%-95.56%)	53.11% (43.86%-63.08%)	2.8347 (2.5871-3.0905)
At least 4 of FATHMM (cancer), FATHMM (missense), Mutation Assessor, PolyPhen-2, PROVEAN	71.34% (68.18%-75.25%)	66.5% (61.9%-71.2%)	79.705% (74.242%-84.851%)	85% (81.13%-89%)	57.91% (52.47%-63.54%)	2.8911 (2.7563-3.0230)	71.46% (63.64%-77.78%)	66.45% (56.72%-75.36%)	80.025% (69.444%-90.25%)	85.1% (76.74%-92.73%)	58.15% (47.17%-69.23%)	2.8971 (2.6296-3.1654)
At least 4 of FATHMM (cancer), FATHMM (missense), Mutation Assessor, PolyPhen-2, SIFT	73.36% (69.7%-76.77%)	70.75% (66.13%-75.37%)	77.852% (72.222%-83.333%)	84.67% (80.77%-88.68%)	60.62% (54.95%-66.3%)	2.9390 (2.7989-3.0759)	73.49% (66.67%-80.81%)	70.71% (61.01%-79.69%)	78.223% (66.667%-89.19%)	84.79% (76.47%-92.45%)	60.87% (49.98%-71.75%)	2.9459 (2.6738-3.2206)
At least 4 of FATHMM (cancer), FATHMM (missense), Mutation Assessor, PolyPhen-2, VEST	73.71% (70.2%-77.27%)	71.29% (66.93%-75.78%)	77.892% (72.499%-83.333%)	84.79% (81.08%-89%)	61.07% (55.56%-66.68%)	2.9504 (2.8124-3.0903)	73.79% (66.67%-80.81%)	71.23% (62.29%-80%)	78.15% (67.422%-88.89%)	84.83% (76.47%-92.31%)	61.28% (50%-72%)	2.9549 (2.6656-3.2295)
At least 4 of FATHMM (cancer), FATHMM (missense), Mutation Assessor, PROVEAN	57.64% (53.54%-61.62%)	37.9% (33.33%-42.64%)	91.773% (88.31%-95.714%)	88.84% (84.09%-94.12%)	46.08% (41.21%-50.35%)	2.6459 (2.5161-2.7679)	57.44% (49.49%-65.66%)	37.47% (27.87%-46.88%)	91.673% (84.609%-97.68%)	88.53% (77.78%-96.78%)	46.07% (37.31%-55.7%)	2.6374 (2.3870-2.9005)
At least 4 of FATHMM (cancer), FATHMM (missense), Mutation Assessor, PROVEAN, SIFT	71.32% (67.68%-74.75%)	66.99% (62.3%-71.54%)	78.794% (73.529%-84.507%)	84.53% (80.41%-88.78%)	57.99% (52.38%-63.37%)	2.8830 (2.7406-3.0213)	71.5% (64.65%-78.79%)	67.05% (57.62%-75.86%)	79.097% (67.568%-90%)	84.63% (76.19%-92.31%)	58.3% (47.06%-69.24%)	2.8908 (2.6118-3.1707)
At least 4 of FATHMM (cancer), FATHMM (missense), Mutation Assessor, PROVEAN, VEST	69.01% (65.15%-72.73%)	62.26% (57.38%-66.94%)	80.658% (75.384%-86.116%)	84.77% (80.68%-89.01%)	55.28% (49.99%-60.75%)	2.8297 (2.6831-2.9719)	69.05% (61.62%-76.77%)	62.14% (52.46%-71.65%)	80.873% (70%-90.91%)	84.79% (75.56%-93.18%)	55.44% (44.44%-66.67%)	2.8325 (2.5467-3.1240)
At least 4 of FATHMM (cancer), FATHMM (missense), Mutation Assessor, SIFT	68.38% (64.65%-71.72%)	56.98% (52%-61.79%)	88.084% (84.211%-92.86%)	89.21% (85.39%-93.51%)	54.21% (49.14%-59.06%)	2.8849 (2.7659-3.0052)	68.29% (61.62%-75.76%)	56.76% (46.88%-66.67%)	88.042% (78.943%-96.55%)	89.08% (80.94%-96.78%)	54.25% (44.44%-64.29%)	2.8813 (2.6178-3.1182)
At least 4 of FATHMM (cancer), FATHMM (missense), Mutation Assessor, SIFT, VEST	72.33% (68.69%-75.76%)	70.74% (66.39%-75.4%)	75.05% (69.225%-81.339%)	83.06% (79.05%-87.5%)	59.73% (53.76%-65.56%)	2.8858 (2.7400-3.0341)	72.52% (65.66%-79.8%)	70.73% (61.54%-79.43%)	75.558% (62.5%-86.84%)	83.25% (74.14%-91.49%)	60.05% (48.84%-71.16%)	2.8958 (2.6022-3.1801)
At least 4 of FATHMM (cancer), FATHMM (missense), Mutation Assessor, VEST	63% (59.09%-66.68%)	49.01% (43.55%-54.17%)	87.166% (82.812%-92%)	86.85% (82.54%-91.55%)	49.71% (44.71%-54.62%)	2.7274 (2.5954-2.8550)	62.9% (55.53%-70.71%)	48.76% (38.46%-59.15%)	87.126% (77.778%-95.25%)	86.68% (76.67%-95.24%)	49.76% (40.32%-59.71%)	2.7233 (2.4539-2.9883)
At least 4 of FATHMM (cancer), FATHMM (missense), MutationTaster, PolyPhen-2	70.74% (67.17%-74.24%)	58.02% (52.8%-62.91%)	92.72% (89.706%-96.001%)	93.23% (90.41%-96.34%)	56.09% (50.85%-60.8%)	3.0006 (2.8901-3.1175)	70.65% (63.64%-77.78%)	57.87% (47.66%-68.12%)	92.537% (85.294%-97.62%)	93.01% (86.11%-97.83%)	56.13% (46.66%-66.15%)	2.9956 (2.7616-3.2211)
At least 4 of FATHMM (cancer), FATHMM (missense), MutationTaster, PolyPhen-2, PROVEAN	78.1% (74.75%-81.31%)	72.84% (68.5%-77.27%)	87.187% (83.071%-91.67%)	90.77% (87.88%-94.12%)	64.99% (59.99%-70%)	3.1578 (3.0370-3.2683)	78.15% (71.72%-84.85%)	72.92% (63.92%-81.43%)	87.075% (78.116%-95%)	90.65% (83.92%-96.23%)	65.21% (55.1%-75.93%)	3.1585 (2.9221-3.3960)
At least 4 of FATHMM (cancer), FATHMM (missense), MutationTaster, PolyPhen-2, SIFT	82.16% (79.29%-85.35%)	79.79% (75.97%-83.74%)	86.231% (81.941%-91.304%)	90.93% (87.96%-94.34%)	71.17% (65.87%-76.4%)	3.2813 (3.1705-3.4019)	82.15% (75.76%-87.88%)	79.74% (71.67%-87.2%)	86.225% (76.467%-94.4%)	90.87% (84.21%-96.49%)	71.28% (60.47%-81.64%)	3.2812 (3.0392-3.5066)
At least 4 of FATHMM (cancer), FATHMM (missense), MutationTaster, PolyPhen-2, VEST	79.12% (75.76%-82.32%)	77.65% (73.44%-81.74%)	81.638% (76.712%-86.767%)	87.97% (84.68%-91.74%)	67.88% (62.07%-73.33%)	3.1514 (3.0184-3.2799)	79.14% (72.73%-85.86%)	77.65% (69.23%-86.16%)	81.647% (71.053%-91.67%)	87.91% (80.36%-94.55%)	68.05% (57.14%-79.56%)	3.1525 (2.8874-3.4107)
At least 4 of FATHMM (cancer), FATHMM (missense), MutationTaster, PROVEAN	60.33% (56.57%-64.14%)	39.49% (34.68%-44.44%)	96.354% (94.203%-98.667%)	94.93% (91.84%-98.15%)	47.94% (43.06%-52.38%)	2.7871 (2.6805-2.8876)	60.15% (52.53%-67.68%)	39.09% (29.03%-48.62%)	96.276% (91.176%-100%)	94.74% (87.5%-100%)	47.94% (38.96%-57.53%)	2.7805 (2.5730-2.9918)
At least 4 of FATHMM (cancer), FATHMM (missense), MutationTaster, PROVEAN, SIFT	77.76% (74.75%-81.31%)	71.26% (66.93%-76%)	88.994% (84.929%-93.243%)	91.8% (88.78%-95%)	64.17% (59.18%-69.44%)	3.1623 (3.0451-3.2818)	77.8% (70.71%-83.84%)	71.27% (61.67%-80.01%)	88.96% (80%-97.22%)	91.74% (85.19%-97.92%)	64.34% (54%-75%)	3.1632 (2.9262-3.3945)
At least 4 of FATHMM (cancer), FATHMM (missense), MutationTaster, PROVEAN, VEST	74.74% (71.21%-78.28%)	67.02% (62.18%-71.88%)	88.09% (84.057%-92.427%)	90.68% (87.37%-94.19%)	60.7% (55.34%-66.34%)	3.0649 (2.9349-3.1884)	74.76% (67.68%-81.82%)	67% (56.92%-76.56%)	88.021% (79.409%-96.88%)	90.57% (83.33%-97.56%)	60.86% (50%-71.7%)	3.0645 (2.8157-3.3211)
At least 4 of FATHMM (cancer), FATHMM (missense), MutationTaster, SIFT	71.4% (67.68%-74.75%)	58.54% (53.6%-63.2%)	93.622% (90.541%-97.183%)	94.07% (91.3%-97.4%)	56.63% (51.61%-61.34%)	3.0287 (2.9193-3.1331)	71.34% (64.65%-78.79%)	58.42% (48.44%-68.42%)	93.483% (86.667%-100%)	93.9% (87.18%-100%)	56.71% (47.27%-67.27%)	3.0251 (2.8150-3.2430)
At least 4 of FATHMM (cancer), FATHMM (missense), MutationTaster, SIFT, VEST	78.75% (75.76%-81.82%)	76.07% (72%-80.47%)	83.379% (78.571%-89.041%)	88.79% (85.58%-92.38%)	66.83% (61.54%-72.53%)	3.1507 (3.0230-3.2746)	78.86% (72.73%-84.85%)	76.02% (67.21%-84.62%)	83.658% (72.5%-92.5%)	88.89% (82%-94.92%)	67.04% (55.56%-77.5%)	3.1561 (2.9080-3.4073)
At least 4 of FATHMM (cancer), FATHMM (missense), MutationTaster, VEST	67.39% (63.64%-71.21%)	52.21% (47.11%-57.03%)	93.622% (90.541%-97.183%)	93.4% (90.28%-97.1%)	53.12% (48.06%-58.02%)	2.9235 (2.8091-3.0338)	67.25% (59.6%-74.75%)	51.94% (42.11%-62.12%)	93.483% (86.667%-100%)	93.19% (85.71%-100%)	53.12% (43.28%-63.24%)	2.9173 (2.6999-3.1483)
At least 4 of FATHMM (cancer), FATHMM (missense), PolyPhen-2, PROVEAN	58.31% (54.55%-62.12%)	39.5% (34.71%-44.36%)	90.821% (87.013%-94.595%)	88.15% (83.33%-93.11%)	46.47% (41.38%-51.03%)	2.6495 (2.5234-2.7757)	58.12% (50.51%-65.66%)	39.04% (29.09%-48.62%)	90.815% (82.857%-97.5%)	87.93% (76.92%-96.77%)	46.47% (37.18%-56.34%)	2.6426 (2.3896-2.8917)
At least 4 of FATHMM (cancer), FATHMM (missense), PolyPhen-2, PROVEAN, SIFT	73.35% (70.2%-76.77%)	70.2% (65.83%-74.8%)	78.787% (73.239%-84.507%)	85.13% (81.31%-89.22%)	60.46% (54.95%-65.96%)	2.9457 (2.8156-3.0762)	73.5% (66.67%-79.8%)	70.21% (61.02%-78.7%)	79.1% (68.421%-89.74%)	85.23% (77.36%-92.86%)	60.73% (50%-71.74%)	2.9528 (2.6946-3.2187)
At least 4 of FATHMM (cancer), FATHMM (missense), PolyPhen-2, PROVEAN, VEST	71.03% (67.17%-74.75%)	65.97% (61.24%-70.74%)	79.747% (74.648%-85.135%)	84.92% (81.05%-89.11%)	57.54% (51.96%-62.96%)	2.8819 (2.7420-3.0238)	71.08% (63.64%-78.79%)	65.9% (56.14%-75.42%)	79.926% (68.571%-89.66%)	84.93% (76.09%-92.6%)	57.72% (47.16%-68.76%)	2.8848 (2.6058-3.1619)
At least 4 of FATHMM (cancer), FATHMM (missense), PolyPhen-2, SIFT	68.36% (64.65%-71.73%)	57.5% (52.5%-62.12%)	87.134% (82.856%-92%)	88.55% (84.81%-92.68%)	54.24% (49.11%-59.13%)	2.8742 (2.7564-2.9996)	68.33% (61.59%-75.76%)	57.31% (47.62%-66.67%)	87.171% (77.143%-95.01%)	88.49% (80.42%-95.56%)	54.32% (44.44%-64.41%)	2.8729 (2.6190-3.1109)
At least 4 of FATHMM (cancer), FATHMM (missense), PolyPhen-2, SIFT, VEST	74.39% (70.71%-77.78%)	75.56% (71.54%-79.69%)	72.358% (66.234%-78.667%)	82.54% (78.69%-86.61%)	63.13% (57.14%-69.05%)	2.9359 (2.7886-3.0759)	74.45% (67.68%-81.82%)	75.45% (67.19%-83.61%)	72.685% (60%-84.85%)	82.59% (74.5%-90.39%)	63.29% (51.28%-75%)	2.9401 (2.6637-3.2295)
At least 4 of FATHMM (cancer), FATHMM (missense), PolyPhen-2, VEST	62.65% (59.09%-66.16%)	50.07% (45%-55.04%)	84.381% (79.71%-89.333%)	84.72% (80.28%-89.48%)	49.42% (44.27%-54.17%)	2.6860 (2.5475-2.8178)	62.59% (55.56%-69.7%)	49.83% (40.28%-59.33%)	84.416% (74.286%-93.94%)	84.62% (75%-93.94%)	49.5% (40.58%-60%)	2.6836 (2.4165-2.9525)
At least 4 of FATHMM (cancer), FATHMM (missense), PROVEAN, SIFT	59.97% (56.06%-63.64%)	41.6% (36.57%-46.46%)	91.726% (88.06%-95.716%)	89.69% (85.45%-94.64%)	47.6% (42.64%-52.08%)	2.7062 (2.5845-2.8265)	59.85% (52.53%-67.68%)	41.23% (31.34%-51.56%)	91.753% (83.784%-100%)	89.58% (80%-100%)	47.64% (38.46%-57.58%)	2.7020 (2.4494-2.9515)
At least 4 of FATHMM (cancer), FATHMM (missense), PROVEAN, SIFT, VEST	69.31% (65.66%-72.73%)	65.42% (60.8%-70.16%)	76.016% (70.27%-82.432%)	82.51% (78.26%-87.13%)	55.97% (50%-61.62%)	2.7991 (2.6554-2.9429)	69.47% (62.63%-76.77%)	65.41% (56.14%-74.58%)	76.375% (63.158%-87.5%)	82.62% (73.08%-90.91%)	56.26% (44.9%-67.92%)	2.8066 (2.5235-3.0916)
At least 4 of FATHMM (cancer), FATHMM (missense), PROVEAN, VEST	56.96% (53.03%-60.61%)	37.37% (32.52%-42.28%)	90.821% (87.013%-94.595%)	87.56% (82.69%-92.73%)	45.61% (40.82%-50%)	2.6137 (2.4821-2.7421)	56.78% (49.49%-64.65%)	36.92% (27.12%-46.67%)	90.815% (82.857%-97.5%)	87.32% (76%-96.55%)	45.62% (36.62%-55.71%)	2.6068 (2.3454-2.8688)
At least 4 of FATHMM (cancer), FATHMM (missense), SIFT, VEST	63.29% (59.6%-67.17%)	51.66% (46.4%-56.25%)	83.379% (78.571%-89.041%)	84.32% (80%-89.19%)	49.94% (44.54%-54.7%)	2.6930 (2.5592-2.8326)	63.32% (55.56%-70.71%)	51.43% (42.37%-61.9%)	83.658% (72.5%-92.5%)	84.4% (75%-92.86%)	50.08% (40.32%-60.66%)	2.6956 (2.4153-2.9619)
At least 4 of FATHMM (cancer), Mutation Assessor, MutationTaster, PolyPhen-2	70% (66.16%-73.74%)	56.89% (52%-61.74%)	92.677% (89.55%-96.055%)	93.07% (90%-96.34%)	55.42% (50.79%-60.17%)	2.9805 (2.8650-3.0942)	70.1% (62.63%-77.78%)	56.96% (46.67%-67.4%)	92.634% (85.294%-100%)	92.98% (86.05%-100%)	55.63% (45.76%-65.46%)	2.9821 (2.7547-3.2152)
At least 4 of FATHMM (cancer), Mutation Assessor, MutationTaster, PolyPhen-2, PROVEAN	73.36% (70.19%-77.27%)	67% (62.4%-71.88%)	84.366% (79.71%-89.189%)	88.11% (84.54%-91.84%)	59.65% (54.45%-65.1%)	2.9912 (2.8634-3.1236)	73.47% (65.66%-79.82%)	67.04% (57.38%-76.19%)	84.496% (75%-94.29%)	88.11% (80.43%-95.65%)	59.9% (49.12%-70.38%)	2.9955 (2.7290-3.2573)
At least 4 of FATHMM (cancer), Mutation Assessor, MutationTaster, PolyPhen-2, SIFT	77.07% (73.74%-80.32%)	74.45% (70.16%-78.86%)	81.587% (76.71%-87.324%)	87.48% (83.96%-91.35%)	64.88% (59.34%-70.46%)	3.0840 (2.9518-3.2188)	77.17% (70.68%-83.84%)	74.47% (65.57%-83.05%)	81.805% (71.429%-91.9%)	87.51% (80%-94.44%)	65.12% (54.05%-76.09%)	3.0891 (2.8157-3.3528)
At least 4 of FATHMM (cancer), Mutation Assessor, MutationTaster, PolyPhen-2, VEST	76.41% (72.73%-79.8%)	74.46% (70.16%-78.91%)	79.781% (74.641%-85.531%)	86.42% (83%-90.48%)	64.37% (59.04%-70.12%)	3.0503 (2.9144-3.1865)	76.48% (69.7%-83.84%)	74.47% (65.52%-83.08%)	79.917% (68.742%-90%)	86.41% (78.57%-93.48%)	64.59% (53.19%-75%)	3.0539 (2.7959-3.3247)
At least 4 of FATHMM (cancer), Mutation Assessor, MutationTaster, PROVEAN	60.97% (57.07%-64.65%)	40.5% (35.48%-45.53%)	96.354% (94.203%-98.667%)	95.05% (92%-98.21%)	48.36% (43.62%-53.1%)	2.8026 (2.6982-2.9128)	60.9% (53.54%-68.69%)	40.27% (29.84%-50.02%)	96.276% (91.176%-100%)	94.88% (87.5%-100%)	48.43% (38.89%-58.11%)	2.7985 (2.5789-3.0132)
At least 4 of FATHMM (cancer), Mutation Assessor, MutationTaster, PROVEAN, SIFT	74.36% (71.2%-77.78%)	68.03% (63.33%-72.66%)	85.292% (80.282%-90.143%)	88.89% (85.29%-92.71%)	60.67% (55.44%-65.98%)	3.0288 (2.9008-3.1594)	74.52% (67.68%-80.83%)	68.17% (58.73%-77.19%)	85.378% (75.756%-94.87%)	88.89% (81.25%-95.74%)	60.98% (50%-71.43%)	3.0342 (2.7730-3.2945)
At least 4 of FATHMM (cancer), Mutation Assessor, MutationTaster, PROVEAN, VEST	71.71% (68.18%-75.27%)	64.37% (59.84%-69.11%)	84.392% (79.704%-89.552%)	87.7% (83.87%-91.86%)	57.8% (52.43%-63.21%)	2.9426 (2.8065-3.0817)	71.74% (64.62%-78.79%)	64.32% (55%-73.68%)	84.441% (74.417%-94.44%)	87.64% (79.49%-95.45%)	57.96% (47.27%-68.52%)	2.9436 (2.6714-3.2184)
At least 4 of FATHMM (cancer), Mutation Assessor, MutationTaster, SIFT	71.71% (68.18%-75.25%)	59.03% (54.1%-63.85%)	93.622% (90.541%-97.183%)	94.12% (91.36%-97.44%)	56.92% (52%-61.79%)	3.0369 (2.9285-3.1559)	71.74% (64.65%-78.79%)	59.05% (49.21%-68.86%)	93.483% (86.667%-100%)	93.96% (87.18%-100%)	57.09% (47.37%-67.8%)	3.0358 (2.8031-3.2684)
At least 4 of FATHMM (cancer), Mutation Assessor, MutationTaster, SIFT, VEST	76.41% (73.23%-79.8%)	73.4% (69.1%-77.6%)	81.612% (76.712%-86.961%)	87.34% (83.65%-91.26%)	63.96% (58.42%-69.39%)	3.0631 (2.9263-3.1968)	76.47% (69.7%-82.83%)	73.39% (64.62%-81.82%)	81.75% (71.053%-91.67%)	87.33% (79.63%-94.44%)	64.15% (53.17%-75%)	3.0661 (2.7937-3.3288)
At least 4 of FATHMM (cancer), Mutation Assessor, MutationTaster, VEST	67.33% (63.64%-71.21%)	52.67% (47.46%-57.7%)	92.677% (89.55%-96.055%)	92.55% (89.39%-96.16%)	53.11% (48.09%-58.2%)	2.9101 (2.7957-3.0323)	67.36% (59.6%-74.75%)	52.62% (42.42%-62.91%)	92.634% (85.294%-100%)	92.44% (84.85%-100%)	53.26% (43.33%-63.5%)	2.9096 (2.6756-3.1575)
At least 4 of FATHMM (cancer), Mutation Assessor, PolyPhen-2, PROVEAN	58.6% (55.04%-62.63%)	41.56% (36.8%-46.62%)	88.043% (83.823%-92.424%)	85.73% (80.65%-91.08%)	46.56% (41.73%-51.13%)	2.6189 (2.4839-2.7567)	58.56% (50.51%-65.68%)	41.32% (31.14%-50.85%)	88.138% (80%-97.06%)	85.64% (74.19%-96.43%)	46.67% (37.5%-56.52%)	2.6176 (2.3467-2.8897)
At least 4 of FATHMM (cancer), Mutation Assessor, PolyPhen-2, PROVEAN, SIFT	71.31% (68.17%-75.25%)	66.98% (62.69%-71.93%)	78.79% (73.437%-84.512%)	84.52% (80.43%-88.78%)	57.99% (52.53%-63.44%)	2.8828 (2.7499-3.0230)	71.51% (63.64%-77.8%)	67.07% (57.14%-75.81%)	79.102% (67.568%-89.75%)	84.64% (76.08%-92.31%)	58.32% (47.17%-69.09%)	2.8912 (2.6173-3.1516)
At least 4 of FATHMM (cancer), Mutation Assessor, PolyPhen-2, PROVEAN, VEST	68.3% (64.65%-72.22%)	62.77% (57.98%-67.77%)	77.854% (72.222%-83.785%)	83.05% (78.72%-87.64%)	54.74% (49.47%-60.58%)	2.7841 (2.6431-2.9343)	68.45% (60.61%-75.76%)	62.73% (53.02%-71.88%)	78.247% (67.499%-89.47%)	83.17% (74%-91.67%)	55.02% (43.86%-65.53%)	2.7917 (2.4992-3.0760)
At least 4 of FATHMM (cancer), Mutation Assessor, PolyPhen-2, SIFT	69.33% (65.66%-73.23%)	60.1% (55.47%-65.15%)	85.281% (80.556%-90.278%)	87.59% (83.69%-91.86%)	55.28% (50%-60.36%)	2.8825 (2.7481-3.0133)	69.42% (61.62%-76.77%)	60.09% (50%-68.86%)	85.402% (76.182%-94.74%)	87.59% (79.16%-95.35%)	55.49% (45.45%-65.45%)	2.8858 (2.6182-3.1537)
At least 4 of FATHMM (cancer), Mutation Assessor, PolyPhen-2, SIFT, VEST	72.37% (68.69%-75.76%)	73.41% (69.23%-78.03%)	70.575% (64.789%-76.712%)	81.18% (77.06%-85.34%)	60.55% (54.65%-66.29%)	2.8571 (2.7099-3.0053)	72.43% (65.66%-79.8%)	73.37% (64.06%-81.97%)	70.791% (58.621%-82.76%)	81.15% (72.58%-89.29%)	60.77% (48.78%-72.34%)	2.8608 (2.5613-3.1474)
At least 4 of FATHMM (cancer), Mutation Assessor, PolyPhen-2, VEST	63.27% (59.6%-67.17%)	52.13% (47.29%-57.38%)	82.531% (77.499%-87.882%)	83.76% (79.22%-88.89%)	49.93% (44.83%-54.87%)	2.6835 (2.5394-2.8322)	63.35% (55.56%-70.71%)	52.09% (41.67%-61.54%)	82.666% (72.5%-92.59%)	83.75% (73.68%-92.69%)	50.13% (40.74%-60.01%)	2.6864 (2.3917-2.9752)
At least 4 of FATHMM (cancer), Mutation Assessor, PROVEAN, SIFT	59.61% (55.56%-63.64%)	43.17% (38.4%-48.25%)	88.024% (83.582%-92.648%)	86.17% (81.03%-91.53%)	47.25% (42.42%-51.88%)	2.6461 (2.5129-2.7838)	59.57% (51.52%-67.68%)	42.89% (32.79%-52.64%)	88.172% (79.301%-97.06%)	86.14% (75%-96.67%)	47.36% (37.87%-57.14%)	2.6457 (2.3698-2.9065)
At least 4 of FATHMM (cancer), Mutation Assessor, PROVEAN, SIFT, VEST	69% (65.15%-72.73%)	63.82% (59.06%-68.75%)	77.926% (72.464%-83.784%)	83.33% (78.95%-88%)	55.47% (50%-60.82%)	2.8055 (2.6675-2.9500)	69.08% (61.62%-76.77%)	63.81% (53.85%-73.34%)	78.087% (66.667%-88.24%)	83.31% (74%-91.84%)	55.69% (45%-66.67%)	2.8090 (2.5240-3.0864)
At least 4 of FATHMM (cancer), Mutation Assessor, PROVEAN, VEST	57.26% (53.03%-61.62%)	39.44% (34.64%-44.26%)	88.068% (83.823%-92.685%)	85.11% (79.66%-90.91%)	45.68% (40.71%-50.36%)	2.5829 (2.4421-2.7235)	57.2% (48.48%-65.66%)	39.19% (28.98%-49.12%)	88.083% (79.31%-97.06%)	84.94% (73.33%-96.43%)	45.77% (36.51%-55.56%)	2.5799 (2.2922-2.8617)
At least 4 of FATHMM (cancer), Mutation Assessor, SIFT, VEST	65.65% (61.62%-69.7%)	54.28% (49.59%-59.09%)	85.307% (80.556%-90.281%)	86.46% (82.19%-91.14%)	51.9% (46.77%-57.14%)	2.7794 (2.6447-2.9187)	65.67% (57.58%-73.74%)	54.19% (44.26%-63.77%)	85.347% (75%-94.74%)	86.39% (77.14%-94.87%)	52.04% (41.82%-62.3%)	2.7798 (2.5024-3.0497)
At least 4 of FATHMM (cancer), MutationTaster, PolyPhen-2, PROVEAN	62.99% (59.09%-66.67%)	43.69% (38.52%-48.47%)	96.354% (94.203%-98.667%)	95.39% (92.59%-98.33%)	49.74% (44.78%-54.36%)	2.8517 (2.7440-2.9622)	62.91% (55.56%-70.71%)	43.45% (33.33%-53.58%)	96.276% (91.176%-100%)	95.23% (88.46%-100%)	49.8% (40.28%-60%)	2.8476 (2.6297-3.0662)
At least 4 of FATHMM (cancer), MutationTaster, PolyPhen-2, PROVEAN, SIFT	75.38% (72.22%-78.79%)	70.19% (65.87%-74.6%)	84.333% (79.167%-89.233%)	88.57% (85.15%-92.55%)	62.07% (56.84%-67.35%)	3.0516 (2.9300-3.1808)	75.51% (68.69%-81.82%)	70.23% (61.29%-78.69%)	84.536% (74.286%-94.6%)	88.64% (81.03%-95.83%)	62.32% (52%-72.73%)	3.0572 (2.8026-3.2997)
At least 4 of FATHMM (cancer), MutationTaster, PolyPhen-2, PROVEAN, VEST	73.37% (69.7%-76.77%)	67.02% (62.5%-71.88%)	84.364% (79.73%-89.19%)	88.11% (84.52%-92.14%)	59.66% (54.37%-65.05%)	2.9915 (2.8657-3.1277)	73.45% (66.67%-80.81%)	67.01% (57.14%-76.19%)	84.488% (75%-93.94%)	88.11% (80%-95.65%)	59.87% (49.06%-70.21%)	2.9948 (2.7280-3.2498)
At least 4 of FATHMM (cancer), MutationTaster, PolyPhen-2, SIFT	73.38% (70.19%-76.77%)	61.68% (56.91%-66.92%)	93.622% (90.541%-97.183%)	94.36% (91.57%-97.53%)	58.56% (53.57%-63.56%)	3.0821 (2.9732-3.1892)	73.43% (66.67%-79.82%)	61.74% (51.61%-70.91%)	93.483% (86.667%-100%)	94.2% (87.88%-100%)	58.74% (49.12%-68.64%)	3.0816 (2.8603-3.3007)
At least 4 of FATHMM (cancer), MutationTaster, PolyPhen-2, SIFT, VEST	77.43% (74.24%-80.81%)	76.07% (71.97%-80.18%)	79.773% (74.648%-85.507%)	86.67% (83.04%-90.65%)	65.85% (60.24%-71.28%)	3.0837 (2.9537-3.2166)	77.46% (70.71%-83.84%)	76.03% (67.69%-84.38%)	79.92% (68.966%-90%)	86.65% (78.95%-94%)	66.01% (54.35%-76.6%)	3.0861 (2.8165-3.3398)
At least 4 of FATHMM (cancer), MutationTaster, PolyPhen-2, VEST	70.35% (66.67%-74.24%)	57.43% (52.75%-62.61%)	92.677% (89.55%-96.055%)	93.13% (90.24%-96.34%)	55.74% (50.8%-60.83%)	2.9897 (2.8759-3.1127)	70.42% (62.63%-77.78%)	57.47% (47.46%-66.67%)	92.634% (85.294%-100%)	93.03% (85.71%-100%)	55.92% (45.76%-66.67%)	2.9905 (2.7536-3.2253)
At least 4 of FATHMM (cancer), MutationTaster, PROVEAN, SIFT	64.32% (60.61%-68.18%)	45.79% (40.77%-51.15%)	96.354% (94.203%-98.667%)	95.59% (92.86%-98.39%)	50.69% (45.77%-55.4%)	2.8842 (2.7804-2.9868)	64.29% (56.57%-71.72%)	45.64% (35.29%-56.06%)	96.276% (91.176%-100%)	95.45% (89.29%-100%)	50.79% (41.18%-60.56%)	2.8816 (2.6764-3.0936)
At least 4 of FATHMM (cancer), MutationTaster, PROVEAN, SIFT, VEST	72.37% (68.69%-76.26%)	65.42% (60.8%-70.08%)	84.389% (79.71%-89.855%)	87.87% (84%-92.13%)	58.53% (53.4%-64.08%)	2.9620 (2.8269-3.0980)	72.43% (64.65%-79.8%)	65.42% (55.73%-74.61%)	84.421% (74.074%-94.12%)	87.82% (79.55%-95.46%)	58.71% (48.08%-69.09%)	2.9637 (2.6924-3.2361)
At least 4 of FATHMM (cancer), MutationTaster, PROVEAN, VEST	62.32% (58.57%-66.16%)	42.63% (37.4%-47.54%)	96.354% (94.203%-98.667%)	95.28% (92.45%-98.28%)	49.27% (44.37%-53.9%)	2.8354 (2.7268-2.9383)	62.23% (54.55%-69.72%)	42.38% (32.72%-52.46%)	96.276% (91.176%-100%)	95.12% (88.46%-100%)	49.34% (40%-59.09%)	2.8311 (2.6251-3.0442)
At least 4 of FATHMM (cancer), MutationTaster, SIFT, VEST	71.05% (67.17%-74.75%)	57.99% (53.17%-62.79%)	93.622% (90.541%-97.183%)	94.02% (91.14%-97.4%)	56.31% (51.22%-61.02%)	3.0193 (2.9052-3.1327)	71.04% (63.64%-78.79%)	57.95% (47.76%-67.74%)	93.483% (86.667%-100%)	93.85% (87.18%-100%)	56.43% (46.55%-67.21%)	3.0171 (2.7868-3.2434)
At least 4 of FATHMM (cancer), PolyPhen-2, PROVEAN, SIFT	60.93% (57.07%-64.66%)	45.8% (40.65%-50.78%)	87.064% (82.609%-91.549%)	85.96% (81.25%-90.91%)	48.16% (43.18%-52.94%)	2.6699 (2.5357-2.8086)	60.98% (53.51%-68.69%)	45.6% (35.38%-55.94%)	87.329% (78.372%-96.77%)	86.05% (75.76%-96.55%)	48.33% (38.89%-58.22%)	2.6731 (2.3855-2.9405)
At least 4 of FATHMM (cancer), PolyPhen-2, PROVEAN, VEST	58.93% (55.04%-63.13%)	42.09% (37.01%-47.11%)	88.04% (83.824%-92.426%)	85.88% (80.95%-91.05%)	46.79% (41.79%-51.47%)	2.6280 (2.4864-2.7635)	58.91% (50.51%-66.69%)	41.86% (32.14%-51.74%)	88.13% (79.412%-96.97%)	85.8% (74.98%-96.43%)	46.9% (37.68%-56.72%)	2.6269 (2.3467-2.9079)
At least 4 of FATHMM (cancer), PolyPhen-2, SIFT, VEST	66.31% (62.63%-70.2%)	56.38% (51.94%-61.42%)	83.467% (78.571%-88.732%)	85.5% (81.25%-90.12%)	52.53% (47.27%-57.63%)	2.7787 (2.6447-2.9208)	66.37% (58.59%-73.74%)	56.37% (46.27%-65.58%)	83.516% (72.973%-93.33%)	85.44% (76.19%-93.94%)	52.72% (42.62%-62.71%)	2.7804 (2.4995-3.0477)
At least 4 of FATHMM (cancer), PROVEAN, SIFT, VEST	59.94% (56.06%-64.14%)	44.21% (39.06%-48.86%)	87.12% (82.667%-91.784%)	85.58% (80.88%-90.63%)	47.46% (42.34%-52.27%)	2.6437 (2.5080-2.7939)	59.92% (51.52%-67.68%)	44% (34.37%-54.24%)	87.214% (77.778%-96.67%)	85.52% (74.19%-95.84%)	47.57% (38.1%-57.97%)	2.6430 (2.3372-2.9118)
At least 4 of FATHMM (missense), Mutation Assessor, MutationTaster, PolyPhen-2	69.04% (65.66%-72.23%)	54.82% (49.59%-59.69%)	93.622% (90.541%-97.183%)	93.7% (90.67%-97.26%)	54.51% (50%-59.17%)	2.9665 (2.8600-3.0760)	68.99% (62.6%-75.76%)	54.7% (44.62%-65%)	93.483% (86.667%-100%)	93.52% (86.49%-100%)	54.59% (45.31%-63.79%)	2.9630 (2.7362-3.1739)
At least 4 of FATHMM (missense), Mutation Assessor, MutationTaster, PROVEAN	58.29% (54.55%-62.12%)	36.27% (31.5%-40.87%)	96.354% (94.203%-98.667%)	94.5% (91.11%-98%)	46.65% (41.89%-51.32%)	2.7377 (2.6371-2.8447)	58.18% (50.51%-65.66%)	35.96% (26.66%-45.9%)	96.276% (91.176%-100%)	94.31% (86.36%-100%)	46.69% (37.66%-55.85%)	2.7324 (2.5117-2.9329)
At least 4 of FATHMM (missense), Mutation Assessor, MutationTaster, SIFT	70.73% (67.17%-74.24%)	57.49% (52.34%-62.2%)	93.622% (90.541%-97.183%)	93.97% (91.14%-97.37%)	56.02% (51.21%-60.8%)	3.0111 (2.9072-3.1238)	70.65% (63.64%-77.78%)	57.33% (47.54%-67.8%)	93.483% (86.667%-100%)	93.8% (86.84%-100%)	56.07% (46.67%-66.07%)	3.0068 (2.7846-3.2151)
At least 4 of FATHMM (missense), Mutation Assessor, MutationTaster, VEST	66.71% (63.13%-70.21%)	51.14% (45.67%-56.06%)	93.622% (90.541%-97.183%)	93.27% (90.16%-97.06%)	52.56% (47.69%-57.35%)	2.9059 (2.7980-3.0182)	66.59% (59.57%-73.74%)	50.9% (40.62%-61.82%)	93.483% (86.667%-100%)	93.07% (85.29%-100%)	52.59% (43.07%-62.32%)	2.9004 (2.6762-3.1176)
At least 4 of FATHMM (missense), Mutation Assessor, PolyPhen-2, PROVEAN	56.27% (52.53%-60.1%)	37.33% (32.77%-42.07%)	88.993% (84.848%-93.058%)	85.43% (80%-90.91%)	45.09% (40.27%-49.65%)	2.5685 (2.4364-2.6981)	56.15% (48.48%-63.64%)	37% (27.27%-46.35%)	88.965% (80.643%-97.22%)	85.2% (73.91%-96.3%)	45.14% (36.11%-55.13%)	2.5631 (2.2840-2.8310)
At least 4 of FATHMM (missense), Mutation Assessor, PolyPhen-2, SIFT	68.37% (64.65%-71.72%)	58.03% (52.94%-62.88%)	86.231% (81.941%-91.026%)	87.94% (83.95%-92.14%)	54.3% (49.12%-59.13%)	2.8650 (2.7424-2.9933)	68.31% (61.62%-75.76%)	57.84% (48.33%-67.65%)	86.228% (76.662%-94.45%)	87.83% (79.54%-95.35%)	54.37% (44.68%-64.29%)	2.8626 (2.6038-3.1073)
At least 4 of FATHMM (missense), Mutation Assessor, PolyPhen-2, VEST	62.99% (59.09%-66.67%)	50.06% (44.91%-55.12%)	85.314% (80.597%-90.281%)	85.5% (81.16%-90.14%)	49.7% (44.44%-54.4%)	2.7057 (2.5705-2.8370)	62.92% (55.56%-70.71%)	49.84% (39.34%-60%)	85.321% (75.676%-94.12%)	85.35% (75.68%-94.12%)	49.78% (40.35%-59.71%)	2.7028 (2.4384-2.9654)
At least 4 of FATHMM (missense), Mutation Assessor, PROVEAN, SIFT	57.61% (53.54%-61.62%)	38.94% (34.38%-43.7%)	89.897% (85.937%-94.118%)	86.96% (82%-92.16%)	45.99% (41.09%-50.38%)	2.6178 (2.4914-2.7468)	57.5% (49.49%-65.66%)	38.58% (28.81%-48.28%)	89.903% (81.571%-97.37%)	86.79% (75.86%-96.43%)	46.03% (36.99%-55.72%)	2.6131 (2.3457-2.8641)
At least 4 of FATHMM (missense), Mutation Assessor, PROVEAN, VEST	55.26% (51.01%-59.09%)	35.2% (30.47%-39.84%)	89.92% (85.937%-94.118%)	85.79% (80.39%-91.84%)	44.52% (39.73%-49.28%)	2.5544 (2.4238-2.6955)	55.14% (47.47%-63.64%)	34.88% (25.67%-44.13%)	89.867% (81.812%-97.37%)	85.52% (73.68%-96.15%)	44.57% (35.52%-54.29%)	2.5484 (2.2702-2.8150)
At least 4 of FATHMM (missense), Mutation Assessor, SIFT, VEST	65.36% (61.62%-69.19%)	52.74% (47.54%-57.85%)	87.159% (83.077%-91.784%)	87.66% (83.56%-92.11%)	51.61% (46.28%-56.56%)	2.7917 (2.6613-2.9217)	65.25% (57.58%-72.73%)	52.47% (42.19%-62.9%)	87.131% (77.419%-94.87%)	87.5% (78.57%-95.24%)	51.64% (41.93%-61.9%)	2.7874 (2.5086-3.0458)
At least 4 of FATHMM (missense), MutationTaster, PolyPhen-2, PROVEAN	58.97% (55.05%-62.63%)	37.34% (32.5%-42.07%)	96.354% (94.203%-98.667%)	94.65% (91.3%-98.04%)	47.07% (42.28%-51.37%)	2.7542 (2.6490-2.8565)	58.83% (51.52%-66.67%)	36.99% (27.53%-46.78%)	96.276% (91.176%-100%)	94.46% (86.96%-100%)	47.1% (38.16%-56.76%)	2.7483 (2.5389-2.9531)
At least 4 of FATHMM (missense), MutationTaster, PolyPhen-2, SIFT	70.39% (67.17%-73.74%)	56.95% (52%-61.91%)	93.622% (90.541%-97.183%)	93.92% (91.02%-97.37%)	55.7% (50.85%-60.47%)	3.0019 (2.8942-3.1119)	70.33% (63.64%-76.77%)	56.82% (46.67%-66.67%)	93.483% (86.667%-100%)	93.75% (86.67%-100%)	55.78% (46.55%-65.46%)	2.9983 (2.7738-3.2143)
At least 4 of FATHMM (missense), MutationTaster, PolyPhen-2, VEST	66.71% (63.13%-70.71%)	51.66% (46.72%-56.56%)	92.727% (89.333%-96%)	92.47% (89.19%-95.83%)	52.59% (47.66%-57.48%)	2.8945 (2.7827-3.0118)	66.58% (58.59%-73.74%)	51.44% (41.53%-61.02%)	92.508% (85.706%-100%)	92.19% (84.85%-100%)	52.6% (43.08%-62.13%)	2.8875 (2.6438-3.1070)
At least 4 of FATHMM (missense), MutationTaster, PROVEAN, SIFT	60.3% (56.55%-64.14%)	39.44% (34.65%-44.1%)	96.354% (94.203%-98.667%)	94.93% (91.84%-98.15%)	47.92% (43.15%-52.41%)	2.7864 (2.6783-2.8874)	60.22% (52.53%-67.7%)	39.18% (29.31%-49.12%)	96.276% (91.176%-100%)	94.76% (87.5%-100%)	47.98% (38.89%-57.36%)	2.7820 (2.5807-2.9943)
At least 4 of FATHMM (missense), MutationTaster, PROVEAN, VEST	58.29% (54.55%-62.12%)	36.28% (31.35%-40.98%)	96.354% (94.203%-98.667%)	94.51% (91.11%-98%)	46.65% (41.72%-51.02%)	2.7379 (2.6322-2.8448)	58.16% (50.51%-65.66%)	35.93% (25.86%-45.76%)	96.276% (91.176%-100%)	94.3% (86.36%-100%)	46.68% (37.5%-56.41%)	2.7319 (2.5153-2.9319)
At least 4 of FATHMM (missense), MutationTaster, SIFT, VEST	68.39% (64.65%-72.22%)	53.79% (48.44%-58.68%)	93.622% (90.541%-97.183%)	93.58% (90.66%-97.22%)	53.95% (49.17%-58.73%)	2.9495 (2.8423-3.0609)	68.28% (60.61%-75.76%)	53.57% (43.93%-63.93%)	93.483% (86.667%-100%)	93.39% (86.11%-100%)	53.98% (44.26%-63.93%)	2.9441 (2.7255-3.1595)
At least 4 of FATHMM (missense), PolyPhen-2, PROVEAN, SIFT	57.6% (53.54%-61.62%)	39.45% (34.62%-44.26%)	88.946% (84.848%-93.058%)	86.06% (81.03%-91.38%)	45.93% (40.88%-50.36%)	2.6040 (2.4805-2.7307)	57.54% (49.49%-65.66%)	39.14% (29.09%-49.15%)	89.045% (80.556%-97.22%)	86% (75%-96.3%)	46.02% (36.76%-56.16%)	2.6020 (2.3455-2.8451)
At least 4 of FATHMM (missense), PolyPhen-2, PROVEAN, VEST	55.25% (51.01%-59.09%)	35.74% (30.47%-40.34%)	88.969% (84.932%-93.056%)	84.85% (79.25%-90.74%)	44.47% (39.46%-49.28%)	2.5403 (2.4066-2.6706)	55.16% (47.47%-63.64%)	35.4% (25.76%-45.45%)	89.009% (80.645%-97.22%)	84.67% (72.72%-95.84%)	44.53% (35.29%-54.67%)	2.5361 (2.2690-2.8066)
At least 4 of FATHMM (missense), PolyPhen-2, SIFT, VEST	62.97% (59.09%-67.17%)	51.65% (46.67%-56.59%)	82.532% (77.631%-87.838%)	83.64% (78.75%-88.47%)	49.68% (44.54%-54.62%)	2.6750 (2.5399-2.8199)	62.95% (54.55%-70.71%)	51.45% (41.27%-61.4%)	82.606% (71.429%-92.31%)	83.55% (73.53%-92.86%)	49.78% (39.99%-59.68%)	2.6739 (2.3866-2.9549)
At least 4 of FATHMM (missense), PROVEAN, SIFT, VEST	56.93% (53.03%-60.61%)	37.86% (32.8%-42.43%)	89.874% (86.11%-94.205%)	86.61% (81.63%-92.31%)	45.55% (40.56%-50%)	2.5989 (2.4687-2.7351)	56.85% (49.49%-64.65%)	37.54% (28.33%-47.62%)	89.948% (81.579%-97.3%)	86.51% (75%-96.3%)	45.63% (36.48%-55.56%)	2.5962 (2.3203-2.8501)
At least 4 of Mutation Assessor, MutationTaster, PolyPhen-2, PROVEAN	60.61% (56.57%-64.15%)	39.93% (34.74%-44.63%)	96.354% (94.203%-98.667%)	94.98% (92%-98.18%)	48.12% (43.36%-52.78%)	2.7939 (2.6890-2.8985)	60.6% (53.51%-68.69%)	39.8% (30.16%-50%)	96.276% (91.176%-100%)	94.83% (87.5%-100%)	48.24% (39.13%-58.11%)	2.7914 (2.5825-2.9991)
At least 4 of Mutation Assessor, MutationTaster, PolyPhen-2, SIFT	72.7% (69.19%-76.26%)	62.19% (57.6%-67.21%)	90.873% (87.342%-94.872%)	92.17% (89.16%-95.61%)	58.16% (53.4%-63.39%)	3.0339 (2.9183-3.1588)	72.78% (65.66%-79.8%)	62.3% (52.23%-71.43%)	90.735% (82.855%-97.3%)	92.02% (84.61%-97.73%)	58.38% (48.21%-68.33%)	3.0343 (2.7893-3.2703)
At least 4 of Mutation Assessor, MutationTaster, PolyPhen-2, VEST	68.01% (64.65%-71.72%)	55.33% (50.39%-60.48%)	89.928% (86.301%-94.122%)	90.47% (87.01%-94.74%)	53.8% (48.74%-58.82%)	2.8952 (2.7773-3.0210)	68.02% (60.61%-74.75%)	55.27% (45%-65.15%)	89.886% (81.081%-97.22%)	90.35% (81.82%-97.3%)	53.94% (43.86%-63.49%)	2.8944 (2.6417-3.1282)
At least 4 of Mutation Assessor, MutationTaster, PROVEAN, SIFT	61.62% (57.58%-65.66%)	41.54% (36.44%-46.34%)	96.354% (94.203%-98.667%)	95.17% (92.31%-98.25%)	48.8% (43.97%-53.57%)	2.8186 (2.7141-2.9263)	61.6% (53.54%-69.7%)	41.38% (31.5%-51.56%)	96.276% (91.176%-100%)	95.01% (88%-100%)	48.9% (39.47%-58.67%)	2.8157 (2.5965-3.0288)
At least 4 of Mutation Assessor, MutationTaster, PROVEAN, VEST	59.6% (55.56%-63.64%)	38.34% (33.33%-43.09%)	96.354% (94.203%-98.667%)	94.78% (91.67%-98.11%)	47.47% (42.76%-52.09%)	2.7695 (2.6629-2.8751)	59.59% (51.52%-67.68%)	38.2% (28.33%-48.39%)	96.276% (91.176%-100%)	94.62% (86.96%-100%)	47.58% (38.03%-57.5%)	2.7667 (2.5458-2.9873)
At least 4 of Mutation Assessor, MutationTaster, SIFT, VEST	70.05% (66.67%-73.74%)	58.01% (53.17%-62.99%)	90.873% (87.342%-94.872%)	91.66% (88.46%-95.35%)	55.58% (50.82%-60.55%)	2.9612 (2.8464-3.0884)	70% (62.63%-76.77%)	57.9% (47.82%-67.74%)	90.735% (82.855%-97.3%)	91.46% (83.71%-97.56%)	55.67% (45.61%-65.57%)	2.9577 (2.7137-3.1879)
At least 4 of Mutation Assessor, PolyPhen-2, PROVEAN, SIFT	58.57% (54.55%-62.63%)	42.6% (37.6%-47.66%)	86.167% (81.429%-90.669%)	84.19% (79.31%-89.4%)	46.47% (41.54%-51.15%)	2.5943 (2.4606-2.7301)	58.62% (50.51%-66.67%)	42.43% (32.2%-52.38%)	86.368% (77.143%-95%)	84.22% (73.33%-93.94%)	46.64% (37.14%-56.72%)	2.5965 (2.3133-2.8638)
At least 4 of Mutation Assessor, PolyPhen-2, PROVEAN, VEST	56.56% (52.53%-60.61%)	38.87% (34.15%-43.75%)	87.139% (82.667%-91.667%)	83.93% (78.57%-89.48%)	45.19% (40.29%-50%)	2.5513 (2.4090-2.6926)	56.58% (48.48%-64.65%)	38.73% (28.57%-48.33%)	87.183% (78.378%-95.46%)	83.82% (72%-93.76%)	45.32% (35.93%-55.22%)	2.5505 (2.2603-2.8275)
At least 4 of Mutation Assessor, PolyPhen-2, SIFT, VEST	64.99% (61.62%-69.19%)	56.94% (52.34%-62.1%)	78.91% (73.846%-84.376%)	82.35% (77.65%-87.21%)	51.45% (46.23%-56.88%)	2.6965 (2.5566-2.8426)	64.97% (56.57%-71.72%)	56.84% (46.66%-66.67%)	78.867% (67.568%-89.48%)	82.17% (72.5%-91.18%)	51.56% (40.81%-61.11%)	2.6945 (2.3891-2.9713)
At least 4 of Mutation Assessor, PROVEAN, SIFT, VEST	56.9% (53.03%-61.11%)	40.48% (35.66%-45.19%)	85.299% (80.519%-90.144%)	82.64% (77.05%-88.06%)	45.32% (40.42%-50%)	2.5373 (2.4018-2.6759)	56.9% (48.48%-64.65%)	40.31% (30.36%-50%)	85.359% (75.676%-94.74%)	82.52% (71.43%-93.55%)	45.45% (36.11%-55.22%)	2.5363 (2.2515-2.8125)
At least 4 of MutationTaster, PolyPhen-2, PROVEAN, SIFT	62.96% (59.09%-66.67%)	43.64% (38.65%-48.44%)	96.354% (94.203%-98.667%)	95.39% (92.45%-98.33%)	49.72% (44.75%-54.35%)	2.8510 (2.7424-2.9563)	62.97% (55.56%-70.71%)	43.55% (33.81%-53.57%)	96.276% (91.176%-100%)	95.24% (88.57%-100%)	49.85% (40.54%-59.75%)	2.8491 (2.6363-3.0693)
At least 4 of MutationTaster, PolyPhen-2, PROVEAN, VEST	61.28% (57.58%-65.15%)	40.99% (35.77%-45.67%)	96.354% (94.203%-98.667%)	95.1% (92.16%-98.22%)	48.57% (43.79%-53.15%)	2.8102 (2.6996-2.9128)	61.28% (53.54%-68.69%)	40.86% (31.57%-50.82%)	96.276% (91.176%-100%)	94.94% (88%-100%)	48.68% (39.44%-58.82%)	2.8077 (2.6058-3.0228)
At least 4 of MutationTaster, PolyPhen-2, SIFT, VEST	71.05% (67.68%-74.75%)	59.58% (54.69%-64.52%)	90.873% (87.342%-94.872%)	91.86% (88.75%-95.45%)	56.52% (51.31%-61.55%)	2.9883 (2.8745-3.1168)	71.04% (63.64%-77.78%)	59.55% (50%-69.49%)	90.735% (82.855%-97.3%)	91.68% (83.72%-97.62%)	56.65% (46.43%-66.67%)	2.9861 (2.7362-3.2174)
At least 4 of MutationTaster, PROVEAN, SIFT, VEST	62.63% (58.59%-66.16%)	43.12% (38.02%-47.97%)	96.354% (94.203%-98.667%)	95.33% (92.45%-98.28%)	49.49% (44.68%-54.17%)	2.8429 (2.7322-2.9431)	62.62% (55.56%-70.71%)	43% (32.84%-53.04%)	96.276% (91.176%-100%)	95.19% (88.89%-100%)	49.61% (40%-59.7%)	2.8407 (2.6331-3.0630)
At least 4 of PolyPhen-2, PROVEAN, SIFT, VEST	58.56% (54.55%-62.12%)	42.58% (37.6%-47.41%)	86.172% (81.69%-90.789%)	84.19% (79.03%-89.47%)	46.46% (41.3%-51.39%)	2.5940 (2.4603-2.7323)	58.65% (51.49%-66.67%)	42.48% (32.84%-52.54%)	86.353% (76.667%-94.74%)	84.23% (72.97%-94.12%)	46.66% (36.98%-56.72%)	2.5972 (2.3113-2.8656)
At least 1 of CHASM (breast), CHASM (lung), CHASM (melanoma), FATHMM (cancer), FATHMM (missense), Mutation Assessor	70.72% (67.17%-74.75%)	98.97% (98.33%-100%)	21.854% (15.999%-27.397%)	68.65% (64.86%-72.63%)	92.46% (85.71%-100%)	2.8194 (2.6862-2.9472)	70.69% (62.63%-77.78%)	98.86% (96.67%-100%)	22.318% (11.101%-34.15%)	68.6% (60.47%-76.14%)	91.95% (75%-100%)	2.8173 (2.5470-3.0446)
At least 1 of CHASM (breast), CHASM (lung), CHASM (melanoma), FATHMM (cancer), FATHMM (missense), Mutation Assessor, MutationTaster	70.41% (66.67%-74.24%)	99.49% (99.16%-100%)	20.123% (14.662%-25.373%)	68.29% (64.48%-72.29%)	95.82% (91.67%-100%)	2.8373 (2.7268-2.9411)	70.29% (62.63%-77.78%)	99.42% (98.21%-100%)	20.296% (9.674%-31.43%)	68.16% (59.99%-75.83%)	95.32% (80%-100%)	2.8322 (2.5548-3.0358)
At least 1 of CHASM (breast), CHASM (lung), CHASM (melanoma), FATHMM (cancer), FATHMM (missense), Mutation Assessor, PolyPhen-2	69.35% (65.66%-73.23%)	99.48% (99.16%-100%)	17.253% (11.94%-22.059%)	67.52% (63.83%-71.59%)	95.03% (90%-100%)	2.7928 (2.6749-2.9040)	69.38% (61.62%-76.77%)	99.44% (98.25%-100%)	17.756% (8.322%-28.13%)	67.48% (59.34%-74.74%)	94.89% (80%-100%)	2.7957 (2.5272-3.0022)
At least 1 of CHASM (breast), CHASM (lung), CHASM (melanoma), FATHMM (cancer), FATHMM (missense), Mutation Assessor, PROVEAN	69.05% (65.15%-72.74%)	98.97% (98.33%-100%)	17.299% (11.94%-22.222%)	67.42% (63.59%-71.35%)	90.65% (81.82%-100%)	2.7434 (2.5975-2.8803)	68.98% (61.59%-76.77%)	98.86% (96.67%-100%)	17.654% (7.895%-28.21%)	67.33% (59.34%-75.01%)	89.98% (66.67%-100%)	2.7383 (2.4324-2.9783)
At least 1 of CHASM (breast), CHASM (lung), CHASM (melanoma), FATHMM (cancer), FATHMM (missense), Mutation Assessor, SIFT	68.01% (64.14%-71.72%)	100% (100%-100%)	12.694% (7.594%-16.901%)	66.45% (62.63%-70.43%)	100% (100%-100%)	2.7914 (2.7276-2.8596)	68.02% (60.61%-75.76%)	100% (100%-100%)	13.144% (4.875%-22.86%)	66.4% (58.51%-74.19%)	100% (100%-100%)	2.7959 (2.6699-2.9313)
At least 1 of CHASM (breast), CHASM (lung), CHASM (melanoma), FATHMM (cancer), FATHMM (missense), Mutation Assessor, VEST	66.01% (62.12%-70.2%)	100% (100%-100%)	7.229% (3.999%-10.606%)	65.08% (61.14%-69.23%)	100% (100%-100%)	2.7232 (2.6698-2.7811)	65.96% (57.58%-73.74%)	100% (100%-100%)	7.525% (0%-14.63%)	64.99% (56.7%-72.92%)	100% (100%-100%)	2.7275 (2.6238-2.8431)
At least 1 of CHASM (breast), CHASM (lung), CHASM (melanoma), FATHMM (cancer), FATHMM (missense), MutationTaster	73.09% (69.7%-76.77%)	99.49% (99.16%-100%)	27.439% (21.333%-33.333%)	70.33% (66.48%-74.3%)	96.9% (93.75%-100%)	2.9417 (2.8374-3.0455)	73.01% (65.66%-79.8%)	99.42% (98.21%-100%)	27.672% (16.655%-40.54%)	70.23% (62.22%-77.78%)	96.54% (87.5%-100%)	2.9387 (2.7094-3.1382)
At least 1 of CHASM (breast), CHASM (lung), CHASM (melanoma), FATHMM (cancer), FATHMM (missense), MutationTaster, PolyPhen-2	71.36% (68.18%-75.25%)	100% (100%-100%)	21.846% (16.418%-27.143%)	68.87% (65.19%-72.87%)	100% (100%-100%)	2.9072 (2.8317-2.9865)	71.41% (63.64%-77.78%)	100% (100%-100%)	22.342% (12.188%-33.33%)	68.85% (60.67%-76.34%)	100% (100%-100%)	2.9119 (2.7665-3.0724)
At least 1 of CHASM (breast), CHASM (lung), CHASM (melanoma), FATHMM (cancer), FATHMM (missense), MutationTaster, PROVEAN	70.42% (66.67%-74.24%)	99.49% (99.16%-100%)	20.149% (14.493%-25.334%)	68.3% (64.52%-72.05%)	95.83% (91.65%-100%)	2.8377 (2.7307-2.9496)	70.27% (62.63%-77.78%)	99.42% (98.21%-100%)	20.214% (9.992%-31.25%)	68.14% (60.23%-75.87%)	95.31% (80%-100%)	2.8309 (2.5688-3.0245)
At least 1 of CHASM (breast), CHASM (lung), CHASM (melanoma), FATHMM (cancer), FATHMM (missense), MutationTaster, SIFT	69.05% (65.66%-72.73%)	100% (100%-100%)	15.531% (10.446%-20.27%)	67.18% (63.39%-71.28%)	100% (100%-100%)	2.8271 (2.7588-2.9040)	68.97% (61.62%-76.77%)	100% (100%-100%)	15.733% (6.891%-25.71%)	67.07% (58.95%-74.75%)	100% (100%-100%)	2.8280 (2.6896-2.9827)
At least 1 of CHASM (breast), CHASM (lung), CHASM (melanoma), FATHMM (cancer), FATHMM (missense), MutationTaster, VEST	67% (63.13%-70.72%)	100% (100%-100%)	9.941% (5.714%-13.638%)	65.75% (61.9%-69.84%)	100% (100%-100%)	2.7569 (2.6979-2.8202)	67.01% (59.57%-74.75%)	100% (100%-100%)	10.361% (2.941%-18.75%)	65.69% (57.45%-73.41%)	100% (100%-100%)	2.7615 (2.6488-2.8915)

At least 1 of CHASM (breast), CHASM (lung), CHASM (melanoma), FATHMM (cancer), FATHMM (missense), PolyPhen-2	71.35% (68.17%-75.25%)	99.48% (99.16%-100%)	22.713% (16.896%-27.782%)	69% (65.36%-72.93%)	96.19% (92.31%-100%)	2.8738 (2.7647-2.9762)	71.44% (63.64%-77.8%)	99.44% (98.25%-100%)	23.35% (12.903%-34.48%)	69.01% (60.92%-76.35%)	96.05% (83.33%-100%)	2.8785 (2.6380-3.0844)
At least 1 of CHASM (breast), CHASM (lung), CHASM (melanoma), FATHMM (cancer), FATHMM (missense), PolyPhen-2, PROVEAN	69.02% (65.64%-72.73%)	99.48% (99.16%-100%)	16.343% (10.959%-21.127%)	67.28% (63.64%-71.28%)	94.76% (88.89%-100%)	2.7787 (2.6534-2.8886)	69.03% (61.62%-75.78%)	99.44% (98.25%-100%)	16.802% (7.309%-27.59%)	67.23% (59.14%-74.47%)	94.6% (75%-100%)	2.7807 (2.4956-2.9857)
At least 1 of CHASM (breast), CHASM (lung), CHASM (melanoma), FATHMM (cancer), FATHMM (missense), PolyPhen-2, SIFT	69.02% (65.66%-72.73%)	100% (100%-100%)	15.467% (10.145%-20%)	67.16% (63.39%-71.28%)	100% (100%-100%)	2.8263 (2.7569-2.8997)	69.02% (61.62%-75.76%)	100% (100%-100%)	15.865% (6.975%-25.81%)	67.1% (58.94%-74.73%)	100% (100%-100%)	2.8297 (2.6952-2.9833)
At least 1 of CHASM (breast), CHASM (lung), CHASM (melanoma), FATHMM (cancer), FATHMM (missense), PolyPhen-2, VEST	67% (63.13%-70.72%)	100% (100%-100%)	9.941% (5.714%-13.638%)	65.75% (61.9%-69.84%)	100% (100%-100%)	2.7569 (2.6979-2.8202)	67.01% (59.57%-74.75%)	100% (100%-100%)	10.361% (2.941%-18.75%)	65.69% (57.45%-73.41%)	100% (100%-100%)	2.7615 (2.6488-2.8915)
At least 1 of CHASM (breast), CHASM (lung), CHASM (melanoma), FATHMM (cancer), FATHMM (missense), PROVEAN	70.74% (67.17%-74.24%)	97.93% (96.72%-99.24%)	23.708% (17.647%-29.335%)	68.94% (65.14%-72.73%)	86.89% (78.57%-95.24%)	2.7747 (2.6301-2.9189)	70.64% (63.64%-77.78%)	97.74% (94.74%-100%)	24.071% (12.903%-35.9%)	68.85% (61.17%-76.67%)	86.01% (66.67%-100%)	2.7667 (2.4544-3.0370)
At least 1 of CHASM (breast), CHASM (lung), CHASM (melanoma), FATHMM (cancer), FATHMM (missense), PROVEAN, SIFT	68.35% (64.65%-72.22%)	100% (100%-100%)	13.607% (8.568%-18.056%)	66.68% (62.96%-70.59%)	100% (100%-100%)	2.8029 (2.7341-2.8696)	68.36% (60.61%-75.76%)	100% (100%-100%)	14.027% (5.405%-24.24%)	66.63% (58.94%-73.96%)	100% (100%-100%)	2.8067 (2.6825-2.9599)
At least 1 of CHASM (breast), CHASM (lung), CHASM (melanoma), FATHMM (cancer), FATHMM (missense), PROVEAN, VEST	65.69% (62.11%-69.7%)	99.5% (99.16%-100%)	7.218% (3.896%-10.256%)	64.97% (60.94%-68.95%)	89.41% (75%-100%)	2.6110 (2.4221-2.7594)	65.59% (57.58%-72.75%)	99.4% (98.25%-100%)	7.551% (0%-15.15%)	64.86% (56.84%-72.73%)	88.22% (50%-100%)	2.6011 (2.1511-2.8326)
At least 1 of CHASM (breast), CHASM (lung), CHASM (melanoma), FATHMM (cancer), FATHMM (missense), SIFT	69.68% (66.16%-73.23%)	100% (100%-100%)	17.263% (11.94%-22.059%)	67.64% (63.78%-71.66%)	100% (100%-100%)	2.8490 (2.7777-2.9223)	69.72% (62.63%-76.77%)	100% (100%-100%)	17.756% (8.108%-28.57%)	67.6% (59.57%-75.27%)	100% (100%-100%)	2.8536 (2.7169-3.0048)
At least 1 of CHASM (breast), CHASM (lung), CHASM (melanoma), FATHMM (cancer), FATHMM (missense), SIFT, VEST	66.36% (62.63%-70.2%)	100% (100%-100%)	8.196% (4.286%-11.594%)	65.32% (61.46%-69.43%)	100% (100%-100%)	2.7352 (2.6799-2.7972)	66.27% (58.59%-73.74%)	100% (100%-100%)	8.362% (2.12%-15.79%)	65.19% (56.84%-72.92%)	100% (100%-100%)	2.7376 (2.6270-2.8596)
At least 1 of CHASM (breast), CHASM (lung), CHASM (melanoma), FATHMM (cancer), FATHMM (missense), VEST	66.68% (63.13%-70.71%)	99.5% (99.16%-100%)	9.941% (5.714%-13.638%)	65.64% (61.78%-69.79%)	92.06% (80%-100%)	2.6714 (2.5195-2.7987)	66.63% (58.59%-73.74%)	99.4% (98.25%-100%)	10.361% (2.941%-18.75%)	65.56% (57.29%-73.4%)	91.14% (66.67%-100%)	2.6652 (2.3159-2.8754)
At least 1 of CHASM (breast), CHASM (lung), CHASM (melanoma), FATHMM (cancer), Mutation Assessor, MutationTaster	74.13% (70.71%-77.78%)	99.49% (99.16%-100%)	30.272% (24.323%-36.235%)	71.16% (67.41%-75.28%)	97.19% (94.74%-100%)	2.9811 (2.8806-3.0919)	73.97% (66.67%-80.81%)	99.42% (98.21%-100%)	30.289% (18.742%-42.31%)	70.99% (62.78%-78.82%)	96.84% (87.5%-100%)	2.9754 (2.7497-3.1688)
At least 1 of CHASM (breast), CHASM (lung), CHASM (melanoma), FATHMM (cancer), Mutation Assessor, MutationTaster, PolyPhen-2	72.74% (69.19%-76.28%)	100% (100%-100%)	25.61% (19.993%-31.081%)	69.92% (66.11%-74.01%)	100% (100%-100%)	2.9553 (2.8803-3.0416)	72.7% (65.63%-79.8%)	100% (100%-100%)	25.827% (14.625%-37.5%)	69.82% (61.79%-77.53%)	100% (100%-100%)	2.9565 (2.8012-3.1156)
At least 1 of CHASM (breast), CHASM (lung), CHASM (melanoma), FATHMM (cancer), Mutation Assessor, MutationTaster, PROVEAN	72.13% (68.69%-75.76%)	99.49% (99.16%-100%)	24.805% (19.231%-30.161%)	69.58% (65.71%-73.63%)	96.59% (93.33%-100%)	2.9047 (2.8048-3.0133)	71.91% (64.65%-78.79%)	99.42% (98.21%-100%)	24.685% (13.786%-36.12%)	69.38% (61.11%-77.02%)	96.15% (85.71%-100%)	2.8963 (2.6549-3.0834)
At least 1 of CHASM (breast), CHASM (lung), CHASM (melanoma), FATHMM (cancer), Mutation Assessor, MutationTaster, SIFT	71.09% (67.68%-74.76%)	100% (100%-100%)	21.112% (15.385%-26.232%)	68.67% (64.84%-72.83%)	100% (100%-100%)	2.8978 (2.8237-2.9775)	70.95% (63.61%-77.78%)	100% (100%-100%)	21.114% (10.808%-32.26%)	68.51% (60.22%-76.09%)	100% (100%-100%)	2.8962 (2.7474-3.0514)
At least 1 of CHASM (breast), CHASM (lung), CHASM (melanoma), FATHMM (cancer), Mutation Assessor, MutationTaster, VEST	67.39% (63.64%-71.21%)	100% (100%-100%)	10.999% (6.849%-15.068%)	66.02% (62.11%-70.16%)	100% (100%-100%)	2.7702 (2.7116-2.8385)	67.24% (59.6%-74.75%)	100% (100%-100%)	11.015% (2.778%-19.44%)	65.86% (57.72%-73.47%)	100% (100%-100%)	2.7694 (2.6467-2.8957)
At least 1 of CHASM (breast), CHASM (lung), CHASM (melanoma), FATHMM (cancer), Mutation Assessor, PolyPhen-2	72.39% (68.69%-75.77%)	98.94% (98.33%-100%)	26.476% (20.548%-32.394%)	69.94% (66.11%-74.01%)	93.54% (88.24%-100%)	2.8890 (2.7668-3.0057)	72.39% (65.63%-79.8%)	98.92% (96.67%-100%)	26.835% (15.148%-38.89%)	69.88% (61.63%-77.53%)	93.53% (80%-100%)	2.8916 (2.6405-3.1110)
At least 1 of CHASM (breast), CHASM (lung), CHASM (melanoma), FATHMM (cancer), Mutation Assessor, PolyPhen-2, PROVEAN	70.73% (67.17%-74.24%)	98.94% (98.33%-100%)	21.93% (16.438%-26.867%)	68.67% (64.97%-72.68%)	92.31% (85.71%-100%)	2.8185 (2.6938-2.9388)	70.67% (63.64%-77.78%)	98.92% (96.67%-100%)	22.142% (11.421%-33.33%)	68.56% (60.44%-76.14%)	92.27% (75%-100%)	2.8189 (2.5570-3.0441)
At least 1 of CHASM (breast), CHASM (lung), CHASM (melanoma), FATHMM (cancer), Mutation Assessor, PolyPhen-2, SIFT	70.73% (67.17%-74.26%)	99.46% (99.16%-100%)	21.052% (15.189%-26.473%)	68.54% (64.67%-72.68%)	95.77% (91.67%-100%)	2.8483 (2.7411-2.9591)	70.66% (63.61%-77.78%)	99.48% (98.25%-100%)	21.208% (10.526%-32.5%)	68.42% (60.21%-76.14%)	95.94% (83.33%-100%)	2.8505 (2.6055-3.0459)
At least 1 of CHASM (breast), CHASM (lung), CHASM (melanoma), FATHMM (cancer), Mutation Assessor, PolyPhen-2, VEST	67.05% (63.64%-70.72%)	100% (100%-100%)	10.073% (5.797%-13.893%)	65.79% (61.98%-69.79%)	100% (100%-100%)	2.7586 (2.7019-2.8237)	66.91% (59.57%-73.74%)	100% (100%-100%)	10.101% (2.632%-17.95%)	65.63% (57.45%-73.2%)	100% (100%-100%)	2.7584 (2.6401-2.8847)
At least 1 of CHASM (breast), CHASM (lung), CHASM (melanoma), FATHMM (cancer), Mutation Assessor, PROVEAN	72.42% (68.69%-76.26%)	98.44% (97.52%-100%)	27.432% (21.33%-33.333%)	70.11% (66.11%-74.14%)	91.03% (85%-100%)	2.8700 (2.7456-2.9902)	72.33% (64.65%-79.8%)	98.34% (95.45%-100%)	27.682% (16.129%-40%)	70% (62.07%-77.78%)	90.67% (75%-100%)	2.8669 (2.6114-3.0861)
At least 1 of CHASM (breast), CHASM (lung), CHASM (melanoma), FATHMM (cancer), Mutation Assessor, PROVEAN, SIFT	70.38% (66.67%-74.24%)	99.46% (99.16%-100%)	20.084% (14.706%-25.352%)	68.27% (64.32%-72.28%)	95.59% (91.67%-100%)	2.8341 (2.7268-2.9411)	70.36% (62.63%-77.78%)	99.48% (98.25%-100%)	20.379% (9.754%-30.56%)	68.19% (60.21%-76.09%)	95.79% (83.25%-100%)	2.8384 (2.5937-3.0446)
At least 1 of CHASM (breast), CHASM (lung), CHASM (melanoma), FATHMM (cancer), Mutation Assessor, PROVEAN, VEST	66.72% (63.13%-70.71%)	100% (100%-100%)	9.189% (5.479%-12.821%)	65.56% (61.7%-69.63%)	100% (100%-100%)	2.7475 (2.6927-2.8109)	66.55% (58.59%-73.74%)	100% (100%-100%)	9.141% (2.562%-16.28%)	65.38% (57.29%-72.92%)	100% (100%-100%)	2.7464 (2.6292-2.8645)
At least 1 of CHASM (breast), CHASM (lung), CHASM (melanoma), FATHMM (cancer), Mutation Assessor, SIFT	71.71% (68.18%-75.25%)	99.46% (99.16%-100%)	23.738% (17.91%-29.577%)	69.28% (65.36%-73.19%)	96.24% (92.86%-100%)	2.8872 (2.7865-2.9960)	71.72% (64.65%-78.79%)	99.48% (98.25%-100%)	24.111% (12.5%-35.48%)	69.22% (61.36%-77.18%)	96.4% (85.71%-100%)	2.8921 (2.6505-3.0867)
At least 1 of CHASM (breast), CHASM (lung), CHASM (melanoma), FATHMM (cancer), Mutation Assessor, SIFT, VEST	66.75% (63.13%-70.71%)	100% (100%-100%)	9.254% (5.479%-12.857%)	65.58% (61.7%-69.63%)	100% (100%-100%)	2.7483 (2.6932-2.8130)	66.5% (58.59%-73.74%)	100% (100%-100%)	9.016% (2.498%-16.67%)	65.35% (57.29%-72.92%)	100% (100%-100%)	2.7449 (2.6270-2.8596)
At least 1 of CHASM (breast), CHASM (lung), CHASM (melanoma), FATHMM (cancer), Mutation Assessor, VEST	67.39% (63.64%-71.21%)	100% (100%-100%)	10.999% (6.849%-15.068%)	66.02% (62.11%-70.16%)	100% (100%-100%)	2.7702 (2.7116-2.8385)	67.24% (59.6%-74.75%)	100% (100%-100%)	11.015% (2.778%-19.44%)	65.86% (57.72%-73.47%)	100% (100%-100%)	2.7694 (2.6467-2.8957)
At least 1 of CHASM (breast), CHASM (lung), CHASM (melanoma), FATHMM (cancer), MutationTaster, PolyPhen-2	75.09% (71.72%-78.79%)	100% (100%-100%)	32.022% (26.027%-37.883%)	71.78% (68.18%-75.72%)	100% (100%-100%)	3.0380 (2.9586-3.1268)	75.07% (67.68%-81.82%)	100% (100%-100%)	32.267% (20%-44.44%)	71.7% (63.73%-79.07%)	100% (100%-100%)	3.0397 (2.8785-3.2101)
At least 1 of CHASM (breast), CHASM (lung), CHASM (melanoma), FATHMM (cancer), MutationTaster, PolyPhen-2, PROVEAN	72.43% (69.19%-75.76%)	100% (100%-100%)	24.741% (18.841%-30.14%)	69.67% (65.92%-73.37%)	100% (100%-100%)	2.9442 (2.8711-3.0258)	72.32% (65.66%-78.79%)	100% (100%-100%)	24.78% (13.886%-35.72%)	69.53% (61.96%-77.02%)	100% (100%-100%)	2.9431 (2.7945-3.1051)
At least 1 of CHASM (breast), CHASM (lung), CHASM (melanoma), FATHMM (cancer), MutationTaster, PolyPhen-2, SIFT	72.43% (69.19%-76.26%)	100% (100%-100%)	24.776% (18.841%-30.558%)	69.68% (65.93%-73.74%)	100% (100%-100%)	2.9446 (2.8645-3.0257)	72.3% (64.65%-79.8%)	100% (100%-100%)	24.782% (13.786%-36.11%)	69.52% (61.36%-76.92%)	100% (100%-100%)	2.9431 (2.7862-3.1099)
At least 1 of CHASM (breast), CHASM (lung), CHASM (melanoma), FATHMM (cancer), MutationTaster, PolyPhen-2, VEST	68.04% (64.65%-71.73%)	100% (100%-100%)	12.785% (8.219%-16.927%)	66.47% (62.63%-70.59%)	100% (100%-100%)	2.7926 (2.7308-2.8613)	67.95% (60.58%-74.75%)	100% (100%-100%)	12.937% (5%-22.22%)	66.35% (58.33%-73.96%)	100% (100%-100%)	2.7932 (2.6693-2.9295)
At least 1 of CHASM (breast), CHASM (lung), CHASM (melanoma), FATHMM (cancer), MutationTaster, PROVEAN	74.15% (70.71%-77.78%)	99.49% (99.16%-100%)	30.339% (23.944%-36%)	71.18% (67.43%-75%)	97.19% (94.74%-100%)	2.9820 (2.8815-3.0861)	73.91% (66.67%-80.81%)	99.42% (98.21%-100%)	30.114% (18.176%-42.86%)	70.94% (63.33%-78.41%)	96.83% (87.5%-100%)	2.9731 (2.7517-3.1614)
At least 1 of CHASM (breast), CHASM (lung), CHASM (melanoma), FATHMM (cancer), MutationTaster, PROVEAN, SIFT	71.44% (68.18%-75.25%)	100% (100%-100%)	22.065% (16.438%-27.536%)	68.93% (65.22%-72.73%)	100% (100%-100%)	2.9100 (2.8364-2.9952)	71.25% (63.64%-77.78%)	100% (100%-100%)	21.904% (11.104%-32.63%)	68.73% (61.11%-76.09%)	100% (100%-100%)	2.9063 (2.7563-3.0653)
At least 1 of CHASM (breast), CHASM (lung), CHASM (melanoma), FATHMM (cancer), MutationTaster, PROVEAN, VEST	67.38% (63.64%-71.21%)	100% (100%-100%)	10.988% (6.942%-15.068%)	66.02% (62.23%-70%)	100% (100%-100%)	2.7700 (2.7101-2.8375)	67.25% (59.6%-74.75%)	100% (100%-100%)	11.041% (2.857%-19.35%)	65.86% (57.89%-73.47%)	100% (100%-100%)	2.7698 (2.6463-2.9007)
At least 1 of CHASM (breast), CHASM (lung), CHASM (melanoma), FATHMM (cancer), MutationTaster, SIFT	73.11% (69.7%-76.77%)	100% (100%-100%)	26.633% (21.125%-32.394%)	70.21% (66.66%-74.16%)	100% (100%-100%)	2.9684 (2.8892-3.0546)	72.96% (65.66%-79.8%)	100% (100%-100%)	26.573% (15.148%-38.24%)	70.03% (61.95%-77.28%)	100% (100%-100%)	2.9660 (2.8091-3.1382)
At least 1 of CHASM (breast), CHASM (lung), CHASM (melanoma), FATHMM (cancer), MutationTaster, SIFT, VEST	67.74% (64.14%-71.72%)	100% (100%-100%)	11.965% (7.246%-16.131%)	66.26% (62.37%-70.37%)	100% (100%-100%)	2.7823 (2.7206-2.8536)	67.55% (59.6%-74.75%)	100% (100%-100%)	11.852% (2.941%-20.59%)	66.07% (57.89%-73.91%)	100% (100%-100%)	2.7800 (2.6531-2.9145)
At least 1 of CHASM (breast), CHASM (lung), CHASM (melanoma), FATHMM (cancer), MutationTaster, VEST	68.38% (64.65%-72.22%)	100% (100%-100%)	13.711% (9.088%-18.182%)	66.71% (62.9%-70.9%)	100% (100%-100%)	2.8042 (2.7395-2.8776)	68.29% (60.61%-75.76%)	100% (100%-100%)	13.851% (5.263%-23.08%)	66.58% (58.33%-74.19%)	100% (100%-100%)	2.8046 (2.6699-2.9473)
At least 1 of CHASM (breast), CHASM (lung), CHASM (melanoma), FATHMM (cancer), PolyPhen-2, PROVEAN	72.08% (68.69%-75.76%)	98.94% (98.33%-100%)	25.607% (19.718%-31.081%)	69.7% (65.92%-73.48%)	93.35% (88.24%-100%)	2.8760 (2.7582-2.9952)	72.01% (64.65%-78.79%)	98.92% (96.67%-100%)	25.788% (14.286%-37.14%)	69.59% (61.8%-77.02%)	93.3% (77.78%-100%)	2.8759 (2.6380-3.0881)
At least 1 of CHASM (breast), CHASM (lung), CHASM (melanoma), FATHMM (cancer), PolyPhen-2, PROVEAN, SIFT	70.74% (67.17%-74.24%)	99.46% (99.16%-100%)	21.075% (15.709%-26.475%)	68.54% (64.84%-72.43%)	95.79% (91.67%-100%)	2.8487 (2.7488-2.9500)	70.64% (63.64%-77.78%)	99.48% (98.25%-100%)	21.122% (9.994%-32.35%)	68.4% (60.67%-75.56%)	95.95% (83.33%-100%)	2.8495 (2.6353-3.0446)
At least 1 of CHASM (breast), CHASM (lung), CHASM (melanoma), FATHMM (cancer), PolyPhen-2, PROVEAN, VEST	67.05% (63.62%-70.71%)	100% (100%-100%)	10.062% (5.797%-13.893%)	65.78% (61.98%-69.68%)	100% (100%-100%)	2.7585 (2.6991-2.8237)	66.92% (59.6%-73.76%)	100% (100%-100%)	10.127% (2.701%-18.19%)	65.63% (57.89%-73.2%)	100% (100%-100%)	2.7585 (2.6379-2.8862)
At least 1 of CHASM (breast), CHASM (lung), CHASM (melanoma), FATHMM (cancer), PolyPhen-2, SIFT	72.41% (69.19%-76.26%)	99.46% (99.16%-100%)	25.643% (19.736%-31.148%)	69.81% (66.11%-73.86%)	96.51% (93.33%-100%)	2.9143 (2.8120-3.0215)	72.35% (64.65%-78.79%)	99.48% (98.25%-100%)	25.791% (14.625%-37.14%)	69.7% (61.36%-77.17%)	96.64% (85.71%-100%)	2.9161 (2.6895-3.1156)
At least 1 of CHASM (breast), CHASM (lung), CHASM (melanoma), FATHMM (cancer), PolyPhen-2, SIFT, VEST	67.4% (63.64%-71.21%)	100% (100%-100%)	11.04% (6.849%-15.002%)	66.03% (62.23%-70.16%)	100% (100%-100%)	2.7707 (2.7116-2.8422)	67.21% (59.6%-74.75%)	100% (100%-100%)	10.938% (2.778%-19.44%)	65.84% (57.45%-73.4%)	100% (100%-100%)	2.7688 (2.6411-2.9007)
At least 1 of CHASM (breast), CHASM (lung), CHASM (melanoma), FATHMM (cancer), PolyPhen-2, VEST	68.04% (64.65%-71.73%)	100% (100%-100%)	12.785% (8.219%-16.927%)	66.47% (62.63%-70.59%)	100% (100%-100%)	2.7926 (2.7308-2.8613)	67.95% (60.58%-74.75%)	100% (100%-100%)	12.937% (5%-22.22%)	66.35% (58.33%-73.96%)	100% (100%-100%)	2.7932 (2.6693-2.9295)
At least 1 of CHASM (breast), CHASM (lung), CHASM (melanoma), FATHMM (cancer), PROVEAN, SIFT	72.07% (68.69%-75.76%)	99.46% (99.16%-100%)	24.692% (19.177%-30.435%)	69.55% (65.92%-73.34%)	96.39% (93.33%-100%)	2.9009 (2.7984-2.9992)	72.03% (64.65%-78.79%)	99.48% (98.25%-100%)	24.901% (13.786%-36.37%)	69.45% (61.95%-76.93%)	96.56% (85.71%-100%)	2.9038 (2.6977-3.0937)
At least 1 of CHASM (breast), CHASM (lung), CHASM (melanoma), FATHMM (cancer), PROVEAN, SIFT, VEST	67.07% (63.13%-71.21%)	100% (100%-100%)	10.135% (5.882%-14.085%)	65.8% (61.9%-69.79%)	100% (100%-100%)	2.7594 (2.7019-2.8237)	66.87% (58.59%-74.75%)	100% (100%-100%)	10.003% (2.63%-18.18%)	65.6% (57.73%-73.4%)	100% (100%-100%)	2.7574 (2.6399-2.8847)
At least 1 of CHASM (breast), CHASM (lung), CHASM (melanoma), FATHMM (cancer), PROVEAN, VEST	67.07% (63.13%-70.71%)	99.5% (99.16%-100%)	10.988% (6.942%-15.068%)	65.9% (62.03%-69.85%)	92.74% (83.33%-100%)	2.6913 (2.5551-2.8179)	66.88% (59.6%-74.75%)	99.4% (98.25%-100%)	11.041% (2.857%-19.35%)	65.73% (57.73%-73.41%)	91.42% (66.67%-100%)	2.6764 (2.3237-2.8915)
At least 1 of CHASM (breast), CHASM (lung), CHASM (melanoma), FATHMM (cancer), SIFT, VEST	67.74% (64.14%-71.72%)	100% (100%-100%)	11.965% (7.246%-16.131%)	66.26% (62.37%-70.37%)	100% (100%-100%)	2.7823 (2.7206-2.8536)	67.55% (59.6%-74.75%)	100% (100%-100%)	11.852% (2.941%-20.59%)	66.07% (57.89%-73.91%)	100% (100%-100%)	2.7800 (2.6531-2.9145)
At least 1 of CHASM (breast), CHASM (lung), CHASM (melanoma), FATHMM (missense), Mutation Assessor, MutationTaster	74.11% (70.71%-77.78%)	99.49% (99.16%-100%)	30.224% (24.631%-36.232%)	71.14% (67.24%-75.14%)	97.18% (94.74%-100%)	2.9804 (2.8824-3.0829)	74% (66.67%-80.81%)	99.42% (98.21%-100%)	30.368% (18.421%-42.42%)	71.02% (62.92%-78.83%)	96.82% (87.5%-100%)	2.9763 (2.7516-3.1748)
At least 1 of CHASM (breast), CHASM (lung), CHASM (melanoma), FATHMM (missense), Mutation Assessor, MutationTaster, PolyPhen-2	72.72% (69.19%-76.26%)	100% (100%-100%)	25.556% (19.718%-31.081%)	69.9% (66.1%-73.89%)	100% (100%-100%)	2.9546 (2.8726-3.0416)	72.74% (65.66%-79.8%)	100% (100%-100%)	25.931% (14.993%-37.51%)	69.85% (61.7%-77.53%)	100% (100%-100%)	2.9578 (2.8011-3.1313)
At least 1 of CHASM (breast), CHASM (lung), CHASM (melanoma), FATHMM (missense), Mutation Assessor, MutationTaster, PROVEAN	71.43% (67.68%-75.25%)	99.49% (99.16%-100%)	22.9% (17.459%-28.788%)	69.05% (65.17%-72.98%)	96.3% (92.86%-100%)	2.8775 (2.7811-2.9844)	71.28% (63.64%-78.79%)	99.42% (98.21%-100%)	22.981% (11.756%-33.33%)	68.9% (60.91%-76.67%)	95.8% (83.33%-100%)	2.8710 (2.6243-3.0628)
At least 1 of CHASM (breast), CHASM (lung), CHASM (melanoma), FATHMM (missense), Mutation Assessor, MutationTaster, SIFT	70.4% (66.67%-74.24%)	100% (100%-100%)	19.231% (14.08%-24.365%)	68.16% (64.17%-72.13%)	100% (100%-100%)	2.8739 (2.8003-2.9500)	70.31% (62.63%-77.78%)	100% (100%-100%)	19.354% (8.824%-30.23%)	68.03% (60.21%-75.87%)	100% (100%-100%)	2.8738 (2.7283-3.0419)
At least 1 of CHASM (breast), CHASM (lung), CHASM (melanoma), FATHMM (missense), Mutation Assessor, MutationTaster, VEST	67.36% (63.64%-71.21%)	100% (100%-100%)	10.914% (6.849%-14.865%)	66% (62.03%-70%)	100% (100%-100%)	2.7691 (2.7081-2.8311)	67.3% (59.6%-74.75%)	100% (100%-100%)	11.152% (2.857%-19.44%)	65.89% (57.89%-73.91%)	100% (100%-100%)	2.7708 (2.6573-2.9007)
At least 1 of CHASM (breast), CHASM (lung), CHASM (melanoma), FATHMM (missense), Mutation Assessor, PolyPhen-2	72.71% (69.19%-76.26%)	99.48% (99.16%-100%)	26.422% (20.541%-32.354%)	70.04% (66.11%-74.01%)	96.7% (93.75%-100%)	2.9265 (2.8236-3.0315)	72.76% (65.66%-79.8%)	99.44% (98.25%-100%)	26.939% (15.785%-38.71%)	70.02% (62.07%-77.78%)	96.55% (85.71%-100%)	2.9296 (2.7163-3.1313)
At least 1 of CHASM (breast), CHASM (lung), CHASM (melanoma), FATHMM (missense), Mutation Assessor, PolyPhen-2, PROVEAN	70.71% (67.17%-74.24%)	99.48% (99.16%-100%)	20.951% (15.278%-26.471%)	68.51% (64.6%-72.43%)	95.88% (91.67%-100%)	2.8482 (2.7392-2.9586)	70.7% (63.64%-77.78%)	99.44% (98.25%-100%)	21.352% (10.25%-32.43%)	68.46% (60.44%-76.34%)	95.68% (83.33%-100%)	2.8494 (2.5975-3.0459)
At least 1 of CHASM (breast), CHASM (lung), CHASM (melanoma), FATHMM (missense), Mutation Assessor, PolyPhen-2, SIFT	70.72% (67.16%-74.75%)	100% (100%-100%)	20.097% (14.662%-25.352%)	68.39% (64.48%-72.43%)	100% (100%-100%)	2.8849 (2.8091-2.9677)	70.68% (63.64%-77.78%)	100% (100%-100%)	20.362% (10.52%-31.43%)	68.3% (60.21%-76.14%)	100% (100%-100%)	2.8867 (2.7417-3.0459)
At least 1 of CHASM (breast), CHASM (lung), CHASM (melanoma), FATHMM (missense), Mutation Assessor, PolyPhen-2, VEST	67.02% (63.13%-70.71%)	100% (100%-100%)	9.982% (5.797%-13.889%)	65.76% (61.78%-69.79%)	100% (100%-100%)	2.7575 (2.6991-2.8192)	66.97% (59.6%-74.75%)	100% (100%-100%)	10.263% (2.703%-18.19%)	65.67% (57.45%-73.68%)	100% (100%-100%)	2.7602 (2.6467-2.8807)
At least 1 of CHASM (breast), CHASM (lung), CHASM (melanoma), FATHMM (missense), Mutation Assessor, PROVEAN	71.07% (67.17%-74.75%)	97.91% (96.69%-99.24%)	24.632% (18.664%-30.435%)	69.2% (65.19%-73.18%)	87.22% (78.95%-95.45%)	2.7897 (2.6389-2.9485)	71% (63.64%-77.78%)	97.78% (94.64%-100%)	25.004% (13.333%-37.14%)	69.11% (61.11%-77.18%)	86.77% (66.67%-100%)	2.7867 (2.4457-3.0576)
At least 1 of CHASM (breast), CHASM (lung), CHASM (melanoma), FATHMM (missense), Mutation Assessor, PROVEAN, SIFT	69.37% (65.66%-73.23%)	99.49% (99.15%-100%)	17.284% (11.594%-22.059%)	67.53% (63.59%-71.51%)	95.12% (90%-100%)	2.7942 (2.6743-2.9079)	69.35% (61.62%-76.77%)	99.43% (98.24%-100%)	17.7% (8.108%-28.57%)	67.46% (59.55%-75.28%)	94.87% (80%-100%)	2.7946 (2.5367-2.9975)
At least 1 of CHASM (breast), CHASM (lung), CHASM (melanoma), FATHMM (missense), Mutation Assessor, PROVEAN, VEST	66.01% (62.12%-69.7%)	100% (100%-100%)	7.238% (3.277%-10.391%)	65.08% (61.14%-69.11%)	100% (100%-100%)	2.7232 (2.6698-2.7784)	65.96% (58.59%-73.74%)	100% (100%-100%)	7.505% (0%-14.29%)	64.98% (56.84%-72.92%)	100% (100%-100%)	2.7268 (2.6238-2.8448)
At least 1 of CHASM (breast), CHASM (lung), CHASM (melanoma), FATHMM (missense), Mutation Assessor, SIFT	70.71% (67.17%-74.75%)	99.49% (99.15%-100%)	20.962% (15.385%-26.156%)	68.52% (64.48%-72.43%)	95.95% (91.67%-100%)	2.8492 (2.7392-2.9537)	70.69% (62.63%-77.78%)	99.43% (98.21%-100%)	21.377% (11.111%-32.5%)	68.46% (60.44%-76.67%)	95.71% (83.33%-100%)	2.8497 (2.6230-3.0565)
At least 1 of CHASM (breast), CHASM (lung), CHASM (melanoma), FATHMM (missense), Mutation Assessor, SIFT, VEST	66.38% (62.63%-70.2%)	100% (100%-100%)	8.237% (4.284%-11.598%)	65.33% (61.34%-69.43%)	100% (100%-100%)	2.7357 (2.6773-2.7949)	66.23% (58.59%-73.74%)	100% (100%-100%)	8.264% (2.326%-15.79%)	65.17% (56.84%-73.2%)	100% (100%-100%)	2.7360 (2.6270-2.8605)
At least 1 of CHASM (breast), CHASM (lung), CHASM (melanoma), FATHMM (missense), Mutation Assessor, VEST	67.36% (63.64%-71.21%)	100% (100%-100%)	10.914% (6.849%-14.865%)	66% (62.03%-70%)	100% (100%-100%)	2.7691 (2.7081-2.8311)	67.3% (59.6%-74.75%)	100% (100%-100%)	11.152% (2.857%-19.44%)	65.89% (57.89%-73.91%)	100% (100%-100%)	2.7708 (2.6573-2.9007)
At least 1 of CHASM (breast), CHASM (lung), CHASM (melanoma), FATHMM (missense), MutationTaster, PolyPhen-2	75.06% (71.72%-78.28%)	100% (100%-100%)	31.939% (25.713%-37.838%)	71.75% (68.05%-75.44%)	100% (100%-100%)	3.0369 (2.9545-3.1268)	75.13% (68.69%-81.82%)	100% (100%-100%)	32.429% (21.05%-44.83%)	71.75% (64.29%-79.07%)	100% (100%-100%)	3.0418 (2.8805-3.2156)
At least 1 of CHASM (breast), CHASM (lung), CHASM (melanoma), FATHMM (missense), MutationTaster, PolyPhen-2, PROVEAN	71.72% (68.18%-75.25%)	100% (100%-100%)	22.81% (17.568%-28.205%)	69.14% (65.38%-72.93%)	100% (100%-100%)	2.9195 (2.8459-2.9966)	71.71% (64.65%-78.79%)	100% (100%-100%)	23.143% (12.5%-34.29%)	69.07% (61.36%-76.67%)	100% (100%-100%)	2.9221 (2.7715-3.0833)
At least 1 of CHASM (breast), CHASM (lung), CHASM (melanoma), FATHMM (missense), MutationTaster, PolyPhen-2, SIFT	71.74% (68.18%-75.25%)	100% (100%-100%)	22.869% (17.143%-28.169%)	69.15% (65.19%-73.22%)	100% (100%-100%)	2.9202 (2.8440-2.9963)	71.68% (64.65%-78.79%)	100% (100%-100%)	23.09% (13.152%-34.48%)	69.05% (60.87%-76.67%)	100% (100%-100%)	2.9214 (2.7804-3.0875)
At least 1 of CHASM (breast), CHASM (lung), CHASM (melanoma), FATHMM (missense), MutationTaster, PolyPhen-2, VEST	68.01% (64.14%-71.72%)	100% (100%-100%)	12.694% (8.219%-16.883%)	66.45% (62.57%-70.53%)	100% (100%-100%)	2.7914 (2.7276-2.8572)	68.02% (60.61%-75.76%)	100% (100%-100%)	13.099% (5%-22.22%)	66.39% (58.33%-74.19%)	100% (100%-100%)	2.7951 (2.6723-2.9319)
At least 1 of CHASM (breast), CHASM (lung), CHASM (melanoma), FATHMM (missense), MutationTaster, PROVEAN	73.11% (69.7%-76.77%)	99.49% (99.16%-100%)	27.482% (21.739%-33.333%)	70.35% (66.48%-74.16%)	96.9% (94.12%-100%)	2.9422 (2.8494-3.0416)	72.98% (65.66%-79.8%)	99.42% (98.21%-100%)	27.563% (15.613%-38.89%)	70.2% (62.22%-78.03%)	96.5% (87.5%-100%)	2.9369 (2.7139-3.1157)
At least 1 of CHASM (breast), CHASM (lung), CHASM (melanoma), FATHMM (missense), MutationTaster, PROVEAN, SIFT	70.4% (66.67%-73.74%)	100% (100%-100%)	19.208% (13.888%-24.286%)	68.16% (64.44%-71.9%)	100% (100%-100%)	2.8736 (2.8018-2.9471)	70.32% (63.64%-77.78%)	100% (100%-100%)	19.353% (9.674%-30.01%)	68.03% (60.43%-75.53%)	100% (100%-100%)	2.8738 (2.7396-3.0245)
At least 1 of CHASM (breast), CHASM (lung), CHASM (melanoma), FATHMM (missense), MutationTaster, PROVEAN, VEST	66.67% (63.13%-70.2%)	100% (100%-100%)	9.036% (5.193%-12.676%)	65.53% (61.58%-69.47%)	100% (100%-100%)	2.7456 (2.6859-2.8041)	66.66% (59.6%-73.74%)	100% (100%-100%)	9.405% (2.632%-17.24%)	65.45% (57.45%-73.2%)	100% (100%-100%)	2.7494 (2.6411-2.8792)
At least 1 of CHASM (breast), CHASM (lung), CHASM (melanoma), FATHMM (missense), MutationTaster, SIFT	72.08% (68.69%-75.76%)	100% (100%-100%)	23.799% (18.053%-28.986%)	69.41% (65.56%-73.37%)	100% (100%-100%)	2.9321 (2.8551-3.0146)	72.01% (64.65%-78.79%)	100% (100%-100%)	23.966% (13.329%-35.29%)	69.3% (61.36%-76.75%)	100% (100%-100%)	2.9326 (2.7844-3.1001)
At least 1 of CHASM (breast), CHASM (lung), CHASM (melanoma), FATHMM (missense), MutationTaster, SIFT, VEST	67.37% (63.64%-71.21%)	100% (100%-100%)	10.949% (6.849%-14.865%)	66.01% (61.98%-70.16%)	100% (100%-100%)	2.7695 (2.7081-2.8341)	67.28% (59.6%-74.75%)	100% (100%-100%)	11.1% (2.941%-19.36%)	65.88% (57.73%-73.96%)	100% (100%-100%)	2.7701 (2.6488-2.9079)
At least 1 of CHASM (breast), CHASM (lung), CHASM (melanoma), FATHMM (missense), MutationTaster, VEST	68.35% (64.65%-72.22%)	100% (100%-100%)	13.626% (8.974%-17.911%)	66.69% (62.77%-70.68%)	100% (100%-100%)	2.8031 (2.7407-2.8696)	68.34% (60.61%-75.76%)	100% (100%-100%)	13.988% (5.263%-22.51%)	66.62% (58.51%-74.47%)	100% (100%-100%)	2.8061 (2.6825-2.9422)
At least 1 of CHASM (breast), CHASM (lung), CHASM (melanoma), FATHMM (missense), PolyPhen-2, PROVEAN	71.71% (68.18%-75.25%)	99.48% (99.16%-100%)	23.676% (17.808%-29.167%)	69.27% (65.54%-73.08%)	96.34% (92.86%-100%)	2.8876 (2.7811-2.9939)	71.74% (64.65%-78.79%)	99.44% (98.25%-100%)	24.152% (13.152%-35.9%)	69.23% (61.54%-76.92%)	96.17% (83.33%-100%)	2.8900 (2.6686-3.0937)
At least 1 of CHASM (breast), CHASM (lung), CHASM (melanoma), FATHMM (missense), PolyPhen-2, PROVEAN, SIFT	70.37% (66.67%-73.74%)	100% (100%-100%)	19.144% (13.333%-23.944%)	68.14% (64.36%-72.04%)	100% (100%-100%)	2.8728 (2.7990-2.9471)	70.36% (63.64%-77.78%)	100% (100%-100%)	19.486% (10%-30.77%)	68.07% (60.22%-75.79%)	100% (100%-100%)	2.8755 (2.7410-3.0419)
At least 1 of CHASM (breast), CHASM (lung), CHASM (melanoma), FATHMM (missense), PolyPhen-2, PROVEAN, VEST	66.67% (63.13%-70.2%)	100% (100%-100%)	9.036% (5.193%-12.676%)	65.53% (61.58%-69.47%)	100% (100%-100%)	2.7456 (2.6859-2.8041)	66.66% (59.6%-73.74%)	100% (100%-100%)	9.405% (2.632%-17.24%)	65.45% (57.45%-73.2%)	100% (100%-100%)	2.7494 (2.6411-2.8792)
At least 1 of CHASM (breast), CHASM (lung), CHASM (melanoma), FATHMM (missense), PolyPhen-2, SIFT	72.05% (68.69%-75.76%)	100% (100%-100%)	23.735% (17.948%-29.167%)	69.39% (65.56%-73.37%)	100% (100%-100%)	2.9313 (2.8551-3.0062)	72.05% (64.65%-78.79%)	100% (100%-100%)	24.099% (13.514%-35.71%)	69.33% (61.29%-76.93%)	100% (100%-100%)	2.9343 (2.7862-3.1001)
At least 1 of CHASM (breast), CHASM (lung), CHASM (melanoma), FATHMM (missense), PolyPhen-2, SIFT, VEST	67.37% (63.64%-71.21%)	100% (100%-100%)	10.949% (6.849%-14.865%)	66.01% (61.98%-70.16%)	100% (100%-100%)	2.7695 (2.7081-2.8341)	67.28% (59.6%-74.75%)	100% (100%-100%)	11.1% (2.941%-19.36%)	65.88% (57.73%-73.96%)	100% (100%-100%)	2.7701 (2.6488-2.9079)
At least 1 of CHASM (breast), CHASM (lung), CHASM (melanoma), FATHMM (missense), PolyPhen-2, VEST	68.01% (64.14%-71.72%)	100% (100%-100%)	12.694% (8.219%-16.883%)	66.45% (62.57%-70.53%)	100% (100%-100%)	2.7914 (2.7276-2.8572)	68.02% (60.61%-75.76%)	100% (100%-100%)	13.099% (5%-22.22%)	66.39% (58.33%-74.19%)	100% (100%-100%)	2.7951 (2.6723-2.9319)
At least 1 of CHASM (breast), CHASM (lung), CHASM (melanoma), FATHMM (missense), PROVEAN, SIFT	71.06% (67.17%-74.75%)	99.49% (99.15%-100%)	21.884% (15.871%-27.027%)	68.77% (64.86%-72.68%)	96.12% (92.31%-100%)	2.8626 (2.7497-2.9677)	71.02% (63.64%-78.79%)	99.43% (98.24%-100%)	22.225% (11.111%-34.09%)	68.7% (60.92%-76.35%)	95.87% (83.33%-100%)	2.8621 (2.6271-3.0667)
At least 1 of CHASM (breast), CHASM (lung), CHASM (melanoma), FATHMM (missense), PROVEAN, SIFT, VEST	66.36% (62.63%-70.2%)	100% (100%-100%)	8.183% (4.225%-11.538%)	65.32% (61.46%-69.27%)	100% (100%-100%)	2.7350 (2.6773-2.7917)	66.27% (58.59%-73.74%)	100% (100%-100%)	8.367% (2.498%-15.79%)	65.19% (57.14%-72.92%)	100% (100%-100%)	2.7371 (2.6292-2.8596)
At least 1 of CHASM (breast), CHASM (lung), CHASM (melanoma), FATHMM (missense), PROVEAN, VEST	66.35% (62.63%-70.2%)	99.5% (99.16%-100%)	9.036% (5.193%-12.676%)	65.41% (61.46%-69.43%)	91.37% (80%-100%)	2.6532 (2.4967-2.7847)	66.28% (58.59%-73.74%)	99.4% (98.25%-100%)	9.405% (2.632%-17.24%)	65.32% (57.29%-73.2%)	90.43% (50%-100%)	2.6461 (2.2292-2.8599)
At least 1 of CHASM (breast), CHASM (lung), CHASM (melanoma), FATHMM (missense), SIFT, VEST	67.37% (63.64%-71.21%)	100% (100%-100%)	10.949% (6.849%-14.865%)	66.01% (61.98%-70.16%)	100% (100%-100%)	2.7695 (2.7081-2.8341)	67.28% (59.6%-74.75%)	100% (100%-100%)	11.1% (2.941%-19.36%)	65.88% (57.73%-73.96%)	100% (100%-100%)	2.7701 (2.6488-2.9079)
At least 1 of CHASM (breast), CHASM (lung), CHASM (melanoma), Mutation Assessor, MutationTaster, PolyPhen-2	77.14% (73.74%-80.3%)	100% (100%-100%)	37.606% (31.081%-43.836%)	73.48% (69.64%-77.33%)	100% (100%-100%)	3.1109 (3.0209-3.2035)	77.04% (70.71%-83.84%)	100% (100%-100%)	37.62% (25%-51.29%)	73.34% (65.85%-80.9%)	100% (100%-100%)	3.1096 (2.9381-3.2986)
At least 1 of CHASM (breast), CHASM (lung), CHASM (melanoma), Mutation Assessor, MutationTaster, PolyPhen-2, PROVEAN	74.8% (71.72%-78.28%)	100% (100%-100%)	31.224% (25%-37.143%)	71.54% (67.65%-75.43%)	100% (100%-100%)	3.0277 (2.9486-3.1156)	74.64% (67.68%-80.81%)	100% (100%-100%)	31.093% (19.344%-43.24%)	71.35% (63.63%-79.07%)	100% (100%-100%)	3.0245 (2.8639-3.2007)
At least 1 of CHASM (breast), CHASM (lung), CHASM (melanoma), Mutation Assessor, MutationTaster, PolyPhen-2, SIFT	73.78% (70.2%-77.27%)	100% (100%-100%)	28.454% (21.913%-34.288%)	70.73% (66.85%-74.72%)	100% (100%-100%)	2.9918 (2.9062-3.0786)	73.65% (66.67%-80.81%)	100% (100%-100%)	28.433% (17.063%-41.03%)	70.57% (62.35%-78.26%)	100% (100%-100%)	2.9900 (2.8287-3.1667)
At least 1 of CHASM (breast), CHASM (lung), CHASM (melanoma), Mutation Assessor, MutationTaster, PolyPhen-2, VEST	68.06% (64.65%-71.72%)	100% (100%-100%)	12.826% (8.333%-17.335%)	66.48% (62.7%-70.43%)	100% (100%-100%)	2.7931 (2.7332-2.8631)	67.92% (60.61%-74.75%)	100% (100%-100%)	12.838% (3.843%-21.62%)	66.32% (58.33%-73.69%)	100% (100%-100%)	2.7920 (2.6688-2.9252)
At least 1 of CHASM (breast), CHASM (lung), CHASM (melanoma), Mutation Assessor, MutationTaster, PROVEAN	76.18% (72.73%-79.8%)	99.49% (99.16%-100%)	35.874% (29.167%-42.031%)	72.84% (68.96%-76.75%)	97.62% (95.45%-100%)	3.0583 (2.9662-3.1600)	75.92% (68.69%-82.83%)	99.42% (98.21%-100%)	35.589% (23.071%-48.57%)	72.59% (64.37%-80.23%)	97.3% (90%-100%)	3.0490 (2.8377-3.2403)
At least 1 of CHASM (breast), CHASM (lung), CHASM (melanoma), Mutation Assessor, MutationTaster, PROVEAN, SIFT	72.77% (69.19%-76.26%)	100% (100%-100%)	25.699% (19.993%-31.25%)	69.94% (65.92%-73.89%)	100% (100%-100%)	2.9564 (2.8797-3.0416)	72.63% (65.66%-79.8%)	100% (100%-100%)	25.675% (14.634%-37.22%)	69.78% (61.8%-77.43%)	100% (100%-100%)	2.9545 (2.7990-3.1276)
At least 1 of CHASM (breast), CHASM (lung), CHASM (melanoma), Mutation Assessor, MutationTaster, PROVEAN, VEST	67.39% (63.64%-71.21%)	100% (100%-100%)	11.007% (7.04%-14.865%)	66.02% (62.11%-70%)	100% (100%-100%)	2.7703 (2.7143-2.8348)	67.24% (59.6%-74.75%)	100% (100%-100%)	10.995% (2.857%-18.75%)	65.85% (57.89%-73.47%)	100% (100%-100%)	2.7690 (2.6531-2.8922)
At least 1 of CHASM (breast), CHASM (lung), CHASM (melanoma), Mutation Assessor, MutationTaster, SIFT	74.45% (71.2%-78.28%)	100% (100%-100%)	30.288% (23.944%-36.238%)	71.27% (67.41%-75.43%)	100% (100%-100%)	3.0155 (2.9316-3.1078)	74.33% (66.67%-80.83%)	100% (100%-100%)	30.292% (18.6%-42.86%)	71.11% (62.92%-79.07%)	100% (100%-100%)	3.0140 (2.8476-3.1997)
At least 1 of CHASM (breast), CHASM (lung), CHASM (melanoma), Mutation Assessor, MutationTaster, SIFT, VEST	67.76% (64.14%-71.72%)	100% (100%-100%)	12.07% (7.576%-16.176%)	66.27% (62.43%-70.22%)	100% (100%-100%)	2.7828 (2.7211-2.8536)	67.52% (59.6%-74.75%)	100% (100%-100%)	11.754% (3.028%-20.46%)	66.04% (58.06%-73.47%)	100% (100%-100%)	2.7783 (2.6531-2.9124)
At least 1 of CHASM (breast), CHASM (lung), CHASM (melanoma), Mutation Assessor, MutationTaster, VEST	68.74% (65.15%-72.73%)	100% (100%-100%)	14.683% (9.996%-19.118%)	66.96% (63.1%-70.97%)	100% (100%-100%)	2.8164 (2.7540-2.8885)	68.58% (60.61%-75.76%)	100% (100%-100%)	14.642% (5.556%-23.68%)	66.79% (58.95%-74.23%)	100% (100%-100%)	2.8143 (2.6825-2.9500)
At least 1 of CHASM (breast), CHASM (lung), CHASM (melanoma), Mutation Assessor, PolyPhen-2, PROVEAN	74.11% (70.71%-77.78%)	98.41% (97.52%-100%)	32.09% (25.675%-38.028%)	71.48% (67.46%-75.43%)	92.11% (86.96%-100%)	2.9408 (2.8202-3.0622)	74% (66.67%-80.81%)	98.4% (95.31%-100%)	32.101% (20%-44.45%)	71.33% (63.53%-79.08%)	92.11% (76.92%-100%)	2.9393 (2.6704-3.1676)
At least 1 of CHASM (breast), CHASM (lung), CHASM (melanoma), Mutation Assessor, PolyPhen-2, PROVEAN, SIFT	71.74% (68.18%-75.25%)	98.93% (98.32%-100%)	24.731% (18.667%-30.263%)	69.44% (65.54%-73.3%)	93.03% (87.5%-100%)	2.8613 (2.7419-2.9873)	71.67% (64.65%-78.79%)	98.95% (96.67%-100%)	24.825% (14.277%-37.21%)	69.32% (61.62%-77.01%)	93.27% (77.78%-100%)	2.8637 (2.5830-3.0785)
At least 1 of CHASM (breast), CHASM (lung), CHASM (melanoma), Mutation Assessor, PolyPhen-2, PROVEAN, VEST	67.05% (63.64%-70.71%)	100% (100%-100%)	10.081% (5.882%-13.699%)	65.79% (61.98%-69.79%)	100% (100%-100%)	2.7587 (2.7039-2.8202)	66.9% (59.6%-73.74%)	100% (100%-100%)	10.081% (2.778%-17.95%)	65.62% (57.6%-73.2%)	100% (100%-100%)	2.7579 (2.6467-2.8764)
At least 1 of CHASM (breast), CHASM (lung), CHASM (melanoma), Mutation Assessor, PolyPhen-2, SIFT	73.42% (69.7%-77.27%)	98.93% (98.32%-100%)	29.32% (22.857%-35.39%)	70.76% (66.86%-74.72%)	94.05% (89.47%-100%)	2.9306 (2.8127-3.0518)	73.36% (65.66%-80.81%)	98.95% (96.67%-100%)	29.441% (17.643%-42.11%)	70.65% (62.5%-78.57%)	94.24% (80%-100%)	2.9328 (2.6746-3.1383)
At least 1 of CHASM (breast), CHASM (lung), CHASM (melanoma), Mutation Assessor, PolyPhen-2, SIFT, VEST	67.42% (63.64%-71.21%)	100% (100%-100%)	11.081% (6.944%-15.068%)	66.04% (62.23%-70.16%)	100% (100%-100%)	2.7712 (2.7110-2.8385)	67.18% (59.6%-74.75%)	100% (100%-100%)	10.839% (2.857%-19.06%)	65.81% (57.61%-73.41%)	100% (100%-100%)	2.7671 (2.6449-2.9007)
At least 1 of CHASM (breast), CHASM (lung), CHASM (melanoma), Mutation Assessor, PolyPhen-2, VEST	68.06% (64.65%-71.72%)	100% (100%-100%)	12.826% (8.333%-17.335%)	66.48% (62.7%-70.43%)	100% (100%-100%)	2.7931 (2.7332-2.8631)	67.92% (60.61%-74.75%)	100% (100%-100%)	12.838% (3.843%-21.62%)	66.32% (58.33%-73.69%)	100% (100%-100%)	2.7920 (2.6688-2.9252)
At least 1 of CHASM (breast), CHASM (lung), CHASM (melanoma), Mutation Assessor, PROVEAN, SIFT	72.39% (68.69%-76.26%)	97.88% (96.72%-99.23%)	28.326% (21.917%-33.824%)	70.25% (66.1%-74.16%)	88.52% (81.82%-96%)	2.8497 (2.7151-2.9873)	72.39% (64.65%-79.8%)	97.86% (94.83%-100%)	28.673% (17.141%-41.03%)	70.19% (62.22%-78.17%)	88.64% (71.43%-100%)	2.8536 (2.5553-3.1110)
At least 1 of CHASM (breast), CHASM (lung), CHASM (melanoma), Mutation Assessor, PROVEAN, SIFT, VEST	66.74% (63.13%-70.71%)	99.46% (99.16%-100%)	10.154% (6.023%-13.889%)	65.68% (61.85%-69.68%)	91.66% (83.33%-100%)	2.6696 (2.5273-2.8020)	66.52% (58.59%-73.74%)	99.48% (98.25%-100%)	9.957% (2.703%-17.65%)	65.47% (57.45%-73.12%)	91.78% (50%-100%)	2.6675 (2.2315-2.8596)
At least 1 of CHASM (breast), CHASM (lung), CHASM (melanoma), Mutation Assessor, PROVEAN, VEST	67.05% (63.64%-70.72%)	99.46% (99.16%-100%)	11.007% (7.04%-14.865%)	65.9% (62.03%-69.84%)	92.24% (83.33%-100%)	2.6861 (2.5613-2.8118)	66.91% (59.57%-74.75%)	99.48% (98.25%-100%)	10.995% (2.857%-18.75%)	65.73% (57.89%-73.4%)	92.54% (66.67%-100%)	2.6879 (2.3159-2.8800)
At least 1 of CHASM (breast), CHASM (lung), CHASM (melanoma), Mutation Assessor, SIFT, VEST	67.08% (63.64%-70.72%)	98.92% (98.33%-100%)	12.007% (7.576%-16.176%)	66.03% (62.16%-70.05%)	86.62% (75%-100%)	2.6358 (2.4767-2.8021)	66.86% (59.57%-73.74%)	98.96% (96.67%-100%)	11.754% (3.028%-20.46%)	65.81% (57.61%-73.4%)	86.8% (50%-100%)	2.6334 (2.2085-2.8847)
At least 1 of CHASM (breast), CHASM (lung), CHASM (melanoma), MutationTaster, PolyPhen-2, PROVEAN	76.49% (73.23%-79.8%)	100% (100%-100%)	35.829% (29.575%-41.892%)	72.93% (69.27%-76.7%)	100% (100%-100%)	3.0876 (3.0018-3.1785)	76.32% (69.7%-82.83%)	100% (100%-100%)	35.634% (23.684%-48.57%)	72.73% (65.42%-80.24%)	100% (100%-100%)	3.0836 (2.9167-3.2665)
At least 1 of CHASM (breast), CHASM (lung), CHASM (melanoma), MutationTaster, PolyPhen-2, PROVEAN, SIFT	73.45% (70.2%-76.77%)	100% (100%-100%)	27.542% (21.212%-32.906%)	70.47% (66.67%-74.3%)	100% (100%-100%)	2.9801 (2.8974-3.0622)	73.3% (66.67%-79.8%)	100% (100%-100%)	27.463% (16.214%-39.48%)	70.29% (62.5%-77.78%)	100% (100%-100%)	2.9775 (2.8290-3.1529)
At least 1 of CHASM (breast), CHASM (lung), CHASM (melanoma), MutationTaster, PolyPhen-2, PROVEAN, VEST	68.05% (64.65%-71.72%)	100% (100%-100%)	12.807% (8.219%-16.901%)	66.48% (62.7%-70.37%)	100% (100%-100%)	2.7929 (2.7301-2.8537)	67.93% (60.61%-74.75%)	100% (100%-100%)	12.875% (4.878%-22.22%)	66.33% (58.51%-73.91%)	100% (100%-100%)	2.7925 (2.6815-2.9319)
At least 1 of CHASM (breast), CHASM (lung), CHASM (melanoma), MutationTaster, PolyPhen-2, SIFT	75.46% (72.22%-78.79%)	100% (100%-100%)	33.044% (26.866%-39.13%)	72.08% (68.23%-76.14%)	100% (100%-100%)	3.0513 (2.9669-3.1382)	75.33% (68.69%-81.82%)	100% (100%-100%)	33.016% (21.208%-45.71%)	71.92% (63.64%-79.55%)	100% (100%-100%)	3.0494 (2.8806-3.2282)
At least 1 of CHASM (breast), CHASM (lung), CHASM (melanoma), MutationTaster, PolyPhen-2, SIFT, VEST	68.41% (64.65%-72.22%)	100% (100%-100%)	13.792% (8.974%-18.056%)	66.73% (62.77%-70.74%)	100% (100%-100%)	2.8052 (2.7407-2.8768)	68.23% (60.61%-75.76%)	100% (100%-100%)	13.675% (5.128%-23.33%)	66.54% (58.51%-74.47%)	100% (100%-100%)	2.8021 (2.6706-2.9470)
At least 1 of CHASM (breast), CHASM (lung), CHASM (melanoma), MutationTaster, PolyPhen-2, VEST	69.39% (65.66%-73.23%)	100% (100%-100%)	16.465% (11.536%-21.127%)	67.43% (63.59%-71.35%)	100% (100%-100%)	2.8389 (2.7734-2.9105)	69.29% (61.62%-76.77%)	100% (100%-100%)	16.569% (7.313%-26.67%)	67.29% (59.55%-75%)	100% (100%-100%)	2.8386 (2.7120-2.9856)
At least 1 of CHASM (breast), CHASM (lung), CHASM (melanoma), MutationTaster, PROVEAN, SIFT	74.46% (71.21%-77.78%)	100% (100%-100%)	30.307% (24.323%-36.111%)	71.27% (67.43%-75%)	100% (100%-100%)	3.0158 (2.9319-3.1001)	74.31% (67.68%-80.81%)	100% (100%-100%)	30.198% (18.182%-42.11%)	71.09% (63.52%-78.82%)	100% (100%-100%)	3.0128 (2.8631-3.1905)
At least 1 of CHASM (breast), CHASM (lung), CHASM (melanoma), MutationTaster, PROVEAN, SIFT, VEST	67.74% (64.14%-71.72%)	100% (100%-100%)	11.953% (7.499%-16%)	66.26% (62.43%-70.16%)	100% (100%-100%)	2.7821 (2.7206-2.8493)	67.55% (59.6%-74.75%)	100% (100%-100%)	11.857% (3.226%-20.46%)	66.07% (58.06%-73.63%)	100% (100%-100%)	2.7801 (2.6597-2.9145)
At least 1 of CHASM (breast), CHASM (lung), CHASM (melanoma), MutationTaster, PROVEAN, VEST	68.39% (64.65%-72.22%)	100% (100%-100%)	13.733% (9.091%-18.056%)	66.72% (62.9%-70.68%)	100% (100%-100%)	2.8045 (2.7373-2.8696)	68.27% (60.61%-75.76%)	100% (100%-100%)	13.79% (5.402%-22.58%)	66.56% (58.69%-74.19%)	100% (100%-100%)	2.8040 (2.6825-2.9496)
At least 1 of CHASM (breast), CHASM (lung), CHASM (melanoma), MutationTaster, SIFT, VEST	68.75% (65.15%-72.73%)	100% (100%-100%)	14.718% (10%-19.119%)	66.97% (63.04%-70.97%)	100% (100%-100%)	2.8169 (2.7503-2.8915)	68.56% (60.61%-75.76%)	100% (100%-100%)	14.59% (5.714%-25%)	66.77% (58.76%-74.5%)	100% (100%-100%)	2.8136 (2.6773-2.9597)
At least 1 of CHASM (breast), CHASM (lung), CHASM (melanoma), PolyPhen-2, PROVEAN, SIFT	73.09% (69.7%-76.77%)	98.93% (98.32%-100%)	28.408% (22.077%-34.211%)	70.5% (66.67%-74.3%)	93.88% (88.89%-100%)	2.9171 (2.8101-3.0345)	73.01% (65.66%-79.8%)	98.95% (96.67%-100%)	28.472% (17.239%-41.18%)	70.37% (62.5%-77.91%)	94.07% (80%-100%)	2.9186 (2.6570-3.1259)
At least 1 of CHASM (breast), CHASM (lung), CHASM (melanoma), PolyPhen-2, PROVEAN, SIFT, VEST	67.4% (63.64%-71.21%)	100% (100%-100%)	11.027% (6.847%-14.866%)	66.03% (62.23%-70%)	100% (100%-100%)	2.7705 (2.7081-2.8385)	67.22% (59.6%-74.75%)	100% (100%-100%)	10.943% (2.857%-19.51%)	65.84% (57.89%-73.4%)	100% (100%-100%)	2.7689 (2.6467-2.9060)
At least 1 of CHASM (breast), CHASM (lung), CHASM (melanoma), PolyPhen-2, PROVEAN, VEST	68.05% (64.65%-71.72%)	100% (100%-100%)	12.807% (8.219%-16.901%)	66.48% (62.7%-70.37%)	100% (100%-100%)	2.7929 (2.7301-2.8537)	67.93% (60.61%-74.75%)	100% (100%-100%)	12.875% (4.878%-22.22%)	66.33% (58.51%-73.91%)	100% (100%-100%)	2.7925 (2.6815-2.9319)
At least 1 of CHASM (breast), CHASM (lung), CHASM (melanoma), PolyPhen-2, SIFT, VEST	68.41% (64.65%-72.22%)	100% (100%-100%)	13.792% (8.974%-18.056%)	66.73% (62.77%-70.74%)	100% (100%-100%)	2.8052 (2.7407-2.8768)	68.23% (60.61%-75.76%)	100% (100%-100%)	13.675% (5.128%-23.33%)	66.54% (58.51%-74.47%)	100% (100%-100%)	2.8021 (2.6706-2.9470)
At least 1 of CHASM (breast), CHASM (lung), CHASM (melanoma), PROVEAN, SIFT, VEST	67.4% (63.64%-71.21%)	99.46% (99.16%-100%)	11.953% (7.499%-16%)	66.14% (62.36%-70%)	92.79% (85.71%-100%)	2.7035 (2.5763-2.8275)	67.23% (59.6%-74.75%)	99.48% (98.25%-100%)	11.857% (3.226%-20.46%)	65.95% (58.06%-73.47%)	92.92% (66.67%-100%)	2.7027 (2.3522-2.8980)
At least 1 of CHASM (breast), CHASM (lung), FATHMM (cancer), FATHMM (missense), Mutation Assessor, MutationTaster	71.74% (68.18%-75.76%)	99.49% (99.16%-100%)	23.758% (17.808%-29.577%)	69.29% (65.54%-73.33%)	96.43% (92.86%-100%)	2.8897 (2.7865-2.9964)	71.67% (64.65%-78.79%)	99.42% (98.21%-100%)	24.023% (13.158%-35.49%)	69.19% (60.87%-76.67%)	96% (83.33%-100%)	2.8867 (2.6421-3.0835)
At least 1 of CHASM (breast), CHASM (lung), FATHMM (cancer), FATHMM (missense), Mutation Assessor, MutationTaster, PolyPhen-2	70.02% (66.67%-73.75%)	100% (100%-100%)	18.184% (12.856%-23.188%)	67.88% (64.13%-72.04%)	100% (100%-100%)	2.8607 (2.7916-2.9407)	70.06% (62.6%-76.77%)	100% (100%-100%)	18.651% (8.571%-29.03%)	67.85% (59.57%-75.53%)	100% (100%-100%)	2.8652 (2.7169-3.0063)
At least 1 of CHASM (breast), CHASM (lung), FATHMM (cancer), FATHMM (missense), Mutation Assessor, MutationTaster, PROVEAN	69.74% (66.16%-73.74%)	99.49% (99.16%-100%)	18.285% (12.857%-23.378%)	67.8% (63.93%-71.74%)	95.41% (90.91%-100%)	2.8098 (2.6939-2.9223)	69.62% (61.62%-76.77%)	99.42% (98.21%-100%)	18.444% (8.328%-29.03%)	67.66% (59.78%-75.29%)	94.82% (80%-100%)	2.8037 (2.5373-3.0048)
At least 1 of CHASM (breast), CHASM (lung), FATHMM (cancer), FATHMM (missense), Mutation Assessor, MutationTaster, SIFT	67.71% (64.14%-71.72%)	100% (100%-100%)	11.876% (7.143%-16%)	66.24% (62.43%-70.37%)	100% (100%-100%)	2.7812 (2.7169-2.8493)	67.61% (59.6%-74.75%)	100% (100%-100%)	12.044% (3.123%-21.05%)	66.12% (58.06%-73.47%)	100% (100%-100%)	2.7820 (2.6582-2.9225)
At least 1 of CHASM (breast), CHASM (lung), FATHMM (cancer), FATHMM (missense), Mutation Assessor, MutationTaster, VEST	66.67% (63.13%-70.71%)	100% (100%-100%)	9.032% (5.195%-12.701%)	65.53% (61.66%-69.64%)	100% (100%-100%)	2.7457 (2.6896-2.8109)	66.66% (58.59%-73.74%)	100% (100%-100%)	9.42% (2.5%-17.5%)	65.46% (57.29%-73.2%)	100% (100%-100%)	2.7498 (2.6292-2.8748)
At least 1 of CHASM (breast), CHASM (lung), FATHMM (cancer), FATHMM (missense), Mutation Assessor, PolyPhen-2	70.01% (66.65%-73.74%)	99.48% (99.16%-100%)	19.051% (13.511%-24.286%)	68% (64.32%-72.05%)	95.48% (90.91%-100%)	2.8201 (2.7118-2.9275)	70.08% (62.63%-76.77%)	99.44% (98.25%-100%)	19.66% (9.368%-29.74%)	68% (59.57%-75.56%)	95.35% (80%-100%)	2.8244 (2.5688-3.0157)
At least 1 of CHASM (breast), CHASM (lung), FATHMM (cancer), FATHMM (missense), Mutation Assessor, PolyPhen-2, PROVEAN	68.68% (65.15%-72.22%)	99.48% (99.16%-100%)	15.416% (10.294%-20.27%)	67.04% (63.31%-71.06%)	94.47% (88.89%-100%)	2.7640 (2.6400-2.8793)	68.7% (61.62%-75.76%)	99.44% (98.25%-100%)	15.905% (5.714%-25.71%)	66.99% (58.89%-74.47%)	94.3% (75%-100%)	2.7664 (2.4700-2.9649)
At least 1 of CHASM (breast), CHASM (lung), FATHMM (cancer), FATHMM (missense), Mutation Assessor, PolyPhen-2, SIFT	68.03% (64.63%-72.22%)	100% (100%-100%)	12.742% (7.689%-17.143%)	66.46% (62.63%-70.53%)	100% (100%-100%)	2.7920 (2.7284-2.8596)	67.99% (59.6%-74.77%)	100% (100%-100%)	13.053% (4.528%-22.51%)	66.37% (58.33%-73.96%)	100% (100%-100%)	2.7946 (2.6699-2.9295)
At least 1 of CHASM (breast), CHASM (lung), FATHMM (cancer), FATHMM (missense), Mutation Assessor, PolyPhen-2, VEST	66.33% (62.63%-70.2%)	100% (100%-100%)	8.114% (4.286%-11.598%)	65.3% (61.46%-69.43%)	100% (100%-100%)	2.7342 (2.6815-2.7950)	66.32% (58.59%-73.74%)	100% (100%-100%)	8.505% (2.381%-15.79%)	65.23% (56.84%-72.92%)	100% (100%-100%)	2.7391 (2.6270-2.8536)
At least 1 of CHASM (breast), CHASM (lung), FATHMM (cancer), FATHMM (missense), Mutation Assessor, PROVEAN	70.38% (66.67%-74.24%)	98.97% (98.33%-100%)	20.933% (15.183%-26.087%)	68.4% (64.6%-72.22%)	92.15% (85.71%-100%)	2.8045 (2.6692-2.9321)	70.35% (62.63%-77.78%)	98.86% (96.67%-100%)	21.386% (10.808%-32.43%)	68.34% (60.46%-76.09%)	91.58% (75%-100%)	2.8017 (2.5250-3.0312)
At least 1 of CHASM (breast), CHASM (lung), FATHMM (cancer), FATHMM (missense), Mutation Assessor, PROVEAN, SIFT	67.68% (64.14%-71.22%)	100% (100%-100%)	11.781% (7.042%-15.789%)	66.22% (62.43%-70.16%)	100% (100%-100%)	2.7800 (2.7144-2.8416)	67.68% (60.58%-74.75%)	100% (100%-100%)	12.194% (3.448%-21.88%)	66.16% (58.16%-73.68%)	100% (100%-100%)	2.7841 (2.6698-2.9247)
At least 1 of CHASM (breast), CHASM (lung), FATHMM (cancer), FATHMM (missense), Mutation Assessor, PROVEAN, VEST	65.67% (62.12%-69.7%)	100% (100%-100%)	6.304% (2.817%-9.377%)	64.86% (61.14%-68.91%)	100% (100%-100%)	2.7118 (2.6597-2.7660)	65.63% (57.58%-72.73%)	100% (100%-100%)	6.631% (0%-13.16%)	64.77% (56.7%-72.16%)	100% (100%-100%)	2.7168 (2.6162-2.8289)
At least 1 of CHASM (breast), CHASM (lung), FATHMM (cancer), FATHMM (missense), Mutation Assessor, SIFT	68.68% (65.15%-72.23%)	100% (100%-100%)	14.524% (9.208%-18.919%)	66.92% (63.1%-70.9%)	100% (100%-100%)	2.8144 (2.7471-2.8857)	68.7% (61.59%-75.76%)	100% (100%-100%)	14.987% (5.878%-25.02%)	66.87% (58.95%-74.47%)	100% (100%-100%)	2.8189 (2.6924-2.9651)
At least 1 of CHASM (breast), CHASM (lung), FATHMM (cancer), FATHMM (missense), Mutation Assessor, SIFT, VEST	65.37% (61.62%-69.19%)	100% (100%-100%)	5.484% (2.631%-8.219%)	64.66% (60.82%-68.72%)	100% (100%-100%)	2.7017 (2.6535-2.7534)	65.22% (57.58%-72.73%)	100% (100%-100%)	5.526% (0%-12.12%)	64.5% (56.25%-72.17%)	100% (100%-100%)	2.7048 (2.6054-2.8051)
At least 1 of CHASM (breast), CHASM (lung), FATHMM (cancer), FATHMM (missense), Mutation Assessor, VEST	66.67% (63.13%-70.71%)	100% (100%-100%)	9.032% (5.195%-12.701%)	65.53% (61.66%-69.64%)	100% (100%-100%)	2.7457 (2.6896-2.8109)	66.66% (58.59%-73.74%)	100% (100%-100%)	9.42% (2.5%-17.5%)	65.46% (57.29%-73.2%)	100% (100%-100%)	2.7498 (2.6292-2.8748)
At least 1 of CHASM (breast), CHASM (lung), FATHMM (cancer), FATHMM (missense), MutationTaster, PolyPhen-2	72.02% (68.69%-75.76%)	100% (100%-100%)	23.644% (17.91%-28.793%)	69.37% (65.73%-73.37%)	100% (100%-100%)	2.9301 (2.8551-3.0133)	72.12% (64.65%-78.79%)	100% (100%-100%)	24.245% (13.509%-35.3%)	69.38% (61.36%-76.67%)	100% (100%-100%)	2.9362 (2.7844-3.1001)
At least 1 of CHASM (breast), CHASM (lung), FATHMM (cancer), FATHMM (missense), MutationTaster, PolyPhen-2, PROVEAN	69.69% (66.16%-73.23%)	100% (100%-100%)	17.274% (12.117%-22.222%)	67.64% (63.98%-71.58%)	100% (100%-100%)	2.8491 (2.7782-2.9236)	69.71% (62.63%-76.77%)	100% (100%-100%)	17.697% (8.108%-28.13%)	67.59% (59.57%-75%)	100% (100%-100%)	2.8529 (2.7120-3.0048)
At least 1 of CHASM (breast), CHASM (lung), FATHMM (cancer), FATHMM (missense), MutationTaster, PolyPhen-2, SIFT	69.04% (65.15%-73.23%)	100% (100%-100%)	15.514% (10.141%-20.27%)	67.18% (63.44%-71.35%)	100% (100%-100%)	2.8269 (2.7557-2.9040)	68.99% (60.61%-76.77%)	100% (100%-100%)	15.781% (5.882%-25.82%)	67.08% (58.94%-74.73%)	100% (100%-100%)	2.8286 (2.6896-2.9827)
At least 1 of CHASM (breast), CHASM (lung), FATHMM (cancer), FATHMM (missense), MutationTaster, PolyPhen-2, VEST	67.33% (63.64%-71.21%)	100% (100%-100%)	10.825% (6.579%-14.493%)	65.98% (62.18%-70.06%)	100% (100%-100%)	2.7680 (2.7080-2.8312)	67.37% (59.6%-74.75%)	100% (100%-100%)	11.341% (3.125%-20%)	65.94% (57.6%-73.47%)	100% (100%-100%)	2.7731 (2.6568-2.9079)
At least 1 of CHASM (breast), CHASM (lung), FATHMM (cancer), FATHMM (missense), MutationTaster, PROVEAN	72.07% (68.69%-75.76%)	99.49% (99.16%-100%)	24.658% (18.919%-30.137%)	69.54% (65.91%-73.34%)	96.56% (92.86%-100%)	2.9025 (2.7996-3.0060)	72.01% (64.65%-78.79%)	99.42% (98.21%-100%)	24.95% (13.889%-36.37%)	69.45% (61.79%-76.75%)	96.18% (85.71%-100%)	2.9000 (2.6768-3.1002)
At least 1 of CHASM (breast), CHASM (lung), FATHMM (cancer), FATHMM (missense), MutationTaster, PROVEAN, SIFT	68.05% (64.65%-71.72%)	100% (100%-100%)	12.788% (8.219%-17.143%)	66.47% (62.7%-70.53%)	100% (100%-100%)	2.7926 (2.7276-2.8596)	67.95% (60.61%-75.76%)	100% (100%-100%)	12.927% (4.878%-22.22%)	66.34% (58.33%-73.69%)	100% (100%-100%)	2.7930 (2.6699-2.9320)
At least 1 of CHASM (breast), CHASM (lung), FATHMM (cancer), FATHMM (missense), MutationTaster, PROVEAN, VEST	66.33% (62.63%-70.2%)	100% (100%-100%)	8.103% (4.225%-11.431%)	65.3% (61.46%-69.27%)	100% (100%-100%)	2.7340 (2.6773-2.7949)	66.33% (58.59%-73.74%)	100% (100%-100%)	8.531% (2.5%-15.91%)	65.23% (57.28%-72.92%)	100% (100%-100%)	2.7387 (2.6270-2.8611)
At least 1 of CHASM (breast), CHASM (lung), FATHMM (cancer), FATHMM (missense), MutationTaster, SIFT	69.38% (65.66%-73.23%)	100% (100%-100%)	16.444% (11.268%-21.333%)	67.42% (63.64%-71.66%)	100% (100%-100%)	2.8386 (2.7665-2.9124)	69.32% (61.62%-76.77%)	100% (100%-100%)	16.657% (7.405%-27.27%)	67.31% (58.95%-75%)	100% (100%-100%)	2.8397 (2.7050-3.0019)
At least 1 of CHASM (breast), CHASM (lung), FATHMM (cancer), FATHMM (missense), MutationTaster, SIFT, VEST	66.36% (62.63%-70.2%)	100% (100%-100%)	8.196% (4.286%-11.594%)	65.32% (61.46%-69.43%)	100% (100%-100%)	2.7352 (2.6799-2.7972)	66.27% (58.59%-73.74%)	100% (100%-100%)	8.362% (2.12%-15.79%)	65.19% (56.84%-72.92%)	100% (100%-100%)	2.7376 (2.6270-2.8596)
At least 1 of CHASM (breast), CHASM (lung), FATHMM (cancer), FATHMM (missense), MutationTaster, VEST	67.66% (64.14%-71.72%)	100% (100%-100%)	11.743% (7.246%-15.789%)	66.21% (62.43%-70.31%)	100% (100%-100%)	2.7795 (2.7162-2.8493)	67.71% (59.6%-74.75%)	100% (100%-100%)	12.256% (3.331%-21.21%)	66.17% (57.89%-73.68%)	100% (100%-100%)	2.7847 (2.6582-2.9180)
At least 1 of CHASM (breast), CHASM (lung), FATHMM (cancer), FATHMM (missense), PolyPhen-2, PROVEAN	69.68% (66.16%-73.23%)	99.48% (99.16%-100%)	18.14% (12.984%-23.188%)	67.76% (64.17%-71.74%)	95.26% (90%-100%)	2.8064 (2.6939-2.9145)	69.73% (62.63%-76.77%)	99.44% (98.25%-100%)	18.706% (8.817%-29.03%)	67.74% (59.78%-75.01%)	95.11% (80%-100%)	2.8100 (2.5373-3.0053)
At least 1 of CHASM (breast), CHASM (lung), FATHMM (cancer), FATHMM (missense), PolyPhen-2, PROVEAN, SIFT	68.02% (64.65%-71.72%)	100% (100%-100%)	12.724% (7.576%-17.105%)	66.46% (62.77%-70.53%)	100% (100%-100%)	2.7918 (2.7244-2.8596)	68% (60.61%-74.75%)	100% (100%-100%)	13.06% (4.997%-23.08%)	66.38% (58.33%-73.91%)	100% (100%-100%)	2.7944 (2.6699-2.9322)
At least 1 of CHASM (breast), CHASM (lung), FATHMM (cancer), FATHMM (missense), PolyPhen-2, PROVEAN, VEST	66.33% (62.63%-70.2%)	100% (100%-100%)	8.103% (4.225%-11.431%)	65.3% (61.46%-69.27%)	100% (100%-100%)	2.7340 (2.6773-2.7949)	66.33% (58.59%-73.74%)	100% (100%-100%)	8.531% (2.5%-15.91%)	65.23% (57.28%-72.92%)	100% (100%-100%)	2.7387 (2.6270-2.8611)
At least 1 of CHASM (breast), CHASM (lung), FATHMM (cancer), FATHMM (missense), PolyPhen-2, SIFT	69.36% (65.66%-73.23%)	100% (100%-100%)	16.38% (10.97%-21.127%)	67.4% (63.49%-71.51%)	100% (100%-100%)	2.8378 (2.7653-2.9124)	69.36% (61.62%-76.77%)	100% (100%-100%)	16.789% (7.692%-27.5%)	67.35% (58.95%-75%)	100% (100%-100%)	2.8414 (2.7080-3.0019)
At least 1 of CHASM (breast), CHASM (lung), FATHMM (cancer), FATHMM (missense), PolyPhen-2, SIFT, VEST	66.36% (62.63%-70.2%)	100% (100%-100%)	8.196% (4.286%-11.594%)	65.32% (61.46%-69.43%)	100% (100%-100%)	2.7352 (2.6799-2.7972)	66.27% (58.59%-73.74%)	100% (100%-100%)	8.362% (2.12%-15.79%)	65.19% (56.84%-72.92%)	100% (100%-100%)	2.7376 (2.6270-2.8596)
At least 1 of CHASM (breast), CHASM (lung), FATHMM (cancer), FATHMM (missense), PolyPhen-2, VEST	67.33% (63.64%-71.21%)	100% (100%-100%)	10.825% (6.579%-14.493%)	65.98% (62.18%-70.06%)	100% (100%-100%)	2.7680 (2.7080-2.8312)	67.37% (59.6%-74.75%)	100% (100%-100%)	11.341% (3.125%-20%)	65.94% (57.6%-73.47%)	100% (100%-100%)	2.7731 (2.6568-2.9079)
At least 1 of CHASM (breast), CHASM (lung), FATHMM (cancer), FATHMM (missense), PROVEAN, SIFT	69.02% (65.66%-72.73%)	100% (100%-100%)	15.436% (9.996%-20.007%)	67.16% (63.39%-70.97%)	100% (100%-100%)	2.8259 (2.7568-2.8957)	69.04% (61.62%-75.76%)	100% (100%-100%)	15.869% (6.452%-26.32%)	67.11% (59.34%-74.74%)	100% (100%-100%)	2.8298 (2.7019-2.9857)
At least 1 of CHASM (breast), CHASM (lung), FATHMM (cancer), FATHMM (missense), PROVEAN, SIFT, VEST	65.69% (62.12%-69.7%)	100% (100%-100%)	6.365% (2.817%-9.459%)	64.87% (61.13%-68.91%)	100% (100%-100%)	2.7124 (2.6595-2.7660)	65.59% (57.58%-72.73%)	100% (100%-100%)	6.513% (0%-13.16%)	64.74% (56.7%-72.17%)	100% (100%-100%)	2.7158 (2.6162-2.8303)
At least 1 of CHASM (breast), CHASM (lung), FATHMM (cancer), FATHMM (missense), PROVEAN, VEST	66.01% (62.12%-69.7%)	99.5% (99.16%-100%)	8.103% (4.225%-11.431%)	65.18% (61.26%-69.11%)	90.47% (75%-100%)	2.6325 (2.4514-2.7743)	65.96% (58.59%-73.74%)	99.4% (98.25%-100%)	8.531% (2.5%-15.91%)	65.1% (56.99%-72.73%)	89.49% (50%-100%)	2.6256 (2.1624-2.8536)
At least 1 of CHASM (breast), CHASM (lung), FATHMM (cancer), FATHMM (missense), SIFT, VEST	66.36% (62.63%-70.2%)	100% (100%-100%)	8.196% (4.286%-11.594%)	65.32% (61.46%-69.43%)	100% (100%-100%)	2.7352 (2.6799-2.7972)	66.27% (58.59%-73.74%)	100% (100%-100%)	8.362% (2.12%-15.79%)	65.19% (56.84%-72.92%)	100% (100%-100%)	2.7376 (2.6270-2.8596)
At least 1 of CHASM (breast), CHASM (lung), FATHMM (cancer), Mutation Assessor, MutationTaster, PolyPhen-2	73.74% (70.2%-77.27%)	100% (100%-100%)	28.331% (22.535%-34.43%)	70.7% (67.04%-74.71%)	100% (100%-100%)	2.9903 (2.9145-3.0811)	73.73% (66.67%-80.81%)	100% (100%-100%)	28.648% (16.667%-40%)	70.63% (62.63%-78.16%)	100% (100%-100%)	2.9928 (2.8290-3.1614)
At least 1 of CHASM (breast), CHASM (lung), FATHMM (cancer), Mutation Assessor, MutationTaster, PolyPhen-2, PROVEAN	72.08% (68.69%-75.76%)	100% (100%-100%)	23.785% (18.31%-29.412%)	69.41% (65.56%-73.33%)	100% (100%-100%)	2.9319 (2.8560-3.0146)	72.01% (64.65%-78.79%)	100% (100%-100%)	23.954% (13.152%-35.14%)	69.3% (61.53%-76.92%)	100% (100%-100%)	2.9325 (2.7805-3.0883)
At least 1 of CHASM (breast), CHASM (lung), FATHMM (cancer), Mutation Assessor, MutationTaster, PolyPhen-2, SIFT	71.09% (67.68%-74.75%)	100% (100%-100%)	21.099% (15.49%-26.471%)	68.67% (64.86%-72.83%)	100% (100%-100%)	2.8976 (2.8212-2.9777)	70.96% (63.64%-77.78%)	100% (100%-100%)	21.123% (10.71%-32.26%)	68.51% (60%-76.09%)	100% (100%-100%)	2.8963 (2.7425-3.0576)
At least 1 of CHASM (breast), CHASM (lung), FATHMM (cancer), Mutation Assessor, MutationTaster, PolyPhen-2, VEST	67.71% (64.14%-71.72%)	100% (100%-100%)	11.881% (7.35%-16.176%)	66.24% (62.43%-70.21%)	100% (100%-100%)	2.7812 (2.7206-2.8536)	67.61% (59.6%-74.75%)	100% (100%-100%)	11.998% (3.223%-20.59%)	66.11% (58.06%-73.68%)	100% (100%-100%)	2.7819 (2.6565-2.9124)
At least 1 of CHASM (breast), CHASM (lung), FATHMM (cancer), Mutation Assessor, MutationTaster, PROVEAN	74.46% (71.2%-77.78%)	99.49% (99.16%-100%)	31.168% (25.352%-37.143%)	71.42% (67.44%-75.28%)	97.26% (94.74%-100%)	2.9934 (2.8941-3.1009)	74.32% (67.68%-80.83%)	99.42% (98.21%-100%)	31.236% (18.421%-43.24%)	71.27% (63.53%-79.07%)	96.92% (88.89%-100%)	2.9885 (2.7667-3.1905)
At least 1 of CHASM (breast), CHASM (lung), FATHMM (cancer), Mutation Assessor, MutationTaster, PROVEAN, SIFT	70.42% (67.16%-74.24%)	100% (100%-100%)	19.28% (14.286%-24.324%)	68.17% (64.32%-72.13%)	100% (100%-100%)	2.8745 (2.8003-2.9500)	70.27% (62.63%-76.79%)	100% (100%-100%)	19.245% (8.571%-29.42%)	68% (59.99%-75.56%)	100% (100%-100%)	2.8726 (2.7289-3.0370)
At least 1 of CHASM (breast), CHASM (lung), FATHMM (cancer), Mutation Assessor, MutationTaster, PROVEAN, VEST	67.39% (63.64%-71.21%)	100% (100%-100%)	10.998% (6.757%-15.068%)	66.02% (62.11%-70%)	100% (100%-100%)	2.7702 (2.7081-2.8348)	67.25% (59.6%-74.75%)	100% (100%-100%)	11.039% (2.857%-20%)	65.86% (57.89%-73.47%)	100% (100%-100%)	2.7696 (2.6467-2.9018)
At least 1 of CHASM (breast), CHASM (lung), FATHMM (cancer), Mutation Assessor, MutationTaster, SIFT	71.76% (68.18%-75.27%)	100% (100%-100%)	22.934% (17.142%-28.378%)	69.17% (65.38%-73.22%)	100% (100%-100%)	2.9210 (2.8440-3.0053)	71.63% (64.62%-78.79%)	100% (100%-100%)	22.978% (11.765%-34.89%)	69.02% (60.86%-76.67%)	100% (100%-100%)	2.9200 (2.7665-3.0866)
At least 1 of CHASM (breast), CHASM (lung), FATHMM (cancer), Mutation Assessor, MutationTaster, SIFT, VEST	66.75% (63.13%-70.71%)	100% (100%-100%)	9.254% (5.479%-12.857%)	65.58% (61.7%-69.63%)	100% (100%-100%)	2.7483 (2.6932-2.8130)	66.5% (58.59%-73.74%)	100% (100%-100%)	9.016% (2.498%-16.67%)	65.35% (57.29%-72.92%)	100% (100%-100%)	2.7449 (2.6270-2.8596)
At least 1 of CHASM (breast), CHASM (lung), FATHMM (cancer), Mutation Assessor, MutationTaster, VEST	68.39% (64.65%-72.22%)	100% (100%-100%)	13.726% (9.208%-18.31%)	66.71% (62.9%-70.75%)	100% (100%-100%)	2.8044 (2.7436-2.8776)	68.28% (60.61%-75.76%)	100% (100%-100%)	13.828% (5.128%-22.86%)	66.57% (58.51%-74.19%)	100% (100%-100%)	2.8045 (2.6722-2.9324)
At least 1 of CHASM (breast), CHASM (lung), FATHMM (cancer), Mutation Assessor, PolyPhen-2, PROVEAN	71.72% (68.18%-75.25%)	98.94% (98.33%-100%)	24.652% (19.114%-30.264%)	69.43% (65.73%-73.45%)	93.1% (87.5%-100%)	2.8612 (2.7467-2.9786)	71.7% (64.65%-78.79%)	98.92% (96.67%-100%)	24.963% (13.333%-36.12%)	69.35% (61.36%-77.01%)	93.07% (77.78%-100%)	2.8630 (2.5897-3.0786)
At least 1 of CHASM (breast), CHASM (lung), FATHMM (cancer), Mutation Assessor, PolyPhen-2, PROVEAN, SIFT	69.73% (66.16%-73.24%)	99.46% (99.16%-100%)	18.311% (13.333%-23.188%)	67.8% (63.98%-71.89%)	95.18% (90.91%-100%)	2.8075 (2.7011-2.9186)	69.63% (62.6%-76.77%)	99.48% (98.25%-100%)	18.399% (8.333%-28.58%)	67.66% (59.34%-75.28%)	95.41% (80%-100%)	2.8095 (2.5623-3.0053)
At least 1 of CHASM (breast), CHASM (lung), FATHMM (cancer), Mutation Assessor, PolyPhen-2, PROVEAN, VEST	67.05% (63.64%-70.71%)	100% (100%-100%)	10.072% (5.968%-13.889%)	65.79% (62.03%-69.79%)	100% (100%-100%)	2.7586 (2.6990-2.8237)	66.91% (59.6%-73.74%)	100% (100%-100%)	10.124% (2.703%-18.18%)	65.63% (57.73%-73.12%)	100% (100%-100%)	2.7588 (2.6461-2.8847)
At least 1 of CHASM (breast), CHASM (lung), FATHMM (cancer), Mutation Assessor, PolyPhen-2, SIFT	71.07% (67.66%-74.75%)	99.46% (99.16%-100%)	21.965% (16.438%-27.539%)	68.79% (65.03%-72.78%)	95.95% (92.31%-100%)	2.8616 (2.7548-2.9749)	71% (63.64%-77.8%)	99.48% (98.25%-100%)	22.131% (11.477%-33.33%)	68.68% (60.67%-76.4%)	96.13% (83.33%-100%)	2.8641 (2.6206-3.0622)
At least 1 of CHASM (breast), CHASM (lung), FATHMM (cancer), Mutation Assessor, PolyPhen-2, SIFT, VEST	66.41% (62.63%-70.2%)	100% (100%-100%)	8.328% (4.348%-11.769%)	65.35% (61.54%-69.43%)	100% (100%-100%)	2.7368 (2.6834-2.7987)	66.17% (58.59%-73.74%)	100% (100%-100%)	8.101% (0%-15.38%)	65.13% (56.99%-72.73%)	100% (100%-100%)	2.7342 (2.6238-2.8448)
At least 1 of CHASM (breast), CHASM (lung), FATHMM (cancer), Mutation Assessor, PolyPhen-2, VEST	67.71% (64.14%-71.72%)	100% (100%-100%)	11.881% (7.35%-16.176%)	66.24% (62.43%-70.21%)	100% (100%-100%)	2.7812 (2.7206-2.8536)	67.61% (59.6%-74.75%)	100% (100%-100%)	11.998% (3.223%-20.59%)	66.11% (58.06%-73.68%)	100% (100%-100%)	2.7819 (2.6565-2.9124)
At least 1 of CHASM (breast), CHASM (lung), FATHMM (cancer), Mutation Assessor, PROVEAN, SIFT	71.38% (67.68%-75.25%)	99.46% (99.16%-100%)	22.824% (17.568%-28.571%)	69.02% (65.19%-73.08%)	96.09% (92.84%-100%)	2.8741 (2.7726-2.9771)	71.38% (63.64%-78.79%)	99.48% (98.25%-100%)	23.162% (11.759%-34.21%)	68.96% (60.86%-76.67%)	96.26% (83.33%-100%)	2.8786 (2.6379-3.0680)
At least 1 of CHASM (breast), CHASM (lung), FATHMM (cancer), Mutation Assessor, PROVEAN, SIFT, VEST	66.41% (62.63%-70.21%)	100% (100%-100%)	8.336% (4.348%-11.765%)	65.35% (61.46%-69.43%)	100% (100%-100%)	2.7369 (2.6815-2.7972)	66.17% (58.56%-73.74%)	100% (100%-100%)	8.103% (2.167%-15.22%)	65.13% (57.14%-72.92%)	100% (100%-100%)	2.7340 (2.6238-2.8536)
At least 1 of CHASM (breast), CHASM (lung), FATHMM (cancer), Mutation Assessor, PROVEAN, VEST	67.39% (63.64%-71.21%)	100% (100%-100%)	10.998% (6.757%-15.068%)	66.02% (62.11%-70%)	100% (100%-100%)	2.7702 (2.7081-2.8348)	67.25% (59.6%-74.75%)	100% (100%-100%)	11.039% (2.857%-20%)	65.86% (57.89%-73.47%)	100% (100%-100%)	2.7696 (2.6467-2.9018)
At least 1 of CHASM (breast), CHASM (lung), FATHMM (cancer), Mutation Assessor, SIFT, VEST	66.75% (63.13%-70.71%)	100% (100%-100%)	9.254% (5.479%-12.857%)	65.58% (61.7%-69.63%)	100% (100%-100%)	2.7483 (2.6932-2.8130)	66.5% (58.59%-73.74%)	100% (100%-100%)	9.016% (2.498%-16.67%)	65.35% (57.29%-72.92%)	100% (100%-100%)	2.7449 (2.6270-2.8596)
At least 1 of CHASM (breast), CHASM (lung), FATHMM (cancer), MutationTaster, PolyPhen-2, PROVEAN	73.42% (70.2%-76.77%)	100% (100%-100%)	27.463% (21.739%-33.333%)	70.45% (66.85%-74.16%)	100% (100%-100%)	2.9791 (2.9053-3.0651)	73.36% (66.67%-79.8%)	100% (100%-100%)	27.601% (15.625%-38.71%)	70.33% (62.96%-77.78%)	100% (100%-100%)	2.9793 (2.8163-3.1435)
At least 1 of CHASM (breast), CHASM (lung), FATHMM (cancer), MutationTaster, PolyPhen-2, PROVEAN, SIFT	71.1% (67.68%-74.75%)	100% (100%-100%)	21.122% (15.714%-26.471%)	68.67% (65%-72.53%)	100% (100%-100%)	2.8979 (2.8265-2.9741)	70.93% (63.64%-77.78%)	100% (100%-100%)	21.037% (10.256%-31.58%)	68.49% (60.67%-75.82%)	100% (100%-100%)	2.8955 (2.7527-3.0451)
At least 1 of CHASM (breast), CHASM (lung), FATHMM (cancer), MutationTaster, PolyPhen-2, PROVEAN, VEST	67.71% (64.14%-71.22%)	100% (100%-100%)	11.871% (7.46%-16.176%)	66.24% (62.5%-70.21%)	100% (100%-100%)	2.7811 (2.7162-2.8496)	67.61% (60.58%-74.75%)	100% (100%-100%)	12.024% (3.125%-21.06%)	66.11% (58.24%-73.47%)	100% (100%-100%)	2.7818 (2.6581-2.9247)
At least 1 of CHASM (breast), CHASM (lung), FATHMM (cancer), MutationTaster, PolyPhen-2, SIFT	72.77% (69.19%-76.77%)	100% (100%-100%)	25.689% (19.696%-31.944%)	69.94% (66.29%-73.89%)	100% (100%-100%)	2.9563 (2.8775-3.0433)	72.64% (64.65%-79.8%)	100% (100%-100%)	25.706% (14.286%-37.21%)	69.79% (61.63%-77.27%)	100% (100%-100%)	2.9549 (2.7947-3.1276)
At least 1 of CHASM (breast), CHASM (lung), FATHMM (cancer), MutationTaster, PolyPhen-2, SIFT, VEST	67.4% (63.64%-71.21%)	100% (100%-100%)	11.04% (6.849%-15.002%)	66.03% (62.23%-70.16%)	100% (100%-100%)	2.7707 (2.7116-2.8422)	67.21% (59.6%-74.75%)	100% (100%-100%)	10.938% (2.778%-19.44%)	65.84% (57.45%-73.4%)	100% (100%-100%)	2.7688 (2.6411-2.9007)
At least 1 of CHASM (breast), CHASM (lung), FATHMM (cancer), MutationTaster, PolyPhen-2, VEST	68.71% (65.15%-72.73%)	100% (100%-100%)	14.593% (9.589%-19.231%)	66.94% (63.1%-70.9%)	100% (100%-100%)	2.8153 (2.7438-2.8885)	68.65% (60.61%-75.76%)	100% (100%-100%)	14.834% (5.714%-25%)	66.84% (58.88%-74.47%)	100% (100%-100%)	2.8169 (2.6815-2.9683)
At least 1 of CHASM (breast), CHASM (lung), FATHMM (cancer), MutationTaster, PROVEAN, SIFT	72.11% (68.69%-75.76%)	100% (100%-100%)	23.888% (18.179%-29.412%)	69.44% (65.75%-73.22%)	100% (100%-100%)	2.9332 (2.8551-3.0146)	71.94% (64.65%-78.79%)	100% (100%-100%)	23.768% (12.5%-35.29%)	69.24% (61.54%-76.67%)	100% (100%-100%)	2.9301 (2.7805-3.1001)
At least 1 of CHASM (breast), CHASM (lung), FATHMM (cancer), MutationTaster, PROVEAN, SIFT, VEST	67.07% (63.13%-71.21%)	100% (100%-100%)	10.135% (5.882%-14.085%)	65.8% (61.9%-69.79%)	100% (100%-100%)	2.7594 (2.7019-2.8237)	66.87% (58.59%-74.75%)	100% (100%-100%)	10.003% (2.63%-18.18%)	65.6% (57.73%-73.4%)	100% (100%-100%)	2.7574 (2.6399-2.8847)
At least 1 of CHASM (breast), CHASM (lung), FATHMM (cancer), MutationTaster, PROVEAN, VEST	68.05% (64.14%-71.72%)	100% (100%-100%)	12.796% (8.333%-17.106%)	66.47% (62.63%-70.43%)	100% (100%-100%)	2.7927 (2.7276-2.8613)	67.95% (60.61%-75.76%)	100% (100%-100%)	12.939% (4.875%-21.88%)	66.34% (58.33%-73.96%)	100% (100%-100%)	2.7931 (2.6698-2.9419)
At least 1 of CHASM (breast), CHASM (lung), FATHMM (cancer), MutationTaster, SIFT, VEST	67.74% (64.14%-71.72%)	100% (100%-100%)	11.965% (7.246%-16.131%)	66.26% (62.37%-70.37%)	100% (100%-100%)	2.7823 (2.7206-2.8536)	67.55% (59.6%-74.75%)	100% (100%-100%)	11.852% (2.941%-20.59%)	66.07% (57.89%-73.91%)	100% (100%-100%)	2.7800 (2.6531-2.9145)
At least 1 of CHASM (breast), CHASM (lung), FATHMM (cancer), PolyPhen-2, PROVEAN, SIFT	71.08% (67.68%-74.75%)	99.46% (99.16%-100%)	21.988% (16.438%-27.536%)	68.8% (65.17%-72.68%)	95.96% (92.31%-100%)	2.8621 (2.7548-2.9676)	70.98% (63.64%-77.78%)	99.48% (98.25%-100%)	22.046% (10.808%-33.33%)	68.66% (60.87%-75.83%)	96.13% (83.33%-100%)	2.8631 (2.6395-3.0575)
At least 1 of CHASM (breast), CHASM (lung), FATHMM (cancer), PolyPhen-2, PROVEAN, SIFT, VEST	66.73% (63.13%-70.71%)	100% (100%-100%)	9.209% (5.332%-12.862%)	65.57% (61.78%-69.48%)	100% (100%-100%)	2.7478 (2.6919-2.8130)	66.53% (58.59%-73.74%)	100% (100%-100%)	9.089% (2.439%-17.07%)	65.37% (57.44%-72.92%)	100% (100%-100%)	2.7464 (2.6292-2.8690)
At least 1 of CHASM (breast), CHASM (lung), FATHMM (cancer), PolyPhen-2, PROVEAN, VEST	67.71% (64.14%-71.22%)	100% (100%-100%)	11.871% (7.46%-16.176%)	66.24% (62.5%-70.21%)	100% (100%-100%)	2.7811 (2.7162-2.8496)	67.61% (60.58%-74.75%)	100% (100%-100%)	12.024% (3.125%-21.06%)	66.11% (58.24%-73.47%)	100% (100%-100%)	2.7818 (2.6581-2.9247)
At least 1 of CHASM (breast), CHASM (lung), FATHMM (cancer), PolyPhen-2, SIFT, VEST	67.4% (63.64%-71.21%)	100% (100%-100%)	11.04% (6.849%-15.002%)	66.03% (62.23%-70.16%)	100% (100%-100%)	2.7707 (2.7116-2.8422)	67.21% (59.6%-74.75%)	100% (100%-100%)	10.938% (2.778%-19.44%)	65.84% (57.45%-73.4%)	100% (100%-100%)	2.7688 (2.6411-2.9007)
At least 1 of CHASM (breast), CHASM (lung), FATHMM (cancer), PROVEAN, SIFT, VEST	67.07% (63.13%-71.21%)	100% (100%-100%)	10.135% (5.882%-14.085%)	65.8% (61.9%-69.79%)	100% (100%-100%)	2.7594 (2.7019-2.8237)	66.87% (58.59%-74.75%)	100% (100%-100%)	10.003% (2.63%-18.18%)	65.6% (57.73%-73.4%)	100% (100%-100%)	2.7574 (2.6399-2.8847)
At least 1 of CHASM (breast), CHASM (lung), FATHMM (missense), Mutation Assessor, MutationTaster, PolyPhen-2	73.38% (70.19%-76.77%)	100% (100%-100%)	27.353% (21.622%-32.877%)	70.42% (66.48%-74.18%)	100% (100%-100%)	2.9777 (2.8975-3.0622)	73.44% (66.67%-79.82%)	100% (100%-100%)	27.834% (16.216%-39.47%)	70.4% (62.5%-78.26%)	100% (100%-100%)	2.9823 (2.8288-3.1508)
At least 1 of CHASM (breast), CHASM (lung), FATHMM (missense), Mutation Assessor, MutationTaster, PolyPhen-2, PROVEAN	71.38% (67.68%-74.75%)	100% (100%-100%)	21.882% (16.216%-27.536%)	68.88% (65%-72.73%)	100% (100%-100%)	2.9076 (2.8305-2.9869)	71.38% (64.65%-78.79%)	100% (100%-100%)	22.247% (11.427%-33.35%)	68.82% (61.11%-76.6%)	100% (100%-100%)	2.9107 (2.7655-3.0724)
At least 1 of CHASM (breast), CHASM (lung), FATHMM (missense), Mutation Assessor, MutationTaster, PolyPhen-2, SIFT	70.74% (67.17%-74.24%)	100% (100%-100%)	20.144% (14.492%-25.352%)	68.41% (64.52%-72.43%)	100% (100%-100%)	2.8855 (2.8087-2.9592)	70.65% (63.64%-77.78%)	100% (100%-100%)	20.278% (10%-31.25%)	68.28% (60.22%-76.4%)	100% (100%-100%)	2.8856 (2.7417-3.0459)
At least 1 of CHASM (breast), CHASM (lung), FATHMM (missense), Mutation Assessor, MutationTaster, PolyPhen-2, VEST	67.34% (63.64%-71.21%)	100% (100%-100%)	10.866% (6.667%-14.865%)	65.99% (62.11%-70%)	100% (100%-100%)	2.7685 (2.7081-2.8341)	67.34% (59.6%-74.75%)	100% (100%-100%)	11.242% (2.857%-19.52%)	65.92% (57.89%-73.68%)	100% (100%-100%)	2.7722 (2.6566-2.9007)
At least 1 of CHASM (breast), CHASM (lung), FATHMM (missense), Mutation Assessor, MutationTaster, PROVEAN	72.42% (68.69%-76.26%)	99.49% (99.16%-100%)	25.618% (20%-31.509%)	69.81% (65.91%-73.75%)	96.68% (93.33%-100%)	2.9160 (2.8195-3.0196)	72.32% (64.65%-79.8%)	99.42% (98.21%-100%)	25.793% (13.629%-37.5%)	69.69% (61.79%-77.42%)	96.25% (85.71%-100%)	2.9115 (2.6769-3.1078)
At least 1 of CHASM (breast), CHASM (lung), FATHMM (missense), Mutation Assessor, MutationTaster, PROVEAN, SIFT	69.39% (65.66%-72.74%)	100% (100%-100%)	16.466% (11.429%-21.215%)	67.43% (63.44%-71.28%)	100% (100%-100%)	2.8389 (2.7702-2.9124)	69.3% (62.6%-76.77%)	100% (100%-100%)	16.601% (7.135%-26.47%)	67.3% (59.34%-75.27%)	100% (100%-100%)	2.8390 (2.7050-2.9869)
At least 1 of CHASM (breast), CHASM (lung), FATHMM (missense), Mutation Assessor, MutationTaster, PROVEAN, VEST	66.34% (62.63%-70.2%)	100% (100%-100%)	8.122% (4.167%-11.594%)	65.3% (61.46%-69.39%)	100% (100%-100%)	2.7342 (2.6799-2.7949)	66.32% (58.59%-73.74%)	100% (100%-100%)	8.485% (2.439%-16.22%)	65.22% (57.14%-72.92%)	100% (100%-100%)	2.7386 (2.6270-2.8596)
At least 1 of CHASM (breast), CHASM (lung), FATHMM (missense), Mutation Assessor, MutationTaster, SIFT	70.74% (67.17%-74.24%)	100% (100%-100%)	20.144% (14.492%-25.352%)	68.41% (64.52%-72.43%)	100% (100%-100%)	2.8855 (2.8087-2.9592)	70.65% (63.64%-77.78%)	100% (100%-100%)	20.278% (10%-31.25%)	68.28% (60.22%-76.4%)	100% (100%-100%)	2.8856 (2.7417-3.0459)
At least 1 of CHASM (breast), CHASM (lung), FATHMM (missense), Mutation Assessor, MutationTaster, SIFT, VEST	66.38% (62.63%-70.2%)	100% (100%-100%)	8.237% (4.284%-11.598%)	65.33% (61.34%-69.43%)	100% (100%-100%)	2.7357 (2.6773-2.7949)	66.23% (58.59%-73.74%)	100% (100%-100%)	8.264% (2.326%-15.79%)	65.17% (56.84%-73.2%)	100% (100%-100%)	2.7360 (2.6270-2.8605)
At least 1 of CHASM (breast), CHASM (lung), FATHMM (missense), Mutation Assessor, MutationTaster, VEST	68.02% (64.65%-71.72%)	100% (100%-100%)	12.716% (8.195%-17.144%)	66.46% (62.57%-70.53%)	100% (100%-100%)	2.7917 (2.7285-2.8570)	68% (60.61%-74.75%)	100% (100%-100%)	13.047% (3.448%-22.22%)	66.38% (58.16%-74.19%)	100% (100%-100%)	2.7945 (2.6706-2.9295)
At least 1 of CHASM (breast), CHASM (lung), FATHMM (missense), Mutation Assessor, PolyPhen-2, PROVEAN	71.03% (67.68%-74.75%)	98.95% (98.32%-100%)	22.749% (16.896%-28.571%)	68.89% (64.97%-72.78%)	92.6% (86.67%-100%)	2.8320 (2.7102-2.9584)	71.07% (63.64%-77.78%)	98.91% (96.67%-100%)	23.255% (11.761%-35%)	68.87% (61.11%-76.34%)	92.57% (75%-100%)	2.8361 (2.5390-3.0565)
At least 1 of CHASM (breast), CHASM (lung), FATHMM (missense), Mutation Assessor, PolyPhen-2, PROVEAN, SIFT	69.37% (65.66%-73.23%)	99.47% (99.16%-100%)	17.332% (11.94%-22.368%)	67.54% (63.58%-71.43%)	94.96% (90%-100%)	2.7930 (2.6794-2.9040)	69.33% (61.62%-76.77%)	99.47% (98.28%-100%)	17.609% (7.895%-28.21%)	67.45% (59.55%-75.53%)	95.05% (80%-100%)	2.7958 (2.5228-2.9964)
At least 1 of CHASM (breast), CHASM (lung), FATHMM (missense), Mutation Assessor, PolyPhen-2, PROVEAN, VEST	66.34% (62.63%-70.2%)	100% (100%-100%)	8.122% (4.167%-11.594%)	65.3% (61.46%-69.39%)	100% (100%-100%)	2.7342 (2.6799-2.7949)	66.32% (58.59%-73.74%)	100% (100%-100%)	8.485% (2.439%-16.22%)	65.22% (57.14%-72.92%)	100% (100%-100%)	2.7386 (2.6270-2.8596)
At least 1 of CHASM (breast), CHASM (lung), FATHMM (missense), Mutation Assessor, PolyPhen-2, SIFT	70.72% (67.17%-74.24%)	99.47% (99.16%-100%)	21.01% (15.382%-26.391%)	68.53% (64.67%-72.53%)	95.81% (91.67%-100%)	2.8482 (2.7392-2.9563)	70.68% (63.64%-77.78%)	99.47% (98.28%-100%)	21.286% (10.811%-32.52%)	68.44% (60.43%-76.4%)	95.91% (83.33%-100%)	2.8510 (2.6213-3.0446)
At least 1 of CHASM (breast), CHASM (lung), FATHMM (missense), Mutation Assessor, PolyPhen-2, SIFT, VEST	66.38% (62.63%-70.2%)	100% (100%-100%)	8.237% (4.284%-11.598%)	65.33% (61.34%-69.43%)	100% (100%-100%)	2.7357 (2.6773-2.7949)	66.23% (58.59%-73.74%)	100% (100%-100%)	8.264% (2.326%-15.79%)	65.17% (56.84%-73.2%)	100% (100%-100%)	2.7360 (2.6270-2.8605)
At least 1 of CHASM (breast), CHASM (lung), FATHMM (missense), Mutation Assessor, PolyPhen-2, VEST	67.34% (63.64%-71.21%)	100% (100%-100%)	10.866% (6.667%-14.865%)	65.99% (62.11%-70%)	100% (100%-100%)	2.7685 (2.7081-2.8341)	67.34% (59.6%-74.75%)	100% (100%-100%)	11.242% (2.857%-19.52%)	65.92% (57.89%-73.68%)	100% (100%-100%)	2.7722 (2.6566-2.9007)
At least 1 of CHASM (breast), CHASM (lung), FATHMM (missense), Mutation Assessor, PROVEAN, SIFT	69.7% (65.66%-73.23%)	98.95% (98.32%-100%)	19.114% (13.043%-24.006%)	67.9% (63.93%-71.82%)	91.36% (84.58%-100%)	2.7733 (2.6345-2.9003)	69.69% (62.63%-77.78%)	98.89% (96.67%-100%)	19.543% (9.67%-30.95%)	67.84% (60.22%-75.82%)	91.16% (75%-100%)	2.7743 (2.4844-3.0048)
At least 1 of CHASM (breast), CHASM (lung), FATHMM (missense), Mutation Assessor, PROVEAN, SIFT, VEST	65.7% (61.62%-69.7%)	100% (100%-100%)	6.384% (2.857%-9.459%)	64.88% (60.94%-68.88%)	100% (100%-100%)	2.7126 (2.6595-2.7702)	65.57% (57.58%-73.74%)	100% (100%-100%)	6.467% (0%-12.91%)	64.73% (56.38%-72.63%)	100% (100%-100%)	2.7150 (2.6162-2.8308)
At least 1 of CHASM (breast), CHASM (lung), FATHMM (missense), Mutation Assessor, PROVEAN, VEST	66.34% (62.63%-70.2%)	100% (100%-100%)	8.122% (4.167%-11.594%)	65.3% (61.46%-69.39%)	100% (100%-100%)	2.7342 (2.6799-2.7949)	66.32% (58.59%-73.74%)	100% (100%-100%)	8.485% (2.439%-16.22%)	65.22% (57.14%-72.92%)	100% (100%-100%)	2.7386 (2.6270-2.8596)
At least 1 of CHASM (breast), CHASM (lung), FATHMM (missense), Mutation Assessor, SIFT, VEST	66.38% (62.63%-70.2%)	100% (100%-100%)	8.237% (4.284%-11.598%)	65.33% (61.34%-69.43%)	100% (100%-100%)	2.7357 (2.6773-2.7949)	66.23% (58.59%-73.74%)	100% (100%-100%)	8.264% (2.326%-15.79%)	65.17% (56.84%-73.2%)	100% (100%-100%)	2.7360 (2.6270-2.8605)
At least 1 of CHASM (breast), CHASM (lung), FATHMM (missense), MutationTaster, PolyPhen-2, PROVEAN	72.38% (69.19%-75.76%)	100% (100%-100%)	24.607% (18.919%-30.14%)	69.64% (65.92%-73.49%)	100% (100%-100%)	2.9424 (2.8640-3.0245)	72.42% (65.66%-78.79%)	100% (100%-100%)	25.047% (13.509%-36.37%)	69.6% (62.1%-77.02%)	100% (100%-100%)	2.9465 (2.7990-3.1156)
At least 1 of CHASM (breast), CHASM (lung), FATHMM (missense), MutationTaster, PolyPhen-2, PROVEAN, SIFT	70.39% (66.67%-73.74%)	100% (100%-100%)	19.191% (13.749%-24.007%)	68.15% (64.28%-71.89%)	100% (100%-100%)	2.8734 (2.8003-2.9486)	70.33% (63.64%-77.78%)	100% (100%-100%)	19.401% (9.668%-30.23%)	68.04% (60.23%-75.83%)	100% (100%-100%)	2.8744 (2.7396-3.0261)
At least 1 of CHASM (breast), CHASM (lung), FATHMM (missense), MutationTaster, PolyPhen-2, PROVEAN, VEST	67% (63.13%-70.71%)	100% (100%-100%)	9.921% (5.714%-13.514%)	65.75% (61.9%-69.63%)	100% (100%-100%)	2.7567 (2.6957-2.8160)	67.02% (59.6%-74.75%)	100% (100%-100%)	10.385% (2.857%-18.75%)	65.7% (57.89%-73.4%)	100% (100%-100%)	2.7612 (2.6525-2.8959)
At least 1 of CHASM (breast), CHASM (lung), FATHMM (missense), MutationTaster, PolyPhen-2, SIFT	72.07% (68.69%-75.76%)	100% (100%-100%)	23.782% (17.904%-29.168%)	69.4% (65.56%-73.33%)	100% (100%-100%)	2.9319 (2.8551-3.0133)	72.02% (64.65%-78.79%)	100% (100%-100%)	24.014% (13.158%-35.72%)	69.31% (61.36%-76.93%)	100% (100%-100%)	2.9332 (2.7844-3.1002)
At least 1 of CHASM (breast), CHASM (lung), FATHMM (missense), MutationTaster, PolyPhen-2, SIFT, VEST	67.37% (63.64%-71.21%)	100% (100%-100%)	10.949% (6.849%-14.865%)	66.01% (61.98%-70.16%)	100% (100%-100%)	2.7695 (2.7081-2.8341)	67.28% (59.6%-74.75%)	100% (100%-100%)	11.1% (2.941%-19.36%)	65.88% (57.73%-73.96%)	100% (100%-100%)	2.7701 (2.6488-2.9079)
At least 1 of CHASM (breast), CHASM (lung), FATHMM (missense), MutationTaster, PolyPhen-2, VEST	68.34% (64.65%-72.22%)	100% (100%-100%)	13.578% (8.955%-17.724%)	66.68% (62.77%-70.68%)	100% (100%-100%)	2.8025 (2.7373-2.8690)	68.38% (60.61%-75.76%)	100% (100%-100%)	14.078% (5.552%-23.33%)	66.64% (58.51%-74.47%)	100% (100%-100%)	2.8072 (2.6856-2.9500)
At least 1 of CHASM (breast), CHASM (lung), FATHMM (missense), MutationTaster, PROVEAN, SIFT	71.07% (67.17%-74.75%)	100% (100%-100%)	21.031% (15.584%-26.158%)	68.65% (64.8%-72.53%)	100% (100%-100%)	2.8968 (2.8239-2.9683)	71% (63.64%-78.79%)	100% (100%-100%)	21.204% (10.804%-31.83%)	68.54% (60.87%-76.14%)	100% (100%-100%)	2.8974 (2.7653-3.0622)
At least 1 of CHASM (breast), CHASM (lung), FATHMM (missense), MutationTaster, PROVEAN, SIFT, VEST	66.36% (62.63%-70.2%)	100% (100%-100%)	8.183% (4.225%-11.538%)	65.32% (61.46%-69.27%)	100% (100%-100%)	2.7350 (2.6773-2.7917)	66.27% (58.59%-73.74%)	100% (100%-100%)	8.367% (2.498%-15.79%)	65.19% (57.14%-72.92%)	100% (100%-100%)	2.7371 (2.6292-2.8596)
At least 1 of CHASM (breast), CHASM (lung), FATHMM (missense), MutationTaster, PROVEAN, VEST	67% (63.13%-70.71%)	100% (100%-100%)	9.921% (5.714%-13.514%)	65.75% (61.9%-69.63%)	100% (100%-100%)	2.7567 (2.6957-2.8160)	67.02% (59.6%-74.75%)	100% (100%-100%)	10.385% (2.857%-18.75%)	65.7% (57.89%-73.4%)	100% (100%-100%)	2.7612 (2.6525-2.8959)
At least 1 of CHASM (breast), CHASM (lung), FATHMM (missense), MutationTaster, SIFT, VEST	67.37% (63.64%-71.21%)	100% (100%-100%)	10.949% (6.849%-14.865%)	66.01% (61.98%-70.16%)	100% (100%-100%)	2.7695 (2.7081-2.8341)	67.28% (59.6%-74.75%)	100% (100%-100%)	11.1% (2.941%-19.36%)	65.88% (57.73%-73.96%)	100% (100%-100%)	2.7701 (2.6488-2.9079)
At least 1 of CHASM (breast), CHASM (lung), FATHMM (missense), PolyPhen-2, PROVEAN, SIFT	70.37% (66.67%-73.74%)	99.47% (99.16%-100%)	20.057% (14.102%-25%)	68.27% (64.32%-72.04%)	95.61% (90.91%-100%)	2.8341 (2.7185-2.9407)	70.37% (63.64%-77.78%)	99.47% (98.28%-100%)	20.409% (10.336%-31.58%)	68.2% (60.44%-76.09%)	95.71% (80%-100%)	2.8379 (2.5793-3.0441)
At least 1 of CHASM (breast), CHASM (lung), FATHMM (missense), PolyPhen-2, PROVEAN, SIFT, VEST	66.36% (62.63%-70.2%)	100% (100%-100%)	8.183% (4.225%-11.538%)	65.32% (61.46%-69.27%)	100% (100%-100%)	2.7350 (2.6773-2.7917)	66.27% (58.59%-73.74%)	100% (100%-100%)	8.367% (2.498%-15.79%)	65.19% (57.14%-72.92%)	100% (100%-100%)	2.7371 (2.6292-2.8596)
At least 1 of CHASM (breast), CHASM (lung), FATHMM (missense), PolyPhen-2, PROVEAN, VEST	67% (63.13%-70.71%)	100% (100%-100%)	9.921% (5.714%-13.514%)	65.75% (61.9%-69.63%)	100% (100%-100%)	2.7567 (2.6957-2.8160)	67.02% (59.6%-74.75%)	100% (100%-100%)	10.385% (2.857%-18.75%)	65.7% (57.89%-73.4%)	100% (100%-100%)	2.7612 (2.6525-2.8959)
At least 1 of CHASM (breast), CHASM (lung), FATHMM (missense), PolyPhen-2, SIFT, VEST	67.37% (63.64%-71.21%)	100% (100%-100%)	10.949% (6.849%-14.865%)	66.01% (61.98%-70.16%)	100% (100%-100%)	2.7695 (2.7081-2.8341)	67.28% (59.6%-74.75%)	100% (100%-100%)	11.1% (2.941%-19.36%)	65.88% (57.73%-73.96%)	100% (100%-100%)	2.7701 (2.6488-2.9079)
At least 1 of CHASM (breast), CHASM (lung), FATHMM (missense), PROVEAN, SIFT, VEST	66.36% (62.63%-70.2%)	100% (100%-100%)	8.183% (4.225%-11.538%)	65.32% (61.46%-69.27%)	100% (100%-100%)	2.7350 (2.6773-2.7917)	66.27% (58.59%-73.74%)	100% (100%-100%)	8.367% (2.498%-15.79%)	65.19% (57.14%-72.92%)	100% (100%-100%)	2.7371 (2.6292-2.8596)
At least 1 of CHASM (breast), CHASM (lung), Mutation Assessor, MutationTaster, PolyPhen-2, PROVEAN	75.8% (72.22%-79.29%)	100% (100%-100%)	33.946% (27.778%-40.299%)	72.36% (68.39%-76.27%)	100% (100%-100%)	3.0630 (2.9781-3.1574)	75.68% (68.69%-82.83%)	100% (100%-100%)	33.914% (21.212%-46.53%)	72.2% (64.44%-80%)	100% (100%-100%)	3.0611 (2.8921-3.2373)
At least 1 of CHASM (breast), CHASM (lung), Mutation Assessor, MutationTaster, PolyPhen-2, PROVEAN, SIFT	72.44% (69.18%-75.76%)	100% (100%-100%)	24.778% (18.838%-30.267%)	69.68% (65.91%-73.48%)	100% (100%-100%)	2.9446 (2.8640-3.0253)	72.3% (65.66%-78.81%)	100% (100%-100%)	24.74% (14.286%-36.67%)	69.52% (61.8%-77.27%)	100% (100%-100%)	2.9426 (2.7990-3.1210)
At least 1 of CHASM (breast), CHASM (lung), Mutation Assessor, MutationTaster, PolyPhen-2, PROVEAN, VEST	67.72% (64.14%-71.22%)	100% (100%-100%)	11.89% (7.35%-16.176%)	66.24% (62.43%-70.21%)	100% (100%-100%)	2.7813 (2.7180-2.8497)	67.6% (60.58%-74.75%)	100% (100%-100%)	11.978% (3.226%-20.59%)	66.1% (58.24%-73.68%)	100% (100%-100%)	2.7813 (2.6582-2.9171)
At least 1 of CHASM (breast), CHASM (lung), Mutation Assessor, MutationTaster, PolyPhen-2, SIFT	74.12% (70.71%-77.78%)	100% (100%-100%)	29.367% (22.857%-35.296%)	71% (67.05%-75%)	100% (100%-100%)	3.0036 (2.9217-3.0901)	73.99% (66.67%-80.81%)	100% (100%-100%)	29.356% (17.647%-41.94%)	70.84% (62.79%-78.65%)	100% (100%-100%)	3.0019 (2.8384-3.1784)
At least 1 of CHASM (breast), CHASM (lung), Mutation Assessor, MutationTaster, PolyPhen-2, SIFT, VEST	67.42% (63.64%-71.21%)	100% (100%-100%)	11.081% (6.944%-15.068%)	66.04% (62.23%-70.16%)	100% (100%-100%)	2.7712 (2.7110-2.8385)	67.18% (59.6%-74.75%)	100% (100%-100%)	10.839% (2.857%-19.06%)	65.81% (57.61%-73.41%)	100% (100%-100%)	2.7671 (2.6449-2.9007)
At least 1 of CHASM (breast), CHASM (lung), Mutation Assessor, MutationTaster, PolyPhen-2, VEST	68.72% (65.15%-72.73%)	100% (100%-100%)	14.634% (9.859%-19.119%)	66.95% (63.1%-70.97%)	100% (100%-100%)	2.8158 (2.7503-2.8915)	68.62% (60.61%-75.76%)	100% (100%-100%)	14.736% (5.556%-24.24%)	66.81% (58.95%-74.23%)	100% (100%-100%)	2.8156 (2.6816-2.9496)
At least 1 of CHASM (breast), CHASM (lung), Mutation Assessor, MutationTaster, PROVEAN, SIFT	73.44% (70.19%-76.77%)	100% (100%-100%)	27.521% (21.739%-33.333%)	70.46% (66.67%-74.29%)	100% (100%-100%)	2.9798 (2.8975-3.0653)	73.32% (66.67%-79.82%)	100% (100%-100%)	27.539% (16.214%-39.39%)	70.31% (62.78%-78.03%)	100% (100%-100%)	2.9785 (2.8235-3.1573)
At least 1 of CHASM (breast), CHASM (lung), Mutation Assessor, MutationTaster, PROVEAN, SIFT, VEST	67.08% (63.64%-70.72%)	100% (100%-100%)	10.154% (6.023%-13.889%)	65.81% (61.98%-69.84%)	100% (100%-100%)	2.7596 (2.7019-2.8231)	66.85% (59.57%-73.74%)	100% (100%-100%)	9.957% (2.703%-17.65%)	65.59% (57.45%-73.2%)	100% (100%-100%)	2.7563 (2.6438-2.8847)
At least 1 of CHASM (breast), CHASM (lung), Mutation Assessor, MutationTaster, PROVEAN, VEST	68.05% (64.65%-71.72%)	100% (100%-100%)	12.816% (8.219%-17.143%)	66.48% (62.63%-70.37%)	100% (100%-100%)	2.7930 (2.7276-2.8631)	67.93% (60.61%-74.75%)	100% (100%-100%)	12.893% (3.568%-21.96%)	66.33% (58.51%-73.96%)	100% (100%-100%)	2.7925 (2.6652-2.9323)
At least 1 of CHASM (breast), CHASM (lung), Mutation Assessor, MutationTaster, SIFT, VEST	67.76% (64.14%-71.72%)	100% (100%-100%)	12.007% (7.576%-16.176%)	66.27% (62.43%-70.22%)	100% (100%-100%)	2.7828 (2.7211-2.8536)	67.52% (59.6%-74.75%)	100% (100%-100%)	11.754% (3.028%-20.46%)	66.04% (58.06%-73.47%)	100% (100%-100%)	2.7783 (2.6531-2.9124)
At least 1 of CHASM (breast), CHASM (lung), Mutation Assessor, PolyPhen-2, PROVEAN, SIFT	71.74% (68.18%-75.25%)	98.4% (97.52%-100%)	25.644% (19.443%-31.429%)	69.59% (65.71%-73.45%)	90.24% (83.33%-100%)	2.8387 (2.6993-2.9683)	71.67% (64.65%-78.79%)	98.42% (95.31%-100%)	25.748% (14.634%-38.24%)	69.47% (61.79%-77.02%)	90.48% (72.73%-100%)	2.8412 (2.5552-3.0861)
At least 1 of CHASM (breast), CHASM (lung), Mutation Assessor, PolyPhen-2, PROVEAN, SIFT, VEST	66.74% (63.13%-70.71%)	100% (100%-100%)	9.228% (5.478%-12.676%)	65.58% (61.78%-69.63%)	100% (100%-100%)	2.7480 (2.6919-2.8089)	66.52% (58.59%-73.74%)	100% (100%-100%)	9.043% (2.632%-16.67%)	65.36% (57.29%-72.92%)	100% (100%-100%)	2.7455 (2.6379-2.8690)
At least 1 of CHASM (breast), CHASM (lung), Mutation Assessor, PolyPhen-2, PROVEAN, VEST	67.72% (64.14%-71.22%)	100% (100%-100%)	11.89% (7.35%-16.176%)	66.24% (62.43%-70.21%)	100% (100%-100%)	2.7813 (2.7180-2.8497)	67.6% (60.58%-74.75%)	100% (100%-100%)	11.978% (3.226%-20.59%)	66.1% (58.24%-73.68%)	100% (100%-100%)	2.7813 (2.6582-2.9171)
At least 1 of CHASM (breast), CHASM (lung), Mutation Assessor, PolyPhen-2, SIFT, VEST	67.42% (63.64%-71.21%)	100% (100%-100%)	11.081% (6.944%-15.068%)	66.04% (62.23%-70.16%)	100% (100%-100%)	2.7712 (2.7110-2.8385)	67.18% (59.6%-74.75%)	100% (100%-100%)	10.839% (2.857%-19.06%)	65.81% (57.61%-73.41%)	100% (100%-100%)	2.7671 (2.6449-2.9007)
At least 1 of CHASM (breast), CHASM (lung), Mutation Assessor, PROVEAN, SIFT, VEST	67.07% (63.64%-70.71%)	99.46% (99.16%-100%)	11.057% (6.944%-15.068%)	65.91% (62.11%-69.85%)	92.28% (83.33%-100%)	2.6871 (2.5584-2.8160)	66.87% (59.6%-73.74%)	99.48% (98.25%-100%)	10.904% (2.778%-19.48%)	65.71% (57.73%-73.49%)	92.52% (66.67%-100%)	2.6864 (2.3279-2.8749)
At least 1 of CHASM (breast), CHASM (lung), MutationTaster, PolyPhen-2, PROVEAN, SIFT	73.79% (70.2%-77.27%)	100% (100%-100%)	28.455% (22.365%-33.847%)	70.73% (67.04%-74.44%)	100% (100%-100%)	2.9919 (2.9062-3.0723)	73.64% (66.67%-80.81%)	100% (100%-100%)	28.387% (17.647%-41.18%)	70.56% (62.92%-78.16%)	100% (100%-100%)	2.9895 (2.8325-3.1687)
At least 1 of CHASM (breast), CHASM (lung), MutationTaster, PolyPhen-2, PROVEAN, SIFT, VEST	67.4% (63.64%-71.21%)	100% (100%-100%)	11.027% (6.847%-14.866%)	66.03% (62.23%-70%)	100% (100%-100%)	2.7705 (2.7081-2.8385)	67.22% (59.6%-74.75%)	100% (100%-100%)	10.943% (2.857%-19.51%)	65.84% (57.89%-73.4%)	100% (100%-100%)	2.7689 (2.6467-2.9060)
At least 1 of CHASM (breast), CHASM (lung), MutationTaster, PolyPhen-2, PROVEAN, VEST	68.72% (65.15%-72.22%)	100% (100%-100%)	14.616% (9.589%-19.118%)	66.94% (63.1%-70.81%)	100% (100%-100%)	2.8156 (2.7474-2.8857)	68.63% (61.62%-75.76%)	100% (100%-100%)	14.773% (5.714%-24.39%)	66.82% (59.13%-74.47%)	100% (100%-100%)	2.8160 (2.6867-2.9649)
At least 1 of CHASM (breast), CHASM (lung), MutationTaster, PolyPhen-2, SIFT, VEST	68.41% (64.65%-72.22%)	100% (100%-100%)	13.792% (8.974%-18.056%)	66.73% (62.77%-70.74%)	100% (100%-100%)	2.8052 (2.7407-2.8768)	68.23% (60.61%-75.76%)	100% (100%-100%)	13.675% (5.128%-23.33%)	66.54% (58.51%-74.47%)	100% (100%-100%)	2.8021 (2.6706-2.9470)
At least 1 of CHASM (breast), CHASM (lung), MutationTaster, PROVEAN, SIFT, VEST	67.74% (64.14%-71.72%)	100% (100%-100%)	11.953% (7.499%-16%)	66.26% (62.43%-70.16%)	100% (100%-100%)	2.7821 (2.7206-2.8493)	67.55% (59.6%-74.75%)	100% (100%-100%)	11.857% (3.226%-20.46%)	66.07% (58.06%-73.63%)	100% (100%-100%)	2.7801 (2.6597-2.9145)
At least 1 of CHASM (breast), CHASM (lung), PolyPhen-2, PROVEAN, SIFT, VEST	67.4% (63.64%-71.21%)	100% (100%-100%)	11.027% (6.847%-14.866%)	66.03% (62.23%-70%)	100% (100%-100%)	2.7705 (2.7081-2.8385)	67.22% (59.6%-74.75%)	100% (100%-100%)	10.943% (2.857%-19.51%)	65.84% (57.89%-73.4%)	100% (100%-100%)	2.7689 (2.6467-2.9060)
At least 1 of CHASM (breast), CHASM (melanoma), FATHMM (cancer), FATHMM (missense), Mutation Assessor, MutationTaster	70.41% (66.67%-74.24%)	99.49% (99.16%-100%)	20.123% (14.662%-25.373%)	68.29% (64.48%-72.29%)	95.82% (91.67%-100%)	2.8373 (2.7268-2.9411)	70.29% (62.63%-77.78%)	99.42% (98.21%-100%)	20.296% (9.674%-31.43%)	68.16% (59.99%-75.83%)	95.32% (80%-100%)	2.8322 (2.5548-3.0358)
At least 1 of CHASM (breast), CHASM (melanoma), FATHMM (cancer), FATHMM (missense), Mutation Assessor, MutationTaster, PolyPhen-2	69.36% (65.66%-73.23%)	100% (100%-100%)	16.387% (10.959%-21.127%)	67.41% (63.73%-71.43%)	100% (100%-100%)	2.8379 (2.7665-2.9163)	69.35% (61.62%-76.77%)	100% (100%-100%)	16.748% (7.405%-27.27%)	67.34% (59.34%-74.73%)	100% (100%-100%)	2.8411 (2.7003-2.9976)
At least 1 of CHASM (breast), CHASM (melanoma), FATHMM (cancer), FATHMM (missense), Mutation Assessor, MutationTaster, PROVEAN	68.74% (65.15%-72.73%)	99.49% (99.16%-100%)	15.567% (10.448%-20.27%)	67.08% (63.1%-71.12%)	94.66% (88.89%-100%)	2.7679 (2.6492-2.8857)	68.58% (60.61%-75.76%)	99.42% (98.21%-100%)	15.631% (6.25%-25.71%)	66.92% (58.95%-74.74%)	93.93% (75%-100%)	2.7592 (2.4571-2.9650)
At least 1 of CHASM (breast), CHASM (melanoma), FATHMM (cancer), FATHMM (missense), Mutation Assessor, MutationTaster, SIFT	67.37% (63.64%-71.21%)	100% (100%-100%)	10.963% (6.577%-14.866%)	66.01% (62.23%-70%)	100% (100%-100%)	2.7697 (2.7080-2.8375)	67.27% (59.6%-74.75%)	100% (100%-100%)	11.121% (2.941%-20%)	65.88% (57.89%-73.41%)	100% (100%-100%)	2.7705 (2.6467-2.9014)
At least 1 of CHASM (breast), CHASM (melanoma), FATHMM (cancer), FATHMM (missense), Mutation Assessor, MutationTaster, VEST	66.01% (62.12%-70.2%)	100% (100%-100%)	7.229% (3.999%-10.606%)	65.08% (61.14%-69.23%)	100% (100%-100%)	2.7232 (2.6698-2.7811)	65.96% (57.58%-73.74%)	100% (100%-100%)	7.525% (0%-14.63%)	64.99% (56.7%-72.92%)	100% (100%-100%)	2.7275 (2.6238-2.8431)
At least 1 of CHASM (breast), CHASM (melanoma), FATHMM (cancer), FATHMM (missense), Mutation Assessor, PolyPhen-2	69.35% (65.66%-73.23%)	99.48% (99.16%-100%)	17.253% (11.94%-22.059%)	67.52% (63.83%-71.59%)	95.03% (90%-100%)	2.7928 (2.6749-2.9040)	69.38% (61.62%-76.77%)	99.44% (98.25%-100%)	17.756% (8.322%-28.13%)	67.48% (59.34%-74.74%)	94.89% (80%-100%)	2.7957 (2.5272-3.0022)
At least 1 of CHASM (breast), CHASM (melanoma), FATHMM (cancer), FATHMM (missense), Mutation Assessor, PolyPhen-2, PROVEAN	68.02% (64.63%-71.72%)	99.48% (99.16%-100%)	13.618% (8.694%-17.951%)	66.57% (62.83%-70.59%)	93.78% (85.71%-100%)	2.7345 (2.6000-2.8519)	68% (60.61%-74.77%)	99.44% (98.25%-100%)	14.002% (5.263%-23.69%)	66.5% (58.33%-73.96%)	93.58% (74.58%-100%)	2.7353 (2.4269-2.9319)
At least 1 of CHASM (breast), CHASM (melanoma), FATHMM (cancer), FATHMM (missense), Mutation Assessor, PolyPhen-2, SIFT	67.69% (64.14%-71.72%)	100% (100%-100%)	11.829% (7.042%-16%)	66.23% (62.43%-70.21%)	100% (100%-100%)	2.7806 (2.7169-2.8496)	67.65% (59.6%-74.75%)	100% (100%-100%)	12.129% (3.333%-21.2%)	66.14% (58.33%-73.68%)	100% (100%-100%)	2.7831 (2.6573-2.9149)
At least 1 of CHASM (breast), CHASM (melanoma), FATHMM (cancer), FATHMM (missense), Mutation Assessor, PolyPhen-2, VEST	66.01% (62.12%-70.2%)	100% (100%-100%)	7.229% (3.999%-10.606%)	65.08% (61.14%-69.23%)	100% (100%-100%)	2.7232 (2.6698-2.7811)	65.96% (57.58%-73.74%)	100% (100%-100%)	7.525% (0%-14.63%)	64.99% (56.7%-72.92%)	100% (100%-100%)	2.7275 (2.6238-2.8431)
At least 1 of CHASM (breast), CHASM (melanoma), FATHMM (cancer), FATHMM (missense), Mutation Assessor, PROVEAN	69.05% (65.15%-72.74%)	98.97% (98.33%-100%)	17.299% (11.94%-22.222%)	67.42% (63.59%-71.35%)	90.65% (81.82%-100%)	2.7434 (2.5975-2.8803)	68.98% (61.59%-76.77%)	98.86% (96.67%-100%)	17.654% (7.895%-28.21%)	67.33% (59.34%-75.01%)	89.98% (66.67%-100%)	2.7383 (2.4324-2.9783)

At least 1 of CHASM (breast), CHASM (melanoma), FATHMM (cancer), FATHMM (missense), Mutation Assessor, PROVEAN, SIFT	67.01% (63.64%-70.71%)	100% (100%-100%)	9.951% (5.479%-13.847%)	65.76% (61.9%-69.79%)	100% (100%-100%)	2.7571 (2.6935-2.8202)	67% (59.6%-73.74%)	100% (100%-100%)	10.351% (2.776%-19.44%)	65.69% (57.73%-73.4%)	100% (100%-100%)	2.7614 (2.6467-2.8915)
At least 1 of CHASM (breast), CHASM (melanoma), FATHMM (cancer), FATHMM (missense), Mutation Assessor, PROVEAN, VEST	65.34% (61.62%-69.19%)	100% (100%-100%)	5.42% (2.597%-8.219%)	64.64% (60.82%-68.72%)	100% (100%-100%)	2.7009 (2.6535-2.7534)	65.27% (57.58%-72.73%)	100% (100%-100%)	5.651% (0%-12.12%)	64.53% (56.25%-72.16%)	100% (100%-100%)	2.7057 (2.6072-2.8051)
At least 1 of CHASM (breast), CHASM (melanoma), FATHMM (cancer), FATHMM (missense), Mutation Assessor, SIFT	68.01% (64.14%-71.72%)	100% (100%-100%)	12.694% (7.594%-16.901%)	66.45% (62.63%-70.43%)	100% (100%-100%)	2.7914 (2.7276-2.8596)	68.02% (60.61%-75.76%)	100% (100%-100%)	13.144% (4.875%-22.86%)	66.4% (58.51%-74.19%)	100% (100%-100%)	2.7959 (2.6699-2.9319)
At least 1 of CHASM (breast), CHASM (melanoma), FATHMM (cancer), FATHMM (missense), Mutation Assessor, SIFT, VEST	65.37% (61.62%-69.19%)	100% (100%-100%)	5.484% (2.631%-8.219%)	64.66% (60.82%-68.72%)	100% (100%-100%)	2.7017 (2.6535-2.7534)	65.22% (57.58%-72.73%)	100% (100%-100%)	5.526% (0%-12.12%)	64.5% (56.25%-72.17%)	100% (100%-100%)	2.7048 (2.6054-2.8051)
At least 1 of CHASM (breast), CHASM (melanoma), FATHMM (cancer), FATHMM (missense), Mutation Assessor, VEST	66.01% (62.12%-70.2%)	100% (100%-100%)	7.229% (3.999%-10.606%)	65.08% (61.14%-69.23%)	100% (100%-100%)	2.7232 (2.6698-2.7811)	65.96% (57.58%-73.74%)	100% (100%-100%)	7.525% (0%-14.63%)	64.99% (56.7%-72.92%)	100% (100%-100%)	2.7275 (2.6238-2.8431)
At least 1 of CHASM (breast), CHASM (melanoma), FATHMM (cancer), FATHMM (missense), MutationTaster, PolyPhen-2	71.36% (68.18%-75.25%)	100% (100%-100%)	21.846% (16.418%-27.143%)	68.87% (65.19%-72.87%)	100% (100%-100%)	2.9072 (2.8317-2.9865)	71.41% (63.64%-77.78%)	100% (100%-100%)	22.342% (12.188%-33.33%)	68.85% (60.67%-76.34%)	100% (100%-100%)	2.9119 (2.7665-3.0724)
At least 1 of CHASM (breast), CHASM (melanoma), FATHMM (cancer), FATHMM (missense), MutationTaster, PolyPhen-2, PROVEAN	69.03% (65.15%-72.73%)	100% (100%-100%)	15.477% (10.39%-20.27%)	67.17% (63.44%-70.97%)	100% (100%-100%)	2.8264 (2.7588-2.8969)	69.01% (61.62%-76.77%)	100% (100%-100%)	15.794% (6.061%-25.82%)	67.09% (59.34%-74.47%)	100% (100%-100%)	2.8288 (2.6952-2.9683)
At least 1 of CHASM (breast), CHASM (melanoma), FATHMM (cancer), FATHMM (missense), MutationTaster, PolyPhen-2, SIFT	68.71% (65.15%-72.73%)	100% (100%-100%)	14.601% (9.459%-19.119%)	66.94% (63.1%-70.98%)	100% (100%-100%)	2.8154 (2.7460-2.8885)	68.65% (60.61%-75.76%)	100% (100%-100%)	14.857% (5.714%-25%)	66.84% (58.69%-74.47%)	100% (100%-100%)	2.8170 (2.6815-2.9650)
At least 1 of CHASM (breast), CHASM (melanoma), FATHMM (cancer), FATHMM (missense), MutationTaster, PolyPhen-2, VEST	67% (63.13%-70.72%)	100% (100%-100%)	9.941% (5.714%-13.638%)	65.75% (61.9%-69.84%)	100% (100%-100%)	2.7569 (2.6979-2.8202)	67.01% (59.57%-74.75%)	100% (100%-100%)	10.361% (2.941%-18.75%)	65.69% (57.45%-73.41%)	100% (100%-100%)	2.7615 (2.6488-2.8915)
At least 1 of CHASM (breast), CHASM (melanoma), FATHMM (cancer), FATHMM (missense), MutationTaster, PROVEAN	70.42% (66.67%-74.24%)	99.49% (99.16%-100%)	20.149% (14.493%-25.334%)	68.3% (64.52%-72.05%)	95.83% (91.65%-100%)	2.8377 (2.7307-2.9496)	70.27% (62.63%-77.78%)	99.42% (98.21%-100%)	20.214% (9.992%-31.25%)	68.14% (60.23%-75.87%)	95.31% (80%-100%)	2.8309 (2.5688-3.0245)
At least 1 of CHASM (breast), CHASM (melanoma), FATHMM (cancer), FATHMM (missense), MutationTaster, PROVEAN, SIFT	67.71% (64.14%-71.21%)	100% (100%-100%)	11.875% (7.143%-16.004%)	66.24% (62.5%-70.16%)	100% (100%-100%)	2.7812 (2.7169-2.8493)	67.61% (60.61%-74.75%)	100% (100%-100%)	12.004% (3.331%-21.06%)	66.11% (58.33%-73.63%)	100% (100%-100%)	2.7816 (2.6597-2.9225)
At least 1 of CHASM (breast), CHASM (melanoma), FATHMM (cancer), FATHMM (missense), MutationTaster, PROVEAN, VEST	66% (62.12%-69.7%)	100% (100%-100%)	7.218% (3.896%-10.256%)	65.08% (61.14%-69.07%)	100% (100%-100%)	2.7230 (2.6679-2.7784)	65.97% (58.59%-73.74%)	100% (100%-100%)	7.551% (0%-15.15%)	64.99% (56.84%-72.92%)	100% (100%-100%)	2.7273 (2.6270-2.8448)
At least 1 of CHASM (breast), CHASM (melanoma), FATHMM (cancer), FATHMM (missense), MutationTaster, SIFT	69.05% (65.66%-72.73%)	100% (100%-100%)	15.531% (10.446%-20.27%)	67.18% (63.39%-71.28%)	100% (100%-100%)	2.8271 (2.7588-2.9040)	68.97% (61.62%-75.76%)	100% (100%-100%)	15.733% (6.891%-25.71%)	67.07% (58.95%-74.73%)	100% (100%-100%)	2.8280 (2.6896-2.9827)
At least 1 of CHASM (breast), CHASM (melanoma), FATHMM (cancer), FATHMM (missense), MutationTaster, SIFT, VEST	66.36% (62.63%-70.2%)	100% (100%-100%)	8.196% (4.286%-11.594%)	65.32% (61.46%-69.43%)	100% (100%-100%)	2.7352 (2.6799-2.7972)	66.27% (58.59%-73.74%)	100% (100%-100%)	8.362% (2.12%-15.79%)	65.19% (56.84%-72.92%)	100% (100%-100%)	2.7376 (2.6270-2.8596)
At least 1 of CHASM (breast), CHASM (melanoma), FATHMM (cancer), FATHMM (missense), MutationTaster, VEST	67% (63.13%-70.72%)	100% (100%-100%)	9.941% (5.714%-13.638%)	65.75% (61.9%-69.84%)	100% (100%-100%)	2.7569 (2.6979-2.8202)	67.01% (59.57%-74.75%)	100% (100%-100%)	10.361% (2.941%-18.75%)	65.69% (57.45%-73.41%)	100% (100%-100%)	2.7615 (2.6488-2.8915)
At least 1 of CHASM (breast), CHASM (melanoma), FATHMM (cancer), FATHMM (missense), PolyPhen-2, PROVEAN	69.02% (65.64%-72.73%)	99.48% (99.16%-100%)	16.343% (10.959%-21.127%)	67.28% (63.64%-71.28%)	94.76% (88.89%-100%)	2.7787 (2.6534-2.8886)	69.03% (61.62%-75.78%)	99.44% (98.25%-100%)	16.802% (7.309%-27.59%)	67.23% (59.14%-74.47%)	94.6% (75%-100%)	2.7807 (2.4956-2.9857)
At least 1 of CHASM (breast), CHASM (melanoma), FATHMM (cancer), FATHMM (missense), PolyPhen-2, PROVEAN, SIFT	67.69% (64.14%-71.72%)	100% (100%-100%)	11.811% (6.944%-15.942%)	66.22% (62.43%-70.21%)	100% (100%-100%)	2.7804 (2.7144-2.8449)	67.66% (59.6%-74.75%)	100% (100%-100%)	12.136% (3.448%-21.88%)	66.14% (58.06%-73.68%)	100% (100%-100%)	2.7829 (2.6597-2.9247)
At least 1 of CHASM (breast), CHASM (melanoma), FATHMM (cancer), FATHMM (missense), PolyPhen-2, PROVEAN, VEST	66% (62.12%-69.7%)	100% (100%-100%)	7.218% (3.896%-10.256%)	65.08% (61.14%-69.07%)	100% (100%-100%)	2.7230 (2.6679-2.7784)	65.97% (58.59%-73.74%)	100% (100%-100%)	7.551% (0%-15.15%)	64.99% (56.84%-72.92%)	100% (100%-100%)	2.7273 (2.6270-2.8448)
At least 1 of CHASM (breast), CHASM (melanoma), FATHMM (cancer), FATHMM (missense), PolyPhen-2, SIFT	69.02% (65.66%-72.73%)	100% (100%-100%)	15.467% (10.145%-20%)	67.16% (63.39%-71.28%)	100% (100%-100%)	2.8263 (2.7569-2.8997)	69.02% (61.62%-75.76%)	100% (100%-100%)	15.865% (6.975%-25.81%)	67.1% (58.94%-74.73%)	100% (100%-100%)	2.8297 (2.6952-2.9833)
At least 1 of CHASM (breast), CHASM (melanoma), FATHMM (cancer), FATHMM (missense), PolyPhen-2, SIFT, VEST	66.36% (62.63%-70.2%)	100% (100%-100%)	8.196% (4.286%-11.594%)	65.32% (61.46%-69.43%)	100% (100%-100%)	2.7352 (2.6799-2.7972)	66.27% (58.59%-73.74%)	100% (100%-100%)	8.362% (2.12%-15.79%)	65.19% (56.84%-72.92%)	100% (100%-100%)	2.7376 (2.6270-2.8596)
At least 1 of CHASM (breast), CHASM (melanoma), FATHMM (cancer), FATHMM (missense), PolyPhen-2, VEST	67% (63.13%-70.72%)	100% (100%-100%)	9.941% (5.714%-13.638%)	65.75% (61.9%-69.84%)	100% (100%-100%)	2.7569 (2.6979-2.8202)	67.01% (59.57%-74.75%)	100% (100%-100%)	10.361% (2.941%-18.75%)	65.69% (57.45%-73.41%)	100% (100%-100%)	2.7615 (2.6488-2.8915)
At least 1 of CHASM (breast), CHASM (melanoma), FATHMM (cancer), FATHMM (missense), PROVEAN, SIFT	68.35% (64.65%-72.22%)	100% (100%-100%)	13.607% (8.568%-18.056%)	66.68% (62.96%-70.59%)	100% (100%-100%)	2.8029 (2.7341-2.8696)	68.36% (60.61%-75.76%)	100% (100%-100%)	14.027% (5.405%-24.24%)	66.63% (58.94%-73.96%)	100% (100%-100%)	2.8067 (2.6825-2.9599)
At least 1 of CHASM (breast), CHASM (melanoma), FATHMM (cancer), FATHMM (missense), PROVEAN, SIFT, VEST	65.69% (62.12%-69.7%)	100% (100%-100%)	6.365% (2.817%-9.459%)	64.87% (61.13%-68.91%)	100% (100%-100%)	2.7124 (2.6595-2.7660)	65.59% (57.58%-72.73%)	100% (100%-100%)	6.513% (0%-13.16%)	64.74% (56.7%-72.17%)	100% (100%-100%)	2.7158 (2.6162-2.8303)
At least 1 of CHASM (breast), CHASM (melanoma), FATHMM (cancer), FATHMM (missense), PROVEAN, VEST	65.69% (62.11%-69.7%)	99.5% (99.16%-100%)	7.218% (3.896%-10.256%)	64.97% (60.94%-68.95%)	89.41% (75%-100%)	2.6110 (2.4221-2.7594)	65.59% (57.58%-72.75%)	99.4% (98.25%-100%)	7.551% (0%-15.15%)	64.86% (56.84%-72.73%)	88.22% (50%-100%)	2.6011 (2.1511-2.8326)
At least 1 of CHASM (breast), CHASM (melanoma), FATHMM (cancer), FATHMM (missense), SIFT, VEST	66.36% (62.63%-70.2%)	100% (100%-100%)	8.196% (4.286%-11.594%)	65.32% (61.46%-69.43%)	100% (100%-100%)	2.7352 (2.6799-2.7972)	66.27% (58.59%-73.74%)	100% (100%-100%)	8.362% (2.12%-15.79%)	65.19% (56.84%-72.92%)	100% (100%-100%)	2.7376 (2.6270-2.8596)
At least 1 of CHASM (breast), CHASM (melanoma), FATHMM (cancer), Mutation Assessor, MutationTaster, PolyPhen-2	73.09% (69.7%-76.77%)	100% (100%-100%)	26.549% (20.833%-32.394%)	70.19% (66.3%-74.3%)	100% (100%-100%)	2.9673 (2.8938-3.0549)	73.02% (65.66%-79.8%)	100% (100%-100%)	26.702% (15.623%-38.1%)	70.07% (61.95%-77.78%)	100% (100%-100%)	2.9678 (2.8090-3.1276)
At least 1 of CHASM (breast), CHASM (melanoma), FATHMM (cancer), Mutation Assessor, MutationTaster, PolyPhen-2, PROVEAN	71.42% (67.68%-75.25%)	100% (100%-100%)	22.003% (16.438%-27.143%)	68.91% (65.19%-72.93%)	100% (100%-100%)	2.9092 (2.8348-2.9869)	71.3% (63.64%-78.79%)	100% (100%-100%)	22.008% (11.429%-32.56%)	68.76% (60.86%-76.4%)	100% (100%-100%)	2.9077 (2.7665-3.0622)
At least 1 of CHASM (breast), CHASM (melanoma), FATHMM (cancer), Mutation Assessor, MutationTaster, PolyPhen-2, SIFT	70.75% (67.17%-74.75%)	100% (100%-100%)	20.186% (14.667%-25.373%)	68.42% (64.6%-72.58%)	100% (100%-100%)	2.8860 (2.8118-2.9678)	70.62% (62.63%-77.78%)	100% (100%-100%)	20.199% (9.368%-31.43%)	68.26% (60%-75.82%)	100% (100%-100%)	2.8846 (2.7390-3.0446)
At least 1 of CHASM (breast), CHASM (melanoma), FATHMM (cancer), Mutation Assessor, MutationTaster, PolyPhen-2, VEST	67.05% (63.64%-70.72%)	100% (100%-100%)	10.073% (5.797%-13.893%)	65.79% (61.98%-69.79%)	100% (100%-100%)	2.7586 (2.7019-2.8237)	66.91% (59.57%-73.74%)	100% (100%-100%)	10.101% (2.632%-17.95%)	65.63% (57.45%-73.2%)	100% (100%-100%)	2.7584 (2.6401-2.8847)
At least 1 of CHASM (breast), CHASM (melanoma), FATHMM (cancer), Mutation Assessor, MutationTaster, PROVEAN	72.81% (69.19%-76.77%)	99.49% (99.16%-100%)	26.682% (20.896%-32.394%)	70.12% (66.29%-74.29%)	96.82% (93.75%-100%)	2.9311 (2.8321-3.0400)	72.56% (64.65%-79.8%)	99.42% (98.21%-100%)	26.438% (14.991%-38.1%)	69.87% (61.63%-77.66%)	96.39% (85.71%-100%)	2.9212 (2.6931-3.1051)
At least 1 of CHASM (breast), CHASM (melanoma), FATHMM (cancer), Mutation Assessor, MutationTaster, PROVEAN, SIFT	69.75% (66.16%-73.74%)	100% (100%-100%)	17.458% (12.5%-22.222%)	67.69% (63.83%-71.82%)	100% (100%-100%)	2.8514 (2.7844-2.9232)	69.58% (61.62%-76.77%)	100% (100%-100%)	17.381% (7.895%-27.27%)	67.5% (59.34%-75.26%)	100% (100%-100%)	2.8490 (2.7143-3.0022)
At least 1 of CHASM (breast), CHASM (melanoma), FATHMM (cancer), Mutation Assessor, MutationTaster, PROVEAN, VEST	66.72% (63.13%-70.71%)	100% (100%-100%)	9.189% (5.479%-12.821%)	65.56% (61.7%-69.63%)	100% (100%-100%)	2.7475 (2.6927-2.8109)	66.55% (58.59%-73.74%)	100% (100%-100%)	9.141% (2.562%-16.28%)	65.38% (57.29%-72.92%)	100% (100%-100%)	2.7464 (2.6292-2.8645)
At least 1 of CHASM (breast), CHASM (melanoma), FATHMM (cancer), Mutation Assessor, MutationTaster, SIFT	71.09% (67.68%-74.76%)	100% (100%-100%)	21.112% (15.385%-26.232%)	68.67% (64.84%-72.83%)	100% (100%-100%)	2.8978 (2.8237-2.9775)	70.95% (63.61%-77.78%)	100% (100%-100%)	21.114% (10.808%-32.26%)	68.51% (60.22%-76.09%)	100% (100%-100%)	2.8962 (2.7474-3.0514)
At least 1 of CHASM (breast), CHASM (melanoma), FATHMM (cancer), Mutation Assessor, MutationTaster, SIFT, VEST	66.75% (63.13%-70.71%)	100% (100%-100%)	9.254% (5.479%-12.857%)	65.58% (61.7%-69.63%)	100% (100%-100%)	2.7483 (2.6932-2.8130)	66.5% (58.59%-73.74%)	100% (100%-100%)	9.016% (2.498%-16.67%)	65.35% (57.29%-72.92%)	100% (100%-100%)	2.7449 (2.6270-2.8596)
At least 1 of CHASM (breast), CHASM (melanoma), FATHMM (cancer), Mutation Assessor, MutationTaster, VEST	67.39% (63.64%-71.21%)	100% (100%-100%)	10.999% (6.849%-15.068%)	66.02% (62.11%-70.16%)	100% (100%-100%)	2.7702 (2.7116-2.8385)	67.24% (59.6%-74.75%)	100% (100%-100%)	11.015% (2.778%-19.44%)	65.86% (57.72%-73.47%)	100% (100%-100%)	2.7694 (2.6467-2.8957)
[illegible]	[illegible]	[illegible]	[illegible]	[illegible]	[illegible]	[illegible]	[illegible]	[illegible]	[illegible]	[illegible]	[illegible]	[illegible]

At least 1 of CHASM (lung), CHASM (melanoma), FATHMM (cancer), Mutation Assessor, PROVEAN, SIFT	70.38% (66.67%-74.24%)	99.46% (99.16%-100%)	20.084% (14.706%-25.352%)	68.27% (64.32%-72.28%)	95.59% (91.67%-100%)	2.8341 (2.7268-2.9411)	70.36% (62.63%-77.78%)	99.48% (98.25%-100%)	20.379% (9.754%-30.56%)	68.19% (60.21%-76.09%)	95.79% (83.25%-100%)	2.8384 (2.5937-3.0446)
At least 1 of CHASM (lung), CHASM (melanoma), FATHMM (cancer), Mutation Assessor, PROVEAN, SIFT, VEST	66.41% (62.63%-70.21%)	100% (100%-100%)	8.336% (4.348%-11.765%)	65.35% (61.46%-69.43%)	100% (100%-100%)	2.7369 (2.6815-2.7972)	66.17% (58.56%-73.74%)	100% (100%-100%)	8.103% (2.167%-15.22%)	65.13% (57.14%-72.92%)	100% (100%-100%)	2.7340 (2.6238-2.8536)
At least 1 of CHASM (lung), CHASM (melanoma), FATHMM (cancer), Mutation Assessor, PROVEAN, VEST	66.72% (63.13%-70.71%)	100% (100%-100%)	9.189% (5.479%-12.821%)	65.56% (61.7%-69.63%)	100% (100%-100%)	2.7475 (2.6927-2.8109)	66.55% (58.59%-73.74%)	100% (100%-100%)	9.141% (2.562%-16.28%)	65.38% (57.29%-72.92%)	100% (100%-100%)	2.7464 (2.6292-2.8645)
At least 1 of CHASM (lung), CHASM (melanoma), FATHMM (cancer), Mutation Assessor, SIFT, VEST	66.75% (63.13%-70.71%)	100% (100%-100%)	9.254% (5.479%-12.857%)	65.58% (61.7%-69.63%)	100% (100%-100%)	2.7483 (2.6932-2.8130)	66.5% (58.59%-73.74%)	100% (100%-100%)	9.016% (2.498%-16.67%)	65.35% (57.29%-72.92%)	100% (100%-100%)	2.7449 (2.6270-2.8596)
At least 1 of CHASM (lung), CHASM (melanoma), FATHMM (cancer), MutationTaster, PolyPhen-2, PROVEAN	72.43% (69.19%-75.76%)	100% (100%-100%)	24.741% (18.841%-30.14%)	69.67% (65.92%-73.37%)	100% (100%-100%)	2.9442 (2.8711-3.0258)	72.32% (65.66%-78.79%)	100% (100%-100%)	24.78% (13.886%-35.72%)	69.53% (61.96%-77.02%)	100% (100%-100%)	2.9431 (2.7945-3.1051)
At least 1 of CHASM (lung), CHASM (melanoma), FATHMM (cancer), MutationTaster, PolyPhen-2, PROVEAN, SIFT	70.76% (67.17%-74.24%)	100% (100%-100%)	20.209% (15.068%-25.352%)	68.42% (64.8%-72.29%)	100% (100%-100%)	2.8863 (2.8153-2.9591)	70.59% (63.64%-77.78%)	100% (100%-100%)	20.114% (9.677%-30.56%)	68.24% (60.44%-75.56%)	100% (100%-100%)	2.8837 (2.7471-3.0416)
At least 1 of CHASM (lung), CHASM (melanoma), FATHMM (cancer), MutationTaster, PolyPhen-2, PROVEAN, VEST	67.05% (63.62%-70.71%)	100% (100%-100%)	10.062% (5.797%-13.893%)	65.78% (61.98%-69.68%)	100% (100%-100%)	2.7585 (2.6991-2.8237)	66.92% (59.6%-73.76%)	100% (100%-100%)	10.127% (2.701%-18.19%)	65.63% (57.89%-73.2%)	100% (100%-100%)	2.7585 (2.6379-2.8862)
At least 1 of CHASM (lung), CHASM (melanoma), FATHMM (cancer), MutationTaster, PolyPhen-2, SIFT	72.43% (69.19%-76.26%)	100% (100%-100%)	24.776% (18.841%-30.558%)	69.68% (65.93%-73.74%)	100% (100%-100%)	2.9446 (2.8645-3.0257)	72.3% (64.65%-78.79%)	100% (100%-100%)	24.782% (13.786%-36.11%)	69.52% (61.36%-76.92%)	100% (100%-100%)	2.9431 (2.7862-3.1099)
At least 1 of CHASM (lung), CHASM (melanoma), FATHMM (cancer), MutationTaster, PolyPhen-2, SIFT, VEST	67.4% (63.64%-71.21%)	100% (100%-100%)	11.04% (6.849%-15.002%)	66.03% (62.23%-70.16%)	100% (100%-100%)	2.7707 (2.7116-2.8422)	67.21% (59.6%-74.75%)	100% (100%-100%)	10.938% (2.778%-19.44%)	65.84% (57.45%-73.4%)	100% (100%-100%)	2.7688 (2.6411-2.9007)
At least 1 of CHASM (lung), CHASM (melanoma), FATHMM (cancer), MutationTaster, PolyPhen-2, VEST	68.04% (64.65%-71.73%)	100% (100%-100%)	12.785% (8.219%-16.927%)	66.47% (62.63%-70.59%)	100% (100%-100%)	2.7926 (2.7308-2.8613)	67.95% (60.58%-74.75%)	100% (100%-100%)	12.937% (5%-22.22%)	66.35% (58.33%-73.96%)	100% (100%-100%)	2.7932 (2.6693-2.9295)
At least 1 of CHASM (lung), CHASM (melanoma), FATHMM (cancer), MutationTaster, PROVEAN, SIFT	71.44% (68.18%-75.25%)	100% (100%-100%)	22.065% (16.438%-27.536%)	68.93% (65.22%-72.73%)	100% (100%-100%)	2.9100 (2.8364-2.9952)	71.25% (63.64%-77.78%)	100% (100%-100%)	21.904% (11.104%-32.63%)	68.73% (61.11%-76.09%)	100% (100%-100%)	2.9063 (2.7563-3.0653)
At least 1 of CHASM (lung), CHASM (melanoma), FATHMM (cancer), MutationTaster, PROVEAN, SIFT, VEST	67.07% (63.13%-71.21%)	100% (100%-100%)	10.135% (5.882%-14.085%)	65.8% (61.9%-69.79%)	100% (100%-100%)	2.7594 (2.7019-2.8237)	66.87% (58.59%-74.75%)	100% (100%-100%)	10.003% (2.63%-18.18%)	65.6% (57.73%-73.4%)	100% (100%-100%)	2.7574 (2.6399-2.8847)
At least 1 of CHASM (lung), CHASM (melanoma), FATHMM (cancer), MutationTaster, PROVEAN, VEST	67.38% (63.64%-71.21%)	100% (100%-100%)	10.988% (6.942%-15.068%)	66.02% (62.23%-70%)	100% (100%-100%)	2.7700 (2.7101-2.8375)	67.25% (59.6%-74.75%)	100% (100%-100%)	11.041% (2.857%-19.35%)	65.86% (57.89%-73.47%)	100% (100%-100%)	2.7698 (2.6463-2.9007)
At least 1 of CHASM (lung), CHASM (melanoma), FATHMM (cancer), MutationTaster, SIFT, VEST	67.74% (64.14%-71.72%)	100% (100%-100%)	11.965% (7.246%-16.131%)	66.26% (62.37%-70.37%)	100% (100%-100%)	2.7823 (2.7206-2.8536)	67.55% (59.6%-74.75%)	100% (100%-100%)	11.852% (2.941%-20.59%)	66.07% (57.89%-73.91%)	100% (100%-100%)	2.7800 (2.6531-2.9145)
At least 1 of CHASM (lung), CHASM (melanoma), FATHMM (cancer), PolyPhen-2, PROVEAN, SIFT	70.74% (67.17%-74.24%)	99.46% (99.16%-100%)	21.075% (15.709%-26.475%)	68.54% (64.84%-72.43%)	95.79% (91.67%-100%)	2.8487 (2.7488-2.9500)	70.64% (63.64%-77.78%)	99.48% (98.25%-100%)	21.122% (9.994%-32.35%)	68.4% (60.67%-75.56%)	95.95% (83.33%-100%)	2.8495 (2.6353-3.0446)
At least 1 of CHASM (lung), CHASM (melanoma), FATHMM (cancer), PolyPhen-2, PROVEAN, SIFT, VEST	66.73% (63.13%-70.71%)	100% (100%-100%)	9.209% (5.332%-12.862%)	65.57% (61.78%-69.48%)	100% (100%-100%)	2.7478 (2.6919-2.8130)	66.53% (58.59%-73.74%)	100% (100%-100%)	9.089% (2.439%-17.07%)	65.37% (57.44%-72.92%)	100% (100%-100%)	2.7464 (2.6292-2.8690)
At least 1 of CHASM (lung), CHASM (melanoma), FATHMM (cancer), PolyPhen-2, PROVEAN, VEST	67.05% (63.62%-70.71%)	100% (100%-100%)	10.062% (5.797%-13.893%)	65.78% (61.98%-69.68%)	100% (100%-100%)	2.7585 (2.6991-2.8237)	66.92% (59.6%-73.76%)	100% (100%-100%)	10.127% (2.701%-18.19%)	65.63% (57.89%-73.2%)	100% (100%-100%)	2.7585 (2.6379-2.8862)
At least 1 of CHASM (lung), CHASM (melanoma), FATHMM (cancer), PolyPhen-2, SIFT, VEST	67.4% (63.64%-71.21%)	100% (100%-100%)	11.04% (6.849%-15.002%)	66.03% (62.23%-70.16%)	100% (100%-100%)	2.7707 (2.7116-2.8422)	67.21% (59.6%-74.75%)	100% (100%-100%)	10.938% (2.778%-19.44%)	65.84% (57.45%-73.4%)	100% (100%-100%)	2.7688 (2.6411-2.9007)
At least 1 of CHASM (lung), CHASM (melanoma), FATHMM (cancer), PROVEAN, SIFT, VEST	67.07% (63.13%-71.21%)	100% (100%-100%)	10.135% (5.882%-14.085%)	65.8% (61.9%-69.79%)	100% (100%-100%)	2.7594 (2.7019-2.8237)	66.87% (58.59%-74.75%)	100% (100%-100%)	10.003% (2.63%-18.18%)	65.6% (57.73%-73.4%)	100% (100%-100%)	2.7574 (2.6399-2.8847)
At least 1 of CHASM (lung), CHASM (melanoma), FATHMM (missense), Mutation Assessor, MutationTaster, PolyPhen-2	72.72% (69.19%-76.26%)	100% (100%-100%)	25.556% (19.718%-31.081%)	69.9% (66.1%-73.89%)	100% (100%-100%)	2.9546 (2.8726-3.0416)	72.74% (65.66%-79.8%)	100% (100%-100%)	25.931% (14.993%-37.51%)	69.85% (61.7%-77.53%)	100% (100%-100%)	2.9578 (2.8011-3.1313)
At least 1 of CHASM (lung), CHASM (melanoma), FATHMM (missense), Mutation Assessor, MutationTaster, PolyPhen-2, PROVEAN	70.72% (67.17%-74.24%)	100% (100%-100%)	20.085% (14.706%-25.352%)	68.39% (64.44%-72.43%)	100% (100%-100%)	2.8848 (2.8138-2.9677)	70.68% (63.64%-77.78%)	100% (100%-100%)	20.343% (9.677%-30.56%)	68.3% (60.22%-76.34%)	100% (100%-100%)	2.8864 (2.7417-3.0446)
[illegible]												

At least 1 of FATHMM (cancer), FATHMM (missense), Mutation Assessor, MutationTaster, PROVEAN, VEST	65.67% (62.12%-69.27%)	100% (100%-100%)	6.304% (2.617%-9.377%)	64.86% (61.14%-68.91%)	100% (100%-100%)	2.7118 (2.6597-2.7660)	65.63% (57.58%-72.73%)	100% (100%-100%)	6.631% (0%-13.16%)	64.77% (56.7%-72.16%)	100% (100%-100%)	2.7168 (2.6162-2.8289)
At least 1 of FATHMM (cancer), FATHMM (missense), Mutation Assessor, MutationTaster, SIFT, VEST	65.37% (61.62%-69.19%)	100% (100%-100%)	5.484% (2.631%-8.219%)	64.66% (60.82%-68.72%)	100% (100%-100%)	2.7017 (2.6535-2.7534)	65.22% (57.58%-72.73%)	100% (100%-100%)	5.526% (0%-12.12%)	64.5% (56.25%-72.17%)	100% (100%-100%)	2.7048 (2.6054-2.8051)
At least 1 of FATHMM (cancer), FATHMM (missense), Mutation Assessor, PolyPhen-2, PROVEAN, SIFT	67.02% (63.13%-71.21%)	100% (100%-100%)	9.999% (5.556%-13.889%)	65.77% (61.98%-69.79%)	100% (100%-100%)	2.7577 (2.6957-2.8202)	66.96% (58.99%-74.75%)	100% (100%-100%)	10.26% (2.703%-19.05%)	65.67% (57.72%-73.2%)	100% (100%-100%)	2.7601 (2.6449-2.8915)
At least 1 of FATHMM (cancer), FATHMM (missense), Mutation Assessor, PolyPhen-2, PROVEAN, SIFT, VEST	65.03% (61.11%-69.19%)	100% (100%-100%)	4.567% (1.428%-7.042%)	64.44% (60.51%-68.53%)	100% (100%-100%)	2.6904 (2.6429-2.7409)	64.89% (56.57%-72.73%)	100% (100%-100%)	4.613% (0%-10.53%)	64.28% (56.12%-72.16%)	100% (100%-100%)	2.6949 (2.6011-2.7917)
At least 1 of FATHMM (cancer), FATHMM (missense), Mutation Assessor, PolyPhen-2, SIFT, VEST	65.67% (62.12%-69.7%)	100% (100%-100%)	6.304% (2.817%-9.377%)	64.86% (61.14%-68.91%)	100% (100%-100%)	2.7118 (2.6597-2.7660)	65.63% (57.58%-72.73%)	100% (100%-100%)	6.631% (0%-13.16%)	64.77% (56.7%-72.16%)	100% (100%-100%)	2.7168 (2.6162-2.8289)
At least 1 of FATHMM (cancer), FATHMM (missense), Mutation Assessor, PROVEAN, SIFT, VEST	65.37% (61.62%-69.19%)	100% (100%-100%)	5.484% (2.631%-8.219%)	64.66% (60.82%-68.72%)	100% (100%-100%)	2.7017 (2.6535-2.7534)	65.22% (57.58%-72.73%)	100% (100%-100%)	5.526% (0%-12.12%)	64.5% (56.25%-72.17%)	100% (100%-100%)	2.7048 (2.6054-2.8051)
At least 1 of FATHMM (cancer), FATHMM (missense), MutationTaster, PolyPhen-2, PROVEAN, SIFT	65.03% (61.11%-69.19%)	100% (100%-100%)	4.567% (1.428%-7.042%)	64.44% (60.51%-68.53%)	100% (100%-100%)	2.6904 (2.6429-2.7409)	64.89% (56.57%-72.73%)	100% (100%-100%)	4.613% (0%-10.53%)	64.28% (56.12%-72.16%)	100% (100%-100%)	2.6949 (2.6011-2.7917)
At least 1 of FATHMM (cancer), FATHMM (missense), MutationTaster, PolyPhen-2, PROVEAN, VEST	67.7% (64.14%-71.72%)	100% (100%-100%)	11.858% (7.042%-16.176%)	66.24% (62.5%-70.22%)	100% (100%-100%)	2.7809 (2.7162-2.8493)	67.62% (59.6%-74.75%)	100% (100%-100%)	12.051% (3.226%-21.63%)	66.12% (58.06%-73.68%)	100% (100%-100%)	2.7821 (2.6597-2.9247)
At least 1 of FATHMM (cancer), FATHMM (missense), MutationTaster, PolyPhen-2, SIFT, VEST	65.69% (62.12%-69.7%)	100% (100%-100%)	6.365% (2.817%-9.459%)	64.87% (61.13%-68.91%)	100% (100%-100%)	2.7124 (2.6595-2.7660)	65.59% (57.58%-72.73%)	100% (100%-100%)	6.513% (0%-13.16%)	64.74% (56.7%-72.17%)	100% (100%-100%)	2.7158 (2.6162-2.8303)
At least 1 of FATHMM (cancer), FATHMM (missense), MutationTaster, PROVEAN, SIFT, VEST	66.33% (62.63%-70.2%)	100% (100%-100%)	8.103% (4.225%-11.431%)	65.3% (61.46%-69.27%)	100% (100%-100%)	2.7340 (2.6773-2.7949)	66.33% (58.59%-73.74%)	100% (100%-100%)	8.531% (2.5%-15.91%)	65.23% (57.28%-72.92%)	100% (100%-100%)	2.7387 (2.6270-2.8611)
At least 1 of FATHMM (cancer), FATHMM (missense), PolyPhen-2, PROVEAN, SIFT, VEST	66.36% (62.63%-70.2%)	100% (100%-100%)	8.196% (4.286%-11.594%)	65.32% (61.46%-69.43%)	100% (100%-100%)	2.7352 (2.6799-2.7972)	66.25% (58.59%-73.74%)	100% (100%-100%)	8.362% (2.12%-15.79%)	65.19% (56.84%-72.92%)	100% (100%-100%)	2.7376 (2.6270-2.8596)
At least 1 of FATHMM (cancer), Mutation Assessor, MutationTaster, PolyPhen-2, PROVEAN, SIFT	65.69% (62.12%-69.7%)	100% (100%-100%)	6.365% (2.817%-9.459%)	64.87% (61.13%-68.91%)	100% (100%-100%)	2.7124 (2.6595-2.7660)	65.59% (57.58%-72.73%)	100% (100%-100%)	6.513% (0%-13.16%)	64.74% (56.7%-72.17%)	100% (100%-100%)	2.7158 (2.6162-2.8303)
At least 1 of FATHMM (cancer), Mutation Assessor, MutationTaster, PolyPhen-2, PROVEAN, VEST	65.69% (62.12%-69.7%)	100% (100%-100%)	6.365% (2.817%-9.459%)	64.87% (61.13%-68.91%)	100% (100%-100%)	2.7124 (2.6595-2.7660)	65.59% (57.58%-72.73%)	100% (100%-100%)	6.513% (0%-13.16%)	64.74% (56.7%-72.17%)	100% (100%-100%)	2.7158 (2.6162-2.8303)
At least 1 of FATHMM (cancer), Mutation Assessor, MutationTaster, PolyPhen-2, SIFT, VEST	69.75% (66.16%-73.24%)	100% (100%-100%)	17.445% (12.5%-21.921%)	67.68% (63.93%-71.66%)	100% (100%-100%)	2.8513 (2.7827-2.9223)	69.59% (62.0%-76.77%)	100% (100%-100%)	17.391% (7.89%-27.27%)	67.51% (59.34%-75%)	100% (100%-100%)	2.8491 (2.7169-3.0048)
At least 1 of FATHMM (cancer), Mutation Assessor, MutationTaster, PROVEAN, SIFT, VEST	66.07% (62.63%-70.2%)	100% (100%-100%)	7.41% (4.108%-10.667%)	65.13% (61.65%-69.17%)	100% (100%-100%)	2.7254 (2.6739-2.7838)	65.83% (57.58%-72.73%)	100% (100%-100%)	7.189% (0%-13.9%)	64.9% (56.84%-72.45%)	100% (100%-100%)	2.7234 (2.6163-2.8322)
At least 1 of FATHMM (cancer), Mutation Assessor, PolyPhen-2, PROVEAN, SIFT, VEST	67.05% (63.64%-70.71%)	100% (100%-100%)	10.072% (5.968%-13.889%)	65.79% (62.03%-69.79%)	100% (100%-100%)	2.7586 (2.6998-2.8227)	66.91% (59.6%-73.74%)	100% (100%-100%)	10.124% (2.703%-18.18%)	65.63% (57.73%-73.12%)	100% (100%-100%)	2.7588 (2.6461-2.8847)
At least 1 of FATHMM (cancer), MutationTaster, PolyPhen-2, PROVEAN, SIFT, VEST	66.41% (62.63%-70.2%)	100% (100%-100%)	8.328% (4.348%-11.768%)	65.35% (61.49%-69.43%)	100% (100%-100%)	2.7369 (2.6834-2.7987)	66.17% (58.59%-73.74%)	100% (100%-100%)	8.101% (0%-15.38%)	65.13% (56.99%-72.73%)	100% (100%-100%)	2.7342 (2.6238-2.8488)
At least 1 of FATHMM (missense), Mutation Assessor, MutationTaster, PolyPhen-2, PROVEAN, SIFT	66.41% (62.63%-70.2%)	100% (100%-100%)	8.336% (4.348%-11.765%)	65.35% (61.46%-69.43%)	100% (100%-100%)	2.7369 (2.6815-2.7972)	66.17% (58.56%-73.74%)	100% (100%-100%)	8.103% (2.167%-15.22%)	65.13% (57.14%-72.92%)	100% (100%-100%)	2.7340 (2.6238-2.8536)
At least 1 of FATHMM (missense), Mutation Assessor, MutationTaster, PolyPhen-2, PROVEAN, VEST	66.07% (62.63%-70.2%)	100% (100%-100%)	7.41% (4.108%-10.667%)	65.13% (61.26%-69.11%)	100% (100%-100%)	2.7254 (2.6739-2.7836)	65.83% (57.58%-72.73%)	100% (100%-100%)	7.189% (0%-13.9%)	64.9% (56.84%-72.45%)	100% (100%-100%)	2.7234 (2.6163-2.8325)
At least 1 of FATHMM (missense), Mutation Assessor, MutationTaster, PolyPhen-2, SIFT, VEST	66.73% (63.13%-70.71%)	100% (100%-100%)	9.209% (5.332%-12.862%)	65.57% (61.78%-69.48%)	100% (100%-100%)	2.7478 (2.6916-2.8130)	66.53% (58.59%-73.74%)	100% (100%-100%)	9.089% (2.439%-17.07%)	65.37% (57.44%-72.92%)	100% (100%-100%)	2.7464 (2.6292-2.8690)
At least 1 of FATHMM (missense), Mutation Assessor, MutationTaster, PROVEAN, SIFT, VEST	69.39% (65.66%-72.74%)	100% (100%-100%)	16.466% (11.429%-21.215%)	67.43% (63.44%-71.28%)	100% (100%-100%)	2.8389 (2.7722-2.9124)	69.3% (62.0%-76.77%)	100% (100%-100%)	16.601% (7.135%-26.47%)	67.3% (59.34%-75.27%)	100% (100%-100%)	2.8390 (2.7050-2.9869)
At least 1 of FATHMM (missense), Mutation Assessor, PolyPhen-2, PROVEAN, SIFT, VEST	65.7% (61.62%-69.7%)	100% (100%-100%)	6.384% (2.857%-9.459%)	64.88% (60.94%-68.88%)	100% (100%-100%)	2.7126 (2.6595-2.7702)	65.57% (57.58%-73.74%)	100% (100%-100%)	6.467% (0%-12.91%)	64.73% (56.38%-72.63%)	100% (100%-100%)	2.7150 (2.6162-2.8308)
At least 1 of FATHMM (missense), MutationTaster, PolyPhen-2, PROVEAN, SIFT, VEST	66.34% (62.63%-70.2%)	100% (100%-100%)	8.122% (4.167%-11.594%)	65.3% (61.46%-69.39%)	100% (100%-100%)	2.7342 (2.6799-2.7949)	66.32% (58.59%-73.74%)	100% (100%-100%)	8.485% (2.439%-16.22%)	65.22% (57.14%-72.92%)	100% (100%-100%)	2.7386 (2.6270-2.8596)
At least 1 of Mutation Assessor, MutationTaster, PolyPhen-2, PROVEAN, SIFT, VEST	66.38% (62.63%-70.2%)	100% (100%-100%)	8.237% (4.284%-11.598%)	65.33% (61.34%-69.43%)	100% (100%-100%)	2.7357 (2.6773-2.7949)	66.23% (58.59%-73.74%)	100% (100%-100%)	8.264% (2.326%-15.79%)	65.17% (56.84%-73.12%)	100% (100%-100%)	2.7360 (2.6270-2.8605)
At least 1 of FATHMM (missense), Mutation Assessor, PROVEAN, SIFT, VEST	65.7% (61.62%-69.7%)	100% (100%-100%)	6.384% (2.857%-9.459%)	64.88% (60.94%-68.88%)	100% (100%-100%)	2.7126 (2.6595-2.7702)	65.57% (57.58%-73.74%)	100% (100%-100%)	6.467% (0%-12.91%)	64.73% (56.38%-72.63%)	100% (100%-100%)	2.7150 (2.6162-2.8308)
At least 1 of FATHMM (missense), Mutation Assessor, PolyPhen-2, SIFT, VEST	65.7% (61.62%-69.7%)	100% (100%-100%)	6.384% (2.857%-9.459%)	64.88% (60.94%-68.88%)	100% (100%-100%)	2.7126 (2.6595-2.7702)	65.57% (57.58%-73.74%)	100% (100%-100%)	6.467% (0%-12.91%)	64.73% (56.38%-72.63%)	100% (100%-100%)	2.7150 (2.6162-2.8308)
At least 1 of FATHMM (missense), MutationTaster, PolyPhen-2, PROVEAN, SIFT, VEST	66.36% (62.63%-70.2%)	100% (100%-100%)	8.183% (4.225%-11.538%)	65.32% (61.46%-69.43%)	100% (100%-100%)	2.7350 (2.6773-2.7917)	66.26% (58.59%-73.74%)	100% (100%-100%)	8.367% (2.498%-15.79%)	65.19% (57.14%-72.92%)	100% (100%-100%)	2.7371 (2.6292-2.8596)
At least 1 of Mutation Assessor, MutationTaster, PolyPhen-2, PROVEAN, SIFT, VEST	63.32% (59.6%-67.17%)	93.55% (91.27%-96.09%)	11.053% (6.7%-14.865%)	64.52% (60.56%-68.65%)	49.8% (35.29%-64.29%)	2.1893 (1.9909-2.3746)	63.25% (55.56%-70.71%)	93.74% (88.33%-98.33%)	10.900% (2.941%-19.58%)	64.36% (56.18%-72.22%)	50.49% (10%-90%)	2.1949 (1.7807-2.5938)
At least 2 of CHASM (breast), CHASM (lung), CHASM (melanoma), FATHMM (cancer), FATHMM (missense), Mutation Assessor	81.82% (78.79%-84.85%)	96.34% (94.49%-98.43%)	56.696% (50%-63.077%)	79.97% (75.77%-82.99%)	89.97% (85.37%-95.45%)	3.2238 (3.1004-3.3457)	81.82% (75.76%-87.88%)	96.14% (91.8%-100%)	57.216% (44.08%-70.59%)	79.41% (71.83%-86.67%)	89.63% (79.17%-100%)	3.2240 (2.9685-3.4729)
At least 2 of CHASM (breast), CHASM (lung), CHASM (melanoma), FATHMM (cancer), FATHMM (missense), Mutation Assessor, MutationTaster	82.15% (78.8%-85.35%)	98.97% (98.33%-100%)	53.058% (46.415%-59.459%)	78.47% (74.84%-82.1%)	96.76% (94.29%-100%)	3.2726 (3.1636-3.3777)	82.1% (75.76%-87.88%)	98.88% (95.74%-100%)	53.688% (40%-66.67%)	78.49% (71.43%-85.57%)	96.48% (89.47%-100%)	3.2732 (3.0611-3.4669)
At least 2 of CHASM (breast), CHASM (lung), CHASM (melanoma), FATHMM (cancer), FATHMM (missense), Mutation Assessor, PolyPhen-2	79.78% (76.77%-83.33%)	98.44% (97.52%-100%)	47.503% (41.026%-54.054%)	76.43% (72.61%-80.13%)	94.62% (90.91%-100%)	3.1698 (3.0588-3.2861)	79.84% (72.73%-85.86%)	98.34% (95.45%-100%)	48.077% (34.286%-60.98%)	76.48% (69.04%-83.75%)	94.41% (85%-100%)	3.1730 (2.9395-3.3910)
At least 2 of CHASM (breast), CHASM (lung), CHASM (melanoma), FATHMM (cancer), FATHMM (missense), Mutation Assessor, PROVEAN	79.13% (75.76%-82.32%)	96.34% (94.49%-98.43%)	49.354% (42.666%-56.164%)	76.69% (73.08%-80.37%)	88.65% (83.33%-94.87%)	3.1104 (2.9823-3.2379)	79.11% (72.73%-85.86%)	96.14% (91.8%-100%)	49.845% (36.357%-62.86%)	76.69% (69.23%-84.21%)	88.27% (76.47%-100%)	3.1056 (2.8493-3.3612)
At least 2 of CHASM (breast), CHASM (lung), CHASM (melanoma), FATHMM (cancer), FATHMM (missense), Mutation Assessor, SIFT	79.47% (76.26%-82.83%)	97.92% (96.69%-99.24%)	47.565% (41.093%-54.055%)	76.35% (72.67%-80.06%)	92.99% (88.89%-97.78%)	3.1482 (3.0293-3.2689)	79.44% (72.73%-85.86%)	97.77% (94.74%-100%)	47.974% (34.286%-60.62%)	76.4% (68.93%-83.75%)	92.64% (82.35%-100%)	3.1470 (2.9056-3.3711)
At least 2 of CHASM (breast), CHASM (lung), CHASM (melanoma), FATHMM (cancer), FATHMM (missense), Mutation Assessor, VEST	74.78% (71.21%-78.28%)	98.47% (97.52%-100%)	33.823% (27.397%-40.250%)	72.01% (68.39%-76%)	92.75% (87.5%-100%)	2.9705 (2.8587-3.0921)	74.68% (67.68%-81.82%)	98.27% (95.38%-100%)	34.167% (21.026%-47.06%)	71.93% (63.95%-79.27%)	91.94% (78.57%-100%)	2.9663 (2.6977-3.2168)
At least 2 of CHASM (breast), CHASM (lung), CHASM (melanoma), FATHMM (cancer), FATHMM (missense), MutationTaster	85.21% (82.32%-87.88%)	97.93% (96.72%-99.24%)	63.185% (56.75%-69.336%)	82.14% (78.95%-85.53%)	94.65% (91.3%-98.08%)	3.3792 (3.2696-3.4848)	85.15% (79.8%-90.91%)	97.74% (94.74%-100%)	63.488% (51.28%-75.76%)	82.13% (75.32%-88.43%)	94.25% (86.36%-100%)	3.3751 (3.1576-3.5853)
At least 2 of CHASM (breast), CHASM (lung), CHASM (melanoma), FATHMM (cancer), FATHMM (missense), MutationTaster, PolyPhen-2	81.11% (78.28%-84.34%)	98.97% (98.33%-100%)	50.371% (43.833%-56.761%)	77.52% (73.91%-81.13%)	96.59% (93.94%-100%)	3.2346 (3.1335-3.3424)	81.1% (74.75%-87.88%)	98.86% (96.67%-100%)	50.596% (37.5%-63.89%)	77.45% (70%-84.52%)	96.3% (88.89%-100%)	3.2321 (3.0134-3.4375)
At least 2 of CHASM (breast), CHASM (lung), CHASM (melanoma), FATHMM (cancer), FATHMM (missense), MutationTaster, PROVEAN	81.48% (78.28%-84.34%)	97.93% (96.72%-99.24%)	53.031% (46.263%-59.459%)	78.29% (74.68%-81.94%)	93.69% (89.47%-97.73%)	3.2295 (3.1170-3.3421)	81.47% (74.75%-87.88%)	97.74% (94.74%-100%)	53.515% (40%-66.67%)	78.31% (70.83%-85.71%)	93.24% (84.21%-100%)	3.2281 (2.9937-3.4519)
[illegible]	[illegible]	[illegible]	[illegible]	[illegible]	[illegible]	[illegible]	[illegible]	[illegible]	[illegible]	[illegible]	[illegible]	[illegible]
At least 2 of CHASM (lung), FATHMM (missense), MutationTaster, PROVEAN, VEST	[illegible]	[illegible]	[illegible]	[illegible]	[illegible]	[illegible]	[illegible]	[illegible]	[illegible]	[illegible]	[illegible]	[illegible]
At least 2 of CHASM (lung), FATHMM (missense), MutationTaster, PROVEAN, SIFT, VEST	[illegible]	[illegible]	[illegible]	[illegible]	[illegible]	[illegible]	[illegible]	[illegible]	[illegible]	[illegible]	[illegible]	[illegible]
At least 2 of CHASM (lung), FATHMM (missense), MutationTaster, PolyPhen-2, PROVEAN, SIFT	[illegible]	[illegible]	[illegible]	[illegible]	[illegible]	[illegible]	[illegible]	[illegible]	[illegible]	[illegible]	[illegible]	[illegible]
At least 2 of CHASM (lung), FATHMM (missense), MutationTaster, PolyPhen-2, PROVEAN, VEST	[illegible]	[illegible]	[illegible]	[illegible]	[illegible]	[illegible]	[illegible]	[illegible]	[illegible]	[illegible]	[illegible]	[illegible]
At least 2 of CHASM (lung), FATHMM (missense), MutationTaster, PolyPhen-2, SIFT, VEST	[illegible]	[illegible]	[illegible]	[illegible]	[illegible]	[illegible]	[illegible]	[illegible]	[illegible]	[illegible]	[illegible]	[illegible]
At least 2 of CHASM (lung), FATHMM (missense), MutationTaster, PROVEAN, SIFT, VEST	[illegible]	[illegible]	[illegible]	[illegible]	[illegible]	[illegible]	[illegible]	[illegible]	[illegible]	[illegible]	[illegible]	[illegible]
At least 2 of CHASM (lung), FATHMM (missense), PolyPhen-2, PROVEAN, SIFT	81.83% (78.79%-84.85%)	98.45% (97.52%-100%)	53.093% (46.575%-59.421%)	78.4% (74.69%-82.05%)	95.19% (91.89%-100%)	3.2512 (3.1342-3.3636)	[illegible]	[illegible]	[illegible]	[illegible]	[illegible]	[illegible]

At least 2 of CHASM (breast), CHASM (lung), FATHMM (missense), MutationTaster, PROVEAN, SIFT, VEST	74.1% (70.71%-77.78%)	100% (100%-100%)	29.304% (23.529%-35.213%)	70.98% (67.23%-74.86%)	100% (100%-100%)	3.0028 (2.9233-3.0892)	74.03% (66.67%-80.81%)	100% (100%-100%)	29.445% (17.239%-41.03%)	70.86% (63.04%-78.26%)	100% (100%-100%)	3.0031 (2.8413-3.1689)
[illegible]	[illegible]	[illegible]	[illegible]	[illegible]	[illegible]	[illegible]	[illegible]	[illegible]	[illegible]	[illegible]	[illegible]	[illegible]

At least 2 of CHASM (lung), CHASM (melanoma), Mutation Assessor, MutationTaster, PolyPhen-2, VEST	79.81% (76.77%-83.33%)	100% (100%-100%)	44.912% (38.459%-51.389%)	75.84% (72.29%-79.75%)	100% (100%-100%)	3.2075 (3.1219-3.3045)	79.77% (72.73%-85.86%)	100% (100%-100%)	45.052% (32.143%-57.9%)	75.74% (67.86%-83.12%)	100% (100%-100%)	3.2079 (3.0356-3.3897)
At least 2 of CHASM (lung), CHASM (melanoma), Mutation Assessor, MutationTaster, PROVEAN, SIFT	79.84% (76.77%-82.83%)	97.37% (95.93%-99.2%)	49.519% (43.077%-56.338%)	76.93% (73.2%-80.65%)	91.59% (87.18%-97.37%)	3.1541 (3.0380-3.2732)	79.72% (73.74%-85.86%)	97.28% (93.65%-100%)	49.589% (36.659%-62.51%)	76.8% (69.33%-84.42%)	91.43% (80%-100%)	3.1510 (2.9054-3.3766)
At least 2 of CHASM (lung), CHASM (melanoma), Mutation Assessor, MutationTaster, PROVEAN, VEST	78.85% (75.76%-82.32%)	98.95% (98.32%-100%)	44.084% (37.681%-50.685%)	75.37% (71.68%-79.25%)	96.06% (93.1%-100%)	3.1447 (3.0455-3.2551)	78.67% (71.72%-84.85%)	98.9% (96.67%-100%)	43.928% (31.247%-57.14%)	75.17% (67.46%-82.5%)	95.91% (87.48%-100%)	3.1396 (2.9190-3.3431)
At least 2 of CHASM (lung), CHASM (melanoma), Mutation Assessor, MutationTaster, SIFT, VEST	75.75% (72.22%-79.29%)	98.92% (98.33%-100%)	35.676% (29.485%-41.892%)	72.67% (68.82%-76.74%)	95.04% (91.67%-100%)	3.0231 (2.9190-3.1382)	75.78% (68.69%-82.83%)	98.96% (96.67%-100%)	36.014% (23.214%-48.94%)	72.63% (64.63%-80.46%)	95.27% (84.62%-100%)	3.0288 (2.7937-3.2403)
At least 2 of CHASM (lung), CHASM (melanoma), Mutation Assessor, PolyPhen-2, PROVEAN, SIFT	77.79% (74.24%-80.81%)	96.83% (95.12%-98.45%)	44.864% (38.233%-50.725%)	75.22% (71.34%-78.92%)	89.1% (83.77%-94.74%)	3.0601 (2.9301-3.1847)	77.76% (71.72%-84.85%)	96.77% (93.22%-100%)	45.136% (32.5%-58.33%)	75.16% (67.53%-82.93%)	89.06% (76.47%-100%)	3.0613 (2.8012-3.3163)
At least 2 of CHASM (lung), CHASM (melanoma), Mutation Assessor, PolyPhen-2, PROVEAN, VEST	76.78% (73.72%-80.3%)	97.87% (96.69%-99.22%)	40.295% (33.333%-46.755%)	73.92% (70.3%-77.71%)	91.63% (86.67%-97.06%)	3.0371 (2.9217-3.1574)	76.75% (69.7%-82.83%)	97.88% (94.92%-100%)	40.465% (27.273%-53.33%)	73.83% (66.25%-81.25%)	91.73% (78.94%-100%)	3.0390 (2.7811-3.2597)
At least 2 of CHASM (lung), CHASM (melanoma), Mutation Assessor, PolyPhen-2, SIFT, VEST	74.71% (71.21%-78.28%)	97.85% (96.72%-99.22%)	34.703% (28.571%-41.027%)	72.15% (68.26%-76.16%)	90.32% (84%-96.55%)	2.9502 (2.8255-3.0848)	74.82% (67.68%-81.82%)	97.91% (94.92%-100%)	35.191% (23.071%-47.5%)	72.16% (63.86%-79.76%)	90.73% (76.92%-100%)	2.9600 (2.6883-3.1913)
At least 2 of CHASM (lung), CHASM (melanoma), Mutation Assessor, PROVEAN, SIFT, VEST	74.03% (70.71%-77.78%)	97.34% (95.93%-99.19%)	33.732% (27.397%-40%)	71.75% (67.84%-75.74%)	88% (81.48%-96%)	2.9082 (2.7816-3.0445)	74.16% (66.67%-80.81%)	97.34% (93.75%-100%)	34.353% (22.5%-46.88%)	71.79% (63.86%-79.53%)	88.3% (72.73%-100%)	2.9178 (2.6230-3.1683)
At least 2 of CHASM (lung), CHASM (melanoma), MutationTaster, PolyPhen-2, PROVEAN, SIFT	79.49% (76.26%-82.83%)	98.93% (98.32%-100%)	45.875% (39.437%-52.056%)	75.96% (72.39%-79.63%)	96.12% (93.33%-100%)	3.1837 (3.0677-3.2724)	79.41% (72.73%-85.86%)	98.95% (96.67%-100%)	45.853% (33.333%-58.83%)	75.82% (68.42%-83.33%)	96.24% (87.5%-100%)	3.1687 (2.9566-3.3788)
At least 2 of CHASM (lung), CHASM (melanoma), MutationTaster, PolyPhen-2, PROVEAN, VEST	78.83% (75.76%-82.32%)	100% (100%-100%)	42.221% (35.821%-48.529%)	74.95% (71.34%-78.79%)	100% (100%-100%)	3.1717 (3.0861-3.2662)	78.71% (71.72%-84.85%)	100% (100%-100%)	42.14% (29.712%-55%)	74.78% (67.09%-82.02%)	100% (100%-100%)	3.1692 (3.0039-3.3514)
At least 2 of CHASM (lung), CHASM (melanoma), MutationTaster, PolyPhen-2, SIFT, VEST	76.78% (73.74%-80.3%)	100% (100%-100%)	36.642% (30.435%-42.466%)	73.18% (69.41%-77.06%)	100% (100%-100%)	3.0982 (3.0140-3.1853)	76.76% (69.7%-83.83%)	100% (100%-100%)	36.824% (25%-48.63%)	73.09% (65.11%-80.46%)	100% (100%-100%)	3.0991 (2.9409-3.2800)
At least 2 of CHASM (lung), CHASM (melanoma), MutationTaster, PROVEAN, SIFT, VEST	75.79% (72.73%-79.29%)	99.46% (99.16%-100%)	34.853% (28.571%-40.58%)	72.52% (68.79%-76.3%)	97.4% (95.45%-100%)	3.0424 (2.9472-3.1435)	75.7% (68.69%-82.83%)	99.49% (98.25%-100%)	34.867% (23.08%-47.5%)	72.38% (64.37%-79.76%)	97.51% (90%-100%)	3.0401 (2.8394-3.2338)
At least 2 of CHASM (lung), CHASM (melanoma), PolyPhen-2, PROVEAN, SIFT, VEST	74.75% (71.72%-78.28%)	98.39% (97.5%-100%)	33.88% (27.778%-39.706%)	72.01% (68.39%-75.86%)	92.41% (87.5%-100%)	2.9669 (2.8558-3.0888)	74.74% (67.68%-80.81%)	98.43% (95.45%-100%)	34.051% (22.857%-46.52%)	71.92% (64.04%-79.12%)	92.65% (80%-100%)	2.9708 (2.7125-3.1785)
At least 2 of CHASM (lung), FATHMM (cancer), FATHMM (missense), Mutation Assessor, MutationTaster, PolyPhen-2	80.81% (77.78%-83.84%)	98.97% (98.33%-100%)	49.4% (42.667%-56.338%)	77.18% (73.38%-80.89%)	96.53% (93.33%-100%)	3.2208 (3.1130-3.3318)	80.81% (74.75%-86.87%)	98.96% (96.49%-100%)	49.805% (36.36%-63.16%)	77.17% (69.62%-84.34%)	96.22% (88.24%-100%)	3.2207 (2.9943-3.4300)
At least 2 of CHASM (lung), FATHMM (cancer), FATHMM (missense), Mutation Assessor, MutationTaster, PROVEAN	82.13% (79.29%-84.86%)	98.97% (98.33%-100%)	52.996% (46.377%-59.459%)	78.45% (75.16%-82.05%)	96.76% (94.29%-100%)	3.2718 (3.1606-3.3733)	82.21% (76.77%-87.88%)	98.96% (96.67%-100%)	53.615% (40%-66.67%)	78.53% (71.42%-85.37%)	96.49% (89.47%-100%)	3.2750 (3.0685-3.4944)
At least 2 of CHASM (lung), FATHMM (cancer), FATHMM (missense), Mutation Assessor, MutationTaster, SIFT	81.47% (78.78%-84.36%)	99.49% (99.16%-100%)	50.301% (43.831%-56.523%)	77.59% (74.07%-81.21%)	98.28% (96.88%-100%)	3.2566 (3.1565-3.3603)	81.51% (75.76%-86.89%)	99.42% (98.21%-100%)	50.744% (37.838%-63.89%)	77.6% (70.24%-84.62%)	98.08% (93.33%-100%)	3.2585 (3.0674-3.4598)
At least 2 of CHASM (lung), FATHMM (cancer), FATHMM (missense), Mutation Assessor, MutationTaster, VEST	75.8% (72.22%-79.29%)	99.49% (99.16%-100%)	34.849% (28.746%-41.026%)	72.53% (68.82%-76.41%)	97.55% (95.24%-100%)	3.0442 (2.9470-3.1458)	75.66% (68.69%-82.83%)	99.49% (98.25%-100%)	34.874% (22.73%-47.5%)	72.38% (64.49%-79.76%)	97.24% (90%-100%)	3.0393 (2.8229-3.2284)
At least 2 of CHASM (lung), FATHMM (cancer), FATHMM (missense), Mutation Assessor, PolyPhen-2, PROVEAN	78.11% (74.75%-81.31%)	97.9% (96.69%-99.22%)	43.862% (36.921%-50.619%)	75.1% (71.34%-78.75%)	92.38% (87.88%-97.22%)	3.0924 (2.9727-3.2056)	78.13% (71.72%-84.85%)	97.8% (94.92%-100%)	44.347% (31.429%-58.34%)	75.1% (67.53%-82.56%)	92.19% (81.25%-100%)	3.0945 (2.8611-3.3149)
At least 2 of CHASM (lung), FATHMM (cancer), FATHMM (missense), Mutation Assessor, PolyPhen-2, SIFT	77.73% (74.75%-80.81%)	98.41% (97.5%-100%)	41.961% (35.382%-47.887%)	74.57% (70.81%-78.44%)	93.87% (89.66%-100%)	3.0881 (2.9759-3.1999)	77.87% (71.72%-83.84%)	98.38% (95.45%-100%)	42.642% (30.295%-55.56%)	74.64% (67.05%-82.05%)	93.93% (83.33%-100%)	3.0960 (2.8714-3.3079)
At least 2 of CHASM (lung), FATHMM (cancer), FATHMM (missense), Mutation Assessor, PolyPhen-2, VEST	73.73% (70.2%-77.27%)	99.48% (99.16%-100%)	29.201% (22.854%-35.135%)	70.84% (67.24%-74.86%)	97.03% (94.44%-100%)	2.9652 (2.8668-3.0705)	73.75% (66.67%-80.81%)	99.44% (98.25%-100%)	29.629% (17.857%-42.42%)	70.81% (63.08%-78.16%)	96.83% (87.5%-100%)	2.9673 (2.7497-3.1623)
At least 2 of CHASM (lung), FATHMM (cancer), FATHMM (missense), Mutation Assessor, PROVEAN, SIFT	78.46% (75.25%-81.82%)	97.39% (95.9%-99.2%)	45.707% (39.239%-52.113%)	75.62% (71.79%-79.25%)	91.02% (86.11%-97.06%)	3.0974 (2.9730-3.2205)	78.44% (71.72%-84.85%)	97.23% (93.65%-100%)	46.173% (33.333%-59.38%)	75.61% (67.95%-83.33%)	90.69% (79.98%-100%)	3.0971 (2.8414-3.3533)
At least 2 of CHASM (lung), FATHMM (cancer), FATHMM (missense), Mutation Assessor, PROVEAN, VEST	74.11% (70.71%-77.78%)	98.47% (97.5%-100%)	31.97% (25.352%-38.03%)	71.45% (67.63%-75.29%)	92.36% (86.96%-100%)	2.9425 (2.8121-3.0721)	74.07% (66.67%-81.82%)	98.46% (95.24%-100%)	32.337% (20%-45.45%)	71.37% (63.53%-78.89%)	91.51% (77.78%-100%)	2.9346 (2.6685-3.1778)
At least 2 of CHASM (lung), FATHMM (cancer), FATHMM (missense), Mutation Assessor, SIFT, VEST	73.36% (70.2%-76.77%)	100% (100%-100%)	27.306% (21.333%-33.333%)	70.4% (66.85%-74.1%)	100% (100%-100%)	2.9771 (2.8974-3.0622)	73.47% (66.67%-79.8%)	100% (100%-100%)	27.945% (16.667%-40%)	70.43% (62.5%-77.53%)	100% (100%-100%)	2.9838 (2.8290-3.1560)
At least 2 of CHASM (lung), FATHMM (cancer), FATHMM (missense), MutationTaster, PolyPhen-2, PROVEAN	80.82% (77.78%-83.84%)	98.97% (98.33%-100%)	49.415% (42.667%-55.718%)	77.19% (73.43%-80.98%)	96.53% (93.33%-100%)	3.2210 (3.1158-3.3307)	80.79% (74.75%-86.87%)	98.86% (96.67%-100%)	49.76% (37.143%-63.16%)	77.16% (69.41%-84.71%)	96.23% (88.89%-100%)	3.2201 (3.0055-3.4296)
At least 2 of CHASM (lung), FATHMM (cancer), FATHMM (missense), MutationTaster, PolyPhen-2, SIFT	79.46% (76.26%-82.83%)	100% (100%-100%)	43.884% (37.179%-50%)	75.59% (71.85%-79.17%)	100% (100%-100%)	3.1938 (3.1050-3.2863)	79.5% (72.73%-85.86%)	100% (100%-100%)	44.088% (31.25%-57.14%)	75.5% (68.24%-82.72%)	100% (100%-100%)	3.1970 (3.0290-3.3671)
At least 2 of CHASM (lung), FATHMM (cancer), FATHMM (missense), MutationTaster, PolyPhen-2, VEST	74.75% (71.21%-78.28%)	100% (100%-100%)	31.083% (25%-36.986%)	71.5% (67.98%-75.42%)	100% (100%-100%)	3.0258 (2.9407-3.1112)	74.75% (67.68%-81.82%)	100% (100%-100%)	31.392% (19.444%-43.75%)	71.44% (63.73%-78.66%)	100% (100%-100%)	3.0283 (2.8639-3.2150)
At least 2 of CHASM (lung), FATHMM (cancer), FATHMM (missense), MutationTaster, PROVEAN, SIFT	81.47% (78.28%-84.85%)	99.49% (99.16%-100%)	50.305% (43.748%-56.757%)	77.59% (74.07%-81.21%)	98.28% (96.88%-100%)	3.2567 (3.1577-3.3579)	81.5% (74.75%-87.88%)	99.42% (98.21%-100%)	50.739% (37.5%-63.89%)	77.6% (70.24%-84.62%)	98.09% (92.86%-100%)	3.2585 (3.0674-3.4579)
At least 2 of CHASM (lung), FATHMM (cancer), FATHMM (missense), MutationTaster, PROVEAN, VEST	75.48% (72.22%-78.79%)	99.49% (99.16%-100%)	34.811% (28.378%-40.909%)	72.42% (68.79%-76.16%)	97.56% (95.24%-100%)	3.0345 (2.9375-3.1322)	75.44% (68.69%-82.83%)	99.49% (98.25%-100%)	34.938% (22.485%-47.74%)	72.28% (64.37%-79.76%)	97.24% (90%-100%)	3.0330 (2.7865-3.2258)
At least 2 of CHASM (lung), FATHMM (cancer), FATHMM (missense), MutationTaster, SIFT, VEST	73.07% (69.7%-76.77%)	100% (100%-100%)	26.515% (20.547%-32.394%)	70.19% (66.48%-74.13%)	100% (100%-100%)	2.9669 (2.8874-3.0488)	73.05% (65.66%-79.8%)	100% (100%-100%)	26.779% (14.812%-39.39%)	70.09% (62.22%-77.53%)	100% (100%-100%)	2.9667 (2.8018-3.1383)
At least 2 of CHASM (lung), FATHMM (cancer), FATHMM (missense), PolyPhen-2, PROVEAN, SIFT	77.42% (74.24%-80.81%)	98.41% (97.5%-100%)	41.04% (34.328%-47.222%)	74.29% (70.66%-78.05%)	93.75% (89.66%-100%)	3.0756 (2.9555-3.1888)	77.49% (70.71%-83.84%)	98.36% (95.45%-100%)	41.589% (29.412%-54.55%)	74.3% (67.06%-81.71%)	93.76% (83.33%-100%)	3.0803 (2.8454-3.3044)
At least 2 of CHASM (lung), FATHMM (cancer), FATHMM (missense), PolyPhen-2, PROVEAN, VEST	72.41% (68.69%-75.76%)	98.98% (98.32%-100%)	26.449% (20.567%-32.395%)	69.94% (66.29%-73.84%)	93.74% (88.24%-100%)	2.8911 (2.7688-3.0036)	72.36% (65.63%-79.8%)	98.85% (96.67%-100%)	26.863% (15.148%-37.14%)	69.88% (62.35%-77.11%)	93.09% (80%-100%)	2.8868 (2.6353-3.1152)
At least 2 of CHASM (lung), FATHMM (cancer), FATHMM (missense), PolyPhen-2, SIFT, VEST	72.37% (69.19%-75.76%)	100% (100%-100%)	24.606% (18.458%-30.137%)	69.64% (65.93%-73.6%)	100% (100%-100%)	2.9424 (2.8640-3.0245)	72.42% (65.66%-79.8%)	100% (100%-100%)	25.081% (13.952%-36.84%)	69.61% (61.8%-76.92%)	100% (100%-100%)	2.9489 (2.7880-3.1156)
At least 2 of CHASM (lung), FATHMM (cancer), FATHMM (missense), PROVEAN, SIFT, VEST	72.05% (68.69%-75.76%)	99.5% (99.16%-100%)	24.506% (18.841%-30.381%)	69.53% (65.88%-73.45%)	96.62% (93.33%-100%)	2.9024 (2.8037-3.0019)	72.05% (64.65%-79.8%)	99.4% (98.25%-100%)	25.08% (13.929%-36.11%)	69.49% (61.9%-76.83%)	96.12% (85.71%-100%)	2.9009 (2.6682-3.1038)
At least 2 of CHASM (lung), FATHMM (cancer), Mutation Assessor, MutationTaster, PolyPhen-2, PROVEAN	81.18% (78.28%-84.34%)	98.44% (97.52%-100%)	51.357% (44.776%-57.746%)	77.77% (74.05%-81.41%)	95% (91.67%-100%)	3.2256 (3.1211-3.3353)	81.06% (74.75%-86.87%)	98.45% (95.16%-100%)	51.42% (38.71%-64.86%)	77.64% (70.37%-84.89%)	94.75% (86.36%-100%)	3.2215 (2.9996-3.4368)
At least 2 of CHASM (lung), FATHMM (cancer), Mutation Assessor, MutationTaster, PolyPhen-2, SIFT	79.1% (76.26%-82.32%)	99.46% (99.16%-100%)	43.891% (37.5%-50%)	75.4% (71.79%-79.04%)	97.93% (96.3%-100%)	3.1669 (3.0693-3.2663)	79.09% (72.73%-85.86%)	99.48% (98.25%-100%)	44.327% (32.255%-57.14%)	75.4% (67.9%-82.72%)	98.01% (92.29%-100%)	3.1721 (2.9614-3.3676)
At least 2 of CHASM (lung), FATHMM (cancer), Mutation Assessor, MutationTaster, PolyPhen-2, VEST	76.42% (73.23%-79.8%)	100% (100%-100%)	35.66% (29.167%-42.029%)	72.87% (69.23%-76.71%)	100% (100%-100%)	3.0851 (3.0068-3.1678)	76.46% (69.7%-82.83%)	100% (100%-100%)	36.05% (22.857%-47.62%)	72.85% (65.1%-80.23%)	100% (100%-100%)	3.0890 (2.9167-3.2622)
At least 2 of CHASM (lung), FATHMM (cancer), Mutation Assessor, MutationTaster, PROVEAN, SIFT	81.8% (78.79%-84.85%)	98.96% (98.33%-100%)	52.129% (45.711%-59.155%)	78.14% (74.51%-81.71%)	96.65% (94.29%-100%)	3.2588 (3.1587-3.3682)	81.86% (75.76%-87.88%)	98.96% (96.61%-100%)	52.615% (38.71%-65.71%)	78.17% (71.04%-85.19%)	96.53% (88.89%-100%)	3.2621 (3.0422-3.4695)
At least 2 of CHASM (lung), FATHMM (cancer), Mutation Assessor, MutationTaster, PROVEAN, VEST	76.78% (73.23%-80.3%)	99.49% (99.16%-100%)	37.512% (31.246%-44.118%)	73.36% (69.59%-77.12%)	97.71% (95.83%-100%)	3.0802 (2.9808-3.1850)	76.74% (69.7%-83.84%)	99.42% (98.21%-100%)	37.779% (24.762%-50%)	73.28% (65.85%-80.95%)	97.44% (90.91%-100%)	3.0792 (2.8564-3.2815)
At least 2 of CHASM (lung), FATHMM (cancer), Mutation Assessor, MutationTaster, SIFT, VEST	75.4% (72.22%-78.79%)	100% (100%-100%)	32.87% (26.761%-39.13%)	72.03% (68.23%-75.9%)	100% (100%-100%)	3.0490 (2.9656-3.1457)	75.46% (68.69%-81.82%)	100% (100%-100%)	33.364% (21.429%-45.71%)	72.03% (64.29%-79.55%)	100% (100%-100%)	3.0539 (2.8805-3.2358)
At least 2 of CHASM (lung), FATHMM (cancer), Mutation Assessor, PolyPhen-2, PROVEAN, SIFT	78.07% (74.75%-81.31%)	97.88% (96.69%-99.22%)	43.82% (37.333%-50%)	75.08% (71.34%-78.88%)	92.28% (87.5%-97.3%)	3.0906 (2.9679-3.2091)	78.2% (71.72%-84.85%)	97.86% (94.83%-100%)	44.454% (32.258%-57.14%)	75.14% (67.53%-82.56%)	92.39% (80%-100%)	3.0984 (2.8494-3.3292)
At least 2 of CHASM (lung), FATHMM (cancer), Mutation Assessor, PolyPhen-2, PROVEAN, VEST	74.05% (70.71%-77.78%)	98.94% (98.33%-100%)	31.006% (24.638%-37.143%)	71.26% (67.61%-75.2%)	94.43% (90%-100%)	2.9565 (2.8422-3.0687)	74.12% (66.67%-81.82%)	98.95% (96.49%-100%)	31.509% (19.17%-44.12%)	71.26% (63.75%-78.57%)	94.42% (83.33%-100%)	2.9610 (2.7075-3.1748)
At least 2 of CHASM (lung), FATHMM (cancer), Mutation Assessor, PolyPhen-2, SIFT, VEST	74.71% (71.21%-78.28%)	99.46% (99.16%-100%)	31.909% (25.676%-38.235%)	71.64% (67.82%-75.71%)	97.17% (94.74%-100%)	3.0018 (2.8969-3.1092)	74.82% (67.68%-81.82%)	99.48% (98.25%-100%)	32.516% (20%-45.45%)	71.67% (63.64%-79.31%)	97.31% (88.89%-100%)	3.0096 (2.7998-3.2139)
At least 2 of CHASM (lung), FATHMM (cancer), Mutation Assessor, PROVEAN, SIFT, VEST	74.01% (70.71%-77.27%)	98.93% (98.32%-100%)	30.921% (24.638%-37.143%)	71.23% (67.63%-75%)	94.34% (90.46%-100%)	2.9540 (2.8425-3.0721)	74.2% (67.68%-80.81%)	98.96% (96.67%-100%)	31.71% (18.919%-44.12%)	71.32% (63.53%-78.41%)	94.67% (81.82%-100%)	2.9666 (2.7124-3.1689)
At least 2 of CHASM (lung), FATHMM (cancer), MutationTaster, PolyPhen-2, PROVEAN, SIFT	78.77% (75.76%-82.32%)	98.89% (98.33%-100%)	42.999% (36.761%-49.296%)	75.11% (71.43%-79.01%)	97.89% (96.3%-100%)	3.1548 (3.0602-3.2574)	78.82% (71.72%-84.85%)	98.48% (98.25%-100%)	43.358% (30.303%-55.88%)	75.09% (67.81%-82.56%)	97.97% (91.67%-100%)	3.1589 (2.9602-3.3530)
At least 2 of CHASM (lung), FATHMM (cancer), MutationTaster, PolyPhen-2, PROVEAN, VEST	75.77% (72.73%-79.29%)	100% (100%-100%)	33.856% (27.692%-39.733%)	72.33% (68.79%-76.1%)	100% (100%-100%)	3.0619 (2.9858-3.1483)	75.74% (68.69%-81.82%)	100% (100%-100%)	34.074% (22.214%-45.95%)	72.25% (64.83%-79.31%)	100% (100%-100%)	3.0632 (2.8974-3.2373)
At least 2 of CHASM (lung), FATHMM (cancer), MutationTaster, PolyPhen-2, SIFT, VEST	75.42% (72.22%-78.79%)	100% (100%-100%)	32.925% (26.923%-39.394%)	72.05% (68.4%-75.94%)	100% (100%-100%)	3.0497 (2.9636-3.1418)	75.42% (68.69%-81.82%)	100% (100%-100%)	33.255% (21.622%-45.95%)	71.99% (63.95%-79.55%)	100% (100%-100%)	3.0525 (2.8805-3.2403)
At least 2 of CHASM (lung), FATHMM (cancer), MutationTaster, PROVEAN, SIFT, VEST	74.42% (71.21%-77.78%)	100% (100%-100%)	30.198% (24.285%-36.367%)	71.24% (67.61%-75.14%)	100% (100%-100%)	3.0144 (2.9322-3.1028)	74.38% (67.68%-80.81%)	100% (100%-100%)	30.406% (17.5%-42.11%)	71.15% (63.53%-78.41%)	100% (100%-100%)	3.0156 (2.8483-3.1853)
At least 2 of CHASM (lung), FATHMM (cancer), PolyPhen-2, PROVEAN, SIFT, VEST	73.73% (70.71%-77.27%)	99.46% (99.16%-100%)	29.225% (23.288%-35.294%)	70.86% (67.23%-74.85%)	96.93% (94.44%-100%)	2.9646 (2.8635-3.0667)	73.76% (66.67%-79.8%)	99.48% (98.25%-100%)	29.599% (17.494%-41.94%)	70.8% (62.92%-78.16%)	97.07% (87.5%-100%)	2.9695 (2.7524-3.1667)
At least 2 of CHASM (lung), FATHMM (missense), Mutation Assessor, MutationTaster, PolyPhen-2, PROVEAN	79.5% (76.26%-82.83%)	98.44% (97.52%-100%)	46.749% (39.726%-53.524%)	76.17% (72.33%-79.87%)	94.55% (90.91%-100%)	3.1590 (3.0453-3.2686)	79.39% (72.73%-85.86%)	98.33% (95.31%-100%)	46.85% (33.333%-60.53%)	76.05% (68.42%-83.33%)	94.25% (85%-100%)	3.1549 (2.9223-3.3635)
At least 2 of CHASM (lung), FATHMM (missense), Mutation Assessor, MutationTaster, PolyPhen-2, SIFT	78.08% (74.75%-81.31%)	99.47% (99.16%-100%)	41.081% (34.327%-47.297%)	74.48% (70.73%-78.32%)	97.82% (96%-100%)	3.1285 (3.0309-3.2285)	78.12% (71.72%-84.85%)	99.47% (98.28%-100%)	41.658% (28.571%-54.29%)	74.53% (66.67%-81.71%)	97.86% (91.67%-100%)	3.1352 (2.9405-3.3320)
At least 2 of CHASM (lung), FATHMM (missense), Mutation Assessor, MutationTaster, PolyPhen-2, VEST	76.43% (73.23%-79.8%)	100% (100%-100%)	35.683% (29.506%-42.029%)	72.89% (69.18%-76.83%)	100% (100%-100%)	3.0857 (3.0018-3.1785)	76.43% (69.7%-82.83%)	100% (100%-100%)	35.968% (23.333%-48.48%)	72.83% (64.77%-80.23%)	100% (100%-100%)	3.0879 (2.9168-3.2722)
At least 2 of CHASM (lung), FATHMM (missense), Mutation Assessor, MutationTaster, PROVEAN, SIFT	79.81% (76.77%-82.83%)	98.46% (97.52%-100%)	47.564% (40.764%-54.545%)	76.45% (72.84%-80%)	94.69% (91.18%-100%)	3.1712 (3.0528-3.2825)	79.78% (73.74%-85.86%)	98.31% (95.24%-100%)	47.953% (35.294%-60.61%)	76.43% (69.14%-83.72%)	94.33% (85%-100%)	3.1702 (2.9434-3.4112)
At least 2 of CHASM (lung), FATHMM (missense), Mutation Assessor, MutationTaster, PROVEAN, VEST	76.49% (73.23%-79.8%)	99.49% (99.16%-100%)	36.704% (30.513%-43.038%)	73.1% (69.4%-77.01%)	97.66% (95.65%-100%)	3.0696 (2.9683-3.1737)	76.32% (69.7%-82.83%)	99.47% (98.21%-100%)	36.663% (24.219%-50%)	72.93% (65.06%-80.23%)	97.35% (90%-100%)	3.0636 (2.8368-3.2662)
At least 2 of CHASM (lung), FATHMM (missense), Mutation Assessor, MutationTaster, SIFT, VEST	74.75% (71.21%-78.28%)	100% (100%-100%)	31.105% (25%-37.179%)	71.51% (67.78%-75.57%)	100% (100%-100%)	3.0261 (2.9383-3.1139)	74.74% (67.68%-81.82%)	100% (100%-100%)	31.387% (18.919%-44.12%)	71.43% (63.22%-79.07%)	100% (100%-100%)	3.0282 (2.8639-3.2136)
At least 2 of CHASM (lung), FATHMM (missense), Mutation Assessor, PolyPhen-2, PROVEAN, SIFT	76.07% (72.73%-79.29%)	97.36% (95.93%-99.19%)	39.245% (32.468%-45.205%)	73.48% (69.51%-77.25%)	89.6% (83.87%-96.67%)	2.9992 (2.8636-3.1259)	76.14% (69.7%-82.83%)	97.29% (93.75%-100%)	39.805% (27.778%-52.64%)	73.5% (65.88%-81.18%)	89.57% (75%-100%)	3.0016 (2.7362-3.2545)
At least 2 of CHASM (lung), FATHMM (missense), Mutation Assessor, PolyPhen-2, PROVEAN, VEST	74.08% (70.71%-77.78%)	98.95% (98.32%-100%)	31.059% (24.638%-36.986%)	71.28% (67.61%-75.14%)	94.48% (90.48%-100%)	2.9570 (2.8469-3.0735)	74.07% (66.67%-80.81%)	98.91% (96.67%-100%)	31.406% (19.442%-42.87%)	71.22% (63.52%-78.41%)	94.41% (82.82%-100%)	2.9595 (2.7136-3.1765)
At least 2 of CHASM (lung), FATHMM (missense), Mutation Assessor, PolyPhen-2, SIFT, VEST	73.72% (70.2%-77.27%)	99.47% (99.16%-100%)	29.195% (22.848%-34.783%)	70.84% (67.22%-74.72%)	96.95% (94.44%-100%)	2.9645 (2.8582-3.0680)	73.77% (66.67%-79.8%)	99.48% (98.25%-100%)	29.679% (17.507%-42.11%)	70.82% (62.97%-78.16%)	97.03% (87.5%-100%)	2.9699 (2.7569-3.1767)
At least 2 of CHASM (lung), FATHMM (missense), Mutation Assessor, PROVEAN, SIFT, VEST	73.05% (69.7%-76.77%)	98.42% (97.5%-100%)	29.175% (23.609%-35.135%)	70.61% (66.86%-74.57%)	91.43% (85.71%-100%)	2.8963 (2.7652-3.0195)	73.1% (65.66%-79.8%)	98.37% (95.45%-100%)	29.693% (17.647%-41.18%)	70.6% (62.92%-77.91%)	91.4% (77.78%-100%)	2.9007 (2.6323-3.1382)
At least 2 of CHASM (lung), FATHMM (missense), MutationTaster, PolyPhen-2, PROVEAN, SIFT	78.11% (74.75%-81.31%)	99.47% (99.16%-100%)	41.169% (34.721%-47.143%)	74.51% (70.79%-78.18%)	97.82% (95.83%-100%)	3.1297 (3.0330-3.2280)	78.12% (71.72%-84.85%)	99.47% (98.28%-100%)	41.457% (29.408%-54.29%)	74.47% (67.07%-81.61%)	97.83% (91.67%-100%)	3.1322 (2.9360-3.3319)
At least 2 of CHASM (lung), FATHMM (missense), MutationTaster, PolyPhen-2, PROVEAN, VEST	75.44% (71.72%-78.79%)	100% (100%-100%)	32.96% (26.76%-39.13%)	72.06% (68.42%-75.88%)	100% (100%-100%)	3.0502 (2.9732-3.1382)	75.39% (68.69%-81.82%)	100% (100%-100%)	33.12% (21.053%-45.45%)	71.96% (64.29%-79.07%)	100% (100%-100%)	3.0507 (2.8847-3.2257)
At least 2 of CHASM (lung), FATHMM (missense), MutationTaster, PolyPhen-2, SIFT, VEST	74.08% (70.71%-77.78%)	100% (100%-100%)	29.255% (23.188%-34.783%)	70.96% (67.22%-74.86%)	100% (100%-100%)	3.0022 (2.9207-3.0862)	74.07% (66.67%-80.81%)	100% (100%-100%)	29.588% (18.415%-42.11%)	70.9% (62.96%-78.41%)	100% (100%-100%)	3.0047 (2.8476-3.1768)
At least 2 of CHASM (lung), FATHMM (missense), MutationTaster, PROVEAN, SIFT, VEST	74.1% (70.71%-77.78%)	100% (100%-100%)	29.304% (23.529%-35.211%)	70.98% (67.22%-74.86%)	100% (100%-100%)	3.0022 (2.9233-3.0882)	74.09% (66.67%-80.81%)	100% (100%-100%)	29.443% (17.241%-41.03%)	70.86% (63.04%-78.41%)	100% (100%-100%)	3.0031 (2.8413-3.1689)
At least 2 of CHASM (lung), FATHMM (missense), PolyPhen-2, PROVEAN, SIFT, VEST	72.06% (68.69%-75.76%)	99.47% (99.16%-100%)	27.404% (21.233%-32.922%)	70.32% (66.67%-74.16%)	96.75% (93.75%-100%)	2.9394 (2.8363-3.0416)	73.06% (65.66%-79.8%)	99.47% (98.28%-100%)	27.735% (16.277%-40%)	70.26% (62.5%-77.53%)	96.79% (85.71%-100%)	2.9425 (2.7164-3.1382)
At least 2 of CHASM (lung), Mutation Assessor, MutationTaster, PolyPhen-2, PROVEAN, SIFT	75.72% (72.22%-79.29%)	91.95% (89.34%-95%)	47.646% (40.901%-54.056%)	75.23% (71.24%-79.22%)	77.4% (70.45%-84.62%)	2.9222 (2.7903-3.0677)	75.84% (68.69%-82.83%)	92.16% (86.21%-96.97%)	47.847% (35.135%-61.54%)	75.2% (67.12%-82.93%)	78.07% (63.62%-91.67%)	2.9328 (2.6434-3.2270)
At least 2 of CHASM (lung), Mutation Assessor, MutationTaster, PolyPhen-2, PROVEAN, VEST	72.7% (69.19%-76.26%)	90.33% (87.6%-93.5%)	42.166% (35.714%-48.592%)	72.88% (68.87%-76.92%)	71.66% (64.29%-79.41%)	2.7717 (2.6232-2.9203)	72.78% (65.66%-79.8%)	90.56% (84.37%-96.37%)	42.244% (29.729%-55.26%)	72.9% (64.29%-80.72%)	72.3% (56.25%-88.24%)	2.7800 (2.4751-3.0958)
At least 2 of CHASM (lung), Mutation Assessor, MutationTaster, PolyPhen-2, SIFT, VEST	73.33% (69.7%-76.77%)	93.55% (91.27%-96.06%)	38.382% (32.05%-44.84%)	72.41% (68.29%-76.36%)	77.51% (69.7%-85.71%)	2.8185 (2.6684-2.9643)	73.53% (66.67%-80.81%)	93.74% (88.33%-98.39%)	38.867% (27.267%-51.61%)	72.45% (64.28%-80.72%)	78.44% (62.5%-93.34%)	2.8350 (2.5401-3.1162)
At least 2 of CHASM (lung), Mutation Assessor, MutationTaster, PROVEAN, SIFT, VEST	72.33% (68.69%-75.77%)	93.01% (90.55%-95.8%)	36.505% (30.302%-42.857%)	71.71% (67.52%-75.61%)	75.64% (67.86%-83.33%)	2.7647 (2.6049-2.9141)	72.52% (65.66%-79.8%)	93.22% (88.06%-98.36%)	36.98% (24.228%-50%)	71.73% (64.19%-79.75%)	76.14% (60%-92.86%)	2.7808 (2.4689-3.0674)
At least 2 of CHASM (lung), Mutation Assessor, PolyPhen-2, PROVEAN, SIFT, VEST	69.3% (65.66%-73.23%)	88.77% (85.77%-91.72%)	35.609% (29.487%-41.429%)	70.49% (66.26%-74.51%)	64.73% (56.41%-73.81%)	2.5956 (2.4312-2.7536)	69.49% (61.62%-76.77%)	88.93% (82.54%-95.16%)	36.104% (24.399%-48.39%)	70.48% (62.19%-78.82%)	65.57% (47.62%-82.35%)	2.6108 (2.2969-2.9188)
At least 2 of CHASM (lung), MutationTaster, PolyPhen-2, PROVEAN, SIFT, VEST	72.36% (68.69%-75.76%)	92.48% (90%-95.28%)	37.56% (31.507%-43.592%)	71.92% (67.79%-75.78%)	74.31% (66.67%-82.22%)	2.7627 (2.6025-2.9051)	72.46% (65.66%-79.8%)	92.69% (86.89%-98.25%)	37.722% (25.712%-50%)	71.86% (64.2%-79.77%)	75.12% (57.89%-90.48%)	2.7742 (2.4819-3.0686)
At least 2 of CHASM (melanoma), FATHMM (cancer), FATHMM (missense), Mutation Assessor, MutationTaster, PolyPhen-2	80.14% (77.28%-83.33%)	98.97% (98.33%-100%)	47.57% (41.096%-54.054%)	76.53% (72.78%-80.31%)	96.44% (93.1%-100%)	3.1950 (3.0863-3.3041)	80.12% (73.74%-85.86%)	98.86% (96.67%-100%)	47.938% (34.286%-60.98%)	76.52% (68.67%-83.93%)	96.09% (87.5%-100%)	3.1941 (2.9761-3.4154)
At least 2 of CHASM (melanoma), FATHMM (cancer), FATHMM (missense), Mutation Assessor, MutationTaster, PROVEAN	80.48% (77.27%-83.84%)	98.97% (98.33%-100%)	48.486% (41.892%-55.074%)	76.87% (73.17%-80.49%)	96.46% (93.75%-100%)	3.2079 (3.1007-3.3139)	80.46% (74.75%-86.87%)	98.86% (96.67%-100%)	48.84% (35.294%-61.77%)	76.84% (69.51%-84.21%)	96.16% (88.24%-100%)	3.2071 (2.9943-3.4207)
At least 2 of CHASM (melanoma), FATHMM (cancer), FATHMM (missense), Mutation Assessor, MutationTaster, SIFT	79.8% (76.77%-82.83%)	99.49% (99.16%-100%)	45.752% (39.423%-52.174%)	76.09% (72.29%-79.76%)	98.12% (96.55%-100%)	3.1939 (3.1036-3.3000)	79.79% (73.74%-85.86%)	99.42% (98.21%-100%)	46.081% (32.498%-58.54%)	75.99% (68.67%-83.53%)	97.91% (92.86%-100%)	3.1940 (2.9869-3.3912)
At least 2 of CHASM (melanoma), FATHMM (cancer), FATHMM (missense), Mutation Assessor, MutationTaster, VEST	74.79% (71.72%-78.28%)	99.49% (99.16%-100%)	32.091% (25.758%-38.15%)	71.7% (68.02%-75.71%)	97.34% (95%-100%)	3.0092 (2.9023-3.1052)	74.66% (67.68%-81.82%)	99.47% (98.21%-100%)	32.14% (20%-44.44%)	71.55% (63.65%-78.98%)	97.02% (88.89%-100%)	3.0051 (2.7770-3.2003)
At least 2 of CHASM (melanoma), FATHMM (cancer), FATHMM (missense), Mutation Assessor, PolyPhen-2, PROVEAN	77.77% (74.75%-80.81%)	98.44% (97.52%-100%)	42.04% (35.065%-48.649%)	74.6% (70.81%-78.18%)	93.96% (90%-100%)	3.0903 (2.9731-3.2015)	77.88% (71.72%-83.84%)	98.42% (95.24%-100%)	42.476% (29.032%-55.82%)	74.59% (67.09%-81.93%)	93.72% (83.33%-100%)	3.0912 (2.8567-3.3153)
At least 2 of CHASM (melanoma), FATHMM (cancer), FATHMM (missense), Mutation Assessor, PolyPhen-2, SIFT	77.41% (74.24%-80.81%)	98.94% (98.33%-100%)	40.1% (33.333%-46.377%)	74.04% (70.15%-77.97%)	95.65% (92.59%-100%)	3.0884 (2.9799-3.1977)	77.51% (70.71%-83.84%)	98.92% (96.67%-100%)	40.743% (28.571%-54.05%)	74.13% (66.28%-81.71%)	95.84% (85.71%-100%)	3.0943 (2.8630-3.3148)
At least 2 of CHASM (melanoma), FATHMM (cancer), FATHMM (missense), Mutation Assessor, PolyPhen-2, VEST	72.72% (69.19%-76.26%)	99.48% (99.16%-100%)	26.446% (20.833%-32.432%)	70.09% (66.3%-74.01%)	96.74% (93.75%-100%)	2.9268 (2.8183-3.0258)	72.69% (65.66%-79.8%)	99.44% (98.25%-100%)	26.885% (14.815%-39.47%)	70.01% (62.22%-77.27%)	96.53% (85.71%-100%)	2.9278 (2.6977-3.1451)
At least 2 of CHASM (melanoma), FATHMM (cancer), FATHMM (missense), Mutation Assessor, PROVEAN, SIFT	77.8% (74.75%-81.31%)	97.92% (96.69%-99.22%)	42.993% (36.486%-49.382%)	74.81% (70.91%-78.57%)	92.3% (87.88%-97.22%)	3.0802 (2.9609-3.2008)	77.77% (70.71%-83.84%)	97.77% (94.73%-100%)	43.344% (30%-57.14%)	74.77% (67.06%-82.36%)	91.9% (80%-100%)	3.0776 (2.8221-3.3200)
At least 2 of CHASM (melanoma), FATHMM (cancer), FATHMM (missense), Mutation Assessor, PROVEAN, VEST	72.42% (69.19%-75.76%)	98.47% (97.52%-100%)	27.37% (21.333%-33.333%)	70.1% (66.43%-73.86%)	91.18% (85%-100%)	2.8714 (2.7419-2.9945)	72.32% (65.66%-79.8%)	98.27% (95.38%-100%)	27.751% (15.152%-39.47%)	70.02% (62.37%-77.65%)	90.27% (75.6%-100%)	2.8624 (2.5644-3.1261)
At least 2 of CHASM (melanoma), FATHMM (cancer), FATHMM (missense), Mutation Assessor, SIFT, VEST	72.03% (68.69%-75.76%)	100% (100%-100%)	23.674% (17.808%-29.412%)	69.38% (65.71%-73.33%)	100% (100%-100%)	2.9305 (2.8538-3.0121)	72.1% (64.65%-78.79%)	100% (100%-100%)	24.207% (12.812%-36.11%)	69.37% (61.54%-76.92%)	100% (100%-100%)	2.9357 (2.7713-3.1014)
At least 2 of CHASM (melanoma), FATHMM (cancer), FATHMM (missense), MutationTaster, PolyPhen-2, PROVEAN	79.83% (76.77%-82.83%)	98.97% (98.33%-100%)	46.719% (40%-53.521%)	76.26% (72.66%-80%)	96.34% (93.55%-100%)	3.1828 (3.0733-3.2833)	79.74% (73.74%-85.86%)	98.86% (96.67%-100%)	46.884% (34.286%-60%)	76.16% (68.35%-83.54%)	96.01% (87.5%-100%)	3.1792 (2.9671-3.4008)
At least 2 of CHASM (melanoma), FATHMM (cancer), FATHMM (missense), MutationTaster, PolyPhen-2, SIFT	79.12% (75.76%-82.32%)	100% (100%-100%)	42.999% (36.486%-49.296%)	75.21% (71.6%-78.82%)	100% (100%-100%)	3.1823 (3.0926-3.2742)	79.1% (72.73%-85.86%)	100% (100%-100%)	43.306% (30.764%-56.42%)	75.17% (67.82%-82.22%)	100% (100%-100%)	3.1848 (3.0169-3.3714)
At least 2 of CHASM (melanoma), FATHMM (cancer), FATHMM (missense), MutationTaster, PolyPhen-2, VEST	73.74% (70.2%-77.27%)	100% (100%-100%)	28.327% (22.077%-33.823%)	70.7% (67.06%-74.58%)	100% (100%-100%)	2.9902 (2.9060-3.0727)	73.69% (66.67%-80.81%)	100% (100%-100%)	28.635% (17.063%-41.67%)	70.63% (62.92%-77.78%)	100% (100%-100%)	2.9918 (2.8278-3.1617)
At least 2 of CHASM (melanoma), FATHMM (cancer), FATHMM (missense), MutationTaster, PROVEAN, SIFT	79.15% (76.26%-82.32%)	99.49% (99.16%-100%)	43.973% (37.681%-50%)	75.43% (71.69%-79.14%)	98.05% (96.43%-100%)	3.1694 (3.0738-3.2732)	79.07% (72.73%-84.85%)	99.42% (98.21%-100%)	44.1% (31.233%-57.1%)	75.34% (67.81%-82.72%)	97.82% (91.67%-100%)	3.1671 (2.9601-3.3715)
At least 2 of CHASM (melanoma), FATHMM (cancer), FATHMM (missense), MutationTaster, PROVEAN, VEST	73.47% (70.2%-76.77%)	98.99% (98.33%-100%)	29.343% (23.077%-35.616%)	70.78% (67.06%-74.58%)	94.64% (90.91%-100%)	2.9362 (2.8219-3.0467)	73.39% (66.67%-79.8%)	98.9% (96.61%-100%)	29.348% (17.143%-41.46%)	70.6% (62.79%-78.16%)	93.58% (80%-100%)	2.9235 (2.6707-3.1420)
At least 2 of CHASM (melanoma), FATHMM (cancer), FATHMM (missense), MutationTaster, SIFT, VEST	72.08% (68.69%-75.76%)	100% (100%-100%)	23.799% (18.31%-29.41%)	69.41% (65.75%-73.33%)	100% (100%-100%)	2.9321 (2.8551-3.0160)	72.01% (64.65%-79.8%)	100% (100%-100%)	23.96% (12.812%-35.14%)	69.3% (61.44%-76.67%)	100% (100%-100%)	2.9326 (2.7715-3.1051)
At least 2 of CHASM (melanoma), FATHMM (cancer), FATHMM (missense), PolyPhen-2, PROVEAN, SIFT	77.09% (73.74%-80.3%)	98.94% (98.33%-100%)	39.208% (32.432%-45.333%)	73.81% (70.17%-77.65%)	95.56% (92.31%-100%)	3.0791 (2.9658-3.1918)	77.16% (70.71%-83.84%)	98.92% (96.67%-100%)	39.681% (27.027%-52.78%)	73.83% (66.28%-81.01%)	95.53% (85.71%-100%)	3.0792 (2.8641-3.3011)
At least 2 of CHASM (melanoma), FATHMM (cancer), FATHMM (missense), PolyPhen-2, PROVEAN, VEST	71.73% (68.18%-75.25%)	98.98% (98.32%-100%)	24.612% (18.841%-30.821%)	69.42% (65.75%-73.18%)	93.31% (87.5%-100%)	2.8632 (2.7386-2.9775)	71.68% (64.65%-78.79%)	98.85% (96.67%-100%)	25.022% (13.158%-37.04%)	69.36% (61.63%-76.67%)	92.62% (77.78%-100%)	2.8585 (2.5847-3.0785)
At least 2 of CHASM (melanoma), FATHMM (cancer), FATHMM (missense), PolyPhen-2, SIFT, VEST	71.72% (68.18%-75.25%)	100% (100%-100%)	22.809% (16.901%-28.169%)	69.16% (65.38%-72.97%)	100% (100%-100%)	2.9194 (2.8403-3.0036)	71.72% (64.65%-78.79%)	100% (100%-100%)	23.178% (12.5%-34.38%)	69.08% (61.36%-76.67%)	100% (100%-100%)	2.9226 (2.7712-3.0861)
At least 2 of CHASM (melanoma), FATHMM (cancer), FATHMM (missense), PROVEAN, SIFT, VEST	71.06% (67.68%-74.75%)	99.5% (99.16%-100%)	21.878% (16.418%-27.451%)	68.71% (65.03%-72.43%)	96.22% (92.86%-100%)	2.8637 (2.7626-2.9669)	71.01% (63.64%-77.78%)	99.4% (98.25%-100%)	22.298% (11.108%-33.33%)	68.7% (61.29%-76.06%)	95.62% (83.33%-100%)	2.8592 (2.6193-3.0671)
At least 2 of CHASM (melanoma), FATHMM (cancer), Mutation Assessor, MutationTaster, PolyPhen-2, PROVEAN	80.19% (77.26%-83.33%)	98.44% (97.52%-100%)	48.653% (42.029%-55.556%)	76.82% (73.08%-80.65%)	94.74% (91.18%-100%)	3.1917 (3.0786-3.2995)	80.02% (73.74%-85.86%)	98.34% (95.45%-100%)	48.567% (35.294%-61.7%)	76.64% (68.83%-84.15%)	94.46% (85.71%-100%)	3.1801 (2.9436-3.3984)
At least 2 of CHASM (melanoma), FATHMM (cancer), Mutation Assessor, MutationTaster, PolyPhen-2, SIFT	78.45% (75.25%-81.82%)	99.46% (99.16%-100%)	42.119% (35.616%-48.485%)	74.87% (71.27%-78.61%)	97.85% (96%-100%)	3.1424 (3.0458-3.2415)	78.45% (71.72%-84.85%)	99.48% (98.25%-100%)	42.364% (30.301%-55.17%)	74.76% (67.07%-82.35%)	97.93% (91.67%-100%)	3.1452 (2.9602-3.3424)
At least 2 of CHASM (melanoma), FATHMM (cancer), Mutation Assessor, MutationTaster, PolyPhen-2, VEST	76.09% (72.73%-79.29%)	100% (100%-100%)	34.748% (28.358%-40.845%)	72.6% (68.96%-76.61%)	100% (100%-100%)	3.0735 (2.9938-3.1615)	76.18% (69.7%-82.83%)	100% (100%-100%)	35.088% (22.581%-47.37%)	72.55% (64.77%-80.22%)	100% (100%-100%)	3.0769 (2.9126-3.2526)
At least 2 of CHASM (melanoma), FATHMM (cancer), Mutation Assessor, MutationTaster, PROVEAN, SIFT	80.49% (77.78%-83.84%)	98.96% (98.33%-100%)	48.508% (41.791%-55.133%)	76.88% (73.17%-80.49%)	96.42% (93.55%-100%)	3.2080 (3.1056-3.3130)	80.44% (73.74%-85.86%)	98.96% (96.67%-100%)	48.781% (35.479%-62.16%)	76.81% (69.33%-84.15%)	96.27% (88.24%-100%)	3.2074 (2.9913-3.4209)
At least 2 of CHASM (melanoma), FATHMM (cancer), Mutation Assessor, MutationTaster, PROVEAN, VEST	75.47% (72.22%-79.29%)	99.49% (99.16%-100%)	33.918% (27.778%-40.541%)	72.25% (68.42%-76.05%)	97.49% (95.24%-100%)	3.0312 (2.9342-3.1395)	75.33% (67.68%-81.82%)	99.49% (98.25%-100%)	33.863% (21.429%-46.88%)	72.1% (64.29%-79.76%)	97.16% (88.89%-100%)	3.0264 (2.8040-3.2257)
At least 2 of CHASM (melanoma), FATHMM (cancer), Mutation Assessor, MutationTaster, SIFT, VEST	73.73% (70.71%-77.27%)	100% (100%-100%)	28.321% (22.388%-34.286%)	70.69% (67.03%-74.62%)	100% (100%-100%)	2.9901 (2.9060-3.0770)	73.83% (66.67%-80.81%)	100% (100%-100%)	28.687% (17.143%-40.62%)	70.64% (62.5%-78.16%)	100% (100%-100%)	2.9933 (2.8290-3.1687)
At least 2 of CHASM (melanoma), FATHMM (cancer), Mutation Assessor, PolyPhen-2, PROVEAN, SIFT	77.42% (74.24%-80.81%)	98.41% (97.52%-100%)	41.133% (34.783%-47.887%)	74.3% (70.37%-78.05%)	93.79% (89.29%-100%)	3.0757 (2.9699-3.1899)	77.48% (70.71%-83.84%)	98.4% (95.31%-100%)	41.572% (28.571%-54.55%)	74.29% (66.67%-82.14%)	93.8% (81.82%-100%)	3.0807 (2.8494-3.3053)
At least 2 of CHASM (melanoma), FATHMM (cancer), Mutation Assessor, PolyPhen-2, PROVEAN, VEST	74.06% (70.71%-77.27%)	98.94% (98.33%-100%)	31.039% (24.658%-36.986%)	71.27% (67.52%-75.14%)	94.44% (90%-100%)	2.9572 (2.8426-3.0664)	74.06% (66.67%-80.81%)	98.9% (96.49%-100%)	31.366% (18.919%-43.59%)	71.24% (63.53%-78.57%)	94.42% (82.82%-100%)	2.9590 (2.7063-3.1760)
At least 2 of CHASM (melanoma), FATHMM (cancer), Mutation Assessor, PolyPhen-2, SIFT, VEST	73.71% (70.2%-77.27%)	99.46% (99.16%-100%)	29.188% (22.972%-35.211%)	70.83% (67.05%-74.72%)	96.92% (94.12%-100%)	2.9640 (2.8607-3.0705)	73.76% (66.67%-80.81%)	99.48% (98.25%-100%)	29.695% (17.5%-42.11%)	70.83% (62.78%-78.41%)	97.04% (87.5%-100%)	2.9704 (2.7541-3.1794)
At least 2 of CHASM (melanoma), FATHMM (cancer), Mutation Assessor, PROVEAN, SIFT, VEST	72.01% (68.69%-75.76%)	98.93% (98.32%-100%)	25.471% (19.178%-31.429%)	69.65% (65.93%-73.48%)	93.22% (88.24%-100%)	2.8727 (2.7582-2.9885)	72.14% (64.65%-78.79%)	98.96% (96.67%-100%)	26.083% (14.286%-37.5%)	69.68% (61.8%-77.01%)	93.61% (77.78%-100%)	2.8832 (2.6180-3.0883)
At least 2 of CHASM (melanoma), FATHMM (cancer), MutationTaster, PolyPhen-2, PROVEAN, SIFT	78.12% (74.75%-81.31%)	98.95% (98.33%-100%)	41.216% (34.722%-47.826%)	74.55% (70.79%-78.11%)	97.84% (96%-100%)	3.1298 (3.0355-3.2284)	78.1% (71.72%-84.85%)	98.99% (96.25%-100%)	41.399% (29.032%-54.29%)	74.45% (66.67%-82.14%)	97.85% (91.67%-100%)	3.1310 (2.9405-3.3201)
At least 2 of CHASM (melanoma), FATHMM (cancer), MutationTaster, PolyPhen-2, PROVEAN, VEST	75.78% (72.73%-79.29%)	100% (100%-100%)	33.885% (27.536%-40.741%)	72.34% (68.79%-76.32%)	100% (100%-100%)	3.0622 (2.9836-3.1498)	75.72% (68.69%-82.83%)	100% (100%-100%)	34.026% (21.869%-45.71%)	72.23% (64.63%-79.55%)	100% (100%-100%)	3.0626 (2.8974-3.2373)
At least 2 of CHASM (melanoma), FATHMM (cancer), MutationTaster, PolyPhen-2, SIFT, VEST	74.42% (71.21%-77.78%)	100% (100%-100%)	30.2% (24.286%-36.111%)	71.24% (67.44%-75.28%)	100% (100%-100%)	3.0143 (2.9293-3.1009)	74.44% (67.68%-81.82%)	100% (100%-100%)	30.418% (18.919%-42.86%)	71.15% (63.22%-78.65%)	100% (100%-100%)	3.0159 (2.8593-3.1917)
At least 2 of CHASM (melanoma), FATHMM (cancer), MutationTaster, PROVEAN, SIFT, VEST	73.09% (69.7%-76.77%)	100% (100%-100%)	26.567% (21.125%-32.353%)	70.19% (66.67%-73.99%)	100% (100%-100%)	2.9676 (2.8893-3.0518)	73% (65.66%-79.8%)	100% (100%-100%)	26.645% (15%-37.84%)	70.06% (62.5%-77.27%)	100% (100%-100%)	2.9670 (2.8089-3.1276)
At least 2 of CHASM (melanoma), FATHMM (cancer), PolyPhen-2, PROVEAN, SIFT, VEST	72.73% (69.68%-76.26%)	99.46% (99.16%-100%)	26.503% (20.548%-32.395%)	70.06% (66.48%-73.99%)	96.62% (93.33%-100%)	2.9265 (2.8263-3.0303)	72.72% (65.66%-79.8%)	99.48% (98.28%-100%)	26.778% (14.706%-38.46%)	69.99% (62.22%-77.27%)	96.76% (85.71%-100%)	2.9300 (2.7164-3.1276)
At least 2 of CHASM (melanoma), FATHMM (missense), Mutation Assessor, MutationTaster, PolyPhen-2, PROVEAN	78.83% (75.76%-82.32%)	98.44% (97.52%-100%)	44.743% (38.235%-51.389%)	75.39% (71.79%-79.14%)	94.34% (90.63%-100%)	3.1316 (3.0212-3.2450)	78.7% (71.72%-84.85%)	98.34% (95.31%-100%)	44.879% (31.579%-58.82%)	75.16% (67.53%-82.72%)	96.02% (84.21%-100%)	3.1291 (2.9060-3.3611)
At least 2 of CHASM (melanoma), FATHMM (missense), Mutation Assessor, MutationTaster, PolyPhen-2, SIFT	78.05% (74.75%-81.31%)	99.47% (99.16%-100%)	40.196% (33.773%-46.479%)	74.3% (70.48%-78.1%)	97.79% (96%-100%)	3.1450 (3.0600-3.2386)	78.17% (71.72%-84.85%)	99.42% (98.28%-100%)	40.678% (28.562%-53.12%)	74.31% (66.67%-81.71%)	97.91% (91.67%-100%)	3.1499 (2.9782-3.3520)
At least 2 of CHASM (melanoma), FATHMM (missense), Mutation Assessor, MutationTaster, PolyPhen-2, VEST	75.42% (72.22%-78.79%)	100% (100%-100%)	32.928% (27.027%-38.893%)	72.05% (68.38%-75.84%)	100% (100%-100%)	3.0499 (2.9665-3.1382)	75.42% (68.69%-81.82%)	100% (100%-100%)	33.212% (21.429%-45.95%)	71.99% (64.29%-79.55%)	100% (100%-100%)	3.0520 (2.8874-3.2429)
At least 2 of CHASM (melanoma), FATHMM (missense), Mutation Assessor, MutationTaster, PROVEAN, SIFT	78.8% (75.76%-82.32%)	98.46% (98.32%-100%)	43.88% (37.333%-50.676%)	75.32% (71.51%-79.14%)	96.14% (93.1%-100%)	3.1435 (3.0322-3.2532)	78.76% (71.72%-84.85%)	98.41% (95.24%-100%)	44.248% (31.25%-57.14%)	75.27% (67.47%-82.56%)	95.77% (86.67%-100%)	3.1435 (2.9192-3.3506)
At least 2 of CHASM (melanoma), FATHMM (missense), Mutation Assessor, MutationTaster, PROVEAN, VEST	75.46% (72.22%-78.79%)	99.49% (99.16%-100%)	33.915% (27.692%-40.54%)	72.25% (68.46%-76.08%)	97.47% (95.24%-100%)	3.0313 (2.9302-3.1360)	75.33% (68.69%-81.82%)	99.42% (98.21%-100%)	33.967% (21.65%-46.88%)	72.1% (64.29%-79.76%)	97.14% (88.89%-100%)	3.0262 (2.8013-3.2286)
At least 2 of CHASM (melanoma), FATHMM (missense), Mutation Assessor, MutationTaster, SIFT, VEST	73.38% (70.2%-76.77%)	100% (100%-100%)	28.389% (22.535%-34.615%)	70.72% (67.02%-74.58%)	100% (100%-100%)	2.9910 (2.9062-3.0770)	73.76% (66.67%-80.81%)	100% (100%-100%)	28.568% (17.143%-41.18%)	70.6% (62.79%-78.16%)	100% (100%-100%)	2.9917 (2.8272-3.1687)
At least 2 of CHASM (melanoma), FATHMM (missense), Mutation Assessor, PolyPhen-2, PROVEAN, SIFT	75.41% (72.22%-78.79%)	97.36% (95.93%-99.19%)	37.444% (30.769%-43.662%)	72.91% (69.23%-76.61%)	89.16% (83.33%-96.3%)	2.9738 (2.8397-3.0977)	75.44% (68.69%-82.83%)	97.29% (93.75%-100%)	37.906% (25.712%-51.43%)	72.89% (65.11%-80.23%)	89.09% (75%-100%)	2.9718 (2.6994-3.2335)
At least 2 of CHASM (melanoma), FATHMM (missense), Mutation Assessor, PolyPhen-2, PROVEAN, VEST	73.74% (70.2%-77.27%)	99.48% (99.16%-100%)	29.222% (23.188%-34.783%)	70.85% (67.12%-74.72%)	97.02% (94.74%-100%)	2.9657 (2.8626-3.0668)	73.75% (66.67%-80.81%)	99.44% (98.25%-100%)	29.564% (17.852%-41.39%)	70.79% (63.04%-77.91%)	96.86% (87.5%-100%)	2.9662 (2.7497-3.1667)
At least 2 of CHASM (melanoma), FATHMM (missense), Mutation Assessor, PolyPhen-2, SIFT, VEST	73.4% (70.2%-76.77%)	100% (100%-100%)	27.398% (21.333%-33.333%)	70.45% (66.67%-74.44%)	100% (100%-100%)	2.9782 (2.8967-3.0653)	73.45% (66.67%-80.81%)	100% (100%-100%)	27.779% (16.667%-40%)	70.43% (62.64%-78.16%)	100% (100%-100%)	2.9818 (2.8187-3.1560)
At least 2 of CHASM (melanoma), FATHMM (missense), Mutation Assessor, PROVEAN, SIFT, VEST	72.06% (68.67%-75.76%)	98.95% (98.32%-100%)	25.558% (20%-31.52%)	69.68% (65.93%-73.63%)	93.37% (88.24%-100%)	2.8758 (2.7581-2.9906)	72.05% (64.65%-79.8%)	98.96% (96.49%-100%)	25.924% (14.634%-37.14%)	69.62% (61.79%-77.27%)	93.78% (77.78%-100%)	2.8776 (2.6065-3.0937)
At least 2 of CHASM (melanoma), FATHMM (missense), MutationTaster, PolyPhen-2, PROVEAN, SIFT	77.76% (74.75%-81.31%)	100% (100%-100%)	39.375% (32.857%-45.07%)	74.04% (70.35%-77.85%)	100% (100%-100%)	3.1342 (3.0478-3.2205)	77.76% (70.71%-83.84%)	100% (100%-100%)	39.55% (27.778%-52.64%)	73.95% (66.28%-81.25%)	100% (100%-100%)	3.1350 (2.9783-3.3200)
At least 2 of CHASM (melanoma), FATHMM (missense), MutationTaster, PolyPhen-2, PROVEAN, VEST	74.76% (71.72%-78.28%)	100% (100%-100%)	31.124% (24.658%-36.77%)	71.51% (67.88%-75.58%)	100% (100%-100%)	3.0259 (2.9425-3.1143)	74.71% (67.68%-81.82%)	100% (100%-100%)	31.287% (20%-43.59%)	71.41% (63.64%-78.82%)	100% (100%-100%)	3.0270 (2.8745-3.2008)
At least 2 of CHASM (melanoma), FATHMM (missense), MutationTaster, PolyPhen-2, SIFT, VEST	73.42% (70.2%-76.77%)	100% (100%-100%)	27.457% (21.617%-32.888%)	70.44% (66.85%-74.44%)	100% (100%-100%)	2.9790 (2.8980-3.0637)	73.37% (66.67%-80.81%)	100% (100%-100%)	27.665% (15.909%-40.63%)	70.34% (62.5%-78.16%)	100% (100%-100%)	2.9801 (2.8262-3.1529)
At least 2 of CHASM (melanoma), FATHMM (missense), MutationTaster, PROVEAN, SIFT, VEST	73.1% (69.7%-76.77%)	100% (100%-100%)	26.565% (20.548%-32.353%)	70.2% (66.48%-74.13%)	100% (100%-100%)	2.9681 (2.8918-3.0530)	73.02% (65.66%-79.8%)	100% (100%-100%)	26.614% (15.151%-38.47%)	70.07% (62.22%-77.38%)	100% (100%-100%)	2.9676 (2.8089-3.1309)
At least 2 of CHASM (melanoma), FATHMM (missense), PolyPhen-2, PROVEAN, SIFT, VEST	72.74% (69.19%-76.26%)	99.46% (99.16%-100%)	25.606% (19.48%-31.081%)	69.92% (66.29%-73.77%)	96.52% (93.33%-100%)	2.9552 (2.8776-3.0370)	72.7% (65.66%-79.8%)	99.48% (98.25%-100%)	25.832% (14.625%-37.5%)	69.82% (61.96%-77.27%)	96.63% (85.71%-100%)	2.9666 (2.8014-3.1223)
At least 2 of CHASM (melanoma), Mutation Assessor, MutationTaster, PolyPhen-2, PROVEAN, SIFT	75.4% (72.22%-78.79%)	92.48% (90%-95.31%)	45.857% (39.059%-52.113%)	74.71% (70.72%-78.67%)	77.92% (70.73%-85.71%)	2.9098 (2.7649-3.0456)	75.46% (68.69%-82.83%)	92.69% (86.56%-98.36%)	45.885% (33.333%-58.82%)	74.61% (66.67%-82.5%)	78.58% (63.62%-93.34%)	2.9172 (2.6603-3.2044)
At least 2 of CHASM (melanoma), Mutation Assessor, MutationTaster, PolyPhen-2, PROVEAN, VEST	72.71% (69.19%-76.26%)	90.35% (87.6%-93.25%)	42.192% (35.616%-48.611%)	72.99% (68.87%-77.01%)	71.79% (64.1%-79.31%)	2.7722 (2.6244-2.9231)	72.79% (65.66%-79.8%)	90.57% (84.38%-96.43%)	42.199% (29.412%-55.88%)	72.86% (64.94%-80.95%)	72.28% (56.25%-88.24%)	2.7791 (2.4725-3.0782)
At least 2 of CHASM (melanoma), Mutation Assessor, MutationTaster, PolyPhen-2, SIFT, VEST	72.34% (68.69%-75.76%)	93.55% (91.27%-96.06%)	35.661% (29.412%-41.667%)	71.54% (67.45%-75.63%)	76.24% (67.74%-84.38%)	2.7696 (2.6211-2.9111)	72.5% (64.65%-79.8%)	93.74% (88.33%-98.39%)	36.045% (24.138%-48.72%)	71.55% (63.29%-79.49%)	77.15% (60%-93.33%)	2.7848 (2.4706-3.0784)
At least 2 of CHASM (melanoma), Mutation Assessor, MutationTaster, PROVEAN, SIFT, VEST	70.67% (67.17%-74.24%)	93.01% (90.56%-95.8%)	32.022% (25.714%-38.028%)	70.29% (66.22%-74.29%)	72.67% (64.29%-81.48%)	2.6794 (2.5260-2.8361)	70.79% (63.64%-77.78%)	93.23% (88.14%-98.25%)	32.395% (20%-45.45%)	70.25% (62.22%-78.21%)	73.46% (55.56%-88.89%)	2.6935 (2.3832-3.0000)
At least 2 of CHASM (melanoma), Mutation Assessor, PolyPhen-2, PROVEAN, SIFT, VEST	68.34% (64.65%-71.72%)	88.8% (85.77%-91.73%)	33.001% (26.765%-39.189%)	69.58% (65.22%-73.89%)	63.03% (53.69%-71.88%)	2.5548 (2.3965-2.7155)	68.45% (61.62%-75.76%)	88.86% (81.97%-94.83%)	33.289% (21.429%-46.15%)	69.49% (60.92%-77.38%)	64.03% (44.44%-80.91%)	2.5541 (2.2278-2.8732)
At least 2 of CHASM (melanoma), MutationTaster, PolyPhen-2, PROVEAN, SIFT, VEST	71.36% (67.68%-74.75%)	92.48% (90%-95.28%)	34.838% (28.765%-40.58%)	71.05% (67.06%-75%)	72.85% (64.71%-81.25%)	2.7122 (2.5676-2.8565)	71.42% (64.65%-78.79%)	92.69% (86.89%-98.25%)	34.906% (23.251%-47.5%)	70.95% (63.09%-78.75%)	73.65% (56.25%-90.48%)	2.7221 (2.4146-3.0145)
At least 2 of FATHMM (cancer), FATHMM (missense), Mutation Assessor, MutationTaster, PolyPhen-2, PROVEAN	78.47% (75.25%-81.82%)	98.97% (98.33%-100%)	43.009% (36.364%-50%)	74.99% (71.43%-78.67%)	96.09% (92.86%-100%)	3.1304 (3.0193-3.2448)	78.41% (71.72%-84.85%)	98.85% (96.67%-100%)	43.207% (30.556%-55.56%)	74.95% (67.07%-82.35%)	95.92% (86.67%-100%)	3.1278 (2.9073-3.3417)
At least 2 of FATHMM (cancer), FATHMM (missense), Mutation Assessor, MutationTaster, PolyPhen-2, SIFT	77.1% (74.24%-80.3%)	99.49% (99.16%-100%)	37.199% (30.986%-43.243%)	73.58% (69.85%-77.37%)	97.63% (95.65%-100%)	3.0946 (2.9976-3.1911)	77.19% (70.71%-83.84%)	99.42% (98.21%-100%)	37.65% (25.641%-50%)	73.6% (65.85%-81.17%)	97.77% (91.67%-100%)	3.1003 (2.9082-3.2985)
At least 2 of FATHMM (cancer), FATHMM (missense), Mutation Assessor, MutationTaster, PolyPhen-2, VEST	73.74% (70.2%-77.27%)	100% (100%-100%)	28.335% (22.059%-34.188%)	70.7% (67.05%-74.58%)	100% (100%-100%)	2.9903 (2.9065-3.0762)	73.74% (66.67%-80.81%)	100% (100%-100%)	28.634% (17.143%-41.03%)	70.63% (62.79%-77.91%)	100% (100%-100%)	2.9927 (2.8325-3.1784)
At least 2 of FATHMM (cancer), FATHMM (missense), Mutation Assessor, MutationTaster, PROVEAN, SIFT	79.79% (76.77%-82.83%)	98.97% (98.33%-100%)	46.567% (40%-53.521%)	76.26% (72.55%-79.87%)	96.33% (93.55%-100%)	3.1823 (3.0732-3.2918)	79.83% (73.74%-85.86%)	98.86% (96.67%-100%)	47.055% (34.287%-60.61%)	76.25% (68.75%-83.76%)	96.11% (87.5%-100%)	3.1828 (2.9744-3.3851)
At least 2 of FATHMM (cancer), FATHMM (missense), Mutation Assessor, MutationTaster, PROVEAN, VEST	74.44% (71.21%-77.78%)	99.49% (99.16%-100%)	31.138% (25.333%-37.671%)	71.43% (67.65%-75.43%)	97.36% (95%-100%)	2.9979 (2.8967-3.0992)	74.41% (66.67%-80.81%)	99.44% (98.25%-100%)	31.407% (20.022%-44.44%)	71.38% (63.36%-78.91%)	97.14% (88.89%-100%)	2.9978 (2.7680-3.1986)
At least 2 of FATHMM (cancer), FATHMM (missense), Mutation Assessor, MutationTaster, SIFT, VEST	72.72% (69.19%-76.26%)	100% (100%-100%)	25.562% (19.718%-31.507%)	69.9% (66.3%-73.77%)	100% (100%-100%)	2.9532 (2.8735-3.0379)	72.77% (65.66%-79.8%)	100% (100%-100%)	25.961% (14.286%-38.1%)	69.88% (61.96%-77.27%)	100% (100%-100%)	2.9566 (2.8013-3.1266)
At least 2 of FATHMM (cancer), FATHMM (missense), Mutation Assessor, PolyPhen-2, PROVEAN, SIFT	76.42% (73.23%-79.8%)	98.41% (97.52%-100%)	37.993% (31.507%-44.595%)	73.39% (69.74%-77.11%)	93.18% (88.24%-100%)	3.0370 (2.9204-3.1517)	76.52% (69.7%-82.83%)	98.39% (95.31%-100%)	38.608% (25.711%-51.35%)	73.45% (65.48%-80.95%)	93.36% (80%-100%)	3.0434 (2.8022-3.2841)
At least 2 of FATHMM (cancer), FATHMM (missense), Mutation Assessor, PolyPhen-2, PROVEAN, VEST	72.39% (69.19%-76.26%)	98.98% (98.32%-100%)	26.485% (20.755%-32.4%)	69.92% (66.16%-73.84%)	93.79% (88.89%-100%)	2.8912 (2.7731-3.0056)	72.39% (64.65%-79.8%)	98.95% (96.49%-100%)	26.889% (15.151%-40%)	69.91% (62.07%-77.38%)	93.52% (80%-100%)	2.8913 (2.6385-3.1277)
At least 2 of FATHMM (cancer), FATHMM (missense), Mutation Assessor, PolyPhen-2, SIFT, VEST	72.36% (68.69%-75.76%)	100% (100%-100%)	24.606% (18.841%-30.556%)	69.63% (65.93%-73.6%)	100% (100%-100%)	2.9424 (2.8640-3.0278)	72.42% (65.66%-79.8%)	100% (100%-100%)	25.07% (13.889%-37.5%)	69.59% (61.54%-77.01%)	100% (100%-100%)	2.9460 (2.7852-3.1221)
At least 2 of FATHMM (cancer), FATHMM (missense), Mutation Assessor, PROVEAN, SIFT, VEST	72.04% (68.69%-75.76%)	99.49% (99.16%-100%)	24.609% (18.571%-30.667%)	69.58% (65.95%-73.48%)	96.53% (93.33%-100%)	2.9017 (2.8065-3.0051)	72.04% (65.66%-78.79%)	99.42% (98.21%-100%)	25.059% (13.513%-36.67%)	69.52% (61.9%-76.74%)	96.12% (85.71%-100%)	2.9006 (2.6864-3.0889)
At least 2 of FATHMM (cancer), FATHMM (missense), MutationTaster, PolyPhen-2, PROVEAN, SIFT	77.77% (74.75%-80.81%)	99.48% (99.16%-100%)	39.324% (32.877%-45.333%)	74.04% (70.37%-77.84%)	97.75% (95.65%-100%)	3.1135 (3.0164-3.2093)	77.79% (70.71%-83.84%)	99.47% (98.28%-100%)	39.605% (27.027%-52.78%)	73.99% (66.27%-81.71%)	97.77% (91.67%-100%)	3.1163 (2.9225-3.3161)
At least 2 of FATHMM (cancer), FATHMM (missense), MutationTaster, PolyPhen-2, PROVEAN, VEST	72.73% (69.19%-76.26%)	100% (100%-100%)	25.583% (19.718%-31.507%)	69.91% (66.3%-73.72%)	100% (100%-100%)	2.9549 (2.8755-3.0357)	72.7% (65.66%-79.8%)	100% (100%-100%)	25.782% (14.634%-37.5%)	69.82% (61.96%-77.27%)	100% (100%-100%)	2.9569 (2.8036-3.1277)
At least 2 of FATHMM (cancer), FATHMM (missense), MutationTaster, PolyPhen-2, SIFT, VEST	72.06% (68.69%-75.25%)	100% (100%-100%)	23.74% (17.908%-29.487%)	69.39% (65.75%-73.33%)	100% (100%-100%)	2.9313 (2.8538-3.0146)	72.02% (64.65%-79.8%)	100% (100%-100%)	24.073% (13.158%-35.42%)	69.33% (61.54%-76.74%)	100% (100%-100%)	2.9340 (2.7843-3.1002)
At least 2 of FATHMM (cancer), FATHMM (missense), MutationTaster, PROVEAN, SIFT, VEST	71.4% (68.18%-75.25%)	100% (100%-100%)	21.94% (16.667%-27.632%)	68.9% (65.3%-72.53%)	100% (100%-100%)	2.9107 (2.8346-2.9880)	71.34% (63.64%-78.79%)	100% (100%-100%)	22.107% (11.111%-33.33%)	68.79% (61.11%-76.14%)	100% (100%-100%)	2.9103 (2.7563-3.0669)
At least 2 of FATHMM (cancer), FATHMM (missense), PolyPhen-2, PROVEAN, SIFT, VEST	71.05% (67.68%-74.75%)	99.46% (99.16%-100%)	20.956% (15.278%-26.389%)	68.63% (65.03%-72.53%)	95.61% (92.31%-100%)	2.8968 (2.8239-2.9698)	71.06% (63.64%-77.78%)	99.48% (98.25%-100%)	21.384% (9.6154%-32.43%)	68.67% (60.71%-76.14%)	95.7% (83.33%-100%)	2.8902 (2.6581-3.0914)
At least 2 of FATHMM (cancer), Mutation Assessor, MutationTaster, PolyPhen-2, PROVEAN, SIFT	75.36% (72.22%-78.28%)	93.02% (90.55%-95.83%)	44.829% (38.028%-51.351%)	74.46% (70.59%-78.55%)	78.78% (71.79%-86.49%)	2.9109 (2.7656-3.0610)	75.54% (68.69%-81.82%)	93.22% (87.72%-98.33%)	45.197% (32.258%-58.54%)	74.48% (67.08%-81.71%)	79.56% (64.71%-94.12%)	2.9245 (2.6343-3.2044)
At least 2 of FATHMM (cancer), Mutation Assessor, MutationTaster, PolyPhen-2, PROVEAN, VEST	69.31% (65.66%-72.73%)	90.35% (87.6%-93.03%)	32.901% (26.761%-39.13%)	69.72% (65.55%-73.79%)	66.35% (57.69%-75%)	2.5957 (2.4503-2.7552)	69.47% (61.62%-76.77%)	90.56% (84.75%-96.49%)	33.22% (20.83%-46.15%)	69.6% (61.36%-77.22%)	67.24% (47.83%-84.21%)	2.6098 (2.3090-2.9047)
At least 2 of FATHMM (cancer), Mutation Assessor, MutationTaster, PolyPhen-2, SIFT, VEST	71.31% (67.68%-74.75%)	93.55% (91.27%-96.06%)	32.867% (26.863%-38.965%)	70.67% (66.58%-74.79%)	74.68% (65.67%-83.33%)	2.7177 (2.5631-2.8621)	71.47% (64.65%-78.79%)	93.74% (88.33%-98.39%)	33.371% (21.429%-46.15%)	70.66% (62.2%-78.75%)	75.72% (58.33%-92.31%)	2.7354 (2.4195-3.0431)
At least 2 of FATHMM (cancer), Mutation Assessor, MutationTaster, PROVEAN, SIFT, VEST	69.68% (66.67%-73.74%)	93.55% (91.27%-96.06%)	29.214% (23.226%-35.616%)	69.62% (65.66%-73.59%)	72.38% (63.33%-81.48%)	2.6471 (2.4959-2.7963)	70.14% (62.63%-77.78%)	93.74% (88.33%-98.39%)	29.624% (17.21%-42.86%)	69.57% (61.7%-77.5%)	73.43% (53.85%-92.31%)	2.6628 (2.3179-2.9682)
At least 2 of FATHMM (cancer), PolyPhen-2, PROVEAN, SIFT, VEST	67.62% (63.64%-71.21%)	90.3% (87.6%-93.02%)	30.183% (24.074%-36.364%)	68.68% (64.58%-72.56%)	62.94% (53.33%-72.41%)	2.5153 (2.3625-2.6651)	67.82% (60.61%-74.75%)	90.32% (84.21%-95.08%)	30.666% (19.048%-43.59%)	68.56% (60%-76.83%)	63.87% (44.44%-81.82%)	2.5217 (2.1997-2.8495)
At least 2 of FATHMM (cancer), MutationTaster, PolyPhen-2, PROVEAN, SIFT, VEST	69.66% (66.16%-73.23%)	92.48% (90%-95.28%)	30.183% (24.324%-36.111%)	69.61% (65.59%-73.68%)	70.05% (61.29%-78.57%)	2.6355 (2.4897-2.7911)	69.74% (62.63%-76.77%)	92.69% (86.89%-98.25%)	30.351% (18.421%-43.59%)	69.5% (60.98%-77.38%)	70.69% (50%-88.89%)	2.6425 (2.3316-2.9487)
At least 2 of FATHMM (missense), Mutation Assessor, MutationTaster, PolyPhen-2, PROVEAN, SIFT	73.33% (69.7%-76.77%)	93.02% (90.68%-95.8%)	38.295% (32.432%-45.07%)	72.88% (68.85%-76.84%)	75.61% (67.57%-83.33%)	2.8142 (2.6672-2.9566)	73.4% (65.66%-80.81%)	93.21% (88.06%-98.33%)	38.734% (26.471%-51.43%)	72.82% (64.93%-81.1%)	77.38% (61.11%-93.33%)	2.8295 (2.5365-3.1050)
At least 2 of FATHMM (missense), Mutation Assessor, MutationTaster, PolyPhen-2, PROVEAN, VEST	68.98% (65.66%-72.73%)	90.37% (87.6%-93.18%)	31.985% (25.676%-38.028%)	69.46% (65.48%-73.7%)	65.58% (56.52%-74.19%)	2.5768 (2.4244-2.7338)	69.11% (62.63%-76.77%)	90.57% (84.75%-96.43%)	32.312% (20%-45.71%)	69.35% (61.25%-77.17%)	66.58% (47.37%-84.21%)	2.5892 (2.2710-2.9052)
At least 2 of FATHMM (missense), Mutation Assessor, MutationTaster, PolyPhen-2, SIFT, VEST	69.99% (66.67%-73.74%)	93.55% (91.27%-96.06%)	30.153% (24.359%-36.986%)	69.87% (65.84%-74.03%)	73.34% (64.29%-82.14%)	2.6657 (2.5152-2.8153)	70.1% (62.63%-76.77%)	93.73% (88.33%-98.39%)	30.532% (18.921%-43.24%)	69.82% (61.73%-77.38%)	74.08% (54.55%-91.67%)	2.6810 (2.3518-2.9960)
At least 2 of FATHMM (missense), Mutation Assessor, MutationTaster, PROVEAN, SIFT, VEST	69% (65.66%-72.73%)	93.02% (90.55%-95.73%)	28.413% (22.222%-34.78%)	69.27% (65.25%-73.46%)	70.48% (61.29%-79.31%)	2.6120 (2.4583-2.7588)	69.18% (61.62%-76.77%)	93.22% (88.06%-98.31%)	28.723% (17.127%-41.19%)	69.15% (60.71%-77.11%)	71.57% (52.63%-88.24%)	2.6249 (2.3210-2.9260)
At least 2 of FATHMM (missense), Mutation Assessor, PolyPhen-2, PROVEAN, SIFT, VEST	67.3% (63.64%-71.21%)	90.38% (87.59%-93.02%)	27.646% (21.918%-33.333%)	68.28% (64.22%-72.29%)	62.23% (52.94%-71.43%)	2.4828 (2.3125-2.6488)	67.41% (59.6%-74.75%)	90.5% (84.21%-96.49%)	27.761% (16.667%-39.53%)	68.25% (60%-76.47%)	63.03% (43.75%-81.82%)	2.4954 (2.1292-2.8295)
At least 2 of FATHMM (missense), MutationTaster, PolyPhen-2, PROVEAN, SIFT, VEST	68.99% (65.66%-72.73%)	92.48% (90%-95.28%)	28.362% (22.535%-34.247%)	69.06% (65.09%-73.1%)	68.82% (59.26%-77.42%)	2.5850 (2.4335-2.7382)	69.09% (61.62%-75.76%)	92.69% (86.89%-98.25%)	28.653% (17.073%-41.03%)	68.98% (60.47%-76.74%)	69.86% (50%-88.89%)	2.5989 (2.2840-2.9057)
At least 2 of Mutation Assessor, MutationTaster, PolyPhen-2, PROVEAN, SIFT, VEST	67.3% (63.13%-70.71%)	86.65% (83.48%-89.96%)	33.841% (27.536%-40.58%)	69.37% (65%-73.67%)	59.47% (50%-68.42%)	2.4525 (2.2801-2.6301)	67.45% (59.6%-74.75%)	86.86% (79.66%-93.75%)	34.169% (21.621%-46.88%)	69.28% (60.47%-77.64%)	60.14% (41.18%-77.78%)	2.4675 (2.0955-2.8046)
At least 3 of (lung), CHASM (melanoma), FATHMM (cancer), FATHMM (missense), Mutation Assessor	85.56% (82.83%-88.38%)	90.49% (87.5%-93.55%)	77.036% (71.617%-82.609%)	87.2% (84.09%-90.7%)	82.41% (77.42%-87.69%)	3.7314 (3.2546-3.9435)	85.58% (80.81%-90.91%)	90.29% (84.13%-96.3%)	77.105% (65.714%-87.5%)	87.13% (80.85%-92.86%)	82.26% (71.87%-92.31%)	3.7078 (3.1242-3.6002)
At least 3 of CHASM (lung), CHASM (melanoma), FATHMM (cancer), FATHMM (missense), MutationTaster	89.96% (87.88%-92.42%)	91.63% (88.89%-94.51%)	86.753% (82.078%-91.071%)	92.55% (90.17%-94.72%)	84.71% (79.55%-89.25%)	3.6128 (3.4493-3.7677)	89.92% (84.85%-94.95%)	91.47% (84.62%-97.06%)	87.13% (77.778%-95.24%)	92.38% (86.89%-97.01%)	84.82% (72.97%-94.29%)	3.6046 (3.2817-3.9100)
At least 3 of CHASM (lung), CHASM (melanoma), FATHMM (cancer), FATHMM (missense), PolyPhen-2	83.9% (81.31%-86.87%)	88.18% (85.09%-91.38%)	75.34% (68.75%-81.25%)	87.76% (84.59%-90.91%)	76.31% (69.86%-82.81%)	3.4555 (3.2817-3.6171)	83.96% (77.78%-89.9%)	88.16% (81.25%-94.83%)	75.639% (61.29%-87.5%)	87.73% (80.88%-94.03%)	76.41% (61.5%-90%)	3.4593 (3.0636-3.8074)
At least 3 of CHASM (lung), CHASM (melanoma), FATHMM (cancer), FATHMM (missense), PROVEAN	84.55% (81.82%-87.37%)	87.84% (84.68%-91.04%)	78.85% (73.61%-84.507%)	87.78% (84.62%-91.12%)	79.06% (73.68%-84.71%)	3.3343 (3.0999-3.5275)	84.43% (77.78%-89.9%)	87.6% (80.65%-93.94%)	78.95% (65.82%-91.18%)	87.58% (80.96%-93.6%)	78.86% (66.61%-90.91%)	3.3239 (2.8749-3.7198)
At least 3 of CHASM (lung), CHASM (melanoma), FATHMM (cancer), FATHMM (missense), SIFT	82.83% (79.8%-85.86%)	90.49% (87.5%-93.33%)	69.525% (63.014%-76%)	83.78% (80.18%-87.35%)	80.89% (74.44%-86.84%)	3.2470 (3.1048-3.4000)	82.81% (76.77%-88.89%)	90.34% (83.33%-96.55%)	69.813% (55%-82.61%)	83.69% (76.12%-90.28%)	80.81% (66.67%-92.86%)	3.2431 (2.9167-3.5463)
At least 3 of CHASM (lung), CHASM (melanoma), FATHMM (cancer), FATHMM (missense), VEST	84.83% (81.31%-87.88%)	91.55% (88.89%-94.4%)	69.67% (63.23%-75.818%)	83.92% (80.54%-87.52%)	82.68% (77.42%-88.33%)	3.2782 (3.1442-3.4107)	84.8% (78.79%-89.9%)	91.43% (84.38%-96.97%)	70.14% (56.25%-82.61%)	83.8% (76.12%-90.79%)	82.48% (69.23%-94.12%)	3.2748 (2.9971-3.5337)
At least 3 of CHASM (lung), CHASM (melanoma), FATHMM (cancer), Mutation Assessor, MutationTaster	90.26% (87.88%-92.93%)	93.65% (91.27%-96.06%)	84.38% (80%-88.89%)	91.1% (88.73%-93.64%)	88.5% (84.5%-92.65%)	3.5774 (3.4693-3.6848)	90.19% (84.85%-94.95%)	93.53% (88.52%-98.33%)	84.44% (74.29%-93.33%)	91.16% (84.85%-96.67%)	88.41% (79.41%-96.67%)	3.5754 (3.3635-3.7716)
At least 3 of CHASM (lung), CHASM (melanoma), FATHMM (cancer), Mutation Assessor, PolyPhen-2	84.88% (81.82%-87.88%)	93.65% (90.7%-96.03%)	71.18% (65.079%-77.143%)	83.9% (80.11%-87.68%)	87.52% (82.67%-92.68%)	3.3728 (3.2455-3.4919)	84.85% (78.79%-90.91%)	93.62% (87.93%-98.36%)	71.49% (56.25%-84.62%)	83.88% (76.47%-90.71%)	87.35% (75%-96.55%)	3.3705 (3.0956-3.6188)
At least 3 of CHASM (lung), CHASM (melanoma), FATHMM (cancer), Mutation Assessor, PROVEAN	85.19% (82.32%-87.88%)	89.39% (86.51%-92.31%)	78.37% (72.857%-83.333%)	87.18% (84.09%-90.32%)	80.75% (75.71%-86.46%)	3.3578 (3.2327-3.4853)	85.25% (79.8%-90.91%)	89.27% (81.82%-95.24%)	78.592% (65.625%-90%)	87.17% (80.6%-93.06%)	80.98% (68.75%-92.86%)	3.3581 (3.1074-3.6116)
At least 3 of CHASM (lung), CHASM (melanoma), FATHMM (cancer), Mutation Assessor, SIFT	85.85% (83.33%-88.89%)	93.12% (90.55%-95.9%)	73.937% (67.521%-79.106%)	85.35% (81.82%-88.86%)	86.76% (81.74%-91.67%)	3.3857 (3.2556-3.5006)	85.85% (80.81%-90.91%)	93% (87.3%-98.31%)	73.42% (62.5%-85.71%)	85.72% (78.69%-92.19%)	85.96% (76%-96.3%)	3.3869 (3.1196-3.6389)
At least 3 of CHASM (lung), CHASM (melanoma), FATHMM (cancer), Mutation Assessor, VEST	85.44% (82.83%-88.38%)	93.65% (91.27%-96.03%)	72.034% (65.975%-78.082%)	84.59% (81.11%-88.07%)	87.58% (82.67%-92.11%)	3.3845 (3.2581-3.5070)	85.39% (79.8%-90.91%)	93.58% (87.72%-98.39%)	72.06% (58.82%-84.62%)	84.59% (77.14%-91.18%)	87.33% (76%-96.77%)	3.3857 (3.1024-3.6439)
At least 3 of CHASM (lung), CHASM (melanoma), FATHMM (cancer), MutationTaster, PolyPhen-2	85.48% (82.83%-88.38%)	92.06% (89.17%-95%)	74.677% (68.254%-80.882%)	85.7% (82.14%-89.17%)	85.12% (79.73%-90.63%)	3.4078 (3.2648-3.5343)	85.43% (78.79%-90.91%)	91.93% (85.71%-98.15%)	74.931% (61.11%-86.96%)	85.59% (78.57%-92.42%)	84.89% (71.87%-96.43%)	3.4065 (3.1262-3.6889)
At least 3 of CHASM (lung), CHASM (melanoma), FATHMM (cancer), MutationTaster, PROVEAN	90.62% (88.38%-92.93%)	94.2% (91.87%-96.75%)	84.423% (79.729%-89.333%)	91.27% (88.89%-94.16%)	89.39% (85.51%-93.33%)	3.5928 (3.4966-3.6967)	90.6% (85.86%-94.95%)	94.04% (89.06%-98.41%)	84.338% (74.286%-93.75%)	91.16% (85.96%-96.49%)	89.2% (80%-97.06%)	3.5875 (3.3816-3.7809)
At least 3 of CHASM (lung), CHASM (melanoma), FATHMM (cancer), MutationTaster, SIFT	90.28% (87.88%-92.93%)	95.23% (93.33%-97.58%)	81.68% (76.812%-87.324%)	89.97% (87.39%-92.73%)	90.87% (87.09%-94.67%)	3.6113 (3.5093-3.7054)	90.27% (84.85%-94.95%)	95.14% (90.16%-100%)	81.808% (70.588%-91.43%)	89.96% (84.06%-95.45%)	90.96% (81.48%-100%)	3.6107 (3.4015-3.8137)
At least 3 of CHASM (lung), CHASM (melanoma), FATHMM (cancer), MutationTaster, VEST	89.61% (87.37%-92.42%)	95.21% (93.22%-97.6%)	79.3% (73.973%-85.135%)	89.2% (86.19%-92.1%)	90.46% (86.11%-94.67%)	3.5869 (3.4800-3.6826)	89.59% (84.85%-93.94%)	95.06% (89.83%-100%)	79.33% (66.67%-90%)	89.17% (82.61%-95.08%)	90.34% (80.77%-100%)	3.5839 (3.3716-3.7885)
At least 3 of CHASM (lung), CHASM (melanoma), FATHMM (cancer), PolyPhen-2, PROVEAN	83.54% (80.3%-86.87%)	93.13% (90.6%-95.9%)	69.539% (60.688%-73.242%)	82.57% (79.17%-86.04%)	84.84% (78.82%-90.02%)	3.2798 (3.1510-3.4054)	83.53% (77.78%-88.89%)	92.97% (87.69%-98.31%)	67.029% (54.545%-79.41%)	82.87% (75.67%-89.86%)	84.29% (71.43%-96.15%)	3.2766 (2.9942-3.5331)
At least 3 of CHASM (lung), CHASM (melanoma), FATHMM (cancer), PolyPhen-2, SIFT	83.55% (80.3%-86.36%)	94.19% (91.87%-96.75%)	65.119% (58.74%-71.642%)	82.38% (78.92%-86.08%)	86.62% (81.29%-92.1%)	3.3002 (3.1754-3.4174)	83.58% (77.78%-88.89%)	94.02% (88.52%-98.44%)	65.499% (51.282%-79.31%)	82.24% (74.62%-89.19%)	86.49% (73.08%-96.55%)	3.3025 (3.0281-3.5539)
At least 3 of CHASM (lung), CHASM (melanoma), FATHMM (cancer), PolyPhen-2, VEST	83.8% (80.81%-86.87%)	95.76% (93.68%-97.84%)	62.6% (55.072%-68.539%)	81.94% (78.24%-85.6%)	89.75% (84.63%-94.29%)	3.3400 (3.2235-3.4560)	83.75% (77.78%-88.89%)	95.68% (90.68%-100%)	62.87% (48.571%-76.92%)	81.89% (73.97%-88.89%)	89.69% (78.13%-100%)	3.3403 (3.0686-3.5977)
At least 3 of CHASM (lung), CHASM (melanoma), FATHMM (cancer), PROVEAN, SIFT	84.19% (81.31%-87.37%)	92.6% (89.99%-95.16%)	69.112% (62.963%-74.853%)	83.85% (80.38%-87.47%)	84.12% (78.47%-89.71%)	3.3111 (3.1741-3.4476)	84.07% (78.79%-89.9%)	92.31% (86.67%-97.14%)	69.1% (55.88%-82.61%)	83.62% (76.71%-90.54%)	83.88% (70.83%-95.65%)	3.3030 (3.0097-3.5749)
At least 3 of CHASM (lung), CHASM (melanoma), FATHMM (cancer), PROVEAN, VEST	83.82% (80.81%-86.87%)	94.19% (92%-96.61%)	65.121% (58.461%-71.831%)	82.95% (79.31%-86.63%)	86.45% (81.02%-91.9%)	3.2861 (3.1528-3.4016)	83.82% (78.79%-88.89%)	94.02% (88.89%-98.44%)	65.343% (52.632%-79.41%)	82.9% (75.47%-89.71%)	86.48% (73.08%-96.77%)	3.2855 (2.9960-3.5573)
At least 3 of CHASM (lung), CHASM (melanoma), FATHMM (cancer), SIFT, VEST	83.53% (80.81%-86.36%)	94.2% (91.87%-96.75%)	64.267% (57.971%-70.833%)	82.39% (78.69%-86.08%)	86.87% (81.58%-92.06%)	3.2896 (3.1604-3.4134)	83.49% (77.78%-88.89%)	94.05% (88.57%-98.39%)	64.581% (50%-77.78%)	82.35% (74.62%-89.71%)	86.7% (73.08%-96.77%)	3.2907 (3.0136-3.5430)
At least 3 of CHASM (lung), CHASM (melanoma), FATHMM (missense), Mutation Assessor, MutationTaster	89.96% (87.37%-92.42%)	92.6% (89.99%-95.26%)	85.342% (80.882%-90.141%)	91.55% (88.97%-94.17%)	87.02% (82.67%-91.89%)	3.5661 (3.4701-3.6687)	89.92% (84.85%-94.95%)	92.5% (86.44%-97.62%)	85.25% (74.194%-95.24%)	91.41% (85.52%-96.72%)	87.03% (76.67%-96.55%)	3.5627 (3.3387-3.7702)
At least 3 of CHASM (lung), CHASM (melanoma), FATHMM (missense), Mutation Assessor, PolyPhen-2	84.58% (81.82%-87.37%)	92.63% (90%-95.2%)	70.603% (64.384%-76.812%)	84.79% (81.25%-88.33%)	84.74% (79.86%-89.93%)	3.3495 (3.2151-3.4813)	84.66% (78.79%-89.9%)	92.38% (86.89%-98.22%)	70.571% (56.25%-83.33%)	84.7% (77.42%-91.38%)	84.4% (71.43%-95.83%)	3.3170 (3.0734-3.5641)
At least 3 of CHASM (lung), CHASM (melanoma), FATHMM (missense), Mutation Assessor, PROVEAN	84.25% (81.31%-87.37%)	88.85% (85.71%-92%)	76.33% (70.37%-81.69%)	86.57% (83.33%-89.86%)	79.58% (73.61%-85.32%)	3.3241 (3.1944-3.4612)	84.24% (78.79%-89.9%)	88.75% (81.25%-95.16%)	76.344% (62.5%-88.24%)	86.56% (79.27%-92.75%)	79.35% (66.67%-91.67%)	3.3195 (3.0398-3.6014)
At least 3 of CHASM (lung), CHASM (melanoma), FATHMM (missense), Mutation Assessor, SIFT	85.9% (83.33%-88.89%)	92.63% (89.92%-95.2%)	74.397% (68.493%-80.82%)	86.22% (82.77%-89.76%)	85.53% (80.14%-90.67%)	3.3951 (3.2611-3.5151)	85.81% (80.81%-90.91%)	92.39% (86.89%-98.25%)	74.5% (60%-86.96%)	86.03% (79.17%-92.42%)	85.25% (73.08%-96.15%)	3.3876 (3.1177-3.6475)
At least 3 of CHASM (lung), CHASM (melanoma), FATHMM (missense), Mutation Assessor, VEST	85.07% (82.32%-87.88%)	93.15% (90.7%-95.77%)	71.221% (64.789%-77.632%)	84.73% (81.18%-88.24%)	85.87% (81.02%-90.91%)	3.3657 (3.2386-3.4945)	85.06% (79.8%-90.91%)	92.96% (87.1%-98.31%)	71.38% (57.143%-84.85%)	84.68% (77.33%-91.03%)	85.45% (73.08%-96.55%)	3.3627 (3.0857-3.6293)
At least 3 of CHASM (lung), CHASM (melanoma), FATHMM (missense), MutationTaster, PolyPhen-2	85.55% (82.32%-88.38%)	91.54% (88.53%-94.35%)	75.311% (69.091%-81.081%)	86.32% (82.95%-89.77%)	84.03% (78.63%-89.7%)	3.4003 (3.2662-3.5336)	85.6% (79.8%-90.91%)	91.4% (84.75%-96.97%)	75.713% (62.5%-88.24%)	86.29% (79.31%-92.75%)	83.89% (71.43%-95.83%)	3.4034 (3.1277-3.6696)
At least 3 of CHASM (lung), CHASM (melanoma), FATHMM (missense), MutationTaster, PROVEAN	89.28% (86.87%-91.92%)	93.71% (91.41%-96.15%)	81.725% (76.389%-87.013%)	89.67% (87.08%-92.59%)	88.6% (84.13%-92.71%)	3.5425 (3.4402-3.6433)	89.22% (83.84%-93.94%)	93.56% (87.88%-98.36%)	81.808% (69.231%-93.1%)	89.58% (82.35%-95.71%)	88.34% (77.78%-96.97%)	3.5404 (3.3150-3.7505)
At least 3 of CHASM (lung), CHASM (melanoma), FATHMM (missense), MutationTaster, SIFT	89.94% (87.37%-92.42%)	95.23% (93.22%-97.6%)	80.996% (75.362%-86.3%)	89.48% (86.7%-92.42%)	90.93% (86.96%-94.74%)	3.5950 (3.4938-3.6927)	89.9% (84.85%-94.95%)	95.1% (89.83%-100%)	81.067% (69.231%-92%)	89.42% (82.61%-95.38%)	90.81% (80.77%-100%)	3.5932 (3.3868-3.8009)
At least 3 of CHASM (lung), CHASM (melanoma), FATHMM (missense), MutationTaster, VEST	90.6% (88.38%-92.93%)	95.23% (93.16%-97.56%)	82.729% (77.465%-87.838%)	90.31% (87.75%-93.02%)	91.06% (87.32%-94.81%)	3.6248 (3.5283-3.7155)	90.59% (85.86%-94.95%)	95.12% (90.16%-100%)	82.804% (72.41%-93.94%)	90.3% (83.87%-95.89%)	90.91% (81.48%-100%)	3.6221 (3.3990-3.8287)
At least 3 of CHASM (lung), CHASM (melanoma), FATHMM (missense), PolyPhen-2, PROVEAN	85.22% (82.32%-87.88%)	91.53% (88.8%-94.31%)	74.311% (68.421%-80.282%)	85.71% (82.22%-89.18%)	83.57% (78.01%-88.89%)	3.3834 (3.2466-3.5090)	85.25% (79.8%-90.91%)	91.42% (84.75%-96.97%)	74.65% (61.29%-87.5%)	85.66% (78.57%-92.31%)	83.54% (70.37%-95.24%)	3.3819 (3.0944-3.6476)
At least 3 of CHASM (lung), CHASM (melanoma), FATHMM (missense), PolyPhen-2, SIFT	84.2% (81.31%-87.37%)	93.12% (90.6%-95.76%)	68.695% (62.319%-75%)	83.77% (80.32%-87.47%)	85.11% (79.71%-90.28%)	3.3287 (3.1994-3.4512)	84.22% (77.78%-89.9%)	93.01% (87.5%-98.31%)	69.015% (55.88%-82.61%)	83.72% (76.12%-90.79%)	85.05% (72%-96.15%)	3.3291 (3.0359-3.5971)
At least 3 of CHASM (lung), CHASM (melanoma), FATHMM (missense), PolyPhen-2, VEST	84.53% (81.82%-87.37%)	94.74% (92.5%-97.08%)	66.72% (60.563%-73.239%)	83.37% (79.83%-86.82%)	87.9% (82.84%-92.75%)	3.3543 (3.2257-3.4756)	84.51% (78.79%-89.9%)	94.62% (89.29%-98.39%)	66.829% (52.778%-80%)	83.38% (75.63%-90.14%)	87.79% (76.19%-96.67%)	3.3532 (3.0759-3.6247)
At least 3 of CHASM (lung), CHASM (melanoma), FATHMM (missense), PROVEAN, SIFT	84.88% (81.82%-87.88%)	92.07% (89.43%-94.79%)	71.968% (65.753%-78.082%)	85.63% (82.03%-89.22%)	83.87% (78.45%-89.36%)	3.3700 (3.2424-3.4958)	84.85% (78.79%-89.9%)	91.92% (85.71%-97.22%)	71.929% (58.33%-84.85%)	85.48% (78.38%-92.21%)	83.75% (70.37%-95.83%)	3.3656 (3.0842-3.6252)
At least 3 of CHASM (lung), CHASM (melanoma), FATHMM (missense), PROVEAN, VEST	85.55% (82.83%-88.38%)	94.73% (92.62%-96.9%)	69.126% (62.687%-75.325%)	84.61% (81.03%-88.15%)	87.89% (82.81%-92.75%)	3.3795 (3.2555-3.4981)	85.58% (80.81%-90.91%)	94.72% (89.83%-100%)	69.405% (55.882%-83.33%)	84.64% (77.33%-91.43%)	87.79% (76.92%-100%)	3.3815 (3.1045-3.6500)
At least 3 of CHASM (lung), CHASM (melanoma), FATHMM (missense), SIFT, VEST	82.87% (79.8%-85.86%)	94.73% (92.62%-96.9%)	62.352% (55.882%-68.493%)	81.31% (77.72%-84.96%)	87.26% (82.35%-92.19%)	3.2566 (3.1385-3.3838)	82.78% (76.77%-88.89%)	94.62% (89.29%-98.39%)	62.454% (50%-75%)	81.22% (73.61%-88.41%)	87.03% (76.19%-96.3%)	3.2526 (2.9959-3.4924)
At least 3 of CHASM (lung), CHASM (melanoma), Mutation Assessor, MutationTaster, PolyPhen-2	88.92% (86.36%-91.41%)	92.07% (89.43%-94.82%)	83.467% (78.378%-88.406%)	90.5% (87.9%-93.24%)	85.86% (81.16%-90.71%)	3.5243 (3.4155-3.6358)	88.92% (83.84%-93.94%)	91.92% (85.71%-97.06%)	83.669% (72.41%-93.75%)	90.51% (84.62%-96.36%)	85.56% (74.07%-95.65%)	3.5168 (3.2819-3.7517)
At least 3 of CHASM (lung), CHASM (melanoma), Mutation Assessor, MutationTaster, PROVEAN	88.95% (86.36%-91.41%)	90.52% (87.5%-93.48%)	86.224% (81.429%-90.909%)	91.97% (89.57%-94.49%)	83.91% (79.17%-88.66%)	3.4919 (3.3818-3.6031)	88.89% (83.84%-93.94%)	90.3% (83.87%-96.3%)	86.237% (75%-96%)	91.89% (86.15%-97.14%)	83.73% (72.73%-93.94%)	3.4849 (3.2478-3.7264)
At least 3 of CHASM (lung), CHASM (melanoma), Mutation Assessor, MutationTaster, SIFT	87.31% (84.85%-89.9%)	96.32% (94.49%-98.4%)	71.727% (65.753%-78.08%)	85.47% (82.22%-88.93%)	91.87% (87.7%-96.1%)	3.4928 (3.3755-3.5979)	87.32% (81.82%-91.92%)	96.18% (91.18%-100%)	71.956% (57.692%-84.62%)	85.48% (78.57%-92%)	91.97% (80.95%-100%)	3.4935 (3.2303-3.7318)
At least 3 of CHASM (lung), CHASM (melanoma), Mutation Assessor, MutationTaster, VEST	88.83% (86.36%-91.41%)	96.82% (95.2%-98.41%)	74.894% (68.919%-80.882%)	87.13% (84.11%-90.32%)	93.13% (89.19%-96.73%)	3.5417 (3.4382-3.6400)	88.77% (83.84%-93.94%)	96.75% (92.31%-100%)	74.98% (60%-88%)	87.07% (80%-93.65%)	93.16% (84%-100%)	3.5387 (3.2825-3.7604)
At least 3 of CHASM (lung), CHASM (melanoma), Mutation Assessor, PolyPhen-2, PROVEAN	83.83% (80.81%-86.87%)	90.49% (87.7%-93.33%)	72.188% (65.753%-78.082%)	85.03% (81.61%-88.68%)	81.35% (75.71%-86.84%)	3.3022 (3.1640-3.4416)	83.84% (77.78%-88.89%)	90.42% (84.13%-96.49%)	72.417% (58.65%-85.29%)	85.03% (77.27%-91.78%)	81.11% (68%-92.59%)	3.3025 (3.0146-3.5901)
At least 3 of CHASM (lung), CHASM (melanoma), Mutation Assessor, PolyPhen-2, SIFT	85.21% (82.32%-88.38%)	94.74% (92.56%-96.9%)	68.506% (61.972%-74.648%)	84.03% (80.62%-87.61%)	88.15% (83.56%-92.86%)	3.3906 (3.2653-3.5132)	85.17% (79.8%-90.91%)	94.63% (89.66%-98.41%)	68.625% (55.88%-81.82%)	83.97% (76.19%-90.91%)	88.06% (76.92%-96.97%)	3.3886 (3.1105-3.6420)
At least 3 of CHASM (lung), CHASM (melanoma), Mutation Assessor, PolyPhen-2, VEST	84.53% (81.82%-87.88%)	95.76% (93.65%-97.87%)	64.757% (57.971%-71.014%)	82.65% (79.08%-86.29%)	89.64% (84.79%-94.52%)	3.3705 (3.2505-3.4861)	84.46% (78.79%-89.9%)	95.65% (90.91%-100%)	64.876% (50%-78.26%)	82.58% (75%-89.55%)	89.51% (77.27%-100%)	3.3661 (3.0929-3.6089)
At least 3 of CHASM (lung), CHASM (melanoma), Mutation Assessor, PROVEAN, SIFT	84.17% (81.31%-87.37%)	92.07% (89.43%-94.7%)	70.176% (63.636%-76.389%)	84.51% (81.05%-88.15%)	83.43% (77.78%-88.89%)	3.3160 (3.1795-3.4418)	84.12% (78.79%-89.9%)	91.93% (85.71%-97.14%)	70.174% (56.25%-83.33%)	84.37% (76.92%-91.18%)	83.24% (69.57%-95.45%)	3.3100 (3.0266-3.5756)
At least 3 of CHASM (lung), CHASM (melanoma), Mutation Assessor, PROVEAN, VEST	85.88% (83.33%-88.89%)	94.73% (92.37%-96.9%)	70.153% (63.889%-76.712%)	84.96% (81.65%-88.45%)	88.14% (83.33%-92.96%)	3.3915 (3.2689-3.5018)	85.86% (80.81%-90.91%)	94.62% (89.06%-98.39%)	70.39% (57.143%-83.33%)	84.9% (77.46%-91.55%)	88.09% (75.76%-96.77%)	3.3913 (3.1120-3.6380)
At least 3 of CHASM (lung), CHASM (melanoma), Mutation Assessor, SIFT, VEST	83.86% (80.81%-86.87%)	95.76% (93.75%-97.6%)	62.818% (56.716%-69.444%)	82% (78.36%-85.49%)	89.32% (84.42%-94.12%)	3.3415 (3.2179-3.4611)	83.89% (77.78%-88.89%)	95.64% (90.91%-100%)	63.201% (48.571%-76.92%)	81.97% (73.97%-88.89%)	89.32% (78.26%-100%)	3.3434 (3.0645-3.5925)
At least 3 of CHASM (lung), CHASM (melanoma), MutationTaster, PolyPhen-2, PROVEAN	88.92% (86.36%-91.41%)	92.07% (89.43%-94.84%)	83.41% (78.082%-88.406%)	90.49% (87.74%-93.28%)	85.85% (80.92%-90.77%)	3.5234 (3.4160-3.6327)	88.91% (83.84%-93.94%)	91.98% (85.94%-97.06%)	83.555% (72.41%-93.33%)	90.49% (84.62%-96.23%)	85.65% (73.33%-96.15%)	3.5200 (3.2824-3.7535)
At least 3 of CHASM (lung), CHASM (melanoma), MutationTaster, PolyPhen-2, SIFT	88.27% (85.86%-90.91%)	96.29% (94.53%-98.36%)	74.236% (68.116%-80.263%)	86.76% (83.67%-89.84%)	92.03% (88.24%-95.95%)	3.5292 (3.4205-3.6305)	88.28% (82.83%-92.93%)	96.18% (91.38%-100%)	74.473% (62.5%-86.96%)	86.76% (80.3%-93.06%)	92.02% (82.61%-100%)	3.5291 (3.2785-3.7693)
At least 3 of CHASM (lung), CHASM (melanoma), MutationTaster, PolyPhen-2, VEST	89.21% (86.87%-91.92%)	96.82% (95.2%-98.41%)	75.988% (70.27%-81.944%)	87.55% (84.67%-90.7%)	93.25% (89.52%-96.63%)	3.5582 (3.4570-3.6549)	89.19% (84.85%-93.94%)	96.72% (92.31%-100%)	76.078% (62.963%-88.46%)	87.53% (81.08%-93.75%)	93.07% (84%-100%)	3.5522 (3.3174-3.7731)
At least 3 of CHASM (lung), CHASM (melanoma), MutationTaster, PROVEAN, SIFT	88.26% (85.86%-90.91%)	95.26% (93.31%-97.48%)	76.059% (70.175%-81.944%)	87.38% (84.38%-90.63%)	90.26% (85.9%-94.52%)	3.5034 (3.3941-3.6081)	88.28% (82.83%-92.93%)	95.13% (90.16%-100%)	76.238% (63.64%-88.89%)	87.38% (80.65%-93.94%)	90.13% (79.31%-100%)	3.5018 (3.2542-3.7355)
At least 3 of CHASM (lung), CHASM (melanoma), MutationTaster, PROVEAN, VEST	89.6% (87.37%-92.42%)	96.3% (94.4%-98.36%)	78.074% (72.603%-83.824%)	88.47% (85.61%-91.47%)	92.36% (88.37%-96%)	3.5616 (3.4608-3.6595)	89.58% (84.85%-93.94%)	96.16% (91.67%-100%)	78.187% (65.52%-90%)	88.44% (81.82%-94.37%)	92.26% (83.33%-100%)	3.5571 (3.3238-3.7809)
At least 3 of CHASM (lung), CHASM (melanoma), MutationTaster, SIFT, VEST	88.96% (86.36%-91.41%)	96.83% (95.12%-98.41%)	75.124% (69.444%-81.159%)	87.3% (84.21%-90.4%)	93.21% (89.33%-96.72%)	3.5496 (3.4463-3.6489)	88.94% (83.84%-93.94%)	96.72% (92.31%-100%)	75.2% (62.5%-87.5%)	87.24% (80.6%-93.75%)	93.1% (84%-100%)	3.5459 (3.2917-3.7748)
At least 3 of CHASM (lung), CHASM (melanoma), PolyPhen-2, PROVEAN, SIFT	84.52% (81.82%-87.37%)	94.2% (92.06%-96.61%)	67.535% (61.111%-73.913%)	83.56% (80.08%-87.13%)	86.93% (81.98%-91.89%)	3.3611 (3.2403-3.4829)	84.55% (78.79%-89.9%)	94.09% (88.89%-98.44%)	67.854% (54.054%-81.48%)	83.52% (76.12%-90.41%)	86.74% (74.07%-96.77%)	3.3609 (3.0705-3.6177)
At least 3 of CHASM (lung), CHASM (melanoma), PolyPhen-2, PROVEAN, VEST	83.87% (81.31%-86.87%)	95.76% (93.7%-97.87%)	62.879% (56.338%-69.014%)	81.99% (78.53%-85.59%)	89.35% (84.29%-94.03%)	3.3419 (3.2201-3.4616)	83.86% (77.78%-89.9%)	95.67% (90.91%-100%)	63.122% (48.571%-76.92%)	81.97% (74.24%-89.04%)	89.29% (77.27%-100%)	3.3422 (3.0576-3.5976)
At least 3 of CHASM (lung), CHASM (melanoma), PolyPhen-2, SIFT, VEST	81.52% (78.79%-84.85%)	95.78% (93.85%-97.67%)	57.668% (51.283%-64.103%)	79.84% (76.22%-83.52%)	88.83% (83.33%-93.94%)	3.2188 (3.0944-3.3411)	81.81% (75.76%-87.88%)	95.65% (90.91%-100%)	58.035% (44.09%-74.04%)	79.64% (72.26%-86.11%)	88.61% (76.19%-100%)	3.2196 (2.9646-3.4787)
At least 3 of CHASM (lung), CHASM (melanoma), PROVEAN, SIFT, VEST	81.83% (78.79%-84.85%)	92.59% (89.9%-95.16%)	62.694% (55.714%-69.118%)	81.61% (77.85%-85.36%)	82.84% (76.47%-88.71%)	3.1866 (3.0453-3.3184)	81.69% (75.76%-87.88%)	92.41% (86.44%-97.37%)	62.659% (48.65%-76.92%)	81.43% (73.68%-88.73%)	82.56% (68.42%-95.24%)	3.1761 (2.8727-3.4687)
At least 3 of CHASM (lung), FATHMM (cancer), FATHMM (missense), Mutation Assessor, MutationTaster	89.6% (87.37%-91.92%)	94.74% (92.43%-97.01%)	80.719% (75.385%-86.567%)	89.41% (86.61%-92.38%)	89.82% (85.45%-93.94%)	3.5675 (3.4642-3.6663)	89.55% (84.85%-93.94%)	94.63% (89.29%-98.44%)	80.765% (68.966%-91.67%)	89.35% (83.08%-95.31%)	89.65% (79.31%-100%)	3.5642 (3.3463-3.7780)
At least 3 of CHASM (lung), FATHMM (cancer), FATHMM (missense), Mutation Assessor, PolyPhen-2	84.18% (81.31%-87.37%)	93.67% (91.34%-96.06%)	67.779% (61.643%-74.286%)	83.46% (79.88%-87.27%)	86.13% (80.79%-91.25%)	3.3283 (3.2041-3.4520)	84.21% (78.79%-89.9%)	93.56% (87.93%-98.39%)	68.07% (54.17%-81.48%)	83.42% (75.71%-90.28%)	85.96% (73.91%-96.43%)	3.3285 (3.0575-3.5823)
At least 3 of CHASM (lung), FATHMM (cancer), FATHMM (missense), Mutation Assessor, PROVEAN	85.86% (83.33%-88.38%)	92.06% (89.34%-94.74%)	75.05% (69.014%-81.25%)	86.53% (83.09%-89.81%)	84.37% (79.25%-89.87%)	3.4165 (3.2847-3.5340)	85.83% (79.8%-90.91%)	91.94% (85.94%-97.22%)	75.128% (62.5%-87.5%)	86.46% (79.37%-92.86%)	84.17% (72%-95.45%)	3.4120 (3.1347-3.6785)
At least 3 of CHASM (lung), FATHMM (cancer), FATHMM (missense), Mutation Assessor, SIFT	83.83% (81.31%-86.87%)	93.13% (90.68%-95.73%)	67.606% (61.333%-74.025%)	83.38% (79.91%-86.96%)	84.82% (79.17%-90.32%)	3.3058 (3.1773-3.4337)	83.85% (77.78%-88.89%)	92.98% (87.1%-98.18%)	68.032% (53.846%-80.77%)	83.38% (75.71%-90.41%)	84.72% (71.42%-96.43%)	3.3035 (3.0283-3.5782)
At least 3 of CHASM (lung), FATHMM (cancer), FATHMM (missense), Mutation Assessor, VEST	83.84% (81.31%-86.36%)	94.72% (92.56%-96.88%)	64.791% (58.108%-71.233%)	82.61% (78.99%-86.3%)	87.65% (82.28%-92.71%)	3.3244 (3.2022-3.4442)	83.84% (77.78%-88.89%)	94.61% (89.47%-98.41%)	65.143% (51.28%-79.31%)	82.59% (75.4%-89.55%)	87.5% (75%-96.55%)	3.3240 (3.0397-3.5747)
At least 3 of CHASM (lung), FATHMM (cancer), FATHMM (missense), MutationTaster, PolyPhen-2	87.92% (85.35%-90.4%)	95.26% (93.33%-97.58%)	75.028% (68.657%-81.159%)	86.94% (83.88%-90.18%)	90.05% (85.59%-94.29%)	3.4803 (3.3672-3.5880)	87.87% (82.83%-92.93%)	95.11% (90.11%-100%)	75.206% (64.29%-86.96%)	86.82% (80%-93.04%)	89.97% (78.26%-100%)	3.4710 (3.2484-3.6922)
At least 3 of CHASM (lung), FATHMM (cancer), FATHMM (missense), MutationTaster, PROVEAN	89.26% (86.87%-91.92%)	93.12% (90.6%-95.87%)	82.576% (77.143%-87.671%)	90.26% (87.6%-92.95%)	87.42% (82.86%-91.86%)	3.5392 (3.4333-3.6383)	89.26% (84.85%-93.94%)	93.04% (87.3%-98.31%)	82.687% (71.43%-92.86%)	90.22% (84.06%-95.71%)	87.32% (76.67%-96.97%)	3.5361 (3.3062-3.7667)
At least 3 of CHASM (lung), FATHMM (cancer), FATHMM (missense), MutationTaster, SIFT	90.27% (88.38%-92.93%)	95.75% (93.75%-97.69%)	80.652% (75.362%-86.301%)	89.67% (86.94%-92.5%)	91.55% (87.88%-95.38%)	3.6133 (3.5193-3.7103)	90.25% (85.86%-94.95%)	95.63% (90.91%-100%)	80.745% (68.966%-92%)	89.59% (83.33%-95.59%)	91.46% (81.48%-100%)	3.6100 (3.3957-3.8138)
At least 3 of CHASM (lung), FATHMM (cancer), FATHMM (missense), MutationTaster, VEST	87.56% (84.85%-89.91%)	96.3% (94.52%-98.33%)	71.461% (65.676%-77.465%)	86.09% (82.9%-89.36%)	91.18% (87.32%-95.52%)	3.4672 (3.3643-3.5712)	87.55% (82.83%-92.93%)	96.18% (91.38%-100%)	71.721% (59.26%-84%)	86.04% (79.37%-92.75%)	91.12% (81.82%-100%)	3.4655 (3.2459-3.6713)
At least 3 of CHASM (lung), FATHMM (cancer), FATHMM (missense), PolyPhen-2, PROVEAN	83.84% (80.81%-86.87%)	92.07% (89.34%-94.74%)	68.915% (62.162%-74.684%)	84.27% (80.77%-87.8%)	83.04% (77.27%-88.89%)	3.3084 (3.1771-3.4341)	83.86% (78.79%-89.9%)	91.94% (85.94%-97.22%)	69.138% (55.172%-82.14%)	84.21% (76.81%-90.91%)	82.9% (69.23%-95%)	3.3076 (3.0220-3.5775)
At least 3 of CHASM (lung), FATHMM (cancer), FATHMM (missense), PolyPhen-2, SIFT	82.2% (79.29%-85.35%)	94.74% (92.56%-96.75%)	59.569% (52.703%-66.216%)	80.81% (77.05%-84.37%)	86.24% (80.6%-91.55%)	3.2383 (3.1202-3.3564)	82.22% (76.77%-87.88%)	94.62% (89.29%-98.41%)	59.88% (45.711%-73.91%)	80.78% (72.73%-87.84%)	86.12% (72.73%-96.43%)	3.2385 (2.9706-3.5033)
At least 3 of CHASM (lung), FATHMM (cancer), FATHMM (missense), PolyPhen-2, VEST	83.19% (80.3%-86.36%)	96.82% (95.12%-98.41%)	59.011% (52.174%-65.714%)	80.76% (77.08%-84.44%)	91.43% (86.67%-95.83%)	3.3164 (3.2002-3.4332)	83.17% (77.78%-88.89%)	96.72% (92.31%-100%)	59.204% (45.71%-73.33%)	80.73% (72.84%-88%)	91.27% (80.95%-100%)	3.3139 (3.0526-3.5584)
At least 3 of CHASM (lung), FATHMM (cancer), FATHMM (missense), PROVEAN, SIFT	83.54% (80.81%-86.87%)	93.13% (90.68%-95.76%)	66.61% (59.722%-73.134%)	83.02% (79.33%-86.52%)	84.6% (78.87%-90.28%)	3.2927 (3.1592-3.4186)	83.53% (77.78%-88.89%)	93.04% (87.3%-98.31%)	66.805% (52.632%-80%)	82.97% (75.71%-90.28%)	84.47% (70.83%-95.83%)	3.2925 (3.0043-3.5497)
At least 3 of CHASM (lung), FATHMM (cancer), FATHMM (missense), PROVEAN, VEST	82.52% (79.29%-85.86%)	95.26% (93.2%-97.44%)	59.853% (53.333%-66.667%)	80.7% (77.02%-84.48%)	87.8% (82.09%-93.06%)	3.2647 (3.1355-3.3888)	82.55% (76.77%-88.89%)	95.14% (90.32%-100%)	60.116% (46.154%-73.33%)	80.7% (72.86%-87.84%)	87.66% (76%-100%)	3.2647 (2.9832-3.5190)
At least 3 of CHASM (lung), FATHMM (cancer), FATHMM (missense), SIFT, VEST	83.51% (80.3%-86.36%)	94.2% (91.87%-96.75%)	65.008% (58.902%-71.429%)	82.95% (79.26%-86.34%)	86.94% (81.69%-92.01%)	3.2859 (3.1571-3.4155)	83.49% (77.78%-88.89%)	94.02% (88.52%-98.41%)	65.183% (51.351%-79.31%)	82.82% (75.64%-90%)	86.4% (74.07%-96.55%)	3.2820 (3.0152-3.5536)
At least 3 of CHASM (lung), FATHMM (cancer), Mutation Assessor, MutationTaster, PolyPhen-2	86.88% (84.34%-89.39%)	94.2% (91.94%-96.72%)	73.957% (68.182%-80%)	86.51% (83.36%-89.74%)	87.88% (83.1%-92.54%)	3.4432 (3.3269-3.5542)	86.86% (81.82%-91.92%)	94.08% (88.89%-98.46%)	74.01% (61.538%-86.21%)	86.48% (79.73%-92.75%)	87.72% (76%-96.88%)	3.4390 (3.1800-3.6707)
At least 3 of CHASM (lung), FATHMM (cancer), Mutation Assessor, MutationTaster, PROVEAN	89.28% (86.87%-91.92%)	93.12% (90.48%-95.73%)	82.512% (77.333%-87.671%)	90.3% (87.37%-93.13%)	87.41% (82.93%-91.89%)	3.5398 (3.4378-3.6418)	89.27% (84.85%-93.94%)	93.04% (87.3%-98.31%)	82.614% (71.43%-92.86%)	90.24% (84.06%-95.71%)	87.3% (76.67%-96.77%)	3.5362 (3.3082-3.7572)
At least 3 of CHASM (lung), FATHMM (cancer), Mutation Assessor, MutationTaster, SIFT	88.6% (86.36%-91.41%)	95.23% (93.16%-97.41%)	77.012% (71.233%-82.692%)	87.87% (85%-90.91%)	90.25% (85.92%-94.29%)	3.5173 (3.4087-3.6140)	88.6% (83.84%-92.93%)	95.14% (90%-100%)	77.141% (64.516%-88.89%)	87.87% (81.16%-94.12%)	90.19% (79.31%-100%)	3.5160 (3.2829-3.7446)
At least 3 of CHASM (lung), FATHMM (cancer), Mutation Assessor, MutationTaster, VEST	87.61% (84.85%-89.9%)	96.3% (94.49%-98.36%)	72.54% (66.216%-78.378%)	86.26% (83.14%-89.59%)	91.52% (87.32%-95.65%)	3.4735 (3.3684-3.5754)	87.58% (82.83%-92.93%)	96.18% (91.38%-100%)	72.795% (60%-85.71%)	86.22% (79.37%-92.65%)	91.35% (81.82%-100%)	3.4688 (3.2439-3.6828)
At least 3 of CHASM (lung), FATHMM (cancer), Mutation Assessor, PolyPhen-2, PROVEAN	85.22% (82.32%-87.88%)	92.06% (89.43%-94.79%)	73.039% (66.667%-79.032%)	85.92% (82.48%-89.36%)	83.86% (78.68%-89.06%)	3.3736 (3.2474-3.4976)	85.23% (79.8%-90.91%)	91.92% (85.71%-97.06%)	73.267% (60%-86.67%)	85.89% (78.87%-92.65%)	83.7% (71.43%-95.83%)	3.3733 (3.0965-3.6407)
At least 3 of CHASM (lung), FATHMM (cancer), Mutation Assessor, PolyPhen-2, SIFT	83.51% (80.3%-86.36%)	94.2% (92.13%-96.75%)	64.4% (58.108%-71.233%)	82.64% (79.17%-86.46%)	86.06% (80.56%-91.38%)	3.2905 (3.1687-3.4131)	83.54% (77.78%-88.89%)	94.09% (88.52%-98.41%)	64.655% (51.43%-78.26%)	82.59% (75%-89.74%)	85.95% (72.73%-96.43%)	3.2913 (3.0206-3.5463)
At least 3 of CHASM (lung), FATHMM (cancer), Mutation Assessor, PolyPhen-2, VEST	83.2% (80.3%-86.36%)	96.29% (94.53%-98.36%)	59.91% (53.425%-66.667%)	81.11% (77.57%-84.78%)	89.95% (85.14%-94.44%)	3.3087 (3.1897-3.4222)	83.23% (77.78%-88.89%)	96.21% (91.53%-100%)	60.174% (46.153%-74.07%)	81.12% (73.49%-88.31%)	89.85% (78.26%-100%)	3.3116 (3.0375-3.5567)
At least 3 of CHASM (lung), FATHMM (cancer), Mutation Assessor, PROVEAN, SIFT	85.86% (83.33%-88.38%)	93.12% (90.63%-95.71%)	72.925% (66.667%-79.412%)	85.99% (82.61%-89.24%)	85.51% (80.28%-90.67%)	3.4047 (3.2810-3.5287)	85.84% (80.81%-90.91%)	93.01% (87.3%-98.31%)	73.099% (59.375%-86.21%)	85.93% (78.87%-92.65%)	85.35% (73.33%-96.43%)	3.4007 (3.1289-3.6578)
At least 3 of CHASM (lung), FATHMM (cancer), Mutation Assessor, PROVEAN, VEST	83.18% (80.3%-86.36%)	94.73% (92.56%-96.9%)	62.404% (55.882%-68.919%)	81.91% (78.3%-85.57%)	86.67% (81.43%-91.78%)	3.2795 (3.1581-3.4024)	83.16% (77.78%-88.89%)	94.61% (89.29%-98.41%)	62.648% (48.485%-76%)	81.86% (74.29%-89.19%)	86.46% (73.91%-96.67%)	3.2780 (3.0137-3.5303)
At least 3 of CHASM (lung), FATHMM (cancer), Mutation Assessor, SIFT, VEST	82.19% (79.29%-85.35%)	96.3% (94.58%-98.39%)	57.025% (50.725%-63.768%)	80% (76.32%-83.8%)	89.58% (84.51%-94.37%)	3.2603 (3.1395-3.3779)	82.18% (76.77%-87.88%)	96.18% (91.53%-100%)	57.244% (43.333%-71.43%)	79.95% (72.06%-87.5%)	89.47% (77.78%-100%)	3.2595 (2.9876-3.5108)
At least 3 of CHASM (lung), FATHMM (cancer), MutationTaster, PolyPhen-2, PROVEAN	88.58% (86.36%-91.41%)	92.58% (90%-95.24%)	81.531% (76.389%-86.667%)	89.84% (87.07%-92.77%)	86.13% (81.25%-90.79%)	3.5017 (3.3904-3.6096)	88.56% (83.84%-93.94%)	92.45% (86.44%-97.78%)	81.605% (70.968%-92.59%)	89.78% (83.56%-95.77%)	85.97% (75%-96.3%)	3.4956 (3.2660-3.7253)
At least 3 of CHASM (lung), FATHMM (cancer), MutationTaster, PolyPhen-2, SIFT	87.6% (85.35%-90.4%)	96.3% (94.49%-98.35%)	72.364% (65.789%-78.481%)	86.05% (82.95%-89.4%)	91.79% (87.5%-95.71%)	3.4749 (3.3679-3.5786)	87.56% (82.83%-92.93%)	96.18% (91.38%-100%)	72.489% (60%-84.62%)	86% (79.1%-92.42%)	91.68% (81.82%-100%)	3.4718 (3.2436-3.6915)
At least 3 of CHASM (lung), FATHMM (cancer), MutationTaster, PolyPhen-2, VEST	88.25% (85.86%-90.91%)	96.3% (94.53%-98.35%)	74.323% (68.421%-80.282%)	86.79% (83.71%-89.83%)	92.07% (88.16%-95.77%)	3.5278 (3.4222-3.6287)	88.24% (83.84%-92.93%)	96.18% (91.67%-100%)	74.456% (61.54%-86.96%)	86.75% (80.3%-93.15%)	91.97% (82.61%-100%)	3.5265 (3.2930-3.7473)
At least 3 of CHASM (lung), FATHMM (cancer), MutationTaster, PROVEAN, SIFT	89.26% (86.87%-91.92%)	95.24% (93.33%-97.52%)	78.978% (73.611%-84.286%)	88.62% (85.71%-91.81%)	90.72% (86.84%-94.74%)	3.5645 (3.4640-3.6637)	89.22% (84.85%-93.94%)	95.1% (89.83%-100%)	79.047% (66.67%-90.48%)	88.54% (81.82%-94.74%)	90.59% (80%-100%)	3.5577 (3.3286-3.7682)
At least 3 of CHASM (lung), FATHMM (cancer), MutationTaster, PROVEAN, VEST	88.58% (86.36%-91.41%)	96.3% (94.57%-98.37%)	75.292% (69.118%-81.159%)	87.29% (84.29%-90.51%)	92.27% (88.41%-95.89%)	3.5363 (3.4341-3.6370)	88.56% (83.84%-93.94%)	96.2% (91.67%-100%)	75.353% (62.5%-87.5%)	87.23% (80.6%-93.65%)	92.13% (82.61%-100%)	3.5317 (3.3006-3.7566)
At least 3 of CHASM (lung), FATHMM (cancer), MutationTaster, SIFT, VEST	87.93% (85.35%-90.4%)	96.83% (95.12%-98.43%)	72.492% (66.216%-78.462%)	86.15% (83.03%-89.39%)	93.01% (88.89%-96.77%)	3.5178 (3.4129-3.6169)	87.89% (82.83%-92.93%)	96.75% (92.06%-100%)	72.597% (60%-85.19%)	86.08% (79.41%-92.54%)	92.87% (83.33%-100%)	3.5133 (3.2793-3.7368)
At least 3 of CHASM (lung), FATHMM (cancer), PolyPhen-2, PROVEAN, SIFT	84.53% (81.82%-87.37%)	93.67% (91.2%-96.12%)	68.634% (62.338%-75%)	83.86% (80.29%-87.37%)	86.16% (80.82%-91.3%)	3.3556 (3.2280-3.4732)	84.53% (78.79%-89.9%)	93.58% (87.93%-98.41%)	68.883% (55.556%-82.61%)	83.81% (76.39%-90.57%)	86.08% (73.08%-96.77%)	3.3549 (3.0791-3.6185)
At least 3 of CHASM (lung), FATHMM (cancer), PolyPhen-2, PROVEAN, VEST	82.84% (79.8%-85.86%)	95.26% (93.39%-97.44%)	60.778% (54.386%-67.606%)	81.2% (77.51%-84.9%)	87.93% (82.35%-92.86%)	3.2786 (3.1578-3.4004)	82.82% (76.77%-88.89%)	95.14% (90.32%-100%)	60.97% (47.059%-75%)	81.14% (73.24%-88.57%)	87.79% (76.19%-100%)	3.2770 (3.0044-3.5212)
At least 3 of CHASM (lung), FATHMM (cancer), PolyPhen-2, SIFT, VEST	82.83% (79.8%-85.86%)	96.29% (94.4%-98.33%)	58.974% (52.113%-65.753%)	80.67% (76.84%-84.44%)	89.96% (84.93%-94.52%)	3.2916 (3.1734-3.4136)	82.84% (76.77%-88.89%)	96.19% (91.38%-100%)	59.169% (45.714%-72.73%)	80.67% (72.97%-88.16%)	89.84% (78.57%-100%)	3.2920 (3.0224-3.5366)
At least 3 of CHASM (lung), FATHMM (cancer), PROVEAN, SIFT, VEST	83.19% (80.3%-86.36%)	96.3% (94.52%-98.39%)	59.919% (53.521%-66.667%)	81.09% (77.36%-84.89%)	90.13% (85.29%-94.67%)	3.3081 (3.1875-3.4270)	83.18% (77.78%-88.89%)	96.18% (91.67%-100%)	60.155% (46.154%-74.07%)	81.08% (73.08%-88.61%)	89.99% (78.26%-100%)	3.3069 (3.0357-3.5631)
At least 3 of CHASM (lung), FATHMM (missense), Mutation Assessor, MutationTaster, PolyPhen-2	87.57% (85.35%-90.4%)	93.66% (91.2%-96.06%)	76.624% (71.233%-82.432%)	87.81% (84.82%-90.91%)	87.01% (82.35%-91.67%)	3.4518 (3.3327-3.5679)	87.53% (82.83%-92.93%)	93.58% (87.72%-98.41%)	76.71% (63.64%-88.89%)	87.76% (80.82%-93.94%)	86.86% (75%-96.67%)	3.4473 (3.1945-3.6878)
At least 3 of CHASM (lung), FATHMM (missense), Mutation Assessor, MutationTaster, PROVEAN	88.28% (85.86%-90.91%)	92.58% (89.92%-95.27%)	80.623% (75.362%-86.111%)	89.53% (86.74%-92.5%)	85.59% (80.52%-90.54%)	3.5011 (3.3889-3.6120)	88.22% (82.83%-92.93%)	92.45% (86.44%-97.78%)	80.772% (69.231%-91.3%)	89.47% (82.67%-95.38%)	85.48% (74.07%-96.15%)	3.4968 (3.2581-3.7217)
At least 3 of CHASM (lung), FATHMM (missense), Mutation Assessor, MutationTaster, SIFT	88.27% (85.86%-90.91%)	95.24% (93.28%-97.56%)	76.023% (70.423%-81.818%)	87.52% (84.46%-90.63%)	90.06% (85.71%-94.44%)	3.5086 (3.4035-3.6108)	88.25% (82.83%-92.93%)	95.14% (89.83%-100%)	76.104% (63.158%-88.46%)	87.48% (80.6%-93.85%)	89.98% (80%-100%)	3.5063 (3.2722-3.7342)
At least 3 of CHASM (lung), FATHMM (missense), Mutation Assessor, MutationTaster, VEST	87.88% (85.35%-90.4%)	96.83% (95.12%-98.41%)	72.373% (66.216%-78.571%)	86.13% (82.95%-89.27%)	92.96% (88.89%-96.72%)	3.5154 (3.4111-3.6170)	87.86% (82.83%-92.93%)	96.75% (92.31%-100%)	72.582% (58.621%-85.71%)	86.13% (79.1%-92.65%)	92.82% (83.33%-100%)	3.5133 (3.2745-3.7323)
At least 3 of CHASM (lung), FATHMM (missense), Mutation Assessor, PolyPhen-2, PROVEAN	84.21% (81.31%-87.37%)	91.01% (88.19%-93.85%)	72.664% (66.216%-78.788%)	85.39% (81.89%-88.85%)	81.88% (76.62%-87.32%)	3.3199 (3.1884-3.4488)	84.23% (78.79%-89.9%)	90.9% (84.62%-96.36%)	72.854% (58.823%-85.71%)	85.35% (78.08%-92.31%)	81.73% (68.75%-93.33%)	3.3192 (3.0268-3.5917)
At least 3 of CHASM (lung), FATHMM (missense), Mutation Assessor, PolyPhen-2, SIFT	84.25% (81.31%-87.37%)	94.73% (92.48%-96.83%)	66.392% (59.722%-72.857%)	82.81% (79.41%-86.38%)	88.03% (83.08%-92.65%)	3.3354 (3.2113-3.4573)	84.27% (78.79%-89.9%)	94.63% (89.47%-98.44%)	66.656% (52.941%-80%)	82.78% (75.32%-89.87%)	87.88% (76%-96.88%)	3.3333 (3.0662-3.5870)
At least 3 of CHASM (lung), FATHMM (missense), Mutation Assessor, PolyPhen-2, VEST	83.52% (80.81%-86.36%)	95.77% (93.65%-97.83%)	62.471% (55.556%-68.919%)	81.49% (77.87%-85.05%)	89.55% (84.42%-94.2%)	3.3157 (3.1926-3.4339)	83.52% (77.78%-88.89%)	95.68% (90.91%-100%)	62.694% (48.485%-76%)	81.46% (73.97%-88.89%)	89.41% (78.26%-100%)	3.3142 (3.0359-3.5696)
At least 3 of CHASM (lung), FATHMM (missense), Mutation Assessor, PROVEAN, SIFT	84.2% (81.31%-86.87%)	92.05% (89.36%-94.74%)	70.552% (64.384%-76.812%)	84.33% (80.84%-87.84%)	83.86% (78.38%-89.04%)	3.3061 (3.1771-3.4342)	84.19% (78.79%-89.9%)	91.93% (85.71%-96.97%)	70.761% (57.143%-83.33%)	84.29% (77.14%-91.04%)	83.75% (70.37%-95.24%)	3.3060 (3.0210-3.5632)
At least 3 of CHASM (lung), FATHMM (missense), Mutation Assessor, PROVEAN, VEST	84.87% (81.82%-87.88%)	94.73% (92.56%-96.9%)	68.17% (61.765%-75%)	83.56% (79.91%-87.01%)	88.38% (83.33%-93.06%)	3.3649 (3.2409-3.4854)	84.86% (78.79%-89.9%)	94.63% (89.66%-98.44%)	68.463% (54.545%-81.48%)	83.53% (76.47%-90.41%)	88.26% (76.92%-96.97%)	3.3634 (3.0853-3.6217)
At least 3 of CHASM (lung), FATHMM (missense), Mutation Assessor, SIFT, VEST	82.52% (79.29%-85.35%)	96.3% (94.49%-98.36%)	57.853% (51.389%-64.286%)	80.38% (76.63%-84.12%)	89.72% (84.62%-94.44%)	3.2752 (3.1578-3.3943)	82.52% (76.77%-87.88%)	96.18% (91.38%-100%)	58.058% (44.118%-71.88%)	80.33% (72.37%-87.67%)	89.64% (77.78%-100%)	3.2740 (3.0004-3.5295)
At least 3 of CHASM (lung), FATHMM (missense), MutationTaster, PolyPhen-2, PROVEAN	88.58% (86.36%-91.41%)	92.06% (89.34%-94.74%)	82.394% (77.027%-87.671%)	90.32% (87.57%-93.06%)	85.39% (80.49%-90.41%)	3.5009 (3.3895-3.6106)	88.58% (83.84%-93.94%)	91.97% (85.71%-97.14%)	82.472% (71.429%-92.86%)	90.3% (84.06%-96%)	85.23% (74.07%-96.15%)	3.4985 (3.2553-3.7313)
At least 3 of CHASM (lung), FATHMM (missense), MutationTaster, PolyPhen-2, SIFT	87.94% (85.35%-90.4%)	96.31% (94.44%-98.35%)	73.255% (67.188%-79.487%)	86.43% (83.33%-89.65%)	91.86% (87.84%-95.83%)	3.4995 (3.3922-3.6032)	87.89% (82.83%-92.93%)	96.18% (91.38%-100%)	73.383% (60.87%-85.71%)	86.36% (79.69%-92.86%)	91.74% (81.82%-100%)	3.4945 (3.2644-3.7142)
At least 3 of CHASM (lung), FATHMM (missense), MutationTaster, PolyPhen-2, VEST	88.91% (86.36%-91.41%)	96.83% (95.16%-98.41%)	75.171% (69.118%-81.159%)	87.3% (84.25%-90.37%)	93.23% (89.47%-96.67%)	3.5469 (3.4479-3.6480)	88.9% (83.84%-93.94%)	96.72% (92.19%-100%)	75.283% (62.963%-87.5%)	87.26% (80.6%-93.65%)	93.07% (84%-100%)	3.5447 (3.3046-3.7669)
At least 3 of CHASM (lung), FATHMM (missense), MutationTaster, PROVEAN, SIFT	87.93% (85.35%-90.4%)	94.72% (92.59%-96.92%)	76.012% (69.863%-81.944%)	87.41% (84.27%-90.62%)	89.17% (84.62%-93.67%)	3.4870 (3.3754-3.5918)	87.89% (82.83%-92.93%)	94.62% (89.47%-98.44%)	76.135% (62.962%-88.46%)	87.36% (80.6%-93.85%)	89.08% (78.57%-100%)	3.4845 (3.2395-3.7111)
At least 3 of CHASM (lung), FATHMM (missense), MutationTaster, PROVEAN, VEST	88.93% (86.36%-91.41%)	96.3% (94.44%-98.35%)	76.102% (70.27%-81.944%)	87.67% (84.7%-90.74%)	92.35% (88.57%-96.15%)	3.5413 (3.4382-3.6430)	88.9% (83.84%-93.94%)	96.2% (91.67%-100%)	76.223% (63.636%-88%)	87.61% (81.16%-93.85%)	92.21% (82.61%-100%)	3.5370 (3.3007-3.7621)
At least 3 of CHASM (lung), FATHMM (missense), MutationTaster, SIFT, VEST	87.93% (85.35%-90.4%)	96.83% (95.16%-98.43%)	72.492% (66.216%-78.667%)	86.15% (83.1%-89.39%)	93.02% (89.04%-96.77%)	3.5179 (3.4143-3.6185)	87.88% (82.83%-92.93%)	96.72% (92.19%-100%)	72.577% (59.259%-85.19%)	86.08% (79.41%-92.54%)	92.87% (83.33%-100%)	3.5133 (3.2830-3.7358)
At least 3 of CHASM (lung), FATHMM (missense), PolyPhen-2, PROVEAN, SIFT	83.86% (80.81%-86.87%)	93.12% (90.68%-95.73%)	67.679% (61.111%-73.973%)	83.49% (79.92%-87.13%)	84.83% (79.45%-90.28%)	3.3136 (3.1826-3.4356)	83.87% (77.78%-88.89%)	93.02% (87.1%-98.28%)	67.916% (54.545%-81.48%)	83.45% (76.06%-90.54%)	84.69% (72%-96%)	3.3127 (3.0272-3.5697)
At least 3 of CHASM (lung), FATHMM (missense), PolyPhen-2, PROVEAN, VEST	83.51% (80.81%-86.36%)	95.24% (93.28%-97.48%)	62.905% (56.338%-69.444%)	81.85% (78.22%-85.48%)	88.44% (83.12%-93.42%)	3.3022 (3.1785-3.4236)	83.49% (77.78%-88.89%)	95.12% (90%-100%)	63.064% (49.091%-76.92%)	81.79% (74.32%-89.04%)	88.31% (76.92%-100%)	3.3000 (3.0268-3.5539)
At least 3 of CHASM (lung), FATHMM (missense), PolyPhen-2, SIFT, VEST	83.18% (80.3%-86.36%)	96.29% (94.44%-98.35%)	59.971% (53.521%-66.667%)	81.12% (77.43%-84.82%)	90.06% (85.33%-94.44%)	3.3084 (3.1856-3.4258)	83.16% (77.78%-88.89%)	96.18% (91.53%-100%)	60.152% (46.154%-74.07%)	81.08% (73.17%-88.41%)	89.91% (78.26%-100%)	3.3066 (3.0349-3.5575)
At least 3 of CHASM (lung), FATHMM (missense), PROVEAN, SIFT, VEST	81.85% (78.79%-84.85%)	94.73% (92.56%-96.88%)	59.649% (53.125%-66.216%)	80.81% (77.02%-84.61%)	87.11% (81.82%-92.42%)	3.2206 (3.0949-3.3452)	81.85% (75.76%-87.88%)	94.56% (89.29%-98.44%)	59.749% (47.054%-72.73%)	80.12% (72.85%-87.32%)	86.52% (75%-96.15%)	3.2206 (2.9449-3.4517)

At least 3 of CHASM (breast), CHASM (lung), FATHMM (missense), Mutation Assessor, PROVEAN, SIFT	84.5% (81.82%-87.37%)	92.59% (90.08%-95.24%)	70.484% (64.706%-76.389%)	84.43% (81.02%-87.88%)	84.63% (79.66%-89.83%)	3.3214 (3.1914-3.4443)	84.54% (78.79%-89.9%)	92.46% (87.03%-97.02%)	70.935% (59.252%-83.33%)	84.52% (77.63%-91.3%)	84.58% (74.19%-93.75%)	3.3249 (3.0665-3.5748)
At least 3 of CHASM (breast), CHASM (lung), FATHMM (missense), Mutation Assessor, PROVEAN, VEST	84.5% (81.81%-87.37%)	93.13% (90.7%-95.9%)	69.582% (63.514%-75.342%)	84.11% (80.69%-87.5%)	85.41% (80.64%-90.48%)	3.3223 (3.1981-3.4447)	84.53% (78.79%-89.9%)	92.99% (87.5%-98.28%)	69.981% (57.574%-81.58%)	84.17% (77.27%-91.05%)	85.35% (75%-95.65%)	3.3250 (3.0755-3.5748)
At least 3 of CHASM (breast), CHASM (lung), FATHMM (missense), Mutation Assessor, SIFT, VEST	81.48% (78.28%-84.34%)	94.73% (92.62%-96.9%)	58.568% (52.113%-65.219%)	79.81% (76.19%-83.44%)	86.55% (81.4%-92.16%)	3.1966 (3.0731-3.3263)	81.48% (75.76%-87.88%)	94.56% (89.85%-98.48%)	58.992% (45.447%-71.88%)	79.83% (72.36%-87.18%)	86.37% (75%-96%)	3.1975 (2.9429-3.4449)
At least 3 of CHASM (breast), CHASM (lung), FATHMM (missense), MutationTaster, PolyPhen-2, PROVEAN	87.58% (85.34%-89.9%)	93.13% (90.7%-95.93%)	77.974% (72.464%-83.333%)	87.97% (84.96%-91.31%)	86.8% (82.35%-91.67%)	3.4588 (3.3518-3.5669)	87.46% (82.83%-91.94%)	92.97% (87.5%-98.25%)	77.952% (65.852%-88.89%)	87.87% (81.24%-93.94%)	86.63% (77.14%-96.15%)	3.4543 (3.2369-3.6637)
At least 3 of CHASM (breast), CHASM (lung), FATHMM (missense), MutationTaster, PolyPhen-2, SIFT	87.25% (84.85%-89.9%)	96.86% (95.16%-98.49%)	70.626% (64.286%-77.027%)	85.08% (81.82%-88.41%)	92.86% (89.29%-96.61%)	3.4542 (3.3499-3.5640)	87.12% (81.82%-91.92%)	96.71% (92.85%-100%)	70.639% (57.887%-82.5%)	84.98% (77.94%-91.55%)	92.62% (84%-100%)	3.4495 (3.2259-3.6620)
At least 3 of CHASM (breast), CHASM (lung), FATHMM (missense), MutationTaster, PolyPhen-2, VEST	86.28% (83.84%-88.89%)	97.94% (96.72%-99.22%)	66.105% (60%-72.603%)	83.32% (80.13%-86.99%)	94.89% (91.67%-98.15%)	3.4226 (3.3229-3.5303)	86.03% (80.81%-90.91%)	97.73% (94.91%-100%)	65.936% (52.774%-78.12%)	83.12% (75.71%-89.75%)	94.44% (86.36%-100%)	3.4123 (3.1891-3.6176)
At least 3 of CHASM (breast), CHASM (lung), FATHMM (missense), MutationTaster, PROVEAN, SIFT	88.56% (86.36%-90.91%)	93.66% (91.34%-96.06%)	79.725% (74.62%-84.932%)	88.88% (86.04%-91.97%)	87.92% (83.61%-92.54%)	3.5019 (3.3962-3.6122)	88.53% (83.84%-92.93%)	93.51% (88.52%-98.36%)	79.947% (69.444%-90%)	88.91% (82.61%-94.45%)	87.79% (78.26%-96.55%)	3.5016 (3.2809-3.7065)
At least 3 of CHASM (breast), CHASM (lung), FATHMM (missense), MutationTaster, PROVEAN, VEST	88.25% (85.86%-90.91%)	94.74% (92.56%-96.88%)	77.027% (71.429%-82.67%)	87.7% (84.72%-91.04%)	89.44% (85.25%-93.75%)	3.4890 (3.3780-3.6006)	88.14% (82.83%-92.93%)	94.56% (90%-98.51%)	77.084% (65.622%-87.88%)	87.64% (81.16%-93.75%)	89.22% (80%-96.97%)	3.4851 (3.2636-3.7045)
At least 3 of CHASM (breast), CHASM (lung), FATHMM (missense), MutationTaster, SIFT, VEST	86.89% (84.34%-89.9%)	96.87% (95.16%-98.47%)	69.629% (63.51%-75.714%)	84.65% (81.29%-88.03%)	92.79% (89.09%-96.55%)	3.4394 (3.3282-3.5456)	86.83% (80.81%-91.92%)	96.68% (92.98%-100%)	69.878% (57.887%-82.5%)	84.65% (77.46%-91.3%)	92.49% (83.87%-100%)	3.4369 (3.2223-3.6550)
At least 3 of CHASM (breast), CHASM (lung), FATHMM (missense), PolyPhen-2, PROVEAN, SIFT	83.16% (80.3%-86.36%)	92.6% (90%-95.31%)	66.847% (60.526%-73.239%)	82.85% (79.31%-86.58%)	83.93% (78.69%-89.66%)	3.2622 (3.1355-3.3997)	83.17% (76.77%-88.89%)	92.45% (86.67%-97.06%)	67.206% (54.286%-80.66%)	82.88% (75.38%-90.14%)	83.85% (72.41%-93.75%)	3.2638 (2.9889-3.5221)
At least 3 of CHASM (breast), CHASM (lung), FATHMM (missense), PolyPhen-2, PROVEAN, VEST	82.86% (79.8%-85.86%)	92.6% (90%-95.31%)	66.022% (59.42%-72.603%)	82.5% (79.26%-86.03%)	83.76% (78.57%-89.47%)	3.2488 (3.1161-3.3840)	82.76% (76.77%-88.89%)	92.45% (86.67%-97.06%)	66.062% (52.5%-78.96%)	82.4% (75%-89.23%)	83.61% (72.41%-93.75%)	3.2453 (2.9754-3.4966)
At least 3 of CHASM (breast), CHASM (lung), FATHMM (missense), PolyPhen-2, SIFT, VEST	81.15% (78.28%-84.34%)	95.79% (93.85%-97.67%)	55.825% (49.315%-62.5%)	78.95% (75.34%-82.69%)	88.45% (83.33%-93.62%)	3.1901 (3.0650-3.3131)	81.14% (74.75%-86.87%)	95.65% (91.52%-100%)	56.18% (42.857%-68.75%)	78.94% (71.43%-85.9%)	88.28% (77.27%-100%)	3.1905 (2.9431-3.4375)
At least 3 of CHASM (breast), CHASM (lung), FATHMM (missense), PROVEAN, SIFT, VEST	81.82% (78.79%-84.85%)	93.13% (90.7%-95.9%)	62.261% (55.714%-68.752%)	81.01% (77.46%-84.72%)	83.97% (78.72%-89.66%)	3.2037 (3.0722-3.3352)	81.82% (75.76%-87.88%)	92.99% (87.5%-98.28%)	62.599% (50%-75%)	81.02% (73.43%-88.06%)	83.9% (72.22%-95.24%)	3.2051 (2.9395-3.4642)
At least 3 of CHASM (breast), CHASM (lung), Mutation Assessor, MutationTaster, PolyPhen-2, PROVEAN	82.44% (79.29%-85.35%)	85.06% (81.45%-88.72%)	77.897% (72.603%-83.562%)	86.93% (83.74%-90.4%)	75.1% (69.74%-80.6%)	3.2499 (3.1243-3.3823)	82.6% (76.77%-88.89%)	85.18% (77.27%-92.31%)	78.168% (67.5%-88.89%)	86.99% (80%-93.45%)	75.48% (63.89%-86.05%)	3.2581 (2.9862-3.4989)
At least 3 of CHASM (breast), CHASM (lung), Mutation Assessor, MutationTaster, PolyPhen-2, SIFT	83.47% (80.3%-86.36%)	89.33% (86.32%-92.5%)	73.326% (67.606%-79.226%)	85.27% (81.68%-88.72%)	79.9% (74.54%-85.48%)	3.2783 (3.1533-3.4046)	83.57% (77.78%-89.9%)	89.41% (83.01%-95.31%)	73.546% (62.061%-84.85%)	85.28% (78.46%-92.19%)	80.21% (69.22%-90.91%)	3.2845 (3.0292-3.5404)
At least 3 of CHASM (breast), CHASM (lung), Mutation Assessor, MutationTaster, PolyPhen-2, VEST	80.13% (77.26%-83.33%)	86.65% (83.33%-90.08%)	68.865% (63.014%-75%)	82.79% (79.07%-86.77%)	74.89% (69.23%-80.7%)	3.1319 (2.9933-3.2699)	80.14% (73.74%-85.88%)	86.8% (80%-93.44%)	68.736% (56.748%-80.49%)	82.63% (75%-89.71%)	75.22% (63.33%-86.67%)	3.1339 (2.8619-3.4051)
At least 3 of CHASM (breast), CHASM (lung), Mutation Assessor, MutationTaster, PROVEAN, SIFT	84.47% (81.82%-87.37%)	88.78% (85.61%-92%)	77.002% (71.795%-82.609%)	86.97% (83.61%-90.3%)	79.89% (74.63%-85.14%)	3.3265 (3.2016-3.4561)	84.6% (78.79%-89.9%)	88.9% (82.14%-94.92%)	77.193% (66.667%-87.89%)	86.99% (80%-93.65%)	80.24% (68.97%-90.62%)	3.3333 (3.0785-3.5748)
At least 3 of CHASM (breast), CHASM (lung), Mutation Assessor, MutationTaster, PROVEAN, VEST	82.14% (79.29%-84.85%)	85.09% (81.6%-88.72%)	77.029% (71.642%-82.61%)	86.49% (83.06%-90.17%)	74.93% (69.7%-80.56%)	3.2353 (3.1129-3.3614)	82.19% (76.77%-87.88%)	85.12% (77.58%-91.94%)	77.159% (66.667%-87.28%)	86.46% (79.1%-93.44%)	75.16% (63.64%-85.71%)	3.2390 (2.9805-3.4859)
At least 3 of CHASM (breast), CHASM (lung), Mutation Assessor, MutationTaster, SIFT, VEST	82.79% (79.8%-85.86%)	89.85% (86.99%-92.91%)	70.591% (64.787%-76.473%)	84.08% (80.45%-87.6%)	80.09% (74.6%-85.48%)	3.2461 (3.1150-3.3702)	82.9% (76.77%-88.89%)	89.97% (83.82%-95.46%)	70.76% (58.824%-82.51%)	84.07% (76.81%-91.3%)	80.46% (69.23%-91.18%)	3.2525 (2.9992-3.5123)
At least 3 of CHASM (breast), CHASM (lung), Mutation Assessor, PolyPhen-2, PROVEAN, SIFT	78.42% (75.25%-81.82%)	85.08% (81.6%-88.71%)	66.899% (60.806%-73.239%)	81.63% (77.86%-85.5%)	72.18% (66.67%-78.12%)	3.0579 (2.9206-3.2045)	78.51% (71.72%-84.85%)	85.13% (78.12%-92.06%)	67.121% (54.831%-79.41%)	81.62% (73.84%-88.89%)	72.5% (60.6%-83.87%)	3.0637 (2.7750-3.3445)
At least 3 of CHASM (breast), CHASM (lung), Mutation Assessor, PolyPhen-2, PROVEAN, VEST	74.72% (71.21%-78.28%)	80.31% (76.38%-84.35%)	65.049% (58.904%-71.233%)	79.89% (75.81%-84%)	65.65% (59.74%-71.64%)	2.9090 (2.7667-3.0510)	74.8% (67.68%-81.82%)	80.3% (72.22%-88.06%)	65.361% (53.328%-77.78%)	79.89% (71.87%-87.7%)	65.93% (53.33%-77.5%)	2.9148 (2.6175-3.1889)
At least 3 of CHASM (breast), CHASM (lung), Mutation Assessor, PolyPhen-2, SIFT, VEST	76.03% (72.73%-79.29%)	87.2% (83.9%-90.37%)	56.709% (50%-63.015%)	77.69% (73.61%-81.43%)	71.94% (65.45%-78%)	2.9353 (2.7838-3.0782)	76.23% (69.7%-82.83%)	87.28% (80.6%-93.65%)	57.248% (44.737%-70.27%)	77.78% (70.15%-85.92%)	72.43% (60%-85.19%)	2.9474 (2.6486-3.2442)
At least 3 of CHASM (breast), CHASM (lung), Mutation Assessor, PROVEAN, SIFT, VEST	77.45% (74.24%-80.81%)	84.07% (80.51%-87.9%)	65.982% (59.722%-72.059%)	81.03% (77.16%-84.73%)	70.56% (64.86%-76.81%)	3.0164 (2.8715-3.1551)	77.43% (70.71%-83.84%)	83.96% (76.18%-90.62%)	66.199% (54.054%-78.57%)	80.99% (73.44%-88.71%)	70.65% (57.69%-82.05%)	3.0180 (2.7373-3.3021)
At least 3 of CHASM (breast), CHASM (lung), MutationTaster, PolyPhen-2, PROVEAN, SIFT	82.78% (79.8%-85.86%)	87.73% (84.5%-91.13%)	74.202% (68.493%-80.007%)	85.46% (82.09%-89.06%)	77.77% (72.31%-83.58%)	3.2517 (3.1283-3.3823)	82.93% (76.77%-88.89%)	87.82% (80.95%-94.12%)	74.534% (62.783%-86.11%)	85.54% (78.33%-92.31%)	78.12% (66.67%-89.29%)	3.2601 (2.9864-3.5042)
At least 3 of CHASM (breast), CHASM (lung), MutationTaster, PolyPhen-2, PROVEAN, VEST	80.13% (76.77%-83.33%)	84.55% (81.15%-88.28%)	72.487% (66.667%-78.691%)	84.16% (80.62%-88%)	73.07% (67.14%-78.87%)	3.1427 (3.0031-3.2755)	80.15% (73.74%-86.87%)	84.61% (76.79%-91.55%)	72.483% (60.526%-84.21%)	84.06% (76.36%-91.38%)	73.31% (61.76%-84.38%)	3.1446 (2.8724-3.4140)
At least 3 of CHASM (breast), CHASM (lung), MutationTaster, PolyPhen-2, SIFT, VEST	79.4% (76.26%-82.34%)	89.8% (86.99%-92.86%)	61.399% (54.795%-68.056%)	80.09% (76.47%-83.92%)	77.69% (71.92%-83.64%)	3.0898 (2.9484-3.2235)	79.59% (73.71%-85.86%)	90.07% (83.87%-95.45%)	61.597% (48.649%-74.36%)	80.1% (72.37%-87.14%)	78.32% (66.67%-90.01%)	3.1008 (2.8249-3.3837)
At least 3 of CHASM (breast), CHASM (lung), MutationTaster, PROVEAN, SIFT, VEST	82.13% (79.29%-84.85%)	87.74% (84.5%-90.91%)	72.416% (66.667%-78.207%)	84.61% (81.25%-88.06%)	77.37% (71.83%-82.61%)	3.2214 (3.0890-3.3491)	82.21% (76.77%-87.88%)	87.79% (81.36%-94.03%)	72.602% (61.111%-84.38%)	84.61% (77.77%-91.53%)	77.62% (66.67%-88.89%)	3.2262 (2.9656-3.4839)
At least 3 of CHASM (breast), CHASM (lung), PolyPhen-2, PROVEAN, SIFT, VEST	74.37% (70.71%-77.78%)	84.56% (81.15%-88.19%)	56.743% (50%-63.239%)	77.17% (73.24%-81.16%)	68.01% (61.82%-74.19%)	2.8649 (2.7177-3.0100)	74.49% (67.68%-81.82%)	84.57% (77.05%-91.43%)	57.146% (43.899%-70.37%)	77.2% (69.12%-84.86%)	68.35% (55.17%-80.77%)	2.8727 (2.5710-3.1760)
At least 3 of CHASM (breast), CHASM (melanoma), FATHMM (cancer), FATHMM (missense), Mutation Assessor, MutationTaster	89.26% (86.87%-91.92%)	96.34% (94.49%-98.43%)	77.006% (71.831%-82.899%)	87.87% (84.96%-91.24%)	92.42% (88.89%-96.61%)	3.5363 (3.4397-3.6446)	89.16% (83.84%-93.94%)	96.14% (91.8%-100%)	77.17% (65.789%-87.18%)	87.84% (81.16%-93.65%)	92.11% (83.87%-100%)	3.5326 (3.3164-3.7333)
At least 3 of CHASM (breast), CHASM (melanoma), FATHMM (cancer), FATHMM (missense), Mutation Assessor, PolyPhen-2	84.21% (81.31%-87.37%)	95.27% (93.39%-97.56%)	65.086% (58.904%-71.062%)	82.51% (78.95%-86.11%)	88.85% (84.31%-94%)	3.3173 (3.1989-3.4391)	84.1% (77.78%-89.9%)	95.08% (90.74%-98.51%)	65.218% (53.125%-77.79%)	82.43% (75%-89.71%)	88.56% (78.26%-96.67%)	3.3129 (3.0722-3.5500)
At least 3 of CHASM (breast), CHASM (melanoma), FATHMM (cancer), FATHMM (missense), Mutation Assessor, PROVEAN	84.5% (81.82%-87.37%)	92.04% (89.43%-95%)	71.443% (65.333%-77.473%)	84.79% (81.43%-88.55%)	83.85% (78.46%-89.83%)	3.3213 (3.1914-3.4438)	84.54% (78.79%-89.9%)	91.97% (86.15%-96.97%)	71.765% (59.365%-84.38%)	84.83% (77.94%-91.94%)	83.92% (73.08%-93.94%)	3.3249 (3.0733-3.5776)
At least 3 of CHASM (breast), CHASM (melanoma), FATHMM (cancer), FATHMM (missense), Mutation Assessor, SIFT	83.17% (80.3%-85.86%)	94.2% (92%-96.72%)	64.088% (57.811%-70.27%)	81.94% (78.38%-85.52%)	86.47% (81.48%-91.8%)	3.2669 (3.1391-3.3908)	83.16% (77.78%-88.89%)	94.04% (89.09%-98.41%)	64.431% (51.429%-77.14%)	81.95% (74.63%-89.19%)	86.32% (75%-96.15%)	3.2674 (3.0149-3.5153)
At least 3 of CHASM (breast), CHASM (melanoma), FATHMM (cancer), FATHMM (missense), Mutation Assessor, VEST	82.49% (79.29%-85.86%)	95.26% (93.33%-97.58%)	60.382% (53.623%-66.667%)	80.61% (76.92%-84.31%)	88.07% (83.33%-93.75%)	3.2433 (3.1191-3.3739)	82.5% (75.76%-88.89%)	95.1% (90.48%-98.53%)	60.856% (48.567%-73.68%)	80.66% (73.01%-87.88%)	87.88% (76.92%-96.43%)	3.2449 (2.9730-3.4987)
At least 3 of CHASM (breast), CHASM (melanoma), FATHMM (cancer), FATHMM (missense), MutationTaster, PolyPhen-2	87.59% (85.35%-90.4%)	97.4% (95.93%-99.19%)	70.626% (64.787%-76.812%)	85.15% (82.07%-88.51%)	94.02% (90.57%-98.11%)	3.4719 (3.3704-3.5828)	87.44% (81.82%-91.94%)	97.22% (93.85%-100%)	70.633% (58.971%-82.35%)	85.04% (78.08%-91.43%)	93.67% (85.71%-100%)	3.4656 (3.2437-3.6675)
At least 3 of CHASM (breast), CHASM (melanoma), FATHMM (cancer), FATHMM (missense), MutationTaster, PROVEAN	88.57% (85.86%-90.91%)	93.65% (91.27%-96.09%)	79.772% (74.667%-85.294%)	88.9% (86.05%-92.19%)	87.9% (83.58%-92.42%)	3.5021 (3.3941-3.6116)	88.53% (83.84%-93.94%)	93.55% (88.4%-98.39%)	79.863% (68.75%-90%)	88.87% (82.54%-94.44%)	87.85% (78.57%-96.88%)	3.5013 (3.2797-3.7251)
At least 3 of CHASM (breast), CHASM (melanoma), FATHMM (cancer), FATHMM (missense), MutationTaster, SIFT	88.23% (85.86%-90.91%)	96.88% (95.16%-98.5%)	73.251% (67.567%-79.173%)	86.24% (83.33%-89.63%)	93.16% (89.47%-96.77%)	3.4953 (3.3935-3.6064)	88.19% (82.83%-92.93%)	96.64% (92.73%-100%)	73.617% (61.111%-85%)	86.3% (79.41%-92.42%)	92.77% (84.61%-100%)	3.4933 (3.2687-3.6970)
At least 3 of CHASM (breast), CHASM (melanoma), FATHMM (cancer), FATHMM (missense), MutationTaster, VEST	87.58% (85.35%-90.4%)	97.93% (96.72%-99.24%)	69.675% (63.636%-76%)	84.82% (81.69%-88.32%)	95.13% (92%-98.25%)	3.4755 (3.3747-3.5804)	87.46% (81.82%-91.92%)	97.74% (94.74%-100%)	69.776% (57.143%-81.82%)	84.75% (77.63%-90.91%)	94.74% (87.5%-100%)	3.4701 (3.2590-3.6644)
At least 3 of CHASM (breast), CHASM (melanoma), FATHMM (cancer), FATHMM (missense), PolyPhen-2, PROVEAN	82.83% (79.8%-85.86%)	92.04% (89.39%-94.74%)	66.9% (60.87%-73.239%)	82.78% (79.41%-86.43%)	82.96% (77.42%-88.34%)	3.2469 (3.1164-3.3774)	82.82% (76.77%-88.89%)	91.96% (86.21%-96.97%)	67.081% (54.825%-79.41%)	82.75% (75.36%-89.71%)	82.98% (71.79%-93.75%)	3.2477 (2.9736-3.5042)
At least 3 of CHASM (breast), CHASM (melanoma), FATHMM (cancer), FATHMM (missense), PolyPhen-2, SIFT	81.85% (78.79%-84.86%)	95.82% (94.02%-97.67%)	57.686% (50.769%-64.104%)	79.66% (76.03%-83.34%)	88.87% (84.09%-94%)	3.2203 (3.0978-3.3462)	81.76% (75.73%-87.88%)	95.59% (91.52%-98.59%)	57.977% (44.828%-71.43%)	79.62% (72.06%-86.67%)	88.47% (76.19%-96.78%)	3.2165 (2.9530-3.4458)
At least 3 of CHASM (breast), CHASM (melanoma), FATHMM (cancer), FATHMM (missense), PolyPhen-2, VEST	83.2% (80.3%-86.36%)	97.4% (95.93%-99.19%)	58.631% (52.174%-64.935%)	80.28% (76.82%-84%)	92.88% (88.89%-97.73%)	3.2919 (3.1828-3.4085)	83.1% (76.77%-88.89%)	97.22% (93.85%-100%)	58.852% (46.332%-71.05%)	80.22% (72.83%-87.14%)	92.5% (83.33%-100%)	3.2879 (3.0516-3.5059)
At least 3 of CHASM (breast), CHASM (melanoma), FATHMM (cancer), FATHMM (missense), PROVEAN, SIFT	81.78% (78.79%-84.85%)	92.04% (89.43%-95%)	64.038% (57.742%-70.423%)	81.57% (77.94%-85.29%)	82.32% (76.56%-88.47%)	3.1997 (3.0688-3.3429)	81.89% (75.76%-87.88%)	91.97% (86.15%-96.97%)	64.528% (51.429%-77.78%)	81.67% (74.28%-88.89%)	82.43% (70.83%-93.55%)	3.2060 (2.9278-3.4640)
At least 3 of CHASM (breast), CHASM (melanoma), FATHMM (cancer), FATHMM (missense), PROVEAN, VEST	82.15% (79.29%-85.35%)	93.11% (90.55%-95.93%)	63.186% (56.757%-69.565%)	81.39% (77.78%-85.11%)	84.14% (78.57%-90.2%)	3.2182 (3.0815-3.3551)	82.17% (75.76%-87.88%)	93.03% (87.69%-98.36%)	63.481% (51.35%-76.32%)	81.4% (73.97%-88.46%)	84.17% (72.73%-95.66%)	3.2208 (2.9567-3.4889)
At least 3 of CHASM (breast), CHASM (melanoma), FATHMM (cancer), FATHMM (missense), SIFT, VEST	81.51% (78.28%-84.85%)	95.81% (93.85%-98.33%)	56.772% (50.684%-63.239%)	79.31% (75.68%-83.11%)	88.68% (83.72%-95%)	3.2057 (3.0826-3.3370)	81.43% (74.75%-87.88%)	95.61% (90.91%-100%)	57.052% (44.73%-69.7%)	79.26% (71.43%-86.25%)	88.35% (76.46%-100%)	3.2027 (2.9288-3.4392)
At least 3 of CHASM (breast), CHASM (melanoma), FATHMM (cancer), Mutation Assessor, MutationTaster, PolyPhen-2	88.24% (85.86%-90.91%)	97.9% (96.72%-99.24%)	71.543% (66.176%-77.778%)	85.61% (82.52%-88.97%)	95.17% (92.16%-98.31%)	3.5022 (3.4063-3.6066)	88.16% (82.83%-92.93%)	97.82% (94.64%-100%)	71.604% (59.522%-82.76%)	85.53% (78.38%-91.67%)	95.03% (87.5%-100%)	3.4998 (3.2898-3.6969)
At least 3 of CHASM (breast), CHASM (melanoma), FATHMM (cancer), Mutation Assessor, MutationTaster, PROVEAN	87.88% (85.35%-90.42%)	94.17% (91.94%-96.75%)	77.017% (71.617%-82.432%)	87.63% (84.62%-90.91%)	88.42% (84.13%-93.44%)	3.4724 (3.3618-3.5892)	87.87% (82.8%-92.93%)	94.11% (89.23%-98.44%)	77.166% (65.789%-87.89%)	87.61% (80.95%-93.85%)	88.43% (78.79%-96.78%)	3.4731 (3.2414-3.6931)
At least 3 of CHASM (breast), CHASM (melanoma), FATHMM (cancer), Mutation Assessor, MutationTaster, SIFT	86.88% (84.34%-89.39%)	96.31% (94.4%-98.4%)	70.587% (64.789%-76.389%)	84.99% (81.69%-88.49%)	91.71% (87.93%-96.37%)	3.4360 (3.3262-3.5487)	86.84% (81.82%-91.92%)	96.2% (92.06%-100%)	70.761% (59.375%-82.35%)	84.95% (77.94%-91.43%)	91.6% (82.13%-100%)	3.4351 (3.2098-3.6549)
At least 3 of CHASM (breast), CHASM (melanoma), FATHMM (cancer), Mutation Assessor, MutationTaster, VEST	87.24% (84.34%-89.9%)	96.86% (95.16%-98.45%)	70.62% (64.863%-76.471%)	85.07% (81.82%-88.51%)	92.86% (89.28%-96.49%)	3.4541 (3.3415-3.5604)	87.13% (81.82%-92.93%)	96.7% (93.22%-100%)	70.7% (58.966%-82.5%)	84.99% (78.08%-91.31%)	92.61% (84.62%-100%)	3.4500 (3.2410-3.6710)
At least 3 of CHASM (breast), CHASM (melanoma), FATHMM (cancer), Mutation Assessor, PolyPhen-2, PROVEAN	84.17% (81.31%-86.87%)	93.1% (90.62%-95.93%)	68.711% (62.5%-75%)	83.73% (80.3%-87.31%)	85.22% (80%-90.62%)	3.3075 (3.1804-3.4325)	84.19% (78.79%-89.9%)	93.04% (87.5%-98.33%)	68.985% (56.66%-81.09%)	83.74% (76.56%-90.41%)	85.27% (73.91%-96.3%)	3.3104 (3.0555-3.5653)
At least 3 of CHASM (breast), CHASM (melanoma), FATHMM (cancer), Mutation Assessor, PolyPhen-2, SIFT	82.85% (79.8%-85.86%)	95.78% (93.75%-97.67%)	60.484% (54.283%-67.124%)	80.73% (77.08%-84.46%)	89.24% (84.61%-94.23%)	3.2623 (3.1419-3.3848)	82.79% (76.77%-88.89%)	95.67% (91.52%-100%)	60.687% (47.368%-73.53%)	80.68% (73.26%-87.85%)	89.12% (77.78%-100%)	3.2615 (3.0122-3.4987)
At least 3 of CHASM (breast), CHASM (melanoma), FATHMM (cancer), Mutation Assessor, PolyPhen-2, VEST	82.53% (79.8%-85.37%)	96.32% (94.49%-98.39%)	58.682% (52.237%-65.278%)	80.12% (76.35%-83.77%)	90.22% (85.71%-95.65%)	3.2535 (3.1363-3.3820)	82.42% (76.74%-87.88%)	96.18% (92.19%-100%)	58.793% (45.934%-71.43%)	80.02% (72.6%-87.32%)	89.98% (80%-100%)	3.2497 (2.9928-3.4772)
At least 3 of CHASM (breast), CHASM (melanoma), FATHMM (cancer), Mutation Assessor, PROVEAN, SIFT	83.16% (80.3%-86.36%)	93.1% (90.55%-95.83%)	65.981% (60%-72.368%)	82.55% (79.14%-86.11%)	84.69% (79.31%-90.32%)	3.2632 (3.1362-3.3890)	83.17% (76.77%-88.89%)	93.05% (87.69%-98.33%)	66.176% (53.846%-77.78%)	82.52% (75.32%-89.19%)	84.77% (73.33%-96%)	3.2652 (3.0103-3.5086)
At least 3 of CHASM (breast), CHASM (melanoma), FATHMM (cancer), Mutation Assessor, PROVEAN, VEST	84.17% (81.31%-86.87%)	93.63% (91.27%-96.06%)	67.813% (61.972%-73.973%)	83.41% (80%-87.05%)	86.03% (81.03%-91.38%)	3.3088 (3.1864-3.4326)	84.18% (78.79%-89.9%)	93.59% (88.52%-98.39%)	68.026% (55.556%-80%)	83.39% (76.32%-90.14%)	86.11% (75%-96.16%)	3.3112 (3.0721-3.5501)
At least 3 of CHASM (breast), CHASM (melanoma), FATHMM (cancer), Mutation Assessor, SIFT, VEST	81.84% (78.79%-84.85%)	95.81% (93.96%-97.73%)	57.687% (51.315%-63.892%)	79.65% (76.19%-83.33%)	88.84% (84.09%-94%)	3.2199 (3.0975-3.3458)	81.77% (75.76%-87.88%)	95.61% (91.18%-98.66%)	58.021% (45.157%-70.46%)	79.62% (72.15%-86.49%)	88.53% (77.27%-96.95%)	3.2177 (2.9606-3.4520)
At least 3 of CHASM (breast), CHASM (melanoma), FATHMM (cancer), MutationTaster, PolyPhen-2, PROVEAN	87.19% (84.85%-89.9%)	95.23% (93.08%-97.56%)	73.296% (67.164%-79.71%)	86.05% (82.86%-89.63%)	89.89% (85.71%-94.74%)	3.4446 (3.3358-3.5637)	87.23% (81.82%-91.92%)	95.18% (90.62%-100%)	73.581% (61.111%-85%)	86.08% (79.03%-92.43%)	89.9% (80.77%-100%)	3.4474 (3.2050-3.6609)
At least 3 of CHASM (breast), CHASM (melanoma), FATHMM (cancer), MutationTaster, PolyPhen-2, SIFT	85.88% (83.33%-88.89%)	97.9% (96.72%-99.24%)	65.102% (59.211%-71.429%)	82.91% (79.49%-86.39%)	94.72% (91.49%-98.11%)	3.4063 (3.3024-3.5136)	85.81% (79.8%-90.91%)	97.82% (94.64%-100%)	65.218% (52.628%-77.14%)	82.83% (75.67%-89.47%)	94.57% (86.66%-100%)	3.4044 (3.1904-3.6074)
At least 3 of CHASM (breast), CHASM (melanoma), FATHMM (cancer), MutationTaster, PolyPhen-2, VEST	84.94% (82.32%-87.88%)	97.93% (96.72%-99.22%)	62.482% (56.162%-69.565%)	81.86% (78.38%-85.62%)	94.6% (91.3%-98%)	3.3687 (3.2610-3.4832)	84.66% (78.79%-89.9%)	97.74% (94.91%-100%)	62.2% (50%-74.38%)	81.61% (74.24%-88.46%)	94.14% (86.21%-100%)	3.3568 (3.1407-3.5679)
At least 3 of CHASM (breast), CHASM (melanoma), FATHMM (cancer), MutationTaster, PROVEAN, SIFT	87.21% (84.85%-89.9%)	94.71% (92.56%-96.97%)	74.221% (68.657%-80%)	86.4% (83.45%-89.71%)	89.03% (84.75%-93.65%)	3.4436 (3.3351-3.5609)	87.21% (81.82%-91.92%)	94.62% (89.7%-98.51%)	74.458% (63.158%-85.29%)	86.42% (79.71%-92.54%)	88.98% (79.17%-96.88%)	3.4447 (3.2152-3.6620)
At least 3 of CHASM (breast), CHASM (melanoma), FATHMM (cancer), MutationTaster, PROVEAN, VEST	87.54% (84.85%-90.4%)	95.76% (93.75%-97.69%)	73.322% (67.164%-79.106%)	86.12% (82.96%-89.58%)	90.91% (87.04%-95.08%)	3.4611 (3.3541-3.5755)	87.55% (81.82%-92.93%)	95.71% (91.38%-100%)	73.532% (61.759%-85.71%)	86.12% (79.1%-92.54%)	90.9% (81.82%-100%)	3.4627 (3.2324-3.6841)
At least 3 of CHASM (breast), CHASM (melanoma), FATHMM (cancer), MutationTaster, SIFT, VEST	84.86% (82.32%-87.88%)	97.38% (95.93%-99.19%)	63.227% (56.944%-69.565%)	82.07% (78.77%-85.62%)	93.31% (89.58%-97.92%)	3.3599 (3.2476-3.4707)	84.82% (78.79%-89.9%)	97.26% (93.75%-100%)	63.46% (51.282%-75.76%)	82.03% (75%-88.57%)	93.12% (84.37%-100%)	3.3587 (3.1403-3.5793)
At least 3 of CHASM (breast), CHASM (melanoma), FATHMM (cancer), PolyPhen-2, PROVEAN, SIFT	82.52% (79.8%-85.86%)	93.65% (91.3%-96.15%)	63.289% (56.944%-69.447%)	81.52% (77.94%-85.31%)	85.22% (79.99%-91.23%)	3.2367 (3.1159-3.3662)	82.43% (75.76%-87.88%)	93.55% (88.33%-98.36%)	63.311% (50%-76.19%)	81.4% (73.97%-88.58%)	85.15% (73.08%-96.15%)	3.2341 (2.9664-3.4769)
At least 3 of CHASM (breast), CHASM (melanoma), FATHMM (cancer), PolyPhen-2, PROVEAN, VEST	81.5% (78.78%-84.85%)	94.69% (92.5%-96.97%)	58.695% (52.172%-65.217%)	79.85% (76.06%-83.56%)	86.48% (81.25%-92.16%)	3.1972 (3.0749-3.3269)	81.44% (74.75%-86.89%)	94.66% (89.83%-98.53%)	58.745% (45.157%-71.43%)	79.75% (72.22%-87.01%)	86.52% (75%-96.43%)	3.1967 (2.9316-3.4393)
At least 3 of CHASM (breast), CHASM (melanoma), FATHMM (cancer), PolyPhen-2, SIFT, VEST	79.44% (76.26%-82.83%)	96.83% (95.2%-98.44%)	49.361% (43.243%-55.714%)	76.78% (73.08%-80.63%)	90.01% (85%-95.24%)	3.1298 (3.0114-3.2529)	79.51% (72.73%-85.86%)	96.76% (93.33%-100%)	49.879% (37.491%-62.22%)	76.81% (69.14%-84.22%)	89.98% (78.95%-100%)	3.1343 (2.8926-3.3675)
At least 3 of CHASM (breast), CHASM (melanoma), FATHMM (cancer), PROVEAN, SIFT, VEST	81.48% (78.79%-84.85%)	94.17% (91.87%-96.77%)	59.543% (52.941%-65.753%)	80.1% (76.76%-83.81%)	85.53% (80%-91.67%)	3.1934 (3.0667-3.3266)	81.48% (74.75%-86.87%)	94.1% (89.06%-98.46%)	59.79% (47.222%-72.51%)	80.07% (72.46%-86.67%)	85.55% (73.68%-95.83%)	3.1950 (2.9254-3.4456)
At least 3 of CHASM (breast), CHASM (melanoma), FATHMM (missense), Mutation Assessor, MutationTaster, PolyPhen-2	85.92% (83.33%-88.89%)	96.31% (94.4%-98.39%)	67.94% (62.119%-73.973%)	83.86% (80.58%-87.34%)	91.43% (87.27%-96.15%)	3.3954 (3.2873-3.5024)	85.74% (79.8%-90.91%)	96.2% (92.19%-100%)	67.755% (55.172%-79.5%)	83.67% (76.39%-90.28%)	91.27% (81.82%-100%)	3.3892 (3.1710-3.6088)
At least 3 of CHASM (breast), CHASM (melanoma), FATHMM (missense), Mutation Assessor, MutationTaster, PROVEAN	89.27% (86.87%-91.92%)	94.21% (91.94%-96.77%)	80.724% (75.714%-86.301%)	89.42% (86.67%-92.7%)	88.98% (85%-93.65%)	3.5334 (3.4294-3.6396)	89.13% (83.84%-93.94%)	94.01% (89.06%-98.44%)	80.726% (69.992%-90.62%)	89.33% (82.86%-95.08%)	88.73% (79.41%-96.88%)	3.5280 (3.3081-3.7368)
At least 3 of CHASM (breast), CHASM (melanoma), FATHMM (missense), Mutation Assessor, MutationTaster, SIFT	86.56% (83.84%-89.39%)	95.23% (93.27%-97.58%)	71.557% (65.753%-77.778%)	85.27% (82.01%-88.65%)	89.68% (85.48%-94.55%)	3.4175 (3.3067-3.5325)	86.47% (80.81%-91.92%)	95.16% (90.62%-98.55%)	71.521% (58.818%-83.33%)	85.16% (78.46%-91.78%)	89.64% (80%-97.14%)	3.4148 (3.1808-3.6392)
At least 3 of CHASM (breast), CHASM (melanoma), FATHMM (missense), Mutation Assessor, MutationTaster, VEST	85.59% (82.83%-88.38%)	96.34% (94.49%-98.39%)	67.009% (60.936%-73.078%)	83.47% (80.14%-86.99%)	91.37% (87.27%-96.15%)	3.3819 (3.2725-3.5003)	85.38% (79.8%-90.91%)	96.15% (92.15%-100%)	66.876% (54.545%-78.95%)	83.28% (76.19%-89.86%)	91.05% (81.82%-100%)	3.3735 (3.1378-3.5925)
At least 3 of CHASM (breast), CHASM (melanoma), FATHMM (missense), Mutation Assessor, PolyPhen-2, PROVEAN	84.57% (81.82%-87.37%)	94.21% (91.94%-96.77%)	67.875% (61.765%-74.286%)	83.53% (80.28%-87.05%)	87.16% (82.45%-92.45%)	3.3278 (3.2054-3.4460)	84.41% (78.79%-89.9%)	94.01% (89.06%-98.44%)	67.893% (55.263%-79.49%)	83.41% (76.47%-90%)	86.87% (75.86%-96.3%)	3.3218 (3.0834-3.5654)
At least 3 of CHASM (breast), CHASM (melanoma), FATHMM (missense), Mutation Assessor, PolyPhen-2, SIFT	82.5% (79.29%-85.86%)	95.23% (93.27%-97.58%)	60.485% (54.049%-66.667%)	80.65% (76.92%-84.4%)	88.02% (83.33%-93.75%)	3.2438 (3.1196-3.3669)	82.47% (75.76%-88.89%)	95.16% (90.62%-98.55%)	60.65% (48.276%-73.33%)	80.59% (73.24%-87.84%)	88% (76.92%-96.78%)	3.2439 (3.0089-3.4894)
At least 3 of CHASM (breast), CHASM (melanoma), FATHMM (missense), Mutation Assessor, PolyPhen-2, VEST	81.17% (78.28%-84.34%)	94.73% (92.62%-96.9%)	57.706% (51.389%-64.288%)	79.48% (75.86%-83.33%)	86.38% (80.95%-91.89%)	3.1829 (3.0610-3.3137)	81.1% (74.75%-86.87%)	94.56% (89.85%-98.48%)	57.969% (44.996%-70.27%)	79.43% (72%-86.49%)	86.17% (74.98%-96%)	3.1813 (2.9110-3.4269)
At least 3 of CHASM (breast), CHASM (melanoma), FATHMM (missense), Mutation Assessor, PROVEAN, SIFT	83.2% (80.3%-85.86%)	93.14% (90.55%-95.8%)	65.988% (59.459%-72.226%)	82.57% (79.29%-85.91%)	84.78% (79.63%-90.16%)	3.2647 (3.1348-3.3901)	83.1% (77.78%-88.89%)	92.95% (87.71%-98.36%)	66.143% (52.778%-78.95%)	82.5% (75.71%-89.23%)	84.57% (73.33%-95.45%)	3.2616 (3.0019-3.5159)
At least 3 of CHASM (breast), CHASM (melanoma), FATHMM (missense), Mutation Assessor, PROVEAN, VEST	82.86% (79.8%-85.86%)	93.68% (91.27%-96.06%)	64.16% (57.965%-70.27%)	81.88% (78.47%-85.21%)	85.45% (80.39%-90.74%)	3.2516 (3.1239-3.3730)	82.76% (76.77%-88.89%)	93.49% (88.52%-98.39%)	64.304% (51.608%-76.92%)	81.81% (75%-88.89%)	85.23% (73.91%-95.83%)	3.2483 (3.0010-3.4968)
At least 3 of CHASM (breast), CHASM (melanoma), FATHMM (missense), Mutation Assessor, SIFT, VEST	80.12% (76.77%-83.33%)	94.73% (92.62%-96.9%)	54.841% (48.61%-61.541%)	78.39% (74.68%-82.12%)	85.77% (80.49%-91.67%)	3.1373 (3.0115-3.2663)	80.17% (73.74%-86.87%)	94.56% (89.85%-98.48%)	55.418% (41.662%-68.19%)	78.45% (70.83%-85.71%)	85.62% (73.68%-95.66%)	3.1406 (2.8661-3.3948)
At least 3 of CHASM (breast), CHASM (melanoma), FATHMM (missense), MutationTaster, PolyPhen-2, PROVEAN	86.6% (83.84%-89.39%)	94.75% (92.68%-96.92%)	72.48% (66.667%-78.264%)	85.62% (82.48%-89.21%)	88.88% (84.48%-93.65%)	3.4174 (3.3047-3.5378)	86.41% (80.81%-91.92%)	94.53% (89.65%-98.46%)	72.416% (60%-83.8%)	85.48% (78.33%-91.89%)	88.55% (78.57%-96.78%)	3.4098 (3.1661-3.6281)
At least 3 of CHASM (breast), CHASM (melanoma), FATHMM (missense), MutationTaster, PolyPhen-2, SIFT	85.23% (82.32%-87.88%)	96.86% (95.16%-98.49%)	65.122% (58.904%-71.233%)	82.77% (79.45%-86.23%)	92.3% (88.37%-96.36%)	3.3705 (3.2565-3.4819)	85.09% (79.8%-90.91%)	96.71% (92.85%-100%)	65.108% (52.778%-77.51%)	82.64% (75.68%-89.19%)	92.06% (82.61%-100%)	3.3652 (3.1378-3.5797)
At least 3 of CHASM (breast), CHASM (melanoma), FATHMM (missense), MutationTaster, PolyPhen-2, VEST	85.61% (82.83%-88.38%)	98.47% (97.52%-100%)	63.38% (57.353%-69.737%)	82.3% (79.02%-85.91%)	96% (93.18%-100%)	3.4015 (3.3057-3.5059)	85.34% (79.8%-90.91%)	98.27% (95.38%-100%)	63.118% (50%-75%)	82.06% (74.68%-88.61%)	95.51% (88.46%-100%)	3.3895 (3.1790-3.5792)
At least 3 of CHASM (breast), CHASM (melanoma), FATHMM (missense), MutationTaster, PROVEAN, SIFT	87.24% (84.84%-89.9%)	94.21% (91.94%-96.77%)	75.17% (69.736%-80.956%)	86.78% (83.8%-89.86%)	88.26% (84.12%-93.22%)	3.4442 (3.3319-3.5566)	87.13% (81.82%-91.94%)	94.01% (89.06%-98.44%)	75.282% (63.631%-86.05%)	86.73% (80.3%-92.96%)	88% (77.78%-96.67%)	3.4403 (3.2064-3.6626)
At least 3 of CHASM (breast), CHASM (melanoma), FATHMM (missense), MutationTaster, PROVEAN, VEST	87.28% (84.85%-89.9%)	95.82% (93.84%-97.69%)	72.509% (66.667%-78.79%)	85.77% (82.61%-89.21%)	90.94% (86.89%-95.08%)	3.4504 (3.3415-3.5637)	87.05% (81.82%-91.92%)	95.59% (91.38%-100%)	72.34% (60%-84.21%)	85.6% (78.57%-92.06%)	90.55% (81.48%-100%)	3.4408 (3.2089-3.6654)
At least 3 of CHASM (breast), CHASM (melanoma), FATHMM (missense), MutationTaster, SIFT, VEST	84.88% (81.82%-87.88%)	97.4% (95.9%-99.2%)	63.222% (56.944%-69.565%)	82.08% (78.81%-85.62%)	93.37% (89.58%-97.92%)	3.3607 (3.2457-3.4708)	84.78% (78.79%-90.91%)	97.21% (93.65%-100%)	63.388% (50%-75.87%)	82.02% (75%-88.74%)	93.03% (84%-100%)	3.3565 (3.1403-3.5802)
At least 3 of CHASM (breast), CHASM (melanoma), FATHMM (missense), PolyPhen-2, PROVEAN, SIFT	82.86% (79.8%-85.86%)	94.21% (91.94%-96.77%)	63.224% (56.944%-69.565%)	81.58% (78.08%-85.24%)	86.35% (81.35%-92%)	3.2537 (3.1299-3.3799)	82.76% (76.77%-88.89%)	94.01% (89.06%-98.44%)	63.413% (50%-75.86%)	81.53% (74.24%-88.73%)	86.07% (74.98%-96.15%)	3.2502 (2.9956-3.4923)
At least 3 of CHASM (breast), CHASM (melanoma), FATHMM (missense), PolyPhen-2, PROVEAN, VEST	81.88% (78.79%-84.85%)	94.21% (91.94%-96.77%)	60.538% (54.283%-66.678%)	80.5% (77.03%-84.14%)	85.82% (80.77%-91.67%)	3.2108 (3.0842-3.3396)	81.7% (75.76%-87.88%)	94.01% (89.06%-98.44%)	60.515% (48.273%-72.98%)	80.35% (72.97%-87.5%)	85.49% (73.91%-96%)	3.2037 (2.9437-3.4516)
At least 3 of CHASM (breast), CHASM (melanoma), FATHMM (missense), PolyPhen-2, SIFT, VEST	79.12% (75.76%-82.32%)	95.79% (93.85%-97.67%)	50.304% (43.66%-56.757%)	76.92% (73.25%-80.72%)	87.35% (81.82%-93.02%)	3.1036 (2.9743-3.2300)	79.12% (72.73%-85.86%)	95.65% (91.52%-100%)	50.703% (38.087%-63.42%)	76.91% (69.23%-84.21%)	87.19% (75%-100%)	3.1046 (2.8457-3.3614)
At least 3 of CHASM (breast), CHASM (melanoma), FATHMM (missense), PROVEAN, SIFT, VEST	80.14% (77.27%-83.33%)	93.68% (91.27%-96.06%)	56.735% (50.685%-62.861%)	78.92% (75.5%-82.58%)	83.85% (78.43%-90%)	3.1318 (3.0088-3.2701)	80.12% (73.74%-85.86%)	93.49% (88.52%-98.39%)	57.112% (44.118%-69.72%)	78.92% (71.43%-85.9%)	83.67% (70.83%-95.45%)	3.1319 (2.8495-3.3773)
At least 3 of CHASM (breast), CHASM (melanoma), Mutation Assessor, MutationTaster, PolyPhen-2, PROVEAN	81.12% (78.28%-84.34%)	86.13% (82.79%-89.6%)	72.458% (66.667%-78.378%)	84.39% (80.83%-88.06%)	75.14% (69.56%-80.95%)	3.1811 (3.0520-3.3198)	81.19% (74.75%-86.87%)	86.24% (79.36%-92.96%)	72.535% (60.526%-83.87%)	84.33% (76.92%-91.43%)	75.46% (63.41%-86.84%)	3.1857 (2.9013-3.4367)
At least 3 of CHASM (breast), CHASM (melanoma), Mutation Assessor, MutationTaster, PolyPhen-2, SIFT	81.46% (78.78%-84.85%)	89.33% (86.32%-92.5%)	67.843% (61.903%-74.195%)	82.77% (79.23%-86.47%)	78.63% (73.01%-84.38%)	3.1857 (3.0600-3.3254)	81.53% (74.75%-87.88%)	89.41% (83.01%-95.31%)	67.988% (55.164%-80.8%)	82.74% (75.71%-89.71%)	78.94% (66.67%-90%)	3.1907 (2.9027-3.4332)
At least 3 of CHASM (breast), CHASM (melanoma), Mutation Assessor, MutationTaster, PolyPhen-2, VEST	78.44% (75.25%-81.82%)	87.18% (83.87%-90.48%)	63.342% (56.578%-70%)	80.43% (76.64%-84.33%)	74.07% (67.8%-80.4%)	3.0503 (2.9099-3.1975)	78.47% (71.72%-84.85%)	87.33% (80.36%-93.75%)	63.247% (50%-75.61%)	80.3% (72.31%-87.5%)	74.43% (61.29%-86.21%)	3.0531 (2.7636-3.3237)
At least 3 of CHASM (breast), CHASM (melanoma), Mutation Assessor, MutationTaster, PROVEAN, SIFT	83.48% (80.3%-86.36%)	88.78% (85.61%-92%)	74.291% (68.488%-79.73%)	85.65% (82.31%-89.06%)	79.31% (73.85%-84.85%)	3.2804 (3.1542-3.4028)	83.55% (77.78%-88.89%)	88.9% (82.14%-94.92%)	74.354% (62.783%-86.49%)	85.6% (78.69%-92.31%)	79.64% (67.85%-90.32%)	3.2850 (3.0342-3.5408)
At least 3 of CHASM (breast), CHASM (melanoma), Mutation Assessor, MutationTaster, PROVEAN, VEST	80.46% (77.78%-83.84%)	85.62% (82.26%-89.06%)	71.533% (65.852%-77.465%)	83.87% (80.47%-87.4%)	74.21% (68.66%-80.26%)	3.1523 (3.0228-3.2901)	80.5% (73.74%-85.86%)	85.66% (78.33%-92.31%)	71.635% (59.986%-83.33%)	83.81% (76.56%-90.62%)	74.45% (62.5%-85.71%)	3.1555 (2.8724-3.4051)
At least 3 of CHASM (breast), CHASM (melanoma), Mutation Assessor, MutationTaster, SIFT, VEST	81.44% (78.79%-84.34%)	90.38% (87.6%-93.55%)	65.992% (60.274%-72.464%)	82.13% (78.52%-85.82%)	79.88% (74.14%-85.94%)	3.1838 (3.0565-3.3187)	81.55% (75.76%-87.88%)	90.5% (84.38%-96.43%)	66.186% (53.12%-77.42%)	82.12% (74.65%-88.89%)	80.28% (68%-91.18%)	3.1908 (2.9028-3.4401)
At least 3 of CHASM (breast), CHASM (melanoma), Mutation Assessor, PolyPhen-2, PROVEAN, SIFT	78.09% (74.75%-81.31%)	86.15% (82.81%-89.57%)	64.154% (57.971%-70.671%)	80.6% (76.81%-84.5%)	72.83% (67.15%-78.79%)	3.0373 (2.8993-3.1720)	78.16% (71.72%-84.85%)	86.19% (78.95%-92.54%)	64.344% (51.429%-76.93%)	80.58% (72.86%-88.06%)	73.12% (60.71%-84.85%)	3.0424 (2.7614-3.3184)
At least 3 of CHASM (breast), CHASM (melanoma), Mutation Assessor, PolyPhen-2, PROVEAN, VEST	73.72% (70.69%-77.27%)	81.38% (77.69%-85.27%)	60.47% (53.846%-67.124%)	78.07% (73.91%-82.09%)	65.26% (59.15%-71.83%)	2.8518 (2.7104-2.9961)	73.77% (66.67%-79.82%)	81.36% (73.33%-88.71%)	60.736% (47.059%-73.17%)	78.04% (69.83%-86.16%)	65.51% (52%-78.12%)	2.8564 (2.5662-3.1339)
At least 3 of CHASM (breast), CHASM (melanoma), Mutation Assessor, PolyPhen-2, SIFT, VEST	75.37% (71.72%-78.79%)	87.2% (83.9%-90.37%)	54.904% (48.485%-61.196%)	76.97% (73.05%-80.85%)	71.28% (64.71%-77.78%)	2.9036 (2.7578-3.0503)	75.53% (68.69%-82.83%)	87.28% (80.6%-93.65%)	55.349% (42.105%-69.24%)	77.02% (69.12%-84.85%)	71.75% (59.09%-84.62%)	2.9140 (2.6198-3.2091)
At least 3 of CHASM (breast), CHASM (melanoma), Mutation Assessor, PROVEAN, SIFT, VEST	76.45% (73.23%-79.8%)	84.07% (80.51%-87.9%)	63.257% (57.232%-69.863%)	79.82% (75.94%-83.59%)	69.67% (63.63%-75.81%)	2.9683 (2.8257-3.1043)	76.4% (69.7%-82.83%)	83.96% (76.18%-90.62%)	63.381% (51.134%-75.87%)	79.73% (71.88%-87.5%)	69.74% (56.67%-81.58%)	2.9682 (2.6944-3.2545)
At least 3 of CHASM (breast), CHASM (melanoma), MutationTaster, PolyPhen-2, PROVEAN, SIFT	81.46% (78.78%-84.34%)	88.8% (85.83%-92%)	68.755% (62.667%-75.009%)	83.09% (79.7%-86.72%)	78.03% (72.41%-84.06%)	3.1868 (3.0626-3.3191)	81.53% (75.76%-86.89%)	88.88% (82.35%-95%)	68.895% (56.25%-80.65%)	83.07% (75.75%-90%)	78.33% (66.67%-88.9%)	3.1917 (2.9344-3.4329)
At least 3 of CHASM (breast), CHASM (melanoma), MutationTaster, PolyPhen-2, PROVEAN, VEST	77.79% (74.75%-81.31%)	86.15% (82.93%-89.6%)	63.343% (56.94%-69.698%)	80.25% (76.51%-84.09%)	72.56% (66.67%-78.79%)	3.0230 (2.8874-3.1722)	77.75% (70.71%-83.84%)	86.2% (78.79%-92.54%)	63.227% (50%-75.76%)	80.09% (72.46%-87.5%)	72.75% (59.99%-84.38%)	3.0228 (2.7104-3.2891)
At least 3 of CHASM (breast), CHASM (melanoma), MutationTaster, PolyPhen-2, SIFT, VEST	78.06% (74.75%-81.31%)	90.33% (87.6%-93.39%)	56.821% (50%-63.517%)	78.34% (74.5%-82.19%)	77.27% (71.15%-83.93%)	3.0277 (2.8925-3.1640)	78.23% (71.72%-84.85%)	90.6% (84.62%-96.43%)	56.99% (44.118%-70.97%)	78.33% (70.42%-86.3%)	77.95% (64.51%-90.63%)	3.0387 (2.7633-3.3094)
At least 3 of CHASM (breast), CHASM (melanoma), MutationTaster, PROVEAN, SIFT, VEST	80.12% (77.27%-82.83%)	88.28% (85.16%-91.41%)	66.016% (60%-72.603%)	81.79% (78.29%-85.4%)	76.51% (70.77%-82.54%)	3.1259 (3.0003-3.2556)	80.16% (74.75%-85.86%)	88.33% (81.67%-94.92%)	66.116% (53.475%-78.38%)	81.73% (74.28%-88.71%)	76.77% (64.52%-89.29%)	3.1294 (2.8698-3.3827)
At least 3 of CHASM (breast), CHASM (melanoma), PolyPhen-2, PROVEAN, SIFT, VEST	74.39% (71.21%-77.78%)	85.63% (82.31%-89.06%)	54.946% (48.557%-61.644%)	76.67% (72.46%-80.58%)	68.87% (62.71%-75%)	2.8611 (2.7201-3.0020)	74.45% (67.68%-80.81%)	85.63% (78.57%-92.19%)	55.234% (41.935%-68.19%)	76.65% (68.83%-84.62%)	69.15% (56%-81.82%)	2.8666 (2.5711-3.1505)
At least 3 of CHASM (breast), FATHMM (cancer), FATHMM (missense), Mutation Assessor, MutationTaster, PolyPhen-2	87.21% (84.85%-89.9%)	97.9% (96.69%-99.22%)	68.717% (63.01%-75%)	84.4% (81.08%-87.92%)	95% (92%-98.18%)	3.4602 (3.3622-3.5645)	87.19% (81.82%-91.92%)	97.8% (94.92%-100%)	68.97% (56.66%-80.65%)	84.4% (77.63%-90.79%)	94.83% (87.5%-100%)	3.4600 (3.2415-3.6595)
At least 3 of CHASM (breast), FATHMM (cancer), FATHMM (missense), Mutation Assessor, MutationTaster, PROVEAN	86.87% (83.84%-89.39%)	94.74% (92.56%-96.88%)	73.262% (67.532%-78.953%)	85.97% (82.71%-89.36%)	88.96% (84.48%-93.33%)	3.4293 (3.3093-3.5460)	86.87% (81.82%-92.93%)	94.55% (90%-98.51%)	73.653% (62.162%-85.71%)	86.03% (79.36%-92.65%)	88.76% (80%-96.88%)	3.4300 (3.1954-3.6710)
At least 3 of CHASM (breast), FATHMM (cancer), FATHMM (missense), Mutation Assessor, MutationTaster, SIFT	86.17% (83.33%-88.89%)	97.39% (95.9%-99.2%)	66.753% (60.811%-72.86%)	83.51% (80.27%-86.93%)	93.68% (90.2%-98%)	3.4133 (3.3080-3.5269)	86.25% (80.81%-91.92%)	97.23% (93.65%-100%)	67.399% (54.831%-79.41%)	83.65% (76.81%-90.28%)	93.44% (85%-100%)	3.4172 (3.1815-3.6367)
At least 3 of CHASM (breast), FATHMM (cancer), FATHMM (missense), Mutation Assessor, MutationTaster, VEST	86.19% (83.84%-88.89%)	98.47% (97.52%-100%)	64.963% (58.902%-71.433%)	82.94% (79.72%-86.53%)	96.09% (93.33%-100%)	3.4246 (3.3216-3.5269)	86.2% (80.81%-90.91%)	98.27% (95.38%-100%)	65.459% (52.632%-77.42%)	83.01% (75.67%-89.47%)	95.66% (88.89%-100%)	3.4239 (3.2255-3.6232)
At least 3 of CHASM (breast), FATHMM (cancer), FATHMM (missense), Mutation Assessor, PolyPhen-2, PROVEAN	84.85% (81.82%-87.88%)	93.67% (91.34%-96.09%)	69.585% (63.38%-75.714%)	84.19% (80.85%-87.86%)	86.41% (81.48%-91.53%)	3.3386 (3.2112-3.4584)	84.85% (78.79%-90.91%)	93.5% (88.33%-98.36%)	69.985% (57.576%-82.25%)	84.25% (76.92%-91.18%)	86.28% (75.86%-96.16%)	3.3401 (3.1001-3.5877)
At least 3 of CHASM (breast), FATHMM (cancer), FATHMM (missense), Mutation Assessor, PolyPhen-2, SIFT	82.49% (79.29%-85.35%)	96.86% (95.16%-98.49%)	57.65% (51.47%-63.889%)	79.82% (76.19%-83.44%)	91.39% (87.17%-96%)	3.2571 (3.1460-3.3778)	82.49% (76.77%-88.89%)	96.71% (92.85%-100%)	58.079% (45.447%-70.59%)	79.83% (72.6%-87.18%)	91.17% (80%-100%)	3.2579 (3.0162-3.4793)
At least 3 of CHASM (breast), FATHMM (cancer), FATHMM (missense), Mutation Assessor, PolyPhen-2, VEST	81.49% (78.78%-84.34%)	97.4% (95.93%-99.2%)	53.979% (47.368%-60.274%)	78.54% (75%-82.17%)	92.33% (88.1%-97.56%)	3.2225 (3.1103-3.3394)	81.46% (75.76%-86.89%)	97.21% (93.65%-100%)	54.418% (41.667%-68.42%)	78.54% (71.42%-85.71%)	91.92% (81.82%-100%)	3.2208 (2.9834-3.4515)
At least 3 of CHASM (breast), FATHMM (cancer), FATHMM (missense), Mutation Assessor, PROVEAN, SIFT	83.48% (80.81%-86.36%)	93.13% (90.83%-95.8%)	66.774% (60.811%-73.239%)	82.9% (79.58%-86.57%)	84.91% (79.63%-90%)	3.2771 (3.1577-3.3976)	83.55% (77.78%-88.89%)	92.98% (87.69%-97.14%)	67.332% (54.286%-79.31%)	83.01% (75.67%-89.4%)	84.83% (74.19%-94.12%)	3.2815 (3.0403-3.5207)
At least 3 of CHASM (breast), FATHMM (cancer), FATHMM (missense), Mutation Assessor, PROVEAN, VEST	82.8% (79.8%-85.86%)	94.2% (92%-96.75%)	63.079% (56.756%-69.565%)	81.52% (78.14%-85.31%)	86.29% (81.13%-91.84%)	3.2509 (3.1260-3.3734)	82.88% (76.77%-88.89%)	94.03% (89.23%-98.41%)	63.715% (51.25%-75.87%)	81.64% (74.19%-88.57%)	86.19% (75.86%-96.3%)	3.2558 (3.0119-3.5035)
At least 3 of CHASM (breast), FATHMM (cancer), FATHMM (missense), Mutation Assessor, SIFT, VEST	80.81% (77.78%-83.84%)	95.81% (93.75%-97.67%)	54.87% (48.57%-61.111%)	78.59% (75%-82.17%)	88.35% (83.33%-93.62%)	3.1762 (3.0524-3.2996)	80.8% (74.75%-86.87%)	95.6% (91.52%-100%)	55.386% (42.857%-67.76%)	78.61% (70.83%-85.53%)	88.03% (76.47%-100%)	3.1762 (2.9248-3.4269)
At least 3 of CHASM (breast), FATHMM (cancer), FATHMM (missense), MutationTaster, PolyPhen-2, PROVEAN	86.54% (83.84%-89.39%)	95.26% (93.33%-97.58%)	71.451% (65.276%-77.465%)	85.23% (82.14%-88.73%)	89.72% (85.25%-94.64%)	3.4166 (3.3067-3.5388)	86.52% (80.81%-91.92%)	95.1% (90.62%-98.53%)	71.737% (60%-83.33%)	85.25% (78.08%-91.43%)	89.54% (80%-97.06%)	3.4163 (3.1871-3.6335)
At least 3 of CHASM (breast), FATHMM (cancer), FATHMM (missense), MutationTaster, PolyPhen-2, SIFT	84.5% (81.82%-87.37%)	97.9% (96.69%-99.22%)	61.306% (55.072%-67.606%)	81.4% (78%-84.83%)	94.42% (91.11%-98%)	3.3503 (3.2402-3.4623)	84.54% (78.79%-89.9%)	97.8% (94.92%-100%)	61.767% (50%-74.29%)	81.45% (74.36%-88.17%)	94.27% (86.19%-100%)	3.3529 (3.1316-3.5631)
At least 3 of CHASM (breast), FATHMM (cancer), FATHMM (missense), MutationTaster, PolyPhen-2, VEST	84.57% (81.82%-87.37%)	98.47% (97.52%-100%)	60.526% (54.167%-67.106%)	81.18% (77.71%-84.93%)	95.82% (92.68%-100%)	3.3599 (3.2533-3.4711)	84.4% (78.79%-89.9%)	98.27% (95.38%-100%)	60.589% (47.5%-72.97%)	81.06% (73.61%-87.84%)	95.32% (88%-100%)	3.3524 (3.1316-3.5655)
At least 3 of CHASM (breast), FATHMM (cancer), FATHMM (missense), MutationTaster, PROVEAN, SIFT	85.18% (82.32%-87.88%)	95.28% (93.22%-97.58%)	67.693% (61.644%-74.286%)	83.61% (80.29%-87.24%)	89.25% (84.62%-94.44%)	3.3584 (3.2369-3.4813)	85.2% (79.8%-90.91%)	95.06% (90.62%-98.57%)	68.243% (55%-80%)	83.72% (76.39%-90.28%)	88.99% (78.78%-96.88%)	3.3601 (3.1239-3.5925)
At least 3 of CHASM (breast), FATHMM (cancer), FATHMM (missense), MutationTaster, PROVEAN, VEST	86.21% (83.33%-88.89%)	96.86% (95.2%-98.48%)	67.769% (61.333%-73.913%)	83.87% (80.58%-87.5%)	92.59% (88.46%-96.43%)	3.4109 (3.2978-3.5177)	86.17% (80.81%-91.92%)	96.69% (92.86%-100%)	68.073% (55.556%-80.01%)	83.88% (76.62%-90.41%)	92.32% (84%-100%)	3.4097 (3.1936-3.6320)
At least 3 of CHASM (breast), FATHMM (cancer), FATHMM (missense), MutationTaster, SIFT, VEST	85.2% (82.82%-88.38%)	98.99% (98.33%-100%)	61.35% (55.072%-68.116%)	81.58% (78.38%-85.33%)	97.24% (95.12%-100%)	3.3916 (3.2894-3.4944)	85.16% (78.79%-89.92%)	98.82% (96.67%-100%)	61.68% (48.714%-75%)	81.57% (74.36%-88%)	96.84% (90.48%-100%)	3.3892 (3.1913-3.5926)
At least 3 of CHASM (breast), FATHMM (cancer), FATHMM (missense), PolyPhen-2, PROVEAN, SIFT	82.16% (79.29%-85.35%)	94.21% (92%-96.77%)	61.311% (55.072%-67.647%)	80.81% (77.24%-84.57%)	85.98% (80.49%-91.5%)	3.2231 (3.0985-3.3563)	82.14% (75.76%-87.88%)	94.01% (89.06%-98.44%)	61.744% (48.571%-74.36%)	80.84% (73.42%-87.84%)	85.75% (74.07%-96.15%)	3.2234 (2.9540-3.4770)
At least 3 of CHASM (breast), FATHMM (cancer), FATHMM (missense), PolyPhen-2, PROVEAN, VEST	81.49% (78.78%-84.34%)	95.26% (93.33%-97.58%)	57.671% (50.725%-63.894%)	79.56% (75.97%-83.44%)	87.57% (82.22%-93.48%)	3.2006 (3.0707-3.3256)	81.46% (75.76%-86.89%)	95.1% (90.62%-98.53%)	58.011% (44.996%-71.88%)	79.55% (71.83%-86.67%)	87.37% (76.19%-96.43%)	3.2004 (2.9465-3.4449)
At least 3 of CHASM (breast), FATHMM (cancer), FATHMM (missense), PolyPhen-2, SIFT, VEST	79.78% (76.77%-82.83%)	97.91% (96.69%-99.23%)	48.434% (42.103%-54.796%)	76.65% (72.9%-80.38%)	93.07% (88.89%-97.56%)	3.1607 (3.0442-3.2754)	79.82% (73.74%-85.86%)	97.79% (94.83%-100%)	48.969% (36.111%-62.5%)	76.68% (69.33%-84.42%)	92.82% (82.35%-100%)	3.1626 (2.9230-3.3998)
At least 3 of CHASM (breast), FATHMM (cancer), FATHMM (missense), PROVEAN, SIFT, VEST	81.82% (78.79%-84.85%)	94.74% (92.56%-96.95%)	59.46% (52.778%-66.197%)	80.16% (76.71%-83.89%)	86.75% (81.48%-92.16%)	3.2111 (3.0812-3.3424)	81.82% (75.76%-87.88%)	94.54% (89.55%-98.51%)	59.934% (46.87%-72.97%)	80.21% (72.46%-87.15%)	86.5% (75.74%-96.3%)	3.2119 (2.9436-3.4644)
At least 3 of CHASM (breast), FATHMM (cancer), Mutation Assessor, MutationTaster, PolyPhen-2, PROVEAN	81.75% (78.79%-84.85%)	87.19% (83.9%-90.62%)	72.343% (66.667%-78.383%)	84.5% (81.06%-88.28%)	76.56% (71.18%-82.2%)	3.2059 (3.0789-3.3440)	81.95% (75.76%-87.88%)	87.31% (80%-93.85%)	72.76% (60.526%-84.38%)	84.61% (77.27%-91.67%)	76.98% (65.52%-88.24%)	3.2165 (2.9468-3.4717)
At least 3 of CHASM (breast), FATHMM (cancer), Mutation Assessor, MutationTaster, PolyPhen-2, SIFT	82.11% (79.29%-85.35%)	90.92% (88.1%-94.03%)	66.886% (61.035%-72.973%)	82.6% (78.94%-86.33%)	81% (75.47%-87.1%)	3.2141 (3.0883-3.3495)	82.24% (75.76%-87.88%)	91.02% (84.75%-96.77%)	67.158% (54.286%-79.41%)	82.62% (75.38%-89.86%)	81.37% (69.23%-92.59%)	3.2217 (2.9504-3.4699)
At least 3 of CHASM (breast), FATHMM (cancer), Mutation Assessor, MutationTaster, PolyPhen-2, VEST	80.13% (77.27%-83.33%)	88.79% (85.71%-92%)	65.157% (59.155%-71.433%)	81.5% (77.86%-85.32%)	77.08% (71.43%-83.08%)	3.1252 (2.9945-3.2636)	80.14% (73.74%-85.86%)	88.89% (82.54%-95.16%)	65.116% (52.632%-76.93%)	81.38% (73.84%-88.71%)	77.37% (64.7%-88.47%)	3.1276 (2.8486-3.3906)
At least 3 of CHASM (breast), FATHMM (cancer), Mutation Assessor, MutationTaster, PROVEAN, SIFT	82.77% (79.8%-85.86%)	89.87% (86.99%-92.97%)	70.505% (64.615%-76.814%)	84.05% (80.45%-87.77%)	80.1% (74.99%-85.94%)	3.2452 (3.1087-3.3790)	82.94% (76.77%-88.89%)	89.93% (83.58%-95.38%)	70.932% (58.328%-82.86%)	84.14% (76.92%-91.18%)	80.44% (68.75%-90.62%)	3.2544 (2.9805-3.5092)
At least 3 of CHASM (breast), FATHMM (cancer), Mutation Assessor, MutationTaster, PROVEAN, VEST	82.12% (79.29%-85.35%)	87.75% (84.55%-90.99%)	72.382% (66.667%-78.378%)	84.6% (81.06%-88.28%)	77.37% (71.88%-82.67%)	3.2210 (3.0919-3.3557)	82.22% (75.76%-87.88%)	87.78% (81.03%-93.94%)	72.692% (60.604%-83.87%)	84.64% (77.05%-91.55%)	77.62% (66.64%-88.64%)	3.2273 (2.9538-3.4769)
At least 3 of CHASM (breast), FATHMM (cancer), Mutation Assessor, MutationTaster, SIFT, VEST	81.08% (78.28%-83.84%)	91.43% (88.71%-94.36%)	63.171% (57.138%-69.565%)	81.1% (77.61%-84.83%)	81.01% (75.41%-87.04%)	3.1671 (3.0353-3.2968)	81.28% (75.76%-86.87%)	91.6% (85.71%-96.88%)	63.577% (51.427%-75.68%)	81.18% (73.68%-88.24%)	81.56% (69.57%-92.59%)	3.1791 (2.9042-3.4296)
At least 3 of CHASM (breast), FATHMM (cancer), Mutation Assessor, PolyPhen-2, PROVEAN, SIFT	78.1% (74.75%-81.31%)	86.7% (83.46%-90.08%)	63.227% (57.143%-69.565%)	80.3% (76.3%-84.18%)	73.33% (67.21%-79.37%)	3.0356 (2.8971-3.1748)	78.15% (71.72%-84.85%)	86.69% (79.62%-93.22%)	63.472% (51.189%-75.77%)	80.27% (72.46%-87.85%)	73.57% (61.29%-85.3%)	3.0400 (2.7524-3.3077)
At least 3 of CHASM (breast), FATHMM (cancer), Mutation Assessor, PolyPhen-2, PROVEAN, VEST	75.41% (72.22%-78.79%)	82.45% (78.81%-86.36%)	63.234% (57.333%-69.444%)	79.49% (75.57%-83.46%)	67.57% (61.54%-73.85%)	2.9274 (2.7916-3.0697)	75.45% (68.69%-81.82%)	82.43% (74.6%-89.48%)	63.462% (51.351%-75.76%)	79.46% (71.64%-87.5%)	67.8% (54.84%-80%)	2.9315 (2.6414-3.2005)
At least 3 of CHASM (breast), FATHMM (cancer), Mutation Assessor, PolyPhen-2, SIFT, VEST	74.65% (71.21%-78.28%)	87.71% (84.3%-90.91%)	52.067% (45.708%-58.333%)	75.98% (72.11%-79.87%)	71.03% (64.15%-77.55%)	2.8680 (2.7223-3.0070)	74.93% (67.68%-81.82%)	87.85% (81.25%-94.12%)	52.749% (40%-65.62%)	76.13% (68.06%-83.63%)	71.72% (58.06%-85%)	2.8845 (2.5856-3.1712)
At least 3 of CHASM (breast), FATHMM (cancer), Mutation Assessor, PROVEAN, SIFT, VEST	75.74% (72.22%-78.79%)	84.61% (81.15%-88.15%)	60.391% (53.968%-66.667%)	78.69% (74.81%-82.48%)	69.43% (63.33%-75.86%)	2.9312 (2.7908-3.0691)	75.79% (69.7%-82.83%)	84.47% (77.05%-91.43%)	60.875% (48.381%-73.53%)	78.74% (70.77%-86.57%)	69.57% (56.67%-81.82%)	2.9365 (2.6500-3.2217)
At least 3 of CHASM (breast), FATHMM (cancer), MutationTaster, PolyPhen-2, PROVEAN, SIFT	81.76% (78.79%-84.85%)	89.32% (86.26%-92.5%)	68.701% (63.01%-75%)	83.15% (79.39%-86.92%)	78.82% (73.21%-84.51%)	3.1999 (3.0697-3.3344)	81.93% (75.76%-87.88%)	89.44% (83.05%-95.38%)	69.035% (56.757%-80.66%)	83.2% (75.76%-90.48%)	79.23% (67.74%-90.32%)	3.2091 (2.9377-3.4643)
At least 3 of CHASM (breast), FATHMM (cancer), MutationTaster, PolyPhen-2, PROVEAN, VEST	79.13% (76.26%-82.32%)	86.69% (83.33%-90.08%)	66.052% (60.267%-72.312%)	81.53% (77.61%-85.61%)	74.16% (68.33%-80%)	3.0843 (2.9492-3.2189)	79.12% (72.73%-84.85%)	86.71% (79.69%-93.44%)	66.073% (54.054%-77.78%)	81.43% (73.53%-88.89%)	74.36% (61.76%-85.71%)	3.0858 (2.8032-3.3566)
At least 3 of CHASM (breast), FATHMM (cancer), MutationTaster, PolyPhen-2, SIFT, VEST	77.71% (74.24%-80.81%)	91.95% (89.31%-94.66%)	53.094% (46.476%-59.155%)	77.22% (73.51%-81.08%)	79.23% (72.92%-86%)	3.0149 (2.8801-3.1435)	77.91% (71.72%-84.85%)	92.16% (86.44%-97.06%)	53.425% (41.173%-66.67%)	77.25% (69.44%-84.72%)	79.93% (66.67%-92.32%)	3.0277 (2.7665-3.2880)
At least 3 of CHASM (breast), FATHMM (cancer), MutationTaster, PROVEAN, SIFT, VEST	81.07% (77.78%-83.84%)	89.86% (86.99%-92.8%)	65.874% (60%-72.464%)	81.99% (78.46%-85.82%)	78.99% (73.44%-85%)	3.1672 (3.0352-3.2954)	81.29% (75.76%-87.88%)	89.94% (83.93%-95.61%)	66.43% (53.846%-77.78%)	82.13% (74.63%-89.39%)	79.41% (67.74%-90.92%)	3.1791 (2.9197-3.4385)
At least 3 of CHASM (breast), FATHMM (cancer), PolyPhen-2, PROVEAN, SIFT, VEST	74.01% (70.71%-77.27%)	86.15% (82.93%-89.6%)	53.007% (46.377%-59.155%)	76.02% (72.22%-79.86%)	68.89% (62%-75%)	2.8406 (2.7008-2.9786)	74.21% (67.68%-80.81%)	86.19% (78.95%-92.86%)	53.604% (41.016%-65.71%)	76.11% (68.42%-83.8%)	69.38% (56%-82.76%)	2.8529 (2.5757-3.1287)
At least 3 of CHASM (breast), FATHMM (missense), Mutation Assessor, MutationTaster, PolyPhen-2, PROVEAN	82.11% (79.29%-84.85%)	86.67% (83.33%-90.17%)	74.213% (68.829%-80%)	85.32% (81.89%-88.89%)	76.3% (70.96%-81.95%)	3.2250 (3.0969-3.3565)	82.25% (76.77%-87.88%)	86.76% (79.66%-93.55%)	74.507% (63.158%-85.71%)	85.37% (78.12%-92.19%)	76.65% (64.71%-87.51%)	3.2329 (2.9564-3.4832)
At least 3 of CHASM (breast), FATHMM (missense), Mutation Assessor, MutationTaster, PolyPhen-2, SIFT	81.79% (78.79%-84.85%)	90.41% (87.69%-93.5%)	66.892% (60.606%-73.244%)	82.52% (78.99%-86.03%)	80.14% (74.58%-86.15%)	3.1996 (3.0657-3.3298)	81.87% (75.76%-87.88%)	90.45% (84.38%-96.08%)	67.115% (54.539%-78.79%)	82.52% (75.38%-89.71%)	80.4% (68.96%-91.3%)	3.2049 (2.9432-3.4615)
At least 3 of CHASM (breast), FATHMM (missense), Mutation Assessor, MutationTaster, PolyPhen-2, VEST	78.78% (75.74%-81.83%)	88.8% (85.71%-92%)	61.461% (55.221%-67.901%)	79.93% (76.19%-83.69%)	76.04% (69.81%-82.26%)	3.0623 (2.9244-3.2042)	78.8% (72.7%-84.87%)	88.89% (82.35%-95.08%)	61.479% (48.267%-73.69%)	79.83% (72.22%-87.15%)	76.34% (62.96%-88.47%)	3.0654 (2.7710-3.3369)
At least 3 of CHASM (breast), FATHMM (missense), Mutation Assessor, MutationTaster, PROVEAN, SIFT	83.11% (80.3%-86.36%)	88.79% (85.71%-92%)	73.284% (67.949%-79.167%)	85.18% (81.54%-88.62%)	79.09% (73.77%-84.85%)	3.2635 (3.1265-3.3893)	83.27% (76.77%-88.89%)	88.88% (82.14%-94.92%)	73.62% (62.069%-85.3%)	85.25% (78.46%-92.42%)	79.45% (67.65%-89.74%)	3.2721 (3.0081-3.5439)
At least 3 of CHASM (breast), FATHMM (missense), Mutation Assessor, MutationTaster, PROVEAN, VEST	81.12% (77.78%-84.34%)	86.7% (83.2%-90.23%)	71.456% (65.333%-77.465%)	84% (80.45%-87.6%)	75.66% (70.15%-81.43%)	3.1781 (3.0397-3.3111)	81.2% (74.75%-87.88%)	86.69% (79.31%-93.22%)	71.756% (60%-83.33%)	84.04% (76.92%-91.05%)	75.88% (64.28%-87.5%)	3.1836 (2.9024-3.4540)
At least 3 of CHASM (breast), FATHMM (missense), Mutation Assessor, MutationTaster, SIFT, VEST	79.72% (76.77%-82.83%)	90.92% (88.14%-93.8%)	60.353% (53.846%-67.124%)	79.86% (76.22%-83.67%)	79.36% (73.21%-85.49%)	3.1050 (2.9627-3.2346)	79.95% (73.74%-85.86%)	91.02% (85.18%-96.72%)	60.916% (46.149%-72.73%)	79.99% (72.22%-87.32%)	79.83% (67.85%-92%)	3.1175 (2.8443-3.3970)
At least 3 of CHASM (breast), FATHMM (missense), Mutation Assessor, PolyPhen-2, PROVEAN, SIFT	78.08% (74.75%-81.31%)	86.15% (82.93%-89.6%)	64.119% (57.575%-70.669%)	80.59% (76.69%-84.33%)	72.82% (67.16%-79.03%)	3.0369 (2.8956-3.1823)	78.17% (71.72%-84.85%)	86.18% (78.95%-92.54%)	64.408% (51.429%-77.42%)	80.6% (73.24%-88.14%)	73.1% (60.71%-84.38%)	3.0429 (2.7506-3.3201)
At least 3 of CHASM (breast), FATHMM (missense), Mutation Assessor, PolyPhen-2, PROVEAN, VEST	73.39% (70.2%-76.77%)	81.92% (78.29%-85.83%)	58.62% (52.113%-64.865%)	77.39% (73.48%-81.54%)	65.22% (59.01%-71.67%)	2.8316 (2.6872-2.9815)	73.43% (66.67%-79.8%)	81.88% (73.77%-89.23%)	58.897% (45.714%-71.05%)	77.36% (69.12%-84.86%)	65.45% (51.72%-77.43%)	2.8360 (2.5350-3.1250)
At least 3 of CHASM (breast), FATHMM (missense), Mutation Assessor, PolyPhen-2, SIFT, VEST	73.31% (69.7%-76.77%)	87.2% (83.9%-90.37%)	49.29% (42.62%-55.714%)	74.83% (70.78%-78.91%)	69.02% (61.82%-75.47%)	2.8034 (2.6434-2.9542)	73.58% (66.67%-80.81%)	87.28% (80.6%-93.65%)	50.038% (37.5%-62.86%)	74.98% (66.67%-82.9%)	69.65% (56.24%-83.33%)	2.8195 (2.5188-3.1342)
At least 3 of CHASM (breast), FATHMM (missense), Mutation Assessor, PROVEAN, SIFT, VEST	74.37% (70.71%-77.78%)	83.54% (79.84%-87.4%)	58.511% (52.174%-65.217%)	77.69% (73.68%-81.68%)	67.28% (60.71%-73.77%)	2.8701 (2.7178-3.0240)	74.48% (67.68%-81.82%)	83.43% (75.76%-90.33%)	59.089% (45.455%-71.8%)	77.78% (69.84%-85.71%)	67.5% (54.54%-80%)	2.8780 (2.5715-3.1989)
At least 3 of CHASM (breast), FATHMM (missense), MutationTaster, PolyPhen-2, PROVEAN, SIFT	81.78% (78.79%-84.85%)	89.34% (86.32%-92.43%)	68.712% (62.857%-74.648%)	83.16% (79.7%-86.72%)	78.85% (73.33%-84.38%)	3.2006 (3.0657-3.3257)	81.89% (75.76%-87.88%)	89.4% (83.04%-95.38%)	68.975% (56.755%-80.56%)	83.19% (76.27%-90.32%)	79.15% (67.55%-90.01%)	3.2071 (2.9494-3.4768)
At least 3 of CHASM (breast), FATHMM (missense), MutationTaster, PolyPhen-2, PROVEAN, VEST	79.14% (75.76%-82.32%)	87.23% (84.03%-90.68%)	65.141% (59.155%-71.233%)	81.23% (77.69%-85.16%)	74.68% (68.75%-80.65%)	3.0827 (2.9451-3.2147)	79.1% (72.73%-85.86%)	87.24% (80%-93.75%)	65.11% (52.937%-77.15%)	81.1% (73.33%-88.41%)	74.84% (62.49%-86.96%)	3.0829 (2.7932-3.3656)
At least 3 of CHASM (breast), FATHMM (missense), MutationTaster, PolyPhen-2, SIFT, VEST	78.06% (74.75%-81.31%)	91.95% (89.34%-94.62%)	54.02% (47.297%-60.611%)	77.57% (73.83%-81.46%)	79.52% (73.47%-86.27%)	3.0306 (2.8888-3.1694)	78.23% (71.72%-84.85%)	92.15% (86.44%-96.97%)	54.314% (41.379%-67.5%)	77.59% (69.86%-84.93%)	80.16% (66.67%-91.67%)	3.0422 (2.7607-3.3188)
At least 3 of CHASM (breast), FATHMM (missense), MutationTaster, PROVEAN, SIFT, VEST	79.76% (76.77%-82.83%)	89.35% (86.29%-92.62%)	63.162% (56.944%-69.622%)	80.75% (77.14%-84.33%)	77.44% (71.19%-83.34%)	3.1071 (2.9589-3.2377)	79.87% (73.74%-85.86%)	89.36% (83.05%-95.38%)	63.537% (50%-75.76%)	80.8% (73.53%-88.24%)	77.7% (65.62%-90%)	3.1139 (2.8270-3.3907)
At least 3 of CHASM (breast), FATHMM (missense), PolyPhen-2, PROVEAN, SIFT, VEST	73.03% (69.7%-76.26%)	86.17% (82.93%-89.52%)	50.292% (43.478%-56.522%)	74.98% (71.23%-78.91%)	67.78% (60.78%-74.47%)	2.7922 (2.6380-2.9341)	73.13% (66.67%-79.8%)	86.15% (79.24%-92.43%)	50.737% (37.838%-62.86%)	75.01% (67.12%-82.44%)	68.12% (54.54%-81.48%)	2.8001 (2.5077-3.0858)
At least 3 of CHASM (breast), Mutation Assessor, MutationTaster, PolyPhen-2, PROVEAN, SIFT	77.4% (74.24%-80.3%)	82.97% (79.36%-86.89%)	67.778% (61.538%-74.031%)	81.66% (77.95%-85.61%)	69.71% (64.06%-75.76%)	3.0211 (2.8829-3.1608)	77.52% (71.72%-83.84%)	82.98% (75.7%-90.16%)	68.12% (55.172%-80%)	81.7% (73.77%-89.06%)	70.01% (57.69%-81.48%)	3.0281 (2.7432-3.3023)
At least 3 of CHASM (breast), Mutation Assessor, MutationTaster, PolyPhen-2, PROVEAN, VEST	76.07% (72.73%-79.29%)	82.41% (78.74%-86.4%)	65.11% (59.015%-71.622%)	80.33% (76.23%-84.38%)	68.16% (62.16%-74.33%)	2.9601 (2.8173-3.1026)	76.14% (69.7%-82.83%)	82.51% (74.6%-89.83%)	65.213% (52.632%-77.42%)	80.27% (72.13%-88.06%)	68.48% (55.55%-80.01%)	2.9646 (2.6793-3.2464)
At least 3 of CHASM (breast), Mutation Assessor, MutationTaster, PolyPhen-2, SIFT, VEST	74.02% (70.71%-77.27%)	83.97% (80.48%-87.69%)	56.813% (50%-63.16%)	77.07% (72.93%-81.02%)	67.21% (61.01%-73.77%)	2.8506 (2.7043-3.0004)	74.18% (67.68%-80.81%)	84.17% (76.66%-91.05%)	57.031% (44.436%-70.27%)	77.06% (69.12%-85.14%)	67.74% (54.54%-80%)	2.8601 (2.5663-3.1463)
At least 3 of CHASM (breast), Mutation Assessor, MutationTaster, PROVEAN, SIFT, VEST	76.4% (73.23%-79.8%)	80.84% (77.17%-84.68%)	68.726% (62.821%-75%)	81.71% (77.87%-85.71%)	67.47% (61.76%-73.44%)	2.9874 (2.8439-3.1285)	76.49% (69.7%-82.83%)	80.86% (73.01%-88.14%)	68.975% (56.757%-80%)	81.72% (74.07%-89.06%)	67.74% (54.76%-78.95%)	2.9929 (2.7089-3.2778)
At least 3 of CHASM (breast), Mutation Assessor, PolyPhen-2, PROVEAN, SIFT, VEST	71.66% (68.18%-75.25%)	80.29% (76.38%-84.17%)	56.739% (50%-63.014%)	76.24% (72.09%-80.45%)	62.47% (56.06%-69.23%)	2.7574 (2.6172-2.9042)	71.83% (64.65%-78.79%)	80.36% (72.41%-88.06%)	57.185% (44.118%-70.27%)	76.3% (68.18%-84.62%)	62.9% (50%-75%)	2.7675 (2.4750-3.0529)
At least 3 of CHASM (breast), MutationTaster, PolyPhen-2, PROVEAN, SIFT, VEST	73.69% (70.2%-77.27%)	83.97% (80.51%-87.7%)	55.902% (49.194%-62.342%)	76.7% (72.59%-80.92%)	66.86% (60.65%-73.44%)	2.8343 (2.6896-2.9775)	73.83% (66.67%-80.81%)	84.17% (76.66%-90.91%)	56.08% (42.857%-69.46%)	76.68% (68.12%-84.51%)	67.35% (53.84%-80%)	2.8428 (2.5502-3.1306)
At least 3 of CHASM (lung), CHASM (melanoma), FATHMM (cancer), FATHMM (missense), Mutation Assessor, MutationTaster	88.92% (86.36%-91.41%)	96.34% (94.49%-98.43%)	76.092% (70.833%-81.693%)	87.45% (84.56%-90.84%)	92.33% (88.88%-96.55%)	3.5221 (3.4219-3.6285)	88.82% (83.84%-93.94%)	96.14% (91.8%-100%)	76.256% (64.861%-86.84%)	87.42% (80.64%-93.15%)	92.03% (83.86%-100%)	3.5184 (3.3076-3.7274)
At least 3 of CHASM (lung), CHASM (melanoma), FATHMM (cancer), FATHMM (missense), Mutation Assessor, PolyPhen-2	83.55% (80.81%-86.36%)	95.27% (93.39%-97.56%)	63.263% (56.757%-69.231%)	81.77% (78.17%-85.52%)	88.57% (83.92%-93.88%)	3.2887 (3.1739-3.4110)	83.41% (77.78%-88.89%)	95.08% (90.74%-98.51%)	63.37% (51.351%-76.48%)	81.67% (74.32%-88.74%)	88.26% (77.27%-96.55%)	3.2838 (3.0404-3.5155)
At least 3 of CHASM (lung), CHASM (melanoma), FATHMM (cancer), FATHMM (missense), Mutation Assessor, PROVEAN	84.84% (81.82%-87.88%)	93.11% (90.68%-95.93%)	70.528% (64.381%-76.715%)	84.53% (81.06%-88.06%)	85.56% (80.64%-90.64%)	3.3372 (3.2119-3.4578)	84.87% (78.79%-90.91%)	93.02% (87.5%-98.31%)	70.851% (58.065%-83.79%)	84.56% (77.61%-91.43%)	85.55% (75%-96%)	3.3399 (3.0921-3.5924)
At least 3 of CHASM (lung), CHASM (melanoma), FATHMM (cancer), FATHMM (missense), Mutation Assessor, SIFT	81.82% (78.79%-84.85%)	94.2% (92%-96.72%)	60.411% (53.623%-66.678%)	80.45% (76.97%-84.14%)	85.76% (80.43%-91.3%)	3.2082 (3.0782-3.3386)	81.81% (75.76%-87.88%)	94.04% (89.09%-98.41%)	60.795% (48.256%-74.29%)	80.46% (72.97%-87.67%)	85.61% (74.07%-96%)	3.2091 (2.9451-3.4650)
At least 3 of CHASM (lung), CHASM (melanoma), FATHMM (cancer), FATHMM (missense), Mutation Assessor, VEST	81.47% (78.28%-84.85%)	95.26% (93.33%-97.58%)	57.62% (50.704%-64.107%)	79.54% (75.97%-83.22%)	87.56% (82.61%-93.62%)	3.1998 (3.0691-3.3321)	81.5% (74.75%-87.88%)	95.1% (90.48%-98.53%)	58.134% (44.828%-71.8%)	79.59% (72%-86.75%)	87.37% (75.84%-96.3%)	3.2019 (2.9294-3.4565)
At least 3 of CHASM (lung), CHASM (melanoma), FATHMM (cancer), FATHMM (missense), MutationTaster, PolyPhen-2	86.92% (84.34%-89.9%)	97.4% (95.93%-99.19%)	68.803% (62.857%-75%)	84.37% (81.21%-87.92%)	93.87% (90.38%-98.08%)	3.4444 (3.3408-3.5600)	86.76% (80.81%-91.92%)	97.22% (93.85%-100%)	68.786% (56.755%-80.56%)	84.24% (77.46%-90.67%)	93.51% (85.19%-100%)	3.4376 (3.2054-3.6434)
At least 3 of CHASM (lung), CHASM (melanoma), FATHMM (cancer), FATHMM (missense), MutationTaster, PROVEAN	88.91% (86.36%-91.41%)	94.72% (92.68%-96.9%)	78.857% (73.611%-84.288%)	88.57% (85.71%-91.73%)	89.62% (85.48%-93.85%)	3.5177 (3.4103-3.6240)	88.85% (83.84%-93.94%)	94.6% (89.85%-98.46%)	78.95% (68.421%-89.19%)	88.53% (82.09%-94.37%)	89.52% (80.65%-97.06%)	3.5160 (3.3080-3.7400)
At least 3 of CHASM (lung), CHASM (melanoma), FATHMM (cancer), FATHMM (missense), MutationTaster, SIFT	86.88% (84.34%-89.39%)	96.88% (95.16%-98.5%)	69.574% (63.768%-75.362%)	84.63% (81.48%-87.84%)	92.82% (89.09%-96.61%)	3.4391 (3.3296-3.5569)	86.84% (81.82%-91.92%)	96.64% (92.73%-100%)	69.98% (58.052%-81.41%)	84.68% (78.12%-91.18%)	92.42% (84%-100%)	3.4373 (3.2121-3.6536)
At least 3 of CHASM (lung), CHASM (melanoma), FATHMM (cancer), FATHMM (missense), MutationTaster, VEST	86.57% (83.84%-89.39%)	97.93% (96.72%-99.24%)	66.913% (60.605%-73.239%)	83.66% (80.41%-87.07%)	94.94% (91.67%-98.18%)	3.4344 (3.3326-3.5431)	86.46% (80.81%-91.92%)	97.74% (94.74%-100%)	67.053% (54.539%-79.49%)	83.59% (76.71%-90.28%)	94.54% (87.1%-100%)	3.4292 (3.2091-3.6392)
At least 3 of CHASM (lung), CHASM (melanoma), FATHMM (cancer), FATHMM (missense), PolyPhen-2, PROVEAN	82.84% (79.8%-85.86%)	93.12% (90.75%-95.8%)	65.076% (58.904%-71.429%)	82.18% (78.72%-86.01%)	84.55% (79.25%-89.83%)	3.2492 (3.1270-3.3856)	82.8% (76.77%-88.89%)	93.01% (87.72%-98.28%)	65.233% (52.478%-77.5%)	82.12% (74.65%-89.04%)	84.48% (73.33%-95.45%)	3.2485 (2.9638-3.4926)
At least 3 of CHASM (lung), CHASM (melanoma), FATHMM (cancer), FATHMM (missense), PolyPhen-2, SIFT	81.51% (78.79%-84.85%)	95.82% (94.02%-97.67%)	56.747% (50%-63.514%)	79.3% (75.82%-83.11%)	88.7% (83.72%-93.88%)	3.2057 (3.0802-3.3326)	81.43% (74.75%-86.87%)	95.59% (91.52%-98.59%)	57.102% (44.112%-70.01%)	79.28% (71.83%-86.25%)	88.31% (76.19%-96.77%)	3.2028 (2.9415-3.4393)
At least 3 of CHASM (lung), CHASM (melanoma), FATHMM (cancer), FATHMM (missense), PolyPhen-2, VEST	82.85% (80.3%-85.86%)	97.4% (95.93%-99.19%)	57.691% (51.47%-64.179%)	79.92% (76.47%-83.79%)	92.77% (88.64%-97.67%)	3.2778 (3.1690-3.3933)	82.78% (76.77%-88.89%)	97.22% (93.85%-100%)	57.977% (45.157%-70.6%)	79.89% (72.22%-86.77%)	92.39% (82.75%-100%)	3.2747 (3.0256-3.4886)
At least 3 of CHASM (lung), CHASM (melanoma), FATHMM (cancer), FATHMM (missense), PROVEAN, SIFT	81.11% (77.78%-84.34%)	93.11% (90.68%-95.93%)	60.361% (53.843%-67.124%)	80.25% (76.62%-84%)	83.52% (78%-89.59%)	3.1724 (3.0450-3.3136)	81.21% (74.75%-87.88%)	93.02% (87.5%-98.31%)	60.892% (47.365%-73.7%)	80.33% (72.5%-87.5%)	83.56% (71.43%-95.24%)	3.1781 (2.8992-3.4353)
At least 3 of CHASM (lung), CHASM (melanoma), FATHMM (cancer), FATHMM (missense), PROVEAN, VEST	81.81% (78.79%-84.85%)	94.18% (92%-96.77%)	60.424% (53.731%-67.106%)	80.45% (76.98%-84.25%)	85.72% (80.39%-91.07%)	3.2078 (3.0775-3.3395)	81.83% (75.76%-87.88%)	94.08% (89.06%-98.41%)	60.758% (47.368%-73.53%)	80.46% (72.97%-87.5%)	85.68% (73.91%-96.15%)	3.2097 (2.9504-3.4758)
At least 3 of CHASM (lung), CHASM (melanoma), FATHMM (cancer), FATHMM (missense), SIFT, VEST	81.51% (78.28%-84.85%)	95.81% (93.85%-98.33%)	56.772% (50.684%-63.239%)	79.31% (75.68%-83.11%)	88.68% (83.72%-95%)	3.2057 (3.0826-3.3370)	81.43% (74.75%-87.88%)	95.61% (90.91%-100%)	57.052% (44.73%-69.7%)	79.26% (71.43%-86.25%)	88.35% (76.46%-100%)	3.2027 (2.9288-3.4392)
At least 3 of CHASM (lung), CHASM (melanoma), FATHMM (cancer), Mutation Assessor, MutationTaster, PolyPhen-2	87.24% (84.85%-89.9%)	97.9% (96.72%-99.24%)	68.815% (63.158%-75%)	84.44% (81.16%-88%)	94.99% (92%-98.25%)	3.4615 (3.3614-3.5693)	87.13% (81.82%-91.92%)	97.82% (94.64%-100%)	68.819% (57.133%-80.56%)	84.32% (77.33%-90.91%)	94.85% (86.96%-100%)	3.4580 (3.2404-3.6620)
At least 3 of CHASM (lung), CHASM (melanoma), FATHMM (cancer), Mutation Assessor, MutationTaster, PROVEAN	87.89% (85.35%-90.4%)	94.72% (92.68%-96.9%)	76.09% (70.584%-81.69%)	87.26% (84.21%-90.37%)	89.28% (85%-93.75%)	3.4735 (3.3647-3.5825)	87.85% (82.83%-92.93%)	94.6% (89.85%-98.46%)	76.264% (65.714%-87.5%)	87.24% (80.6%-93.55%)	89.19% (79.41%-96.97%)	3.4729 (3.2557-3.6930)
At least 3 of CHASM (lung), CHASM (melanoma), FATHMM (cancer), Mutation Assessor, MutationTaster, SIFT	86.56% (83.84%-89.39%)	96.31% (94.4%-98.4%)	69.701% (63.768%-75.362%)	84.6% (81.25%-88%)	91.62% (87.93%-96.36%)	3.4223 (3.3126-3.5338)	86.48% (80.81%-91.92%)	96.2% (92.06%-100%)	69.791% (58.333%-81.58%)	84.52% (77.63%-91.3%)	91.49% (81.82%-100%)	3.4201 (3.1922-3.6447)
At least 3 of CHASM (lung), CHASM (melanoma), FATHMM (cancer), Mutation Assessor, MutationTaster, VEST	86.58% (83.84%-89.39%)	96.86% (95.16%-98.45%)	68.82% (62.857%-75%)	84.3% (80.95%-87.86%)	92.69% (88.89%-96.49%)	3.4266 (3.3148-3.5387)	86.46% (80.81%-91.92%)	96.7% (93.22%-100%)	68.817% (57.133%-81.08%)	84.17% (77.03%-90.91%)	92.42% (84%-100%)	3.4211 (3.2020-3.6482)
At least 3 of CHASM (lung), CHASM (melanoma), FATHMM (cancer), Mutation Assessor, PolyPhen-2, PROVEAN	83.51% (80.8%-86.87%)	93.65% (91.34%-96.09%)	65.983% (59.74%-71.832%)	82.64% (79.14%-86.11%)	85.74% (80.7%-91.07%)	3.2801 (3.1588-3.4058)	83.48% (76.77%-88.91%)	93.54% (88.33%-98.36%)	66.199% (54.28%-78.58%)	82.6% (75.38%-89.71%)	85.68% (74.19%-96.15%)	3.2802 (3.0219-3.5232)
At least 3 of CHASM (lung), CHASM (melanoma), FATHMM (cancer), Mutation Assessor, PolyPhen-2, SIFT	82.17% (79.29%-85.35%)	95.78% (93.75%-97.67%)	58.632% (52.172%-65.083%)	80.01% (76.19%-83.79%)	88.93% (84%-94%)	3.2336 (3.1121-3.3614)	82.13% (75.76%-87.88%)	95.67% (91.52%-100%)	58.896% (45.714%-71.88%)	79.97% (72.46%-86.67%)	88.83% (77.77%-100%)	3.2336 (2.9845-3.4728)
At least 3 of CHASM (lung), CHASM (melanoma), FATHMM (cancer), Mutation Assessor, PolyPhen-2, VEST	82.19% (79.29%-85.35%)	96.32% (94.49%-98.39%)	57.745% (51.428%-64.105%)	79.76% (76.1%-83.45%)	90.08% (85.71%-95.56%)	3.2391 (3.1208-3.3663)	82.09% (75.76%-87.88%)	96.18% (92.19%-100%)	57.915% (44.737%-70.27%)	79.68% (72.22%-87.18%)	89.84% (79.17%-100%)	3.2361 (2.9822-3.4567)
At least 3 of CHASM (lung), CHASM (melanoma), FATHMM (cancer), Mutation Assessor, PROVEAN, SIFT	83.19% (80.3%-86.36%)	93.65% (91.27%-96.06%)	65.095% (58.974%-71.429%)	82.27% (78.83%-85.82%)	85.56% (80.39%-90.91%)	3.2657 (3.1442-3.3904)	83.12% (76.77%-88.89%)	93.55% (88.52%-98.39%)	65.206% (52.632%-77.14%)	82.19% (75%-89.04%)	85.51% (74.07%-96.15%)	3.2645 (3.0090-3.5045)
At least 3 of CHASM (lung), CHASM (melanoma), FATHMM (cancer), Mutation Assessor, PROVEAN, VEST	83.86% (80.81%-86.87%)	94.18% (92%-96.75%)	66.03% (59.74%-71.832%)	82.73% (79.31%-86.23%)	86.77% (82%-92.31%)	3.2969 (3.1750-3.4177)	83.8% (77.78%-89.9%)	94.08% (89.09%-98.41%)	66.143% (54.286%-78.95%)	82.66% (75.38%-89.39%)	86.72% (76%-96.3%)	3.2961 (3.0434-3.5371)
At least 3 of CHASM (lung), CHASM (melanoma), FATHMM (cancer), Mutation Assessor, SIFT, VEST	80.82% (77.78%-83.84%)	95.81% (93.96%-97.73%)	54.895% (48.569%-61.538%)	78.6% (75%-82.35%)	88.34% (83.33%-93.75%)	3.1764 (3.0516-3.3025)	80.79% (74.75%-86.87%)	95.61% (91.18%-98.66%)	55.354% (42.416%-68.42%)	78.6% (71.05%-85.51%)	88.04% (76.19%-96.95%)	3.1760 (2.9200-3.4169)
At least 3 of CHASM (lung), CHASM (melanoma), FATHMM (cancer), MutationTaster, PolyPhen-2, PROVEAN	86.54% (83.84%-89.39%)	95.78% (93.85%-97.67%)	70.569% (64.7%-76.926%)	84.91% (81.62%-88.51%)	90.63% (86.44%-94.92%)	3.4188 (3.3143-3.5378)	86.52% (80.81%-91.92%)	95.67% (91.53%-100%)	70.796% (58.327%-82.51%)	84.9% (77.63%-91.78%)	90.52% (81.81%-100%)	3.4189 (3.1755-3.6320)
At least 3 of CHASM (lung), CHASM (melanoma), FATHMM (cancer), MutationTaster, PolyPhen-2, SIFT	85.2% (82.32%-87.88%)	97.9% (96.72%-99.24%)	63.249% (57.138%-69.565%)	82.16% (78.76%-85.71%)	94.57% (91.3%-98.08%)	3.3788 (3.2732-3.4886)	85.15% (79.8%-90.91%)	97.82% (94.64%-100%)	63.427% (51.345%-75.68%)	82.1% (75%-89.04%)	94.43% (86.36%-100%)	3.3777 (3.1570-3.5876)
At least 3 of CHASM (lung), CHASM (melanoma), FATHMM (cancer), MutationTaster, PolyPhen-2, VEST	84.6% (81.82%-87.88%)	97.93% (96.72%-99.22%)	61.545% (55.224%-68.116%)	81.49% (78%-85.23%)	94.52% (91.11%-98%)	3.3549 (3.2491-3.4702)	84.34% (77.78%-89.9%)	97.74% (94.91%-100%)	61.322% (48.482%-74.19%)	81.26% (73.61%-88.16%)	94.06% (86.19%-100%)	3.3438 (3.1199-3.5538)
At least 3 of CHASM (lung), CHASM (melanoma), FATHMM (cancer), MutationTaster, PROVEAN, SIFT	87.23% (84.85%-89.9%)	95.26% (93.33%-97.56%)	73.335% (67.606%-79.167%)	86.07% (83.09%-89.29%)	89.95% (85.71%-94.83%)	3.4461 (3.3407-3.5630)	87.16% (81.82%-91.92%)	95.11% (90.62%-98.53%)	73.488% (62.5%-84.38%)	86.03% (79.45%-92.19%)	89.77% (80%-96.97%)	3.4440 (3.2183-3.6549)
At least 3 of CHASM (lung), CHASM (melanoma), FATHMM (cancer), MutationTaster, PROVEAN, VEST	87.23% (84.34%-89.9%)	96.31% (94.44%-98.37%)	71.522% (65.332%-77.781%)	85.39% (82.14%-88.97%)	91.81% (87.93%-96.43%)	3.4503 (3.3413-3.5670)	87.17% (81.82%-92.93%)	96.21% (92.31%-100%)	71.649% (58.974%-83.79%)	85.34% (78.26%-91.78%)	91.67% (82.76%-100%)	3.4487 (3.2221-3.6736)
At least 3 of CHASM (lung), CHASM (melanoma), FATHMM (cancer), MutationTaster, SIFT, VEST	83.84% (80.81%-86.87%)	97.38% (95.93%-99.19%)	60.436% (54.545%-67.124%)	80.97% (77.62%-84.77%)	93.03% (89.13%-97.83%)	3.3181 (3.2031-3.4363)	83.83% (77.78%-89.9%)	97.26% (93.75%-100%)	60.794% (48.384%-73.53%)	80.97% (73.42%-87.81%)	92.83% (84%-100%)	3.3186 (3.0886-3.5487)
At least 3 of CHASM (lung), CHASM (melanoma), FATHMM (cancer), PolyPhen-2, PROVEAN, SIFT	82.19% (79.29%-85.35%)	94.19% (92.06%-96.77%)	61.437% (55.127%-67.95%)	80.85% (77.24%-84.72%)	85.96% (80.77%-91.84%)	3.2245 (3.1022-3.3619)	82.08% (75.76%-87.88%)	94.05% (89.06%-98.39%)	61.52% (48.387%-74.29%)	80.75% (73.13%-87.7%)	85.78% (73.91%-96%)	3.2209 (2.9494-3.4640)
At least 3 of CHASM (lung), CHASM (melanoma), FATHMM (cancer), PolyPhen-2, PROVEAN, VEST	81.51% (78.79%-84.85%)	95.24% (93.28%-97.58%)	57.758% (51.389%-64.103%)	79.58% (75.97%-83.23%)	87.53% (82.35%-93.48%)	3.2011 (3.0802-3.3289)	81.43% (74.75%-87.88%)	95.15% (90.62%-98.55%)	57.867% (44.737%-70.59%)	79.49% (72%-86.59%)	87.44% (76%-96.3%)	3.1995 (2.9289-3.4359)
At least 3 of CHASM (lung), CHASM (melanoma), FATHMM (cancer), PolyPhen-2, SIFT, VEST	79.09% (75.76%-82.32%)	96.83% (95.2%-98.44%)	48.422% (42.105%-54.667%)	76.45% (72.73%-80.26%)	89.83% (84.62%-95.12%)	3.1153 (2.9926-3.2403)	79.18% (72.73%-85.86%)	96.76% (93.33%-100%)	49.004% (35.897%-61.29%)	76.5% (68.75%-83.95%)	89.81% (78.56%-100%)	3.1208 (2.8626-3.3641)
At least 3 of CHASM (lung), CHASM (melanoma), FATHMM (cancer), PROVEAN, SIFT, VEST	80.81% (77.78%-83.84%)	94.72% (92.62%-96.95%)	56.751% (50.685%-63.293%)	79.11% (75.81%-82.8%)	86.15% (80.85%-92%)	3.1673 (3.0398-3.3079)	80.81% (74.75%-86.87%)	94.59% (89.66%-98.48%)	57.123% (44.118%-69.7%)	79.11% (71.42%-85.92%)	86.05% (74.98%-96%)	3.1687 (2.9018-3.4171)
At least 3 of CHASM (lung), CHASM (melanoma), FATHMM (missense), Mutation Assessor, MutationTaster, PolyPhen-2	85.23% (82.32%-87.88%)	96.31% (94.4%-98.39%)	66.075% (60.256%-72.222%)	83.08% (79.72%-86.49%)	91.21% (87.04%-96.08%)	3.3667 (3.2558-3.4788)	85.09% (79.8%-90.91%)	96.2% (92.19%-100%)	65.994% (52.778%-77.78%)	82.92% (75.71%-89.55%)	91.06% (81.48%-100%)	3.3617 (3.1268-3.5842)
At least 3 of CHASM (lung), CHASM (melanoma), FATHMM (missense), Mutation Assessor, MutationTaster, PROVEAN	88.93% (86.36%-91.41%)	94.74% (92.68%-96.85%)	78.882% (73.846%-84.286%)	88.58% (85.71%-91.73%)	89.66% (85.71%-93.85%)	3.5186 (3.4209-3.6253)	88.81% (83.84%-93.94%)	94.56% (90.16%-98.46%)	78.91% (68.571%-89.47%)	88.5% (82.09%-94.37%)	89.44% (80.54%-96.97%)	3.5142 (3.3032-3.7119)
At least 3 of CHASM (lung), CHASM (melanoma), FATHMM (missense), Mutation Assessor, MutationTaster, SIFT	85.54% (82.83%-88.38%)	95.23% (93.27%-97.58%)	68.766% (62.5%-75%)	84.06% (80.58%-87.59%)	89.31% (85%-94.34%)	3.3737 (3.2600-3.4893)	85.49% (79.8%-90.91%)	95.16% (90.62%-98.55%)	68.855% (56.246%-81.09%)	83.99% (76.92%-90.67%)	89.28% (79.98%-97.14%)	3.3729 (3.1375-3.6064)
At least 3 of CHASM (lung), CHASM (melanoma), FATHMM (missense), Mutation Assessor, MutationTaster, VEST	84.9% (82.32%-87.88%)	96.34% (94.49%-98.39%)	65.133% (58.973%-71.254%)	82.69% (79.33%-86.21%)	91.15% (86.96%-96%)	3.3531 (3.2388-3.4709)	84.74% (78.79%-89.9%)	96.15% (92.15%-100%)	65.123% (52.628%-77.5%)	82.55% (75.36%-89.23%)	90.82% (81.47%-100%)	3.3465 (3.1094-3.5717)
At least 3 of CHASM (lung), CHASM (melanoma), FATHMM (missense), Mutation Assessor, PolyPhen-2, PROVEAN	84.21% (81.31%-86.88%)	94.74% (92.68%-96.85%)	66.01% (60.274%-71.878%)	82.82% (79.43%-86.33%)	87.89% (83.33%-92.86%)	3.3145 (3.1968-3.4342)	84.1% (78.76%-89.9%)	94.56% (90.16%-98.46%)	66.112% (54.286%-78.13%)	82.73% (75.76%-89.4%)	87.64% (76.92%-96.43%)	3.3104 (3.0688-3.5421)
At least 3 of CHASM (lung), CHASM (melanoma), FATHMM (missense), Mutation Assessor, PolyPhen-2, SIFT	81.83% (78.79%-84.85%)	95.23% (93.27%-97.58%)	58.66% (52.055%-65.386%)	79.93% (76.03%-83.87%)	87.69% (82.61%-93.48%)	3.2152 (3.0861-3.3408)	81.79% (75.76%-87.88%)	95.16% (90.62%-98.55%)	58.805% (45.161%-71.43%)	79.86% (72.22%-87.18%)	87.66% (76%-96.67%)	3.2149 (2.9528-3.4641)
At least 3 of CHASM (lung), CHASM (melanoma), FATHMM (missense), Mutation Assessor, PolyPhen-2, VEST	80.5% (77.77%-83.84%)	94.73% (92.62%-96.9%)	55.881% (49.275%-62.5%)	78.78% (75.16%-82.67%)	85.99% (80.49%-91.67%)	3.1539 (3.0309-3.2893)	80.42% (73.74%-85.88%)	94.56% (89.85%-98.48%)	56.124% (42.857%-68.58%)	78.71% (71.05%-86.25%)	85.78% (73.68%-96%)	3.1518 (2.8747-3.3948)
At least 3 of CHASM (lung), CHASM (melanoma), FATHMM (missense), Mutation Assessor, PROVEAN, SIFT	82.51% (79.29%-85.35%)	93.67% (91.27%-96.06%)	63.196% (56.919%-69.444%)	81.48% (78.01%-84.93%)	85.23% (80%-90.74%)	3.2358 (3.1049-3.3614)	82.46% (76.77%-88.89%)	93.5% (88.52%-98.39%)	63.476% (51.351%-75.76%)	81.46% (74.32%-88.57%)	85.07% (73.91%-96%)	3.2352 (2.9793-3.4891)
At least 3 of CHASM (lung), CHASM (melanoma), FATHMM (missense), Mutation Assessor, PROVEAN, VEST	82.51% (79.8%-85.35%)	94.2% (92%-96.72%)	62.283% (56.059%-68.493%)	81.2% (77.62%-84.72%)	86.13% (81.13%-91.31%)	3.2381 (3.1142-3.3590)	82.46% (76.77%-88.89%)	94.04% (89.09%-98.41%)	62.551% (50%-75%)	81.17% (73.91%-88.31%)	85.97% (75%-96%)	3.2373 (2.9928-3.4764)
At least 3 of CHASM (lung), CHASM (melanoma), FATHMM (missense), Mutation Assessor, SIFT, VEST	79.79% (76.77%-82.83%)	94.73% (92.62%-96.9%)	53.956% (47.498%-60.811%)	78.06% (74.32%-81.76%)	85.57% (80.43%-91.49%)	3.1231 (2.9969-3.2538)	79.81% (73.74%-85.86%)	94.56% (89.85%-98.48%)	54.448% (41.173%-67.65%)	78.08% (70.42%-85.34%)	85.4% (73.07%-95.65%)	3.1250 (2.8560-3.3820)
At least 3 of CHASM (lung), CHASM (melanoma), FATHMM (missense), MutationTaster, PolyPhen-2, PROVEAN	86.24% (83.84%-88.89%)	95.27% (93.39%-97.56%)	70.616% (64.865%-76.923%)	84.87% (81.62%-88.36%)	89.64% (85.41%-94.55%)	3.4039 (3.2963-3.5223)	86.1% (80.81%-90.91%)	95.08% (90.74%-98.51%)	70.636% (58.06%-82.06%)	84.77% (77.94%-91.31%)	89.34% (79.31%-96.88%)	3.3982 (3.1571-3.6138)
At least 3 of CHASM (lung), CHASM (melanoma), FATHMM (missense), MutationTaster, PolyPhen-2, SIFT	84.56% (81.82%-87.37%)	96.86% (95.16%-98.49%)	63.297% (56.944%-69.444%)	82.03% (78.76%-85.62%)	92.1% (88%-96.23%)	3.3428 (3.2323-3.4566)	84.41% (78.79%-89.9%)	96.71% (92.85%-100%)	63.263% (51.351%-75.76%)	81.89% (74.67%-88.47%)	91.84% (82.6%-100%)	3.3370 (3.0973-3.5583)
At least 3 of CHASM (lung), CHASM (melanoma), FATHMM (missense), MutationTaster, PolyPhen-2, VEST	84.94% (82.32%-87.88%)	98.47% (97.52%-100%)	61.555% (55.26%-67.569%)	81.58% (78.2%-85.16%)	95.89% (93.02%-100%)	3.3749 (3.2743-3.4850)	84.66% (78.79%-89.9%)	98.27% (95.38%-100%)	61.273% (48.387%-73.33%)	81.32% (73.97%-88%)	95.38% (88%-100%)	3.3624 (3.1474-3.5679)
At least 3 of CHASM (lung), CHASM (melanoma), FATHMM (missense), MutationTaster, PROVEAN, SIFT	86.55% (83.84%-89.39%)	94.74% (92.68%-96.85%)	72.379% (66.667%-78.261%)	85.57% (82.48%-88.89%)	88.83% (84.48%-93.44%)	3.4152 (3.3018-3.5306)	86.5% (80.81%-91.92%)	94.56% (90.16%-98.46%)	72.616% (60.712%-84.38%)	85.58% (78.87%-92.31%)	88.62% (78.57%-96.77%)	3.4137 (3.1771-3.6427)
At least 3 of CHASM (lung), CHASM (melanoma), FATHMM (missense), MutationTaster, PROVEAN, VEST	86.92% (84.34%-89.39%)	96.34% (94.44%-98.4%)	70.633% (64.553%-77.143%)	85.02% (81.82%-88.44%)	91.78% (87.93%-96.36%)	3.4377 (3.3291-3.5498)	86.76% (80.81%-91.92%)	96.14% (92.06%-100%)	70.587% (57.576%-82.86%)	84.89% (77.77%-91.55%)	91.44% (82.76%-100%)	3.4306 (3.2039-3.6447)
At least 3 of CHASM (lung), CHASM (melanoma), FATHMM (missense), MutationTaster, SIFT, VEST	84.56% (81.82%-87.37%)	97.4% (95.9%-99.2%)	62.337% (56%-68.188%)	81.73% (78.32%-85.16%)	93.29% (89.58%-97.87%)	3.3475 (3.2331-3.4623)	84.42% (78.79%-89.9%)	97.21% (93.65%-100%)	62.419% (50%-75%)	81.63% (74.62%-88.41%)	92.93% (84%-100%)	3.3419 (3.1185-3.5630)
At least 3 of CHASM (lung), CHASM (melanoma), FATHMM (missense), PolyPhen-2, PROVEAN, SIFT	82.52% (79.8%-85.35%)	94.74% (92.68%-96.85%)	61.399% (55.069%-67.607%)	80.93% (77.55%-84.72%)	87.09% (82%-92.31%)	3.2416 (3.1188-3.3690)	82.43% (76.77%-88.89%)	94.56% (90.16%-98.46%)	61.568% (48.387%-74.36%)	80.86% (73.42%-87.68%)	86.84% (75%-96.3%)	3.2383 (2.9845-3.4764)
At least 3 of CHASM (lung), CHASM (melanoma), FATHMM (missense), PolyPhen-2, PROVEAN, VEST	81.54% (78.79%-84.85%)	94.74% (92.68%-96.85%)	58.714% (52.172%-65.335%)	79.87% (76.39%-83.67%)	86.58% (81.48%-92.16%)	3.1990 (3.0774-3.3279)	81.36% (74.75%-86.87%)	94.56% (90.16%-98.46%)	58.671% (45.7%-71.44%)	79.71% (72.29%-86.67%)	86.27% (74.07%-96%)	3.1921 (2.9277-3.4348)
At least 3 of CHASM (lung), CHASM (melanoma), FATHMM (missense), PolyPhen-2, SIFT, VEST	79.12% (75.76%-82.32%)	95.79% (93.85%-97.67%)	50.304% (43.66%-56.757%)	76.92% (73.25%-80.72%)	87.35% (81.82%-93.02%)	3.1036 (2.9743-3.2300)	79.12% (72.73%-85.86%)	95.65% (91.52%-100%)	50.703% (38.087%-63.42%)	76.91% (69.23%-84.21%)	87.19% (75%-100%)	3.1046 (2.8457-3.3614)
At least 3 of CHASM (lung), CHASM (melanoma), FATHMM (missense), PROVEAN, SIFT, VEST	80.15% (77.27%-83.33%)	94.2% (92%-96.72%)	55.849% (49.315%-62.162%)	78.67% (75.16%-82.43%)	84.78% (79.17%-90.48%)	3.1350 (3.0120-3.2701)	80.11% (73.74%-85.86%)	94.04% (89.09%-98.41%)	56.142% (42.857%-68.75%)	78.64% (71.01%-85.71%)	84.61% (72%-95.65%)	3.1343 (2.8661-3.3830)
At least 3 of CHASM (lung), CHASM (melanoma), Mutation Assessor, MutationTaster, PolyPhen-2, PROVEAN	80.78% (77.78%-83.84%)	86.13% (82.79%-89.6%)	71.543% (65.714%-77.612%)	83.95% (80.31%-87.79%)	74.9% (69.23%-80.82%)	3.1652 (3.0385-3.3042)	80.86% (74.75%-86.87%)	86.24% (79.36%-92.96%)	71.622% (59.365%-82.86%)	83.89% (76.12%-91.05%)	75.23% (63.33%-86.49%)	3.1698 (2.8890-3.4139)
At least 3 of CHASM (lung), CHASM (melanoma), Mutation Assessor, MutationTaster, PolyPhen-2, SIFT	80.11% (76.77%-83.33%)	89.33% (86.32%-92.5%)	64.178% (57.746%-70.274%)	81.17% (77.61%-84.89%)	77.68% (71.93%-83.64%)	3.1236 (2.9855-3.2636)	80.18% (73.74%-86.87%)	89.41% (83.01%-95.31%)	64.325% (51.429%-77.14%)	81.13% (73.85%-88.24%)	78.01% (65.62%-89.29%)	3.1287 (2.8486-3.4073)
At least 3 of CHASM (lung), CHASM (melanoma), Mutation Assessor, MutationTaster, PolyPhen-2, VEST	77.44% (74.24%-80.81%)	87.18% (83.87%-90.48%)	60.592% (54.286%-66.678%)	79.27% (75.36%-83.1%)	73.22% (66.67%-79.7%)	3.0026 (2.8587-3.1471)	77.45% (70.71%-83.84%)	87.33% (80.36%-93.75%)	60.497% (47.616%-72.73%)	79.13% (71.01%-86.96%)	73.58% (60%-85.71%)	3.0054 (2.7062-3.2799)
At least 3 of CHASM (lung), CHASM (melanoma), Mutation Assessor, MutationTaster, PROVEAN, SIFT	83.14% (80.29%-85.86%)	88.78% (85.61%-92%)	73.364% (67.568%-79.167%)	85.21% (81.75%-88.64%)	79.1% (73.61%-84.62%)	3.2647 (3.1381-3.3908)	83.22% (77.78%-88.91%)	88.9% (82.14%-94.92%)	73.451% (62.5%-85.29%)	85.17% (78.12%-91.94%)	79.45% (67.85%-90%)	3.2697 (3.0205-3.5231)
At least 3 of CHASM (lung), CHASM (melanoma), Mutation Assessor, MutationTaster, PROVEAN, VEST	79.78% (76.77%-82.83%)	85.62% (82.26%-89.06%)	69.691% (63.997%-75.642%)	83% (79.37%-86.57%)	73.71% (68.18%-79.69%)	3.1202 (2.9903-3.2591)	79.83% (73.74%-85.86%)	85.66% (78.33%-92.31%)	69.8% (57.895%-82.35%)	82.95% (75.41%-90.28%)	73.96% (61.76%-84.85%)	3.1238 (2.8527-3.3871)
At least 3 of CHASM (lung), CHASM (melanoma), Mutation Assessor, MutationTaster, SIFT, VEST	80.1% (77.26%-83.33%)	90.38% (87.6%-93.55%)	62.315% (56.164%-68.832%)	80.57% (76.81%-84.29%)	78.94% (72.73%-85%)	3.1220 (2.9908-3.2533)	80.21% (73.74%-85.86%)	90.5% (84.38%-96.43%)	62.549% (50%-75%)	80.56% (73.13%-87.85%)	79.37% (66.67%-90.91%)	3.1298 (2.8549-3.3882)
At least 3 of CHASM (lung), CHASM (melanoma), Mutation Assessor, PolyPhen-2, PROVEAN, SIFT	76.75% (73.23%-79.8%)	86.15% (82.81%-89.57%)	60.489% (54.164%-66.667%)	79.03% (75.36%-82.84%)	71.65% (65.52%-77.78%)	2.9732 (2.8281-3.1126)	76.81% (70.71%-83.84%)	86.19% (78.95%-92.54%)	60.681% (47.368%-73.53%)	78.99% (71.43%-86.37%)	71.96% (60%-83.87%)	2.9783 (2.6984-3.2688)
At least 3 of CHASM (lung), CHASM (melanoma), Mutation Assessor, PolyPhen-2, PROVEAN, VEST	72.71% (69.19%-76.26%)	81.38% (77.69%-85.27%)	57.72% (51.389%-64%)	76.89% (72.87%-80.88%)	64.2% (57.63%-70.49%)	2.8019 (2.6509-2.9413)	72.76% (65.66%-79.8%)	81.36% (73.33%-88.71%)	57.986% (44.818%-70.97%)	76.85% (68.42%-84.85%)	64.46% (51.35%-77.78%)	2.8066 (2.5189-3.0976)
At least 3 of CHASM (lung), CHASM (melanoma), Mutation Assessor, PolyPhen-2, SIFT, VEST	75.02% (71.7%-78.28%)	87.2% (83.9%-90.37%)	53.965% (47.436%-60.257%)	76.61% (72.66%-80.56%)	70.92% (64.15%-77.36%)	2.8869 (2.7350-3.0345)	75.21% (68.69%-81.84%)	87.28% (80.6%-93.65%)	54.474% (41.662%-67.65%)	76.68% (68.65%-84.72%)	71.42% (58.33%-84.38%)	2.8985 (2.6088-3.1950)
At least 3 of CHASM (lung), CHASM (melanoma), Mutation Assessor, PROVEAN, SIFT, VEST	75.1% (71.72%-78.28%)	84.07% (80.51%-87.9%)	59.581% (53.333%-66.197%)	78.24% (74.24%-82.22%)	68.39% (62.3%-75%)	2.9029 (2.7547-3.0456)	75.05% (68.69%-81.82%)	83.96% (76.18%-90.62%)	59.745% (46.852%-72.51%)	78.16% (70.15%-86.16%)	68.48% (54.84%-80.65%)	2.9035 (2.6120-3.1940)
At least 3 of CHASM (lung), CHASM (melanoma), MutationTaster, PolyPhen-2, PROVEAN, SIFT	80.11% (77.26%-82.84%)	88.8% (85.83%-92%)	65.09% (58.82%-71.429%)	81.48% (77.86%-85.29%)	77.08% (71.19%-83.05%)	3.1244 (2.9931-3.2546)	80.18% (74.72%-85.88%)	88.88% (82.35%-95%)	65.231% (51.724%-77.78%)	81.44% (73.91%-88.74%)	77.39% (65.38%-88.89%)	3.1293 (2.8672-3.3871)
At least 3 of CHASM (lung), CHASM (melanoma), MutationTaster, PolyPhen-2, PROVEAN, VEST	76.78% (73.74%-80.3%)	86.15% (82.93%-89.6%)	60.593% (54.052%-66.667%)	79.08% (75.18%-83.09%)	71.67% (65.51%-77.78%)	2.9749 (2.8359-3.1163)	76.74% (69.7%-82.83%)	86.2% (78.79%-92.54%)	60.477% (48.387%-73.68%)	78.91% (71.21%-86.76%)	71.87% (59.08%-83.87%)	2.9747 (2.6858-3.2538)
At least 3 of CHASM (lung), CHASM (melanoma), MutationTaster, PolyPhen-2, SIFT, VEST	77.71% (74.75%-80.81%)	90.33% (87.6%-93.39%)	55.882% (48.717%-62.319%)	77.98% (74.1%-81.95%)	76.98% (70.69%-83.67%)	3.0117 (2.8796-3.1489)	77.91% (71.72%-83.84%)	90.6% (84.62%-96.43%)	56.115% (42.857%-70.27%)	77.99% (69.86%-85.53%)	77.68% (63.99%-90.48%)	3.0239 (2.7385-3.2807)
At least 3 of CHASM (lung), CHASM (melanoma), MutationTaster, PROVEAN, SIFT, VEST	78.77% (75.76%-81.82%)	88.28% (85.16%-91.41%)	62.339% (56%-68.833%)	80.21% (76.47%-83.94%)	75.47% (69.35%-81.67%)	3.0629 (2.9260-3.1880)	78.82% (72.73%-84.85%)	88.33% (81.67%-94.92%)	62.48% (48.716%-75%)	80.16% (72.58%-87.5%)	75.74% (63.88%-88.46%)	3.0671 (2.8132-3.3345)
At least 3 of CHASM (lung), CHASM (melanoma), PolyPhen-2, PROVEAN, SIFT, VEST	74.05% (70.71%-77.27%)	85.63% (82.31%-89.06%)	54.007% (47.434%-60.611%)	76.3% (72.22%-80.42%)	68.49% (62.07%-74.58%)	2.8443 (2.7041-2.9826)	74.13% (67.68%-80.81%)	85.63% (78.57%-92.19%)	54.359% (40.623%-67.65%)	76.3% (68.49%-84.38%)	68.8% (55.56%-81.49%)	2.8509 (2.5658-3.1392)
At least 3 of CHASM (lung), FATHMM (cancer), FATHMM (missense), Mutation Assessor, MutationTaster, PolyPhen-2	85.87% (83.33%-88.4%)	97.9% (96.69%-99.22%)	65.052% (59.089%-71.233%)	82.89% (79.58%-86.49%)	94.73% (91.49%-98.15%)	3.4057 (3.3033-3.5099)	85.84% (80.78%-90.91%)	97.8% (94.92%-100%)	65.307% (52.774%-76.94%)	82.87% (75.9%-89.48%)	94.55% (86.36%-100%)	3.4053 (3.1928-3.6131)
At least 3 of CHASM (lung), FATHMM (cancer), FATHMM (missense), Mutation Assessor, MutationTaster, PROVEAN	86.53% (83.84%-89.39%)	94.74% (92.56%-96.88%)	72.334% (66.234%-78.38%)	85.55% (82.14%-88.97%)	88.83% (84.21%-93.33%)	3.4146 (3.2957-3.5344)	86.53% (80.81%-91.92%)	94.55% (90%-98.51%)	72.751% (60.526%-84.62%)	85.62% (78.79%-92.42%)	88.64% (79.31%-96.88%)	3.4156 (3.1734-3.6582)
At least 3 of CHASM (lung), FATHMM (cancer), FATHMM (missense), Mutation Assessor, MutationTaster, SIFT	85.15% (82.32%-87.88%)	97.39% (95.9%-99.2%)	63.991% (57.688%-70.425%)	82.38% (79.02%-85.92%)	93.42% (89.8%-97.92%)	3.3718 (3.2623-3.4917)	85.25% (79.8%-90.91%)	97.23% (93.65%-100%)	64.676% (51.515%-77.14%)	82.52% (75.36%-89.33%)	93.18% (84.61%-100%)	3.3761 (3.1348-3.5944)
At least 3 of CHASM (lung), FATHMM (cancer), FATHMM (missense), Mutation Assessor, MutationTaster, VEST	84.85% (82.32%-87.88%)	98.47% (97.52%-100%)	61.287% (54.545%-68.001%)	81.48% (78.15%-85.16%)	95.87% (93.02%-100%)	3.3710 (3.2667-3.4772)	84.85% (78.79%-90.91%)	98.27% (95.38%-100%)	61.823% (48.571%-74.2%)	81.54% (74.32%-88.16%)	95.41% (88.24%-100%)	3.3705 (3.1518-3.5785)
At least 3 of CHASM (lung), FATHMM (cancer), FATHMM (missense), Mutation Assessor, PolyPhen-2, PROVEAN	83.5% (80.8%-86.36%)	93.67% (91.34%-96.09%)	65.92% (59.994%-72.059%)	82.62% (78.98%-86.43%)	85.77% (80.39%-91.07%)	3.2797 (3.1581-3.4081)	83.5% (77.78%-88.89%)	93.5% (88.33%-98.36%)	66.322% (54.054%-78.96%)	82.65% (75%-89.86%)	85.63% (75%-95.84%)	3.2810 (3.0299-3.5336)
At least 3 of CHASM (lung), FATHMM (cancer), FATHMM (missense), Mutation Assessor, PolyPhen-2, SIFT	81.81% (78.79%-84.85%)	96.86% (95.16%-98.49%)	55.796% (49.315%-62.162%)	79.12% (75.5%-82.89%)	91.13% (86.67%-95.92%)	3.2289 (3.1146-3.3535)	81.83% (75.76%-87.88%)	96.71% (92.85%-100%)	56.29% (42.857%-69.44%)	79.15% (71.62%-86.42%)	90.91% (80%-100%)	3.2306 (2.9822-3.4598)
At least 3 of CHASM (lung), FATHMM (cancer), FATHMM (missense), Mutation Assessor, PolyPhen-2, VEST	81.15% (78.28%-84.34%)	97.4% (95.93%-99.2%)	53.04% (46.377%-59.421%)	78.2% (74.52%-81.87%)	92.2% (88.1%-97.5%)	3.2084 (3.0932-3.3281)	81.14% (74.75%-86.87%)	97.21% (93.65%-100%)	53.542% (40.527%-66.67%)	78.22% (70.73%-85.53%)	91.8% (81.48%-100%)	3.2076 (2.9742-3.4314)
At least 3 of CHASM (lung), FATHMM (cancer), FATHMM (missense), Mutation Assessor, PROVEAN, SIFT	82.47% (79.29%-85.37%)	93.13% (90.83%-95.8%)	64.011% (57.891%-70.423%)	81.74% (78.23%-85.34%)	84.35% (79.25%-89.8%)	3.2323 (3.1080-3.3648)	82.54% (76.74%-88.89%)	92.98% (87.69%-97.14%)	64.609% (51.515%-76.92%)	81.85% (74.65%-88.57%)	84.28% (73.52%-93.75%)	3.2372 (2.9671-3.4859)
At least 3 of CHASM (lung), FATHMM (cancer), FATHMM (missense), Mutation Assessor, PROVEAN, VEST	81.45% (78.28%-84.36%)	94.2% (92%-96.75%)	59.402% (53.239%-66.177%)	80.05% (76.51%-83.89%)	85.56% (80%-91.12%)	3.1921 (3.0667-3.3253)	81.54% (75.73%-87.88%)	94.03% (89.23%-98.41%)	60.079% (47.054%-72.51%)	80.16% (72.37%-87.32%)	85.47% (73.91%-96%)	3.1975 (2.9370-3.4517)
At least 3 of CHASM (lung), FATHMM (cancer), FATHMM (missense), Mutation Assessor, SIFT, VEST	80.48% (77.27%-83.35%)	95.81% (93.75%-97.67%)	53.955% (47.684%-60.563%)	78.25% (74.68%-81.88%)	88.18% (82.98%-93.48%)	3.1619 (3.0387-3.2878)	80.46% (74.72%-86.87%)	95.6% (91.52%-100%)	54.472% (41.935%-66.67%)	78.27% (70.83%-85.33%)	87.85% (76.19%-100%)	3.1619 (2.8981-3.4080)
At least 3 of CHASM (lung), FATHMM (cancer), FATHMM (missense), MutationTaster, PolyPhen-2, PROVEAN	85.2% (82.32%-87.88%)	95.26% (93.33%-97.58%)	67.786% (61.903%-73.973%)	83.64% (80.28%-87.23%)	89.22% (84.61%-94.34%)	3.3591 (3.2419-3.4730)	85.16% (79.8%-90.91%)	95.1% (90.62%-98.53%)	68.074% (55.556%-80%)	83.64% (76.47%-90.28%)	89.03% (79.31%-96.88%)	3.3586 (3.1229-3.5881)
At least 3 of CHASM (lung), FATHMM (cancer), FATHMM (missense), MutationTaster, PolyPhen-2, SIFT	83.82% (81.31%-86.87%)	97.9% (96.69%-99.22%)	59.452% (52.857%-65.753%)	80.68% (77.18%-84.31%)	94.26% (90.7%-97.92%)	3.3229 (3.2140-3.4332)	83.88% (77.78%-88.89%)	97.8% (94.92%-100%)	59.979% (47.365%-72.73%)	80.75% (73.61%-88%)	94.1% (85.71%-100%)	3.3263 (3.1014-3.5422)
At least 3 of CHASM (lung), FATHMM (cancer), FATHMM (missense), MutationTaster, PolyPhen-2, VEST	84.22% (81.31%-87.37%)	98.47% (97.52%-100%)	59.587% (53.241%-66.216%)	80.82% (77.4%-84.52%)	95.75% (92.86%-100%)	3.3463 (3.2447-3.4532)	84.08% (77.78%-89.9%)	98.27% (95.38%-100%)	59.714% (46.659%-72.22%)	80.72% (73.33%-87.5%)	95.25% (87.99%-100%)	3.3395 (3.1258-3.5459)
At least 3 of CHASM (lung), FATHMM (cancer), FATHMM (missense), MutationTaster, PROVEAN, SIFT	84.16% (81.31%-87.37%)	95.28% (93.22%-97.58%)	64.93% (58.664%-71.646%)	82.45% (79.08%-86.11%)	88.85% (84%-94.12%)	3.3151 (3.1927-3.4375)	84.2% (77.78%-89.9%)	95.06% (90.62%-98.57%)	65.52% (51.613%-77.78%)	82.56% (75.32%-89.4%)	88.58% (78.26%-96.77%)	3.3172 (3.0718-3.5579)
At least 3 of CHASM (lung), FATHMM (cancer), FATHMM (missense), MutationTaster, PROVEAN, VEST	84.86% (82.32%-87.88%)	96.86% (95.2%-98.48%)	64.092% (57.534%-70.59%)	82.35% (79.05%-86.09%)	92.2% (88%-96.36%)	3.3550 (3.2405-3.4758)	84.83% (78.79%-89.9%)	96.69% (92.86%-100%)	64.437% (51.427%-76.93%)	82.36% (75%-89.04%)	91.92% (83.32%-100%)	3.3541 (3.1121-3.5776)
At least 3 of CHASM (lung), FATHMM (cancer), FATHMM (missense), MutationTaster, SIFT, VEST	84.86% (82.32%-87.88%)	98.99% (98.33%-100%)	60.436% (54.043%-67.568%)	81.23% (77.92%-84.97%)	97.2% (95.12%-100%)	3.3785 (3.2752-3.4848)	84.82% (78.79%-89.9%)	98.82% (96.67%-100%)	60.766% (47.368%-73.54%)	81.21% (73.75%-87.84%)	96.8% (90.48%-100%)	3.3760 (3.1678-3.5852)
At least 3 of CHASM (lung), FATHMM (cancer), FATHMM (missense), PolyPhen-2, PROVEAN, SIFT	81.48% (78.28%-84.85%)	94.21% (92%-96.77%)	59.457% (53.118%-66.216%)	80.07% (76.55%-83.89%)	85.6% (80.38%-91.31%)	3.1934 (3.0636-3.3291)	81.48% (74.75%-87.88%)	94.01% (89.06%-98.44%)	59.956% (46.87%-72.73%)	80.12% (72.6%-87.5%)	85.38% (73.68%-96%)	3.1947 (2.9231-3.4518)
At least 3 of CHASM (lung), FATHMM (cancer), FATHMM (missense), PolyPhen-2, PROVEAN, VEST	81.15% (78.28%-84.34%)	95.26% (93.33%-97.58%)	56.732% (50%-63.514%)	79.2% (75.5%-83.01%)	87.39% (81.82%-93.33%)	3.1858 (3.0587-3.3161)	81.14% (74.75%-86.87%)	95.1% (90.62%-98.53%)	57.136% (43.902%-70.74%)	79.21% (71.42%-86.67%)	87.2% (76%-96.43%)	3.1865 (2.9288-3.4393)
At least 3 of CHASM (lung), FATHMM (cancer), FATHMM (missense), PolyPhen-2, SIFT, VEST	79.78% (76.77%-82.83%)	97.91% (96.69%-99.23%)	48.434% (42.103%-54.796%)	76.65% (72.9%-80.38%)	93.07% (88.89%-97.56%)	3.1607 (3.0442-3.2754)	79.82% (73.74%-85.86%)	97.79% (94.83%-100%)	48.969% (36.111%-62.5%)	76.68% (69.33%-84.42%)	92.82% (82.35%-100%)	3.1626 (2.9230-3.3998)
At least 3 of CHASM (lung), FATHMM (cancer), FATHMM (missense), PROVEAN, SIFT, VEST	81.48% (78.28%-84.85%)	94.74% (92.56%-96.95%)	58.545% (52.113%-64.874%)	79.81% (76.35%-83.67%)	86.57% (81.25%-92.16%)	3.1966 (3.0675-3.3231)	81.48% (74.75%-87.88%)	94.54% (89.55%-98.51%)	59.021% (45.714%-72.23%)	79.84% (72.06%-86.67%)	86.33% (75%-96.15%)	3.1973 (2.9308-3.4487)
At least 3 of CHASM (lung), FATHMM (cancer), Mutation Assessor, MutationTaster, PolyPhen-2, PROVEAN	81.43% (78.28%-84.34%)	87.19% (83.9%-90.62%)	71.457% (65.753%-77.465%)	84.08% (80.49%-87.79%)	76.34% (70.69%-81.95%)	3.1907 (3.0605-3.3255)	81.59% (75.76%-87.88%)	87.31% (80%-93.85%)	71.791% (60%-83.33%)	84.14% (76.92%-91.05%)	76.74% (64.86%-88.24%)	3.1998 (2.9295-3.4625)
At least 3 of CHASM (lung), FATHMM (cancer), Mutation Assessor, MutationTaster, PolyPhen-2, SIFT	81.43% (78.28%-84.34%)	90.92% (88.1%-94.03%)	65.021% (58.904%-71.233%)	81.8% (78.17%-85.51%)	80.56% (75%-86.79%)	3.1830 (3.0575-3.3167)	81.58% (75.76%-87.88%)	91.02% (84.75%-96.77%)	65.378% (52.632%-77.5%)	81.85% (74.65%-89.23%)	80.97% (69.22%-92.31%)	3.1922 (2.9153-3.4439)
At least 3 of CHASM (lung), FATHMM (cancer), Mutation Assessor, MutationTaster, PolyPhen-2, VEST	79.11% (76.25%-82.32%)	88.79% (85.71%-92%)	62.377% (56.061%-68.58%)	80.31% (76.51%-84.17%)	76.3% (70.31%-82.46%)	3.0778 (2.9385-3.2200)	79.15% (72.73%-84.85%)	88.89% (82.54%-95.16%)	62.422% (50%-75%)	80.22% (72.6%-87.82%)	76.63% (63.89%-88.24%)	3.0817 (2.8033-3.3518)
At least 3 of CHASM (lung), FATHMM (cancer), Mutation Assessor, MutationTaster, PROVEAN, SIFT	82.11% (79.29%-85.35%)	89.87% (86.99%-92.97%)	68.693% (62.821%-75%)	83.23% (79.69%-86.92%)	79.69% (74.19%-85.48%)	3.2148 (3.0851-3.3450)	82.25% (75.76%-87.88%)	89.93% (83.58%-95.38%)	69.06% (57.143%-80.65%)	83.29% (75.76%-90.48%)	80.02% (68.56%-90.32%)	3.2229 (2.9525-3.4796)
At least 3 of CHASM (lung), FATHMM (cancer), Mutation Assessor, MutationTaster, PROVEAN, VEST	81.46% (78.79%-84.34%)	87.75% (84.55%-90.99%)	70.569% (64.863%-76.715%)	83.75% (80.15%-87.5%)	76.92% (71.64%-82.35%)	3.1899 (3.0629-3.3220)	81.53% (75.76%-87.88%)	87.78% (81.03%-93.94%)	70.819% (58.333%-82.5%)	83.75% (76.39%-90.77%)	77.17% (65.71%-88.46%)	3.1952 (2.9295-3.4402)
At least 3 of CHASM (lung), FATHMM (cancer), Mutation Assessor, MutationTaster, SIFT, VEST	80.39% (77.27%-83.33%)	91.43% (88.71%-94.36%)	61.294% (55.072%-67.606%)	80.33% (76.71%-84.17%)	80.54% (74.99%-86.54%)	3.1359 (3.0026-3.2665)	80.64% (74.75%-86.87%)	91.6% (85.71%-96.88%)	61.824% (48.571%-73.69%)	80.45% (72.72%-87.5%)	81.13% (69.22%-92.31%)	3.1501 (2.8844-3.4094)
At least 3 of CHASM (lung), FATHMM (cancer), Mutation Assessor, PolyPhen-2, PROVEAN, SIFT	77.42% (74.24%-80.81%)	86.7% (83.46%-90.08%)	61.362% (55.128%-67.614%)	79.51% (75.56%-83.34%)	72.74% (66.67%-78.95%)	3.0031 (2.8648-3.1448)	77.49% (70.71%-83.84%)	86.69% (79.62%-93.22%)	61.691% (48.78%-74.08%)	79.51% (71.83%-87.33%)	73.01% (60.71%-84.86%)	3.0090 (2.7091-3.2903)

At least 3 of CHASM (lung), FATHMM (cancer), Mutation Assessor, PolyPhen-2, PROVEAN, VEST	74.39% (71.21%-77.78%)	82.45% (78.81%-86.36%)	60.454% (54.286%-66.667%)	78.28% (74.24%-82.36%)	66.58% (60.61%-72.73%)	2.8776 (2.7427-3.0202)	74.46% (67.68%-80.81%)	82.43% (74.6%-89.48%)	60.768% (48.387%-72.73%)	78.27% (70.15%-86.16%)	66.85% (53.85%-79.41%)	2.8832 (2.5925-3.1551)
At least 3 of CHASM (lung), FATHMM (cancer), Mutation Assessor, PolyPhen-2, SIFT, VEST	73.97% (70.2%-77.27%)	87.71% (84.3%-90.91%)	50.213% (43.75%-56.34%)	75.28% (71.33%-79.17%)	70.28% (63.27%-76.79%)	2.8349 (2.6780-2.9807)	74.27% (67.68%-81.82%)	87.85% (81.25%-94.12%)	50.96% (38.889%-64.1%)	75.44% (67.61%-83.12%)	71.02% (57.14%-84.62%)	2.8528 (2.5661-3.1568)
At least 3 of CHASM (lung), FATHMM (cancer), Mutation Assessor, PROVEAN, SIFT, VEST	75.05% (71.7%-78.28%)	84.61% (81.15%-88.15%)	58.514% (52.174%-65.153%)	77.91% (73.91%-81.75%)	68.75% (62.71%-75.41%)	2.8979 (2.7513-3.0447)	75.15% (68.69%-81.84%)	84.47% (77.05%-91.43%)	59.121% (46.422%-71.8%)	78% (69.84%-86.15%)	68.94% (55.88%-81.48%)	2.9053 (2.6081-3.2044)
At least 3 of CHASM (lung), FATHMM (cancer), MutationTaster, PolyPhen-2, PROVEAN, SIFT	81.08% (77.78%-83.84%)	89.32% (86.26%-92.5%)	66.837% (61.035%-73.239%)	82.32% (78.68%-86.03%)	78.36% (72.72%-84.32%)	3.1683 (3.0386-3.2989)	81.27% (75.76%-87.88%)	89.44% (83.05%-95.38%)	67.255% (55.168%-79.41%)	82.41% (75%-89.71%)	78.8% (66.67%-90.01%)	3.1791 (2.9057-3.4364)
At least 3 of CHASM (lung), FATHMM (cancer), MutationTaster, PolyPhen-2, PROVEAN, VEST	78.11% (74.75%-81.31%)	86.69% (83.33%-90.08%)	63.272% (56.944%-69.567%)	80.32% (76.47%-84.44%)	73.33% (67.27%-79.31%)	3.0360 (2.9015-3.1792)	78.13% (71.72%-84.85%)	86.71% (79.69%-93.44%)	63.38% (50%-75.68%)	80.25% (72.3%-87.88%)	73.56% (60.71%-85.19%)	3.0390 (2.7503-3.3114)
At least 3 of CHASM (lung), FATHMM (cancer), MutationTaster, PolyPhen-2, SIFT, VEST	77.03% (73.74%-80.3%)	91.95% (89.31%-94.66%)	51.241% (44.595%-57.58%)	76.53% (72.79%-80.42%)	78.64% (72.34%-85.42%)	2.9835 (2.8511-3.1120)	77.25% (70.71%-83.84%)	92.16% (86.44%-97.06%)	51.636% (39.394%-65.52%)	76.58% (68.83%-84.15%)	79.38% (65%-92.31%)	2.9976 (2.7309-3.2688)
At least 3 of CHASM (lung), FATHMM (cancer), MutationTaster, PROVEAN, SIFT, VEST	80.39% (77.27%-83.33%)	89.86% (86.99%-92.8%)	63.997% (57.746%-70.425%)	81.19% (77.54%-85%)	78.51% (72.88%-84.75%)	3.1356 (3.0012-3.2618)	80.64% (74.75%-86.87%)	89.94% (83.93%-95.61%)	64.677% (51.613%-76.92%)	81.37% (73.85%-88.41%)	78.97% (67.55%-90.92%)	3.1495 (2.8979-3.4139)
At least 3 of CHASM (lung), FATHMM (cancer), PolyPhen-2, PROVEAN, SIFT, VEST	73.33% (69.7%-76.77%)	86.15% (82.93%-89.6%)	51.153% (44.872%-57.534%)	75.3% (71.43%-79.29%)	68.12% (61.22%-74.55%)	2.8073 (2.6591-2.9485)	73.54% (66.67%-80.81%)	86.19% (78.95%-92.86%)	51.815% (39.279%-64.1%)	75.42% (67.57%-83.1%)	68.66% (54.99%-82.15%)	2.8209 (2.5359-3.1221)
At least 3 of CHASM (lung), FATHMM (missense), Mutation Assessor, MutationTaster, PolyPhen-2, PROVEAN	81.43% (78.28%-84.34%)	86.67% (83.33%-90.17%)	72.361% (66.667%-78.261%)	84.43% (80.83%-88.1%)	75.84% (70.15%-81.69%)	3.1930 (3.0582-3.3216)	81.59% (75.76%-87.88%)	86.76% (79.66%-93.55%)	72.716% (60.969%-84.21%)	84.5% (77.05%-91.53%)	76.21% (64.1%-87.11%)	3.2019 (2.9430-3.4625)
At least 3 of CHASM (lung), FATHMM (missense), Mutation Assessor, MutationTaster, PolyPhen-2, SIFT	80.45% (77.27%-83.33%)	90.41% (87.69%-93.5%)	63.226% (56.713%-69.569%)	80.95% (77.3%-84.73%)	79.23% (73.33%-85.25%)	3.1381 (2.9950-3.2710)	80.52% (74.75%-86.87%)	90.45% (84.38%-96.08%)	63.455% (51.189%-75.87%)	80.94% (73.42%-88.24%)	79.51% (67.85%-90.62%)	3.1435 (2.8706-3.4169)
At least 3 of CHASM (lung), FATHMM (missense), Mutation Assessor, MutationTaster, PolyPhen-2, VEST	77.77% (74.75%-81.31%)	88.8% (85.71%-92%)	58.71% (52.174%-65.278%)	78.8% (75%-82.71%)	75.19% (68.97%-81.48%)	3.0150 (2.8761-3.1580)	77.79% (70.71%-83.84%)	88.89% (82.35%-95.08%)	58.731% (45.161%-70.97%)	78.7% (71.01%-86.11%)	75.51% (62.5%-88%)	3.0182 (2.7174-3.2903)
At least 3 of CHASM (lung), FATHMM (missense), Mutation Assessor, MutationTaster, PROVEAN, SIFT	82.09% (79.29%-84.85%)	88.79% (85.71%-92%)	70.48% (64.789%-76.812%)	83.87% (80.16%-87.5%)	78.44% (73.01%-84.29%)	3.2159 (3.0812-3.3470)	82.29% (76.77%-87.88%)	88.88% (82.14%-94.92%)	70.964% (58.532%-82.94%)	84.01% (76.92%-91.31%)	78.84% (66.67%-89.29%)	3.2270 (2.9631-3.4859)
At least 3 of CHASM (lung), FATHMM (missense), Mutation Assessor, MutationTaster, PROVEAN, VEST	79.75% (76.77%-82.83%)	86.7% (83.2%-90.23%)	67.738% (61.644%-74.026%)	82.29% (78.63%-85.94%)	74.66% (68.96%-80.6%)	3.1138 (2.9731-3.2504)	79.89% (73.74%-85.86%)	86.69% (79.31%-93.22%)	68.187% (55.261%-80.56%)	82.38% (75%-89.71%)	74.93% (62.86%-86.67%)	3.1218 (2.8508-3.3971)
At least 3 of CHASM (lung), FATHMM (missense), Mutation Assessor, MutationTaster, SIFT, VEST	79.06% (75.76%-82.32%)	90.92% (88.14%-93.8%)	58.552% (52.172%-65.217%)	79.14% (75.34%-82.98%)	78.87% (72.72%-85%)	3.0747 (2.9336-3.2043)	79.25% (72.73%-85.86%)	91.02% (85.18%-96.72%)	59.032% (45.447%-71.88%)	79.22% (71.64%-86.67%)	79.33% (66.67%-91.67%)	3.0860 (2.8048-3.3613)
At least 3 of CHASM (lung), FATHMM (missense), Mutation Assessor, PolyPhen-2, PROVEAN, SIFT	76.74% (73.23%-80.3%)	86.15% (82.93%-89.6%)	60.453% (53.846%-66.667%)	79.02% (75.18%-82.84%)	71.64% (65.57%-77.78%)	2.9727 (2.8262-3.1155)	76.82% (69.7%-83.84%)	86.18% (78.95%-92.54%)	60.747% (48.276%-74.08%)	79.02% (71.43%-86.76%)	71.94% (60%-83.33%)	2.9788 (2.6768-3.2783)
At least 3 of CHASM (lung), FATHMM (missense), Mutation Assessor, PolyPhen-2, PROVEAN, VEST	72.38% (69.19%-75.76%)	81.92% (78.29%-85.83%)	55.868% (49.333%-62.319%)	76.24% (72.22%-80.15%)	64.13% (57.63%-70.77%)	2.7816 (2.6329-2.9240)	72.42% (65.66%-79.79%)	81.88% (73.77%-89.23%)	56.15% (43.243%-68.76%)	76.21% (68.25%-84.06%)	64.37% (51.43%-77.14%)	2.7861 (2.4879-3.0834)
At least 3 of CHASM (lung), FATHMM (missense), Mutation Assessor, PolyPhen-2, SIFT, VEST	73.31% (69.7%-76.77%)	87.2% (83.9%-90.37%)	49.29% (42.62%-55.714%)	74.83% (70.78%-78.91%)	69.02% (61.82%-75.47%)	2.8034 (2.6434-2.9542)	73.58% (66.67%-80.81%)	87.28% (80.6%-93.65%)	50.038% (37.5%-62.86%)	74.98% (66.67%-82.9%)	69.65% (56.24%-83.33%)	2.8195 (2.5188-3.1342)
At least 3 of CHASM (lung), FATHMM (missense), Mutation Assessor, PROVEAN, SIFT, VEST	73.71% (70.2%-77.27%)	83.54% (79.84%-87.4%)	56.711% (50.649%-63.384%)	76.94% (72.93%-81.02%)	66.59% (60%-73.33%)	2.8378 (2.6858-2.9856)	73.79% (66.67%-80.81%)	83.43% (75.76%-90.33%)	57.206% (43.581%-70.27%)	76.98% (68.92%-85.07%)	66.79% (52.93%-79.31%)	2.8441 (2.5454-3.1519)
At least 3 of CHASM (lung), FATHMM (missense), MutationTaster, PolyPhen-2, PROVEAN, SIFT	80.44% (77.27%-83.33%)	89.34% (86.32%-92.43%)	65.045% (58.904%-71.233%)	81.55% (78.01%-85.21%)	77.92% (72.13%-83.61%)	3.1385 (2.9977-3.2710)	80.54% (74.75%-86.87%)	89.4% (83.04%-95.38%)	65.314% (52.632%-77.42%)	81.56% (74.24%-88.89%)	78.24% (66.67%-89.66%)	3.1451 (2.8781-3.4178)
At least 3 of CHASM (lung), FATHMM (missense), MutationTaster, PolyPhen-2, PROVEAN, VEST	78.13% (74.75%-81.31%)	87.23% (84.03%-90.68%)	62.389% (56.162%-68.919%)	80.04% (76.09%-83.94%)	73.86% (67.74%-80.01%)	3.0351 (2.8899-3.1696)	78.09% (71.72%-84.85%)	87.24% (80%-93.75%)	62.362% (50%-75%)	79.91% (72.06%-87.5%)	74.02% (61.54%-86.36%)	3.0353 (2.7628-3.3237)
At least 3 of CHASM (lung), FATHMM (missense), MutationTaster, PolyPhen-2, SIFT, VEST	78.06% (74.75%-81.31%)	91.95% (89.34%-94.62%)	54.02% (47.297%-60.611%)	77.57% (73.83%-81.46%)	79.52% (73.47%-86.27%)	3.0306 (2.8888-3.1694)	78.23% (71.72%-84.85%)	92.15% (86.44%-96.97%)	54.314% (41.379%-67.5%)	77.59% (69.86%-84.93%)	80.16% (66.67%-91.67%)	3.0422 (2.7607-3.3188)
At least 3 of CHASM (lung), FATHMM (missense), MutationTaster, PROVEAN, SIFT, VEST	79.1% (75.76%-82.32%)	89.35% (86.29%-92.62%)	61.362% (55.071%-67.606%)	80% (76.26%-83.69%)	76.93% (70.69%-83.05%)	3.0764 (2.9376-3.2089)	79.17% (72.73%-85.86%)	89.36% (83.05%-95.38%)	61.654% (48.571%-75%)	80% (72.73%-87.34%)	77.17% (64.7%-89.66%)	3.0818 (2.8073-3.3613)
At least 3 of CHASM (lung), FATHMM (missense), PolyPhen-2, PROVEAN, SIFT, VEST	73.03% (69.7%-76.26%)	86.17% (82.93%-89.52%)	50.292% (43.478%-56.522%)	74.98% (71.23%-78.91%)	67.78% (60.78%-74.47%)	2.7922 (2.6380-2.9341)	73.13% (66.67%-79.8%)	86.15% (79.24%-92.43%)	50.737% (37.838%-62.86%)	75.01% (67.12%-82.44%)	68.12% (54.54%-81.48%)	2.8001 (2.5077-3.0858)
At least 3 of CHASM (lung), Mutation Assessor, MutationTaster, PolyPhen-2, PROVEAN, SIFT	76.73% (73.23%-79.8%)	82.97% (79.36%-86.89%)	65.954% (59.722%-72.467%)	80.82% (76.86%-84.79%)	69.13% (63.23%-75.34%)	2.9887 (2.8419-3.1347)	76.84% (70.71%-83.84%)	82.98% (75.7%-90.16%)	66.273% (53.125%-78.79%)	80.84% (72.88%-88.52%)	69.43% (57.13%-80.77%)	2.9952 (2.7019-3.2779)
At least 3 of CHASM (lung), Mutation Assessor, MutationTaster, PolyPhen-2, PROVEAN, VEST	75.07% (71.72%-78.28%)	82.41% (78.74%-86.4%)	62.372% (56.164%-68.753%)	79.11% (75%-83.08%)	67.22% (60.94%-73.62%)	2.9111 (2.7655-3.0541)	75.12% (68.69%-81.82%)	82.51% (74.6%-89.83%)	62.452% (50%-75.76%)	79.02% (71.21%-86.76%)	67.54% (54.54%-80%)	2.9152 (2.6282-3.2062)
At least 3 of CHASM (lung), Mutation Assessor, MutationTaster, PolyPhen-2, SIFT, VEST	73% (69.7%-76.26%)	83.97% (80.48%-87.69%)	54.032% (47.297%-60.274%)	75.95% (71.85%-80%)	66.09% (59.65%-72.88%)	2.8005 (2.6515-2.9452)	73.19% (66.67%-79.8%)	84.17% (76.66%-91.05%)	54.34% (41.929%-67.74%)	75.97% (67.65%-84.13%)	66.68% (53.12%-79.17%)	2.8117 (2.5262-3.1103)
At least 3 of CHASM (lung), Mutation Assessor, MutationTaster, PROVEAN, SIFT, VEST	75.05% (71.72%-78.28%)	80.84% (77.17%-84.68%)	65.037% (58.664%-71.233%)	79.99% (75.97%-83.72%)	66.25% (60.29%-72.37%)	2.9211 (2.7734-3.0611)	75.16% (68.69%-81.82%)	80.86% (73.01%-88.14%)	65.35% (52.941%-77.78%)	80.01% (72.13%-87.93%)	66.55% (53.33%-78.13%)	2.9277 (2.6376-3.2161)
At least 3 of CHASM (lung), Mutation Assessor, PolyPhen-2, PROVEAN, SIFT, VEST	70.64% (67.17%-73.75%)	80.29% (76.38%-84.17%)	53.958% (47.436%-60.563%)	75.09% (70.99%-79.26%)	61.29% (54.84%-68.25%)	2.7063 (2.5614-2.8479)	70.84% (64.62%-77.78%)	80.36% (72.41%-88.06%)	54.494% (41.935%-67.5%)	75.18% (66.67%-83.58%)	61.78% (48.48%-74.19%)	2.7181 (2.4237-3.0023)
At least 3 of CHASM (lung), MutationTaster, PolyPhen-2, PROVEAN, SIFT, VEST	72.67% (69.19%-76.26%)	83.97% (80.51%-87.7%)	53.121% (46.154%-59.42%)	75.59% (71.53%-79.72%)	65.72% (59.26%-72.42%)	2.7840 (2.6333-2.9231)	72.84% (65.66%-79.8%)	84.17% (76.66%-90.91%)	53.389% (40.625%-66.67%)	75.6% (67.12%-83.59%)	66.27% (52.77%-78.8%)	2.7942 (2.4909-3.0852)
At least 3 of CHASM (melanoma), FATHMM (cancer), FATHMM (missense), Mutation Assessor, MutationTaster, PolyPhen-2	84.88% (82.32%-87.88%)	98.44% (97.52%-100%)	61.445% (55.072%-67.659%)	81.53% (78.15%-85.14%)	95.79% (93.02%-100%)	3.3720 (3.2727-3.4772)	84.78% (78.79%-89.9%)	98.34% (95.45%-100%)	61.49% (48.714%-74.29%)	81.42% (74.65%-88.16%)	95.58% (88.24%-100%)	3.3683 (3.1510-3.5717)
At least 3 of CHASM (melanoma), FATHMM (cancer), FATHMM (missense), Mutation Assessor, MutationTaster, PROVEAN	86.87% (84.34%-89.39%)	96.34% (94.49%-98.43%)	70.498% (64.384%-76.814%)	84.96% (81.69%-88.49%)	91.77% (87.93%-96.36%)	3.4357 (3.3275-3.5512)	86.86% (81.82%-91.92%)	96.14% (91.8%-100%)	70.916% (58.327%-83.33%)	85.01% (77.92%-91.43%)	91.47% (82.61%-100%)	3.4354 (3.2019-3.6549)
At least 3 of CHASM (melanoma), FATHMM (cancer), FATHMM (missense), Mutation Assessor, MutationTaster, SIFT	84.51% (81.82%-87.37%)	97.92% (96.69%-99.24%)	61.315% (54.926%-67.607%)	81.4% (77.92%-85.03%)	94.47% (91.11%-98.04%)	3.3511 (3.2413-3.4619)	84.52% (78.79%-89.9%)	97.77% (94.73%-100%)	61.749% (48.571%-74.36%)	81.44% (74.32%-88.57%)	94.18% (85.71%-100%)	3.3513 (3.1359-3.5723)
At least 3 of CHASM (melanoma), FATHMM (cancer), FATHMM (missense), Mutation Assessor, MutationTaster, VEST	83.18% (80.3%-86.36%)	98.47% (97.52%-100%)	56.742% (50.649%-63.38%)	79.74% (76.13%-83.34%)	95.55% (92.5%-100%)	3.3050 (3.1991-3.4177)	83.13% (76.77%-88.89%)	98.27% (95.38%-100%)	57.129% (43.746%-69.7%)	79.73% (72.6%-86.84%)	95.05% (86.96%-100%)	3.3018 (3.0801-3.5199)
At least 3 of CHASM (melanoma), FATHMM (cancer), FATHMM (missense), Mutation Assessor, PolyPhen-2, PROVEAN	83.2% (80.3%-86.36%)	95.27% (93.39%-97.56%)	62.312% (55.844%-68.831%)	81.38% (77.85%-84.93%)	88.41% (83.67%-93.75%)	3.2738 (3.1490-3.3938)	83.1% (76.77%-88.89%)	95.08% (90.74%-98.51%)	62.509% (50%-75.76%)	81.32% (73.97%-88.58%)	88.12% (77.27%-96.55%)	3.2702 (3.0181-3.5233)
At least 3 of CHASM (melanoma), FATHMM (cancer), FATHMM (missense), Mutation Assessor, PolyPhen-2, SIFT	80.81% (77.78%-83.84%)	96.86% (95.16%-98.49%)	53.063% (46.664%-59.376%)	78.11% (74.34%-81.82%)	90.71% (86.05%-95.65%)	3.1874 (3.0739-3.3117)	80.8% (74.75%-86.87%)	96.71% (92.85%-100%)	53.483% (40%-66.67%)	78.11% (70.51%-85.53%)	90.49% (78.95%-100%)	3.1879 (2.9390-3.4200)
At least 3 of CHASM (melanoma), FATHMM (cancer), FATHMM (missense), Mutation Assessor, PolyPhen-2, VEST	81.16% (78.28%-84.34%)	97.93% (96.72%-99.22%)	52.142% (45.588%-58.826%)	77.96% (74.21%-81.7%)	93.59% (89.74%-97.67%)	3.2163 (3.1061-3.3325)	81.12% (74.75%-86.87%)	97.74% (94.91%-100%)	52.589% (39.987%-65.12%)	77.96% (70.51%-85.19%)	93.14% (83.33%-100%)	3.2143 (2.9709-3.4293)
At least 3 of CHASM (melanoma), FATHMM (cancer), FATHMM (missense), Mutation Assessor, PROVEAN, SIFT	81.5% (78.28%-84.34%)	94.21% (92%-96.67%)	59.515% (53.422%-66.198%)	80.09% (76.67%-83.79%)	85.59% (80.35%-90.91%)	3.1941 (3.0720-3.3237)	81.45% (75.76%-87.88%)	94.02% (89.23%-98.41%)	59.82% (45.946%-72.22%)	80.07% (72.37%-86.84%)	85.37% (73.91%-96%)	3.1928 (2.9309-3.4496)
At least 3 of CHASM (melanoma), FATHMM (cancer), FATHMM (missense), Mutation Assessor, PROVEAN, VEST	81.5% (78.28%-84.85%)	95.8% (93.91%-97.74%)	56.748% (50.649%-63.019%)	79.3% (75.68%-82.99%)	88.67% (83.99%-94%)	3.2052 (3.0838-3.3325)	81.45% (74.75%-87.88%)	95.62% (91.18%-98.66%)	57.11% (44.436%-69.24%)	79.28% (71.79%-86.59%)	88.38% (77.26%-96.85%)	3.2039 (2.9530-3.4530)
At least 3 of CHASM (melanoma), FATHMM (cancer), FATHMM (missense), Mutation Assessor, SIFT, VEST	79.47% (76.26%-82.83%)	96.34% (94.49%-98.39%)	50.299% (43.477%-56.757%)	77.02% (73.42%-80.65%)	88.84% (83.72%-95%)	3.1251 (3.0039-3.2589)	79.44% (72.73%-85.86%)	96.13% (92.18%-100%)	50.759% (37.493%-63.64%)	77.01% (69.33%-84.21%)	88.46% (76.46%-100%)	3.1236 (2.8316-3.3681)
At least 3 of CHASM (melanoma), FATHMM (cancer), FATHMM (missense), MutationTaster, PolyPhen-2, PROVEAN	85.22% (82.32%-88.38%)	97.4% (95.93%-99.19%)	64.142% (57.971%-70.588%)	82.45% (79.02%-86.11%)	93.45% (89.58%-97.96%)	3.3744 (3.2668-3.4916)	85.12% (78.79%-90.91%)	97.22% (93.85%-100%)	64.338% (51.427%-76.92%)	82.4% (75.3%-89.33%)	93.09% (84.21%-100%)	3.3705 (3.1423-3.5928)
At least 3 of CHASM (melanoma), FATHMM (cancer), FATHMM (missense), MutationTaster, PolyPhen-2, SIFT	81.83% (78.79%-84.85%)	98.44% (97.52%-100%)	53.101% (46.575%-59.459%)	78.4% (74.84%-81.99%)	95.16% (91.89%-100%)	3.2509 (3.1443-3.3624)	81.8% (75.76%-87.88%)	98.34% (95.45%-100%)	53.401% (40.623%-66.67%)	78.37% (71.05%-85.19%)	94.95% (86.66%-100%)	3.2505 (3.0235-3.4679)
At least 3 of CHASM (melanoma), FATHMM (cancer), FATHMM (missense), MutationTaster, PolyPhen-2, VEST	82.89% (79.8%-85.86%)	98.47% (97.52%-100%)	55.937% (50%-62.319%)	79.44% (75.84%-83.22%)	95.49% (92.5%-100%)	3.2934 (3.1925-3.4033)	82.71% (76.77%-88.89%)	98.27% (95.38%-100%)	56.003% (43.234%-68.3%)	79.31% (71.79%-86.3%)	94.96% (87.49%-100%)	3.2854 (3.0714-3.4913)
At least 3 of CHASM (melanoma), FATHMM (cancer), FATHMM (missense), MutationTaster, PROVEAN, SIFT	84.85% (82.32%-87.88%)	96.88% (95.16%-98.5%)	64.038% (57.742%-70.423%)	82.33% (78.85%-86.02%)	92.25% (88.1%-96.37%)	3.3550 (3.2405-3.4707)	84.84% (78.79%-89.9%)	96.64% (92.73%-100%)	64.528% (51.429%-77.78%)	82.4% (75.32%-89.47%)	91.83% (82.61%-100%)	3.3540 (3.1186-3.5797)
At least 3 of CHASM (melanoma), FATHMM (cancer), FATHMM (missense), MutationTaster, PROVEAN, VEST	84.2% (81.31%-87.37%)	97.93% (96.72%-99.24%)	60.429% (54.054%-66.667%)	81.06% (77.42%-84.67%)	94.42% (90.91%-97.96%)	3.3385 (3.2266-3.4493)	84.13% (77.78%-89.9%)	97.74% (94.74%-100%)	60.739% (48.384%-73.17%)	81.04% (73.68%-88.24%)	94% (85.71%-100%)	3.3353 (3.1095-3.5579)
At least 3 of CHASM (melanoma), FATHMM (cancer), FATHMM (missense), MutationTaster, SIFT, VEST	81.84% (78.79%-84.85%)	98.99% (98.33%-100%)	52.187% (45.714%-58.571%)	78.17% (74.53%-81.94%)	96.77% (94.29%-100%)	3.2611 (3.1638-3.3664)	81.77% (75.76%-87.88%)	98.82% (96.67%-100%)	52.476% (40%-65.71%)	78.11% (70.88%-85%)	96.31% (88.89%-100%)	3.2573 (3.0476-3.4598)
At least 3 of CHASM (melanoma), FATHMM (cancer), FATHMM (missense), PolyPhen-2, PROVEAN, SIFT	80.84% (77.78%-83.84%)	95.82% (94.02%-97.67%)	54.941% (48.648%-61.251%)	78.62% (75%-82.31%)	88.38% (83.33%-93.75%)	3.1776 (3.0539-3.3086)	80.74% (74.75%-86.87%)	95.59% (91.52%-98.59%)	55.212% (42.094%-68.75%)	78.56% (71.05%-85.9%)	87.96% (75.98%-96.67%)	3.1732 (2.9054-3.4168)
At least 3 of CHASM (melanoma), FATHMM (cancer), FATHMM (missense), PolyPhen-2, PROVEAN, VEST	81.18% (78.28%-84.34%)	97.4% (95.93%-99.19%)	53.124% (46.476%-59.459%)	78.23% (74.53%-81.94%)	92.2% (87.8%-97.5%)	3.2095 (3.0929-3.3279)	81.08% (74.75%-86.87%)	97.22% (93.85%-100%)	53.343% (40%-66.67%)	78.15% (70.67%-85.37%)	91.78% (81.24%-100%)	3.2049 (2.9607-3.4399)
At least 3 of CHASM (melanoma), FATHMM (cancer), FATHMM (missense), PolyPhen-2, SIFT, VEST	79.11% (75.76%-82.32%)	98.44% (97.5%-100%)	45.675% (39.129%-52.114%)	75.81% (72.12%-79.5%)	94.44% (90.62%-100%)	3.1436 (3.0348-3.2541)	79.15% (72.73%-85.86%)	98.32% (95.45%-100%)	46.217% (33.333%-59.38%)	75.83% (68.67%-83.33%)	94.14% (84.62%-100%)	3.1451 (2.9189-3.3675)
At least 3 of CHASM (melanoma), FATHMM (cancer), FATHMM (missense), PROVEAN, SIFT, VEST	79.83% (76.77%-83.33%)	96.35% (94.49%-98.4%)	51.269% (44.869%-57.746%)	77.37% (73.85%-81.13%)	89.04% (84.09%-95.24%)	3.1402 (3.0159-3.2647)	79.73% (72.73%-85.86%)	96.13% (92.06%-100%)	51.541% (38.228%-64.3%)	77.3% (69.73%-84.29%)	88.59% (76.47%-100%)	3.1356 (2.8737-3.3838)
At least 3 of CHASM (melanoma), FATHMM (cancer), Mutation Assessor, MutationTaster, PolyPhen-2, PROVEAN	80.76% (77.78%-83.84%)	88.26% (85.24%-91.48%)	67.79% (61.644%-74.286%)	82.57% (78.91%-86.16%)	76.95% (71.21%-83.1%)	3.1557 (3.0159-3.3036)	80.91% (74.75%-86.87%)	88.37% (81.67%-94.65%)	68.094% (55%-80.01%)	82.61% (75.36%-89.86%)	77.34% (64.86%-88.89%)	3.1642 (2.8754-3.4260)
At least 3 of CHASM (melanoma), FATHMM (cancer), Mutation Assessor, MutationTaster, PolyPhen-2, SIFT	80.44% (77.27%-83.84%)	91.45% (88.71%-94.4%)	61.392% (55.072%-67.655%)	80.38% (76.47%-84.06%)	80.61% (74.55%-87.27%)	3.1382 (3.0089-3.2779)	80.54% (73.74%-86.87%)	91.56% (85.71%-96.88%)	61.631% (48.485%-74.29%)	80.37% (72.86%-87.84%)	81% (68%-92.59%)	3.1456 (2.8586-3.3989)
At least 3 of CHASM (melanoma), FATHMM (cancer), Mutation Assessor, MutationTaster, PolyPhen-2, VEST	77.78% (74.75%-81.31%)	88.79% (85.71%-92%)	58.746% (52.306%-65.217%)	78.82% (75%-82.73%)	75.2% (69.09%-81.63%)	3.0155 (2.8837-3.1593)	77.77% (70.71%-83.84%)	88.89% (82.54%-95.16%)	58.667% (45.161%-71.43%)	78.67% (70.83%-86.3%)	75.49% (61.54%-87.51%)	3.0173 (2.7083-3.2872)
At least 3 of CHASM (melanoma), FATHMM (cancer), Mutation Assessor, MutationTaster, PROVEAN, SIFT	80.77% (77.78%-83.84%)	90.4% (87.7%-93.44%)	64.129% (57.967%-70.667%)	81.33% (77.7%-85%)	79.45% (73.68%-85.46%)	3.1531 (3.0170-3.2883)	80.87% (74.75%-86.87%)	90.46% (84.38%-95.65%)	64.41% (51.351%-76.48%)	81.34% (73.97%-88.74%)	79.78% (67.74%-90.62%)	3.1600 (2.8904-3.4195)
At least 3 of CHASM (melanoma), FATHMM (cancer), Mutation Assessor, MutationTaster, PROVEAN, VEST	79.78% (76.77%-82.83%)	87.75% (84.55%-90.99%)	65.99% (60.274%-72.222%)	81.69% (77.86%-85.29%)	75.71% (70%-81.43%)	3.1114 (2.9822-3.2504)	79.84% (73.74%-85.86%)	87.78% (81.03%-93.94%)	66.19% (53.846%-77.78%)	81.66% (74.29%-88.89%)	75.96% (63.33%-87.5%)	3.1158 (2.8316-3.3710)
At least 3 of CHASM (melanoma), FATHMM (cancer), Mutation Assessor, MutationTaster, SIFT, VEST	79.07% (76.26%-82.32%)	91.43% (88.71%-94.36%)	57.692% (51.315%-64.286%)	78.89% (75.18%-82.73%)	79.57% (73.58%-86.01%)	3.0758 (2.9409-3.2145)	79.24% (72.73%-84.85%)	91.6% (85.71%-96.88%)	58.018% (45%-70.27%)	78.92% (71.64%-86.3%)	80.14% (66.67%-92%)	3.0867 (2.7965-3.3501)
At least 3 of CHASM (melanoma), FATHMM (cancer), Mutation Assessor, PolyPhen-2, PROVEAN, SIFT	76.76% (73.23%-79.81%)	87.23% (84.12%-90.55%)	58.665% (52.239%-65.153%)	78.49% (74.64%-82.61%)	72.66% (66.1%-78.95%)	2.9705 (2.8240-3.1116)	76.77% (70.68%-83.84%)	87.22% (80.36%-93.55%)	58.833% (45.714%-71.43%)	78.42% (70.42%-86.57%)	72.87% (60.71%-85.71%)	2.9735 (2.6764-3.2582)
At least 3 of CHASM (melanoma), FATHMM (cancer), Mutation Assessor, PolyPhen-2, PROVEAN, VEST	73.07% (69.7%-76.28%)	83.51% (80.16%-87.22%)	55.013% (48.683%-61.336%)	76.24% (72.06%-80.43%)	65.87% (59.02%-72.42%)	2.8064 (2.6559-2.9563)	73.06% (66.64%-79.8%)	83.5% (75.75%-90.39%)	55.134% (42.101%-67.65%)	76.14% (68.11%-84.51%)	66.07% (52%-79.43%)	2.8085 (2.4985-3.1044)
At least 3 of CHASM (melanoma), FATHMM (cancer), Mutation Assessor, PolyPhen-2, SIFT, VEST	73.32% (69.7%-76.77%)	88.24% (85%-91.34%)	47.506% (41.026%-53.623%)	74.4% (70.39%-78.53%)	70.05% (62.75%-77.08%)	2.8020 (2.6410-2.9535)	73.56% (66.67%-80.81%)	88.39% (81.96%-95%)	48.114% (35.893%-61.12%)	74.5% (66.22%-82.9%)	70.76% (56.24%-85.71%)	2.8177 (2.5193-3.1379)
At least 3 of CHASM (melanoma), FATHMM (cancer), Mutation Assessor, PROVEAN, SIFT, VEST	74.74% (71.21%-77.78%)	85.15% (81.75%-88.7%)	56.741% (50.667%-63.514%)	77.29% (73.38%-81.29%)	68.84% (62.26%-75.52%)	2.8802 (2.7305-3.0174)	74.76% (68.69%-81.82%)	85.01% (77.78%-91.67%)	57.169% (44.112%-70.01%)	77.3% (69.12%-85.08%)	68.97% (55.55%-82.14%)	2.8845 (2.5964-3.1708)
At least 3 of CHASM (melanoma), FATHMM (cancer), MutationTaster, PolyPhen-2, PROVEAN, SIFT	79.77% (76.77%-82.83%)	90.38% (87.6%-93.55%)	61.423% (55.263%-67.606%)	80.2% (76.26%-83.92%)	78.71% (72.88%-84.91%)	3.1071 (2.9793-3.2376)	79.85% (73.74%-85.86%)	90.5% (84.37%-96.49%)	61.572% (48.571%-74.19%)	80.16% (72.6%-87.84%)	79.11% (66.67%-90.91%)	3.1134 (2.8423-3.3710)
At least 3 of CHASM (melanoma), FATHMM (cancer), MutationTaster, PolyPhen-2, PROVEAN, VEST	76.12% (72.73%-79.8%)	87.22% (84%-90.4%)	56.931% (50.685%-63.38%)	77.78% (73.76%-81.69%)	72.04% (66.07%-78.43%)	2.9398 (2.8042-3.0896)	76.04% (68.69%-82.83%)	87.25% (80.33%-93.65%)	56.785% (43.331%-69.46%)	77.59% (69.56%-85.53%)	72.19% (59.07%-85%)	2.9382 (2.6350-3.2090)
At least 3 of CHASM (melanoma), FATHMM (cancer), MutationTaster, PolyPhen-2, SIFT, VEST	76.37% (73.23%-79.8%)	91.95% (89.31%-94.66%)	49.425% (42.857%-55.563%)	75.86% (71.9%-79.87%)	78.03% (71.43%-84.91%)	2.9526 (2.8145-3.0822)	76.56% (69.7%-82.83%)	92.16% (86.44%-97.06%)	49.753% (37.493%-62.5%)	75.89% (68.05%-83.79%)	78.77% (64%-92.32%)	2.9657 (2.6846-3.2378)
At least 3 of CHASM (melanoma), FATHMM (cancer), MutationTaster, PROVEAN, SIFT, VEST	77.74% (74.75%-80.81%)	89.86% (86.99%-92.8%)	56.775% (50.617%-63.235%)	78.23% (74.48%-82.19%)	76.42% (70.37%-82.98%)	3.0130 (2.8761-3.1449)	77.85% (71.72%-83.84%)	89.94% (83.93%-95.61%)	57.105% (44.825%-69.77%)	78.25% (70.27%-85.72%)	76.83% (63.64%-90%)	3.0212 (2.7404-3.2891)
At least 3 of CHASM (melanoma), FATHMM (cancer), PolyPhen-2, PROVEAN, SIFT, VEST	73.35% (69.7%-76.77%)	87.21% (84.03%-90.55%)	49.363% (43.239%-55.409%)	74.86% (70.83%-78.77%)	69.08% (62%-75.93%)	2.8052 (2.6575-2.9439)	73.51% (66.67%-80.81%)	87.26% (80.33%-93.65%)	49.885% (37.208%-62.5%)	74.92% (67.12%-83.1%)	69.56% (55.54%-83.33%)	2.8163 (2.5323-3.1221)
At least 3 of CHASM (melanoma), FATHMM (missense), Mutation Assessor, MutationTaster, PolyPhen-2, PROVEAN	78.77% (75.76%-82.32%)	86.67% (83.33%-90.17%)	65.114% (58.902%-71.233%)	81.11% (77.44%-84.8%)	73.86% (67.86%-80%)	3.0675 (2.9297-3.2105)	78.82% (71.72%-84.85%)	86.76% (79.66%-93.55%)	65.179% (51.724%-77.14%)	81.04% (73.53%-88.41%)	74.17% (62.06%-86.21%)	3.0715 (2.7813-3.3366)
At least 3 of CHASM (melanoma), FATHMM (missense), Mutation Assessor, MutationTaster, PolyPhen-2, SIFT	79.1% (75.76%-82.32%)	90.41% (87.69%-93.5%)	59.552% (53.03%-65.789%)	79.44% (75.69%-83.1%)	78.22% (72.22%-84.62%)	3.0763 (2.9420-3.2057)	79.17% (72.73%-85.86%)	90.45% (84.38%-96.08%)	59.771% (46.875%-72.52%)	79.42% (72%-87.02%)	78.52% (66.64%-90.32%)	3.0816 (2.8132-3.3462)
At least 3 of CHASM (melanoma), FATHMM (missense), Mutation Assessor, MutationTaster, PolyPhen-2, VEST	78.09% (75.24%-81.31%)	89.33% (86.4%-92.37%)	58.665% (52.172%-65.217%)	78.89% (75%-82.76%)	76.07% (70%-82.46%)	3.0295 (2.8954-3.1683)	78.16% (71.72%-83.86%)	89.42% (83.33%-95.31%)	58.817% (45.161%-71.79%)	78.84% (71.01%-86.67%)	76.43% (62.85%-88.89%)	3.0350 (2.7502-3.3060)
At least 3 of CHASM (melanoma), FATHMM (missense), Mutation Assessor, MutationTaster, PROVEAN, SIFT	80.11% (76.77%-83.33%)	88.79% (85.71%-92%)	65.081% (58.9%-71.233%)	81.47% (77.78%-85.08%)	77.06% (71.19%-83.05%)	3.1241 (2.9835-3.2574)	80.19% (73.74%-86.87%)	88.88% (82.14%-94.92%)	65.225% (52.778%-77.51%)	81.44% (74.24%-88.74%)	77.4% (65.22%-88.57%)	3.1295 (2.8538-3.4073)
At least 3 of CHASM (melanoma), FATHMM (missense), Mutation Assessor, MutationTaster, PROVEAN, VEST	77.44% (74.24%-80.81%)	87.23% (84%-90.62%)	60.496% (54.167%-66.667%)	79.24% (75.37%-82.98%)	73.27% (67.24%-79.66%)	3.0024 (2.8609-3.1419)	77.45% (70.71%-83.84%)	87.22% (79.99%-93.65%)	60.644% (48.132%-73.08%)	79.18% (71.64%-86.49%)	73.47% (60%-85.71%)	3.0051 (2.7112-3.2800)
At least 3 of CHASM (melanoma), FATHMM (missense), Mutation Assessor, MutationTaster, SIFT, VEST	77.37% (74.24%-80.3%)	91.45% (88.8%-94.4%)	53.006% (46.575%-59.722%)	77.09% (73.33%-80.8%)	78.21% (71.99%-85.11%)	2.9976 (2.8542-3.1385)	77.59% (71.72%-83.84%)	91.55% (85.71%-96.88%)	53.592% (39.391%-65.86%)	77.2% (69.56%-84.93%)	78.76% (65.38%-91.67%)	3.0111 (2.7146-3.2860)
At least 3 of CHASM (melanoma), FATHMM (missense), Mutation Assessor, PolyPhen-2, PROVEAN, SIFT	75.74% (72.22%-78.79%)	86.15% (82.93%-89.6%)	57.712% (51.388%-64.288%)	77.89% (73.94%-81.88%)	70.69% (64.29%-76.79%)	2.9245 (2.7823-3.0631)	75.8% (69.7%-82.83%)	86.18% (78.95%-92.54%)	57.952% (44.737%-70.59%)	77.86% (70%-85.71%)	70.98% (58.33%-83.33%)	2.9297 (2.6486-3.2153)
At least 3 of CHASM (melanoma), FATHMM (missense), Mutation Assessor, PolyPhen-2, PROVEAN, VEST	71.71% (68.18%-75.25%)	81.92% (78.29%-85.83%)	54.036% (47.616%-60.494%)	75.5% (71.53%-79.7%)	63.35% (56.67%-70.18%)	2.7481 (2.5992-2.9008)	71.74% (64.65%-78.79%)	81.88% (73.77%-89.23%)	54.305% (41.374%-67.5%)	75.46% (67.14%-83.58%)	63.59% (50%-76.67%)	2.7524 (2.4346-3.0415)
At least 3 of CHASM (melanoma), FATHMM (missense), Mutation Assessor, PolyPhen-2, SIFT, VEST	71.97% (68.18%-75.76%)	87.2% (83.9%-90.37%)	45.628% (39.13%-52.174%)	73.5% (69.39%-77.7%)	67.34% (59.99%-74.42%)	2.7366 (2.5730-2.8935)	72.22% (64.65%-79.8%)	87.28% (80.6%-93.65%)	46.339% (33.333%-59.39%)	73.62% (65.33%-81.94%)	68% (52.63%-82.61%)	2.7524 (2.4348-3.0687)
At least 3 of CHASM (melanoma), FATHMM (missense), Mutation Assessor, PROVEAN, SIFT, VEST	72.36% (68.69%-75.76%)	83.54% (79.84%-87.4%)	53.015% (46.753%-59.459%)	75.46% (71.32%-79.56%)	65.07% (58.33%-71.93%)	2.7708 (2.6167-2.9086)	72.45% (65.66%-79.8%)	83.43% (75.76%-90.33%)	53.566% (40%-65.71%)	75.51% (67.6%-83.34%)	65.32% (51.72%-78.12%)	2.7783 (2.4867-3.0756)
At least 3 of CHASM (melanoma), FATHMM (missense), MutationTaster, PolyPhen-2, PROVEAN, SIFT	79.11% (76.25%-81.83%)	89.34% (86.32%-92.43%)	61.408% (54.795%-67.655%)	80.01% (76.26%-83.69%)	76.92% (70.91%-83.02%)	3.0768 (2.9394-3.1989)	79.16% (73.71%-84.87%)	89.4% (83.04%-95.38%)	61.548% (48.384%-74.38%)	79.97% (72.46%-87.5%)	77.22% (65.22%-88.89%)	3.0814 (2.8258-3.3506)
At least 3 of CHASM (melanoma), FATHMM (missense), MutationTaster, PolyPhen-2, PROVEAN, VEST	77.12% (73.74%-80.3%)	87.76% (84.68%-91.06%)	58.725% (52.174%-64.865%)	78.61% (74.81%-82.58%)	73.51% (67.24%-80%)	2.9860 (2.8484-3.1299)	77.07% (70.71%-83.84%)	87.77% (81.24%-93.94%)	58.681% (45.714%-72.22%)	78.47% (70.83%-86.11%)	73.67% (60.71%-86.21%)	2.9860 (2.6897-3.2571)
At least 3 of CHASM (melanoma), FATHMM (missense), MutationTaster, PolyPhen-2, SIFT, VEST	77.04% (73.74%-80.3%)	92.48% (90%-95.28%)	50.318% (43.662%-56.528%)	76.3% (72.55%-80.25%)	79.48% (73.17%-86.37%)	2.9858 (2.8461-3.1235)	77.24% (70.71%-83.84%)	92.69% (86.89%-98.15%)	50.704% (37.5%-63.64%)	76.34% (68.42%-83.57%)	80.21% (65.21%-93.33%)	2.9994 (2.7158-3.2638)
At least 3 of CHASM (melanoma), FATHMM (missense), MutationTaster, PROVEAN, SIFT, VEST	76.75% (73.74%-79.8%)	89.89% (86.89%-92.97%)	54.016% (47.826%-60.811%)	77.17% (73.47%-80.85%)	75.56% (69.23%-82.35%)	2.9663 (2.8267-3.1026)	76.81% (70.71%-82.83%)	89.89% (83.33%-95.71%)	54.301% (41.379%-66.67%)	77.15% (69.86%-84.42%)	75.84% (61.9%-89.66%)	2.9719 (2.6841-3.2421)
At least 3 of CHASM (melanoma), FATHMM (missense), PolyPhen-2, PROVEAN, SIFT, VEST	71.69% (68.18%-75.25%)	86.17% (82.93%-89.52%)	46.639% (40.271%-53.088%)	73.63% (69.72%-77.55%)	66.11% (59.09%-72.92%)	2.7255 (2.5741-2.8646)	71.77% (64.65%-78.79%)	86.15% (79.24%-92.43%)	47.026% (34.286%-60%)	73.63% (65.75%-81.43%)	66.45% (52.17%-80.02%)	2.7326 (2.4355-3.0331)
At least 3 of CHASM (melanoma), Mutation Assessor, MutationTaster, PolyPhen-2, PROVEAN, SIFT	75.06% (71.72%-78.28%)	82.97% (79.36%-86.89%)	61.39% (55.072%-68.056%)	78.79% (74.8%-82.95%)	67.58% (61.54%-73.91%)	2.9073 (2.7636-3.0558)	75.13% (68.69%-81.82%)	82.98% (75.7%-90.16%)	61.63% (48.485%-74.2%)	78.77% (70.77%-86.57%)	67.86% (54.83%-80%)	2.9124 (2.6174-3.1852)
At least 3 of CHASM (melanoma), Mutation Assessor, MutationTaster, PolyPhen-2, PROVEAN, VEST	73.73% (70.2%-77.27%)	82.94% (79.48%-86.96%)	57.807% (51.27%-64.18%)	77.27% (73.13%-81.34%)	66.22% (59.68%-73.13%)	2.8423 (2.6939-2.9875)	73.75% (66.67%-80.81%)	83.04% (74.99%-90.32%)	57.801% (44.444%-70.74%)	77.15% (69.01%-85.25%)	66.51% (52.77%-79.41%)	2.8450 (2.5471-3.1405)
At least 3 of CHASM (melanoma), Mutation Assessor, MutationTaster, PolyPhen-2, SIFT, VEST	72.68% (69.19%-76.26%)	84.5% (80.95%-88.33%)	52.241% (45.455%-58.336%)	75.36% (71.22%-79.56%)	66.09% (59.26%-73.22%)	2.7820 (2.6330-2.9277)	72.82% (65.66%-79.8%)	84.71% (77.05%-91.67%)	52.413% (40%-65.74%)	75.33% (67.14%-83.57%)	66.64% (52.17%-80.77%)	2.7909 (2.4901-3.0825)
At least 3 of CHASM (melanoma), Mutation Assessor, MutationTaster, PROVEAN, SIFT, VEST	74.05% (70.71%-77.27%)	81.37% (77.78%-85.27%)	61.396% (55.263%-67.648%)	78.47% (74.42%-82.58%)	65.58% (59.32%-72.13%)	2.8681 (2.7213-3.0120)	74.12% (67.68%-80.81%)	81.4% (73.21%-88.52%)	61.613% (48.571%-74.19%)	78.44% (70.49%-86.37%)	65.86% (52.78%-78.12%)	2.8731 (2.5754-3.1592)
At least 3 of CHASM (melanoma), Mutation Assessor, PolyPhen-2, PROVEAN, SIFT, VEST	70.32% (66.67%-73.74%)	80.29% (76.38%-84.17%)	53.071% (46.154%-59.421%)	74.73% (70.5%-78.95%)	60.89% (54.24%-67.74%)	2.6899 (2.5424-2.8400)	70.48% (63.64%-77.78%)	80.36% (72.41%-88.06%)	53.51% (41.016%-66.67%)	74.78% (66.2%-83.33%)	61.34% (48.15%-74.19%)	2.6999 (2.3978-2.9903)
At least 3 of CHASM (melanoma), MutationTaster, PolyPhen-2, PROVEAN, SIFT, VEST	72.35% (69.18%-75.76%)	84.5% (81.1%-88.28%)	51.338% (44.872%-57.579%)	75.02% (70.8%-79.17%)	65.71% (59.01%-72.55%)	2.7657 (2.6209-2.9176)	72.47% (65.66%-78.81%)	84.7% (76.67%-91.53%)	51.45% (37.931%-65%)	74.96% (66.67%-83.12%)	66.22% (52%-80%)	2.7733 (2.4702-3.0664)
At least 3 of FATHMM (cancer), FATHMM (missense), Mutation Assessor, MutationTaster, PolyPhen-2, PROVEAN	81.09% (78.28%-83.85%)	88.26% (85.04%-91.41%)	68.686% (62.853%-75%)	82.97% (79.36%-86.72%)	77.19% (71.43%-83.1%)	3.1711 (3.0434-3.3067)	81.25% (75.73%-86.87%)	88.36% (81.96%-95%)	69.04% (56.757%-80.78%)	83.04% (75.38%-90.54%)	77.57% (65.37%-88.89%)	3.1802 (2.8993-3.4339)
At least 3 of FATHMM (cancer), FATHMM (missense), Mutation Assessor, MutationTaster, PolyPhen-2, SIFT	79.41% (76.26%-82.32%)	91.46% (88.71%-94.35%)	58.579% (52.495%-64.868%)	79.24% (75.54%-82.89%)	79.87% (73.58%-86.27%)	3.0915 (2.9523-3.2250)	79.56% (73.74%-85.86%)	91.54% (85.94%-96.88%)	58.967% (45.714%-71.43%)	79.29% (71.83%-86.96%)	80.29% (67.74%-92.59%)	3.1010 (2.8180-3.3750)
At least 3 of FATHMM (cancer), FATHMM (missense), Mutation Assessor, MutationTaster, PolyPhen-2, VEST	76.39% (73.23%-79.8%)	89.33% (86.4%-92.37%)	54.022% (47.824%-60.294%)	77.06% (72.97%-80.99%)	74.54% (68%-81.26%)	2.9495 (2.8033-3.0905)	76.51% (69.7%-82.83%)	89.42% (83.33%-95.31%)	54.347% (40%-66.69%)	77.06% (69.23%-85.08%)	74.98% (60.87%-88%)	2.9581 (2.6655-3.2416)
At least 3 of FATHMM (cancer), FATHMM (missense), Mutation Assessor, MutationTaster, PROVEAN, SIFT	81.07% (77.78%-84.34%)	90.41% (87.6%-93.5%)	64.922% (59.091%-71.622%)	81.67% (77.94%-85.42%)	79.66% (74.14%-85.49%)	3.1667 (3.0345-3.3036)	81.29% (74.75%-87.88%)	90.44% (84.38%-96.3%)	65.566% (52.5%-77.14%)	81.84% (74.32%-89.04%)	80.02% (67.86%-90.93%)	3.1785 (2.8969-3.4399)
At least 3 of FATHMM (cancer), FATHMM (missense), Mutation Assessor, MutationTaster, PROVEAN, VEST	78.73% (75.76%-81.82%)	88.83% (85.83%-92%)	61.277% (55.071%-67.606%)	79.86% (76.26%-83.57%)	76.03% (69.81%-81.97%)	3.0600 (2.9249-3.1941)	78.89% (72.73%-84.85%)	88.82% (82.45%-94.92%)	61.823% (48.485%-74.29%)	79.97% (72.46%-87.15%)	76.34% (64%-87.88%)	3.0696 (2.7710-3.3359)
At least 3 of FATHMM (cancer), FATHMM (missense), Mutation Assessor, MutationTaster, SIFT, VEST	79.07% (76.26%-82.32%)	93.04% (90.6%-95.8%)	54.896% (48.571%-61.431%)	78.1% (74.65%-81.94%)	82.04% (76%-88.64%)	3.0808 (2.9459-3.2200)	79.24% (72.73%-84.85%)	93.16% (87.93%-98.31%)	55.328% (42.421%-67.76%)	78.16% (70.66%-85.19%)	82.55% (69.57%-94.74%)	3.0920 (2.8050-3.3476)
At least 3 of FATHMM (cancer), FATHMM (missense), Mutation Assessor, PolyPhen-2, PROVEAN, SIFT	75.4% (72.22%-78.79%)	87.24% (84.03%-90.76%)	54.912% (48.611%-60.87%)	76.99% (73.05%-80.85%)	71.34% (64.91%-78%)	2.9048 (2.7558-3.0494)	75.47% (68.69%-81.82%)	87.2% (80%-93.55%)	55.309% (42.857%-67.65%)	77% (69.12%-84.73%)	71.61% (59.08%-84.38%)	2.9112 (2.6175-3.2099)
At least 3 of FATHMM (cancer), FATHMM (missense), Mutation Assessor, PolyPhen-2, PROVEAN, VEST	71.69% (68.18%-75.25%)	84.05% (80.65%-87.8%)	50.317% (43.657%-56.522%)	74.52% (70.63%-78.57%)	64.6% (57.69%-71.43%)	2.7349 (2.5822-2.8817)	71.76% (64.65%-78.79%)	84% (76.27%-90.74%)	50.735% (37.5%-64.29%)	74.53% (66.2%-82.61%)	64.9% (50%-78.13%)	2.7416 (2.4445-3.0517)
At least 3 of FATHMM (cancer), FATHMM (missense), Mutation Assessor, PolyPhen-2, SIFT, VEST	73.66% (70.2%-77.27%)	89.33% (86.4%-92.31%)	46.554% (40%-52.705%)	74.29% (70.27%-78.29%)	71.62% (64.29%-78.72%)	2.8179 (2.6507-2.9634)	73.9% (66.67%-80.81%)	89.41% (83.05%-95.31%)	47.253% (34.99%-60.61%)	74.41% (66.22%-82.28%)	72.29% (57.89%-86.36%)	2.8337 (2.5305-3.1511)
At least 3 of FATHMM (cancer), FATHMM (missense), Mutation Assessor, PROVEAN, SIFT, VEST	73.72% (70.2%-77.27%)	85.16% (81.82%-88.71%)	53.93% (47.367%-60.257%)	76.17% (72.14%-80.14%)	67.76% (61.11%-74.58%)	2.8302 (2.6839-2.9778)	73.77% (66.67%-80.81%)	84.98% (77.59%-91.67%)	54.51% (41.654%-67.57%)	76.22% (68.25%-84.06%)	67.9% (53.84%-80.78%)	2.8361 (2.5336-3.1227)
At least 3 of FATHMM (cancer), FATHMM (missense), MutationTaster, PolyPhen-2, PROVEAN, SIFT	79.06% (75.76%-82.32%)	90.39% (87.5%-93.55%)	59.467% (53.422%-65.753%)	79.41% (75.54%-83.33%)	78.16% (72.22%-84.75%)	3.0742 (2.9350-3.2095)	79.25% (72.73%-85.86%)	90.49% (84.13%-96.67%)	59.95% (47.365%-72.73%)	79.5% (71.64%-87.14%)	78.64% (65.52%-90.63%)	3.0858 (2.8086-3.3644)
At least 3 of FATHMM (cancer), FATHMM (missense), MutationTaster, PolyPhen-2, PROVEAN, VEST	76.08% (72.73%-79.29%)	87.76% (84.68%-91.06%)	55.885% (49.315%-62.166%)	77.47% (73.57%-81.48%)	72.53% (66.07%-78.95%)	2.9365 (2.7920-3.0720)	76.12% (69.7%-82.83%)	87.77% (81.24%-93.94%)	56.106% (43.74%-68.97%)	77.43% (69.44%-85.29%)	72.79% (59.26%-85.71%)	2.9410 (2.6631-3.2341)
At least 3 of FATHMM (cancer), FATHMM (missense), MutationTaster, PolyPhen-2, SIFT, VEST	76.36% (73.23%-79.8%)	92.48% (90%-95.28%)	48.476% (41.788%-54.93%)	75.63% (71.79%-79.62%)	78.87% (72.5%-86.12%)	2.9546 (2.8173-3.0957)	76.57% (69.7%-82.83%)	92.69% (86.89%-98.15%)	48.899% (36.357%-62.16%)	75.69% (67.57%-83.34%)	79.63% (64.71%-93.11%)	2.9690 (2.6894-3.2387)
At least 3 of FATHMM (cancer), FATHMM (missense), MutationTaster, PROVEAN, SIFT, VEST	78.4% (75.25%-81.82%)	90.94% (88.13%-93.8%)	56.713% (50%-63.235%)	78.41% (74.82%-82.14%)	78.36% (72.22%-84.78%)	3.0443 (2.9089-3.1734)	78.55% (71.72%-84.85%)	90.99% (85%-96.72%)	57.194% (44.444%-70.28%)	78.49% (70.97%-85.92%)	78.75% (66.67%-91.67%)	3.0541 (2.7848-3.3282)
At least 3 of FATHMM (cancer), FATHMM (missense), PolyPhen-2, PROVEAN, SIFT, VEST	72.68% (69.19%-76.26%)	87.76% (84.73%-91.2%)	46.608% (40.271%-52.703%)	73.97% (69.87%-77.93%)	68.78% (61.7%-75.62%)	2.7711 (2.6193-2.9103)	72.82% (65.66%-79.8%)	87.77% (80.95%-93.94%)	47.121% (34.483%-59.54%)	74.01% (66.22%-82.05%)	69.22% (54.55%-83.33%)	2.7812 (2.4936-3.0770)
At least 3 of FATHMM (cancer), Mutation Assessor, MutationTaster, PolyPhen-2, PROVEAN, SIFT	76.07% (72.73%-79.29%)	84.02% (80.64%-87.88%)	62.325% (56.162%-68.919%)	79.4% (75.37%-83.45%)	69.29% (63.23%-75.72%)	2.9503 (2.8098-3.0928)	76.14% (69.7%-82.83%)	84.07% (76.46%-90.91%)	62.525% (50%-75%)	79.37% (71.23%-87.5%)	69.6% (56%-82.15%)	2.9557 (2.6623-3.2373)
At least 3 of FATHMM (cancer), Mutation Assessor, MutationTaster, PolyPhen-2, PROVEAN, VEST	75.09% (72.21%-78.28%)	84.02% (80.49%-87.81%)	59.654% (53.521%-65.832%)	78.26% (74.28%-82.44%)	68.34% (62.06%-75%)	2.9027 (2.7698-3.0450)	75.07% (68.69%-80.83%)	84.08% (76.27%-91.05%)	59.607% (47.368%-72.23%)	78.12% (70%-85.92%)	68.58% (55.17%-81.25%)	2.9039 (2.6173-3.1692)
At least 3 of FATHMM (cancer), Mutation Assessor, MutationTaster, PolyPhen-2, SIFT, VEST	72.33% (69.19%-75.76%)	86.11% (82.79%-89.6%)	48.487% (42.029%-54.795%)	74.29% (70.42%-78.32%)	66.88% (60%-74.07%)	2.7578 (2.6052-2.8996)	72.52% (65.66%-78.79%)	86.27% (78.95%-92.73%)	48.894% (36.665%-61.76%)	74.33% (66.2%-82.19%)	67.5% (53.85%-81.49%)	2.7700 (2.4890-3.0735)
At least 3 of FATHMM (cancer), Mutation Assessor, MutationTaster, PROVEAN, SIFT, VEST	74.69% (71.21%-77.78%)	82.96% (79.51%-86.89%)	60.391% (53.968%-66.667%)	78.36% (74.42%-82.27%)	67.2% (60.94%-73.85%)	2.8891 (2.7427-3.0290)	74.86% (68.69%-81.82%)	83.01% (75.38%-89.83%)	60.875% (48.381%-73.53%)	78.45% (70.49%-86.36%)	67.61% (54.29%-80.02%)	2.8994 (2.6102-3.1848)
At least 3 of FATHMM (cancer), Mutation Assessor, PolyPhen-2, PROVEAN, SIFT, VEST	70.28% (66.67%-73.74%)	81.34% (77.68%-85.25%)	51.149% (44.595%-57.333%)	74.22% (70.07%-78.42%)	61.33% (54.24%-68.34%)	2.6804 (2.5306-2.8215)	70.55% (63.64%-77.78%)	81.45% (73.44%-88.89%)	51.833% (40%-64.71%)	74.36% (66.15%-82.61%)	61.95% (48.14%-75.86%)	2.6960 (2.4083-2.9969)
At least 3 of FATHMM (cancer), MutationTaster, PolyPhen-2, PROVEAN, SIFT, VEST	72.33% (68.69%-75.76%)	85.58% (82.35%-89.17%)	49.427% (42.857%-55.556%)	74.53% (70.63%-78.62%)	66.47% (59.57%-73.34%)	2.7600 (2.6088-2.8996)	72.5% (65.66%-79.8%)	85.74% (78.46%-92.19%)	49.749% (37.771%-61.77%)	74.54% (66.22%-82.35%)	67.05% (53.57%-80.02%)	2.7708 (2.4909-3.0736)
At least 3 of FATHMM (missense), Mutation Assessor, MutationTaster, PolyPhen-2, PROVEAN, SIFT	75.06% (71.72%-78.28%)	82.97% (79.37%-86.92%)	61.377% (54.998%-67.608%)	78.79% (74.81%-82.68%)	67.58% (61.54%-74.19%)	2.9072 (2.7601-3.0542)	75.13% (68.69%-81.82%)	82.97% (75.38%-89.86%)	61.639% (48.571%-74.29%)	78.77% (70.77%-86.68%)	67.84% (54.29%-79.41%)	2.9122 (2.6214-3.2062)
At least 3 of FATHMM (missense), Mutation Assessor, MutationTaster, PolyPhen-2, PROVEAN, VEST	73.73% (70.2%-77.27%)	84.02% (80.34%-87.9%)	55.936% (49.315%-62.342%)	76.73% (72.79%-80.74%)	66.94% (60.66%-73.77%)	2.8362 (2.6930-2.9793)	73.75% (66.67%-80.81%)	84.07% (76.47%-90.91%)	56.017% (42.424%-68.57%)	76.63% (68.18%-84.21%)	67.22% (53.33%-80.56%)	2.8394 (2.5368-3.1266)
At least 3 of FATHMM (missense), Mutation Assessor, MutationTaster, PolyPhen-2, SIFT, VEST	71.64% (68.18%-75.25%)	86.12% (82.9%-89.68%)	46.605% (40%-52.941%)	73.6% (69.59%-77.93%)	66.01% (58.7%-72.92%)	2.7234 (2.5691-2.8754)	71.87% (64.65%-78.79%)	86.26% (78.95%-92.73%)	47.158% (34.286%-60.61%)	73.69% (65%-81.94%)	66.68% (52.17%-80%)	2.7379 (2.4420-3.0414)
At least 3 of FATHMM (missense), Mutation Assessor, MutationTaster, PROVEAN, SIFT, VEST	72.67% (69.19%-76.26%)	81.91% (78.29%-85.83%)	56.671% (50%-63.514%)	76.58% (72.46%-80.6%)	64.44% (57.81%-71.02%)	2.7960 (2.6450-2.9501)	72.84% (65.66%-79.8%)	81.89% (73.97%-89.29%)	57.266% (42.857%-69.7%)	76.69% (68.75%-84.85%)	64.81% (51.39%-77.79%)	2.8066 (2.4941-3.1092)
At least 3 of FATHMM (missense), Mutation Assessor, PolyPhen-2, PROVEAN, SIFT, VEST	67.6% (64.14%-71.21%)	80.3% (76.42%-84.21%)	45.627% (38.887%-51.389%)	71.86% (67.63%-76.09%)	57.25% (50%-64.41%)	2.5504 (2.3936-2.6966)	67.84% (60.61%-74.75%)	80.34% (72.41%-88.06%)	46.344% (34.286%-58.98%)	71.98% (63.51%-80.88%)	57.88% (43.74%-72.41%)	2.5655 (2.2737-2.8795)
At least 3 of FATHMM (missense), MutationTaster, PolyPhen-2, PROVEAN, SIFT, VEST	71.33% (68.18%-74.75%)	85.58% (82.26%-89.06%)	46.663% (40%-52.78%)	73.5% (69.66%-77.55%)	65.18% (58%-72%)	2.7093 (2.5609-2.8554)	71.49% (64.65%-77.78%)	85.73% (78.33%-92.19%)	47.008% (34.483%-60%)	73.52% (65.28%-81.43%)	65.77% (51.85%-80%)	2.7203 (2.4198-3.0238)
At least 3 of Mutation Assessor, MutationTaster, PolyPhen-2, PROVEAN, SIFT, VEST	71.34% (68.18%-74.75%)	81.33% (77.69%-85.37%)	54.062% (47.367%-60.811%)	75.38% (71.11%-79.41%)	62.61% (56.06%-69.36%)	2.7337 (2.5835-2.8812)	71.47% (64.65%-77.78%)	81.48% (73.33%-88.89%)	54.277% (41.662%-67.65%)	75.35% (67.16%-83.82%)	63.06% (50%-76.32%)	2.7417 (2.4413-3.0397)
At least 4 of CHASM (breast), CHASM (lung), CHASM (melanoma), FATHMM (cancer), FATHMM (missense), Mutation Assessor	86.58% (83.84%-89.39%)	85.19% (81.66%-88.8%)	88.971% (85.071%-93.153%)	93.04% (90.74%-95.69%)	77.66% (72.62%-82.76%)	3.4487 (3.3466-3.5636)	86.43% (80.81%-91.92%)	84.91% (77.78%-92.07%)	89.003% (80.554%-97.14%)	92.99% (87.5%-98.11%)	77.5% (67.35%-87.8%)	3.4440 (3.2153-3.6515)
At least 4 of CHASM (breast), CHASM (lung), CHASM (melanoma), FATHMM (cancer), FATHMM (missense), MutationTaster	86.92% (84.34%-89.9%)	85.71% (82.26%-89.34%)	88.992% (85.071%-93.243%)	93.09% (90.76%-95.8%)	78.28% (73.17%-83.54%)	3.4607 (3.3562-3.5768)	86.77% (80.81%-91.92%)	85.47% (78.12%-92.43%)	88.976% (80.488%-96.97%)	93.01% (87.27%-98.11%)	78.13% (67.5%-88.1%)	3.4559 (3.2293-3.6661)
At least 4 of CHASM (breast), CHASM (lung), CHASM (melanoma), FATHMM (cancer), FATHMM (missense), PolyPhen-2	85.57% (82.83%-88.38%)	85.18% (81.6%-88.89%)	86.228% (81.941%-91.304%)	91.45% (88.98%-94.6%)	77.1% (71.79%-82.28%)	3.3996 (3.2900-3.5211)	85.43% (79.8%-90.91%)	84.94% (77.61%-92.07%)	86.236% (76.471%-94.74%)	91.38% (85.25%-96.49%)	76.97% (65.85%-87.23%)	3.3952 (3.1530-3.6213)
At least 4 of CHASM (breast), CHASM (lung), CHASM (melanoma), FATHMM (cancer), FATHMM (missense), PROVEAN	83.89% (80.81%-86.87%)	79.9% (75.97%-84%)	90.796% (87.324%-94.595%)	93.76% (91.34%-96.3%)	72.32% (66.67%-77.65%)	3.3677 (3.2582-3.4851)	83.73% (77.78%-89.9%)	79.55% (71.43%-87.69%)	90.86% (82.857%-97.37%)	93.74% (88.46%-98.22%)	72.16% (61.36%-82.93%)	3.3630 (3.1288-3.5871)
At least 4 of CHASM (breast), CHASM (lung), CHASM (melanoma), FATHMM (cancer), FATHMM (missense), SIFT	86.57% (83.84%-89.39%)	85.71% (82.26%-89.34%)	88.045% (83.824%-92.54%)	92.54% (90.09%-95.5%)	78.1% (72.94%-83.33%)	3.4440 (3.3397-3.5594)	86.45% (80.81%-91.92%)	85.47% (78.12%-92.43%)	88.095% (78.947%-96.67%)	92.51% (86.89%-97.96%)	77.97% (67.44%-87.81%)	3.4404 (3.2149-3.6484)
At least 4 of CHASM (breast), CHASM (lung), CHASM (melanoma), FATHMM (cancer), FATHMM (missense), VEST	85.9% (83.33%-88.89%)	85.19% (81.67%-88.8%)	87.13% (82.926%-91.781%)	91.97% (89.57%-94.87%)	77.29% (72.09%-82.5%)	3.4158 (3.3081-3.5377)	85.77% (79.8%-90.91%)	84.92% (77.78%-91.94%)	87.182% (78.049%-95%)	91.93% (85.96%-96.61%)	77.14% (66.67%-87.18%)	3.4117 (3.1660-3.6270)
At least 4 of CHASM (breast), CHASM (lung), CHASM (melanoma), FATHMM (cancer), Mutation Assessor, MutationTaster	87.92% (85.35%-90.4%)	86.24% (82.81%-89.77%)	90.821% (87.179%-94.668%)	94.2% (92.11%-96.64%)	79.25% (74.12%-84.62%)	3.5052 (3.4073-3.6116)	87.79% (82.83%-92.93%)	86.01% (78.69%-92.98%)	90.825% (82.925%-97.44%)	94.15% (88.68%-98.28%)	79.12% (69.44%-89.13%)	3.5010 (3.2870-3.7056)
At least 4 of CHASM (breast), CHASM (lung), CHASM (melanoma), FATHMM (cancer), Mutation Assessor, PolyPhen-2	86.57% (83.84%-89.39%)	85.71% (82.17%-89.34%)	88.039% (84.058%-92.647%)	92.53% (90.16%-95.58%)	78.09% (72.84%-83.14%)	3.4437 (3.3415-3.5574)	86.47% (80.81%-91.92%)	85.48% (78.12%-92.66%)	88.128% (80%-96.56%)	92.52% (86.79%-98.04%)	77.99% (67.57%-88.24%)	3.4411 (3.2197-3.6389)
At least 4 of CHASM (breast), CHASM (lung), CHASM (melanoma), FATHMM (cancer), Mutation Assessor, PROVEAN	83.55% (80.81%-86.87%)	79.9% (75.91%-83.97%)	89.853% (85.915%-94.203%)	93.16% (90.74%-96.12%)	72.12% (66.67%-77.53%)	3.3503 (3.2447-3.4757)	83.4% (76.77%-88.89%)	79.54% (71.43%-87.5%)	89.998% (81.818%-97.37%)	93.18% (87.5%-98.11%)	71.95% (60.98%-82.61%)	3.3467 (3.1003-3.5600)
At least 4 of CHASM (breast), CHASM (lung), CHASM (melanoma), FATHMM (cancer), Mutation Assessor, SIFT	86.55% (83.84%-89.39%)	85.69% (82.31%-89.52%)	88.022% (84.058%-92.308%)	92.52% (90.08%-95.5%)	78.07% (72.84%-83.14%)	3.4431 (3.3342-3.5569)	86.5% (80.81%-91.92%)	85.51% (78.12%-92.54%)	88.152% (79.485%-96.88%)	92.53% (86.79%-98.04%)	78.03% (67.5%-88.1%)	3.4423 (3.2152-3.6601)
At least 4 of CHASM (breast), CHASM (lung), CHASM (melanoma), FATHMM (cancer), Mutation Assessor, VEST	86.58% (83.84%-89.39%)	85.72% (82.31%-89.17%)	88.061% (84.058%-92.427%)	92.55% (90.16%-95.69%)	78.11% (72.84%-83.33%)	3.4443 (3.3428-3.5593)	86.44% (80.81%-91.92%)	85.46% (78.33%-92.54%)	88.075% (79.412%-95.37%)	92.49% (86.21%-96.95%)	77.96% (67.44%-88.1%)	3.4399 (3.2127-3.6446)
At least 4 of CHASM (breast), CHASM (lung), CHASM (melanoma), FATHMM (cancer), MutationTaster, PolyPhen-2	87.58% (85.35%-90.4%)	86.23% (82.68%-89.76%)	89.919% (86.111%-94.12%)	93.67% (91.45%-96.49%)	79.07% (74.03%-84%)	3.4888 (3.3883-3.6039)	87.46% (81.82%-91.92%)	86.03% (78.94%-93.1%)	89.878% (81.818%-97.3%)	93.58% (87.93%-98.22%)	78.98% (69.04%-88.89%)	3.4847 (3.2644-3.6775)
At least 4 of CHASM (breast), CHASM (lung), CHASM (melanoma), FATHMM (cancer), MutationTaster, PROVEAN	84.57% (81.82%-87.37%)	80.42% (76.47%-84.43%)	91.726% (88.235%-95.654%)	94.38% (92.08%-97.14%)	73.05% (67.78%-78.26%)	3.3958 (3.2957-3.5100)	84.4% (78.79%-89.9%)	80.09% (71.87%-87.88%)	91.768% (84.211%-97.67%)	94.35% (89.09%-98.31%)	72.89% (61.9%-83.34%)	3.3911 (3.1619-3.5952)
At least 4 of CHASM (breast), CHASM (lung), CHASM (melanoma), FATHMM (cancer), MutationTaster, SIFT	87.91% (85.35%-90.4%)	86.76% (83.33%-90.37%)	89.894% (86.111%-94.286%)	93.69% (91.53%-96.55%)	79.71% (74.7%-84.71%)	3.5006 (3.4007-3.6090)	87.81% (82.83%-92.93%)	86.56% (79.37%-93.22%)	89.922% (81.25%-97.3%)	93.65% (87.93%-98.21%)	79.62% (69.44%-89.74%)	3.4975 (3.2737-3.6971)
At least 4 of CHASM (breast), CHASM (lung), CHASM (melanoma), FATHMM (cancer), MutationTaster, VEST	87.92% (85.35%-90.4%)	86.77% (83.47%-90.16%)	89.894% (86.111%-94.286%)	93.69% (91.53%-96.52%)	79.72% (74.99%-84.71%)	3.5007 (3.3997-3.6020)	87.8% (82.83%-92.93%)	86.55% (79.69%-93.22%)	89.922% (81.25%-97.3%)	93.65% (87.76%-98.21%)	79.6% (69.44%-89.74%)	3.4973 (3.2945-3.6890)
At least 4 of CHASM (breast), CHASM (lung), CHASM (melanoma), FATHMM (cancer), PolyPhen-2, PROVEAN	83.88% (80.81%-86.87%)	80.42% (76.47%-84.43%)	89.853% (85.915%-94.203%)	93.2% (90.74%-96.12%)	72.64% (67.42%-77.91%)	3.3612 (3.2523-3.4778)	83.75% (77.78%-89.9%)	80.09% (71.87%-87.88%)	89.998% (81.818%-97.37%)	93.22% (87.5%-98.15%)	72.51% (60.98%-83.33%)	3.3582 (3.1238-3.5756)
At least 4 of CHASM (breast), CHASM (lung), CHASM (melanoma), FATHMM (cancer), PolyPhen-2, SIFT	86.22% (83.84%-88.89%)	86.23% (82.68%-89.76%)	86.21% (81.69%-91.176%)	91.53% (88.98%-94.69%)	78.36% (73.17%-83.54%)	3.4234 (3.3159-3.5369)	86.14% (80.81%-90.91%)	86.03% (78.94%-93.1%)	86.275% (76.471%-94.74%)	91.5% (85.45%-96.61%)	78.29% (67.57%-88.89%)	3.4210 (3.1927-3.6328)
At least 4 of CHASM (breast), CHASM (lung), CHASM (melanoma), FATHMM (cancer), PolyPhen-2, VEST	85.57% (82.83%-88.38%)	85.7% (82.2%-89.31%)	85.334% (80.822%-90.411%)	90.99% (88.29%-94.17%)	77.55% (72.15%-82.72%)	3.3958 (3.2835-3.5191)	85.43% (79.8%-90.91%)	85.49% (78.12%-92.54%)	85.285% (75.595%-94.29%)	90.89% (84.48%-96.43%)	77.43% (66.67%-87.8%)	3.3910 (3.1415-3.6141)
At least 4 of CHASM (breast), CHASM (lung), CHASM (melanoma), FATHMM (cancer), PROVEAN, SIFT	83.54% (80.81%-86.87%)	80.42% (76.47%-84.43%)	88.926% (84.932%-93.058%)	92.62% (90.18%-95.45%)	72.44% (67.05%-77.78%)	3.3441 (3.2308-3.4688)	83.42% (76.77%-88.89%)	80.09% (71.87%-87.88%)	89.096% (80.643%-97.22%)	92.65% (86.95%-98.04%)	72.31% (60.98%-82.99%)	3.3415 (3.0918-3.5728)
At least 4 of CHASM (breast), CHASM (lung), CHASM (melanoma), FATHMM (cancer), PROVEAN, VEST	83.56% (80.81%-86.36%)	79.9% (76.15%-84%)	89.875% (86.111%-94.03%)	93.17% (90.65%-96.04%)	72.12% (66.67%-77.42%)	3.3507 (3.2393-3.4652)	83.39% (77.78%-88.89%)	79.55% (71.19%-87.27%)	89.946% (81.818%-97.3%)	93.15% (87.5%-98.15%)	71.95% (60.97%-82.61%)	3.3460 (3.1118-3.5604)
At least 4 of CHASM (breast), CHASM (lung), CHASM (melanoma), FATHMM (cancer), SIFT, VEST	86.58% (83.84%-89.39%)	86.24% (82.9%-89.76%)	87.159% (82.894%-91.781%)	92.07% (89.57%-95.04%)	78.56% (73.42%-83.75%)	3.4403 (3.3378-3.5553)	86.44% (80.81%-91.92%)	86.02% (78.69%-92.66%)	87.126% (77.778%-95%)	91.99% (86.15%-96.83%)	78.44% (68.18%-88.89%)	3.4357 (3.2017-3.6422)
At least 4 of CHASM (breast), CHASM (lung), CHASM (melanoma), FATHMM (missense), Mutation Assessor, MutationTaster	88.61% (86.36%-90.91%)	87.33% (84%-90.77%)	90.821% (87.179%-94.668%)	94.27% (92.24%-96.69%)	80.57% (75.61%-85.71%)	3.5299 (3.4330-3.6318)	88.44% (83.84%-92.93%)	87.03% (80.3%-93.65%)	90.825% (82.925%-97.44%)	94.22% (88.88%-98.28%)	80.35% (70.45%-90.24%)	3.5243 (3.3158-3.7150)
At least 4 of CHASM (breast), CHASM (lung), CHASM (melanoma), FATHMM (missense), Mutation Assessor, PolyPhen-2	86.92% (84.34%-89.39%)	86.26% (82.64%-89.76%)	88.056% (84%-92.539%)	92.59% (90.09%-95.54%)	78.76% (73.68%-84%)	3.4566 (3.3531-3.5685)	86.77% (81.82%-91.92%)	85.97% (78.79%-93.22%)	88.089% (79.412%-96.97%)	92.53% (87.03%-98.08%)	78.58% (68.42%-88.89%)	3.4518 (3.2293-3.6632)
At least 4 of CHASM (breast), CHASM (lung), CHASM (melanoma), FATHMM (missense), Mutation Assessor, PROVEAN	83.56% (80.81%-86.36%)	79.9% (75.61%-84%)	89.875% (86.111%-94.203%)	93.17% (90.65%-96.08%)	72.12% (66.67%-77.43%)	3.3507 (3.2424-3.4688)	83.39% (77.78%-88.89%)	79.54% (71.21%-88.14%)	89.946% (81.812%-97.3%)	93.15% (87.5%-98.15%)	71.95% (61.22%-82.98%)	3.3458 (3.1119-3.5584)
At least 4 of CHASM (breast), CHASM (lung), CHASM (melanoma), FATHMM (missense), Mutation Assessor, SIFT	86.91% (84.34%-89.39%)	86.24% (82.9%-89.76%)	88.043% (83.996%-92.188%)	92.58% (90.09%-95.5%)	78.74% (73.81%-84.15%)	3.4561 (3.3556-3.5641)	86.79% (81.82%-91.92%)	86% (78.69%-92.86%)	88.1% (79.412%-96.88%)	92.55% (87.03%-98%)	78.61% (68.42%-88.57%)	3.4526 (3.2373-3.6601)
At least 4 of CHASM (breast), CHASM (lung), CHASM (melanoma), FATHMM (missense), Mutation Assessor, VEST	87.26% (84.85%-89.9%)	86.8% (83.33%-90.32%)	88.056% (84.144%-92.537%)	92.63% (90.27%-95.61%)	79.42% (74.67%-84.42%)	3.4691 (3.3628-3.5825)	87.09% (81.82%-91.92%)	86.49% (79.37%-93.33%)	88.089% (79.4%-95.35%)	92.58% (86.89%-96.92%)	79.19% (69.05%-89.19%)	3.4635 (3.2327-3.6711)
At least 4 of CHASM (breast), CHASM (lung), CHASM (melanoma), FATHMM (missense), MutationTaster, PolyPhen-2	87.59% (84.85%-90.4%)	86.78% (83.33%-90.17%)	89.004% (84.932%-93.591%)	93.17% (90.83%-96.1%)	79.57% (74.7%-84.81%)	3.4852 (3.3820-3.6008)	87.44% (81.82%-92.93%)	86.53% (79.66%-93.34%)	88.965% (80.476%-97.22%)	93.08% (87.04%-98.18%)	79.41% (68.75%-89.19%)	3.4798 (3.2497-3.6837)
At least 4 of CHASM (breast), CHASM (lung), CHASM (melanoma), FATHMM (missense), MutationTaster, PROVEAN	84.9% (82.32%-87.88%)	80.95% (76.98%-85.04%)	91.726% (88.235%-95.654%)	94.42% (92.16%-97.17%)	73.59% (68.23%-78.89%)	3.4068 (3.3055-3.5183)	84.74% (78.79%-89.9%)	80.63% (72.41%-88.71%)	91.768% (84.211%-97.67%)	94.39% (89.09%-98.33%)	73.44% (62.79%-84.09%)	3.4022 (3.1762-3.6066)
At least 4 of CHASM (breast), CHASM (lung), CHASM (melanoma), FATHMM (missense), MutationTaster, SIFT	88.6% (86.36%-90.91%)	87.85% (84.55%-91.13%)	89.894% (86.111%-94.286%)	93.76% (91.6%-96.52%)	81.06% (76.31%-86.05%)	3.5256 (3.4284-3.6260)	88.46% (83.84%-92.93%)	87.59% (80.95%-94.03%)	89.922% (81.25%-97.3%)	93.72% (88.32%-98.25%)	80.88% (71.05%-90.48%)	3.5211 (3.3201-3.7133)
At least 4 of CHASM (breast), CHASM (lung), CHASM (melanoma), FATHMM (missense), MutationTaster, VEST	87.93% (85.35%-90.4%)	87.32% (84%-90.98%)	88.98% (84.932%-93.336%)	93.2% (90.91%-96%)	80.24% (75.6%-85.53%)	3.4974 (3.3976-3.6090)	87.78% (82.83%-92.93%)	87.04% (79.99%-93.65%)	89.009% (80.476%-97.14%)	93.15% (87.27%-98.15%)	80.04% (69.76%-89.76%)	3.4924 (3.2721-3.6967)
At least 4 of CHASM (breast), CHASM (lung), CHASM (melanoma), FATHMM (missense), PolyPhen-2, PROVEAN	83.89% (80.81%-86.87%)	80.95% (76.98%-85.04%)	88.96% (84.848%-93.151%)	92.69% (90.18%-95.5%)	72.99% (67.44%-78.31%)	3.3559 (3.2457-3.4702)	83.74% (77.78%-89.9%)	80.63% (72.41%-88.71%)	89.032% (80.556%-97.22%)	92.66% (86.79%-98.08%)	72.84% (62%-83.72%)	3.3516 (3.1249-3.5839)
At least 4 of CHASM (breast), CHASM (lung), CHASM (melanoma), FATHMM (missense), PolyPhen-2, SIFT	85.56% (82.83%-88.38%)	86.78% (83.33%-90.17%)	83.453% (78.571%-88.235%)	90.07% (87.2%-93.16%)	78.5% (73.33%-84.21%)	3.3880 (3.2710-3.5115)	85.44% (79.8%-90.91%)	86.53% (79.66%-93.34%)	83.52% (73.529%-93.11%)	90.02% (83.93%-96.08%)	78.35% (67.5%-88.89%)	3.3842 (3.1401-3.6180)
At least 4 of CHASM (breast), CHASM (lung), CHASM (melanoma), FATHMM (missense), PolyPhen-2, VEST	85.24% (82.32%-87.88%)	86.25% (82.76%-89.77%)	83.471% (78.571%-88.407%)	90.03% (87.29%-93.13%)	77.84% (72.84%-83.54%)	3.3759 (3.2554-3.4975)	85.08% (79.8%-90.91%)	85.99% (78.79%-92.99%)	83.483% (73.333%-93.11%)	89.95% (83.86%-96.08%)	77.67% (66.67%-88.38%)	3.3708 (3.1285-3.6067)
At least 4 of CHASM (breast), CHASM (lung), CHASM (melanoma), FATHMM (missense), PROVEAN, SIFT	83.55% (80.81%-86.36%)	80.42% (76.33%-84.5%)	88.948% (85.13%-93.058%)	92.64% (90.09%-95.45%)	72.44% (67.03%-77.78%)	3.3445 (3.2391-3.4621)	83.41% (77.78%-88.89%)	80.09% (71.88%-88.52%)	89.043% (80.556%-97.14%)	92.63% (86.79%-98.08%)	72.3% (61.54%-83.33%)	3.3407 (3.1028-3.5566)
At least 4 of CHASM (breast), CHASM (lung), CHASM (melanoma), FATHMM (missense), PROVEAN, VEST	83.56% (80.81%-86.36%)	80.43% (76.38%-84.55%)	88.961% (85.134%-93.245%)	92.65% (90.1%-95.54%)	72.45% (67.03%-77.78%)	3.3448 (3.2354-3.4576)	83.39% (77.78%-88.89%)	80.08% (71.87%-88.34%)	89.033% (80.476%-97.14%)	92.62% (86.27%-98.04%)	72.28% (61.53%-83.67%)	3.3402 (3.1202-3.5708)
At least 4 of CHASM (breast), CHASM (lung), CHASM (melanoma), FATHMM (missense), SIFT, VEST	85.9% (83.33%-88.89%)	87.32% (84%-90.98%)	83.441% (78.664%-88.406%)	90.12% (87.39%-93.22%)	79.2% (74.32%-84.51%)	3.4008 (3.2827-3.5171)	85.77% (79.8%-90.91%)	87.04% (79.99%-93.65%)	83.548% (73.529%-92.31%)	90.09% (83.87%-95.39%)	79.01% (68.42%-89.2%)	3.3969 (3.1624-3.6214)
At least 4 of CHASM (breast), CHASM (lung), CHASM (melanoma), Mutation Assessor, MutationTaster, PolyPhen-2	83.83% (80.81%-86.87%)	78.73% (74.44%-82.82%)	92.646% (89.548%-96.001%)	94.87% (92.66%-97.25%)	71.6% (66.32%-76.67%)	3.3785 (3.2699-3.4867)	83.85% (77.78%-89.9%)	78.67% (70.3%-87.1%)	92.688% (85.366%-100%)	94.86% (89.47%-100%)	71.73% (61.36%-82.23%)	3.3795 (3.1658-3.5966)
At least 4 of CHASM (breast), CHASM (lung), CHASM (melanoma), Mutation Assessor, MutationTaster, PROVEAN	79.46% (76.26%-82.83%)	71.31% (66.41%-76%)	93.55% (90.537%-97.102%)	95.03% (92.71%-97.85%)	65.36% (59.63%-70.48%)	3.2525 (3.1466-3.3632)	79.45% (72.73%-85.86%)	71.17% (61.66%-81.03%)	93.631% (86.842%-100%)	95.04% (89.36%-100%)	65.46% (54.72%-76.6%)	3.2531 (3.0348-3.4685)
At least 4 of CHASM (breast), CHASM (lung), CHASM (melanoma), Mutation Assessor, MutationTaster, SIFT	86.19% (83.33%-88.89%)	82.45% (78.74%-86.36%)	92.646% (89.548%-96.001%)	95.09% (92.98%-97.37%)	75.33% (70.24%-80.44%)	3.4552 (3.3515-3.5583)	86.21% (80.81%-91.92%)	82.42% (74.54%-89.83%)	92.688% (85.366%-100%)	95.08% (90%-100%)	75.47% (65.11%-85.42%)	3.4567 (3.2546-3.6675)
At least 4 of CHASM (breast), CHASM (lung), CHASM (melanoma), Mutation Assessor, MutationTaster, VEST	83.84% (80.81%-86.87%)	78.74% (74.44%-83.19%)	92.646% (89.548%-96.001%)	94.87% (92.66%-97.25%)	71.6% (66.29%-76.92%)	3.3787 (3.2746-3.4923)	83.83% (77.78%-89.9%)	78.66% (69.7%-86.97%)	92.688% (85.366%-100%)	94.86% (89.58%-100%)	71.71% (61.22%-82.35%)	3.3791 (3.1554-3.5919)
At least 4 of CHASM (breast), CHASM (lung), CHASM (melanoma), Mutation Assessor, PolyPhen-2, PROVEAN	76.09% (72.73%-79.31%)	67.61% (62.7%-72.22%)	90.75% (87.143%-94.521%)	92.67% (89.8%-95.7%)	61.84% (56.48%-66.99%)	3.1287 (3.0160-3.2472)	76.1% (69.67%-82.83%)	67.41% (58.21%-77.42%)	90.959% (83.333%-97.44%)	92.75% (86.36%-97.96%)	61.94% (51.06%-72.88%)	3.1307 (2.8879-3.3542)
At least 4 of CHASM (breast), CHASM (lung), CHASM (melanoma), Mutation Assessor, PolyPhen-2, SIFT	82.16% (79.29%-85.35%)	78.74% (74.59%-82.81%)	88.054% (84.057%-92.54%)	91.93% (89.19%-95.15%)	70.56% (65.17%-75.56%)	3.2929 (3.1744-3.4079)	82.15% (75.76%-87.88%)	78.65% (70%-86.77%)	88.091% (79.412%-96.77%)	91.9% (85.99%-97.96%)	70.66% (60.47%-81.14%)	3.2930 (3.0590-3.5330)
At least 4 of CHASM (breast), CHASM (lung), CHASM (melanoma), Mutation Assessor, PolyPhen-2, VEST	79.17% (75.76%-82.32%)	73.99% (69.23%-78.63%)	88.106% (84.057%-92.754%)	91.49% (88.57%-95%)	66.21% (60.67%-71.43%)	3.1980 (3.0852-3.3234)	79.04% (72.73%-85.86%)	73.79% (64.99%-83.05%)	88.004% (78.947%-96.67%)	91.35% (84.09%-97.78%)	66.21% (55.76%-77.5%)	3.1935 (2.9417-3.4208)
At least 4 of CHASM (breast), CHASM (lung), CHASM (melanoma), Mutation Assessor, PROVEAN, SIFT	76.44% (73.23%-79.8%)	68.15% (63.49%-73.02%)	90.75% (87.143%-94.521%)	92.72% (90%-95.74%)	62.24% (56.86%-67.31%)	3.1386 (3.0300-3.2591)	76.42% (69.7%-82.83%)	67.92% (57.81%-77.42%)	90.959% (83.333%-97.44%)	92.8% (86.36%-97.96%)	62.31% (52%-72.41%)	3.1400 (2.9015-3.3668)
At least 4 of CHASM (breast), CHASM (lung), CHASM (melanoma), Mutation Assessor, PROVEAN, VEST	75.1% (71.72%-78.79%)	64.96% (59.68%-69.68%)	92.627% (89.33%-96%)	93.84% (91.21%-96.67%)	60.46% (55.26%-65.49%)	3.1189 (3.0124-3.2379)	75.05% (67.68%-81.82%)	64.73% (55%-74.66%)	92.712% (85.366%-100%)	93.85% (87.5%-100%)	60.51% (50%-70.91%)	3.1181 (2.8806-3.3303)
At least 4 of CHASM (breast), CHASM (lung), CHASM (melanoma), Mutation Assessor, SIFT, VEST	81.85% (78.79%-84.85%)	77.71% (73.6%-81.97%)	89.004% (84.932%-93.245%)	92.44% (89.72%-95.45%)	69.78% (64.58%-75.26%)	3.2894 (3.1777-3.4103)	81.75% (75.76%-87.88%)	77.54% (68.85%-85.49%)	88.942% (80.488%-97.22%)	92.34% (85.96%-98.04%)	69.77% (59.17%-80.01%)	3.2859 (3.0444-3.5099)
At least 4 of CHASM (breast), CHASM (lung), CHASM (melanoma), MutationTaster, PolyPhen-2, PROVEAN	79.12% (75.76%-82.32%)	70.76% (65.85%-75.41%)	93.55% (90.537%-97.102%)	94.99% (92.71%-97.85%)	64.93% (59.59%-70%)	3.2423 (3.1357-3.3530)	79.14% (72.73%-85.86%)	70.68% (61.29%-80.6%)	93.631% (86.842%-100%)	95.01% (89.13%-100%)	65.07% (54.39%-75.56%)	3.2439 (3.0189-3.4611)
At least 4 of CHASM (breast), CHASM (lung), CHASM (melanoma), MutationTaster, PolyPhen-2, SIFT	85.51% (82.83%-88.38%)	81.38% (77.42%-85.48%)	92.646% (89.548%-96.001%)	95.03% (92.86%-97.32%)	74.22% (69.15%-79.39%)	3.4328 (3.3309-3.5440)	85.54% (79.8%-90.91%)	81.36% (73.01%-89.06%)	92.688% (85.366%-100%)	95.02% (89.8%-100%)	74.38% (63.82%-84.44%)	3.4345 (3.2183-3.6388)
At least 4 of CHASM (breast), CHASM (lung), CHASM (melanoma), MutationTaster, PolyPhen-2, VEST	82.48% (79.29%-85.86%)	77.66% (73.39%-82.12%)	90.814% (87.179%-94.668%)	93.59% (91.26%-96.33%)	70.17% (64.89%-75.53%)	3.3223 (3.2116-3.4425)	82.51% (75.76%-88.89%)	77.63% (68.75%-85.97%)	90.855% (82.925%-97.44%)	93.57% (87.71%-98.11%)	70.33% (59.57%-80.85%)	3.3239 (3.0875-3.5469)
At least 4 of CHASM (breast), CHASM (lung), CHASM (melanoma), MutationTaster, PROVEAN, SIFT	81.15% (78.27%-84.34%)	73.43% (68.75%-78.13%)	94.49% (91.667%-97.333%)	95.84% (93.75%-98%)	67.29% (62%-72.38%)	3.3104 (3.2095-3.4231)	81.14% (74.75%-86.89%)	73.33% (63.93%-83.05%)	94.503% (88.235%-100%)	95.81% (90.91%-100%)	67.4% (56.9%-78.18%)	3.3105 (3.0964-3.5205)
At least 4 of CHASM (breast), CHASM (lung), CHASM (melanoma), MutationTaster, PROVEAN, VEST	77.77% (74.75%-80.81%)	68.63% (63.71%-73.64%)	93.55% (90.537%-97.102%)	94.84% (92.47%-97.78%)	63.31% (58.12%-68.52%)	3.2033 (3.0992-3.3133)	77.8% (71.72%-83.84%)	68.56% (58.72%-78.12%)	93.631% (86.842%-100%)	94.86% (88.89%-100%)	63.46% (52.94%-74%)	3.2052 (2.9863-3.4161)
At least 4 of CHASM (breast), CHASM (lung), CHASM (melanoma), MutationTaster, SIFT, VEST	85.18% (82.32%-87.88%)	81.39% (77.52%-85.37%)	91.719% (88.406%-95.833%)	94.44% (92.23%-97.22%)	74.03% (68.82%-79.31%)	3.4158 (3.3139-3.5238)	85.2% (79.8%-90.91%)	81.35% (73.02%-89.23%)	91.785% (83.784%-97.56%)	94.45% (88.89%-98.31%)	74.17% (63.82%-84.45%)	3.4175 (3.2049-3.6247)
At least 4 of CHASM (breast), CHASM (lung), CHASM (melanoma), PolyPhen-2, PROVEAN, SIFT	78.11% (74.75%-81.31%)	71.32% (66.39%-76%)	89.832% (86.111%-93.591%)	92.38% (89.58%-95.45%)	64.44% (58.95%-69.61%)	3.1798 (3.0642-3.2982)	78.12% (71.72%-84.85%)	71.15% (61.9%-80.88%)	90.043% (82.141%-97.3%)	92.47% (86.49%-97.96%)	64.55% (54%-75.47%)	3.1821 (2.9407-3.4161)
At least 4 of CHASM (breast), CHASM (lung), CHASM (melanoma), PolyPhen-2, PROVEAN, VEST	75.76% (72.22%-79.29%)	66.53% (61.47%-71.2%)	91.712% (88.406%-95.833%)	93.28% (90.48%-96.55%)	61.32% (56.31%-66.37%)	3.1284 (3.0232-3.2439)	75.75% (68.69%-82.83%)	66.37% (56.67%-76.28%)	91.798% (83.784%-97.62%)	93.29% (86.84%-98.08%)	61.41% (50.88%-71.7%)	3.1287 (2.8991-3.3485)
At least 4 of CHASM (breast), CHASM (lung), CHASM (melanoma), PolyPhen-2, SIFT, VEST	79.83% (76.77%-83.33%)	78.22% (74.01%-82.4%)	82.607% (77.918%-88.06%)	88.6% (85.32%-92.31%)	68.69% (63.1%-74.19%)	3.1812 (3.0580-3.3127)	79.74% (72.73%-85.86%)	78.11% (69.81%-86.37%)	82.483% (71.995%-92.31%)	88.45% (81.03%-94.83%)	68.73% (57.5%-80.01%)	3.1777 (2.9177-3.4266)
At least 4 of CHASM (breast), CHASM (lung), CHASM (melanoma), PROVEAN, SIFT, VEST	77.12% (74.24%-80.81%)	68.69% (64%-73.73%)	91.7% (88.406%-95.775%)	93.47% (90.82%-96.67%)	62.88% (57.84%-67.93%)	3.1674 (3.0619-3.2859)	77.07% (69.7%-82.83%)	68.45% (58.33%-77.78%)	91.809% (84.202%-97.56%)	93.49% (87.23%-98.08%)	62.92% (52.45%-73.33%)	3.1667 (2.9281-3.3745)
At least 4 of CHASM (breast), CHASM (lung), FATHMM (cancer), FATHMM (missense), Mutation Assessor, MutationTaster	88.96% (86.36%-91.41%)	88.38% (85.04%-91.74%)	89.937% (86.301%-94.032%)	93.82% (91.8%-96.55%)	81.76% (76.92%-86.67%)	3.5390 (3.4370-3.6410)	88.75% (83.84%-93.94%)	88.1% (81.35%-94.92%)	89.834% (81.818%-97.22%)	93.7% (88.33%-98.18%)	81.52% (71.79%-91.43%)	3.5316 (3.3212-3.7242)
At least 4 of CHASM (breast), CHASM (lung), FATHMM (cancer), FATHMM (missense), Mutation Assessor, PolyPhen-2	86.6% (83.84%-89.39%)	87.85% (84.37%-91.27%)	84.421% (79.71%-89.335%)	90.7% (88.09%-93.7%)	80.08% (75%-85.19%)	3.4306 (3.3214-3.5412)	86.4% (80.81%-91.92%)	87.58% (80.64%-94.2%)	84.349% (74.194%-93.75%)	90.58% (84.37%-96.3%)	79.86% (69.44%-90%)	3.4236 (3.2028-3.6437)
At least 4 of CHASM (breast), CHASM (lung), FATHMM (cancer), FATHMM (missense), Mutation Assessor, PROVEAN	82.93% (79.8%-85.86%)	80.47% (76.61%-84.5%)	87.181% (82.857%-91.667%)	91.57% (88.79%-94.64%)	72.09% (66.67%-77.38%)	3.3131 (3.1901-3.4345)	82.62% (76.77%-88.89%)	79.99% (71.43%-88.14%)	87.088% (78.116%-94.87%)	91.41% (85.18%-96.67%)	71.75% (60.47%-82.98%)	3.3024 (3.0563-3.5472)
At least 4 of CHASM (breast), CHASM (lung), FATHMM (cancer), FATHMM (missense), Mutation Assessor, SIFT	86.25% (83.84%-89.39%)	87.85% (84.25%-91.2%)	83.471% (78.873%-88.611%)	90.19% (87.5%-93.44%)	79.91% (74.67%-85.08%)	3.4142 (3.3042-3.5346)	86.08% (79.8%-90.91%)	87.57% (80.88%-94.37%)	83.491% (73.171%-92.11%)	90.11% (83.58%-95.24%)	79.68% (69.23%-90%)	3.4085 (3.1591-3.6265)
At least 4 of CHASM (breast), CHASM (lung), FATHMM (cancer), FATHMM (missense), Mutation Assessor, VEST	86.6% (83.84%-89.39%)	88.38% (85.04%-91.74%)	83.494% (78.571%-88.462%)	90.25% (87.5%-93.5%)	80.62% (75.64%-85.71%)	3.4275 (3.3181-3.5455)	86.4% (80.81%-91.92%)	88.1% (81.35%-94.92%)	83.447% (73.529%-92.31%)	90.14% (83.61%-95.39%)	80.38% (70.27%-90.62%)	3.4207 (3.1899-3.6314)
At least 4 of CHASM (breast), CHASM (lung), FATHMM (cancer), FATHMM (missense), MutationTaster, PolyPhen-2	89.3% (87.36%-91.92%)	89.45% (86.44%-92.62%)	89.034% (85.29%-93.243%)	93.38% (91.13%-95.97%)	83% (78.21%-87.84%)	3.5486 (3.4521-3.6562)	89.08% (83.84%-92.95%)	89.17% (82.81%-95.16%)	88.888% (80.476%-96.97%)	93.23% (87.69%-98.18%)	82.73% (73.68%-92.11%)	3.5402 (3.3228-3.7180)
At least 4 of CHASM (breast), CHASM (lung), FATHMM (cancer), FATHMM (missense), MutationTaster, PROVEAN	85.62% (82.83%-88.38%)	82.06% (78.33%-86.05%)	91.762% (88.406%-95.59%)	94.51% (92.38%-97.22%)	74.74% (69.41%-79.79%)	3.4308 (3.3248-3.5431)	85.33% (79.8%-90.91%)	81.6% (73.43%-89.47%)	91.692% (84.211%-97.5%)	94.4% (88.89%-98.28%)	74.41% (64%-84.78%)	3.4210 (3.1968-3.6352)
At least 4 of CHASM (breast), CHASM (lung), FATHMM (cancer), FATHMM (missense), MutationTaster, SIFT	88.95% (86.36%-91.41%)	88.9% (85.83%-92.19%)	89.01% (85.294%-93.336%)	93.33% (91.13%-95.93%)	82.28% (77.63%-87.18%)	3.5352 (3.4330-3.6438)	88.77% (83.84%-93.94%)	88.66% (81.97%-95.08%)	88.932% (80.488%-96.97%)	93.22% (87.69%-98.15%)	82.08% (72.22%-91.67%)	3.5289 (3.3089-3.7233)
At least 4 of CHASM (breast), CHASM (lung), FATHMM (cancer), FATHMM (missense), MutationTaster, VEST	89.29% (86.87%-91.92%)	89.44% (86.4%-92.68%)	89.01% (85.294%-93.336%)	93.36% (91.2%-95.97%)	82.99% (78.2%-87.84%)	3.5480 (3.4486-3.6528)	89.11% (83.84%-93.94%)	89.18% (82.76%-95.24%)	88.932% (80.488%-96.97%)	93.26% (87.69%-98.15%)	82.77% (72.73%-91.9%)	3.5414 (3.3246-3.7411)
At least 4 of CHASM (breast), CHASM (lung), FATHMM (cancer), FATHMM (missense), PolyPhen-2, PROVEAN	83.59% (80.81%-86.36%)	82.06% (78.33%-86.05%)	86.23% (81.69%-90.667%)	91.16% (88.29%-94.02%)	73.55% (68.18%-79.01%)	3.3300 (3.2031-3.4491)	83.32% (77.78%-88.89%)	81.6% (73.43%-89.47%)	86.23% (76.316%-94.74%)	91.06% (84.91%-96.61%)	73.22% (62%-84.22%)	3.3211 (3.0826-3.5664)
At least 4 of CHASM (breast), CHASM (lung), FATHMM (cancer), FATHMM (missense), PolyPhen-2, SIFT	85.56% (82.83%-88.38%)	88.37% (85.04%-91.67%)	80.698% (75.713%-86.301%)	88.79% (85.83%-92.13%)	80.06% (74.68%-85.33%)	3.3792 (3.2679-3.5020)	85.44% (79.8%-90.91%)	88.13% (81.53%-94.74%)	80.772% (69.697%-90.91%)	88.73% (82.09%-94.92%)	79.89% (69.05%-90%)	3.3753 (3.1285-3.5984)
At least 4 of CHASM (breast), CHASM (lung), FATHMM (cancer), FATHMM (missense), PolyPhen-2, VEST	84.56% (81.82%-87.37%)	88.37% (85.04%-91.67%)	77.97% (72.599%-83.784%)	87.4% (84.38%-90.77%)	79.51% (74.29%-84.94%)	3.3325 (3.2147-3.4621)	84.41% (78.79%-89.9%)	88.13% (81.53%-94.74%)	77.976% (66.667%-88.64%)	87.3% (80.33%-93.55%)	79.32% (68.42%-89.3%)	3.3272 (3.0637-3.5635)
At least 4 of CHASM (breast), CHASM (lung), FATHMM (cancer), FATHMM (missense), PROVEAN, SIFT	82.91% (79.8%-85.86%)	80.99% (77.1%-85.04%)	86.207% (81.69%-90.67%)	91.04% (88.18%-94.02%)	72.41% (67.04%-77.78%)	3.3064 (3.1838-3.4243)	82.67% (76.77%-88.89%)	80.55% (72.13%-88.52%)	86.266% (76.917%-94.6%)	90.98% (84.91%-96.55%)	72.13% (60.78%-83.33%)	3.2992 (3.0586-3.5471)
At least 4 of CHASM (breast), CHASM (lung), FATHMM (cancer), FATHMM (missense), PROVEAN, VEST	82.91% (79.8%-85.86%)	81.52% (77.6%-85.49%)	85.303% (80.822%-90%)	90.56% (87.72%-93.69%)	72.76% (67.07%-78.16%)	3.3015 (3.1784-3.4266)	82.66% (76.77%-88.89%)	81.08% (72.88%-88.89%)	85.328% (75.756%-94.44%)	90.47% (84.21%-96.36%)	72.47% (61.36%-83.72%)	3.2935 (3.0397-3.5429)
At least 4 of CHASM (breast), CHASM (lung), FATHMM (cancer), FATHMM (missense), SIFT, VEST	84.52% (81.82%-87.37%)	88.9% (85.83%-92.19%)	76.932% (71.429%-83.099%)	86.96% (83.85%-90.4%)	80.05% (74.63%-85.34%)	3.3284 (3.2102-3.4555)	84.49% (78.79%-89.9%)	88.66% (81.97%-95.08%)	77.287% (65.514%-87.81%)	87.02% (80%-93.33%)	79.91% (68.96%-90.62%)	3.3288 (3.0765-3.5656)
At least 4 of CHASM (breast), CHASM (lung), FATHMM (cancer), Mutation Assessor, MutationTaster, PolyPhen-2	83.82% (80.81%-86.87%)	79.77% (75.59%-84.25%)	90.816% (87.324%-94.522%)	93.75% (91.51%-96.4%)	72.2% (67.02%-77.17%)	3.3654 (3.2590-3.4835)	83.87% (77.78%-89.9%)	79.8% (70.76%-88.14%)	90.849% (82.927%-97.37%)	93.72% (88%-98.15%)	72.4% (62.22%-82.93%)	3.3677 (3.1341-3.5870)
At least 4 of CHASM (breast), CHASM (lung), FATHMM (cancer), Mutation Assessor, MutationTaster, PROVEAN	80.49% (77.27%-83.84%)	73.45% (68.64%-78.13%)	92.665% (89.394%-96.105%)	94.54% (92.16%-97.09%)	66.87% (61.38%-71.88%)	3.2752 (3.1615-3.3850)	80.43% (73.74%-86.87%)	73.3% (64.06%-83.08%)	92.64% (85.366%-100%)	94.47% (88.88%-100%)	66.93% (56.52%-78.01%)	3.2734 (3.0545-3.5167)
At least 4 of CHASM (breast), CHASM (lung), FATHMM (cancer), Mutation Assessor, MutationTaster, SIFT	86.53% (83.84%-89.39%)	83.5% (79.84%-87.2%)	91.761% (88.406%-95.652%)	94.6% (92.52%-97.3%)	76.29% (71.11%-81.02%)	3.4615 (3.3553-3.5728)	86.54% (80.81%-91.92%)	83.51% (75.86%-90.91%)	91.697% (84.375%-97.5%)	94.52% (88.89%-98.31%)	76.44% (66.67%-86.67%)	3.4617 (3.2442-3.6769)
At least 4 of CHASM (breast), CHASM (lung), FATHMM (cancer), Mutation Assessor, MutationTaster, VEST	83.82% (80.81%-86.87%)	79.77% (75.61%-84.25%)	90.816% (87.324%-94.522%)	93.75% (91.43%-96.4%)	72.2% (67.02%-77.17%)	3.3654 (3.2590-3.4761)	83.87% (77.78%-89.9%)	79.79% (70.49%-87.93%)	90.849% (82.927%-97.37%)	93.72% (88%-98.18%)	72.4% (62%-82.93%)	3.3676 (3.1455-3.5798)
At least 4 of CHASM (breast), CHASM (lung), FATHMM (cancer), Mutation Assessor, PolyPhen-2, PROVEAN	75.1% (71.21%-78.28%)	69.74% (65.08%-74.44%)	84.354% (79.452%-89.189%)	88.51% (85%-92.23%)	61.72% (56.31%-67.01%)	3.0433 (2.9015-3.1698)	75.06% (68.69%-82.83%)	69.54% (60.29%-79.11%)	84.502% (74.984%-94.44%)	88.5% (80.95%-95.83%)	61.79% (51.16%-72.92%)	3.0433 (2.7836-3.3201)
At least 4 of CHASM (breast), CHASM (lung), FATHMM (cancer), Mutation Assessor, PolyPhen-2, SIFT	81.14% (77.78%-84.34%)	80.33% (76.38%-84.38%)	82.535% (77.462%-87.5%)	88.83% (85.47%-92.17%)	70.82% (65.38%-75.9%)	3.2251 (3.0904-3.3506)	81.16% (74.75%-87.88%)	80.27% (71.67%-88.14%)	82.646% (72.973%-92.5%)	88.8% (81.82%-95.08%)	70.97% (60.87%-82.22%)	3.2269 (2.9698-3.4975)
At least 4 of CHASM (breast), CHASM (lung), FATHMM (cancer), Mutation Assessor, PolyPhen-2, VEST	77.45% (74.24%-80.81%)	75.55% (71.32%-80%)	80.724% (75.362%-85.942%)	87.14% (83.5%-90.91%)	65.63% (60.22%-70.93%)	3.0905 (2.9566-3.2260)	77.43% (70.71%-83.84%)	75.46% (66.67%-84.32%)	80.782% (70.27%-91.18%)	87.06% (79.24%-94.23%)	65.74% (55%-76.93%)	3.0904 (2.8161-3.3613)
At least 4 of CHASM (breast), CHASM (lung), FATHMM (cancer), Mutation Assessor, PROVEAN, SIFT	75.78% (72.22%-79.29%)	70.83% (66.13%-75.4%)	84.335% (79.167%-89.189%)	88.66% (85.1%-92.23%)	62.58% (56.99%-67.71%)	3.0641 (2.9325-3.1891)	75.71% (68.69%-82.83%)	70.54% (61.01%-79.42%)	84.536% (74.984%-94.74%)	88.67% (81.25%-95.92%)	62.58% (51.16%-73.47%)	3.0633 (2.8032-3.3281)
At least 4 of CHASM (breast), CHASM (lung), FATHMM (cancer), Mutation Assessor, PROVEAN, VEST	73.76% (70.2%-77.27%)	67.62% (62.81%-72.44%)	84.38% (79.167%-89.333%)	88.21% (84.44%-92.01%)	60.11% (54.72%-65.35%)	3.0032 (2.8617-3.1332)	73.69% (66.67%-80.81%)	67.4% (57.81%-76.93%)	84.448% (74.28%-94.45%)	88.15% (80.39%-95.74%)	60.15% (50%-71.43%)	3.0015 (2.7413-3.2775)
At least 4 of CHASM (breast), CHASM (lung), FATHMM (cancer), Mutation Assessor, SIFT, VEST	80.15% (76.77%-83.33%)	79.29% (75.4%-83.47%)	81.633% (76.712%-87.143%)	88.18% (84.82%-91.74%)	69.51% (63.86%-75%)	3.1862 (3.0485-3.3229)	80.11% (73.74%-86.87%)	79.17% (70.49%-86.89%)	81.689% (70.588%-91.43%)	88.12% (81.03%-94.83%)	69.57% (58.53%-80.49%)	3.1854 (2.9112-3.4524)
At least 4 of CHASM (breast), CHASM (lung), FATHMM (cancer), MutationTaster, PolyPhen-2, PROVEAN	80.48% (77.27%-83.84%)	73.44% (68.75%-78.12%)	92.665% (89.394%-96.105%)	94.54% (92.22%-97.09%)	66.86% (61.54%-72.05%)	3.2750 (3.1631-3.3883)	80.45% (73.74%-86.87%)	73.32% (63.79%-83.05%)	92.64% (85.366%-100%)	94.47% (88.89%-100%)	66.94% (56.36%-78.01%)	3.2738 (3.0473-3.5008)
At least 4 of CHASM (breast), CHASM (lung), FATHMM (cancer), MutationTaster, PolyPhen-2, SIFT	85.86% (83.33%-88.89%)	82.96% (79.34%-87.1%)	90.876% (87.324%-94.667%)	94.02% (91.74%-96.55%)	75.53% (70.37%-80.72%)	3.4338 (3.3241-3.5468)	85.85% (79.8%-90.91%)	82.99% (74.57%-90.32%)	90.727% (82.855%-97.37%)	93.88% (88.33%-98.25%)	75.69% (65.61%-85.71%)	3.4329 (3.2129-3.6437)
At least 4 of CHASM (breast), CHASM (lung), FATHMM (cancer), MutationTaster, PolyPhen-2, VEST	83.82% (80.81%-86.87%)	80.28% (76.27%-84.68%)	89.931% (86.111%-94.118%)	93.23% (90.83%-96.19%)	72.51% (67.42%-77.53%)	3.3595 (3.2493-3.4744)	83.88% (77.78%-89.9%)	80.38% (71.43%-88.14%)	89.879% (82.039%-97.3%)	93.15% (87.27%-98.11%)	72.77% (62.49%-82.99%)	3.3617 (3.1394-3.5870)
At least 4 of CHASM (breast), CHASM (lung), FATHMM (cancer), MutationTaster, PROVEAN, SIFT	82.17% (78.79%-85.35%)	75.56% (70.97%-80.16%)	93.605% (90.539%-97.102%)	95.33% (93.14%-97.96%)	68.9% (63.54%-74.19%)	3.3340 (3.2221-3.4475)	82.12% (75.76%-88.89%)	75.46% (66.09%-84.62%)	93.512% (86.667%-100%)	95.23% (90.2%-100%)	68.98% (58.69%-79.6%)	3.3318 (3.1017-3.5538)
At least 4 of CHASM (breast), CHASM (lung), FATHMM (cancer), MutationTaster, PROVEAN, VEST	79.12% (75.76%-82.32%)	71.29% (66.41%-75.97%)	92.665% (89.394%-96.105%)	94.38% (91.95%-97%)	65.12% (59.8%-70.37%)	3.2346 (3.1210-3.3433)	79.13% (72.73%-85.86%)	71.23% (61.66%-80.95%)	92.64% (85.366%-100%)	94.32% (88.46%-100%)	65.25% (54.55%-76.47%)	3.2344 (3.0169-3.4665)
At least 4 of CHASM (breast), CHASM (lung), FATHMM (cancer), MutationTaster, SIFT, VEST	85.52% (82.83%-88.38%)	82.96% (79.23%-87.02%)	89.948% (86.301%-94.205%)	93.45% (90.99%-96.4%)	75.34% (70%-80.44%)	3.4169 (3.3085-3.5313)	85.52% (79.8%-90.91%)	82.99% (75%-90.33%)	89.825% (81.579%-97.14%)	93.33% (87.27%-98.21%)	75.51% (65.79%-85.71%)	3.4165 (3.1939-3.6366)
At least 4 of CHASM (breast), CHASM (lung), FATHMM (cancer), PolyPhen-2, PROVEAN, SIFT	76.77% (73.23%-80.3%)	73.45% (68.79%-78.13%)	82.49% (77.462%-87.5%)	87.88% (84.31%-91.51%)	64.26% (58.58%-69.7%)	3.0808 (2.9435-3.2101)	76.77% (69.7%-83.84%)	73.28% (63.33%-82.36%)	82.724% (72.5%-92.11%)	87.92% (80.39%-94.55%)	64.36% (53.19%-75.61%)	3.0828 (2.8287-3.3597)
At least 4 of CHASM (breast), CHASM (lung), FATHMM (cancer), PolyPhen-2, PROVEAN, VEST	74.42% (70.71%-77.78%)	69.19% (64.34%-73.98%)	83.466% (78.571%-88.41%)	87.86% (84.21%-91.75%)	61.04% (55.67%-66.33%)	3.0156 (2.8790-3.1452)	74.38% (67.68%-81.82%)	69.04% (59.64%-78.69%)	83.524% (73.529%-93.19%)	87.79% (80%-95.24%)	61.13% (50.92%-72.34%)	3.0149 (2.7534-3.2909)
At least 4 of CHASM (breast), CHASM (lung), FATHMM (cancer), PolyPhen-2, SIFT, VEST	80.11% (76.77%-83.33%)	80.32% (76.56%-84.5%)	79.741% (74.627%-85.507%)	87.27% (83.93%-90.91%)	70.1% (64.63%-75.61%)	3.1744 (3.0350-3.3087)	80.18% (73.74%-86.87%)	80.28% (71.43%-88.14%)	79.95% (68.421%-90.32%)	87.3% (80%-94.23%)	70.28% (59.52%-81.08%)	3.1782 (2.9052-3.4567)
At least 4 of CHASM (breast), CHASM (lung), FATHMM (cancer), PROVEAN, SIFT, VEST	75.11% (71.72%-78.79%)	71.34% (66.67%-76%)	81.619% (76.469%-87.143%)	87.03% (83.33%-90.91%)	62.23% (56.56%-67.68%)	3.0222 (2.8839-3.1575)	75.03% (67.68%-81.82%)	71.12% (61.53%-80.6%)	81.706% (70.588%-91.67%)	86.96% (79.24%-94.23%)	62.26% (51.02%-73.81%)	3.0204 (2.7513-3.2930)
At least 4 of CHASM (breast), CHASM (lung), FATHMM (missense), Mutation Assessor, MutationTaster, PolyPhen-2	84.52% (81.82%-87.37%)	80.33% (76.27%-84.38%)	91.761% (88.406%-95.652%)	94.4% (92.16%-97.17%)	72.96% (67.82%-77.9%)	3.3945 (3.2921-3.5045)	84.49% (78.79%-89.9%)	80.27% (71.88%-87.88%)	91.697% (84.375%-97.5%)	94.32% (88.68%-98.28%)	73.07% (63.04%-82.99%)	3.3936 (3.1734-3.6006)
At least 4 of CHASM (breast), CHASM (lung), FATHMM (missense), Mutation Assessor, MutationTaster, PROVEAN	80.18% (77.27%-83.33%)	72.95% (68.46%-77.6%)	92.665% (89.394%-96.105%)	94.5% (92.16%-97.06%)	66.47% (61%-71.43%)	3.2659 (3.1602-3.3748)	80.04% (73.74%-85.86%)	72.68% (63.33%-81.97%)	92.64% (85.366%-100%)	94.43% (88.64%-100%)	66.43% (55.81%-77.09%)	3.2618 (3.0454-3.4781)
At least 4 of CHASM (breast), CHASM (lung), FATHMM (missense), Mutation Assessor, MutationTaster, SIFT	86.89% (84.34%-89.39%)	84.06% (80.34%-87.79%)	91.761% (88.406%-95.652%)	94.64% (92.52%-97.3%)	76.91% (71.74%-81.72%)	3.4737 (3.3727-3.5729)	86.84% (81.82%-91.92%)	83.98% (76.27%-91.38%)	91.697% (84.375%-97.5%)	94.56% (89.09%-98.33%)	76.97% (67.39%-87.18%)	3.4720 (3.2761-3.6756)
At least 4 of CHASM (breast), CHASM (lung), FATHMM (missense), Mutation Assessor, MutationTaster, VEST	84.86% (82.32%-87.88%)	80.86% (76.92%-84.88%)	91.761% (88.406%-95.652%)	94.44% (92.23%-97.2%)	73.5% (68.47%-78.41%)	3.4056 (3.3055-3.5122)	84.83% (78.79%-89.9%)	80.8% (72.41%-88.71%)	91.697% (84.375%-97.5%)	94.35% (88.89%-98.28%)	73.6% (63.41%-83.73%)	3.4045 (3.1906-3.6046)
At least 4 of CHASM (breast), CHASM (lung), FATHMM (missense), Mutation Assessor, PolyPhen-2, PROVEAN	75.14% (71.72%-78.79%)	69.25% (64.29%-74.19%)	85.304% (80.822%-90.278%)	89.07% (85.71%-92.71%)	61.61% (56%-66.67%)	3.0523 (2.9274-3.1797)	74.98% (67.68%-81.82%)	68.92% (59.38%-78.34%)	85.329% (75.676%-94.44%)	88.98% (81.81%-95.84%)	61.55% (50.98%-72.73%)	3.0477 (2.7850-3.2947)
At least 4 of CHASM (breast), CHASM (lung), FATHMM (missense), Mutation Assessor, PolyPhen-2, SIFT	81.49% (78.28%-84.34%)	80.35% (76.51%-84.38%)	83.432% (78.667%-88.571%)	89.35% (86.11%-92.79%)	71.07% (65.48%-76.06%)	3.2421 (3.1189-3.3626)	81.47% (75.76%-87.88%)	80.21% (71.93%-87.72%)	83.565% (73.529%-93.33%)	89.35% (82.76%-96%)	71.13% (61.36%-81.82%)	3.2425 (3.0022-3.4889)
At least 4 of CHASM (breast), CHASM (lung), FATHMM (missense), Mutation Assessor, PolyPhen-2, VEST	78.47% (75.25%-81.31%)	76.11% (71.9%-80.3%)	82.541% (77.465%-87.838%)	88.29% (84.82%-91.89%)	66.65% (61.17%-71.88%)	3.1359 (3.0091-3.2583)	78.41% (72.73%-84.85%)	75.94% (67.69%-84.38%)	82.615% (71.875%-92.31%)	88.24% (80.95%-94.74%)	66.69% (56.41%-77.78%)	3.1348 (2.8888-3.3837)
At least 4 of CHASM (breast), CHASM (lung), FATHMM (missense), Mutation Assessor, PROVEAN, SIFT	76.5% (73.23%-79.8%)	70.87% (66.41%-75.59%)	86.209% (81.818%-91.045%)	89.89% (86.6%-93.62%)	63.13% (57.73%-68.37%)	3.1010 (2.9839-3.2274)	76.3% (69.7%-82.83%)	70.46% (60.94%-79.7%)	86.267% (77.143%-94.74%)	89.82% (82.69%-96.23%)	63% (52.38%-73.58%)	3.0954 (2.8410-3.3242)
At least 4 of CHASM (breast), CHASM (lung), FATHMM (missense), Mutation Assessor, PROVEAN, VEST	74.13% (70.71%-77.78%)	67.13% (62.5%-71.91%)	86.232% (81.69%-90.912%)	89.4% (86.02%-93.26%)	60.27% (54.81%-65.38%)	3.0303 (2.9058-3.1581)	73.96% (66.67%-80.81%)	66.78% (57.35%-75.93%)	86.231% (76.471%-94.87%)	89.29% (81.82%-96.15%)	60.21% (50%-71.15%)	3.0251 (2.7685-3.2725)
At least 4 of CHASM (breast), CHASM (lung), FATHMM (missense), Mutation Assessor, SIFT, VEST	80.82% (77.78%-83.84%)	79.85% (75.97%-84%)	82.494% (77.465%-87.838%)	88.75% (85.45%-92.04%)	70.31% (64.99%-75.86%)	3.2140 (3.0948-3.3424)	80.78% (74.75%-86.87%)	79.64% (71.18%-87.5%)	82.69% (71.875%-92.5%)	88.77% (82.14%-95.08%)	70.31% (59.46%-80.49%)	3.2141 (2.9557-3.4560)
At least 4 of CHASM (breast), CHASM (lung), FATHMM (missense), MutationTaster, PolyPhen-2, PROVEAN	80.51% (77.27%-83.84%)	73.47% (68.8%-78.05%)	92.665% (89.394%-96.105%)	94.54% (92.22%-97.09%)	66.9% (61.54%-72%)	3.2758 (3.1682-3.3874)	80.39% (73.74%-86.87%)	73.24% (64.06%-82.54%)	92.64% (85.366%-100%)	94.47% (88.64%-100%)	66.88% (56.25%-77.56%)	3.2723 (3.0462-3.4884)
At least 4 of CHASM (breast), CHASM (lung), FATHMM (missense), MutationTaster, PolyPhen-2, SIFT	85.85% (82.83%-88.89%)	82.97% (79.2%-86.82%)	90.824% (87.324%-94.595%)	93.99% (91.82%-96.52%)	75.53% (70.59%-80.46%)	3.4332 (3.3251-3.5402)	85.87% (79.8%-91.92%)	82.96% (74.6%-90.62%)	90.819% (82.927%-97.37%)	93.94% (88.37%-98.22%)	75.68% (65.85%-86.05%)	3.4340 (3.2184-3.6482)
At least 4 of CHASM (breast), CHASM (lung), FATHMM (missense), MutationTaster, PolyPhen-2, VEST	83.17% (80.3%-86.36%)	79.78% (75.78%-83.74%)	89.014% (84.932%-93.245%)	92.62% (90%-95.5%)	71.8% (66.67%-76.74%)	3.3322 (3.2191-3.4425)	83.16% (76.77%-88.89%)	79.77% (71.67%-87.69%)	88.931% (80.556%-97.14%)	92.52% (86.27%-98.08%)	71.96% (61.9%-82.5%)	3.3319 (3.1077-3.5562)
At least 4 of CHASM (breast), CHASM (lung), FATHMM (missense), MutationTaster, PROVEAN, SIFT	81.86% (78.79%-84.85%)	75.06% (70.54%-79.84%)	93.605% (90.539%-97.102%)	95.3% (93.14%-97.94%)	68.47% (63.16%-73.68%)	3.3245 (3.2171-3.4351)	81.73% (75.76%-87.88%)	74.84% (65.08%-83.58%)	93.512% (86.667%-100%)	95.2% (90.2%-100%)	68.44% (58.14%-78.95%)	3.3199 (3.0992-3.5347)
At least 4 of CHASM (breast), CHASM (lung), FATHMM (missense), MutationTaster, PROVEAN, VEST	79.15% (76.26%-82.32%)	71.33% (66.67%-76%)	92.665% (89.394%-96.105%)	94.39% (92%-97%)	65.15% (59.8%-70.11%)	3.2353 (3.1290-3.3430)	79.07% (72.73%-84.85%)	71.14% (61.66%-80.3%)	92.64% (85.366%-100%)	94.32% (88.37%-100%)	65.19% (54.39%-75.56%)	3.2330 (3.0124-3.4468)
At least 4 of CHASM (breast), CHASM (lung), FATHMM (missense), MutationTaster, SIFT, VEST	85.51% (82.83%-88.38%)	83.51% (79.83%-87.2%)	88.957% (85.29%-93.151%)	92.9% (90.48%-95.69%)	75.73% (70.59%-80.68%)	3.4109 (3.3011-3.5234)	85.55% (79.8%-90.91%)	83.49% (75.47%-91.23%)	89.042% (80.556%-96.97%)	92.9% (87.09%-98.11%)	75.89% (65.91%-86.79%)	3.4133 (3.1903-3.6313)
At least 4 of CHASM (breast), CHASM (lung), FATHMM (missense), PolyPhen-2, PROVEAN, SIFT	77.13% (73.74%-80.3%)	72.96% (68.29%-77.62%)	84.32% (79.452%-89.045%)	88.95% (85.71%-92.45%)	64.34% (58.7%-69.57%)	3.1057 (2.9847-3.2364)	77.05% (70.71%-83.84%)	72.65% (63.16%-81.67%)	84.531% (75%-93.94%)	88.98% (82%-95.83%)	64.33% (54%-75%)	3.1050 (2.8470-3.3479)
At least 4 of CHASM (breast), CHASM (lung), FATHMM (missense), PolyPhen-2, PROVEAN, VEST	74.44% (71.21%-77.78%)	69.23% (64.29%-74.02%)	83.428% (78.664%-88.464%)	87.84% (84.16%-91.75%)	61.06% (55.45%-66.34%)	3.0156 (2.8855-3.1416)	74.36% (67.68%-80.81%)	68.96% (59.38%-78.88%)	83.582% (73.529%-93.75%)	87.82% (80%-95.35%)	61.09% (50%-72.1%)	3.0145 (2.7610-3.2728)
At least 4 of CHASM (breast), CHASM (lung), FATHMM (missense), PolyPhen-2, SIFT, VEST	79.12% (75.76%-82.32%)	80.34% (76.23%-84.25%)	77.004% (71.617%-82.61%)	85.8% (82.2%-89.52%)	69.38% (63.63%-74.7%)	3.1252 (2.9920-3.2633)	79.13% (72.73%-85.86%)	80.25% (72.13%-87.88%)	77.16% (64.865%-87.88%)	85.79% (78.18%-92.86%)	69.5% (57.89%-80.95%)	3.1270 (2.8536-3.3931)
At least 4 of CHASM (breast), CHASM (lung), FATHMM (missense), PROVEAN, SIFT, VEST	75.45% (72.22%-78.79%)	70.85% (66.14%-75.59%)	83.381% (78.571%-88.41%)	88.06% (84.69%-91.84%)	62.33% (56.7%-67.65%)	3.0462 (2.9162-3.1787)	75.36% (68.69%-81.82%)	70.5% (60.94%-80%)	83.657% (73.529%-93.55%)	88.11% (80.7%-95.01%)	62.31% (51.06%-73.08%)	3.0458 (2.7859-3.3073)
At least 4 of CHASM (breast), CHASM (lung), Mutation Assessor, MutationTaster, PolyPhen-2, PROVEAN	78.81% (75.25%-82.32%)	70.8% (66.12%-75.66%)	92.665% (89.394%-96.105%)	94.35% (91.92%-97%)	64.73% (59.61%-69.64%)	3.2254 (3.1121-3.3349)	78.74% (71.72%-85.86%)	70.61% (61.02%-80.01%)	92.64% (85.366%-100%)	94.28% (88.37%-100%)	64.76% (54.55%-75.47%)	3.2229 (3.0029-3.4506)
At least 4 of CHASM (breast), CHASM (lung), Mutation Assessor, MutationTaster, PolyPhen-2, SIFT	83.83% (80.81%-86.87%)	80.29% (76.42%-84.3%)	89.933% (86.301%-94.288%)	93.24% (90.74%-96.23%)	72.53% (67.73%-77.78%)	3.3599 (3.2494-3.4774)	83.86% (77.78%-89.9%)	80.34% (72.13%-87.93%)	89.863% (81.081%-97.22%)	93.14% (87.23%-98.15%)	72.73% (62.22%-82.5%)	3.3607 (3.1249-3.5815)
At least 4 of CHASM (breast), CHASM (lung), Mutation Assessor, MutationTaster, PolyPhen-2, VEST	81.83% (78.79%-84.85%)	77.68% (73.6%-81.9%)	88.988% (85.29%-93.243%)	92.42% (89.8%-95.46%)	69.75% (64.77%-75.27%)	3.2884 (3.1746-3.4061)	81.8% (75.76%-87.88%)	77.59% (69.35%-85.72%)	89.014% (80.488%-97.06%)	92.36% (85.71%-97.96%)	69.84% (58.97%-79.55%)	3.2880 (3.0542-3.5099)
At least 4 of CHASM (breast), CHASM (lung), Mutation Assessor, MutationTaster, PROVEAN, SIFT	77.49% (74.24%-80.81%)	68.16% (63.41%-73.11%)	93.605% (90.539%-97.102%)	94.85% (92.39%-97.75%)	62.97% (57.94%-67.89%)	3.1958 (3.0891-3.3068)	77.35% (70.71%-83.84%)	67.91% (58.06%-77.27%)	93.512% (86.667%-100%)	94.73% (89.13%-100%)	62.94% (52.72%-73.21%)	3.1909 (2.9658-3.4024)
At least 4 of CHASM (breast), CHASM (lung), Mutation Assessor, MutationTaster, PROVEAN, VEST	76.11% (72.73%-79.8%)	66.53% (61.72%-71.3%)	92.665% (89.394%-96.105%)	94.01% (91.46%-96.81%)	61.56% (56.36%-66.67%)	3.1476 (3.0343-3.2638)	76.06% (68.69%-82.83%)	66.37% (56.9%-76.12%)	92.64% (85.366%-100%)	93.93% (87.76%-100%)	61.62% (50.98%-72.01%)	3.1457 (2.9142-3.3713)
At least 4 of CHASM (breast), CHASM (lung), Mutation Assessor, MutationTaster, SIFT, VEST	82.51% (79.8%-85.35%)	78.22% (74.38%-82.4%)	89.933% (86.301%-94.288%)	93.07% (90.53%-96.15%)	70.49% (65.56%-75.76%)	3.3171 (3.2126-3.4311)	82.45% (76.77%-87.88%)	78.11% (69.84%-86.15%)	89.863% (81.081%-97.22%)	92.96% (86.67%-98.08%)	70.53% (60%-80.44%)	3.3146 (3.0825-3.5254)
At least 4 of CHASM (breast), CHASM (lung), Mutation Assessor, PolyPhen-2, PROVEAN, SIFT	73.07% (69.7%-76.77%)	67.63% (62.99%-72.44%)	82.479% (77.462%-87.5%)	86.97% (83.16%-90.82%)	59.57% (53.85%-64.76%)	2.9664 (2.8300-3.1004)	73.05% (65.66%-79.8%)	67.38% (57.81%-76.79%)	82.733% (72.967%-92.5%)	87.01% (79.24%-94.45%)	59.64% (48.84%-70.84%)	2.9676 (2.7019-3.2395)
At least 4 of CHASM (breast), CHASM (lung), Mutation Assessor, PolyPhen-2, PROVEAN, VEST	73.77% (70.2%-77.27%)	68.16% (63.33%-73.11%)	83.451% (78.571%-88.71%)	87.69% (83.91%-91.67%)	60.25% (54.71%-65.66%)	2.9955 (2.8558-3.1315)	73.68% (66.67%-80.81%)	67.9% (58.33%-77.59%)	83.547% (73.684%-93.33%)	87.63% (79.63%-95.24%)	60.27% (48.98%-71.43%)	2.9934 (2.7258-3.2591)
At least 4 of CHASM (breast), CHASM (lung), Mutation Assessor, PolyPhen-2, SIFT, VEST	76.46% (73.23%-79.8%)	76.64% (72.66%-80.8%)	76.147% (70.667%-81.69%)	84.74% (80.9%-88.5%)	65.34% (59.52%-71.09%)	3.0287 (2.8902-3.1698)	76.37% (69.7%-82.83%)	76.49% (68.25%-84.75%)	76.148% (65.375%-87.19%)	84.61% (77.05%-92.19%)	65.36% (53.48%-76.6%)	3.0260 (2.7374-3.2988)
At least 4 of CHASM (breast), CHASM (lung), Mutation Assessor, PROVEAN, SIFT, VEST	72.07% (68.69%-75.76%)	66.54% (61.83%-71.54%)	81.611% (76.469%-86.486%)	86.22% (82.42%-90.2%)	58.52% (52.83%-63.81%)	2.9289 (2.7872-3.0580)	72.03% (64.65%-78.79%)	66.35% (56.36%-75.82%)	81.723% (71.786%-91.89%)	86.16% (77.99%-93.75%)	58.59% (47.83%-69.39%)	2.9283 (2.6694-3.2034)
At least 4 of CHASM (breast), CHASM (lung), MutationTaster, PolyPhen-2, PROVEAN, SIFT	79.82% (76.77%-82.84%)	71.85% (67.16%-76.61%)	93.605% (90.539%-97.102%)	95.1% (92.86%-97.85%)	65.79% (60.57%-71.03%)	3.2635 (3.1566-3.3754)	79.74% (73.71%-85.86%)	71.69% (62.49%-80.95%)	93.512% (86.667%-100%)	94.99% (89.79%-100%)	65.83% (55.55%-76.09%)	3.2603 (3.0408-3.4780)
At least 4 of CHASM (breast), CHASM (lung), MutationTaster, PolyPhen-2, PROVEAN, VEST	78.12% (74.75%-81.31%)	69.71% (65.08%-74.4%)	92.665% (89.394%-96.105%)	94.26% (91.76%-96.97%)	63.89% (58.72%-68.93%)	3.2053 (3.0925-3.3160)	78.1% (71.72%-84.85%)	69.6% (60.32%-79.1%)	92.64% (85.366%-100%)	94.2% (88.23%-100%)	63.98% (53.7%-74.08%)	3.2042 (2.9834-3.4331)
At least 4 of CHASM (breast), CHASM (lung), MutationTaster, PolyPhen-2, SIFT, VEST	83.18% (80.3%-86.36%)	80.32% (76.67%-84.38%)	88.11% (84.058%-92.857%)	92.11% (89.47%-95.24%)	72.15% (67.39%-77.27%)	3.3269 (3.2150-3.4445)	83.14% (76.77%-88.89%)	80.28% (72.31%-87.5%)	88.015% (79.409%-95.46%)	91.98% (85.71%-96.72%)	72.24% (62.16%-82.5%)	3.3252 (3.0959-3.5504)
At least 4 of CHASM (breast), CHASM (lung), MutationTaster, PROVEAN, SIFT, VEST	78.15% (74.75%-81.31%)	69.2% (64.46%-73.77%)	93.605% (90.539%-97.102%)	94.93% (92.55%-97.75%)	63.74% (58.82%-68.81%)	3.2147 (3.1044-3.3262)	78.05% (71.72%-84.85%)	69.02% (60%-78.46%)	93.512% (86.667%-100%)	94.81% (89.57%-100%)	63.76% (53.06%-74%)	3.2110 (2.9863-3.4356)
At least 4 of CHASM (breast), CHASM (lung), PolyPhen-2, PROVEAN, SIFT, VEST	73.41% (69.7%-76.77%)	69.21% (64.29%-74.02%)	80.661% (75.384%-85.916%)	86.09% (82.18%-90.1%)	60.24% (54.44%-65.69%)	2.9619 (2.8218-3.0952)	73.39% (66.67%-80.81%)	69.01% (59.38%-78.46%)	80.869% (70%-91.18%)	86.09% (78%-93.88%)	60.32% (48.94%-71.75%)	2.9630 (2.6966-3.2450)
At least 4 of CHASM (breast), CHASM (melanoma), FATHMM (cancer), FATHMM (missense), Mutation Assessor, MutationTaster	88.61% (86.36%-90.91%)	88.89% (85.71%-92%)	88.111% (84.207%-92.308%)	92.82% (90.55%-95.69%)	82.11% (77.5%-86.84%)	3.5193 (3.4191-3.6183)	88.44% (83.84%-92.93%)	88.69% (82.45%-95.08%)	87.968% (78.947%-95.12%)	92.69% (87.27%-96.92%)	81.96% (72.41%-91.43%)	3.5131 (3.3133-3.7134)
At least 4 of CHASM (breast), CHASM (melanoma), FATHMM (cancer), FATHMM (missense), Mutation Assessor, PolyPhen-2	85.23% (82.32%-87.89%)	88.36% (85.12%-91.67%)	79.806% (74.648%-85.338%)	88.33% (85.37%-91.73%)	79.86% (74.67%-85.29%)	3.3635 (3.2437-3.4876)	85.1% (79.77%-90.91%)	88.16% (81.35%-94.92%)	79.815% (68.571%-89.74%)	88.24% (81.36%-94.03%)	79.75% (69.23%-90.32%)	3.3597 (3.1240-3.6007)
At least 4 of CHASM (breast), CHASM (melanoma), FATHMM (cancer), FATHMM (missense), Mutation Assessor, PROVEAN	84.88% (82.32%-87.88%)	85.15% (81.75%-88.8%)	84.4% (79.994%-89.333%)	90.42% (87.7%-93.39%)	76.68% (71.25%-81.95%)	3.3664 (3.2504-3.4839)	84.79% (78.79%-89.9%)	85.01% (77.42%-91.94%)	84.392% (74.357%-93.75%)	90.34% (84.21%-96.23%)	76.67% (65.79%-87.18%)	3.3640 (3.1345-3.5904)
At least 4 of CHASM (breast), CHASM (melanoma), FATHMM (cancer), FATHMM (missense), Mutation Assessor, SIFT	85.2% (82.32%-87.88%)	87.81% (84.61%-91.2%)	80.668% (75.713%-85.915%)	88.71% (85.93%-92%)	79.29% (73.97%-84.51%)	3.3648 (3.2472-3.4832)	85.16% (79.8%-90.91%)	87.66% (80.95%-93.85%)	80.83% (69.992%-90.33%)	88.71% (81.97%-94.65%)	79.28% (68.57%-89.47%)	3.3649 (3.1269-3.6002)
At least 4 of CHASM (breast), CHASM (melanoma), FATHMM (cancer), FATHMM (missense), Mutation Assessor, VEST	85.91% (83.33%-88.38%)	88.36% (85.12%-91.67%)	81.657% (76.809%-87.016%)	89.29% (86.44%-92.68%)	80.24% (75.31%-85.53%)	3.3955 (3.2861-3.5125)	85.76% (80.81%-90.91%)	88.15% (81.36%-94.92%)	81.612% (70.588%-91.67%)	89.18% (82.45%-95%)	80.09% (69.56%-90.33%)	3.3903 (3.1553-3.6099)
At least 4 of CHASM (breast), CHASM (melanoma), FATHMM (cancer), FATHMM (missense), MutationTaster, PolyPhen-2	88.61% (86.36%-91.41%)	89.43% (86.4%-92.59%)	87.185% (83.099%-91.892%)	92.35% (90.08%-95.16%)	82.68% (78.08%-87.5%)	3.5165 (3.4184-3.6231)	88.43% (82.83%-92.93%)	89.2% (83.05%-95.24%)	87.061% (77.143%-94.87%)	92.22% (86.21%-96.72%)	82.48% (72.97%-91.89%)	3.5096 (3.2873-3.7035)
At least 4 of CHASM (breast), CHASM (melanoma), FATHMM (cancer), FATHMM (missense), MutationTaster, PROVEAN	87.25% (84.85%-89.9%)	86.22% (82.9%-89.76%)	89.032% (85.294%-93.333%)	93.15% (90.91%-95.83%)	78.89% (73.81%-84%)	3.4729 (3.3628-3.5836)	87.11% (81.82%-91.92%)	86.06% (78.69%-92.65%)	88.882% (79.987%-96.88%)	93.01% (87.3%-98.11%)	78.81% (68.42%-88.89%)	3.4676 (3.2497-3.6756)
At least 4 of CHASM (breast), CHASM (melanoma), FATHMM (cancer), FATHMM (missense), MutationTaster, SIFT	87.93% (85.35%-90.4%)	88.89% (85.71%-92%)	86.258% (82.049%-90.909%)	91.8% (89.38%-94.83%)	81.79% (77.03%-86.67%)	3.4874 (3.3841-3.5973)	87.78% (82.83%-92.93%)	88.69% (82.45%-95.08%)	86.158% (76.316%-94.45%)	91.68% (85.71%-96.61%)	81.65% (71.43%-91.43%)	3.4819 (3.2544-3.6802)
At least 4 of CHASM (breast), CHASM (melanoma), FATHMM (cancer), FATHMM (missense), MutationTaster, VEST	88.27% (85.86%-90.91%)	89.43% (86.29%-92.62%)	86.258% (82.049%-90.909%)	91.84% (89.43%-94.87%)	82.52% (78.08%-87.5%)	3.5005 (3.4001-3.6099)	88.1% (82.83%-92.93%)	89.21% (82.81%-95.24%)	86.158% (76.316%-94.45%)	91.72% (85.71%-96.67%)	82.34% (72.22%-91.18%)	3.4943 (3.2745-3.6969)
At least 4 of CHASM (breast), CHASM (melanoma), FATHMM (cancer), FATHMM (missense), PolyPhen-2, PROVEAN	84.2% (81.31%-86.87%)	85.68% (82.26%-89.35%)	81.62% (76.812%-86.957%)	88.97% (86.04%-92.37%)	76.73% (71.23%-82.19%)	3.3300 (3.2062-3.4491)	84.13% (78.79%-89.9%)	85.53% (78.12%-92.19%)	81.68% (71.419%-91.18%)	88.91% (82.26%-94.92%)	76.7% (65.85%-87.18%)	3.3283 (3.0889-3.5685)
At least 4 of CHASM (breast), CHASM (melanoma), FATHMM (cancer), FATHMM (missense), PolyPhen-2, SIFT	84.54% (81.82%-87.37%)	88.36% (85.12%-91.67%)	77.93% (72.857%-83.582%)	87.38% (84.25%-90.83%)	79.48% (74.29%-85.08%)	3.3314 (3.2147-3.4581)	84.46% (78.79%-89.9%)	88.16% (81.35%-94.92%)	78.039% (66.667%-88.38%)	87.34% (80.65%-93.65%)	79.38% (68.57%-90.24%)	3.3292 (3.0825-3.5655)
At least 4 of CHASM (breast), CHASM (melanoma), FATHMM (cancer), FATHMM (missense), PolyPhen-2, VEST	83.23% (80.3%-86.36%)	87.83% (84.5%-91.13%)	75.26% (69.565%-81.339%)	85.99% (82.76%-89.68%)	78.16% (73.13%-83.82%)	3.2724 (3.1461-3.4024)	83.04% (76.77%-88.89%)	87.62% (80.95%-94.03%)	75.128% (62.857%-86.21%)	85.82% (78.33%-92.31%)	77.99% (66.67%-88.9%)	3.2656 (3.0022-3.5282)

At least 4 of CHASM (breast), CHASM (melanoma), FATHMM (cancer), FATHMM (missense), PROVEAN, SIFT	84.18% (81.31%-86.87%)	85.15% (81.75%-88.8%)	82.5% (77.465%-87.328%)	89.38% (86.44%-92.5%)	76.27% (70.89%-81.58%)	3.3329 (3.2155-3.4528)	84.16% (78.79%-89.9%)	85.01% (77.42%-91.94%)	82.663% (72.727%-92.11%)	89.39% (83.33%-95.08%)	76.29% (65.71%-86.84%)	3.3335 (3.0917-3.5707)
At least 4 of CHASM (breast), CHASM (melanoma), FATHMM (cancer), FATHMM (missense), PROVEAN, VEST	84.54% (81.82%-87.37%)	85.15% (81.66%-88.8%)	83.472% (78.571%-88.575%)	89.91% (86.99%-93.22%)	76.49% (71.43%-82.05%)	3.3502 (3.2346-3.4674)	84.45% (78.79%-89.9%)	85% (77.42%-91.94%)	83.477% (73.529%-93.11%)	89.84% (83.33%-96.08%)	76.45% (65.71%-86.84%)	3.3477 (3.1105-3.5805)
At least 4 of CHASM (breast), CHASM (melanoma), FATHMM (cancer), FATHMM (missense), SIFT, VEST	84.53% (81.82%-87.37%)	88.36% (85.12%-91.67%)	77.882% (72.308%-83.785%)	87.36% (84.42%-90.91%)	79.48% (74.32%-84.93%)	3.3308 (3.2048-3.4562)	84.48% (78.79%-89.9%)	88.15% (81.36%-94.92%)	78.128% (65.789%-88.65%)	87.39% (80.3%-93.45%)	79.38% (68.57%-89.75%)	3.3305 (3.0780-3.5730)
At least 4 of CHASM (breast), CHASM (melanoma), FATHMM (cancer), Mutation Assessor, MutationTaster, PolyPhen-2	84.13% (81.31%-87.37%)	82.4% (78.45%-86.36%)	87.132% (82.894%-91.667%)	91.71% (88.99%-94.78%)	74.12% (68.83%-79.31%)	3.3536 (3.2424-3.4735)	84.26% (77.78%-89.9%)	82.53% (74.6%-90.32%)	87.205% (78.123%-95%)	91.71% (85.24%-96.72%)	74.43% (63.41%-84.62%)	3.3587 (3.1193-3.5853)
At least 4 of CHASM (breast), CHASM (melanoma), FATHMM (cancer), Mutation Assessor, MutationTaster, PROVEAN	82.44% (79.29%-85.86%)	78.12% (73.77%-82.68%)	89.889% (86.072%-94.205%)	93.04% (90.57%-96.04%)	70.39% (64.89%-75.91%)	3.3144 (3.2026-3.4376)	82.6% (75.76%-88.89%)	78.3% (68.85%-86.9%)	89.927% (82.045%-97.37%)	93.03% (87.27%-98.08%)	70.75% (60%-81.58%)	3.3201 (3.0814-3.5471)
At least 4 of CHASM (breast), CHASM (melanoma), FATHMM (cancer), Mutation Assessor, MutationTaster, SIFT	87.16% (84.34%-89.9%)	85.58% (82.17%-89.23%)	89.889% (86.072%-94.205%)	93.6% (91.3%-96.4%)	78.29% (73.03%-83.33%)	3.4736 (3.3658-3.5809)	87.3% (81.82%-92.93%)	85.75% (78.12%-92.31%)	89.927% (82.045%-97.37%)	93.6% (88.46%-98.25%)	78.64% (68.29%-88.64%)	3.4792 (3.2658-3.7036)
At least 4 of CHASM (breast), CHASM (melanoma), FATHMM (cancer), Mutation Assessor, MutationTaster, VEST	84.82% (82.32%-87.88%)	82.4% (78.63%-86.29%)	88.983% (84.929%-93.243%)	92.82% (90.35%-95.61%)	74.52% (69.32%-79.78%)	3.3873 (3.2790-3.5047)	84.91% (78.79%-89.9%)	82.51% (74.6%-89.83%)	89.002% (80.554%-97.22%)	92.79% (87.03%-98.11%)	74.8% (64.28%-84.62%)	3.3911 (3.1571-3.6040)
At least 4 of CHASM (breast), CHASM (melanoma), FATHMM (cancer), Mutation Assessor, PolyPhen-2, PROVEAN	76.72% (73.72%-80.3%)	73.88% (69.42%-78.46%)	81.612% (76.469%-86.887%)	87.42% (83.93%-91.07%)	64.38% (58.94%-70%)	3.0729 (2.9476-3.2078)	76.87% (69.7%-82.85%)	74.02% (64.81%-82.82%)	81.729% (71.429%-92.11%)	87.42% (80%-94.44%)	64.72% (53.99%-75.61%)	3.0788 (2.7962-3.3344)
At least 4 of CHASM (breast), CHASM (melanoma), FATHMM (cancer), Mutation Assessor, PolyPhen-2, SIFT	81.11% (78.28%-84.34%)	82.4% (78.57%-86.36%)	78.864% (73.433%-84.058%)	87.08% (83.62%-90.52%)	72.17% (66.67%-77.38%)	3.2052 (3.0764-3.3276)	81.21% (74.75%-86.87%)	82.5% (74.28%-89.83%)	78.955% (68.571%-89.74%)	87.07% (80%-93.65%)	72.48% (61.11%-83.72%)	3.2100 (2.9662-3.4665)
At least 4 of CHASM (breast), CHASM (melanoma), FATHMM (cancer), Mutation Assessor, PolyPhen-2, VEST	77.76% (74.75%-80.82%)	77.65% (73.44%-81.89%)	77.942% (72.368%-83.583%)	85.89% (82.35%-89.66%)	66.86% (61.45%-72.5%)	3.0834 (2.9526-3.2153)	77.81% (71.69%-83.84%)	77.65% (69.35%-86.21%)	78.07% (67.5%-89.47%)	85.86% (78.18%-92.98%)	67.05% (55.81%-78.38%)	3.0864 (2.8228-3.3477)
At least 4 of CHASM (breast), CHASM (melanoma), FATHMM (cancer), Mutation Assessor, PROVEAN, SIFT	77.73% (74.73%-81.31%)	74.96% (70.63%-79.67%)	82.504% (77.273%-87.5%)	88.11% (84.55%-91.67%)	65.59% (60.21%-70.97%)	3.1116 (2.9865-3.2478)	77.88% (70.71%-83.86%)	75.05% (65.5%-83.87%)	82.69% (72.5%-92.59%)	88.15% (81.13%-95.08%)	65.9% (54.9%-76.48%)	3.1180 (2.8471-3.3778)
At least 4 of CHASM (breast), CHASM (melanoma), FATHMM (cancer), Mutation Assessor, PROVEAN, VEST	75.38% (72.22%-79.29%)	71.77% (67.44%-76.52%)	81.616% (76.385%-86.957%)	87.1% (83.33%-91%)	62.58% (57.29%-68.13%)	3.0306 (2.8988-3.1691)	75.5% (67.68%-81.82%)	71.86% (62.29%-80.65%)	81.727% (71.053%-92.11%)	87.09% (79.59%-94.34%)	62.87% (52%-73.34%)	3.0355 (2.7609-3.2965)
At least 4 of CHASM (breast), CHASM (melanoma), FATHMM (cancer), Mutation Assessor, SIFT, VEST	79.78% (76.77%-82.83%)	80.84% (77.05%-84.8%)	77.941% (72.365%-83.562%)	86.37% (82.91%-90%)	70.17% (64.47%-75.95%)	3.1532 (3.0252-3.2756)	79.84% (73.74%-85.86%)	80.86% (72.58%-88.24%)	78.05% (67.5%-89.19%)	86.35% (79.25%-93.22%)	70.39% (58.97%-81.4%)	3.1565 (2.9106-3.4093)
At least 4 of CHASM (breast), CHASM (melanoma), FATHMM (cancer), MutationTaster, PolyPhen-2, PROVEAN	81.43% (78.28%-84.85%)	77.06% (72.65%-81.82%)	88.987% (84.929%-93.243%)	92.36% (89.72%-95.28%)	69.18% (63.83%-74.74%)	3.2759 (3.1670-3.4050)	81.58% (74.75%-87.88%)	77.24% (67.69%-85.94%)	88.981% (80.645%-97.22%)	92.33% (86.2%-97.96%)	69.53% (58%-80.01%)	3.2808 (3.0238-3.5029)
At least 4 of CHASM (breast), CHASM (melanoma), FATHMM (cancer), MutationTaster, PolyPhen-2, SIFT	86.17% (83.33%-88.89%)	85.07% (81.51%-88.8%)	88.06% (84%-92.65%)	92.49% (90%-95.65%)	77.33% (72.29%-82.43%)	3.4295 (3.3189-3.5383)	86.25% (80.81%-91.92%)	85.17% (77.58%-92.06%)	88.074% (79.301%-96.88%)	92.47% (86.21%-98.04%)	77.6% (66.67%-87.51%)	3.4331 (3.2088-3.6567)
At least 4 of CHASM (breast), CHASM (melanoma), FATHMM (cancer), MutationTaster, PolyPhen-2, VEST	83.8% (80.81%-86.87%)	82.39% (78.69%-86.4%)	86.237% (81.944%-91.183%)	91.19% (88.39%-94.44%)	73.91% (69.04%-79.07%)	3.3373 (3.2231-3.4620)	83.91% (77.78%-89.9%)	82.54% (74.58%-89.86%)	86.229% (76.471%-94.74%)	91.14% (85.19%-96.55%)	74.22% (63.99%-84.21%)	3.3413 (3.0965-3.5746)
At least 4 of CHASM (breast), CHASM (melanoma), FATHMM (cancer), MutationTaster, PROVEAN, SIFT	83.79% (80.81%-86.87%)	79.72% (75.42%-84%)	90.829% (87.143%-94.522%)	93.76% (91.43%-96.33%)	72.15% (66.67%-77.27%)	3.3646 (3.2537-3.4834)	83.93% (77.78%-89.9%)	79.91% (70.96%-88.24%)	90.799% (83.333%-97.44%)	93.72% (88.24%-98.18%)	72.51% (61.9%-82.99%)	3.3694 (3.1275-3.5927)
At least 4 of CHASM (breast), CHASM (melanoma), FATHMM (cancer), MutationTaster, PROVEAN, VEST	81.09% (77.78%-84.34%)	75.45% (70.99%-80%)	90.838% (87.324%-94.668%)	93.44% (90.91%-96.19%)	68.16% (62.77%-73.53%)	3.2789 (3.1650-3.3982)	81.25% (74.75%-87.88%)	75.67% (66.67%-84.48%)	90.777% (82.5%-97.44%)	93.38% (87.71%-98.11%)	68.52% (57.45%-79.55%)	3.2834 (3.0371-3.5027)
At least 4 of CHASM (breast), CHASM (melanoma), FATHMM (cancer), MutationTaster, SIFT, VEST	86.51% (83.84%-88.89%)	85.07% (81.6%-88.8%)	88.985% (85.134%-93.243%)	93.04% (90.6%-95.76%)	77.52% (72.37%-82.56%)	3.4461 (3.3419-3.5545)	86.58% (81.82%-91.92%)	85.16% (77.77%-92.07%)	88.968% (80%-97.06%)	93% (87.27%-98.18%)	77.76% (68.07%-87.88%)	3.4489 (3.2372-3.6633)
At least 4 of CHASM (breast), CHASM (melanoma), FATHMM (cancer), PolyPhen-2, PROVEAN, SIFT	78.04% (74.75%-81.82%)	77.08% (72.73%-81.6%)	79.7% (74.286%-85.135%)	86.78% (83.33%-90.44%)	66.8% (61.25%-72.41%)	3.1036 (2.9714-3.2478)	78.26% (70.71%-84.85%)	77.2% (67.74%-85.94%)	80.025% (69.444%-90.01%)	86.91% (79.66%-94%)	67.19% (56.09%-77.78%)	3.1132 (2.8407-3.3747)
At least 4 of CHASM (breast), CHASM (melanoma), FATHMM (cancer), PolyPhen-2, PROVEAN, VEST	75.36% (72.22%-78.79%)	72.29% (67.96%-76.74%)	80.662% (75.362%-86.486%)	86.6% (83%-90.66%)	62.74% (57.45%-68.29%)	3.0229 (2.8934-3.1676)	75.54% (68.69%-81.82%)	72.42% (63.16%-81.03%)	80.866% (69.444%-91.18%)	86.67% (78.84%-94.12%)	63.09% (51.84%-73.91%)	3.0304 (2.7455-3.2868)
At least 4 of CHASM (breast), CHASM (melanoma), FATHMM (cancer), PolyPhen-2, SIFT, VEST	79.12% (76.25%-82.32%)	81.9% (78.05%-85.82%)	74.296% (68.35%-80.282%)	84.64% (80.95%-88.43%)	70.37% (64.79%-75.95%)	3.1121 (2.9805-3.2479)	79.14% (72.73%-84.87%)	81.91% (73.77%-89.66%)	74.337% (62.5%-86.21%)	84.57% (76.92%-92%)	70.56% (58.82%-82.05%)	3.1138 (2.8367-3.3778)
[illegible]	[illegible]	[illegible]	[illegible]	[illegible]	[illegible]	[illegible]	[illegible]	[illegible]	[illegible]	[illegible]	[illegible]	[illegible]

**At least 4 of CHASM (melanoma), FATHMM (cancer), FATHMM (missense), MutationTaster, PolyPhen-2, SIFT**	85.17% (82.32%-87.89%)	88.3% (85.16%-91.67%)	79.754% (74.647%-85.295%)	88.3% (85.25%-91.6%)	79.77% (74.32%-85.25%)	3.3612 (3.2435-3.4911)	85.21% (79.77%-90.91%)	88.28% (81.25%-94.83%)	79.902% (68.75%-90%)	88.3% (81.81%-94.29%)	79.9% (69.99%-90.32%)	3.3638 (3.0989-3.5924)
**At least 4 of CHASM (melanoma), FATHMM (cancer), FATHMM (missense), MutationTaster, PolyPhen-2, VEST**	82.16% (79.28%-85.35%)	86.69% (83.33%-90.08%)	74.327% (68.571%-80.007%)	85.38% (81.88%-88.98%)	76.35% (70.77%-82.09%)	3.2274 (3.0989-3.3679)	82.14% (75.76%-87.9%)	86.72% (79.69%-93.55%)	74.249% (62.5%-85%)	85.26% (77.94%-92.31%)	76.52% (65.5%-87.5%)	3.2275 (2.9581-3.4875)
**At least 4 of CHASM (melanoma), FATHMM (cancer), FATHMM (missense), MutationTaster, PROVEAN, SIFT**	83.79% (80.81%-86.36%)	83.46% (79.67%-87.6%)	84.362% (80%-89.189%)	90.23% (87.27%-93.22%)	74.69% (69.13%-80%)	3.3273 (3.2084-3.4381)	83.93% (78.79%-89.9%)	83.6% (75%-91.53%)	84.451% (75%-93.11%)	90.23% (84.2%-96.3%)	75.04% (64.44%-86.11%)	3.3332 (3.1061-3.5746)
**At least 4 of CHASM (melanoma), FATHMM (cancer), FATHMM (missense), MutationTaster, PROVEAN, VEST**	82.11% (79.28%-84.85%)	80.78% (76.61%-84.68%)	84.394% (79.729%-89.71%)	89.95% (86.84%-93.46%)	71.76% (66.29%-77.01%)	3.2689 (3.1547-3.3848)	82.24% (76.77%-87.9%)	80.96% (73.13%-89.07%)	84.394% (74.194%-93.75%)	89.92% (83.02%-96.23%)	72.11% (61.9%-82.86%)	3.2739 (3.0398-3.5046)
**At least 4 of CHASM (melanoma), FATHMM (cancer), FATHMM (missense), MutationTaster, SIFT, VEST**	84.48% (81.82%-87.37%)	87.75% (84.68%-91.13%)	78.804% (73.416%-84.848%)	87.75% (84.68%-91.2%)	78.83% (73.61%-84.29%)	3.3313 (3.2099-3.4561)	84.58% (78.79%-89.9%)	87.77% (81.03%-93.94%)	79.045% (66.667%-89.66%)	87.81% (80.95%-93.75%)	79.04% (68.42%-89.19%)	3.3366 (3.0812-3.5749)
**At least 4 of CHASM (melanoma), FATHMM (cancer), FATHMM (missense), PolyPhen-2, PROVEAN, SIFT**	78.06% (74.75%-81.31%)	80.82% (76.74%-85.12%)	73.287% (67.532%-78.947%)	83.95% (80.33%-87.72%)	68.85% (63.15%-74.33%)	3.0691 (2.9299-3.2018)	78.21% (71.72%-84.85%)	80.88% (71.88%-88.89%)	73.583% (61.528%-85.29%)	84.02% (76.27%-91.38%)	69.19% (58.06%-80.49%)	3.0768 (2.8040-3.3562)
**At least 4 of CHASM (melanoma), FATHMM (cancer), FATHMM (missense), PolyPhen-2, PROVEAN, VEST**	75.04% (71.72%-78.28%)	77.09% (73.02%-81.25%)	71.495% (65.67%-77.337%)	82.38% (78.33%-86.33%)	64.35% (58.44%-70%)	2.9531 (2.8102-3.0848)	75.17% (68.69%-81.82%)	77.19% (68.33%-85.25%)	71.663% (60%-83.33%)	82.38% (74.58%-90.16%)	64.67% (52.63%-76.32%)	2.9590 (2.6931-3.2352)
**At least 4 of CHASM (melanoma), FATHMM (cancer), FATHMM (missense), PolyPhen-2, SIFT, VEST**	76.76% (73.72%-79.81%)	84.59% (81.25%-88.24%)	63.212% (56.944%-69.447%)	79.9% (76.15%-83.94%)	70.34% (64.06%-76.2%)	2.9804 (2.8321-3.1165)	76.79% (70.68%-82.85%)	84.53% (77.19%-91.53%)	63.457% (50%-75.76%)	79.88% (71.67%-87.5%)	70.52% (58.06%-82.35%)	2.9838 (2.7020-3.2798)
**At least 4 of CHASM (melanoma), FATHMM (cancer), FATHMM (missense), PROVEAN, SIFT, VEST**	76.03% (72.73%-79.29%)	78.7% (74.6%-82.93%)	71.388% (65.664%-77.468%)	82.63% (78.81%-86.72%)	65.98% (59.73%-71.61%)	2.9870 (2.8421-3.1221)	76.23% (69.7%-82.83%)	78.74% (70.37%-86.89%)	71.869% (58.824%-83.33%)	82.78% (74.58%-90.33%)	66.34% (54.76%-79.07%)	2.9972 (2.7197-3.2778)
**At least 4 of CHASM (melanoma), FATHMM (cancer), Mutation Assessor, MutationTaster, PolyPhen-2, PROVEAN**	79.08% (76.26%-82.32%)	78.68% (74.62%-82.79%)	79.765% (74.667%-85.338%)	87.05% (83.64%-90.76%)	68.39% (62.82%-73.86%)	3.1389 (3.0170-3.2689)	79.21% (72.73%-84.85%)	78.78% (70.49%-86.67%)	79.934% (68.75%-90.33%)	87.07% (79.66%-93.88%)	68.71% (57.14%-79.49%)	3.1449 (2.8797-3.3871)
**At least 4 of CHASM (melanoma), FATHMM (cancer), Mutation Assessor, MutationTaster, PolyPhen-2, SIFT**	79.43% (76.26%-82.32%)	81.88% (78.03%-85.72%)	75.189% (69.736%-81.081%)	85.09% (81.51%-88.89%)	70.59% (65.33%-76.32%)	3.1274 (3.0009-3.2621)	79.53% (73.74%-85.86%)	81.97% (74.19%-89.23%)	75.326% (63.889%-86.21%)	85.07% (77.42%-91.94%)	70.9% (58.82%-81.58%)	3.1326 (2.8583-3.3889)
**At least 4 of CHASM (melanoma), FATHMM (cancer), Mutation Assessor, MutationTaster, PolyPhen-2, VEST**	78.45% (75.25%-81.82%)	80.86% (77.34%-84.8%)	74.295% (68.571%-80.282%)	84.47% (80.83%-88.33%)	69.18% (63.75%-75%)	3.0880 (2.9565-3.2235)	78.45% (71.72%-84.85%)	80.82% (72.87%-88.14%)	74.37% (62.5%-85.71%)	84.39% (76.66%-91.67%)	69.33% (57.14%-80.57%)	3.0891 (2.8144-3.3443)
**At least 4 of CHASM (melanoma), FATHMM (cancer), Mutation Assessor, MutationTaster, PROVEAN, SIFT**	78.41% (75.25%-81.82%)	75.49% (71.31%-79.68%)	83.443% (78.462%-88.407%)	88.74% (85.44%-92.23%)	66.31% (60.71%-71.74%)	3.1399 (3.0147-3.2707)	78.54% (71.72%-84.85%)	75.6% (67.19%-83.61%)	83.562% (73.529%-93.11%)	88.76% (81.63%-95.35%)	66.6% (55.56%-77.55%)	3.1452 (2.8867-3.3878)
**At least 4 of CHASM (melanoma), FATHMM (cancer), Mutation Assessor, MutationTaster, PROVEAN, VEST**	77.04% (73.74%-80.3%)	74.94% (70.87%-79.37%)	80.672% (75.362%-86.111%)	87.02% (83.48%-90.83%)	65.06% (59.77%-70.65%)	3.0769 (2.9462-3.2107)	77.23% (70.71%-83.84%)	75.09% (66.18%-83.08%)	80.878% (70.27%-91.43%)	87.07% (79.25%-94.45%)	65.42% (54.35%-76.19%)	3.0845 (2.8117-3.3459)
**At least 4 of CHASM (melanoma), FATHMM (cancer), Mutation Assessor, MutationTaster, SIFT, VEST**	77.76% (74.24%-80.81%)	79.26% (75.4%-83.33%)	75.168% (69.619%-81.081%)	84.66% (80.99%-88.6%)	67.71% (62.03%-73.69%)	3.0679 (2.9327-3.1987)	77.81% (71.72%-84.85%)	79.22% (71.15%-87.1%)	75.368% (64.097%-86.49%)	84.65% (77.05%-91.8%)	67.88% (55.88%-78.58%)	3.0711 (2.8113-3.3388)
**At least 4 of CHASM (melanoma), FATHMM (cancer), Mutation Assessor, PolyPhen-2, PROVEAN, SIFT**	73.68% (70.2%-77.27%)	74.42% (70.09%-78.63%)	72.382% (66.667%-78.261%)	82.33% (78.57%-86.33%)	62.07% (56.25%-67.86%)	2.9120 (2.7787-3.0544)	73.86% (66.67%-80.81%)	74.54% (66.1%-83.05%)	72.643% (60.513%-84.21%)	82.39% (74.55%-89.83%)	62.43% (50%-73.81%)	2.9200 (2.6351-3.1848)
**At least 4 of CHASM (melanoma), FATHMM (cancer), Mutation Assessor, PolyPhen-2, PROVEAN, VEST**	74.7% (71.21%-77.78%)	75.51% (71.43%-80%)	73.287% (67.567%-78.953%)	83.01% (79.13%-86.96%)	63.38% (57.64%-69.05%)	2.9519 (2.8098-3.0877)	74.85% (68.69%-81.82%)	75.55% (66.67%-83.82%)	73.599% (61.538%-85.01%)	83.08% (75%-90.48%)	63.68% (51.43%-75%)	2.9591 (2.6858-3.2344)
**At least 4 of CHASM (melanoma), FATHMM (cancer), Mutation Assessor, PolyPhen-2, SIFT, VEST**	73.74% (70.2%-77.27%)	78.22% (74.4%-82.26%)	65.988% (60%-72.063%)	79.9% (75.83%-83.87%)	63.67% (57.89%-70.27%)	2.8778 (2.7310-3.0241)	73.73% (66.67%-80.81%)	78.12% (69.84%-85.92%)	66.195% (53.846%-78.79%)	79.85% (71.87%-88.14%)	63.79% (51.28%-75.68%)	2.8795 (2.5789-3.1730)
**At least 4 of CHASM (melanoma), FATHMM (cancer), Mutation Assessor, PROVEAN, SIFT, VEST**	72.67% (69.19%-76.26%)	73.87% (69.77%-78.23%)	70.582% (64.865%-76.812%)	81.28% (77.31%-85.22%)	60.97% (55.13%-66.68%)	2.8671 (2.7235-3.0077)	72.84% (65.66%-79.8%)	74.04% (65.08%-82.46%)	70.738% (58.974%-81.83%)	81.28% (73.21%-89.1%)	61.35% (49.97%-73.17%)	2.8740 (2.5924-3.1517)
**At least 4 of CHASM (melanoma), FATHMM (cancer), MutationTaster, PolyPhen-2, PROVEAN, SIFT**	79.76% (76.77%-82.83%)	79.76% (75.86%-83.87%)	79.737% (74.323%-85.135%)	87.19% (83.93%-90.83%)	69.5% (64.19%-75%)	3.1618 (3.0395-3.2944)	79.88% (73.74%-85.86%)	79.82% (71.43%-87.5%)	79.95% (69.046%-90%)	87.24% (79.66%-93.75%)	69.78% (58.33%-80.43%)	3.1679 (2.8993-3.4094)
**At least 4 of CHASM (melanoma), FATHMM (cancer), MutationTaster, PolyPhen-2, PROVEAN, VEST**	78.38% (75.25%-81.82%)	78.66% (74.8%-82.79%)	77.898% (72.368%-83.784%)	86.02% (82.57%-89.91%)	67.86% (62.03%-73.69%)	3.1044 (2.9754-3.2369)	78.59% (71.72%-84.85%)	78.83% (70.49%-86.67%)	78.152% (66.667%-89.2%)	86.1% (78.43%-93.22%)	68.26% (56.82%-79.55%)	3.1134 (2.8424-3.3723)
**At least 4 of CHASM (melanoma), FATHMM (cancer), MutationTaster, PolyPhen-2, SIFT, VEST**	77.77% (74.24%-81.31%)	82.44% (78.91%-86.4%)	69.703% (63.514%-75.715%)	82.47% (78.62%-86.36%)	69.65% (64.1%-75.36%)	3.0426 (2.9035-3.1822)	77.79% (70.71%-84.85%)	82.45% (74.54%-89.4%)	69.788% (57.143%-81.82%)	82.4% (74.6%-90.01%)	69.86% (57.58%-80.65%)	3.0449 (2.7614-3.3308)
**At least 4 of CHASM (melanoma), FATHMM (cancer), MutationTaster, PROVEAN, SIFT, VEST**	78.75% (75.76%-81.82%)	77.64% (73.5%-81.75%)	80.665% (75.362%-86.301%)	87.41% (84.21%-90.99%)	67.59% (62.2%-73.08%)	3.1331 (3.0043-3.2545)	78.86% (72.73%-84.85%)	77.69% (69.35%-85.25%)	80.847% (69.986%-90.91%)	87.45% (80.36%-94.23%)	67.85% (56.52%-78.57%)	3.1383 (2.8948-3.3882)
**At least 4 of CHASM (melanoma), FATHMM (cancer), PolyPhen-2, PROVEAN, SIFT, VEST**	72.71% (69.19%-76.26%)	76.58% (72.58%-80.8%)	66.016% (60%-72.506%)	79.58% (75.61%-83.74%)	61.97% (55.84%-67.95%)	2.8414 (2.6920-2.9903)	72.76% (65.66%-79.8%)	76.62% (67.79%-84.62%)	66.12% (53.328%-78.96%)	79.52% (71.42%-87.72%)	62.21% (50%-74.36%)	2.8446 (2.5442-3.1332)
**At least 4 of CHASM (melanoma), FATHMM (missense), Mutation Assessor, MutationTaster, PolyPhen-2, PROVEAN**	80.47% (77.27%-83.84%)	79.26% (75.41%-83.21%)	82.548% (77.778%-87.504%)	88.7% (85.45%-92.24%)	69.72% (64.37%-75.29%)	3.2024 (3.0780-3.3315)	80.47% (73.74%-86.87%)	79.21% (70.96%-86.89%)	82.589% (72.722%-91.89%)	88.65% (81.82%-94.83%)	69.84% (58.69%-80.44%)	3.2030 (2.9511-3.4451)
**At least 4 of CHASM (melanoma), FATHMM (missense), Mutation Assessor, MutationTaster, PolyPhen-2, SIFT**	79.1% (76.25%-82.32%)	80.8% (76.74%-84.8%)	76.16% (70.767%-82.098%)	85.42% (81.9%-89.08%)	69.65% (64.1%-75.64%)	3.1203 (2.9931-3.2588)	79.16% (72.73%-84.87%)	80.92% (73.02%-88.46%)	76.099% (64.861%-87.1%)	85.32% (77.97%-92.54%)	69.94% (57.89%-80.96%)	3.1228 (2.8499-3.3737)
**At least 4 of CHASM (melanoma), FATHMM (missense), Mutation Assessor, MutationTaster, PolyPhen-2, VEST**	76.14% (73.22%-79.29%)	79.27% (75.41%-83.33%)	70.713% (64.863%-76.817%)	82.39% (78.51%-86.29%)	66.36% (60.53%-72.73%)	2.9874 (2.8521-3.1311)	76.01% (69.7%-81.84%)	79.2% (70.91%-86.57%)	70.488% (58.06%-82.5%)	82.16% (74.19%-89.86%)	66.39% (53.85%-77.78%)	2.9824 (2.6947-3.2522)
**At least 4 of CHASM (melanoma), FATHMM (missense), Mutation Assessor, MutationTaster, PROVEAN, SIFT**	79.45% (76.26%-82.34%)	76.07% (71.9%-80.17%)	85.296% (80.881%-90.141%)	89.95% (86.67%-93.33%)	67.33% (62.07%-72.83%)	3.1864 (3.0639-3.3076)	79.48% (73.71%-85.86%)	76.03% (67.8%-84.48%)	85.345% (76.316%-94.29%)	89.91% (83.02%-96.3%)	67.47% (56.52%-78.05%)	3.1876 (2.9419-3.4332)
**At least 4 of CHASM (melanoma), FATHMM (missense), Mutation Assessor, MutationTaster, PROVEAN, VEST**	78.11% (74.75%-81.31%)	74.46% (70.31%-78.63%)	84.4% (79.729%-89.479%)	89.19% (85.98%-92.78%)	65.65% (60.44%-71.28%)	3.1371 (3.0150-3.2635)	78.13% (71.72%-84.85%)	74.45% (66.03%-83.05%)	84.385% (74.359%-93.75%)	89.12% (82.14%-95.92%)	65.81% (54.54%-76.09%)	3.1377 (2.8882-3.3834)
**At least 4 of CHASM (melanoma), FATHMM (missense), Mutation Assessor, MutationTaster, SIFT, VEST**	77.45% (74.24%-80.81%)	77.66% (73.6%-81.74%)	77.084% (71.791%-82.814%)	85.42% (81.65%-88.99%)	66.62% (60.98%-72.5%)	3.0678 (2.9426-3.2142)	77.42% (70.71%-83.84%)	77.64% (69.49%-85.71%)	76.994% (65.706%-88.1%)	85.28% (77.78%-92.86%)	66.74% (54.99%-77.78%)	3.0665 (2.7773-3.3167)
**At least 4 of CHASM (melanoma), FATHMM (missense), Mutation Assessor, PolyPhen-2, PROVEAN, SIFT**	74.73% (71.21%-78.28%)	75.53% (71.43%-79.69%)	73.336% (67.188%-78.953%)	83.05% (79.31%-86.84%)	63.42% (57.65%-69.41%)	2.9533 (2.8119-3.0904)	74.78% (67.68%-81.82%)	75.51% (67.19%-83.61%)	73.483% (62.493%-85.3%)	83.02% (75.44%-90.91%)	63.6% (51.35%-75%)	2.9561 (2.6853-3.2337)
**At least 4 of CHASM (melanoma), FATHMM (missense), Mutation Assessor, PolyPhen-2, PROVEAN, VEST**	74.42% (71.21%-77.78%)	76.09% (71.9%-80.47%)	71.52% (65.714%-77.337%)	82.21% (78.26%-85.98%)	63.37% (57.69%-69.51%)	2.9319 (2.7914-3.0640)	74.39% (67.68%-80.81%)	75.98% (67.19%-84.06%)	71.617% (60%-83.88%)	82.13% (74.07%-89.83%)	63.47% (51.22%-75%)	2.9320 (2.6542-3.2150)
**At least 4 of CHASM (melanoma), FATHMM (missense), Mutation Assessor, PolyPhen-2, SIFT, VEST**	73.06% (69.7%-76.26%)	77.15% (73.08%-81.4%)	65.981% (60%-72.464%)	79.68% (75.65%-83.79%)	62.54% (56.41%-68.68%)	2.8535 (2.7040-2.9946)	73.08% (66.67%-79.8%)	77.07% (68.25%-84.85%)	66.187% (54.049%-77.78%)	79.64% (71.18%-87.5%)	62.71% (50%-74.43%)	2.8561 (2.5506-3.1460)
**At least 4 of CHASM (melanoma), FATHMM (missense), Mutation Assessor, PROVEAN, SIFT, VEST**	73.04% (69.7%-76.26%)	74.45% (70.08%-78.74%)	70.582% (64.706%-76.715%)	81.4% (77.5%-85.32%)	61.5% (55.7%-67.47%)	2.8794 (2.7350-3.0182)	73.11% (66.67%-79.8%)	74.47% (66.06%-83.05%)	70.734% (57.895%-82.51%)	81.37% (73.68%-89.09%)	61.74% (50%-73.17%)	2.8832 (2.5980-3.1743)
**At least 4 of CHASM (melanoma), FATHMM (missense), MutationTaster, PolyPhen-2, PROVEAN, SIFT**	79.8% (76.77%-82.83%)	79.8% (75.97%-83.87%)	79.785% (74.627%-85.299%)	87.22% (83.76%-90.76%)	69.55% (63.95%-75.29%)	3.1635 (3.0363-3.2981)	79.8% (73.74%-85.86%)	79.74% (71.66%-87.16%)	79.837% (68.571%-90.24%)	87.17% (80%-93.88%)	69.66% (58.82%-80.49%)	3.1641 (2.8834-3.4220)
**At least 4 of CHASM (melanoma), FATHMM (missense), MutationTaster, PolyPhen-2, PROVEAN, VEST**	77.45% (74.24%-80.81%)	78.19% (74.22%-82.31%)	76.172% (70.419%-82.26%)	85.02% (81.51%-88.89%)	66.88% (61.18%-72.73%)	3.0626 (2.9277-3.1984)	77.42% (70.71%-83.84%)	78.18% (69.55%-86.21%)	76.045% (63.889%-87.5%)	84.87% (77.19%-91.94%)	67% (55.55%-78.26%)	3.0609 (2.7845-3.3292)
**At least 4 of CHASM (melanoma), FATHMM (missense), MutationTaster, PolyPhen-2, SIFT, VEST**	77.13% (74.24%-80.3%)	81.39% (77.78%-85.37%)	69.737% (63.765%-76.058%)	82.3% (78.46%-86.32%)	68.43% (62.82%-74.32%)	3.0186 (2.8877-3.1596)	77.06% (70.71%-82.83%)	81.34% (73.33%-88.53%)	69.655% (56.757%-81.25%)	82.15% (74.13%-89.39%)	68.51% (56.67%-79.43%)	3.0166 (2.7293-3.2746)
**At least 4 of CHASM (melanoma), FATHMM (missense), MutationTaster, PROVEAN, SIFT, VEST**	78.45% (75.25%-81.82%)	76.61% (72.44%-80.65%)	81.628% (76.623%-86.957%)	87.83% (84.26%-91.67%)	66.87% (61.45%-72.53%)	3.1293 (3.0019-3.2725)	78.45% (71.72%-84.85%)	76.54% (68.33%-84.75%)	81.647% (70.588%-91.43%)	87.76% (80.39%-94.74%)	66.97% (55.56%-78%)	3.1292 (2.8489-3.3849)
**At least 4 of CHASM (melanoma), FATHMM (missense), PolyPhen-2, PROVEAN, SIFT, VEST**	73.39% (69.7%-76.77%)	76.61% (72.58%-80.8%)	67.807% (61.641%-73.974%)	80.45% (76.38%-84.43%)	62.64% (56.76%-68.83%)	2.8751 (2.7298-3.0129)	73.42% (66.67%-80.81%)	76.54% (68.25%-84.51%)	68.03% (55.882%-80.66%)	80.43% (72.22%-88.53%)	62.8% (50%-74.36%)	2.8780 (2.6043-3.1729)
**At least 4 of CHASM (melanoma), Mutation Assessor, MutationTaster, PolyPhen-2, PROVEAN, SIFT**	77.08% (73.74%-80.3%)	76.56% (72.65%-80.8%)	77.976% (72.854%-83.562%)	85.73% (81.89%-89.57%)	65.8% (60%-71.61%)	3.0606 (2.9274-3.1956)	77.15% (70.71%-83.84%)	76.65% (68.18%-84.62%)	77.987% (66.667%-88.64%)	85.66% (78.18%-93.1%)	66.05% (54.35%-77.42%)	3.0634 (2.7937-3.3346)
**At least 4 of CHASM (melanoma), Mutation Assessor, MutationTaster, PolyPhen-2, PROVEAN, VEST**	75.08% (71.72%-78.28%)	75.54% (71.31%-79.84%)	74.278% (68.569%-80.264%)	83.54% (79.82%-87.61%)	63.72% (58.23%-69.51%)	2.9708 (2.8285-3.1136)	75.09% (68.69%-81.82%)	75.49% (66.67%-83.61%)	74.397% (62.5%-85.01%)	83.48% (75.44%-91.07%)	63.88% (52.38%-75.01%)	2.9726 (2.6870-3.2545)
**At least 4 of CHASM (melanoma), Mutation Assessor, MutationTaster, PolyPhen-2, SIFT, VEST**	74.73% (71.21%-78.28%)	77.65% (73.77%-81.9%)	69.664% (63.997%-75.714%)	81.57% (77.59%-85.72%)	64.32% (58.11%-70.37%)	2.9321 (2.7865-3.0811)	74.79% (67.68%-81.82%)	77.65% (68.85%-85.71%)	69.85% (57.143%-82.06%)	81.54% (73.44%-89.1%)	64.55% (51.43%-76.19%)	2.9360 (2.6386-3.2218)
**At least 4 of CHASM (melanoma), Mutation Assessor, MutationTaster, PROVEAN, SIFT, VEST**	74.73% (71.21%-78.28%)	73.91% (69.75%-78.23%)	76.15% (70.665%-81.69%)	84.27% (80.37%-88.18%)	62.79% (57.14%-68.61%)	2.9712 (2.8342-3.1068)	74.78% (67.68%-81.82%)	73.97% (65.08%-82.46%)	76.141% (65%-87.1%)	84.17% (76.36%-91.8%)	63.02% (51.16%-74.36%)	2.9730 (2.7066-3.2450)
**At least 4 of CHASM (melanoma), Mutation Assessor, PolyPhen-2, PROVEAN, SIFT, VEST**	71.37% (67.68%-74.75%)	75% (70.87%-79.51%)	65.086% (59.15%-71.622%)	78.79% (74.59%-83.19%)	60.09% (54.05%-66.25%)	2.7896 (2.6411-2.9324)	71.4% (64.65%-78.79%)	74.98% (66.15%-83.08%)	65.233% (52.5%-77.42%)	78.72% (70%-86.89%)	60.29% (47.22%-72.22%)	2.7922 (2.4985-3.0890)
**At least 4 of CHASM (melanoma), MutationTaster, PolyPhen-2, PROVEAN, SIFT, VEST**	75.43% (72.22%-78.79%)	77.67% (73.77%-81.75%)	71.53% (65.753%-77.616%)	82.51% (78.63%-86.56%)	64.94% (59.26%-70.89%)	2.9666 (2.8234-3.1023)	75.41% (68.69%-81.82%)	77.61% (69.23%-85.49%)	71.602% (59.375%-83.33%)	82.43% (74.5%-90%)	65.06% (52.63%-76.92%)	2.9671 (2.6904-3.2465)
**At least 4 of FATHMM (cancer), FATHMM (missense), Mutation Assessor, MutationTaster, PolyPhen-2, PROVEAN**	78.41% (75.25%-81.31%)	78.72% (74.8%-82.68%)	77.858% (72.222%-83.333%)	86.01% (82.5%-89.82%)	67.91% (62.34%-73.75%)	3.1049 (2.9753-3.2299)	78.54% (72.73%-84.85%)	78.71% (70.31%-86.67%)	78.23% (67.645%-89.19%)	86.11% (78.33%-92.98%)	68.17% (56.52%-79.07%)	3.1122 (2.8636-3.3766)
**At least 4 of FATHMM (cancer), FATHMM (missense), Mutation Assessor, MutationTaster, PolyPhen-2, SIFT**	79.41% (76.26%-82.83%)	82.97% (79.37%-86.92%)	73.255% (67.532%-79.453%)	84.29% (80.67%-88.03%)	71.33% (66.2%-77.34%)	3.1185 (2.9886-3.2504)	79.56% (72.73%-85.86%)	82.97% (75.38%-89.86%)	73.68% (61.532%-85%)	84.39% (76.92%-91.67%)	71.61% (59.46%-82.5%)	3.1265 (2.8439-3.3957)
**At least 4 of FATHMM (cancer), FATHMM (missense), Mutation Assessor, MutationTaster, PolyPhen-2, VEST**	78.79% (75.76%-81.82%)	81.94% (78.46%-85.94%)	73.345% (67.991%-79.458%)	84.16% (80.64%-88%)	70.14% (64.38%-76%)	3.0959 (2.9619-3.2308)	78.78% (72.73%-84.85%)	81.85% (73.68%-89.06%)	73.481% (61.286%-84.62%)	84.11% (76.27%-91.23%)	70.23% (58.33%-82.05%)	3.0967 (2.8232-3.3495)
**At least 4 of FATHMM (cancer), FATHMM (missense), Mutation Assessor, MutationTaster, PROVEAN, SIFT**	77.41% (74.24%-80.81%)	76.61% (72.58%-80.95%)	78.794% (73.529%-84.507%)	86.2% (82.52%-90%)	66.08% (60.24%-71.74%)	3.0768 (2.9381-3.2124)	77.5% (70.71%-83.84%)	76.55% (67.74%-84.51%)	79.097% (67.568%-90%)	86.28% (78.57%-93.33%)	66.27% (54.76%-77.15%)	3.0819 (2.8117-3.3425)
**At least 4 of FATHMM (cancer), FATHMM (missense), Mutation Assessor, MutationTaster, PROVEAN, VEST**	76.72% (73.23%-79.8%)	74.99% (70.97%-79.2%)	79.713% (74.286%-85.294%)	86.47% (82.88%-90.43%)	64.83% (59.09%-70.45%)	3.0599 (2.9257-3.1944)	76.86% (70.71%-83.84%)	75% (66.65%-83.34%)	80.024% (69.444%-90.62%)	86.56% (78.57%-93.65%)	65.1% (53.65%-76.6%)	3.0668 (2.7944-3.3282)
**At least 4 of FATHMM (cancer), FATHMM (missense), Mutation Assessor, MutationTaster, SIFT, VEST**	76.38% (73.23%-79.8%)	79.29% (75.4%-83.33%)	71.356% (65.714%-77.632%)	82.72% (78.99%-86.92%)	66.58% (60.56%-72.6%)	2.9994 (2.8553-3.1403)	76.52% (69.7%-82.83%)	79.16% (71.15%-86.96%)	71.962% (58.971%-83.33%)	82.89% (74.6%-90.2%)	66.8% (54.84%-78.13%)	3.0081 (2.7373-3.2903)
**At least 4 of FATHMM (cancer), FATHMM (missense), Mutation Assessor, PolyPhen-2, PROVEAN, SIFT**	75.71% (72.22%-79.29%)	75.54% (71.65%-79.84%)	75.996% (70.27%-81.821%)	84.48% (80.7%-88.5%)	64.24% (58.33%-69.88%)	3.0026 (2.8623-3.1363)	75.85% (68.69%-82.83%)	75.49% (66.67%-83.34%)	76.429% (64.286%-88.24%)	84.62% (76.56%-92.45%)	64.49% (53.32%-76.09%)	3.0103 (2.7376-3.2873)
**At least 4 of FATHMM (cancer), FATHMM (missense), Mutation Assessor, PolyPhen-2, PROVEAN, VEST**	75.38% (72.22%-78.79%)	75.55% (71.54%-80%)	75.092% (69.442%-81.081%)	83.99% (80.18%-88.07%)	63.98% (58.02%-69.88%)	2.9860 (2.8470-3.1227)	75.49% (68.69%-81.82%)	75.48% (66.67%-83.61%)	75.493% (63.158%-87.18%)	84.09% (76.36%-91.53%)	64.2% (52.63%-75.56%)	2.9926 (2.7185-3.2761)
**At least 4 of FATHMM (cancer), FATHMM (missense), Mutation Assessor, PolyPhen-2, SIFT, VEST**	72.04% (68.18%-75.25%)	78.24% (74.13%-82.17%)	61.308% (55.263%-67.606%)	77.76% (73.6%-81.82%)	61.97% (55.71%-68.5%)	2.7927 (2.6406-2.9413)	72.08% (65.66%-79.8%)	78.07% (69.86%-85.92%)	61.777% (50%-74.29%)	77.8% (69.56%-85.94%)	62.13% (48.39%-74.29%)	2.7978 (2.4912-3.1057)
**At least 4 of FATHMM (cancer), FATHMM (missense), Mutation Assessor, PROVEAN, SIFT, VEST**	72.99% (69.68%-76.26%)	74.45% (70.4%-78.86%)	70.451% (64.378%-77.027%)	81.33% (77.39%-85.71%)	61.46% (55.4%-67.44%)	2.8770 (2.7277-3.0191)	73.21% (66.67%-79.82%)	74.47% (66.03%-82.55%)	70.999% (57.692%-82.76%)	81.51% (73.13%-89.47%)	61.83% (50%-73.17%)	2.8881 (2.6044-3.1773)
**At least 4 of FATHMM (cancer), FATHMM (missense), MutationTaster, PolyPhen-2, PROVEAN, SIFT**	79.1% (76.26%-82.32%)	79.79% (75.97%-83.74%)	77.884% (72.499%-83.751%)	86.19% (82.73%-89.91%)	69.04% (63.41%-74.68%)	3.1290 (3.0024-3.2589)	79.17% (72.73%-84.85%)	79.74% (71.67%-87.72%)	78.154% (65.852%-88.89%)	86.24% (78.94%-93.1%)	69.23% (57.89%-80.49%)	3.1336 (2.8775-3.3890)
**At least 4 of FATHMM (cancer), FATHMM (missense), MutationTaster, PolyPhen-2, PROVEAN, VEST**	78.42% (75.25%-81.31%)	79.23% (75.38%-83.2%)	77.005% (71.429%-83.099%)	85.63% (82.14%-89.57%)	68.2% (62.34%-73.75%)	3.1007 (2.9707-3.2269)	78.51% (72.73%-84.85%)	79.27% (70.77%-87.5%)	77.157% (65.517%-88.24%)	85.63% (77.97%-92.73%)	68.45% (57.77%-80.01%)	3.1051 (2.8488-3.3729)
**At least 4 of FATHMM (cancer), FATHMM (missense), MutationTaster, PolyPhen-2, SIFT, VEST**	77.42% (74.24%-80.81%)	83.52% (80.15%-87.12%)	66.866% (60.87%-72.98%)	81.34% (77.68%-85.27%)	70.12% (63.89%-75.76%)	3.0184 (2.8773-3.1522)	77.48% (70.71%-83.84%)	83.48% (76.19%-90.63%)	67.167% (54.539%-78.95%)	81.35% (73.61%-88.89%)	70.32% (58.06%-82.5%)	3.0231 (2.7471-3.2980)
**At least 4 of FATHMM (cancer), FATHMM (missense), MutationTaster, PROVEAN, SIFT, VEST**	77.07% (73.74%-80.3%)	77.68% (73.6%-81.82%)	76.016% (70.27%-82.432%)	84.85% (81.35%-88.89%)	66.32% (60.26%-72.01%)	3.0487 (2.9041-3.1842)	77.17% (70.71%-83.84%)	77.6% (69.23%-85.96%)	76.375% (63.158%-87.5%)	84.94% (76.66%-92.31%)	66.51% (54.84%-78.12%)	3.0542 (2.7909-3.3442)
**At least 4 of FATHMM (cancer), FATHMM (missense), PolyPhen-2, PROVEAN, SIFT, VEST**	73.02% (69.7%-76.26%)	76.61% (72.73%-80.65%)	66.804% (60.81%-73.239%)	79.96% (75.86%-84.03%)	62.29% (56%-68.42%)	2.8567 (2.7039-2.9946)	73.15% (66.67%-79.8%)	76.54% (68.25%-84.62%)	67.278% (54.839%-80%)	80.06% (71.7%-87.94%)	62.55% (50%-75%)	2.8643 (2.5861-3.1554)
**At least 4 of FATHMM (cancer), Mutation Assessor, MutationTaster, PolyPhen-2, PROVEAN, SIFT**	76.72% (73.23%-80.3%)	77.66% (73.81%-81.82%)	75.095% (69.511%-81.159%)	84.35% (80.7%-88.39%)	66.03% (60.23%-71.79%)	3.0313 (2.8893-3.1704)	76.86% (69.7%-83.84%)	77.64% (69.23%-85.48%)	75.506% (63.624%-86.84%)	84.46% (76.66%-91.8%)	66.29% (54.75%-77.78%)	3.0390 (2.7525-3.3202)
**At least 4 of FATHMM (cancer), Mutation Assessor, MutationTaster, PolyPhen-2, PROVEAN, VEST**	76.06% (72.73%-79.29%)	76.6% (72.58%-80.8%)	75.105% (69.444%-81.159%)	84.17% (80.34%-88.24%)	64.99% (59.03%-70.79%)	3.0087 (2.8680-3.1517)	76.17% (69.7%-82.83%)	76.56% (68.25%-84.75%)	75.499% (64.701%-87.18%)	84.27% (76.27%-92%)	65.24% (53.18%-76.93%)	3.0156 (2.7319-3.2949)
**At least 4 of FATHMM (cancer), Mutation Assessor, MutationTaster, PolyPhen-2, SIFT, VEST**	75.73% (72.22%-79.29%)	78.72% (74.8%-82.81%)	70.575% (64.789%-76.712%)	82.22% (78.33%-86.09%)	65.73% (59.76%-71.79%)	2.9724 (2.8254-3.1122)	75.8% (68.69%-82.83%)	78.72% (70.31%-86.37%)	70.791% (58.621%-82.76%)	82.21% (74.24%-90%)	65.96% (53.19%-77.78%)	2.9768 (2.6859-3.2636)
**At least 4 of FATHMM (cancer), Mutation Assessor, MutationTaster, PROVEAN, SIFT, VEST**	74.72% (71.21%-78.28%)	75% (70.87%-79.13%)	74.232% (68.829%-80.282%)	83.42% (79.46%-87.5%)	63.19% (57.5%-69.05%)	2.9585 (2.8190-3.0943)	74.8% (67.68%-81.82%)	74.98% (66.67%-83.08%)	74.49% (62.5%-85.42%)	83.44% (75%-91.07%)	63.42% (52.17%-74.01%)	2.9632 (2.6943-3.2401)
**At least 4 of FATHMM (cancer), Mutation Assessor, PolyPhen-2, PROVEAN, SIFT, VEST**	71.72% (68.18%-75.25%)	75.56% (71.54%-79.69%)	65.073% (59.091%-71.605%)	78.9% (74.77%-83.05%)	60.63% (54.55%-66.67%)	2.8016 (2.6488-2.9456)	71.71% (64.65%-78.79%)	75.45% (67.17%-83.1%)	65.282% (52.5%-77.28%)	78.84% (70.58%-86.89%)	60.76% (48.65%-72.5%)	2.8033 (2.5047-3.1073)
**At least 4 of FATHMM (cancer), MutationTaster, PolyPhen-2, PROVEAN, SIFT, VEST**	76.07% (72.73%-79.29%)	78.19% (74.19%-82.17%)	72.389% (66.667%-78.467%)	83.04% (79.17%-87.1%)	65.75% (59.99%-71.43%)	2.9937 (2.8579-3.1381)	76.15% (69.7%-82.83%)	78.17% (69.84%-86.21%)	72.671% (60%-83.87%)	83.07% (75%-90.74%)	65.98% (54.05%-77.5%)	2.9989 (2.7103-3.2637)
**At least 4 of FATHMM (cancer), PolyPhen-2, PROVEAN, SIFT, VEST**	69.01% (65.66%-72.73%)	64.91% (60.31%-69.67%)	76.084% (70.311%-82.355%)	82.43% (78.12%-87.1%)	55.63% (50%-61%)	2.7905 (2.6495-2.9428)	69.06% (61.62%-75.76%)	64.84% (55.38%-73.77%)	76.268% (63.889%-87.81%)	82.42% (73.33%-91.07%)	55.81% (44.9%-66.67%)	2.7934 (2.4938-3.0668)
**At least 4 of FATHMM (missense), Mutation Assessor, MutationTaster, PolyPhen-2, PROVEAN**	74.09% (70.71%-77.29%)	67.04% (62.3%-71.77%)	86.261% (81.941%-91.178%)	89.41% (85.87%-93.11%)	60.22% (54.95%-65.46%)	3.0293 (2.9017-3.1593)	74.04% (67.65%-80.81%)	66.95% (57.38%-75.76%)	86.171% (76.923%-94.44%)	89.26% (81.81%-96%)	60.3% (50%-70.92%)	3.0268 (2.7695-3.2825)
**At least 4 of FATHMM (missense), Mutation Assessor, MutationTaster, PolyPhen-2, PROVEAN, SIFT**	77.74% (74.73%-81.31%)	77.65% (73.77%-81.95%)	77.896% (72.599%-83.562%)	85.86% (82.14%-89.63%)	66.83% (61.17%-72.83%)	3.0824 (2.9425-3.2199)	77.85% (70.71%-83.86%)	77.67% (68.85%-85.48%)	78.148% (66.667%-88.89%)	85.91% (78.57%-93.1%)	67.08% (55.31%-78.06%)	3.0880 (2.8132-3.3608)
**At least 4 of FATHMM (missense), Mutation Assessor, MutationTaster, PolyPhen-2, PROVEAN, VEST**	75.74% (72.22%-79.29%)	75.55% (71.43%-79.7%)	76.065% (70.769%-81.693%)	84.51% (80.73%-88.5%)	64.27% (58.69%-70.11%)	3.0040 (2.8630-3.1460)	75.79% (68.69%-82.83%)	75.48% (67.17%-83.61%)	76.304% (64.865%-86.85%)	84.53% (76.66%-91.8%)	64.45% (52.5%-75.61%)	3.0075 (2.7195-3.2779)
**At least 4 of FATHMM (missense), Mutation Assessor, MutationTaster, PolyPhen-2, SIFT**	76.42% (73.23%-79.8%)	72.33% (67.96%-76.86%)	83.482% (78.688%-88.736%)	88.33% (84.69%-91.84%)	63.56% (58.06%-69.15%)	3.0771 (2.9505-3.2101)	76.46% (69.7%-82.83%)	72.35% (63.07%-81.36%)	83.48% (72.967%-93.55%)	88.26% (81.24%-95.83%)	63.75% (52.17%-74.42%)	3.0783 (2.8152-3.3344)
**At least 4 of FATHMM (missense), Mutation Assessor, MutationTaster, PolyPhen-2, SIFT, VEST**	74.74% (71.21%-78.28%)	77.67% (73.6%-81.89%)	69.671% (63.633%-75.677%)	81.58% (77.78%-85.59%)	64.34% (58.33%-70.37%)	2.9326 (2.7884-3.0793)	74.75% (67.68%-81.82%)	77.61% (68.96%-85.92%)	69.807% (57.5%-81.82%)	81.52% (73.77%-89.29%)	64.49% (51.72%-76.92%)	2.9343 (2.6380-3.2149)
**At least 4 of FATHMM (missense), Mutation Assessor, MutationTaster, PolyPhen-2, VEST**	74.75% (71.21%-78.28%)	71.28% (66.94%-75.97%)	80.724% (75.362%-85.916%)	86.48% (82.69%-90.29%)	61.92% (56.32%-67.42%)	3.0040 (2.8678-3.1369)	74.75% (67.68%-81.82%)	71.24% (61.76%-80%)	80.749% (70.27%-90.92%)	86.39% (78.72%-93.88%)	62.06% (51.11%-72.92%)	3.0044 (2.7349-3.2762)
**At least 4 of FATHMM (missense), Mutation Assessor, MutationTaster, PROVEAN, SIFT**	75.41% (72.22%-78.79%)	68.6% (64%-73.17%)	87.166% (83.072%-91.667%)	90.24% (86.81%-93.88%)	61.62% (56.43%-67.29%)	3.0763 (2.9557-3.2023)	75.44% (68.69%-81.82%)	68.61% (59.68%-77.78%)	87.11% (78.372%-95.01%)	90.14% (82.98%-96.3%)	61.78% (50.98%-72.42%)	3.0764 (2.8242-3.3200)
**At least 4 of FATHMM (missense), Mutation Assessor, MutationTaster, PROVEAN, SIFT, VEST**	75.06% (71.72%-78.3%)	74.46% (70.08%-78.86%)	76.1% (70.767%-82.051%)	84.34% (80.36%-88.24%)	63.27% (57.64%-69.05%)	2.9817 (2.8417-3.1205)	75.13% (68.66%-81.82%)	74.47% (65.62%-83.05%)	76.236% (63.889%-87.51%)	84.32% (76.36%-92.31%)	63.49% (52.27%-74.42%)	2.9851 (2.7071-3.2626)
**At least 4 of FATHMM (missense), Mutation Assessor, MutationTaster, PROVEAN, VEST**	73.1% (69.7%-76.77%)	64.95% (60.32%-69.6%)	87.189% (83.07%-91.667%)	89.76% (86.21%-93.48%)	58.99% (53.57%-64.22%)	3.0089 (2.8812-3.1477)	72.99% (65.66%-79.8%)	64.76% (55.38%-73.85%)	87.074% (78.116%-95.25%)	89.58% (82.22%-96.15%)	59.01% (48.27%-69.09%)	3.0042 (2.7398-3.2570)
**At least 4 of FATHMM (missense), Mutation Assessor, MutationTaster, SIFT, VEST**	75.75% (72.22%-79.29%)	70.74% (66.39%-75.21%)	84.409% (79.729%-89.554%)	88.69% (85.11%-92.31%)	62.52% (56.99%-68.09%)	3.0637 (2.9343-3.1944)	75.77% (68.69%-82.83%)	70.73% (61.54%-79.31%)	84.383% (74.359%-93.75%)	88.6% (81.25%-95.92%)	62.68% (51.91%-73.47%)	3.0639 (2.8012-3.3210)
**At least 4 of FATHMM (missense), Mutation Assessor, PolyPhen-2, PROVEAN, SIFT**	72.02% (68.69%-75.76%)	67.03% (62.4%-71.67%)	80.645% (75.362%-85.942%)	85.69% (81.63%-89.8%)	58.58% (53.19%-64%)	2.9195 (2.7833-3.0523)	72.12% (64.65%-78.79%)	66.97% (57.38%-75.87%)	80.896% (70.58%-90.91%)	85.74% (77.27%-93.62%)	58.79% (48.08%-69.39%)	2.9240 (2.6565-3.1970)
**At least 4 of FATHMM (missense), Mutation Assessor, PolyPhen-2, PROVEAN, SIFT, VEST**	72.38% (69.19%-75.76%)	75.55% (71.54%-79.7%)	66.897% (60.811%-72.973%)	79.78% (75.83%-83.61%)	61.27% (55.26%-67.5%)	2.8350 (2.6892-2.9795)	72.41% (65.66%-78.79%)	75.47% (66.67%-83.33%)	67.107% (54.989%-79.41%)	79.74% (71.92%-87.72%)	61.44% (48.78%-73.17%)	2.8376 (2.5515-3.1242)
**At least 4 of FATHMM (missense), Mutation Assessor, PolyPhen-2, PROVEAN, VEST**	69.36% (65.66%-73.23%)	62.81% (58.12%-67.72%)	80.654% (75.362%-85.938%)	84.88% (80.85%-89.13%)	55.64% (50.45%-61.32%)	2.8399 (2.7058-2.9842)	69.37% (61.62%-76.77%)	62.64% (53.03%-72.06%)	80.875% (70.27%-90.91%)	84.89% (75.61%-92.86%)	55.77% (45.15%-66.07%)	2.8417 (2.5602-3.1076)
**At least 4 of FATHMM (missense), Mutation Assessor, PolyPhen-2, SIFT, VEST**	71.37% (67.68%-74.75%)	71.28% (66.93%-75.83%)	71.511% (65.618%-77.466%)	81.23% (77.06%-85.45%)	59.02% (53.01%-64.84%)	2.8303 (2.6734-2.9755)	71.4% (64.65%-78.79%)	71.25% (61.9%-80.01%)	71.63% (58.818%-83.33%)	81.17% (72.55%-89.29%)	59.2% (46.94%-70.84%)	2.8325 (2.5394-3.1276)
**At least 4 of FATHMM (missense), Mutation Assessor, PROVEAN, SIFT, VEST**	69.7% (66.16%-73.74%)	64.4% (59.66%-69.11%)	78.849% (73.75%-84.51%)	84.04% (79.79%-88.3%)	56.16% (50.51%-61.76%)	2.8345 (2.6910-2.9788)	69.69% (61.62%-76.77%)	64.24% (54.71%-73.77%)	78.985% (67.568%-88.9%)	83.98% (75.51%-92.16%)	56.27% (45.1%-67.27%)	2.8348 (2.5467-3.1223)
**At least 4 of FATHMM (missense), MutationTaster, PolyPhen-2, PROVEAN, SIFT**	77.1% (73.74%-80.81%)	71.81% (67.42%-76.38%)	86.214% (81.815%-91.026%)	90.01% (86.73%-93.48%)	63.88% (58.24%-69.23%)	3.1192 (2.9933-3.2472)	77.12% (69.7%-83.84%)	71.77% (62.5%-80.33%)	86.252% (76.923%-94.6%)	89.97% (83.05%-96.23%)	64.03% (53.48%-75%)	3.1202 (2.8651-3.3693)
**At least 4 of FATHMM (missense), MutationTaster, PolyPhen-2, PROVEAN, SIFT, VEST**	76.1% (72.73%-79.8%)	77.68% (73.64%-81.76%)	73.353% (67.493%-79.412%)	83.45% (79.65%-87.39%)	65.52% (59.76%-71.27%)	3.0001 (2.8600-3.1510)	76.08% (68.69%-82.83%)	77.6% (69.23%-85.94%)	73.447% (61.111%-85.29%)	83.39% (75.81%-90.91%)	65.62% (53.84%-77.5%)	3.0005 (2.7016-3.2707)
**At least 4 of FATHMM (missense), MutationTaster, PolyPhen-2, PROVEAN, VEST**	74.1% (70.71%-77.78%)	67.59% (63.03%-72.36%)	85.342% (80.597%-90.144%)	88.86% (85.39%-92.63%)	60.36% (54.9%-65.98%)	3.0215 (2.8838-3.1517)	74.02% (66.67%-80.81%)	67.45% (57.89%-76.47%)	85.241% (75.676%-94.29%)	88.71% (80.95%-95.74%)	60.41% (50%-70.91%)	3.0181 (2.7522-3.2889)
**At least 4 of FATHMM (missense), MutationTaster, PolyPhen-2, SIFT, VEST**	76.79% (73.23%-80.3%)	75.56% (71.31%-79.83%)	78.887% (73.134%-84.618%)	86.09% (82.41%-89.91%)	65.12% (59.34%-70.93%)	3.0566 (2.9144-3.2010)	76.73% (69.7%-83.84%)	75.45% (67.15%-83.61%)	78.884% (67.5%-89.75%)	85.99% (78.26%-93.22%)	65.17% (52.93%-76.19%)	3.0550 (2.7618-3.3418)
**At least 4 of FATHMM (missense), MutationTaster, PROVEAN, SIFT, VEST**	74.75% (71.21%-78.28%)	67.57% (62.9%-72.22%)	87.142% (82.857%-91.67%)	90.09% (86.67%-93.68%)	60.85% (55.45%-66.34%)	3.0564 (2.9278-3.1919)	74.75% (67.68%-81.82%)	67.49% (58.33%-76.67%)	87.154% (77.778%-95%)	90.02% (82.93%-96.23%)	60.97% (50%-71.43%)	3.0564 (2.7855-3.3109)
**At least 4 of FATHMM (missense), PolyPhen-2, PROVEAN, SIFT, VEST**	70.37% (66.67%-73.75%)	66.53% (62.01%-71.32%)	76.998% (71.25%-82.72%)	83.34% (79%-87.74%)	57.08% (51.55%-62.64%)	2.8395 (2.6911-2.9798)	70.37% (63.61%-77.78%)	66.38% (56.92%-75.01%)	77.17% (65.712%-88.24%)	83.31% (74.47%-92%)	57.21% (46.15%-68%)	2.8407 (2.5610-3.1315)
**At least 4 of Mutation Assessor, MutationTaster, PolyPhen-2, PROVEAN, SIFT**	74.03% (70.71%-77.78%)	70.17% (65.62%-74.61%)	80.686% (75.676%-86.301%)	86.26% (82.35%-90.29%)	61.01% (55.45%-66.67%)	2.9813 (2.8513-3.1226)	74.16% (66.67%-80.81%)	70.26% (61.53%-79.66%)	80.827% (69.691%-90.91%)	86.27% (78.18%-94%)	61.3% (50%-72%)	2.9865 (2.7042-3.2379)
**At least 4 of Mutation Assessor, MutationTaster, PolyPhen-2, PROVEAN, SIFT, VEST**	73.37% (69.7%-76.77%)	74.99% (70.83%-79.23%)	70.567% (64.789%-76.566%)	81.5% (77.36%-85.45%)	62% (56.25%-67.95%)	2.8905 (2.7495-3.0301)	73.46% (66.67%-80.81%)	75% (66.67%-83.08%)	70.797% (58.614%-82.76%)	81.49% (73.08%-89.48%)	62.25% (50%-73.68%)	2.8954 (2.6143-3.1756)
**At least 4 of Mutation Assessor, MutationTaster, PolyPhen-2, PROVEAN, VEST**	71.73% (68.18%-75.25%)	66.53% (62.09%-71.1%)	80.713% (75.713%-86.154%)	85.64% (81.72%-90%)	58.24% (53.06%-63.83%)	2.9111 (2.7774-3.0571)	71.7% (64.65%-78.79%)	66.39% (57.14%-75.41%)	80.792% (69.444%-90.92%)	85.55% (76.78%-93.34%)	58.34% (46.94%-68.42%)	2.9107 (2.6224-3.1736)
**At least 4 of Mutation Assessor, MutationTaster, PolyPhen-2, SIFT, VEST**	74.72% (71.21%-78.28%)	72.83% (68.29%-77.42%)	77.966% (72.603%-83.582%)	85.1% (81.25%-89.11%)	62.4% (56.52%-68.13%)	2.9831 (2.8434-3.1238)	74.81% (67.68%-81.82%)	72.93% (63.64%-81.69%)	78.019% (66.667%-88.9%)	85.04% (77.08%-92.45%)	62.68% (51.16%-74.36%)	2.9867 (2.7001-3.2625)
**At least 4 of Mutation Assessor, MutationTaster, PROVEAN, SIFT, VEST**	72.41% (68.69%-76.26%)	68.12% (63.49%-72.58%)	79.818% (74.647%-85.139%)	85.37% (81.25%-89.36%)	59.15% (53.76%-64.76%)	2.9245 (2.7911-3.0602)	72.36% (64.65%-79.8%)	67.99% (58.73%-77.28%)	79.817% (68.966%-90.33%)	85.24% (76.74%-93.48%)	59.23% (47.92%-70%)	2.9228 (2.6428-3.1969)
**At least 4 of Mutation Assessor, PolyPhen-2, PROVEAN, SIFT, VEST**	68.01% (64.65%-71.72%)	65.98% (61.29%-70.87%)	71.512% (65.753%-77.465%)	80.02% (75.73%-84.31%)	54.87% (49.47%-60.42%)	2.7238 (2.5814-2.8693)	68.02% (60.61%-74.75%)	65.87% (56.25%-75.01%)	71.645% (59.459%-83.33%)	79.93% (71.15%-88.46%)	55.02% (43.48%-65.22%)	2.7247 (2.4285-3.0083)
**At least 4 of MutationTaster, PolyPhen-2, PROVEAN, SIFT, VEST**	72.74% (69.68%-76.26%)	68.13% (63.71%-72.58%)	80.691% (75.713%-86.364%)	85.92% (82%-90.32%)	59.42% (54.26%-65.01%)	2.9416 (2.8110-3.0787)	72.71% (65.66%-78.81%)	67.96% (59.01%-76.79%)	80.811% (69.691%-91.18%)	85.86% (77.36%-93.62%)	59.5% (48.21%-69.39%)	2.9413 (2.6653-3.1995)
**At least 5 of CHASM (breast), CHASM (lung), CHASM (melanoma), FATHMM (cancer), FATHMM (missense)**	77.18% (73.74%-80.3%)	66.65% (61.9%-71.43%)	95.379% (92.857%-98.592%)	96.15% (94.12%-98.84%)	62.32% (56.9%-67.59%)	3.2050 (3.0985-3.3132)	76.96% (70.71%-83.84%)	66.16% (56.14%-75.45%)	95.474% (89.474%-100%)	96.17% (90.91%-100%)	62.18% (51.79%-72.89%)	3.1998 (2.9849-3.4180)
**At least 5 of CHASM (breast), CHASM (lung), CHASM (melanoma), FATHMM (cancer), FATHMM (missense), Mutation Assessor**	79.18% (76.26%-82.34%)	70.85% (66.11%-75.61%)	93.582% (90.411%-97.183%)	95.02% (92.63%-97.83%)	65% (59.62%-70.11%)	3.2445 (3.1403-3.3568)	79.01% (72.7%-84.85%)	70.52% (60.71%-80.28%)	93.559% (86.842%-100%)	94.95% (89.58%-100%)	64.91% (54.16%-75.47%)	3.2394 (3.0156-3.4527)
**At least 5 of CHASM (breast), CHASM (lung), CHASM (melanoma), FATHMM (cancer), FATHMM (missense), MutationTaster**	81.21% (78.28%-84.34%)	74.05% (69.42%-78.69%)	93.582% (90.411%-97.183%)	95.23% (92.93%-97.92%)	67.59% (62.26%-72.92%)	3.3045 (3.1989-3.4205)	81.02% (74.75%-86.87%)	73.7% (64.61%-82.54%)	93.559% (86.842%-100%)	95.16% (90%-100%)	67.46% (57.14%-78.43%)	3.2988 (3.0725-3.5121)
**At least 5 of CHASM (breast), CHASM (lung), CHASM (melanoma), FATHMM (cancer), FATHMM (missense), PolyPhen-2**	81.21% (78.28%-84.34%)	74.05% (69.42%-78.69%)	93.582% (90.411%-97.183%)	95.23% (92.93%-97.92%)	67.59% (62.26%-72.92%)	3.3045 (3.1989-3.4205)	81.02% (74.75%-86.87%)	73.7% (64.61%-82.54%)	93.559% (86.842%-100%)	95.16% (90%-100%)	67.46% (57.14%-78.43%)	3.2988 (3.0725-3.5121)
**At least 5 of CHASM (breast), CHASM (lung), CHASM (melanoma), FATHMM (cancer), FATHMM (missense), PROVEAN**	79.86% (76.77%-83.33%)	71.41% (66.92%-76.04%)	94.469% (91.549%-97.333%)	95.71% (93.55%-97.94%)	65.65% (60.2%-70.88%)	3.2725 (3.1678-3.3849)	79.67% (72.73%-85.86%)	70.99% (61.66%-80.33%)	94.535% (88.235%-100%)	95.71% (90.69%-100%)	65.51% (55.1%-76.47%)	3.2675 (3.0434-3.4852)
**At least 5 of CHASM (breast), CHASM (lung), CHASM (melanoma), FATHMM (cancer), FATHMM (missense), SIFT**	80.18% (77.27%-83.33%)	72.97% (68.5%-77.5%)	92.654% (89.394%-96%)	94.5% (92.16%-96.97%)	66.47% (61.29%-71.85%)	3.2659 (3.1586-3.3854)	80.04% (73.74%-85.86%)	72.67% (63.64%-81.67%)	92.657% (85.714%-100%)	94.44% (89.13%-100%)	66.4% (55.55%-77.09%)	3.2616 (3.0185-3.4780)
**At least 5 of CHASM (breast), CHASM (lung), CHASM (melanoma), FATHMM (cancer), FATHMM (missense), VEST**	80.87% (77.78%-84.34%)	74.05% (69.42%-78.69%)	92.654% (89.394%-96%)	94.57% (92.22%-97.03%)	67.37% (62.1%-72.82%)	3.2865 (3.1795-3.4037)	80.69% (73.74%-86.87%)	73.7% (64.61%-82.54%)	92.657% (85.714%-100%)	94.52% (89.13%-100%)	67.25% (56.52%-78.26%)	3.2812 (3.0426-3.4991)
**At least 5 of CHASM (breast), CHASM (lung), CHASM (melanoma), FATHMM (cancer), Mutation Assessor**	65.68% (61.62%-69.19%)	47.44% (42.37%-52.76%)	97.203% (95.588%-100%)	96.7% (94.74%-100%)	51.68% (46.81%-56.2%)	2.9303 (2.8383-3.0355)	65.62% (58.59%-73.74%)	47.13% (36.92%-57.35%)	97.337% (92.5%-100%)	96.82% (91.17%-100%)	51.75% (42.62%-61.67%)	2.9304 (2.7204-3.1285)
**At least 5 of CHASM (breast), CHASM (lung), CHASM (melanoma), FATHMM (cancer), Mutation Assessor, MutationTaster**	76.1% (72.73%-79.8%)	65.47% (60.66%-70.4%)	94.485% (91.667%-97.368%)	95.35% (92.94%-97.75%)	61.28% (55.83%-66.38%)	3.1658 (3.0602-3.2827)	76.08% (68.69%-82.83%)	65.32% (55.56%-75.01%)	94.508% (88.09%-100%)	95.33% (90%-100%)	61.37% (50%-71.74%)	3.1653 (2.9358-3.3792)
**At least 5 of CHASM (breast), CHASM (lung), CHASM (melanoma), FATHMM (cancer), Mutation Assessor, PolyPhen-2**	73.09% (69.7%-76.77%)	60.71% (55.73%-65.87%)	94.485% (91.667%-97.368%)	95.01% (92.5%-97.59%)	58.18% (53.04%-63.03%)	3.0838 (2.9797-3.1898)	73.01% (65.66%-79.8%)	60.47% (50%-70.19%)	94.508% (88.09%-100%)	94.97% (89.19%-100%)	58.22% (48.21%-68.63%)	3.0816 (2.8665-3.3006)
**At least 5 of CHASM (breast), CHASM (lung), CHASM (melanoma), FATHMM (cancer), Mutation Assessor, PROVEAN**	70.73% (67.17%-74.24%)	55.95% (51.14%-60.94%)	96.293% (94.203%-98.667%)	96.31% (94.2%-98.67%)	55.84% (50.81%-60.47%)	3.0439 (2.9482-3.1503)	70.65% (63.64%-77.78%)	55.63% (45.61%-65.63%)	96.398% (91.176%-100%)	96.37% (91.18%-100%)	55.87% (46.66%-66.13%)	3.0427 (2.8282-3.2421)
**At least 5 of CHASM (breast), CHASM (lung), CHASM (melanoma), FATHMM (cancer), Mutation Assessor, SIFT**	74.78% (71.72%-78.28%)	63.39% (58.53%-68.29%)	94.485% (91.667%-97.368%)	95.21% (92.77%-97.7%)	59.88% (54.7%-64.96%)	3.1296 (3.0311-3.2432)	74.67% (67.68%-80.81%)	63.1% (53.12%-72.42%)	94.508% (88.09%-100%)	95.17% (89.47%-100%)	59.88% (49.18%-70.37%)	3.1265 (2.9007-3.3277)
**At least 5 of CHASM (breast), CHASM (lung), CHASM (melanoma), FATHMM (cancer), Mutation Assessor, VEST**	72.09% (68.69%-75.76%)	59.13% (54.33%-64.4%)	94.485% (91.667%-97.368%)	94.88% (92.31%-97.53%)	57.21% (52.14%-62.19%)	3.0570 (2.9531-3.1623)	71.99% (64.65%-78.79%)	58.85% (48.53%-68.66%)	94.508% (88.09%-100%)	94.84% (88.89%-100%)	57.24% (46.77%-66.68%)	3.0543 (2.8401-3.2700)
**At least 5 of CHASM (breast), CHASM (lung), CHASM (melanoma), FATHMM (cancer), MutationTaster**	70.69% (66.67%-74.24%)	54.81% (49.61%-60%)	98.143% (97.015%-100%)	98.08% (96.88%-100%)	55.68% (50.74%-60.61%)	3.0671 (2.9682-3.1667)	70.75% (63.64%-78.79%)	54.74% (44.26%-65%)	98.209% (94.118%-100%)	98.13% (93.94%-100%)	55.85% (46.27%-66.15%)	3.0693 (2.8821-3.2723)
**At least 5 of CHASM (breast), CHASM (lung), CHASM (melanoma), FATHMM (cancer), MutationTaster, PolyPhen-2**	76.11% (72.73%-79.29%)	65.48% (60.66%-70.49%)	94.485% (91.667%-97.368%)	95.35% (93.02%-97.78%)	61.29% (56.07%-66.36%)	3.1661 (3.0595-3.2792)	76.06% (69.7%-82.83%)	65.29% (55.22%-75.39%)	94.508% (88.09%-100%)	95.33% (89.99%-100%)	61.35% (50.94%-71.67%)	3.1648 (2.9348-3.3810)
**At least 5 of CHASM (breast), CHASM (lung), CHASM (melanoma), FATHMM (cancer), MutationTaster, PROVEAN**	74.77% (71.21%-78.28%)	61.77% (56.92%-66.67%)	97.233% (95.588%-100%)	97.48% (95.95%-100%)	59.53% (54.54%-64.46%)	3.1601 (3.0634-3.2595)	74.71% (67.68%-81.82%)	61.55% (51.46%-71.22%)	97.269% (92.303%-100%)	97.49% (93.18%-100%)	59.59% (49.18%-69.84%)	3.1590 (2.9546-3.3600)
**At least 5 of CHASM (breast), CHASM (lung), CHASM (melanoma), FATHMM (cancer), MutationTaster, SIFT**	77.79% (74.75%-81.31%)	67.59% (62.9%-72.5%)	95.425% (92.958%-98.592%)	96.23% (94.18%-98.85%)	63.01% (57.8%-67.96%)	3.2225 (3.1250-3.3301)	77.75% (70.71%-83.84%)	67.46% (57.37%-77.05%)	95.38% (89.469%-100%)	96.16% (91.11%-100%)	63.08% (52.94%-73.47%)	3.2208 (3.0097-3.4310)
**At least 5 of CHASM (breast), CHASM (lung), CHASM (melanoma), FATHMM (cancer), MutationTaster, VEST**	74.42% (70.71%-77.78%)	62.8% (57.85%-68.15%)	94.485% (91.667%-97.368%)	95.17% (92.68%-97.67%)	59.5% (54.24%-64.49%)	3.1196 (3.0156-3.2287)	74.4% (67.68%-81.82%)	62.67% (52.31%-72.73%)	94.508% (88.09%-100%)	95.14% (89.47%-100%)	59.61% (49.15%-70%)	3.1193 (2.9038-3.3390)
**At least 5 of CHASM (breast), CHASM (lung), CHASM (melanoma), FATHMM (cancer), PolyPhen-2**	68.69% (64.65%-72.22%)	52.19% (46.97%-57.5%)	97.203% (95.588%-100%)	97% (95.24%-100%)	54.04% (49.22%-58.52%)	3.0044 (2.9091-3.1072)	68.68% (61.62%-76.77%)	51.98% (41.17%-62.12%)	97.337% (92.5%-100%)	97.11% (92%-100%)	54.15% (45.31%-64.18%)	3.0058 (2.7948-3.2095)
**At least 5 of CHASM (breast), CHASM (lung), CHASM (melanoma), FATHMM (cancer), PolyPhen-2, PROVEAN**	72.75% (69.19%-76.26%)	59.14% (53.97%-64%)	96.293% (94.203%-98.667%)	96.5% (94.52%-98.73%)	57.68% (52.59%-62.5%)	3.0961 (2.9968-3.2015)	72.67% (65.66%-79.8%)	58.83% (49.12%-68.75%)	96.398% (91.176%-100%)	96.56% (91.67%-100%)	57.71% (48.21%-67.74%)	3.0950 (2.8849-3.2969)
**At least 5 of CHASM (breast), CHASM (lung), CHASM (melanoma), FATHMM (cancer), PolyPhen-2, SIFT**	76.12% (72.73%-79.29%)	65.49% (60.5%-70.4%)	94.485% (91.667%-97.368%)	95.35% (92.94%-97.78%)	61.29% (56.25%-66.36%)	3.1662 (3.0614-3.2733)	76.05% (69.7%-82.83%)	65.27% (55.36%-74.63%)	94.508% (88.09%-100%)	95.33% (89.74%-100%)	61.33% (50.94%-71.7%)	3.1644 (2.9522-3.3710)
**At least 5 of CHASM (breast), CHASM (lung), CHASM (melanoma), FATHMM (cancer), PolyPhen-2, VEST**	73.44% (70.2%-76.77%)	61.26% (56.25%-66.15%)	94.485% (91.667%-97.368%)	95.05% (92.5%-97.62%)	58.52% (53.78%-63.48%)	3.0932 (2.9888-3.2012)	73.32% (66.67%-79.8%)	60.96% (50.91%-71.19%)	94.508% (88.09%-100%)	95.01% (89.19%-100%)	58.52% (48.28%-68.42%)	3.0900 (2.8765-3.3010)
**At least 5 of CHASM (breast), CHASM (lung), CHASM (melanoma), FATHMM (cancer), PROVEAN**	61.3% (57.58%-65.15%)	39.45% (34.43%-44.26%)	99.084% (98.485%-100%)	98.67% (97.73%-100%)	48.62% (43.75%-52.98%)	2.8583 (2.7711-2.9411)	61.23% (53.54%-68.69%)	39.17% (29.73%-49.18%)	99.083% (96.774%-100%)	98.66% (95.24%-100%)	48.69% (39.74%-58.44%)	2.8560 (2.6907-3.0380)
**At least 5 of CHASM (breast), CHASM (lung), CHASM (melanoma), FATHMM (cancer), PROVEAN, SIFT**	73.11% (69.7%-76.77%)	59.15% (54.26%-64.29%)	97.233% (95.588%-100%)	97.37% (95.77%-100%)	57.92% (52.89%-62.6%)	3.1167 (3.0207-3.2163)	72.98% (65.66%-79.8%)	58.81% (48.39%-68.34%)	97.269% (92.303%-100%)	97.37% (92.85%-100%)	57.91% (48.28%-67.8%)	3.1136 (2.9117-3.3010)
**At least 5 of CHASM (breast), CHASM (lung), CHASM (melanoma), FATHMM (cancer), PROVEAN, VEST**	68.39% (64.65%-71.72%)	52.25% (47.15%-57.26%)	96.293% (94.203%-98.667%)	96.06% (93.85%-98.57%)	53.84% (48.85%-58.46%)	2.9844 (2.8868-3.0806)	68.27% (61.62%-75.76%)	51.86% (42.02%-61.77%)	96.398% (91.176%-100%)	96.13% (90.62%-100%)	53.85% (44.25%-63.77%)	2.9822 (2.7801-3.1871)
**At least 5 of CHASM (breast), CHASM (lung), CHASM (melanoma), FATHMM (cancer), SIFT**	71.41% (68.17%-74.75%)	55.94% (50.82%-61.11%)	98.143% (97.015%-100%)	98.12% (96.92%-100%)	56.3% (51.22%-60.94%)	3.0850 (2.9926-3.1806)	71.33% (64.65%-77.8%)	55.65% (45.16%-65.72%)	98.209% (94.118%-100%)	98.16% (94.12%-100%)	56.34% (46.87%-66.15%)	3.0836 (2.8917-3.2718)
**At least 5 of CHASM (breast), CHASM (lung), CHASM (melanoma), FATHMM (cancer), SIFT, VEST**	74.44% (71.21%-77.78%)	63.38% (58.53%-68.55%)	93.557% (90.537%-97.183%)	94.45% (91.86%-97.65%)	59.64% (54.62%-64.91%)	3.1102 (3.0116-3.2261)	74.35% (67.68%-80.81%)	63.11% (53.02%-72.58%)	93.606% (86.842%-100%)	94.43% (87.8%-100%)	59.66% (49.12%-69.82%)	3.1081 (2.8764-3.3140)
**At least 5 of CHASM (breast), CHASM (lung), CHASM (melanoma), FATHMM (cancer), VEST**	63.65% (60.1%-67.17%)	44.24% (38.98%-49.21%)	97.203% (95.588%-100%)	96.48% (94.34%-100%)	50.21% (45.33%-54.68%)	2.8813 (2.7823-2.9773)	63.61% (56.57%-70.71%)	43.95% (33.85%-54.1%)	97.337% (92.5%-100%)	96.61% (90.91%-100%)	50.3% (41.17%-60.27%)	2.8819 (2.6907-3.0714)
**At least 5 of CHASM (breast), CHASM (lung), CHASM (melanoma), FATHMM (missense), Mutation Assessor**	65.35% (61.62%-69.19%)	46.92% (41.88%-52.03%)	97.203% (95.588%-100%)	96.67% (94.64%-100%)	51.44% (46.56%-55.97%)	2.9223 (2.8281-3.0243)	65.26% (57.58%-72.73%)	46.56% (36.36%-56.45%)	97.337% (92.5%-100%)	96.78% (91.17%-100%)	51.49% (42.65%-61.54%)	2.9217 (2.7235-3.1137)
**At least 5 of CHASM (breast), CHASM (lung), CHASM (melanoma), FATHMM (missense), Mutation Assessor, MutationTaster**	73.41% (70.19%-76.78%)	61.21% (56.1%-66.14%)	94.485% (91.667%-97.368%)	95.05% (92.5%-97.65%)	58.49% (53.1%-63.33%)	3.0923 (2.9894-3.2018)	73.39% (66.64%-79.82%)	61.07% (50.82%-71.19%)	94.508% (88.09%-100%)	95.02% (88.89%-100%)	58.59% (48.27%-69.09%)	3.0919 (2.8713-3.2998)
**At least 5 of CHASM (breast), CHASM (lung), CHASM (melanoma), FATHMM (missense), Mutation Assessor, PolyPhen-2**	70.39% (66.67%-74.24%)	56.45% (51.22%-61.6%)	94.485% (91.667%-97.368%)	94.65% (91.89%-97.44%)	55.65% (50.41%-60.33%)	3.0124 (2.9075-3.1205)	70.33% (62.63%-77.78%)	56.21% (46.03%-66.67%)	94.508% (88.09%-100%)	94.61% (88.24%-100%)	55.71% (46.15%-66.1%)	3.0104 (2.7898-3.2191)
**At least 5 of CHASM (breast), CHASM (lung), CHASM (melanoma), FATHMM (missense), Mutation Assessor, PROVEAN**	68.36% (64.65%-71.72%)	52.2% (46.87%-57.03%)	96.293% (94.203%-98.667%)	96.06% (93.85%-98.57%)	53.81% (48.9%-58.46%)	2.9836 (2.8855-3.0865)	68.33% (61.62%-75.76%)	51.95% (42.1%-62.12%)	96.398% (91.176%-100%)	96.13% (90.62%-100%)	53.9% (44.62%-64.06%)	2.9838 (2.7758-3.1804)
**At least 5 of CHASM (breast), CHASM (lung), CHASM (melanoma), FATHMM (missense), Mutation Assessor, SIFT**	72.42% (68.69%-75.76%)	59.66% (54.33%-64.57%)	94.485% (91.667%-97.368%)	94.92% (92.31%-97.59%)	57.53% (52.5%-62.61%)	3.0660 (2.9616-3.1732)	72.32% (65.66%-79.8%)	59.38% (49.23%-70%)	94.508% (88.09%-100%)	94.88% (88.89%-100%)	57.55% (47.37%-67.8%)	3.0632 (2.8418-3.2725)
**At least 5 of CHASM (breast), CHASM (lung), CHASM (melanoma), FATHMM (missense), Mutation Assessor, VEST**	69.39% (65.66%-72.73%)	54.87% (49.22%-59.85%)	94.485% (91.667%-97.368%)	94.51% (91.67%-97.37%)	54.77% (49.99%-59.54%)	2.9863 (2.8809-3.0943)	69.3% (62.63%-76.77%)	54.6% (44.26%-65.52%)	94.508% (88.09%-100%)	94.46% (87.88%-100%)	54.81% (45%-64.91%)	2.9838 (2.7615-3.1902)
**At least 5 of CHASM (breast), CHASM (lung), CHASM (melanoma), FATHMM (missense), MutationTaster**	68.68% (65.14%-72.22%)	51.64% (46.46%-56.92%)	98.143% (97.015%-100%)	97.96% (96.67%-100%)	54% (49.21%-58.73%)	3.0175 (2.9193-3.1176)	68.7% (61.62%-75.78%)	51.49% (40.68%-61.91%)	98.209% (94.118%-100%)	98.02% (93.55%-100%)	54.13% (44.29%-64.07%)	3.0185 (2.8175-3.2190)
**At least 5 of CHASM (breast), CHASM (lung), CHASM (melanoma), FATHMM (missense), MutationTaster, PolyPhen-2**	75.09% (71.72%-78.3%)	63.87% (58.82%-68.91%)	94.485% (91.667%-97.368%)	95.24% (92.86%-97.7%)	60.2% (55%-65.26%)	3.1380 (3.0311-3.2457)	75.07% (68.66%-81.82%)	63.72% (53.33%-74.14%)	94.508% (88.09%-100%)	95.22% (89.74%-100%)	60.3% (50%-70.92%)	3.1375 (2.9110-3.3530)
**At least 5 of CHASM (breast), CHASM (lung), CHASM (melanoma), FATHMM (missense), MutationTaster, PROVEAN**	73.4% (69.7%-76.77%)	59.61% (54.33%-64.8%)	97.233% (95.588%-100%)	97.39% (95.83%-100%)	58.2% (53.17%-62.93%)	3.1243 (3.0246-3.2282)	73.41% (66.67%-80.81%)	59.48% (49.18%-69.85%)	97.269% (92.303%-100%)	97.41% (93.02%-100%)	58.33% (48.39%-68.85%)	3.1249 (2.9166-3.3286)
**At least 5 of CHASM (breast), CHASM (lung), CHASM (melanoma), FATHMM (missense), MutationTaster, SIFT**	76.44% (73.23%-79.8%)	65.45% (60.47%-70.54%)	95.425% (92.958%-98.592%)	96.11% (93.98%-98.81%)	61.5% (56.36%-66.38%)	3.1849 (3.0847-3.2975)	76.42% (69.7%-82.83%)	65.35% (55%-75.41%)	95.38% (89.469%-100%)	96.04% (90.9%-100%)	61.61% (51.72%-72.23%)	3.1838 (2.9615-3.3911)
**At least 5 of CHASM (breast), CHASM (lung), CHASM (melanoma), FATHMM (missense), MutationTaster, VEST**	73.4% (69.7%-76.77%)	61.2% (56.2%-66.41%)	94.485% (91.667%-97.368%)	95.05% (92.5%-97.62%)	58.48% (53.33%-63.39%)	3.0920 (2.9866-3.2008)	73.41% (66.67%-80.81%)	61.1% (50.77%-71.19%)	94.508% (88.09%-100%)	95.03% (89.19%-100%)	58.61% (48.43%-69.23%)	3.0925 (2.8769-3.3080)
**At least 5 of CHASM (breast), CHASM (lung), CHASM (melanoma), FATHMM (missense), PolyPhen-2**	66.68% (62.63%-70.21%)	49.03% (44.09%-54.33%)	97.203% (95.588%-100%)	96.81% (94.92%-100%)	52.45% (47.62%-57.04%)	2.9549 (2.8569-3.0600)	66.63% (59.57%-74.75%)	48.73% (38.1%-59.09%)	97.337% (92.5%-100%)	96.92% (91.43%-100%)	52.53% (43.28%-62.32%)	2.9552 (2.7470-3.1604)
**At least 5 of CHASM (breast), CHASM (lung), CHASM (melanoma), FATHMM (missense), PolyPhen-2, PROVEAN**	71.39% (67.68%-74.75%)	56.98% (51.72%-62.3%)	96.293% (94.203%-98.667%)	96.38% (94.29%-98.68%)	56.42% (51.56%-61.11%)	3.0606 (2.9631-3.1633)	71.37% (64.65%-78.79%)	56.77% (46.03%-66.67%)	96.398% (91.176%-100%)	96.44% (91.43%-100%)	56.51% (46.97%-66.67%)	3.0612 (2.8553-3.2470)
**At least 5 of CHASM (breast), CHASM (lung), CHASM (melanoma), FATHMM (missense), PolyPhen-2, SIFT**	74.76% (71.21%-78.28%)	63.35% (58.27%-68.22%)	94.485% (91.667%-97.368%)	95.21% (92.68%-97.7%)	59.86% (54.62%-64.66%)	3.1290 (3.0272-3.2398)	74.72% (67.68%-81.82%)	63.17% (53.23%-72.89%)	94.508% (88.09%-100%)	95.18% (89.47%-100%)	59.93% (50%-70.37%)	3.1278 (2.9075-3.3390)
**At least 5 of CHASM (breast), CHASM (lung), CHASM (melanoma), FATHMM (missense), PolyPhen-2, VEST**	72.08% (68.69%-75.76%)	59.65% (54.47%-64.75%)	93.57% (90.537%-97.184%)	94.13% (91.36%-97.47%)	57.29% (52.42%-62.07%)	3.0464 (2.9455-3.1587)	71.99% (64.65%-78.79%)	59.39% (49.21%-69.85%)	93.595% (86.662%-100%)	94.08% (87.49%-100%)	57.32% (47.37%-67.27%)	3.0439 (2.8188-3.2474)
**At least 5 of CHASM (breast), CHASM (lung), CHASM (melanoma), FATHMM (missense), PROVEAN**	59.31% (55.05%-63.13%)	36.3% (31.45%-40.94%)	99.084% (98.485%-100%)	98.56% (97.5%-100%)	47.36% (42.48%-51.68%)	2.8130 (2.7289-2.8945)	59.17% (51.52%-67.68%)	35.9% (26.31%-45.9%)	99.083% (96.774%-100%)	98.54% (94.74%-100%)	47.38% (38.66%-56.96%)	2.8090 (2.6399-2.9812)
**At least 5 of CHASM (breast), CHASM (lung), CHASM (melanoma), FATHMM (missense), PROVEAN, SIFT**	72.07% (68.69%-75.76%)	57.52% (52.42%-62.6%)	97.233% (95.588%-100%)	97.29% (95.65%-100%)	56.97% (52.03%-61.79%)	3.0901 (2.9949-3.1895)	72.01% (64.65%-78.79%)	57.28% (46.96%-67.21%)	97.269% (92.303%-100%)	97.31% (92.5%-100%)	57.02% (47.54%-66.67%)	3.0887 (2.8852-3.2764)
**At least 5 of CHASM (breast), CHASM (lung), CHASM (melanoma), FATHMM (missense), PROVEAN, VEST**	67.02% (63.13%-70.21%)	50.09% (44.71%-55.2%)	96.293% (94.203%-98.667%)	95.9% (93.55%-98.53%)	52.74% (47.79%-57.35%)	2.9502 (2.8506-3.0473)	66.97% (60.58%-74.75%)	49.8% (39.62%-60.94%)	96.398% (91.176%-100%)	95.97% (90%-100%)	52.81% (43.47%-62.72%)	2.9498 (2.7520-3.1592)
**At least 5 of CHASM (breast), CHASM (lung), CHASM (melanoma), FATHMM (missense), SIFT**	69.74% (66.16%-73.23%)	53.31% (48.36%-58.4%)	98.143% (97.015%-100%)	98.03% (96.77%-100%)	54.87% (50%-59.54%)	3.0434 (2.9529-3.1424)	69.62% (62.63%-76.77%)	52.94% (42.42%-62.72%)	98.209% (94.118%-100%)	98.07% (93.94%-100%)	54.87% (45.71%-64.62%)	3.0409 (2.8473-3.2408)
**At least 5 of CHASM (breast), CHASM (lung), CHASM (melanoma), FATHMM (missense), SIFT, VEST**	73.08% (69.7%-76.77%)	61.24% (56.15%-66.16%)	93.557% (90.537%-97.183%)	94.26% (91.57%-97.59%)	58.26% (53.28%-63.39%)	3.0732 (2.9721-3.1868)	73.02% (65.66%-79.8%)	61.01% (50.82%-70.97%)	93.606% (86.842%-100%)	94.25% (87.23%-100%)	58.32% (48.15%-68.43%)	3.0718 (2.8451-3.2777)
**At least 5 of CHASM (breast), CHASM (lung), CHASM (melanoma), FATHMM (missense), VEST**	61.64% (57.58%-65.15%)	41.08% (35.71%-46.09%)	97.203% (95.588%-100%)	96.22% (93.88%-100%)	48.83% (43.97%-53.24%)	2.8332 (2.7361-2.9293)	61.56% (54.55%-69.7%)	40.7% (30.3%-50.89%)	97.337% (92.5%-100%)	96.35% (90%-100%)	48.89% (39.99%-58.73%)	2.8328 (2.6449-3.0248)
**At least 5 of CHASM (breast), CHASM (lung), CHASM (melanoma), Mutation Assessor, MutationTaster**	62.62% (59.08%-66.17%)	42.61% (37.5%-47.58%)	97.222% (95.588%-100%)	96.37% (94.12%-100%)	49.49% (44.76%-54.11%)	2.8569 (2.7586-2.9575)	62.64% (55.53%-69.72%)	42.43% (32.68%-52.94%)	97.294% (92.303%-100%)	96.42% (90.46%-100%)	49.62% (40.27%-59.16%)	2.8576 (2.6478-3.0534)
**At least 5 of CHASM (breast), CHASM (lung), CHASM (melanoma), Mutation Assessor, MutationTaster, PolyPhen-2**	72.42% (68.69%-75.76%)	59.65% (54.4%-64.57%)	94.485% (91.667%-97.368%)	94.92% (92.31%-97.56%)	57.52% (52.54%-62.5%)	3.0658 (2.9619-3.1728)	72.34% (65.66%-79.8%)	59.4% (49.23%-69.7%)	94.508% (88.09%-100%)	94.89% (88.89%-100%)	57.57% (47.54%-67.8%)	3.0637 (2.8479-3.2726)
**At least 5 of CHASM (breast), CHASM (lung), CHASM (melanoma), Mutation Assessor, MutationTaster, PROVEAN**	69.04% (65.64%-72.22%)	53.26% (48.33%-58.26%)	96.311% (94.286%-98.667%)	96.15% (93.94%-98.63%)	54.37% (49.6%-58.96%)	3.0009 (2.8996-3.1055)	69% (62.63%-75.78%)	53.04% (42.62%-63.33%)	96.355% (91.176%-100%)	96.15% (90.32%-100%)	54.45% (44.82%-64.52%)	3.0000 (2.7934-3.2116)
**At least 5 of CHASM (breast), CHASM (lung), CHASM (melanoma), Mutation Assessor, MutationTaster, SIFT**	72.09% (68.69%-75.76%)	58.59% (53.49%-63.57%)	95.425% (92.958%-98.592%)	95.68% (93.24%-98.67%)	57.13% (52.07%-61.99%)	3.0683 (2.9669-3.1757)	71.98% (64.65%-78.79%)	58.32% (48.27%-68.66%)	95.38% (89.469%-100%)	95.59% (89.74%-100%)	57.15% (47.36%-67.24%)	3.0644 (2.8446-3.2727)
**At least 5 of CHASM (breast), CHASM (lung), CHASM (melanoma), Mutation Assessor, MutationTaster, VEST**	70.39% (66.67%-73.74%)	56.45% (51.59%-61.42%)	94.485% (91.667%-97.368%)	94.65% (91.89%-97.44%)	55.65% (50.81%-60.35%)	3.0124 (2.9068-3.1201)	70.33% (63.64%-77.78%)	56.22% (45.9%-66.13%)	94.508% (88.09%-100%)	94.62% (88.37%-100%)	55.71% (45.76%-65.62%)	3.0107 (2.7932-3.2174)
**At least 5 of CHASM (breast), CHASM (lung), CHASM (melanoma), Mutation Assessor, PolyPhen-2**	63.63% (59.6%-67.68%)	44.75% (39.67%-50%)	96.282% (94.284%-98.667%)	95.42% (92.73%-98.33%)	50.2% (45.32%-54.61%)	2.8664 (2.7660-2.9755)	63.64% (55.56%-71.72%)	44.52% (34.38%-54.55%)	96.423% (91.176%-100%)	95.53% (89.28%-100%)	50.32% (41.17%-60%)	2.8679 (2.6485-3.0693)
**At least 5 of CHASM (breast), CHASM (lung), CHASM (melanoma), Mutation Assessor, PolyPhen-2, PROVEAN**	68.71% (65.15%-72.22%)	53.28% (48.46%-58.4%)	95.372% (92.955%-98.571%)	95.22% (92.65%-98.55%)	54.14% (49.21%-58.65%)	2.9802 (2.8793-3.0858)	68.64% (61.62%-75.76%)	52.98% (42.61%-62.72%)	95.484% (89.651%-100%)	95.27% (88.89%-100%)	54.2% (44.64%-64.07%)	2.9794 (2.7672-3.1872)
**At least 5 of CHASM (breast), CHASM (lung), CHASM (melanoma), Mutation Assessor, PolyPhen-2, SIFT**	70.74% (67.17%-74.24%)	57.01% (51.94%-62.2%)	94.485% (91.667%-97.368%)	94.7% (92%-97.47%)	55.97% (50.83%-60.8%)	3.0217 (2.9183-3.1327)	70.63% (63.64%-77.78%)	56.7% (46.42%-66.67%)	94.508% (88.09%-100%)	94.66% (88.56%-100%)	55.99% (45.9%-66.14%)	3.0186 (2.7846-3.2191)
**At least 5 of CHASM (breast), CHASM (lung), CHASM (melanoma), Mutation Assessor, PolyPhen-2, VEST**	71.07% (67.68%-74.75%)	57.53% (52.34%-62.7%)	94.485% (91.667%-97.368%)	94.75% (92%-97.47%)	56.27% (51.26%-61.16%)	3.0303 (2.9282-3.1420)	70.99% (63.64%-77.78%)	57.26% (46.77%-67.21%)	94.508% (88.09%-100%)	94.71% (88.57%-100%)	56.31% (46.54%-66.14%)	3.0279 (2.8027-3.2406)
**At least 5 of CHASM (breast), CHASM (lung), CHASM (melanoma), Mutation Assessor, PROVEAN**	57.94% (54.04%-61.62%)	34.15% (29.41%-38.71%)	99.084% (98.485%-100%)	98.47% (97.37%-100%)	46.53% (41.83%-50.94%)	2.7823 (2.6972-2.8732)	57.85% (50.51%-65.66%)	33.82% (24.56%-43.75%)	99.083% (96.774%-100%)	98.44% (94.12%-100%)	46.59% (38.09%-56%)	2.7793 (2.6022-2.9550)
**At least 5 of CHASM (breast), CHASM (lung), CHASM (melanoma), Mutation Assessor, PROVEAN, SIFT**	69.72% (66.16%-73.23%)	54.33% (49.21%-59.5%)	96.311% (94.286%-98.667%)	96.22% (94.03%-98.65%)	54.95% (50%-59.68%)	3.0181 (2.9162-3.1191)	69.66% (62.63%-76.77%)	54.07% (43.74%-64.41%)	96.355% (91.176%-100%)	96.22% (90.62%-100%)	55.01% (45.16%-64.91%)	3.0166 (2.8150-3.2191)
**At least 5 of CHASM (breast), CHASM (lung), CHASM (melanoma), Mutation Assessor, PROVEAN, VEST**	66.03% (62.12%-69.7%)	49.05% (44.09%-54.03%)	95.372% (92.955%-98.571%)	94.83% (92.06%-98.41%)	51.99% (47.18%-56.62%)	2.9124 (2.8091-3.0222)	65.92% (58.59%-73.74%)	48.67% (38.59%-58.74%)	95.484% (89.651%-100%)	94.88% (88.24%-100%)	52.02% (42.42%-61.67%)	2.9105 (2.6915-3.1176)
**At least 5 of CHASM (breast), CHASM (lung), CHASM (melanoma), Mutation Assessor, SIFT**	64.66% (61.1%-68.18%)	45.82% (40.74%-50.81%)	97.222% (95.588%-100%)	96.61% (94.55%-100%)	50.93% (46.1%-55.47%)	2.9059 (2.8126-3.0076)	64.62% (57.58%-71.74%)	45.57% (35.38%-55.39%)	97.294% (92.303%-100%)	96.65% (90.91%-100%)	51.02% (41.79%-60.56%)	2.9053 (2.7008-3.1008)
**At least 5 of CHASM (breast), CHASM (lung), CHASM (melanoma), Mutation Assessor, SIFT, VEST**	70.4% (66.67%-74.24%)	56.47% (51.26%-61.72%)	94.485% (91.667%-97.368%)	94.65% (91.89%-97.44%)	55.66% (50.79%-60.47%)	3.0126 (2.9097-3.1203)	70.31% (62.63%-77.78%)	56.19% (45.45%-66.13%)	94.508% (88.09%-100%)	94.61% (88.24%-100%)	55.7% (45.45%-65.57%)	3.0101 (2.7901-3.2169)
**At least 5 of CHASM (breast), CHASM (lung), CHASM (melanoma), Mutation Assessor, VEST**	59.6% (55.56%-63.64%)	38.38% (33.33%-43.55%)	96.282% (94.284%-98.667%)	94.7% (91.67%-98.11%)	47.47% (42.76%-52.03%)	2.7683 (2.6681-2.8734)	59.59% (51.52%-67.68%)	38.11% (27.69%-48.33%)	96.423% (91.176%-100%)	94.82% (86.96%-100%)	47.59% (38.36%-56.92%)	2.7695 (2.5576-2.9727)
**At least 5 of CHASM (breast), CHASM (lung), CHASM (melanoma), MutationTaster, PolyPhen-2**	65.97% (62.12%-69.7%)	47.9% (42.52%-52.85%)	97.222% (95.588%-100%)	96.76% (94.74%-100%)	51.91% (47.06%-56.34%)	2.9378 (2.8387-3.0368)	66.03% (58.59%-73.74%)	47.81% (37.7%-58.06%)	97.294% (92.303%-100%)	96.81% (91.67%-100%)	52.07% (42.86%-61.9%)	2.9398 (2.7425-3.1420)
**At least 5 of CHASM (breast), CHASM (lung), CHASM (melanoma), MutationTaster, PolyPhen-2, PROVEAN**	72.07% (68.18%-75.25%)	58.05% (52.94%-63.08%)	96.311% (94.286%-98.667%)	96.46% (94.44%-98.73%)	57.04% (52.14%-61.72%)	3.0785 (2.9737-3.1764)	72.02% (65.66%-79.8%)	57.82% (47.69%-67.74%)	96.355% (91.176%-100%)	96.47% (91.18%-100%)	57.11% (47.46%-67.24%)	3.0775 (2.8795-3.2969)
**At least 5 of CHASM (breast), CHASM (lung), CHASM (melanoma), MutationTaster, PolyPhen-2, SIFT**	74.44% (71.21%-77.78%)	62.3% (57.14%-67.2%)	95.425% (92.958%-98.592%)	95.93% (93.67%-98.75%)	59.41% (54.47%-64.35%)	3.1306 (3.0311-3.2412)	74.36% (67.68%-80.81%)	62.08% (51.79%-72.41%)	95.38% (89.469%-100%)	95.85% (90.48%-100%)	59.45% (49.23%-69.65%)	3.1276 (2.9169-3.3307)
**At least 5 of CHASM (breast), CHASM (lung), CHASM (melanoma), MutationTaster, PolyPhen-2, VEST**	72.08% (68.69%-75.25%)	59.12% (54.19%-64%)	94.485% (91.667%-97.368%)	94.88% (92.31%-97.53%)	57.21% (52.14%-61.79%)	3.0570 (2.9489-3.1610)	71.99% (65.66%-78.79%)	58.85% (48.44%-68.87%)	94.508% (88.09%-100%)	94.85% (88.89%-100%)	57.24% (47.37%-67.24%)	3.0545 (2.8451-3.2723)
**At least 5 of CHASM (breast), CHASM (lung), CHASM (melanoma), MutationTaster, PROVEAN**	59.27% (55.05%-62.64%)	36.24% (31.45%-41.22%)	99.084% (98.485%-100%)	98.56% (97.5%-100%)	47.34% (42.57%-51.63%)	2.8122 (2.7237-2.8945)	59.23% (52.5%-67.68%)	36.01% (26.23%-45.9%)	99.083% (96.774%-100%)	98.54% (94.74%-100%)	47.43% (38.88%-57.14%)	2.8106 (2.6450-2.9919)
**At least 5 of CHASM (breast), CHASM (lung), CHASM (melanoma), MutationTaster, PROVEAN, SIFT**	70.39% (66.67%-73.74%)	55.4% (50.4%-60.31%)	96.311% (94.286%-98.667%)	96.29% (94.12%-98.68%)	55.53% (50.39%-60.31%)	3.0353 (2.9356-3.1371)	70.33% (63.64%-77.78%)	55.14% (45.16%-65.08%)	96.355% (91.176%-100%)	96.29% (90.62%-100%)	55.58% (45.9%-65.67%)	3.0337 (2.8250-3.2426)
**At least 5 of CHASM (breast), CHASM (lung), CHASM (melanoma), MutationTaster, PROVEAN, VEST**	68.05% (64.63%-71.72%)	51.7% (46.56%-56.82%)	96.311% (94.286%-98.667%)	96.04% (93.75%-98.59%)	53.56% (48.82%-58.21%)	2.9761 (2.8758-3.0802)	67.94% (60.61%-74.77%)	51.37% (40.98%-61.67%)	96.355% (91.176%-100%)	96.04% (90%-100%)	53.59% (43.93%-63.02%)	2.9736 (2.7672-3.1774)
**At least 5 of CHASM (breast), CHASM (lung), CHASM (melanoma), MutationTaster, SIFT**	66.66% (62.63%-70.2%)	48.99% (43.65%-54.33%)	97.222% (95.588%-100%)	96.82% (94.83%-100%)	52.43% (47.44%-57.04%)	2.9546 (2.8607-3.0579)	66.67% (59.6%-74.75%)	48.82% (37.88%-59.38%)	97.294% (92.303%-100%)	96.87% (91.67%-100%)	52.56% (43.28%-62.69%)	2.9554 (2.7470-3.1583)
**At least 5 of CHASM (breast), CHASM (lung), CHASM (melanoma), MutationTaster, SIFT, VEST**	72.08% (68.69%-75.27%)	58.58% (53.6%-63.57%)	95.425% (92.958%-98.592%)	95.68% (93.24%-98.67%)	57.13% (52.1%-61.98%)	3.0681 (2.9654-3.1737)	72% (65.63%-78.79%)	58.35% (48.28%-68.18%)	95.38% (89.469%-100%)	95.6% (89.74%-100%)	57.17% (47.36%-67.24%)	3.0649 (2.8479-3.2725)
**At least 5 of CHASM (breast), CHASM (lung), CHASM (melanoma), MutationTaster, VEST**	62.63% (58.59%-66.16%)	42.62% (37.21%-48%)	97.222% (95.588%-100%)	96.37% (94.12%-100%)	49.5% (44.76%-54%)	2.8571 (2.7602-2.9586)	62.62% (55.56%-70.71%)	42.4% (31.75%-52.94%)	97.294% (92.303%-100%)	96.41% (90.32%-100%)	49.61% (40.28%-59.68%)	2.8572 (2.6549-3.0571)
**At least 5 of CHASM (breast), CHASM (lung), CHASM (melanoma), PolyPhen-2, PROVEAN**	59.28% (55.56%-63.13%)	36.26% (31.2%-40.99%)	99.084% (98.485%-100%)	98.56% (97.5%-100%)	47.34% (42.4%-51.66%)	2.8125 (2.7264-2.8984)	59.21% (51.52%-66.67%)	35.96% (26.32%-46.03%)	99.083% (96.774%-100%)	98.53% (94.73%-100%)	47.41% (38.89%-56.96%)	2.8099 (2.6359-2.9811)
**At least 5 of CHASM (breast), CHASM (lung), CHASM (melanoma), PolyPhen-2, PROVEAN, SIFT**	70.06% (66.67%-73.23%)	54.87% (50%-60%)	96.311% (94.286%-98.667%)	96.26% (94.03%-98.67%)	55.24% (50.38%-59.85%)	3.0268 (2.9240-3.1244)	69.99% (63.64%-76.77%)	54.6% (44.26%-64.52%)	96.355% (91.176%-100%)	96.26% (90.62%-100%)	55.29% (45.76%-65.15%)	3.0251 (2.8223-3.2420)
**At least 5 of CHASM (breast), CHASM (lung), CHASM (melanoma), PolyPhen-2, PROVEAN, VEST**	67.72% (64.13%-71.21%)	51.72% (46.4%-56.59%)	95.372% (92.955%-98.571%)	95.08% (92.42%-98.51%)	53.33% (48.46%-57.89%)	2.9550 (2.8513-3.0555)	67.59% (60.61%-74.77%)	51.32% (41.38%-61.67%)	95.484% (89.651%-100%)	95.14% (88.57%-100%)	53.33% (43.55%-63.02%)	2.9527 (2.7496-3.1669)
**At least 5 of CHASM (breast), CHASM (lung), CHASM (melanoma), PolyPhen-2, SIFT**	66.34% (62.63%-70.2%)	48.48% (43.1%-53.66%)	97.222% (95.588%-100%)	96.79% (94.83%-100%)	52.18% (47.14%-56.52%)	2.9467 (2.8427-3.0453)	66.31% (58.59%-73.74%)	48.24% (37.5%-59.02%)	97.294% (92.303%-100%)	96.83% (91.43%-100%)	52.28% (43.08%-62.3%)	2.9465 (2.7496-3.1583)
**At least 5 of CHASM (breast), CHASM (lung), CHASM (melanoma), PolyPhen-2, SIFT, VEST**	72.09% (68.69%-75.76%)	59.14% (54.26%-64.06%)	94.485% (91.667%-97.368%)	94.88% (92.31%-97.53%)	57.22% (52.17%-62.18%)	3.0573 (2.9497-3.1716)	71.97% (64.65%-78.79%)	58.83% (48.44%-68.66%)	94.508% (88.09%-100%)	94.84% (88.89%-100%)	57.22% (47.27%-67.19%)	3.0540 (2.8282-3.2715)
**At least 5 of CHASM (breast), CHASM (lung), CHASM (melanoma), PolyPhen-2, VEST**	61.61% (57.58%-65.16%)	41.55% (36.29%-46.77%)	96.282% (94.284%-98.667%)	95.08% (92.31%-98.25%)	48.79% (43.88%-53.15%)	2.8171 (2.7112-2.9183)	61.63% (54.52%-69.7%)	41.35% (31.15%-51.61%)	96.423% (91.176%-100%)	95.21% (88%-100%)	48.93% (40.27%-58.67%)	2.8191 (2.6115-3.0275)
**At least 5 of CHASM (breast), CHASM (lung), CHASM (melanoma), PROVEAN, SIFT**	61.3% (57.58%-65.15%)	39.45% (34.43%-44.26%)	99.084% (98.485%-100%)	98.67% (97.73%-100%)	48.62% (43.75%-52.98%)	2.8583 (2.7711-2.9411)	61.23% (53.54%-68.69%)	39.17% (29.73%-49.18%)	99.083% (96.774%-100%)	98.66% (95.24%-100%)	48.69% (39.74%-58.44%)	2.8560 (2.6907-3.0380)
**At least 5 of CHASM (breast), CHASM (lung), CHASM (melanoma), PROVEAN, SIFT, VEST**	67.03% (63.13%-70.2%)	50.09% (44.92%-55.12%)	96.311% (94.286%-98.667%)	95.92% (93.55%-98.55%)	52.74% (47.73%-57.46%)	2.9506 (2.8516-3.0545)	66.95% (60.61%-74.75%)	49.79% (39.68%-60%)	96.355% (91.176%-100%)	95.92% (89.66%-100%)	52.79% (42.86%-62.69%)	2.9485 (2.7396-3.1543)
**At least 5 of CHASM (breast), CHASM (lung), CHASM (melanoma), PROVEAN, VEST**	58.61% (54.55%-62.12%)	35.2% (30.3%-39.84%)	99.084% (98.485%-100%)	98.52% (97.44%-100%)	46.93% (42.1%-51.3%)	2.7973 (2.7133-2.8819)	58.54% (51.52%-66.67%)	34.91% (25.36%-45.45%)	99.083% (96.774%-100%)	98.5% (94.44%-100%)	47.01% (38.35%-56.25%)	2.7949 (2.6207-2.9724)
**At least 5 of CHASM (breast), CHASM (lung), CHASM (melanoma), SIFT, VEST**	62.64% (58.59%-66.16%)	42.63% (37.4%-47.97%)	97.222% (95.588%-100%)	96.37% (94.12%-100%)	49.5% (44.75%-53.9%)	2.8572 (2.7597-2.9531)	62.6% (55.56%-70.71%)	42.37% (32.25%-52.46%)	97.294% (92.303%-100%)	96.41% (90.62%-100%)	49.6% (40.3%-59.16%)	2.8567 (2.6635-3.0463)
**At least 5 of CHASM (breast), CHASM (lung), FATHMM (cancer), FATHMM (missense), Mutation Assessor**	65.02% (61.11%-68.69%)	46.41% (41.32%-51.54%)	97.203% (95.588%-100%)	96.63% (94.64%-100%)	51.19% (46.32%-55.71%)	2.9144 (2.8208-3.0157)	64.91% (57.58%-72.73%)	46% (36.07%-56.06%)	97.337% (92.5%-100%)	96.75% (90.9%-100%)	51.23% (42.25%-61.29%)	2.9131 (2.7047-3.1009)
**At least 5 of CHASM (breast), CHASM (lung), FATHMM (cancer), FATHMM (missense), Mutation Assessor, MutationTaster**	74.78% (71.21%-78.28%)	63.38% (58.46%-68.55%)	94.471% (91.664%-97.368%)	95.2% (92.77%-97.73%)	59.88% (54.7%-64.66%)	3.1293 (3.0274-3.2428)	74.69% (67.68%-81.82%)	63.1% (52.46%-73.13%)	94.545% (88.235%-100%)	95.2% (89.47%-100%)	59.9% (50%-70.37%)	3.1274 (2.9068-3.3390)
**At least 5 of CHASM (breast), CHASM (lung), FATHMM (cancer), FATHMM (missense), Mutation Assessor, PolyPhen-2**	71.77% (68.18%-75.25%)	58.63% (53.72%-63.71%)	94.471% (91.664%-97.368%)	94.83% (92.31%-97.56%)	56.91% (51.69%-61.72%)	3.0484 (2.9476-3.1590)	71.62% (64.65%-78.79%)	58.25% (48.26%-68.33%)	94.545% (88.235%-100%)	94.82% (88.37%-100%)	56.89% (47.16%-67.21%)	3.0450 (2.8223-3.2472)
**At least 5 of CHASM (breast), CHASM (lung), FATHMM (cancer), FATHMM (missense), Mutation Assessor, PROVEAN**	69.05% (65.66%-72.73%)	53.84% (48.85%-59.06%)	95.358% (92.857%-98.592%)	95.25% (92.75%-98.53%)	54.44% (49.61%-59.02%)	2.9888 (2.8915-3.0935)	68.96% (61.62%-75.76%)	53.47% (42.86%-62.97%)	95.521% (89.474%-100%)	95.34% (88.89%-100%)	54.47% (45%-64.18%)	2.9880 (2.7783-3.1877)
**At least 5 of CHASM (breast), CHASM (lung), FATHMM (cancer), FATHMM (missense), Mutation Assessor, SIFT**	73.45% (70.19%-77.27%)	61.28% (56.2%-66.16%)	94.471% (91.664%-97.368%)	95.04% (92.59%-97.67%)	58.53% (53.39%-63.56%)	3.0932 (2.9883-3.2130)	73.31% (65.66%-79.82%)	60.91% (50.82%-70.97%)	94.545% (88.235%-100%)	95.03% (88.88%-100%)	58.5% (48.39%-68.97%)	3.0899 (2.8538-3.2992)
**At least 5 of CHASM (breast), CHASM (lung), FATHMM (cancer), FATHMM (missense), Mutation Assessor, VEST**	70.43% (67.17%-74.24%)	56.52% (51.61%-61.6%)	94.471% (91.664%-97.368%)	94.64% (92%-97.5%)	55.69% (50.81%-60.33%)	3.0132 (2.9068-3.1286)	70.25% (62.63%-76.77%)	56.08% (45.45%-65.68%)	94.545% (88.235%-100%)	94.63% (87.88%-100%)	55.64% (46.27%-65.57%)	3.0090 (2.7901-3.2153)
**At least 5 of CHASM (breast), CHASM (lung), FATHMM (cancer), FATHMM (missense), MutationTaster**	68.7% (65.15%-72.22%)	51.67% (46.46%-57.02%)	98.143% (97.015%-100%)	97.97% (96.67%-100%)	54.02% (48.87%-58.78%)	3.0180 (2.9194-3.1179)	68.65% (61.62%-75.76%)	51.43% (40.68%-61.91%)	98.209% (94.118%-100%)	98.02% (93.74%-100%)	54.1% (44.29%-64.18%)	3.0175 (2.8168-3.2182)
**At least 5 of CHASM (breast), CHASM (lung), FATHMM (cancer), FATHMM (missense), MutationTaster, PolyPhen-2**	76.12% (72.73%-79.8%)	65.5% (60.63%-70.31%)	94.471% (91.664%-97.368%)	95.34% (93.02%-97.78%)	61.3% (56.14%-66.11%)	3.1661 (3.0661-3.2818)	76.05% (68.69%-82.83%)	65.26% (55.38%-75%)	94.545% (88.235%-100%)	95.35% (89.74%-100%)	61.34% (50.98%-71.93%)	3.1650 (2.9332-3.3713)
**At least 5 of CHASM (breast), CHASM (lung), FATHMM (cancer), FATHMM (missense), MutationTaster, PROVEAN**	73.74% (70.2%-77.27%)	60.69% (55.74%-66.12%)	96.298% (94.203%-98.684%)	96.59% (94.59%-98.78%)	58.63% (53.39%-63.39%)	3.1221 (3.0226-3.2267)	73.73% (66.67%-80.81%)	60.5% (50%-70.15%)	96.392% (90.909%-100%)	96.64% (91.67%-100%)	58.72% (49.09%-69.23%)	3.1226 (2.9169-3.3297)
**At least 5 of CHASM (breast), CHASM (lung), FATHMM (cancer), FATHMM (missense), MutationTaster, SIFT**	77.81% (74.75%-81.31%)	67.63% (62.81%-72.66%)	95.411% (92.958%-98.571%)	96.22% (94.12%-98.86%)	63.03% (57.8%-67.89%)	3.2229 (3.1262-3.3292)	77.71% (70.71%-83.84%)	67.38% (57.35%-77.05%)	95.417% (89.474%-100%)	96.18% (90.91%-100%)	63.04% (52.94%-73.34%)	3.2202 (3.0136-3.4219)
**At least 5 of CHASM (breast), CHASM (lung), FATHMM (cancer), FATHMM (missense), MutationTaster, VEST**	74.09% (70.71%-77.27%)	62.29% (57.69%-67.46%)	94.471% (91.664%-97.368%)	95.12% (92.68%-97.67%)	59.17% (53.98%-64.04%)	3.1105 (3.0071-3.2164)	74.05% (67.68%-80.81%)	62.09% (51.56%-71.67%)	94.545% (88.235%-100%)	95.13% (89.19%-100%)	59.24% (49.18%-69.65%)	3.1101 (2.8964-3.3172)
**At least 5 of CHASM (breast), CHASM (lung), FATHMM (cancer), FATHMM (missense), PolyPhen-2**	66.7% (63.13%-70.2%)	49.06% (43.85%-54.03%)	97.203% (95.588%-100%)	96.81% (94.92%-100%)	52.46% (47.66%-57.04%)	2.9554 (2.8516-3.0583)	66.59% (59.6%-73.74%)	48.67% (38.71%-59.1%)	97.337% (92.5%-100%)	96.92% (91.43%-100%)	52.49% (43.28%-62.3%)	2.9541 (2.7470-3.1616)
**At least 5 of CHASM (breast), CHASM (lung), FATHMM (cancer), FATHMM (missense), PolyPhen-2, PROVEAN**	71.73% (68.18%-75.25%)	58.06% (53.17%-63.2%)	95.358% (92.857%-98.592%)	95.58% (93.24%-98.63%)	56.81% (51.69%-61.48%)	3.0581 (2.9553-3.1605)	71.69% (64.65%-78.79%)	57.79% (46.97%-67.74%)	95.521% (89.474%-100%)	95.68% (90%-100%)	56.87% (47.46%-66.67%)	3.0586 (2.8483-3.2684)
**At least 5 of CHASM (breast), CHASM (lung), FATHMM (cancer), FATHMM (missense), PolyPhen-2, SIFT**	76.13% (72.73%-79.8%)	65.52% (60.46%-70.49%)	94.471% (91.664%-97.368%)	95.34% (93.02%-97.8%)	61.32% (56.14%-66.09%)	3.1665 (3.0595-3.2738)	76.02% (68.69%-82.83%)	65.2% (54.84%-75%)	94.545% (88.235%-100%)	95.35% (89.47%-100%)	61.3% (51.72%-71.93%)	3.1639 (2.9467-3.3824)
**At least 5 of CHASM (breast), CHASM (lung), FATHMM (cancer), FATHMM (missense), PolyPhen-2, VEST**	73.11% (69.7%-76.77%)	60.75% (55.74%-65.83%)	94.471% (91.664%-97.368%)	95% (92.5%-97.67%)	58.2% (53.33%-63.03%)	3.0842 (2.9774-3.1899)	72.97% (65.66%-79.8%)	60.38% (50%-70.32%)	94.545% (88.235%-100%)	95% (88.57%-100%)	58.17% (48.28%-68.33%)	3.0809 (2.8623-3.2885)
**At least 5 of CHASM (breast), CHASM (lung), FATHMM (cancer), FATHMM (missense), PROVEAN**	59.65% (55.56%-63.13%)	36.85% (32.23%-41.6%)	99.084% (98.485%-100%)	98.58% (97.56%-100%)	47.57% (42.76%-51.97%)	2.8209 (2.7343-2.9046)	59.48% (52.53%-67.68%)	36.39% (26.66%-46.16%)	99.083% (96.774%-100%)	98.56% (94.74%-100%)	47.58% (38.67%-57.33%)	2.8161 (2.6428-2.9919)
**At least 5 of CHASM (breast), CHASM (lung), FATHMM (cancer), FATHMM (missense), PROVEAN, SIFT**	72.77% (69.19%-76.26%)	59.16% (54.16%-64.41%)	96.298% (94.203%-98.684%)	96.51% (94.44%-98.77%)	57.69% (52.59%-62.3%)	3.0965 (2.9941-3.2014)	72.65% (65.66%-79.8%)	58.79% (48.33%-68.85%)	96.392% (90.909%-100%)	96.54% (91.18%-100%)	57.68% (48.28%-67.21%)	3.0941 (2.8806-3.2982)
**At least 5 of CHASM (breast), CHASM (lung), FATHMM (cancer), FATHMM (missense), PROVEAN, VEST**	67.03% (63.13%-70.21%)	50.65% (45.45%-55.91%)	95.358% (92.857%-98.592%)	94.97% (92.31%-98.44%)	52.78% (47.76%-57.35%)	2.9375 (2.8345-3.0427)	66.94% (60.58%-74.75%)	50.27% (40%-60.61%)	95.521% (89.474%-100%)	95.07% (88.46%-100%)	52.82% (43.54%-62.32%)	2.9368 (2.7235-3.1410)
**At least 5 of CHASM (breast), CHASM (lung), FATHMM (cancer), FATHMM (missense), SIFT**	69.41% (65.66%-73.23%)	52.79% (47.62%-57.94%)	98.143% (97.015%-100%)	98.01% (96.77%-100%)	54.59% (49.62%-59.23%)	3.0353 (2.9437-3.1304)	69.26% (61.62%-76.77%)	52.38% (42.3%-62.12%)	98.209% (94.118%-100%)	98.05% (93.75%-100%)	54.58% (45.45%-64.41%)	3.0322 (2.8416-3.2191)
**At least 5 of CHASM (breast), CHASM (lung), FATHMM (cancer), FATHMM (missense), SIFT, VEST**	74.12% (70.71%-77.78%)	62.89% (57.76%-67.97%)	93.544% (90.541%-97.222%)	94.39% (91.76%-97.59%)	59.31% (54.17%-64.35%)	3.1014 (2.9967-3.2155)	73.97% (66.67%-80.81%)	62.5% (51.79%-72.41%)	93.643% (86.662%-100%)	94.4% (88.09%-100%)	59.27% (49.15%-69.64%)	3.0981 (2.8677-3.3080)
**At least 5 of CHASM (breast), CHASM (lung), FATHMM (cancer), FATHMM (missense), VEST**	61.67% (57.58%-65.15%)	41.11% (36.06%-46.16%)	97.203% (95.588%-100%)	96.22% (93.88%-100%)	48.84% (44%-53.29%)	2.8337 (2.7376-2.9291)	61.52% (54.55%-69.7%)	40.64% (30.3%-50.75%)	97.337% (92.5%-100%)	96.35% (90%-100%)	48.86% (39.71%-58.67%)	2.8318 (2.6474-3.0270)
**At least 5 of CHASM (breast), CHASM (lung), FATHMM (cancer), Mutation Assessor, MutationTaster**	63.31% (59.09%-67.17%)	43.17% (37.9%-48.1%)	98.143% (97.015%-100%)	97.58% (96%-100%)	49.97% (45.2%-54.55%)	2.8886 (2.7997-2.9823)	63.27% (55.56%-71.72%)	42.91% (32.81%-53.45%)	98.209% (94.118%-100%)	97.64% (92.59%-100%)	50.06% (40.85%-59.73%)	2.8881 (2.7035-3.0759)
**At least 5 of CHASM (breast), CHASM (lung), FATHMM (cancer), Mutation Assessor, MutationTaster, PolyPhen-2**	73.43% (70.2%-76.77%)	61.79% (56.91%-66.94%)	93.55% (90.537%-97.102%)	94.3% (91.67%-97.56%)	58.61% (53.33%-63.64%)	3.0826 (2.9752-3.1898)	73.34% (66.67%-79.8%)	61.49% (51.56%-71.43%)	93.631% (86.842%-100%)	94.3% (87.8%-100%)	58.63% (49.01%-69.36%)	3.0806 (2.8666-3.2996)
**At least 5 of CHASM (breast), CHASM (lung), FATHMM (cancer), Mutation Assessor, MutationTaster, PROVEAN**	69.72% (66.16%-73.23%)	54.34% (48.84%-59.4%)	96.298% (94.203%-98.684%)	96.21% (93.94%-98.67%)	54.95% (50%-59.4%)	3.0180 (2.9156-3.1237)	69.66% (62.63%-76.77%)	54.06% (43.86%-64.29%)	96.392% (90.909%-100%)	96.26% (90.32%-100%)	55.01% (45.16%-65.08%)	3.0172 (2.8050-3.2182)
**At least 5 of CHASM (breast), CHASM (lung), FATHMM (cancer), Mutation Assessor, MutationTaster, SIFT**	72.76% (69.19%-76.26%)	60.19% (55.04%-65.32%)	94.49% (91.667%-97.333%)	94.97% (92.41%-97.59%)	57.85% (52.59%-62.81%)	3.0750 (2.9642-3.1875)	72.66% (65.66%-79.8%)	59.92% (49.25%-69.7%)	94.503% (88.235%-100%)	94.91% (88.89%-100%)	57.88% (47.69%-68.34%)	3.0721 (2.8431-3.2984)
**At least 5 of CHASM (breast), CHASM (lung), FATHMM (cancer), Mutation Assessor, MutationTaster, VEST**	71.07% (67.68%-74.75%)	58.07% (53.08%-62.99%)	93.55% (90.537%-97.102%)	93.96% (91.36%-97.37%)	56.34% (51.24%-61.35%)	3.0192 (2.9075-3.1307)	70.98% (63.64%-77.78%)	57.77% (47.62%-67.8%)	93.631% (86.842%-100%)	93.96% (86.84%-100%)	56.37% (46.55%-66.67%)	3.0173 (2.7937-3.2420)
**At least 5 of CHASM (breast), CHASM (lung), FATHMM (cancer), Mutation Assessor, PolyPhen-2**	64.33% (60.61%-68.18%)	45.31% (40.16%-50.41%)	97.203% (95.588%-100%)	96.56% (94.54%-100%)	50.69% (45.83%-55.07%)	2.8976 (2.8035-2.9998)	64.27% (56.57%-71.72%)	45% (34.42%-55.17%)	97.337% (92.5%-100%)	96.68% (90.62%-100%)	50.77% (41.77%-60.56%)	2.8979 (2.6890-3.0882)
**At least 5 of CHASM (breast), CHASM (lung), FATHMM (cancer), Mutation Assessor, PolyPhen-2, PROVEAN**	69.39% (65.66%-73.23%)	54.37% (49.6%-59.38%)	95.358% (92.857%-98.592%)	95.29% (92.75%-98.55%)	54.72% (49.61%-59.4%)	2.9974 (2.8994-3.1112)	69.3% (61.62%-76.77%)	54.01% (43.75%-63.93%)	95.521% (89.474%-100%)	95.38% (89.19%-100%)	54.76% (45.31%-64.62%)	2.9967 (2.7701-3.1917)
**At least 5 of CHASM (breast), CHASM (lung), FATHMM (cancer), Mutation Assessor, PolyPhen-2, SIFT**	71.41% (67.68%-75.25%)	58.61% (53.72%-63.71%)	93.55% (90.537%-97.102%)	94.01% (91.36%-97.4%)	56.66% (51.6%-61.74%)	3.0282 (2.9209-3.1450)	71.32% (63.64%-78.79%)	58.3% (48.44%-68.18%)	93.631% (86.842%-100%)	94% (87.18%-100%)	56.68% (46.77%-67.21%)	3.0262 (2.7948-3.2420)
**At least 5 of CHASM (breast), CHASM (lung), FATHMM (cancer), Mutation Assessor, PolyPhen-2, VEST**	71.76% (68.18%-75.25%)	59.15% (54.09%-64.04%)	93.55% (90.537%-97.102%)	94.06% (91.36%-97.44%)	56.98% (52.07%-61.98%)	3.0374 (2.9324-3.1469)	71.64% (64.65%-78.79%)	58.81% (48.48%-69.45%)	93.631% (86.842%-100%)	94.06% (87.18%-100%)	56.99% (46.77%-67.21%)	3.0349 (2.8149-3.2441)
**At least 5 of CHASM (breast), CHASM (lung), FATHMM (cancer), Mutation Assessor, PROVEAN**	58.63% (54.55%-62.63%)	35.22% (30.51%-40%)	99.084% (98.485%-100%)	98.52% (97.44%-100%)	46.94% (42.21%-51.32%)	2.7977 (2.7129-2.8868)	58.51% (50.51%-66.67%)	34.85% (25.39%-44.45%)	99.083% (96.774%-100%)	98.49% (94.44%-100%)	46.98% (38.16%-56.41%)	2.7941 (2.6126-2.9704)
**At least 5 of CHASM (breast), CHASM (lung), FATHMM (cancer), Mutation Assessor, PROVEAN, SIFT**	70.05% (66.16%-73.74%)	54.87% (49.61%-59.85%)	96.298% (94.203%-98.684%)	96.24% (94.03%-98.68%)	55.24% (50.4%-60%)	3.0264 (2.9250-3.1336)	70% (62.63%-77.78%)	54.6% (44.26%-64.91%)	96.392% (90.909%-100%)	96.29% (90.62%-100%)	55.3% (45.31%-65.08%)	3.0259 (2.8168-3.2198)
**At least 5 of CHASM (breast), CHASM (lung), FATHMM (cancer), Mutation Assessor, PROVEAN, VEST**	66.38% (62.63%-69.71%)	49.61% (44.44%-54.69%)	95.358% (92.857%-98.592%)	94.87% (92.06%-98.41%)	52.26% (47.41%-56.92%)	2.9210 (2.8178-3.0297)	66.23% (59.57%-73.74%)	49.15% (39.06%-59.68%)	95.521% (89.474%-100%)	94.96% (88%-100%)	52.26% (42.86%-61.9%)	2.9189 (2.6999-3.1306)
**At least 5 of CHASM (breast), CHASM (lung), FATHMM (cancer), Mutation Assessor, SIFT**	65.35% (61.62%-69.19%)	46.38% (41.18%-51.59%)	98.143% (97.015%-100%)	97.74% (96.3%-100%)	51.43% (46.72%-56.12%)	2.9369 (2.8506-3.0347)	65.26% (57.58%-72.73%)	46.04% (36.06%-56.14%)	98.209% (94.118%-100%)	97.79% (93.1%-100%)	51.47% (42.25%-61.11%)	2.9351 (2.7470-3.1176)
**At least 5 of CHASM (breast), CHASM (lung), FATHMM (cancer), Mutation Assessor, SIFT, VEST**	70.73% (67.17%-74.24%)	57.54% (52.38%-62.88%)	93.55% (90.537%-97.102%)	93.91% (91.14%-97.37%)	56.03% (50.83%-60.98%)	3.0102 (2.8977-3.1206)	70.65% (63.64%-77.78%)	57.24% (46.77%-67.25%)	93.631% (86.842%-100%)	93.9% (86.84%-100%)	56.07% (46.03%-66.17%)	3.0084 (2.7872-3.2336)
**At least 5 of CHASM (breast), CHASM (lung), FATHMM (cancer), Mutation Assessor, VEST**	60.29% (56.06%-64.14%)	38.94% (33.61%-44.17%)	97.203% (95.588%-100%)	96.02% (93.62%-100%)	47.94% (43.24%-52.67%)	2.8010 (2.7018-2.8959)	60.22% (52.53%-68.69%)	38.59% (28.35%-49.02%)	97.337% (92.5%-100%)	96.16% (89.29%-100%)	48.02% (38.67%-57.53%)	2.8010 (2.6080-3.0043)
**At least 5 of CHASM (breast), CHASM (lung), FATHMM (cancer), MutationTaster, PolyPhen-2**	67.02% (63.13%-70.71%)	49.01% (43.8%-54.1%)	98.143% (97.015%-100%)	97.86% (96.49%-100%)	52.68% (47.76%-57.14%)	2.9769 (2.8852-3.0692)	66.98% (59.6%-74.75%)	48.78% (38.46%-59.02%)	98.209% (94.118%-100%)	97.92% (93.33%-100%)	52.77% (43.55%-62.5%)	2.9767 (2.7919-3.1638)
**At least 5 of CHASM (breast), CHASM (lung), FATHMM (cancer), MutationTaster, PolyPhen-2, PROVEAN**	72.4% (68.69%-75.76%)	58.58% (53.28%-63.28%)	96.298% (94.203%-98.684%)	96.47% (94.44%-98.75%)	57.35% (52.42%-62.2%)	3.0870 (2.9851-3.1877)	72.36% (65.66%-79.8%)	58.35% (49.04%-68.75%)	96.392% (90.909%-100%)	96.53% (91.42%-100%)	57.43% (47.99%-67.74%)	3.0869 (2.8911-3.2918)
**At least 5 of CHASM (breast), CHASM (lung), FATHMM (cancer), MutationTaster, PolyPhen-2, SIFT**	75.45% (72.22%-78.8%)	64.44% (59.5%-69.53%)	94.49% (91.667%-97.333%)	95.29% (92.86%-97.73%)	60.58% (55.45%-65.74%)	3.1480 (3.0417-3.2593)	75.35% (68.66%-81.82%)	64.18% (54.1%-73.85%)	94.503% (88.235%-100%)	95.24% (89.74%-100%)	60.6% (50.8%-71.43%)	3.1451 (2.9254-3.3606)
**At least 5 of CHASM (breast), CHASM (lung), FATHMM (cancer), MutationTaster, PolyPhen-2, VEST**	72.42% (68.69%-75.76%)	60.19% (55.12%-65.32%)	93.55% (90.537%-97.102%)	94.16% (91.57%-97.47%)	57.61% (52.5%-62.5%)	3.0552 (2.9469-3.1605)	72.33% (65.66%-79.8%)	59.91% (50%-70%)	93.631% (86.842%-100%)	94.16% (87.5%-100%)	57.65% (47.62%-68.42%)	3.0535 (2.8380-3.2726)
**At least 5 of CHASM (breast), CHASM (lung), FATHMM (cancer), MutationTaster, PROVEAN**	60.3% (56.06%-64.14%)	37.87% (33.06%-42.86%)	99.084% (98.485%-100%)	98.62% (97.62%-100%)	47.98% (43.22%-52.32%)	2.8356 (2.7488-2.9221)	60.2% (52.53%-68.69%)	37.54% (27.58%-47.37%)	99.083% (96.774%-100%)	98.6% (95%-100%)	48.03% (39.24%-57.69%)	2.8326 (2.6663-3.0133)
**At least 5 of CHASM (breast), CHASM (lung), FATHMM (cancer), MutationTaster, PROVEAN, SIFT**	71.07% (67.68%-74.75%)	56.48% (51.24%-61.48%)	96.298% (94.203%-98.684%)	96.35% (94.2%-98.7%)	56.14% (51.2%-61.16%)	3.0526 (2.9548-3.1603)	70.98% (63.64%-77.78%)	56.16% (46.03%-66.18%)	96.392% (90.909%-100%)	96.39% (90.91%-100%)	56.16% (46.29%-66.15%)	3.0511 (2.8317-3.2434)
**At least 5 of CHASM (breast), CHASM (lung), FATHMM (cancer), MutationTaster, PROVEAN, VEST**	68.05% (64.14%-71.72%)	51.71% (46.51%-56.7%)	96.298% (94.203%-98.684%)	96.02% (93.75%-98.59%)	53.56% (48.51%-58.34%)	2.9759 (2.8727-3.0809)	67.94% (60.61%-75.76%)	51.35% (40.98%-62.26%)	96.392% (90.909%-100%)	96.07% (90%-100%)	53.59% (43.86%-63.5%)	2.9741 (2.7672-3.1877)
**At least 5 of CHASM (breast), CHASM (lung), FATHMM (cancer), MutationTaster, SIFT**	67.36% (63.64%-70.72%)	49.55% (44.44%-54.69%)	98.143% (97.015%-100%)	97.88% (96.49%-100%)	52.94% (48.03%-57.46%)	2.9851 (2.8932-3.0823)	67.31% (60.58%-74.75%)	49.3% (38.98%-59.68%)	98.209% (94.118%-100%)	97.93% (93.54%-100%)	53.02% (43.83%-63.24%)	2.9846 (2.7948-3.1679)
**At least 5 of CHASM (breast), CHASM (lung), FATHMM (cancer), MutationTaster, SIFT, VEST**	72.77% (69.19%-76.26%)	60.2% (55.2%-65.08%)	94.49% (91.667%-97.333%)	94.97% (92.41%-97.59%)	57.86% (52.84%-62.81%)	3.0752 (2.9679-3.1875)	72.65% (65.66%-79.8%)	59.9% (50%-69.84%)	94.503% (88.235%-100%)	94.92% (88.89%-100%)	57.87% (47.54%-68.33%)	3.0719 (2.8479-3.2917)
**At least 5 of CHASM (breast), CHASM (lung), FATHMM (cancer), MutationTaster, VEST**	63.67% (59.6%-67.17%)	43.74% (38.33%-48.82%)	98.143% (97.015%-100%)	97.61% (96.08%-100%)	50.22% (45.32%-54.8%)	2.8971 (2.8044-2.9868)	63.56% (56.57%-71.72%)	43.37% (33.32%-53.97%)	98.209% (94.118%-100%)	97.66% (92.31%-100%)	50.27% (40.91%-60.01%)	2.8951 (2.7142-3.0830)
**At least 5 of CHASM (breast), CHASM (lung), FATHMM (cancer), PolyPhen-2, PROVEAN**	60.32% (56.57%-64.14%)	37.89% (32.82%-42.86%)	99.084% (98.485%-100%)	98.62% (97.62%-100%)	47.99% (43.15%-52.38%)	2.8358 (2.7520-2.9203)	60.18% (52.53%-67.68%)	37.49% (27.27%-47.46%)	99.083% (96.774%-100%)	98.59% (94.74%-100%)	48.02% (39.24%-57.54%)	2.8319 (2.6663-3.0062)
**At least 5 of CHASM (breast), CHASM (lung), FATHMM (cancer), PolyPhen-2, PROVEAN, SIFT**	70.74% (67.17%-74.24%)	55.95% (50.83%-60.98%)	96.298% (94.203%-98.684%)	96.31% (94.12%-98.7%)	55.84% (50.81%-60.63%)	3.0440 (2.9484-3.1470)	70.64% (63.64%-77.78%)	55.63% (45.9%-65.62%)	96.392% (90.909%-100%)	96.35% (90.62%-100%)	55.87% (46.03%-65.68%)	3.0424 (2.8383-3.2434)
**At least 5 of CHASM (breast), CHASM (lung), FATHMM (cancer), PolyPhen-2, PROVEAN, VEST**	67.72% (63.64%-71.21%)	51.73% (46.4%-56.59%)	95.358% (92.857%-98.592%)	95.07% (92.42%-98.46%)	53.33% (48.41%-57.9%)	2.9549 (2.8515-3.0640)	67.59% (60.61%-75.76%)	51.3% (41.66%-61.9%)	95.521% (89.474%-100%)	95.16% (88.88%-100%)	53.34% (43.94%-63.24%)	2.9532 (2.7309-3.1603)
**At least 5 of CHASM (breast), CHASM (lung), FATHMM (cancer), PolyPhen-2, SIFT**	67.03% (63.13%-70.71%)	49.04% (43.9%-54.33%)	98.143% (97.015%-100%)	97.86% (96.49%-100%)	52.69% (47.73%-57.25%)	2.9773 (2.8831-3.0736)	66.94% (59.6%-74.75%)	48.72% (38.1%-58.93%)	98.209% (94.118%-100%)	97.91% (93.33%-100%)	52.74% (43.47%-62.69%)	2.9757 (2.7898-3.1667)
**At least 5 of CHASM (breast), CHASM (lung), FATHMM (cancer), PolyPhen-2, SIFT, VEST**	72.45% (69.19%-75.76%)	60.76% (55.83%-65.63%)	92.664% (89.392%-96.003%)	93.47% (90.59%-96.51%)	57.73% (52.73%-62.61%)	3.0462 (2.9324-3.1568)	72.27% (65.66%-78.79%)	60.37% (50%-70%)	92.661% (85.714%-100%)	93.37% (86.67%-100%)	57.67% (48.15%-67.29%)	3.0408 (2.8188-3.2711)
**At least 5 of CHASM (breast), CHASM (lung), FATHMM (cancer), PolyPhen-2, VEST**	62.65% (58.59%-66.16%)	42.66% (37.69%-48%)	97.203% (95.588%-100%)	96.35% (94.12%-100%)	49.51% (44.6%-53.85%)	2.8573 (2.7602-2.9513)	62.58% (55.56%-70.71%)	42.32% (31.82%-52.54%)	97.337% (92.5%-100%)	96.49% (90.62%-100%)	49.58% (40.32%-59.16%)	2.8573 (2.6717-3.0533)
**At least 5 of CHASM (breast), CHASM (lung), FATHMM (cancer), PROVEAN, SIFT**	61.98% (58.08%-65.66%)	40.53% (35.66%-45.24%)	99.084% (98.485%-100%)	98.71% (97.78%-100%)	49.07% (44.29%-53.38%)	2.8739 (2.7880-2.9546)	61.89% (54.55%-69.7%)	40.2% (30.5%-50.77%)	99.083% (96.774%-100%)	98.69% (95.24%-100%)	49.12% (40.28%-58.67%)	2.8710 (2.7079-3.0453)
**At least 5 of CHASM (breast), CHASM (lung), FATHMM (cancer), PROVEAN, SIFT, VEST**	67.38% (63.64%-70.71%)	50.65% (45.53%-55.47%)	96.298% (94.203%-98.684%)	95.94% (93.65%-98.59%)	53.02% (48.09%-57.78%)	2.9591 (2.8609-3.0675)	67.26% (60.61%-74.75%)	50.27% (40.57%-60.66%)	96.392% (90.909%-100%)	95.99% (89.65%-100%)	53.04% (43.47%-62.86%)	2.9569 (2.7435-3.1606)
**At least 5 of CHASM (breast), CHASM (lung), FATHMM (cancer), PROVEAN, VEST**	59.64% (55.56%-63.64%)	36.82% (32.03%-41.41%)	99.084% (98.485%-100%)	98.58% (97.56%-100%)	47.56% (42.85%-51.97%)	2.8206 (2.7354-2.9046)	59.51% (51.52%-67.68%)	36.44% (26.78%-46.16%)	99.083% (96.774%-100%)	98.56% (94.74%-100%)	47.6% (38.67%-56.97%)	2.8168 (2.6467-2.9942)
**At least 5 of CHASM (breast), CHASM (lung), FATHMM (cancer), SIFT, VEST**	63.33% (59.6%-66.68%)	43.19% (38.13%-48.41%)	98.143% (97.015%-100%)	97.58% (96%-100%)	49.98% (45.18%-54.42%)	2.8890 (2.7949-2.9797)	63.24% (56.54%-70.71%)	42.85% (32.35%-53.13%)	98.209% (94.118%-100%)	97.63% (92.31%-100%)	50.04% (40.79%-60%)	2.8872 (2.7047-3.0756)
**At least 5 of CHASM (breast), CHASM (lung), FATHMM (missense), Mutation Assessor, MutationTaster**	62.3% (58.59%-66.16%)	41.57% (36.51%-46.46%)	98.143% (97.015%-100%)	97.49% (95.83%-100%)	49.28% (44.52%-53.79%)	2.8648 (2.7742-2.9602)	62.26% (54.55%-69.7%)	41.31% (31.74%-51.61%)	98.209% (94.118%-100%)	97.55% (92%-100%)	49.37% (40.28%-58.9%)	2.8644 (2.6768-3.0458)
**At least 5 of CHASM (breast), CHASM (lung), FATHMM (missense), Mutation Assessor, MutationTaster, PolyPhen-2**	71.1% (67.68%-74.75%)	58.11% (52.94%-63.2%)	93.55% (90.537%-97.102%)	93.96% (91.14%-97.4%)	56.37% (51.24%-61.16%)	3.0199 (2.9160-3.1327)	70.93% (63.64%-77.78%)	57.68% (47.54%-67.8%)	93.631% (86.842%-100%)	93.95% (86.84%-100%)	56.32% (46.43%-66.67%)	3.0158 (2.7899-3.2233)
**At least 5 of CHASM (breast), CHASM (lung), FATHMM (missense), Mutation Assessor, MutationTaster, PROVEAN**	67.69% (64.14%-71.21%)	51.14% (45.97%-56.2%)	96.298% (94.203%-98.684%)	95.98% (93.65%-98.57%)	53.27% (48.51%-57.86%)	2.9670 (2.8633-3.0684)	67.64% (60.61%-74.75%)	50.87% (40.68%-61.02%)	96.392% (90.909%-100%)	96.04% (90%-100%)	53.34% (43.66%-62.69%)	2.9665 (2.7627-3.1762)
**At least 5 of CHASM (breast), CHASM (lung), FATHMM (missense), Mutation Assessor, MutationTaster, SIFT**	70.76% (67.17%-74.24%)	57.04% (51.64%-62.4%)	94.49% (91.667%-97.333%)	94.7% (92%-97.47%)	55.99% (50.88%-60.8%)	3.0222 (2.9159-3.1315)	70.59% (63.64%-77.78%)	56.64% (46.15%-66.68%)	94.503% (88.235%-100%)	94.64% (88.24%-100%)	55.95% (46.03%-66.13%)	3.0173 (2.7901-3.2451)
**At least 5 of CHASM (breast), CHASM (lung), FATHMM (missense), Mutation Assessor, MutationTaster, VEST**	68.74% (65.15%-72.73%)	54.39% (49.56%-59.68%)	93.55% (90.537%-97.102%)	93.58% (90.77%-97.19%)	54.26% (49.23%-58.91%)	2.9578 (2.8531-3.0743)	68.57% (60.61%-75.76%)	53.95% (43.1%-63.94%)	93.631% (86.842%-100%)	93.56% (85.71%-100%)	54.23% (44.26%-64.52%)	2.9538 (2.7263-3.1637)
**At least 5 of CHASM (breast), CHASM (lung), FATHMM (missense), Mutation Assessor, PolyPhen-2**	63.32% (59.6%-67.17%)	43.72% (38.52%-48.8%)	97.203% (95.588%-100%)	96.44% (94.34%-100%)	49.97% (45.21%-54.41%)	2.8733 (2.7796-2.9774)	63.26% (55.56%-70.71%)	43.4% (33.33%-53.45%)	97.337% (92.5%-100%)	96.56% (90.47%-100%)	50.05% (41.25%-59.72%)	2.8735 (2.6635-3.0652)
**At least 5 of CHASM (breast), CHASM (lung), FATHMM (missense), Mutation Assessor, PolyPhen-2, PROVEAN**	67.37% (63.64%-71.21%)	51.17% (46.28%-56.35%)	95.358% (92.857%-98.592%)	95.01% (92.31%-98.44%)	53.04% (48.09%-57.58%)	2.9459 (2.8508-3.0553)	67.29% (59.6%-74.75%)	50.82% (40%-60.66%)	95.521% (89.474%-100%)	95.11% (88.88%-100%)	53.09% (43.55%-62.72%)	2.9455 (2.7268-3.1559)
**At least 5 of CHASM (breast), CHASM (lung), FATHMM (missense), Mutation Assessor, PolyPhen-2, SIFT**	69.42% (65.66%-73.23%)	55.46% (50.41%-60.48%)	93.55% (90.537%-97.102%)	93.69% (90.79%-97.3%)	54.85% (50%-59.55%)	2.9755 (2.8666-3.0887)	69.25% (61.62%-76.77%)	55.02% (45.31%-65.16%)	93.631% (86.842%-100%)	93.67% (86.48%-100%)	54.81% (45.16%-64.79%)	2.9713 (2.7496-3.1877)
**At least 5 of CHASM (breast), CHASM (lung), FATHMM (missense), Mutation Assessor, PolyPhen-2, VEST**	69.42% (65.66%-73.23%)	55.47% (50.39%-60.77%)	93.55% (90.537%-97.102%)	93.69% (90.79%-97.3%)	54.85% (50%-59.66%)	2.9756 (2.8677-3.0934)	69.23% (61.62%-76.77%)	55% (44.44%-65.16%)	93.631% (86.842%-100%)	93.67% (86.11%-100%)	54.8% (45.16%-64.62%)	2.9710 (2.7384-3.1871)
**At least 5 of CHASM (breast), CHASM (lung), FATHMM (missense), Mutation Assessor, PROVEAN**	57.29% (53.03%-61.11%)	33.11% (28.23%-37.8%)	99.084% (98.485%-100%)	98.43% (97.3%-100%)	46.14% (41.45%-50.33%)	2.7676 (2.6829-2.8590)	57.14% (49.49%-65.66%)	32.69% (23.43%-42.62%)	99.083% (96.774%-100%)	98.39% (94.12%-100%)	46.17% (37.66%-55.56%)	2.7634 (2.5827-2.9402)
**At least 5 of CHASM (breast), CHASM (lung), FATHMM (missense), Mutation Assessor, PROVEAN, SIFT**	68.03% (64.65%-71.22%)	51.67% (46.61%-56.8%)	96.298% (94.203%-98.684%)	96.02% (93.65%-98.59%)	53.54% (48.84%-58.21%)	2.9753 (2.8752-3.0742)	67.99% (61.59%-74.75%)	51.42% (40.98%-61.67%)	96.392% (90.909%-100%)	96.07% (90%-100%)	53.62% (44.12%-63.08%)	2.9750 (2.7737-3.1799)
**At least 5 of CHASM (breast), CHASM (lung), FATHMM (missense), Mutation Assessor, PROVEAN, VEST**	64.35% (60.61%-68.18%)	46.42% (41.32%-51.61%)	95.358% (92.857%-98.592%)	94.53% (91.53%-98.28%)	50.72% (45.99%-55.4%)	2.8703 (2.7692-2.9773)	64.22% (56.57%-71.72%)	45.96% (35.93%-56.14%)	95.521% (89.474%-100%)	94.64% (87.1%-100%)	50.74% (41.42%-60%)	2.8686 (2.6529-3.0684)
**At least 5 of CHASM (breast), CHASM (lung), FATHMM (missense), Mutation Assessor, SIFT**	64.34% (60.61%-68.18%)	44.79% (39.52%-49.61%)	98.143% (97.015%-100%)	97.66% (96.15%-100%)	50.69% (45.93%-55.25%)	2.9128 (2.8247-3.0097)	64.25% (56.57%-71.72%)	44.44% (34.43%-54.69%)	98.209% (94.118%-100%)	97.71% (92.59%-100%)	50.74% (41.43%-60.3%)	2.9111 (2.7228-3.0929)
**At least 5 of CHASM (breast), CHASM (lung), FATHMM (missense), Mutation Assessor, SIFT, VEST**	68.74% (65.15%-72.73%)	54.39% (49.21%-59.68%)	93.55% (90.537%-97.102%)	93.58% (90.67%-97.22%)	54.21% (49.59%-58.91%)	2.9577 (2.8538-3.0748)	68.58% (60.61%-75.76%)	53.96% (43.33%-64.41%)	93.631% (86.842%-100%)	93.56% (86.1%-100%)	54.24% (44.61%-63.93%)	2.9539 (2.7233-3.1637)
**At least 5 of CHASM (breast), CHASM (lung), FATHMM (missense), Mutation Assessor, VEST**	59.28% (55.05%-63.13%)	37.35% (32.26%-42.52%)	97.203% (95.588%-100%)	95.85% (93.33%-100%)	47.29% (42.57%-51.72%)	2.7770 (2.6785-2.8750)	59.21% (51.52%-67.68%)	36.99% (26.56%-46.97%)	97.337% (92.5%-100%)	96% (88.89%-100%)	47.38% (38.36%-56.76%)	2.7770 (2.5790-2.9728)
**At least 5 of CHASM (breast), CHASM (lung), FATHMM (missense), MutationTaster, PolyPhen-2**	64.33% (60.1%-67.68%)	44.77% (39.52%-49.61%)	98.143% (97.015%-100%)	97.66% (96.08%-100%)	50.68% (45.77%-55.22%)	2.9125 (2.8171-3.0049)	64.28% (57.58%-72.73%)	44.49% (34.43%-54.84%)	98.209% (94.118%-100%)	97.72% (92.86%-100%)	50.77% (41.43%-60.29%)	2.9119 (2.7309-3.1021)

At least 5 of CHASM (breast), CHASM (lung), FATHMM (missense), MutationTaster, PolyPhen-2, PROVEAN	71.38% (67.68%-74.75%)	56.97% (51.72%-61.91%)	96.298% (94.203%-98.684%)	96.38% (94.29%-98.7%)	56.42% (51.53%-61.02%)	3.0606 (2.9592-3.1589)	71.37% (64.65%-78.79%)	56.78% (46.55%-67.16%)	96.392% (90.909%-100%)	96.44% (91.18%-100%)	56.52% (47.27%-66.67%)	3.0612 (2.8668-3.2711)
At least 5 of CHASM (breast), CHASM (lung), FATHMM (missense), MutationTaster, PolyPhen-2, SIFT	74.47% (71.21%-78.28%)	62.88% (57.81%-68.04%)	94.49% (91.667%-97.333%)	95.17% (92.68%-97.7%)	59.55% (54.39%-64.46%)	3.1209 (3.0156-3.2287)	74.3% (66.67%-80.81%)	62.51% (52.36%-72.58%)	94.503% (88.235%-100%)	95.12% (89.19%-100%)	59.51% (50%-70.18%)	3.1163 (2.8965-3.3303)
At least 5 of CHASM (breast), CHASM (lung), FATHMM (missense), MutationTaster, PolyPhen-2, VEST	71.77% (68.18%-75.25%)	59.16% (54.33%-64.29%)	93.55% (90.537%-97.102%)	94.07% (91.36%-97.4%)	56.99% (52%-61.67%)	3.0377 (2.9338-3.1463)	71.62% (64.65%-78.79%)	58.77% (48.38%-68.34%)	93.631% (86.842%-100%)	94.06% (87.18%-100%)	56.96% (47.37%-67.24%)	3.0343 (2.8122-3.2450)
At least 5 of CHASM (breast), CHASM (lung), FATHMM (missense), MutationTaster, PROVEAN	57.62% (53.54%-61.62%)	33.64% (29.03%-38.4%)	99.084% (98.485%-100%)	98.45% (97.3%-100%)	46.34% (41.56%-50.65%)	2.7752 (2.6884-2.8595)	57.48% (49.49%-65.66%)	33.23% (23.81%-42.65%)	99.083% (96.774%-100%)	98.43% (94.12%-100%)	46.37% (37.66%-55.85%)	2.7712 (2.6021-2.9515)
At least 5 of CHASM (breast), CHASM (lung), FATHMM (missense), MutationTaster, PROVEAN, SIFT	70.39% (67.17%-73.74%)	55.4% (50%-60.77%)	96.298% (94.203%-98.684%)	96.28% (94.12%-98.67%)	55.53% (50.42%-60.31%)	3.0351 (2.9385-3.1374)	70.33% (63.64%-76.77%)	55.12% (44.44%-65.62%)	96.392% (90.909%-100%)	96.33% (90.91%-100%)	55.59% (46.03%-65.22%)	3.0343 (2.8308-3.2337)
At least 5 of CHASM (breast), CHASM (lung), FATHMM (missense), MutationTaster, PROVEAN, VEST	67.03% (63.64%-70.71%)	50.1% (44.63%-55.39%)	96.298% (94.203%-98.684%)	95.9% (93.55%-98.55%)	52.74% (48%-57.36%)	2.9504 (2.8516-3.0508)	66.95% (59.6%-73.74%)	49.78% (39.06%-60.32%)	96.392% (90.909%-100%)	95.96% (89.66%-100%)	52.8% (43.08%-62.12%)	2.9493 (2.7472-3.1559)
At least 5 of CHASM (breast), CHASM (lung), FATHMM (missense), MutationTaster, SIFT	65% (61.11%-68.69%)	45.83% (40.5%-50.82%)	98.143% (97.015%-100%)	97.71% (96.23%-100%)	51.17% (46.27%-55.71%)	2.9286 (2.8325-3.0227)	64.94% (57.58%-72.73%)	45.55% (35.29%-55.88%)	98.209% (94.118%-100%)	97.77% (92.86%-100%)	51.24% (42.03%-61.29%)	2.9277 (2.7394-3.1181)
At least 5 of CHASM (breast), CHASM (lung), FATHMM (missense), MutationTaster, SIFT, VEST	71.78% (68.18%-75.25%)	58.64% (53.66%-64.06%)	94.49% (91.667%-97.333%)	94.84% (92.31%-97.53%)	56.92% (52%-61.67%)	3.0489 (2.9377-3.1590)	71.6% (64.65%-78.79%)	58.23% (47.06%-67.8%)	94.503% (88.235%-100%)	94.78% (88.57%-100%)	56.87% (47.06%-67.21%)	3.0439 (2.8250-3.2667)
At least 5 of CHASM (breast), CHASM (lung), FATHMM (missense), MutationTaster, VEST	60.98% (57.07%-64.65%)	39.49% (34.42%-44.36%)	98.143% (97.015%-100%)	97.36% (95.65%-100%)	48.4% (43.79%-52.98%)	2.8339 (2.7385-2.9274)	60.86% (53.54%-68.69%)	39.09% (29.5%-49.15%)	98.209% (94.118%-100%)	97.41% (91.67%-100%)	48.44% (39.13%-58.33%)	2.8316 (2.6424-3.0224)
At least 5 of CHASM (breast), CHASM (lung), FATHMM (missense), PolyPhen-2, PROVEAN	57.64% (53.54%-61.11%)	33.66% (28.8%-38.58%)	99.084% (98.485%-100%)	98.45% (97.3%-100%)	46.35% (41.51%-50.63%)	2.7754 (2.6909-2.8595)	57.46% (50.51%-65.66%)	33.19% (23.33%-43.33%)	99.083% (96.774%-100%)	98.42% (94.44%-100%)	46.35% (37.83%-55.7%)	2.7704 (2.5942-2.9437)
At least 5 of CHASM (breast), CHASM (lung), FATHMM (missense), PolyPhen-2, PROVEAN, SIFT	70.06% (66.16%-73.74%)	54.88% (49.61%-60.16%)	96.298% (94.203%-98.684%)	96.24% (94.03%-98.67%)	55.24% (50.37%-59.84%)	3.0266 (2.9286-3.1247)	69.99% (62.63%-77.78%)	54.59% (44.12%-64.71%)	96.392% (90.909%-100%)	96.29% (90.62%-100%)	55.29% (45.76%-65.52%)	3.0257 (2.8270-3.2190)
At least 5 of CHASM (breast), CHASM (lung), FATHMM (missense), PolyPhen-2, PROVEAN, VEST	66.7% (63.12%-70.2%)	50.12% (44.88%-55.28%)	95.358% (92.857%-98.592%)	94.92% (92.19%-98.41%)	52.51% (47.69%-57.03%)	2.9291 (2.8221-3.0339)	66.6% (59.6%-73.76%)	49.73% (39.34%-60%)	95.521% (89.474%-100%)	95.02% (88.57%-100%)	52.55% (42.86%-62.13%)	2.9281 (2.7137-3.1394)
At least 5 of CHASM (breast), CHASM (lung), FATHMM (missense), PolyPhen-2, SIFT	64.68% (60.61%-68.18%)	45.32% (40%-50.4%)	98.143% (97.015%-100%)	97.69% (96.15%-100%)	50.94% (46.04%-55.4%)	2.9209 (2.8271-3.0176)	64.58% (57.58%-72.73%)	44.97% (34.43%-55.71%)	98.209% (94.118%-100%)	97.74% (92.59%-100%)	50.98% (42.03%-60.81%)	2.9190 (2.7393-3.1137)
At least 5 of CHASM (breast), CHASM (lung), FATHMM (missense), PolyPhen-2, SIFT, VEST	71.44% (67.68%-75.25%)	59.2% (54.16%-64.35%)	92.612% (89.333%-96%)	93.26% (90.36%-96.43%)	56.76% (51.75%-61.6%)	3.0184 (2.9072-3.1305)	71.25% (63.64%-78.79%)	58.7% (48.44%-68.85%)	92.753% (85.714%-100%)	93.28% (86.11%-100%)	56.69% (46.67%-66.67%)	3.0142 (2.7892-3.2430)
At least 5 of CHASM (breast), CHASM (lung), FATHMM (missense), PolyPhen-2, VEST	59.96% (55.56%-63.64%)	38.42% (33.08%-43.41%)	97.203% (95.588%-100%)	95.97% (93.48%-100%)	47.72% (42.86%-52.03%)	2.7931 (2.6966-2.8918)	59.88% (52.53%-68.69%)	38.04% (28.12%-48.44%)	97.337% (92.5%-100%)	96.11% (89.47%-100%)	47.79% (39.12%-57.54%)	2.7928 (2.6013-2.9874)
At least 5 of CHASM (breast), CHASM (lung), FATHMM (missense), PROVEAN, SIFT	59.31% (55.05%-63.13%)	36.3% (31.45%-40.94%)	99.084% (98.485%-100%)	98.56% (97.5%-100%)	47.36% (42.48%-51.68%)	2.8130 (2.7289-2.8945)	59.17% (51.52%-67.68%)	35.9% (26.31%-45.9%)	99.083% (96.774%-100%)	98.54% (94.74%-100%)	47.38% (38.66%-56.96%)	2.8090 (2.6399-2.9812)
At least 5 of CHASM (breast), CHASM (lung), FATHMM (missense), PROVEAN, SIFT, VEST	66.7% (63.13%-70.2%)	49.57% (44.19%-54.55%)	96.298% (94.203%-98.684%)	95.86% (93.44%-98.55%)	52.48% (47.66%-57.15%)	2.9421 (2.8421-3.0473)	66.61% (59.6%-73.74%)	49.23% (39.34%-59.68%)	96.392% (90.909%-100%)	95.91% (89.29%-100%)	52.52% (43.07%-62.07%)	2.9406 (2.7273-3.1409)
At least 5 of CHASM (breast), CHASM (lung), FATHMM (missense), PROVEAN, VEST	56.96% (53.03%-60.61%)	32.6% (27.56%-37.4%)	99.084% (98.485%-100%)	98.4% (97.22%-100%)	45.95% (41.03%-50.31%)	2.7603 (2.6755-2.8417)	56.79% (49.49%-64.65%)	32.13% (22.38%-42.11%)	99.083% (96.774%-100%)	98.37% (94.11%-100%)	45.97% (37.5%-55.56%)	2.7555 (2.5898-2.9321)
At least 5 of CHASM (breast), CHASM (lung), FATHMM (missense), SIFT, VEST	60.98% (57.07%-64.65%)	39.48% (34.39%-44.63%)	98.143% (97.015%-100%)	97.36% (95.65%-100%)	48.4% (43.62%-52.9%)	2.8338 (2.7400-2.9221)	60.87% (53.54%-68.69%)	39.1% (28.84%-50%)	98.209% (94.118%-100%)	97.41% (91.67%-100%)	48.45% (39.7%-58.11%)	2.8317 (2.6536-3.0201)
At least 5 of CHASM (breast), CHASM (lung), Mutation Assessor, MutationTaster, PolyPhen-2	61.95% (58.08%-65.66%)	41.55% (36.29%-46.72%)	97.222% (95.588%-100%)	96.28% (94%-100%)	49.03% (44.36%-53.57%)	2.8408 (2.7452-2.9417)	61.96% (54.55%-69.7%)	41.36% (31.14%-51.56%)	97.294% (92.303%-100%)	96.33% (90%-100%)	49.16% (39.99%-58.57%)	2.8414 (2.6321-3.0350)
At least 5 of CHASM (breast), CHASM (lung), Mutation Assessor, MutationTaster, PolyPhen-2, PROVEAN	68.37% (64.65%-71.72%)	52.74% (47.62%-57.94%)	95.377% (92.958%-98.529%)	95.17% (92.54%-98.53%)	53.86% (48.84%-58.46%)	2.9715 (2.8695-3.0773)	68.31% (61.62%-75.76%)	52.46% (42.19%-62.71%)	95.478% (90%-100%)	95.21% (88.89%-100%)	53.93% (44.44%-63.93%)	2.9708 (2.7579-3.1857)
At least 5 of CHASM (breast), CHASM (lung), Mutation Assessor, MutationTaster, PolyPhen-2, SIFT	71.77% (68.18%-75.25%)	58.62% (53.49%-63.71%)	94.49% (91.667%-97.333%)	94.84% (92.21%-97.53%)	56.91% (51.93%-61.79%)	3.0487 (2.9421-3.1592)	71.61% (64.65%-78.79%)	58.26% (48.39%-68.25%)	94.503% (88.235%-100%)	94.78% (88.57%-100%)	56.89% (46.77%-67.19%)	3.0443 (2.8164-3.2715)
At least 5 of CHASM (breast), CHASM (lung), Mutation Assessor, MutationTaster, PolyPhen-2, VEST	71.08% (67.68%-74.75%)	58.09% (52.89%-63.2%)	93.55% (90.537%-97.102%)	93.96% (91.36%-97.4%)	56.35% (51.24%-61.16%)	3.0195 (2.9115-3.1314)	70.96% (63.64%-77.78%)	57.74% (48.26%-67.8%)	93.631% (86.842%-100%)	93.96% (87.18%-100%)	56.35% (46.55%-66.67%)	3.0168 (2.7918-3.2408)
At least 5 of CHASM (breast), CHASM (lung), Mutation Assessor, MutationTaster, PROVEAN	56.93% (53.03%-60.62%)	32.55% (27.78%-37.1%)	99.084% (98.485%-100%)	98.4% (97.22%-100%)	45.94% (41.25%-50.32%)	2.7597 (2.6710-2.8483)	56.84% (49.47%-64.65%)	32.22% (22.95%-42.11%)	99.083% (96.774%-100%)	98.37% (93.75%-100%)	46% (37.33%-55.43%)	2.7568 (2.5770-2.9401)
At least 5 of CHASM (breast), CHASM (lung), Mutation Assessor, MutationTaster, PROVEAN, SIFT	69.02% (65.15%-72.22%)	53.78% (48.41%-58.78%)	95.377% (92.958%-98.529%)	95.26% (92.65%-98.55%)	54.41% (49.59%-58.92%)	2.9882 (2.8851-3.0944)	69.02% (62.63%-76.77%)	53.58% (43.75%-64.18%)	95.478% (90%-100%)	95.3% (89.12%-100%)	54.52% (44.61%-64.62%)	2.9889 (2.7734-3.1933)
At least 5 of CHASM (breast), CHASM (lung), Mutation Assessor, MutationTaster, PROVEAN, VEST	66.37% (62.63%-69.71%)	49.59% (44.44%-54.84%)	95.377% (92.958%-98.529%)	94.88% (92.06%-98.41%)	52.25% (47.61%-56.93%)	2.9211 (2.8174-3.0286)	66.24% (59.57%-73.74%)	49.19% (39.06%-59.68%)	95.478% (90%-100%)	94.91% (88.24%-100%)	52.27% (42.64%-61.97%)	2.9185 (2.6999-3.1321)
At least 5 of CHASM (breast), CHASM (lung), Mutation Assessor, MutationTaster, SIFT	62.97% (59.09%-66.67%)	43.16% (38.16%-48.03%)	97.222% (95.588%-100%)	96.41% (94.23%-100%)	49.73% (45%-54.35%)	2.8652 (2.7700-2.9665)	62.95% (55.56%-70.71%)	42.93% (32.79%-53.34%)	97.294% (92.303%-100%)	96.45% (90.47%-100%)	49.84% (40.3%-59.43%)	2.8651 (2.6548-3.0571)
At least 5 of CHASM (breast), CHASM (lung), Mutation Assessor, MutationTaster, SIFT, VEST	70.75% (67.17%-74.24%)	57.01% (52.03%-62.1%)	94.49% (91.667%-97.333%)	94.7% (92.11%-97.47%)	55.97% (50.83%-60.8%)	3.0218 (2.9115-3.1308)	70.63% (63.64%-77.78%)	56.69% (46.67%-66.67%)	94.503% (88.235%-100%)	94.65% (88.24%-100%)	55.98% (46.03%-66.1%)	3.0182 (2.7937-3.2430)
At least 5 of CHASM (breast), CHASM (lung), Mutation Assessor, MutationTaster, VEST	59.27% (55.05%-63.13%)	37.33% (31.97%-42.62%)	97.222% (95.588%-100%)	95.87% (93.33%-100%)	47.29% (42.58%-51.75%)	2.7771 (2.6749-2.8778)	59.23% (51.52%-67.68%)	37.04% (26.86%-48.28%)	97.294% (92.303%-100%)	95.91% (88.89%-100%)	47.39% (38.16%-56.97%)	2.7763 (2.5726-2.9874)
At least 5 of CHASM (breast), CHASM (lung), Mutation Assessor, PolyPhen-2, PROVEAN	57.61% (53.54%-61.62%)	33.62% (28.68%-38.17%)	99.084% (98.485%-100%)	98.45% (97.3%-100%)	46.33% (41.72%-50.65%)	2.7748 (2.6884-2.8595)	57.51% (49.49%-65.66%)	33.27% (24.14%-42.86%)	99.083% (96.774%-100%)	98.42% (94.12%-100%)	46.39% (37.66%-55.84%)	2.7716 (2.5942-2.9438)
At least 5 of CHASM (breast), CHASM (lung), Mutation Assessor, PolyPhen-2, PROVEAN, SIFT	68.7% (65.15%-72.22%)	53.27% (48.06%-58.4%)	95.377% (92.958%-98.529%)	95.22% (92.54%-98.55%)	54.14% (49.22%-58.78%)	2.9800 (2.8755-3.0901)	68.66% (61.62%-75.76%)	53.01% (42.62%-63.49%)	95.478% (90%-100%)	95.25% (88.89%-100%)	54.21% (44.29%-64.06%)	2.9795 (2.7672-3.1902)
At least 5 of CHASM (breast), CHASM (lung), Mutation Assessor, PolyPhen-2, PROVEAN, VEST	65.35% (61.6%-69.19%)	48.53% (43.33%-53.6%)	94.437% (91.667%-97.333%)	93.78% (90.77%-97.06%)	51.48% (46.87%-55.91%)	2.8823 (2.7709-2.9929)	65.25% (57.58%-72.75%)	48.13% (37.93%-58.62%)	94.607% (88.569%-100%)	93.86% (86.21%-100%)	51.53% (42.37%-60.94%)	2.8813 (2.6573-3.1010)
At least 5 of CHASM (breast), CHASM (lung), Mutation Assessor, PolyPhen-2, SIFT	63.99% (60.1%-67.68%)	44.76% (39.83%-50%)	97.222% (95.588%-100%)	96.53% (94.34%-100%)	50.45% (45.51%-54.93%)	2.8897 (2.7937-2.9922)	63.94% (56.57%-71.72%)	44.49% (33.87%-54.55%)	97.294% (92.303%-100%)	96.58% (90.62%-100%)	50.53% (41.42%-60.27%)	2.8890 (2.6778-3.0882)
At least 5 of CHASM (breast), CHASM (lung), Mutation Assessor, PolyPhen-2, SIFT, VEST	70.75% (67.17%-74.24%)	57.55% (52.5%-62.7%)	93.55% (90.537%-97.102%)	93.91% (91.18%-97.37%)	56.04% (50.85%-60.87%)	3.0105 (2.9054-3.1301)	70.63% (63.64%-77.78%)	57.21% (46.97%-67.19%)	93.631% (86.842%-100%)	93.9% (86.84%-100%)	56.05% (46.29%-66.1%)	3.0079 (2.7737-3.2191)
At least 5 of CHASM (breast), CHASM (lung), Mutation Assessor, PolyPhen-2, VEST	58.93% (55.05%-62.63%)	37.32% (31.93%-42.4%)	96.282% (94.284%-98.667%)	94.55% (91.49%-98.04%)	47.05% (42.46%-51.39%)	2.7521 (2.6492-2.8592)	58.91% (51.52%-66.67%)	37.04% (26.78%-47.69%)	96.423% (91.176%-100%)	94.68% (86.96%-100%)	47.16% (38.02%-56.52%)	2.7530 (2.5339-2.9550)
At least 5 of CHASM (breast), CHASM (lung), Mutation Assessor, PROVEAN, SIFT	58.63% (54.55%-62.63%)	35.22% (30.51%-40%)	99.084% (98.485%-100%)	98.52% (97.44%-100%)	46.94% (42.21%-51.32%)	2.7977 (2.7129-2.8868)	58.51% (50.51%-66.67%)	34.85% (25.39%-44.45%)	99.083% (96.774%-100%)	98.49% (94.44%-100%)	46.98% (38.16%-56.41%)	2.7941 (2.6126-2.9704)
At least 5 of CHASM (breast), CHASM (lung), Mutation Assessor, PROVEAN, SIFT, VEST	66.03% (62.12%-69.7%)	49.05% (43.75%-54.03%)	95.377% (92.958%-98.529%)	94.83% (92.06%-98.41%)	51.99% (47.28%-56.62%)	2.9124 (2.8060-3.0178)	65.92% (58.59%-73.74%)	48.67% (38.8%-59.02%)	95.478% (90%-100%)	94.86% (88.24%-100%)	52.02% (42.19%-61.54%)	2.9103 (2.6895-3.1264)
At least 5 of CHASM (breast), CHASM (lung), Mutation Assessor, PROVEAN, VEST	56.59% (52.53%-60.61%)	32.02% (27.34%-36.64%)	99.084% (98.485%-100%)	98.37% (97.14%-100%)	45.74% (40.99%-50%)	2.7521 (2.6646-2.8407)	56.51% (48.48%-64.65%)	31.7% (22.27%-41.18%)	99.083% (96.774%-100%)	98.35% (93.75%-100%)	45.81% (37.18%-55.26%)	2.7493 (2.5723-2.9315)
At least 5 of CHASM (breast), CHASM (lung), Mutation Assessor, SIFT, VEST	60.29% (56.57%-64.14%)	38.93% (34.11%-44.09%)	97.222% (95.588%-100%)	96.04% (93.62%-100%)	47.94% (43.36%-52.45%)	2.8012 (2.7081-2.8993)	60.22% (52.53%-67.68%)	38.61% (28.35%-49.12%)	97.294% (92.303%-100%)	96.07% (89.29%-100%)	48.02% (39.13%-57.5%)	2.7999 (2.6027-2.9874)
At least 5 of CHASM (breast), CHASM (lung), MutationTaster, PolyPhen-2, PROVEAN	58.28% (54.04%-62.12%)	34.69% (29.84%-39.54%)	99.084% (98.485%-100%)	98.5% (97.37%-100%)	46.73% (41.94%-51%)	2.7900 (2.7008-2.8752)	58.18% (50.51%-66.67%)	34.33% (24.55%-43.95%)	99.083% (96.774%-100%)	98.47% (94.44%-100%)	46.79% (38.15%-56.25%)	2.7867 (2.6186-2.9714)
At least 5 of CHASM (breast), CHASM (lung), MutationTaster, PolyPhen-2, PROVEAN, SIFT	70.05% (66.67%-73.23%)	55.4% (50.39%-60.17%)	95.377% (92.958%-98.529%)	95.39% (92.86%-98.57%)	55.29% (50.39%-60%)	3.0146 (2.9117-3.1196)	70% (63.64%-76.77%)	55.14% (45.58%-65.52%)	95.478% (90%-100%)	95.43% (89.47%-100%)	55.36% (45.76%-65.62%)	3.0141 (2.8027-3.2170)
At least 5 of CHASM (breast), CHASM (lung), MutationTaster, PolyPhen-2, PROVEAN, VEST	67.38% (63.64%-70.71%)	51.18% (45.74%-56.45%)	95.377% (92.958%-98.529%)	95.03% (92.31%-98.48%)	53.05% (48.12%-57.78%)	2.9464 (2.8397-3.0509)	67.27% (60.58%-74.75%)	50.81% (40.62%-61.67%)	95.478% (90%-100%)	95.07% (88.46%-100%)	53.08% (43.28%-62.9%)	2.9444 (2.7268-3.1641)
At least 5 of CHASM (breast), CHASM (lung), MutationTaster, PolyPhen-2, SIFT	64.31% (60.59%-68.18%)	45.28% (40.16%-50.4%)	97.222% (95.588%-100%)	96.57% (94.44%-100%)	50.68% (45.92%-55.07%)	2.8975 (2.8004-2.9972)	64.3% (56.57%-71.74%)	45.07% (34.38%-55.17%)	97.294% (92.303%-100%)	96.62% (90.91%-100%)	50.79% (41.79%-60.56%)	2.8978 (2.6973-3.0969)
At least 5 of CHASM (breast), CHASM (lung), MutationTaster, PolyPhen-2, SIFT, VEST	72.45% (68.69%-75.76%)	59.71% (54.76%-64.52%)	94.49% (91.667%-97.333%)	94.93% (92.41%-97.59%)	57.56% (52.65%-62.39%)	3.0668 (2.9598-3.1740)	72.27% (65.66%-79.8%)	59.29% (50%-69.49%)	94.503% (88.235%-100%)	94.87% (88.88%-100%)	57.5% (47.69%-67.8%)	3.0616 (2.8430-3.2760)
At least 5 of CHASM (breast), CHASM (lung), MutationTaster, PolyPhen-2, VEST	61.63% (57.58%-65.66%)	41.04% (35.77%-46.22%)	97.222% (95.588%-100%)	96.23% (93.88%-100%)	48.82% (44.05%-53.15%)	2.8332 (2.7345-2.9358)	61.59% (53.54%-69.7%)	40.77% (30.51%-51.43%)	97.294% (92.303%-100%)	96.28% (90%-100%)	48.91% (39.73%-58.57%)	2.8326 (2.6282-3.0315)
At least 5 of CHASM (breast), CHASM (lung), MutationTaster, PROVEAN, SIFT	59.95% (56.06%-63.64%)	37.32% (32.54%-42.4%)	99.084% (98.485%-100%)	98.6% (97.62%-100%)	47.76% (42.95%-52.13%)	2.8277 (2.7394-2.9118)	59.89% (52.53%-67.68%)	37.04% (26.98%-46.88%)	99.083% (96.774%-100%)	98.58% (94.74%-100%)	47.84% (38.96%-57.58%)	2.8255 (2.6606-3.0099)
At least 5 of CHASM (breast), CHASM (lung), MutationTaster, PROVEAN, SIFT, VEST	66.69% (63.13%-70.2%)	50.1% (45%-55.12%)	95.377% (92.958%-98.529%)	94.93% (92.19%-98.41%)	52.5% (47.73%-57.14%)	2.9291 (2.8276-3.0366)	66.62% (59.6%-73.74%)	49.78% (39.34%-60.01%)	95.478% (90%-100%)	94.97% (88.57%-100%)	52.56% (42.86%-62.12%)	2.9278 (2.7128-3.1394)
At least 5 of CHASM (breast), CHASM (lung), MutationTaster, PROVEAN, VEST	58.29% (54.04%-62.12%)	34.69% (29.8%-39.2%)	99.084% (98.485%-100%)	98.5% (97.43%-100%)	46.74% (41.88%-50.98%)	2.7901 (2.7023-2.8707)	58.17% (50.51%-66.67%)	34.33% (24.99%-44.07%)	99.083% (96.774%-100%)	98.47% (94.44%-100%)	46.79% (38.16%-56.41%)	2.7867 (2.6165-2.9714)
At least 5 of CHASM (breast), CHASM (lung), MutationTaster, SIFT, VEST	61.97% (58.08%-65.66%)	41.58% (36%-46.83%)	97.222% (95.588%-100%)	96.28% (94%-100%)	49.04% (44.23%-53.38%)	2.8412 (2.7451-2.9383)	61.92% (54.55%-69.7%)	41.3% (30.51%-52.31%)	97.294% (92.303%-100%)	96.31% (90.32%-100%)	49.14% (39.73%-58.91%)	2.8404 (2.6445-3.0405)
At least 5 of CHASM (breast), CHASM (lung), PolyPhen-2, PROVEAN, SIFT	59.7% (56.06%-63.64%)	37.34% (32.26%-42.31%)	99.084% (98.485%-100%)	98.6% (97.56%-100%)	47.77% (42.86%-52.06%)	2.8279 (2.7438-2.9147)	59.86% (52.53%-67.68%)	37% (27.12%-46.88%)	99.083% (96.774%-100%)	98.57% (94.74%-100%)	47.82% (39.13%-57.53%)	2.8248 (2.6599-2.9963)
At least 5 of CHASM (breast), CHASM (lung), PolyPhen-2, PROVEAN, SIFT, VEST	66.71% (63.12%-70.2%)	50.12% (45%-55.2%)	95.377% (92.958%-98.529%)	94.93% (92.19%-98.44%)	52.52% (47.73%-57.03%)	2.9295 (2.8280-3.0366)	66.58% (59.6%-73.76%)	49.73% (39.68%-60.32%)	95.478% (90%-100%)	94.96% (88.57%-100%)	52.53% (42.86%-62.3%)	2.9270 (2.7142-3.1409)
At least 5 of CHASM (breast), CHASM (lung), PolyPhen-2, PROVEAN, VEST	57.95% (54.04%-61.62%)	34.15% (29.17%-38.84%)	99.084% (98.485%-100%)	98.47% (97.37%-100%)	46.53% (41.72%-50.94%)	2.7824 (2.6953-2.8707)	57.85% (50.51%-65.66%)	33.81% (24.61%-43.64%)	99.083% (96.774%-100%)	98.44% (94.44%-100%)	46.59% (37.97%-56%)	2.7792 (2.6029-2.9597)
At least 5 of CHASM (breast), CHASM (lung), PolyPhen-2, SIFT, VEST	61.29% (57.07%-65.15%)	40.5% (35.2%-45.67%)	97.222% (95.588%-100%)	96.18% (93.87%-100%)	48.59% (43.83%-52.82%)	2.8250 (2.7257-2.9249)	61.26% (53.54%-69.7%)	40.25% (29.69%-50.79%)	97.294% (92.303%-100%)	96.22% (89.99%-100%)	48.69% (39.73%-58.34%)	2.8246 (2.6257-3.0276)
At least 5 of CHASM (breast), CHASM (lung), PROVEAN, SIFT, VEST	59.29% (55.05%-63.13%)	36.27% (31.4%-40.98%)	99.084% (98.485%-100%)	98.56% (97.5%-100%)	47.35% (42.57%-51.66%)	2.8127 (2.7248-2.8965)	59.19% (51.52%-67.68%)	35.94% (26.56%-45.9%)	99.083% (96.774%-100%)	98.54% (94.74%-100%)	47.41% (38.66%-56.94%)	2.8097 (2.6428-2.9917)
At least 5 of CHASM (breast), CHASM (melanoma), FATHMM (cancer), FATHMM (missense), Mutation Assessor	64.69% (60.61%-68.69%)	46.41% (41.32%-51.54%)	96.293% (94.203%-98.667%)	95.59% (93.1%-98.41%)	50.96% (46.16%-55.56%)	2.8924 (2.7954-2.9998)	64.56% (56.57%-72.73%)	46% (36.07%-56.06%)	96.398% (91.176%-100%)	95.65% (89.65%-100%)	50.98% (41.79%-61.11%)	2.8903 (2.6766-3.0882)
At least 5 of CHASM (breast), CHASM (melanoma), FATHMM (cancer), FATHMM (missense), Mutation Assessor, MutationTaster	77.75% (74.75%-81.31%)	68.05% (63.08%-73.17%)	94.502% (91.667%-97.368%)	95.54% (93.33%-97.92%)	63.11% (57.8%-68.27%)	3.2120 (3.1152-3.3271)	77.84% (70.71%-83.84%)	68.12% (57.81%-77.94%)	94.479% (88.235%-100%)	95.49% (90%-100%)	63.35% (52.94%-74.14%)	3.2145 (2.9883-3.4163)
At least 5 of CHASM (breast), CHASM (melanoma), FATHMM (cancer), FATHMM (missense), Mutation Assessor, PolyPhen-2	74.06% (70.71%-77.78%)	62.76% (57.72%-67.72%)	93.579% (90.411%-97.26%)	94.42% (91.76%-97.59%)	59.24% (54.05%-64.04%)	3.1000 (2.9896-3.2149)	74.11% (66.67%-80.81%)	62.74% (52.46%-72.58%)	93.559% (86.111%-100%)	94.37% (87.88%-100%)	59.41% (50%-70%)	3.1009 (2.8750-3.3228)
At least 5 of CHASM (breast), CHASM (melanoma), FATHMM (cancer), FATHMM (missense), Mutation Assessor, PROVEAN	69.38% (65.66%-72.74%)	54.34% (48.82%-59.17%)	95.37% (92.857%-98.611%)	95.31% (92.75%-98.57%)	54.71% (49.61%-59.38%)	2.9973 (2.8915-3.1067)	69.32% (62.6%-76.77%)	54.05% (44.26%-64.71%)	95.478% (88.889%-100%)	95.38% (89.19%-100%)	54.77% (45.16%-64.62%)	2.9968 (2.7709-3.2133)
At least 5 of CHASM (breast), CHASM (melanoma), FATHMM (cancer), FATHMM (missense), Mutation Assessor, SIFT	76.43% (73.23%-79.8%)	66.5% (61.47%-71.31%)	93.579% (90.411%-97.26%)	94.71% (92.13%-97.7%)	61.77% (56.52%-66.67%)	3.1657 (3.0574-3.2814)	76.44% (69.7%-82.83%)	66.44% (57.13%-76.27%)	93.559% (86.111%-100%)	94.67% (88.63%-100%)	61.9% (51.85%-72.58%)	3.1656 (2.9378-3.3837)
At least 5 of CHASM (breast), CHASM (melanoma), FATHMM (cancer), FATHMM (missense), Mutation Assessor, VEST	73.39% (69.7%-77.27%)	61.71% (56.66%-66.93%)	93.579% (90.411%-97.26%)	94.33% (91.66%-97.53%)	58.57% (53.57%-63.39%)	3.0819 (2.9732-3.1990)	73.42% (65.66%-80.81%)	61.65% (51.46%-71.83%)	93.559% (86.111%-100%)	94.28% (87.8%-100%)	58.71% (49.06%-69.23%)	3.0820 (2.8483-3.3005)
At least 5 of CHASM (breast), CHASM (melanoma), FATHMM (cancer), FATHMM (missense), MutationTaster	68.02% (64.14%-71.72%)	51.12% (45.86%-56.25%)	97.233% (95.588%-100%)	96.97% (95.08%-100%)	53.5% (48.51%-58.33%)	2.9883 (2.8868-3.0929)	68% (60.61%-75.76%)	50.93% (40.62%-61.29%)	97.269% (92.303%-100%)	96.98% (91.89%-100%)	53.6% (43.86%-63.8%)	2.9879 (2.7831-3.1902)
At least 5 of CHASM (breast), CHASM (melanoma), FATHMM (cancer), FATHMM (missense), MutationTaster, PolyPhen-2	79.09% (75.76%-82.32%)	70.7% (66.1%-75.61%)	93.599% (90.411%-97.222%)	95.02% (92.63%-97.85%)	64.89% (59.81%-70%)	3.2421 (3.1353-3.3578)	79.19% (72.73%-85.86%)	70.81% (60.93%-80.3%)	93.533% (86.479%-100%)	94.94% (89.13%-100%)	65.15% (54.71%-75.86%)	3.2443 (3.0176-3.4653)
At least 5 of CHASM (breast), CHASM (melanoma), FATHMM (cancer), FATHMM (missense), MutationTaster, PROVEAN	75.08% (71.72%-78.3%)	62.27% (57.26%-67.48%)	97.233% (95.588%-100%)	97.5% (96%-100%)	59.85% (54.74%-64.91%)	3.1685 (3.0679-3.2706)	75.09% (68.66%-81.82%)	62.14% (51.67%-72.13%)	97.269% (92.303%-100%)	97.51% (93.33%-100%)	59.97% (50%-70%)	3.1689 (2.9712-3.3770)
At least 5 of CHASM (breast), CHASM (melanoma), FATHMM (cancer), FATHMM (missense), MutationTaster, SIFT	81.11% (77.78%-84.34%)	72.81% (68.1%-77.52%)	95.442% (92.958%-98.592%)	96.51% (94.62%-98.92%)	67.01% (61.76%-72.12%)	3.3177 (3.2225-3.4229)	81.22% (74.75%-87.88%)	72.96% (63.33%-82.36%)	95.351% (89.283%-100%)	96.42% (91.84%-100%)	67.29% (56.9%-78.18%)	3.3203 (3.1112-3.5309)
At least 5 of CHASM (breast), CHASM (melanoma), FATHMM (cancer), FATHMM (missense), MutationTaster, VEST	78.07% (74.75%-81.82%)	68.56% (63.78%-73.64%)	94.502% (91.667%-97.368%)	95.57% (93.26%-97.9%)	63.49% (58.33%-68.32%)	3.2211 (3.1263-3.3302)	78.21% (70.71%-84.85%)	68.71% (58.62%-78.46%)	94.479% (88.235%-100%)	95.53% (90%-100%)	63.78% (53.85%-74.07%)	3.2251 (3.0110-3.4289)
At least 5 of CHASM (breast), CHASM (melanoma), FATHMM (cancer), FATHMM (missense), PolyPhen-2	66.02% (62.12%-69.7%)	48.51% (43.44%-53.6%)	96.293% (94.203%-98.667%)	95.77% (93.33%-98.48%)	51.96% (47.06%-56.52%)	2.9254 (2.8266-3.0300)	65.94% (58.59%-73.74%)	48.17% (37.87%-58.62%)	96.398% (91.176%-100%)	95.84% (89.99%-100%)	52.01% (42.86%-61.76%)	2.9242 (2.7142-3.1285)
At least 5 of CHASM (breast), CHASM (melanoma), FATHMM (cancer), FATHMM (missense), PolyPhen-2, PROVEAN	72.73% (69.19%-76.26%)	59.64% (54.54%-64.84%)	95.37% (92.857%-98.611%)	95.71% (93.42%-98.68%)	57.75% (52.84%-62.5%)	3.0846 (2.9811-3.1981)	72.71% (65.66%-79.8%)	59.42% (49.15%-69.81%)	95.478% (88.889%-100%)	95.78% (90.24%-100%)	57.83% (48.21%-67.8%)	3.0851 (2.8640-3.2982)
At least 5 of CHASM (breast), CHASM (melanoma), FATHMM (cancer), FATHMM (missense), PolyPhen-2, SIFT	78.42% (75.25%-81.82%)	70.18% (65.12%-75.2%)	92.652% (89.231%-96.725%)	94.29% (91.67%-97.65%)	64.25% (59%-69.39%)	3.2137 (3.1017-3.3282)	78.52% (71.72%-84.85%)	70.25% (60.38%-80.3%)	92.652% (84.848%-100%)	94.27% (88.37%-100%)	64.5% (54.17%-75.44%)	3.2168 (2.9839-3.4468)
At least 5 of CHASM (breast), CHASM (melanoma), FATHMM (cancer), FATHMM (missense), PolyPhen-2, VEST	76.08% (72.73%-79.8%)	66.48% (61.79%-71.54%)	92.652% (89.231%-96.725%)	94% (91.3%-97.47%)	61.52% (56.19%-66.67%)	3.1465 (3.0382-3.2617)	76.13% (68.69%-82.83%)	66.47% (56.14%-75.86%)	92.652% (84.848%-100%)	93.97% (87.76%-100%)	61.7% (51.72%-72.23%)	3.1480 (2.9289-3.3693)
At least 5 of CHASM (breast), CHASM (melanoma), FATHMM (cancer), FATHMM (missense), PROVEAN	58.97% (55.04%-62.63%)	36.3% (31.45%-40.94%)	98.173% (97.015%-100%)	97.17% (95.24%-100%)	47.13% (42.21%-51.61%)	2.7877 (2.6957-2.8815)	58.82% (51.52%-66.69%)	35.9% (26.31%-45.9%)	98.144% (94.118%-100%)	97.09% (90.48%-100%)	47.15% (38.36%-56.79%)	2.7827 (2.5878-2.9704)
At least 5 of CHASM (breast), CHASM (melanoma), FATHMM (cancer), FATHMM (missense), PROVEAN, SIFT	73.42% (70.19%-76.77%)	60.18% (55%-65.12%)	96.309% (94.203%-98.702%)	96.58% (94.67%-98.77%)	58.31% (53.39%-63.2%)	3.1138 (3.0116-3.2193)	73.36% (66.67%-79.82%)	59.93% (50%-70.15%)	96.35% (90.625%-100%)	96.6% (91.67%-100%)	58.36% (48.53%-68.43%)	3.1124 (2.9043-3.3254)
At least 5 of CHASM (breast), CHASM (melanoma), FATHMM (cancer), FATHMM (missense), PROVEAN, VEST	68.37% (64.65%-72.22%)	52.75% (47.15%-58.02%)	95.37% (92.857%-98.611%)	95.18% (92.65%-98.51%)	53.86% (48.8%-58.4%)	2.9716 (2.8695-3.0812)	68.31% (60.61%-75.76%)	52.45% (41.67%-63.08%)	95.478% (88.889%-100%)	95.25% (89.18%-100%)	53.92% (44.44%-63.79%)	2.9710 (2.7496-3.1763)
At least 5 of CHASM (breast), CHASM (melanoma), FATHMM (cancer), FATHMM (missense), SIFT	69.08% (65.64%-72.73%)	52.79% (47.62%-57.94%)	97.233% (95.588%-100%)	97.06% (95.31%-100%)	54.36% (49.59%-58.96%)	3.0144 (2.9199-3.1176)	68.92% (61.62%-75.76%)	52.38% (42.3%-62.12%)	97.269% (92.303%-100%)	97.06% (91.89%-100%)	54.34% (44.93%-64.18%)	3.0105 (2.8004-3.2078)
At least 5 of CHASM (breast), CHASM (melanoma), FATHMM (cancer), FATHMM (missense), SIFT, VEST	77.08% (73.74%-80.3%)	68.6% (63.71%-73.39%)	91.725% (88.403%-95.778%)	93.48% (90.8%-96.7%)	62.82% (57.52%-67.96%)	3.1663 (3.0555-3.2821)	77.16% (70.71%-83.84%)	68.62% (58.73%-78.12%)	91.75% (83.711%-97.56%)	93.47% (87.18%-98.04%)	63.03% (53.06%-73.47%)	3.1687 (2.9348-3.3909)
At least 5 of CHASM (breast), CHASM (melanoma), FATHMM (cancer), FATHMM (missense), VEST	60.98% (57.07%-64.65%)	40.56% (35.29%-45.6%)	96.293% (94.203%-98.667%)	94.99% (92%-98.18%)	48.37% (43.45%-52.82%)	2.8021 (2.7017-2.9036)	60.86% (53.54%-68.69%)	40.14% (30%-50.02%)	96.398% (91.176%-100%)	95.06% (88%-100%)	48.41% (39.13%-58.21%)	2.8001 (2.5918-3.0019)
At least 5 of CHASM (breast), CHASM (melanoma), FATHMM (cancer), Mutation Assessor, MutationTaster	62.3% (58.59%-66.16%)	42.09% (36.89%-46.88%)	97.233% (95.588%-100%)	96.34% (94.12%-100%)	49.27% (44.53%-53.85%)	2.8493 (2.7498-2.9505)	62.27% (54.55%-69.7%)	41.87% (32.2%-52.38%)	97.269% (92.303%-100%)	96.36% (90.31%-100%)	49.37% (40%-58.83%)	2.8487 (2.6448-3.0489)
At least 5 of CHASM (breast), CHASM (melanoma), FATHMM (cancer), Mutation Assessor, MutationTaster, PolyPhen-2	76.08% (72.73%-79.8%)	65.96% (61.24%-70.87%)	93.581% (90.537%-97.183%)	94.67% (92.13%-97.68%)	61.39% (56.25%-66.38%)	3.1560 (3.0478-3.2726)	76.12% (68.69%-82.83%)	65.93% (55.74%-75.86%)	93.565% (86.838%-100%)	94.62% (88.57%-100%)	61.56% (51.65%-72%)	3.1568 (2.9233-3.3712)
At least 5 of CHASM (breast), CHASM (melanoma), FATHMM (cancer), Mutation Assessor, MutationTaster, PROVEAN	70.73% (67.17%-74.24%)	55.4% (50%-60.47%)	97.233% (95.588%-100%)	97.2% (95.52%-100%)	55.77% (50.79%-60.31%)	3.0559 (2.9608-3.1538)	70.66% (63.64%-77.78%)	55.13% (45.16%-65.62%)	97.269% (92.303%-100%)	97.21% (92.5%-100%)	55.82% (46.55%-65.62%)	3.0543 (2.8580-3.2441)
At least 5 of CHASM (breast), CHASM (melanoma), FATHMM (cancer), Mutation Assessor, MutationTaster, SIFT	76.1% (72.73%-79.8%)	65.43% (60.63%-70.49%)	94.52% (91.667%-97.368%)	95.38% (92.94%-97.8%)	61.27% (56.03%-66.36%)	3.1660 (3.0583-3.2818)	76.09% (68.69%-82.83%)	65.38% (55%-75%)	94.436% (88.232%-100%)	95.28% (89.74%-100%)	61.39% (51.72%-71.43%)	3.1648 (2.9342-3.3837)
At least 5 of CHASM (breast), CHASM (melanoma), FATHMM (cancer), Mutation Assessor, MutationTaster, VEST	74.39% (70.71%-77.78%)	63.29% (58.47%-68.03%)	93.581% (90.537%-97.183%)	94.46% (91.95%-97.59%)	59.59% (54.39%-64.6%)	3.1092 (3.0009-3.2279)	74.44% (67.68%-81.82%)	63.26% (53.33%-72.58%)	93.565% (86.838%-100%)	94.41% (88.37%-100%)	59.76% (50%-70%)	3.1102 (2.8764-3.3304)
At least 5 of CHASM (breast), CHASM (melanoma), FATHMM (cancer), Mutation Assessor, PolyPhen-2	63.31% (59.6%-67.17%)	44.23% (39.16%-49.21%)	96.293% (94.203%-98.667%)	95.38% (92.73%-98.31%)	49.97% (45.32%-54.43%)	2.8587 (2.7620-2.9673)	63.28% (55.56%-70.71%)	43.96% (33.87%-54.55%)	96.398% (91.176%-100%)	95.46% (89.29%-100%)	50.06% (41.09%-59.42%)	2.8588 (2.6381-3.0513)
At least 5 of CHASM (breast), CHASM (melanoma), FATHMM (cancer), Mutation Assessor, PolyPhen-2, PROVEAN	69.72% (66.16%-73.23%)	55.43% (50.77%-60.66%)	94.421% (91.549%-97.368%)	94.5% (91.78%-97.4%)	55.06% (50%-59.69%)	2.9941 (2.8859-3.1137)	69.66% (62.63%-76.77%)	55.08% (45%-65%)	94.628% (87.879%-100%)	94.64% (88.24%-100%)	55.11% (45.61%-65.08%)	2.9946 (2.7638-3.2144)
At least 5 of CHASM (breast), CHASM (melanoma), FATHMM (cancer), Mutation Assessor, PolyPhen-2, SIFT	73.72% (70.2%-77.29%)	63.32% (58.4%-68.29%)	91.708% (88.233%-95.652%)	92.36% (90.12%-96.43%)	59.12% (53.84%-63.89%)	3.0711 (2.9582-3.2001)	73.76% (66.64%-80.81%)	63.23% (53.12%-73.02%)	91.795% (84.611%-97.73%)	92.98% (86.36%-98.09%)	59.27% (49.06%-69.64%)	3.0728 (2.8192-3.2996)
At least 5 of CHASM (breast), CHASM (melanoma), FATHMM (cancer), Mutation Assessor, PolyPhen-2, VEST	74.07% (70.71%-77.78%)	63.83% (58.91%-68.8%)	91.747% (88.406%-95.775%)	93.05% (90.22%-96.39%)	59.47% (54.39%-64.55%)	3.0810 (2.9724-3.2023)	74.09% (66.67%-80.81%)	63.79% (53.97%-73.45%)	91.718% (84.202%-97.56%)	92.98% (86.66%-97.96%)	59.63% (49.21%-69.65%)	3.0812 (2.8447-3.2992)
At least 5 of CHASM (breast), CHASM (melanoma), FATHMM (cancer), Mutation Assessor, PROVEAN	57.61% (53.54%-61.62%)	34.15% (29.41%-38.71%)	98.173% (97.015%-100%)	97% (94.87%-100%)	46.3% (41.61%-50.65%)	2.7562 (2.6614-2.8492)	57.51% (49.49%-65.66%)	33.82% (24.56%-43.75%)	98.144% (94.118%-100%)	96.9% (90%-100%)	46.35% (37.66%-55.7%)	2.7522 (2.5611-2.9437)
At least 5 of CHASM (breast), CHASM (melanoma), FATHMM (cancer), Mutation Assessor, PROVEAN, SIFT	70.73% (67.17%-74.24%)	56.47% (51.2%-61.6%)	95.361% (92.857%-98.592%)	95.47% (93.06%-98.59%)	55.89% (50.86%-60.63%)	3.0319 (2.9280-3.1420)	70.67% (63.64%-77.78%)	56.17% (45.9%-66.67%)	95.5% (89.474%-100%)	95.56% (89.74%-100%)	55.94% (46.15%-65.58%)	3.0317 (2.8125-3.2421)
At least 5 of CHASM (breast), CHASM (melanoma), FATHMM (cancer), Mutation Assessor, PROVEAN, VEST	67.39% (63.64%-70.71%)	51.2% (45.76%-56.35%)	95.37% (92.857%-98.611%)	95.04% (92.42%-98.46%)	53.06% (48.15%-57.58%)	2.9466 (2.8423-3.0574)	67.25% (60.61%-74.75%)	50.77% (40.32%-61.19%)	95.478% (88.889%-100%)	95.1% (88.89%-100%)	53.06% (43.55%-62.9%)	2.9440 (2.7189-3.1495)
At least 5 of CHASM (breast), CHASM (melanoma), FATHMM (cancer), Mutation Assessor, SIFT	64.34% (60.61%-68.18%)	45.31% (40%-50.39%)	97.233% (95.588%-100%)	96.59% (94.55%-100%)	50.69% (45.99%-55.25%)	2.8982 (2.8034-3.0008)	64.26% (56.57%-71.72%)	45.01% (34.91%-55.17%)	97.269% (92.303%-100%)	96.6% (90.91%-100%)	50.75% (41.43%-60.29%)	2.8963 (2.6907-3.0918)
At least 5 of CHASM (breast), CHASM (melanoma), FATHMM (cancer), Mutation Assessor, SIFT, VEST	74.07% (70.71%-77.78%)	63.31% (58.33%-68.46%)	92.657% (89.331%-96%)	93.72% (90.91%-96.59%)	59.36% (54.21%-64.41%)	3.0904 (2.9751-3.2112)	74.09% (66.67%-80.81%)	63.25% (52.54%-73.02%)	92.645% (85.364%-100%)	93.67% (87.5%-100%)	59.5% (49.15%-70%)	3.0907 (2.8533-3.3211)
At least 5 of CHASM (breast), CHASM (melanoma), FATHMM (cancer), Mutation Assessor, VEST	59.28% (55.05%-63.13%)	37.87% (33.05%-42.97%)	96.293% (94.203%-98.667%)	94.65% (91.49%-98.08%)	47.27% (42.57%-51.97%)	2.7607 (2.6537-2.8682)	59.22% (51.52%-67.68%)	37.55% (27.41%-47.55%)	96.398% (91.176%-100%)	94.74% (86.96%-100%)	47.36% (38.27%-56.94%)	2.7604 (2.5384-2.9727)
At least 5 of CHASM (breast), CHASM (melanoma), FATHMM (cancer), MutationTaster, PolyPhen-2	65.65% (61.62%-69.7%)	47.38% (42.15%-52.42%)	97.233% (95.588%-100%)	96.74% (94.74%-100%)	51.66% (46.76%-56.12%)	2.9302 (2.8317-3.0300)	65.57% (57.58%-73.74%)	47.24% (37.29%-57.63%)	97.269% (92.303%-100%)	96.76% (91.3%-100%)	51.8% (42.42%-61.76%)	2.9307 (2.7273-3.1308)
At least 5 of CHASM (breast), CHASM (melanoma), FATHMM (cancer), MutationTaster, PolyPhen-2, PROVEAN	74.09% (70.2%-77.27%)	60.71% (55.81%-65.62%)	97.233% (95.588%-100%)	97.43% (95.89%-100%)	58.87% (53.96%-63.64%)	3.1424 (3.0424-3.2416)	74.03% (67.68%-81.82%)	60.47% (50.77%-70.31%)	97.269% (92.303%-100%)	97.45% (93.18%-100%)	58.92% (49.18%-69.64%)	3.1412 (2.9519-3.3543)
At least 5 of CHASM (breast), CHASM (melanoma), FATHMM (cancer), MutationTaster, PolyPhen-2, SIFT	78.43% (75%-81.82%)	69.12% (64.57%-73.98%)	94.52% (91.667%-97.368%)	95.62% (93.33%-97.9%)	63.91% (58.89%-69.23%)	3.2317 (3.1302-3.3483)	78.49% (71.72%-84.85%)	69.17% (59.38%-78.57%)	94.436% (88.232%-100%)	95.53% (90.24%-100%)	64.11% (53.99%-74.07%)	3.2324 (3.0033-3.4377)
At least 5 of CHASM (breast), CHASM (melanoma), FATHMM (cancer), MutationTaster, PolyPhen-2, VEST	76.08% (72.73%-79.31%)	65.95% (61.16%-70.73%)	93.581% (90.537%-97.183%)	94.67% (92.21%-97.67%)	61.39% (56.31%-66.36%)	3.1559 (3.0552-3.2725)	76.13% (69.7%-82.83%)	65.94% (56.6%-75.4%)	93.565% (86.838%-100%)	94.62% (88.64%-100%)	61.56% (51.79%-71.88%)	3.1569 (2.9314-3.3677)
At least 5 of CHASM (breast), CHASM (melanoma), FATHMM (cancer), MutationTaster, PROVEAN	58.94% (55.05%-62.63%)	36.24% (31.45%-41.22%)	98.173% (97.015%-100%)	97.17% (95.24%-100%)	47.11% (42.28%-51.35%)	2.7869 (2.6932-2.8770)	58.89% (51.52%-66.67%)	36.01% (26.23%-45.9%)	98.144% (94.118%-100%)	97.09% (90.48%-100%)	47.19% (38.36%-56.94%)	2.7843 (2.6011-2.9812)
At least 5 of CHASM (breast), CHASM (melanoma), FATHMM (cancer), MutationTaster, PROVEAN, SIFT	72.42% (69.19%-75.76%)	58.06% (53.12%-63.28%)	97.233% (95.588%-100%)	97.32% (95.71%-100%)	57.28% (52.31%-62.1%)	3.0989 (3.0051-3.2004)	72.34% (65.66%-78.79%)	57.79% (47.54%-67.69%)	97.269% (92.303%-100%)	97.33% (92.68%-100%)	57.32% (47.54%-67.32%)	3.0971 (2.8965-3.2966)
At least 5 of CHASM (breast), CHASM (melanoma), FATHMM (cancer), MutationTaster, PROVEAN, VEST	70.07% (66.67%-73.74%)	54.36% (49.16%-59.53%)	97.233% (95.588%-100%)	97.14% (95.38%-100%)	55.2% (50.39%-59.85%)	3.0394 (2.9444-3.1367)	69.96% (62.63%-76.77%)	54.02% (44.06%-64.29%)	97.269% (92.303%-100%)	97.16% (92.31%-100%)	55.22% (45.45%-64.79%)	3.0367 (2.8423-3.2358)
At least 5 of CHASM (breast), CHASM (melanoma), FATHMM (cancer), MutationTaster, SIFT	66.34% (62.63%-70.2%)	48.47% (43.2%-53.66%)	97.233% (95.588%-100%)	96.81% (94.83%-100%)	52.18% (47.26%-56.82%)	2.9469 (2.8493-3.0480)	66.31% (58.59%-73.74%)	48.26% (37.7%-58.62%)	97.269% (92.303%-100%)	96.82% (91.43%-100%)	52.28% (42.86%-62.5%)	2.9463 (2.7462-3.1428)
At least 5 of CHASM (breast), CHASM (melanoma), FATHMM (cancer), MutationTaster, SIFT, VEST	76.42% (73.23%-79.8%)	65.94% (61.42%-70.64%)	94.52% (91.667%-97.368%)	95.41% (93.02%-97.83%)	61.61% (56.52%-66.67%)	3.1749 (3.0699-3.2875)	76.46% (69.7%-82.83%)	65.96% (56.25%-74.66%)	94.436% (88.232%-100%)	95.32% (89.74%-100%)	61.8% (51.72%-71.88%)	3.1752 (2.9480-3.3878)
At least 5 of CHASM (breast), CHASM (melanoma), FATHMM (cancer), MutationTaster, VEST	62.31% (58.59%-66.16%)	42.11% (36.8%-47.29%)	97.233% (95.588%-100%)	96.34% (94%-100%)	49.27% (44.52%-53.79%)	2.8496 (2.7516-2.9514)	62.25% (54.55%-69.7%)	41.84% (31.15%-52.31%)	97.269% (92.303%-100%)	96.36% (90%-100%)	49.36% (40%-59.22%)	2.8483 (2.6381-3.0441)
At least 5 of CHASM (breast), CHASM (melanoma), FATHMM (cancer), PolyPhen-2, PROVEAN	58.95% (55.05%-62.63%)	36.26% (31.2%-40.99%)	98.173% (97.015%-100%)	97.17% (95.24%-100%)	47.11% (42.18%-51.35%)	2.7872 (2.6908-2.8819)	58.87% (51.52%-66.67%)	35.96% (26.32%-46.03%)	98.144% (94.118%-100%)	97.08% (90.48%-100%)	47.18% (38.36%-56.96%)	2.7836 (2.5898-2.9760)
At least 5 of CHASM (breast), CHASM (melanoma), FATHMM (cancer), PolyPhen-2, PROVEAN, SIFT	71.4% (67.68%-74.76%)	57.53% (52.5%-62.61%)	95.361% (92.857%-98.592%)	95.55% (93.24%-98.63%)	56.49% (51.61%-61.36%)	3.0493 (2.9477-3.1583)	71.35% (64.62%-78.79%)	57.26% (47.54%-67.19%)	95.5% (89.474%-100%)	95.64% (90%-100%)	56.56% (46.55%-66.67%)	3.0495 (2.8328-3.2667)
At least 5 of CHASM (breast), CHASM (melanoma), FATHMM (cancer), PolyPhen-2, PROVEAN, VEST	69.41% (65.66%-72.73%)	54.38% (49.24%-59.36%)	95.37% (92.857%-98.611%)	95.32% (92.86%-98.53%)	54.73% (49.63%-59.35%)	2.9980 (2.8980-3.1058)	69.27% (62.63%-76.77%)	53.97% (44.28%-64.29%)	95.478% (88.889%-100%)	95.38% (89.47%-100%)	54.73% (45%-64.71%)	2.9956 (2.7783-3.1937)
At least 5 of CHASM (breast), CHASM (melanoma), FATHMM (cancer), PolyPhen-2, SIFT	66.02% (62.12%-69.7%)	47.96% (42.64%-53.23%)	97.233% (95.588%-100%)	96.77% (94.83%-100%)	51.94% (47.01%-56.35%)	2.9390 (2.8443-3.0424)	65.95% (58.59%-73.74%)	47.68% (37.1%-58.06%)	97.269% (92.303%-100%)	96.78% (91.3%-100%)	52% (43.07%-61.9%)	2.9373 (2.7297-3.1310)
At least 5 of CHASM (breast), CHASM (melanoma), FATHMM (cancer), PolyPhen-2, SIFT, VEST	75.41% (72.22%-78.79%)	65.97% (61.42%-70.87%)	91.731% (88.402%-95.775%)	93.25% (90.53%-96.51%)	60.92% (55.66%-66.06%)	3.1187 (3.0068-3.2352)	75.44% (68.69%-81.82%)	65.91% (55.93%-75.01%)	91.738% (83.784%-97.62%)	93.21% (86.67%-98%)	61.06% (50.91%-71.43%)	3.1193 (2.8805-3.3395)
At least 5 of CHASM (breast), CHASM (melanoma), FATHMM (cancer), PolyPhen-2, VEST	61.28% (57.07%-65.15%)	41.04% (35.71%-46.28%)	96.293% (94.203%-98.667%)	95.04% (92.16%-98.21%)	48.57% (43.62%-52.86%)	2.8094 (2.7036-2.9097)	61.27% (53.54%-69.7%)	40.79% (30.5%-50.92%)	96.398% (91.176%-100%)	95.14% (88%-100%)	48.69% (39.71%-58.67%)	2.8101 (2.5988-3.0201)
At least 5 of CHASM (breast), CHASM (melanoma), FATHMM (cancer), PROVEAN, SIFT	60.97% (57.07%-64.65%)	39.45% (34.43%-44.26%)	98.173% (97.015%-100%)	97.39% (95.65%-100%)	48.39% (43.45%-52.74%)	2.8341 (2.7425-2.9233)	60.89% (53.54%-68.69%)	39.17% (29.73%-49.18%)	98.144% (94.118%-100%)	97.32% (91.3%-100%)	48.46% (39.44%-58.33%)	2.8309 (2.6427-3.0161)
At least 5 of CHASM (breast), CHASM (melanoma), FATHMM (cancer), PROVEAN, SIFT, VEST	68.72% (65.15%-72.22%)	52.75% (47.58%-57.85%)	96.309% (94.203%-98.702%)	96.12% (93.94%-98.61%)	54.11% (49.21%-58.78%)	2.9929 (2.8911-3.0963)	68.63% (61.62%-75.76%)	52.44% (42.37%-62.69%)	96.35% (90.625%-100%)	96.14% (90.62%-100%)	54.14% (44.61%-63.77%)	2.9907 (2.7769-3.1917)
At least 5 of CHASM (breast), CHASM (melanoma), FATHMM (cancer), PROVEAN, VEST	58.28% (54.04%-62.12%)	35.2% (30.3%-39.84%)	98.173% (97.015%-100%)	97.09% (95.12%-100%)	46.7% (42%-50.98%)	2.7716 (2.6760-2.8613)	58.2% (50.51%-66.67%)	34.91% (25.36%-45.45%)	98.144% (94.118%-100%)	97% (90%-100%)	46.77% (37.97%-56.01%)	2.7682 (2.5791-2.9598)
At least 5 of CHASM (breast), CHASM (melanoma), FATHMM (cancer), SIFT, VEST	62.32% (58.08%-66.16%)	42.12% (37.1%-47.2%)	97.233% (95.588%-100%)	96.34% (94%-100%)	49.28% (44.53%-53.79%)	2.8497 (2.7532-2.9480)	62.24% (54.55%-70.71%)	41.81% (31.74%-51.61%)	97.269% (92.303%-100%)	96.35% (90%-100%)	49.35% (40.3%-59.09%)	2.8478 (2.6443-3.0445)
At least 5 of CHASM (breast), CHASM (melanoma), FATHMM (missense), Mutation Assessor, MutationTaster	61.97% (58.08%-65.66%)	41.57% (36.51%-46.46%)	97.233% (95.588%-100%)	96.3% (94%-100%)	49.04% (44.29%-53.57%)	2.8415 (2.7451-2.9424)	61.92% (54.55%-69.7%)	41.31% (31.74%-51.61%)	97.269% (92.303%-100%)	96.31% (89.99%-100%)	49.13% (40%-58.67%)	2.8402 (2.6331-3.0426)
At least 5 of CHASM (breast), CHASM (melanoma), FATHMM (missense), Mutation Assessor, MutationTaster, PolyPhen-2	74.05% (70.71%-77.27%)	62.75% (57.5%-67.5%)	93.581% (90.537%-97.183%)	94.41% (91.78%-97.56%)	59.23% (54.17%-64.29%)	3.0997 (2.9943-3.2144)	74.13% (67.68%-80.81%)	62.77% (52.94%-73.24%)	93.565% (86.838%-100%)	94.37% (88.1%-100%)	59.44% (50%-69.64%)	3.1014 (2.8765-3.3165)
At least 5 of CHASM (breast), CHASM (melanoma), FATHMM (missense), Mutation Assessor, MutationTaster, PROVEAN	68.68% (65.15%-72.22%)	52.17% (47.15%-57.26%)	97.233% (95.588%-100%)	97.03% (95.24%-100%)	54.04% (49.23%-58.65%)	3.0047 (2.9088-3.1003)	68.69% (61.62%-75.76%)	52.01% (41.82%-62.26%)	97.269% (92.303%-100%)	97.05% (91.89%-100%)	54.15% (44.61%-63.64%)	3.0049 (2.8065-3.1937)
At least 5 of CHASM (breast), CHASM (melanoma), FATHMM (missense), Mutation Assessor, MutationTaster, SIFT	74.74% (71.21%-78.28%)	63.29% (58.14%-68%)	94.52% (91.667%-97.368%)	95.23% (92.85%-97.73%)	59.83% (54.86%-64.66%)	3.1287 (3.0243-3.2436)	74.77% (67.68%-81.82%)	63.29% (53.57%-73.34%)	94.436% (88.232%-100%)	95.13% (89.36%-100%)	59.99% (50%-70.01%)	3.1285 (2.9002-3.3368)
At least 5 of CHASM (breast), CHASM (melanoma), FATHMM (missense), Mutation Assessor, MutationTaster, VEST	72.7% (69.19%-76.26%)	60.62% (55.46%-65.83%)	93.581% (90.537%-97.183%)	94.23% (91.57%-97.47%)	57.88% (52.94%-62.71%)	3.0631 (2.9577-3.1752)	72.78% (65.66%-79.8%)	60.65% (50%-71.19%)	93.565% (86.838%-100%)	94.19% (87.8%-100%)	58.08% (48.21%-67.93%)	3.0649 (2.8402-3.2745)
At least 5 of CHASM (breast), CHASM (melanoma), FATHMM (missense), Mutation Assessor, PolyPhen-2	62.98% (59.09%-66.67%)	43.72% (38.52%-48.8%)	96.293% (94.203%-98.667%)	95.33% (92.73%-98.31%)	49.74% (45%-54.29%)	2.8507 (2.7520-2.9622)	62.92% (55.56%-70.71%)	43.4% (33.33%-53.45%)	96.398% (91.176%-100%)	95.41% (88.89%-100%)	49.81% (40.85%-59.16%)	2.8502 (2.6286-3.0472)
At least 5 of CHASM (breast), CHASM (melanoma), FATHMM (missense), Mutation Assessor, PolyPhen-2, PROVEAN	67.68% (64.14%-71.21%)	52.19% (47.2%-57.15%)	94.442% (91.667%-97.368%)	94.21% (91.3%-97.18%)	53.33% (48.41%-57.85%)	2.9417 (2.8341-3.0553)	67.67% (60.61%-74.75%)	51.96% (41.82%-61.9%)	94.575% (88.09%-100%)	94.3% (87.88%-100%)	53.43% (44.26%-63.48%)	2.9427 (2.7128-3.1595)
At least 5 of CHASM (breast), CHASM (melanoma), FATHMM (missense), Mutation Assessor, PolyPhen-2, SIFT	72.04% (68.18%-75.76%)	60.64% (55.55%-65.35%)	91.73% (88.235%-95.775%)	92.69% (89.61%-96.2%)	57.41% (52.29%-62.4%)	3.0247 (2.9038-3.1435)	72.09% (64.65%-79.8%)	60.61% (50.82%-70.42%)	91.742% (84.211%-97.62%)	92.66% (85.42%-97.87%)	57.58% (47.46%-67.93%)	3.0259 (2.7892-3.2708)
At least 5 of CHASM (breast), CHASM (melanoma), FATHMM (missense), Mutation Assessor, PolyPhen-2, VEST	72.37% (68.69%-75.76%)	60.62% (55.47%-65.57%)	92.657% (89.331%-96%)	93.46% (90.59%-96.43%)	57.65% (52.63%-62.5%)	3.0439 (2.9314-3.1590)	72.44% (65.66%-79.8%)	60.63% (50.79%-70.77%)	92.645% (85.364%-100%)	93.42% (87.09%-100%)	57.84% (48.15%-67.86%)	3.0453 (2.8157-3.2746)
At least 5 of CHASM (breast), CHASM (melanoma), FATHMM (missense), Mutation Assessor, PROVEAN	56.95% (53.02%-61.11%)	33.11% (28.23%-37.8%)	98.173% (97.015%-100%)	96.91% (94.74%-100%)	45.91% (41.14%-50.32%)	2.7411 (2.6487-2.8347)	56.8% (48.48%-64.67%)	32.69% (23.43%-42.62%)	98.144% (94.118%-100%)	96.8% (89.47%-100%)	45.94% (37.18%-55.41%)	2.7357 (2.5444-2.9204)
At least 5 of CHASM (breast), CHASM (melanoma), FATHMM (missense), Mutation Assessor, PROVEAN, SIFT	68.69% (65.15%-72.22%)	53.24% (48.09%-58.27%)	95.382% (92.857%-98.592%)	95.23% (92.65%-98.51%)	54.12% (49.21%-58.73%)	2.9798 (2.8793-3.0863)	68.68% (61.62%-75.76%)	53.05% (42.62%-63.33%)	95.447% (89.467%-100%)	95.28% (89.19%-100%)	54.22% (44.77%-63.94%)	2.9800 (2.7672-3.1763)
At least 5 of CHASM (breast), CHASM (melanoma), FATHMM (missense), Mutation Assessor, PROVEAN, VEST	65.34% (61.62%-69.19%)	47.97% (42.74%-53.13%)	95.37% (92.857%-98.611%)	94.72% (91.94%-98.36%)	51.46% (46.62%-56.2%)	2.8951 (2.7935-3.0054)	65.28% (57.58%-72.73%)	47.65% (37.5%-57.82%)	95.478% (88.889%-100%)	94.8% (87.88%-100%)	51.52% (42.19%-61.11%)	2.8945 (2.6642-3.1028)
At least 5 of CHASM (breast), CHASM (melanoma), FATHMM (missense), Mutation Assessor, SIFT	64.01% (60.1%-67.68%)	44.79% (39.52%-49.61%)	97.233% (95.588%-100%)	96.55% (94.44%-100%)	50.46% (45.71%-55.1%)	2.8903 (2.7929-2.9891)	63.9% (56.57%-71.72%)	44.44% (34.43%-54.69%)	97.269% (92.303%-100%)	96.56% (90.62%-100%)	50.5% (41.43%-60%)	2.8877 (2.6907-3.0794)
At least 5 of CHASM (breast), CHASM (melanoma), FATHMM (missense), Mutation Assessor, SIFT, VEST	72.71% (69.19%-76.26%)	61.16% (55.91%-66.14%)	92.657% (89.331%-96%)	93.51% (90.67%-96.47%)	57.98% (52.89%-62.93%)	3.0531 (2.9387-3.1728)	72.77% (65.66%-79.8%)	61.16% (51.46%-71.22%)	92.645% (85.364%-100%)	93.47% (87.17%-100%)	58.16% (48.27%-68.34%)	3.0543 (2.8158-3.2776)
At least 5 of CHASM (breast), CHASM (melanoma), FATHMM (missense), Mutation Assessor, VEST	58.95% (55.05%-62.63%)	37.35% (32.26%-42.52%)	96.293% (94.203%-98.667%)	94.58% (91.49%-98.04%)	47.06% (42.48%-51.68%)	2.7528 (2.6486-2.8565)	58.87% (51.52%-66.67%)	36.99% (26.56%-46.97%)	96.398% (91.176%-100%)	94.67% (86.96%-100%)	47.14% (38.03%-56.41%)	2.7519 (2.5384-2.9548)
At least 5 of CHASM (breast), CHASM (melanoma), FATHMM (missense), MutationTaster, PolyPhen-2	63.64% (59.6%-67.17%)	44.22% (39.02%-49.19%)	97.233% (95.588%-100%)	96.51% (94.34%-100%)	50.2% (45.51%-54.79%)	2.8816 (2.7812-2.9797)	63.62% (56.57%-71.72%)	44% (33.9%-54.1%)	97.269% (92.303%-100%)	96.53% (90.62%-100%)	50.3% (41.09%-59.73%)	2.8810 (2.6747-3.0756)
At least 5 of CHASM (breast), CHASM (melanoma), FATHMM (missense), MutationTaster, PolyPhen-2, PROVEAN	73.05% (69.7%-76.26%)	59.06% (53.97%-64.46%)	97.233% (95.588%-100%)	97.37% (95.77%-100%)	57.87% (52.99%-62.6%)	3.1154 (3.0163-3.2122)	73.08% (66.67%-79.8%)	58.97% (47.76%-69.35%)	97.269% (92.303%-100%)	97.39% (93.02%-100%)	58.02% (48.33%-67.87%)	3.1164 (2.9289-3.3266)
At least 5 of CHASM (breast), CHASM (melanoma), FATHMM (missense), MutationTaster, PolyPhen-2, SIFT	77.74% (74.24%-81.31%)	68.03% (63.11%-72.95%)	94.52% (91.667%-97.368%)	95.55% (93.26%-97.87%)	63.1% (57.94%-68.11%)	3.2120 (3.1096-3.3252)	77.85% (70.71%-84.85%)	68.16% (58.21%-77.78%)	94.436% (88.232%-100%)	95.47% (90.24%-100%)	63.37% (53.57%-73.33%)	3.2143 (2.9885-3.4263)
At least 5 of CHASM (breast), CHASM (melanoma), FATHMM (missense), MutationTaster, PolyPhen-2, VEST	75.72% (72.22%-78.8%)	65.39% (60.47%-70.31%)	93.581% (90.537%-97.183%)	94.63% (92.13%-97.65%)	61% (55.96%-65.77%)	3.1460 (3.0422-3.2583)	75.83% (69.67%-82.83%)	65.46% (55.56%-75.41%)	93.565% (86.838%-100%)	94.59% (88.88%-100%)	61.23% (51.06%-71.43%)	3.1485 (2.9288-3.3633)
At least 5 of CHASM (breast), CHASM (melanoma), FATHMM (missense), MutationTaster, PROVEAN	56.94% (53.03%-60.61%)	33.09% (28.33%-37.7%)	98.173% (97.015%-100%)	96.91% (94.74%-100%)	45.91% (41.18%-50.32%)	2.7408 (2.6486-2.8325)	56.82% (49.49%-64.65%)	32.73% (23.72%-42.62%)	98.144% (94.118%-100%)	96.81% (90%-100%)	45.95% (37.18%-55.41%)	2.7365 (2.5444-2.9268)
At least 5 of CHASM (breast), CHASM (melanoma), FATHMM (missense), MutationTaster, PROVEAN, SIFT	71.71% (68.18%-75.25%)	56.94% (51.64%-61.91%)	97.233% (95.588%-100%)	97.27% (95.59%-100%)	56.64% (51.67%-61.16%)	3.0809 (2.9860-3.1804)	71.73% (64.65%-78.79%)	56.82% (46.42%-66.68%)	97.269% (92.303%-100%)	97.29% (92.5%-100%)	56.77% (47.46%-66.67%)	3.0814 (2.8798-3.2723)
At least 5 of CHASM (breast), CHASM (melanoma), FATHMM (missense), MutationTaster, PROVEAN, VEST	69.03% (65.15%-72.73%)	52.72% (46.87%-58.14%)	97.233% (95.588%-100%)	97.06% (95.24%-100%)	54.32% (49.59%-58.99%)	3.0133 (2.9177-3.1085)	69.01% (61.62%-76.77%)	52.52% (41.66%-63.34%)	97.269% (92.303%-100%)	97.08% (92.31%-100%)	54.42% (44.83%-64.29%)	3.0129 (2.8276-3.2132)
At least 5 of CHASM (breast), CHASM (melanoma), FATHMM (missense), MutationTaster, SIFT	64.67% (61.11%-68.69%)	45.83% (40.5%-50.82%)	97.233% (95.588%-100%)	96.63% (94.55%-100%)	50.94% (46.04%-55.63%)	2.9063 (2.8034-3.0139)	64.6% (56.57%-71.72%)	45.55% (35.29%-55.88%)	97.269% (92.303%-100%)	96.64% (90.9%-100%)	51% (41.54%-60.88%)	2.9046 (2.6999-3.1137)
At least 5 of CHASM (breast), CHASM (melanoma), FATHMM (missense), MutationTaster, SIFT, VEST	76.06% (72.73%-79.29%)	65.38% (60.46%-70.25%)	94.52% (91.667%-97.368%)	95.38% (92.94%-97.78%)	61.23% (56.25%-66.35%)	3.1651 (3.0591-3.2760)	76.16% (69.7%-82.83%)	65.49% (55.56%-75.81%)	94.436% (88.232%-100%)	95.29% (89.74%-100%)	61.47% (51.67%-71.7%)	3.1668 (2.9420-3.3810)
At least 5 of CHASM (breast), CHASM (melanoma), FATHMM (missense), MutationTaster, VEST	60.3% (56.06%-64.14%)	38.94% (33.84%-44%)	97.233% (95.588%-100%)	96.06% (93.48%-100%)	47.94% (43.24%-52.42%)	2.8017 (2.7049-2.9067)	60.2% (52.53%-68.69%)	38.59% (28.57%-48.62%)	97.269% (92.303%-100%)	96.06% (89.29%-100%)	48% (38.66%-57.75%)	2.7993 (2.5893-2.9943)
At least 5 of CHASM (breast), CHASM (melanoma), FATHMM (missense), PolyPhen-2, PROVEAN	56.95% (53.03%-60.61%)	33.11% (28.12%-37.88%)	98.173% (97.015%-100%)	96.91% (94.74%-100%)	45.91% (41.14%-50.32%)	2.7410 (2.6456-2.8331)	56.8% (49.49%-64.65%)	32.69% (22.95%-42.86%)	98.144% (94.118%-100%)	96.8% (89.47%-100%)	45.93% (37.5%-55.56%)	2.7357 (2.5483-2.9262)
At least 5 of CHASM (breast), CHASM (melanoma), FATHMM (missense), PolyPhen-2, PROVEAN, SIFT	70.7% (67.17%-74.24%)	56.42% (51.51%-61.48%)	95.382% (92.857%-98.592%)	95.49% (93.06%-98.59%)	55.87% (50.82%-60.51%)	3.0315 (2.9287-3.1371)	70.72% (63.64%-77.78%)	56.28% (46.03%-66.67%)	95.447% (89.467%-100%)	95.54% (90%-100%)	55.99% (46.42%-65.61%)	3.0326 (2.8188-3.2430)
At least 5 of CHASM (breast), CHASM (melanoma), FATHMM (missense), PolyPhen-2, PROVEAN, VEST	68.37% (64.65%-72.22%)	52.74% (47.29%-57.85%)	95.37% (92.857%-98.611%)	95.18% (92.65%-98.51%)	53.86% (48.85%-58.54%)	2.9714 (2.8727-3.0812)	68.32% (60.61%-75.76%)	52.46% (41.79%-63.08%)	95.478% (88.889%-100%)	95.25% (89.18%-100%)	53.93% (44.26%-63.8%)	2.9713 (2.7496-3.1675)
At least 5 of CHASM (breast), CHASM (melanoma), FATHMM (missense), PolyPhen-2, SIFT	64.35% (60.61%-68.18%)	45.32% (40%-50.4%)	97.233% (95.588%-100%)	96.59% (94.44%-100%)	50.7% (45.86%-55.3%)	2.8985 (2.8016-2.9991)	64.24% (56.57%-71.72%)	44.97% (34.43%-55.71%)	97.269% (92.303%-100%)	96.6% (90.9%-100%)	50.74% (41.67%-60.56%)	2.8958 (2.6907-3.0929)
At least 5 of CHASM (breast), CHASM (melanoma), FATHMM (missense), PolyPhen-2, SIFT, VEST	74.06% (70.71%-77.78%)	64.88% (60.16%-69.6%)	89.919% (85.937%-94.288%)	91.76% (88.61%-95.29%)	59.69% (54.37%-64.82%)	3.0624 (2.9408-3.1919)	74.11% (66.67%-80.81%)	64.9% (55.17%-74.58%)	89.857% (81.246%-97.37%)	91.68% (84.78%-97.78%)	59.87% (49.98%-70.59%)	3.0630 (2.8132-3.3080)
At least 5 of CHASM (breast), CHASM (melanoma), FATHMM (missense), PolyPhen-2, VEST	59.28% (55.05%-62.63%)	37.87% (32.5%-42.97%)	96.293% (94.203%-98.667%)	94.65% (91.49%-98.08%)	47.27% (42.38%-51.63%)	2.7608 (2.6587-2.8651)	59.22% (52.54%-67.68%)	37.54% (27.12%-48.22%)	96.398% (91.176%-100%)	94.74% (87.49%-100%)	47.35% (38.57%-57.14%)	2.7603 (2.5458-2.9655)
At least 5 of CHASM (breast), CHASM (melanoma), FATHMM (missense), PROVEAN, SIFT	58.97% (55.04%-62.63%)	36.3% (31.45%-40.94%)	98.173% (97.015%-100%)	97.17% (95.24%-100%)	47.13% (42.21%-51.61%)	2.7877 (2.6957-2.8815)	58.82% (51.52%-66.69%)	35.9% (26.31%-45.9%)	98.144% (94.118%-100%)	97.09% (90.48%-100%)	47.15% (38.36%-56.79%)	2.7827 (2.5878-2.9704)
At least 5 of CHASM (breast), CHASM (melanoma), FATHMM (missense), PROVEAN, SIFT, VEST	68.01% (64.65%-71.72%)	51.64% (46.15%-56.8%)	96.309% (94.203%-98.702%)	96.04% (93.75%-98.57%)	53.53% (48.53%-58.09%)	2.9752 (2.8767-3.0802)	68.01% (60.61%-74.75%)	51.47% (41.17%-62.13%)	96.35% (90.625%-100%)	96.07% (90.32%-100%)	53.64% (44.44%-63.28%)	2.9753 (2.7701-3.1815)
At least 5 of CHASM (breast), CHASM (melanoma), FATHMM (missense), PROVEAN, VEST	56.28% (52.02%-60.1%)	32.05% (27.13%-36.72%)	98.173% (97.015%-100%)	96.81% (94.59%-100%)	45.52% (40.88%-49.7%)	2.7255 (2.6318-2.8196)	56.13% (48.48%-64.65%)	31.63% (22.22%-41.54%)	98.144% (94.118%-100%)	96.71% (89.47%-100%)	45.55% (37.03%-55.13%)	2.7203 (2.5283-2.9097)
At least 5 of CHASM (breast), CHASM (melanoma), FATHMM (missense), SIFT, VEST	60.65% (56.57%-64.14%)	39.48% (34.39%-44.63%)	97.233% (95.588%-100%)	96.11% (93.62%-100%)	48.17% (43.45%-52.67%)	2.8099 (2.7129-2.9118)	60.53% (53.54%-68.69%)	39.1% (28.84%-50%)	97.269% (92.303%-100%)	96.11% (89.29%-100%)	48.21% (38.96%-57.9%)	2.8069 (2.5996-3.0100)
At least 5 of CHASM (breast), CHASM (melanoma), Mutation Assessor, MutationTaster, PolyPhen-2	60.93% (57.07%-64.65%)	40.47% (35.29%-45.46%)	96.311% (94.286%-98.667%)	94.99% (92%-98.21%)	48.34% (43.66%-52.82%)	2.8012 (2.6985-2.9099)	60.96% (53.54%-68.69%)	40.32% (30.3%-50.79%)	96.355% (91.176%-100%)	95% (87.5%-100%)	48.48% (39.19%-57.81%)	2.8016 (2.5793-3.0050)
At least 5 of CHASM (breast), CHASM (melanoma), Mutation Assessor, MutationTaster, PolyPhen-2, PROVEAN	69.71% (66.16%-73.23%)	54.32% (49.19%-59.68%)	96.311% (94.286%-98.667%)	96.22% (94.03%-98.67%)	54.94% (50%-59.52%)	3.0180 (2.9224-3.1205)	69.67% (62.63%-76.77%)	54.1% (43.55%-64.29%)	96.355% (91.176%-100%)	96.23% (90.62%-100%)	55.02% (45.76%-65.08%)	3.0171 (2.8096-3.2174)
At least 5 of CHASM (breast), CHASM (melanoma), Mutation Assessor, MutationTaster, PolyPhen-2, SIFT	75.76% (72.22%-79.29%)	64.91% (60%-69.67%)	94.52% (91.667%-97.368%)	95.35% (92.94%-97.78%)	60.91% (55.66%-65.77%)	3.1568 (3.0547-3.2709)	75.75% (68.69%-82.83%)	64.84% (54.79%-74.58%)	94.436% (88.232%-100%)	95.24% (89.47%-100%)	61.02% (50.98%-71.19%)	3.1553 (2.9326-3.3657)
At least 5 of CHASM (breast), CHASM (melanoma), Mutation Assessor, MutationTaster, PolyPhen-2, VEST	75.06% (71.72%-78.28%)	64.35% (59.38%-69.11%)	93.581% (90.537%-97.183%)	94.55% (92.05%-97.62%)	60.29% (55.24%-65.42%)	3.1276 (3.0176-3.2398)	75.13% (68.69%-81.82%)	64.36% (54.68%-74.14%)	93.565% (86.838%-100%)	94.5% (88.37%-100%)	60.48% (50.8%-70.49%)	3.1291 (2.9068-3.3530)
At least 5 of CHASM (breast), CHASM (melanoma), Mutation Assessor, MutationTaster, PROVEAN	55.92% (51.52%-59.6%)	31.48% (26.72%-36%)	98.173% (97.015%-100%)	96.75% (94.44%-100%)	45.31% (40.62%-49.69%)	2.7171 (2.6219-2.8085)	55.84% (48.48%-64.65%)	31.19% (22.06%-40.99%)	98.144% (94.118%-100%)	96.65% (89.47%-100%)	45.39% (36.49%-54.8%)	2.7137 (2.5268-2.9058)
At least 5 of CHASM (breast), CHASM (melanoma), Mutation Assessor, MutationTaster, PROVEAN, SIFT	70.71% (67.17%-74.24%)	55.9% (50.78%-61.11%)	96.311% (94.286%-98.667%)	96.33% (94.2%-98.68%)	55.81% (50.85%-60.47%)	3.0435 (2.9485-3.1469)	70.69% (63.64%-77.78%)	55.71% (45.45%-65.63%)	96.355% (91.176%-100%)	96.34% (90.91%-100%)	55.91% (46.15%-65.67%)	3.0431 (2.8381-3.2430)
At least 5 of CHASM (breast), CHASM (melanoma), Mutation Assessor, MutationTaster, PROVEAN, VEST	68.05% (64.65%-71.72%)	51.69% (46.4%-56.8%)	96.311% (94.286%-98.667%)	96.04% (93.75%-98.59%)	53.56% (48.84%-58.27%)	2.9760 (2.8754-3.0806)	67.95% (60.61%-74.75%)	51.36% (41.07%-61.76%)	96.355% (91.176%-100%)	96.04% (90.31%-100%)	53.59% (43.74%-63.24%)	2.9735 (2.7688-3.1682)
At least 5 of CHASM (breast), CHASM (melanoma), Mutation Assessor, MutationTaster, SIFT	61.95% (58.08%-65.66%)	42.08% (36.8%-46.92%)	96.311% (94.286%-98.667%)	95.18% (92.31%-98.31%)	49.02% (44.37%-53.79%)	2.8259 (2.7248-2.9311)	61.95% (54.55%-69.7%)	41.89% (32.2%-52.24%)	96.355% (91.176%-100%)	95.18% (88%-100%)	49.14% (39.72%-58.57%)	2.8257 (2.6087-3.0280)
At least 5 of CHASM (breast), CHASM (melanoma), Mutation Assessor, MutationTaster, SIFT, VEST	75.75% (72.71%-79.29%)	64.89% (60%-69.7%)	94.52% (91.667%-97.368%)	95.34% (92.94%-97.78%)	60.89% (55.96%-65.77%)	3.1565 (3.0478-3.2711)	75.77% (68.69%-81.84%)	64.88% (55%-74.58%)	94.436% (88.232%-100%)	95.25% (89.74%-100%)	61.04% (51.55%-70.97%)	3.1560 (2.9314-3.3680)
At least 5 of CHASM (breast), CHASM (melanoma), Mutation Assessor, MutationTaster, VEST	58.26% (54.04%-62.12%)	36.25% (31.01%-41.47%)	96.311% (94.286%-98.667%)	94.44% (91.11%-98.04%)	46.63% (41.89%-51.3%)	2.7363 (2.6282-2.8432)	58.23% (50.51%-66.67%)	36.01% (25.67%-46.88%)	96.355% (91.176%-100%)	94.43% (86.36%-100%)	46.74% (37.5%-56.41%)	2.7353 (2.5120-2.9494)
At least 5 of CHASM (breast), CHASM (melanoma), Mutation Assessor, PolyPhen-2, PROVEAN	56.59% (52.53%-60.61%)	32.54% (27.59%-37.01%)	98.173% (97.015%-100%)	96.85% (94.59%-100%)	45.7% (41.13%-50%)	2.7327 (2.6393-2.8271)	56.51% (48.48%-64.65%)	32.24% (22.95%-42.37%)	98.144% (94.118%-100%)	96.76% (89.99%-100%)	45.77% (37.18%-55.01%)	2.7290 (2.5430-2.9162)
At least 5 of CHASM (breast), CHASM (melanoma), Mutation Assessor, PolyPhen-2, PROVEAN, SIFT	69.37% (65.66%-72.73%)	55.39% (50.4%-60.63%)	93.512% (90.411%-97.143%)	93.66% (90.67%-97.18%)	54.8% (49.61%-59.5%)	2.9737 (2.8598-3.0867)	69.35% (62.63%-76.77%)	55.14% (44.77%-65.08%)	93.683% (87.09%-100%)	93.76% (87.18%-100%)	54.89% (45.16%-65%)	2.9747 (2.7520-3.2132)
At least 5 of CHASM (breast), CHASM (melanoma), Mutation Assessor, PolyPhen-2, PROVEAN, VEST	66.69% (63.13%-70.2%)	50.63% (45.45%-55.82%)	94.448% (91.667%-97.368%)	94.04% (91.04%-97.18%)	52.53% (47.69%-57.14%)	2.9164 (2.8078-3.0286)	66.62% (59.6%-73.74%)	50.3% (39.68%-60.32%)	94.564% (88.095%-100%)	94.1% (87.09%-100%)	52.58% (43.28%-62.5%)	2.9155 (2.6873-3.1297)
At least 5 of CHASM (breast), CHASM (melanoma), Mutation Assessor, PolyPhen-2, SIFT	62.97% (59.09%-66.67%)	43.69% (38.33%-48.78%)	96.311% (94.286%-98.667%)	95.34% (92.59%-98.33%)	49.73% (45.07%-54.29%)	2.8507 (2.7461-2.9602)	62.95% (55.56%-70.71%)	43.46% (32.79%-53.34%)	96.355% (91.176%-100%)	95.35% (88.46%-100%)	49.83% (40.57%-59.7%)	2.8499 (2.6320-3.0652)
At least 5 of CHASM (breast), CHASM (melanoma), Mutation Assessor, PolyPhen-2, SIFT, VEST	74.08% (70.71%-77.78%)	64.36% (59.37%-69.29%)	90.866% (87.324%-94.805%)	92.42% (89.53%-95.56%)	59.59% (54.45%-64.55%)	3.0724 (2.9595-3.1935)	74.07% (66.67%-80.81%)	64.33% (54.55%-74.14%)	90.721% (82.5%-97.37%)	92.26% (85.71%-97.83%)	59.72% (50%-69.82%)	3.0703 (2.8241-3.2966)
At least 5 of CHASM (breast), CHASM (melanoma), Mutation Assessor, PolyPhen-2, VEST	57.91% (54.04%-61.62%)	36.24% (31.03%-41.27%)	95.372% (92.955%-98.571%)	93.12% (89.58%-97.87%)	46.39% (41.67%-50.98%)	2.7113 (2.5983-2.8215)	57.91% (50.51%-65.66%)	36% (26.15%-46.16%)	95.484% (89.651%-100%)	93.2% (84.21%-100%)	46.51% (37.33%-55.84%)	2.7120 (2.4855-2.9286)
At least 5 of CHASM (breast), CHASM (melanoma), Mutation Assessor, PROVEAN, SIFT	57.61% (53.54%-61.62%)	34.15% (29.41%-38.71%)	98.173% (97.015%-100%)	97% (94.87%-100%)	46.3% (41.61%-50.65%)	2.7562 (2.6614-2.8492)	57.51% (49.49%-65.66%)	33.82% (24.56%-43.75%)	98.144% (94.118%-100%)	96.9% (90%-100%)	46.35% (37.66%-55.7%)	2.7522 (2.5611-2.9437)
At least 5 of CHASM (breast), CHASM (melanoma), Mutation Assessor, PROVEAN, SIFT, VEST	67.71% (64.14%-71.21%)	51.7% (46.51%-56.69%)	95.388% (92.958%-98.592%)	95.1% (92.53%-98.46%)	53.32% (48.46%-58.02%)	2.9551 (2.8582-3.0675)	67.6% (60.61%-74.75%)	51.35% (40.98%-61.67%)	95.436% (89.189%-100%)	95.1% (88.89%-100%)	53.34% (43.55%-62.86%)	2.9522 (2.7360-3.1515)
At least 5 of CHASM (breast), CHASM (melanoma), Mutation Assessor, PROVEAN, VEST	55.58% (51.52%-59.6%)	30.94% (26.19%-35.48%)	98.173% (97.015%-100%)	96.7% (94.44%-100%)	45.12% (40.38%-49.38%)	2.7093 (2.6162-2.8036)	55.51% (47.47%-63.64%)	30.66% (21.43%-40.36%)	98.144% (94.118%-100%)	96.6% (88.89%-100%)	45.2% (36.47%-54.55%)	2.7060 (2.5100-2.8925)
At least 5 of CHASM (breast), CHASM (melanoma), Mutation Assessor, SIFT, VEST	59.28% (55.56%-63.13%)	37.85% (33.06%-42.97%)	96.311% (94.286%-98.667%)	94.67% (91.49%-98.08%)	47.27% (42.66%-51.97%)	2.7610 (2.6557-2.8707)	59.23% (51.52%-66.67%)	37.57% (27.41%-47.46%)	96.355% (91.176%-100%)	94.66% (86.96%-100%)	47.36% (38.23%-56.72%)	2.7594 (2.5378-2.9676)
At least 5 of CHASM (breast), CHASM (melanoma), MutationTaster, PolyPhen-2, PROVEAN	56.92% (52.53%-60.61%)	33.06% (27.96%-37.88%)	98.173% (97.015%-100%)	96.9% (94.74%-100%)	45.89% (41.14%-50%)	2.7403 (2.6428-2.8315)	56.87% (49.49%-65.66%)	32.8% (23.43%-42.86%)	98.144% (94.118%-100%)	96.81% (90%-100%)	45.98% (37.5%-55.56%)	2.7373 (2.5458-2.9329)
At least 5 of CHASM (breast), CHASM (melanoma), MutationTaster, PolyPhen-2, PROVEAN, SIFT	71.72% (68.18%-74.76%)	57.49% (52.5%-62.4%)	96.311% (94.286%-98.667%)	96.42% (94.37%-98.72%)	56.72% (51.94%-61.29%)	3.0694 (2.9714-3.1708)	71.71% (65.63%-78.79%)	57.33% (47.46%-67.19%)	96.355% (91.176%-100%)	96.44% (91.18%-100%)	56.8% (47.27%-66.57%)	3.0694 (2.8669-3.2718)
At least 5 of CHASM (breast), CHASM (melanoma), MutationTaster, PolyPhen-2, PROVEAN, VEST	69.73% (66.16%-73.23%)	54.35% (49.18%-59.65%)	96.311% (94.286%-98.667%)	96.23% (94.12%-98.65%)	54.96% (50.39%-59.85%)	3.0185 (2.9169-3.1181)	69.63% (62.63%-76.77%)	54.04% (43.75%-64.18%)	96.355% (91.176%-100%)	96.23% (90.62%-100%)	54.99% (45.44%-64.52%)	3.0162 (2.8183-3.2174)
At least 5 of CHASM (breast), CHASM (melanoma), MutationTaster, PolyPhen-2, SIFT	63.3% (59.09%-67.17%)	44.2% (39.02%-49.19%)	96.311% (94.286%-98.667%)	95.4% (92.73%-98.36%)	49.96% (45.18%-54.35%)	2.8586 (2.7546-2.9645)	63.31% (55.56%-71.72%)	44.04% (33.9%-54.1%)	96.355% (91.176%-100%)	95.4% (88.46%-100%)	50.08% (40.91%-59.72%)	2.8588 (2.6427-3.0697)
At least 5 of CHASM (breast), CHASM (melanoma), MutationTaster, PolyPhen-2, SIFT, VEST	76.76% (73.74%-80.3%)	66.48% (61.98%-71.2%)	94.52% (91.667%-97.368%)	95.45% (93.1%-97.83%)	61.99% (57.14%-67.27%)	3.1845 (3.0808-3.2960)	76.78% (69.7%-82.83%)	66.47% (56.89%-75.38%)	94.436% (88.232%-100%)	95.36% (89.79%-100%)	62.14% (51.79%-71.93%)	3.1841 (2.9619-3.4022)
At least 5 of CHASM (breast), CHASM (melanoma), MutationTaster, PolyPhen-2, VEST	60.27% (56.06%-64.14%)	39.42% (34.15%-44.54%)	96.311% (94.286%-98.667%)	94.87% (91.84%-98.18%)	47.9% (43.24%-52.29%)	2.7850 (2.6799-2.8987)	60.28% (52.53%-68.69%)	39.24% (29.02%-50%)	96.355% (91.176%-100%)	94.87% (87.49%-100%)	48.03% (39.13%-57.75%)	2.7850 (2.5586-2.9953)
At least 5 of CHASM (breast), CHASM (melanoma), MutationTaster, PROVEAN, SIFT	58.94% (55.05%-62.63%)	36.24% (31.45%-41.22%)	98.173% (97.015%-100%)	97.17% (95.24%-100%)	47.11% (42.28%-51.35%)	2.7869 (2.6932-2.8770)	58.89% (51.52%-66.67%)	36.01% (26.23%-45.9%)	98.144% (94.118%-100%)	97.09% (90.48%-100%)	47.19% (38.36%-56.94%)	2.7843 (2.6011-2.9812)
At least 5 of CHASM (breast), CHASM (melanoma), MutationTaster, PROVEAN, SIFT, VEST	68.7% (65.15%-72.22%)	52.72% (47.33%-57.9%)	96.311% (94.286%-98.667%)	96.11% (93.85%-98.61%)	54.09% (49.25%-58.87%)	2.9923 (2.8911-3.0938)	68.67% (61.62%-75.76%)	52.51% (41.94%-62.72%)	96.355% (91.176%-100%)	96.12% (90.32%-100%)	54.18% (44.44%-63.77%)	2.9917 (2.7898-3.1933)
At least 5 of CHASM (breast), CHASM (melanoma), MutationTaster, PROVEAN, VEST	56.92% (52.53%-60.61%)	33.06% (28.33%-37.82%)	98.173% (97.015%-100%)	96.9% (94.74%-100%)	45.89% (41.14%-50.31%)	2.7403 (2.6428-2.8331)	56.86% (49.49%-65.66%)	32.8% (23.21%-42.43%)	98.144% (94.118%-100%)	96.81% (89.99%-100%)	45.98% (37.18%-55.56%)	2.7373 (2.5445-2.9331)
At least 5 of CHASM (breast), CHASM (melanoma), MutationTaster, SIFT, VEST	60.95% (57.07%-64.65%)	40.5% (35.2%-45.74%)	96.311% (94.286%-98.667%)	95% (92%-98.21%)	48.35% (43.59%-52.7%)	2.8016 (2.7001-2.9073)	60.92% (53.54%-68.69%)	40.26% (29.68%-50.85%)	96.355% (91.176%-100%)	94.99% (87.5%-100%)	48.46% (39.13%-58.34%)	2.8007 (2.5891-3.0043)
At least 5 of CHASM (breast), CHASM (melanoma), PolyPhen-2, PROVEAN, SIFT	58.95% (55.05%-62.63%)	36.26% (31.2%-40.99%)	98.173% (97.015%-100%)	97.17% (95.24%-100%)	47.11% (42.18%-51.35%)	2.7872 (2.6908-2.8819)	58.87% (51.52%-66.67%)	35.96% (26.32%-46.03%)	98.144% (94.118%-100%)	97.08% (90.48%-100%)	47.18% (38.36%-56.96%)	2.7836 (2.5898-2.9760)
At least 5 of CHASM (breast), CHASM (melanoma), PolyPhen-2, PROVEAN, SIFT, VEST	68.37% (64.65%-71.72%)	52.74% (47.54%-57.6%)	95.388% (92.958%-98.592%)	95.19% (92.54%-98.51%)	53.86% (48.9%-58.46%)	2.9719 (2.8669-3.0759)	68.3% (61.62%-75.76%)	52.46% (41.94%-62.69%)	95.436% (89.189%-100%)	95.2% (89.19%-100%)	53.91% (44.44%-63.5%)	2.9701 (2.7520-3.1829)
At least 5 of CHASM (breast), CHASM (melanoma), PolyPhen-2, PROVEAN, VEST	56.58% (52.53%-60.61%)	32.52% (27.56%-37.12%)	98.173% (97.015%-100%)	96.85% (94.59%-100%)	45.7% (40.88%-50%)	2.7324 (2.6354-2.8252)	56.54% (49.49%-64.65%)	32.27% (22.8%-42.03%)	98.144% (94.118%-100%)	96.76% (89.47%-100%)	45.78% (37.18%-55.41%)	2.7296 (2.5435-2.9262)
At least 5 of CHASM (breast), CHASM (melanoma), PolyPhen-2, SIFT, VEST	60.27% (56.06%-64.14%)	39.43% (34.15%-44.7%)	96.311% (94.286%-98.667%)	94.87% (91.84%-98.18%)	47.91% (43.15%-52.2%)	2.7851 (2.6783-2.8945)	60.26% (52.53%-68.69%)	39.21% (28.81%-50%)	96.355% (91.176%-100%)	94.87% (86.96%-100%)	48.02% (39.24%-57.54%)	2.7846 (2.5605-2.9979)
At least 5 of CHASM (breast), CHASM (melanoma), PROVEAN, SIFT, VEST	58.28% (54.04%-62.12%)	35.2% (30.3%-39.84%)	98.173% (97.015%-100%)	97.09% (95.12%-100%)	46.7% (42%-50.98%)	2.7716 (2.6760-2.8613)	58.2% (50.51%-66.67%)	34.91% (25.36%-45.45%)	98.144% (94.118%-100%)	97% (90%-100%)	46.77% (37.97%-56.01%)	2.7682 (2.5791-2.9598)
At least 5 of CHASM (breast), FATHMM (cancer), FATHMM (missense), Mutation Assessor, MutationTaster	61.64% (57.58%-65.66%)	41.06% (35.88%-45.9%)	97.233% (95.588%-100%)	96.25% (94%-100%)	48.82% (44.14%-53.42%)	2.8337 (2.7371-2.9306)	61.56% (53.54%-69.7%)	40.75% (31.15%-51.47%)	97.269% (92.303%-100%)	96.26% (89.99%-100%)	48.89% (39.73%-58.46%)	2.8317 (2.6322-3.0286)
At least 5 of CHASM (breast), FATHMM (cancer), FATHMM (missense), Mutation Assessor, MutationTaster, PolyPhen-2	75.44% (72.22%-78.79%)	65.46% (60.66%-70.16%)	92.682% (89.474%-96.001%)	93.93% (91.36%-96.81%)	60.82% (55.77%-65.8%)	3.1289 (3.0170-3.2412)	75.38% (68.69%-81.82%)	65.31% (55.56%-74.66%)	92.611% (85.294%-100%)	93.82% (87.5%-100%)	60.88% (50.91%-70.69%)	3.1261 (2.9016-3.3530)
At least 5 of CHASM (breast), FATHMM (cancer), FATHMM (missense), Mutation Assessor, MutationTaster, PROVEAN	69.07% (65.66%-72.73%)	53.83% (48.74%-59.06%)	95.413% (92.955%-98.53%)	95.31% (92.65%-98.55%)	54.45% (49.61%-59.06%)	2.9899 (2.8845-3.0942)	68.93% (61.62%-75.76%)	53.48% (42.62%-63.34%)	95.402% (89.655%-100%)	95.24% (89.19%-100%)	54.44% (44.93%-64.18%)	2.9857 (2.7709-3.1884)
At least 5 of CHASM (breast), FATHMM (cancer), FATHMM (missense), Mutation Assessor, MutationTaster, SIFT	75.44% (72.22%-78.79%)	64.92% (60.15%-69.67%)	93.622% (90.541%-97.183%)	94.62% (92.05%-97.67%)	60.69% (55.56%-65.72%)	3.1386 (3.0311-3.2532)	75.38% (68.69%-81.82%)	64.8% (54.84%-74.51%)	93.483% (86.667%-100%)	94.46% (88.37%-100%)	60.75% (50.85%-70.92%)	3.1349 (2.9068-3.3536)
At least 5 of CHASM (breast), FATHMM (cancer), FATHMM (missense), Mutation Assessor, MutationTaster, VEST	73.42% (70.2%-76.77%)	62.28% (57.48%-67.2%)	92.682% (89.474%-96.001%)	93.64% (90.91%-96.63%)	58.69% (53.57%-63.56%)	3.0729 (2.9695-3.1856)	73.36% (66.67%-79.8%)	62.11% (52.31%-71.64%)	92.611% (85.294%-100%)	93.52% (86.84%-100%)	58.75% (49.09%-68.97%)	3.0700 (2.8469-3.2850)
At least 5 of CHASM (breast), FATHMM (cancer), FATHMM (missense), Mutation Assessor, PolyPhen-2	62.66% (58.59%-66.16%)	43.2% (37.93%-48.07%)	96.293% (94.203%-98.667%)	95.28% (92.59%-98.28%)	49.51% (44.68%-54%)	2.8428 (2.7439-2.9508)	62.57% (55.56%-70.71%)	42.84% (32.79%-53.03%)	96.398% (91.176%-100%)	95.35% (88.89%-100%)	49.56% (40.84%-59.09%)	2.8415 (2.6268-3.0406)
At least 5 of CHASM (breast), FATHMM (cancer), FATHMM (missense), Mutation Assessor, PolyPhen-2, PROVEAN	67.06% (63.64%-70.71%)	53.86% (48.78%-59.06%)	89.892% (85.915%-94.366%)	90.21% (86.42%-94.37%)	52.98% (47.62%-57.94%)	2.8694 (2.7472-2.9896)	66.88% (59.6%-73.74%)	53.43% (42.86%-63.24%)	89.927% (81.471%-97.37%)	90.11% (82.5%-97.44%)	52.95% (43.28%-63.24%)	2.8642 (2.6184-3.1072)
At least 5 of CHASM (breast), FATHMM (cancer), FATHMM (missense), Mutation Assessor, PolyPhen-2, SIFT	72.07% (68.69%-75.76%)	62.81% (58.14%-67.48%)	88.071% (84%-92.756%)	90.11% (86.66%-94.05%)	57.8% (52.43%-62.84%)	2.9878 (2.8703-3.1161)	72.02% (64.65%-78.79%)	62.65% (52.83%-72.41%)	88.065% (78.782%-96.78%)	90.03% (82.35%-97.37%)	57.87% (48%-67.93%)	2.9861 (2.7311-3.2155)
At least 5 of CHASM (breast), FATHMM (cancer), FATHMM (missense), Mutation Assessor, PolyPhen-2, VEST	71.74% (68.18%-75.25%)	62.82% (58.14%-67.69%)	87.166% (82.812%-92%)	89.44% (85.88%-93.26%)	57.55% (52.29%-62.63%)	2.9697 (2.8537-3.0916)	71.66% (64.65%-78.79%)	62.63% (53.03%-71.88%)	87.126% (77.778%-95.25%)	89.32% (81.4%-96%)	57.6% (47.46%-67.8%)	2.9667 (2.7147-3.1939)
At least 5 of CHASM (breast), FATHMM (cancer), FATHMM (missense), Mutation Assessor, PROVEAN	56.95% (53.02%-61.11%)	33.11% (28.23%-37.8%)	98.173% (97.015%-100%)	96.91% (94.74%-100%)	45.91% (41.14%-50.32%)	2.7411 (2.6487-2.8347)	56.8% (48.48%-64.67%)	32.69% (23.43%-42.62%)	98.144% (94.118%-100%)	96.8% (89.47%-100%)	45.94% (37.18%-55.41%)	2.7357 (2.5444-2.9204)
At least 5 of CHASM (breast), FATHMM (cancer), FATHMM (missense), Mutation Assessor, PROVEAN, SIFT	68.06% (64.65%-71.72%)	54.35% (49.16%-59.53%)	91.737% (88.233%-95.776%)	91.92% (88.24%-95.83%)	53.75% (48.74%-58.73%)	2.9177 (2.8059-3.0324)	67.93% (60.61%-74.75%)	54.03% (43.54%-64.18%)	91.737% (83.784%-100%)	91.84% (84.21%-100%)	53.76% (43.93%-63.33%)	2.9136 (2.6827-3.1341)
At least 5 of CHASM (breast), FATHMM (cancer), FATHMM (missense), Mutation Assessor, PROVEAN, VEST	64.05% (60.1%-67.68%)	49.1% (44.06%-54.33%)	89.892% (85.915%-94.366%)	89.36% (85.24%-93.85%)	50.53% (45.52%-55.31%)	2.7889 (2.6609-2.9068)	63.82% (56.57%-71.72%)	48.57% (37.74%-58.34%)	89.927% (81.471%-97.37%)	89.23% (80.95%-97.3%)	50.47% (40.98%-60.32%)	2.7820 (2.5431-3.0334)
At least 5 of CHASM (breast), FATHMM (cancer), FATHMM (missense), Mutation Assessor, SIFT	63.68% (60.09%-67.18%)	44.27% (39.17%-49.18%)	97.233% (95.588%-100%)	96.51% (94.34%-100%)	50.23% (45.45%-54.8%)	2.8825 (2.7882-2.9827)	63.55% (56.54%-70.73%)	43.88% (34.36%-53.52%)	97.269% (92.303%-100%)	96.52% (90.47%-100%)	50.25% (41.27%-59.72%)	2.8791 (2.6777-3.0711)
At least 5 of CHASM (breast), FATHMM (cancer), FATHMM (missense), Mutation Assessor, SIFT, VEST	71.73% (68.18%-75.25%)	62.27% (57.48%-67.2%)	88.071% (84%-92.756%)	90.03% (86.59%-94.05%)	57.45% (52.34%-62.39%)	2.9782 (2.8601-3.1017)	71.69% (64.65%-78.79%)	62.12% (52.3%-71.93%)	88.065% (78.782%-96.78%)	89.95% (82.22%-97.37%)	57.53% (47.98%-67.74%)	2.9767 (2.7338-3.2152)
At least 5 of CHASM (breast), FATHMM (cancer), FATHMM (missense), Mutation Assessor, VEST	58.62% (54.55%-62.63%)	36.83% (31.62%-41.86%)	96.293% (94.203%-98.667%)	94.51% (91.3%-98%)	46.86% (42.21%-51.35%)	2.7449 (2.6387-2.8492)	58.51% (50.51%-66.67%)	36.43% (26.47%-46.56%)	96.398% (91.176%-100%)	94.59% (86.96%-100%)	46.91% (37.84%-56.25%)	2.7433 (2.5314-2.9494)
At least 5 of CHASM (breast), FATHMM (cancer), FATHMM (missense), MutationTaster, PolyPhen-2	63.67% (59.6%-67.17%)	44.25% (39.06%-49.23%)	97.233% (95.588%-100%)	96.51% (94.34%-100%)	50.22% (45.32%-54.86%)	2.8821 (2.7823-2.9790)	63.58% (56.57%-71.72%)	43.93% (33.85%-54.39%)	97.269% (92.303%-100%)	96.52% (90.62%-100%)	50.27% (40.91%-59.73%)	2.8800 (2.6778-3.0806)
At least 5 of CHASM (breast), FATHMM (cancer), FATHMM (missense), MutationTaster, PolyPhen-2, PROVEAN	73.43% (69.7%-76.77%)	60.71% (55.64%-65.93%)	95.413% (92.955%-98.53%)	95.82% (93.42%-98.68%)	58.42% (53.33%-63.16%)	3.1036 (2.9941-3.2062)	73.34% (66.67%-80.81%)	60.46% (50%-70.59%)	95.402% (89.655%-100%)	95.77% (90.48%-100%)	58.45% (48.97%-68.97%)	3.1008 (2.9001-3.3170)
At least 5 of CHASM (breast), FATHMM (cancer), FATHMM (missense), MutationTaster, PolyPhen-2, SIFT	79.13% (76.26%-82.32%)	70.75% (66.14%-75.41%)	93.622% (90.541%-97.183%)	95.04% (92.68%-97.85%)	64.93% (60%-69.91%)	3.2435 (3.1415-3.3566)	79.1% (72.73%-85.86%)	70.7% (61.11%-79.69%)	93.483% (86.667%-100%)	94.9% (89.36%-100%)	65.04% (55.17%-75%)	3.2412 (3.0189-3.4505)
At least 5 of CHASM (breast), FATHMM (cancer), FATHMM (missense), MutationTaster, PolyPhen-2, VEST	76.44% (73.23%-79.8%)	67.03% (62.5%-71.88%)	92.682% (89.474%-96.001%)	94.06% (91.56%-96.91%)	61.92% (56.76%-66.97%)	3.1570 (3.0458-3.2707)	76.42% (69.7%-82.83%)	66.96% (56.92%-75.86%)	92.611% (85.294%-100%)	93.96% (87.5%-100%)	62.04% (52.08%-72.41%)	3.1557 (2.9326-3.3750)
At least 5 of CHASM (breast), FATHMM (cancer), FATHMM (missense), MutationTaster, PROVEAN	57.29% (53.03%-61.11%)	33.64% (29.03%-38.4%)	98.173% (97.015%-100%)	96.96% (94.87%-100%)	46.11% (41.4%-50.33%)	2.7488 (2.6540-2.8403)	57.14% (49.49%-65.66%)	33.23% (23.81%-42.65%)	98.144% (94.118%-100%)	96.86% (90%-100%)	46.14% (37.33%-55.56%)	2.7437 (2.5529-2.9401)
At least 5 of CHASM (breast), FATHMM (cancer), FATHMM (missense), MutationTaster, PROVEAN, SIFT	72.1% (68.69%-75.76%)	58.61% (53.6%-63.78%)	95.413% (92.955%-98.53%)	95.67% (93.24%-98.67%)	57.14% (52.1%-61.79%)	3.0683 (2.9638-3.1741)	71.97% (64.65%-78.79%)	58.29% (47.76%-68.12%)	95.402% (89.655%-100%)	95.61% (90%-100%)	57.14% (47.46%-67.17%)	3.0644 (2.8499-3.2723)
At least 5 of CHASM (breast), FATHMM (cancer), FATHMM (missense), MutationTaster, PROVEAN, VEST	68.73% (65.15%-72.22%)	53.3% (47.93%-58.68%)	95.413% (92.955%-98.53%)	95.26% (92.65%-98.48%)	54.16% (49.23%-58.82%)	2.9814 (2.8720-3.0866)	68.59% (61.62%-75.76%)	52.95% (42.37%-63.64%)	95.402% (89.655%-100%)	95.19% (89.29%-100%)	54.17% (44.44%-64.29%)	2.9770 (2.7667-3.1917)
At least 5 of CHASM (breast), FATHMM (cancer), FATHMM (missense), MutationTaster, SIFT	64.34% (60.61%-68.18%)	45.32% (40%-50.39%)	97.233% (95.588%-100%)	96.59% (94.44%-100%)	50.7% (45.86%-55.32%)	2.8984 (2.7989-3.0021)	64.24% (56.57%-71.72%)	44.99% (35.18%-55.01%)	97.269% (92.303%-100%)	96.6% (90.9%-100%)	50.74% (41.18%-60.3%)	2.8960 (2.6860-3.1007)
At least 5 of CHASM (breast), FATHMM (cancer), FATHMM (missense), MutationTaster, SIFT, VEST	76.78% (73.74%-80.3%)	67.04% (62.39%-71.78%)	93.622% (90.541%-97.183%)	94.78% (92.31%-97.75%)	62.17% (57.02%-66.98%)	3.1761 (3.0711-3.2861)	76.73% (69.7%-82.83%)	66.95% (56.92%-76.27%)	93.483% (86.667%-100%)	94.63% (88.64%-100%)	62.25% (52.3%-72.55%)	3.1731 (2.9572-3.3853)
At least 5 of CHASM (breast), FATHMM (cancer), FATHMM (missense), MutationTaster, VEST	60.32% (56.06%-64.14%)	38.97% (33.88%-44%)	97.233% (95.588%-100%)	96.06% (93.48%-100%)	47.96% (43.24%-52.63%)	2.8023 (2.7029-2.9041)	60.16% (52.53%-68.69%)	38.53% (28.57%-48.39%)	97.269% (92.303%-100%)	96.06% (89.29%-100%)	47.97% (38.57%-57.75%)	2.7983 (2.5918-2.9971)
At least 5 of CHASM (breast), FATHMM (cancer), FATHMM (missense), PolyPhen-2, PROVEAN	57.3% (53.54%-61.11%)	33.66% (28.8%-38.58%)	98.173% (97.015%-100%)	96.96% (94.87%-100%)	46.12% (41.33%-50.33%)	2.7491 (2.6548-2.8428)	57.11% (49.49%-64.65%)	33.19% (23.33%-43.33%)	98.144% (94.118%-100%)	96.85% (89.99%-100%)	46.12% (37.5%-55.7%)	2.7430 (2.5507-2.9292)
At least 5 of CHASM (breast), FATHMM (cancer), FATHMM (missense), PolyPhen-2, PROVEAN, SIFT	70.07% (66.67%-73.74%)	58.08% (52.89%-63.12%)	90.786% (86.957%-94.667%)	91.6% (88.16%-95.12%)	55.6% (50.44%-60.53%)	2.9607 (2.8358-3.0847)	69.97% (62.63%-76.77%)	57.75% (47.54%-67.12%)	90.879% (82.345%-97.5%)	91.6% (84.61%-97.73%)	55.63% (45.99%-65.45%)	2.9586 (2.7129-3.2036)
At least 5 of CHASM (breast), FATHMM (cancer), FATHMM (missense), PolyPhen-2, PROVEAN, VEST	66.38% (62.63%-70.2%)	53.33% (48.06%-58.68%)	88.941% (84.808%-93.243%)	89.29% (85.5%-93.33%)	52.43% (47.15%-57.14%)	2.8399 (2.7135-2.9632)	66.23% (58.59%-73.74%)	52.89% (42.37%-63.16%)	89.069% (80%-97.22%)	89.26% (80.65%-97.14%)	52.43% (42.65%-62.72%)	2.8365 (2.5869-3.0754)
At least 5 of CHASM (breast), FATHMM (cancer), FATHMM (missense), PolyPhen-2, SIFT	64.02% (60.1%-67.68%)	44.81% (39.52%-50%)	97.233% (95.588%-100%)	96.55% (94.44%-100%)	50.47% (45.65%-55%)	2.8906 (2.7937-2.9882)	63.88% (56.57%-71.72%)	44.41% (33.93%-54.69%)	97.269% (92.303%-100%)	96.56% (90.48%-100%)	50.48% (41.54%-60.27%)	2.8872 (2.6842-3.0807)
At least 5 of CHASM (breast), FATHMM (cancer), FATHMM (missense), PolyPhen-2, SIFT, VEST	74.08% (70.71%-77.78%)	67.07% (62.4%-71.77%)	86.194% (81.69%-91.18%)	89.37% (85.88%-93.33%)	60.22% (54.81%-65.62%)	3.0285 (2.9073-3.1564)	74.06% (66.67%-80.81%)	66.89% (57.37%-76.19%)	86.299% (76.471%-94.74%)	89.36% (81.82%-95.92%)	60.3% (50%-71.15%)	3.0285 (2.7737-3.2771)
At least 5 of CHASM (breast), FATHMM (cancer), FATHMM (missense), PolyPhen-2, VEST	59.3% (55.05%-63.13%)	37.9% (32.75%-42.86%)	96.293% (94.203%-98.667%)	94.65% (91.49%-98.08%)	47.28% (42.48%-51.62%)	2.7613 (2.6572-2.8605)	59.18% (51.52%-67.68%)	37.48% (27.49%-47.62%)	96.398% (91.176%-100%)	94.73% (86.96%-100%)	47.32% (38.57%-56.94%)	2.7593 (2.5548-2.9704)
At least 5 of CHASM (breast), FATHMM (cancer), FATHMM (missense), PROVEAN, SIFT	58.97% (55.04%-62.63%)	36.3% (31.45%-40.94%)	98.173% (97.015%-100%)	97.17% (95.24%-100%)	47.13% (42.21%-51.61%)	2.7877 (2.6957-2.8815)	58.82% (51.52%-66.69%)	35.9% (26.31%-45.9%)	98.144% (94.118%-100%)	97.09% (90.48%-100%)	47.15% (38.36%-56.79%)	2.7827 (2.5878-2.9704)
At least 5 of CHASM (breast), FATHMM (cancer), FATHMM (missense), PROVEAN, SIFT, VEST	66.71% (63.13%-70.2%)	52.78% (47.54%-57.85%)	90.786% (86.957%-94.667%)	90.83% (87.14%-94.6%)	52.65% (47.49%-57.38%)	2.8704 (2.7497-2.9903)	66.58% (59.6%-73.74%)	52.4% (41.94%-62.69%)	90.879% (82.345%-97.5%)	90.81% (83.33%-97.56%)	52.66% (43.1%-62.5%)	2.8675 (2.6187-3.1071)
At least 5 of CHASM (breast), FATHMM (cancer), FATHMM (missense), PROVEAN, VEST	56.63% (52.53%-60.1%)	32.6% (27.56%-37.4%)	98.173% (97.015%-100%)	96.86% (94.74%-100%)	45.72% (41.02%-50%)	2.7335 (2.6393-2.8308)	56.44% (49.49%-64.65%)	32.13% (22.38%-42.11%)	98.144% (94.118%-100%)	96.75% (90%-100%)	45.73% (37.18%-55.26%)	2.7276 (2.5294-2.9200)
At least 5 of CHASM (breast), FATHMM (cancer), FATHMM (missense), SIFT, VEST	60.32% (56.06%-64.14%)	38.97% (34.09%-43.91%)	97.233% (95.588%-100%)	96.06% (93.62%-100%)	47.95% (43.33%-52.38%)	2.8021 (2.7059-2.9032)	60.17% (52.53%-68.69%)	38.54% (28.33%-48.39%)	97.269% (92.303%-100%)	96.06% (89.29%-100%)	47.98% (38.75%-57.58%)	2.7984 (2.5893-2.9965)
At least 5 of CHASM (breast), FATHMM (cancer), Mutation Assessor, MutationTaster, PolyPhen-2	61.63% (57.58%-65.15%)	41.03% (35.71%-46.03%)	97.233% (95.588%-100%)	96.25% (93.88%-100%)	48.81% (44.14%-53.33%)	2.8333 (2.7326-2.9319)	61.59% (54.55%-69.7%)	40.8% (30.64%-51.43%)	97.269% (92.303%-100%)	96.27% (90%-100%)	48.91% (39.73%-58.33%)	2.8325 (2.6322-3.0350)
At least 5 of CHASM (breast), FATHMM (cancer), Mutation Assessor, MutationTaster, PolyPhen-2, PROVEAN	71.07% (67.17%-74.75%)	56.99% (51.9%-62.12%)	95.413% (92.955%-98.53%)	95.55% (92.96%-98.59%)	56.2% (51.22%-61.16%)	3.0416 (2.9367-3.1551)	70.98% (63.64%-78.79%)	56.74% (45.9%-67.19%)	95.402% (89.655%-100%)	95.49% (89.66%-100%)	56.24% (46.66%-66.13%)	3.0387 (2.8199-3.2457)
At least 5 of CHASM (breast), FATHMM (cancer), Mutation Assessor, MutationTaster, PolyPhen-2, SIFT	76.76% (73.23%-80.3%)	67.54% (62.9%-72.44%)	92.701% (89.609%-96%)	94.11% (91.67%-96.84%)	62.29% (57.01%-67.31%)	3.1665 (3.0556-3.2902)	76.78% (69.7%-82.83%)	67.56% (57.73%-76.93%)	92.569% (85.294%-97.78%)	93.97% (87.75%-98.11%)	62.45% (52.72%-72.73%)	3.1654 (2.9134-3.3878)
At least 5 of CHASM (breast), FATHMM (cancer), Mutation Assessor, MutationTaster, PolyPhen-2, VEST	75.73% (72.22%-79.29%)	67% (62.4%-71.78%)	90.816% (87.324%-94.522%)	92.65% (89.89%-95.75%)	61.42% (56.31%-66.36%)	3.1189 (3.0041-3.2396)	75.81% (68.69%-82.83%)	67.03% (57.63%-76.27%)	90.849% (82.927%-97.37%)	92.61% (86.05%-97.92%)	61.63% (51.71%-72.34%)	3.1212 (2.8795-3.3542)
At least 5 of CHASM (breast), FATHMM (cancer), Mutation Assessor, MutationTaster, PROVEAN	56.6% (52.53%-60.61%)	32.55% (27.78%-37.1%)	98.173% (97.015%-100%)	96.85% (94.59%-100%)	45.71% (41.06%-50.01%)	2.7329 (2.6393-2.8252)	56.5% (48.48%-64.65%)	32.22% (22.95%-42.11%)	98.144% (94.118%-100%)	96.75% (89.99%-100%)	45.77% (36.98%-55.08%)	2.7289 (2.5417-2.9205)
At least 5 of CHASM (breast), FATHMM (cancer), Mutation Assessor, MutationTaster, PROVEAN, SIFT	71.73% (68.18%-75.25%)	58.02% (52.8%-62.91%)	95.413% (92.955%-98.53%)	95.63% (93.15%-98.63%)	56.8% (51.97%-61.54%)	3.0586 (2.9509-3.1715)	71.69% (64.65%-78.79%)	57.86% (48.33%-68.34%)	95.402% (89.655%-100%)	95.58% (89.99%-100%)	56.89% (47.17%-67.16%)	3.0572 (2.8401-3.2718)
At least 5 of CHASM (breast), FATHMM (cancer), Mutation Assessor, MutationTaster, PROVEAN, VEST	69.08% (65.15%-72.73%)	53.84% (48.76%-58.92%)	95.413% (92.955%-98.53%)	95.3% (92.64%-98.51%)	54.45% (49.6%-59.35%)	2.9901 (2.8819-3.1028)	68.92% (61.62%-76.77%)	53.46% (43.55%-63.8%)	95.402% (89.655%-100%)	95.23% (89.29%-100%)	54.44% (44.62%-64.29%)	2.9853 (2.7640-3.1939)
At least 5 of CHASM (breast), FATHMM (cancer), Mutation Assessor, MutationTaster, SIFT	62.65% (58.59%-66.17%)	42.64% (37.5%-47.54%)	97.233% (95.588%-100%)	96.39% (94.12%-100%)	49.5% (44.68%-54.11%)	2.8576 (2.7602-2.9542)	62.59% (55.53%-70.71%)	42.37% (32.76%-53.03%)	97.269% (92.303%-100%)	96.4% (90.32%-100%)	49.58% (40.28%-59.42%)	2.8562 (2.6582-3.0545)
At least 5 of CHASM (breast), FATHMM (cancer), Mutation Assessor, MutationTaster, SIFT, VEST	76.08% (72.73%-79.29%)	66.46% (61.67%-71.2%)	92.701% (89.609%-96%)	94.03% (91.58%-96.78%)	61.52% (56.36%-66.37%)	3.1471 (3.0380-3.2631)	76.12% (69.7%-82.83%)	66.52% (56.92%-76.27%)	92.569% (85.294%-97.78%)	93.88% (87.5%-98.08%)	61.7% (51.85%-72.23%)	3.1467 (2.9064-3.3708)
At least 5 of CHASM (breast), FATHMM (cancer), Mutation Assessor, MutationTaster, VEST	58.95% (54.55%-62.63%)	36.81% (31.4%-41.94%)	97.233% (95.588%-100%)	95.84% (93.18%-100%)	47.09% (42.46%-51.72%)	2.7697 (2.6685-2.8714)	58.87% (51.52%-67.68%)	36.48% (26.23%-47.15%)	97.269% (92.303%-100%)	95.84% (88.46%-100%)	47.16% (38.15%-56.94%)	2.7675 (2.5611-2.9727)
At least 5 of CHASM (breast), FATHMM (cancer), Mutation Assessor, PolyPhen-2, PROVEAN	57.28% (53.03%-61.11%)	33.62% (28.68%-38.17%)	98.173% (97.015%-100%)	96.95% (94.87%-100%)	46.1% (41.4%-50.35%)	2.7485 (2.6577-2.8463)	57.17% (49.49%-65.66%)	33.27% (24.14%-42.86%)	98.144% (94.118%-100%)	96.85% (90%-100%)	46.15% (37.5%-55.41%)	2.7442 (2.5547-2.9286)
At least 5 of CHASM (breast), FATHMM (cancer), Mutation Assessor, PolyPhen-2, PROVEAN, SIFT	68.69% (65.15%-72.73%)	57.52% (52.46%-62.61%)	88.007% (83.784%-92.501%)	89.24% (85.33%-93.51%)	54.51% (49.53%-59.51%)	2.8927 (2.7593-3.0174)	68.68% (60.61%-75.76%)	57.28% (47.06%-67.25%)	88.202% (79.301%-97.06%)	89.28% (81.25%-97.3%)	54.61% (43.86%-64.91%)	2.8937 (2.6458-3.1594)
At least 5 of CHASM (breast), FATHMM (cancer), Mutation Assessor, PolyPhen-2, PROVEAN, VEST	65.36% (61.62%-69.19%)	52.78% (47.62%-57.94%)	87.111% (82.665%-91.667%)	87.62% (83.33%-92.11%)	51.62% (46.55%-56.67%)	2.7913 (2.6550-2.9326)	65.24% (57.58%-72.73%)	52.4% (41.93%-62.72%)	87.242% (78.125%-96.88%)	87.56% (78.12%-96.77%)	51.65% (41.54%-61.54%)	2.7885 (2.5031-3.0686)
At least 5 of CHASM (breast), FATHMM (cancer), Mutation Assessor, PolyPhen-2, SIFT	63.66% (60.1%-67.68%)	44.25% (39.17%-49.22%)	97.233% (95.588%-100%)	96.51% (94.34%-100%)	50.22% (45.39%-54.61%)	2.8821 (2.7850-2.9855)	63.58% (55.56%-70.71%)	43.93% (33.33%-53.85%)	97.269% (92.303%-100%)	96.52% (90.48%-100%)	50.27% (41.18%-60%)	2.8799 (2.6716-3.0806)
At least 5 of CHASM (breast), FATHMM (cancer), Mutation Assessor, PolyPhen-2, SIFT, VEST	73.03% (69.7%-76.77%)	66.47% (61.67%-71.43%)	84.369% (79.71%-89.333%)	88.03% (84.38%-91.96%)	59.27% (53.85%-64.65%)	2.9814 (2.8508-3.1194)	73.13% (65.66%-79.8%)	66.51% (56.66%-76.19%)	84.466% (74.357%-94.29%)	88.02% (80%-95.56%)	59.51% (48.93%-70.59%)	2.9850 (2.7088-3.2489)
At least 5 of CHASM (breast), FATHMM (cancer), Mutation Assessor, PolyPhen-2, VEST	58.61% (54.55%-62.63%)	36.81% (31.45%-41.74%)	96.293% (94.203%-98.667%)	94.5% (91.3%-98%)	46.85% (42.18%-51.35%)	2.7445 (2.6382-2.8513)	58.55% (50.51%-66.67%)	36.48% (26.66%-47.07%)	96.398% (91.176%-100%)	94.59% (86.96%-100%)	46.93% (37.97%-56.34%)	2.7440 (2.5277-2.9525)
At least 5 of CHASM (breast), FATHMM (cancer), Mutation Assessor, PROVEAN, SIFT	58.29% (54.04%-62.12%)	35.22% (30.51%-40%)	98.173% (97.015%-100%)	97.09% (95.12%-100%)	46.71% (42%-51.01%)	2.7719 (2.6797-2.8675)	58.16% (50.51%-66.67%)	34.85% (25.39%-44.45%)	98.144% (94.118%-100%)	96.99% (90.46%-100%)	46.75% (37.97%-56%)	2.7673 (2.5722-2.9550)
At least 5 of CHASM (breast), FATHMM (cancer), Mutation Assessor, PROVEAN, SIFT, VEST	66.03% (62.12%-69.7%)	53.3% (48%-58.27%)	88.032% (83.562%-92.754%)	88.51% (84.06%-93.06%)	52.16% (47.11%-57.14%)	2.8200 (2.6876-2.9507)	65.92% (58.59%-73.74%)	52.95% (42.86%-63.77%)	88.148% (79.301%-97.06%)	88.46% (79.41%-97.14%)	52.19% (42.1%-62.12%)	2.8175 (2.5584-3.0807)
At least 5 of CHASM (breast), FATHMM (cancer), Mutation Assessor, PROVEAN, VEST	56.26% (52.02%-60.1%)	32.02% (27.34%-36.64%)	98.173% (97.015%-100%)	96.81% (94.59%-100%)	45.51% (40.79%-50%)	2.7250 (2.6323-2.8201)	56.17% (48.48%-64.65%)	31.7% (22.22%-41.27%)	98.144% (94.118%-100%)	96.7% (89.47%-100%)	45.57% (36.71%-54.88%)	2.7211 (2.5282-2.9118)
At least 5 of CHASM (breast), FATHMM (cancer), Mutation Assessor, SIFT, VEST	59.97% (56.06%-63.64%)	38.41% (33.33%-43.55%)	97.233% (95.588%-100%)	96% (93.48%-100%)	47.73% (43.04%-52.38%)	2.7938 (2.6982-2.8959)	59.86% (52.53%-67.68%)	38.05% (28.12%-48.28%)	97.269% (92.303%-100%)	96.01% (88.89%-100%)	47.78% (38.67%-57.14%)	2.7910 (2.5878-2.9874)
At least 5 of CHASM (breast), FATHMM (cancer), MutationTaster, PolyPhen-2, PROVEAN	57.95% (54.04%-61.62%)	34.69% (29.84%-39.54%)	98.173% (97.015%-100%)	97.04% (95%-100%)	46.5% (41.87%-50.97%)	2.7641 (2.6696-2.8567)	57.83% (50.51%-65.66%)	34.33% (24.55%-43.95%)	98.144% (94.118%-100%)	96.94% (90.46%-100%)	46.55% (37.66%-56%)	2.7597 (2.5676-2.9516)
At least 5 of CHASM (breast), FATHMM (cancer), MutationTaster, PolyPhen-2, PROVEAN, SIFT	73.08% (69.7%-76.77%)	60.16% (55.12%-64.84%)	95.413% (92.955%-98.53%)	95.78% (93.33%-98.67%)	58.08% (52.94%-62.99%)	3.0943 (2.9903-3.2054)	73.03% (65.66%-79.8%)	59.97% (50%-69.71%)	95.402% (89.655%-100%)	95.73% (90.32%-100%)	58.14% (48.33%-68.34%)	3.0924 (2.8752-3.3005)
At least 5 of CHASM (breast), FATHMM (cancer), MutationTaster, PolyPhen-2, PROVEAN, VEST	70.41% (66.67%-73.74%)	55.95% (50.82%-61.16%)	95.413% (92.955%-98.53%)	95.47% (92.86%-98.57%)	55.61% (50.78%-60.48%)	3.0244 (2.9148-3.1336)	70.29% (63.64%-77.78%)	55.64% (45.2%-66.1%)	95.402% (89.655%-100%)	95.41% (89.74%-100%)	55.63% (45.31%-65.57%)	3.0207 (2.8003-3.2388)
At least 5 of CHASM (breast), FATHMM (cancer), MutationTaster, PolyPhen-2, SIFT	63.99% (60.1%-67.68%)	44.76% (39.68%-50%)	97.233% (95.588%-100%)	96.55% (94.34%-100%)	50.45% (45.65%-54.93%)	2.8899 (2.7917-2.9921)	63.94% (56.57%-71.72%)	44.51% (34.32%-54.42%)	97.269% (92.303%-100%)	96.56% (90.48%-100%)	50.53% (41.43%-60.27%)	2.8887 (2.6851-3.0929)
At least 5 of CHASM (breast), FATHMM (cancer), MutationTaster, PolyPhen-2, SIFT, VEST	78.12% (74.75%-81.31%)	69.68% (65.29%-74.22%)	92.701% (89.609%-96%)	94.29% (91.95%-96.94%)	63.88% (58.71%-68.94%)	3.2055 (3.0977-3.3242)	78.1% (71.72%-84.85%)	69.65% (60.34%-78.57%)	92.569% (85.294%-97.78%)	94.14% (88%-98.22%)	64% (53.84%-74.51%)	3.2036 (2.9588-3.4197)
At least 5 of CHASM (breast), FATHMM (cancer), MutationTaster, PolyPhen-2, VEST	61.31% (57.07%-65.15%)	40.53% (35.24%-45.6%)	97.233% (95.588%-100%)	96.2% (93.75%-100%)	48.6% (43.79%-53.1%)	2.8257 (2.7253-2.9249)	61.22% (53.54%-69.7%)	40.21% (30.16%-50.79%)	97.269% (92.303%-100%)	96.21% (89.66%-100%)	48.67% (39.39%-58.21%)	2.8236 (2.6268-3.0270)
At least 5 of CHASM (breast), FATHMM (cancer), MutationTaster, PROVEAN, SIFT	59.62% (55.56%-63.13%)	37.32% (32.54%-42.4%)	98.173% (97.015%-100%)	97.25% (95.35%-100%)	47.53% (42.76%-51.95%)	2.8027 (2.7073-2.8938)	59.55% (52.53%-67.68%)	37.04% (26.98%-46.88%)	98.144% (94.118%-100%)	97.17% (90.91%-100%)	47.6% (38.67%-57.33%)	2.7995 (2.6113-2.9917)
At least 5 of CHASM (breast), FATHMM (cancer), MutationTaster, PROVEAN, SIFT, VEST	69.73% (66.16%-73.23%)	54.87% (49.62%-59.85%)	95.413% (92.955%-98.53%)	95.39% (92.75%-98.55%)	55.01% (50%-59.85%)	3.0068 (2.8985-3.1200)	69.64% (62.63%-76.77%)	54.61% (44.59%-64.71%)	95.402% (89.655%-100%)	95.32% (89.47%-100%)	55.05% (45.16%-65.08%)	3.0038 (2.7758-3.2152)
At least 5 of CHASM (breast), FATHMM (cancer), MutationTaster, PROVEAN, VEST	57.95% (54.04%-61.62%)	34.69% (29.84%-39.2%)	98.173% (97.015%-100%)	97.04% (95%-100%)	46.51% (41.67%-50.97%)	2.7641 (2.6696-2.8580)	57.83% (50.51%-65.66%)	34.33% (24.99%-44.07%)	98.144% (94.118%-100%)	96.95% (90%-100%)	46.55% (37.97%-56.25%)	2.7597 (2.5674-2.9562)
At least 5 of CHASM (breast), FATHMM (cancer), MutationTaster, SIFT, VEST	61.64% (57.58%-65.16%)	41.06% (35.83%-46.34%)	97.233% (95.588%-100%)	96.25% (93.88%-100%)	48.83% (44%-53.15%)	2.8337 (2.7376-2.9320)	61.56% (54.52%-69.7%)	40.74% (30.16%-50.88%)	97.269% (92.303%-100%)	96.26% (89.99%-100%)	48.89% (39.71%-58.67%)	2.8316 (2.6207-3.0270)
At least 5 of CHASM (breast), FATHMM (cancer), PolyPhen-2, PROVEAN, SIFT	59.63% (55.56%-63.13%)	37.34% (32.26%-42.31%)	98.173% (97.015%-100%)	97.25% (95.35%-100%)	47.54% (42.75%-51.97%)	2.8030 (2.7074-2.8984)	59.52% (52.53%-67.68%)	37% (27.12%-46.88%)	98.144% (94.118%-100%)	97.16% (90.91%-100%)	47.58% (38.66%-56.96%)	2.7988 (2.6027-2.9886)
At least 5 of CHASM (breast), FATHMM (cancer), PolyPhen-2, PROVEAN, SIFT, VEST	67.03% (63.13%-70.72%)	54.89% (49.62%-59.84%)	88.005% (83.778%-92.754%)	88.78% (84.81%-93.33%)	53.01% (47.83%-57.99%)	2.8469 (2.7079-2.9811)	66.95% (59.57%-74.75%)	54.55% (44.44%-64.82%)	88.194% (78.788%-97.06%)	88.8% (80%-97.06%)	53.07% (42.86%-63.16%)	2.8462 (2.5760-3.1152)
At least 5 of CHASM (breast), FATHMM (cancer), PolyPhen-2, PROVEAN, VEST	57.61% (53.54%-61.12%)	34.15% (29.17%-38.84%)	98.173% (97.015%-100%)	97% (94.87%-100%)	46.3% (41.45%-50.64%)	2.7562 (2.6560-2.8492)	57.5% (50.48%-65.66%)	33.81% (24.61%-43.64%)	98.144% (94.118%-100%)	96.9% (90%-100%)	46.35% (37.66%-55.84%)	2.7520 (2.5605-2.9513)
At least 5 of CHASM (breast), FATHMM (cancer), PolyPhen-2, SIFT, VEST	60.97% (57.07%-64.65%)	39.99% (34.85%-45.04%)	97.233% (95.588%-100%)	96.16% (93.75%-100%)	48.38% (43.54%-52.74%)	2.8175 (2.7152-2.9178)	60.9% (53.54%-68.69%)	39.69% (29.4%-50%)	97.269% (92.303%-100%)	96.16% (89.29%-100%)	48.45% (39.7%-58.34%)	2.8157 (2.6125-3.0202)
At least 5 of CHASM (breast), FATHMM (cancer), PROVEAN, SIFT, VEST	58.96% (55.05%-62.63%)	36.27% (31.4%-40.98%)	98.173% (97.015%-100%)	97.17% (95.24%-100%)	47.12% (42.28%-51.35%)	2.7874 (2.6948-2.8815)	58.85% (51.52%-66.67%)	35.94% (26.56%-45.9%)	98.144% (94.118%-100%)	97.08% (90.48%-100%)	47.17% (38.15%-56.76%)	2.7834 (2.5893-2.9791)
At least 5 of CHASM (breast), FATHMM (missense), Mutation Assessor, MutationTaster, PolyPhen-2	60.62% (56.57%-64.15%)	39.44% (34.17%-44.26%)	97.233% (95.588%-100%)	96.1% (93.62%-100%)	48.15% (43.54%-52.45%)	2.8092 (2.7130-2.9096)	60.58% (53.51%-68.69%)	39.2% (29.51%-50%)	97.269% (92.303%-100%)	96.12% (89.65%-100%)	48.25% (39.44%-57.75%)	2.8084 (2.6062-3.0001)

At least 5 of CHASM (breast), FATHMM (missense), Mutation Assessor, MutationTaster, PolyPhen-2, PROVEAN	68.72% (65.15%-72.22%)	53.27% (48.03%-58.27%)	95.413% (92.955%-98.53%)	95.26% (92.54%-98.51%)	[illegible]	[illegible]	[illegible]	[illegible]	[illegible]	[illegible]	[illegible]	[illegible]
At least 5 of CHASM (breast), FATHMM (missense), Mutation Assessor, MutationTaster, PolyPhen-2, SIFT	75.12% (71.72%-78.79%)	64.95% (60.32%-69.63%)	92.701% (89.609%-96%)	93.9% (91.36%-96.7%)	[illegible]	[illegible]	[illegible]	[illegible]	[illegible]	[illegible]	[illegible]	[illegible]
At least 5 of CHASM (breast), FATHMM (missense), Mutation Assessor, MutationTaster, PolyPhen-2, VEST	74.1% (70.71%-77.78%)	63.88% (59.23%-68.66%)	91.761% (88.406%-95.652%)	93.06% (90.36%-96.47%)	[illegible]	[illegible]	[illegible]	[illegible]	[illegible]	[illegible]	[illegible]	[illegible]
At least 5 of CHASM (breast), FATHMM (missense), Mutation Assessor, MutationTaster, PROVEAN	55.26% (51.01%-59.09%)	30.44% (25.78%-34.92%)	98.173% (97.015%-100%)	96.65% (94.29%-100%)	[illegible]	[illegible]	[illegible]	[illegible]	[illegible]	[illegible]	[illegible]	[illegible]
At least 5 of CHASM (breast), FATHMM (missense), Mutation Assessor, MutationTaster, PROVEAN, SIFT	69.37% (65.66%-72.73%)	54.3% (49.19%-59.2%)	95.413% (92.955%-98.53%)	95.34% (92.75%-98.53%)	[illegible]	[illegible]	[illegible]	[illegible]	[illegible]	[illegible]	[illegible]	[illegible]
At least 5 of CHASM (breast), FATHMM (missense), Mutation Assessor, MutationTaster, PROVEAN, VEST	66.72% (63.13%-70.2%)	50.12% (45.08%-55.3%)	95.413% (92.955%-98.53%)	94.98% (92.06%-98.39%)	[illegible]	[illegible]	[illegible]	[illegible]	[illegible]	[illegible]	[illegible]	[illegible]
At least 5 of CHASM (breast), FATHMM (missense), Mutation Assessor, MutationTaster, SIFT	61.64% (57.58%-65.66%)	41.04% (36.06%-45.8%)	97.233% (95.588%-100%)	96.25% (93.88%-100%)	[illegible]	[illegible]	[illegible]	[illegible]	[illegible]	[illegible]	[illegible]	[illegible]
At least 5 of CHASM (breast), FATHMM (missense), Mutation Assessor, MutationTaster, SIFT, VEST	74.78% (71.21%-78.28%)	64.41% (59.5%-69.11%)	92.701% (89.609%-96%)	93.85% (91.3%-96.7%)	[illegible]	[illegible]	[illegible]	[illegible]	[illegible]	[illegible]	[illegible]	[illegible]
At least 5 of CHASM (breast), FATHMM (missense), Mutation Assessor, MutationTaster, VEST	57.94% (54.04%-61.63%)	35.21% (30.25%-40.32%)	97.233% (95.588%-100%)	95.66% (92.86%-100%)	[illegible]	[illegible]	[illegible]	[illegible]	[illegible]	[illegible]	[illegible]	[illegible]
At least 5 of CHASM (breast), FATHMM (missense), Mutation Assessor, PolyPhen-2, PROVEAN	55.94% (52.02%-59.6%)	31.51% (26.45%-36.07%)	98.173% (97.015%-100%)	96.76% (94.44%-100%)	[illegible]	[illegible]	[illegible]	[illegible]	[illegible]	[illegible]	[illegible]	[illegible]
At least 5 of CHASM (breast), FATHMM (missense), Mutation Assessor, PolyPhen-2, PROVEAN, SIFT	66.69% (63.13%-70.2%)	53.8% (48.74%-59.02%)	88.957% (84.808%-93.151%)	89.39% (85.51%-93.42%)	[illegible]	[illegible]	[illegible]	[illegible]	[illegible]	[illegible]	[illegible]	[illegible]
At least 5 of CHASM (breast), FATHMM (missense), Mutation Assessor, PolyPhen-2, PROVEAN, VEST	63.34% (59.6%-67.17%)	49.05% (44.19%-54.1%)	88.04% (83.784%-92.647%)	87.64% (83.33%-92.31%)	[illegible]	[illegible]	[illegible]	[illegible]	[illegible]	[illegible]	[illegible]	[illegible]
At least 5 of CHASM (breast), FATHMM (missense), Mutation Assessor, PolyPhen-2, SIFT	62.65% (58.59%-66.16%)	42.65% (37.4%-47.62%)	97.233% (95.588%-100%)	96.39% (94.12%-100%)	[illegible]	[illegible]	[illegible]	[illegible]	[illegible]	[illegible]	[illegible]	[illegible]
At least 5 of CHASM (breast), FATHMM (missense), Mutation Assessor, PolyPhen-2, SIFT, VEST	71.73% (68.18%-75.25%)	63.88% (59.16%-68.8%)	85.298% (80.822%-90.278%)	88.25% (84.61%-92.14%)	[illegible]	[illegible]	[illegible]	[illegible]	[illegible]	[illegible]	[illegible]	[illegible]
At least 5 of CHASM (breast), FATHMM (missense), Mutation Assessor, PolyPhen-2, VEST	57.59% (53.54%-61.11%)	35.21% (29.92%-40%)	96.293% (94.203%-98.667%)	94.27% (90.91%-97.92%)	[illegible]	[illegible]	[illegible]	[illegible]	[illegible]	[illegible]	[illegible]	[illegible]
At least 5 of CHASM (breast), FATHMM (missense), Mutation Assessor, PROVEAN, SIFT	56.95% (53.02%-61.11%)	33.11% (28.23%-37.8%)	98.173% (97.015%-100%)	96.91% (94.74%-100%)	[illegible]	[illegible]	[illegible]	[illegible]	[illegible]	[illegible]	[illegible]	[illegible]
At least 5 of CHASM (breast), FATHMM (missense), Mutation Assessor, PROVEAN, SIFT, VEST	64.35% (60.61%-68.18%)	49.58% (44.44%-54.76%)	89.884% (85.937%-94.286%)	89.45% (85.29%-93.85%)	[illegible]	[illegible]	[illegible]	[illegible]	[illegible]	[illegible]	[illegible]	[illegible]
At least 5 of CHASM (breast), FATHMM (missense), Mutation Assessor, PROVEAN, VEST	54.92% (51%-58.6%)	29.9% (25%-34.17%)	98.173% (97.015%-100%)	96.59% (94.28%-100%)	[illegible]	[illegible]	[illegible]	[illegible]	[illegible]	[illegible]	[illegible]	[illegible]
At least 5 of CHASM (breast), FATHMM (missense), Mutation Assessor, SIFT, VEST	58.96% (55.05%-62.63%)	36.82% (31.75%-41.94%)	97.233% (95.588%-100%)	95.84% (93.33%-100%)	[illegible]	[illegible]	[illegible]	[illegible]	[illegible]	[illegible]	[illegible]	[illegible]
At least 5 of CHASM (breast), FATHMM (missense), MutationTaster, PolyPhen-2, PROVEAN	55.27% (51.01%-59.09%)	30.46% (25.4%-35.16%)	98.173% (97.015%-100%)	96.65% (94.29%-100%)	[illegible]	[illegible]	[illegible]	[illegible]	[illegible]	[illegible]	[illegible]	[illegible]
At least 5 of CHASM (breast), FATHMM (missense), MutationTaster, PolyPhen-2, PROVEAN, SIFT	72.07% (68.69%-75.25%)	58.56% (53.28%-63.57%)	95.413% (92.955%-98.53%)	95.67% (93.24%-98.65%)	[illegible]	[illegible]	[illegible]	[illegible]	[illegible]	[illegible]	[illegible]	[illegible]
At least 5 of CHASM (breast), FATHMM (missense), MutationTaster, PolyPhen-2, PROVEAN, VEST	69.06% (65.15%-72.73%)	53.81% (48.78%-59.06%)	95.413% (92.955%-98.53%)	95.3% (92.65%-98.51%)	[illegible]	[illegible]	[illegible]	[illegible]	[illegible]	[illegible]	[illegible]	[illegible]
At least 5 of CHASM (breast), FATHMM (missense), MutationTaster, PolyPhen-2, SIFT	61.64% (57.58%-65.15%)	41.05% (35.83%-45.97%)	97.233% (95.588%-100%)	96.25% (93.88%-100%)	[illegible]	[illegible]	[illegible]	[illegible]	[illegible]	[illegible]	[illegible]	[illegible]
[illegible]	[illegible]	[illegible]	[illegible]	[illegible]	[illegible]	[illegible]	[illegible]	[illegible]	[illegible]	[illegible]	[illegible]	[illegible]

**At least 5 of CHASM (lung), FATHMM (missense), Mutation Assessor, MutationTaster, PolyPhen-2**	63.34% (59.6%-67.17%)	44.26% (38.98%-49.21%)	96.322% (94.203%-98.685%)	95.41% (92.59%-98.39%)	49.98% (45.32%-54.41%)	2.8597 (2.7630-2.9608)	63.23% (55.56%-70.71%)	43.91% (33.87%-54.1%)	96.356% (91.111%-100%)	95.39% (88%-100%)	50.03% (41.09%-59.38%)	2.8568 (2.6518-3.0516)
**At least 5 of CHASM (lung), FATHMM (missense), Mutation Assessor, MutationTaster, PolyPhen-2, PROVEAN**	71.45% (68.17%-75.25%)	58.1% (53.12%-63.34%)	94.526% (91.667%-97.436%)	94.83% (92.11%-97.56%)	56.61% (51.56%-61.48%)	3.0407 (2.9346-3.1502)	71.24% (63.64%-77.8%)	57.7% (47.36%-67.21%)	94.426% (88.235%-100%)	94.67% (88.23%-100%)	56.54% (46.87%-66.67%)	3.0335 (2.8167-3.2463)
**At least 5 of CHASM (lung), FATHMM (missense), Mutation Assessor, MutationTaster, PolyPhen-2, SIFT**	76.46% (73.23%-79.8%)	67.07% (62.29%-71.77%)	92.701% (89.609%-96%)	94.08% (91.67%-96.77%)	61.95% (57.14%-66.97%)	3.1579 (3.0503-3.2746)	76.37% (69.7%-82.83%)	66.9% (57.38%-76.27%)	92.569% (85.294%-97.78%)	93.92% (87.8%-98.08%)	61.98% (51.92%-72.1%)	3.1537 (2.9135-3.3657)
**At least 5 of CHASM (lung), FATHMM (missense), Mutation Assessor, MutationTaster, PolyPhen-2, VEST**	75.78% (72.22%-79.29%)	66.52% (61.79%-71.21%)	91.761% (88.406%-95.652%)	93.32% (90.7%-96.63%)	61.32% (56.36%-66.37%)	3.1292 (3.0205-3.2490)	75.72% (68.69%-82.83%)	66.39% (56.72%-75.87%)	91.697% (84.375%-97.5%)	93.21% (86.54%-97.96%)	61.39% (50.98%-71.43%)	3.1269 (2.8891-3.3487)
**At least 5 of CHASM (lung), FATHMM (missense), Mutation Assessor, MutationTaster, PROVEAN**	56.26% (52.02%-60.1%)	32.03% (27.42%-36.59%)	98.149% (97.015%-100%)	96.76% (94.44%-100%)	45.51% (40.91%-49.69%)	2.7245 (2.6353-2.8167)	56.17% (48.48%-64.65%)	31.67% (22.72%-40.98%)	98.206% (94.282%-100%)	96.8% (88.89%-100%)	45.58% (36.84%-54.55%)	2.7225 (2.5340-2.9032)
**At least 5 of CHASM (lung), FATHMM (missense), Mutation Assessor, MutationTaster, PROVEAN, SIFT**	72.11% (68.69%-75.76%)	59.13% (54.1%-64.29%)	94.526% (91.667%-97.436%)	94.92% (92.21%-97.59%)	57.22% (52.38%-62.16%)	3.0580 (2.9502-3.1678)	71.95% (64.65%-78.79%)	58.82% (48.53%-68.85%)	94.426% (88.235%-100%)	94.77% (88.37%-100%)	57.2% (47.46%-67.19%)	3.0522 (2.8251-3.2667)
**At least 5 of CHASM (lung), FATHMM (missense), Mutation Assessor, MutationTaster, PROVEAN, VEST**	69.79% (66.16%-73.24%)	55.47% (50.4%-60.63%)	94.526% (91.667%-97.436%)	94.6% (91.78%-97.44%)	55.11% (50%-60.16%)	2.9971 (2.8929-3.1042)	69.52% (62.6%-76.77%)	54.97% (44.26%-65.08%)	94.426% (88.235%-100%)	94.43% (87.5%-100%)	55% (44.78%-65.08%)	2.9882 (2.7702-3.2069)
**At least 5 of CHASM (lung), FATHMM (missense), Mutation Assessor, MutationTaster, SIFT**	64.36% (60.61%-67.68%)	45.86% (40.62%-50.44%)	96.322% (94.203%-98.685%)	95.57% (92.86%-98.44%)	50.71% (45.99%-55.32%)	2.8847 (2.7884-2.9867)	64.22% (57.58%-71.72%)	45.48% (35.59%-55.74%)	96.356% (91.111%-100%)	95.54% (88.46%-100%)	50.73% (41.79%-60.27%)	2.8810 (2.6768-3.0697)
**At least 5 of CHASM (lung), FATHMM (missense), Mutation Assessor, MutationTaster, SIFT, VEST**	76.45% (73.23%-79.8%)	67.05% (62.39%-71.77%)	92.701% (89.609%-96%)	94.08% (91.67%-96.78%)	61.93% (56.76%-66.99%)	3.1576 (3.0489-3.2755)	76.39% (69.7%-82.83%)	66.94% (57.58%-76.12%)	92.569% (85.294%-97.78%)	93.93% (88%-98.11%)	62% (51.72%-72.22%)	3.1544 (2.9211-3.3812)
**At least 5 of CHASM (lung), FATHMM (missense), Mutation Assessor, MutationTaster, VEST**	60.33% (56.57%-64.14%)	39.51% (34.19%-44.53%)	96.322% (94.203%-98.685%)	94.89% (91.84%-98.21%)	47.94% (43.24%-52.41%)	2.7866 (2.6882-2.8921)	60.15% (52.53%-67.68%)	39.05% (28.81%-49.15%)	96.356% (91.111%-100%)	94.83% (86.36%-100%)	47.95% (39.19%-57.33%)	2.7819 (2.5650-2.9850)
**At least 5 of CHASM (lung), FATHMM (missense), Mutation Assessor, PolyPhen-2, PROVEAN**	56.93% (53.03%-60.61%)	33.09% (28.33%-37.69%)	98.149% (97.015%-100%)	96.86% (94.74%-100%)	45.9% (41.18%-50%)	2.7400 (2.6445-2.8325)	56.84% (49.49%-64.65%)	32.72% (23.43%-42.37%)	98.206% (94.282%-100%)	96.89% (89.47%-100%)	45.96% (37.5%-55.13%)	2.7378 (2.5444-2.9286)
**At least 5 of CHASM (lung), FATHMM (missense), Mutation Assessor, PolyPhen-2, PROVEAN, SIFT**	69.42% (65.66%-72.73%)	58.62% (53.6%-63.64%)	88.07% (83.784%-92.647%)	89.47% (85.71%-93.75%)	55.18% (49.99%-60.34%)	2.9134 (2.7885-3.0406)	69.24% (62.63%-76.77%)	58.25% (48.13%-68.18%)	88.053% (78.947%-97.06%)	89.34% (81.25%-97.22%)	55.13% (44.83%-65.39%)	2.9077 (2.6525-3.1576)
**At least 5 of CHASM (lung), FATHMM (missense), Mutation Assessor, PolyPhen-2, PROVEAN, VEST**	66.41% (62.63%-70.2%)	54.41% (49.57%-59.68%)	87.153% (82.667%-91.892%)	87.98% (84%-92.31%)	52.51% (47.24%-57.63%)	2.8205 (2.6897-2.9482)	66.17% (58.59%-73.74%)	53.91% (43.55%-64.06%)	87.146% (77.771%-95.56%)	87.81% (78.94%-95.35%)	52.42% (42.59%-62.69%)	2.8129 (2.5574-3.0687)
**At least 5 of CHASM (lung), FATHMM (missense), Mutation Assessor, PolyPhen-2, SIFT**	65.37% (61.62%-69.19%)	47.47% (41.94%-52.42%)	96.322% (94.203%-98.685%)	95.71% (93.22%-98.51%)	51.47% (46.67%-55.97%)	2.9097 (2.8064-3.0113)	65.21% (57.58%-72.73%)	47.05% (36.92%-57.81%)	96.356% (91.111%-100%)	95.67% (88.57%-100%)	51.46% (42.65%-60.87%)	2.9054 (2.6961-3.1071)
**At least 5 of CHASM (lung), FATHMM (missense), Mutation Assessor, PolyPhen-2, SIFT, VEST**	73.06% (69.7%-76.77%)	66.52% (61.79%-71.31%)	84.36% (79.729%-89.189%)	88.03% (84.38%-91.84%)	59.31% (54%-64.71%)	2.9822 (2.8590-3.1167)	73.07% (65.66%-79.8%)	66.4% (56.92%-75.87%)	84.467% (74.286%-93.75%)	88.01% (80%-95.56%)	59.43% (48.94%-69.82%)	2.9831 (2.7110-3.2264)
**At least 5 of CHASM (lung), FATHMM (missense), Mutation Assessor, PolyPhen-2, VEST**	59.98% (56.06%-63.64%)	39.51% (34.1%-44.53%)	95.382% (92.857%-98.611%)	93.67% (90.38%-98.08%)	47.7% (43.06%-51.8%)	2.7625 (2.6535-2.8667)	59.83% (52.53%-67.68%)	39.05% (28.81%-49.31%)	95.485% (89.182%-100%)	93.69% (84.99%-100%)	47.72% (39.39%-56.76%)	2.7595 (2.5502-2.9728)
**At least 5 of CHASM (lung), FATHMM (missense), Mutation Assessor, PROVEAN, SIFT**	57.95% (54.04%-61.62%)	34.7% (29.92%-39.47%)	98.149% (97.015%-100%)	97% (95%-100%)	46.5% (41.72%-50.67%)	2.7635 (2.6674-2.8549)	57.84% (50.51%-65.66%)	34.3% (25%-44.07%)	98.206% (94.282%-100%)	97.02% (90%-100%)	46.55% (37.97%-55.72%)	2.7608 (2.5768-2.9515)
**At least 5 of CHASM (lung), FATHMM (missense), Mutation Assessor, PROVEAN, SIFT, VEST**	67.42% (63.64%-71.21%)	54.93% (50%-60%)	88.998% (84.932%-93.333%)	89.62% (85.71%-93.67%)	53.31% (48%-58.34%)	2.8686 (2.7477-2.9908)	67.18% (59.6%-74.75%)	54.46% (44.06%-64.06%)	88.956% (80%-97.14%)	89.45% (81.08%-97.3%)	53.23% (43.28%-63.34%)	2.8609 (2.6142-3.1031)
**At least 5 of CHASM (lung), FATHMM (missense), Mutation Assessor, PROVEAN, VEST**	55.58% (51.52%-59.6%)	30.97% (26.23%-35.48%)	98.149% (97.015%-100%)	96.65% (94.29%-100%)	45.12% (40.38%-49.38%)	2.7089 (2.6162-2.8039)	55.5% (47.47%-63.64%)	30.6% (21.43%-39.71%)	98.206% (94.282%-100%)	96.68% (88.89%-100%)	45.19% (36.59%-54.55%)	2.7068 (2.5121-2.8924)
**At least 5 of CHASM (lung), FATHMM (missense), Mutation Assessor, SIFT, VEST**	61.34% (57.58%-65.15%)	41.11% (35.71%-46.1%)	96.322% (94.203%-98.685%)	95.08% (92.16%-98.28%)	48.61% (43.97%-52.86%)	2.8112 (2.7130-2.9168)	61.15% (53.54%-68.69%)	40.62% (30.16%-50.86%)	96.356% (91.111%-100%)	95.02% (86.96%-100%)	48.6% (40%-57.58%)	2.8059 (2.5959-3.0043)
**At least 5 of CHASM (lung), FATHMM (missense), MutationTaster, PolyPhen-2, PROVEAN**	56.27% (52.53%-60.1%)	32.04% (26.98%-36.8%)	98.149% (97.015%-100%)	96.76% (94.59%-100%)	45.51% (40.88%-49.68%)	2.7247 (2.6360-2.8155)	56.15% (48.48%-63.64%)	31.63% (22.22%-41.27%)	98.206% (94.282%-100%)	96.79% (89.47%-100%)	45.56% (37.18%-54.88%)	2.7219 (2.5282-2.9015)
**At least 5 of CHASM (lung), FATHMM (missense), MutationTaster, PolyPhen-2, PROVEAN, SIFT**	74.8% (71.72%-78.28%)	63.39% (58.59%-68.22%)	94.526% (91.667%-97.436%)	95.24% (92.77%-97.73%)	59.89% (54.87%-64.71%)	3.1305 (3.0208-3.2377)	74.64% (67.68%-80.81%)	63.08% (53.22%-72.58%)	94.426% (88.235%-100%)	95.11% (89.19%-100%)	59.85% (50%-70.18%)	3.1247 (2.9053-3.3440)
**At least 5 of CHASM (lung), FATHMM (missense), MutationTaster, PolyPhen-2, PROVEAN, VEST**	72.13% (68.69%-75.76%)	59.17% (54.4%-64.07%)	94.526% (91.667%-97.436%)	94.92% (92.31%-97.59%)	57.24% (52.1%-62.19%)	3.0586 (2.9441-3.1715)	71.91% (64.65%-78.79%)	58.76% (49.11%-68.42%)	94.426% (88.235%-100%)	94.76% (88.37%-100%)	57.17% (46.87%-67.19%)	3.0512 (2.8381-3.2723)

At least 5 of FATHMM (missense), Mutation Assessor, PolyPhen-2, SIFT, VEST	63.33% (59.6%-67.17%)	49.53% (44.44%-54.47%)	87.159% (83.077%-91.784%)	86.96% (82.61%-91.55%)	49.97% (45%-54.7%)	2.7363 (2.6071-2.8728)	63.25% (55.56%-70.71%)	49.3% (39.06%-59.09%)	87.131% (77.419%-94.87%)	86.8% (77.5%-94.87%)	50.03% (40.68%-60%)	2.7326 (2.4653-2.9883)
At least 5 of FATHMM (missense), Mutation Assessor, PROVEAN, SIFT, VEST	55.59% (51.52%-59.6%)	35.2% (30.47%-39.84%)	90.825% (87.013%-94.737%)	86.9% (81.81%-92.59%)	44.77% (40%-49.32%)	2.5771 (2.4489-2.7070)	55.49% (47.47%-63.64%)	34.88% (25.67%-44.13%)	90.806% (82.759%-97.56%)	86.7% (75%-96.3%)	44.83% (35.62%-54.32%)	2.5721 (2.2915-2.8272)
At least 5 of FATHMM (missense), MutationTaster, PolyPhen-2, PROVEAN, SIFT	58.28% (54.55%-62.12%)	36.25% (31.2%-40.91%)	96.354% (94.203%-98.667%)	94.5% (91.11%-98%)	46.64% (41.72%-51.01%)	2.7375 (2.6322-2.8413)	58.19% (50.51%-65.66%)	35.98% (26.15%-46.16%)	96.276% (91.176%-100%)	94.31% (86.36%-100%)	46.7% (37.84%-56.34%)	2.7327 (2.5230-2.9359)
At least 5 of FATHMM (missense), MutationTaster, PolyPhen-2, PROVEAN, SIFT, VEST	73.41% (70.2%-77.27%)	65.46% (60.8%-70.25%)	87.142% (82.857%-91.67%)	89.8% (86.21%-93.48%)	59.33% (53.92%-64.49%)	3.0173 (2.8908-3.1554)	73.38% (65.66%-79.8%)	65.33% (55.93%-74.6%)	87.154% (77.778%-95%)	89.72% (82.61%-96.15%)	59.42% (49.12%-69.64%)	3.0163 (2.7392-3.2638)
At least 5 of FATHMM (missense), MutationTaster, PolyPhen-2, PROVEAN, VEST	56.6% (52.53%-60.61%)	33.6% (28.57%-38.58%)	96.354% (94.203%-98.667%)	94.09% (90.48%-97.87%)	45.63% (40.91%-50%)	2.6968 (2.5893-2.8025)	56.5% (48.48%-64.65%)	33.29% (23.88%-42.86%)	96.276% (91.176%-100%)	93.87% (85%-100%)	45.68% (36.84%-55.26%)	2.6913 (2.4673-2.9032)
At least 5 of FATHMM (missense), MutationTaster, PolyPhen-2, SIFT, VEST	65.68% (62.12%-69.7%)	49.51% (44.27%-54.55%)	93.622% (90.541%-97.183%)	93.07% (89.85%-96.97%)	51.75% (46.92%-56.39%)	2.8795 (2.7712-2.9932)	65.61% (57.58%-72.73%)	49.35% (39.28%-59.38%)	93.483% (86.667%-100%)	92.86% (84.85%-100%)	51.81% (42.65%-61.43%)	2.8750 (2.6475-3.0929)
At least 5 of FATHMM (missense), MutationTaster, PROVEAN, SIFT, VEST	57.95% (54.04%-61.62%)	35.73% (30.77%-40.48%)	96.354% (94.203%-98.667%)	94.43% (91.11%-97.96%)	46.44% (41.61%-51.01%)	2.7295 (2.6254-2.8353)	57.84% (50.51%-65.66%)	35.43% (25.45%-45.61%)	96.276% (91.176%-100%)	94.23% (86.36%-100%)	46.49% (37.5%-56%)	2.7242 (2.5105-2.9305)
At least 5 of FATHMM (missense), PolyPhen-2, PROVEAN, SIFT, VEST	55.23% (51.01%-59.09%)	35.19% (30.08%-40%)	89.874% (86.11%-94.205%)	85.73% (80.39%-91.67%)	44.51% (39.58%-48.98%)	2.5530 (2.4254-2.6871)	55.19% (47.47%-63.64%)	34.91% (25.36%-45%)	89.948% (81.579%-97.3%)	85.63% (73.91%-96.15%)	44.6% (35.61%-54.67%)	2.5509 (2.2725-2.8037)
At least 5 of Mutation Assessor, MutationTaster, PolyPhen-2, PROVEAN, SIFT	60.61% (56.57%-64.15%)	39.93% (34.74%-44.63%)	96.354% (94.203%-98.667%)	94.98% (92%-98.18%)	48.12% (43.36%-52.78%)	2.7939 (2.6890-2.8985)	60.6% (53.51%-68.69%)	39.8% (30.16%-50%)	96.276% (91.176%-100%)	94.83% (87.5%-100%)	48.24% (39.13%-58.11%)	2.7914 (2.5825-2.9991)
At least 5 of Mutation Assessor, MutationTaster, PolyPhen-2, PROVEAN, SIFT, VEST	71.72% (68.18%-75.76%)	65.45% (60.66%-70.08%)	82.563% (77.612%-87.879%)	86.64% (82.61%-90.91%)	58.01% (52.88%-63.54%)	2.9267 (2.7967-3.0707)	71.71% (63.64%-78.79%)	65.35% (55.77%-75%)	82.579% (71.795%-92.31%)	86.54% (78.18%-94.12%)	58.13% (46.94%-68.42%)	2.9261 (2.6430-3.1860)
At least 5 of Mutation Assessor, MutationTaster, PolyPhen-2, PROVEAN, VEST	58.58% (54.55%-62.13%)	36.74% (31.7%-41.54%)	96.354% (94.203%-98.667%)	94.57% (91.3%-98.04%)	46.83% (42.14%-51.63%)	2.7449 (2.6413-2.8499)	58.59% (51.49%-66.67%)	36.62% (26.98%-46.78%)	96.276% (91.176%-100%)	94.4% (86.66%-100%)	46.95% (37.68%-56.58%)	2.7425 (2.5322-2.9520)
At least 5 of Mutation Assessor, MutationTaster, PolyPhen-2, SIFT, VEST	68.02% (64.65%-71.72%)	54.8% (49.99%-59.84%)	90.873% (87.342%-94.872%)	91.21% (87.88%-95.06%)	53.76% (48.82%-58.82%)	2.9065 (2.7910-3.0316)	68% (60.61%-74.75%)	54.74% (44.44%-64.62%)	90.735% (82.855%-97.3%)	91.01% (82.85%-97.37%)	53.87% (44.07%-63.49%)	2.9036 (2.6561-3.1332)
At least 5 of Mutation Assessor, MutationTaster, PROVEAN, SIFT, VEST	59.6% (55.56%-63.64%)	38.34% (33.33%-43.09%)	96.354% (94.203%-98.667%)	94.78% (91.67%-98.11%)	47.47% (42.76%-52.09%)	2.7695 (2.6629-2.8751)	59.59% (51.52%-67.68%)	38.2% (28.33%-48.39%)	96.276% (91.176%-100%)	94.62% (86.96%-100%)	47.58% (38.03%-57.5%)	2.7667 (2.5458-2.9873)
At least 5 of Mutation Assessor, PolyPhen-2, PROVEAN, SIFT, VEST	56.89% (53.03%-61.11%)	38.87% (34.15%-43.75%)	88.044% (83.784%-92.754%)	84.9% (79.66%-90.57%)	45.44% (40.69%-50.35%)	2.5725 (2.4337-2.7077)	56.92% (48.48%-64.65%)	38.73% (28.57%-48.33%)	88.121% (78.947%-96.97%)	84.83% (73.08%-96%)	45.59% (36.11%-55.41%)	2.5727 (2.2913-2.8545)
At least 5 of MutationTaster, PolyPhen-2, PROVEAN, SIFT, VEST	60.93% (57.07%-64.65%)	40.44% (35.24%-45.16%)	96.354% (94.203%-98.667%)	95.04% (92%-98.21%)	48.34% (43.54%-52.94%)	2.8018 (2.6915-2.9054)	60.96% (53.54%-68.69%)	40.37% (30.88%-50.77%)	96.276% (91.176%-100%)	94.88% (87.5%-100%)	48.48% (39.13%-58.34%)	2.8001 (2.5956-3.0187)
At least 6 of CHASM (breast), CHASM (lung), CHASM (melanoma), FATHMM (cancer), FATHMM (missense), Mutation Assessor	65.02% (61.11%-68.69%)	46.41% (41.32%-51.54%)	97.203% (95.588%-100%)	96.63% (94.64%-100%)	51.19% (46.32%-55.71%)	2.9144 (2.8208-3.0157)	64.91% (57.58%-72.73%)	46% (36.07%-56.06%)	97.337% (92.5%-100%)	96.75% (90.9%-100%)	51.23% (42.25%-61.29%)	2.9131 (2.7047-3.1009)
At least 6 of CHASM (breast), CHASM (lung), CHASM (melanoma), FATHMM (cancer), FATHMM (missense), MutationTaster	68.35% (64.65%-72.22%)	51.12% (45.86%-56.25%)	98.143% (97.015%-100%)	97.94% (96.67%-100%)	53.73% (48.85%-58.46%)	3.0095 (2.9091-3.1073)	68.34% (60.61%-75.76%)	50.93% (40.62%-61.29%)	98.209% (94.118%-100%)	98% (93.55%-100%)	53.84% (44.26%-63.8%)	3.0098 (2.8129-3.2079)
At least 6 of CHASM (breast), CHASM (lung), CHASM (melanoma), FATHMM (cancer), FATHMM (missense), PolyPhen-2	66.36% (62.63%-70.2%)	48.51% (43.44%-53.6%)	97.203% (95.588%-100%)	96.78% (94.92%-100%)	52.2% (47.37%-56.82%)	2.9469 (2.8493-3.0495)	66.28% (58.59%-73.74%)	48.17% (37.87%-58.62%)	97.337% (92.5%-100%)	96.89% (91.43%-100%)	52.25% (43.1%-61.91%)	2.9465 (2.7425-3.1420)
At least 6 of CHASM (breast), CHASM (lung), CHASM (melanoma), FATHMM (cancer), FATHMM (missense), PROVEAN	59.31% (55.05%-63.13%)	36.3% (31.45%-40.94%)	99.084% (98.485%-100%)	98.56% (97.5%-100%)	47.36% (42.48%-51.68%)	2.8130 (2.7289-2.8945)	59.17% (51.52%-67.68%)	35.9% (26.31%-45.9%)	99.083% (96.774%-100%)	98.54% (94.74%-100%)	47.38% (38.66%-56.96%)	2.8090 (2.6399-2.9812)
At least 6 of CHASM (breast), CHASM (lung), CHASM (melanoma), FATHMM (cancer), FATHMM (missense), SIFT	69.41% (65.66%-73.23%)	52.79% (47.62%-57.94%)	98.143% (97.015%-100%)	98.01% (96.77%-100%)	54.59% (49.62%-59.23%)	3.0353 (2.9437-3.1304)	69.26% (61.62%-76.77%)	52.38% (42.3%-62.12%)	98.209% (94.118%-100%)	98.05% (93.75%-100%)	54.58% (45.45%-64.41%)	3.0322 (2.8416-3.2191)
At least 6 of CHASM (breast), CHASM (lung), CHASM (melanoma), FATHMM (cancer), FATHMM (missense), VEST	61.32% (57.07%-64.66%)	40.56% (35.29%-45.6%)	97.203% (95.588%-100%)	96.17% (93.88%-100%)	48.61% (43.75%-53.06%)	2.8254 (2.7272-2.9185)	61.21% (54.52%-69.7%)	40.14% (30%-50.02%)	97.337% (92.5%-100%)	96.3% (90%-100%)	48.65% (39.44%-58.44%)	2.8244 (2.6346-3.0201)
At least 6 of CHASM (breast), CHASM (lung), CHASM (melanoma), FATHMM (cancer), Mutation Assessor, MutationTaster	62.63% (59.08%-66.67%)	42.09% (36.89%-46.88%)	98.143% (97.015%-100%)	97.52% (95.92%-100%)	49.5% (44.83%-54.06%)	2.8725 (2.7838-2.9656)	62.62% (54.55%-69.72%)	41.87% (32.2%-52.38%)	98.209% (94.118%-100%)	97.58% (92.31%-100%)	49.61% (40.28%-59.16%)	2.8727 (2.6890-3.0553)
At least 6 of CHASM (breast), CHASM (lung), CHASM (melanoma), FATHMM (cancer), Mutation Assessor, PolyPhen-2	63.64% (60.09%-67.18%)	44.23% (39.16%-49.21%)	97.203% (95.588%-100%)	96.48% (94.34%-100%)	50.2% (45.39%-54.62%)	2.8811 (2.7906-2.9845)	63.62% (56.54%-70.73%)	43.96% (33.87%-53.85%)	97.337% (92.5%-100%)	96.61% (90.47%-100%)	50.3% (41.33%-59.72%)	2.8821 (2.6742-3.0671)
At least 6 of CHASM (breast), CHASM (lung), CHASM (melanoma), FATHMM (cancer), Mutation Assessor, PROVEAN	57.94% (54.04%-61.62%)	34.15% (29.41%-38.71%)	99.084% (98.485%-100%)	98.47% (97.37%-100%)	46.53% (41.83%-50.94%)	2.7823 (2.6972-2.8732)	57.85% (50.51%-65.66%)	33.82% (24.56%-43.75%)	99.083% (96.774%-100%)	98.44% (94.12%-100%)	46.59% (38.09%-56%)	2.7793 (2.6022-2.9550)
At least 6 of CHASM (breast), CHASM (lung), CHASM (melanoma), FATHMM (cancer), Mutation Assessor, SIFT	64.67% (61.11%-68.18%)	45.31% (40%-50.39%)	98.143% (97.015%-100%)	97.69% (96.15%-100%)	50.93% (46.15%-55.32%)	2.9206 (2.8324-3.0204)	64.6% (57.58%-71.72%)	45.01% (34.91%-55.17%)	98.209% (94.118%-100%)	97.74% (92.86%-100%)	50.99% (41.79%-60.56%)	2.9195 (2.7337-3.1020)
At least 6 of CHASM (breast), CHASM (lung), CHASM (melanoma), FATHMM (cancer), Mutation Assessor, VEST	59.61% (55.56%-63.64%)	37.87% (33.05%-42.97%)	97.203% (95.588%-100%)	95.91% (93.48%-100%)	47.5% (42.76%-52.03%)	2.7848 (2.6861-2.8813)	59.57% (51.52%-67.68%)	37.55% (27.41%-47.55%)	97.337% (92.5%-100%)	96.06% (89.28%-100%)	47.6% (38.36%-57.14%)	2.7854 (2.5867-2.9887)
At least 6 of CHASM (breast), CHASM (lung), CHASM (melanoma), FATHMM (cancer), MutationTaster, PolyPhen-2	65.98% (62.12%-69.7%)	47.38% (42.15%-52.42%)	98.143% (97.015%-100%)	97.79% (96.36%-100%)	51.9% (47.06%-56.3%)	2.9521 (2.8601-3.0474)	66.01% (58.59%-73.74%)	47.24% (37.29%-57.63%)	98.209% (94.118%-100%)	97.85% (93.1%-100%)	52.04% (42.86%-61.77%)	2.9534 (2.7672-3.1420)
At least 6 of CHASM (breast), CHASM (lung), CHASM (melanoma), FATHMM (cancer), MutationTaster, PROVEAN	59.27% (55.05%-62.64%)	36.24% (31.45%-41.22%)	99.084% (98.485%-100%)	98.56% (97.5%-100%)	47.34% (42.57%-51.63%)	2.8122 (2.7237-2.8945)	59.23% (52.5%-67.68%)	36.01% (26.23%-45.9%)	99.083% (96.774%-100%)	98.54% (94.74%-100%)	47.43% (38.88%-57.14%)	2.8106 (2.6450-2.9919)
At least 6 of CHASM (breast), CHASM (lung), CHASM (melanoma), FATHMM (cancer), MutationTaster, SIFT	66.67% (63.12%-70.2%)	48.47% (43.2%-53.66%)	98.143% (97.015%-100%)	97.83% (96.43%-100%)	52.42% (47.45%-56.95%)	2.9686 (2.8737-3.0675)	66.65% (59.6%-73.76%)	48.26% (37.7%-58.62%)	98.209% (94.118%-100%)	97.89% (93.1%-100%)	52.52% (43.48%-62.5%)	2.9688 (2.7776-3.1654)
At least 6 of CHASM (breast), CHASM (lung), CHASM (melanoma), FATHMM (cancer), MutationTaster, VEST	62.64% (58.59%-66.67%)	42.11% (36.8%-47.29%)	98.143% (97.015%-100%)	97.52% (95.92%-100%)	49.51% (44.68%-54%)	2.8727 (2.7814-2.9676)	62.6% (54.55%-70.71%)	41.84% (31.15%-52.31%)	98.209% (94.118%-100%)	97.58% (92.31%-100%)	49.6% (40.3%-59.42%)	2.8723 (2.6834-3.0671)
At least 6 of CHASM (breast), CHASM (lung), CHASM (melanoma), FATHMM (cancer), PolyPhen-2, PROVEAN	59.28% (55.56%-63.13%)	36.26% (31.2%-40.99%)	99.084% (98.485%-100%)	98.56% (97.5%-100%)	47.34% (42.4%-51.66%)	2.8125 (2.7264-2.8984)	59.21% (51.52%-66.67%)	35.96% (26.32%-46.03%)	99.083% (96.774%-100%)	98.53% (94.73%-100%)	47.41% (38.89%-56.96%)	2.8099 (2.6359-2.9811)
At least 6 of CHASM (breast), CHASM (lung), CHASM (melanoma), FATHMM (cancer), PolyPhen-2, SIFT	66.35% (62.63%-70.2%)	47.96% (42.64%-53.23%)	98.143% (97.015%-100%)	97.81% (96.36%-100%)	52.17% (47.14%-56.52%)	2.9608 (2.8626-3.0562)	66.29% (58.59%-73.74%)	47.68% (37.1%-58.06%)	98.209% (94.118%-100%)	97.86% (93.1%-100%)	52.24% (43.28%-61.98%)	2.9599 (2.7701-3.1589)
At least 6 of CHASM (breast), CHASM (lung), CHASM (melanoma), FATHMM (cancer), PolyPhen-2, VEST	61.62% (57.58%-65.16%)	41.04% (35.71%-46.28%)	97.203% (95.588%-100%)	96.21% (93.88%-100%)	48.81% (43.88%-53.1%)	2.8326 (2.7370-2.9296)	61.61% (54.52%-69.7%)	40.79% (30.5%-50.92%)	97.337% (92.5%-100%)	96.36% (90.31%-100%)	48.93% (40%-58.67%)	2.8341 (2.6433-3.0275)
At least 6 of CHASM (breast), CHASM (lung), CHASM (melanoma), FATHMM (cancer), PROVEAN, SIFT	61.3% (57.58%-65.15%)	39.45% (34.43%-44.26%)	99.084% (98.485%-100%)	98.67% (97.73%-100%)	48.62% (43.75%-52.98%)	2.8583 (2.7711-2.9411)	61.23% (53.54%-68.69%)	39.17% (29.73%-49.18%)	99.083% (96.774%-100%)	98.66% (95.24%-100%)	48.69% (39.74%-58.44%)	2.8560 (2.6907-3.0380)
At least 6 of CHASM (breast), CHASM (lung), CHASM (melanoma), FATHMM (cancer), PROVEAN, VEST	58.61% (54.55%-62.12%)	35.2% (30.3%-39.84%)	99.084% (98.485%-100%)	98.52% (97.44%-100%)	46.93% (42.1%-51.3%)	2.7973 (2.7133-2.8819)	58.54% (51.52%-66.67%)	34.91% (25.36%-45.45%)	99.083% (96.774%-100%)	98.5% (94.44%-100%)	47.01% (38.35%-56.25%)	2.7949 (2.6207-2.9724)
At least 6 of CHASM (breast), CHASM (lung), CHASM (melanoma), FATHMM (cancer), SIFT, VEST	62.65% (58.59%-66.16%)	42.12% (37.1%-47.2%)	98.143% (97.015%-100%)	97.52% (95.92%-100%)	49.51% (44.68%-53.9%)	2.8729 (2.7814-2.9639)	62.58% (55.56%-70.71%)	41.81% (31.74%-51.61%)	98.209% (94.118%-100%)	97.57% (92%-100%)	49.59% (40.58%-59.16%)	2.8718 (2.6890-3.0662)
At least 6 of CHASM (breast), CHASM (lung), CHASM (melanoma), FATHMM (missense), Mutation Assessor, MutationTaster	62.3% (58.59%-66.16%)	41.57% (36.51%-46.46%)	98.143% (97.015%-100%)	97.49% (95.83%-100%)	49.28% (44.52%-53.79%)	2.8648 (2.7742-2.9602)	62.26% (54.55%-69.7%)	41.31% (31.74%-51.61%)	98.209% (94.118%-100%)	97.55% (92%-100%)	49.37% (40.28%-58.9%)	2.8644 (2.6768-3.0458)
At least 6 of CHASM (breast), CHASM (lung), CHASM (melanoma), FATHMM (missense), Mutation Assessor, PolyPhen-2	63.32% (59.6%-67.17%)	43.72% (38.52%-48.8%)	97.203% (95.588%-100%)	96.44% (94.34%-100%)	49.97% (45.21%-54.41%)	2.8733 (2.7796-2.9774)	63.26% (55.56%-70.71%)	43.4% (33.33%-53.45%)	97.337% (92.5%-100%)	96.56% (90.47%-100%)	50.05% (41.25%-59.72%)	2.8735 (2.6635-3.0652)
At least 6 of CHASM (breast), CHASM (lung), CHASM (melanoma), FATHMM (missense), Mutation Assessor, PROVEAN	57.29% (53.03%-61.11%)	33.11% (28.23%-37.8%)	99.084% (98.485%-100%)	98.43% (97.3%-100%)	46.14% (41.45%-50.33%)	2.7676 (2.6829-2.8590)	57.14% (49.49%-65.66%)	32.69% (23.43%-42.62%)	99.083% (96.774%-100%)	98.39% (94.12%-100%)	46.17% (37.66%-55.56%)	2.7634 (2.5827-2.9402)
At least 6 of CHASM (breast), CHASM (lung), CHASM (melanoma), FATHMM (missense), Mutation Assessor, SIFT	64.34% (60.61%-68.18%)	44.79% (39.52%-49.61%)	98.143% (97.015%-100%)	97.66% (96.15%-100%)	50.69% (45.93%-55.25%)	2.9128 (2.8247-3.0097)	64.25% (56.57%-71.72%)	44.44% (34.43%-54.69%)	98.209% (94.118%-100%)	97.71% (92.59%-100%)	50.74% (41.43%-60.3%)	2.9111 (2.7228-3.0929)
At least 6 of CHASM (breast), CHASM (lung), CHASM (melanoma), FATHMM (missense), Mutation Assessor, VEST	59.28% (55.05%-63.13%)	37.35% (32.26%-42.52%)	97.203% (95.588%-100%)	95.85% (93.33%-100%)	47.29% (42.57%-51.72%)	2.7770 (2.6785-2.8750)	59.21% (51.52%-67.68%)	36.99% (26.56%-46.97%)	97.337% (92.5%-100%)	96% (88.89%-100%)	47.38% (38.36%-56.76%)	2.7770 (2.5790-2.9728)
At least 6 of CHASM (breast), CHASM (lung), CHASM (melanoma), FATHMM (missense), MutationTaster, PolyPhen-2	63.98% (60.1%-67.68%)	44.22% (39.02%-49.19%)	98.143% (97.015%-100%)	97.63% (96.08%-100%)	50.44% (45.59%-54.93%)	2.9043 (2.8079-2.9973)	63.96% (56.57%-71.72%)	44% (33.9%-54.1%)	98.209% (94.118%-100%)	97.7% (92.59%-100%)	50.54% (41.43%-60%)	2.9045 (2.7223-3.0969)
At least 6 of CHASM (breast), CHASM (lung), CHASM (melanoma), FATHMM (missense), MutationTaster, PROVEAN	57.27% (53.03%-61.11%)	33.09% (28.33%-37.7%)	99.084% (98.485%-100%)	98.43% (97.22%-100%)	46.13% (41.4%-50.33%)	2.7674 (2.6797-2.8519)	57.17% (49.49%-65.66%)	32.73% (23.72%-42.62%)	99.083% (96.774%-100%)	98.41% (94.12%-100%)	46.19% (37.66%-55.56%)	2.7641 (2.5988-2.9468)
At least 6 of CHASM (breast), CHASM (lung), CHASM (melanoma), FATHMM (missense), MutationTaster, SIFT	65% (61.11%-68.69%)	45.83% (40.5%-50.82%)	98.143% (97.015%-100%)	97.71% (96.23%-100%)	51.17% (46.27%-55.71%)	2.9286 (2.8325-3.0227)	64.94% (57.58%-72.73%)	45.55% (35.29%-55.88%)	98.209% (94.118%-100%)	97.77% (92.86%-100%)	51.24% (42.03%-61.29%)	2.9277 (2.7394-3.1181)
At least 6 of CHASM (breast), CHASM (lung), CHASM (melanoma), FATHMM (missense), MutationTaster, VEST	60.64% (56.57%-64.65%)	38.94% (33.84%-44%)	98.143% (97.015%-100%)	97.32% (95.56%-100%)	48.18% (43.51%-52.67%)	2.8258 (2.7281-2.9173)	60.55% (52.53%-68.69%)	38.59% (28.57%-48.62%)	98.209% (94.118%-100%)	97.38% (91.67%-100%)	48.24% (38.89%-58.11%)	2.8243 (2.6361-3.0201)
At least 6 of CHASM (breast), CHASM (lung), CHASM (melanoma), FATHMM (missense), PolyPhen-2, PROVEAN	57.29% (53.54%-61.11%)	33.11% (28.12%-37.88%)	99.084% (98.485%-100%)	98.43% (97.3%-100%)	46.14% (41.25%-50.32%)	2.7676 (2.6810-2.8542)	57.14% (49.49%-64.65%)	32.69% (22.95%-42.86%)	99.083% (96.774%-100%)	98.4% (94.12%-100%)	46.17% (37.5%-55.56%)	2.7634 (2.5933-2.9331)
At least 6 of CHASM (breast), CHASM (lung), CHASM (melanoma), FATHMM (missense), PolyPhen-2, SIFT	64.68% (60.61%-68.18%)	45.32% (40%-50.4%)	98.143% (97.015%-100%)	97.69% (96.15%-100%)	50.94% (46.04%-55.4%)	2.9209 (2.8271-3.0176)	64.58% (57.58%-72.73%)	44.97% (34.43%-55.71%)	98.209% (94.118%-100%)	97.74% (92.59%-100%)	50.98% (42.03%-60.81%)	2.9190 (2.7393-3.1137)
At least 6 of CHASM (breast), CHASM (lung), CHASM (melanoma), FATHMM (missense), PolyPhen-2, VEST	59.61% (55.56%-63.13%)	37.87% (32.5%-42.97%)	97.203% (95.588%-100%)	95.91% (93.48%-100%)	47.5% (42.66%-51.68%)	2.7848 (2.6883-2.8819)	59.56% (52.53%-67.68%)	37.54% (27.12%-48.22%)	97.337% (92.5%-100%)	96.06% (89.28%-100%)	47.6% (38.75%-57.34%)	2.7853 (2.5893-2.9811)
At least 6 of CHASM (breast), CHASM (lung), CHASM (melanoma), FATHMM (missense), PROVEAN, SIFT	59.31% (55.05%-63.13%)	36.3% (31.45%-40.94%)	99.084% (98.485%-100%)	98.56% (97.5%-100%)	47.36% (42.48%-51.68%)	2.8130 (2.7289-2.8945)	59.17% (51.52%-67.68%)	35.9% (26.31%-45.9%)	99.083% (96.774%-100%)	98.54% (94.74%-100%)	47.38% (38.66%-56.96%)	2.8090 (2.6399-2.9812)
At least 6 of CHASM (breast), CHASM (lung), CHASM (melanoma), FATHMM (missense), PROVEAN, VEST	56.61% (52.53%-60.11%)	32.05% (27.13%-36.72%)	99.084% (98.485%-100%)	98.38% (97.14%-100%)	45.75% (40.91%-50%)	2.7525 (2.6650-2.8335)	56.47% (49.47%-64.65%)	31.63% (22.22%-41.54%)	99.083% (96.774%-100%)	98.35% (93.75%-100%)	45.79% (37.18%-55.41%)	2.7485 (2.5807-2.9314)
At least 6 of CHASM (breast), CHASM (lung), CHASM (melanoma), FATHMM (missense), SIFT, VEST	60.98% (57.07%-64.65%)	39.48% (34.39%-44.63%)	98.143% (97.015%-100%)	97.36% (95.65%-100%)	48.4% (43.62%-52.9%)	2.8338 (2.7400-2.9221)	60.87% (53.54%-68.69%)	39.1% (28.84%-50%)	98.209% (94.118%-100%)	97.41% (91.67%-100%)	48.45% (39.7%-58.11%)	2.8317 (2.6536-3.0201)
At least 6 of CHASM (breast), CHASM (lung), CHASM (melanoma), Mutation Assessor, MutationTaster, PolyPhen-2	61.27% (57.07%-65.15%)	40.47% (35.29%-45.46%)	97.222% (95.588%-100%)	96.18% (93.75%-100%)	48.58% (43.97%-52.98%)	2.8245 (2.7321-2.9248)	61.3% (53.54%-69.7%)	40.32% (30.3%-50.79%)	97.294% (92.303%-100%)	96.24% (90%-100%)	48.72% (39.44%-58.34%)	2.8258 (2.6207-3.0183)
At least 6 of CHASM (breast), CHASM (lung), CHASM (melanoma), Mutation Assessor, MutationTaster, PROVEAN	56.25% (52.02%-60.1%)	31.48% (26.72%-36%)	99.084% (98.485%-100%)	98.34% (97.14%-100%)	45.54% (40.91%-50%)	2.7445 (2.6573-2.8315)	56.19% (48.48%-64.65%)	31.19% (22.06%-40.99%)	99.083% (96.774%-100%)	98.32% (93.75%-100%)	45.62% (37.04%-55%)	2.7421 (2.5668-2.9234)
At least 6 of CHASM (breast), CHASM (lung), CHASM (melanoma), Mutation Assessor, MutationTaster, SIFT	62.29% (58.59%-66.16%)	42.08% (36.8%-46.92%)	97.222% (95.588%-100%)	96.32% (94%-100%)	49.26% (44.6%-53.85%)	2.8488 (2.7516-2.9494)	62.3% (54.55%-69.7%)	41.89% (32.2%-52.24%)	97.294% (92.303%-100%)	96.37% (90.31%-100%)	49.39% (39.99%-58.9%)	2.8494 (2.6448-3.0441)
At least 6 of CHASM (breast), CHASM (lung), CHASM (melanoma), Mutation Assessor, MutationTaster, VEST	58.59% (54.55%-62.63%)	36.25% (31.01%-41.47%)	97.222% (95.588%-100%)	95.75% (93.18%-100%)	46.87% (42.21%-51.33%)	2.7609 (2.6607-2.8613)	58.57% (50.51%-66.67%)	36.01% (25.67%-46.88%)	97.294% (92.303%-100%)	95.8% (88.46%-100%)	46.98% (37.8%-56.52%)	2.7608 (2.5586-2.9676)
At least 6 of CHASM (breast), CHASM (lung), CHASM (melanoma), Mutation Assessor, PolyPhen-2, PROVEAN	56.93% (53.03%-60.61%)	32.54% (27.59%-37.01%)	99.084% (98.485%-100%)	98.4% (97.22%-100%)	45.93% (41.18%-50.31%)	2.7595 (2.6744-2.8466)	56.86% (49.49%-64.65%)	32.24% (22.95%-42.37%)	99.083% (96.774%-100%)	98.37% (94.11%-100%)	46% (37.33%-55.26%)	2.7569 (2.5807-2.9286)
At least 6 of CHASM (breast), CHASM (lung), CHASM (melanoma), Mutation Assessor, PolyPhen-2, SIFT	63.31% (59.6%-67.17%)	43.69% (38.33%-48.78%)	97.222% (95.588%-100%)	96.45% (94.23%-100%)	49.96% (45.14%-54.35%)	2.8732 (2.7786-2.9726)	63.29% (55.56%-70.71%)	43.46% (32.79%-53.34%)	97.294% (92.303%-100%)	96.5% (90.48%-100%)	50.07% (40.91%-59.75%)	2.8732 (2.6718-3.0677)
At least 6 of CHASM (breast), CHASM (lung), CHASM (melanoma), Mutation Assessor, PolyPhen-2, VEST	58.25% (54.04%-62.12%)	36.24% (31.03%-41.27%)	96.282% (94.284%-98.667%)	94.4% (91.3%-98%)	46.62% (41.89%-51.01%)	2.7355 (2.6347-2.8427)	58.26% (50.51%-66.67%)	36% (26.15%-46.16%)	96.423% (91.176%-100%)	94.53% (86.36%-100%)	46.76% (37.66%-55.85%)	2.7372 (2.5198-2.9361)
At least 6 of CHASM (breast), CHASM (lung), CHASM (melanoma), Mutation Assessor, PROVEAN, SIFT	57.94% (54.04%-61.62%)	34.15% (29.41%-38.71%)	99.084% (98.485%-100%)	98.47% (97.37%-100%)	46.53% (41.83%-50.94%)	2.7823 (2.6972-2.8732)	57.85% (50.51%-65.66%)	33.82% (24.56%-43.75%)	99.083% (96.774%-100%)	98.44% (94.12%-100%)	46.59% (38.09%-56%)	2.7793 (2.6022-2.9550)
At least 6 of CHASM (breast), CHASM (lung), CHASM (melanoma), Mutation Assessor, PROVEAN, VEST	55.91% (51.52%-59.6%)	30.94% (26.19%-35.48%)	99.084% (98.485%-100%)	98.32% (97.06%-100%)	45.35% (40.64%-49.69%)	2.7369 (2.6515-2.8254)	55.86% (48.48%-64.65%)	30.66% (21.43%-40.36%)	99.083% (96.774%-100%)	98.29% (93.75%-100%)	45.43% (36.9%-54.88%)	2.7347 (2.5591-2.9135)
At least 6 of CHASM (breast), CHASM (lung), CHASM (melanoma), Mutation Assessor, SIFT, VEST	59.61% (55.56%-63.64%)	37.85% (33.06%-42.97%)	97.222% (95.588%-100%)	95.93% (93.48%-100%)	47.5% (42.76%-52.03%)	2.7850 (2.6902-2.8832)	59.57% (51.52%-67.68%)	37.57% (27.41%-47.46%)	97.294% (92.303%-100%)	95.97% (89.28%-100%)	47.6% (38.46%-56.92%)	2.7844 (2.5855-2.9746)
At least 6 of CHASM (breast), CHASM (lung), CHASM (melanoma), MutationTaster, PolyPhen-2, PROVEAN	57.25% (53.03%-61.11%)	33.06% (27.96%-37.88%)	99.084% (98.485%-100%)	98.42% (97.22%-100%)	46.12% (41.29%-50.32%)	2.7669 (2.6793-2.8483)	57.21% (49.49%-65.66%)	32.8% (23.43%-42.86%)	99.083% (96.774%-100%)	98.4% (94.12%-100%)	46.21% (37.8%-55.56%)	2.7650 (2.6022-2.9468)
At least 6 of CHASM (breast), CHASM (lung), CHASM (melanoma), MutationTaster, PolyPhen-2, SIFT	63.63% (59.6%-67.17%)	44.2% (39.02%-49.19%)	97.222% (95.588%-100%)	96.49% (94.23%-100%)	50.19% (45.39%-54.55%)	2.8810 (2.7822-2.9791)	63.65% (56.57%-71.72%)	44.04% (33.9%-54.1%)	97.294% (92.303%-100%)	96.54% (90.91%-100%)	50.33% (41.17%-60.01%)	2.8820 (2.6849-3.0759)
At least 6 of CHASM (breast), CHASM (lung), CHASM (melanoma), MutationTaster, PolyPhen-2, VEST	60.6% (56.57%-64.14%)	39.42% (34.15%-44.54%)	97.222% (95.588%-100%)	96.08% (93.62%-100%)	48.14% (43.45%-52.41%)	2.8086 (2.7112-2.9115)	60.62% (53.54%-68.69%)	39.24% (29.02%-50%)	97.294% (92.303%-100%)	96.13% (89.66%-100%)	48.28% (39.39%-57.81%)	2.8094 (2.6024-3.0029)
At least 6 of CHASM (breast), CHASM (lung), CHASM (melanoma), MutationTaster, PROVEAN, SIFT	59.27% (55.05%-62.64%)	36.24% (31.45%-41.22%)	99.084% (98.485%-100%)	98.56% (97.5%-100%)	47.34% (42.57%-51.63%)	2.8122 (2.7237-2.8945)	59.23% (52.5%-67.68%)	36.01% (26.23%-45.9%)	99.083% (96.774%-100%)	98.54% (94.74%-100%)	47.43% (38.88%-57.14%)	2.8106 (2.6450-2.9919)
At least 6 of CHASM (breast), CHASM (lung), CHASM (melanoma), MutationTaster, PROVEAN, VEST	57.26% (53.03%-61.11%)	33.06% (28.33%-37.82%)	99.084% (98.485%-100%)	98.42% (97.3%-100%)	46.12% (41.29%-50.32%)	2.7669 (2.6782-2.8500)	57.21% (49.49%-65.66%)	32.8% (23.21%-42.43%)	99.083% (96.774%-100%)	98.4% (94.12%-100%)	46.21% (37.66%-55.7%)	2.7650 (2.5942-2.9468)
At least 6 of CHASM (breast), CHASM (lung), CHASM (melanoma), MutationTaster, SIFT, VEST	61.29% (57.58%-65.15%)	40.5% (35.2%-45.74%)	97.222% (95.588%-100%)	96.18% (93.75%-100%)	48.59% (43.84%-52.98%)	2.8249 (2.7272-2.9203)	61.27% (53.54%-68.69%)	40.26% (29.68%-50.85%)	97.294% (92.303%-100%)	96.22% (90%-100%)	48.7% (39.44%-58.34%)	2.8248 (2.6284-3.0215)
At least 6 of CHASM (breast), CHASM (lung), CHASM (melanoma), PolyPhen-2, PROVEAN, SIFT	59.28% (55.56%-63.13%)	36.26% (31.2%-40.99%)	99.084% (98.485%-100%)	98.56% (97.5%-100%)	47.34% (42.4%-51.66%)	2.8125 (2.7264-2.8984)	59.21% (51.52%-66.67%)	35.96% (26.32%-46.03%)	99.083% (96.774%-100%)	98.53% (94.73%-100%)	47.41% (38.89%-56.96%)	2.8099 (2.6359-2.9811)
At least 6 of CHASM (breast), CHASM (lung), CHASM (melanoma), PolyPhen-2, PROVEAN, VEST	56.91% (53.03%-60.61%)	32.52% (27.56%-37.12%)	99.084% (98.485%-100%)	98.4% (97.22%-100%)	45.92% (40.99%-50%)	2.7593 (2.6743-2.8483)	56.88% (49.49%-65.66%)	32.27% (22.8%-42.03%)	99.083% (96.774%-100%)	98.37% (94.12%-100%)	46.02% (37.5%-55.56%)	2.7575 (2.5772-2.9319)
At least 6 of CHASM (breast), CHASM (lung), CHASM (melanoma), PolyPhen-2, SIFT, VEST	60.61% (56.57%-64.14%)	39.43% (34.15%-44.7%)	97.222% (95.588%-100%)	96.08% (93.62%-100%)	48.14% (43.45%-52.35%)	2.8087 (2.7106-2.9058)	60.61% (53.54%-68.69%)	39.21% (28.81%-50%)	97.294% (92.303%-100%)	96.13% (89.99%-100%)	48.26% (39.44%-57.58%)	2.8090 (2.6061-3.0019)
At least 6 of CHASM (breast), CHASM (lung), CHASM (melanoma), PROVEAN, SIFT, VEST	58.61% (54.55%-62.12%)	35.2% (30.3%-39.84%)	99.084% (98.485%-100%)	98.52% (97.44%-100%)	46.93% (42.1%-51.3%)	2.7973 (2.7133-2.8819)	58.54% (51.52%-66.67%)	34.91% (25.36%-45.45%)	99.083% (96.774%-100%)	98.5% (94.44%-100%)	47.01% (38.35%-56.25%)	2.7949 (2.6207-2.9724)
At least 6 of CHASM (breast), CHASM (lung), FATHMM (cancer), FATHMM (missense), Mutation Assessor, MutationTaster	61.98% (58.08%-65.66%)	41.06% (35.88%-45.9%)	98.143% (97.015%-100%)	97.45% (95.74%-100%)	49.06% (44.44%-53.47%)	2.8571 (2.7662-2.9504)	61.91% (54.55%-69.7%)	40.75% (31.15%-51.47%)	98.209% (94.118%-100%)	97.52% (92%-100%)	49.13% (40%-58.67%)	2.8560 (2.6721-3.0426)
At least 6 of CHASM (breast), CHASM (lung), FATHMM (cancer), FATHMM (missense), Mutation Assessor, PolyPhen-2	62.99% (59.09%-66.67%)	43.2% (37.93%-48.07%)	97.203% (95.588%-100%)	96.4% (94.23%-100%)	49.74% (45%-54.23%)	2.8654 (2.7707-2.9695)	62.91% (55.56%-70.71%)	42.84% (32.79%-53.03%)	97.337% (92.5%-100%)	96.52% (90.32%-100%)	49.8% (41.1%-59.7%)	2.8650 (2.6552-3.0516)
At least 6 of CHASM (breast), CHASM (lung), FATHMM (cancer), FATHMM (missense), Mutation Assessor, PROVEAN	57.29% (53.03%-61.11%)	33.11% (28.23%-37.8%)	99.084% (98.485%-100%)	98.43% (97.3%-100%)	46.14% (41.45%-50.33%)	2.7676 (2.6829-2.8590)	57.14% (49.49%-65.66%)	32.69% (23.43%-42.62%)	99.083% (96.774%-100%)	98.39% (94.12%-100%)	46.17% (37.66%-55.56%)	2.7634 (2.5827-2.9402)
At least 6 of CHASM (breast), CHASM (lung), FATHMM (cancer), FATHMM (missense), Mutation Assessor, SIFT	64.01% (60.1%-67.68%)	44.27% (39.17%-49.18%)	98.143% (97.015%-100%)	97.63% (96.08%-100%)	50.46% (45.71%-54.93%)	2.9051 (2.8166-2.9982)	63.89% (56.57%-71.72%)	43.88% (34.36%-53.52%)	98.209% (94.118%-100%)	97.69% (92.59%-100%)	50.49% (41.43%-60%)	2.9026 (2.7177-3.0828)
At least 6 of CHASM (breast), CHASM (lung), FATHMM (cancer), FATHMM (missense), Mutation Assessor, VEST	58.96% (55.05%-62.63%)	36.83% (31.62%-41.86%)	97.203% (95.588%-100%)	95.8% (93.33%-100%)	47.09% (42.28%-51.63%)	2.7692 (2.6722-2.8660)	58.86% (51.52%-66.67%)	36.43% (26.47%-46.56%)	97.337% (92.5%-100%)	95.94% (88.89%-100%)	47.15% (38.16%-56.41%)	2.7686 (2.5748-2.9602)
At least 6 of CHASM (breast), CHASM (lung), FATHMM (cancer), FATHMM (missense), MutationTaster, PolyPhen-2	64% (60.1%-67.68%)	44.25% (39.06%-49.23%)	98.143% (97.015%-100%)	97.63% (96.08%-100%)	50.45% (45.71%-54.93%)	2.9048 (2.8101-2.9973)	63.92% (56.57%-71.72%)	43.93% (33.85%-54.39%)	98.209% (94.118%-100%)	97.69% (92.59%-100%)	50.51% (41.1%-60%)	2.9035 (2.7227-3.0929)
At least 6 of CHASM (breast), CHASM (lung), FATHMM (cancer), FATHMM (missense), MutationTaster, PROVEAN	57.62% (53.54%-61.62%)	33.64% (29.03%-38.4%)	99.084% (98.485%-100%)	98.45% (97.3%-100%)	46.34% (41.56%-50.65%)	2.7752 (2.6884-2.8595)	57.48% (49.49%-65.66%)	33.23% (23.81%-42.65%)	99.083% (96.774%-100%)	98.43% (94.12%-100%)	46.37% (37.66%-55.85%)	2.7712 (2.6021-2.9515)
At least 6 of CHASM (breast), CHASM (lung), FATHMM (cancer), FATHMM (missense), MutationTaster, SIFT	64.68% (61.1%-68.69%)	45.32% (40%-50.39%)	98.143% (97.015%-100%)	97.69% (96.23%-100%)	50.93% (46.04%-55.4%)	2.9208 (2.8247-3.0189)	64.59% (56.57%-71.74%)	44.99% (35.18%-55.01%)	98.209% (94.118%-100%)	97.74% (92.86%-100%)	50.98% (42.02%-60.88%)	2.9192 (2.7306-3.1167)
At least 6 of CHASM (breast), CHASM (lung), FATHMM (cancer), FATHMM (missense), MutationTaster, VEST	60.66% (56.57%-64.65%)	38.97% (33.88%-44%)	98.143% (97.015%-100%)	97.32% (95.56%-100%)	48.19% (43.51%-52.7%)	2.8263 (2.7326-2.9184)	60.51% (52.53%-68.69%)	38.53% (28.57%-48.39%)	98.209% (94.118%-100%)	97.38% (91.67%-100%)	48.21% (38.89%-57.9%)	2.8233 (2.6306-3.0160)
At least 6 of CHASM (breast), CHASM (lung), FATHMM (cancer), FATHMM (missense), PolyPhen-2, PROVEAN	57.64% (53.54%-61.11%)	33.66% (28.8%-38.58%)	99.084% (98.485%-100%)	98.45% (97.3%-100%)	46.35% (41.51%-50.63%)	2.7754 (2.6909-2.8595)	57.46% (50.51%-65.66%)	33.19% (23.33%-43.33%)	99.083% (96.774%-100%)	98.42% (94.44%-100%)	46.35% (37.83%-55.7%)	2.7704 (2.5942-2.9437)
At least 6 of CHASM (breast), CHASM (lung), FATHMM (cancer), FATHMM (missense), PolyPhen-2, SIFT	64.35% (60.61%-68.18%)	44.81% (39.52%-50%)	98.143% (97.015%-100%)	97.66% (96.15%-100%)	50.7% (45.77%-55.15%)	2.9131 (2.8168-3.0090)	64.22% (56.57%-71.72%)	44.41% (33.93%-54.69%)	98.209% (94.118%-100%)	97.71% (92.59%-100%)	50.72% (41.93%-60.56%)	2.9105 (2.7227-3.1049)
At least 6 of CHASM (breast), CHASM (lung), FATHMM (cancer), FATHMM (missense), PolyPhen-2, VEST	59.63% (55.56%-63.13%)	37.9% (32.75%-42.86%)	97.203% (95.588%-100%)	95.91% (93.48%-100%)	47.52% (42.66%-51.75%)	2.7854 (2.6879-2.8817)	59.52% (52.53%-67.68%)	37.48% (27.69%-47.62%)	97.337% (92.5%-100%)	96.06% (89.29%-100%)	47.57% (38.67%-57.33%)	2.7844 (2.5867-2.9767)
At least 6 of CHASM (breast), CHASM (lung), FATHMM (cancer), FATHMM (missense), PROVEAN, SIFT	59.31% (55.05%-63.13%)	36.3% (31.45%-40.94%)	99.084% (98.485%-100%)	98.56% (97.5%-100%)	47.36% (42.48%-51.68%)	2.8130 (2.7289-2.8945)	59.17% (51.52%-67.68%)	35.9% (26.31%-45.9%)	99.083% (96.774%-100%)	98.54% (94.74%-100%)	47.38% (38.66%-56.96%)	2.8090 (2.6399-2.9812)
At least 6 of CHASM (breast), CHASM (lung), FATHMM (cancer), FATHMM (missense), PROVEAN, VEST	56.96% (53.03%-60.61%)	32.6% (27.56%-37.4%)	99.084% (98.485%-100%)	98.4% (97.22%-100%)	45.95% (41.03%-50.31%)	2.7603 (2.6755-2.8417)	56.79% (49.49%-64.65%)	32.13% (22.38%-42.11%)	99.083% (96.774%-100%)	98.37% (94.11%-100%)	45.97% (37.5%-55.56%)	2.7555 (2.5898-2.9321)
At least 6 of CHASM (breast), CHASM (lung), FATHMM (cancer), FATHMM (missense), SIFT, VEST	60.65% (56.57%-64.15%)	38.97% (34.09%-43.91%)	98.143% (97.015%-100%)	97.32% (95.56%-100%)	48.19% (43.45%-52.67%)	2.8262 (2.7342-2.9161)	60.52% (53.51%-68.69%)	38.54% (28.33%-48.39%)	98.209% (94.118%-100%)	97.38% (91.3%-100%)	48.22% (39.19%-57.69%)	2.8234 (2.6444-3.0125)
At least 6 of CHASM (breast), CHASM (lung), FATHMM (cancer), Mutation Assessor, MutationTaster, PolyPhen-2	61.96% (58.08%-65.66%)	41.03% (35.71%-46.03%)	98.143% (97.015%-100%)	97.45% (95.74%-100%)	49.05% (44.55%-53.47%)	2.8568 (2.7657-2.9535)	61.94% (54.55%-69.7%)	40.8% (30.64%-51.43%)	98.209% (94.118%-100%)	97.52% (92%-100%)	49.15% (40%-58.44%)	2.8568 (2.6668-3.0427)
At least 6 of CHASM (breast), CHASM (lung), FATHMM (cancer), Mutation Assessor, MutationTaster, PROVEAN	56.93% (53.03%-60.62%)	32.55% (27.78%-37.1%)	99.084% (98.485%-100%)	98.4% (97.22%-100%)	45.94% (41.25%-50.32%)	2.7597 (2.6710-2.8483)	56.84% (49.47%-64.65%)	32.22% (22.95%-42.11%)	99.083% (96.774%-100%)	98.37% (93.75%-100%)	46% (37.33%-55.43%)	2.7568 (2.5770-2.9401)
At least 6 of CHASM (breast), CHASM (lung), FATHMM (cancer), Mutation Assessor, MutationTaster, SIFT	62.98% (59.09%-66.67%)	42.64% (37.5%-47.54%)	98.143% (97.015%-100%)	97.55% (95.92%-100%)	49.74% (45.03%-54.29%)	2.8807 (2.7900-2.9728)	62.93% (55.56%-70.71%)	42.37% (32.76%-53.03%)	98.209% (94.118%-100%)	97.61% (92.31%-100%)	49.82% (40.84%-59.42%)	2.8801 (2.6998-3.0684)
At least 6 of CHASM (breast), CHASM (lung), FATHMM (cancer), Mutation Assessor, MutationTaster, VEST	59.28% (55.05%-63.13%)	36.81% (31.4%-41.94%)	98.143% (97.015%-100%)	97.17% (95.24%-100%)	47.32% (42.58%-51.97%)	2.7944 (2.7027-2.8855)	59.21% (51.52%-67.68%)	36.48% (26.23%-47.15%)	98.209% (94.118%-100%)	97.23% (91.3%-100%)	47.4% (38.23%-56.96%)	2.7932 (2.6087-2.9887)
At least 6 of CHASM (breast), CHASM (lung), FATHMM (cancer), Mutation Assessor, PolyPhen-2, PROVEAN	57.61% (53.54%-61.62%)	33.62% (28.68%-38.17%)	99.084% (98.485%-100%)	98.45% (97.3%-100%)	46.33% (41.72%-50.65%)	2.7748 (2.6884-2.8595)	57.51% (49.49%-65.66%)	33.27% (24.14%-42.86%)	99.083% (96.774%-100%)	98.42% (94.12%-100%)	46.39% (37.66%-55.84%)	2.7716 (2.5942-2.9438)
At least 6 of CHASM (breast), CHASM (lung), FATHMM (cancer), Mutation Assessor, PolyPhen-2, SIFT	64% (60.1%-67.68%)	44.25% (39.17%-49.22%)	98.143% (97.015%-100%)	97.63% (96.08%-100%)	50.45% (45.52%-54.93%)	2.9047 (2.8129-3.0038)	63.92% (56.57%-71.72%)	43.93% (33.33%-53.85%)	98.209% (94.118%-100%)	97.69% (92.59%-100%)	50.51% (41.43%-60.3%)	2.9035 (2.7219-3.0929)
At least 6 of CHASM (breast), CHASM (lung), FATHMM (cancer), Mutation Assessor, PolyPhen-2, VEST	58.94% (55.05%-62.63%)	36.81% (31.45%-41.74%)	97.203% (95.588%-100%)	95.8% (93.18%-100%)	47.08% (42.36%-51.63%)	2.7689 (2.6686-2.8648)	58.89% (51.52%-66.67%)	36.48% (26.66%-47.07%)	97.337% (92.5%-100%)	95.95% (88.88%-100%)	47.18% (38.16%-56.58%)	2.7694 (2.5785-2.9704)
At least 6 of CHASM (breast), CHASM (lung), FATHMM (cancer), Mutation Assessor, PROVEAN, SIFT	58.63% (54.55%-62.63%)	35.22% (30.51%-40%)	99.084% (98.485%-100%)	98.52% (97.44%-100%)	46.94% (42.21%-51.32%)	2.7977 (2.7129-2.8868)	58.51% (50.51%-66.67%)	34.85% (25.39%-44.45%)	99.083% (96.774%-100%)	98.49% (94.44%-100%)	46.98% (38.16%-56.41%)	2.7941 (2.6126-2.9704)
At least 6 of CHASM (breast), CHASM (lung), FATHMM (cancer), Mutation Assessor, PROVEAN, VEST	56.59% (52.53%-60.61%)	32.02% (27.34%-36.64%)	99.084% (98.485%-100%)	98.37% (97.14%-100%)	45.74% (40.99%-50%)	2.7521 (2.6646-2.8407)	56.51% (48.48%-64.65%)	31.7% (22.22%-41.27%)	99.083% (96.774%-100%)	98.35% (93.75%-100%)	45.81% (37.18%-55.26%)	2.7493 (2.5723-2.9315)
At least 6 of CHASM (breast), CHASM (lung), FATHMM (cancer), Mutation Assessor, SIFT, VEST	60.3% (56.55%-64.14%)	38.41% (33.33%-43.55%)	98.143% (97.015%-100%)	97.28% (95.45%-100%)	47.96% (43.24%-52.63%)	2.8180 (2.7265-2.9130)	60.2% (52.53%-67.7%)	38.05% (28.12%-48.28%)	98.209% (94.118%-100%)	97.34% (91.66%-100%)	48.02% (38.67%-57.5%)	2.8162 (2.6305-3.0020)
At least 6 of CHASM (breast), CHASM (lung), FATHMM (cancer), MutationTaster, PolyPhen-2, PROVEAN	58.28% (54.04%-62.12%)	34.69% (29.84%-39.54%)	99.084% (98.485%-100%)	98.5% (97.37%-100%)	46.73% (41.94%-51%)	2.7900 (2.7008-2.8752)	58.18% (50.51%-66.67%)	34.33% (24.55%-43.95%)	99.083% (96.774%-100%)	98.47% (94.44%-100%)	46.79% (38.15%-56.25%)	2.7867 (2.6186-2.9714)
At least 6 of CHASM (breast), CHASM (lung), FATHMM (cancer), MutationTaster, PolyPhen-2, SIFT	64.32% (60.61%-68.18%)	44.76% (39.68%-50%)	98.143% (97.015%-100%)	97.66% (96.15%-100%)	50.68% (45.93%-55.17%)	2.9124 (2.8175-3.0090)	64.28% (56.57%-71.72%)	44.51% (34.32%-54.42%)	98.209% (94.118%-100%)	97.72% (92.59%-100%)	50.77% (41.67%-60.3%)	2.9121 (2.7257-3.1047)
At least 6 of CHASM (breast), CHASM (lung), FATHMM (cancer), MutationTaster, PolyPhen-2, VEST	61.64% (57.58%-65.15%)	40.53% (35.24%-45.6%)	98.143% (97.015%-100%)	97.42% (95.74%-100%)	48.84% (44.05%-53.15%)	2.8493 (2.7572-2.9416)	61.57% (54.55%-69.7%)	40.21% (30.16%-50.79%)	98.209% (94.118%-100%)	97.48% (92%-100%)	48.91% (39.71%-58.57%)	2.8481 (2.6637-3.0449)
At least 6 of CHASM (breast), CHASM (lung), FATHMM (cancer), MutationTaster, PROVEAN, SIFT	59.95% (56.06%-63.64%)	37.32% (32.54%-42.4%)	99.084% (98.485%-100%)	98.6% (97.62%-100%)	47.76% (42.95%-52.03%)	2.8277 (2.7394-2.9118)	59.89% (52.53%-67.68%)	37.04% (26.98%-46.88%)	99.083% (96.774%-100%)	98.58% (94.74%-100%)	47.84% (38.96%-57.58%)	2.8255 (2.6606-3.0099)
At least 6 of CHASM (breast), CHASM (lung), FATHMM (cancer), MutationTaster, PROVEAN, VEST	58.29% (54.04%-62.12%)	34.69% (29.84%-39.2%)	99.084% (98.485%-100%)	98.5% (97.43%-100%)	46.74% (41.88%-50.98%)	2.7901 (2.7023-2.8707)	58.17% (50.51%-66.67%)	34.33% (24.99%-44.07%)	99.083% (96.774%-100%)	98.47% (94.44%-100%)	46.79% (38.16%-56.41%)	2.7867 (2.6186-2.9677)
At least 6 of CHASM (breast), CHASM (lung), FATHMM (cancer), MutationTaster, SIFT, VEST	61.98% (58.08%-65.66%)	41.06% (35.83%-46.34%)	98.143% (97.015%-100%)	97.45% (95.74%-100%)	49.06% (44.3%-53.47%)	2.8572 (2.7614-2.9494)	61.9% (54.55%-69.7%)	40.74% (30.16%-50.88%)	98.209% (94.118%-100%)	97.51% (92%-100%)	49.13% (40%-58.91%)	2.8559 (2.6721-3.0533)
At least 6 of CHASM (breast), CHASM (lung), FATHMM (cancer), PolyPhen-2, PROVEAN, SIFT	59.97% (56.06%-63.64%)	37.34% (32.26%-42.31%)	99.084% (98.485%-100%)	98.6% (97.56%-100%)	47.77% (42.86%-52.06%)	2.8279 (2.7438-2.9147)	59.86% (52.53%-67.68%)	37% (27.12%-46.88%)	99.083% (96.774%-100%)	98.57% (94.74%-100%)	47.82% (39.13%-57.53%)	2.8248 (2.6599-2.9963)
At least 6 of CHASM (breast), CHASM (lung), FATHMM (cancer), PolyPhen-2, PROVEAN, VEST	57.95% (54.04%-61.62%)	34.15% (29.17%-38.84%)	99.084% (98.485%-100%)	98.47% (97.37%-100%)	46.53% (41.72%-50.94%)	2.7824 (2.6953-2.8707)	57.85% (50.51%-65.66%)	33.81% (24.61%-43.64%)	99.083% (96.774%-100%)	98.44% (94.44%-100%)	46.59% (37.97%-56%)	2.7792 (2.6029-2.9597)
At least 6 of CHASM (breast), CHASM (lung), FATHMM (cancer), PolyPhen-2, SIFT, VEST	61.3% (57.07%-65.15%)	39.99% (34.85%-45.04%)	98.143% (97.015%-100%)	97.39% (95.65%-100%)	48.61% (43.84%-52.98%)	2.8413 (2.7400-2.9343)	61.24% (53.54%-69.7%)	39.69% (29.4%-50%)	98.209% (94.118%-100%)	97.45% (91.67%-100%)	48.69% (39.72%-58.44%)	2.8403 (2.6537-3.0441)
At least 6 of CHASM (breast), CHASM (lung), FATHMM (cancer), PROVEAN, SIFT, VEST	59.29% (55.05%-63.13%)	36.27% (31.4%-40.98%)	99.084% (98.485%-100%)	98.56% (97.5%-100%)	47.35% (42.57%-51.66%)	2.8127 (2.7248-2.8965)	59.19% (51.52%-67.68%)	35.94% (26.56%-45.9%)	99.083% (96.774%-100%)	98.54% (94.74%-100%)	47.41% (38.66%-56.94%)	2.8097 (2.6428-2.9917)
At least 6 of CHASM (breast), CHASM (lung), FATHMM (missense), Mutation Assessor, MutationTaster, PolyPhen-2	60.95% (57.07%-64.65%)	39.44% (34.17%-44.26%)	98.143% (97.015%-100%)	97.35% (95.56%-100%)	48.38% (43.62%-52.74%)	2.8331 (2.7405-2.9268)	60.93% (53.54%-68.69%)	39.2% (29.51%-50%)	98.209% (94.118%-100%)	97.43% (91.67%-100%)	48.49% (39.73%-57.97%)	2.8332 (2.6487-3.0180)
At least 6 of CHASM (breast), CHASM (lung), FATHMM (missense), Mutation Assessor, MutationTaster, PROVEAN	55.59% (51.52%-59.6%)	30.44% (25.78%-34.92%)	99.084% (98.485%-100%)	98.29% (96.97%-100%)	45.17% (40.51%-49.38%)	2.7299 (2.6432-2.8168)	55.48% (47.47%-63.64%)	30.06% (21.05%-39.66%)	99.083% (96.774%-100%)	98.26% (93.33%-100%)	45.22% (36.48%-54.43%)	2.7263 (2.5496-2.9001)
At least 6 of CHASM (breast), CHASM (lung), FATHMM (missense), Mutation Assessor, MutationTaster, SIFT	61.97% (58.08%-65.66%)	41.04% (36.06%-45.8%)	98.143% (97.015%-100%)	97.45% (95.74%-100%)	49.05% (44.28%-53.47%)	2.8569 (2.7680-2.9480)	61.92% (54.55%-69.7%)	40.77% (31.25%-50.85%)	98.209% (94.118%-100%)	97.52% (92%-100%)	49.14% (40.28%-58.67%)	2.8563 (2.6757-3.0381)
At least 6 of CHASM (breast), CHASM (lung), FATHMM (missense), Mutation Assessor, MutationTaster, VEST	58.27% (54.04%-62.12%)	35.21% (30.25%-40.32%)	98.143% (97.015%-100%)	97.04% (95.12%-100%)	46.7% (42.04%-51.3%)	2.7710 (2.6788-2.8650)	58.2% (50.51%-66.67%)	34.88% (24.99%-45%)	98.209% (94.118%-100%)	97.11% (90.91%-100%)	46.78% (37.66%-56.41%)	2.7698 (2.5839-2.9643)
At least 6 of CHASM (breast), CHASM (lung), FATHMM (missense), Mutation Assessor, PolyPhen-2, PROVEAN	56.27% (52.02%-60.1%)	31.51% (26.45%-36.07%)	99.084% (98.485%-100%)	98.35% (97.06%-100%)	45.55% (40.88%-49.69%)	2.7449 (2.6572-2.8294)	56.15% (48.48%-64.65%)	31.11% (22.03%-41.27%)	99.083% (96.774%-100%)	98.31% (93.75%-100%)	45.6% (36.84%-54.88%)	2.7410 (2.5675-2.9144)
At least 6 of CHASM (breast), CHASM (lung), FATHMM (missense), Mutation Assessor, PolyPhen-2, SIFT	62.99% (59.09%-66.67%)	42.65% (37.4%-47.62%)	98.143% (97.015%-100%)	97.55% (95.92%-100%)	49.74% (44.9%-54.17%)	2.8808 (2.7893-2.9782)	62.91% (55.56%-70.71%)	42.33% (31.74%-52.94%)	98.209% (94.118%-100%)	97.61% (92.31%-100%)	49.81% (40.85%-59.47%)	2.8796 (2.6907-3.0684)
At least 6 of CHASM (breast), CHASM (lung), FATHMM (missense), Mutation Assessor, PolyPhen-2, VEST	57.93% (54.04%-61.62%)	35.21% (29.92%-40%)	97.203% (95.588%-100%)	95.61% (93.02%-100%)	46.46% (41.72%-50.97%)	2.7449 (2.6511-2.8466)	57.88% (50.51%-66.67%)	34.88% (25%-45.17%)	97.337% (92.5%-100%)	95.77% (88.24%-100%)	46.56% (37.83%-55.84%)	2.7454 (2.5417-2.9315)
At least 6 of CHASM (breast), CHASM (lung), FATHMM (missense), Mutation Assessor, PROVEAN, SIFT	57.29% (53.03%-61.11%)	33.11% (28.23%-37.8%)	99.084% (98.485%-100%)	98.43% (97.3%-100%)	46.14% (41.45%-50.33%)	2.7676 (2.6829-2.8590)	57.14% (49.49%-65.66%)	32.69% (23.43%-42.62%)	99.083% (96.774%-100%)	98.39% (94.12%-100%)	46.17% (37.66%-55.56%)	2.7634 (2.5827-2.9402)
At least 6 of CHASM (breast), CHASM (lung), FATHMM (missense), Mutation Assessor, PROVEAN, VEST	55.26% (51.01%-59.09%)	29.9% (25%-34.17%)	99.084% (98.485%-100%)	98.26% (96.97%-100%)	44.98% (40.26%-49.37%)	2.7223 (2.6401-2.8105)	55.15% (47.47%-63.64%)	29.54% (20.63%-39.29%)	99.083% (96.774%-100%)	98.23% (93.33%-100%)	45.04% (36.36%-54.22%)	2.7188 (2.5417-2.8827)
At least 6 of CHASM (breast), CHASM (lung), FATHMM (missense), Mutation Assessor, SIFT, VEST	59.29% (55.05%-63.13%)	36.82% (31.75%-41.94%)	98.143% (97.015%-100%)	97.17% (95.24%-100%)	47.32% (42.57%-51.7%)	2.7945 (2.7092-2.8855)	59.19% (51.52%-67.68%)	36.45% (26.15%-46.67%)	98.209% (94.118%-100%)	97.23% (90.91%-100%)	47.38% (38.57%-56.79%)	2.7927 (2.6125-2.9791)
At least 6 of CHASM (breast), CHASM (lung), FATHMM (missense), MutationTaster, PolyPhen-2, PROVEAN	55.61% (51.52%-59.09%)	30.46% (25.4%-35.16%)	99.084% (98.485%-100%)	98.29% (96.97%-100%)	45.18% (40.6%-49.37%)	2.7301 (2.6416-2.8155)	55.46% (48.48%-63.64%)	30.03% (20.69%-40.32%)	99.083% (96.774%-100%)	98.26% (93.33%-100%)	45.21% (36.84%-54.55%)	2.7258 (2.5495-2.9015)
At least 6 of CHASM (breast), CHASM (lung), FATHMM (missense), MutationTaster, PolyPhen-2, SIFT	61.97% (58.08%-65.66%)	41.05% (35.83%-45.97%)	98.143% (97.015%-100%)	97.45% (95.74%-100%)	49.05% (44.36%-53.42%)	2.8570 (2.7602-2.9513)	61.92% (54.55%-69.7%)	40.76% (31.15%-50.79%)	98.209% (94.118%-100%)	97.52% (92%-100%)	49.14% (40.28%-58.46%)	2.8563 (2.6721-3.0459)
At least 6 of CHASM (breast), CHASM (lung), FATHMM (missense), MutationTaster, PolyPhen-2, VEST	58.95% (55.05%-62.64%)	36.28% (31.09%-41.27%)	98.143% (97.015%-100%)	97.13% (95.24%-100%)	47.11% (42.38%-51.59%)	2.7867 (2.6919-2.8768)	58.86% (51.49%-66.67%)	35.93% (26.23%-45.9%)	98.209% (94.118%-100%)	97.19% (90.91%-100%)	47.18% (38.15%-56.72%)	2.7851 (2.5974-2.9774)
At least 6 of CHASM (breast), CHASM (lung), FATHMM (missense), MutationTaster, PROVEAN, SIFT	57.27% (53.03%-61.11%)	33.09% (28.33%-37.7%)	99.084% (98.485%-100%)	98.43% (97.22%-100%)	46.13% (41.4%-50.33%)	2.7674 (2.6797-2.8519)	57.17% (49.49%-65.66%)	32.73% (23.72%-42.62%)	99.083% (96.774%-100%)	98.41% (94.12%-100%)	46.19% (37.66%-55.56%)	2.7641 (2.5988-2.9468)
At least 6 of CHASM (breast), CHASM (lung), FATHMM (missense), MutationTaster, PROVEAN, VEST	55.61% (51.52%-59.6%)	30.46% (25.76%-35.2%)	99.084% (98.485%-100%)	98.29% (97.06%-100%)	45.18% (40.51%-49.4%)	2.7302 (2.6428-2.8150)	55.45% (47.47%-63.64%)	30.02% (20.63%-39.71%)	99.083% (96.774%-100%)	98.26% (93.33%-100%)	45.21% (36.59%-54.76%)	2.7258 (2.5570-2.9069)
At least 6 of CHASM (breast), CHASM (lung), FATHMM (missense), MutationTaster, SIFT, VEST	59.63% (55.56%-63.64%)	37.35% (32.26%-42.5%)	98.143% (97.015%-100%)	97.21% (95.35%-100%)	47.53% (42.86%-51.95%)	2.8023 (2.7094-2.8965)	59.54% (51.52%-67.68%)	36.99% (26.98%-47.14%)	98.209% (94.118%-100%)	97.27% (91.3%-100%)	47.6% (38.46%-57.34%)	2.8007 (2.6088-2.9944)
At least 6 of CHASM (breast), CHASM (lung), FATHMM (missense), PolyPhen-2, PROVEAN, SIFT	57.29% (53.54%-61.11%)	33.11% (28.12%-37.88%)	99.084% (98.485%-100%)	98.43% (97.3%-100%)	46.14% (41.25%-50.32%)	2.7676 (2.6810-2.8542)	57.14% (49.49%-64.65%)	32.69% (22.95%-42.86%)	99.083% (96.774%-100%)	98.4% (94.12%-100%)	46.17% (37.5%-55.56%)	2.7634 (2.5933-2.9331)
At least 6 of CHASM (breast), CHASM (lung), FATHMM (missense), PolyPhen-2, PROVEAN, VEST	55.27% (51.01%-59.09%)	29.92% (25%-34.65%)	99.084% (98.485%-100%)	98.26% (96.97%-100%)	44.99% (40.13%-49.1%)	2.7225 (2.6376-2.8066)	55.13% (47.47%-63.64%)	29.5% (20.31%-39.34%)	99.083% (96.774%-100%)	98.23% (93.33%-100%)	45.02% (36.71%-54.32%)	2.7183 (2.5444-2.8965)
At least 6 of CHASM (breast), CHASM (lung), FATHMM (missense), PolyPhen-2, SIFT, VEST	58.95% (55.05%-62.63%)	36.27% (31.15%-41.22%)	98.143% (97.015%-100%)	97.13% (95.24%-100%)	47.11% (42.28%-51.35%)	2.7866 (2.6933-2.8788)	58.87% (51.52%-66.67%)	35.94% (25.81%-45.76%)	98.209% (94.118%-100%)	97.19% (90.91%-100%)	47.19% (38.66%-56.58%)	2.7853 (2.6003-2.9704)
At least 6 of CHASM (breast), CHASM (lung), FATHMM (missense), PROVEAN, SIFT, VEST	56.61% (52.53%-60.11%)	32.05% (27.13%-36.72%)	99.084% (98.485%-100%)	98.38% (97.14%-100%)	45.75% (40.91%-50%)	2.7525 (2.6650-2.8335)	56.47% (49.47%-64.65%)	31.63% (22.22%-41.54%)	99.083% (96.774%-100%)	98.35% (93.75%-100%)	45.79% (37.18%-55.41%)	2.7485 (2.5807-2.9314)
At least 6 of CHASM (breast), CHASM (lung), Mutation Assessor, MutationTaster, PolyPhen-2, PROVEAN	55.92% (51.52%-59.6%)	30.95% (26.02%-35.65%)	99.084% (98.485%-100%)	98.32% (97.06%-100%)	45.35% (40.67%-49.69%)	2.7370 (2.6492-2.8246)	55.85% (48.48%-64.65%)	30.64% (21.31%-40.62%)	99.083% (96.774%-100%)	98.29% (93.75%-100%)	45.43% (36.71%-54.67%)	2.7345 (2.5590-2.9144)
At least 6 of CHASM (breast), CHASM (lung), Mutation Assessor, MutationTaster, PolyPhen-2, SIFT	61.62% (57.58%-65.16%)	41.02% (35.88%-46.03%)	97.222% (95.588%-100%)	96.23% (93.88%-100%)	48.81% (44.05%-53.33%)	2.8328 (2.7371-2.9327)	61.62% (54.52%-69.7%)	40.82% (30.65%-50.86%)	97.294% (92.303%-100%)	96.28% (90%-100%)	48.93% (39.44%-58.21%)	2.8332 (2.6258-3.0229)
At least 6 of CHASM (breast), CHASM (lung), Mutation Assessor, MutationTaster, PolyPhen-2, VEST	57.92% (53.54%-61.62%)	35.19% (29.84%-40.32%)	97.222% (95.588%-100%)	95.63% (92.86%-100%)	46.46% (41.72%-50.68%)	2.7450 (2.6428-2.8492)	57.9% (50.51%-66.67%)	34.93% (24.61%-45.62%)	97.294% (92.303%-100%)	95.67% (88%-100%)	46.57% (37.66%-55.85%)	2.7447 (2.5305-2.9494)
At least 6 of CHASM (breast), CHASM (lung), Mutation Assessor, MutationTaster, PROVEAN, SIFT	56.93% (53.03%-60.62%)	32.55% (27.78%-37.1%)	99.084% (98.485%-100%)	98.4% (97.22%-100%)	45.94% (41.25%-50.32%)	2.7597 (2.6710-2.8483)	56.84% (49.47%-64.65%)	32.22% (22.95%-42.11%)	99.083% (96.774%-100%)	98.37% (93.75%-100%)	46% (37.33%-55.43%)	2.7568 (2.5770-2.9401)
At least 6 of CHASM (breast), CHASM (lung), Mutation Assessor, MutationTaster, PROVEAN, VEST	55.24% (51.01%-59.09%)	29.88% (25.19%-34.43%)	99.084% (98.485%-100%)	98.26% (96.97%-100%)	44.97% (40.25%-49.37%)	2.7220 (2.6342-2.8090)	55.18% (47.47%-63.64%)	29.59% (20.63%-39.07%)	99.083% (96.774%-100%)	98.23% (93.33%-100%)	45.06% (36.25%-54.32%)	2.7196 (2.5468-2.9015)
At least 6 of CHASM (breast), CHASM (lung), Mutation Assessor, MutationTaster, SIFT, VEST	58.94% (55.04%-62.63%)	36.8% (31.62%-42.02%)	97.222% (95.588%-100%)	95.81% (93.18%-100%)	47.08% (42.46%-51.61%)	2.7691 (2.6728-2.8729)	58.89% (51.52%-66.69%)	36.5% (26.55%-47.07%)	97.294% (92.303%-100%)	95.85% (88.88%-100%)	47.17% (38.16%-56.58%)	2.7682 (2.5541-2.9706)
At least 6 of CHASM (breast), CHASM (lung), Mutation Assessor, PolyPhen-2, PROVEAN, SIFT	57.61% (53.54%-61.62%)	33.62% (28.68%-38.17%)	99.084% (98.485%-100%)	98.45% (97.3%-100%)	46.33% (41.72%-50.65%)	2.7748 (2.6884-2.8595)	57.51% (49.49%-65.66%)	33.27% (24.14%-42.86%)	99.083% (96.774%-100%)	98.42% (94.12%-100%)	46.39% (37.66%-55.84%)	2.7716 (2.5942-2.9438)
At least 6 of CHASM (breast), CHASM (lung), Mutation Assessor, PolyPhen-2, PROVEAN, VEST	55.58% (51.52%-59.6%)	30.41% (25.58%-34.92%)	99.084% (98.485%-100%)	98.29% (96.97%-100%)	45.16% (40.38%-49.38%)	2.7294 (2.6432-2.8156)	55.51% (47.47%-63.64%)	30.12% (21.05%-39.68%)	99.083% (96.774%-100%)	98.26% (93.33%-100%)	45.24% (36.71%-54.67%)	2.7270 (2.5529-2.9001)
At least 6 of CHASM (breast), CHASM (lung), Mutation Assessor, PolyPhen-2, SIFT, VEST	58.94% (55.05%-62.63%)	36.79% (31.62%-41.86%)	97.222% (95.588%-100%)	95.81% (93.18%-100%)	47.08% (42.38%-51.63%)	2.7691 (2.6729-2.8689)	58.89% (51.52%-66.67%)	36.5% (26.46%-46.97%)	97.294% (92.303%-100%)	95.86% (88.46%-100%)	47.17% (38.15%-56.52%)	2.7683 (2.5611-2.9606)
At least 6 of CHASM (breast), CHASM (lung), Mutation Assessor, PROVEAN, SIFT, VEST	56.59% (52.53%-60.61%)	32.02% (27.34%-36.64%)	99.084% (98.485%-100%)	98.37% (97.14%-100%)	45.74% (40.99%-50%)	2.7521 (2.6646-2.8407)	56.51% (48.48%-64.65%)	31.7% (22.22%-41.27%)	99.083% (96.774%-100%)	98.35% (93.75%-100%)	45.81% (37.18%-55.26%)	2.7493 (2.5723-2.9315)
At least 6 of CHASM (breast), CHASM (lung), MutationTaster, PolyPhen-2, PROVEAN, SIFT	57.94% (54.04%-61.62%)	34.14% (29.17%-39.17%)	99.084% (98.485%-100%)	98.47% (97.3%-100%)	46.53% (41.72%-50.96%)	2.7822 (2.6945-2.8636)	57.86% (50.51%-65.66%)	33.84% (24.13%-43.75%)	99.083% (96.774%-100%)	98.45% (94.44%-100%)	46.6% (37.97%-56.17%)	2.7797 (2.6171-2.9606)
At least 6 of CHASM (breast), CHASM (lung), MutationTaster, PolyPhen-2, PROVEAN, VEST	56.59% (52.53%-60.11%)	32.02% (27.2%-36.8%)	99.084% (98.485%-100%)	98.37% (97.14%-100%)	45.74% (40.91%-50%)	2.7521 (2.6646-2.8372)	56.51% (49.47%-64.65%)	31.7% (22.38%-41.27%)	99.083% (96.774%-100%)	98.34% (93.75%-100%)	45.81% (37.18%-55.41%)	2.7494 (2.5770-2.9330)
At least 6 of CHASM (breast), CHASM (lung), MutationTaster, PolyPhen-2, SIFT, VEST	59.94% (56.06%-63.64%)	38.37% (33.08%-43.41%)	97.222% (95.588%-100%)	95.98% (93.48%-100%)	47.71% (42.95%-52.03%)	2.7928 (2.6909-2.8945)	59.93% (52.53%-67.68%)	38.14% (27.93%-48.53%)	97.294% (92.303%-100%)	96.02% (89.47%-100%)	47.83% (38.96%-57.53%)	2.7928 (2.5890-2.9943)
At least 6 of CHASM (breast), CHASM (lung), MutationTaster, PROVEAN, SIFT, VEST	57.94% (53.54%-61.62%)	34.14% (29.27%-38.89%)	99.084% (98.485%-100%)	98.47% (97.37%-100%)	46.53% (41.72%-50.67%)	2.7822 (2.6946-2.8637)	57.86% (50.51%-66.67%)	33.83% (24.24%-43.75%)	99.083% (96.774%-100%)	98.45% (94.44%-100%)	46.6% (38.02%-56.25%)	2.7797 (2.6170-2.9643)
At least 6 of CHASM (breast), CHASM (lung), PolyPhen-2, PROVEAN, SIFT, VEST	57.6% (53.54%-61.11%)	33.6% (28.57%-38.4%)	99.084% (98.485%-100%)	98.45% (97.3%-100%)	46.32% (41.45%-50.64%)	2.7746 (2.6884-2.8595)	57.53% (50.51%-65.66%)	33.31% (24.19%-42.86%)	99.083% (96.774%-100%)	98.42% (94.12%-100%)	46.4% (37.84%-55.84%)	2.7722 (2.6012-2.9513)
At least 6 of CHASM (breast), CHASM (melanoma), FATHMM (cancer), FATHMM (missense), Mutation Assessor, MutationTaster	61.64% (57.58%-65.66%)	41.06% (35.88%-45.9%)	97.233% (95.588%-100%)	96.25% (94%-100%)	48.82% (44.14%-53.42%)	2.8337 (2.7371-2.9306)	61.56% (53.54%-69.7%)	40.75% (31.15%-51.47%)	97.269% (92.303%-100%)	96.26% (89.99%-100%)	48.89% (39.73%-58.46%)	2.8317 (2.6322-3.0286)
At least 6 of CHASM (breast), CHASM (melanoma), FATHMM (cancer), FATHMM (missense), Mutation Assessor, PolyPhen-2	62.66% (58.59%-66.16%)	43.2% (37.93%-48.07%)	96.293% (94.203%-98.667%)	95.28% (92.59%-98.28%)	49.51% (44.68%-54%)	2.8428 (2.7439-2.9508)	62.57% (55.56%-70.71%)	42.84% (32.79%-53.03%)	96.398% (91.176%-100%)	95.35% (88.89%-100%)	49.56% (40.84%-59.09%)	2.8415 (2.6268-3.0406)
At least 6 of CHASM (breast), CHASM (melanoma), FATHMM (cancer), FATHMM (missense), Mutation Assessor, PROVEAN	56.95% (53.02%-61.11%)	33.11% (28.23%-37.8%)	98.173% (97.015%-100%)	96.91% (94.74%-100%)	45.91% (41.14%-50.32%)	2.7411 (2.6487-2.8347)	56.8% (48.48%-64.67%)	32.69% (23.43%-42.62%)	98.144% (94.118%-100%)	96.8% (89.47%-100%)	45.94% (37.18%-55.41%)	2.7357 (2.5444-2.9204)
At least 6 of CHASM (breast), CHASM (melanoma), FATHMM (cancer), FATHMM (missense), Mutation Assessor, SIFT	63.68% (60.09%-67.18%)	44.27% (39.17%-49.18%)	97.233% (95.588%-100%)	96.51% (94.34%-100%)	50.23% (45.45%-54.8%)	2.8825 (2.7882-2.9827)	63.55% (56.54%-70.73%)	43.88% (34.36%-53.52%)	97.269% (92.303%-100%)	96.52% (90.47%-100%)	50.25% (41.27%-59.72%)	2.8791 (2.6777-3.0711)
At least 6 of CHASM (breast), CHASM (melanoma), FATHMM (cancer), FATHMM (missense), Mutation Assessor, VEST	58.62% (54.55%-62.63%)	36.83% (31.62%-41.86%)	96.293% (94.203%-98.667%)	94.51% (91.3%-98%)	46.86% (42.21%-51.35%)	2.7449 (2.6387-2.8492)	58.51% (50.51%-66.67%)	36.43% (26.47%-46.56%)	96.398% (91.176%-100%)	94.59% (86.96%-100%)	46.91% (37.84%-56.25%)	2.7433 (2.5314-2.9494)
At least 6 of CHASM (breast), CHASM (melanoma), FATHMM (cancer), FATHMM (missense), MutationTaster, PolyPhen-2	63.32% (59.58%-67.17%)	43.7% (38.46%-48.82%)	97.233% (95.588%-100%)	96.47% (94.23%-100%)	49.97% (45.19%-54.55%)	2.8738 (2.7744-2.9725)	63.26% (55.56%-70.73%)	43.44% (33.33%-53.85%)	97.269% (92.303%-100%)	96.48% (90.62%-100%)	50.05% (40.85%-59.68%)	2.8724 (2.6717-3.0697)
At least 6 of CHASM (breast), CHASM (melanoma), FATHMM (cancer), FATHMM (missense), MutationTaster, PROVEAN	56.94% (53.03%-60.61%)	33.09% (28.33%-37.7%)	98.173% (97.015%-100%)	96.91% (94.74%-100%)	45.91% (41.18%-50.32%)	2.7408 (2.6486-2.8325)	56.82% (49.49%-64.65%)	32.73% (23.72%-42.62%)	98.144% (94.118%-100%)	96.81% (90%-100%)	45.95% (37.18%-55.41%)	2.7365 (2.5444-2.9268)
At least 6 of CHASM (breast), CHASM (melanoma), FATHMM (cancer), FATHMM (missense), MutationTaster, SIFT	64.34% (60.61%-68.18%)	45.32% (40%-50.39%)	97.233% (95.588%-100%)	96.59% (94.44%-100%)	50.7% (45.86%-55.32%)	2.8984 (2.7989-3.0021)	64.24% (56.57%-71.72%)	44.99% (35.18%-55.01%)	97.269% (92.303%-100%)	96.6% (90.9%-100%)	50.74% (41.18%-60.3%)	2.8960 (2.6860-3.1007)
At least 6 of CHASM (breast), CHASM (melanoma), FATHMM (cancer), FATHMM (missense), MutationTaster, VEST	59.97% (56.06%-63.64%)	38.42% (33.06%-43.31%)	97.233% (95.588%-100%)	96.01% (93.47%-100%)	47.73% (42.95%-52.32%)	2.7940 (2.6995-2.8969)	59.85% (52.53%-67.68%)	38.03% (28.07%-47.76%)	97.269% (92.303%-100%)	96.01% (89.29%-100%)	47.77% (38.36%-57.53%)	2.7908 (2.5807-2.9825)
At least 6 of CHASM (breast), CHASM (melanoma), FATHMM (cancer), FATHMM (missense), PolyPhen-2, PROVEAN	56.95% (53.03%-60.61%)	33.11% (28.12%-37.88%)	98.173% (97.015%-100%)	96.91% (94.74%-100%)	45.91% (41.14%-50%)	2.7410 (2.6456-2.8331)	56.8% (49.49%-64.65%)	32.69% (22.95%-42.86%)	98.144% (94.118%-100%)	96.8% (89.47%-100%)	45.93% (37.5%-55.56%)	2.7357 (2.5483-2.9262)
At least 6 of CHASM (breast), CHASM (melanoma), FATHMM (cancer), FATHMM (missense), PolyPhen-2, SIFT	64.02% (60.1%-67.68%)	44.81% (39.52%-50%)	97.233% (95.588%-100%)	96.55% (94.44%-100%)	50.47% (45.65%-55%)	2.8906 (2.7937-2.9882)	63.88% (56.57%-71.72%)	44.41% (33.93%-54.69%)	97.269% (92.303%-100%)	96.56% (90.48%-100%)	50.48% (41.54%-60.27%)	2.8872 (2.6842-3.0807)
At least 6 of CHASM (breast), CHASM (melanoma), FATHMM (cancer), FATHMM (missense), PolyPhen-2, VEST	58.95% (54.55%-62.63%)	37.35% (32%-42.4%)	96.293% (94.203%-98.667%)	94.58% (91.49%-98.04%)	47.06% (42.21%-51.35%)	2.7529 (2.6482-2.8560)	58.86% (51.52%-67.68%)	36.98% (26.86%-47.54%)	96.398% (91.176%-100%)	94.67% (86.96%-100%)	47.13% (38.35%-56.76%)	2.7517 (2.5389-2.9605)
At least 6 of CHASM (breast), CHASM (melanoma), FATHMM (cancer), FATHMM (missense), PROVEAN, SIFT	58.97% (55.04%-62.63%)	36.3% (31.45%-40.94%)	98.173% (97.015%-100%)	97.17% (95.24%-100%)	47.13% (42.21%-51.61%)	2.7877 (2.6957-2.8815)	58.82% (51.52%-66.69%)	35.9% (26.31%-45.9%)	98.144% (94.118%-100%)	97.09% (90.48%-100%)	47.15% (38.36%-56.79%)	2.7827 (2.5878-2.9704)
At least 6 of CHASM (breast), CHASM (melanoma), FATHMM (cancer), FATHMM (missense), PROVEAN, VEST	56.28% (52.02%-60.1%)	32.05% (27.13%-36.72%)	98.173% (97.015%-100%)	96.81% (94.59%-100%)	45.52% (40.88%-49.7%)	2.7255 (2.6318-2.8196)	56.13% (48.48%-64.65%)	31.63% (22.22%-41.54%)	98.144% (94.118%-100%)	96.71% (89.47%-100%)	45.55% (37.03%-55.13%)	2.7203 (2.5283-2.9097)
At least 6 of CHASM (breast), CHASM (melanoma), FATHMM (cancer), FATHMM (missense), SIFT, VEST	60.32% (56.06%-64.14%)	38.97% (34.09%-43.91%)	97.233% (95.588%-100%)	96.06% (93.62%-100%)	47.95% (43.33%-52.38%)	2.8021 (2.7059-2.9032)	60.17% (52.53%-68.69%)	38.54% (28.33%-48.39%)	97.269% (92.303%-100%)	96.06% (89.29%-100%)	47.98% (38.75%-57.58%)	2.7984 (2.5893-2.9965)
At least 6 of CHASM (breast), CHASM (melanoma), FATHMM (cancer), Mutation Assessor, MutationTaster, PolyPhen-2	60.94% (57.07%-64.65%)	39.96% (34.88%-44.8%)	97.233% (95.588%-100%)	96.15% (93.75%-100%)	48.36% (43.75%-52.78%)	2.8170 (2.7151-2.9166)	60.94% (53.54%-68.69%)	39.76% (30.13%-50%)	97.269% (92.303%-100%)	96.17% (89.65%-100%)	48.48% (39.44%-57.69%)	2.8169 (2.6125-3.0183)
At least 6 of CHASM (breast), CHASM (melanoma), FATHMM (cancer), Mutation Assessor, MutationTaster, PROVEAN	55.92% (51.52%-59.6%)	31.48% (26.72%-36%)	98.173% (97.015%-100%)	96.75% (94.44%-100%)	45.31% (40.62%-49.69%)	2.7171 (2.6219-2.8085)	55.84% (48.48%-64.65%)	31.19% (22.06%-40.99%)	98.144% (94.118%-100%)	96.65% (89.47%-100%)	45.39% (36.49%-54.8%)	2.7137 (2.5268-2.9058)
At least 6 of CHASM (breast), CHASM (melanoma), FATHMM (cancer), Mutation Assessor, MutationTaster, SIFT	61.96% (58.08%-65.66%)	41.56% (36.44%-46.34%)	97.233% (95.588%-100%)	96.3% (94%-100%)	49.04% (44.36%-53.58%)	2.8413 (2.7452-2.9394)	61.93% (54.55%-69.7%)	41.33% (31.74%-51.57%)	97.269% (92.303%-100%)	96.31% (90.31%-100%)	49.14% (39.74%-58.57%)	2.8405 (2.6361-3.0426)
At least 6 of CHASM (breast), CHASM (melanoma), FATHMM (cancer), Mutation Assessor, MutationTaster, VEST	58.27% (54.04%-62.12%)	35.73% (30.47%-40.83%)	97.233% (95.588%-100%)	95.72% (92.86%-100%)	46.67% (41.96%-51.3%)	2.7535 (2.6540-2.8545)	58.21% (50.51%-66.67%)	35.44% (25.36%-45.9%)	97.269% (92.303%-100%)	95.72% (88.46%-100%)	46.76% (37.5%-56.34%)	2.7519 (2.5445-2.9515)
At least 6 of CHASM (breast), CHASM (melanoma), FATHMM (cancer), Mutation Assessor, PolyPhen-2, PROVEAN	56.59% (52.53%-60.61%)	32.54% (27.59%-37.01%)	98.173% (97.015%-100%)	96.85% (94.59%-100%)	45.7% (41.13%-50%)	2.7327 (2.6393-2.8271)	56.51% (48.48%-64.65%)	32.24% (22.95%-42.37%)	98.144% (94.118%-100%)	96.76% (89.99%-100%)	45.77% (37.18%-55.01%)	2.7290 (2.5430-2.9162)
At least 6 of CHASM (breast), CHASM (melanoma), FATHMM (cancer), Mutation Assessor, PolyPhen-2, SIFT	62.98% (59.09%-66.67%)	43.17% (38.1%-48.36%)	97.233% (95.588%-100%)	96.43% (94.23%-100%)	49.74% (45.03%-54.23%)	2.8657 (2.7686-2.9665)	62.93% (55.56%-70.71%)	42.9% (32.79%-53.03%)	97.269% (92.303%-100%)	96.44% (90.47%-100%)	49.81% (40.85%-59.42%)	2.8642 (2.6529-3.0630)
At least 6 of CHASM (breast), CHASM (melanoma), FATHMM (cancer), Mutation Assessor, PolyPhen-2, VEST	57.92% (54.03%-61.62%)	35.73% (30.58%-40.62%)	96.293% (94.203%-98.667%)	94.35% (91.11%-97.96%)	46.42% (41.67%-50.99%)	2.7279 (2.6225-2.8353)	57.89% (50.51%-65.68%)	35.44% (25.42%-45.46%)	96.398% (91.176%-100%)	94.44% (86.36%-100%)	46.53% (37.5%-55.84%)	2.7281 (2.5104-2.9425)
At least 6 of CHASM (breast), CHASM (melanoma), FATHMM (cancer), Mutation Assessor, PROVEAN, SIFT	57.61% (53.54%-61.62%)	34.15% (29.41%-38.71%)	98.173% (97.015%-100%)	97% (94.87%-100%)	46.3% (41.61%-50.65%)	2.7562 (2.6614-2.8492)	57.51% (49.49%-65.66%)	33.82% (24.56%-43.75%)	98.144% (94.118%-100%)	96.9% (90%-100%)	46.35% (37.66%-55.7%)	2.7522 (2.5611-2.9437)
At least 6 of CHASM (breast), CHASM (melanoma), FATHMM (cancer), Mutation Assessor, PROVEAN, VEST	55.58% (51.52%-59.6%)	30.94% (26.19%-35.48%)	98.173% (97.015%-100%)	96.7% (94.44%-100%)	45.12% (40.38%-49.38%)	2.7093 (2.6162-2.8036)	55.51% (47.47%-63.64%)	30.66% (21.43%-40.36%)	98.144% (94.118%-100%)	96.6% (88.89%-100%)	45.2% (36.47%-54.55%)	2.7060 (2.5100-2.8925)
At least 6 of CHASM (breast), CHASM (melanoma), FATHMM (cancer), Mutation Assessor, SIFT, VEST	59.29% (55.05%-63.13%)	37.34% (32.31%-42.28%)	97.233% (95.588%-100%)	95.89% (93.33%-100%)	47.3% (42.48%-51.95%)	2.7776 (2.6815-2.8750)	59.21% (51.52%-67.68%)	37.01% (26.86%-46.97%)	97.269% (92.303%-100%)	95.9% (88.89%-100%)	47.37% (38.27%-56.92%)	2.7755 (2.5768-2.9704)
At least 6 of CHASM (breast), CHASM (melanoma), FATHMM (cancer), MutationTaster, PolyPhen-2, PROVEAN	56.92% (52.53%-60.61%)	33.06% (27.96%-37.88%)	98.173% (97.015%-100%)	96.9% (94.74%-100%)	45.89% (41.14%-50%)	2.7403 (2.6428-2.8315)	56.87% (49.49%-65.66%)	32.8% (23.43%-42.86%)	98.144% (94.118%-100%)	96.81% (90%-100%)	45.98% (37.5%-55.56%)	2.7373 (2.5458-2.9329)
At least 6 of CHASM (breast), CHASM (melanoma), FATHMM (cancer), MutationTaster, PolyPhen-2, SIFT	63.31% (59.6%-67.17%)	43.68% (38.52%-48.45%)	97.233% (95.588%-100%)	96.47% (94.23%-100%)	49.96% (45.19%-54.41%)	2.8735 (2.7760-2.9755)	63.29% (55.56%-70.71%)	43.47% (33.33%-53.85%)	97.269% (92.303%-100%)	96.48% (90.48%-100%)	50.07% (40.91%-60%)	2.8730 (2.6705-3.0697)
At least 6 of CHASM (breast), CHASM (melanoma), FATHMM (cancer), MutationTaster, PolyPhen-2, VEST	60.28% (56.06%-64.14%)	38.9% (33.84%-44%)	97.233% (95.588%-100%)	96.05% (93.48%-100%)	47.93% (43.15%-52.32%)	2.8011 (2.7058-2.9002)	60.26% (52.53%-68.69%)	38.68% (28.33%-49.15%)	97.269% (92.303%-100%)	96.07% (89.28%-100%)	48.04% (38.89%-57.69%)	2.8006 (2.5981-2.9998)
At least 6 of CHASM (breast), CHASM (melanoma), FATHMM (cancer), MutationTaster, PROVEAN, SIFT	58.94% (55.05%-62.63%)	36.24% (31.45%-41.22%)	98.173% (97.015%-100%)	97.17% (95.24%-100%)	47.11% (42.28%-51.35%)	2.7869 (2.6932-2.8770)	58.89% (51.52%-66.67%)	36.01% (26.23%-45.9%)	98.144% (94.118%-100%)	97.09% (90.48%-100%)	47.19% (38.36%-56.94%)	2.7843 (2.6011-2.9812)
At least 6 of CHASM (breast), CHASM (melanoma), FATHMM (cancer), MutationTaster, PROVEAN, VEST	56.92% (52.53%-60.61%)	33.06% (28.33%-37.82%)	98.173% (97.015%-100%)	96.9% (94.74%-100%)	45.89% (41.14%-50.31%)	2.7403 (2.6428-2.8331)	56.86% (49.49%-65.66%)	32.8% (23.21%-42.43%)	98.144% (94.118%-100%)	96.81% (89.99%-100%)	45.98% (37.18%-55.56%)	2.7373 (2.5445-2.9331)
At least 6 of CHASM (breast), CHASM (melanoma), FATHMM (cancer), MutationTaster, SIFT, VEST	60.96% (57.07%-64.65%)	39.98% (34.71%-45.24%)	97.233% (95.588%-100%)	96.15% (93.75%-100%)	48.37% (43.62%-52.74%)	2.8174 (2.7238-2.9221)	60.9% (53.54%-68.69%)	39.7% (29.03%-50%)	97.269% (92.303%-100%)	96.16% (89.47%-100%)	48.46% (39.43%-58.21%)	2.8159 (2.6021-3.0043)
At least 6 of CHASM (breast), CHASM (melanoma), FATHMM (cancer), PolyPhen-2, PROVEAN, SIFT	58.95% (55.05%-62.63%)	36.26% (31.2%-40.99%)	98.173% (97.015%-100%)	97.17% (95.24%-100%)	47.11% (42.18%-51.35%)	2.7872 (2.6908-2.8819)	58.87% (51.52%-66.67%)	35.96% (26.32%-46.03%)	98.144% (94.118%-100%)	97.08% (90.48%-100%)	47.18% (38.36%-56.96%)	2.7836 (2.5898-2.9760)
At least 6 of CHASM (breast), CHASM (melanoma), FATHMM (cancer), PolyPhen-2, PROVEAN, VEST	56.58% (52.53%-60.11%)	32.52% (27.56%-37.12%)	98.173% (97.015%-100%)	96.85% (94.59%-100%)	45.7% (40.88%-50%)	2.7324 (2.6354-2.8252)	56.54% (49.47%-65.66%)	32.27% (22.8%-42.03%)	98.144% (94.118%-100%)	96.76% (89.47%-100%)	45.78% (37.18%-55.41%)	2.7296 (2.5435-2.9262)
At least 6 of CHASM (breast), CHASM (melanoma), FATHMM (cancer), PolyPhen-2, SIFT, VEST	60.28% (56.06%-64.14%)	38.91% (33.87%-44%)	97.233% (95.588%-100%)	96.05% (93.61%-100%)	47.93% (43.15%-52.08%)	2.8013 (2.7018-2.9055)	60.24% (52.53%-68.69%)	38.65% (28.57%-48.54%)	97.269% (92.303%-100%)	96.07% (88.89%-100%)	48.02% (39.39%-57.75%)	2.8001 (2.5820-3.0000)
At least 6 of CHASM (breast), CHASM (melanoma), FATHMM (cancer), PROVEAN, SIFT, VEST	58.28% (54.04%-62.12%)	35.2% (30.3%-39.84%)	98.173% (97.015%-100%)	97.09% (95.12%-100%)	46.7% (42%-50.98%)	2.7716 (2.6760-2.8613)	58.2% (50.51%-66.67%)	34.91% (25.36%-45.45%)	98.144% (94.118%-100%)	97% (90%-100%)	46.77% (37.97%-56.01%)	2.7682 (2.5791-2.9598)
At least 6 of CHASM (breast), CHASM (melanoma), FATHMM (missense), Mutation Assessor, MutationTaster, PolyPhen-2	60.62% (56.57%-64.15%)	39.44% (34.17%-44.26%)	97.233% (95.588%-100%)	96.1% (93.62%-100%)	48.15% (43.54%-52.45%)	2.8092 (2.7130-2.9096)	60.58% (53.51%-68.69%)	39.2% (29.51%-50%)	97.269% (92.303%-100%)	96.12% (89.65%-100%)	48.25% (39.44%-57.75%)	2.8084 (2.6062-3.0001)
At least 6 of CHASM (breast), CHASM (melanoma), FATHMM (missense), Mutation Assessor, MutationTaster, PROVEAN	55.26% (51.01%-59.09%)	30.44% (25.78%-34.92%)	98.173% (97.015%-100%)	96.65% (94.29%-100%)	44.94% (40.26%-49.37%)	2.7020 (2.6064-2.7962)	55.13% (47.47%-63.64%)	30.06% (21.05%-39.66%)	98.144% (94.118%-100%)	96.53% (88.89%-100%)	44.99% (36.25%-54.29%)	2.6973 (2.5072-2.8877)
At least 6 of CHASM (breast), CHASM (melanoma), FATHMM (missense), Mutation Assessor, MutationTaster, SIFT	61.64% (57.58%-65.66%)	41.04% (36.06%-45.8%)	97.233% (95.588%-100%)	96.25% (93.88%-100%)	48.82% (44.14%-53.38%)	2.8335 (2.7361-2.9327)	61.58% (53.54%-69.7%)	40.77% (31.25%-50.85%)	97.269% (92.303%-100%)	96.27% (89.66%-100%)	48.9% (40%-58.46%)	2.8320 (2.6318-3.0229)
At least 6 of CHASM (breast), CHASM (melanoma), FATHMM (missense), Mutation Assessor, MutationTaster, VEST	57.94% (54.04%-61.63%)	35.21% (30.25%-40.32%)	97.233% (95.588%-100%)	95.66% (92.86%-100%)	46.47% (41.83%-50.99%)	2.7457 (2.6450-2.8479)	57.86% (50.48%-65.66%)	34.88% (24.99%-45%)	97.269% (92.303%-100%)	95.66% (88.24%-100%)	46.54% (37.5%-56.25%)	2.7435 (2.5340-2.9437)
At least 6 of CHASM (breast), CHASM (melanoma), FATHMM (missense), Mutation Assessor, PolyPhen-2, PROVEAN	55.94% (52.02%-59.6%)	31.51% (26.45%-36.07%)	98.173% (97.015%-100%)	96.76% (94.44%-100%)	45.32% (40.62%-49.66%)	2.7176 (2.6219-2.8091)	55.8% (48.48%-63.64%)	31.11% (22.03%-41.27%)	98.144% (94.118%-100%)	96.64% (88.89%-100%)	45.36% (36.71%-54.67%)	2.7126 (2.5188-2.9015)
At least 6 of CHASM (breast), CHASM (melanoma), FATHMM (missense), Mutation Assessor, PolyPhen-2, SIFT	62.65% (58.59%-66.16%)	42.65% (37.4%-47.62%)	97.233% (95.588%-100%)	96.39% (94.12%-100%)	49.51% (44.68%-53.95%)	2.8578 (2.7573-2.9548)	62.57% (55.56%-70.71%)	42.33% (31.74%-52.94%)	97.269% (92.303%-100%)	96.4% (90.31%-100%)	49.57% (40.84%-59.15%)	2.8557 (2.6557-3.0572)
At least 6 of CHASM (breast), CHASM (melanoma), FATHMM (missense), Mutation Assessor, PolyPhen-2, VEST	57.59% (53.54%-61.11%)	35.21% (29.92%-40%)	96.293% (94.203%-98.667%)	94.27% (90.91%-97.92%)	46.23% (41.45%-50.66%)	2.7200 (2.6169-2.8237)	57.54% (50.51%-65.66%)	34.88% (25%-45.17%)	96.398% (91.176%-100%)	94.36% (86.36%-100%)	46.32% (37.5%-55.56%)	2.7196 (2.5065-2.9239)
At least 6 of CHASM (breast), CHASM (melanoma), FATHMM (missense), Mutation Assessor, PROVEAN, SIFT	56.95% (53.02%-61.11%)	33.11% (28.23%-37.8%)	98.173% (97.015%-100%)	96.91% (94.74%-100%)	45.91% (41.14%-50.32%)	2.7411 (2.6487-2.8347)	56.8% (48.48%-64.67%)	32.69% (23.43%-42.62%)	98.144% (94.118%-100%)	96.8% (89.47%-100%)	45.94% (37.18%-55.41%)	2.7357 (2.5444-2.9204)
At least 6 of CHASM (breast), CHASM (melanoma), FATHMM (missense), Mutation Assessor, PROVEAN, VEST	54.92% (51%-58.6%)	29.9% (25%-34.17%)	98.173% (97.015%-100%)	96.59% (94.28%-100%)	44.75% (40%-49.07%)	2.6942 (2.6012-2.7901)	54.8% (47.45%-62.65%)	29.54% (20.63%-39.29%)	98.144% (94.118%-100%)	96.48% (88.89%-100%)	44.8% (36.25%-54.22%)	2.6896 (2.4882-2.8770)
At least 6 of CHASM (breast), CHASM (melanoma), FATHMM (missense), Mutation Assessor, SIFT, VEST	58.96% (55.05%-62.63%)	36.82% (31.75%-41.94%)	97.233% (95.588%-100%)	95.84% (93.33%-100%)	47.09% (42.38%-51.63%)	2.7698 (2.6728-2.8675)	58.85% (51.52%-66.67%)	36.45% (26.15%-46.67%)	97.269% (92.303%-100%)	95.84% (88.46%-100%)	47.15% (38.03%-56.58%)	2.7671 (2.5649-2.9643)
At least 6 of CHASM (breast), CHASM (melanoma), FATHMM (missense), MutationTaster, PolyPhen-2, PROVEAN	54.92% (50.51%-58.59%)	29.91% (24.99%-34.43%)	98.173% (97.015%-100%)	96.59% (94.12%-100%)	44.75% (40.12%-49.04%)	2.6942 (2.5978-2.7867)	54.8% (47.47%-63.64%)	29.53% (20.63%-39.34%)	98.144% (94.118%-100%)	96.47% (88.89%-100%)	44.8% (36.36%-54.32%)	2.6895 (2.4928-2.8804)
At least 6 of CHASM (breast), CHASM (melanoma), FATHMM (missense), MutationTaster, PolyPhen-2, SIFT	61.64% (57.58%-65.15%)	41.05% (35.83%-45.97%)	97.233% (95.588%-100%)	96.25% (93.88%-100%)	48.82% (44.14%-53.24%)	2.8335 (2.7357-2.9343)	61.57% (54.55%-69.7%)	40.76% (31.15%-50.79%)	97.269% (92.303%-100%)	96.26% (89.66%-100%)	48.9% (39.99%-58.21%)	2.8319 (2.6297-3.0275)
At least 6 of CHASM (breast), CHASM (melanoma), FATHMM (missense), MutationTaster, PolyPhen-2, VEST	58.27% (54.04%-62.12%)	35.73% (30.53%-40.63%)	97.233% (95.588%-100%)	95.72% (92.86%-100%)	46.67% (42%-51.01%)	2.7535 (2.6511-2.8559)	58.21% (50.51%-66.67%)	35.43% (25.67%-45.46%)	97.269% (92.303%-100%)	95.73% (88.24%-100%)	46.75% (37.83%-56.25%)	2.7518 (2.5346-2.9522)
At least 6 of CHASM (breast), CHASM (melanoma), FATHMM (missense), MutationTaster, PROVEAN, SIFT	56.94% (53.03%-60.61%)	33.09% (28.33%-37.7%)	98.173% (97.015%-100%)	96.91% (94.74%-100%)	45.91% (41.18%-50.32%)	2.7408 (2.6486-2.8325)	56.82% (49.49%-64.65%)	32.73% (23.72%-42.62%)	98.144% (94.118%-100%)	96.81% (90%-100%)	45.95% (37.18%-55.41%)	2.7365 (2.5444-2.9268)
At least 6 of CHASM (breast), CHASM (melanoma), FATHMM (missense), MutationTaster, PROVEAN, VEST	54.93% (50.51%-58.6%)	29.91% (25%-34.71%)	98.173% (97.015%-100%)	96.59% (94.12%-100%)	44.75% (40%-49.07%)	2.6943 (2.5987-2.7899)	54.8% (47.45%-63.64%)	29.53% (20%-39.35%)	98.144% (94.118%-100%)	96.48% (88.89%-100%)	44.8% (36.14%-54.43%)	2.6895 (2.4874-2.8877)
At least 6 of CHASM (breast), CHASM (melanoma), FATHMM (missense), MutationTaster, SIFT, VEST	59.29% (55.05%-63.13%)	37.35% (32.26%-42.5%)	97.233% (95.588%-100%)	95.89% (93.33%-100%)	47.3% (42.76%-51.7%)	2.7778 (2.6789-2.8819)	59.19% (51.52%-67.68%)	36.99% (26.98%-47.14%)	97.269% (92.303%-100%)	95.9% (88.89%-100%)	47.36% (38.03%-56.97%)	2.7752 (2.5649-2.9714)
At least 6 of CHASM (breast), CHASM (melanoma), FATHMM (missense), PolyPhen-2, PROVEAN, SIFT	56.95% (53.03%-60.61%)	33.11% (28.12%-37.88%)	98.173% (97.015%-100%)	96.91% (94.74%-100%)	45.91% (41.14%-50%)	2.7410 (2.6456-2.8331)	56.8% (49.49%-64.65%)	32.69% (22.95%-42.86%)	98.144% (94.118%-100%)	96.8% (89.47%-100%)	45.93% (37.5%-55.56%)	2.7357 (2.5483-2.9262)
At least 6 of CHASM (breast), CHASM (melanoma), FATHMM (missense), PolyPhen-2, PROVEAN, VEST	54.58% (50.51%-58.08%)	29.37% (24.41%-33.87%)	98.173% (97.015%-100%)	96.53% (94.12%-100%)	44.56% (39.87%-48.75%)	2.6864 (2.5893-2.7776)	54.47% (47.47%-62.63%)	29% (20%-38.72%)	98.144% (94.118%-100%)	96.41% (88.24%-100%)	44.62% (36.25%-54.22%)	2.6818 (2.4891-2.8732)
At least 6 of CHASM (breast), CHASM (melanoma), FATHMM (missense), PolyPhen-2, SIFT, VEST	58.61% (54.55%-62.13%)	36.27% (31.15%-41.22%)	97.233% (95.588%-100%)	95.78% (93.18%-100%)	46.88% (42.07%-51.32%)	2.7616 (2.6635-2.8632)	58.53% (51.49%-66.67%)	35.94% (25.81%-45.76%)	97.269% (92.303%-100%)	95.79% (88.24%-100%)	46.95% (38.16%-56.41%)	2.7594 (2.5499-2.9596)
At least 6 of CHASM (breast), CHASM (melanoma), FATHMM (missense), PROVEAN, SIFT, VEST	56.28% (52.02%-60.1%)	32.05% (27.13%-36.72%)	98.173% (97.015%-100%)	96.81% (94.59%-100%)	45.52% (40.88%-49.7%)	2.7255 (2.6318-2.8196)	56.13% (48.48%-64.65%)	31.63% (22.22%-41.54%)	98.144% (94.118%-100%)	96.71% (89.47%-100%)	45.55% (37.03%-55.13%)	2.7203 (2.5283-2.9097)
At least 6 of CHASM (breast), CHASM (melanoma), Mutation Assessor, MutationTaster, PolyPhen-2, PROVEAN	54.9% (50.51%-58.59%)	29.87% (25%-34.4%)	98.173% (97.015%-100%)	96.58% (94.12%-100%)	44.74% (40%-49.06%)	2.6936 (2.5978-2.7866)	54.85% (47.47%-63.64%)	29.61% (20.63%-39.66%)	98.144% (94.118%-100%)	96.48% (88.89%-100%)	44.83% (36.25%-54.22%)	2.6906 (2.5045-2.8770)
At least 6 of CHASM (breast), CHASM (melanoma), Mutation Assessor, MutationTaster, PolyPhen-2, SIFT	60.6% (56.57%-64.65%)	39.94% (35%-44.8%)	96.311% (94.286%-98.667%)	94.93% (92%-98.18%)	48.12% (43.45%-52.67%)	2.7930 (2.6907-2.9024)	60.62% (52.53%-68.69%)	39.78% (29.69%-50%)	96.355% (91.176%-100%)	94.94% (87.5%-100%)	48.25% (39.06%-57.53%)	2.7933 (2.5704-2.9964)
At least 6 of CHASM (breast), CHASM (melanoma), Mutation Assessor, MutationTaster, PolyPhen-2, VEST	56.9% (52.53%-61.11%)	34.11% (28.92%-39.06%)	96.311% (94.286%-98.667%)	94.11% (90.69%-97.92%)	45.81% (41.22%-50.01%)	2.7035 (2.5983-2.8125)	56.9% (48.48%-65.66%)	33.9% (23.81%-44.27%)	96.355% (91.176%-100%)	94.11% (85.7%-100%)	45.93% (37.32%-55.27%)	2.7029 (2.4813-2.9118)
At least 6 of CHASM (breast), CHASM (melanoma), Mutation Assessor, MutationTaster, PROVEAN, SIFT	55.92% (51.52%-59.6%)	31.48% (26.72%-36%)	98.173% (97.015%-100%)	96.75% (94.44%-100%)	45.31% (40.62%-49.69%)	2.7171 (2.6219-2.8085)	55.84% (48.48%-64.65%)	31.19% (22.06%-40.99%)	98.144% (94.118%-100%)	96.65% (89.47%-100%)	45.39% (36.49%-54.8%)	2.7137 (2.5268-2.9058)
At least 6 of CHASM (breast), CHASM (melanoma), Mutation Assessor, MutationTaster, PROVEAN, VEST	54.22% (50%-58.08%)	28.8% (24%-33.33%)	98.173% (97.015%-100%)	96.46% (93.94%-100%)	44.37% (39.63%-48.75%)	2.6781 (2.5835-2.7729)	54.18% (46.46%-62.63%)	28.55% (19.64%-38.46%)	98.144% (94.118%-100%)	96.35% (88.24%-100%)	44.46% (35.8%-53.85%)	2.6751 (2.4808-2.8688)
At least 6 of CHASM (breast), CHASM (melanoma), Mutation Assessor, MutationTaster, SIFT, VEST	57.92% (54.04%-61.62%)	35.72% (30.64%-40.8%)	96.311% (94.286%-98.667%)	94.36% (90.91%-98%)	46.43% (41.72%-50.97%)	2.7282 (2.6230-2.8347)	57.89% (50.51%-65.66%)	35.47% (25.4%-45.76%)	96.355% (91.176%-100%)	94.35% (86.36%-100%)	46.53% (37.33%-56%)	2.7270 (2.5064-2.9336)
At least 6 of CHASM (breast), CHASM (melanoma), Mutation Assessor, PolyPhen-2, PROVEAN, SIFT	56.59% (52.53%-60.61%)	32.54% (27.59%-37.01%)	98.173% (97.015%-100%)	96.85% (94.59%-100%)	45.7% (41.13%-50%)	2.7327 (2.6393-2.8271)	56.51% (48.48%-64.65%)	32.24% (22.95%-42.37%)	98.144% (94.118%-100%)	96.76% (89.99%-100%)	45.77% (37.18%-55.01%)	2.7290 (2.5430-2.9162)
At least 6 of CHASM (breast), CHASM (melanoma), Mutation Assessor, PolyPhen-2, PROVEAN, VEST	54.56% (50.51%-58.59%)	29.33% (24.39%-33.6%)	98.173% (97.015%-100%)	96.52% (93.94%-100%)	44.55% (39.88%-48.77%)	2.6858 (2.5909-2.7820)	54.52% (46.46%-62.63%)	29.08% (20%-38.98%)	98.144% (94.118%-100%)	96.42% (88.89%-100%)	44.64% (36.14%-54.22%)	2.6828 (2.4891-2.8689)
At least 6 of CHASM (breast), CHASM (melanoma), Mutation Assessor, PolyPhen-2, SIFT, VEST	57.92% (54.04%-61.62%)	35.71% (30.65%-40.63%)	96.311% (94.286%-98.667%)	94.36% (91.11%-98%)	46.42% (41.83%-50.69%)	2.7282 (2.6223-2.8393)	57.89% (50.51%-65.66%)	35.46% (25.67%-45.62%)	96.355% (91.176%-100%)	94.36% (86.35%-100%)	46.53% (37.66%-55.56%)	2.7271 (2.5022-2.9401)
At least 6 of CHASM (breast), CHASM (melanoma), Mutation Assessor, PROVEAN, SIFT, VEST	55.58% (51.52%-59.6%)	30.94% (26.19%-35.48%)	98.173% (97.015%-100%)	96.7% (94.44%-100%)	45.12% (40.38%-49.38%)	2.7093 (2.6162-2.8036)	55.51% (47.47%-63.64%)	30.66% (21.43%-40.36%)	98.144% (94.118%-100%)	96.6% (88.89%-100%)	45.2% (36.47%-54.55%)	2.7060 (2.5100-2.8925)
At least 6 of CHASM (breast), CHASM (melanoma), MutationTaster, PolyPhen-2, PROVEAN, SIFT	56.92% (52.53%-60.61%)	33.06% (27.96%-37.88%)	98.173% (97.015%-100%)	96.9% (94.74%-100%)	45.89% (41.14%-50%)	2.7403 (2.6428-2.8315)	56.87% (49.49%-65.66%)	32.8% (23.43%-42.86%)	98.144% (94.118%-100%)	96.81% (90%-100%)	45.98% (37.5%-55.56%)	2.7373 (2.5458-2.9329)
At least 6 of CHASM (breast), CHASM (melanoma), MutationTaster, PolyPhen-2, PROVEAN, VEST	55.23% (51.01%-59.09%)	30.39% (25.4%-35%)	98.173% (97.015%-100%)	96.64% (94.29%-100%)	44.92% (40.13%-49.07%)	2.7012 (2.6037-2.7902)	55.2% (47.47%-63.64%)	30.17% (20.97%-40.32%)	98.144% (94.118%-100%)	96.54% (88.89%-100%)	45.03% (36.58%-54.67%)	2.6988 (2.5070-2.8965)
At least 6 of CHASM (breast), CHASM (melanoma), MutationTaster, PolyPhen-2, SIFT, VEST	58.92% (55.04%-62.63%)	37.29% (32.5%-42.28%)	96.311% (94.286%-98.667%)	94.59% (91.3%-98.11%)	47.04% (42.38%-51.35%)	2.7523 (2.6468-2.8594)	58.93% (51.52%-66.69%)	37.1% (27.12%-47.46%)	96.355% (91.176%-100%)	94.59% (86.66%-100%)	47.17% (38.57%-56.94%)	2.7522 (2.5347-2.9602)
At least 6 of CHASM (breast), CHASM (melanoma), MutationTaster, PROVEAN, SIFT, VEST	56.92% (52.53%-60.61%)	33.06% (28.33%-37.82%)	98.173% (97.015%-100%)	96.9% (94.74%-100%)	45.89% (41.14%-50.31%)	2.7403 (2.6428-2.8331)	56.86% (49.49%-65.66%)	32.8% (23.21%-42.43%)	98.144% (94.118%-100%)	96.81% (89.99%-100%)	45.98% (37.18%-55.56%)	2.7373 (2.5445-2.9331)
At least 6 of CHASM (breast), CHASM (melanoma), PolyPhen-2, PROVEAN, SIFT, VEST	56.58% (52.53%-60.11%)	32.52% (27.56%-37.12%)	98.173% (97.015%-100%)	96.85% (94.59%-100%)	45.7% (40.88%-50%)	2.7324 (2.6354-2.8252)	56.54% (49.47%-65.66%)	32.27% (22.8%-42.03%)	98.144% (94.118%-100%)	96.76% (89.47%-100%)	45.78% (37.18%-55.41%)	2.7296 (2.5435-2.9262)
At least 6 of CHASM (breast), FATHMM (cancer), FATHMM (missense), Mutation Assessor, MutationTaster, PolyPhen-2	60.29% (56.57%-64.14%)	38.92% (33.6%-43.75%)	97.233% (95.588%-100%)	96.06% (93.62%-100%)	47.94% (43.36%-52.38%)	2.8014 (2.7026-2.8986)	60.23% (52.53%-67.68%)	38.64% (29.23%-49.18%)	97.269% (92.303%-100%)	96.07% (89.47%-100%)	48.02% (39.19%-57.33%)	2.7999 (2.6026-2.9953)
At least 6 of CHASM (breast), FATHMM (cancer), FATHMM (missense), Mutation Assessor, MutationTaster, PROVEAN	55.26% (51.01%-59.09%)	30.44% (25.78%-34.92%)	98.173% (97.015%-100%)	96.65% (94.29%-100%)	44.94% (40.26%-49.37%)	2.7020 (2.6064-2.7962)	55.13% (47.47%-63.64%)	30.06% (21.05%-39.66%)	98.144% (94.118%-100%)	96.53% (88.89%-100%)	44.99% (36.25%-54.29%)	2.6973 (2.5072-2.8877)
At least 6 of CHASM (breast), FATHMM (cancer), FATHMM (missense), Mutation Assessor, MutationTaster, SIFT	61.31% (57.58%-65.15%)	40.53% (35.48%-45.16%)	97.233% (95.588%-100%)	96.21% (93.88%-100%)	48.6% (43.87%-53.1%)	2.8257 (2.7273-2.9222)	61.22% (53.54%-68.69%)	40.21% (30.77%-50.75%)	97.269% (92.303%-100%)	96.22% (89.66%-100%)	48.66% (39.47%-58.33%)	2.8235 (2.6280-3.0183)
At least 6 of CHASM (breast), FATHMM (cancer), FATHMM (missense), Mutation Assessor, MutationTaster, VEST	57.61% (53.54%-61.62%)	34.7% (29.84%-39.84%)	97.233% (95.588%-100%)	95.59% (92.68%-100%)	46.27% (41.67%-50.68%)	2.7380 (2.6387-2.8393)	57.5% (49.49%-65.66%)	34.32% (24.19%-44.07%)	97.269% (92.303%-100%)	95.59% (88%-100%)	46.33% (37.35%-55.72%)	2.7351 (2.5277-2.9286)
At least 6 of CHASM (breast), FATHMM (cancer), FATHMM (missense), Mutation Assessor, PolyPhen-2, PROVEAN	55.94% (52.02%-59.6%)	31.51% (26.45%-36.07%)	98.173% (97.015%-100%)	96.76% (94.44%-100%)	45.32% (40.62%-49.69%)	2.7176 (2.6219-2.8091)	55.8% (48.48%-63.64%)	31.11% (22.03%-41.27%)	98.144% (94.118%-100%)	96.64% (88.89%-100%)	45.36% (36.71%-54.67%)	2.7126 (2.5188-2.9015)
At least 6 of CHASM (breast), FATHMM (cancer), FATHMM (missense), Mutation Assessor, PolyPhen-2, SIFT	62.33% (58.59%-66.16%)	42.14% (36.97%-47.06%)	97.233% (95.588%-100%)	96.34% (94.12%-100%)	49.29% (44.44%-53.68%)	2.8500 (2.7524-2.9480)	62.22% (54.55%-69.7%)	41.77% (31.58%-51.67%)	97.269% (92.303%-100%)	96.35% (90.31%-100%)	49.32% (40.58%-58.91%)	2.8472 (2.6448-3.0445)
At least 6 of CHASM (breast), FATHMM (cancer), FATHMM (missense), Mutation Assessor, PolyPhen-2, VEST	57.27% (53.03%-61.11%)	34.69% (29.54%-39.52%)	96.293% (94.203%-98.667%)	94.19% (90.91%-97.87%)	46.03% (41.45%-50.36%)	2.7120 (2.6045-2.8168)	57.18% (49.49%-65.66%)	34.32% (24.99%-44.78%)	96.398% (91.176%-100%)	94.28% (86.36%-100%)	46.1% (37.33%-55.41%)	2.7109 (2.4960-2.9234)
At least 6 of CHASM (breast), FATHMM (cancer), FATHMM (missense), Mutation Assessor, PROVEAN, SIFT	56.95% (53.02%-61.11%)	33.11% (28.23%-37.8%)	98.173% (97.015%-100%)	96.91% (94.74%-100%)	45.91% (41.14%-50.32%)	2.7411 (2.6487-2.8347)	56.8% (48.48%-64.67%)	32.69% (23.43%-42.62%)	98.144% (94.118%-100%)	96.8% (89.47%-100%)	45.94% (37.18%-55.41%)	2.7357 (2.5444-2.9204)
At least 6 of CHASM (breast), FATHMM (cancer), FATHMM (missense), Mutation Assessor, PROVEAN, VEST	54.92% (51%-58.6%)	29.9% (25%-34.17%)	98.173% (97.015%-100%)	96.59% (94.28%-100%)	44.75% (40%-49.07%)	2.6942 (2.6012-2.7901)	54.8% (47.45%-62.65%)	29.54% (20.63%-39.29%)	98.144% (94.118%-100%)	96.48% (88.89%-100%)	44.8% (36.25%-54.22%)	2.6896 (2.4882-2.8770)
At least 6 of CHASM (breast), FATHMM (cancer), FATHMM (missense), Mutation Assessor, SIFT, VEST	58.63% (54.55%-62.63%)	36.3% (31.15%-41.27%)	97.233% (95.588%-100%)	95.78% (93.18%-100%)	46.89% (42.17%-51.33%)	2.7621 (2.6646-2.8613)	58.5% (50.51%-66.67%)	35.89% (26.15%-45.62%)	97.269% (92.303%-100%)	95.78% (88.46%-100%)	46.92% (37.97%-56.34%)	2.7586 (2.5531-2.9520)
At least 6 of CHASM (breast), FATHMM (cancer), FATHMM (missense), MutationTaster, PolyPhen-2, PROVEAN	55.27% (51.01%-59.09%)	30.46% (25.4%-35.16%)	98.173% (97.015%-100%)	96.65% (94.29%-100%)	44.95% (40.37%-49.08%)	2.7022 (2.6044-2.7941)	55.11% (47.47%-63.64%)	30.03% (20.69%-40.32%)	98.144% (94.118%-100%)	96.53% (88.89%-100%)	44.97% (36.36%-54.55%)	2.6967 (2.5063-2.8924)
At least 6 of CHASM (breast), FATHMM (cancer), FATHMM (missense), MutationTaster, PolyPhen-2, SIFT	61.31% (57.58%-65.15%)	40.53% (35.43%-45.24%)	97.233% (95.588%-100%)	96.2% (93.88%-100%)	48.6% (43.88%-53.02%)	2.8257 (2.7253-2.9236)	61.22% (53.54%-68.69%)	40.2% (30.36%-50%)	97.269% (92.303%-100%)	96.21% (89.65%-100%)	48.66% (39.7%-58.11%)	2.8234 (2.6230-3.0201)
At least 6 of CHASM (breast), FATHMM (cancer), FATHMM (missense), MutationTaster, PolyPhen-2, VEST	58.29% (54.04%-62.12%)	35.77% (30.71%-40.77%)	97.233% (95.588%-100%)	95.72% (92.86%-100%)	46.68% (42.04%-51.01%)	2.7540 (2.6513-2.8583)	58.16% (50.51%-66.67%)	35.37% (25.67%-45.31%)	97.269% (92.303%-100%)	95.72% (88.46%-100%)	46.72% (37.83%-56.25%)	2.7508 (2.5389-2.9532)
At least 6 of CHASM (breast), FATHMM (cancer), FATHMM (missense), MutationTaster, PROVEAN, SIFT	56.94% (53.03%-60.61%)	33.09% (28.33%-37.7%)	98.173% (97.015%-100%)	96.91% (94.74%-100%)	45.91% (41.18%-50.32%)	2.7408 (2.6486-2.8325)	56.82% (49.49%-64.65%)	32.73% (23.72%-42.62%)	98.144% (94.118%-100%)	96.81% (90%-100%)	45.95% (37.18%-55.41%)	2.7365 (2.5444-2.9268)
At least 6 of CHASM (breast), FATHMM (cancer), FATHMM (missense), MutationTaster, PROVEAN, VEST	55.27% (51.01%-59.09%)	30.46% (25.76%-35.2%)	98.173% (97.015%-100%)	96.65% (94.29%-100%)	44.95% (40.25%-49.36%)	2.7023 (2.6074-2.7963)	55.11% (47.47%-63.64%)	30.02% (20.63%-39.71%)	98.144% (94.118%-100%)	96.53% (88.89%-100%)	44.97% (36.36%-54.55%)	2.6967 (2.5031-2.8955)
At least 6 of CHASM (breast), FATHMM (cancer), FATHMM (missense), MutationTaster, SIFT, VEST	58.97% (55.05%-62.63%)	36.83% (31.74%-41.94%)	97.233% (95.588%-100%)	95.84% (93.18%-100%)	47.1% (42.57%-51.63%)	2.7700 (2.6759-2.8720)	58.84% (51.52%-66.67%)	36.43% (26.23%-46.38%)	97.269% (92.303%-100%)	95.84% (88.88%-100%)	47.14% (38.03%-56.41%)	2.7667 (2.5540-2.9606)
At least 6 of CHASM (breast), FATHMM (cancer), FATHMM (missense), PolyPhen-2, PROVEAN, SIFT	56.95% (53.03%-60.61%)	33.11% (28.12%-37.88%)	98.173% (97.015%-100%)	96.91% (94.74%-100%)	45.91% (41.14%-50%)	2.7410 (2.6456-2.8331)	56.8% (49.49%-64.65%)	32.69% (22.95%-42.86%)	98.144% (94.118%-100%)	96.8% (89.47%-100%)	45.93% (37.5%-55.56%)	2.7357 (2.5483-2.9262)
At least 6 of CHASM (breast), FATHMM (cancer), FATHMM (missense), PolyPhen-2, PROVEAN, VEST	54.93% (51.01%-58.59%)	29.92% (25%-34.65%)	98.173% (97.015%-100%)	96.59% (94.12%-100%)	44.76% (40%-49.04%)	2.6944 (2.5987-2.7876)	54.78% (47.47%-62.63%)	29.5% (20.31%-39.34%)	98.144% (94.118%-100%)	96.47% (88.89%-100%)	44.79% (36.25%-54.32%)	2.6890 (2.4967-2.8770)
At least 6 of CHASM (breast), FATHMM (cancer), FATHMM (missense), PolyPhen-2, SIFT, VEST	58.29% (54.04%-62.12%)	35.76% (30.65%-40.8%)	97.233% (95.588%-100%)	95.72% (93.02%-100%)	46.68% (41.83%-50.98%)	2.7539 (2.6513-2.8549)	58.17% (50.51%-66.67%)	35.38% (25.4%-45.32%)	97.269% (92.303%-100%)	95.72% (88.23%-100%)	46.73% (38.16%-56.34%)	2.7510 (2.5440-2.9524)
At least 6 of CHASM (breast), FATHMM (cancer), FATHMM (missense), PROVEAN, SIFT, VEST	56.28% (52.02%-60.1%)	32.05% (27.13%-36.72%)	98.173% (97.015%-100%)	96.81% (94.59%-100%)	45.52% (40.88%-49.7%)	2.7255 (2.6318-2.8196)	56.13% (48.48%-64.65%)	31.63% (22.22%-41.54%)	98.144% (94.118%-100%)	96.71% (89.47%-100%)	45.55% (37.03%-55.13%)	2.7203 (2.5283-2.9097)
At least 6 of CHASM (breast), FATHMM (cancer), Mutation Assessor, MutationTaster, PolyPhen-2, PROVEAN	55.58% (51.52%-59.6%)	30.95% (26.02%-35.65%)	98.173% (97.015%-100%)	96.7% (94.29%-100%)	45.12% (40.52%-49.38%)	2.7094 (2.6149-2.8012)	55.5% (47.47%-63.64%)	30.64% (21.31%-40.62%)	98.144% (94.118%-100%)	96.59% (89.47%-100%)	45.19% (36.58%-54.55%)	2.7057 (2.5146-2.8955)
At least 6 of CHASM (breast), FATHMM (cancer), Mutation Assessor, MutationTaster, PolyPhen-2, SIFT	61.29% (57.07%-65.15%)	40.5% (35.2%-45.6%)	97.233% (95.588%-100%)	96.2% (93.75%-100%)	48.59% (43.97%-53.1%)	2.8253 (2.7273-2.9239)	61.25% (53.54%-69.7%)	40.26% (30%-50.77%)	97.269% (92.303%-100%)	96.22% (89.66%-100%)	48.69% (39.7%-57.9%)	2.8243 (2.6250-3.0201)
At least 6 of CHASM (breast), FATHMM (cancer), Mutation Assessor, MutationTaster, PolyPhen-2, VEST	57.6% (53.54%-61.62%)	34.67% (29.27%-39.67%)	97.233% (95.588%-100%)	95.59% (92.68%-100%)	46.26% (41.61%-50.64%)	2.7376 (2.6377-2.8396)	57.53% (49.49%-65.66%)	34.37% (24.24%-45.1%)	97.269% (92.303%-100%)	95.6% (88%-100%)	46.35% (37.5%-55.85%)	2.7358 (2.5219-2.9438)
At least 6 of CHASM (breast), FATHMM (cancer), Mutation Assessor, MutationTaster, PROVEAN, SIFT	56.6% (52.53%-60.61%)	32.55% (27.78%-37.1%)	98.173% (97.015%-100%)	96.85% (94.59%-100%)	45.71% (41.06%-50.01%)	2.7329 (2.6393-2.8252)	56.5% (48.48%-64.65%)	32.22% (22.95%-42.11%)	98.144% (94.118%-100%)	96.75% (89.99%-100%)	45.77% (36.98%-55.08%)	2.7289 (2.5417-2.9205)
At least 6 of CHASM (breast), FATHMM (cancer), Mutation Assessor, MutationTaster, PROVEAN, VEST	54.91% (50.51%-58.6%)	29.88% (25.19%-34.43%)	98.173% (97.015%-100%)	96.58% (94.12%-100%)	44.74% (40.12%-49.08%)	2.6938 (2.5987-2.7892)	54.83% (47.45%-63.64%)	29.59% (20.63%-39.07%)	98.144% (94.118%-100%)	96.47% (88.89%-100%)	44.82% (36%-54.22%)	2.6903 (2.5025-2.8826)
At least 6 of CHASM (breast), FATHMM (cancer), Mutation Assessor, MutationTaster, SIFT, VEST	58.62% (54.55%-62.63%)	36.28% (31.01%-41.32%)	97.233% (95.588%-100%)	95.78% (93.02%-100%)	46.88% (42.18%-51.37%)	2.7617 (2.6630-2.8615)	58.53% (50.51%-66.67%)	35.94% (26.15%-46.67%)	97.269% (92.303%-100%)	95.78% (88.46%-100%)	46.95% (38.03%-56.41%)	2.7593 (2.5541-2.9561)
At least 6 of CHASM (breast), FATHMM (cancer), Mutation Assessor, PolyPhen-2, PROVEAN, SIFT	57.28% (53.03%-61.11%)	33.62% (28.68%-38.17%)	98.173% (97.015%-100%)	96.95% (94.87%-100%)	46.1% (41.4%-50.35%)	2.7485 (2.6577-2.8463)	57.17% (49.49%-65.66%)	33.27% (24.14%-42.86%)	98.144% (94.118%-100%)	96.85% (90%-100%)	46.15% (37.5%-55.41%)	2.7442 (2.5547-2.9286)
At least 6 of CHASM (breast), FATHMM (cancer), Mutation Assessor, PolyPhen-2, PROVEAN, VEST	55.24% (51.01%-59.09%)	30.41% (25.58%-34.92%)	98.173% (97.015%-100%)	96.64% (94.29%-100%)	44.93% (40.13%-49.08%)	2.7016 (2.6072-2.7987)	55.17% (47.47%-63.64%)	30.12% (21.05%-39.68%)	98.144% (94.118%-100%)	96.53% (88.89%-100%)	45.01% (36.47%-54.43%)	2.6980 (2.5028-2.8889)
At least 6 of CHASM (breast), FATHMM (cancer), Mutation Assessor, PolyPhen-2, SIFT, VEST	58.61% (54.55%-62.13%)	36.28% (31.01%-41.13%)	97.233% (95.588%-100%)	95.78% (93.02%-100%)	46.88% (42.18%-51.33%)	2.7617 (2.6623-2.8625)	58.53% (51.49%-66.67%)	35.94% (25.76%-46.88%)	97.269% (92.303%-100%)	95.78% (88.23%-100%)	46.95% (38.15%-56.41%)	2.7594 (2.5490-2.9654)
At least 6 of CHASM (breast), FATHMM (cancer), Mutation Assessor, PROVEAN, SIFT, VEST	56.26% (52.02%-60.1%)	32.02% (27.34%-36.64%)	98.173% (97.015%-100%)	96.81% (94.59%-100%)	45.51% (40.79%-50%)	2.7250 (2.6323-2.8201)	56.17% (48.48%-64.65%)	31.7% (22.22%-41.27%)	98.144% (94.118%-100%)	96.7% (89.47%-100%)	45.57% (36.71%-54.88%)	2.7211 (2.5282-2.9118)
At least 6 of CHASM (breast), FATHMM (cancer), MutationTaster, PolyPhen-2, PROVEAN, SIFT	57.6% (53.54%-61.11%)	34.14% (29.17%-39.17%)	98.173% (97.015%-100%)	97% (94.87%-100%)	46.3% (41.61%-50.66%)	2.7560 (2.6599-2.8483)	57.52% (50.51%-65.66%)	33.84% (24.13%-43.75%)	98.144% (94.118%-100%)	96.9% (90%-100%)	46.36% (37.66%-56%)	2.7525 (2.5548-2.9459)
At least 6 of CHASM (breast), FATHMM (cancer), MutationTaster, PolyPhen-2, PROVEAN, VEST	56.26% (52.02%-60.1%)	32.02% (27.2%-36.8%)	98.173% (97.015%-100%)	96.8% (94.59%-100%)	45.51% (40.76%-50%)	2.7250 (2.6273-2.8196)	56.17% (48.48%-64.65%)	31.7% (22.38%-41.27%)	98.144% (94.118%-100%)	96.7% (89.47%-100%)	45.57% (37.04%-55.26%)	2.7211 (2.5282-2.9161)
At least 6 of CHASM (breast), FATHMM (cancer), MutationTaster, PolyPhen-2, SIFT, VEST	59.61% (55.56%-63.64%)	37.85% (32.54%-42.75%)	97.233% (95.588%-100%)	95.95% (93.33%-100%)	47.5% (42.76%-51.7%)	2.7854 (2.6846-2.8873)	59.56% (51.52%-67.68%)	37.58% (27.12%-48.33%)	97.269% (92.303%-100%)	95.95% (88.89%-100%)	47.59% (38.75%-57.53%)	2.7839 (2.5749-2.9881)
At least 6 of CHASM (breast), FATHMM (cancer), MutationTaster, PROVEAN, SIFT, VEST	57.61% (53.54%-61.12%)	34.14% (29.27%-38.89%)	98.173% (97.015%-100%)	97% (94.87%-100%)	46.3% (41.45%-50.65%)	2.7561 (2.6598-2.8493)	57.52% (50.48%-65.66%)	33.83% (24.24%-43.75%)	98.144% (94.118%-100%)	96.91% (90%-100%)	46.36% (37.5%-56%)	2.7525 (2.5590-2.9495)
At least 6 of CHASM (breast), FATHMM (cancer), PolyPhen-2, PROVEAN, SIFT, VEST	57.26% (53.03%-61.11%)	33.6% (28.57%-38.4%)	98.173% (97.015%-100%)	96.95% (94.87%-100%)	46.09% (41.18%-50.33%)	2.7482 (2.6512-2.8422)	57.19% (49.49%-65.66%)	33.31% (24.19%-42.86%)	98.144% (94.118%-100%)	96.85% (90%-100%)	46.17% (37.5%-55.56%)	2.7448 (2.5529-2.9402)
At least 6 of CHASM (breast), FATHMM (missense), Mutation Assessor, MutationTaster, PolyPhen-2, PROVEAN	54.24% (50%-58.08%)	28.84% (24.16%-33.33%)	98.173% (97.015%-100%)	96.46% (93.94%-100%)	44.38% (39.73%-48.75%)	2.6785 (2.5807-2.7732)	54.14% (46.46%-62.63%)	28.48% (19.66%-37.94%)	98.144% (94.118%-100%)	96.35% (88.24%-100%)	44.44% (35.8%-53.85%)	2.6741 (2.4800-2.8688)
At least 6 of CHASM (breast), FATHMM (missense), Mutation Assessor, MutationTaster, PolyPhen-2, SIFT	60.28% (56.55%-64.14%)	38.91% (33.87%-43.65%)	97.233% (95.588%-100%)	96.05% (93.48%-100%)	47.93% (43.26%-52.38%)	2.8012 (2.7026-2.8993)	60.25% (52.53%-67.7%)	38.66% (29.03%-48.49%)	97.269% (92.303%-100%)	96.07% (89.28%-100%)	48.03% (39.19%-57.69%)	2.8003 (2.5996-2.9953)
At least 6 of CHASM (breast), FATHMM (missense), Mutation Assessor, MutationTaster, PolyPhen-2, VEST	56.59% (52.53%-60.61%)	33.08% (28.1%-37.91%)	97.233% (95.588%-100%)	95.39% (92.31%-100%)	45.66% (41.06%-50%)	2.7136 (2.6102-2.8150)	56.52% (48.48%-64.65%)	32.77% (22.95%-42.86%)	97.269% (92.303%-100%)	95.4% (87.5%-100%)	45.75% (37.04%-54.88%)	2.7119 (2.5027-2.9162)
At least 6 of CHASM (breast), FATHMM (missense), Mutation Assessor, MutationTaster, PROVEAN, SIFT	55.26% (51.01%-59.09%)	30.44% (25.78%-34.92%)	98.173% (97.015%-100%)	96.65% (94.29%-100%)	44.94% (40.26%-49.37%)	2.7020 (2.6064-2.7962)	55.13% (47.47%-63.64%)	30.06% (21.05%-39.66%)	98.144% (94.118%-100%)	96.53% (88.89%-100%)	44.99% (36.25%-54.29%)	2.6973 (2.5072-2.8877)
At least 6 of CHASM (breast), FATHMM (missense), Mutation Assessor, MutationTaster, PROVEAN, VEST	53.57% (49.49%-57.58%)	27.77% (22.9%-32.23%)	98.173% (97.015%-100%)	96.34% (93.75%-100%)	44.01% (39.37%-48.41%)	2.6629 (2.5695-2.7588)	53.47% (45.45%-61.62%)	27.43% (18.96%-36.93%)	98.144% (94.118%-100%)	96.21% (88.24%-100%)	44.07% (35.44%-53.57%)	2.6586 (2.4640-2.8458)

At least 6 of CHASM (breast), FATHMM (missense), Mutation Assessor, MutationTaster, SIFT, VEST	57.61% (53.54%-61.62%)	34.68% (29.69%-39.84%)	97.233% (95.588%-100%)	95.59% (92.86%-100%)	46.26% (41.67%-50.67%)	2.7377 (2.6387-2.8372)	57.52% (49.49%-65.66%)	34.34% (24.24%-44.44%)	97.269% (92.303%-100%)	95.59% (88%-100%)	46.33% (37.5%-55.89%)	2.7354 (2.5278-2.9268)
At least 6 of CHASM (breast), FATHMM (missense), Mutation Assessor, PolyPhen-2, PROVEAN, SIFT	55.94% (52.02%-59.6%)	31.51% (26.45%-36.07%)	98.173% (97.015%-100%)	96.76% (94.44%-100%)	45.32% (40.62%-49.66%)	2.7176 (2.6219-2.8091)	55.8% (48.48%-63.64%)	31.11% (22.03%-41.27%)	98.144% (94.118%-100%)	96.64% (88.89%-100%)	45.36% (36.71%-54.67%)	2.7126 (2.5188-2.9015)
At least 6 of CHASM (breast), FATHMM (missense), Mutation Assessor, PolyPhen-2, PROVEAN, VEST	53.91% (49.99%-57.58%)	28.3% (23.25%-32.54%)	98.173% (97.015%-100%)	96.4% (93.75%-100%)	44.19% (39.39%-48.45%)	2.6707 (2.5747-2.7652)	53.81% (46.46%-61.62%)	27.96% (19.4%-38.1%)	98.144% (94.118%-100%)	96.28% (88.24%-100%)	44.25% (35.8%-53.66%)	2.6664 (2.4711-2.8542)
At least 6 of CHASM (breast), FATHMM (missense), Mutation Assessor, PolyPhen-2, SIFT, VEST	57.6% (53.54%-61.11%)	34.68% (29.51%-39.52%)	97.233% (95.588%-100%)	95.59% (92.86%-100%)	46.26% (41.56%-50.66%)	2.7377 (2.6415-2.8372)	57.52% (49.49%-65.66%)	34.34% (24.59%-44.07%)	97.269% (92.303%-100%)	95.6% (87.5%-100%)	46.33% (37.65%-55.56%)	2.7354 (2.5292-2.9286)
At least 6 of CHASM (breast), FATHMM (missense), Mutation Assessor, PROVEAN, SIFT, VEST	54.92% (51%-58.6%)	29.9% (25%-34.17%)	98.173% (97.015%-100%)	96.59% (94.28%-100%)	44.75% (40%-49.07%)	2.6942 (2.6012-2.7901)	54.8% (47.47%-63.64%)	29.54% (20.63%-39.29%)	98.144% (94.118%-100%)	96.48% (88.89%-100%)	44.8% (36.25%-54.22%)	2.6896 (2.4882-2.8804)
At least 6 of CHASM (breast), FATHMM (missense), MutationTaster, PolyPhen-2, PROVEAN, SIFT	54.92% (50.51%-58.59%)	29.91% (24.99%-34.43%)	98.173% (97.015%-100%)	96.59% (94.12%-100%)	44.75% (40.12%-49.04%)	2.6942 (2.5978-2.7867)	54.8% (47.47%-63.64%)	29.53% (20.63%-39.34%)	98.144% (94.118%-100%)	96.47% (88.89%-100%)	44.8% (36.36%-54.43%)	2.6895 (2.4928-2.8804)
At least 6 of CHASM (breast), FATHMM (missense), MutationTaster, PolyPhen-2, PROVEAN, VEST	53.58% (48.99%-57.58%)	27.79% (22.73%-32.26%)	98.173% (97.015%-100%)	96.34% (93.75%-100%)	44.02% (39.39%-48.17%)	2.6631 (2.5651-2.7588)	53.45% (45.45%-62.63%)	27.39% (18.45%-37.5%)	98.144% (94.118%-100%)	96.21% (88.24%-100%)	44.06% (35.44%-53.66%)	2.6580 (2.4534-2.8542)
At least 6 of CHASM (breast), FATHMM (missense), MutationTaster, PolyPhen-2, SIFT, VEST	57.26% (53.03%-61.11%)	34.14% (29.17%-38.93%)	97.233% (95.588%-100%)	95.53% (92.66%-100%)	46.06% (41.56%-50.33%)	2.7296 (2.6279-2.8325)	57.2% (49.49%-65.66%)	33.83% (23.73%-43.75%)	97.269% (92.303%-100%)	95.53% (87.5%-100%)	46.14% (37.5%-55.56%)	2.7277 (2.5105-2.9330)
At least 6 of CHASM (breast), FATHMM (missense), MutationTaster, PROVEAN, SIFT, VEST	54.93% (50.51%-58.6%)	29.91% (25%-34.71%)	98.173% (97.015%-100%)	96.59% (94.12%-100%)	44.75% (40.12%-49.07%)	2.6943 (2.5987-2.7899)	54.8% (47.47%-63.64%)	29.53% (20.63%-39.34%)	98.144% (94.118%-100%)	96.48% (88.89%-100%)	44.8% (36.14%-54.43%)	2.6895 (2.4874-2.8877)
At least 6 of CHASM (breast), FATHMM (missense), PolyPhen-2, PROVEAN, SIFT, VEST	54.58% (50.51%-58.08%)	29.37% (24.41%-33.87%)	98.173% (97.015%-100%)	96.53% (94.12%-100%)	44.56% (39.87%-48.75%)	2.6864 (2.5893-2.7776)	54.47% (47.47%-62.63%)	29% (20%-38.72%)	98.144% (94.118%-100%)	96.41% (88.24%-100%)	44.62% (36.25%-54.22%)	2.6818 (2.4891-2.8732)
At least 6 of CHASM (breast), Mutation Assessor, MutationTaster, PolyPhen-2, PROVEAN, SIFT	55.58% (51.52%-59.6%)	30.95% (26.02%-35.65%)	98.173% (97.015%-100%)	96.7% (94.29%-100%)	45.12% (40.52%-49.38%)	2.7094 (2.6149-2.8012)	55.5% (47.47%-63.64%)	30.64% (21.31%-40.62%)	98.144% (94.118%-100%)	96.59% (89.47%-100%)	45.19% (36.58%-54.55%)	2.7057 (2.5146-2.8955)
At least 6 of CHASM (breast), Mutation Assessor, MutationTaster, PolyPhen-2, PROVEAN, VEST	53.89% (49.49%-58.08%)	28.28% (23.25%-32.8%)	98.173% (97.015%-100%)	96.4% (93.75%-100%)	44.19% (39.51%-48.45%)	2.6703 (2.5748-2.7645)	53.84% (45.45%-61.62%)	28.01% (19.14%-37.93%)	98.144% (94.118%-100%)	96.28% (88.24%-100%)	44.27% (35.53%-53.85%)	2.6671 (2.4740-2.8610)
At least 6 of CHASM (breast), Mutation Assessor, MutationTaster, PolyPhen-2, SIFT, VEST	57.25% (53.03%-61.11%)	34.66% (29.41%-39.53%)	96.311% (94.286%-98.667%)	94.2% (90.7%-97.96%)	46.02% (41.35%-50.33%)	2.7119 (2.6044-2.8177)	57.21% (49.49%-65.66%)	34.39% (24.24%-45.17%)	96.355% (91.176%-100%)	94.18% (85.71%-100%)	46.12% (37.33%-55.7%)	2.7105 (2.4968-2.9207)
At least 6 of CHASM (breast), Mutation Assessor, MutationTaster, PROVEAN, SIFT, VEST	54.91% (50.51%-58.6%)	29.88% (25.19%-34.43%)	98.173% (97.015%-100%)	96.58% (94.12%-100%)	44.74% (40.12%-49.08%)	2.6938 (2.6012-2.7888)	54.83% (47.47%-63.64%)	29.59% (20.63%-39.07%)	98.144% (94.118%-100%)	96.47% (88.89%-100%)	44.83% (36.14%-54.22%)	2.6903 (2.5025-2.8826)
At least 6 of CHASM (breast), Mutation Assessor, PolyPhen-2, PROVEAN, SIFT, VEST	55.24% (51.01%-59.09%)	30.41% (25.58%-34.92%)	98.173% (97.015%-100%)	96.64% (94.29%-100%)	44.93% (40.13%-49.08%)	2.7016 (2.6072-2.7987)	55.17% (47.47%-63.64%)	30.12% (21.05%-39.68%)	98.144% (94.118%-100%)	96.53% (88.89%-100%)	45.01% (36.47%-54.43%)	2.6980 (2.5028-2.8869)
At least 6 of CHASM (breast), MutationTaster, PolyPhen-2, PROVEAN, SIFT, VEST	55.91% (51.52%-59.6%)	31.47% (26.56%-36.22%)	98.173% (97.015%-100%)	96.75% (94.44%-100%)	45.31% (40.49%-49.66%)	2.7170 (2.6162-2.8098)	55.86% (48.48%-64.65%)	31.2% (21.88%-41.07%)	98.144% (94.118%-100%)	96.65% (89.47%-100%)	45.4% (36.84%-55%)	2.7119 (2.5200-2.9161)
At least 6 of CHASM (lung), CHASM (melanoma), FATHMM (cancer), FATHMM (missense), Mutation Assessor, MutationTaster	63.33% (59.6%-67.17%)	43.71% (38.46%-48.78%)	97.256% (95.588%-100%)	96.5% (94.23%-100%)	49.98% (45.24%-54.55%)	2.8744 (2.7756-2.9748)	63.24% (55.56%-71.71%)	43.42% (33.33%-53.57%)	97.226% (92.303%-100%)	96.42% (89.29%-100%)	50.03% (40.74%-59.49%)	2.8711 (2.6768-3.0570)
At least 6 of CHASM (lung), CHASM (melanoma), FATHMM (cancer), FATHMM (missense), Mutation Assessor, PolyPhen-2	64.34% (60.61%-68.18%)	45.85% (40.65%-50.82%)	96.317% (94.203%-98.667%)	95.56% (92.98%-98.41%)	50.71% (45.99%-55.15%)	2.8843 (2.7817-2.9876)	64.24% (56.57%-71.72%)	45.51% (35.71%-56.06%)	96.362% (91.176%-100%)	95.55% (89.18%-100%)	50.75% (41.78%-60.32%)	2.8818 (2.6778-3.0918)
At least 6 of CHASM (lung), CHASM (melanoma), FATHMM (cancer), FATHMM (missense), Mutation Assessor, PROVEAN	57.96% (54.04%-61.62%)	34.17% (29.31%-38.76%)	99.084% (98.485%-100%)	98.47% (97.37%-100%)	46.54% (41.97%-51.04%)	2.7827 (2.6873-2.8760)	57.82% (50.51%-65.66%)	33.76% (24.56%-43.86%)	99.083% (96.774%-100%)	98.44% (94.12%-100%)	46.57% (38.16%-56.1%)	2.7795 (2.6029-2.9561)
At least 6 of CHASM (lung), CHASM (melanoma), FATHMM (cancer), FATHMM (missense), Mutation Assessor, SIFT	65.37% (61.62%-69.19%)	46.92% (41.54%-51.97%)	97.256% (95.588%-100%)	96.73% (94.74%-100%)	51.45% (46.72%-55.93%)	2.9206 (2.8235-3.0225)	65.29% (57.58%-73.74%)	46.59% (36.36%-56.92%)	97.226% (92.303%-100%)	96.65% (90.91%-100%)	51.46% (42.11%-60.81%)	2.9189 (2.7177-3.1115)
At least 6 of CHASM (lung), CHASM (melanoma), FATHMM (cancer), FATHMM (missense), Mutation Assessor, VEST	60.31% (56.57%-64.14%)	39.48% (34.19%-44.36%)	96.317% (94.203%-98.667%)	94.88% (91.84%-98.18%)	47.93% (43.12%-52.38%)	2.7861 (2.6879-2.8907)	60.19% (52.53%-67.68%)	39.1% (29.31%-49.18%)	96.362% (91.176%-100%)	94.87% (86.96%-100%)	47.97% (38.96%-57.54%)	2.7830 (2.5784-2.9802)
At least 6 of CHASM (lung), CHASM (melanoma), FATHMM (cancer), FATHMM (missense), MutationTaster, PolyPhen-2	65.01% (61.11%-68.69%)	46.35% (41.09%-51.54%)	97.256% (95.588%-100%)	96.69% (94.64%-100%)	51.18% (46.38%-55.71%)	2.9148 (2.8208-3.0129)	64.94% (57.58%-72.73%)	46.11% (35.59%-56.14%)	97.226% (92.303%-100%)	96.62% (90.91%-100%)	51.25% (41.98%-60.81%)	2.9122 (2.7146-3.1062)
At least 6 of CHASM (lung), CHASM (melanoma), FATHMM (cancer), FATHMM (missense), MutationTaster, PROVEAN	57.95% (54.04%-61.62%)	34.15% (29.36%-38.89%)	99.084% (98.485%-100%)	98.47% (97.37%-100%)	46.53% (41.77%-50.94%)	2.7824 (2.6973-2.8669)	57.84% (50.51%-65.66%)	33.8% (24.59%-43.64%)	99.083% (96.774%-100%)	98.46% (94.44%-100%)	46.58% (37.97%-56.1%)	2.7792 (2.6047-2.9562)
At least 6 of CHASM (lung), CHASM (melanoma), FATHMM (cancer), FATHMM (missense), MutationTaster, SIFT	66.03% (62.12%-69.7%)	47.97% (42.75%-52.89%)	97.256% (95.588%-100%)	96.8% (94.74%-100%)	51.95% (47.14%-56.72%)	2.9397 (2.8439-3.0405)	65.92% (58.59%-73.74%)	47.66% (37.76%-57.81%)	97.233% (92.105%-100%)	96.73% (90.91%-100%)	51.98% (42.85%-61.92%)	2.9360 (2.7471-3.1337)
At least 6 of CHASM (lung), CHASM (melanoma), FATHMM (cancer), FATHMM (missense), MutationTaster, VEST	61.66% (57.58%-65.15%)	41.07% (35.83%-45.97%)	97.256% (95.588%-100%)	96.28% (93.88%-100%)	48.84% (44.13%-53.19%)	2.8345 (2.7382-2.9343)	61.52% (54.55%-69.7%)	40.71% (31.03%-50.79%)	97.233% (92.105%-100%)	96.19% (89.66%-100%)	48.86% (39.71%-58.21%)	2.8299 (2.6330-3.0201)
At least 6 of CHASM (lung), CHASM (melanoma), FATHMM (cancer), FATHMM (missense), PolyPhen-2, PROVEAN	57.96% (54.04%-61.62%)	34.17% (29.17%-38.76%)	99.084% (98.485%-100%)	98.47% (97.37%-100%)	46.54% (41.72%-50.96%)	2.7827 (2.6973-2.8637)	57.82% (50.51%-65.66%)	33.76% (24.24%-43.75%)	99.083% (96.774%-100%)	98.45% (94.44%-100%)	46.57% (38.16%-56.34%)	2.7785 (2.6126-2.9561)
At least 6 of CHASM (lung), CHASM (melanoma), FATHMM (cancer), FATHMM (missense), PolyPhen-2, SIFT	65.71% (62.12%-69.19%)	47.46% (42.28%-52.46%)	97.256% (95.588%-100%)	96.76% (94.74%-100%)	51.7% (46.87%-56.12%)	2.9318 (2.8363-3.0286)	65.55% (57.58%-72.73%)	47.08% (36.91%-57.38%)	97.233% (92.105%-100%)	96.68% (91.18%-100%)	51.7% (42.55%-61.97%)	2.9271 (2.7297-3.1176)
At least 6 of CHASM (lung), CHASM (melanoma), FATHMM (cancer), FATHMM (missense), PolyPhen-2, VEST	60.64% (56.57%-64.15%)	40% (34.96%-45.08%)	96.317% (94.203%-98.667%)	94.95% (92%-98.11%)	48.14% (43.33%-52.41%)	2.7941 (2.6932-2.8958)	60.54% (53.53%-68.69%)	39.69% (30%-50%)	96.362% (91.176%-100%)	94.96% (87.5%-100%)	48.2% (39.29%-57.59%)	2.7916 (2.5928-2.9937)
At least 6 of CHASM (lung), CHASM (melanoma), FATHMM (cancer), FATHMM (missense), PROVEAN, SIFT	59.98% (56.06%-63.64%)	37.36% (32.52%-42.02%)	99.084% (98.485%-100%)	98.6% (97.62%-100%)	47.77% (42.86%-52%)	2.8282 (2.7469-2.9117)	59.84% (52.53%-67.68%)	36.96% (27.42%-46.78%)	99.083% (96.774%-100%)	98.58% (94.74%-100%)	47.8% (39.19%-57.69%)	2.8243 (2.6580-2.9963)
At least 6 of CHASM (lung), CHASM (melanoma), FATHMM (cancer), FATHMM (missense), PROVEAN, VEST	57.28% (53.03%-61.11%)	33.11% (28.23%-37.88%)	99.084% (98.485%-100%)	98.43% (97.22%-100%)	46.14% (41.36%-50.62%)	2.7676 (2.6804-2.8530)	57.15% (49.49%-65.66%)	32.7% (23.46%-42.39%)	99.083% (96.774%-100%)	98.4% (94.12%-100%)	46.18% (37.66%-55.56%)	2.7636 (2.5947-2.9459)
At least 6 of CHASM (lung), CHASM (melanoma), FATHMM (cancer), FATHMM (missense), SIFT, VEST	61.67% (58.07%-65.15%)	41.61% (36.51%-46.67%)	95.351% (92.903%-98.667%)	95.15% (92.9%-98.28%)	48.83% (44%-53.38%)	2.8192 (2.7227-2.9200)	61.52% (54.55%-68.71%)	41.21% (31.08%-50.85%)	96.331% (91.176%-100%)	95.07% (87.87%-100%)	48.84% (39.74%-58.34%)	2.8145 (2.6070-3.0176)
At least 6 of CHASM (lung), CHASM (melanoma), FATHMM (cancer), Mutation Assessor, MutationTaster, PolyPhen-2	63.31% (59.6%-67.17%)	43.67% (38.52%-48.74%)	97.256% (95.588%-100%)	96.5% (94.3%-100%)	49.96% (45.11%-54.61%)	2.8739 (2.7796-2.9668)	63.28% (55.56%-70.71%)	43.49% (33.33%-53.44%)	97.233% (92.105%-100%)	96.43% (90.47%-100%)	50.07% (40.85%-59.47%)	2.8722 (2.6784-3.0678)
At least 6 of CHASM (lung), CHASM (melanoma), FATHMM (cancer), Mutation Assessor, MutationTaster, PROVEAN	57.6% (53.54%-61.62%)	33.6% (28.93%-38.4%)	99.084% (98.485%-100%)	98.45% (97.3%-100%)	46.33% (41.77%-50.65%)	2.7746 (2.6884-2.8616)	57.53% (50.48%-65.66%)	33.31% (23.81%-42.86%)	99.083% (96.774%-100%)	98.43% (94.12%-100%)	46.4% (37.5%-56%)	2.7722 (2.5989-2.9468)
At least 6 of CHASM (lung), CHASM (melanoma), FATHMM (cancer), Mutation Assessor, MutationTaster, SIFT	64.33% (60.61%-68.18%)	45.28% (40.17%-50%)	97.256% (95.588%-100%)	96.62% (94.54%-100%)	50.69% (45.92%-55.4%)	2.8984 (2.8044-2.9950)	64.27% (56.57%-71.72%)	45.06% (35.48%-55.01%)	97.233% (92.105%-100%)	96.55% (90.62%-100%)	50.77% (41.43%-60.32%)	2.8961 (2.7031-3.0858)
At least 6 of CHASM (lung), CHASM (melanoma), FATHMM (cancer), Mutation Assessor, MutationTaster, VEST	60.63% (56.57%-64.65%)	39.45% (34.37%-44.44%)	97.256% (95.588%-100%)	96.1% (93.62%-100%)	48.16% (43.48%-52.94%)	2.8100 (2.7163-2.9111)	60.55% (53.54%-68.69%)	39.17% (29.03%-49.21%)	97.233% (92.105%-100%)	96.04% (88.89%-100%)	48.23% (38.96%-57.89%)	2.8068 (2.6057-3.0103)
At least 6 of CHASM (lung), CHASM (melanoma), FATHMM (cancer), Mutation Assessor, PolyPhen-2, PROVEAN	58.28% (54.53%-62.12%)	34.67% (29.83%-39.2%)	99.084% (98.485%-100%)	98.5% (97.22%-100%)	46.72% (42.15%-50.99%)	2.7898 (2.7062-2.8734)	58.2% (50.51%-65.66%)	34.36% (25%-44.45%)	99.083% (96.774%-100%)	98.47% (94.44%-100%)	46.79% (37.84%-56.1%)	2.7870 (2.6173-2.9561)
At least 6 of CHASM (lung), CHASM (melanoma), FATHMM (cancer), Mutation Assessor, PolyPhen-2, SIFT	65.35% (62.11%-69.19%)	46.89% (41.8%-52.07%)	96.317% (94.203%-98.667%)	95.73% (93.1%-98.41%)	51.43% (46.72%-55.93%)	2.9230 (2.8263-3.0236)	65.27% (57.58%-71.74%)	46.63% (36.07%-56.9%)	96.362% (91.176%-100%)	95.66% (90.91%-100%)	51.49% (42.42%-61.11%)	2.9201 (2.7205-3.1088)
At least 6 of CHASM (lung), CHASM (melanoma), FATHMM (cancer), Mutation Assessor, PolyPhen-2, VEST	60.29% (56.06%-64.14%)	39.45% (34.42%-44.35%)	96.317% (94.203%-98.667%)	94.88% (92%-98.18%)	47.91% (43.15%-52.41%)	2.7856 (2.6828-2.8893)	60.2% (52.53%-67.68%)	39.09% (29.51%-49.15%)	96.362% (91.176%-100%)	94.89% (87.5%-100%)	48% (38.89%-57.34%)	2.7842 (2.5785-2.9928)
At least 6 of CHASM (lung), CHASM (melanoma), FATHMM (cancer), Mutation Assessor, PROVEAN, SIFT	59.29% (55.05%-63.13%)	36.27% (31.41%-41.13%)	99.084% (98.485%-100%)	98.56% (97.5%-100%)	47.35% (42.58%-51.66%)	2.8127 (2.7301-2.8984)	59.19% (51.52%-67.68%)	35.94% (26.08%-45.76%)	99.083% (96.774%-100%)	98.54% (94.44%-100%)	47.4% (38.67%-56.79%)	2.8096 (2.6354-2.9811)
At least 6 of CHASM (lung), CHASM (melanoma), FATHMM (cancer), Mutation Assessor, PROVEAN, VEST	57.26% (53.03%-61.11%)	33.07% (28.35%-37.82%)	99.084% (98.485%-100%)	98.42% (97.3%-100%)	46.13% (41.26%-50.64%)	2.7670 (2.6821-2.8522)	57.2% (49.49%-65.66%)	32.78% (23.43%-42.37%)	99.083% (96.774%-100%)	98.4% (94.12%-100%)	46.2% (37.35%-55.56%)	2.7647 (2.5989-2.9401)
At least 6 of CHASM (lung), CHASM (melanoma), FATHMM (cancer), Mutation Assessor, SIFT, VEST	61.65% (58.08%-65.66%)	41.05% (36.07%-46.03%)	97.256% (95.588%-100%)	96.28% (93.88%-100%)	48.84% (44.06%-53.52%)	2.8343 (2.7361-2.9358)	61.55% (53.54%-68.69%)	40.74% (30.88%-50.79%)	97.233% (92.105%-100%)	96.19% (89.66%-100%)	48.88% (39.74%-58.44%)	2.8306 (2.6369-3.0187)
At least 6 of CHASM (lung), CHASM (melanoma), FATHMM (cancer), MutationTaster, PolyPhen-2, PROVEAN	58.6% (54.55%-62.12%)	35.19% (30.16%-40.15%)	99.084% (98.485%-100%)	98.52% (97.44%-100%)	46.93% (42.11%-51.01%)	2.7971 (2.7129-2.8785)	58.55% (51.49%-66.67%)	34.92% (25.39%-45.16%)	99.083% (96.774%-100%)	98.5% (94.44%-100%)	47.01% (38.35%-56.58%)	2.7951 (2.6312-2.9727)
At least 6 of CHASM (lung), CHASM (melanoma), FATHMM (cancer), MutationTaster, PolyPhen-2, SIFT	65.67% (62.12%-69.19%)	47.4% (42.28%-52.42%)	97.256% (95.588%-100%)	96.76% (94.64%-100%)	51.68% (46.96%-56.3%)	2.9309 (2.8360-3.0286)	65.63% (58.59%-72.73%)	47.2% (36.36%-57.14%)	97.233% (92.105%-100%)	96.7% (90.91%-100%)	51.76% (42.62%-61.73%)	2.9290 (2.7383-3.1181)
At least 6 of CHASM (lung), CHASM (melanoma), FATHMM (cancer), MutationTaster, PolyPhen-2, VEST	62.64% (59.08%-66.67%)	42.62% (37.5%-47.62%)	97.256% (95.588%-100%)	96.41% (94.12%-100%)	49.5% (44.68%-53.93%)	2.8579 (2.7656-2.9572)	62.6% (54.55%-70.71%)	42.41% (32.42%-52.63%)	97.233% (92.105%-100%)	96.34% (90%-100%)	49.59% (40.58%-59.15%)	2.8557 (2.6578-3.0589)
At least 6 of CHASM (lung), CHASM (melanoma), FATHMM (cancer), MutationTaster, PROVEAN, SIFT	60.62% (56.57%-64.14%)	38.37% (33.33%-43.51%)	99.084% (98.485%-100%)	98.64% (97.67%-100%)	48.18% (43.48%-52.9%)	2.8428 (2.7622-2.9270)	60.58% (53.54%-68.69%)	38.13% (27.86%-48.33%)	99.083% (96.774%-100%)	98.62% (95%-100%)	48.27% (39.7%-57.75%)	2.8410 (2.6786-3.0315)
At least 6 of CHASM (lung), CHASM (melanoma), FATHMM (cancer), MutationTaster, PROVEAN, VEST	58.61% (54.55%-62.63%)	35.19% (30.3%-40%)	99.084% (98.485%-100%)	98.52% (97.44%-100%)	46.93% (42.15%-51.3%)	2.7972 (2.7086-2.8824)	58.55% (50.51%-65.66%)	34.92% (25%-44.83%)	99.083% (96.774%-100%)	98.5% (94.44%-100%)	47.01% (38.27%-56.34%)	2.7951 (2.6300-2.9681)
At least 6 of CHASM (lung), CHASM (melanoma), FATHMM (cancer), MutationTaster, SIFT, VEST	63.33% (59.6%-67.17%)	43.7% (38.52%-48.8%)	97.256% (95.588%-100%)	96.5% (94.34%-100%)	49.98% (45.21%-54.42%)	2.8743 (2.7790-2.9762)	63.25% (55.56%-70.71%)	43.43% (33.32%-53.46%)	97.233% (92.105%-100%)	96.42% (90%-100%)	50.04% (40.96%-59.73%)	2.8712 (2.6722-3.0630)
At least 6 of CHASM (lung), CHASM (melanoma), FATHMM (cancer), PolyPhen-2, PROVEAN, SIFT	60.63% (56.57%-64.14%)	38.39% (33.33%-43.2%)	99.084% (98.485%-100%)	98.64% (97.67%-100%)	48.19% (43.23%-52.63%)	2.8430 (2.7623-2.9236)	60.55% (53.54%-68.69%)	38.08% (28.33%-48.33%)	99.083% (96.774%-100%)	98.62% (95%-100%)	48.25% (39.72%-57.69%)	2.8403 (2.6606-3.0215)
At least 6 of CHASM (lung), CHASM (melanoma), FATHMM (cancer), PolyPhen-2, PROVEAN, VEST	58.26% (54.04%-61.62%)	34.66% (29.83%-39.2%)	99.084% (98.485%-100%)	98.49% (97.37%-100%)	46.72% (42.11%-51.16%)	2.7895 (2.7059-2.8707)	58.22% (51.52%-65.66%)	34.42% (24.59%-44.64%)	99.083% (96.774%-100%)	98.47% (94.44%-100%)	46.81% (38.23%-56.58%)	2.7876 (2.6185-2.9606)
At least 6 of CHASM (lung), CHASM (melanoma), FATHMM (cancer), PolyPhen-2, SIFT, VEST	62.65% (58.59%-66.67%)	42.63% (37.7%-47.97%)	97.256% (95.588%-100%)	96.41% (94.12%-100%)	49.51% (44.69%-54.05%)	2.8580 (2.7663-2.9564)	62.58% (54.55%-70.71%)	42.38% (32.13%-52.38%)	97.233% (92.105%-100%)	96.33% (89.66%-100%)	49.58% (40.58%-59.42%)	2.8552 (2.6576-3.0472)
At least 6 of CHASM (lung), CHASM (melanoma), FATHMM (cancer), PROVEAN, SIFT, VEST	59.96% (56.06%-63.64%)	37.33% (32.45%-42.07%)	99.084% (98.485%-100%)	98.6% (97.62%-100%)	47.76% (42.86%-52.14%)	2.8277 (2.7428-2.9090)	59.88% (52.53%-67.68%)	37.02% (27.42%-46.77%)	99.083% (96.774%-100%)	98.58% (94.74%-100%)	47.83% (39.13%-57.53%)	2.8252 (2.6643-3.0043)
At least 6 of CHASM (lung), CHASM (melanoma), FATHMM (missense), Mutation Assessor, MutationTaster, PolyPhen-2	62.64% (59.09%-66.67%)	42.62% (37.47%-47.54%)	97.256% (95.588%-100%)	96.41% (94.12%-100%)	49.5% (44.87%-54.12%)	2.8580 (2.7621-2.9548)	62.59% (54.55%-69.7%)	42.39% (32.79%-52.46%)	97.233% (92.105%-100%)	96.34% (90%-100%)	49.59% (40.58%-59.15%)	2.8555 (2.6622-3.0441)
At least 6 of CHASM (lung), CHASM (melanoma), FATHMM (missense), Mutation Assessor, MutationTaster, PROVEAN	56.27% (52.02%-60.1%)	31.5% (26.72%-36%)	99.084% (98.485%-100%)	98.35% (97.14%-100%)	45.55% (40.91%-50%)	2.7448 (2.6599-2.8314)	56.15% (48.48%-64.65%)	31.13% (22.03%-40.68%)	99.083% (96.774%-100%)	98.32% (93.75%-100%)	45.6% (36.98%-54.88%)	2.7413 (2.5668-2.9144)
At least 6 of CHASM (lung), CHASM (melanoma), FATHMM (missense), Mutation Assessor, MutationTaster, SIFT	63.69% (60.1%-67.17%)	44.23% (39.02%-49.17%)	97.256% (95.588%-100%)	96.54% (94.34%-100%)	50.21% (45.39%-54.69%)	2.8824 (2.7863-2.9785)	63.58% (55.56%-71.72%)	43.96% (34.26%-54.17%)	97.233% (92.105%-100%)	96.46% (90.32%-100%)	50.27% (41.23%-59.73%)	2.8793 (2.6860-3.0684)
At least 6 of CHASM (lung), CHASM (melanoma), FATHMM (missense), Mutation Assessor, MutationTaster, VEST	59.97% (56.06%-63.64%)	38.4% (33.33%-43.41%)	97.256% (95.588%-100%)	96.03% (93.48%-100%)	47.73% (43.04%-52.43%)	2.7942 (2.6933-2.8918)	59.86% (52.53%-67.68%)	38.08% (28.33%-47.78%)	97.233% (92.105%-100%)	95.93% (88.89%-100%)	47.77% (38.66%-57.14%)	2.7902 (2.5998-2.9963)
At least 6 of CHASM (lung), CHASM (melanoma), FATHMM (missense), Mutation Assessor, PolyPhen-2, PROVEAN	56.94% (53.03%-60.61%)	32.57% (27.59%-37.01%)	99.084% (98.485%-100%)	98.4% (97.22%-100%)	45.94% (41.25%-50.31%)	2.7599 (2.6765-2.8448)	56.82% (49.49%-64.65%)	32.18% (22.95%-41.94%)	99.083% (96.774%-100%)	98.37% (94.12%-100%)	45.98% (37.33%-55.7%)	2.7561 (2.5802-2.9286)
At least 6 of CHASM (lung), CHASM (melanoma), FATHMM (missense), Mutation Assessor, PolyPhen-2, SIFT	64.68% (61.11%-68.19%)	45.84% (40.32%-50.81%)	97.256% (95.588%-100%)	96.65% (94.55%-100%)	50.94% (46.15%-55.56%)	2.9069 (2.8113-3.0036)	64.58% (57.58%-71.72%)	45.53% (35.09%-56.06%)	97.233% (92.105%-100%)	96.58% (90.91%-100%)	50.98% (41.69%-60.53%)	2.9032 (2.7047-3.0945)
At least 6 of CHASM (lung), CHASM (melanoma), FATHMM (missense), Mutation Assessor, PolyPhen-2, VEST	59.62% (55.56%-63.64%)	38.4% (33.07%-43.34%)	96.317% (94.203%-98.667%)	94.75% (91.84%-98.11%)	47.49% (42.85%-51.7%)	2.7695 (2.6709-2.8696)	59.54% (51.52%-67.68%)	38.07% (28.33%-48.44%)	96.362% (91.176%-100%)	94.74% (86.96%-100%)	47.57% (38.89%-56.76%)	2.7673 (2.5616-2.9670)
At least 6 of CHASM (lung), CHASM (melanoma), FATHMM (missense), Mutation Assessor, PROVEAN, SIFT	57.96% (54.04%-61.62%)	34.17% (29.31%-38.76%)	99.084% (98.485%-100%)	98.47% (97.37%-100%)	46.54% (41.97%-51.04%)	2.7827 (2.6973-2.8707)	57.82% (50.51%-65.66%)	33.76% (24.56%-43.86%)	99.083% (96.774%-100%)	98.44% (94.12%-100%)	46.57% (38.16%-56.1%)	2.7795 (2.6029-2.9561)
At least 6 of CHASM (lung), CHASM (melanoma), FATHMM (missense), Mutation Assessor, PROVEAN, VEST	55.93% (51.52%-59.6%)	30.97% (26.23%-35.48%)	99.084% (98.485%-100%)	98.32% (97.06%-100%)	45.36% (40.66%-49.69%)	2.7373 (2.6531-2.8246)	55.82% (48.48%-64.65%)	30.6% (21.43%-39.71%)	99.083% (96.774%-100%)	98.29% (93.74%-100%)	45.41% (36.71%-54.55%)	2.7339 (2.5591-2.9069)
At least 6 of CHASM (lung), CHASM (melanoma), FATHMM (missense), MutationTaster, PolyPhen-2, PROVEAN	60.99% (57.07%-64.65%)	40.01% (34.74%-45%)	97.256% (95.588%-100%)	96.18% (93.75%-100%)	48.39% (43.71%-52.94%)	2.8183 (2.7245-2.9155)	60.86% (53.54%-68.69%)	39.64% (29.69%-50%)	97.233% (92.105%-100%)	96.09% (88.89%-100%)	48.42% (39.44%-57.89%)	2.8139 (2.6170-3.0043)
At least 6 of CHASM (lung), CHASM (melanoma), FATHMM (missense), MutationTaster, PolyPhen-2, PROVEAN	55.93% (52.01%-59.6%)	30.97% (26.15%-35.42%)	99.084% (98.485%-100%)	98.32% (97.06%-100%)	45.36% (40.64%-49.69%)	2.7373 (2.6515-2.8201)	55.82% (48.48%-63.64%)	30.59% (21.54%-40.51%)	99.083% (96.774%-100%)	98.29% (93.75%-100%)	45.41% (36.84%-54.88%)	2.7338 (2.5693-2.9093)
At least 6 of CHASM (lung), CHASM (melanoma), FATHMM (missense), MutationTaster, PolyPhen-2, SIFT	63.66% (60.1%-67.17%)	44.23% (39.19%-49.18%)	97.256% (95.588%-100%)	96.54% (94.34%-100%)	50.21% (45.39%-54.65%)	2.8824 (2.7867-2.9751)	63.58% (55.56%-71.72%)	43.96% (34.38%-53.85%)	97.233% (92.105%-100%)	96.46% (90.32%-100%)	50.27% (41.17%-59.52%)	2.8793 (2.6961-3.0693)
At least 6 of CHASM (lung), CHASM (melanoma), FATHMM (missense), MutationTaster, PolyPhen-2, VEST	60.3% (56.57%-64.14%)	38.92% (33.61%-43.8%)	97.256% (95.588%-100%)	96.08% (93.48%-100%)	47.94% (43.33%-52.29%)	2.8020 (2.7085-2.9036)	60.21% (52.53%-67.68%)	38.63% (28.78%-49.15%)	97.233% (92.105%-100%)	95.99% (88.89%-100%)	48.01% (39.13%-57.53%)	2.7986 (2.5981-2.9943)
At least 6 of CHASM (lung), CHASM (melanoma), FATHMM (missense), MutationTaster, PROVEAN, SIFT	57.95% (54.04%-61.62%)	34.15% (29.36%-38.89%)	99.084% (98.485%-100%)	98.47% (97.37%-100%)	46.53% (41.77%-50.94%)	2.7824 (2.6973-2.8669)	57.84% (50.51%-65.66%)	33.8% (24.59%-43.64%)	99.083% (96.774%-100%)	98.46% (94.44%-100%)	46.58% (37.97%-56.1%)	2.7792 (2.6047-2.9562)
At least 6 of CHASM (lung), CHASM (melanoma), FATHMM (missense), MutationTaster, PROVEAN, VEST	55.93% (51.52%-59.6%)	30.97% (26.23%-35.56%)	99.084% (98.485%-100%)	98.32% (97.06%-100%)	45.36% (40.66%-49.69%)	2.7374 (2.6510-2.8201)	55.81% (48.48%-64.65%)	30.59% (21.31%-40.3%)	99.083% (96.774%-100%)	98.3% (93.75%-100%)	45.41% (36.71%-54.55%)	2.7338 (2.5668-2.9118)
At least 6 of CHASM (lung), CHASM (melanoma), FATHMM (missense), MutationTaster, SIFT, VEST	61.32% (57.58%-65.15%)	40.53% (35.54%-45.53%)	97.256% (95.588%-100%)	96.22% (93.88%-100%)	48.61% (43.93%-53.1%)	2.8263 (2.7355-2.9221)	61.2% (53.54%-68.69%)	40.19% (30.64%-50%)	97.233% (92.105%-100%)	96.14% (89.47%-100%)	48.64% (39.47%-57.89%)	2.8221 (2.6268-3.0160)
At least 6 of CHASM (lung), CHASM (melanoma), FATHMM (missense), PolyPhen-2, PROVEAN, SIFT	57.96% (54.04%-61.62%)	34.17% (29.17%-38.76%)	99.084% (98.485%-100%)	98.47% (97.37%-100%)	46.54% (41.72%-50.96%)	2.7827 (2.6973-2.8637)	57.82% (50.51%-65.66%)	33.76% (24.24%-43.75%)	99.083% (96.774%-100%)	98.45% (94.44%-100%)	46.57% (38.16%-56.34%)	2.7785 (2.6126-2.9561)
At least 6 of CHASM (lung), CHASM (melanoma), FATHMM (missense), PolyPhen-2, PROVEAN, VEST	55.59% (51.52%-59.09%)	30.43% (25.4%-34.96%)	99.084% (98.485%-100%)	98.29% (96.97%-100%)	45.17% (40.38%-49.37%)	2.7297 (2.6457-2.8155)	55.49% (48.48%-63.64%)	30.07% (21.05%-39.71%)	99.083% (96.774%-100%)	98.26% (93.33%-100%)	45.22% (36.71%-54.67%)	2.7264 (2.5548-2.9001)
At least 6 of CHASM (lung), CHASM (melanoma), FATHMM (missense), PolyPhen-2, SIFT, VEST	60.64% (57.06%-64.65%)	39.46% (34.17%-44.53%)	97.256% (95.588%-100%)	96.13% (93.62%-100%)	48.16% (43.42%-52.41%)	2.8102 (2.7158-2.9067)	60.54% (52.53%-67.68%)	39.14% (29.23%-49.06%)	97.233% (92.105%-100%)	96.04% (88.89%-100%)	48.21% (39.24%-57.75%)	2.8062 (2.6069-3.0070)
At least 6 of CHASM (lung), CHASM (melanoma), FATHMM (missense), PROVEAN, SIFT, VEST	57.28% (53.03%-61.11%)	33.11% (28.23%-37.88%)	99.084% (98.485%-100%)	98.43% (97.22%-100%)	46.14% (41.36%-50.62%)	2.7676 (2.6821-2.8500)	57.15% (49.49%-65.66%)	32.7% (22.97%-42.43%)	99.083% (96.774%-100%)	98.4% (94.12%-100%)	46.18% (37.66%-55.84%)	2.7636 (2.5947-2.9459)
At least 6 of CHASM (lung), CHASM (melanoma), Mutation Assessor, MutationTaster, PolyPhen-2, PROVEAN	56.58% (52.53%-60.1%)	32.04% (27.12%-36.51%)	99.084% (98.485%-100%)	98.37% (97.14%-100%)	45.73% (41.14%-50%)	2.7519 (2.6658-2.8385)	56.53% (49.49%-64.65%)	31.73% (22.41%-41.38%)	99.083% (96.774%-100%)	98.35% (93.75%-100%)	45.82% (37.33%-55.13%)	2.7498 (2.5745-2.9223)
At least 6 of CHASM (lung), CHASM (melanoma), Mutation Assessor, MutationTaster, PolyPhen-2, SIFT	63.3% (59.6%-67.17%)	44.2% (38.98%-49.18%)	96.317% (94.203%-98.684%)	95.42% (92.73%-98.39%)	49.96% (45.16%-54.68%)	2.8591 (2.7611-2.9621)	63.29% (55.56%-70.71%)	44.03% (34.43%-54.1%)	96.355% (90.909%-100%)	95.35% (88.46%-100%)	50.07% (40.85%-59.7%)	2.8577 (2.6652-3.0513)
At least 6 of CHASM (lung), CHASM (melanoma), Mutation Assessor, MutationTaster, PolyPhen-2, VEST	59.61% (55.56%-63.64%)	38.37% (33.33%-43.45%)	96.335% (94.286%-98.684%)	94.76% (91.67%-98.15%)	47.48% (42.85%-51.9%)	2.7694 (2.6682-2.8731)	59.57% (51.52%-67.68%)	38.15% (28.12%-47.76%)	96.319% (90.909%-100%)	94.67% (86.36%-100%)	47.57% (38.66%-56.76%)	2.7671 (2.5507-2.9676)
At least 6 of CHASM (lung), CHASM (melanoma), Mutation Assessor, MutationTaster, PROVEAN, SIFT	57.6% (53.54%-61.12%)	33.6% (28.93%-38.4%)	99.084% (98.485%-100%)	98.45% (97.3%-100%)	46.33% (41.77%-50.65%)	2.7746 (2.6884-2.8616)	57.53% (50.48%-65.66%)	33.31% (23.81%-42.86%)	99.083% (96.774%-100%)	98.43% (94.12%-100%)	46.4% (37.5%-56%)	2.7722 (2.5989-2.9468)
At least 6 of CHASM (lung), CHASM (melanoma), Mutation Assessor, MutationTaster, PROVEAN, VEST	55.91% (52.01%-59.6%)	30.93% (26.09%-35.54%)	99.084% (98.485%-100%)	98.32% (97.06%-100%)	45.35% (40.66%-49.69%)	2.7368 (2.6537-2.8246)	55.86% (48.48%-64.65%)	30.67% (21.62%-40.3%)	99.083% (96.774%-100%)	98.3% (93.75%-100%)	45.44% (36.84%-54.67%)	2.7349 (2.5679-2.9093)
At least 6 of CHASM (lung), CHASM (melanoma), Mutation Assessor, MutationTaster, SIFT, VEST	60.63% (57.06%-64.65%)	39.97% (35.2%-45.16%)	96.335% (94.286%-98.684%)	94.96% (92.2%-98.21%)	48.14% (43.59%-52.81%)	2.7941 (2.6943-2.8958)	60.56% (52.53%-67.68%)	39.72% (29.69%-49.15%)	96.319% (90.909%-100%)	94.87% (86.96%-100%)	48.21% (39.13%-57.34%)	2.7911 (2.5782-2.9971)
At least 6 of CHASM (lung), CHASM (melanoma), Mutation Assessor, PolyPhen-2, PROVEAN, SIFT	58.28% (54.53%-62.12%)	34.67% (29.83%-39.2%)	99.084% (98.485%-100%)	98.5% (97.22%-100%)	46.73% (42.11%-51.04%)	2.7898 (2.7062-2.8734)	58.2% (50.51%-66.67%)	34.36% (24.59%-44.83%)	99.083% (96.774%-100%)	98.47% (94.44%-100%)	46.79% (38.03%-56.34%)	2.7870 (2.6171-2.9561)
At least 6 of CHASM (lung), CHASM (melanoma), Mutation Assessor, PolyPhen-2, PROVEAN, VEST	56.2% (52.53%-60.1%)	31.46% (26.67%-35.77%)	99.084% (98.485%-100%)	98.35% (97.06%-100%)	45.54% (40.76%-50%)	2.7443 (2.6620-2.8293)	56.2% (48.48%-63.64%)	31.2% (22.58%-40.99%)	99.083% (96.774%-100%)	98.32% (93.75%-100%)	45.63% (37.04%-55.13%)	2.7423 (2.5695-2.9070)
At least 6 of CHASM (lung), CHASM (melanoma), Mutation Assessor, PolyPhen-2, SIFT, VEST	60.63% (57.07%-64.65%)	39.97% (34.88%-45.16%)	96.335% (94.286%-98.684%)	94.96% (92%-98.25%)	48.14% (43.33%-52.45%)	2.7940 (2.6965-2.8987)	60.57% (52.53%-68.69%)	39.71% (29.03%-50%)	96.319% (90.909%-100%)	94.87% (87.06%-100%)	48.21% (39.13%-57.81%)	2.7912 (2.5783-2.9962)
At least 6 of CHASM (lung), CHASM (melanoma), Mutation Assessor, PROVEAN, SIFT, VEST	57.26% (53.03%-61.11%)	33.07% (28.35%-37.82%)	99.084% (98.485%-100%)	98.42% (97.3%-100%)	46.13% (41.26%-50.64%)	2.7670 (2.6821-2.8522)	57.2% (49.49%-65.66%)	32.78% (23.43%-42.37%)	99.083% (96.774%-100%)	98.4% (94.12%-100%)	46.2% (37.35%-55.56%)	2.7647 (2.5989-2.9401)
At least 6 of CHASM (lung), CHASM (melanoma), MutationTaster, PolyPhen-2, PROVEAN, SIFT	58.6% (54.55%-62.12%)	35.19% (30.16%-40.15%)	99.084% (98.485%-100%)	98.52% (97.44%-100%)	46.93% (42.11%-51.01%)	2.7971 (2.7129-2.8785)	58.55% (51.49%-66.67%)	34.92% (25.39%-45.16%)	99.083% (96.774%-100%)	98.5% (94.44%-100%)	47.01% (38.35%-56.58%)	2.7951 (2.6312-2.9727)
At least 6 of CHASM (lung), CHASM (melanoma), MutationTaster, PolyPhen-2, PROVEAN, VEST	56.91% (53.03%-60.61%)	32.52% (27.69%-37.1%)	99.084% (98.485%-100%)	98.4% (97.22%-100%)	45.92% (41.14%-50%)	2.7592 (2.6740-2.8428)	56.89% (49.49%-64.65%)	32.29% (22.81%-42.2%)	99.083% (96.774%-100%)	98.38% (94.12%-100%)	46.02% (37.5%-55.71%)	2.7577 (2.5898-2.9351)
At least 6 of CHASM (lung), CHASM (melanoma), MutationTaster, PolyPhen-2, SIFT, VEST	61.62% (58.08%-65.66%)	41.55% (36.58%-46.77%)	96.335% (94.286%-98.684%)	95.1% (92.31%-98.28%)	48.8% (43.96%-53.19%)	2.8182 (2.7215-2.9231)	61.6% (53.54%-68.69%)	41.33% (31.15%-51.43%)	96.319% (90.909%-100%)	95.06% (88%-100%)	48.9% (40%-58.33%)	2.8162 (2.6069-3.0083)
At least 6 of CHASM (lung), CHASM (melanoma), MutationTaster, PROVEAN, SIFT, VEST	58.61% (54.55%-62.63%)	35.19% (30.3%-40%)	99.084% (98.485%-100%)	98.52% (97.44%-100%)	46.93% (42.15%-51.3%)	2.7972 (2.7086-2.8824)	58.55% (51.49%-66.67%)	34.92% (25%-44.83%)	99.083% (96.774%-100%)	98.5% (94.44%-100%)	47.01% (38.27%-56.34%)	2.7951 (2.6300-2.9681)
At least 6 of CHASM (lung), CHASM (melanoma), PolyPhen-2, PROVEAN, SIFT, VEST	58.26% (54.04%-61.62%)	34.66% (29.83%-39.2%)	99.084% (98.485%-100%)	98.49% (97.37%-100%)	46.72% (42.11%-51.16%)	2.7895 (2.7059-2.8707)	58.22% (51.52%-65.66%)	34.42% (24.59%-44.64%)	99.083% (96.774%-100%)	98.47% (94.44%-100%)	46.81% (38.23%-56.58%)	2.7876 (2.6185-2.9606)
At least 6 of CHASM (lung), FATHMM (cancer), FATHMM (missense), Mutation Assessor, MutationTaster, PolyPhen-2	62.32% (58.59%-66.16%)	42.65% (37.47%-47.58%)	96.322% (94.203%-98.667%)	95.25% (92.45%-98.33%)	49.27% (44.6%-53.93%)	2.8350 (2.7356-2.9367)	62.23% (54.55%-69.7%)	42.33% (32.26%-53.03%)	96.356% (91.111%-100%)	95.22% (87.5%-100%)	49.33% (40.3%-59.09%)	2.8324 (2.6279-3.0225)
At least 6 of CHASM (lung), FATHMM (cancer), FATHMM (missense), Mutation Assessor, MutationTaster, PROVEAN	56.26% (52.02%-60.1%)	32.03% (27.42%-36.59%)	98.173% (97.015%-100%)	96.74% (94.44%-100%)	45.51% (40.91%-50%)	2.7245 (2.6353-2.8167)	56.17% (48.48%-64.65%)	31.67% (22.76%-40.98%)	98.206% (94.282%-100%)	96.8% (88.89%-100%)	45.58% (36.84%-54.93%)	2.7225 (2.5340-2.9032)
At least 6 of CHASM (lung), FATHMM (cancer), FATHMM (missense), Mutation Assessor, MutationTaster, SIFT	63.34% (59.6%-67.17%)	44.26% (38.98%-49.18%)	96.322% (94.203%-98.667%)	95.38% (92.73%-98.36%)	49.9% (45.25%-54.61%)	2.8591 (2.7612-2.9584)	63.25% (55.56%-71.72%)	43.9% (34.43%-54.1%)	96.356% (91.111%-100%)	95.38% (88.46%-100%)	50.02% (41.09%-59.15%)	2.8566 (2.6473-3.0483)
At least 6 of CHASM (lung), FATHMM (cancer), FATHMM (missense), Mutation Assessor, MutationTaster, VEST	59.31% (55.56%-63.13%)	37.91% (32.79%-42.75%)	96.322% (94.203%-98.667%)	94.68% (91.49%-98.11%)	47.29% (42.66%-51.72%)	2.7620 (2.6637-2.8688)	59.15% (51.52%-66.67%)	37.47% (27.69%-47.62%)	96.356% (91.111%-100%)	94.63% (85.71%-100%)	47.31% (38.35%-56.76%)	2.7577 (2.5404-2.9564)
At least 6 of CHASM (lung), FATHMM (cancer), FATHMM (missense), Mutation Assessor, PolyPhen-2, PROVEAN	56.93% (53.03%-60.61%)	33.09% (28.33%-37.69%)	98.173% (97.015%-100%)	96.86% (94.74%-100%)	45.87% (41.22%-50.31%)	2.7400 (2.6533-2.8261)	56.84% (49.49%-64.65%)	32.73% (23.33%-42.37%)	98.206% (94.282%-100%)	96.89% (89.47%-100%)	45.95% (37.5%-55.56%)	2.7378 (2.5444-2.9286)
At least 6 of CHASM (lung), FATHMM (cancer), FATHMM (missense), Mutation Assessor, PolyPhen-2, SIFT	64.36% (60.61%-68.18%)	46.37% (40.98%-51.15%)	95.382% (92.857%-98.611%)	94.56% (91.38%-97.92%)	50.56% (45.73%-55.19%)	2.8847 (2.7899-2.9872)	64.24% (56.57%-72.73%)	45.97% (35.19%-56.25%)	95.393% (90%-100%)	94.53% (86.67%-100%)	50.61% (41.18%-60%)	2.8808 (2.6748-3.0857)
At least 6 of CHASM (lung), FATHMM (cancer), FATHMM (missense), Mutation Assessor, PolyPhen-2, VEST	58.57% (55.05%-62.64%)	37.9% (32.56%-42.2%)	95.382% (92.857%-98.611%)	93.42% (89.99%-96.97%)	47.04% (42.28%-51.36%)	2.7374 (2.6277-2.8421)	58.84% (50.51%-66.67%)	37.47% (27.87%-48.33%)	95.485% (89.189%-100%)	93.44% (84%-100%)	47.08% (38.2%-56.34%)	2.7348 (2.5121-2.9360)
At least 6 of CHASM (lung), FATHMM (cancer), FATHMM (missense), Mutation Assessor, PROVEAN, SIFT	57.59% (54.04%-61.62%)	34.7% (29.56%-39.02%)	98.173% (97.015%-100%)	97% (95%-100%)	46.5% (41.77%-50.67%)	2.7635 (2.6674-2.8535)	57.48% (50.51%-65.66%)	34.23% (24.59%-44.07%)	98.206% (94.282%-100%)	97.02% (90%-100%)	46.58% (37.79%-55.7%)	2.7608 (2.5722-2.9438)
At least 6 of CHASM (lung), FATHMM (cancer), FATHMM (missense), Mutation Assessor, PROVEAN, VEST	55.58% (51.52%-59.6%)	30.97% (26.23%-35.48%)	98.173% (97.015%-100%)	96.63% (94.12%-100%)	45.12% (40.38%-49.38%)	2.7089 (2.6162-2.8039)	55.5% (47.47%-63.64%)	30.6% (21.43%-39.71%)	98.206% (94.282%-100%)	96.68% (88.89%-100%)	45.19% (36.71%-54.43%)	2.7068 (2.5121-2.8924)
At least 6 of CHASM (lung), FATHMM (cancer), FATHMM (missense), Mutation Assessor, SIFT, VEST	60.33% (56.57%-64.14%)	39.51% (34.48%-44.35%)	96.322% (94.203%-98.667%)	94.89% (91.84%-98.18%)	47.94% (43.19%-52.48%)	2.7866 (2.6863-2.8923)	60.15% (52.53%-67.68%)	39.04% (29.31%-49.12%)	96.356% (91.111%-100%)	94.83% (86.36%-100%)	47.94% (39.19%-57.14%)	2.7817 (2.5676-2.9751)
At least 6 of CHASM (lung), FATHMM (cancer), FATHMM (missense), MutationTaster, PolyPhen-2, PROVEAN	56.7% (52.53%-60.61%)	32.04% (26.36%-36.8%)	98.173% (97.015%-100%)	96.79% (94.59%-100%)	45.51% (40.86%-49.68%)	2.7247 (2.6302-2.8155)	56.15% (48.48%-64.65%)	31.64% (22.58%-41.54%)	98.206% (94.282%-100%)	96.79% (88.89%-100%)	45.56% (37.18%-54.88%)	2.7219 (2.5282-2.9015)
At least 6 of CHASM (lung), FATHMM (cancer), FATHMM (missense), MutationTaster, PolyPhen-2, SIFT	63.34% (59.6%-67.17%)	44.26% (39.06%-49.18%)	96.322% (94.203%-98.667%)	95.41% (92.59%-98.36%)	49.99% (45.16%-54.59%)	2.8592 (2.7560-2.9594)	63.23% (55.56%-70.71%)	43.9% (33.87%-53.85%)	96.356% (91.111%-100%)	95.38% (88.46%-100%)	50.02% (41.1%-59.73%)	2.8565 (2.6548-3.0563)
At least 6 of CHASM (lung), FATHMM (cancer), FATHMM (missense), MutationTaster, PolyPhen-2, VEST	59.99% (56.06%-63.64%)	38.98% (33.85%-43.94%)	96.322% (94.203%-98.667%)	94.82% (91.67%-98.08%)	47.72% (42.95%-52.07%)	2.7784 (2.6813-2.8813)	59.82% (52.53%-67.68%)	38.52% (29.23%-48.59%)	96.356% (91.111%-100%)	94.77% (86.36%-100%)	47.73% (38.89%-57.14%)	2.7772 (2.5623-2.9722)
At least 6 of CHASM (lung), FATHMM (cancer), FATHMM (missense), MutationTaster, PROVEAN, SIFT	57.94% (54.04%-61.62%)	34.68% (29.84%-39.47%)	98.173% (97.015%-100%)	97% (94.87%-100%)	46.5% (41.82%-51.03%)	2.7633 (2.6735-2.8535)	57.47% (50.51%-65.66%)	34.19% (24.59%-44.07%)	98.206% (94.282%-100%)	97.03% (90%-100%)	46.57% (37.86%-56.1%)	2.7616 (2.5709-2.9438)
At least 6 of CHASM (lung), FATHMM (cancer), FATHMM (missense), MutationTaster, PROVEAN, VEST	55.59% (52.01%-59.6%)	31.52% (26.77%-36.22%)	98.173% (97.015%-100%)	96.7% (94.44%-100%)	45.32% (40.64%-49.69%)	2.7171 (2.6275-2.8075)	55.48% (48.48%-63.64%)	31.11% (21.54%-40.63%)	98.206% (94.282%-100%)	96.73% (88.89%-100%)	45.37% (36.84%-54.67%)	2.7139 (2.5202-2.9058)
At least 6 of CHASM (lung), FATHMM (cancer), FATHMM (missense), SIFT, VEST	60.99% (56.57%-64.65%)	40% (35%-45%)	96.322% (94.203%-98.667%)	94.95% (92%-98.25%)	48.16% (43.49%-52.75%)	2.7943 (2.6932-2.8956)	60.85% (52.53%-68.69%)	39.59% (29.69%-49.12%)	96.356% (91.111%-100%)	94.9% (86.96%-100%)	48.16% (39.13%-57.53%)	2.7900 (2.5759-2.9852)
At least 6 of CHASM (lung), FATHMM (cancer), Mutation Assessor, MutationTaster, PolyPhen-2, PROVEAN	57.25% (53.54%-61.11%)	34.15% (29.23%-38.76%)	98.173% (97.015%-100%)	97% (95%-100%)	46.46% (41.92%-50.85%)	2.7620 (2.6716-2.8522)	57.19% (50.51%-65.66%)	33.93% (24.59%-43.55%)	98.206% (94.282%-100%)	97.02% (90%-100%)	46.55% (37.8%-55.7%)	2.7609 (2.5707-2.9421)
At least 6 of CHASM (lung), FATHMM (cancer), Mutation Assessor, MutationTaster, PolyPhen-2, SIFT	63.69% (60.1%-67.17%)	44.79% (39.53%-49.76%)	96.322% (94.203%-98.667%)	95.42% (92.73%-98.33%)	50.21% (45.45%-54.84%)	2.8677 (2.7696-2.9665)	63.64% (55.56%-70.71%)	44.58% (34.43%-55%)	96.355% (91.111%-100%)	95.41% (88.89%-100%)	50.31% (41.1%-60%)	2.8658 (2.6644-3.0645)
At least 6 of CHASM (lung), FATHMM (cancer), Mutation Assessor, MutationTaster, PolyPhen-2, VEST	59.6% (56.06%-63.64%)	38.41% (33.07%-43.41%)	96.322% (94.203%-98.667%)	94.75% (91.84%-98.15%)	47.49% (42.86%-51.82%)	2.7708 (2.6710-2.8707)	59.55% (51.52%-67.68%)	38.2% (28.57%-48.33%)	96.355% (91.111%-100%)	94.72% (86.96%-100%)	47.58% (38.81%-56.95%)	2.7695 (2.5572-2.9679)
At least 6 of CHASM (lung), FATHMM (cancer), Mutation Assessor, MutationTaster, PROVEAN, SIFT	57.95% (54.04%-61.62%)	35.2% (30.16%-40.23%)	98.173% (97.015%-100%)	97.05% (95%-100%)	46.7% (42.05%-51.16%)	2.7711 (2.6810-2.8616)	57.88% (50.51%-65.66%)	34.97% (25.4%-45%)	98.206% (94.282%-100%)	97.07% (90.48%-100%)	46.79% (38.03%-56.25%)	2.7693 (2.5837-2.9568)
At least 6 of CHASM (lung), FATHMM (cancer), Mutation Assessor, MutationTaster, PROVEAN, VEST	56.25% (52.53%-60.1%)	32.55% (27.69%-37.12%)	98.173% (97.015%-100%)	96.81% (94.59%-100%)	45.89% (41.22%-50.31%)	2.7319 (2.6410-2.8197)	56.18% (48.48%-64.65%)	32.32% (23.33%-41.94%)	98.206% (94.282%-100%)	96.83% (88.89%-100%)	45.96% (37.18%-55.7%)	2.7300 (2.5348-2.9144)
At least 6 of CHASM (lung), FATHMM (cancer), Mutation Assessor, MutationTaster, SIFT, VEST	61.66% (57.58%-65.66%)	41.6% (36.51%-46.62%)	96.322% (94.203%-98.667%)	95.13% (92.16%-98.28%)	48.83% (44.14%-53.38%)	2.8155 (2.7146-2.9152)	61.61% (53.54%-69.7%)	41.24% (31.25%-52.08%)	96.355% (91.111%-100%)	95.09% (86.96%-100%)	48.86% (39.73%-58.33%)	2.8155 (2.6061-3.0207)
At least 6 of CHASM (lung), FATHMM (cancer), Mutation Assessor, PolyPhen-2, PROVEAN, SIFT	58.29% (54.55%-62.12%)	35.74% (30.83%-40.65%)	98.173% (97.015%-100%)	97.11% (95.12%-100%)	46.89% (42.22%-51.28%)	2.7789 (2.6898-2.8708)	58.21% (51.52%-66.67%)	35.51% (25.86%-45.31%)	98.206% (94.282%-100%)	97.1% (90.48%-100%)	46.98% (38.36%-56.34%)	2.7769 (2.5964-2.9668)
At least 6 of CHASM (lung), FATHMM (cancer), Mutation Assessor, PolyPhen-2, PROVEAN, VEST	56.92% (53.03%-61.11%)	33.07% (28.57%-37.69%)	98.173% (97.015%-100%)	96.86% (94.74%-100%)	46.08% (41.55%-50.31%)	2.7396 (2.6519-2.8291)	56.85% (49.49%-64.65%)	32.87% (23.73%-42.62%)	98.206% (94.282%-100%)	96.88% (89.47%-100%)	46.16% (37.5%-55.41%)	2.7386 (2.5432-2.9205)
At least 6 of CHASM (lung), FATHMM (cancer), Mutation Assessor, PolyPhen-2, SIFT, VEST	61.65% (58.08%-65.66%)	41.6% (36.44%-46.77%)	96.322% (94.203%-98.667%)	95.13% (92.31%-98.28%)	48.82% (44.04%-53.45%)	2.8137 (2.7170-2.9139)	61.54% (53.54%-69.7%)	41.24% (31.15%-51.61%)	96.355% (91.111%-100%)	95.09% (86.96%-100%)	48.86% (39.74%-58.44%)	2.8155 (2.6043-3.0223)
At least 6 of CHASM (lung), FATHMM (cancer), Mutation Assessor, PROVEAN, SIFT, VEST	57.93% (54.04%-61.62%)	34.68% (29.56%-39.47%)	98.173% (97.015%-100%)	97% (94.87%-100%)	46.5% (41.8%-50.83%)	2.7632 (2.6751-2.8516)	57.84% (50.51%-65.66%)	34.43% (24.59%-44.26%)	98.206% (94.282%-100%)	97% (90%-100%)	46.58% (37.97%-55.84%)	2.7612 (2.5769-2.9480)
At least 6 of CHASM (lung), FATHMM (cancer), MutationTaster, PolyPhen-2, PROVEAN, SIFT	59.28% (55.05%-63.13%)	37.33% (32.56%-42.22%)	98.173% (97.015%-100%)	97.23% (95.35%-100%)	47.44% (42.86%-51.85%)	2.8019 (2.7175-2.8938)	59.2% (51.52%-67.68%)	37.06% (27.12%-46.88%)	98.206% (94.282%-100%)	97.24% (90.91%-100%)	47.52% (38.67%-56.76%)	2.8011 (2.6186-2.9867)
At least 6 of CHASM (lung), FATHMM (cancer), MutationTaster, PolyPhen-2, PROVEAN, VEST	57.6% (53.54%-61.62%)	34.18% (29.17%-39.02%)	98.173% (97.015%-100%)	96.97% (95%-100%)	46.47% (41.72%-50.64%)	2.7551 (2.6630-2.8481)	57.54% (49.49%-65.66%)	33.93% (24.24%-43.86%)	98.206% (94.282%-100%)	96.98% (89.47%-100%)	46.58% (37.84%-55.84%)	2.7538 (2.5698-2.9468)
At least 6 of CHASM (lung), FATHMM (cancer), MutationTaster, PolyPhen-2, SIFT, VEST	62.65% (59.08%-66.67%)	43.18% (38.1%-48.06%)	96.322% (94.203%-98.667%)	95.3% (92.45%-98.36%)	49.5% (44.76%-54.11%)	2.8430 (2.7458-2.9522)	62.57% (54.55%-70.71%)	42.88% (32.79%-52.63%)	96.355% (91.111%-100%)	95.26% (87.5%-100%)	49.57% (40.3%-59.42%)	2.8407 (2.6182-3.0441)
At least 6 of CHASM (lung), FATHMM (cancer), MutationTaster, PROVEAN, SIFT, VEST	59.29% (55.05%-63.13%)	36.8% (31.78%-41.6%)	98.173% (97.015%-100%)	97.17% (95.12%-100%)	47.32% (42.57%-51.7%)	2.7943 (2.6973-2.8853)	59.22% (51.52%-67.68%)	36.5% (26.56%-46.43%)	98.206% (94.282%-100%)	97.2% (90.48%-100%)	47.4% (38.69%-57.14%)	2.7920 (2.6062-2.9758)
At least 6 of CHASM (lung), FATHMM (cancer), PolyPhen-2, PROVEAN, SIFT, VEST	58.94% (55.05%-62.63%)	36.26% (31.2%-40.91%)	98.173% (97.015%-100%)	97.13% (95.12%-100%)	47.13% (42.43%-51.54%)	2.7863 (2.6932-2.8813)	58.89% (51.52%-67.68%)	35.98% (26.15%-45.9%)	98.206% (94.282%-100%)	97.15% (90.48%-100%)	47.21% (38.46%-56.58%)	2.7853 (2.5982-2.9706)
At least 6 of CHASM (lung), FATHMM (missense), Mutation Assessor, MutationTaster, PolyPhen-2, PROVEAN	55.24% (51.01%-59.09%)	30.42% (25.81%-34.88%)	98.173% (97.015%-100%)	96.6% (94.12%-100%)	44.93% (40.26%-49.06%)	2.7010 (2.6112-2.7941)	55.18% (47.47%-63.64%)	30.09% (21.21%-39.66%)	98.206% (94.282%-100%)	96.61% (88.89%-100%)	45.01% (36.71%-54.17%)	2.6995 (2.5100-2.8788)
At least 6 of CHASM (lung), FATHMM (missense), Mutation Assessor, MutationTaster, PolyPhen-2, SIFT	63% (59.09%-66.67%)	43.73% (38.33%-48.51%)	96.322% (94.203%-98.667%)	95.36% (92.59%-98.36%)	49.79% (45.14%-54.29%)	2.8515 (2.7546-2.9534)	62.89% (55.56%-70.71%)	43.37% (33.33%-53.73%)	96.355% (91.111%-100%)	95.33% (88%-100%)	49.84% (40.54%-59.09%)	2.8484 (2.6433-3.0459)
At least 6 of CHASM (lung), FATHMM (missense), Mutation Assessor, MutationTaster, PolyPhen-2, VEST	58.97% (55.05%-63.13%)	37.37% (32.06%-42.4%)	96.322% (94.203%-98.667%)	94.61% (91.49%-98.04%)	47.1% (42.49%-51.47%)	2.7542 (2.6496-2.8559)	58.85% (50.51%-66.67%)	37.01% (27.12%-47.37%)	96.355% (91.111%-100%)	94.55% (85.71%-100%)	47.12% (38.16%-56.76%)	2.7504 (2.5352-2.9596)
At least 6 of CHASM (lung), FATHMM (missense), Mutation Assessor, MutationTaster, PROVEAN, SIFT	56.26% (52.02%-60.1%)	32.03% (27.42%-36.59%)	98.173% (97.015%-100%)	96.74% (94.44%-100%)	45.51% (40.91%-50%)	2.7245 (2.6353-2.8167)	56.17% (48.48%-64.65%)	31.67% (22.76%-40.98%)	98.206% (94.282%-100%)	96.8% (88.89%-100%)	45.58% (36.84%-54.93%)	2.7225 (2.5340-2.9032)
At least 6 of CHASM (lung), FATHMM (missense), Mutation Assessor, MutationTaster, PROVEAN, VEST	54.23% (50%-58.08%)	28.83% (24.19%-33.33%)	98.173% (97.015%-100%)	96.41% (93.94%-100%)	44.35% (39.87%-48.76%)	2.6773 (2.5897-2.7696)	54.16% (46.46%-62.63%)	28.49% (19.67%-37.7%)	98.206% (94.282%-100%)	96.43% (88.24%-100%)	44.42% (35.9%-53.66%)	2.6752 (2.4856-2.8657)
At least 6 of CHASM (lung), FATHMM (missense), Mutation Assessor, MutationTaster, SIFT, VEST	59.99% (56.06%-63.64%)	38.98% (33.89%-43.94%)	96.322% (94.203%-98.667%)	94.82% (91.84%-98.15%)	47.72% (43.06%-52.12%)	2.7784 (2.6764-2.8774)	59.81% (52.53%-67.68%)	38.56% (28.81%-48.39%)	96.355% (91.111%-100%)	94.78% (86.36%-100%)	47.73% (38.96%-56.76%)	2.7736 (2.5629-2.9701)
At least 6 of CHASM (lung), FATHMM (missense), Mutation Assessor, PolyPhen-2, PROVEAN, SIFT	56.93% (53.03%-60.61%)	33.09% (28.33%-37.69%)	98.173% (97.015%-100%)	96.86% (94.74%-100%)	45.87% (41.22%-50.31%)	2.7400 (2.6533-2.8261)	56.84% (49.49%-64.65%)	32.73% (23.33%-42.37%)	98.206% (94.282%-100%)	96.89% (89.47%-100%)	45.95% (37.5%-55.56%)	2.7378 (2.5444-2.9286)
At least 6 of CHASM (lung), FATHMM (missense), Mutation Assessor, PolyPhen-2, PROVEAN, VEST	54.57% (50.51%-58.59%)	29.36% (24.6%-33.87%)	98.173% (97.015%-100%)	96.48% (93.94%-100%)	44.54% (39.87%-48.75%)	2.6859 (2.5928-2.7784)	54.5% (46.46%-62.63%)	29.03% (20%-38.71%)	98.206% (94.282%-100%)	96.49% (88.24%-100%)	44.61% (35.9%-53.85%)	2.6837 (2.4900-2.8808)
At least 6 of CHASM (lung), FATHMM (missense), Mutation Assessor, PolyPhen-2, SIFT, VEST	59.99% (56.06%-63.64%)	38.98% (33.85%-43.94%)	96.322% (94.203%-98.667%)	94.82% (91.67%-98.08%)	47.72% (42.95%-52.07%)	2.7784 (2.6813-2.8813)	59.82% (52.53%-67.68%)	38.52% (29.23%-48.59%)	96.356% (91.111%-100%)	94.77% (86.36%-100%)	47.73% (38.89%-57.14%)	2.7736 (2.5623-2.9722)
At least 6 of CHASM (lung), FATHMM (missense), Mutation Assessor, PROVEAN, SIFT, VEST	55.25% (51.01%-59.09%)	30.45% (25.81%-35.16%)	98.173% (97.015%-100%)	96.61% (94.12%-100%)	44.94% (40.25%-49.38%)	2.7012 (2.6063-2.7934)	55.13% (47.47%-63.64%)	30.07% (21.05%-39.34%)	98.206% (94.282%-100%)	96.65% (88.89%-100%)	45% (36.36%-54.43%)	2.6986 (2.5008-2.8912)
At least 6 of CHASM (lung), FATHMM (missense), MutationTaster, PolyPhen-2, PROVEAN, SIFT	56.93% (53.03%-60.61%)	33.09% (28.23%-37.88%)	98.173% (97.015%-100%)	96.86% (94.74%-100%)	45.87% (41.14%-50.31%)	2.7401 (2.6461-2.8261)	56.83% (49.49%-64.65%)	32.7% (23.33%-42.59%)	98.206% (94.282%-100%)	96.89% (89.47%-100%)	45.94% (37.33%-55.41%)	2.7376 (2.5444-2.9286)
At least 6 of CHASM (lung), FATHMM (missense), MutationTaster, PolyPhen-2, PROVEAN, VEST	54.92% (51.01%-59.09%)	29.9% (25.19%-34.43%)	98.173% (97.015%-100%)	96.54% (94.12%-100%)	44.73% (40.13%-49.07%)	2.6936 (2.6012-2.7852)	54.84% (47.47%-62.63%)	29.57% (20.69%-39.34%)	98.206% (94.282%-100%)	96.57% (88.24%-100%)	44.8% (36.14%-54.32%)	2.6923 (2.4950-2.8867)
At least 6 of CHASM (lung), FATHMM (missense), MutationTaster, PolyPhen-2, SIFT, VEST	61% (57.07%-64.65%)	40.55% (35.38%-45.6%)	96.322% (94.203%-98.667%)	95.04% (92.16%-98.25%)	48.27% (43.4%-52.94%)	2.8020 (2.7017-2.9046)	60.89% (52.53%-68.69%)	40.2% (30.51%-50.79%)	96.355% (91.111%-100%)	95.01% (87.5%-100%)	48.31% (38.96%-57.69%)	2.7990 (2.5937-3.0026)
At least 6 of CHASM (lung), FATHMM (missense), MutationTaster, PROVEAN, SIFT, VEST	55.59% (52.01%-59.6%)	31.52% (26.77%-36.22%)	98.173% (97.015%-100%)	96.7% (94.44%-100%)	45.32% (40.64%-49.69%)	2.7171 (2.6275-2.8075)	55.48% (48.48%-63.64%)	31.11% (21.54%-40.63%)	98.206% (94.282%-100%)	96.73% (88.89%-100%)	45.37% (36.84%-54.67%)	2.7139 (2.5202-2.9058)
At least 6 of CHASM (lung), FATHMM (missense), PolyPhen-2, PROVEAN, SIFT, VEST	55.25% (51.52%-59.09%)	30.45% (25.63%-34.88%)	98.173% (97.015%-100%)	96.6% (94.29%-100%)	44.94% (40.25%-49.38%)	2.7011 (2.6144-2.7924)	55.17% (47.47%-63.64%)	30.15% (21.05%-39.34%)	98.206% (94.282%-100%)	96.62% (88.89%-100%)	45.02% (36.25%-54.43%)	2.6993 (2.5058-2.8924)
At least 6 of CHASM (lung), Mutation Assessor, MutationTaster, PolyPhen-2, PROVEAN, SIFT	57.59% (54.04%-61.62%)	34.13% (29.23%-38.76%)	98.173% (97.015%-100%)	96.96% (94.87%-100%)	46.28% (41.72%-50.68%)	2.7555 (2.6710-2.8462)	57.56% (50.51%-65.66%)	33.98% (24.59%-43.86%)	98.162% (94.282%-100%)	96.96% (90%-100%)	46.38% (37.5%-55.7%)	2.7547 (2.5657-2.9423)
At least 6 of CHASM (lung), Mutation Assessor, MutationTaster, PolyPhen-2, PROVEAN, VEST	55.56% (51.52%-59.6%)	30.93% (26.19%-35.71%)	98.173% (97.015%-100%)	96.65% (94.29%-100%)	45.11% (40.51%-49.69%)	2.7084 (2.6182-2.8002)	55.5% (47.47%-63.64%)	30.76% (21.82%-40.32%)	98.162% (94.282%-100%)	96.67% (88.89%-100%)	45.23% (36.84%-54.67%)	2.7079 (2.5100-2.8889)
At least 6 of CHASM (lung), Mutation Assessor, MutationTaster, PolyPhen-2, SIFT, VEST	60.98% (57.07%-64.65%)	41.07% (35.66%-45.97%)	95.4% (92.857%-98.571%)	93.91% (90.74%-97.5%)	48.11% (43.33%-52.8%)	2.7811 (2.6769-2.8834)	60.94% (53.54%-68.69%)	40.89% (31.03%-50.79%)	95.442% (89.996%-100%)	93.86% (85.19%-100%)	48.19% (38.96%-57.53%)	2.7784 (2.5603-2.9857)
At least 6 of CHASM (lung), Mutation Assessor, MutationTaster, PROVEAN, SIFT, VEST	56.59% (52.53%-60.61%)	32.54% (27.69%-37.31%)	98.173% (97.015%-100%)	96.81% (94.59%-100%)	45.69% (41.04%-50.15%)	2.7321 (2.6421-2.8236)	56.5% (48.48%-64.65%)	32.33% (23.33%-42.62%)	98.162% (94.282%-100%)	96.8% (88.89%-100%)	45.79% (36.99%-55%)	2.7309 (2.5414-2.9223)
At least 6 of CHASM (lung), Mutation Assessor, PolyPhen-2, PROVEAN, SIFT, VEST	56.92% (53.03%-60.61%)	33.06% (28.33%-37.69%)	98.173% (97.015%-100%)	96.85% (94.74%-100%)	45.87% (41.29%-50.31%)	2.7397 (2.6515-2.8291)	56.84% (49.49%-64.65%)	32.85% (23.33%-42.37%)	98.162% (94.282%-100%)	96.84% (89.47%-100%)	45.97% (37.18%-55.41%)	2.7384 (2.5446-2.9286)
At least 6 of CHASM (lung), MutationTaster, PolyPhen-2, PROVEAN, SIFT, VEST	57.93% (54.04%-62.12%)	34.67% (29.83%-39.52%)	98.173% (97.015%-100%)	97% (94.87%-100%)	46.48% (41.93%-50.94%)	2.7631 (2.6716-2.8535)	57.85% (50.51%-65.66%)	34.45% (25%-44.44%)	98.162% (94.282%-100%)	96.98% (90%-100%)	46.57% (37.98%-55.84%)	2.7616 (2.5736-2.9554)
At least 6 of CHASM (melanoma), FATHMM (cancer), FATHMM (missense), Mutation Assessor, MutationTaster, PolyPhen-2	65.65% (62.12%-69.19%)	48.42% (43.25%-53.46%)	95.442% (92.958%-98.592%)	94.84% (92.06%-98.33%)	51.67% (46.94%-56.3%)	2.9039 (2.7973-3.0102)	65.67% (58.59%-73.74%)	48.23% (37.5%-58.73%)	95.351% (89.189%-100%)	94.71% (87.88%-100%)	51.83% (42.62%-61.64%)	2.9025 (2.6859-3.1015)
At least 6 of CHASM (melanoma), FATHMM (cancer), FATHMM (missense), Mutation Assessor, MutationTaster, PROVEAN	57.62% (53.54%-61.62%)	34.7% (29.76%-38.76%)	98.173% (97.015%-100%)	97% (94.87%-100%)	46.3% (41.6%-50.68%)	2.7564 (2.6632-2.8465)	57.49% (49.49%-65.66%)	33.79% (24.14%-43.75%)	98.163% (94.444%-100%)	96.91% (90%-100%)	46.34% (37.5%-55.56%)	2.7518 (2.5592-2.9402)
At least 6 of CHASM (melanoma), FATHMM (cancer), FATHMM (missense), Mutation Assessor, MutationTaster, SIFT	66.33% (62.63%-69.7%)	50.56% (45.31%-55.74%)	94.442% (91.781%-97.183%)	93.79% (90.7%-96.88%)	51.96% (47.14%-56.72%)	2.9025 (2.7980-3.0066)	66.27% (58.59%-73.74%)	50.4% (40%-61.02%)	94.367% (88.235%-100%)	93.68% (86.21%-100%)	52.06% (42.66%-61.8%)	2.8998 (2.6758-3.1214)
At least 6 of CHASM (melanoma), FATHMM (cancer), FATHMM (missense), Mutation Assessor, MutationTaster, VEST	63.31% (59.09%-67.17%)	44.73% (39.39%-49.62%)	95.442% (92.958%-98.592%)	94.44% (91.38%-98.15%)	50.1% (45.33%-54.79%)	2.8452 (2.7410-2.9505)	63.24% (55.56%-71.72%)	44.51% (34.38%-55%)	95.351% (89.189%-100%)	94.28% (86.96%-100%)	50.16% (40.54%-59.74%)	2.8427 (2.6207-3.0597)
At least 6 of CHASM (melanoma), FATHMM (cancer), FATHMM (missense), Mutation Assessor, PolyPhen-2, PROVEAN	57.96% (54.04%-61.62%)	35.24% (30.16%-40%)	97.223% (95.521%-100%)	95.9% (93.33%-100%)	46.47% (41.77%-50.96%)	2.7464 (2.6512-2.8384)	57.82% (50.51%-65.66%)	34.84% (25%-44.62%)	97.224% (92.105%-100%)	95.59% (88.89%-100%)	46.51% (37.8%-55.7%)	2.7426 (2.5494-2.9366)
At least 6 of CHASM (melanoma), FATHMM (cancer), FATHMM (missense), Mutation Assessor, PolyPhen-2, SIFT	67.02% (63.64%-71.21%)	51.63% (46.46%-56.88%)	94.485% (91.667%-97.222%)	93.91% (90.91%-97.14%)	52.4% (47.65%-57.26%)	2.9242 (2.8172-3.0336)	67% (59.6%-74.75%)	51.46% (40.91%-62.26%)	94.409% (88.235%-100%)	94.09% (86.67%-100%)	52.41% (42.86%-62.16%)	2.9214 (2.6958-3.1485)
At least 6 of CHASM (melanoma), FATHMM (cancer), FATHMM (missense), Mutation Assessor, PolyPhen-2, VEST	62.29% (58.59%-66.16%)	44.19% (39.06%-49.24%)	93.579% (90.411%-96.97%)	92.25% (88.52%-95.91%)	49.3% (44.6%-53.93%)	2.7969 (2.6865-2.9074)	62.24% (54.55%-69.7%)	43.96% (33.33%-54.24%)	93.559% (86.486%-100%)	92.18% (83.33%-100%)	49.37% (40.26%-58.97%)	2.7942 (2.5588-3.0126)
At least 6 of CHASM (melanoma), FATHMM (cancer), FATHMM (missense), Mutation Assessor, PROVEAN, SIFT	58.97% (55.05%-62.63%)	36.83% (31.75%-41.53%)	97.223% (95.521%-100%)	96.02% (93.62%-100%)	47.04% (42.32%-51.53%)	2.7688 (2.6754-2.8632)	58.86% (51.52%-66.67%)	36.44% (26.67%-46.15%)	97.224% (92.105%-100%)	95.96% (88.89%-100%)	47.09% (38.04%-56.58%)	2.7650 (2.5665-2.9549)
At least 6 of CHASM (melanoma), FATHMM (cancer), FATHMM (missense), Mutation Assessor, PROVEAN, VEST	56.94% (53.03%-61.11%)	33.62% (28.39%-38.02%)	98.173% (97.015%-100%)	96.88% (94.59%-100%)	45.94% (41.35%-50.31%)	2.7412 (2.6563-2.8291)	56.8% (49.49%-64.65%)	33.26% (24.14%-43.33%)	98.163% (94.444%-100%)	96.84% (89.47%-100%)	45.99% (37.33%-55.56%)	2.7375 (2.5549-2.9294)
At least 6 of CHASM (melanoma), FATHMM (cancer), FATHMM (missense), Mutation Assessor, SIFT, VEST	63.99% (60.1%-67.69%)	46.33% (40.94%-51.2%)	94.518% (91.667%-97.263%)	93.58% (90.33%-97.06%)	50.37% (45.77%-55.04%)	2.8462 (2.7382-2.9570)	63.92% (56.57%-71.72%)	46.19% (35.94%-56.06%)	94.431% (88.235%-100%)	93.44% (85.71%-100%)	50.44% (41.13%-60%)	2.8432 (2.6284-3.0526)
At least 6 of CHASM (melanoma), FATHMM (cancer), FATHMM (missense), MutationTaster, PolyPhen-2, PROVEAN	57.61% (53.54%-61.62%)	34.15% (29.31%-39.02%)	98.173% (97.015%-100%)	96.95% (94.74%-100%)	46.3% (41.77%-50.68%)	2.7564 (2.6632-2.8442)	57.49% (49.49%-65.66%)	33.79% (24.14%-43.55%)	98.163% (94.444%-100%)	96.91% (90%-100%)	46.34% (37.5%-55.56%)	2.7518 (2.5576-2.9402)
At least 6 of CHASM (melanoma), FATHMM (cancer), FATHMM (missense), MutationTaster, PolyPhen-2, SIFT	67% (63.64%-70.71%)	50.55% (45.38%-55.37%)	95.442% (92.958%-98.592%)	95.04% (92.42%-98.39%)	52.74% (48%-57.58%)	2.9378 (2.8359-3.0378)	67.01% (59.6%-73.74%)	50.48% (40%-60.66%)	95.351% (89.189%-100%)	94.93% (87.88%-100%)	52.87% (43.06%-62.5%)	2.9362 (2.7329-3.1375)
At least 6 of CHASM (melanoma), FATHMM (cancer), FATHMM (missense), MutationTaster, PolyPhen-2, VEST	63.66% (60.1%-67.17%)	45.25% (40.16%-50.39%)	95.442% (92.958%-98.592%)	94.5% (91.53%-98.15%)	50.32% (45.52%-54.9%)	2.8535 (2.7508-2.9573)	63.6% (55.56%-71.72%)	45.15% (35%-55.56%)	95.351% (89.189%-100%)	94.36% (86.36%-100%)	50.38% (41.1%-59.74%)	2.8510 (2.6351-3.0668)
At least 6 of CHASM (melanoma), FATHMM (cancer), FATHMM (missense), MutationTaster, PROVEAN, SIFT	59.33% (55.56%-63.13%)	37.88% (32.77%-42.62%)	97.223% (95.521%-100%)	96.11% (93.75%-100%)	47.24% (42.62%-51.7%)	2.7773 (2.6826-2.8707)	59.21% (51.52%-66.67%)	37.5% (27.87%-47.69%)	97.224% (92.105%-100%)	96.04% (89.47%-100%)	47.27% (38.46%-56.76%)	2.7734 (2.5791-2.9631)
At least 6 of CHASM (melanoma), FATHMM (cancer), FATHMM (missense), MutationTaster, PROVEAN, VEST	57.28% (53.54%-61.11%)	33.63% (28.46%-38.4%)	98.173% (97.015%-100%)	96.91% (94.74%-100%)	46.11% (41.56%-50.33%)	2.7488 (2.6610-2.8392)	57.16% (49.49%-64.65%)	33.26% (23.73%-42.86%)	98.163% (94.444%-100%)	96.88% (89.47%-100%)	46.16% (37.5%-55.26%)	2.7447 (2.5564-2.9310)
At least 6 of CHASM (melanoma), FATHMM (cancer), FATHMM (missense), SIFT, VEST	64.65% (61.11%-68.69%)	47.38% (42.15%-52.42%)	94.485% (91.667%-97.222%)	93.7% (90.48%-97.06%)	50.74% (45.99%-55.32%)	2.8625 (2.7521-2.9710)	64.57% (57.58%-71.72%)	47.15% (36.84%-57.58%)	94.409% (88.235%-100%)	93.56% (85.71%-100%)	50.77% (41.67%-60.81%)	2.8590 (2.6362-3.0816)
At least 6 of CHASM (melanoma), FATHMM (cancer), Mutation Assessor, MutationTaster, PolyPhen-2, PROVEAN	59.3% (55.56%-63.13%)	37.34% (32.33%-42.11%)	97.223% (95.521%-100%)	96.11% (93.75%-100%)	47.21% (42.58%-51.64%)	2.7749 (2.6806-2.8692)	59.23% (51.52%-67.68%)	37.14% (27.27%-47.06%)	97.224% (92.105%-100%)	96.04% (89.47%-100%)	47.29% (38.46%-56.76%)	2.7725 (2.5770-2.9601)
At least 6 of CHASM (melanoma), FATHMM (cancer), Mutation Assessor, MutationTaster, PolyPhen-2, SIFT	67.02% (63.64%-71.21%)	51.63% (46.46%-56.88%)	94.485% (91.667%-97.222%)	93.91% (90.91%-97.14%)	52.4% (47.65%-57.26%)	2.9242 (2.8172-3.0336)	66.93% (59.6%-74.75%)	51.41% (40.91%-62.5%)	94.409% (88.235%-100%)	93.78% (86.21%-100%)	52.47% (43.06%-62.32%)	2.9214 (2.6958-3.1485)
At least 6 of CHASM (melanoma), FATHMM (cancer), Mutation Assessor, MutationTaster, PolyPhen-2, VEST	62.98% (59.09%-66.67%)	44.75% (39.55%-49.61%)	93.579% (90.411%-96.97%)	92.38% (88.89%-96.15%)	49.64% (44.93%-54.36%)	2.8047 (2.6989-2.9154)	62.9% (54.55%-70.71%)	44.5% (34.43%-55.17%)	93.559% (86.486%-100%)	92.28% (83.33%-100%)	49.71% (40.54%-59.22%)	2.8019 (2.5756-3.0264)
At least 6 of CHASM (melanoma), FATHMM (cancer), Mutation Assessor, MutationTaster, PROVEAN, SIFT	59.28% (55.56%-63.13%)	37.32% (32.31%-42.19%)	97.223% (95.521%-100%)	96.05% (93.62%-100%)	47.21% (42.55%-51.7%)	2.7745 (2.6799-2.8696)	59.21% (51.52%-67.68%)	37.12% (27.12%-47.06%)	97.224% (92.105%-100%)	96% (88.89%-100%)	47.28% (38.36%-56.76%)	2.7717 (2.5814-2.9630)
At least 6 of CHASM (melanoma), FATHMM (cancer), Mutation Assessor, MutationTaster, PROVEAN, VEST	57.61% (53.54%-61.62%)	34.72% (29.83%-39.34%)	97.223% (95.521%-100%)	95.85% (93.18%-100%)	46.29% (41.56%-50.68%)	2.7307 (2.6362-2.8238)	57.52% (49.49%-65.66%)	34.54% (24.59%-44.83%)	97.224% (92.105%-100%)	95.79% (88.24%-100%)	46.37% (37.5%-55.84%)	2.7285 (2.5314-2.9225)
At least 6 of CHASM (melanoma), FATHMM (cancer), Mutation Assessor, MutationTaster, SIFT, VEST	63.65% (59.6%-67.17%)	46.24% (40.94%-51.16%)	93.579% (90.411%-96.97%)	92.53% (89.06%-96.23%)	50.01% (45.2%-54.41%)	2.8227 (2.7083-2.9307)	63.57% (55.56%-70.71%)	45.98% (35.48%-56.45%)	93.559% (86.486%-100%)	92.42% (83.33%-100%)	50.07% (40.79%-59.73%)	2.8195 (2.5888-3.0433)
At least 6 of CHASM (melanoma), FATHMM (cancer), Mutation Assessor, PolyPhen-2, PROVEAN, SIFT	60.31% (56.57%-64.14%)	38.91% (33.85%-43.7%)	97.223% (95.521%-100%)	96.19% (93.88%-100%)	47.8% (43.14%-52.29%)	2.7962 (2.7019-2.8904)	60.24% (52.53%-67.68%)	38.68% (28.79%-48.44%)	97.224% (92.105%-100%)	96.14% (89.47%-100%)	47.87% (39.06%-57.34%)	2.7934 (2.5960-2.9857)
At least 6 of CHASM (melanoma), FATHMM (cancer), Mutation Assessor, PolyPhen-2, PROVEAN, VEST	58.27% (54.53%-62.12%)	35.22% (30.3%-40%)	98.173% (97.015%-100%)	97% (94.87%-100%)	46.69% (42.07%-51.16%)	2.7711 (2.6828-2.8616)	58.22% (50.51%-65.66%)	35.03% (25.4%-44.83%)	98.163% (94.444%-100%)	96.96% (90%-100%)	46.78% (38.03%-56.1%)	2.7690 (2.5838-2.9534)
At least 6 of CHASM (melanoma), FATHMM (cancer), Mutation Assessor, PolyPhen-2, SIFT, VEST	63.64% (60.1%-67.17%)	46.24% (41.13%-51.52%)	93.579% (90.411%-96.97%)	92.53% (89.06%-96.36%)	50% (45.04%-54.59%)	2.8220 (2.7077-2.9342)	63.57% (55.56%-71.72%)	45.98% (35.48%-56.45%)	93.559% (86.486%-100%)	92.42% (83.33%-100%)	50.07% (40.54%-59.74%)	2.8195 (2.5882-3.0466)
At least 6 of CHASM (melanoma), FATHMM (cancer), Mutation Assessor, PROVEAN, SIFT, VEST	59.29% (55.05%-63.13%)	37.33% (32.06%-42.28%)	97.223% (95.521%-100%)	96.06% (93.62%-100%)	47.21% (42.48%-51.72%)	2.7747 (2.6780-2.8679)	59.21% (51.52%-67.68%)	37.11% (27.27%-47.06%)	97.224% (92.105%-100%)	96% (88.89%-100%)	47.28% (38.46%-56.58%)	2.7717 (2.5775-2.9630)
At least 6 of CHASM (melanoma), FATHMM (cancer), MutationTaster, PolyPhen-2, PROVEAN, SIFT	60.64% (56.57%-64.65%)	39.43% (34.21%-44.44%)	97.223% (95.521%-100%)	96.24% (93.88%-100%)	48.01% (43.32%-52.56%)	2.8041 (2.7063-2.8992)	60.56% (53.54%-68.69%)	39.2% (29.31%-49.23%)	97.224% (92.105%-100%)	96.18% (90%-100%)	48.08% (39.19%-57.53%)	2.8012 (2.6058-2.9979)
At least 6 of CHASM (melanoma), FATHMM (cancer), MutationTaster, PolyPhen-2, PROVEAN, VEST	58.61% (54.55%-62.63%)	35.75% (30.83%-40.65%)	98.173% (97.015%-100%)	97.05% (95%-100%)	46.89% (42.15%-51.28%)	2.7790 (2.6908-2.8708)	58.54% (50.51%-66.67%)	35.54% (25.81%-45.45%)	98.163% (94.444%-100%)	97.02% (90%-100%)	46.96% (38.16%-56.34%)	2.7767 (2.5897-2.9640)
At least 6 of CHASM (melanoma), FATHMM (cancer), MutationTaster, PolyPhen-2, SIFT, VEST	64.33% (60.61%-68.18%)	46.81% (41.6%-51.69%)	94.518% (91.667%-97.263%)	93.65% (90.48%-97.06%)	50.56% (45.89%-55.22%)	2.8542 (2.7456-2.9621)	64.25% (56.57%-71.72%)	46.54% (36.51%-56.45%)	94.431% (88.235%-100%)	93.5% (85.71%-100%)	50.62% (41.33%-60.53%)	2.8507 (2.6311-3.0645)
At least 6 of CHASM (melanoma), FATHMM (cancer), MutationTaster, PROVEAN, SIFT, VEST	59.63% (55.56%-63.64%)	37.87% (32.79%-42.75%)	97.223% (95.521%-100%)	96.11% (93.75%-100%)	47.4% (42.77%-51.97%)	2.7826 (2.6850-2.8786)	59.56% (51.52%-67.68%)	37.65% (27.87%-47.54%)	97.224% (92.105%-100%)	96.05% (89.47%-100%)	47.48% (38.46%-56.76%)	2.7797 (2.5848-2.9706)
At least 6 of CHASM (melanoma), FATHMM (cancer), PolyPhen-2, PROVEAN, SIFT, VEST	60.31% (56.57%-64.14%)	38.91% (33.85%-43.94%)	97.223% (95.521%-100%)	96.19% (93.88%-100%)	47.8% (43.04%-52.21%)	2.7962 (2.7017-2.8901)	60.24% (52.53%-67.68%)	38.68% (29.03%-48.48%)	97.224% (92.105%-100%)	96.13% (89.47%-100%)	47.88% (39.13%-57.14%)	2.7935 (2.5989-2.9886)
At least 6 of CHASM (melanoma), FATHMM (missense), Mutation Assessor, MutationTaster, PolyPhen-2, PROVEAN	56.27% (52.02%-60.1%)	32.04% (27.27%-36.51%)	98.173% (97.015%-100%)	96.79% (94.59%-100%)	45.51% (40.91%-49.69%)	2.7247 (2.6343-2.8155)	56.22% (48.48%-64.65%)	31.88% (22.58%-41.94%)	98.163% (94.444%-100%)	96.76% (88.89%-100%)	45.61% (36.99%-55%)	2.7237 (2.5347-2.9144)
At least 6 of CHASM (melanoma), FATHMM (missense), Mutation Assessor, MutationTaster, PolyPhen-2, SIFT	66% (62.12%-69.7%)	50.01% (44.62%-55.12%)	94.442% (91.781%-97.183%)	93.73% (90.57%-96.97%)	51.77% (46.97%-56.47%)	2.8947 (2.7895-3.0036)	65.95% (58.59%-73.74%)	49.83% (39.39%-60.71%)	94.367% (88.235%-100%)	93.62% (86.21%-100%)	51.87% (42.62%-61.64%)	2.8921 (2.6742-3.1149)
At least 6 of CHASM (melanoma), FATHMM (missense), Mutation Assessor, MutationTaster, PolyPhen-2, VEST	62.29% (58.59%-66.16%)	42.62% (37.5%-48.12%)	95.442% (92.958%-98.592%)	94.24% (90.91%-97.92%)	49.46% (44.63%-53.96%)	2.8216 (2.7173-2.9239)	62.22% (54.55%-69.7%)	42.39% (32.26%-52.63%)	95.351% (89.189%-100%)	94.08% (86.36%-100%)	49.51% (40.26%-58.97%)	2.8191 (2.5960-3.0392)
At least 6 of CHASM (melanoma), FATHMM (missense), Mutation Assessor, MutationTaster, PROVEAN, SIFT	57.62% (53.54%-61.62%)	34.7% (29.76%-38.76%)	98.173% (97.015%-100%)	97% (94.87%-100%)	46.3% (41.6%-50.68%)	2.7564 (2.6632-2.8465)	57.49% (49.49%-65.66%)	33.79% (24.14%-43.75%)	98.163% (94.444%-100%)	96.91% (90%-100%)	46.34% (37.5%-55.56%)	2.7518 (2.5592-2.9402)
At least 6 of CHASM (melanoma), FATHMM (missense), Mutation Assessor, MutationTaster, PROVEAN, VEST	55.58% (51.52%-59.6%)	31.01% (26.04%-35.48%)	98.173% (97.015%-100%)	96.65% (94.29%-100%)	45.13% (40.38%-49.38%)	2.7091 (2.6135-2.8005)	55.47% (47.47%-63.64%)	30.66% (21.54%-40.32%)	98.163% (94.444%-100%)	96.6% (88.24%-100%)	45.19% (36.59%-54.55%)	2.7045 (2.5135-2.8967)
At least 6 of CHASM (melanoma), FATHMM (missense), Mutation Assessor, MutationTaster, SIFT, VEST	63.65% (60.1%-67.17%)	45.26% (40%-50.42%)	95.442% (92.958%-98.592%)	94.5% (91.49%-98.11%)	50.32% (45.65%-55.07%)	2.8535 (2.7480-2.9578)	63.57% (55.56%-71.72%)	45.04% (34.85%-55.74%)	95.351% (89.189%-100%)	94.34% (86.96%-100%)	50.37% (40.85%-60%)	2.8502 (2.6342-3.0623)
At least 6 of CHASM (melanoma), FATHMM (missense), Mutation Assessor, PolyPhen-2, PROVEAN, SIFT	57.96% (54.04%-61.62%)	35.24% (30.16%-40%)	97.223% (95.521%-100%)	95.9% (93.33%-100%)	46.47% (41.77%-50.96%)	2.7464 (2.6512-2.8384)	57.82% (50.51%-65.66%)	34.84% (25%-44.62%)	97.224% (92.105%-100%)	95.59% (88.89%-100%)	46.51% (37.8%-55.7%)	2.7426 (2.5494-2.9366)
At least 6 of CHASM (melanoma), FATHMM (missense), Mutation Assessor, PolyPhen-2, PROVEAN, VEST	55.92% (52.01%-59.6%)	31.53% (26.67%-36.22%)	98.173% (97.015%-100%)	96.7% (94.29%-100%)	45.33% (40.66%-49.69%)	2.7174 (2.6249-2.8078)	55.8% (47.47%-63.64%)	31.16% (21.88%-41.27%)	98.163% (94.444%-100%)	96.65% (88.89%-100%)	45.38% (36.36%-54.67%)	2.7126 (2.5211-2.9050)
At least 6 of CHASM (melanoma), FATHMM (missense), Mutation Assessor, PolyPhen-2, SIFT, VEST	63.99% (60.1%-67.69%)	46.33% (40.94%-51.2%)	94.518% (91.667%-97.263%)	93.58% (90.33%-97.06%)	50.37% (45.77%-55.04%)	2.8462 (2.7382-2.9570)	63.92% (56.57%-71.72%)	46.19% (35.94%-56.06%)	94.431% (88.235%-100%)	93.44% (85.71%-100%)	50.44% (41.13%-60%)	2.8432 (2.6284-3.0526)
At least 6 of CHASM (melanoma), FATHMM (missense), Mutation Assessor, PROVEAN, SIFT, VEST	56.94% (53.03%-61.11%)	33.62% (28.39%-38.02%)	98.173% (97.015%-100%)	96.88% (94.59%-100%)	45.94% (41.35%-50.31%)	2.7412 (2.6563-2.8291)	56.8% (49.49%-64.65%)	33.26% (24.14%-43.33%)	98.163% (94.444%-100%)	96.84% (89.47%-100%)	45.99% (37.33%-55.56%)	2.7375 (2.5549-2.9294)
At least 6 of CHASM (melanoma), FATHMM (missense), MutationTaster, PolyPhen-2, PROVEAN, SIFT	57.61% (53.54%-61.62%)	34.15% (29.31%-39.02%)	98.173% (97.015%-100%)	96.95% (94.74%-100%)	46.3% (41.77%-50.68%)	2.7564 (2.6632-2.8442)	57.49% (49.49%-65.66%)	33.79% (24.14%-43.55%)	98.163% (94.444%-100%)	96.91% (90%-100%)	46.34% (37.5%-55.56%)	2.7518 (2.5576-2.9402)
At least 6 of CHASM (melanoma), FATHMM (missense), MutationTaster, PolyPhen-2, PROVEAN, VEST	55.93% (51.52%-59.6%)	31.5% (26.67%-36.07%)	98.173% (97.015%-100%)	96.7% (94.44%-100%)	45.32% (40.64%-49.69%)	2.7173 (2.6274-2.8098)	55.82% (48.48%-63.64%)	31.13% (21.88%-40.98%)	98.163% (94.444%-100%)	96.65% (88.89%-100%)	45.38% (36.84%-54.88%)	2.7127 (2.5251-2.9015)
At least 6 of CHASM (melanoma), FATHMM (missense), MutationTaster, PolyPhen-2, SIFT, VEST	64.68% (61.11%-68.18%)	47.35% (42.06%-52.38%)	94.518% (91.667%-97.263%)	93.7% (90.57%-97.06%)	50.75% (45.95%-55.3%)	2.8623 (2.7537-2.9680)	64.6% (56.57%-71.72%)	47.14% (37.1%-57.38%)	94.431% (88.235%-100%)	93.56% (85.71%-100%)	50.81% (41.56%-60.53%)	2.8590 (2.6317-3.0785)
At least 6 of CHASM (melanoma), FATHMM (missense), MutationTaster, PROVEAN, SIFT, VEST	57.28% (53.54%-61.11%)	33.63% (28.46%-38.4%)	98.173% (97.015%-100%)	96.91% (94.74%-100%)	46.11% (41.56%-50.33%)	2.7488 (2.6610-2.8392)	57.16% (49.49%-64.65%)	33.26% (23.73%-42.86%)	98.163% (94.444%-100%)	96.88% (89.47%-100%)	46.16% (37.5%-55.26%)	2.7447 (2.5564-2.9310)
At least 6 of CHASM (melanoma), FATHMM (missense), PolyPhen-2, PROVEAN, SIFT, VEST	57.62% (53.54%-61.62%)	34.7% (29.76%-39.47%)	97.223% (95.521%-100%)	95.85% (93.18%-100%)	46.29% (41.56%-50.68%)	2.7390 (2.6434-2.8340)	57.49% (49.49%-65.66%)	34.33% (24.59%-44.26%)	97.224% (92.105%-100%)	95.78% (88.24%-100%)	46.33% (37.5%-55.7%)	2.7354 (2.5440-2.9286)
At least 6 of CHASM (melanoma), Mutation Assessor, MutationTaster, PolyPhen-2, PROVEAN, SIFT	59.3% (55.56%-63.13%)	37.34% (32.33%-42.11%)	97.223% (95.521%-100%)	96.11% (93.75%-100%)	47.21% (42.58%-51.64%)	2.7749 (2.6806-2.8692)	59.23% (51.52%-67.68%)	37.14% (27.27%-47.06%)	97.224% (92.105%-100%)	96.04% (89.47%-100%)	47.29% (38.46%-56.76%)	2.7725 (2.5770-2.9601)
At least 6 of CHASM (melanoma), Mutation Assessor, MutationTaster, PolyPhen-2, PROVEAN, VEST	57.25% (53.03%-61.11%)	33.66% (28.79%-38.46%)	97.223% (95.521%-100%)	95.79% (93.18%-100%)	46.1% (41.38%-50.68%)	2.7229 (2.6312-2.8156)	57.19% (49.49%-65.66%)	33.48% (23.81%-43.33%)	97.224% (92.105%-100%)	95.73% (88.24%-100%)	46.18% (37.33%-55.56%)	2.7208 (2.5227-2.9144)
At least 6 of CHASM (melanoma), Mutation Assessor, MutationTaster, PolyPhen-2, SIFT, VEST	63.31% (59.6%-67.17%)	45.7% (40.48%-50.79%)	93.579% (90.411%-96.97%)	92.47% (89.06%-96.3%)	49.83% (45.1%-54.41%)	2.8150 (2.7056-2.9272)	63.24% (55.56%-71.72%)	45.45% (35%-55.74%)	93.559% (86.486%-100%)	92.35% (83.33%-100%)	49.89% (40.79%-59.47%)	2.8118 (2.5832-3.0400)
At least 6 of CHASM (melanoma), Mutation Assessor, MutationTaster, PROVEAN, SIFT, VEST	58.61% (54.55%-62.63%)	36.27% (31.2%-41.13%)	97.223% (95.521%-100%)	95.98% (93.48%-100%)	46.84% (42.07%-51.33%)	2.7587 (2.6641-2.8532)	58.55% (51.52%-66.67%)	36.08% (26.23%-46.03%)	97.224% (92.105%-100%)	95.92% (88.89%-100%)	46.92% (38.16%-56.41%)	2.7562 (2.5609-2.9495)
At least 6 of CHASM (melanoma), Mutation Assessor, PolyPhen-2, PROVEAN, SIFT, VEST	59.29% (55.05%-63.13%)	37.33% (32.06%-42.28%)	97.223% (95.521%-100%)	96.06% (93.62%-100%)	47.21% (42.48%-51.72%)	2.7747 (2.6780-2.8679)	59.21% (51.52%-67.68%)	37.11% (27.27%-47.06%)	97.224% (92.105%-100%)	96% (88.89%-100%)	47.28% (38.46%-56.58%)	2.7717 (2.5775-2.9630)
At least 6 of CHASM (melanoma), MutationTaster, PolyPhen-2, PROVEAN, SIFT, VEST	59.63% (55.56%-63.64%)	37.87% (32.79%-42.75%)	97.223% (95.521%-100%)	96.11% (93.75%-100%)	47.4% (42.77%-51.97%)	2.7826 (2.6850-2.8786)	59.56% (51.52%-67.68%)	37.65% (27.87%-47.54%)	97.224% (92.105%-100%)	96.05% (89.47%-100%)	47.48% (38.46%-56.76%)	2.7797 (2.5848-2.9706)
At least 6 of FATHMM (cancer), FATHMM (missense), Mutation Assessor, MutationTaster, PolyPhen-2, PROVEAN	56.61% (52.53%-60.1%)	33.62% (28.68%-38.21%)	96.354% (94.203%-98.667%)	94.1% (90.48%-97.87%)	45.64% (40.82%-50%)	2.6971 (2.5920-2.7976)	56.55% (48.48%-64.65%)	33.26% (24.14%-43.1%)	96.276% (91.176%-100%)	93.88% (85.71%-100%)	45.61% (36.99%-54.93%)	2.6909 (2.4728-2.8903)
At least 6 of FATHMM (cancer), FATHMM (missense), Mutation Assessor, MutationTaster, PolyPhen-2, SIFT	66.34% (62.63%-69.7%)	50.56% (45.38%-55.81%)	93.622% (90.541%-96.97%)	92.98% (89.71%-96.67%)	52.27% (47.6%-56.69%)	2.8966 (2.7885-3.0025)	66.3% (58.59%-73.74%)	50.44% (40.63%-60.87%)	93.571% (86.486%-100%)	93.02% (85.19%-100%)	52.36% (42.86%-62.32%)	2.8929 (2.6705-3.1181)
At least 6 of FATHMM (cancer), FATHMM (missense), Mutation Assessor, MutationTaster, PolyPhen-2, VEST	62.3% (58.59%-66.16%)	44.2% (38.89%-49.21%)	93.622% (90.541%-96.97%)	92.24% (88.52%-96.15%)	49.31% (44.69%-53.93%)	2.7967 (2.6903-2.9025)	62.23% (54.55%-70.71%)	43.9% (33.87%-54.24%)	93.571% (86.486%-100%)	92.17% (83.33%-100%)	49.35% (40.26%-58.97%)	2.7932 (2.5705-3.0200)
At least 6 of FATHMM (cancer), FATHMM (missense), Mutation Assessor, MutationTaster, PROVEAN, SIFT	57.59% (54.04%-61.62%)	35.23% (30.4%-40.15%)	96.354% (94.203%-98.667%)	94.5% (91.11%-98.11%)	46.25% (41.67%-50.64%)	2.7194 (2.6218-2.8143)	57.5% (49.49%-65.66%)	34.84% (25.4%-44.62%)	96.276% (91.176%-100%)	94.38% (86.36%-100%)	46.27% (37.5%-55.41%)	2.7139 (2.5053-2.9130)
At least 6 of FATHMM (cancer), FATHMM (missense), Mutation Assessor, MutationTaster, PROVEAN, VEST	55.58% (51.52%-59.6%)	32.06% (27.27%-36.84%)	96.354% (94.203%-98.667%)	93.9% (90.24%-97.78%)	45.06% (40.38%-49.38%)	2.6735 (2.5721-2.7727)	55.51% (47.47%-63.64%)	31.69% (22.41%-41.67%)	96.276% (91.176%-100%)	93.71% (84.62%-100%)	45.07% (36.36%-54.43%)	2.6678 (2.4534-2.8671)
At least 6 of FATHMM (cancer), FATHMM (missense), Mutation Assessor, MutationTaster, SIFT, VEST	63.99% (60.1%-67.68%)	46.8% (41.74%-51.94%)	92.692% (89.474%-96.104%)	91.5% (87.69%-95.45%)	50.24% (45.61%-54.81%)	2.8174 (2.7067-2.9324)	63.91% (56.57%-71.72%)	46.5% (36.07%-56.9%)	92.644% (85%-100%)	91.42% (82.35%-100%)	50.28% (41.03%-59.73%)	2.8133 (2.5770-3.0467)
At least 6 of FATHMM (cancer), FATHMM (missense), Mutation Assessor, PolyPhen-2, PROVEAN, SIFT	57.93% (53.54%-61.62%)	35.77% (30.65%-40.63%)	96.354% (94.203%-98.667%)	94.59% (91.49%-98.11%)	46.45% (41.75%-50.91%)	2.7271 (2.6248-2.8254)	57.84% (50.51%-65.66%)	35.39% (25.81%-45.31%)	96.276% (91.176%-100%)	94.47% (86.36%-100%)	46.47% (37.5%-55.7%)	2.7220 (2.5128-2.9249)
At least 6 of FATHMM (cancer), FATHMM (missense), Mutation Assessor, PolyPhen-2, PROVEAN, VEST	54.92% (50.51%-58.59%)	31.01% (26.12%-35.71%)	96.354% (94.203%-98.667%)	93.72% (90%-97.73%)	44.67% (39.87%-48.96%)	2.6579 (2.5608-2.7576)	54.85% (46.46%-62.63%)	30.64% (21.54%-40.35%)	96.276% (91.176%-100%)	93.52% (84.62%-100%)	44.68% (36.14%-53.85%)	2.6521 (2.4383-2.8613)
At least 6 of FATHMM (cancer), FATHMM (missense), Mutation Assessor, PolyPhen-2, SIFT, VEST	62.97% (59.09%-66.67%)	45.26% (40.16%-50.39%)	92.692% (89.474%-96.104%)	91.26% (87.5%-95.45%)	49.63% (45.07%-54.17%)	2.7924 (2.6804-2.9029)	62.9% (55.56%-70.71%)	44.95% (35%-55.56%)	92.644% (85%-100%)	91.18% (82.35%-100%)	49.67% (40.26%-59.15%)	2.7878 (2.5560-3.0189)
At least 6 of FATHMM (cancer), FATHMM (missense), Mutation Assessor, PROVEAN, SIFT, VEST	55.59% (51.52%-59.6%)	32.06% (27.03%-36.72%)	96.354% (94.203%-98.667%)	93.9% (90.24%-97.78%)	45.06% (40.32%-49.36%)	2.6733 (2.5732-2.7696)	55.5% (47.47%-63.64%)	31.69% (22.41%-41.54%)	96.276% (91.176%-100%)	93.71% (84.62%-100%)	45.07% (36.36%-54.43%)	2.6677 (2.4532-2.8746)
At least 6 of FATHMM (cancer), FATHMM (missense), MutationTaster, PolyPhen-2, PROVEAN, SIFT	57.96% (54.04%-61.62%)	35.76% (30.77%-40.68%)	96.354% (94.203%-98.667%)	94.59% (91.3%-98.08%)	46.47% (41.86%-51.02%)	2.7274 (2.6288-2.8267)	57.87% (50.51%-65.66%)	35.38% (25.4%-45.31%)	96.276% (91.176%-100%)	94.46% (86.36%-100%)	46.5% (37.68%-55.84%)	2.7221 (2.5088-2.9198)
At least 6 of FATHMM (cancer), FATHMM (missense), MutationTaster, PolyPhen-2, PROVEAN, VEST	55.93% (52.02%-59.6%)	32.6% (27.69%-37.5%)	96.354% (94.203%-98.667%)	94.03% (90.48%-97.83%)	45.27% (40.58%-49.68%)	2.6815 (2.5826-2.7797)	55.84% (47.47%-63.64%)	32.23% (23.08%-42.19%)	96.276% (91.176%-100%)	93.85% (85.19%-100%)	45.28% (36.59%-54.55%)	2.6759 (2.4627-2.8800)
At least 6 of FATHMM (cancer), FATHMM (missense), MutationTaster, PolyPhen-2, SIFT, VEST	64.34% (60.61%-68.18%)	47.35% (42.28%-52.59%)	92.692% (89.474%-96.104%)	91.59% (87.93%-95.59%)	50.45% (45.81%-55%)	2.8257 (2.7122-2.9380)	64.26% (56.57%-71.72%)	47.05% (36.67%-57.63%)	92.644% (85%-100%)	91.51% (82.35%-100%)	50.49% (41.1%-60%)	2.8216 (2.5895-3.0501)
At least 6 of FATHMM (cancer), FATHMM (missense), MutationTaster, PROVEAN, SIFT, VEST	56.26% (52.02%-60.1%)	33.14% (28.13%-37.98%)	96.354% (94.203%-98.667%)	94.14% (90.7%-97.87%)	45.45% (40.88%-49.85%)	2.6894 (2.5923-2.7888)	56.17% (48.48%-64.65%)	32.77% (23.33%-42.62%)	96.276% (91.176%-100%)	93.97% (85.71%-100%)	45.47% (36.71%-54.55%)	2.6838 (2.4717-2.8815)
At least 6 of FATHMM (cancer), FATHMM (missense), PolyPhen-2, PROVEAN, SIFT, VEST	54.57% (50.51%-58.59%)	30.47% (25.64%-35.24%)	96.354% (94.203%-98.667%)	93.62% (89.74%-97.56%)	44.48% (39.74%-48.99%)	2.6498 (2.5500-2.7528)	54.49% (46.46%-62.63%)	30.08% (21.05%-39.68%)	96.276% (91.176%-100%)	93.42% (84%-100%)	44.49% (35.96%-53.57%)	2.6440 (2.4290-2.8501)
At least 6 of FATHMM (cancer), Mutation Assessor, MutationTaster, PolyPhen-2, PROVEAN, SIFT	57.94% (53.54%-61.62%)	35.23% (30.3%-40%)	96.354% (94.203%-98.667%)	94.5% (91.3%-98%)	46.45% (41.87%-50.96%)	2.7270 (2.6275-2.8217)	57.9% (49.49%-65.66%)	35.04% (25.81%-45.16%)	96.276% (91.176%-100%)	94.38% (86.36%-100%)	46.51% (37.65%-55.56%)	2.7226 (2.5124-2.9261)
At least 6 of FATHMM (cancer), Mutation Assessor, MutationTaster, PolyPhen-2, PROVEAN, VEST	55.59% (51.52%-59.6%)	31.5% (26.61%-36.36%)	96.354% (94.203%-98.667%)	93.81% (90.48%-97.73%)	45.06% (40.38%-49.38%)	2.6738 (2.5783-2.7743)	55.55% (47.47%-63.64%)	31.33% (22.22%-41.67%)	96.276% (91.176%-100%)	93.66% (84.62%-100%)	45.11% (36.36%-54.43%)	2.6691 (2.4625-2.8754)
At least 6 of FATHMM (cancer), Mutation Assessor, MutationTaster, PolyPhen-2, SIFT, VEST	63.65% (59.6%-67.68%)	46.82% (41.35%-51.97%)	91.699% (88.312%-95.238%)	90.39% (86.21%-94.64%)	49.92% (45.26%-54.42%)	2.7887 (2.6761-2.9088)	63.61% (55.56%-71.72%)	46.66% (36.51%-57.14%)	91.596% (83.871%-100%)	90.26% (80.77%-100%)	49.96% (40.54%-59.49%)	2.7845 (2.5411-3.0135)
At least 6 of FATHMM (cancer), Mutation Assessor, MutationTaster, PROVEAN, SIFT, VEST	56.59% (52.53%-60.61%)	32.62% (27.78%-37.41%)	96.354% (94.203%-98.667%)	94.02% (90.48%-97.83%)	45.64% (41.03%-50%)	2.6971 (2.6012-2.7936)	56.54% (48.48%-64.65%)	32.43% (23.33%-42.86%)	96.276% (91.176%-100%)	93.87% (85.71%-100%)	45.69% (36.99%-55.13%)	2.6924 (2.4790-2.8954)
At least 6 of FATHMM (cancer), Mutation Assessor, PolyPhen-2, PROVEAN, SIFT, VEST	56.91% (52.53%-60.61%)	33.16% (28.1%-37.88%)	96.354% (94.203%-98.667%)	94.11% (90.7%-97.92%)	45.83% (41.18%-50.15%)	2.7049 (2.6070-2.8030)	56.87% (48.48%-64.65%)	32.98% (23.73%-42.86%)	96.276% (91.176%-100%)	93.99% (85.71%-100%)	45.88% (37.18%-55.13%)	2.7003 (2.4919-2.9050)
At least 6 of FATHMM (cancer), MutationTaster, PolyPhen-2, PROVEAN, SIFT, VEST	57.25% (53.54%-61.11%)	33.68% (28.68%-38.52%)	96.354% (94.203%-98.667%)	94.24% (90.91%-98%)	46.02% (41.35%-50.47%)	2.7128 (2.6128-2.8095)	57.21% (49.49%-65.66%)	33.52% (24.14%-43.55%)	96.276% (91.176%-100%)	94.12% (85.71%-100%)	46.08% (37.33%-55.41%)	2.7084 (2.4974-2.9106)
At least 6 of FATHMM (missense), Mutation Assessor, MutationTaster, PolyPhen-2, PROVEAN, SIFT	54.58% (50.51%-58.59%)	29.88% (25%-34.51%)	96.354% (94.203%-98.667%)	93.51% (89.74%-97.44%)	44.29% (39.61%-48.68%)	2.6418 (2.5406-2.7414)	54.46% (46.46%-62.63%)	29.51% (20.31%-39.34%)	96.276% (91.176%-100%)	93.3% (84%-100%)	44.29% (35.8%-53.66%)	2.6359 (2.4186-2.8410)
At least 6 of FATHMM (missense), Mutation Assessor, MutationTaster, PolyPhen-2, PROVEAN, VEST	51.89% (47.98%-56.06%)	26.11% (21.47%-30.71%)	95.426% (92.958%-98.611%)	91.13% (86.49%-96.15%)	42.72% (38.1%-47.08%)	2.5717 (2.4657-2.6777)	51.82% (43.43%-60.61%)	25.71% (17.24%-35.29%)	95.408% (89.474%-100%)	90.99% (80%-100%)	42.74% (34.12%-51.85%)	2.5667 (2.3466-2.7769)
At least 6 of FATHMM (missense), Mutation Assessor, MutationTaster, PolyPhen-2, SIFT, VEST	61.66% (57.58%-65.66%)	43.13% (37.69%-48.12%)	92.692% (89.474%-96.104%)	90.95% (87.1%-95.16%)	48.89% (44.22%-53.49%)	2.7657 (2.6540-2.8754)	61.56% (53.54%-69.7%)	42.79% (32.26%-53.45%)	92.656% (85%-100%)	90.85% (81.25%-100%)	48.9% (39.73%-58.44%)	2.7604 (2.5283-2.9854)
At least 6 of FATHMM (missense), Mutation Assessor, MutationTaster, PROVEAN, SIFT, VEST	53.23% (49.49%-57.07%)	27.75% (23.08%-32.56%)	96.354% (94.203%-98.667%)	93.16% (89.19%-97.3%)	43.53% (38.93%-47.95%)	2.6111 (2.5129-2.7069)	53.15% (44.44%-61.62%)	27.36% (18.97%-36.51%)	96.276% (91.176%-100%)	92.92% (82.61%-100%)	43.53% (35%-52.56%)	2.6055 (2.3847-2.8161)
At least 6 of FATHMM (missense), Mutation Assessor, PolyPhen-2, PROVEAN, SIFT, VEST	52.91% (48.99%-56.57%)	27.2% (22.48%-31.75%)	96.354% (94.203%-98.667%)	93.05% (89.19%-97.22%)	43.35% (38.76%-47.62%)	2.6033 (2.5050-2.6982)	52.82% (44.44%-61.62%)	26.82% (18.18%-36.21%)	96.276% (91.176%-100%)	92.8% (82.61%-100%)	43.35% (34.88%-52.38%)	2.5977 (2.3796-2.8051)
At least 6 of FATHMM (missense), MutationTaster, PolyPhen-2, PROVEAN, SIFT, VEST	54.23% (50%-58.08%)	29.87% (24.81%-34.43%)	95.426% (92.958%-98.611%)	92.25% (88.1%-96.61%)	44.07% (39.39%-48.44%)	2.6225 (2.5166-2.7267)	54.15% (45.45%-62.63%)	29.49% (20.34%-39.06%)	95.408% (89.474%-100%)	92.09% (81.82%-100%)	44.09% (35.37%-53.25%)	2.6181 (2.3917-2.8367)
At least 6 of Mutation Assessor, MutationTaster, PolyPhen-2, PROVEAN, SIFT, VEST	54.92% (51.01%-58.59%)	30.94% (26.05%-35.66%)	95.426% (92.958%-98.611%)	92.52% (88.46%-96.72%)	44.44% (39.74%-48.84%)	2.6380 (2.5349-2.7428)	54.87% (46.46%-62.63%)	30.77% (21.31%-40.98%)	95.408% (89.474%-100%)	92.4% (82.61%-100%)	44.49% (35.9%-53.57%)	2.6345 (2.4069-2.8562)
At least 1 of CHASM (lung), CHASM (melanoma), FATHMM (cancer), FATHMM (missense), Mutation Assessor, MutationTaster, PolyPhen-2	69.36% (65.66%-73.23%)	100% (100%-100%)	16.387% (9.091%-24.127%)	67.41% (63.73%-71.43%)	100% (100%-100%)	2.8379 (2.7605-2.9168)	69.34% (62.63%-76.77%)	100% (100%-100%)	16.748% (7.405%-27.27%)	67.34% (59.34%-74.74%)	100% (100%-100%)	2.8360 (2.7003-2.9781)
At least 1 of CHASM (lung), CHASM (melanoma), FATHMM (cancer), FATHMM (missense), Mutation Assessor, MutationTaster, PROVEAN	67.02% (63.13%-70.71%)	100% (100%-100%)	12.752% (6.165%-19.61%)	66.03% (62.24%-69.93%)	100% (100%-100%)	2.7793 (2.7064-2.8566)	66.99% (59.6%-74.75%)	100% (100%-100%)	13.015% (3.704%-23.92%)	65.96% (58.04%-73.47%)	100% (100%-100%)	2.7772 (2.6381-2.9266)
At least 1 of CHASM (lung), CHASM (melanoma), FATHMM (cancer), FATHMM (missense), Mutation Assessor, MutationTaster, PolyPhen-2, PROVEAN, SIFT, VEST	65.03% (61.11%-68.69%)	100% (100%-100%)	8.226% (4.165%-13.115%)	64.99% (61.37%-68.78%)	100% (100%-100%)	2.7296 (2.6707-2.7935)	64.99% (57.58%-72.73%)	100% (100%-100%)	8.328% (2.128%-16.667%)	64.93% (57.3%-72.63%)	100% (100%-100%)	2.7283 (2.6200-2.8464)
At least 1 of CHASM (lung), CHASM (melanoma), FATHMM (cancer), FATHMM (missense), Mutation Assessor, MutationTaster, SIFT	66.02% (62.12%-69.19%)	100% (100%-100%)	8.226% (4.165%-13.115%)	65.54% (61.78%-69.1%)	100% (100%-100%)	2.7545 (2.6909-2.8173)	65.98% (58.59%-73.74%)	100% (100%-100%)	8.375% (2.128%-16.667%)	65.48% (57.61%-73.2%)	100% (100%-100%)	2.7532 (2.6333-2.8727)
At least 1 of CHASM (lung), CHASM (melanoma), FATHMM (cancer), FATHMM (missense), Mutation Assessor, MutationTaster, VEST	64.68% (60.61%-68.69%)	100% (100%-100%)	5.42% (2.597%-8.219%)	64.64% (60.89%-68.48%)	100% (100%-100%)	2.7087 (2.6535-2.7638)	64.65% (57.58%-72.73%)	100% (100%-100%)	5.651% (0%-12.12%)	64.53% (56.25%-72.16%)	100% (100%-100%)	2.7057 (2.6072-2.8051)
At least 1 of CHASM (lung), CHASM (melanoma), FATHMM (cancer), FATHMM (missense), Mutation Assessor, PolyPhen-2, PROVEAN	67.37% (63.64%-71.21%)	100% (100%-100%)	13.716% (7.143%-21.127%)	66.28% (62.5%-70.22%)	100% (100%-100%)	2.7880 (2.7123-2.8706)	67.33% (59.6%-74.75%)	100% (100%-100%)	14.106% (4.762%-24.39%)	66.21% (58.33%-73.68%)	100% (100%-100%)	2.7859 (2.6439-2.9337)
At least 1 of CHASM (lung), CHASM (melanoma), FATHMM (cancer), FATHMM (missense), Mutation Assessor, PolyPhen-2, SIFT	66.02% (62.12%-69.7%)	100% (100%-100%)	9.162% (4.478%-14.493%)	65.58% (61.78%-69.23%)	100% (100%-100%)	2.7538 (2.6908-2.8236)	65.99% (58.59%-73.74%)	100% (100%-100%)	9.416% (2.174%-18.182%)	65.51% (57.61%-73.2%)	100% (100%-100%)	2.7521 (2.6271-2.8790)
At least 1 of CHASM (lung), CHASM (melanoma), FATHMM (cancer), FATHMM (missense), Mutation Assessor, PolyPhen-2, VEST	64.68% (60.61%-68.69%)	100% (100%-100%)	6.373% (3.077%-10.169%)	64.73% (60.84%-68.38%)	100% (100%-100%)	2.7117 (2.6549-2.7711)	64.65% (57.58%-72.73%)	100% (100%-100%)	6.565% (0%-14.29%)	64.62% (56.52%-72.34%)	100% (100%-100%)	2.7106 (2.6031-2.8257)
At least 1 of CHASM (lung), CHASM (melanoma), FATHMM (cancer), FATHMM (missense), Mutation Assessor, PROVEAN, SIFT	65.03% (61.11%-68.69%)	100% (100%-100%)	7.293% (3.226%-11.765%)	65.08% (61.37%-68.78%)	100% (100%-100%)	2.7311 (2.6707-2.7935)	64.99% (57.58%-72.73%)	100% (100%-100%)	7.379% (2.128%-14.815%)	64.95% (57.3%-72.63%)	100% (100%-100%)	2.7289 (2.6200-2.8464)
At least 1 of CHASM (lung), CHASM (melanoma), FATHMM (cancer), FATHMM (missense), Mutation Assessor, PROVEAN, VEST	65.01% (61.11%-68.69%)	100% (100%-100%)	7.293% (3.226%-11.765%)	64.99% (61.37%-68.78%)	100% (100%-100%)	2.7296 (2.6707-2.7935)	64.98% (57.58%-72.73%)	100% (100%-100%)	7.468% (2.128%-14.815%)	64.92% (57.3%-72.63%)	100% (100%-100%)	2.7283 (2.6200-2.8464)
At least 1 of CHASM (lung), CHASM (melanoma), FATHMM (cancer), FATHMM (missense), Mutation Assessor, SIFT, VEST	64.35% (60.1%-68.19%)	100% (100%-100%)	4.465% (1.754%-7.692%)	64.44% (60.73%-68.13%)	100% (100%-100%)	2.6903 (2.6452-2.7456)	64.33% (56.57%-72.73%)	100% (100%-100%)	4.682% (0%-10.81%)	64.37% (56.04%-72.04%)	100% (100%-100%)	2.6890 (2.5922-2.7800)
At least 1 of CHASM (lung), CHASM (melanoma), FATHMM (cancer), FATHMM (missense), MutationTaster, PolyPhen-2, PROVEAN	67.36% (63.64%-71.21%)	100% (100%-100%)	13.716% (7.143%-21.127%)	66.28% (62.5%-70.22%)	100% (100%-100%)	2.7880 (2.7123-2.8706)	67.33% (59.6%-74.75%)	100% (100%-100%)	14.106% (4.762%-24.39%)	66.21% (58.33%-73.68%)	100% (100%-100%)	2.7859 (2.6439-2.9337)
At least 1 of CHASM (lung), CHASM (melanoma), FATHMM (cancer), FATHMM (missense), MutationTaster, PolyPhen-2, SIFT	66.02% (62.12%-69.7%)	100% (100%-100%)	10.121% (4.762%-15.873%)	65.64% (61.78%-69.23%)	100% (100%-100%)	2.7563 (2.6883-2.8261)	65.99% (58.59%-73.74%)	100% (100%-100%)	10.361% (2.222%-20%)	65.57% (57.61%-73.2%)	100% (100%-100%)	2.7541 (2.6271-2.8837)
At least 1 of CHASM (lung), CHASM (melanoma), FATHMM (cancer), FATHMM (missense), MutationTaster, PolyPhen-2, VEST	64.68% (60.61%-68.69%)	100% (100%-100%)	7.301% (3.4%-11.765%)	64.82% (60.84%-68.38%)	100% (100%-100%)	2.7142 (2.6549-2.7748)	64.65% (57.58%-72.73%)	100% (100%-100%)	7.517% (0%-15.38%)	64.71% (56.52%-72.34%)	100% (100%-100%)	2.7126 (2.6031-2.8257)
At least 1 of CHASM (lung), CHASM (melanoma), FATHMM (cancer), FATHMM (missense), MutationTaster, PROVEAN, SIFT	65.01% (61.11%-68.69%)	100% (100%-100%)	8.226% (4.165%-13.115%)	65.04% (61.37%-68.78%)	100% (100%-100%)	2.7316 (2.6707-2.7969)	64.98% (57.58%-72.73%)	100% (100%-100%)	8.415% (2.128%-16.667%)	64.97% (57.3%-72.63%)	100% (100%-100%)	2.7303 (2.6200-2.8485)
At least 1 of CHASM (lung), CHASM (melanoma), FATHMM (cancer), FATHMM (missense), MutationTaster, PROVEAN, VEST	65.01% (61.11%-68.69%)	100% (100%-100%)	8.226% (4.165%-13.115%)	64.99% (61.37%-68.78%)	100% (100%-100%)	2.7296 (2.6707-2.7935)	64.98% (57.58%-72.73%)	100% (100%-100%)	8.415% (2.128%-16.667%)	64.92% (57.3%-72.63%)	100% (100%-100%)	2.7283 (2.6200-2.8464)
At least 1 of CHASM (lung), CHASM (melanoma), FATHMM (cancer), FATHMM (missense), MutationTaster, SIFT, VEST	64.35% (60.1%-68.19%)	100% (100%-100%)	5.419% (2.353%-8.955%)	64.53% (60.73%-68.13%)	100% (100%-100%)	2.6927 (2.6452-2.7456)	64.33% (56.57%-72.73%)	100% (100%-100%)	5.526% (0%-12.12%)	64.46% (56.04%-72.04%)	100% (100%-100%)	2.6910 (2.5922-2.7853)
At least 1 of CHASM (lung), CHASM (melanoma), FATHMM (cancer), FATHMM (missense), PolyPhen-2, PROVEAN, SIFT	65.03% (61.11%-68.69%)	100% (100%-100%)	8.226% (4.165%-13.115%)	65.04% (61.37%-68.78%)	100% (100%-100%)	2.7316 (2.6707-2.7969)	64.99% (57.58%-72.73%)	100% (100%-100%)	8.415% (2.128%-16.667%)	64.97% (57.3%-72.63%)	100% (100%-100%)	2.7303 (2.6200-2.8485)
At least 1 of CHASM (lung), CHASM (melanoma), FATHMM (cancer), FATHMM (missense), PolyPhen-2, PROVEAN, VEST	65.01% (61.11%-68.69%)	100% (100%-100%)	8.226% (4.165%-13.115%)	64.99% (61.37%-68.78%)	100% (100%-100%)	2.7296 (2.6707-2.7935)	64.98% (57.58%-72.73%)	100% (100%-100%)	8.415% (2.128%-16.667%)	64.92% (57.3%-72.63%)	100% (100%-100%)	2.7283 (2.6200-2.8464)
At least 1 of CHASM (lung), CHASM (melanoma), FATHMM (cancer), FATHMM (missense), PolyPhen-2, SIFT, VEST	64.35% (60.1%-68.19%)	100% (100%-100%)	5.419% (2.353%-8.955%)	64.53% (60.73%-68.13%)	100% (100%-100%)	2.6927 (2.6452-2.7456)	64.33% (56.57%-72.73%)	100% (100%-100%)	5.526% (0%-12.12%)	64.46% (56.04%-72.04%)	100% (100%-100%)	2.6910 (2.5922-2.7853)
At least 1 of CHASM (lung), CHASM (melanoma), FATHMM (cancer), FATHMM (missense), PROVEAN, SIFT, VEST	64.35% (60.1%-68.19%)	100% (100%-100%)	5.419% (2.353%-8.955%)	64.44% (60.73%-68.13%)	100% (100%-100%)	2.6903 (2.6452-2.7456)	64.33% (56.57%-72.73%)	100% (100%-100%)	5.526% (0%-12.12%)	64.37% (56.04%-72.04%)	100% (100%-100%)	2.6890 (2.5922-2.7800)
At least 1 of CHASM (lung), CHASM (melanoma), FATHMM (cancer), Mutation Assessor, MutationTaster, PolyPhen-2, PROVEAN	67.01% (63.13%-70.71%)	100% (100%-100%)	12.752% (6.165%-19.61%)	66.03% (62.24%-69.93%)	100% (100%-100%)	2.7793 (2.7064-2.8566)	66.99% (59.6%-74.75%)	100% (100%-100%)	13.015% (3.704%-23.92%)	65.96% (58.04%-73.47%)	100% (100%-100%)	2.7772 (2.6381-2.9266)
At least 1 of CHASM (lung), CHASM (melanoma), FATHMM (cancer), Mutation Assessor, MutationTaster, PolyPhen-2, SIFT	66.02% (62.12%-69.7%)	100% (100%-100%)	9.162% (4.478%-14.493%)	65.58% (61.78%-69.23%)	100% (100%-100%)	2.7538 (2.6908-2.8236)	65.99% (58.59%-73.74%)	100% (100%-100%)	9.416% (2.174%-18.182%)	65.51% (57.61%-73.2%)	100% (100%-100%)	2.7521 (2.6271-2.8790)
At least 1 of CHASM (lung), CHASM (melanoma), FATHMM (cancer), Mutation Assessor, MutationTaster, PolyPhen-2, VEST	64.68% (60.61%-68.69%)	100% (100%-100%)	6.373% (3.077%-10.169%)	64.73% (60.84%-68.38%)	100% (100%-100%)	2.7117 (2.6549-2.7711)	64.65% (57.58%-72.73%)	100% (100%-100%)	6.565% (0%-14.29%)	64.62% (56.52%-72.34%)	100% (100%-100%)	2.7106 (2.6031-2.8257)
At least 1 of CHASM (lung), CHASM (melanoma), FATHMM (cancer), Mutation Assessor, MutationTaster, PROVEAN, SIFT	65.01% (61.11%-68.69%)	100% (100%-100%)	7.293% (3.226%-11.765%)	64.99% (61.37%-68.78%)	100% (100%-100%)	2.7296 (2.6707-2.7935)	64.98% (57.58%-72.73%)	100% (100%-100%)	7.468% (2.128%-14.815%)	64.92% (57.3%-72.63%)	100% (100%-100%)	2.7283 (2.6200-2.8464)
At least 1 of CHASM (lung), CHASM (melanoma), FATHMM (cancer), Mutation Assessor, MutationTaster, PROVEAN, VEST	65.01% (61.11%-68.69%)	100% (100%-100%)	7.293% (3.226%-11.765%)	64.99% (61.37%-68.78%)	100% (100%-100%)	2.7296 (2.6707-2.7935)	64.98% (57.58%-72.73%)	100% (100%-100%)	7.468% (2.128%-14.815%)	64.92% (57.3%-72.63%)	100% (100%-100%)	2.7283 (2.6200-2.8464)
At least 1 of CHASM (lung), CHASM (melanoma), FATHMM (cancer), Mutation Assessor, MutationTaster, SIFT, VEST	64.35% (60.1%-68.19%)	100% (100%-100%)	4.465% (1.754%-7.692%)	64.44% (60.73%-68.13%)	100% (100%-100%)	2.6903 (2.6452-2.7456)	64.33% (56.57%-72.73%)	100% (100%-100%)	4.682% (0%-10.81%)	64.37% (56.04%-72.04%)	100% (100%-100%)	2.6890 (2.5922-2.7800)
At least 1 of CHASM (lung), CHASM (melanoma), FATHMM (cancer), Mutation Assessor, PolyPhen-2, PROVEAN, SIFT	65.03% (61.11%-68.69%)	100% (100%-100%)	7.293% (3.226%-11.765%)	65.08% (61.37%-68.78%)	100% (100%-100%)	2.7311 (2.6707-2.7935)	64.99% (57.58%-72.73%)	100% (100%-100%)	7.379% (2.128%-14.815%)	64.95% (57.3%-72.63%)	100% (100%-100%)	2.7289 (2.6200-2.8464)
At least 1 of CHASM (lung), CHASM (melanoma), FATHMM (cancer), Mutation Assessor, PolyPhen-2, PROVEAN, VEST	65.01% (61.11%-68.69%)	100% (100%-100%)	7.293% (3.226%-11.765%)	64.99% (61.37%-68.78%)	100% (100%-100%)	2.7296 (2.6707-2.7935)	64.98% (57.58%-72.73%)	100% (100%-100%)	7.468% (2.128%-14.815%)	64.92% (57.3%-72.63%)	100% (100%-100%)	2.7283 (2.6200-2.8464)
At least 1 of CHASM (lung), CHASM (melanoma), FATHMM (cancer), Mutation Assessor, PolyPhen-2, SIFT, VEST	64.35% (60.1%-68.19%)	100% (100%-100%)	4.465% (1.754%-7.692%)	64.44% (60.73%-68.13%)	100% (100%-100%)	2.6903 (2.6452-2.7456)	64.33% (56.57%-72.73%)	100% (100%-100%)	4.682% (0%-10.81%)	64.37% (56.04%-72.04%)	100% (100%-100%)	2.6890 (2.5922-2.7800)
At least 1 of CHASM (lung), CHASM (melanoma), FATHMM (cancer), Mutation Assessor, PROVEAN, SIFT, VEST	64.35% (60.1%-68.19%)	100% (100%-100%)	5.42% (2.597%-8.219%)	64.44% (60.73%-68.13%)	100% (100%-100%)	2.6903 (2.6452-2.7456)	64.33% (56.57%-72.73%)	100% (100%-100%)	5.651% (0%-12.12%)	64.37% (56.04%-72.04%)	100% (100%-100%)	2.6890 (2.5922-2.7800)
At least 1 of CHASM (lung), CHASM (melanoma), FATHMM (cancer), MutationTaster, PolyPhen-2, PROVEAN, SIFT	65.03% (61.11%-68.69%)	100% (100%-100%)	8.226% (4.165%-13.115%)	65.04% (61.37%-68.78%)	100% (100%-100%)	2.7316 (2.6707-2.7969)	64.99% (57.58%-72.73%)	100% (100%-100%)	8.415% (2.128%-16.667%)	64.97% (57.3%-72.63%)	100% (100%-100%)	2.7303 (2.6200-2.8485)
At least 1 of CHASM (lung), CHASM (melanoma), FATHMM (cancer), MutationTaster, PolyPhen-2, PROVEAN, VEST	65.01% (61.11%-68.69%)	100% (100%-100%)	8.226% (4.165%-13.115%)	64.99% (61.37%-68.78%)	100% (100%-100%)	2.7296 (2.6707-2.7935)	64.98% (57.58%-72.73%)	100% (100%-100%)	8.415% (2.128%-16.667%)	64.92% (57.3%-72.63%)	100% (100%-100%)	2.7283 (2.6200-2.8464)
At least 1 of CHASM (lung), CHASM (melanoma), FATHMM (cancer), MutationTaster, PolyPhen-2, SIFT, VEST	64.35% (60.1%-68.19%)	100% (100%-100%)	5.419% (2.353%-8.955%)	64.53% (60.73%-68.13%)	100% (100%-100%)	2.6927 (2.6452-2.7456)	64.33% (56.57%-72.73%)	100% (100%-100%)	5.526% (0%-12.12%)	64.46% (56.04%-72.04%)	100% (100%-100%)	2.6910 (2.5922-2.7853)
At least 1 of CHASM (lung), CHASM (melanoma), FATHMM (cancer), MutationTaster, PROVEAN, SIFT, VEST	64.35% (60.1%-68.19%)	100% (100%-100%)	5.42% (2.597%-8.219%)	64.44% (60.73%-68.13%)	100% (100%-100%)	2.6903 (2.6452-2.7456)	64.33% (56.57%-72.73%)	100% (100%-100%)	5.651% (0%-12.12%)	64.37% (56.04%-72.04%)	100% (100%-100%)	2.6890 (2.5922-2.7800)
At least 1 of CHASM (lung), CHASM (melanoma), FATHMM (cancer), PolyPhen-2, PROVEAN, SIFT, VEST	64.35% (60.1%-68.19%)	100% (100%-100%)	5.42% (2.597%-8.219%)	64.44% (60.73%-68.13%)	100% (100%-100%)	2.6903 (2.6452-2.7456)	64.33% (56.57%-72.73%)	100% (100%-100%)	5.651% (0%-12.12%)	64.37% (56.04%-72.04%)	100% (100%-100%)	2.6890 (2.5922-2.7800)

At least 1 of CHASM (breast), CHASM (lung), CHASM (melanoma), FATHMM (cancer), FATHMM (missense), Mutation Assessor, SIFT, VEST	65.37% (61.62%-69.19%)	100% (100%-100%)	5.484% (2.631%-8.219%)	64.66% (60.82%-68.72%)	100% (100%-100%)	2.7017 (2.6535-2.7534)	65.22% (57.58%-72.73%)	100% (100%-100%)	5.526% (0%-12.12%)	64.5% (56.25%-72.17%)	100% (100%-100%)	2.7048 (2.6054-2.8051)
At least 1 of CHASM (breast), CHASM (lung), CHASM (melanoma), FATHMM (cancer), FATHMM (missense), MutationTaster, PolyPhen-2, PROVEAN	69.03% (65.15%-72.73%)	100% (100%-100%)	15.477% (10.39%-20.27%)	67.17% (63.44%-70.97%)	100% (100%-100%)	2.8264 (2.7588-2.8969)	69.01% (61.62%-76.77%)	100% (100%-100%)	15.794% (6.061%-25.82%)	67.09% (59.34%-74.47%)	100% (100%-100%)	2.8288 (2.6952-2.9683)
At least 1 of CHASM (breast), CHASM (lung), CHASM (melanoma), FATHMM (cancer), FATHMM (missense), MutationTaster, PolyPhen-2, PROVEAN, SIFT	67.37% (63.64%-71.21%)	100% (100%-100%)	10.945% (6.491%-14.866%)	66% (62.23%-70%)	100% (100%-100%)	2.7695 (2.7080-2.8341)	67.28% (59.6%-74.75%)	100% (100%-100%)	11.128% (2.941%-20.46%)	65.88% (57.89%-73.41%)	100% (100%-100%)	2.7706 (2.6573-2.9079)
At least 1 of CHASM (breast), CHASM (lung), CHASM (melanoma), FATHMM (cancer), FATHMM (missense), MutationTaster, PolyPhen-2, PROVEAN, SIFT, VEST	65.69% (62.12%-69.7%)	100% (100%-100%)	6.365% (2.817%-9.459%)	64.87% (61.13%-68.91%)	100% (100%-100%)	2.7124 (2.6595-2.7660)	65.59% (57.58%-72.73%)	100% (100%-100%)	6.513% (0%-13.16%)	64.74% (56.7%-72.17%)	100% (100%-100%)	2.7158 (2.6162-2.8303)
At least 1 of CHASM (breast), CHASM (lung), CHASM (melanoma), FATHMM (cancer), FATHMM (missense), MutationTaster, PolyPhen-2, PROVEAN, VEST	66% (62.12%-69.7%)	100% (100%-100%)	7.218% (3.896%-10.256%)	65.08% (61.14%-69.07%)	100% (100%-100%)	2.7230 (2.6679-2.7784)	65.97% (58.59%-73.74%)	100% (100%-100%)	7.551% (0%-15.15%)	64.99% (56.84%-72.92%)	100% (100%-100%)	2.7273 (2.6270-2.8448)
At least 1 of CHASM (breast), CHASM (lung), CHASM (melanoma), FATHMM (cancer), FATHMM (missense), MutationTaster, PolyPhen-2, SIFT	68.71% (65.15%-72.73%)	100% (100%-100%)	14.601% (9.459%-19.119%)	66.94% (63.1%-70.98%)	100% (100%-100%)	2.8154 (2.7460-2.8885)	68.65% (60.61%-75.76%)	100% (100%-100%)	14.857% (5.714%-25%)	66.84% (58.69%-74.47%)	100% (100%-100%)	2.8170 (2.6815-2.9650)
At least 1 of CHASM (breast), CHASM (lung), CHASM (melanoma), FATHMM (cancer), FATHMM (missense), MutationTaster, PolyPhen-2, SIFT, VEST	66.36% (62.63%-70.2%)	100% (100%-100%)	8.196% (4.286%-11.594%)	65.32% (61.46%-69.43%)	100% (100%-100%)	2.7352 (2.6799-2.7972)	66.27% (58.59%-73.74%)	100% (100%-100%)	8.362% (2.12%-15.79%)	65.19% (56.84%-72.92%)	100% (100%-100%)	2.7376 (2.6270-2.8596)
At least 1 of CHASM (breast), CHASM (lung), CHASM (melanoma), FATHMM (cancer), FATHMM (missense), MutationTaster, PolyPhen-2, VEST	67% (63.13%-70.72%)	100% (100%-100%)	9.941% (5.714%-13.638%)	65.75% (61.9%-69.84%)	100% (100%-100%)	2.7569 (2.6979-2.8202)	67.01% (59.57%-74.75%)	100% (100%-100%)	10.361% (2.941%-18.75%)	65.69% (57.45%-73.41%)	100% (100%-100%)	2.7615 (2.6488-2.8915)
At least 1 of CHASM (breast), CHASM (lung), CHASM (melanoma), FATHMM (cancer), FATHMM (missense), MutationTaster, PROVEAN, SIFT	67.71% (64.14%-71.21%)	100% (100%-100%)	11.875% (7.143%-16.004%)	66.24% (62.5%-70.16%)	100% (100%-100%)	2.7812 (2.7169-2.8493)	67.61% (60.61%-74.75%)	100% (100%-100%)	12.004% (3.331%-21.06%)	66.11% (58.33%-73.63%)	100% (100%-100%)	2.7816 (2.6597-2.9225)
At least 1 of CHASM (breast), CHASM (lung), CHASM (melanoma), FATHMM (cancer), FATHMM (missense), MutationTaster, PROVEAN, SIFT, VEST	65.69% (62.12%-69.7%)	100% (100%-100%)	6.365% (2.817%-9.459%)	64.87% (61.13%-68.91%)	100% (100%-100%)	2.7124 (2.6595-2.7660)	65.59% (57.58%-72.73%)	100% (100%-100%)	6.513% (0%-13.16%)	64.74% (56.7%-72.17%)	100% (100%-100%)	2.7158 (2.6162-2.8303)
At least 1 of CHASM (breast), CHASM (lung), CHASM (melanoma), FATHMM (cancer), FATHMM (missense), MutationTaster, PROVEAN, VEST	66% (62.12%-69.7%)	100% (100%-100%)	7.218% (3.896%-10.256%)	65.08% (61.14%-69.07%)	100% (100%-100%)	2.7230 (2.6679-2.7784)	65.97% (58.59%-73.74%)	100% (100%-100%)	7.551% (0%-15.15%)	64.99% (56.84%-72.92%)	100% (100%-100%)	2.7273 (2.6270-2.8448)
At least 1 of CHASM (breast), CHASM (lung), CHASM (melanoma), FATHMM (cancer), FATHMM (missense), MutationTaster, SIFT, VEST	66.36% (62.63%-70.2%)	100% (100%-100%)	8.196% (4.286%-11.594%)	65.32% (61.46%-69.43%)	100% (100%-100%)	2.7352 (2.6799-2.7972)	66.27% (58.59%-73.74%)	100% (100%-100%)	8.362% (2.12%-15.79%)	65.19% (56.84%-72.92%)	100% (100%-100%)	2.7376 (2.6270-2.8596)
At least 1 of CHASM (breast), CHASM (lung), CHASM (melanoma), FATHMM (cancer), FATHMM (missense), PolyPhen-2, PROVEAN, SIFT	67.69% (64.14%-71.72%)	100% (100%-100%)	11.811% (6.944%-15.942%)	66.22% (62.43%-70.21%)	100% (100%-100%)	2.7804 (2.7144-2.8449)	67.66% (59.6%-74.75%)	100% (100%-100%)	12.136% (3.448%-21.88%)	66.14% (58.06%-73.68%)	100% (100%-100%)	2.7829 (2.6597-2.9247)
At least 1 of CHASM (breast), CHASM (lung), CHASM (melanoma), FATHMM (cancer), FATHMM (missense), PolyPhen-2, PROVEAN, SIFT, VEST	65.69% (62.12%-69.7%)	100% (100%-100%)	6.365% (2.817%-9.459%)	64.87% (61.13%-68.91%)	100% (100%-100%)	2.7124 (2.6595-2.7660)	65.59% (57.58%-72.73%)	100% (100%-100%)	6.513% (0%-13.16%)	64.74% (56.7%-72.17%)	100% (100%-100%)	2.7158 (2.6162-2.8303)
At least 1 of CHASM (breast), CHASM (lung), CHASM (melanoma), FATHMM (cancer), FATHMM (missense), PolyPhen-2, PROVEAN, VEST	66% (62.12%-69.7%)	100% (100%-100%)	7.218% (3.896%-10.256%)	65.08% (61.14%-69.07%)	100% (100%-100%)	2.7230 (2.6679-2.7784)	65.97% (58.59%-73.74%)	100% (100%-100%)	7.551% (0%-15.15%)	64.99% (56.84%-72.92%)	100% (100%-100%)	2.7273 (2.6270-2.8448)
At least 1 of CHASM (breast), CHASM (lung), CHASM (melanoma), FATHMM (cancer), FATHMM (missense), PolyPhen-2, SIFT, VEST	66.36% (62.63%-70.2%)	100% (100%-100%)	8.196% (4.286%-11.594%)	65.32% (61.46%-69.43%)	100% (100%-100%)	2.7352 (2.6799-2.7972)	66.27% (58.59%-73.74%)	100% (100%-100%)	8.362% (2.12%-15.79%)	65.19% (56.84%-72.92%)	100% (100%-100%)	2.7376 (2.6270-2.8596)
At least 1 of CHASM (breast), CHASM (lung), CHASM (melanoma), FATHMM (cancer), FATHMM (missense), PROVEAN, SIFT, VEST	65.69% (62.12%-69.7%)	100% (100%-100%)	6.365% (2.817%-9.459%)	64.87% (61.13%-68.91%)	100% (100%-100%)	2.7124 (2.6595-2.7660)	65.59% (57.58%-72.73%)	100% (100%-100%)	6.513% (0%-13.16%)	64.74% (56.7%-72.17%)	100% (100%-100%)	2.7158 (2.6162-2.8303)
At least 1 of CHASM (breast), CHASM (lung), CHASM (melanoma), FATHMM (cancer), Mutation Assessor, MutationTaster, PolyPhen-2, PROVEAN	71.08% (67.68%-74.75%)	100% (100%-100%)	21.064% (15.582%-26.089%)	68.66% (64.84%-72.78%)	100% (100%-100%)	2.8972 (2.8266-2.9762)	70.97% (63.64%-77.78%)	100% (100%-100%)	21.133% (10.71%-31.72%)	68.52% (60.63%-76.14%)	100% (100%-100%)	2.8965 (2.7527-3.0459)
At least 1 of CHASM (breast), CHASM (lung), CHASM (melanoma), FATHMM (cancer), Mutation Assessor, MutationTaster, PolyPhen-2, PROVEAN, SIFT	69.42% (65.66%-73.23%)	100% (100%-100%)	16.532% (11.59%-21.127%)	67.44% (63.64%-71.51%)	100% (100%-100%)	2.8397 (2.7734-2.9105)	69.25% (61.62%-76.77%)	100% (100%-100%)	16.467% (7.143%-26.19%)	67.26% (59.14%-74.74%)	100% (100%-100%)	2.8374 (2.7118-2.9834)
At least 1 of CHASM (breast), CHASM (lung), CHASM (melanoma), FATHMM (cancer), Mutation Assessor, MutationTaster, PolyPhen-2, PROVEAN, SIFT, VEST	66.07% (62.63%-70.2%)	100% (100%-100%)	7.41% (4.108%-10.667%)	65.13% (61.26%-69.11%)	100% (100%-100%)	2.7254 (2.6739-2.7836)	65.83% (57.58%-72.73%)	100% (100%-100%)	7.189% (0%-13.9%)	64.9% (56.84%-72.45%)	100% (100%-100%)	2.7234 (2.6163-2.8325)
At least 1 of CHASM (breast), CHASM (lung), CHASM (melanoma), FATHMM (cancer), Mutation Assessor, MutationTaster, PolyPhen-2, PROVEAN, VEST	66.39% (62.63%-70.2%)	100% (100%-100%)	8.264% (4.348%-11.594%)	65.34% (61.46%-69.43%)	100% (100%-100%)	2.7360 (2.6815-2.7949)	66.22% (58.59%-73.74%)	100% (100%-100%)	8.227% (2.326%-15.62%)	65.16% (57.14%-72.92%)	100% (100%-100%)	2.7356 (2.6279-2.8536)
At least 1 of CHASM (breast), CHASM (lung), CHASM (melanoma), FATHMM (cancer), Mutation Assessor, MutationTaster, PolyPhen-2, SIFT	70.75% (67.17%-74.75%)	100% (100%-100%)	20.186% (14.667%-25.373%)	68.42% (64.6%-72.58%)	100% (100%-100%)	2.8860 (2.8118-2.9678)	70.62% (62.63%-77.78%)	100% (100%-100%)	20.199% (9.368%-31.43%)	68.26% (60%-75.82%)	100% (100%-100%)	2.8846 (2.7390-3.0446)
At least 1 of CHASM (breast), CHASM (lung), CHASM (melanoma), FATHMM (cancer), Mutation Assessor, MutationTaster, PolyPhen-2, SIFT, VEST	66.41% (62.63%-70.2%)	100% (100%-100%)	8.328% (4.348%-11.769%)	65.35% (61.54%-69.43%)	100% (100%-100%)	2.7368 (2.6834-2.7987)	66.17% (58.59%-73.74%)	100% (100%-100%)	8.101% (0%-15.38%)	65.13% (56.99%-72.73%)	100% (100%-100%)	2.7342 (2.6238-2.8448)
At least 1 of CHASM (breast), CHASM (lung), CHASM (melanoma), FATHMM (cancer), Mutation Assessor, MutationTaster, PolyPhen-2, VEST	67.05% (63.64%-70.72%)	100% (100%-100%)	10.073% (5.797%-13.893%)	65.79% (61.98%-69.79%)	100% (100%-100%)	2.7586 (2.7019-2.8237)	66.91% (59.57%-74.75%)	100% (100%-100%)	10.101% (2.632%-17.95%)	65.63% (57.45%-73.2%)	100% (100%-100%)	2.7584 (2.6401-2.8847)
At least 1 of CHASM (breast), CHASM (lung), CHASM (melanoma), FATHMM (cancer), Mutation Assessor, MutationTaster, PROVEAN, SIFT	69.75% (66.16%-73.74%)	100% (100%-100%)	17.458% (12.5%-22.222%)	67.69% (63.83%-71.82%)	100% (100%-100%)	2.8514 (2.7844-2.9232)	69.58% (61.62%-76.77%)	100% (100%-100%)	17.381% (7.895%-27.27%)	67.5% (59.34%-75.26%)	100% (100%-100%)	2.8490 (2.7143-3.0022)
At least 1 of CHASM (breast), CHASM (lung), CHASM (melanoma), FATHMM (cancer), Mutation Assessor, MutationTaster, PROVEAN, SIFT, VEST	66.41% (62.63%-70.21%)	100% (100%-100%)	8.336% (4.348%-11.765%)	65.35% (61.46%-69.43%)	100% (100%-100%)	2.7369 (2.6815-2.7972)	66.17% (58.56%-73.74%)	100% (100%-100%)	8.103% (2.167%-15.22%)	65.13% (57.14%-72.92%)	100% (100%-100%)	2.7340 (2.6238-2.8536)
At least 1 of CHASM (breast), CHASM (lung), CHASM (melanoma), FATHMM (cancer), Mutation Assessor, MutationTaster, PROVEAN, VEST	66.72% (63.13%-70.71%)	100% (100%-100%)	9.189% (5.479%-12.821%)	65.56% (61.7%-69.63%)	100% (100%-100%)	2.7475 (2.6927-2.8109)	66.55% (58.59%-73.74%)	100% (100%-100%)	9.141% (2.562%-16.28%)	65.38% (57.29%-72.92%)	100% (100%-100%)	2.7464 (2.6292-2.8645)
At least 1 of CHASM (breast), CHASM (lung), CHASM (melanoma), FATHMM (cancer), Mutation Assessor, MutationTaster, SIFT, VEST	66.75% (63.13%-70.71%)	100% (100%-100%)	9.254% (5.479%-12.857%)	65.58% (61.7%-69.63%)	100% (100%-100%)	2.7483 (2.6932-2.8130)	66.5% (58.59%-73.74%)	100% (100%-100%)	9.016% (2.498%-16.67%)	65.35% (57.29%-72.92%)	100% (100%-100%)	2.7449 (2.6270-2.8596)
At least 1 of CHASM (breast), CHASM (lung), CHASM (melanoma), FATHMM (cancer), Mutation Assessor, PolyPhen-2, PROVEAN, SIFT	69.39% (65.66%-73.23%)	99.46% (99.16%-100%)	17.398% (12.162%-21.921%)	67.55% (63.78%-71.59%)	94.94% (90%-100%)	2.7936 (2.6749-2.9053)	69.29% (61.62%-76.77%)	99.48% (98.25%-100%)	17.475% (8.108%-27.91%)	67.41% (59.34%-75%)	95.16% (80%-100%)	2.7953 (2.5272-2.9857)
At least 1 of CHASM (breast), CHASM (lung), CHASM (melanoma), FATHMM (cancer), Mutation Assessor, PolyPhen-2, PROVEAN, SIFT, VEST	66.07% (62.63%-70.2%)	100% (100%-100%)	7.41% (4.108%-10.667%)	65.13% (61.26%-69.11%)	100% (100%-100%)	2.7254 (2.6739-2.7836)	65.83% (57.58%-72.73%)	100% (100%-100%)	7.189% (0%-13.9%)	64.9% (56.84%-72.45%)	100% (100%-100%)	2.7234 (2.6163-2.8325)
At least 1 of CHASM (breast), CHASM (lung), CHASM (melanoma), FATHMM (cancer), Mutation Assessor, PolyPhen-2, PROVEAN, VEST	66.39% (62.63%-70.2%)	100% (100%-100%)	8.264% (4.348%-11.594%)	65.34% (61.46%-69.43%)	100% (100%-100%)	2.7360 (2.6815-2.7949)	66.22% (58.59%-73.74%)	100% (100%-100%)	8.227% (2.326%-15.62%)	65.16% (57.14%-72.92%)	100% (100%-100%)	2.7356 (2.6279-2.8536)
At least 1 of CHASM (breast), CHASM (lung), CHASM (melanoma), FATHMM (cancer), Mutation Assessor, PolyPhen-2, SIFT, VEST	66.41% (62.63%-70.2%)	100% (100%-100%)	8.328% (4.348%-11.769%)	65.35% (61.54%-69.43%)	100% (100%-100%)	2.7368 (2.6834-2.7987)	66.17% (58.59%-73.74%)	100% (100%-100%)	8.101% (0%-15.38%)	65.13% (56.99%-72.73%)	100% (100%-100%)	2.7342 (2.6238-2.8448)
At least 1 of CHASM (breast), CHASM (lung), CHASM (melanoma), FATHMM (cancer), Mutation Assessor, PROVEAN, SIFT, VEST	66.41% (62.63%-70.21%)	100% (100%-100%)	8.336% (4.348%-11.765%)	65.35% (61.46%-69.43%)	100% (100%-100%)	2.7369 (2.6815-2.7972)	66.17% (58.56%-73.74%)	100% (100%-100%)	8.103% (2.167%-15.22%)	65.13% (57.14%-72.92%)	100% (100%-100%)	2.7340 (2.6238-2.8536)
At least 1 of CHASM (breast), CHASM (lung), CHASM (melanoma), FATHMM (cancer), MutationTaster, PolyPhen-2, PROVEAN, SIFT	70.76% (67.17%-74.24%)	100% (100%-100%)	20.209% (15.068%-25.352%)	68.42% (64.8%-72.29%)	100% (100%-100%)	2.8863 (2.8153-2.9591)	70.59% (63.64%-77.78%)	100% (100%-100%)	20.114% (9.677%-30.56%)	68.24% (60.44%-75.56%)	100% (100%-100%)	2.8837 (2.7471-3.0416)
At least 1 of CHASM (breast), CHASM (lung), CHASM (melanoma), FATHMM (cancer), MutationTaster, PolyPhen-2, PROVEAN, SIFT, VEST	66.73% (63.13%-70.71%)	100% (100%-100%)	9.209% (5.332%-12.862%)	65.57% (61.78%-69.48%)	100% (100%-100%)	2.7478 (2.6919-2.8130)	66.53% (58.59%-73.74%)	100% (100%-100%)	9.089% (2.439%-17.07%)	65.37% (57.44%-72.92%)	100% (100%-100%)	2.7464 (2.6292-2.8690)
At least 1 of CHASM (breast), CHASM (lung), CHASM (melanoma), FATHMM (cancer), MutationTaster, PolyPhen-2, PROVEAN, VEST	67.05% (63.62%-70.71%)	100% (100%-100%)	10.062% (5.797%-13.893%)	65.78% (61.98%-69.68%)	100% (100%-100%)	2.7585 (2.6991-2.8237)	66.92% (59.6%-73.76%)	100% (100%-100%)	10.127% (2.701%-18.19%)	65.63% (57.89%-73.2%)	100% (100%-100%)	2.7585 (2.6379-2.8862)
At least 1 of CHASM (breast), CHASM (lung), CHASM (melanoma), FATHMM (cancer), MutationTaster, PolyPhen-2, SIFT, VEST	67.4% (63.64%-71.21%)	100% (100%-100%)	11.04% (6.849%-15.002%)	66.03% (62.23%-70.16%)	100% (100%-100%)	2.7707 (2.7116-2.8422)	67.21% (59.6%-74.75%)	100% (100%-100%)	10.938% (2.778%-19.44%)	65.84% (57.45%-73.4%)	100% (100%-100%)	2.7688 (2.6411-2.9007)
At least 1 of CHASM (breast), CHASM (lung), CHASM (melanoma), FATHMM (cancer), MutationTaster, PROVEAN, SIFT, VEST	67.07% (63.13%-71.21%)	100% (100%-100%)	10.135% (5.882%-14.085%)	65.8% (61.9%-69.79%)	100% (100%-100%)	2.7594 (2.7019-2.8237)	66.87% (58.59%-74.75%)	100% (100%-100%)	10.003% (2.63%-18.18%)	65.6% (57.73%-73.4%)	100% (100%-100%)	2.7574 (2.6399-2.8847)
At least 1 of CHASM (breast), CHASM (lung), CHASM (melanoma), FATHMM (cancer), PolyPhen-2, PROVEAN, SIFT, VEST	66.73% (63.13%-70.71%)	100% (100%-100%)	9.209% (5.332%-12.862%)	65.57% (61.78%-69.48%)	100% (100%-100%)	2.7478 (2.6919-2.8130)	66.53% (58.59%-73.74%)	100% (100%-100%)	9.089% (2.439%-17.07%)	65.37% (57.44%-72.92%)	100% (100%-100%)	2.7464 (2.6292-2.8690)
At least 1 of CHASM (breast), CHASM (lung), CHASM (melanoma), FATHMM (missense), Mutation Assessor, MutationTaster, PolyPhen-2, PROVEAN	70.72% (67.17%-74.24%)	100% (100%-100%)	20.085% (14.706%-25.352%)	68.39% (64.44%-72.43%)	100% (100%-100%)	2.8848 (2.8138-2.9677)	70.68% (63.64%-77.78%)	100% (100%-100%)	20.343% (9.677%-30.56%)	68.3% (60.22%-76.34%)	100% (100%-100%)	2.8864 (2.7417-3.0446)
At least 1 of CHASM (breast), CHASM (lung), CHASM (melanoma), FATHMM (missense), Mutation Assessor, MutationTaster, PolyPhen-2, PROVEAN, SIFT	69.06% (65.15%-72.73%)	100% (100%-100%)	15.553% (10.523%-20.27%)	67.19% (63.29%-71.12%)	100% (100%-100%)	2.8274 (2.7588-2.8997)	68.96% (61.62%-76.77%)	100% (100%-100%)	15.677% (6.452%-25.64%)	67.05% (59.14%-75%)	100% (100%-100%)	2.8273 (2.6927-2.9691)
At least 1 of CHASM (breast), CHASM (lung), CHASM (melanoma), FATHMM (missense), Mutation Assessor, MutationTaster, PolyPhen-2, PROVEAN, SIFT, VEST	65.7% (61.62%-69.7%)	100% (100%-100%)	6.384% (2.857%-9.459%)	64.88% (60.94%-68.88%)	100% (100%-100%)	2.7126 (2.6595-2.7702)	65.57% (57.58%-73.74%)	100% (100%-100%)	6.467% (0%-12.91%)	64.73% (56.38%-72.63%)	100% (100%-100%)	2.7150 (2.6162-2.8308)
At least 1 of CHASM (breast), CHASM (lung), CHASM (melanoma), FATHMM (missense), Mutation Assessor, MutationTaster, PolyPhen-2, PROVEAN, VEST	66.01% (62.12%-69.7%)	100% (100%-100%)	7.238% (3.277%-10.391%)	65.08% (61.14%-69.11%)	100% (100%-100%)	2.7232 (2.6698-2.7784)	65.96% (58.59%-73.74%)	100% (100%-100%)	7.505% (0%-14.29%)	64.98% (56.84%-72.92%)	100% (100%-100%)	2.7268 (2.6238-2.8448)
At least 1 of CHASM (breast), CHASM (lung), CHASM (melanoma), FATHMM (missense), Mutation Assessor, MutationTaster, PolyPhen-2, SIFT	70.4% (66.67%-74.24%)	100% (100%-100%)	19.231% (14.08%-24.365%)	68.16% (64.17%-72.13%)	100% (100%-100%)	2.8739 (2.8003-2.9500)	70.31% (62.63%-77.78%)	100% (100%-100%)	19.354% (8.824%-30.23%)	68.03% (60.21%-75.87%)	100% (100%-100%)	2.8738 (2.7283-3.0419)
At least 1 of CHASM (breast), CHASM (lung), CHASM (melanoma), FATHMM (missense), Mutation Assessor, MutationTaster, PolyPhen-2, SIFT, VEST	66.38% (62.63%-70.2%)	100% (100%-100%)	8.237% (4.284%-11.598%)	65.33% (61.43%-69.43%)	100% (100%-100%)	2.7357 (2.6773-2.7949)	66.23% (58.59%-73.74%)	100% (100%-100%)	8.264% (2.326%-15.79%)	65.17% (56.84%-73.2%)	100% (100%-100%)	2.7360 (2.6270-2.8605)
At least 1 of CHASM (breast), CHASM (lung), CHASM (melanoma), FATHMM (missense), Mutation Assessor, MutationTaster, PolyPhen-2, VEST	67.02% (63.13%-70.71%)	100% (100%-100%)	9.982% (5.797%-13.889%)	65.76% (61.78%-69.79%)	100% (100%-100%)	2.7575 (2.6991-2.8192)	66.97% (59.6%-74.75%)	100% (100%-100%)	10.263% (2.703%-18.19%)	65.67% (57.45%-73.68%)	100% (100%-100%)	2.7602 (2.6467-2.8807)
At least 1 of CHASM (breast), CHASM (lung), CHASM (melanoma), FATHMM (missense), Mutation Assessor, MutationTaster, PROVEAN, SIFT	69.06% (65.15%-72.73%)	100% (100%-100%)	15.553% (10.523%-20.27%)	67.19% (63.29%-71.12%)	100% (100%-100%)	2.8274 (2.7588-2.8997)	68.96% (61.62%-76.77%)	100% (100%-100%)	15.677% (6.452%-25.64%)	67.05% (59.14%-75%)	100% (100%-100%)	2.8273 (2.6927-2.9691)
At least 1 of CHASM (breast), CHASM (lung), CHASM (melanoma), FATHMM (missense), Mutation Assessor, MutationTaster, PROVEAN, SIFT, VEST	65.7% (61.62%-69.7%)	100% (100%-100%)	6.384% (2.857%-9.459%)	64.88% (60.94%-68.88%)	100% (100%-100%)	2.7126 (2.6595-2.7702)	65.57% (57.58%-73.74%)	100% (100%-100%)	6.467% (0%-12.91%)	64.73% (56.38%-72.63%)	100% (100%-100%)	2.7150 (2.6162-2.8308)
At least 1 of CHASM (breast), CHASM (lung), CHASM (melanoma), FATHMM (missense), Mutation Assessor, MutationTaster, PROVEAN, VEST	66.01% (62.12%-69.7%)	100% (100%-100%)	7.238% (3.277%-10.391%)	65.08% (61.14%-69.11%)	100% (100%-100%)	2.7232 (2.6698-2.7784)	65.96% (58.59%-73.74%)	100% (100%-100%)	7.505% (0%-14.29%)	64.98% (56.84%-72.92%)	100% (100%-100%)	2.7268 (2.6238-2.8448)
At least 1 of CHASM (breast), CHASM (lung), CHASM (melanoma), FATHMM (missense), Mutation Assessor, MutationTaster, SIFT, VEST	66.38% (62.63%-70.2%)	100% (100%-100%)	8.237% (4.284%-11.598%)	65.33% (61.34%-69.43%)	100% (100%-100%)	2.7357 (2.6773-2.7949)	66.23% (58.59%-73.74%)	100% (100%-100%)	8.264% (2.326%-15.79%)	65.17% (56.84%-73.2%)	100% (100%-100%)	2.7360 (2.6270-2.8605)
At least 1 of CHASM (breast), CHASM (lung), CHASM (melanoma), FATHMM (missense), Mutation Assessor, PolyPhen-2, PROVEAN, SIFT	69.38% (65.66%-73.23%)	100% (100%-100%)	16.419% (10.958%-21.127%)	67.41% (63.44%-71.35%)	100% (100%-100%)	2.8383 (2.7685-2.9124)	69.33% (61.62%-76.77%)	100% (100%-100%)	16.685% (7.5%-27.59%)	67.32% (59.34%-75.27%)	100% (100%-100%)	2.8401 (2.7081-2.9863)
At least 1 of CHASM (breast), CHASM (lung), CHASM (melanoma), FATHMM (missense), Mutation Assessor, PolyPhen-2, PROVEAN, SIFT, VEST	65.7% (61.62%-69.7%)	100% (100%-100%)	6.384% (2.857%-9.459%)	64.88% (60.94%-68.88%)	100% (100%-100%)	2.7126 (2.6595-2.7702)	65.57% (57.58%-73.74%)	100% (100%-100%)	6.467% (0%-12.91%)	64.73% (56.38%-72.63%)	100% (100%-100%)	2.7150 (2.6162-2.8308)
At least 1 of CHASM (breast), CHASM (lung), CHASM (melanoma), FATHMM (missense), Mutation Assessor, PolyPhen-2, PROVEAN, VEST	66.01% (62.12%-69.7%)	100% (100%-100%)	7.238% (3.277%-10.391%)	65.08% (61.14%-69.11%)	100% (100%-100%)	2.7232 (2.6698-2.7784)	65.96% (58.59%-73.74%)	100% (100%-100%)	7.505% (0%-14.29%)	64.98% (56.84%-72.92%)	100% (100%-100%)	2.7268 (2.6238-2.8448)
At least 1 of CHASM (breast), CHASM (lung), CHASM (melanoma), FATHMM (missense), Mutation Assessor, PolyPhen-2, SIFT, VEST	66.38% (62.63%-70.2%)	100% (100%-100%)	8.237% (4.284%-11.598%)	65.33% (61.34%-69.43%)	100% (100%-100%)	2.7357 (2.6773-2.7949)	66.23% (58.59%-73.74%)	100% (100%-100%)	8.264% (2.326%-15.79%)	65.17% (56.84%-73.2%)	100% (100%-100%)	2.7360 (2.6270-2.8605)
At least 1 of CHASM (breast), CHASM (lung), CHASM (melanoma), FATHMM (missense), Mutation Assessor, PROVEAN, SIFT, VEST	65.7% (61.62%-69.7%)	100% (100%-100%)	6.384% (2.857%-9.459%)	64.88% (60.94%-68.88%)	100% (100%-100%)	2.7126 (2.6595-2.7702)	65.57% (57.58%-73.74%)	100% (100%-100%)	6.467% (0%-12.91%)	64.73% (56.38%-72.63%)	100% (100%-100%)	2.7150 (2.6162-2.8308)
At least 1 of CHASM (breast), CHASM (lung), CHASM (melanoma), FATHMM (missense), MutationTaster, PolyPhen-2, PROVEAN, SIFT	70.06% (66.16%-73.74%)	100% (100%-100%)	18.278% (12.987%-22.976%)	67.91% (64.13%-71.74%)	100% (100%-100%)	2.8618 (2.7876-2.9321)	69.99% (62.63%-77.78%)	100% (100%-100%)	18.477% (8.824%-29.41%)	67.79% (60%-75.28%)	100% (100%-100%)	2.8627 (2.7282-3.0245)
At least 1 of CHASM (breast), CHASM (lung), CHASM (melanoma), FATHMM (missense), MutationTaster, PolyPhen-2, PROVEAN, SIFT, VEST	66.36% (62.63%-70.2%)	100% (100%-100%)	8.183% (4.225%-11.538%)	65.32% (61.46%-69.27%)	100% (100%-100%)	2.7350 (2.6773-2.7917)	66.27% (58.59%-73.74%)	100% (100%-100%)	8.367% (2.498%-15.79%)	65.19% (57.14%-72.92%)	100% (100%-100%)	2.7371 (2.6292-2.8596)
At least 1 of CHASM (breast), CHASM (lung), CHASM (melanoma), FATHMM (missense), MutationTaster, PolyPhen-2, PROVEAN, VEST	66.67% (63.13%-70.2%)	100% (100%-100%)	9.036% (5.193%-12.676%)	65.53% (61.58%-69.47%)	100% (100%-100%)	2.7456 (2.6859-2.8041)	66.66% (59.6%-73.74%)	100% (100%-100%)	9.405% (2.632%-17.24%)	65.45% (57.45%-73.2%)	100% (100%-100%)	2.7494 (2.6411-2.8792)
At least 1 of CHASM (breast), CHASM (lung), CHASM (melanoma), FATHMM (missense), MutationTaster, PolyPhen-2, SIFT, VEST	67.37% (63.64%-71.21%)	100% (100%-100%)	10.949% (6.849%-14.865%)	66.01% (62.23%-70.16%)	100% (100%-100%)	2.7695 (2.7081-2.8341)	67.28% (59.6%-74.75%)	100% (100%-100%)	11.1% (2.941%-19.36%)	65.88% (57.73%-73.96%)	100% (100%-100%)	2.7701 (2.6488-2.9079)
At least 1 of CHASM (breast), CHASM (lung), CHASM (melanoma), FATHMM (missense), MutationTaster, PROVEAN, SIFT, VEST	66.36% (62.63%-70.2%)	100% (100%-100%)	8.183% (4.225%-11.538%)	65.32% (61.46%-69.27%)	100% (100%-100%)	2.7350 (2.6773-2.7917)	66.27% (58.59%-73.74%)	100% (100%-100%)	8.367% (2.498%-15.79%)	65.19% (57.14%-72.92%)	100% (100%-100%)	2.7371 (2.6292-2.8596)
At least 1 of CHASM (breast), CHASM (lung), CHASM (melanoma), FATHMM (missense), PolyPhen-2, PROVEAN, SIFT, VEST	66.36% (62.63%-70.2%)	100% (100%-100%)	8.183% (4.225%-11.538%)	65.32% (61.46%-69.27%)	100% (100%-100%)	2.7350 (2.6773-2.7917)	66.27% (58.59%-73.74%)	100% (100%-100%)	8.367% (2.498%-15.79%)	65.19% (57.14%-72.92%)	100% (100%-100%)	2.7371 (2.6292-2.8596)
At least 1 of CHASM (breast), CHASM (lung), CHASM (melanoma), Mutation Assessor, MutationTaster, PolyPhen-2, PROVEAN, SIFT	72.1% (68.69%-75.76%)	100% (100%-100%)	23.865% (17.806%-29.335%)	69.43% (65.56%-73.22%)	100% (100%-100%)	2.9329 (2.8551-3.0154)	71.96% (64.65%-78.79%)	100% (100%-100%)	23.816% (13.505%-35.49%)	69.26% (61.36%-76.93%)	100% (100%-100%)	2.9307 (2.7804-3.0937)
At least 1 of CHASM (breast), CHASM (lung), CHASM (melanoma), Mutation Assessor, MutationTaster, PolyPhen-2, PROVEAN, SIFT, VEST	66.74% (63.13%-70.71%)	100% (100%-100%)	9.228% (5.478%-12.676%)	65.58% (61.78%-69.63%)	100% (100%-100%)	2.7480 (2.6919-2.8089)	66.52% (58.59%-73.74%)	100% (100%-100%)	9.043% (2.632%-16.67%)	65.36% (57.29%-72.92%)	100% (100%-100%)	2.7455 (2.6379-2.8690)
At least 1 of CHASM (breast), CHASM (lung), CHASM (melanoma), Mutation Assessor, MutationTaster, PolyPhen-2, PROVEAN, VEST	67.05% (63.64%-70.71%)	100% (100%-100%)	10.081% (5.882%-13.699%)	65.79% (61.98%-69.79%)	100% (100%-100%)	2.7587 (2.7039-2.8202)	66.9% (59.6%-73.74%)	100% (100%-100%)	10.081% (2.778%-17.95%)	65.62% (57.6%-73.2%)	100% (100%-100%)	2.7579 (2.6467-2.8764)
At least 1 of CHASM (breast), CHASM (lung), CHASM (melanoma), Mutation Assessor, MutationTaster, PolyPhen-2, SIFT, VEST	67.42% (63.64%-71.21%)	100% (100%-100%)	11.081% (6.944%-15.068%)	66.04% (62.23%-70.16%)	100% (100%-100%)	2.7712 (2.7110-2.8385)	67.18% (59.6%-74.75%)	100% (100%-100%)	10.839% (2.857%-19.06%)	65.81% (57.61%-73.41%)	100% (100%-100%)	2.7671 (2.6449-2.9007)
At least 1 of CHASM (breast), CHASM (lung), CHASM (melanoma), Mutation Assessor, MutationTaster, PROVEAN, SIFT, VEST	67.08% (63.64%-70.72%)	100% (100%-100%)	10.154% (6.023%-13.889%)	65.81% (61.98%-69.84%)	100% (100%-100%)	2.7596 (2.7019-2.8231)	66.85% (59.57%-73.74%)	100% (100%-100%)	9.957% (2.703%-17.65%)	65.59% (57.45%-73.2%)	100% (100%-100%)	2.7563 (2.6438-2.8847)
At least 1 of CHASM (breast), CHASM (lung), CHASM (melanoma), Mutation Assessor, PolyPhen-2, PROVEAN, SIFT, VEST	66.74% (63.13%-70.71%)	100% (100%-100%)	9.228% (5.478%-12.676%)	65.58% (61.78%-69.63%)	100% (100%-100%)	2.7480 (2.6919-2.8089)	66.52% (58.59%-73.74%)	100% (100%-100%)	9.043% (2.632%-16.67%)	65.36% (57.29%-72.92%)	100% (100%-100%)	2.7455 (2.6379-2.8690)
At least 1 of CHASM (breast), CHASM (lung), CHASM (melanoma), MutationTaster, PolyPhen-2, PROVEAN, SIFT, VEST	67.4% (63.64%-71.21%)	100% (100%-100%)	11.027% (6.847%-14.866%)	66.03% (62.23%-70%)	100% (100%-100%)	2.7705 (2.7081-2.8385)	67.22% (59.6%-74.75%)	100% (100%-100%)	10.943% (2.857%-19.51%)	65.84% (57.89%-73.4%)	100% (100%-100%)	2.7689 (2.6467-2.9060)
At least 1 of CHASM (breast), CHASM (lung), FATHMM (cancer), FATHMM (missense), Mutation Assessor, MutationTaster, PolyPhen-2, PROVEAN	68.69% (65.15%-72.23%)	100% (100%-100%)	14.549% (9.459%-19.178%)	66.93% (63.16%-70.9%)	100% (100%-100%)	2.8148 (2.7503-2.8885)	68.68% (61.59%-75.76%)	100% (100%-100%)	14.897% (5.71%-24.41%)	66.85% (58.88%-74.23%)	100% (100%-100%)	2.8177 (2.6856-2.9516)
At least 1 of CHASM (breast), CHASM (lung), FATHMM (cancer), FATHMM (missense), Mutation Assessor, MutationTaster, PolyPhen-2, PROVEAN, SIFT	66.7% (63.13%-70.71%)	100% (100%-100%)	9.133% (5.125%-12.857%)	65.55% (61.78%-69.63%)	100% (100%-100%)	2.7468 (2.6871-2.8059)	66.59% (58.59%-73.74%)	100% (100%-100%)	9.251% (2.5%-18.18%)	65.41% (57.29%-72.92%)	100% (100%-100%)	2.7477 (2.6360-2.8755)
At least 1 of CHASM (breast), CHASM (lung), FATHMM (cancer), FATHMM (missense), Mutation Assessor, MutationTaster, PolyPhen-2, PROVEAN, SIFT, VEST	65.03% (61.11%-69.19%)	100% (100%-100%)	4.567% (1.428%-7.042%)	64.44% (60.51%-68.53%)	100% (100%-100%)	2.6904 (2.6429-2.7409)	64.89% (56.57%-72.73%)	100% (100%-100%)	4.613% (0%-10.53%)	64.28% (56.12%-72.16%)	100% (100%-100%)	2.6949 (2.6011-2.7917)
At least 1 of CHASM (breast), CHASM (lung), FATHMM (cancer), FATHMM (missense), Mutation Assessor, MutationTaster, PolyPhen-2, PROVEAN, VEST	65.67% (62.12%-69.7%)	100% (100%-100%)	6.304% (2.817%-9.377%)	64.86% (61.14%-68.91%)	100% (100%-100%)	2.7118 (2.6597-2.7660)	65.63% (57.58%-72.73%)	100% (100%-100%)	6.631% (0%-13.16%)	64.77% (56.7%-72.16%)	100% (100%-100%)	2.7168 (2.6162-2.8289)
At least 1 of CHASM (breast), CHASM (lung), FATHMM (cancer), FATHMM (missense), Mutation Assessor, MutationTaster, PolyPhen-2, SIFT	67.71% (64.14%-71.72%)	100% (100%-100%)	11.876% (7.143%-16%)	66.24% (62.43%-70.37%)	100% (100%-100%)	2.7812 (2.7169-2.8493)	67.61% (59.6%-74.75%)	100% (100%-100%)	12.044% (3.123%-21.05%)	66.12% (58.06%-73.47%)	100% (100%-100%)	2.7820 (2.6582-2.9225)
At least 1 of CHASM (breast), CHASM (lung), FATHMM (cancer), FATHMM (missense), Mutation Assessor, MutationTaster, PolyPhen-2, SIFT, VEST	65.37% (61.62%-69.19%)	100% (100%-100%)	5.484% (2.631%-8.219%)	64.66% (60.82%-68.72%)	100% (100%-100%)	2.7017 (2.6535-2.7534)	65.22% (57.58%-72.73%)	100% (100%-100%)	5.526% (0%-12.12%)	64.5% (56.25%-72.17%)	100% (100%-100%)	2.7048 (2.6054-2.8051)
At least 1 of CHASM (breast), CHASM (lung), FATHMM (cancer), FATHMM (missense), Mutation Assessor, MutationTaster, PolyPhen-2, VEST	66.33% (62.63%-70.2%)	100% (100%-100%)	8.114% (4.286%-11.598%)	65.3% (61.46%-69.43%)	100% (100%-100%)	2.7342 (2.6815-2.7950)	66.32% (58.59%-73.74%)	100% (100%-100%)	8.505% (2.381%-15.79%)	65.23% (56.84%-72.92%)	100% (100%-100%)	2.7391 (2.6270-2.8536)
At least 1 of CHASM (breast), CHASM (lung), FATHMM (cancer), FATHMM (missense), Mutation Assessor, MutationTaster, PROVEAN, SIFT	66.7% (63.13%-70.71%)	100% (100%-100%)	9.133% (5.125%-12.857%)	65.55% (61.78%-69.63%)	100% (100%-100%)	2.7468 (2.6871-2.8059)	66.59% (58.59%-73.74%)	100% (100%-100%)	9.251% (2.5%-18.18%)	65.41% (57.29%-72.92%)	100% (100%-100%)	2.7477 (2.6360-2.8755)
At least 1 of CHASM (breast), CHASM (lung), FATHMM (cancer), FATHMM (missense), Mutation Assessor, MutationTaster, PROVEAN, SIFT, VEST	65.03% (61.11%-69.19%)	100% (100%-100%)	4.567% (1.428%-7.042%)	64.44% (60.51%-68.53%)	100% (100%-100%)	2.6904 (2.6429-2.7409)	64.89% (56.57%-72.73%)	100% (100%-100%)	4.613% (0%-10.53%)	64.28% (56.12%-72.16%)	100% (100%-100%)	2.6949 (2.6011-2.7917)
At least 1 of CHASM (breast), CHASM (lung), FATHMM (cancer), FATHMM (missense), Mutation Assessor, MutationTaster, PROVEAN, VEST	65.67% (62.12%-69.7%)	100% (100%-100%)	6.304% (2.817%-9.377%)	64.86% (61.14%-68.91%)	100% (100%-100%)	2.7118 (2.6597-2.7660)	65.63% (57.58%-72.73%)	100% (100%-100%)	6.631% (0%-13.16%)	64.77% (56.7%-72.16%)	100% (100%-100%)	2.7168 (2.6162-2.8289)
At least 1 of CHASM (breast), CHASM (lung), FATHMM (cancer), FATHMM (missense), Mutation Assessor, MutationTaster, SIFT, VEST	65.37% (61.62%-69.19%)	100% (100%-100%)	5.484% (2.631%-8.219%)	64.66% (60.82%-68.72%)	100% (100%-100%)	2.7017 (2.6535-2.7534)	65.22% (57.58%-72.73%)	100% (100%-100%)	5.526% (0%-12.12%)	64.5% (56.25%-72.17%)	100% (100%-100%)	2.7048 (2.6054-2.8051)
At least 1 of CHASM (breast), CHASM (lung), FATHMM (cancer), FATHMM (missense), Mutation Assessor, PolyPhen-2, PROVEAN, SIFT	67.02% (63.13%-71.21%)	100% (100%-100%)	9.999% (5.556%-13.889%)	65.77% (61.98%-69.79%)	100% (100%-100%)	2.7577 (2.6957-2.8202)	66.96% (58.59%-74.75%)	100% (100%-100%)	10.26% (2.703%-19.05%)	65.67% (57.72%-73.2%)	100% (100%-100%)	2.7601 (2.6449-2.8915)
At least 1 of CHASM (breast), CHASM (lung), FATHMM (cancer), FATHMM (missense), Mutation Assessor, PolyPhen-2, PROVEAN, SIFT, VEST	65.03% (61.11%-69.19%)	100% (100%-100%)	4.567% (1.428%-7.042%)	64.44% (60.51%-68.53%)	100% (100%-100%)	2.6904 (2.6429-2.7409)	64.89% (56.57%-72.73%)	100% (100%-100%)	4.613% (0%-10.53%)	64.28% (56.12%-72.16%)	100% (100%-100%)	2.6949 (2.6011-2.7917)
At least 1 of CHASM (breast), CHASM (lung), FATHMM (cancer), FATHMM (missense), Mutation Assessor, PolyPhen-2, PROVEAN, VEST	65.67% (62.12%-69.7%)	100% (100%-100%)	6.304% (2.817%-9.377%)	64.86% (61.14%-68.91%)	100% (100%-100%)	2.7118 (2.6597-2.7660)	65.63% (57.58%-72.73%)	100% (100%-100%)	6.631% (0%-13.16%)	64.77% (56.7%-72.16%)	100% (100%-100%)	2.7168 (2.6162-2.8289)
At least 1 of CHASM (breast), CHASM (lung), FATHMM (cancer), FATHMM (missense), Mutation Assessor, PolyPhen-2, SIFT, VEST	65.37% (61.62%-69.19%)	100% (100%-100%)	5.484% (2.631%-8.219%)	64.66% (60.82%-68.72%)	100% (100%-100%)	2.7017 (2.6535-2.7534)	65.22% (57.58%-72.73%)	100% (100%-100%)	5.526% (0%-12.12%)	64.5% (56.25%-72.17%)	100% (100%-100%)	2.7048 (2.6054-2.8051)
At least 1 of CHASM (breast), CHASM (lung), FATHMM (cancer), FATHMM (missense), Mutation Assessor, PROVEAN, SIFT, VEST	65.03% (61.11%-69.19%)	100% (100%-100%)	4.567% (1.428%-7.042%)	64.44% (60.51%-68.53%)	100% (100%-100%)	2.6904 (2.6429-2.7409)	64.89% (56.57%-72.73%)	100% (100%-100%)	4.613% (0%-10.53%)	64.28% (56.12%-72.16%)	100% (100%-100%)	2.6949 (2.6011-2.7917)
At least 1 of CHASM (breast), CHASM (lung), FATHMM (cancer), FATHMM (missense), MutationTaster, PolyPhen-2, PROVEAN, SIFT	67.7% (64.14%-71.72%)	100% (100%-100%)	11.858% (7.042%-16.176%)	66.24% (62.5%-70.22%)	100% (100%-100%)	2.7809 (2.7162-2.8493)	67.62% (59.6%-74.75%)	100% (100%-100%)	12.051% (3.226%-21.63%)	66.12% (58.06%-73.68%)	100% (100%-100%)	2.7821 (2.6597-2.9247)
At least 1 of CHASM (breast), CHASM (lung), FATHMM (cancer), FATHMM (missense), MutationTaster, PolyPhen-2, PROVEAN, SIFT, VEST	65.69% (62.12%-69.7%)	100% (100%-100%)	6.365% (2.817%-9.459%)	64.87% (61.13%-68.91%)	100% (100%-100%)	2.7124 (2.6595-2.7660)	65.59% (57.58%-72.73%)	100% (100%-100%)	6.513% (0%-13.16%)	64.74% (56.7%-72.17%)	100% (100%-100%)	2.7158 (2.6162-2.8303)
At least 1 of CHASM (breast), CHASM (lung), FATHMM (cancer), FATHMM (missense), MutationTaster, PolyPhen-2, PROVEAN, VEST	66.33% (62.63%-70.2%)	100% (100%-100%)	8.103% (4.225%-11.431%)	65.3% (61.46%-69.27%)	100% (100%-100%)	2.7340 (2.6773-2.7949)	66.33% (58.59%-73.74%)	100% (100%-100%)	8.531% (2.5%-15.91%)	65.23% (57.28%-72.92%)	100% (100%-100%)	2.7387 (2.6270-2.8611)
At least 1 of CHASM (breast), CHASM (lung), FATHMM (cancer), FATHMM (missense), MutationTaster, PolyPhen-2, SIFT, VEST	66.36% (62.63%-70.2%)	100% (100%-100%)	8.196% (4.286%-11.594%)	65.32% (61.46%-69.43%)	100% (100%-100%)	2.7352 (2.6799-2.7972)	66.27% (58.59%-73.74%)	100% (100%-100%)	8.362% (2.12%-15.79%)	65.19% (56.84%-72.92%)	100% (100%-100%)	2.7376 (2.6270-2.8596)
At least 1 of CHASM (breast), CHASM (lung), FATHMM (cancer), FATHMM (missense), MutationTaster, PROVEAN, SIFT, VEST	65.69% (62.12%-69.7%)	100% (100%-100%)	6.365% (2.817%-9.459%)	64.87% (61.13%-68.91%)	100% (100%-100%)	2.7124 (2.6595-2.7660)	65.59% (57.58%-72.73%)	100% (100%-100%)	6.513% (0%-13.16%)	64.74% (56.7%-72.17%)	100% (100%-100%)	2.7158 (2.6162-2.8303)
At least 1 of CHASM (breast), CHASM (lung), FATHMM (cancer), FATHMM (missense), PolyPhen-2, PROVEAN, SIFT, VEST	65.69% (62.12%-69.7%)	100% (100%-100%)	6.365% (2.817%-9.459%)	64.87% (61.13%-68.91%)	100% (100%-100%)	2.7124 (2.6595-2.7660)	65.59% (57.58%-72.73%)	100% (100%-100%)	6.513% (0%-13.16%)	64.74% (56.7%-72.17%)	100% (100%-100%)	2.7158 (2.6162-2.8303)
At least 1 of CHASM (breast), CHASM (lung), FATHMM (cancer), Mutation Assessor, MutationTaster, PolyPhen-2, PROVEAN, SIFT	69.75% (66.16%-73.24%)	100% (100%-100%)	17.445% (12.5%-21.921%)	67.68% (63.93%-71.66%)	100% (100%-100%)	2.8513 (2.7827-2.9223)	69.59% (62.6%-76.77%)	100% (100%-100%)	17.391% (7.89%-27.27%)	67.51% (59.34%-75%)	100% (100%-100%)	2.8491 (2.7169-3.0048)
At least 1 of CHASM (breast), CHASM (lung), FATHMM (cancer), Mutation Assessor, MutationTaster, PolyPhen-2, PROVEAN, SIFT, VEST	66.07% (62.63%-70.2%)	100% (100%-100%)	7.41% (4.108%-10.667%)	65.13% (61.26%-69.11%)	100% (100%-100%)	2.7254 (2.6739-2.7836)	65.83% (57.58%-72.73%)	100% (100%-100%)	7.189% (0%-13.9%)	64.9% (56.84%-72.45%)	100% (100%-100%)	2.7234 (2.6163-2.8325)
At least 1 of CHASM (breast), CHASM (lung), FATHMM (cancer), Mutation Assessor, MutationTaster, PolyPhen-2, PROVEAN, VEST	67.05% (63.64%-70.71%)	100% (100%-100%)	10.072% (5.968%-13.889%)	65.79% (62.03%-69.79%)	100% (100%-100%)	2.7586 (2.6990-2.8237)	66.91% (59.6%-73.74%)	100% (100%-100%)	10.124% (2.703%-18.18%)	65.63% (57.73%-73.12%)	100% (100%-100%)	2.7588 (2.6461-2.8847)
At least 1 of CHASM (breast), CHASM (lung), FATHMM (cancer), Mutation Assessor, MutationTaster, PolyPhen-2, SIFT, VEST	66.41% (62.63%-70.2%)	100% (100%-100%)	8.328% (4.348%-11.769%)	65.35% (61.54%-69.43%)	100% (100%-100%)	2.7368 (2.6834-2.7987)	66.17% (58.59%-73.74%)	100% (100%-100%)	8.101% (0%-15.38%)	65.13% (56.99%-72.73%)	100% (100%-100%)	2.7342 (2.6238-2.8448)
At least 1 of CHASM (breast), CHASM (lung), FATHMM (cancer), Mutation Assessor, MutationTaster, PROVEAN, SIFT, VEST	66.41% (62.63%-70.21%)	100% (100%-100%)	8.336% (4.348%-11.765%)	65.35% (61.46%-69.43%)	100% (100%-100%)	2.7369 (2.6815-2.7972)	66.17% (58.56%-73.74%)	100% (100%-100%)	8.103% (2.167%-15.22%)	65.13% (57.14%-72.92%)	100% (100%-100%)	2.7340 (2.6238-2.8536)
At least 1 of CHASM (breast), CHASM (lung), FATHMM (cancer), Mutation Assessor, PolyPhen-2, PROVEAN, SIFT, VEST	66.07% (62.63%-70.2%)	100% (100%-100%)	7.41% (4.108%-10.667%)	65.13% (61.26%-69.11%)	100% (100%-100%)	2.7254 (2.6739-2.7836)	65.83% (57.58%-72.73%)	100% (100%-100%)	7.189% (0%-13.9%)	64.9% (56.84%-72.45%)	100% (100%-100%)	2.7234 (2.6163-2.8325)
At least 1 of CHASM (breast), CHASM (lung), FATHMM (cancer), MutationTaster, PolyPhen-2, PROVEAN, SIFT, VEST	66.73% (63.13%-70.71%)	100% (100%-100%)	9.209% (5.332%-12.862%)	65.57% (61.78%-69.48%)	100% (100%-100%)	2.7478 (2.6919-2.8130)	66.53% (58.59%-73.74%)	100% (100%-100%)	9.089% (2.439%-17.07%)	65.37% (57.44%-72.92%)	100% (100%-100%)	2.7464 (2.6292-2.8690)
At least 1 of CHASM (breast), CHASM (lung), FATHMM (missense), Mutation Assessor, MutationTaster, PolyPhen-2, PROVEAN, SIFT	69.39% (65.66%-72.74%)	100% (100%-100%)	16.466% (11.429%-21.215%)	67.43% (63.44%-71.28%)	100% (100%-100%)	2.8389 (2.7702-2.9124)	69.3% (62.6%-76.77%)	100% (100%-100%)	16.601% (7.135%-26.47%)	67.3% (59.34%-75.27%)	100% (100%-100%)	2.8390 (2.7050-2.9869)
At least 1 of CHASM (breast), CHASM (lung), FATHMM (missense), Mutation Assessor, MutationTaster, PolyPhen-2, PROVEAN, SIFT, VEST	65.7% (61.62%-69.7%)	100% (100%-100%)	6.384% (2.857%-9.459%)	64.88% (60.94%-68.88%)	100% (100%-100%)	2.7126 (2.6595-2.7702)	65.57% (57.58%-73.74%)	100% (100%-100%)	6.467% (0%-12.91%)	64.73% (56.38%-72.63%)	100% (100%-100%)	2.7150 (2.6162-2.8308)
At least 1 of CHASM (breast), CHASM (lung), FATHMM (missense), Mutation Assessor, MutationTaster, PolyPhen-2, PROVEAN, VEST	66.34% (62.63%-70.2%)	100% (100%-100%)	8.122% (4.167%-11.594%)	65.3% (61.46%-69.39%)	100% (100%-100%)	2.7342 (2.6799-2.7949)	66.32% (58.59%-73.74%)	100% (100%-100%)	8.485% (2.439%-16.22%)	65.22% (57.14%-72.92%)	100% (100%-100%)	2.7386 (2.6270-2.8605)
At least 1 of CHASM (breast), CHASM (lung), FATHMM (missense), Mutation Assessor, MutationTaster, PolyPhen-2, SIFT, VEST	66.38% (62.63%-70.2%)	100% (100%-100%)	8.237% (4.284%-11.598%)	65.33% (61.34%-69.43%)	100% (100%-100%)	2.7357 (2.6773-2.7949)	66.23% (58.59%-73.74%)	100% (100%-100%)	8.264% (2.326%-15.79%)	65.17% (56.84%-73.2%)	100% (100%-100%)	2.7360 (2.6270-2.8605)
At least 1 of CHASM (breast), CHASM (lung), FATHMM (missense), Mutation Assessor, MutationTaster, PROVEAN, SIFT, VEST	65.7% (61.62%-69.7%)	100% (100%-100%)	6.384% (2.857%-9.459%)	64.88% (60.94%-68.88%)	100% (100%-100%)	2.7126 (2.6595-2.7702)	65.57% (57.58%-73.74%)	100% (100%-100%)	6.467% (0%-12.91%)	64.73% (56.38%-72.63%)	100% (100%-100%)	2.7150 (2.6162-2.8308)
At least 1 of CHASM (breast), CHASM (lung), FATHMM (missense), Mutation Assessor, PolyPhen-2, PROVEAN, SIFT, VEST	65.7% (61.62%-69.7%)	100% (100%-100%)	6.384% (2.857%-9.459%)	64.88% (60.94%-68.88%)	100% (100%-100%)	2.7126 (2.6595-2.7702)	65.57% (57.58%-73.74%)	100% (100%-100%)	6.467% (0%-12.91%)	64.73% (56.38%-72.63%)	100% (100%-100%)	2.7150 (2.6162-2.8308)
At least 1 of CHASM (breast), CHASM (lung), FATHMM (missense), MutationTaster, PolyPhen-2, PROVEAN, SIFT, VEST	66.36% (62.63%-70.2%)	100% (100%-100%)	8.183% (4.225%-11.538%)	65.32% (61.46%-69.27%)	100% (100%-100%)	2.7350 (2.6773-2.7917)	66.27% (58.59%-73.74%)	100% (100%-100%)	8.367% (2.498%-15.79%)	65.19% (57.14%-72.92%)	100% (100%-100%)	2.7371 (2.6292-2.8596)
At least 1 of CHASM (breast), CHASM (lung), Mutation Assessor, MutationTaster, PolyPhen-2, PROVEAN, SIFT, VEST	66.74% (63.13%-70.71%)	100% (100%-100%)	9.228% (5.478%-12.676%)	65.58% (61.78%-69.63%)	100% (100%-100%)	2.7480 (2.6919-2.8089)	66.52% (58.59%-73.74%)	100% (100%-100%)	9.043% (2.632%-16.67%)	65.36% (57.29%-72.92%)	100% (100%-100%)	2.7455 (2.6379-2.8690)
At least 1 of CHASM (breast), CHASM (melanoma), FATHMM (cancer), FATHMM (missense), Mutation Assessor, MutationTaster, PolyPhen-2, PROVEAN	68.03% (64.63%-71.72%)	100% (100%-100%)	12.752% (8.105%-16.901%)	66.46% (62.77%-70.43%)	100% (100%-100%)	2.7922 (2.7269-2.8597)	67.98% (60.61%-74.77%)	100% (100%-100%)	12.994% (5%-22.58%)	66.36% (58.33%-73.91%)	100% (100%-100%)	2.7938 (2.6706-2.9319)
At least 1 of CHASM (breast), CHASM (melanoma), FATHMM (cancer), FATHMM (missense), Mutation Assessor, MutationTaster, PolyPhen-2, PROVEAN, SIFT	66.37% (62.63%-70.2%)	100% (100%-100%)	8.22% (4.165%-11.594%)	65.33% (61.54%-69.43%)	100% (100%-100%)	2.7355 (2.6764-2.7972)	66.25% (58.59%-73.74%)	100% (100%-100%)	8.328% (2.439%-16.29%)	65.18% (56.84%-72.92%)	100% (100%-100%)	2.7366 (2.6238-2.8596)
At least 1 of CHASM (breast), CHASM (melanoma), FATHMM (cancer), FATHMM (missense), Mutation Assessor, MutationTaster, PolyPhen-2, PROVEAN, SIFT, VEST	65.03% (61.11%-69.19%)	100% (100%-100%)	4.567% (1.428%-7.042%)	64.44% (60.51%-68.53%)	100% (100%-100%)	2.6904 (2.6429-2.7409)	64.89% (56.57%-72.73%)	100% (100%-100%)	4.613% (0%-10.53%)	64.28% (56.12%-72.16%)	100% (100%-100%)	2.6949 (2.6011-2.7917)
At least 1 of CHASM (breast), CHASM (melanoma), FATHMM (cancer), FATHMM (missense), Mutation Assessor, MutationTaster, PolyPhen-2, PROVEAN, VEST	65.34% (61.62%-69.19%)	100% (100%-100%)	5.42% (2.597%-8.219%)	64.64% (60.82%-68.72%)	100% (100%-100%)	2.7009 (2.6535-2.7534)	65.27% (57.58%-72.73%)	100% (100%-100%)	5.651% (0%-12.12%)	64.53% (56.25%-72.16%)	100% (100%-100%)	2.7057 (2.6072-2.8051)
At least 1 of CHASM (breast), CHASM (melanoma), FATHMM (cancer), FATHMM (missense), Mutation Assessor, MutationTaster, PolyPhen-2, SIFT	67.37% (63.64%-71.21%)	100% (100%-100%)	10.963% (6.577%-14.866%)	66.01% (62.23%-70%)	100% (100%-100%)	2.7697 (2.7080-2.8375)	67.27% (59.6%-74.75%)	100% (100%-100%)	11.121% (2.941%-20%)	65.88% (57.89%-73.41%)	100% (100%-100%)	2.7705 (2.6467-2.9014)
At least 1 of CHASM (breast), CHASM (melanoma), FATHMM (cancer), FATHMM (missense), Mutation Assessor, MutationTaster, PolyPhen-2, SIFT, VEST	65.37% (61.62%-69.19%)	100% (100%-100%)	5.484% (2.631%-8.219%)	64.66% (60.82%-68.72%)	100% (100%-100%)	2.7017 (2.6535-2.7534)	65.22% (57.58%-72.73%)	100% (100%-100%)	5.526% (0%-12.12%)	64.5% (56.25%-72.17%)	100% (100%-100%)	2.7048 (2.6054-2.8051)
At least 1 of CHASM (breast), CHASM (melanoma), FATHMM (cancer), FATHMM (missense), Mutation Assessor, MutationTaster, PolyPhen-2, VEST	66.01% (62.12%-70.2%)	100% (100%-100%)	7.229% (3.999%-10.606%)	65.08% (61.14%-69.23%)	100% (100%-100%)	2.7232 (2.6698-2.7811)	65.96% (57.58%-73.74%)	100% (100%-100%)	7.525% (0%-14.63%)	64.99% (56.7%-72.92%)	100% (100%-100%)	2.7275 (2.6238-2.8431)
At least 1 of CHASM (breast), CHASM (melanoma), FATHMM (cancer), FATHMM (missense), Mutation Assessor, MutationTaster, PROVEAN, SIFT	66.37% (62.63%-70.2%)	100% (100%-100%)	8.22% (4.165%-11.594%)	65.33% (61.54%-69.43%)	100% (100%-100%)	2.7355 (2.6764-2.7972)	66.25% (58.59%-73.74%)	100% (100%-100%)	8.328% (2.439%-16.29%)	65.18% (56.84%-72.92%)	100% (100%-100%)	2.7366 (2.6238-2.8596)
At least 1 of CHASM (breast), CHASM (melanoma), FATHMM (cancer), FATHMM (missense), Mutation Assessor, MutationTaster, PROVEAN, SIFT, VEST	65.03% (61.11%-69.19%)	100% (100%-100%)	4.567% (1.428%-7.042%)	64.44% (60.51%-68.53%)	100% (100%-100%)	2.6904 (2.6429-2.7409)	64.89% (56.57%-72.73%)	100% (100%-100%)	4.613% (0%-10.53%)	64.28% (56.12%-72.16%)	100% (100%-100%)	2.6949 (2.6011-2.7917)
At least 1 of CHASM (breast), CHASM (melanoma), FATHMM (cancer), FATHMM (missense), Mutation Assessor, MutationTaster, PROVEAN, VEST	65.34% (61.62%-69.19%)	100% (100%-100%)	5.42% (2.597%-8.219%)	64.64% (60.82%-68.72%)	100% (100%-100%)	2.7009 (2.6535-2.7534)	65.27% (57.58%-72.73%)	100% (100%-100%)	5.651% (0%-12.12%)	64.53% (56.25%-72.16%)	100% (100%-100%)	2.7057 (2.6072-2.8051)
At least 1 of CHASM (breast), CHASM (melanoma), FATHMM (cancer), FATHMM (missense), Mutation Assessor, MutationTaster, SIFT, VEST	65.37% (61.62%-69.19%)	100% (100%-100%)	5.484% (2.631%-8.219%)	64.66% (60.82%-68.72%)	100% (100%-100%)	2.7017 (2.6535-2.7534)	65.22% (57.58%-72.73%)	100% (100%-100%)	5.526% (0%-12.12%)	64.5% (56.25%-72.17%)	100% (100%-100%)	2.7048 (2.6054-2.8051)
At least 1 of CHASM (breast), CHASM (melanoma), FATHMM (cancer), FATHMM (missense), Mutation Assessor, PolyPhen-2, PROVEAN, SIFT	66.69% (63.13%-70.71%)	100% (100%-100%)	9.086% (4.684%-12.676%)	65.54% (61.78%-69.47%)	100% (100%-100%)	2.7463 (2.6871-2.8091)	66.62% (58.59%-73.74%)	100% (100%-100%)	9.336% (2.564%-18.18%)	65.43% (57.45%-72.92%)	100% (100%-100%)	2.7490 (2.6352-2.8754)
At least 1 of CHASM (breast), CHASM (melanoma), FATHMM (cancer), FATHMM (missense), Mutation Assessor, PolyPhen-2, PROVEAN, SIFT, VEST	65.03% (61.11%-69.19%)	100% (100%-100%)	4.567% (1.428%-7.042%)	64.44% (60.51%-68.53%)	100% (100%-100%)	2.6904 (2.6429-2.7409)	64.89% (56.57%-72.73%)	100% (100%-100%)	4.613% (0%-10.53%)	64.28% (56.12%-72.16%)	100% (100%-100%)	2.6949 (2.6011-2.7917)
At least 1 of CHASM (breast), CHASM (melanoma), FATHMM (cancer), FATHMM (missense), Mutation Assessor, PolyPhen-2, PROVEAN, VEST	65.34% (61.62%-69.19%)	100% (100%-100%)	5.42% (2.597%-8.219%)	64.64% (60.82%-68.72%)	100% (100%-100%)	2.7009 (2.6535-2.7534)	65.27% (57.58%-72.73%)	100% (100%-100%)	5.651% (0%-12.12%)	64.53% (56.25%-72.16%)	100% (100%-100%)	2.7057 (2.6072-2.8051)
At least 1 of CHASM (breast), CHASM (melanoma), FATHMM (cancer), FATHMM (missense), Mutation Assessor, PolyPhen-2, SIFT, VEST	65.37% (61.62%-69.19%)	100% (100%-100%)	5.484% (2.631%-8.219%)	64.66% (60.82%-68.72%)	100% (100%-100%)	2.7017 (2.6535-2.7534)	65.22% (57.58%-72.73%)	100% (100%-100%)	5.526% (0%-12.12%)	64.5% (56.25%-72.17%)	100% (100%-100%)	2.7048 (2.6054-2.8051)
At least 1 of CHASM (breast), CHASM (melanoma), FATHMM (cancer), FATHMM (missense), Mutation Assessor, PROVEAN, SIFT, VEST	65.03% (61.11%-69.19%)	100% (100%-100%)	4.567% (1.428%-7.042%)	64.44% (60.51%-68.53%)	100% (100%-100%)	2.6904 (2.6429-2.7409)	64.89% (56.57%-72.73%)	100% (100%-100%)	4.613% (0%-10.53%)	64.28% (56.12%-72.16%)	100% (100%-100%)	2.6949 (2.6011-2.7917)
At least 1 of CHASM (breast), CHASM (melanoma), FATHMM (cancer), FATHMM (missense), MutationTaster, PolyPhen-2, PROVEAN, SIFT	67.37% (63.64%-71.21%)	100% (100%-100%)	10.945% (6.491%-14.866%)	66% (62.23%-70%)	100% (100%-100%)	2.7695 (2.7080-2.8341)	67.28% (59.6%-74.75%)	100% (100%-100%)	11.128% (2.941%-20.46%)	65.88% (57.89%-73.41%)	100% (100%-100%)	2.7706 (2.6573-2.9079)
At least 1 of CHASM (breast), CHASM (melanoma), FATHMM (cancer), FATHMM (missense), MutationTaster, PolyPhen-2, PROVEAN, SIFT, VEST	65.69% (62.12%-69.7%)	100% (100%-100%)	6.365% (2.817%-9.459%)	64.87% (61.13%-68.91%)	100% (100%-100%)	2.7124 (2.6595-2.7660)	65.59% (57.58%-72.73%)	100% (100%-100%)	6.513% (0%-13.16%)	64.74% (56.7%-72.17%)	100% (100%-100%)	2.7158 (2.6162-2.8303)
At least 1 of CHASM (breast), CHASM (melanoma), FATHMM (cancer), FATHMM (missense), MutationTaster, PolyPhen-2, PROVEAN, VEST	66% (62.12%-69.7%)	100% (100%-100%)	7.218% (3.896%-10.256%)	65.08% (61.14%-69.07%)	100% (100%-100%)	2.7230 (2.6679-2.7784)	65.97% (58.59%-73.74%)	100% (100%-100%)	7.551% (0%-15.15%)	64.99% (56.84%-72.92%)	100% (100%-100%)	2.7273 (2.6270-2.8448)
At least 1 of CHASM (breast), CHASM (melanoma), FATHMM (cancer), FATHMM (missense), MutationTaster, PolyPhen-2, SIFT, VEST	66.36% (62.63%-70.2%)	100% (100%-100%)	8.196% (4.286%-11.594%)	65.32% (61.46%-69.43%)	100% (100%-100%)	2.7352 (2.6799-2.7972)	66.27% (58.59%-73.74%)	100% (100%-100%)	8.362% (2.12%-15.79%)	65.19% (56.84%-72.92%)	100% (100%-100%)	2.7376 (2.6270-2.8596)
At least 1 of CHASM (breast), CHASM (melanoma), FATHMM (cancer), FATHMM (missense), MutationTaster, PROVEAN, SIFT, VEST	65.69% (62.12%-69.7%)	100% (100%-100%)	6.365% (2.817%-9.459%)	64.87% (61.13%-68.91%)	100% (100%-100%)	2.7124 (2.6595-2.7660)	65.59% (57.58%-72.73%)	100% (100%-100%)	6.513% (0%-13.16%)	64.74% (56.7%-72.17%)	100% (100%-100%)	2.7158 (2.6162-2.8303)
At least 1 of CHASM (breast), CHASM (melanoma), FATHMM (cancer), FATHMM (missense), PolyPhen-2, PROVEAN, SIFT, VEST	65.69% (62.12%-69.7%)	100% (100%-100%)	6.365% (2.817%-9.459%)	64.87% (61.13%-68.91%)	100% (100%-100%)	2.7124 (2.6595-2.7660)	65.59% (57.58%-72.73%)	100% (100%-100%)	6.513% (0%-13.16%)	64.74% (56.7%-72.17%)	100% (100%-100%)	2.7158 (2.6162-2.8303)
At least 1 of CHASM (breast), CHASM (melanoma), FATHMM (cancer), Mutation Assessor, MutationTaster, PolyPhen-2, PROVEAN, SIFT	69.42% (65.66%-73.23%)	100% (100%-100%)	16.532% (11.59%-21.127%)	67.44% (63.64%-71.51%)	100% (100%-100%)	2.8397 (2.7734-2.9105)	69.25% (61.62%-76.77%)	100% (100%-100%)	16.467% (7.143%-26.19%)	67.26% (59.14%-74.74%)	100% (100%-100%)	2.8374 (2.7118-2.9834)
At least 1 of CHASM (breast), CHASM (melanoma), FATHMM (cancer), Mutation Assessor, MutationTaster, PolyPhen-2, PROVEAN, SIFT, VEST	66.07% (62.63%-70.2%)	100% (100%-100%)	7.41% (4.108%-10.667%)	65.13% (61.26%-69.11%)	100% (100%-100%)	2.7254 (2.6739-2.7836)	65.83% (57.58%-72.73%)	100% (100%-100%)	7.189% (0%-13.9%)	64.9% (56.84%-72.45%)	100% (100%-100%)	2.7234 (2.6163-2.8325)
At least 1 of CHASM (breast), CHASM (melanoma), FATHMM (cancer), Mutation Assessor, MutationTaster, PolyPhen-2, PROVEAN, VEST	66.39% (62.63%-70.2%)	100% (100%-100%)	8.264% (4.348%-11.594%)	65.34% (61.46%-69.43%)	100% (100%-100%)	2.7360 (2.6815-2.7949)	66.22% (58.59%-73.74%)	100% (100%-100%)	8.227% (2.326%-15.62%)	65.16% (57.14%-72.92%)	100% (100%-100%)	2.7356 (2.6279-2.8536)
At least 1 of CHASM (breast), CHASM (melanoma), FATHMM (cancer), Mutation Assessor, MutationTaster, PolyPhen-2, SIFT, VEST	66.41% (62.63%-70.2%)	100% (100%-100%)	8.328% (4.348%-11.769%)	65.35% (61.54%-69.43%)	100% (100%-100%)	2.7368 (2.6834-2.7987)	66.17% (58.59%-73.74%)	100% (100%-100%)	8.101% (0%-15.38%)	65.13% (56.99%-72.73%)	100% (100%-100%)	2.7342 (2.6238-2.8448)
At least 1 of CHASM (breast), CHASM (melanoma), FATHMM (cancer), Mutation Assessor, MutationTaster, PROVEAN, SIFT, VEST	66.41% (62.63%-70.21%)	100% (100%-100%)	8.336% (4.348%-11.765%)	65.35% (61.46%-69.43%)	100% (100%-100%)	2.7369 (2.6815-2.7972)	66.17% (58.56%-73.74%)	100% (100%-100%)	8.103% (2.167%-15.22%)	65.13% (57.14%-72.92%)	100% (100%-100%)	2.7340 (2.6238-2.8536)
At least 1 of CHASM (breast), CHASM (melanoma), FATHMM (cancer), Mutation Assessor, PolyPhen-2, PROVEAN, SIFT, VEST	66.07% (62.63%-70.2%)	100% (100%-100%)	7.41% (4.108%-10.667%)	65.13% (61.26%-69.11%)	100% (100%-100%)	2.7254 (2.6739-2.7836)	65.83% (57.58%-72.73%)	100% (100%-100%)	7.189% (0%-13.9%)	64.9% (56.84%-72.45%)	100% (100%-100%)	2.7234 (2.6163-2.8325)
At least 1 of CHASM (breast), CHASM (melanoma), FATHMM (cancer), MutationTaster, PolyPhen-2, PROVEAN, SIFT, VEST	66.73% (63.13%-70.71%)	100% (100%-100%)	9.209% (5.332%-12.862%)	65.57% (61.78%-69.48%)	100% (100%-100%)	2.7478 (2.6919-2.8130)	66.53% (58.59%-73.74%)	100% (100%-100%)	9.089% (2.439%-17.07%)	65.37% (57.44%-72.92%)	100% (100%-100%)	2.7464 (2.6292-2.8690)
At least 1 of CHASM (breast), CHASM (melanoma), FATHMM (missense), Mutation Assessor, MutationTaster, PolyPhen-2, PROVEAN, SIFT	69.06% (65.15%-72.73%)	100% (100%-100%)	15.553% (10.523%-20.27%)	67.19% (63.29%-71.12%)	100% (100%-100%)	2.8274 (2.7588-2.8997)	68.96% (61.62%-76.77%)	100% (100%-100%)	15.677% (6.452%-25.64%)	67.05% (59.14%-75%)	100% (100%-100%)	2.8273 (2.6927-2.9691)
At least 1 of CHASM (breast), CHASM (melanoma), FATHMM (missense), Mutation Assessor, MutationTaster, PolyPhen-2, PROVEAN, SIFT, VEST	65.7% (61.62%-69.7%)	100% (100%-100%)	6.384% (2.857%-9.459%)	64.88% (60.94%-68.88%)	100% (100%-100%)	2.7126 (2.6595-2.7702)	65.57% (57.58%-73.74%)	100% (100%-100%)	6.467% (0%-12.91%)	64.73% (56.38%-72.63%)	100% (100%-100%)	2.7150 (2.6162-2.8308)
At least 1 of CHASM (breast), CHASM (melanoma), FATHMM (missense), Mutation Assessor, MutationTaster, PolyPhen-2, PROVEAN, VEST	66.01% (62.12%-69.7%)	100% (100%-100%)	7.238% (3.277%-10.391%)	65.08% (61.14%-69.11%)	100% (100%-100%)	2.7232 (2.6698-2.7784)	65.96% (58.59%-73.74%)	100% (100%-100%)	7.505% (0%-14.29%)	64.98% (56.84%-72.92%)	100% (100%-100%)	2.7268 (2.6238-2.8448)
At least 1 of CHASM (breast), CHASM (melanoma), FATHMM (missense), Mutation Assessor, MutationTaster, PolyPhen-2, SIFT, VEST	66.38% (62.63%-70.2%)	100% (100%-100%)	8.237% (4.284%-11.598%)	65.33% (61.34%-69.43%)	100% (100%-100%)	2.7357 (2.6773-2.7949)	66.23% (58.59%-73.74%)	100% (100%-100%)	8.264% (2.326%-15.79%)	65.17% (56.84%-73.2%)	100% (100%-100%)	2.7360 (2.6270-2.8605)
At least 1 of CHASM (breast), CHASM (melanoma), FATHMM (missense), Mutation Assessor, MutationTaster, PROVEAN, SIFT, VEST	65.7% (61.62%-69.7%)	100% (100%-100%)	6.384% (2.857%-9.459%)	64.88% (60.94%-68.88%)	100% (100%-100%)	2.7126 (2.6595-2.7702)	65.57% (57.58%-73.74%)	100% (100%-100%)	6.467% (0%-12.91%)	64.73% (56.38%-72.63%)	100% (100%-100%)	2.7150 (2.6162-2.8308)
At least 1 of CHASM (breast), CHASM (melanoma), FATHMM (missense), Mutation Assessor, PolyPhen-2, PROVEAN, SIFT, VEST	65.7% (61.62%-69.7%)	100% (100%-100%)	6.384% (2.857%-9.459%)	64.88% (60.94%-68.88%)	100% (100%-100%)	2.7126 (2.6595-2.7702)	65.57% (57.58%-73.74%)	100% (100%-100%)	6.467% (0%-12.91%)	64.73% (56.38%-72.63%)	100% (100%-100%)	2.7150 (2.6162-2.8308)
At least 1 of CHASM (breast), CHASM (melanoma), FATHMM (missense), MutationTaster, PolyPhen-2, PROVEAN, SIFT, VEST	66.36% (62.63%-70.2%)	100% (100%-100%)	8.183% (4.225%-11.538%)	65.32% (61.46%-69.27%)	100% (100%-100%)	2.7350 (2.6773-2.7917)	66.27% (58.59%-73.74%)	100% (100%-100%)	8.367% (2.498%-15.79%)	65.19% (57.14%-72.92%)	100% (100%-100%)	2.7371 (2.6292-2.8596)
At least 1 of CHASM (breast), CHASM (melanoma), Mutation Assessor, MutationTaster, PolyPhen-2, PROVEAN, SIFT, VEST	66.74% (63.13%-70.71%)	100% (100%-100%)	9.228% (5.478%-12.676%)	65.58% (61.78%-69.63%)	100% (100%-100%)	2.7480 (2.6919-2.8089)	66.52% (58.59%-73.74%)	100% (100%-100%)	9.043% (2.632%-16.67%)	65.36% (57.29%-72.92%)	100% (100%-100%)	2.7455 (2.6379-2.8690)
At least 1 of CHASM (breast), FATHMM (cancer), FATHMM (missense), Mutation Assessor, MutationTaster, PolyPhen-2, PROVEAN, SIFT	66.7% (63.13%-70.71%)	100% (100%-100%)	9.133% (5.125%-12.857%)	65.55% (61.78%-69.63%)	100% (100%-100%)	2.7468 (2.6871-2.8059)	66.59% (58.59%-73.74%)	100% (100%-100%)	9.251% (2.5%-18.18%)	65.41% (57.29%-72.92%)	100% (100%-100%)	2.7477 (2.6360-2.8755)
At least 1 of CHASM (breast), FATHMM (cancer), FATHMM (missense), Mutation Assessor, MutationTaster, PolyPhen-2, PROVEAN, SIFT, VEST	65.03% (61.11%-69.19%)	100% (100%-100%)	4.567% (1.428%-7.042%)	64.44% (60.51%-68.53%)	100% (100%-100%)	2.6904 (2.6429-2.7409)	64.89% (56.57%-72.73%)	100% (100%-100%)	4.613% (0%-10.53%)	64.28% (56.12%-72.16%)	100% (100%-100%)	2.6949 (2.6011-2.7917)
At least 1 of CHASM (breast), FATHMM (cancer), FATHMM (missense), Mutation Assessor, MutationTaster, PolyPhen-2, PROVEAN, VEST	65.67% (62.12%-69.7%)	100% (100%-100%)	6.304% (2.817%-9.377%)	64.86% (61.14%-68.91%)	100% (100%-100%)	2.7118 (2.6597-2.7660)	65.63% (57.58%-72.73%)	100% (100%-100%)	6.631% (0%-13.16%)	64.77% (56.7%-72.16%)	100% (100%-100%)	2.7168 (2.6162-2.8289)
At least 1 of CHASM (breast), FATHMM (cancer), FATHMM (missense), Mutation Assessor, MutationTaster, PolyPhen-2, SIFT, VEST	65.37% (61.62%-69.19%)	100% (100%-100%)	5.484% (2.631%-8.219%)	64.66% (60.82%-68.72%)	100% (100%-100%)	2.7017 (2.6535-2.7534)	65.22% (57.58%-72.73%)	100% (100%-100%)	5.526% (0%-12.12%)	64.5% (56.25%-72.17%)	100% (100%-100%)	2.7048 (2.6054-2.8051)
At least 1 of CHASM (breast), FATHMM (cancer), FATHMM (missense), Mutation Assessor, MutationTaster, PROVEAN, SIFT, VEST	65.03% (61.11%-69.19%)	100% (100%-100%)	4.567% (1.428%-7.042%)	64.44% (60.51%-68.53%)	100% (100%-100%)	2.6904 (2.6429-2.7409)	64.89% (56.57%-72.73%)	100% (100%-100%)	4.613% (0%-10.53%)	64.28% (56.12%-72.16%)	100% (100%-100%)	2.6949 (2.6011-2.7917)
At least 1 of CHASM (breast), FATHMM (cancer), FATHMM (missense), Mutation Assessor, PolyPhen-2, PROVEAN, SIFT, VEST	65.03% (61.11%-69.19%)	100% (100%-100%)	4.567% (1.428%-7.042%)	64.44% (60.51%-68.53%)	100% (100%-100%)	2.6904 (2.6429-2.7409)	64.89% (56.57%-72.73%)	100% (100%-100%)	4.613% (0%-10.53%)	64.28% (56.12%-72.16%)	100% (100%-100%)	2.6949 (2.6011-2.7917)
At least 1 of CHASM (breast), FATHMM (cancer), FATHMM (missense), MutationTaster, PolyPhen-2, PROVEAN, SIFT, VEST	65.69% (62.12%-69.7%)	100% (100%-100%)	6.365% (2.817%-9.459%)	64.87% (61.13%-68.91%)	100% (100%-100%)	2.7124 (2.6595-2.7660)	65.59% (57.58%-72.73%)	100% (100%-100%)	6.513% (0%-13.16%)	64.74% (56.7%-72.17%)	100% (100%-100%)	2.7158 (2.6162-2.8303)
At least 1 of CHASM (breast), FATHMM (cancer), Mutation Assessor, MutationTaster, PolyPhen-2, PROVEAN, SIFT, VEST	66.07% (62.63%-70.2%)	100% (100%-100%)	7.41% (4.108%-10.667%)	65.13% (61.26%-69.11%)	100% (100%-100%)	2.7254 (2.6739-2.7836)	65.83% (57.58%-72.73%)	100% (100%-100%)	7.189% (0%-13.9%)	64.9% (56.84%-72.45%)	100% (100%-100%)	2.7234 (2.6163-2.8325)
At least 1 of CHASM (breast), FATHMM (missense), Mutation Assessor, MutationTaster, PolyPhen-2, PROVEAN, SIFT, VEST	65.7% (61.62%-69.7%)	100% (100%-100%)	6.384% (2.857%-9.459%)	64.88% (60.94%-68.88%)	100% (100%-100%)	2.7126 (2.6595-2.7702)	65.57% (57.58%-73.74%)	100% (100%-100%)	6.467% (0%-12.91%)	64.73% (56.38%-72.63%)	100% (100%-100%)	2.7150 (2.6162-2.8308)
At least 1 of CHASM (lung), CHASM (melanoma), FATHMM (cancer), FATHMM (missense), Mutation Assessor, MutationTaster, PolyPhen-2, PROVEAN	68.03% (64.63%-71.72%)	100% (100%-100%)	12.752% (8.105%-16.901%)	66.46% (62.77%-70.43%)	100% (100%-100%)	2.7922 (2.7269-2.8597)	67.98% (60.61%-74.77%)	100% (100%-100%)	12.994% (5%-22.58%)	66.36% (58.33%-73.91%)	100% (100%-100%)	2.7938 (2.6706-2.9319)
At least 1 of CHASM (lung), CHASM (melanoma), FATHMM (cancer), FATHMM (missense), Mutation Assessor, MutationTaster, PolyPhen-2, PROVEAN, SIFT	66.37% (62.63%-70.2%)	100% (100%-100%)	8.22% (4.165%-11.594%)	65.33% (61.54%-69.43%)	100% (100%-100%)	2.7355 (2.6764-2.7972)	66.25% (58.59%-73.74%)	100% (100%-100%)	8.328% (2.439%-16.29%)	65.18% (56.84%-72.92%)	100% (100%-100%)	2.7366 (2.6238-2.8596)
At least 1 of CHASM (lung), CHASM (melanoma), FATHMM (cancer), FATHMM (missense), Mutation Assessor, MutationTaster, PolyPhen-2, PROVEAN, SIFT, VEST	65.03% (61.11%-69.19%)	100% (100%-100%)	4.567% (1.428%-7.042%)	64.44% (60.51%-68.53%)	100% (100%-100%)	2.6904 (2.6429-2.7409)	64.89% (56.57%-72.73%)	100% (100%-100%)	4.613% (0%-10.53%)	64.28% (56.12%-72.16%)	100% (100%-100%)	2.6949 (2.6011-2.7917)
At least 1 of CHASM (lung), CHASM (melanoma), FATHMM (cancer), FATHMM (missense), Mutation Assessor, MutationTaster, PolyPhen-2, PROVEAN, VEST	65.34% (61.62%-69.19%)	100% (100%-100%)	5.42% (2.597%-8.219%)	64.64% (60.82%-68.72%)	100% (100%-100%)	2.7009 (2.6535-2.7534)	65.27% (57.58%-72.73%)	100% (100%-100%)	5.651% (0%-12.12%)	64.53% (56.25%-72.16%)	100% (100%-100%)	2.7057 (2.6072-2.8051)
At least 1 of CHASM (lung), CHASM (melanoma), FATHMM (cancer), FATHMM (missense), Mutation Assessor, MutationTaster, PolyPhen-2, SIFT	67.37% (63.64%-71.21%)	100% (100%-100%)	10.963% (6.577%-14.866%)	66.01% (62.23%-70%)	100% (100%-100%)	2.7697 (2.7080-2.8375)	67.27% (59.6%-74.75%)	100% (100%-100%)	11.121% (2.941%-20%)	65.88% (57.89%-73.41%)	100% (100%-100%)	2.7705 (2.6467-2.9014)
At least 1 of CHASM (lung), CHASM (melanoma), FATHMM (cancer), FATHMM (missense), Mutation Assessor, MutationTaster, PolyPhen-2, SIFT, VEST	65.37% (61.62%-69.19%)	100% (100%-100%)	5.484% (2.631%-8.219%)	64.66% (60.82%-68.72%)	100% (100%-100%)	2.7017 (2.6535-2.7534)	65.22% (57.58%-72.73%)	100% (100%-100%)	5.526% (0%-12.12%)	64.5% (56.25%-72.17%)	100% (100%-100%)	2.7048 (2.6054-2.8051)
At least 1 of CHASM (lung), CHASM (melanoma), FATHMM (cancer), FATHMM (missense), Mutation Assessor, MutationTaster, PolyPhen-2, VEST	66.01% (62.12%-70.2%)	100% (100%-100%)	7.229% (3.999%-10.606%)	65.08% (61.14%-69.23%)	100% (100%-100%)	2.7232 (2.6698-2.7811)	65.96% (57.58%-73.74%)	100% (100%-100%)	7.525% (0%-14.63%)	64.99% (56.7%-72.92%)	100% (100%-100%)	2.7275 (2.6238-2.8431)
At least 1 of CHASM (lung), CHASM (melanoma), FATHMM (cancer), FATHMM (missense), Mutation Assessor, MutationTaster, PROVEAN, SIFT	66.37% (62.63%-70.2%)	100% (100%-100%)	8.22% (4.165%-11.594%)	65.33% (61.54%-69.43%)	100% (100%-100%)	2.7355 (2.6764-2.7972)	66.25% (58.59%-73.74%)	100% (100%-100%)	8.328% (2.439%-16.29%)	65.18% (56.84%-72.92%)	100% (100%-100%)	2.7366 (2.6238-2.8596)
At least 1 of CHASM (lung), CHASM (melanoma), FATHMM (cancer), FATHMM (missense), Mutation Assessor, MutationTaster, PROVEAN, SIFT, VEST	65.03% (61.11%-69.19%)	100% (100%-100%)	4.567% (1.428%-7.042%)	64.44% (60.51%-68.53%)	100% (100%-100%)	2.6904 (2.6429-2.7409)	64.89% (56.57%-72.73%)	100% (100%-100%)	4.613% (0%-10.53%)	64.28% (56.12%-72.16%)	100% (100%-100%)	2.6949 (2.6011-2.7917)
At least 1 of CHASM (lung), CHASM (melanoma), FATHMM (cancer), FATHMM (missense), Mutation Assessor, MutationTaster, PROVEAN, VEST	65.34% (61.62%-69.19%)	100% (100%-100%)	5.42% (2.597%-8.219%)	64.64% (60.82%-68.72%)	100% (100%-100%)	2.7009 (2.6535-2.7534)	65.27% (57.58%-72.73%)	100% (100%-100%)	5.651% (0%-12.12%)	64.53% (56.25%-72.16%)	100% (100%-100%)	2.7057 (2.6072-2.8051)
At least 1 of CHASM (lung), CHASM (melanoma), FATHMM (cancer), FATHMM (missense), Mutation Assessor, MutationTaster, SIFT, VEST	65.37% (61.62%-69.19%)	100% (100%-100%)	5.484% (2.631%-8.219%)	64.66% (60.82%-68.72%)	100% (100%-100%)	2.7017 (2.6535-2.7534)	65.22% (57.58%-72.73%)	100% (100%-100%)	5.526% (0%-12.12%)	64.5% (56.25%-72.17%)	100% (100%-100%)	2.7048 (2.6054-2.8051)
At least 1 of CHASM (lung), CHASM (melanoma), FATHMM (cancer), FATHMM (missense), Mutation Assessor, PolyPhen-2, PROVEAN, SIFT	66.69% (63.13%-70.71%)	100% (100%-100%)	9.086% (4.684%-12.676%)	65.54% (61.78%-69.47%)	100% (100%-100%)	2.7463 (2.6871-2.8091)	66.62% (58.59%-73.74%)	100% (100%-100%)	9.336% (2.564%-18.18%)	65.43% (57.45%-72.92%)	100% (100%-100%)	2.7490 (2.6352-2.8754)
At least 1 of CHASM (lung), CHASM (melanoma), FATHMM (cancer), FATHMM (missense), Mutation Assessor, PolyPhen-2, PROVEAN, SIFT, VEST	65.03% (61.11%-69.19%)	100% (100%-100%)	4.567% (1.428%-7.042%)	64.44% (60.51%-68.53%)	100% (100%-100%)	2.6904 (2.6429-2.7409)	64.89% (56.57%-72.73%)	100% (100%-100%)	4.613% (0%-10.53%)	64.28% (56.12%-72.16%)	100% (100%-100%)	2.6949 (2.6011-2.7917)
At least 1 of CHASM (lung), CHASM (melanoma), FATHMM (cancer), FATHMM (missense), Mutation Assessor, PolyPhen-2, PROVEAN, VEST	65.34% (61.62%-69.19%)	100% (100%-100%)	5.42% (2.597%-8.219%)	64.64% (60.82%-68.72%)	100% (100%-100%)	2.7009 (2.6535-2.7534)	65.27% (57.58%-72.73%)	100% (100%-100%)	5.651% (0%-12.12%)	64.53% (56.25%-72.16%)	100% (100%-100%)	2.7057 (2.6072-2.8051)
At least 1 of CHASM (lung), CHASM (melanoma), FATHMM (cancer), FATHMM (missense), Mutation Assessor, PolyPhen-2, SIFT, VEST	65.37% (61.62%-69.19%)	100% (100%-100%)	5.484% (2.631%-8.219%)	64.66% (60.82%-68.72%)	100% (100%-100%)	2.7017 (2.6535-2.7534)	65.22% (57.58%-72.73%)	100% (100%-100%)	5.526% (0%-12.12%)	64.5% (56.25%-72.17%)	100% (100%-100%)	2.7048 (2.6054-2.8051)
At least 1 of CHASM (lung), CHASM (melanoma), FATHMM (cancer), FATHMM (missense), Mutation Assessor, PROVEAN, SIFT, VEST	65.03% (61.11%-69.19%)	100% (100%-100%)	4.567% (1.428%-7.042%)	64.44% (60.51%-68.53%)	100% (100%-100%)	2.6904 (2.6429-2.7409)	64.89% (56.57%-72.73%)	100% (100%-100%)	4.613% (0%-10.53%)	64.28% (56.12%-72.16%)	100% (100%-100%)	2.6949 (2.6011-2.7917)
At least 1 of CHASM (lung), CHASM (melanoma), FATHMM (cancer), FATHMM (missense), MutationTaster, PolyPhen-2, PROVEAN, SIFT	67.37% (63.64%-71.21%)	100% (100%-100%)	10.945% (6.491%-14.866%)	66% (62.23%-70%)	100% (100%-100%)	2.7695 (2.7080-2.8341)	67.28% (59.6%-74.75%)	100% (100%-100%)	11.128% (2.941%-20.46%)	65.88% (57.89%-73.41%)	100% (100%-100%)	2.7706 (2.6573-2.9079)
At least 1 of CHASM (lung), CHASM (melanoma), FATHMM (cancer), FATHMM (missense), MutationTaster, PolyPhen-2, PROVEAN, SIFT, VEST	65.69% (62.12%-69.7%)	100% (100%-100%)	6.365% (2.817%-9.459%)	64.87% (61.13%-68.91%)	100% (100%-100%)	2.7124 (2.6595-2.7660)	65.59% (57.58%-72.73%)	100% (100%-100%)	6.513% (0%-13.16%)	64.74% (56.7%-72.17%)	100% (100%-100%)	2.7158 (2.6162-2.8303)
At least 1 of CHASM (lung), CHASM (melanoma), FATHMM (cancer), FATHMM (missense), MutationTaster, PolyPhen-2, PROVEAN, VEST	66% (62.12%-69.7%)	100% (100%-100%)	7.218% (3.896%-10.256%)	65.08% (61.14%-69.07%)	100% (100%-100%)	2.7230 (2.6679-2.7784)	65.97% (58.59%-73.74%)	100% (100%-100%)	7.551% (0%-15.15%)	64.99% (56.84%-72.92%)	100% (100%-100%)	2.7273 (2.6270-2.8448)
At least 1 of CHASM (lung), CHASM (melanoma), FATHMM (cancer), FATHMM (missense), MutationTaster, PolyPhen-2, SIFT, VEST	66.36% (62.63%-70.2%)	100% (100%-100%)	8.196% (4.286%-11.594%)	65.32% (61.46%-69.43%)	100% (100%-100%)	2.7352 (2.6799-2.7972)	66.27% (58.59%-73.74%)	100% (100%-100%)	8.362% (2.12%-15.79%)	65.19% (56.84%-72.92%)	100% (100%-100%)	2.7376 (2.6270-2.8596)
At least 1 of CHASM (lung), CHASM (melanoma), FATHMM (cancer), FATHMM (missense), MutationTaster, PROVEAN, SIFT, VEST	65.69% (62.12%-69.7%)	100% (100%-100%)	6.365% (2.817%-9.459%)	64.87% (61.13%-68.91%)	100% (100%-100%)	2.7124 (2.6595-2.7660)	65.59% (57.58%-72.73%)	100% (100%-100%)	6.513% (0%-13.16%)	64.74% (56.7%-72.17%)	100% (100%-100%)	2.7158 (2.6162-2.8303)
At least 1 of CHASM (lung), CHASM (melanoma), FATHMM (cancer), FATHMM (missense), PolyPhen-2, PROVEAN, SIFT, VEST	65.69% (62.12%-69.7%)	100% (100%-100%)	6.365% (2.817%-9.459%)	64.87% (61.13%-68.91%)	100% (100%-100%)	2.7124 (2.6595-2.7660)	65.59% (57.58%-72.73%)	100% (100%-100%)	6.513% (0%-13.16%)	64.74% (56.7%-72.17%)	100% (100%-100%)	2.7158 (2.6162-2.8303)
At least 1 of CHASM (lung), CHASM (melanoma), FATHMM (cancer), Mutation Assessor, MutationTaster, PolyPhen-2, PROVEAN, SIFT	69.42% (65.66%-73.23%)	100% (100%-100%)	16.532% (11.59%-21.127%)	67.44% (63.64%-71.51%)	100% (100%-100%)	2.8397 (2.7734-2.9105)	69.25% (61.62%-76.77%)	100% (100%-100%)	16.467% (7.143%-26.19%)	67.26% (59.14%-74.74%)	100% (100%-100%)	2.8374 (2.7118-2.9834)
At least 1 of CHASM (lung), CHASM (melanoma), FATHMM (cancer), Mutation Assessor, MutationTaster, PolyPhen-2, PROVEAN, SIFT, VEST	66.07% (62.63%-70.2%)	100% (100%-100%)	7.41% (4.108%-10.667%)	65.13% (61.26%-69.11%)	100% (100%-100%)	2.7254 (2.6739-2.7836)	65.83% (57.58%-72.73%)	100% (100%-100%)	7.189% (0%-13.9%)	64.9% (56.84%-72.45%)	100% (100%-100%)	2.7234 (2.6163-2.8325)
At least 1 of CHASM (lung), CHASM (melanoma), FATHMM (cancer), Mutation Assessor, MutationTaster, PolyPhen-2, PROVEAN, VEST	66.39% (62.63%-70.2%)	100% (100%-100%)	8.264% (4.348%-11.594%)	65.34% (61.46%-69.43%)	100% (100%-100%)	2.7360 (2.6815-2.7949)	66.22% (58.59%-73.74%)	100% (100%-100%)	8.227% (2.326%-15.62%)	65.16% (57.14%-72.92%)	100% (100%-100%)	2.7356 (2.6279-2.8536)
At least 1 of CHASM (lung), CHASM (melanoma), FATHMM (cancer), Mutation Assessor, MutationTaster, PolyPhen-2, SIFT, VEST	66.41% (62.63%-70.2%)	100% (100%-100%)	8.328% (4.348%-11.769%)	65.35% (61.54%-69.43%)	100% (100%-100%)	2.7368 (2.6834-2.7987)	66.17% (58.59%-73.74%)	100% (100%-100%)	8.101% (0%-15.38%)	65.13% (56.99%-72.73%)	100% (100%-100%)	2.7342 (2.6238-2.8448)
At least 1 of CHASM (lung), CHASM (melanoma), FATHMM (cancer), Mutation Assessor, MutationTaster, PROVEAN, SIFT, VEST	66.41% (62.63%-70.21%)	100% (100%-100%)	8.336% (4.348%-11.765%)	65.35% (61.46%-69.43%)	100% (100%-100%)	2.7369 (2.6815-2.7972)	66.17% (58.56%-73.74%)	100% (100%-100%)	8.103% (2.167%-15.22%)	65.13% (57.14%-72.92%)	100% (100%-100%)	2.7340 (2.6238-2.8536)
At least 1 of CHASM (lung), CHASM (melanoma), FATHMM (cancer), Mutation Assessor, PolyPhen-2, PROVEAN, SIFT, VEST	66.07% (62.63%-70.2%)	100% (100%-100%)	7.41% (4.108%-10.667%)	65.13% (61.26%-69.11%)	100% (100%-100%)	2.7254 (2.6739-2.7836)	65.83% (57.58%-72.73%)	100% (100%-100%)	7.189% (0%-13.9%)	64.9% (56.84%-72.45%)	100% (100%-100%)	2.7234 (2.6163-2.8325)
At least 1 of CHASM (lung), CHASM (melanoma), FATHMM (cancer), MutationTaster, PolyPhen-2, PROVEAN, SIFT, VEST	66.73% (63.13%-70.71%)	100% (100%-100%)	9.209% (5.332%-12.862%)	65.57% (61.78%-69.48%)	100% (100%-100%)	2.7478 (2.6919-2.8130)	66.53% (58.59%-73.74%)	100% (100%-100%)	9.089% (2.439%-17.07%)	65.37% (57.44%-72.92%)	100% (100%-100%)	2.7464 (2.6292-2.8690)
At least 1 of CHASM (lung), CHASM (melanoma), FATHMM (missense), Mutation Assessor, MutationTaster, PolyPhen-2, PROVEAN, SIFT	69.06% (65.15%-72.73%)	100% (100%-100%)	15.553% (10.523%-20.27%)	67.19% (63.29%-71.12%)	100% (100%-100%)	2.8274 (2.7588-2.8997)	68.96% (61.62%-76.77%)	100% (100%-100%)	15.677% (6.452%-25.64%)	67.05% (59.14%-75%)	100% (100%-100%)	2.8273 (2.6927-2.9691)
At least 1 of CHASM (lung), CHASM (melanoma), FATHMM (missense), Mutation Assessor, MutationTaster, PolyPhen-2, PROVEAN, SIFT, VEST	65.7% (61.62%-69.7%)	100% (100%-100%)	6.384% (2.857%-9.459%)	64.88% (60.94%-68.88%)	100% (100%-100%)	2.7126 (2.6595-2.7702)	65.57% (57.58%-73.74%)	100% (100%-100%)	6.467% (0%-12.91%)	64.73% (56.38%-72.63%)	100% (100%-100%)	2.7150 (2.6162-2.8308)
At least 1 of CHASM (lung), CHASM (melanoma), FATHMM (missense), Mutation Assessor, MutationTaster, PolyPhen-2, PROVEAN, VEST	66.01% (62.12%-69.7%)	100% (100%-100%)	7.238% (3.277%-10.391%)	65.08% (61.14%-69.11%)	100% (100%-100%)	2.7232 (2.6698-2.7784)	65.96% (58.59%-73.74%)	100% (100%-100%)	7.505% (0%-14.29%)	64.98% (56.84%-72.92%)	100% (100%-100%)	2.7268 (2.6238-2.8448)
At least 1 of CHASM (lung), CHASM (melanoma), FATHMM (missense), Mutation Assessor, MutationTaster, PolyPhen-2, SIFT, VEST	66.38% (62.63%-70.2%)	100% (100%-100%)	8.237% (4.284%-11.598%)	65.33% (61.34%-69.43%)	100% (100%-100%)	2.7357 (2.6773-2.7949)	66.23% (58.59%-73.74%)	100% (100%-100%)	8.264% (2.326%-15.79%)	65.17% (56.84%-73.2%)	100% (100%-100%)	2.7360 (2.6270-2.8605)
At least 1 of CHASM (lung), CHASM (melanoma), FATHMM (missense), Mutation Assessor, MutationTaster, PROVEAN, SIFT, VEST	65.7% (61.62%-69.7%)	100% (100%-100%)	6.384% (2.857%-9.459%)	64.88% (60.94%-68.88%)	100% (100%-100%)	2.7126 (2.6595-2.7702)	65.57% (57.58%-73.74%)	100% (100%-100%)	6.467% (0%-12.91%)	64.73% (56.38%-72.63%)	100% (100%-100%)	2.7150 (2.6162-2.8308)
At least 1 of CHASM (lung), CHASM (melanoma), FATHMM (missense), Mutation Assessor, PolyPhen-2, PROVEAN, SIFT, VEST	65.7% (61.62%-69.7%)	100% (100%-100%)	6.384% (2.857%-9.459%)	64.88% (60.94%-68.88%)	100% (100%-100%)	2.7126 (2.6595-2.7702)	65.57% (57.58%-73.74%)	100% (100%-100%)	6.467% (0%-12.91%)	64.73% (56.38%-72.63%)	100% (100%-100%)	2.7150 (2.6162-2.8308)
At least 1 of CHASM (lung), CHASM (melanoma), FATHMM (missense), MutationTaster, PolyPhen-2, PROVEAN, SIFT, VEST	66.36% (62.63%-70.2%)	100% (100%-100%)	8.183% (4.225%-11.538%)	65.32% (61.46%-69.27%)	100% (100%-100%)	2.7350 (2.6773-2.7917)	66.27% (58.59%-73.74%)	100% (100%-100%)	8.367% (2.498%-15.79%)	65.19% (57.14%-72.92%)	100% (100%-100%)	2.7371 (2.6292-2.8596)
At least 1 of CHASM (lung), CHASM (melanoma), Mutation Assessor, MutationTaster, PolyPhen-2, PROVEAN, SIFT, VEST	66.74% (63.13%-70.71%)	100% (100%-100%)	9.228% (5.478%-12.676%)	65.58% (61.78%-69.63%)	100% (100%-100%)	2.7480 (2.6919-2.8089)	66.52% (58.59%-73.74%)	100% (100%-100%)	9.043% (2.632%-16.67%)	65.36% (57.29%-72.92%)	100% (100%-100%)	2.7455 (2.6379-2.8690)
At least 1 of CHASM (lung), FATHMM (cancer), FATHMM (missense), Mutation Assessor, MutationTaster, PolyPhen-2, PROVEAN, SIFT	66.7% (63.13%-70.71%)	100% (100%-100%)	9.133% (5.125%-12.857%)	65.55% (61.78%-69.63%)	100% (100%-100%)	2.7468 (2.6871-2.8059)	66.59% (58.59%-73.74%)	100% (100%-100%)	9.251% (2.5%-18.18%)	65.41% (57.29%-72.92%)	100% (100%-100%)	2.7477 (2.6360-2.8755)
At least 1 of CHASM (lung), FATHMM (cancer), FATHMM (missense), Mutation Assessor, MutationTaster, PolyPhen-2, PROVEAN, SIFT, VEST	65.03% (61.11%-69.19%)	100% (100%-100%)	4.567% (1.428%-7.042%)	64.44% (60.51%-68.53%)	100% (100%-100%)	2.6904 (2.6429-2.7409)	64.89% (56.57%-72.73%)	100% (100%-100%)	4.613% (0%-10.53%)	64.28% (56.12%-72.16%)	100% (100%-100%)	2.6949 (2.6011-2.7917)
At least 1 of CHASM (lung), FATHMM (cancer), FATHMM (missense), Mutation Assessor, MutationTaster, PolyPhen-2, PROVEAN, VEST	65.67% (62.12%-69.7%)	100% (100%-100%)	6.304% (2.817%-9.377%)	64.86% (61.14%-68.91%)	100% (100%-100%)	2.7118 (2.6597-2.7660)	65.63% (57.58%-72.73%)	100% (100%-100%)	6.631% (0%-13.16%)	64.77% (56.7%-72.16%)	100% (100%-100%)	2.7168 (2.6162-2.8289)
At least 1 of CHASM (lung), FATHMM (cancer), FATHMM (missense), Mutation Assessor, MutationTaster, PolyPhen-2, SIFT, VEST	65.37% (61.62%-69.19%)	100% (100%-100%)	5.484% (2.631%-8.219%)	64.66% (60.82%-68.72%)	100% (100%-100%)	2.7017 (2.6535-2.7534)	65.22% (57.58%-72.73%)	100% (100%-100%)	5.526% (0%-12.12%)	64.5% (56.25%-72.17%)	100% (100%-100%)	2.7048 (2.6054-2.8051)
At least 1 of CHASM (lung), FATHMM (cancer), FATHMM (missense), Mutation Assessor, MutationTaster, PROVEAN, SIFT, VEST	65.03% (61.11%-69.19%)	100% (100%-100%)	4.567% (1.428%-7.042%)	64.44% (60.51%-68.53%)	100% (100%-100%)	2.6904 (2.6429-2.7409)	64.89% (56.57%-72.73%)	100% (100%-100%)	4.613% (0%-10.53%)	64.28% (56.12%-72.16%)	100% (100%-100%)	2.6949 (2.6011-2.7917)
At least 1 of CHASM (lung), FATHMM (cancer), FATHMM (missense), Mutation Assessor, PolyPhen-2, PROVEAN, SIFT, VEST	65.03% (61.11%-69.19%)	100% (100%-100%)	4.567% (1.428%-7.042%)	64.44% (60.51%-68.53%)	100% (100%-100%)	2.6904 (2.6429-2.7409)	64.89% (56.57%-72.73%)	100% (100%-100%)	4.613% (0%-10.53%)	64.28% (56.12%-72.16%)	100% (100%-100%)	2.6949 (2.6011-2.7917)
At least 1 of CHASM (lung), FATHMM (cancer), FATHMM (missense), MutationTaster, PolyPhen-2, PROVEAN, SIFT, VEST	65.69% (62.12%-69.7%)	100% (100%-100%)	6.365% (2.817%-9.459%)	64.87% (61.13%-68.91%)	100% (100%-100%)	2.7124 (2.6595-2.7660)	65.59% (57.58%-72.73%)	100% (100%-100%)	6.513% (0%-13.16%)	64.74% (56.7%-72.17%)	100% (100%-100%)	2.7158 (2.6162-2.8303)
At least 1 of CHASM (lung), FATHMM (cancer), Mutation Assessor, MutationTaster, PolyPhen-2, PROVEAN, SIFT, VEST	66.07% (62.63%-70.2%)	100% (100%-100%)	7.41% (4.108%-10.667%)	65.13% (61.26%-69.11%)	100% (100%-100%)	2.7254 (2.6739-2.7836)	65.83% (57.58%-72.73%)	100% (100%-100%)	7.189% (0%-13.9%)	64.9% (56.84%-72.45%)	100% (100%-100%)	2.7234 (2.6163-2.8325)
At least 1 of CHASM (lung), FATHMM (missense), Mutation Assessor, MutationTaster, PolyPhen-2, PROVEAN, SIFT, VEST	65.7% (61.62%-69.7%)	100% (100%-100%)	6.384% (2.857%-9.459%)	64.88% (60.94%-68.88%)	100% (100%-100%)	2.7126 (2.6595-2.7702)	65.57% (57.58%-73.74%)	100% (100%-100%)	6.467% (0%-12.91%)	64.73% (56.38%-72.63%)	100% (100%-100%)	2.7150 (2.6162-2.8308)
At least 1 of CHASM (melanoma), FATHMM (cancer), FATHMM (missense), Mutation Assessor, MutationTaster, PolyPhen-2, PROVEAN, SIFT	66.37% (62.63%-70.2%)	100% (100%-100%)	8.22% (4.165%-11.594%)	65.33% (61.54%-69.43%)	100% (100%-100%)	2.7355 (2.6764-2.7972)	66.25% (58.59%-73.74%)	100% (100%-100%)	8.328% (2.439%-16.29%)	65.18% (56.84%-72.92%)	100% (100%-100%)	2.7366 (2.6238-2.8596)
At least 1 of CHASM (melanoma), FATHMM (cancer), FATHMM (missense), Mutation Assessor, MutationTaster, PolyPhen-2, PROVEAN, SIFT, VEST	65.03% (61.11%-69.19%)	100% (100%-100%)	4.567% (1.428%-7.042%)	64.44% (60.51%-68.53%)	100% (100%-100%)	2.6904 (2.6429-2.7409)	64.89% (56.57%-72.73%)	100% (100%-100%)	4.613% (0%-10.53%)	64.28% (56.12%-72.16%)	100% (100%-100%)	2.6949 (2.6011-2.7917)
At least 1 of CHASM (melanoma), FATHMM (cancer), FATHMM (missense), Mutation Assessor, MutationTaster, PolyPhen-2, PROVEAN, VEST	65.34% (61.62%-69.19%)	100% (100%-100%)	5.42% (2.597%-8.219%)	64.64% (60.82%-68.72%)	100% (100%-100%)	2.7009 (2.6535-2.7534)	65.27% (57.58%-72.73%)	100% (100%-100%)	5.651% (0%-12.12%)	64.53% (56.25%-72.16%)	100% (100%-100%)	2.7057 (2.6072-2.8051)
At least 1 of CHASM (melanoma), FATHMM (cancer), FATHMM (missense), Mutation Assessor, MutationTaster, PolyPhen-2, SIFT, VEST	65.37% (61.62%-69.19%)	100% (100%-100%)	5.484% (2.631%-8.219%)	64.66% (60.82%-68.72%)	100% (100%-100%)	2.7017 (2.6535-2.7534)	65.22% (57.58%-72.73%)	100% (100%-100%)	5.526% (0%-12.12%)	64.5% (56.25%-72.17%)	100% (100%-100%)	2.7048 (2.6054-2.8051)
At least 1 of CHASM (melanoma), FATHMM (cancer), FATHMM (missense), Mutation Assessor, MutationTaster, PROVEAN, SIFT, VEST	65.03% (61.11%-69.19%)	100% (100%-100%)	4.567% (1.428%-7.042%)	64.44% (60.51%-68.53%)	100% (100%-100%)	2.6904 (2.6429-2.7409)	64.89% (56.57%-72.73%)	100% (100%-100%)	4.613% (0%-10.53%)	64.28% (56.12%-72.16%)	100% (100%-100%)	2.6949 (2.6011-2.7917)
At least 1 of CHASM (melanoma), FATHMM (cancer), FATHMM (missense), Mutation Assessor, PolyPhen-2, PROVEAN, SIFT, VEST	65.03% (61.11%-69.19%)	100% (100%-100%)	4.567% (1.428%-7.042%)	64.44% (60.51%-68.53%)	100% (100%-100%)	2.6904 (2.6429-2.7409)	64.89% (56.57%-72.73%)	100% (100%-100%)	4.613% (0%-10.53%)	64.28% (56.12%-72.16%)	100% (100%-100%)	2.6949 (2.6011-2.7917)
At least 1 of CHASM (melanoma), FATHMM (cancer), FATHMM (missense), MutationTaster, PolyPhen-2, PROVEAN, SIFT, VEST	65.69% (62.12%-69.7%)	100% (100%-100%)	6.365% (2.817%-9.459%)	64.87% (61.13%-68.91%)	100% (100%-100%)	2.7124 (2.6595-2.7660)	65.59% (57.58%-72.73%)	100% (100%-100%)	6.513% (0%-13.16%)	64.74% (56.7%-72.17%)	100% (100%-100%)	2.7158 (2.6162-2.8303)
At least 1 of CHASM (melanoma), FATHMM (cancer), Mutation Assessor, MutationTaster, PolyPhen-2, PROVEAN, SIFT, VEST	66.07% (62.63%-70.2%)	100% (100%-100%)	7.41% (4.108%-10.667%)	65.13% (61.26%-69.11%)	100% (100%-100%)	2.7254 (2.6739-2.7836)	65.83% (57.58%-72.73%)	100% (100%-100%)	7.189% (0%-13.9%)	64.9% (56.84%-72.45%)	100% (100%-100%)	2.7234 (2.6163-2.8325)
At least 1 of CHASM (melanoma), FATHMM (missense), Mutation Assessor, MutationTaster, PolyPhen-2, PROVEAN, SIFT, VEST	65.7% (61.62%-69.7%)	100% (100%-100%)	6.384% (2.857%-9.459%)	64.88% (60.94%-68.88%)	100% (100%-100%)	2.7126 (2.6595-2.7702)	65.57% (57.58%-73.74%)	100% (100%-100%)	6.467% (0%-12.91%)	64.73% (56.38%-72.63%)	100% (100%-100%)	2.7150 (2.6162-2.8308)
At least 1 of FATHMM (cancer), FATHMM (missense), Mutation Assessor, MutationTaster, PolyPhen-2, PROVEAN, SIFT, VEST	65.03% (61.11%-69.19%)	100% (100%-100%)	4.567% (1.428%-7.042%)	64.44% (60.51%-68.53%)	100% (100%-100%)	2.6904 (2.6429-2.7409)	64.89% (56.57%-72.73%)	100% (100%-100%)	4.613% (0%-10.53%)	64.28% (56.12%-72.16%)	100% (100%-100%)	2.6949 (2.6011-2.7917)
At least 10 of CHASM (breast), CHASM (lung), CHASM (melanoma), FATHMM (cancer), FATHMM (missense), Mutation Assessor, MutationTaster, PolyPhen-2, PROVEAN, SIFT	54.58% (50.51%-58.59%)	28.84% (24.16%-33.33%)	99.084% (98.485%-100%)	98.2% (96.77%-100%)	44.61% (40%-48.78%)	2.7072 (2.6204-2.7928)	54.48% (46.46%-62.63%)	28.48% (19.66%-37.94%)	99.083% (96.774%-100%)	98.17% (93.33%-100%)	44.67% (36.24%-53.85%)	2.7040 (2.5284-2.8770)
At least 10 of CHASM (breast), CHASM (lung), CHASM (melanoma), FATHMM (cancer), FATHMM (missense), Mutation Assessor, MutationTaster, PolyPhen-2, PROVEAN, SIFT, VEST	61.99% (58.08%-65.66%)	41.61% (36.58%-46.77%)	97.233% (95.588%-100%)	96.3% (94%-100%)	49.06% (44.3%-53.43%)	2.8420 (2.7469-2.9431)	61.87% (54.55%-69.7%)	41.23% (30.88%-50.96%)	97.269% (92.303%-100%)	96.31% (89.66%-100%)	49.09% (40%-58.33%)	2.8390 (2.6297-3.0303)
At least 10 of CHASM (breast), CHASM (lung), CHASM (melanoma), FATHMM (cancer), FATHMM (missense), Mutation Assessor, MutationTaster, PolyPhen-2, PROVEAN, VEST	52.89% (48.48%-56.58%)	26.17% (21.21%-30.47%)	99.084% (98.485%-100%)	98.02% (96.43%-100%)	43.7% (39.13%-47.88%)	2.6696 (2.5823-2.7562)	52.82% (45.43%-61.62%)	25.85% (17.3%-35.5%)	99.083% (96.774%-100%)	97.98% (92.31%-100%)	43.78% (35.37%-53.09%)	2.6669 (2.4926-2.8388)
At least 10 of CHASM (breast), CHASM (lung), CHASM (melanoma), FATHMM (cancer), FATHMM (missense), Mutation Assessor, MutationTaster, PolyPhen-2, SIFT, VEST	56.26% (52.02%-60.1%)	32.03% (27.05%-36.72%)	98.143% (97.015%-100%)	96.76% (94.59%-100%)	45.51% (40.79%-49.69%)	2.7244 (2.6333-2.8156)	56.17% (48.48%-64.65%)	31.67% (22.22%-42.03%)	98.209% (94.118%-100%)	96.83% (89.47%-100%)	45.58% (37.04%-55.13%)	2.7229 (2.5374-2.9065)
At least 10 of CHASM (breast), CHASM (lung), CHASM (melanoma), FATHMM (cancer), FATHMM (missense), Mutation Assessor, MutationTaster, PROVEAN, SIFT, VEST	53.9% (49.49%-57.58%)	27.77% (22.9%-32.23%)	99.084% (98.485%-100%)	98.13% (96.67%-100%)	44.24% (39.52%-48.49%)	2.6922 (2.6043-2.7788)	53.81% (46.46%-62.63%)	27.43% (18.96%-36.93%)	99.083% (96.774%-100%)	98.1% (92.86%-100%)	44.31% (35.71%-53.57%)	2.6892 (2.5121-2.8688)
At least 10 of CHASM (breast), CHASM (lung), CHASM (melanoma), FATHMM (cancer), FATHMM (missense), Mutation Assessor, PolyPhen-2, PROVEAN, SIFT, VEST	54.24% (50%-58.08%)	28.3% (23.25%-32.54%)	99.084% (98.485%-100%)	98.16% (96.77%-100%)	44.42% (39.64%-48.7%)	2.6997 (2.6126-2.7840)	54.15% (46.46%-62.63%)	27.96% (19.4%-38.1%)	99.083% (96.774%-100%)	98.13% (92.86%-100%)	44.49% (36.14%-53.85%)	2.6966 (2.5200-2.8731)
At least 10 of CHASM (breast), CHASM (lung), CHASM (melanoma), FATHMM (cancer), FATHMM (missense), MutationTaster, PolyPhen-2, PROVEAN, SIFT, VEST	53.56% (49.48%-57.07%)	27.24% (22.22%-31.67%)	99.084% (98.485%-100%)	98.09% (96.55%-100%)	44.06% (39.41%-48.17%)	2.6847 (2.5940-2.7681)	53.48% (46.46%-61.64%)	26.9% (17.86%-36.84%)	99.083% (96.774%-100%)	98.06% (92.86%-100%)	44.13% (35.8%-53.75%)	2.6817 (2.5081-2.8637)
At least 10 of CHASM (breast), CHASM (lung), CHASM (melanoma), FATHMM (cancer), Mutation Assessor, MutationTaster, PolyPhen-2, PROVEAN, SIFT, VEST	53.54% (49.48%-57.58%)	27.2% (22.22%-31.75%)	99.084% (98.485%-100%)	98.09% (96.55%-100%)	44.05% (39.37%-48.19%)	2.6842 (2.5990-2.7732)	53.53% (45.45%-61.64%)	26.97% (17.91%-37.29%)	99.083% (96.774%-100%)	98.06% (92.31%-100%)	44.15% (35.63%-53.57%)	2.6827 (2.5030-2.8578)
At least 10 of CHASM (breast), CHASM (lung), CHASM (melanoma), FATHMM (missense), Mutation Assessor, MutationTaster, PolyPhen-2, PROVEAN, SIFT, VEST	52.89% (48.48%-56.58%)	26.17% (21.21%-30.47%)	99.084% (98.485%-100%)	98.02% (96.43%-100%)	43.7% (39.13%-47.88%)	2.6696 (2.5823-2.7562)	52.82% (45.43%-61.62%)	25.85% (17.3%-35.5%)	99.083% (96.774%-100%)	97.98% (92.31%-100%)	43.78% (35.37%-53.09%)	2.6669 (2.4926-2.8388)
At least 10 of CHASM (breast), CHASM (lung), FATHMM (cancer), FATHMM (missense), Mutation Assessor, MutationTaster, PolyPhen-2, PROVEAN, SIFT, VEST	52.89% (48.48%-56.58%)	26.17% (21.21%-30.47%)	99.084% (98.485%-100%)	98.02% (96.43%-100%)	43.7% (39.13%-47.88%)	2.6696 (2.5823-2.7562)	52.82% (45.43%-61.62%)	25.85% (17.3%-35.5%)	99.083% (96.774%-100%)	97.98% (92.31%-100%)	43.78% (35.37%-53.09%)	2.6669 (2.4926-2.8388)
At least 10 of CHASM (breast), CHASM (melanoma), FATHMM (cancer), FATHMM (missense), Mutation Assessor, MutationTaster, PolyPhen-2, PROVEAN, SIFT, VEST	52.55% (48.48%-56.57%)	26.17% (21.21%-30.47%)	98.173% (97.015%-100%)	96.12% (93.33%-100%)	43.47% (38.79%-47.83%)	2.6393 (2.5443-2.7363)	52.47% (44.44%-60.61%)	25.85% (17.3%-35.5%)	98.144% (94.118%-100%)	95.99% (87.5%-100%)	43.54% (34.94%-53.09%)	2.6353 (2.4342-2.8270)
At least 10 of CHASM (lung), CHASM (melanoma), FATHMM (cancer), FATHMM (missense), Mutation Assessor, MutationTaster, PolyPhen-2, PROVEAN, SIFT, VEST	53.56% (49.49%-57.07%)	27.23% (22.58%-31.5%)	99.084% (98.485%-100%)	98.09% (96.55%-100%)	44.05% (39.39%-48.39%)	2.6846 (2.5990-2.7690)	53.49% (45.45%-61.64%)	26.91% (18.33%-36.51%)	99.083% (96.774%-100%)	98.06% (92.86%-100%)	44.13% (35.61%-53.57%)	2.6819 (2.5036-2.8542)
At least 11 of CHASM (breast), CHASM (lung), CHASM (melanoma), FATHMM (cancer), FATHMM (missense), Mutation Assessor, MutationTaster, PolyPhen-2, PROVEAN, SIFT, VEST	52.89% (48.48%-56.58%)	26.17% (21.21%-30.47%)	99.084% (98.485%-100%)	98.02% (96.43%-100%)	43.7% (39.13%-47.88%)	2.6696 (2.5823-2.7562)	52.82% (45.43%-61.62%)	25.85% (17.3%-35.5%)	99.083% (96.774%-100%)	97.98% (92.31%-100%)	43.78% (35.37%-53.09%)	2.6669 (2.4926-2.8388)
At least 2 of CHASM (breast), CHASM (lung), CHASM (melanoma), FATHMM (cancer), FATHMM (missense), Mutation Assessor, MutationTaster, PolyPhen-2	79.8% (76.77%-82.84%)	98.97% (98.33%-100%)	46.638% (40.253%-53.034%)	76.23% (72.33%-80%)	96.33% (93.55%-100%)	3.1817 (3.0734-3.2927)	79.8% (73.71%-85.86%)	98.86% (96.67%-100%)	47.062% (33.333%-60%)	76.22% (68.42%-83.72%)	96.02% (87.5%-100%)	3.1817 (2.9663-3.3963)
At least 2 of CHASM (breast), CHASM (lung), CHASM (melanoma), FATHMM (cancer), FATHMM (missense), Mutation Assessor, MutationTaster, PolyPhen-2, PROVEAN	77.45% (74.24%-80.81%)	98.97% (98.33%-100%)	40.236% (33.783%-46.671%)	74.12% (70.3%-77.91%)	95.77% (92.59%-100%)	3.0909 (2.9780-3.2026)	77.42% (70.71%-83.84%)	98.86% (96.67%-100%)	40.586% (27.584%-53.49%)	74.07% (66.67%-81.48%)	95.41% (85.71%-100%)	3.0892 (2.8564-3.3079)
At least 2 of CHASM (breast), CHASM (lung), CHASM (melanoma), FATHMM (cancer), FATHMM (missense), Mutation Assessor, MutationTaster, PolyPhen-2, PROVEAN, SIFT	76.42% (73.23%-79.8%)	100% (100%-100%)	35.644% (28.986%-41.895%)	72.88% (69.14%-76.71%)	100% (100%-100%)	3.0852 (2.9997-3.1717)	76.45% (69.7%-82.83%)	100% (100%-100%)	36.008% (23.525%-48.78%)	72.84% (65.12%-80.24%)	100% (100%-100%)	3.0885 (2.9279-3.2720)
At least 2 of CHASM (breast), CHASM (lung), CHASM (melanoma), FATHMM (cancer), FATHMM (missense), Mutation Assessor, MutationTaster, PolyPhen-2, PROVEAN, SIFT, VEST	69.39% (65.66%-73.23%)	100% (100%-100%)	16.451% (11.268%-21.429%)	67.42% (63.78%-71.28%)	100% (100%-100%)	2.8387 (2.7675-2.9137)	69.31% (61.62%-76.77%)	100% (100%-100%)	16.608% (7.137%-27.28%)	67.3% (59.57%-74.73%)	100% (100%-100%)	2.8391 (2.7019-2.9863)
At least 2 of CHASM (breast), CHASM (lung), CHASM (melanoma), FATHMM (cancer), FATHMM (missense), Mutation Assessor, MutationTaster, PolyPhen-2, PROVEAN, VEST	71.39% (67.68%-74.75%)	100% (100%-100%)	21.906% (16.216%-27.397%)	68.89% (65.19%-72.63%)	100% (100%-100%)	2.9079 (2.8316-2.9869)	71.36% (64.65%-78.79%)	100% (100%-100%)	22.177% (11.111%-33.33%)	68.81% (61.36%-76.14%)	100% (100%-100%)	2.9099 (2.7652-3.0785)
At least 2 of CHASM (breast), CHASM (lung), CHASM (melanoma), FATHMM (cancer), FATHMM (missense), Mutation Assessor, MutationTaster, PolyPhen-2, SIFT	77.76% (74.24%-80.81%)	100% (100%-100%)	39.295% (32.857%-45.455%)	74.01% (70.3%-77.91%)	100% (100%-100%)	3.1331 (3.0433-3.2217)	77.82% (71.72%-84.85%)	100% (100%-100%)	39.728% (27.773%-53.12%)	74.01% (66.26%-81.71%)	100% (100%-100%)	3.1374 (2.9683-3.3201)
At least 2 of CHASM (breast), CHASM (lung), CHASM (melanoma), FATHMM (cancer), FATHMM (missense), Mutation Assessor, MutationTaster, PolyPhen-2, SIFT, VEST	71.06% (67.68%-74.75%)	100% (100%-100%)	21.016% (15.493%-26.391%)	68.64% (64.84%-72.53%)	100% (100%-100%)	2.8966 (2.8191-2.9775)	71.02% (63.64%-77.78%)	100% (100%-100%)	21.269% (10.811%-33.33%)	68.55% (60.86%-76.09%)	100% (100%-100%)	2.8982 (2.7523-3.0622)
At least 2 of CHASM (breast), CHASM (lung), CHASM (melanoma), FATHMM (cancer), FATHMM (missense), Mutation Assessor, MutationTaster, PolyPhen-2, VEST	72.73% (69.19%-76.26%)	100% (100%-100%)	25.579% (19.443%-31.081%)	69.91% (66.2%-73.86%)	100% (100%-100%)	2.9549 (2.8711-3.0357)	72.72% (65.66%-79.8%)	100% (100%-100%)	25.877% (14.286%-38.47%)	69.84% (62.1%-77.01%)	100% (100%-100%)	2.9572 (2.8050-3.1383)
At least 2 of CHASM (breast), CHASM (lung), CHASM (melanoma), FATHMM (cancer), FATHMM (missense), Mutation Assessor, MutationTaster, PROVEAN	79.46% (76.26%-82.32%)	98.97% (98.33%-100%)	45.724% (39.189%-52.056%)	75.92% (72.22%-79.5%)	96.26% (93.33%-100%)	3.1688 (3.0583-3.2725)	79.46% (73.74%-85.86%)	98.86% (96.67%-100%)	46.117% (32.5%-59.46%)	75.9% (68.6%-83.33%)	95.94% (87.5%-100%)	3.1683 (2.9500-3.3973)
At least 2 of CHASM (breast), CHASM (lung), CHASM (melanoma), FATHMM (cancer), FATHMM (missense), Mutation Assessor, MutationTaster, PROVEAN, SIFT	78.13% (74.75%-81.31%)	99.49% (99.16%-100%)	41.181% (34.374%-47.446%)	74.52% (70.73%-78.24%)	97.91% (96.15%-100%)	3.1311 (3.0374-3.2386)	78.09% (71.72%-84.85%)	99.42% (98.21%-100%)	41.45% (28.571%-54.55%)	74.46% (67.06%-81.82%)	97.67% (91.67%-100%)	3.1299 (2.9190-3.3355)
At least 2 of CHASM (breast), CHASM (lung), CHASM (melanoma), FATHMM (cancer), FATHMM (missense), Mutation Assessor, MutationTaster, PROVEAN, SIFT, VEST	69.73% (66.16%-73.23%)	100% (100%-100%)	17.377% (12.304%-22.226%)	67.67% (63.83%-71.43%)	100% (100%-100%)	2.8504 (2.7735-2.9310)	69.64% (62.63%-76.77%)	100% (100%-100%)	17.522% (7.687%-28.14%)	67.54% (60%-75.01%)	100% (100%-100%)	2.8507 (2.7080-3.0159)
At least 2 of CHASM (breast), CHASM (lung), CHASM (melanoma), FATHMM (cancer), FATHMM (missense), Mutation Assessor, MutationTaster, PROVEAN, VEST	72.44% (68.69%-76.26%)	99.49% (99.16%-100%)	25.647% (19.718%-31.579%)	69.82% (65.93%-73.6%)	96.69% (93.33%-100%)	2.9165 (2.8079-3.0232)	72.3% (64.65%-79.8%)	99.42% (98.21%-100%)	25.728% (13.786%-37.84%)	69.68% (62.22%-77.28%)	96.24% (85.71%-100%)	2.9107 (2.6662-3.1078)
At least 2 of CHASM (breast), CHASM (lung), CHASM (melanoma), FATHMM (cancer), FATHMM (missense), Mutation Assessor, MutationTaster, SIFT	79.8% (76.77%-82.83%)	99.49% (99.16%-100%)	45.752% (39.436%-52.174%)	76.03% (72.29%-79.76%)	98.12% (96.55%-100%)	3.1939 (3.1016-3.3000)	79.79% (73.74%-85.86%)	99.42% (98.21%-100%)	46.081% (32.498%-58.54%)	75.99% (68.67%-83.53%)	97.91% (92.84%-100%)	3.1940 (2.9874-3.3816)
At least 2 of CHASM (breast), CHASM (lung), CHASM (melanoma), FATHMM (cancer), FATHMM (missense), Mutation Assessor, MutationTaster, SIFT, VEST	71.4% (67.68%-75.25%)	100% (100%-100%)	21.942% (16.437%-27.536%)	68.9% (65.17%-72.88%)	100% (100%-100%)	2.9084 (2.8288-2.9952)	71.35% (63.64%-78.79%)	100% (100%-100%)	22.184% (11.421%-34.29%)	68.8% (60.87%-76.4%)	100% (100%-100%)	2.9099 (2.7557-3.0786)
At least 2 of CHASM (breast), CHASM (lung), CHASM (melanoma), FATHMM (cancer), FATHMM (missense), Mutation Assessor, MutationTaster, VEST	74.79% (71.72%-78.28%)	99.49% (99.16%-100%)	32.091% (25.714%-38.235%)	71.7% (68.02%-75.71%)	97.34% (95%-100%)	3.0062 (2.9062-3.1052)	74.65% (67.68%-80.81%)	99.42% (98.21%-100%)	32.144% (19.355%-44.44%)	71.55% (63.63%-78.82%)	97% (88.89%-100%)	3.0011 (2.7770-3.2035)
At least 2 of CHASM (breast), CHASM (lung), CHASM (melanoma), FATHMM (cancer), FATHMM (missense), Mutation Assessor, PolyPhen-2, PROVEAN	77.43% (74.24%-80.81%)	98.44% (97.52%-100%)	41.101% (34.327%-47.887%)	74.29% (70.59%-77.91%)	93.83% (89.66%-100%)	3.0766 (2.9617-3.1905)	77.46% (70.71%-83.84%)	98.34% (95.45%-100%)	41.6% (28.571%-54.55%)	74.3% (67.05%-81.71%)	93.59% (83.33%-100%)	3.0783 (2.8487-3.2928)
At least 2 of CHASM (breast), CHASM (lung), CHASM (melanoma), FATHMM (cancer), FATHMM (missense), Mutation Assessor, PolyPhen-2, PROVEAN, SIFT	76.07% (72.73%-79.29%)	98.94% (98.33%-100%)	36.509% (29.73%-42.671%)	72.94% (69.14%-76.65%)	95.24% (91.67%-100%)	3.0363 (2.9276-3.1435)	76.14% (69.7%-82.83%)	98.92% (96.67%-100%)	37.022% (23.68%-50.03%)	72.95% (65.43%-80.72%)	95.22% (84.62%-100%)	3.0411 (2.8085-3.2519)
At least 2 of CHASM (breast), CHASM (lung), CHASM (melanoma), FATHMM (cancer), FATHMM (missense), Mutation Assessor, PolyPhen-2, PROVEAN, SIFT, VEST	69.71% (66.16%-73.23%)	100% (100%-100%)	17.317% (12.162%-22.059%)	67.65% (63.98%-71.43%)	100% (100%-100%)	2.8497 (2.7742-2.9235)	69.68% (62.63%-76.77%)	100% (100%-100%)	17.616% (7.89%-28.14%)	67.57% (59.99%-74.74%)	100% (100%-100%)	2.8519 (2.7142-3.0062)
At least 2 of CHASM (breast), CHASM (lung), CHASM (melanoma), FATHMM (cancer), FATHMM (missense), Mutation Assessor, PolyPhen-2, PROVEAN, VEST	71.38% (68.18%-74.75%)	99.48% (99.16%-100%)	22.772% (16.923%-28.378%)	69.02% (65.36%-72.83%)	96.2% (92.31%-100%)	2.8746 (2.7705-2.9771)	71.39% (64.65%-77.78%)	99.44% (98.25%-100%)	23.185% (11.765%-34.38%)	68.97% (61.54%-76.35%)	95.99% (83.33%-100%)	2.8759 (2.6270-3.0833)
At least 2 of CHASM (breast), CHASM (lung), CHASM (melanoma), FATHMM (cancer), FATHMM (missense), Mutation Assessor, PolyPhen-2, SIFT	77.41% (74.24%-80.81%)	98.94% (98.33%-100%)	40.16% (33.333%-46.377%)	74.09% (70.24%-77.78%)	95.65% (92.59%-100%)	3.0884 (2.9799-3.1976)	77.51% (70.71%-83.84%)	98.92% (96.67%-100%)	40.743% (28.571%-54.05%)	74.13% (66.28%-81.71%)	95.64% (85.71%-100%)	3.0943 (2.8630-3.3077)
At least 2 of CHASM (breast), CHASM (lung), CHASM (melanoma), FATHMM (cancer), FATHMM (missense), Mutation Assessor, PolyPhen-2, SIFT, VEST	71.38% (67.68%-75.25%)	100% (100%-100%)	21.882% (15.942%-27.276%)	68.88% (65.19%-72.83%)	100% (100%-100%)	2.9076 (2.8288-2.9869)	71.39% (63.64%-78.79%)	100% (100%-100%)	22.278% (11.429%-34.29%)	68.83% (60.87%-76.14%)	100% (100%-100%)	2.9111 (2.7652-3.0785)
At least 2 of CHASM (breast), CHASM (lung), CHASM (melanoma), FATHMM (cancer), FATHMM (missense), Mutation Assessor, PolyPhen-2, VEST	72.72% (69.19%-76.26%)	99.48% (99.16%-100%)	26.446% (20.289%-32.052%)	70.05% (66.48%-74.01%)	96.71% (93.75%-100%)	2.9268 (2.8163-3.0256)	72.74% (65.66%-79.8%)	99.44% (98.25%-100%)	26.885% (15.143%-39.48%)	70.01% (62.22%-77.27%)	96.53% (85.71%-100%)	2.9287 (2.6977-3.1451)
At least 2 of CHASM (breast), CHASM (lung), CHASM (melanoma), FATHMM (cancer), FATHMM (missense), Mutation Assessor, PROVEAN, SIFT	77.8% (74.75%-81.31%)	97.92% (96.69%-99.24%)	42.993% (36.486%-49.383%)	74.81% (70.91%-78.57%)	92.3% (87.88%-97.22%)	3.0802 (2.9609-3.2026)	77.74% (70.71%-83.84%)	97.77% (94.73%-100%)	43.344% (30%-57.14%)	74.76% (67.09%-82.36%)	91.9% (80%-100%)	3.0776 (2.8221-3.3200)
At least 2 of CHASM (breast), CHASM (lung), CHASM (melanoma), FATHMM (cancer), FATHMM (missense), Mutation Assessor, PROVEAN, SIFT, VEST	70.36% (66.67%-74.24%)	100% (100%-100%)	19.108% (13.697%-24.324%)	68.13% (64.48%-71.89%)	100% (100%-100%)	2.8724 (2.7986-2.9559)	70.39% (62.63%-77.78%)	100% (100%-100%)	19.545% (9.084%-30.56%)	68.09% (60.44%-75.53%)	100% (100%-100%)	2.8763 (2.7277-3.0257)
At least 2 of CHASM (breast), CHASM (lung), CHASM (melanoma), FATHMM (cancer), FATHMM (missense), Mutation Assessor, PROVEAN, VEST	72.42% (69.19%-75.76%)	98.47% (97.52%-100%)	27.379% (21.333%-33.333%)	70.1% (66.47%-73.86%)	91.18% (85%-100%)	2.8714 (2.7416-3.0045)	72.32% (65.66%-78.79%)	98.27% (95.38%-100%)	27.751% (15.148%-40%)	70.02% (62.35%-77.65%)	90.2% (74.94%-100%)	2.8624 (2.5564-3.1156)
At least 2 of CHASM (breast), CHASM (lung), CHASM (melanoma), FATHMM (cancer), FATHMM (missense), Mutation Assessor, SIFT, VEST	72.03% (68.69%-75.76%)	100% (100%-100%)	23.674% (17.808%-29.412%)	69.38% (65.71%-73.3%)	100% (100%-100%)	2.9305 (2.8538-3.0157)	72.1% (64.65%-78.79%)	100% (100%-100%)	24.207% (12.812%-36.11%)	69.37% (61.54%-76.92%)	100% (100%-100%)	2.9357 (2.7743-3.1051)
At least 2 of CHASM (breast), CHASM (lung), CHASM (melanoma), FATHMM (cancer), FATHMM (missense), MutationTaster, PolyPhen-2, PROVEAN	79.48% (76.26%-82.83%)	98.97% (98.33%-100%)	45.779% (39.13%-52.113%)	75.94% (72.33%-79.52%)	96.26% (93.55%-100%)	3.1696 (3.0636-3.2744)	79.42% (72.73%-85.86%)	98.86% (96.67%-100%)	46.009% (34.189%-58.98%)	75.87% (68.35%-83.12%)	95.94% (87.5%-100%)	3.1667 (2.9553-3.3817)
At least 2 of CHASM (breast), CHASM (lung), CHASM (melanoma), FATHMM (cancer), FATHMM (missense), MutationTaster, PolyPhen-2, PROVEAN, SIFT	77.45% (74.24%-80.81%)	100% (100%-100%)	38.44% (31.944%-44.595%)	73.75% (70.12%-77.71%)	100% (100%-100%)	3.1219 (3.0341-3.2093)	77.43% (70.71%-83.84%)	100% (100%-100%)	38.667% (26.316%-51.61%)	73.68% (65.87%-80.95%)	100% (100%-100%)	3.1234 (2.9570-3.3163)
At least 2 of CHASM (breast), CHASM (lung), CHASM (melanoma), FATHMM (cancer), FATHMM (missense), MutationTaster, PolyPhen-2, PROVEAN, SIFT, VEST	70.06% (66.67%-73.74%)	100% (100%-100%)	18.29% (13.043%-23.191%)	67.91% (64.28%-71.74%)	100% (100%-100%)	2.8620 (2.7899-2.9407)	69.98% (62.63%-76.77%)	100% (100%-100%)	18.445% (8.328%-29.17%)	67.79% (60%-75.27%)	100% (100%-100%)	2.8623 (2.7211-3.0245)
At least 2 of CHASM (breast), CHASM (lung), CHASM (melanoma), FATHMM (cancer), FATHMM (missense), MutationTaster, PolyPhen-2, PROVEAN, VEST	72.06% (68.69%-75.76%)	100% (100%-100%)	23.745% (17.808%-29.412%)	69.4% (65.75%-73.08%)	100% (100%-100%)	2.9314 (2.8521-3.0116)	72.04% (64.65%-78.79%)	100% (100%-100%)	24.013% (12.5%-36.11%)	69.32% (61.8%-76.6%)	100% (100%-100%)	2.9333 (2.7805-3.1078)
At least 2 of CHASM (breast), CHASM (lung), CHASM (melanoma), FATHMM (cancer), FATHMM (missense), MutationTaster, PolyPhen-2, SIFT	79.12% (76.25%-82.32%)	100% (100%-100%)	42.999% (36.486%-49.296%)	75.21% (71.6%-78.82%)	100% (100%-100%)	3.1821 (3.0926-3.2732)	79.14% (72.73%-84.87%)	100% (100%-100%)	43.308% (30.764%-56.42%)	75.17% (67.82%-82.5%)	100% (100%-100%)	3.1848 (3.0169-3.3719)

At least 2 of CHASM (breast), CHASM (lung), CHASM (melanoma), FATHMM (cancer), FATHMM (missense), MutationTaster, PolyPhen-2, SIFT, VEST	71.4% (68.18%-75.25%)	100% (100%-100%)	21.942% (16.216%-27.397%)	68.9% (65.19%-72.68%)	100% (100%-100%)	2.9084 (2.8311-2.9869)	71.35% (63.64%-77.78%)	100% (100%-100%)	22.17% (11.429%-33.35%)	68.8% (60.87%-76.09%)	100% (100%-100%)	2.9097 (2.7607-3.0774)
At least 2 of CHASM (breast), CHASM (lung), CHASM (melanoma), FATHMM (cancer), FATHMM (missense), MutationTaster, PolyPhen-2, VEST	73.74% (70.2%-77.27%)	100% (100%-100%)	28.327% (22.077%-33.832%)	70.7% (67.05%-74.58%)	100% (100%-100%)	2.9902 (2.9060-3.0727)	73.73% (66.67%-80.81%)	100% (100%-100%)	28.635% (17.063%-41.67%)	70.63% (62.99%-77.78%)	100% (100%-100%)	2.9781 (2.8413-3.1748)
At least 2 of CHASM (breast), CHASM (lung), CHASM (melanoma), FATHMM (cancer), FATHMM (missense), MutationTaster, PROVEAN, SIFT	79.15% (76.26%-82.32%)	99.49% (99.16%-100%)	43.973% (37.681%-50%)	75.43% (71.69%-79.14%)	98.05% (96.43%-100%)	3.1694 (3.0718-3.2732)	79.07% (72.73%-84.85%)	99.42% (98.21%-100%)	44.14% (31.233%-57.15%)	75.34% (67.81%-82.75%)	97.82% (91.67%-100%)	3.1671 (2.9601-3.3715)
[illegible]	[illegible]	[illegible]	[illegible]	[illegible]	[illegible]	[illegible]	[illegible]	[illegible]	[illegible]	[illegible]	[illegible]	[illegible]

At least 2 of CHASM (lung), CHASM (melanoma), FATHMM (cancer), MutationTaster, PolyPhen-2, SIFT, VEST	74.42% (71.21%-78.28%)	100% (100%-100%)	30.2% (24.286%-36.111%)	[illegible]	[illegible]	[illegible]	[illegible]	[illegible]	[illegible]	[illegible]	[illegible]	[illegible]
At least 2 of CHASM (lung), CHASM (melanoma), FATHMM (cancer), MutationTaster, PROVEAN, SIFT, VEST	73.09% (69.7%-76.77%)	100% (100%-100%)	26.567% (21.125%-32.353%)	[illegible]	[illegible]	[illegible]	[illegible]	[illegible]	[illegible]	[illegible]	[illegible]	[illegible]
At least 2 of CHASM (lung), CHASM (melanoma), FATHMM (cancer), PolyPhen-2, PROVEAN, SIFT, VEST	72.73% (69.68%-76.26%)	99.46% (99.16%-100%)	26.503% (20.548%-32.395%)	[illegible]	[illegible]	[illegible]	[illegible]	[illegible]	[illegible]	[illegible]	[illegible]	[illegible]
At least 2 of CHASM (lung), CHASM (melanoma), FATHMM (missense), Mutation Assessor, MutationTaster, PolyPhen-2, PROVEAN	79.16% (76.26%-82.32%)	98.97% (98.33%-100%)	44.899% (38.155%-51.391%)	[illegible]	[illegible]	[illegible]	[illegible]	[illegible]	[illegible]	[illegible]	[illegible]	[illegible]
At least 2 of CHASM (lung), CHASM (melanoma), FATHMM (missense), Mutation Assessor, MutationTaster, PolyPhen-2, PROVEAN, SIFT	76.76% (73.74%-79.8%)	100% (100%-100%)	36.579% (29.87%-42.667%)	[illegible]	[illegible]	[illegible]	[illegible]	[illegible]	[illegible]	[illegible]	[illegible]	[illegible]
At least 2 of CHASM (lung), CHASM (melanoma), FATHMM (missense), Mutation Assessor, MutationTaster, PolyPhen-2, PROVEAN, SIFT, VEST	71.75% (68.18%-75.25%)	100% (100%-100%)	22.901% (17.566%-28.359%)	[illegible]	[illegible]	[illegible]	[illegible]	[illegible]	[illegible]	[illegible]	[illegible]	[illegible]
At least 2 of CHASM (lung), CHASM (melanoma), FATHMM (missense), Mutation Assessor, MutationTaster, PolyPhen-2, PROVEAN, VEST	73.75% (70.2%-77.27%)	100% (100%-100%)	28.356% (22.534%-33.803%)	[illegible]	[illegible]	[illegible]	[illegible]	[illegible]	[illegible]	[illegible]	[illegible]	[illegible]
At least 2 of CHASM (lung), CHASM (melanoma), FATHMM (missense), Mutation Assessor, MutationTaster, PolyPhen-2, SIFT	78.09% (74.75%-81.31%)	100% (100%-100%)	40.196% (33.773%-46.479%)	[illegible]	[illegible]	[illegible]	[illegible]	[illegible]	[illegible]	[illegible]	[illegible]	[illegible]
At least 2 of CHASM (lung), CHASM (melanoma), FATHMM (missense), Mutation Assessor, MutationTaster, PolyPhen-2, SIFT, VEST	73.08% (69.7%-76.77%)	100% (100%-100%)	26.531% (20.774%-32.309%)	[illegible]	[illegible]	[illegible]	[illegible]	[illegible]	[illegible]	[illegible]	[illegible]	[illegible]
At least 2 of CHASM (lung), CHASM (melanoma), FATHMM (missense), Mutation Assessor, MutationTaster, PolyPhen-2, VEST	75.42% (72.22%-78.79%)	100% (100%-100%)	32.928% (27.023%-38.893%)	[illegible]	[illegible]	[illegible]	[illegible]	[illegible]	[illegible]	[illegible]	[illegible]	[illegible]
At least 2 of CHASM (lung), CHASM (melanoma), FATHMM (missense), Mutation Assessor, MutationTaster, PROVEAN, SIFT	78.48% (75.25%-81.82%)	98.98% (98.32%-100%)	43.024% (36.707%-49.296%)	[illegible]	[illegible]	[illegible]	[illegible]	[illegible]	[illegible]	[illegible]	[illegible]	[illegible]
At least 2 of CHASM (lung), CHASM (melanoma), FATHMM (missense), Mutation Assessor, MutationTaster, PROVEAN, SIFT, VEST	72.09% (68.69%-75.76%)	100% (100%-100%)	23.826% (18.307%-29.414%)	[illegible]	[illegible]	[illegible]	[illegible]	[illegible]	[illegible]	[illegible]	[illegible]	[illegible]
At least 2 of CHASM (lung), CHASM (melanoma), FATHMM (missense), Mutation Assessor, MutationTaster, PROVEAN, VEST	75.14% (71.72%-78.79%)	99.49% (99.16%-100%)	33.029% (27.269%-39.13%)	[illegible]	[illegible]	[illegible]	[illegible]	[illegible]	[illegible]	[illegible]	[illegible]	[illegible]
At least 2 of CHASM (lung), CHASM (melanoma), FATHMM (missense), Mutation Assessor, MutationTaster, SIFT, VEST	73.76% (70.2%-77.27%)	100% (100%-100%)	28.389% (22.535%-34.617%)	[illegible]	[illegible]	[illegible]	[illegible]	[illegible]	[illegible]	[illegible]	[illegible]	[illegible]
At least 2 of CHASM (lung), CHASM (melanoma), FATHMM (missense), Mutation Assessor, PolyPhen-2, PROVEAN, SIFT	75.41% (71.72%-78.79%)	97.36% (95.93%-99.19%)	37.444% (30.769%-43.481%)	[illegible]	[illegible]	[illegible]	[illegible]	[illegible]	[illegible]	[illegible]	[illegible]	[illegible]
At least 2 of CHASM (lung), CHASM (melanoma), FATHMM (missense), Mutation Assessor, PolyPhen-2, PROVEAN, SIFT, VEST	72.07% (68.69%-75.76%)	100% (100%-100%)	23.767% (17.91%-29.233%)	[illegible]	[illegible]	[illegible]	[illegible]	[illegible]	[illegible]	[illegible]	[illegible]	[illegible]
At least 2 of CHASM (lung), CHASM (melanoma), FATHMM (missense), Mutation Assessor, PolyPhen-2, PROVEAN, VEST	73.74% (70.2%-77.27%)	99.48% (99.16%-100%)	29.222% (23.188%-34.783%)	[illegible]	[illegible]	[illegible]	[illegible]	[illegible]	[illegible]	[illegible]	[illegible]	[illegible]
At least 2 of CHASM (lung), CHASM (melanoma), FATHMM (missense), Mutation Assessor, PolyPhen-2, SIFT, VEST	73.4% (70.2%-76.77%)	100% (100%-100%)	27.398% (21.426%-33.333%)	[illegible]	[illegible]	[illegible]	[illegible]	[illegible]	[illegible]	[illegible]	[illegible]	[illegible]
At least 2 of CHASM (lung), CHASM (melanoma), FATHMM (missense), Mutation Assessor, PROVEAN, SIFT, VEST	72.06% (68.67%-75.76%)	98.95% (98.32%-100%)	25.558% (20%-31.152%)	[illegible]	[illegible]	[illegible]	[illegible]	[illegible]	[illegible]	[illegible]	[illegible]	[illegible]
At least 2 of CHASM (lung), CHASM (melanoma), FATHMM (missense), MutationTaster, PolyPhen-2, PROVEAN, SIFT	77.79% (74.75%-81.31%)	100% (100%-100%)	39.375% (32.857%-45.07%)	[illegible]	[illegible]	[illegible]	[illegible]	[illegible]	[illegible]	[illegible]	[illegible]	[illegible]
At least 2 of CHASM (lung), CHASM (melanoma), FATHMM (missense), MutationTaster, PolyPhen-2, PROVEAN, SIFT, VEST	72.42% (69.19%-75.77%)	100% (100%-100%)	24.74% (18.917%-30%)	[illegible]	[illegible]	[illegible]	[illegible]	[illegible]	[illegible]	[illegible]	[illegible]	[illegible]
[illegible]	[illegible]	[illegible]	[illegible]	[illegible]	[illegible]	[illegible]	[illegible]	[illegible]	[illegible]	[illegible]	[illegible]	[illegible]

**At least 3 of CHASM (breast), CHASM (lung), CHASM (melanoma), FATHMM (missense), PolyPhen-2, PROVEAN, SIFT**	82.52% (79.8%-85.35%)	94.74% (92.68%-96.85%)	61.399% (55.069%-67.607%)	80.93% (77.55%-84.72%)	87.09% (82%-92.31%)	3.2416 (3.1188-3.3690)	82.43% (76.77%-87.88%)	94.56% (90.16%-98.46%)	61.568% (48.387%-74.36%)	80.86% (73.42%-87.68%)	86.84% (75%-96.3%)	3.2383 (2.9845-3.4764)
**At least 3 of CHASM (breast), CHASM (lung), CHASM (melanoma), FATHMM (missense), PolyPhen-2, PROVEAN, SIFT, VEST**	77.46% (74.24%-80.81%)	96.32% (94.49%-98.39%)	44.814% (38.462%-51.251%)	75.11% (71.52%-78.85%)	87.58% (81.82%-94.44%)	3.0384 (2.9068-3.1712)	77.41% (70.71%-83.84%)	96.17% (92.19%-100%)	45.163% (32.557%-57.58%)	75.07% (67.5%-82.05%)	87.32% (75%-100%)	3.0372 (2.7714-3.2902)
**At least 3 of CHASM (breast), CHASM (lung), CHASM (melanoma), FATHMM (missense), PolyPhen-2, PROVEAN, VEST**	81.54% (78.79%-84.85%)	94.74% (92.68%-96.85%)	58.714% (52.172%-65.335%)	79.87% (76.39%-83.67%)	86.58% (81.48%-92%)	3.1990 (3.0774-3.3279)	81.36% (74.75%-86.87%)	94.56% (90.16%-98.46%)	58.671% (45.7%-71.44%)	79.71% (72.29%-86.67%)	86.27% (74.07%-96%)	3.1921 (2.9277-3.4348)
**At least 3 of CHASM (breast), CHASM (lung), CHASM (melanoma), FATHMM (missense), PolyPhen-2, SIFT, VEST**	79.12% (75.76%-82.32%)	95.79% (93.85%-97.67%)	50.304% (43.66%-56.757%)	76.92% (73.25%-80.72%)	87.35% (81.82%-93.02%)	3.1036 (2.9743-3.2300)	79.12% (72.73%-85.86%)	95.65% (91.52%-100%)	50.703% (38.087%-63.42%)	76.91% (69.23%-84.21%)	87.19% (75%-100%)	3.1046 (2.8457-3.3614)
**At least 3 of CHASM (breast), CHASM (lung), CHASM (melanoma), FATHMM (missense), PROVEAN, SIFT, VEST**	80.15% (77.27%-83.33%)	94.2% (92%-96.72%)	55.849% (49.315%-62.162%)	78.67% (75.16%-82.43%)	84.78% (79.17%-90.48%)	3.1350 (3.0120-3.2701)	80.11% (73.74%-85.86%)	94.04% (89.09%-98.41%)	56.142% (42.857%-68.75%)	78.64% (71.01%-85.71%)	84.61% (72%-95.65%)	3.1343 (2.8661-3.3830)
**At least 3 of CHASM (breast), CHASM (lung), CHASM (melanoma), Mutation Assessor, MutationTaster, PolyPhen-2, PROVEAN**	86.23% (83.84%-88.89%)	95.77% (93.7%-97.64%)	69.719% (63.768%-76.056%)	84.54% (81.21%-88.28%)	90.52% (86.27%-94.83%)	3.4055 (3.2920-3.5150)	86.14% (80.81%-90.91%)	95.68% (91.8%-100%)	69.759% (57.773%-81.26%)	84.44% (77.03%-90.91%)	90.43% (80.95%-100%)	3.4030 (3.1710-3.6246)
**At least 3 of CHASM (breast), CHASM (lung), CHASM (melanoma), Mutation Assessor, MutationTaster, PolyPhen-2, PROVEAN, SIFT**	81.51% (78.28%-84.85%)	96.32% (94.4%-98.39%)	55.901% (50%-62.319%)	79.06% (75.32%-82.8%)	89.78% (85%-95.45%)	3.2106 (3.0944-3.3331)	81.43% (74.75%-87.88%)	96.19% (92.19%-100%)	56.103% (42.857%-68.57%)	78.99% (71.42%-86.42%)	89.59% (78.95%-100%)	3.2087 (2.9569-3.4399)
**At least 3 of CHASM (breast), CHASM (lung), CHASM (melanoma), Mutation Assessor, MutationTaster, PolyPhen-2, PROVEAN, SIFT, VEST**	79.8% (76.77%-82.83%)	98.39% (97.5%-100%)	47.645% (41.025%-54.054%)	76.47% (72.73%-80.13%)	94.48% (90.91%-100%)	3.1698 (3.0563-3.2785)	79.8% (73.74%-85.86%)	98.43% (95.45%-100%)	47.836% (34.997%-61.12%)	76.4% (68.83%-83.75%)	94.67% (85.71%-100%)	3.1735 (2.9452-3.3942)
**At least 3 of CHASM (breast), CHASM (lung), CHASM (melanoma), Mutation Assessor, MutationTaster, PolyPhen-2, PROVEAN, VEST**	81.19% (78.28%-84.34%)	97.36% (95.93%-99.19%)	53.232% (46.57%-59.466%)	78.26% (74.51%-81.94%)	92.11% (87.8%-97.56%)	3.2096 (3.0955-3.3281)	81.05% (74.75%-86.87%)	97.3% (93.75%-100%)	53.188% (40%-66.67%)	78.1% (70.66%-85.53%)	91.99% (81.25%-100%)	3.2057 (2.9661-3.4293)
**At least 3 of CHASM (breast), CHASM (lung), CHASM (melanoma), Mutation Assessor, MutationTaster, PolyPhen-2, SIFT**	83.53% (80.81%-86.87%)	95.78% (93.75%-97.67%)	62.354% (56.336%-68.831%)	81.48% (78%-85.04%)	89.53% (84.91%-94.34%)	3.2914 (3.1743-3.4149)	83.44% (76.77%-88.89%)	95.67% (91.52%-100%)	62.462% (48.718%-75%)	81.38% (73.91%-88.41%)	89.41% (78.95%-100%)	3.2892 (3.0403-3.5240)
**At least 3 of CHASM (breast), CHASM (lung), CHASM (melanoma), Mutation Assessor, MutationTaster, PolyPhen-2, SIFT, VEST**	80.13% (77.26%-83.33%)	97.85% (96.72%-99.22%)	49.478% (42.857%-55.882%)	77% (73.29%-80.77%)	93.01% (88.89%-97.5%)	3.1735 (3.0507-3.2828)	80.15% (73.74%-85.88%)	97.91% (94.92%-100%)	49.675% (36.838%-62.5%)	76.95% (69.33%-84.52%)	93.27% (83.33%-100%)	3.1780 (2.9403-3.4155)
**At least 3 of CHASM (breast), CHASM (lung), CHASM (melanoma), Mutation Assessor, MutationTaster, PolyPhen-2, VEST**	82.88% (79.8%-85.86%)	96.82% (95.12%-98.48%)	58.767% (52.855%-65.278%)	80.23% (76.66%-83.87%)	91.45% (87.23%-96%)	3.2728 (3.1595-3.3953)	82.73% (76.77%-88.89%)	96.78% (92.86%-100%)	58.633% (45.946%-70.59%)	80.05% (72.6%-87.18%)	91.4% (80.95%-100%)	3.2686 (3.0122-3.4914)
**At least 3 of CHASM (breast), CHASM (lung), CHASM (melanoma), Mutation Assessor, MutationTaster, PROVEAN, SIFT**	86.55% (83.84%-89.39%)	95.23% (93.27%-97.58%)	71.54% (65.705%-77.143%)	85.26% (82.07%-88.49%)	89.68% (85.45%-94.44%)	3.4172 (3.3047-3.5305)	86.49% (80.81%-91.92%)	95.16% (90.62%-98.55%)	71.588% (60.604%-83.78%)	85.17% (78.26%-91.78%)	89.64% (80%-97.22%)	3.4157 (3.1832-3.6444)
**At least 3 of CHASM (breast), CHASM (lung), CHASM (melanoma), Mutation Assessor, MutationTaster, PROVEAN, SIFT, VEST**	81.83% (78.79%-84.85%)	96.83% (95.12%-98.49%)	55.895% (49.333%-62.319%)	79.15% (75.5%-82.8%)	91.08% (86.67%-95.83%)	3.2295 (3.1103-3.3535)	81.8% (75.76%-87.88%)	96.76% (92.85%-100%)	56.114% (42.857%-69.23%)	79.09% (71.79%-86.49%)	91.03% (80%-100%)	3.2300 (2.9859-3.4598)
**At least 3 of CHASM (breast), CHASM (lung), CHASM (melanoma), Mutation Assessor, MutationTaster, PROVEAN, VEST**	85.22% (82.32%-87.88%)	95.26% (93.39%-97.56%)	67.867% (61.837%-73.531%)	83.67% (80.28%-87.14%)	89.24% (84.91%-94.34%)	3.3605 (3.2519-3.4820)	85.11% (79.8%-90.91%)	95.1% (90.62%-98.51%)	67.956% (56.406%-80%)	83.57% (76.56%-90.41%)	89.03% (79.31%-96.67%)	3.3565 (3.1186-3.5755)
**At least 3 of CHASM (breast), CHASM (lung), CHASM (melanoma), Mutation Assessor, MutationTaster, SIFT, VEST**	83.51% (80.81%-86.36%)	96.83% (95.12%-98.49%)	60.49% (54.167%-66.667%)	80.9% (77.4%-84.62%)	91.7% (87.5%-96.08%)	3.2992 (3.1862-3.4162)	83.48% (77.78%-88.89%)	96.76% (92.85%-100%)	60.686% (47.5%-72.97%)	80.85% (73.42%-87.67%)	91.65% (81.82%-100%)	3.2995 (3.0667-3.5232)
**At least 3 of CHASM (breast), CHASM (lung), CHASM (melanoma), Mutation Assessor, PolyPhen-2, PROVEAN, SIFT**	81.83% (78.79%-84.85%)	95.23% (93.27%-97.58%)	58.665% (52.495%-65.083%)	79.93% (76.39%-83.56%)	87.69% (82.69%-93.34%)	3.2152 (3.0949-3.3408)	81.78% (75.76%-87.88%)	95.16% (90.62%-98.55%)	58.818% (45.714%-71.43%)	79.85% (72.36%-86.84%)	87.67% (76.19%-96.67%)	3.2150 (2.9541-3.4531)
**At least 3 of CHASM (breast), CHASM (lung), CHASM (melanoma), Mutation Assessor, PolyPhen-2, PROVEAN, SIFT, VEST**	79.44% (76.26%-82.83%)	96.29% (94.4%-98.39%)	50.308% (43.662%-56.757%)	77.01% (73.25%-80.77%)	88.69% (83.33%-94.74%)	3.1229 (2.9906-3.2569)	79.51% (72.73%-85.86%)	96.25% (92.19%-100%)	50.772% (37.838%-63.89%)	77.03% (69.51%-84.62%)	88.78% (77.26%-100%)	3.1283 (2.8524-3.3911)
**At least 3 of CHASM (breast), CHASM (lung), CHASM (melanoma), Mutation Assessor, PolyPhen-2, PROVEAN, VEST**	80.5% (77.27%-83.84%)	94.73% (92.62%-96.9%)	55.895% (49.315%-62.319%)	78.78% (75%-82.4%)	86% (80.49%-91.84%)	3.1541 (3.0261-3.2847)	80.41% (73.74%-86.87%)	94.56% (89.85%-98.48%)	56.123% (42.857%-68.76%)	78.7% (71.05%-86.08%)	85.79% (74.07%-95.83%)	3.1518 (2.8952-3.4081)
**At least 3 of CHASM (breast), CHASM (lung), CHASM (melanoma), Mutation Assessor, PolyPhen-2, SIFT, VEST**	80.11% (76.77%-83.33%)	96.29% (94.4%-98.39%)	52.141% (45.455%-58.571%)	77.67% (74%-81.37%)	89.04% (83.78%-95%)	3.1514 (3.0224-3.2825)	80.18% (73.74%-86.87%)	96.25% (92.19%-100%)	52.61% (40%-65.71%)	77.7% (70.12%-85%)	89.12% (77.27%-100%)	3.1568 (2.9020-3.4081)
**At least 3 of CHASM (breast), CHASM (lung), CHASM (melanoma), Mutation Assessor, PROVEAN, SIFT, VEST**	81.18% (77.78%-84.34%)	94.73% (92.62%-96.9%)	57.756% (51.389%-64.384%)	79.5% (75.84%-83.33%)	86.39% (81.25%-92%)	3.1837 (3.0543-3.3150)	81.06% (74.75%-87.88%)	94.56% (89.85%-98.48%)	57.882% (44.73%-70.46%)	79.39% (71.83%-86.77%)	86.15% (75%-96%)	3.1798 (2.9057-3.4398)
**At least 3 of CHASM (breast), CHASM (lung), CHASM (melanoma), MutationTaster, PolyPhen-2, PROVEAN, SIFT**	84.21% (81.31%-87.37%)	96.32% (94.4%-98.39%)	63.266% (56.919%-69.569%)	81.93% (78.57%-85.53%)	90.86% (86.54%-95.92%)	3.3237 (3.2107-3.4415)	84.11% (77.78%-89.9%)	96.19% (92.19%-100%)	63.368% (50%-76.32%)	81.84% (74.64%-88.57%)	90.67% (80.77%-100%)	3.3207 (3.0887-3.5422)
**At least 3 of CHASM (breast), CHASM (lung), CHASM (melanoma), MutationTaster, PolyPhen-2, PROVEAN, SIFT, VEST**	80.14% (77.27%-83.33%)	98.39% (97.5%-100%)	48.575% (42.029%-55.072%)	76.79% (73.12%-80.5%)	94.58% (91.18%-100%)	3.1833 (3.0677-3.2942)	80.13% (73.74%-85.86%)	98.43% (95.45%-100%)	48.712% (35.714%-61.76%)	76.71% (69.13%-83.95%)	94.77% (85.71%-100%)	3.1862 (2.9638-3.4082)
**At least 3 of CHASM (breast), CHASM (lung), CHASM (melanoma), MutationTaster, PolyPhen-2, PROVEAN, VEST**	82.9% (80.3%-85.86%)	96.86% (95.2%-98.47%)	58.769% (52.174%-65.278%)	80.24% (76.82%-84.08%)	91.55% (87.23%-95.92%)	3.2742 (3.1598-3.3970)	82.68% (76.77%-87.88%)	96.7% (92.98%-100%)	58.614% (45.714%-71.43%)	80.04% (72.6%-86.84%)	91.2% (80.95%-100%)	3.2655 (3.0236-3.4968)
**At least 3 of CHASM (breast), CHASM (lung), CHASM (melanoma), MutationTaster, PolyPhen-2, SIFT, VEST**	81.81% (78.79%-84.85%)	97.85% (96.72%-99.22%)	54.058% (47.497%-60.301%)	78.64% (75%-82.39%)	93.57% (89.74%-97.73%)	3.2412 (3.1270-3.3525)	81.84% (75.76%-87.88%)	97.91% (94.92%-100%)	54.252% (41.461%-66.67%)	78.6% (70.89%-85.55%)	93.8% (84.21%-100%)	3.2456 (3.0134-3.4642)
**At least 3 of CHASM (breast), CHASM (lung), CHASM (melanoma), MutationTaster, PROVEAN, SIFT, VEST**	83.21% (80.3%-85.87%)	96.33% (94.49%-98.39%)	60.515% (54.054%-66.667%)	80.84% (77.48%-84.52%)	90.51% (86.05%-95.74%)	3.2819 (3.1666-3.3982)	83.08% (77.75%-88.89%)	96.17% (92.18%-100%)	60.617% (47.497%-74.08%)	80.73% (73.33%-87.5%)	90.24% (80%-100%)	3.2776 (3.0482-3.5068)
[illegible]	[illegible]	[illegible]	[illegible]	[illegible]	[illegible]	[illegible]	[illegible]	[illegible]	[illegible]	[illegible]	[illegible]	[illegible]

At least 3 of CHASM (lung), CHASM (melanoma), FATHMM (cancer), FATHMM (missense), Mutation Assessor, MutationTaster, PolyPhen-2, PROVEAN	83.2% (80.3%-85.86%)	98.44% (97.52%-100%)	56.849% (50.649%-63.38%)	79.77% (76.1%-83.44%)	95.46% (92.5%-100%)	3.3052 (3.2001-3.4069)	83.1% (77.78%-88.89%)	98.34% (95.45%-100%)	56.933% (44.73%-70%)	79.67% (72.5%-87.02%)	95.24% (87.5%-100%)	3.3018 (3.0896-3.5155)
At least 3 of CHASM (lung), CHASM (melanoma), FATHMM (cancer), FATHMM (missense), Mutation Assessor, MutationTaster, PolyPhen-2, PROVEAN, SIFT	79.47% (76.26%-82.83%)	98.44% (97.52%-100%)	46.669% (40.277%-53.425%)	76.14% (72.56%-79.87%)	94.53% (90.91%-100%)	3.1577 (3.0474-3.2683)	79.45% (72.73%-85.86%)	98.34% (95.45%-100%)	46.997% (33.333%-60%)	76.1% (68.83%-83.34%)	94.3% (84.99%-100%)	3.1573 (2.9222-3.3712)
At least 3 of CHASM (lung), CHASM (melanoma), FATHMM (cancer), FATHMM (missense), Mutation Assessor, MutationTaster, PolyPhen-2, PROVEAN, SIFT, VEST	78.09% (74.75%-81.31%)	100% (100%-100%)	40.194% (33.784%-46.253%)	74.3% (70.73%-78.24%)	100% (100%-100%)	3.1449 (3.0583-3.2345)	78.17% (71.72%-84.85%)	100% (100%-100%)	40.683% (28.571%-53.13%)	74.31% (66.66%-81.71%)	100% (100%-100%)	3.1500 (2.9827-3.3354)
At least 3 of CHASM (lung), CHASM (melanoma), FATHMM (cancer), FATHMM (missense), Mutation Assessor, MutationTaster, PolyPhen-2, PROVEAN, VEST	78.8% (75.76%-82.32%)	98.97% (98.33%-100%)	43.934% (37.495%-50%)	75.32% (71.6%-79.27%)	96.12% (93.1%-100%)	3.1434 (3.0369-3.2547)	78.75% (71.72%-84.85%)	98.86% (96.67%-100%)	44.238% (31.707%-57.5%)	75.26% (67.47%-82.56%)	95.79% (87.5%-100%)	3.1415 (2.9222-3.3587)
At least 3 of CHASM (lung), CHASM (melanoma), FATHMM (cancer), FATHMM (missense), Mutation Assessor, MutationTaster, PolyPhen-2, SIFT	80.8% (77.78%-83.84%)	98.44% (97.52%-100%)	50.312% (43.836%-56.757%)	77.4% (73.86%-81.13%)	94.9% (91.66%-100%)	3.2105 (3.0989-3.3224)	80.82% (74.75%-86.87%)	98.34% (95.45%-100%)	50.714% (37.143%-63.64%)	77.4% (70%-84.42%)	94.69% (85.71%-100%)	3.2114 (2.9819-3.4296)
At least 3 of CHASM (lung), CHASM (melanoma), FATHMM (cancer), FATHMM (missense), Mutation Assessor, MutationTaster, PolyPhen-2, SIFT, VEST	78.42% (75.25%-81.82%)	100% (100%-100%)	41.105% (34.722%-47.222%)	74.59% (70.91%-78.45%)	100% (100%-100%)	3.1570 (3.0705-3.2460)	78.51% (71.72%-84.85%)	100% (100%-100%)	41.622% (29.032%-54.55%)	74.62% (66.67%-81.93%)	100% (100%-100%)	3.1624 (3.0018-3.3489)
At least 3 of CHASM (lung), CHASM (melanoma), FATHMM (cancer), FATHMM (missense), Mutation Assessor, MutationTaster, PolyPhen-2, VEST	81.15% (78.28%-84.34%)	98.97% (98.33%-100%)	50.338% (43.662%-56.758%)	77.51% (73.75%-81.29%)	96.59% (93.94%-100%)	3.2341 (3.1255-3.3441)	81.13% (74.75%-86.87%)	98.86% (96.67%-100%)	50.699% (37.143%-63.34%)	77.48% (70%-84.81%)	96.3% (88.89%-100%)	3.2334 (3.0092-3.4419)
At least 3 of CHASM (lung), CHASM (melanoma), FATHMM (cancer), FATHMM (missense), Mutation Assessor, MutationTaster, PROVEAN	86.54% (83.84%-89.39%)	96.34% (94.49%-98.43%)	69.583% (63.38%-75.714%)	84.56% (81.21%-88.11%)	91.67% (87.75%-96.3%)	3.4216 (3.3106-3.5344)	86.52% (80.81%-91.92%)	96.14% (91.8%-100%)	70.003% (57.576%-82.76%)	84.61% (77.46%-91.31%)	91.37% (82.61%-100%)	3.4212 (3.1916-3.6434)
At least 3 of CHASM (lung), CHASM (melanoma), FATHMM (cancer), FATHMM (missense), Mutation Assessor, MutationTaster, PROVEAN, SIFT	81.49% (78.28%-84.34%)	97.92% (96.69%-99.24%)	53.065% (46.575%-59.42%)	78.29% (74.69%-81.87%)	93.67% (89.74%-97.73%)	3.2295 (3.1184-3.3439)	81.47% (75.76%-87.88%)	97.77% (94.73%-100%)	53.501% (40%-66.67%)	78.29% (71.05%-85.33%)	93.35% (84.21%-100%)	3.2291 (2.9943-3.4531)
At least 3 of CHASM (lung), CHASM (melanoma), FATHMM (cancer), FATHMM (missense), Mutation Assessor, MutationTaster, PROVEAN, SIFT, VEST	79.46% (76.26%-82.83%)	99.49% (99.16%-100%)	44.805% (38.23%-51.391%)	75.71% (72.12%-79.5%)	98.08% (96.43%-100%)	3.1809 (3.0826-3.2865)	79.47% (72.73%-85.86%)	99.42% (98.21%-100%)	45.211% (32.258%-58.34%)	75.7% (68.35%-82.76%)	97.87% (92.31%-100%)	3.1819 (2.9782-3.3816)
At least 3 of CHASM (lung), CHASM (melanoma), FATHMM (cancer), FATHMM (missense), Mutation Assessor, MutationTaster, PROVEAN, VEST	80.84% (77.78%-83.84%)	98.47% (97.52%-100%)	50.346% (43.746%-56.583%)	77.42% (73.78%-81.1%)	95.01% (91.43%-100%)	3.2125 (3.1021-3.3204)	80.75% (74.75%-86.87%)	98.27% (95.38%-100%)	50.659% (37.829%-63.89%)	77.36% (70.13%-84.62%)	94.45% (85.71%-100%)	3.2074 (2.9937-3.4225)
At least 3 of CHASM (lung), CHASM (melanoma), FATHMM (cancer), FATHMM (missense), Mutation Assessor, MutationTaster, SIFT	83.16% (80.3%-86.36%)	97.92% (96.69%-99.24%)	57.638% (50.768%-63.889%)	79.99% (76.58%-83.54%)	94.14% (90.48%-97.92%)	3.2968 (3.1864-3.4162)	83.17% (76.77%-88.89%)	97.77% (94.73%-100%)	58.113% (45.161%-71.44%)	80.02% (72.73%-87.18%)	93.84% (85%-100%)	3.2973 (3.0651-3.5244)
At least 3 of CHASM (lung), CHASM (melanoma), FATHMM (cancer), FATHMM (missense), Mutation Assessor, MutationTaster, SIFT, VEST	80.8% (78.27%-83.84%)	99.49% (99.16%-100%)	48.487% (41.889%-55.072%)	76.96% (73.42%-80.5%)	98.22% (96.77%-100%)	3.2316 (3.1369-3.3391)	80.82% (74.75%-85.88%)	99.42% (98.21%-100%)	48.865% (35.484%-61.54%)	76.94% (69.73%-83.75%)	98.02% (92.86%-100%)	3.2324 (3.0238-3.4205)
At least 3 of CHASM (lung), CHASM (melanoma), FATHMM (cancer), FATHMM (missense), Mutation Assessor, MutationTaster, VEST	82.17% (79.29%-85.35%)	98.47% (97.52%-100%)	53.98% (47.54%-60.53%)	78.72% (75.16%-82.43%)	95.33% (92.11%-100%)	3.2651 (3.1587-3.3725)	82.13% (75.76%-87.88%)	98.27% (95.38%-100%)	54.406% (40.623%-67.57%)	78.72% (71.43%-85.72%)	94.81% (86.67%-100%)	3.2620 (3.0415-3.4822)
At least 3 of CHASM (lung), CHASM (melanoma), FATHMM (cancer), FATHMM (missense), Mutation Assessor, PolyPhen-2, PROVEAN	82.53% (79.8%-85.35%)	95.27% (93.39%-97.56%)	60.489% (54.052%-66.667%)	80.65% (76.97%-84.35%)	88.11% (83.02%-93.62%)	3.2452 (3.1285-3.3725)	82.42% (76.77%-87.88%)	95.08% (90.74%-98.51%)	60.661% (48.276%-74.29%)	80.57% (73.24%-88.16%)	87.8% (76.19%-96.43%)	3.2411 (2.9853-3.4832)
At least 3 of CHASM (lung), CHASM (melanoma), FATHMM (cancer), FATHMM (missense), Mutation Assessor, PolyPhen-2, PROVEAN, SIFT	79.13% (76.25%-82.32%)	96.86% (95.16%-98.49%)	48.481% (41.791%-55.405%)	76.47% (72.73%-80.13%)	89.92% (84.85%-95.24%)	3.1173 (2.9903-3.2469)	79.11% (72.73%-84.87%)	96.71% (92.85%-100%)	48.891% (35.132%-62.16%)	76.45% (68.83%-83.75%)	89.69% (77.78%-100%)	3.1174 (2.8541-3.3681)
At least 3 of CHASM (lung), CHASM (melanoma), FATHMM (cancer), FATHMM (missense), Mutation Assessor, PolyPhen-2, PROVEAN, SIFT, VEST	77.74% (74.75%-80.81%)	98.94% (98.33%-100%)	41.059% (34.722%-47.222%)	74.38% (70.73%-78.18%)	95.75% (92.59%-100%)	3.1013 (2.9923-3.2122)	77.86% (71.72%-83.84%)	98.92% (96.67%-100%)	41.698% (29.032%-54.55%)	74.44% (67.05%-81.71%)	95.74% (85.71%-100%)	3.1079 (2.8907-3.3159)
At least 3 of CHASM (lung), CHASM (melanoma), FATHMM (cancer), FATHMM (missense), Mutation Assessor, PolyPhen-2, PROVEAN, VEST	78.47% (75.25%-81.82%)	97.93% (96.72%-99.22%)	44.799% (37.838%-51.317%)	75.42% (71.77%-79.14%)	92.62% (88.24%-97.3%)	3.1077 (2.9936-3.2272)	78.42% (71.72%-84.85%)	97.74% (94.91%-100%)	45.253% (32.258%-58.54%)	75.39% (67.85%-82.72%)	92.11% (81.25%-100%)	3.1049 (2.8626-3.3272)
At least 3 of CHASM (lung), CHASM (melanoma), FATHMM (cancer), FATHMM (missense), Mutation Assessor, PolyPhen-2, SIFT	80.47% (77.27%-83.84%)	96.86% (95.16%-98.49%)	52.124% (45.333%-58.333%)	77.77% (74.03%-81.41%)	90.56% (85.71%-95.56%)	3.1731 (3.0555-3.2968)	80.48% (73.74%-86.87%)	96.71% (92.85%-100%)	52.608% (39.474%-65.86%)	77.79% (70.27%-85.19%)	90.35% (78.56%-100%)	3.1745 (2.9145-3.4110)
At least 3 of CHASM (lung), CHASM (melanoma), FATHMM (cancer), FATHMM (missense), Mutation Assessor, PolyPhen-2, SIFT, VEST	78.07% (74.75%-81.31%)	98.94% (98.33%-100%)	41.971% (35.614%-48.01%)	74.67% (70.83%-78.45%)	95.83% (92.86%-100%)	3.1142 (3.0081-3.2221)	78.2% (71.72%-84.85%)	98.92% (96.67%-100%)	42.637% (29.722%-55.56%)	74.74% (67.07%-82.36%)	95.83% (86.67%-100%)	3.1213 (2.8967-3.3273)
At least 3 of CHASM (lung), CHASM (melanoma), FATHMM (cancer), FATHMM (missense), Mutation Assessor, PolyPhen-2, VEST	80.81% (77.78%-83.84%)	97.93% (96.72%-99.22%)	51.203% (44.615%-57.971%)	77.63% (73.88%-81.37%)	93.48% (89.47%-97.62%)	3.2025 (3.0950-3.3179)	80.8% (74.75%-86.87%)	97.74% (94.91%-100%)	51.714% (38.71%-64.52%)	77.65% (70.13%-84.81%)	93.02% (83.33%-100%)	3.2013 (2.9636-3.4171)
At least 3 of CHASM (lung), CHASM (melanoma), FATHMM (cancer), FATHMM (missense), Mutation Assessor, PROVEAN, SIFT	80.49% (77.27%-83.33%)	94.74% (92.68%-96.85%)	55.838% (49.333%-62.319%)	78.77% (75.17%-82.47%)	85.99% (80.76%-91.67%)	3.1533 (3.0297-3.2847)	80.44% (74.75%-86.87%)	94.56% (90.16%-98.46%)	56.184% (42.857%-68.97%)	78.74% (71.05%-85.71%)	85.75% (73.68%-95.83%)	3.1524 (2.8843-3.4016)
At least 3 of CHASM (lung), CHASM (melanoma), FATHMM (cancer), FATHMM (missense), Mutation Assessor, PROVEAN, SIFT, VEST	78.47% (75.25%-81.82%)	96.88% (95.2%-98.47%)	46.617% (40%-53.427%)	75.83% (72.22%-79.5%)	89.65% (84.62%-95%)	3.0898 (2.9698-3.2154)	78.42% (71.72%-84.85%)	96.65% (92.98%-100%)	47.105% (34.209%-60%)	75.82% (68.29%-83.12%)	89.15% (76.91%-100%)	3.0872 (2.8284-3.3334)
At least 3 of CHASM (lung), CHASM (melanoma), FATHMM (cancer), FATHMM (missense), Mutation Assessor, PROVEAN, VEST	80.48% (77.27%-83.35%)	95.8% (93.91%-97.74%)	53.986% (47.295%-60.3%)	78.26% (74.68%-81.94%)	88.16% (83.33%-93.88%)	3.1621 (3.0353-3.2928)	80.45% (74.72%-86.87%)	95.62% (91.18%-98.66%)	54.387% (41.924%-67.44%)	78.25% (70.67%-85.37%)	87.86% (76.19%-96.85%)	3.1612 (2.9015-3.4089)
At least 3 of CHASM (lung), CHASM (melanoma), FATHMM (cancer), FATHMM (missense), Mutation Assessor, SIFT, VEST	79.47% (76.26%-82.83%)	96.34% (94.49%-98.39%)	50.299% (43.477%-56.757%)	77.02% (73.42%-80.65%)	88.84% (83.72%-95%)	3.1251 (3.0039-3.2589)	79.44% (72.73%-85.86%)	96.13% (92.18%-100%)	50.759% (37.493%-63.64%)	77.01% (69.33%-84.21%)	88.46% (76.46%-100%)	3.1236 (2.8316-3.3681)
At least 3 of CHASM (lung), CHASM (melanoma), FATHMM (cancer), FATHMM (missense), MutationTaster, PolyPhen-2, PROVEAN	84.55% (81.82%-87.37%)	97.4% (95.93%-99.19%)	62.319% (55.841%-69.014%)	81.72% (78.23%-85.43%)	93.27% (89.36%-97.87%)	3.3471 (3.2350-3.4652)	84.44% (78.79%-89.9%)	97.22% (93.85%-100%)	62.491% (50%-75%)	81.65% (74.32%-88.89%)	92.9% (84%-100%)	3.3426 (3.1057-3.5655)
At least 3 of CHASM (lung), CHASM (melanoma), FATHMM (cancer), FATHMM (missense), MutationTaster, PolyPhen-2, PROVEAN, SIFT	80.48% (77.27%-83.84%)	98.44% (97.52%-100%)	49.417% (42.857%-55.697%)	77.09% (73.42%-80.8%)	94.82% (91.43%-100%)	3.1976 (3.0883-3.3082)	80.46% (73.74%-86.87%)	98.34% (95.45%-100%)	49.761% (36.838%-62.86%)	77.06% (69.73%-84.21%)	94.6% (85.71%-100%)	3.1976 (2.9637-3.4199)
At least 3 of CHASM (lung), CHASM (melanoma), FATHMM (cancer), FATHMM (missense), MutationTaster, PolyPhen-2, PROVEAN, SIFT, VEST	78.78% (75.76%-82.32%)	100% (100%-100%)	42.077% (35.616%-48.111%)	74.91% (71.34%-78.7%)	100% (100%-100%)	3.1698 (3.0860-3.2591)	78.81% (71.72%-84.85%)	100% (100%-100%)	42.405% (30%-54.85%)	74.87% (67.44%-81.93%)	100% (100%-100%)	3.1728 (3.0060-3.3530)
At least 3 of CHASM (lung), CHASM (melanoma), FATHMM (cancer), FATHMM (missense), MutationTaster, PolyPhen-2, PROVEAN, VEST	80.52% (77.77%-83.84%)	98.47% (97.52%-100%)	49.492% (42.666%-55.844%)	77.12% (73.58%-80.98%)	94.93% (91.43%-100%)	3.2002 (3.0883-3.3126)	80.36% (73.74%-85.88%)	98.27% (95.38%-100%)	49.619% (37.143%-62.5%)	76.99% (69.33%-83.95%)	94.35% (85.71%-100%)	3.1923 (2.9638-3.4163)
At least 3 of CHASM (lung), CHASM (melanoma), FATHMM (cancer), FATHMM (missense), MutationTaster, PolyPhen-2, SIFT	81.48% (78.79%-84.34%)	98.44% (97.52%-100%)	52.162% (45.205%-59.02%)	78.06% (74.52%-81.65%)	95.07% (91.89%-100%)	3.2373 (3.1278-3.3491)	81.48% (75.76%-86.87%)	98.34% (95.45%-100%)	52.525% (39.391%-66.67%)	78.05% (70.88%-85%)	94.87% (86.36%-100%)	3.2378 (3.0131-3.4582)
At least 3 of CHASM (lung), CHASM (melanoma), FATHMM (cancer), FATHMM (missense), MutationTaster, PolyPhen-2, SIFT, VEST	79.78% (76.77%-82.83%)	100% (100%-100%)	44.81% (38.353%-51.351%)	75.8% (72.19%-79.52%)	100% (100%-100%)	3.2061 (3.1189-3.2987)	79.84% (73.74%-85.86%)	100% (100%-100%)	45.202% (32.432%-58.33%)	75.8% (68.6%-82.95%)	100% (100%-100%)	3.2100 (3.0416-3.3998)
At least 3 of CHASM (lung), CHASM (melanoma), FATHMM (cancer), FATHMM (missense), MutationTaster, PolyPhen-2, VEST	82.54% (79.8%-85.86%)	98.47% (97.52%-100%)	54.998% (48.683%-61.336%)	79.09% (75.64%-82.8%)	95.42% (92.31%-100%)	3.2798 (3.1788-3.3933)	82.39% (75.76%-87.88%)	98.27% (95.38%-100%)	55.128% (42.101%-67.65%)	78.98% (71.6%-85.9%)	94.88% (86.96%-100%)	3.2726 (3.0468-3.4822)
At least 3 of CHASM (lung), CHASM (melanoma), FATHMM (cancer), FATHMM (missense), MutationTaster, PROVEAN, SIFT	83.51% (80.81%-86.36%)	96.88% (95.16%-98.5%)	60.361% (53.843%-67.124%)	80.87% (77.33%-84.52%)	91.82% (87.5%-96.08%)	3.2993 (3.1859-3.4177)	83.49% (77.78%-88.89%)	96.64% (92.73%-100%)	60.892% (47.365%-73.7%)	80.93% (73.33%-88%)	91.38% (81.82%-100%)	3.2985 (3.0555-3.5304)
At least 3 of CHASM (lung), CHASM (melanoma), FATHMM (cancer), FATHMM (missense), MutationTaster, PROVEAN, SIFT, VEST	79.83% (76.77%-83.33%)	98.99% (98.33%-100%)	46.684% (40.299%-52.946%)	76.25% (72.67%-79.88%)	96.4% (93.55%-100%)	3.1833 (3.0816-3.2915)	79.74% (72.73%-85.86%)	98.82% (96.67%-100%)	46.964% (34.284%-60%)	76.18% (68.75%-83.33%)	95.9% (87.5%-100%)	3.1787 (2.9587-3.3829)
At least 3 of CHASM (lung), CHASM (melanoma), FATHMM (cancer), FATHMM (missense), MutationTaster, PROVEAN, VEST	83.18% (80.3%-86.36%)	97.93% (96.72%-99.24%)	57.667% (51.351%-64.706%)	80% (76.51%-83.68%)	94.17% (90.48%-97.92%)	3.2977 (3.1862-3.4142)	83.13% (76.77%-88.89%)	97.74% (94.74%-100%)	58.016% (44.737%-71.06%)	79.99% (72.37%-86.84%)	93.73% (85.18%-100%)	3.2948 (3.0685-3.5154)
At least 3 of CHASM (lung), CHASM (melanoma), FATHMM (cancer), FATHMM (missense), MutationTaster, SIFT, VEST	81.84% (78.79%-84.85%)	98.99% (98.33%-100%)	52.187% (45.714%-58.571%)	78.17% (74.53%-81.94%)	96.77% (94.29%-100%)	3.2611 (3.1638-3.3664)	81.77% (75.76%-87.88%)	98.82% (96.67%-100%)	52.476% (40%-65.71%)	78.11% (70.88%-85%)	96.31% (88.89%-100%)	3.2573 (3.0476-3.4598)
At least 3 of CHASM (lung), CHASM (melanoma), FATHMM (cancer), FATHMM (missense), PolyPhen-2, PROVEAN, SIFT	80.5% (77.27%-83.84%)	95.82% (94.02%-97.67%)	54.002% (47.367%-60.294%)	78.27% (74.67%-82.05%)	88.2% (82.93%-93.62%)	3.1629 (3.0379-3.2913)	80.42% (73.74%-86.87%)	95.59% (91.52%-98.59%)	54.337% (41.173%-67.65%)	78.23% (70.51%-85.33%)	87.79% (75%-96.55%)	3.1595 (2.9015-3.4079)
At least 3 of CHASM (lung), CHASM (melanoma), FATHMM (cancer), FATHMM (missense), PolyPhen-2, PROVEAN, SIFT, VEST	78.11% (75.25%-81.31%)	98.44% (97.5%-100%)	42.942% (36.486%-49.276%)	74.9% (71.34%-78.57%)	94.11% (90%-100%)	3.1039 (2.9933-3.2144)	78.12% (71.72%-83.84%)	98.32% (95.45%-100%)	43.42% (31.424%-56.25%)	74.89% (67.47%-82.05%)	93.78% (83.33%-100%)	3.1041 (2.8783-3.3254)
At least 3 of CHASM (lung), CHASM (melanoma), FATHMM (cancer), FATHMM (missense), PolyPhen-2, PROVEAN, VEST	80.83% (77.78%-84.34%)	97.4% (95.93%-99.19%)	52.185% (45.33%-58.752%)	77.89% (74.21%-81.46%)	92.07% (87.5%-97.44%)	3.1954 (3.0785-3.3158)	80.76% (73.74%-86.87%)	97.22% (93.85%-100%)	52.468% (39.024%-65.87%)	77.83% (70.27%-85%)	91.65% (80.95%-100%)	3.1917 (2.9363-3.4207)
At least 3 of CHASM (lung), CHASM (melanoma), FATHMM (cancer), FATHMM (missense), PolyPhen-2, SIFT, VEST	79.11% (75.76%-82.32%)	98.44% (97.5%-100%)	45.675% (39.129%-52.114%)	75.81% (72.12%-79.5%)	94.44% (90.62%-100%)	3.1436 (3.0348-3.2541)	79.15% (72.73%-85.86%)	98.32% (95.45%-100%)	46.217% (33.333%-59.38%)	75.83% (68.67%-83.33%)	94.14% (84.62%-100%)	3.1451 (2.9189-3.3675)
At least 3 of CHASM (lung), CHASM (melanoma), FATHMM (cancer), FATHMM (missense), PROVEAN, SIFT, VEST	79.83% (76.77%-83.33%)	96.35% (94.49%-98.4%)	51.269% (44.869%-57.746%)	77.37% (73.85%-81.13%)	89.04% (84.09%-95.24%)	3.1402 (3.0159-3.2647)	79.73% (72.73%-85.86%)	96.13% (92.06%-100%)	51.541% (38.228%-64.3%)	77.3% (69.73%-84.29%)	88.59% (76.47%-100%)	3.1356 (2.8737-3.3838)
At least 3 of CHASM (lung), CHASM (melanoma), FATHMM (cancer), Mutation Assessor, MutationTaster, PolyPhen-2, PROVEAN	85.2% (82.32%-87.88%)	97.9% (96.72%-99.24%)	63.238% (57.333%-69.565%)	82.16% (78.72%-85.71%)	94.57% (91.3%-98.08%)	3.3787 (3.2760-3.4856)	85.16% (79.8%-90.91%)	97.82% (94.64%-100%)	63.446% (51.282%-75.02%)	82.11% (75%-89.19%)	94.44% (86.36%-100%)	3.3781 (3.1614-3.5789)
At least 3 of CHASM (lung), CHASM (melanoma), FATHMM (cancer), Mutation Assessor, MutationTaster, PolyPhen-2, PROVEAN, SIFT	81.84% (78.79%-84.85%)	97.9% (96.72%-99.24%)	54.072% (47.823%-60.811%)	78.66% (74.84%-82.39%)	93.71% (89.74%-97.78%)	3.2434 (3.1353-3.3624)	81.78% (75.76%-87.88%)	97.82% (94.64%-100%)	54.259% (41.176%-67.5%)	78.58% (71.05%-86.25%)	93.55% (84.19%-100%)	3.2421 (3.0055-3.4644)
At least 3 of CHASM (lung), CHASM (melanoma), FATHMM (cancer), Mutation Assessor, MutationTaster, PolyPhen-2, PROVEAN, SIFT, VEST	78.77% (75.76%-81.82%)	99.46% (99.16%-100%)	42.982% (36.507%-49.275%)	75.1% (71.43%-78.7%)	97.89% (96.29%-100%)	3.1544 (3.0581-3.2533)	78.83% (72.73%-84.85%)	99.48% (98.25%-100%)	43.387% (31.429%-56.1%)	75.09% (67.5%-82.5%)	97.97% (91.67%-100%)	3.1593 (2.9602-3.3530)
At least 3 of CHASM (lung), CHASM (melanoma), FATHMM (cancer), Mutation Assessor, MutationTaster, PolyPhen-2, PROVEAN, VEST	80.87% (77.78%-83.85%)	98.44% (97.52%-100%)	50.491% (43.937%-56.944%)	77.46% (73.81%-81.37%)	94.92% (91.67%-100%)	3.2131 (3.1057-3.3247)	80.69% (74.72%-86.87%)	98.34% (95.45%-100%)	50.404% (37.836%-62.97%)	77.28% (69.41%-84.62%)	94.66% (85.71%-100%)	3.2068 (2.9829-3.4206)
At least 3 of CHASM (lung), CHASM (melanoma), FATHMM (cancer), Mutation Assessor, MutationTaster, PolyPhen-2, SIFT	83.17% (80.3%-86.36%)	97.9% (96.72%-99.24%)	57.715% (51.351%-64.107%)	80.01% (76.28%-83.65%)	94.08% (90.48%-97.92%)	3.2971 (3.1864-3.4138)	83.15% (76.77%-88.89%)	97.82% (94.64%-100%)	57.976% (44.828%-70.97%)	79.97% (72.6%-87.35%)	93.94% (85%-100%)	3.2970 (3.0686-3.5124)
At least 3 of CHASM (lung), CHASM (melanoma), FATHMM (cancer), Mutation Assessor, MutationTaster, PolyPhen-2, SIFT, VEST	78.77% (75.76%-81.82%)	99.46% (99.16%-100%)	42.982% (36.507%-49.275%)	75.1% (71.43%-78.7%)	97.89% (96.29%-100%)	3.1544 (3.0581-3.2533)	78.83% (72.73%-84.85%)	99.48% (98.25%-100%)	43.387% (31.429%-56.1%)	75.09% (67.5%-82.5%)	97.97% (91.67%-100%)	3.1593 (2.9602-3.3530)
At least 3 of CHASM (lung), CHASM (melanoma), FATHMM (cancer), Mutation Assessor, MutationTaster, PolyPhen-2, VEST	82.88% (79.8%-85.86%)	98.44% (97.52%-100%)	55.984% (49.984%-62.5%)	79.45% (75.84%-83.23%)	95.39% (92.31%-100%)	3.2926 (3.1868-3.4035)	82.73% (76.77%-88.89%)	98.34% (95.45%-100%)	55.926% (42.49%-68.42%)	79.29% (71.79%-86.49%)	95.15% (86.96%-100%)	3.2870 (3.0609-3.5067)
At least 3 of CHASM (lung), CHASM (melanoma), FATHMM (cancer), Mutation Assessor, MutationTaster, PROVEAN, SIFT	83.87% (80.81%-86.87%)	96.85% (95.16%-98.48%)	61.42% (55.378%-67.607%)	81.27% (77.78%-84.93%)	91.86% (88%-96.08%)	3.3140 (3.2010-3.4269)	83.78% (77.78%-89.9%)	96.72% (92.86%-100%)	61.577% (48.649%-74.19%)	81.19% (73.91%-88.06%)	91.66% (81.82%-100%)	3.3115 (3.0765-3.5431)
At least 3 of CHASM (lung), CHASM (melanoma), FATHMM (cancer), Mutation Assessor, MutationTaster, PROVEAN, SIFT, VEST	80.79% (77.78%-83.84%)	98.42% (97.52%-100%)	50.306% (44.115%-56.761%)	77.4% (73.86%-80.99%)	94.84% (91.43%-100%)	3.2097 (3.1095-3.3247)	80.85% (74.75%-86.87%)	98.38% (95.31%-100%)	50.774% (37.5%-63.34%)	77.42% (70%-84.62%)	94.81% (85.71%-100%)	3.2138 (2.9871-3.4154)
At least 3 of CHASM (lung), CHASM (melanoma), FATHMM (cancer), Mutation Assessor, MutationTaster, PROVEAN, VEST	84.56% (81.82%-87.37%)	97.4% (95.93%-99.19%)	62.365% (56.164%-68.186%)	81.73% (78.17%-85.14%)	93.27% (89.58%-97.87%)	3.3477 (3.2402-3.4563)	84.42% (78.79%-89.9%)	97.22% (93.85%-100%)	62.443% (50%-75%)	81.61% (74.64%-88.75%)	92.92% (84.21%-100%)	3.3420 (3.1230-3.5578)
At least 3 of CHASM (lung), CHASM (melanoma), FATHMM (cancer), Mutation Assessor, MutationTaster, SIFT, VEST	81.46% (78.78%-84.85%)	97.88% (96.69%-99.23%)	53.076% (46.575%-60%)	78.29% (74.84%-81.99%)	93.54% (89.74%-97.73%)	3.2279 (3.1155-3.3457)	81.52% (74.75%-86.89%)	97.86% (94.83%-100%)	53.488% (40%-65.81%)	78.3% (70.89%-85.33%)	93.59% (84.21%-100%)	3.2324 (2.9966-3.4487)
At least 3 of CHASM (lung), CHASM (melanoma), FATHMM (cancer), Mutation Assessor, PolyPhen-2, PROVEAN, SIFT	81.17% (78.28%-84.34%)	96.32% (94.4%-98.39%)	54.989% (48.611%-61.538%)	78.72% (74.84%-82.58%)	89.63% (84.78%-95.35%)	3.1965 (3.0719-3.3209)	81.09% (74.75%-86.87%)	96.19% (92.19%-100%)	55.178% (42.424%-67.74%)	78.64% (71.05%-86.08%)	89.42% (78.95%-100%)	3.1943 (2.9389-3.4486)
At least 3 of CHASM (lung), CHASM (melanoma), FATHMM (cancer), Mutation Assessor, PolyPhen-2, PROVEAN, SIFT, VEST	78.4% (75.25%-81.82%)	97.87% (96.69%-99.22%)	44.742% (38.233%-51.284%)	75.38% (71.6%-79.14%)	92.39% (87.88%-97.3%)	3.1039 (2.9799-3.2220)	78.55% (71.72%-84.85%)	97.88% (94.92%-100%)	45.376% (32.353%-58.33%)	75.46% (67.9%-82.93%)	92.56% (81.25%-100%)	3.1126 (2.8720-3.3506)
At least 3 of CHASM (lung), CHASM (melanoma), FATHMM (cancer), Mutation Assessor, PolyPhen-2, PROVEAN, VEST	80.51% (77.77%-83.84%)	96.86% (95.2%-98.47%)	52.251% (45.827%-58.827%)	77.81% (74.17%-81.65%)	90.59% (85.71%-95.45%)	3.1752 (3.0563-3.2996)	80.39% (73.74%-85.88%)	96.7% (92.98%-100%)	52.393% (39.474%-65%)	77.7% (69.88%-84.93%)	90.25% (78.95%-100%)	3.1704 (2.9206-3.4016)
At least 3 of CHASM (lung), CHASM (melanoma), FATHMM (cancer), Mutation Assessor, PolyPhen-2, SIFT, VEST	78.06% (74.75%-81.31%)	97.33% (95.9%-99.19%)	44.742% (38.233%-51.284%)	75.28% (71.52%-79.02%)	90.65% (85.71%-97.06%)	3.0800 (2.9548-3.2056)	78.22% (71.72%-84.85%)	97.36% (93.85%-100%)	45.376% (32.353%-58.33%)	75.36% (67.6%-82.93%)	90.92% (78.55%-100%)	3.0901 (2.8328-3.3354)
At least 3 of CHASM (lung), CHASM (melanoma), FATHMM (cancer), Mutation Assessor, PROVEAN, SIFT, VEST	79.8% (76.77%-83.33%)	95.81% (93.96%-97.73%)	52.125% (45.83%-58.906%)	77.58% (74%-81.46%)	87.8% (82.61%-93.48%)	3.1331 (3.0116-3.2673)	79.79% (72.73%-85.86%)	95.61% (91.18%-98.66%)	52.639% (39.472%-64.86%)	77.59% (69.86%-84.62%)	87.5% (76%-96.85%)	3.1334 (2.8587-3.3750)
At least 3 of CHASM (lung), CHASM (melanoma), FATHMM (cancer), MutationTaster, PolyPhen-2, PROVEAN, SIFT	83.19% (80.3%-86.36%)	97.9% (96.72%-99.24%)	57.747% (51.429%-64.064%)	80.02% (76.47%-83.77%)	94.09% (90.48%-97.92%)	3.2976 (3.1921-3.4132)	83.12% (76.77%-88.89%)	97.82% (94.64%-100%)	57.918% (45%-70.59%)	79.95% (72.5%-87.02%)	93.94% (85%-100%)	3.2962 (3.0673-3.5055)
At least 3 of CHASM (lung), CHASM (melanoma), FATHMM (cancer), MutationTaster, PolyPhen-2, PROVEAN, SIFT, VEST	79.45% (76.26%-82.83%)	99.46% (99.16%-100%)	44.84% (38.356%-51.351%)	75.71% (72.12%-79.5%)	97.98% (96.43%-100%)	3.1800 (3.0826-3.2745)	79.48% (72.73%-85.86%)	99.48% (98.25%-100%)	45.157% (32.432%-57.58%)	75.68% (68.29%-82.93%)	98.06% (92.31%-100%)	3.1837 (2.9996-3.3792)
At least 3 of CHASM (lung), CHASM (melanoma), FATHMM (cancer), MutationTaster, PolyPhen-2, PROVEAN, VEST	81.9% (78.79%-85.35%)	97.93% (96.72%-99.22%)	54.169% (47.826%-60.61%)	78.7% (75.15%-82.47%)	93.82% (90%-97.73%)	3.2462 (3.1377-3.3587)	81.66% (74.75%-87.88%)	97.74% (94.91%-100%)	54.044% (41.461%-66.67%)	78.49% (71.05%-85.71%)	93.32% (84.21%-100%)	3.2359 (2.9993-3.4487)
At least 3 of CHASM (lung), CHASM (melanoma), FATHMM (cancer), MutationTaster, PolyPhen-2, SIFT, VEST	80.12% (77.27%-83.33%)	99.46% (99.16%-100%)	46.661% (40.278%-52.941%)	76.33% (72.73%-80.12%)	98.06% (96.55%-100%)	3.2051 (3.1118-3.3044)	80.17% (73.74%-85.86%)	99.48% (98.25%-100%)	47.015% (35%-60%)	76.31% (68.75%-83.72%)	98.13% (92.84%-100%)	3.2093 (3.0213-3.3998)
At least 3 of CHASM (lung), CHASM (melanoma), FATHMM (cancer), MutationTaster, PROVEAN, SIFT, VEST	81.15% (78.28%-84.34%)	97.92% (96.72%-99.23%)	52.159% (45.833%-58.904%)	77.97% (74.37%-81.65%)	93.55% (89.74%-97.67%)	3.2159 (3.1103-3.3325)	81.14% (74.75%-86.87%)	97.78% (94.83%-100%)	52.575% (38.884%-65.02%)	77.96% (70.67%-84.81%)	93.26% (83.98%-100%)	3.2158 (2.9823-3.4240)
At least 3 of CHASM (lung), CHASM (melanoma), FATHMM (cancer), PolyPhen-2, PROVEAN, SIFT, VEST	78.77% (75.76%-82.32%)	97.37% (95.93%-99.19%)	46.6% (40.534%-52.946%)	75.92% (72.15%-79.63%)	91.11% (86.11%-97.14%)	3.1100 (2.9865-3.2337)	78.83% (71.72%-84.85%)	97.28% (93.75%-100%)	47.146% (34.871%-59.54%)	75.95% (68.42%-83.33%)	90.98% (80%-100%)	3.1136 (2.8694-3.3514)
At least 3 of CHASM (lung), CHASM (melanoma), FATHMM (missense), Mutation Assessor, MutationTaster, PolyPhen-2, PROVEAN	83.53% (80.81%-86.36%)	96.31% (94.4%-98.39%)	61.425% (55.071%-67.568%)	81.19% (77.63%-84.77%)	90.61% (86.27%-95.75%)	3.2953 (3.1835-3.4091)	83.45% (77.78%-88.89%)	96.2% (92.19%-100%)	61.536% (48.571%-74.2%)	81.1% (73.97%-88.16%)	90.47% (80%-100%)	3.2930 (3.0656-3.5310)
At least 3 of CHASM (lung), CHASM (melanoma), FATHMM (missense), Mutation Assessor, MutationTaster, PolyPhen-2, PROVEAN, SIFT	80.17% (77.26%-83.33%)	96.86% (95.16%-98.49%)	51.306% (44.869%-57.971%)	77.47% (73.75%-81.17%)	90.43% (85.37%-95.56%)	3.1606 (3.0384-3.2913)	80.07% (73.74%-85.88%)	96.71% (92.85%-100%)	51.501% (37.5%-64.52%)	77.39% (70.23%-84.62%)	90.15% (78.57%-100%)	3.1575 (2.8965-3.4079)
At least 3 of CHASM (lung), CHASM (melanoma), FATHMM (missense), Mutation Assessor, MutationTaster, PolyPhen-2, PROVEAN, SIFT, VEST	78.09% (74.75%-81.31%)	100% (100%-100%)	40.194% (33.784%-46.253%)	74.3% (70.73%-78.24%)	100% (100%-100%)	3.1449 (3.0583-3.2345)	78.17% (71.72%-84.85%)	100% (100%-100%)	40.683% (28.571%-53.13%)	74.31% (66.66%-81.71%)	100% (100%-100%)	3.1500 (2.9827-3.3354)
At least 3 of CHASM (lung), CHASM (melanoma), FATHMM (missense), Mutation Assessor, MutationTaster, PolyPhen-2, PROVEAN, VEST	80.85% (77.78%-84.34%)	98.97% (98.33%-100%)	49.522% (43.239%-56.252%)	77.22% (73.58%-80.98%)	96.54% (93.94%-100%)	3.2225 (3.1218-3.3337)	80.72% (73.74%-86.87%)	98.86% (96.67%-100%)	49.582% (36.357%-62.5%)	77.09% (69.62%-84.15%)	96.22% (88.24%-100%)	3.2175 (2.9913-3.4223)
At least 3 of CHASM (lung), CHASM (melanoma), FATHMM (missense), Mutation Assessor, MutationTaster, PolyPhen-2, SIFT	81.85% (78.79%-84.85%)	96.86% (95.16%-98.49%)	55.903% (49.314%-62.5%)	79.16% (75.33%-82.9%)	91.14% (86.67%-95.92%)	3.2306 (3.1091-3.3535)	81.75% (75.76%-87.88%)	96.71% (92.85%-100%)	56.062% (42.848%-68.59%)	79.07% (71.79%-86.26%)	90.89% (80%-100%)	3.2273 (2.9720-3.4770)
At least 3 of CHASM (lung), CHASM (melanoma), FATHMM (missense), Mutation Assessor, MutationTaster, PolyPhen-2, SIFT, VEST	79.09% (75.76%-82.32%)	100% (100%-100%)	42.939% (36.486%-49.296%)	75.19% (71.51%-79.04%)	100% (100%-100%)	3.1813 (3.0941-3.2721)	79.19% (72.73%-85.86%)	100% (100%-100%)	43.461% (30.549%-56.25%)	75.22% (67.47%-82.72%)	100% (100%-100%)	3.1868 (3.0110-3.3657)
At least 3 of CHASM (lung), CHASM (melanoma), FATHMM (missense), Mutation Assessor, MutationTaster, PolyPhen-2, VEST	82.86% (80.3%-85.86%)	98.97% (98.33%-100%)	55.016% (48.61%-61.541%)	79.18% (75.48%-82.9%)	96.87% (94.59%-100%)	3.3004 (3.1996-3.4159)	82.76% (76.77%-87.88%)	98.86% (96.67%-100%)	55.109% (41.929%-67.5%)	79.07% (71.62%-86.08%)	96.58% (89.47%-100%)	3.2963 (3.0622-3.4949)
At least 3 of CHASM (lung), CHASM (melanoma), FATHMM (missense), Mutation Assessor, MutationTaster, PROVEAN, SIFT	82.84% (79.8%-85.86%)	95.77% (93.7%-97.64%)	60.466% (54.054%-66.667%)	80.73% (77.14%-84.25%)	89.22% (84.44%-94.12%)	3.2619 (3.1380-3.3794)	82.81% (76.77%-88.89%)	95.68% (91.8%-100%)	60.696% (48.276%-73.53%)	80.69% (73.33%-87.88%)	89.14% (78.57%-100%)	3.2620 (3.0186-3.5158)
At least 3 of CHASM (lung), CHASM (melanoma), FATHMM (missense), Mutation Assessor, MutationTaster, PROVEAN, SIFT, VEST	79.46% (76.26%-82.83%)	98.44% (97.5%-100%)	46.645% (40.26%-52.945%)	76.13% (72.56%-79.76%)	94.55% (90.91%-100%)	3.1577 (3.0524-3.2754)	79.46% (72.73%-85.86%)	98.33% (95.31%-100%)	47.044% (34.286%-60.02%)	76.12% (68.83%-83.54%)	94.3% (83.33%-100%)	3.1579 (2.9201-3.3713)
At least 3 of CHASM (lung), CHASM (melanoma), FATHMM (missense), Mutation Assessor, MutationTaster, PROVEAN, VEST	82.19% (79.29%-85.35%)	96.87% (95.2%-98.49%)	56.796% (50.668%-63.019%)	79.49% (75.97%-83.23%)	91.32% (86.84%-95.92%)	3.2449 (3.1283-3.3667)	82.08% (75.76%-87.88%)	96.67% (92.85%-100%)	57.028% (44.118%-69.7%)	79.42% (71.83%-86.67%)	90.91% (80%-100%)	3.2403 (2.9983-3.4695)
At least 3 of CHASM (lung), CHASM (melanoma), FATHMM (missense), Mutation Assessor, MutationTaster, SIFT, VEST	80.46% (77.77%-83.84%)	97.9% (96.69%-99.24%)	50.296% (43.932%-56.761%)	77.3% (73.72%-81.01%)	93.28% (89.19%-97.62%)	3.1878 (3.0686-3.3068)	80.5% (73.74%-85.88%)	97.81% (94.64%-100%)	50.759% (37.143%-63.64%)	77.32% (69.88%-84.62%)	93.16% (83.33%-100%)	3.1905 (2.9449-3.4207)
At least 3 of CHASM (lung), CHASM (melanoma), FATHMM (missense), Mutation Assessor, PolyPhen-2, PROVEAN, SIFT	80.49% (77.27%-83.35%)	95.77% (93.7%-97.64%)	54.063% (47.826%-60.811%)	78.28% (74.52%-82.05%)	88.1% (82.93%-93.48%)	3.1622 (3.0356-3.2909)	80.43% (74.72%-86.87%)	95.68% (91.8%-100%)	54.243% (41.161%-66.67%)	78.21% (70.51%-85.53%)	88.01% (76.19%-100%)	3.1614 (2.9018-3.4168)
At least 3 of CHASM (lung), CHASM (melanoma), FATHMM (missense), Mutation Assessor, PolyPhen-2, PROVEAN, SIFT, VEST	76.39% (72.73%-79.8%)	96.83% (95.12%-98.45%)	41.059% (34.722%-47.222%)	73.96% (70.19%-77.85%)	88.21% (82.35%-94.29%)	3.0006 (2.8676-3.1318)	76.5% (69.7%-83.84%)	96.77% (93.22%-100%)	41.698% (29.032%-54.55%)	74.01% (66.27%-81.48%)	88.29% (75%-100%)	3.0077 (2.7365-3.2747)
At least 3 of CHASM (lung), CHASM (melanoma), FATHMM (missense), Mutation Assessor, PolyPhen-2, PROVEAN, VEST	78.83% (75.76%-82.32%)	95.27% (93.33%-97.56%)	50.387% (43.827%-56.945%)	76.85% (73.08%-80.77%)	86.05% (80.49%-92.5%)	3.0856 (2.9517-3.2196)	78.71% (71.72%-84.85%)	95.08% (90.77%-98.53%)	50.597% (37.135%-62.97%)	76.76% (68.92%-84.21%)	85.74% (72.73%-95.65%)	3.0817 (2.8186-3.3472)
At least 3 of CHASM (lung), CHASM (melanoma), FATHMM (missense), Mutation Assessor, PolyPhen-2, SIFT, VEST	77.06% (73.74%-80.3%)	96.29% (94.4%-98.39%)	43.804% (37.313%-50.016%)	74.76% (70.97%-78.75%)	87.22% (81.25%-94.12%)	3.0207 (2.8847-3.1552)	77.2% (70.71%-83.84%)	96.25% (92.19%-100%)	44.476% (31.429%-57.15%)	74.84% (67.07%-82.28%)	87.38% (73.33%-100%)	3.0294 (2.7404-3.2893)
At least 3 of CHASM (lung), CHASM (melanoma), FATHMM (missense), Mutation Assessor, PROVEAN, SIFT, VEST	78.46% (75.25%-81.82%)	94.73% (92.62%-96.9%)	50.305% (43.996%-56.758%)	76.72% (73.08%-80.52%)	84.68% (79.07%-90.91%)	3.0644 (2.9345-3.1990)	78.44% (71.72%-84.85%)	94.56% (89.85%-98.48%)	50.733% (37.143%-63.34%)	76.71% (69.23%-84%)	84.5% (71.43%-95.45%)	3.0651 (2.7716-3.3289)
At least 3 of CHASM (lung), CHASM (melanoma), FATHMM (missense), MutationTaster, PolyPhen-2, PROVEAN, SIFT	82.53% (79.29%-85.86%)	96.86% (95.16%-98.49%)	57.759% (51.351%-64.11%)	79.86% (76.43%-83.45%)	91.41% (86.96%-96%)	3.2588 (3.1432-3.3776)	82.41% (75.76%-88.89%)	96.71% (92.85%-100%)	57.84% (44.828%-70.47%)	79.75% (72.5%-86.67%)	91.15% (80.77%-100%)	3.2545 (3.0090-3.4831)
At least 3 of CHASM (lung), CHASM (melanoma), FATHMM (missense), MutationTaster, PolyPhen-2, PROVEAN, SIFT, VEST	79.12% (76.26%-82.32%)	100% (100%-100%)	43.004% (36.616%-49.183%)	75.21% (71.76%-78.95%)	100% (100%-100%)	3.1821 (3.0969-3.2702)	79.14% (72.73%-84.85%)	100% (100%-100%)	43.3% (31.25%-55.88%)	75.17% (67.5%-81.93%)	100% (100%-100%)	3.1847 (3.0169-3.3647)
At least 3 of CHASM (lung), CHASM (melanoma), FATHMM (missense), MutationTaster, PolyPhen-2, PROVEAN, VEST	82.57% (79.8%-85.86%)	98.47% (97.52%-100%)	55.076% (48.683%-61.765%)	79.12% (75.5%-82.9%)	95.42% (92.31%-100%)	3.2809 (3.1782-3.3912)	82.33% (75.76%-87.88%)	98.27% (95.38%-100%)	54.973% (42.498%-67.66%)	78.93% (71.25%-85.92%)	94.86% (86.96%-100%)	3.2703 (3.0468-3.4822)
At least 3 of CHASM (lung), CHASM (melanoma), FATHMM (missense), MutationTaster, PolyPhen-2, SIFT, VEST	81.13% (78.28%-84.34%)	100% (100%-100%)	48.494% (41.889%-54.795%)	77.05% (73.62%-80.75%)	100% (100%-100%)	3.2554 (3.1676-3.3483)	81.18% (74.75%-86.87%)	100% (100%-100%)	48.84% (35.484%-61.77%)	77.04% (69.51%-84.15%)	100% (100%-100%)	3.2588 (3.0881-3.4415)
At least 3 of CHASM (lung), CHASM (melanoma), FATHMM (missense), MutationTaster, PROVEAN, SIFT, VEST	80.85% (77.78%-83.84%)	97.94% (96.72%-99.24%)	51.306% (44.775%-57.812%)	77.67% (74.21%-81.25%)	93.52% (89.47%-97.73%)	3.2043 (3.0932-3.3224)	80.72% (74.75%-86.87%)	97.73% (94.64%-100%)	51.468% (38.228%-64.52%)	77.56% (70.37%-84.62%)	93.01% (82.61%-100%)	3.1978 (2.9587-3.4199)
At least 3 of CHASM (lung), CHASM (melanoma), FATHMM (missense), PolyPhen-2, PROVEAN, SIFT, VEST	77.46% (74.24%-80.81%)	96.32% (94.49%-98.39%)	44.814% (38.462%-51.251%)	75.11% (71.52%-78.85%)	87.58% (81.82%-94.44%)	3.0384 (2.9068-3.1712)	77.41% (70.71%-83.84%)	96.17% (92.19%-100%)	45.163% (32.557%-57.58%)	75.07% (67.5%-82.05%)	87.32% (75%-100%)	3.0372 (2.7714-3.2902)
At least 3 of CHASM (lung), CHASM (melanoma), Mutation Assessor, MutationTaster, PolyPhen-2, PROVEAN, SIFT	78.09% (74.75%-81.31%)	89.87% (86.99%-92.97%)	57.725% (51.514%-64.286%)	78.61% (74.82%-82.55%)	76.73% (70.37%-83.34%)	3.0293 (2.8937-3.1683)	78.16% (71.72%-84.85%)	89.93% (83.58%-95.38%)	57.967% (44.737%-70.27%)	78.59% (70.59%-86.12%)	77.07% (63.99%-89.29%)	3.0355 (2.7494-3.3018)
At least 3 of CHASM (lung), CHASM (melanoma), Mutation Assessor, MutationTaster, PolyPhen-2, PROVEAN, SIFT, VEST	76.38% (72.73%-79.8%)	91.94% (89.34%-95%)	49.469% (42.852%-55.882%)	75.88% (71.92%-79.74%)	78.03% (71.11%-85.37%)	2.9532 (2.8131-3.0888)	76.54% (69.7%-83.84%)	92.17% (86.36%-96.97%)	49.699% (37.135%-62.86%)	75.87% (68%-83.78%)	78.76% (64.99%-92.31%)	2.9650 (2.6853-3.2427)
At least 3 of CHASM (lung), CHASM (melanoma), Mutation Assessor, MutationTaster, PolyPhen-2, PROVEAN, VEST	75.75% (72.22%-79.29%)	87.72% (84.55%-90.99%)	55.057% (48.528%-61.646%)	77.14% (73.1%-80.99%)	72.17% (65.45%-78.95%)	2.9208 (2.7688-3.0655)	75.77% (68.69%-82.83%)	87.85% (81.25%-94.03%)	55.051% (42.105%-67.57%)	77.02% (69.23%-84.85%)	72.55% (58.61%-85.71%)	2.9248 (2.6377-3.2005)
At least 3 of CHASM (lung), CHASM (melanoma), Mutation Assessor, MutationTaster, PolyPhen-2, SIFT, VEST	76.71% (73.23%-79.8%)	91.4% (88.72%-94.35%)	51.302% (44.737%-57.501%)	76.44% (72.48%-80.54%)	77.54% (70.73%-84.32%)	2.9669 (2.8226-3.1035)	76.88% (70.71%-83.84%)	91.65% (85.71%-96.88%)	51.538% (39.279%-64.52%)	76.44% (68.42%-84.51%)	78.28% (65%-91.31%)	2.9791 (2.6928-3.2730)
At least 3 of CHASM (lung), CHASM (melanoma), Mutation Assessor, MutationTaster, PROVEAN, SIFT, VEST	78.41% (75.25%-81.33%)	90.38% (87.6%-93.55%)	57.719% (51.35%-64.288%)	78.7% (74.83%-82.52%)	77.64% (71.43%-84.32%)	3.0444 (2.9090-3.1804)	78.53% (72.7%-84.85%)	90.5% (84.38%-96.43%)	57.977% (44.737%-70.74%)	78.7% (71.23%-86.37%)	78.1% (65.38%-90.32%)	3.0528 (2.7609-3.3245)
At least 3 of CHASM (lung), CHASM (melanoma), Mutation Assessor, PolyPhen-2, PROVEAN, SIFT, VEST	74.35% (70.71%-77.78%)	87.2% (83.9%-90.37%)	52.132% (44.928%-58.464%)	75.9% (71.94%-79.86%)	70.21% (63.46%-76.48%)	2.8544 (2.7025-3.0026)	74.53% (67.68%-81.82%)	87.28% (80.6%-93.65%)	52.635% (40%-66.67%)	75.96% (68.05%-84%)	70.72% (57.69%-83.87%)	2.8660 (2.5710-3.1735)
At least 3 of CHASM (lung), CHASM (melanoma), MutationTaster, PolyPhen-2, PROVEAN, SIFT, VEST	76.04% (72.73%-79.29%)	90.87% (88.19%-93.8%)	50.399% (43.662%-56.757%)	76.01% (72%-80%)	76.16% (69.39%-83.33%)	2.9344 (2.7926-3.0738)	76.2% (69.7%-82.83%)	91.12% (85.18%-96.67%)	50.575% (37.5%-64.52%)	75.98% (68%-83.79%)	76.87% (62.49%-90.48%)	2.9455 (2.6653-3.2213)
At least 3 of CHASM (lung), FATHMM (cancer), FATHMM (missense), Mutation Assessor, MutationTaster, PolyPhen-2, PROVEAN	85.19% (82.32%-87.88%)	97.9% (96.69%-99.22%)	63.197% (56.944%-69.231%)	82.14% (78.67%-85.82%)	94.58% (91.3%-98.08%)	3.3782 (3.2695-3.4853)	85.18% (79.8%-90.91%)	97.8% (94.92%-100%)	63.513% (51.427%-75.76%)	82.14% (75%-89.04%)	94.41% (86.35%-100%)	3.3786 (3.1643-3.5960)
At least 3 of CHASM (lung), FATHMM (cancer), FATHMM (missense), Mutation Assessor, MutationTaster, PolyPhen-2, PROVEAN, SIFT	80.48% (77.27%-83.84%)	97.9% (96.69%-99.22%)	50.339% (43.836%-56.583%)	77.32% (73.75%-81.01%)	93.29% (89.19%-97.56%)	3.1885 (3.0767-3.3049)	80.46% (73.74%-86.87%)	97.8% (94.92%-100%)	50.683% (37.5%-63.89%)	77.29% (70%-84.42%)	93.09% (83.33%-100%)	3.1887 (2.9638-3.4199)
At least 3 of CHASM (lung), FATHMM (cancer), FATHMM (missense), Mutation Assessor, MutationTaster, PolyPhen-2, PROVEAN, SIFT, VEST	78.43% (75.25%-81.82%)	100% (100%-100%)	41.118% (34.721%-47.222%)	74.6% (70.93%-78.36%)	100% (100%-100%)	3.1571 (3.0712-3.2516)	78.5% (71.72%-84.85%)	100% (100%-100%)	41.601% (29.262%-54.29%)	74.61% (66.67%-81.93%)	100% (100%-100%)	3.1621 (2.9956-3.3483)
At least 3 of CHASM (lung), FATHMM (cancer), FATHMM (missense), Mutation Assessor, MutationTaster, PolyPhen-2, PROVEAN, VEST	79.48% (76.26%-82.83%)	98.97% (98.33%-100%)	45.772% (39.13%-52.001%)	75.94% (72.22%-79.75%)	96.27% (93.55%-100%)	3.1694 (3.0616-3.2804)	79.43% (72.73%-85.86%)	98.86% (96.67%-100%)	46.063% (33.333%-58.83%)	75.88% (68.29%-83.14%)	95.94% (88.22%-100%)	3.1674 (2.9564-3.3816)
At least 3 of CHASM (lung), FATHMM (cancer), FATHMM (missense), Mutation Assessor, MutationTaster, PolyPhen-2, SIFT	82.15% (79.29%-85.35%)	97.9% (96.69%-99.22%)	54.9% (48.571%-61.251%)	78.96% (75.31%-82.67%)	93.81% (90%-97.78%)	3.2558 (3.1460-3.3683)	82.17% (75.76%-87.88%)	97.8% (94.92%-100%)	55.316% (42.424%-67.76%)	78.97% (71.95%-86.25%)	93.63% (84.62%-100%)	3.2573 (3.0252-3.4817)
At least 3 of CHASM (lung), FATHMM (cancer), FATHMM (missense), Mutation Assessor, MutationTaster, PolyPhen-2, SIFT, VEST	79.09% (75.76%-82.32%)	100% (100%-100%)	42.948% (36.486%-49.276%)	75.19% (71.51%-79.05%)	100% (100%-100%)	3.1814 (3.0937-3.2734)	79.18% (72.73%-85.86%)	100% (100%-100%)	43.456% (31.034%-56.41%)	75.21% (67.47%-82.72%)	100% (100%-100%)	3.1867 (3.0154-3.3715)
At least 3 of CHASM (lung), FATHMM (cancer), FATHMM (missense), Mutation Assessor, MutationTaster, PolyPhen-2, VEST	81.49% (78.28%-84.85%)	98.97% (98.33%-100%)	51.258% (44.772%-57.897%)	77.83% (74.05%-81.53%)	96.65% (94.12%-100%)	3.2471 (3.1409-3.3570)	81.47% (74.75%-87.88%)	98.86% (96.67%-100%)	51.608% (38.235%-64.71%)	77.8% (70.24%-85.19%)	96.36% (88.89%-100%)	3.2463 (3.0237-3.4598)
At least 3 of CHASM (lung), FATHMM (cancer), FATHMM (missense), Mutation Assessor, MutationTaster, PROVEAN, SIFT	83.81% (80.81%-86.87%)	97.39% (95.9%-99.2%)	60.336% (54.167%-66.667%)	80.94% (77.48%-84.62%)	93.05% (89.13%-97.78%)	3.3171 (3.2067-3.4400)	83.89% (77.78%-89.9%)	97.23% (93.65%-100%)	60.98% (48.482%-73.33%)	81.04% (73.61%-87.84%)	92.8% (83.98%-100%)	3.3205 (3.0766-3.5404)
At least 3 of CHASM (lung), FATHMM (cancer), FATHMM (missense), Mutation Assessor, MutationTaster, PROVEAN, SIFT, VEST	81.14% (78.28%-84.34%)	99.49% (99.16%-100%)	49.394% (42.852%-55.715%)	77.27% (73.72%-81.01%)	98.25% (96.77%-100%)	3.2441 (3.1411-3.3480)	81.16% (74.75%-86.87%)	99.42% (98.21%-100%)	49.813% (37.135%-63.42%)	77.27% (70%-84.34%)	98.05% (93.32%-100%)	3.2456 (3.0441-3.4520)
At least 3 of CHASM (lung), FATHMM (cancer), FATHMM (missense), Mutation Assessor, MutationTaster, PROVEAN, VEST	82.83% (79.8%-85.86%)	98.47% (97.52%-100%)	55.776% (49.275%-62.162%)	79.38% (75.95%-83.01%)	95.48% (92.31%-100%)	3.2911 (3.1864-3.3961)	82.83% (76.77%-88.89%)	98.27% (95.38%-100%)	56.324% (43.583%-69.05%)	79.43% (72.22%-86.49%)	94.99% (87.5%-100%)	3.2901 (3.0802-3.4968)
At least 3 of CHASM (lung), FATHMM (cancer), FATHMM (missense), Mutation Assessor, MutationTaster, SIFT, VEST	82.15% (79.29%-85.35%)	99.49% (99.16%-100%)	52.157% (45.711%-58.571%)	78.24% (74.84%-81.88%)	98.34% (96.97%-100%)	3.2823 (3.1850-3.3864)	82.17% (75.76%-87.88%)	99.42% (98.21%-100%)	52.551% (40%-65.52%)	78.24% (71.05%-85.33%)	98.15% (93.33%-100%)	3.2836 (3.0785-3.4733)
At least 3 of CHASM (lung), FATHMM (cancer), FATHMM (missense), Mutation Assessor, PolyPhen-2, PROVEAN, SIFT	80.14% (76.77%-83.33%)	96.86% (95.16%-98.49%)	51.234% (44.591%-57.746%)	77.45% (73.75%-81.17%)	90.41% (85.71%-95.56%)	3.1595 (3.0350-3.2877)	80.13% (73.74%-86.87%)	96.71% (92.85%-100%)	51.658% (38.884%-64.86%)	77.44% (70%-84.62%)	90.18% (78.94%-100%)	3.1598 (2.9055-3.4080)
At least 3 of CHASM (lung), FATHMM (cancer), FATHMM (missense), Mutation Assessor, PolyPhen-2, PROVEAN, SIFT, VEST	78.07% (74.75%-81.31%)	98.41% (97.5%-100%)	42.9% (36.232%-48.649%)	74.87% (71.25%-78.66%)	93.99% (90.31%-100%)	3.1018 (2.9914-3.2154)	78.2% (71.72%-84.85%)	98.38% (95.45%-100%)	43.535% (31.575%-56.76%)	74.93% (67.46%-82.28%)	94.03% (83.33%-100%)	3.1088 (2.8764-3.3319)
At least 3 of CHASM (lung), FATHMM (cancer), FATHMM (missense), Mutation Assessor, PolyPhen-2, PROVEAN, VEST	79.14% (75.76%-82.32%)	97.4% (95.93%-99.2%)	47.554% (40.58%-53.849%)	76.25% (72.67%-80%)	91.38% (86.83%-97.22%)	3.1259 (3.0063-3.2403)	79.1% (72.73%-85.86%)	97.21% (93.65%-100%)	47.997% (35.132%-61.76%)	76.23% (68.74%-83.34%)	90.94% (80%-100%)	3.1238 (2.8948-3.3675)
At least 3 of CHASM (lung), FATHMM (cancer), FATHMM (missense), Mutation Assessor, PolyPhen-2, SIFT, VEST	78.74% (75.76%-81.82%)	98.41% (97.5%-100%)	44.73% (38.158%-50.725%)	75.48% (71.95%-79.27%)	94.22% (90.32%-100%)	3.1285 (3.0145-3.2409)	78.88% (72.73%-84.85%)	98.38% (95.45%-100%)	45.39% (32.43%-58.34%)	75.56% (67.9%-82.72%)	94.26% (83.33%-100%)	3.1359 (2.8966-3.3530)
[illegible]	[illegible]	[illegible]	[illegible]	[illegible]	[illegible]	[illegible]	[illegible]	[illegible]	[illegible]	[illegible]	[illegible]	[illegible]

At least 4 of CHASM (breast), CHASM (lung), CHASM (melanoma), FATHMM (cancer), Mutation Assessor, PolyPhen-2, SIFT	86.23% (83.33%-88.89%)	92.59% (90%-95.28%)	75.227% (69.565%-80.823%)	86.6% (83.33%-89.86%)	85.46% (80.65%-90.33%)	3.3987 (3.2767-3.5149)	86.13% (80.81%-91.92%)	92.46% (86.88%-97.06%)	75.219% (64.276%-87.1%)	86.49% (79.69%-93.22%)	85.36% (74.19%-94.29%)	3.3954 (3.1521-3.6444)
At least 4 of CHASM (breast), CHASM (lung), CHASM (melanoma), FATHMM (cancer), Mutation Assessor, PolyPhen-2, SIFT, VEST	83.2% (80.3%-86.36%)	95.28% (93.33%-97.58%)	62.312% (56.164%-68.75%)	81.38% (77.7%-85.21%)	88.42% (83.67%-93.75%)	3.2739 (3.1535-3.3964)	83.1% (76.77%-88.89%)	95.07% (90.62%-98.53%)	62.541% (50%-75%)	81.32% (73.97%-88.46%)	88.12% (77.78%-96.55%)	3.2705 (3.0252-3.5134)
At least 4 of CHASM (breast), CHASM (lung), CHASM (melanoma), FATHMM (cancer), Mutation Assessor, PolyPhen-2, VEST	85.55% (82.83%-88.38%)	92.59% (90%-95.28%)	73.391% (67.606%-79.221%)	85.74% (82.44%-89.23%)	85.15% (80.33%-90.16%)	3.3687 (3.2491-3.4888)	85.46% (79.8%-90.91%)	92.46% (86.88%-97.06%)	73.421% (62.152%-85.19%)	85.65% (78.57%-92.42%)	85.07% (74.19%-94.29%)	3.3660 (3.1193-3.6116)
At least 4 of CHASM (breast), CHASM (lung), CHASM (melanoma), FATHMM (cancer), Mutation Assessor, PROVEAN, SIFT	85.86% (82.83%-88.4%)	89.39% (86.51%-92.63%)	79.752% (74.286%-84.932%)	88.42% (85.48%-91.34%)	81.3% (76.06%-86.67%)	3.3886 (3.2667-3.5077)	85.86% (80.78%-91.92%)	89.3% (82.54%-95.16%)	79.924% (69.992%-90%)	88.42% (82.45%-94.45%)	81.34% (70.59%-91.67%)	3.3898 (3.1538-3.6416)
At least 4 of CHASM (breast), CHASM (lung), CHASM (melanoma), FATHMM (cancer), Mutation Assessor, PROVEAN, SIFT, VEST	84.2% (81.31%-86.87%)	93.65% (91.27%-96.06%)	67.872% (61.972%-73.973%)	83.44% (80%-86.81%)	86.07% (81.13%-91.23%)	3.3103 (3.1912-3.4300)	84.12% (78.79%-89.9%)	93.55% (88.52%-98.39%)	67.905% (55.263%-80%)	83.34% (76.39%-90.14%)	86.01% (75%-96.16%)	3.3080 (3.0602-3.5521)
At least 4 of CHASM (breast), CHASM (lung), CHASM (melanoma), FATHMM (cancer), Mutation Assessor, PROVEAN, VEST	85.53% (82.32%-88.38%)	89.39% (86.51%-92.63%)	78.865% (73.416%-84.06%)	87.97% (84.92%-91.2%)	81.13% (75.76%-86.67%)	3.3735 (3.2435-3.4989)	85.5% (79.8%-91.92%)	89.3% (82.54%-95.16%)	78.961% (68.571%-89.74%)	87.93% (81.16%-94.12%)	81.15% (70.58%-91.43%)	3.3734 (3.1193-3.6244)
At least 4 of CHASM (breast), CHASM (lung), CHASM (melanoma), FATHMM (cancer), Mutation Assessor, SIFT, VEST	86.57% (83.84%-89.39%)	93.12% (90.55%-95.84%)	75.231% (69.511%-80.771%)	86.67% (83.33%-89.92%)	86.36% (81.67%-91.23%)	3.4138 (3.2942-3.5271)	86.46% (80.81%-91.92%)	93% (87.69%-98.33%)	75.217% (63.883%-87.19%)	86.56% (79.71%-93.55%)	86.25% (75.86%-96.43%)	3.4102 (3.1755-3.6495)
At least 4 of CHASM (breast), CHASM (lung), CHASM (melanoma), FATHMM (cancer), MutationTaster, PolyPhen-2, PROVEAN	88.58% (86.36%-91.41%)	89.39% (86.51%-92.74%)	87.162% (83.099%-91.549%)	92.33% (90%-95.16%)	82.62% (77.63%-87.84%)	3.5151 (3.4106-3.6281)	88.5% (82.83%-92.93%)	89.29% (82.54%-95.16%)	87.118% (78.125%-94.87%)	92.25% (86.21%-96.77%)	82.6% (72.5%-92.31%)	3.5125 (3.2929-3.7118)
At least 4 of CHASM (breast), CHASM (lung), CHASM (melanoma), FATHMM (cancer), MutationTaster, PolyPhen-2, PROVEAN, SIFT	87.22% (84.84%-89.9%)	94.19% (92.06%-96.77%)	75.16% (69.86%-80.887%)	86.77% (83.82%-90.08%)	88.22% (83.64%-93.22%)	3.4435 (3.3347-3.5627)	87.17% (81.82%-91.94%)	94.05% (89.06%-98.39%)	75.343% (63.889%-86.11%)	86.75% (80%-92.76%)	88.08% (78.26%-96.77%)	3.4422 (3.1981-3.6645)
At least 4 of CHASM (breast), CHASM (lung), CHASM (melanoma), FATHMM (cancer), MutationTaster, PolyPhen-2, PROVEAN, SIFT, VEST	82.53% (79.8%-85.86%)	96.86% (95.2%-98.47%)	57.747% (51.429%-64.064%)	79.85% (76.16%-83.55%)	91.41% (87.18%-95.83%)	3.2587 (3.1458-3.3785)	82.42% (75.76%-87.88%)	96.7% (92.98%-100%)	57.918% (45%-70.59%)	79.76% (72.22%-87.01%)	91.1% (80.95%-100%)	3.2548 (3.0105-3.4814)
At least 4 of CHASM (breast), CHASM (lung), CHASM (melanoma), FATHMM (cancer), MutationTaster, PolyPhen-2, PROVEAN, VEST	87.22% (84.85%-89.9%)	95.24% (93.28%-97.58%)	73.347% (67.523%-79.71%)	86.07% (82.84%-89.71%)	89.91% (85.71%-94.74%)	3.4457 (3.3413-3.5594)	87.18% (81.82%-91.92%)	95.15% (90.62%-98.55%)	73.503% (61.101%-85.19%)	86.04% (78.67%-92.65%)	89.85% (80%-97.06%)	3.4454 (3.2054-3.6549)
At least 4 of CHASM (breast), CHASM (lung), CHASM (melanoma), FATHMM (cancer), MutationTaster, PolyPhen-2, SIFT	89.94% (87.88%-92.42%)	93.13% (90.62%-95.9%)	84.423% (79.729%-89.333%)	91.18% (88.64%-94.12%)	87.68% (83.33%-92.21%)	3.5642 (3.4668-3.6765)	89.81% (84.85%-93.94%)	92.97% (87.5%-98.31%)	84.338% (74.286%-93.94%)	91.07% (85%-96.49%)	87.53% (78.12%-96.55%)	3.5591 (3.3331-3.7519)
At least 4 of CHASM (breast), CHASM (lung), CHASM (melanoma), FATHMM (cancer), MutationTaster, PolyPhen-2, SIFT, VEST	85.22% (82.32%-88.38%)	96.32% (94.49%-98.39%)	66.027% (59.722%-72.464%)	83.05% (79.59%-86.72%)	91.22% (87.23%-96.08%)	3.3662 (3.2537-3.4846)	85.12% (78.79%-90.91%)	96.18% (92.19%-100%)	66.134% (52.778%-78.38%)	82.97% (75.34%-89.86%)	90.98% (81.48%-100%)	3.3626 (3.1171-3.5880)
At least 4 of CHASM (breast), CHASM (lung), CHASM (melanoma), FATHMM (cancer), MutationTaster, PolyPhen-2, VEST	89.6% (87.37%-91.92%)	94.18% (91.87%-96.77%)	81.686% (76.812%-87.144%)	89.89% (87.12%-93.08%)	89.04% (85%-93.65%)	3.5479 (3.4458-3.6582)	89.48% (84.85%-93.94%)	94.08% (88.89%-98.46%)	81.58% (70.968%-91.43%)	89.76% (83.33%-95.38%)	88.96% (80%-97.06%)	3.5438 (3.3325-3.7457)
At least 4 of CHASM (breast), CHASM (lung), CHASM (melanoma), FATHMM (cancer), MutationTaster, PROVEAN, SIFT	88.57% (86.36%-91.41%)	89.39% (86.51%-92.63%)	87.138% (83.099%-91.667%)	92.32% (90%-95.12%)	82.61% (77.63%-87.65%)	3.5145 (3.4118-3.6241)	88.53% (82.83%-92.93%)	89.3% (82.54%-95.16%)	87.162% (78.257%-94.74%)	92.28% (86.44%-96.77%)	82.62% (72.5%-92.11%)	3.5136 (3.2893-3.7118)
At least 4 of CHASM (breast), CHASM (lung), CHASM (melanoma), FATHMM (cancer), MutationTaster, PROVEAN, SIFT, VEST	88.23% (85.86%-90.91%)	94.72% (92.62%-96.95%)	77.015% (71.233%-82.814%)	87.69% (84.85%-90.91%)	89.41% (85.48%-93.85%)	3.4884 (3.3794-3.5979)	88.18% (82.83%-92.93%)	94.59% (89.66%-98.48%)	77.142% (65.789%-88.1%)	87.66% (81.16%-93.55%)	89.29% (80%-96.88%)	3.4869 (3.2686-3.7029)
At least 4 of CHASM (breast), CHASM (lung), CHASM (melanoma), FATHMM (cancer), MutationTaster, PROVEAN, VEST	89.25% (86.87%-91.92%)	89.92% (86.99%-93.18%)	88.087% (84.286%-92.537%)	92.89% (90.7%-95.69%)	83.48% (78.67%-88.37%)	3.5437 (3.4448-3.6546)	89.18% (83.84%-93.94%)	89.83% (83.33%-95.39%)	88.012% (78.788%-95%)	92.8% (87.5%-96.92%)	83.47% (73.68%-92.5%)	3.5411 (3.3246-3.7294)
At least 4 of CHASM (breast), CHASM (lung), CHASM (melanoma), FATHMM (cancer), MutationTaster, SIFT, VEST	91.3% (89.39%-93.43%)	94.2% (91.87%-96.75%)	86.275% (81.944%-91.176%)	92.23% (89.92%-95.12%)	89.59% (85.71%-94.03%)	3.6229 (3.5314-3.7227)	91.14% (86.87%-94.95%)	94.04% (89.06%-98.46%)	86.135% (76.471%-94.59%)	92.1% (86.36%-96.83%)	89.42% (80.65%-97.06%)	3.6169 (3.4088-3.7943)
At least 4 of CHASM (breast), CHASM (lung), CHASM (melanoma), FATHMM (cancer), PolyPhen-2, PROVEAN, SIFT	84.17% (81.31%-87.37%)	88.85% (85.83%-92.25%)	76.063% (70.665%-81.821%)	86.52% (83.33%-90.08%)	79.79% (74.32%-85.51%)	3.3123 (3.1887-3.4448)	84.19% (77.78%-89.9%)	88.77% (81.67%-94.92%)	76.289% (65%-87.18%)	86.54% (79.66%-92.98%)	79.86% (68.42%-90.62%)	3.3145 (3.0434-3.5635)
At least 4 of CHASM (breast), CHASM (lung), CHASM (melanoma), FATHMM (cancer), PolyPhen-2, PROVEAN, SIFT, VEST	82.18% (79.29%-85.86%)	93.65% (91.34%-96.09%)	62.339% (55.997%-69.014%)	81.13% (77.46%-84.97%)	85.05% (80%-90.57%)	3.2217 (3.0961-3.3614)	82.1% (74.75%-87.88%)	93.53% (88.33%-98.36%)	62.466% (50%-75%)	81.05% (73.61%-88.32%)	84.93% (73.08%-95.65%)	3.2198 (2.9371-3.4758)
At least 4 of CHASM (breast), CHASM (lung), CHASM (melanoma), FATHMM (cancer), PolyPhen-2, PROVEAN, VEST	84.18% (81.31%-87.37%)	88.85% (85.83%-92.25%)	76.111% (70.77%-82.196%)	86.54% (83.21%-90.32%)	79.8% (74.63%-85.71%)	3.3131 (3.1806-3.4513)	84.16% (77.78%-89.9%)	88.77% (81.67%-94.92%)	76.217% (63.624%-87.88%)	86.5% (79.03%-93.22%)	79.84% (68.29%-90.63%)	3.3133 (3.0286-3.5708)
At least 4 of CHASM (breast), CHASM (lung), CHASM (melanoma), FATHMM (cancer), PolyPhen-2, SIFT, VEST	84.88% (81.82%-87.88%)	93.13% (90.62%-95.9%)	70.619% (64.384%-76.712%)	84.57% (81.12%-88.15%)	85.62% (80.79%-90.91%)	3.3394 (3.2142-3.4681)	84.78% (79.78%-90.91%)	92.97% (87.5%-98.31%)	70.682% (58.333%-82.5%)	84.48% (77.02%-91.43%)	85.45% (74.98%-95.84%)	3.3359 (3.0746-3.5884)
At least 4 of CHASM (breast), CHASM (lung), CHASM (melanoma), FATHMM (cancer), PROVEAN, SIFT, VEST	84.86% (81.82%-87.88%)	89.39% (86.51%-92.63%)	77.015% (71.233%-82.814%)	87.05% (83.97%-90.44%)	80.76% (75.63%-86.36%)	3.3422 (3.2178-3.4701)	84.83% (78.79%-90.91%)	89.3% (82.54%-95.16%)	77.142% (65.789%-88.1%)	87.02% (80.3%-93.33%)	80.79% (69.44%-91.31%)	3.3425 (3.0874-3.5905)
At least 4 of CHASM (breast), CHASM (lung), CHASM (melanoma), FATHMM (missense), Mutation Assessor, MutationTaster, PolyPhen-2	90.3% (88.38%-92.42%)	92.63% (90%-95.2%)	86.269% (82.049%-90.789%)	92.1% (89.76%-94.86%)	87.14% (82.86%-91.55%)	3.5815 (3.4876-3.6799)	90.1% (85.86%-93.94%)	92.38% (87.1%-97.06%)	86.152% (76.923%-94.44%)	91.97% (86.44%-96.77%)	86.85% (77.78%-94.87%)	3.5735 (3.3780-3.7500)
At least 4 of CHASM (breast), CHASM (lung), CHASM (melanoma), FATHMM (missense), Mutation Assessor, MutationTaster, PolyPhen-2, PROVEAN	89.27% (86.87%-91.92%)	94.74% (92.68%-96.85%)	79.809% (74.648%-85.294%)	89.03% (86.15%-92.25%)	89.77% (85.94%-93.94%)	3.5334 (3.4364-3.6383)	89.14% (83.84%-93.94%)	94.56% (90.16%-98.46%)	79.812% (69.231%-90%)	88.94% (82.35%-94.6%)	89.55% (80.65%-96.97%)	3.5286 (3.3193-3.7252)
At least 4 of CHASM (breast), CHASM (lung), CHASM (melanoma), FATHMM (missense), Mutation Assessor, MutationTaster, PolyPhen-2, PROVEAN, SIFT	84.87% (82.32%-87.88%)	95.77% (93.7%-97.64%)	66.001% (60%-71.878%)	82.97% (79.59%-86.3%)	90.04% (85.71%-94.64%)	3.3478 (3.2340-3.4582)	84.81% (78.79%-89.9%)	95.68% (91.8%-100%)	66.145% (54.054%-77.79%)	82.9% (76.12%-89.56%)	89.95% (80%-100%)	3.3468 (3.1229-3.5725)
[illegible]	[illegible]	[illegible]	[illegible]	[illegible]	[illegible]	[illegible]	[illegible]	[illegible]	[illegible]	[illegible]	[illegible]	[illegible]

At least 4 of CHASM (breast), CHASM (melanoma), FATHMM (missense), Mutation Assessor, MutationTaster, PROVEAN, SIFT	85.84% (83.33%-88.38%)	86.69% (83.46%-90.32%)	84.356% (79.73%-89.066%)	90.55% (87.72%-93.39%)	78.58% (73.33%-83.79%)	3.4018 (3.2869-3.5171)	85.89% (80.81%-90.91%)	86.69% (79.41%-93.34%)	84.474% (75.542%-93.75%)	90.56% (84.62%-96.49%)	78.75% (68.17%-88.57%)	3.4047 (3.1688-3.6270)
At least 4 of CHASM (breast), CHASM (melanoma), FATHMM (missense), Mutation Assessor, MutationTaster, PROVEAN, SIFT, VEST	81.13% (77.78%-84.34%)	88.28% (85.27%-91.54%)	68.753% (62.662%-75%)	83.01% (79.41%-86.51%)	77.25% (71.43%-82.86%)	3.1730 (3.0385-3.3032)	81.17% (74.75%-87.88%)	88.31% (81.54%-94.03%)	68.9% (56.757%-81.09%)	82.97% (76.06%-90.16%)	77.48% (65.79%-88.1%)	3.1765 (2.8993-3.4424)
At least 4 of CHASM (breast), CHASM (melanoma), FATHMM (missense), Mutation Assessor, MutationTaster, PROVEAN, VEST	84.17% (81.31%-87.37%)	84.03% (80.34%-87.69%)	84.4% (79.729%-89.479%)	90.31% (87.39%-93.5%)	75.35% (70%-80.72%)	3.3409 (3.2196-3.4643)	84.19% (77.78%-89.9%)	84.04% (76.36%-91.05%)	84.385% (74.359%-93.75%)	90.24% (84.13%-96.43%)	75.52% (64.7%-85.71%)	3.3419 (3.1021-3.5655)
At least 4 of CHASM (breast), CHASM (melanoma), FATHMM (missense), Mutation Assessor, MutationTaster, SIFT, VEST	84.18% (81.31%-86.87%)	88.28% (85.27%-91.54%)	77.084% (71.791%-82.814%)	86.95% (83.59%-90.3%)	79.2% (73.85%-84.51%)	3.3152 (3.1953-3.4390)	84.16% (78.79%-89.9%)	88.31% (81.54%-94.03%)	76.906% (65.706%-88.1%)	86.83% (80.3%-93.65%)	79.36% (68.57%-89.19%)	3.3148 (3.0564-3.5572)
At least 4 of CHASM (breast), CHASM (melanoma), FATHMM (missense), Mutation Assessor, PolyPhen-2, PROVEAN, SIFT	80.13% (76.77%-83.33%)	84.06% (80.47%-87.69%)	73.336% (67.188%-78.953%)	84.5% (81.1%-88%)	72.69% (67.11%-78.38%)	3.1458 (3.0088-3.2755)	80.14% (73.74%-86.87%)	83.98% (76.47%-90.91%)	73.483% (62.493%-85.3%)	84.47% (77.78%-91.67%)	72.8% (61.29%-83.72%)	3.1473 (2.8814-3.4128)
At least 4 of CHASM (breast), CHASM (melanoma), FATHMM (missense), Mutation Assessor, PolyPhen-2, PROVEAN, SIFT, VEST	76.76% (73.74%-79.8%)	85.65% (82.26%-89.26%)	61.397% (55.378%-67.656%)	79.32% (75.56%-83.09%)	71.22% (65.08%-77.05%)	2.9759 (2.8365-3.1077)	76.77% (70.71%-82.83%)	85.6% (78.46%-91.94%)	61.6% (48.387%-74.2%)	79.27% (71.43%-86.76%)	71.4% (59.26%-83.33%)	2.9787 (2.7004-3.2571)
At least 4 of CHASM (breast), CHASM (melanoma), FATHMM (missense), Mutation Assessor, PolyPhen-2, PROVEAN, VEST	77.1% (73.74%-80.3%)	80.32% (76.38%-84.38%)	71.52% (65.714%-77.337%)	82.98% (79.2%-86.67%)	67.77% (62.16%-73.97%)	3.0259 (2.8896-3.1532)	77.11% (70.71%-83.84%)	80.28% (71.88%-88.06%)	71.617% (60%-83.88%)	82.92% (75.41%-90.39%)	67.92% (55.55%-80%)	3.0275 (2.7638-3.3032)
At least 4 of CHASM (breast), CHASM (melanoma), FATHMM (missense), Mutation Assessor, PolyPhen-2, SIFT, VEST	78.44% (75.25%-81.31%)	85.65% (82.26%-89.26%)	65.981% (60%-72.464%)	81.32% (77.69%-85.19%)	72.68% (66.67%-78.46%)	3.0562 (2.9215-3.1847)	78.47% (72.73%-84.85%)	85.6% (78.46%-91.94%)	66.187% (54.049%-77.78%)	81.29% (73.44%-88.57%)	72.84% (60.71%-84.38%)	3.0591 (2.7965-3.3240)
At least 4 of CHASM (breast), CHASM (melanoma), FATHMM (missense), Mutation Assessor, PROVEAN, SIFT, VEST	78.13% (74.75%-81.31%)	82.48% (78.81%-86.4%)	70.582% (64.706%-76.715%)	82.9% (79.34%-86.56%)	69.98% (64.1%-76%)	3.0594 (2.9188-3.1977)	78.09% (71.72%-84.85%)	82.35% (74.28%-89.56%)	70.734% (57.895%-82.51%)	82.85% (75.41%-90%)	70.03% (57.58%-81.48%)	3.0596 (2.7786-3.3423)
At least 4 of CHASM (breast), CHASM (melanoma), FATHMM (missense), MutationTaster, PolyPhen-2, PROVEAN, SIFT	83.82% (80.81%-86.87%)	86.7% (83.33%-90.25%)	78.845% (73.611%-84.507%)	87.64% (84.43%-90.91%)	77.42% (72%-82.86%)	3.3060 (3.1803-3.4358)	83.87% (77.78%-89.9%)	86.69% (79.66%-93.34%)	78.965% (68.282%-89.47%)	87.63% (80.95%-93.94%)	77.6% (66.67%-88.37%)	3.3088 (3.0520-3.5522)
At least 4 of CHASM (breast), CHASM (melanoma), FATHMM (missense), MutationTaster, PolyPhen-2, PROVEAN, SIFT, VEST	79.13% (76.26%-82.32%)	88.3% (85.25%-91.41%)	63.264% (56.578%-69.333%)	80.61% (76.81%-84.5%)	75.78% (69.84%-81.82%)	3.0795 (2.9445-3.2068)	79.12% (72.73%-84.85%)	88.28% (81.67%-94.45%)	63.355% (50%-75.76%)	80.53% (73.13%-88%)	75.94% (63.88%-87.88%)	3.0810 (2.8239-3.3455)
At least 4 of CHASM (breast), CHASM (melanoma), FATHMM (missense), MutationTaster, PolyPhen-2, PROVEAN, VEST	80.82% (77.78%-83.84%)	83.5% (79.67%-87.29%)	76.172% (70.419%-82.26%)	85.84% (82.54%-89.57%)	72.75% (67.09%-78.38%)	3.1827 (3.0520-3.3127)	80.78% (74.75%-86.87%)	83.51% (75.41%-90.91%)	76.045% (63.889%-87.5%)	85.7% (78.33%-92.46%)	72.89% (61.29%-84.09%)	3.1815 (2.9139-3.4424)
At least 4 of CHASM (breast), CHASM (melanoma), FATHMM (missense), MutationTaster, PolyPhen-2, SIFT, VEST	81.16% (78.28%-83.84%)	87.76% (84.55%-91.06%)	69.737% (63.765%-76.058%)	83.38% (79.7%-87.2%)	76.72% (71.21%-82.26%)	3.1760 (3.0454-3.3040)	81.11% (75.76%-86.87%)	87.76% (81.25%-93.85%)	69.655% (56.757%-81.25%)	83.24% (75.76%-90.14%)	76.86% (65.62%-87.5%)	3.1752 (2.9184-3.4297)
At least 4 of CHASM (breast), CHASM (melanoma), FATHMM (missense), MutationTaster, PROVEAN, SIFT, VEST	83.82% (80.81%-86.87%)	85.62% (82.11%-89.15%)	80.688% (75.384%-86.486%)	88.47% (85.34%-91.87%)	76.45% (71.08%-81.82%)	3.3123 (3.1859-3.4357)	83.88% (77.78%-89.9%)	85.65% (78.33%-92.54%)	80.775% (69.992%-90.91%)	88.46% (81.67%-94.92%)	76.65% (65.79%-87.18%)	3.3153 (3.0627-3.5634)
At least 4 of CHASM (breast), CHASM (melanoma), FATHMM (missense), PolyPhen-2, PROVEAN, SIFT, VEST	77.77% (74.75%-80.81%)	83.52% (80%-87.2%)	67.807% (61.641%-73.974%)	81.77% (78.03%-85.4%)	70.42% (64.61%-76.47%)	3.0352 (2.8954-3.1674)	77.8% (71.72%-83.84%)	83.47% (75.86%-90.32%)	68.03% (55.882%-80.66%)	81.76% (74.19%-89.4%)	70.59% (58.82%-81.82%)	3.0385 (2.7680-3.3112)
At least 4 of CHASM (breast), CHASM (melanoma), Mutation Assessor, MutationTaster, PolyPhen-2, PROVEAN, SIFT	80.11% (76.77%-83.33%)	81.88% (78.05%-85.83%)	77.037% (71.795%-82.503%)	86.04% (82.5%-89.57%)	71.09% (65.85%-76.83%)	3.1606 (3.0286-3.2977)	80.18% (73.74%-86.87%)	81.95% (74.57%-89.29%)	77.116% (65.625%-88.24%)	86% (78.95%-93.22%)	71.36% (59.52%-82.35%)	3.1644 (2.8847-3.4222)
At least 4 of CHASM (breast), CHASM (melanoma), Mutation Assessor, MutationTaster, PolyPhen-2, PROVEAN, SIFT, VEST	75.39% (72.22%-78.79%)	82.43% (78.69%-86.4%)	63.224% (57.143%-69.623%)	79.49% (75.57%-83.47%)	67.54% (61.64%-73.91%)	2.9268 (2.7893-3.0719)	75.48% (68.69%-81.82%)	82.46% (74.6%-89.4%)	63.465% (50%-75.76%)	79.47% (71.64%-87.3%)	67.85% (54.83%-80%)	2.9325 (2.6281-3.2019)
At least 4 of CHASM (breast), CHASM (melanoma), Mutation Assessor, MutationTaster, PolyPhen-2, PROVEAN, VEST	78.1% (74.75%-81.31%)	80.31% (76.52%-84.25%)	74.278% (68.569%-80.264%)	84.37% (80.7%-88.18%)	68.58% (63.16%-74.32%)	3.0754 (2.9380-3.2088)	78.14% (71.72%-84.85%)	80.31% (72.31%-87.88%)	74.397% (62.5%-85.01%)	84.32% (76.66%-91.53%)	68.77% (57.14%-79.56%)	3.0780 (2.8054-3.3436)
At least 4 of CHASM (breast), CHASM (melanoma), Mutation Assessor, MutationTaster, PolyPhen-2, SIFT, VEST	77.75% (74.75%-80.81%)	82.43% (78.69%-86.4%)	69.664% (63.997%-75.714%)	82.45% (78.63%-86.4%)	69.63% (64.28%-75.68%)	3.0417 (2.9035-3.1805)	77.83% (71.72%-83.84%)	82.46% (74.6%-89.4%)	69.85% (57.143%-82.06%)	82.43% (74.63%-89.66%)	69.9% (57.5%-80.96%)	3.0464 (2.7554-3.3167)
At least 4 of CHASM (breast), CHASM (melanoma), Mutation Assessor, MutationTaster, PROVEAN, SIFT, VEST	77.79% (74.75%-81.31%)	79.27% (75.42%-83.07%)	75.21% (69.444%-80.822%)	84.68% (80.99%-88.39%)	67.73% (62.19%-73.49%)	3.0689 (2.9336-3.2126)	77.76% (70.71%-83.84%)	79.2% (71.18%-86.89%)	75.269% (64.097%-86.84%)	84.6% (77.08%-91.67%)	67.83% (55.56%-78.58%)	3.0690 (2.7791-3.3308)
At least 4 of CHASM (breast), CHASM (melanoma), Mutation Assessor, PolyPhen-2, PROVEAN, SIFT, VEST	73.74% (70.2%-77.27%)	78.74% (74.8%-82.93%)	65.086% (59.15%-71.622%)	79.59% (75.61%-83.72%)	63.9% (57.97%-70%)	2.8732 (2.7323-3.0157)	73.74% (66.67%-80.81%)	78.68% (70.37%-86.57%)	65.233% (52.5%-77.42%)	79.52% (71.7%-87.3%)	64.05% (51.52%-76.19%)	2.8748 (2.5860-3.1626)
At least 4 of CHASM (breast), CHASM (melanoma), MutationTaster, PolyPhen-2, PROVEAN, SIFT, VEST	77.44% (74.24%-80.81%)	81.39% (77.78%-85.37%)	70.59% (64.787%-76.718%)	82.71% (79.16%-86.56%)	68.69% (63.16%-74.37%)	3.0338 (2.9024-3.1722)	77.45% (70.71%-83.84%)	81.34% (73.33%-88.53%)	70.73% (58.614%-82.35%)	82.67% (75%-90%)	68.83% (57.13%-80%)	3.0358 (2.7664-3.3032)
At least 4 of CHASM (breast), FATHMM (cancer), FATHMM (missense), Mutation Assessor, MutationTaster, PolyPhen-2, PROVEAN	81.43% (78.28%-84.34%)	84.02% (80.34%-87.71%)	76.937% (71.233%-82.609%)	86.3% (82.91%-90%)	73.59% (68.12%-78.75%)	3.2085 (3.0793-3.3417)	81.58% (75.76%-87.88%)	84.06% (76.27%-91.38%)	77.316% (66.646%-88.24%)	86.4% (79.3%-93.11%)	73.89% (62.86%-84.85%)	3.2167 (2.9436-3.4665)
At least 4 of CHASM (breast), FATHMM (cancer), FATHMM (missense), Mutation Assessor, MutationTaster, PolyPhen-2, PROVEAN, SIFT	83.44% (80.3%-86.36%)	90.41% (87.69%-93.5%)	71.4% (65.67%-77.923%)	84.53% (81.06%-88.19%)	81.16% (76.12%-86.79%)	3.2749 (3.1510-3.4106)	83.62% (77.78%-89.9%)	90.45% (84.38%-96.08%)	71.886% (58.971%-83.79%)	84.66% (77.77%-91.78%)	81.46% (70%-91.67%)	3.2846 (3.0110-3.5390)
At least 4 of CHASM (breast), FATHMM (cancer), FATHMM (missense), Mutation Assessor, MutationTaster, PolyPhen-2, PROVEAN, SIFT, VEST	78.08% (74.75%-81.31%)	91.46% (88.71%-94.35%)	54.935% (48.611%-61.117%)	77.82% (74.15%-81.63%)	78.82% (72.34%-85.42%)	3.0303 (2.8907-3.1693)	78.19% (71.72%-84.85%)	91.54% (85.94%-96.88%)	55.25% (42.105%-67.65%)	77.83% (70%-84.93%)	79.25% (66.63%-92%)	3.0387 (2.7523-3.3140)
At least 4 of CHASM (breast), FATHMM (cancer), FATHMM (missense), Mutation Assessor, MutationTaster, PolyPhen-2, PROVEAN, VEST	81.45% (78.79%-84.34%)	87.76% (84.55%-91.05%)	70.545% (64.933%-76.562%)	83.74% (80.15%-87.41%)	76.92% (71.42%-82.61%)	3.1897 (3.0618-3.3224)	81.54% (75.76%-86.87%)	87.76% (81.03%-93.94%)	70.824% (58.607%-82.35%)	83.77% (76.39%-90.77%)	77.14% (65.5%-88.24%)	3.1950 (2.9153-3.4424)
At least 4 of CHASM (breast), FATHMM (cancer), FATHMM (missense), Mutation Assessor, MutationTaster, PolyPhen-2, SIFT	84.12% (81.31%-86.87%)	90.41% (87.69%-93.5%)	73.255% (67.532%-79.453%)	85.39% (82.03%-88.89%)	81.55% (76.56%-87.04%)	3.3060 (3.1825-3.4363)	84.28% (78.79%-89.9%)	90.45% (84.38%-96.08%)	73.68% (61.532%-85%)	85.5% (78.46%-92.06%)	81.84% (70.59%-92%)	3.3146 (3.0418-3.5600)
At least 4 of CHASM (breast), FATHMM (cancer), FATHMM (missense), Mutation Assessor, MutationTaster, PolyPhen-2, SIFT, VEST	79.75% (76.77%-82.83%)	91.46% (88.71%-94.35%)	59.51% (53.425%-65.789%)	79.61% (76.03%-83.33%)	80.12% (74%-86.54%)	3.1070 (2.9713-3.2425)	79.89% (73.74%-85.86%)	91.54% (85.94%-96.88%)	59.857% (46.667%-72.5%)	79.65% (72.22%-86.96%)	80.53% (68%-92.6%)	3.1157 (2.8261-3.3830)
At least 4 of CHASM (breast), FATHMM (cancer), FATHMM (missense), Mutation Assessor, MutationTaster, PolyPhen-2, VEST	82.48% (79.8%-85.35%)	87.76% (84.55%-91.05%)	73.345% (67.991%-79.458%)	85.06% (81.68%-88.71%)	77.61% (72.46%-83.1%)	3.2377 (3.1152-3.3688)	82.52% (76.77%-87.88%)	87.76% (81.03%-93.94%)	73.481% (61.286%-84.62%)	85.03% (77.78%-91.78%)	77.79% (66.67%-88.57%)	3.2406 (2.9756-3.4836)
At least 4 of CHASM (breast), FATHMM (cancer), FATHMM (missense), Mutation Assessor, MutationTaster, PROVEAN, SIFT	83.11% (80.3%-85.87%)	86.68% (83.47%-90.32%)	76.933% (71.233%-82.67%)	86.66% (83.33%-90.17%)	76.97% (71.43%-82.35%)	3.2725 (3.1444-3.3992)	83.27% (77.75%-88.89%)	86.72% (79.68%-93.22%)	77.311% (65.714%-88.89%)	86.78% (80%-93.55%)	77.25% (66.67%-88.24%)	3.2806 (3.0197-3.5283)
At least 4 of CHASM (breast), FATHMM (cancer), FATHMM (missense), Mutation Assessor, MutationTaster, PROVEAN, SIFT, VEST	81.42% (78.28%-84.34%)	89.9% (86.92%-92.91%)	66.757% (60.868%-73.077%)	82.38% (78.83%-85.92%)	79.28% (73.44%-85.01%)	3.1832 (3.0498-3.3172)	81.6% (75.76%-87.88%)	89.86% (83.61%-95.45%)	67.402% (55%-79.41%)	82.54% (75%-89.55%)	79.51% (67.74%-90.62%)	3.1931 (2.9115-3.4459)
At least 4 of CHASM (breast), FATHMM (cancer), FATHMM (missense), Mutation Assessor, MutationTaster, PROVEAN, VEST	82.45% (79.29%-85.35%)	84.55% (80.8%-88.19%)	78.792% (73.413%-84.286%)	87.33% (84.03%-90.91%)	74.69% (69.23%-79.73%)	3.2537 (3.1196-3.3801)	82.58% (76.77%-88.89%)	84.59% (77.05%-92.07%)	79.11% (68.571%-89.75%)	87.42% (80.3%-93.85%)	74.97% (64.71%-86.49%)	3.2609 (2.9991-3.5186)
At least 4 of CHASM (breast), FATHMM (cancer), FATHMM (missense), Mutation Assessor, MutationTaster, SIFT, VEST	82.77% (79.8%-85.37%)	89.36% (86.29%-92.43%)	71.356% (65.714%-77.632%)	84.36% (81.06%-88.15%)	79.52% (74.19%-84.85%)	3.2460 (3.1143-3.3716)	82.95% (77.75%-88.89%)	89.34% (83.05%-95.31%)	71.962% (58.971%-83.33%)	84.54% (77.05%-91.07%)	79.76% (68.75%-90.01%)	3.2560 (2.9990-3.5148)
At least 4 of CHASM (breast), FATHMM (cancer), FATHMM (missense), Mutation Assessor, PolyPhen-2, PROVEAN, SIFT	80.43% (77.27%-83.84%)	83.51% (80.16%-87.4%)	75.075% (69.444%-81.081%)	85.28% (81.82%-88.89%)	72.48% (66.67%-77.78%)	3.1635 (3.0271-3.2973)	80.56% (73.74%-86.87%)	83.48% (75.41%-90.32%)	75.515% (63.636%-87.1%)	85.41% (78.33%-92.45%)	72.71% (61.9%-83.78%)	3.1712 (2.9106-3.4363)
At least 4 of CHASM (breast), FATHMM (cancer), FATHMM (missense), Mutation Assessor, PolyPhen-2, PROVEAN, SIFT, VEST	75.74% (72.22%-79.29%)	86.73% (83.61%-90.23%)	56.733% (50%-63.014%)	77.61% (73.76%-81.48%)	71.21% (64.91%-77.59%)	2.9228 (2.7810-3.0602)	75.79% (68.69%-82.83%)	86.62% (79.37%-92.65%)	57.171% (44.444%-70%)	77.63% (69.7%-85.29%)	71.38% (58.33%-84.38%)	2.9280 (2.6441-3.2127)
At least 4 of CHASM (breast), FATHMM (cancer), FATHMM (missense), Mutation Assessor, PolyPhen-2, PROVEAN, VEST	77.73% (74.24%-80.81%)	79.78% (76.03%-83.71%)	74.171% (68.493%-80.264%)	84.23% (80.64%-88.14%)	67.96% (62.34%-73.75%)	3.0614 (2.9214-3.1908)	77.88% (71.72%-84.85%)	79.78% (71.7%-87.5%)	74.578% (62.5%-86.49%)	84.34% (76.67%-91.67%)	68.24% (56.76%-79.49%)	3.0694 (2.8126-3.3472)
At least 4 of CHASM (breast), FATHMM (cancer), FATHMM (missense), Mutation Assessor, PolyPhen-2, SIFT, VEST	77.08% (73.74%-80.3%)	86.19% (82.95%-89.6%)	61.308% (55.263%-67.606%)	79.39% (75.71%-83.21%)	71.98% (66.09%-77.97%)	2.9886 (2.8522-3.1314)	77.16% (70.71%-83.84%)	86.1% (78.95%-92.31%)	61.777% (50%-74.29%)	79.44% (71.67%-86.84%)	72.17% (60%-84.38%)	2.9949 (2.7042-3.2746)
At least 4 of CHASM (breast), FATHMM (cancer), FATHMM (missense), Mutation Assessor, PROVEAN, SIFT, VEST	77.39% (74.24%-80.81%)	81.93% (78.22%-85.95%)	69.53% (63.633%-76%)	82.3% (78.62%-86.21%)	69% (63.07%-74.67%)	3.0276 (2.8898-3.1668)	77.54% (70.71%-83.84%)	81.86% (73.77%-89.06%)	70.084% (57.5%-81.25%)	82.44% (74.6%-89.83%)	69.24% (57.49%-80.56%)	3.0363 (2.7444-3.3034)
At least 4 of CHASM (breast), FATHMM (cancer), FATHMM (missense), MutationTaster, PolyPhen-2, PROVEAN, SIFT	82.79% (79.8%-85.86%)	86.69% (83.33%-90.32%)	76.023% (70.423%-81.818%)	86.21% (83.05%-89.92%)	76.78% (71.23%-81.95%)	3.2571 (3.1267-3.3848)	82.91% (76.77%-88.89%)	86.7% (79.36%-93.65%)	76.368% (65%-87.5%)	86.3% (79.03%-92.86%)	77.02% (66.67%-88.24%)	3.2639 (3.0081-3.5184)
At least 4 of CHASM (breast), FATHMM (cancer), FATHMM (missense), MutationTaster, PolyPhen-2, PROVEAN, SIFT, VEST	79.42% (76.26%-82.32%)	89.89% (86.89%-92.91%)	61.312% (55.072%-67.606%)	80.07% (76.43%-83.7%)	77.82% (71.7%-83.93%)	3.0908 (2.9563-3.2221)	79.55% (73.74%-85.86%)	89.9% (83.58%-95.52%)	61.76% (50%-74.42%)	80.13% (72.73%-87.32%)	78.11% (65.38%-90.32%)	3.0990 (2.8188-3.3723)
At least 4 of CHASM (breast), FATHMM (cancer), FATHMM (missense), MutationTaster, PolyPhen-2, PROVEAN, VEST	81.45% (78.28%-84.34%)	84.55% (80.95%-88.24%)	76.084% (70.588%-81.944%)	85.94% (82.64%-89.68%)	74.01% (68.49%-79.49%)	3.2059 (3.0719-3.3391)	81.54% (75.76%-87.88%)	84.61% (76.92%-91.94%)	76.242% (64.286%-87.19%)	85.95% (78.46%-92.42%)	74.29% (63.15%-85.37%)	3.2109 (2.9472-3.4822)
At least 4 of CHASM (breast), FATHMM (cancer), FATHMM (missense), MutationTaster, PolyPhen-2, SIFT, VEST	81.45% (78.28%-84.34%)	89.89% (86.89%-92.91%)	66.866% (60.87%-72.98%)	82.43% (78.99%-86.23%)	79.28% (73.44%-85.08%)	3.1845 (3.0474-3.3084)	81.54% (75.76%-87.88%)	89.9% (83.58%-95.52%)	67.167% (54.539%-78.95%)	82.45% (75%-89.4%)	79.51% (66.67%-90.91%)	3.1903 (2.9236-3.4622)
At least 4 of CHASM (breast), FATHMM (cancer), FATHMM (missense), MutationTaster, PROVEAN, SIFT, VEST	82.1% (78.79%-85.35%)	86.69% (83.46%-90.32%)	74.155% (68.18%-80.303%)	85.3% (81.89%-88.98%)	76.32% (70.42%-81.69%)	3.2247 (3.0873-3.3537)	82.26% (75.76%-88.89%)	86.71% (79.37%-93.45%)	74.589% (62.493%-85.3%)	85.42% (78.12%-92.19%)	76.61% (65.62%-88.24%)	3.2333 (2.9699-3.4970)
At least 4 of CHASM (breast), FATHMM (cancer), FATHMM (missense), PolyPhen-2, PROVEAN, SIFT, VEST	77.06% (73.74%-80.3%)	83.52% (79.84%-87.2%)	65.883% (59.739%-71.831%)	80.89% (77.1%-84.73%)	69.81% (63.64%-75.48%)	3.0010 (2.8566-3.1403)	77.19% (70.71%-83.84%)	83.47% (75.81%-90.74%)	66.364% (54.044%-78.79%)	80.99% (73.02%-88.53%)	70.07% (58.33%-82.35%)	3.0089 (2.7346-3.2890)
At least 4 of CHASM (breast), FATHMM (cancer), Mutation Assessor, MutationTaster, PolyPhen-2, PROVEAN, SIFT	79.4% (76.25%-82.32%)	82.43% (78.74%-86.36%)	74.156% (68.353%-80.264%)	84.65% (80.99%-88.6%)	70.94% (65.47%-76.63%)	3.1218 (2.9845-3.2572)	79.58% (73.74%-85.86%)	82.45% (74.6%-89.83%)	74.634% (62.5%-86.11%)	84.79% (77.19%-91.94%)	71.26% (60%-82.15%)	3.1314 (2.8521-3.4073)
At least 4 of CHASM (breast), FATHMM (cancer), Mutation Assessor, MutationTaster, PolyPhen-2, PROVEAN, SIFT, VEST	76.74% (73.23%-79.8%)	83.48% (79.84%-87.2%)	65.073% (59.091%-71.605%)	80.51% (76.56%-84.5%)	69.5% (63.64%-75.71%)	2.9856 (2.8493-3.1221)	76.83% (70.71%-83.84%)	83.56% (76.18%-90.62%)	65.282% (52.5%-77.28%)	80.49% (72.58%-88.14%)	69.83% (57.14%-81.82%)	2.9917 (2.7127-3.2548)
[illegible]	[illegible]	[illegible]	[illegible]	[illegible]	[illegible]	[illegible]	[illegible]	[illegible]	[illegible]	[illegible]	[illegible]	[illegible]	[illegible]

At least 5 of CHASM (breast), CHASM (lung), CHASM (melanoma), FATHMM (cancer), FATHMM (missense), Mutation Assessor, PolyPhen-2	86.58% (83.84%-89.39%)	84.66% (80.95%-88.33%)	89.898% (86.072%-94.032%)	93.55% (91.3%-96.36%)	77.23% (72.09%-82.28%)	3.4533 (3.3543-3.5636)	86.43% (80.81%-91.92%)	84.38% (77.05%-91.94%)	89.905% (81.812%-97.37%)	93.49% (88.14%-98.18%)	77.07% (66.67%-87.23%)	3.4485 (3.2286-3.6515)
At least 5 of CHASM (breast), CHASM (lung), CHASM (melanoma), FATHMM (cancer), FATHMM (missense), Mutation Assessor, PolyPhen-2, PROVEAN	85.56% (82.83%-88.38%)	86.77% (83.33%-90.4%)	83.479% (78.871%-88.41%)	90.08% (87.39%-93.22%)	78.5% (73.24%-83.78%)	3.3882 (3.2718-3.5086)	85.44% (79.8%-90.91%)	86.55% (79.1%-93.45%)	83.478% (73.333%-92.31%)	90% (83.64%-95.31%)	78.39% (67.74%-88.89%)	3.3841 (3.1431-3.6135)
At least 5 of CHASM (breast), CHASM (lung), CHASM (melanoma), FATHMM (cancer), FATHMM (missense), Mutation Assessor, PolyPhen-2, PROVEAN, SIFT	85.84% (82.83%-88.89%)	92.04% (89.43%-95.04%)	75.106% (69.564%-80.771%)	86.48% (83.33%-89.78%)	84.52% (79.66%-89.48%)	3.3814 (3.2562-3.5039)	85.9% (79.8%-91.92%)	91.97% (86.15%-96.92%)	75.438% (64.097%-86.85%)	86.54% (79.71%-92.86%)	84.59% (74.29%-94.12%)	3.3854 (3.1383-3.6276)
At least 5 of CHASM (breast), CHASM (lung), CHASM (melanoma), FATHMM (cancer), FATHMM (missense), Mutation Assessor, PolyPhen-2, PROVEAN, SIFT, VEST	84.21% (81.31%-86.88%)	94.2% (92%-96.72%)	66.931% (60.811%-72.727%)	83.12% (79.73%-86.62%)	86.97% (82.14%-92.07%)	3.3122 (3.1883-3.4364)	84.11% (78.76%-89.9%)	94.04% (89.09%-98.41%)	67.04% (54.996%-79.49%)	83.04% (75.76%-90%)	86.78% (76.65%-96.3%)	3.3090 (3.0610-3.5534)
At least 5 of CHASM (breast), CHASM (lung), CHASM (melanoma), FATHMM (cancer), FATHMM (missense), Mutation Assessor, PolyPhen-2, PROVEAN, VEST	85.17% (82.32%-88.38%)	92.04% (89.43%-95.04%)	73.281% (67.143%-79.174%)	85.63% (82.39%-89.15%)	84.2% (79.31%-89.23%)	3.3514 (3.2260-3.4779)	85.22% (78.79%-90.91%)	91.97% (86.15%-96.92%)	73.597% (61.759%-85.71%)	85.68% (78.46%-92.31%)	84.26% (74.07%-93.94%)	3.3551 (3.1055-3.6054)
At least 5 of CHASM (breast), CHASM (lung), CHASM (melanoma), FATHMM (cancer), FATHMM (missense), Mutation Assessor, PolyPhen-2, SIFT	86.89% (84.34%-89.9%)	89.95% (86.99%-93.02%)	81.596% (76.812%-86.669%)	89.42% (86.61%-92.54%)	82.46% (77.46%-87.5%)	3.4343 (3.3263-3.5505)	86.82% (80.81%-91.92%)	89.76% (83.6%-95.52%)	81.725% (71.419%-91.18%)	89.41% (83.05%-95.16%)	82.34% (72.41%-91.89%)	3.4323 (3.2044-3.6483)
At least 5 of CHASM (breast), CHASM (lung), CHASM (melanoma), FATHMM (cancer), FATHMM (missense), Mutation Assessor, PolyPhen-2, SIFT, VEST	84.55% (81.82%-87.37%)	94.2% (92%-96.72%)	67.855% (61.762%-73.913%)	83.52% (80.27%-87.07%)	87.12% (82.35%-92.16%)	3.3269 (3.2050-3.4489)	84.44% (78.79%-89.9%)	94.04% (89.09%-98.41%)	67.944% (55.556%-80%)	83.43% (76.31%-90.14%)	86.93% (76.67%-96.3%)	3.3235 (3.0722-3.5656)
At least 5 of CHASM (breast), CHASM (lung), CHASM (melanoma), FATHMM (cancer), FATHMM (missense), Mutation Assessor, PolyPhen-2, VEST	86.58% (83.84%-89.39%)	89.95% (86.99%-93.02%)	80.743% (75.714%-86.303%)	88.99% (86.13%-92.25%)	82.31% (77.46%-87.32%)	3.4199 (3.3081-3.5413)	86.43% (80.81%-91.92%)	89.76% (83.6%-95.52%)	80.699% (70%-90.62%)	88.88% (81.97%-94.83%)	82.16% (72%-91.67%)	3.4149 (3.1758-3.6437)
At least 5 of CHASM (breast), CHASM (lung), CHASM (melanoma), FATHMM (cancer), FATHMM (missense), Mutation Assessor, PROVEAN	83.57% (80.81%-86.36%)	78.85% (74.62%-83.06%)	91.724% (88.158%-95.776%)	94.28% (91.96%-97.06%)	71.5% (66.27%-76.67%)	3.3635 (3.2569-3.4804)	83.37% (77.78%-88.89%)	78.46% (70.17%-86.89%)	91.763% (83.784%-97.78%)	94.24% (88.89%-98.31%)	71.3% (60.78%-82.22%)	3.3577 (3.1272-3.5728)
At least 5 of CHASM (breast), CHASM (lung), CHASM (melanoma), FATHMM (cancer), FATHMM (missense), Mutation Assessor, PROVEAN, SIFT	85.56% (82.83%-88.38%)	86.77% (83.33%-90.4%)	83.457% (78.788%-88.406%)	90.07% (87.2%-93.16%)	78.5% (73.08%-84%)	3.3879 (3.2710-3.5052)	85.45% (79.8%-90.91%)	86.55% (79.37%-93.22%)	83.514% (73.81%-92.5%)	90.02% (83.93%-95.39%)	78.39% (68.09%-88.89%)	3.3846 (3.1516-3.6179)
At least 5 of CHASM (breast), CHASM (lung), CHASM (melanoma), FATHMM (cancer), FATHMM (missense), Mutation Assessor, PROVEAN, SIFT, VEST	85.84% (82.83%-88.4%)	92.57% (90.08%-95.28%)	74.183% (68.493%-80.007%)	86.11% (82.96%-89.36%)	85.25% (80.33%-90.41%)	3.3812 (3.2581-3.5025)	85.9% (80.78%-91.92%)	92.5% (86.89%-97.06%)	74.533% (62.5%-86.11%)	86.19% (79.69%-92.65%)	85.3% (75%-94.44%)	3.3852 (3.1405-3.6267)
At least 5 of CHASM (breast), CHASM (lung), CHASM (melanoma), FATHMM (cancer), FATHMM (missense), Mutation Assessor, PROVEAN, VEST	86.25% (83.84%-88.89%)	87.3% (84%-91.06%)	84.428% (79.71%-89.233%)	90.65% (87.93%-93.65%)	79.37% (74.07%-84.62%)	3.4175 (3.3082-3.5329)	86.08% (80.81%-90.91%)	87.08% (79.69%-93.65%)	84.328% (75%-93.75%)	90.52% (84%-96.43%)	79.23% (68.57%-89.19%)	3.4116 (3.1789-3.6281)
At least 5 of CHASM (breast), CHASM (lung), CHASM (melanoma), FATHMM (cancer), FATHMM (missense), Mutation Assessor, SIFT	85.9% (83.33%-88.89%)	84.11% (80.47%-88%)	88.971% (85.071%-93.153%)	92.95% (90.6%-95.65%)	76.42% (71.08%-81.82%)	3.4246 (3.3218-3.5362)	85.78% (79.8%-90.91%)	83.88% (76.19%-91.23%)	89.003% (80.554%-97.14%)	92.91% (87.27%-98.08%)	76.32% (65.85%-86.84%)	3.4212 (3.1968-3.6276)
At least 5 of CHASM (breast), CHASM (lung), CHASM (melanoma), FATHMM (cancer), FATHMM (missense), Mutation Assessor, SIFT, VEST	87.92% (85.35%-90.4%)	90.49% (87.5%-93.55%)	83.472% (78.871%-88.732%)	90.45% (87.88%-93.55%)	83.55% (78.87%-88.57%)	3.4796 (3.3776-3.5891)	87.8% (82.83%-92.93%)	90.29% (84.13%-96.49%)	83.478% (72.973%-92.31%)	90.38% (84.28%-95.45%)	83.39% (73.52%-92.86%)	3.4754 (3.2520-3.6888)
At least 5 of CHASM (breast), CHASM (lung), CHASM (melanoma), FATHMM (cancer), FATHMM (missense), Mutation Assessor, VEST	86.25% (83.84%-88.89%)	84.67% (80.95%-88.24%)	88.971% (85.071%-93.153%)	93% (90.68%-95.65%)	77.05% (71.95%-82.28%)	3.4369 (3.3326-3.5508)	86.09% (80.81%-90.91%)	84.37% (77.05%-91.67%)	89.003% (80.554%-97.14%)	92.95% (87.5%-98.08%)	76.87% (65.96%-87.18%)	3.4319 (3.2017-3.6384)
At least 5 of CHASM (breast), CHASM (lung), CHASM (melanoma), FATHMM (cancer), FATHMM (missense), MutationTaster, PolyPhen-2	86.92% (84.34%-89.9%)	85.18% (81.6%-88.89%)	89.919% (86.111%-94.12%)	93.59% (91.3%-96.43%)	77.83% (72.72%-82.9%)	3.4652 (3.3594-3.5794)	86.77% (80.81%-91.92%)	84.94% (77.61%-92.07%)	89.878% (81.818%-97.3%)	93.5% (87.72%-98.21%)	77.69% (67.5%-87.8%)	3.4601 (3.2377-3.6708)
At least 5 of CHASM (breast), CHASM (lung), CHASM (melanoma), FATHMM (cancer), FATHMM (missense), MutationTaster, PolyPhen-2, PROVEAN	87.6% (85.35%-90.4%)	87.31% (84%-90.83%)	88.111% (84.207%-92.308%)	92.7% (90.35%-95.61%)	80.06% (75%-85.14%)	3.4818 (3.3809-3.5965)	87.42% (81.82%-91.92%)	87.07% (79.69%-93.65%)	87.968% (78.947%-95.12%)	92.56% (87.1%-96.83%)	79.89% (69.69%-89.74%)	3.4749 (3.2507-3.6711)
At least 5 of CHASM (breast), CHASM (lung), CHASM (melanoma), FATHMM (cancer), FATHMM (missense), MutationTaster, PolyPhen-2, PROVEAN, SIFT	88.22% (85.86%-90.91%)	92.58% (90%-95.28%)	80.677% (75.713%-85.915%)	89.23% (86.36%-92.48%)	86.28% (81.54%-91.04%)	3.4877 (3.3852-3.5959)	88.2% (82.83%-92.93%)	92.49% (86.95%-97.06%)	80.802% (70.264%-90.62%)	89.22% (82.86%-95.08%)	86.27% (76.67%-94.87%)	3.4878 (3.2686-3.7000)
At least 5 of CHASM (breast), CHASM (lung), CHASM (melanoma), FATHMM (cancer), FATHMM (missense), MutationTaster, PolyPhen-2, PROVEAN, SIFT, VEST	85.89% (83.33%-88.89%)	95.28% (93.39%-97.56%)	69.654% (63.768%-75.758%)	84.45% (80.99%-88.11%)	89.52% (85.45%-94.45%)	3.3890 (3.2746-3.5036)	85.79% (79.8%-90.91%)	95.07% (90.77%-98.51%)	69.849% (57.689%-82.05%)	84.41% (77.14%-91.43%)	89.2% (79.16%-96.77%)	3.3853 (3.1452-3.6123)
At least 5 of CHASM (breast), CHASM (lung), CHASM (melanoma), FATHMM (cancer), FATHMM (missense), MutationTaster, PolyPhen-2, PROVEAN, VEST	88.24% (85.86%-90.91%)	92.58% (90%-95.28%)	80.734% (75.676%-86.301%)	89.26% (86.36%-92.54%)	86.29% (81.81%-91.18%)	3.4886 (3.3830-3.5973)	88.16% (82.83%-92.93%)	92.49% (86.95%-97.06%)	80.703% (69.691%-90.32%)	89.18% (82.61%-95.08%)	86.25% (76.66%-94.74%)	3.4862 (3.2628-3.6969)
At least 5 of CHASM (breast), CHASM (lung), CHASM (melanoma), FATHMM (cancer), FATHMM (missense), MutationTaster, PolyPhen-2, SIFT	88.6% (86.36%-90.92%)	89.95% (86.99%-93.02%)	86.258% (82.049%-90.909%)	91.89% (89.52%-94.92%)	83.25% (78.38%-88.06%)	3.5134 (3.4167-3.6230)	88.45% (83.81%-92.93%)	89.76% (83.6%-95.52%)	86.158% (76.316%-94.45%)	91.77% (85.71%-96.67%)	83.09% (73.17%-92.31%)	3.5078 (3.2901-3.7000)
At least 5 of CHASM (breast), CHASM (lung), CHASM (melanoma), FATHMM (cancer), FATHMM (missense), MutationTaster, PolyPhen-2, SIFT, VEST	88.27% (85.86%-90.91%)	94.74% (92.62%-96.9%)	77.069% (71.617%-83.099%)	87.72% (84.73%-90.98%)	89.45% (85.48%-93.85%)	3.4898 (3.3849-3.6124)	88.11% (82.83%-92.93%)	94.55% (89.85%-98.48%)	77.031% (65.619%-87.5%)	87.61% (80.65%-93.55%)	89.19% (79.31%-96.88%)	3.4838 (3.2415-3.6931)
At least 5 of CHASM (breast), CHASM (lung), CHASM (melanoma), FATHMM (cancer), FATHMM (missense), MutationTaster, PolyPhen-2, VEST	88.27% (85.86%-90.91%)	89.95% (86.99%-93.02%)	85.343% (80.882%-90.281%)	91.39% (88.89%-94.36%)	83.1% (78.2%-88%)	3.4978 (3.3941-3.6134)	88.11% (82.83%-92.93%)	89.76% (83.6%-95.52%)	85.245% (75%-94.12%)	91.27% (85.19%-96.55%)	82.94% (73.07%-92.31%)	3.4922 (3.2637-3.7000)
At least 5 of CHASM (breast), CHASM (lung), CHASM (melanoma), FATHMM (cancer), FATHMM (missense), MutationTaster, PROVEAN	84.57% (81.82%-87.37%)	79.9% (75.97%-84%)	92.647% (89.392%-96.053%)	94.95% (92.79%-97.3%)	72.72% (67.39%-77.91%)	3.4021 (3.2945-3.5128)	84.39% (78.79%-89.9%)	79.55% (71.43%-87.69%)	92.682% (85.706%-100%)	94.91% (89.65%-100%)	72.55% (62.21%-82.99%)	3.3970 (3.1761-3.6117)
[illegible]	[illegible]	[illegible]	[illegible]	[illegible]	[illegible]	[illegible]	[illegible]	[illegible]	[illegible]	[illegible]	[illegible]	[illegible]

At least 5 of CHASM (breast), CHASM (melanoma), FATHMM (cancer), FATHMM (missense), Mutation Assessor, MutationTaster, PROVEAN, VEST	83.81% (80.81%-86.87%)	81.86% (77.87%-85.82%)	87.18% (82.812%-91.784%)	91.7% (88.89%-94.69%)	73.54% (68.29%-78.72%)	3.3428 (3.2293-3.4586)	83.89% (77.78%-89.9%)	82.01% (73.77%-90%)	87.094% (78.042%-95.26%)	91.61% (85.45%-96.95%)	73.86% (63.41%-84.22%)	3.3457 (3.1087-3.5741)
At least 5 of CHASM (breast), CHASM (melanoma), FATHMM (cancer), FATHMM (missense), Mutation Assessor, MutationTaster, SIFT	84.84% (81.82%-87.88%)	81.36% (77.39%-85.37%)	90.838% (87.324%-94.668%)	93.89% (91.59%-96.43%)	73.82% (68.29%-78.89%)	3.3990 (3.2852-3.5085)	84.87% (78.79%-90.91%)	81.4% (73.21%-89.4%)	90.777% (82.5%-97.44%)	93.82% (88.46%-98.22%)	74.02% (64%-84.32%)	3.4002 (3.1771-3.6293)
At least 5 of CHASM (breast), CHASM (melanoma), FATHMM (cancer), FATHMM (missense), Mutation Assessor, MutationTaster, SIFT, VEST	86.85% (84.34%-89.39%)	87.75% (84.68%-91.13%)	85.297% (80.882%-90.363%)	91.17% (88.43%-94.22%)	80.11% (75%-85.19%)	3.4433 (3.3317-3.5577)	86.9% (81.82%-91.92%)	87.78% (81.03%-93.85%)	85.341% (75.674%-94.29%)	91.15% (85.07%-96.67%)	80.3% (69.44%-89.74%)	3.4457 (3.2099-3.6645)
At least 5 of CHASM (breast), CHASM (melanoma), FATHMM (cancer), FATHMM (missense), Mutation Assessor, MutationTaster, VEST	82.83% (79.8%-85.86%)	78.19% (73.81%-82.68%)	90.838% (87.324%-94.668%)	93.66% (91.26%-96.3%)	70.67% (65.51%-75.79%)	3.3335 (3.2233-3.4490)	82.83% (76.77%-88.89%)	78.17% (69.23%-86.96%)	90.777% (82.5%-97.44%)	93.58% (87.99%-98.18%)	70.82% (60.78%-81.4%)	3.3335 (3.1040-3.5538)
At least 5 of CHASM (breast), CHASM (melanoma), FATHMM (cancer), FATHMM (missense), Mutation Assessor, PolyPhen-2, PROVEAN	76.75% (73.74%-80.3%)	71.26% (66.92%-75.81%)	86.225% (81.944%-90.77%)	89.94% (86.91%-93.27%)	63.44% (58.09%-68.42%)	3.1087 (2.9880-3.2390)	76.81% (69.7%-82.83%)	71.27% (62.07%-80.33%)	86.255% (77.143%-94.59%)	89.9% (82.98%-96.08%)	63.65% (53.06%-74.47%)	3.1108 (2.8571-3.3434)
At least 5 of CHASM (breast), CHASM (melanoma), FATHMM (cancer), FATHMM (missense), Mutation Assessor, PolyPhen-2, PROVEAN, SIFT	80.08% (76.77%-83.33%)	81.35% (77.42%-85.5%)	77.858% (72.306%-83.339%)	86.4% (83.05%-89.92%)	70.72% (65%-76%)	3.1633 (3.0280-3.3014)	80.25% (73.74%-86.87%)	81.42% (72.88%-89.29%)	78.203% (67.566%-89.2%)	86.51% (79.62%-93.22%)	71.07% (60.52%-82.23%)	3.1720 (2.9013-3.4424)
At least 5 of CHASM (breast), CHASM (melanoma), FATHMM (cancer), FATHMM (missense), Mutation Assessor, PolyPhen-2, PROVEAN, SIFT, VEST	80.13% (77.27%-83.33%)	85.11% (81.82%-88.71%)	71.493% (65.714%-77.778%)	83.77% (80.31%-87.41%)	73.53% (67.61%-79.17%)	3.1391 (3.0033-3.2694)	80.15% (73.74%-85.86%)	85.07% (77.61%-91.67%)	71.664% (59.252%-83.33%)	83.75% (76.56%-90.63%)	73.69% (62.07%-85%)	3.1418 (2.8724-3.4093)
At least 5 of CHASM (breast), CHASM (melanoma), FATHMM (cancer), FATHMM (missense), Mutation Assessor, PolyPhen-2, PROVEAN, VEST	77.38% (74.24%-80.81%)	77.62% (73.48%-81.82%)	76.948% (71.424%-82.61%)	85.34% (81.74%-89.19%)	66.54% (60.92%-72.29%)	3.0644 (2.9265-3.1898)	77.57% (71.72%-83.84%)	77.72% (68.52%-86.21%)	77.276% (65.714%-88.38%)	85.45% (78.12%-92.73%)	66.91% (55.81%-78.58%)	3.0735 (2.8146-3.3472)
At least 5 of CHASM (breast), CHASM (melanoma), FATHMM (cancer), FATHMM (missense), Mutation Assessor, PolyPhen-2, SIFT	80.45% (77.27%-83.35%)	78.19% (73.81%-82.44%)	84.347% (79.73%-89.613%)	89.63% (86.54%-93.07%)	69.11% (63.86%-74.42%)	3.2127 (3.0873-3.3340)	80.51% (74.72%-86.87%)	78.16% (69.56%-86.76%)	84.491% (73.81%-93.94%)	89.65% (83.05%-95.92%)	69.3% (59.09%-80.01%)	3.2161 (2.9714-3.4675)
At least 5 of CHASM (breast), CHASM (melanoma), FATHMM (cancer), FATHMM (missense), Mutation Assessor, PolyPhen-2, SIFT, VEST	80.46% (77.27%-83.33%)	85.11% (81.82%-88.71%)	72.417% (66.667%-78.574%)	84.22% (80.65%-87.88%)	73.78% (68.06%-79.41%)	3.1552 (3.0208-3.2844)	80.49% (74.75%-86.87%)	85.07% (77.61%-91.67%)	72.569% (60%-84.38%)	84.19% (77.14%-91.18%)	73.93% (62.16%-85.3%)	3.1576 (2.8968-3.4221)
At least 5 of CHASM (breast), CHASM (melanoma), FATHMM (cancer), FATHMM (missense), Mutation Assessor, PolyPhen-2, VEST	77.8% (74.75%-80.82%)	73.97% (69.6%-78.46%)	84.409% (79.71%-89.554%)	89.14% (85.85%-92.86%)	65.22% (59.99%-70.46%)	3.1274 (3.0040-3.2474)	77.74% (71.69%-83.84%)	73.84% (64.9%-82.69%)	84.378% (74.267%-93.94%)	89.04% (82%-95.83%)	65.28% (54.99%-75.61%)	3.1254 (2.8795-3.3660)
At least 5 of CHASM (breast), CHASM (melanoma), FATHMM (cancer), FATHMM (missense), Mutation Assessor, PROVEAN, SIFT	78.1% (74.75%-81.82%)	72.87% (68.55%-77.78%)	87.129% (82.856%-91.667%)	90.73% (87.63%-94.06%)	65.01% (59.78%-70.21%)	3.1574 (3.0412-3.2858)	78.14% (70.71%-84.85%)	72.85% (63.23%-81.82%)	87.194% (78.378%-95.57%)	90.71% (84.09%-96.46%)	65.19% (54.71%-76%)	3.1594 (2.9054-3.3920)
At least 5 of CHASM (breast), CHASM (melanoma), FATHMM (cancer), FATHMM (missense), Mutation Assessor, PROVEAN, SIFT, VEST	78.74% (75.76%-81.83%)	80.31% (76.27%-84.45%)	76.007% (70.42%-81.69%)	85.27% (81.74%-89.08%)	69.07% (63.29%-75%)	3.1065 (2.9674-3.2478)	78.89% (72.7%-84.85%)	80.32% (71.88%-88.06%)	76.396% (64.861%-87.5%)	85.39% (77.97%-92.42%)	69.36% (57.57%-80.56%)	3.1146 (2.8312-3.3853)
At least 5 of CHASM (breast), CHASM (melanoma), FATHMM (cancer), FATHMM (missense), Mutation Assessor, PROVEAN, VEST	76.1% (72.73%-79.29%)	69.15% (64.57%-73.81%)	88.101% (84%-92.756%)	90.95% (87.78%-94.51%)	62.29% (56.86%-67.37%)	3.1049 (2.9861-3.2337)	76.09% (69.7%-82.83%)	69.12% (59.37%-78.26%)	88.008% (78.943%-96.78%)	90.82% (83.72%-97.56%)	62.43% (52%-72.92%)	3.1038 (2.8477-3.3426)
At least 5 of CHASM (breast), CHASM (melanoma), FATHMM (cancer), FATHMM (missense), Mutation Assessor, SIFT, VEST	79.8% (76.77%-82.83%)	76.62% (72.36%-80.99%)	85.297% (80.882%-90.363%)	90.01% (87%-93.52%)	67.85% (62.64%-73.26%)	3.1978 (3.0731-3.3249)	79.78% (73.74%-85.86%)	76.52% (67.64%-84.85%)	85.341% (75.674%-94.29%)	89.98% (83.02%-96.15%)	67.94% (57.45%-78.43%)	3.1977 (2.9420-3.4404)
At least 5 of CHASM (breast), CHASM (melanoma), FATHMM (cancer), FATHMM (missense), MutationTaster, PolyPhen-2, PROVEAN	80.79% (77.78%-84.34%)	74.97% (70.73%-79.67%)	90.857% (87.324%-94.737%)	93.41% (90.91%-96.26%)	67.74% (62.63%-73.04%)	3.2698 (3.1613-3.3927)	80.84% (73.74%-86.87%)	75.04% (65.62%-83.61%)	90.745% (82.5%-97.37%)	93.31% (87.27%-98.08%)	67.95% (57.14%-78.26%)	3.2704 (3.0351-3.4884)
At least 5 of CHASM (breast), CHASM (melanoma), FATHMM (cancer), FATHMM (missense), MutationTaster, PolyPhen-2, PROVEAN, SIFT	83.79% (80.81%-86.87%)	83.99% (80.33%-87.91%)	83.428% (78.873%-88.732%)	89.76% (86.88%-92.99%)	75.09% (69.62%-80.26%)	3.3227 (3.2020-3.4454)	83.94% (77.78%-89.9%)	84.13% (76.27%-91.53%)	83.568% (73.684%-92.11%)	89.79% (83.33%-95.16%)	75.46% (65%-86.49%)	3.3295 (3.0851-3.5770)
At least 5 of CHASM (breast), CHASM (melanoma), FATHMM (cancer), FATHMM (missense), MutationTaster, PolyPhen-2, PROVEAN, SIFT, VEST	82.8% (79.8%-85.86%)	87.76% (84.68%-91.2%)	74.216% (68.655%-80.282%)	85.48% (82.11%-89.15%)	77.82% (72.31%-83.58%)	3.2528 (3.1220-3.3789)	82.88% (76.77%-88.89%)	87.76% (80.95%-93.85%)	74.473% (62.848%-85.71%)	85.52% (78.33%-92.31%)	78.01% (66.67%-88.89%)	3.2576 (2.9908-3.5113)
At least 5 of CHASM (breast), CHASM (melanoma), FATHMM (cancer), FATHMM (missense), MutationTaster, PolyPhen-2, PROVEAN, VEST	82.12% (79.29%-85.35%)	80.79% (76.72%-84.88%)	84.4% (79.993%-89.859%)	89.96% (86.92%-93.46%)	71.77% (66.67%-76.83%)	3.2692 (3.1534-3.3907)	82.23% (75.76%-87.88%)	80.95% (72.13%-89.06%)	84.382% (73.684%-93.55%)	89.91% (83.33%-96.08%)	72.09% (61.54%-82.98%)	3.2733 (3.0248-3.5030)
At least 5 of CHASM (breast), CHASM (melanoma), FATHMM (cancer), FATHMM (missense), MutationTaster, PolyPhen-2, SIFT	83.84% (80.81%-86.87%)	80.84% (76.74%-84.92%)	89.009% (85.075%-93.336%)	92.71% (90%-95.69%)	72.88% (67.74%-78.02%)	3.3544 (3.2486-3.4684)	83.84% (77.78%-89.9%)	80.85% (72.22%-89.06%)	88.924% (80%-97.06%)	92.62% (86.54%-98.08%)	73.04% (62.79%-83.73%)	3.3544 (3.1250-3.5728)
At least 5 of CHASM (breast), CHASM (melanoma), FATHMM (cancer), FATHMM (missense), MutationTaster, PolyPhen-2, SIFT, VEST	85.18% (82.32%-87.88%)	87.22% (84%-90.62%)	81.631% (76.56%-87.328%)	89.15% (86.15%-92.62%)	78.71% (73.91%-84%)	3.3671 (3.2487-3.4939)	85.2% (79.8%-90.91%)	87.24% (80.33%-93.65%)	81.655% (70.27%-91.43%)	89.1% (82.45%-95.16%)	78.87% (68.42%-88.64%)	3.3686 (3.1077-3.6116)
At least 5 of CHASM (breast), CHASM (melanoma), FATHMM (cancer), FATHMM (missense), MutationTaster, PolyPhen-2, VEST	81.48% (78.79%-84.85%)	77.12% (72.8%-81.54%)	89.009% (85.075%-93.336%)	92.39% (89.72%-95.54%)	69.23% (64.13%-74.47%)	3.2775 (3.1705-3.3978)	81.49% (74.75%-86.87%)	77.12% (67.92%-85.71%)	88.924% (80%-97.06%)	92.29% (86%-98%)	69.39% (59.08%-80%)	3.2773 (3.0352-3.4880)
At least 5 of CHASM (breast), CHASM (melanoma), FATHMM (cancer), FATHMM (missense), MutationTaster, PROVEAN, SIFT	82.82% (79.8%-85.86%)	77.09% (72.87%-81.67%)	92.699% (89.333%-96%)	94.81% (92.52%-97.2%)	70.07% (64.89%-75.27%)	3.3468 (3.2388-3.4588)	82.85% (76.77%-88.89%)	77.17% (67.8%-85.94%)	92.563% (85.366%-100%)	94.7% (89.28%-100%)	70.29% (60%-80.44%)	3.3471 (3.1269-3.5664)
At least 5 of CHASM (breast), CHASM (melanoma), FATHMM (cancer), FATHMM (missense), MutationTaster, PROVEAN, SIFT, VEST	84.12% (81.31%-86.87%)	83.45% (79.53%-87.5%)	85.28% (80.822%-90.278%)	90.75% (87.93%-93.97%)	74.88% (69.23%-80%)	3.3436 (3.2197-3.4587)	84.28% (78.79%-89.9%)	83.62% (75.44%-91.23%)	85.364% (75.676%-94.44%)	90.77% (84.38%-96.49%)	75.26% (64.86%-86.67%)	3.3500 (3.1176-3.5942)
At least 5 of CHASM (breast), CHASM (melanoma), FATHMM (cancer), FATHMM (missense), MutationTaster, PROVEAN, VEST	79.77% (76.77%-83.33%)	72.83% (68.46%-77.6%)	91.759% (88.235%-95.775%)	93.86% (91.3%-96.84%)	66.14% (61%-71.43%)	3.2460 (3.1371-3.3620)	79.85% (72.73%-85.86%)	72.93% (63.07%-81.67%)	91.692% (84.211%-97.68%)	93.79% (87.93%-98.18%)	66.39% (56%-76.74%)	3.2480 (3.0182-3.4577)
At least 5 of CHASM (breast), CHASM (melanoma), FATHMM (cancer), FATHMM (missense), MutationTaster, SIFT, VEST	83.49% (80.81%-86.36%)	80.31% (76.15%-84.5%)	88.985% (85.134%-93.243%)	92.66% (90.09%-95.54%)	72.33% (67.05%-77.66%)	3.3429 (3.2299-3.4566)	83.52% (77.78%-88.89%)	80.31% (71.67%-88.34%)	88.968% (80%-97.06%)	92.61% (86.66%-98.08%)	72.5% (62%-83.33%)	3.3439 (3.1183-3.5664)
[illegible]	[illegible]	[illegible]	[illegible]	[illegible]	[illegible]	[illegible]	[illegible]	[illegible]	[illegible]	[illegible]	[illegible]	[illegible]

At least 5 of CHASM (melanoma), FATHMM (cancer), FATHMM (missense), Mutation Assessor, MutationTaster, PolyPhen-2, PROVEAN, SIFT, VEST	77.1% (73.74%-80.3%)	82.46% (78.86%-86.36%)	67.817% (61.972%-74.026%)	81.58% (77.86%-85.39%)	69.11% (63.63%-75%)	3.0097 (2.8720-3.1510)	77.11% (70.71%-83.84%)	82.39% (74.6%-89.55%)	68.035% (55.548%-80%)	81.55% (74.07%-89.06%)	69.25% (56.67%-80.49%)	3.0122 (2.7239-3.2780)
At least 5 of CHASM (melanoma), FATHMM (cancer), FATHMM (missense), Mutation Assessor, MutationTaster, PolyPhen-2, PROVEAN, VEST	78.41% (75.25%-81.82%)	80.32% (76.61%-84.25%)	75.1% (69.694%-81.081%)	84.8% (81.2%-88.6%)	68.81% (63.16%-74.67%)	3.0903 (2.9551-3.2232)	78.54% (71.72%-84.85%)	80.3% (72.13%-87.88%)	75.481% (63.624%-87.1%)	84.89% (77.42%-91.84%)	69.07% (57.13%-80%)	3.0974 (2.8311-3.3662)
[illegible]	[illegible]	[illegible]	[illegible]	[illegible]	[illegible]	[illegible]	[illegible]	[illegible]	[illegible]	[illegible]	[illegible]	[illegible]

Page 41

**At least 6 of CHASM (breast), CHASM (lung), FATHMM (cancer), Mutation Assessor, MutationTaster, PolyPhen-2, PROVEAN, SIFT, VEST**	78.43% (75.25%-81.82%)	74.44% (70.34%-78.86%)	85.304% (80.282%-90.278%)	89.75% (86.41%-93.27%)	65.88% (60.46%-71.28%)	3.1538 (3.0267-3.2823)	78.5% (71.72%-84.85%)	74.49% (65.15%-82.82%)	85.348% (75.676%-94.74%)	89.72% (82.69%-96.23%)	66.09% (55.1%-76.47%)	3.1566 (2.8951-3.4079)
**At least 6 of CHASM (breast), CHASM (lung), FATHMM (cancer), Mutation Assessor, MutationTaster, PolyPhen-2, PROVEAN, VEST**	74.44% (70.71%-77.78%)	63.9% (58.97%-68.8%)	92.665% (89.394%-96.105%)	93.77% (91.21%-96.71%)	59.75% (54.7%-64.66%)	3.1009 (2.9855-3.2143)	74.35% (67.68%-81.82%)	63.67% (53.97%-73.69%)	92.64% (85.366%-100%)	93.69% (87.23%-100%)	59.78% (50%-70.37%)	3.0978 (2.8752-3.3304)
**At least 6 of CHASM (breast), CHASM (lung), FATHMM (cancer), Mutation Assessor, MutationTaster, PolyPhen-2, SIFT**	71.08% (67.68%-74.75%)	57.54% (52.46%-62.41%)	94.49% (91.667%-97.333%)	94.75% (92.11%-97.5%)	56.28% (51.22%-61.21%)	3.0305 (2.9246-3.1445)	70.97% (63.64%-77.78%)	57.24% (47.45%-67.19%)	94.503% (88.235%-100%)	94.68% (88.24%-100%)	56.3% (46.55%-66.67%)	3.0273 (2.7971-3.2430)
**At least 6 of CHASM (breast), CHASM (lung), FATHMM (cancer), Mutation Assessor, MutationTaster, PolyPhen-2, SIFT, VEST**	79.14% (75.76%-82.32%)	71.84% (67.44%-76.34%)	91.761% (88.406%-95.652%)	93.78% (91.35%-96.84%)	65.34% (60%-70.41%)	3.2272 (3.1127-3.3464)	79.09% (72.73%-85.86%)	71.72% (62.5%-80.88%)	91.697% (84.375%-97.5%)	93.68% (87.23%-98.08%)	65.4% (55.32%-76%)	3.2249 (2.9899-3.4506)
**At least 6 of CHASM (breast), CHASM (lung), FATHMM (cancer), Mutation Assessor, MutationTaster, PolyPhen-2, VEST**	70.4% (66.67%-74.24%)	57% (51.94%-62.1%)	93.55% (90.537%-97.102%)	93.86% (91.14%-97.33%)	55.72% (50.76%-60.49%)	3.0013 (2.8940-3.1159)	70.32% (62.63%-77.78%)	56.72% (46.77%-66.67%)	93.631% (86.842%-100%)	93.86% (86.84%-100%)	55.77% (45.9%-66.1%)	2.9997 (2.7757-3.2154)
**At least 6 of CHASM (breast), CHASM (lung), FATHMM (cancer), Mutation Assessor, MutationTaster, PROVEAN, SIFT**	69.03% (65.15%-72.73%)	53.26% (47.93%-58.26%)	96.298% (94.203%-98.684%)	96.13% (93.85%-98.65%)	54.37% (49.61%-58.92%)	3.0006 (2.8996-3.1056)	69% (61.62%-76.77%)	53.02% (43.07%-63.5%)	96.392% (90.909%-100%)	96.19% (90.24%-100%)	54.46% (44.61%-64.41%)	3.0006 (2.7920-3.2132)
**At least 6 of CHASM (breast), CHASM (lung), FATHMM (cancer), Mutation Assessor, MutationTaster, PROVEAN, SIFT, VEST**	74.1% (70.71%-77.78%)	62.81% (57.93%-67.74%)	93.605% (90.539%-97.102%)	94.44% (91.95%-97.56%)	59.28% (54.2%-64.29%)	3.1014 (2.9889-3.2153)	74.02% (66.67%-80.81%)	62.65% (52.54%-72.47%)	93.512% (86.667%-100%)	94.3% (88.1%-100%)	59.33% (49.12%-69.5%)	3.0979 (2.8640-3.3254)
**At least 6 of CHASM (breast), CHASM (lung), FATHMM (cancer), Mutation Assessor, MutationTaster, PROVEAN, VEST**	66.38% (62.63%-70.2%)	49.08% (43.9%-54.2%)	96.298% (94.203%-98.684%)	95.82% (93.33%-98.53%)	52.24% (47.44%-56.92%)	2.9344 (2.8292-3.0425)	66.22% (58.59%-73.74%)	48.63% (38.33%-58.93%)	96.392% (90.909%-100%)	95.86% (89.29%-100%)	52.23% (42.86%-61.91%)	2.9311 (2.7177-3.1394)
**At least 6 of CHASM (breast), CHASM (lung), FATHMM (cancer), Mutation Assessor, MutationTaster, SIFT, VEST**	70.06% (66.16%-73.74%)	55.93% (51.14%-61.11%)	94.49% (91.667%-97.333%)	94.61% (91.89%-97.44%)	55.36% (50.39%-60.17%)	3.0038 (2.8942-3.1167)	69.98% (62.63%-77.78%)	55.68% (45.16%-65.52%)	94.503% (88.235%-100%)	94.55% (87.88%-100%)	55.41% (45.45%-65.63%)	3.0014 (2.7756-3.2196)
**At least 6 of CHASM (breast), CHASM (lung), FATHMM (cancer), Mutation Assessor, PolyPhen-2, PROVEAN, SIFT**	68.71% (65.15%-72.23%)	52.75% (47.73%-57.94%)	96.298% (94.203%-98.684%)	96.1% (93.84%-98.63%)	54.1% (49.22%-58.73%)	2.9925 (2.8915-3.1038)	68.64% (61.59%-75.76%)	52.45% (42.18%-62.51%)	96.392% (90.909%-100%)	96.14% (90.31%-100%)	54.15% (44.44%-64.06%)	2.9913 (2.7702-3.1902)
**At least 6 of CHASM (breast), CHASM (lung), FATHMM (cancer), Mutation Assessor, PolyPhen-2, PROVEAN, SIFT, VEST**	72.08% (68.18%-75.76%)	63.91% (58.91%-68.8%)	86.208% (81.429%-90.67%)	88.9% (85.23%-92.86%)	58.01% (52.68%-63.21%)	2.9703 (2.8366-3.0977)	72% (64.65%-79.8%)	63.65% (53.84%-73.77%)	86.291% (76.667%-95%)	88.85% (80.56%-95.83%)	58.04% (47.27%-68.97%)	2.9683 (2.7012-3.2254)
**At least 6 of CHASM (breast), CHASM (lung), FATHMM (cancer), Mutation Assessor, PolyPhen-2, PROVEAN, VEST**	65.36% (61.62%-69.19%)	48.01% (42.86%-53.05%)	95.358% (92.857%-98.592%)	94.7% (91.8%-98.33%)	51.48% (46.67%-56.06%)	2.8955 (2.7934-3.0083)	65.23% (57.58%-72.73%)	47.57% (37.7%-57.81%)	95.521% (89.474%-100%)	94.8% (87.88%-100%)	51.5% (42.63%-61.02%)	2.8938 (2.6682-3.1031)
**At least 6 of CHASM (breast), CHASM (lung), FATHMM (cancer), Mutation Assessor, PolyPhen-2, SIFT, VEST**	70.06% (66.16%-73.74%)	56.47% (51.24%-61.65%)	93.55% (90.537%-97.102%)	93.8% (91.03%-97.33%)	55.42% (50.41%-60.34%)	2.9923 (2.8845-3.1073)	69.99% (62.63%-77.78%)	56.19% (45.9%-66.13%)	93.631% (86.842%-100%)	93.79% (86.66%-100%)	55.47% (45.76%-65.64%)	2.9909 (2.7601-3.2069)
**At least 6 of CHASM (breast), CHASM (lung), FATHMM (cancer), Mutation Assessor, PROVEAN, SIFT, VEST**	66.04% (62.12%-69.7%)	48.53% (43.22%-53.54%)	96.298% (94.203%-98.684%)	95.77% (93.33%-98.53%)	51.97% (47.1%-56.62%)	2.9258 (2.8168-3.0345)	65.9% (58.59%-73.74%)	48.11% (38.23%-58.62%)	96.392% (90.909%-100%)	95.81% (88.89%-100%)	51.98% (42.25%-61.54%)	2.9230 (2.7016-3.1409)
**At least 6 of CHASM (breast), CHASM (lung), FATHMM (cancer), MutationTaster, PolyPhen-2, PROVEAN, SIFT**	70.06% (66.67%-73.23%)	54.88% (50%-59.69%)	96.298% (94.203%-98.684%)	96.24% (94.03%-98.68%)	55.24% (50.39%-59.85%)	3.0267 (2.9280-3.1306)	69.98% (63.64%-76.77%)	54.58% (45.16%-64.62%)	96.392% (90.909%-100%)	96.29% (90.62%-100%)	55.29% (45.61%-65.15%)	3.0255 (2.8182-3.2183)
**At least 6 of CHASM (breast), CHASM (lung), FATHMM (cancer), MutationTaster, PolyPhen-2, PROVEAN, SIFT, VEST**	76.12% (72.73%-79.31%)	66.01% (60.98%-70.74%)	93.605% (90.539%-97.102%)	94.69% (92.22%-97.65%)	61.43% (56.31%-66.67%)	3.1573 (3.0439-3.2686)	76.04% (69.67%-82.83%)	65.84% (56.36%-75.41%)	93.512% (86.667%-100%)	94.57% (88.88%-100%)	61.47% (51.02%-72.34%)	3.1538 (2.9313-3.3892)
**At least 6 of CHASM (breast), CHASM (lung), FATHMM (cancer), MutationTaster, PolyPhen-2, PROVEAN, VEST**	67.39% (63.62%-71.21%)	50.66% (45.24%-55.66%)	96.298% (94.203%-98.684%)	95.95% (93.55%-98.57%)	53.03% (48.09%-57.58%)	2.9594 (2.8516-3.0640)	67.25% (59.6%-74.77%)	50.25% (40.29%-61.02%)	96.392% (90.909%-100%)	95.99% (89.99%-100%)	53.03% (43.54%-62.91%)	2.9566 (2.7470-3.1684)
**At least 6 of CHASM (breast), CHASM (lung), FATHMM (cancer), MutationTaster, PolyPhen-2, SIFT, VEST**	71.76% (68.18%-75.25%)	58.62% (53.54%-63.71%)	94.49% (91.667%-97.333%)	94.84% (92.31%-97.56%)	56.91% (51.69%-61.74%)	3.0486 (2.9377-3.1592)	71.62% (64.65%-78.79%)	58.28% (48.33%-68.18%)	94.503% (88.235%-100%)	94.78% (88.56%-100%)	56.89% (47.17%-67.27%)	3.0445 (2.8189-3.2718)
**At least 6 of CHASM (breast), CHASM (lung), FATHMM (cancer), MutationTaster, PROVEAN, SIFT, VEST**	66.7% (63.13%-70.2%)	49.58% (44.44%-54.69%)	96.298% (94.203%-98.684%)	95.86% (93.44%-98.55%)	52.49% (47.62%-57.14%)	2.9423 (2.8416-3.0472)	66.59% (59.6%-73.74%)	49.22% (39.13%-59.38%)	96.392% (90.909%-100%)	95.9% (89.29%-100%)	52.52% (43.07%-62.32%)	2.9403 (2.7296-3.1409)
**At least 6 of CHASM (breast), CHASM (lung), FATHMM (cancer), PolyPhen-2, PROVEAN, SIFT, VEST**	66.72% (62.63%-70.2%)	49.61% (44.44%-54.55%)	96.298% (94.203%-98.684%)	95.86% (93.44%-98.55%)	52.5% (47.69%-57.25%)	2.9427 (2.8416-3.0493)	66.56% (59.6%-74.75%)	49.16% (39.06%-59.38%)	96.392% (90.909%-100%)	95.9% (89.47%-100%)	52.49% (42.86%-62.3%)	2.9395 (2.7223-3.1409)
**At least 6 of CHASM (breast), CHASM (lung), FATHMM (missense), Mutation Assessor, MutationTaster, PolyPhen-2, PROVEAN**	66.35% (62.63%-70.2%)	49.03% (44.09%-54.33%)	96.298% (94.203%-98.684%)	95.82% (93.33%-98.51%)	52.22% (47.41%-56.72%)	2.9336 (2.8339-3.0366)	66.28% (58.59%-73.74%)	48.72% (38.32%-58.93%)	96.392% (90.909%-100%)	95.87% (89.99%-100%)	52.28% (43.05%-61.67%)	2.9325 (2.7235-3.1331)
**At least 6 of CHASM (breast), CHASM (lung), FATHMM (missense), Mutation Assessor, MutationTaster, PolyPhen-2, PROVEAN, SIFT**	75.49% (72.22%-78.79%)	65% (60.16%-69.84%)	93.605% (90.539%-97.102%)	94.62% (92.13%-97.65%)	60.74% (55.45%-65.49%)	3.1396 (3.0294-3.2494)	75.29% (68.69%-81.82%)	64.64% (54.68%-74.58%)	93.512% (86.667%-100%)	94.48% (88.57%-100%)	60.65% (50.8%-70.97%)	3.1328 (2.9115-3.3580)
**At least 6 of CHASM (breast), CHASM (lung), FATHMM (missense), Mutation Assessor, MutationTaster, PolyPhen-2, PROVEAN, SIFT, VEST**	79.12% (76.25%-82.32%)	75% (70.87%-79.23%)	86.232% (81.818%-90.912%)	90.4% (87.25%-93.81%)	66.61% (61.05%-71.91%)	3.1824 (3.0628-3.3116)	79.14% (72.73%-84.87%)	74.98% (66.18%-83.1%)	86.227% (76.923%-94.59%)	90.34% (83.64%-96.36%)	66.76% (56%-77.78%)	3.1830 (2.9181-3.4205)
**At least 6 of CHASM (breast), CHASM (lung), FATHMM (missense), Mutation Assessor, MutationTaster, PolyPhen-2, PROVEAN, VEST**	74.13% (70.69%-77.27%)	63.4% (58.4%-68.55%)	92.665% (89.394%-96.105%)	93.73% (91.11%-96.71%)	59.43% (54.05%-64.49%)	3.0922 (2.9733-3.2079)	73.96% (67.68%-80.83%)	63.05% (53.12%-72.86%)	92.64% (85.366%-100%)	93.64% (87.18%-100%)	59.38% (49.05%-70.18%)	3.0870 (2.8534-3.3211)
**At least 6 of CHASM (breast), CHASM (lung), FATHMM (missense), Mutation Assessor, MutationTaster, PolyPhen-2, SIFT**	69.09% (65.64%-72.73%)	54.39% (49.21%-59.52%)	94.49% (91.667%-97.333%)	94.46% (91.67%-97.37%)	54.51% (49.6%-59.35%)	2.9785 (2.8705-3.0934)	68.9% (61.62%-75.78%)	53.96% (43.75%-64.15%)	94.503% (88.235%-100%)	94.38% (87.5%-100%)	54.47% (44.64%-64.41%)	2.9731 (2.7361-3.1872)
**At least 6 of CHASM (breast), CHASM (lung), FATHMM (missense), Mutation Assessor, MutationTaster, PolyPhen-2, SIFT, VEST**	79.16% (75.76%-82.32%)	71.87% (67.46%-76.04%)	91.761% (88.406%-95.652%)	93.78% (91.4%-96.88%)	65.36% (60.19%-70.37%)	3.2277 (3.1165-3.3415)	79.05% (72.73%-85.86%)	71.66% (63.16%-80.65%)	91.697% (84.375%-97.5%)	93.68% (87.27%-98.08%)	65.35% (55.1%-75.62%)	3.2238 (2.9941-3.4468)
**At least 6 of CHASM (breast), CHASM (lung), FATHMM (missense), Mutation Assessor, MutationTaster, PolyPhen-2, VEST**	68.07% (64.65%-71.72%)	53.32% (48.33%-58.46%)	93.55% (90.537%-97.102%)	93.46% (90.54%-97.18%)	53.69% (48.78%-58.34%)	2.9402 (2.8373-3.0605)	67.91% (60.61%-74.75%)	52.9% (42.19%-62.91%)	93.631% (86.842%-100%)	93.44% (85.71%-100%)	53.67% (43.94%-63.64%)	2.9365 (2.6999-3.1428)
**At least 6 of CHASM (breast), CHASM (lung), FATHMM (missense), Mutation Assessor, MutationTaster, PROVEAN, SIFT**	67.01% (63.13%-70.71%)	50.06% (45.04%-54.92%)	96.298% (94.203%-98.684%)	95.9% (93.55%-98.55%)	52.73% (48.09%-57.45%)	2.9499 (2.8467-3.0532)	66.99% (59.6%-74.75%)	49.84% (39.65%-60.01%)	96.392% (90.909%-100%)	95.96% (89.66%-100%)	52.82% (43.28%-62.3%)	2.9501 (2.7437-3.1542)
**At least 6 of CHASM (breast), CHASM (lung), FATHMM (missense), Mutation Assessor, MutationTaster, PROVEAN, SIFT, VEST**	74.12% (70.71%-77.78%)	62.85% (57.69%-67.83%)	93.605% (90.539%-97.102%)	94.44% (91.95%-97.56%)	59.31% (54.05%-64.29%)	3.1020 (2.9893-3.2158)	73.97% (66.67%-80.81%)	62.56% (52.63%-72.73%)	93.512% (86.667%-100%)	94.3% (88.23%-100%)	59.28% (49.09%-69.5%)	3.0966 (2.8751-3.3273)
**At least 6 of CHASM (breast), CHASM (lung), FATHMM (missense), Mutation Assessor, MutationTaster, PROVEAN, VEST**	64.36% (60.61%-68.18%)	45.88% (40.65%-51.18%)	96.298% (94.203%-98.684%)	95.54% (92.86%-98.44%)	50.72% (45.99%-55.32%)	2.8844 (2.7831-2.9886)	64.21% (56.57%-71.72%)	45.44% (34.92%-55.88%)	96.392% (90.909%-100%)	95.59% (88.89%-100%)	50.73% (41.1%-60%)	2.8815 (2.6747-3.0796)
**At least 6 of CHASM (breast), CHASM (lung), FATHMM (missense), Mutation Assessor, MutationTaster, SIFT, VEST**	68.07% (64.65%-71.72%)	52.78% (47.58%-58.06%)	94.49% (91.667%-97.333%)	94.3% (91.43%-97.3%)	53.65% (48.8%-58.27%)	2.9522 (2.8470-3.0641)	67.91% (60.61%-74.75%)	52.39% (41.66%-62.72%)	94.503% (88.235%-100%)	94.23% (87.1%-100%)	53.63% (43.93%-63.49%)	2.9476 (2.7137-3.1602)
**At least 6 of CHASM (breast), CHASM (lung), FATHMM (missense), Mutation Assessor, PolyPhen-2, PROVEAN, SIFT**	66.69% (63.12%-70.2%)	49.56% (44.35%-54.69%)	96.298% (94.203%-98.684%)	95.86% (93.44%-98.53%)	52.47% (47.69%-57.14%)	2.9419 (2.8397-3.0445)	66.63% (59.6%-73.76%)	49.26% (38.88%-59.38%)	96.392% (90.909%-100%)	95.91% (89.66%-100%)	52.54% (43.1%-62.3%)	2.9410 (2.7269-3.1559)
**At least 6 of CHASM (breast), CHASM (lung), FATHMM (missense), Mutation Assessor, PolyPhen-2, PROVEAN, SIFT, VEST**	72.45% (68.69%-75.76%)	63.95% (58.73%-68.94%)	87.136% (82.856%-91.784%)	89.58% (86.05%-93.33%)	58.3% (52.83%-63.48%)	2.9896 (2.8594-3.1121)	72.27% (65.66%-79.8%)	63.56% (53.33%-73.85%)	87.17% (78.042%-95.57%)	89.49% (81.82%-96.37%)	58.23% (48%-68.97%)	2.9846 (2.7318-3.2430)
**At least 6 of CHASM (breast), CHASM (lung), FATHMM (missense), Mutation Assessor, PolyPhen-2, PROVEAN, VEST**	63.34% (59.6%-67.17%)	44.82% (39.53%-50%)	95.358% (92.857%-98.592%)	94.35% (91.23%-98.21%)	49.99% (45.38%-54.68%)	2.8451 (2.7450-2.9574)	63.22% (55.56%-70.71%)	44.38% (33.87%-54.69%)	95.521% (89.474%-100%)	94.45% (87.09%-100%)	50.02% (40.85%-59.42%)	2.8438 (2.6184-3.0516)
**At least 6 of CHASM (breast), CHASM (lung), FATHMM (missense), Mutation Assessor, PolyPhen-2, SIFT, VEST**	68.06% (64.63%-71.72%)	53.32% (48.06%-58.4%)	93.55% (90.537%-97.102%)	93.46% (90.48%-97.18%)	53.68% (48.82%-58.4%)	2.9401 (2.8357-3.0556)	67.92% (60.61%-74.77%)	52.91% (42.59%-63.24%)	93.631% (86.842%-100%)	93.43% (85.71%-100%)	53.68% (44.06%-63.49%)	2.9366 (2.7025-3.1575)
[illegible]	[illegible]	[illegible]	[illegible]	[illegible]	[illegible]	[illegible]	[illegible]	[illegible]	[illegible]	[illegible]	[illegible]	[illegible]

**At least 6 of CHASM (lung), CHASM (melanoma), FATHMM (missense), Mutation Assessor, MutationTaster, PROVEAN, SIFT, VEST**	77.1% (73.74%-80.32%)	68.61% (63.79%-73.23%)	91.778% (88.406%-95.714%)	93.52% (90.91%-96.63%)	62.84% (57.69%-68.14%)	3.1674 (3.0556-3.2833)	77.11% (70.68%-83.84%)	68.61% (59.25%-77.97%)	91.649% (84.371%-97.56%)	93.39% (87.17%-98.04%)	62.99% (52.17%-73.59%)	3.1664 (2.9348-3.3944)
**At least 6 of CHASM (lung), CHASM (melanoma), FATHMM (missense), Mutation Assessor, MutationTaster, PROVEAN, VEST**	67.04% (63.64%-70.71%)	50.62% (45.45%-55.83%)	95.411% (92.958%-98.571%)	95.02% (92.3%-98.48%)	52.78% (48.06%-57.58%)	2.9383 (2.8402-3.0429)	66.93% (59.6%-73.74%)	50.31% (39.68%-60.57%)	95.417% (89.474%-100%)	94.97% (87.87%-100%)	52.81% (42.86%-62.5%)	2.9351 (2.7166-3.1337)
**At least 6 of CHASM (lung), CHASM (melanoma), FATHMM (missense), Mutation Assessor, MutationTaster, SIFT, VEST**	73.41% (69.7%-77.27%)	61.73% (56.35%-66.67%)	93.586% (90.541%-97.144%)	94.33% (91.76%-97.53%)	58.58% (53.72%-63.64%)	3.0823 (2.9721-3.2008)	73.39% (65.66%-80.81%)	61.62% (51.61%-72.23%)	93.559% (86.838%-100%)	94.26% (87.8%-100%)	58.69% (49.09%-68.86%)	3.0813 (2.8467-3.3073)
**At least 6 of CHASM (lung), CHASM (melanoma), FATHMM (missense), Mutation Assessor, PolyPhen-2, PROVEAN, SIFT**	69.04% (65.66%-72.73%)	54.85% (49.61%-59.85%)	93.56% (90.411%-97.184%)	93.64% (90.67%-97.22%)	54.51% (49.58%-59.5%)	2.9657 (2.8545-3.0813)	68.99% (61.62%-75.76%)	54.63% (44.26%-64.62%)	93.595% (86.486%-100%)	93.62% (86.67%-100%)	54.59% (44.77%-64.52%)	2.9643 (2.7310-3.1871)
**At least 6 of CHASM (lung), CHASM (melanoma), FATHMM (missense), Mutation Assessor, PolyPhen-2, PROVEAN, SIFT, VEST**	74.75% (71.21%-78.28%)	68.63% (64.23%-73.17%)	85.305% (80.881%-90.278%)	88.98% (85.57%-92.71%)	61.13% (55.66%-66.67%)	3.0406 (2.9163-3.1695)	74.75% (67.68%-81.82%)	68.55% (59.37%-77.78%)	85.324% (75.862%-94.12%)	88.92% (81.63%-95.83%)	61.26% (50.89%-72%)	3.0406 (2.7757-3.2895)
**At least 6 of CHASM (lung), CHASM (melanoma), FATHMM (missense), Mutation Assessor, PolyPhen-2, PROVEAN, VEST**	65.68% (62.12%-69.7%)	49.56% (44.44%-54.84%)	93.548% (90.278%-97.183%)	93% (89.83%-96.97%)	51.75% (46.88%-56.39%)	2.8786 (2.7723-2.9950)	65.61% (57.58%-72.73%)	49.25% (38.46%-59.38%)	93.626% (86.667%-100%)	93.01% (84.85%-100%)	51.81% (42.19%-61.9%)	2.8770 (2.6359-3.1007)
**At least 6 of CHASM (lung), CHASM (melanoma), FATHMM (missense), Mutation Assessor, PolyPhen-2, SIFT, VEST**	72.39% (68.69%-76.26%)	61.2% (56.25%-66.13%)	91.723% (88.235%-95.714%)	92.75% (89.74%-96.34%)	57.76% (52.89%-62.81%)	3.0343 (2.9212-3.1587)	72.39% (64.65%-79.8%)	61.07% (50.91%-71.19%)	91.768% (84.375%-97.62%)	92.73% (85.71%-97.92%)	57.88% (47.54%-67.92%)	3.0345 (2.7869-3.2557)
**At least 6 of CHASM (lung), CHASM (melanoma), FATHMM (missense), Mutation Assessor, PROVEAN, SIFT, VEST**	66.71% (63.13%-70.2%)	50.63% (45.45%-55.65%)	94.488% (91.549%-97.368%)	94.08% (91.04%-97.22%)	52.54% (47.69%-57.35%)	2.9173 (2.8156-3.0286)	66.59% (59.6%-73.74%)	50.29% (40%-60.56%)	94.497% (87.879%-100%)	94.03% (86.67%-100%)	52.55% (42.86%-62.32%)	2.9138 (2.6872-3.1177)
**At least 6 of CHASM (lung), CHASM (melanoma), FATHMM (missense), MutationTaster, PolyPhen-2, PROVEAN, SIFT**	72.39% (69.19%-75.76%)	59.06% (54.16%-64%)	95.411% (92.958%-98.571%)	95.7% (93.42%-98.72%)	57.41% (52.5%-62.07%)	3.0758 (2.9774-3.1757)	72.4% (65.66%-78.79%)	58.97% (48.48%-68.97%)	95.417% (89.474%-100%)	95.67% (89.47%-100%)	57.54% (47.62%-67.27%)	3.0759 (2.8749-3.2723)
**At least 6 of CHASM (lung), CHASM (melanoma), FATHMM (missense), MutationTaster, PolyPhen-2, PROVEAN, SIFT, VEST**	78.13% (74.75%-81.31%)	70.23% (65.62%-74.8%)	91.778% (88.406%-95.714%)	93.66% (91.21%-96.7%)	64.07% (59%-69.47%)	3.1974 (3.0887-3.3130)	78.08% (71.72%-84.85%)	70.14% (60.94%-79.03%)	91.649% (84.371%-97.56%)	93.53% (87.49%-98.04%)	64.15% (53.33%-74.51%)	3.1947 (2.9640-3.4144)
**At least 6 of CHASM (lung), CHASM (melanoma), FATHMM (missense), MutationTaster, PolyPhen-2, PROVEAN, VEST**	69.73% (66.16%-73.23%)	54.87% (50%-59.84%)	95.411% (92.958%-98.571%)	95.39% (92.86%-98.61%)	55.01% (50%-59.83%)	3.0067 (2.9105-3.1159)	69.64% (62.63%-76.77%)	54.6% (44.64%-64.52%)	95.417% (89.474%-100%)	95.34% (88.89%-100%)	55.05% (45.31%-64.52%)	3.0041 (2.7908-3.2133)
**At least 6 of CHASM (lung), CHASM (melanoma), FATHMM (missense), MutationTaster, PolyPhen-2, SIFT, VEST**	75.42% (72.22%-78.79%)	64.91% (60%-69.6%)	93.586% (90.541%-97.144%)	94.59% (92.1%-97.62%)	60.67% (55.75%-65.82%)	3.1376 (3.0284-3.2533)	75.42% (68.69%-81.82%)	64.83% (55.17%-74.58%)	93.559% (86.838%-100%)	94.53% (88.37%-100%)	60.8% (50.85%-71.15%)	3.1371 (2.9056-3.3605)
**At least 6 of CHASM (lung), CHASM (melanoma), FATHMM (missense), MutationTaster, PROVEAN, SIFT, VEST**	69.03% (65.66%-72.73%)	53.77% (48.71%-58.73%)	95.411% (92.958%-98.571%)	95.3% (92.75%-98.59%)	54.41% (49.59%-59.2%)	2.9888 (2.8904-3.0942)	69.01% (61.62%-75.76%)	53.6% (43.55%-63.64%)	95.417% (89.474%-100%)	95.26% (88.46%-100%)	54.52% (44.77%-64.18%)	2.9880 (2.7757-3.1917)
**At least 6 of CHASM (lung), CHASM (melanoma), FATHMM (missense), PolyPhen-2, PROVEAN, SIFT, VEST**	68.71% (65.15%-72.22%)	53.79% (48.71%-58.73%)	94.488% (91.549%-97.368%)	94.41% (91.66%-97.37%)	54.18% (49.18%-58.82%)	2.9687 (2.8633-3.0848)	68.65% (61.62%-75.76%)	53.55% (43.33%-63.94%)	94.497% (87.879%-100%)	94.37% (87.18%-100%)	54.24% (45%-64.18%)	2.9667 (2.7362-3.1738)
**At least 6 of CHASM (lung), CHASM (melanoma), Mutation Assessor, MutationTaster, PolyPhen-2, PROVEAN, SIFT**	70.72% (67.17%-74.24%)	56.97% (51.61%-62.12%)	94.49% (91.667%-97.333%)	94.7% (92.11%-97.5%)	55.95% (50.82%-60.5%)	3.0210 (2.9170-3.1311)	70.68% (63.64%-77.78%)	56.78% (46.3%-67.19%)	94.503% (88.235%-100%)	94.65% (88.23%-100%)	56.03% (46.55%-66.13%)	3.0196 (2.7919-3.2198)
**At least 6 of CHASM (lung), CHASM (melanoma), Mutation Assessor, MutationTaster, PolyPhen-2, PROVEAN, SIFT, VEST**	78.1% (74.75%-81.31%)	70.19% (65.85%-75%)	91.778% (88.406%-95.714%)	93.66% (91.11%-96.7%)	64.03% (58.82%-69.23%)	3.1965 (3.0856-3.3130)	78.14% (71.72%-84.85%)	70.24% (61.11%-79.03%)	91.649% (84.371%-97.56%)	93.53% (87.5%-98.04%)	64.21% (53.7%-74.07%)	3.1963 (2.9644-3.4233)
**At least 6 of CHASM (lung), CHASM (melanoma), Mutation Assessor, MutationTaster, PolyPhen-2, PROVEAN, VEST**	68.04% (64.65%-71.72%)	52.74% (47.66%-57.81%)	94.49% (91.667%-97.333%)	94.3% (91.55%-97.3%)	53.63% (48.8%-58.46%)	2.9516 (2.8501-3.0640)	67.95% (60.61%-74.75%)	52.46% (42.42%-62.71%)	94.503% (88.235%-100%)	94.25% (87.18%-100%)	53.67% (43.93%-63.49%)	2.9488 (2.7166-3.1586)
**At least 6 of CHASM (lung), CHASM (melanoma), Mutation Assessor, MutationTaster, PolyPhen-2, SIFT, VEST**	75.42% (72.22%-78.79%)	64.91% (60%-69.92%)	93.586% (90.541%-97.144%)	94.59% (92.05%-97.65%)	60.67% (55.37%-65.71%)	3.1376 (3.0309-3.2545)	75.42% (68.69%-81.82%)	64.83% (54.84%-74.6%)	93.559% (86.838%-100%)	94.53% (88.37%-100%)	60.8% (50.91%-70.91%)	3.1372 (2.9086-3.3563)
**At least 6 of CHASM (lung), CHASM (melanoma), Mutation Assessor, MutationTaster, PROVEAN, SIFT, VEST**	68.72% (65.64%-72.22%)	53.82% (48.8%-58.87%)	94.49% (91.667%-97.333%)	94.41% (91.67%-97.37%)	54.2% (49.22%-59.06%)	2.9691 (2.8675-3.0765)	68.61% (61.62%-74.77%)	53.5% (43.07%-63.49%)	94.503% (88.235%-100%)	94.35% (87.49%-100%)	54.22% (44.44%-63.94%)	2.9658 (2.7469-3.1606)
[illegible]	[illegible]	[illegible]	[illegible]	[illegible]	[illegible]	[illegible]	[illegible]	[illegible]	[illegible]	[illegible]	[illegible]	[illegible]

At least 7 of CHASM (breast), CHASM (lung), CHASM (melanoma), Mutation Assessor, MutationTaster, PolyPhen-2, VEST	57.24% (53.03%-61.11%)	34.11% (28.92%-39.06%)	97.222% (95.588%-100%)	95.5% (92.68%-100%)	46.05% (41.45%-50.33%)	2.7288 (2.6293-2.8325)	57.24% (49.49%-65.66%)	33.9% (23.81%-44.27%)	97.294% (92.303%-100%)	95.55% (87.99%-100%)	46.17% (37.5%-55.41%)	2.7291 (2.5121-2.9262)
At least 7 of CHASM (breast), CHASM (lung), CHASM (melanoma), Mutation Assessor, MutationTaster, PROVEAN, SIFT	56.25% (52.02%-60.1%)	31.48% (26.72%-36%)	99.084% (98.485%-100%)	98.34% (97.14%-100%)	45.54% (40.91%-50%)	2.7445 (2.6573-2.8315)	56.19% (48.48%-64.65%)	31.19% (22.06%-40.99%)	99.083% (96.774%-100%)	98.32% (93.75%-100%)	45.62% (37.04%-55%)	2.7421 (2.5668-2.9234)
At least 7 of CHASM (breast), CHASM (lung), CHASM (melanoma), Mutation Assessor, MutationTaster, PROVEAN, SIFT, VEST	65.7% (62.12%-69.19%)	47.99% (42.86%-52.94%)	96.311% (94.286%-98.667%)	95.75% (93.33%-98.48%)	51.71% (46.97%-56.43%)	2.9176 (2.8154-3.0237)	65.57% (58.59%-72.73%)	47.61% (37.7%-57.38%)	96.355% (91.176%-100%)	95.74% (89.29%-100%)	51.73% (41.79%-61.43%)	2.9143 (2.7016-3.1181)
At least 7 of CHASM (breast), CHASM (lung), CHASM (melanoma), Mutation Assessor, MutationTaster, PROVEAN, VEST	54.56% (50.51%-58.59%)	28.8% (24%-33.33%)	99.084% (98.485%-100%)	98.19% (96.77%-100%)	44.6% (39.88%-49.03%)	2.7068 (2.6209-2.7949)	54.52% (46.46%-62.63%)	28.55% (19.64%-38.46%)	99.083% (96.774%-100%)	98.17% (92.86%-100%)	44.7% (36.11%-54.12%)	2.7050 (2.5268-2.8825)
At least 7 of CHASM (breast), CHASM (lung), CHASM (melanoma), Mutation Assessor, MutationTaster, SIFT, VEST	58.26% (54.04%-62.12%)	35.72% (30.64%-40.8%)	97.222% (95.588%-100%)	95.69% (93.02%-100%)	46.66% (41.96%-51.01%)	2.7529 (2.6559-2.8513)	58.24% (50.51%-66.67%)	35.47% (25.4%-45.76%)	97.294% (92.303%-100%)	95.74% (88.46%-100%)	46.77% (37.8%-56.41%)	2.7527 (2.5541-2.9494)
At least 7 of CHASM (breast), CHASM (lung), CHASM (melanoma), Mutation Assessor, PolyPhen-2, PROVEAN, SIFT	56.93% (53.03%-60.61%)	32.54% (27.59%-37.01%)	99.084% (98.485%-100%)	98.4% (97.22%-100%)	45.93% (41.18%-50.31%)	2.7595 (2.6744-2.8466)	56.86% (49.49%-64.65%)	32.24% (22.95%-42.37%)	99.083% (96.774%-100%)	98.37% (94.11%-100%)	46% (37.33%-55.56%)	2.7569 (2.5807-2.9286)
At least 7 of CHASM (breast), CHASM (lung), CHASM (melanoma), Mutation Assessor, PolyPhen-2, PROVEAN, SIFT, VEST	65.02% (61.11%-68.69%)	46.92% (41.86%-52.28%)	96.311% (94.286%-98.667%)	95.65% (93.1%-98.46%)	51.21% (46.56%-55.71%)	2.9009 (2.8011-3.0073)	64.9% (57.58%-72.73%)	46.55% (36.06%-56.45%)	96.355% (91.176%-100%)	95.64% (88.89%-100%)	51.23% (41.79%-60.61%)	2.8978 (2.6842-3.1072)
At least 7 of CHASM (breast), CHASM (lung), CHASM (melanoma), Mutation Assessor, PolyPhen-2, PROVEAN, VEST	54.89% (50.51%-58.59%)	29.33% (24.39%-33.6%)	99.084% (98.485%-100%)	98.23% (96.88%-100%)	44.78% (40%-49.06%)	2.7142 (2.6286-2.8020)	54.86% (47.47%-63.64%)	29.08% (20%-38.98%)	99.083% (96.774%-100%)	98.2% (93.33%-100%)	44.88% (36.48%-54.22%)	2.7124 (2.5326-2.8876)
At least 7 of CHASM (breast), CHASM (lung), CHASM (melanoma), Mutation Assessor, PolyPhen-2, SIFT, VEST	58.26% (54.55%-62.12%)	35.71% (30.65%-40.63%)	97.222% (95.588%-100%)	95.69% (93.02%-100%)	46.66% (42%-50.99%)	2.7529 (2.6548-2.8501)	58.24% (50.51%-65.66%)	35.46% (25.67%-45.62%)	97.294% (92.303%-100%)	95.74% (88.46%-100%)	46.77% (38.15%-55.85%)	2.7527 (2.5523-2.9462)
At least 7 of CHASM (breast), CHASM (lung), CHASM (melanoma), Mutation Assessor, PROVEAN, SIFT, VEST	55.91% (51.52%-59.6%)	30.94% (26.19%-35.48%)	99.084% (98.485%-100%)	98.32% (97.06%-100%)	45.35% (40.64%-49.69%)	2.7369 (2.6515-2.8254)	55.86% (48.48%-64.65%)	30.66% (21.43%-40.36%)	99.083% (96.774%-100%)	98.29% (93.75%-100%)	45.43% (36.9%-54.88%)	2.7347 (2.5591-2.9135)
At least 7 of CHASM (breast), CHASM (lung), CHASM (melanoma), MutationTaster, PolyPhen-2, PROVEAN, SIFT	57.25% (53.03%-61.11%)	33.06% (27.96%-37.88%)	99.084% (98.485%-100%)	98.42% (97.22%-100%)	46.12% (41.29%-50.32%)	2.7669 (2.6793-2.8483)	57.21% (49.49%-65.66%)	32.8% (23.43%-42.86%)	99.083% (96.774%-100%)	98.4% (94.12%-100%)	46.21% (37.8%-55.56%)	2.7650 (2.6022-2.9468)
At least 7 of CHASM (breast), CHASM (lung), CHASM (melanoma), MutationTaster, PolyPhen-2, PROVEAN, SIFT, VEST	65.69% (62.12%-69.19%)	47.98% (42.96%-53.03%)	96.311% (94.286%-98.667%)	95.74% (93.22%-98.48%)	51.71% (46.97%-56.25%)	2.9174 (2.8146-3.0243)	65.58% (58.59%-72.73%)	47.63% (37.5%-57.63%)	96.355% (91.176%-100%)	95.74% (89.29%-100%)	51.74% (42.19%-61.54%)	2.9147 (2.7024-3.1305)
At least 7 of CHASM (breast), CHASM (lung), CHASM (melanoma), MutationTaster, PolyPhen-2, PROVEAN, VEST	55.56% (51.52%-59.6%)	30.39% (25.4%-35%)	99.084% (98.485%-100%)	98.29% (96.97%-100%)	45.15% (40.38%-49.35%)	2.7291 (2.6431-2.8168)	55.54% (47.47%-63.64%)	30.17% (20.97%-40.32%)	99.083% (96.774%-100%)	98.26% (93.33%-100%)	45.26% (36.84%-54.88%)	2.7277 (2.5529-2.9058)
At least 7 of CHASM (breast), CHASM (lung), CHASM (melanoma), MutationTaster, PolyPhen-2, SIFT, VEST	59.25% (55.05%-63.13%)	37.29% (32.5%-42.28%)	97.222% (95.588%-100%)	95.87% (93.33%-100%)	47.28% (42.57%-51.63%)	2.7766 (2.6797-2.8811)	59.27% (51.52%-67.68%)	37.1% (27.12%-47.46%)	97.294% (92.303%-100%)	95.91% (89.29%-100%)	47.41% (38.67%-57.14%)	2.7772 (2.5675-2.9722)
At least 7 of CHASM (breast), CHASM (lung), CHASM (melanoma), MutationTaster, PROVEAN, SIFT, VEST	57.26% (53.03%-61.11%)	33.06% (28.33%-37.82%)	99.084% (98.485%-100%)	98.42% (97.3%-100%)	46.12% (41.29%-50.32%)	2.7669 (2.6782-2.8500)	57.21% (49.49%-65.66%)	32.8% (23.21%-42.43%)	99.083% (96.774%-100%)	98.4% (94.12%-100%)	46.21% (37.66%-55.7%)	2.7650 (2.5942-2.9468)
At least 7 of CHASM (breast), CHASM (lung), CHASM (melanoma), PolyPhen-2, PROVEAN, SIFT, VEST	56.91% (53.03%-60.61%)	32.52% (27.56%-37.12%)	99.084% (98.485%-100%)	98.4% (97.22%-100%)	45.92% (40.99%-50%)	2.7593 (2.6743-2.8483)	56.88% (49.49%-64.65%)	32.27% (22.8%-42.03%)	99.083% (96.774%-100%)	98.37% (94.12%-100%)	46.02% (37.5%-55.56%)	2.7575 (2.5772-2.9319)
At least 7 of CHASM (breast), CHASM (lung), FATHMM (cancer), FATHMM (missense), Mutation Assessor, MutationTaster, PolyPhen-2	60.62% (56.57%-64.65%)	38.92% (33.6%-43.75%)	98.143% (97.015%-100%)	97.32% (95.56%-100%)	48.17% (43.54%-52.67%)	2.8255 (2.7345-2.9203)	60.57% (52.53%-68.69%)	38.64% (29.23%-49.18%)	98.209% (94.118%-100%)	97.39% (91.67%-100%)	48.26% (39.47%-57.69%)	2.8249 (2.6397-3.0062)
At least 7 of CHASM (breast), CHASM (lung), FATHMM (cancer), FATHMM (missense), Mutation Assessor, MutationTaster, PolyPhen-2, PROVEAN	66.03% (62.12%-69.7%)	48.52% (43.55%-53.66%)	96.298% (94.203%-98.684%)	95.77% (93.33%-98.48%)	51.96% (47.1%-56.49%)	2.9255 (2.8223-3.0286)	65.93% (58.59%-73.74%)	48.16% (37.87%-58.62%)	96.392% (90.909%-100%)	95.82% (89.66%-100%)	52% (42.65%-61.43%)	2.9237 (2.7223-3.1297)
At least 7 of CHASM (breast), CHASM (lung), FATHMM (cancer), FATHMM (missense), Mutation Assessor, MutationTaster, PolyPhen-2, PROVEAN, SIFT	74.48% (71.21%-77.78%)	62.88% (57.85%-67.97%)	94.526% (91.667%-97.436%)	95.21% (92.68%-97.75%)	59.56% (54.39%-64.41%)	3.1217 (3.0160-3.2298)	74.28% (67.68%-80.81%)	62.51% (52.38%-72.58%)	94.426% (88.235%-100%)	95.06% (88.89%-100%)	59.48% (49.21%-69.64%)	3.1148 (2.8983-3.3297)
At least 7 of CHASM (breast), CHASM (lung), FATHMM (cancer), FATHMM (missense), Mutation Assessor, MutationTaster, PolyPhen-2, PROVEAN, SIFT, VEST	79.11% (75.76%-82.32%)	73.91% (69.77%-78.36%)	88.085% (84%-92.756%)	91.47% (88.46%-94.9%)	66.13% (60.87%-71.43%)	3.1961 (3.0767-3.3182)	79.15% (72.73%-85.86%)	73.96% (65%-82.36%)	88.032% (79.31%-97.06%)	91.39% (84.91%-97.83%)	66.33% (55.56%-76.6%)	3.1971 (2.9498-3.4348)
At least 7 of CHASM (breast), CHASM (lung), FATHMM (cancer), FATHMM (missense), Mutation Assessor, MutationTaster, PolyPhen-2, PROVEAN, VEST	73.12% (69.7%-76.77%)	61.28% (56.2%-66.16%)	93.587% (90.411%-97.102%)	94.29% (91.57%-97.53%)	58.3% (53.21%-63.33%)	3.0746 (2.9606-3.1872)	72.95% (65.66%-79.8%)	60.92% (50.77%-70.77%)	93.555% (86.667%-100%)	94.2% (87.5%-100%)	58.26% (48.33%-68.52%)	3.0693 (2.8430-3.3007)
At least 7 of CHASM (breast), CHASM (lung), FATHMM (cancer), FATHMM (missense), Mutation Assessor, MutationTaster, PolyPhen-2, SIFT	68.73% (65.15%-72.73%)	53.3% (48.03%-58.4%)	95.411% (92.958%-98.571%)	95.25% (92.65%-98.55%)	54.16% (49.23%-58.87%)	2.9813 (2.8776-3.0938)	68.59% (60.61%-75.76%)	52.94% (42.59%-63.16%)	95.417% (89.474%-100%)	95.19% (88.57%-100%)	54.16% (44.77%-64.04%)	2.9771 (2.7512-3.1872)
At least 7 of CHASM (breast), CHASM (lung), FATHMM (cancer), FATHMM (missense), Mutation Assessor, MutationTaster, PolyPhen-2, SIFT, VEST	77.82% (74.75%-81.31%)	69.75% (64.95%-74.24%)	91.761% (88.406%-95.652%)	93.6% (91.11%-96.77%)	63.69% (58.42%-68.69%)	3.1880 (3.0723-3.3074)	77.7% (70.71%-83.84%)	69.52% (60.34%-79.42%)	91.697% (84.375%-97.5%)	93.49% (86.96%-98%)	63.69% (53.06%-74.08%)	3.1840 (2.9499-3.4114)
At least 7 of CHASM (breast), CHASM (lung), FATHMM (cancer), FATHMM (missense), Mutation Assessor, MutationTaster, PolyPhen-2, VEST	67.71% (64.14%-71.72%)	52.24% (47.15%-57.6%)	94.471% (91.664%-97.368%)	94.23% (91.3%-97.3%)	53.36% (48.46%-58.02%)	2.9430 (2.8374-3.0505)	67.6% (59.6%-74.75%)	51.88% (40.62%-61.9%)	94.545% (88.235%-100%)	94.23% (87.1%-100%)	53.38% (43.75%-63.49%)	2.9404 (2.7273-3.1575)
At least 7 of CHASM (breast), CHASM (lung), FATHMM (cancer), FATHMM (missense), Mutation Assessor, MutationTaster, PROVEAN	55.59% (51.52%-59.6%)	30.44% (25.78%-34.92%)	99.084% (98.485%-100%)	98.29% (96.97%-100%)	45.17% (40.51%-49.38%)	2.7299 (2.6432-2.8168)	55.48% (47.47%-63.64%)	30.06% (21.05%-39.66%)	99.083% (96.774%-100%)	98.26% (93.33%-100%)	45.22% (36.48%-54.43%)	2.7263 (2.5496-2.9001)
At least 7 of CHASM (breast), CHASM (lung), FATHMM (cancer), FATHMM (missense), Mutation Assessor, MutationTaster, PROVEAN, SIFT	66.68% (63.13%-70.2%)	49.55% (44.53%-54.4%)	96.298% (94.203%-98.684%)	95.86% (93.44%-98.53%)	52.47% (47.76%-57.25%)	2.9417 (2.8392-3.0427)	66.64% (59.6%-73.74%)	49.28% (39.34%-59.38%)	96.392% (90.909%-100%)	95.91% (89.66%-100%)	52.54% (43.1%-61.76%)	2.9413 (2.7258-3.1484)
At least 7 of CHASM (breast), CHASM (lung), FATHMM (cancer), FATHMM (missense), Mutation Assessor, MutationTaster, PROVEAN, SIFT, VEST	73.11% (69.7%-76.77%)	60.73% (55.65%-65.87%)	94.526% (91.667%-97.436%)	95.04% (92.41%-97.67%)	58.2% (52.99%-63.16%)	3.0849 (2.9756-3.1980)	72.96% (65.66%-79.8%)	60.43% (50%-70.69%)	94.426% (88.235%-100%)	94.9% (88.64%-100%)	58.17% (48.27%-68.34%)	3.0793 (2.8538-3.3006)
At least 7 of CHASM (breast), CHASM (lung), FATHMM (cancer), FATHMM (missense), Mutation Assessor, MutationTaster, PROVEAN, VEST	64.03% (60.1%-67.68%)	45.37% (40.32%-50.78%)	96.298% (94.203%-98.684%)	95.49% (92.86%-98.41%)	50.48% (45.86%-55.08%)	2.8764 (2.7756-2.9790)	63.86% (56.57%-71.72%)	44.88% (34.43%-55%)	96.392% (90.909%-100%)	95.53% (88.88%-100%)	50.47% (40.91%-59.47%)	2.8728 (2.6682-3.0780)
At least 7 of CHASM (breast), CHASM (lung), FATHMM (cancer), FATHMM (missense), Mutation Assessor, MutationTaster, SIFT	61.64% (58.07%-65.15%)	40.53% (35.48%-45.16%)	98.143% (97.015%-100%)	97.42% (95.74%-100%)	48.83% (44.16%-53.2%)	2.8493 (2.7597-2.9390)	61.57% (54.55%-68.71%)	40.21% (30.77%-50.75%)	98.209% (94.118%-100%)	97.49% (92%-100%)	48.9% (40%-58.44%)	2.8480 (2.6637-3.0276)
At least 7 of CHASM (breast), CHASM (lung), FATHMM (cancer), FATHMM (missense), Mutation Assessor, MutationTaster, SIFT, VEST	67.71% (64.14%-71.21%)	51.69% (46.46%-56.91%)	95.411% (92.958%-98.571%)	95.12% (92.42%-98.53%)	53.32% (48.48%-57.94%)	2.9554 (2.8546-3.0675)	67.6% (60.61%-74.75%)	51.38% (40.32%-61.54%)	95.417% (89.474%-100%)	95.06% (88.24%-100%)	53.35% (43.55%-63.23%)	2.9520 (2.7273-3.1576)
At least 7 of CHASM (breast), CHASM (lung), FATHMM (cancer), FATHMM (missense), Mutation Assessor, MutationTaster, VEST	57.95% (54.04%-61.62%)	34.7% (29.84%-39.84%)	98.143% (97.015%-100%)	97% (95%-100%)	46.5% (41.83%-50.98%)	2.7634 (2.6696-2.8549)	57.84% (50.51%-65.66%)	34.32% (24.19%-44.07%)	98.209% (94.118%-100%)	97.06% (90.48%-100%)	46.56% (37.5%-56.17%)	2.7615 (2.5772-2.9494)
At least 7 of CHASM (breast), CHASM (lung), FATHMM (cancer), FATHMM (missense), Mutation Assessor, PolyPhen-2, PROVEAN	56.27% (52.02%-60.1%)	31.51% (26.45%-36.07%)	99.084% (98.485%-100%)	98.35% (97.06%-100%)	45.55% (40.88%-49.69%)	2.7449 (2.6572-2.8294)	56.15% (48.48%-64.65%)	31.11% (22.03%-41.27%)	99.083% (96.774%-100%)	98.31% (93.75%-100%)	45.6% (36.84%-54.88%)	2.7410 (2.5675-2.9144)
At least 7 of CHASM (breast), CHASM (lung), FATHMM (cancer), FATHMM (missense), Mutation Assessor, PolyPhen-2, PROVEAN, SIFT	66.36% (62.63%-70.2%)	49.04% (44.09%-54.27%)	96.298% (94.203%-98.684%)	95.81% (93.33%-98.51%)	52.22% (47.41%-56.72%)	2.9337 (2.8301-3.0367)	66.27% (58.59%-73.74%)	48.7% (38.33%-58.73%)	96.392% (90.909%-100%)	95.86% (89.66%-100%)	52.26% (42.86%-62.12%)	2.9321 (2.7235-3.1375)
At least 7 of CHASM (breast), CHASM (lung), FATHMM (cancer), FATHMM (missense), Mutation Assessor, PolyPhen-2, PROVEAN, SIFT, VEST	72.12% (68.18%-75.76%)	61.82% (56.59%-66.94%)	89.91% (86.076%-94.366%)	91.38% (87.95%-95.18%)	57.66% (52.38%-62.73%)	3.0077 (2.8859-3.1285)	71.93% (64.65%-79.8%)	61.44% (50.82%-71.67%)	89.89% (81.577%-97.3%)	91.27% (83.71%-97.73%)	57.6% (47.54%-68.42%)	3.0019 (2.7580-3.2427)
At least 7 of CHASM (breast), CHASM (lung), FATHMM (cancer), FATHMM (missense), Mutation Assessor, PolyPhen-2, PROVEAN, VEST	63.01% (59.09%-66.67%)	44.3% (39.06%-49.6%)	95.358% (92.857%-98.592%)	94.29% (91.07%-98.18%)	49.75% (45.18%-54.35%)	2.8370 (2.7343-2.9472)	62.87% (55.56%-70.71%)	43.82% (33.33%-54.24%)	95.521% (89.474%-100%)	94.38% (86.96%-100%)	49.77% (40.79%-59.1%)	2.8350 (2.6126-3.0449)
At least 7 of CHASM (breast), CHASM (lung), FATHMM (cancer), FATHMM (missense), Mutation Assessor, PolyPhen-2, SIFT	62.66% (59.08%-66.16%)	42.14% (36.97%-47.06%)	98.143% (97.015%-100%)	97.52% (95.92%-100%)	49.52% (44.6%-53.85%)	2.8731 (2.7824-2.9704)	62.56% (55.56%-70.72%)	41.77% (31.58%-51.67%)	98.209% (94.118%-100%)	97.57% (92.31%-100%)	49.56% (40.85%-59.46%)	2.8712 (2.6877-3.0489)
At least 7 of CHASM (breast), CHASM (lung), FATHMM (cancer), FATHMM (missense), Mutation Assessor, PolyPhen-2, SIFT, VEST	67.71% (64.14%-71.72%)	52.23% (46.92%-57.38%)	94.471% (91.664%-97.368%)	94.23% (91.3%-97.3%)	53.35% (48.48%-58.06%)	2.9429 (2.8413-3.0539)	67.61% (59.6%-74.75%)	51.89% (41.38%-61.98%)	94.545% (88.235%-100%)	94.22% (86.84%-100%)	53.39% (44.12%-63.02%)	2.9405 (2.7112-3.1426)
At least 7 of CHASM (breast), CHASM (lung), FATHMM (cancer), FATHMM (missense), Mutation Assessor, PolyPhen-2, VEST	57.6% (53.54%-61.11%)	34.69% (29.54%-39.52%)	97.203% (95.588%-100%)	95.55% (92.86%-100%)	46.26% (41.45%-50.66%)	2.7371 (2.6387-2.8372)	57.53% (50.51%-65.66%)	34.32% (24.99%-44.78%)	97.337% (92.5%-100%)	95.71% (88.24%-100%)	46.34% (37.66%-55.84%)	2.7370 (2.5322-2.9262)
At least 7 of CHASM (breast), CHASM (lung), FATHMM (cancer), FATHMM (missense), Mutation Assessor, PROVEAN, SIFT	57.29% (53.03%-61.11%)	33.11% (28.23%-37.8%)	99.084% (98.485%-100%)	98.43% (97.3%-100%)	46.14% (41.45%-50.33%)	2.7676 (2.6829-2.8590)	57.14% (49.49%-65.66%)	32.69% (23.43%-42.62%)	99.083% (96.774%-100%)	98.39% (94.12%-100%)	46.17% (37.66%-55.56%)	2.7634 (2.5827-2.9402)
At least 7 of CHASM (breast), CHASM (lung), FATHMM (cancer), FATHMM (missense), Mutation Assessor, PROVEAN, SIFT, VEST	63.69% (60.1%-67.17%)	44.82% (39.67%-50%)	96.298% (94.203%-98.684%)	95.44% (92.73%-98.39%)	50.23% (45.65%-54.86%)	2.8679 (2.7646-2.9715)	63.53% (56.57%-70.71%)	44.37% (34.42%-54.11%)	96.392% (90.909%-100%)	95.48% (88.46%-100%)	50.24% (40.91%-59.42%)	2.8648 (2.6561-3.0693)
At least 7 of CHASM (breast), CHASM (lung), FATHMM (cancer), FATHMM (missense), Mutation Assessor, PROVEAN, VEST	55.26% (51.01%-59.09%)	29.9% (25%-34.17%)	99.084% (98.485%-100%)	98.26% (96.97%-100%)	44.98% (40.26%-49.37%)	2.7223 (2.6401-2.8105)	55.15% (47.47%-63.64%)	29.54% (20.63%-39.29%)	99.083% (96.774%-100%)	98.23% (93.33%-100%)	45.04% (36.36%-54.22%)	2.7188 (2.5417-2.8827)
At least 7 of CHASM (breast), CHASM (lung), FATHMM (cancer), FATHMM (missense), Mutation Assessor, SIFT, VEST	58.96% (55.05%-62.63%)	36.3% (31.15%-41.27%)	98.143% (97.015%-100%)	97.13% (95.24%-100%)	47.12% (42.31%-51.61%)	2.7870 (2.6954-2.8786)	58.84% (51.52%-66.67%)	35.89% (26.15%-45.62%)	98.209% (94.118%-100%)	97.19% (90.91%-100%)	47.16% (38.35%-56.58%)	2.7845 (2.6012-2.9727)
At least 7 of CHASM (breast), CHASM (lung), FATHMM (cancer), FATHMM (missense), MutationTaster, PolyPhen-2, PROVEAN	55.61% (51.52%-59.09%)	30.46% (25.4%-35.16%)	99.084% (98.485%-100%)	98.29% (96.97%-100%)	45.18% (40.6%-49.37%)	2.7301 (2.6416-2.8155)	55.46% (48.48%-63.64%)	30.03% (20.69%-40.32%)	99.083% (96.774%-100%)	98.26% (93.33%-100%)	45.21% (36.84%-54.55%)	2.7258 (2.5495-2.9015)
At least 7 of CHASM (breast), CHASM (lung), FATHMM (cancer), FATHMM (missense), MutationTaster, PolyPhen-2, PROVEAN, SIFT	69.05% (65.66%-72.23%)	53.29% (48.39%-58.54%)	96.298% (94.203%-98.684%)	96.14% (93.85%-98.63%)	54.38% (49.59%-58.92%)	3.0011 (2.9033-3.1027)	68.97% (62.6%-75.76%)	52.98% (42.62%-62.9%)	96.392% (90.909%-100%)	96.19% (90.32%-100%)	54.43% (45%-64.18%)	2.9999 (2.7980-3.1933)
At least 7 of CHASM (breast), CHASM (lung), FATHMM (cancer), FATHMM (missense), MutationTaster, PolyPhen-2, PROVEAN, SIFT, VEST	74.8% (71.21%-78.28%)	63.39% (58.54%-68.22%)	94.526% (91.667%-97.436%)	95.24% (92.77%-97.75%)	59.89% (54.78%-64.96%)	3.1305 (3.0167-3.2388)	74.64% (67.68%-81.82%)	63.09% (53.12%-73.21%)	94.426% (88.235%-100%)	95.11% (89.12%-100%)	59.86% (49.98%-70.37%)	3.1248 (2.9075-3.3542)
At least 7 of CHASM (breast), CHASM (lung), FATHMM (cancer), FATHMM (missense), MutationTaster, PolyPhen-2, PROVEAN, VEST	66.04% (62.12%-69.7%)	48.54% (43.3%-53.79%)	96.298% (94.203%-98.684%)	95.78% (93.33%-98.51%)	51.98% (47.29%-56.52%)	2.9259 (2.8238-3.0297)	65.9% (58.59%-73.74%)	48.11% (37.31%-58.82%)	96.392% (90.909%-100%)	95.82% (89.65%-100%)	51.98% (42.42%-61.29%)	2.9231 (2.7177-3.1321)
At least 7 of CHASM (breast), CHASM (lung), FATHMM (cancer), FATHMM (missense), MutationTaster, PolyPhen-2, SIFT	61.64% (57.58%-65.15%)	40.53% (35.43%-45.24%)	98.143% (97.015%-100%)	97.42% (95.74%-100%)	48.83% (44.14%-53.11%)	2.8493 (2.7586-2.9410)	61.56% (54.55%-69.7%)	40.2% (30.36%-50%)	98.209% (94.118%-100%)	97.48% (91.67%-100%)	48.9% (40%-58.21%)	2.8480 (2.6643-3.0315)
At least 7 of CHASM (breast), CHASM (lung), FATHMM (cancer), FATHMM (missense), MutationTaster, PolyPhen-2, SIFT, VEST	70.42% (66.67%-73.75%)	55.97% (51.16%-61.11%)	95.411% (92.958%-98.571%)	95.47% (92.96%-98.63%)	55.62% (50.78%-60.31%)	3.0247 (2.9170-3.1309)	70.26% (63.61%-77.78%)	55.59% (44.83%-65.57%)	95.417% (89.474%-100%)	95.42% (89.18%-100%)	55.59% (46.03%-65.58%)	3.0201 (2.8149-3.2430)
At least 7 of CHASM (breast), CHASM (lung), FATHMM (cancer), FATHMM (missense), MutationTaster, PolyPhen-2, VEST	58.62% (54.55%-62.63%)	35.77% (30.71%-40.77%)	98.143% (97.015%-100%)	97.09% (95.12%-100%)	46.91% (42.28%-51.32%)	2.7791 (2.6815-2.8720)	58.51% (50.51%-66.67%)	35.37% (25.67%-45.31%)	98.209% (94.118%-100%)	97.15% (90.91%-100%)	46.96% (38.03%-56.41%)	2.7769 (2.5898-2.9728)
At least 7 of CHASM (breast), CHASM (lung), FATHMM (cancer), FATHMM (missense), MutationTaster, PROVEAN, SIFT	57.27% (53.03%-61.11%)	33.09% (28.33%-37.7%)	99.084% (98.485%-100%)	98.43% (97.22%-100%)	46.13% (41.4%-50.33%)	2.7674 (2.6797-2.8519)	57.17% (49.49%-65.66%)	32.73% (23.72%-42.62%)	99.083% (96.774%-100%)	98.41% (94.12%-100%)	46.19% (37.66%-55.56%)	2.7641 (2.5988-2.9468)
At least 7 of CHASM (breast), CHASM (lung), FATHMM (cancer), FATHMM (missense), MutationTaster, PROVEAN, SIFT, VEST	65.69% (62.12%-69.19%)	47.99% (42.97%-53.13%)	96.298% (94.203%-98.684%)	95.73% (93.22%-98.49%)	51.71% (46.92%-56.3%)	2.9172 (2.8168-3.0202)	65.58% (58.59%-72.73%)	47.62% (37.31%-57.81%)	96.392% (90.909%-100%)	95.78% (89.29%-100%)	51.74% (42.62%-61.19%)	2.9153 (2.7177-3.1168)
At least 7 of CHASM (breast), CHASM (lung), FATHMM (cancer), FATHMM (missense), MutationTaster, PROVEAN, VEST	55.61% (51.52%-59.6%)	30.46% (25.76%-35.2%)	99.084% (98.485%-100%)	98.29% (97.06%-100%)	45.18% (40.51%-49.4%)	2.7302 (2.6428-2.8150)	55.45% (47.47%-63.64%)	30.02% (20.63%-39.71%)	99.083% (96.774%-100%)	98.26% (93.33%-100%)	45.21% (36.59%-54.76%)	2.7258 (2.5570-2.9069)
At least 7 of CHASM (breast), CHASM (lung), FATHMM (cancer), FATHMM (missense), MutationTaster, SIFT, VEST	59.3% (55.05%-63.13%)	36.83% (31.74%-41.94%)	98.143% (97.015%-100%)	97.17% (95.35%-100%)	47.33% (42.67%-51.73%)	2.7947 (2.7001-2.8856)	59.18% (51.52%-67.68%)	36.43% (26.23%-46.38%)	98.209% (94.118%-100%)	97.23% (90.91%-100%)	47.38% (38.35%-56.76%)	2.7924 (2.6078-2.9919)
At least 7 of CHASM (breast), CHASM (lung), FATHMM (cancer), FATHMM (missense), PolyPhen-2, PROVEAN, SIFT	57.29% (53.54%-61.11%)	33.11% (28.12%-37.88%)	99.084% (98.485%-100%)	98.43% (97.3%-100%)	46.14% (41.25%-50.32%)	2.7676 (2.6810-2.8542)	57.14% (49.49%-64.65%)	32.69% (22.95%-42.86%)	99.083% (96.774%-100%)	98.4% (94.12%-100%)	46.17% (37.5%-55.56%)	2.7634 (2.5933-2.9331)
At least 7 of CHASM (breast), CHASM (lung), FATHMM (cancer), FATHMM (missense), PolyPhen-2, PROVEAN, SIFT, VEST	65.71% (62.12%-69.19%)	48.01% (42.86%-53.23%)	96.298% (94.203%-98.684%)	95.73% (93.22%-98.51%)	51.72% (46.97%-56.39%)	2.9176 (2.8159-3.0230)	65.57% (58.59%-72.73%)	47.57% (37.49%-57.81%)	96.392% (90.909%-100%)	95.77% (89.28%-100%)	51.72% (42.65%-61.19%)	2.9145 (2.6985-3.1176)
At least 7 of CHASM (breast), CHASM (lung), FATHMM (cancer), FATHMM (missense), PolyPhen-2, PROVEAN, VEST	55.27% (51.01%-59.09%)	29.92% (25%-34.65%)	99.084% (98.485%-100%)	98.26% (96.97%-100%)	44.99% (40.13%-49.1%)	2.7225 (2.6376-2.8066)	55.13% (47.47%-63.64%)	29.5% (20.31%-39.34%)	99.083% (96.774%-100%)	98.23% (93.33%-100%)	45.02% (36.71%-54.32%)	2.7183 (2.5444-2.8965)
At least 7 of CHASM (breast), CHASM (lung), FATHMM (cancer), FATHMM (missense), PolyPhen-2, SIFT, VEST	58.62% (54.55%-62.12%)	35.76% (30.65%-40.8%)	98.143% (97.015%-100%)	97.09% (95.12%-100%)	46.91% (42.11%-51.28%)	2.7790 (2.6862-2.8707)	58.52% (51.52%-66.67%)	35.38% (25.4%-45.32%)	98.209% (94.118%-100%)	97.15% (90.9%-100%)	46.97% (38.35%-56.58%)	2.7770 (2.5942-2.9643)
At least 7 of CHASM (breast), CHASM (lung), FATHMM (cancer), FATHMM (missense), PROVEAN, SIFT, VEST	56.61% (52.53%-60.11%)	32.05% (27.13%-36.72%)	99.084% (98.485%-100%)	98.38% (97.14%-100%)	45.75% (40.91%-50%)	2.7525 (2.6650-2.8335)	56.47% (49.47%-64.65%)	31.63% (22.22%-41.54%)	99.083% (96.774%-100%)	98.35% (93.75%-100%)	45.79% (37.18%-55.41%)	2.7485 (2.5807-2.9314)
At least 7 of CHASM (breast), CHASM (lung), FATHMM (cancer), Mutation Assessor, MutationTaster, PolyPhen-2, PROVEAN	55.92% (51.52%-59.6%)	30.95% (26.02%-35.65%)	99.084% (98.485%-100%)	98.32% (97.06%-100%)	45.35% (40.67%-49.69%)	2.7370 (2.6492-2.8246)	55.85% (48.48%-64.65%)	30.64% (21.31%-40.62%)	99.083% (96.774%-100%)	98.29% (93.75%-100%)	45.43% (36.71%-54.67%)	2.7345 (2.5590-2.9144)
At least 7 of CHASM (breast), CHASM (lung), FATHMM (cancer), Mutation Assessor, MutationTaster, PolyPhen-2, PROVEAN, SIFT	67.69% (64.13%-71.21%)	51.15% (45.96%-56.15%)	96.298% (94.203%-98.684%)	95.98% (93.65%-98.57%)	53.27% (48.48%-57.94%)	2.9670 (2.8648-3.0704)	67.64% (60.61%-74.77%)	50.87% (40.98%-61.3%)	96.392% (90.909%-100%)	96.03% (90%-100%)	53.34% (43.54%-63.08%)	2.9663 (2.7472-3.1679)
At least 7 of CHASM (breast), CHASM (lung), FATHMM (cancer), Mutation Assessor, MutationTaster, PolyPhen-2, PROVEAN, SIFT, VEST	73.78% (70.2%-77.27%)	62.3% (57.36%-67.44%)	93.605% (90.539%-97.102%)	94.39% (91.86%-97.53%)	58.95% (53.85%-63.8%)	3.0925 (2.9858-3.2030)	73.66% (66.67%-80.81%)	62.07% (51.78%-72.14%)	93.512% (86.667%-100%)	94.25% (87.88%-100%)	58.96% (49.09%-69.23%)	3.0880 (2.8522-3.3080)
At least 7 of CHASM (breast), CHASM (lung), FATHMM (cancer), Mutation Assessor, MutationTaster, PolyPhen-2, PROVEAN, VEST	65.37% (61.62%-69.19%)	47.48% (42.4%-52.46%)	96.298% (94.203%-98.684%)	95.68% (93.1%-98.46%)	51.47% (46.67%-56.03%)	2.9092 (2.8052-3.0212)	65.22% (57.58%-72.73%)	47.05% (36.84%-57.14%)	96.392% (90.909%-100%)	95.72% (89.29%-100%)	51.48% (42.25%-61.19%)	2.9064 (2.6835-3.1154)
At least 7 of CHASM (breast), CHASM (lung), FATHMM (cancer), Mutation Assessor, MutationTaster, PolyPhen-2, SIFT	61.63% (57.58%-65.15%)	40.5% (35.2%-45.6%)	98.143% (97.015%-100%)	97.42% (95.74%-100%)	48.82% (44.14%-53.29%)	2.8489 (2.7585-2.9451)	61.6% (54.55%-69.7%)	40.26% (30%-50.77%)	98.209% (94.118%-100%)	97.49% (92%-100%)	48.93% (39.99%-58.11%)	2.8488 (2.6628-3.0350)
At least 7 of CHASM (breast), CHASM (lung), FATHMM (cancer), Mutation Assessor, MutationTaster, PolyPhen-2, SIFT, VEST	69.38% (65.66%-73.23%)	54.86% (49.99%-60%)	94.49% (91.667%-97.333%)	94.51% (91.78%-97.37%)	54.76% (49.62%-59.68%)	2.9862 (2.8792-3.1026)	69.32% (61.62%-76.77%)	54.63% (44.26%-64.79%)	94.503% (88.235%-100%)	94.45% (87.88%-100%)	54.83% (44.77%-65.08%)	2.9841 (2.7520-3.2037)
At least 7 of CHASM (breast), CHASM (lung), FATHMM (cancer), Mutation Assessor, MutationTaster, PolyPhen-2, VEST	57.93% (53.54%-61.62%)	34.67% (29.27%-39.67%)	98.143% (97.015%-100%)	97% (94.87%-100%)	46.49% (41.77%-50.94%)	2.7631 (2.6674-2.8545)	57.88% (50.51%-65.66%)	34.37% (24.24%-45.1%)	98.209% (94.118%-100%)	97.07% (90.48%-100%)	46.59% (37.68%-55.88%)	2.7623 (2.5708-2.9562)
At least 7 of CHASM (breast), CHASM (lung), FATHMM (cancer), Mutation Assessor, MutationTaster, PROVEAN, SIFT	56.93% (53.03%-60.62%)	32.55% (27.78%-37.1%)	99.084% (98.485%-100%)	98.4% (97.22%-100%)	45.94% (41.25%-50.32%)	2.7597 (2.6710-2.8483)	56.84% (49.47%-64.65%)	32.22% (22.95%-42.11%)	99.083% (96.774%-100%)	98.37% (93.75%-100%)	46% (37.33%-55.43%)	2.7568 (2.5770-2.9401)
At least 7 of CHASM (breast), CHASM (lung), FATHMM (cancer), Mutation Assessor, MutationTaster, PROVEAN, SIFT, VEST	65.7% (62.11%-69.19%)	48% (42.75%-52.94%)	96.298% (94.203%-98.684%)	95.73% (93.22%-98.51%)	51.71% (46.92%-56.39%)	2.9174 (2.8135-3.0226)	65.57% (58.59%-72.75%)	47.59% (37.31%-57.81%)	96.392% (90.909%-100%)	95.77% (88.89%-100%)	51.73% (42.19%-61.54%)	2.9149 (2.6985-3.1306)
At least 7 of CHASM (breast), CHASM (lung), FATHMM (cancer), Mutation Assessor, MutationTaster, PROVEAN, VEST	55.24% (51.01%-59.09%)	29.88% (25.19%-34.43%)	99.084% (98.485%-100%)	98.26% (96.97%-100%)	44.97% (40.25%-49.37%)	2.7220 (2.6342-2.8090)	55.18% (47.47%-63.64%)	29.59% (20.63%-39.07%)	99.083% (96.774%-100%)	98.23% (93.33%-100%)	45.06% (36.25%-54.32%)	2.7196 (2.5468-2.9015)
At least 7 of CHASM (breast), CHASM (lung), FATHMM (cancer), Mutation Assessor, MutationTaster, SIFT, VEST	58.95% (55.04%-62.64%)	36.28% (31.01%-41.32%)	98.143% (97.015%-100%)	97.13% (95.24%-100%)	47.11% (42.4%-51.66%)	2.7866 (2.6944-2.8800)	58.87% (51.49%-66.69%)	35.94% (26.15%-46.67%)	98.209% (94.118%-100%)	97.19% (90.91%-100%)	47.19% (38.16%-56.76%)	2.7853 (2.5981-2.9727)
At least 7 of CHASM (breast), CHASM (lung), FATHMM (cancer), Mutation Assessor, PolyPhen-2, PROVEAN, SIFT	57.61% (53.54%-61.62%)	33.62% (28.68%-38.17%)	99.084% (98.485%-100%)	98.45% (97.3%-100%)	46.33% (41.72%-50.65%)	2.7748 (2.6884-2.8595)	57.51% (49.49%-65.66%)	33.27% (24.14%-42.86%)	99.083% (96.774%-100%)	98.42% (94.12%-100%)	46.39% (37.66%-55.84%)	2.7716 (2.5942-2.9438)
At least 7 of CHASM (breast), CHASM (lung), FATHMM (cancer), Mutation Assessor, PolyPhen-2, PROVEAN, SIFT, VEST	65.02% (61.11%-68.69%)	46.93% (41.86%-52%)	96.298% (94.203%-98.684%)	95.64% (93.1%-98.46%)	51.21% (46.38%-55.8%)	2.9007 (2.7961-3.0114)	64.9% (57.58%-72.73%)	46.53% (36.36%-56.67%)	96.392% (90.909%-100%)	95.67% (88.89%-100%)	51.23% (41.79%-60.61%)	2.8983 (2.6764-3.1154)
At least 7 of CHASM (breast), CHASM (lung), FATHMM (cancer), Mutation Assessor, PolyPhen-2, PROVEAN, VEST	55.58% (51.52%-59.6%)	30.41% (25.58%-34.92%)	99.084% (98.485%-100%)	98.29% (96.97%-100%)	45.16% (40.38%-49.38%)	2.7294 (2.6432-2.8156)	55.51% (47.47%-63.64%)	30.12% (21.05%-39.68%)	99.083% (96.774%-100%)	98.26% (93.33%-100%)	45.24% (36.71%-54.67%)	2.7270 (2.5529-2.9001)
At least 7 of CHASM (breast), CHASM (lung), FATHMM (cancer), Mutation Assessor, PolyPhen-2, SIFT, VEST	58.95% (55.05%-62.63%)	36.28% (31.01%-41.13%)	98.143% (97.015%-100%)	97.13% (95.24%-100%)	47.11% (42.36%-51.63%)	2.7866 (2.6911-2.8800)	58.87% (51.52%-66.67%)	35.94% (25.76%-46.88%)	98.209% (94.118%-100%)	97.19% (90.91%-100%)	47.19% (38.16%-56.58%)	2.7853 (2.6003-2.9728)
At least 7 of CHASM (breast), CHASM (lung), FATHMM (cancer), Mutation Assessor, PROVEAN, SIFT, VEST	56.59% (52.53%-60.61%)	32.02% (27.34%-36.64%)	99.084% (98.485%-100%)	98.37% (97.14%-100%)	45.74% (40.99%-50%)	2.7521 (2.6646-2.8407)	56.51% (48.48%-64.65%)	31.7% (22.22%-41.27%)	99.083% (96.774%-100%)	98.35% (93.75%-100%)	45.81% (37.18%-55.26%)	2.7493 (2.5723-2.9315)
At least 7 of CHASM (breast), CHASM (lung), FATHMM (cancer), MutationTaster, PolyPhen-2, PROVEAN, SIFT	57.94% (54.04%-61.62%)	34.14% (29.17%-39.17%)	99.084% (98.485%-100%)	98.47% (97.3%-100%)	46.53% (41.72%-50.96%)	2.7822 (2.6945-2.8636)	57.86% (50.51%-65.66%)	33.84% (24.13%-43.75%)	99.083% (96.774%-100%)	98.45% (94.44%-100%)	46.6% (37.97%-56.17%)	2.7797 (2.6171-2.9606)
At least 7 of CHASM (breast), CHASM (lung), FATHMM (cancer), MutationTaster, PolyPhen-2, PROVEAN, SIFT, VEST	66.04% (62.12%-69.7%)	48.54% (43.54%-53.6%)	96.298% (94.203%-98.684%)	95.77% (93.33%-98.51%)	51.98% (47.24%-56.52%)	2.9259 (2.8273-3.0333)	65.9% (58.59%-73.74%)	48.11% (37.83%-58.06%)	96.392% (90.909%-100%)	95.81% (89.29%-100%)	51.98% (42.62%-61.64%)	2.9230 (2.7078-3.1332)
At least 7 of CHASM (breast), CHASM (lung), FATHMM (cancer), MutationTaster, PolyPhen-2, PROVEAN, VEST	56.59% (52.53%-60.11%)	32.02% (27.2%-36.8%)	99.084% (98.485%-100%)	98.37% (97.14%-100%)	45.74% (40.91%-50%)	2.7521 (2.6646-2.8372)	56.51% (49.49%-64.65%)	31.7% (22.38%-41.27%)	99.083% (96.774%-100%)	98.34% (93.75%-100%)	45.81% (37.18%-55.41%)	2.7494 (2.5770-2.9330)
At least 7 of CHASM (breast), CHASM (lung), FATHMM (cancer), MutationTaster, PolyPhen-2, SIFT, VEST	59.95% (56.05%-63.64%)	37.85% (32.54%-42.75%)	98.143% (97.015%-100%)	97.24% (95.35%-100%)	47.74% (42.95%-52%)	2.8098 (2.7144-2.9023)	59.91% (52.53%-67.7%)	37.58% (27.12%-48.33%)	98.209% (94.118%-100%)	97.3% (91.3%-100%)	47.83% (38.96%-57.75%)	2.8093 (2.6207-3.0153)
At least 7 of CHASM (breast), CHASM (lung), FATHMM (cancer), MutationTaster, PROVEAN, SIFT, VEST	57.94% (53.54%-61.62%)	34.14% (29.27%-38.89%)	99.084% (98.485%-100%)	98.47% (97.37%-100%)	46.53% (41.72%-50.67%)	2.7822 (2.6946-2.8637)	57.86% (50.51%-66.67%)	33.83% (24.24%-43.75%)	99.083% (96.774%-100%)	98.45% (94.44%-100%)	46.6% (38.02%-56.25%)	2.7797 (2.6170-2.9643)
At least 7 of CHASM (breast), CHASM (lung), FATHMM (cancer), PolyPhen-2, PROVEAN, SIFT, VEST	57.6% (53.54%-61.11%)	33.6% (28.57%-38.4%)	99.084% (98.485%-100%)	98.45% (97.3%-100%)	46.32% (41.45%-50.64%)	2.7746 (2.6884-2.8595)	57.53% (50.51%-65.66%)	33.31% (24.19%-42.86%)	99.083% (96.774%-100%)	98.42% (94.12%-100%)	46.4% (37.84%-55.84%)	2.7722 (2.6012-2.9513)
At least 7 of CHASM (breast), CHASM (lung), FATHMM (missense), Mutation Assessor, MutationTaster, PolyPhen-2, PROVEAN	54.58% (50.51%-58.59%)	28.84% (24.16%-33.33%)	99.084% (98.485%-100%)	98.2% (96.77%-100%)	44.61% (40%-48.78%)	2.7072 (2.6204-2.7928)	54.48% (46.46%-62.63%)	28.48% (19.66%-37.94%)	99.083% (96.774%-100%)	98.17% (93.33%-100%)	44.67% (36.24%-53.85%)	2.7040 (2.5284-2.8770)
At least 7 of CHASM (breast), CHASM (lung), FATHMM (missense), Mutation Assessor, MutationTaster, PolyPhen-2, PROVEAN, SIFT	65.67% (61.62%-69.19%)	47.95% (42.85%-53.04%)	96.298% (94.203%-98.684%)	95.73% (93.22%-98.48%)	51.69% (46.97%-56.3%)	2.9167 (2.8164-3.0201)	65.63% (58.59%-73.74%)	47.68% (37.28%-58.07%)	96.392% (90.909%-100%)	95.78% (89.66%-100%)	51.78% (42.62%-61.2%)	2.9163 (2.7025-3.1178)
At least 7 of CHASM (breast), CHASM (lung), FATHMM (missense), Mutation Assessor, MutationTaster, PolyPhen-2, PROVEAN, SIFT, VEST	73.8% (70.2%-77.27%)	62.34% (57.02%-67.44%)	93.605% (90.539%-97.102%)	94.4% (91.86%-97.53%)	58.98% (53.72%-64.04%)	3.0932 (2.9770-3.2045)	73.61% (66.67%-80.81%)	61.99% (51.67%-72.31%)	93.512% (86.667%-100%)	94.25% (88.1%-100%)	58.91% (48.97%-69.24%)	3.0866 (2.8522-3.3020)
At least 7 of CHASM (breast), CHASM (lung), FATHMM (missense), Mutation Assessor, MutationTaster, PolyPhen-2, PROVEAN, VEST	63.34% (59.6%-67.17%)	44.28% (39.2%-49.61%)	96.298% (94.203%-98.684%)	95.39% (92.59%-98.36%)	49.99% (45.32%-54.48%)	2.8596 (2.7569-2.9655)	63.21% (55.56%-70.71%)	43.86% (33.33%-54.24%)	96.392% (90.909%-100%)	95.43% (88.88%-100%)	50.02% (40.84%-59.38%)	2.8570 (2.6480-3.0652)
At least 7 of CHASM (breast), CHASM (lung), FATHMM (missense), Mutation Assessor, MutationTaster, PolyPhen-2, SIFT	60.61% (56.57%-64.14%)	38.91% (33.87%-43.65%)	98.143% (97.015%-100%)	97.32% (95.45%-100%)	48.16% (43.53%-52.48%)	2.8253 (2.7357-2.9166)	60.59% (53.54%-68.69%)	38.66% (29.03%-48.49%)	98.209% (94.118%-100%)	97.39% (91.67%-100%)	48.26% (39.71%-57.89%)	2.8252 (2.6457-3.0062)
At least 7 of CHASM (breast), CHASM (lung), FATHMM (missense), Mutation Assessor, MutationTaster, PolyPhen-2, SIFT, VEST	67.39% (63.64%-71.21%)	51.71% (46.46%-56.8%)	94.49% (91.667%-97.333%)	94.19% (91.3%-97.22%)	53.09% (48.09%-57.6%)	2.9348 (2.8285-3.0505)	67.25% (59.6%-74.75%)	51.34% (41.26%-61.67%)	94.503% (88.235%-100%)	94.12% (87.1%-100%)	53.09% (43.33%-63.08%)	2.9306 (2.6986-3.1430)
At least 7 of CHASM (breast), CHASM (lung), FATHMM (missense), Mutation Assessor, MutationTaster, PolyPhen-2, VEST	56.92% (53.02%-60.61%)	33.08% (28.1%-37.91%)	98.143% (97.015%-100%)	96.86% (94.74%-100%)	45.89% (41.29%-50.32%)	2.7397 (2.6428-2.8315)	56.87% (49.49%-64.64%)	32.77% (22.95%-42.86%)	98.209% (94.118%-100%)	96.93% (90%-100%)	45.99% (37.18%-55.13%)	2.7390 (2.5541-2.9286)
At least 7 of CHASM (breast), CHASM (lung), FATHMM (missense), Mutation Assessor, MutationTaster, PROVEAN, SIFT	55.59% (51.52%-59.6%)	30.44% (25.78%-34.92%)	99.084% (98.485%-100%)	98.29% (96.97%-100%)	45.17% (40.51%-49.38%)	2.7299 (2.6432-2.8168)	55.48% (47.47%-63.64%)	30.06% (21.05%-39.66%)	99.083% (96.774%-100%)	98.26% (93.33%-100%)	45.22% (36.48%-54.43%)	2.7263 (2.5496-2.9001)
At least 7 of CHASM (breast), CHASM (lung), FATHMM (missense), Mutation Assessor, MutationTaster, PROVEAN, SIFT, VEST	63.67% (60.1%-67.18%)	44.8% (39.67%-50%)	96.298% (94.203%-98.684%)	95.44% (92.73%-98.39%)	50.22% (45.58%-54.86%)	2.8676 (2.7654-2.9725)	63.56% (56.54%-70.71%)	44.41% (34.36%-54.39%)	96.392% (90.909%-100%)	95.49% (88.46%-100%)	50.26% (40.91%-59.42%)	2.8655 (2.6538-3.0693)
At least 7 of CHASM (breast), CHASM (lung), FATHMM (missense), Mutation Assessor, MutationTaster, PROVEAN, VEST	53.9% (49.49%-57.58%)	27.77% (22.9%-32.23%)	99.084% (98.485%-100%)	98.13% (96.67%-100%)	44.24% (39.52%-48.49%)	2.6922 (2.6043-2.7788)	53.81% (46.46%-62.63%)	27.43% (18.96%-36.93%)	99.083% (96.774%-100%)	98.1% (92.86%-100%)	44.31% (35.71%-53.57%)	2.6892 (2.5121-2.8688)
At least 7 of CHASM (breast), CHASM (lung), FATHMM (missense), Mutation Assessor, MutationTaster, SIFT, VEST	57.94% (54.04%-61.62%)	34.68% (29.69%-39.84%)	98.143% (97.015%-100%)	97% (95%-100%)	46.5% (41.83%-50.98%)	2.7632 (2.6722-2.8549)	57.86% (50.51%-65.66%)	34.34% (24.24%-44.44%)	98.209% (94.118%-100%)	97.06% (90.48%-100%)	46.57% (37.72%-56.25%)	2.7618 (2.5772-2.9468)
At least 7 of CHASM (breast), CHASM (lung), FATHMM (missense), Mutation Assessor, PolyPhen-2, PROVEAN, SIFT	56.27% (52.02%-60.1%)	31.51% (26.45%-36.07%)	99.084% (98.485%-100%)	98.35% (97.06%-100%)	45.55% (40.88%-49.69%)	2.7449 (2.6572-2.8294)	56.15% (48.48%-64.65%)	31.11% (22.03%-41.27%)	99.083% (96.774%-100%)	98.31% (93.75%-100%)	45.6% (36.84%-54.88%)	2.7410 (2.5675-2.9144)
At least 7 of CHASM (breast), CHASM (lung), FATHMM (missense), Mutation Assessor, PolyPhen-2, PROVEAN, SIFT, VEST	63% (59.09%-66.67%)	43.74% (38.52%-48.82%)	96.298% (94.203%-98.684%)	95.33% (92.45%-98.33%)	49.75% (45.07%-54.29%)	2.8511 (2.7484-2.9589)	62.89% (55.56%-70.71%)	43.35% (33.33%-53.45%)	96.392% (90.909%-100%)	95.38% (88.46%-100%)	49.79% (40.62%-59.69%)	2.8490 (2.6286-3.0546)
At least 7 of CHASM (breast), CHASM (lung), FATHMM (missense), Mutation Assessor, PolyPhen-2, PROVEAN, VEST	54.24% (50%-58.08%)	28.3% (23.25%-32.54%)	99.084% (98.485%-100%)	98.16% (96.77%-100%)	44.42% (39.64%-48.7%)	2.6997 (2.6126-2.7840)	54.15% (46.46%-62.63%)	27.96% (19.4%-38.1%)	99.083% (96.774%-100%)	98.13% (92.86%-100%)	44.49% (36.14%-53.85%)	2.6966 (2.5200-2.8731)
At least 7 of CHASM (breast), CHASM (lung), FATHMM (missense), Mutation Assessor, PolyPhen-2, SIFT, VEST	57.94% (54.04%-61.62%)	34.68% (29.51%-39.52%)	98.143% (97.015%-100%)	97% (95%-100%)	46.5% (41.78%-50.96%)	2.7632 (2.6693-2.8561)	57.86% (50.51%-65.66%)	34.34% (24.59%-44.07%)	98.209% (94.118%-100%)	97.07% (90.48%-100%)	46.57% (37.97%-55.84%)	2.7619 (2.5785-2.9468)
At least 7 of CHASM (breast), CHASM (lung), FATHMM (missense), Mutation Assessor, PROVEAN, SIFT, VEST	55.26% (51.01%-59.09%)	29.9% (25%-34.17%)	99.084% (98.485%-100%)	98.26% (96.97%-100%)	44.98% (40.26%-49.37%)	2.7223 (2.6401-2.8105)	55.15% (47.47%-63.64%)	29.54% (20.63%-39.29%)	99.083% (96.774%-100%)	98.23% (93.33%-100%)	45.04% (36.36%-54.22%)	2.7188 (2.5417-2.8827)
At least 7 of CHASM (breast), CHASM (lung), FATHMM (missense), MutationTaster, PolyPhen-2, PROVEAN, SIFT	55.26% (51.01%-59.09%)	29.91% (24.99%-34.43%)	99.084% (98.485%-100%)	98.26% (96.97%-100%)	44.98% (40.13%-49.07%)	2.7223 (2.6348-2.8042)	55.14% (47.47%-63.64%)	29.53% (20.63%-39.34%)	99.083% (96.774%-100%)	98.23% (93.33%-100%)	45.03% (36.75%-54.55%)	2.7188 (2.5444-2.8965)
At least 7 of CHASM (breast), CHASM (lung), FATHMM (missense), MutationTaster, PolyPhen-2, PROVEAN, SIFT, VEST	65.36% (61.62%-69.19%)	47.46% (42.19%-52.8%)	96.298% (94.203%-98.684%)	95.68% (93.1%-98.48%)	51.46% (46.72%-56.03%)	2.9090 (2.8046-3.0120)	65.24% (57.58%-72.73%)	47.07% (36.66%-57.58%)	96.392% (90.909%-100%)	95.73% (88.89%-100%)	51.49% (42.25%-61.11%)	2.9068 (2.6972-3.1168)
At least 7 of CHASM (breast), CHASM (lung), FATHMM (missense), MutationTaster, PolyPhen-2, PROVEAN, VEST	53.91% (49.49%-57.58%)	27.79% (22.73%-32.26%)	99.084% (98.485%-100%)	98.13% (96.67%-100%)	44.24% (39.64%-48.45%)	2.6924 (2.6041-2.7769)	53.79% (46.46%-62.63%)	27.39% (18.45%-37.5%)	99.083% (96.774%-100%)	98.1% (92.86%-100%)	44.3% (36.05%-53.85%)	2.6887 (2.5154-2.8658)
At least 7 of CHASM (breast), CHASM (lung), FATHMM (missense), MutationTaster, PolyPhen-2, SIFT, VEST	57.59% (53.54%-61.62%)	34.14% (29.17%-38.93%)	98.143% (97.015%-100%)	96.95% (94.87%-100%)	46.29% (41.56%-50.63%)	2.7553 (2.6580-2.8500)	57.54% (49.49%-65.66%)	33.83% (23.73%-43.75%)	98.209% (94.118%-100%)	97.02% (90.48%-100%)	46.38% (37.66%-55.85%)	2.7544 (2.5652-2.9494)
At least 7 of CHASM (breast), CHASM (lung), FATHMM (missense), MutationTaster, PROVEAN, SIFT, VEST	55.26% (51.01%-59.09%)	29.91% (25%-34.71%)	99.084% (98.485%-100%)	98.26% (96.97%-100%)	44.98% (40.25%-49.37%)	2.7224 (2.6319-2.8039)	55.14% (47.47%-63.64%)	29.53% (20%-39.35%)	99.083% (96.774%-100%)	98.24% (93.33%-100%)	45.03% (36.47%-54.55%)	2.7188 (2.5506-2.9033)
At least 7 of CHASM (breast), CHASM (lung), FATHMM (missense), PolyPhen-2, PROVEAN, SIFT, VEST	54.92% (51.01%-58.59%)	29.37% (24.41%-33.87%)	99.084% (98.485%-100%)	98.23% (96.88%-100%)	44.79% (39.88%-49.06%)	2.7148 (2.6286-2.7977)	54.81% (47.47%-62.63%)	29% (20%-38.72%)	99.083% (96.774%-100%)	98.2% (93.33%-100%)	44.85% (36.25%-54.22%)	2.7114 (2.5435-2.8805)
At least 7 of CHASM (breast), CHASM (lung), Mutation Assessor, MutationTaster, PolyPhen-2, PROVEAN, SIFT	55.92% (51.52%-59.6%)	30.95% (26.02%-35.65%)	99.084% (98.485%-100%)	98.32% (97.06%-100%)	45.35% (40.67%-49.69%)	2.7370 (2.6492-2.8246)	55.85% (48.48%-64.65%)	30.64% (21.31%-40.62%)	99.083% (96.774%-100%)	98.29% (93.75%-100%)	45.43% (36.71%-54.67%)	2.7345 (2.5590-2.9144)
At least 7 of CHASM (breast), CHASM (lung), Mutation Assessor, MutationTaster, PolyPhen-2, PROVEAN, SIFT, VEST	64.67% (60.61%-68.69%)	46.91% (41.86%-52.18%)	95.377% (92.958%-98.529%)	94.6% (91.66%-98.36%)	50.96% (46.27%-55.48%)	2.8785 (2.7712-2.9867)	64.59% (56.57%-72.73%)	46.57% (36.07%-56.46%)	95.478% (90%-100%)	94.64% (87.5%-100%)	51.02% (41.66%-60.87%)	2.8770 (2.6589-3.0902)
At least 7 of CHASM (breast), CHASM (lung), Mutation Assessor, MutationTaster, PolyPhen-2, PROVEAN, VEST	54.22% (50%-58.08%)	28.28% (23.25%-32.8%)	99.084% (98.485%-100%)	98.16% (96.77%-100%)	44.41% (39.63%-48.75%)	2.6994 (2.6150-2.7841)	54.18% (46.46%-62.63%)	28.01% (19.04%-37.93%)	99.083% (96.774%-100%)	98.13% (92.86%-100%)	44.51% (35.8%-53.85%)	2.6973 (2.5268-2.8705)
At least 7 of CHASM (breast), CHASM (lung), Mutation Assessor, MutationTaster, PolyPhen-2, SIFT, VEST	57.58% (53.54%-61.11%)	34.66% (29.41%-39.53%)	97.222% (95.588%-100%)	95.57% (92.68%-100%)	46.25% (41.51%-50.64%)	2.7370 (2.6387-2.8394)	57.56% (50.51%-65.66%)	34.39% (24.24%-45.17%)	97.294% (92.303%-100%)	95.61% (87.5%-100%)	46.36% (37.35%-55.7%)	2.7366 (2.5188-2.9329)
At least 7 of CHASM (breast), CHASM (lung), Mutation Assessor, MutationTaster, PROVEAN, SIFT, VEST	55.24% (51.01%-59.09%)	29.88% (25.19%-34.43%)	99.084% (98.485%-100%)	98.26% (96.97%-100%)	44.97% (40.25%-49.37%)	2.7220 (2.6342-2.8090)	55.18% (47.47%-63.64%)	29.59% (20.63%-39.07%)	99.083% (96.774%-100%)	98.23% (93.33%-100%)	45.06% (36.25%-54.32%)	2.7196 (2.5468-2.9015)
At least 7 of CHASM (breast), CHASM (lung), Mutation Assessor, PolyPhen-2, PROVEAN, SIFT, VEST	55.58% (51.52%-59.6%)	30.41% (25.58%-34.92%)	99.084% (98.485%-100%)	98.29% (96.97%-100%)	45.16% (40.38%-49.38%)	2.7294 (2.6432-2.8156)	55.51% (47.47%-63.64%)	30.12% (21.05%-39.68%)	99.083% (96.774%-100%)	98.26% (93.33%-100%)	45.24% (36.71%-54.67%)	2.7270 (2.5529-2.9001)
At least 7 of CHASM (breast), CHASM (lung), MutationTaster, PolyPhen-2, PROVEAN, SIFT, VEST	56.24% (52.02%-60.1%)	31.47% (26.56%-36.22%)	99.084% (98.485%-100%)	98.34% (97.06%-100%)	45.54% (40.74%-49.69%)	2.7443 (2.6567-2.8274)	56.2% (48.48%-64.65%)	31.2% (21.88%-41.07%)	99.083% (96.774%-100%)	98.32% (93.75%-100%)	45.63% (37.14%-55.27%)	2.7424 (2.5723-2.9234)
At least 7 of CHASM (breast), CHASM (melanoma), FATHMM (cancer), FATHMM (missense), Mutation Assessor, MutationTaster, PolyPhen-2	60.29% (56.57%-64.14%)	38.92% (33.6%-43.75%)	97.233% (95.588%-100%)	96.06% (93.62%-100%)	47.94% (43.36%-52.38%)	2.8014 (2.7026-2.8986)	60.23% (52.53%-67.68%)	38.64% (29.23%-49.18%)	97.269% (92.303%-100%)	96.07% (89.47%-100%)	48.02% (39.19%-57.33%)	2.7999 (2.6026-2.9953)
At least 7 of CHASM (breast), CHASM (melanoma), FATHMM (cancer), FATHMM (missense), Mutation Assessor, MutationTaster, PolyPhen-2, PROVEAN	66.69% (63.13%-70.2%)	49.02% (43.9%-54.03%)	97.233% (95.588%-100%)	96.84% (94.92%-100%)	52.45% (47.65%-57.04%)	2.9555 (2.8628-3.0584)	66.62% (59.6%-73.74%)	48.74% (38.71%-58.93%)	97.269% (92.303%-100%)	96.86% (91.43%-100%)	52.51% (43.47%-62.32%)	2.9538 (2.7516-3.1483)
At least 7 of CHASM (breast), CHASM (melanoma), FATHMM (cancer), FATHMM (missense), Mutation Assessor, MutationTaster, PolyPhen-2, PROVEAN, SIFT	76.08% (72.73%-79.29%)	66.47% (62.02%-70.97%)	92.699% (89.333%-96%)	94.03% (91.46%-96.77%)	61.52% (56.25%-66.36%)	3.1472 (3.0415-3.2597)	76.12% (69.7%-82.83%)	66.5% (57.14%-75.42%)	92.563% (85.366%-100%)	93.9% (87.75%-100%)	61.69% (51.78%-72.13%)	3.1466 (2.9249-3.3682)
At least 7 of CHASM (breast), CHASM (melanoma), FATHMM (cancer), FATHMM (missense), Mutation Assessor, MutationTaster, PolyPhen-2, PROVEAN, SIFT, VEST	79.11% (75.76%-82.32%)	73.91% (69.77%-78.36%)	88.085% (84%-92.756%)	91.47% (88.46%-94.85%)	66.13% (60.82%-71.43%)	3.1959 (3.0751-3.3225)	79.16% (72.73%-85.86%)	73.97% (64.4%-82.76%)	88.032% (79.31%-97.06%)	91.4% (84.91%-97.83%)	66.34% (55.55%-77.08%)	3.1974 (2.9498-3.4378)
At least 7 of CHASM (breast), CHASM (melanoma), FATHMM (cancer), FATHMM (missense), Mutation Assessor, MutationTaster, PolyPhen-2, PROVEAN, VEST	74.74% (71.21%-78.28%)	64.89% (60.31%-69.6%)	91.759% (88.235%-95.775%)	93.16% (90.36%-96.51%)	60.19% (55.04%-65.18%)	3.1000 (2.9875-3.2245)	74.76% (67.68%-81.82%)	64.86% (55%-74.19%)	91.692% (84.211%-97.68%)	93.07% (86.36%-97.96%)	60.33% (50%-70.18%)	3.0995 (2.8507-3.3228)
At least 7 of CHASM (breast), CHASM (melanoma), FATHMM (cancer), FATHMM (missense), Mutation Assessor, MutationTaster, PolyPhen-2, SIFT	71.71% (68.18%-75.25%)	57.99% (52.84%-62.9%)	95.442% (92.958%-98.592%)	95.65% (93.33%-98.65%)	56.78% (52%-61.67%)	3.0586 (2.9595-3.1680)	71.72% (64.65%-78.79%)	57.94% (48.21%-68.33%)	95.351% (89.283%-100%)	95.54% (89.47%-100%)	56.92% (47.37%-66.68%)	3.0574 (2.8414-3.2667)
At least 7 of CHASM (breast), CHASM (melanoma), FATHMM (cancer), FATHMM (missense), Mutation Assessor, MutationTaster, PolyPhen-2, SIFT, VEST	77.12% (73.74%-80.3%)	69.17% (64.57%-73.65%)	90.838% (87.324%-94.668%)	92.89% (90.22%-95.88%)	63.02% (57.8%-68.04%)	3.1592 (3.0474-3.2790)	77.08% (70.71%-83.84%)	69.07% (59.37%-78.47%)	90.777% (82.5%-97.44%)	92.79% (86.54%-97.96%)	63.1% (52.83%-73.47%)	3.1574 (2.9138-3.3846)
At least 7 of CHASM (breast), CHASM (melanoma), FATHMM (cancer), FATHMM (missense), Mutation Assessor, MutationTaster, PolyPhen-2, VEST	70.68% (67.17%-74.24%)	56.89% (51.93%-61.94%)	94.502% (91.667%-97.368%)	94.71% (92.11%-97.5%)	55.91% (50.88%-60.51%)	3.0201 (2.9190-3.1313)	70.76% (63.64%-77.78%)	56.93% (46.77%-67.21%)	94.479% (88.235%-100%)	94.66% (88.37%-100%)	56.11% (46.43%-66.1%)	3.0218 (2.8027-3.2196)
At least 7 of CHASM (breast), CHASM (melanoma), FATHMM (cancer), FATHMM (missense), Mutation Assessor, MutationTaster, PROVEAN	55.26% (51.01%-59.09%)	30.44% (25.78%-34.92%)	98.173% (97.015%-100%)	96.65% (94.29%-100%)	44.94% (40.26%-49.37%)	2.7020 (2.6064-2.7962)	55.13% (47.47%-63.64%)	30.06% (21.05%-39.66%)	98.144% (94.118%-100%)	96.53% (88.89%-100%)	44.99% (36.25%-54.29%)	2.6973 (2.5072-2.8877)
At least 7 of CHASM (breast), CHASM (melanoma), FATHMM (cancer), FATHMM (missense), Mutation Assessor, MutationTaster, PROVEAN, SIFT	67.69% (64.14%-71.21%)	50.6% (45.53%-55.37%)	97.233% (95.588%-100%)	96.94% (95.08%-100%)	53.24% (48.48%-57.89%)	2.9801 (2.8858-3.0806)	67.64% (60.61%-74.75%)	50.35% (40.31%-60.66%)	97.269% (92.303%-100%)	96.96% (91.67%-100%)	53.31% (43.94%-62.69%)	2.9789 (2.7757-3.1762)
At least 7 of CHASM (breast), CHASM (melanoma), FATHMM (cancer), FATHMM (missense), Mutation Assessor, MutationTaster, PROVEAN, SIFT, VEST	74.73% (71.21%-78.28%)	64.33% (59.69%-69.3%)	92.699% (89.333%-96%)	93.84% (91.14%-96.7%)	60.05% (55.04%-65.14%)	3.1091 (2.9986-3.2251)	74.79% (67.68%-81.82%)	64.4% (54.54%-73.77%)	92.563% (85.366%-100%)	93.71% (87.5%-100%)	60.25% (50%-70.22%)	3.1093 (2.8765-3.3348)
At least 7 of CHASM (breast), CHASM (melanoma), FATHMM (cancer), FATHMM (missense), Mutation Assessor, MutationTaster, PROVEAN, VEST	65.03% (61.11%-68.69%)	46.4% (41.13%-51.56%)	97.233% (95.588%-100%)	96.67% (94.64%-100%)	51.2% (46.51%-55.88%)	2.9150 (2.8166-3.0160)	64.89% (57.58%-72.73%)	46.01% (35.29%-56.46%)	97.269% (92.303%-100%)	96.68% (90.91%-100%)	51.22% (41.89%-60.66%)	2.9117 (2.7153-3.1031)
At least 7 of CHASM (breast), CHASM (melanoma), FATHMM (cancer), FATHMM (missense), Mutation Assessor, MutationTaster, SIFT	61.31% (57.58%-65.15%)	40.53% (35.48%-45.16%)	97.233% (95.588%-100%)	96.21% (93.88%-100%)	48.6% (43.87%-53.1%)	2.8257 (2.7273-2.9222)	61.22% (53.54%-68.69%)	40.21% (30.77%-50.75%)	97.269% (92.303%-100%)	96.22% (89.66%-100%)	48.66% (39.47%-58.33%)	2.8235 (2.6280-3.0183)
At least 7 of CHASM (breast), CHASM (melanoma), FATHMM (cancer), FATHMM (missense), Mutation Assessor, MutationTaster, SIFT, VEST	71.36% (67.68%-75.25%)	57.43% (52.38%-62.5%)	95.442% (92.958%-98.592%)	95.61% (93.24%-98.65%)	56.46% (51.61%-61.21%)	3.0495 (2.9479-3.1587)	71.41% (63.64%-78.79%)	57.44% (46.97%-67.8%)	95.351% (89.283%-100%)	95.5% (89.66%-100%)	56.63% (46.88%-66.67%)	3.0493 (2.8416-3.2671)
At least 7 of CHASM (breast), CHASM (melanoma), FATHMM (cancer), FATHMM (missense), Mutation Assessor, MutationTaster, VEST	57.61% (53.54%-61.62%)	34.7% (29.84%-39.84%)	97.233% (95.588%-100%)	95.59% (92.68%-100%)	46.27% (41.67%-50.68%)	2.7380 (2.6387-2.8393)	57.5% (49.49%-65.66%)	34.32% (24.19%-44.07%)	97.269% (92.303%-100%)	95.59% (88%-100%)	46.33% (37.35%-55.72%)	2.7351 (2.5277-2.9286)
At least 7 of CHASM (breast), CHASM (melanoma), FATHMM (cancer), FATHMM (missense), Mutation Assessor, PolyPhen-2, PROVEAN	55.94% (52.02%-59.6%)	31.51% (26.45%-36.07%)	98.173% (97.015%-100%)	96.76% (94.44%-100%)	45.32% (40.62%-49.66%)	2.7176 (2.6219-2.8091)	55.8% (48.48%-63.64%)	31.11% (22.03%-41.27%)	98.144% (94.118%-100%)	96.64% (88.89%-100%)	45.36% (36.71%-54.67%)	2.7126 (2.5188-2.9015)
At least 7 of CHASM (breast), CHASM (melanoma), FATHMM (cancer), FATHMM (missense), Mutation Assessor, PolyPhen-2, PROVEAN, SIFT	67.03% (63.13%-70.71%)	50.1% (45.04%-55.2%)	96.309% (94.203%-98.702%)	95.92% (93.55%-98.55%)	52.74% (47.79%-57.25%)	2.9507 (2.8512-3.0595)	66.94% (59.6%-74.75%)	49.78% (39.68%-59.65%)	96.35% (90.625%-100%)	95.93% (89.99%-100%)	52.78% (43.66%-62.5%)	2.9484 (2.7256-3.1582)
At least 7 of CHASM (breast), CHASM (melanoma), FATHMM (cancer), FATHMM (missense), Mutation Assessor, PolyPhen-2, PROVEAN, SIFT, VEST	73.73% (70.2%-77.27%)	64.89% (60.17%-69.77%)	89.006% (84.928%-93.243%)	91.08% (87.8%-94.51%)	59.45% (54.28%-64.55%)	3.0442 (2.9252-3.1646)	73.76% (66.67%-80.81%)	64.88% (54.84%-74.19%)	88.947% (80.556%-97.14%)	90.98% (84.09%-97.67%)	59.62% (50%-69.82%)	3.0442 (2.8044-3.2825)
At least 7 of CHASM (breast), CHASM (melanoma), FATHMM (cancer), FATHMM (missense), Mutation Assessor, PolyPhen-2, PROVEAN, VEST	63.67% (59.6%-67.68%)	45.33% (40.16%-50.4%)	95.37% (92.857%-98.611%)	94.43% (91.38%-98.28%)	50.22% (45.39%-54.75%)	2.8535 (2.7456-2.9647)	63.57% (55.56%-71.72%)	44.95% (34.48%-55.23%)	95.478% (88.889%-100%)	94.5% (87.5%-100%)	50.26% (41.43%-59.73%)	2.8519 (2.6280-3.0700)
At least 7 of CHASM (breast), CHASM (melanoma), FATHMM (cancer), FATHMM (missense), Mutation Assessor, PolyPhen-2, SIFT	62.33% (58.59%-66.16%)	42.14% (36.97%-47.06%)	97.233% (95.588%-100%)	96.34% (94.12%-100%)	49.29% (44.44%-53.68%)	2.8500 (2.7524-2.9480)	62.22% (54.55%-69.7%)	41.77% (31.58%-51.67%)	97.269% (92.303%-100%)	96.35% (90.31%-100%)	49.32% (40.58%-58.91%)	2.8472 (2.6448-3.0445)
At least 7 of CHASM (breast), CHASM (melanoma), FATHMM (cancer), FATHMM (missense), Mutation Assessor, PolyPhen-2, SIFT, VEST	70.69% (67.17%-74.24%)	57.44% (52.42%-62.6%)	93.579% (90.411%-97.26%)	93.93% (91.03%-97.37%)	55.98% (50.88%-60.87%)	3.0093 (2.8982-3.1297)	70.75% (63.64%-77.78%)	57.43% (46.97%-67.61%)	93.559% (86.111%-100%)	93.89% (86.95%-100%)	56.16% (46.55%-66.1%)	3.0104 (2.7658-3.2421)
At least 7 of CHASM (breast), CHASM (melanoma), FATHMM (cancer), FATHMM (missense), Mutation Assessor, PolyPhen-2, VEST	57.27% (53.03%-61.11%)	34.69% (29.54%-39.52%)	96.293% (94.203%-98.667%)	94.19% (90.91%-97.87%)	46.03% (41.45%-50.36%)	2.7120 (2.6045-2.8168)	57.18% (49.49%-65.66%)	34.32% (24.99%-44.78%)	96.398% (91.176%-100%)	94.28% (86.36%-100%)	46.1% (37.33%-55.41%)	2.7109 (2.4960-2.9234)
At least 7 of CHASM (breast), CHASM (melanoma), FATHMM (cancer), FATHMM (missense), Mutation Assessor, PROVEAN, SIFT	56.95% (53.02%-61.11%)	33.11% (28.23%-37.8%)	98.173% (97.015%-100%)	96.91% (94.74%-100%)	45.91% (41.14%-50.32%)	2.7411 (2.6487-2.8347)	56.8% (48.48%-64.67%)	32.69% (23.43%-42.62%)	98.144% (94.118%-100%)	96.8% (89.47%-100%)	45.94% (37.18%-55.41%)	2.7357 (2.5444-2.9204)
At least 7 of CHASM (breast), CHASM (melanoma), FATHMM (cancer), FATHMM (missense), Mutation Assessor, PROVEAN, SIFT, VEST	64.69% (61.11%-68.19%)	46.4% (41.38%-51.25%)	96.309% (94.203%-98.702%)	95.61% (93.1%-98.44%)	50.96% (46.15%-55.56%)	2.8929 (2.7923-2.9989)	64.55% (57.55%-71.72%)	45.99% (35.71%-55.75%)	96.35% (90.625%-100%)	95.62% (89.18%-100%)	50.97% (41.79%-60.61%)	2.8892 (2.6764-3.0934)
At least 7 of CHASM (breast), CHASM (melanoma), FATHMM (cancer), FATHMM (missense), Mutation Assessor, PROVEAN, VEST	54.92% (51%-58.6%)	29.9% (25%-34.17%)	98.173% (97.015%-100%)	96.59% (94.28%-100%)	44.75% (40%-49.07%)	2.6942 (2.6012-2.7901)	54.8% (47.45%-62.65%)	29.54% (20.63%-39.29%)	98.144% (94.118%-100%)	96.48% (88.89%-100%)	44.8% (36.25%-54.22%)	2.6896 (2.4882-2.8770)
At least 7 of CHASM (breast), CHASM (melanoma), FATHMM (cancer), FATHMM (missense), Mutation Assessor, SIFT, VEST	58.63% (54.55%-62.63%)	36.3% (31.15%-41.27%)	97.233% (95.588%-100%)	95.78% (93.18%-100%)	46.89% (42.17%-51.33%)	2.7621 (2.6646-2.8613)	58.5% (50.51%-66.67%)	35.89% (26.15%-45.62%)	97.269% (92.303%-100%)	95.78% (88.46%-100%)	46.92% (37.97%-56.34%)	2.7586 (2.5531-2.9520)
At least 7 of CHASM (breast), CHASM (melanoma), FATHMM (cancer), FATHMM (missense), MutationTaster, PolyPhen-2, PROVEAN	54.92% (50.51%-58.59%)	29.91% (24.99%-34.43%)	98.173% (97.015%-100%)	96.59% (94.12%-100%)	44.75% (40.12%-49.04%)	2.6942 (2.5978-2.7867)	54.8% (47.47%-63.64%)	29.53% (20.63%-39.34%)	98.144% (94.118%-100%)	96.47% (88.89%-100%)	44.8% (36.36%-54.32%)	2.6895 (2.4928-2.8804)
At least 7 of CHASM (breast), CHASM (melanoma), FATHMM (cancer), FATHMM (missense), MutationTaster, PolyPhen-2, PROVEAN, SIFT	70.04% (66.16%-73.23%)	54.31% (49.22%-59.2%)	97.233% (95.588%-100%)	97.14% (95.38%-100%)	55.17% (50.37%-59.84%)	3.0386 (2.9441-3.1363)	70.02% (63.64%-77.78%)	54.12% (44.26%-63.64%)	97.269% (92.303%-100%)	97.16% (92.31%-100%)	55.26% (45.9%-65.22%)	3.0381 (2.8416-3.2436)
At least 7 of CHASM (breast), CHASM (melanoma), FATHMM (cancer), FATHMM (missense), MutationTaster, PolyPhen-2, PROVEAN, SIFT, VEST	76.09% (72.73%-79.8%)	66.48% (62.02%-70.99%)	92.699% (89.333%-96%)	94.03% (91.49%-96.78%)	61.53% (56.36%-66.67%)	3.1475 (3.0409-3.2659)	76.09% (68.69%-82.83%)	66.47% (56.72%-75.41%)	92.563% (85.366%-100%)	93.9% (87.75%-100%)	61.67% (51.72%-71.93%)	3.1460 (2.9056-3.3606)
At least 7 of CHASM (breast), CHASM (melanoma), FATHMM (cancer), FATHMM (missense), MutationTaster, PolyPhen-2, PROVEAN, VEST	67.71% (64.13%-71.21%)	50.64% (45.38%-55.81%)	97.233% (95.588%-100%)	96.94% (95.08%-100%)	53.26% (48.44%-57.78%)	2.9807 (2.8855-3.0770)	67.6% (60.61%-74.75%)	50.29% (39.69%-61.02%)	97.269% (92.303%-100%)	96.95% (91.89%-100%)	53.28% (43.75%-63.03%)	2.9780 (2.7868-3.1683)
At least 7 of CHASM (breast), CHASM (melanoma), FATHMM (cancer), FATHMM (missense), MutationTaster, PolyPhen-2, SIFT	61.31% (57.58%-65.15%)	40.53% (35.43%-45.24%)	97.233% (95.588%-100%)	96.2% (93.88%-100%)	48.6% (43.88%-53.02%)	2.8257 (2.7253-2.9236)	61.22% (53.54%-68.69%)	40.2% (30.36%-50%)	97.269% (92.303%-100%)	96.21% (89.65%-100%)	48.66% (39.7%-58.11%)	2.8234 (2.6230-3.0201)
At least 7 of CHASM (breast), CHASM (melanoma), FATHMM (cancer), FATHMM (missense), MutationTaster, PolyPhen-2, SIFT, VEST	73.72% (70.2%-77.27%)	61.15% (56.35%-66.13%)	95.442% (92.958%-98.592%)	95.87% (93.67%-98.73%)	58.69% (53.72%-63.48%)	3.1115 (3.0067-3.2152)	73.78% (66.67%-80.81%)	61.19% (50.88%-70.97%)	95.351% (89.283%-100%)	95.77% (90.24%-100%)	58.88% (49.15%-69.09%)	3.1118 (2.9042-3.3271)
At least 7 of CHASM (breast), CHASM (melanoma), FATHMM (cancer), FATHMM (missense), MutationTaster, PolyPhen-2, VEST	57.94% (53.54%-61.62%)	35.22% (30%-40%)	97.233% (95.588%-100%)	95.66% (92.86%-100%)	46.47% (41.77%-50.92%)	2.7458 (2.6458-2.8492)	57.85% (50.51%-66.67%)	34.87% (25.36%-45.16%)	97.269% (92.303%-100%)	95.66% (88%-100%)	46.53% (37.66%-56.06%)	2.7434 (2.5219-2.9468)
At least 7 of CHASM (breast), CHASM (melanoma), FATHMM (cancer), FATHMM (missense), MutationTaster, PROVEAN, SIFT	56.94% (53.03%-60.61%)	33.09% (28.33%-37.7%)	98.173% (97.015%-100%)	96.91% (94.74%-100%)	45.91% (41.14%-50.3%)	2.7408 (2.6486-2.8325)	56.82% (49.49%-64.65%)	32.73% (23.72%-42.62%)	98.144% (94.118%-100%)	96.81% (90%-100%)	45.95% (37.18%-55.41%)	2.7365 (2.5464-2.9204)
At least 7 of CHASM (breast), CHASM (melanoma), FATHMM (cancer), FATHMM (missense), MutationTaster, PROVEAN, SIFT, VEST	67.02% (63.13%-70.71%)	49.54% (43.9%-54.69%)	97.233% (95.588%-100%)	96.87% (95%-100%)	52.71% (47.79%-57.15%)	2.9635 (2.8675-3.0592)	66.97% (59.6%-74.75%)	49.3% (38.98%-60.29%)	97.269% (92.303%-100%)	96.89% (91.66%-100%)	52.79% (43.55%-62.69%)	2.9625 (2.7690-3.1602)
At least 7 of CHASM (breast), CHASM (melanoma), FATHMM (cancer), FATHMM (missense), MutationTaster, PROVEAN, VEST	54.93% (50.51%-58.6%)	29.91% (25%-34.71%)	98.173% (97.015%-100%)	96.59% (94.12%-100%)	44.75% (40%-49.07%)	2.6943 (2.5987-2.7899)	54.8% (47.45%-63.64%)	29.53% (20%-39.35%)	98.144% (94.118%-100%)	96.48% (88.89%-100%)	44.8% (36.14%-54.43%)	2.6895 (2.4874-2.8877)
At least 7 of CHASM (breast), CHASM (melanoma), FATHMM (cancer), FATHMM (missense), MutationTaster, SIFT, VEST	58.97% (55.05%-62.63%)	36.83% (31.74%-41.94%)	97.233% (95.588%-100%)	95.84% (93.18%-100%)	47.1% (42.57%-51.63%)	2.7700 (2.6759-2.8700)	58.84% (51.52%-66.67%)	36.43% (26.23%-46.38%)	97.269% (92.303%-100%)	95.84% (88.88%-100%)	47.14% (38.03%-56.41%)	2.7667 (2.5540-2.9606)
At least 7 of CHASM (breast), CHASM (melanoma), FATHMM (cancer), FATHMM (missense), PolyPhen-2, PROVEAN, SIFT	56.95% (53.03%-60.61%)	33.11% (28.12%-37.88%)	98.173% (97.015%-100%)	96.91% (94.74%-100%)	45.91% (41.14%-50.32%)	2.7410 (2.6456-2.8331)	56.8% (49.49%-64.65%)	32.69% (22.95%-42.86%)	98.144% (94.118%-100%)	96.8% (89.47%-100%)	45.93% (37.5%-55.56%)	2.7357 (2.5483-2.9262)
At least 7 of CHASM (breast), CHASM (melanoma), FATHMM (cancer), FATHMM (missense), PolyPhen-2, PROVEAN, SIFT, VEST	66.7% (63.13%-70.2%)	49.56% (44.16%-54.4%)	96.309% (94.203%-98.702%)	95.88% (93.55%-98.53%)	52.48% (47.45%-57.03%)	2.9423 (2.8416-3.0459)	66.61% (59.6%-73.74%)	49.25% (39.29%-60%)	96.35% (90.625%-100%)	95.9% (90%-100%)	52.52% (43.48%-62.32%)	2.9402 (2.7297-3.1428)
At least 7 of CHASM (breast), CHASM (melanoma), FATHMM (cancer), FATHMM (missense), PolyPhen-2, PROVEAN, VEST	54.58% (50.51%-58.08%)	29.37% (24.41%-33.87%)	98.173% (97.015%-100%)	96.53% (94.12%-100%)	44.56% (39.87%-48.75%)	2.6864 (2.5893-2.7776)	54.47% (47.47%-62.63%)	29% (20%-38.72%)	98.144% (94.118%-100%)	96.41% (88.24%-100%)	44.62% (36.25%-54.22%)	2.6818 (2.4891-2.8732)
At least 7 of CHASM (breast), CHASM (melanoma), FATHMM (cancer), FATHMM (missense), PolyPhen-2, SIFT, VEST	58.29% (54.04%-62.12%)	35.76% (30.65%-40.8%)	97.233% (95.588%-100%)	95.72% (93.02%-100%)	46.68% (41.83%-50.98%)	2.7539 (2.6513-2.8549)	58.17% (50.51%-66.67%)	35.38% (25.4%-45.32%)	97.269% (92.303%-100%)	95.72% (88.23%-100%)	46.73% (38.16%-56.34%)	2.7510 (2.5440-2.9524)
At least 7 of CHASM (breast), CHASM (melanoma), FATHMM (cancer), FATHMM (missense), PROVEAN, SIFT, VEST	56.28% (52.02%-60.1%)	32.05% (27.13%-36.72%)	98.173% (97.015%-100%)	96.81% (94.59%-100%)	45.52% (40.88%-49.87%)	2.7255 (2.6318-2.8196)	56.13% (48.48%-64.65%)	31.63% (22.22%-41.54%)	98.144% (94.118%-100%)	96.71% (89.47%-100%)	45.55% (37.03%-55.13%)	2.7203 (2.5283-2.9097)
At least 7 of CHASM (breast), CHASM (melanoma), FATHMM (cancer), Mutation Assessor, MutationTaster, PolyPhen-2, PROVEAN	54.9% (50.51%-58.59%)	29.87% (25%-34.4%)	98.173% (97.015%-100%)	96.58% (94.12%-100%)	44.74% (40%-49.06%)	2.6936 (2.5978-2.7866)	54.85% (47.47%-63.64%)	29.61% (20.63%-39.66%)	98.144% (94.118%-100%)	96.48% (88.89%-100%)	44.83% (36.25%-54.22%)	2.6906 (2.5045-2.8770)
At least 7 of CHASM (breast), CHASM (melanoma), FATHMM (cancer), Mutation Assessor, MutationTaster, PolyPhen-2, PROVEAN, SIFT	69.06% (65.64%-72.23%)	52.75% (47.62%-58.14%)	97.233% (95.588%-100%)	97.06% (95.24%-100%)	54.34% (49.25%-58.92%)	3.0139 (2.9207-3.1118)	68.96% (62.6%-75.78%)	52.44% (42.42%-62.91%)	97.269% (92.303%-100%)	97.07% (92.11%-100%)	54.38% (45%-64.62%)	3.0115 (2.8166-3.2095)
At least 7 of CHASM (breast), CHASM (melanoma), FATHMM (cancer), Mutation Assessor, MutationTaster, PolyPhen-2, PROVEAN, SIFT, VEST	75.05% (71.72%-78.79%)	65.37% (60.48%-70.31%)	91.778% (88.406%-95.714%)	93.22% (90.59%-96.47%)	60.52% (55.45%-65.74%)	3.1089 (2.9935-3.2277)	75.15% (67.68%-81.82%)	65.51% (55.55%-75.41%)	91.649% (84.371%-97.56%)	93.09% (86.49%-97.92%)	60.77% (50%-70.91%)	3.1102 (2.8736-3.3381)
At least 7 of CHASM (breast), CHASM (melanoma), FATHMM (cancer), Mutation Assessor, MutationTaster, PolyPhen-2, PROVEAN, VEST	66.71% (63.13%-70.2%)	49.06% (43.85%-54.27%)	97.233% (95.588%-100%)	96.85% (94.92%-100%)	52.47% (47.73%-56.84%)	2.9561 (2.8607-3.0553)	66.57% (59.6%-73.74%)	48.66% (39.09%-58.83%)	97.269% (92.303%-100%)	96.85% (91.43%-100%)	52.48% (43.47%-62.32%)	2.9526 (2.7509-3.1543)
At least 7 of CHASM (breast), CHASM (melanoma), FATHMM (cancer), Mutation Assessor, MutationTaster, PolyPhen-2, SIFT	60.61% (56.57%-64.65%)	39.43% (34.4%-44.26%)	97.233% (95.588%-100%)	96.1% (93.62%-100%)	48.14% (43.51%-52.67%)	2.8090 (2.7099-2.9058)	60.6% (52.53%-68.69%)	39.22% (29.21%-49.21%)	97.269% (92.303%-100%)	96.12% (89.47%-100%)	48.26% (39.43%-57.53%)	2.8087 (2.6062-3.0053)
At least 7 of CHASM (breast), CHASM (melanoma), FATHMM (cancer), Mutation Assessor, MutationTaster, PolyPhen-2, SIFT, VEST	72.71% (69.19%-76.26%)	60.1% (55.04%-65.39%)	94.52% (91.667%-97.368%)	94.99% (92.41%-97.65%)	57.81% (52.68%-62.71%)	3.0741 (2.9638-3.1867)	72.76% (65.66%-79.8%)	60.1% (49.98%-70%)	94.436% (88.232%-100%)	94.89% (88.64%-100%)	57.98% (48.27%-68.34%)	3.0740 (2.8469-3.2997)
At least 7 of CHASM (breast), CHASM (melanoma), FATHMM (cancer), Mutation Assessor, MutationTaster, PolyPhen-2, VEST	56.91% (52.53%-60.62%)	33.59% (28.45%-38.4%)	97.233% (95.588%-100%)	95.46% (92.5%-100%)	45.85% (41.22%-50.31%)	2.7214 (2.6199-2.8242)	56.88% (49.47%-65.66%)	33.34% (23.53%-43.56%)	97.269% (92.303%-100%)	95.47% (87.5%-100%)	45.96% (37.33%-55.41%)	2.7203 (2.5065-2.9233)
At least 7 of CHASM (breast), CHASM (melanoma), FATHMM (cancer), Mutation Assessor, MutationTaster, PROVEAN, SIFT	55.92% (51.52%-59.6%)	31.48% (26.72%-36%)	98.173% (97.015%-100%)	96.75% (94.44%-100%)	45.31% (40.62%-49.69%)	2.7171 (2.6219-2.8085)	55.84% (48.48%-64.65%)	31.19% (22.06%-40.99%)	98.144% (94.118%-100%)	96.65% (89.47%-100%)	45.39% (36.49%-54.8%)	2.7137 (2.5268-2.9058)
At least 7 of CHASM (breast), CHASM (melanoma), FATHMM (cancer), Mutation Assessor, MutationTaster, PROVEAN, SIFT, VEST	67.39% (63.64%-70.71%)	50.13% (44.88%-55.09%)	97.233% (95.588%-100%)	96.91% (95%-100%)	53% (48.12%-57.58%)	2.9727 (2.8746-3.0683)	67.23% (60.61%-74.75%)	49.71% (39.39%-59.71%)	97.269% (92.303%-100%)	96.92% (91.66%-100%)	52.99% (43.55%-62.5%)	2.9688 (2.7769-3.1616)
At least 7 of CHASM (breast), CHASM (melanoma), FATHMM (cancer), Mutation Assessor, MutationTaster, PROVEAN, VEST	54.22% (50%-58.08%)	28.8% (24%-33.33%)	98.173% (97.015%-100%)	96.46% (93.94%-100%)	44.37% (39.63%-48.75%)	2.6781 (2.5835-2.7729)	54.18% (46.46%-62.63%)	28.55% (19.64%-38.46%)	98.144% (94.118%-100%)	96.35% (88.24%-100%)	44.46% (35.8%-53.85%)	2.6751 (2.4808-2.8688)
At least 7 of CHASM (breast), CHASM (melanoma), FATHMM (cancer), Mutation Assessor, SIFT, VEST	57.93% (54.04%-61.63%)	35.2% (30.08%-40.31%)	97.233% (95.588%-100%)	95.65% (92.86%-100%)	46.46% (41.72%-50.97%)	2.7455 (2.6482-2.8448)	57.87% (50.48%-65.66%)	34.9% (25%-45.32%)	97.269% (92.303%-100%)	95.66% (88.24%-100%)	46.55% (37.5%-56.16%)	2.7438 (2.5388-2.9401)
At least 7 of CHASM (breast), CHASM (melanoma), FATHMM (cancer), Mutation Assessor, PolyPhen-2, PROVEAN, SIFT	56.59% (52.53%-60.61%)	32.54% (27.59%-37.01%)	98.173% (97.015%-100%)	96.85% (94.59%-100%)	45.7% (41.13%-50%)	2.7327 (2.6393-2.8271)	56.51% (48.48%-64.65%)	32.24% (22.95%-42.37%)	98.144% (94.118%-100%)	96.76% (89.99%-100%)	45.77% (37.18%-55.01%)	2.7290 (2.5430-2.9162)
At least 7 of CHASM (breast), CHASM (melanoma), FATHMM (cancer), Mutation Assessor, PolyPhen-2, PROVEAN, SIFT, VEST	66.38% (62.63%-70.2%)	49.06% (44.09%-54.27%)	96.309% (94.203%-98.702%)	95.84% (93.44%-98.51%)	52.23% (47.37%-56.82%)	2.9345 (2.8292-3.0415)	66.23% (58.59%-73.74%)	48.65% (38.1%-58.73%)	96.35% (90.625%-100%)	95.84% (89.66%-100%)	52.23% (43.28%-62.4%)	2.9306 (2.7166-3.1409)
At least 7 of CHASM (breast), CHASM (melanoma), FATHMM (cancer), Mutation Assessor, PolyPhen-2, PROVEAN, VEST	54.56% (50.51%-58.59%)	29.33% (24.39%-33.6%)	98.173% (97.015%-100%)	96.52% (93.94%-100%)	44.55% (39.88%-48.77%)	2.6858 (2.5909-2.7820)	54.52% (46.46%-62.63%)	29.08% (20%-38.98%)	98.144% (94.118%-100%)	96.42% (88.89%-100%)	44.64% (36.14%-54.22%)	2.6828 (2.4891-2.8689)
At least 7 of CHASM (breast), CHASM (melanoma), FATHMM (cancer), Mutation Assessor, PolyPhen-2, SIFT, VEST	57.93% (54.04%-61.62%)	35.2% (30.08%-39.85%)	97.233% (95.588%-100%)	95.66% (92.86%-100%)	46.46% (41.77%-50.98%)	2.7455 (2.6468-2.8431)	57.87% (50.51%-65.66%)	34.9% (25.36%-45.31%)	97.269% (92.303%-100%)	95.67% (88%-100%)	46.55% (37.8%-55.89%)	2.7439 (2.5388-2.9462)
At least 7 of CHASM (breast), CHASM (melanoma), FATHMM (cancer), Mutation Assessor, PROVEAN, SIFT, VEST	55.58% (51.52%-59.6%)	30.94% (26.19%-35.48%)	98.173% (97.015%-100%)	96.7% (94.44%-100%)	45.12% (40.38%-49.38%)	2.7093 (2.6162-2.8036)	55.51% (47.47%-63.64%)	30.66% (21.43%-40.36%)	98.144% (94.118%-100%)	96.6% (88.89%-100%)	45.2% (36.47%-54.55%)	2.7060 (2.5100-2.8925)
At least 7 of CHASM (breast), CHASM (melanoma), FATHMM (cancer), MutationTaster, PolyPhen-2, PROVEAN, SIFT	56.92% (52.53%-60.61%)	33.06% (27.96%-37.88%)	98.173% (97.015%-100%)	96.9% (94.74%-100%)	45.89% (41.14%-50%)	2.7403 (2.6428-2.8315)	56.87% (49.49%-65.66%)	32.8% (23.43%-42.86%)	98.144% (94.118%-100%)	96.81% (90%-100%)	45.98% (37.5%-55.56%)	2.7373 (2.5458-2.9329)
At least 7 of CHASM (breast), CHASM (melanoma), FATHMM (cancer), MutationTaster, PolyPhen-2, PROVEAN, SIFT, VEST	67.72% (64.14%-71.21%)	50.64% (45.45%-55.81%)	97.233% (95.588%-100%)	96.94% (95.08%-100%)	53.26% (48.48%-57.69%)	2.9807 (2.8777-3.0742)	67.6% (60.61%-74.75%)	50.29% (39.99%-60.34%)	97.269% (92.303%-100%)	96.95% (91.67%-100%)	53.28% (44.07%-63.02%)	2.9778 (2.7831-3.1862)
At least 7 of CHASM (breast), CHASM (melanoma), FATHMM (cancer), MutationTaster, PolyPhen-2, PROVEAN, VEST	55.23% (51.01%-59.09%)	30.39% (25.4%-35%)	98.173% (97.015%-100%)	96.64% (94.29%-100%)	44.92% (40.13%-49.07%)	2.7012 (2.6037-2.7902)	55.2% (47.47%-63.64%)	30.17% (20.97%-40.32%)	98.144% (94.118%-100%)	96.54% (88.89%-100%)	45.03% (36.58%-54.67%)	2.6988 (2.5070-2.8965)
At least 7 of CHASM (breast), CHASM (melanoma), FATHMM (cancer), MutationTaster, PolyPhen-2, SIFT, VEST	58.93% (55.05%-62.63%)	36.78% (31.71%-41.86%)	97.233% (95.588%-100%)	95.83% (93.18%-100%)	47.08% (42.36%-51.3%)	2.7692 (2.6675-2.8746)	58.91% (51.52%-66.67%)	36.54% (26.56%-46.67%)	97.269% (92.303%-100%)	95.84% (88.46%-100%)	47.18% (38.57%-56.94%)	2.7684 (2.5507-2.9705)
At least 7 of CHASM (breast), CHASM (melanoma), FATHMM (cancer), MutationTaster, PROVEAN, SIFT, VEST	56.92% (52.53%-60.61%)	33.06% (28.33%-37.82%)	98.173% (97.015%-100%)	96.9% (94.74%-100%)	45.89% (41.14%-50.31%)	2.7403 (2.6428-2.8331)	56.86% (49.49%-65.66%)	32.8% (23.21%-42.43%)	98.144% (94.118%-100%)	96.81% (89.99%-100%)	45.98% (37.18%-55.56%)	2.7373 (2.5445-2.9331)
At least 7 of CHASM (breast), CHASM (melanoma), FATHMM (cancer), PolyPhen-2, PROVEAN, SIFT, VEST	56.58% (52.53%-60.11%)	32.52% (27.56%-37.12%)	98.173% (97.015%-100%)	96.85% (94.59%-100%)	45.7% (40.88%-50%)	2.7324 (2.6354-2.8252)	56.54% (49.49%-64.65%)	32.27% (22.8%-42.03%)	98.144% (94.118%-100%)	96.76% (89.47%-100%)	45.78% (37.18%-55.41%)	2.7296 (2.5435-2.9262)
At least 7 of CHASM (breast), CHASM (melanoma), FATHMM (missense), Mutation Assessor, MutationTaster, PolyPhen-2, PROVEAN	54.24% (50%-58.08%)	28.84% (24.16%-33.33%)	98.173% (97.015%-100%)	96.46% (93.94%-100%)	44.38% (39.73%-48.75%)	2.6785 (2.5807-2.7732)	54.14% (46.46%-62.63%)	28.48% (19.66%-37.94%)	98.144% (94.118%-100%)	96.35% (88.24%-100%)	44.44% (35.8%-53.85%)	2.6741 (2.4800-2.8688)
At least 7 of CHASM (breast), CHASM (melanoma), FATHMM (missense), Mutation Assessor, MutationTaster, PolyPhen-2, PROVEAN, SIFT	67.01% (63.62%-70.71%)	49.52% (44.44%-54.62%)	97.233% (95.588%-100%)	96.87% (94.92%-100%)	52.7% (47.83%-57.14%)	2.9633 (2.8701-3.0663)	66.99% (59.6%-73.76%)	49.32% (38.88%-59.38%)	97.269% (92.303%-100%)	96.9% (91.43%-100%)	52.79% (43.66%-62.5%)	2.9628 (2.7569-3.1592)
At least 7 of CHASM (breast), CHASM (melanoma), FATHMM (missense), Mutation Assessor, MutationTaster, PolyPhen-2, PROVEAN, SIFT, VEST	74.73% (71.21%-78.28%)	64.87% (60%-69.57%)	91.778% (88.406%-95.714%)	93.17% (90.48%-96.43%)	60.17% (55.14%-65.18%)	3.0999 (2.9863-3.2179)	74.78% (67.68%-81.82%)	64.91% (55.38%-74.6%)	91.649% (84.371%-97.56%)	93.04% (86.49%-97.92%)	60.36% (50%-70.37%)	3.0996 (2.8681-3.3286)
At least 7 of CHASM (breast), CHASM (melanoma), FATHMM (missense), Mutation Assessor, MutationTaster, PolyPhen-2, PROVEAN, VEST	64.67% (60.61%-68.19%)	45.83% (40.77%-51.18%)	97.233% (95.588%-100%)	96.63% (94.55%-100%)	50.94% (46.15%-55.56%)	2.9063 (2.8113-3.0090)	64.6% (57.55%-72.73%)	45.55% (35%-55.74%)	97.269% (92.303%-100%)	96.65% (90.9%-100%)	51.01% (41.78%-60.56%)	2.9047 (2.6973-3.1027)
At least 7 of CHASM (breast), CHASM (melanoma), FATHMM (missense), Mutation Assessor, MutationTaster, PolyPhen-2, SIFT	60.28% (56.55%-64.14%)	38.91% (33.87%-43.65%)	97.233% (95.588%-100%)	96.05% (93.63%-100%)	47.93% (43.26%-52.38%)	2.8012 (2.7026-2.8993)	60.25% (52.53%-67.7%)	38.66% (29.03%-48.49%)	97.269% (92.303%-100%)	96.07% (89.28%-100%)	48.03% (39.19%-57.69%)	2.8003 (2.5996-2.9953)
At least 7 of CHASM (breast), CHASM (melanoma), FATHMM (missense), Mutation Assessor, MutationTaster, PolyPhen-2, SIFT, VEST	71.02% (67.17%-74.75%)	57.42% (52.13%-62.4%)	94.52% (91.667%-97.368%)	94.77% (92%-97.5%)	56.21% (51.28%-60.98%)	3.0292 (2.9209-3.1446)	71.1% (63.64%-78.79%)	57.48% (47.46%-67.81%)	94.436% (88.232%-100%)	94.67% (88.57%-100%)	56.42% (46.67%-66.67%)	3.0300 (2.8027-3.2457)
At least 7 of CHASM (breast), CHASM (melanoma), FATHMM (missense), Mutation Assessor, MutationTaster, PolyPhen-2, VEST	56.59% (52.53%-60.61%)	33.08% (28.1%-37.91%)	97.233% (95.588%-100%)	95.39% (92.31%-100%)	45.66% (41.06%-50%)	2.7136 (2.6102-2.8150)	56.52% (48.48%-64.65%)	32.77% (22.95%-42.86%)	97.269% (92.303%-100%)	95.4% (87.5%-100%)	45.75% (37.04%-54.88%)	2.7119 (2.5027-2.9162)
At least 7 of CHASM (breast), CHASM (melanoma), FATHMM (missense), Mutation Assessor, MutationTaster, PROVEAN, SIFT	55.26% (51.01%-59.09%)	30.44% (25.78%-34.92%)	98.173% (97.015%-100%)	96.65% (94.29%-100%)	44.94% (40.26%-49.37%)	2.7020 (2.6064-2.7962)	55.13% (47.47%-63.64%)	30.06% (21.05%-39.66%)	98.144% (94.118%-100%)	96.53% (88.89%-100%)	44.99% (36.25%-54.29%)	2.6973 (2.5072-2.8877)
At least 7 of CHASM (breast), CHASM (melanoma), FATHMM (missense), Mutation Assessor, MutationTaster, PROVEAN, SIFT, VEST	65.35% (61.62%-69.19%)	46.9% (41.54%-52%)	97.233% (95.588%-100%)	96.71% (94.64%-100%)	51.43% (46.62%-56.03%)	2.9227 (2.8248-3.0209)	65.26% (57.58%-72.73%)	46.59% (36.2%-56.72%)	97.269% (92.303%-100%)	96.72% (91.17%-100%)	51.48% (42.25%-60.87%)	2.9206 (2.7235-3.1181)
At least 7 of CHASM (breast), CHASM (melanoma), FATHMM (missense), Mutation Assessor, MutationTaster, PROVEAN, VEST	53.57% (49.49%-57.58%)	27.77% (22.9%-32.23%)	98.173% (97.015%-100%)	96.34% (93.75%-100%)	44.01% (39.37%-48.41%)	2.6629 (2.5695-2.7588)	53.47% (45.45%-61.62%)	27.43% (18.96%-36.93%)	98.144% (94.118%-100%)	96.21% (88.24%-100%)	44.07% (35.44%-53.57%)	2.6586 (2.4640-2.8458)
At least 7 of CHASM (breast), CHASM (melanoma), FATHMM (missense), Mutation Assessor, MutationTaster, SIFT, VEST	57.61% (53.54%-61.62%)	34.68% (29.69%-39.84%)	97.233% (95.588%-100%)	95.59% (92.86%-100%)	46.26% (41.67%-50.67%)	2.7377 (2.6387-2.8372)	57.52% (49.49%-65.66%)	34.34% (24.24%-44.44%)	97.269% (92.303%-100%)	95.59% (88%-100%)	46.33% (37.5%-55.89%)	2.7354 (2.5278-2.9268)
At least 7 of CHASM (breast), CHASM (melanoma), FATHMM (missense), Mutation Assessor, PolyPhen-2, PROVEAN, SIFT	55.94% (52.02%-59.6%)	31.51% (26.45%-36.07%)	98.173% (97.015%-100%)	96.76% (94.44%-100%)	45.32% (40.62%-49.66%)	2.7176 (2.6219-2.8091)	55.8% (48.48%-63.64%)	31.11% (22.03%-41.27%)	98.144% (94.118%-100%)	96.64% (88.89%-100%)	45.36% (36.71%-54.67%)	2.7126 (2.5188-2.9015)
At least 7 of CHASM (breast), CHASM (melanoma), FATHMM (missense), Mutation Assessor, PolyPhen-2, PROVEAN, SIFT, VEST	64.33% (60.61%-68.18%)	45.83% (40.8%-50.82%)	96.309% (94.203%-98.702%)	95.56% (92.98%-98.41%)	50.7% (45.8%-55.25%)	2.8840 (2.7805-2.9887)	64.26% (56.57%-71.72%)	45.53% (35.59%-55.38%)	96.35% (90.625%-100%)	95.58% (88.89%-100%)	50.76% (41.54%-60.29%)	2.8821 (2.6562-3.0863)
At least 7 of CHASM (breast), CHASM (melanoma), FATHMM (missense), Mutation Assessor, PolyPhen-2, PROVEAN, VEST	53.91% (49.99%-57.58%)	28.3% (23.25%-32.54%)	98.173% (97.015%-100%)	96.4% (93.75%-100%)	44.19% (39.39%-48.45%)	2.6707 (2.5747-2.7652)	53.81% (46.46%-61.64%)	27.96% (19.4%-38.1%)	98.144% (94.118%-100%)	96.28% (88.24%-100%)	44.25% (35.8%-53.66%)	2.6664 (2.4711-2.8542)
At least 7 of CHASM (breast), CHASM (melanoma), FATHMM (missense), Mutation Assessor, PolyPhen-2, SIFT, VEST	57.6% (53.54%-61.11%)	34.68% (29.51%-39.52%)	97.233% (95.588%-100%)	95.59% (92.86%-100%)	46.26% (41.56%-50.66%)	2.7377 (2.6415-2.8372)	57.52% (50.51%-65.66%)	34.34% (24.59%-44.07%)	97.269% (92.303%-100%)	95.6% (87.5%-100%)	46.33% (37.66%-55.66%)	2.7354 (2.5292-2.9286)
At least 7 of CHASM (breast), CHASM (melanoma), FATHMM (missense), Mutation Assessor, PROVEAN, SIFT, VEST	54.92% (51%-58.6%)	29.9% (25%-34.17%)	98.173% (97.015%-100%)	96.59% (94.28%-100%)	44.75% (40%-49.07%)	2.6942 (2.6012-2.7901)	54.8% (47.45%-62.65%)	29.54% (20.63%-39.29%)	98.144% (94.118%-100%)	96.48% (88.89%-100%)	44.8% (36.25%-54.22%)	2.6896 (2.4882-2.8770)
At least 7 of CHASM (breast), CHASM (melanoma), FATHMM (missense), MutationTaster, PolyPhen-2, PROVEAN, SIFT	54.92% (50.51%-58.59%)	29.91% (24.99%-34.43%)	98.173% (97.015%-100%)	96.59% (94.12%-100%)	44.75% (40.12%-49.04%)	2.6942 (2.5978-2.7867)	54.8% (47.47%-63.64%)	29.53% (20.63%-39.34%)	98.144% (94.118%-100%)	96.47% (88.89%-100%)	44.8% (36.36%-54.32%)	2.6895 (2.4928-2.8804)
At least 7 of CHASM (breast), CHASM (melanoma), FATHMM (missense), MutationTaster, PolyPhen-2, PROVEAN, SIFT, VEST	67.01% (63.13%-70.71%)	49.53% (44.07%-54.78%)	97.233% (95.588%-100%)	96.87% (95%-100%)	52.7% (47.79%-57.25%)	2.9634 (2.8682-3.0574)	66.99% (59.6%-74.75%)	49.31% (38.98%-60.29%)	97.269% (92.303%-100%)	96.9% (91.67%-100%)	52.8% (43.55%-62.3%)	2.9628 (2.7701-3.1616)
At least 7 of CHASM (breast), CHASM (melanoma), FATHMM (missense), MutationTaster, PolyPhen-2, PROVEAN, VEST	53.23% (48.99%-57.07%)	27.24% (22.22%-31.67%)	98.173% (97.015%-100%)	96.27% (93.55%-100%)	43.83% (39.16%-48.15%)	2.6550 (2.5570-2.7531)	53.13% (45.45%-61.62%)	26.9% (17.86%-36.84%)	98.144% (94.118%-100%)	96.14% (87.5%-100%)	43.89% (35.37%-53.57%)	2.6507 (2.4524-2.8441)
At least 7 of CHASM (breast), CHASM (melanoma), FATHMM (missense), MutationTaster, PolyPhen-2, SIFT, VEST	57.26% (53.03%-61.11%)	34.14% (29.17%-38.93%)	97.233% (95.588%-100%)	95.53% (92.68%-100%)	46.06% (41.56%-50.33%)	2.7296 (2.6279-2.8325)	57.2% (49.49%-65.66%)	33.83% (23.73%-43.75%)	97.269% (92.303%-100%)	95.53% (87.5%-100%)	46.14% (37.5%-55.56%)	2.7277 (2.5105-2.9330)
At least 7 of CHASM (breast), CHASM (melanoma), FATHMM (missense), MutationTaster, PROVEAN, SIFT, VEST	54.93% (50.51%-58.6%)	29.91% (25%-34.71%)	98.173% (97.015%-100%)	96.59% (94.12%-100%)	44.75% (40%-49.07%)	2.6943 (2.5987-2.7899)	54.8% (47.45%-63.64%)	29.53% (20%-39.35%)	98.144% (94.118%-100%)	96.48% (88.89%-100%)	44.8% (36.14%-54.43%)	2.6895 (2.4874-2.8877)
At least 7 of CHASM (breast), CHASM (melanoma), FATHMM (missense), PolyPhen-2, PROVEAN, SIFT, VEST	54.58% (50.51%-58.08%)	29.37% (24.41%-33.87%)	98.173% (97.015%-100%)	96.53% (94.12%-100%)	44.56% (39.87%-48.75%)	2.6864 (2.5893-2.7776)	54.47% (47.47%-62.63%)	29% (20%-38.72%)	98.144% (94.118%-100%)	96.41% (88.24%-100%)	44.62% (36.25%-54.22%)	2.6818 (2.4891-2.8732)
At least 7 of CHASM (breast), CHASM (melanoma), Mutation Assessor, MutationTaster, PolyPhen-2, PROVEAN, SIFT	54.9% (50.51%-58.59%)	29.87% (25%-34.4%)	98.173% (97.015%-100%)	96.58% (94.12%-100%)	44.74% (40%-49.06%)	2.6936 (2.5978-2.7866)	54.85% (47.47%-63.64%)	29.61% (20.63%-39.66%)	98.144% (94.118%-100%)	96.48% (88.89%-100%)	44.83% (36.25%-54.22%)	2.6906 (2.5045-2.8770)
At least 7 of CHASM (breast), CHASM (melanoma), Mutation Assessor, MutationTaster, PolyPhen-2, PROVEAN, SIFT, VEST	66.7% (63.13%-70.2%)	49.56% (44.54%-54.69%)	96.311% (94.286%-98.667%)	95.88% (93.44%-98.53%)	52.48% (47.73%-57.04%)	2.9423 (2.8410-3.0448)	66.61% (59.6%-73.74%)	49.24% (38.46%-58.94%)	96.355% (91.176%-100%)	95.88% (89.66%-100%)	52.52% (43.33%-62.12%)	2.9400 (2.7395-3.1420)
At least 7 of CHASM (breast), CHASM (melanoma), Mutation Assessor, MutationTaster, PolyPhen-2, PROVEAN, VEST	53.21% (48.99%-57.07%)	27.2% (22.22%-31.75%)	98.173% (97.015%-100%)	96.26% (93.55%-100%)	43.82% (39.13%-48.13%)	2.6545 (2.5570-2.7450)	53.18% (45.45%-61.62%)	26.97% (17.91%-37.29%)	98.144% (94.118%-100%)	96.14% (88.24%-100%)	43.92% (35.44%-53.17%)	2.6518 (2.4560-2.8453)
At least 7 of CHASM (breast), CHASM (melanoma), Mutation Assessor, MutationTaster, PolyPhen-2, SIFT, VEST	56.57% (52.53%-60.61%)	33.58% (28.57%-38.4%)	96.311% (94.286%-98.667%)	94.03% (90.48%-97.87%)	45.61% (41.02%-50%)	2.6953 (2.5890-2.8034)	56.56% (48.48%-64.65%)	33.36% (23.72%-43.55%)	96.355% (91.176%-100%)	94.02% (85%-100%)	45.73% (36.99%-55.13%)	2.6946 (2.4751-2.9033)
At least 7 of CHASM (breast), CHASM (melanoma), Mutation Assessor, MutationTaster, PROVEAN, SIFT, VEST	54.22% (50%-58.08%)	28.8% (24%-33.33%)	98.173% (97.015%-100%)	96.46% (93.94%-100%)	44.37% (39.63%-48.75%)	2.6781 (2.5835-2.7729)	54.18% (46.46%-62.63%)	28.55% (19.64%-38.46%)	98.144% (94.118%-100%)	96.35% (88.24%-100%)	44.46% (35.8%-53.85%)	2.6751 (2.4808-2.8688)
At least 7 of CHASM (breast), CHASM (melanoma), Mutation Assessor, PolyPhen-2, PROVEAN, SIFT, VEST	54.56% (50.51%-58.59%)	29.33% (24.39%-33.6%)	98.173% (97.015%-100%)	96.52% (93.94%-100%)	44.55% (39.88%-48.77%)	2.6858 (2.5909-2.7820)	54.52% (46.46%-62.63%)	29.08% (20%-38.98%)	98.144% (94.118%-100%)	96.42% (88.89%-100%)	44.64% (36.14%-54.22%)	2.6828 (2.4891-2.8689)
At least 7 of CHASM (breast), CHASM (melanoma), MutationTaster, PolyPhen-2, PROVEAN, SIFT, VEST	55.23% (51.01%-59.09%)	30.39% (25.4%-35%)	98.173% (97.015%-100%)	96.64% (94.29%-100%)	44.92% (40.13%-49.07%)	2.7012 (2.6037-2.7902)	55.2% (47.47%-63.64%)	30.17% (20.97%-40.32%)	98.144% (94.118%-100%)	96.54% (88.89%-100%)	45.03% (36.58%-54.67%)	2.6988 (2.5070-2.8965)
At least 7 of CHASM (breast), FATHMM (cancer), FATHMM (missense), Mutation Assessor, MutationTaster, PolyPhen-2, PROVEAN	54.24% (50%-58.08%)	28.84% (24.16%-33.33%)	98.173% (97.015%-100%)	96.46% (93.94%-100%)	44.38% (39.73%-48.75%)	2.6785 (2.5807-2.7732)	54.14% (46.46%-62.63%)	28.48% (19.66%-37.94%)	98.144% (94.118%-100%)	96.35% (88.24%-100%)	44.44% (35.8%-53.85%)	2.6741 (2.4800-2.8688)
At least 7 of CHASM (breast), FATHMM (cancer), FATHMM (missense), Mutation Assessor, MutationTaster, PolyPhen-2, PROVEAN, SIFT	67.05% (63.64%-70.71%)	50.63% (45.53%-55.81%)	95.413% (92.955%-98.53%)	95.02% (92.19%-98.41%)	52.78% (48.06%-57.46%)	2.9385 (2.8301-3.0493)	66.92% (59.6%-74.75%)	50.29% (40%-60.66%)	95.402% (89.655%-100%)	94.95% (88.57%-100%)	52.79% (43.55%-62.5%)	2.9344 (2.7166-3.1484)
At least 7 of CHASM (breast), FATHMM (cancer), FATHMM (missense), Mutation Assessor, MutationTaster, PolyPhen-2, PROVEAN, SIFT, VEST	76.41% (73.23%-79.8%)	68.58% (63.93%-73.23%)	89.946% (85.937%-94.286%)	92.19% (89.01%-95.65%)	62.34% (57.14%-67.65%)	3.1305 (3.0131-3.2474)	76.47% (69.7%-82.83%)	68.67% (59.09%-77.97%)	89.818% (81.476%-97.37%)	92.06% (85.36%-97.96%)	62.56% (52.08%-72.73%)	3.1310 (2.8915-3.3629)
At least 7 of CHASM (breast), FATHMM (cancer), FATHMM (missense), Mutation Assessor, MutationTaster, PolyPhen-2, PROVEAN, VEST	64.72% (61.11%-68.69%)	46.96% (41.86%-52.27%)	95.413% (92.955%-98.53%)	94.65% (91.53%-98.31%)	50.99% (46.15%-55.64%)	2.8802 (2.7772-2.9925)	64.5% (56.57%-71.72%)	46.47% (35.71%-56.93%)	95.402% (89.655%-100%)	94.55% (87.88%-100%)	50.95% (41.67%-60.61%)	2.8737 (2.6437-3.0811)
At least 7 of CHASM (breast), FATHMM (cancer), FATHMM (missense), Mutation Assessor, MutationTaster, PolyPhen-2, SIFT	59.95% (56.06%-63.64%)	38.39% (33.33%-43.09%)	97.233% (95.588%-100%)	96% (93.48%-100%)	47.72% (43.14%-52.06%)	2.7935 (2.6948-2.8918)	59.89% (52.53%-67.68%)	38.1% (28.33%-48.33%)	97.269% (92.303%-100%)	96.02% (89.28%-100%)	47.8% (38.96%-57.15%)	2.7918 (2.5918-2.9887)
At least 7 of CHASM (breast), FATHMM (cancer), FATHMM (missense), Mutation Assessor, MutationTaster, PolyPhen-2, SIFT, VEST	71.73% (68.18%-75.25%)	59.06% (53.9%-63.94%)	93.622% (90.541%-97.183%)	94.12% (91.36%-97.47%)	56.94% (52.07%-61.67%)	3.0375 (2.9332-3.1505)	71.7% (64.65%-78.79%)	58.98% (49.04%-68.85%)	93.483% (86.667%-100%)	93.95% (87.18%-100%)	57.05% (47.37%-67.44%)	3.0346 (2.8131-3.2432)
At least 7 of CHASM (breast), FATHMM (cancer), FATHMM (missense), Mutation Assessor, MutationTaster, PolyPhen-2, VEST	56.26% (52.02%-60.1%)	32.56% (27.35%-37.3%)	97.233% (95.588%-100%)	95.32% (92.31%-100%)	45.47% (40.79%-50%)	2.7058 (2.6045-2.8053)	56.17% (48.48%-64.65%)	32.21% (22.58%-42.37%)	97.269% (92.303%-100%)	95.32% (86.96%-100%)	45.54% (36.99%-54.88%)	2.7034 (2.4859-2.9059)
At least 7 of CHASM (breast), FATHMM (cancer), FATHMM (missense), Mutation Assessor, MutationTaster, PROVEAN, SIFT	55.26% (51.01%-59.09%)	30.44% (25.78%-34.92%)	98.173% (97.015%-100%)	96.65% (94.29%-100%)	44.94% (40.26%-49.37%)	2.7020 (2.6064-2.7962)	55.13% (47.47%-63.64%)	30.06% (21.05%-39.66%)	98.144% (94.118%-100%)	96.53% (88.89%-100%)	44.99% (36.25%-54.29%)	2.6973 (2.5072-2.8877)
At least 7 of CHASM (breast), FATHMM (cancer), FATHMM (missense), Mutation Assessor, MutationTaster, PROVEAN, SIFT, VEST	65.05% (61.6%-68.69%)	47.49% (42.4%-52.5%)	95.413% (92.955%-98.53%)	94.71% (91.67%-98.33%)	51.24% (46.56%-55.8%)	2.8885 (2.7838-2.9986)	64.85% (57.58%-71.74%)	47.02% (37.1%-56.92%)	95.402% (89.655%-100%)	94.62% (88%-100%)	51.2% (42.02%-60.61%)	2.8824 (2.6609-3.0903)
At least 7 of CHASM (breast), FATHMM (cancer), FATHMM (missense), Mutation Assessor, MutationTaster, PROVEAN, VEST	53.57% (49.49%-57.58%)	27.77% (22.9%-32.23%)	98.173% (97.015%-100%)	96.34% (93.75%-100%)	44.01% (39.37%-48.41%)	2.6629 (2.5695-2.7588)	53.47% (45.45%-61.62%)	27.43% (18.96%-36.93%)	98.144% (94.118%-100%)	96.21% (88.24%-100%)	44.07% (35.44%-53.57%)	2.6586 (2.4640-2.8458)
At least 7 of CHASM (breast), FATHMM (cancer), FATHMM (missense), Mutation Assessor, MutationTaster, SIFT, VEST	57.28% (53.52%-61.11%)	34.17% (29.13%-39.2%)	97.233% (95.588%-100%)	95.53% (92.68%-100%)	46.07% (41.56%-50.64%)	2.7300 (2.6322-2.8291)	57.16% (49.49%-64.67%)	33.78% (23.73%-43.55%)	97.269% (92.303%-100%)	95.52% (88%-100%)	46.12% (37.33%-55.56%)	2.7269 (2.5201-2.9200)
At least 7 of CHASM (breast), FATHMM (cancer), FATHMM (missense), Mutation Assessor, PolyPhen-2, PROVEAN, SIFT	55.94% (52.02%-59.6%)	31.51% (26.45%-36.07%)	98.173% (97.015%-100%)	96.76% (94.44%-100%)	45.32% (40.62%-49.66%)	2.7176 (2.6219-2.8091)	55.8% (48.48%-63.64%)	31.11% (22.03%-41.27%)	98.144% (94.118%-100%)	96.64% (88.89%-100%)	45.36% (36.71%-54.67%)	2.7126 (2.5188-2.9015)
At least 7 of CHASM (breast), FATHMM (cancer), FATHMM (missense), Mutation Assessor, PolyPhen-2, PROVEAN, SIFT, VEST	63.03% (59.09%-66.67%)	46.42% (41.32%-51.24%)	91.737% (88.233%-95.776%)	90.67% (86.21%-95.24%)	49.75% (44.88%-54.55%)	2.7858 (2.6615-2.9098)	62.83% (55.56%-70.71%)	45.96% (35.93%-55.94%)	91.737% (83.784%-100%)	90.53% (81.81%-100%)	49.73% (40.3%-59.7%)	2.7795 (2.5339-3.0229)
At least 7 of CHASM (breast), FATHMM (cancer), FATHMM (missense), Mutation Assessor, PolyPhen-2, PROVEAN, VEST	53.91% (49.99%-57.58%)	28.3% (23.25%-32.54%)	98.173% (97.015%-100%)	96.4% (93.75%-100%)	44.19% (39.39%-48.45%)	2.6707 (2.5747-2.7652)	53.81% (46.46%-61.64%)	27.96% (19.4%-38.1%)	98.144% (94.118%-100%)	96.28% (88.24%-100%)	44.25% (35.8%-53.66%)	2.6664 (2.4711-2.8542)
At least 7 of CHASM (breast), FATHMM (cancer), FATHMM (missense), Mutation Assessor, PolyPhen-2, SIFT, VEST	57.28% (53.03%-61.11%)	34.16% (29.03%-38.89%)	97.233% (95.588%-100%)	95.53% (92.68%-100%)	46.07% (41.29%-50.33%)	2.7300 (2.6322-2.8274)	57.16% (49.49%-65.66%)	33.78% (24.58%-43.86%)	97.269% (92.303%-100%)	95.53% (87.5%-100%)	46.12% (37.5%-55.41%)	2.7270 (2.5231-2.9262)
At least 7 of CHASM (breast), FATHMM (cancer), FATHMM (missense), Mutation Assessor, PROVEAN, SIFT, VEST	54.92% (51%-58.6%)	29.9% (25%-34.17%)	98.173% (97.015%-100%)	96.59% (94.28%-100%)	44.75% (40%-49.07%)	2.6942 (2.6012-2.7901)	54.8% (47.45%-62.65%)	29.54% (20.63%-39.29%)	98.144% (94.118%-100%)	96.48% (88.89%-100%)	44.8% (36.25%-54.22%)	2.6896 (2.4882-2.8770)
At least 7 of CHASM (breast), FATHMM (cancer), FATHMM (missense), MutationTaster, PolyPhen-2, PROVEAN, SIFT	54.92% (50.51%-58.59%)	29.91% (24.99%-34.43%)	98.173% (97.015%-100%)	96.59% (94.12%-100%)	44.75% (40.12%-49.04%)	2.6942 (2.5978-2.7867)	54.8% (47.47%-63.64%)	29.53% (20.63%-39.34%)	98.144% (94.118%-100%)	96.47% (88.89%-100%)	44.8% (36.36%-54.32%)	2.6895 (2.4928-2.8804)
At least 7 of CHASM (breast), FATHMM (cancer), FATHMM (missense), MutationTaster, PolyPhen-2, PROVEAN, SIFT, VEST	67.06% (63.13%-70.71%)	50.66% (45.38%-55.91%)	95.413% (92.955%-98.53%)	95.03% (92.19%-98.39%)	52.8% (48.03%-57.36%)	2.9390 (2.8328-3.0471)	66.88% (59.6%-73.74%)	50.24% (40%-60.72%)	95.402% (89.655%-100%)	94.94% (88.89%-100%)	52.77% (43.47%-62.42%)	2.9335 (2.7166-3.1516)
At least 7 of CHASM (breast), FATHMM (cancer), FATHMM (missense), MutationTaster, PolyPhen-2, PROVEAN, VEST	53.58% (48.99%-57.58%)	27.79% (22.73%-32.26%)	98.173% (97.015%-100%)	96.34% (93.75%-100%)	44.02% (39.39%-48.17%)	2.6631 (2.5651-2.7588)	53.45% (45.45%-62.63%)	27.39% (18.45%-37.5%)	98.144% (94.118%-100%)	96.21% (88.24%-100%)	44.06% (35.44%-53.66%)	2.6580 (2.4534-2.8542)
At least 7 of CHASM (breast), FATHMM (cancer), FATHMM (missense), MutationTaster, PolyPhen-2, SIFT, VEST	56.93% (52.53%-60.61%)	33.62% (28.57%-38.53%)	97.233% (95.588%-100%)	95.46% (92.5%-100%)	45.86% (41.2%-50%)	2.7218 (2.6194-2.8252)	56.84% (49.49%-64.65%)	33.27% (23.21%-43.11%)	97.269% (92.303%-100%)	95.46% (87.5%-100%)	45.93% (37.5%-55.56%)	2.7193 (2.5065-2.9262)
At least 7 of CHASM (breast), FATHMM (cancer), FATHMM (missense), MutationTaster, PROVEAN, SIFT, VEST	54.93% (50.51%-58.6%)	29.91% (25%-34.71%)	98.173% (97.015%-100%)	96.59% (94.12%-100%)	44.75% (40%-49.07%)	2.6943 (2.5987-2.7899)	54.8% (47.45%-63.64%)	29.53% (20%-39.35%)	98.144% (94.118%-100%)	96.48% (88.89%-100%)	44.8% (36.14%-54.43%)	2.6895 (2.4874-2.8877)
At least 7 of CHASM (breast), FATHMM (cancer), FATHMM (missense), PolyPhen-2, PROVEAN, SIFT, VEST	54.58% (50.51%-58.08%)	29.37% (24.41%-33.87%)	98.173% (97.015%-100%)	96.53% (94.12%-100%)	44.56% (39.87%-48.75%)	2.6864 (2.5893-2.7776)	54.47% (47.47%-62.63%)	29% (20%-38.72%)	98.144% (94.118%-100%)	96.41% (88.24%-100%)	44.62% (36.25%-54.22%)	2.6818 (2.4891-2.8732)
At least 7 of CHASM (breast), FATHMM (cancer), Mutation Assessor, MutationTaster, PolyPhen-2, PROVEAN, SIFT	55.58% (51.52%-59.6%)	30.95% (26.02%-35.65%)	98.173% (97.015%-100%)	96.7% (94.29%-100%)	45.12% (40.52%-49.38%)	2.7094 (2.6149-2.8012)	55.5% (47.47%-63.64%)	30.64% (21.31%-40.62%)	98.144% (94.118%-100%)	96.59% (89.47%-100%)	45.19% (36.58%-54.55%)	2.7057 (2.5146-2.8955)
At least 7 of CHASM (breast), FATHMM (cancer), Mutation Assessor, MutationTaster, PolyPhen-2, PROVEAN, SIFT, VEST	67.38% (63.64%-71.21%)	51.16% (45.9%-56.2%)	95.413% (92.955%-98.53%)	95.07% (92.19%-98.46%)	53.05% (48.41%-57.81%)	2.9469 (2.8347-3.0574)	67.26% (59.6%-74.75%)	50.84% (40.62%-60.87%)	95.402% (89.655%-100%)	94.99% (88.89%-100%)	53.08% (43.28%-62.9%)	2.9431 (2.7238-3.1654)
At least 7 of CHASM (breast), FATHMM (cancer), Mutation Assessor, MutationTaster, PolyPhen-2, PROVEAN, VEST	53.89% (49.49%-58.08%)	28.28% (23.25%-32.8%)	98.173% (97.015%-100%)	96.4% (93.75%-100%)	44.19% (39.51%-48.47%)	2.6703 (2.5738-2.7653)	53.84% (45.45%-62.63%)	28.01% (19.04%-37.93%)	98.144% (94.118%-100%)	96.28% (88.24%-100%)	44.27% (35.52%-53.85%)	2.6671 (2.4740-2.8610)
At least 7 of CHASM (breast), FATHMM (cancer), Mutation Assessor, MutationTaster, PolyPhen-2, SIFT, VEST	57.26% (53.03%-61.11%)	34.14% (28.69%-38.84%)	97.233% (95.588%-100%)	95.52% (92.5%-100%)	46.06% (41.4%-50.33%)	2.7296 (2.6279-2.8325)	57.19% (49.49%-65.66%)	33.83% (23.81%-44.62%)	97.269% (92.303%-100%)	95.53% (87.5%-100%)	46.14% (37.35%-55.7%)	2.7277 (2.5189-2.9287)
At least 7 of CHASM (breast), FATHMM (cancer), Mutation Assessor, MutationTaster, PROVEAN, SIFT, VEST	54.91% (50.51%-58.6%)	29.88% (25.19%-34.43%)	98.173% (97.015%-100%)	96.58% (94.12%-100%)	44.74% (40.12%-49.08%)	2.6938 (2.5987-2.7892)	54.83% (47.45%-63.64%)	29.59% (20.63%-39.07%)	98.144% (94.118%-100%)	96.47% (88.89%-100%)	44.82% (36%-54.22%)	2.6903 (2.5025-2.8826)
At least 7 of CHASM (breast), FATHMM (cancer), Mutation Assessor, PolyPhen-2, PROVEAN, SIFT, VEST	55.24% (51.01%-59.09%)	30.41% (25.58%-34.92%)	98.173% (97.015%-100%)	96.64% (94.29%-100%)	44.93% (40.13%-49.08%)	2.7016 (2.6072-2.7987)	55.17% (47.47%-63.64%)	30.12% (21.05%-39.68%)	98.144% (94.118%-100%)	96.53% (88.89%-100%)	45.01% (36.47%-54.43%)	2.6980 (2.5028-2.8889)
At least 7 of CHASM (breast), FATHMM (cancer), MutationTaster, PolyPhen-2, PROVEAN, SIFT, VEST	55.91% (51.52%-59.6%)	31.47% (26.56%-36.22%)	98.173% (97.015%-100%)	96.75% (94.44%-100%)	45.31% (40.52%-49.68%)	2.7170 (2.6162-2.8091)	55.86% (48.48%-64.65%)	31.2% (21.88%-41.07%)	98.144% (94.118%-100%)	96.65% (89.47%-100%)	45.4% (36.84%-55%)	2.7139 (2.5200-2.9161)
At least 7 of CHASM (breast), FATHMM (missense), Mutation Assessor, MutationTaster, PolyPhen-2, PROVEAN, SIFT	54.24% (50%-58.08%)	28.84% (24.16%-33.33%)	98.173% (97.015%-100%)	96.46% (93.94%-100%)	44.38% (39.73%-48.75%)	2.6785 (2.5807-2.7732)	54.14% (46.46%-62.63%)	28.48% (19.66%-37.94%)	98.144% (94.118%-100%)	96.35% (88.24%-100%)	44.44% (35.8%-53.85%)	2.6741 (2.4800-2.8688)
At least 7 of CHASM (breast), FATHMM (missense), Mutation Assessor, MutationTaster, PolyPhen-2, PROVEAN, SIFT, VEST	65.02% (61.62%-68.69%)	47.44% (42.4%-52.42%)	95.413% (92.955%-98.53%)	94.71% (91.67%-98.33%)	51.22% (46.38%-55.97%)	2.8877 (2.7838-2.9982)	64.91% (57.58%-71.72%)	47.11% (37.1%-57.15%)	95.402% (89.655%-100%)	94.62% (88%-100%)	51.25% (41.79%-60.61%)	2.8838 (2.6562-3.0935)
At least 7 of CHASM (breast), FATHMM (missense), Mutation Assessor, MutationTaster, PolyPhen-2, PROVEAN, VEST	52.55% (48.48%-56.57%)	26.17% (21.21%-30.47%)	98.173% (97.015%-100%)	96.12% (93.33%-100%)	43.47% (38.79%-47.83%)	2.6393 (2.5443-2.7363)	52.47% (44.44%-60.61%)	25.85% (17.31%-35.5%)	98.144% (94.118%-100%)	95.99% (87.5%-100%)	43.54% (34.94%-53.09%)	2.6352 (2.4342-2.8270)
At least 7 of CHASM (breast), FATHMM (missense), Mutation Assessor, MutationTaster, PolyPhen-2, SIFT, VEST	56.25% (52.02%-60.1%)	32.55% (27.64%-37.3%)	97.233% (95.588%-100%)	95.32% (92.31%-100%)	45.47% (40.79%-49.68%)	2.7056 (2.6045-2.8036)	56.18% (48.48%-64.65%)	32.23% (22.95%-42.37%)	97.269% (92.303%-100%)	95.32% (87.49%-100%)	45.55% (36.99%-54.88%)	2.7037 (2.4927-2.9018)
At least 7 of CHASM (breast), FATHMM (missense), Mutation Assessor, MutationTaster, PROVEAN, SIFT, VEST	53.57% (49.49%-57.58%)	27.77% (22.9%-32.23%)	98.173% (97.015%-100%)	96.34% (93.75%-100%)	44.01% (39.37%-48.41%)	2.6629 (2.5695-2.7588)	53.47% (45.45%-61.62%)	27.43% (18.96%-36.93%)	98.144% (94.118%-100%)	96.21% (88.24%-100%)	44.07% (35.44%-53.57%)	2.6586 (2.4640-2.8458)
At least 7 of CHASM (breast), FATHMM (missense), Mutation Assessor, PolyPhen-2, PROVEAN, SIFT, VEST	53.91% (49.99%-57.58%)	28.3% (23.25%-32.54%)	98.173% (97.015%-100%)	96.4% (93.75%-100%)	44.19% (39.39%-48.45%)	2.6707 (2.5747-2.7652)	53.81% (46.46%-61.64%)	27.96% (19.4%-38.1%)	98.144% (94.118%-100%)	96.28% (88.24%-100%)	44.25% (35.8%-53.66%)	2.6664 (2.4711-2.8542)
At least 7 of CHASM (breast), FATHMM (missense), MutationTaster, PolyPhen-2, PROVEAN, SIFT, VEST	53.23% (48.99%-57.07%)	27.24% (22.22%-31.67%)	98.173% (97.015%-100%)	96.27% (93.55%-100%)	43.83% (39.16%-48.15%)	2.6550 (2.5570-2.7531)	53.13% (45.45%-61.62%)	26.9% (17.86%-36.84%)	98.144% (94.118%-100%)	96.14% (87.5%-100%)	43.89% (35.37%-53.57%)	2.6507 (2.4524-2.8441)
At least 7 of CHASM (breast), Mutation Assessor, MutationTaster, PolyPhen-2, PROVEAN, SIFT, VEST	53.89% (49.49%-58.08%)	28.28% (23.25%-32.8%)	98.173% (97.015%-100%)	96.4% (93.75%-100%)	44.19% (39.51%-48.47%)	2.6703 (2.5738-2.7653)	53.84% (45.45%-62.63%)	28.01% (19.04%-37.93%)	98.144% (94.118%-100%)	96.28% (88.24%-100%)	44.27% (35.52%-53.85%)	2.6671 (2.4740-2.8610)
At least 7 of CHASM (lung), CHASM (melanoma), FATHMM (cancer), FATHMM (missense), Mutation Assessor, MutationTaster, PolyPhen-2	61.98% (58.08%-65.66%)	41.57% (36.44%-46.72%)	97.256% (95.588%-100%)	96.32% (94%-100%)	49.05% (44.36%-53.47%)	2.8420 (2.7469-2.9394)	61.91% (54.55%-69.7%)	41.31% (31.75%-51.57%)	97.233% (92.126%-100%)	96.25% (89.99%-100%)	49.12% (40.28%-58.67%)	2.8391 (2.6518-3.0287)
At least 7 of CHASM (lung), CHASM (melanoma), FATHMM (cancer), FATHMM (missense), Mutation Assessor, MutationTaster, PolyPhen-2, PROVEAN	68.04% (64.14%-71.72%)	52.2% (47.15%-57.26%)	95.411% (92.958%-98.571%)	95.16% (92.54%-98.55%)	53.59% (48.82%-58.46%)	2.9636 (2.8600-3.0742)	67.96% (60.61%-75.76%)	51.95% (42.19%-62.07%)	95.417% (89.474%-100%)	95.11% (88.24%-100%)	53.64% (44.12%-63.49%)	2.9612 (2.7394-3.1654)
At least 7 of CHASM (lung), CHASM (melanoma), FATHMM (cancer), FATHMM (missense), Mutation Assessor, MutationTaster, PolyPhen-2, PROVEAN, SIFT	77.11% (73.74%-80.81%)	68.09% (63.57%-72.73%)	92.699% (89.333%-96%)	94.16% (91.67%-96.84%)	62.69% (57.41%-67.86%)	3.1764 (3.0653-3.2943)	77.09% (69.7%-83.84%)	68.05% (58.33%-77.42%)	92.563% (85.366%-100%)	94.03% (88%-100%)	62.8% (52.62%-73.33%)	3.1744 (2.9349-3.3970)
At least 7 of CHASM (lung), CHASM (melanoma), FATHMM (cancer), FATHMM (missense), Mutation Assessor, MutationTaster, PolyPhen-2, PROVEAN, SIFT, VEST	79.78% (76.77%-82.83%)	74.98% (70.94%-79.23%)	88.085% (84%-92.756%)	91.58% (88.66%-94.9%)	67.06% (61.7%-72.28%)	3.2171 (3.0991-3.3389)	79.83% (73.74%-85.86%)	75.02% (66.07%-83.08%)	88.032% (79.31%-97.06%)	91.51% (84.91%-97.87%)	67.25% (56.82%-77.78%)	3.2181 (2.9765-3.4554)
At least 7 of CHASM (lung), CHASM (melanoma), FATHMM (cancer), FATHMM (missense), Mutation Assessor, MutationTaster, PolyPhen-2, PROVEAN, VEST	76.1% (72.73%-79.8%)	67.04% (62.39%-71.9%)	91.759% (88.235%-95.775%)	93.37% (90.7%-96.63%)	61.69% (56.48%-66.97%)	3.1386 (3.0215-3.2578)	76.08% (68.69%-82.83%)	66.95% (57.14%-76.12%)	91.692% (84.211%-97.68%)	93.27% (86.67%-98.04%)	61.79% (51.72%-72.22%)	3.1370 (2.9007-3.3693)
At least 7 of CHASM (lung), CHASM (melanoma), FATHMM (cancer), FATHMM (missense), Mutation Assessor, MutationTaster, PolyPhen-2, SIFT	73.08% (69.7%-76.77%)	60.68% (55.46%-65.83%)	94.507% (91.667%-97.333%)	95.02% (92.59%-97.67%)	58.16% (53.23%-63.11%)	3.0836 (2.9803-3.1996)	73.04% (65.66%-79.8%)	60.53% (50.73%-70.59%)	94.474% (88.235%-100%)	94.95% (88.88%-100%)	58.25% (48.39%-68.52%)	3.0820 (2.8500-3.2984)
At least 7 of CHASM (lung), CHASM (melanoma), FATHMM (cancer), FATHMM (missense), Mutation Assessor, MutationTaster, PolyPhen-2, SIFT, VEST	78.46% (75.25%-81.82%)	71.3% (66.93%-75.78%)	90.838% (87.324%-94.668%)	93.08% (90.43%-96%)	64.67% (59.22%-69.81%)	3.1989 (3.0821-3.3170)	78.43% (71.72%-84.85%)	71.2% (61.9%-80%)	90.777% (82.5%-97.44%)	93% (86.95%-98%)	64.76% (54.5%-75.44%)	3.1974 (2.9639-3.4331)
At least 7 of CHASM (lung), CHASM (melanoma), FATHMM (cancer), FATHMM (missense), Mutation Assessor, MutationTaster, PolyPhen-2, VEST	71.71% (68.18%-75.25%)	59.06% (54.03%-64.52%)	93.567% (90.541%-97.184%)	94.07% (91.36%-97.4%)	56.93% (51.96%-61.74%)	3.0363 (2.9318-3.1459)	71.74% (64.65%-78.79%)	58.98% (48.33%-68.97%)	93.602% (86.667%-100%)	94.06% (87.49%-100%)	57.08% (47.46%-67.24%)	3.0372 (2.8100-3.2473)
At least 7 of CHASM (lung), CHASM (melanoma), FATHMM (cancer), FATHMM (missense), Mutation Assessor, MutationTaster, PROVEAN	56.27% (52.02%-60.1%)	31.5% (26.72%-36%)	99.084% (98.485%-100%)	98.35% (97.14%-100%)	45.55% (40.91%-50%)	2.7448 (2.6599-2.8314)	56.15% (48.48%-64.65%)	31.13% (22.03%-40.32%)	99.083% (96.774%-100%)	98.32% (93.75%-100%)	45.6% (36.98%-54.88%)	2.7413 (2.5668-2.9144)
At least 7 of CHASM (lung), CHASM (melanoma), FATHMM (cancer), FATHMM (missense), Mutation Assessor, MutationTaster, PROVEAN, SIFT	69.04% (65.66%-72.73%)	53.79% (48.8%-58.73%)	95.411% (92.958%-98.571%)	95.3% (92.75%-98.59%)	54.42% (49.61%-59.26%)	2.9891 (2.8970-3.0938)	68.99% (61.62%-75.76%)	53.56% (43.55%-63.79%)	95.417% (89.474%-100%)	95.25% (88.57%-100%)	54.49% (44.64%-64.18%)	2.9872 (2.7737-3.1871)
At least 7 of CHASM (lung), CHASM (melanoma), FATHMM (cancer), FATHMM (missense), Mutation Assessor, MutationTaster, PROVEAN, SIFT, VEST	76.09% (72.73%-79.8%)	66.47% (61.83%-71.32%)	92.699% (89.333%-96%)	94.03% (91.46%-96.78%)	61.53% (56.19%-66.97%)	3.1473 (3.0316-3.2675)	76.11% (68.69%-82.83%)	66.49% (56.67%-75.86%)	92.563% (85.366%-100%)	93.89% (87.5%-100%)	61.69% (51.61%-72.22%)	3.1464 (2.9056-3.3678)
At least 7 of CHASM (lung), CHASM (melanoma), FATHMM (cancer), FATHMM (missense), Mutation Assessor, MutationTaster, PROVEAN, VEST	66.04% (62.63%-69.7%)	49.05% (43.85%-54.1%)	95.411% (92.958%-98.571%)	94.87% (92.06%-98.44%)	51.99% (47.29%-56.82%)	2.9133 (2.8159-3.0247)	65.89% (58.59%-72.73%)	48.67% (38.7%-58.62%)	95.417% (89.474%-100%)	94.81% (87.5%-100%)	52% (42.25%-61.67%)	2.9089 (2.6804-3.1154)

**At least 7 of CHASM (lung), CHASM (melanoma), FATHMM (cancer), FATHMM (missense), Mutation Assessor, MutationTaster, SIFT**	63% (59.09%-66.67%)	43.18% (38.09%-48%)	97.256% (95.588%-100%)	96.46% (94.23%-100%)	49.75% (44.93%-54.29%)	2.8664 (2.7722-2.9634)	62.9% (55.56%-70.71%)	42.88% (33.33%-52.94%)	97.233% (92.105%-100%)	96.38% (90%-100%)	49.79% (40.79%-59.09%)	2.8629 (2.6681-3.0488)
**At least 7 of CHASM (lung), CHASM (melanoma), FATHMM (cancer), FATHMM (missense), Mutation Assessor, MutationTaster, SIFT, VEST**	72.39% (68.69%-76.26%)	59.6% (54.61%-64.84%)	94.507% (91.667%-97.333%)	94.94% (92.41%-97.65%)	57.5% (52.5%-62.5%)	3.0654 (2.9618-3.1849)	72.39% (64.65%-79.8%)	59.49% (49.15%-70.18%)	94.474% (88.235%-100%)	94.87% (88.64%-100%)	57.62% (47.69%-67.86%)	3.0645 (2.8401-3.2728)
**At least 7 of CHASM (lung), CHASM (melanoma), FATHMM (cancer), FATHMM (missense), Mutation Assessor, MutationTaster, VEST**	59.3% (55.05%-63.13%)	37.35% (32.23%-42.4%)	97.256% (95.588%-100%)	95.92% (93.33%-100%)	47.31% (42.58%-51.95%)	2.7783 (2.6788-2.8735)	59.18% (51.52%-67.68%)	36.99% (26.98%-46.88%)	97.233% (92.105%-100%)	95.82% (88.46%-100%)	47.35% (38.15%-56.76%)	2.7739 (2.5824-2.9728)
**At least 7 of CHASM (lung), CHASM (melanoma), FATHMM (cancer), FATHMM (missense), Mutation Assessor, PolyPhen-2, PROVEAN**	56.94% (53.03%-60.61%)	32.57% (27.59%-37.01%)	99.084% (98.485%-100%)	98.4% (97.22%-100%)	45.94% (41.25%-50.01%)	2.7599 (2.6755-2.8448)	56.82% (49.49%-64.65%)	32.18% (22.95%-41.94%)	99.083% (96.774%-100%)	98.37% (94.12%-100%)	45.98% (37.33%-55.27%)	2.7561 (2.5880-2.9286)
**At least 7 of CHASM (lung), CHASM (melanoma), FATHMM (cancer), FATHMM (missense), Mutation Assessor, PolyPhen-2, PROVEAN, SIFT**	68.38% (65.15%-72.22%)	53.28% (48.15%-58.27%)	94.488% (91.549%-97.368%)	94.36% (91.43%-97.4%)	53.91% (48.85%-58.59%)	2.9603 (2.8572-3.0720)	68.29% (60.61%-74.75%)	52.99% (43.1%-63.08%)	94.497% (87.879%-100%)	94.3% (86.67%-100%)	53.94% (44.26%-63.93%)	2.9573 (2.7339-3.1629)
**At least 7 of CHASM (lung), CHASM (melanoma), FATHMM (cancer), FATHMM (missense), Mutation Assessor, PolyPhen-2, PROVEAN, SIFT, VEST**	75.09% (71.72%-78.28%)	67.03% (62.31%-71.88%)	89.006% (84.928%-93.243%)	91.34% (88.23%-94.63%)	60.96% (55.56%-66.34%)	3.0834 (2.9649-3.2023)	75.08% (68.69%-81.82%)	66.97% (56.92%-76.56%)	88.947% (80.556%-97.14%)	91.24% (84.44%-97.73%)	61.08% (50.83%-71.7%)	3.0823 (2.8374-3.3200)
**At least 7 of CHASM (lung), CHASM (melanoma), FATHMM (cancer), FATHMM (missense), Mutation Assessor, PolyPhen-2, PROVEAN, VEST**	64.69% (61.11%-68.19%)	47.99% (42.97%-53.17%)	93.548% (90.278%-97.183%)	92.79% (89.47%-96.88%)	50.99% (46.09%-55.64%)	2.8531 (2.7448-2.9723)	64.57% (57.55%-71.72%)	47.61% (37.5%-57.97%)	93.626% (86.667%-100%)	92.78% (84.61%-100%)	51.01% (41.54%-60.87%)	2.8504 (2.6108-3.0620)
**At least 7 of CHASM (lung), CHASM (melanoma), FATHMM (cancer), FATHMM (missense), Mutation Assessor, PolyPhen-2, SIFT**	64.01% (60.61%-67.69%)	44.78% (39.31%-50%)	97.256% (95.588%-100%)	96.58% (94.44%-100%)	50.46% (45.71%-54.79%)	2.8908 (2.7944-2.9892)	63.89% (56.54%-70.71%)	44.45% (34.42%-55%)	97.233% (92.105%-100%)	96.5% (90.48%-100%)	50.49% (41.54%-60%)	2.8867 (2.6905-3.0796)
**At least 7 of CHASM (lung), CHASM (melanoma), FATHMM (cancer), FATHMM (missense), Mutation Assessor, PolyPhen-2, SIFT, VEST**	71.71% (68.18%-75.76%)	59.6% (54.4%-64.75%)	92.644% (89.33%-96.155%)	93.34% (90.36%-96.51%)	57.02% (52%-61.99%)	3.0260 (2.9205-3.1450)	71.72% (63.64%-78.79%)	59.48% (49.21%-69.49%)	92.682% (85.287%-100%)	93.32% (86.49%-100%)	57.14% (47.27%-67.27%)	3.0263 (2.7829-3.2432)
**At least 7 of CHASM (lung), CHASM (melanoma), FATHMM (cancer), FATHMM (missense), Mutation Assessor, PolyPhen-2, VEST**	58.96% (55.05%-62.63%)	37.34% (32.26%-42.15%)	96.317% (94.203%-98.667%)	94.61% (91.49%-98.08%)	47.06% (42.28%-51.41%)	2.7533 (2.6546-2.8583)	58.86% (51.52%-66.67%)	36.99% (27.42%-47.37%)	96.362% (91.176%-100%)	94.59% (86.36%-100%)	47.12% (38.35%-56.34%)	2.7507 (2.5421-2.9547)
**At least 7 of CHASM (lung), CHASM (melanoma), FATHMM (cancer), FATHMM (missense), Mutation Assessor, PROVEAN, SIFT**	57.96% (54.04%-61.62%)	34.17% (29.31%-38.76%)	99.084% (98.485%-100%)	98.47% (97.37%-100%)	46.54% (41.77%-50.67%)	2.7827 (2.6973-2.8707)	57.82% (50.51%-65.66%)	33.76% (24.56%-43.86%)	99.083% (96.774%-100%)	98.44% (94.12%-100%)	46.57% (38.16%-56%)	2.7785 (2.6029-2.9561)
**At least 7 of CHASM (lung), CHASM (melanoma), FATHMM (cancer), FATHMM (missense), Mutation Assessor, PROVEAN, SIFT, VEST**	65.71% (62.12%-69.7%)	49.06% (44%-53.97%)	94.488% (91.549%-97.368%)	93.9% (90.77%-97.14%)	51.75% (46.92%-56.59%)	2.8920 (2.7900-3.0061)	65.55% (57.58%-72.73%)	48.65% (38.8%-58.73%)	94.497% (87.879%-100%)	93.84% (86.11%-100%)	51.74% (42.19%-61.54%)	2.8873 (2.6526-3.0883)
**At least 7 of CHASM (lung), CHASM (melanoma), FATHMM (cancer), FATHMM (missense), Mutation Assessor, PROVEAN, VEST**	55.93% (51.52%-59.6%)	30.97% (26.23%-35.48%)	99.084% (98.485%-100%)	98.32% (97.06%-100%)	45.36% (40.64%-49.69%)	2.7373 (2.6545-2.8246)	55.82% (48.48%-64.65%)	30.6% (21.43%-39.71%)	99.083% (96.774%-100%)	98.29% (93.74%-100%)	45.41% (36.71%-54.55%)	2.7339 (2.5591-2.9069)
**At least 7 of CHASM (lung), CHASM (melanoma), FATHMM (cancer), FATHMM (missense), Mutation Assessor, SIFT, VEST**	60.32% (56.57%-64.14%)	38.95% (33.88%-43.8%)	97.256% (95.588%-100%)	96.09% (93.62%-100%)	47.95% (43.14%-52.38%)	2.8025 (2.7068-2.8984)	60.17% (52.53%-67.68%)	38.56% (28.78%-48.39%)	97.233% (92.105%-100%)	95.98% (88.46%-100%)	47.97% (39.13%-57.14%)	2.7975 (2.5988-2.9925)
**At least 7 of CHASM (lung), CHASM (melanoma), FATHMM (cancer), FATHMM (missense), MutationTaster, PolyPhen-2, PROVEAN**	55.93% (52.01%-59.6%)	30.97% (26.15%-35.61%)	99.084% (98.485%-100%)	98.32% (97.06%-100%)	45.36% (40.64%-49.68%)	2.7373 (2.6515-2.8201)	55.82% (48.48%-63.66%)	30.59% (21.54%-40.36%)	99.083% (96.774%-100%)	98.29% (93.75%-100%)	45.41% (37.18%-54.88%)	2.7338 (2.5665-2.9059)
**At least 7 of CHASM (lung), CHASM (melanoma), FATHMM (cancer), FATHMM (missense), MutationTaster, PolyPhen-2, PROVEAN, SIFT**	71.39% (68.18%-74.75%)	57.49% (52.8%-62.4%)	95.411% (92.958%-98.571%)	95.59% (93.24%-98.68%)	56.49% (51.61%-61.29%)	3.0498 (2.9507-3.1595)	71.36% (64.65%-77.78%)	57.33% (47.62%-66.67%)	95.417% (89.474%-100%)	95.55% (89.19%-100%)	56.58% (46.67%-66.67%)	3.0487 (2.8282-3.2435)
**At least 7 of CHASM (lung), CHASM (melanoma), FATHMM (cancer), FATHMM (missense), MutationTaster, PolyPhen-2, PROVEAN, SIFT, VEST**	77.45% (74.24%-80.81%)	68.63% (64.17%-73.39%)	92.699% (89.333%-96%)	94.21% (91.67%-96.88%)	63.09% (57.94%-68.23%)	3.1863 (3.0761-3.3023)	77.41% (70.71%-83.84%)	68.56% (58.72%-77.78%)	92.563% (85.366%-100%)	94.07% (87.88%-100%)	63.18% (52.83%-73.59%)	3.1837 (2.9579-3.4023)
**At least 7 of CHASM (lung), CHASM (melanoma), FATHMM (cancer), FATHMM (missense), MutationTaster, PolyPhen-2, PROVEAN, VEST**	68.73% (65.15%-72.22%)	53.29% (48.41%-58.4%)	95.411% (92.958%-98.571%)	95.26% (92.65%-98.57%)	54.16% (49.23%-58.78%)	2.9812 (2.8838-3.0934)	68.6% (61.62%-75.76%)	52.96% (42.86%-62.86%)	95.417% (89.474%-100%)	95.2% (88.57%-100%)	54.17% (44.44%-63.79%)	2.9775 (2.7521-3.1683)
**At least 7 of CHASM (lung), CHASM (melanoma), FATHMM (cancer), FATHMM (missense), MutationTaster, PolyPhen-2, SIFT**	63% (59.09%-66.67%)	43.18% (38.13%-48.03%)	97.256% (95.588%-100%)	96.46% (94.23%-100%)	49.75% (45%-54.06%)	2.8664 (2.7707-2.9608)	62.89% (55.56%-70.71%)	42.88% (33.32%-53.12%)	97.233% (92.105%-100%)	96.38% (90.31%-100%)	49.79% (40.85%-59.16%)	2.8628 (2.6746-3.0514)
**At least 7 of CHASM (lung), CHASM (melanoma), FATHMM (cancer), FATHMM (missense), MutationTaster, PolyPhen-2, SIFT, VEST**	74.75% (71.21%-78.28%)	63.31% (58.46%-68.25%)	94.507% (91.667%-97.333%)	95.22% (92.77%-97.75%)	59.84% (54.78%-64.82%)	3.1288 (3.0240-3.2432)	74.75% (67.68%-81.82%)	63.24% (53.23%-73.34%)	94.474% (88.235%-100%)	95.15% (89.36%-100%)	59.97% (50%-70.18%)	3.1284 (2.9042-3.3518)
**At least 7 of CHASM (lung), CHASM (melanoma), FATHMM (cancer), FATHMM (missense), MutationTaster, PolyPhen-2, VEST**	59.63% (55.56%-63.64%)	37.87% (32.77%-42.74%)	97.256% (95.588%-100%)	95.98% (93.33%-100%)	47.51% (42.76%-51.8%)	2.7862 (2.6906-2.8876)	59.53% (51.52%-67.68%)	37.55% (28.12%-48.33%)	97.233% (92.105%-100%)	95.88% (88.89%-100%)	47.57% (38.57%-57.14%)	2.7823 (2.5790-2.9727)
**At least 7 of CHASM (lung), CHASM (melanoma), FATHMM (cancer), FATHMM (missense), MutationTaster, PROVEAN, SIFT**	57.95% (54.04%-61.62%)	34.15% (29.36%-38.89%)	99.084% (98.485%-100%)	98.47% (97.37%-100%)	46.53% (41.77%-50.94%)	2.7824 (2.6973-2.8669)	57.84% (50.51%-65.66%)	33.8% (24.59%-43.64%)	99.083% (96.774%-100%)	98.46% (94.44%-100%)	46.58% (37.97%-56.1%)	2.7792 (2.6047-2.9562)
**At least 7 of CHASM (lung), CHASM (melanoma), FATHMM (cancer), FATHMM (missense), MutationTaster, PROVEAN, SIFT, VEST**	68.03% (64.65%-71.72%)	52.19% (46.82%-57.03%)	95.411% (92.958%-98.571%)	95.16% (92.54%-98.55%)	53.58% (48.8%-58.21%)	2.9635 (2.8662-3.0741)	67.97% (60.61%-74.75%)	51.96% (41.94%-62.12%)	95.417% (89.474%-100%)	95.12% (88.24%-100%)	53.65% (44.11%-63.49%)	2.9615 (2.7397-3.1602)
**At least 7 of CHASM (lung), CHASM (melanoma), FATHMM (cancer), FATHMM (missense), MutationTaster, PROVEAN, VEST**	55.93% (51.52%-59.6%)	30.97% (26.19%-35.71%)	99.084% (98.485%-100%)	98.32% (97.06%-100%)	45.36% (40.64%-49.69%)	2.7374 (2.6510-2.8201)	55.81% (48.48%-64.65%)	30.59% (21.31%-40.3%)	99.083% (96.774%-100%)	98.3% (93.75%-100%)	45.41% (36.71%-54.88%)	2.7338 (2.5668-2.9118)
**At least 7 of CHASM (lung), CHASM (melanoma), FATHMM (cancer), FATHMM (missense), MutationTaster, SIFT, VEST**	60.65% (57.06%-64.15%)	39.48% (34.43%-44.53%)	97.256% (95.588%-100%)	96.14% (93.75%-100%)	48.17% (43.45%-52.7%)	2.8104 (2.7179-2.9099)	60.51% (53.51%-67.7%)	39.11% (29.31%-48.53%)	97.233% (92.105%-100%)	96.04% (89.28%-100%)	48.2% (39.06%-57.53%)	2.8057 (2.6112-2.9963)
**At least 7 of CHASM (lung), CHASM (melanoma), FATHMM (cancer), FATHMM (missense), PolyPhen-2, PROVEAN, SIFT**	57.96% (54.04%-61.62%)	34.17% (29.17%-38.76%)	99.084% (98.485%-100%)	98.47% (97.37%-100%)	46.54% (41.72%-50.66%)	2.7827 (2.6973-2.8637)	57.82% (50.51%-65.66%)	33.76% (24.24%-43.75%)	99.083% (96.774%-100%)	98.45% (94.44%-100%)	46.57% (38.16%-56.34%)	2.7785 (2.6126-2.9561)
**At least 7 of CHASM (lung), CHASM (melanoma), FATHMM (cancer), FATHMM (missense), PolyPhen-2, PROVEAN, SIFT, VEST**	67.71% (64.14%-71.21%)	52.22% (47.15%-57.38%)	94.488% (91.549%-97.368%)	94.25% (91.3%-97.33%)	53.35% (48.39%-58.02%)	2.9431 (2.8399-3.0556)	67.61% (60.61%-74.75%)	51.91% (41.66%-61.97%)	94.497% (87.879%-100%)	94.2% (86.67%-100%)	53.38% (43.94%-63.24%)	2.9399 (2.7047-3.1483)
**At least 7 of CHASM (lung), CHASM (melanoma), FATHMM (cancer), FATHMM (missense), PolyPhen-2, PROVEAN, VEST**	55.59% (51.52%-59.09%)	30.43% (25.4%-34.96%)	99.084% (98.485%-100%)	98.29% (96.97%-100%)	45.17% (40.38%-49.37%)	2.7297 (2.6450-2.8132)	55.49% (48.48%-63.64%)	30.07% (21.05%-39.71%)	99.083% (96.774%-100%)	98.26% (93.33%-100%)	45.22% (36.71%-54.67%)	2.7264 (2.5548-2.9001)
**At least 7 of CHASM (lung), CHASM (melanoma), FATHMM (cancer), FATHMM (missense), PolyPhen-2, SIFT, VEST**	59.97% (56.06%-63.65%)	38.41% (33.33%-43.34%)	97.256% (95.588%-100%)	96.03% (93.48%-100%)	47.73% (42.95%-52%)	2.7943 (2.7029-2.8932)	59.85% (52.5%-67.68%)	38.05% (28.33%-48.39%)	97.233% (92.105%-100%)	95.93% (88.46%-100%)	47.77% (38.96%-57.33%)	2.7899 (2.5941-2.9768)
**At least 7 of CHASM (lung), CHASM (melanoma), FATHMM (cancer), FATHMM (missense), PROVEAN, SIFT, VEST**	57.28% (53.03%-61.11%)	33.11% (28.23%-37.88%)	99.084% (98.485%-100%)	98.43% (97.22%-100%)	46.14% (41.36%-50.32%)	2.7676 (2.6821-2.8500)	57.15% (49.49%-65.66%)	32.7% (22.97%-42.43%)	99.083% (96.774%-100%)	98.4% (94.12%-100%)	46.18% (37.66%-55.84%)	2.7636 (2.5947-2.9459)
**At least 7 of CHASM (lung), CHASM (melanoma), FATHMM (cancer), Mutation Assessor, MutationTaster, PolyPhen-2, PROVEAN**	56.58% (52.53%-60.1%)	32% (27.12%-36.51%)	99.084% (98.485%-100%)	98.37% (97.14%-100%)	45.73% (41.14%-50%)	2.7519 (2.6658-2.8385)	56.53% (49.49%-64.65%)	31.73% (22.41%-41.38%)	99.083% (96.774%-100%)	98.35% (93.75%-100%)	45.82% (37.33%-55.13%)	2.7498 (2.5745-2.9233)
**At least 7 of CHASM (lung), CHASM (melanoma), FATHMM (cancer), Mutation Assessor, MutationTaster, PolyPhen-2, PROVEAN, SIFT**	70.06% (66.67%-73.74%)	55.39% (50.39%-60.66%)	95.411% (92.958%-98.571%)	95.43% (92.96%-98.61%)	55.3% (50.38%-60%)	3.0153 (2.9166-3.1209)	69.98% (62.63%-76.77%)	55.14% (44.61%-65.52%)	95.417% (89.474%-100%)	95.37% (89.18%-100%)	55.35% (45.76%-66.1%)	3.0128 (2.7941-3.2169)
**At least 7 of CHASM (lung), CHASM (melanoma), FATHMM (cancer), Mutation Assessor, MutationTaster, PolyPhen-2, PROVEAN, SIFT, VEST**	76.41% (72.73%-79.8%)	67.51% (62.88%-72.58%)	91.778% (88.406%-95.714%)	93.42% (90.82%-96.59%)	62.03% (56.73%-67.27%)	3.1475 (3.0343-3.2686)	76.47% (69.7%-83.84%)	67.6% (58.06%-77.05%)	91.649% (84.371%-97.56%)	93.29% (86.96%-98%)	62.25% (51.92%-72.88%)	3.1479 (2.9107-3.3780)
**At least 7 of CHASM (lung), CHASM (melanoma), FATHMM (cancer), Mutation Assessor, MutationTaster, PolyPhen-2, PROVEAN, VEST**	67.39% (63.64%-71.21%)	51.17% (46.21%-56.25%)	95.411% (92.958%-98.571%)	95.07% (92.31%-98.53%)	53.06% (48.12%-57.78%)	2.9471 (2.8440-3.0556)	67.25% (59.6%-74.75%)	50.82% (40.91%-61.02%)	95.417% (89.474%-100%)	95.01% (87.88%-100%)	53.07% (43.74%-63.08%)	2.9431 (2.7223-3.1582)
**At least 7 of CHASM (lung), CHASM (melanoma), FATHMM (cancer), Mutation Assessor, MutationTaster, PolyPhen-2, SIFT**	62.97% (59.09%-66.67%)	43.14% (38.1%-48.39%)	97.256% (95.588%-100%)	96.45% (94.23%-100%)	49.73% (44.93%-54.29%)	2.8658 (2.7707-2.9622)	62.94% (55.56%-70.71%)	42.95% (32.79%-52.94%)	97.233% (92.105%-100%)	96.39% (90.32%-100%)	49.83% (40.58%-59.38%)	2.8640 (2.6717-3.0513)
**At least 7 of CHASM (lung), CHASM (melanoma), FATHMM (cancer), Mutation Assessor, MutationTaster, PolyPhen-2, SIFT, VEST**	73.73% (70.2%-77.27%)	62.24% (57.26%-67.46%)	93.586% (90.541%-97.144%)	94.37% (91.76%-97.53%)	58.91% (53.64%-63.79%)	3.0910 (2.9811-3.2112)	73.76% (66.67%-80.81%)	62.2% (51.85%-71.93%)	93.559% (86.838%-100%)	94.31% (87.88%-100%)	59.06% (49.18%-69.7%)	3.0912 (2.8521-3.3141)
**At least 7 of CHASM (lung), CHASM (melanoma), FATHMM (cancer), Mutation Assessor, MutationTaster, PolyPhen-2, VEST**	59.28% (55.56%-63.13%)	37.31% (32.28%-42.19%)	97.256% (95.588%-100%)	95.92% (93.33%-100%)	47.29% (42.67%-51.7%)	2.7778 (2.6768-2.8771)	59.22% (51.52%-66.67%)	37.06% (27.42%-47.14%)	97.233% (92.105%-100%)	95.83% (88.46%-100%)	47.38% (38.46%-56.76%)	2.7751 (2.5708-2.9728)
**At least 7 of CHASM (lung), CHASM (melanoma), FATHMM (cancer), Mutation Assessor, MutationTaster, PROVEAN, SIFT**	57.6% (53.54%-61.12%)	33.6% (28.93%-38.4%)	99.084% (98.485%-100%)	98.45% (97.3%-100%)	46.33% (41.77%-50.65%)	2.7746 (2.6884-2.8616)	57.53% (50.48%-65.66%)	33.31% (23.63%-42.86%)	99.083% (96.774%-100%)	98.43% (94.12%-100%)	46.4% (37.5%-56%)	2.7722 (2.5989-2.9468)
**At least 7 of CHASM (lung), CHASM (melanoma), FATHMM (cancer), Mutation Assessor, MutationTaster, PROVEAN, SIFT, VEST**	68.07% (64.65%-71.72%)	52.25% (47.15%-57.26%)	95.411% (92.958%-98.571%)	95.16% (92.42%-98.55%)	53.61% (48.8%-58.33%)	2.9643 (2.8614-3.0742)	67.91% (60.61%-74.75%)	51.86% (42.19%-61.77%)	95.417% (89.474%-100%)	95.1% (88.24%-100%)	53.6% (44.11%-63.09%)	2.9598 (2.7457-3.1654)
**At least 7 of CHASM (lung), CHASM (melanoma), FATHMM (cancer), Mutation Assessor, MutationTaster, PROVEAN, VEST**	55.91% (52.01%-59.61%)	30.93% (26.23%-35.54%)	99.084% (98.485%-100%)	98.32% (97.06%-100%)	45.35% (40.65%-49.69%)	2.7368 (2.6537-2.8246)	55.86% (48.46%-63.66%)	30.67% (21.62%-40.3%)	99.083% (96.774%-100%)	98.3% (93.75%-100%)	45.44% (36.59%-54.76%)	2.7349 (2.5589-2.9059)
**At least 7 of CHASM (lung), CHASM (melanoma), FATHMM (cancer), Mutation Assessor, MutationTaster, SIFT, VEST**	60.3% (56.57%-64.14%)	38.92% (33.87%-43.75%)	97.256% (95.588%-100%)	96.08% (93.62%-100%)	47.94% (43.35%-52.74%)	2.8020 (2.7024-2.9036)	60.21% (52.53%-67.68%)	38.63% (28.98%-48.48%)	97.233% (92.105%-100%)	95.99% (88.89%-100%)	48% (38.88%-57.58%)	2.7986 (2.5918-2.9980)
**At least 7 of CHASM (lung), CHASM (melanoma), FATHMM (cancer), Mutation Assessor, PolyPhen-2, PROVEAN, SIFT**	58.28% (54.53%-62.12%)	34.67% (29.83%-39.2%)	99.084% (98.485%-100%)	98.5% (97.37%-100%)	46.73% (42.1%-50.99%)	2.7898 (2.7082-2.8734)	58.2% (50.51%-65.68%)	34.36% (25%-44.45%)	99.083% (96.774%-100%)	98.47% (94.44%-100%)	46.79% (37.84%-56.1%)	2.7870 (2.6171-2.9561)
**At least 7 of CHASM (lung), CHASM (melanoma), FATHMM (cancer), Mutation Assessor, PolyPhen-2, PROVEAN, SIFT, VEST**	67.05% (63.13%-70.71%)	51.18% (45.97%-56.14%)	94.488% (91.549%-97.368%)	94.14% (91.17%-97.3%)	52.82% (47.76%-57.6%)	2.9262 (2.8178-3.0381)	66.91% (59.6%-74.75%)	50.8% (40.91%-60.87%)	94.497% (87.879%-100%)	94.07% (86.66%-100%)	52.81% (43.28%-62.69%)	2.9218 (2.6873-3.1420)
**At least 7 of CHASM (lung), CHASM (melanoma), FATHMM (cancer), Mutation Assessor, PolyPhen-2, PROVEAN, VEST**	56.24% (52.53%-60.1%)	31.46% (26.67%-35.77%)	99.084% (98.485%-100%)	98.35% (97.06%-100%)	45.54% (40.76%-50%)	2.7443 (2.6620-2.8293)	56.2% (48.48%-63.64%)	31.2% (22.58%-40.99%)	99.083% (96.774%-100%)	98.32% (93.75%-100%)	45.63% (37.04%-55.13%)	2.7423 (2.5695-2.9070)
**At least 7 of CHASM (lung), CHASM (melanoma), FATHMM (cancer), Mutation Assessor, PolyPhen-2, SIFT, VEST**	60.3% (56.57%-64.14%)	38.92% (33.88%-43.94%)	97.256% (95.588%-100%)	96.08% (93.62%-100%)	47.94% (43.15%-52.41%)	2.8019 (2.7051-2.9036)	60.22% (52.53%-67.68%)	38.63% (28.33%-48.39%)	97.233% (92.105%-100%)	95.99% (88.46%-100%)	48% (39.19%-57.53%)	2.7986 (2.5898-2.9918)
**At least 7 of CHASM (lung), CHASM (melanoma), FATHMM (cancer), Mutation Assessor, PROVEAN, SIFT, VEST**	57.26% (53.03%-61.11%)	33.07% (28.35%-37.82%)	99.084% (98.485%-100%)	98.42% (97.3%-100%)	46.13% (41.25%-50.64%)	2.7670 (2.6821-2.8522)	57.2% (49.49%-65.66%)	32.78% (23.43%-42.37%)	99.083% (96.774%-100%)	98.4% (94.12%-100%)	46.2% (37.35%-55.56%)	2.7647 (2.5989-2.9401)
**At least 7 of CHASM (lung), CHASM (melanoma), FATHMM (cancer), MutationTaster, PolyPhen-2, PROVEAN, SIFT**	58.6% (54.55%-62.13%)	35.19% (30.16%-40.15%)	99.084% (98.485%-100%)	98.52% (97.44%-100%)	46.93% (42.11%-51.01%)	2.7971 (2.7129-2.8785)	58.55% (51.49%-66.67%)	34.92% (25.39%-45.16%)	99.083% (96.774%-100%)	98.5% (94.44%-100%)	47.01% (38.35%-56.58%)	2.7951 (2.6312-2.9727)
**At least 7 of CHASM (lung), CHASM (melanoma), FATHMM (cancer), MutationTaster, PolyPhen-2, PROVEAN, SIFT, VEST**	68.39% (65.14%-72.22%)	52.76% (47.93%-57.94%)	95.411% (92.958%-98.571%)	95.21% (92.54%-98.57%)	53.87% (48.92%-58.54%)	2.9725 (2.8724-3.0823)	68.27% (60.61%-74.77%)	52.44% (41.94%-62.5%)	95.417% (89.474%-100%)	95.15% (88.24%-100%)	53.9% (44.26%-63.64%)	2.9691 (2.7462-3.1740)
**At least 7 of CHASM (lung), CHASM (melanoma), FATHMM (cancer), MutationTaster, PolyPhen-2, PROVEAN, VEST**	56.91% (53.03%-60.61%)	32.52% (27.56%-37.12%)	99.084% (98.485%-100%)	98.4% (97.22%-100%)	45.92% (41.14%-50%)	2.7592 (2.6740-2.8428)	56.89% (49.49%-64.65%)	32.29% (22.81%-42.2%)	99.083% (96.774%-100%)	98.38% (94.12%-100%)	46.02% (37.5%-55.7%)	2.7577 (2.5898-2.9315)
**At least 7 of CHASM (lung), CHASM (melanoma), FATHMM (cancer), MutationTaster, PolyPhen-2, SIFT, VEST**	61.29% (57.58%-65.15%)	40.49% (35.65%-45.6%)	97.256% (95.588%-100%)	96.23% (93.88%-100%)	48.59% (43.84%-53.02%)	2.8257 (2.7321-2.9296)	61.25% (53.54%-68.69%)	40.27% (30.15%-50%)	97.233% (92.105%-100%)	96.15% (89.29%-100%)	48.68% (39.7%-58.33%)	2.8233 (2.6185-3.0161)
**At least 7 of CHASM (lung), CHASM (melanoma), FATHMM (cancer), MutationTaster, PROVEAN, SIFT, VEST**	58.61% (54.55%-62.63%)	35.19% (30.3%-40%)	99.084% (98.485%-100%)	98.52% (97.44%-100%)	46.93% (42.1%-51.3%)	2.7972 (2.7086-2.8824)	58.55% (50.51%-66.67%)	34.92% (25%-44.83%)	99.083% (96.774%-100%)	98.5% (94.44%-100%)	47.01% (38.27%-56.76%)	2.7951 (2.6306-2.9811)
**At least 7 of CHASM (lung), CHASM (melanoma), FATHMM (cancer), PolyPhen-2, PROVEAN, SIFT, VEST**	58.26% (54.04%-61.63%)	34.65% (29.83%-39.2%)	99.084% (98.485%-100%)	98.49% (97.37%-100%)	46.72% (41.72%-50.98%)	2.7895 (2.7059-2.8707)	58.22% (51.49%-66.67%)	34.39% (25%-44.45%)	99.083% (96.774%-100%)	98.47% (94.44%-100%)	46.81% (38.23%-56.58%)	2.7876 (2.6186-2.9606)
**At least 7 of CHASM (lung), CHASM (melanoma), FATHMM (missense), Mutation Assessor, MutationTaster, PolyPhen-2, PROVEAN**	55.25% (51.01%-59.09%)	29.9% (25.2%-34.43%)	99.084% (98.485%-100%)	98.26% (96.97%-100%)	44.98% (40.38%-49.08%)	2.7222 (2.6385-2.8081)	55.16% (47.47%-63.64%)	29.55% (20.63%-39.07%)	99.083% (96.774%-100%)	98.23% (93.33%-100%)	45.04% (36.62%-54.55%)	2.7190 (2.5417-2.8924)
[illegible]	[illegible]	[illegible]	[illegible]	[illegible]	[illegible]	[illegible]	[illegible]	[illegible]	[illegible]	[illegible]	[illegible]	[illegible]

At least 8 of CHASM (breast), CHASM (lung), CHASM (melanoma), Mutation Assessor, MutationTaster, PolyPhen-2, PROVEAN, SIFT, VEST	64.68% (61.11%-68.69%)	46.39% (41.32%-51.61%)	96.311% (94.286%-98.667%)	95.61% (92.98%-98.44%)	50.96% (46.21%-55.4%)	2.8926 (2.7910-2.9991)	64.57% (56.57%-71.72%)	46.03% (35.48%-55.88%)	96.355% (91.176%-100%)	95.6% (88.89%-100%)	50.99% (41.66%-60.61%)	2.8897 (2.6742-3.0936)
At least 8 of CHASM (breast), CHASM (lung), CHASM (melanoma), Mutation Assessor, MutationTaster, PolyPhen-2, PROVEAN, VEST	53.54% (49.48%-57.58%)	27.2% (22.22%-31.75%)	99.084% (98.485%-100%)	98.09% (96.55%-100%)	44.05% (39.37%-48.19%)	2.6842 (2.5990-2.7732)	53.53% (45.45%-61.64%)	26.97% (17.91%-37.29%)	99.083% (96.774%-100%)	98.06% (92.31%-100%)	44.15% (35.63%-53.57%)	2.6827 (2.5030-2.8578)
At least 8 of CHASM (breast), CHASM (lung), CHASM (melanoma), Mutation Assessor, MutationTaster, PolyPhen-2, SIFT, VEST	56.9% (53.03%-60.61%)	33.58% (28.57%-38.4%)	97.222% (95.588%-100%)	95.43% (92.5%-100%)	45.85% (41.18%-50%)	2.7208 (2.6232-2.8234)	56.9% (49.49%-64.65%)	33.36% (23.72%-43.55%)	97.294% (92.303%-100%)	95.48% (87.5%-100%)	45.97% (37.33%-55.27%)	2.7210 (2.5070-2.9119)
At least 8 of CHASM (breast), CHASM (lung), CHASM (melanoma), Mutation Assessor, MutationTaster, PROVEAN, SIFT, VEST	54.56% (50.51%-58.59%)	28.8% (24%-33.33%)	99.084% (98.485%-100%)	98.19% (96.77%-100%)	44.6% (39.88%-49.03%)	2.7068 (2.6209-2.7949)	54.52% (46.46%-62.63%)	28.55% (19.64%-38.46%)	99.083% (96.774%-100%)	98.17% (92.86%-100%)	44.7% (36.11%-54.12%)	2.7050 (2.5268-2.8825)
At least 8 of CHASM (breast), CHASM (lung), CHASM (melanoma), Mutation Assessor, PolyPhen-2, PROVEAN, SIFT, VEST	54.89% (50.51%-58.59%)	29.33% (24.39%-33.6%)	99.084% (98.485%-100%)	98.23% (96.88%-100%)	44.78% (40%-49.06%)	2.7142 (2.6286-2.8020)	54.86% (47.47%-63.64%)	29.08% (20%-38.98%)	99.083% (96.774%-100%)	98.2% (93.33%-100%)	44.88% (36.48%-54.22%)	2.7124 (2.5326-2.8876)
At least 8 of CHASM (breast), CHASM (lung), CHASM (melanoma), MutationTaster, PolyPhen-2, PROVEAN, SIFT, VEST	55.56% (51.52%-59.6%)	30.39% (25.4%-35%)	99.084% (98.485%-100%)	98.29% (96.97%-100%)	45.15% (40.38%-49.35%)	2.7291 (2.6431-2.8168)	55.54% (47.47%-63.64%)	30.17% (20.97%-40.32%)	99.083% (96.774%-100%)	98.26% (93.33%-100%)	45.26% (36.84%-54.88%)	2.7277 (2.5529-2.9058)
At least 8 of CHASM (breast), CHASM (lung), FATHMM (cancer), FATHMM (missense), Mutation Assessor, MutationTaster, PolyPhen-2, PROVEAN	54.58% (50.51%-58.59%)	28.84% (24.16%-33.33%)	99.084% (98.485%-100%)	98.2% (96.77%-100%)	44.61% (40%-48.78%)	2.7072 (2.6204-2.7928)	54.48% (46.46%-62.63%)	28.48% (19.66%-37.94%)	99.083% (96.774%-100%)	98.17% (93.33%-100%)	44.67% (36.24%-53.85%)	2.7040 (2.5284-2.8770)
At least 8 of CHASM (breast), CHASM (lung), FATHMM (cancer), FATHMM (missense), Mutation Assessor, MutationTaster, PolyPhen-2, PROVEAN, SIFT	65.34% (61.62%-69.19%)	47.44% (42.31%-52.51%)	96.298% (94.203%-98.684%)	95.68% (93.1%-98.46%)	51.45% (46.72%-56.06%)	2.9086 (2.8056-3.0097)	65.27% (57.58%-72.73%)	47.12% (36.92%-57.15%)	96.392% (90.909%-100%)	95.73% (89.29%-100%)	51.51% (42.25%-61.02%)	2.9075 (2.7009-3.1116)
At least 8 of CHASM (breast), CHASM (lung), FATHMM (cancer), FATHMM (missense), Mutation Assessor, MutationTaster, PolyPhen-2, PROVEAN, SIFT, VEST	72.79% (69.19%-76.26%)	60.21% (55.28%-65.6%)	94.526% (91.667%-97.436%)	95% (92.41%-97.65%)	57.88% (52.76%-63.03%)	3.0763 (2.9638-3.1862)	72.6% (65.66%-79.8%)	59.86% (49.09%-70.18%)	94.426% (88.235%-100%)	94.85% (88.37%-100%)	57.82% (47.62%-68.26%)	3.0696 (2.8479-3.2998)
At least 8 of CHASM (breast), CHASM (lung), FATHMM (cancer), FATHMM (missense), Mutation Assessor, MutationTaster, PolyPhen-2, PROVEAN, VEST	63.02% (59.09%-66.67%)	43.76% (38.46%-49.17%)	96.298% (94.203%-98.684%)	95.34% (92.59%-98.33%)	49.76% (45.14%-54.29%)	2.8516 (2.7483-2.9576)	62.86% (55.56%-70.71%)	43.3% (32.79%-53.34%)	96.392% (90.909%-100%)	95.37% (88.46%-100%)	49.77% (40.58%-59.15%)	2.8483 (2.6295-3.0531)
At least 8 of CHASM (breast), CHASM (lung), FATHMM (cancer), FATHMM (missense), Mutation Assessor, MutationTaster, PolyPhen-2, SIFT	60.29% (56.57%-64.14%)	38.39% (33.33%-43.09%)	98.143% (97.015%-100%)	97.28% (95.45%-100%)	47.95% (43.26%-52.35%)	2.8177 (2.7281-2.9114)	60.23% (52.53%-67.68%)	38.1% (28.33%-48.33%)	98.209% (94.118%-100%)	97.35% (91.66%-100%)	48.04% (39.47%-57.53%)	2.8170 (2.6354-2.9953)
At least 8 of CHASM (breast), CHASM (lung), FATHMM (cancer), FATHMM (missense), Mutation Assessor, MutationTaster, PolyPhen-2, SIFT, VEST	67.04% (63.13%-70.71%)	50.62% (45.31%-55.97%)	95.411% (92.958%-98.571%)	95.02% (92.3%-98.48%)	52.78% (48%-57.46%)	2.9383 (2.8398-3.0505)	66.94% (59.6%-74.75%)	50.33% (39.65%-60.72%)	95.417% (89.474%-100%)	94.96% (88%-100%)	52.82% (43.33%-62.91%)	2.9352 (2.7140-3.1375)
At least 8 of CHASM (breast), CHASM (lung), FATHMM (cancer), FATHMM (missense), Mutation Assessor, MutationTaster, PolyPhen-2, VEST	56.59% (52.53%-60.61%)	32.56% (27.35%-37.3%)	98.143% (97.015%-100%)	96.81% (94.59%-100%)	45.7% (41.06%-50%)	2.7322 (2.6368-2.8227)	56.51% (48.48%-64.65%)	32.21% (22.58%-42.37%)	98.209% (94.118%-100%)	96.88% (90%-100%)	45.78% (37.04%-55.13%)	2.7308 (2.5444-2.9262)
At least 8 of CHASM (breast), CHASM (lung), FATHMM (cancer), FATHMM (missense), Mutation Assessor, MutationTaster, PROVEAN, SIFT	55.59% (51.52%-59.6%)	30.44% (25.78%-34.92%)	99.084% (98.485%-100%)	98.29% (96.97%-100%)	45.17% (40.51%-49.38%)	2.7299 (2.6432-2.8168)	55.48% (47.47%-63.64%)	30.06% (21.05%-39.66%)	99.083% (96.774%-100%)	98.26% (93.33%-100%)	45.22% (36.48%-54.43%)	2.7263 (2.5496-2.9001)
At least 8 of CHASM (breast), CHASM (lung), FATHMM (cancer), FATHMM (missense), Mutation Assessor, MutationTaster, PROVEAN, SIFT, VEST	63.35% (59.6%-67.17%)	44.29% (39.34%-49.22%)	96.298% (94.203%-98.684%)	95.39% (92.72%-98.39%)	49.99% (45.39%-54.61%)	2.8596 (2.7535-2.9642)	63.2% (55.56%-70.71%)	43.85% (33.82%-53.85%)	96.392% (90.909%-100%)	95.43% (88.46%-100%)	50% (40.84%-59.16%)	2.8568 (2.6512-3.0671)
At least 8 of CHASM (breast), CHASM (lung), FATHMM (cancer), FATHMM (missense), Mutation Assessor, MutationTaster, PROVEAN, VEST	53.9% (49.49%-57.58%)	27.77% (22.9%-32.23%)	99.084% (98.485%-100%)	98.13% (96.67%-100%)	44.24% (39.52%-48.49%)	2.6922 (2.6043-2.7788)	53.81% (46.46%-62.63%)	27.43% (18.96%-36.93%)	99.083% (96.774%-100%)	98.1% (92.86%-100%)	44.31% (35.71%-53.57%)	2.6892 (2.5121-2.8688)
At least 8 of CHASM (breast), CHASM (lung), FATHMM (cancer), FATHMM (missense), Mutation Assessor, MutationTaster, SIFT, VEST	57.61% (53.54%-61.62%)	34.17% (29.13%-39.2%)	98.143% (97.015%-100%)	96.96% (94.87%-100%)	46.3% (41.56%-50.67%)	2.7557 (2.6638-2.8483)	57.51% (49.49%-65.66%)	33.78% (23.73%-43.55%)	98.209% (94.118%-100%)	97.02% (90.48%-100%)	46.36% (37.5%-55.84%)	2.7536 (2.5695-2.9438)
At least 8 of CHASM (breast), CHASM (lung), FATHMM (cancer), FATHMM (missense), Mutation Assessor, PolyPhen-2, PROVEAN, SIFT	56.27% (52.02%-60.1%)	31.51% (26.45%-36.07%)	99.084% (98.485%-100%)	98.35% (97.06%-100%)	45.55% (40.88%-49.69%)	2.7449 (2.6572-2.8294)	56.15% (48.48%-64.65%)	31.11% (22.03%-41.27%)	99.083% (96.774%-100%)	98.31% (93.75%-100%)	45.6% (36.84%-54.88%)	2.7410 (2.5675-2.9144)
At least 8 of CHASM (breast), CHASM (lung), FATHMM (cancer), FATHMM (missense), Mutation Assessor, PolyPhen-2, PROVEAN, SIFT, VEST	62.67% (59.09%-66.16%)	43.22% (38.09%-48.39%)	96.298% (94.203%-98.684%)	95.28% (92.45%-98.33%)	49.52% (44.93%-54.11%)	2.8432 (2.7394-2.9508)	62.53% (55.56%-69.7%)	42.79% (32.78%-52.94%)	96.392% (90.909%-100%)	95.32% (88.46%-100%)	49.54% (40.32%-58.67%)	2.8404 (2.6251-3.0458)
At least 8 of CHASM (breast), CHASM (lung), FATHMM (cancer), FATHMM (missense), Mutation Assessor, PolyPhen-2, PROVEAN, VEST	54.24% (50%-58.08%)	28.3% (23.25%-32.54%)	99.084% (98.485%-100%)	98.16% (96.77%-100%)	44.42% (39.64%-48.7%)	2.6997 (2.6126-2.7840)	54.15% (46.46%-62.63%)	27.96% (19.4%-38.1%)	99.083% (96.774%-100%)	98.13% (92.86%-100%)	44.49% (36.14%-53.85%)	2.6966 (2.5200-2.8731)
At least 8 of CHASM (breast), CHASM (lung), FATHMM (cancer), FATHMM (missense), Mutation Assessor, PolyPhen-2, SIFT, VEST	57.61% (53.54%-61.12%)	34.16% (29.03%-38.89%)	98.143% (97.015%-100%)	96.96% (94.87%-100%)	46.3% (41.56%-50.65%)	2.7556 (2.6629-2.8466)	57.51% (50.48%-65.66%)	33.78% (24.58%-43.86%)	98.209% (94.118%-100%)	97.02% (90.48%-100%)	46.36% (37.66%-55.84%)	2.7537 (2.5695-2.9336)
At least 8 of CHASM (breast), CHASM (lung), FATHMM (cancer), FATHMM (missense), Mutation Assessor, PROVEAN, SIFT, VEST	55.26% (51.01%-59.09%)	29.9% (25%-34.17%)	99.084% (98.485%-100%)	98.26% (96.97%-100%)	44.98% (40.26%-49.37%)	2.7223 (2.6401-2.8105)	55.15% (47.47%-63.64%)	29.54% (20.63%-39.29%)	99.083% (96.774%-100%)	98.23% (93.33%-100%)	45.04% (36.36%-54.22%)	2.7188 (2.5417-2.8827)
[illegible]	[illegible]	[illegible]	[illegible]	[illegible]	[illegible]	[illegible]	[illegible]	[illegible]	[illegible]	[illegible]	[illegible]	[illegible]

NPV, negative predictive value; PPV, positive predictive value.
