# Supplementary material for: Benchmarking mutation effect prediction algorithms using functionally validated cancer-related missense mutations
Source: Genome Biol. 2014 Oct 28;15(10):484. doi: 10.1186/s13059-014-0484-1 (PMC4232638; doi:10.1186/s13059-014-0484-1)
Supplement: Additional file 19: — Number of mutation effect prediction algorithm combinations that outperform single predictors and meta-predictors. [file 13059_2014_484_MOESM19_ESM.pdf]

Additional file 19: Number of mutation effect prediction algorithm combinations that outperform single predictors and meta-predictors.

Compared to single predictors		All mutations (n=989)			Without COSMIC mutations (n=297)		
		Subset 1	Subset 2	Intersection	Subset 1	Subset 2	Intersection
**Accuracy**	Mean	1950	1860	1854	869	834	789
	Statistically significant	146	6	6	15	1	1
**Sensitivity**	Mean	4353	4311	4311	4665	4663	4629
	Statistically significant	3231	2860	2860	3514	2764	2764
**Specificity**	Mean	3971	3873	3865	2722	2695	2567
	Statistically significant	1880	886	886	384	0	0
**PPV**	Mean	3689	3664	3662	2075	2052	2040
	Statistically significant	121	0	0	0	0	0
**NPV**	Mean	4444	4428	4428	4738	4735	4727
	Statistically significant	2579	1721	1721	2997	2054	2054
**Composite score**	Mean	1530	1487	1483	804	797	789
	Statistically significant	69	5	5	7	1	1

Compared to meta-predictors		All mutations (n=989)			Without COSMIC mutations (n=297)		
		Subset 1	Subset 2	Intersection	Subset 1	Subset 2	Intersection
**Accuracy**	Mean	1666	1604	1604	789	783	783
	Statistically significant	89	3	3	15	1	1
**Sensitivity**	Mean	4958	4945	4945	3344	3137	3137
	Statistically significant	4474	4270	4270	1788	1788	1788
**Specificity**	Mean	3864	3864	3864	2239	2239	2239
	Statistically significant	1880	886	886	0	0	0
**PPV**	Mean	3273	3230	3229	1017	1001	991
	Statistically significant	6	0	0	0	0	0
**NPV**	Mean	5380	5361	5361	4809	4827	4805
	Statistically significant	4539	3920	3920	2939	2026	2026
**Composite score**	Mean	794	761	761	466	459	453
	Statistically significant	6	1	1	4	0	0

NPV, negative predictive value; PPV, positive predictive value.
