# Supplementary material for: Benchmarking mutation effect prediction algorithms using functionally validated cancer-related missense mutations
Source: Genome Biol. 2014 Oct 28;15(10):484. doi: 10.1186/s13059-014-0484-1 (PMC4232638; doi:10.1186/s13059-014-0484-1)
Supplement: Additional file 21: — Top five mutation effect prediction algorithm combinations ranked by either accuracy or composite score separately for subsets 1 and 2 using all 989 single nucleotide variants for which functional data are available and the corresponding best performing single and meta-predictors. [file 13059_2014_484_MOESM21_ESM.pdf]

**Additional file 21: Top 5 mutation effect prediction algorithm combinations ranked by either accuracy or composite score separately for subsets 1 and 2 using all 989 single nucleotide variants for which functional data are available and the corresponding best performing single and meta-predictors.**

| | Ranking | Mutation effect prediction algorithm combination | Accuracy (95% CI) | Sensitivity (95% CI) | Specificity (95% CI) | PPV (95% CI) | NPV (95% CI) | Composite score (95% CI) |
|---|---|---|---|---|---|---|---|---|
| Subset 1 | By accuracy | At least 1 of CHASM (breast), MutationTaster | 95.46% (94.54%-96.51%) | 99.88% (99.82%-100%) | 68.61% (63.09%-74.73%) | 95.07% (94.11%-96.13%) | 98.98% (98.25%-100%) | 3.6255 (3.5584-3.6982) |
| | | At least 2 of CHASM (breast), CHASM (lung), MutationTaster, SIFT | 95.16% (94.23%-96.21%) | 99.07% (98.59%-99.48%) | 71.47% (66%-77%) | 95.47% (94.54%-96.48%) | 92.69% (89.39%-96.05%) | 3.5870 (3.5056-3.6697) |
| | | At least 2 of CHASM (breast), CHASM (lung), Mutation Assessor, MutationTaster | 95.08% (94.23%-96.05%) | 98.49% (98.01%-99.12%) | 74.36% (69.23%-79.78%) | 95.89% (94.99%-96.91%) | 89.04% (85.19%-93.33%) | 3.5777 (3.4957-3.6589) |
| | | At least 1 of CHASM (breast), CHASM (lung), MutationTaster | 94.95% (93.93%-96.05%) | 99.88% (99.82%-100%) | 64.99% (59.3%-70.84%) | 94.54% (93.49%-95.67%) | 98.93% (98.11%-100%) | 3.5834 (3.5144-3.6572) |
| | | At least 1 of CHASM (lung), MutationTaster | 94.95% (93.93%-96.05%) | 99.88% (99.82%-100%) | 64.99% (59.3%-70.84%) | 94.54% (93.49%-95.67%) | 98.93% (98.11%-100%) | 3.5834 (3.5144-3.6572) |
| | | FATHMM (cancer) [best single predictor] | 91% (89.18%-92.62%) | 97.88% (96.87%-98.82%) | 49.29% (41.08%-57.24%) | 92.13% (90.26%-93.72%) | 79.31% (70.59%-87.66%) | 3.1860 (3.0326-3.3292) |
| | | CanDrA (lung) [best meta-predictor] | 91.28% (89.54%-93.04%) | 92.86% (91.05%-94.62%) | 80.95% (73.64%-87.32%) | 96.97% (95.75%-98.03%) | 63.35% (56.1%-71.01%) | 3.3413 (3.2103-3.4747) |
| | By composite score | At least 1 of CHASM (breast), MutationTaster | 95.46% (94.54%-96.51%) | 99.88% (99.82%-100%) | 68.61% (63.09%-74.73%) | 95.07% (94.11%-96.13%) | 98.98% (98.25%-100%) | 3.6255 (3.5584-3.6982) |
| | | At least 2 of CHASM (breast), CHASM (lung), MutationTaster, SIFT | 95.16% (94.23%-96.21%) | 99.07% (98.59%-99.48%) | 71.47% (66%-77%) | 95.47% (94.54%-96.48%) | 92.69% (89.39%-96.05%) | 3.5870 (3.5056-3.6697) |
| | | At least 1 of CHASM (breast), CHASM (lung), MutationTaster | 94.95% (93.93%-96.05%) | 99.88% (99.82%-100%) | 64.99% (59.3%-70.84%) | 94.54% (93.49%-95.67%) | 98.93% (98.11%-100%) | 3.5834 (3.5144-3.6572) |
| | | At least 1 of CHASM (lung), MutationTaster | 94.95% (93.93%-96.05%) | 99.88% (99.82%-100%) | 64.99% (59.3%-70.84%) | 94.54% (93.49%-95.67%) | 98.93% (98.11%-100%) | 3.5834 (3.5144-3.6572) |
| | | At least 2 of CHASM (breast), CHASM (lung), Mutation Assessor, MutationTaster | 95.08% (94.23%-96.05%) | 98.49% (98.01%-99.12%) | 74.36% (69.23%-79.78%) | 95.89% (94.99%-96.91%) | 89.04% (85.19%-93.33%) | 3.5777 (3.4957-3.6589) |
| | | CHASM (lung) [best single predictor] | 90.09% (88.27%-91.81%) | 92.23% (90.4%-94.04%) | 77.14% (69.93%-83.58%) | 96.07% (94.74%-97.31%) | 62.07% (54.72%-69.38%) | 3.2751 (3.1418-3.3988) |
| | | CanDrA (lung) [best meta-predictor] | 91.28% (89.54%-93.04%) | 92.86% (91.05%-94.62%) | 80.95% (73.64%-87.32%) | 96.97% (95.75%-98.03%) | 63.35% (56.1%-71.01%) | 3.3413 (3.2103-3.4747) |
| Subset 2 | By accuracy | At least 1 of CHASM (breast), MutationTaster | 95.43% (93.33%-97.27%) | 99.88% (99.64%-100%) | 68.5% (57.14%-79.55%) | 95.05% (92.95%-96.98%) | 98.93% (96.15%-100%) | 3.6236 (3.4841-3.7573) |
| | | At least 2 of CHASM (breast), CHASM (lung), MutationTaster, SIFT | 95.11% (93.03%-96.97%) | 99.03% (98.18%-100%) | 71.35% (60%-82%) | 95.44% (93.38%-97.29%) | 92.42% (85.29%-100%) | 3.5825 (3.4147-3.7387) |
| | | At least 2 of CHASM (breast), CHASM (lung), Mutation Assessor, MutationTaster | 94.98% (93.03%-96.67%) | 98.42% (97.16%-99.33%) | 74.13% (64%-84.62%) | 95.84% (93.77%-97.61%) | 88.58% (80%-96.02%) | 3.5699 (3.4077-3.7313) |
| | | At least 1 of CHASM (breast), CHASM (lung), MutationTaster | 94.94% (92.73%-96.97%) | 99.88% (99.64%-100%) | 65.05% (53.19%-76.6%) | 94.54% (92.26%-96.64%) | 98.87% (96%-100%) | 3.5834 (3.4389-3.7270) |
| | | At least 1 of CHASM (lung), MutationTaster | 94.94% (92.73%-96.97%) | 99.88% (99.64%-100%) | 65.05% (53.19%-76.6%) | 94.54% (92.26%-96.64%) | 98.87% (96%-100%) | 3.5834 (3.4389-3.7270) |
| | | FATHMM (cancer) [best single predictor] | 91% (89.18%-92.62%) | 97.88% (96.87%-98.82%) | 49.29% (41.08%-57.24%) | 92.13% (90.26%-93.72%) | 79.31% (70.59%-87.66%) | 3.1860 (3.0326-3.3292) |
| | | CanDrA (lung) [best meta-predictor] | 91.28% (89.54%-93.04%) | 92.86% (91.05%-94.62%) | 80.95% (73.64%-87.32%) | 96.97% (95.75%-98.03%) | 63.35% (56.1%-71.01%) | 3.3413 (3.2103-3.4747) |
| | By composite score | At least 1 of CHASM (breast), MutationTaster | 95.43% (93.33%-97.27%) | 99.88% (99.64%-100%) | 68.5% (57.14%-79.55%) | 95.05% (92.95%-96.98%) | 98.93% (96.15%-100%) | 3.6236 (3.4841-3.7573) |
| | | At least 1 of CHASM (breast), CHASM (lung), MutationTaster | 94.94% (92.73%-96.97%) | 99.88% (99.64%-100%) | 65.05% (53.19%-76.6%) | 94.54% (92.26%-96.64%) | 98.87% (96%-100%) | 3.5834 (3.4389-3.7270) |
| | | At least 1 of CHASM (lung), MutationTaster | 94.94% (92.73%-96.97%) | 99.88% (99.64%-100%) | 65.05% (53.19%-76.6%) | 94.54% (92.26%-96.64%) | 98.87% (96%-100%) | 3.5834 (3.4389-3.7270) |
| | | At least 2 of CHASM (breast), CHASM (lung), MutationTaster, SIFT | 95.11% (93.03%-96.97%) | 99.03% (98.18%-100%) | 71.35% (60%-82%) | 95.44% (93.38%-97.29%) | 92.42% (85.29%-100%) | 3.5825 (3.4147-3.7387) |
| | | At least 2 of CHASM (breast), CHASM (lung), Mutation Assessor, MutationTaster | 94.98% (93.03%-96.67%) | 98.42% (97.16%-99.33%) | 74.13% (64%-84.62%) | 95.84% (93.77%-97.61%) | 88.58% (80%-96.02%) | 3.5699 (3.4077-3.7313) |
| | | CHASM (lung) [best single predictor] | 90.09% (88.27%-91.81%) | 92.23% (90.4%-94.04%) | 77.14% (69.93%-83.58%) | 96.07% (94.74%-97.31%) | 62.07% (54.72%-69.38%) | 3.2751 (3.1418-3.3988) |
| | | CanDrA (lung) [best meta-predictor] | 91.28% (89.54%-93.04%) | 92.86% (91.05%-94.62%) | 80.95% (73.64%-87.32%) | 96.97% (95.75%-98.03%) | 63.35% (56.1%-71.01%) | 3.3413 (3.2103-3.4747) |
