# Supplementary material for: Benchmarking mutation effect prediction algorithms using functionally validated cancer-related missense mutations
Source: Genome Biol. 2014 Oct 28;15(10):484. doi: 10.1186/s13059-014-0484-1 (PMC4232638; doi:10.1186/s13059-014-0484-1)
Supplement: Additional file 22: — Top five mutation effect prediction algorithm combinations ranked by either accuracy or composite score separately for subsets 1 and 2 using all 297 single nucleotide variants not included in the COSMIC database for which functional data are available and the corresponding best performing single and meta-predictors. [file 13059_2014_484_MOESM22_ESM.pdf]

Additional file 22: Top 5 mutation effect prediction algorithm combinations ranked by either accuracy or composite score separately for subsets 1 and 2 using all 297 single nucleotide variants for which functional data are available not included in the COSMIC database and the corresponding best performing single and meta-predictors.

	Ranking	Mutation effect prediction algorithm combination	Accuracy (95% CI)	Sensitivity (95% CI)	Specificity (95% CI)	PPV (95% CI)	NPV (95% CI)	Composite score (95% CI)
Subset 1	By accuracy	At least 1 of CHASM (breast), MutationTaster	93.34% (91.41%-95.45%)	99.49% (99.16%-100%)	82.72% (78.08%-88%)	90.87% (88.32%-93.99%)	98.95% (98.15%-100%)	3.7203 (3.6502-3.8042)
		At least 2 of CHASM (breast), CHASM (lung), Mutation Assessor, MutationTaster	91.95% (89.9%-93.95%)	94.71% (92.56%-96.9%)	87.16% (83.33%-91.89%)	92.73% (90.48%-95.49%)	90.52% (86.76%-94.44%)	3.6512 (3.5626-3.7463)
		At least 2 of CHASM (breast), CHASM (lung), MutationTaster, SIFT	91.95% (89.9%-93.94%)	96.3% (94.4%-98.39%)	84.42% (80%-89.34%)	91.44% (88.98%-94.29%)	92.96% (89.55%-96.83%)	3.6512 (3.5640-3.7456)
		At least 1 of CHASM (breast), CHASM (lung), MutationTaster	91.66% (89.9%-93.94%)	99.49% (99.16%-100%)	78.11% (73.13%-83.78%)	88.71% (86.03%-91.85%)	98.89% (98.04%-100%)	3.6520 (3.5777-3.7447)
		At least 1 of CHASM (lung), MutationTaster	91.66% (89.9%-93.94%)	99.49% (99.16%-100%)	78.11% (73.13%-83.78%)	88.71% (86.03%-91.85%)	98.89% (98.04%-100%)	3.6520 (3.5777-3.7447)
		MutationTaster [best single predictor]	84.51% (80.13%-88.55%)	84.04% (78.53%-89.25%)	85.32% (78.18%-91.75%)	90.8% (86.05%-95%)	75.61% (67.42%-82.88%)	3.3578 (3.1879-3.5244)
		CanDrA (lung) [best meta-predictor]	84.7% (80.5%-88.65%)	79.12% (73.21%-84.57%)	94.95% (89.81%-98.94%)	96.64% (93.29%-99.31%)	71.21% (63.41%-78.3%)	3.4193 (3.2675-3.5628)
	By composite score	At least 1 of CHASM (breast), MutationTaster	93.34% (91.41%-95.45%)	99.49% (99.16%-100%)	82.72% (78.08%-88%)	90.87% (88.32%-93.99%)	98.95% (98.15%-100%)	3.7203 (3.6502-3.8042)
		At least 1 of CHASM (breast), CHASM (lung), MutationTaster	91.66% (89.9%-93.94%)	99.49% (99.16%-100%)	78.11% (73.13%-83.78%)	88.71% (86.03%-91.85%)	98.89% (98.04%-100%)	3.6520 (3.5777-3.7447)
		At least 1 of CHASM (lung), MutationTaster	91.66% (89.9%-93.94%)	99.49% (99.16%-100%)	78.11% (73.13%-83.78%)	88.71% (86.03%-91.85%)	98.89% (98.04%-100%)	3.6520 (3.5777-3.7447)
		At least 2 of CHASM (breast), CHASM (lung), Mutation Assessor, MutationTaster	91.95% (89.9%-93.95%)	94.71% (92.56%-96.9%)	87.16% (83.33%-91.89%)	92.73% (90.48%-95.49%)	90.52% (86.76%-94.44%)	3.6512 (3.5626-3.7463)
		At least 2 of CHASM (breast), CHASM (lung), MutationTaster, SIFT	91.95% (89.9%-93.94%)	96.3% (94.4%-98.39%)	84.42% (80%-89.34%)	91.44% (88.98%-94.29%)	92.96% (89.55%-96.83%)	3.6512 (3.5640-3.7456)
		MutationTaster [best single predictor]	84.51% (80.13%-88.55%)	84.04% (78.53%-89.25%)	85.32% (78.18%-91.75%)	90.8% (86.05%-95%)	75.61% (67.42%-82.88%)	3.3578 (3.1879-3.5244)
		CanDrA (lung) [best meta-predictor]	84.7% (80.5%-88.65%)	79.12% (73.21%-84.57%)	94.95% (89.81%-98.94%)	96.64% (93.29%-99.31%)	71.21% (63.41%-78.3%)	3.4193 (3.2675-3.5628)
Subset 2	By accuracy	At least 1 of CHASM (breast), MutationTaster	93.11% (88.89%-96.97%)	99.42% (98.21%-100%)	82.29% (72.21%-91.67%)	90.59% (84.38%-95.65%)	98.81% (96%-100%)	3.7111 (3.5515-3.8634)
		At least 2 of CHASM (breast), CHASM (lung), MutationTaster, SIFT	91.87% (87.88%-95.96%)	96.23% (92.19%-100%)	84.37% (74.98%-93.55%)	91.35% (85.25%-96.55%)	92.9% (85.7%-100%)	3.6485 (3.4633-3.8216)
		At least 2 of CHASM (breast), CHASM (lung), Mutation Assessor, MutationTaster	91.86% (87.85%-95.96%)	94.6% (89.83%-98.51%)	87.15% (78.12%-94.59%)	92.66% (86.66%-96.97%)	90.43% (82.05%-97.37%)	3.6484 (3.4479-3.8254)
		At least 1 of CHASM (breast), CHASM (lung), MutationTaster	91.44% (86.87%-94.95%)	99.42% (98.21%-100%)	77.75% (65.85%-87.5%)	88.45% (81.82%-93.85%)	98.75% (95.83%-100%)	3.6436 (3.4653-3.7927)
		At least 1 of CHASM (lung), MutationTaster	91.44% (86.87%-94.95%)	99.42% (98.21%-100%)	77.75% (65.85%-87.5%)	88.45% (81.82%-93.85%)	98.75% (95.83%-100%)	3.6436 (3.4653-3.7927)
		MutationTaster [best single predictor]	84.51% (80.13%-88.55%)	84.04% (78.53%-89.25%)	85.32% (78.18%-91.75%)	90.8% (86.05%-95%)	75.61% (67.42%-82.88%)	3.3578 (3.1879-3.5244)
		CanDrA (lung) [best meta-predictor]	84.7% (80.5%-88.65%)	79.12% (73.21%-84.57%)	94.95% (89.81%-98.94%)	96.64% (93.29%-99.31%)	71.21% (63.41%-78.3%)	3.4193 (3.2675-3.5628)
	By composite score	At least 1 of CHASM (breast), MutationTaster	93.11% (88.89%-96.97%)	99.42% (98.21%-100%)	82.29% (72.21%-91.67%)	90.59% (84.38%-95.65%)	98.81% (96%-100%)	3.7111 (3.5515-3.8634)
		At least 2 of CHASM (breast), CHASM (lung), MutationTaster, SIFT	91.87% (87.88%-95.96%)	96.23% (92.19%-100%)	84.37% (74.98%-93.55%)	91.35% (85.25%-96.55%)	92.9% (85.7%-100%)	3.6485 (3.4633-3.8216)
		At least 2 of CHASM (breast), CHASM (lung), Mutation Assessor, MutationTaster	91.86% (87.85%-95.96%)	94.6% (89.83%-98.51%)	87.15% (78.12%-94.59%)	92.66% (86.66%-96.97%)	90.43% (82.05%-97.37%)	3.6484 (3.4479-3.8254)
		At least 1 of CHASM (breast), CHASM (lung), MutationTaster	91.44% (86.87%-94.95%)	99.42% (98.21%-100%)	77.75% (65.85%-87.5%)	88.45% (81.82%-93.85%)	98.75% (95.83%-100%)	3.6436 (3.4653-3.7927)
		At least 1 of CHASM (lung), MutationTaster	91.44% (86.87%-94.95%)	99.42% (98.21%-100%)	77.75% (65.85%-87.5%)	88.45% (81.82%-93.85%)	98.75% (95.83%-100%)	3.6436 (3.4653-3.7927)
		MutationTaster [best single predictor]	84.51% (80.13%-88.55%)	84.04% (78.53%-89.25%)	85.32% (78.18%-91.75%)	90.8% (86.05%-95%)	75.61% (67.42%-82.88%)	3.3578 (3.1879-3.5244)
		CanDrA (lung) [best meta-predictor]	84.7% (80.5%-88.65%)	79.12% (73.21%-84.57%)	94.95% (89.81%-98.94%)	96.64% (93.29%-99.31%)	71.21% (63.41%-78.3%)	3.4193 (3.2675-3.5628)

NPV, negative predictive value; PPV, positive predictive value.
