# Supplementary material for: Benchmarking mutation effect prediction algorithms using functionally validated cancer-related missense mutations
Source: Genome Biol. 2014 Oct 28;15(10):484. doi: 10.1186/s13059-014-0484-1 (PMC4232638; doi:10.1186/s13059-014-0484-1)

Additional file 23

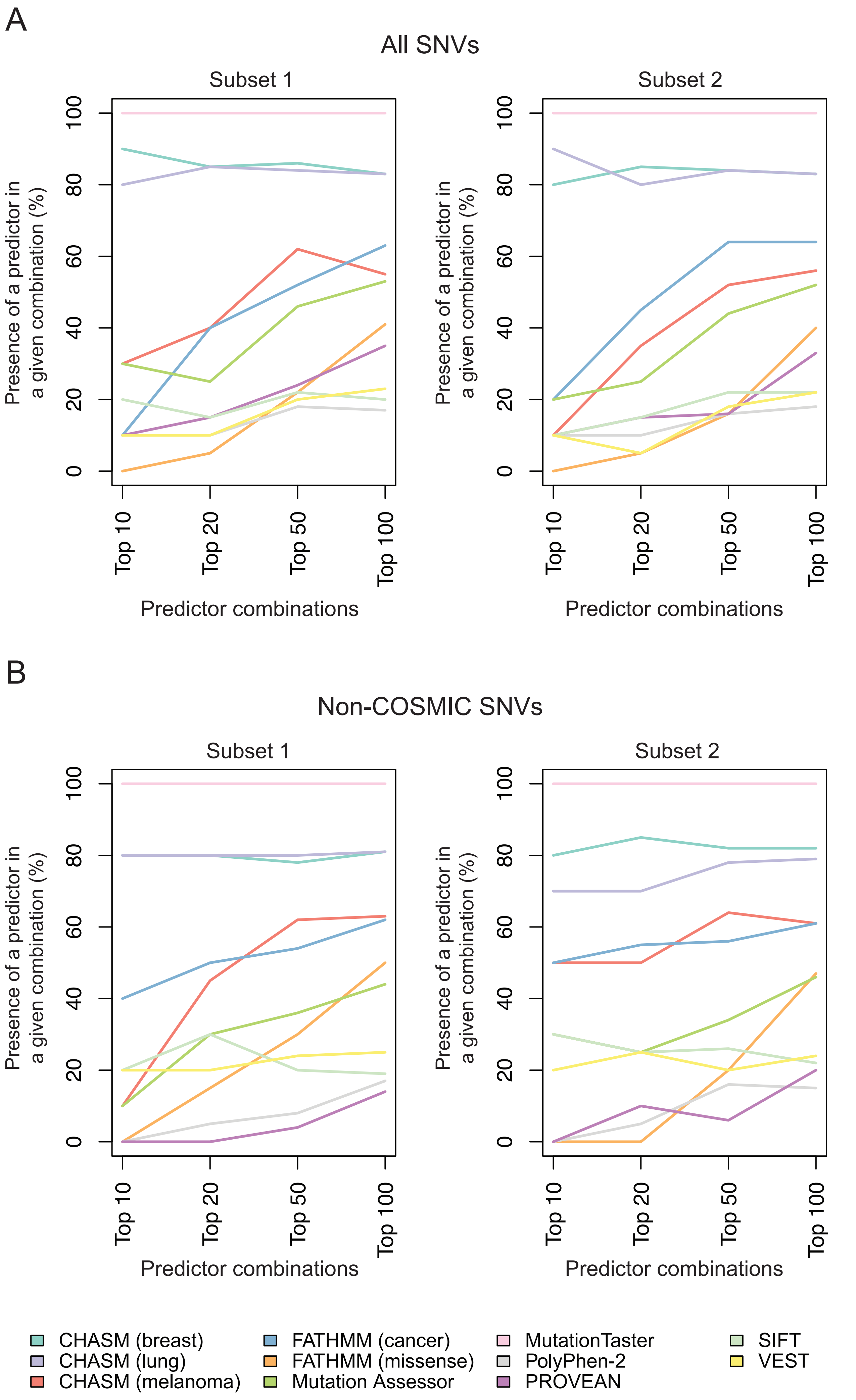

Supplement: Additional file 23: — Recurrence of individual mutation effect prediction algorithms in top performing mutation effect prediction algorithm combinations ranked by accuracy. The top 10, top 20, top 50, and top 100 combinations of mutation effect prediction algorithms were defined using the non-neutral (n = 849) and neutral (n = 140) single nucleotide variants (SNVs) included the entire dataset and ranked according to accuracy. The frequency of each single mutation effect predictor present in these top combinations was determined in subset 1 and subset 2 (A). The top 10, top 20, top 50, and top 100 combinations of mutation effect prediction algorithms were defined using the non-neutral (n = 188) and neutral (n = 109) SNVs not present in the COSMIC database and ranked according to accuracy. The frequency of each single mutation effect predictor present in these top combinations was determined in subset 1 and subset 2 (B). [file 13059_2014_484_MOESM23_ESM.pdf]
