# Supplementary material for: Benchmarking mutation effect prediction algorithms using functionally validated cancer-related missense mutations
Source: Genome Biol. 2014 Oct 28;15(10):484. doi: 10.1186/s13059-014-0484-1 (PMC4232638; doi:10.1186/s13059-014-0484-1)
Supplement: Additional file 25: — Optimal p and n using all 989 functionally defined non-neutral or neutral single nucleotide variants included in this dataset. [file 13059_2014_484_MOESM25_ESM.pdf]

Additional file 25: Optimal *p* and *n* using all 989 functionally defined non-neutral or neutral single nucleotide variants included in this dataset.

A	*n*	*p*	Mutation effect prediction algorithms	Accuracy (subset 1)	Sensitivity (subset 1)	Specificity (subset 1)	PPV (subset 1)
Subset 1	1	2	At least 1 of CHASM (breast), MutationTaster	95.46% (94.54%-96.51%)	99.88% (99.82%-100%)	68.61% (63.09%-74.73%)	95.07% (94.11%-96.13%)
	2	4	At least 2 of CHASM (breast), CHASM (lung), MutationTaster, SIFT	95.16% (94.23%-96.21%)	99.07% (98.59%-99.48%)	71.47% (66%-77%)	95.47% (94.54%-96.48%)
	3	6	At least 3 of CHASM (breast), CHASM (lung), CHASM (melanoma), Mutation Assessor, MutationTaster, PROVEAN	94.67% (93.78%-95.75%)	97.9% (97.31%-98.58%)	75.07% (70.1%-80.41%)	95.97% (95.08%-97.04%)
	4	7	At least 4 of CHASM (breast), CHASM (lung), CHASM (melanoma), FATHMM (cancer), Mutation Assessor, MutationTaster, SIFT	94.36% (93.32%-95.45%)	97.66% (97%-98.41%)	74.37% (69.05%-79.8%)	95.85% (94.97%-96.88%)
	5	9	At least 5 of CHASM (breast), CHASM (lung), CHASM (melanoma), FATHMM (cancer), FATHMM (missense), Mutation Assessor, MutationTaster, SIFT, VEST	94.16% (93.17%-95.3%)	97.78% (97.15%-98.42%)	72.22% (67.03%-77.66%)	95.52% (94.61%-96.56%)
	6	10	At least 6 of CHASM (breast), CHASM (lung), CHASM (melanoma), FATHMM (cancer), FATHMM (missense), Mutation Assessor, MutationTaster, PolyPhen-2, SIFT, VEST	91.93% (90.74%-93.17%)	94.49% (93.45%-95.45%)	76.46% (71.58%-81.32%)	96.05% (95.14%-96.98%)
	7	11	At least 7 of CHASM (breast), CHASM (lung), CHASM (melanoma), FATHMM (cancer), FATHMM (missense), Mutation Assessor, MutationTaster, PolyPhen-2, PROVEAN, SIFT, VEST	88.58% (87.25%-89.98%)	89.75% (88.41%-91.27%)	81.44% (77.08%-86.17%)	96.7% (95.9%-97.56%)
	8	11	At least 8 of CHASM (breast), CHASM (lung), CHASM (melanoma), FATHMM (cancer), FATHMM (missense), Mutation Assessor, MutationTaster, PolyPhen-2, PROVEAN, SIFT, VEST	83.35% (81.64%-85.13%)	83.18% (81.45%-84.93%)	84.36% (80.41%-89%)	96.99% (96.11%-97.93%)
	9	11	At least 9 of CHASM (breast), CHASM (lung), CHASM (melanoma), FATHMM (cancer), FATHMM (missense), Mutation Assessor, MutationTaster, PolyPhen-2, PROVEAN, SIFT, VEST	78.7% (76.78%-80.58%)	76.83% (74.82%-78.97%)	90.07% (86.67%-93.62%)	97.91% (97.23%-98.66%)
	10	11	At least 10 of CHASM (breast), CHASM (lung), CHASM (melanoma), FATHMM (cancer), FATHMM (missense), Mutation Assessor, MutationTaster, PolyPhen-2, PROVEAN, SIFT, VEST	69.88% (67.83%-71.78%)	66.21% (64.12%-68.32%)	92.16% (89.01%-95.6%)	98.09% (97.33%-98.94%)
	11	11	At least 11 of CHASM (breast), CHASM (lung), CHASM (melanoma), FATHMM (cancer), FATHMM (missense), Mutation Assessor, MutationTaster, PolyPhen-2, PROVEAN, SIFT, VEST	54.46% (52.35%-56.6%)	47.77% (45.36%-50.26%)	95.03% (92.71%-97.8%)	98.31% (97.45%-99.26%)

B	*n*	*p*	Mutation effect prediction algorithms	Accuracy (subset 1)	Sensitivity (subset 1)	Specificity (subset 1)	PPV (subset 1)
Subset 1	1	2	At least 1 of CHASM (breast), MutationTaster	95.46% (94.54%-96.51%)	99.88% (99.82%-100%)	68.61% (63.09%-74.73%)	95.07% (94.11%-96.13%)
	1	3	At least 1 of CHASM (breast), CHASM (lung), MutationTaster	94.95% (93.93%-96.05%)	99.88% (99.82%-100%)	64.99% (59.3%-70.84%)	94.54% (93.49%-95.67%)
	2	4	At least 2 of CHASM (breast), CHASM (lung), MutationTaster, SIFT	95.16% (94.23%-96.21%)	99.07% (98.59%-99.48%)	71.47% (66%-77%)	95.47% (94.54%-96.48%)
	2	5	At least 2 of CHASM (breast), CHASM (lung), CHASM (melanoma), MutationTaster, PROVEAN	94.47% (93.47%-95.6%)	98.37% (97.86%-98.95%)	70.79% (65.26%-76.35%)	95.33% (94.35%-96.39%)
	3	6	At least 3 of CHASM (breast), CHASM (lung), CHASM (melanoma), Mutation Assessor, MutationTaster, PROVEAN	94.67% (93.78%-95.75%)	97.9% (97.31%-98.58%)	75.07% (70.1%-80.41%)	95.97% (95.08%-97.04%)
	4	7	At least 4 of CHASM (breast), CHASM (lung), CHASM (melanoma), FATHMM (cancer), Mutation Assessor, MutationTaster, SIFT	94.36% (93.32%-95.45%)	97.66% (97%-98.41%)	74.37% (69.05%-79.8%)	95.85% (94.97%-96.88%)
	4	8	At least 4 of CHASM (breast), CHASM (lung), CHASM (melanoma), FATHMM (missense), Mutation Assessor, MutationTaster, PROVEAN, VEST	94.17% (93.17%-95.3%)	98.14% (97.52%-98.77%)	70.07% (64.58%-75.82%)	95.21% (94.22%-96.25%)
	5	9	At least 5 of CHASM (breast), CHASM (lung), CHASM (melanoma), FATHMM (cancer), FATHMM (missense), Mutation Assessor, MutationTaster, SIFT, VEST	94.16% (93.17%-95.3%)	97.78% (97.15%-98.42%)	72.22% (67.03%-77.66%)	95.52% (94.61%-96.56%)
	5	10	At least 5 of CHASM (breast), CHASM (lung), CHASM (melanoma), FATHMM (cancer), FATHMM (missense), Mutation Assessor, MutationTaster, PolyPhen-2, PROVEAN, SIFT	93.46% (92.41%-94.69%)	98.26% (97.69%-98.78%)	64.39% (58.62%-70.1%)	94.36% (93.28%-95.45%)
	5	11	At least 5 of CHASM (breast), CHASM (lung), CHASM (melanoma), FATHMM (cancer), FATHMM (missense), Mutation Assessor, MutationTaster, PolyPhen-2, PROVEAN, SIFT, VEST	92.44% (91.2%-93.63%)	98.49% (97.9%-99.1%)	55.73% (50%-61.7%)	93.1% (91.91%-94.3%)

NPV, negative predictive value; PPV, positive predictive value.
