# Supplementary material for: Benchmarking mutation effect prediction algorithms using functionally validated cancer-related missense mutations
Source: Genome Biol. 2014 Oct 28;15(10):484. doi: 10.1186/s13059-014-0484-1 (PMC4232638; doi:10.1186/s13059-014-0484-1)
Supplement: Additional file 27: — Optimal p and n using all 297 functionally defined non-neutral or neutral single nucleotide variants not included in the COSMIC database. [file 13059_2014_484_MOESM27_ESM.pdf]

Additional file 27: Optimal *p* and *n* using all 297 functionally defined non-neutral or neutral single nucleotide variants not included in the COSMIC database.

A	*n*	*p*	Mutation effect predictor algorithm combination	Accuracy (subset 1)	Sensitivity (subset 1)	Specificity (subset 1)	PPV (subset 1)	NPV (subset 1)
Subset 1	1	2	At least 1 of CHASM (breast), MutationTaster	93.34% (91.41%-95.45%)	99.49% (99.16%-100%)	82.72% (78.08%-88%)	90.87% (88.32%-93.99%)	98.95% (98.15%-100%)
	2	4	At least 2 of CHASM (breast), CHASM (lung), Mutation Assessor, MutationTaster	91.95% (89.9%-93.95%)	94.71% (92.56%-96.9%)	87.16% (83.33%-91.89%)	92.73% (90.48%-95.49%)	90.52% (86.76%-94.44%)
	3	5	At least 3 of CHASM (breast), CHASM (lung), FATHMM (missense), Mutation Assessor, MutationTaster	90.64% (88.38%-92.93%)	91.04% (88.28%-94.22%)	89.94% (86.3%-94.03%)	93.99% (92%-96.67%)	85.32% (81.01%-90.14%)
	4	7	At least 4 of CHASM (breast), CHASM (lung), CHASM (melanoma), FATHMM (cancer), MutationTaster, SIFT, VEST	91.3% (89.39%-93.43%)	94.2% (91.87%-96.75%)	86.27% (81.94%-91.18%)	92.23% (89.92%-95.12%)	89.59% (85.71%-94.03%)
	5	9	At least 5 of CHASM (breast), CHASM (lung), CHASM (melanoma), FATHMM (cancer), FATHMM (missense), Mutation Assessor, MutationTaster, SIFT, VEST	90.27% (88.37%-92.93%)	94.2% (92%-96.72%)	83.47% (78.87%-88.73%)	90.79% (88.28%-93.85%)	89.28% (85.33%-93.65%)
	6	10	At least 6 of CHASM (breast), CHASM (lung), CHASM (melanoma), FATHMM (cancer), FATHMM (missense), Mutation Assessor, MutationTaster, PolyPhen-2, SIFT, VEST	86.85% (84.34%-89.39%)	87.75% (84.68%-91.13%)	85.3% (80.88%-90.36%)	91.17% (88.43%-94.22%)	80.11% (75%-85.19%)
	7	11	At least 7 of CHASM (breast), CHASM (lung), CHASM (melanoma), FATHMM (cancer), FATHMM (missense), Mutation Assessor, MutationTaster, PolyPhen-2, PROVEAN, SIFT, VEST	82.13% (79.29%-84.86%)	78.69% (74.4%-82.81%)	88.08% (84%-92.76%)	91.95% (89.19%-95.15%)	70.51% (65.47%-75.82%)
	8	11	At least 8 of CHASM (breast), CHASM (lung), CHASM (melanoma), FATHMM (cancer), FATHMM (missense), Mutation Assessor, MutationTaster, PolyPhen-2, PROVEAN, SIFT, VEST	77.45% (74.24%-80.81%)	69.7% (65.08%-74.22%)	90.84% (87.32%-94.67%)	92.94% (90.22%-95.92%)	63.43% (58.16%-68.63%)
	9	11	At least 9 of CHASM (breast), CHASM (lung), CHASM (melanoma), FATHMM (cancer), FATHMM (missense), Mutation Assessor, MutationTaster, PolyPhen-2, PROVEAN, SIFT, VEST	71.4% (67.68%-74.75%)	57.51% (52.34%-62.79%)	95.41% (92.96%-98.57%)	95.59% (93.24%-98.67%)	56.5% (51.54%-61.29%)
	10	11	At least 10 of CHASM (breast), CHASM (lung), CHASM (melanoma), FATHMM (cancer), FATHMM (missense), Mutation Assessor, MutationTaster, PolyPhen-2, PROVEAN, SIFT, VEST	61.99% (58.08%-65.66%)	41.61% (36.58%-46.77%)	97.23% (95.59%-100%)	96.3% (94%-100%)	49.06% (44.3%-53.43%)
	11	11	At least 11 of CHASM (breast), CHASM (lung), CHASM (melanoma), FATHMM (cancer), FATHMM (missense), Mutation Assessor, MutationTaster, PolyPhen-2, PROVEAN, SIFT, VEST	52.89% (48.48%-56.58%)	26.17% (21.21%-30.47%)	99.08% (98.48%-100%)	98.02% (96.43%-100%)	43.7% (39.13%-47.88%)

B	*n*	*p*	Mutation effect predictor algorithm combination	Accuracy (subset 1)	Sensitivity (subset 1)	Specificity (subset 1)	PPV (subset 1)	NPV (subset 1)
Subset 1	1	2	At least 1 of CHASM (breast), MutationTaster	93.34% (91.41%-95.45%)	99.49% (99.16%-100%)	82.72% (78.08%-88%)	90.87% (88.32%-93.99%)	98.95% (98.15%-100%)
	1	3	At least 1 of CHASM (breast), CHASM (lung), MutationTaster	91.66% (89.9%-93.94%)	99.49% (99.16%-100%)	78.11% (73.13%-83.78%)	88.71% (86.03%-91.85%)	98.89% (98.04%-100%)
	2	4	At least 2 of CHASM (breast), CHASM (lung), Mutation Assessor, MutationTaster	91.95% (89.9%-93.95%)	94.71% (92.56%-96.9%)	87.16% (83.33%-91.89%)	92.73% (90.48%-95.49%)	90.52% (86.76%-94.44%)
	3	5	At least 3 of CHASM (breast), CHASM (lung), FATHMM (missense), Mutation Assessor, MutationTaster	90.64% (88.38%-92.93%)	91.04% (88.28%-94.22%)	89.94% (86.3%-94.03%)	93.99% (92%-96.67%)	85.32% (81.01%-90.14%)
	3	6	At least 3 of CHASM (breast), CHASM (lung), CHASM (melanoma), FATHMM (cancer), MutationTaster, SIFT	90.62% (88.38%-92.93%)	94.2% (91.87%-96.75%)	84.42% (79.73%-89.33%)	91.27% (88.8%-94.16%)	89.39% (85.51%-93.85%)
	4	7	At least 4 of CHASM (breast), CHASM (lung), CHASM (melanoma), FATHMM (cancer), MutationTaster, SIFT, VEST	91.3% (89.39%-93.43%)	94.2% (91.87%-96.75%)	86.27% (81.94%-91.18%)	92.23% (89.92%-95.12%)	89.59% (85.71%-94.03%)
	5	8	At least 5 of CHASM (breast), CHASM (lung), CHASM (melanoma), FATHMM (cancer), FATHMM (missense), Mutation Assessor, MutationTaster, SIFT	89.61% (87.37%-91.92%)	90.49% (87.5%-93.55%)	88.09% (84.29%-92.54%)	92.93% (90.75%-95.73%)	84.27% (79.73%-89.04%)
	5	9	At least 5 of CHASM (breast), CHASM (lung), CHASM (melanoma), FATHMM (cancer), FATHMM (missense), Mutation Assessor, MutationTaster, SIFT, VEST	90.27% (88.37%-92.93%)	94.2% (92%-96.72%)	83.47% (78.87%-88.73%)	90.79% (88.28%-93.85%)	89.28% (85.33%-93.65%)
	5	10	At least 5 of CHASM (breast), CHASM (lung), CHASM (melanoma), FATHMM (cancer), FATHMM (missense), Mutation Assessor, MutationTaster, PolyPhen-2, PROVEAN, SIFT	87.22% (84.85%-89.9%)	94.74% (92.68%-96.85%)	74.2% (68.57%-80.01%)	86.4% (83.21%-89.71%)	89.08% (84.9%-93.55%)
	5	11	At least 5 of CHASM (breast), CHASM (lung), CHASM (melanoma), FATHMM (cancer), FATHMM (missense), Mutation Assessor, MutationTaster, PolyPhen-2, PROVEAN, SIFT, VEST	83.88% (80.81%-86.87%)	95.82% (94.02%-97.69%)	63.26% (57.14%-69.02%)	81.84% (78.32%-85.43%)	89.74% (85.41%-94.44%)
