# Supplementary material for: Benchmarking mutation effect prediction algorithms using functionally validated cancer-related missense mutations
Source: Genome Biol. 2014 Oct 28;15(10):484. doi: 10.1186/s13059-014-0484-1 (PMC4232638; doi:10.1186/s13059-014-0484-1)
Supplement: Additional file 28: — Residual single nucleotide variants in the set of 989 functionally defined non-neutral and neutral single nucleotide variants and in the subset of 297 single nucleotide variants not present in the COSMIC database. [file 13059_2014_484_MOESM28_ESM.pdf]

Additional file 28: Residual single nucleotide mutations in the set of 989 functionally defined non-neutral and neutral single nucleotide variants and in the subset of 297 single nucleotide variants not present in the COSMIC database.

Mutation prediction algorithm	Mutations from the training set of mutation function prediction algorithm included in the dataset of 989 non-neutral or neutral single nucleotide variants (n)	Mutations from the training set of mutation function prediction algorithm included in the dataset of 989 non-neutral or neutral single nucleotide variants after exclusion of COSMIC single nucleotide mutations (n)
**CHASM (breast)**	570	51
**CHASM (lung)**	570	51
**CHASM (melanoma)**	570	51
**FATHMM (cancer)**	682	68
**FATHMM (missense)**	158	48
**PolyPhen-2**	14	4
