# Supplementary material for: Benchmarking mutation effect prediction algorithms using functionally validated cancer-related missense mutations
Source: Genome Biol. 2014 Oct 28;15(10):484. doi: 10.1186/s13059-014-0484-1 (PMC4232638; doi:10.1186/s13059-014-0484-1)

Additional file 4

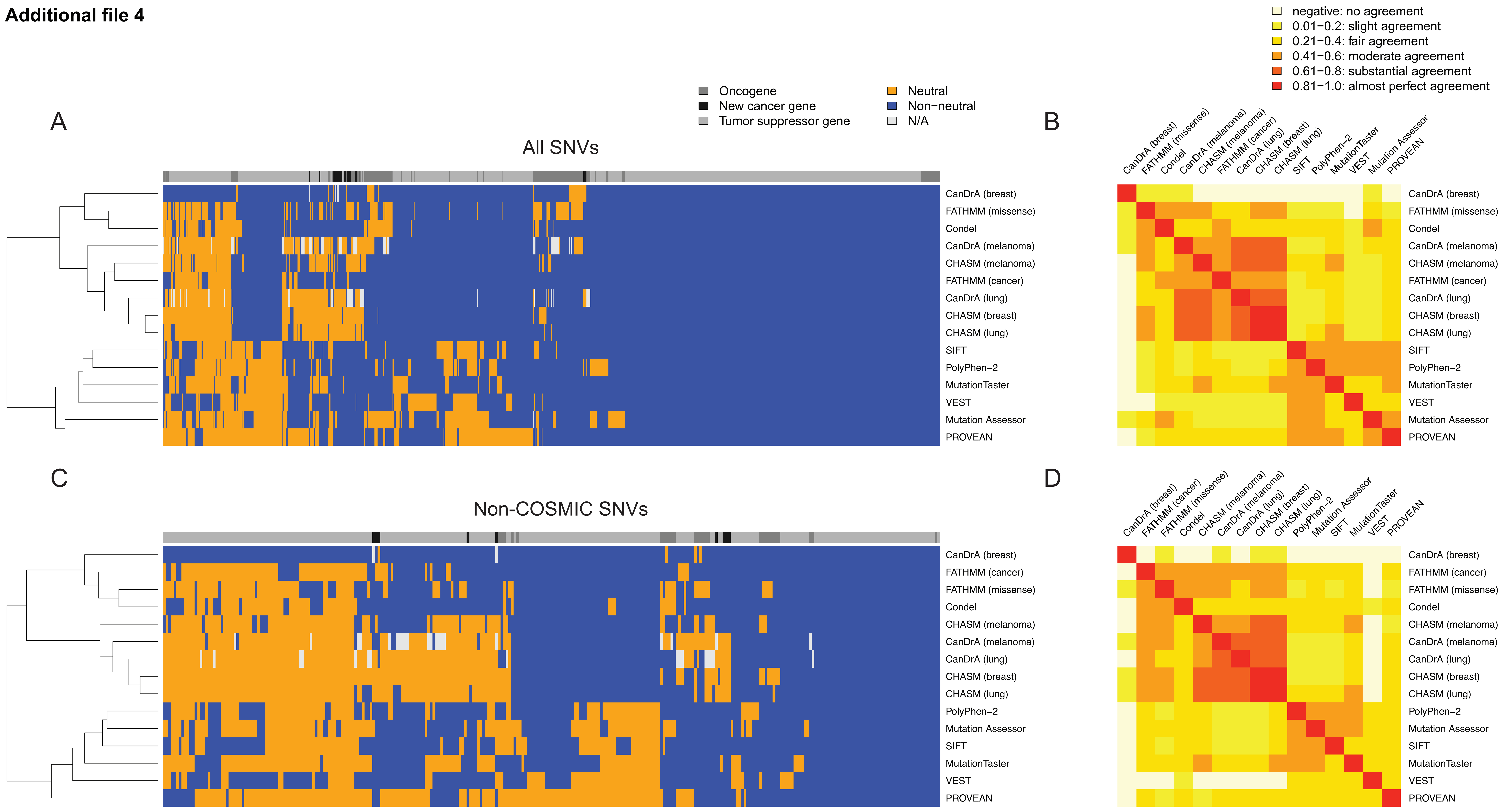

Supplement: Additional file 4: — Inter-rater agreement between 15 mutation effect prediction algorithms for the single nucleotide variants for which functional data are available. Hierarchical clustering of the calls made by 15 mutation effect prediction algorithms using (A) all 989 single nucleotide variants (SNVs) for which functional data are available, and (C) the subset of 297 SNVs not present in the COSMIC database. The unweighted Cohen’s Kappa coefficient was computed for each pair of predictors using (B) all 989 SNVs and (D) the subset of 297 SNVs not present in the COSMIC database. The ranges of unweighted Kappa values and their corresponding colors are indicated in the color key. [file 13059_2014_484_MOESM4_ESM.pdf]
